# Transcriptomic and proteomic signatures following AS03-adjuvanted Influenza A/H7N9 vaccine

**DOI:** 10.3389/fimmu.2026.1786231

**Published:** 2026-08-07

**Authors:** C. Buddy Creech, Kristen J. Steenbergen, Casey E. Gelber, Leigh M. Howard, Kevin L. Schey, Kristie Lindsey Rose, Sandra M. Yoder, Kathryn M. Edwards, Johannes B. Goll

**Affiliations:** 1 Vanderbilt Vaccine Research Program, Department of Pediatrics, Vanderbilt University Medical Center, Nashville, TN, United States; 2 Department of Biomedical Data Science and Bioinformatics, The Emmes Company, LLC, Rockville, MD, United States; 3 Department of Biochemistry, Vanderbilt University, Nashville, TN, United States

**Keywords:** adjuvants, AS03, avian influenza, H7N9, influenza, influenza vaccine, proteomics, systems biology

## Abstract

**Introduction:**

Vaccines targeting avian influenza virus A/H7N9 are poorly immunogenic. While the immune responses can be improved with oil-in-water emulsion adjuvants such as Adjuvant System 03 (AS03), the cellular mechanisms underpinning the adjuvant effect are incompletely characterized and poorly understood.

**Methods:**

We enrolled 30 healthy adult participants and used RNA sequencing and quantitative proteomics to characterize the response to two doses of the influenza A/H7N9 vaccine, with and without AS03, in six immune cell types. These responses were compared to those seen after administration of an unadjuvanted seasonal in uenza A/H3N2 variant vaccine to identify signatures unique to adjuvanted influenza vaccines and correlated with later antibody responses. Transcriptomic and proteomic analyses revealed that.

**Results:**

AS03-adjuvanted vaccine was associated with upregulation of immune pathways in innate immune cells within 24h following vaccination for phagocytosis, antigen presentation and processing, inflammasome activation, NK-cell mediated cytotoxicity, IgA production, and interferon-response pathways. Moreover, while major histocompatibility complex (MHC I and II) upregulation was observed across multiple immune cell types, MHCII gene transcription was also increased in the neutrophil compartment, generating the hypothesis that neutrophils may play a more important role in antigen presentation than previously understood.

**Discussion:**

Taken together, these data provide a more complete mechanistic understanding of oil-in-water adjuvants and their role in enhancing the immune response for pandemic influenza preparedness.

**Clinical Trial Registration:**

https://clinicaltrials.gov/study/NCT02921997?term=NCT02921997&viewType, idientifier NCT02921997.

## Introduction

Since its emergence in Southeast Asia in 2013, influenza A/H7N9 continues to cause sporadic infections in humans and is associated with a high-case fatality rate (1). The virus holds significant pandemic potential since the avian population is rarely symptomatic from A/H7N9 infection (making avian disease surveillance difficult), and there is little preexisting immunity to the virus in the human population (2); however, its current inability to sustain efficient human-to-human transmission has limited its spread (3).

Unfortunately, as with other avian influenza strains, the immunogenicity of the inactivated influenza A/H7N9 vaccine is poor. Higher doses of hemagglutinin (HA), a major target of the protective immune response, generate higher antibody titers (4); however, clinical trials of influenza A/H5N1, A/H7N7, and A/H7N9 vaccines have demonstrated that avian HAs are substantially less immunogenic than seasonal influenza vaccines, even when administered in very large dosages (5–7).

The use of adjuvants is an alternative to increasing HA content or administering additional doses of vaccine. Adjuvants increase both the cellular and humoral responses at a given dose of antigen, decrease the amount of antigen needed in the vaccine, and typically improve immunogenicity among those that generally respond poorly to influenza antigens (e.g., the elderly or immunocompromised) (8–11). While the addition of adjuvants has sometimes been associated with increased injection site reactogenicity and occasionally systemic reactogenicity, the overall safety of novel oil-in-water adjuvants such as MF59D and AS03 is reassuring (12–16).

The mechanisms of action by which oil-in-water emulsion adjuvants enhance the immune response to vaccination are not completely understood (17, 18). Murine and recent human data suggest that AS03 works locally to stimulate immune effectors (19) and induces the local production of monocyte-, dendritic cell (DC)–, and neutrophil-recruiting chemokines, as well as the pro-inflammatory cytokines interleukin (IL)–6, IL-1β, IL-1α, tumor necrosis factor (TNF), and interferon (IFN)–gamma (20). AS03 also appears to promote recruitment of antigen-loaded monocytes to draining lymph nodes (19). AS03 is unique among adjuvants in that it includes α-tocopherol, a highly bioavailable form of Vitamin E. The presence of α-tocopherol appears to be necessary to induce local cytokine responses and stimulate antigen-specific adaptive responses, but a more precise understanding of the mechanism of action in humans is needed. Elucidating the mechanisms by which AS03 augments the immune response to influenza vaccine in humans is important not only in developing more efficient vaccine regimens but also in determining the safety and the potential for adverse events after adjuvanted vaccine. Toward this goal, we assessed differences in gene expression and protein abundance in immune cells and/or bulk PBMCs and metabolites in serum and urine following H5N1 vaccination with and without AS03 adjuvant in a previous study (21–24).

Here, we extend our prior systems biology approach to assess early gene and protein signatures expressed in response to influenza A/H7N9 and influenza A/H3N2 variant vaccines. Transcriptomics and proteomics data were generated for six major immune cell compartments (monocytes, dendritic, neutrophil, NK, B, and T cells) to characterize differential responses unique to each vaccine. Results were correlated with serologic responses (hemagglutination inhibition [HAI] and neutralizing [Neut] antibody assays) to understand which of these signals were associated with serum antibody responses. We integrated these results with cytokine data to develop a systems biology framework of the human immune response to these vaccines.

## Results

### Clinical trial characteristics, serologic responses, and cytokine profiles

**Figure 1:**
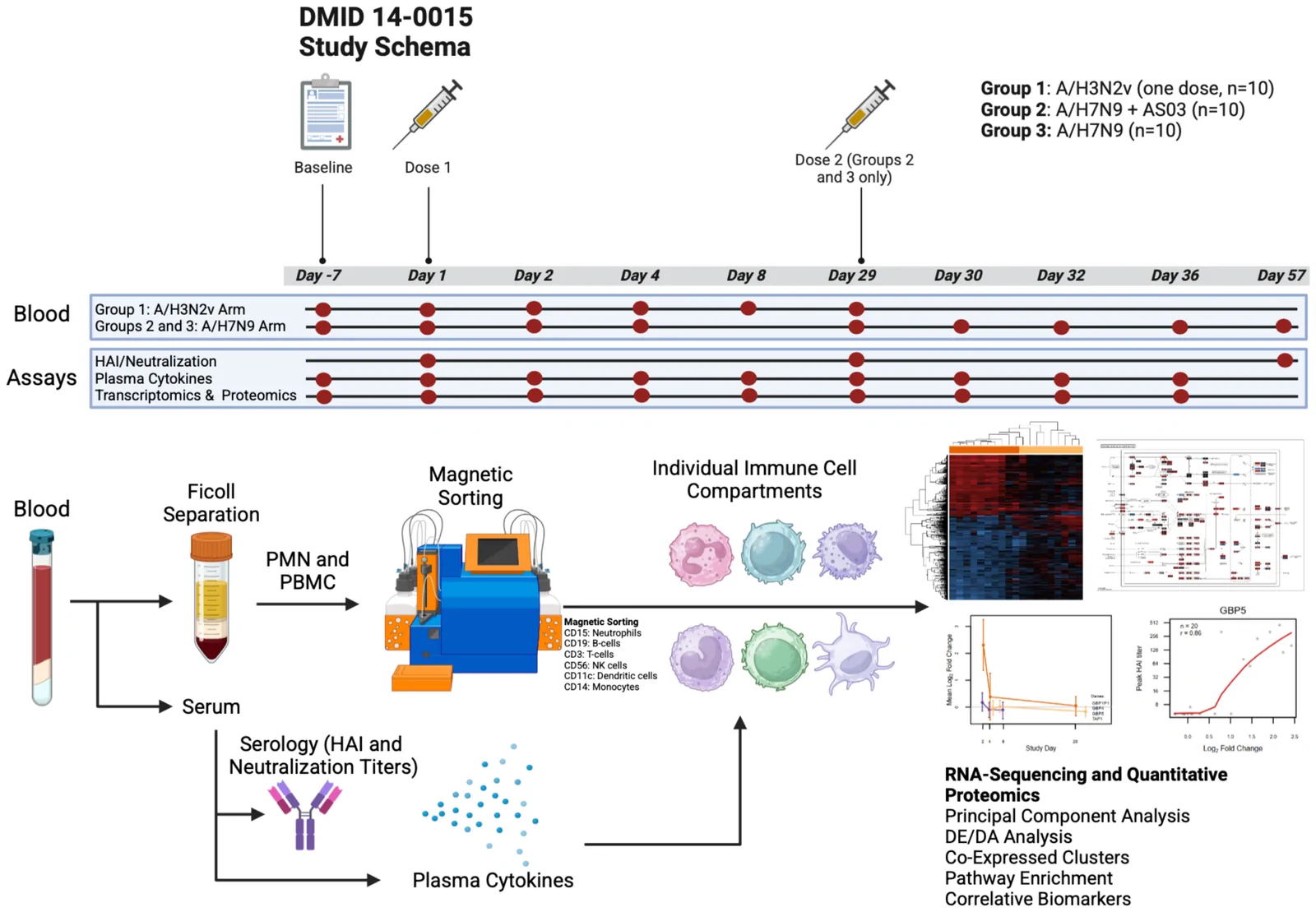
Study schema. Participants received either one dose of unadjuvanted influenza A/H3N2v or two doses (28 days apart) of influenza A/H7N9, with or without AS03 adjuvant. Days are labeled according to the CDISC standard with the day of first vaccination represented as Day 1 (24h after first vaccination as Day 2, 72h after first-vaccination as Day 4, etc.). HAI and Neut antibody assays were performed on samples collected prior to first vaccination and 28 days after each dose. Serum concentrations, immune-cell specific gene expression, and protein abundance was measured pre-vaccination for all groups at 1, 3, and 7 days after each vaccination.

An overview of the study design is provided in **Figure 1**. Thirty participants were enrolled, 19 females and 11 males. All participants were White and non-Hispanic with a median age of 28 years (**Supplementary Tables 1A**, **S1B**). Participants received either one dose of unadjuvanted influenza A/H3N2v (porcine strain of influenza) or two doses (28 days apart) of influenza A/H7N9, with or without AS03 adjuvant. HAI and Neut antibody assays were performed on samples collected prior to the first vaccination and 28 days after each dose. Serum cytokines were measured pre-vaccination for all groups and 1, 3, and 7 days after each vaccination. At the same time points, monocytes, dendritic, neutrophil, NK, B, and T cells were analyzed using RNA-seq and quantitative LC-MS/MS proteomics.

**Figure 2:**
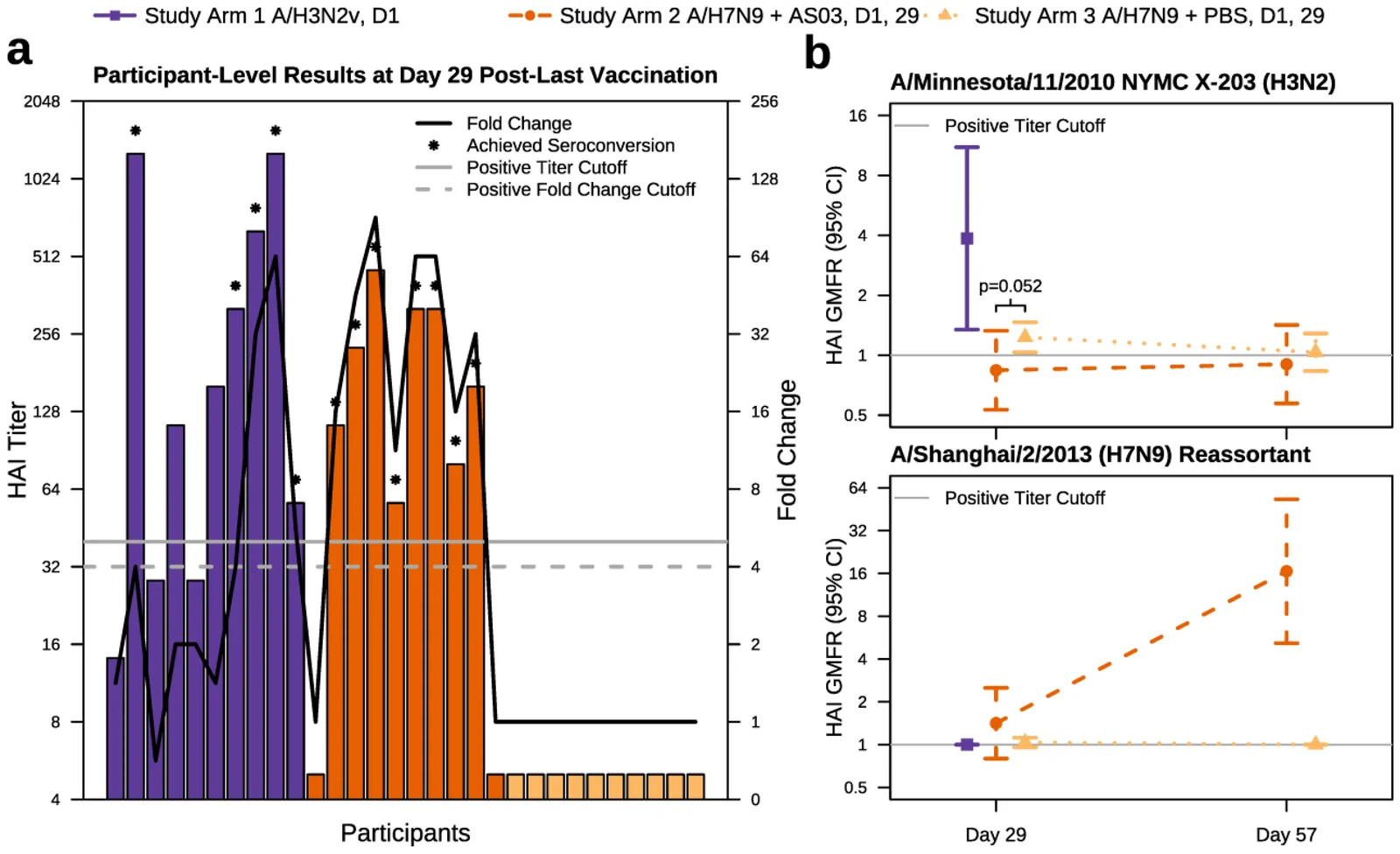
Hemagglutination Inhibition (HAI) results. **(A)** Barplot of participant-level HAI results at Day 29 post-last vaccination in the intent-to-treat population. Seroconversion is defined as a post-vaccination HAI titer ≥1:40 or ≥4-fold rise in post-vaccination HAI titer if baseline was ≥1:40. **(B)** HAI titer geometric mean fold rise (GMFR) and associated 95% CI by study arm and study day in the intent-to-treat population. Error bars indicate 95% confidence intervals based on 1000 bootstrap replicates. p: *p*-value of upper one-sided Welch’s two-sample t-test comparing adjuvanted vs. unadjuvanted H7N9 arm HAI fold changes at Day 29.

Seroconversion was defined as a postvaccination HAI titer ≥1:40 (or minimum fourfold rise in titer if baseline HAI titer ≥1:10). No participants who received two doses of unadjuvanted A/H7N9 vaccine achieved seroconversion based on HAI titer, compared to 5/10 (50%) of A/H3N2v recipients and 8/10 (80%) of A/H7N9 + AS03 recipients (**Figure 2A**). Corresponding neutralizing antibody results are shown in **Supplementary Figure 1**. Seroconversion based on neutralizing antibody titer was similarly defined; no recipients of the unadjuvanted A/H7N9 vaccine seroconverted, compared to 3/10 (30%) among A/H3N2v recipients and all 10 participants receiving the AS03-adjuvanted A/H7N9 vaccine (**Supplementary Figure 1A**).

To evaluate generation of within clade and cross-clade reactive antibodies, we compared HAI geometric mean titers (GMT) and geometric mean fold rises (GMFR) against A/H3N2v and A/H7N9 antigens between all groups (**Figure 2B**). Both homologous and heterologous immune responses were measured. As expected, for recipients of the A/H3N2 vaccine, in which seasonal immunization and repeat exposure likely provide substantial immunologic memory, HAI GMFRs were increased 3.9-fold from baseline one month after vaccination (Day 29). A 1.4-fold increase from baseline in A/H3N2-specific HAI was also observed for the non-adjuvanted A/H7N9 vaccine group. This increase was statistically significant with the lower 95% confidence interval (CI) bound exceeding a fold change of 1. Similar cross-reactivity was not seen for the adjuvanted A/H7N9 vaccine group. A direct comparison between antibody fold changes against A/H3N2 for the adjuvanted and unadjuvanted fold changes was marginally significant (*p* = 0.052, one-sided t-test). In contrast, the adjuvanted A/H7N9 group achieved an average HAI titer increase of 16.6-fold against the A/H7N9 antigen, whereas neither the unadjuvanted nor seasonal vaccines induced responses above baseline against the H7N9 vaccine antigen.

Next, we assessed concentrations of 24 cytokines in serum over time (**Figure 3**; **Supplementary Figures 2**–**S23**). Median changes in serum cytokine concentrations, overall, were strongest 24h after the second dose of adjuvanted A/H7N9 vaccine (**Figure 3A**). Highest median changes were seen for IP-10 (FC = 4.5), ITAC (FC = 2.3), IFN-γ (FC = 1.8), IL-6 (FC = 1.8), TNF (FC = 1.6), MIP1-β (FC = 1.6), and IFN-β (FC = 1.2), each with a lower 95% CI of the median exceeding a fold change of 1. In contrast, the seasonal A/H3N2v vaccine and unadjuvanted A/H7N9 groups did not show statistically significant changes in serum cytokine levels.

**Figure 3:**
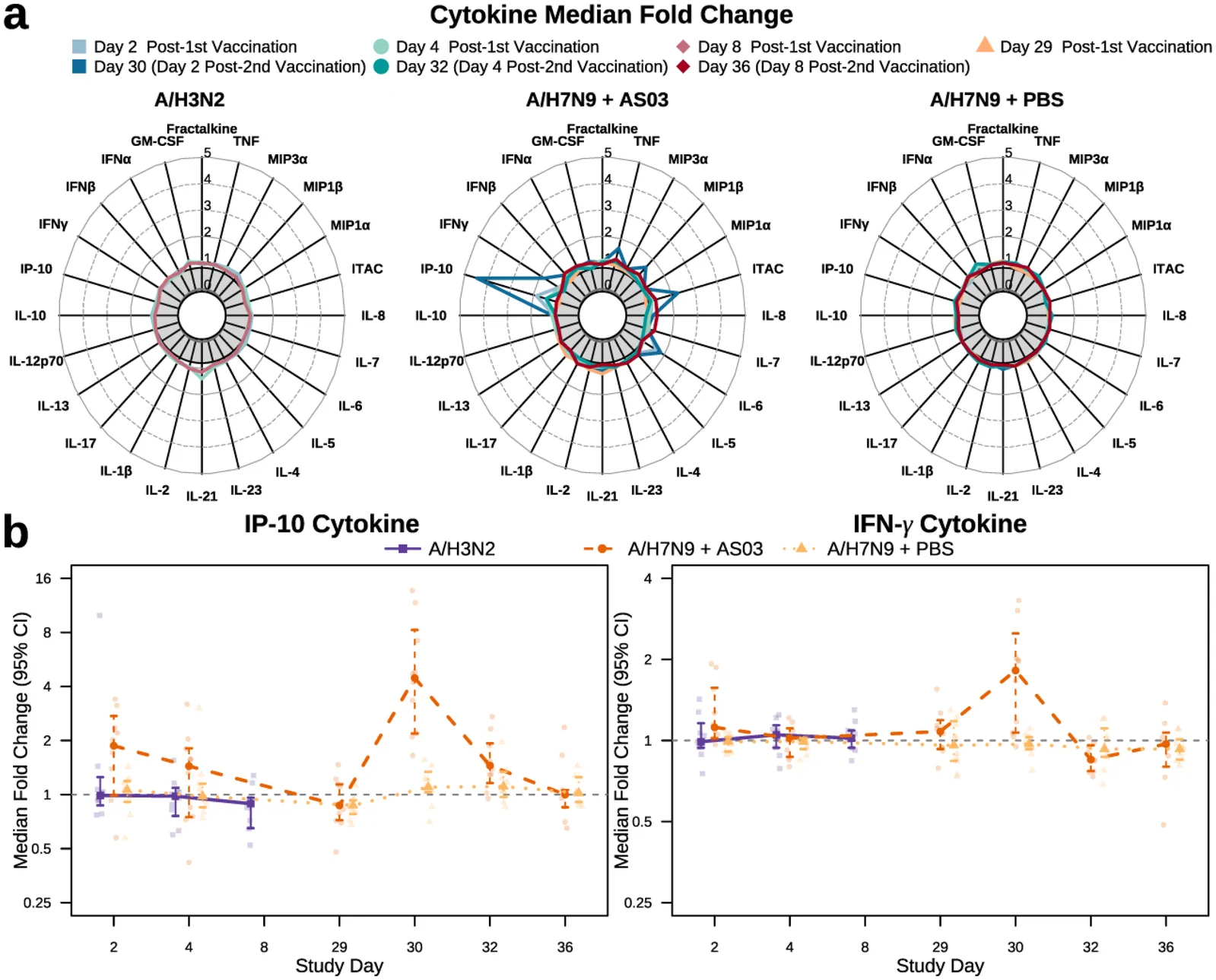
Inflammatory cytokine concentrations were increased in serum 24h after adjuvanted A/H7N9 vaccination. **(A)** Spider plot summarizing median fold change for 24 cytokine/chemokines by study arm and day in the intent-to-treat population. **(B)** Time trend of median fold change of IP-10 and IFN-g and associated 95% CI by study arm and post-vaccination day in the intent-to-treat population. Error bars indicate 95% confidence intervals based on 1000 bootstrap replicates.

### Adjuvanted vaccination induced changes in gene expression and protein abundance in innate immune cells 24h after adjuvanted vaccination

**Figure 4:**
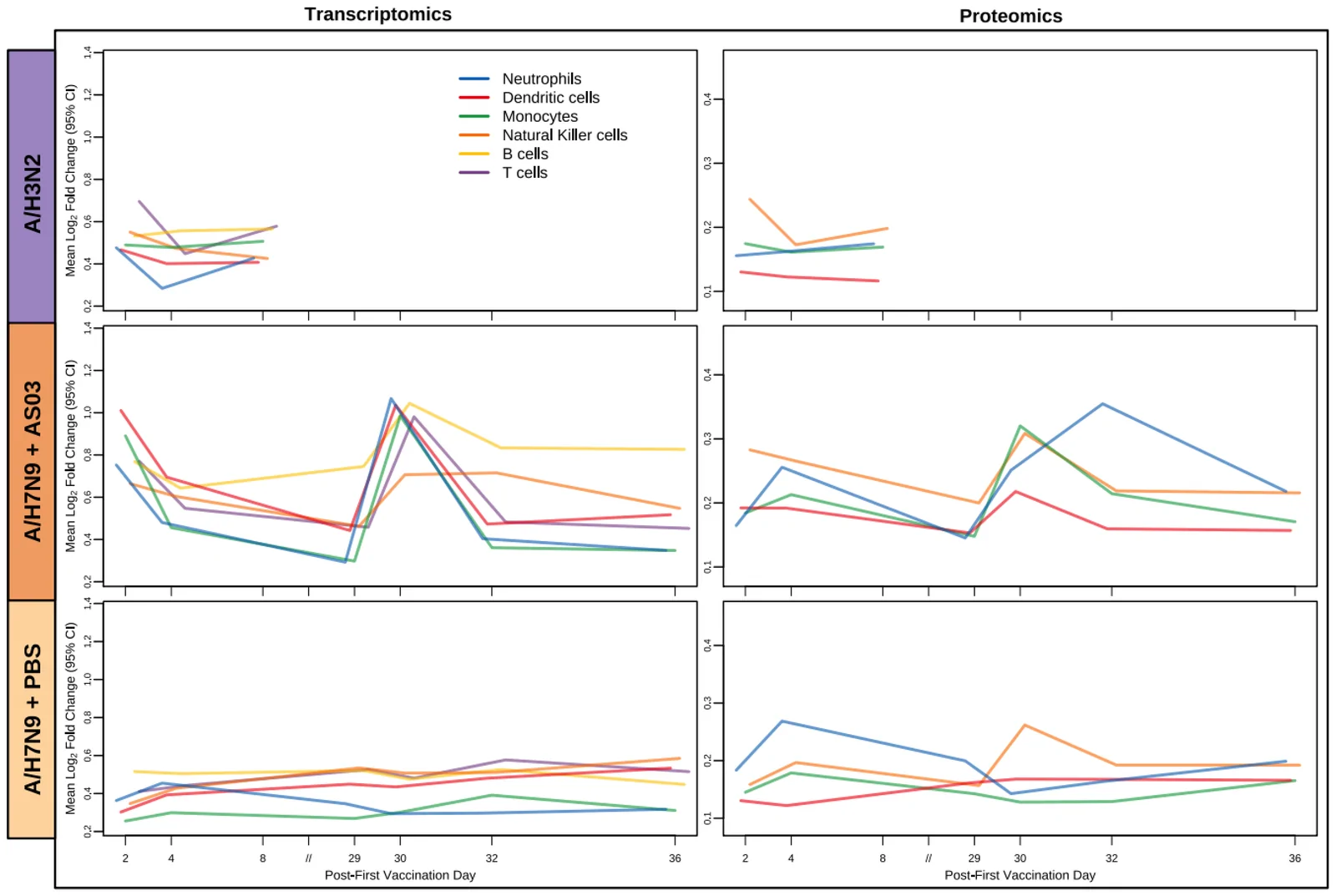
Adjuvanted vaccination induced changes in gene expression and protein abundance in innate immune cells early after adjuvanted A/H7N9 vaccination. Time trend plot of the mean of mean absolute log_2_ fold change of all DE genes (shown on the left) and DA proteins (shown on the right) identified for a certain cell type.

Across all cell types, 1,209 of 1,232 (98.2%) RNA samples from 30 participants were successfully processed and analyzed. Approximately 10,000–14,000 genes were quantified above the minimum expression threshold within each cell type. Next, we assessed differential expression (DE) compared to pre-vaccination at each of the post-vaccination time points using negative binomial generalized linear models (**Supplementary Tables 2**–**S41**). Across all time points and study arms, DE gene counts were highest in neutrophils (2,294), followed by dendritic cells (2,048) and monocytes (1,488), while NK cells (716), B cells (424), and T cells (315) had relatively fewer DE genes (**Table 1**). Of these genes, nearly all (99.8%) were identified for the adjuvanted A/H7N9 vaccine arm. Peak average absolute fold change responses for DE genes in this arm were seen 24h after each dose for monocytes, dendritic cells, and neutrophils, while NK cells reached peak responses 72h after the second dose (**Figure 4**).

**Table 1:** Number of identified differentially expressed genes and differentially abundant proteins.

Cell type	# differentially expressed genes	# differentially Abundant proteins
Neutrophils	2294	53
Dendritic cells	2048	978
Monocytes	1488	158
Natural Killer cells	716	20
B cells	424	2
T cells	315	1

Across all cell types, 1,043 of 1,260 (84.6%) samples from 30 participants were successfully analyzed by quantitative LC/MS-MS, and 7,821 unique proteins were identified in at least one sample. Approximately 1,700–3,600 proteins were quantified and analyzed within each cell type. To identify differentially abundant (DA) proteins compared to pre-vaccination at each of the post-vaccination time point, we used linear models (**Supplementary Tables 42**–**S70**). DA counts were highest among dendritic cells (978) and monocytes (158), followed by neutrophils (53), NK cells (20), B cells (3), and T cells (1) (**Table 1**). As with DE genes, DA proteins were almost exclusively observed (99.9%) in the AS03-adjuvanted A/H7N9 vaccine arm. Average absolute fold changes for DA proteins for cells with >5 DA proteins are presented in **Figure 4**. In contrast to DE gene expression, peak DA protein responses were seen later for neutrophils, reaching peak DA responses 72h following each dose, and monocytes, occurring 72h after the first dose. Dendritic cells showed similar trends in both the gene and protein responses with peak activation 24h after each dose.

### Innate immune signaling pathways were activated 24h–72h after receipt of the adjuvanted A/H7N9 vaccine

**Figure 5:**
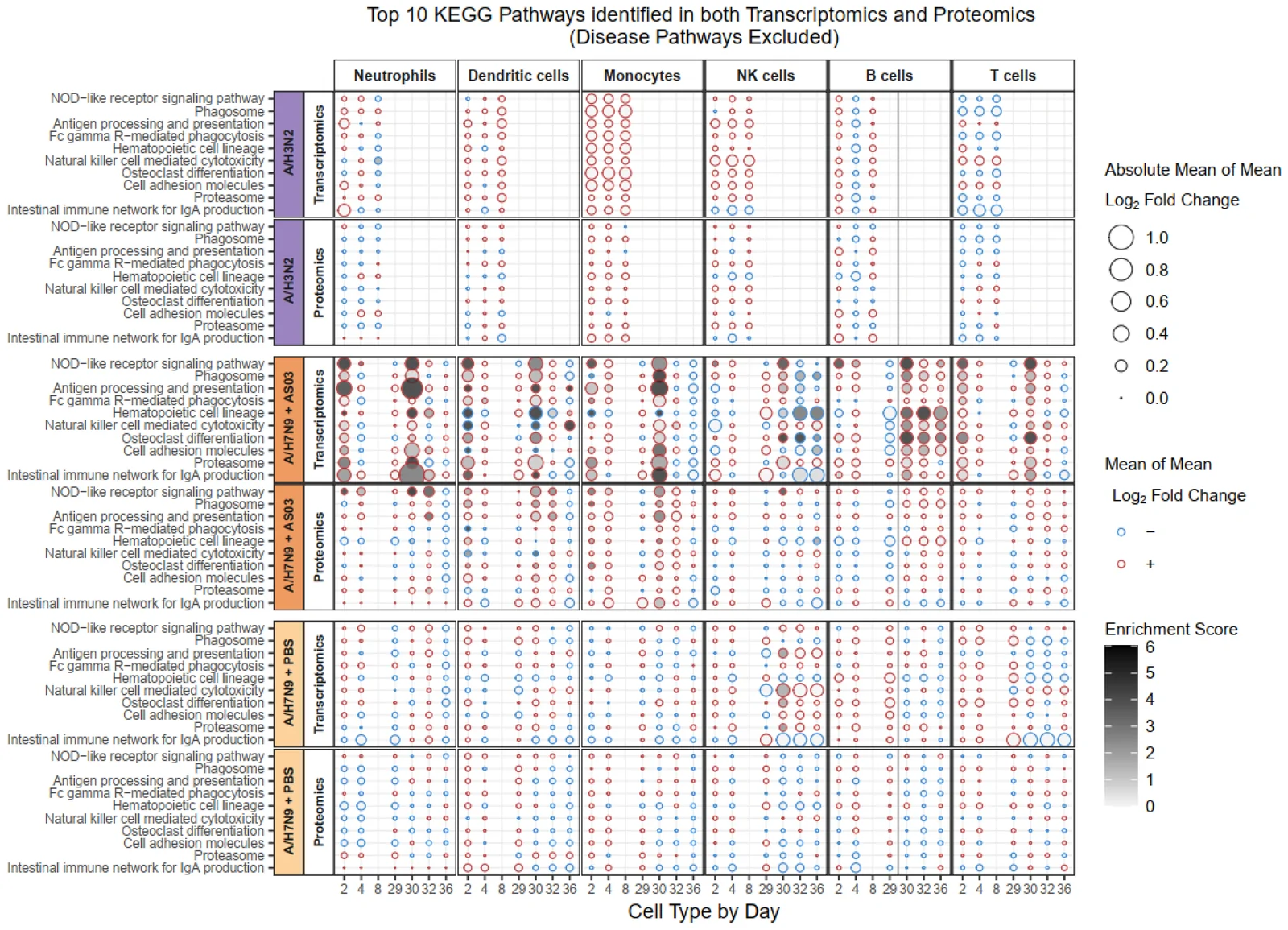
Innate immune signaling pathways were activated 24h–72h after receipt of adjuvanted A/H7N9 vaccine. Bubble plot summarizing activation and enrichment of the top 10 most enriched KEGG pathways across study arms, time, and assay. Pathways were selected based on the maximum difference of the enrichment score (−log_10_ enrichment *p*-value) between study arms. The pathways were ranked in descending order by the sum of the absolute maximum delta values from both the proteomics and transcriptomics analyses after scaling the scores within assay. Log_2_ fold change represents the absolute mean of the mean log_2_ fold change of all genes/proteins in a pathway. In the event of ranking ties, the pathway with the higher absolute mean of mean log_2_ fold change value was selected. Blue circle outlines indicate a fold change <0 and red outlines a fold change ≥0.

To characterize DE/DA signals from a functional standpoint, we conducted pathway enrichment analysis (**Figure 5**; **Supplementary Tables 71**–**S185**). Greater absolute means of the mean log_2_ fold changes were observed in the transcriptomic dataset compared to the proteomic dataset. This difference is consistent with known biological and technical factors, including post-transcriptional regulatory mechanisms like translational control, protein stability, and the inherently lower sensitivity of proteomic platforms relative to transcriptomic assays.

Enrichment was strongest for the NOD-like receptor signaling pathway, followed by phagosome, antigen processing and presentation, FcγR-mediated phagocytosis, hematopoietic cell lineage, NK-cell–mediated cytotoxicity, osteoclast differentiation, cell adhesion molecules, proteasome, and intestinal immune network for IgA production pathways among recipients of the AS03-adjuvanted A/H7N9 vaccine. Enrichment was seen primarily at 24h after each vaccination in innate immune cells, with higher responses seen following the second vaccination. In contrast, minimal pathway enrichment was observed among unadjuvanted A/H7N9 or A/H3N2v vaccine recipients. DA pathway enrichment mirrored those identified by DE genes. Reactome pathway analysis revealed enrichment of IFN-α, IFN-β, and IFN-γ signaling for both transcriptomics (all cell types) and proteomics (dendritic cells, monocytes, and neutrophils) among AS03-adjuvanted A/H7N9 recipients 24h after each dose (**Supplementary Figure 24**). Enrichment of GO processes related to innate immunity was also observed across all cell types after receipt of the AS03-adjuvanted A/H7N9 vaccine, including innate immune responses, defense responses, immune effector responses, response to cytokines, defense response to viruses, and cytokine mediated signaling pathways (**Supplementary Figure 25**). Enrichment of blood transcription modules involved in viral sensing and immunity, innate antiviral responses, and type I interferon responses were observed across all cell types by transcriptomics and in dendritic cells, monocytes, and neutrophils by proteomics, but only among recipients of the AS03-adjuvanted A/H7N9 vaccine (**Supplementary Figure 26**).

Next, to understand the kinetics of pathway activation in AS03-adjuvanted A/H7N9 recipients, we visualized the mean of the mean log_2_ fold change of all genes/proteins mapped to top enriched pathways over time (**Supplementary Figures 27**–**S38**). For NOD-like signaling, interferon signaling, and antigen processing and presentation, all immune cells showed similar pathway activation trends based on gene expression, with the highest increases for innate immune cells 24h after either dose of adjuvanted A/H7N9 with higher responses post-boost (**Supplementary Figures 27**, **S30**, **S29**). Peak activation of these pathways on the protein level occurred 24h later in neutrophils following either dose and for monocytes following the first dose, relative to gene expression. Additionally, dendric cells and monocytes maintained a sustained, although decreased, protein response 72h following the second dose. Following the booster dose of vaccine, the NOD-like signaling pathway was activated in B cells and activation persisted longer than for other cell types on both the gene expression and proteomics levels.

### Cell proliferation signatures were activated in T cells 24h after vaccination and in NK cells and B cells 72h after the second dose of adjuvanted A/H7N9 vaccination

**Figure 6:**
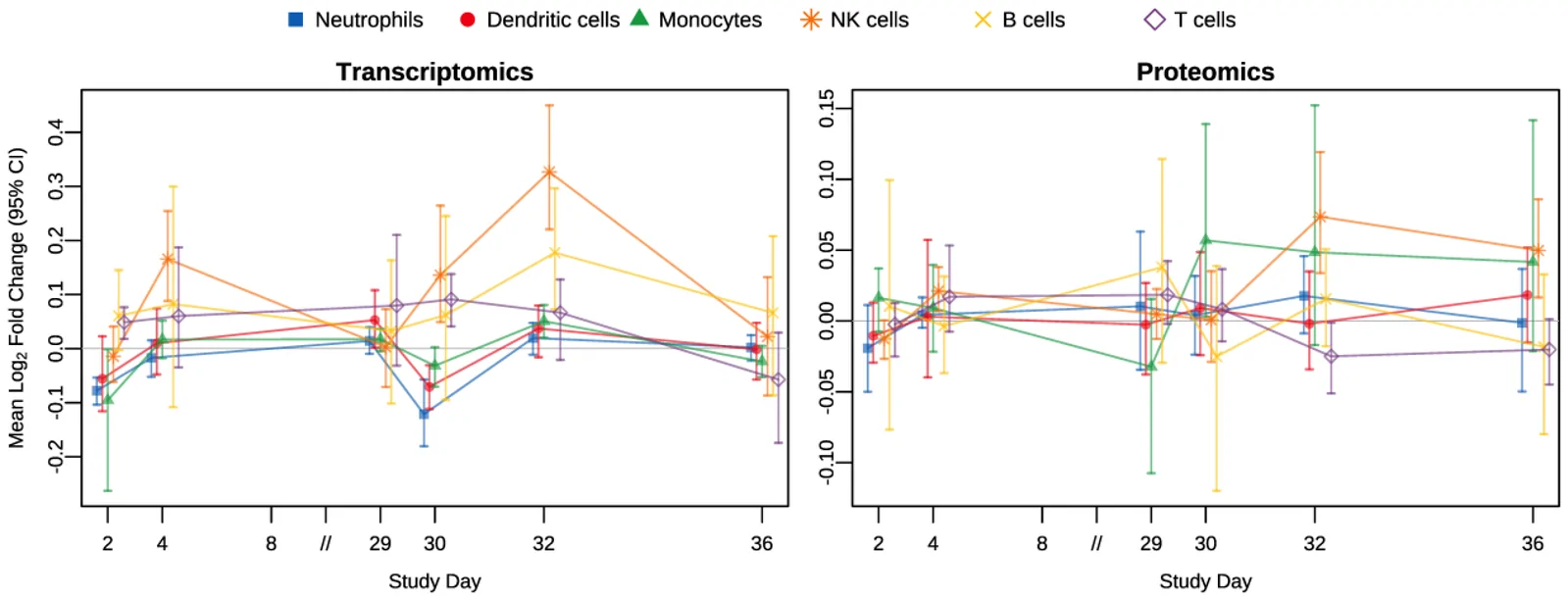
Cell cycle activity was increased in T cells 24h after vaccination and in NK cells and B cells 72h after second dose of adjuvanted A/H7N9 vaccination. Mean of mean log_2_ fold change and associated 95% CI of log_2_ fold change of all genes/proteins in the KEGG cell cycle pathway following adjuvanted A/H7N9 vaccine. Error bars indicate 95% confidence intervals based on 1000 bootstrap replicates.

As a surrogate measure of cell proliferation, time trends for the KEGG cell cycle pathway, which was one of the significantly enriched pathways in the adjuvanted A/H7N9 arm, were assessed (**Figure 6**). The earliest activation of the cell cycle pathway was seen on the gene expression level in T cells 24h after dose one. This signal was followed by stronger activation in NK cells 72h after the first dose, followed by increased activation 24h and 72h after the booster dose. To a lesser extent, the cell cycle pathway was activated in T cells at 24h and in B cells at 72h post-booster dose. On the proteomics level, the cell cycle pathway was activated most strongly in NK cells 72h after the boost and continued to be activated through one week after the second vaccination.

Conversely, neutrophils showed a reduction in cell cycle activity 24h after each dose on the transcriptomics level, but no changes were observed on the proteomics level. No perturbation of this pathway was seen for the A/H3N2v arm (**Supplementary Figure 39**). In the unadjuvanted A/H7N9 group, cell cycle-related gene expression was increased in T and B cells 72h after the first dose and remained higher for B cells one week after the first vaccination. These signals were not seen on the proteomics level.

### Neutrophils displayed a unique major histocompatibility class II gene expression profile following two doses of adjuvanted A/H7N9 vaccine

**Figure 7:**
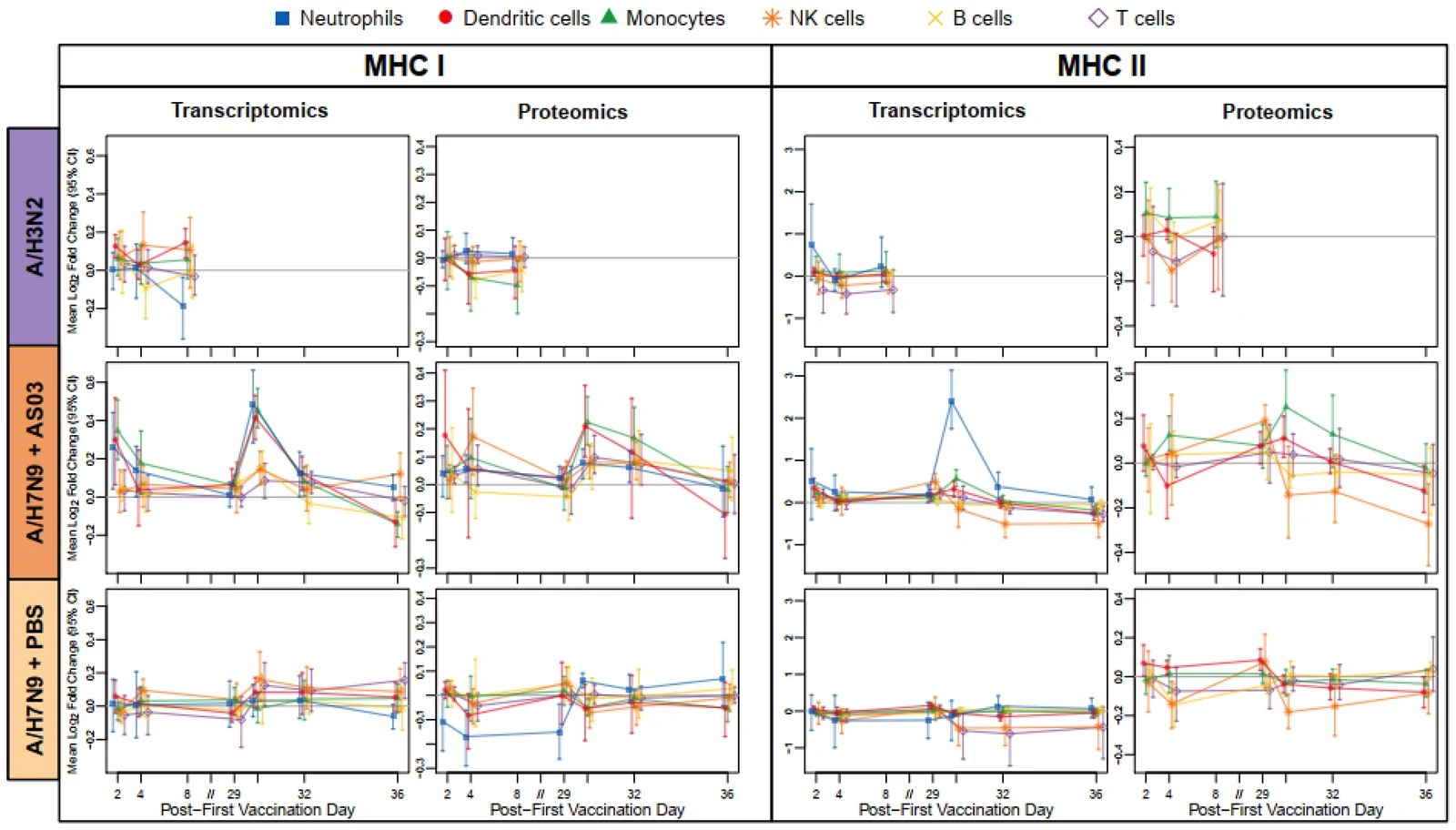
Major histocompatibility class I and II gene and protein expression was activated following adjuvanted A/H7N9 vaccine. Mean of mean log_2_ fold change and associated 95% CI of log_2_ fold change of all MHC I/II genes/proteins following adjuvanted A/H7N9 vaccine. Error bars indicate 95% confidence intervals based on 1000 bootstrap replicates. MHC I gene set includes HLA-A, HLA-B, HLA-C, HLA-E, HLA-F, and HLA-G. MHC II gene set includes HLA-DMA, HLA-DMB, HLA-DOA, HLA-DOB, HLA-DPA1, HLA-DPB1, HLA-DQA1, HLA-DQA2, HLA-DQB1, HLA-DRA, HLA-DRB1, HLA-DRB3, HLA-DRB4, HLA-DRB5. MHC II proteins were absent from neutrophil proteomics analysis dataset.

Next, we investigated the enrichment of antigen processing and presentation signal further by visualizing average MHC-I and MHC-II gene expression and protein abundance over time (**Figure 7**). Following A/H3N2v receipt, we saw increased expression of MHC-I genes among dendritic cells 24h following immunization and one week later. In contrast, average MHC-I gene expression was reduced one week following immunization in the neutrophil compartment. No substantial changes in MHC-II gene expression were seen. Following receipt of AS03-adjuvanted A/H7N9 vaccine, average MHC-I gene expression was increased in neutrophils, monocytes, and dendritic cells 24h after both doses of vaccine. Following the second vaccination, MHC-I genes in B-cells and NK cells were induced, though not as strongly as in innate immune cell subsets. Similarly, MHC-II gene expression was increased among monocytes and dendritic cells following the first dose of adjuvanted A/H7N9vaccine. Within 24h of the second dose of vaccine, we observed a strong increase in MHC-II expression among neutrophils (greater than twofold), which persisted at decreasing levels through 72h post-second immunization. Increased responses were also seen in dendritic cells and monocytes at 24h post-second vaccination.

Proteomics analysis mirrored that of the transcriptome (**Figure 7**). The seasonal H3N2v vaccine induced minimal changes in MHC-I or MHC-II protein abundance. The unadjuvanted A/H7N9 vaccine led to decreased abundance of MHC-I in neutrophils following the first vaccination and slightly increased abundance following the second vaccination. Unadjuvanted A/H7N9 vaccine also led to decreased abundance of NK cells 24h after the first vaccination. Following receipt of the AS03-adjuvanted vaccine, an increase in MHC-I protein was seen in dendritic cells (at 24h) and NK cells (at 72h) following the first dose. Increased abundance of MHC-I peptides was also seen after booster immunization in all cell types except B cells, with sustained increases through 72h in neutrophils, monocytes, and NK cells. MHC-II protein abundance was higher in NK cells just prior to the boost (1 month after the first dose) and decreased 7 days after the second immunization. Induction of MHC-II protein was seen 72h post-first dose for monocytes and 24h after the second dose for both monocytes and dendritic cells. For neutrophils, MHC II protein was not sufficiently detected for analysis and thus was not reported. The kinetics of MHC peptide abundance showed a longer lasting peak response following the second dose compared to MHC gene expression.

### CD38 and IgD expression were increased among recipients of the AS03-adjuvanted A/H7N9 vaccine

To identify subpopulations of B cells activated in response to vaccination, we evaluated gene expression and differential protein abundance of CD19, CD27, IgD, and CD38 (**Supplementary Figure 40**). Gene expression of IgD (characteristic of naive/non-switched memory B cells) increased following the first dose of AS03-adjuvanted A/H7N9 vaccine, peaking at 72h post-immunization, followed by decreased expression 24h following the second vaccination. In addition, gene expression of CD38 (a marker of memory B cells) increased 24h after the first dose of vaccine and appeared to increase after the second dose as well, though changes in CD38 were not statistically significant. In the seasonal A/H3N2v arm, gene expression of CD19 (a marker of B-cell development) and associated protein abundance increased from pre-vaccination 24h after the vaccination.

### Increased expression and abundance of interferon-induced guanylate binding proteins are a dominant feature of the response to the AS03-adjuvanted influenza A/H7N9 vaccine and correlated with peak HAI titer

**Figure 8:**
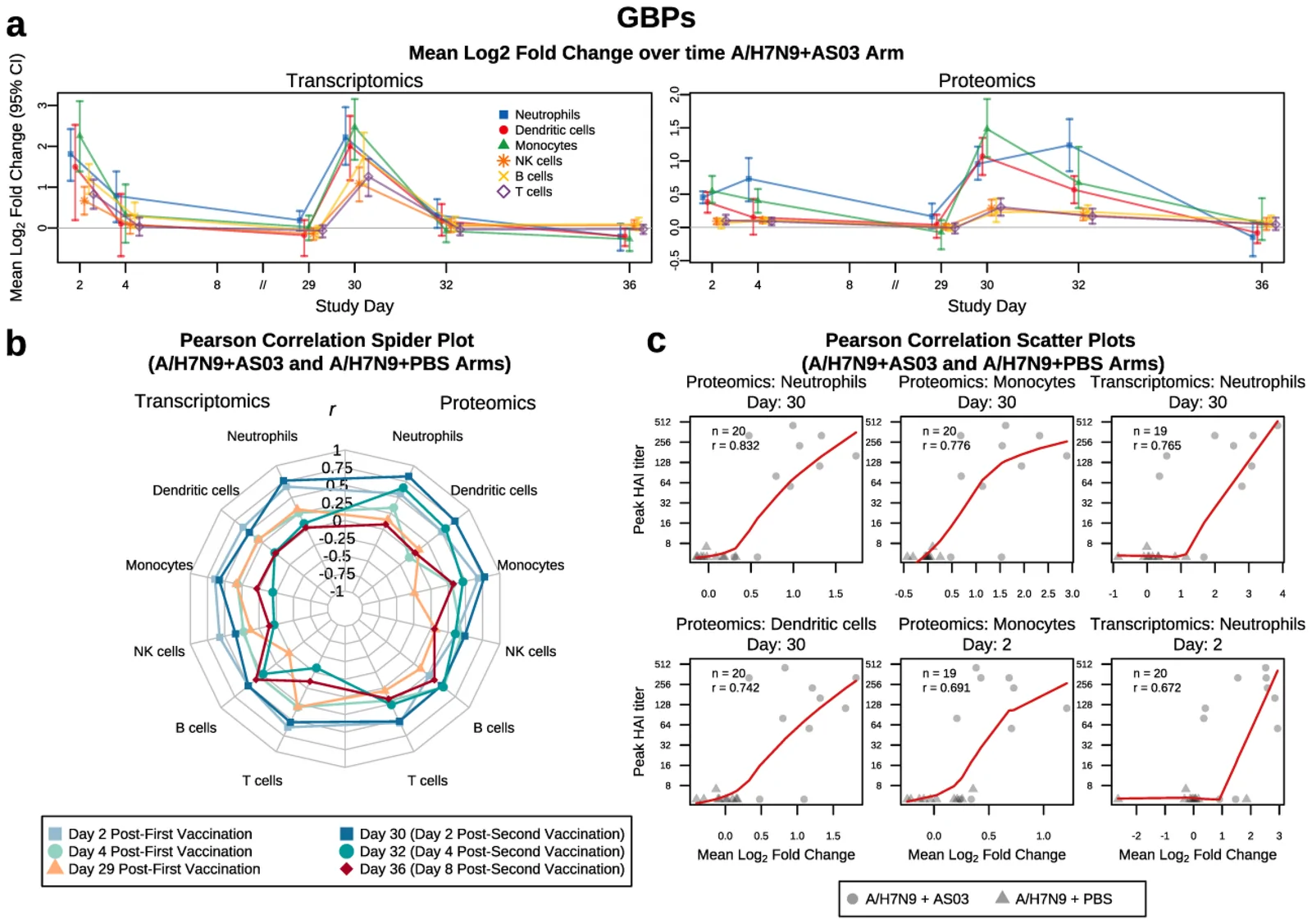
Increased interferon-induced guanylate binding (GBP) activity following AS03-adjuvanted influenza A/H7N9 vaccine and correlation with peak HAI titer. **(A)** Time trends plot of mean of mean *log*2 fold change of GBP genes and proteins for each cell type following adjuvanted A/H7N9 vaccine. Error bars indicate 95% confidence intervals based on 1000 bootstrap replicates. GBP features include GBP1, GBP2, GBP3, GBP4, and GBP5. **(B)** Spider plot summarizing Pearson correlation between the mean of mean *log*2 fold change of all present GBP genes/proteins and *log*_2_ HAI peak titer. **(C)** Scatterplots summarizing the Pearson correlation for the top 6 correlated events across omics assays, cell types and days. In red: locally weighted scatterplot smoothing (LOWESS) curve.

To identify signatures that correlated best with peak HAI titer, we conducted regularized linear regression analysis for each cell type and time point across the two A/H7N9 vaccine arms. We used peak log_2_ HAI titer as the dependent variable and log_2_ fold change in gene expression and protein abundance as predictor variables (**Supplementary Table 186**). Among predictive features, guanylate binding protein (GBP) 1-5, MHC-I, MHC-II, STAT3, STAT1, SOCS3, PSME2, IFIT, FCγR1A, IRF9, and TAP1 were selected for further characterization based on selection prevalence in these models and results of previous studies (22) (**Supplementary Figures 42**–**S56**). Guanylate binding protein (GBP)–related interferon-induced antiviral genes *GBP5*, *GBP1*, *and GBP2* were selected most frequently to predict peak HAI titer (**Supplementary Table 186**). Time trends for the average GBP response and associated correlations with HAI peak titers are presented in **Figure 8**. Individual results are presented in **Supplementary Figures 47**–**S51**. On average, GBPs were significantly upregulated in the AS03-adjuvanted group, peaking 24h after either dose on the gene expression level. This was also seen on the proteomics level, albeit with a delayed peak response for neutrophils and more persistent response in all cell types after the second dose (**Figure 8A**). Pearson correlation *r*-values > 0.5 were considered representative of a moderate positive correlation, and values > 0.7 were considered a strong correlation. Overall, Pearson correlation with peak HAI titer was strongest 24h after either dose with *r*-values > 0.5 for most cell types (**Figure 8B**). GBP protein increases in neutrophils, monocytes, and dendritic cells achieving the highest correlations ranging from 0.67 to 0.83 (**Figure 8C**). Among individual GBPs, GBP5 achieved the overall highest correlation 24h post-second vaccination in neutrophils on the proteomics (*r* = 0.86) and transcriptomics levels (*r* = 0.81) (**Supplementary Figure 51**). In addition to GBP, IFIT abundance in dendritic cells [*r* = 0.84 (proteomics)] was correlated with peak HAI titer (**Supplementary Figure 53**). Change in MHC-I gene and protein (monocytes, Day 30, *r* ≥ 0.74), PSME2 protein (monocytes, Day 30, *r* = 0.78), and FcGγR1A protein (monocytes, *r* = 0.76) and gene expression (neutrophils, *r* = 0.75) also showed strong positive correlation with peak HAI titer (**Supplementary Figures 42**, **S52**, **S54**). Taken together, these data suggest that expression and synthesis of MHC genes and proteins, as well as interferon-response elements that were increased after each dose of vaccine, were associated with eventual production of influenza-specific immune responses, as measured by HAI titer.

## Discussion

To overcome the poor antigenicity of avian influenza vaccines, novel adjuvants are needed. In this study, we interrogated the cellular response to AS03 adjuvant in the context of the influenza A/H7N9 vaccine. In addition, we compared the transcriptomic and proteomic responses to the A/H7N9 vaccine to a monovalent seasonal influenza vaccine (A/H3N2v-unadjuvanted) to determine whether changes in cellular responses were due to the AS03 adjuvant or to hemagglutinin with sufficient antigenicity.

While both the adjuvanted A/H7N9 vaccine and unadjuvanted A/H3N2v vaccine induced HAI responses against the homologous vaccine antigen, the unadjuvanted A/H7N9 vaccine failed to induce a serologic response. Moreover, recipients of the unadjuvanted A/H7N9 vaccine had a 40% increase in HAI titer to the cross-clade A/H3N2v antigen; this may suggest that the unadjuvanted A/H7N9 vaccine leads principally to a weak cross-clade memory response rather than the induction of naïve B-cells capable of generating a robust homologous response. The precise mechanism for this memory response, and the pathways that govern it, remain to be elucidated.

Overall, the transcriptomic and proteomic response to the unadjuvanted A/H3N2v vaccine largely mirrored that of the unadjuvanted A/H7N9 vaccine; specifically, neither vaccine evoked substantial changes in gene expression or protein abundance. In contrast, the addition of AS03 led to the upregulation of immune pathways responsible for NOD-like signaling, interferon signaling, phagocytosis, antigen presentation and processing, NK-cell–mediated cytotoxicity, and IgA production, among others. Additionally, cytokine profiling showed a distinct pro-inflammatory phenotype, including increased levels of IP-10, IFNγ, and IL-6. Concomitantly, HAI responses to influenza A/H7 were significantly higher in recipients of the AS03-adjuvanted A/H7N9 vaccine, despite the use of a very low dose of antigen (3.75 mcg). Across analyses, the second vaccination consistently elicited a stronger and more sustained immune response compared to the first, as reflected by increased magnitude of gene and protein changes, enhanced pathway activation, and greater coordination across cell types, particularly in the AS03-adjuvanted arm. These coordinated differences across cell types and molecular layers are consistent with a priming effect from the initial dose, resulting in an amplified recall response upon boosting, and are consistent with a “prime-boost” effect. Consistent with this, following the second vaccination, we observed upregulation of CD38 and concomitant downregulation in CD19, which suggests that memory B-cells were engaged to a greater extent than naïve B-cells.

In previous work with the influenza A/H5N1 vaccine, we identified upregulation of MHC class I and II genes and enrichment of MHC class I and II antigen presentation following receipt of the AS03-adjuvanted vaccine (22, 23). Specifically, we identified increased expression of MHC I genes in neutrophils, monocytes, and dendritic cells after the first and second vaccinations, significant enrichment of MHC II sub-pathway genes in neutrophils, and a greater than fourfold increase in MHC-II gene expression within neutrophils in the 24h following the second immunization. Additionally, IFNγ expression, JAK-STAT signaling, and MHC class II transactivator (CTIIA) gene expression were upregulated in neutrophils following the second vaccination, consistent with true induction of MHC II. It has become clearer in the past decade that neutrophils are not merely innate effector cells but also highly versatile members of the adaptive immune system. Recent evidence confirms their role as antigen-presenting cells to CD4+ T cells. Vono et al. have shown in both *in-vitro* and *ex-vivo* models that neutrophils are capable of engaging memory CD4+ T cells, resulting in HLA-DR–dependent T-cell proliferation (25). While HLA-DR expression is typically absent in neutrophils, MHC-II class genes can be induced under the right conditions, including the presence of antigen and antigen-specific memory CD4+ T cells. In the current study, the greatest upregulation of MHC class II genes was observed after the second dose of the vaccine. Whether this is an adjuvant-specific effect or the result of successful adjuvant-induced T-cell responses, including memory CD4+ T-cell responses following the first vaccination, remains unknown.

We also observed robust NK-cell activation approximately 72h following each dose of AS03-adjuvanted vaccine. NK cells are lymphocytes with direct cytotoxic and cytokine-producing functions that also play a critical regulatory role in the innate and adaptive response to cellular stress or infection (26). NK cells primed by type 1 IFN, IL-15, IL-12, and IL-18 produce IFNγ and TNF, leading to activation of T cells, dendritic cells, and macrophages. These activated NK cells also control the inflammatory response through direct cytotoxic mechanisms on hyperactivated macrophages, immature dendritic cells, and activated CD4+ T cells. Therefore, we hypothesize that upon vaccination, NK cells are primed by stress signals from neighboring cells induced by AS03; in response, three processes may occur. First, NK cells expand and produce pro-inflammatory cytokines that will activate other innate effector cells, as well as B and T cells. The observed concomitant increase in expression of IgD, a marker of naïve B cells, is consistent with this hypothesis and has been shown to correlate with influenza antibody responses (27). Second, NK cells upregulate pathways for antiviral responses, antigen presentation and processing, and production of antimicrobial pathways. Third, NK cells activate cellular signals for senescence by approximately 72h after vaccination, leading to subsequent downregulation of interferon signaling and cellular division. Consistent with this, following the second vaccination, we observed upregulation of CD38 and concomitant downregulation in CD19, which suggests that memory B cells were engaged to a greater extent than naïve B cells, leading to a prime-boost response (**Supplementary Figure 41**).

Responses within GBPs are of particular interest given their role in the innate response to viral infections (28–31). GBPs are IFN-inducible proteins that have both direct antiviral activity and regulatory roles in innate immune responses. GBP1, GBP2, GBP3, and GBP5 work via distinct mechanisms to reduce viral replication, block viral release, and inhibit nuclear delivery of DNA viruses. Moreover, GBP5 promotes IFN expression in the context of viral infection. In our study, GBP5 expression in monocytes one day after the second vaccination showed the highest log_2_ fold change (>3.0) from baseline; DA of GBP5 was also observed in neutrophils, peaking 72h after each vaccination. Change in GBP5 protein in neutrophils one day after the second vaccination showed the highest correlation with peak HAI titer (*r* = 0.86). Not only were the interferon-inducible GBPs expressed differentially after vaccination, but also other interferon-inducible genes and proteins, such as IFIT1-3, STAT1, STAT3, and SOCS3. Thus, it appears that a principal mechanism of action for AS03, and potentially other oil-in-water emulsion adjuvants, may be the induction of interferons, triggering a pro-inflammatory state in which immune effector cells are recruited and pathways for antigen presentation and processing are enriched. Our analysis also suggests that these early interferon signaling-related signatures as well as antigen presentation and processing signatures (MHC-1 and PSME2 protein) correlate strongly with subsequent peak serum antibody responses. These findings are compelling given their convergence with the work of Tasdighian et al., who identified a core innate signature (including interferon pathway-related gene expression and IP-10) for AS01_B_, AS01_E,_ and AS03 adjuvanted hepatitis B vaccines that predicted the antibody response magnitude and quality consistently over time (32). These data also suggest that expression of interferon-inducible genes and proteins may be a broader, potentially universal mode of action for AS03 and not merely influenza specific.

Our findings are consistent with other systems biology studies of AS03-adjuvanted avian influenza vaccines. Wimmers et al. evaluated the epigenomic and transcriptomic changes seen after AS03-adjuvanted A/H5N1 vaccine using ATAC-Seq and RNA-seq (33). Myeloid cells were found to have persistent, global epigenomic changes at 3–4 weeks following vaccination that persisted at low levels for 6 months following immunization. These changes led to alterations in function, including interferon-responsiveness and cellular differentiation within monocytes that had heterologous effects for other viruses. For example, PBMCs from individuals vaccinated with AS03-adjuvanted A/H5N1 more readily impaired replication of Dengue and Zika viruses, which belong to completely unrelated viral families to influenza. Similar heterologous protection was not observed among PBMCs from individuals vaccinated with seasonal influenza virus vaccine. Cortese et al. have also shown upregulation in interferon response genes, dendritic cell activation, and monocyte activation based on blood transcription modules (BTM) (20).

Three key limitations should be considered in this work. First, the comparison of seasonal influenza vaccine to avian influenza vaccine responses is confounded by the number of doses administered (1 vs. 2), the role of the memory response to seasonal influenza vaccine or to repeated natural exposure to influenza virus, and the role of antigenic imprinting, though the latter may be diminished since seasonal H3 and avian H7 both belong to HA group 1 (34). Second, we did not explore the potential role of epigenetic changes within immune cells that may contribute to changes in gene expression and protein production following the second vaccination. Therefore, we are unable to determine precisely whether changes in immune cell function are due solely to adjuvant exposure at the time of the second dose or due to persistent changes following the first immunization. Future work should include prime-boost strategies in which the second dose of vaccine is unadjuvanted. Third, as a single-center, US-based study in white, non-Hispanic adults, our findings may not be generalizable across all races, ethnicities, and geographic locations.

**Figure 9:**
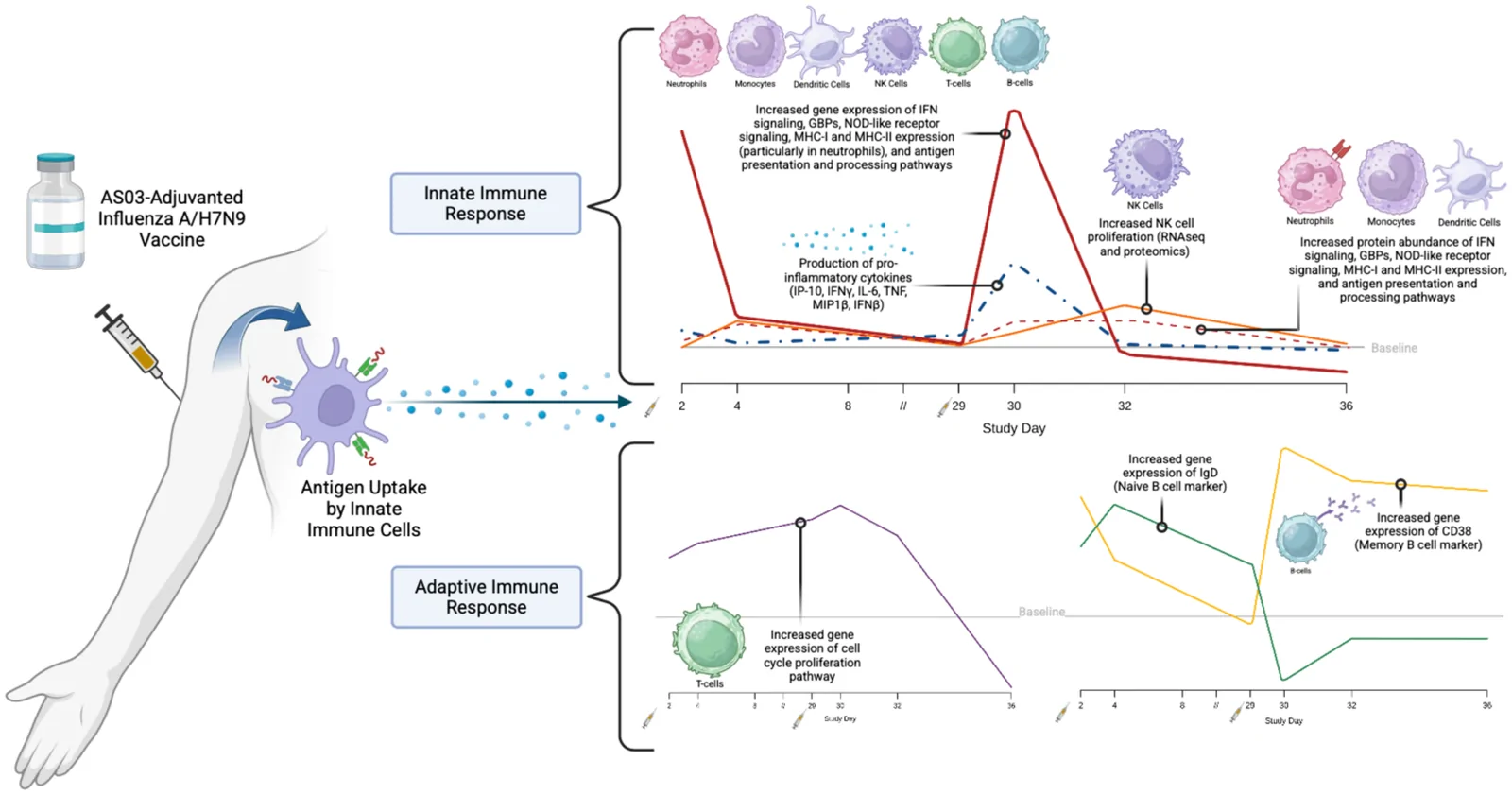
Proposed effect of AS03 adjuvant in the response to Influenza A/H7N9 vaccine.

Our results demonstrate that the AS03 adjuvant, containing α-tocopherol and squalene, induces a potent antibody response to influenza A/H7N9 in a diverse, yet integrated fashion, leading to early changes in serum cytokine concentrations, activation of innate immune cells, and NK and B-cell-specific signals (**Figure 9**). Enhanced antigen presentation and processing (potentially among neutrophils as well), activation of the inflammasome, and induction of a robust interferon response were seen consistently across recipients of the adjuvanted vaccine. Our approach provides a framework by which other adjuvant-antigen combinations can be evaluated, not only for the influenza vaccine but for other vaccine platforms as well.

## Methods

### Study design

We conducted a single-center, randomized, partially blinded, Phase II, prospective study in 30 healthy male and non-pregnant female subjects aged 18 to 49 years old designed to compare the immunogenicity of three influenza vaccines—influenza A/H7N9, influenza A/H7N9 with AS03 adjuvant, and influenza A/H3N2v. The study used a systems biology approach to assess early gene and protein signatures expressed in response to vaccination. Multi-omics data were integrated with serologic immunogenicity [hemagglutination inhibition (HAI) and neutralizing (Neut) antibody assays], cytokine data, and reactogenicity data to develop a systems biology model of the human immune response to unadjuvanted inactivated influenza A/H3N2v virus vaccine and inactivated influenza A/H7N9 virus vaccine given with and without AS03 adjuvant.

Primary objectives of this study were to assess the serum HAI response to influenza A/H7N9 antigen and to identify DE genes in human immune cells following the vaccination of influenza A/H7N9 compared to baseline pre-vaccination assessments. Primary outcome measures include the percentage of participants achieving seroconversion based on HAI titer levels (seroconversion criteria described above in the Results section) and the number of DE genes based on RNA expression determined by RNA-seq analysis. Secondary objectives included comparing cytokine/chemokine profiles at specific time points, assessing neutralizing antibody responses to influenza A/H7N9, and identifying DE genes following the vaccination of influenza A/H3N2. Secondary outcomes were measured by plasma cytokine/chemokine measurements at each study visit, percentage of participants achieving seroconversion based on neutralizing antibody levels, and the number of DE genes based on RNA expression determined by RNA-seq analysis. Tertiary and exploratory objectives included identifying DA proteins in human immune cells following the vaccination of influenza A/H7N9 compared to baseline pre-vaccination assessments and were measured by the number of DA proteins based on protein abundance determined by quantitative proteomics. Additional objectives and further detail can be found in the study protocol available on ClinicalTrials.gov (NCT02921997).

The sample size in the Phase II study is not based on a formal hypothesis, but instead on practical considerations with the goal of gathering enough information to evaluate immune system responses to the vaccine and adjuvant on the molecular level. Study recruitment commenced on 1 November 2016, and the final study follow-up visit occurred on 28 February 2018.

### Participant eligibility criteria

Participants were in good general health and had no immunocompromising conditions; individuals with a history of Guillain-Barre Syndrome, narcolepsy, seizures, recent use of corticosteroids or immunosuppressive medications, or recent receipt of a non-study vaccination were excluded.

### Treatment arms

After obtaining written informed consent, 30 participants were randomized (1:1:1) to one of three treatment (vaccine) arms. The randomization code was prepared by statisticians at the Statistics and Data Coordinating Center (SDCC), and the SDCC’s AdvantageEDC_SM_ (Electronic Data Capture System) assigned each participant to a treatment arm after demographic and eligibility data were entered into the system.

The first vaccine group (*n* = 10) received one intramuscular dose of 15 mcg influenza A/H3N2v (A/Minnesota/11/2010, Sanofi Pasteur). The second vaccine group (*n* = 10) received two intramuscular doses, 28 days apart, of 3.75 mcg influenza A/H7N9 + AS03, and the third vaccine group (*n* = 10) received two intramuscular doses, 28 days apart, of 3.75 mcg influenza A/H7N9 without adjuvant (A/Shanghai/2/2013, manufactured by Sanofi Pasteur; AS03 was provided by the Biomedical Advanced Research and Development Authority (BARDA). Both influenza vaccines were monovalent, inactivated, split influenza virus vaccines produced in embryonated chicken eggs, and HA content was confirmed with reversed phase-high performance liquid chromatography. After randomization, no participant was lost to follow-up. Dosing of adjuvanted A/H7N9 vaccine was set at 3.75 mcg HA, which is consistent with other pre-pandemic clinical trials; studies of adjuvanted 3.75 mcg HA vaccine have shown consistently suitable responses to immunization meeting the Committee for Medicine Products for Human Use criteria for seroconversion (35).

### Adverse event assessment

Reactogenicity was measured by the occurrence of solicited injection site and systemic reactions from the time of each study vaccination through 8 days after each study vaccination. Unsolicited non-serious adverse events (AE’s) were collected from the time of each study vaccination through approximately 28 days after each study vaccination. Serious AEs (SAEs) and medically attended AEs (MAAEs), including new-onset chronic medical conditions (NOCMCs) and potentially immune-mediated medical conditions (PIMMCs), were collected from the time of the first study vaccination through approximately 1 month (A/H3N2v arm) and 12 months (A/H7N9 arms) after the last study vaccination.

### Blood sample collection

Venous blood samples (approximately 90 ml per sample) were collected at the Vanderbilt Vaccine Research Program (Nashville, TN) from participants one week prior to vaccination and again immediately prior to the first study vaccination. These samples were repeated on Days 2 and 4 post-vaccination for all participants and on Day 8 for A/H3N2v-vaccinated participants for systems biology studies (cytokine and chemokine levels, immune cell activation status, and transcriptome and proteome profiles of major blood immune cells). Additionally, participants in the two A/H7N9 treatment arms had blood samples collected immediately prior to the second vaccination (Day 29) and repeated on Days 30, 32, and 36 for systems biology studies (**Figure 1**).

Hemagglutination inhibition (HAI) assays (36) were conducted on sera derived from venous blood samples collected from participants on Day 1 (immediately prior to the first study vaccination) and on Days 29 and 57 (A/H7N9-vaccinated subjects only) after the first study vaccination.

### Masking procedures

This was a partially blinded clinical study, as the unadjuvanted A/H3N2v vaccine treatment arm was open-label to avoid the need for placebo administration on Day 29. Investigators, study personnel, and laboratory technicians remained blinded to the allocation of A/H7N9 with or without AS03.

The unblinded study vaccine administrator was a study personnel member credentialed to administer vaccines but was not involved in study-related assessments and did not have participant contact for data collection following study vaccine administration.

Laboratory personnel performing antibody, cytokine/chemokine, and RNA-Seq assays were masked to A/H7N9 and A/H3N2v treatment groups, participant ID, and study visit for all study specimens. For proteomics assays, A/H7N9 treatment groups and study visits were masked. Participant IDs were replaced by random IDs generated by the Statistics and Data Coordinating Center (SDCC) to allow batch-processing specimens obtained from the same participant. In all cases, the SDCC generated random specimen picklists for each laboratory to determine which specimens were tested. After all participants completed Day 57, the database was locked to facilitate data analysis.

### Immune cell separation

Peripheral blood mononuclear cells (PBMC) and neutrophils (PMN) were recovered from phlebotomy samples and processed immediately. Following standard Ficoll separation of PMN (and subsequent neutrophil purification with microbeads coated with CD15), magnetic sorting (AutoMACS Pro) was used to isolate individual immune cells using microbeads coated with CD19 (B cells), CD3 (T cells), CD56 (NK cells), CD11c (dendritic cells), and CD14 (monocytes). During optimization, flow cytometric analysis confirmed >99% purity of each immune cell compartment (37).

### RNA sequencing and data processing

RNA was extracted from PBMC using the Promega SimplyRNA Cell Kit according to the manufacturer’s protocol. Polyadenylated RNAs were captured using NEBNext Magnetic Oligo d(T)25 Beads, and the NEBNext mRNA Library Prep Reagent Set for Illumina (New England BioLabs Inc., Ipswich, MA, USA) was used to prepare individually bar-coded libraries as per the manufacturer’s recommended protocol. Accurate quantification for sequencing applications was determined using the qPCR-based KAPA Biosystems Library Quantification Kit (Kapa Biosystems, Inc., Woburn, MA, USA). Paired-end sequencing (25 million, 75-bp, paired-end reads) was performed on an Illumina NovaSeq sequencer (Illumina, Inc., San Diego, CA, USA) as per the manufacturer’s recommended protocol.

Human reference genome assembly (GRCh38), gene models, and associated gene annotation information were obtained from the ENSEMBL database (Version 99) (38). The genomic reference was built by merging all human chromosomes except for X and Y chromosomes (to avoid gender-specific effects). Sequencing quality was reviewed with FastQC. Adapter sequences and low-quality 5′ ending sequences were removed from raw sequencing reads using Trimmomatic software (Version 0.39) (39). Following adapter removal, sequence reads were aligned to the reference transcriptome/genome using HISAT2 splice-aware sequence aligner (Version 2.2.0) (40). For each sample, the quality of reference alignments was evaluated using the RSeQC software (Version 3.0.1) (41). Gene expression quantification was conducted on the gene level using Subread (Version 2.0.0) (42) counting mapped paired reads to obtain fragment counts per gene.

### Proteomics

After cell lysis, proteins were reduced, alkylated, and precipitated with cold acetone overnight. For B cells and NK cells, lysate volumes containing 10 µg of protein were prepared by precipitation. For neutrophils, T cells, monocytes, and dendritic cells, lysate volumes containing 25 µg were prepared. Proteins were reconstituted in 100 mM TEAB (pH 8.0), digested with Promega Gold trypsin overnight at 37 °C, and peptides were labeled with TMT isobaric mass tagging reagents (TMT10plex, ThermoFisher Scientific, Waltham, Massachusetts). For cell type–specific common controls, lysate aliquots of each cell type were prepared in parallel and were precipitated, digested, and labeled alongside experimental samples for each TMT experiment. Digestion and labeling efficiencies were estimated for each sample by LC-MS/MS analysis. TMT-labeled samples were pooled and analyzed by LC-MS/MS (MudPIT) using a Q Exactive Plus mass spectrometer (ThermoFisher Scientific). The Q Exactive instrument was operated using data-dependent acquisition, with HCD MS/MS acquired after each MS1 scan on the 15 most abundant ions. For identification of peptides, tandem mass spectra were searched in Proteome Discoverer 2.2 (ThermoFisher Scientific) using SequestHT for database searching against a subset of the UniprotKB protein database (Uniprot release 2017_08) containing *Homo sapiens* protein sequences. Proteins were quantified using the Reporter-Based Quan Consensus workflow in Proteome Discover (PD) without scaling and normalization, and results were filtered for proteins with a minimum of two unique peptides identified.

### Statistical analysis

For transcriptomic analysis, the *edgeR* package (Version 3.26.8) (43) was used to correct systematic sample differences in fragment counts using the TMM normalization method and to calculate moderated log_2_ counts per million (LCPM) for each gene. For proteomic analysis, to correct systemic sample differences between experiments, protein log_2_ ratios were normalized within cell types to align the medians of the log_2_ protein ratio (LPR) distributions. Missing observations were imputed using the k-nearest neighbor algorithm implemented in the *impute* R package (Version 1.58.0). Only proteins with at least 85% non-missing observations in both pre- and post-vaccination samples were used as input for imputation and downstream analysis.

For each participant, the mean of pre-first vaccination samples (Day -7 and Day 1) determined the baseline for the first vaccination for all study arms. The pre-second vaccination sample (Day 29) determined the baseline for the second vaccination for A/H7N9-vaccinated participants only. For both transcriptomics and proteomics, the log_2_ fold changes per gene or protein were calculated for each participant within each immune cell type. DE genes were identified for each study arm and post-vaccination day within each immune cell type by fitting negative binomial generalized linear models as implemented in *edgeR* after the exclusion of outlying samples and lowly expressed genes. In a similar approach for proteomics, DA proteins were identified by applying linear models by the *limma* R package (Version 3.40.2) (44), which uses a moderated t-statistic for hypothesis testing that leverages information across all proteins using a Bayesian model that improves power to detect DA proteins compared to the standard t-statistic.

To control for testing multiple genes, the false-discovery rate (FDR) is based on the Benjamini-Hochberg procedure as implemented using the *p.adjust* R function (45). Genes with an absolute fold change (effect size) of ≥1.5 compared to pre-vaccination and an FDR-adjusted *p*-value < 0.05 were considered significantly DE. A similar approach was taken in proteomics, and proteins with an FDR-adjusted *p*-value <0.15 were considered DA. Gene set enrichment analysis to identify significantly enriched KEGG (version 5.0+), MsigDB (version 7.1+), Reactome, and Blood Transcription Modules (BTM) functional pathways was carried out using the *goseq* R package (version 1.36.0) accounting for gene length bias and multiple testing (46–49). An FDR-adjusted *p*-value ≤0.05 and <.15 were considered significantly enriched for transcriptomics and proteomics, respectively. After the trial commenced, the *p*-value significance threshold for the proteomics analysis was adjusted from <0.05 to <0.15. This change was made to increase the statistical power given the sample size, ensuring the detection of meaningful differences in protein abundance and pathway enrichment. To reduce the risk of false discovery, findings were interpreted in the context of effect size, transcriptomic concordance, and pathway-level validation to ensure biological relevance and to prevent overinterpretation of the results.

### Data-driven approach to pathway selection

The union of enriched pathways across all cell types for each pathway database was collated. For each pathway, the maximum absolute mean fold change for each cell at each day was calculated, as well as the maximum absolute enrichment score (−log_10_ FDR) delta value between each treatment arm comparison. The maximum delta scores were scaled within ‘omics type. Pathways were then ranked in descending order based on the sum of the proteomics and transcriptomics maximum delta score, with the mean of mean absolute fold change determining ties.

### Regularized linear regression analysis

For each cell type and post-vaccination day, a regularized linear regression model was fit to determine changes in DE gene expression and DA protein abundance that best predict log_2_ peak HAI titer in subjects from combined A/H7N9 Study Arms: Study Arm 2 (A/H7N9 + AS03, D1, 29) and Study Arm 3 (A/H7N9 + PBS, D1, 29). Peak titer was defined as a continuous variable representing the maximum HAI titer reported across post-first vaccination (Day 29) and post-second vaccination (Day 57). To avoid overfitting (n ≪ #genes and collinearity among genes) and to facilitate variable selection, an elastic net regularization step (combination of L1 Lasso and L2 ridge penalization) was applied. Leave-one-out cross-validation was used to determine the optimum regularization parameters λ and α that minimize the model deviance mean squared error.

## Data Availability

The transcriptomics data has been deposited with NCBI GEO, accession number GSE286042. Proteomics data were submitted to MassIVE; the ProteomeExchange ID for the data is PXD070689.
