# Supplementary material for: Transcriptomic and proteomic signatures following AS03-adjuvanted Influenza A/H7N9 vaccine
Source: Front Immunol. 2026 Aug 7;17:1786231. doi: 10.3389/fimmu.2026.1786231 (PMC13494703; doi:10.3389/fimmu.2026.1786231)

Confidential/Proprietary Information



**Figure S24:** Reactome pathways signals after vaccination. Bubble plot summarizing activation and enrichment of the top 10 most enriched Reactome pathways across study arms, time points and assays. Pathways were selected based on the maximum difference of the enrichment score ($-log_{10}$ enrichment p-value) between study arms. The pathways were ranked in descending order by the sum of the absolute maximum delta values from both the proteomics and transcriptomics analyses after scaling the scores within assay. The $log_2$ fold change represents the absolute mean of the mean $log_2$ fold changes of all genes/proteins in a pathway. In the event of ranking ties, the pathway with the higher absolute mean of mean $log_2$ fold change value was selected. Circle outlines in blue indicate a fold change < 0 and red outlines indicate a fold change $\geq 0$.

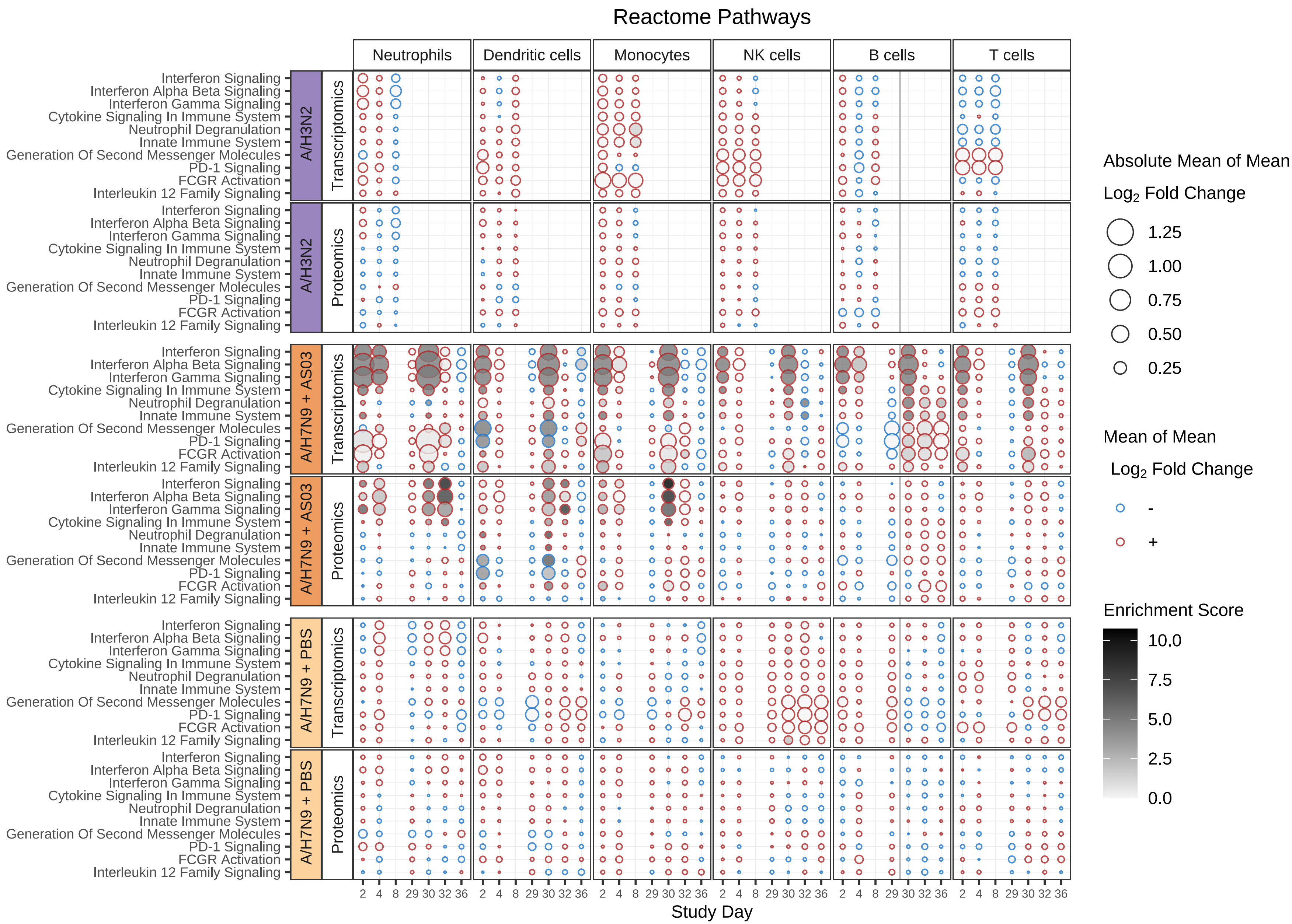

**Figure S25:** GO processes signals after vaccination. Bubble plot summarizing activation and enrichment of the top 10 most enriched GO processes across study arms, time points and assays. Pathways were selected based on the maximum difference of the enrichment score (-$log_{10}$ enrichment p-value) between study arms. The pathways were ranked in descending order by the sum of the absolute maximum delta values from both the proteomics and transcriptomics analyses after scaling the scores within assay. The $log_2$ fold change represents the absolute mean of the mean $log_2$ fold changes of all genes/proteins in a pathway. In the event of ranking ties, the pathway with the higher absolute mean of mean $log_2$ fold change value was selected. Circle outlines in blue indicate a fold change < 0 and red outlines indicate a fold change ≥ 0.

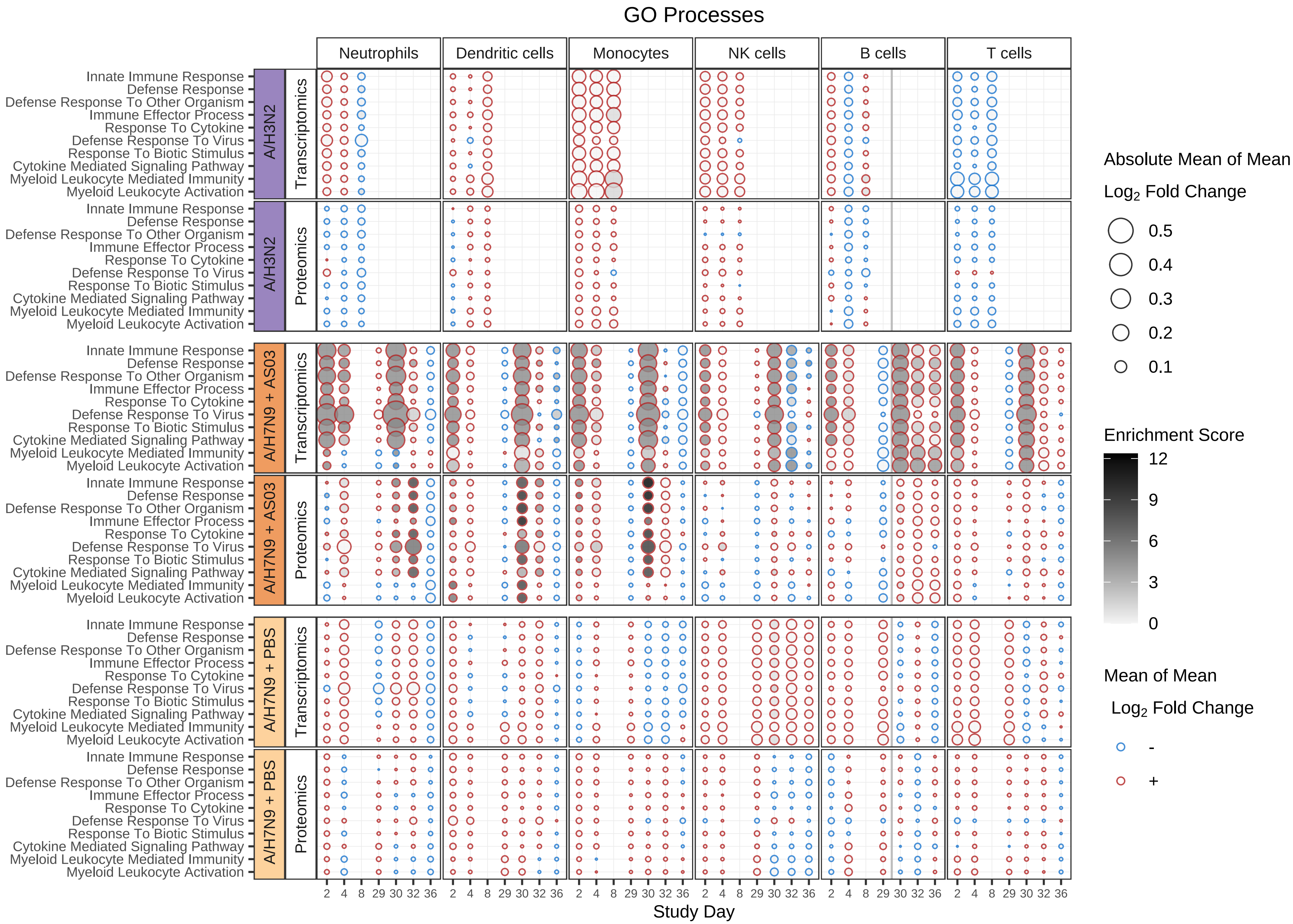

Confidential/Proprietary Information



**Figure S26:** Blood transcription modules signals after vaccination. Bubble plot summarizing activation and enrichment of the top 10 most enriched Blood transcription modules across study arms, time points and assays. Pathways were selected based on the maximum difference of the enrichment score (-$log_{10}$ enrichment p-value) between study arms. The pathways were ranked in descending order by the sum of the absolute maximum delta values from both the proteomics and transcriptomics analyses after scaling the scores within assay. The $log_2$ fold change represents the absolute mean of the mean $log_2$ fold changes of all genes/proteins in a pathway. In the event of ranking ties, the pathway with the higher absolute mean of mean $log_2$ fold change value was selected. Circle outlines in blue indicate a fold change < 0 and red outlines indicate a fold change ≥ 0.

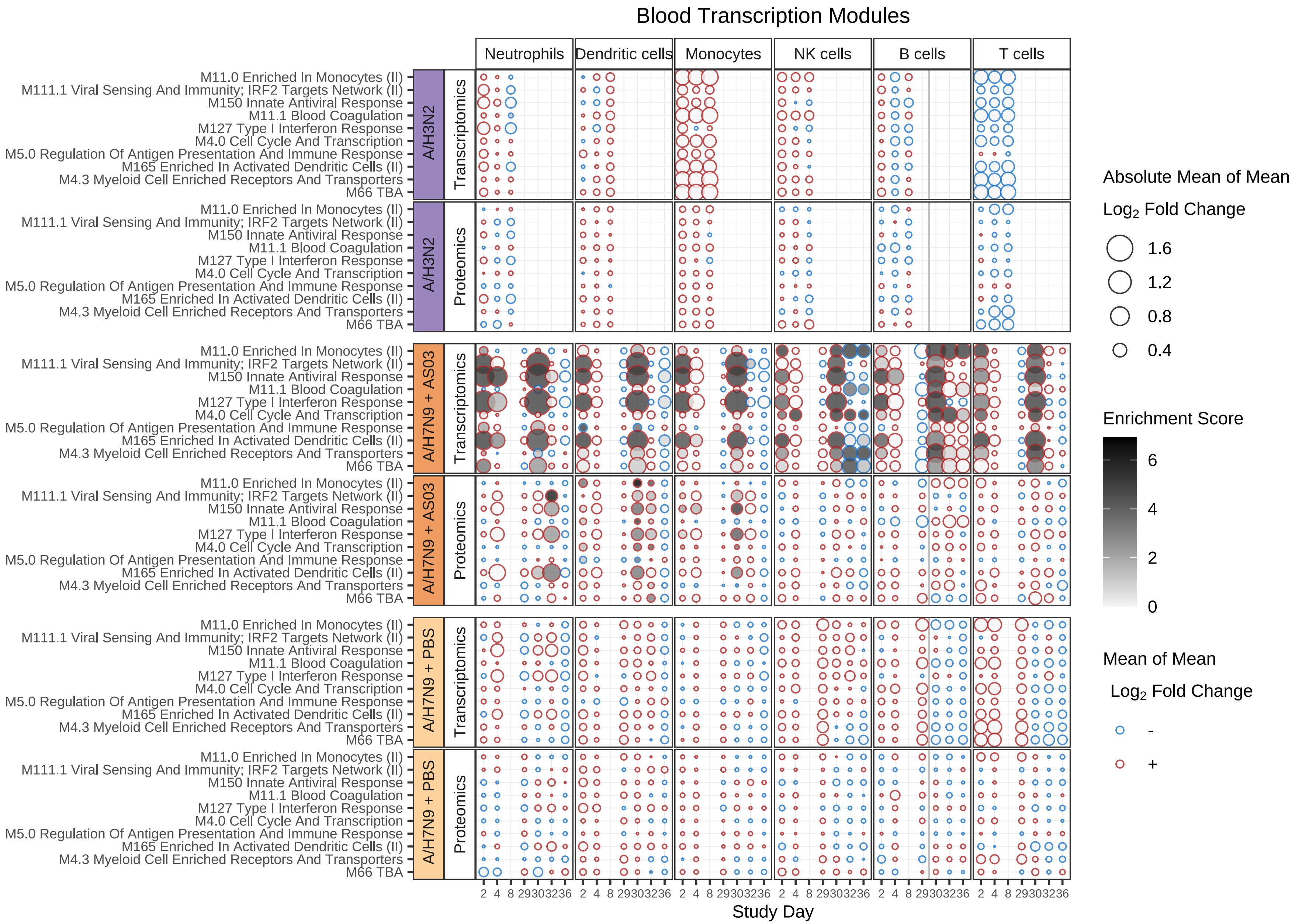

Supplement: Supplementary file 1 [file Image1.pdf]
