# Supplementary material for: Transcriptomic and proteomic signatures following AS03-adjuvanted Influenza A/H7N9 vaccine
Source: Front Immunol. 2026 Aug 7;17:1786231. doi: 10.3389/fimmu.2026.1786231 (PMC13494703; doi:10.3389/fimmu.2026.1786231)

Figure S43: MHC I activity following vaccination and correlation with peak HAI titer. MHC I features include HLA-A, HLA-B, HLA-C, HLA-E, HLA-F, HLA-G. a) Time trends plot of mean of mean log_2 fold change of MHC I genes and proteins for each cell type following adjuvanted A/H7N9 vaccine. Error bars indicate 95% confidence intervals based on 1000 bootstrap replicates. b) Spider plot summarizing Pearson correlation between the mean of mean log_2 fold change of all present MHC I genes/proteins and log_2 HAI peak titer. c) Scatterplots summarizing the Pearson correlation for the top 6 correlated events across omics assays, cell types and days. In red: locally weighted scatterplot smoothing (LOWESS) curve.

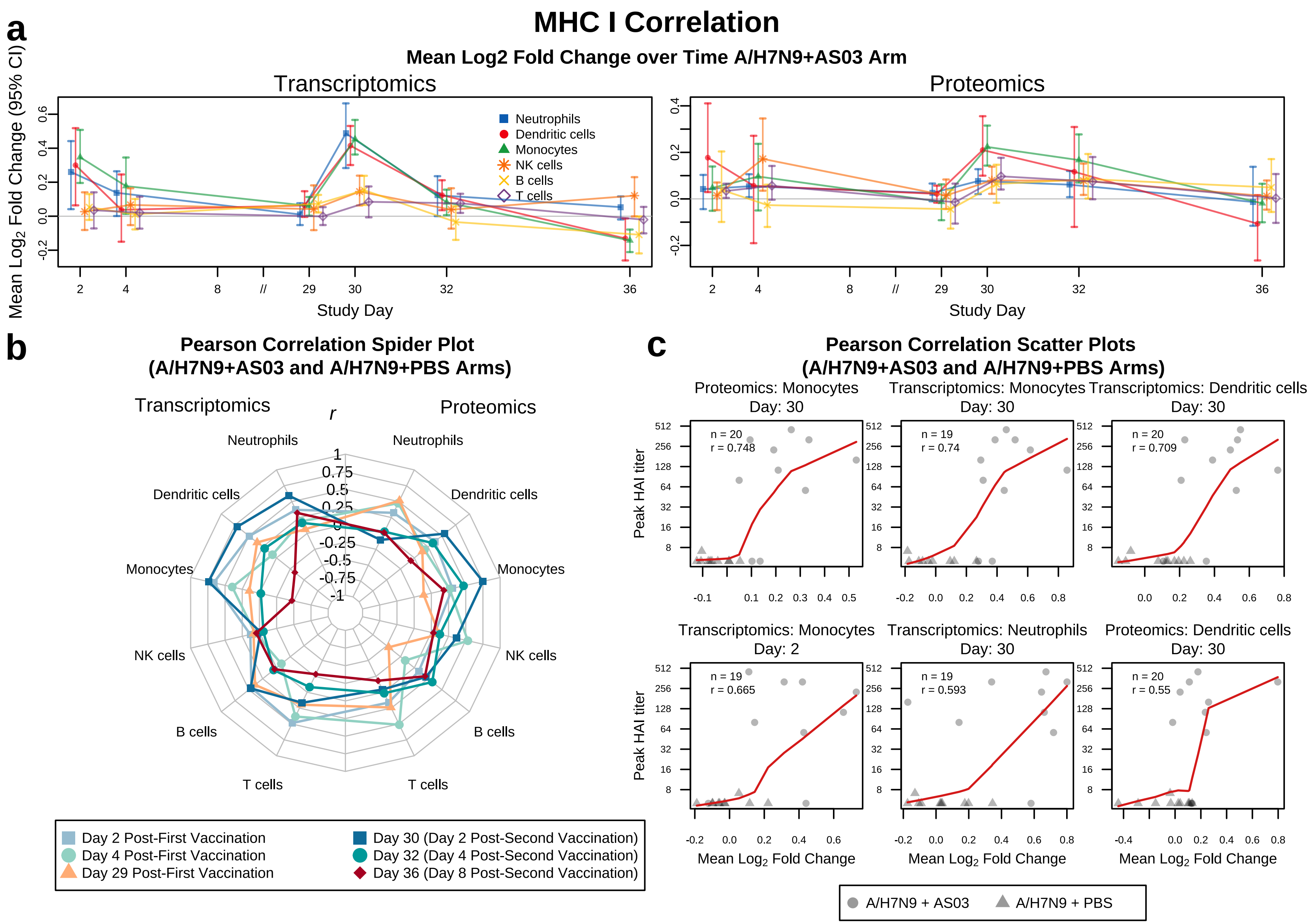

Confidential/Proprietary Information

Figure S44: MHC II activity following vaccination and correlation with peak HAI titer. MHC II features include HLA-DMA, HLA-DMB, HLA-DOA, HLA-DOB, HLA-DPA1, HLA-DPB1, HLA-DQA1, HLA-DQA2, HLA-DQB1, HLA-DRA, HLA-DRB1, HLA-DRB5. a) Time trends plot of mean of mean log_2 fold change of MHC II genes and proteins for each cell type following adjuvanted A/H7N9 vaccine. Error bars indicate 95% confidence intervals based on 1000 bootstrap replicates. b) Spider plot summarizing Pearson correlation between the mean of mean log_2 fold change of all present MHC II genes/proteins and log_2 HAI peak titer. c) Scatterplots summarizing the Pearson correlation for the top 6 correlated events across omics assays, cell types and days. In red: locally weighted scatterplot smoothing (LOWESS) curve.

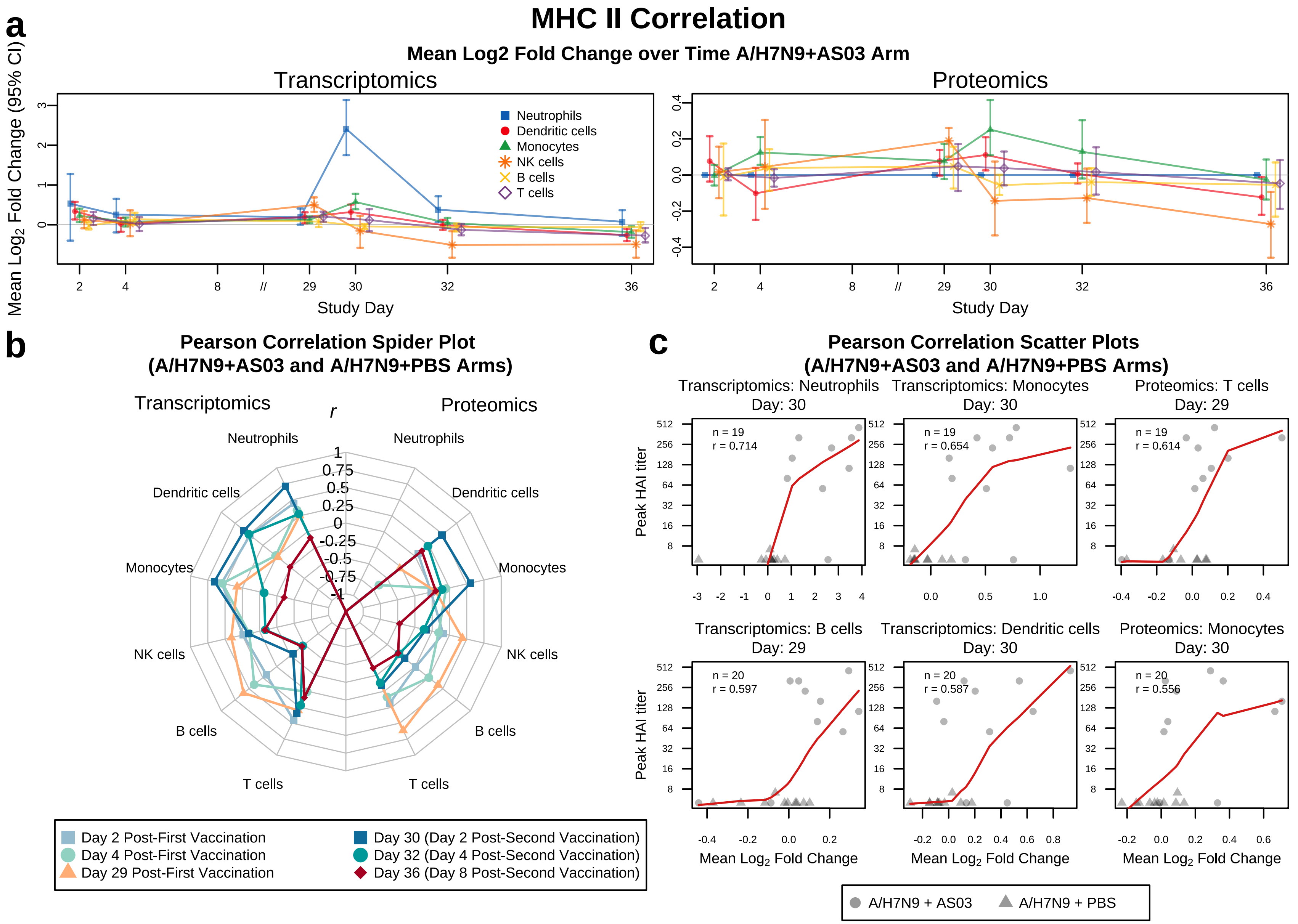

Figure S45: STAT1 activity following vaccination and correlation with peak HAI titer. STAT1 not present in proteomics analysis data. a) Time trends plot of mean of mean log_2 fold change of STAT1 genes and proteins for each cell type following adjuvanted A/H7N9 vaccine. Error bars indicate 95% confidence intervals based on 1000 bootstrap replicates. b) Spider plot summarizing Pearson correlation between the mean of mean log_2 fold change of all present STAT1 genes/proteins and log_2 HAI peak titer. c) Scatterplots summarizing the Pearson correlation for the top 6 correlated events across omics assays, cell types and days. In red: locally weighted scatterplot smoothing (LOWESS) curve.

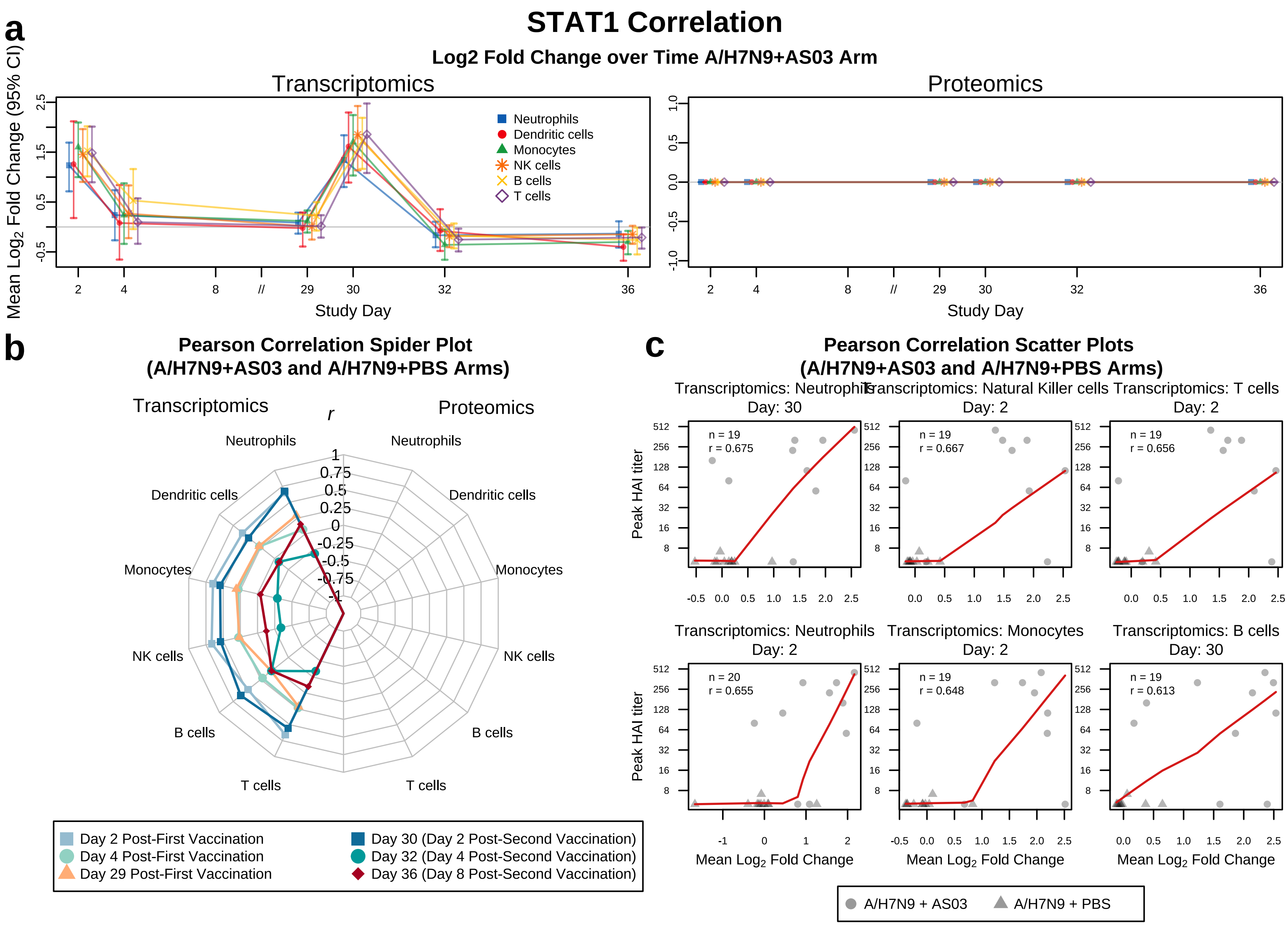

Figure S46: CIITA activity following vaccination and correlation with peak HAI titer. CIITA not present in proteomics analysis data. a) Time trends plot of mean of mean log_2 fold change of CIITA genes and proteins for each cell type following adjuvanted A/H7N9 vaccine. Error bars indicate 95% confidence intervals based on 1000 bootstrap replicates. b) Spider plot summarizing Pearson correlation between the mean of mean log_2 fold change of all present CIITA genes/proteins and log_2 HAI peak titer. c) Scatterplots summarizing the Pearson correlation for the top 6 correlated events across omics assays, cell types and days. In red: locally weighted scatterplot smoothing (LOWESS) curve.

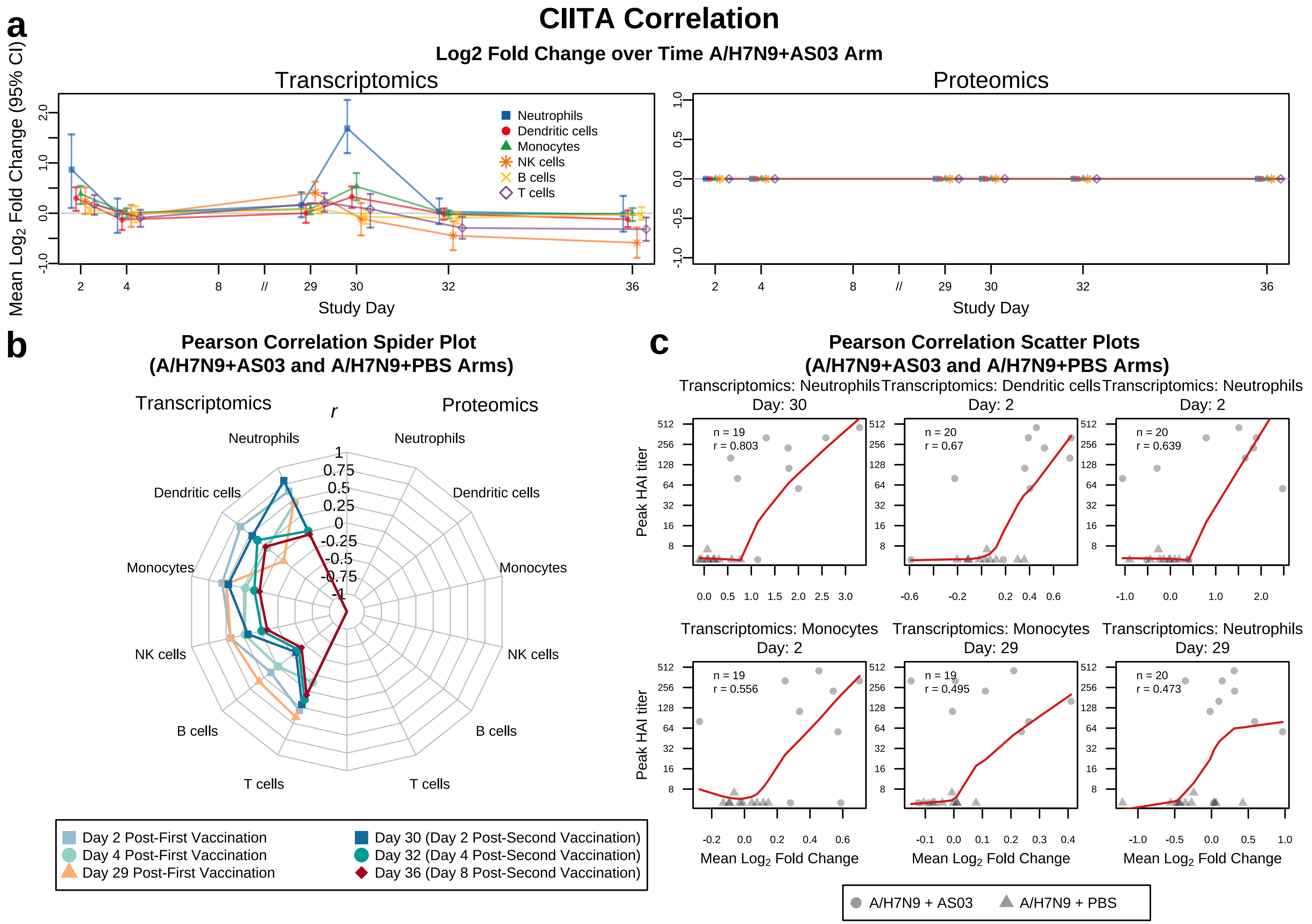

Figure S47: STAT3 activity following vaccination and correlation with peak HAI titer. a) Time trends plot of mean of mean log_2 fold change of STAT3 genes and proteins for each cell type following adjuvanted A/H7N9 vaccine. Error bars indicate 95% confidence intervals based on 1000 bootstrap replicates. b) Spider plot summarizing Pearson correlation between the mean of mean log_2 fold change of all present STAT3 genes/proteins and log_2 HAI peak titer. c) Scatterplots summarizing the Pearson correlation for the top 6 correlated events across omics assays, cell types and days. In red: locally weighted scatterplot smoothing (LOWESS) curve.

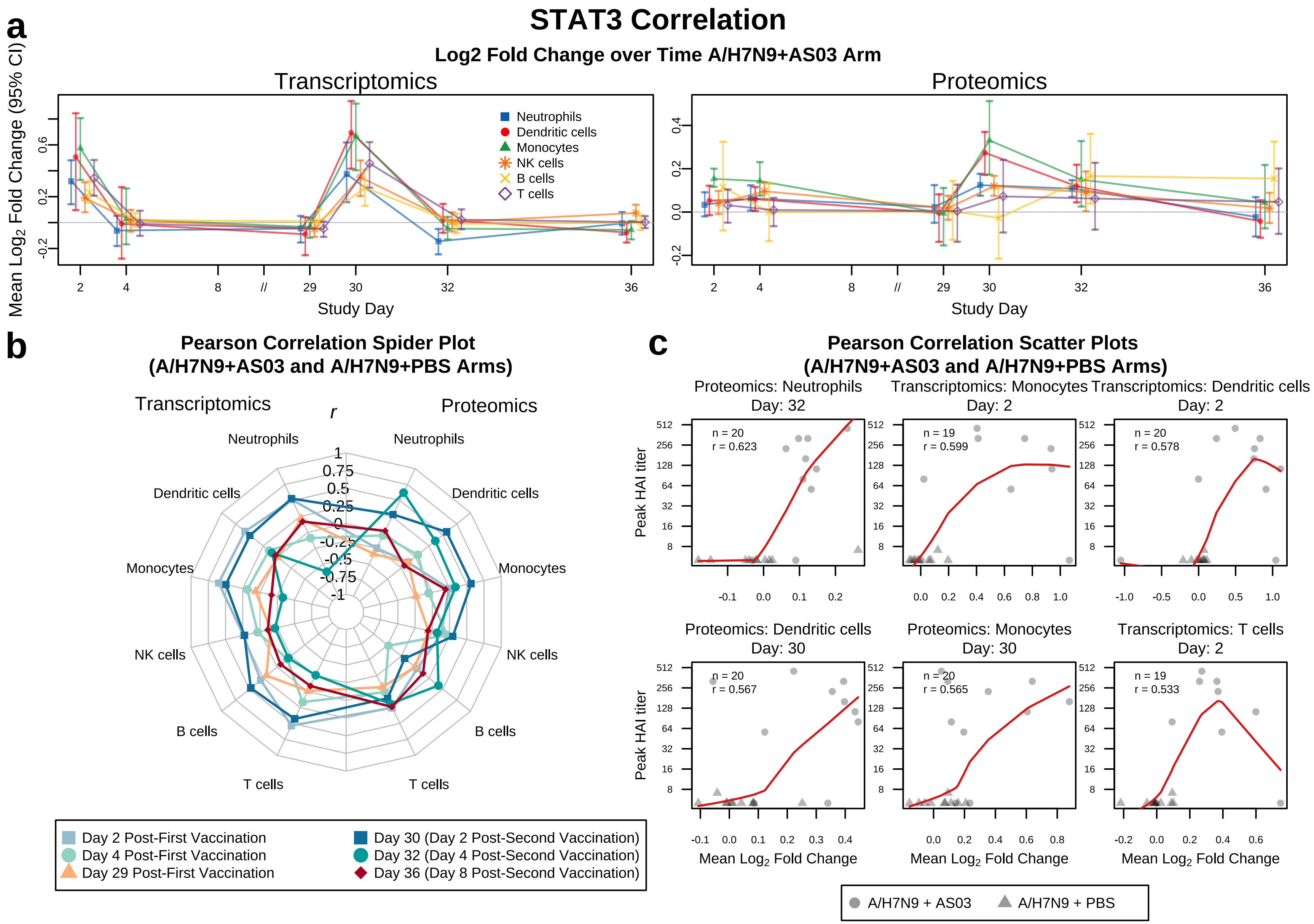

Figure S48: SOCS3 activity following vaccination and correlation with peak HAI titer. SOCS3 not present in proteomics analysis data. a) Time trends plot of mean of mean log_2 fold change of SOCS3 genes and proteins for each cell type following adjuvanted A/H7N9 vaccine. Error bars indicate 95% confidence intervals based on 1000 bootstrap replicates. b) Spider plot summarizing Pearson correlation between the mean of mean log_2 fold change of all present SOCS3 genes/proteins and log_2 HAI peak titer. c) Scatterplots summarizing the Pearson correlation for the top 6 correlated events across omics assays, cell types and days. In red: locally weighted scatterplot smoothing (LOWESS) curve.

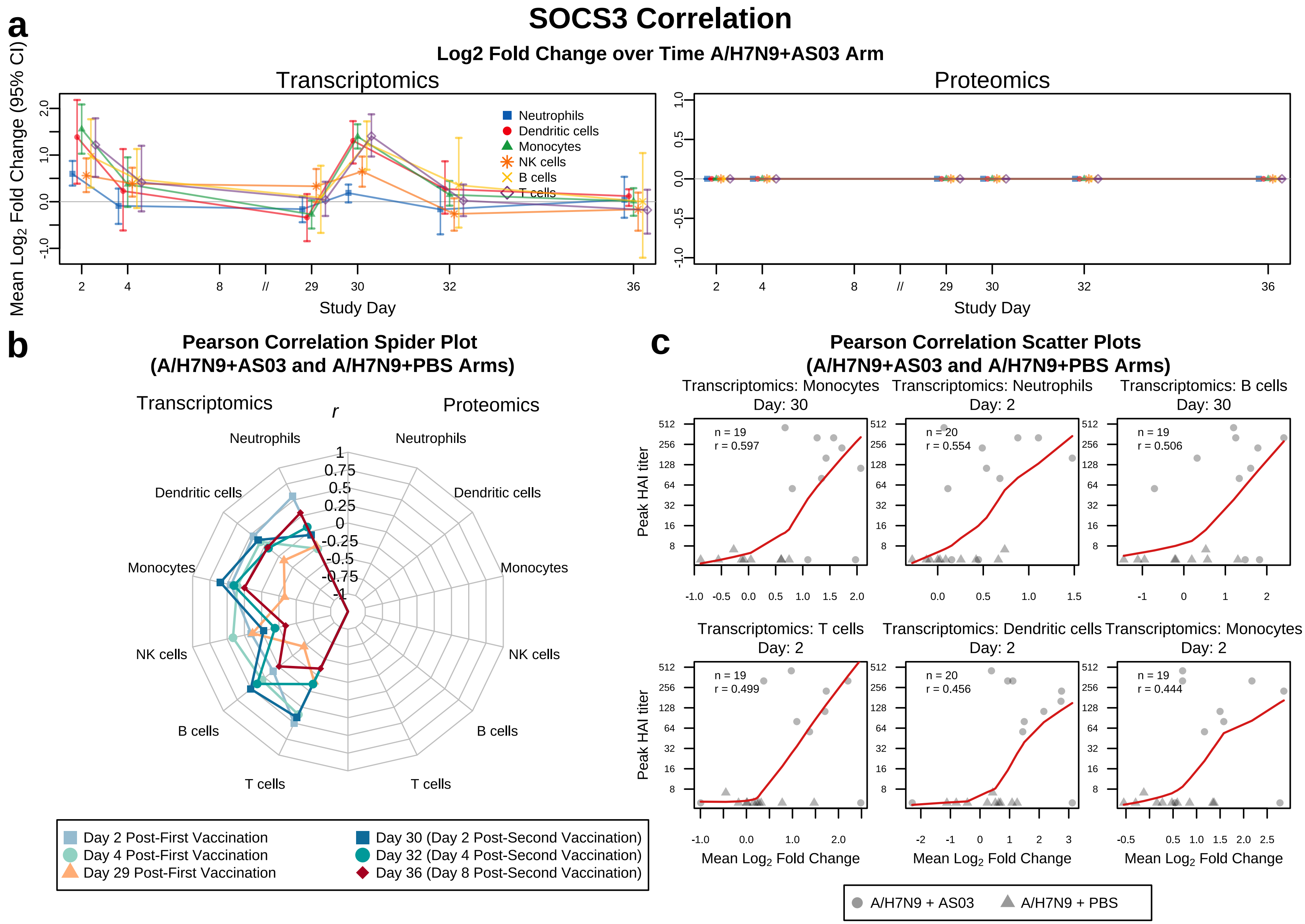

Figure S49: GBP1 activity following vaccination and correlation with peak HAI titer. a) Time trends plot of mean of mean log_2 fold change of GBP1 genes and proteins for each cell type following adjuvanted A/H7N9 vaccine. Error bars indicate 95% confidence intervals based on 1000 bootstrap replicates. b) Spider plot summarizing Pearson correlation between the mean of mean log_2 fold change of all present GBP1 genes/proteins and log_2 HAI peak titer. c) Scatterplots summarizing the Pearson correlation for the top 6 correlated events across omics assays, cell types and days. In red: locally weighted scatterplot smoothing (LOWESS) curve.

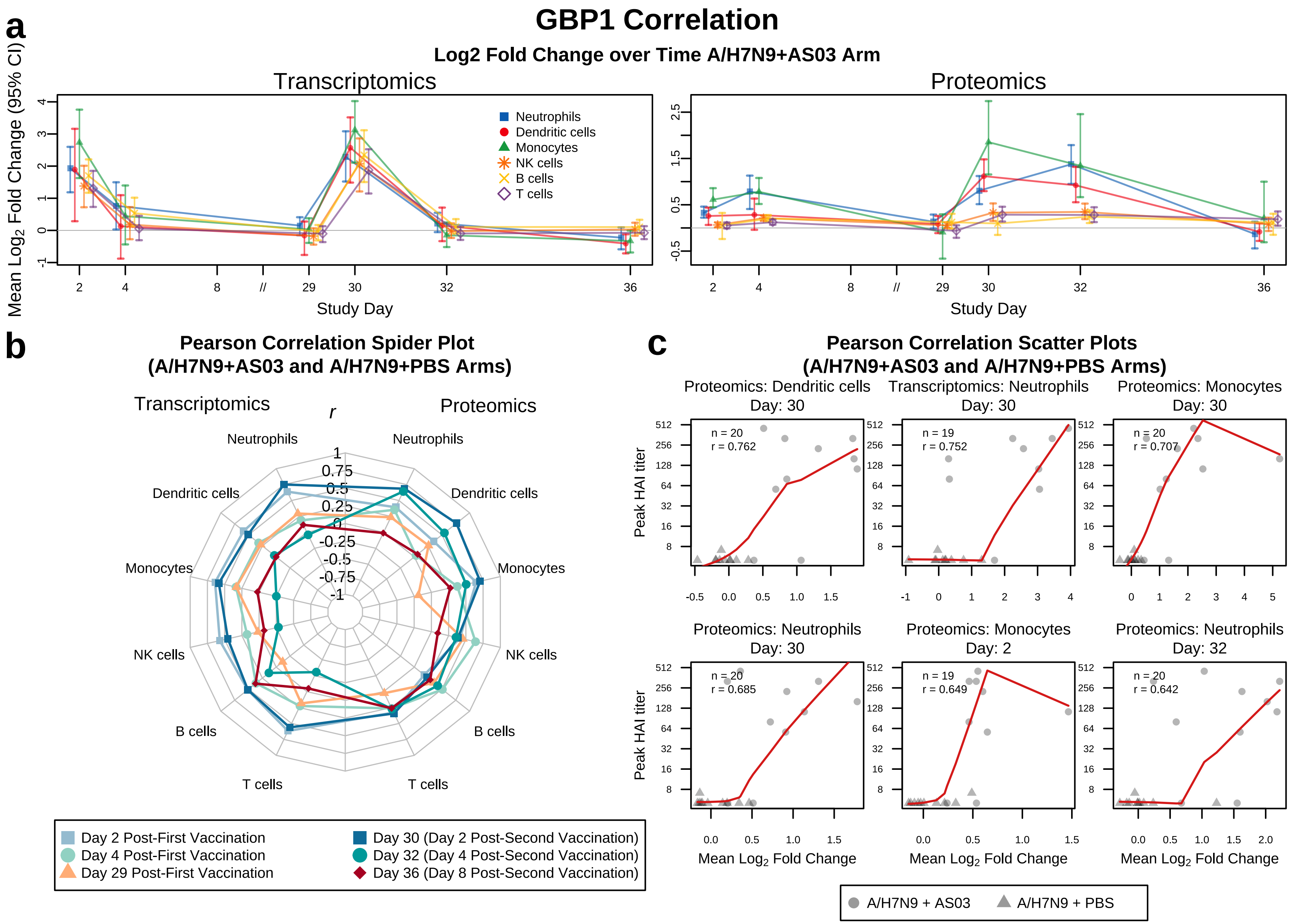

Figure S50: GBP2 activity following vaccination and correlation with peak HAI titer. a) Time trends plot of mean of mean log_2 fold change of GBP2 genes and proteins for each cell type following adjuvanted A/H7N9 vaccine. Error bars indicate 95% confidence intervals based on 1000 bootstrap replicates. b) Spider plot summarizing Pearson correlation between the mean of mean log_2 fold change of all present GBP2 genes/proteins and log_2 HAI peak titer. c) Scatterplots summarizing the Pearson correlation for the top 6 correlated events across omics assays, cell types and days. In red: locally weighted scatterplot smoothing (LOWESS) curve.

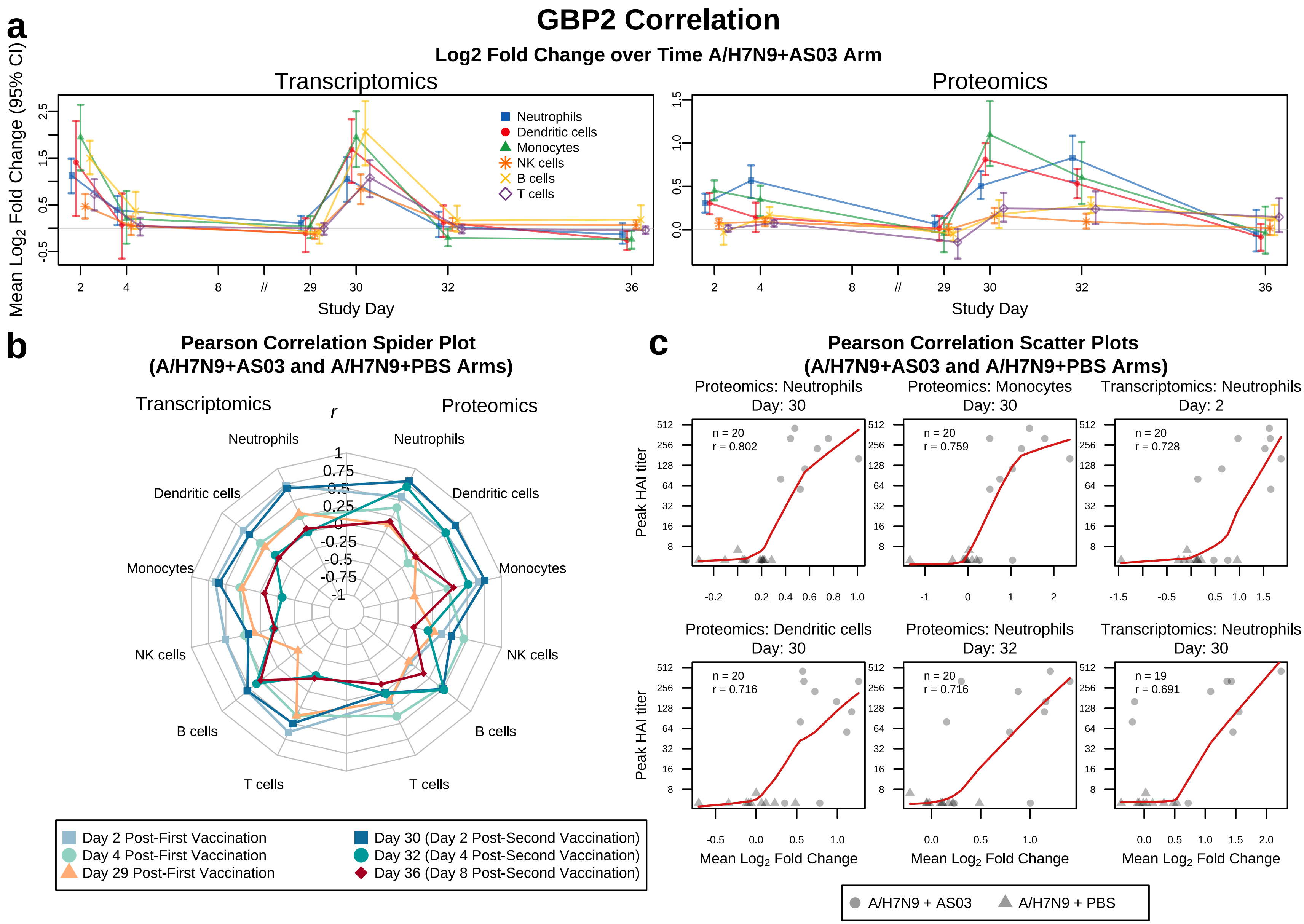

Figure S51: GBP3 activity following vaccination and correlation with peak HAI titer. GBP3 not present in proteomics analysis data. a) Time trends plot of mean of mean log_2 fold change of GBP3 genes and proteins for each cell type following adjuvanted A/H7N9 vaccine. Error bars indicate 95% confidence intervals based on 1000 bootstrap replicates. b) Spider plot summarizing Pearson correlation between the mean of mean log_2 fold change of all present GBP3 genes/proteins and log_2 HAI peak titer. c) Scatterplots summarizing the Pearson correlation for the top 6 correlated events across omics assays, cell types and days. In red: locally weighted scatterplot smoothing (LOWESS) curve.

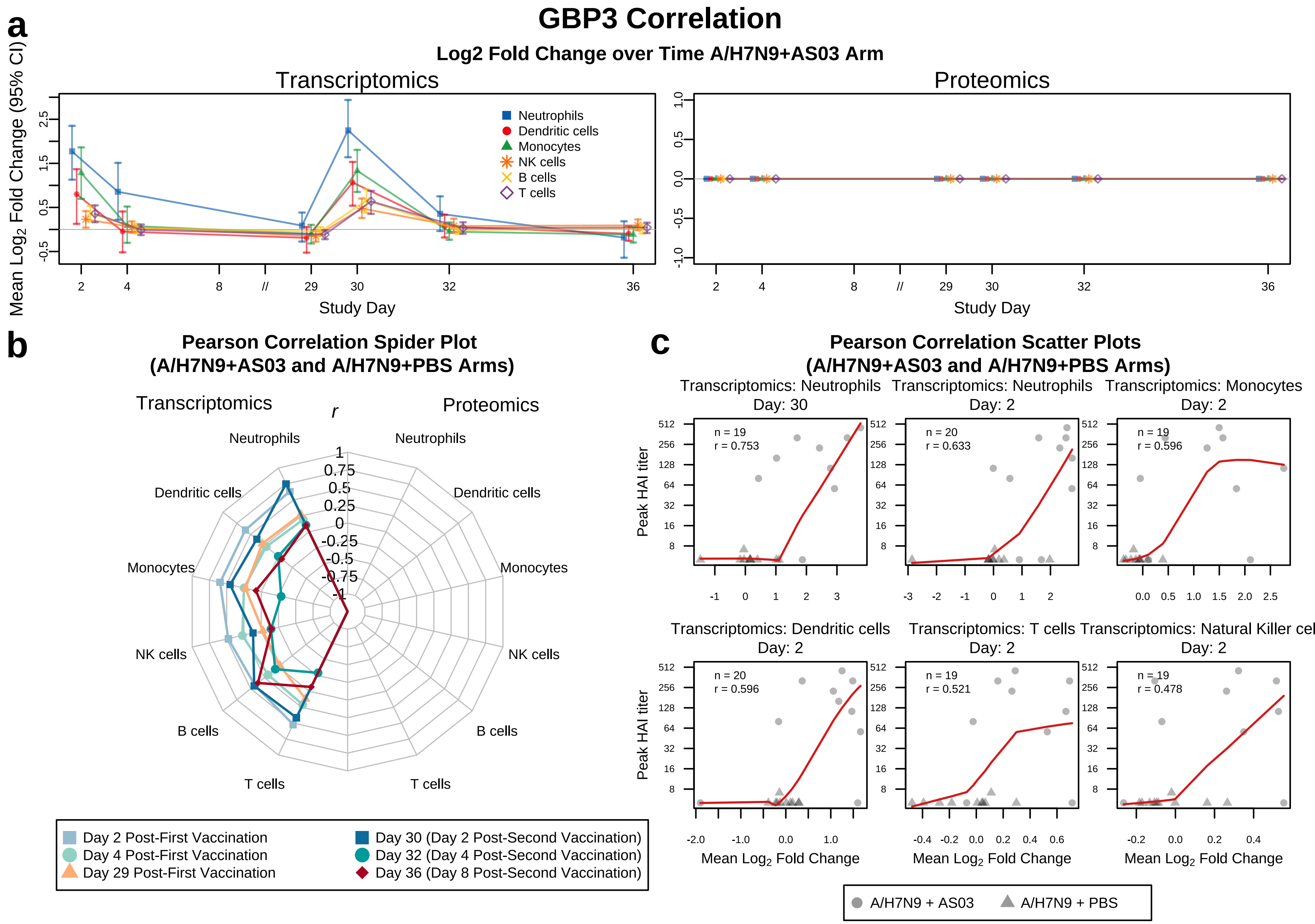

Confidential/Proprietary Information

Figure S52: GBP4 activity following vaccination and correlation with peak HAI titer. a) Time trends plot of mean of mean log_2 fold change of GBP4 genes and proteins for each cell type following adjuvanted A/H7N9 vaccine. Error bars indicate 95% confidence intervals based on 1000 bootstrap replicates. b) Spider plot summarizing Pearson correlation between the mean of mean log_2 fold change of all present GBP4 genes/proteins and log_2 HAI peak titer. c) Scatterplots summarizing the Pearson correlation for the top 6 correlated events across omics assays, cell types and days. In red: locally weighted scatterplot smoothing (LOWESS) curve.

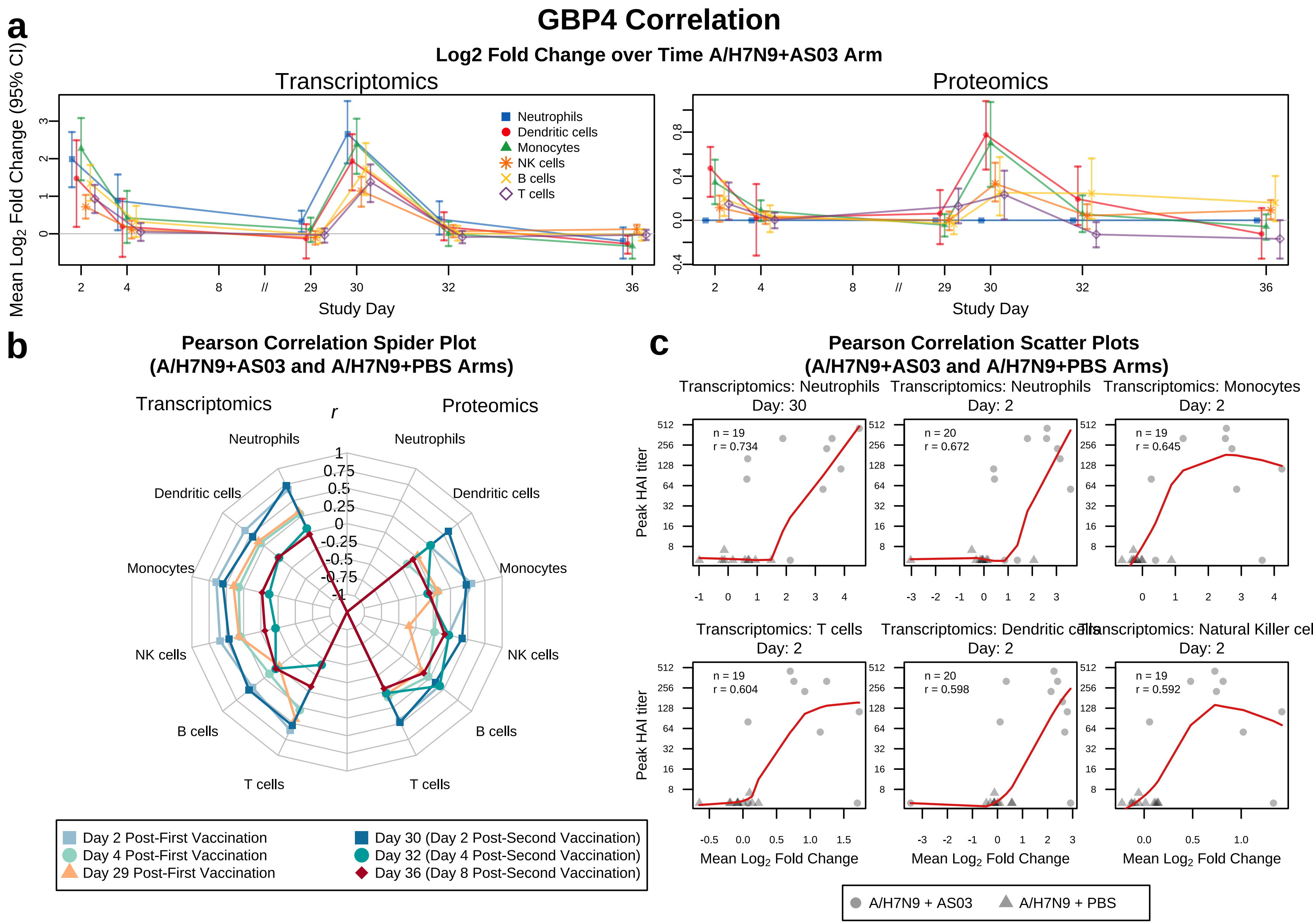

Figure S53: GBP5 activity following vaccination and correlation with peak HAI titer. a) Time trends plot of mean of mean log_2 fold change of GBP5 genes and proteins for each cell type following adjuvanted A/H7N9 vaccine. Error bars indicate 95% confidence intervals based on 1000 bootstrap replicates. b) Spider plot summarizing Pearson correlation between the mean of mean log_2 fold change of all present GBP5 genes/proteins and log_2 HAI peak titer. c) Scatterplots summarizing the Pearson correlation for the top 6 correlated events across omics assays, cell types and days. In red: locally weighted scatterplot smoothing (LOWESS) curve.

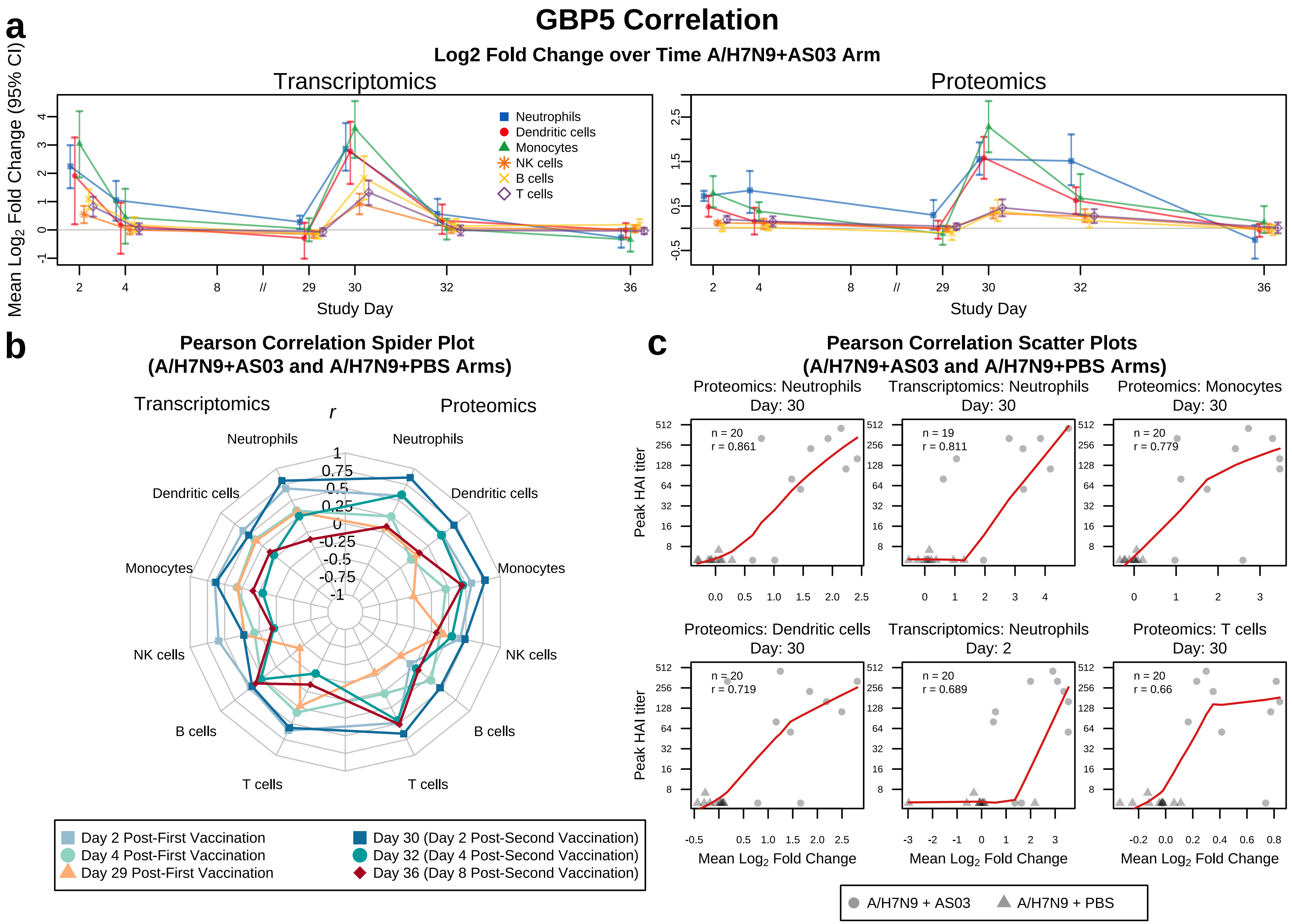

Confidential/Proprietary Information

Figure S54: PSME2 activity following vaccination and correlation with peak HAI titer. a) Time trends plot of mean of mean log_2 fold change of PSME2 genes and proteins for each cell type following adjuvanted A/H7N9 vaccine. Error bars indicate 95% confidence intervals based on 1000 bootstrap replicates. b) Spider plot summarizing Pearson correlation between the mean of mean log_2 fold change of all present PSME2 genes/proteins and log_2 HAI peak titer. c) Scatterplots summarizing the Pearson correlation for the top 6 correlated events across omics assays, cell types and days. In red: locally weighted scatterplot smoothing (LOWESS) curve.

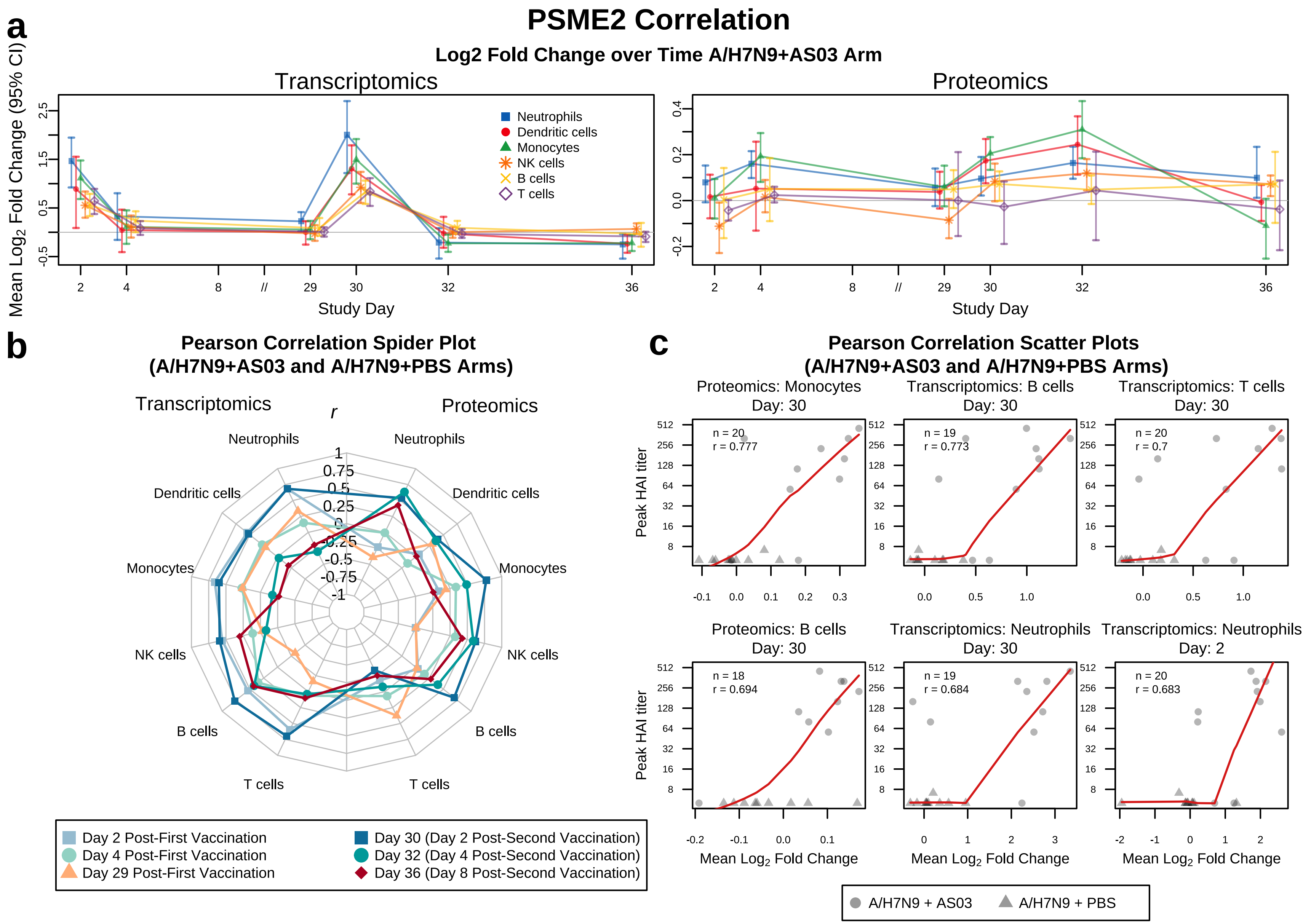

Figure S55: IFIT activity following vaccination and correlation with peak HAI titer. IFIT features include IFIT3, IFIT2, IFIT1, IFIT5. a) Time trends plot of mean of mean log_2 fold change of IFIT genes and proteins for each cell type following adjuvanted A/H7N9 vaccine. Error bars indicate 95% confidence intervals based on 1000 bootstrap replicates. b) Spider plot summarizing Pearson correlation between the mean of mean log_2 fold change of all present IFIT genes/proteins and log_2 HAI peak titer. c) Scatterplots summarizing the Pearson correlation for the top 6 correlated events across omics assays, cell types and days. In red: locally weighted scatterplot smoothing (LOWESS) curve.

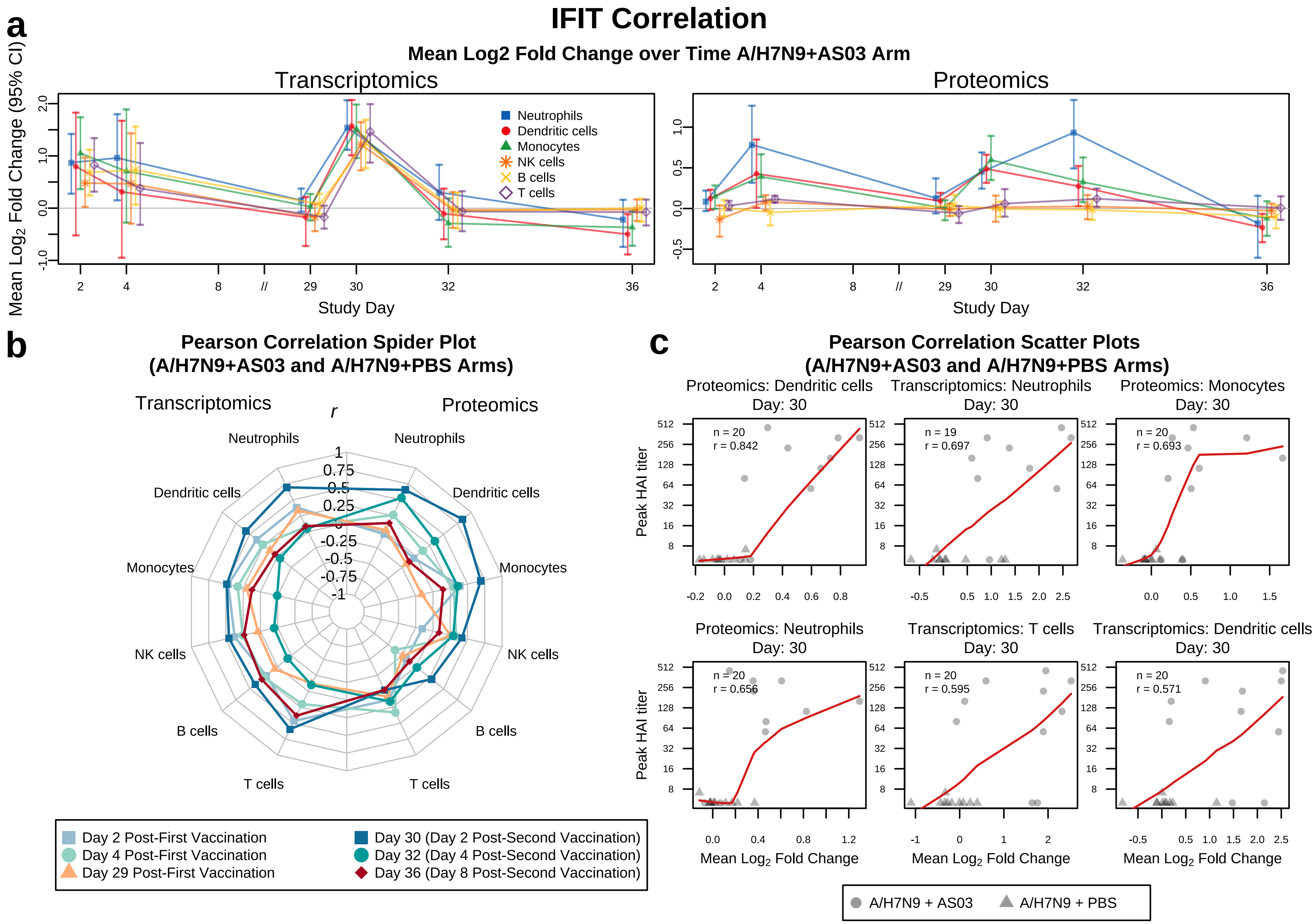

Confidential/Proprietary Information

Figure S56: FCGR1A activity following vaccination and correlation with peak HAI titer. a) Time trends plot of mean of mean log_2 fold change of FCGR1A genes and proteins for each cell type following adjuvanted A/H7N9 vaccine. Error bars indicate 95% confidence intervals based on 1000 bootstrap replicates. b) Spider plot summarizing Pearson correlation between the mean of mean log_2 fold change of all present FCGR1A genes/proteins and log_2 HAI peak titer. c) Scatterplots summarizing the Pearson correlation for the top 6 correlated events across omics assays, cell types and days. In red: locally weighted scatterplot smoothing (LOWESS) curve.

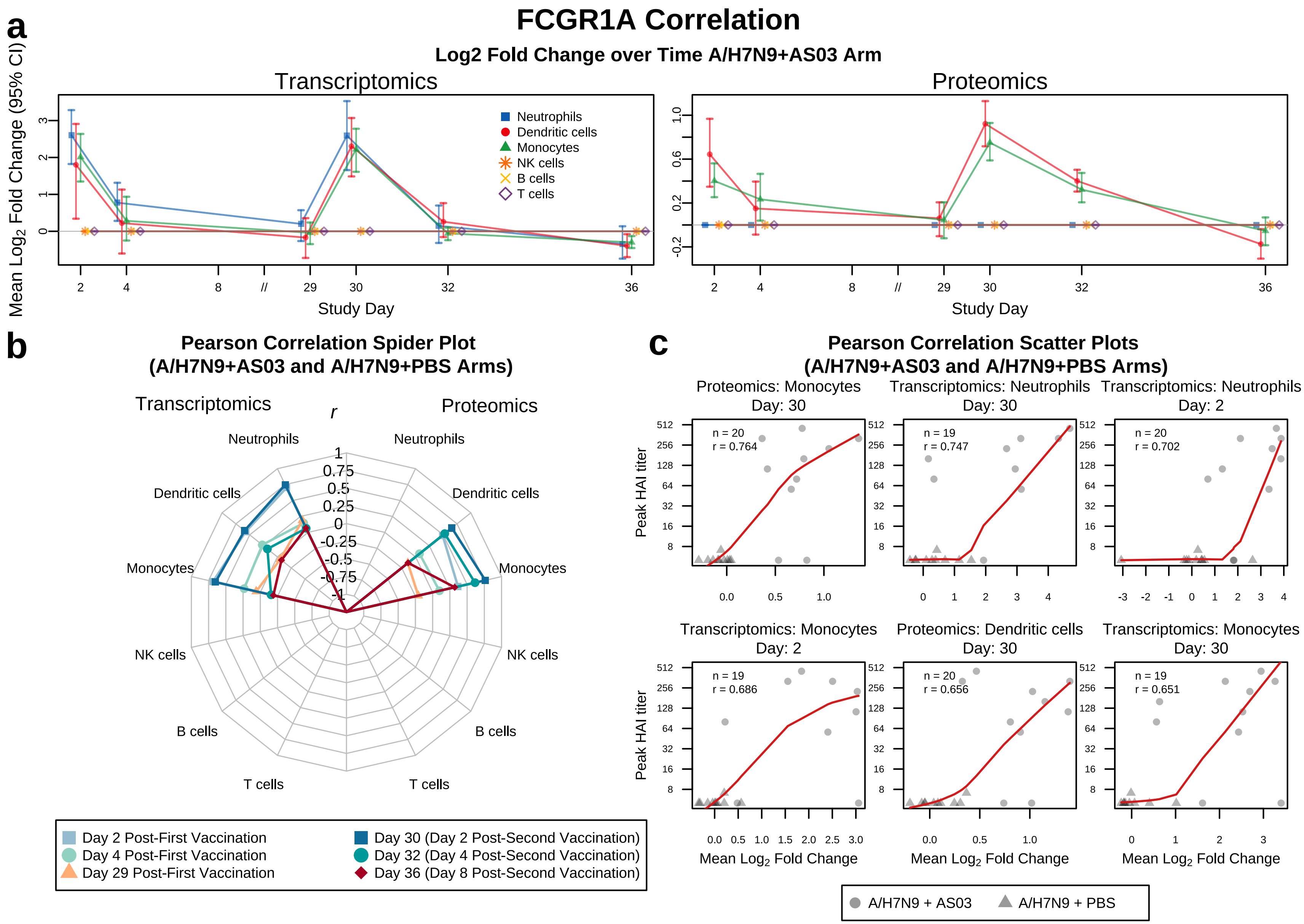

Figure S57: IRF9 activity following vaccination and correlation with peak HAI titer. a) Time trends plot of mean of mean log_2 fold change of IRF9 genes and proteins for each cell type following adjuvanted A/H7N9 vaccine. Error bars indicate 95% confidence intervals based on 1000 bootstrap replicates. b) Spider plot summarizing Pearson correlation between the mean of mean log_2 fold change of all present IRF9 genes/proteins and log_2 HAI peak titer. c) Scatterplots summarizing the Pearson correlation for the top 6 correlated events across omics assays, cell types and days. In red: locally weighted scatterplot smoothing (LOWESS) curve.

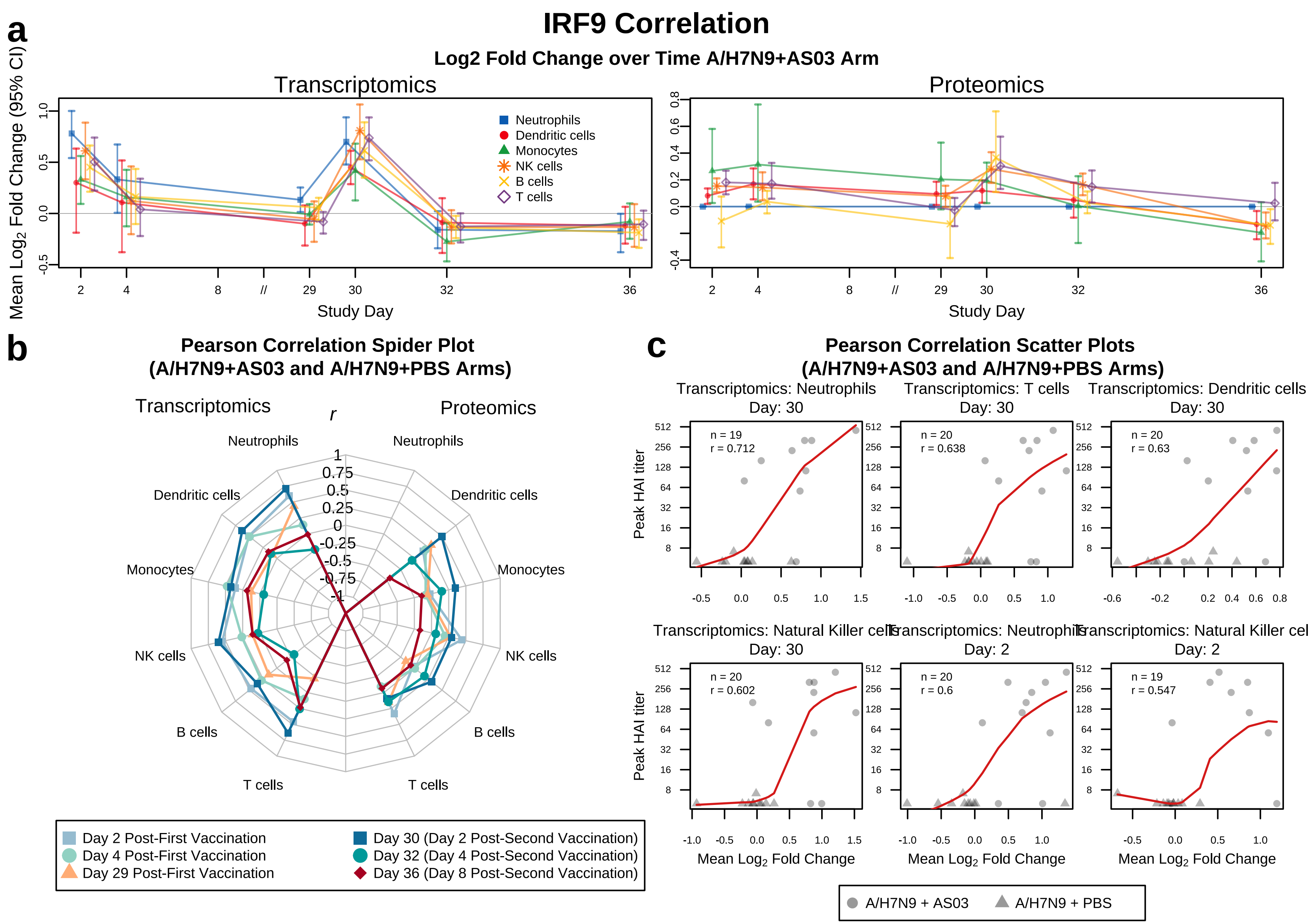

Figure S58: TAP1 activity following vaccination and correlation with peak HAI titer. a) Time trends plot of mean of mean log_2 fold change of TAP1 genes and proteins for each cell type following adjuvanted A/H7N9 vaccine. Error bars indicate 95% confidence intervals based on 1000 bootstrap replicates. b) Spider plot summarizing Pearson correlation between the mean of mean log_2 fold change of all present TAP1 genes/proteins and log_2 HAI peak titer. c) Scatterplots summarizing the Pearson correlation for the top 6 correlated events across omics assays, cell types and days. In red: locally weighted scatterplot smoothing (LOWESS) curve.

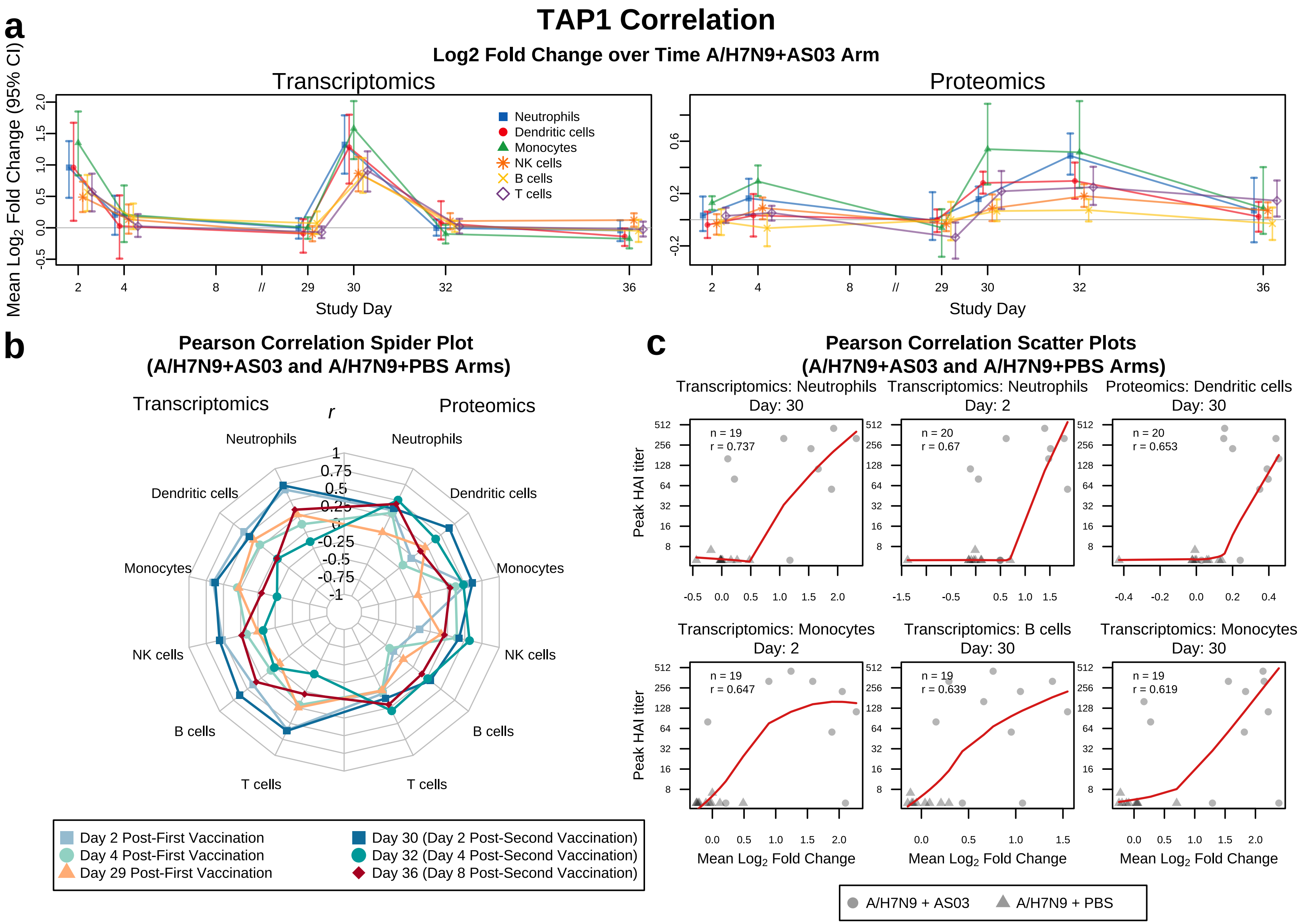

Supplement: Supplementary file 2 [file Image2.pdf]
