# Supplementary material for: Transcriptomic and proteomic signatures following AS03-adjuvanted Influenza A/H7N9 vaccine
Source: Front Immunol. 2026 Aug 7;17:1786231. doi: 10.3389/fimmu.2026.1786231 (PMC13494703; doi:10.3389/fimmu.2026.1786231)
Supplement: Supplementary file 3 [file SupplementaryFile1.pdf]

Supplementary materials for

Transcriptomic and Proteomic Signatures Following AS03-adjuvanted Influenza A/H7N9 Vaccine

C. Buddy Creech, Kristen J. Steenbergen, Casey E. Gelber,
Leigh M. Howard, Kevin Schey, Kristie Lindsey Rose,
Sandra Yoder, Kathryn Edwards, Johannes B. Goll

September 11, 2025

List of Figures

Figure S1 Neutralizing Antibody Results . . . 14
Figure S2 Inflammatory Fractalkine concentration response after vaccination . . . 15
Figure S3 Inflammatory GM-CSF concentration response after vaccination . . . 16
Figure S4 Inflammatory Interleukin 1β concentration response after vaccination . . . 17
Figure S5 Inflammatory Interleukin 2 concentration response after vaccination . . . 18
Figure S6 Inflammatory Interleukin 4 concentration response after vaccination . . . 19
Figure S7 Inflammatory Interleukin 5 concentration response after vaccination . . . 20
Figure S8 Inflammatory Interleukin 6 concentration response after vaccination . . . 21
Figure S9 Inflammatory Interleukin 7 concentration response after vaccination . . . 22
Figure S10 Inflammatory Interleukin 8 concentration response after vaccination . . . 23
Figure S11 Inflammatory Interleukin 10 concentration response after vaccination . . . 24
Figure S12 Inflammatory Interleukin 12, p70 concentration response after vaccination . . . 25
Figure S13 Inflammatory Interleukin 13 concentration response after vaccination . . . 26
Figure S14 Inflammatory Interleukin 17 concentration response after vaccination . . . 27
Figure S15 Inflammatory Interleukin 21 concentration response after vaccination . . . 28
Figure S16 Inflammatory Interleukin 23 concentration response after vaccination . . . 29
Figure S17 Inflammatory Interferon-Inducible T-cell α Chemoattractant concentration response after vaccination . . . 30
Figure S18 Inflammatory Macrophage Inflammatory Protein-1 α concentration response after vaccination 31
Figure S19 Inflammatory Macrophage Inflammatory Protein-1 β concentration response after vaccination 32
Figure S20 Inflammatory Macrophage Inflammatory Protein-3 α concentration response after vaccination 33
Figure S21 Inflammatory Tumor Necrosis Factor concentration response after vaccination . . . 34
Figure S22 Inflammatory Interferon α concentration response after vaccination . . . 35
Figure S23 Inflammatory Interferon β concentration response after vaccination . . . 36
Figure S24 Reactome pathways signals after vaccination . . . 37
Figure S25 GO processes signals after vaccination . . . 38
Figure S26 Blood transcription modules signals after vaccination . . . 39
Figure S27 Top pathway time trend for KEGG NOD-like receptor signaling pathway . . . 40
Figure S28 Top pathway time trend for KEGG Phagosome . . . 41
Figure S29 Top pathway time trend for KEGG Antigen processing and presentation . . . 42
Figure S30 Top pathway time trend for Reactome Interferon Signaling . . . 43
Figure S31 Top pathway time trend for Reactome Interferon Alpha Beta Signaling . . . 44
Figure S32 Top pathway time trend for Reactome Interferon Gamma Signaling . . . 45
Figure S33 Top pathway time trend for GO Innate Immune Response . . . 46
Figure S34 Top pathway time trend for GO Defense Response . . . 47
Figure S35 Top pathway time trend for GO Defense Response To Other Organism . . . 48
Figure S36 Top pathway time trend for M11.0 Enriched In Monocytes (II) . . . 49
Figure S37 Top pathway time trend for M111.1 Viral Sensing And Immunity . . . 50
Figure S38 Top pathway time trend for M150 Innate Antiviral Response . . . 51

Figure S39 KEGG cell cycle time trend 52
Figure S40 KEGG JAK-STAT signaling pathway time trend 53
Figure S41 B-cell population marker time trend 54
Figure S42 GBP genes and protein time trend 55
Figure S43 MHC I activity following vaccination and correlation with peak HAI titer 56
Figure S44 MHC II activity following vaccination and correlation with peak HAI titer 57
Figure S45 STAT1 activity following vaccination and correlation with peak HAI titer 58
Figure S46 CIITA activity following vaccination and correlation with peak HAI titer 59
Figure S47 STAT3 activity following vaccination and correlation with peak HAI titer 60
Figure S48 SOCS3 activity following vaccination and correlation with peak HAI titer 61
Figure S49 GBP1 activity following vaccination and correlation with peak HAI titer 62
Figure S50 GBP2 activity following vaccination and correlation with peak HAI titer 63
Figure S51 GBP3 activity following vaccination and correlation with peak HAI titer 64
Figure S52 GBP4 activity following vaccination and correlation with peak HAI titer 65
Figure S53 GBP5 activity following vaccination and correlation with peak HAI titer 66
Figure S54 PSME2 activity following vaccination and correlation with peak HAI titer 67
Figure S55 IFIT activity following vaccination and correlation with peak HAI titer 68
Figure S56 FCGR1A activity following vaccination and correlation with peak HAI titer 69
Figure S57 IRF9 activity following vaccination and correlation with peak HAI titer 70
Figure S58 TAP1 activity following vaccination and correlation with peak HAI titer 71

List of Tables

Table S1A Summary of Categorical Demographic and Baseline Characteristics by Treatment Group - All Enrolled Participants. 72
Table S1B Summary of Continuous Demographic and Baseline Characteristics by Treatment Group - All Enrolled Participants. 73
Table S2 Genes differentially expressed at Day 8 compared to pre-first vaccination (Study Arm 1 A/H3N2v, D1 , Monocytes). 74
Table S3 Genes differentially expressed at Day 2 compared to pre-first vaccination (Study Arm 2 A/H7N9+AS03, D1, 29, Monocytes). 74
Table S4 Genes differentially expressed at Day 4 compared to pre-first vaccination (Study Arm 2 A/H7N9+AS03, D1, 29, Monocytes). 109
Table S5 Genes differentially expressed at Day 30 compared to pre-second vaccination (Study Arm 2 A/H7N9+AS03, D1, 29, Monocytes). 112
Table S6 Genes differentially expressed at Day 32 compared to pre-second vaccination (Study Arm 2 A/H7N9+AS03, D1, 29, Monocytes). 193
Table S7 Genes differentially expressed at Day 36 compared to pre-second vaccination (Study Arm 2 A/H7N9+AS03, D1, 29, Monocytes). 194
Table S8 Genes differentially expressed at Day 8 compared to pre-first vaccination (Study Arm 1 A/H3N2v, D1 , Dendritic cells). 195
Table S9 Genes differentially expressed at Day 2 compared to pre-first vaccination (Study Arm 2 A/H7N9+AS03, D1, 29, Dendritic cells). 195
Table S10 Genes differentially expressed at Day 4 compared to pre-first vaccination (Study Arm 2 A/H7N9+AS03, D1, 29, Dendritic cells). 258
Table S11 Genes differentially expressed at Day 29 compared to pre-first vaccination (Study Arm 2 A/H7N9+AS03, D1, 29, Dendritic cells). 259
Table S12 Genes differentially expressed at Day 30 compared to pre-second vaccination (Study Arm 2 A/H7N9+AS03, D1, 29, Dendritic cells). 259
Table S13 Genes differentially expressed at Day 32 compared to pre-second vaccination (Study Arm 2 A/H7N9+AS03, D1, 29, Dendritic cells). 372
Table S14 Genes differentially expressed at Day 36 compared to pre-second vaccination (Study Arm 2 A/H7N9+AS03, D1, 29, Dendritic cells). 374
Table S15 Genes differentially expressed at Day 8 compared to pre-first vaccination (Study Arm 1 A/H3N2v, D1 , Neutrophils). 384
Table S16 Genes differentially expressed at Day 2 compared to pre-first vaccination (Study Arm 2 A/H7N9+AS03, D1, 29, Neutrophils). 386
Table S17 Genes differentially expressed at Day 4 compared to pre-first vaccination (Study Arm 2 A/H7N9+AS03, D1, 29, Neutrophils). 449
Table S18 Genes differentially expressed at Day 30 compared to pre-second vaccination (Study Arm 2 A/H7N9+AS03, D1, 29, Neutrophils). 451

Table S19 Genes differentially expressed at Day 32 compared to pre-second vaccination (Study Arm 2 A/H7N9+AS03, D1, 29, Neutrophils). 567
Table S20 Genes differentially expressed at Day 8 compared to pre-first vaccination (Study Arm 1 A/H3N2v, D1 , NK cells). 577
Table S21 Genes differentially expressed at Day 2 compared to pre-first vaccination (Study Arm 2 A/H7N9+AS03, D1, 29, NK cells). 577
Table S22 Genes differentially expressed at Day 4 compared to pre-first vaccination (Study Arm 2 A/H7N9+AS03, D1, 29, NK cells). 581
Table S23 Genes differentially expressed at Day 29 compared to pre-first vaccination (Study Arm 2 A/H7N9+AS03, D1, 29, NK cells). 585
Table S24 Genes differentially expressed at Day 30 compared to pre-second vaccination (Study Arm 2 A/H7N9+AS03, D1, 29, NK cells). 586
Table S25 Genes differentially expressed at Day 32 compared to pre-second vaccination (Study Arm 2 A/H7N9+AS03, D1, 29, NK cells). 603
Table S26 Genes differentially expressed at Day 36 compared to pre-second vaccination (Study Arm 2 A/H7N9+AS03, D1, 29, NK cells). 626
Table S27 Genes differentially expressed at Day 30 compared to pre-second vaccination (Study Arm 3 A/H7N9 + PBS, D1, 29, NK cells). 636
Table S28 Genes differentially expressed at Day 8 compared to pre-first vaccination (Study Arm 1 A/H3N2v, D1 , B cells). 636
Table S29 Genes differentially expressed at Day 2 compared to pre-first vaccination (Study Arm 2 A/H7N9+AS03, D1, 29, B cells). 637
Table S30 Genes differentially expressed at Day 4 compared to pre-first vaccination (Study Arm 2 A/H7N9+AS03, D1, 29, B cells). 640
Table S31 Genes differentially expressed at Day 29 compared to pre-first vaccination (Study Arm 2 A/H7N9+AS03, D1, 29, B cells). 640
Table S32 Genes differentially expressed at Day 4 compared to pre-first vaccination (Study Arm 3 A/H7N9 + PBS, D1, 29, B cells). 640
Table S33 Genes differentially expressed at Day 30 compared to pre-second vaccination (Study Arm 2 A/H7N9+AS03, D1, 29, B cells). 641
Table S34 Genes differentially expressed at Day 32 compared to pre-second vaccination (Study Arm 2 A/H7N9+AS03, D1, 29, B cells). 660
Table S35 Genes differentially expressed at Day 36 compared to pre-second vaccination (Study Arm 2 A/H7N9+AS03, D1, 29, B cells). 666
Table S36 Genes differentially expressed at Day 2 compared to pre-first vaccination (Study Arm 2 A/H7N9+AS03, D1, 29, T cells). 670
Table S37 Genes differentially expressed at Day 29 compared to pre-first vaccination (Study Arm 2 A/H7N9+AS03, D1, 29, T cells). 675
Table S38 Genes differentially expressed at Day 4 compared to pre-first vaccination (Study Arm 3 A/H7N9 + PBS, D1, 29, T cells). 676

Table S39 Genes differentially expressed at Day 30 compared to pre-second vaccination (Study Arm 2 A/H7N9+AS03, D1, 29, T cells). . . . 676
Table S40 Genes differentially expressed at Day 32 compared to pre-second vaccination (Study Arm 2 A/H7N9+AS03, D1, 29, T cells). . . . 693
Table S41 Genes differentially expressed at Day 36 compared to pre-second vaccination (Study Arm 2 A/H7N9+AS03, D1, 29, T cells). . . . 693
Table S42 Proteins differentially abundant at Day 2 compared to pre-first vaccination (Study Arm 2 (A/H7N9 + AS03 D1, 29), Neutrophils). . . . 694
Table S43 Proteins differentially abundant at Day 2 compared to pre-first vaccination (Study Arm 1 (A/H3N2v, D1) versus Study Arm 2 (A/H7N9 + AS03 D1, 29), Neutrophils). . . . 695
Table S44 Proteins differentially abundant at Day 2 compared to pre-first vaccination (Study Arm 2 (A/H7N9 + AS03 D1, 29) versus Study Arm 3 (A/H7N9 + PBS, D1, 29), Neutrophils). . . . 695
Table S45 Proteins differentially abundant at Day 4 compared to pre-first vaccination (Study Arm 2 (A/H7N9 + AS03 D1, 29), Neutrophils). . . . 695
Table S46 Proteins differentially abundant at Day 30 compared to pre-second vaccination (Study Arm 2 (A/H7N9 + AS03 D1, 29), Neutrophils). . . . 696
Table S47 Proteins differentially abundant at Day 30 compared to pre-second vaccination (Study Arm 2 (A/H7N9 + AS03 D1, 29) versus Study Arm 3 (A/H7N9 + PBS, D1, 29), Neutrophils). . . . 697
Table S48 Proteins differentially abundant at Day 32 compared to pre-second vaccination (Study Arm 2 (A/H7N9 + AS03 D1, 29), Neutrophils). . . . 698
Table S49 Proteins differentially abundant at Day 32 compared to pre-second vaccination (Study Arm 2 (A/H7N9 + AS03 D1, 29) versus Study Arm 3 (A/H7N9 + PBS, D1, 29), Neutrophils). . . . 704
Table S50 Proteins differentially abundant at Day 30 compared to pre-second vaccination (Study Arm 2 (A/H7N9 + AS03 D1, 29), T cells). . . . 706
Table S51 Proteins differentially abundant at Day 2 compared to pre-first vaccination (Study Arm 1 (A/H3N2v, D1), Dendritic cells). . . . 706
Table S52 Proteins differentially abundant at Day 2 compared to pre-first vaccination (Study Arm 2 (A/H7N9 + AS03 D1, 29), Dendritic cells). . . . 706
Table S53 Proteins differentially abundant at Day 2 compared to pre-first vaccination (Study Arm 1 (A/H3N2v, D1) versus Study Arm 2 (A/H7N9 + AS03 D1, 29), Dendritic cells). . . . 755
Table S54 Proteins differentially abundant at Day 2 compared to pre-first vaccination (Study Arm 2 (A/H7N9 + AS03 D1, 29) versus Study Arm 3 (A/H7N9 + PBS, D1, 29), Dendritic cells). . . . 764
Table S55 Proteins differentially abundant at Day 30 compared to pre-second vaccination (Study Arm 2 (A/H7N9 + AS03 D1, 29), Dendritic cells). . . . 765
Table S56 Proteins differentially abundant at Day 30 compared to pre-second vaccination (Study Arm 2 (A/H7N9 + AS03 D1, 29) versus Study Arm 3 (A/H7N9 + PBS, D1, 29), Dendritic cells). . . . 819
Table S57 Proteins differentially abundant at Day 32 compared to pre-second vaccination (Study Arm 2 (A/H7N9 + AS03 D1, 29), Dendritic cells). . . . 823
Table S58 Proteins differentially abundant at Day 2 compared to pre-first vaccination (Study Arm 2 (A/H7N9 + AS03 D1, 29), Monocytes). . . . 825

Table S59 Proteins differentially abundant at Day 2 compared to pre-first vaccination (Study Arm 2 (A/H7N9 + AS03 D1, 29) versus Study Arm 3 (A/H7N9 + PBS, D1, 29), Monocytes). 832
Table S60 Proteins differentially abundant at Day 4 compared to pre-first vaccination (Study Arm 2 (A/H7N9 + AS03 D1, 29), Monocytes). 832
Table S61 Proteins differentially abundant at Day 4 compared to pre-first vaccination (Study Arm 1 (A/H3N2v, D1) versus Study Arm 2 (A/H7N9 + AS03 D1, 29), Monocytes). 833
Table S62 Proteins differentially abundant at Day 30 compared to pre-second vaccination (Study Arm 2 (A/H7N9 + AS03 D1, 29), Monocytes). 833
Table S63 Proteins differentially abundant at Day 30 compared to pre-second vaccination (Study Arm 2 (A/H7N9 + AS03 D1, 29) versus Study Arm 3 (A/H7N9 + PBS, D1, 29), Monocytes). 846
Table S64 Proteins differentially abundant at Day 32 compared to pre-second vaccination (Study Arm 2 (A/H7N9 + AS03 D1, 29) versus Study Arm 3 (A/H7N9 + PBS, D1, 29), Monocytes). 853
Table S65 Proteins differentially abundant at Day 4 compared to pre-first vaccination (Study Arm 3 (A/H7N9 + PBS, D1, 29), B cells). 854
Table S66 Proteins differentially abundant at Day 29 compared to pre-first vaccination (Study Arm 2 (A/H7N9 + AS03 D1, 29) versus Study Arm 3 (A/H7N9 + PBS, D1, 29), B cells). 855
Table S67 Proteins differentially abundant at Day 30 compared to pre-second vaccination (Study Arm 2 (A/H7N9 + AS03 D1, 29), B cells). 855
Table S68 Proteins differentially abundant at Day 4 compared to pre-first vaccination (Study Arm 2 (A/H7N9 + AS03 D1, 29), Natural Killer cells). 855
Table S69 Proteins differentially abundant at Day 30 compared to pre-second vaccination (Study Arm 2 (A/H7N9 + AS03 D1, 29), Natural Killer cells). 856
Table S70 Proteins differentially abundant at Day 36 compared to pre-second vaccination (Study Arm 2 (A/H7N9 + AS03 D1, 29), Natural Killer cells). 857
Table S71 KEGG Pathways enriched in differentially expressed genes at Day 2 compared to pre-First Vaccination (Monocytes, Study Arm 2 A/H7N9+AS03, D1, 29). 858
Table S72 MSigDB Reactome Pathways enriched in differentially expressed genes at Day 2 compared to pre-First Vaccination (Monocytes, Study Arm 2 A/H7N9+AS03, D1, 29). 858
Table S73 MSigDB GO Biological Processes enriched in differentially expressed genes at Day 2 compared to pre-First Vaccination (Monocytes, Study Arm 2 A/H7N9+AS03, D1, 29). 859
Table S74 Blood Transcription Modules enriched in differentially expressed genes at Day 2 compared to pre-First Vaccination (Monocytes, Study Arm 2 A/H7N9+AS03, D1, 29). 865
Table S75 KEGG Pathways enriched in differentially expressed genes at Day 4 compared to pre-First Vaccination (Monocytes, Study Arm 2 A/H7N9+AS03, D1, 29). 866
Table S76 MSigDB Reactome Pathways enriched in differentially expressed genes at Day 4 compared to pre-First Vaccination (Monocytes, Study Arm 2 A/H7N9+AS03, D1, 29). 866
Table S77 MSigDB GO Biological Processes enriched in differentially expressed genes at Day 4 compared to pre-First Vaccination (Monocytes, Study Arm 2 A/H7N9+AS03, D1, 29). 866
Table S78 Blood Transcription Modules enriched in differentially expressed genes at Day 4 compared to pre-First Vaccination (Monocytes, Study Arm 2 A/H7N9+AS03, D1, 29). 867

Table S79 KEGG Pathways enriched in differentially expressed genes at Day 30 compared to pre-Second Vaccination (Monocytes, Study Arm 2 A/H7N9+AS03, D1, 29). 867
Table S80 MSigDB Reactome Pathways enriched in differentially expressed genes at Day 30 compared to pre-Second Vaccination (Monocytes, Study Arm 2 A/H7N9+AS03, D1, 29). 868
Table S81 MSigDB GO Biological Processes enriched in differentially expressed genes at Day 30 compared to pre-Second Vaccination (Monocytes, Study Arm 2 A/H7N9+AS03, D1, 29). 869
Table S82 Blood Transcription Modules enriched in differentially expressed genes at Day 30 compared to pre-Second Vaccination (Monocytes, Study Arm 2 A/H7N9+AS03, D1, 29). 881
Table S83 KEGG Pathways enriched in differentially expressed genes at Day 32 compared to pre-Second Vaccination (Monocytes, Study Arm 2 A/H7N9+AS03, D1, 29). 882
Table S84 MSigDB Reactome Pathways enriched in differentially expressed genes at Day 32 compared to pre-Second Vaccination (Monocytes, Study Arm 2 A/H7N9+AS03, D1, 29). 882
Table S85 MSigDB GO Biological Processes enriched in differentially expressed genes at Day 32 compared to pre-Second Vaccination (Monocytes, Study Arm 2 A/H7N9+AS03, D1, 29). 882
Table S86 Blood Transcription Modules enriched in differentially expressed genes at Day 32 compared to pre-Second Vaccination (Monocytes, Study Arm 2 A/H7N9+AS03, D1, 29). 883
Table S87 KEGG Pathways enriched in differentially expressed genes at Day 2 compared to pre-First Vaccination (Dendritic cells, Study Arm 2 A/H7N9+AS03, D1, 29). 883
Table S88 MSigDB Reactome Pathways enriched in differentially expressed genes at Day 2 compared to pre-First Vaccination (Dendritic cells, Study Arm 2 A/H7N9+AS03, D1, 29). 884
Table S89 MSigDB GO Biological Processes enriched in differentially expressed genes at Day 2 compared to pre-First Vaccination (Dendritic cells, Study Arm 2 A/H7N9+AS03, D1, 29). 885
Table S90 Blood Transcription Modules enriched in differentially expressed genes at Day 2 compared to pre-First Vaccination (Dendritic cells, Study Arm 2 A/H7N9+AS03, D1, 29). 897
Table S91 KEGG Pathways enriched in differentially expressed genes at Day 8 compared to pre-First Vaccination (Dendritic cells, Study Arm 1 A/H3N2v, D1). 898
Table S92 MSigDB Reactome Pathways enriched in differentially expressed genes at Day 8 compared to pre-First Vaccination (Dendritic cells, Study Arm 1 A/H3N2v, D1). 898
Table S93 MSigDB GO Biological Processes enriched in differentially expressed genes at Day 8 compared to pre-First Vaccination (Dendritic cells, Study Arm 1 A/H3N2v, D1). 898
Table S94 KEGG Pathways enriched in differentially expressed genes at Day 30 compared to pre-Second Vaccination (Dendritic cells, Study Arm 2 A/H7N9+AS03, D1, 29). 899
Table S95 MSigDB Reactome Pathways enriched in differentially expressed genes at Day 30 compared to pre-Second Vaccination (Dendritic cells, Study Arm 2 A/H7N9+AS03, D1, 29). 900
Table S96 MSigDB GO Biological Processes enriched in differentially expressed genes at Day 30 compared to pre-Second Vaccination (Dendritic cells, Study Arm 2 A/H7N9+AS03, D1, 29). . . . 901
Table S97 Blood Transcription Modules enriched in differentially expressed genes at Day 30 compared to pre-Second Vaccination (Dendritic cells, Study Arm 2 A/H7N9+AS03, D1, 29). 911
Table S98 KEGG Pathways enriched in differentially expressed genes at Day 32 compared to pre-Second Vaccination (Dendritic cells, Study Arm 2 A/H7N9+AS03, D1, 29). 913

Table S99 MSigDB Reactome Pathways enriched in differentially expressed genes at Day 32 compared to pre-Second Vaccination (Dendritic cells, Study Arm 2 A/H7N9+AS03, D1, 29). 913
Table S100 MSigDB GO Biological Processes enriched in differentially expressed genes at Day 32 compared to pre-Second Vaccination (Dendritic cells, Study Arm 2 A/H7N9+AS03, D1, 29). . . . 914
Table S101 Blood Transcription Modules enriched in differentially expressed genes at Day 32 compared to pre-Second Vaccination (Dendritic cells, Study Arm 2 A/H7N9+AS03, D1, 29). 914
Table S102 KEGG Pathways enriched in differentially expressed genes at Day 36 compared to pre-Second Vaccination (Dendritic cells, Study Arm 2 A/H7N9+AS03, D1, 29). 914
Table S103 MSigDB Reactome Pathways enriched in differentially expressed genes at Day 36 compared to pre-Second Vaccination (Dendritic cells, Study Arm 2 A/H7N9+AS03, D1, 29). 915
Table S104 Blood Transcription Modules enriched in differentially expressed genes at Day 36 compared to pre-Second Vaccination (Dendritic cells, Study Arm 2 A/H7N9+AS03, D1, 29). 915
Table S105 KEGG Pathways enriched in differentially expressed genes at Day 2 compared to pre-First Vaccination (Neutrophils, Study Arm 2 A/H7N9+AS03, D1, 29). 915
Table S106 MSigDB Reactome Pathways enriched in differentially expressed genes at Day 2 compared to pre-First Vaccination (Neutrophils, Study Arm 2 A/H7N9+AS03, D1, 29). 916
Table S107 MSigDB GO Biological Processes enriched in differentially expressed genes at Day 2 compared to pre-First Vaccination (Neutrophils, Study Arm 2 A/H7N9+AS03, D1, 29). 916
Table S108 Blood Transcription Modules enriched in differentially expressed genes at Day 2 compared to pre-First Vaccination (Neutrophils, Study Arm 2 A/H7N9+AS03, D1, 29). 923
Table S109 MSigDB Reactome Pathways enriched in differentially expressed genes at Day 4 compared to pre-First Vaccination (Neutrophils, Study Arm 2 A/H7N9+AS03, D1, 29). 924
Table S110 MSigDB GO Biological Processes enriched in differentially expressed genes at Day 4 compared to pre-First Vaccination (Neutrophils, Study Arm 2 A/H7N9+AS03, D1, 29). 924
Table S111 Blood Transcription Modules enriched in differentially expressed genes at Day 4 compared to pre-First Vaccination (Neutrophils, Study Arm 2 A/H7N9+AS03, D1, 29). 925
Table S112 KEGG Pathways enriched in differentially expressed genes at Day 30 compared to pre-Second Vaccination (Neutrophils, Study Arm 2 A/H7N9+AS03, D1, 29). 925
Table S113 MSigDB Reactome Pathways enriched in differentially expressed genes at Day 30 compared to pre-Second Vaccination (Neutrophils, Study Arm 2 A/H7N9+AS03, D1, 29). 926
Table S114 MSigDB GO Biological Processes enriched in differentially expressed genes at Day 30 compared to pre-Second Vaccination (Neutrophils, Study Arm 2 A/H7N9+AS03, D1, 29). 927
Table S115 Blood Transcription Modules enriched in differentially expressed genes at Day 30 compared to pre-Second Vaccination (Neutrophils, Study Arm 2 A/H7N9+AS03, D1, 29). 933
Table S116 MSigDB Reactome Pathways enriched in differentially expressed genes at Day 2 compared to pre-First Vaccination (NK cells, Study Arm 2 A/H7N9+AS03, D1, 29). 933
Table S117 MSigDB GO Biological Processes enriched in differentially expressed genes at Day 2 compared to pre-First Vaccination (NK cells, Study Arm 2 A/H7N9+AS03, D1, 29). 933
Table S118 Blood Transcription Modules enriched in differentially expressed genes at Day 2 compared to pre-First Vaccination (NK cells, Study Arm 2 A/H7N9+AS03, D1, 29). 935

Table S119 KEGG Pathways enriched in differentially expressed genes at Day 4 compared to pre-First Vaccination (NK cells, Study Arm 2 A/H7N9+AS03, D1, 29). 936
Table S120 MSigDB Reactome Pathways enriched in differentially expressed genes at Day 4 compared to pre-First Vaccination (NK cells, Study Arm 2 A/H7N9+AS03, D1, 29). 936
Table S121 MSigDB GO Biological Processes enriched in differentially expressed genes at Day 4 compared to pre-First Vaccination (NK cells, Study Arm 2 A/H7N9+AS03, D1, 29). 938
Table S122 Blood Transcription Modules enriched in differentially expressed genes at Day 4 compared to pre-First Vaccination (NK cells, Study Arm 2 A/H7N9+AS03, D1, 29). 942
Table S123 KEGG Pathways enriched in differentially expressed genes at Day 30 compared to pre-Second Vaccination (NK cells, Study Arm 2 A/H7N9+AS03, D1, 29). 942
Table S124 MSigDB Reactome Pathways enriched in differentially expressed genes at Day 30 compared to pre-Second Vaccination (NK cells, Study Arm 2 A/H7N9+AS03, D1, 29). 943
Table S125 MSigDB GO Biological Processes enriched in differentially expressed genes at Day 30 compared to pre-Second Vaccination (NK cells, Study Arm 2 A/H7N9+AS03, D1, 29). 943
Table S126 Blood Transcription Modules enriched in differentially expressed genes at Day 30 compared to pre-Second Vaccination (NK cells, Study Arm 2 A/H7N9+AS03, D1, 29). 945
Table S127 KEGG Pathways enriched in differentially expressed genes at Day 32 compared to pre-Second Vaccination (NK cells, Study Arm 2 A/H7N9+AS03, D1, 29). 946
Table S128 MSigDB Reactome Pathways enriched in differentially expressed genes at Day 32 compared to pre-Second Vaccination (NK cells, Study Arm 2 A/H7N9+AS03, D1, 29). 947
Table S129 MSigDB GO Biological Processes enriched in differentially expressed genes at Day 32 compared to pre-Second Vaccination (NK cells, Study Arm 2 A/H7N9+AS03, D1, 29). 949
Table S130 Blood Transcription Modules enriched in differentially expressed genes at Day 32 compared to pre-Second Vaccination (NK cells, Study Arm 2 A/H7N9+AS03, D1, 29). 954
Table S131 Blood Transcription Modules enriched in differentially expressed genes at Day 36 compared to pre-Second Vaccination (NK cells, Study Arm 2 A/H7N9+AS03, D1, 29). 955
Table S132 KEGG Pathways enriched in differentially expressed genes at Day 2 compared to pre-First Vaccination (B cells, Study Arm 2 A/H7N9+AS03, D1, 29). 956
Table S133 MSigDB Reactome Pathways enriched in differentially expressed genes at Day 2 compared to pre-First Vaccination (B cells, Study Arm 2 A/H7N9+AS03, D1, 29). 956
Table S134 MSigDB GO Biological Processes enriched in differentially expressed genes at Day 2 compared to pre-First Vaccination (B cells, Study Arm 2 A/H7N9+AS03, D1, 29). 956
Table S135 Blood Transcription Modules enriched in differentially expressed genes at Day 2 compared to pre-First Vaccination (B cells, Study Arm 2 A/H7N9+AS03, D1, 29). 957
Table S136 KEGG Pathways enriched in differentially expressed genes at Day 8 compared to pre-First Vaccination (B cells, Study Arm 1 A/H3N2v, D1). 958
Table S137 MSigDB Reactome Pathways enriched in differentially expressed genes at Day 8 compared to pre-First Vaccination (B cells, Study Arm 1 A/H3N2v, D1). 958
Table S138 MSigDB GO Biological Processes enriched in differentially expressed genes at Day 8 compared to pre-First Vaccination (B cells, Study Arm 1 A/H3N2v, D1). 958

Table S139 KEGG Pathways enriched in differentially expressed genes at Day 30 compared to pre-Second Vaccination (B cells, Study Arm 2 A/H7N9+AS03, D1, 29). 959
Table S140 MSigDB Reactome Pathways enriched in differentially expressed genes at Day 30 compared to pre-Second Vaccination (B cells, Study Arm 2 A/H7N9+AS03, D1, 29). 960
Table S141 MSigDB GO Biological Processes enriched in differentially expressed genes at Day 30 compared to pre-Second Vaccination (B cells, Study Arm 2 A/H7N9+AS03, D1, 29). 961
Table S142 Blood Transcription Modules enriched in differentially expressed genes at Day 30 compared to pre-Second Vaccination (B cells, Study Arm 2 A/H7N9+AS03, D1, 29). 972
Table S143 KEGG Pathways enriched in differentially expressed genes at Day 32 compared to pre-Second Vaccination (B cells, Study Arm 2 A/H7N9+AS03, D1, 29). 973
Table S144 MSigDB Reactome Pathways enriched in differentially expressed genes at Day 32 compared to pre-Second Vaccination (B cells, Study Arm 2 A/H7N9+AS03, D1, 29). 973
Table S145 MSigDB GO Biological Processes enriched in differentially expressed genes at Day 32 compared to pre-Second Vaccination (B cells, Study Arm 2 A/H7N9+AS03, D1, 29). 974
Table S146 Blood Transcription Modules enriched in differentially expressed genes at Day 32 compared to pre-Second Vaccination (B cells, Study Arm 2 A/H7N9+AS03, D1, 29). 976
Table S147 Blood Transcription Modules enriched in differentially expressed genes at Day 36 compared to pre-Second Vaccination (B cells, Study Arm 2 A/H7N9+AS03, D1, 29). 977
Table S148 KEGG Pathways enriched in differentially expressed genes at Day 2 compared to pre-First Vaccination (T cells, Study Arm 2 A/H7N9+AS03, D1, 29). 977
Table S149 MSigDB Reactome Pathways enriched in differentially expressed genes at Day 2 compared to pre-First Vaccination (T cells, Study Arm 2 A/H7N9+AS03, D1, 29). 978
Table S150 MSigDB GO Biological Processes enriched in differentially expressed genes at Day 2 compared to pre-First Vaccination (T cells, Study Arm 2 A/H7N9+AS03, D1, 29). 978
Table S151 Blood Transcription Modules enriched in differentially expressed genes at Day 2 compared to pre-First Vaccination (T cells, Study Arm 2 A/H7N9+AS03, D1, 29). 980
Table S152 KEGG Pathways enriched in differentially expressed genes at Day 30 compared to pre-Second Vaccination (T cells, Study Arm 2 A/H7N9+AS03, D1, 29). 980
Table S153 MSigDB Reactome Pathways enriched in differentially expressed genes at Day 30 compared to pre-Second Vaccination (T cells, Study Arm 2 A/H7N9+AS03, D1, 29). 981
Table S154 MSigDB GO Biological Processes enriched in differentially expressed genes at Day 30 compared to pre-Second Vaccination (T cells, Study Arm 2 A/H7N9+AS03, D1, 29). 981
Table S155 Blood Transcription Modules enriched in differentially expressed genes at Day 30 compared to pre-Second Vaccination (T cells, Study Arm 2 A/H7N9+AS03, D1, 29). 990
Table S156 KEGG pathways enriched in differentially abundant proteins at Day 2 compared to pre-first vaccination (Neutrophils, Study Arm 2 (A/H7N9 + AS03 D1, 29)). 990
Table S157 KEGG pathways enriched in differentially abundant proteins at Day 30 compared to pre-second vaccination (Neutrophils, Study Arm 2 (A/H7N9 + AS03 D1, 29)). 991
Table S158 KEGG pathways enriched in differentially abundant proteins at Day 32 compared to pre-second vaccination (Neutrophils, Study Arm 2 (A/H7N9 + AS03 D1, 29)). 992

Table S159 KEGG pathways enriched in differentially abundant proteins at Day 2 compared to pre-first vaccination (Dendritic cells, Study Arm 2 (A/H7N9 + AS03 D1, 29)). 993
Table S160 KEGG pathways enriched in differentially abundant proteins at Day 30 compared to pre-second vaccination (Dendritic cells, Study Arm 2 (A/H7N9 + AS03 D1, 29)). 994
Table S161 KEGG pathways enriched in differentially abundant proteins at Day 2 compared to pre-first vaccination (Monocytes, Study Arm 2 (A/H7N9 + AS03 D1, 29)). 995
Table S162 KEGG pathways enriched in differentially abundant proteins at Day 30 compared to pre-second vaccination (Monocytes, Study Arm 2 (A/H7N9 + AS03 D1, 29)). 996
Table S163 KEGG pathways enriched in differentially abundant proteins at Day 30 compared to pre-second vaccination (Natural Killer cells, Study Arm 2 (A/H7N9 + AS03 D1, 29)). 997
Table S164 Blood Transcription Modules enriched in differentially abundant proteins at Day 32 compared to pre-second vaccination (Neutrophils, Study Arm 2 (A/H7N9 + AS03 D1, 29)). 998
Table S165 Blood Transcription Modules enriched in differentially abundant proteins at Day 2 compared to pre-first vaccination (Dendritic cells, Study Arm 2 (A/H7N9 + AS03 D1, 29)). 999
Table S166 Blood Transcription Modules enriched in differentially abundant proteins at Day 30 compared to pre-second vaccination (Dendritic cells, Study Arm 2 (A/H7N9 + AS03 D1, 29)). 1000
Table S167 Blood Transcription Modules enriched in differentially abundant proteins at Day 32 compared to pre-second vaccination (Dendritic cells, Study Arm 2 (A/H7N9 + AS03 D1, 29)). 1001
Table S168 Blood Transcription Modules enriched in differentially abundant proteins at Day 30 compared to pre-second vaccination (Monocytes, Study Arm 2 (A/H7N9 + AS03 D1, 29)). 1002
Table S169 MSigDB Reactome pathways enriched in differentially abundant proteins at Day 2 compared to pre-first vaccination (Neutrophils, Study Arm 2 (A/H7N9 + AS03 D1, 29)). 1003
Table S170 MSigDB Reactome pathways enriched in differentially abundant proteins at Day 30 compared to pre-second vaccination (Neutrophils, Study Arm 2 (A/H7N9 + AS03 D1, 29)). 1004
Table S171 MSigDB Reactome pathways enriched in differentially abundant proteins at Day 32 compared to pre-second vaccination (Neutrophils, Study Arm 2 (A/H7N9 + AS03 D1, 29)). 1005
Table S172 MSigDB Reactome pathways enriched in differentially abundant proteins at Day 2 compared to pre-first vaccination (Dendritic cells, Study Arm 2 (A/H7N9 + AS03 D1, 29)). 1006
Table S173 MSigDB Reactome pathways enriched in differentially abundant proteins at Day 30 compared to pre-second vaccination (Dendritic cells, Study Arm 2 (A/H7N9 + AS03 D1, 29)). 1007
Table S174 MSigDB Reactome pathways enriched in differentially abundant proteins at Day 32 compared to pre-second vaccination (Dendritic cells, Study Arm 2 (A/H7N9 + AS03 D1, 29)). 1008
Table S175 MSigDB Reactome pathways enriched in differentially abundant proteins at Day 2 compared to pre-first vaccination (Monocytes, Study Arm 2 (A/H7N9 + AS03 D1, 29)). 1009
Table S176 MSigDB Reactome pathways enriched in differentially abundant proteins at Day 30 compared to pre-second vaccination (Monocytes, Study Arm 2 (A/H7N9 + AS03 D1, 29)). 1010
Table S177 MSigDB Reactome pathways enriched in differentially abundant proteins at Day 30 compared to pre-second vaccination (Natural Killer cells, Study Arm 2 (A/H7N9 + AS03 D1, 29)). . . . 1011
Table S178 MSigDB GO Biological Process pathways enriched in differentially abundant proteins at Day 2 compared to pre-first vaccination (Neutrophils, Study Arm 2 (A/H7N9 + AS03 D1, 29)). . . 1012

Table S179 MSigDB GO Biological Process pathways enriched in differentially abundant proteins at Day 30 compared to pre-second vaccination (Neutrophils, Study Arm 2 (A/H7N9 + AS03 D1, 29)). 1013
Table S180 MSigDB GO Biological Process pathways enriched in differentially abundant proteins at Day 32 compared to pre-second vaccination (Neutrophils, Study Arm 2 (A/H7N9 + AS03 D1, 29)). 1014
Table S181 MSigDB GO Biological Process pathways enriched in differentially abundant proteins at Day 2 compared to pre-first vaccination (Dendritic cells, Study Arm 2 (A/H7N9 + AS03 D1, 29)). 1016
Table S182 MSigDB GO Biological Process pathways enriched in differentially abundant proteins at Day 30 compared to pre-second vaccination (Dendritic cells, Study Arm 2 (A/H7N9 + AS03 D1, 29)). 1019
Table S183 MSigDB GO Biological Process pathways enriched in differentially abundant proteins at Day 32 compared to pre-second vaccination (Dendritic cells, Study Arm 2 (A/H7N9 + AS03 D1, 29)). 1021
Table S184 MSigDB GO Biological Process pathways enriched in differentially abundant proteins at Day 2 compared to pre-first vaccination (Monocytes, Study Arm 2 (A/H7N9 + AS03 D1, 29)). . . . 1022
Table S185 MSigDB GO Biological Process pathways enriched in differentially abundant proteins at Day 30 compared to pre-second vaccination (Monocytes, Study Arm 2 (A/H7N9 + AS03 D1, 29)). 1023
Table S186 Overlap in top 50 genes that best predict peak HAI titer response for both transcriptomics and proteomics (Combined A/H7N9 Study Arms). 1024

Figure S1: Neutralizing Antibody Results. a) Barplot of participant-level neutralization (Nt) titer results at Day 29 post-last vaccination in the intent-to-treat population. Seroconversion is defined as a post-vaccination Nt titer $\geq$ 1:40 or $\geq$ 4-fold rise in post-vaccination Nt titer if the baseline was $\geq$ 1:40. b) Nt titer geometric mean fold rise (GMFR) and associated 95% CI by study arm and post-vaccination day in the intent-to-treat population. Error bars indicate 95% confidence intervals based on 1000 bootstrap replicates. p: p-value of upper one-sided Welch's two-sample t-test comparing adjuvanted vs. unadjuvanted H7N9 arm Nt fold changes at Day 29.

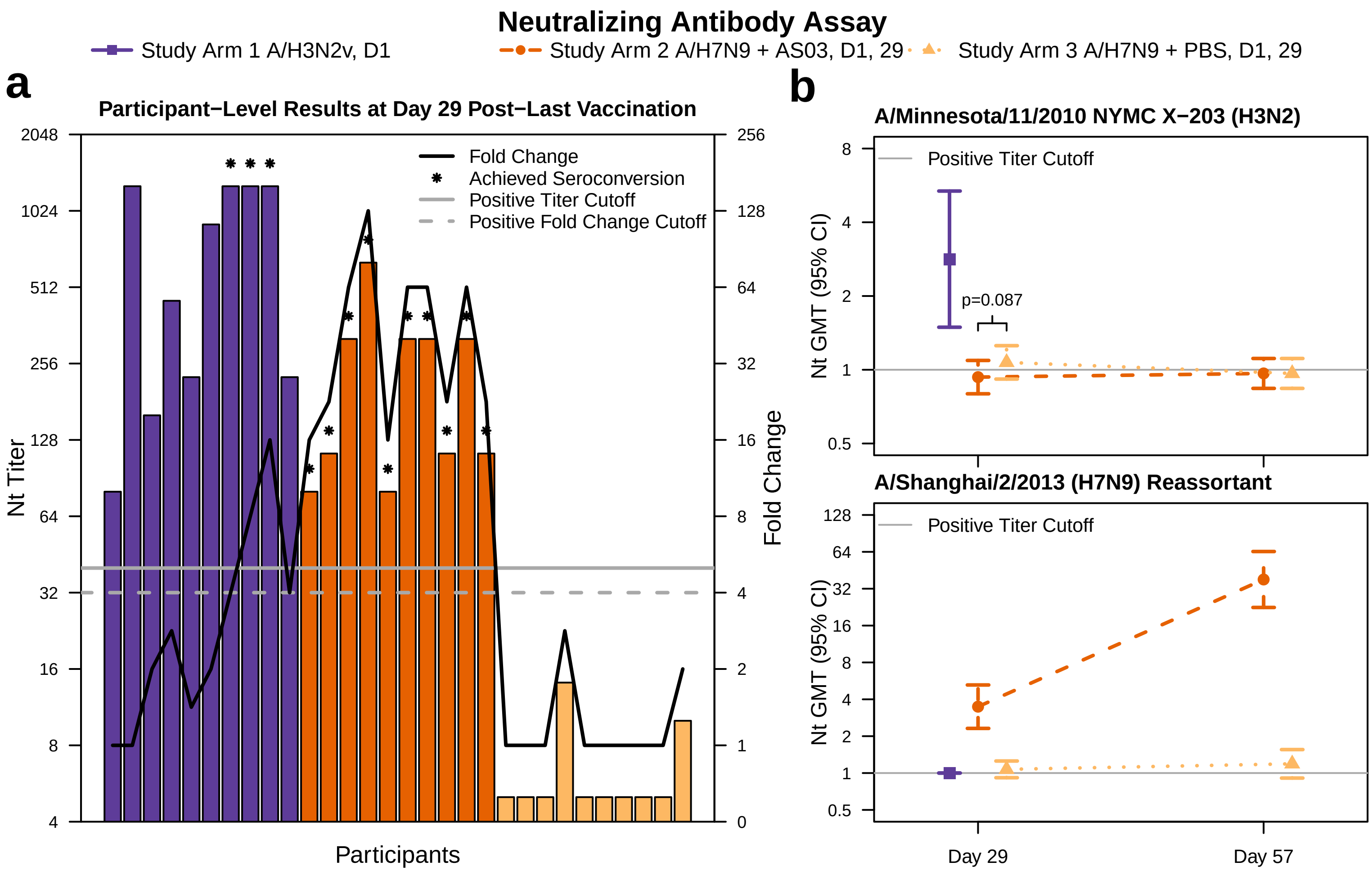

Figure S2: Inflammatory Fractalkine concentration response after vaccination. Time trend of median fold change of Fractalkine and associated 95% CI by study arm and post-vaccination day in the intent-to-treat population. Error bars indicate 95% confidence intervals based on 1000 bootstrap replicates.

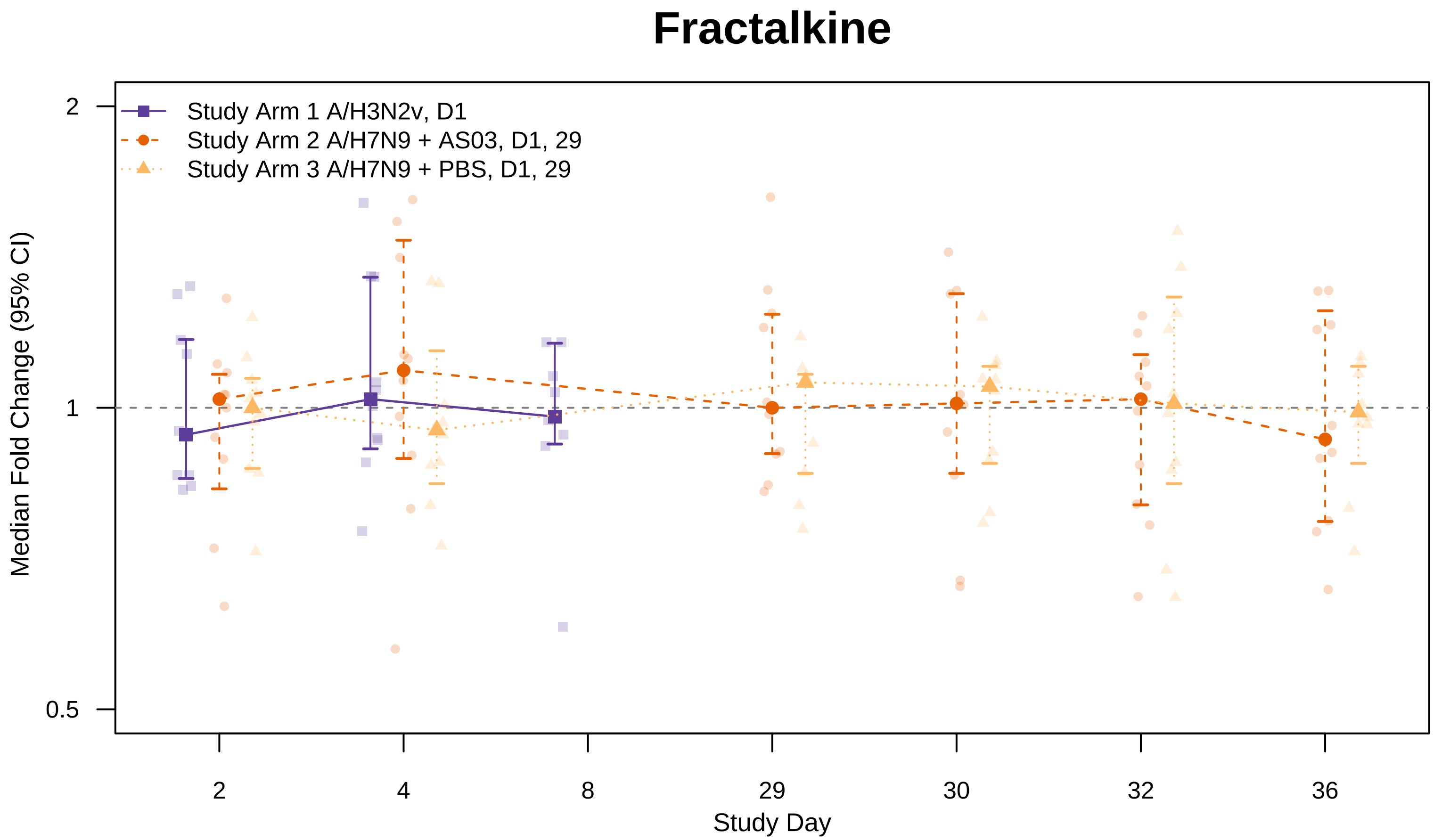

Figure S3: Inflammatory GM-CSF concentration response after vaccination. Time trend of median fold change of GM-CSF and associated 95% CI by study arm and post-vaccination day in the intent-to-treat population. Error bars indicate 95% confidence intervals based on 1000 bootstrap replicates.

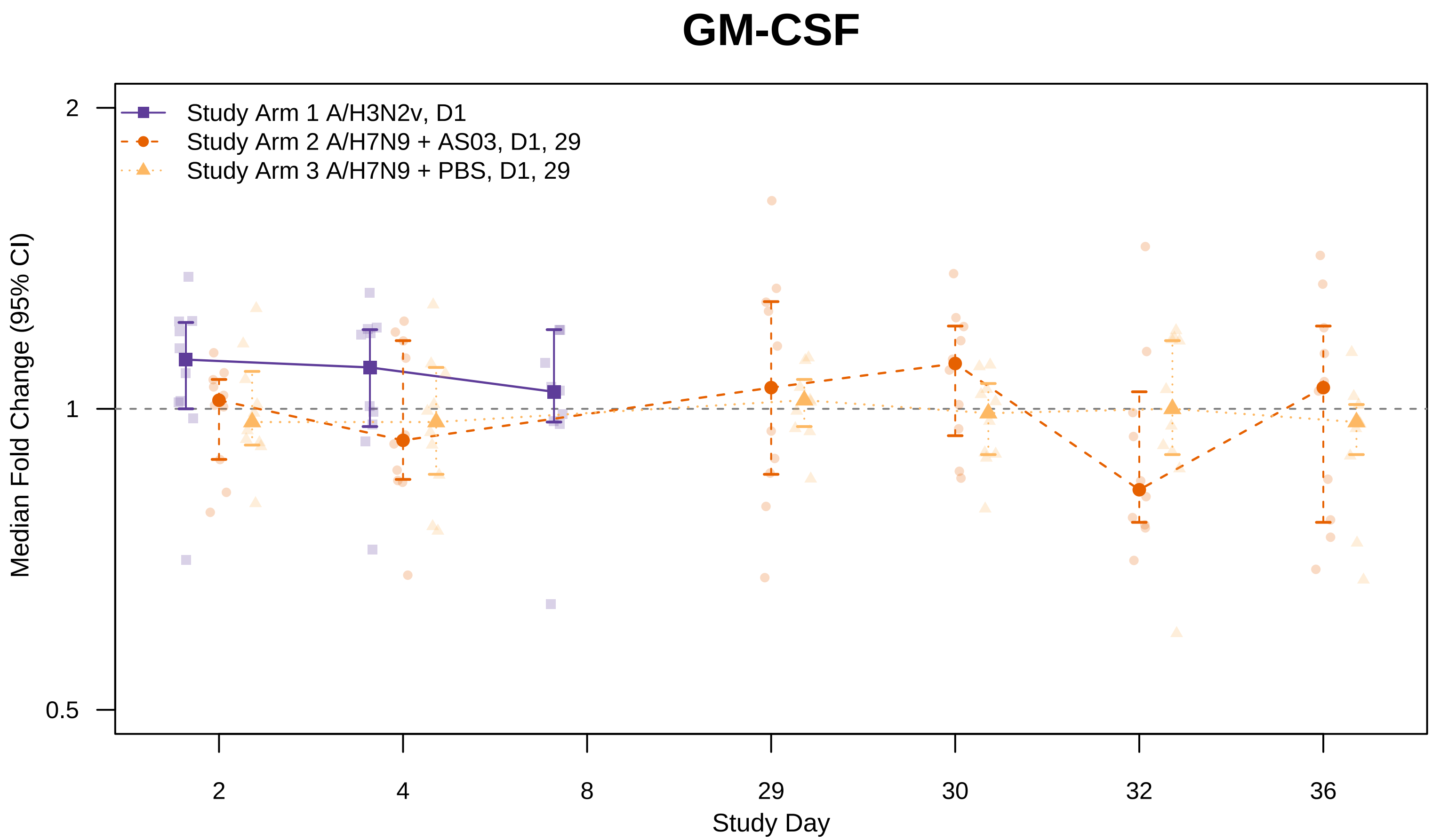

Figure S4: Inflammatory Interleukin 1β concentration response after vaccination. Time trend of median fold change of Interleukin 1β and associated 95% CI by study arm and post-vaccination day in the intent-to-treat population. Error bars indicate 95% confidence intervals based on 1000 bootstrap replicates.

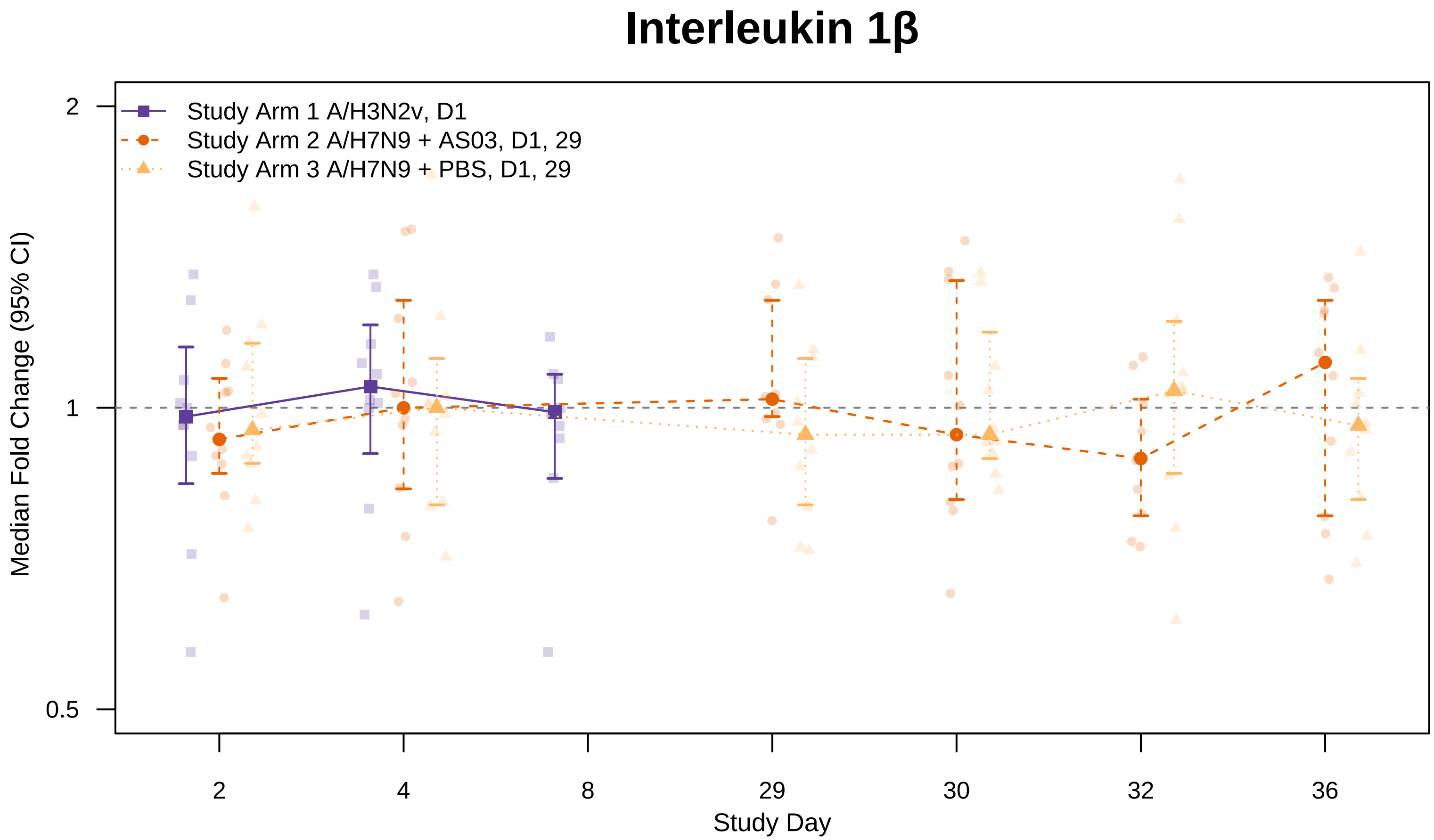

Confidential/Proprietary Information

Figure S5: Inflammatory Interleukin 2 concentration response after vaccination. Time trend of median fold change of Interleukin 2 and associated 95% CI by study arm and post-vaccination day in the intent-to-treat population. Error bars indicate 95% confidence intervals based on 1000 bootstrap replicates.

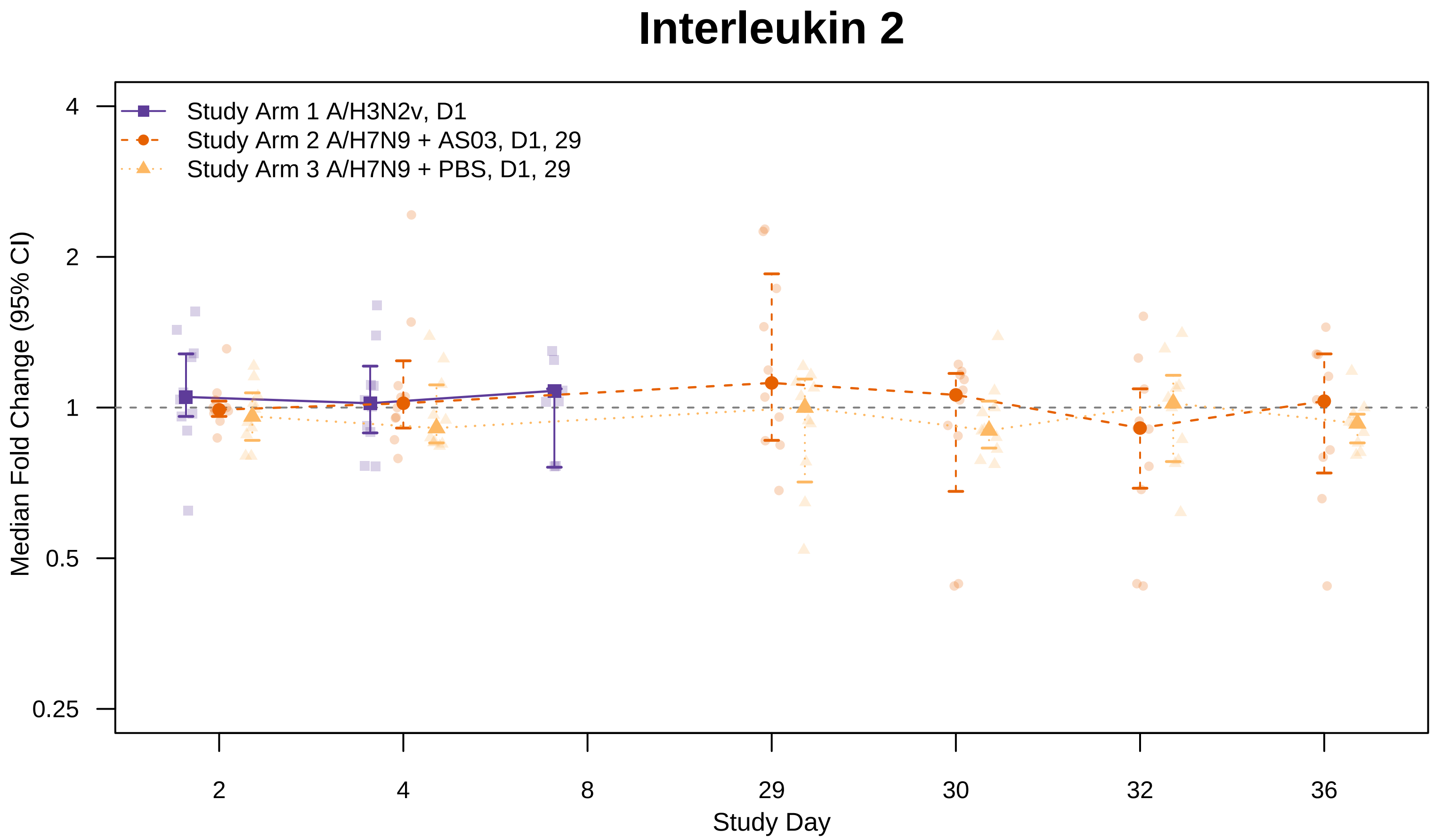

Confidential/Proprietary Information

Figure S6: Inflammatory Interleukin 4 concentration response after vaccination. Time trend of median fold change of Interleukin 4 and associated 95% CI by study arm and post-vaccination day in the intent-to-treat population. Error bars indicate 95% confidence intervals based on 1000 bootstrap replicates.

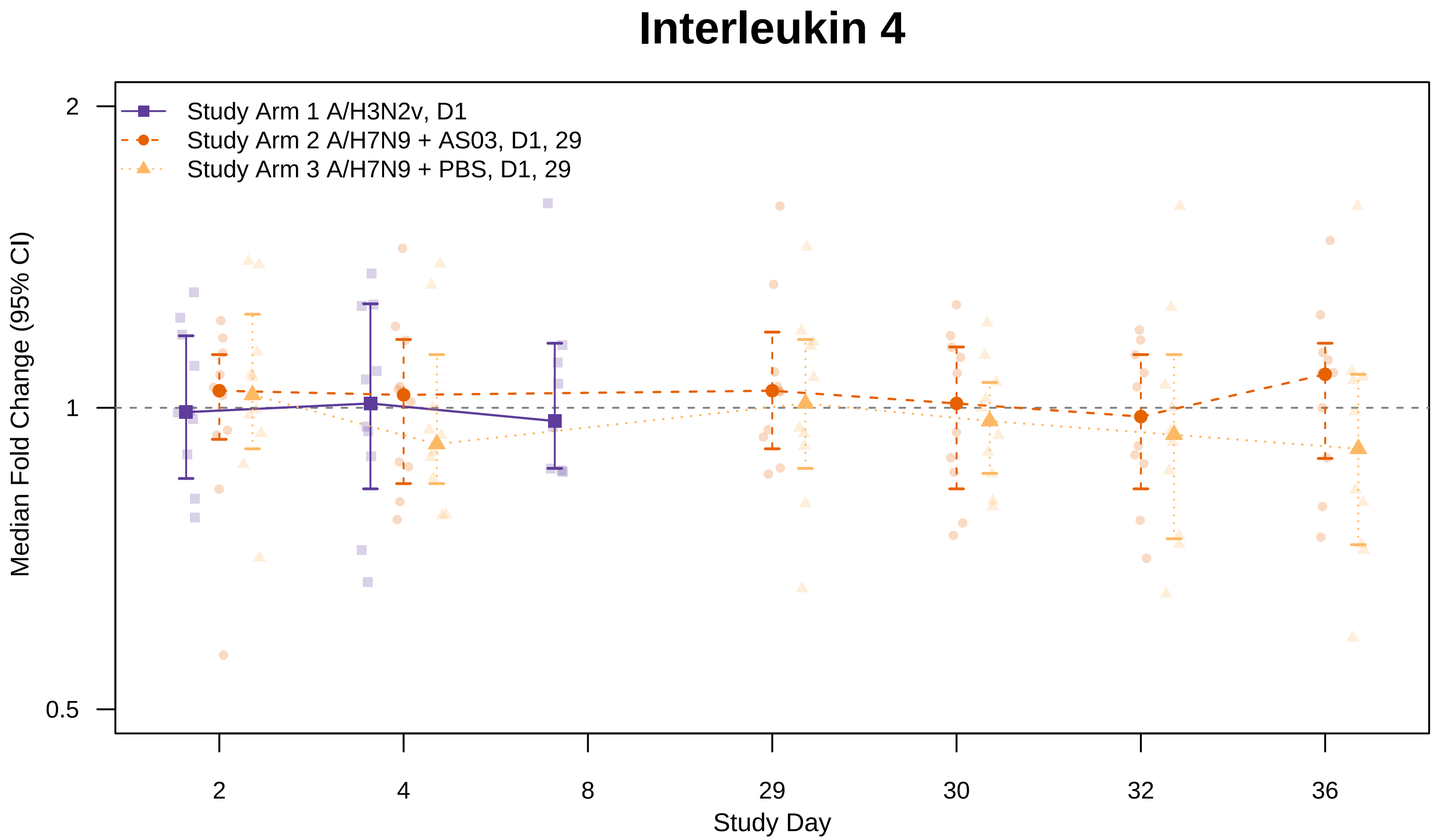

Figure S7: Inflammatory Interleukin 5 concentration response after vaccination. Time trend of median fold change of Interleukin 5 and associated 95% CI by study arm and post-vaccination day in the intent-to-treat population. Error bars indicate 95% confidence intervals based on 1000 bootstrap replicates.

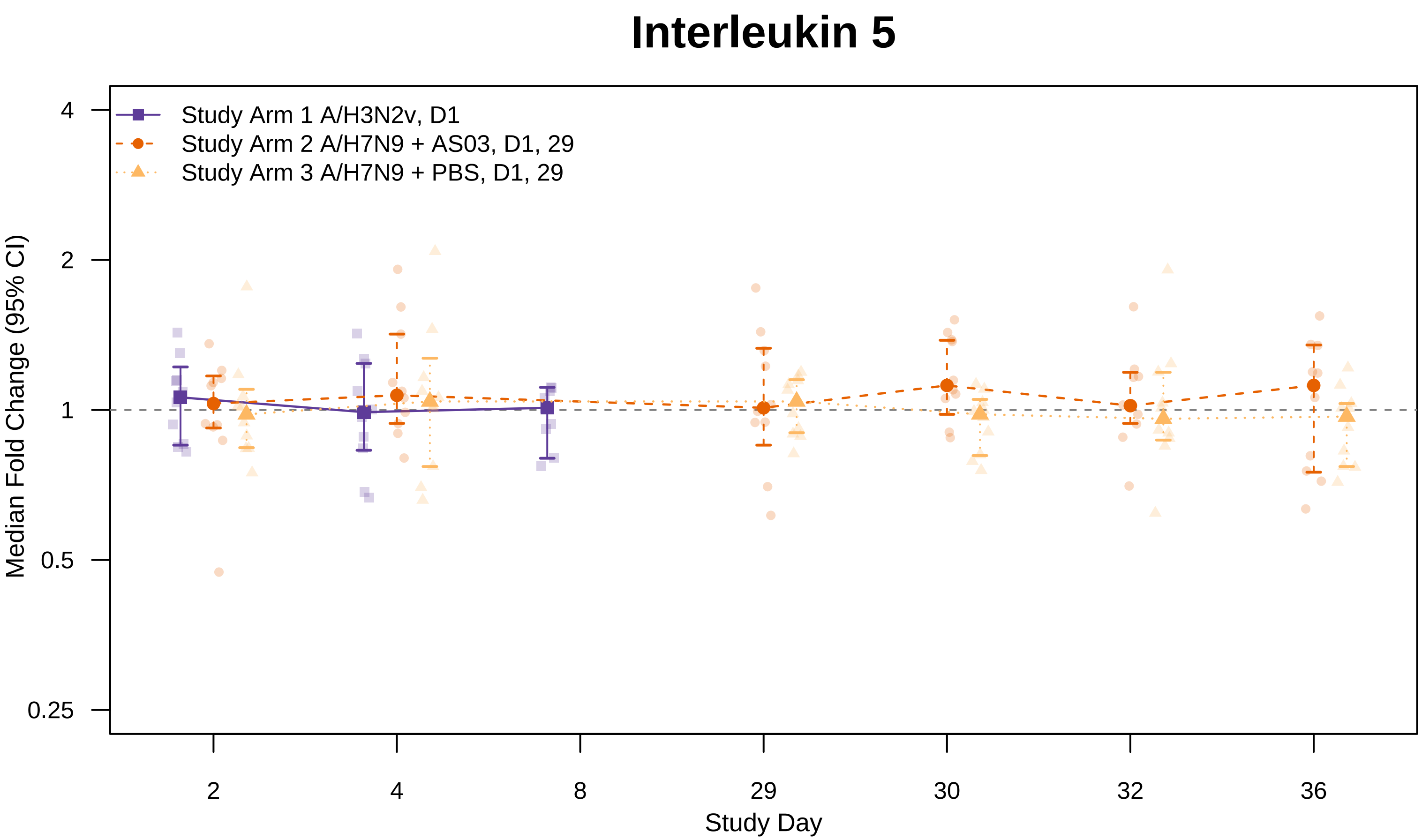

Figure S8: Inflammatory Interleukin 6 concentration response after vaccination. Time trend of median fold change of Interleukin 6 and associated 95% CI by study arm and post-vaccination day in the intent-to-treat population. Error bars indicate 95% confidence intervals based on 1000 bootstrap replicates.

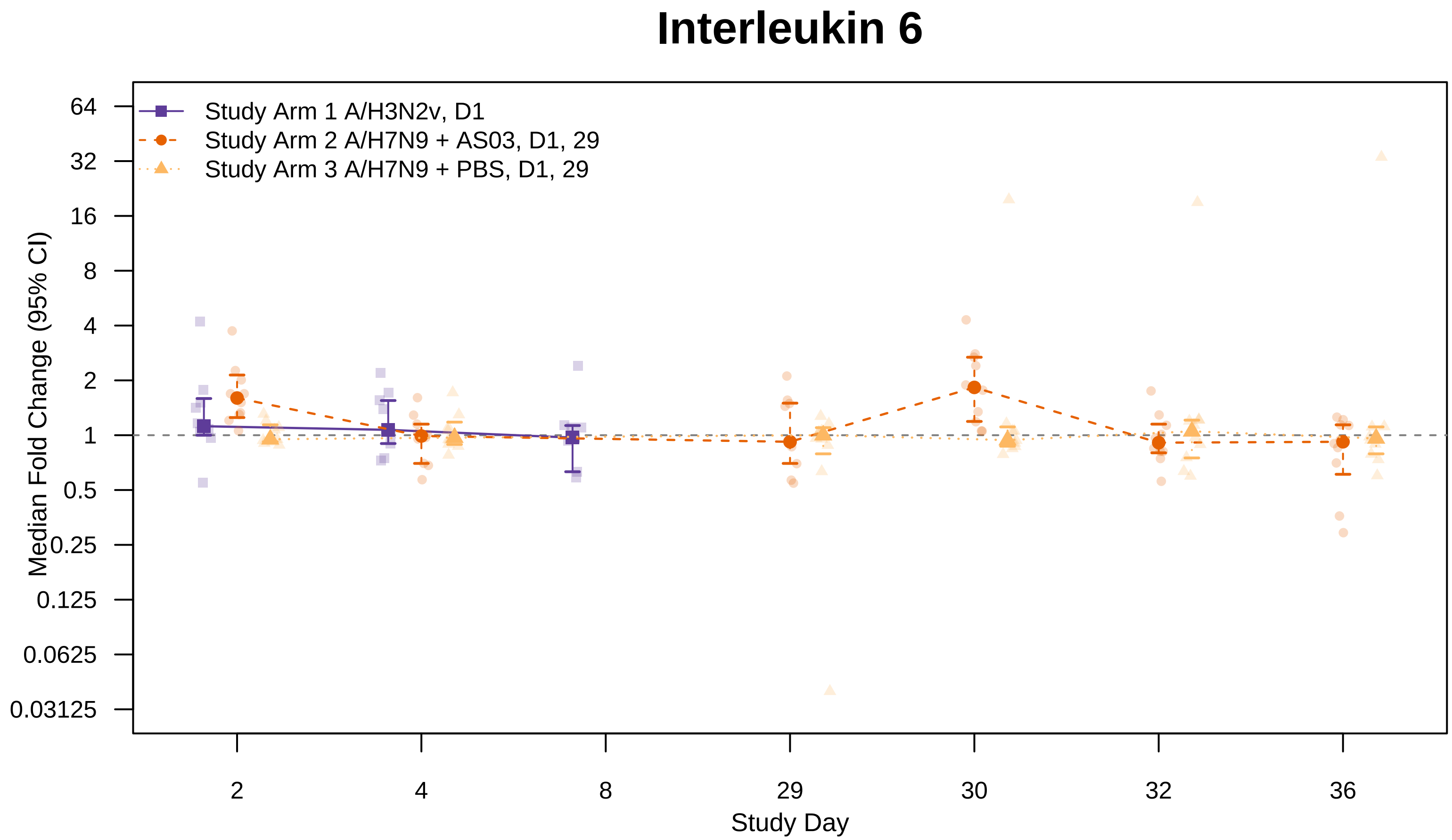

Figure S9: Inflammatory Interleukin 7 concentration response after vaccination. Time trend of median fold change of Interleukin 7 and associated 95% CI by study arm and post-vaccination day in the intent-to-treat population. Error bars indicate 95% confidence intervals based on 1000 bootstrap replicates.

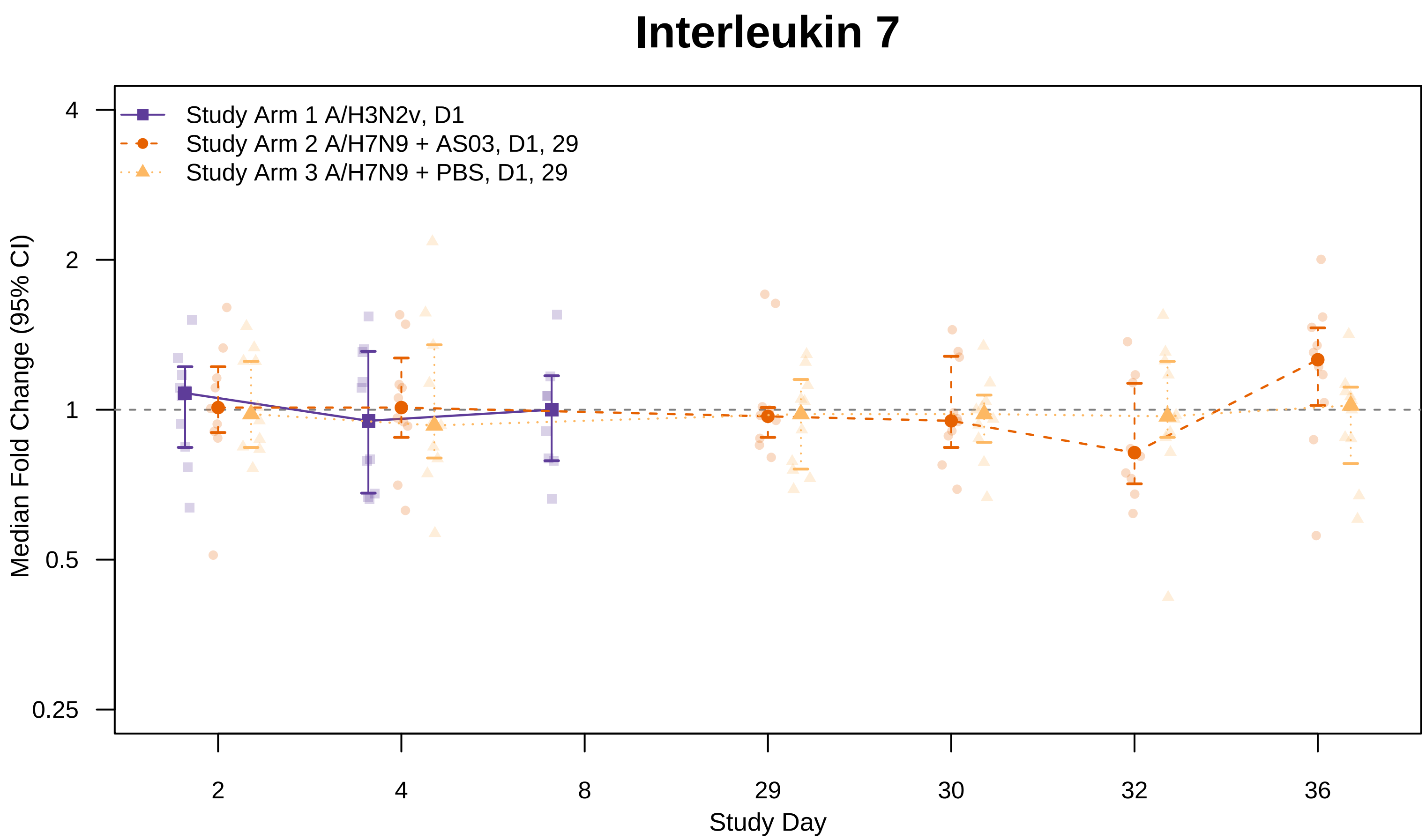

Figure S10: Inflammatory Interleukin 8 concentration response after vaccination. Time trend of median fold change of Interleukin 8 and associated 95% CI by study arm and post-vaccination day in the intent-to-treat population. Error bars indicate 95% confidence intervals based on 1000 bootstrap replicates.

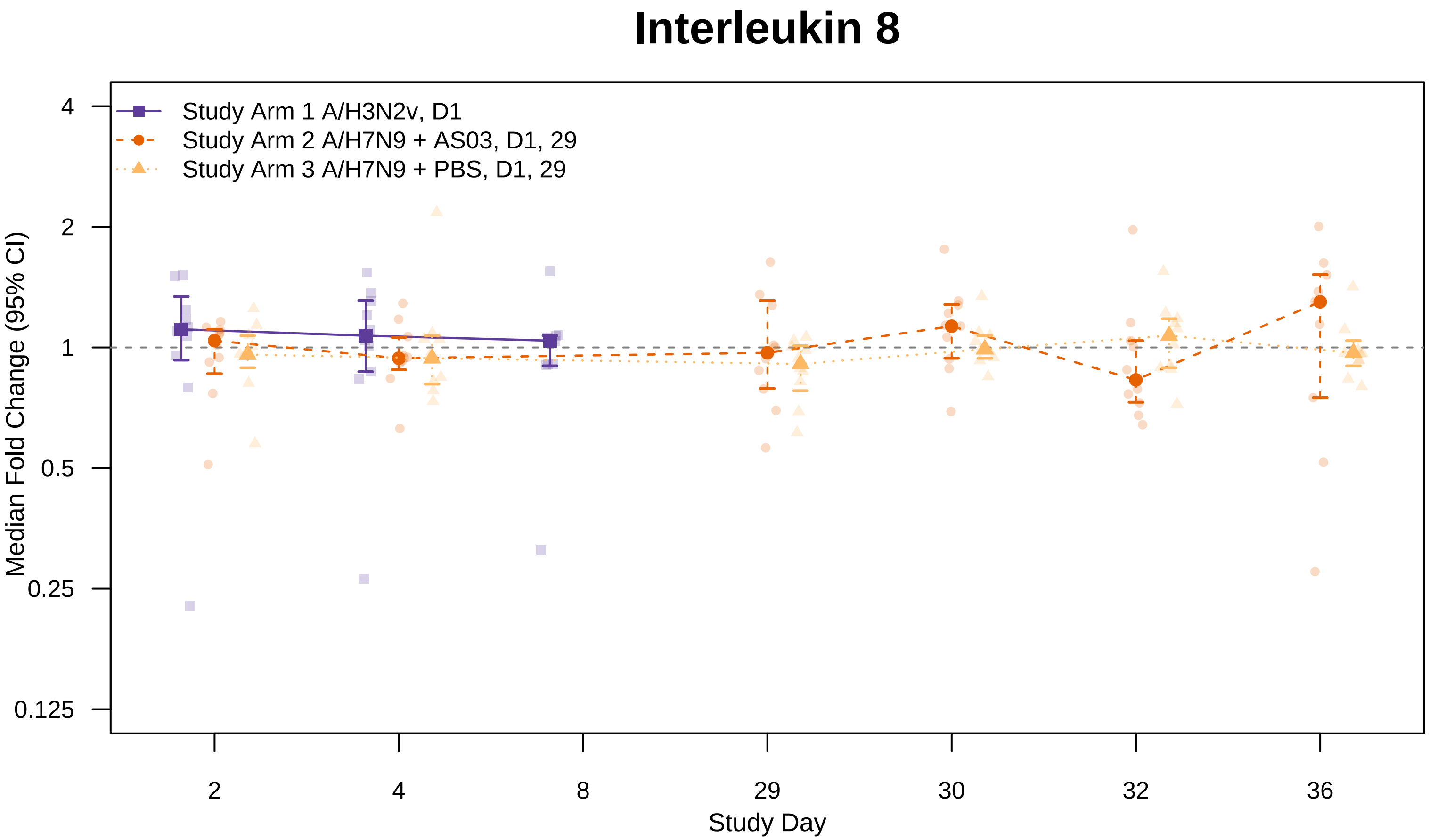

Figure S11: Inflammatory Interleukin 10 concentration response after vaccination. Time trend of median fold change of Interleukin 10 and associated 95% CI by study arm and post-vaccination day in the intent-to-treat population. Error bars indicate 95% confidence intervals based on 1000 bootstrap replicates.

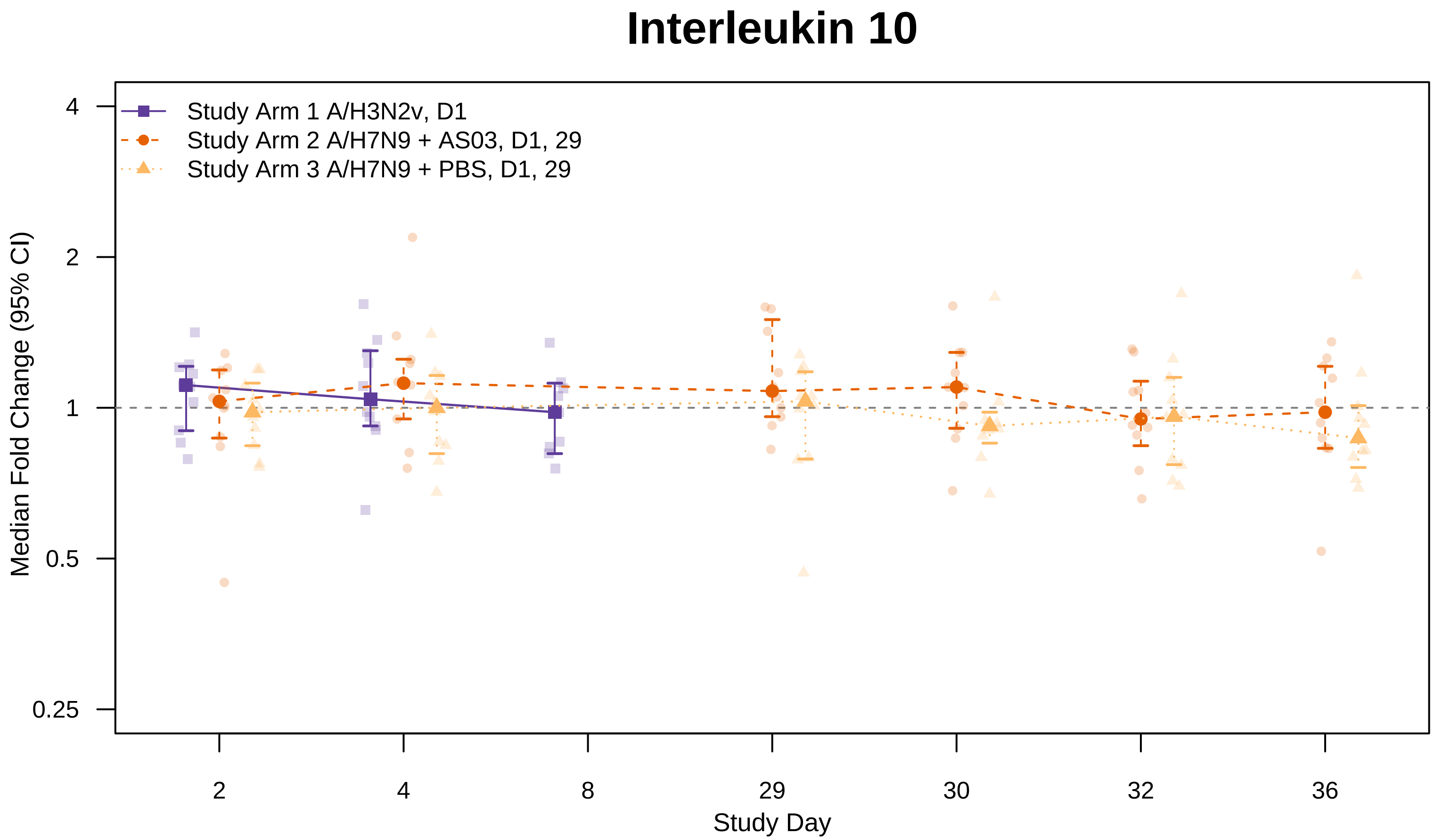

Figure S12: Inflammatory Interleukin 12, p70 concentration response after vaccination. Time trend of median fold change of Interleukin 12, p70 and associated 95% CI by study arm and post-vaccination day in the intent-to-treat population. Error bars indicate 95% confidence intervals based on 1000 bootstrap replicates.

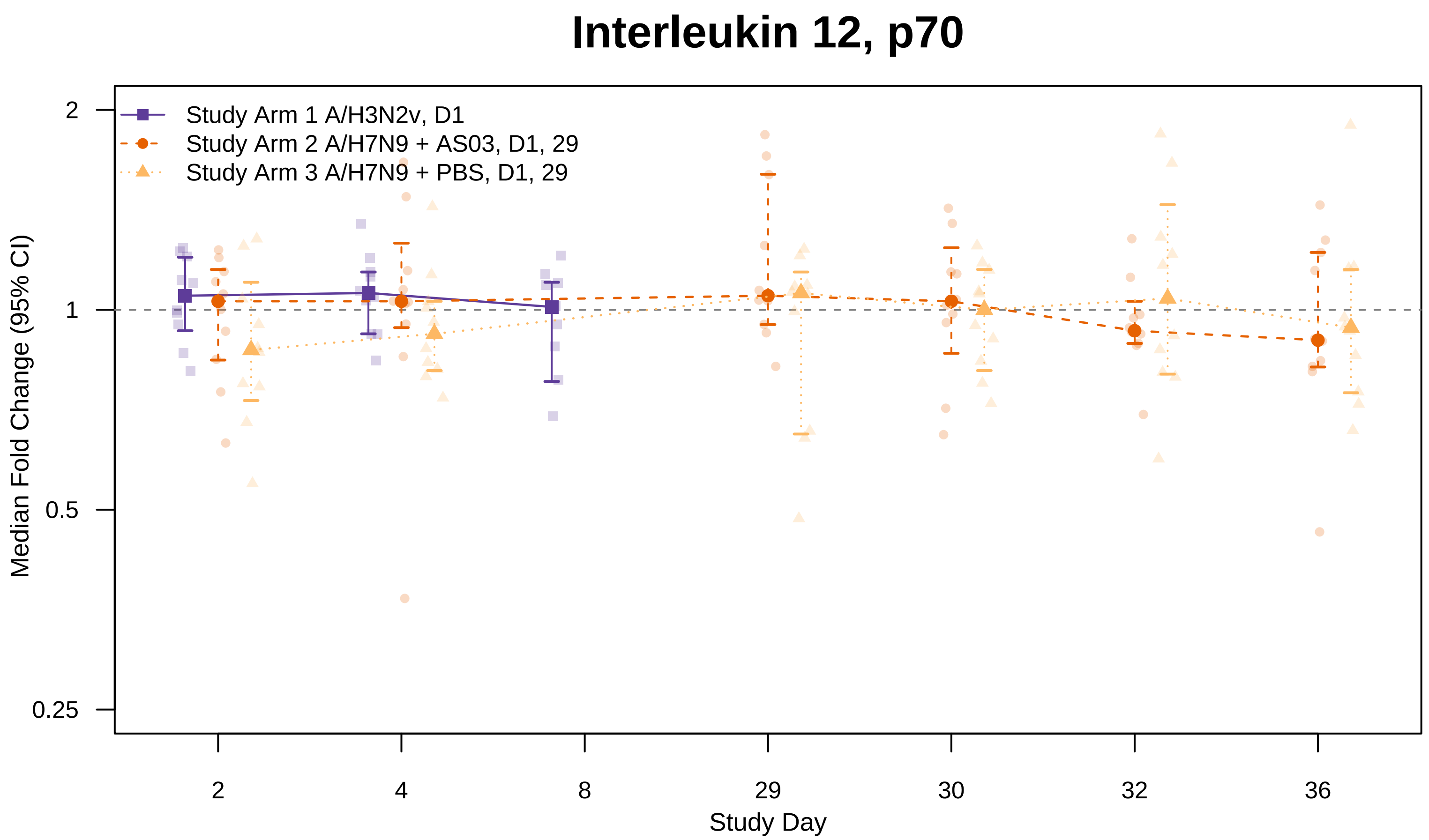

Figure S13: Inflammatory Interleukin 13 concentration response after vaccination. Time trend of median fold change of Interleukin 13 and associated 95% CI by study arm and post-vaccination day in the intent-to-treat population. Error bars indicate 95% confidence intervals based on 1000 bootstrap replicates.

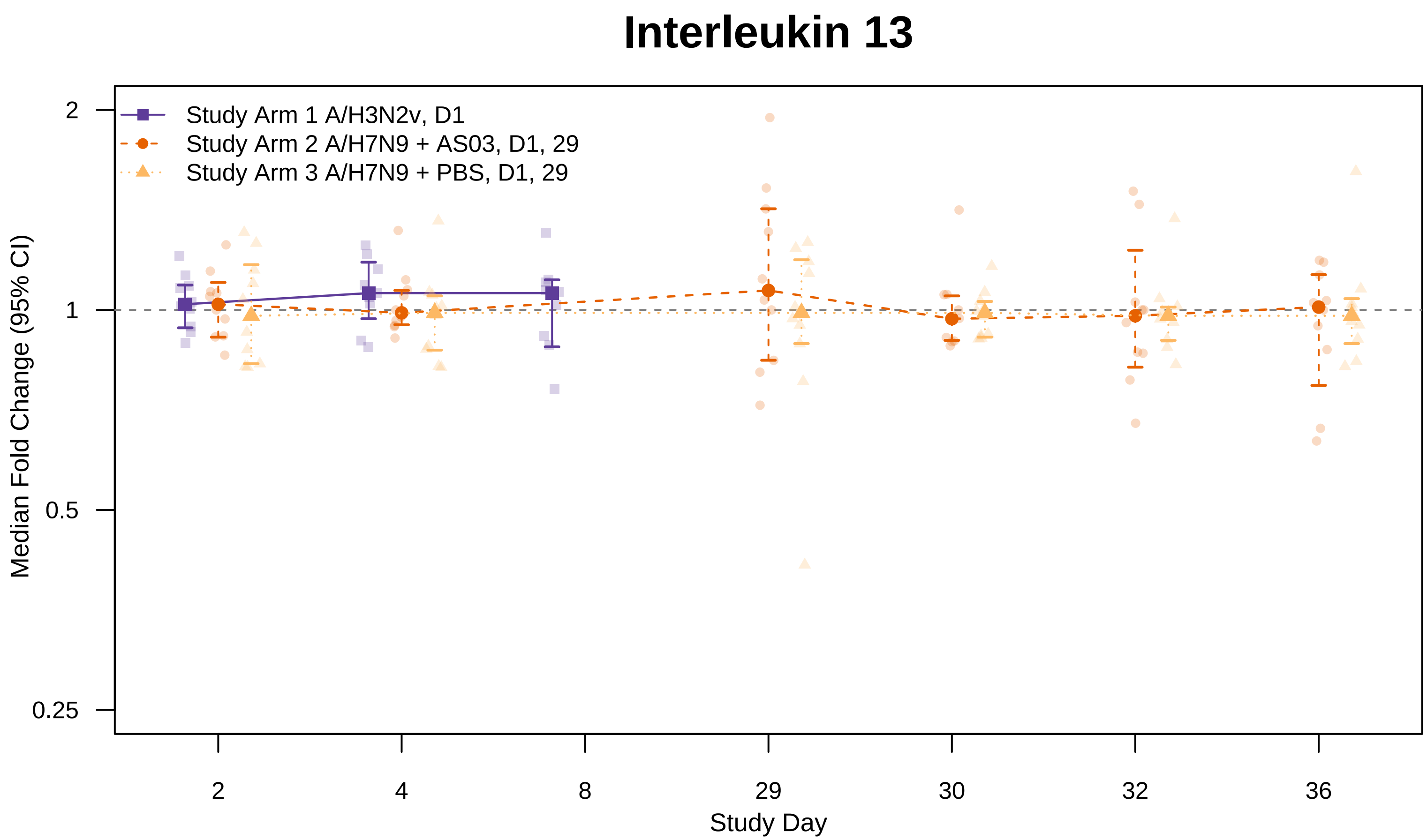

Figure S14: Inflammatory Interleukin 17 concentration response after vaccination. Time trend of median fold change of Interleukin 17 and associated 95% CI by study arm and post-vaccination day in the intent-to-treat population. Error bars indicate 95% confidence intervals based on 1000 bootstrap replicates.

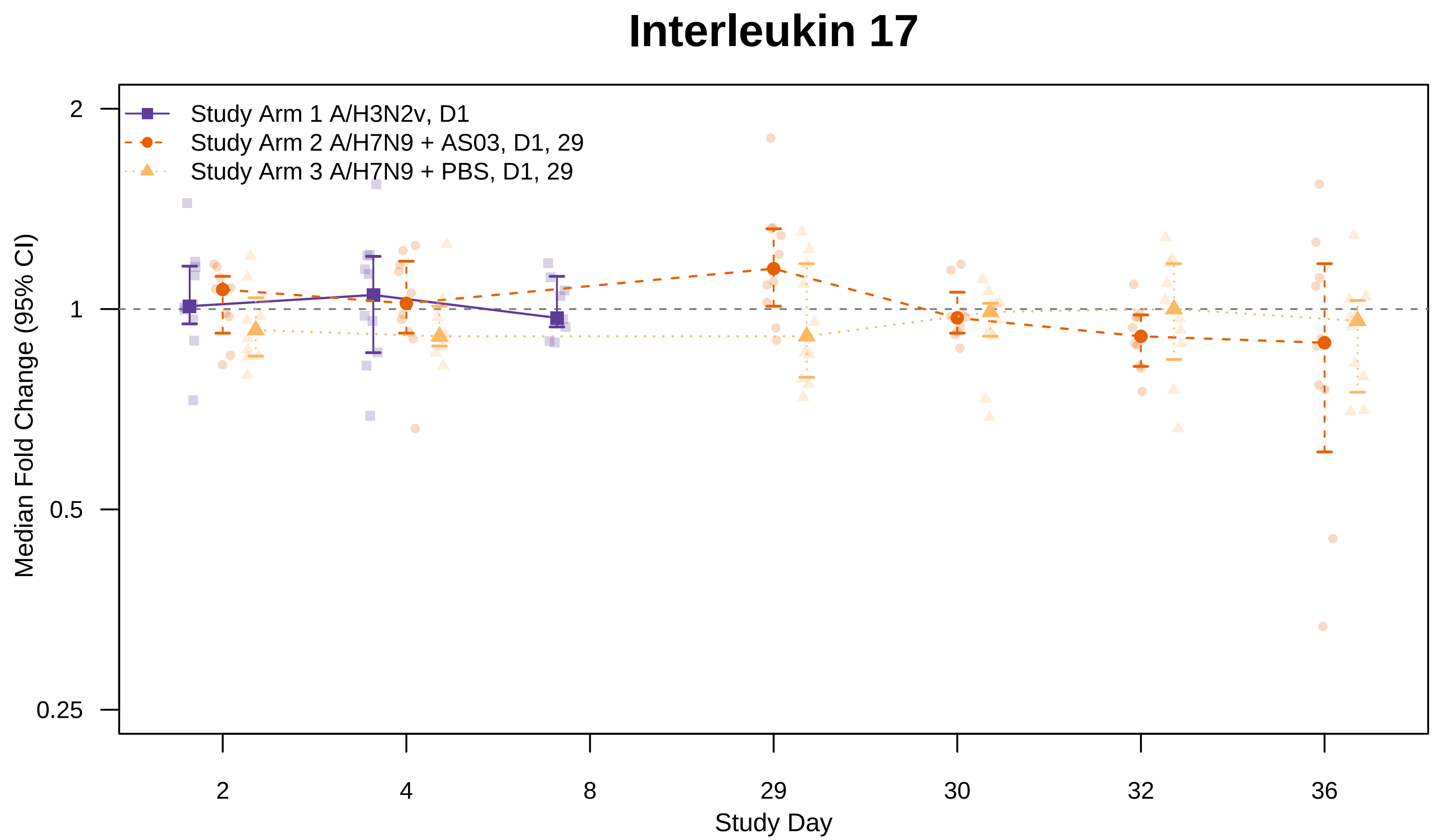

Figure S15: Inflammatory Interleukin 21 concentration response after vaccination. Time trend of median fold change of Interleukin 21 and associated 95% CI by study arm and post-vaccination day in the intent-to-treat population. Error bars indicate 95% confidence intervals based on 1000 bootstrap replicates.

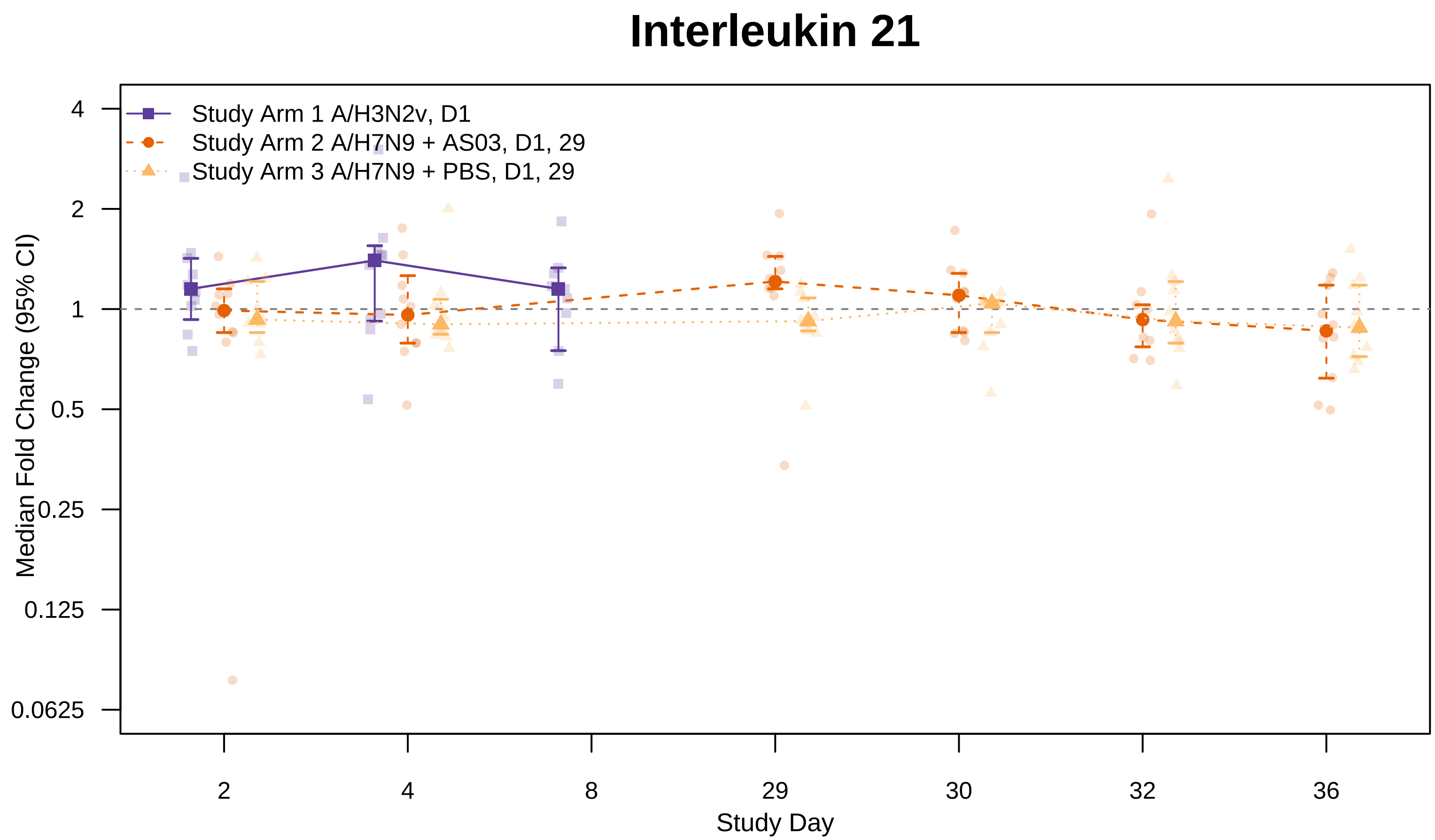

Confidential/Proprietary Information

Figure S16: Inflammatory Interleukin 23 concentration response after vaccination. Time trend of median fold change of Interleukin 23 and associated 95% CI by study arm and post-vaccination day in the intent-to-treat population. Error bars indicate 95% confidence intervals based on 1000 bootstrap replicates.

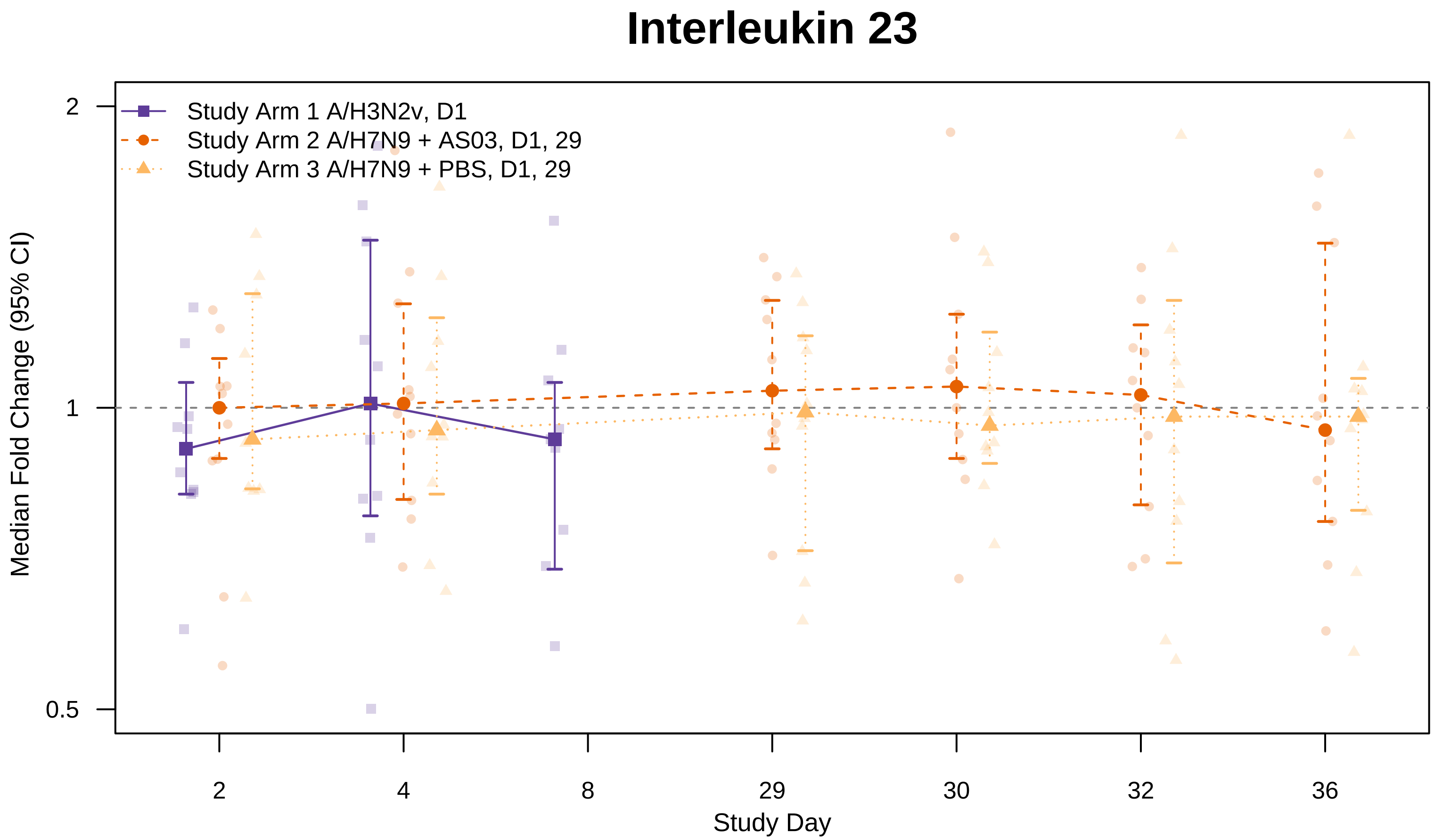

Figure S17: Inflammatory Interferon-Inducible T-cell α Chemoattractant concentration response after vaccination. Time trend of median fold change of Interferon-Inducible T-cell α Chemoattractant and associated 95% CI by study arm and post-vaccination day in the intent-to-treat population. Error bars indicate 95% confidence intervals based on 1000 bootstrap replicates.

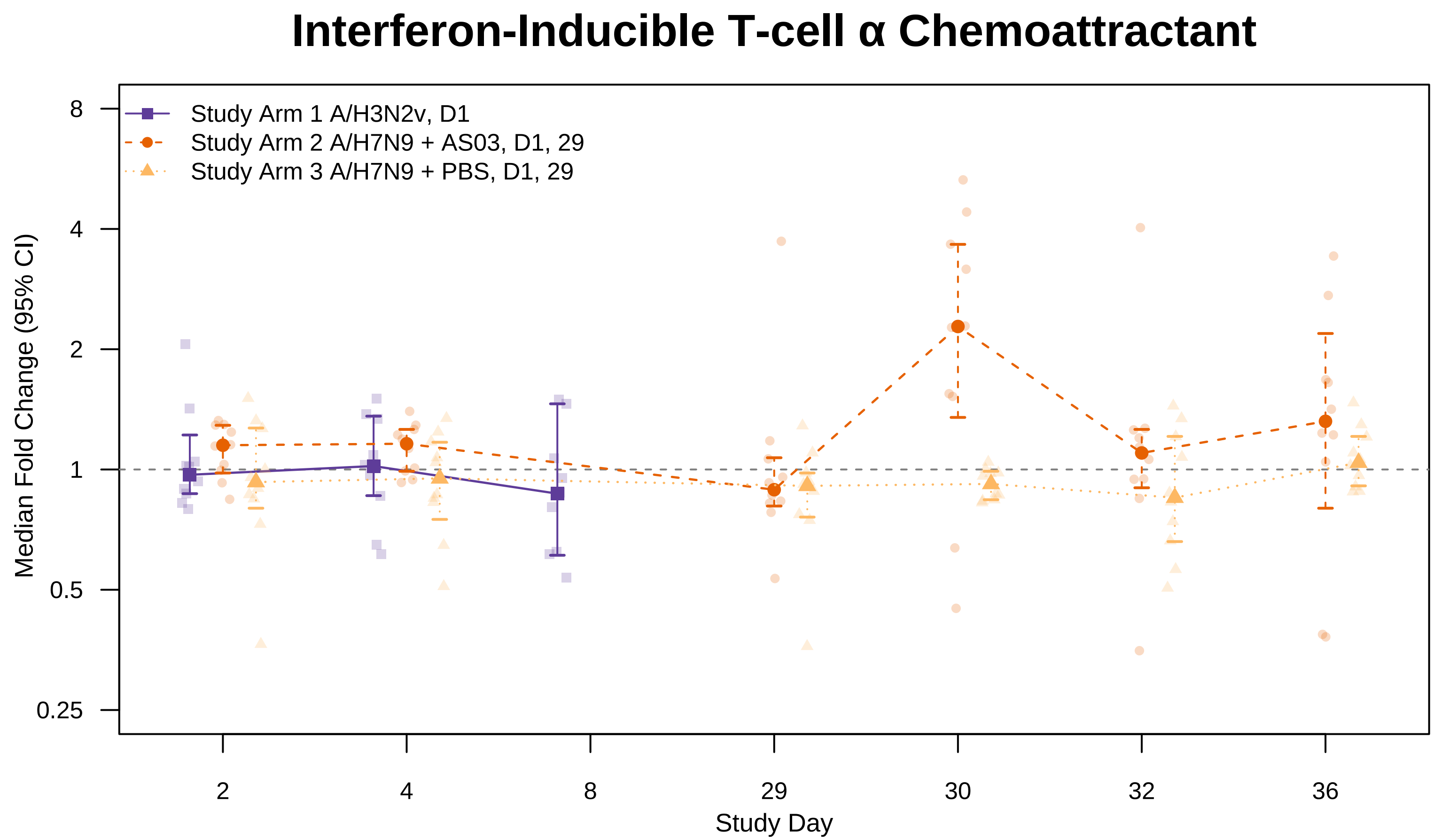

Confidential/Proprietary Information

Figure S18: Inflammatory Macrophage Inflammatory Protein-1 α concentration response after vaccination. Time trend of median fold change of Macrophage Inflammatory Protein-1 α and associated 95% CI by study arm and post-vaccination day in the intent-to-treat population. Error bars indicate 95% confidence intervals based on 1000 bootstrap replicates.

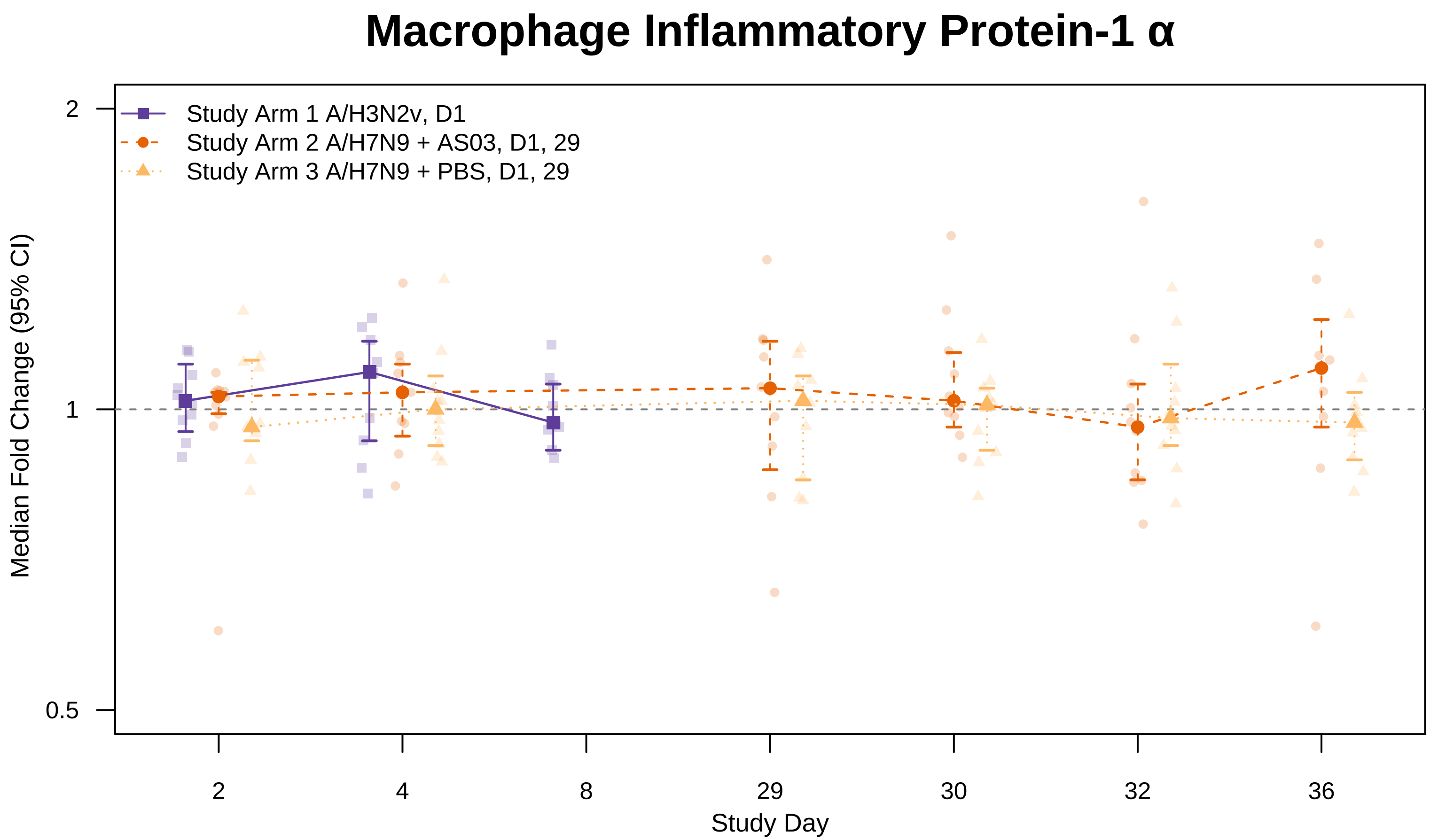

Figure S19: Inflammatory Macrophage Inflammatory Protein-1 β concentration response after vaccination. Time trend of median fold change of Macrophage Inflammatory Protein-1 β and associated 95% CI by study arm and post-vaccination day in the intent-to-treat population. Error bars indicate 95% confidence intervals based on 1000 bootstrap replicates.

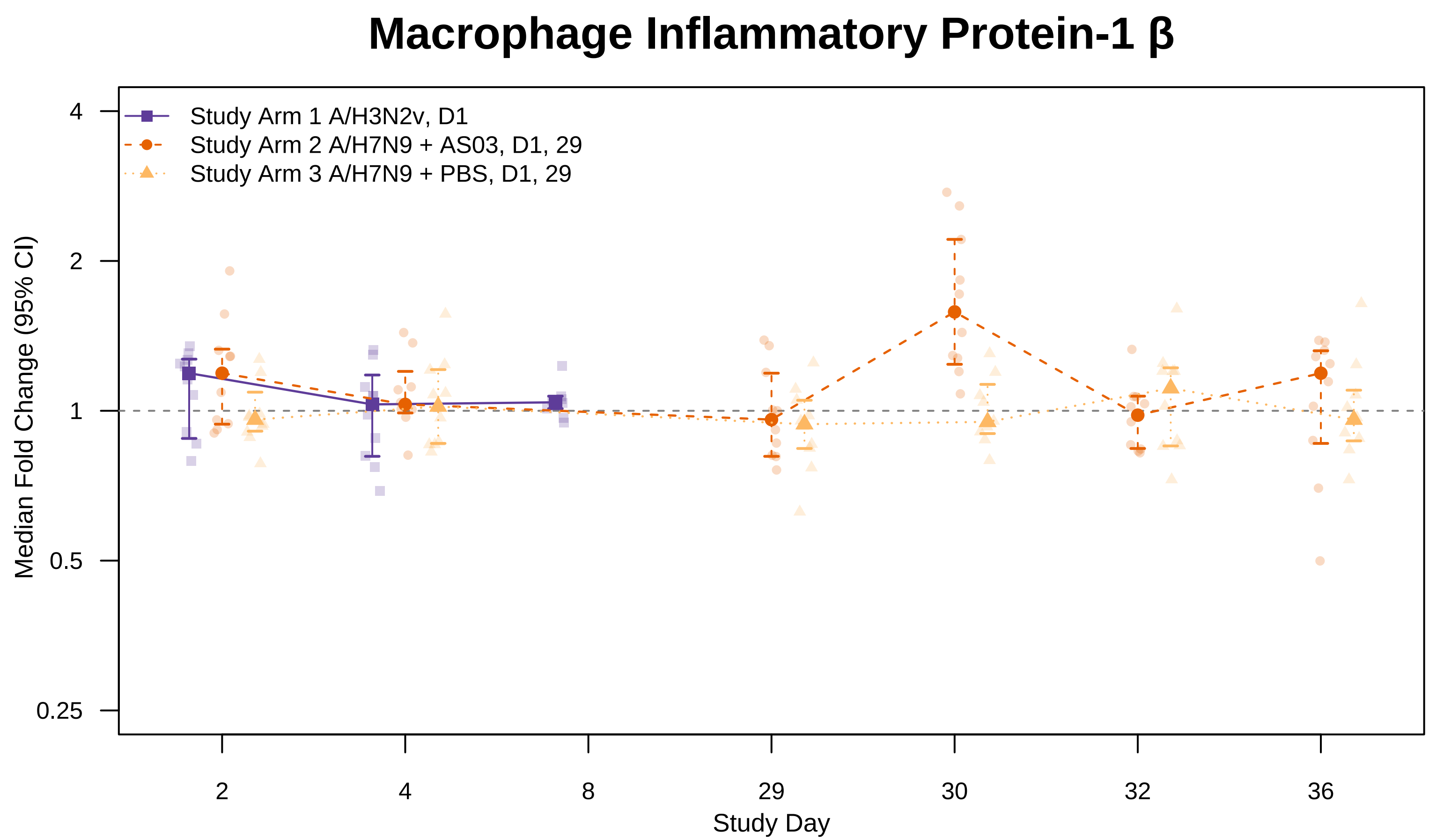

Figure S20: Inflammatory Macrophage Inflammatory Protein-3 α concentration response after vaccination. Time trend of median fold change of Macrophage Inflammatory Protein-3 α and associated 95% CI by study arm and post-vaccination day in the intent-to-treat population. Error bars indicate 95% confidence intervals based on 1000 bootstrap replicates.

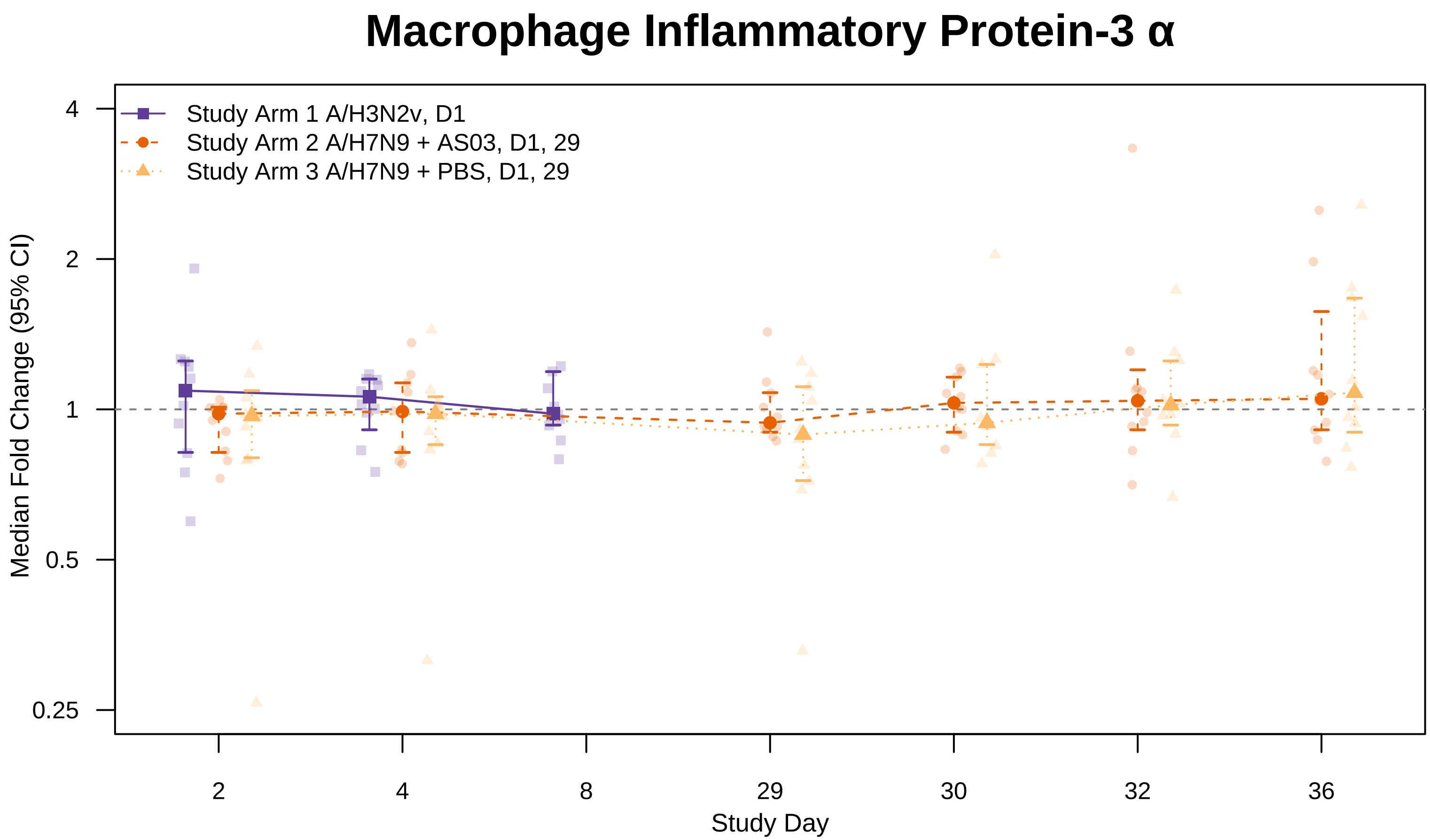

Figure S21: Inflammatory Tumor Necrosis Factor concentration response after vaccination. Time trend of median fold change of Tumor Necrosis Factor and associated 95% CI by study arm and post-vaccination day in the intent-to-treat population. Error bars indicate 95% confidence intervals based on 1000 bootstrap replicates.

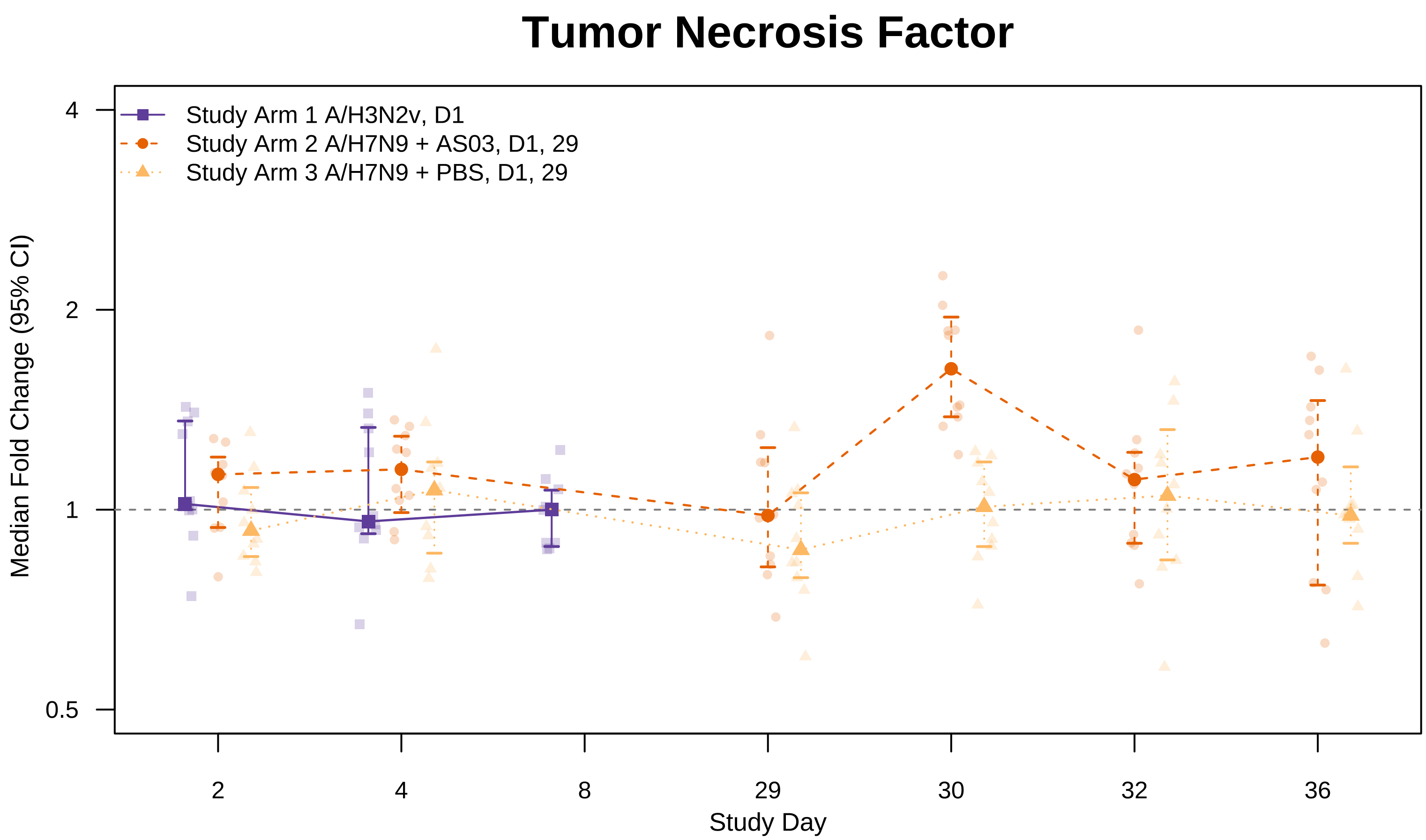

Confidential/Proprietary Information

Figure S22: Inflammatory Interferon α concentration response after vaccination. Time trend of median fold change of Interferon α and associated 95% CI by study arm and post-vaccination day in the intent-to-treat population. Error bars indicate 95% confidence intervals based on 1000 bootstrap replicates.

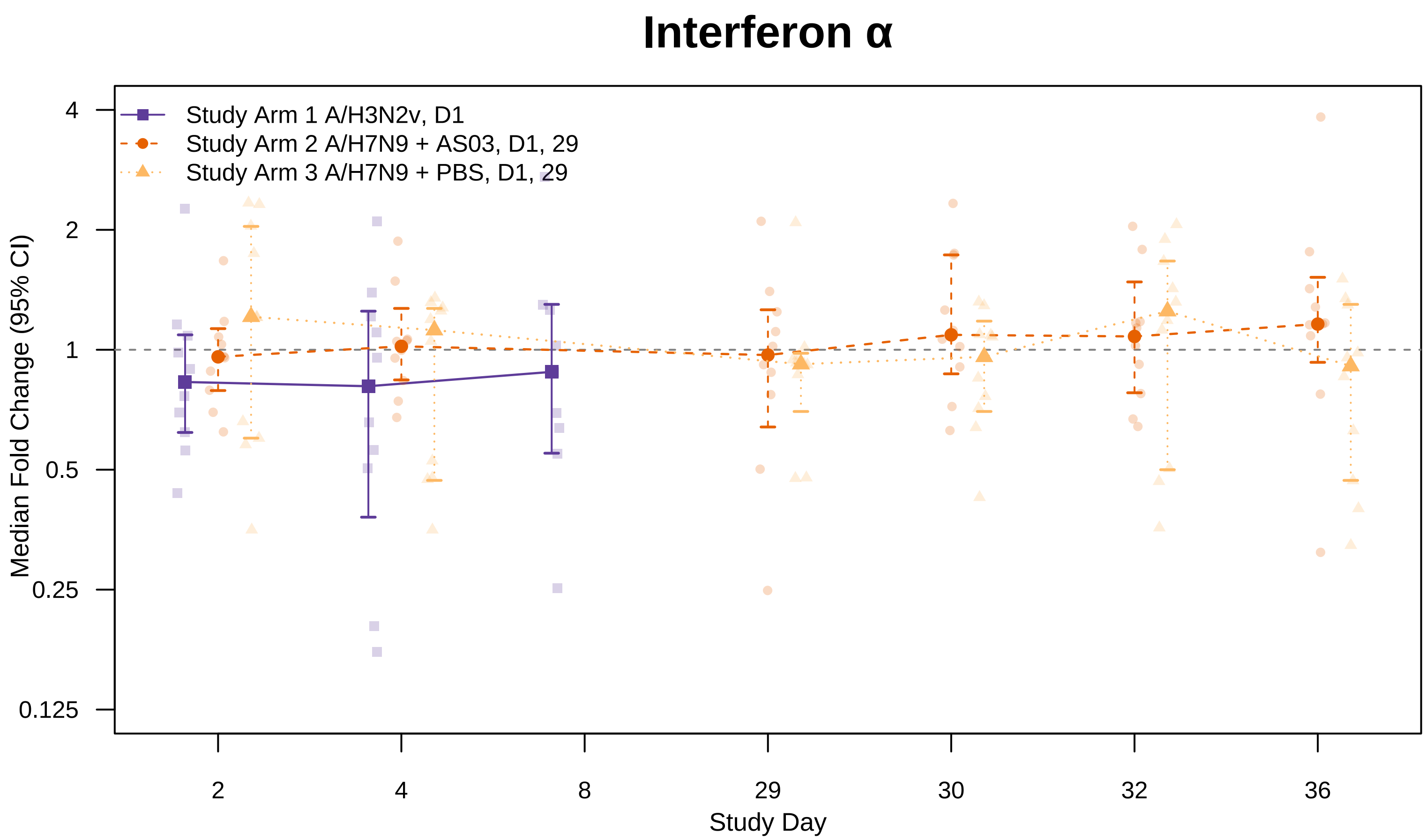

Figure S23: Inflammatory Interferon β concentration response after vaccination. Time trend of median fold change of Interferon β and associated 95% CI by study arm and post-vaccination day in the intent-to-treat population. Error bars indicate 95% confidence intervals based on 1000 bootstrap replicates.

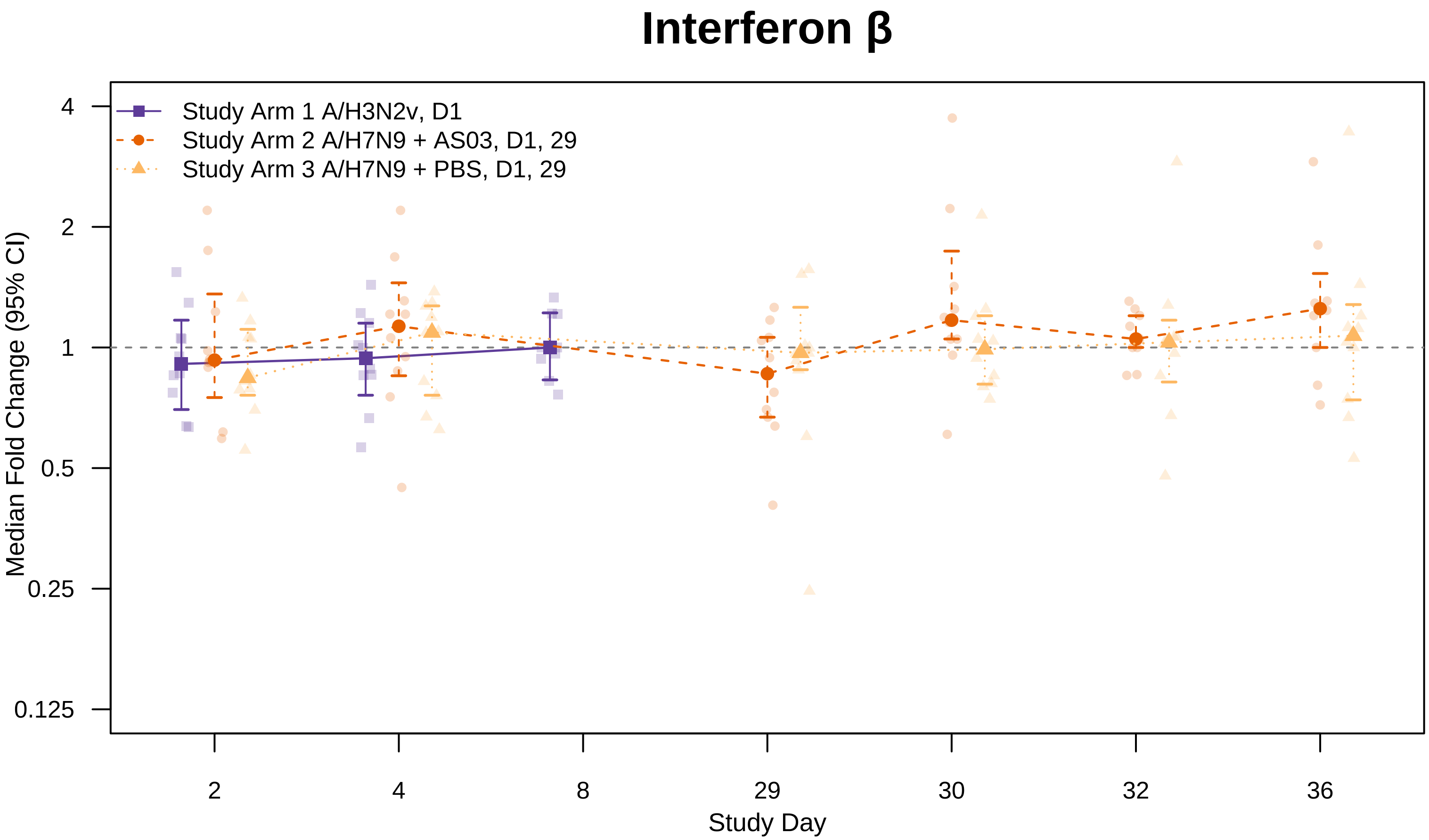

Figure S27: Top pathway time trend for KEGG NOD-like receptor signaling pathway. Mean of mean log_2 fold change and associated 95% CI of log_2 fold change of all genes/proteins in the pathway. Error bars indicate 95% confidence intervals based on 1000 bootstrap replicates.

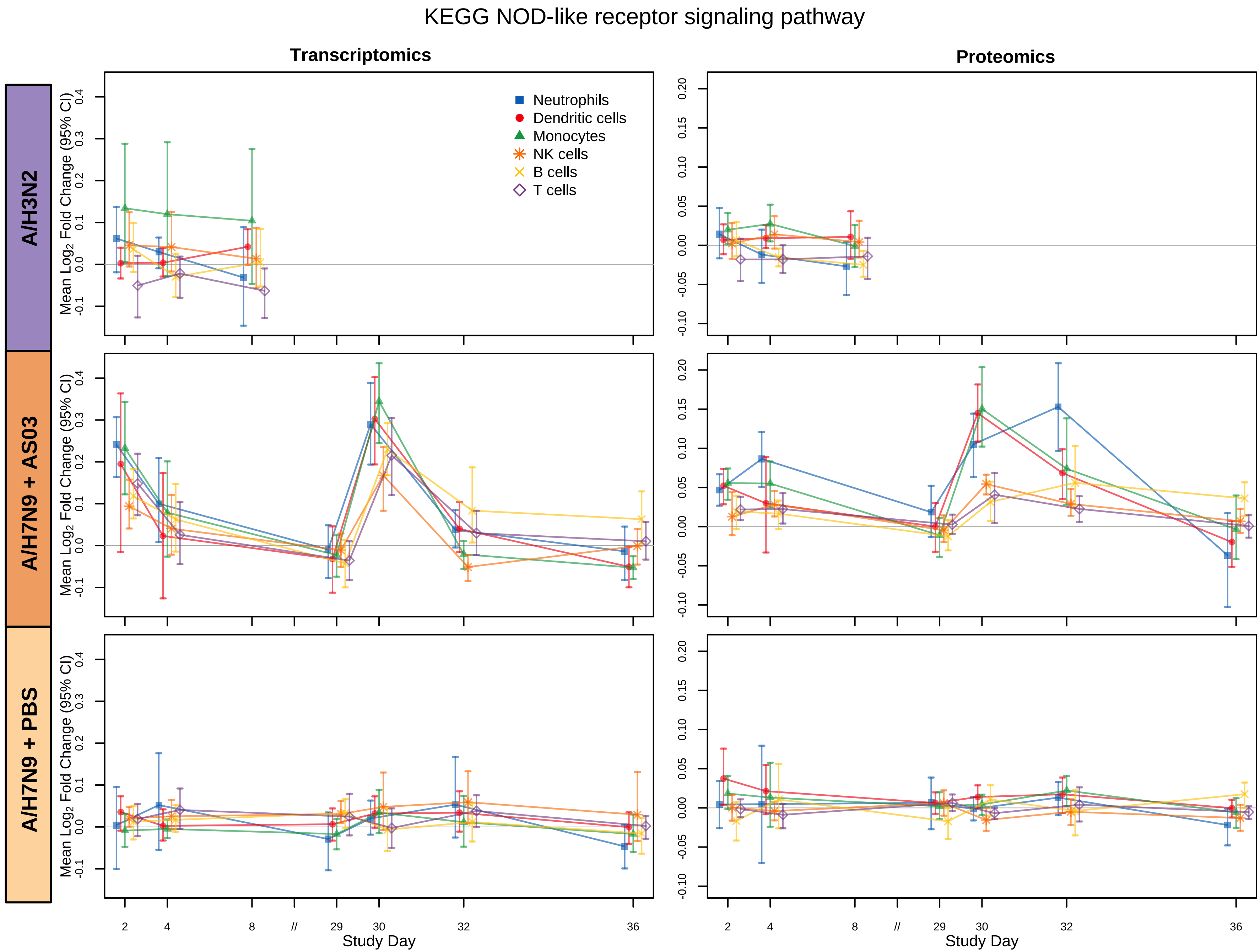

Figure S28: Top pathway time trend for KEGG Phagosome. Mean of mean log_2 fold change and associated 95% CI of log_2 fold change of all genes/proteins in the pathway. Error bars indicate 95% confidence intervals based on 1000 bootstrap replicates.

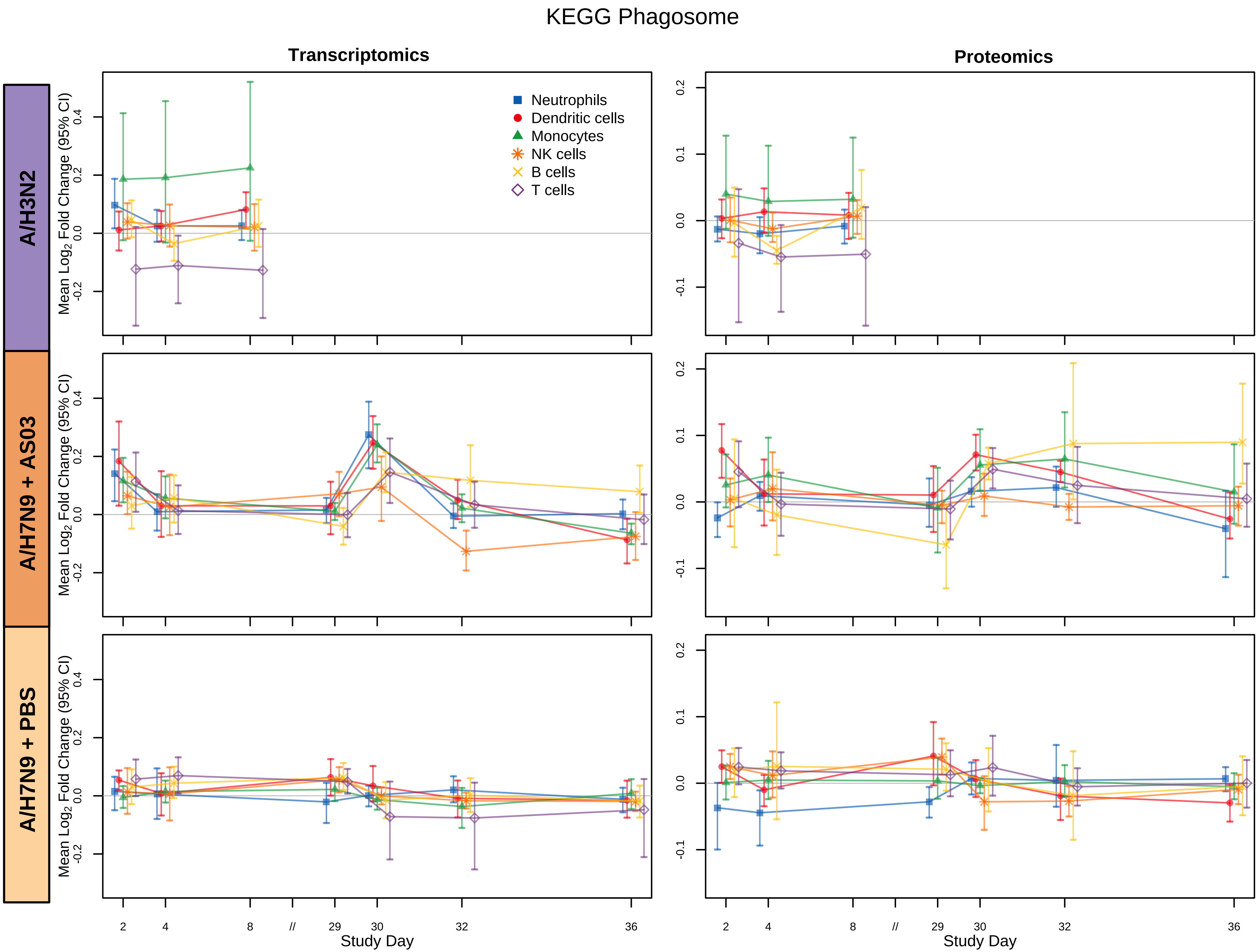

Figure S29: Top pathway time trend for KEGG Antigen processing and presentation. Mean of mean log_2 fold change and associated 95% CI of log_2 fold change of all genes/proteins in the pathway. Error bars indicate 95% confidence intervals based on 1000 bootstrap replicates.

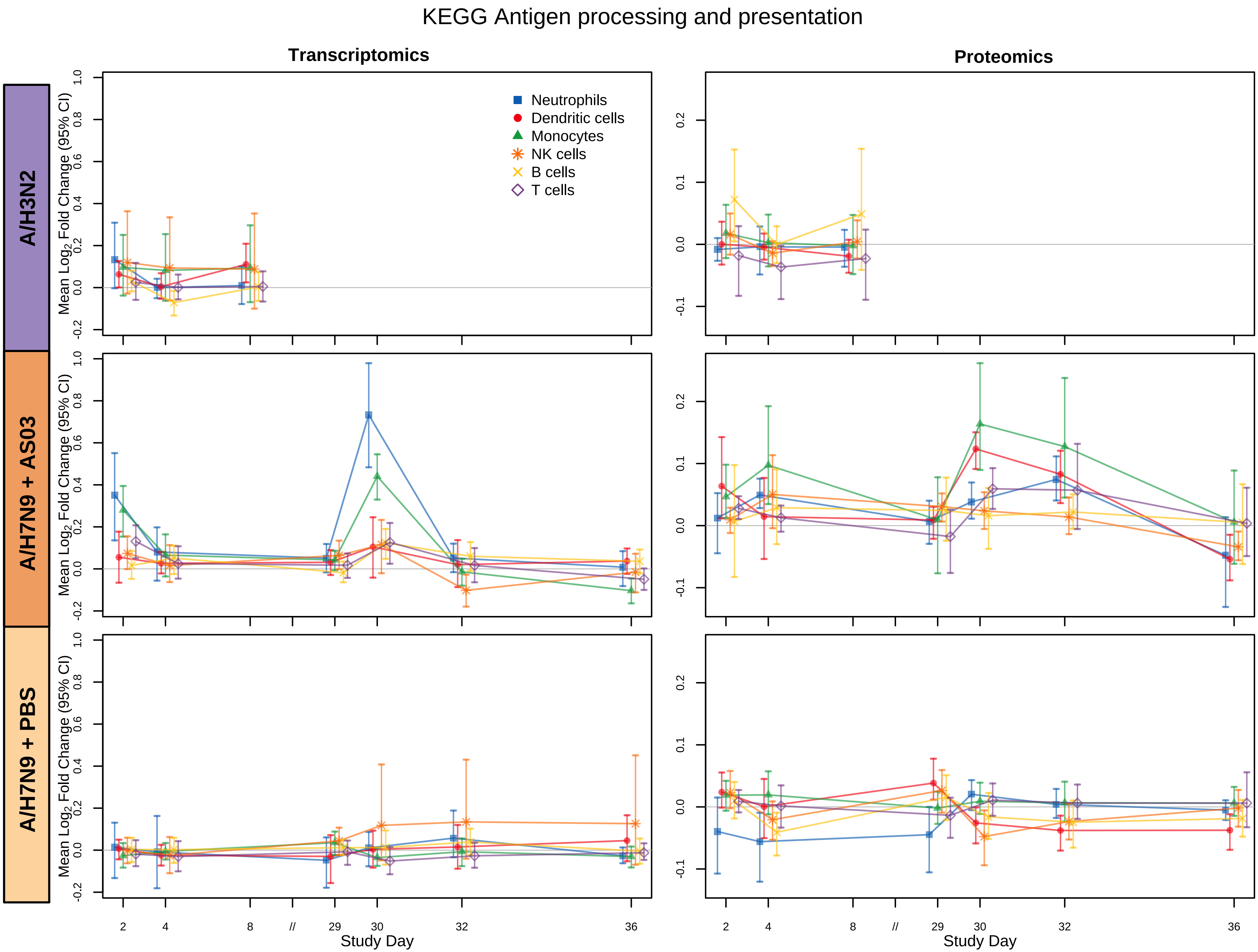

Confidential/Proprietary Information

Figure S30: Top pathway time trend for Reactome Interferon Signaling. Mean of mean log_2 fold change and associated 95% CI of log_2 fold change of all genes/proteins in the pathway. Error bars indicate 95% confidence intervals based on 1000 bootstrap replicates.

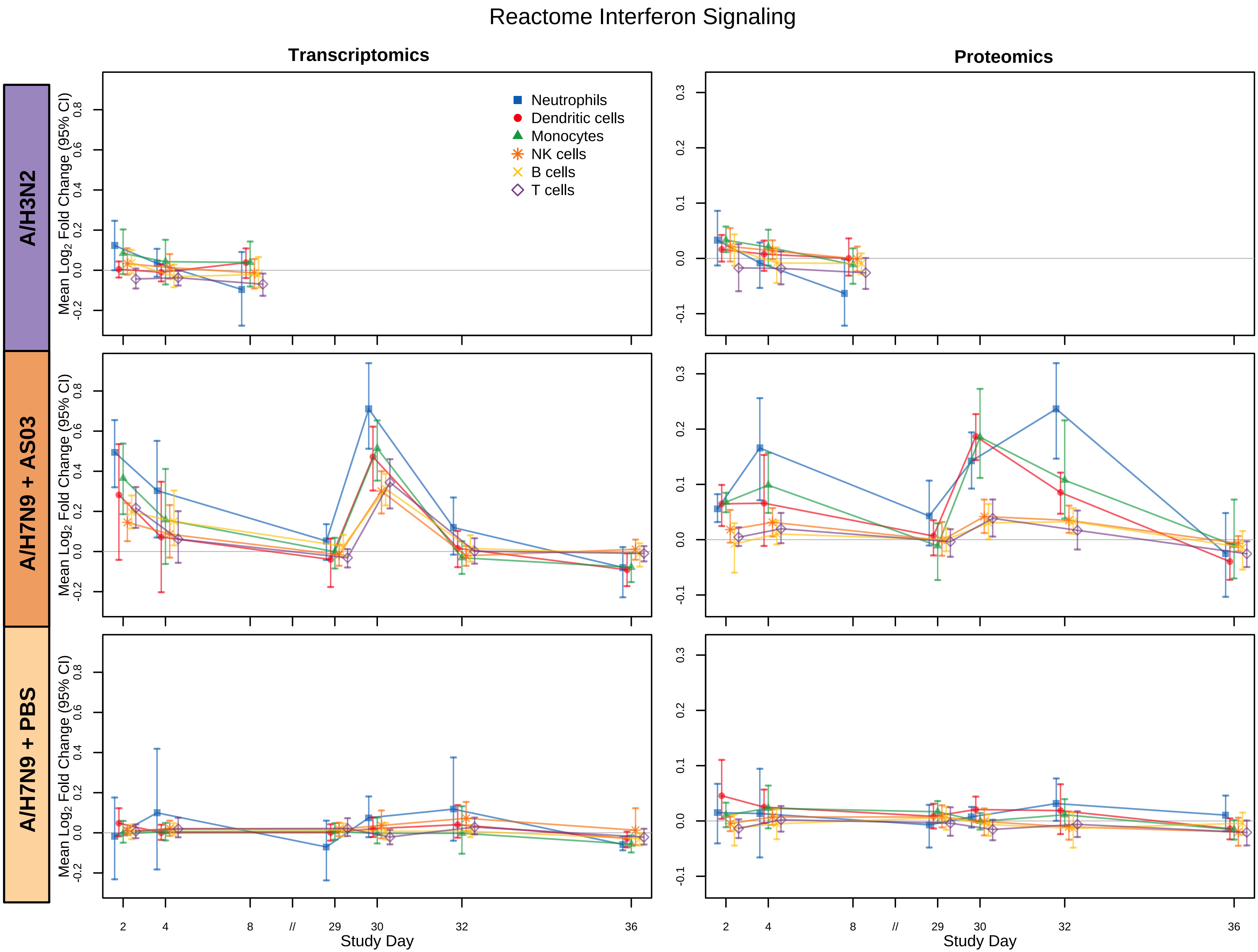

Figure S31: Top pathway time trend for Reactome Interferon Alpha Beta Signaling. Mean of mean log_2 fold change and associated 95% CI of log_2 fold change of all genes/proteins in the pathway. Error bars indicate 95% confidence intervals based on 1000 bootstrap replicates.

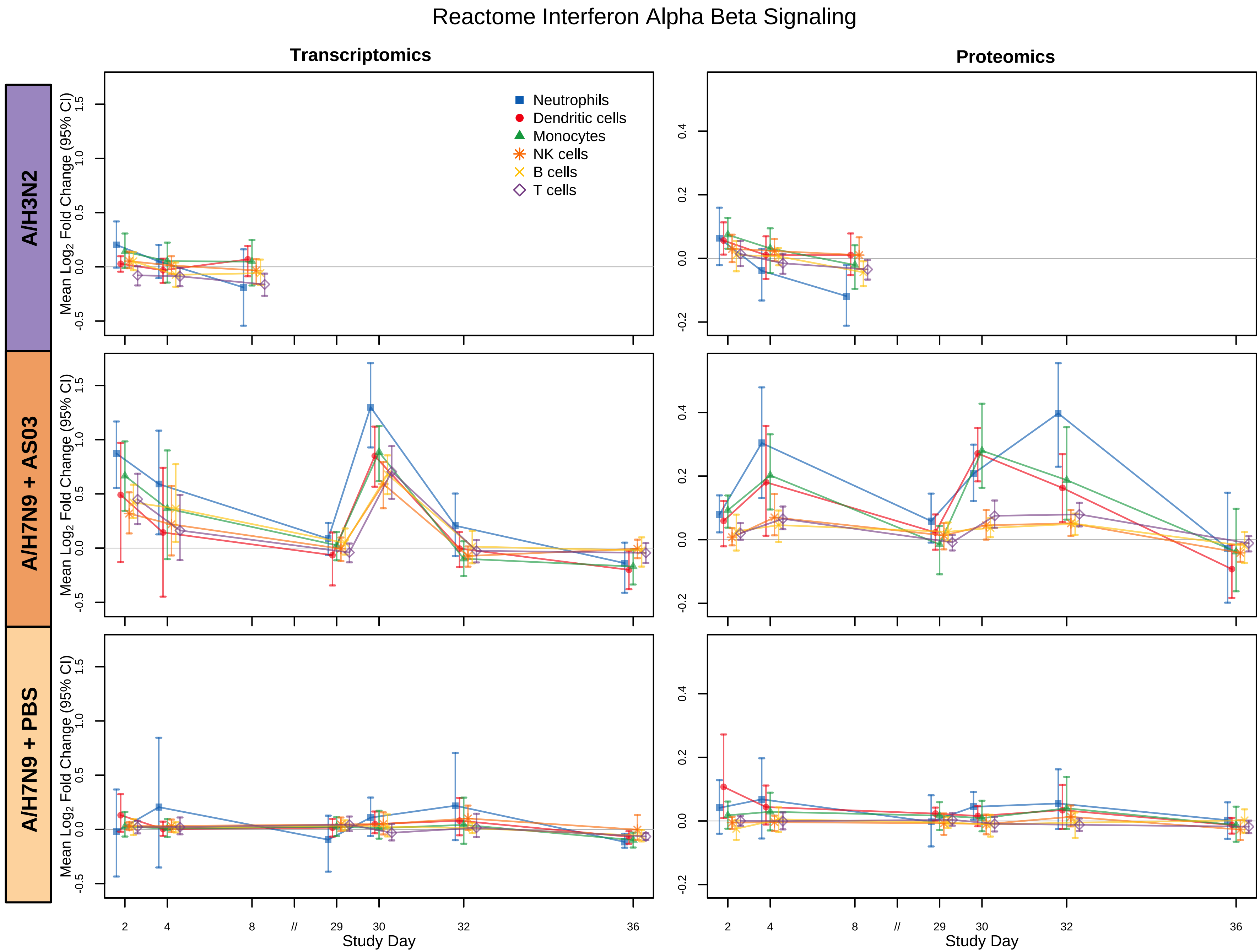

Figure S32: Top pathway time trend for Reactome Interferon Gamma Signaling. Mean of mean log_2 fold change and associated 95% CI of log_2 fold change of all genes/proteins in the pathway. Error bars indicate 95% confidence intervals based on 1000 bootstrap replicates.

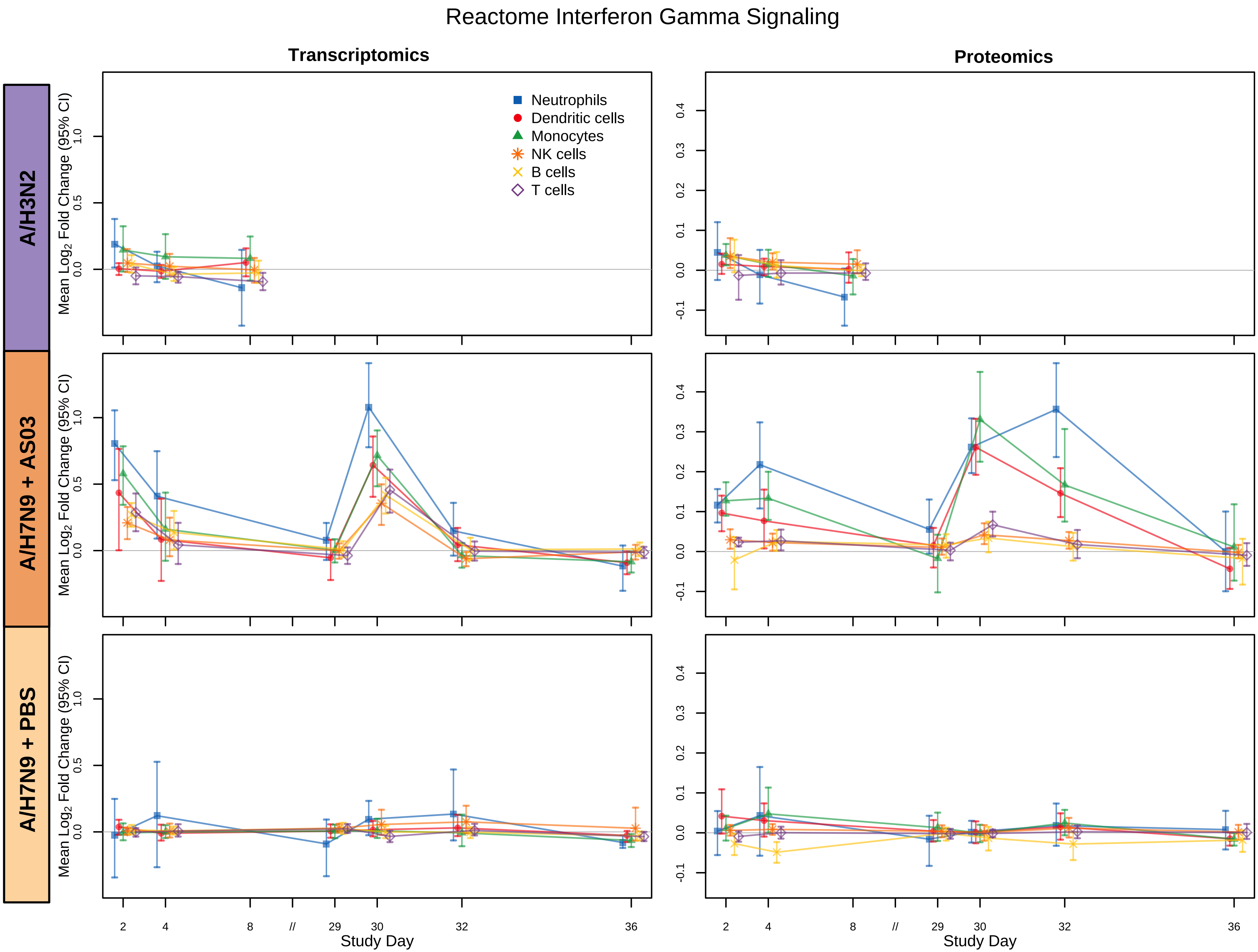

Figure S33: Top pathway time trend for GO Innate Immune Response. Mean of mean log_2 fold change and associated 95% CI of log_2 fold change of all genes/proteins in the pathway. Error bars indicate 95% confidence intervals based on 1000 bootstrap replicates.

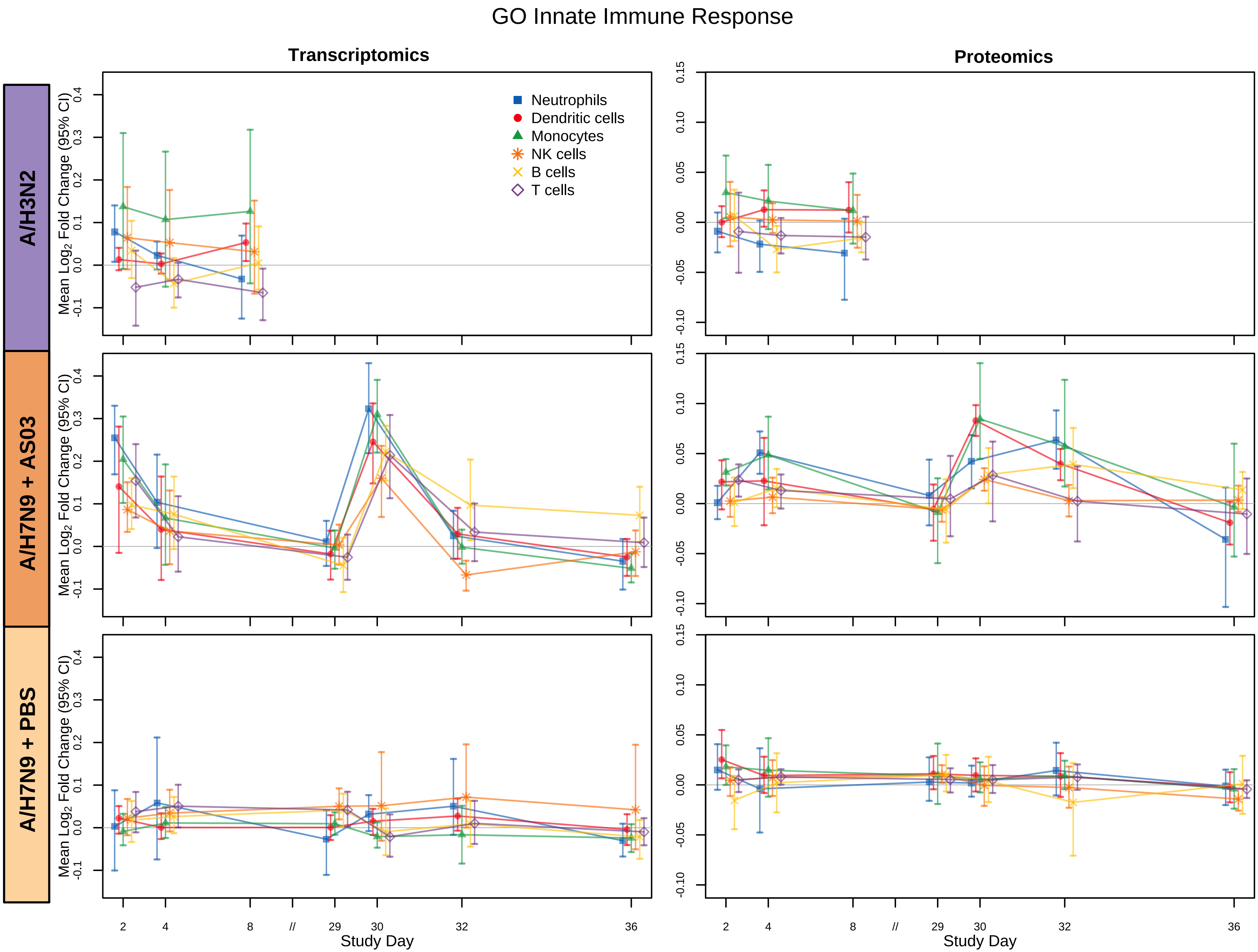

Confidential/Proprietary Information

Figure S34: Top pathway time trend for GO Defense Response. Mean of mean log_2 fold change and associated 95% CI of log_2 fold change of all genes/proteins in the pathway. Error bars indicate 95% confidence intervals based on 1000 bootstrap replicates.

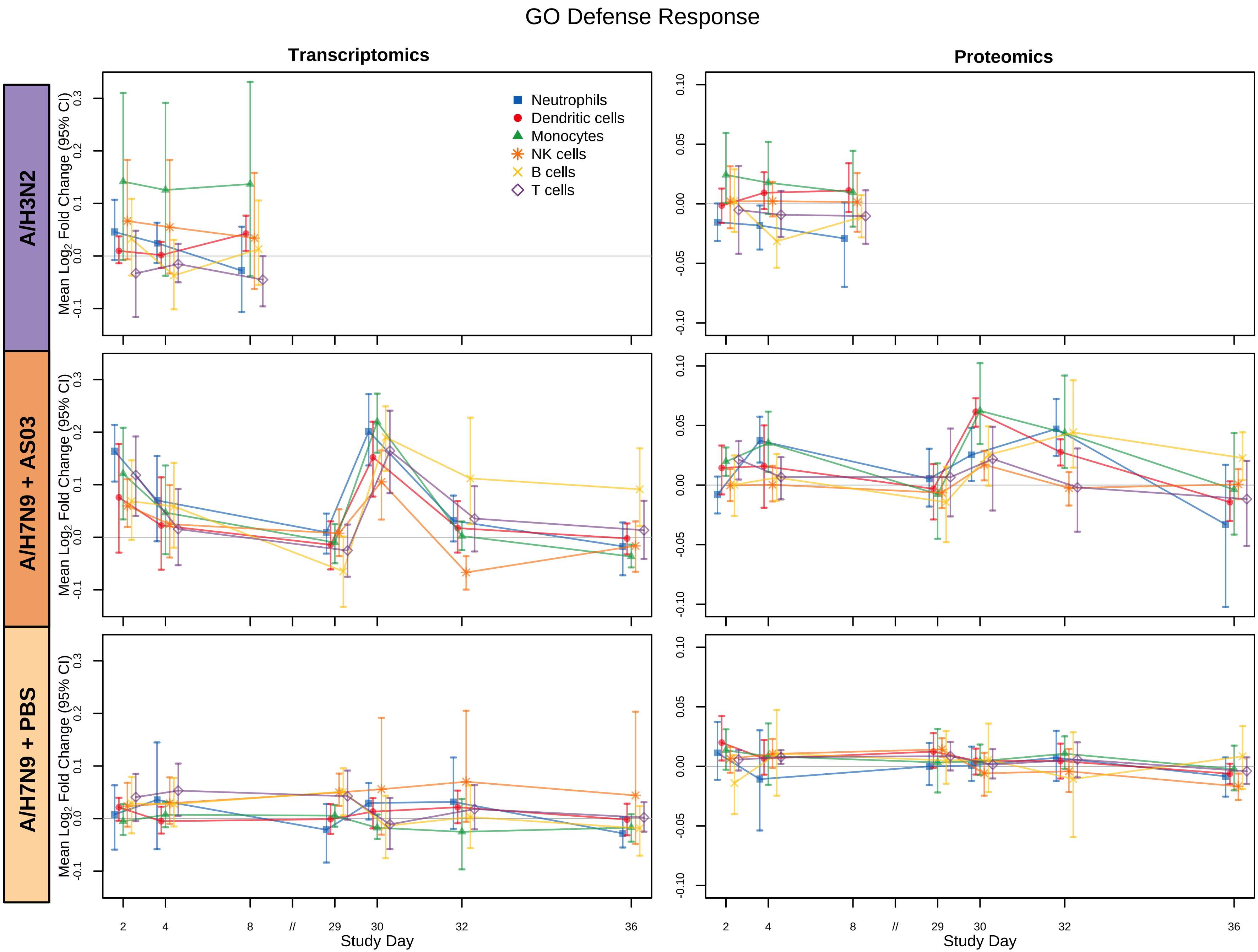

Confidential/Proprietary Information

Figure S35: Top pathway time trend for GO Defense Response To Other Organism. Mean of mean log_2 fold change and associated 95% CI of log_2 fold change of all genes/proteins in the pathway. Error bars indicate 95% confidence intervals based on 1000 bootstrap replicates.

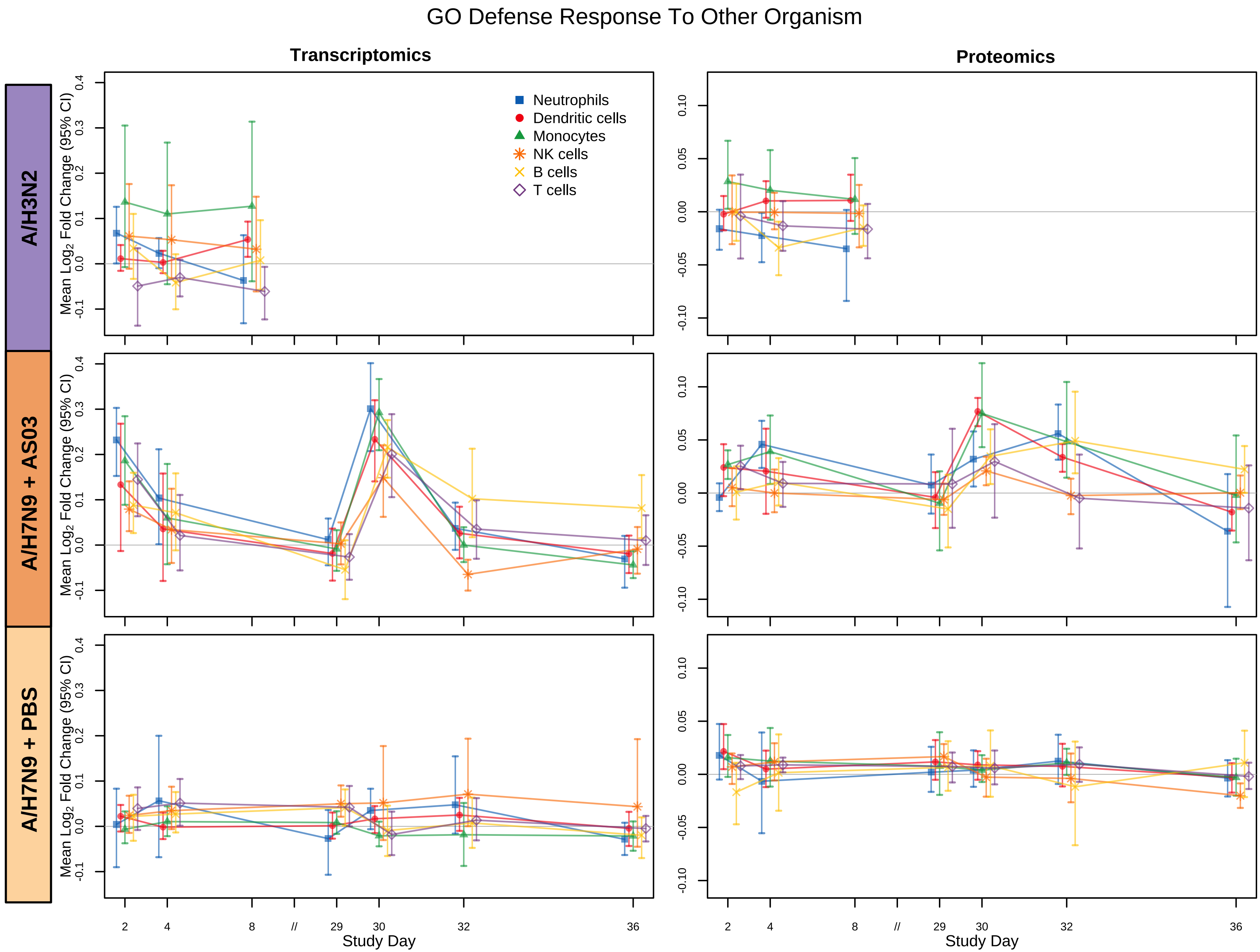

Figure S36: Top pathway time trend for M11.0 Enriched In Monocytes (II). Mean of mean log_2 fold change and associated 95% CI of log_2 fold change of all genes/proteins in the pathway. Error bars indicate 95% confidence intervals based on 1000 bootstrap replicates.

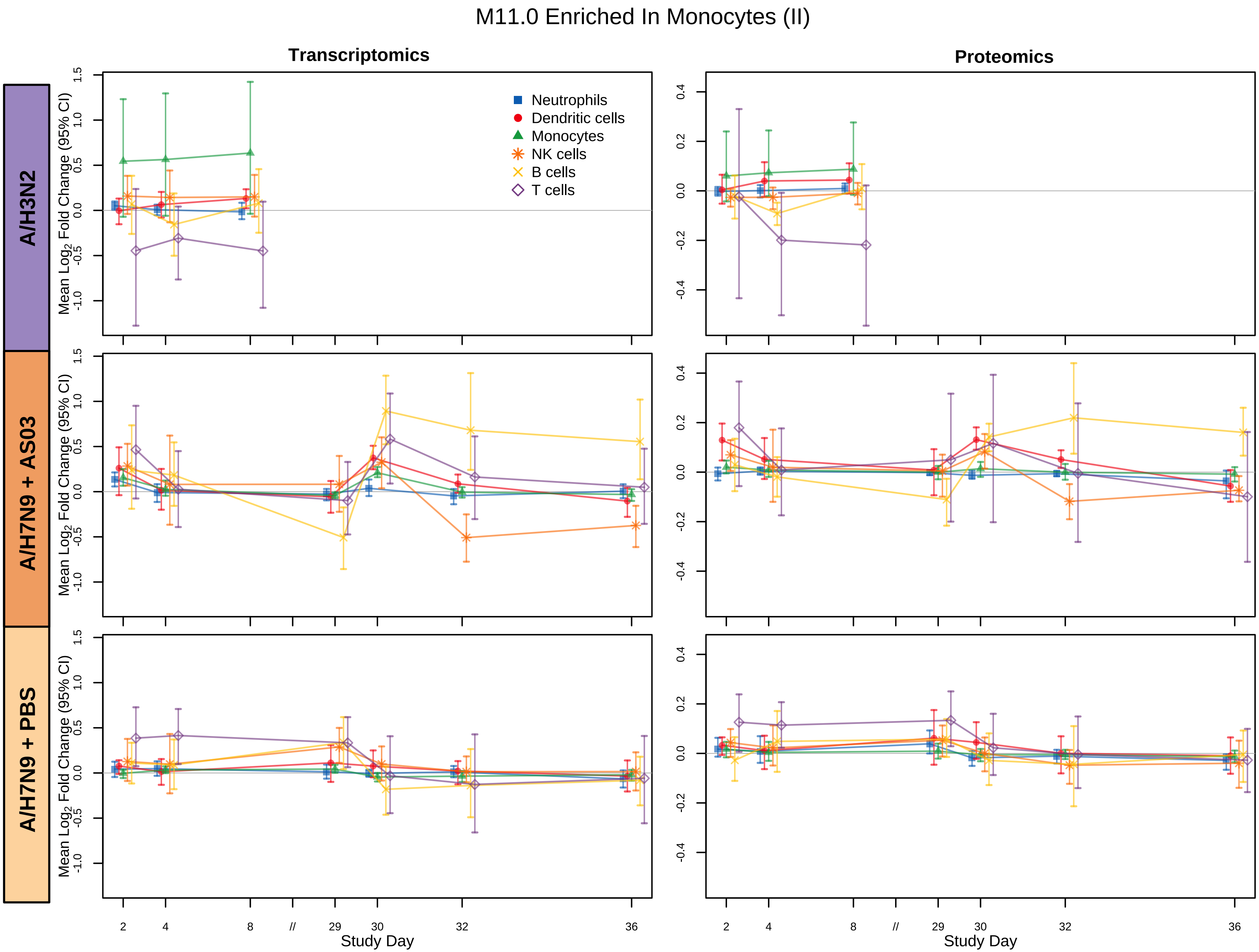

Figure S37: Top pathway time trend for M111.1 Viral Sensing And Immunity; IRF2 Targets Network (II). Mean of mean log_2 fold change and associated 95% CI of log_2 fold change of all genes/proteins in the pathway. Error bars indicate 95% confidence intervals based on 1000 bootstrap replicates.

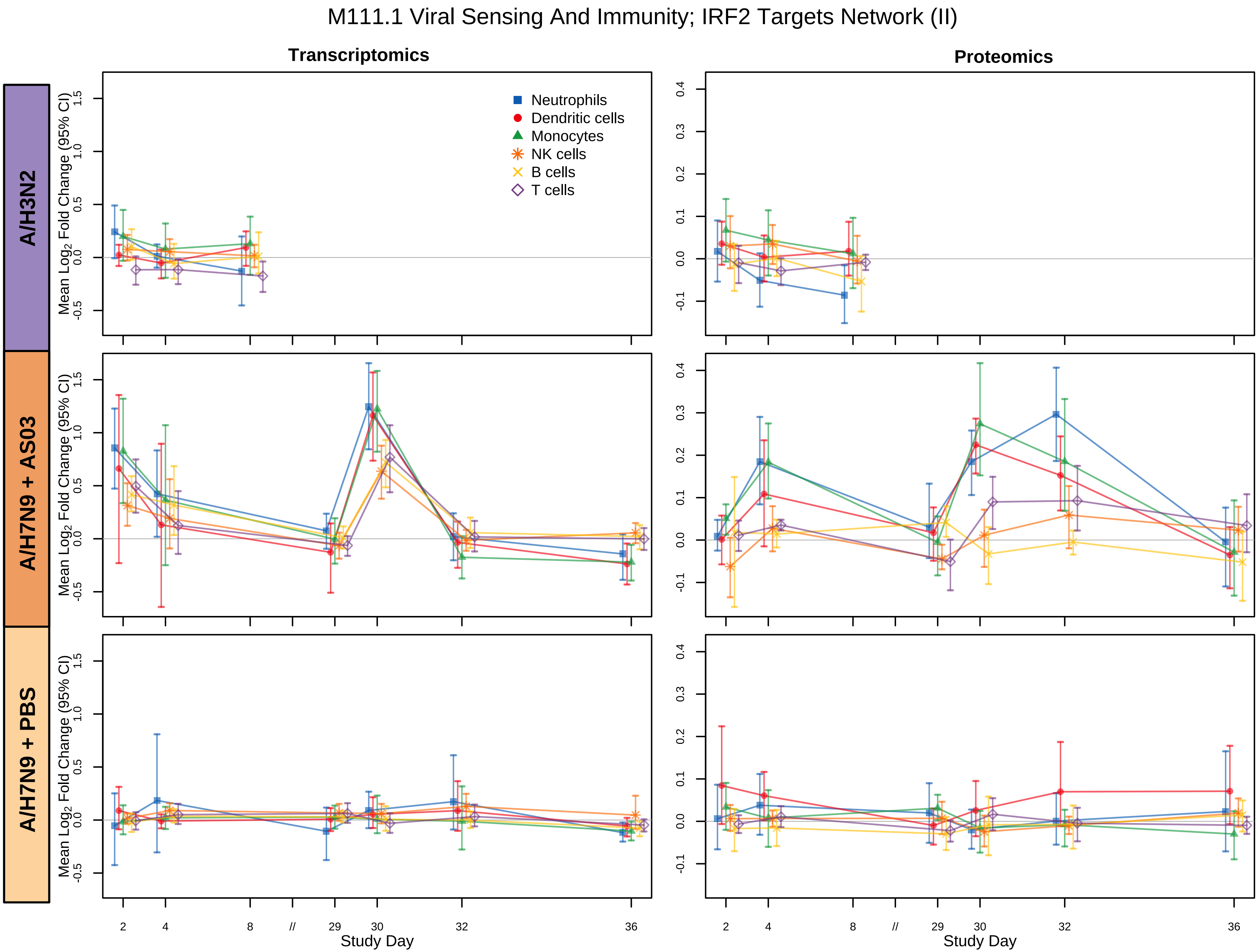

Figure S38: Top pathway time trend for M150 Innate Antiviral Response. Mean of mean log_2 fold change and associated 95% CI of log_2 fold change of all genes/proteins in the pathway. Error bars indicate 95% confidence intervals based on 1000 bootstrap replicates.

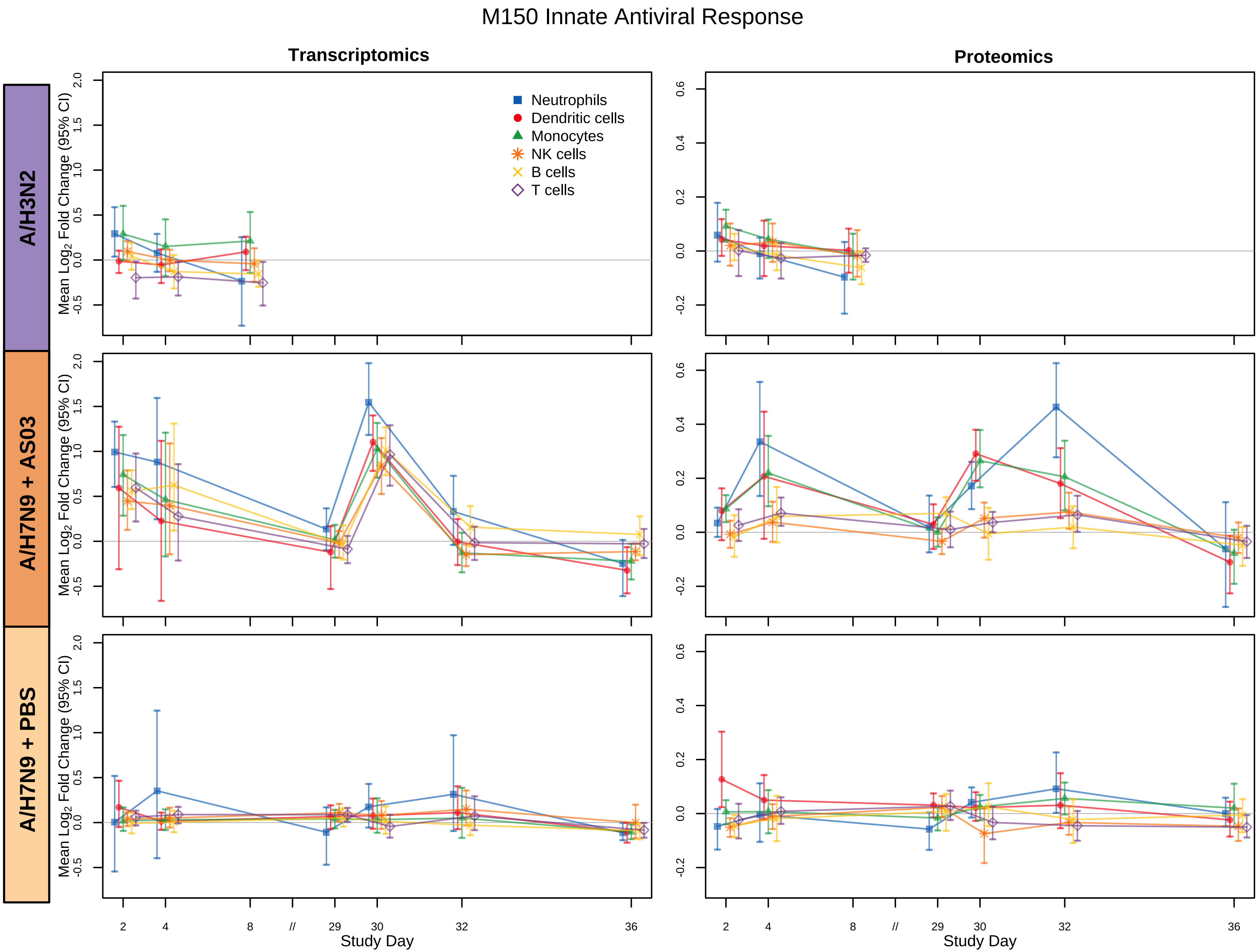

Figure S39: KEGG cell cycle time trend. Mean of mean log_2 fold change and associated 95% CI of log_2 fold change of all genes/proteins in the pathway. Error bars indicate 95% confidence intervals based on 1000 bootstrap replicates.

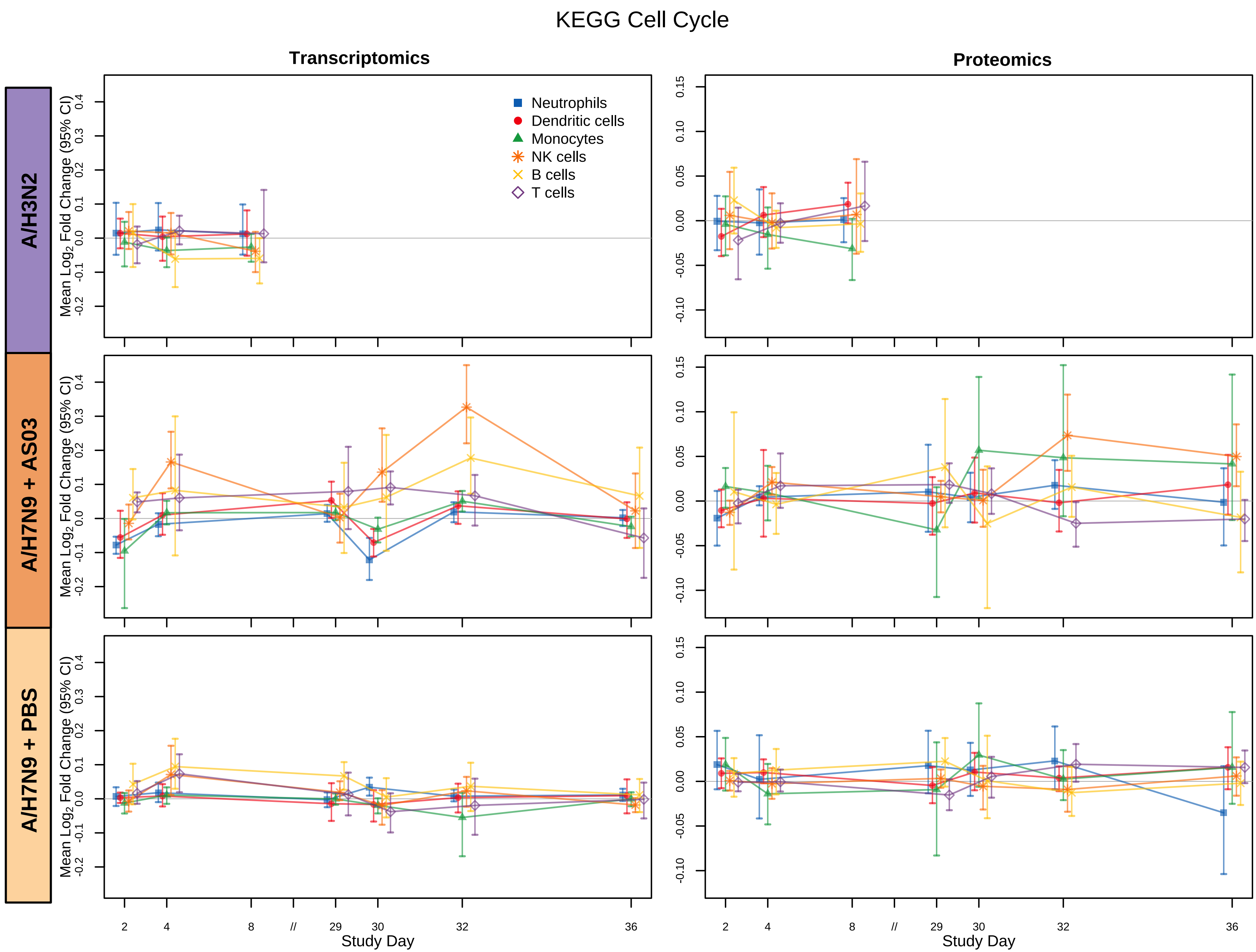

Figure S40: KEGG JAK-STAT signaling pathway time trend. Mean of mean log_2 fold change and associated 95% CI of log_2 fold change of all genes/proteins in the pathway. Error bars indicate 95% confidence intervals based on 1000 bootstrap replicates.

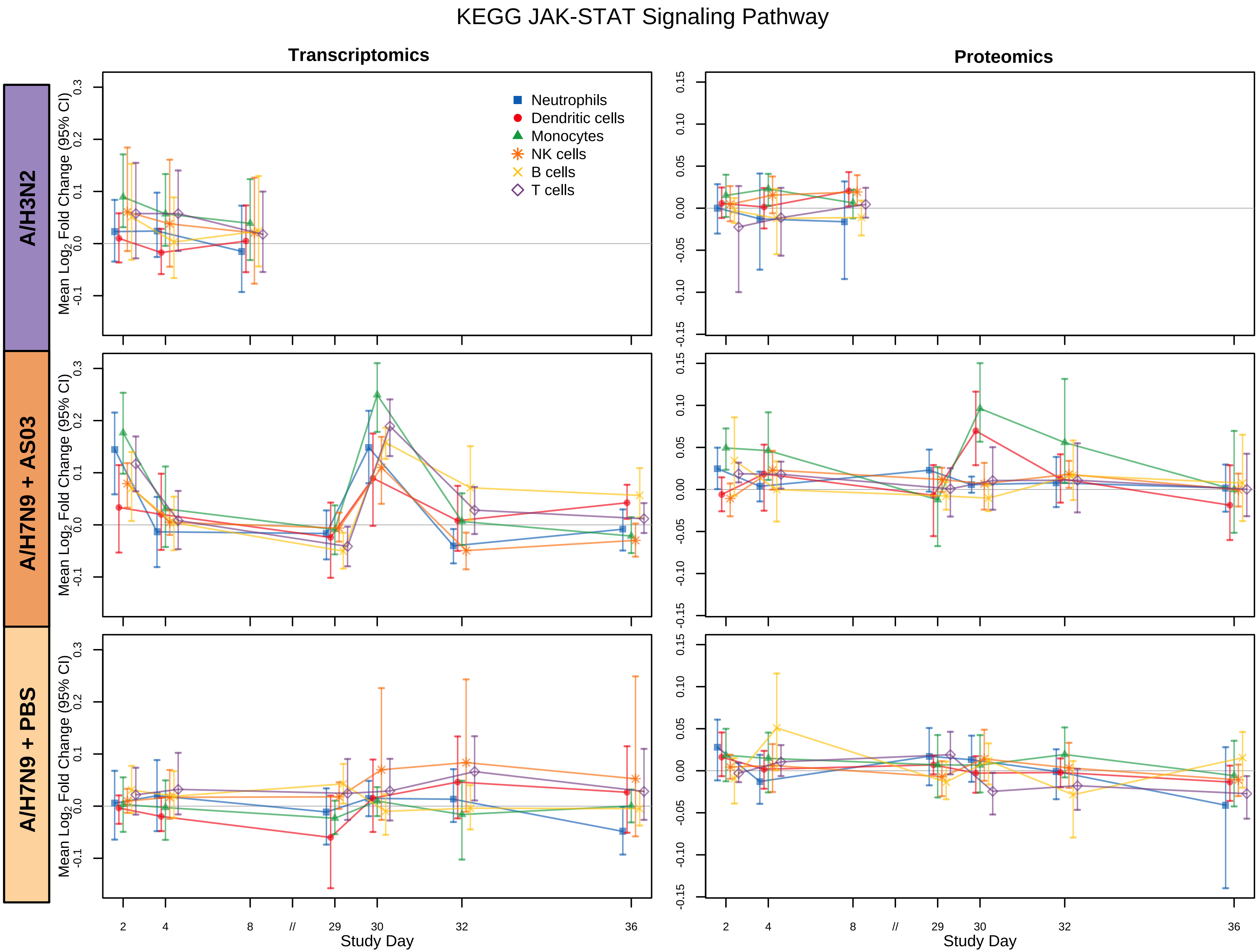

Figure S41: B-cell population marker time trend. Mean of mean log_2 fold change and associated 95% CI of log_2 fold change of each B-cell marker. Error bars indicate 95% confidence intervals based on 1000 bootstrap replicates. B-cell markers included in the analyses were CD19, CD27, IgD, and CD38. No signals were detected for CD27 and IgD in proteomics analysis data.

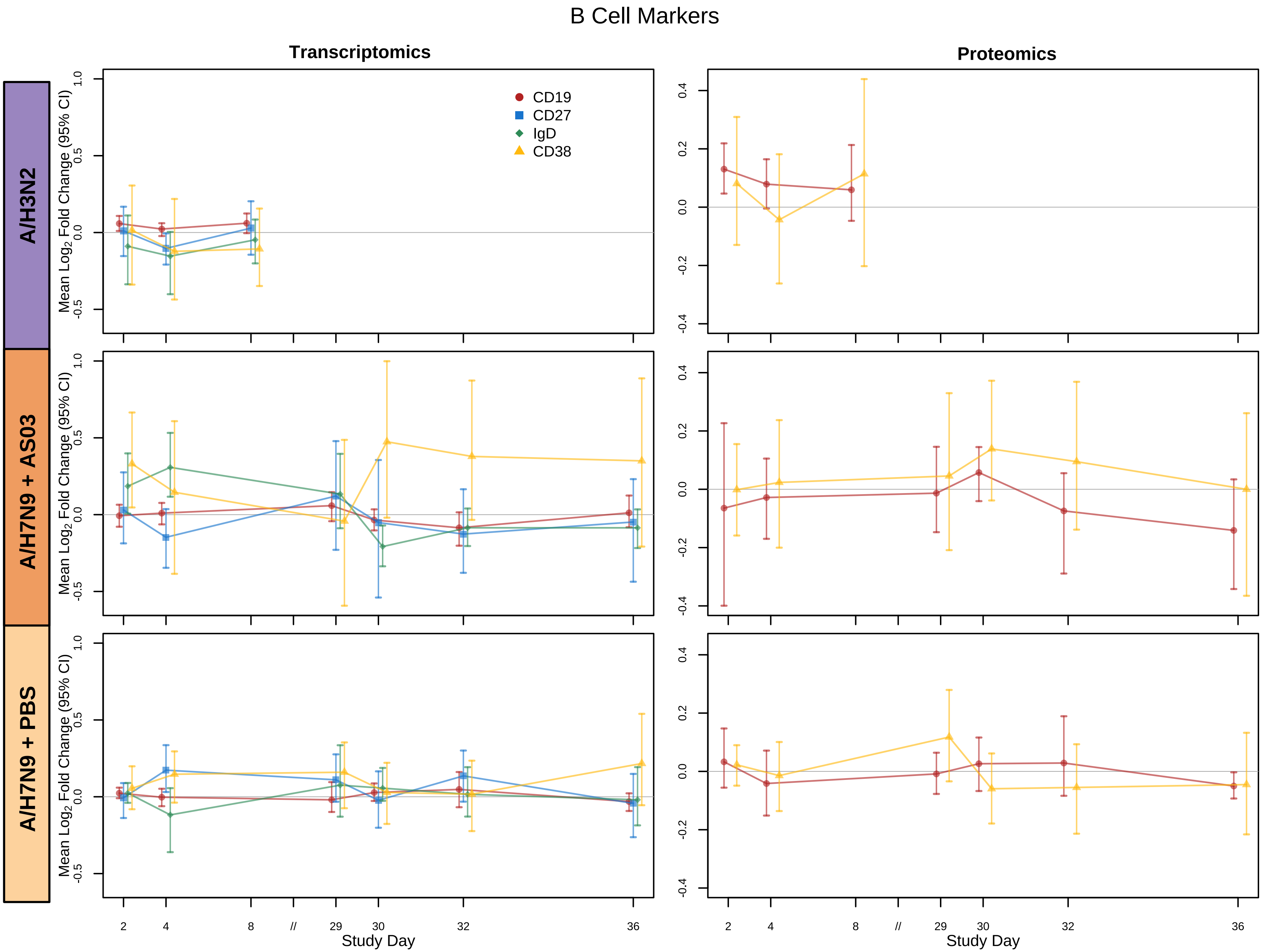

Figure S42: GBP genes and protein time trend. Mean of mean log_2 fold change and associated 95% CI of log_2 fold change of GBP genes/proteins. Error bars indicate 95% confidence intervals based on 1000 bootstrap replicates. GBP features include GBP1, GBP2, GBP3, GBP4, and GBP5.

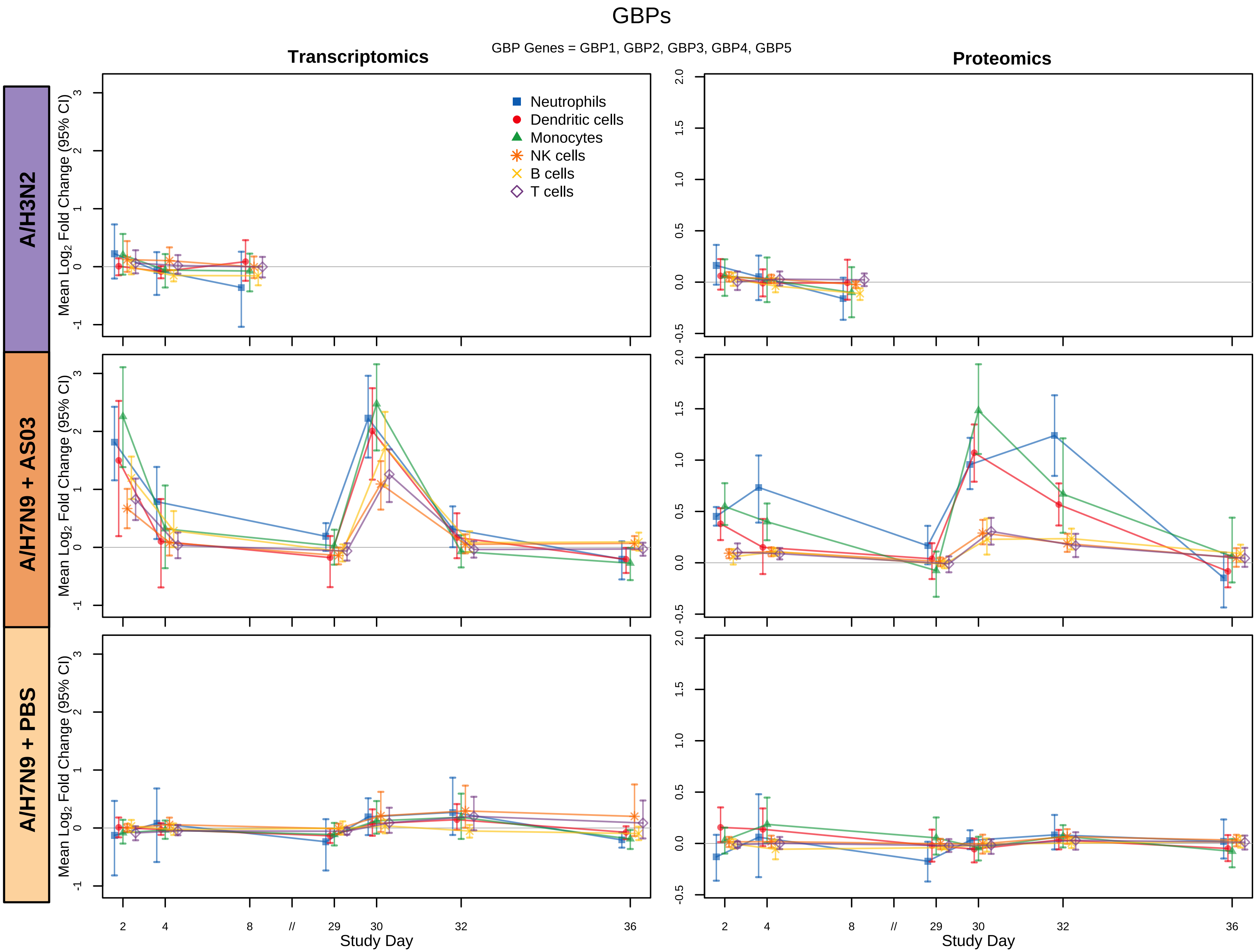

Table S1A: Summary of Categorical Demographic and Baseline Characteristics by Treatment Group - All Enrolled Participants.

		Study Arm 1 A/H3N2v, D1 (N=10)		Study Arm 2 A/H7N9 + AS03, D1, 29 (N=10)		Study Arm 3 A/H7N9 + PBS, D1, 29 (N=10)		All Participants (N=30)	
Variable	Characteristic	n	%	n	%	n	%	n	%
Sex	Male	3	30	3	30	5	50	11	37
	Female	7	70	7	70	5	50	19	63
Ethnicity	Not Hispanic or Latino	10	100	10	100	10	100	30	100
	Hispanic or Latino	0	-	0	-	0	-	0	-
Race	American Indian or Alaska Native	0	-	0	-	0	-	0	-
	Asian	0	-	0	-	0	-	0	-
	Black or African American	0	-	0	-	0	-	0	-
	Native Hawaiian or Other Pacific Islander	0	-	0	-	0	-	0	-
	White	10	100	10	100	10	100	30	100
	Multi-Racial	0	-	0	-	0	-	0	-

Table S1B: Summary of Continuous Demographic and Baseline Characteristics by Treatment Group - All Enrolled Participants.

Variable	Statistic	Study Arm 1 A/H3N2v, D1 (N=10)	Study Arm 2 A/H7N9 + AS03, D1, 29 (N=10)	Study Arm 3 A/H7N9 + PBS, D1, 29 (N=10)	All Subjects (N=30)
Age (years)	Mean	31.0	28.0	33.7	30.9
	Standard Deviation	7.1	6.2	8.3	7.4
	Median	29	28	33	28
	Minimum	22	20	21	20
	Maximum	47	40	48	48
BMI	Mean	24.125	21.885	28.453	24.821
	Standard Deviation	5.609	2.520	10.768	7.443
	Median	21.52	21.37	25.54	22.24
	Minimum	19.40	18.61	19.43	18.61
	Maximum	36.58	25.78	56.23	56.23

Table S2: Genes differentially expressed at Day 8 compared to pre-first vaccination (Study Arm 1 A/H3N2v, D1 , Monocytes). Genes are sorted by descending absolute log_2 fold change. Gene model summaries and annotations are based on Ensembl Version 99.

Ensembl Gene ID	Ensembl Gene Name	Ensembl Gene Description	Gene Type	log_2 Fold Change (Day 8 vs. Pre-First Vaccination)	Average log_2 CPM	Likelihood Ratio Test Statistic	P-Value	FDR Adjusted P-Value
ENSG00000244734	HBB	hemoglobin subunit beta [Source:HGNC Symbol;Acc:HGNC:4827]	protein coding	2.03	1.93	21.44	<0.0001	0.047

Table S3: Genes differentially expressed at Day 2 compared to pre-first vaccination (Study Arm 2 A/H7N9+AS03, D1, 29, Monocytes). Genes are sorted by descending absolute log_2 fold change. Gene model summaries and annotations are based on Ensembl Version 99.

Ensembl Gene ID	Ensembl Gene Name	Ensembl Gene Description	Gene Type	log_2 Fold Change (Day 2 vs. Pre-First Vaccination)	Average log_2 CPM	Likelihood Ratio Test Statistic	P-Value	FDR Adjusted P-Value
ENSG00000225492	GBP1P1	guanylate binding protein 1 pseudogene 1 [Source:HGNC Symbol;Acc:HGNC:39561]	transcribed unprocessed pseudogene	3.89	2.09	73.21	<0.0001	<0.0001
ENSG00000152766	ANKRD22	ankyrin repeat domain 22 [Source:HGNC Symbol;Acc:HGNC:28321]	protein coding	3.55	4.29	90.92	<0.0001	<0.0001
ENSG00000154451	GBP5	guanylate binding protein 5 [Source:HGNC Symbol;Acc:HGNC:19895]	protein coding	3.37	7.66	86.59	<0.0001	<0.0001
ENSG00000120217	CD274	CD274 molecule [Source:HGNC Symbol;Acc:HGNC:17635]	protein coding	3.25	1.42	69.25	<0.0001	<0.0001
ENSG00000010030	ETV7	ETS variant transcription factor 7 [Source:HGNC Symbol;Acc:HGNC:18160]	protein coding	3.24	3.37	81.38	<0.0001	<0.0001
ENSG00000117228	GBP1	guanylate binding protein 1 [Source:HGNC Symbol;Acc:HGNC:4182]	protein coding	3.00	7.29	84.78	<0.0001	<0.0001
ENSG00000100336	APOL4	apolipoprotein L4 [Source:HGNC Symbol;Acc:HGNC:14867]	protein coding	3.00	2.51	104.94	<0.0001	<0.0001
ENSG00000140511	HAPLN3	hyaluronan and proteoglycan link protein 3 [Source:HGNC Symbol;Acc:HGNC:21446]	protein coding	2.64	2.67	66.64	<0.0001	<0.0001
ENSG00000149131	SERPING1	serpin family G member 1 [Source:HGNC Symbol;Acc:HGNC:1228]	protein coding	2.63	5.03	64.70	<0.0001	<0.0001
ENSG00000108950	FAM20A	FAM20A golgi associated secretory pathway pseudokinase [Source:HGNC Symbol;Acc:HGNC:23015]	protein coding	2.56	2.50	89.59	<0.0001	<0.0001
ENSG00000183762	KREMEN1	kringle containing transmembrane protein 1 [Source:HGNC Symbol;Acc:HGNC:17550]	protein coding	2.52	2.82	125.57	<0.0001	<0.0001

Table S3: Genes differentially expressed at Day 2 compared to pre-first vaccination (Study Arm 2 A/H7N9+AS03, D1, 29, Monocytes). Genes are sorted by descending absolute log_2 fold change. Gene model summaries and annotations are based on Ensembl Version 99 (continued.)

Ensembl Gene ID	Ensembl Gene Name	Ensembl Gene Description	Gene Type	log_2 Fold Change (Day 2 vs. Pre-First Vaccination)	Average log_2 CPM	Likelihood Ratio Test Statistic	P-Value	FDR Adjusted P-Value
ENSG00000265531	FCGR1CP	Fc fragment of IgG receptor Ic, pseudogene [Source:HGNC Symbol;Acc:HGNC:3615]	unprocessed pseudogene	2.43	2.49	98.50	<0.0001	<0.0001
ENSG00000026751	SLAMF7	SLAM family member 7 [Source:HGNC Symbol;Acc:HGNC:21394]	protein coding	2.43	5.20	94.01	<0.0001	<0.0001
ENSG00000162654	GBP4	guanylate binding protein 4 [Source:HGNC Symbol;Acc:HGNC:20480]	protein coding	2.40	6.84	79.40	<0.0001	<0.0001
ENSG00000120885	CLU	clusterin [Source:HGNC Symbol;Acc:HGNC:2095]	protein coding	2.39	3.29	65.66	<0.0001	<0.0001
ENSG00000169245	CXCL10	C-X-C motif chemokine ligand 10 [Source:HGNC Symbol;Acc:HGNC:10637]	protein coding	2.22	4.19	34.72	<0.0001	<0.0001
ENSG00000168062	BATF2	basic leucine zipper ATF-like transcription factor 2 [Source:HGNC Symbol;Acc:HGNC:25163]	protein coding	2.18	3.38	68.04	<0.0001	<0.0001
ENSG00000198019	FCGR1B	Fc fragment of IgG receptor Ib [Source:HGNC Symbol;Acc:HGNC:3614]	protein coding	2.13	4.09	100.49	<0.0001	<0.0001
ENSG00000158714	SLAMF8	SLAM family member 8 [Source:HGNC Symbol;Acc:HGNC:21391]	protein coding	2.12	2.57	100.95	<0.0001	<0.0001
ENSG00000150337	FCGR1A	Fc fragment of IgG receptor Ia [Source:HGNC Symbol;Acc:HGNC:3613]	protein coding	2.09	5.59	112.57	<0.0001	<0.0001
ENSG00000233029		novel transcript	lncRNA	2.07	0.72	80.95	<0.0001	<0.0001
ENSG00000162645	GBP2	guanylate binding protein 2 [Source:HGNC Symbol;Acc:HGNC:4183]	protein coding	2.04	8.15	94.29	<0.0001	<0.0001
ENSG00000185885	IFITM1	interferon induced transmembrane protein 1 [Source:HGNC Symbol;Acc:HGNC:5412]	protein coding	1.98	4.22	33.51	<0.0001	<0.0001
ENSG00000188820	CALHM6	calcium homeostasis modulator family member 6 [Source:HGNC Symbol;Acc:HGNC:33391]	protein coding	1.83	5.47	68.66	<0.0001	<0.0001
ENSG00000174944	P2RY14	purinergic receptor P2Y14 [Source:HGNC Symbol;Acc:HGNC:16442]	protein coding	1.82	0.95	33.00	<0.0001	<0.0001
ENSG00000168899	VAMP5	vesicle associated membrane protein 5 [Source:HGNC Symbol;Acc:HGNC:12646]	protein coding	1.76	5.92	96.49	<0.0001	<0.0001
ENSG00000108387	SEPTIN4	septin 4 [Source:HGNC Symbol;Acc:HGNC:9165]	protein coding	1.72	2.17	51.00	<0.0001	<0.0001
ENSG00000115415	STAT1	signal transducer and activator of transcription 1 [Source:HGNC Symbol;Acc:HGNC:11362]	protein coding	1.64	8.79	78.11	<0.0001	<0.0001
ENSG00000164509	IL31RA	interleukin 31 receptor A [Source:HGNC Symbol;Acc:HGNC:18969]	protein coding	1.63	1.92	49.37	<0.0001	<0.0001

Table S3: Genes differentially expressed at Day 2 compared to pre-first vaccination (Study Arm 2 A/H7N9+AS03, D1, 29, Monocytes). Genes are sorted by descending absolute log_2 fold change. Gene model summaries and annotations are based on Ensembl Version 99 (continued.)

Ensembl Gene ID	Ensembl Gene Name	Ensembl Gene Description	Gene Type	log_2 Fold Change (Day 2 vs. Pre-First Vaccination)	Average log_2 CPM	Likelihood Ratio Test Statistic	P-Value	FDR Adjusted P-Value
ENSG00000002549	LAP3	leucine aminopeptidase 3 [Source:HGNC Symbol;Acc:HGNC:18449]	protein coding	1.61	7.95	69.80	<0.0001	<0.0001
ENSG00000119917	IFIT3	interferon induced protein with tetratricopeptide repeats 3 [Source:HGNC Symbol;Acc:HGNC:5411]	protein coding	1.58	6.35	30.66	<0.0001	<0.0001
ENSG00000119922	IFIT2	interferon induced protein with tetratricopeptide repeats 2 [Source:HGNC Symbol;Acc:HGNC:5409]	protein coding	1.57	6.16	26.90	<0.0001	<0.0001
ENSG00000275395	FCGBP	Fc fragment of IgG binding protein [Source:HGNC Symbol;Acc:HGNC:13572]	protein coding	-1.55	2.16	56.80	<0.0001	<0.0001
ENSG00000162772	ATF3	activating transcription factor 3 [Source:HGNC Symbol;Acc:HGNC:785]	protein coding	1.54	3.39	23.87	<0.0001	<0.0001
ENSG00000136231	IGF2BP3	insulin like growth factor 2 mRNA binding protein 3 [Source:HGNC Symbol;Acc:HGNC:28868]	protein coding	1.52	0.86	55.77	<0.0001	<0.0001
ENSG00000234518	PTGES3P1	prostaglandin E synthase 3 pseudogene 1 [Source:HGNC Symbol;Acc:HGNC:43824]	processed pseudogene	1.48	2.76	70.93	<0.0001	<0.0001
ENSG00000140105	WARS1	tryptophanyl-tRNA synthetase 1 [Source:HGNC Symbol;Acc:HGNC:12729]	protein coding	1.47	9.29	82.42	<0.0001	<0.0001
ENSG00000184557	SOCS3	suppressor of cytokine signaling 3 [Source:HGNC Symbol;Acc:HGNC:19391]	protein coding	1.46	6.39	30.77	<0.0001	<0.0001
ENSG00000100342	APOL1	apolipoprotein L1 [Source:HGNC Symbol;Acc:HGNC:618]	protein coding	1.46	5.50	81.71	<0.0001	<0.0001
ENSG00000115594	IL1R1	interleukin 1 receptor type 1 [Source:HGNC Symbol;Acc:HGNC:5993]	protein coding	-1.45	2.62	58.20	<0.0001	<0.0001
ENSG00000145365	TIFA	TRAF interacting protein with forkhead associated domain [Source:HGNC Symbol;Acc:HGNC:19075]	protein coding	1.44	3.29	73.27	<0.0001	<0.0001
ENSG00000137801	THBS1	thrombospondin 1 [Source:HGNC Symbol;Acc:HGNC:11785]	protein coding	-1.42	6.76	23.86	<0.0001	<0.0001
ENSG00000142089	IFITM3	interferon induced transmembrane protein 3 [Source:HGNC Symbol;Acc:HGNC:5414]	protein coding	1.42	7.37	42.98	<0.0001	<0.0001
ENSG00000179639	FCER1A	Fc fragment of IgE receptor Ia [Source:HGNC Symbol;Acc:HGNC:3609]	protein coding	-1.41	3.70	25.32	<0.0001	<0.0001
ENSG00000231528	FAM225A	family with sequence similarity 225 member A [Source:HGNC Symbol;Acc:HGNC:27855]	lncRNA	1.40	0.39	42.62	<0.0001	<0.0001
ENSG00000181036	FCRL6	Fc receptor like 6 [Source:HGNC Symbol;Acc:HGNC:31910]	protein coding	1.40	0.92	23.97	<0.0001	<0.0001
ENSG00000273314		novel transcript, antisense to CUL1	lncRNA	1.40	0.99	59.67	<0.0001	<0.0001

Table S3: Genes differentially expressed at Day 2 compared to pre-first vaccination (Study Arm 2 A/H7N9+AS03, D1, 29, Monocytes). Genes are sorted by descending absolute log_2 fold change. Gene model summaries and annotations are based on Ensembl Version 99 (continued.)

Ensembl Gene ID	Ensembl Gene Name	Ensembl Gene Description	Gene Type	log_2 Fold Change (Day 2 vs. Pre-First Vaccination)	Average log_2 CPM	Likelihood Ratio Test Statistic	P-Value	FDR Adjusted P-Value
ENSG00000168394	TAP1	transporter 1, ATP binding cassette subfamily B member [Source:HGNC Symbol;Acc:HGNC:43]	protein coding	1.39	8.04	79.66	<0.0001	<0.0001
ENSG00000198959	TGM2	transglutaminase 2 [Source:HGNC Symbol;Acc:HGNC:11778]	protein coding	1.39	2.96	53.84	<0.0001	<0.0001
ENSG00000137193	PIM1	Pim-1 proto-oncogene, serine/threonine kinase [Source:HGNC Symbol;Acc:HGNC:8986]	protein coding	1.39	6.43	50.15	<0.0001	<0.0001
ENSG00000163568	AIM2	absent in melanoma 2 [Source:HGNC Symbol;Acc:HGNC:357]	protein coding	1.39	3.89	52.15	<0.0001	<0.0001
ENSG00000166278	C2	complement C2 [Source:HGNC Symbol;Acc:HGNC:1248]	protein coding	1.38	2.80	65.74	<0.0001	<0.0001
ENSG00000238000		proteasome (prosome, macropain) activator subunit 2 (PA28 beta) (PSME2) pseudogene	processed pseudogene	1.37	0.60	50.37	<0.0001	<0.0001
ENSG00000175445	LPL	lipoprotein lipase [Source:HGNC Symbol;Acc:HGNC:6677]	protein coding	-1.37	1.27	25.56	<0.0001	<0.0001
ENSG00000151693	ASAP2	ArfGAP with SH3 domain, ankyrin repeat and PH domain 2 [Source:HGNC Symbol;Acc:HGNC:2721]	protein coding	1.36	2.02	98.67	<0.0001	<0.0001
ENSG00000126262	FFAR2	free fatty acid receptor 2 [Source:HGNC Symbol;Acc:HGNC:4501]	protein coding	1.36	3.63	45.10	<0.0001	<0.0001
ENSG00000158156	XKR8	XK related 8 [Source:HGNC Symbol;Acc:HGNC:25508]	protein coding	1.36	5.51	65.48	<0.0001	<0.0001
ENSG00000133321	PLAAT4	phospholipase A and acyltransferase 4 [Source:HGNC Symbol;Acc:HGNC:9869]	protein coding	1.35	4.94	79.16	<0.0001	<0.0001
ENSG00000236567	TCF3P1	transcription factor 3 pseudogene 1 [Source:HGNC Symbol;Acc:HGNC:49742]	processed pseudogene	1.34	1.13	26.30	<0.0001	<0.0001
ENSG00000256262	USP30-AS1	USP30 antisense RNA 1 [Source:HGNC Symbol;Acc:HGNC:40909]	lncRNA	1.34	0.81	48.01	<0.0001	<0.0001
ENSG00000117226	GBP3	guanylate binding protein 3 [Source:HGNC Symbol;Acc:HGNC:4184]	protein coding	1.33	5.25	67.19	<0.0001	<0.0001
ENSG00000162614	NEXN	nexilin F-actin binding protein [Source:HGNC Symbol;Acc:HGNC:29557]	protein coding	1.33	3.54	37.06	<0.0001	<0.0001
ENSG00000197272	IL27	interleukin 27 [Source:HGNC Symbol;Acc:HGNC:19157]	protein coding	1.32	2.37	54.20	<0.0001	<0.0001
ENSG00000168209	DDIT4	DNA damage inducible transcript 4 [Source:HGNC Symbol;Acc:HGNC:24944]	protein coding	-1.31	3.03	7.29	0.0069	0.0221

Table S3: Genes differentially expressed at Day 2 compared to pre-first vaccination (Study Arm 2 A/H7N9+AS03, D1, 29, Monocytes). Genes are sorted by descending absolute log_2 fold change. Gene model summaries and annotations are based on Ensembl Version 99 (continued.)

Ensembl Gene ID	Ensembl Gene Name	Ensembl Gene Description	Gene Type	log_2 Fold Change (Day 2 vs. Pre-First Vaccination)	Average log_2 CPM	Likelihood Ratio Test Statistic	P-Value	FDR Adjusted P-Value
ENSG00000133106	EPSTI1	epithelial stromal interaction 1 [Source:HGNC Symbol;Acc:HGNC:16465]	protein coding	1.31	6.37	37.71	<0.0001	<0.0001
ENSG00000162512	SDC3	syndecan 3 [Source:HGNC Symbol;Acc:HGNC:10660]	protein coding	1.30	4.42	65.46	<0.0001	<0.0001
ENSG00000187608	ISG15	ISG15 ubiquitin like modifier [Source:HGNC Symbol;Acc:HGNC:4053]	protein coding	1.30	6.07	23.08	<0.0001	<0.0001
ENSG00000177409	SAMD9L	sterile alpha motif domain containing 9 like [Source:HGNC Symbol;Acc:HGNC:1349]	protein coding	1.29	7.01	47.28	<0.0001	<0.0001
ENSG00000214872	SMTNL1	smoothelin like 1 [Source:HGNC Symbol;Acc:HGNC:32394]	protein coding	1.29	1.47	35.31	<0.0001	<0.0001
ENSG00000156587	UBE2L6	ubiquitin conjugating enzyme E2 L6 [Source:HGNC Symbol;Acc:HGNC:12490]	protein coding	1.28	7.48	61.48	<0.0001	<0.0001
ENSG00000116663	FBXO6	F-box protein 6 [Source:HGNC Symbol;Acc:HGNC:13585]	protein coding	1.28	5.10	61.87	<0.0001	<0.0001
ENSG00000254017	IGHEP2	immunoglobulin heavy constant epsilon P2 (pseudogene) [Source:HGNC Symbol;Acc:HGNC:5524]	IG C pseudogene	1.27	0.65	21.04	<0.0001	<0.0001
ENSG00000196220	SRGAP3	SLIT-ROBO Rho GTPase activating protein 3 [Source:HGNC Symbol;Acc:HGNC:19744]	protein coding	-1.27	1.64	74.80	<0.0001	<0.0001
ENSG00000138496	PARP9	poly(ADP-ribose) polymerase family member 9 [Source:HGNC Symbol;Acc:HGNC:24118]	protein coding	1.26	7.20	58.60	<0.0001	<0.0001
ENSG00000225131	PSME2P2	proteasome activator subunit 2 pseudogene 2 [Source:HGNC Symbol;Acc:HGNC:30160]	processed pseudogene	1.26	0.79	53.72	<0.0001	<0.0001
ENSG00000137959	IFI44L	interferon induced protein 44 like [Source:HGNC Symbol;Acc:HGNC:17817]	protein coding	1.26	6.68	21.03	<0.0001	<0.0001
ENSG00000174370	C11orf45	chromosome 11 open reading frame 45 [Source:HGNC Symbol;Acc:HGNC:28584]	protein coding	-1.26	0.85	47.32	<0.0001	<0.0001
ENSG00000258521		novel transcript, antisense to WARS	lncRNA	1.25	0.60	27.53	<0.0001	<0.0001
ENSG00000029153	ARNTL2	aryl hydrocarbon receptor nuclear translocator like 2 [Source:HGNC Symbol;Acc:HGNC:18984]	protein coding	1.25	1.61	64.37	<0.0001	<0.0001
ENSG00000179044	EXOC3L1	exocyst complex component 3 like 1 [Source:HGNC Symbol;Acc:HGNC:27540]	protein coding	1.24	2.49	36.06	<0.0001	<0.0001
ENSG00000221963	APOL6	apolipoprotein L6 [Source:HGNC Symbol;Acc:HGNC:14870]	protein coding	1.21	7.05	46.79	<0.0001	<0.0001
ENSG00000138119	MYOF	myoferlin [Source:HGNC Symbol;Acc:HGNC:3656]	protein coding	1.20	6.82	101.96	<0.0001	<0.0001

Table S3: Genes differentially expressed at Day 2 compared to pre-first vaccination (Study Arm 2 A/H7N9+AS03, D1, 29, Monocytes). Genes are sorted by descending absolute log_2 fold change. Gene model summaries and annotations are based on Ensembl Version 99 (continued.)

Ensembl Gene ID	Ensembl Gene Name	Ensembl Gene Description	Gene Type	log_2 Fold Change (Day 2 vs. Pre-First Vaccination)	Average log_2 CPM	Likelihood Ratio Test Statistic	P-Value	FDR Adjusted P-Value
ENSG00000158270	COLEC12	collectin subfamily member 12 [Source:HGNC Symbol;Acc:HGNC:16016]	protein coding	-1.20	1.40	29.69	<0.0001	<0.0001
ENSG00000172232	AZU1	azurocidin 1 [Source:HGNC Symbol;Acc:HGNC:913]	protein coding	-1.20	2.06	69.62	<0.0001	<0.0001
ENSG00000121858	TNFSF10	TNF superfamily member 10 [Source:HGNC Symbol;Acc:HGNC:11925]	protein coding	1.20	8.12	54.00	<0.0001	<0.0001
ENSG00000144218	AFF3	AF4/FMR2 family member 3 [Source:HGNC Symbol;Acc:HGNC:6473]	protein coding	-1.19	3.52	82.62	<0.0001	<0.0001
ENSG00000163814	CDCP1	CUB domain containing protein 1 [Source:HGNC Symbol;Acc:HGNC:24357]	protein coding	1.18	0.78	55.56	<0.0001	<0.0001
ENSG00000131979	GCH1	GTP cyclohydrolase 1 [Source:HGNC Symbol;Acc:HGNC:4193]	protein coding	1.18	6.83	62.93	<0.0001	<0.0001
ENSG00000173369	C1QB	complement C1q B chain [Source:HGNC Symbol;Acc:HGNC:1242]	protein coding	1.17	2.73	35.25	<0.0001	<0.0001
ENSG00000111181	SLC6A12	solute carrier family 6 member 12 [Source:HGNC Symbol;Acc:HGNC:11045]	protein coding	1.16	4.04	104.82	<0.0001	<0.0001
ENSG00000004468	CD38	CD38 molecule [Source:HGNC Symbol;Acc:HGNC:1667]	protein coding	1.16	4.02	61.04	<0.0001	<0.0001
ENSG00000268758	ADGRE4P	adhesion G protein-coupled receptor E4, pseudogene [Source:HGNC Symbol;Acc:HGNC:19240]	transcribed unprocessed pseudogene	-1.16	2.93	45.33	<0.0001	<0.0001
ENSG00000125347	IRF1	interferon regulatory factor 1 [Source:HGNC Symbol;Acc:HGNC:6116]	protein coding	1.16	8.20	70.63	<0.0001	<0.0001
ENSG00000139211	AMIGO2	adhesion molecule with Ig like domain 2 [Source:HGNC Symbol;Acc:HGNC:24073]	protein coding	1.15	1.37	48.78	<0.0001	<0.0001
ENSG00000123610	TNFAIP6	TNF alpha induced protein 6 [Source:HGNC Symbol;Acc:HGNC:11898]	protein coding	1.15	1.50	40.81	<0.0001	<0.0001
ENSG00000103485	QPRT	quinolinate phosphoribosyltransferase [Source:HGNC Symbol;Acc:HGNC:9755]	protein coding	-1.14	2.35	56.43	<0.0001	<0.0001
ENSG00000125148	MT2A	metallothionein 2A [Source:HGNC Symbol;Acc:HGNC:7406]	protein coding	1.13	4.99	54.76	<0.0001	<0.0001
ENSG00000055130	CUL1	cullin 1 [Source:HGNC Symbol;Acc:HGNC:2551]	protein coding	1.13	6.48	80.42	<0.0001	<0.0001

Table S3: Genes differentially expressed at Day 2 compared to pre-first vaccination (Study Arm 2 A/H7N9+AS03, D1, 29, Monocytes). Genes are sorted by descending absolute log_2 fold change. Gene model summaries and annotations are based on Ensembl Version 99 (continued.)

Ensembl Gene ID	Ensembl Gene Name	Ensembl Gene Description	Gene Type	log_2 Fold Change (Day 2 vs. Pre-First Vaccination)	Average log_2 CPM	Likelihood Ratio Test Statistic	P-Value	FDR Adjusted P-Value
ENSG00000100911	PSME2	proteasome activator subunit 2 [Source:HGNC Symbol;Acc:HGNC:9569]	protein coding	1.13	7.67	69.38	<0.0001	<0.0001
ENSG00000141574	SECTM1	secreted and transmembrane 1 [Source:HGNC Symbol;Acc:HGNC:10707]	protein coding	1.13	8.51	83.70	<0.0001	<0.0001
ENSG00000137965	IFI44	interferon induced protein 44 [Source:HGNC Symbol;Acc:HGNC:16938]	protein coding	1.12	6.48	26.40	<0.0001	<0.0001
ENSG00000145287	PLAC8	placenta associated 8 [Source:HGNC Symbol;Acc:HGNC:19254]	protein coding	1.12	6.44	60.74	<0.0001	<0.0001
ENSG00000164292	RHOBTB3	Rho related BTB domain containing 3 [Source:HGNC Symbol;Acc:HGNC:18757]	protein coding	1.12	0.65	49.35	<0.0001	<0.0001
ENSG00000049089	COL9A2	collagen type IX alpha 2 chain [Source:HGNC Symbol;Acc:HGNC:2218]	protein coding	-1.11	4.98	101.90	<0.0001	<0.0001
ENSG00000108691	CCL2	C-C motif chemokine ligand 2 [Source:HGNC Symbol;Acc:HGNC:10618]	protein coding	1.11	1.27	6.12	0.0133	0.0374
ENSG00000135114	OASL	2'-5'-oligoadenylate synthetase like [Source:HGNC Symbol;Acc:HGNC:8090]	protein coding	1.11	4.62	22.30	<0.0001	<0.0001
ENSG00000108679	LGALS3BP	galectin 3 binding protein [Source:HGNC Symbol;Acc:HGNC:6564]	protein coding	1.10	5.02	33.12	<0.0001	<0.0001
ENSG00000179593	ALOX15B	arachidonate 15-lipoxygenase type B [Source:HGNC Symbol;Acc:HGNC:434]	protein coding	-1.10	3.11	42.06	<0.0001	<0.0001
ENSG00000082293	COL19A1	collagen type XIX alpha 1 chain [Source:HGNC Symbol;Acc:HGNC:2196]	protein coding	-1.09	1.34	21.11	<0.0001	<0.0001
ENSG00000068079	IFI35	interferon induced protein 35 [Source:HGNC Symbol;Acc:HGNC:5399]	protein coding	1.09	6.33	43.22	<0.0001	<0.0001
ENSG00000213557		ribosomal protein L31 (RPL31) pseudogene	processed pseudogene	-1.08	2.61	69.94	<0.0001	<0.0001
ENSG00000171621	SPSB1	splA/ryanodine receptor domain and SOCS box containing 1 [Source:HGNC Symbol;Acc:HGNC:30628]	protein coding	1.08	0.63	29.08	<0.0001	<0.0001
ENSG00000020577	SAMD4A	sterile alpha motif domain containing 4A [Source:HGNC Symbol;Acc:HGNC:23023]	protein coding	1.07	4.56	34.05	<0.0001	<0.0001
ENSG00000134321	RSAD2	radical S-adenosyl methionine domain containing 2 [Source:HGNC Symbol;Acc:HGNC:30908]	protein coding	1.07	4.85	16.58	<0.0001	0.0004
ENSG00000172159	FRMD3	FERM domain containing 3 [Source:HGNC Symbol;Acc:HGNC:24125]	protein coding	1.07	3.45	75.52	<0.0001	<0.0001

Table S3: Genes differentially expressed at Day 2 compared to pre-first vaccination (Study Arm 2 A/H7N9+AS03, D1, 29, Monocytes). Genes are sorted by descending absolute log_2 fold change. Gene model summaries and annotations are based on Ensembl Version 99 (continued.)

Ensembl Gene ID	Ensembl Gene Name	Ensembl Gene Description	Gene Type	log_2 Fold Change (Day 2 vs. Pre-First Vaccination)	Average log_2 CPM	Likelihood Ratio Test Statistic	P-Value	FDR Adjusted P-Value
ENSG00000154102	C16orf74	chromosome 16 open reading frame 74 [Source:HGNC Symbol;Acc:HGNC:23362]	protein coding	-1.07	3.13	70.94	<0.0001	<0.0001
ENSG00000136960	ENPP2	ectonucleotide pyrophosphatase/phosphodiesterase 2 [Source:HGNC Symbol;Acc:HGNC:3357]	protein coding	1.07	0.64	25.20	<0.0001	<0.0001
ENSG00000184221	OLIG1	oligodendrocyte transcription factor 1 [Source:HGNC Symbol;Acc:HGNC:16983]	protein coding	-1.06	5.72	54.77	<0.0001	<0.0001
ENSG00000240065	PSMB9	proteasome 20S subunit beta 9 [Source:HGNC Symbol;Acc:HGNC:9546]	protein coding	1.05	7.53	55.94	<0.0001	<0.0001
ENSG00000173193	PARP14	poly(ADP-ribose) polymerase family member 14 [Source:HGNC Symbol;Acc:HGNC:29232]	protein coding	1.05	8.04	38.98	<0.0001	<0.0001
ENSG00000159189	C1QC	complement C1q C chain [Source:HGNC Symbol;Acc:HGNC:1245]	protein coding	1.05	1.97	21.79	<0.0001	<0.0001
ENSG00000258581		NADH dehydrogenase (ubiquinone) 1 beta subcomplex, 3, 12kDa (NDUFB3) pseudogene	processed pseudogene	1.05	0.36	24.15	<0.0001	<0.0001
ENSG00000146072	TNFRSF21	TNF receptor superfamily member 21 [Source:HGNC Symbol;Acc:HGNC:13469]	protein coding	-1.04	0.47	13.58	0.0002	0.0013
ENSG00000137757	CASP5	caspase 5 [Source:HGNC Symbol;Acc:HGNC:1506]	protein coding	1.04	2.49	51.16	<0.0001	<0.0001
ENSG00000134326	CMPK2	cytidine/uridine monophosphate kinase 2 [Source:HGNC Symbol;Acc:HGNC:27015]	protein coding	1.04	5.52	20.41	<0.0001	<0.0001
ENSG00000128284	APOL3	apolipoprotein L3 [Source:HGNC Symbol;Acc:HGNC:14868]	protein coding	1.03	7.40	74.67	<0.0001	<0.0001
ENSG00000182782	HCAR2	hydroxycarboxylic acid receptor 2 [Source:HGNC Symbol;Acc:HGNC:24827]	protein coding	1.03	2.00	10.01	0.0016	0.0066
ENSG00000156265	MAP3K7CL	MAP3K7 C-terminal like [Source:HGNC Symbol;Acc:HGNC:16457]	protein coding	1.03	4.87	32.28	<0.0001	<0.0001
ENSG00000135362	PRR5L	proline rich 5 like [Source:HGNC Symbol;Acc:HGNC:25878]	protein coding	1.03	3.74	56.73	<0.0001	<0.0001
ENSG00000185022	MAFF	MAF bZIP transcription factor F [Source:HGNC Symbol;Acc:HGNC:6780]	protein coding	1.03	1.62	8.17	0.0043	0.0148
ENSG00000115155	OTOF	otoferlin [Source:HGNC Symbol;Acc:HGNC:8515]	protein coding	1.03	1.00	10.53	0.0012	0.0052
ENSG00000166428	PLD4	phospholipase D family member 4 [Source:HGNC Symbol;Acc:HGNC:23792]	protein coding	-1.03	5.08	44.57	<0.0001	<0.0001

Confidential/Proprietary Information

Table S3: Genes differentially expressed at Day 2 compared to pre-first vaccination (Study Arm 2 A/H7N9+AS03, D1, 29, Monocytes). Genes are sorted by descending absolute log_2 fold change. Gene model summaries and annotations are based on Ensembl Version 99 (continued.)

Ensembl Gene ID	Ensembl Gene Name	Ensembl Gene Description	Gene Type	log_2 Fold Change (Day 2 vs. Pre-First Vaccination)	Average log_2 CPM	Likelihood Ratio Test Statistic	P-Value	FDR Adjusted P-Value
ENSG00000135636	DYSF	dysferlin [Source:HGNC Symbol;Acc:HGNC:3097]	protein coding	1.02	7.86	91.29	<0.0001	<0.0001
ENSG00000183087	GAS6	growth arrest specific 6 [Source:HGNC Symbol;Acc:HGNC:4168]	protein coding	1.02	4.27	54.27	<0.0001	<0.0001
ENSG00000128335	APOL2	apolipoprotein L2 [Source:HGNC Symbol;Acc:HGNC:619]	protein coding	1.02	6.26	63.61	<0.0001	<0.0001
ENSG00000148175	STOM	stomatin [Source:HGNC Symbol;Acc:HGNC:3383]	protein coding	1.01	5.98	76.41	<0.0001	<0.0001
ENSG00000138821	SLC39A8	solute carrier family 39 member 8 [Source:HGNC Symbol;Acc:HGNC:20862]	protein coding	1.01	3.10	47.22	<0.0001	<0.0001
ENSG00000138134	STAMBPL1	STAM binding protein like 1 [Source:HGNC Symbol;Acc:HGNC:24105]	protein coding	1.01	1.22	29.65	<0.0001	<0.0001
ENSG00000267534	S1PR2	sphingosine-1-phosphate receptor 2 [Source:HGNC Symbol;Acc:HGNC:3169]	protein coding	1.01	1.43	34.24	<0.0001	<0.0001
ENSG00000185880	TRIM69	tripartite motif containing 69 [Source:HGNC Symbol;Acc:HGNC:17857]	protein coding	1.00	5.30	70.46	<0.0001	<0.0001
ENSG00000185507	IRF7	interferon regulatory factor 7 [Source:HGNC Symbol;Acc:HGNC:6122]	protein coding	1.00	7.37	34.11	<0.0001	<0.0001
ENSG00000204267	TAP2	transporter 2, ATP binding cassette subfamily B member [Source:HGNC Symbol;Acc:HGNC:44]	protein coding	1.00	6.14	57.62	<0.0001	<0.0001
ENSG00000187621	TCL6	T cell leukemia/lymphoma 6 [Source:HGNC Symbol;Acc:HGNC:13463]	lncRNA	-0.99	0.70	21.36	<0.0001	<0.0001
ENSG00000182557	SPNS3	sphingolipid transporter 3 (putative) [Source:HGNC Symbol;Acc:HGNC:28433]	protein coding	-0.99	3.36	75.34	<0.0001	<0.0001
ENSG00000118513	MYB	MYB proto-oncogene, transcription factor [Source:HGNC Symbol;Acc:HGNC:7545]	protein coding	-0.99	2.07	48.46	<0.0001	<0.0001
ENSG00000173762	CD7	CD7 molecule [Source:HGNC Symbol;Acc:HGNC:1695]	protein coding	0.99	3.55	10.33	0.0013	0.0057
ENSG00000164116	GUCY1A1	guanylate cyclase 1 soluble subunit alpha 1 [Source:HGNC Symbol;Acc:HGNC:4685]	protein coding	0.99	0.69	24.67	<0.0001	<0.0001
ENSG00000138646	HERC5	HECT and RLD domain containing E3 ubiquitin protein ligase 5 [Source:HGNC Symbol;Acc:HGNC:24368]	protein coding	0.99	4.74	15.96	<0.0001	0.0005
ENSG00000101017	CD40	CD40 molecule [Source:HGNC Symbol;Acc:HGNC:11919]	protein coding	0.98	4.70	80.99	<0.0001	<0.0001
ENSG00000137628	DDX60	DExD/H-box helicase 60 [Source:HGNC Symbol;Acc:HGNC:25942]	protein coding	0.98	5.23	31.90	<0.0001	<0.0001

Table S3: Genes differentially expressed at Day 2 compared to pre-first vaccination (Study Arm 2 A/H7N9+AS03, D1, 29, Monocytes). Genes are sorted by descending absolute log_2 fold change. Gene model summaries and annotations are based on Ensembl Version 99 (continued.)

Ensembl Gene ID	Ensembl Gene Name	Ensembl Gene Description	Gene Type	log_2 Fold Change (Day 2 vs. Pre-First Vaccination)	Average log_2 CPM	Likelihood Ratio Test Statistic	P-Value	FDR Adjusted P-Value
ENSG00000225886		novel transcript, antisense to IFI6	lncRNA	0.98	1.14	12.43	0.0004	0.0022
ENSG00000169031	COL4A3	collagen type IV alpha 3 chain [Source:HGNC Symbol;Acc:HGNC:2204]	protein coding	-0.98	0.84	20.70	<0.0001	<0.0001
ENSG00000087589	CASS4	Cas scaffold protein family member 4 [Source:HGNC Symbol;Acc:HGNC:15878]	protein coding	-0.97	3.29	53.82	<0.0001	<0.0001
ENSG00000156113	KCNMA1	potassium calcium-activated channel subfamily M alpha 1 [Source:HGNC Symbol;Acc:HGNC:6284]	protein coding	0.97	1.26	19.67	<0.0001	<0.0001
ENSG00000096996	IL12RB1	interleukin 12 receptor subunit beta 1 [Source:HGNC Symbol;Acc:HGNC:5971]	protein coding	0.97	5.18	90.90	<0.0001	<0.0001
ENSG00000116824	CD2	CD2 molecule [Source:HGNC Symbol;Acc:HGNC:1639]	protein coding	-0.97	1.61	8.85	0.0029	0.0109
ENSG00000154736	ADAMTS5	ADAM metallopeptidase with thrombospondin type 1 motif 5 [Source:HGNC Symbol;Acc:HGNC:221]	protein coding	-0.97	2.84	31.67	<0.0001	<0.0001
ENSG00000111331	OAS3	2'-5'-oligoadenylate synthetase 3 [Source:HGNC Symbol;Acc:HGNC:8088]	protein coding	0.97	7.99	20.09	<0.0001	<0.0001
ENSG00000182389	CACNB4	calcium voltage-gated channel auxiliary subunit beta 4 [Source:HGNC Symbol;Acc:HGNC:1404]	protein coding	-0.96	1.57	38.41	<0.0001	<0.0001
ENSG00000237940	LINC01238	long intergenic non-protein coding RNA 1238 [Source:HGNC Symbol;Acc:HGNC:49795]	lncRNA	-0.95	0.62	29.25	<0.0001	<0.0001
ENSG00000287733		novel transcript	lncRNA	0.95	0.87	26.54	<0.0001	<0.0001
ENSG00000229754	CXCR2P1	C-X-C motif chemokine receptor 2 pseudogene 1 [Source:HGNC Symbol;Acc:HGNC:6028]	transcribed unprocessed pseudogene	0.95	5.14	25.37	<0.0001	<0.0001
ENSG00000269954		novel transcript, antisense to BLK	lncRNA	-0.95	1.77	21.79	<0.0001	<0.0001
ENSG00000111335	OAS2	2'-5'-oligoadenylate synthetase 2 [Source:HGNC Symbol;Acc:HGNC:8087]	protein coding	0.94	7.55	26.63	<0.0001	<0.0001
ENSG00000144354	CDCA7	cell division cycle associated 7 [Source:HGNC Symbol;Acc:HGNC:14628]	protein coding	-0.94	0.89	31.56	<0.0001	<0.0001
ENSG00000198785	GRIN3A	glutamate ionotropic receptor NMDA type subunit 3A [Source:HGNC Symbol;Acc:HGNC:16767]	protein coding	0.94	1.65	38.65	<0.0001	<0.0001
ENSG00000139572	GPR84	G protein-coupled receptor 84 [Source:HGNC Symbol;Acc:HGNC:4535]	protein coding	0.94	1.85	39.16	<0.0001	<0.0001
ENSG00000165806	CASP7	caspase 7 [Source:HGNC Symbol;Acc:HGNC:1508]	protein coding	0.93	4.71	81.58	<0.0001	<0.0001

Table S3: Genes differentially expressed at Day 2 compared to pre-first vaccination (Study Arm 2 A/H7N9+AS03, D1, 29, Monocytes). Genes are sorted by descending absolute log_2 fold change. Gene model summaries and annotations are based on Ensembl Version 99 (continued.)

Ensembl Gene ID	Ensembl Gene Name	Ensembl Gene Description	Gene Type	log_2 Fold Change (Day 2 vs. Pre-First Vaccination)	Average log_2 CPM	Likelihood Ratio Test Statistic	P-Value	FDR Adjusted P-Value
ENSG00000126709	IFI6	interferon alpha inducible protein 6 [Source:HGNC Symbol;Acc:HGNC:4054]	protein coding	0.93	7.28	15.31	<0.0001	0.0006
ENSG00000140859	KIFC3	kinesin family member C3 [Source:HGNC Symbol;Acc:HGNC:6326]	protein coding	0.93	1.19	18.68	<0.0001	0.0001
ENSG00000182541	LIMK2	LIM domain kinase 2 [Source:HGNC Symbol;Acc:HGNC:6614]	protein coding	0.93	5.47	63.32	<0.0001	<0.0001
ENSG00000184226	PCDH9	protocadherin 9 [Source:HGNC Symbol;Acc:HGNC:8661]	protein coding	-0.93	1.33	17.21	<0.0001	0.0003
ENSG00000213366	GSTM2	glutathione S-transferase mu 2 [Source:HGNC Symbol;Acc:HGNC:4634]	protein coding	-0.92	2.18	51.18	<0.0001	<0.0001
ENSG00000173110	HSPA6	heat shock protein family A (Hsp70) member 6 [Source:HGNC Symbol;Acc:HGNC:5239]	protein coding	0.92	5.60	52.14	<0.0001	<0.0001
ENSG00000106624	AEBP1	AE binding protein 1 [Source:HGNC Symbol;Acc:HGNC:303]	protein coding	-0.92	0.73	16.00	<0.0001	0.0005
ENSG00000130487	KLHDC7B	kelch domain containing 7B [Source:HGNC Symbol;Acc:HGNC:25145]	protein coding	0.92	3.90	41.51	<0.0001	<0.0001
ENSG00000171049	FPR2	formyl peptide receptor 2 [Source:HGNC Symbol;Acc:HGNC:3827]	protein coding	0.92	6.04	33.20	<0.0001	<0.0001
ENSG00000159958	TNFRSF13C	TNF receptor superfamily member 13C [Source:HGNC Symbol;Acc:HGNC:17755]	protein coding	-0.92	3.70	23.29	<0.0001	<0.0001
ENSG00000135144	DTX1	deltex E3 ubiquitin ligase 1 [Source:HGNC Symbol;Acc:HGNC:3060]	protein coding	-0.91	1.14	14.35	0.0002	0.001
ENSG00000158488	CD1E	CD1e molecule [Source:HGNC Symbol;Acc:HGNC:1638]	protein coding	-0.91	1.22	27.84	<0.0001	<0.0001
ENSG00000111186	WNT5B	Wnt family member 5B [Source:HGNC Symbol;Acc:HGNC:16265]	protein coding	-0.91	2.38	30.57	<0.0001	<0.0001
ENSG00000187912	CLEC17A	C-type lectin domain containing 17A [Source:HGNC Symbol;Acc:HGNC:34520]	protein coding	-0.91	2.13	37.64	<0.0001	<0.0001
ENSG00000136514	RTP4	receptor transporter protein 4 [Source:HGNC Symbol;Acc:HGNC:23992]	protein coding	0.91	3.08	26.15	<0.0001	<0.0001
ENSG00000136573	BLK	BLK proto-oncogene, Src family tyrosine kinase [Source:HGNC Symbol;Acc:HGNC:1057]	protein coding	-0.91	4.10	27.98	<0.0001	<0.0001
ENSG00000134470	IL15RA	interleukin 15 receptor subunit alpha [Source:HGNC Symbol;Acc:HGNC:5978]	protein coding	0.91	4.35	69.04	<0.0001	<0.0001
ENSG00000106976	DNM1	dynamin 1 [Source:HGNC Symbol;Acc:HGNC:2972]	protein coding	-0.91	2.14	41.97	<0.0001	<0.0001

Table S3: Genes differentially expressed at Day 2 compared to pre-first vaccination (Study Arm 2 A/H7N9+AS03, D1, 29, Monocytes). Genes are sorted by descending absolute log_2 fold change. Gene model summaries and annotations are based on Ensembl Version 99 (continued.)

Ensembl Gene ID	Ensembl Gene Name	Ensembl Gene Description	Gene Type	log_2 Fold Change (Day 2 vs. Pre-First Vaccination)	Average log_2 CPM	Likelihood Ratio Test Statistic	P-Value	FDR Adjusted P-Value
ENSG00000188549	CCDC9B	coiled-coil domain containing 9B [Source:HGNC Symbol;Acc:HGNC:33488]	protein coding	-0.91	2.85	47.32	<0.0001	<0.0001
ENSG00000228058	LINC01736	long intergenic non-protein coding RNA 1736 [Source:HGNC Symbol;Acc:HGNC:52524]	lncRNA	-0.90	0.33	20.51	<0.0001	<0.0001
ENSG00000163251	FZD5	frizzled class receptor 5 [Source:HGNC Symbol;Acc:HGNC:4043]	protein coding	0.90	2.02	46.85	<0.0001	<0.0001
ENSG00000164330	EBF1	EBF transcription factor 1 [Source:HGNC Symbol;Acc:HGNC:3126]	protein coding	-0.90	0.42	19.72	<0.0001	<0.0001
ENSG00000177989	ODF3B	outer dense fiber of sperm tails 3B [Source:HGNC Symbol;Acc:HGNC:34388]	protein coding	0.90	8.51	42.81	<0.0001	<0.0001
ENSG00000185745	IFIT1	interferon induced protein with tetratricopeptide repeats 1 [Source:HGNC Symbol;Acc:HGNC:5407]	protein coding	0.90	5.26	9.66	0.0019	0.0076
ENSG00000083454	P2RX5	purinergic receptor P2X 5 [Source:HGNC Symbol;Acc:HGNC:8536]	protein coding	-0.89	2.91	42.92	<0.0001	<0.0001
ENSG00000197121	PGAP1	post-GPI attachment to proteins inositol deacylase 1 [Source:HGNC Symbol;Acc:HGNC:25712]	protein coding	0.89	1.77	13.89	0.0002	0.0012
ENSG00000174080	CTSF	cathepsin F [Source:HGNC Symbol;Acc:HGNC:2531]	protein coding	-0.89	1.63	30.84	<0.0001	<0.0001
ENSG00000155363	MOV10	Mov10 RISC complex RNA helicase [Source:HGNC Symbol;Acc:HGNC:7200]	protein coding	0.89	5.77	39.51	<0.0001	<0.0001
ENSG00000287252		novel transcript	lncRNA	-0.89	0.51	20.44	<0.0001	<0.0001
ENSG00000007516	BAIAP3	BAI1 associated protein 3 [Source:HGNC Symbol;Acc:HGNC:948]	protein coding	-0.88	2.06	32.14	<0.0001	<0.0001
ENSG00000105639	JAK3	Janus kinase 3 [Source:HGNC Symbol;Acc:HGNC:6193]	protein coding	0.88	6.77	68.73	<0.0001	<0.0001
ENSG00000170581	STAT2	signal transducer and activator of transcription 2 [Source:HGNC Symbol;Acc:HGNC:11363]	protein coding	0.88	8.79	41.43	<0.0001	<0.0001
ENSG00000197705	KLHL14	kelch like family member 14 [Source:HGNC Symbol;Acc:HGNC:29266]	protein coding	-0.88	0.72	18.72	<0.0001	0.0001
ENSG00000149927	DOC2A	double C2 domain alpha [Source:HGNC Symbol;Acc:HGNC:2985]	protein coding	-0.88	0.47	35.55	<0.0001	<0.0001
ENSG00000091972	CD200	CD200 molecule [Source:HGNC Symbol;Acc:HGNC:7203]	protein coding	-0.88	0.94	11.66	0.0006	0.0031
ENSG00000284194	SCO2	SCO cytochrome c oxidase assembly protein 2 [Source:HGNC Symbol;Acc:HGNC:10604]	protein coding	0.88	5.50	32.59	<0.0001	<0.0001

Table S3: Genes differentially expressed at Day 2 compared to pre-first vaccination (Study Arm 2 A/H7N9+AS03, D1, 29, Monocytes). Genes are sorted by descending absolute log_2 fold change. Gene model summaries and annotations are based on Ensembl Version 99 (continued.)

Ensembl Gene ID	Ensembl Gene Name	Ensembl Gene Description	Gene Type	log_2 Fold Change (Day 2 vs. Pre-First Vaccination)	Average log_2 CPM	Likelihood Ratio Test Statistic	P-Value	FDR Adjusted P-Value
ENSG00000136235	GPNMB	glycoprotein nmb [Source:HGNC Symbol;Acc:HGNC:4462]	protein coding	-0.88	1.48	24.91	<0.0001	<0.0001
ENSG00000112182	BACH2	BTB domain and CNC homolog 2 [Source:HGNC Symbol;Acc:HGNC:14078]	protein coding	-0.87	2.44	21.82	<0.0001	<0.0001
ENSG00000073111	MCM2	minichromosome maintenance complex component 2 [Source:HGNC Symbol;Acc:HGNC:6944]	protein coding	-0.87	1.17	33.89	<0.0001	<0.0001
ENSG00000113368	LMNB1	lamin B1 [Source:HGNC Symbol;Acc:HGNC:6637]	protein coding	0.87	6.67	45.81	<0.0001	<0.0001
ENSG00000075399	VPS9D1	VPS9 domain containing 1 [Source:HGNC Symbol;Acc:HGNC:13526]	protein coding	0.87	5.83	77.40	<0.0001	<0.0001
ENSG00000151623	NR3C2	nuclear receptor subfamily 3 group C member 2 [Source:HGNC Symbol;Acc:HGNC:7979]	protein coding	-0.87	0.52	22.27	<0.0001	<0.0001
ENSG00000229644	NAMPTP1	nicotinamide phosphoribosyltransferase pseudogene 1 [Source:HGNC Symbol;Acc:HGNC:17633]	processed pseudogene	0.87	3.85	26.59	<0.0001	<0.0001
ENSG00000211892	IGHG4	immunoglobulin heavy constant gamma 4 (G4m marker) [Source:HGNC Symbol;Acc:HGNC:5528]	IG C gene	-0.86	1.76	9.87	0.0017	0.0069
ENSG00000154258	ABCA9	ATP binding cassette subfamily A member 9 [Source:HGNC Symbol;Acc:HGNC:39]	protein coding	-0.86	0.30	27.06	<0.0001	<0.0001
ENSG00000132530	XAF1	XIAP associated factor 1 [Source:HGNC Symbol;Acc:HGNC:30932]	protein coding	0.86	6.12	24.07	<0.0001	<0.0001
ENSG00000169136	ATF5	activating transcription factor 5 [Source:HGNC Symbol;Acc:HGNC:790]	protein coding	0.86	5.66	46.89	<0.0001	<0.0001
ENSG00000257017	HP	haptoglobin [Source:HGNC Symbol;Acc:HGNC:5141]	protein coding	0.86	3.23	46.85	<0.0001	<0.0001
ENSG00000142798	HSPG2	heparan sulfate proteoglycan 2 [Source:HGNC Symbol;Acc:HGNC:5273]	protein coding	-0.86	0.84	25.79	<0.0001	<0.0001
ENSG00000168016	TRANK1	tetratricopeptide repeat and ankyrin repeat containing 1 [Source:HGNC Symbol;Acc:HGNC:29011]	protein coding	0.86	5.61	48.07	<0.0001	<0.0001
ENSG00000133121	STARD13	StAR related lipid transfer domain containing 13 [Source:HGNC Symbol;Acc:HGNC:19164]	protein coding	-0.85	0.61	27.06	<0.0001	<0.0001
ENSG00000135842	NIBAN1	niban apoptosis regulator 1 [Source:HGNC Symbol;Acc:HGNC:16784]	protein coding	0.85	6.74	62.06	<0.0001	<0.0001
ENSG00000111696	NT5DC3	5'-nucleotidase domain containing 3 [Source:HGNC Symbol;Acc:HGNC:30826]	protein coding	-0.85	2.79	38.82	<0.0001	<0.0001

Table S3: Genes differentially expressed at Day 2 compared to pre-first vaccination (Study Arm 2 A/H7N9+AS03, D1, 29, Monocytes). Genes are sorted by descending absolute log_2 fold change. Gene model summaries and annotations are based on Ensembl Version 99 (continued.)

Ensembl Gene ID	Ensembl Gene Name	Ensembl Gene Description	Gene Type	log_2 Fold Change (Day 2 vs. Pre-First Vaccination)	Average log_2 CPM	Likelihood Ratio Test Statistic	P-Value	FDR Adjusted P-Value
ENSG00000138166	DUSP5	dual specificity phosphatase 5 [Source:HGNC Symbol;Acc:HGNC:3071]	protein coding	0.85	3.86	23.68	<0.0001	<0.0001
ENSG00000242779	ZNF702P	zinc finger protein 702, pseudogene [Source:HGNC Symbol;Acc:HGNC:25775]	transcribed processed pseudogene	0.85	2.00	14.73	0.0001	0.0008
ENSG00000148926	ADM	adrenomedullin [Source:HGNC Symbol;Acc:HGNC:259]	protein coding	0.85	3.87	14.58	0.0001	0.0009
ENSG00000118922	KLF12	Kruppel like factor 12 [Source:HGNC Symbol;Acc:HGNC:6346]	protein coding	-0.85	3.17	37.60	<0.0001	<0.0001
ENSG00000159618	ADGRG5	adhesion G protein-coupled receptor G5 [Source:HGNC Symbol;Acc:HGNC:19010]	protein coding	-0.85	1.54	15.00	0.0001	0.0007
ENSG00000164867	NOS3	nitric oxide synthase 3 [Source:HGNC Symbol;Acc:HGNC:7876]	protein coding	-0.84	0.03	27.20	<0.0001	<0.0001
ENSG00000166532	RIMKLB	ribosomal modification protein rimK like family member B [Source:HGNC Symbol;Acc:HGNC:29228]	protein coding	0.84	4.12	45.13	<0.0001	<0.0001
ENSG00000272941		novel transcript, antisense to C7orf49	lncRNA	0.84	0.21	22.01	<0.0001	<0.0001
ENSG00000025708	TYMP	thymidine phosphorylase [Source:HGNC Symbol;Acc:HGNC:3148]	protein coding	0.84	10.63	43.72	<0.0001	<0.0001
ENSG00000144668	ITGA9	integrin subunit alpha 9 [Source:HGNC Symbol;Acc:HGNC:6145]	protein coding	-0.83	0.75	30.00	<0.0001	<0.0001
ENSG00000168913	ENHO	energy homeostasis associated [Source:HGNC Symbol;Acc:HGNC:24838]	protein coding	-0.83	0.75	14.56	0.0001	0.0009
ENSG00000183134	PTGDR2	prostaglandin D2 receptor 2 [Source:HGNC Symbol;Acc:HGNC:4502]	protein coding	-0.83	1.99	10.72	0.0011	0.0048
ENSG00000112096	SOD2	superoxide dismutase 2 [Source:HGNC Symbol;Acc:HGNC:11180]	protein coding	0.83	8.40	66.23	<0.0001	<0.0001
ENSG00000121895	TMEM156	transmembrane protein 156 [Source:HGNC Symbol;Acc:HGNC:26260]	protein coding	-0.83	1.20	25.92	<0.0001	<0.0001
ENSG00000235621	LINC00494	long intergenic non-protein coding RNA 494 [Source:HGNC Symbol;Acc:HGNC:27657]	lncRNA	-0.82	0.71	13.21	0.0003	0.0016
ENSG00000236783	RPS15AP27	ribosomal protein S15a pseudogene 27 [Source:HGNC Symbol;Acc:HGNC:37011]	processed pseudogene	-0.82	0.59	21.76	<0.0001	<0.0001
ENSG00000287839		novel transcript	lncRNA	0.82	3.56	84.30	<0.0001	<0.0001

Table S3: Genes differentially expressed at Day 2 compared to pre-first vaccination (Study Arm 2 A/H7N9+AS03, D1, 29, Monocytes). Genes are sorted by descending absolute log_2 fold change. Gene model summaries and annotations are based on Ensembl Version 99 (continued.)

Ensembl Gene ID	Ensembl Gene Name	Ensembl Gene Description	Gene Type	log_2 Fold Change (Day 2 vs. Pre-First Vaccination)	Average log_2 CPM	Likelihood Ratio Test Statistic	P-Value	FDR Adjusted P-Value
ENSG00000163840	DTX3L	deltex E3 ubiquitin ligase 3L [Source:HGNC Symbol;Acc:HGNC:30323]	protein coding	0.82	6.42	38.92	<0.0001	<0.0001
ENSG00000157551	KCNJ15	potassium inwardly rectifying channel subfamily J member 15 [Source:HGNC Symbol;Acc:HGNC:6261]	protein coding	0.82	3.37	22.40	<0.0001	<0.0001
ENSG00000238105	GOLGA2P5	GOLGA2 pseudogene 5 [Source:HGNC Symbol;Acc:HGNC:25315]	transcribed unprocessed pseudogene	-0.82	0.66	15.23	<0.0001	0.0006
ENSG00000175322	ZNF519	zinc finger protein 519 [Source:HGNC Symbol;Acc:HGNC:30574]	protein coding	-0.82	0.98	28.39	<0.0001	<0.0001
ENSG00000105835	NAMPT	nicotinamide phosphoribosyltransferase [Source:HGNC Symbol;Acc:HGNC:30092]	protein coding	0.82	7.24	21.81	<0.0001	<0.0001
ENSG00000247982	LINC00926	long intergenic non-protein coding RNA 926 [Source:HGNC Symbol;Acc:HGNC:27514]	lncRNA	-0.82	5.24	18.15	<0.0001	0.0002
ENSG00000120278	PLEKHG1	pleckstrin homology and RhoGEF domain containing G1 [Source:HGNC Symbol;Acc:HGNC:20884]	protein coding	-0.81	1.25	13.64	0.0002	0.0013
ENSG00000099834	CDHR5	cadherin related family member 5 [Source:HGNC Symbol;Acc:HGNC:7521]	protein coding	0.81	1.16	20.05	<0.0001	<0.0001
ENSG00000225217	HSPA7	heat shock protein family A (Hsp70) member 7 [Source:HGNC Symbol;Acc:HGNC:5240]	unprocessed pseudogene	0.81	6.19	59.00	<0.0001	<0.0001
ENSG00000144645	OSBPL10	oxysterol binding protein like 10 [Source:HGNC Symbol;Acc:HGNC:16395]	protein coding	-0.81	2.05	24.36	<0.0001	<0.0001
ENSG00000166483	WEE1	WEE1 G2 checkpoint kinase [Source:HGNC Symbol;Acc:HGNC:12761]	protein coding	-0.81	0.52	16.54	<0.0001	0.0004
ENSG00000162433	AK4	adenylate kinase 4 [Source:HGNC Symbol;Acc:HGNC:363]	protein coding	0.81	1.79	51.23	<0.0001	<0.0001
ENSG00000165795	NDRG2	NDRG family member 2 [Source:HGNC Symbol;Acc:HGNC:14460]	protein coding	-0.81	3.09	40.38	<0.0001	<0.0001
ENSG00000226777	FAM30A	family with sequence similarity 30 member A [Source:HGNC Symbol;Acc:HGNC:19955]	lncRNA	-0.80	3.61	20.44	<0.0001	<0.0001
ENSG00000152229	PSTPIP2	proline-serine-threonine phosphatase interacting protein 2 [Source:HGNC Symbol;Acc:HGNC:9581]	protein coding	0.80	6.48	41.62	<0.0001	<0.0001
ENSG00000224429	LINC00539	long intergenic non-protein coding RNA 539 [Source:HGNC Symbol;Acc:HGNC:43672]	lncRNA	-0.80	0.81	23.62	<0.0001	<0.0001

Table S3: Genes differentially expressed at Day 2 compared to pre-first vaccination (Study Arm 2 A/H7N9+AS03, D1, 29, Monocytes). Genes are sorted by descending absolute log_2 fold change. Gene model summaries and annotations are based on Ensembl Version 99 (continued.)

Ensembl Gene ID	Ensembl Gene Name	Ensembl Gene Description	Gene Type	log_2 Fold Change (Day 2 vs. Pre-First Vaccination)	Average log_2 CPM	Likelihood Ratio Test Statistic	P-Value	FDR Adjusted P-Value
ENSG00000129673	AANAT	aralkylamine N-acetyltransferase [Source:HGNC Symbol;Acc:HGNC:19]	protein coding	0.80	1.55	28.94	<0.0001	<0.0001
ENSG00000124181	PLCG1	phospholipase C gamma 1 [Source:HGNC Symbol;Acc:HGNC:9065]	protein coding	-0.80	2.55	10.59	0.0011	0.0051
ENSG00000141837	CACNA1A	calcium voltage-gated channel subunit alpha1 A [Source:HGNC Symbol;Acc:HGNC:1388]	protein coding	0.80	0.97	18.01	<0.0001	0.0002
ENSG00000186265	BTLA	B and T lymphocyte associated [Source:HGNC Symbol;Acc:HGNC:21087]	protein coding	-0.80	1.94	14.13	0.0002	0.0011
ENSG00000175262	C1orf127	chromosome 1 open reading frame 127 [Source:HGNC Symbol;Acc:HGNC:26730]	protein coding	-0.80	1.80	31.36	<0.0001	<0.0001
ENSG00000173821	RNF213	ring finger protein 213 [Source:HGNC Symbol;Acc:HGNC:14539]	protein coding	0.79	9.51	33.42	<0.0001	<0.0001
ENSG00000152672	CLEC4F	C-type lectin domain family 4 member F [Source:HGNC Symbol;Acc:HGNC:25357]	protein coding	-0.79	2.98	12.09	0.0005	0.0026
ENSG00000090975	PITPNM2	phosphatidylinositol transfer protein membrane associated 2 [Source:HGNC Symbol;Acc:HGNC:21044]	protein coding	-0.79	2.43	32.00	<0.0001	<0.0001
ENSG00000143344	RGL1	ral guanine nucleotide dissociation stimulator like 1 [Source:HGNC Symbol;Acc:HGNC:30281]	protein coding	0.79	3.33	20.31	<0.0001	<0.0001
ENSG00000128438	TBC1D27P	TBC1 domain family member 27, pseudogene [Source:HGNC Symbol;Acc:HGNC:28104]	transcribed unprocessed pseudogene	-0.78	1.44	18.46	<0.0001	0.0002
ENSG00000019169	MARCO	macrophage receptor with collagenous structure [Source:HGNC Symbol;Acc:HGNC:6895]	protein coding	0.78	5.84	62.61	<0.0001	<0.0001
ENSG00000142583	SLC2A5	solute carrier family 2 member 5 [Source:HGNC Symbol;Acc:HGNC:11010]	protein coding	-0.78	1.32	24.30	<0.0001	<0.0001
ENSG00000131797	CLUHP3	clustered mitochondria homolog pseudogene 3 [Source:HGNC Symbol;Acc:HGNC:28447]	transcribed unprocessed pseudogene	-0.78	0.94	9.61	0.0019	0.0078
ENSG00000183496	MEX3B	mex-3 RNA binding family member B [Source:HGNC Symbol;Acc:HGNC:25297]	protein coding	-0.78	1.28	27.64	<0.0001	<0.0001
ENSG00000149564	ESAM	endothelial cell adhesion molecule [Source:HGNC Symbol;Acc:HGNC:17474]	protein coding	-0.78	1.17	11.24	0.0008	0.0038

Table S3: Genes differentially expressed at Day 2 compared to pre-first vaccination (Study Arm 2 A/H7N9+AS03, D1, 29, Monocytes). Genes are sorted by descending absolute log_2 fold change. Gene model summaries and annotations are based on Ensembl Version 99 (continued.)

Ensembl Gene ID	Ensembl Gene Name	Ensembl Gene Description	Gene Type	log_2 Fold Change (Day 2 vs. Pre-First Vaccination)	Average log_2 CPM	Likelihood Ratio Test Statistic	P-Value	FDR Adjusted P-Value
ENSG00000177455	CD19	CD19 molecule [Source:HGNC Symbol;Acc:HGNC:1633]	protein coding	-0.78	3.31	20.14	<0.0001	<0.0001
ENSG00000156738	MS4A1	membrane spanning 4-domains A1 [Source:HGNC Symbol;Acc:HGNC:7315]	protein coding	-0.78	5.64	18.06	<0.0001	0.0002
ENSG00000263069	RNF213-AS1	RNF213 antisense RNA 1 [Source:HGNC Symbol;Acc:HGNC:54402]	lncRNA	0.78	3.27	20.87	<0.0001	<0.0001
ENSG00000174749	FAM241A	family with sequence similarity 241 member A [Source:HGNC Symbol;Acc:HGNC:26813]	protein coding	0.77	4.84	62.04	<0.0001	<0.0001
ENSG00000161405	IKZF3	IKAROS family zinc finger 3 [Source:HGNC Symbol;Acc:HGNC:13178]	protein coding	-0.77	3.12	15.55	<0.0001	0.0006
ENSG00000110987	BCL7A	BAF chromatin remodeling complex subunit BCL7A [Source:HGNC Symbol;Acc:HGNC:1004]	protein coding	-0.77	2.33	21.83	<0.0001	<0.0001
ENSG00000157601	MX1	MX dynamin like GTPase 1 [Source:HGNC Symbol;Acc:HGNC:7532]	protein coding	0.77	7.58	12.80	0.0003	0.0019
ENSG00000182240	BACE2	beta-secretase 2 [Source:HGNC Symbol;Acc:HGNC:934]	protein coding	-0.77	0.75	10.69	0.0011	0.0049
ENSG00000211955	IGHV3-33	immunoglobulin heavy variable 3-33 [Source:HGNC Symbol;Acc:HGNC:5596]	IG V gene	-0.77	0.78	13.22	0.0003	0.0016
ENSG00000135047	CTSL	cathepsin L [Source:HGNC Symbol;Acc:HGNC:2537]	protein coding	0.77	4.69	36.67	<0.0001	<0.0001
ENSG00000279805		TEC	TEC	0.77	0.67	23.74	<0.0001	<0.0001
ENSG00000279278		novel transcript	lncRNA	-0.77	1.64	17.14	<0.0001	0.0003
ENSG00000140853	NLRC5	NLR family CARD domain containing 5 [Source:HGNC Symbol;Acc:HGNC:29933]	protein coding	0.77	7.94	62.77	<0.0001	<0.0001
ENSG00000236901	MIR600HG	MIR600 host gene [Source:HGNC Symbol;Acc:HGNC:23642]	lncRNA	-0.77	1.78	18.33	<0.0001	0.0002
ENSG00000271270	TMCC1-AS1	TMCC1 antisense RNA 1 (head to head) [Source:HGNC Symbol;Acc:HGNC:49060]	lncRNA	-0.77	0.76	14.98	0.0001	0.0007
ENSG00000128383	APOBEC3A	apolipoprotein B mRNA editing enzyme catalytic subunit 3A [Source:HGNC Symbol;Acc:HGNC:17343]	protein coding	0.77	7.58	20.61	<0.0001	<0.0001
ENSG00000171766	GATM	glycine amidinotransferase [Source:HGNC Symbol;Acc:HGNC:4175]	protein coding	-0.77	1.49	30.40	<0.0001	<0.0001
ENSG00000132274	TRIM22	tripartite motif containing 22 [Source:HGNC Symbol;Acc:HGNC:16379]	protein coding	0.77	8.53	32.92	<0.0001	<0.0001
ENSG00000123609	NMI	N-myc and STAT interactor [Source:HGNC Symbol;Acc:HGNC:7854]	protein coding	0.77	6.23	47.25	<0.0001	<0.0001

Table S3: Genes differentially expressed at Day 2 compared to pre-first vaccination (Study Arm 2 A/H7N9+AS03, D1, 29, Monocytes). Genes are sorted by descending absolute log_2 fold change. Gene model summaries and annotations are based on Ensembl Version 99 (continued.)

Ensembl Gene ID	Ensembl Gene Name	Ensembl Gene Description	Gene Type	log_2 Fold Change (Day 2 vs. Pre-First Vaccination)	Average log_2 CPM	Likelihood Ratio Test Statistic	P-Value	FDR Adjusted P-Value
ENSG00000106484	MEST	mesoderm specific transcript [Source:HGNC Symbol;Acc:HGNC:7028]	protein coding	-0.77	0.79	39.75	<0.0001	<0.0001
ENSG00000187583	PLEKHN1	pleckstrin homology domain containing N1 [Source:HGNC Symbol;Acc:HGNC:25284]	protein coding	0.77	1.53	28.24	<0.0001	<0.0001
ENSG00000185339	TCN2	transcobalamin 2 [Source:HGNC Symbol;Acc:HGNC:11653]	protein coding	0.77	5.24	47.37	<0.0001	<0.0001
ENSG00000096968	JAK2	Janus kinase 2 [Source:HGNC Symbol;Acc:HGNC:6192]	protein coding	0.77	7.50	60.60	<0.0001	<0.0001
ENSG00000211964	IGHV3-48	immunoglobulin heavy variable 3-48 [Source:HGNC Symbol;Acc:HGNC:5606]	IG V gene	-0.77	0.54	7.04	0.008	0.0247
ENSG00000232931	LINC00342	long intergenic non-protein coding RNA 342 [Source:HGNC Symbol;Acc:HGNC:42470]	lncRNA	-0.77	2.97	31.79	<0.0001	<0.0001
ENSG00000254719		guanine nucleotide binding protein (G protein) pseudogene	processed pseudogene	0.76	0.56	26.51	<0.0001	<0.0001
ENSG00000103569	AQP9	aquaporin 9 [Source:HGNC Symbol;Acc:HGNC:643]	protein coding	0.76	6.97	32.82	<0.0001	<0.0001
ENSG00000005471	ABCB4	ATP binding cassette subfamily B member 4 [Source:HGNC Symbol;Acc:HGNC:45]	protein coding	-0.76	0.56	14.19	0.0002	0.001
ENSG00000133317	LGALS12	galectin 12 [Source:HGNC Symbol;Acc:HGNC:15788]	protein coding	-0.76	3.84	21.09	<0.0001	<0.0001
ENSG00000182118	FAM89A	family with sequence similarity 89 member A [Source:HGNC Symbol;Acc:HGNC:25057]	protein coding	0.76	2.16	40.84	<0.0001	<0.0001
ENSG00000114127	XRN1	5'-3' exoribonuclease 1 [Source:HGNC Symbol;Acc:HGNC:30654]	protein coding	0.76	6.00	41.61	<0.0001	<0.0001
ENSG00000206567		novel transcript	lncRNA	-0.76	0.58	32.24	<0.0001	<0.0001
ENSG00000138031	ADCY3	adenylate cyclase 3 [Source:HGNC Symbol;Acc:HGNC:234]	protein coding	0.76	3.14	25.51	<0.0001	<0.0001
ENSG00000196092	PAX5	paired box 5 [Source:HGNC Symbol;Acc:HGNC:8619]	protein coding	-0.76	4.22	16.73	<0.0001	0.0003
ENSG00000224078	SNHG14	small nucleolar RNA host gene 14 [Source:HGNC Symbol;Acc:HGNC:37462]	lncRNA	-0.76	2.89	36.72	<0.0001	<0.0001
ENSG00000272398	CD24	CD24 molecule [Source:HGNC Symbol;Acc:HGNC:1645]	protein coding	-0.76	2.10	11.36	0.0008	0.0036
ENSG00000132386	SERPINF1	serpin family F member 1 [Source:HGNC Symbol;Acc:HGNC:8824]	protein coding	-0.76	2.84	29.44	<0.0001	<0.0001

Table S3: Genes differentially expressed at Day 2 compared to pre-first vaccination (Study Arm 2 A/H7N9+AS03, D1, 29, Monocytes). Genes are sorted by descending absolute log_2 fold change. Gene model summaries and annotations are based on Ensembl Version 99 (continued.)

Ensembl Gene ID	Ensembl Gene Name	Ensembl Gene Description	Gene Type	log_2 Fold Change (Day 2 vs. Pre-First Vaccination)	Average log_2 CPM	Likelihood Ratio Test Statistic	P-Value	FDR Adjusted P-Value
ENSG00000205413	SAMD9	sterile alpha motif domain containing 9 [Source:HGNC Symbol;Acc:HGNC:1348]	protein coding	0.76	5.91	25.53	<0.0001	<0.0001
ENSG00000061918	GUCY1B1	guanylate cyclase 1 soluble subunit beta 1 [Source:HGNC Symbol;Acc:HGNC:4687]	protein coding	0.76	0.53	15.16	<0.0001	0.0007
ENSG00000160932	LY6E	lymphocyte antigen 6 family member E [Source:HGNC Symbol;Acc:HGNC:6727]	protein coding	0.76	7.78	14.86	0.0001	0.0008
ENSG00000115267	IFIH1	interferon induced with helicase C domain 1 [Source:HGNC Symbol;Acc:HGNC:18873]	protein coding	0.75	5.87	28.76	<0.0001	<0.0001
ENSG00000137502	RAB30	RAB30, member RAS oncogene family [Source:HGNC Symbol;Acc:HGNC:9770]	protein coding	-0.75	1.92	18.07	<0.0001	0.0002
ENSG00000172878	METAP1D	methionyl aminopeptidase type 1D, mitochondrial [Source:HGNC Symbol;Acc:HGNC:32583]	protein coding	-0.75	1.22	28.12	<0.0001	<0.0001
ENSG00000268027		novel transcript, sense intronic to CEACAM21	lncRNA	-0.75	0.79	13.47	0.0002	0.0014
ENSG00000168427	KLHL30	kelch like family member 30 [Source:HGNC Symbol;Acc:HGNC:24770]	protein coding	-0.75	0.45	11.79	0.0006	0.003
ENSG00000019102	VSIG2	V-set and immunoglobulin domain containing 2 [Source:HGNC Symbol;Acc:HGNC:17149]	protein coding	-0.75	2.81	36.15	<0.0001	<0.0001
ENSG00000211668	IGLV2-11	immunoglobulin lambda variable 2-11 [Source:HGNC Symbol;Acc:HGNC:5887]	IG V gene	-0.75	0.90	6.86	0.0088	0.0269
ENSG00000072840	EVC	EvC ciliary complex subunit 1 [Source:HGNC Symbol;Acc:HGNC:3497]	protein coding	-0.75	1.22	20.16	<0.0001	<0.0001
ENSG00000172508	CARNS1	carnosine synthase 1 [Source:HGNC Symbol;Acc:HGNC:29268]	protein coding	-0.75	1.47	23.68	<0.0001	<0.0001
ENSG00000235750	KIAA0040	KIAA0040 [Source:HGNC Symbol;Acc:HGNC:28950]	protein coding	0.75	6.41	48.05	<0.0001	<0.0001
ENSG00000135604	STX11	syntaxin 11 [Source:HGNC Symbol;Acc:HGNC:11429]	protein coding	0.75	8.35	43.43	<0.0001	<0.0001
ENSG00000124215	CDH26	cadherin 26 [Source:HGNC Symbol;Acc:HGNC:15902]	protein coding	-0.75	1.49	31.20	<0.0001	<0.0001
ENSG00000140464	PML	promyelocytic leukemia [Source:HGNC Symbol;Acc:HGNC:9113]	protein coding	0.75	6.66	30.34	<0.0001	<0.0001
ENSG00000126368	NR1D1	nuclear receptor subfamily 1 group D member 1 [Source:HGNC Symbol;Acc:HGNC:7962]	protein coding	-0.75	2.06	12.65	0.0004	0.002
ENSG00000204264	PSMB8	proteasome 20S subunit beta 8 [Source:HGNC Symbol;Acc:HGNC:9545]	protein coding	0.75	7.72	57.46	<0.0001	<0.0001

Table S3: Genes differentially expressed at Day 2 compared to pre-first vaccination (Study Arm 2 A/H7N9+AS03, D1, 29, Monocytes). Genes are sorted by descending absolute log_2 fold change. Gene model summaries and annotations are based on Ensembl Version 99 (continued.)

Ensembl Gene ID	Ensembl Gene Name	Ensembl Gene Description	Gene Type	log_2 Fold Change (Day 2 vs. Pre-First Vaccination)	Average log_2 CPM	Likelihood Ratio Test Statistic	P-Value	FDR Adjusted P-Value
ENSG00000244682	FCGR2C	Fc fragment of IgG receptor IIc (gene/pseudogene) [Source:HGNC Symbol;Acc:HGNC:15626]	polymorphic pseudogene	0.75	6.55	51.27	<0.0001	<0.0001
ENSG00000130202	NECTIN2	nectin cell adhesion molecule 2 [Source:HGNC Symbol;Acc:HGNC:9707]	protein coding	0.74	4.77	48.30	<0.0001	<0.0001
ENSG00000028116	VRK2	VRK serine/threonine kinase 2 [Source:HGNC Symbol;Acc:HGNC:12719]	protein coding	0.74	4.72	56.90	<0.0001	<0.0001
ENSG00000166002	SMCO4	single-pass membrane protein with coiled-coil domains 4 [Source:HGNC Symbol;Acc:HGNC:24810]	protein coding	0.74	6.10	73.12	<0.0001	<0.0001
ENSG00000106537	TSPAN13	tetraspanin 13 [Source:HGNC Symbol;Acc:HGNC:21643]	protein coding	-0.74	1.66	11.74	0.0006	0.003
ENSG00000111452	ADGRD1	adhesion G protein-coupled receptor D1 [Source:HGNC Symbol;Acc:HGNC:19893]	protein coding	-0.74	4.33	33.72	<0.0001	<0.0001
ENSG00000038945	MSR1	macrophage scavenger receptor 1 [Source:HGNC Symbol;Acc:HGNC:7376]	protein coding	0.74	3.99	25.15	<0.0001	<0.0001
ENSG00000164715	LMTK2	lemur tyrosine kinase 2 [Source:HGNC Symbol;Acc:HGNC:17880]	protein coding	0.74	5.43	50.10	<0.0001	<0.0001
ENSG00000171992	SYNPO	synaptopodin [Source:HGNC Symbol;Acc:HGNC:30672]	protein coding	-0.74	1.58	13.10	0.0003	0.0016
ENSG00000188822	CNR2	cannabinoid receptor 2 [Source:HGNC Symbol;Acc:HGNC:2160]	protein coding	-0.74	1.46	14.90	0.0001	0.0007
ENSG00000245904		novel transcript, antisense to BTG1	lncRNA	-0.73	2.12	36.82	<0.0001	<0.0001
ENSG00000012124	CD22	CD22 molecule [Source:HGNC Symbol;Acc:HGNC:1643]	protein coding	-0.73	5.06	16.37	<0.0001	0.0004
ENSG00000198087	CD2AP	CD2 associated protein [Source:HGNC Symbol;Acc:HGNC:14258]	protein coding	0.73	4.42	35.08	<0.0001	<0.0001
ENSG00000166405	RIC3	RIC3 acetylcholine receptor chaperone [Source:HGNC Symbol;Acc:HGNC:30338]	protein coding	-0.73	0.55	13.00	0.0003	0.0017
ENSG00000064932	SBNO2	strawberry notch homolog 2 [Source:HGNC Symbol;Acc:HGNC:29158]	protein coding	0.73	8.06	86.77	<0.0001	<0.0001
ENSG00000143297	FCRL5	Fc receptor like 5 [Source:HGNC Symbol;Acc:HGNC:18508]	protein coding	-0.73	3.14	12.54	0.0004	0.0021
ENSG00000109099	PMP22	peripheral myelin protein 22 [Source:HGNC Symbol;Acc:HGNC:9118]	protein coding	-0.73	0.86	16.64	<0.0001	0.0003
ENSG00000133105	RXFP2	relaxin family peptide receptor 2 [Source:HGNC Symbol;Acc:HGNC:17318]	protein coding	-0.73	2.64	36.89	<0.0001	<0.0001

Table S3: Genes differentially expressed at Day 2 compared to pre-first vaccination (Study Arm 2 A/H7N9+AS03, D1, 29, Monocytes). Genes are sorted by descending absolute log_2 fold change. Gene model summaries and annotations are based on Ensembl Version 99 (continued.)

Ensembl Gene ID	Ensembl Gene Name	Ensembl Gene Description	Gene Type	log_2 Fold Change (Day 2 vs. Pre-First Vaccination)	Average log_2 CPM	Likelihood Ratio Test Statistic	P-Value	FDR Adjusted P-Value
ENSG00000132846	ZBED3	zinc finger BED-type containing 3 [Source:HGNC Symbol;Acc:HGNC:20711]	protein coding	-0.73	3.36	61.13	<0.0001	<0.0001
ENSG00000141854	MISP3	MISP family member 3 [Source:HGNC Symbol;Acc:HGNC:26963]	protein coding	-0.73	1.06	18.94	<0.0001	0.0001
ENSG00000100721	TCL1A	T cell leukemia/lymphoma 1A [Source:HGNC Symbol;Acc:HGNC:11648]	protein coding	-0.73	3.69	14.28	0.0002	0.001
ENSG00000145349	CAMK2D	calcium/calmodulin dependent protein kinase II delta [Source:HGNC Symbol;Acc:HGNC:1462]	protein coding	0.73	5.32	62.69	<0.0001	<0.0001
ENSG00000107201	DDX58	DExD/H-box helicase 58 [Source:HGNC Symbol;Acc:HGNC:19102]	protein coding	0.73	5.57	20.50	<0.0001	<0.0001
ENSG00000143869	GDF7	growth differentiation factor 7 [Source:HGNC Symbol;Acc:HGNC:4222]	protein coding	-0.73	0.76	12.07	0.0005	0.0026
ENSG00000263366	ABHD17AP5	abhydrolase domain containing 17A pseudogene 5 [Source:HGNC Symbol;Acc:HGNC:34043]	processed pseudogene	-0.73	0.86	31.06	<0.0001	<0.0001
ENSG00000132622	HSPA12B	heat shock protein family A (Hsp70) member 12B [Source:HGNC Symbol;Acc:HGNC:16193]	protein coding	-0.73	1.61	15.32	<0.0001	0.0006
ENSG00000276672		novel transcript, sense intronic to RFC3	lncRNA	-0.73	0.21	15.99	<0.0001	0.0005
ENSG00000013374	NUB1	negative regulator of ubiquitin like proteins 1 [Source:HGNC Symbol;Acc:HGNC:17623]	protein coding	0.73	6.50	55.94	<0.0001	<0.0001
ENSG00000158517	NCF1	neutrophil cytosolic factor 1 [Source:HGNC Symbol;Acc:HGNC:7660]	protein coding	0.73	8.32	59.06	<0.0001	<0.0001
ENSG00000104972	LILRB1	leukocyte immunoglobulin like receptor B1 [Source:HGNC Symbol;Acc:HGNC:6605]	protein coding	0.73	8.47	68.07	<0.0001	<0.0001
ENSG00000128203	ASPHD2	aspartate beta-hydroxylase domain containing 2 [Source:HGNC Symbol;Acc:HGNC:30437]	protein coding	0.73	4.39	60.90	<0.0001	<0.0001
ENSG00000128218	VPREB3	V-set pre-B cell surrogate light chain 3 [Source:HGNC Symbol;Acc:HGNC:12710]	protein coding	-0.73	0.97	10.55	0.0012	0.0052
ENSG00000112290	WASF1	WASP family member 1 [Source:HGNC Symbol;Acc:HGNC:12732]	protein coding	0.73	1.77	36.27	<0.0001	<0.0001
ENSG00000076554	TPD52	tumor protein D52 [Source:HGNC Symbol;Acc:HGNC:12005]	protein coding	-0.72	1.65	15.96	<0.0001	0.0005
ENSG00000004799	PDK4	pyruvate dehydrogenase kinase 4 [Source:HGNC Symbol;Acc:HGNC:8812]	protein coding	-0.72	6.37	8.08	0.0045	0.0153
ENSG00000130589	HELZ2	helicase with zinc finger 2 [Source:HGNC Symbol;Acc:HGNC:30021]	protein coding	0.72	7.27	19.38	<0.0001	0.0001

Table S3: Genes differentially expressed at Day 2 compared to pre-first vaccination (Study Arm 2 A/H7N9+AS03, D1, 29, Monocytes). Genes are sorted by descending absolute log_2 fold change. Gene model summaries and annotations are based on Ensembl Version 99 (continued.)

Ensembl Gene ID	Ensembl Gene Name	Ensembl Gene Description	Gene Type	log_2 Fold Change (Day 2 vs. Pre-First Vaccination)	Average log_2 CPM	Likelihood Ratio Test Statistic	P-Value	FDR Adjusted P-Value
ENSG00000107742	SPOCK2	SPARC (osteonectin), cwcv and kazal like domains proteoglycan 2 [Source:HGNC Symbol;Acc:HGNC:13564]	protein coding	-0.72	3.26	6.15	0.0131	0.0369
ENSG00000228340	MIR646HG	MIR646 host gene [Source:HGNC Symbol;Acc:HGNC:27659]	lncRNA	-0.72	2.98	35.58	<0.0001	<0.0001
ENSG00000162747	FCGR3B	Fc fragment of IgG receptor IIIb [Source:HGNC Symbol;Acc:HGNC:3620]	protein coding	0.72	1.27	7.55	0.006	0.0197
ENSG00000170271	FAXDC2	fatty acid hydroxylase domain containing 2 [Source:HGNC Symbol;Acc:HGNC:1334]	protein coding	-0.72	2.13	53.58	<0.0001	<0.0001
ENSG00000165178	NCF1C	neutrophil cytosolic factor 1C pseudogene [Source:HGNC Symbol;Acc:HGNC:32523]	unprocessed pseudogene	0.72	7.31	64.48	<0.0001	<0.0001
ENSG00000100100	PIK3IP1	phosphoinositide-3-kinase interacting protein 1 [Source:HGNC Symbol;Acc:HGNC:24942]	protein coding	-0.72	5.58	48.63	<0.0001	<0.0001
ENSG00000213373	LINC00671	long intergenic non-protein coding RNA 671 [Source:HGNC Symbol;Acc:HGNC:44339]	lncRNA	-0.72	1.17	27.36	<0.0001	<0.0001
ENSG00000157734	SNX22	sorting nexin 22 [Source:HGNC Symbol;Acc:HGNC:16315]	protein coding	-0.72	3.11	21.29	<0.0001	<0.0001
ENSG00000189350	TOGARAM2	TOG array regulator of axonemal microtubules 2 [Source:HGNC Symbol;Acc:HGNC:33715]	protein coding	-0.72	1.73	19.07	<0.0001	0.0001
ENSG00000088543	C3orf18	chromosome 3 open reading frame 18 [Source:HGNC Symbol;Acc:HGNC:24837]	protein coding	-0.72	0.78	21.15	<0.0001	<0.0001
ENSG00000120210	INSL6	insulin like 6 [Source:HGNC Symbol;Acc:HGNC:6089]	protein coding	0.72	0.45	11.74	0.0006	0.003
ENSG00000134755	DSC2	desmocollin 2 [Source:HGNC Symbol;Acc:HGNC:3036]	protein coding	0.72	4.81	32.94	<0.0001	<0.0001
ENSG00000133739	LRRCC1	leucine rich repeat and coiled-coil centrosomal protein 1 [Source:HGNC Symbol;Acc:HGNC:29373]	protein coding	0.72	1.91	36.75	<0.0001	<0.0001
ENSG00000242616	GNG10	G protein subunit gamma 10 [Source:HGNC Symbol;Acc:HGNC:4402]	protein coding	0.72	2.69	35.43	<0.0001	<0.0001
ENSG00000065911	MTHFD2	methylenetetrahydrofolate dehydrogenase (NADP+ dependent) 2, methenyltetrahydrofolate cyclohydrolase [Source:HGNC Symbol;Acc:HGNC:7434]	protein coding	0.72	5.81	74.73	<0.0001	<0.0001
ENSG00000141542	RAB40B	RAB40B, member RAS oncogene family [Source:HGNC Symbol;Acc:HGNC:18284]	protein coding	-0.72	1.36	29.99	<0.0001	<0.0001
ENSG00000042980	ADAM28	ADAM metallopeptidase domain 28 [Source:HGNC Symbol;Acc:HGNC:206]	protein coding	-0.72	4.27	50.29	<0.0001	<0.0001

Table S3: Genes differentially expressed at Day 2 compared to pre-first vaccination (Study Arm 2 A/H7N9+AS03, D1, 29, Monocytes). Genes are sorted by descending absolute log_2 fold change. Gene model summaries and annotations are based on Ensembl Version 99 (continued.)

Ensembl Gene ID	Ensembl Gene Name	Ensembl Gene Description	Gene Type	log_2 Fold Change (Day 2 vs. Pre-First Vaccination)	Average log_2 CPM	Likelihood Ratio Test Statistic	P-Value	FDR Adjusted P-Value
ENSG00000179750	APOBEC3B	apolipoprotein B mRNA editing enzyme catalytic subunit 3B [Source:HGNC Symbol;Acc:HGNC:17352]	protein coding	0.71	1.06	9.96	0.0016	0.0067
ENSG00000281398	SNHG4	small nucleolar RNA host gene 4 [Source:HGNC Symbol;Acc:HGNC:32964]	lncRNA	-0.71	1.55	29.93	<0.0001	<0.0001
ENSG00000140379	BCL2A1	BCL2 related protein A1 [Source:HGNC Symbol;Acc:HGNC:991]	protein coding	0.71	5.56	35.48	<0.0001	<0.0001
ENSG00000099958	DERL3	derlin 3 [Source:HGNC Symbol;Acc:HGNC:14236]	protein coding	-0.71	0.30	10.49	0.0012	0.0053
ENSG00000013364	MVP	major vault protein [Source:HGNC Symbol;Acc:HGNC:7531]	protein coding	0.71	8.65	79.98	<0.0001	<0.0001
ENSG00000103528	SYT17	synaptotagmin 17 [Source:HGNC Symbol;Acc:HGNC:24119]	protein coding	-0.71	1.11	15.96	<0.0001	0.0005
ENSG00000215533	LINC00189	long intergenic non-protein coding RNA 189 [Source:HGNC Symbol;Acc:HGNC:18461]	lncRNA	0.71	0.90	19.88	<0.0001	<0.0001
ENSG00000278869		novel transcript	lncRNA	0.71	0.41	17.13	<0.0001	0.0003
ENSG00000165138	ANKS6	ankyrin repeat and sterile alpha motif domain containing 6 [Source:HGNC Symbol;Acc:HGNC:26724]	protein coding	-0.71	1.08	30.37	<0.0001	<0.0001
ENSG00000163661	PTX3	pentraxin 3 [Source:HGNC Symbol;Acc:HGNC:9692]	protein coding	-0.71	2.83	45.79	<0.0001	<0.0001
ENSG00000069188	SDK2	sidekick cell adhesion molecule 2 [Source:HGNC Symbol;Acc:HGNC:19308]	protein coding	-0.71	0.50	11.28	0.0008	0.0038
ENSG00000145911	N4BP3	NEDD4 binding protein 3 [Source:HGNC Symbol;Acc:HGNC:29852]	protein coding	-0.71	0.60	12.95	0.0003	0.0018
ENSG00000163565	IFI16	interferon gamma inducible protein 16 [Source:HGNC Symbol;Acc:HGNC:5395]	protein coding	0.71	7.73	33.95	<0.0001	<0.0001
ENSG00000158715	SLC45A3	solute carrier family 45 member 3 [Source:HGNC Symbol;Acc:HGNC:8642]	protein coding	-0.71	1.29	6.85	0.0089	0.027
ENSG00000203710	CR1	complement C3b/C4b receptor 1 (Knops blood group) [Source:HGNC Symbol;Acc:HGNC:2334]	protein coding	0.71	6.84	65.44	<0.0001	<0.0001
ENSG00000173706	HEG1	heart development protein with EGF like domains 1 [Source:HGNC Symbol;Acc:HGNC:29227]	protein coding	0.71	4.09	32.39	<0.0001	<0.0001
ENSG00000100027	YPEL1	yippee like 1 [Source:HGNC Symbol;Acc:HGNC:12845]	protein coding	-0.70	0.72	24.98	<0.0001	<0.0001
ENSG00000169116	PARM1	prostate androgen-regulated mucin-like protein 1 [Source:HGNC Symbol;Acc:HGNC:24536]	protein coding	-0.70	0.91	13.83	0.0002	0.0012

Table S3: Genes differentially expressed at Day 2 compared to pre-first vaccination (Study Arm 2 A/H7N9+AS03, D1, 29, Monocytes). Genes are sorted by descending absolute log_2 fold change. Gene model summaries and annotations are based on Ensembl Version 99 (continued.)

Ensembl Gene ID	Ensembl Gene Name	Ensembl Gene Description	Gene Type	log_2 Fold Change (Day 2 vs. Pre-First Vaccination)	Average log_2 CPM	Likelihood Ratio Test Statistic	P-Value	FDR Adjusted P-Value
ENSG00000086300	SNX10	sorting nexin 10 [Source:HGNC Symbol;Acc:HGNC:14974]	protein coding	0.70	7.16	59.58	<0.0001	<0.0001
ENSG00000035664	DAPK2	death associated protein kinase 2 [Source:HGNC Symbol;Acc:HGNC:2675]	protein coding	-0.70	1.22	12.84	0.0003	0.0019
ENSG00000188313	PLSCR1	phospholipid scramblase 1 [Source:HGNC Symbol;Acc:HGNC:9092]	protein coding	0.70	7.00	28.19	<0.0001	<0.0001
ENSG00000269404	SPIB	Spi-B transcription factor [Source:HGNC Symbol;Acc:HGNC:11242]	protein coding	-0.70	2.22	16.88	<0.0001	0.0003
ENSG00000205846	CLEC6A	C-type lectin domain containing 6A [Source:HGNC Symbol;Acc:HGNC:14556]	protein coding	0.70	2.77	38.90	<0.0001	<0.0001
ENSG00000133818	RRAS2	RAS related 2 [Source:HGNC Symbol;Acc:HGNC:17271]	protein coding	-0.70	0.55	11.56	0.0007	0.0033
ENSG00000211959	IGHV4-39	immunoglobulin heavy variable 4-39 [Source:HGNC Symbol;Acc:HGNC:5651]	IG V gene	-0.70	0.44	6.00	0.0143	0.0395
ENSG00000154274	C4orf19	chromosome 4 open reading frame 19 [Source:HGNC Symbol;Acc:HGNC:25618]	protein coding	-0.70	0.37	10.08	0.0015	0.0063
ENSG00000167483	NIBAN3	niban apoptosis regulator 3 [Source:HGNC Symbol;Acc:HGNC:24130]	protein coding	-0.70	5.00	17.58	<0.0001	0.0002
ENSG00000198286	CARD11	caspase recruitment domain family member 11 [Source:HGNC Symbol;Acc:HGNC:16393]	protein coding	-0.70	3.17	9.63	0.0019	0.0077
ENSG00000211445	GPX3	glutathione peroxidase 3 [Source:HGNC Symbol;Acc:HGNC:4555]	protein coding	-0.70	1.55	22.89	<0.0001	<0.0001
ENSG00000010278	CD9	CD9 molecule [Source:HGNC Symbol;Acc:HGNC:1709]	protein coding	-0.70	3.90	13.92	0.0002	0.0012
ENSG00000143226	FCGR2A	Fc fragment of IgG receptor IIa [Source:HGNC Symbol;Acc:HGNC:3616]	protein coding	0.69	7.95	56.69	<0.0001	<0.0001
ENSG00000230910		novel transcript	lncRNA	-0.69	3.17	22.01	<0.0001	<0.0001
ENSG00000124256	ZBP1	Z-DNA binding protein 1 [Source:HGNC Symbol;Acc:HGNC:16176]	protein coding	0.69	4.29	12.52	0.0004	0.0021
ENSG00000082438	COBLL1	cordon-bleu WH2 repeat protein like 1 [Source:HGNC Symbol;Acc:HGNC:23571]	protein coding	-0.69	1.63	17.12	<0.0001	0.0003
ENSG00000162694	EXTL2	exostosin like glycosyltransferase 2 [Source:HGNC Symbol;Acc:HGNC:3516]	protein coding	-0.69	0.88	20.61	<0.0001	<0.0001
ENSG00000211893	IGHG2	immunoglobulin heavy constant gamma 2 (G2m marker) [Source:HGNC Symbol;Acc:HGNC:5526]	IG C gene	-0.69	4.72	9.38	0.0022	0.0087

Table S3: Genes differentially expressed at Day 2 compared to pre-first vaccination (Study Arm 2 A/H7N9+AS03, D1, 29, Monocytes). Genes are sorted by descending absolute log_2 fold change. Gene model summaries and annotations are based on Ensembl Version 99 (continued.)

Ensembl Gene ID	Ensembl Gene Name	Ensembl Gene Description	Gene Type	log_2 Fold Change (Day 2 vs. Pre-First Vaccination)	Average log_2 CPM	Likelihood Ratio Test Statistic	P-Value	FDR Adjusted P-Value
ENSG00000213626	LBH	LBH regulator of WNT signaling pathway [Source:HGNC Symbol;Acc:HGNC:29532]	protein coding	-0.69	3.55	13.05	0.0003	0.0017
ENSG00000255587	RAB44	RAB44, member RAS oncogene family [Source:HGNC Symbol;Acc:HGNC:21068]	protein coding	-0.69	5.96	64.04	<0.0001	<0.0001
ENSG00000134243	SORT1	sortilin 1 [Source:HGNC Symbol;Acc:HGNC:11186]	protein coding	0.69	7.12	42.77	<0.0001	<0.0001
ENSG00000099860	GADD45B	growth arrest and DNA damage inducible beta [Source:HGNC Symbol;Acc:HGNC:4096]	protein coding	0.69	6.58	26.49	<0.0001	<0.0001
ENSG00000072858	SIDT1	SID1 transmembrane family member 1 [Source:HGNC Symbol;Acc:HGNC:25967]	protein coding	-0.69	2.10	23.52	<0.0001	<0.0001
ENSG00000158481	CD1C	CD1c molecule [Source:HGNC Symbol;Acc:HGNC:1636]	protein coding	-0.69	4.58	49.47	<0.0001	<0.0001
ENSG00000115339	GALNT3	polypeptide N-acetylgalactosaminyltransferase 3 [Source:HGNC Symbol;Acc:HGNC:4125]	protein coding	0.69	3.77	46.53	<0.0001	<0.0001
ENSG00000023902	PLEKHO1	pleckstrin homology domain containing O1 [Source:HGNC Symbol;Acc:HGNC:24310]	protein coding	0.68	8.89	47.22	<0.0001	<0.0001
ENSG00000008441	NFIX	nuclear factor I X [Source:HGNC Symbol;Acc:HGNC:7788]	protein coding	0.68	4.56	53.46	<0.0001	<0.0001
ENSG00000181690	PLAG1	PLAG1 zinc finger [Source:HGNC Symbol;Acc:HGNC:9045]	protein coding	-0.68	0.25	19.77	<0.0001	<0.0001
ENSG00000205220	PSMB10	proteasome 20S subunit beta 10 [Source:HGNC Symbol;Acc:HGNC:9538]	protein coding	0.68	6.81	54.37	<0.0001	<0.0001
ENSG00000168734	PKIG	cAMP-dependent protein kinase inhibitor gamma [Source:HGNC Symbol;Acc:HGNC:9019]	protein coding	-0.68	1.69	20.63	<0.0001	<0.0001
ENSG00000238160	LINC02863	long intergenic non-protein coding RNA 2863 [Source:HGNC Symbol;Acc:HGNC:41290]	lncRNA	-0.68	0.46	20.79	<0.0001	<0.0001
ENSG00000131355	ADGRE3	adhesion G protein-coupled receptor E3 [Source:HGNC Symbol;Acc:HGNC:23647]	protein coding	-0.68	4.64	23.08	<0.0001	<0.0001
ENSG00000179240	GVQW3	GVQW motif containing 3 [Source:HGNC Symbol;Acc:HGNC:51239]	protein coding	-0.68	1.39	17.66	<0.0001	0.0002
ENSG00000258476	LINC02207	long intergenic non-protein coding RNA 2207 [Source:HGNC Symbol;Acc:HGNC:53073]	lncRNA	-0.68	1.83	23.03	<0.0001	<0.0001
ENSG00000160111	CPAMD8	C3 and PZP like alpha-2-macroglobulin domain containing 8 [Source:HGNC Symbol;Acc:HGNC:23228]	protein coding	-0.68	5.36	43.66	<0.0001	<0.0001
ENSG00000062524	LTK	leukocyte receptor tyrosine kinase [Source:HGNC Symbol;Acc:HGNC:6721]	protein coding	-0.68	0.64	6.76	0.0093	0.0281

Table S3: Genes differentially expressed at Day 2 compared to pre-first vaccination (Study Arm 2 A/H7N9+AS03, D1, 29, Monocytes). Genes are sorted by descending absolute log_2 fold change. Gene model summaries and annotations are based on Ensembl Version 99 (continued.)

Ensembl Gene ID	Ensembl Gene Name	Ensembl Gene Description	Gene Type	log_2 Fold Change (Day 2 vs. Pre-First Vaccination)	Average log_2 CPM	Likelihood Ratio Test Statistic	P-Value	FDR Adjusted P-Value
ENSG00000130787	HIP1R	huntingtin interacting protein 1 related [Source:HGNC Symbol;Acc:HGNC:18415]	protein coding	-0.68	3.52	15.92	<0.0001	0.0005
ENSG00000146215	CRIP3	cysteine rich protein 3 [Source:HGNC Symbol;Acc:HGNC:17751]	protein coding	-0.68	1.28	13.97	0.0002	0.0011
ENSG00000163606	CD200R1	CD200 receptor 1 [Source:HGNC Symbol;Acc:HGNC:24235]	protein coding	-0.68	2.56	19.46	<0.0001	0.0001
ENSG00000160710	ADAR	adenosine deaminase RNA specific [Source:HGNC Symbol;Acc:HGNC:225]	protein coding	0.68	8.73	39.77	<0.0001	<0.0001
ENSG00000160856	FCRL3	Fc receptor like 3 [Source:HGNC Symbol;Acc:HGNC:18506]	protein coding	-0.67	3.24	11.01	0.0009	0.0042
ENSG00000255045		novel transcript	lncRNA	-0.67	0.77	20.81	<0.0001	<0.0001
ENSG00000197536	IRF1-AS1	IRF1 antisense RNA 1 [Source:HGNC Symbol;Acc:HGNC:33838]	lncRNA	0.67	4.72	29.54	<0.0001	<0.0001
ENSG00000026103	FAS	Fas cell surface death receptor [Source:HGNC Symbol;Acc:HGNC:11920]	protein coding	0.67	4.96	38.62	<0.0001	<0.0001
ENSG00000135148	TRAFD1	TRAF-type zinc finger domain containing 1 [Source:HGNC Symbol;Acc:HGNC:24808]	protein coding	0.67	6.84	53.48	<0.0001	<0.0001
ENSG00000161647	MPP3	membrane palmitoylated protein 3 [Source:HGNC Symbol;Acc:HGNC:7221]	protein coding	0.67	0.68	18.08	<0.0001	0.0002
ENSG00000188282	RUFY4	RUN and FYVE domain containing 4 [Source:HGNC Symbol;Acc:HGNC:24804]	protein coding	0.67	4.04	20.05	<0.0001	<0.0001
ENSG00000137078	SIT1	signaling threshold regulating transmembrane adaptor 1 [Source:HGNC Symbol;Acc:HGNC:17710]	protein coding	-0.67	0.82	7.11	0.0076	0.0239
ENSG00000157445	CACNA2D3	calcium voltage-gated channel auxiliary subunit alpha2delta 3 [Source:HGNC Symbol;Acc:HGNC:15460]	protein coding	-0.67	4.23	40.21	<0.0001	<0.0001
ENSG00000111912	NCOA7	nuclear receptor coactivator 7 [Source:HGNC Symbol;Acc:HGNC:21081]	protein coding	0.67	4.96	27.88	<0.0001	<0.0001
ENSG00000170542	SERPINB9	serpin family B member 9 [Source:HGNC Symbol;Acc:HGNC:8955]	protein coding	0.67	7.19	43.32	<0.0001	<0.0001
ENSG00000173372	C1QA	complement C1q A chain [Source:HGNC Symbol;Acc:HGNC:1241]	protein coding	0.67	3.97	18.57	<0.0001	0.0001
ENSG00000141096	DPEP3	dipeptidase 3 [Source:HGNC Symbol;Acc:HGNC:23029]	protein coding	-0.67	0.49	20.99	<0.0001	<0.0001
ENSG00000286248		novel transcript, antisense to P2RX7	lncRNA	0.67	0.82	28.11	<0.0001	<0.0001
ENSG00000211966	IGHV5-51	immunoglobulin heavy variable 5-51 [Source:HGNC Symbol;Acc:HGNC:5659]	IG V gene	-0.67	1.31	10.93	0.0009	0.0044

Table S3: Genes differentially expressed at Day 2 compared to pre-first vaccination (Study Arm 2 A/H7N9+AS03, D1, 29, Monocytes). Genes are sorted by descending absolute log_2 fold change. Gene model summaries and annotations are based on Ensembl Version 99 (continued.)

Ensembl Gene ID	Ensembl Gene Name	Ensembl Gene Description	Gene Type	log_2 Fold Change (Day 2 vs. Pre-First Vaccination)	Average log_2 CPM	Likelihood Ratio Test Statistic	P-Value	FDR Adjusted P-Value
ENSG00000176659	C20orf197	chromosome 20 open reading frame 197 [Source:HGNC Symbol;Acc:HGNC:26601]	lncRNA	-0.67	2.32	29.73	<0.0001	<0.0001
ENSG00000124374	PAIP2B	poly(A) binding protein interacting protein 2B [Source:HGNC Symbol;Acc:HGNC:29200]	protein coding	-0.67	0.70	20.97	<0.0001	<0.0001
ENSG00000185201	IFITM2	interferon induced transmembrane protein 2 [Source:HGNC Symbol;Acc:HGNC:5413]	protein coding	0.67	7.51	32.61	<0.0001	<0.0001
ENSG00000213949	ITGA1	integrin subunit alpha 1 [Source:HGNC Symbol;Acc:HGNC:6134]	protein coding	0.67	1.21	27.33	<0.0001	<0.0001
ENSG00000165905	LARGE2	LARGE xylosyl- and glucuronyltransferase 2 [Source:HGNC Symbol;Acc:HGNC:16522]	protein coding	-0.67	1.87	10.56	0.0012	0.0052
ENSG00000182580	EPHB3	EPH receptor B3 [Source:HGNC Symbol;Acc:HGNC:3394]	protein coding	-0.67	2.69	10.79	0.001	0.0046
ENSG00000101096	NFATC2	nuclear factor of activated T cells 2 [Source:HGNC Symbol;Acc:HGNC:7776]	protein coding	-0.67	1.68	7.89	0.005	0.0168
ENSG00000059378	PARP12	poly(ADP-ribose) polymerase family member 12 [Source:HGNC Symbol;Acc:HGNC:21919]	protein coding	0.67	6.62	28.58	<0.0001	<0.0001
ENSG00000261798		novel transcript, antisense to BMP8B	lncRNA	-0.67	0.15	10.43	0.0012	0.0054
ENSG00000235888		novel transcript	lncRNA	-0.66	0.67	17.24	<0.0001	0.0003
ENSG00000189007	ADAT2	adenosine deaminase tRNA specific 2 [Source:HGNC Symbol;Acc:HGNC:21172]	protein coding	-0.66	2.87	36.13	<0.0001	<0.0001
ENSG00000143847	PPFIA4	PTPRF interacting protein alpha 4 [Source:HGNC Symbol;Acc:HGNC:9248]	protein coding	-0.66	4.25	27.79	<0.0001	<0.0001
ENSG00000145390	USP53	ubiquitin specific peptidase 53 [Source:HGNC Symbol;Acc:HGNC:29255]	protein coding	-0.66	0.60	11.47	0.0007	0.0034
ENSG00000125246	CLYBL	citrate lyase beta like [Source:HGNC Symbol;Acc:HGNC:18355]	protein coding	-0.66	0.82	24.85	<0.0001	<0.0001
ENSG00000156535	CD109	CD109 molecule [Source:HGNC Symbol;Acc:HGNC:21685]	protein coding	-0.66	0.61	18.67	<0.0001	0.0001
ENSG00000138755	CXCL9	C-X-C motif chemokine ligand 9 [Source:HGNC Symbol;Acc:HGNC:7098]	protein coding	0.66	1.25	8.56	0.0034	0.0125
ENSG00000072134	EPN2	epsin 2 [Source:HGNC Symbol;Acc:HGNC:18639]	protein coding	-0.66	0.30	22.40	<0.0001	<0.0001
ENSG00000169313	P2RY12	purinergic receptor P2Y12 [Source:HGNC Symbol;Acc:HGNC:18124]	protein coding	0.66	1.54	18.53	<0.0001	0.0002
ENSG00000089127	OAS1	2'-5'-oligoadenylate synthetase 1 [Source:HGNC Symbol;Acc:HGNC:8086]	protein coding	0.66	7.66	23.15	<0.0001	<0.0001

Table S3: Genes differentially expressed at Day 2 compared to pre-first vaccination (Study Arm 2 A/H7N9+AS03, D1, 29, Monocytes). Genes are sorted by descending absolute log_2 fold change. Gene model summaries and annotations are based on Ensembl Version 99 (continued.)

Ensembl Gene ID	Ensembl Gene Name	Ensembl Gene Description	Gene Type	log_2 Fold Change (Day 2 vs. Pre-First Vaccination)	Average log_2 CPM	Likelihood Ratio Test Statistic	P-Value	FDR Adjusted P-Value
ENSG00000086061	DNAJA1	DnaJ heat shock protein family (Hsp40) member A1 [Source:HGNC Symbol;Acc:HGNC:5229]	protein coding	0.66	7.25	44.30	<0.0001	<0.0001
ENSG00000164308	ERAP2	endoplasmic reticulum aminopeptidase 2 [Source:HGNC Symbol;Acc:HGNC:29499]	protein coding	0.66	5.96	42.54	<0.0001	<0.0001
ENSG00000224934		novel transcript	lncRNA	-0.66	0.88	12.75	0.0004	0.0019
ENSG00000099204	ABLIM1	actin binding LIM protein 1 [Source:HGNC Symbol;Acc:HGNC:78]	protein coding	-0.66	2.50	7.73	0.0054	0.0181
ENSG00000111371	SLC38A1	solute carrier family 38 member 1 [Source:HGNC Symbol;Acc:HGNC:13447]	protein coding	-0.66	3.79	11.97	0.0005	0.0027
ENSG00000130940	CASZ1	castor zinc finger 1 [Source:HGNC Symbol;Acc:HGNC:26002]	protein coding	0.66	2.41	33.40	<0.0001	<0.0001
ENSG00000182048	TRPC2	transient receptor potential cation channel subfamily C member 2 (pseudogene) [Source:HGNC Symbol;Acc:HGNC:12334]	transcribed unprocessed pseudogene	-0.66	0.36	19.83	<0.0001	<0.0001
ENSG00000162894	FCMR	Fc fragment of IgM receptor [Source:HGNC Symbol;Acc:HGNC:14315]	protein coding	-0.66	4.70	14.25	0.0002	0.001
ENSG00000115419	GLS	glutaminase [Source:HGNC Symbol;Acc:HGNC:4331]	protein coding	0.66	5.79	55.56	<0.0001	<0.0001
ENSG00000125731	SH2D3A	SH2 domain containing 3A [Source:HGNC Symbol;Acc:HGNC:16885]	protein coding	-0.65	1.07	9.51	0.002	0.0082
ENSG00000082074	FYB1	FYN binding protein 1 [Source:HGNC Symbol;Acc:HGNC:4036]	protein coding	0.65	9.12	70.20	<0.0001	<0.0001
ENSG00000075275	CELSR1	cadherin EGF LAG seven-pass G-type receptor 1 [Source:HGNC Symbol;Acc:HGNC:1850]	protein coding	-0.65	1.72	18.04	<0.0001	0.0002
ENSG00000116133	DHCR24	24-dehydrocholesterol reductase [Source:HGNC Symbol;Acc:HGNC:2859]	protein coding	0.65	2.64	16.39	<0.0001	0.0004
ENSG00000099250	NRP1	neuropilin 1 [Source:HGNC Symbol;Acc:HGNC:8004]	protein coding	0.65	2.68	22.55	<0.0001	<0.0001
ENSG00000118971	CCND2	cyclin D2 [Source:HGNC Symbol;Acc:HGNC:1583]	protein coding	-0.65	3.36	33.56	<0.0001	<0.0001
ENSG00000198133	TMEM229B	transmembrane protein 229B [Source:HGNC Symbol;Acc:HGNC:20130]	protein coding	0.65	5.08	49.51	<0.0001	<0.0001
ENSG00000254709	IGLL5	immunoglobulin lambda like polypeptide 5 [Source:HGNC Symbol;Acc:HGNC:38476]	protein coding	-0.65	0.76	9.04	0.0026	0.01

Table S3: Genes differentially expressed at Day 2 compared to pre-first vaccination (Study Arm 2 A/H7N9+AS03, D1, 29, Monocytes). Genes are sorted by descending absolute log_2 fold change. Gene model summaries and annotations are based on Ensembl Version 99 (continued.)

Ensembl Gene ID	Ensembl Gene Name	Ensembl Gene Description	Gene Type	log_2 Fold Change (Day 2 vs. Pre-First Vaccination)	Average log_2 CPM	Likelihood Ratio Test Statistic	P-Value	FDR Adjusted P-Value
ENSG00000254528		novel transcript, antisense to FXYD6	lncRNA	-0.65	1.00	16.66	<0.0001	0.0003
ENSG00000205056	LINC02397	long intergenic non-protein coding RNA 2397 [Source:HGNC Symbol;Acc:HGNC:53323]	lncRNA	-0.65	0.68	6.98	0.0082	0.0254
ENSG00000171729	TMEM51	transmembrane protein 51 [Source:HGNC Symbol;Acc:HGNC:25488]	protein coding	0.65	2.81	31.52	<0.0001	<0.0001
ENSG00000104951	IL4I1	interleukin 4 induced 1 [Source:HGNC Symbol;Acc:HGNC:19094]	protein coding	0.65	3.31	16.30	<0.0001	0.0004
ENSG00000235513	L3MBTL2-AS1	L3MBTL2 antisense RNA 1 [Source:HGNC Symbol;Acc:HGNC:40847]	lncRNA	0.65	0.86	20.94	<0.0001	<0.0001
ENSG00000047579	DTNBP1	dystrobrevin binding protein 1 [Source:HGNC Symbol;Acc:HGNC:17328]	protein coding	0.65	4.47	76.34	<0.0001	<0.0001
ENSG00000135318	NT5E	5'-nucleotidase ecto [Source:HGNC Symbol;Acc:HGNC:8021]	protein coding	-0.65	1.32	11.63	0.0006	0.0032
ENSG00000160298	C21orf58	chromosome 21 open reading frame 58 [Source:HGNC Symbol;Acc:HGNC:1300]	protein coding	-0.65	0.93	20.42	<0.0001	<0.0001
ENSG00000171860	C3AR1	complement C3a receptor 1 [Source:HGNC Symbol;Acc:HGNC:1319]	protein coding	0.64	6.62	19.73	<0.0001	<0.0001
ENSG00000152377	SPOCK1	SPARC (osteonectin), cwcv and kazal like domains proteoglycan 1 [Source:HGNC Symbol;Acc:HGNC:11251]	protein coding	-0.64	1.96	32.70	<0.0001	<0.0001
ENSG00000057294	PKP2	plakophilin 2 [Source:HGNC Symbol;Acc:HGNC:9024]	protein coding	-0.64	1.44	16.72	<0.0001	0.0003
ENSG00000240505	TNFRSF13B	TNF receptor superfamily member 13B [Source:HGNC Symbol;Acc:HGNC:18153]	protein coding	-0.64	1.36	12.49	0.0004	0.0022
ENSG00000170989	S1PR1	sphingosine-1-phosphate receptor 1 [Source:HGNC Symbol;Acc:HGNC:3165]	protein coding	-0.64	2.17	6.60	0.0102	0.0303
ENSG00000100095	SEZ6L	seizure related 6 homolog like [Source:HGNC Symbol;Acc:HGNC:10763]	protein coding	-0.64	2.82	35.17	<0.0001	<0.0001
ENSG00000089041	P2RX7	purinergic receptor P2X 7 [Source:HGNC Symbol;Acc:HGNC:8537]	protein coding	0.64	6.15	72.98	<0.0001	<0.0001
ENSG00000184988	TMEM106A	transmembrane protein 106A [Source:HGNC Symbol;Acc:HGNC:28288]	protein coding	0.64	4.96	47.27	<0.0001	<0.0001
ENSG00000111886	GABRR2	gamma-aminobutyric acid type A receptor rho2 subunit [Source:HGNC Symbol;Acc:HGNC:4091]	protein coding	0.64	2.18	26.04	<0.0001	<0.0001
ENSG00000204653	ASPDH	aspartate dehydrogenase domain containing [Source:HGNC Symbol;Acc:HGNC:33856]	protein coding	-0.64	1.19	21.22	<0.0001	<0.0001

Table S3: Genes differentially expressed at Day 2 compared to pre-first vaccination (Study Arm 2 A/H7N9+AS03, D1, 29, Monocytes). Genes are sorted by descending absolute log_2 fold change. Gene model summaries and annotations are based on Ensembl Version 99 (continued.)

Ensembl Gene ID	Ensembl Gene Name	Ensembl Gene Description	Gene Type	log_2 Fold Change (Day 2 vs. Pre-First Vaccination)	Average log_2 CPM	Likelihood Ratio Test Statistic	P-Value	FDR Adjusted P-Value
ENSG00000198892	SHISA4	shisa family member 4 [Source:HGNC Symbol;Acc:HGNC:27139]	protein coding	0.64	0.96	19.08	<0.0001	0.0001
ENSG00000113140	SPARC	secreted protein acidic and cysteine rich [Source:HGNC Symbol;Acc:HGNC:11219]	protein coding	-0.64	3.11	20.70	<0.0001	<0.0001
ENSG00000041357	PSMA4	proteasome 20S subunit alpha 4 [Source:HGNC Symbol;Acc:HGNC:9533]	protein coding	0.64	7.14	43.74	<0.0001	<0.0001
ENSG00000168546	GFRA2	GDNF family receptor alpha 2 [Source:HGNC Symbol;Acc:HGNC:4244]	protein coding	-0.64	2.64	21.38	<0.0001	<0.0001
ENSG00000143153	ATP1B1	ATPase Na+/K+ transporting subunit beta 1 [Source:HGNC Symbol;Acc:HGNC:804]	protein coding	0.64	2.28	31.67	<0.0001	<0.0001
ENSG00000089169	RPH3A	rabphilin 3A [Source:HGNC Symbol;Acc:HGNC:17056]	protein coding	-0.64	4.73	39.31	<0.0001	<0.0001
ENSG00000099954	CECR2	CECR2 histone acetyl-lysine reader [Source:HGNC Symbol;Acc:HGNC:1840]	protein coding	-0.64	1.18	21.92	<0.0001	<0.0001
ENSG00000182487	NCF1B	neutrophil cytosolic factor 1B pseudogene [Source:HGNC Symbol;Acc:HGNC:32522]	transcribed unprocessed pseudogene	0.64	6.41	56.31	<0.0001	<0.0001
ENSG00000150347	ARID5B	AT-rich interaction domain 5B [Source:HGNC Symbol;Acc:HGNC:17362]	protein coding	0.64	2.93	17.74	<0.0001	0.0002
ENSG00000283696		novel transcript	lncRNA	0.64	0.23	23.47	<0.0001	<0.0001
ENSG00000211897	IGHG3	immunoglobulin heavy constant gamma 3 (G3m marker) [Source:HGNC Symbol;Acc:HGNC:5527]	IG C gene	-0.64	3.19	5.72	0.0168	0.0448
ENSG00000081052	COL4A4	collagen type IV alpha 4 chain [Source:HGNC Symbol;Acc:HGNC:2206]	protein coding	-0.63	0.62	11.13	0.0008	0.004
ENSG00000196141	SPATS2L	spermatogenesis associated serine rich 2 like [Source:HGNC Symbol;Acc:HGNC:24574]	protein coding	0.63	3.02	13.09	0.0003	0.0017
ENSG00000105369	CD79A	CD79a molecule [Source:HGNC Symbol;Acc:HGNC:1698]	protein coding	-0.63	5.26	15.95	<0.0001	0.0005
ENSG00000250397		novel transcript	lncRNA	-0.63	0.99	18.37	<0.0001	0.0002
ENSG00000211679	IGLC3	immunoglobulin lambda constant 3 (Kern-Oz+ marker) [Source:HGNC Symbol;Acc:HGNC:5857]	IG C gene	-0.63	3.37	7.21	0.0073	0.0229
ENSG00000224411	HSP90AA2P	heat shock protein 90 alpha family class A member 2, pseudogene [Source:HGNC Symbol;Acc:HGNC:5256]	processed pseudogene	0.63	2.13	26.63	<0.0001	<0.0001

Table S3: Genes differentially expressed at Day 2 compared to pre-first vaccination (Study Arm 2 A/H7N9+AS03, D1, 29, Monocytes). Genes are sorted by descending absolute log_2 fold change. Gene model summaries and annotations are based on Ensembl Version 99 (continued.)

Ensembl Gene ID	Ensembl Gene Name	Ensembl Gene Description	Gene Type	log_2 Fold Change (Day 2 vs. Pre-First Vaccination)	Average log_2 CPM	Likelihood Ratio Test Statistic	P-Value	FDR Adjusted P-Value
ENSG00000143106	PSMA5	proteasome 20S subunit alpha 5 [Source:HGNC Symbol;Acc:HGNC:9534]	protein coding	0.63	6.26	42.69	<0.0001	<0.0001
ENSG00000104518	GSDMD	gasdermin D [Source:HGNC Symbol;Acc:HGNC:25697]	protein coding	0.63	7.52	51.92	<0.0001	<0.0001
ENSG00000211899	IGHM	immunoglobulin heavy constant mu [Source:HGNC Symbol;Acc:HGNC:5541]	IG C gene	-0.63	7.92	13.23	0.0003	0.0016
ENSG00000127585	FBXL16	F-box and leucine rich repeat protein 16 [Source:HGNC Symbol;Acc:HGNC:14150]	protein coding	-0.63	1.71	24.30	<0.0001	<0.0001
ENSG00000129484	PARP2	poly(ADP-ribose) polymerase 2 [Source:HGNC Symbol;Acc:HGNC:272]	protein coding	-0.63	2.53	41.47	<0.0001	<0.0001
ENSG00000128694	OSGEPL1	O-sialoglycoprotein endopeptidase like 1 [Source:HGNC Symbol;Acc:HGNC:23075]	protein coding	-0.63	1.88	24.83	<0.0001	<0.0001
ENSG00000178038	ALS2CL	ALS2 C-terminal like [Source:HGNC Symbol;Acc:HGNC:20605]	protein coding	-0.63	3.53	33.14	<0.0001	<0.0001
ENSG00000184588	PDE4B	phosphodiesterase 4B [Source:HGNC Symbol;Acc:HGNC:8781]	protein coding	0.63	4.52	21.79	<0.0001	<0.0001
ENSG00000110777	POU2AF1	POU class 2 homeobox associating factor 1 [Source:HGNC Symbol;Acc:HGNC:9211]	protein coding	-0.63	3.40	13.42	0.0002	0.0014
ENSG00000287737		novel transcript	lncRNA	-0.63	0.91	7.74	0.0054	0.0181
ENSG00000241860		novel transcript	lncRNA	0.63	0.47	7.14	0.0076	0.0237
ENSG00000137103	TMEM8B	transmembrane protein 8B [Source:HGNC Symbol;Acc:HGNC:21427]	protein coding	-0.63	4.13	46.38	<0.0001	<0.0001
ENSG00000226321	CROCC2	ciliary rootlet coiled-coil, rootletin family member 2 [Source:HGNC Symbol;Acc:HGNC:51677]	protein coding	-0.63	3.75	12.60	0.0004	0.0021
ENSG00000171954	CYP4F22	cytochrome P450 family 4 subfamily F member 22 [Source:HGNC Symbol;Acc:HGNC:26820]	protein coding	0.63	1.79	11.08	0.0009	0.0041
ENSG00000135525	MAP7	microtubule associated protein 7 [Source:HGNC Symbol;Acc:HGNC:6869]	protein coding	-0.62	2.18	26.40	<0.0001	<0.0001
ENSG00000168421	RHOH	ras homolog family member H [Source:HGNC Symbol;Acc:HGNC:686]	protein coding	-0.62	1.83	9.79	0.0018	0.0072
ENSG00000258130		coiled-coil domain containing 101 (CCDC101) pseudogene	transcribed unprocessed pseudogene	-0.62	0.62	22.19	<0.0001	<0.0001
ENSG00000233461		novel transcript	lncRNA	0.62	1.58	31.91	<0.0001	<0.0001

Table S3: Genes differentially expressed at Day 2 compared to pre-first vaccination (Study Arm 2 A/H7N9+AS03, D1, 29, Monocytes). Genes are sorted by descending absolute log_2 fold change. Gene model summaries and annotations are based on Ensembl Version 99 (continued.)

Ensembl Gene ID	Ensembl Gene Name	Ensembl Gene Description	Gene Type	log_2 Fold Change (Day 2 vs. Pre-First Vaccination)	Average log_2 CPM	Likelihood Ratio Test Statistic	P-Value	FDR Adjusted P-Value
ENSG00000233184		novel transcript	lncRNA	-0.62	1.24	21.97	<0.0001	<0.0001
ENSG00000090447	TFAP4	transcription factor AP-4 [Source:HGNC Symbol;Acc:HGNC:11745]	protein coding	-0.62	4.26	50.47	<0.0001	<0.0001
ENSG00000187741	FANCA	FA complementation group A [Source:HGNC Symbol;Acc:HGNC:3582]	protein coding	0.62	3.54	47.05	<0.0001	<0.0001
ENSG00000203747	FCGR3A	Fc fragment of IgG receptor IIIa [Source:HGNC Symbol;Acc:HGNC:3619]	protein coding	0.62	7.76	19.03	<0.0001	0.0001
ENSG00000118420	UBE3D	ubiquitin protein ligase E3D [Source:HGNC Symbol;Acc:HGNC:21381]	protein coding	-0.62	0.43	14.73	0.0001	0.0008
ENSG00000169258	GPRIN1	G protein regulated inducer of neurite outgrowth 1 [Source:HGNC Symbol;Acc:HGNC:24835]	protein coding	0.62	0.25	14.55	0.0001	0.0009
ENSG00000258810		novel transcript, antisense to RNASE1	lncRNA	-0.62	1.64	18.18	<0.0001	0.0002
ENSG00000101194	SLC17A9	solute carrier family 17 member 9 [Source:HGNC Symbol;Acc:HGNC:16192]	protein coding	-0.62	5.29	56.19	<0.0001	<0.0001
ENSG00000169413	RNASE6	ribonuclease A family member k6 [Source:HGNC Symbol;Acc:HGNC:10048]	protein coding	-0.62	6.64	35.80	<0.0001	<0.0001
ENSG00000116691	MIIP	migration and invasion inhibitory protein [Source:HGNC Symbol;Acc:HGNC:25715]	protein coding	0.62	6.62	48.06	<0.0001	<0.0001
ENSG00000287576		novel transcript	lncRNA	0.62	1.21	14.02	0.0002	0.0011
ENSG00000244879	GABPB1-AS1	GABPB1 antisense RNA 1 [Source:HGNC Symbol;Acc:HGNC:44157]	lncRNA	-0.62	5.43	33.41	<0.0001	<0.0001
ENSG00000026559	KCNG1	potassium voltage-gated channel modifier subfamily G member 1 [Source:HGNC Symbol;Acc:HGNC:6248]	protein coding	-0.62	0.71	5.87	0.0154	0.0418
ENSG00000196912	ANKRD36B	ankyrin repeat domain 36B [Source:HGNC Symbol;Acc:HGNC:29333]	protein coding	-0.62	0.93	18.89	<0.0001	0.0001
ENSG00000100814	CCNB1IP1	cyclin B1 interacting protein 1 [Source:HGNC Symbol;Acc:HGNC:19437]	protein coding	-0.62	3.16	46.78	<0.0001	<0.0001
ENSG00000115392	FANCL	FA complementation group L [Source:HGNC Symbol;Acc:HGNC:20748]	protein coding	0.62	2.89	40.45	<0.0001	<0.0001
ENSG00000268903		novel pseudogene	processed pseudogene	0.62	3.35	24.45	<0.0001	<0.0001
ENSG00000143801	PSEN2	presenilin 2 [Source:HGNC Symbol;Acc:HGNC:9509]	protein coding	0.61	3.67	49.39	<0.0001	<0.0001
ENSG00000124766	SOX4	SRY-box transcription factor 4 [Source:HGNC Symbol;Acc:HGNC:11200]	protein coding	-0.61	5.48	31.49	<0.0001	<0.0001

Table S3: Genes differentially expressed at Day 2 compared to pre-first vaccination (Study Arm 2 A/H7N9+AS03, D1, 29, Monocytes). Genes are sorted by descending absolute log_2 fold change. Gene model summaries and annotations are based on Ensembl Version 99 (continued.)

Ensembl Gene ID	Ensembl Gene Name	Ensembl Gene Description	Gene Type	log_2 Fold Change (Day 2 vs. Pre-First Vaccination)	Average log_2 CPM	Likelihood Ratio Test Statistic	P-Value	FDR Adjusted P-Value
ENSG00000163221	S100A12	S100 calcium binding protein A12 [Source:HGNC Symbol;Acc:HGNC:10489]	protein coding	0.61	8.06	41.51	<0.0001	<0.0001
ENSG00000133313	CNDP2	carnosine dipeptidase 2 [Source:HGNC Symbol;Acc:HGNC:24437]	protein coding	0.61	7.67	34.64	<0.0001	<0.0001
ENSG00000115590	IL1R2	interleukin 1 receptor type 2 [Source:HGNC Symbol;Acc:HGNC:5994]	protein coding	-0.61	1.93	11.01	0.0009	0.0042
ENSG00000116711	PLA2G4A	phospholipase A2 group IVA [Source:HGNC Symbol;Acc:HGNC:9035]	protein coding	0.61	3.50	17.94	<0.0001	0.0002
ENSG00000174501	ANKRD36C	ankyrin repeat domain 36C [Source:HGNC Symbol;Acc:HGNC:32946]	protein coding	-0.61	1.82	27.86	<0.0001	<0.0001
ENSG00000205930	C21orf62-AS1	C21orf62 antisense RNA 1 [Source:HGNC Symbol;Acc:HGNC:1290]	lncRNA	-0.61	0.19	12.28	0.0005	0.0024
ENSG00000160469	BRSK1	BR serine/threonine kinase 1 [Source:HGNC Symbol;Acc:HGNC:18994]	protein coding	0.61	0.95	29.78	<0.0001	<0.0001
ENSG00000181656	GPR88	G protein-coupled receptor 88 [Source:HGNC Symbol;Acc:HGNC:4539]	protein coding	-0.61	0.37	10.26	0.0014	0.0058
ENSG00000076604	TRAF4	TNF receptor associated factor 4 [Source:HGNC Symbol;Acc:HGNC:12034]	protein coding	-0.61	2.73	17.29	<0.0001	0.0003
ENSG00000184574	LPAR5	lysophosphatidic acid receptor 5 [Source:HGNC Symbol;Acc:HGNC:13307]	protein coding	-0.61	0.63	9.27	0.0023	0.009
ENSG00000183734	ASCL2	achaete-scute family bHLH transcription factor 2 [Source:HGNC Symbol;Acc:HGNC:739]	protein coding	0.61	4.93	41.75	<0.0001	<0.0001
ENSG00000146859	TMEM140	transmembrane protein 140 [Source:HGNC Symbol;Acc:HGNC:21870]	protein coding	0.61	4.55	38.30	<0.0001	<0.0001
ENSG00000180096	SEPTIN1	septin 1 [Source:HGNC Symbol;Acc:HGNC:2879]	protein coding	-0.61	3.34	13.21	0.0003	0.0016
ENSG00000143546	S100A8	S100 calcium binding protein A8 [Source:HGNC Symbol;Acc:HGNC:10498]	protein coding	0.61	11.17	38.47	<0.0001	<0.0001
ENSG00000167680	SEMA6B	semaphorin 6B [Source:HGNC Symbol;Acc:HGNC:10739]	protein coding	0.61	2.57	16.82	<0.0001	0.0003
ENSG00000130702	LAMA5	laminin subunit alpha 5 [Source:HGNC Symbol;Acc:HGNC:6485]	protein coding	-0.61	2.18	18.29	<0.0001	0.0002
ENSG00000125730	C3	complement C3 [Source:HGNC Symbol;Acc:HGNC:1318]	protein coding	-0.61	3.16	28.09	<0.0001	<0.0001
ENSG00000108771	DHX58	DExH-box helicase 58 [Source:HGNC Symbol;Acc:HGNC:29517]	protein coding	0.61	5.77	18.52	<0.0001	0.0002

Table S3: Genes differentially expressed at Day 2 compared to pre-first vaccination (Study Arm 2 A/H7N9+AS03, D1, 29, Monocytes). Genes are sorted by descending absolute log_2 fold change. Gene model summaries and annotations are based on Ensembl Version 99 (continued.)

Ensembl Gene ID	Ensembl Gene Name	Ensembl Gene Description	Gene Type	log_2 Fold Change (Day 2 vs. Pre-First Vaccination)	Average log_2 CPM	Likelihood Ratio Test Statistic	P-Value	FDR Adjusted P-Value
ENSG00000067057	PFKP	phosphofructokinase, platelet [Source:HGNC Symbol;Acc:HGNC:8878]	protein coding	0.60	4.96	47.23	<0.0001	<0.0001
ENSG00000271856	LINC01215	long intergenic non-protein coding RNA 1215 [Source:HGNC Symbol;Acc:HGNC:49651]	lncRNA	-0.60	1.60	9.89	0.0017	0.0069
ENSG00000269981		novel pseudogene	processed pseudogene	0.60	3.34	23.96	<0.0001	<0.0001
ENSG00000167106	FAM102A	family with sequence similarity 102 member A [Source:HGNC Symbol;Acc:HGNC:31419]	protein coding	-0.60	3.59	18.79	<0.0001	0.0001
ENSG00000172183	ISG20	interferon stimulated exonuclease gene 20 [Source:HGNC Symbol;Acc:HGNC:6130]	protein coding	0.60	4.75	10.69	0.0011	0.0049
ENSG00000275131		phosphodiesterase 4D interacting protein (PDE4DIP) pseudogene	transcribed unprocessed pseudogene	-0.60	2.80	42.28	<0.0001	<0.0001
ENSG00000112137	PHACTR1	phosphatase and actin regulator 1 [Source:HGNC Symbol;Acc:HGNC:20990]	protein coding	-0.60	3.05	17.84	<0.0001	0.0002
ENSG00000160789	LMNA	lamin A/C [Source:HGNC Symbol;Acc:HGNC:6636]	protein coding	-0.60	4.79	16.83	<0.0001	0.0003
ENSG00000287263		novel transcript	lncRNA	0.60	4.45	28.38	<0.0001	<0.0001
ENSG00000135821	GLUL	glutamate-ammonia ligase [Source:HGNC Symbol;Acc:HGNC:4341]	protein coding	0.60	9.17	31.70	<0.0001	<0.0001
ENSG00000087237	CETP	cholesteryl ester transfer protein [Source:HGNC Symbol;Acc:HGNC:1869]	protein coding	0.60	1.29	19.30	<0.0001	0.0001
ENSG00000145685	LHFPL2	LHFPL tetraspan subfamily member 2 [Source:HGNC Symbol;Acc:HGNC:6588]	protein coding	0.60	4.96	37.52	<0.0001	<0.0001
ENSG00000130653	PNPLA7	patatin like phospholipase domain containing 7 [Source:HGNC Symbol;Acc:HGNC:24768]	protein coding	-0.60	2.82	28.37	<0.0001	<0.0001
ENSG00000137198	GMPR	guanosine monophosphate reductase [Source:HGNC Symbol;Acc:HGNC:4376]	protein coding	0.60	2.17	5.91	0.015	0.041
ENSG00000154134	ROBO3	roundabout guidance receptor 3 [Source:HGNC Symbol;Acc:HGNC:13433]	protein coding	-0.60	2.03	30.78	<0.0001	<0.0001
ENSG00000259772		uncharacterized LOC283710 [Source:NCBI gene;Acc:283710]	lncRNA	-0.60	1.23	21.28	<0.0001	<0.0001

Table S3: Genes differentially expressed at Day 2 compared to pre-first vaccination (Study Arm 2 A/H7N9+AS03, D1, 29, Monocytes). Genes are sorted by descending absolute log_2 fold change. Gene model summaries and annotations are based on Ensembl Version 99 (continued.)

Ensembl Gene ID	Ensembl Gene Name	Ensembl Gene Description	Gene Type	log_2 Fold Change (Day 2 vs. Pre-First Vaccination)	Average log_2 CPM	Likelihood Ratio Test Statistic	P-Value	FDR Adjusted P-Value
ENSG00000170525	PFKFB3	6-phosphofructo-2-kinase/fructose-2,6-biphosphatase 3 [Source:HGNC Symbol;Acc:HGNC:8874]	protein coding	0.60	6.41	15.94	<0.0001	0.0005
ENSG00000156127	BATF	basic leucine zipper ATF-like transcription factor [Source:HGNC Symbol;Acc:HGNC:958]	protein coding	0.60	4.05	55.11	<0.0001	<0.0001
ENSG00000130164	LDLR	low density lipoprotein receptor [Source:HGNC Symbol;Acc:HGNC:6547]	protein coding	0.59	5.08	20.29	<0.0001	<0.0001
ENSG00000261373	VPS9D1-AS1	VPS9D1 antisense RNA 1 [Source:HGNC Symbol;Acc:HGNC:48915]	lncRNA	0.59	2.76	22.52	<0.0001	<0.0001
ENSG00000159339	PADI4	peptidyl arginine deiminase 4 [Source:HGNC Symbol;Acc:HGNC:18368]	protein coding	-0.59	7.25	37.15	<0.0001	<0.0001
ENSG00000127951	FGL2	fibrinogen like 2 [Source:HGNC Symbol;Acc:HGNC:3696]	protein coding	0.59	10.80	46.06	<0.0001	<0.0001
ENSG00000135899	SP110	SP110 nuclear body protein [Source:HGNC Symbol;Acc:HGNC:5401]	protein coding	0.59	6.81	33.92	<0.0001	<0.0001
ENSG00000100473	COCH	cochlin [Source:HGNC Symbol;Acc:HGNC:2180]	protein coding	-0.59	0.65	12.07	0.0005	0.0026
ENSG00000140543	DET1	DET1 partner of COP1 E3 ubiquitin ligase [Source:HGNC Symbol;Acc:HGNC:25477]	protein coding	-0.59	1.03	15.83	<0.0001	0.0005
ENSG00000109501	WFS1	wolframin ER transmembrane glycoprotein [Source:HGNC Symbol;Acc:HGNC:12762]	protein coding	-0.59	0.92	19.81	<0.0001	<0.0001
ENSG00000167984	NLRC3	NLR family CARD domain containing 3 [Source:HGNC Symbol;Acc:HGNC:29889]	protein coding	-0.59	2.17	5.48	0.0192	0.0499
ENSG00000274422		novel transcript	lncRNA	-0.59	1.12	12.25	0.0005	0.0024
ENSG00000123700	KCNJ2	potassium inwardly rectifying channel subfamily J member 2 [Source:HGNC Symbol;Acc:HGNC:6263]	protein coding	0.59	3.07	8.87	0.0029	0.0108
ENSG00000211598	IGKV4-1	immunoglobulin kappa variable 4-1 [Source:HGNC Symbol;Acc:HGNC:5834]	IG V gene	-0.59	2.55	10.22	0.0014	0.006
ENSG00000155254	MARVELD1	MARVEL domain containing 1 [Source:HGNC Symbol;Acc:HGNC:28674]	protein coding	-0.59	6.17	38.01	<0.0001	<0.0001
ENSG00000135424	ITGA7	integrin subunit alpha 7 [Source:HGNC Symbol;Acc:HGNC:6143]	protein coding	0.59	1.63	15.38	<0.0001	0.0006
ENSG00000107282	APBA1	amyloid beta precursor protein binding family A member 1 [Source:HGNC Symbol;Acc:HGNC:578]	protein coding	-0.59	1.42	27.36	<0.0001	<0.0001
ENSG00000176533	GNG7	G protein subunit gamma 7 [Source:HGNC Symbol;Acc:HGNC:4410]	protein coding	-0.59	3.70	39.38	<0.0001	<0.0001

Table S3: Genes differentially expressed at Day 2 compared to pre-first vaccination (Study Arm 2 A/H7N9+AS03, D1, 29, Monocytes). Genes are sorted by descending absolute log_2 fold change. Gene model summaries and annotations are based on Ensembl Version 99 (continued.)

Ensembl Gene ID	Ensembl Gene Name	Ensembl Gene Description	Gene Type	log_2 Fold Change (Day 2 vs. Pre-First Vaccination)	Average log_2 CPM	Likelihood Ratio Test Statistic	P-Value	FDR Adjusted P-Value
ENSG00000248791		pleckstrin homology domain containing, family A (phosphoinositide binding specific) member 1 (PLEKHA1) pseudogene	processed pseudogene	-0.59	0.59	7.34	0.0067	0.0216
ENSG00000197381	ADARB1	adenosine deaminase RNA specific B1 [Source:HGNC Symbol;Acc:HGNC:226]	protein coding	0.59	3.84	19.87	<0.0001	<0.0001
ENSG00000133315	MACROD1	mono-ADP ribosylhydrolase 1 [Source:HGNC Symbol;Acc:HGNC:29598]	protein coding	-0.59	1.09	21.89	<0.0001	<0.0001
ENSG00000111224	PARP11	poly(ADP-ribose) polymerase family member 11 [Source:HGNC Symbol;Acc:HGNC:1186]	protein coding	0.59	3.95	29.06	<0.0001	<0.0001
ENSG00000059588	TARBP1	TAR (HIV-1) RNA binding protein 1 [Source:HGNC Symbol;Acc:HGNC:11568]	protein coding	-0.59	4.92	33.91	<0.0001	<0.0001
ENSG00000135740	SLC9A5	solute carrier family 9 member A5 [Source:HGNC Symbol;Acc:HGNC:11078]	protein coding	-0.59	0.41	19.91	<0.0001	<0.0001
ENSG00000132199	ENOSF1	enolase superfamily member 1 [Source:HGNC Symbol;Acc:HGNC:30365]	protein coding	-0.59	3.21	36.45	<0.0001	<0.0001
ENSG00000175505	CLCF1	cardiotrophin like cytokine factor 1 [Source:HGNC Symbol;Acc:HGNC:17412]	protein coding	-0.59	2.20	14.15	0.0002	0.001

Table S4: Genes differentially expressed at Day 4 compared to pre-first vaccination (Study Arm 2 A/H7N9+AS03, D1, 29, Monocytes). Genes are sorted by descending absolute log_2 fold change. Gene model summaries and annotations are based on Ensembl Version 99.

Ensembl Gene ID	Ensembl Gene Name	Ensembl Gene Description	Gene Type	log_2 Fold Change (Day 4 vs. Pre-First Vaccination)	Average log_2 CPM	Likelihood Ratio Test Statistic	P-Value	FDR Adjusted P-Value
ENSG00000120885	CLU	clusterin [Source:HGNC Symbol;Acc:HGNC:2095]	protein coding	1.51	3.13	16.55	<0.0001	0.0185
ENSG00000088827	SIGLEC1	sialic acid binding Ig like lectin 1 [Source:HGNC Symbol;Acc:HGNC:11127]	protein coding	1.49	7.24	17.60	<0.0001	0.0157
ENSG00000137959	IFI44L	interferon induced protein 44 like [Source:HGNC Symbol;Acc:HGNC:17817]	protein coding	1.26	6.69	12.81	0.0003	0.0477
ENSG00000142089	IFITM3	interferon induced transmembrane protein 3 [Source:HGNC Symbol;Acc:HGNC:5414]	protein coding	1.06	7.33	13.46	0.0002	0.0429
ENSG00000275395	FCGBP	Fc fragment of IgG binding protein [Source:HGNC Symbol;Acc:HGNC:13572]	protein coding	-1.06	2.29	18.02	<0.0001	0.0157

Table S4: Genes differentially expressed at Day 4 compared to pre-first vaccination (Study Arm 2 A/H7N9+AS03, D1, 29, Monocytes). Genes are sorted by descending absolute log_2 fold change. Gene model summaries and annotations are based on Ensembl Version 99 (continued.)

Ensembl Gene ID	Ensembl Gene Name	Ensembl Gene Description	Gene Type	log_2 Fold Change (Day 4 vs. Pre-First Vaccination)	Average log_2 CPM	Likelihood Ratio Test Statistic	P-Value	FDR Adjusted P-Value
ENSG00000173369	C1QB	complement C1q B chain [Source:HGNC Symbol;Acc:HGNC:1242]	protein coding	1.04	2.67	17.59	<0.0001	0.0157
ENSG00000225964	NRIR	negative regulator of interferon response [Source:HGNC Symbol;Acc:HGNC:51269]	lncRNA	1.04	1.53	15.80	<0.0001	0.0211
ENSG00000156113	KCNMA1	potassium calcium-activated channel subfamily M alpha 1 [Source:HGNC Symbol;Acc:HGNC:6284]	protein coding	0.98	1.28	14.71	0.0001	0.0293
ENSG00000108679	LGALS3BP	galectin 3 binding protein [Source:HGNC Symbol;Acc:HGNC:6564]	protein coding	0.95	4.98	18.41	<0.0001	0.0153
ENSG00000160932	LY6E	lymphocyte antigen 6 family member E [Source:HGNC Symbol;Acc:HGNC:6727]	protein coding	0.93	7.84	12.95	0.0003	0.0473
ENSG00000159189	C1QC	complement C1q C chain [Source:HGNC Symbol;Acc:HGNC:1245]	protein coding	0.91	1.90	12.90	0.0003	0.0476
ENSG00000171954	CYP4F22	cytochrome P450 family 4 subfamily F member 22 [Source:HGNC Symbol;Acc:HGNC:26820]	protein coding	0.89	1.83	15.18	<0.0001	0.0245
ENSG00000173372	C1QA	complement C1q A chain [Source:HGNC Symbol;Acc:HGNC:1241]	protein coding	0.86	4.01	22.02	<0.0001	0.0077
ENSG00000188157	AGRN	agrin [Source:HGNC Symbol;Acc:HGNC:329]	protein coding	0.82	4.92	13.76	0.0002	0.0404
ENSG00000169031	COL4A3	collagen type IV alpha 3 chain [Source:HGNC Symbol;Acc:HGNC:2204]	protein coding	-0.80	0.81	12.84	0.0003	0.0476
ENSG00000175544	CABP4	calcium binding protein 4 [Source:HGNC Symbol;Acc:HGNC:1386]	protein coding	0.79	0.88	16.54	<0.0001	0.0185
ENSG00000164330	EBF1	EBF transcription factor 1 [Source:HGNC Symbol;Acc:HGNC:3126]	protein coding	-0.77	0.40	16.34	<0.0001	0.0189
ENSG00000188290	HES4	hes family bHLH transcription factor 4 [Source:HGNC Symbol;Acc:HGNC:24149]	protein coding	0.71	3.47	15.35	<0.0001	0.0234
ENSG00000110079	MS4A4A	membrane spanning 4-domains A4A [Source:HGNC Symbol;Acc:HGNC:13371]	protein coding	0.70	3.93	24.75	<0.0001	0.0077
ENSG00000179593	ALOX15B	arachidonate 15-lipoxygenase type B [Source:HGNC Symbol;Acc:HGNC:434]	protein coding	-0.69	3.11	18.65	<0.0001	0.0145
ENSG00000138166	DUSP5	dual specificity phosphatase 5 [Source:HGNC Symbol;Acc:HGNC:3071]	protein coding	0.68	3.81	16.30	<0.0001	0.0189
ENSG00000129757	CDKN1C	cyclin dependent kinase inhibitor 1C [Source:HGNC Symbol;Acc:HGNC:1786]	protein coding	0.68	5.95	13.20	0.0003	0.0462
ENSG00000166278	C2	complement C2 [Source:HGNC Symbol;Acc:HGNC:1248]	protein coding	0.68	2.65	13.00	0.0003	0.0473

Table S4: Genes differentially expressed at Day 4 compared to pre-first vaccination (Study Arm 2 A/H7N9+AS03, D1, 29, Monocytes). Genes are sorted by descending absolute log_2 fold change. Gene model summaries and annotations are based on Ensembl Version 99 (continued.)

Ensembl Gene ID	Ensembl Gene Name	Ensembl Gene Description	Gene Type	log_2 Fold Change (Day 4 vs. Pre-First Vaccination)	Average log_2 CPM	Likelihood Ratio Test Statistic	P-Value	FDR Adjusted P-Value
ENSG00000135047	CTSL	cathepsin L [Source:HGNC Symbol;Acc:HGNC:2537]	protein coding	0.66	4.67	22.21	<0.0001	0.0077
ENSG00000173706	HEG1	heart development protein with EGF like domains 1 [Source:HGNC Symbol;Acc:HGNC:29227]	protein coding	0.66	4.07	20.12	<0.0001	0.0108
ENSG00000187621	TCL6	T cell leukemia/lymphoma 6 [Source:HGNC Symbol;Acc:HGNC:13463]	lncRNA	-0.66	0.65	13.11	0.0003	0.0462
ENSG00000180155	LYNX1	Ly6/neurotoxin 1 [Source:HGNC Symbol;Acc:HGNC:29604]	protein coding	0.65	3.42	17.86	<0.0001	0.0157
ENSG00000130487	KLHDC7B	kelch domain containing 7B [Source:HGNC Symbol;Acc:HGNC:25145]	protein coding	0.64	3.84	14.26	0.0002	0.0348
ENSG00000166165	CKB	creatine kinase B [Source:HGNC Symbol;Acc:HGNC:1991]	protein coding	0.64	2.42	17.38	<0.0001	0.0164
ENSG00000133216	EPHB2	EPH receptor B2 [Source:HGNC Symbol;Acc:HGNC:3393]	protein coding	0.63	4.35	17.56	<0.0001	0.0157
ENSG00000100985	MMP9	matrix metallopeptidase 9 [Source:HGNC Symbol;Acc:HGNC:7176]	protein coding	-0.62	1.59	13.98	0.0002	0.0379
ENSG00000206190	ATP10A	ATPase phospholipid transporting 10A (putative) [Source:HGNC Symbol;Acc:HGNC:13542]	protein coding	0.61	4.43	13.79	0.0002	0.0404
ENSG00000112182	BACH2	BTB domain and CNC homolog 2 [Source:HGNC Symbol;Acc:HGNC:14078]	protein coding	-0.61	2.40	12.66	0.0004	0.0492
ENSG00000136573	BLK	BLK proto-oncogene, Src family tyrosine kinase [Source:HGNC Symbol;Acc:HGNC:1057]	protein coding	-0.60	4.08	13.65	0.0002	0.0411
ENSG00000124762	CDKN1A	cyclin dependent kinase inhibitor 1A [Source:HGNC Symbol;Acc:HGNC:1784]	protein coding	0.59	5.98	13.12	0.0003	0.0462
ENSG00000196839	ADA	adenosine deaminase [Source:HGNC Symbol;Acc:HGNC:186]	protein coding	0.59	3.94	19.06	<0.0001	0.0136

Table S5: Genes differentially expressed at Day 30 compared to pre-second vaccination (Study Arm 2 A/H7N9+AS03, D1, 29, Monocytes). Genes are sorted by descending absolute log_2 fold change. Gene model summaries and annotations are based on Ensembl Version 99.

Ensembl Gene ID	Ensembl Gene Name	Ensembl Gene Description	Gene Type	log_2 Fold Change (Day 30 vs. Pre-Second Vaccination)	Average log_2 CPM	Likelihood Ratio Test Statistic	P-Value	FDR Adjusted P-Value
ENSG00000152766	ANKRD22	ankyrin repeat domain 22 [Source:HGNC Symbol;Acc:HGNC:28321]	protein coding	4.17	5.88	98.68	<0.0001	<0.0001
ENSG00000225492	GBP1P1	guanylate binding protein 1 pseudogene 1 [Source:HGNC Symbol;Acc:HGNC:39561]	transcribed unprocessed pseudogene	4.12	3.49	91.35	<0.0001	<0.0001
ENSG00000120217	CD274	CD274 molecule [Source:HGNC Symbol;Acc:HGNC:17635]	protein coding	4.07	3.00	75.19	<0.0001	<0.0001
ENSG00000154451	GBP5	guanylate binding protein 5 [Source:HGNC Symbol;Acc:HGNC:19895]	protein coding	3.71	8.75	97.21	<0.0001	<0.0001
ENSG00000100336	APOL4	apolipoprotein L4 [Source:HGNC Symbol;Acc:HGNC:14867]	protein coding	3.57	4.18	97.19	<0.0001	<0.0001
ENSG00000010030	ETV7	ETS variant transcription factor 7 [Source:HGNC Symbol;Acc:HGNC:18160]	protein coding	3.40	4.49	71.60	<0.0001	<0.0001
ENSG00000117228	GBP1	guanylate binding protein 1 [Source:HGNC Symbol;Acc:HGNC:4182]	protein coding	3.24	8.26	84.39	<0.0001	<0.0001
ENSG00000149131	SERPING1	serpin family G member 1 [Source:HGNC Symbol;Acc:HGNC:1228]	protein coding	3.22	6.24	93.05	<0.0001	<0.0001
ENSG00000140511	HAPLN3	hyaluronan and proteoglycan link protein 3 [Source:HGNC Symbol;Acc:HGNC:21446]	protein coding	3.14	4.02	66.62	<0.0001	<0.0001
ENSG00000174944	P2RY14	purinergic receptor P2Y14 [Source:HGNC Symbol;Acc:HGNC:16442]	protein coding	3.12	2.37	60.08	<0.0001	<0.0001
ENSG00000198959	TGM2	transglutaminase 2 [Source:HGNC Symbol;Acc:HGNC:11778]	protein coding	3.05	4.53	82.92	<0.0001	<0.0001
ENSG00000026751	SLAMF7	SLAM family member 7 [Source:HGNC Symbol;Acc:HGNC:21394]	protein coding	3.02	6.48	124.88	<0.0001	<0.0001
ENSG00000169245	CXCL10	C-X-C motif chemokine ligand 10 [Source:HGNC Symbol;Acc:HGNC:10637]	protein coding	2.91	4.99	66.23	<0.0001	<0.0001
ENSG00000120885	CLU	clusterin [Source:HGNC Symbol;Acc:HGNC:2095]	protein coding	2.86	3.96	125.13	<0.0001	<0.0001
ENSG00000108950	FAM20A	FAM20A golgi associated secretory pathway pseudokinase [Source:HGNC Symbol;Acc:HGNC:23015]	protein coding	2.76	2.81	113.93	<0.0001	<0.0001
ENSG00000168062	BATF2	basic leucine zipper ATF-like transcription factor 2 [Source:HGNC Symbol;Acc:HGNC:25163]	protein coding	2.76	4.18	102.60	<0.0001	<0.0001

Table S5: Genes differentially expressed at Day 30 compared to pre-second vaccination (Study Arm 2 A/H7N9+AS03, D1, 29, Monocytes). Genes are sorted by descending absolute log_2 fold change. Gene model summaries and annotations are based on Ensembl Version 99 (continued.)

Ensembl Gene ID	Ensembl Gene Name	Ensembl Gene Description	Gene Type	log_2 Fold Change (Day 30 vs. Pre-Second Vaccination)	Average log_2 CPM	Likelihood Ratio Test Statistic	P-Value	FDR Adjusted P-Value
ENSG00000158714	SLAMF8	SLAM family member 8 [Source:HGNC Symbol;Acc:HGNC:21391]	protein coding	2.73	3.41	148.28	<0.0001	<0.0001
ENSG00000183762	KREMEN1	kringle containing transmembrane protein 1 [Source:HGNC Symbol;Acc:HGNC:17550]	protein coding	2.70	3.65	87.84	<0.0001	<0.0001
ENSG00000122877	EGR2	early growth response 2 [Source:HGNC Symbol;Acc:HGNC:3239]	protein coding	2.64	5.11	63.91	<0.0001	<0.0001
ENSG00000108691	CCL2	C-C motif chemokine ligand 2 [Source:HGNC Symbol;Acc:HGNC:10618]	protein coding	2.64	2.26	30.67	<0.0001	<0.0001
ENSG00000108387	SEPTIN4	septin 4 [Source:HGNC Symbol;Acc:HGNC:9165]	protein coding	2.63	3.41	65.43	<0.0001	<0.0001
ENSG00000265531	FCGR1CP	Fc fragment of IgG receptor Ic, pseudogene [Source:HGNC Symbol;Acc:HGNC:3615]	unprocessed pseudogene	2.61	3.26	79.17	<0.0001	<0.0001
ENSG00000115155	OTOF	otoferlin [Source:HGNC Symbol;Acc:HGNC:8515]	protein coding	2.50	2.66	194.05	<0.0001	<0.0001
ENSG00000185885	IFITM1	interferon induced transmembrane protein 1 [Source:HGNC Symbol;Acc:HGNC:5412]	protein coding	2.45	5.24	107.99	<0.0001	<0.0001
ENSG00000164509	IL31RA	interleukin 31 receptor A [Source:HGNC Symbol;Acc:HGNC:18969]	protein coding	2.43	2.59	108.58	<0.0001	<0.0001
ENSG00000162654	GBP4	guanylate binding protein 4 [Source:HGNC Symbol;Acc:HGNC:20480]	protein coding	2.42	7.44	87.43	<0.0001	<0.0001
ENSG00000179639	FCER1A	Fc fragment of IgE receptor Ia [Source:HGNC Symbol;Acc:HGNC:3609]	protein coding	-2.42	3.59	40.02	<0.0001	<0.0001
ENSG00000188820	CALHM6	calcium homeostasis modulator family member 6 [Source:HGNC Symbol;Acc:HGNC:33391]	protein coding	2.36	6.30	138.84	<0.0001	<0.0001
ENSG00000258521		novel transcript, antisense to WARS	lncRNA	2.35	1.76	48.39	<0.0001	<0.0001
ENSG00000233029		novel transcript	lncRNA	2.33	1.44	44.95	<0.0001	<0.0001
ENSG00000168899	VAMP5	vesicle associated membrane protein 5 [Source:HGNC Symbol;Acc:HGNC:12646]	protein coding	2.32	6.80	119.89	<0.0001	<0.0001
ENSG00000004468	CD38	CD38 molecule [Source:HGNC Symbol;Acc:HGNC:1667]	protein coding	2.28	5.04	82.27	<0.0001	<0.0001
ENSG00000198019	FCGR1B	Fc fragment of IgG receptor Ib [Source:HGNC Symbol;Acc:HGNC:3614]	protein coding	2.25	4.69	91.04	<0.0001	<0.0001
ENSG00000231528	FAM225A	family with sequence similarity 225 member A [Source:HGNC Symbol;Acc:HGNC:27855]	lncRNA	2.25	1.16	129.46	<0.0001	<0.0001

Table S5: Genes differentially expressed at Day 30 compared to pre-second vaccination (Study Arm 2 A/H7N9+AS03, D1, 29, Monocytes). Genes are sorted by descending absolute log_2 fold change. Gene model summaries and annotations are based on Ensembl Version 99 (continued.)

Ensembl Gene ID	Ensembl Gene Name	Ensembl Gene Description	Gene Type	log_2 Fold Change (Day 30 vs. Pre-Second Vaccination)	Average log_2 CPM	Likelihood Ratio Test Statistic	P-Value	FDR Adjusted P-Value
ENSG00000150337	FCGR1A	Fc fragment of IgG receptor Ia [Source:HGNC Symbol;Acc:HGNC:3613]	protein coding	2.24	6.22	112.93	<0.0001	<0.0001
ENSG00000115594	IL1R1	interleukin 1 receptor type 1 [Source:HGNC Symbol;Acc:HGNC:5993]	protein coding	-2.23	2.26	112.66	<0.0001	<0.0001
ENSG00000162772	ATF3	activating transcription factor 3 [Source:HGNC Symbol;Acc:HGNC:785]	protein coding	2.23	4.03	59.94	<0.0001	<0.0001
ENSG00000185022	MAFF	MAF bZIP transcription factor F [Source:HGNC Symbol;Acc:HGNC:6780]	protein coding	2.21	2.85	37.22	<0.0001	<0.0001
ENSG00000179388	EGR3	early growth response 3 [Source:HGNC Symbol;Acc:HGNC:3240]	protein coding	2.19	4.42	31.27	<0.0001	<0.0001
ENSG00000182782	HCAR2	hydroxycarboxylic acid receptor 2 [Source:HGNC Symbol;Acc:HGNC:24827]	protein coding	2.19	2.61	29.97	<0.0001	<0.0001
ENSG00000126262	FFAR2	free fatty acid receptor 2 [Source:HGNC Symbol;Acc:HGNC:4501]	protein coding	2.13	4.61	103.35	<0.0001	<0.0001
ENSG00000166278	C2	complement C2 [Source:HGNC Symbol;Acc:HGNC:1248]	protein coding	2.10	3.60	159.90	<0.0001	<0.0001
ENSG00000140105	WARS1	tryptophanyl-tRNA synthetase 1 [Source:HGNC Symbol;Acc:HGNC:12729]	protein coding	2.07	10.05	81.33	<0.0001	<0.0001
ENSG00000139572	GPR84	G protein-coupled receptor 84 [Source:HGNC Symbol;Acc:HGNC:4535]	protein coding	2.07	2.59	67.23	<0.0001	<0.0001
ENSG00000133321	PLAAT4	phospholipase A and acyltransferase 4 [Source:HGNC Symbol;Acc:HGNC:9869]	protein coding	2.06	5.80	94.50	<0.0001	<0.0001
ENSG00000156113	KCNMA1	potassium calcium-activated channel subfamily M alpha 1 [Source:HGNC Symbol;Acc:HGNC:6284]	protein coding	2.06	2.34	107.31	<0.0001	<0.0001
ENSG00000196220	SRGAP3	SLIT-ROBO Rho GTPase activating protein 3 [Source:HGNC Symbol;Acc:HGNC:19744]	protein coding	-2.01	1.51	70.18	<0.0001	<0.0001
ENSG00000162645	GBP2	guanylate binding protein 2 [Source:HGNC Symbol;Acc:HGNC:4183]	protein coding	1.98	8.57	83.84	<0.0001	<0.0001
ENSG00000181036	FCRL6	Fc receptor like 6 [Source:HGNC Symbol;Acc:HGNC:31910]	protein coding	1.97	1.04	79.04	<0.0001	<0.0001
ENSG00000119922	IFIT2	interferon induced protein with tetratricopeptide repeats 2 [Source:HGNC Symbol;Acc:HGNC:5409]	protein coding	1.97	6.70	52.41	<0.0001	<0.0001
ENSG00000002549	LAP3	leucine aminopeptidase 3 [Source:HGNC Symbol;Acc:HGNC:18449]	protein coding	1.96	8.63	82.13	<0.0001	<0.0001
ENSG00000119917	IFIT3	interferon induced protein with tetratricopeptide repeats 3 [Source:HGNC Symbol;Acc:HGNC:5411]	protein coding	1.96	6.94	71.54	<0.0001	<0.0001

Table S5: Genes differentially expressed at Day 30 compared to pre-second vaccination (Study Arm 2 A/H7N9+AS03, D1, 29, Monocytes). Genes are sorted by descending absolute log_2 fold change. Gene model summaries and annotations are based on Ensembl Version 99 (continued.)

Ensembl Gene ID	Ensembl Gene Name	Ensembl Gene Description	Gene Type	log_2 Fold Change (Day 30 vs. Pre-Second Vaccination)	Average log_2 CPM	Likelihood Ratio Test Statistic	P-Value	FDR Adjusted P-Value
ENSG00000236567	TCF3P1	transcription factor 3 pseudogene 1 [Source:HGNC Symbol;Acc:HGNC:49742]	processed pseudogene	1.94	1.90	36.21	<0.0001	<0.0001
ENSG00000145365	TIFA	TRAF interacting protein with forkhead associated domain [Source:HGNC Symbol;Acc:HGNC:19075]	protein coding	1.94	3.85	65.13	<0.0001	<0.0001
ENSG00000168209	DDIT4	DNA damage inducible transcript 4 [Source:HGNC Symbol;Acc:HGNC:24944]	protein coding	-1.93	3.67	28.67	<0.0001	<0.0001
ENSG00000173369	C1QB	complement C1q B chain [Source:HGNC Symbol;Acc:HGNC:1242]	protein coding	1.92	3.61	90.39	<0.0001	<0.0001
ENSG00000108679	LGALS3BP	galectin 3 binding protein [Source:HGNC Symbol;Acc:HGNC:6564]	protein coding	1.91	5.84	135.70	<0.0001	<0.0001
ENSG00000125148	MT2A	metallothionein 2A [Source:HGNC Symbol;Acc:HGNC:7406]	protein coding	1.91	5.72	138.96	<0.0001	<0.0001
ENSG00000138755	CXCL9	C-X-C motif chemokine ligand 9 [Source:HGNC Symbol;Acc:HGNC:7098]	protein coding	1.90	1.84	57.88	<0.0001	<0.0001
ENSG00000158270	COLEC12	collectin subfamily member 12 [Source:HGNC Symbol;Acc:HGNC:16016]	protein coding	-1.86	1.30	110.52	<0.0001	<0.0001
ENSG00000010278	CD9	CD9 molecule [Source:HGNC Symbol;Acc:HGNC:1709]	protein coding	-1.86	4.02	50.78	<0.0001	<0.0001
ENSG00000197272	IL27	interleukin 27 [Source:HGNC Symbol;Acc:HGNC:19157]	protein coding	1.85	2.79	80.82	<0.0001	<0.0001
ENSG00000125538	IL1B	interleukin 1 beta [Source:HGNC Symbol;Acc:HGNC:5992]	protein coding	1.85	5.40	38.81	<0.0001	<0.0001
ENSG00000174370	C11orf45	chromosome 11 open reading frame 45 [Source:HGNC Symbol;Acc:HGNC:28584]	protein coding	-1.83	0.79	65.25	<0.0001	<0.0001
ENSG00000184221	OLIG1	oligodendrocyte transcription factor 1 [Source:HGNC Symbol;Acc:HGNC:16983]	protein coding	-1.83	5.60	67.95	<0.0001	<0.0001
ENSG00000229754	CXCR2P1	C-X-C motif chemokine receptor 2 pseudogene 1 [Source:HGNC Symbol;Acc:HGNC:6028]	transcribed unprocessed pseudogene	1.83	5.91	179.67	<0.0001	<0.0001
ENSG00000163568	AIM2	absent in melanoma 2 [Source:HGNC Symbol;Acc:HGNC:357]	protein coding	1.82	4.42	113.57	<0.0001	<0.0001
ENSG00000275395	FCGBP	Fc fragment of IgG binding protein [Source:HGNC Symbol;Acc:HGNC:13572]	protein coding	-1.82	1.89	120.90	<0.0001	<0.0001

Table S5: Genes differentially expressed at Day 30 compared to pre-second vaccination (Study Arm 2 A/H7N9+AS03, D1, 29, Monocytes). Genes are sorted by descending absolute log_2 fold change. Gene model summaries and annotations are based on Ensembl Version 99 (continued.)

Ensembl Gene ID	Ensembl Gene Name	Ensembl Gene Description	Gene Type	log_2 Fold Change (Day 30 vs. Pre-Second Vaccination)	Average log_2 CPM	Likelihood Ratio Test Statistic	P-Value	FDR Adjusted P-Value
ENSG00000254017	IGHEP2	immunoglobulin heavy constant epsilon P2 (pseudogene) [Source:HGNC Symbol;Acc:HGNC:5524]	IG C pseudogene	1.80	1.36	25.49	<0.0001	<0.0001
ENSG00000159189	C1QC	complement C1q C chain [Source:HGNC Symbol;Acc:HGNC:1245]	protein coding	1.80	2.83	66.97	<0.0001	<0.0001
ENSG00000268758	ADGRE4P	adhesion G protein-coupled receptor E4, pseudogene [Source:HGNC Symbol;Acc:HGNC:19240]	transcribed unprocessed pseudogene	-1.80	2.86	39.23	<0.0001	<0.0001
ENSG00000142303	ADAMTS10	ADAM metallopeptidase with thrombospondin type 1 motif 10 [Source:HGNC Symbol;Acc:HGNC:13201]	protein coding	-1.80	1.52	63.36	<0.0001	<0.0001
ENSG00000139211	AMIGO2	adhesion molecule with Ig like domain 2 [Source:HGNC Symbol;Acc:HGNC:24073]	protein coding	1.79	1.79	80.02	<0.0001	<0.0001
ENSG00000142089	IFITM3	interferon induced transmembrane protein 3 [Source:HGNC Symbol;Acc:HGNC:5414]	protein coding	1.79	7.96	128.63	<0.0001	<0.0001
ENSG00000136231	IGF2BP3	insulin like growth factor 2 mRNA binding protein 3 [Source:HGNC Symbol;Acc:HGNC:28868]	protein coding	1.78	1.27	52.68	<0.0001	<0.0001
ENSG00000089169	RPH3A	rabphilin 3A [Source:HGNC Symbol;Acc:HGNC:17056]	protein coding	-1.77	4.53	24.56	<0.0001	<0.0001
ENSG00000111181	SLC6A12	solute carrier family 6 member 12 [Source:HGNC Symbol;Acc:HGNC:11045]	protein coding	1.77	4.69	114.27	<0.0001	<0.0001
ENSG00000255398	HCAR3	hydroxycarboxylic acid receptor 3 [Source:HGNC Symbol;Acc:HGNC:16824]	protein coding	1.77	1.97	17.21	<0.0001	0.0002
ENSG00000100342	APOL1	apolipoprotein L1 [Source:HGNC Symbol;Acc:HGNC:618]	protein coding	1.76	6.01	78.13	<0.0001	<0.0001
ENSG00000145287	PLAC8	placenta associated 8 [Source:HGNC Symbol;Acc:HGNC:19254]	protein coding	1.75	7.08	89.87	<0.0001	<0.0001
ENSG00000115415	STAT1	signal transducer and activator of transcription 1 [Source:HGNC Symbol;Acc:HGNC:11362]	protein coding	1.74	9.30	62.65	<0.0001	<0.0001
ENSG00000258581		NADH dehydrogenase (ubiquinone) 1 beta subcomplex, 3, 12kDa (NDUFB3) pseudogene	processed pseudogene	1.74	1.14	44.49	<0.0001	<0.0001
ENSG00000154188	ANGPT1	angiopoietin 1 [Source:HGNC Symbol;Acc:HGNC:484]	protein coding	-1.73	1.46	56.13	<0.0001	<0.0001
ENSG00000020577	SAMD4A	sterile alpha motif domain containing 4A [Source:HGNC Symbol;Acc:HGNC:23023]	protein coding	1.73	5.16	75.94	<0.0001	<0.0001

Confidential/Proprietary Information

Table S5: Genes differentially expressed at Day 30 compared to pre-second vaccination (Study Arm 2 A/H7N9+AS03, D1, 29, Monocytes). Genes are sorted by descending absolute log_2 fold change. Gene model summaries and annotations are based on Ensembl Version 99 (continued.)

Ensembl Gene ID	Ensembl Gene Name	Ensembl Gene Description	Gene Type	log_2 Fold Change (Day 30 vs. Pre-Second Vaccination)	Average log_2 CPM	Likelihood Ratio Test Statistic	P-Value	FDR Adjusted P-Value
ENSG00000273314		novel transcript, antisense to CUL1	lncRNA	1.72	1.44	65.81	<0.0001	<0.0001
ENSG00000156265	MAP3K7CL	MAP3K7 C-terminal like [Source:HGNC Symbol;Acc:HGNC:16457]	protein coding	1.72	5.26	93.35	<0.0001	<0.0001
ENSG00000138119	MYOF	myoferlin [Source:HGNC Symbol;Acc:HGNC:3656]	protein coding	1.72	7.54	71.49	<0.0001	<0.0001
ENSG00000137801	THBS1	thrombospondin 1 [Source:HGNC Symbol;Acc:HGNC:11785]	protein coding	-1.72	5.91	23.78	<0.0001	<0.0001
ENSG00000073756	PTGS2	prostaglandin-endoperoxide synthase 2 [Source:HGNC Symbol;Acc:HGNC:9605]	protein coding	1.72	5.14	43.18	<0.0001	<0.0001
ENSG00000087589	CASS4	Cas scaffold protein family member 4 [Source:HGNC Symbol;Acc:HGNC:15878]	protein coding	-1.72	3.01	50.47	<0.0001	<0.0001
ENSG00000131979	GCH1	GTP cyclohydrolase 1 [Source:HGNC Symbol;Acc:HGNC:4193]	protein coding	1.69	7.32	93.18	<0.0001	<0.0001
ENSG00000164292	RHOBTB3	Rho related BTB domain containing 3 [Source:HGNC Symbol;Acc:HGNC:18757]	protein coding	1.67	1.06	74.15	<0.0001	<0.0001
ENSG00000154258	ABCA9	ATP binding cassette subfamily A member 9 [Source:HGNC Symbol;Acc:HGNC:39]	protein coding	-1.66	0.15	47.47	<0.0001	<0.0001
ENSG00000172159	FRMD3	FERM domain containing 3 [Source:HGNC Symbol;Acc:HGNC:24125]	protein coding	1.66	4.00	112.02	<0.0001	<0.0001
ENSG00000162512	SDC3	syndecan 3 [Source:HGNC Symbol;Acc:HGNC:10660]	protein coding	1.64	4.81	147.71	<0.0001	<0.0001
ENSG00000133106	EPSTI1	epithelial stromal interaction 1 [Source:HGNC Symbol;Acc:HGNC:16465]	protein coding	1.64	6.92	85.07	<0.0001	<0.0001
ENSG00000174080	CTSF	cathepsin F [Source:HGNC Symbol;Acc:HGNC:2531]	protein coding	-1.63	1.65	41.77	<0.0001	<0.0001
ENSG00000221963	APOL6	apolipoprotein L6 [Source:HGNC Symbol;Acc:HGNC:14870]	protein coding	1.63	7.47	75.45	<0.0001	<0.0001
ENSG00000166033	HTRA1	HtrA serine peptidase 1 [Source:HGNC Symbol;Acc:HGNC:9476]	protein coding	-1.62	2.12	72.07	<0.0001	<0.0001
ENSG00000101017	CD40	CD40 molecule [Source:HGNC Symbol;Acc:HGNC:11919]	protein coding	1.62	5.26	143.68	<0.0001	<0.0001
ENSG00000099834	CDHR5	cadherin related family member 5 [Source:HGNC Symbol;Acc:HGNC:7521]	protein coding	1.61	1.74	60.25	<0.0001	<0.0001
ENSG00000196549	MME	membrane metalloendopeptidase [Source:HGNC Symbol;Acc:HGNC:7154]	protein coding	-1.60	0.38	34.36	<0.0001	<0.0001

Table S5: Genes differentially expressed at Day 30 compared to pre-second vaccination (Study Arm 2 A/H7N9+AS03, D1, 29, Monocytes). Genes are sorted by descending absolute log_2 fold change. Gene model summaries and annotations are based on Ensembl Version 99 (continued.)

Ensembl Gene ID	Ensembl Gene Name	Ensembl Gene Description	Gene Type	log_2 Fold Change (Day 30 vs. Pre-Second Vaccination)	Average log_2 CPM	Likelihood Ratio Test Statistic	P-Value	FDR Adjusted P-Value
ENSG00000136960	ENPP2	ectonucleotide pyrophosphatase/phosphodiesterase 2 [Source:HGNC Symbol;Acc:HGNC:3357]	protein coding	1.60	0.97	55.14	<0.0001	<0.0001
ENSG00000198785	GRIN3A	glutamate ionotropic receptor NMDA type subunit 3A [Source:HGNC Symbol;Acc:HGNC:16767]	protein coding	1.60	2.08	86.31	<0.0001	<0.0001
ENSG00000168394	TAP1	transporter 1, ATP binding cassette subfamily B member [Source:HGNC Symbol;Acc:HGNC:43]	protein coding	1.60	8.47	91.33	<0.0001	<0.0001
ENSG00000238000		proteasome (prosome, macropain) activator subunit 2 (PA28 beta) (PSME2) pseudogene	processed pseudogene	1.59	1.11	54.03	<0.0001	<0.0001
ENSG00000137959	IFI44L	interferon induced protein 44 like [Source:HGNC Symbol;Acc:HGNC:17817]	protein coding	1.58	7.18	87.97	<0.0001	<0.0001
ENSG00000234518	PTGES3P1	prostaglandin E synthase 3 pseudogene 1 [Source:HGNC Symbol;Acc:HGNC:43824]	processed pseudogene	1.57	3.12	84.05	<0.0001	<0.0001
ENSG00000154102	C16orf74	chromosome 16 open reading frame 74 [Source:HGNC Symbol;Acc:HGNC:23362]	protein coding	-1.57	2.97	68.14	<0.0001	<0.0001
ENSG00000214872	SMTNL1	smoothelin like 1 [Source:HGNC Symbol;Acc:HGNC:32394]	protein coding	1.57	1.99	53.77	<0.0001	<0.0001
ENSG00000144218	AFF3	AF4/FMR2 family member 3 [Source:HGNC Symbol;Acc:HGNC:6473]	protein coding	-1.56	3.40	116.82	<0.0001	<0.0001
ENSG00000225131	PSME2P2	proteasome activator subunit 2 pseudogene 2 [Source:HGNC Symbol;Acc:HGNC:30160]	processed pseudogene	1.53	1.39	59.33	<0.0001	<0.0001
ENSG00000162614	NEXN	nexilin F-actin binding protein [Source:HGNC Symbol;Acc:HGNC:29557]	protein coding	1.53	3.91	60.79	<0.0001	<0.0001
ENSG00000128284	APOL3	apolipoprotein L3 [Source:HGNC Symbol;Acc:HGNC:14868]	protein coding	1.52	7.85	82.36	<0.0001	<0.0001
ENSG00000183087	GAS6	growth arrest specific 6 [Source:HGNC Symbol;Acc:HGNC:4168]	protein coding	1.51	4.80	72.41	<0.0001	<0.0001
ENSG00000171631	P2RY6	pyrimidinergic receptor P2Y6 [Source:HGNC Symbol;Acc:HGNC:8543]	protein coding	1.51	2.05	65.08	<0.0001	<0.0001
ENSG00000158156	XKR8	XK related 8 [Source:HGNC Symbol;Acc:HGNC:25508]	protein coding	1.51	5.94	85.25	<0.0001	<0.0001
ENSG00000177409	SAMD9L	sterile alpha motif domain containing 9 like [Source:HGNC Symbol;Acc:HGNC:1349]	protein coding	1.51	7.39	60.18	<0.0001	<0.0001

Table S5: Genes differentially expressed at Day 30 compared to pre-second vaccination (Study Arm 2 A/H7N9+AS03, D1, 29, Monocytes). Genes are sorted by descending absolute log_2 fold change. Gene model summaries and annotations are based on Ensembl Version 99 (continued.)

Ensembl Gene ID	Ensembl Gene Name	Ensembl Gene Description	Gene Type	log_2 Fold Change (Day 30 vs. Pre-Second Vaccination)	Average log_2 CPM	Likelihood Ratio Test Statistic	P-Value	FDR Adjusted P-Value
ENSG00000100911	PSME2	proteasome activator subunit 2 [Source:HGNC Symbol;Acc:HGNC:9569]	protein coding	1.51	8.15	89.03	<0.0001	<0.0001
ENSG00000137193	PIM1	Pim-1 proto-oncogene, serine/threonine kinase [Source:HGNC Symbol;Acc:HGNC:8986]	protein coding	1.50	6.74	79.10	<0.0001	<0.0001
ENSG00000138821	SLC39A8	solute carrier family 39 member 8 [Source:HGNC Symbol;Acc:HGNC:20862]	protein coding	1.50	3.65	53.40	<0.0001	<0.0001
ENSG00000091972	CD200	CD200 molecule [Source:HGNC Symbol;Acc:HGNC:7203]	protein coding	-1.50	0.57	38.83	<0.0001	<0.0001
ENSG00000134321	RSAD2	radical S-adenosyl methionine domain containing 2 [Source:HGNC Symbol;Acc:HGNC:30908]	protein coding	1.49	5.31	54.99	<0.0001	<0.0001
ENSG00000152672	CLEC4F	C-type lectin domain family 4 member F [Source:HGNC Symbol;Acc:HGNC:25357]	protein coding	-1.49	3.17	45.78	<0.0001	<0.0001
ENSG00000182580	EPHB3	EPH receptor B3 [Source:HGNC Symbol;Acc:HGNC:3394]	protein coding	-1.48	2.45	55.41	<0.0001	<0.0001
ENSG00000123610	TNFAIP6	TNF alpha induced protein 6 [Source:HGNC Symbol;Acc:HGNC:11898]	protein coding	1.47	1.74	58.97	<0.0001	<0.0001
ENSG00000254709	IGLL5	immunoglobulin lambda like polypeptide 5 [Source:HGNC Symbol;Acc:HGNC:38476]	protein coding	-1.47	0.64	59.11	<0.0001	<0.0001
ENSG00000157168	NRG1	neuregulin 1 [Source:HGNC Symbol;Acc:HGNC:7997]	protein coding	-1.46	4.54	33.46	<0.0001	<0.0001
ENSG00000160789	LMNA	lamin A/C [Source:HGNC Symbol;Acc:HGNC:6636]	protein coding	-1.46	4.69	54.69	<0.0001	<0.0001
ENSG00000171049	FPR2	formyl peptide receptor 2 [Source:HGNC Symbol;Acc:HGNC:3827]	protein coding	1.45	6.33	71.74	<0.0001	<0.0001
ENSG00000135636	DYSF	dysferlin [Source:HGNC Symbol;Acc:HGNC:3097]	protein coding	1.45	8.21	91.37	<0.0001	<0.0001
ENSG00000163814	CDCP1	CUB domain containing protein 1 [Source:HGNC Symbol;Acc:HGNC:24357]	protein coding	1.45	0.96	80.84	<0.0001	<0.0001
ENSG00000029153	ARNTL2	aryl hydrocarbon receptor nuclear translocator like 2 [Source:HGNC Symbol;Acc:HGNC:18984]	protein coding	1.44	2.09	37.38	<0.0001	<0.0001
ENSG00000160111	CPAMD8	C3 and PZP like alpha-2-macroglobulin domain containing 8 [Source:HGNC Symbol;Acc:HGNC:23228]	protein coding	-1.44	5.21	110.19	<0.0001	<0.0001
ENSG00000165905	LARGE2	LARGE xylosyl- and glucuronyltransferase 2 [Source:HGNC Symbol;Acc:HGNC:16522]	protein coding	-1.44	1.58	44.23	<0.0001	<0.0001
ENSG00000179044	EXOC3L1	exocyst complex component 3 like 1 [Source:HGNC Symbol;Acc:HGNC:27540]	protein coding	1.44	2.89	88.42	<0.0001	<0.0001

Table S5: Genes differentially expressed at Day 30 compared to pre-second vaccination (Study Arm 2 A/H7N9+AS03, D1, 29, Monocytes). Genes are sorted by descending absolute log_2 fold change. Gene model summaries and annotations are based on Ensembl Version 99 (continued.)

Ensembl Gene ID	Ensembl Gene Name	Ensembl Gene Description	Gene Type	log_2 Fold Change (Day 30 vs. Pre-Second Vaccination)	Average log_2 CPM	Likelihood Ratio Test Statistic	P-Value	FDR Adjusted P-Value
ENSG00000211955	IGHV3-33	immunoglobulin heavy variable 3-33 [Source:HGNC Symbol;Acc:HGNC:5596]	IG V gene	-1.43	0.42	40.80	<0.0001	<0.0001
ENSG00000109099	PMP22	peripheral myelin protein 22 [Source:HGNC Symbol;Acc:HGNC:9118]	protein coding	-1.43	0.68	32.84	<0.0001	<0.0001
ENSG00000187608	ISG15	ISG15 ubiquitin like modifier [Source:HGNC Symbol;Acc:HGNC:4053]	protein coding	1.43	6.50	73.75	<0.0001	<0.0001
ENSG00000116663	FBXO6	F-box protein 6 [Source:HGNC Symbol;Acc:HGNC:13585]	protein coding	1.42	5.45	109.92	<0.0001	<0.0001
ENSG00000182541	LIMK2	LIM domain kinase 2 [Source:HGNC Symbol;Acc:HGNC:6614]	protein coding	1.42	5.83	84.08	<0.0001	<0.0001
ENSG00000135114	OASL	2'-5'-oligoadenylate synthetase like [Source:HGNC Symbol;Acc:HGNC:8090]	protein coding	1.41	5.04	84.95	<0.0001	<0.0001
ENSG00000154856	APCDD1	APC down-regulated 1 [Source:HGNC Symbol;Acc:HGNC:15718]	protein coding	-1.40	2.73	79.73	<0.0001	<0.0001
ENSG00000055130	CUL1	cullin 1 [Source:HGNC Symbol;Acc:HGNC:2551]	protein coding	1.40	6.87	77.77	<0.0001	<0.0001
ENSG00000116824	CD2	CD2 molecule [Source:HGNC Symbol;Acc:HGNC:1639]	protein coding	-1.40	1.23	26.02	<0.0001	<0.0001
ENSG00000136573	BLK	BLK proto-oncogene, Src family tyrosine kinase [Source:HGNC Symbol;Acc:HGNC:1057]	protein coding	-1.40	3.83	71.20	<0.0001	<0.0001
ENSG00000184557	SOCS3	suppressor of cytokine signaling 3 [Source:HGNC Symbol;Acc:HGNC:19391]	protein coding	1.40	6.37	104.59	<0.0001	<0.0001
ENSG00000121858	TNFSF10	TNF superfamily member 10 [Source:HGNC Symbol;Acc:HGNC:11925]	protein coding	1.39	8.43	66.17	<0.0001	<0.0001
ENSG00000160856	FCRL3	Fc receptor like 3 [Source:HGNC Symbol;Acc:HGNC:18506]	protein coding	-1.39	2.78	38.42	<0.0001	<0.0001
ENSG00000138496	PARP9	poly(ADP-ribose) polymerase family member 9 [Source:HGNC Symbol;Acc:HGNC:24118]	protein coding	1.39	7.55	65.68	<0.0001	<0.0001
ENSG00000156738	MS4A1	membrane spanning 4-domains A1 [Source:HGNC Symbol;Acc:HGNC:7315]	protein coding	-1.39	5.39	41.56	<0.0001	<0.0001
ENSG00000156587	UBE2L6	ubiquitin conjugating enzyme E2 L6 [Source:HGNC Symbol;Acc:HGNC:12490]	protein coding	1.39	7.85	86.58	<0.0001	<0.0001
ENSG00000125347	IRF1	interferon regulatory factor 1 [Source:HGNC Symbol;Acc:HGNC:6116]	protein coding	1.39	8.65	65.60	<0.0001	<0.0001
ENSG00000256262	USP30-AS1	USP30 antisense RNA 1 [Source:HGNC Symbol;Acc:HGNC:40909]	lncRNA	1.39	1.36	54.40	<0.0001	<0.0001

Table S5: Genes differentially expressed at Day 30 compared to pre-second vaccination (Study Arm 2 A/H7N9+AS03, D1, 29, Monocytes). Genes are sorted by descending absolute log_2 fold change. Gene model summaries and annotations are based on Ensembl Version 99 (continued.)

Ensembl Gene ID	Ensembl Gene Name	Ensembl Gene Description	Gene Type	log_2 Fold Change (Day 30 vs. Pre-Second Vaccination)	Average log_2 CPM	Likelihood Ratio Test Statistic	P-Value	FDR Adjusted P-Value
ENSG00000164116	GUCY1A1	guanylate cyclase 1 soluble subunit alpha 1 [Source:HGNC Symbol;Acc:HGNC:4685]	protein coding	1.39	1.28	27.84	<0.0001	<0.0001
ENSG00000169031	COL4A3	collagen type IV alpha 3 chain [Source:HGNC Symbol;Acc:HGNC:2204]	protein coding	-1.38	0.63	44.89	<0.0001	<0.0001
ENSG00000128438	TBC1D27P	TBC1 domain family member 27, pseudogene [Source:HGNC Symbol;Acc:HGNC:28104]	transcribed unprocessed pseudogene	-1.38	1.33	55.06	<0.0001	<0.0001
ENSG00000247982	LINC00926	long intergenic non-protein coding RNA 926 [Source:HGNC Symbol;Acc:HGNC:27514]	lncRNA	-1.37	4.99	57.17	<0.0001	<0.0001
ENSG00000236783	RPS15AP27	ribosomal protein S15a pseudogene 27 [Source:HGNC Symbol;Acc:HGNC:37011]	processed pseudogene	-1.37	0.47	45.03	<0.0001	<0.0001
ENSG00000211898	IGHD	immunoglobulin heavy constant delta [Source:HGNC Symbol;Acc:HGNC:5480]	IG C gene	-1.37	4.86	67.00	<0.0001	<0.0001
ENSG00000082293	COL19A1	collagen type XIX alpha 1 chain [Source:HGNC Symbol;Acc:HGNC:2196]	protein coding	-1.36	1.07	49.39	<0.0001	<0.0001
ENSG00000188282	RUFY4	RUN and FYVE domain containing 4 [Source:HGNC Symbol;Acc:HGNC:24804]	protein coding	1.36	4.54	105.14	<0.0001	<0.0001
ENSG00000240505	TNFRSF13B	TNF receptor superfamily member 13B [Source:HGNC Symbol;Acc:HGNC:18153]	protein coding	-1.36	1.26	56.59	<0.0001	<0.0001
ENSG00000197121	PGAP1	post-GPI attachment to proteins inositol deacylase 1 [Source:HGNC Symbol;Acc:HGNC:25712]	protein coding	1.35	2.14	34.50	<0.0001	<0.0001
ENSG00000243466	IGKV1-5	immunoglobulin kappa variable 1-5 [Source:HGNC Symbol;Acc:HGNC:5741]	IG V gene	-1.35	1.68	27.62	<0.0001	<0.0001
ENSG00000269954		novel transcript, antisense to BLK	lncRNA	-1.35	1.55	47.47	<0.0001	<0.0001
ENSG00000134326	CMPK2	cytidine/uridine monophosphate kinase 2 [Source:HGNC Symbol;Acc:HGNC:27015]	protein coding	1.34	5.95	79.48	<0.0001	<0.0001
ENSG00000100721	TCL1A	T cell leukemia/lymphoma 1A [Source:HGNC Symbol;Acc:HGNC:11648]	protein coding	-1.34	3.45	33.62	<0.0001	<0.0001
ENSG00000133739	LRRCC1	leucine rich repeat and coiled-coil centrosomal protein 1 [Source:HGNC Symbol;Acc:HGNC:29373]	protein coding	1.34	2.38	59.33	<0.0001	<0.0001
ENSG00000162804	SNED1	sushi, nidogen and EGF like domains 1 [Source:HGNC Symbol;Acc:HGNC:24696]	protein coding	-1.34	3.68	80.76	<0.0001	<0.0001

Confidential/Proprietary Information

Table S5: Genes differentially expressed at Day 30 compared to pre-second vaccination (Study Arm 2 A/H7N9+AS03, D1, 29, Monocytes). Genes are sorted by descending absolute log_2 fold change. Gene model summaries and annotations are based on Ensembl Version 99 (continued.)

Ensembl Gene ID	Ensembl Gene Name	Ensembl Gene Description	Gene Type	log_2 Fold Change (Day 30 vs. Pre-Second Vaccination)	Average log_2 CPM	Likelihood Ratio Test Statistic	P-Value	FDR Adjusted P-Value
ENSG00000151693	ASAP2	ArfGAP with SH3 domain, ankyrin repeat and PH domain 2 [Source:HGNC Symbol;Acc:HGNC:2721]	protein coding	1.34	2.31	49.97	<0.0001	<0.0001
ENSG00000117226	GBP3	guanylate binding protein 3 [Source:HGNC Symbol;Acc:HGNC:4184]	protein coding	1.34	5.42	66.46	<0.0001	<0.0001
ENSG00000135318	NT5E	5'-nucleotidase ecto [Source:HGNC Symbol;Acc:HGNC:8021]	protein coding	-1.32	1.09	53.37	<0.0001	<0.0001
ENSG00000205846	CLEC6A	C-type lectin domain containing 6A [Source:HGNC Symbol;Acc:HGNC:14556]	protein coding	1.32	2.95	82.64	<0.0001	<0.0001
ENSG00000151623	NR3C2	nuclear receptor subfamily 3 group C member 2 [Source:HGNC Symbol;Acc:HGNC:7979]	protein coding	-1.32	0.41	48.96	<0.0001	<0.0001
ENSG00000100100	PIK3IP1	phosphoinositide-3-kinase interacting protein 1 [Source:HGNC Symbol;Acc:HGNC:24942]	protein coding	-1.32	5.55	78.62	<0.0001	<0.0001
ENSG00000007516	BAIAP3	BAI1 associated protein 3 [Source:HGNC Symbol;Acc:HGNC:948]	protein coding	-1.32	1.98	56.97	<0.0001	<0.0001
ENSG00000170525	PFKFB3	6-phosphofructo-2-kinase/fructose-2,6-biphosphatase 3 [Source:HGNC Symbol;Acc:HGNC:8874]	protein coding	1.32	6.85	148.99	<0.0001	<0.0001
ENSG00000120738	EGR1	early growth response 1 [Source:HGNC Symbol;Acc:HGNC:3238]	protein coding	1.32	8.37	7.14	0.0075	0.0174
ENSG00000169116	PARM1	prostate androgen-regulated mucin-like protein 1 [Source:HGNC Symbol;Acc:HGNC:24536]	protein coding	-1.32	0.83	60.43	<0.0001	<0.0001
ENSG00000184226	PCDH9	protocadherin 9 [Source:HGNC Symbol;Acc:HGNC:8661]	protein coding	-1.31	1.26	33.00	<0.0001	<0.0001
ENSG00000173193	PARP14	poly(ADP-ribose) polymerase family member 14 [Source:HGNC Symbol;Acc:HGNC:29232]	protein coding	1.31	8.36	57.46	<0.0001	<0.0001
ENSG00000144645	OSBPL10	oxysterol binding protein like 10 [Source:HGNC Symbol;Acc:HGNC:16395]	protein coding	-1.31	1.94	59.73	<0.0001	<0.0001
ENSG00000106976	DNM1	dynamin 1 [Source:HGNC Symbol;Acc:HGNC:2972]	protein coding	-1.31	1.97	44.32	<0.0001	<0.0001
ENSG00000135144	DTX1	deltex E3 ubiquitin ligase 1 [Source:HGNC Symbol;Acc:HGNC:3060]	protein coding	-1.31	0.73	37.21	<0.0001	<0.0001
ENSG00000131355	ADGRE3	adhesion G protein-coupled receptor E3 [Source:HGNC Symbol;Acc:HGNC:23647]	protein coding	-1.31	4.43	35.21	<0.0001	<0.0001
ENSG00000175262	C1orf127	chromosome 1 open reading frame 127 [Source:HGNC Symbol;Acc:HGNC:26730]	protein coding	-1.31	1.71	66.96	<0.0001	<0.0001
ENSG00000211945	IGHV1-18	immunoglobulin heavy variable 1-18 [Source:HGNC Symbol;Acc:HGNC:5549]	IG V gene	-1.30	0.28	28.65	<0.0001	<0.0001

Table S5: Genes differentially expressed at Day 30 compared to pre-second vaccination (Study Arm 2 A/H7N9+AS03, D1, 29, Monocytes). Genes are sorted by descending absolute log_2 fold change. Gene model summaries and annotations are based on Ensembl Version 99 (continued.)

Ensembl Gene ID	Ensembl Gene Name	Ensembl Gene Description	Gene Type	log_2 Fold Change (Day 30 vs. Pre-Second Vaccination)	Average log_2 CPM	Likelihood Ratio Test Statistic	P-Value	FDR Adjusted P-Value
ENSG00000152229	PSTPIP2	proline-serine-threonine phosphatase interacting protein 2 [Source:HGNC Symbol;Acc:HGNC:9581]	protein coding	1.30	6.86	79.30	<0.0001	<0.0001
ENSG00000137628	DDX60	DExD/H-box helicase 60 [Source:HGNC Symbol;Acc:HGNC:25942]	protein coding	1.30	5.59	74.42	<0.0001	<0.0001
ENSG00000068079	IFI35	interferon induced protein 35 [Source:HGNC Symbol;Acc:HGNC:5399]	protein coding	1.30	6.66	91.16	<0.0001	<0.0001
ENSG00000124256	ZBP1	Z-DNA binding protein 1 [Source:HGNC Symbol;Acc:HGNC:16176]	protein coding	1.30	4.83	71.98	<0.0001	<0.0001
ENSG00000213366	GSTM2	glutathione S-transferase mu 2 [Source:HGNC Symbol;Acc:HGNC:4634]	protein coding	-1.30	2.20	44.96	<0.0001	<0.0001
ENSG00000211897	IGHG3	immunoglobulin heavy constant gamma 3 (G3m marker) [Source:HGNC Symbol;Acc:HGNC:5527]	IG C gene	-1.30	3.25	19.52	<0.0001	<0.0001
ENSG00000138646	HERC5	HECT and RLD domain containing E3 ubiquitin protein ligase 5 [Source:HGNC Symbol;Acc:HGNC:24368]	protein coding	1.30	5.15	45.95	<0.0001	<0.0001
ENSG00000154736	ADAMTS5	ADAM metallopeptidase with thrombospondin type 1 motif 5 [Source:HGNC Symbol;Acc:HGNC:221]	protein coding	-1.29	2.59	74.46	<0.0001	<0.0001
ENSG00000134470	IL15RA	interleukin 15 receptor subunit alpha [Source:HGNC Symbol;Acc:HGNC:5978]	protein coding	1.29	4.64	111.00	<0.0001	<0.0001
ENSG00000185507	IRF7	interferon regulatory factor 7 [Source:HGNC Symbol;Acc:HGNC:6122]	protein coding	1.29	7.67	91.49	<0.0001	<0.0001
ENSG00000187912	CLEC17A	C-type lectin domain containing 17A [Source:HGNC Symbol;Acc:HGNC:34520]	protein coding	-1.29	1.80	64.67	<0.0001	<0.0001
ENSG00000123240	OPTN	optineurin [Source:HGNC Symbol;Acc:HGNC:17142]	protein coding	1.29	2.82	40.93	<0.0001	<0.0001
ENSG00000206567		novel transcript	lncRNA	-1.29	0.33	58.58	<0.0001	<0.0001
ENSG00000128383	APOBEC3A	apolipoprotein B mRNA editing enzyme catalytic subunit 3A [Source:HGNC Symbol;Acc:HGNC:17343]	protein coding	1.29	7.96	126.85	<0.0001	<0.0001
ENSG00000236901	MIR600HG	MIR600 host gene [Source:HGNC Symbol;Acc:HGNC:23642]	lncRNA	-1.28	1.59	48.16	<0.0001	<0.0001
ENSG00000211668	IGLV2-11	immunoglobulin lambda variable 2-11 [Source:HGNC Symbol;Acc:HGNC:5887]	IG V gene	-1.28	1.02	13.31	0.0003	0.0009
ENSG00000168546	GFRA2	GDNF family receptor alpha 2 [Source:HGNC Symbol;Acc:HGNC:4244]	protein coding	-1.28	2.67	52.38	<0.0001	<0.0001
ENSG00000113368	LMNB1	lamin B1 [Source:HGNC Symbol;Acc:HGNC:6637]	protein coding	1.28	6.96	64.24	<0.0001	<0.0001

Confidential/Proprietary Information

Table S5: Genes differentially expressed at Day 30 compared to pre-second vaccination (Study Arm 2 A/H7N9+AS03, D1, 29, Monocytes). Genes are sorted by descending absolute log_2 fold change. Gene model summaries and annotations are based on Ensembl Version 99 (continued.)

Ensembl Gene ID	Ensembl Gene Name	Ensembl Gene Description	Gene Type	log_2 Fold Change (Day 30 vs. Pre-Second Vaccination)	Average log_2 CPM	Likelihood Ratio Test Statistic	P-Value	FDR Adjusted P-Value
ENSG00000090975	PITPNM2	phosphatidylinositol transfer protein membrane associated 2 [Source:HGNC Symbol;Acc:HGNC:21044]	protein coding	-1.28	2.23	82.61	<0.0001	<0.0001
ENSG00000235621	LINC00494	long intergenic non-protein coding RNA 494 [Source:HGNC Symbol;Acc:HGNC:27657]	lncRNA	-1.27	0.48	30.00	<0.0001	<0.0001
ENSG00000111912	NCOA7	nuclear receptor coactivator 7 [Source:HGNC Symbol;Acc:HGNC:21081]	protein coding	1.27	5.44	71.28	<0.0001	<0.0001
ENSG00000168016	TRANK1	tetratricopeptide repeat and ankyrin repeat containing 1 [Source:HGNC Symbol;Acc:HGNC:29011]	protein coding	1.27	5.96	67.80	<0.0001	<0.0001
ENSG00000185339	TCN2	transcobalamin 2 [Source:HGNC Symbol;Acc:HGNC:11653]	protein coding	1.27	5.67	187.18	<0.0001	<0.0001
ENSG00000172232	AZU1	azurocidin 1 [Source:HGNC Symbol;Acc:HGNC:913]	protein coding	-1.27	1.95	32.05	<0.0001	<0.0001
ENSG00000130487	KLHDC7B	kelch domain containing 7B [Source:HGNC Symbol;Acc:HGNC:25145]	protein coding	1.26	4.33	177.21	<0.0001	<0.0001
ENSG00000162894	FCMR	Fc fragment of IgM receptor [Source:HGNC Symbol;Acc:HGNC:14315]	protein coding	-1.26	4.41	46.11	<0.0001	<0.0001
ENSG00000185880	TRIM69	tripartite motif containing 69 [Source:HGNC Symbol;Acc:HGNC:17857]	protein coding	1.26	5.59	127.53	<0.0001	<0.0001
ENSG00000035664	DAPK2	death associated protein kinase 2 [Source:HGNC Symbol;Acc:HGNC:2675]	protein coding	-1.26	0.86	26.95	<0.0001	<0.0001
ENSG00000099204	ABLIM1	actin binding LIM protein 1 [Source:HGNC Symbol;Acc:HGNC:78]	protein coding	-1.26	2.18	33.42	<0.0001	<0.0001
ENSG00000082438	COBLL1	cordon-bleu WH2 repeat protein like 1 [Source:HGNC Symbol;Acc:HGNC:23571]	protein coding	-1.26	1.42	70.09	<0.0001	<0.0001
ENSG00000079308	TNS1	tensin 1 [Source:HGNC Symbol;Acc:HGNC:11973]	protein coding	1.26	3.40	72.48	<0.0001	<0.0001
ENSG00000287252		novel transcript	lncRNA	-1.26	0.47	51.27	<0.0001	<0.0001
ENSG00000138166	DUSP5	dual specificity phosphatase 5 [Source:HGNC Symbol;Acc:HGNC:3071]	protein coding	1.26	4.15	42.09	<0.0001	<0.0001
ENSG00000148175	STOM	stomatin [Source:HGNC Symbol;Acc:HGNC:3383]	protein coding	1.25	6.31	93.63	<0.0001	<0.0001
ENSG00000069188	SDK2	sidekick cell adhesion molecule 2 [Source:HGNC Symbol;Acc:HGNC:19308]	protein coding	-1.25	-0.01	35.03	<0.0001	<0.0001
ENSG00000162747	FCGR3B	Fc fragment of IgG receptor IIIb [Source:HGNC Symbol;Acc:HGNC:3620]	protein coding	1.25	1.64	88.58	<0.0001	<0.0001

Table S5: Genes differentially expressed at Day 30 compared to pre-second vaccination (Study Arm 2 A/H7N9+AS03, D1, 29, Monocytes). Genes are sorted by descending absolute log_2 fold change. Gene model summaries and annotations are based on Ensembl Version 99 (continued.)

Ensembl Gene ID	Ensembl Gene Name	Ensembl Gene Description	Gene Type	log_2 Fold Change (Day 30 vs. Pre-Second Vaccination)	Average log_2 CPM	Likelihood Ratio Test Statistic	P-Value	FDR Adjusted P-Value
ENSG00000130787	HIP1R	huntingtin interacting protein 1 related [Source:HGNC Symbol;Acc:HGNC:18415]	protein coding	-1.25	3.31	54.44	<0.0001	<0.0001
ENSG00000172183	ISG20	interferon stimulated exonuclease gene 20 [Source:HGNC Symbol;Acc:HGNC:6130]	protein coding	1.25	5.23	33.77	<0.0001	<0.0001
ENSG00000211653	IGLV1-40	immunoglobulin lambda variable 1-40 [Source:HGNC Symbol;Acc:HGNC:5877]	IG V gene	-1.24	1.12	24.71	<0.0001	<0.0001
ENSG00000159958	TNFRSF13C	TNF receptor superfamily member 13C [Source:HGNC Symbol;Acc:HGNC:17755]	protein coding	-1.24	3.46	45.15	<0.0001	<0.0001
ENSG00000166483	WEE1	WEE1 G2 checkpoint kinase [Source:HGNC Symbol;Acc:HGNC:12761]	protein coding	-1.24	0.32	43.05	<0.0001	<0.0001
ENSG00000167483	NIBAN3	niban apoptosis regulator 3 [Source:HGNC Symbol;Acc:HGNC:24130]	protein coding	-1.23	4.72	46.67	<0.0001	<0.0001
ENSG00000105369	CD79A	CD79a molecule [Source:HGNC Symbol;Acc:HGNC:1698]	protein coding	-1.23	5.03	55.46	<0.0001	<0.0001
ENSG00000166405	RIC3	RIC3 acetylcholine receptor chaperone [Source:HGNC Symbol;Acc:HGNC:30338]	protein coding	-1.23	0.23	27.90	<0.0001	<0.0001
ENSG00000096996	IL12RB1	interleukin 12 receptor subunit beta 1 [Source:HGNC Symbol;Acc:HGNC:5971]	protein coding	1.22	5.56	112.16	<0.0001	<0.0001
ENSG00000240065	PSMB9	proteasome 20S subunit beta 9 [Source:HGNC Symbol;Acc:HGNC:9546]	protein coding	1.22	7.87	105.08	<0.0001	<0.0001
ENSG00000226321	CROCC2	ciliary rootlet coiled-coil, rootletin family member 2 [Source:HGNC Symbol;Acc:HGNC:51677]	protein coding	-1.22	3.69	33.57	<0.0001	<0.0001
ENSG00000125900	SIRPD	signal regulatory protein delta [Source:HGNC Symbol;Acc:HGNC:16248]	protein coding	1.22	2.24	56.60	<0.0001	<0.0001
ENSG00000168913	ENHO	energy homeostasis associated [Source:HGNC Symbol;Acc:HGNC:24838]	protein coding	-1.22	0.87	59.17	<0.0001	<0.0001
ENSG00000128335	APOL2	apolipoprotein L2 [Source:HGNC Symbol;Acc:HGNC:619]	protein coding	1.22	6.56	57.77	<0.0001	<0.0001
ENSG00000136235	GPNMB	glycoprotein nmb [Source:HGNC Symbol;Acc:HGNC:4462]	protein coding	-1.22	1.29	29.93	<0.0001	<0.0001
ENSG00000111452	ADGRD1	adhesion G protein-coupled receptor D1 [Source:HGNC Symbol;Acc:HGNC:19893]	protein coding	-1.22	4.18	73.02	<0.0001	<0.0001
ENSG00000145349	CAMK2D	calcium/calmodulin dependent protein kinase II delta [Source:HGNC Symbol;Acc:HGNC:1462]	protein coding	1.22	5.70	69.53	<0.0001	<0.0001
ENSG00000143869	GDF7	growth differentiation factor 7 [Source:HGNC Symbol;Acc:HGNC:4222]	protein coding	-1.22	0.44	41.34	<0.0001	<0.0001

Table S5: Genes differentially expressed at Day 30 compared to pre-second vaccination (Study Arm 2 A/H7N9+AS03, D1, 29, Monocytes). Genes are sorted by descending absolute log_2 fold change. Gene model summaries and annotations are based on Ensembl Version 99 (continued.)

Ensembl Gene ID	Ensembl Gene Name	Ensembl Gene Description	Gene Type	log_2 Fold Change (Day 30 vs. Pre-Second Vaccination)	Average log_2 CPM	Likelihood Ratio Test Statistic	P-Value	FDR Adjusted P-Value
ENSG00000182389	CACNB4	calcium voltage-gated channel auxiliary subunit beta 4 [Source:HGNC Symbol;Acc:HGNC:1404]	protein coding	-1.22	1.39	65.24	<0.0001	<0.0001
ENSG00000261798		novel transcript, antisense to BMP8B	lncRNA	-1.22	0.05	24.67	<0.0001	<0.0001
ENSG00000186265	BTLA	B and T lymphocyte associated [Source:HGNC Symbol;Acc:HGNC:21087]	protein coding	-1.22	1.69	46.24	<0.0001	<0.0001
ENSG00000131797	CLUHP3	clustered mitochondria homolog pseudogene 3 [Source:HGNC Symbol;Acc:HGNC:28447]	transcribed unprocessed pseudogene	-1.22	0.50	32.68	<0.0001	<0.0001
ENSG00000184979	USP18	ubiquitin specific peptidase 18 [Source:HGNC Symbol;Acc:HGNC:12616]	protein coding	1.22	2.50	46.93	<0.0001	<0.0001
ENSG00000211966	IGHV5-51	immunoglobulin heavy variable 5-51 [Source:HGNC Symbol;Acc:HGNC:5659]	IG V gene	-1.22	1.21	35.60	<0.0001	<0.0001
ENSG00000213373	LINC00671	long intergenic non-protein coding RNA 671 [Source:HGNC Symbol;Acc:HGNC:44339]	lncRNA	-1.21	1.04	38.07	<0.0001	<0.0001
ENSG00000019102	VSIG2	V-set and immunoglobulin domain containing 2 [Source:HGNC Symbol;Acc:HGNC:17149]	protein coding	-1.21	2.68	42.65	<0.0001	<0.0001
ENSG00000260260	SNHG19	small nucleolar RNA host gene 19 [Source:HGNC Symbol;Acc:HGNC:49574]	lncRNA	-1.21	1.26	60.54	<0.0001	<0.0001
ENSG00000137965	IFI44	interferon induced protein 44 [Source:HGNC Symbol;Acc:HGNC:16938]	protein coding	1.21	6.77	73.09	<0.0001	<0.0001
ENSG00000145911	N4BP3	NEDD4 binding protein 3 [Source:HGNC Symbol;Acc:HGNC:29852]	protein coding	-1.21	0.14	39.64	<0.0001	<0.0001
ENSG00000238105	GOLGA2P5	GOLGA2 pseudogene 5 [Source:HGNC Symbol;Acc:HGNC:25315]	transcribed unprocessed pseudogene	-1.21	0.47	26.01	<0.0001	<0.0001
ENSG00000159674	SPON2	spondin 2 [Source:HGNC Symbol;Acc:HGNC:11253]	protein coding	1.21	1.79	51.27	<0.0001	<0.0001
ENSG00000185745	IFIT1	interferon induced protein with tetratricopeptide repeats 1 [Source:HGNC Symbol;Acc:HGNC:5407]	protein coding	1.20	5.63	36.12	<0.0001	<0.0001
ENSG00000197353	LYPD2	LY6/PLAUR domain containing 2 [Source:HGNC Symbol;Acc:HGNC:25215]	protein coding	-1.20	0.94	26.23	<0.0001	<0.0001

Table S5: Genes differentially expressed at Day 30 compared to pre-second vaccination (Study Arm 2 A/H7N9+AS03, D1, 29, Monocytes). Genes are sorted by descending absolute log_2 fold change. Gene model summaries and annotations are based on Ensembl Version 99 (continued.)

Ensembl Gene ID	Ensembl Gene Name	Ensembl Gene Description	Gene Type	log_2 Fold Change (Day 30 vs. Pre-Second Vaccination)	Average log_2 CPM	Likelihood Ratio Test Statistic	P-Value	FDR Adjusted P-Value
ENSG00000038945	MSR1	macrophage scavenger receptor 1 [Source:HGNC Symbol;Acc:HGNC:7376]	protein coding	1.20	4.47	134.87	<0.0001	<0.0001
ENSG00000134247	PTGFRN	prostaglandin F2 receptor inhibitor [Source:HGNC Symbol;Acc:HGNC:9601]	protein coding	-1.20	2.58	61.48	<0.0001	<0.0001
ENSG00000133985	TTC9	tetratricopeptide repeat domain 9 [Source:HGNC Symbol;Acc:HGNC:20267]	protein coding	-1.20	3.60	61.26	<0.0001	<0.0001
ENSG00000111331	OAS3	2'-5'-oligoadenylate synthetase 3 [Source:HGNC Symbol;Acc:HGNC:8088]	protein coding	1.20	8.36	61.69	<0.0001	<0.0001
ENSG00000177455	CD19	CD19 molecule [Source:HGNC Symbol;Acc:HGNC:1633]	protein coding	-1.20	3.04	48.45	<0.0001	<0.0001
ENSG00000065911	MTHFD2	methylenetetrahydrofolate dehydrogenase (NADP+ dependent) 2, methenyltetrahydrofolate cyclohydrolase [Source:HGNC Symbol;Acc:HGNC:7434]	protein coding	1.20	6.14	161.19	<0.0001	<0.0001
ENSG00000126709	IFI6	interferon alpha inducible protein 6 [Source:HGNC Symbol;Acc:HGNC:4054]	protein coding	1.20	7.71	80.40	<0.0001	<0.0001
ENSG00000230910		novel transcript	lncRNA	-1.20	2.95	46.30	<0.0001	<0.0001
ENSG00000163251	FZD5	frizzled class receptor 5 [Source:HGNC Symbol;Acc:HGNC:4043]	protein coding	1.20	2.27	73.16	<0.0001	<0.0001
ENSG00000141574	SECTM1	secreted and transmembrane 1 [Source:HGNC Symbol;Acc:HGNC:10707]	protein coding	1.19	8.82	113.57	<0.0001	<0.0001
ENSG00000105835	NAMPT	nicotinamide phosphoribosyltransferase [Source:HGNC Symbol;Acc:HGNC:30092]	protein coding	1.19	7.65	53.31	<0.0001	<0.0001
ENSG00000279805		TEC	TEC	1.19	1.05	32.78	<0.0001	<0.0001
ENSG00000012124	CD22	CD22 molecule [Source:HGNC Symbol;Acc:HGNC:1643]	protein coding	-1.19	4.73	46.30	<0.0001	<0.0001
ENSG00000143434	SEMA6C	semaphorin 6C [Source:HGNC Symbol;Acc:HGNC:10740]	protein coding	-1.19	1.83	44.43	<0.0001	<0.0001
ENSG00000121895	TMEM156	transmembrane protein 156 [Source:HGNC Symbol;Acc:HGNC:26260]	protein coding	-1.19	0.95	35.85	<0.0001	<0.0001
ENSG00000158517	NCF1	neutrophil cytosolic factor 1 [Source:HGNC Symbol;Acc:HGNC:7660]	protein coding	1.19	8.77	58.46	<0.0001	<0.0001
ENSG00000213557		ribosomal protein L31 (RPL31) pseudogene	processed pseudogene	-1.19	2.38	69.56	<0.0001	<0.0001
ENSG00000138134	STAMBPL1	STAM binding protein like 1 [Source:HGNC Symbol;Acc:HGNC:24105]	protein coding	1.19	1.40	32.74	<0.0001	<0.0001

Table S5: Genes differentially expressed at Day 30 compared to pre-second vaccination (Study Arm 2 A/H7N9+AS03, D1, 29, Monocytes). Genes are sorted by descending absolute log_2 fold change. Gene model summaries and annotations are based on Ensembl Version 99 (continued.)

Ensembl Gene ID	Ensembl Gene Name	Ensembl Gene Description	Gene Type	log_2 Fold Change (Day 30 vs. Pre-Second Vaccination)	Average log_2 CPM	Likelihood Ratio Test Statistic	P-Value	FDR Adjusted P-Value
ENSG00000174749	FAM241A	family with sequence similarity 241 member A [Source:HGNC Symbol;Acc:HGNC:26813]	protein coding	1.19	5.13	87.92	<0.0001	<0.0001
ENSG00000287733		novel transcript	lncRNA	1.19	0.99	33.50	<0.0001	<0.0001
ENSG00000188822	CNR2	cannabinoid receptor 2 [Source:HGNC Symbol;Acc:HGNC:2160]	protein coding	-1.19	1.18	35.50	<0.0001	<0.0001
ENSG00000143297	FCRL5	Fc receptor like 5 [Source:HGNC Symbol;Acc:HGNC:18508]	protein coding	-1.19	2.89	42.33	<0.0001	<0.0001
ENSG00000137103	TMEM8B	transmembrane protein 8B [Source:HGNC Symbol;Acc:HGNC:21427]	protein coding	-1.18	3.97	91.04	<0.0001	<0.0001
ENSG00000162591	MEGF6	multiple EGF like domains 6 [Source:HGNC Symbol;Acc:HGNC:3232]	protein coding	-1.18	4.71	80.53	<0.0001	<0.0001
ENSG00000211663	IGLV3-19	immunoglobulin lambda variable 3-19 [Source:HGNC Symbol;Acc:HGNC:5903]	IG V gene	-1.18	0.53	20.59	<0.0001	<0.0001
ENSG00000225886		novel transcript, antisense to IFI6	lncRNA	1.18	1.55	26.96	<0.0001	<0.0001
ENSG00000157734	SNX22	sorting nexin 22 [Source:HGNC Symbol;Acc:HGNC:16315]	protein coding	-1.17	2.86	62.26	<0.0001	<0.0001
ENSG00000229759	MRPS18AP1	mitochondrial ribosomal protein S18A pseudogene 1 [Source:HGNC Symbol;Acc:HGNC:29740]	processed pseudogene	-1.17	0.17	46.77	<0.0001	<0.0001
ENSG00000211947	IGHV3-21	immunoglobulin heavy variable 3-21 [Source:HGNC Symbol;Acc:HGNC:5586]	IG V gene	-1.17	0.69	37.61	<0.0001	<0.0001
ENSG00000233392		novel transcript	lncRNA	-1.17	4.54	26.57	<0.0001	<0.0001
ENSG00000242779	ZNF702P	zinc finger protein 702, pseudogene [Source:HGNC Symbol;Acc:HGNC:25775]	transcribed processed pseudogene	1.17	1.99	55.88	<0.0001	<0.0001
ENSG00000158481	CD1C	CD1c molecule [Source:HGNC Symbol;Acc:HGNC:1636]	protein coding	-1.17	4.47	67.47	<0.0001	<0.0001
ENSG00000165806	CASP7	caspase 7 [Source:HGNC Symbol;Acc:HGNC:1508]	protein coding	1.17	5.01	76.03	<0.0001	<0.0001
ENSG00000163534	FCRL1	Fc receptor like 1 [Source:HGNC Symbol;Acc:HGNC:18509]	protein coding	-1.17	4.51	53.54	<0.0001	<0.0001
ENSG00000226777	FAM30A	family with sequence similarity 30 member A [Source:HGNC Symbol;Acc:HGNC:19955]	lncRNA	-1.17	3.44	79.42	<0.0001	<0.0001
ENSG00000196092	PAX5	paired box 5 [Source:HGNC Symbol;Acc:HGNC:8619]	protein coding	-1.17	4.01	34.71	<0.0001	<0.0001

Table S5: Genes differentially expressed at Day 30 compared to pre-second vaccination (Study Arm 2 A/H7N9+AS03, D1, 29, Monocytes). Genes are sorted by descending absolute log_2 fold change. Gene model summaries and annotations are based on Ensembl Version 99 (continued.)

Ensembl Gene ID	Ensembl Gene Name	Ensembl Gene Description	Gene Type	log_2 Fold Change (Day 30 vs. Pre-Second Vaccination)	Average log_2 CPM	Likelihood Ratio Test Statistic	P-Value	FDR Adjusted P-Value
ENSG00000211892	IGHG4	immunoglobulin heavy constant gamma 4 (G4m marker) [Source:HGNC Symbol;Acc:HGNC:5528]	IG C gene	-1.17	1.69	24.33	<0.0001	<0.0001
ENSG00000166428	PLD4	phospholipase D family member 4 [Source:HGNC Symbol;Acc:HGNC:23792]	protein coding	-1.17	5.15	23.27	<0.0001	<0.0001
ENSG00000229644	NAMPTP1	nicotinamide phosphoribosyltransferase pseudogene 1 [Source:HGNC Symbol;Acc:HGNC:17633]	processed pseudogene	1.17	4.29	51.15	<0.0001	<0.0001
ENSG00000269404	SPIB	Spi-B transcription factor [Source:HGNC Symbol;Acc:HGNC:11242]	protein coding	-1.16	1.92	32.62	<0.0001	<0.0001
ENSG00000083454	P2RX5	purinergic receptor P2X 5 [Source:HGNC Symbol;Acc:HGNC:8536]	protein coding	-1.16	2.66	88.68	<0.0001	<0.0001
ENSG00000169136	ATF5	activating transcription factor 5 [Source:HGNC Symbol;Acc:HGNC:790]	protein coding	1.16	6.02	86.53	<0.0001	<0.0001
ENSG00000204267	TAP2	transporter 2, ATP binding cassette subfamily B member [Source:HGNC Symbol;Acc:HGNC:44]	protein coding	1.16	6.41	85.97	<0.0001	<0.0001
ENSG00000136514	RTP4	receptor transporter protein 4 [Source:HGNC Symbol;Acc:HGNC:23992]	protein coding	1.16	3.26	41.59	<0.0001	<0.0001
ENSG00000135047	CTSL	cathepsin L [Source:HGNC Symbol;Acc:HGNC:2537]	protein coding	1.16	5.13	130.92	<0.0001	<0.0001
ENSG00000140379	BCL2A1	BCL2 related protein A1 [Source:HGNC Symbol;Acc:HGNC:991]	protein coding	1.16	5.89	126.86	<0.0001	<0.0001
ENSG00000128218	VPREB3	V-set pre-B cell surrogate light chain 3 [Source:HGNC Symbol;Acc:HGNC:12710]	protein coding	-1.16	0.63	22.71	<0.0001	<0.0001
ENSG00000111674	ENO2	enolase 2 [Source:HGNC Symbol;Acc:HGNC:3353]	protein coding	-1.16	0.24	21.17	<0.0001	<0.0001
ENSG00000211896	IGHG1	immunoglobulin heavy constant gamma 1 (G1m marker) [Source:HGNC Symbol;Acc:HGNC:5525]	IG C gene	-1.15	5.01	33.41	<0.0001	<0.0001
ENSG00000164715	LMTK2	lemur tyrosine kinase 2 [Source:HGNC Symbol;Acc:HGNC:17880]	protein coding	1.15	5.66	70.00	<0.0001	<0.0001
ENSG00000111110	PPM1H	protein phosphatase, Mg2+/Mn2+ dependent 1H [Source:HGNC Symbol;Acc:HGNC:18583]	protein coding	-1.15	3.10	58.36	<0.0001	<0.0001
ENSG00000100985	MMP9	matrix metallopeptidase 9 [Source:HGNC Symbol;Acc:HGNC:7176]	protein coding	-1.15	1.33	52.39	<0.0001	<0.0001
ENSG00000100473	COCH	cochlin [Source:HGNC Symbol;Acc:HGNC:2180]	protein coding	-1.15	0.43	36.36	<0.0001	<0.0001

Table S5: Genes differentially expressed at Day 30 compared to pre-second vaccination (Study Arm 2 A/H7N9+AS03, D1, 29, Monocytes). Genes are sorted by descending absolute log_2 fold change. Gene model summaries and annotations are based on Ensembl Version 99 (continued.)

Ensembl Gene ID	Ensembl Gene Name	Ensembl Gene Description	Gene Type	log_2 Fold Change (Day 30 vs. Pre-Second Vaccination)	Average log_2 CPM	Likelihood Ratio Test Statistic	P-Value	FDR Adjusted P-Value
ENSG00000158488	CD1E	CD1e molecule [Source:HGNC Symbol;Acc:HGNC:1638]	protein coding	-1.15	1.12	25.56	<0.0001	<0.0001
ENSG00000183134	PTGDR2	prostaglandin D2 receptor 2 [Source:HGNC Symbol;Acc:HGNC:4502]	protein coding	-1.15	2.01	17.45	<0.0001	0.0001
ENSG00000179240	GVQW3	GVQW motif containing 3 [Source:HGNC Symbol;Acc:HGNC:51239]	protein coding	-1.15	1.22	53.77	<0.0001	<0.0001
ENSG00000139626	ITGB7	integrin subunit beta 7 [Source:HGNC Symbol;Acc:HGNC:6162]	protein coding	1.15	5.26	40.57	<0.0001	<0.0001
ENSG00000205056	LINC02397	long intergenic non-protein coding RNA 2397 [Source:HGNC Symbol;Acc:HGNC:53323]	lncRNA	-1.15	0.48	34.67	<0.0001	<0.0001
ENSG00000138031	ADCY3	adenylate cyclase 3 [Source:HGNC Symbol;Acc:HGNC:234]	protein coding	1.15	3.46	26.07	<0.0001	<0.0001
ENSG00000157601	MX1	MX dynamin like GTPase 1 [Source:HGNC Symbol;Acc:HGNC:7532]	protein coding	1.14	7.91	54.13	<0.0001	<0.0001
ENSG00000159753	CARMIL2	capping protein regulator and myosin 1 linker 2 [Source:HGNC Symbol;Acc:HGNC:27089]	protein coding	-1.14	2.73	39.55	<0.0001	<0.0001
ENSG00000137757	CASP5	caspase 5 [Source:HGNC Symbol;Acc:HGNC:1506]	protein coding	1.14	2.76	93.40	<0.0001	<0.0001
ENSG00000141542	RAB40B	RAB40B, member RAS oncogene family [Source:HGNC Symbol;Acc:HGNC:18284]	protein coding	-1.14	1.23	48.96	<0.0001	<0.0001
ENSG00000108244	KRT23	keratin 23 [Source:HGNC Symbol;Acc:HGNC:6438]	protein coding	-1.14	1.09	51.83	<0.0001	<0.0001
ENSG00000141854	MISP3	MISP family member 3 [Source:HGNC Symbol;Acc:HGNC:26963]	protein coding	-1.14	0.97	42.52	<0.0001	<0.0001
ENSG00000107742	SPOCK2	SPARC (osteonectin), cwcv and kazal like domains proteoglycan 2 [Source:HGNC Symbol;Acc:HGNC:13564]	protein coding	-1.14	2.81	22.46	<0.0001	<0.0001
ENSG00000005471	ABCB4	ATP binding cassette subfamily B member 4 [Source:HGNC Symbol;Acc:HGNC:45]	protein coding	-1.13	0.27	33.70	<0.0001	<0.0001
ENSG00000120278	PLEKHG1	pleckstrin homology and RhoGEF domain containing G1 [Source:HGNC Symbol;Acc:HGNC:20884]	protein coding	-1.13	1.05	27.14	<0.0001	<0.0001
ENSG00000171408	PDE7B	phosphodiesterase 7B [Source:HGNC Symbol;Acc:HGNC:8792]	protein coding	-1.13	0.28	26.94	<0.0001	<0.0001
ENSG00000160932	LY6E	lymphocyte antigen 6 family member E [Source:HGNC Symbol;Acc:HGNC:6727]	protein coding	1.13	8.25	88.50	<0.0001	<0.0001
ENSG00000111335	OAS2	2'-5'-oligoadenylate synthetase 2 [Source:HGNC Symbol;Acc:HGNC:8087]	protein coding	1.13	7.86	63.02	<0.0001	<0.0001

Table S5: Genes differentially expressed at Day 30 compared to pre-second vaccination (Study Arm 2 A/H7N9+AS03, D1, 29, Monocytes). Genes are sorted by descending absolute log_2 fold change. Gene model summaries and annotations are based on Ensembl Version 99 (continued.)

Ensembl Gene ID	Ensembl Gene Name	Ensembl Gene Description	Gene Type	log_2 Fold Change (Day 30 vs. Pre-Second Vaccination)	Average log_2 CPM	Likelihood Ratio Test Statistic	P-Value	FDR Adjusted P-Value
ENSG00000165178	NCF1C	neutrophil cytosolic factor 1C pseudogene [Source:HGNC Symbol;Acc:HGNC:32523]	unprocessed pseudogene	1.13	7.61	53.77	<0.0001	<0.0001
ENSG00000103528	SYT17	synaptotagmin 17 [Source:HGNC Symbol;Acc:HGNC:24119]	protein coding	-1.12	1.24	35.08	<0.0001	<0.0001
ENSG00000245904		novel transcript, antisense to BTG1	lncRNA	-1.12	2.02	60.22	<0.0001	<0.0001
ENSG00000130589	HELZ2	helicase with zinc finger 2 [Source:HGNC Symbol;Acc:HGNC:30021]	protein coding	1.12	7.60	54.02	<0.0001	<0.0001
ENSG00000139832	RAB20	RAB20, member RAS oncogene family [Source:HGNC Symbol;Acc:HGNC:18260]	protein coding	1.11	5.43	98.01	<0.0001	<0.0001
ENSG00000270550	IGHV3-30	immunoglobulin heavy variable 3-30 [Source:HGNC Symbol;Acc:HGNC:5591]	IG V gene	-1.11	0.17	23.08	<0.0001	<0.0001
ENSG00000075399	VPS9D1	VPS9 domain containing 1 [Source:HGNC Symbol;Acc:HGNC:13526]	protein coding	1.11	6.12	89.34	<0.0001	<0.0001
ENSG00000286162		novel transcript	lncRNA	1.11	1.45	18.45	<0.0001	<0.0001
ENSG00000197119	SLC25A29	solute carrier family 25 member 29 [Source:HGNC Symbol;Acc:HGNC:20116]	protein coding	-1.11	5.98	49.27	<0.0001	<0.0001
ENSG00000169313	P2RY12	purinergic receptor P2Y12 [Source:HGNC Symbol;Acc:HGNC:18124]	protein coding	1.11	1.78	43.19	<0.0001	<0.0001
ENSG00000140932	CMTM2	CKLF like MARVEL transmembrane domain containing 2 [Source:HGNC Symbol;Acc:HGNC:19173]	protein coding	-1.11	1.70	36.52	<0.0001	<0.0001
ENSG00000188483	IER5L	immediate early response 5 like [Source:HGNC Symbol;Acc:HGNC:23679]	protein coding	-1.11	4.74	35.60	<0.0001	<0.0001
ENSG00000125869	LAMP5	lysosomal associated membrane protein family member 5 [Source:HGNC Symbol;Acc:HGNC:16097]	protein coding	1.10	0.82	19.72	<0.0001	<0.0001
ENSG00000115339	GALNT3	polypeptide N-acetylgalactosaminyltransferase 3 [Source:HGNC Symbol;Acc:HGNC:4125]	protein coding	1.10	3.92	78.14	<0.0001	<0.0001
ENSG00000211651	IGLV1-44	immunoglobulin lambda variable 1-44 [Source:HGNC Symbol;Acc:HGNC:5879]	IG V gene	-1.10	0.86	16.78	<0.0001	0.0002
ENSG00000078804	TP53INP2	tumor protein p53 inducible nuclear protein 2 [Source:HGNC Symbol;Acc:HGNC:16104]	protein coding	1.10	2.94	31.20	<0.0001	<0.0001
ENSG00000150637	CD226	CD226 molecule [Source:HGNC Symbol;Acc:HGNC:16961]	protein coding	1.10	0.74	20.08	<0.0001	<0.0001
ENSG00000144668	ITGA9	integrin subunit alpha 9 [Source:HGNC Symbol;Acc:HGNC:6145]	protein coding	-1.10	0.57	28.68	<0.0001	<0.0001

Table S5: Genes differentially expressed at Day 30 compared to pre-second vaccination (Study Arm 2 A/H7N9+AS03, D1, 29, Monocytes). Genes are sorted by descending absolute log_2 fold change. Gene model summaries and annotations are based on Ensembl Version 99 (continued.)

Ensembl Gene ID	Ensembl Gene Name	Ensembl Gene Description	Gene Type	log_2 Fold Change (Day 30 vs. Pre-Second Vaccination)	Average log_2 CPM	Likelihood Ratio Test Statistic	P-Value	FDR Adjusted P-Value
ENSG00000173821	RNF213	ring finger protein 213 [Source:HGNC Symbol;Acc:HGNC:14539]	protein coding	1.10	9.76	52.88	<0.0001	<0.0001
ENSG00000267534	S1PR2	sphingosine-1-phosphate receptor 2 [Source:HGNC Symbol;Acc:HGNC:3169]	protein coding	1.10	1.77	41.71	<0.0001	<0.0001
ENSG00000255045		novel transcript	lncRNA	-1.10	0.71	33.77	<0.0001	<0.0001
ENSG00000135362	PRR5L	proline rich 5 like [Source:HGNC Symbol;Acc:HGNC:25878]	protein coding	1.09	4.11	44.67	<0.0001	<0.0001
ENSG00000141096	DPEP3	dipeptidase 3 [Source:HGNC Symbol;Acc:HGNC:23029]	protein coding	-1.09	0.45	28.05	<0.0001	<0.0001
ENSG00000177989	ODF3B	outer dense fiber of sperm tails 3B [Source:HGNC Symbol;Acc:HGNC:34388]	protein coding	1.09	8.83	63.78	<0.0001	<0.0001
ENSG00000143153	ATP1B1	ATPase Na+/K+ transporting subunit beta 1 [Source:HGNC Symbol;Acc:HGNC:804]	protein coding	1.09	2.60	68.05	<0.0001	<0.0001
ENSG00000173706	HEG1	heart development protein with EGF like domains 1 [Source:HGNC Symbol;Acc:HGNC:29227]	protein coding	1.09	4.41	90.59	<0.0001	<0.0001
ENSG00000238160	LINC02863	long intergenic non-protein coding RNA 2863 [Source:HGNC Symbol;Acc:HGNC:41290]	lncRNA	-1.09	0.29	40.83	<0.0001	<0.0001
ENSG00000161640	SIGLEC11	sialic acid binding Ig like lectin 11 [Source:HGNC Symbol;Acc:HGNC:15622]	protein coding	1.09	2.59	45.38	<0.0001	<0.0001
ENSG00000100181	TPTEP1	TPTE pseudogene 1 [Source:HGNC Symbol;Acc:HGNC:43648]	transcribed unprocessed pseudogene	-1.09	2.61	64.58	<0.0001	<0.0001
ENSG00000114127	XRN1	5'-3' exoribonuclease 1 [Source:HGNC Symbol;Acc:HGNC:30654]	protein coding	1.09	6.24	61.25	<0.0001	<0.0001
ENSG00000161714	PLCD3	phospholipase C delta 3 [Source:HGNC Symbol;Acc:HGNC:9061]	protein coding	-1.09	2.65	65.51	<0.0001	<0.0001
ENSG00000110987	BCL7A	BAF chromatin remodeling complex subunit BCL7A [Source:HGNC Symbol;Acc:HGNC:1004]	protein coding	-1.09	2.14	55.36	<0.0001	<0.0001
ENSG00000197705	KLHL14	kelch like family member 14 [Source:HGNC Symbol;Acc:HGNC:29266]	protein coding	-1.09	0.53	37.41	<0.0001	<0.0001
ENSG00000140464	PML	promyelocytic leukemia [Source:HGNC Symbol;Acc:HGNC:9113]	protein coding	1.09	6.96	72.56	<0.0001	<0.0001
ENSG00000196743	GM2A	GM2 ganglioside activator [Source:HGNC Symbol;Acc:HGNC:4367]	protein coding	1.09	7.54	75.93	<0.0001	<0.0001

Table S5: Genes differentially expressed at Day 30 compared to pre-second vaccination (Study Arm 2 A/H7N9+AS03, D1, 29, Monocytes). Genes are sorted by descending absolute log_2 fold change. Gene model summaries and annotations are based on Ensembl Version 99 (continued.)

Ensembl Gene ID	Ensembl Gene Name	Ensembl Gene Description	Gene Type	log_2 Fold Change (Day 30 vs. Pre-Second Vaccination)	Average log_2 CPM	Likelihood Ratio Test Statistic	P-Value	FDR Adjusted P-Value
ENSG00000268027		novel transcript, sense intronic to CEACAM21	lncRNA	-1.09	0.54	41.93	<0.0001	<0.0001
ENSG00000211772	TRBC2	T cell receptor beta constant 2 [Source:HGNC Symbol;Acc:HGNC:12157]	TR C gene	-1.08	1.91	21.81	<0.0001	<0.0001
ENSG00000186567	CEACAM19	CEA cell adhesion molecule 19 [Source:HGNC Symbol;Acc:HGNC:31951]	protein coding	-1.08	2.64	51.47	<0.0001	<0.0001
ENSG00000132704	FCRL2	Fc receptor like 2 [Source:HGNC Symbol;Acc:HGNC:14875]	protein coding	-1.08	2.89	39.99	<0.0001	<0.0001
ENSG00000186529	CYP4F3	cytochrome P450 family 4 subfamily F member 3 [Source:HGNC Symbol;Acc:HGNC:2646]	protein coding	-1.08	1.40	27.32	<0.0001	<0.0001
ENSG00000133313	CNDP2	carnosine dipeptidase 2 [Source:HGNC Symbol;Acc:HGNC:24437]	protein coding	1.08	8.00	71.16	<0.0001	<0.0001
ENSG00000132386	SERPINF1	serpin family F member 1 [Source:HGNC Symbol;Acc:HGNC:8824]	protein coding	-1.08	2.72	29.53	<0.0001	<0.0001
ENSG00000170382	LRRN2	leucine rich repeat neuronal 2 [Source:HGNC Symbol;Acc:HGNC:16914]	protein coding	1.08	2.78	21.88	<0.0001	<0.0001
ENSG00000112182	BACH2	BTB domain and CNC homolog 2 [Source:HGNC Symbol;Acc:HGNC:14078]	protein coding	-1.08	2.20	33.61	<0.0001	<0.0001
ENSG00000025708	TYMP	thymidine phosphorylase [Source:HGNC Symbol;Acc:HGNC:3148]	protein coding	1.08	11.00	71.05	<0.0001	<0.0001
ENSG00000172508	CARNS1	carnosine synthase 1 [Source:HGNC Symbol;Acc:HGNC:29268]	protein coding	-1.07	1.28	37.28	<0.0001	<0.0001
ENSG00000132185	FCRLA	Fc receptor like A [Source:HGNC Symbol;Acc:HGNC:18504]	protein coding	-1.07	2.53	25.80	<0.0001	<0.0001
ENSG00000142798	HSPG2	heparan sulfate proteoglycan 2 [Source:HGNC Symbol;Acc:HGNC:5273]	protein coding	-1.07	0.73	39.67	<0.0001	<0.0001
ENSG00000124491	F13A1	coagulation factor XIII A chain [Source:HGNC Symbol;Acc:HGNC:3531]	protein coding	-1.07	7.75	26.17	<0.0001	<0.0001
ENSG00000274471		hect domain and RLD 2 (HERC2) pseudogene	transcribed unprocessed pseudogene	-1.07	1.19	55.77	<0.0001	<0.0001
ENSG00000159713	TPPP3	tubulin polymerization promoting protein family member 3 [Source:HGNC Symbol;Acc:HGNC:24162]	protein coding	-1.07	5.10	66.40	<0.0001	<0.0001
ENSG00000107282	APBA1	amyloid beta precursor protein binding family A member 1 [Source:HGNC Symbol;Acc:HGNC:578]	protein coding	-1.07	1.41	30.18	<0.0001	<0.0001

Table S5: Genes differentially expressed at Day 30 compared to pre-second vaccination (Study Arm 2 A/H7N9+AS03, D1, 29, Monocytes). Genes are sorted by descending absolute log_2 fold change. Gene model summaries and annotations are based on Ensembl Version 99 (continued.)

Ensembl Gene ID	Ensembl Gene Name	Ensembl Gene Description	Gene Type	log_2 Fold Change (Day 30 vs. Pre-Second Vaccination)	Average log_2 CPM	Likelihood Ratio Test Statistic	P-Value	FDR Adjusted P-Value
ENSG00000284194	SCO2	SCO cytochrome c oxidase assembly protein 2 [Source:HGNC Symbol;Acc:HGNC:10604]	protein coding	1.07	5.78	62.76	<0.0001	<0.0001
ENSG00000132530	XAF1	XIAP associated factor 1 [Source:HGNC Symbol;Acc:HGNC:30932]	protein coding	1.06	6.30	47.18	<0.0001	<0.0001
ENSG00000180644	PRF1	perforin 1 [Source:HGNC Symbol;Acc:HGNC:9360]	protein coding	1.06	1.30	12.96	0.0003	0.0011
ENSG00000272398	CD24	CD24 molecule [Source:HGNC Symbol;Acc:HGNC:1645]	protein coding	-1.06	1.87	18.42	<0.0001	<0.0001
ENSG00000182487	NCF1B	neutrophil cytosolic factor 1B pseudogene [Source:HGNC Symbol;Acc:HGNC:32522]	transcribed unprocessed pseudogene	1.06	6.71	49.28	<0.0001	<0.0001
ENSG00000262097	LINC02185	long intergenic non-protein coding RNA 2185 [Source:HGNC Symbol;Acc:HGNC:53047]	lncRNA	-1.06	0.49	17.06	<0.0001	0.0002
ENSG00000128203	ASPHD2	aspartate beta-hydroxylase domain containing 2 [Source:HGNC Symbol;Acc:HGNC:30437]	protein coding	1.06	4.66	107.61	<0.0001	<0.0001
ENSG00000158470	B4GALT5	beta-1,4-galactosyltransferase 5 [Source:HGNC Symbol;Acc:HGNC:928]	protein coding	1.06	6.41	90.57	<0.0001	<0.0001
ENSG00000142583	SLC2A5	solute carrier family 2 member 5 [Source:HGNC Symbol;Acc:HGNC:11010]	protein coding	-1.06	1.27	41.66	<0.0001	<0.0001
ENSG00000110777	POU2AF1	POU class 2 homeobox associating factor 1 [Source:HGNC Symbol;Acc:HGNC:9211]	protein coding	-1.05	3.18	40.26	<0.0001	<0.0001
ENSG00000263069	RNF213-AS1	RNF213 antisense RNA 1 [Source:HGNC Symbol;Acc:HGNC:54402]	lncRNA	1.05	3.58	44.95	<0.0001	<0.0001
ENSG00000161405	IKZF3	IKAROS family zinc finger 3 [Source:HGNC Symbol;Acc:HGNC:13178]	protein coding	-1.05	2.73	32.89	<0.0001	<0.0001
ENSG00000241351	IGKV3-11	immunoglobulin kappa variable 3-11 [Source:HGNC Symbol;Acc:HGNC:5815]	IG V gene	-1.05	2.62	17.23	<0.0001	0.0002
ENSG00000170989	S1PR1	sphingosine-1-phosphate receptor 1 [Source:HGNC Symbol;Acc:HGNC:3165]	protein coding	-1.05	1.73	27.92	<0.0001	<0.0001
ENSG00000168734	PKIG	cAMP-dependent protein kinase inhibitor gamma [Source:HGNC Symbol;Acc:HGNC:9019]	protein coding	-1.05	1.55	48.14	<0.0001	<0.0001
ENSG00000057294	PKP2	plakophilin 2 [Source:HGNC Symbol;Acc:HGNC:9024]	protein coding	-1.05	1.27	25.91	<0.0001	<0.0001

Table S5: Genes differentially expressed at Day 30 compared to pre-second vaccination (Study Arm 2 A/H7N9+AS03, D1, 29, Monocytes). Genes are sorted by descending absolute log_2 fold change. Gene model summaries and annotations are based on Ensembl Version 99 (continued.)

Ensembl Gene ID	Ensembl Gene Name	Ensembl Gene Description	Gene Type	log_2 Fold Change (Day 30 vs. Pre-Second Vaccination)	Average log_2 CPM	Likelihood Ratio Test Statistic	P-Value	FDR Adjusted P-Value
ENSG00000158715	SLC45A3	solute carrier family 45 member 3 [Source:HGNC Symbol;Acc:HGNC:8642]	protein coding	-1.05	0.80	14.91	0.0001	0.0004
ENSG00000049089	COL9A2	collagen type IX alpha 2 chain [Source:HGNC Symbol;Acc:HGNC:2218]	protein coding	-1.04	4.94	61.02	<0.0001	<0.0001
ENSG00000186187	ZNRF1	zinc and ring finger 1 [Source:HGNC Symbol;Acc:HGNC:18452]	protein coding	-1.04	3.83	50.80	<0.0001	<0.0001
ENSG00000145431	PDGFC	platelet derived growth factor C [Source:HGNC Symbol;Acc:HGNC:8801]	protein coding	-1.04	4.08	42.54	<0.0001	<0.0001
ENSG00000274422		novel transcript	lncRNA	-1.04	1.00	34.28	<0.0001	<0.0001
ENSG00000188906	LRRK2	leucine rich repeat kinase 2 [Source:HGNC Symbol;Acc:HGNC:18618]	protein coding	1.04	8.78	81.64	<0.0001	<0.0001
ENSG00000172878	METAP1D	methionyl aminopeptidase type 1D, mitochondrial [Source:HGNC Symbol;Acc:HGNC:32583]	protein coding	-1.04	1.00	51.04	<0.0001	<0.0001
ENSG00000096968	JAK2	Janus kinase 2 [Source:HGNC Symbol;Acc:HGNC:6192]	protein coding	1.04	7.78	63.54	<0.0001	<0.0001
ENSG00000121797	CCRL2	C-C motif chemokine receptor like 2 [Source:HGNC Symbol;Acc:HGNC:1612]	protein coding	1.03	2.88	54.11	<0.0001	<0.0001
ENSG00000198711	SSBP3-AS1	SSBP3 antisense RNA 1 [Source:HGNC Symbol;Acc:HGNC:32328]	lncRNA	-1.03	1.00	34.23	<0.0001	<0.0001
ENSG00000006607	FARP2	FERM, ARH/RhoGEF and pleckstrin domain protein 2 [Source:HGNC Symbol;Acc:HGNC:16460]	protein coding	1.03	4.24	51.34	<0.0001	<0.0001
ENSG00000164330	EBF1	EBF transcription factor 1 [Source:HGNC Symbol;Acc:HGNC:3126]	protein coding	-1.03	0.27	23.32	<0.0001	<0.0001
ENSG00000163823	CCR1	C-C motif chemokine receptor 1 [Source:HGNC Symbol;Acc:HGNC:1602]	protein coding	1.03	7.45	47.37	<0.0001	<0.0001
ENSG00000159714	ZDHHC1	zinc finger DHHC-type containing 1 [Source:HGNC Symbol;Acc:HGNC:17916]	protein coding	-1.03	4.07	42.97	<0.0001	<0.0001
ENSG00000232926		ribosomal protein L8 (RPL8) pseudogene	processed pseudogene	-1.03	1.14	39.11	<0.0001	<0.0001
ENSG00000180096	SEPTIN1	septin 1 [Source:HGNC Symbol;Acc:HGNC:2879]	protein coding	-1.03	3.09	58.91	<0.0001	<0.0001
ENSG00000235888		novel transcript	lncRNA	-1.03	0.62	36.39	<0.0001	<0.0001
ENSG00000149927	DOC2A	double C2 domain alpha [Source:HGNC Symbol;Acc:HGNC:2985]	protein coding	-1.03	0.39	27.92	<0.0001	<0.0001

Table S5: Genes differentially expressed at Day 30 compared to pre-second vaccination (Study Arm 2 A/H7N9+AS03, D1, 29, Monocytes). Genes are sorted by descending absolute log_2 fold change. Gene model summaries and annotations are based on Ensembl Version 99 (continued.)

Ensembl Gene ID	Ensembl Gene Name	Ensembl Gene Description	Gene Type	log_2 Fold Change (Day 30 vs. Pre-Second Vaccination)	Average log_2 CPM	Likelihood Ratio Test Statistic	P-Value	FDR Adjusted P-Value
ENSG00000167261	DPEP2	dipeptidase 2 [Source:HGNC Symbol;Acc:HGNC:23028]	protein coding	-1.02	7.00	46.46	<0.0001	<0.0001
ENSG00000120210	INSL6	insulin like 6 [Source:HGNC Symbol;Acc:HGNC:6089]	protein coding	1.02	0.72	20.17	<0.0001	<0.0001
ENSG00000005379	TSPOAP1	TSPO associated protein 1 [Source:HGNC Symbol;Acc:HGNC:16831]	protein coding	1.02	3.73	24.25	<0.0001	<0.0001
ENSG00000005448	WDR54	WD repeat domain 54 [Source:HGNC Symbol;Acc:HGNC:25770]	protein coding	-1.02	1.86	40.44	<0.0001	<0.0001
ENSG00000182240	BACE2	beta-secretase 2 [Source:HGNC Symbol;Acc:HGNC:934]	protein coding	-1.02	0.42	32.04	<0.0001	<0.0001
ENSG00000104951	IL4I1	interleukin 4 induced 1 [Source:HGNC Symbol;Acc:HGNC:19094]	protein coding	1.02	3.74	50.66	<0.0001	<0.0001
ENSG00000198286	CARD11	caspase recruitment domain family member 11 [Source:HGNC Symbol;Acc:HGNC:16393]	protein coding	-1.01	2.88	30.24	<0.0001	<0.0001
ENSG00000263366	ABHD17AP5	abhydrolase domain containing 17A pseudogene 5 [Source:HGNC Symbol;Acc:HGNC:34043]	processed pseudogene	-1.01	0.66	24.22	<0.0001	<0.0001
ENSG00000154262	ABCA6	ATP binding cassette subfamily A member 6 [Source:HGNC Symbol;Acc:HGNC:36]	protein coding	-1.01	0.55	32.63	<0.0001	<0.0001
ENSG00000257017	HP	haptoglobin [Source:HGNC Symbol;Acc:HGNC:5141]	protein coding	1.01	3.28	40.35	<0.0001	<0.0001
ENSG00000179750	APOBEC3B	apolipoprotein B mRNA editing enzyme catalytic subunit 3B [Source:HGNC Symbol;Acc:HGNC:17352]	protein coding	1.01	1.60	26.85	<0.0001	<0.0001
ENSG00000275131		phosphodiesterase 4D interacting protein (PDE4DIP) pseudogene	transcribed unprocessed pseudogene	-1.01	2.64	75.52	<0.0001	<0.0001
ENSG00000076554	TPD52	tumor protein D52 [Source:HGNC Symbol;Acc:HGNC:12005]	protein coding	-1.01	1.45	53.30	<0.0001	<0.0001
ENSG00000232931	LINC00342	long intergenic non-protein coding RNA 342 [Source:HGNC Symbol;Acc:HGNC:42470]	lncRNA	-1.01	2.73	56.56	<0.0001	<0.0001
ENSG00000103485	QPRT	quinolinate phosphoribosyltransferase [Source:HGNC Symbol;Acc:HGNC:9755]	protein coding	-1.01	2.28	33.90	<0.0001	<0.0001
ENSG00000152778	IFIT5	interferon induced protein with tetratricopeptide repeats 5 [Source:HGNC Symbol;Acc:HGNC:13328]	protein coding	1.01	4.48	70.99	<0.0001	<0.0001

Confidential/Proprietary Information

Table S5: Genes differentially expressed at Day 30 compared to pre-second vaccination (Study Arm 2 A/H7N9+AS03, D1, 29, Monocytes). Genes are sorted by descending absolute log_2 fold change. Gene model summaries and annotations are based on Ensembl Version 99 (continued.)

Ensembl Gene ID	Ensembl Gene Name	Ensembl Gene Description	Gene Type	log_2 Fold Change (Day 30 vs. Pre-Second Vaccination)	Average log_2 CPM	Likelihood Ratio Test Statistic	P-Value	FDR Adjusted P-Value
ENSG00000259436		novel transcript, antisense to POU2F2	lncRNA	-1.01	3.19	58.02	<0.0001	<0.0001
ENSG00000205413	SAMD9	sterile alpha motif domain containing 9 [Source:HGNC Symbol;Acc:HGNC:1348]	protein coding	1.01	6.11	43.58	<0.0001	<0.0001
ENSG00000106624	AEBP1	AE binding protein 1 [Source:HGNC Symbol;Acc:HGNC:303]	protein coding	-1.01	0.57	25.77	<0.0001	<0.0001
ENSG00000213626	LBH	LBH regulator of WNT signaling pathway [Source:HGNC Symbol;Acc:HGNC:29532]	protein coding	-1.01	3.23	31.95	<0.0001	<0.0001
ENSG00000211893	IGHG2	immunoglobulin heavy constant gamma 2 (G2m marker) [Source:HGNC Symbol;Acc:HGNC:5526]	IG C gene	-1.00	4.75	17.67	<0.0001	0.0001
ENSG00000150347	ARID5B	AT-rich interaction domain 5B [Source:HGNC Symbol;Acc:HGNC:17362]	protein coding	1.00	3.24	38.26	<0.0001	<0.0001
ENSG00000111199	TRPV4	transient receptor potential cation channel subfamily V member 4 [Source:HGNC Symbol;Acc:HGNC:18083]	protein coding	1.00	2.71	35.17	<0.0001	<0.0001
ENSG00000139193	CD27	CD27 molecule [Source:HGNC Symbol;Acc:HGNC:11922]	protein coding	-1.00	0.95	23.11	<0.0001	<0.0001
ENSG00000099985	OSM	oncostatin M [Source:HGNC Symbol;Acc:HGNC:8506]	protein coding	1.00	2.53	17.89	<0.0001	0.0001
ENSG00000240219		novel transcript	lncRNA	1.00	1.39	17.40	<0.0001	0.0001
ENSG00000128594	LRRC4	leucine rich repeat containing 4 [Source:HGNC Symbol;Acc:HGNC:15586]	protein coding	0.99	3.85	30.43	<0.0001	<0.0001
ENSG00000242324		PTPNS (protein tyrosine phosphatase, non-receptor type substrate 1) protein family pseudogene	unprocessed pseudogene	0.99	2.18	25.02	<0.0001	<0.0001
ENSG00000106537	TSPAN13	tetraspanin 13 [Source:HGNC Symbol;Acc:HGNC:21643]	protein coding	-0.99	1.38	19.28	<0.0001	<0.0001
ENSG00000163635	ATXN7	ataxin 7 [Source:HGNC Symbol;Acc:HGNC:10560]	protein coding	0.99	2.27	54.19	<0.0001	<0.0001
ENSG00000211899	IGHM	immunoglobulin heavy constant mu [Source:HGNC Symbol;Acc:HGNC:5541]	IG C gene	-0.98	7.74	47.39	<0.0001	<0.0001
ENSG00000124772	CPNE5	copine 5 [Source:HGNC Symbol;Acc:HGNC:2318]	protein coding	-0.98	2.80	46.12	<0.0001	<0.0001
ENSG00000111371	SLC38A1	solute carrier family 38 member 1 [Source:HGNC Symbol;Acc:HGNC:13447]	protein coding	-0.98	3.53	57.63	<0.0001	<0.0001
ENSG00000143126	CELSR2	cadherin EGF LAG seven-pass G-type receptor 2 [Source:HGNC Symbol;Acc:HGNC:3231]	protein coding	-0.98	2.37	36.48	<0.0001	<0.0001

Table S5: Genes differentially expressed at Day 30 compared to pre-second vaccination (Study Arm 2 A/H7N9+AS03, D1, 29, Monocytes). Genes are sorted by descending absolute log_2 fold change. Gene model summaries and annotations are based on Ensembl Version 99 (continued.)

Ensembl Gene ID	Ensembl Gene Name	Ensembl Gene Description	Gene Type	log_2 Fold Change (Day 30 vs. Pre-Second Vaccination)	Average log_2 CPM	Likelihood Ratio Test Statistic	P-Value	FDR Adjusted P-Value
ENSG00000163840	DTX3L	deltex E3 ubiquitin ligase 3L [Source:HGNC Symbol;Acc:HGNC:30323]	protein coding	0.98	6.60	38.27	<0.0001	<0.0001
ENSG00000115267	IFIH1	interferon induced with helicase C domain 1 [Source:HGNC Symbol;Acc:HGNC:18873]	protein coding	0.98	6.08	54.19	<0.0001	<0.0001
ENSG00000162599	NFIA	nuclear factor I A [Source:HGNC Symbol;Acc:HGNC:7784]	protein coding	-0.98	2.89	34.43	<0.0001	<0.0001
ENSG00000130164	LDLR	low density lipoprotein receptor [Source:HGNC Symbol;Acc:HGNC:6547]	protein coding	0.98	5.32	40.23	<0.0001	<0.0001
ENSG00000143847	PPFIA4	PTPRF interacting protein alpha 4 [Source:HGNC Symbol;Acc:HGNC:9248]	protein coding	-0.98	4.26	56.55	<0.0001	<0.0001
ENSG00000187210	GCNT1	glucosaminyl (N-acetyl) transferase 1 [Source:HGNC Symbol;Acc:HGNC:4203]	protein coding	0.98	4.80	49.68	<0.0001	<0.0001
ENSG00000251453	HAUS1P1	HAUS augmin like complex subunit 1 pseudogene 1 [Source:HGNC Symbol;Acc:HGNC:43760]	processed pseudogene	-0.98	1.08	24.16	<0.0001	<0.0001
ENSG00000129116	PALLD	palladin, cytoskeletal associated protein [Source:HGNC Symbol;Acc:HGNC:17068]	protein coding	-0.98	3.51	26.52	<0.0001	<0.0001
ENSG00000258130		coiled-coil domain containing 101 (CCDC101) pseudogene	transcribed unprocessed pseudogene	-0.98	0.57	33.59	<0.0001	<0.0001
ENSG00000287737		novel transcript	lncRNA	-0.98	0.80	14.92	0.0001	0.0004
ENSG00000154319	FAM167A	family with sequence similarity 167 member A [Source:HGNC Symbol;Acc:HGNC:15549]	protein coding	-0.98	0.77	26.91	<0.0001	<0.0001
ENSG00000126368	NR1D1	nuclear receptor subfamily 1 group D member 1 [Source:HGNC Symbol;Acc:HGNC:7962]	protein coding	-0.97	2.14	17.13	<0.0001	0.0002
ENSG00000072858	SIDT1	SID1 transmembrane family member 1 [Source:HGNC Symbol;Acc:HGNC:25967]	protein coding	-0.97	1.84	48.28	<0.0001	<0.0001
ENSG00000067057	PFKP	phosphofructokinase, platelet [Source:HGNC Symbol;Acc:HGNC:8878]	protein coding	0.97	5.25	43.09	<0.0001	<0.0001
ENSG00000061918	GUCY1B1	guanylate cyclase 1 soluble subunit beta 1 [Source:HGNC Symbol;Acc:HGNC:4687]	protein coding	0.97	0.85	17.36	<0.0001	0.0001
ENSG00000170581	STAT2	signal transducer and activator of transcription 2 [Source:HGNC Symbol;Acc:HGNC:11363]	protein coding	0.97	8.97	47.73	<0.0001	<0.0001

Table S5: Genes differentially expressed at Day 30 compared to pre-second vaccination (Study Arm 2 A/H7N9+AS03, D1, 29, Monocytes). Genes are sorted by descending absolute log_2 fold change. Gene model summaries and annotations are based on Ensembl Version 99 (continued.)

Ensembl Gene ID	Ensembl Gene Name	Ensembl Gene Description	Gene Type	log_2 Fold Change (Day 30 vs. Pre-Second Vaccination)	Average log_2 CPM	Likelihood Ratio Test Statistic	P-Value	FDR Adjusted P-Value
ENSG00000171766	GATM	glycine amidinotransferase [Source:HGNC Symbol;Acc:HGNC:4175]	protein coding	-0.97	1.55	48.52	<0.0001	<0.0001
ENSG00000198087	CD2AP	CD2 associated protein [Source:HGNC Symbol;Acc:HGNC:14258]	protein coding	0.97	4.66	59.47	<0.0001	<0.0001
ENSG00000041357	PSMA4	proteasome 20S subunit alpha 4 [Source:HGNC Symbol;Acc:HGNC:9533]	protein coding	0.97	7.40	86.75	<0.0001	<0.0001
ENSG00000132274	TRIM22	tripartite motif containing 22 [Source:HGNC Symbol;Acc:HGNC:16379]	protein coding	0.97	8.75	65.34	<0.0001	<0.0001
ENSG00000026559	KCNG1	potassium voltage-gated channel modifier subfamily G member 1 [Source:HGNC Symbol;Acc:HGNC:6248]	protein coding	-0.96	0.27	13.09	0.0003	0.001
ENSG00000205220	PSMB10	proteasome 20S subunit beta 10 [Source:HGNC Symbol;Acc:HGNC:9538]	protein coding	0.96	7.08	79.75	<0.0001	<0.0001
ENSG00000232629	HLA-DQB2	major histocompatibility complex, class II, DQ beta 2 [Source:HGNC Symbol;Acc:HGNC:4945]	protein coding	0.96	3.13	46.01	<0.0001	<0.0001
ENSG00000135604	STX11	syntaxin 11 [Source:HGNC Symbol;Acc:HGNC:11429]	protein coding	0.96	8.61	78.87	<0.0001	<0.0001
ENSG00000137098	SPAG8	sperm associated antigen 8 [Source:HGNC Symbol;Acc:HGNC:14105]	protein coding	-0.96	0.94	39.23	<0.0001	<0.0001
ENSG00000151726	ACSL1	acyl-CoA synthetase long chain family member 1 [Source:HGNC Symbol;Acc:HGNC:3569]	protein coding	0.96	8.15	59.66	<0.0001	<0.0001
ENSG00000112096	SOD2	superoxide dismutase 2 [Source:HGNC Symbol;Acc:HGNC:11180]	protein coding	0.96	8.58	51.29	<0.0001	<0.0001
ENSG00000211959	IGHV4-39	immunoglobulin heavy variable 4-39 [Source:HGNC Symbol;Acc:HGNC:5651]	IG V gene	-0.96	0.41	20.32	<0.0001	<0.0001
ENSG00000157551	KCNJ15	potassium inwardly rectifying channel subfamily J member 15 [Source:HGNC Symbol;Acc:HGNC:6261]	protein coding	0.96	3.34	23.40	<0.0001	<0.0001
ENSG00000214595	EML6	EMAP like 6 [Source:HGNC Symbol;Acc:HGNC:35412]	protein coding	-0.96	1.12	40.37	<0.0001	<0.0001
ENSG00000100027	YPEL1	yippee like 1 [Source:HGNC Symbol;Acc:HGNC:12845]	protein coding	-0.96	0.60	32.93	<0.0001	<0.0001
ENSG00000211949	IGHV3-23	immunoglobulin heavy variable 3-23 [Source:HGNC Symbol;Acc:HGNC:5588]	IG V gene	-0.96	2.59	23.09	<0.0001	<0.0001
ENSG00000211934	IGHV1-2	immunoglobulin heavy variable 1-2 [Source:HGNC Symbol;Acc:HGNC:5550]	IG V gene	-0.95	0.53	26.75	<0.0001	<0.0001
ENSG00000135424	ITGA7	integrin subunit alpha 7 [Source:HGNC Symbol;Acc:HGNC:6143]	protein coding	0.95	2.07	35.30	<0.0001	<0.0001

Table S5: Genes differentially expressed at Day 30 compared to pre-second vaccination (Study Arm 2 A/H7N9+AS03, D1, 29, Monocytes). Genes are sorted by descending absolute log_2 fold change. Gene model summaries and annotations are based on Ensembl Version 99 (continued.)

Ensembl Gene ID	Ensembl Gene Name	Ensembl Gene Description	Gene Type	log_2 Fold Change (Day 30 vs. Pre-Second Vaccination)	Average log_2 CPM	Likelihood Ratio Test Statistic	P-Value	FDR Adjusted P-Value
ENSG00000268403		novel transcript, antisense to ZNF143	lncRNA	-0.95	0.05	19.24	<0.0001	<0.0001
ENSG00000141527	CARD14	caspase recruitment domain family member 14 [Source:HGNC Symbol;Acc:HGNC:16446]	protein coding	-0.95	2.25	42.66	<0.0001	<0.0001
ENSG00000211943	IGHV3-15	immunoglobulin heavy variable 3-15 [Source:HGNC Symbol;Acc:HGNC:5582]	IG V gene	-0.95	0.77	16.00	<0.0001	0.0003
ENSG00000187741	FANCA	FA complementation group A [Source:HGNC Symbol;Acc:HGNC:3582]	protein coding	0.95	3.77	84.43	<0.0001	<0.0001
ENSG00000272941		novel transcript, antisense to C7orf49	lncRNA	0.95	0.35	29.39	<0.0001	<0.0001
ENSG00000173110	HSPA6	heat shock protein family A (Hsp70) member 6 [Source:HGNC Symbol;Acc:HGNC:5239]	protein coding	0.95	5.65	56.05	<0.0001	<0.0001
ENSG00000163606	CD200R1	CD200 receptor 1 [Source:HGNC Symbol;Acc:HGNC:24235]	protein coding	-0.95	2.40	27.32	<0.0001	<0.0001
ENSG00000158555	GDPD5	glycerophosphodiester phosphodiesterase domain containing 5 [Source:HGNC Symbol;Acc:HGNC:28804]	protein coding	0.95	5.98	88.52	<0.0001	<0.0001
ENSG00000090447	TFAP4	transcription factor AP-4 [Source:HGNC Symbol;Acc:HGNC:11745]	protein coding	-0.95	4.13	62.57	<0.0001	<0.0001
ENSG00000173372	C1QA	complement C1q A chain [Source:HGNC Symbol;Acc:HGNC:1241]	protein coding	0.95	4.58	31.18	<0.0001	<0.0001
ENSG00000177675	CD163L1	CD163 molecule like 1 [Source:HGNC Symbol;Acc:HGNC:30375]	protein coding	-0.95	0.60	24.44	<0.0001	<0.0001
ENSG00000138670	RASGEF1B	RasGEF domain family member 1B [Source:HGNC Symbol;Acc:HGNC:24881]	protein coding	0.95	5.46	11.69	0.0006	0.002
ENSG00000163629	PTPN13	protein tyrosine phosphatase non-receptor type 13 [Source:HGNC Symbol;Acc:HGNC:9646]	protein coding	-0.95	1.22	36.01	<0.0001	<0.0001
ENSG00000091262	ABCC6	ATP binding cassette subfamily C member 6 [Source:HGNC Symbol;Acc:HGNC:57]	protein coding	-0.95	3.93	63.96	<0.0001	<0.0001
ENSG00000239951	IGKV3-20	immunoglobulin kappa variable 3-20 [Source:HGNC Symbol;Acc:HGNC:5817]	IG V gene	-0.94	2.52	25.59	<0.0001	<0.0001
ENSG00000272734	ADIRF-AS1	ADIRF antisense RNA 1 [Source:HGNC Symbol;Acc:HGNC:45127]	lncRNA	-0.94	2.52	75.21	<0.0001	<0.0001
ENSG00000086300	SNX10	sorting nexin 10 [Source:HGNC Symbol;Acc:HGNC:14974]	protein coding	0.94	7.40	69.62	<0.0001	<0.0001
ENSG00000132963	POMP	proteasome maturation protein [Source:HGNC Symbol;Acc:HGNC:20330]	protein coding	0.94	5.75	87.36	<0.0001	<0.0001
ENSG00000111186	WNT5B	Wnt family member 5B [Source:HGNC Symbol;Acc:HGNC:16265]	protein coding	-0.94	2.06	28.90	<0.0001	<0.0001

Table S5: Genes differentially expressed at Day 30 compared to pre-second vaccination (Study Arm 2 A/H7N9+AS03, D1, 29, Monocytes). Genes are sorted by descending absolute log_2 fold change. Gene model summaries and annotations are based on Ensembl Version 99 (continued.)

Ensembl Gene ID	Ensembl Gene Name	Ensembl Gene Description	Gene Type	log_2 Fold Change (Day 30 vs. Pre-Second Vaccination)	Average log_2 CPM	Likelihood Ratio Test Statistic	P-Value	FDR Adjusted P-Value
ENSG00000112232	KHDRBS2	KH RNA binding domain containing, signal transduction associated 2 [Source:HGNC Symbol;Acc:HGNC:18114]	protein coding	-0.94	0.11	27.93	<0.0001	<0.0001
ENSG00000260086		novel transcript	lncRNA	-0.94	0.58	24.28	<0.0001	<0.0001
ENSG00000167984	NLRC3	NLR family CARD domain containing 3 [Source:HGNC Symbol;Acc:HGNC:29889]	protein coding	-0.94	1.77	26.81	<0.0001	<0.0001
ENSG00000136895	GARNL3	GTPase activating Rap/RanGAP domain like 3 [Source:HGNC Symbol;Acc:HGNC:25425]	protein coding	-0.94	0.26	19.44	<0.0001	<0.0001
ENSG00000023171	GRAMD1B	GRAM domain containing 1B [Source:HGNC Symbol;Acc:HGNC:29214]	protein coding	0.94	5.19	59.99	<0.0001	<0.0001
ENSG00000115419	GLS	glutaminase [Source:HGNC Symbol;Acc:HGNC:4331]	protein coding	0.94	5.99	54.39	<0.0001	<0.0001
ENSG00000279278		novel transcript	lncRNA	-0.94	1.51	21.50	<0.0001	<0.0001
ENSG00000228486	C2orf92	chromosome 2 open reading frame 92 [Source:HGNC Symbol;Acc:HGNC:49272]	protein coding	-0.94	1.68	40.96	<0.0001	<0.0001
ENSG00000164050	PLXNB1	plexin B1 [Source:HGNC Symbol;Acc:HGNC:9103]	protein coding	-0.94	2.06	58.46	<0.0001	<0.0001
ENSG00000124215	CDH26	cadherin 26 [Source:HGNC Symbol;Acc:HGNC:15902]	protein coding	-0.94	1.43	56.86	<0.0001	<0.0001
ENSG00000211677	IGLC2	immunoglobulin lambda constant 2 [Source:HGNC Symbol;Acc:HGNC:5856]	IG C gene	-0.94	4.22	21.56	<0.0001	<0.0001
ENSG00000183734	ASCL2	achaete-scute family bHLH transcription factor 2 [Source:HGNC Symbol;Acc:HGNC:739]	protein coding	0.93	5.23	83.58	<0.0001	<0.0001
ENSG00000125731	SH2D3A	SH2 domain containing 3A [Source:HGNC Symbol;Acc:HGNC:16885]	protein coding	-0.93	0.81	27.48	<0.0001	<0.0001
ENSG00000116711	PLA2G4A	phospholipase A2 group IVA [Source:HGNC Symbol;Acc:HGNC:9035]	protein coding	0.93	3.55	50.37	<0.0001	<0.0001
ENSG00000103569	AQP9	aquaporin 9 [Source:HGNC Symbol;Acc:HGNC:643]	protein coding	0.93	7.04	69.49	<0.0001	<0.0001
ENSG00000187240	DYNC2H1	dynein cytoplasmic 2 heavy chain 1 [Source:HGNC Symbol;Acc:HGNC:2962]	protein coding	-0.93	1.15	28.70	<0.0001	<0.0001
ENSG00000254719		guanine nucleotide binding protein (G protein) pseudogene	processed pseudogene	0.93	0.76	40.53	<0.0001	<0.0001
ENSG00000160298	C21orf58	chromosome 21 open reading frame 58 [Source:HGNC Symbol;Acc:HGNC:1300]	protein coding	-0.93	0.77	33.72	<0.0001	<0.0001

Table S5: Genes differentially expressed at Day 30 compared to pre-second vaccination (Study Arm 2 A/H7N9+AS03, D1, 29, Monocytes). Genes are sorted by descending absolute log_2 fold change. Gene model summaries and annotations are based on Ensembl Version 99 (continued.)

Ensembl Gene ID	Ensembl Gene Name	Ensembl Gene Description	Gene Type	log_2 Fold Change (Day 30 vs. Pre-Second Vaccination)	Average log_2 CPM	Likelihood Ratio Test Statistic	P-Value	FDR Adjusted P-Value
ENSG00000130558	OLFM1	olfactomedin 1 [Source:HGNC Symbol;Acc:HGNC:17187]	protein coding	-0.93	4.53	45.97	<0.0001	<0.0001
ENSG00000135519	KCNH3	potassium voltage-gated channel subfamily H member 3 [Source:HGNC Symbol;Acc:HGNC:6252]	protein coding	-0.93	4.63	60.37	<0.0001	<0.0001
ENSG00000140104	CLBA1	clathrin binding box of aftiphilin containing 1 [Source:HGNC Symbol;Acc:HGNC:20126]	protein coding	-0.93	0.57	33.04	<0.0001	<0.0001
ENSG00000244437	IGKV3-15	immunoglobulin kappa variable 3-15 [Source:HGNC Symbol;Acc:HGNC:5816]	IG V gene	-0.92	1.33	9.92	0.0016	0.0046
ENSG00000123609	NMI	N-myc and STAT interactor [Source:HGNC Symbol;Acc:HGNC:7854]	protein coding	0.92	6.44	71.39	<0.0001	<0.0001
ENSG00000118922	KLF12	Kruppel like factor 12 [Source:HGNC Symbol;Acc:HGNC:6346]	protein coding	-0.92	3.09	46.75	<0.0001	<0.0001
ENSG00000182557	SPNS3	sphingolipid transporter 3 (putative) [Source:HGNC Symbol;Acc:HGNC:28433]	protein coding	-0.92	3.30	40.11	<0.0001	<0.0001
ENSG00000169994	MYO7B	myosin VIIB [Source:HGNC Symbol;Acc:HGNC:7607]	protein coding	0.92	4.44	21.61	<0.0001	<0.0001
ENSG00000211598	IGKV4-1	immunoglobulin kappa variable 4-1 [Source:HGNC Symbol;Acc:HGNC:5834]	IG V gene	-0.92	2.51	26.02	<0.0001	<0.0001
ENSG00000108106	UBE2S	ubiquitin conjugating enzyme E2 S [Source:HGNC Symbol;Acc:HGNC:17895]	protein coding	0.92	4.68	109.53	<0.0001	<0.0001
ENSG00000028116	VRK2	VRK serine/threonine kinase 2 [Source:HGNC Symbol;Acc:HGNC:12719]	protein coding	0.92	4.99	79.06	<0.0001	<0.0001
ENSG00000204103	MAFB	MAF bZIP transcription factor B [Source:HGNC Symbol;Acc:HGNC:6408]	protein coding	0.92	8.10	48.43	<0.0001	<0.0001
ENSG00000253785		pyrophosphatase (inorganic) 1 (PPA1) pseudogene	processed pseudogene	0.92	0.82	36.60	<0.0001	<0.0001
ENSG00000184588	PDE4B	phosphodiesterase 4B [Source:HGNC Symbol;Acc:HGNC:8781]	protein coding	0.92	4.69	67.76	<0.0001	<0.0001
ENSG00000136689	IL1RN	interleukin 1 receptor antagonist [Source:HGNC Symbol;Acc:HGNC:6000]	protein coding	0.92	6.24	45.99	<0.0001	<0.0001
ENSG00000224307		novel transcript	lncRNA	-0.91	3.64	48.57	<0.0001	<0.0001
ENSG00000232442	MHENCR	melanoma highly expressed competing endogenous lncRNA for miR-425 and miR-489 [Source:HGNC Symbol;Acc:HGNC:53110]	lncRNA	-0.91	4.51	58.92	<0.0001	<0.0001

Table S5: Genes differentially expressed at Day 30 compared to pre-second vaccination (Study Arm 2 A/H7N9+AS03, D1, 29, Monocytes). Genes are sorted by descending absolute log_2 fold change. Gene model summaries and annotations are based on Ensembl Version 99 (continued.)

Ensembl Gene ID	Ensembl Gene Name	Ensembl Gene Description	Gene Type	log_2 Fold Change (Day 30 vs. Pre-Second Vaccination)	Average log_2 CPM	Likelihood Ratio Test Statistic	P-Value	FDR Adjusted P-Value
ENSG00000130702	LAMA5	laminin subunit alpha 5 [Source:HGNC Symbol;Acc:HGNC:6485]	protein coding	-0.91	2.03	49.42	<0.0001	<0.0001
ENSG00000233461		novel transcript	lncRNA	0.91	1.77	31.53	<0.0001	<0.0001
ENSG00000204264	PSMB8	proteasome 20S subunit beta 8 [Source:HGNC Symbol;Acc:HGNC:9545]	protein coding	0.91	7.95	91.54	<0.0001	<0.0001
ENSG00000234290		novel transcript	lncRNA	0.91	3.17	32.03	<0.0001	<0.0001
ENSG00000261373	VPS9D1-AS1	VPS9D1 antisense RNA 1 [Source:HGNC Symbol;Acc:HGNC:48915]	lncRNA	0.91	2.89	33.54	<0.0001	<0.0001
ENSG00000088827	SIGLEC1	sialic acid binding Ig like lectin 1 [Source:HGNC Symbol;Acc:HGNC:11127]	protein coding	0.91	7.36	32.48	<0.0001	<0.0001
ENSG00000261655		novel transcript, overlapping to GPR20	lncRNA	-0.91	0.43	12.02	0.0005	0.0017
ENSG00000189007	ADAT2	adenosine deaminase tRNA specific 2 [Source:HGNC Symbol;Acc:HGNC:21172]	protein coding	-0.91	2.73	49.07	<0.0001	<0.0001
ENSG00000270157		novel transcript	lncRNA	-0.91	0.76	33.36	<0.0001	<0.0001
ENSG00000173599	PC	pyruvate carboxylase [Source:HGNC Symbol;Acc:HGNC:8636]	protein coding	-0.91	3.29	47.90	<0.0001	<0.0001
ENSG00000123700	KCNJ2	potassium inwardly rectifying channel subfamily J member 2 [Source:HGNC Symbol;Acc:HGNC:6263]	protein coding	0.91	3.17	20.91	<0.0001	<0.0001
ENSG00000054219	LY75	lymphocyte antigen 75 [Source:HGNC Symbol;Acc:HGNC:6729]	protein coding	0.91	4.32	44.39	<0.0001	<0.0001
ENSG00000168778	TCTN2	tectonic family member 2 [Source:HGNC Symbol;Acc:HGNC:25774]	protein coding	-0.91	0.06	25.83	<0.0001	<0.0001
ENSG00000013374	NUB1	negative regulator of ubiquitin like proteins 1 [Source:HGNC Symbol;Acc:HGNC:17623]	protein coding	0.91	6.71	60.77	<0.0001	<0.0001
ENSG00000275993	SIK1B	salt inducible kinase 1B (putative) [Source:HGNC Symbol;Acc:HGNC:52389]	protein coding	0.90	2.46	14.29	0.0002	0.0006
ENSG00000271856	LINC01215	long intergenic non-protein coding RNA 1215 [Source:HGNC Symbol;Acc:HGNC:49651]	lncRNA	-0.90	1.45	45.99	<0.0001	<0.0001
ENSG00000225964	NRIR	negative regulator of interferon response [Source:HGNC Symbol;Acc:HGNC:51269]	lncRNA	0.90	1.71	18.25	<0.0001	<0.0001
ENSG00000103942	HOMER2	homer scaffold protein 2 [Source:HGNC Symbol;Acc:HGNC:17513]	protein coding	-0.90	1.73	65.89	<0.0001	<0.0001
ENSG00000118420	UBE3D	ubiquitin protein ligase E3D [Source:HGNC Symbol;Acc:HGNC:21381]	protein coding	-0.90	0.36	33.11	<0.0001	<0.0001
ENSG00000166016	ABTB2	ankyrin repeat and BTB domain containing 2 [Source:HGNC Symbol;Acc:HGNC:23842]	protein coding	0.90	2.55	24.62	<0.0001	<0.0001

Confidential/Proprietary Information

Table S5: Genes differentially expressed at Day 30 compared to pre-second vaccination (Study Arm 2 A/H7N9+AS03, D1, 29, Monocytes). Genes are sorted by descending absolute log_2 fold change. Gene model summaries and annotations are based on Ensembl Version 99 (continued.)

Ensembl Gene ID	Ensembl Gene Name	Ensembl Gene Description	Gene Type	log_2 Fold Change (Day 30 vs. Pre-Second Vaccination)	Average log_2 CPM	Likelihood Ratio Test Statistic	P-Value	FDR Adjusted P-Value
ENSG00000211666	IGLV2-14	immunoglobulin lambda variable 2-14 [Source:HGNC Symbol;Acc:HGNC:5888]	IG V gene	-0.90	1.82	14.78	0.0001	0.0005
ENSG00000213949	ITGA1	integrin subunit alpha 1 [Source:HGNC Symbol;Acc:HGNC:6134]	protein coding	0.90	1.44	28.50	<0.0001	<0.0001
ENSG00000188343	FAM92A	family with sequence similarity 92 member A [Source:HGNC Symbol;Acc:HGNC:30452]	protein coding	0.90	1.83	26.93	<0.0001	<0.0001
ENSG00000135643	KCNMB4	potassium calcium-activated channel subfamily M regulatory beta subunit 4 [Source:HGNC Symbol;Acc:HGNC:6289]	protein coding	-0.89	1.45	29.99	<0.0001	<0.0001
ENSG00000251259		novel transcript	lncRNA	-0.89	1.28	29.61	<0.0001	<0.0001
ENSG00000287576		novel transcript	lncRNA	0.89	1.35	45.77	<0.0001	<0.0001
ENSG00000113140	SPARC	secreted protein acidic and cysteine rich [Source:HGNC Symbol;Acc:HGNC:11219]	protein coding	-0.89	3.10	66.92	<0.0001	<0.0001
ENSG00000165030	NFIL3	nuclear factor, interleukin 3 regulated [Source:HGNC Symbol;Acc:HGNC:7787]	protein coding	0.89	5.25	48.39	<0.0001	<0.0001
ENSG00000170542	SERPINB9	serpin family B member 9 [Source:HGNC Symbol;Acc:HGNC:8955]	protein coding	0.89	7.37	50.50	<0.0001	<0.0001
ENSG00000156535	CD109	CD109 molecule [Source:HGNC Symbol;Acc:HGNC:21685]	protein coding	-0.89	0.44	30.40	<0.0001	<0.0001
ENSG00000065833	ME1	malic enzyme 1 [Source:HGNC Symbol;Acc:HGNC:6983]	protein coding	-0.89	1.83	21.93	<0.0001	<0.0001
ENSG00000188313	PLSCR1	phospholipid scramblase 1 [Source:HGNC Symbol;Acc:HGNC:9092]	protein coding	0.89	7.15	65.99	<0.0001	<0.0001
ENSG00000148680	HTR7	5-hydroxytryptamine receptor 7 [Source:HGNC Symbol;Acc:HGNC:5302]	protein coding	-0.89	0.97	29.37	<0.0001	<0.0001
ENSG00000166750	SLFN5	schlafen family member 5 [Source:HGNC Symbol;Acc:HGNC:28286]	protein coding	0.89	5.65	42.66	<0.0001	<0.0001
ENSG00000204136	GGTA1P	glycoprotein alpha-galactosyltransferase 1, pseudogene [Source:HGNC Symbol;Acc:HGNC:4253]	transcribed unitary pseudogene	-0.89	4.29	41.39	<0.0001	<0.0001
ENSG00000137078	SIT1	signaling threshold regulating transmembrane adaptor 1 [Source:HGNC Symbol;Acc:HGNC:17710]	protein coding	-0.89	0.50	13.92	0.0002	0.0007
ENSG00000211964	IGHV3-48	immunoglobulin heavy variable 3-48 [Source:HGNC Symbol;Acc:HGNC:5606]	IG V gene	-0.89	0.61	11.16	0.0008	0.0026
ENSG00000235513	L3MBTL2-AS1	L3MBTL2 antisense RNA 1 [Source:HGNC Symbol;Acc:HGNC:40847]	lncRNA	0.89	1.08	15.99	<0.0001	0.0003

Table S5: Genes differentially expressed at Day 30 compared to pre-second vaccination (Study Arm 2 A/H7N9+AS03, D1, 29, Monocytes). Genes are sorted by descending absolute log_2 fold change. Gene model summaries and annotations are based on Ensembl Version 99 (continued.)

Ensembl Gene ID	Ensembl Gene Name	Ensembl Gene Description	Gene Type	log_2 Fold Change (Day 30 vs. Pre-Second Vaccination)	Average log_2 CPM	Likelihood Ratio Test Statistic	P-Value	FDR Adjusted P-Value
ENSG00000287839		novel transcript	lncRNA	0.88	3.75	68.62	<0.0001	<0.0001
ENSG00000172728	FUT10	fucosyltransferase 10 [Source:HGNC Symbol;Acc:HGNC:19234]	protein coding	-0.88	1.25	40.51	<0.0001	<0.0001
ENSG00000133460	SLC2A11	solute carrier family 2 member 11 [Source:HGNC Symbol;Acc:HGNC:14239]	protein coding	-0.88	2.20	38.66	<0.0001	<0.0001
ENSG00000162433	AK4	adenylate kinase 4 [Source:HGNC Symbol;Acc:HGNC:363]	protein coding	0.88	2.12	29.84	<0.0001	<0.0001
ENSG00000073060	SCARB1	scavenger receptor class B member 1 [Source:HGNC Symbol;Acc:HGNC:1664]	protein coding	-0.88	5.07	81.01	<0.0001	<0.0001
ENSG00000188404	SELL	selectin L [Source:HGNC Symbol;Acc:HGNC:10720]	protein coding	0.88	9.77	39.35	<0.0001	<0.0001
ENSG00000157827	FMNL2	formin like 2 [Source:HGNC Symbol;Acc:HGNC:18267]	protein coding	0.88	4.10	52.38	<0.0001	<0.0001
ENSG00000272555		novel transcript	lncRNA	-0.88	0.54	37.38	<0.0001	<0.0001
ENSG00000211592	IGKC	immunoglobulin kappa constant [Source:HGNC Symbol;Acc:HGNC:5716]	IG C gene	-0.88	6.90	19.74	<0.0001	<0.0001
ENSG00000284930		novel transcript	lncRNA	-0.88	0.68	36.58	<0.0001	<0.0001
ENSG00000177425	PAWR	pro-apoptotic WT1 regulator [Source:HGNC Symbol;Acc:HGNC:8614]	protein coding	-0.88	0.17	16.38	<0.0001	0.0002
ENSG00000071967	CYBRD1	cytochrome b reductase 1 [Source:HGNC Symbol;Acc:HGNC:20797]	protein coding	-0.88	6.04	45.20	<0.0001	<0.0001
ENSG00000162694	EXTL2	exostosin like glycosyltransferase 2 [Source:HGNC Symbol;Acc:HGNC:3516]	protein coding	-0.88	0.64	32.26	<0.0001	<0.0001
ENSG00000187621	TCL6	T cell leukemia/lymphoma 6 [Source:HGNC Symbol;Acc:HGNC:13463]	lncRNA	-0.88	0.60	19.78	<0.0001	<0.0001
ENSG00000182809	CRIP2	cysteine rich protein 2 [Source:HGNC Symbol;Acc:HGNC:2361]	protein coding	-0.88	1.15	19.43	<0.0001	<0.0001
ENSG00000105699	LSR	lipolysis stimulated lipoprotein receptor [Source:HGNC Symbol;Acc:HGNC:29572]	protein coding	-0.87	1.61	31.21	<0.0001	<0.0001
ENSG00000134954	ETS1	ETS proto-oncogene 1, transcription factor [Source:HGNC Symbol;Acc:HGNC:3488]	protein coding	-0.87	4.48	38.62	<0.0001	<0.0001
ENSG00000069849	ATP1B3	ATPase Na+/K+ transporting subunit beta 3 [Source:HGNC Symbol;Acc:HGNC:806]	protein coding	0.87	6.72	85.40	<0.0001	<0.0001
ENSG00000171992	SYNPO	synaptopodin [Source:HGNC Symbol;Acc:HGNC:30672]	protein coding	-0.87	1.33	15.29	<0.0001	0.0004

Table S5: Genes differentially expressed at Day 30 compared to pre-second vaccination (Study Arm 2 A/H7N9+AS03, D1, 29, Monocytes). Genes are sorted by descending absolute log_2 fold change. Gene model summaries and annotations are based on Ensembl Version 99 (continued.)

Ensembl Gene ID	Ensembl Gene Name	Ensembl Gene Description	Gene Type	log_2 Fold Change (Day 30 vs. Pre-Second Vaccination)	Average log_2 CPM	Likelihood Ratio Test Statistic	P-Value	FDR Adjusted P-Value
ENSG00000010818	HIVEP2	HIVEP zinc finger 2 [Source:HGNC Symbol;Acc:HGNC:4921]	protein coding	0.87	4.16	37.97	<0.0001	<0.0001
ENSG00000181649	PHLDA2	pleckstrin homology like domain family A member 2 [Source:HGNC Symbol;Acc:HGNC:12385]	protein coding	0.87	1.23	8.04	0.0046	0.0113
ENSG00000159433	STARD9	StAR related lipid transfer domain containing 9 [Source:HGNC Symbol;Acc:HGNC:19162]	protein coding	-0.87	1.03	39.90	<0.0001	<0.0001
ENSG00000104312	RIPK2	receptor interacting serine/threonine kinase 2 [Source:HGNC Symbol;Acc:HGNC:10020]	protein coding	0.87	5.53	51.70	<0.0001	<0.0001
ENSG00000253716	MINCR	MYC-induced long non-coding RNA [Source:HGNC Symbol;Acc:HGNC:51653]	lncRNA	-0.87	1.52	45.28	<0.0001	<0.0001
ENSG00000137767	SQOR	sulfide quinone oxidoreductase [Source:HGNC Symbol;Acc:HGNC:20390]	protein coding	0.87	5.96	91.86	<0.0001	<0.0001
ENSG00000013016	EHD3	EH domain containing 3 [Source:HGNC Symbol;Acc:HGNC:3244]	protein coding	-0.87	2.51	38.21	<0.0001	<0.0001
ENSG00000204653	ASPDH	aspartate dehydrogenase domain containing [Source:HGNC Symbol;Acc:HGNC:33856]	protein coding	-0.87	1.17	52.53	<0.0001	<0.0001
ENSG00000237638	LINC02245	long intergenic non-protein coding RNA 2245 [Source:HGNC Symbol;Acc:HGNC:53134]	lncRNA	-0.87	1.85	24.30	<0.0001	<0.0001
ENSG00000186918	ZNF395	zinc finger protein 395 [Source:HGNC Symbol;Acc:HGNC:18737]	protein coding	-0.87	4.83	103.19	<0.0001	<0.0001
ENSG00000219607	PPP1R3G	protein phosphatase 1 regulatory subunit 3G [Source:HGNC Symbol;Acc:HGNC:14945]	protein coding	-0.87	1.31	38.85	<0.0001	<0.0001
ENSG00000143801	PSEN2	presenilin 2 [Source:HGNC Symbol;Acc:HGNC:9509]	protein coding	0.87	3.91	61.95	<0.0001	<0.0001
ENSG00000273373		novel transcript, antisense to SLC16A4	lncRNA	-0.87	1.60	21.67	<0.0001	<0.0001
ENSG00000059378	PARP12	poly(ADP-ribose) polymerase family member 12 [Source:HGNC Symbol;Acc:HGNC:21919]	protein coding	0.87	6.85	66.83	<0.0001	<0.0001
ENSG00000072134	EPN2	epsin 2 [Source:HGNC Symbol;Acc:HGNC:18639]	protein coding	-0.86	0.17	21.37	<0.0001	<0.0001
ENSG00000283696		novel transcript	lncRNA	0.86	0.38	18.61	<0.0001	<0.0001
ENSG00000149564	ESAM	endothelial cell adhesion molecule [Source:HGNC Symbol;Acc:HGNC:17474]	protein coding	-0.86	1.02	11.55	0.0007	0.0022
ENSG00000214826	DDX12P	DEAD/H-box helicase 12, pseudogene [Source:HGNC Symbol;Acc:HGNC:2737]	unprocessed pseudogene	-0.86	1.27	26.69	<0.0001	<0.0001

Table S5: Genes differentially expressed at Day 30 compared to pre-second vaccination (Study Arm 2 A/H7N9+AS03, D1, 29, Monocytes). Genes are sorted by descending absolute log_2 fold change. Gene model summaries and annotations are based on Ensembl Version 99 (continued.)

Ensembl Gene ID	Ensembl Gene Name	Ensembl Gene Description	Gene Type	log_2 Fold Change (Day 30 vs. Pre-Second Vaccination)	Average log_2 CPM	Likelihood Ratio Test Statistic	P-Value	FDR Adjusted P-Value
ENSG00000110799	VWF	von Willebrand factor [Source:HGNC Symbol;Acc:HGNC:12726]	protein coding	-0.86	1.46	31.66	<0.0001	<0.0001
ENSG00000204622	HLA-J	major histocompatibility complex, class I, J (pseudogene) [Source:HGNC Symbol;Acc:HGNC:4967]	transcribed unprocessed pseudogene	0.86	1.19	9.86	0.0017	0.0048
ENSG00000129673	AANAT	aralkylamine N-acetyltransferase [Source:HGNC Symbol;Acc:HGNC:19]	protein coding	0.86	1.74	27.34	<0.0001	<0.0001
ENSG00000173611	SCAI	suppressor of cancer cell invasion [Source:HGNC Symbol;Acc:HGNC:26709]	protein coding	-0.86	2.57	75.87	<0.0001	<0.0001
ENSG00000137502	RAB30	RAB30, member RAS oncogene family [Source:HGNC Symbol;Acc:HGNC:9770]	protein coding	-0.86	1.75	35.20	<0.0001	<0.0001
ENSG00000075391	RASAL2	RAS protein activator like 2 [Source:HGNC Symbol;Acc:HGNC:9874]	protein coding	-0.86	1.30	37.49	<0.0001	<0.0001
ENSG00000253368	TRNP1	TMF1 regulated nuclear protein 1 [Source:HGNC Symbol;Acc:HGNC:34348]	protein coding	-0.86	1.43	24.21	<0.0001	<0.0001
ENSG00000204852	TCTN1	tectonic family member 1 [Source:HGNC Symbol;Acc:HGNC:26113]	protein coding	-0.86	2.29	61.31	<0.0001	<0.0001
ENSG00000117322	CR2	complement C3d receptor 2 [Source:HGNC Symbol;Acc:HGNC:2336]	protein coding	-0.86	1.34	12.22	0.0005	0.0016
ENSG00000211679	IGLC3	immunoglobulin lambda constant 3 (Kern-Oz+ marker) [Source:HGNC Symbol;Acc:HGNC:5857]	IG C gene	-0.86	3.51	15.61	<0.0001	0.0003
ENSG00000179331	RAB39A	RAB39A, member RAS oncogene family [Source:HGNC Symbol;Acc:HGNC:16521]	protein coding	0.86	3.03	61.11	<0.0001	<0.0001
ENSG00000165209	STRBP	spermatid perinuclear RNA binding protein [Source:HGNC Symbol;Acc:HGNC:16462]	protein coding	-0.86	2.88	67.54	<0.0001	<0.0001
ENSG00000224429	LINC00539	long intergenic non-protein coding RNA 539 [Source:HGNC Symbol;Acc:HGNC:43672]	lncRNA	-0.86	0.58	21.71	<0.0001	<0.0001
ENSG00000174306	ZHX3	zinc fingers and homeoboxes 3 [Source:HGNC Symbol;Acc:HGNC:15935]	protein coding	-0.86	3.40	60.24	<0.0001	<0.0001
ENSG00000163611	SPICE1	spindle and centriole associated protein 1 [Source:HGNC Symbol;Acc:HGNC:25083]	protein coding	-0.86	0.83	43.69	<0.0001	<0.0001
ENSG00000287537		novel transcript	lncRNA	-0.86	0.68	23.21	<0.0001	<0.0001
ENSG00000183508	TENT5C	terminal nucleotidyltransferase 5C [Source:HGNC Symbol;Acc:HGNC:24712]	protein coding	-0.86	1.66	22.20	<0.0001	<0.0001

Table S5: Genes differentially expressed at Day 30 compared to pre-second vaccination (Study Arm 2 A/H7N9+AS03, D1, 29, Monocytes). Genes are sorted by descending absolute log_2 fold change. Gene model summaries and annotations are based on Ensembl Version 99 (continued.)

Ensembl Gene ID	Ensembl Gene Name	Ensembl Gene Description	Gene Type	log_2 Fold Change (Day 30 vs. Pre-Second Vaccination)	Average log_2 CPM	Likelihood Ratio Test Statistic	P-Value	FDR Adjusted P-Value
ENSG00000269834	ZNF528-AS1	ZNF528 antisense RNA 1 [Source:HGNC Symbol;Acc:HGNC:51305]	lncRNA	-0.86	0.23	21.09	<0.0001	<0.0001
ENSG00000042317		spermatogenesis associated 7 [Source:NCBI gene;Acc:55812]	protein coding	-0.86	1.22	36.27	<0.0001	<0.0001
ENSG00000148429	USP6NL	USP6 N-terminal like [Source:HGNC Symbol;Acc:HGNC:16858]	protein coding	0.86	4.90	50.51	<0.0001	<0.0001
ENSG00000109501	WFS1	wolframin ER transmembrane glycoprotein [Source:HGNC Symbol;Acc:HGNC:12762]	protein coding	-0.86	0.96	39.96	<0.0001	<0.0001
ENSG00000253328	SUMO2P19	SUMO2 pseudogene 19 [Source:HGNC Symbol;Acc:HGNC:49357]	unprocessed pseudogene	-0.86	0.15	19.54	<0.0001	<0.0001
ENSG00000026103	FAS	Fas cell surface death receptor [Source:HGNC Symbol;Acc:HGNC:11920]	protein coding	0.85	5.11	55.01	<0.0001	<0.0001
ENSG00000270605		novel transcript	lncRNA	-0.85	0.48	29.09	<0.0001	<0.0001
ENSG00000185201	IFITM2	interferon induced transmembrane protein 2 [Source:HGNC Symbol;Acc:HGNC:5413]	protein coding	0.85	7.75	114.66	<0.0001	<0.0001
ENSG00000107201	DDX58	DExD/H-box helicase 58 [Source:HGNC Symbol;Acc:HGNC:19102]	protein coding	0.85	5.76	30.78	<0.0001	<0.0001
ENSG00000228340	MIR646HG	MIR646 host gene [Source:HGNC Symbol;Acc:HGNC:27659]	lncRNA	-0.85	2.80	44.47	<0.0001	<0.0001
ENSG00000182118	FAM89A	family with sequence similarity 89 member A [Source:HGNC Symbol;Acc:HGNC:25057]	protein coding	0.85	2.25	40.92	<0.0001	<0.0001
ENSG00000096093	EFHC1	EF-hand domain containing 1 [Source:HGNC Symbol;Acc:HGNC:16406]	protein coding	-0.85	2.91	68.77	<0.0001	<0.0001
ENSG00000146215	CRIP3	cysteine rich protein 3 [Source:HGNC Symbol;Acc:HGNC:17751]	protein coding	-0.85	1.27	25.17	<0.0001	<0.0001
ENSG00000175592	FOSL1	FOS like 1, AP-1 transcription factor subunit [Source:HGNC Symbol;Acc:HGNC:13718]	protein coding	0.85	1.83	5.80	0.016	0.0337
ENSG00000130202	NECTIN2	nectin cell adhesion molecule 2 [Source:HGNC Symbol;Acc:HGNC:9707]	protein coding	0.85	4.88	67.82	<0.0001	<0.0001
ENSG00000247134		novel transcript	lncRNA	-0.85	1.65	27.53	<0.0001	<0.0001
ENSG00000184465	WDR27	WD repeat domain 27 [Source:HGNC Symbol;Acc:HGNC:21248]	protein coding	-0.85	3.45	63.26	<0.0001	<0.0001
ENSG00000145685	LHFPL2	LHFPL tetraspan subfamily member 2 [Source:HGNC Symbol;Acc:HGNC:6588]	protein coding	0.85	5.14	55.35	<0.0001	<0.0001

Table S5: Genes differentially expressed at Day 30 compared to pre-second vaccination (Study Arm 2 A/H7N9+AS03, D1, 29, Monocytes). Genes are sorted by descending absolute log_2 fold change. Gene model summaries and annotations are based on Ensembl Version 99 (continued.)

Ensembl Gene ID	Ensembl Gene Name	Ensembl Gene Description	Gene Type	log_2 Fold Change (Day 30 vs. Pre-Second Vaccination)	Average log_2 CPM	Likelihood Ratio Test Statistic	P-Value	FDR Adjusted P-Value
ENSG00000135525	MAP7	microtubule associated protein 7 [Source:HGNC Symbol;Acc:HGNC:6869]	protein coding	-0.85	2.22	44.77	<0.0001	<0.0001
ENSG00000042980	ADAM28	ADAM metallopeptidase domain 28 [Source:HGNC Symbol;Acc:HGNC:206]	protein coding	-0.84	4.18	84.72	<0.0001	<0.0001
ENSG00000092010	PSME1	proteasome activator subunit 1 [Source:HGNC Symbol;Acc:HGNC:9568]	protein coding	0.84	8.28	67.45	<0.0001	<0.0001
ENSG00000035720	STAP1	signal transducing adaptor family member 1 [Source:HGNC Symbol;Acc:HGNC:24133]	protein coding	-0.84	1.28	18.66	<0.0001	<0.0001
ENSG00000090339	ICAM1	intercellular adhesion molecule 1 [Source:HGNC Symbol;Acc:HGNC:5344]	protein coding	0.84	6.37	47.09	<0.0001	<0.0001
ENSG00000258810		novel transcript, antisense to RNASE1	lncRNA	-0.84	1.43	21.68	<0.0001	<0.0001
ENSG00000197506	SLC28A3	solute carrier family 28 member 3 [Source:HGNC Symbol;Acc:HGNC:16484]	protein coding	-0.84	1.14	10.79	0.001	0.0031
ENSG00000013364	MVP	major vault protein [Source:HGNC Symbol;Acc:HGNC:7531]	protein coding	0.84	8.85	60.58	<0.0001	<0.0001
ENSG00000180767	CHST13	carbohydrate sulfotransferase 13 [Source:HGNC Symbol;Acc:HGNC:21755]	protein coding	-0.84	4.98	49.70	<0.0001	<0.0001
ENSG00000180385	EMC3-AS1	EMC3 antisense RNA 1 [Source:HGNC Symbol;Acc:HGNC:49223]	transcribed unprocessed pseudogene	-0.84	-0.03	23.10	<0.0001	<0.0001
ENSG00000138002	IFT172	intraflagellar transport 172 [Source:HGNC Symbol;Acc:HGNC:30391]	protein coding	-0.84	3.36	43.90	<0.0001	<0.0001
ENSG00000111224	PARP11	poly(ADP-ribose) polymerase family member 11 [Source:HGNC Symbol;Acc:HGNC:1186]	protein coding	0.84	4.13	50.71	<0.0001	<0.0001
ENSG00000085449	WDFY1	WD repeat and FYVE domain containing 1 [Source:HGNC Symbol;Acc:HGNC:20451]	protein coding	0.84	6.07	73.36	<0.0001	<0.0001
ENSG00000180354	MTURN	maturin, neural progenitor differentiation regulator homolog [Source:HGNC Symbol;Acc:HGNC:25457]	protein coding	-0.84	4.06	73.92	<0.0001	<0.0001
ENSG00000166002	SMCO4	single-pass membrane protein with coiled-coil domains 4 [Source:HGNC Symbol;Acc:HGNC:24810]	protein coding	0.84	6.27	104.02	<0.0001	<0.0001
ENSG00000135245	HILPDA	hypoxia inducible lipid droplet associated [Source:HGNC Symbol;Acc:HGNC:28859]	protein coding	-0.84	0.54	22.49	<0.0001	<0.0001
ENSG00000183496	MEX3B	mex-3 RNA binding family member B [Source:HGNC Symbol;Acc:HGNC:25297]	protein coding	-0.84	1.14	31.91	<0.0001	<0.0001

Table S5: Genes differentially expressed at Day 30 compared to pre-second vaccination (Study Arm 2 A/H7N9+AS03, D1, 29, Monocytes). Genes are sorted by descending absolute log_2 fold change. Gene model summaries and annotations are based on Ensembl Version 99 (continued.)

Ensembl Gene ID	Ensembl Gene Name	Ensembl Gene Description	Gene Type	log_2 Fold Change (Day 30 vs. Pre-Second Vaccination)	Average log_2 CPM	Likelihood Ratio Test Statistic	P-Value	FDR Adjusted P-Value
ENSG00000223704	LINC01422	long intergenic non-protein coding RNA 1422 [Source:HGNC Symbol;Acc:HGNC:50728]	lncRNA	-0.84	0.33	22.15	<0.0001	<0.0001
ENSG00000124762	CDKN1A	cyclin dependent kinase inhibitor 1A [Source:HGNC Symbol;Acc:HGNC:1784]	protein coding	0.84	6.43	58.30	<0.0001	<0.0001
ENSG00000100321	SYNGR1	synaptogyrin 1 [Source:HGNC Symbol;Acc:HGNC:11498]	protein coding	-0.84	4.51	50.30	<0.0001	<0.0001
ENSG00000169413	RNASE6	ribonuclease A family member k6 [Source:HGNC Symbol;Acc:HGNC:10048]	protein coding	-0.84	6.53	35.00	<0.0001	<0.0001
ENSG00000144354	CDCA7	cell division cycle associated 7 [Source:HGNC Symbol;Acc:HGNC:14628]	protein coding	-0.84	0.64	23.04	<0.0001	<0.0001
ENSG00000170476	MZB1	marginal zone B and B1 cell specific protein [Source:HGNC Symbol;Acc:HGNC:30125]	protein coding	-0.84	2.73	20.77	<0.0001	<0.0001
ENSG00000170456	DENND5B	DENN domain containing 5B [Source:HGNC Symbol;Acc:HGNC:28338]	protein coding	-0.83	1.42	39.56	<0.0001	<0.0001
ENSG00000079263	SP140	SP140 nuclear body protein [Source:HGNC Symbol;Acc:HGNC:17133]	protein coding	0.83	4.29	40.61	<0.0001	<0.0001
ENSG00000124201	ZNFX1	zinc finger NFX1-type containing 1 [Source:HGNC Symbol;Acc:HGNC:29271]	protein coding	0.83	6.52	58.93	<0.0001	<0.0001
ENSG00000007038	PRSS21	serine protease 21 [Source:HGNC Symbol;Acc:HGNC:9485]	protein coding	-0.83	1.94	23.77	<0.0001	<0.0001
ENSG00000170917	NUDT6	nudix hydrolase 6 [Source:HGNC Symbol;Acc:HGNC:8053]	protein coding	-0.83	1.09	32.85	<0.0001	<0.0001
ENSG00000128694	OSGEPL1	O-sialoglycoprotein endopeptidase like 1 [Source:HGNC Symbol;Acc:HGNC:23075]	protein coding	-0.83	1.75	32.92	<0.0001	<0.0001
ENSG00000180817	PPA1	inorganic pyrophosphatase 1 [Source:HGNC Symbol;Acc:HGNC:9226]	protein coding	0.83	5.93	73.62	<0.0001	<0.0001
ENSG00000171604	CXXC5	CXXC finger protein 5 [Source:HGNC Symbol;Acc:HGNC:26943]	protein coding	-0.83	5.45	52.82	<0.0001	<0.0001
ENSG00000248323	LUCAT1	lung cancer associated transcript 1 [Source:HGNC Symbol;Acc:HGNC:48498]	lncRNA	-0.83	3.62	42.27	<0.0001	<0.0001
ENSG00000007129	CEACAM21	CEA cell adhesion molecule 21 [Source:HGNC Symbol;Acc:HGNC:28834]	protein coding	0.83	2.27	32.75	<0.0001	<0.0001
ENSG00000167106	FAM102A	family with sequence similarity 102 member A [Source:HGNC Symbol;Acc:HGNC:31419]	protein coding	-0.83	3.40	50.30	<0.0001	<0.0001
ENSG00000189419	SPATA41	spermatogenesis associated 41 [Source:HGNC Symbol;Acc:HGNC:48613]	lncRNA	-0.83	0.28	17.78	<0.0001	0.0001

Confidential/Proprietary Information

Table S5: Genes differentially expressed at Day 30 compared to pre-second vaccination (Study Arm 2 A/H7N9+AS03, D1, 29, Monocytes). Genes are sorted by descending absolute log_2 fold change. Gene model summaries and annotations are based on Ensembl Version 99 (continued.)

Ensembl Gene ID	Ensembl Gene Name	Ensembl Gene Description	Gene Type	log_2 Fold Change (Day 30 vs. Pre-Second Vaccination)	Average log_2 CPM	Likelihood Ratio Test Statistic	P-Value	FDR Adjusted P-Value
ENSG00000126353	CCR7	C-C motif chemokine receptor 7 [Source:HGNC Symbol;Acc:HGNC:1608]	protein coding	-0.83	1.77	12.07	0.0005	0.0017
ENSG00000243811	APOBEC3D	apolipoprotein B mRNA editing enzyme catalytic subunit 3D [Source:HGNC Symbol;Acc:HGNC:17354]	protein coding	0.83	0.44	39.06	<0.0001	<0.0001
ENSG00000124374	PAIP2B	poly(A) binding protein interacting protein 2B [Source:HGNC Symbol;Acc:HGNC:29200]	protein coding	-0.83	0.60	30.29	<0.0001	<0.0001
ENSG00000161643	SIGLEC16	sialic acid binding Ig like lectin 16 (gene/pseudogene) [Source:HGNC Symbol;Acc:HGNC:24851]	transcribed unprocessed pseudogene	0.82	4.14	49.26	<0.0001	<0.0001
ENSG00000008323	PLEKHG6	pleckstrin homology and RhoGEF domain containing G6 [Source:HGNC Symbol;Acc:HGNC:25562]	protein coding	0.82	0.58	17.09	<0.0001	0.0002
ENSG00000113070	HBEGF	heparin binding EGF like growth factor [Source:HGNC Symbol;Acc:HGNC:3059]	protein coding	0.82	3.78	9.01	0.0027	0.0071
ENSG00000114770	ABCC5	ATP binding cassette subfamily C member 5 [Source:HGNC Symbol;Acc:HGNC:56]	protein coding	-0.82	5.48	56.95	<0.0001	<0.0001
ENSG00000213420	GPC2	glypican 2 [Source:HGNC Symbol;Acc:HGNC:4450]	protein coding	-0.82	0.34	24.81	<0.0001	<0.0001
ENSG00000175445	LPL	lipoprotein lipase [Source:HGNC Symbol;Acc:HGNC:6677]	protein coding	-0.82	0.99	9.46	0.0021	0.0058
ENSG00000132846	ZBED3	zinc finger BED-type containing 3 [Source:HGNC Symbol;Acc:HGNC:20711]	protein coding	-0.82	3.23	52.59	<0.0001	<0.0001
ENSG00000086061	DNAJA1	DnaJ heat shock protein family (Hsp40) member A1 [Source:HGNC Symbol;Acc:HGNC:5229]	protein coding	0.82	7.36	49.37	<0.0001	<0.0001
ENSG00000262877		novel transcript	lncRNA	-0.82	3.55	48.66	<0.0001	<0.0001
ENSG00000225975	LINC01534	long intergenic non-protein coding RNA 1534 [Source:HGNC Symbol;Acc:HGNC:51281]	lncRNA	-0.82	0.93	30.20	<0.0001	<0.0001
ENSG00000116133	DHCR24	24-dehydrocholesterol reductase [Source:HGNC Symbol;Acc:HGNC:2859]	protein coding	0.82	2.88	16.98	<0.0001	0.0002
ENSG00000231711	LINC00899	long intergenic non-protein coding RNA 899 [Source:HGNC Symbol;Acc:HGNC:48583]	lncRNA	-0.82	2.59	38.49	<0.0001	<0.0001
ENSG00000260314	MRC1	mannose receptor C-type 1 [Source:HGNC Symbol;Acc:HGNC:7228]	protein coding	0.82	1.72	18.89	<0.0001	<0.0001
ENSG00000136867	SLC31A2	solute carrier family 31 member 2 [Source:HGNC Symbol;Acc:HGNC:11017]	protein coding	0.82	4.79	72.22	<0.0001	<0.0001

Table S5: Genes differentially expressed at Day 30 compared to pre-second vaccination (Study Arm 2 A/H7N9+AS03, D1, 29, Monocytes). Genes are sorted by descending absolute log_2 fold change. Gene model summaries and annotations are based on Ensembl Version 99 (continued.)

Ensembl Gene ID	Ensembl Gene Name	Ensembl Gene Description	Gene Type	log_2 Fold Change (Day 30 vs. Pre-Second Vaccination)	Average log_2 CPM	Likelihood Ratio Test Statistic	P-Value	FDR Adjusted P-Value
ENSG00000226950	DANCR	differentiation antagonizing non-protein coding RNA [Source:HGNC Symbol;Acc:HGNC:28964]	lncRNA	-0.81	2.82	63.75	<0.0001	<0.0001
ENSG00000130653	PNPLA7	patatin like phospholipase domain containing 7 [Source:HGNC Symbol;Acc:HGNC:24768]	protein coding	-0.81	2.73	30.29	<0.0001	<0.0001
ENSG00000149548	CCDC15	coiled-coil domain containing 15 [Source:HGNC Symbol;Acc:HGNC:25798]	protein coding	-0.81	0.02	26.26	<0.0001	<0.0001
ENSG00000127951	FGL2	fibrinogen like 2 [Source:HGNC Symbol;Acc:HGNC:3696]	protein coding	0.81	10.98	49.57	<0.0001	<0.0001
ENSG00000205930	C21orf62-AS1	C21orf62 antisense RNA 1 [Source:HGNC Symbol;Acc:HGNC:1290]	lncRNA	-0.81	0.10	21.23	<0.0001	<0.0001
ENSG00000224078	SNHG14	small nucleolar RNA host gene 14 [Source:HGNC Symbol;Acc:HGNC:37462]	lncRNA	-0.81	2.76	57.46	<0.0001	<0.0001
ENSG00000179094	PER1	period circadian regulator 1 [Source:HGNC Symbol;Acc:HGNC:8845]	protein coding	-0.81	7.66	18.78	<0.0001	<0.0001
ENSG00000185950	IRS2	insulin receptor substrate 2 [Source:HGNC Symbol;Acc:HGNC:6126]	protein coding	-0.81	7.43	36.37	<0.0001	<0.0001
ENSG00000089127	OAS1	2'-5'-oligoadenylate synthetase 1 [Source:HGNC Symbol;Acc:HGNC:8086]	protein coding	0.81	7.84	53.80	<0.0001	<0.0001
ENSG00000173295	FAM86B3P	family with sequence similarity 86 member B3, pseudogene [Source:HGNC Symbol;Acc:HGNC:44371]	transcribed unprocessed pseudogene	-0.81	0.90	36.90	<0.0001	<0.0001
ENSG00000055332	EIF2AK2	eukaryotic translation initiation factor 2 alpha kinase 2 [Source:HGNC Symbol;Acc:HGNC:9437]	protein coding	0.81	6.21	53.44	<0.0001	<0.0001
ENSG00000110660	SLC35F2	solute carrier family 35 member F2 [Source:HGNC Symbol;Acc:HGNC:23615]	protein coding	-0.81	0.11	24.35	<0.0001	<0.0001
ENSG00000231389	HLA-DPA1	major histocompatibility complex, class II, DP alpha 1 [Source:HGNC Symbol;Acc:HGNC:4938]	protein coding	0.81	10.30	61.78	<0.0001	<0.0001
ENSG00000162572	SCNN1D	sodium channel epithelial 1 delta subunit [Source:HGNC Symbol;Acc:HGNC:10601]	protein coding	-0.81	2.27	32.20	<0.0001	<0.0001
ENSG00000286931		novel transcript	lncRNA	-0.81	0.53	25.17	<0.0001	<0.0001
ENSG00000119333	WDR34	WD repeat domain 34 [Source:HGNC Symbol;Acc:HGNC:28296]	protein coding	-0.81	1.74	47.41	<0.0001	<0.0001
ENSG00000178104	PDE4DIP	phosphodiesterase 4D interacting protein [Source:HGNC Symbol;Acc:HGNC:15580]	protein coding	-0.81	4.13	79.82	<0.0001	<0.0001

Table S5: Genes differentially expressed at Day 30 compared to pre-second vaccination (Study Arm 2 A/H7N9+AS03, D1, 29, Monocytes). Genes are sorted by descending absolute log_2 fold change. Gene model summaries and annotations are based on Ensembl Version 99 (continued.)

Ensembl Gene ID	Ensembl Gene Name	Ensembl Gene Description	Gene Type	log_2 Fold Change (Day 30 vs. Pre-Second Vaccination)	Average log_2 CPM	Likelihood Ratio Test Statistic	P-Value	FDR Adjusted P-Value
ENSG00000140853	NLRC5	NLR family CARD domain containing 5 [Source:HGNC Symbol;Acc:HGNC:29933]	protein coding	0.81	8.10	46.35	<0.0001	<0.0001
ENSG00000089041	P2RX7	purinergic receptor P2X 7 [Source:HGNC Symbol;Acc:HGNC:8537]	protein coding	0.81	6.38	70.44	<0.0001	<0.0001
ENSG00000124766	SOX4	SRY-box transcription factor 4 [Source:HGNC Symbol;Acc:HGNC:11200]	protein coding	-0.81	5.42	52.18	<0.0001	<0.0001
ENSG00000108846	ABCC3	ATP binding cassette subfamily C member 3 [Source:HGNC Symbol;Acc:HGNC:54]	protein coding	-0.81	5.40	31.19	<0.0001	<0.0001
ENSG00000167074	TEF	TEF transcription factor, PAR bZIP family member [Source:HGNC Symbol;Acc:HGNC:11722]	protein coding	-0.80	3.08	43.75	<0.0001	<0.0001
ENSG00000119227	PIGZ	phosphatidylinositol glycan anchor biosynthesis class Z [Source:HGNC Symbol;Acc:HGNC:30596]	protein coding	-0.80	1.72	29.72	<0.0001	<0.0001
ENSG00000211659	IGLV3-25	immunoglobulin lambda variable 3-25 [Source:HGNC Symbol;Acc:HGNC:5908]	IG V gene	-0.80	0.69	9.58	0.002	0.0054
ENSG00000133121	STARD13	StAR related lipid transfer domain containing 13 [Source:HGNC Symbol;Acc:HGNC:19164]	protein coding	-0.80	0.57	13.87	0.0002	0.0007
ENSG00000181381	DDX60L	DExD/H-box 60 like [Source:HGNC Symbol;Acc:HGNC:26429]	protein coding	0.80	7.29	54.26	<0.0001	<0.0001
ENSG00000133056	PIK3C2B	phosphatidylinositol-4-phosphate 3-kinase catalytic subunit type 2 beta [Source:HGNC Symbol;Acc:HGNC:8972]	protein coding	-0.80	2.62	48.03	<0.0001	<0.0001
ENSG00000226491	FTOP1	FTO pseudogene 1 [Source:HGNC Symbol;Acc:HGNC:43759]	processed pseudogene	-0.80	1.27	12.72	0.0004	0.0012
ENSG00000140543	DET1	DET1 partner of COP1 E3 ubiquitin ligase [Source:HGNC Symbol;Acc:HGNC:25477]	protein coding	-0.80	1.00	26.94	<0.0001	<0.0001
ENSG00000197780	TAF13	TATA-box binding protein associated factor 13 [Source:HGNC Symbol;Acc:HGNC:11546]	protein coding	0.80	3.51	92.07	<0.0001	<0.0001
ENSG00000225783	MIAT	myocardial infarction associated transcript [Source:HGNC Symbol;Acc:HGNC:33425]	lncRNA	-0.80	1.46	13.29	0.0003	0.0009
ENSG00000287431		novel transcript	lncRNA	-0.80	2.44	25.47	<0.0001	<0.0001
ENSG00000168874	ATOH8	atonal bHLH transcription factor 8 [Source:HGNC Symbol;Acc:HGNC:24126]	protein coding	-0.80	3.41	27.73	<0.0001	<0.0001
ENSG00000157654	PALM2AKAP2	PALM2 and AKAP2 fusion [Source:HGNC Symbol;Acc:HGNC:33529]	protein coding	0.80	3.49	9.71	0.0018	0.0051
ENSG00000149260	CAPN5	calpain 5 [Source:HGNC Symbol;Acc:HGNC:1482]	protein coding	-0.80	0.87	42.72	<0.0001	<0.0001

Table S5: Genes differentially expressed at Day 30 compared to pre-second vaccination (Study Arm 2 A/H7N9+AS03, D1, 29, Monocytes). Genes are sorted by descending absolute log_2 fold change. Gene model summaries and annotations are based on Ensembl Version 99 (continued.)

Ensembl Gene ID	Ensembl Gene Name	Ensembl Gene Description	Gene Type	log_2 Fold Change (Day 30 vs. Pre-Second Vaccination)	Average log_2 CPM	Likelihood Ratio Test Statistic	P-Value	FDR Adjusted P-Value
ENSG00000133661	SFTPD	surfactant protein D [Source:HGNC Symbol;Acc:HGNC:10803]	protein coding	-0.80	0.50	14.73	0.0001	0.0005
ENSG00000116016	EPAS1	endothelial PAS domain protein 1 [Source:HGNC Symbol;Acc:HGNC:3374]	protein coding	0.80	1.46	28.90	<0.0001	<0.0001
ENSG00000145247	OCIAD2	OCIA domain containing 2 [Source:HGNC Symbol;Acc:HGNC:28685]	protein coding	-0.80	0.31	30.28	<0.0001	<0.0001
ENSG00000023902	PLEKHO1	pleckstrin homology domain containing O1 [Source:HGNC Symbol;Acc:HGNC:24310]	protein coding	0.80	9.08	60.79	<0.0001	<0.0001
ENSG00000204366	ZBTB12	zinc finger and BTB domain containing 12 [Source:HGNC Symbol;Acc:HGNC:19066]	protein coding	-0.80	0.12	25.42	<0.0001	<0.0001
ENSG00000196141	SPATS2L	spermatogenesis associated serine rich 2 like [Source:HGNC Symbol;Acc:HGNC:24574]	protein coding	0.80	3.21	28.18	<0.0001	<0.0001
ENSG00000213073		calcium binding protein P22 (CHP) pseudogene	transcribed processed pseudogene	0.80	2.22	28.94	<0.0001	<0.0001
ENSG00000105711	SCN1B	sodium voltage-gated channel beta subunit 1 [Source:HGNC Symbol;Acc:HGNC:10586]	protein coding	0.80	0.39	18.65	<0.0001	<0.0001
ENSG00000206530	CFAP44	cilia and flagella associated protein 44 [Source:HGNC Symbol;Acc:HGNC:25631]	protein coding	-0.79	1.65	30.03	<0.0001	<0.0001
ENSG00000225231	LINC02470	long intergenic non-protein coding RNA 2470 [Source:HGNC Symbol;Acc:HGNC:53409]	lncRNA	-0.79	2.54	19.89	<0.0001	<0.0001
ENSG00000266402	SNHG25	small nucleolar RNA host gene 25 [Source:HGNC Symbol;Acc:HGNC:51534]	lncRNA	-0.79	0.62	22.10	<0.0001	<0.0001
ENSG00000197536	IRF1-AS1	IRF1 antisense RNA 1 [Source:HGNC Symbol;Acc:HGNC:33838]	lncRNA	0.79	4.92	34.16	<0.0001	<0.0001
ENSG00000204623	ZNRD1ASP	zinc ribbon domain containing 1 antisense, pseudogene [Source:HGNC Symbol;Acc:HGNC:13924]	transcribed unitary pseudogene	-0.79	3.03	41.57	<0.0001	<0.0001
ENSG00000215533	LINC00189	long intergenic non-protein coding RNA 189 [Source:HGNC Symbol;Acc:HGNC:18461]	lncRNA	0.79	0.79	33.23	<0.0001	<0.0001
ENSG00000099958	DERL3	derlin 3 [Source:HGNC Symbol;Acc:HGNC:14236]	protein coding	-0.79	0.43	11.62	0.0007	0.0021
ENSG00000164211	STARD4	StAR related lipid transfer domain containing 4 [Source:HGNC Symbol;Acc:HGNC:18058]	protein coding	0.79	4.06	48.79	<0.0001	<0.0001

Table S5: Genes differentially expressed at Day 30 compared to pre-second vaccination (Study Arm 2 A/H7N9+AS03, D1, 29, Monocytes). Genes are sorted by descending absolute log_2 fold change. Gene model summaries and annotations are based on Ensembl Version 99 (continued.)

Ensembl Gene ID	Ensembl Gene Name	Ensembl Gene Description	Gene Type	log_2 Fold Change (Day 30 vs. Pre-Second Vaccination)	Average log_2 CPM	Likelihood Ratio Test Statistic	P-Value	FDR Adjusted P-Value
ENSG00000173715	C11orf80	chromosome 11 open reading frame 80 [Source:HGNC Symbol;Acc:HGNC:26197]	protein coding	-0.79	0.41	30.79	<0.0001	<0.0001
ENSG00000102524	TNFSF13B	TNF superfamily member 13b [Source:HGNC Symbol;Acc:HGNC:11929]	protein coding	0.79	7.85	72.93	<0.0001	<0.0001
ENSG00000170271	FAXDC2	fatty acid hydroxylase domain containing 2 [Source:HGNC Symbol;Acc:HGNC:1334]	protein coding	-0.79	2.02	28.99	<0.0001	<0.0001
ENSG00000139318	DUSP6	dual specificity phosphatase 6 [Source:HGNC Symbol;Acc:HGNC:3072]	protein coding	0.79	8.91	29.30	<0.0001	<0.0001
ENSG00000001561	ENPP4	ectonucleotide pyrophosphatase/phosphodiesterase 4 [Source:HGNC Symbol;Acc:HGNC:3359]	protein coding	0.79	2.06	30.68	<0.0001	<0.0001
ENSG00000105516	DBP	D-box binding PAR bZIP transcription factor [Source:HGNC Symbol;Acc:HGNC:2697]	protein coding	-0.79	4.92	32.47	<0.0001	<0.0001
ENSG00000127399	LRRC61	leucine rich repeat containing 61 [Source:HGNC Symbol;Acc:HGNC:21704]	protein coding	0.79	1.71	32.19	<0.0001	<0.0001
ENSG00000155287	SLC25A28	solute carrier family 25 member 28 [Source:HGNC Symbol;Acc:HGNC:23472]	protein coding	0.79	6.02	68.38	<0.0001	<0.0001
ENSG00000250397		novel transcript	lncRNA	-0.79	1.01	16.49	<0.0001	0.0002
ENSG00000164430	CGAS	cyclic GMP-AMP synthase [Source:HGNC Symbol;Acc:HGNC:21367]	protein coding	0.79	4.93	38.29	<0.0001	<0.0001
ENSG00000088543	C3orf18	chromosome 3 open reading frame 18 [Source:HGNC Symbol;Acc:HGNC:24837]	protein coding	-0.79	0.74	21.60	<0.0001	<0.0001
ENSG00000174669	SLC29A2	solute carrier family 29 member 2 [Source:HGNC Symbol;Acc:HGNC:11004]	protein coding	-0.79	0.78	19.61	<0.0001	<0.0001
ENSG00000100368	CSF2RB	colony stimulating factor 2 receptor beta common subunit [Source:HGNC Symbol;Acc:HGNC:2436]	protein coding	0.79	8.40	61.13	<0.0001	<0.0001
ENSG00000197696	NMB	neuromedin B [Source:HGNC Symbol;Acc:HGNC:7842]	protein coding	-0.79	0.08	20.66	<0.0001	<0.0001
ENSG00000143224	PPOX	protoporphyrinogen oxidase [Source:HGNC Symbol;Acc:HGNC:9280]	protein coding	-0.79	4.35	69.11	<0.0001	<0.0001
ENSG00000183486	MX2	MX dynamin like GTPase 2 [Source:HGNC Symbol;Acc:HGNC:7533]	protein coding	0.78	7.86	45.28	<0.0001	<0.0001
ENSG00000171621	SPSB1	splA/ryanodine receptor domain and SOCS box containing 1 [Source:HGNC Symbol;Acc:HGNC:30628]	protein coding	0.78	0.74	17.30	<0.0001	0.0001
ENSG00000112299	VNN1	vanin 1 [Source:HGNC Symbol;Acc:HGNC:12705]	protein coding	-0.78	4.94	15.32	<0.0001	0.0004

Table S5: Genes differentially expressed at Day 30 compared to pre-second vaccination (Study Arm 2 A/H7N9+AS03, D1, 29, Monocytes). Genes are sorted by descending absolute log_2 fold change. Gene model summaries and annotations are based on Ensembl Version 99 (continued.)

Ensembl Gene ID	Ensembl Gene Name	Ensembl Gene Description	Gene Type	log_2 Fold Change (Day 30 vs. Pre-Second Vaccination)	Average log_2 CPM	Likelihood Ratio Test Statistic	P-Value	FDR Adjusted P-Value
ENSG00000184271	POU6F1	POU class 6 homeobox 1 [Source:HGNC Symbol;Acc:HGNC:9224]	protein coding	-0.78	2.55	46.05	<0.0001	<0.0001
ENSG00000135929	CYP27A1	cytochrome P450 family 27 subfamily A member 1 [Source:HGNC Symbol;Acc:HGNC:2605]	protein coding	-0.78	7.03	35.67	<0.0001	<0.0001
ENSG00000004799	PDK4	pyruvate dehydrogenase kinase 4 [Source:HGNC Symbol;Acc:HGNC:8812]	protein coding	-0.78	6.36	12.07	0.0005	0.0017
ENSG00000148841	ITPRIP	inositol 1,4,5-trisphosphate receptor interacting protein [Source:HGNC Symbol;Acc:HGNC:29370]	protein coding	0.78	6.57	49.30	<0.0001	<0.0001
ENSG00000171130	ATP6V0E2	ATPase H+ transporting V0 subunit e2 [Source:HGNC Symbol;Acc:HGNC:21723]	protein coding	-0.78	2.89	28.51	<0.0001	<0.0001
ENSG00000176659	C20orf197	chromosome 20 open reading frame 197 [Source:HGNC Symbol;Acc:HGNC:26601]	lncRNA	-0.78	2.25	41.59	<0.0001	<0.0001
ENSG00000082781	ITGB5	integrin subunit beta 5 [Source:HGNC Symbol;Acc:HGNC:6160]	protein coding	-0.78	1.28	25.69	<0.0001	<0.0001
ENSG00000100226	GTPBP1	GTP binding protein 1 [Source:HGNC Symbol;Acc:HGNC:4669]	protein coding	0.78	6.25	65.89	<0.0001	<0.0001
ENSG00000076555	ACACB	acetyl-CoA carboxylase beta [Source:HGNC Symbol;Acc:HGNC:85]	protein coding	-0.78	3.13	51.03	<0.0001	<0.0001
ENSG00000143891	GALM	galactose mutarotase [Source:HGNC Symbol;Acc:HGNC:24063]	protein coding	0.78	2.78	22.42	<0.0001	<0.0001
ENSG00000274292		novel transcript	lncRNA	-0.78	2.03	33.45	<0.0001	<0.0001
ENSG00000174721	FGFBP3	fibroblast growth factor binding protein 3 [Source:HGNC Symbol;Acc:HGNC:23428]	protein coding	-0.78	1.34	33.56	<0.0001	<0.0001
ENSG00000262580		novel transcript, antisense to CARD14	lncRNA	-0.78	4.90	42.11	<0.0001	<0.0001
ENSG00000167004	PDIA3	protein disulfide isomerase family A member 3 [Source:HGNC Symbol;Acc:HGNC:4606]	protein coding	0.78	8.11	53.05	<0.0001	<0.0001
ENSG00000092200	RPGRIP1	RPGR interacting protein 1 [Source:HGNC Symbol;Acc:HGNC:13436]	protein coding	-0.78	3.22	62.30	<0.0001	<0.0001
ENSG00000132199	ENOSF1	enolase superfamily member 1 [Source:HGNC Symbol;Acc:HGNC:30365]	protein coding	-0.77	3.02	42.81	<0.0001	<0.0001
ENSG00000226174	TEX22	testis expressed 22 [Source:HGNC Symbol;Acc:HGNC:40026]	protein coding	-0.77	1.57	25.42	<0.0001	<0.0001
ENSG00000105808	RASA4	RAS p21 protein activator 4 [Source:HGNC Symbol;Acc:HGNC:23181]	protein coding	-0.77	1.23	27.35	<0.0001	<0.0001
ENSG00000164308	ERAP2	endoplasmic reticulum aminopeptidase 2 [Source:HGNC Symbol;Acc:HGNC:29499]	protein coding	0.77	5.98	53.94	<0.0001	<0.0001

Table S5: Genes differentially expressed at Day 30 compared to pre-second vaccination (Study Arm 2 A/H7N9+AS03, D1, 29, Monocytes). Genes are sorted by descending absolute log_2 fold change. Gene model summaries and annotations are based on Ensembl Version 99 (continued.)

Ensembl Gene ID	Ensembl Gene Name	Ensembl Gene Description	Gene Type	log_2 Fold Change (Day 30 vs. Pre-Second Vaccination)	Average log_2 CPM	Likelihood Ratio Test Statistic	P-Value	FDR Adjusted P-Value
ENSG00000199753	SNORD104	small nucleolar RNA, C/D box 104 [Source:HGNC Symbol;Acc:HGNC:32768]	snoRNA	-0.77	1.78	48.25	<0.0001	<0.0001
ENSG00000154146	NRGN	neurogranin [Source:HGNC Symbol;Acc:HGNC:8000]	protein coding	-0.77	7.37	44.05	<0.0001	<0.0001
ENSG00000142046		transmembrane protein 91 [Source:NCBI gene;Acc:641649]	protein coding	-0.77	5.18	48.41	<0.0001	<0.0001
ENSG00000285632		novel transcript	lncRNA	-0.77	0.56	31.86	<0.0001	<0.0001
ENSG00000168421	RHOH	ras homolog family member H [Source:HGNC Symbol;Acc:HGNC:686]	protein coding	-0.77	1.50	17.02	<0.0001	0.0002
ENSG00000104267	CA2	carbonic anhydrase 2 [Source:HGNC Symbol;Acc:HGNC:1373]	protein coding	0.77	3.23	74.13	<0.0001	<0.0001
ENSG00000144655	CSRNP1	cysteine and serine rich nuclear protein 1 [Source:HGNC Symbol;Acc:HGNC:14300]	protein coding	0.77	6.66	24.45	<0.0001	<0.0001
ENSG00000100095	SEZ6L	seizure related 6 homolog like [Source:HGNC Symbol;Acc:HGNC:10763]	protein coding	-0.77	2.90	42.51	<0.0001	<0.0001
ENSG00000062524	LTK	leukocyte receptor tyrosine kinase [Source:HGNC Symbol;Acc:HGNC:6721]	protein coding	-0.77	0.59	19.11	<0.0001	<0.0001
ENSG00000171860	C3AR1	complement C3a receptor 1 [Source:HGNC Symbol;Acc:HGNC:1319]	protein coding	0.77	6.92	62.10	<0.0001	<0.0001
ENSG00000005249	PRKAR2B	protein kinase cAMP-dependent type II regulatory subunit beta [Source:HGNC Symbol;Acc:HGNC:9392]	protein coding	0.77	3.80	35.55	<0.0001	<0.0001
ENSG00000164649	CDCA7L	cell division cycle associated 7 like [Source:HGNC Symbol;Acc:HGNC:30777]	protein coding	-0.77	2.31	41.85	<0.0001	<0.0001
ENSG00000163864	NMNAT3	nicotinamide nucleotide adenylyltransferase 3 [Source:HGNC Symbol;Acc:HGNC:20989]	protein coding	-0.77	1.74	40.65	<0.0001	<0.0001
ENSG00000152475	ZNF837	zinc finger protein 837 [Source:HGNC Symbol;Acc:HGNC:25164]	protein coding	-0.77	0.90	26.72	<0.0001	<0.0001
ENSG00000211956	IGHV4-34	immunoglobulin heavy variable 4-34 [Source:HGNC Symbol;Acc:HGNC:5650]	IG V gene	-0.77	0.57	11.23	0.0008	0.0025
ENSG00000181004	BBS12	Bardet-Biedl syndrome 12 [Source:HGNC Symbol;Acc:HGNC:26648]	protein coding	0.77	0.85	11.97	0.0005	0.0018
ENSG00000148926	ADM	adrenomedullin [Source:HGNC Symbol;Acc:HGNC:259]	protein coding	0.77	4.07	20.26	<0.0001	<0.0001
ENSG00000081052	COL4A4	collagen type IV alpha 4 chain [Source:HGNC Symbol;Acc:HGNC:2206]	protein coding	-0.77	0.49	21.70	<0.0001	<0.0001

Table S5: Genes differentially expressed at Day 30 compared to pre-second vaccination (Study Arm 2 A/H7N9+AS03, D1, 29, Monocytes). Genes are sorted by descending absolute log_2 fold change. Gene model summaries and annotations are based on Ensembl Version 99 (continued.)

Ensembl Gene ID	Ensembl Gene Name	Ensembl Gene Description	Gene Type	log_2 Fold Change (Day 30 vs. Pre-Second Vaccination)	Average log_2 CPM	Likelihood Ratio Test Statistic	P-Value	FDR Adjusted P-Value
ENSG00000282936		novel protein	protein coding	-0.77	0.83	22.58	<0.0001	<0.0001
ENSG00000166582	CENPV	centromere protein V [Source:HGNC Symbol;Acc:HGNC:29920]	protein coding	-0.76	1.34	22.91	<0.0001	<0.0001
ENSG00000127585	FBXL16	F-box and leucine rich repeat protein 16 [Source:HGNC Symbol;Acc:HGNC:14150]	protein coding	-0.76	1.66	29.23	<0.0001	<0.0001
ENSG00000232354	VIPR1-AS1	VIPR1 antisense RNA 1 [Source:HGNC Symbol;Acc:HGNC:40610]	lncRNA	-0.76	2.04	35.32	<0.0001	<0.0001
ENSG00000187607	ZNF286A	zinc finger protein 286A [Source:HGNC Symbol;Acc:HGNC:13501]	protein coding	-0.76	0.26	16.10	<0.0001	0.0003
ENSG00000167600	CYP2S1	cytochrome P450 family 2 subfamily S member 1 [Source:HGNC Symbol;Acc:HGNC:15654]	protein coding	-0.76	4.65	50.39	<0.0001	<0.0001
ENSG00000237499	WAKMAR2	wound and keratinocyte migration associated lncRNA 2 [Source:HGNC Symbol;Acc:HGNC:53754]	lncRNA	0.76	2.60	40.90	<0.0001	<0.0001
ENSG00000104972	LILRB1	leukocyte immunoglobulin like receptor B1 [Source:HGNC Symbol;Acc:HGNC:6605]	protein coding	0.76	8.59	99.68	<0.0001	<0.0001
ENSG00000107438	PDLIM1	PDZ and LIM domain 1 [Source:HGNC Symbol;Acc:HGNC:2067]	protein coding	-0.76	2.42	43.99	<0.0001	<0.0001
ENSG00000196912	ANKRD36B	ankyrin repeat domain 36B [Source:HGNC Symbol;Acc:HGNC:29333]	protein coding	-0.76	0.84	32.99	<0.0001	<0.0001
ENSG00000100276	RASL10A	RAS like family 10 member A [Source:HGNC Symbol;Acc:HGNC:16954]	protein coding	-0.76	0.55	20.70	<0.0001	<0.0001
ENSG00000259448	LINC02352	long intergenic non-protein coding RNA 2352 [Source:HGNC Symbol;Acc:HGNC:53274]	lncRNA	-0.76	2.26	27.83	<0.0001	<0.0001
ENSG00000237940	LINC01238	long intergenic non-protein coding RNA 1238 [Source:HGNC Symbol;Acc:HGNC:49795]	lncRNA	-0.76	0.45	12.76	0.0004	0.0012
ENSG00000143819	EPHX1	epoxide hydrolase 1 [Source:HGNC Symbol;Acc:HGNC:3401]	protein coding	-0.76	4.53	74.22	<0.0001	<0.0001
ENSG00000137834	SMAD6	SMAD family member 6 [Source:HGNC Symbol;Acc:HGNC:6772]	protein coding	-0.76	1.01	23.59	<0.0001	<0.0001
ENSG00000136048	DRAM1	DNA damage regulated autophagy modulator 1 [Source:HGNC Symbol;Acc:HGNC:25645]	protein coding	0.76	5.54	69.62	<0.0001	<0.0001
ENSG00000104518	GSDMD	gasdermin D [Source:HGNC Symbol;Acc:HGNC:25697]	protein coding	0.76	7.68	59.46	<0.0001	<0.0001
ENSG00000285906		Novel transcript, antisense to HRH1	lncRNA	0.76	1.51	37.94	<0.0001	<0.0001

Table S5: Genes differentially expressed at Day 30 compared to pre-second vaccination (Study Arm 2 A/H7N9+AS03, D1, 29, Monocytes). Genes are sorted by descending absolute log_2 fold change. Gene model summaries and annotations are based on Ensembl Version 99 (continued.)

Ensembl Gene ID	Ensembl Gene Name	Ensembl Gene Description	Gene Type	log_2 Fold Change (Day 30 vs. Pre-Second Vaccination)	Average log_2 CPM	Likelihood Ratio Test Statistic	P-Value	FDR Adjusted P-Value
ENSG00000171236	LRG1	leucine rich alpha-2-glycoprotein 1 [Source:HGNC Symbol;Acc:HGNC:29480]	protein coding	0.76	3.53	31.67	<0.0001	<0.0001
ENSG00000179082	C9orf106	chromosome 9 open reading frame 106 (putative) [Source:HGNC Symbol;Acc:HGNC:31370]	lncRNA	-0.76	1.97	27.62	<0.0001	<0.0001
ENSG00000135378	PRRG4	proline rich and Gla domain 4 [Source:HGNC Symbol;Acc:HGNC:30799]	protein coding	0.76	5.05	68.85	<0.0001	<0.0001
ENSG00000090661	CERS4	ceramide synthase 4 [Source:HGNC Symbol;Acc:HGNC:23747]	protein coding	-0.75	4.11	63.51	<0.0001	<0.0001
ENSG00000279833		novel transcript, antisense to CBX7	lncRNA	-0.75	2.14	31.78	<0.0001	<0.0001
ENSG00000146859	TMEM140	transmembrane protein 140 [Source:HGNC Symbol;Acc:HGNC:21870]	protein coding	0.75	4.64	32.48	<0.0001	<0.0001
ENSG00000196689	TRPV1	transient receptor potential cation channel subfamily V member 1 [Source:HGNC Symbol;Acc:HGNC:12716]	protein coding	-0.75	1.68	28.21	<0.0001	<0.0001
ENSG00000211895	IGHA1	immunoglobulin heavy constant alpha 1 [Source:HGNC Symbol;Acc:HGNC:5478]	IG C gene	-0.75	6.99	9.90	0.0017	0.0047
ENSG00000123416	TUBA1B	tubulin alpha 1b [Source:HGNC Symbol;Acc:HGNC:18809]	protein coding	0.75	6.35	54.66	<0.0001	<0.0001
ENSG00000197019	SERTAD1	SERTA domain containing 1 [Source:HGNC Symbol;Acc:HGNC:17932]	protein coding	0.75	3.92	53.11	<0.0001	<0.0001
ENSG00000143546	S100A8	S100 calcium binding protein A8 [Source:HGNC Symbol;Acc:HGNC:10498]	protein coding	0.75	11.32	32.44	<0.0001	<0.0001
ENSG00000105402	NAPA	NSF attachment protein alpha [Source:HGNC Symbol;Acc:HGNC:7641]	protein coding	0.75	6.70	62.44	<0.0001	<0.0001
ENSG00000137198	GMPR	guanosine monophosphate reductase [Source:HGNC Symbol;Acc:HGNC:4376]	protein coding	0.75	2.40	19.02	<0.0001	<0.0001
ENSG00000105875	WDR91	WD repeat domain 91 [Source:HGNC Symbol;Acc:HGNC:24997]	protein coding	-0.75	5.42	59.35	<0.0001	<0.0001
ENSG00000111696	NT5DC3	5'-nucleotidase domain containing 3 [Source:HGNC Symbol;Acc:HGNC:30826]	protein coding	-0.75	2.39	26.10	<0.0001	<0.0001
ENSG00000272316		novel transcript	lncRNA	-0.75	1.56	41.60	<0.0001	<0.0001
ENSG00000182749	PAQR7	progestin and adipoQ receptor family member 7 [Source:HGNC Symbol;Acc:HGNC:23146]	protein coding	-0.75	4.43	46.32	<0.0001	<0.0001
ENSG00000107960	STN1	STN1 subunit of CST complex [Source:HGNC Symbol;Acc:HGNC:26200]	protein coding	0.75	4.63	41.44	<0.0001	<0.0001
ENSG00000115392	FANCL	FA complementation group L [Source:HGNC Symbol;Acc:HGNC:20748]	protein coding	0.75	3.06	35.75	<0.0001	<0.0001

Table S5: Genes differentially expressed at Day 30 compared to pre-second vaccination (Study Arm 2 A/H7N9+AS03, D1, 29, Monocytes). Genes are sorted by descending absolute log_2 fold change. Gene model summaries and annotations are based on Ensembl Version 99 (continued.)

Ensembl Gene ID	Ensembl Gene Name	Ensembl Gene Description	Gene Type	log_2 Fold Change (Day 30 vs. Pre-Second Vaccination)	Average log_2 CPM	Likelihood Ratio Test Statistic	P-Value	FDR Adjusted P-Value
ENSG00000119915	ELOVL3	ELOVL fatty acid elongase 3 [Source:HGNC Symbol;Acc:HGNC:18047]	protein coding	0.75	0.72	16.11	<0.0001	0.0003
ENSG00000183023	SLC8A1	solute carrier family 8 member A1 [Source:HGNC Symbol;Acc:HGNC:11068]	protein coding	0.75	6.34	81.32	<0.0001	<0.0001
ENSG00000062598	ELMO2	engulfment and cell motility 2 [Source:HGNC Symbol;Acc:HGNC:17233]	protein coding	0.75	6.03	55.58	<0.0001	<0.0001
ENSG00000173200	PARP15	poly(ADP-ribose) polymerase family member 15 [Source:HGNC Symbol;Acc:HGNC:26876]	protein coding	-0.75	3.36	43.90	<0.0001	<0.0001
ENSG00000198105	ZNF248	zinc finger protein 248 [Source:HGNC Symbol;Acc:HGNC:13041]	protein coding	-0.74	2.44	25.60	<0.0001	<0.0001
ENSG00000090659	CD209	CD209 molecule [Source:HGNC Symbol;Acc:HGNC:1641]	protein coding	0.74	2.63	29.11	<0.0001	<0.0001
ENSG00000059588	TARBP1	TAR (HIV-1) RNA binding protein 1 [Source:HGNC Symbol;Acc:HGNC:11568]	protein coding	-0.74	4.86	48.12	<0.0001	<0.0001
ENSG00000057657	PRDM1	PR/SET domain 1 [Source:HGNC Symbol;Acc:HGNC:9346]	protein coding	0.74	2.86	38.52	<0.0001	<0.0001
ENSG00000082074	FYB1	FYN binding protein 1 [Source:HGNC Symbol;Acc:HGNC:4036]	protein coding	0.74	9.27	65.09	<0.0001	<0.0001
ENSG00000239713	APOBEC3G	apolipoprotein B mRNA editing enzyme catalytic subunit 3G [Source:HGNC Symbol;Acc:HGNC:17357]	protein coding	0.74	5.62	69.19	<0.0001	<0.0001
ENSG00000030110	BAK1	BCL2 antagonist/killer 1 [Source:HGNC Symbol;Acc:HGNC:949]	protein coding	0.74	5.69	91.82	<0.0001	<0.0001
ENSG00000197081	IGF2R	insulin like growth factor 2 receptor [Source:HGNC Symbol;Acc:HGNC:5467]	protein coding	0.74	8.66	54.05	<0.0001	<0.0001
ENSG00000213066	FGFR1OP	FGFR1 oncogene partner [Source:HGNC Symbol;Acc:HGNC:17012]	protein coding	-0.74	2.90	43.33	<0.0001	<0.0001
ENSG00000285954		novel transcript	lncRNA	-0.74	0.06	20.28	<0.0001	<0.0001
ENSG00000135899	SP110	SP110 nuclear body protein [Source:HGNC Symbol;Acc:HGNC:5401]	protein coding	0.74	6.99	75.24	<0.0001	<0.0001
ENSG00000261884		novel transcript	protein coding	0.74	4.13	37.93	<0.0001	<0.0001
ENSG00000163464	CXCR1	C-X-C motif chemokine receptor 1 [Source:HGNC Symbol;Acc:HGNC:6026]	protein coding	0.74	1.13	16.00	<0.0001	0.0003
ENSG00000135148	TRAFD1	TRAF-type zinc finger domain containing 1 [Source:HGNC Symbol;Acc:HGNC:24808]	protein coding	0.74	6.97	48.21	<0.0001	<0.0001

Table S5: Genes differentially expressed at Day 30 compared to pre-second vaccination (Study Arm 2 A/H7N9+AS03, D1, 29, Monocytes). Genes are sorted by descending absolute log_2 fold change. Gene model summaries and annotations are based on Ensembl Version 99 (continued.)

Ensembl Gene ID	Ensembl Gene Name	Ensembl Gene Description	Gene Type	log_2 Fold Change (Day 30 vs. Pre-Second Vaccination)	Average log_2 CPM	Likelihood Ratio Test Statistic	P-Value	FDR Adjusted P-Value
ENSG00000088305	DNMT3B	DNA methyltransferase 3 beta [Source:HGNC Symbol;Acc:HGNC:2979]	protein coding	-0.74	0.06	17.78	<0.0001	0.0001
ENSG00000124181	PLCG1	phospholipase C gamma 1 [Source:HGNC Symbol;Acc:HGNC:9065]	protein coding	-0.74	2.22	18.57	<0.0001	<0.0001
ENSG00000082397	EPB41L3	erythrocyte membrane protein band 4.1 like 3 [Source:HGNC Symbol;Acc:HGNC:3380]	protein coding	0.74	7.24	75.08	<0.0001	<0.0001
ENSG00000159202	UBE2Z	ubiquitin conjugating enzyme E2 Z [Source:HGNC Symbol;Acc:HGNC:25847]	protein coding	0.74	6.92	72.54	<0.0001	<0.0001
ENSG00000198133	TMEM229B	transmembrane protein 229B [Source:HGNC Symbol;Acc:HGNC:20130]	protein coding	0.74	5.25	50.52	<0.0001	<0.0001
ENSG00000109654	TRIM2	tripartite motif containing 2 [Source:HGNC Symbol;Acc:HGNC:15974]	protein coding	-0.74	1.07	27.89	<0.0001	<0.0001
ENSG00000122643	NT5C3A	5’-nucleotidase, cytosolic IIIA [Source:HGNC Symbol;Acc:HGNC:17820]	protein coding	0.74	4.98	75.74	<0.0001	<0.0001
ENSG00000276728		novel transcript	lncRNA	-0.74	1.33	24.93	<0.0001	<0.0001
ENSG00000170915	PAQR8	progestin and adipoQ receptor family member 8 [Source:HGNC Symbol;Acc:HGNC:15708]	protein coding	0.74	4.91	31.69	<0.0001	<0.0001
ENSG00000174684	B4GAT1	beta-1,4-glucuronyltransferase 1 [Source:HGNC Symbol;Acc:HGNC:15685]	protein coding	-0.73	1.24	20.86	<0.0001	<0.0001
ENSG00000075275	CELSR1	cadherin EGF LAG seven-pass G-type receptor 1 [Source:HGNC Symbol;Acc:HGNC:1850]	protein coding	-0.73	1.48	21.23	<0.0001	<0.0001
ENSG00000198892	SHISA4	shisa family member 4 [Source:HGNC Symbol;Acc:HGNC:27139]	protein coding	0.73	1.24	28.93	<0.0001	<0.0001
ENSG00000198604	BAZ1A	bromodomain adjacent to zinc finger domain 1A [Source:HGNC Symbol;Acc:HGNC:960]	protein coding	0.73	7.11	48.12	<0.0001	<0.0001
ENSG00000211673	IGLV3-1	immunoglobulin lambda variable 3-1 [Source:HGNC Symbol;Acc:HGNC:5896]	IG V gene	-0.73	0.91	9.44	0.0021	0.0058
ENSG00000235750	KIAA0040	KIAA0040 [Source:HGNC Symbol;Acc:HGNC:28950]	protein coding	0.73	6.49	31.20	<0.0001	<0.0001
ENSG00000204650	LINC02210	long intergenic non-protein coding RNA 2210 [Source:HGNC Symbol;Acc:HGNC:26327]	transcribed unitary pseudogene	-0.73	1.31	30.59	<0.0001	<0.0001
ENSG00000118513	MYB	MYB proto-oncogene, transcription factor [Source:HGNC Symbol;Acc:HGNC:7545]	protein coding	-0.73	1.97	12.54	0.0004	0.0013

Table S5: Genes differentially expressed at Day 30 compared to pre-second vaccination (Study Arm 2 A/H7N9+AS03, D1, 29, Monocytes). Genes are sorted by descending absolute log_2 fold change. Gene model summaries and annotations are based on Ensembl Version 99 (continued.)

Ensembl Gene ID	Ensembl Gene Name	Ensembl Gene Description	Gene Type	log_2 Fold Change (Day 30 vs. Pre-Second Vaccination)	Average log_2 CPM	Likelihood Ratio Test Statistic	P-Value	FDR Adjusted P-Value
ENSG00000172575	RASGRP1	RAS guanyl releasing protein 1 [Source:HGNC Symbol;Acc:HGNC:9878]	protein coding	-0.73	0.86	16.35	<0.0001	0.0002
ENSG00000168813	ZNF507	zinc finger protein 507 [Source:HGNC Symbol;Acc:HGNC:23783]	protein coding	-0.73	1.07	42.69	<0.0001	<0.0001
ENSG00000164048	ZNF589	zinc finger protein 589 [Source:HGNC Symbol;Acc:HGNC:16747]	protein coding	-0.73	4.86	44.24	<0.0001	<0.0001
ENSG00000159216	RUNX1	RUNX family transcription factor 1 [Source:HGNC Symbol;Acc:HGNC:10471]	protein coding	0.73	6.54	69.20	<0.0001	<0.0001
ENSG00000167208	SNX20	sorting nexin 20 [Source:HGNC Symbol;Acc:HGNC:30390]	protein coding	0.73	6.88	49.52	<0.0001	<0.0001
ENSG00000286813		novel transcript, sense intronic to ST20-MTHFSand MTHFS	lncRNA	-0.73	0.97	18.08	<0.0001	0.0001
ENSG00000179344	HLA-DQB1	major histocompatibility complex, class II, DQ beta 1 [Source:HGNC Symbol;Acc:HGNC:4944]	protein coding	0.73	8.74	64.74	<0.0001	<0.0001
ENSG00000059804	SLC2A3	solute carrier family 2 member 3 [Source:HGNC Symbol;Acc:HGNC:11007]	protein coding	0.73	7.59	52.86	<0.0001	<0.0001
ENSG00000114812	VIPR1	vasoactive intestinal peptide receptor 1 [Source:HGNC Symbol;Acc:HGNC:12694]	protein coding	-0.73	5.90	67.37	<0.0001	<0.0001
ENSG00000115109	EPB41L5	erythrocyte membrane protein band 4.1 like 5 [Source:HGNC Symbol;Acc:HGNC:19819]	protein coding	0.73	1.46	14.30	0.0002	0.0006
ENSG00000146425	DYNLT1	dynein light chain Tctex-type 1 [Source:HGNC Symbol;Acc:HGNC:11697]	protein coding	0.73	6.36	58.59	<0.0001	<0.0001
ENSG00000225032		novel transcript	lncRNA	-0.73	1.79	20.20	<0.0001	<0.0001
ENSG00000171051	FPR1	formyl peptide receptor 1 [Source:HGNC Symbol;Acc:HGNC:3826]	protein coding	0.73	8.82	62.52	<0.0001	<0.0001
ENSG00000211890	IGHA2	immunoglobulin heavy constant alpha 2 (A2m marker) [Source:HGNC Symbol;Acc:HGNC:5479]	IG C gene	-0.73	4.80	7.20	0.0073	0.017
ENSG00000166068	SPRED1	sprouty related EVH1 domain containing 1 [Source:HGNC Symbol;Acc:HGNC:20249]	protein coding	-0.73	3.68	36.23	<0.0001	<0.0001
ENSG00000170667	RASA4B	RAS p21 protein activator 4B [Source:HGNC Symbol;Acc:HGNC:35202]	protein coding	-0.73	2.66	24.13	<0.0001	<0.0001
ENSG00000177732	SOX12	SRY-box transcription factor 12 [Source:HGNC Symbol;Acc:HGNC:11198]	protein coding	-0.73	2.70	34.70	<0.0001	<0.0001
ENSG00000212694	LINC01089	long intergenic non-protein coding RNA 1089 [Source:HGNC Symbol;Acc:HGNC:27886]	lncRNA	-0.73	4.47	38.13	<0.0001	<0.0001
ENSG00000105639	JAK3	Janus kinase 3 [Source:HGNC Symbol;Acc:HGNC:6193]	protein coding	0.73	6.82	54.23	<0.0001	<0.0001

Table S5: Genes differentially expressed at Day 30 compared to pre-second vaccination (Study Arm 2 A/H7N9+AS03, D1, 29, Monocytes). Genes are sorted by descending absolute log_2 fold change. Gene model summaries and annotations are based on Ensembl Version 99 (continued.)

Ensembl Gene ID	Ensembl Gene Name	Ensembl Gene Description	Gene Type	log_2 Fold Change (Day 30 vs. Pre-Second Vaccination)	Average log_2 CPM	Likelihood Ratio Test Statistic	P-Value	FDR Adjusted P-Value
ENSG00000132465	JCHAIN	joining chain of multimeric IgA and IgM [Source:HGNC Symbol;Acc:HGNC:5713]	protein coding	-0.73	5.34	10.46	0.0012	0.0036
ENSG00000135862	LAMC1	laminin subunit gamma 1 [Source:HGNC Symbol;Acc:HGNC:6492]	protein coding	-0.73	2.83	52.41	<0.0001	<0.0001
ENSG00000138190	EXOC6	exocyst complex component 6 [Source:HGNC Symbol;Acc:HGNC:23196]	protein coding	0.73	4.39	56.65	<0.0001	<0.0001
ENSG00000256223	ZNF10	zinc finger protein 10 [Source:HGNC Symbol;Acc:HGNC:12879]	protein coding	-0.73	0.15	15.90	<0.0001	0.0003
ENSG00000198355	PIM3	Pim-3 proto-oncogene, serine/threonine kinase [Source:HGNC Symbol;Acc:HGNC:19310]	protein coding	0.73	7.54	21.75	<0.0001	<0.0001
ENSG00000138030	KHK	ketohexokinase [Source:HGNC Symbol;Acc:HGNC:6315]	protein coding	-0.73	1.83	16.51	<0.0001	0.0002
ENSG00000175265	GOLGA8A	golgin A8 family member A [Source:HGNC Symbol;Acc:HGNC:31972]	protein coding	-0.73	2.70	30.10	<0.0001	<0.0001
ENSG00000137414	FAM8A1	family with sequence similarity 8 member A1 [Source:HGNC Symbol;Acc:HGNC:16372]	protein coding	0.73	5.43	58.33	<0.0001	<0.0001
ENSG00000166578	IQCD	IQ motif containing D [Source:HGNC Symbol;Acc:HGNC:25168]	protein coding	-0.73	1.02	22.15	<0.0001	<0.0001
ENSG00000143771	CNIH4	cornichon family AMPA receptor auxiliary protein 4 [Source:HGNC Symbol;Acc:HGNC:25013]	protein coding	0.72	5.16	106.56	<0.0001	<0.0001
ENSG00000204161	TMEM273	transmembrane protein 273 [Source:HGNC Symbol;Acc:HGNC:27274]	protein coding	0.72	3.27	36.07	<0.0001	<0.0001
ENSG00000116191	RALGPS2	Ral GEF with PH domain and SH3 binding motif 2 [Source:HGNC Symbol;Acc:HGNC:30279]	protein coding	-0.72	4.25	50.66	<0.0001	<0.0001
ENSG00000115525	ST3GAL5	ST3 beta-galactoside alpha-2,3-sialyltransferase 5 [Source:HGNC Symbol;Acc:HGNC:10872]	protein coding	0.72	6.20	51.56	<0.0001	<0.0001
ENSG00000153208	MERTK	MER proto-oncogene, tyrosine kinase [Source:HGNC Symbol;Acc:HGNC:7027]	protein coding	-0.72	4.51	17.71	<0.0001	0.0001
ENSG00000166710	B2M	beta-2-microglobulin [Source:HGNC Symbol;Acc:HGNC:914]	protein coding	0.72	12.66	46.79	<0.0001	<0.0001
ENSG00000179593	ALOX15B	arachidonate 15-lipoxygenase type B [Source:HGNC Symbol;Acc:HGNC:434]	protein coding	-0.72	3.08	12.10	0.0005	0.0017
ENSG00000129484	PARP2	poly(ADP-ribose) polymerase 2 [Source:HGNC Symbol;Acc:HGNC:272]	protein coding	-0.72	2.42	26.47	<0.0001	<0.0001
ENSG00000186818	LILRB4	leukocyte immunoglobulin like receptor B4 [Source:HGNC Symbol;Acc:HGNC:6608]	protein coding	0.72	7.10	109.90	<0.0001	<0.0001

Table S5: Genes differentially expressed at Day 30 compared to pre-second vaccination (Study Arm 2 A/H7N9+AS03, D1, 29, Monocytes). Genes are sorted by descending absolute log_2 fold change. Gene model summaries and annotations are based on Ensembl Version 99 (continued.)

Ensembl Gene ID	Ensembl Gene Name	Ensembl Gene Description	Gene Type	log_2 Fold Change (Day 30 vs. Pre-Second Vaccination)	Average log_2 CPM	Likelihood Ratio Test Statistic	P-Value	FDR Adjusted P-Value
ENSG00000288025		novel transcript, antisense to SCRG1	lncRNA	-0.72	0.27	16.08	<0.0001	0.0003
ENSG00000117009	KMO	kynurenine 3-monooxygenase [Source:HGNC Symbol;Acc:HGNC:6381]	protein coding	0.72	3.77	45.22	<0.0001	<0.0001
ENSG00000185477	GPRIN3	GPRIN family member 3 [Source:HGNC Symbol;Acc:HGNC:27733]	protein coding	-0.72	4.28	42.97	<0.0001	<0.0001
ENSG00000204287	HLA-DRA	major histocompatibility complex, class II, DR alpha [Source:HGNC Symbol;Acc:HGNC:4947]	protein coding	0.72	11.48	46.15	<0.0001	<0.0001
ENSG00000247092	SNHG10	small nucleolar RNA host gene 10 [Source:HGNC Symbol;Acc:HGNC:27510]	lncRNA	-0.72	2.20	42.23	<0.0001	<0.0001
ENSG00000234741	GAS5	growth arrest specific 5 [Source:HGNC Symbol;Acc:HGNC:16355]	lncRNA	-0.72	5.96	41.28	<0.0001	<0.0001
ENSG00000108474	PIGL	phosphatidylinositol glycan anchor biosynthesis class L [Source:HGNC Symbol;Acc:HGNC:8966]	protein coding	-0.72	3.48	36.76	<0.0001	<0.0001
ENSG00000106392	C1GALT1	core 1 synthase, glycoprotein-N-acetylgalactosamine 3-beta-galactosyltransferase 1 [Source:HGNC Symbol;Acc:HGNC:24337]	protein coding	0.72	5.54	64.55	<0.0001	<0.0001
ENSG00000128394	APOBEC3F	apolipoprotein B mRNA editing enzyme catalytic subunit 3F [Source:HGNC Symbol;Acc:HGNC:17356]	protein coding	0.72	3.75	40.79	<0.0001	<0.0001
ENSG00000115956	PLEK	pleckstrin [Source:HGNC Symbol;Acc:HGNC:9070]	protein coding	0.72	8.79	52.04	<0.0001	<0.0001
ENSG00000105499	PLA2G4C	phospholipase A2 group IVC [Source:HGNC Symbol;Acc:HGNC:9037]	protein coding	0.72	2.85	37.43	<0.0001	<0.0001
ENSG00000158485	CD1B	CD1b molecule [Source:HGNC Symbol;Acc:HGNC:1635]	protein coding	-0.72	0.61	16.13	<0.0001	0.0003
ENSG00000237440	ZNF737	zinc finger protein 737 [Source:HGNC Symbol;Acc:HGNC:32468]	protein coding	-0.72	1.00	16.45	<0.0001	0.0002
ENSG00000166546	BEAN1	brain expressed associated with NEDD4 1 [Source:HGNC Symbol;Acc:HGNC:24160]	protein coding	-0.72	1.51	21.44	<0.0001	<0.0001
ENSG00000262155	LINC02175	long intergenic non-protein coding RNA 2175 [Source:HGNC Symbol;Acc:HGNC:27550]	lncRNA	-0.72	0.28	16.04	<0.0001	0.0003
ENSG00000188372	ZP3	zona pellucida glycoprotein 3 [Source:HGNC Symbol;Acc:HGNC:13189]	protein coding	-0.72	1.97	34.00	<0.0001	<0.0001
ENSG00000165272	AQP3	aquaporin 3 (Gill blood group) [Source:HGNC Symbol;Acc:HGNC:636]	protein coding	-0.72	0.63	7.75	0.0054	0.013
ENSG00000187474	FPR3	formyl peptide receptor 3 [Source:HGNC Symbol;Acc:HGNC:3828]	protein coding	-0.72	2.97	14.88	0.0001	0.0004

Table S5: Genes differentially expressed at Day 30 compared to pre-second vaccination (Study Arm 2 A/H7N9+AS03, D1, 29, Monocytes). Genes are sorted by descending absolute log_2 fold change. Gene model summaries and annotations are based on Ensembl Version 99 (continued.)

Ensembl Gene ID	Ensembl Gene Name	Ensembl Gene Description	Gene Type	log_2 Fold Change (Day 30 vs. Pre-Second Vaccination)	Average log_2 CPM	Likelihood Ratio Test Statistic	P-Value	FDR Adjusted P-Value
ENSG00000224796	RPL32P1	ribosomal protein L32 pseudogene 1 [Source:HGNC Symbol;Acc:HGNC:10339]	processed pseudogene	0.72	2.41	11.13	0.0009	0.0026
ENSG00000112249	ASCC3	activating signal cointegrator 1 complex subunit 3 [Source:HGNC Symbol;Acc:HGNC:18697]	protein coding	0.72	5.36	48.59	<0.0001	<0.0001
ENSG00000110841	PPFIBP1	PPFIA binding protein 1 [Source:HGNC Symbol;Acc:HGNC:9249]	protein coding	-0.72	0.75	24.64	<0.0001	<0.0001
ENSG00000197555	SIPA1L1	signal induced proliferation associated 1 like 1 [Source:HGNC Symbol;Acc:HGNC:20284]	protein coding	0.72	6.16	50.94	<0.0001	<0.0001
ENSG00000177556	ATOX1	antioxidant 1 copper chaperone [Source:HGNC Symbol;Acc:HGNC:798]	protein coding	0.72	4.52	57.03	<0.0001	<0.0001
ENSG00000155363	MOV10	Mov10 RISC complex RNA helicase [Source:HGNC Symbol;Acc:HGNC:7200]	protein coding	0.72	5.97	41.36	<0.0001	<0.0001
ENSG00000164136	IL15	interleukin 15 [Source:HGNC Symbol;Acc:HGNC:5977]	protein coding	0.72	5.38	43.31	<0.0001	<0.0001
ENSG00000164691	TAGAP	T cell activation RhoGTPase activating protein [Source:HGNC Symbol;Acc:HGNC:15669]	protein coding	0.72	6.89	22.13	<0.0001	<0.0001
ENSG00000267254	ZNF790-AS1	ZNF790 antisense RNA 1 [Source:HGNC Symbol;Acc:HGNC:27617]	lncRNA	-0.71	0.40	21.23	<0.0001	<0.0001
ENSG00000275832	ARHGAP23	Rho GTPase activating protein 23 [Source:HGNC Symbol;Acc:HGNC:29293]	protein coding	0.71	1.15	22.87	<0.0001	<0.0001
ENSG00000129538	RNASE1	ribonuclease A family member 1, pancreatic [Source:HGNC Symbol;Acc:HGNC:10044]	protein coding	-0.71	0.34	12.47	0.0004	0.0014
ENSG00000275210		novel transcript	lncRNA	0.71	2.41	38.56	<0.0001	<0.0001
ENSG00000271270	TMCC1-AS1	TMCC1 antisense RNA 1 (head to head) [Source:HGNC Symbol;Acc:HGNC:49060]	lncRNA	-0.71	0.69	23.69	<0.0001	<0.0001
ENSG00000132109	TRIM21	tripartite motif containing 21 [Source:HGNC Symbol;Acc:HGNC:11312]	protein coding	0.71	5.88	27.15	<0.0001	<0.0001
ENSG00000171954	CYP4F22	cytochrome P450 family 4 subfamily F member 22 [Source:HGNC Symbol;Acc:HGNC:26820]	protein coding	0.71	2.24	15.66	<0.0001	0.0003
ENSG00000243156	MICAL3	microtubule associated monooxygenase, calponin and LIM domain containing 3 [Source:HGNC Symbol;Acc:HGNC:24694]	protein coding	-0.71	2.16	27.75	<0.0001	<0.0001
ENSG00000279447		TEC	TEC	-0.71	0.11	15.86	<0.0001	0.0003
ENSG00000134256	CD101	CD101 molecule [Source:HGNC Symbol;Acc:HGNC:5949]	protein coding	-0.71	5.59	27.43	<0.0001	<0.0001

Table S5: Genes differentially expressed at Day 30 compared to pre-second vaccination (Study Arm 2 A/H7N9+AS03, D1, 29, Monocytes). Genes are sorted by descending absolute log_2 fold change. Gene model summaries and annotations are based on Ensembl Version 99 (continued.)

Ensembl Gene ID	Ensembl Gene Name	Ensembl Gene Description	Gene Type	log_2 Fold Change (Day 30 vs. Pre-Second Vaccination)	Average log_2 CPM	Likelihood Ratio Test Statistic	P-Value	FDR Adjusted P-Value
ENSG00000175414	ARL10	ADP ribosylation factor like GTPase 10 [Source:HGNC Symbol;Acc:HGNC:22042]	protein coding	-0.71	0.77	26.65	<0.0001	<0.0001
ENSG00000131242	RAB11FIP4	RAB11 family interacting protein 4 [Source:HGNC Symbol;Acc:HGNC:30267]	protein coding	0.71	6.02	75.81	<0.0001	<0.0001
ENSG00000185442	FAM174B	family with sequence similarity 174 member B [Source:HGNC Symbol;Acc:HGNC:34339]	protein coding	-0.71	0.23	15.57	<0.0001	0.0003
ENSG00000135976	ANKRD36	ankyrin repeat domain 36 [Source:HGNC Symbol;Acc:HGNC:24079]	protein coding	-0.71	2.02	30.12	<0.0001	<0.0001
ENSG00000109762	SNX25	sorting nexin 25 [Source:HGNC Symbol;Acc:HGNC:21883]	protein coding	-0.71	1.11	28.80	<0.0001	<0.0001
ENSG00000147883	CDKN2B	cyclin dependent kinase inhibitor 2B [Source:HGNC Symbol;Acc:HGNC:1788]	protein coding	-0.71	2.96	17.55	<0.0001	0.0001
ENSG00000108771	DHX58	DExH-box helicase 58 [Source:HGNC Symbol;Acc:HGNC:29517]	protein coding	0.71	5.97	57.92	<0.0001	<0.0001
ENSG00000064932	SBNO2	strawberry notch homolog 2 [Source:HGNC Symbol;Acc:HGNC:29158]	protein coding	0.71	8.15	52.56	<0.0001	<0.0001
ENSG00000283633		novel transcript	lncRNA	-0.71	1.94	23.49	<0.0001	<0.0001
ENSG00000166532	RIMKLB	ribosomal modification protein rimK like family member B [Source:HGNC Symbol;Acc:HGNC:29228]	protein coding	0.71	4.28	18.00	<0.0001	0.0001
ENSG00000086065	CHMP5	charged multivesicular body protein 5 [Source:HGNC Symbol;Acc:HGNC:26942]	protein coding	0.71	5.61	89.21	<0.0001	<0.0001
ENSG00000065054	SLC9A3R2	SLC9A3 regulator 2 [Source:HGNC Symbol;Acc:HGNC:11076]	protein coding	-0.71	2.45	30.98	<0.0001	<0.0001
ENSG00000244879	GABPB1-AS1	GABPB1 antisense RNA 1 [Source:HGNC Symbol;Acc:HGNC:44157]	lncRNA	-0.71	5.43	60.24	<0.0001	<0.0001
ENSG00000165804	ZNF219	zinc finger protein 219 [Source:HGNC Symbol;Acc:HGNC:13011]	protein coding	-0.70	3.64	42.54	<0.0001	<0.0001
ENSG00000283674		novel transcript	lncRNA	-0.70	0.63	15.80	<0.0001	0.0003
ENSG00000188549	CCDC9B	coiled-coil domain containing 9B [Source:HGNC Symbol;Acc:HGNC:33488]	protein coding	-0.70	2.80	24.64	<0.0001	<0.0001
ENSG00000254838	GVINP1	GTPase, very large interferon inducible pseudogene 1 [Source:HGNC Symbol;Acc:HGNC:25813]	transcribed unprocessed pseudogene	0.70	4.89	43.96	<0.0001	<0.0001

Confidential/Proprietary Information

Table S5: Genes differentially expressed at Day 30 compared to pre-second vaccination (Study Arm 2 A/H7N9+AS03, D1, 29, Monocytes). Genes are sorted by descending absolute log_2 fold change. Gene model summaries and annotations are based on Ensembl Version 99 (continued.)

Ensembl Gene ID	Ensembl Gene Name	Ensembl Gene Description	Gene Type	log_2 Fold Change (Day 30 vs. Pre-Second Vaccination)	Average log_2 CPM	Likelihood Ratio Test Statistic	P-Value	FDR Adjusted P-Value
ENSG00000153044	CENPH	centromere protein H [Source:HGNC Symbol;Acc:HGNC:17268]	protein coding	-0.70	0.61	21.22	<0.0001	<0.0001
ENSG00000180867	PDIA3P1	protein disulfide isomerase family A member 3 pseudogene 1 [Source:HGNC Symbol;Acc:HGNC:4607]	transcribed processed pseudogene	0.70	4.51	47.90	<0.0001	<0.0001
ENSG00000130304	SLC27A1	solute carrier family 27 member 1 [Source:HGNC Symbol;Acc:HGNC:10995]	protein coding	-0.70	6.35	68.67	<0.0001	<0.0001
ENSG00000156381	ANKRD9	ankyrin repeat domain 9 [Source:HGNC Symbol;Acc:HGNC:20096]	protein coding	0.70	2.02	23.46	<0.0001	<0.0001
ENSG00000179241	LDLRAD3	low density lipoprotein receptor class A domain containing 3 [Source:HGNC Symbol;Acc:HGNC:27046]	protein coding	-0.70	4.06	36.11	<0.0001	<0.0001
ENSG00000160216	AGPAT3	1-acylglycerol-3-phosphate O-acyltransferase 3 [Source:HGNC Symbol;Acc:HGNC:326]	protein coding	0.70	8.06	83.73	<0.0001	<0.0001
ENSG00000085465	OVGP1	oviductal glycoprotein 1 [Source:HGNC Symbol;Acc:HGNC:8524]	protein coding	-0.70	0.51	15.14	<0.0001	0.0004
ENSG00000116717	GADD45A	growth arrest and DNA damage inducible alpha [Source:HGNC Symbol;Acc:HGNC:4095]	protein coding	-0.70	3.06	19.58	<0.0001	<0.0001
ENSG00000087253	LPCAT2	lysophosphatidylcholine acyltransferase 2 [Source:HGNC Symbol;Acc:HGNC:26032]	protein coding	0.70	6.72	64.62	<0.0001	<0.0001
ENSG00000180530	NRIP1	nuclear receptor interacting protein 1 [Source:HGNC Symbol;Acc:HGNC:8001]	protein coding	0.70	5.66	41.93	<0.0001	<0.0001
ENSG00000225138	SLC9A3-AS1	SLC9A3 antisense RNA 1 [Source:HGNC Symbol;Acc:HGNC:40550]	lncRNA	-0.70	3.35	34.54	<0.0001	<0.0001
ENSG00000182621	PLCB1	phospholipase C beta 1 [Source:HGNC Symbol;Acc:HGNC:15917]	protein coding	-0.70	4.54	33.37	<0.0001	<0.0001
ENSG00000173334	TRIB1	tribbles pseudokinase 1 [Source:HGNC Symbol;Acc:HGNC:16891]	protein coding	0.70	6.41	11.09	0.0009	0.0027
ENSG00000152223	EPG5	ectopic P-granules autophagy protein 5 homolog [Source:HGNC Symbol;Acc:HGNC:29331]	protein coding	0.70	5.72	43.30	<0.0001	<0.0001
ENSG00000143344	RGL1	ral guanine nucleotide dissociation stimulator like 1 [Source:HGNC Symbol;Acc:HGNC:30281]	protein coding	0.70	3.61	31.43	<0.0001	<0.0001
ENSG00000273066		novel transcript	lncRNA	-0.70	1.00	17.93	<0.0001	0.0001
ENSG00000181690	PLAG1	PLAG1 zinc finger [Source:HGNC Symbol;Acc:HGNC:9045]	protein coding	-0.70	0.05	19.07	<0.0001	<0.0001

Table S5: Genes differentially expressed at Day 30 compared to pre-second vaccination (Study Arm 2 A/H7N9+AS03, D1, 29, Monocytes). Genes are sorted by descending absolute log_2 fold change. Gene model summaries and annotations are based on Ensembl Version 99 (continued.)

Ensembl Gene ID	Ensembl Gene Name	Ensembl Gene Description	Gene Type	log_2 Fold Change (Day 30 vs. Pre-Second Vaccination)	Average log_2 CPM	Likelihood Ratio Test Statistic	P-Value	FDR Adjusted P-Value
ENSG00000124813	RUNX2	RUNX family transcription factor 2 [Source:HGNC Symbol;Acc:HGNC:10472]	protein coding	-0.70	3.91	54.18	<0.0001	<0.0001
ENSG00000229671	LINC01150	long intergenic non-protein coding RNA 1150 [Source:HGNC Symbol;Acc:HGNC:49469]	lncRNA	-0.70	0.72	21.97	<0.0001	<0.0001
ENSG00000073331	ALPK1	alpha kinase 1 [Source:HGNC Symbol;Acc:HGNC:20917]	protein coding	0.70	5.51	38.53	<0.0001	<0.0001
ENSG00000103056	SMPD3	sphingomyelin phosphodiesterase 3 [Source:HGNC Symbol;Acc:HGNC:14240]	protein coding	0.70	2.85	36.47	<0.0001	<0.0001
ENSG00000176533	GNG7	G protein subunit gamma 7 [Source:HGNC Symbol;Acc:HGNC:4410]	protein coding	-0.70	3.59	37.18	<0.0001	<0.0001
ENSG00000172037	LAMB2	laminin subunit beta 2 [Source:HGNC Symbol;Acc:HGNC:6487]	protein coding	-0.70	4.63	62.18	<0.0001	<0.0001
ENSG00000134575	ACP2	acid phosphatase 2, lysosomal [Source:HGNC Symbol;Acc:HGNC:123]	polymorphic pseudogene	0.70	5.34	138.61	<0.0001	<0.0001
ENSG00000265511		novel transcript	lncRNA	-0.70	0.50	18.82	<0.0001	<0.0001
ENSG00000196954	CASP4	caspase 4 [Source:HGNC Symbol;Acc:HGNC:1505]	protein coding	0.70	7.70	85.22	<0.0001	<0.0001
ENSG00000257093	DENND11	DENN domain containing 11 [Source:HGNC Symbol;Acc:HGNC:29472]	protein coding	-0.70	4.85	54.45	<0.0001	<0.0001
ENSG00000221988	PPT2	palmitoyl-protein thioesterase 2 [Source:HGNC Symbol;Acc:HGNC:9326]	protein coding	-0.70	0.06	16.19	<0.0001	0.0002
ENSG00000139618	BRCA2	BRCA2 DNA repair associated [Source:HGNC Symbol;Acc:HGNC:1101]	protein coding	0.70	1.38	21.03	<0.0001	<0.0001
ENSG00000181631	P2RY13	purinergic receptor P2Y13 [Source:HGNC Symbol;Acc:HGNC:4537]	protein coding	0.70	7.04	25.31	<0.0001	<0.0001
ENSG00000165716	DIPK1B	divergent protein kinase domain 1B [Source:HGNC Symbol;Acc:HGNC:28290]	protein coding	-0.70	1.45	42.88	<0.0001	<0.0001
ENSG00000133818	RRAS2	RAS related 2 [Source:HGNC Symbol;Acc:HGNC:17271]	protein coding	-0.70	0.23	12.09	0.0005	0.0017
ENSG00000100979	PLTP	phospholipid transfer protein [Source:HGNC Symbol;Acc:HGNC:9093]	protein coding	-0.70	1.07	16.41	<0.0001	0.0002
ENSG00000237541	HLA-DQA2	major histocompatibility complex, class II, DQ alpha 2 [Source:HGNC Symbol;Acc:HGNC:4943]	protein coding	0.70	4.59	37.92	<0.0001	<0.0001
ENSG00000272849		novel transcript	lncRNA	-0.69	2.45	28.19	<0.0001	<0.0001

Confidential/Proprietary Information

Table S5: Genes differentially expressed at Day 30 compared to pre-second vaccination (Study Arm 2 A/H7N9+AS03, D1, 29, Monocytes). Genes are sorted by descending absolute log_2 fold change. Gene model summaries and annotations are based on Ensembl Version 99 (continued.)

Ensembl Gene ID	Ensembl Gene Name	Ensembl Gene Description	Gene Type	log_2 Fold Change (Day 30 vs. Pre-Second Vaccination)	Average log_2 CPM	Likelihood Ratio Test Statistic	P-Value	FDR Adjusted P-Value
ENSG00000145687	SSBP2	single stranded DNA binding protein 2 [Source:HGNC Symbol;Acc:HGNC:15831]	protein coding	-0.69	4.28	49.02	<0.0001	<0.0001
ENSG00000287562		novel transcript	lncRNA	-0.69	0.68	14.07	0.0002	0.0007
ENSG00000267041	ZNF850	zinc finger protein 850 [Source:HGNC Symbol;Acc:HGNC:27994]	protein coding	-0.69	0.56	20.47	<0.0001	<0.0001
ENSG00000145390	USP53	ubiquitin specific peptidase 53 [Source:HGNC Symbol;Acc:HGNC:29255]	protein coding	-0.69	0.47	13.58	0.0002	0.0008
ENSG00000213923	CSNK1E	casein kinase 1 epsilon [Source:HGNC Symbol;Acc:HGNC:2453]	protein coding	-0.69	3.90	57.59	<0.0001	<0.0001
ENSG00000123685	BATF3	basic leucine zipper ATF-like transcription factor 3 [Source:HGNC Symbol;Acc:HGNC:28915]	protein coding	0.69	3.27	36.55	<0.0001	<0.0001
ENSG00000152465	NMT2	N-myristoyltransferase 2 [Source:HGNC Symbol;Acc:HGNC:7858]	protein coding	-0.69	0.94	28.04	<0.0001	<0.0001
ENSG00000041880	PARP3	poly(ADP-ribose) polymerase family member 3 [Source:HGNC Symbol;Acc:HGNC:273]	protein coding	0.69	3.67	46.66	<0.0001	<0.0001
ENSG00000286116		FAS antisense RNA 1	lncRNA	0.69	0.30	8.38	0.0038	0.0096
ENSG00000109046	WSB1	WD repeat and SOCS box containing 1 [Source:HGNC Symbol;Acc:HGNC:19221]	protein coding	0.69	8.28	57.76	<0.0001	<0.0001
ENSG00000174600	CMKLR1	chemerin chemokine-like receptor 1 [Source:HGNC Symbol;Acc:HGNC:2121]	protein coding	0.69	5.19	33.43	<0.0001	<0.0001
ENSG00000203965	EFCAB7	EF-hand calcium binding domain 7 [Source:HGNC Symbol;Acc:HGNC:29379]	protein coding	-0.69	0.88	21.51	<0.0001	<0.0001
ENSG00000196116	TDRD7	tudor domain containing 7 [Source:HGNC Symbol;Acc:HGNC:30831]	protein coding	0.69	5.20	52.12	<0.0001	<0.0001
ENSG00000287837		novel transcript	lncRNA	-0.69	1.23	24.78	<0.0001	<0.0001
ENSG00000057935	MTA3	metastasis associated 1 family member 3 [Source:HGNC Symbol;Acc:HGNC:23784]	protein coding	-0.69	2.47	49.18	<0.0001	<0.0001
ENSG00000134897	BIVM	basic, immunoglobulin-like variable motif containing [Source:HGNC Symbol;Acc:HGNC:16034]	protein coding	-0.69	0.85	27.08	<0.0001	<0.0001
ENSG00000124225	PMEPA1	prostate transmembrane protein, androgen induced 1 [Source:HGNC Symbol;Acc:HGNC:14107]	protein coding	-0.69	0.76	22.24	<0.0001	<0.0001
ENSG00000233695	GAS6-AS1	GAS6 antisense RNA 1 [Source:HGNC Symbol;Acc:HGNC:39826]	lncRNA	-0.69	4.15	28.04	<0.0001	<0.0001
ENSG00000122224	LY9	lymphocyte antigen 9 [Source:HGNC Symbol;Acc:HGNC:6730]	protein coding	-0.69	1.97	34.18	<0.0001	<0.0001

Confidential/Proprietary Information

Table S5: Genes differentially expressed at Day 30 compared to pre-second vaccination (Study Arm 2 A/H7N9+AS03, D1, 29, Monocytes). Genes are sorted by descending absolute log_2 fold change. Gene model summaries and annotations are based on Ensembl Version 99 (continued.)

Ensembl Gene ID	Ensembl Gene Name	Ensembl Gene Description	Gene Type	log_2 Fold Change (Day 30 vs. Pre-Second Vaccination)	Average log_2 CPM	Likelihood Ratio Test Statistic	P-Value	FDR Adjusted P-Value
ENSG00000108773	KAT2A	lysine acetyltransferase 2A [Source:HGNC Symbol;Acc:HGNC:4201]	protein coding	-0.69	5.42	72.15	<0.0001	<0.0001
ENSG00000070501	POLB	DNA polymerase beta [Source:HGNC Symbol;Acc:HGNC:9174]	protein coding	0.69	4.46	54.98	<0.0001	<0.0001
ENSG00000228060	PABPC4-AS1	PABPC4 antisense RNA 1 [Source:HGNC Symbol;Acc:HGNC:40424]	lncRNA	-0.69	0.16	8.76	0.0031	0.008
ENSG00000167552	TUBA1A	tubulin alpha 1a [Source:HGNC Symbol;Acc:HGNC:20766]	protein coding	0.69	7.65	20.17	<0.0001	<0.0001
ENSG00000122547	EEPD1	endonuclease/exonuclease/phosphatase family domain containing 1 [Source:HGNC Symbol;Acc:HGNC:22223]	protein coding	-0.69	4.56	40.87	<0.0001	<0.0001
ENSG00000211660	IGLV2-23	immunoglobulin lambda variable 2-23 [Source:HGNC Symbol;Acc:HGNC:5890]	IG V gene	-0.69	0.86	8.46	0.0036	0.0093
ENSG00000166025	AMOTL1	angiomotin like 1 [Source:HGNC Symbol;Acc:HGNC:17811]	protein coding	-0.69	1.51	39.52	<0.0001	<0.0001
ENSG00000259772		uncharacterized LOC283710 [Source:NCBI gene;Acc:283710]	lncRNA	-0.69	1.10	20.95	<0.0001	<0.0001
ENSG00000165997	ARL5B	ADP ribosylation factor like GTPase 5B [Source:HGNC Symbol;Acc:HGNC:23052]	protein coding	0.69	4.43	28.49	<0.0001	<0.0001
ENSG00000158106	RHPN1	rhophilin Rho GTPase binding protein 1 [Source:HGNC Symbol;Acc:HGNC:19973]	protein coding	-0.69	1.07	16.58	<0.0001	0.0002
ENSG00000187583	PLEKHN1	pleckstrin homology domain containing N1 [Source:HGNC Symbol;Acc:HGNC:25284]	protein coding	0.69	1.71	26.07	<0.0001	<0.0001
ENSG00000179021	C3orf38	chromosome 3 open reading frame 38 [Source:HGNC Symbol;Acc:HGNC:28384]	protein coding	0.69	4.90	64.39	<0.0001	<0.0001
ENSG00000213064	SFT2D2	SFT2 domain containing 2 [Source:HGNC Symbol;Acc:HGNC:25140]	protein coding	0.69	6.53	52.72	<0.0001	<0.0001
ENSG00000176222	ZNF404	zinc finger protein 404 [Source:HGNC Symbol;Acc:HGNC:19417]	protein coding	-0.69	0.21	15.38	<0.0001	0.0004
ENSG00000272669		novel transcript, antisense to SUN2	lncRNA	0.69	1.16	21.55	<0.0001	<0.0001
ENSG00000267348	GEMIN7-AS1	GEMIN7 antisense RNA 1 [Source:HGNC Symbol;Acc:HGNC:53773]	lncRNA	-0.69	1.84	36.40	<0.0001	<0.0001
ENSG00000173762	CD7	CD7 molecule [Source:HGNC Symbol;Acc:HGNC:1695]	protein coding	0.69	3.68	7.63	0.0058	0.0138
ENSG00000159784	FAM131B	family with sequence similarity 131 member B [Source:HGNC Symbol;Acc:HGNC:22202]	protein coding	-0.69	1.11	21.08	<0.0001	<0.0001
ENSG00000287265		novel transcript, antisense to DUSP22	lncRNA	-0.69	2.32	29.98	<0.0001	<0.0001

Table S5: Genes differentially expressed at Day 30 compared to pre-second vaccination (Study Arm 2 A/H7N9+AS03, D1, 29, Monocytes). Genes are sorted by descending absolute log_2 fold change. Gene model summaries and annotations are based on Ensembl Version 99 (continued.)

Ensembl Gene ID	Ensembl Gene Name	Ensembl Gene Description	Gene Type	log_2 Fold Change (Day 30 vs. Pre-Second Vaccination)	Average log_2 CPM	Likelihood Ratio Test Statistic	P-Value	FDR Adjusted P-Value
ENSG00000161929	SCIMP	SLP adaptor and CSK interacting membrane protein [Source:HGNC Symbol;Acc:HGNC:33504]	protein coding	0.68	7.57	63.42	<0.0001	<0.0001
ENSG00000273669		novel transcript	lncRNA	-0.68	1.06	16.40	<0.0001	0.0002
ENSG00000104814	MAP4K1	mitogen-activated protein kinase kinase kinase kinase 1 [Source:HGNC Symbol;Acc:HGNC:6863]	protein coding	-0.68	5.52	62.18	<0.0001	<0.0001
ENSG00000277715		novel transcript, antisense to CKAP4	lncRNA	-0.68	0.67	19.60	<0.0001	<0.0001
ENSG00000228784	LINC00954	long intergenic non-protein coding RNA 954 [Source:HGNC Symbol;Acc:HGNC:48668]	lncRNA	-0.68	2.94	32.78	<0.0001	<0.0001
ENSG00000154978	VOPP1	VOPP1 WW domain binding protein [Source:HGNC Symbol;Acc:HGNC:34518]	protein coding	0.68	5.86	31.06	<0.0001	<0.0001
ENSG00000141837	CACNA1A	calcium voltage-gated channel subunit alpha1 A [Source:HGNC Symbol;Acc:HGNC:1388]	protein coding	0.68	1.17	16.17	<0.0001	0.0002
ENSG00000110047	EHD1	EH domain containing 1 [Source:HGNC Symbol;Acc:HGNC:3242]	protein coding	0.68	7.49	32.74	<0.0001	<0.0001
ENSG00000105854	PON2	paraoxonase 2 [Source:HGNC Symbol;Acc:HGNC:9205]	protein coding	-0.68	3.27	62.76	<0.0001	<0.0001
ENSG00000224934		novel transcript	lncRNA	-0.68	0.69	21.16	<0.0001	<0.0001
ENSG00000278922		novel transcript	TEC	-0.68	1.40	30.53	<0.0001	<0.0001
ENSG00000160710	ADAR	adenosine deaminase RNA specific [Source:HGNC Symbol;Acc:HGNC:225]	protein coding	0.68	8.84	47.18	<0.0001	<0.0001
ENSG00000139344	AMDHD1	amidohydrolase domain containing 1 [Source:HGNC Symbol;Acc:HGNC:28577]	protein coding	-0.68	0.82	24.26	<0.0001	<0.0001
ENSG00000118503	TNFAIP3	TNF alpha induced protein 3 [Source:HGNC Symbol;Acc:HGNC:11896]	protein coding	0.68	7.35	26.91	<0.0001	<0.0001
ENSG00000176531	PHLDB3	pleckstrin homology like domain family B member 3 [Source:HGNC Symbol;Acc:HGNC:30499]	protein coding	-0.68	2.65	37.02	<0.0001	<0.0001
ENSG00000111913	RIPOR2	RHO family interacting cell polarization regulator 2 [Source:HGNC Symbol;Acc:HGNC:13872]	protein coding	0.68	8.73	46.46	<0.0001	<0.0001
ENSG00000269934		novel transcript, antisense to OBSCN	lncRNA	-0.68	0.98	24.72	<0.0001	<0.0001
ENSG00000133466	C1QTNF6	C1q and TNF related 6 [Source:HGNC Symbol;Acc:HGNC:14343]	protein coding	-0.68	1.48	33.09	<0.0001	<0.0001
ENSG00000140022	STON2	stonin 2 [Source:HGNC Symbol;Acc:HGNC:30652]	protein coding	-0.68	1.79	24.37	<0.0001	<0.0001
ENSG00000163975	MELTF	melanotransferrin [Source:HGNC Symbol;Acc:HGNC:7037]	protein coding	-0.68	0.41	11.57	0.0007	0.0021

Table S5: Genes differentially expressed at Day 30 compared to pre-second vaccination (Study Arm 2 A/H7N9+AS03, D1, 29, Monocytes). Genes are sorted by descending absolute log_2 fold change. Gene model summaries and annotations are based on Ensembl Version 99 (continued.)

Ensembl Gene ID	Ensembl Gene Name	Ensembl Gene Description	Gene Type	log_2 Fold Change (Day 30 vs. Pre-Second Vaccination)	Average log_2 CPM	Likelihood Ratio Test Statistic	P-Value	FDR Adjusted P-Value
ENSG00000159228	CBR1	carbonyl reductase 1 [Source:HGNC Symbol;Acc:HGNC:1548]	protein coding	0.68	5.19	81.38	<0.0001	<0.0001
ENSG00000148450	MSRB2	methionine sulfoxide reductase B2 [Source:HGNC Symbol;Acc:HGNC:17061]	protein coding	0.68	4.61	72.04	<0.0001	<0.0001
ENSG00000261997		novel transcript	lncRNA	0.68	1.88	17.96	<0.0001	0.0001
ENSG00000165795	NDRG2	NDRG family member 2 [Source:HGNC Symbol;Acc:HGNC:14460]	protein coding	-0.68	3.12	31.30	<0.0001	<0.0001
ENSG00000160190	SLC37A1	solute carrier family 37 member 1 [Source:HGNC Symbol;Acc:HGNC:11024]	protein coding	0.68	4.98	59.36	<0.0001	<0.0001
ENSG00000140280	LYSMD2	LysM domain containing 2 [Source:HGNC Symbol;Acc:HGNC:28571]	protein coding	0.68	5.42	66.03	<0.0001	<0.0001
ENSG00000111206	FOXM1	forkhead box M1 [Source:HGNC Symbol;Acc:HGNC:3818]	protein coding	-0.68	0.19	18.00	<0.0001	0.0001
ENSG00000233901	LINC01503	long intergenic non-protein coding RNA 1503 [Source:HGNC Symbol;Acc:HGNC:51184]	lncRNA	-0.68	3.80	33.69	<0.0001	<0.0001
ENSG00000198805	PNP	purine nucleoside phosphorylase [Source:HGNC Symbol;Acc:HGNC:7892]	protein coding	0.68	5.57	34.42	<0.0001	<0.0001
ENSG00000168310	IRF2	interferon regulatory factor 2 [Source:HGNC Symbol;Acc:HGNC:6117]	protein coding	0.68	6.73	44.28	<0.0001	<0.0001
ENSG00000213047	DENND1B	DENN domain containing 1B [Source:HGNC Symbol;Acc:HGNC:28404]	protein coding	0.68	3.72	45.16	<0.0001	<0.0001
ENSG00000278811	LINC00624	long intergenic non-protein coding RNA 624 [Source:HGNC Symbol;Acc:HGNC:44254]	lncRNA	-0.67	0.31	21.24	<0.0001	<0.0001
ENSG00000162836	ACP6	acid phosphatase 6, lysophosphatidic [Source:HGNC Symbol;Acc:HGNC:29609]	protein coding	-0.67	1.99	31.37	<0.0001	<0.0001
ENSG00000182048	TRPC2	transient receptor potential cation channel subfamily C member 2 (pseudogene) [Source:HGNC Symbol;Acc:HGNC:12334]	transcribed unprocessed pseudogene	-0.67	0.29	12.04	0.0005	0.0017
ENSG00000245552		novel transcript, antisense to SESN3	lncRNA	-0.67	0.13	15.31	<0.0001	0.0004
ENSG00000115159	GPD2	glycerol-3-phosphate dehydrogenase 2 [Source:HGNC Symbol;Acc:HGNC:4456]	protein coding	0.67	5.36	54.92	<0.0001	<0.0001
ENSG00000271335		novel transcript, antisense to CCNY	lncRNA	-0.67	0.87	17.68	<0.0001	0.0001
ENSG00000130303	BST2	bone marrow stromal cell antigen 2 [Source:HGNC Symbol;Acc:HGNC:1119]	protein coding	0.67	7.62	58.03	<0.0001	<0.0001

Table S5: Genes differentially expressed at Day 30 compared to pre-second vaccination (Study Arm 2 A/H7N9+AS03, D1, 29, Monocytes). Genes are sorted by descending absolute log_2 fold change. Gene model summaries and annotations are based on Ensembl Version 99 (continued.)

Ensembl Gene ID	Ensembl Gene Name	Ensembl Gene Description	Gene Type	log_2 Fold Change (Day 30 vs. Pre-Second Vaccination)	Average log_2 CPM	Likelihood Ratio Test Statistic	P-Value	FDR Adjusted P-Value
ENSG00000243753	HLA-L	major histocompatibility complex, class I, L (pseudogene) [Source:HGNC Symbol;Acc:HGNC:4970]	transcribed unprocessed pseudogene	0.67	2.96	34.89	<0.0001	<0.0001
ENSG00000258733	LINC02328	long intergenic non-protein coding RNA 2328 [Source:HGNC Symbol;Acc:HGNC:53248]	lncRNA	0.67	1.45	29.46	<0.0001	<0.0001
ENSG00000223865	HLA-DPB1	major histocompatibility complex, class II, DP beta 1 [Source:HGNC Symbol;Acc:HGNC:4940]	protein coding	0.67	9.65	56.65	<0.0001	<0.0001
ENSG00000185101	ANO9	anoctamin 9 [Source:HGNC Symbol;Acc:HGNC:20679]	protein coding	-0.67	2.76	27.68	<0.0001	<0.0001
ENSG00000279861		TEC	TEC	0.67	2.85	15.41	<0.0001	0.0004
ENSG00000101194	SLC17A9	solute carrier family 17 member 9 [Source:HGNC Symbol;Acc:HGNC:16192]	protein coding	-0.67	5.28	41.06	<0.0001	<0.0001
ENSG00000238005		novel transcript	lncRNA	-0.67	1.00	12.74	0.0004	0.0012
ENSG00000168610	STAT3	signal transducer and activator of transcription 3 [Source:HGNC Symbol;Acc:HGNC:11364]	protein coding	0.67	8.21	46.70	<0.0001	<0.0001
ENSG00000279019		TEC	TEC	-0.67	1.09	20.36	<0.0001	<0.0001
ENSG00000232344		ribosomal protein S2 (RPS2) pseudogene	processed pseudogene	-0.67	0.25	17.47	<0.0001	0.0001
ENSG00000123933	MXD4	MAX dimerization protein 4 [Source:HGNC Symbol;Acc:HGNC:13906]	protein coding	-0.67	7.09	42.73	<0.0001	<0.0001
ENSG00000010704	HFE	homeostatic iron regulator [Source:HGNC Symbol;Acc:HGNC:4886]	protein coding	-0.67	4.22	23.57	<0.0001	<0.0001
ENSG00000280734	LINC01232	long intergenic non-protein coding RNA 1232 [Source:HGNC Symbol;Acc:HGNC:49755]	lncRNA	0.67	2.18	28.82	<0.0001	<0.0001
ENSG00000100814	CCNB1IP1	cyclin B1 interacting protein 1 [Source:HGNC Symbol;Acc:HGNC:19437]	protein coding	-0.67	3.10	40.37	<0.0001	<0.0001
ENSG00000286989		novel transcript	lncRNA	-0.67	1.81	20.48	<0.0001	<0.0001
ENSG00000168056	LTBP3	latent transforming growth factor beta binding protein 3 [Source:HGNC Symbol;Acc:HGNC:6716]	protein coding	-0.67	4.25	43.81	<0.0001	<0.0001
ENSG00000009724	MASP2	mannan binding lectin serine peptidase 2 [Source:HGNC Symbol;Acc:HGNC:6902]	protein coding	-0.67	2.51	26.01	<0.0001	<0.0001
ENSG00000145949	MYLK4	myosin light chain kinase family member 4 [Source:HGNC Symbol;Acc:HGNC:27972]	protein coding	-0.67	1.54	24.14	<0.0001	<0.0001

Table S5: Genes differentially expressed at Day 30 compared to pre-second vaccination (Study Arm 2 A/H7N9+AS03, D1, 29, Monocytes). Genes are sorted by descending absolute log_2 fold change. Gene model summaries and annotations are based on Ensembl Version 99 (continued.)

Ensembl Gene ID	Ensembl Gene Name	Ensembl Gene Description	Gene Type	log_2 Fold Change (Day 30 vs. Pre-Second Vaccination)	Average log_2 CPM	Likelihood Ratio Test Statistic	P-Value	FDR Adjusted P-Value
ENSG00000231607	DLEU2	deleted in lymphocytic leukemia 2 [Source:HGNC Symbol;Acc:HGNC:13748]	lncRNA	0.67	4.30	46.39	<0.0001	<0.0001
ENSG00000155254	MARVELD1	MARVEL domain containing 1 [Source:HGNC Symbol;Acc:HGNC:28674]	protein coding	-0.67	6.14	48.42	<0.0001	<0.0001
ENSG00000211662	IGLV3-21	immunoglobulin lambda variable 3-21 [Source:HGNC Symbol;Acc:HGNC:5905]	IG V gene	-0.67	1.22	9.69	0.0018	0.0052
ENSG00000081059	TCF7	transcription factor 7 [Source:HGNC Symbol;Acc:HGNC:11639]	protein coding	-0.67	1.74	5.14	0.0233	0.0466
ENSG00000172331	BPGM	bisphosphoglycerate mutase [Source:HGNC Symbol;Acc:HGNC:1093]	protein coding	0.67	3.78	40.43	<0.0001	<0.0001
ENSG00000198520	ARMH1	armadillo like helical domain containing 1 [Source:HGNC Symbol;Acc:HGNC:34345]	protein coding	0.67	5.71	35.48	<0.0001	<0.0001
ENSG00000183814	LIN9	lin-9 DREAM MuvB core complex component [Source:HGNC Symbol;Acc:HGNC:30830]	protein coding	-0.67	0.96	25.25	<0.0001	<0.0001
ENSG00000171798	KNDC1	kinase non-catalytic C-lobe domain containing 1 [Source:HGNC Symbol;Acc:HGNC:29374]	protein coding	-0.67	2.60	14.43	0.0001	0.0006
ENSG00000221926	TRIM16	tripartite motif containing 16 [Source:HGNC Symbol;Acc:HGNC:17241]	protein coding	-0.67	1.48	25.89	<0.0001	<0.0001
ENSG00000101400	SNTA1	syntrophin alpha 1 [Source:HGNC Symbol;Acc:HGNC:11167]	protein coding	-0.67	0.50	19.13	<0.0001	<0.0001
ENSG00000233016	SNHG7	small nucleolar RNA host gene 7 [Source:HGNC Symbol;Acc:HGNC:28254]	lncRNA	-0.67	5.14	31.88	<0.0001	<0.0001
ENSG00000184574	LPAR5	lysophosphatidic acid receptor 5 [Source:HGNC Symbol;Acc:HGNC:13307]	protein coding	-0.66	0.40	19.30	<0.0001	<0.0001
ENSG00000047579	DTNBP1	dystrobrevin binding protein 1 [Source:HGNC Symbol;Acc:HGNC:17328]	protein coding	0.66	4.68	58.91	<0.0001	<0.0001
ENSG00000196329	GIMAP5	GTPase, IMAP family member 5 [Source:HGNC Symbol;Acc:HGNC:18005]	protein coding	0.66	2.92	12.58	0.0004	0.0013
ENSG00000175730	BAK1P1	BCL2 antagonist/killer 1 pseudogene 1 [Source:HGNC Symbol;Acc:HGNC:996]	processed pseudogene	0.66	0.92	23.89	<0.0001	<0.0001
ENSG00000135541	AHI1	Abelson helper integration site 1 [Source:HGNC Symbol;Acc:HGNC:21575]	protein coding	-0.66	0.80	22.13	<0.0001	<0.0001
ENSG00000107957	SH3PXD2A	SH3 and PX domains 2A [Source:HGNC Symbol;Acc:HGNC:23664]	protein coding	-0.66	3.59	43.69	<0.0001	<0.0001

Confidential/Proprietary Information

Table S5: Genes differentially expressed at Day 30 compared to pre-second vaccination (Study Arm 2 A/H7N9+AS03, D1, 29, Monocytes). Genes are sorted by descending absolute log_2 fold change. Gene model summaries and annotations are based on Ensembl Version 99 (continued.)

Ensembl Gene ID	Ensembl Gene Name	Ensembl Gene Description	Gene Type	log_2 Fold Change (Day 30 vs. Pre-Second Vaccination)	Average log_2 CPM	Likelihood Ratio Test Statistic	P-Value	FDR Adjusted P-Value
ENSG00000168268	NT5DC2	5'-nucleotidase domain containing 2 [Source:HGNC Symbol;Acc:HGNC:25717]	protein coding	-0.66	3.55	40.86	<0.0001	<0.0001
ENSG00000166263	STXBP4	syntaxin binding protein 4 [Source:HGNC Symbol;Acc:HGNC:19694]	protein coding	-0.66	0.76	23.67	<0.0001	<0.0001
ENSG00000167281	RBFOX3	RNA binding fox-1 homolog 3 [Source:HGNC Symbol;Acc:HGNC:27097]	protein coding	-0.66	0.80	8.88	0.0029	0.0076
ENSG00000137364	TPMT	thiopurine S-methyltransferase [Source:HGNC Symbol;Acc:HGNC:12014]	protein coding	0.66	5.65	68.78	<0.0001	<0.0001
ENSG00000277443	MARCKS	myristoylated alanine rich protein kinase C substrate [Source:HGNC Symbol;Acc:HGNC:6759]	protein coding	0.66	8.24	34.65	<0.0001	<0.0001
ENSG00000104921	FCER2	Fc fragment of IgE receptor II [Source:HGNC Symbol;Acc:HGNC:3612]	protein coding	-0.66	4.37	20.71	<0.0001	<0.0001
ENSG00000283297	TEX52	testis expressed 52 [Source:HGNC Symbol;Acc:HGNC:53643]	protein coding	-0.66	0.20	13.86	0.0002	0.0007
ENSG00000163874	ZC3H12A	zinc finger CCCH-type containing 12A [Source:HGNC Symbol;Acc:HGNC:26259]	protein coding	0.66	4.71	27.58	<0.0001	<0.0001
ENSG00000003400	CASP10	caspase 10 [Source:HGNC Symbol;Acc:HGNC:1500]	protein coding	0.66	6.05	39.98	<0.0001	<0.0001
ENSG00000197381	ADARB1	adenosine deaminase RNA specific B1 [Source:HGNC Symbol;Acc:HGNC:226]	protein coding	0.66	3.89	19.63	<0.0001	<0.0001
ENSG00000196684	HSH2D	hematopoietic SH2 domain containing [Source:HGNC Symbol;Acc:HGNC:24920]	protein coding	0.66	6.72	57.76	<0.0001	<0.0001
ENSG00000223534	HLA-DQB1-AS1	HLA-DQB1 antisense RNA 1 [Source:HGNC Symbol;Acc:HGNC:39762]	lncRNA	0.66	2.62	9.03	0.0026	0.0071
ENSG00000156869	FRRS1	ferric chelate reductase 1 [Source:HGNC Symbol;Acc:HGNC:27622]	protein coding	0.66	1.88	27.53	<0.0001	<0.0001
ENSG00000117632	STMN1	stathmin 1 [Source:HGNC Symbol;Acc:HGNC:6510]	protein coding	-0.66	1.57	15.65	<0.0001	0.0003
ENSG00000043462	LCP2	lymphocyte cytosolic protein 2 [Source:HGNC Symbol;Acc:HGNC:6529]	protein coding	0.66	8.06	74.22	<0.0001	<0.0001
ENSG00000276758		leukocyte immunoglobulin-like receptor, subfamily A member pseudogene	unprocessed pseudogene	0.66	2.41	32.60	<0.0001	<0.0001
ENSG00000242616	GNG10	G protein subunit gamma 10 [Source:HGNC Symbol;Acc:HGNC:4402]	protein coding	0.66	2.80	35.45	<0.0001	<0.0001

Table S5: Genes differentially expressed at Day 30 compared to pre-second vaccination (Study Arm 2 A/H7N9+AS03, D1, 29, Monocytes). Genes are sorted by descending absolute log_2 fold change. Gene model summaries and annotations are based on Ensembl Version 99 (continued.)

Ensembl Gene ID	Ensembl Gene Name	Ensembl Gene Description	Gene Type	log_2 Fold Change (Day 30 vs. Pre-Second Vaccination)	Average log_2 CPM	Likelihood Ratio Test Statistic	P-Value	FDR Adjusted P-Value
ENSG00000163235	TGFA	transforming growth factor alpha [Source:HGNC Symbol;Acc:HGNC:11765]	protein coding	-0.66	1.87	15.92	<0.0001	0.0003
ENSG00000125246	CLYBL	citrate lyase beta like [Source:HGNC Symbol;Acc:HGNC:18355]	protein coding	-0.65	0.77	13.07	0.0003	0.0011
ENSG00000189350	TOGARAM2	TOG array regulator of axonemal microtubules 2 [Source:HGNC Symbol;Acc:HGNC:33715]	protein coding	-0.65	1.42	13.75	0.0002	0.0008
ENSG00000072840	EVC	EvC ciliary complex subunit 1 [Source:HGNC Symbol;Acc:HGNC:3497]	protein coding	-0.65	1.12	12.98	0.0003	0.0011
ENSG00000100228	RAB36	RAB36, member RAS oncogene family [Source:HGNC Symbol;Acc:HGNC:9775]	protein coding	-0.65	0.36	16.67	<0.0001	0.0002
ENSG00000196735	HLA-DQA1	major histocompatibility complex, class II, DQ alpha 1 [Source:HGNC Symbol;Acc:HGNC:4942]	protein coding	0.65	6.92	53.66	<0.0001	<0.0001
ENSG00000181826	RELL1	RELT like 1 [Source:HGNC Symbol;Acc:HGNC:27379]	protein coding	-0.65	4.67	28.92	<0.0001	<0.0001
ENSG00000106991	ENG	endoglin [Source:HGNC Symbol;Acc:HGNC:3349]	protein coding	-0.65	6.23	37.45	<0.0001	<0.0001
ENSG00000007312	CD79B	CD79b molecule [Source:HGNC Symbol;Acc:HGNC:1699]	protein coding	-0.65	4.76	34.93	<0.0001	<0.0001
ENSG00000143106	PSMA5	proteasome 20S subunit alpha 5 [Source:HGNC Symbol;Acc:HGNC:9534]	protein coding	0.65	6.41	63.00	<0.0001	<0.0001
ENSG00000125430	HS3ST3B1	heparan sulfate-glucosamine 3-sulfotransferase 3B1 [Source:HGNC Symbol;Acc:HGNC:5198]	protein coding	0.65	0.46	15.51	<0.0001	0.0003
ENSG00000186654	PRR5	proline rich 5 [Source:HGNC Symbol;Acc:HGNC:31682]	protein coding	-0.65	1.43	18.64	<0.0001	<0.0001
ENSG00000106785	TRIM14	tripartite motif containing 14 [Source:HGNC Symbol;Acc:HGNC:16283]	protein coding	0.65	6.81	65.33	<0.0001	<0.0001
ENSG00000120896	SORBS3	sorbin and SH3 domain containing 3 [Source:HGNC Symbol;Acc:HGNC:30907]	protein coding	-0.65	5.16	29.85	<0.0001	<0.0001
ENSG00000087301	TXNDC16	thioredoxin domain containing 16 [Source:HGNC Symbol;Acc:HGNC:19965]	protein coding	-0.65	3.73	34.73	<0.0001	<0.0001
ENSG00000285938		BCL6 antisense 1	protein coding	0.65	2.93	14.36	0.0002	0.0006
ENSG00000211938	IGHV3-7	immunoglobulin heavy variable 3-7 [Source:HGNC Symbol;Acc:HGNC:5620]	IG V gene	-0.65	1.27	7.79	0.0052	0.0128
ENSG00000159339	PADI4	peptidyl arginine deiminase 4 [Source:HGNC Symbol;Acc:HGNC:18368]	protein coding	-0.65	7.15	28.76	<0.0001	<0.0001

Table S5: Genes differentially expressed at Day 30 compared to pre-second vaccination (Study Arm 2 A/H7N9+AS03, D1, 29, Monocytes). Genes are sorted by descending absolute log_2 fold change. Gene model summaries and annotations are based on Ensembl Version 99 (continued.)

Ensembl Gene ID	Ensembl Gene Name	Ensembl Gene Description	Gene Type	log_2 Fold Change (Day 30 vs. Pre-Second Vaccination)	Average log_2 CPM	Likelihood Ratio Test Statistic	P-Value	FDR Adjusted P-Value
ENSG00000168675	LDLRAD4	low density lipoprotein receptor class A domain containing 4 [Source:HGNC Symbol;Acc:HGNC:1224]	protein coding	-0.65	5.12	58.63	<0.0001	<0.0001
ENSG00000196476	C20orf96	chromosome 20 open reading frame 96 [Source:HGNC Symbol;Acc:HGNC:16227]	protein coding	-0.65	2.64	28.04	<0.0001	<0.0001
ENSG00000280254		TEC	TEC	-0.65	1.47	21.76	<0.0001	<0.0001
ENSG00000251247	ZNF345	zinc finger protein 345 [Source:HGNC Symbol;Acc:HGNC:16367]	protein coding	-0.65	1.98	29.73	<0.0001	<0.0001
ENSG00000286248		novel transcript, antisense to P2RX7	lncRNA	0.65	1.00	16.17	<0.0001	0.0002
ENSG00000128268	MGAT3	beta-1,4-mannosyl-glycoprotein 4-beta-N-acetylglucosaminyltransferase [Source:HGNC Symbol;Acc:HGNC:7046]	protein coding	-0.65	0.49	11.23	0.0008	0.0025
ENSG00000164845	FAM86FP	family with sequence similarity 86 member F, pseudogene [Source:HGNC Symbol;Acc:HGNC:42357]	transcribed unprocessed pseudogene	-0.65	1.15	25.27	<0.0001	<0.0001
ENSG00000111424	VDR	vitamin D receptor [Source:HGNC Symbol;Acc:HGNC:12679]	polymorphic pseudogene	0.65	5.32	97.07	<0.0001	<0.0001
ENSG00000281005	LINC00921	long intergenic non-protein coding RNA 921 [Source:HGNC Symbol;Acc:HGNC:26830]	lncRNA	-0.65	3.48	40.56	<0.0001	<0.0001
ENSG00000159131	GART	phosphoribosylglycinamide formyltransferase, phosphoribosylglycinamide synthetase, phosphoribosylaminoimidazole synthetase [Source:HGNC Symbol;Acc:HGNC:4163]	protein coding	0.65	5.34	49.19	<0.0001	<0.0001
ENSG00000248008	NRAV	negative regulator of antiviral response [Source:HGNC Symbol;Acc:HGNC:48588]	lncRNA	-0.65	0.17	16.26	<0.0001	0.0002
ENSG00000065427	KARS1	lysyl-tRNA synthetase 1 [Source:HGNC Symbol;Acc:HGNC:6215]	protein coding	0.65	7.21	59.78	<0.0001	<0.0001
ENSG00000105967	TFEC	transcription factor EC [Source:HGNC Symbol;Acc:HGNC:11754]	protein coding	0.65	7.26	44.42	<0.0001	<0.0001
ENSG00000183723	CMTM4	CKLF like MARVEL transmembrane domain containing 4 [Source:HGNC Symbol;Acc:HGNC:19175]	protein coding	-0.65	4.56	41.54	<0.0001	<0.0001
ENSG00000163947	ARHGEF3	Rho guanine nucleotide exchange factor 3 [Source:HGNC Symbol;Acc:HGNC:683]	protein coding	0.65	6.38	53.15	<0.0001	<0.0001
ENSG00000136816	TOR1B	torsin family 1 member B [Source:HGNC Symbol;Acc:HGNC:11995]	protein coding	0.65	5.83	51.56	<0.0001	<0.0001

Table S5: Genes differentially expressed at Day 30 compared to pre-second vaccination (Study Arm 2 A/H7N9+AS03, D1, 29, Monocytes). Genes are sorted by descending absolute log_2 fold change. Gene model summaries and annotations are based on Ensembl Version 99 (continued.)

Ensembl Gene ID	Ensembl Gene Name	Ensembl Gene Description	Gene Type	log_2 Fold Change (Day 30 vs. Pre-Second Vaccination)	Average log_2 CPM	Likelihood Ratio Test Statistic	P-Value	FDR Adjusted P-Value
ENSG00000115414	FN1	fibronectin 1 [Source:HGNC Symbol;Acc:HGNC:3778]	protein coding	0.65	2.13	5.98	0.0145	0.031
ENSG00000095539	SEMA4G	semaphorin 4G [Source:HGNC Symbol;Acc:HGNC:10735]	protein coding	-0.65	1.39	17.31	<0.0001	0.0001
ENSG00000197558	SSPO	SCO-spondin [Source:HGNC Symbol;Acc:HGNC:21998]	transcribed unitary pseudogene	-0.65	4.27	32.55	<0.0001	<0.0001
ENSG00000244560		zinc finger protein pseudogene	transcribed unprocessed pseudogene	-0.65	1.73	34.06	<0.0001	<0.0001
ENSG00000164403	SHROOM1	shroom family member 1 [Source:HGNC Symbol;Acc:HGNC:24084]	protein coding	-0.64	5.15	49.06	<0.0001	<0.0001
ENSG00000231925	TAPBP	TAP binding protein [Source:HGNC Symbol;Acc:HGNC:11566]	protein coding	0.64	8.71	52.71	<0.0001	<0.0001
ENSG00000235194	PPP1R3E	protein phosphatase 1 regulatory subunit 3E [Source:HGNC Symbol;Acc:HGNC:14943]	protein coding	-0.64	3.66	53.11	<0.0001	<0.0001
ENSG00000173166	RAPH1	Ras association (RalGDS/AF-6) and pleckstrin homology domains 1 [Source:HGNC Symbol;Acc:HGNC:14436]	protein coding	-0.64	1.27	15.95	<0.0001	0.0003
ENSG00000226328	NUP50-DT	NUP50 divergent transcript [Source:HGNC Symbol;Acc:HGNC:50502]	lncRNA	0.64	4.67	46.67	<0.0001	<0.0001
ENSG00000107554	DNMBP	dynamin binding protein [Source:HGNC Symbol;Acc:HGNC:30373]	protein coding	-0.64	4.02	30.13	<0.0001	<0.0001
ENSG00000236345	SCAT8	S-phase cancer associated transcript 8 [Source:HGNC Symbol;Acc:HGNC:40967]	lncRNA	-0.64	1.52	23.43	<0.0001	<0.0001
ENSG00000076513	ANKRD13A	ankyrin repeat domain 13A [Source:HGNC Symbol;Acc:HGNC:21268]	protein coding	0.64	7.37	65.90	<0.0001	<0.0001
ENSG00000162104	ADCY9	adenylate cyclase 9 [Source:HGNC Symbol;Acc:HGNC:240]	protein coding	-0.64	5.09	60.06	<0.0001	<0.0001
ENSG00000101596	SMCHD1	structural maintenance of chromosomes flexible hinge domain containing 1 [Source:HGNC Symbol;Acc:HGNC:29090]	protein coding	0.64	7.74	33.84	<0.0001	<0.0001
ENSG00000154274	C4orf19	chromosome 4 open reading frame 19 [Source:HGNC Symbol;Acc:HGNC:25618]	protein coding	-0.64	0.32	9.09	0.0026	0.0069

Table S5: Genes differentially expressed at Day 30 compared to pre-second vaccination (Study Arm 2 A/H7N9+AS03, D1, 29, Monocytes). Genes are sorted by descending absolute log_2 fold change. Gene model summaries and annotations are based on Ensembl Version 99 (continued.)

Ensembl Gene ID	Ensembl Gene Name	Ensembl Gene Description	Gene Type	log_2 Fold Change (Day 30 vs. Pre-Second Vaccination)	Average log_2 CPM	Likelihood Ratio Test Statistic	P-Value	FDR Adjusted P-Value
ENSG00000196396	PTPN1	protein tyrosine phosphatase non-receptor type 1 [Source:HGNC Symbol;Acc:HGNC:9642]	protein coding	0.64	6.45	63.54	<0.0001	<0.0001
ENSG00000232677	LINC00665	long intergenic non-protein coding RNA 665 [Source:HGNC Symbol;Acc:HGNC:44323]	lncRNA	-0.64	0.67	24.20	<0.0001	<0.0001
ENSG00000159128	IFNGR2	interferon gamma receptor 2 [Source:HGNC Symbol;Acc:HGNC:5440]	protein coding	0.64	8.10	67.95	<0.0001	<0.0001
ENSG00000169047	IRS1	insulin receptor substrate 1 [Source:HGNC Symbol;Acc:HGNC:6125]	protein coding	-0.64	2.40	32.53	<0.0001	<0.0001
ENSG00000105374	NKG7	natural killer cell granule protein 7 [Source:HGNC Symbol;Acc:HGNC:7830]	protein coding	0.64	3.61	18.94	<0.0001	<0.0001
ENSG00000186017	ZNF566	zinc finger protein 566 [Source:HGNC Symbol;Acc:HGNC:25919]	protein coding	-0.64	1.32	25.97	<0.0001	<0.0001
ENSG00000121413	ZSCAN18	zinc finger and SCAN domain containing 18 [Source:HGNC Symbol;Acc:HGNC:21037]	protein coding	-0.64	2.02	36.34	<0.0001	<0.0001
ENSG00000141858	SAMD1	sterile alpha motif domain containing 1 [Source:HGNC Symbol;Acc:HGNC:17958]	protein coding	-0.64	5.25	43.50	<0.0001	<0.0001
ENSG00000100628	ASB2	ankyrin repeat and SOCS box containing 2 [Source:HGNC Symbol;Acc:HGNC:16012]	protein coding	0.64	0.91	10.46	0.0012	0.0036
ENSG00000214870		novel transcript	lncRNA	-0.64	0.25	10.66	0.0011	0.0033
ENSG00000153896	ZNF599	zinc finger protein 599 [Source:HGNC Symbol;Acc:HGNC:26408]	protein coding	-0.64	0.12	14.38	0.0001	0.0006
ENSG00000163462	TRIM46	tripartite motif containing 46 [Source:HGNC Symbol;Acc:HGNC:19019]	protein coding	0.64	0.61	12.37	0.0004	0.0015
ENSG00000107338	SHB	SH2 domain containing adaptor protein B [Source:HGNC Symbol;Acc:HGNC:10838]	protein coding	-0.64	0.28	12.48	0.0004	0.0014
ENSG00000157796	WDR19	WD repeat domain 19 [Source:HGNC Symbol;Acc:HGNC:18340]	protein coding	-0.64	3.03	31.93	<0.0001	<0.0001
ENSG00000214274	ANG	angiogenin [Source:HGNC Symbol;Acc:HGNC:483]	protein coding	-0.64	2.00	15.43	<0.0001	0.0003
ENSG00000206418	RAB12	RAB12, member RAS oncogene family [Source:HGNC Symbol;Acc:HGNC:31332]	protein coding	0.64	4.93	51.47	<0.0001	<0.0001
ENSG00000173281	PPP1R3B	protein phosphatase 1 regulatory subunit 3B [Source:HGNC Symbol;Acc:HGNC:14942]	protein coding	0.64	3.05	19.96	<0.0001	<0.0001
ENSG00000169258	GPRIN1	G protein regulated inducer of neurite outgrowth 1 [Source:HGNC Symbol;Acc:HGNC:24835]	protein coding	0.64	0.40	13.93	0.0002	0.0007

Table S5: Genes differentially expressed at Day 30 compared to pre-second vaccination (Study Arm 2 A/H7N9+AS03, D1, 29, Monocytes). Genes are sorted by descending absolute log_2 fold change. Gene model summaries and annotations are based on Ensembl Version 99 (continued.)

Ensembl Gene ID	Ensembl Gene Name	Ensembl Gene Description	Gene Type	log_2 Fold Change (Day 30 vs. Pre-Second Vaccination)	Average log_2 CPM	Likelihood Ratio Test Statistic	P-Value	FDR Adjusted P-Value
ENSG00000140563	MCTP2	multiple C2 and transmembrane domain containing 2 [Source:HGNC Symbol;Acc:HGNC:25636]	protein coding	0.64	3.88	24.19	<0.0001	<0.0001
ENSG00000156110	ADK	adenosine kinase [Source:HGNC Symbol;Acc:HGNC:257]	protein coding	-0.64	4.23	54.09	<0.0001	<0.0001
ENSG00000161996	WDR90	WD repeat domain 90 [Source:HGNC Symbol;Acc:HGNC:26960]	protein coding	-0.64	3.85	39.75	<0.0001	<0.0001
ENSG00000163220	S100A9	S100 calcium binding protein A9 [Source:HGNC Symbol;Acc:HGNC:10499]	protein coding	0.64	12.63	26.02	<0.0001	<0.0001
ENSG00000281398	SNHG4	small nucleolar RNA host gene 4 [Source:HGNC Symbol;Acc:HGNC:32964]	lncRNA	-0.64	1.55	18.20	<0.0001	<0.0001
ENSG00000188707	ZBED6CL	ZBED6 C-terminal like [Source:HGNC Symbol;Acc:HGNC:21720]	protein coding	0.64	3.56	33.39	<0.0001	<0.0001
ENSG00000255717	SNHG1	small nucleolar RNA host gene 1 [Source:HGNC Symbol;Acc:HGNC:32688]	lncRNA	-0.64	5.16	55.69	<0.0001	<0.0001
ENSG00000069493	CLEC2D	C-type lectin domain family 2 member D [Source:HGNC Symbol;Acc:HGNC:14351]	protein coding	-0.64	4.42	49.52	<0.0001	<0.0001
ENSG00000197122	SRC	SRC proto-oncogene, non-receptor tyrosine kinase [Source:HGNC Symbol;Acc:HGNC:11283]	protein coding	0.64	6.88	59.37	<0.0001	<0.0001
ENSG00000122970	IFT81	intraflagellar transport 81 [Source:HGNC Symbol;Acc:HGNC:14313]	protein coding	-0.64	0.57	17.84	<0.0001	0.0001
ENSG00000127586	CHTF18	chromosome transmission fidelity factor 18 [Source:HGNC Symbol;Acc:HGNC:18435]	protein coding	-0.64	4.00	39.23	<0.0001	<0.0001
ENSG00000280077		TEC	TEC	-0.64	2.53	27.71	<0.0001	<0.0001
ENSG00000134243	SORT1	sortilin 1 [Source:HGNC Symbol;Acc:HGNC:11186]	protein coding	0.64	7.32	64.01	<0.0001	<0.0001
ENSG00000248487	ABHD14A	abhydrolase domain containing 14A [Source:HGNC Symbol;Acc:HGNC:24538]	protein coding	-0.63	0.81	16.22	<0.0001	0.0002
ENSG00000148339	SLC25A25	solute carrier family 25 member 25 [Source:HGNC Symbol;Acc:HGNC:20663]	protein coding	0.63	4.77	60.46	<0.0001	<0.0001
ENSG00000234511	C5orf58	chromosome 5 open reading frame 58 [Source:HGNC Symbol;Acc:HGNC:37272]	protein coding	0.63	1.38	11.34	0.0008	0.0024
ENSG00000279283		novel transcript	TEC	-0.63	1.31	17.06	<0.0001	0.0002
ENSG00000235106	BRD3OS	BRD3 opposite strand [Source:HGNC Symbol;Acc:HGNC:24742]	protein coding	-0.63	2.71	33.94	<0.0001	<0.0001
ENSG00000125245	GPR18	G protein-coupled receptor 18 [Source:HGNC Symbol;Acc:HGNC:4472]	protein coding	0.63	0.84	14.91	0.0001	0.0004

Table S5: Genes differentially expressed at Day 30 compared to pre-second vaccination (Study Arm 2 A/H7N9+AS03, D1, 29, Monocytes). Genes are sorted by descending absolute log_2 fold change. Gene model summaries and annotations are based on Ensembl Version 99 (continued.)

Ensembl Gene ID	Ensembl Gene Name	Ensembl Gene Description	Gene Type	log_2 Fold Change (Day 30 vs. Pre-Second Vaccination)	Average log_2 CPM	Likelihood Ratio Test Statistic	P-Value	FDR Adjusted P-Value
ENSG00000104660	LEPROTL1	leptin receptor overlapping transcript like 1 [Source:HGNC Symbol;Acc:HGNC:6555]	protein coding	0.63	5.76	60.18	<0.0001	<0.0001
ENSG00000228058	LINC01736	long intergenic non-protein coding RNA 1736 [Source:HGNC Symbol;Acc:HGNC:52524]	lncRNA	-0.63	0.24	8.20	0.0042	0.0105
ENSG00000131943	C19orf12	chromosome 19 open reading frame 12 [Source:HGNC Symbol;Acc:HGNC:25443]	protein coding	0.63	3.95	43.53	<0.0001	<0.0001
ENSG00000157216	SSBP3	single stranded DNA binding protein 3 [Source:HGNC Symbol;Acc:HGNC:15674]	protein coding	-0.63	5.67	56.75	<0.0001	<0.0001
ENSG00000137265	IRF4	interferon regulatory factor 4 [Source:HGNC Symbol;Acc:HGNC:6119]	protein coding	-0.63	4.61	40.24	<0.0001	<0.0001
ENSG00000233452	STXBP5-AS1	STXBP5 antisense RNA 1 [Source:HGNC Symbol;Acc:HGNC:44183]	lncRNA	-0.63	0.85	17.33	<0.0001	0.0001
ENSG00000073605	GSDMB	gasdermin B [Source:HGNC Symbol;Acc:HGNC:23690]	protein coding	-0.63	3.11	35.70	<0.0001	<0.0001
ENSG00000174738	NR1D2	nuclear receptor subfamily 1 group D member 2 [Source:HGNC Symbol;Acc:HGNC:7963]	protein coding	-0.63	4.79	38.82	<0.0001	<0.0001
ENSG00000166704	ZNF606	zinc finger protein 606 [Source:HGNC Symbol;Acc:HGNC:25879]	protein coding	-0.63	2.63	15.07	0.0001	0.0004
ENSG00000085662	AKR1B1	aldo-keto reductase family 1 member B [Source:HGNC Symbol;Acc:HGNC:381]	protein coding	-0.63	5.48	57.10	<0.0001	<0.0001
ENSG00000215912	TTC34	tetratricopeptide repeat domain 34 [Source:HGNC Symbol;Acc:HGNC:34297]	protein coding	-0.63	1.24	11.59	0.0007	0.0021
ENSG00000150907	FOXO1	forkhead box O1 [Source:HGNC Symbol;Acc:HGNC:3819]	protein coding	-0.63	4.35	49.01	<0.0001	<0.0001
ENSG00000236144	TMEM147-AS1	TMEM147 antisense RNA 1 [Source:HGNC Symbol;Acc:HGNC:51273]	lncRNA	-0.63	3.36	34.58	<0.0001	<0.0001
ENSG00000049449	RCN1	reticulocalbin 1 [Source:HGNC Symbol;Acc:HGNC:9934]	protein coding	0.63	4.01	36.23	<0.0001	<0.0001
ENSG00000156127	BATF	basic leucine zipper ATF-like transcription factor [Source:HGNC Symbol;Acc:HGNC:958]	protein coding	0.63	4.07	32.57	<0.0001	<0.0001
ENSG00000140386	SCAPER	S-phase cyclin A associated protein in the ER [Source:HGNC Symbol;Acc:HGNC:13081]	protein coding	0.63	3.92	38.86	<0.0001	<0.0001
ENSG00000260425		novel transcript, antisense to GNPTG	lncRNA	-0.63	0.22	11.61	0.0007	0.0021
ENSG00000181744	DIPK2A	divergent protein kinase domain 2A [Source:HGNC Symbol;Acc:HGNC:28490]	protein coding	0.63	5.29	42.86	<0.0001	<0.0001

Table S5: Genes differentially expressed at Day 30 compared to pre-second vaccination (Study Arm 2 A/H7N9+AS03, D1, 29, Monocytes). Genes are sorted by descending absolute log_2 fold change. Gene model summaries and annotations are based on Ensembl Version 99 (continued.)

Ensembl Gene ID	Ensembl Gene Name	Ensembl Gene Description	Gene Type	log_2 Fold Change (Day 30 vs. Pre-Second Vaccination)	Average log_2 CPM	Likelihood Ratio Test Statistic	P-Value	FDR Adjusted P-Value
ENSG00000196724	ZNF418	zinc finger protein 418 [Source:HGNC Symbol;Acc:HGNC:20647]	protein coding	-0.63	1.15	22.74	<0.0001	<0.0001
ENSG00000119383	PTPA	protein phosphatase 2 phosphatase activator [Source:HGNC Symbol;Acc:HGNC:9308]	protein coding	0.63	6.99	56.79	<0.0001	<0.0001
ENSG00000187109	NAP1L1	nucleosome assembly protein 1 like 1 [Source:HGNC Symbol;Acc:HGNC:7637]	protein coding	-0.63	8.51	34.38	<0.0001	<0.0001
ENSG00000167851	CD300A	CD300a molecule [Source:HGNC Symbol;Acc:HGNC:19319]	protein coding	0.63	7.72	76.51	<0.0001	<0.0001
ENSG00000019169	MARCO	macrophage receptor with collagenous structure [Source:HGNC Symbol;Acc:HGNC:6895]	protein coding	0.63	6.10	47.85	<0.0001	<0.0001
ENSG00000162994	CLHC1	clathrin heavy chain linker domain containing 1 [Source:HGNC Symbol;Acc:HGNC:26453]	protein coding	-0.63	1.64	34.70	<0.0001	<0.0001
ENSG00000052126	PLEKHA5	pleckstrin homology domain containing A5 [Source:HGNC Symbol;Acc:HGNC:30036]	protein coding	-0.63	0.93	13.13	0.0003	0.001
ENSG00000174125	TLR1	toll like receptor 1 [Source:HGNC Symbol;Acc:HGNC:11847]	protein coding	0.63	6.46	51.15	<0.0001	<0.0001
ENSG00000129667	RHBDF2	rhomboid 5 homolog 2 [Source:HGNC Symbol;Acc:HGNC:20788]	protein coding	0.63	7.71	53.07	<0.0001	<0.0001
ENSG00000287115		novel transcript	lncRNA	-0.63	0.97	18.53	<0.0001	<0.0001
ENSG00000038210	PI4K2B	phosphatidylinositol 4-kinase type 2 beta [Source:HGNC Symbol;Acc:HGNC:18215]	protein coding	0.63	4.38	31.30	<0.0001	<0.0001
ENSG00000169252	ADRB2	adrenoceptor beta 2 [Source:HGNC Symbol;Acc:HGNC:286]	protein coding	0.63	3.95	6.81	0.009	0.0205
ENSG00000185252	ZNF74	zinc finger protein 74 [Source:HGNC Symbol;Acc:HGNC:13144]	protein coding	-0.63	3.15	31.37	<0.0001	<0.0001
ENSG00000114737	CISH	cytokine inducible SH2 containing protein [Source:HGNC Symbol;Acc:HGNC:1984]	protein coding	0.62	4.21	28.26	<0.0001	<0.0001
ENSG00000057019	DCBLD2	discoidin, CUB and LCCL domain containing 2 [Source:HGNC Symbol;Acc:HGNC:24627]	protein coding	-0.62	1.32	16.90	<0.0001	0.0002
ENSG00000004399	PLXND1	plexin D1 [Source:HGNC Symbol;Acc:HGNC:9107]	protein coding	-0.62	8.50	31.57	<0.0001	<0.0001
ENSG00000279873	LINC01126	long intergenic non-protein coding RNA 1126 [Source:HGNC Symbol;Acc:HGNC:49275]	lncRNA	-0.62	1.99	19.38	<0.0001	<0.0001
ENSG00000154358	OBSCN	obscurin, cytoskeletal calmodulin and titin-interacting RhoGEF [Source:HGNC Symbol;Acc:HGNC:15719]	protein coding	-0.62	2.77	33.12	<0.0001	<0.0001

Table S5: Genes differentially expressed at Day 30 compared to pre-second vaccination (Study Arm 2 A/H7N9+AS03, D1, 29, Monocytes). Genes are sorted by descending absolute log_2 fold change. Gene model summaries and annotations are based on Ensembl Version 99 (continued.)

Ensembl Gene ID	Ensembl Gene Name	Ensembl Gene Description	Gene Type	log_2 Fold Change (Day 30 vs. Pre-Second Vaccination)	Average log_2 CPM	Likelihood Ratio Test Statistic	P-Value	FDR Adjusted P-Value
ENSG00000222041	CYTOR	cytoskeleton regulator RNA [Source:HGNC Symbol;Acc:HGNC:28717]	lncRNA	0.62	3.59	49.81	<0.0001	<0.0001
ENSG00000100714	MTHFD1	methylenetetrahydrofolate dehydrogenase, cyclohydrolase and formyltetrahydrofolate synthetase 1 [Source:HGNC Symbol;Acc:HGNC:7432]	protein coding	0.62	4.94	40.15	<0.0001	<0.0001
ENSG00000144802	NFKBIZ	NFKB inhibitor zeta [Source:HGNC Symbol;Acc:HGNC:29805]	protein coding	0.62	7.90	15.23	<0.0001	0.0004
ENSG00000178685	PARP10	poly(ADP-ribose) polymerase family member 10 [Source:HGNC Symbol;Acc:HGNC:25895]	protein coding	0.62	7.61	52.00	<0.0001	<0.0001
ENSG00000114739	ACVR2B	activin A receptor type 2B [Source:HGNC Symbol;Acc:HGNC:174]	protein coding	-0.62	1.36	23.82	<0.0001	<0.0001
ENSG00000151376	ME3	malic enzyme 3 [Source:HGNC Symbol;Acc:HGNC:6985]	protein coding	-0.62	0.88	14.59	0.0001	0.0005
ENSG00000196388	INCA1	inhibitor of CDK, cyclin A1 interacting protein 1 [Source:HGNC Symbol;Acc:HGNC:32224]	protein coding	-0.62	2.47	19.32	<0.0001	<0.0001
ENSG00000119013	NDUFB3	NADH:ubiquinone oxidoreductase subunit B3 [Source:HGNC Symbol;Acc:HGNC:7698]	protein coding	0.62	5.03	45.73	<0.0001	<0.0001
ENSG00000261526		novel transcript	lncRNA	-0.62	2.14	12.87	0.0003	0.0012
ENSG00000211445	GPX3	glutathione peroxidase 3 [Source:HGNC Symbol;Acc:HGNC:4555]	protein coding	-0.62	1.42	9.34	0.0022	0.0061
ENSG00000134574	DDB2	damage specific DNA binding protein 2 [Source:HGNC Symbol;Acc:HGNC:2718]	protein coding	0.62	5.18	87.36	<0.0001	<0.0001
ENSG00000180539	C9orf139	chromosome 9 open reading frame 139 [Source:HGNC Symbol;Acc:HGNC:31426]	lncRNA	-0.62	5.92	27.29	<0.0001	<0.0001
ENSG00000138036	DYNC2LI1	dynein cytoplasmic 2 light intermediate chain 1 [Source:HGNC Symbol;Acc:HGNC:24595]	protein coding	-0.62	1.32	22.09	<0.0001	<0.0001
ENSG00000079215	SLC1A3	solute carrier family 1 member 3 [Source:HGNC Symbol;Acc:HGNC:10941]	protein coding	0.62	1.97	15.97	<0.0001	0.0003
ENSG00000075568	TMEM131	transmembrane protein 131 [Source:HGNC Symbol;Acc:HGNC:30366]	protein coding	0.62	6.73	40.27	<0.0001	<0.0001
ENSG00000121807	CCR2	C-C motif chemokine receptor 2 [Source:HGNC Symbol;Acc:HGNC:1603]	protein coding	0.62	7.90	12.56	0.0004	0.0013
ENSG00000149527	PLCH2	phospholipase C eta 2 [Source:HGNC Symbol;Acc:HGNC:29037]	protein coding	-0.62	1.39	19.48	<0.0001	<0.0001
ENSG00000203666	EFCAB2	EF-hand calcium binding domain 2 [Source:HGNC Symbol;Acc:HGNC:28166]	protein coding	0.62	2.39	17.57	<0.0001	0.0001

Table S5: Genes differentially expressed at Day 30 compared to pre-second vaccination (Study Arm 2 A/H7N9+AS03, D1, 29, Monocytes). Genes are sorted by descending absolute log_2 fold change. Gene model summaries and annotations are based on Ensembl Version 99 (continued.)

Ensembl Gene ID	Ensembl Gene Name	Ensembl Gene Description	Gene Type	log_2 Fold Change (Day 30 vs. Pre-Second Vaccination)	Average log_2 CPM	Likelihood Ratio Test Statistic	P-Value	FDR Adjusted P-Value
ENSG00000270001		novel transcript	lncRNA	-0.62	0.22	13.14	0.0003	0.001
ENSG00000144120	TMEM177	transmembrane protein 177 [Source:HGNC Symbol;Acc:HGNC:28143]	protein coding	-0.62	0.75	11.92	0.0006	0.0018
ENSG00000154640	BTG3	BTG anti-proliferation factor 3 [Source:HGNC Symbol;Acc:HGNC:1132]	protein coding	0.62	1.03	23.27	<0.0001	<0.0001
ENSG00000070718	AP3M2	adaptor related protein complex 3 subunit mu 2 [Source:HGNC Symbol;Acc:HGNC:570]	protein coding	-0.62	2.29	32.92	<0.0001	<0.0001
ENSG00000179840	PIK3CD-AS1	PIK3CD antisense RNA 1 [Source:HGNC Symbol;Acc:HGNC:32346]	lncRNA	0.62	1.12	23.71	<0.0001	<0.0001
ENSG00000187764	SEMA4D	semaphorin 4D [Source:HGNC Symbol;Acc:HGNC:10732]	protein coding	0.62	7.98	75.84	<0.0001	<0.0001
ENSG00000227507	LTB	lymphotoxin beta [Source:HGNC Symbol;Acc:HGNC:6711]	protein coding	-0.62	5.37	38.35	<0.0001	<0.0001
ENSG00000119686	FLVCR2	feline leukemia virus subgroup C cellular receptor family member 2 [Source:HGNC Symbol;Acc:HGNC:20105]	protein coding	0.62	5.16	60.71	<0.0001	<0.0001
ENSG00000139410	SDSL	serine dehydratase like [Source:HGNC Symbol;Acc:HGNC:30404]	protein coding	0.62	2.64	31.89	<0.0001	<0.0001
ENSG00000185100	ADSS1	adenylosuccinate synthase 1 [Source:HGNC Symbol;Acc:HGNC:20093]	protein coding	-0.62	1.91	21.44	<0.0001	<0.0001
ENSG00000111962	UST	uronyl 2-sulfotransferase [Source:HGNC Symbol;Acc:HGNC:17223]	protein coding	0.62	1.25	16.73	<0.0001	0.0002
ENSG00000198417	MT1F	metallothionein 1F [Source:HGNC Symbol;Acc:HGNC:7398]	protein coding	0.62	2.48	28.16	<0.0001	<0.0001
ENSG00000165490	DDIAS	DNA damage induced apoptosis suppressor [Source:HGNC Symbol;Acc:HGNC:26351]	protein coding	0.62	3.57	33.76	<0.0001	<0.0001
ENSG00000076641	PAG1	phosphoprotein membrane anchor with glycosphingolipid microdomains 1 [Source:HGNC Symbol;Acc:HGNC:30043]	protein coding	0.62	6.22	33.27	<0.0001	<0.0001
ENSG00000180061	TMEM150B	transmembrane protein 150B [Source:HGNC Symbol;Acc:HGNC:34415]	protein coding	0.61	5.78	39.93	<0.0001	<0.0001
ENSG00000178921	PFAS	phosphoribosylformylglycinamidine synthase [Source:HGNC Symbol;Acc:HGNC:8863]	protein coding	-0.61	4.34	55.39	<0.0001	<0.0001
ENSG00000186318	BACE1	beta-secretase 1 [Source:HGNC Symbol;Acc:HGNC:933]	protein coding	-0.61	3.65	43.66	<0.0001	<0.0001
ENSG00000154655	L3MBTL4	L3MBTL histone methyl-lysine binding protein 4 [Source:HGNC Symbol;Acc:HGNC:26677]	protein coding	0.61	1.92	26.58	<0.0001	<0.0001

Table S5: Genes differentially expressed at Day 30 compared to pre-second vaccination (Study Arm 2 A/H7N9+AS03, D1, 29, Monocytes). Genes are sorted by descending absolute log_2 fold change. Gene model summaries and annotations are based on Ensembl Version 99 (continued.)

Ensembl Gene ID	Ensembl Gene Name	Ensembl Gene Description	Gene Type	log_2 Fold Change (Day 30 vs. Pre-Second Vaccination)	Average log_2 CPM	Likelihood Ratio Test Statistic	P-Value	FDR Adjusted P-Value
ENSG00000034510	TMSB10	thymosin beta 10 [Source:HGNC Symbol;Acc:HGNC:11879]	protein coding	0.61	10.58	40.23	<0.0001	<0.0001
ENSG00000111837	MAK	male germ cell associated kinase [Source:HGNC Symbol;Acc:HGNC:6816]	protein coding	-0.61	1.82	15.43	<0.0001	0.0003
ENSG00000225792		novel transcript	lncRNA	0.61	1.86	11.99	0.0005	0.0017
ENSG00000257270		novel transcript, antisense to MTA1	lncRNA	-0.61	1.19	22.85	<0.0001	<0.0001
ENSG00000168405	CMAHP	cytidine monophospho-N-acetylneuraminic acid hydroxylase, pseudogene [Source:HGNC Symbol;Acc:HGNC:2098]	transcribed unitary pseudogene	0.61	3.85	43.39	<0.0001	<0.0001
ENSG00000163354	DCST2	DC-STAMP domain containing 2 [Source:HGNC Symbol;Acc:HGNC:26562]	protein coding	-0.61	0.74	13.47	0.0002	0.0009
ENSG00000137310	TCF19	transcription factor 19 [Source:HGNC Symbol;Acc:HGNC:11629]	protein coding	-0.61	1.39	16.73	<0.0001	0.0002
ENSG00000163818	LZTFL1	leucine zipper transcription factor like 1 [Source:HGNC Symbol;Acc:HGNC:6741]	protein coding	-0.61	2.77	34.14	<0.0001	<0.0001
ENSG00000163006	CCDC138	coiled-coil domain containing 138 [Source:HGNC Symbol;Acc:HGNC:26531]	protein coding	-0.61	0.15	9.64	0.0019	0.0053
ENSG00000105649	RAB3A	RAB3A, member RAS oncogene family [Source:HGNC Symbol;Acc:HGNC:9777]	protein coding	-0.61	1.30	8.49	0.0036	0.0091
ENSG00000242125	SNHG3	small nucleolar RNA host gene 3 [Source:HGNC Symbol;Acc:HGNC:10118]	lncRNA	-0.61	4.47	46.26	<0.0001	<0.0001
ENSG00000184988	TMEM106A	transmembrane protein 106A [Source:HGNC Symbol;Acc:HGNC:28288]	protein coding	0.61	5.09	28.24	<0.0001	<0.0001
ENSG00000104626	ERI1	exoribonuclease 1 [Source:HGNC Symbol;Acc:HGNC:23994]	protein coding	0.61	3.88	32.64	<0.0001	<0.0001
ENSG00000163913	IFT122	intraflagellar transport 122 [Source:HGNC Symbol;Acc:HGNC:13556]	protein coding	-0.61	3.91	46.41	<0.0001	<0.0001
ENSG00000224411	HSP90AA2P	heat shock protein 90 alpha family class A member 2, pseudogene [Source:HGNC Symbol;Acc:HGNC:5256]	processed pseudogene	0.61	2.28	26.51	<0.0001	<0.0001
ENSG00000071242	RPS6KA2	ribosomal protein S6 kinase A2 [Source:HGNC Symbol;Acc:HGNC:10431]	protein coding	-0.61	2.94	33.01	<0.0001	<0.0001
ENSG00000197165	SULT1A2	sulfotransferase family 1A member 2 [Source:HGNC Symbol;Acc:HGNC:11454]	protein coding	-0.61	1.13	19.89	<0.0001	<0.0001

Table S5: Genes differentially expressed at Day 30 compared to pre-second vaccination (Study Arm 2 A/H7N9+AS03, D1, 29, Monocytes). Genes are sorted by descending absolute log_2 fold change. Gene model summaries and annotations are based on Ensembl Version 99 (continued.)

Ensembl Gene ID	Ensembl Gene Name	Ensembl Gene Description	Gene Type	log_2 Fold Change (Day 30 vs. Pre-Second Vaccination)	Average log_2 CPM	Likelihood Ratio Test Statistic	P-Value	FDR Adjusted P-Value
ENSG00000093134	VNN3	vanin 3 [Source:HGNC Symbol;Acc:HGNC:16431]	protein coding	-0.61	3.82	20.86	<0.0001	<0.0001
ENSG00000166503	HDGFL3	HDGF like 3 [Source:HGNC Symbol;Acc:HGNC:24937]	protein coding	-0.61	3.92	30.27	<0.0001	<0.0001
ENSG00000119699	TGFB3	transforming growth factor beta 3 [Source:HGNC Symbol;Acc:HGNC:11769]	protein coding	-0.61	0.54	17.20	<0.0001	0.0002
ENSG00000163565	IFI16	interferon gamma inducible protein 16 [Source:HGNC Symbol;Acc:HGNC:5395]	protein coding	0.61	7.83	28.28	<0.0001	<0.0001
ENSG00000128512	DOCK4	dedicator of cytokinesis 4 [Source:HGNC Symbol;Acc:HGNC:19192]	protein coding	0.61	2.88	28.70	<0.0001	<0.0001
ENSG00000257335	MGAM	maltase-glucoamylase [Source:HGNC Symbol;Acc:HGNC:7043]	protein coding	0.61	3.46	11.89	0.0006	0.0018
ENSG00000196693	ZNF33B	zinc finger protein 33B [Source:HGNC Symbol;Acc:HGNC:13097]	protein coding	-0.61	4.21	37.25	<0.0001	<0.0001
ENSG00000104549	SQLE	squalene epoxidase [Source:HGNC Symbol;Acc:HGNC:11279]	protein coding	0.61	3.61	17.88	<0.0001	0.0001
ENSG00000142867	BCL10	BCL10 immune signaling adaptor [Source:HGNC Symbol;Acc:HGNC:989]	protein coding	0.61	4.46	48.96	<0.0001	<0.0001
ENSG00000179909	ZNF154	zinc finger protein 154 [Source:HGNC Symbol;Acc:HGNC:12939]	protein coding	-0.61	1.39	20.36	<0.0001	<0.0001
ENSG00000168807	SNTB2	syntrophin beta 2 [Source:HGNC Symbol;Acc:HGNC:11169]	protein coding	0.61	4.08	44.56	<0.0001	<0.0001
ENSG00000114450	GNB4	G protein subunit beta 4 [Source:HGNC Symbol;Acc:HGNC:20731]	protein coding	0.61	6.27	57.36	<0.0001	<0.0001
ENSG00000215252	GOLGA8B	golgin A8 family member B [Source:HGNC Symbol;Acc:HGNC:31973]	protein coding	-0.61	2.23	19.60	<0.0001	<0.0001
ENSG00000163221	S100A12	S100 calcium binding protein A12 [Source:HGNC Symbol;Acc:HGNC:10489]	protein coding	0.61	8.15	26.14	<0.0001	<0.0001
ENSG00000267002		novel transcript	lncRNA	-0.61	2.15	22.44	<0.0001	<0.0001
ENSG00000135916	ITM2C	integral membrane protein 2C [Source:HGNC Symbol;Acc:HGNC:6175]	protein coding	-0.61	3.50	11.48	0.0007	0.0022
ENSG00000182685	BRICD5	BRICHOS domain containing 5 [Source:HGNC Symbol;Acc:HGNC:28309]	protein coding	-0.61	3.06	27.92	<0.0001	<0.0001
ENSG00000135404	CD63	CD63 molecule [Source:HGNC Symbol;Acc:HGNC:1692]	protein coding	0.61	8.13	56.87	<0.0001	<0.0001

Table S5: Genes differentially expressed at Day 30 compared to pre-second vaccination (Study Arm 2 A/H7N9+AS03, D1, 29, Monocytes). Genes are sorted by descending absolute log_2 fold change. Gene model summaries and annotations are based on Ensembl Version 99 (continued.)

Ensembl Gene ID	Ensembl Gene Name	Ensembl Gene Description	Gene Type	log_2 Fold Change (Day 30 vs. Pre-Second Vaccination)	Average log_2 CPM	Likelihood Ratio Test Statistic	P-Value	FDR Adjusted P-Value
ENSG00000172893	DHCR7	7-dehydrocholesterol reductase [Source:HGNC Symbol;Acc:HGNC:2860]	protein coding	0.61	1.99	10.43	0.0012	0.0036
ENSG00000167202	TBC1D2B	TBC1 domain family member 2B [Source:HGNC Symbol;Acc:HGNC:29183]	protein coding	0.61	6.81	54.76	<0.0001	<0.0001
ENSG00000100197	CYP2D6	cytochrome P450 family 2 subfamily D member 6 [Source:HGNC Symbol;Acc:HGNC:2625]	protein coding	-0.61	1.40	19.67	<0.0001	<0.0001
ENSG00000164867	NOS3	nitric oxide synthase 3 [Source:HGNC Symbol;Acc:HGNC:7876]	protein coding	-0.61	0.19	14.68	0.0001	0.0005
ENSG00000109756	RAPGEF2	Rap guanine nucleotide exchange factor 2 [Source:HGNC Symbol;Acc:HGNC:16854]	protein coding	0.61	5.94	43.37	<0.0001	<0.0001
ENSG00000140043	PTGR2	prostaglandin reductase 2 [Source:HGNC Symbol;Acc:HGNC:20149]	protein coding	-0.61	0.66	15.14	0.0001	0.0004
ENSG00000101577	LPIN2	lipin 2 [Source:HGNC Symbol;Acc:HGNC:14450]	protein coding	0.61	6.20	32.31	<0.0001	<0.0001
ENSG00000182742	HOXB4	homeobox B4 [Source:HGNC Symbol;Acc:HGNC:5115]	protein coding	0.61	0.39	11.66	0.0006	0.002
ENSG00000268575		novel transcript, readthrough between SLC35E2 and CDK11A	lncRNA	-0.61	1.72	20.62	<0.0001	<0.0001
ENSG00000123146	ADGRE5	adhesion G protein-coupled receptor E5 [Source:HGNC Symbol;Acc:HGNC:1711]	protein coding	0.61	9.49	44.94	<0.0001	<0.0001
ENSG00000173801	JUP	junction plakoglobin [Source:HGNC Symbol;Acc:HGNC:6207]	protein coding	-0.61	6.29	31.40	<0.0001	<0.0001
ENSG00000101278	RPS10P5	ribosomal protein S10 pseudogene 5 [Source:HGNC Symbol;Acc:HGNC:15795]	processed pseudogene	-0.61	1.35	16.94	<0.0001	0.0002
ENSG00000172260	NEGR1	neuronal growth regulator 1 [Source:HGNC Symbol;Acc:HGNC:17302]	protein coding	-0.60	1.09	11.75	0.0006	0.002
ENSG00000004838	ZMYND10	zinc finger MYND-type containing 10 [Source:HGNC Symbol;Acc:HGNC:19412]	protein coding	-0.60	0.86	12.76	0.0004	0.0012
ENSG00000001630	CYP51A1	cytochrome P450 family 51 subfamily A member 1 [Source:HGNC Symbol;Acc:HGNC:2649]	protein coding	0.60	1.37	15.53	<0.0001	0.0003
ENSG00000159685	CHCHD6	coiled-coil-helix-coiled-coil-helix domain containing 6 [Source:HGNC Symbol;Acc:HGNC:28184]	protein coding	-0.60	0.68	19.31	<0.0001	<0.0001

Table S5: Genes differentially expressed at Day 30 compared to pre-second vaccination (Study Arm 2 A/H7N9+AS03, D1, 29, Monocytes). Genes are sorted by descending absolute log_2 fold change. Gene model summaries and annotations are based on Ensembl Version 99 (continued.)

Ensembl Gene ID	Ensembl Gene Name	Ensembl Gene Description	Gene Type	log_2 Fold Change (Day 30 vs. Pre-Second Vaccination)	Average log_2 CPM	Likelihood Ratio Test Statistic	P-Value	FDR Adjusted P-Value
ENSG00000215158		Putative beta-glucuronidase-like protein FLJ75287 pseudogene	transcribed unprocessed pseudogene	-0.60	1.08	15.04	0.0001	0.0004
ENSG00000175048	ZDHHC14	zinc finger DHHC-type containing 14 [Source:HGNC Symbol;Acc:HGNC:20341]	protein coding	-0.60	3.05	38.15	<0.0001	<0.0001
ENSG00000267174		novel transcript	lncRNA	-0.60	2.77	29.77	<0.0001	<0.0001
ENSG00000287263		novel transcript	lncRNA	0.60	4.53	25.24	<0.0001	<0.0001
ENSG00000223705	NSUN5P1	NSUN5 pseudogene 1 [Source:HGNC Symbol;Acc:HGNC:19146]	transcribed unprocessed pseudogene	-0.60	4.45	51.68	<0.0001	<0.0001
ENSG00000114978	MOB1A	MOB kinase activator 1A [Source:HGNC Symbol;Acc:HGNC:16015]	protein coding	0.60	8.52	56.90	<0.0001	<0.0001
ENSG00000106211	HSPB1	heat shock protein family B (small) member 1 [Source:HGNC Symbol;Acc:HGNC:5246]	protein coding	0.60	3.78	31.12	<0.0001	<0.0001
ENSG00000133624	ZNF767P	zinc finger family member 767, pseudogene [Source:HGNC Symbol;Acc:HGNC:21884]	transcribed unprocessed pseudogene	-0.60	4.23	58.81	<0.0001	<0.0001
ENSG00000138760	SCARB2	scavenger receptor class B member 2 [Source:HGNC Symbol;Acc:HGNC:1665]	protein coding	0.60	7.30	78.63	<0.0001	<0.0001
ENSG00000224616	RTCA-AS1	RTCA antisense RNA 1 [Source:HGNC Symbol;Acc:HGNC:50573]	lncRNA	-0.60	1.45	21.91	<0.0001	<0.0001
ENSG00000249859	PVT1	Pvt1 oncogene [Source:HGNC Symbol;Acc:HGNC:9709]	lncRNA	-0.60	3.65	29.89	<0.0001	<0.0001
ENSG00000204856	FAM216A	family with sequence similarity 216 member A [Source:HGNC Symbol;Acc:HGNC:30180]	protein coding	-0.60	2.46	20.73	<0.0001	<0.0001
ENSG00000165533	TTC8	tetratricopeptide repeat domain 8 [Source:HGNC Symbol;Acc:HGNC:20087]	protein coding	-0.60	0.85	13.64	0.0002	0.0008
ENSG00000181019	NQO1	NAD(P)H quinone dehydrogenase 1 [Source:HGNC Symbol;Acc:HGNC:2874]	protein coding	-0.60	0.48	11.40	0.0007	0.0023
ENSG00000154330	PGM5	phosphoglucomutase 5 [Source:HGNC Symbol;Acc:HGNC:8908]	protein coding	-0.60	2.09	10.61	0.0011	0.0033

Table S5: Genes differentially expressed at Day 30 compared to pre-second vaccination (Study Arm 2 A/H7N9+AS03, D1, 29, Monocytes). Genes are sorted by descending absolute log_2 fold change. Gene model summaries and annotations are based on Ensembl Version 99 (continued.)

Ensembl Gene ID	Ensembl Gene Name	Ensembl Gene Description	Gene Type	log_2 Fold Change (Day 30 vs. Pre-Second Vaccination)	Average log_2 CPM	Likelihood Ratio Test Statistic	P-Value	FDR Adjusted P-Value
ENSG00000105281	SLC1A5	solute carrier family 1 member 5 [Source:HGNC Symbol;Acc:HGNC:10943]	protein coding	0.60	6.55	47.14	<0.0001	<0.0001
ENSG00000068028	RASSF1	Ras association domain family member 1 [Source:HGNC Symbol;Acc:HGNC:9882]	protein coding	-0.60	6.86	62.66	<0.0001	<0.0001
ENSG00000157445	CACNA2D3	calcium voltage-gated channel auxiliary subunit alpha2delta 3 [Source:HGNC Symbol;Acc:HGNC:15460]	protein coding	-0.60	4.06	39.37	<0.0001	<0.0001
ENSG00000183784	DOCK8-AS1	DOCK8 antisense RNA 1 [Source:HGNC Symbol;Acc:HGNC:26436]	lncRNA	0.60	1.90	26.61	<0.0001	<0.0001
ENSG00000127954	STEAP4	STEAP4 metalloreductase [Source:HGNC Symbol;Acc:HGNC:21923]	protein coding	0.60	5.73	12.39	0.0004	0.0014
ENSG00000270091		novel transcript	lncRNA	-0.60	1.05	13.07	0.0003	0.0011
ENSG00000196126	HLA-DRB1	major histocompatibility complex, class II, DR beta 1 [Source:HGNC Symbol;Acc:HGNC:4948]	protein coding	0.60	9.58	49.20	<0.0001	<0.0001
ENSG00000106701	FSD1L	fibronectin type III and SPRY domain containing 1 like [Source:HGNC Symbol;Acc:HGNC:13753]	protein coding	-0.60	2.97	41.87	<0.0001	<0.0001
ENSG00000089220	PEBP1	phosphatidylethanolamine binding protein 1 [Source:HGNC Symbol;Acc:HGNC:8630]	protein coding	-0.60	5.27	57.91	<0.0001	<0.0001
ENSG00000066923	STAG3	stromal antigen 3 [Source:HGNC Symbol;Acc:HGNC:11356]	protein coding	-0.60	1.00	16.69	<0.0001	0.0002
ENSG00000247572	CKMT2-AS1	CKMT2 antisense RNA 1 [Source:HGNC Symbol;Acc:HGNC:48997]	lncRNA	-0.60	1.29	28.68	<0.0001	<0.0001
ENSG00000125730	C3	complement C3 [Source:HGNC Symbol;Acc:HGNC:1318]	protein coding	-0.60	3.24	24.31	<0.0001	<0.0001
ENSG00000245025		novel transcript, antisense to RHOBTB2	lncRNA	0.60	0.21	8.98	0.0027	0.0072
ENSG00000113583	C5orf15	chromosome 5 open reading frame 15 [Source:HGNC Symbol;Acc:HGNC:20656]	protein coding	0.60	6.02	49.34	<0.0001	<0.0001
ENSG00000162063	CCNF	cyclin F [Source:HGNC Symbol;Acc:HGNC:1591]	protein coding	-0.60	0.71	14.42	0.0001	0.0006
ENSG00000196352	CD55	CD55 molecule (Cromer blood group) [Source:HGNC Symbol;Acc:HGNC:2665]	protein coding	0.60	7.46	64.09	<0.0001	<0.0001
ENSG00000183426	NPIPA1	nuclear pore complex interacting protein family member A1 [Source:HGNC Symbol;Acc:HGNC:7909]	protein coding	-0.60	1.11	24.29	<0.0001	<0.0001

Table S5: Genes differentially expressed at Day 30 compared to pre-second vaccination (Study Arm 2 A/H7N9+AS03, D1, 29, Monocytes). Genes are sorted by descending absolute log_2 fold change. Gene model summaries and annotations are based on Ensembl Version 99 (continued.)

Ensembl Gene ID	Ensembl Gene Name	Ensembl Gene Description	Gene Type	log_2 Fold Change (Day 30 vs. Pre-Second Vaccination)	Average log_2 CPM	Likelihood Ratio Test Statistic	P-Value	FDR Adjusted P-Value
ENSG00000244627	TPTEP2	TPTE pseudogene 2 [Source:HGNC Symbol;Acc:HGNC:53828]	transcribed unprocessed pseudogene	-0.60	1.91	22.40	<0.0001	<0.0001
ENSG00000074181	NOTCH3	notch receptor 3 [Source:HGNC Symbol;Acc:HGNC:7883]	protein coding	-0.59	0.40	6.16	0.0131	0.0283
ENSG00000108733	PEX12	peroxisomal biogenesis factor 12 [Source:HGNC Symbol;Acc:HGNC:8854]	protein coding	0.59	0.72	5.84	0.0156	0.033
ENSG00000163507	CIP2A	cell proliferation regulating inhibitor of protein phosphatase 2A [Source:HGNC Symbol;Acc:HGNC:29302]	protein coding	-0.59	1.32	14.72	0.0001	0.0005
ENSG00000182774	RPS17	ribosomal protein S17 [Source:HGNC Symbol;Acc:HGNC:10397]	protein coding	-0.59	3.26	25.38	<0.0001	<0.0001
ENSG00000286190		uncharacterized LOC728392 [Source:NCBI gene;Acc:728392]	protein coding	-0.59	5.33	32.02	<0.0001	<0.0001
ENSG00000176595	KBTBD11	kelch repeat and BTB domain containing 11 [Source:HGNC Symbol;Acc:HGNC:29104]	protein coding	-0.59	6.09	46.40	<0.0001	<0.0001
ENSG00000237513		novel transcript	lncRNA	-0.59	1.34	17.80	<0.0001	0.0001
ENSG00000246263	UBR5-AS1	UBR5 antisense RNA 1 [Source:HGNC Symbol;Acc:HGNC:51661]	lncRNA	-0.59	2.76	42.63	<0.0001	<0.0001
ENSG00000281103	TRG-AS1	T cell receptor gamma locus antisense RNA 1 [Source:HGNC Symbol;Acc:HGNC:48974]	lncRNA	-0.59	3.41	23.50	<0.0001	<0.0001
ENSG00000166780	BMERB1	bMERB domain containing 1 [Source:HGNC Symbol;Acc:HGNC:19213]	protein coding	-0.59	0.29	14.54	0.0001	0.0005
ENSG00000229980	TOB1-AS1	TOB1 antisense RNA 1 [Source:HGNC Symbol;Acc:HGNC:44340]	lncRNA	-0.59	0.06	9.80	0.0017	0.0049
ENSG00000183048	SLC25A10	solute carrier family 25 member 10 [Source:HGNC Symbol;Acc:HGNC:10980]	protein coding	-0.59	0.09	10.93	0.0009	0.0029
ENSG00000123136	DDX39A	DExD-box helicase 39A [Source:HGNC Symbol;Acc:HGNC:17821]	protein coding	0.59	6.88	28.42	<0.0001	<0.0001
ENSG00000153885	KCTD15	potassium channel tetramerization domain containing 15 [Source:HGNC Symbol;Acc:HGNC:23297]	protein coding	-0.59	5.47	47.71	<0.0001	<0.0001
ENSG00000186648	CARMIL3	capping protein regulator and myosin 1 linker 3 [Source:HGNC Symbol;Acc:HGNC:20272]	protein coding	-0.59	1.46	15.96	<0.0001	0.0003
ENSG00000146192	FGD2	FYVE, RhoGEF and PH domain containing 2 [Source:HGNC Symbol;Acc:HGNC:3664]	protein coding	0.59	9.14	61.65	<0.0001	<0.0001

Table S5: Genes differentially expressed at Day 30 compared to pre-second vaccination (Study Arm 2 A/H7N9+AS03, D1, 29, Monocytes). Genes are sorted by descending absolute log_2 fold change. Gene model summaries and annotations are based on Ensembl Version 99 (continued.)

Ensembl Gene ID	Ensembl Gene Name	Ensembl Gene Description	Gene Type	log_2 Fold Change (Day 30 vs. Pre-Second Vaccination)	Average log_2 CPM	Likelihood Ratio Test Statistic	P-Value	FDR Adjusted P-Value
ENSG00000268061	NAPA-AS1	NAPA antisense RNA 1 [Source:HGNC Symbol;Acc:HGNC:44118]	lncRNA	0.59	0.35	6.98	0.0082	0.0188
ENSG00000100596	SPTLC2	serine palmitoyltransferase long chain base subunit 2 [Source:HGNC Symbol;Acc:HGNC:11278]	protein coding	0.59	7.77	50.27	<0.0001	<0.0001
ENSG00000047365	ARAP2	ArfGAP with RhoGAP domain, ankyrin repeat and PH domain 2 [Source:HGNC Symbol;Acc:HGNC:16924]	protein coding	0.59	5.91	41.34	<0.0001	<0.0001
ENSG00000154153	RETREG1	reticulophagy regulator 1 [Source:HGNC Symbol;Acc:HGNC:25964]	protein coding	0.59	2.55	21.79	<0.0001	<0.0001
ENSG00000278974		tec	TEC	0.59	0.35	16.91	<0.0001	0.0002
ENSG00000143498	TAF1A	TATA-box binding protein associated factor, RNA polymerase I subunit A [Source:HGNC Symbol;Acc:HGNC:11532]	protein coding	0.59	2.28	22.58	<0.0001	<0.0001
ENSG00000162739	SLAMF6	SLAM family member 6 [Source:HGNC Symbol;Acc:HGNC:21392]	protein coding	-0.59	1.93	10.02	0.0015	0.0044
ENSG00000186230	ZNF749	zinc finger protein 749 [Source:HGNC Symbol;Acc:HGNC:32783]	protein coding	-0.59	1.01	18.27	<0.0001	<0.0001
ENSG00000166086	JAM3	junctional adhesion molecule 3 [Source:HGNC Symbol;Acc:HGNC:15532]	protein coding	-0.59	0.47	10.15	0.0014	0.0041
ENSG00000132122	SPATA6	spermatogenesis associated 6 [Source:HGNC Symbol;Acc:HGNC:18309]	protein coding	-0.59	3.66	16.05	<0.0001	0.0003
ENSG00000106123	EPHB6	EPH receptor B6 [Source:HGNC Symbol;Acc:HGNC:3396]	protein coding	-0.59	5.31	62.61	<0.0001	<0.0001
ENSG00000234912	SNHG20	small nucleolar RNA host gene 20 [Source:HGNC Symbol;Acc:HGNC:33099]	lncRNA	-0.59	3.05	29.22	<0.0001	<0.0001
ENSG00000238042	LINC02257	long intergenic non-protein coding RNA 2257 [Source:HGNC Symbol;Acc:HGNC:53159]	lncRNA	-0.59	0.27	10.75	0.001	0.0032
ENSG00000255833	TIFAB	TIFA inhibitor [Source:HGNC Symbol;Acc:HGNC:34024]	protein coding	0.59	1.54	10.14	0.0015	0.0042
ENSG00000267296	CEBPA-DT	CEBPA divergent transcript [Source:HGNC Symbol;Acc:HGNC:25710]	lncRNA	-0.59	2.91	29.15	<0.0001	<0.0001
ENSG00000255163	HSPE1P18	heat shock protein family E (Hsp10) member 1 pseudogene 18 [Source:HGNC Symbol;Acc:HGNC:49337]	processed pseudogene	-0.59	2.87	28.53	<0.0001	<0.0001
ENSG00000112149	CD83	CD83 molecule [Source:HGNC Symbol;Acc:HGNC:1703]	protein coding	0.59	4.61	5.79	0.0161	0.0339

Table S5: Genes differentially expressed at Day 30 compared to pre-second vaccination (Study Arm 2 A/H7N9+AS03, D1, 29, Monocytes). Genes are sorted by descending absolute log_2 fold change. Gene model summaries and annotations are based on Ensembl Version 99 (continued.)

Ensembl Gene ID	Ensembl Gene Name	Ensembl Gene Description	Gene Type	log_2 Fold Change (Day 30 vs. Pre-Second Vaccination)	Average log_2 CPM	Likelihood Ratio Test Statistic	P-Value	FDR Adjusted P-Value
ENSG00000198848	CES1	carboxylesterase 1 [Source:HGNC Symbol;Acc:HGNC:1863]	protein coding	0.59	6.85	29.86	<0.0001	<0.0001
ENSG00000124243	BCAS4	breast carcinoma amplified sequence 4 [Source:HGNC Symbol;Acc:HGNC:14367]	protein coding	-0.59	1.74	25.98	<0.0001	<0.0001
ENSG00000143630	HCN3	hyperpolarization activated cyclic nucleotide gated potassium channel 3 [Source:HGNC Symbol;Acc:HGNC:19183]	protein coding	-0.59	0.94	19.86	<0.0001	<0.0001
ENSG00000163412	EIF4E3	eukaryotic translation initiation factor 4E family member 3 [Source:HGNC Symbol;Acc:HGNC:31837]	protein coding	0.59	6.42	56.97	<0.0001	<0.0001
ENSG00000131400	NAPSA	napsin A aspartic peptidase [Source:HGNC Symbol;Acc:HGNC:13395]	protein coding	-0.59	0.07	10.27	0.0014	0.0039
ENSG00000198743	SLC5A3	solute carrier family 5 member 3 [Source:HGNC Symbol;Acc:HGNC:11038]	protein coding	-0.59	4.08	24.40	<0.0001	<0.0001
ENSG00000146072	TNFRSF21	TNF receptor superfamily member 21 [Source:HGNC Symbol;Acc:HGNC:13469]	protein coding	-0.59	0.35	5.28	0.0216	0.0436
ENSG00000182197	EXT1	exostosin glycosyltransferase 1 [Source:HGNC Symbol;Acc:HGNC:3512]	protein coding	0.59	5.27	48.02	<0.0001	<0.0001
ENSG00000214029	ZNF891	zinc finger protein 891 [Source:HGNC Symbol;Acc:HGNC:38709]	protein coding	-0.59	1.99	26.83	<0.0001	<0.0001
ENSG00000168679	SLC16A4	solute carrier family 16 member 4 [Source:HGNC Symbol;Acc:HGNC:10925]	protein coding	-0.59	0.33	10.37	0.0013	0.0037
ENSG00000118965	WDR35	WD repeat domain 35 [Source:HGNC Symbol;Acc:HGNC:29250]	protein coding	-0.59	1.51	19.72	<0.0001	<0.0001
ENSG00000135821	GLUL	glutamate-ammonia ligase [Source:HGNC Symbol;Acc:HGNC:4341]	protein coding	0.59	9.28	44.39	<0.0001	<0.0001
ENSG00000130813	SHFL	shiftless antiviral inhibitor of ribosomal frameshifting [Source:HGNC Symbol;Acc:HGNC:25649]	protein coding	0.59	5.81	36.65	<0.0001	<0.0001
ENSG00000138443	ABI2	abl interactor 2 [Source:HGNC Symbol;Acc:HGNC:24011]	protein coding	-0.59	3.30	35.19	<0.0001	<0.0001

Table S6: Genes differentially expressed at Day 32 compared to pre-second vaccination (Study Arm 2 A/H7N9+AS03, D1, 29, Monocytes). Genes are sorted by descending absolute log_2 fold change. Gene model summaries and annotations are based on Ensembl Version 99.

Ensembl Gene ID	Ensembl Gene Name	Ensembl Gene Description	Gene Type	log_2 Fold Change (Day 32 vs. Pre-Second Vaccination)	Average log_2 CPM	Likelihood Ratio Test Statistic	P-Value	FDR Adjusted P-Value
ENSG00000173369	C1QB	complement C1q B chain [Source:HGNC Symbol;Acc:HGNC:1242]	protein coding	1.24	3.21	39.48	<0.0001	<0.0001
ENSG00000156113	KCNMA1	potassium calcium-activated channel subfamily M alpha 1 [Source:HGNC Symbol;Acc:HGNC:6284]	protein coding	1.23	1.92	28.26	<0.0001	0.0001
ENSG00000159189	C1QC	complement C1q C chain [Source:HGNC Symbol;Acc:HGNC:1245]	protein coding	1.17	2.39	23.28	<0.0001	0.001
ENSG00000043591	ADRB1	adrenoceptor beta 1 [Source:HGNC Symbol;Acc:HGNC:285]	protein coding	-1.12	0.28	18.55	<0.0001	0.0069
ENSG00000171954	CYP4F22	cytochrome P450 family 4 subfamily F member 22 [Source:HGNC Symbol;Acc:HGNC:26820]	protein coding	1.07	2.34	23.06	<0.0001	0.0011
ENSG00000108950	FAM20A	FAM20A golgi associated secretory pathway pseudokinase [Source:HGNC Symbol;Acc:HGNC:23015]	protein coding	1.03	1.82	30.64	<0.0001	<0.0001
ENSG00000175544	CABP4	calcium binding protein 4 [Source:HGNC Symbol;Acc:HGNC:1386]	protein coding	0.99	1.13	36.41	<0.0001	<0.0001
ENSG00000120885	CLU	clusterin [Source:HGNC Symbol;Acc:HGNC:2095]	protein coding	0.98	2.73	53.68	<0.0001	<0.0001
ENSG00000173372	C1QA	complement C1q A chain [Source:HGNC Symbol;Acc:HGNC:1241]	protein coding	0.91	4.53	25.58	<0.0001	0.0004
ENSG00000183134	PTGDR2	prostaglandin D2 receptor 2 [Source:HGNC Symbol;Acc:HGNC:4502]	protein coding	0.89	2.56	28.42	<0.0001	0.0001
ENSG00000169715	MT1E	metallothionein 1E [Source:HGNC Symbol;Acc:HGNC:7397]	protein coding	0.81	0.68	37.14	<0.0001	<0.0001
ENSG00000129757	CDKN1C	cyclin dependent kinase inhibitor 1C [Source:HGNC Symbol;Acc:HGNC:1786]	protein coding	0.79	6.33	31.05	<0.0001	<0.0001
ENSG00000169245	CXCL10	C-X-C motif chemokine ligand 10 [Source:HGNC Symbol;Acc:HGNC:10637]	protein coding	-0.79	2.95	15.67	<0.0001	0.0212
ENSG00000166165	CKB	creatine kinase B [Source:HGNC Symbol;Acc:HGNC:1991]	protein coding	0.76	2.71	27.94	<0.0001	0.0001
ENSG00000203747	FCGR3A	Fc fragment of IgG receptor IIIa [Source:HGNC Symbol;Acc:HGNC:3619]	protein coding	0.76	8.17	20.23	<0.0001	0.0034
ENSG00000128438	TBC1D27P	TBC1 domain family member 27, pseudogene [Source:HGNC Symbol;Acc:HGNC:28104]	transcribed unprocessed pseudogene	-0.73	1.51	15.23	<0.0001	0.0231

Table S6: Genes differentially expressed at Day 32 compared to pre-second vaccination (Study Arm 2 A/H7N9+AS03, D1, 29, Monocytes). Genes are sorted by descending absolute log_2 fold change. Gene model summaries and annotations are based on Ensembl Version 99 (continued.)

Ensembl Gene ID	Ensembl Gene Name	Ensembl Gene Description	Gene Type	log_2 Fold Change (Day 32 vs. Pre-Second Vaccination)	Average log_2 CPM	Likelihood Ratio Test Statistic	P-Value	FDR Adjusted P-Value
ENSG00000196839	ADA	adenosine deaminase [Source:HGNC Symbol;Acc:HGNC:186]	protein coding	0.70	4.24	25.57	<0.0001	0.0004
ENSG00000240505	TNFRSF13B	TNF receptor superfamily member 13B [Source:HGNC Symbol;Acc:HGNC:18153]	protein coding	-0.68	1.46	15.29	<0.0001	0.0231
ENSG00000103522	IL21R	interleukin 21 receptor [Source:HGNC Symbol;Acc:HGNC:6006]	protein coding	0.66	2.13	16.71	<0.0001	0.0144
ENSG00000166278	C2	complement C2 [Source:HGNC Symbol;Acc:HGNC:1248]	protein coding	0.65	2.88	19.58	<0.0001	0.0045
ENSG00000162745	OLFML2B	olfactomedin like 2B [Source:HGNC Symbol;Acc:HGNC:24558]	protein coding	0.65	2.07	26.84	<0.0001	0.0002
ENSG00000180155	LYNX1	Ly6/neurotoxin 1 [Source:HGNC Symbol;Acc:HGNC:29604]	protein coding	0.62	3.52	22.58	<0.0001	0.0012
ENSG00000262097	LINC02185	long intergenic non-protein coding RNA 2185 [Source:HGNC Symbol;Acc:HGNC:53047]	lncRNA	0.62	0.71	13.03	0.0003	0.0499
ENSG00000135424	ITGA7	integrin subunit alpha 7 [Source:HGNC Symbol;Acc:HGNC:6143]	protein coding	0.61	1.83	20.82	<0.0001	0.0026
ENSG00000159496	RGL4	ral guanine nucleotide dissociation stimulator like 4 [Source:HGNC Symbol;Acc:HGNC:31911]	protein coding	0.59	0.46	14.57	0.0001	0.0289
ENSG00000144645	OSBPL10	oxysterol binding protein like 10 [Source:HGNC Symbol;Acc:HGNC:16395]	protein coding	-0.59	2.10	13.32	0.0003	0.0448

Table S7: Genes differentially expressed at Day 36 compared to pre-second vaccination (Study Arm 2 A/H7N9+AS03, D1, 29, Monocytes). Genes are sorted by descending absolute log_2 fold change. Gene model summaries and annotations are based on Ensembl Version 99.

Ensembl Gene ID	Ensembl Gene Name	Ensembl Gene Description	Gene Type	log_2 Fold Change (Day 36 vs. Pre-Second Vaccination)	Average log_2 CPM	Likelihood Ratio Test Statistic	P-Value	FDR Adjusted P-Value
ENSG00000211896	IGHG1	immunoglobulin heavy constant gamma 1 (G1m marker) [Source:HGNC Symbol;Acc:HGNC:5525]	IG C gene	1.56	6.24	88.98	<0.0001	<0.0001
ENSG00000211673	IGLV3-1	immunoglobulin lambda variable 3-1 [Source:HGNC Symbol;Acc:HGNC:5896]	IG V gene	1.35	1.89	43.17	<0.0001	<0.0001
ENSG00000211897	IGHG3	immunoglobulin heavy constant gamma 3 (G3m marker) [Source:HGNC Symbol;Acc:HGNC:5527]	IG C gene	1.06	4.18	20.89	<0.0001	0.0209

Table S7: Genes differentially expressed at Day 36 compared to pre-second vaccination (Study Arm 2 A/H7N9+AS03, D1, 29, Monocytes). Genes are sorted by descending absolute log_2 fold change. Gene model summaries and annotations are based on Ensembl Version 99 (continued.)

Ensembl Gene ID	Ensembl Gene Name	Ensembl Gene Description	Gene Type	log_2 Fold Change (Day 36 vs. Pre-Second Vaccination)	Average log_2 CPM	Likelihood Ratio Test Statistic	P-Value	FDR Adjusted P-Value
ENSG00000004799	PDK4	pyruvate dehydrogenase kinase 4 [Source:HGNC Symbol;Acc:HGNC:8812]	protein coding	-0.72	6.34	19.63	<0.0001	0.0279

Table S8: Genes differentially expressed at Day 8 compared to pre-first vaccination (Study Arm 1 A/H3N2v, D1 , Dendritic cells). Genes are sorted by descending absolute log_2 fold change. Gene model summaries and annotations are based on Ensembl Version 99.

Ensembl Gene ID	Ensembl Gene Name	Ensembl Gene Description	Gene Type	log_2 Fold Change (Day 8 vs. Pre-First Vaccination)	Average log_2 CPM	Likelihood Ratio Test Statistic	P-Value	FDR Adjusted P-Value
ENSG00000206172	HBA1	hemoglobin subunit alpha 1 [Source:HGNC Symbol;Acc:HGNC:4823]	protein coding	4.25	5.39	23.19	<0.0001	0.0202
ENSG00000188536	HBA2	hemoglobin subunit alpha 2 [Source:HGNC Symbol;Acc:HGNC:4824]	protein coding	2.84	5.67	20.16	<0.0001	0.049

Table S9: Genes differentially expressed at Day 2 compared to pre-first vaccination (Study Arm 2 A/H7N9+AS03, D1, 29, Dendritic cells). Genes are sorted by descending absolute log_2 fold change. Gene model summaries and annotations are based on Ensembl Version 99.

Ensembl Gene ID	Ensembl Gene Name	Ensembl Gene Description	Gene Type	log_2 Fold Change (Day 2 vs. Pre-First Vaccination)	Average log_2 CPM	Likelihood Ratio Test Statistic	P-Value	FDR Adjusted P-Value
ENSG00000152766	ANKRD22	ankyrin repeat domain 22 [Source:HGNC Symbol;Acc:HGNC:28321]	protein coding	3.98	4.16	82.30	<0.0001	<0.0001
ENSG00000225492	GBP1P1	guanylate binding protein 1 pseudogene 1 [Source:HGNC Symbol;Acc:HGNC:39561]	transcribed unprocessed pseudogene	3.83	2.16	74.94	<0.0001	<0.0001
ENSG00000010030	ETV7	ETS variant transcription factor 7 [Source:HGNC Symbol;Acc:HGNC:18160]	protein coding	3.22	3.32	66.93	<0.0001	<0.0001
ENSG00000100336	APOL4	apolipoprotein L4 [Source:HGNC Symbol;Acc:HGNC:14867]	protein coding	3.10	2.62	81.70	<0.0001	<0.0001
ENSG00000149131	SERPING1	serpin family G member 1 [Source:HGNC Symbol;Acc:HGNC:1228]	protein coding	2.77	4.85	57.43	<0.0001	<0.0001

Table S9: Genes differentially expressed at Day 2 compared to pre-first vaccination (Study Arm 2 A/H7N9+AS03, D1, 29, Dendritic cells). Genes are sorted by descending absolute log_2 fold change. Gene model summaries and annotations are based on Ensembl Version 99 (continued.)

Ensembl Gene ID	Ensembl Gene Name	Ensembl Gene Description	Gene Type	log_2 Fold Change (Day 2 vs. Pre-First Vaccination)	Average log_2 CPM	Likelihood Ratio Test Statistic	P-Value	FDR Adjusted P-Value
ENSG00000265531	FCGR1CP	Fc fragment of IgG receptor Ic, pseudogene [Source:HGNC Symbol;Acc:HGNC:3615]	unprocessed pseudogene	2.76	2.04	76.96	<0.0001	<0.0001
ENSG00000183762	KREMEN1	kringle containing transmembrane protein 1 [Source:HGNC Symbol;Acc:HGNC:17550]	protein coding	2.65	2.61	84.29	<0.0001	<0.0001
ENSG00000154451	GBP5	guanylate binding protein 5 [Source:HGNC Symbol;Acc:HGNC:19895]	protein coding	2.61	7.88	79.74	<0.0001	<0.0001
ENSG00000169245	CXCL10	C-X-C motif chemokine ligand 10 [Source:HGNC Symbol;Acc:HGNC:10637]	protein coding	2.58	4.16	47.89	<0.0001	<0.0001
ENSG00000117228	GBP1	guanylate binding protein 1 [Source:HGNC Symbol;Acc:HGNC:4182]	protein coding	2.53	7.41	85.01	<0.0001	<0.0001
ENSG00000120217	CD274	CD274 molecule [Source:HGNC Symbol;Acc:HGNC:17635]	protein coding	2.45	2.01	60.69	<0.0001	<0.0001
ENSG00000198019	FCGR1B	Fc fragment of IgG receptor Ib [Source:HGNC Symbol;Acc:HGNC:3614]	protein coding	2.34	3.66	80.03	<0.0001	<0.0001
ENSG00000131203	IDO1	indoleamine 2,3-dioxygenase 1 [Source:HGNC Symbol;Acc:HGNC:6059]	protein coding	2.33	2.14	40.72	<0.0001	<0.0001
ENSG00000150337	FCGR1A	Fc fragment of IgG receptor Ia [Source:HGNC Symbol;Acc:HGNC:3613]	protein coding	2.30	5.02	73.81	<0.0001	<0.0001
ENSG00000168062	BATF2	basic leucine zipper ATF-like transcription factor 2 [Source:HGNC Symbol;Acc:HGNC:25163]	protein coding	2.24	3.32	64.15	<0.0001	<0.0001
ENSG00000164509	IL31RA	interleukin 31 receptor A [Source:HGNC Symbol;Acc:HGNC:18969]	protein coding	2.01	1.34	55.58	<0.0001	<0.0001
ENSG00000198829	SUCNR1	succinate receptor 1 [Source:HGNC Symbol;Acc:HGNC:4542]	protein coding	1.96	0.67	101.34	<0.0001	<0.0001
ENSG00000162654	GBP4	guanylate binding protein 4 [Source:HGNC Symbol;Acc:HGNC:20480]	protein coding	1.91	7.16	73.51	<0.0001	<0.0001
ENSG00000108387	SEPTIN4	septin 4 [Source:HGNC Symbol;Acc:HGNC:9165]	protein coding	1.83	1.77	46.21	<0.0001	<0.0001
ENSG00000080573	COL5A3	collagen type V alpha 3 chain [Source:HGNC Symbol;Acc:HGNC:14864]	protein coding	-1.82	2.41	19.92	<0.0001	0.0001
ENSG00000064300	NGFR	nerve growth factor receptor [Source:HGNC Symbol;Acc:HGNC:7809]	protein coding	1.81	0.67	47.50	<0.0001	<0.0001
ENSG00000258521		novel transcript, antisense to WARS	lncRNA	1.81	1.07	57.09	<0.0001	<0.0001
ENSG00000002549	LAP3	leucine aminopeptidase 3 [Source:HGNC Symbol;Acc:HGNC:18449]	protein coding	1.79	7.84	61.40	<0.0001	<0.0001

Table S9: Genes differentially expressed at Day 2 compared to pre-first vaccination (Study Arm 2 A/H7N9+AS03, D1, 29, Dendritic cells). Genes are sorted by descending absolute log_2 fold change. Gene model summaries and annotations are based on Ensembl Version 99 (continued.)

Ensembl Gene ID	Ensembl Gene Name	Ensembl Gene Description	Gene Type	log_2 Fold Change (Day 2 vs. Pre-First Vaccination)	Average log_2 CPM	Likelihood Ratio Test Statistic	P-Value	FDR Adjusted P-Value
ENSG00000162645	GBP2	guanylate binding protein 2 [Source:HGNC Symbol;Acc:HGNC:4183]	protein coding	1.77	8.15	80.86	<0.0001	<0.0001
ENSG00000119917	IFIT3	interferon induced protein with tetratricopeptide repeats 3 [Source:HGNC Symbol;Acc:HGNC:5411]	protein coding	1.73	6.40	35.25	<0.0001	<0.0001
ENSG00000100346	CACNA1I	calcium voltage-gated channel subunit alpha1 I [Source:HGNC Symbol;Acc:HGNC:1396]	protein coding	-1.69	3.38	19.77	<0.0001	0.0001
ENSG00000198959	TGM2	transglutaminase 2 [Source:HGNC Symbol;Acc:HGNC:11778]	protein coding	1.68	3.17	61.47	<0.0001	<0.0001
ENSG00000112394	SLC16A10	solute carrier family 16 member 10 [Source:HGNC Symbol;Acc:HGNC:17027]	protein coding	-1.67	0.79	20.39	<0.0001	0.0001
ENSG00000119922	IFIT2	interferon induced protein with tetratricopeptide repeats 2 [Source:HGNC Symbol;Acc:HGNC:5409]	protein coding	1.67	6.09	36.99	<0.0001	<0.0001
ENSG00000168899	VAMP5	vesicle associated membrane protein 5 [Source:HGNC Symbol;Acc:HGNC:12646]	protein coding	1.67	6.03	77.28	<0.0001	<0.0001
ENSG00000254017	IGHEP2	immunoglobulin heavy constant epsilon P2 (pseudogene) [Source:HGNC Symbol;Acc:HGNC:5524]	IG C pseudogene	1.65	0.65	40.16	<0.0001	<0.0001
ENSG00000197272	IL27	interleukin 27 [Source:HGNC Symbol;Acc:HGNC:19157]	protein coding	1.63	1.95	54.93	<0.0001	<0.0001
ENSG00000188820	CALHM6	calcium homeostasis modulator family member 6 [Source:HGNC Symbol;Acc:HGNC:33391]	protein coding	1.60	5.84	56.27	<0.0001	<0.0001
ENSG00000236567	TCF3P1	transcription factor 3 pseudogene 1 [Source:HGNC Symbol;Acc:HGNC:49742]	processed pseudogene	1.59	1.19	37.25	<0.0001	<0.0001
ENSG00000162512	SDC3	syndecan 3 [Source:HGNC Symbol;Acc:HGNC:10660]	protein coding	1.59	4.65	55.87	<0.0001	<0.0001
ENSG00000115415	STAT1	signal transducer and activator of transcription 1 [Source:HGNC Symbol;Acc:HGNC:11362]	protein coding	1.59	8.67	60.18	<0.0001	<0.0001
ENSG00000140105	WARS1	tryptophanyl-tRNA synthetase 1 [Source:HGNC Symbol;Acc:HGNC:12729]	protein coding	1.59	9.55	69.69	<0.0001	<0.0001
ENSG00000171680	PLEKHG5	pleckstrin homology and RhoGEF domain containing G5 [Source:HGNC Symbol;Acc:HGNC:29105]	protein coding	-1.55	1.30	21.72	<0.0001	<0.0001
ENSG00000120885	CLU	clusterin [Source:HGNC Symbol;Acc:HGNC:2095]	protein coding	1.55	2.84	49.73	<0.0001	<0.0001
ENSG00000184557	SOCS3	suppressor of cytokine signaling 3 [Source:HGNC Symbol;Acc:HGNC:19391]	protein coding	1.54	5.80	35.63	<0.0001	<0.0001

Table S9: Genes differentially expressed at Day 2 compared to pre-first vaccination (Study Arm 2 A/H7N9+AS03, D1, 29, Dendritic cells). Genes are sorted by descending absolute log_2 fold change. Gene model summaries and annotations are based on Ensembl Version 99 (continued.)

Ensembl Gene ID	Ensembl Gene Name	Ensembl Gene Description	Gene Type	log_2 Fold Change (Day 2 vs. Pre-First Vaccination)	Average log_2 CPM	Likelihood Ratio Test Statistic	P-Value	FDR Adjusted P-Value
ENSG00000037280	FLT4	fms related tyrosine kinase 4 [Source:HGNC Symbol;Acc:HGNC:3767]	protein coding	-1.53	2.68	20.84	<0.0001	<0.0001
ENSG00000162614	NEXN	nexilin F-actin binding protein [Source:HGNC Symbol;Acc:HGNC:29557]	protein coding	1.52	3.07	41.64	<0.0001	<0.0001
ENSG00000164398	ACSL6	acyl-CoA synthetase long chain family member 6 [Source:HGNC Symbol;Acc:HGNC:16496]	protein coding	-1.51	1.42	16.37	<0.0001	0.0005
ENSG00000286010		novel transcript	lncRNA	-1.50	2.48	26.56	<0.0001	<0.0001
ENSG00000282780	TRBJ1-6	T cell receptor beta joining 1-6 [Source:HGNC Symbol;Acc:HGNC:12167]	TR J gene	-1.50	0.86	17.51	<0.0001	0.0003
ENSG00000158714	SLAMF8	SLAM family member 8 [Source:HGNC Symbol;Acc:HGNC:21391]	protein coding	1.50	2.97	70.79	<0.0001	<0.0001
ENSG00000133106	EPSTI1	epithelial stromal interaction 1 [Source:HGNC Symbol;Acc:HGNC:16465]	protein coding	1.48	6.41	39.31	<0.0001	<0.0001
ENSG00000168685	IL7R	interleukin 7 receptor [Source:HGNC Symbol;Acc:HGNC:6024]	protein coding	-1.48	7.63	14.74	0.0001	0.001
ENSG00000125084	WNT1	Wnt family member 1 [Source:HGNC Symbol;Acc:HGNC:12774]	protein coding	-1.48	0.53	25.78	<0.0001	<0.0001
ENSG00000175643	RMI2	RecQ mediated genome instability 2 [Source:HGNC Symbol;Acc:HGNC:28349]	protein coding	1.46	0.45	27.80	<0.0001	<0.0001
ENSG00000162772	ATF3	activating transcription factor 3 [Source:HGNC Symbol;Acc:HGNC:785]	protein coding	1.46	3.63	45.41	<0.0001	<0.0001
ENSG00000223534	HLA-DQB1-AS1	HLA-DQB1 antisense RNA 1 [Source:HGNC Symbol;Acc:HGNC:39762]	lncRNA	1.45	2.64	14.31	0.0002	0.0012
ENSG00000137959	IFI44L	interferon induced protein 44 like [Source:HGNC Symbol;Acc:HGNC:17817]	protein coding	1.44	6.42	19.81	<0.0001	0.0001
ENSG00000273314		novel transcript, antisense to CUL1	lncRNA	1.43	1.02	69.69	<0.0001	<0.0001
ENSG00000143365	RORC	RAR related orphan receptor C [Source:HGNC Symbol;Acc:HGNC:10260]	protein coding	-1.43	2.33	15.67	<0.0001	0.0007
ENSG00000135960	EDAR	ectodysplasin A receptor [Source:HGNC Symbol;Acc:HGNC:2895]	protein coding	-1.43	0.93	15.39	<0.0001	0.0008
ENSG00000169884	WNT10B	Wnt family member 10B [Source:HGNC Symbol;Acc:HGNC:12775]	protein coding	-1.42	1.94	24.77	<0.0001	<0.0001
ENSG00000221957	KIR2DS4	killer cell immunoglobulin like receptor, two Ig domains and short cytoplasmic tail 4 [Source:HGNC Symbol;Acc:HGNC:6336]	polymorphic pseudogene	-1.42	1.52	36.55	<0.0001	<0.0001

Table S9: Genes differentially expressed at Day 2 compared to pre-first vaccination (Study Arm 2 A/H7N9+AS03, D1, 29, Dendritic cells). Genes are sorted by descending absolute log_2 fold change. Gene model summaries and annotations are based on Ensembl Version 99 (continued.)

Ensembl Gene ID	Ensembl Gene Name	Ensembl Gene Description	Gene Type	log_2 Fold Change (Day 2 vs. Pre-First Vaccination)	Average log_2 CPM	Likelihood Ratio Test Statistic	P-Value	FDR Adjusted P-Value
ENSG00000165929	TC2N	tandem C2 domains, nuclear [Source:HGNC Symbol;Acc:HGNC:19859]	protein coding	-1.41	5.03	18.55	<0.0001	0.0002
ENSG00000179044	EXOC3L1	exocyst complex component 3 like 1 [Source:HGNC Symbol;Acc:HGNC:27540]	protein coding	1.41	2.50	37.95	<0.0001	<0.0001
ENSG00000166278	C2	complement C2 [Source:HGNC Symbol;Acc:HGNC:1248]	protein coding	1.41	2.86	47.42	<0.0001	<0.0001
ENSG00000156265	MAP3K7CL	MAP3K7 C-terminal like [Source:HGNC Symbol;Acc:HGNC:16457]	protein coding	1.41	4.40	53.82	<0.0001	<0.0001
ENSG00000258581		NADH dehydrogenase (ubiquinone) 1 beta subcomplex, 3, 12kDa (NDUFB3) pseudogene	processed pseudogene	1.39	0.79	40.04	<0.0001	<0.0001
ENSG00000184613	NELL2	neural EGFL like 2 [Source:HGNC Symbol;Acc:HGNC:7751]	protein coding	-1.39	4.31	14.63	0.0001	0.001
ENSG00000172159	FRMD3	FERM domain containing 3 [Source:HGNC Symbol;Acc:HGNC:24125]	protein coding	1.38	3.22	78.44	<0.0001	<0.0001
ENSG00000170486	KRT72	keratin 72 [Source:HGNC Symbol;Acc:HGNC:28932]	protein coding	-1.38	2.91	18.26	<0.0001	0.0002
ENSG00000236213		novel transcript	lncRNA	-1.38	0.53	19.82	<0.0001	0.0001
ENSG00000232021	LEF1-AS1	LEF1 antisense RNA 1 [Source:HGNC Symbol;Acc:HGNC:40339]	lncRNA	-1.37	0.67	11.51	0.0007	0.0037
ENSG00000228314	CYP4F29P	cytochrome P450 family 4 subfamily F member 29, pseudogene [Source:HGNC Symbol;Acc:HGNC:2647]	transcribed unprocessed pseudogene	-1.37	2.49	26.59	<0.0001	<0.0001
ENSG00000173114	LRRN3	leucine rich repeat neuronal 3 [Source:HGNC Symbol;Acc:HGNC:17200]	protein coding	-1.36	2.47	13.10	0.0003	0.0019
ENSG00000245164	LINC00861	long intergenic non-protein coding RNA 861 [Source:HGNC Symbol;Acc:HGNC:45133]	lncRNA	-1.36	6.15	16.70	<0.0001	0.0005
ENSG00000138795	LEF1	lymphoid enhancer binding factor 1 [Source:HGNC Symbol;Acc:HGNC:6551]	protein coding	-1.36	5.23	13.54	0.0002	0.0016
ENSG00000170074	FAM153A	family with sequence similarity 153 member A [Source:HGNC Symbol;Acc:HGNC:29940]	protein coding	-1.36	2.37	14.95	0.0001	0.0009
ENSG00000126262	FFAR2	free fatty acid receptor 2 [Source:HGNC Symbol;Acc:HGNC:4501]	protein coding	1.36	3.59	37.90	<0.0001	<0.0001

Table S9: Genes differentially expressed at Day 2 compared to pre-first vaccination (Study Arm 2 A/H7N9+AS03, D1, 29, Dendritic cells). Genes are sorted by descending absolute log_2 fold change. Gene model summaries and annotations are based on Ensembl Version 99 (continued.)

Ensembl Gene ID	Ensembl Gene Name	Ensembl Gene Description	Gene Type	log_2 Fold Change (Day 2 vs. Pre-First Vaccination)	Average log_2 CPM	Likelihood Ratio Test Statistic	P-Value	FDR Adjusted P-Value
ENSG00000067141	NEO1	neogenin 1 [Source:HGNC Symbol;Acc:HGNC:7754]	protein coding	-1.35	1.99	26.87	<0.0001	<0.0001
ENSG00000269086		novel transcript	lncRNA	-1.35	1.06	15.43	<0.0001	0.0007
ENSG00000260711		novel transcript, intronic to CATSPERB	lncRNA	-1.35	1.40	15.55	<0.0001	0.0007
ENSG00000280135		TEC	TEC	-1.34	2.24	18.64	<0.0001	0.0002
ENSG00000121858	TNFSF10	TNF superfamily member 10 [Source:HGNC Symbol;Acc:HGNC:11925]	protein coding	1.33	7.86	48.81	<0.0001	<0.0001
ENSG00000116106	EPHA4	EPH receptor A4 [Source:HGNC Symbol;Acc:HGNC:3388]	protein coding	-1.33	2.16	19.37	<0.0001	0.0002
ENSG00000281181		novel transcript, similar to YY1 associated myogenesis RNA 1 YAM1	lncRNA	1.33	4.43	8.58	0.0034	0.0127
ENSG00000069667	RORA	RAR related orphan receptor A [Source:HGNC Symbol;Acc:HGNC:10258]	protein coding	-1.33	5.24	22.22	<0.0001	<0.0001
ENSG00000162545	CAMK2N1	calcium/calmodulin dependent protein kinase II inhibitor 1 [Source:HGNC Symbol;Acc:HGNC:24190]	protein coding	-1.33	0.59	17.00	<0.0001	0.0004
ENSG00000256039	LINC02446	long intergenic non-protein coding RNA 2446 [Source:HGNC Symbol;Acc:HGNC:53378]	lncRNA	-1.33	2.44	13.15	0.0003	0.0019
ENSG00000113319	RASGRF2	Ras protein specific guanine nucleotide releasing factor 2 [Source:HGNC Symbol;Acc:HGNC:9876]	protein coding	-1.32	3.04	17.36	<0.0001	0.0004
ENSG00000124507	PACSIN1	protein kinase C and casein kinase substrate in neurons 1 [Source:HGNC Symbol;Acc:HGNC:8570]	protein coding	-1.32	0.96	24.82	<0.0001	<0.0001
ENSG00000108950	FAM20A	FAM20A golgi associated secretory pathway pseudokinase [Source:HGNC Symbol;Acc:HGNC:23015]	protein coding	1.32	3.25	56.36	<0.0001	<0.0001
ENSG00000034053	APBA2	amyloid beta precursor protein binding family A member 2 [Source:HGNC Symbol;Acc:HGNC:579]	protein coding	-1.32	4.64	19.18	<0.0001	0.0002
ENSG00000187017	ESPN	espin [Source:HGNC Symbol;Acc:HGNC:13281]	protein coding	-1.31	0.89	13.75	0.0002	0.0014
ENSG00000135636	DYSF	dysferlin [Source:HGNC Symbol;Acc:HGNC:3097]	protein coding	1.31	7.04	52.27	<0.0001	<0.0001
ENSG00000065717	TLE2	TLE family member 2, transcriptional corepressor [Source:HGNC Symbol;Acc:HGNC:11838]	protein coding	-1.31	2.17	16.82	<0.0001	0.0004
ENSG00000113088	GZMK	granzyme K [Source:HGNC Symbol;Acc:HGNC:4711]	protein coding	-1.31	2.70	18.52	<0.0001	0.0002
ENSG00000152495	CAMK4	calcium/calmodulin dependent protein kinase IV [Source:HGNC Symbol;Acc:HGNC:1464]	protein coding	-1.31	4.36	13.70	0.0002	0.0015

Table S9: Genes differentially expressed at Day 2 compared to pre-first vaccination (Study Arm 2 A/H7N9+AS03, D1, 29, Dendritic cells). Genes are sorted by descending absolute log_2 fold change. Gene model summaries and annotations are based on Ensembl Version 99 (continued.)

Ensembl Gene ID	Ensembl Gene Name	Ensembl Gene Description	Gene Type	log_2 Fold Change (Day 2 vs. Pre-First Vaccination)	Average log_2 CPM	Likelihood Ratio Test Statistic	P-Value	FDR Adjusted P-Value
ENSG00000142173	COL6A2	collagen type VI alpha 2 chain [Source:HGNC Symbol;Acc:HGNC:2212]	protein coding	-1.31	5.00	20.10	<0.0001	0.0001
ENSG00000214872	SMTNL1	smoothelin like 1 [Source:HGNC Symbol;Acc:HGNC:32394]	protein coding	1.31	1.71	32.41	<0.0001	<0.0001
ENSG00000100342	APOL1	apolipoprotein L1 [Source:HGNC Symbol;Acc:HGNC:618]	protein coding	1.31	5.60	60.94	<0.0001	<0.0001
ENSG00000211770	TRBJ2-6	T cell receptor beta joining 2-6 [Source:HGNC Symbol;Acc:HGNC:12174]	TR J gene	-1.30	0.48	18.73	<0.0001	0.0002
ENSG00000146285	SCML4	Scm polycomb group protein like 4 [Source:HGNC Symbol;Acc:HGNC:21397]	protein coding	-1.30	4.28	20.28	<0.0001	0.0001
ENSG00000120915	EPHX2	epoxide hydrolase 2 [Source:HGNC Symbol;Acc:HGNC:3402]	protein coding	-1.30	2.78	15.36	<0.0001	0.0008
ENSG00000007516	BAIAP3	BAI1 associated protein 3 [Source:HGNC Symbol;Acc:HGNC:948]	protein coding	-1.30	3.02	37.48	<0.0001	<0.0001
ENSG00000213809	KLRK1	killer cell lectin like receptor K1 [Source:HGNC Symbol;Acc:HGNC:18788]	protein coding	-1.30	2.44	15.36	<0.0001	0.0008
ENSG00000107742	SPOCK2	SPARC (osteonectin), cwcv and kazal like domains proteoglycan 2 [Source:HGNC Symbol;Acc:HGNC:13564]	protein coding	-1.30	7.47	18.88	<0.0001	0.0002
ENSG00000256069	A2MP1	alpha-2-macroglobulin pseudogene 1 [Source:HGNC Symbol;Acc:HGNC:8]	transcribed unprocessed pseudogene	-1.29	1.14	16.48	<0.0001	0.0005
ENSG00000150687	PRSS23	serine protease 23 [Source:HGNC Symbol;Acc:HGNC:14370]	protein coding	-1.29	3.82	20.24	<0.0001	0.0001
ENSG00000204789	ZNF204P	zinc finger protein 204, pseudogene [Source:HGNC Symbol;Acc:HGNC:12995]	processed pseudogene	-1.29	0.56	16.03	<0.0001	0.0006
ENSG00000225783	MIAT	myocardial infarction associated transcript [Source:HGNC Symbol;Acc:HGNC:33425]	lncRNA	-1.29	5.51	17.30	<0.0001	0.0004
ENSG00000225079	FTH1P22	ferritin heavy chain 1 pseudogene 22 [Source:HGNC Symbol;Acc:HGNC:37640]	processed pseudogene	-1.28	0.57	21.75	<0.0001	<0.0001
ENSG00000163568	AIM2	absent in melanoma 2 [Source:HGNC Symbol;Acc:HGNC:357]	protein coding	1.28	3.66	43.23	<0.0001	<0.0001

Table S9: Genes differentially expressed at Day 2 compared to pre-first vaccination (Study Arm 2 A/H7N9+AS03, D1, 29, Dendritic cells). Genes are sorted by descending absolute log_2 fold change. Gene model summaries and annotations are based on Ensembl Version 99 (continued.)

Ensembl Gene ID	Ensembl Gene Name	Ensembl Gene Description	Gene Type	log_2 Fold Change (Day 2 vs. Pre-First Vaccination)	Average log_2 CPM	Likelihood Ratio Test Statistic	P-Value	FDR Adjusted P-Value
ENSG00000138496	PARP9	poly(ADP-ribose) polymerase family member 9 [Source:HGNC Symbol;Acc:HGNC:24118]	protein coding	1.28	7.19	49.17	<0.0001	<0.0001
ENSG00000122877	EGR2	early growth response 2 [Source:HGNC Symbol;Acc:HGNC:3239]	protein coding	1.27	4.83	9.88	0.0017	0.0073
ENSG00000262655	SPON1	spondin 1 [Source:HGNC Symbol;Acc:HGNC:11252]	protein coding	-1.27	0.53	17.53	<0.0001	0.0003
ENSG00000282173	TRBJ1-5	T cell receptor beta joining 1-5 [Source:HGNC Symbol;Acc:HGNC:12166]	TR J gene	-1.27	0.84	13.69	0.0002	0.0015
ENSG00000272498		novel transcript, antisense to RFTN1	lncRNA	-1.27	0.43	21.36	<0.0001	<0.0001
ENSG00000174171		novel transcript, antisense to SPTBN5	lncRNA	-1.27	2.92	18.90	<0.0001	0.0002
ENSG00000142089	IFITM3	interferon induced transmembrane protein 3 [Source:HGNC Symbol;Acc:HGNC:5414]	protein coding	1.27	7.88	42.35	<0.0001	<0.0001
ENSG00000211753	TRBV28	T cell receptor beta variable 28 [Source:HGNC Symbol;Acc:HGNC:12209]	TR V gene	-1.27	1.21	13.25	0.0003	0.0018
ENSG00000013725	CD6	CD6 molecule [Source:HGNC Symbol;Acc:HGNC:1691]	protein coding	-1.26	6.23	16.94	<0.0001	0.0004
ENSG00000234389		novel transcript	lncRNA	-1.26	1.78	24.41	<0.0001	<0.0001
ENSG00000284194	SCO2	SCO cytochrome c oxidase assembly protein 2 [Source:HGNC Symbol;Acc:HGNC:10604]	protein coding	1.26	5.34	40.38	<0.0001	<0.0001
ENSG00000211746	TRBV19	T cell receptor beta variable 19 [Source:HGNC Symbol;Acc:HGNC:12194]	TR V gene	-1.26	1.07	10.62	0.0011	0.0053
ENSG00000186994	KANK3	KN motif and ankyrin repeat domains 3 [Source:HGNC Symbol;Acc:HGNC:24796]	protein coding	-1.26	1.24	23.15	<0.0001	<0.0001
ENSG00000260807	CEROX1	cytoplasmic endogenous regulator of oxidative phosphorylation 1 [Source:HGNC Symbol;Acc:HGNC:53928]	lncRNA	-1.26	2.85	11.28	0.0008	0.004
ENSG00000176083	ZNF683	zinc finger protein 683 [Source:HGNC Symbol;Acc:HGNC:28495]	protein coding	-1.26	3.27	12.01	0.0005	0.003
ENSG00000245648		novel transcript, antisense to KLRK1	lncRNA	-1.26	3.51	21.05	<0.0001	<0.0001
ENSG00000183813	CCR4	C-C motif chemokine receptor 4 [Source:HGNC Symbol;Acc:HGNC:1605]	protein coding	-1.25	1.68	8.62	0.0033	0.0125
ENSG00000006555	TTC22	tetratricopeptide repeat domain 22 [Source:HGNC Symbol;Acc:HGNC:26067]	protein coding	-1.25	2.94	22.94	<0.0001	<0.0001
ENSG00000081059	TCF7	transcription factor 7 [Source:HGNC Symbol;Acc:HGNC:11639]	protein coding	-1.25	6.92	13.30	0.0003	0.0017
ENSG00000172116	CD8B	CD8b molecule [Source:HGNC Symbol;Acc:HGNC:1707]	protein coding	-1.25	4.38	15.09	0.0001	0.0008

Table S9: Genes differentially expressed at Day 2 compared to pre-first vaccination (Study Arm 2 A/H7N9+AS03, D1, 29, Dendritic cells). Genes are sorted by descending absolute log_2 fold change. Gene model summaries and annotations are based on Ensembl Version 99 (continued.)

Ensembl Gene ID	Ensembl Gene Name	Ensembl Gene Description	Gene Type	log_2 Fold Change (Day 2 vs. Pre-First Vaccination)	Average log_2 CPM	Likelihood Ratio Test Statistic	P-Value	FDR Adjusted P-Value
ENSG00000173258	ZNF483	zinc finger protein 483 [Source:HGNC Symbol;Acc:HGNC:23384]	protein coding	-1.25	0.98	16.48	<0.0001	0.0005
ENSG00000081377	CDC14B	cell division cycle 14B [Source:HGNC Symbol;Acc:HGNC:1719]	protein coding	-1.25	1.54	22.43	<0.0001	<0.0001
ENSG00000281106	TMEM272	transmembrane protein 272 [Source:HGNC Symbol;Acc:HGNC:26737]	protein coding	-1.25	1.08	11.22	0.0008	0.0041
ENSG00000255819	KLRC4-KLRK1	KLRC4-KLRK1 readthrough [Source:HGNC Symbol;Acc:HGNC:48357]	protein coding	-1.25	1.30	18.88	<0.0001	0.0002
ENSG00000144290	SLC4A10	solute carrier family 4 member 10 [Source:HGNC Symbol;Acc:HGNC:13811]	protein coding	-1.25	3.06	15.86	<0.0001	0.0006
ENSG00000131634	TMEM204	transmembrane protein 204 [Source:HGNC Symbol;Acc:HGNC:14158]	protein coding	-1.25	3.91	18.01	<0.0001	0.0003
ENSG00000170989	S1PR1	sphingosine-1-phosphate receptor 1 [Source:HGNC Symbol;Acc:HGNC:3165]	protein coding	-1.24	5.36	15.69	<0.0001	0.0007
ENSG00000148600	CDHR1	cadherin related family member 1 [Source:HGNC Symbol;Acc:HGNC:14550]	protein coding	-1.24	1.11	18.29	<0.0001	0.0002
ENSG00000104490	NCALD	neurocalcin delta [Source:HGNC Symbol;Acc:HGNC:7655]	protein coding	-1.24	2.99	22.38	<0.0001	<0.0001
ENSG00000275395	FCGBP	Fc fragment of IgG binding protein [Source:HGNC Symbol;Acc:HGNC:13572]	protein coding	-1.24	3.98	35.04	<0.0001	<0.0001
ENSG00000029153	ARNTL2	aryl hydrocarbon receptor nuclear translocator like 2 [Source:HGNC Symbol;Acc:HGNC:18984]	protein coding	1.24	1.40	50.29	<0.0001	<0.0001
ENSG00000160654	CD3G	CD3g molecule [Source:HGNC Symbol;Acc:HGNC:1675]	protein coding	-1.24	4.78	12.27	0.0005	0.0027
ENSG00000235532	LINC00402	long intergenic non-protein coding RNA 402 [Source:HGNC Symbol;Acc:HGNC:42732]	lncRNA	-1.24	1.82	11.30	0.0008	0.004
ENSG00000271109		novel transcript	lncRNA	-1.24	1.49	16.24	<0.0001	0.0005
ENSG00000253755	IGHGP	immunoglobulin heavy constant gamma P (non-functional) [Source:HGNC Symbol;Acc:HGNC:5529]	IG C pseudogene	-1.23	0.86	7.90	0.0049	0.0171
ENSG00000178562	CD28	CD28 molecule [Source:HGNC Symbol;Acc:HGNC:1653]	protein coding	-1.23	3.97	10.52	0.0012	0.0055
ENSG00000111181	SLC6A12	solute carrier family 6 member 12 [Source:HGNC Symbol;Acc:HGNC:11045]	protein coding	1.23	3.84	56.25	<0.0001	<0.0001
ENSG00000229852		novel transcript, antisense to KHDC1	lncRNA	-1.23	0.43	18.44	<0.0001	0.0002
ENSG00000170962	PDGFD	platelet derived growth factor D [Source:HGNC Symbol;Acc:HGNC:30620]	protein coding	-1.23	2.19	19.50	<0.0001	0.0002

Table S9: Genes differentially expressed at Day 2 compared to pre-first vaccination (Study Arm 2 A/H7N9+AS03, D1, 29, Dendritic cells). Genes are sorted by descending absolute log_2 fold change. Gene model summaries and annotations are based on Ensembl Version 99 (continued.)

Ensembl Gene ID	Ensembl Gene Name	Ensembl Gene Description	Gene Type	log_2 Fold Change (Day 2 vs. Pre-First Vaccination)	Average log_2 CPM	Likelihood Ratio Test Statistic	P-Value	FDR Adjusted P-Value
ENSG00000126838	PZP	PZP alpha-2-macroglobulin like [Source:HGNC Symbol;Acc:HGNC:9750]	protein coding	-1.23	0.75	16.87	<0.0001	0.0004
ENSG00000127152	BCL11B	BAF chromatin remodeling complex subunit BCL11B [Source:HGNC Symbol;Acc:HGNC:13222]	protein coding	-1.22	5.41	15.19	<0.0001	0.0008
ENSG00000141469	SLC14A1	solute carrier family 14 member 1 (Kidd blood group) [Source:HGNC Symbol;Acc:HGNC:10918]	protein coding	-1.22	2.11	16.56	<0.0001	0.0005
ENSG00000197077	KIAA1671	KIAA1671 [Source:HGNC Symbol;Acc:HGNC:29345]	protein coding	-1.22	1.94	15.25	<0.0001	0.0008
ENSG00000155265	GOLGA7B	golgin A7 family member B [Source:HGNC Symbol;Acc:HGNC:31668]	protein coding	-1.22	2.67	14.85	0.0001	0.0009
ENSG00000113263	ITK	IL2 inducible T cell kinase [Source:HGNC Symbol;Acc:HGNC:6171]	protein coding	-1.21	5.92	14.78	0.0001	0.001
ENSG00000158156	XKR8	XK related 8 [Source:HGNC Symbol;Acc:HGNC:25508]	protein coding	1.21	5.25	50.70	<0.0001	<0.0001
ENSG00000185920	PTCH1	patched 1 [Source:HGNC Symbol;Acc:HGNC:9585]	protein coding	-1.21	3.79	17.08	<0.0001	0.0004
ENSG00000088387	DOCK9	dedicator of cytokinesis 9 [Source:HGNC Symbol;Acc:HGNC:14132]	protein coding	-1.21	4.06	17.32	<0.0001	0.0004
ENSG00000168824	NSG1	neuronal vesicle trafficking associated 1 [Source:HGNC Symbol;Acc:HGNC:18790]	protein coding	-1.21	2.66	13.84	0.0002	0.0014
ENSG00000126217	MCF2L	MCF.2 cell line derived transforming sequence like [Source:HGNC Symbol;Acc:HGNC:14576]	protein coding	-1.21	2.09	15.65	<0.0001	0.0007
ENSG00000271503	CCL5	C-C motif chemokine ligand 5 [Source:HGNC Symbol;Acc:HGNC:10632]	protein coding	-1.21	7.11	19.89	<0.0001	0.0001
ENSG00000182010	RTKN2	rhotekin 2 [Source:HGNC Symbol;Acc:HGNC:19364]	protein coding	-1.21	1.63	13.31	0.0003	0.0017
ENSG00000020577	SAMD4A	sterile alpha motif domain containing 4A [Source:HGNC Symbol;Acc:HGNC:23023]	protein coding	1.21	4.84	37.60	<0.0001	<0.0001
ENSG00000198851	CD3E	CD3e molecule [Source:HGNC Symbol;Acc:HGNC:1674]	protein coding	-1.21	6.68	13.15	0.0003	0.0019
ENSG00000215788	TNFRSF25	TNF receptor superfamily member 25 [Source:HGNC Symbol;Acc:HGNC:11910]	protein coding	-1.21	5.45	17.45	<0.0001	0.0003
ENSG00000137441	FGFBP2	fibroblast growth factor binding protein 2 [Source:HGNC Symbol;Acc:HGNC:29451]	protein coding	-1.21	5.50	19.48	<0.0001	0.0002
ENSG00000181754	AMIGO1	adhesion molecule with Ig like domain 1 [Source:HGNC Symbol;Acc:HGNC:20824]	protein coding	-1.20	2.98	13.30	0.0003	0.0017

Table S9: Genes differentially expressed at Day 2 compared to pre-first vaccination (Study Arm 2 A/H7N9+AS03, D1, 29, Dendritic cells). Genes are sorted by descending absolute log_2 fold change. Gene model summaries and annotations are based on Ensembl Version 99 (continued.)

Ensembl Gene ID	Ensembl Gene Name	Ensembl Gene Description	Gene Type	log_2 Fold Change (Day 2 vs. Pre-First Vaccination)	Average log_2 CPM	Likelihood Ratio Test Statistic	P-Value	FDR Adjusted P-Value
ENSG00000153563	CD8A	CD8a molecule [Source:HGNC Symbol;Acc:HGNC:1706]	protein coding	-1.20	6.21	15.99	<0.0001	0.0006
ENSG00000232869	TRBV29-1	T cell receptor beta variable 29-1 [Source:HGNC Symbol;Acc:HGNC:12210]	TR V gene	-1.20	1.45	10.67	0.0011	0.0052
ENSG00000151623	NR3C2	nuclear receptor subfamily 3 group C member 2 [Source:HGNC Symbol;Acc:HGNC:7979]	protein coding	-1.20	2.48	26.29	<0.0001	<0.0001
ENSG00000062524	LTK	leukocyte receptor tyrosine kinase [Source:HGNC Symbol;Acc:HGNC:6721]	protein coding	-1.20	4.04	29.45	<0.0001	<0.0001
ENSG00000258875		novel transcript, antisense to GPR68	lncRNA	-1.20	0.54	18.23	<0.0001	0.0003
ENSG00000172005	MAL	mal, T cell differentiation protein [Source:HGNC Symbol;Acc:HGNC:6817]	protein coding	-1.20	3.59	11.40	0.0007	0.0038
ENSG00000156510	HKDC1	hexokinase domain containing 1 [Source:HGNC Symbol;Acc:HGNC:23302]	protein coding	-1.20	0.76	11.57	0.0007	0.0036
ENSG00000092096	SLC22A17	solute carrier family 22 member 17 [Source:HGNC Symbol;Acc:HGNC:23095]	protein coding	-1.20	1.15	12.61	0.0004	0.0023
ENSG00000156453	PCDH1	protocadherin 1 [Source:HGNC Symbol;Acc:HGNC:8655]	protein coding	-1.19	1.43	18.77	<0.0001	0.0002
ENSG00000111796	KLRB1	killer cell lectin like receptor B1 [Source:HGNC Symbol;Acc:HGNC:6373]	protein coding	-1.19	4.84	20.26	<0.0001	0.0001
ENSG00000228956	SATB1-AS1	SATB1 antisense RNA 1 [Source:HGNC Symbol;Acc:HGNC:50687]	lncRNA	-1.19	2.42	18.81	<0.0001	0.0002
ENSG00000173369	C1QB	complement C1q B chain [Source:HGNC Symbol;Acc:HGNC:1242]	protein coding	1.19	4.17	36.40	<0.0001	<0.0001
ENSG00000267554		RAB32, member RAS oncogene family (RAB32) pseudogene	processed pseudogene	-1.18	1.98	25.07	<0.0001	<0.0001
ENSG00000133067	LGR6	leucine rich repeat containing G protein-coupled receptor 6 [Source:HGNC Symbol;Acc:HGNC:19719]	protein coding	-1.18	2.86	15.83	<0.0001	0.0006
ENSG00000148468	FAM171A1	family with sequence similarity 171 member A1 [Source:HGNC Symbol;Acc:HGNC:23522]	protein coding	-1.18	0.85	13.12	0.0003	0.0019
ENSG00000105088	OLFM2	olfactomedin 2 [Source:HGNC Symbol;Acc:HGNC:17189]	protein coding	-1.18	1.05	11.02	0.0009	0.0045
ENSG00000088881	EBF4	EBF family member 4 [Source:HGNC Symbol;Acc:HGNC:29278]	protein coding	-1.18	1.69	22.69	<0.0001	<0.0001
ENSG00000124203	ZNF831	zinc finger protein 831 [Source:HGNC Symbol;Acc:HGNC:16167]	protein coding	-1.18	4.30	20.19	<0.0001	0.0001

Table S9: Genes differentially expressed at Day 2 compared to pre-first vaccination (Study Arm 2 A/H7N9+AS03, D1, 29, Dendritic cells). Genes are sorted by descending absolute log_2 fold change. Gene model summaries and annotations are based on Ensembl Version 99 (continued.)

Ensembl Gene ID	Ensembl Gene Name	Ensembl Gene Description	Gene Type	log_2 Fold Change (Day 2 vs. Pre-First Vaccination)	Average log_2 CPM	Likelihood Ratio Test Statistic	P-Value	FDR Adjusted P-Value
ENSG00000161381	PLXDC1	plexin domain containing 1 [Source:HGNC Symbol;Acc:HGNC:20945]	protein coding	-1.18	3.19	14.18	0.0002	0.0012
ENSG00000188322	SBK1	SH3 domain binding kinase 1 [Source:HGNC Symbol;Acc:HGNC:17699]	protein coding	-1.18	4.52	17.17	<0.0001	0.0004
ENSG00000162892	IL24	interleukin 24 [Source:HGNC Symbol;Acc:HGNC:11346]	protein coding	-1.18	0.67	15.88	<0.0001	0.0006
ENSG00000198756	COLGALT2	collagen beta(1-O)galactosyltransferase 2 [Source:HGNC Symbol;Acc:HGNC:16790]	protein coding	-1.18	1.11	35.56	<0.0001	<0.0001
ENSG00000156587	UBE2L6	ubiquitin conjugating enzyme E2 L6 [Source:HGNC Symbol;Acc:HGNC:12490]	protein coding	1.18	7.43	47.96	<0.0001	<0.0001
ENSG00000089012	SIRPG	signal regulatory protein gamma [Source:HGNC Symbol;Acc:HGNC:15757]	protein coding	-1.18	2.71	10.06	0.0015	0.0067
ENSG00000187608	ISG15	ISG15 ubiquitin like modifier [Source:HGNC Symbol;Acc:HGNC:4053]	protein coding	1.18	6.03	24.05	<0.0001	<0.0001
ENSG00000087258	GNAO1	G protein subunit alpha o1 [Source:HGNC Symbol;Acc:HGNC:4389]	protein coding	-1.18	0.76	14.35	0.0002	0.0011
ENSG00000134539	KLRD1	killer cell lectin like receptor D1 [Source:HGNC Symbol;Acc:HGNC:6378]	protein coding	-1.18	6.14	18.23	<0.0001	0.0003
ENSG00000163519	TRAT1	T cell receptor associated transmembrane adaptor 1 [Source:HGNC Symbol;Acc:HGNC:30698]	protein coding	-1.18	3.26	12.10	0.0005	0.0029
ENSG00000282939	TRBV7-2	T cell receptor beta variable 7-2 [Source:HGNC Symbol;Acc:HGNC:12236]	TR V gene	-1.18	0.98	17.37	<0.0001	0.0004
ENSG00000168229	PTGDR	prostaglandin D2 receptor [Source:HGNC Symbol;Acc:HGNC:9591]	protein coding	-1.18	3.83	18.86	<0.0001	0.0002
ENSG00000112655	PTK7	protein tyrosine kinase 7 (inactive) [Source:HGNC Symbol;Acc:HGNC:9618]	protein coding	-1.17	1.03	27.47	<0.0001	<0.0001
ENSG00000244720		5'-nucleotidase, cytosolic III (NT5C3) pseudogene	processed pseudogene	-1.17	0.76	14.38	0.0001	0.0011
ENSG00000168394	TAP1	transporter 1, ATP binding cassette subfamily B member [Source:HGNC Symbol;Acc:HGNC:43]	protein coding	1.17	8.15	56.09	<0.0001	<0.0001
ENSG00000183691	NOG	noggin [Source:HGNC Symbol;Acc:HGNC:7866]	protein coding	-1.17	2.48	8.52	0.0035	0.013
ENSG00000197057	DTHD1	death domain containing 1 [Source:HGNC Symbol;Acc:HGNC:37261]	protein coding	-1.17	2.42	17.86	<0.0001	0.0003

Table S9: Genes differentially expressed at Day 2 compared to pre-first vaccination (Study Arm 2 A/H7N9+AS03, D1, 29, Dendritic cells). Genes are sorted by descending absolute log_2 fold change. Gene model summaries and annotations are based on Ensembl Version 99 (continued.)

Ensembl Gene ID	Ensembl Gene Name	Ensembl Gene Description	Gene Type	log_2 Fold Change (Day 2 vs. Pre-First Vaccination)	Average log_2 CPM	Likelihood Ratio Test Statistic	P-Value	FDR Adjusted P-Value
ENSG00000144824	PHLDB2	pleckstrin homology like domain family B member 2 [Source:HGNC Symbol;Acc:HGNC:29573]	protein coding	-1.17	1.66	19.48	<0.0001	0.0002
ENSG00000181847	TIGIT	T cell immunoreceptor with Ig and ITIM domains [Source:HGNC Symbol;Acc:HGNC:26838]	protein coding	-1.17	3.78	19.50	<0.0001	0.0002
ENSG00000111674	ENO2	enolase 2 [Source:HGNC Symbol;Acc:HGNC:3353]	protein coding	-1.17	3.99	14.70	0.0001	0.001
ENSG00000230006	ANKRD36BP2	ankyrin repeat domain 36B pseudogene 2 [Source:HGNC Symbol;Acc:HGNC:33607]	transcribed unprocessed pseudogene	-1.17	1.54	14.34	0.0002	0.0011
ENSG00000131686	CA6	carbonic anhydrase 6 [Source:HGNC Symbol;Acc:HGNC:1380]	protein coding	-1.17	0.97	11.44	0.0007	0.0038
ENSG00000235576	LINC01871	long intergenic non-protein coding RNA 1871 [Source:HGNC Symbol;Acc:HGNC:52690]	lncRNA	-1.17	1.34	14.21	0.0002	0.0012
ENSG00000211751	TRBC1	T cell receptor beta constant 1 [Source:HGNC Symbol;Acc:HGNC:12156]	TR C gene	-1.17	5.66	12.49	0.0004	0.0024
ENSG00000177989	ODF3B	outer dense fiber of sperm tails 3B [Source:HGNC Symbol;Acc:HGNC:34388]	protein coding	1.17	8.21	38.52	<0.0001	<0.0001
ENSG00000164483	SAMD3	sterile alpha motif domain containing 3 [Source:HGNC Symbol;Acc:HGNC:21574]	protein coding	-1.17	4.13	19.63	<0.0001	0.0001
ENSG00000140092	FBLN5	fibulin 5 [Source:HGNC Symbol;Acc:HGNC:3602]	protein coding	-1.17	0.72	12.33	0.0004	0.0026
ENSG00000172673	THEMIS	thymocyte selection associated [Source:HGNC Symbol;Acc:HGNC:21569]	protein coding	-1.17	3.28	10.94	0.0009	0.0046
ENSG00000134321	RSAD2	radical S-adenosyl methionine domain containing 2 [Source:HGNC Symbol;Acc:HGNC:30908]	protein coding	1.17	4.72	18.95	<0.0001	0.0002
ENSG00000277734	TRAC	T cell receptor alpha constant [Source:HGNC Symbol;Acc:HGNC:12029]	TR C gene	-1.16	6.00	10.44	0.0012	0.0057
ENSG00000148488	ST8SIA6	ST8 alpha-N-acetyl-neuraminide alpha-2,8-sialyltransferase 6 [Source:HGNC Symbol;Acc:HGNC:23317]	protein coding	-1.16	1.46	24.94	<0.0001	<0.0001
ENSG00000153283	CD96	CD96 molecule [Source:HGNC Symbol;Acc:HGNC:16892]	protein coding	-1.16	4.97	18.39	<0.0001	0.0002
ENSG00000157985	AGAP1	ArfGAP with GTPase domain, ankyrin repeat and PH domain 1 [Source:HGNC Symbol;Acc:HGNC:16922]	protein coding	-1.16	2.89	15.46	<0.0001	0.0007

Table S9: Genes differentially expressed at Day 2 compared to pre-first vaccination (Study Arm 2 A/H7N9+AS03, D1, 29, Dendritic cells). Genes are sorted by descending absolute log_2 fold change. Gene model summaries and annotations are based on Ensembl Version 99 (continued.)

Ensembl Gene ID	Ensembl Gene Name	Ensembl Gene Description	Gene Type	log_2 Fold Change (Day 2 vs. Pre-First Vaccination)	Average log_2 CPM	Likelihood Ratio Test Statistic	P-Value	FDR Adjusted P-Value
ENSG00000234518	PTGES3P1	prostaglandin E synthase 3 pseudogene 1 [Source:HGNC Symbol;Acc:HGNC:43824]	processed pseudogene	1.16	3.00	53.11	<0.0001	<0.0001
ENSG00000197880	MDS2	myelodysplastic syndrome 2 translocation associated [Source:HGNC Symbol;Acc:HGNC:29633]	lncRNA	-1.16	0.64	9.29	0.0023	0.0094
ENSG00000286885		novel transcript	lncRNA	-1.16	0.36	23.08	<0.0001	<0.0001
ENSG00000115085	ZAP70	zeta chain of T cell receptor associated protein kinase 70 [Source:HGNC Symbol;Acc:HGNC:12858]	protein coding	-1.16	7.73	17.65	<0.0001	0.0003
ENSG00000080854	IGSF9B	immunoglobulin superfamily member 9B [Source:HGNC Symbol;Acc:HGNC:32326]	protein coding	-1.16	3.30	11.83	0.0006	0.0032
ENSG00000185745	IFIT1	interferon induced protein with tetratricopeptide repeats 1 [Source:HGNC Symbol;Acc:HGNC:5407]	protein coding	1.16	5.17	15.88	<0.0001	0.0006
ENSG00000132849	PATJ	PATJ crumbs cell polarity complex component [Source:HGNC Symbol;Acc:HGNC:28881]	protein coding	-1.15	2.78	16.20	<0.0001	0.0006
ENSG00000101004	NINL	ninein like [Source:HGNC Symbol;Acc:HGNC:29163]	protein coding	-1.15	0.71	21.31	<0.0001	<0.0001
ENSG00000109956	B3GAT1	beta-1,3-glucuronyltransferase 1 [Source:HGNC Symbol;Acc:HGNC:921]	protein coding	-1.15	3.31	14.71	0.0001	0.001
ENSG00000211772	TRBC2	T cell receptor beta constant 2 [Source:HGNC Symbol;Acc:HGNC:12157]	TR C gene	-1.15	6.38	15.74	<0.0001	0.0007
ENSG00000211689	TRGC1	T cell receptor gamma constant 1 [Source:HGNC Symbol;Acc:HGNC:12275]	TR C gene	-1.15	3.60	14.84	0.0001	0.0009
ENSG00000121753	ADGRB2	adhesion G protein-coupled receptor B2 [Source:HGNC Symbol;Acc:HGNC:944]	protein coding	-1.15	1.18	12.89	0.0003	0.0021
ENSG00000167779	IGFBP6	insulin like growth factor binding protein 6 [Source:HGNC Symbol;Acc:HGNC:5475]	protein coding	-1.15	0.53	34.71	<0.0001	<0.0001
ENSG00000243836	WDR86-AS1	WDR86 antisense RNA 1 [Source:HGNC Symbol;Acc:HGNC:41186]	lncRNA	-1.15	1.19	16.67	<0.0001	0.0005
ENSG00000099204	ABLIM1	actin binding LIM protein 1 [Source:HGNC Symbol;Acc:HGNC:78]	protein coding	-1.15	5.68	17.37	<0.0001	0.0004
ENSG00000135114	OASL	2'-5'-oligoadenylate synthetase like [Source:HGNC Symbol;Acc:HGNC:8090]	protein coding	1.15	4.45	23.06	<0.0001	<0.0001
ENSG00000134954	ETS1	ETS proto-oncogene 1, transcription factor [Source:HGNC Symbol;Acc:HGNC:3488]	protein coding	-1.15	8.15	20.68	<0.0001	<0.0001
ENSG00000278030	TRBV7-9	T cell receptor beta variable 7-9 [Source:HGNC Symbol;Acc:HGNC:12243]	TR V gene	-1.15	1.26	12.86	0.0003	0.0021

Table S9: Genes differentially expressed at Day 2 compared to pre-first vaccination (Study Arm 2 A/H7N9+AS03, D1, 29, Dendritic cells). Genes are sorted by descending absolute log_2 fold change. Gene model summaries and annotations are based on Ensembl Version 99 (continued.)

Ensembl Gene ID	Ensembl Gene Name	Ensembl Gene Description	Gene Type	log_2 Fold Change (Day 2 vs. Pre-First Vaccination)	Average log_2 CPM	Likelihood Ratio Test Statistic	P-Value	FDR Adjusted P-Value
ENSG00000206052	DOK6	docking protein 6 [Source:HGNC Symbol;Acc:HGNC:28301]	protein coding	-1.15	0.61	15.68	<0.0001	0.0007
ENSG00000115523	GNLY	granulysin [Source:HGNC Symbol;Acc:HGNC:4414]	protein coding	-1.15	8.46	21.54	<0.0001	<0.0001
ENSG00000162676	GFI1	growth factor independent 1 transcriptional repressor [Source:HGNC Symbol;Acc:HGNC:4237]	protein coding	-1.15	3.45	22.44	<0.0001	<0.0001
ENSG00000151882	CCL28	C-C motif chemokine ligand 28 [Source:HGNC Symbol;Acc:HGNC:17700]	protein coding	-1.15	1.29	20.74	<0.0001	<0.0001
ENSG00000145365	TIFA	TRAF interacting protein with forkhead associated domain [Source:HGNC Symbol;Acc:HGNC:19075]	protein coding	1.15	3.47	45.17	<0.0001	<0.0001
ENSG00000125347	IRF1	interferon regulatory factor 1 [Source:HGNC Symbol;Acc:HGNC:6116]	protein coding	1.15	8.25	57.23	<0.0001	<0.0001
ENSG00000073861	TBX21	T-box transcription factor 21 [Source:HGNC Symbol;Acc:HGNC:11599]	protein coding	-1.14	5.63	18.10	<0.0001	0.0003
ENSG00000021300	PLEKHB1	pleckstrin homology domain containing B1 [Source:HGNC Symbol;Acc:HGNC:19079]	protein coding	-1.14	2.73	12.28	0.0005	0.0027
ENSG00000135318	NT5E	5'-nucleotidase ecto [Source:HGNC Symbol;Acc:HGNC:8021]	protein coding	-1.14	2.60	15.51	<0.0001	0.0007
ENSG00000122188	LAX1	lymphocyte transmembrane adaptor 1 [Source:HGNC Symbol;Acc:HGNC:26005]	protein coding	-1.14	3.37	17.42	<0.0001	0.0003
ENSG00000135127	BICDL1	BICD family like cargo adaptor 1 [Source:HGNC Symbol;Acc:HGNC:28095]	protein coding	-1.14	3.85	16.02	<0.0001	0.0006
ENSG00000233355	CHRM3-AS2	CHRM3 antisense RNA 2 [Source:HGNC Symbol;Acc:HGNC:43725]	lncRNA	-1.14	4.12	11.77	0.0006	0.0033
ENSG00000172508	CARNS1	carnosine synthase 1 [Source:HGNC Symbol;Acc:HGNC:29268]	protein coding	-1.14	3.87	22.08	<0.0001	<0.0001
ENSG00000229754	CXCR2P1	C-X-C motif chemokine receptor 2 pseudogene 1 [Source:HGNC Symbol;Acc:HGNC:6028]	transcribed unprocessed pseudogene	1.14	5.40	32.03	<0.0001	<0.0001
ENSG00000107485	GATA3	GATA binding protein 3 [Source:HGNC Symbol;Acc:HGNC:4172]	protein coding	-1.13	3.85	14.60	0.0001	0.001
ENSG00000246223	LINC01550	long intergenic non-protein coding RNA 1550 [Source:HGNC Symbol;Acc:HGNC:20111]	lncRNA	-1.13	1.76	10.48	0.0012	0.0056

Table S9: Genes differentially expressed at Day 2 compared to pre-first vaccination (Study Arm 2 A/H7N9+AS03, D1, 29, Dendritic cells). Genes are sorted by descending absolute log_2 fold change. Gene model summaries and annotations are based on Ensembl Version 99 (continued.)

Ensembl Gene ID	Ensembl Gene Name	Ensembl Gene Description	Gene Type	log_2 Fold Change (Day 2 vs. Pre-First Vaccination)	Average log_2 CPM	Likelihood Ratio Test Statistic	P-Value	FDR Adjusted P-Value
ENSG00000124181	PLCG1	phospholipase C gamma 1 [Source:HGNC Symbol;Acc:HGNC:9065]	protein coding	-1.13	6.22	16.56	<0.0001	0.0005
ENSG00000205336	ADGRG1	adhesion G protein-coupled receptor G1 [Source:HGNC Symbol;Acc:HGNC:4512]	protein coding	-1.13	6.56	16.92	<0.0001	0.0004
ENSG00000160185	UBASH3A	ubiquitin associated and SH3 domain containing A [Source:HGNC Symbol;Acc:HGNC:12462]	protein coding	-1.13	3.76	11.25	0.0008	0.0041
ENSG00000167094	TTC16	tetratricopeptide repeat domain 16 [Source:HGNC Symbol;Acc:HGNC:26536]	protein coding	-1.13	3.62	13.31	0.0003	0.0017
ENSG00000180739	S1PR5	sphingosine-1-phosphate receptor 5 [Source:HGNC Symbol;Acc:HGNC:14299]	protein coding	-1.13	5.49	16.40	<0.0001	0.0005
ENSG00000197635	DPP4	dipeptidyl peptidase 4 [Source:HGNC Symbol;Acc:HGNC:3009]	protein coding	-1.13	3.34	15.02	0.0001	0.0009
ENSG00000142303	ADAMTS10	ADAM metallopeptidase with thrombospondin type 1 motif 10 [Source:HGNC Symbol;Acc:HGNC:13201]	protein coding	-1.13	4.69	25.69	<0.0001	<0.0001
ENSG00000030419	IKZF2	IKAROS family zinc finger 2 [Source:HGNC Symbol;Acc:HGNC:13177]	protein coding	-1.13	3.78	25.45	<0.0001	<0.0001
ENSG00000140545	MFGE8	milk fat globule-EGF factor 8 protein [Source:HGNC Symbol;Acc:HGNC:7036]	protein coding	-1.13	3.26	22.24	<0.0001	<0.0001
ENSG00000137965	IFI44	interferon induced protein 44 [Source:HGNC Symbol;Acc:HGNC:16938]	protein coding	1.13	6.16	20.09	<0.0001	0.0001
ENSG00000138378	STAT4	signal transducer and activator of transcription 4 [Source:HGNC Symbol;Acc:HGNC:11365]	protein coding	-1.13	4.57	20.30	<0.0001	0.0001
ENSG00000157404	KIT	KIT proto-oncogene, receptor tyrosine kinase [Source:HGNC Symbol;Acc:HGNC:6342]	protein coding	-1.12	0.87	32.37	<0.0001	<0.0001
ENSG00000069702	TGFBR3	transforming growth factor beta receptor 3 [Source:HGNC Symbol;Acc:HGNC:11774]	protein coding	-1.12	4.52	18.71	<0.0001	0.0002
ENSG00000167286	CD3D	CD3d molecule [Source:HGNC Symbol;Acc:HGNC:1673]	protein coding	-1.12	4.86	11.70	0.0006	0.0034
ENSG00000231793	DOC2GP	double C2 domain gamma, pseudogene [Source:HGNC Symbol;Acc:HGNC:37962]	transcribed unitary pseudogene	-1.12	1.82	20.29	<0.0001	0.0001
ENSG00000211771	TRBJ2-7	T cell receptor beta joining 2-7 [Source:HGNC Symbol;Acc:HGNC:12175]	TR J gene	-1.12	0.64	14.14	0.0002	0.0012
ENSG00000112796	ENPP5	ectonucleotide pyrophosphatase/phosphodiesterase family member 5 [Source:HGNC Symbol;Acc:HGNC:13717]	protein coding	-1.12	1.15	15.60	<0.0001	0.0007

Table S9: Genes differentially expressed at Day 2 compared to pre-first vaccination (Study Arm 2 A/H7N9+AS03, D1, 29, Dendritic cells). Genes are sorted by descending absolute log_2 fold change. Gene model summaries and annotations are based on Ensembl Version 99 (continued.)

Ensembl Gene ID	Ensembl Gene Name	Ensembl Gene Description	Gene Type	log_2 Fold Change (Day 2 vs. Pre-First Vaccination)	Average log_2 CPM	Likelihood Ratio Test Statistic	P-Value	FDR Adjusted P-Value
ENSG00000091409	ITGA6	integrin subunit alpha 6 [Source:HGNC Symbol;Acc:HGNC:6142]	protein coding	-1.12	4.78	18.16	<0.0001	0.0003
ENSG00000099282	TSPAN15	tetraspanin 15 [Source:HGNC Symbol;Acc:HGNC:23298]	protein coding	-1.12	0.77	22.56	<0.0001	<0.0001
ENSG00000138646	HERC5	HECT and RLD domain containing E3 ubiquitin protein ligase 5 [Source:HGNC Symbol;Acc:HGNC:24368]	protein coding	1.12	4.69	22.47	<0.0001	<0.0001
ENSG00000182866	LCK	LCK proto-oncogene, Src family tyrosine kinase [Source:HGNC Symbol;Acc:HGNC:6524]	protein coding	-1.12	6.47	15.01	0.0001	0.0009
ENSG00000196557	CACNA1H	calcium voltage-gated channel subunit alpha1 H [Source:HGNC Symbol;Acc:HGNC:1395]	protein coding	-1.12	1.23	13.55	0.0002	0.0016
ENSG00000167106	FAM102A	family with sequence similarity 102 member A [Source:HGNC Symbol;Acc:HGNC:31419]	protein coding	-1.12	6.08	17.12	<0.0001	0.0004
ENSG00000198821	CD247	CD247 molecule [Source:HGNC Symbol;Acc:HGNC:1677]	protein coding	-1.12	6.69	16.69	<0.0001	0.0005
ENSG00000139880	CDH24	cadherin 24 [Source:HGNC Symbol;Acc:HGNC:14265]	protein coding	-1.12	0.44	19.00	<0.0001	0.0002
ENSG00000221890	NPTXR	neuronal pentraxin receptor [Source:HGNC Symbol;Acc:HGNC:7954]	protein coding	-1.12	2.07	16.59	<0.0001	0.0005
ENSG00000221963	APOL6	apolipoprotein L6 [Source:HGNC Symbol;Acc:HGNC:14870]	protein coding	1.12	7.26	40.07	<0.0001	<0.0001
ENSG00000176928	GCNT4	glucosaminyl (N-acetyl) transferase 4 [Source:HGNC Symbol;Acc:HGNC:17973]	protein coding	-1.12	1.87	11.07	0.0009	0.0044
ENSG00000177409	SAMD9L	sterile alpha motif domain containing 9 like [Source:HGNC Symbol;Acc:HGNC:1349]	protein coding	1.11	7.21	37.06	<0.0001	<0.0001
ENSG00000100351	GRAP2	GRB2 related adaptor protein 2 [Source:HGNC Symbol;Acc:HGNC:4563]	protein coding	-1.11	4.65	16.18	<0.0001	0.0006
ENSG00000161405	IKZF3	IKAROS family zinc finger 3 [Source:HGNC Symbol;Acc:HGNC:13178]	protein coding	-1.11	5.55	17.58	<0.0001	0.0003
ENSG00000247287		novel transcript, antisense to PRKCH	lncRNA	-1.11	1.69	13.01	0.0003	0.002
ENSG00000126353	CCR7	C-C motif chemokine receptor 7 [Source:HGNC Symbol;Acc:HGNC:1608]	protein coding	-1.11	5.17	9.90	0.0017	0.0072
ENSG00000198780	FAM169A	family with sequence similarity 169 member A [Source:HGNC Symbol;Acc:HGNC:29138]	protein coding	-1.11	1.16	19.78	<0.0001	0.0001
ENSG00000185760	KCNQ5	potassium voltage-gated channel subfamily Q member 5 [Source:HGNC Symbol;Acc:HGNC:6299]	protein coding	-1.11	0.68	15.44	<0.0001	0.0007

Table S9: Genes differentially expressed at Day 2 compared to pre-first vaccination (Study Arm 2 A/H7N9+AS03, D1, 29, Dendritic cells). Genes are sorted by descending absolute log_2 fold change. Gene model summaries and annotations are based on Ensembl Version 99 (continued.)

Ensembl Gene ID	Ensembl Gene Name	Ensembl Gene Description	Gene Type	log_2 Fold Change (Day 2 vs. Pre-First Vaccination)	Average log_2 CPM	Likelihood Ratio Test Statistic	P-Value	FDR Adjusted P-Value
ENSG00000186049	KRT73	keratin 73 [Source:HGNC Symbol;Acc:HGNC:28928]	protein coding	-1.11	1.55	14.77	0.0001	0.001
ENSG00000008517	IL32	interleukin 32 [Source:HGNC Symbol;Acc:HGNC:16830]	protein coding	-1.10	6.13	14.43	0.0001	0.0011
ENSG00000183542	KLRC4	killer cell lectin like receptor C4 [Source:HGNC Symbol;Acc:HGNC:6377]	protein coding	-1.10	0.67	14.24	0.0002	0.0012
ENSG00000163564	PYHIN1	pyrin and HIN domain family member 1 [Source:HGNC Symbol;Acc:HGNC:28894]	protein coding	-1.10	4.02	18.44	<0.0001	0.0002
ENSG00000182183	SHISAL2A	shisa like 2A [Source:HGNC Symbol;Acc:HGNC:28757]	protein coding	-1.10	2.51	16.52	<0.0001	0.0005
ENSG00000134326	CMPK2	cytidine/uridine monophosphate kinase 2 [Source:HGNC Symbol;Acc:HGNC:27015]	protein coding	1.10	5.42	21.06	<0.0001	<0.0001
ENSG00000137801	THBS1	thrombospondin 1 [Source:HGNC Symbol;Acc:HGNC:11785]	protein coding	-1.10	5.26	15.06	0.0001	0.0009
ENSG00000163508	EOMES	eomesodermin [Source:HGNC Symbol;Acc:HGNC:3372]	protein coding	-1.10	4.00	12.65	0.0004	0.0023
ENSG00000101017	CD40	CD40 molecule [Source:HGNC Symbol;Acc:HGNC:11919]	protein coding	1.10	4.76	84.42	<0.0001	<0.0001
ENSG00000211734	TRBV5-1	T cell receptor beta variable 5-1 [Source:HGNC Symbol;Acc:HGNC:12218]	TR V gene	-1.10	0.86	7.66	0.0057	0.019
ENSG00000167984	NLRC3	NLR family CARD domain containing 3 [Source:HGNC Symbol;Acc:HGNC:29889]	protein coding	-1.10	5.85	20.18	<0.0001	0.0001
ENSG00000166289	PLEKHF1	pleckstrin homology and FYVE domain containing 1 [Source:HGNC Symbol;Acc:HGNC:20764]	protein coding	-1.10	3.91	14.04	0.0002	0.0013
ENSG00000225886		novel transcript, antisense to IFI6	lncRNA	1.09	1.11	16.53	<0.0001	0.0005
ENSG00000128383	APOBEC3A	apolipoprotein B mRNA editing enzyme catalytic subunit 3A [Source:HGNC Symbol;Acc:HGNC:17343]	protein coding	1.09	7.41	32.04	<0.0001	<0.0001
ENSG00000213626	LBH	LBH regulator of WNT signaling pathway [Source:HGNC Symbol;Acc:HGNC:29532]	protein coding	-1.09	6.36	15.57	<0.0001	0.0007
ENSG00000211747	TRBV20-1	T cell receptor beta variable 20-1 [Source:HGNC Symbol;Acc:HGNC:12196]	TR V gene	-1.09	1.97	10.98	0.0009	0.0046
ENSG00000156475	PPP2R2B	protein phosphatase 2 regulatory subunit Bbeta [Source:HGNC Symbol;Acc:HGNC:9305]	protein coding	-1.09	2.31	17.42	<0.0001	0.0003
ENSG00000151612	ZNF827	zinc finger protein 827 [Source:HGNC Symbol;Acc:HGNC:27193]	protein coding	-1.09	2.51	18.16	<0.0001	0.0003

Table S9: Genes differentially expressed at Day 2 compared to pre-first vaccination (Study Arm 2 A/H7N9+AS03, D1, 29, Dendritic cells). Genes are sorted by descending absolute log_2 fold change. Gene model summaries and annotations are based on Ensembl Version 99 (continued.)

Ensembl Gene ID	Ensembl Gene Name	Ensembl Gene Description	Gene Type	log_2 Fold Change (Day 2 vs. Pre-First Vaccination)	Average log_2 CPM	Likelihood Ratio Test Statistic	P-Value	FDR Adjusted P-Value
ENSG00000134545	KLRC1	killer cell lectin like receptor C1 [Source:HGNC Symbol;Acc:HGNC:6374]	protein coding	-1.09	2.40	13.47	0.0002	0.0016
ENSG00000115594	IL1R1	interleukin 1 receptor type 1 [Source:HGNC Symbol;Acc:HGNC:5993]	protein coding	-1.09	2.54	20.08	<0.0001	0.0001
ENSG00000085563	ABCB1	ATP binding cassette subfamily B member 1 [Source:HGNC Symbol;Acc:HGNC:40]	protein coding	-1.08	3.38	17.73	<0.0001	0.0003
ENSG00000074966	TXK	TXK tyrosine kinase [Source:HGNC Symbol;Acc:HGNC:12434]	protein coding	-1.08	4.62	18.40	<0.0001	0.0002
ENSG00000159189	C1QC	complement C1q C chain [Source:HGNC Symbol;Acc:HGNC:1245]	protein coding	1.08	3.08	23.27	<0.0001	<0.0001
ENSG00000267046	E2F3P1	E2F transcription factor 3 pseudogene 1 [Source:HGNC Symbol;Acc:HGNC:3116]	processed pseudogene	-1.08	0.82	13.86	0.0002	0.0014
ENSG00000168209	DDIT4	DNA damage inducible transcript 4 [Source:HGNC Symbol;Acc:HGNC:24944]	protein coding	-1.08	4.79	13.10	0.0003	0.0019
ENSG00000007402	CACNA2D2	calcium voltage-gated channel auxiliary subunit alpha2delta 2 [Source:HGNC Symbol;Acc:HGNC:1400]	protein coding	-1.08	3.41	21.96	<0.0001	<0.0001
ENSG00000125148	MT2A	metallothionein 2A [Source:HGNC Symbol;Acc:HGNC:7406]	protein coding	1.08	5.13	41.21	<0.0001	<0.0001
ENSG00000100911	PSME2	proteasome activator subunit 2 [Source:HGNC Symbol;Acc:HGNC:9569]	protein coding	1.08	7.75	50.78	<0.0001	<0.0001
ENSG00000238105	GOLGA2P5	GOLGA2 pseudogene 5 [Source:HGNC Symbol;Acc:HGNC:25315]	transcribed unprocessed pseudogene	-1.08	2.79	17.34	<0.0001	0.0004
ENSG00000168646	AXIN2	axin 2 [Source:HGNC Symbol;Acc:HGNC:904]	protein coding	-1.07	1.90	10.09	0.0015	0.0066
ENSG00000157551	KCNJ15	potassium inwardly rectifying channel subfamily J member 15 [Source:HGNC Symbol;Acc:HGNC:6261]	protein coding	1.07	2.42	27.30	<0.0001	<0.0001
ENSG00000256948		novel transcript	lncRNA	1.07	0.51	36.61	<0.0001	<0.0001
ENSG00000237943	PRKCQ-AS1	PRKCQ antisense RNA 1 [Source:HGNC Symbol;Acc:HGNC:44689]	lncRNA	-1.07	4.10	14.57	0.0001	0.001
ENSG00000176105	YES1	YES proto-oncogene 1, Src family tyrosine kinase [Source:HGNC Symbol;Acc:HGNC:12841]	protein coding	-1.07	1.62	17.69	<0.0001	0.0003

Table S9: Genes differentially expressed at Day 2 compared to pre-first vaccination (Study Arm 2 A/H7N9+AS03, D1, 29, Dendritic cells). Genes are sorted by descending absolute log_2 fold change. Gene model summaries and annotations are based on Ensembl Version 99 (continued.)

Ensembl Gene ID	Ensembl Gene Name	Ensembl Gene Description	Gene Type	log_2 Fold Change (Day 2 vs. Pre-First Vaccination)	Average log_2 CPM	Likelihood Ratio Test Statistic	P-Value	FDR Adjusted P-Value
ENSG00000238241	CCR12P	C-C motif chemokine receptor 12, pseudogene [Source:HGNC Symbol;Acc:HGNC:39812]	transcribed unprocessed pseudogene	-1.07	1.42	11.85	0.0006	0.0032
ENSG00000146674	IGFBP3	insulin like growth factor binding protein 3 [Source:HGNC Symbol;Acc:HGNC:5472]	protein coding	-1.07	0.85	8.66	0.0033	0.0123
ENSG00000154734	ADAMTS1	ADAM metallopeptidase with thrombospondin type 1 motif 1 [Source:HGNC Symbol;Acc:HGNC:217]	protein coding	-1.07	1.99	12.10	0.0005	0.0029
ENSG00000173193	PARP14	poly(ADP-ribose) polymerase family member 14 [Source:HGNC Symbol;Acc:HGNC:29232]	protein coding	1.07	8.04	38.51	<0.0001	<0.0001
ENSG00000149582	TMEM25	transmembrane protein 25 [Source:HGNC Symbol;Acc:HGNC:25890]	protein coding	-1.07	1.71	14.55	0.0001	0.001
ENSG00000168280	KIF5C	kinesin family member 5C [Source:HGNC Symbol;Acc:HGNC:6325]	protein coding	-1.07	1.97	24.69	<0.0001	<0.0001
ENSG00000286640		SET domain and mariner transposase fusion gene (SETMAR) pseudogene	transcribed unprocessed pseudogene	-1.06	0.40	16.32	<0.0001	0.0005
ENSG00000131979	GCH1	GTP cyclohydrolase 1 [Source:HGNC Symbol;Acc:HGNC:4193]	protein coding	1.06	7.03	46.25	<0.0001	<0.0001
ENSG00000172215	CXCR6	C-X-C motif chemokine receptor 6 [Source:HGNC Symbol;Acc:HGNC:16647]	protein coding	-1.06	1.66	8.02	0.0046	0.0163
ENSG00000234663	LINC01934	long intergenic non-protein coding RNA 1934 [Source:HGNC Symbol;Acc:HGNC:52757]	lncRNA	-1.06	1.14	11.68	0.0006	0.0034
ENSG00000214402	LCNL1	lipocalin like 1 [Source:HGNC Symbol;Acc:HGNC:34436]	protein coding	-1.06	0.63	16.03	<0.0001	0.0006
ENSG00000151693	ASAP2	ArfGAP with SH3 domain, ankyrin repeat and PH domain 2 [Source:HGNC Symbol;Acc:HGNC:2721]	protein coding	1.06	2.13	37.41	<0.0001	<0.0001
ENSG00000168672	LRATD2	LRAT domain containing 2 [Source:HGNC Symbol;Acc:HGNC:24166]	protein coding	-1.06	2.08	15.38	<0.0001	0.0008
ENSG00000225131	PSME2P2	proteasome activator subunit 2 pseudogene 2 [Source:HGNC Symbol;Acc:HGNC:30160]	processed pseudogene	1.06	0.78	33.16	<0.0001	<0.0001

Table S9: Genes differentially expressed at Day 2 compared to pre-first vaccination (Study Arm 2 A/H7N9+AS03, D1, 29, Dendritic cells). Genes are sorted by descending absolute log_2 fold change. Gene model summaries and annotations are based on Ensembl Version 99 (continued.)

Ensembl Gene ID	Ensembl Gene Name	Ensembl Gene Description	Gene Type	log_2 Fold Change (Day 2 vs. Pre-First Vaccination)	Average log_2 CPM	Likelihood Ratio Test Statistic	P-Value	FDR Adjusted P-Value
ENSG00000128284	APOL3	apolipoprotein L3 [Source:HGNC Symbol;Acc:HGNC:14868]	protein coding	1.06	7.47	64.51	<0.0001	<0.0001
ENSG00000131797	CLUHP3	clustered mitochondria homolog pseudogene 3 [Source:HGNC Symbol;Acc:HGNC:28447]	transcribed unprocessed pseudogene	-1.06	3.78	20.63	<0.0001	<0.0001
ENSG00000116824	CD2	CD2 molecule [Source:HGNC Symbol;Acc:HGNC:1639]	protein coding	-1.06	5.36	19.87	<0.0001	0.0001
ENSG00000196220	SRGAP3	SLIT-ROBO Rho GTPase activating protein 3 [Source:HGNC Symbol;Acc:HGNC:19744]	protein coding	-1.06	1.74	36.46	<0.0001	<0.0001
ENSG00000101298	SNPH	syntaphilin [Source:HGNC Symbol;Acc:HGNC:15931]	protein coding	-1.06	2.61	15.15	<0.0001	0.0008
ENSG00000182782	HCAR2	hydroxycarboxylic acid receptor 2 [Source:HGNC Symbol;Acc:HGNC:24827]	protein coding	1.06	1.80	31.12	<0.0001	<0.0001
ENSG00000054654	SYNE2	spectrin repeat containing nuclear envelope protein 2 [Source:HGNC Symbol;Acc:HGNC:17084]	protein coding	-1.06	6.54	16.97	<0.0001	0.0004
ENSG00000065675	PRKCQ	protein kinase C theta [Source:HGNC Symbol;Acc:HGNC:9410]	protein coding	-1.05	3.69	16.25	<0.0001	0.0005
ENSG00000068079	IFI35	interferon induced protein 35 [Source:HGNC Symbol;Acc:HGNC:5399]	protein coding	1.05	6.27	39.34	<0.0001	<0.0001
ENSG00000026751	SLAMF7	SLAM family member 7 [Source:HGNC Symbol;Acc:HGNC:21394]	protein coding	1.05	6.47	40.09	<0.0001	<0.0001
ENSG00000143842	SOX13	SRY-box transcription factor 13 [Source:HGNC Symbol;Acc:HGNC:11192]	protein coding	-1.05	2.62	26.11	<0.0001	<0.0001
ENSG00000116663	FBXO6	F-box protein 6 [Source:HGNC Symbol;Acc:HGNC:13585]	protein coding	1.05	5.16	50.71	<0.0001	<0.0001
ENSG00000184545	DUSP8	dual specificity phosphatase 8 [Source:HGNC Symbol;Acc:HGNC:3074]	protein coding	-1.05	1.54	15.36	<0.0001	0.0008
ENSG00000141293	SKAP1	src kinase associated phosphoprotein 1 [Source:HGNC Symbol;Acc:HGNC:15605]	protein coding	-1.05	4.32	16.72	<0.0001	0.0005
ENSG00000025708	TYMP	thymidine phosphorylase [Source:HGNC Symbol;Acc:HGNC:3148]	protein coding	1.05	10.46	39.68	<0.0001	<0.0001
ENSG00000105289	TJP3	tight junction protein 3 [Source:HGNC Symbol;Acc:HGNC:11829]	protein coding	-1.05	1.37	10.66	0.0011	0.0052

Table S9: Genes differentially expressed at Day 2 compared to pre-first vaccination (Study Arm 2 A/H7N9+AS03, D1, 29, Dendritic cells). Genes are sorted by descending absolute log_2 fold change. Gene model summaries and annotations are based on Ensembl Version 99 (continued.)

Ensembl Gene ID	Ensembl Gene Name	Ensembl Gene Description	Gene Type	log_2 Fold Change (Day 2 vs. Pre-First Vaccination)	Average log_2 CPM	Likelihood Ratio Test Statistic	P-Value	FDR Adjusted P-Value
ENSG00000159753	CARMIL2	capping protein regulator and myosin 1 linker 2 [Source:HGNC Symbol;Acc:HGNC:27089]	protein coding	-1.05	5.52	17.26	<0.0001	0.0004
ENSG00000187862	TTC24	tetratricopeptide repeat domain 24 [Source:HGNC Symbol;Acc:HGNC:32348]	protein coding	-1.05	1.65	19.51	<0.0001	0.0002
ENSG00000178498	DTX3	deltex E3 ubiquitin ligase 3 [Source:HGNC Symbol;Acc:HGNC:24457]	protein coding	-1.05	3.64	19.31	<0.0001	0.0002
ENSG00000149527	PLCH2	phospholipase C eta 2 [Source:HGNC Symbol;Acc:HGNC:29037]	protein coding	-1.05	4.67	29.25	<0.0001	<0.0001
ENSG00000174500	GCSAM	germinal center associated signaling and motility [Source:HGNC Symbol;Acc:HGNC:20253]	protein coding	-1.04	2.15	12.56	0.0004	0.0024
ENSG00000115607	IL18RAP	interleukin 18 receptor accessory protein [Source:HGNC Symbol;Acc:HGNC:5989]	protein coding	-1.04	4.53	18.67	<0.0001	0.0002
ENSG00000172575	RASGRP1	RAS guanyl releasing protein 1 [Source:HGNC Symbol;Acc:HGNC:9878]	protein coding	-1.04	4.84	15.78	<0.0001	0.0006
ENSG00000171596	NMUR1	neuromedin U receptor 1 [Source:HGNC Symbol;Acc:HGNC:4518]	protein coding	-1.04	3.74	16.74	<0.0001	0.0004
ENSG00000262312		novel transcript, antisense to NLRC3	lncRNA	-1.04	2.13	16.13	<0.0001	0.0006
ENSG00000100450	GZMH	granzyme H [Source:HGNC Symbol;Acc:HGNC:4710]	protein coding	-1.04	5.34	12.98	0.0003	0.002
ENSG00000123610	TNFAIP6	TNF alpha induced protein 6 [Source:HGNC Symbol;Acc:HGNC:11898]	protein coding	1.04	0.67	21.44	<0.0001	<0.0001
ENSG00000027869	SH2D2A	SH2 domain containing 2A [Source:HGNC Symbol;Acc:HGNC:10821]	protein coding	-1.04	4.37	14.66	0.0001	0.001
ENSG00000171476	HOPX	HOP homeobox [Source:HGNC Symbol;Acc:HGNC:24961]	protein coding	-1.04	4.29	18.11	<0.0001	0.0003
ENSG00000116983	HPCAL4	hippocalcin like 4 [Source:HGNC Symbol;Acc:HGNC:18212]	protein coding	-1.04	1.10	10.43	0.0012	0.0057
ENSG00000110448	CD5	CD5 molecule [Source:HGNC Symbol;Acc:HGNC:1685]	protein coding	-1.04	5.24	15.56	<0.0001	0.0007
ENSG00000125731	SH2D3A	SH2 domain containing 3A [Source:HGNC Symbol;Acc:HGNC:16885]	protein coding	-1.03	3.78	15.56	<0.0001	0.0007
ENSG00000238000		proteasome (prosome, macropain) activator subunit 2 (PA28 beta) (PSME2) pseudogene	processed pseudogene	1.03	0.67	30.21	<0.0001	<0.0001
ENSG00000174946	GPR171	G protein-coupled receptor 171 [Source:HGNC Symbol;Acc:HGNC:30057]	protein coding	-1.03	2.63	10.84	0.001	0.0048

Table S9: Genes differentially expressed at Day 2 compared to pre-first vaccination (Study Arm 2 A/H7N9+AS03, D1, 29, Dendritic cells). Genes are sorted by descending absolute log_2 fold change. Gene model summaries and annotations are based on Ensembl Version 99 (continued.)

Ensembl Gene ID	Ensembl Gene Name	Ensembl Gene Description	Gene Type	log_2 Fold Change (Day 2 vs. Pre-First Vaccination)	Average log_2 CPM	Likelihood Ratio Test Statistic	P-Value	FDR Adjusted P-Value
ENSG00000185697	MYBL1	MYB proto-oncogene like 1 [Source:HGNC Symbol;Acc:HGNC:7547]	protein coding	-1.03	5.05	17.40	<0.0001	0.0003
ENSG00000261801	LOXL1-AS1	LOXL1 antisense RNA 1 [Source:HGNC Symbol;Acc:HGNC:44169]	lncRNA	-1.03	0.78	13.20	0.0003	0.0018
ENSG00000165272	AQP3	aquaporin 3 (Gill blood group) [Source:HGNC Symbol;Acc:HGNC:636]	protein coding	-1.03	4.31	13.14	0.0003	0.0019
ENSG00000169583	CLIC3	chloride intracellular channel 3 [Source:HGNC Symbol;Acc:HGNC:2064]	protein coding	-1.03	3.74	19.76	<0.0001	0.0001
ENSG00000205045	SLFN12L	schlafen family member 12 like [Source:HGNC Symbol;Acc:HGNC:33920]	protein coding	-1.03	2.94	17.83	<0.0001	0.0003
ENSG00000227191	TRGC2	T cell receptor gamma constant 2 [Source:HGNC Symbol;Acc:HGNC:12276]	TR C gene	-1.03	3.35	14.04	0.0002	0.0013
ENSG00000151150	ANK3	ankyrin 3 [Source:HGNC Symbol;Acc:HGNC:494]	protein coding	-1.03	1.94	10.04	0.0015	0.0068
ENSG00000143167	GPA33	glycoprotein A33 [Source:HGNC Symbol;Acc:HGNC:4445]	protein coding	-1.03	2.35	22.18	<0.0001	<0.0001
ENSG00000162894	FCMR	Fc fragment of IgM receptor [Source:HGNC Symbol;Acc:HGNC:14315]	protein coding	-1.03	6.53	16.04	<0.0001	0.0006
ENSG00000143515	ATP8B2	ATPase phospholipid transporting 8B2 [Source:HGNC Symbol;Acc:HGNC:13534]	protein coding	-1.03	6.03	15.02	0.0001	0.0009
ENSG00000278195	SSTR3	somatostatin receptor 3 [Source:HGNC Symbol;Acc:HGNC:11332]	protein coding	-1.03	1.41	12.78	0.0004	0.0022
ENSG00000139187	KLRG1	killer cell lectin like receptor G1 [Source:HGNC Symbol;Acc:HGNC:6380]	protein coding	-1.03	3.75	18.18	<0.0001	0.0003
ENSG00000182463	TSHZ2	teashirt zinc finger homeobox 2 [Source:HGNC Symbol;Acc:HGNC:13010]	protein coding	-1.02	1.29	7.53	0.0061	0.0201
ENSG00000240403	KIR3DL2	killer cell immunoglobulin like receptor, three Ig domains and long cytoplasmic tail 2 [Source:HGNC Symbol;Acc:HGNC:6339]	protein coding	-1.02	1.40	26.81	<0.0001	<0.0001
ENSG00000145390	USP53	ubiquitin specific peptidase 53 [Source:HGNC Symbol;Acc:HGNC:29255]	protein coding	-1.02	3.01	16.74	<0.0001	0.0004
ENSG00000135426	TESPA1	thymocyte expressed, positive selection associated 1 [Source:HGNC Symbol;Acc:HGNC:29109]	protein coding	-1.02	4.71	16.55	<0.0001	0.0005
ENSG00000119608	PROX2	prospero homeobox 2 [Source:HGNC Symbol;Acc:HGNC:26715]	protein coding	-1.02	0.44	26.57	<0.0001	<0.0001
ENSG00000273179		novel transcript, antisense to SPON2	lncRNA	-1.02	2.21	13.07	0.0003	0.0019

Table S9: Genes differentially expressed at Day 2 compared to pre-first vaccination (Study Arm 2 A/H7N9+AS03, D1, 29, Dendritic cells). Genes are sorted by descending absolute log_2 fold change. Gene model summaries and annotations are based on Ensembl Version 99 (continued.)

Ensembl Gene ID	Ensembl Gene Name	Ensembl Gene Description	Gene Type	log_2 Fold Change (Day 2 vs. Pre-First Vaccination)	Average log_2 CPM	Likelihood Ratio Test Statistic	P-Value	FDR Adjusted P-Value
ENSG00000138119	MYOF	myoferlin [Source:HGNC Symbol;Acc:HGNC:3656]	protein coding	1.02	7.25	54.18	<0.0001	<0.0001
ENSG00000141574	SECTM1	secreted and transmembrane 1 [Source:HGNC Symbol;Acc:HGNC:10707]	protein coding	1.02	8.50	47.17	<0.0001	<0.0001
ENSG00000181652	ATG9B	autophagy related 9B [Source:HGNC Symbol;Acc:HGNC:21899]	protein coding	-1.02	1.02	12.92	0.0003	0.002
ENSG00000188282	RUFY4	RUN and FYVE domain containing 4 [Source:HGNC Symbol;Acc:HGNC:24804]	protein coding	1.02	3.83	32.79	<0.0001	<0.0001
ENSG00000196407	THEM5	thioesterase superfamily member 5 [Source:HGNC Symbol;Acc:HGNC:26755]	protein coding	-1.01	0.79	9.78	0.0018	0.0076
ENSG00000180096	SEPTIN1	septin 1 [Source:HGNC Symbol;Acc:HGNC:2879]	protein coding	-1.01	6.08	18.07	<0.0001	0.0003
ENSG00000197540	GZMM	granzyme M [Source:HGNC Symbol;Acc:HGNC:4712]	protein coding	-1.01	5.29	16.18	<0.0001	0.0006
ENSG00000106624	AEBP1	AE binding protein 1 [Source:HGNC Symbol;Acc:HGNC:303]	protein coding	-1.01	2.60	17.64	<0.0001	0.0003
ENSG00000213071	LPAL2	lipoprotein(a) like 2, pseudogene [Source:HGNC Symbol;Acc:HGNC:21210]	transcribed unprocessed pseudogene	-1.01	1.51	15.21	<0.0001	0.0008
ENSG00000215533	LINC00189	long intergenic non-protein coding RNA 189 [Source:HGNC Symbol;Acc:HGNC:18461]	lncRNA	1.01	0.53	27.66	<0.0001	<0.0001
ENSG00000134470	IL15RA	interleukin 15 receptor subunit alpha [Source:HGNC Symbol;Acc:HGNC:5978]	protein coding	1.01	4.13	49.79	<0.0001	<0.0001
ENSG00000226791	LINC02611	long intergenic non-protein coding RNA 2611 [Source:HGNC Symbol;Acc:HGNC:54060]	lncRNA	-1.01	0.50	14.87	0.0001	0.0009
ENSG00000096092	TMEM14A	transmembrane protein 14A [Source:HGNC Symbol;Acc:HGNC:21076]	protein coding	-1.01	0.82	14.83	0.0001	0.0009
ENSG00000113368	LMNB1	lamin B1 [Source:HGNC Symbol;Acc:HGNC:6637]	protein coding	1.01	6.18	42.53	<0.0001	<0.0001
ENSG00000103569	AQP9	aquaporin 9 [Source:HGNC Symbol;Acc:HGNC:643]	protein coding	1.01	6.15	29.73	<0.0001	<0.0001
ENSG00000152229	PSTPIP2	proline-serine-threonine phosphatase interacting protein 2 [Source:HGNC Symbol;Acc:HGNC:9581]	protein coding	1.01	6.47	50.33	<0.0001	<0.0001

Table S9: Genes differentially expressed at Day 2 compared to pre-first vaccination (Study Arm 2 A/H7N9+AS03, D1, 29, Dendritic cells). Genes are sorted by descending absolute log_2 fold change. Gene model summaries and annotations are based on Ensembl Version 99 (continued.)

Ensembl Gene ID	Ensembl Gene Name	Ensembl Gene Description	Gene Type	log_2 Fold Change (Day 2 vs. Pre-First Vaccination)	Average log_2 CPM	Likelihood Ratio Test Statistic	P-Value	FDR Adjusted P-Value
ENSG00000163251	FZD5	frizzled class receptor 5 [Source:HGNC Symbol;Acc:HGNC:4043]	protein coding	1.01	1.63	33.06	<0.0001	<0.0001
ENSG00000158517	NCF1	neutrophil cytosolic factor 1 [Source:HGNC Symbol;Acc:HGNC:7660]	protein coding	1.00	7.72	39.78	<0.0001	<0.0001
ENSG00000198785	GRIN3A	glutamate ionotropic receptor NMDA type subunit 3A [Source:HGNC Symbol;Acc:HGNC:16767]	protein coding	1.00	1.89	39.24	<0.0001	<0.0001
ENSG00000162433	AK4	adenylate kinase 4 [Source:HGNC Symbol;Acc:HGNC:363]	protein coding	1.00	1.17	35.33	<0.0001	<0.0001
ENSG00000262823		novel transcript, antisense to SPNS3	lncRNA	-1.00	0.83	25.26	<0.0001	<0.0001
ENSG00000267731		novel transcript	lncRNA	-1.00	0.67	13.25	0.0003	0.0018
ENSG00000173208	ABCD2	ATP binding cassette subfamily D member 2 [Source:HGNC Symbol;Acc:HGNC:66]	protein coding	-1.00	2.08	8.37	0.0038	0.0139
ENSG00000143185	XCL2	X-C motif chemokine ligand 2 [Source:HGNC Symbol;Acc:HGNC:10646]	protein coding	-1.00	1.31	11.32	0.0008	0.004
ENSG00000196586	MYO6	myosin VI [Source:HGNC Symbol;Acc:HGNC:7605]	protein coding	-1.00	1.56	11.54	0.0007	0.0036
ENSG00000171817	ZNF540	zinc finger protein 540 [Source:HGNC Symbol;Acc:HGNC:25331]	protein coding	-1.00	0.94	10.57	0.0011	0.0054
ENSG00000134755	DSC2	desmocollin 2 [Source:HGNC Symbol;Acc:HGNC:3036]	protein coding	1.00	4.02	37.88	<0.0001	<0.0001
ENSG00000145911	N4BP3	NEDD4 binding protein 3 [Source:HGNC Symbol;Acc:HGNC:29852]	protein coding	-1.00	1.58	11.84	0.0006	0.0032
ENSG00000055130	CUL1	cullin 1 [Source:HGNC Symbol;Acc:HGNC:2551]	protein coding	1.00	6.53	60.90	<0.0001	<0.0001
ENSG00000185507	IRF7	interferon regulatory factor 7 [Source:HGNC Symbol;Acc:HGNC:6122]	protein coding	1.00	7.35	32.66	<0.0001	<0.0001
ENSG00000109452	INPP4B	inositol polyphosphate-4-phosphatase type II B [Source:HGNC Symbol;Acc:HGNC:6075]	protein coding	-1.00	3.46	9.06	0.0026	0.0103
ENSG00000090975	PITPNM2	phosphatidylinositol transfer protein membrane associated 2 [Source:HGNC Symbol;Acc:HGNC:21044]	protein coding	-1.00	4.46	28.44	<0.0001	<0.0001
ENSG00000007264	MATK	megakaryocyte-associated tyrosine kinase [Source:HGNC Symbol;Acc:HGNC:6906]	protein coding	-0.99	5.75	16.73	<0.0001	0.0005
ENSG00000179344	HLA-DQB1	major histocompatibility complex, class II, DQ beta 1 [Source:HGNC Symbol;Acc:HGNC:4944]	protein coding	0.99	8.73	17.80	<0.0001	0.0003
ENSG00000154358	OBSCN	obscurin, cytoskeletal calmodulin and titin-interacting RhoGEF [Source:HGNC Symbol;Acc:HGNC:15719]	protein coding	-0.99	5.20	16.92	<0.0001	0.0004

Table S9: Genes differentially expressed at Day 2 compared to pre-first vaccination (Study Arm 2 A/H7N9+AS03, D1, 29, Dendritic cells). Genes are sorted by descending absolute log_2 fold change. Gene model summaries and annotations are based on Ensembl Version 99 (continued.)

Ensembl Gene ID	Ensembl Gene Name	Ensembl Gene Description	Gene Type	log_2 Fold Change (Day 2 vs. Pre-First Vaccination)	Average log_2 CPM	Likelihood Ratio Test Statistic	P-Value	FDR Adjusted P-Value
ENSG00000006432	MAP3K9	mitogen-activated protein kinase kinase kinase 9 [Source:HGNC Symbol;Acc:HGNC:6861]	protein coding	-0.99	1.77	14.86	0.0001	0.0009
ENSG00000100311	PDGFB	platelet derived growth factor subunit B [Source:HGNC Symbol;Acc:HGNC:8800]	protein coding	-0.99	1.38	8.10	0.0044	0.0157
ENSG00000172543	CTSW	cathepsin W [Source:HGNC Symbol;Acc:HGNC:2546]	protein coding	-0.99	6.53	19.55	<0.0001	0.0001
ENSG00000198286	CARD11	caspase recruitment domain family member 11 [Source:HGNC Symbol;Acc:HGNC:16393]	protein coding	-0.99	6.17	19.39	<0.0001	0.0002
ENSG00000148516	ZEB1	zinc finger E-box binding homeobox 1 [Source:HGNC Symbol;Acc:HGNC:11642]	protein coding	-0.99	2.74	18.56	<0.0001	0.0002
ENSG00000111331	OAS3	2'-5'-oligoadenylate synthetase 3 [Source:HGNC Symbol;Acc:HGNC:8088]	protein coding	0.99	7.98	18.37	<0.0001	0.0002
ENSG00000150045	KLRF1	killer cell lectin like receptor F1 [Source:HGNC Symbol;Acc:HGNC:13342]	protein coding	-0.98	4.49	21.49	<0.0001	<0.0001
ENSG00000164530	PI16	peptidase inhibitor 16 [Source:HGNC Symbol;Acc:HGNC:21245]	protein coding	-0.98	1.28	10.17	0.0014	0.0064
ENSG00000139572	GPR84	G protein-coupled receptor 84 [Source:HGNC Symbol;Acc:HGNC:4535]	protein coding	0.98	1.35	34.72	<0.0001	<0.0001
ENSG00000162739	SLAMF6	SLAM family member 6 [Source:HGNC Symbol;Acc:HGNC:21392]	protein coding	-0.98	4.84	17.79	<0.0001	0.0003
ENSG00000162383	SLC1A7	solute carrier family 1 member 7 [Source:HGNC Symbol;Acc:HGNC:10945]	protein coding	-0.98	1.74	10.16	0.0014	0.0064
ENSG00000135083	CCNJL	cyclin J like [Source:HGNC Symbol;Acc:HGNC:25876]	protein coding	-0.98	1.18	15.68	<0.0001	0.0007
ENSG00000101082	SLA2	Src like adaptor 2 [Source:HGNC Symbol;Acc:HGNC:17329]	protein coding	-0.97	3.73	15.80	<0.0001	0.0006
ENSG00000088538	DOCK3	dedicator of cytokinesis 3 [Source:HGNC Symbol;Acc:HGNC:2989]	protein coding	-0.97	0.88	20.05	<0.0001	0.0001
ENSG00000137501	SYTL2	synaptotagmin like 2 [Source:HGNC Symbol;Acc:HGNC:15585]	protein coding	-0.97	4.27	18.03	<0.0001	0.0003
ENSG00000175445	LPL	lipoprotein lipase [Source:HGNC Symbol;Acc:HGNC:6677]	protein coding	-0.97	2.10	13.04	0.0003	0.0019
ENSG00000185339	TCN2	transcobalamin 2 [Source:HGNC Symbol;Acc:HGNC:11653]	protein coding	0.97	4.89	43.73	<0.0001	<0.0001
ENSG00000180644	PRF1	perforin 1 [Source:HGNC Symbol;Acc:HGNC:9360]	protein coding	-0.97	8.10	13.84	0.0002	0.0014

Table S9: Genes differentially expressed at Day 2 compared to pre-first vaccination (Study Arm 2 A/H7N9+AS03, D1, 29, Dendritic cells). Genes are sorted by descending absolute log_2 fold change. Gene model summaries and annotations are based on Ensembl Version 99 (continued.)

Ensembl Gene ID	Ensembl Gene Name	Ensembl Gene Description	Gene Type	log_2 Fold Change (Day 2 vs. Pre-First Vaccination)	Average log_2 CPM	Likelihood Ratio Test Statistic	P-Value	FDR Adjusted P-Value
ENSG00000171049	FPR2	formyl peptide receptor 2 [Source:HGNC Symbol;Acc:HGNC:3827]	protein coding	0.97	5.47	34.27	<0.0001	<0.0001
ENSG00000163600	ICOS	inducible T cell costimulator [Source:HGNC Symbol;Acc:HGNC:5351]	protein coding	-0.97	1.88	9.24	0.0024	0.0095
ENSG00000157601	MX1	MX dynamin like GTPase 1 [Source:HGNC Symbol;Acc:HGNC:7532]	protein coding	0.96	7.44	15.62	<0.0001	0.0007
ENSG00000236609	ZNF853	zinc finger protein 853 [Source:HGNC Symbol;Acc:HGNC:21767]	protein coding	-0.96	2.11	18.56	<0.0001	0.0002
ENSG00000165178	NCF1C	neutrophil cytosolic factor 1C pseudogene [Source:HGNC Symbol;Acc:HGNC:32523]	unprocessed pseudogene	0.96	6.66	38.78	<0.0001	<0.0001
ENSG00000255026		novel transcript	lncRNA	-0.96	4.96	13.62	0.0002	0.0015
ENSG00000175265	GOLGA8A	golgin A8 family member A [Source:HGNC Symbol;Acc:HGNC:31972]	protein coding	-0.96	4.89	20.15	<0.0001	0.0001
ENSG00000158856	DMTN	dematin actin binding protein [Source:HGNC Symbol;Acc:HGNC:3382]	protein coding	-0.96	2.46	15.24	<0.0001	0.0008
ENSG00000167861	HID1	HID1 domain containing [Source:HGNC Symbol;Acc:HGNC:15736]	protein coding	-0.96	0.65	12.01	0.0005	0.003
ENSG00000154027	AK5	adenylate kinase 5 [Source:HGNC Symbol;Acc:HGNC:365]	protein coding	-0.96	2.25	14.86	0.0001	0.0009
ENSG00000149328	GLB1L2	galactosidase beta 1 like 2 [Source:HGNC Symbol;Acc:HGNC:25129]	protein coding	-0.96	0.75	10.82	0.001	0.0049
ENSG00000105255	FSD1	fibronectin type III and SPRY domain containing 1 [Source:HGNC Symbol;Acc:HGNC:13745]	protein coding	-0.96	1.15	13.70	0.0002	0.0015
ENSG00000105717	PBX4	PBX homeobox 4 [Source:HGNC Symbol;Acc:HGNC:13403]	protein coding	-0.96	2.20	14.16	0.0002	0.0012
ENSG00000137078	SIT1	signaling threshold regulating transmembrane adaptor 1 [Source:HGNC Symbol;Acc:HGNC:17710]	protein coding	-0.96	3.44	10.12	0.0015	0.0066
ENSG00000173068	BNC2	basonuclin 2 [Source:HGNC Symbol;Acc:HGNC:30988]	protein coding	-0.96	1.07	12.16	0.0005	0.0028
ENSG00000162496	DHRS3	dehydrogenase/reductase 3 [Source:HGNC Symbol;Acc:HGNC:17693]	protein coding	-0.96	3.45	21.46	<0.0001	<0.0001
ENSG00000272282	LINC02084	long intergenic non-protein coding RNA 2084 [Source:HGNC Symbol;Acc:HGNC:52934]	lncRNA	-0.96	1.65	16.44	<0.0001	0.0005
ENSG00000160318	CLDND2	claudin domain containing 2 [Source:HGNC Symbol;Acc:HGNC:28511]	protein coding	-0.95	2.75	20.72	<0.0001	<0.0001

Table S9: Genes differentially expressed at Day 2 compared to pre-first vaccination (Study Arm 2 A/H7N9+AS03, D1, 29, Dendritic cells). Genes are sorted by descending absolute log_2 fold change. Gene model summaries and annotations are based on Ensembl Version 99 (continued.)

Ensembl Gene ID	Ensembl Gene Name	Ensembl Gene Description	Gene Type	log_2 Fold Change (Day 2 vs. Pre-First Vaccination)	Average log_2 CPM	Likelihood Ratio Test Statistic	P-Value	FDR Adjusted P-Value
ENSG00000125648	SLC25A23	solute carrier family 25 member 23 [Source:HGNC Symbol;Acc:HGNC:19375]	protein coding	-0.95	2.42	13.70	0.0002	0.0015
ENSG00000145649	GZMA	granzyme A [Source:HGNC Symbol;Acc:HGNC:4708]	protein coding	-0.95	5.36	13.44	0.0002	0.0016
ENSG00000128335	APOL2	apolipoprotein L2 [Source:HGNC Symbol;Acc:HGNC:619]	protein coding	0.95	6.49	52.07	<0.0001	<0.0001
ENSG00000189430	NCR1	natural cytotoxicity triggering receptor 1 [Source:HGNC Symbol;Acc:HGNC:6731]	protein coding	-0.95	2.81	16.53	<0.0001	0.0005
ENSG00000184221	OLIG1	oligodendrocyte transcription factor 1 [Source:HGNC Symbol;Acc:HGNC:16983]	protein coding	-0.95	5.40	56.25	<0.0001	<0.0001
ENSG00000141576	RNF157	ring finger protein 157 [Source:HGNC Symbol;Acc:HGNC:29402]	protein coding	-0.95	2.86	9.10	0.0026	0.0101
ENSG00000179593	ALOX15B	arachidonate 15-lipoxygenase type B [Source:HGNC Symbol;Acc:HGNC:434]	protein coding	-0.95	2.36	25.81	<0.0001	<0.0001
ENSG00000182487	NCF1B	neutrophil cytosolic factor 1B pseudogene [Source:HGNC Symbol;Acc:HGNC:32522]	transcribed unprocessed pseudogene	0.95	5.74	39.36	<0.0001	<0.0001
ENSG00000107317	PTGDS	prostaglandin D2 synthase [Source:HGNC Symbol;Acc:HGNC:9592]	protein coding	-0.95	3.19	19.68	<0.0001	0.0001
ENSG00000139116	KIF21A	kinesin family member 21A [Source:HGNC Symbol;Acc:HGNC:19349]	protein coding	-0.95	2.19	12.21	0.0005	0.0027
ENSG00000198846	TOX	thymocyte selection associated high mobility group box [Source:HGNC Symbol;Acc:HGNC:18988]	protein coding	-0.95	2.12	17.61	<0.0001	0.0003
ENSG00000117226	GBP3	guanylate binding protein 3 [Source:HGNC Symbol;Acc:HGNC:4184]	protein coding	0.95	5.36	48.92	<0.0001	<0.0001
ENSG00000257524		novel protein	protein coding	-0.95	1.67	24.16	<0.0001	<0.0001
ENSG00000211694	TRGV10	T cell receptor gamma variable 10 (non-functional) [Source:HGNC Symbol;Acc:HGNC:12285]	TR V gene	-0.95	1.61	13.01	0.0003	0.002
ENSG00000181104	F2R	coagulation factor II thrombin receptor [Source:HGNC Symbol;Acc:HGNC:3537]	protein coding	-0.95	3.72	14.93	0.0001	0.0009
ENSG00000273272		novel transcript	lncRNA	0.95	0.62	12.78	0.0004	0.0022
ENSG00000145247	OCIAD2	OCIA domain containing 2 [Source:HGNC Symbol;Acc:HGNC:28685]	protein coding	-0.95	2.83	17.24	<0.0001	0.0004

Table S9: Genes differentially expressed at Day 2 compared to pre-first vaccination (Study Arm 2 A/H7N9+AS03, D1, 29, Dendritic cells). Genes are sorted by descending absolute log_2 fold change. Gene model summaries and annotations are based on Ensembl Version 99 (continued.)

Ensembl Gene ID	Ensembl Gene Name	Ensembl Gene Description	Gene Type	log_2 Fold Change (Day 2 vs. Pre-First Vaccination)	Average log_2 CPM	Likelihood Ratio Test Statistic	P-Value	FDR Adjusted P-Value
ENSG00000246526	LINC002481	long intergenic non-protein coding RNA 2481 [Source:HGNC Symbol;Acc:HGNC:27958]	lncRNA	-0.94	2.08	15.98	<0.0001	0.0006
ENSG00000100027	YPEL1	yippee like 1 [Source:HGNC Symbol;Acc:HGNC:12845]	protein coding	-0.94	2.88	22.37	<0.0001	<0.0001
ENSG00000158321	AUTS2	activator of transcription and developmental regulator AUTS2 [Source:HGNC Symbol;Acc:HGNC:14262]	protein coding	-0.94	4.11	22.04	<0.0001	<0.0001
ENSG00000188389	PDCD1	programmed cell death 1 [Source:HGNC Symbol;Acc:HGNC:8760]	protein coding	-0.94	1.26	11.14	0.0008	0.0043
ENSG00000132744	ACY3	aminoacylase 3 [Source:HGNC Symbol;Acc:HGNC:24104]	protein coding	-0.94	1.56	24.85	<0.0001	<0.0001
ENSG00000106992	AK1	adenylate kinase 1 [Source:HGNC Symbol;Acc:HGNC:361]	protein coding	-0.94	1.30	22.12	<0.0001	<0.0001
ENSG00000120210	INSL6	insulin like 6 [Source:HGNC Symbol;Acc:HGNC:6089]	protein coding	0.94	0.46	18.49	<0.0001	0.0002
ENSG00000126709	IFI6	interferon alpha inducible protein 6 [Source:HGNC Symbol;Acc:HGNC:4054]	protein coding	0.94	7.03	14.28	0.0002	0.0012
ENSG00000183087	GAS6	growth arrest specific 6 [Source:HGNC Symbol;Acc:HGNC:4168]	protein coding	0.94	4.79	64.46	<0.0001	<0.0001
ENSG00000267369		TAF5-like RNA polymerase II, p300/CBP-associated factor (PCAF)-associated factor, 65kDa (TAF5L) pseudogene	processed pseudogene	-0.94	2.51	12.69	0.0004	0.0022
ENSG00000168661	ZNF30	zinc finger protein 30 [Source:HGNC Symbol;Acc:HGNC:13090]	protein coding	-0.94	0.98	15.63	<0.0001	0.0007
ENSG00000157570	TSPAN18	tetraspanin 18 [Source:HGNC Symbol;Acc:HGNC:20660]	protein coding	-0.94	2.60	11.02	0.0009	0.0045
ENSG00000174749	FAM241A	family with sequence similarity 241 member A [Source:HGNC Symbol;Acc:HGNC:26813]	protein coding	0.93	4.70	54.60	<0.0001	<0.0001
ENSG00000156966	B3GNT7	UDP-GlcNAc:betaGal beta-1,3-N-acetylglucosaminyltransferase 7 [Source:HGNC Symbol;Acc:HGNC:18811]	protein coding	-0.93	2.23	32.16	<0.0001	<0.0001
ENSG00000242779	ZNF702P	zinc finger protein 702, pseudogene [Source:HGNC Symbol;Acc:HGNC:25775]	transcribed processed pseudogene	0.93	2.04	25.12	<0.0001	<0.0001
ENSG00000072195	SPEG	striated muscle enriched protein kinase [Source:HGNC Symbol;Acc:HGNC:16901]	protein coding	-0.93	1.74	9.36	0.0022	0.0091

Table S9: Genes differentially expressed at Day 2 compared to pre-first vaccination (Study Arm 2 A/H7N9+AS03, D1, 29, Dendritic cells). Genes are sorted by descending absolute log_2 fold change. Gene model summaries and annotations are based on Ensembl Version 99 (continued.)

Ensembl Gene ID	Ensembl Gene Name	Ensembl Gene Description	Gene Type	log_2 Fold Change (Day 2 vs. Pre-First Vaccination)	Average log_2 CPM	Likelihood Ratio Test Statistic	P-Value	FDR Adjusted P-Value
ENSG00000211893	IGHG2	immunoglobulin heavy constant gamma 2 (G2m marker) [Source:HGNC Symbol;Acc:HGNC:5526]	IG C gene	-0.93	5.34	14.36	0.0002	0.0011
ENSG00000112378	PERP	p53 apoptosis effector related to PMP22 [Source:HGNC Symbol;Acc:HGNC:17637]	protein coding	-0.93	0.45	11.14	0.0008	0.0043
ENSG00000133818	RRAS2	RAS related 2 [Source:HGNC Symbol;Acc:HGNC:17271]	protein coding	-0.93	2.61	14.16	0.0002	0.0012
ENSG00000204475	NCR3	natural cytotoxicity triggering receptor 3 [Source:HGNC Symbol;Acc:HGNC:19077]	protein coding	-0.93	3.65	16.13	<0.0001	0.0006
ENSG00000137628	DDX60	DExD/H-box helicase 60 [Source:HGNC Symbol;Acc:HGNC:25942]	protein coding	0.93	5.49	33.65	<0.0001	<0.0001
ENSG00000126243	LRFN3	leucine rich repeat and fibronectin type III domain containing 3 [Source:HGNC Symbol;Acc:HGNC:28370]	protein coding	-0.93	2.00	11.36	0.0008	0.0039
ENSG00000166313	APBB1	amyloid beta precursor protein binding family B member 1 [Source:HGNC Symbol;Acc:HGNC:581]	protein coding	-0.93	4.41	16.52	<0.0001	0.0005
ENSG00000287576		novel transcript	lncRNA	0.93	0.89	42.66	<0.0001	<0.0001
ENSG00000287733		novel transcript	lncRNA	0.93	0.93	30.22	<0.0001	<0.0001
ENSG00000116299	KIAA1324	KIAA1324 [Source:HGNC Symbol;Acc:HGNC:29618]	protein coding	-0.93	1.57	10.98	0.0009	0.0046
ENSG00000117560	FASLG	Fas ligand [Source:HGNC Symbol;Acc:HGNC:11936]	protein coding	-0.93	2.12	7.27	0.007	0.0225
ENSG00000100385	IL2RB	interleukin 2 receptor subunit beta [Source:HGNC Symbol;Acc:HGNC:6009]	protein coding	-0.93	7.15	17.10	<0.0001	0.0004
ENSG00000211966	IGHV5-51	immunoglobulin heavy variable 5-51 [Source:HGNC Symbol;Acc:HGNC:5659]	IG V gene	-0.92	1.99	9.62	0.0019	0.0081
ENSG00000174080	CTSF	cathepsin F [Source:HGNC Symbol;Acc:HGNC:2531]	protein coding	-0.92	3.11	22.38	<0.0001	<0.0001
ENSG00000125498	KIR2DL1	killer cell immunoglobulin like receptor, two Ig domains and long cytoplasmic tail 1 [Source:HGNC Symbol;Acc:HGNC:6329]	protein coding	-0.92	0.81	13.19	0.0003	0.0018
ENSG00000168421	RHOH	ras homolog family member H [Source:HGNC Symbol;Acc:HGNC:686]	protein coding	-0.92	4.31	14.85	0.0001	0.0009
ENSG00000224078	SNHG14	small nucleolar RNA host gene 14 [Source:HGNC Symbol;Acc:HGNC:37462]	lncRNA	-0.92	4.54	21.53	<0.0001	<0.0001
ENSG00000119714	GPR68	G protein-coupled receptor 68 [Source:HGNC Symbol;Acc:HGNC:4519]	protein coding	-0.92	2.96	12.18	0.0005	0.0028
ENSG00000268027		novel transcript, sense intronic to CEACAM21	lncRNA	-0.92	3.33	12.66	0.0004	0.0023

Table S9: Genes differentially expressed at Day 2 compared to pre-first vaccination (Study Arm 2 A/H7N9+AS03, D1, 29, Dendritic cells). Genes are sorted by descending absolute log_2 fold change. Gene model summaries and annotations are based on Ensembl Version 99 (continued.)

Ensembl Gene ID	Ensembl Gene Name	Ensembl Gene Description	Gene Type	log_2 Fold Change (Day 2 vs. Pre-First Vaccination)	Average log_2 CPM	Likelihood Ratio Test Statistic	P-Value	FDR Adjusted P-Value
ENSG00000054793	ATP9A	ATPase phospholipid transporting 9A (putative) [Source:HGNC Symbol;Acc:HGNC:13540]	protein coding	-0.92	1.20	18.84	<0.0001	0.0002
ENSG00000077984	CST7	cystatin F [Source:HGNC Symbol;Acc:HGNC:2479]	protein coding	-0.92	5.56	16.60	<0.0001	0.0005
ENSG00000130595	TNNT3	troponin T3, fast skeletal type [Source:HGNC Symbol;Acc:HGNC:11950]	protein coding	-0.92	1.56	20.41	<0.0001	0.0001
ENSG00000184574	LPAR5	lysophosphatidic acid receptor 5 [Source:HGNC Symbol;Acc:HGNC:13307]	protein coding	-0.91	2.81	15.11	0.0001	0.0008
ENSG00000160183	TMPRSS3	transmembrane serine protease 3 [Source:HGNC Symbol;Acc:HGNC:11877]	protein coding	0.91	1.31	21.13	<0.0001	<0.0001
ENSG00000154760	SLFN13	schlafen family member 13 [Source:HGNC Symbol;Acc:HGNC:26481]	protein coding	-0.91	4.38	23.68	<0.0001	<0.0001
ENSG00000166483	WEE1	WEE1 G2 checkpoint kinase [Source:HGNC Symbol;Acc:HGNC:12761]	protein coding	-0.91	1.74	16.33	<0.0001	0.0005
ENSG00000169918	OTUD7A	OTU deubiquitinase 7A [Source:HGNC Symbol;Acc:HGNC:20718]	protein coding	-0.91	0.87	14.08	0.0002	0.0013
ENSG00000168785	TSPAN5	tetraspanin 5 [Source:HGNC Symbol;Acc:HGNC:17753]	protein coding	-0.91	2.17	13.60	0.0002	0.0015
ENSG00000211829	TRDC	T cell receptor delta constant [Source:HGNC Symbol;Acc:HGNC:12253]	TR C gene	-0.91	3.98	10.08	0.0015	0.0067
ENSG00000099834	CDHR5	cadherin related family member 5 [Source:HGNC Symbol;Acc:HGNC:7521]	protein coding	0.91	1.21	25.86	<0.0001	<0.0001
ENSG00000140511	HAPLN3	hyaluronan and proteoglycan link protein 3 [Source:HGNC Symbol;Acc:HGNC:21446]	protein coding	0.91	4.48	16.52	<0.0001	0.0005
ENSG00000186827	TNFRSF4	TNF receptor superfamily member 4 [Source:HGNC Symbol;Acc:HGNC:11918]	protein coding	-0.91	2.07	12.46	0.0004	0.0025
ENSG00000149294	NCAM1	neural cell adhesion molecule 1 [Source:HGNC Symbol;Acc:HGNC:7656]	protein coding	-0.91	3.68	14.84	0.0001	0.0009
ENSG00000137198	GMPR	guanosine monophosphate reductase [Source:HGNC Symbol;Acc:HGNC:4376]	protein coding	0.91	2.02	13.72	0.0002	0.0015
ENSG00000234338		endogenous retroviral family W, env(C7), member 2 (ERVWE2) pseudogene	processed pseudogene	-0.91	0.41	15.45	<0.0001	0.0007
ENSG00000139193	CD27	CD27 molecule [Source:HGNC Symbol;Acc:HGNC:11922]	protein coding	-0.91	3.83	10.18	0.0014	0.0064

Table S9: Genes differentially expressed at Day 2 compared to pre-first vaccination (Study Arm 2 A/H7N9+AS03, D1, 29, Dendritic cells). Genes are sorted by descending absolute log_2 fold change. Gene model summaries and annotations are based on Ensembl Version 99 (continued.)

Ensembl Gene ID	Ensembl Gene Name	Ensembl Gene Description	Gene Type	log_2 Fold Change (Day 2 vs. Pre-First Vaccination)	Average log_2 CPM	Likelihood Ratio Test Statistic	P-Value	FDR Adjusted P-Value
ENSG00000211821	TRDV2	T cell receptor delta variable 2 [Source:HGNC Symbol;Acc:HGNC:12263]	TR V gene	-0.91	1.38	6.06	0.0139	0.038
ENSG00000221946	FXYD7	FXYD domain containing ion transport regulator 7 [Source:HGNC Symbol;Acc:HGNC:4034]	protein coding	-0.91	0.77	14.91	0.0001	0.0009
ENSG00000279059		TEC	TEC	-0.90	1.33	24.51	<0.0001	<0.0001
ENSG00000269954		novel transcript, antisense to BLK	lncRNA	-0.90	2.12	9.34	0.0022	0.0092
ENSG00000071575	TRIB2	tribbles pseudokinase 2 [Source:HGNC Symbol;Acc:HGNC:30809]	protein coding	-0.90	5.49	17.09	<0.0001	0.0004
ENSG00000211955	IGHV3-33	immunoglobulin heavy variable 3-33 [Source:HGNC Symbol;Acc:HGNC:5596]	IG V gene	-0.90	1.44	8.25	0.0041	0.0147
ENSG00000205930	C21orf62-AS1	C21orf62 antisense RNA 1 [Source:HGNC Symbol;Acc:HGNC:1290]	lncRNA	-0.90	1.24	17.56	<0.0001	0.0003
ENSG00000237945	LINC00649	long intergenic non-protein coding RNA 649 [Source:HGNC Symbol;Acc:HGNC:44305]	lncRNA	-0.90	3.86	12.99	0.0003	0.002
ENSG00000159618	ADGRG5	adhesion G protein-coupled receptor G5 [Source:HGNC Symbol;Acc:HGNC:19010]	protein coding	-0.90	4.36	19.85	<0.0001	0.0001
ENSG00000258926		novel transcript, antisense to PRKCH	lncRNA	-0.90	1.74	16.47	<0.0001	0.0005
ENSG00000100453	GZMB	granzyme B [Source:HGNC Symbol;Acc:HGNC:4709]	protein coding	-0.90	5.74	13.17	0.0003	0.0018
ENSG00000286786		novel transcript, antisense to RASGRP1	lncRNA	-0.90	1.53	15.85	<0.0001	0.0006
ENSG00000116667	C1orf21	chromosome 1 open reading frame 21 [Source:HGNC Symbol;Acc:HGNC:15494]	protein coding	-0.90	3.36	17.47	<0.0001	0.0003
ENSG00000088827	SIGLEC1	sialic acid binding Ig like lectin 1 [Source:HGNC Symbol;Acc:HGNC:11127]	protein coding	0.90	6.64	8.38	0.0038	0.0139
ENSG00000169136	ATF5	activating transcription factor 5 [Source:HGNC Symbol;Acc:HGNC:790]	protein coding	0.90	5.65	40.24	<0.0001	<0.0001
ENSG00000240065	PSMB9	proteasome 20S subunit beta 9 [Source:HGNC Symbol;Acc:HGNC:9546]	protein coding	0.90	7.67	41.14	<0.0001	<0.0001
ENSG00000142156	COL6A1	collagen type VI alpha 1 chain [Source:HGNC Symbol;Acc:HGNC:2211]	protein coding	-0.90	1.65	12.04	0.0005	0.0029
ENSG00000196169	KIF19	kinesin family member 19 [Source:HGNC Symbol;Acc:HGNC:26735]	protein coding	-0.90	0.85	7.85	0.0051	0.0175
ENSG00000112182	BACH2	BTB domain and CNC homolog 2 [Source:HGNC Symbol;Acc:HGNC:14078]	protein coding	-0.90	4.04	13.08	0.0003	0.0019
ENSG00000271447	MMP28	matrix metallopeptidase 28 [Source:HGNC Symbol;Acc:HGNC:14366]	protein coding	-0.89	0.55	7.92	0.0049	0.0169

Table S9: Genes differentially expressed at Day 2 compared to pre-first vaccination (Study Arm 2 A/H7N9+AS03, D1, 29, Dendritic cells). Genes are sorted by descending absolute log_2 fold change. Gene model summaries and annotations are based on Ensembl Version 99 (continued.)

Ensembl Gene ID	Ensembl Gene Name	Ensembl Gene Description	Gene Type	log_2 Fold Change (Day 2 vs. Pre-First Vaccination)	Average log_2 CPM	Likelihood Ratio Test Statistic	P-Value	FDR Adjusted P-Value
ENSG00000168813	ZNF507	zinc finger protein 507 [Source:HGNC Symbol;Acc:HGNC:23783]	protein coding	-0.89	3.46	16.87	<0.0001	0.0004
ENSG00000141622	RNF165	ring finger protein 165 [Source:HGNC Symbol;Acc:HGNC:31696]	protein coding	-0.89	2.19	17.37	<0.0001	0.0004
ENSG00000142798	HSPG2	heparan sulfate proteoglycan 2 [Source:HGNC Symbol;Acc:HGNC:5273]	protein coding	-0.89	2.13	29.75	<0.0001	<0.0001
ENSG00000125430	HS3ST3B1	heparan sulfate-glucosamine 3-sulfotransferase 3B1 [Source:HGNC Symbol;Acc:HGNC:5198]	protein coding	-0.89	3.03	16.31	<0.0001	0.0005
ENSG00000182809	CRIP2	cysteine rich protein 2 [Source:HGNC Symbol;Acc:HGNC:2361]	protein coding	-0.89	2.85	18.34	<0.0001	0.0002
ENSG00000159433	STARD9	StAR related lipid transfer domain containing 9 [Source:HGNC Symbol;Acc:HGNC:19162]	protein coding	-0.89	3.23	19.17	<0.0001	0.0002
ENSG00000184860	SDR42E1	short chain dehydrogenase/reductase family 42E, member 1 [Source:HGNC Symbol;Acc:HGNC:29834]	protein coding	-0.89	1.46	12.95	0.0003	0.002
ENSG00000237989	LINC01679	long intergenic non-protein coding RNA 1679 [Source:HGNC Symbol;Acc:HGNC:52469]	lncRNA	0.88	0.50	20.92	<0.0001	<0.0001
ENSG00000105835	NAMPT	nicotinamide phosphoribosyltransferase [Source:HGNC Symbol;Acc:HGNC:30092]	protein coding	0.88	7.30	31.62	<0.0001	<0.0001
ENSG00000101096	NFATC2	nuclear factor of activated T cells 2 [Source:HGNC Symbol;Acc:HGNC:7776]	protein coding	-0.88	5.28	19.65	<0.0001	0.0001
ENSG00000108679	LGALS3BP	galectin 3 binding protein [Source:HGNC Symbol;Acc:HGNC:6564]	protein coding	0.88	5.18	20.96	<0.0001	<0.0001
ENSG00000167664	TMIGD2	transmembrane and immunoglobulin domain containing 2 [Source:HGNC Symbol;Acc:HGNC:28324]	protein coding	-0.88	2.94	12.47	0.0004	0.0025
ENSG00000027075	PRKCH	protein kinase C eta [Source:HGNC Symbol;Acc:HGNC:9403]	protein coding	-0.88	5.91	18.72	<0.0001	0.0002
ENSG00000248334	WHAMMP2	WAS protein homolog associated with actin, golgi membranes and microtubules pseudogene 2 [Source:HGNC Symbol;Acc:HGNC:32360]	transcribed unprocessed pseudogene	-0.88	2.48	23.23	<0.0001	<0.0001
ENSG00000112096	SOD2	superoxide dismutase 2 [Source:HGNC Symbol;Acc:HGNC:11180]	protein coding	0.88	8.01	43.95	<0.0001	<0.0001
ENSG00000171033	PKIA	cAMP-dependent protein kinase inhibitor alpha [Source:HGNC Symbol;Acc:HGNC:9017]	protein coding	-0.88	1.61	10.10	0.0015	0.0066

Table S9: Genes differentially expressed at Day 2 compared to pre-first vaccination (Study Arm 2 A/H7N9+AS03, D1, 29, Dendritic cells). Genes are sorted by descending absolute log_2 fold change. Gene model summaries and annotations are based on Ensembl Version 99 (continued.)

Ensembl Gene ID	Ensembl Gene Name	Ensembl Gene Description	Gene Type	log_2 Fold Change (Day 2 vs. Pre-First Vaccination)	Average log_2 CPM	Likelihood Ratio Test Statistic	P-Value	FDR Adjusted P-Value
ENSG00000139832	RAB20	RAB20, member RAS oncogene family [Source:HGNC Symbol;Acc:HGNC:18260]	protein coding	0.88	4.80	31.09	<0.0001	<0.0001
ENSG00000160856	FCRL3	Fc receptor like 3 [Source:HGNC Symbol;Acc:HGNC:18506]	protein coding	-0.88	5.03	13.76	0.0002	0.0014
ENSG00000168056	LTBP3	latent transforming growth factor beta binding protein 3 [Source:HGNC Symbol;Acc:HGNC:6716]	protein coding	-0.88	6.27	20.17	<0.0001	0.0001
ENSG00000130787	HIP1R	huntingtin interacting protein 1 related [Source:HGNC Symbol;Acc:HGNC:18415]	protein coding	-0.88	4.96	13.77	0.0002	0.0014
ENSG00000170629	DPY19L2P2	DPY19L2 pseudogene 2 [Source:HGNC Symbol;Acc:HGNC:21764]	transcribed unprocessed pseudogene	-0.88	1.17	9.46	0.0021	0.0087
ENSG00000158106	RHPN1	rhophilin Rho GTPase binding protein 1 [Source:HGNC Symbol;Acc:HGNC:19973]	protein coding	-0.88	3.67	14.06	0.0002	0.0013
ENSG00000120833	SOCS2	suppressor of cytokine signaling 2 [Source:HGNC Symbol;Acc:HGNC:19382]	protein coding	-0.88	2.59	16.93	<0.0001	0.0004
ENSG00000238121	LINC00426	long intergenic non-protein coding RNA 426 [Source:HGNC Symbol;Acc:HGNC:42761]	lncRNA	-0.87	1.96	11.63	0.0007	0.0035
ENSG00000113758	DBN1	drebrin 1 [Source:HGNC Symbol;Acc:HGNC:2695]	protein coding	-0.87	2.99	25.73	<0.0001	<0.0001
ENSG00000111371	SLC38A1	solute carrier family 38 member 1 [Source:HGNC Symbol;Acc:HGNC:13447]	protein coding	-0.87	6.59	20.27	<0.0001	0.0001
ENSG00000159674	SPON2	spondin 2 [Source:HGNC Symbol;Acc:HGNC:11253]	protein coding	-0.87	6.84	13.89	0.0002	0.0014
ENSG00000111335	OAS2	2'-5'-oligoadenylate synthetase 2 [Source:HGNC Symbol;Acc:HGNC:8087]	protein coding	0.87	7.57	23.12	<0.0001	<0.0001
ENSG00000176049	JAKMIP2	janus kinase and microtubule interacting protein 2 [Source:HGNC Symbol;Acc:HGNC:29067]	protein coding	-0.87	1.45	14.41	0.0001	0.0011
ENSG00000175505	CLCF1	cardiotrophin like cytokine factor 1 [Source:HGNC Symbol;Acc:HGNC:17412]	protein coding	-0.87	3.17	46.60	<0.0001	<0.0001
ENSG00000127954	STEAP4	STEAP4 metalloreductase [Source:HGNC Symbol;Acc:HGNC:21923]	protein coding	0.87	4.75	44.80	<0.0001	<0.0001
ENSG00000116661	FBXO2	F-box protein 2 [Source:HGNC Symbol;Acc:HGNC:13581]	protein coding	-0.87	0.84	9.44	0.0021	0.0088

Table S9: Genes differentially expressed at Day 2 compared to pre-first vaccination (Study Arm 2 A/H7N9+AS03, D1, 29, Dendritic cells). Genes are sorted by descending absolute log_2 fold change. Gene model summaries and annotations are based on Ensembl Version 99 (continued.)

Ensembl Gene ID	Ensembl Gene Name	Ensembl Gene Description	Gene Type	log_2 Fold Change (Day 2 vs. Pre-First Vaccination)	Average log_2 CPM	Likelihood Ratio Test Statistic	P-Value	FDR Adjusted P-Value
ENSG00000211892	IGHG4	immunoglobulin heavy constant gamma 4 (G4m marker) [Source:HGNC Symbol;Acc:HGNC:5528]	IG C gene	-0.87	2.18	6.71	0.0096	0.0287
ENSG00000159899	NPR2	natriuretic peptide receptor 2 [Source:HGNC Symbol;Acc:HGNC:7944]	protein coding	-0.87	0.95	16.76	<0.0001	0.0004
ENSG00000186891	TNFRSF18	TNF receptor superfamily member 18 [Source:HGNC Symbol;Acc:HGNC:11914]	protein coding	-0.87	2.53	16.90	<0.0001	0.0004
ENSG00000165806	CASP7	caspase 7 [Source:HGNC Symbol;Acc:HGNC:1508]	protein coding	0.87	4.77	45.73	<0.0001	<0.0001
ENSG00000133321	PLAAT4	phospholipase A and acyltransferase 4 [Source:HGNC Symbol;Acc:HGNC:9869]	protein coding	0.87	5.56	49.72	<0.0001	<0.0001
ENSG00000112290	WASF1	WASP family member 1 [Source:HGNC Symbol;Acc:HGNC:12732]	protein coding	0.87	1.27	43.98	<0.0001	<0.0001
ENSG00000166405	RIC3	RIC3 acetylcholine receptor chaperone [Source:HGNC Symbol;Acc:HGNC:30338]	protein coding	-0.87	2.22	9.83	0.0017	0.0074
ENSG00000118922	KLF12	Kruppel like factor 12 [Source:HGNC Symbol;Acc:HGNC:6346]	protein coding	-0.87	5.27	32.89	<0.0001	<0.0001
ENSG00000115604	IL18R1	interleukin 18 receptor 1 [Source:HGNC Symbol;Acc:HGNC:5988]	protein coding	-0.87	3.68	30.58	<0.0001	<0.0001
ENSG00000188313	PLSCR1	phospholipid scramblase 1 [Source:HGNC Symbol;Acc:HGNC:9092]	protein coding	0.87	6.64	30.56	<0.0001	<0.0001
ENSG00000257017	HP	haptoglobin [Source:HGNC Symbol;Acc:HGNC:5141]	protein coding	0.87	2.21	23.19	<0.0001	<0.0001
ENSG00000111863	ADTRP	androgen dependent TFPI regulating protein [Source:HGNC Symbol;Acc:HGNC:21214]	protein coding	-0.87	1.38	7.59	0.0059	0.0196
ENSG00000274767		novel transcript, antisense CCL3L3	lncRNA	-0.86	1.02	22.14	<0.0001	<0.0001
ENSG00000183134	PTGDR2	prostaglandin D2 receptor 2 [Source:HGNC Symbol;Acc:HGNC:4502]	protein coding	-0.86	4.58	25.37	<0.0001	<0.0001
ENSG00000211896	IGHG1	immunoglobulin heavy constant gamma 1 (G1m marker) [Source:HGNC Symbol;Acc:HGNC:5525]	IG C gene	-0.86	5.90	5.53	0.0187	0.0482
ENSG00000128536	CDHR3	cadherin related family member 3 [Source:HGNC Symbol;Acc:HGNC:26308]	protein coding	-0.86	0.45	12.39	0.0004	0.0025
ENSG00000185880	TRIM69	tripartite motif containing 69 [Source:HGNC Symbol;Acc:HGNC:17857]	protein coding	0.86	5.49	45.61	<0.0001	<0.0001
ENSG00000175899	A2M	alpha-2-macroglobulin [Source:HGNC Symbol;Acc:HGNC:7]	protein coding	-0.86	1.72	18.03	<0.0001	0.0003

Table S9: Genes differentially expressed at Day 2 compared to pre-first vaccination (Study Arm 2 A/H7N9+AS03, D1, 29, Dendritic cells). Genes are sorted by descending absolute log_2 fold change. Gene model summaries and annotations are based on Ensembl Version 99 (continued.)

Ensembl Gene ID	Ensembl Gene Name	Ensembl Gene Description	Gene Type	log_2 Fold Change (Day 2 vs. Pre-First Vaccination)	Average log_2 CPM	Likelihood Ratio Test Statistic	P-Value	FDR Adjusted P-Value
ENSG00000170581	STAT2	signal transducer and activator of transcription 2 [Source:HGNC Symbol;Acc:HGNC:11363]	protein coding	0.86	8.72	35.21	<0.0001	<0.0001
ENSG00000112294	ALDH5A1	aldehyde dehydrogenase 5 family member A1 [Source:HGNC Symbol;Acc:HGNC:408]	protein coding	-0.86	2.28	9.07	0.0026	0.0103
ENSG00000166002	SMCO4	single-pass membrane protein with coiled-coil domains 4 [Source:HGNC Symbol;Acc:HGNC:24810]	protein coding	0.86	5.81	56.07	<0.0001	<0.0001
ENSG00000119514	GALNT12	polypeptide N-acetylgalactosaminyltransferase 12 [Source:HGNC Symbol;Acc:HGNC:19877]	protein coding	-0.86	1.76	13.83	0.0002	0.0014
ENSG00000096968	JAK2	Janus kinase 2 [Source:HGNC Symbol;Acc:HGNC:6192]	protein coding	0.86	7.38	45.14	<0.0001	<0.0001
ENSG00000185101	ANO9	anoctamin 9 [Source:HGNC Symbol;Acc:HGNC:20679]	protein coding	-0.86	5.10	17.14	<0.0001	0.0004
ENSG00000211698	TRGV4	T cell receptor gamma variable 4 [Source:HGNC Symbol;Acc:HGNC:12289]	TR V gene	-0.86	1.16	12.15	0.0005	0.0028
ENSG00000080493	SLC4A4	solute carrier family 4 member 4 [Source:HGNC Symbol;Acc:HGNC:11030]	protein coding	-0.86	0.85	13.91	0.0002	0.0014
ENSG00000114107	CEP70	centrosomal protein 70 [Source:HGNC Symbol;Acc:HGNC:29972]	protein coding	-0.86	0.44	10.29	0.0013	0.0061
ENSG00000135736	CCDC102A	coiled-coil domain containing 102A [Source:HGNC Symbol;Acc:HGNC:28097]	protein coding	-0.85	2.55	12.65	0.0004	0.0023
ENSG00000038945	MSR1	macrophage scavenger receptor 1 [Source:HGNC Symbol;Acc:HGNC:7376]	protein coding	0.85	4.44	39.74	<0.0001	<0.0001
ENSG00000205846	CLEC6A	C-type lectin domain containing 6A [Source:HGNC Symbol;Acc:HGNC:14556]	protein coding	0.85	1.82	25.19	<0.0001	<0.0001
ENSG00000211945	IGHV1-18	immunoglobulin heavy variable 1-18 [Source:HGNC Symbol;Acc:HGNC:5549]	IG V gene	-0.85	1.25	7.59	0.0059	0.0196
ENSG00000186854	TRABD2A	TraB domain containing 2A [Source:HGNC Symbol;Acc:HGNC:27013]	protein coding	-0.85	4.83	10.93	0.0009	0.0047
ENSG00000105855	ITGB8	integrin subunit beta 8 [Source:HGNC Symbol;Acc:HGNC:6163]	protein coding	-0.85	0.46	11.84	0.0006	0.0032
ENSG00000123453	SARDH	sarcosine dehydrogenase [Source:HGNC Symbol;Acc:HGNC:10536]	protein coding	-0.85	0.85	7.85	0.0051	0.0175
ENSG00000185338	SOCS1	suppressor of cytokine signaling 1 [Source:HGNC Symbol;Acc:HGNC:19383]	protein coding	0.85	1.79	9.19	0.0024	0.0098
ENSG00000221866	PLXNA4	plexin A4 [Source:HGNC Symbol;Acc:HGNC:9102]	protein coding	-0.85	0.63	12.10	0.0005	0.0029

Table S9: Genes differentially expressed at Day 2 compared to pre-first vaccination (Study Arm 2 A/H7N9+AS03, D1, 29, Dendritic cells). Genes are sorted by descending absolute log_2 fold change. Gene model summaries and annotations are based on Ensembl Version 99 (continued.)

Ensembl Gene ID	Ensembl Gene Name	Ensembl Gene Description	Gene Type	log_2 Fold Change (Day 2 vs. Pre-First Vaccination)	Average log_2 CPM	Likelihood Ratio Test Statistic	P-Value	FDR Adjusted P-Value
ENSG00000204381	LAYN	layilin [Source:HGNC Symbol;Acc:HGNC:29471]	protein coding	-0.85	0.46	14.55	0.0001	0.001
ENSG00000174807	CD248	CD248 molecule [Source:HGNC Symbol;Acc:HGNC:18219]	protein coding	-0.85	1.34	6.27	0.0123	0.0346
ENSG00000110318	CEP126	centrosomal protein 126 [Source:HGNC Symbol;Acc:HGNC:29264]	protein coding	-0.85	1.54	18.19	<0.0001	0.0003
ENSG00000171916	LGALS9C	galectin 9C [Source:HGNC Symbol;Acc:HGNC:33874]	protein coding	-0.84	0.92	17.57	<0.0001	0.0003
ENSG00000117090	SLAMF1	signaling lymphocytic activation molecule family member 1 [Source:HGNC Symbol;Acc:HGNC:10903]	protein coding	-0.84	2.99	11.26	0.0008	0.0041
ENSG00000108370	RGS9	regulator of G protein signaling 9 [Source:HGNC Symbol;Acc:HGNC:10004]	protein coding	-0.84	1.73	11.24	0.0008	0.0041
ENSG00000164114	MAP9	microtubule associated protein 9 [Source:HGNC Symbol;Acc:HGNC:26118]	protein coding	-0.84	0.74	11.08	0.0009	0.0044
ENSG00000247774	PCED1B-AS1	PCED1B antisense RNA 1 [Source:HGNC Symbol;Acc:HGNC:44166]	lncRNA	-0.84	5.45	14.43	0.0001	0.0011
ENSG00000255987	TOMM20P2	TOMM20 pseudogene 2 [Source:HGNC Symbol;Acc:HGNC:50519]	processed pseudogene	-0.84	0.63	10.02	0.0015	0.0068
ENSG00000198885	ITPRIPL1	ITPRIP like 1 [Source:HGNC Symbol;Acc:HGNC:29371]	protein coding	-0.84	2.49	15.28	<0.0001	0.0008
ENSG00000069020	MAST4	microtubule associated serine/threonine kinase family member 4 [Source:HGNC Symbol;Acc:HGNC:19037]	protein coding	-0.84	2.77	12.86	0.0003	0.0021
ENSG00000179639	FCER1A	Fc fragment of IgE receptor Ia [Source:HGNC Symbol;Acc:HGNC:3609]	protein coding	-0.84	6.25	25.45	<0.0001	<0.0001
ENSG00000170525	PFKFB3	6-phosphofructo-2-kinase/fructose-2,6-biphosphatase 3 [Source:HGNC Symbol;Acc:HGNC:8874]	protein coding	0.84	6.33	26.04	<0.0001	<0.0001
ENSG00000175164	ABO	ABO, alpha 1-3-N-acetylgalactosaminyltransferase and alpha 1-3-galactosyltransferase [Source:HGNC Symbol;Acc:HGNC:79]	protein coding	0.84	1.93	12.62	0.0004	0.0023
ENSG00000264522	OTUD7B	OTU deubiquitinase 7B [Source:HGNC Symbol;Acc:HGNC:16683]	protein coding	-0.84	1.87	13.92	0.0002	0.0014
ENSG00000135604	STX11	syntaxin 11 [Source:HGNC Symbol;Acc:HGNC:11429]	protein coding	0.83	8.01	33.79	<0.0001	<0.0001
ENSG00000260910	LINC00565	long intergenic non-protein coding RNA 565 [Source:HGNC Symbol;Acc:HGNC:43709]	lncRNA	-0.83	0.64	6.02	0.0141	0.0386

Table S9: Genes differentially expressed at Day 2 compared to pre-first vaccination (Study Arm 2 A/H7N9+AS03, D1, 29, Dendritic cells). Genes are sorted by descending absolute log_2 fold change. Gene model summaries and annotations are based on Ensembl Version 99 (continued.)

Ensembl Gene ID	Ensembl Gene Name	Ensembl Gene Description	Gene Type	log_2 Fold Change (Day 2 vs. Pre-First Vaccination)	Average log_2 CPM	Likelihood Ratio Test Statistic	P-Value	FDR Adjusted P-Value
ENSG00000122224	LY9	lymphocyte antigen 9 [Source:HGNC Symbol;Acc:HGNC:6730]	protein coding	-0.83	4.40	15.57	<0.0001	0.0007
ENSG00000164136	IL15	interleukin 15 [Source:HGNC Symbol;Acc:HGNC:5977]	protein coding	0.83	4.94	35.89	<0.0001	<0.0001
ENSG00000105374	NKG7	natural killer cell granule protein 7 [Source:HGNC Symbol;Acc:HGNC:7830]	protein coding	-0.83	7.38	16.11	<0.0001	0.0006
ENSG00000109680	TBC1D19	TBC1 domain family member 19 [Source:HGNC Symbol;Acc:HGNC:25624]	protein coding	-0.83	0.63	24.44	<0.0001	<0.0001
ENSG00000144199	FAHD2B	fumarylacetoacetate hydrolase domain containing 2B [Source:HGNC Symbol;Acc:HGNC:25318]	protein coding	-0.83	0.81	15.80	<0.0001	0.0006
ENSG00000100100	PIK3IP1	phosphoinositide-3-kinase interacting protein 1 [Source:HGNC Symbol;Acc:HGNC:24942]	protein coding	-0.83	6.89	23.76	<0.0001	<0.0001
ENSG00000171101	SIGLEC17P	sialic acid binding Ig like lectin 17, pseudogene [Source:HGNC Symbol;Acc:HGNC:15604]	transcribed unprocessed pseudogene	-0.83	2.90	22.50	<0.0001	<0.0001
ENSG00000240445	FOXO3B	forkhead box O3B [Source:HGNC Symbol;Acc:HGNC:3822]	protein coding	-0.83	0.99	16.97	<0.0001	0.0004
ENSG00000179240	GVQW3	GVQW motif containing 3 [Source:HGNC Symbol;Acc:HGNC:51239]	protein coding	-0.83	2.22	19.02	<0.0001	0.0002
ENSG00000116254	CHD5	chromodomain helicase DNA binding protein 5 [Source:HGNC Symbol;Acc:HGNC:16816]	protein coding	-0.83	1.22	23.94	<0.0001	<0.0001
ENSG00000072163	LIMS2	LIM zinc finger domain containing 2 [Source:HGNC Symbol;Acc:HGNC:16084]	protein coding	-0.83	0.78	11.33	0.0008	0.004
ENSG00000041353	RAB27B	RAB27B, member RAS oncogene family [Source:HGNC Symbol;Acc:HGNC:9767]	protein coding	-0.83	1.57	13.15	0.0003	0.0019
ENSG00000130202	NECTIN2	nectin cell adhesion molecule 2 [Source:HGNC Symbol;Acc:HGNC:9707]	protein coding	0.82	4.42	46.87	<0.0001	<0.0001
ENSG00000146021	KLHL3	kelch like family member 3 [Source:HGNC Symbol;Acc:HGNC:6354]	protein coding	-0.82	2.57	13.71	0.0002	0.0015
ENSG00000184986	TMEM121	transmembrane protein 121 [Source:HGNC Symbol;Acc:HGNC:20511]	protein coding	-0.82	0.37	11.30	0.0008	0.004
ENSG00000197093	GAL3ST4	galactose-3-O-sulfotransferase 4 [Source:HGNC Symbol;Acc:HGNC:24145]	protein coding	-0.82	1.96	10.00	0.0016	0.0069

Table S9: Genes differentially expressed at Day 2 compared to pre-first vaccination (Study Arm 2 A/H7N9+AS03, D1, 29, Dendritic cells). Genes are sorted by descending absolute log_2 fold change. Gene model summaries and annotations are based on Ensembl Version 99 (continued.)

Ensembl Gene ID	Ensembl Gene Name	Ensembl Gene Description	Gene Type	log_2 Fold Change (Day 2 vs. Pre-First Vaccination)	Average log_2 CPM	Likelihood Ratio Test Statistic	P-Value	FDR Adjusted P-Value
ENSG00000004468	CD38	CD38 molecule [Source:HGNC Symbol;Acc:HGNC:1667]	protein coding	0.82	4.67	32.08	<0.0001	<0.0001
ENSG00000139714	MORN3	MORN repeat containing 3 [Source:HGNC Symbol;Acc:HGNC:29807]	protein coding	-0.82	1.49	16.93	<0.0001	0.0004
ENSG00000163485	ADORA1	adenosine A1 receptor [Source:HGNC Symbol;Acc:HGNC:262]	protein coding	-0.82	1.14	8.63	0.0033	0.0124
ENSG00000170128	GPR25	G protein-coupled receptor 25 [Source:HGNC Symbol;Acc:HGNC:4480]	protein coding	-0.82	1.10	7.45	0.0064	0.0209
ENSG00000185436	IFNLR1	interferon lambda receptor 1 [Source:HGNC Symbol;Acc:HGNC:18584]	protein coding	-0.82	0.90	6.84	0.0089	0.0273
ENSG00000105426	PTPRS	protein tyrosine phosphatase receptor type S [Source:HGNC Symbol;Acc:HGNC:9681]	protein coding	-0.82	0.37	7.49	0.0062	0.0205
ENSG00000272941		novel transcript, antisense to C7orf49	lncRNA	0.82	0.25	26.70	<0.0001	<0.0001
ENSG00000258352		interferon induced transmembrane protein 3 (1-8U) (IFITM3) pseudogene	transcribed processed pseudogene	-0.82	0.85	10.89	0.001	0.0048
ENSG00000267074		novel transcript, sense intronic SLFN12L	lncRNA	-0.82	1.79	10.29	0.0013	0.0061
ENSG00000256262	USP30-AS1	USP30 antisense RNA 1 [Source:HGNC Symbol;Acc:HGNC:40909]	lncRNA	0.82	1.53	29.47	<0.0001	<0.0001
ENSG00000163814	CDCP1	CUB domain containing protein 1 [Source:HGNC Symbol;Acc:HGNC:24357]	protein coding	0.82	1.51	30.58	<0.0001	<0.0001
ENSG00000136848	DAB2IP	DAB2 interacting protein [Source:HGNC Symbol;Acc:HGNC:17294]	protein coding	-0.82	0.54	15.30	<0.0001	0.0008
ENSG00000144476	ACKR3	atypical chemokine receptor 3 [Source:HGNC Symbol;Acc:HGNC:23692]	protein coding	-0.82	1.67	22.10	<0.0001	<0.0001
ENSG00000196139	AKR1C3	aldo-keto reductase family 1 member C3 [Source:HGNC Symbol;Acc:HGNC:386]	protein coding	-0.82	2.26	11.81	0.0006	0.0032
ENSG00000164512	ANKRD55	ankyrin repeat domain 55 [Source:HGNC Symbol;Acc:HGNC:25681]	protein coding	-0.81	0.96	11.46	0.0007	0.0037
ENSG00000169031	COL4A3	collagen type IV alpha 3 chain [Source:HGNC Symbol;Acc:HGNC:2204]	protein coding	-0.81	0.97	7.48	0.0062	0.0206
ENSG00000154165	GPR15	G protein-coupled receptor 15 [Source:HGNC Symbol;Acc:HGNC:4469]	protein coding	-0.81	0.67	6.73	0.0095	0.0285
ENSG00000141696	P3H4	prolyl 3-hydroxylase family member 4 (inactive) [Source:HGNC Symbol;Acc:HGNC:16946]	protein coding	-0.81	1.37	16.38	<0.0001	0.0005

Table S9: Genes differentially expressed at Day 2 compared to pre-first vaccination (Study Arm 2 A/H7N9+AS03, D1, 29, Dendritic cells). Genes are sorted by descending absolute log_2 fold change. Gene model summaries and annotations are based on Ensembl Version 99 (continued.)

Ensembl Gene ID	Ensembl Gene Name	Ensembl Gene Description	Gene Type	log_2 Fold Change (Day 2 vs. Pre-First Vaccination)	Average log_2 CPM	Likelihood Ratio Test Statistic	P-Value	FDR Adjusted P-Value
ENSG00000130649	CYP2E1	cytochrome P450 family 2 subfamily E member 1 [Source:HGNC Symbol;Acc:HGNC:2631]	protein coding	-0.81	0.34	21.64	<0.0001	<0.0001
ENSG00000179715	PCED1B	PC-esterase domain containing 1B [Source:HGNC Symbol;Acc:HGNC:28255]	protein coding	-0.81	4.45	10.02	0.0016	0.0069
ENSG00000196172	ZNF681	zinc finger protein 681 [Source:HGNC Symbol;Acc:HGNC:26457]	protein coding	-0.81	0.82	10.32	0.0013	0.006
ENSG00000235162	C12orf75	chromosome 12 open reading frame 75 [Source:HGNC Symbol;Acc:HGNC:35164]	protein coding	-0.81	3.45	19.82	<0.0001	0.0001
ENSG00000232931	LINC00342	long intergenic non-protein coding RNA 342 [Source:HGNC Symbol;Acc:HGNC:42470]	lncRNA	-0.81	4.21	16.57	<0.0001	0.0005
ENSG00000072858	SIDT1	SID1 transmembrane family member 1 [Source:HGNC Symbol;Acc:HGNC:25967]	protein coding	-0.81	4.08	16.62	<0.0001	0.0005
ENSG00000148926	ADM	adrenomedullin [Source:HGNC Symbol;Acc:HGNC:259]	protein coding	0.81	3.46	11.16	0.0008	0.0042
ENSG00000279805		TEC	TEC	0.81	0.79	26.44	<0.0001	<0.0001
ENSG00000189409	MMP23B	matrix metallopeptidase 23B [Source:HGNC Symbol;Acc:HGNC:7171]	protein coding	-0.80	0.93	10.17	0.0014	0.0064
ENSG00000186654	PRR5	proline rich 5 [Source:HGNC Symbol;Acc:HGNC:31682]	protein coding	-0.80	4.61	27.06	<0.0001	<0.0001
ENSG00000287839		novel transcript	lncRNA	0.80	3.42	43.54	<0.0001	<0.0001
ENSG00000213420	GPC2	glypican 2 [Source:HGNC Symbol;Acc:HGNC:4450]	protein coding	-0.80	1.72	16.12	<0.0001	0.0006
ENSG00000165905	LARGE2	LARGE xylosyl- and glucuronyltransferase 2 [Source:HGNC Symbol;Acc:HGNC:16522]	protein coding	-0.80	2.62	7.33	0.0068	0.022
ENSG00000204186	ZDBF2	zinc finger DBF-type containing 2 [Source:HGNC Symbol;Acc:HGNC:29313]	protein coding	-0.80	1.45	10.80	0.001	0.0049
ENSG00000155792	DEPTOR	DEP domain containing MTOR interacting protein [Source:HGNC Symbol;Acc:HGNC:22953]	protein coding	-0.80	1.62	16.06	<0.0001	0.0006
ENSG00000136573	BLK	BLK proto-oncogene, Src family tyrosine kinase [Source:HGNC Symbol;Acc:HGNC:1057]	protein coding	-0.80	4.49	8.15	0.0043	0.0153
ENSG00000232022	FAAHP1	fatty acid amide hydrolase pseudogene 1 [Source:HGNC Symbol;Acc:HGNC:50679]	transcribed unprocessed pseudogene	-0.80	0.95	8.63	0.0033	0.0125

Table S9: Genes differentially expressed at Day 2 compared to pre-first vaccination (Study Arm 2 A/H7N9+AS03, D1, 29, Dendritic cells). Genes are sorted by descending absolute log_2 fold change. Gene model summaries and annotations are based on Ensembl Version 99 (continued.)

Ensembl Gene ID	Ensembl Gene Name	Ensembl Gene Description	Gene Type	log_2 Fold Change (Day 2 vs. Pre-First Vaccination)	Average log_2 CPM	Likelihood Ratio Test Statistic	P-Value	FDR Adjusted P-Value
ENSG00000163599	CTLA4	cytotoxic T-lymphocyte associated protein 4 [Source:HGNC Symbol;Acc:HGNC:2505]	protein coding	-0.80	0.79	8.17	0.0042	0.0152
ENSG00000145287	PLAC8	placenta associated 8 [Source:HGNC Symbol;Acc:HGNC:19254]	protein coding	0.80	6.96	35.54	<0.0001	<0.0001
ENSG00000233461		novel transcript	lncRNA	0.80	1.06	34.01	<0.0001	<0.0001
ENSG00000279141		long intergenic non-protein coding RNA 1451	lncRNA	-0.80	1.56	25.52	<0.0001	<0.0001
ENSG00000197121	PGAP1	post-GPI attachment to proteins inositol deacylase 1 [Source:HGNC Symbol;Acc:HGNC:25712]	protein coding	0.80	2.02	16.91	<0.0001	0.0004
ENSG00000229644	NAMPTP1	nicotinamide phosphoribosyltransferase pseudogene 1 [Source:HGNC Symbol;Acc:HGNC:17633]	processed pseudogene	0.80	3.96	29.44	<0.0001	<0.0001
ENSG00000008277	ADAM22	ADAM metallopeptidase domain 22 [Source:HGNC Symbol;Acc:HGNC:201]	protein coding	-0.80	1.04	11.86	0.0006	0.0032
ENSG00000136960	ENPP2	ectonucleotide pyrophosphatase/phosphodiesterase 2 [Source:HGNC Symbol;Acc:HGNC:3357]	protein coding	0.80	0.96	19.38	<0.0001	0.0002
ENSG00000123609	NMI	N-myc and STAT interactor [Source:HGNC Symbol;Acc:HGNC:7854]	protein coding	0.80	6.13	40.06	<0.0001	<0.0001
ENSG00000185567	AHNAK2	AHNAK nucleoprotein 2 [Source:HGNC Symbol;Acc:HGNC:20125]	protein coding	-0.80	1.29	21.31	<0.0001	<0.0001
ENSG00000236790	LINC00299	long intergenic non-protein coding RNA 299 [Source:HGNC Symbol;Acc:HGNC:27940]	lncRNA	-0.80	2.64	20.80	<0.0001	<0.0001
ENSG00000096433	ITPR3	inositol 1,4,5-trisphosphate receptor type 3 [Source:HGNC Symbol;Acc:HGNC:6182]	protein coding	-0.80	5.56	17.43	<0.0001	0.0003
ENSG00000130487	KLHDC7B	kelch domain containing 7B [Source:HGNC Symbol;Acc:HGNC:25145]	protein coding	0.80	4.10	25.04	<0.0001	<0.0001
ENSG00000183508	TENT5C	terminal nucleotidyltransferase 5C [Source:HGNC Symbol;Acc:HGNC:24712]	protein coding	-0.80	3.43	9.99	0.0016	0.0069
ENSG00000243772	KIR2DL3	killer cell immunoglobulin like receptor, two Ig domains and long cytoplasmic tail 3 [Source:HGNC Symbol;Acc:HGNC:6331]	protein coding	-0.80	0.66	9.51	0.002	0.0086
ENSG00000254415	SIGLEC14	sialic acid binding Ig like lectin 14 [Source:HGNC Symbol;Acc:HGNC:32926]	protein coding	0.80	6.52	11.36	0.0008	0.0039
ENSG00000211695	TRGV9	T cell receptor gamma variable 9 [Source:HGNC Symbol;Acc:HGNC:12295]	TR V gene	-0.79	1.90	8.22	0.0042	0.0149
ENSG00000176749	CDK5R1	cyclin dependent kinase 5 regulatory subunit 1 [Source:HGNC Symbol;Acc:HGNC:1775]	protein coding	-0.79	3.28	31.49	<0.0001	<0.0001

Table S9: Genes differentially expressed at Day 2 compared to pre-first vaccination (Study Arm 2 A/H7N9+AS03, D1, 29, Dendritic cells). Genes are sorted by descending absolute log_2 fold change. Gene model summaries and annotations are based on Ensembl Version 99 (continued.)

Ensembl Gene ID	Ensembl Gene Name	Ensembl Gene Description	Gene Type	log_2 Fold Change (Day 2 vs. Pre-First Vaccination)	Average log_2 CPM	Likelihood Ratio Test Statistic	P-Value	FDR Adjusted P-Value
ENSG00000167633	KIR3DL1	killer cell immunoglobulin like receptor, three Ig domains and long cytoplasmic tail 1 [Source:HGNC Symbol;Acc:HGNC:6338]	protein coding	-0.79	1.95	11.70	0.0006	0.0034
ENSG00000088179	PTPN4	protein tyrosine phosphatase non-receptor type 4 [Source:HGNC Symbol;Acc:HGNC:9656]	protein coding	-0.79	5.04	17.18	<0.0001	0.0004
ENSG00000156804	FBXO32	F-box protein 32 [Source:HGNC Symbol;Acc:HGNC:16731]	protein coding	-0.79	3.29	19.48	<0.0001	0.0002
ENSG00000175414	ARL10	ADP ribosylation factor like GTPase 10 [Source:HGNC Symbol;Acc:HGNC:22042]	protein coding	-0.79	2.72	17.01	<0.0001	0.0004
ENSG00000197816	CCDC180	coiled-coil domain containing 180 [Source:HGNC Symbol;Acc:HGNC:29303]	protein coding	-0.79	0.41	8.94	0.0028	0.0109
ENSG00000010310	GIPR	gastric inhibitory polypeptide receptor [Source:HGNC Symbol;Acc:HGNC:4271]	protein coding	-0.79	3.69	19.35	<0.0001	0.0002
ENSG00000091972	CD200	CD200 molecule [Source:HGNC Symbol;Acc:HGNC:7203]	protein coding	-0.79	1.37	6.20	0.0128	0.0355
ENSG00000164241	C5orf63	chromosome 5 open reading frame 63 [Source:HGNC Symbol;Acc:HGNC:40051]	protein coding	-0.79	1.68	12.90	0.0003	0.002
ENSG00000159164	SV2A	synaptic vesicle glycoprotein 2A [Source:HGNC Symbol;Acc:HGNC:20566]	protein coding	-0.79	0.45	8.52	0.0035	0.0131
ENSG00000204267	TAP2	transporter 2, ATP binding cassette subfamily B member [Source:HGNC Symbol;Acc:HGNC:44]	protein coding	0.79	6.40	46.21	<0.0001	<0.0001
ENSG00000174669	SLC29A2	solute carrier family 29 member 2 [Source:HGNC Symbol;Acc:HGNC:11004]	protein coding	-0.79	2.27	15.84	<0.0001	0.0006
ENSG00000171219	CDC42BPG	CDC42 binding protein kinase gamma [Source:HGNC Symbol;Acc:HGNC:29829]	protein coding	-0.79	2.27	11.90	0.0006	0.0031
ENSG00000156113	KCNMA1	potassium calcium-activated channel subfamily M alpha 1 [Source:HGNC Symbol;Acc:HGNC:6284]	protein coding	0.79	2.22	13.75	0.0002	0.0014
ENSG00000151726	ACSL1	acyl-CoA synthetase long chain family member 1 [Source:HGNC Symbol;Acc:HGNC:3569]	protein coding	0.79	7.38	29.82	<0.0001	<0.0001
ENSG00000105926	MPP6	membrane palmitoylated protein 6 [Source:HGNC Symbol;Acc:HGNC:18167]	protein coding	-0.79	1.86	12.94	0.0003	0.002
ENSG00000075399	VPS9D1	VPS9 domain containing 1 [Source:HGNC Symbol;Acc:HGNC:13526]	protein coding	0.78	5.78	43.80	<0.0001	<0.0001
ENSG00000115155	OTOF	otoferlin [Source:HGNC Symbol;Acc:HGNC:8515]	protein coding	0.78	1.18	6.44	0.0112	0.0321

Table S9: Genes differentially expressed at Day 2 compared to pre-first vaccination (Study Arm 2 A/H7N9+AS03, D1, 29, Dendritic cells). Genes are sorted by descending absolute log_2 fold change. Gene model summaries and annotations are based on Ensembl Version 99 (continued.)

Ensembl Gene ID	Ensembl Gene Name	Ensembl Gene Description	Gene Type	log_2 Fold Change (Day 2 vs. Pre-First Vaccination)	Average log_2 CPM	Likelihood Ratio Test Statistic	P-Value	FDR Adjusted P-Value
ENSG00000185198	PRSS57	serine protease 57 [Source:HGNC Symbol;Acc:HGNC:31397]	protein coding	-0.78	0.50	18.06	<0.0001	0.0003
ENSG00000115687	PASK	PAS domain containing serine/threonine kinase [Source:HGNC Symbol;Acc:HGNC:17270]	protein coding	-0.78	4.58	19.26	<0.0001	0.0002
ENSG00000211699	TRGV3	T cell receptor gamma variable 3 [Source:HGNC Symbol;Acc:HGNC:12288]	TR V gene	-0.78	1.03	12.14	0.0005	0.0028
ENSG00000214279	SCART1	scavenger receptor family member expressed on T cells 1 [Source:HGNC Symbol;Acc:HGNC:32411]	protein coding	-0.78	4.45	27.42	<0.0001	<0.0001
ENSG00000086300	SNX10	sorting nexin 10 [Source:HGNC Symbol;Acc:HGNC:14974]	protein coding	0.78	6.86	37.71	<0.0001	<0.0001
ENSG00000163840	DTX3L	deltex E3 ubiquitin ligase 3L [Source:HGNC Symbol;Acc:HGNC:30323]	protein coding	0.78	6.46	31.71	<0.0001	<0.0001
ENSG00000132185	FCRLA	Fc receptor like A [Source:HGNC Symbol;Acc:HGNC:18504]	protein coding	-0.78	3.11	6.37	0.0116	0.0331
ENSG00000127585	FBXL16	F-box and leucine rich repeat protein 16 [Source:HGNC Symbol;Acc:HGNC:14150]	protein coding	-0.78	2.88	16.67	<0.0001	0.0005
ENSG00000233306	TRGV2	T cell receptor gamma variable 2 [Source:HGNC Symbol;Acc:HGNC:12287]	TR V gene	-0.78	0.54	9.68	0.0019	0.0079
ENSG00000104290	FZD3	frizzled class receptor 3 [Source:HGNC Symbol;Acc:HGNC:4041]	protein coding	-0.78	0.71	16.22	<0.0001	0.0005
ENSG00000170522	ELOVL6	ELOVL fatty acid elongase 6 [Source:HGNC Symbol;Acc:HGNC:15829]	protein coding	-0.78	2.04	12.77	0.0004	0.0022
ENSG00000154914	USP43	ubiquitin specific peptidase 43 [Source:HGNC Symbol;Acc:HGNC:20072]	protein coding	-0.78	0.48	23.71	<0.0001	<0.0001
ENSG00000118242	MREG	melanoregulin [Source:HGNC Symbol;Acc:HGNC:25478]	protein coding	-0.78	1.05	19.80	<0.0001	0.0001
ENSG00000172840	PDP2	pyruvate dehyrogenase phosphatase catalytic subunit 2 [Source:HGNC Symbol;Acc:HGNC:30263]	protein coding	-0.78	1.39	10.74	0.001	0.005
ENSG00000181649	PHLDA2	pleckstrin homology like domain family A member 2 [Source:HGNC Symbol;Acc:HGNC:12385]	protein coding	0.77	0.49	9.78	0.0018	0.0076
ENSG00000081985	IL12RB2	interleukin 12 receptor subunit beta 2 [Source:HGNC Symbol;Acc:HGNC:5972]	protein coding	-0.77	1.79	11.66	0.0006	0.0035
ENSG00000161298	ZNF382	zinc finger protein 382 [Source:HGNC Symbol;Acc:HGNC:17409]	protein coding	-0.77	1.50	14.14	0.0002	0.0012
ENSG00000242616	GNG10	G protein subunit gamma 10 [Source:HGNC Symbol;Acc:HGNC:4402]	protein coding	0.77	2.37	30.18	<0.0001	<0.0001

Table S9: Genes differentially expressed at Day 2 compared to pre-first vaccination (Study Arm 2 A/H7N9+AS03, D1, 29, Dendritic cells). Genes are sorted by descending absolute log_2 fold change. Gene model summaries and annotations are based on Ensembl Version 99 (continued.)

Ensembl Gene ID	Ensembl Gene Name	Ensembl Gene Description	Gene Type	log_2 Fold Change (Day 2 vs. Pre-First Vaccination)	Average log_2 CPM	Likelihood Ratio Test Statistic	P-Value	FDR Adjusted P-Value
ENSG00000172232	AZU1	azurocidin 1 [Source:HGNC Symbol;Acc:HGNC:913]	protein coding	-0.77	1.35	24.14	<0.0001	<0.0001
ENSG00000215252	GOLGA8B	golgin A8 family member B [Source:HGNC Symbol;Acc:HGNC:31973]	protein coding	-0.77	4.73	13.76	0.0002	0.0014
ENSG00000139289	PHLDA1	pleckstrin homology like domain family A member 1 [Source:HGNC Symbol;Acc:HGNC:8933]	protein coding	-0.77	0.73	14.58	0.0001	0.001
ENSG00000165572	KBTBD6	kelch repeat and BTB domain containing 6 [Source:HGNC Symbol;Acc:HGNC:25340]	protein coding	-0.77	1.91	11.22	0.0008	0.0041
ENSG00000117394	SLC2A1	solute carrier family 2 member 1 [Source:HGNC Symbol;Acc:HGNC:11005]	protein coding	-0.77	4.23	20.51	<0.0001	0.0001
ENSG00000134709	HOOK1	hook microtubule tethering protein 1 [Source:HGNC Symbol;Acc:HGNC:19884]	protein coding	-0.77	1.38	7.41	0.0065	0.0212
ENSG00000205056	LINC02397	long intergenic non-protein coding RNA 2397 [Source:HGNC Symbol;Acc:HGNC:53323]	lncRNA	-0.77	1.12	6.00	0.0143	0.0389
ENSG00000159445	THEM4	thioesterase superfamily member 4 [Source:HGNC Symbol;Acc:HGNC:17947]	protein coding	-0.77	4.17	15.11	0.0001	0.0008
ENSG00000213366	GSTM2	glutathione S-transferase mu 2 [Source:HGNC Symbol;Acc:HGNC:4634]	protein coding	-0.76	3.45	18.98	<0.0001	0.0002
ENSG00000115267	IFIH1	interferon induced with helicase C domain 1 [Source:HGNC Symbol;Acc:HGNC:18873]	protein coding	0.76	5.80	26.69	<0.0001	<0.0001
ENSG00000140464	PML	promyelocytic leukemia [Source:HGNC Symbol;Acc:HGNC:9113]	protein coding	0.76	6.80	30.12	<0.0001	<0.0001
ENSG00000189350	TOGARAM2	TOG array regulator of axonemal microtubules 2 [Source:HGNC Symbol;Acc:HGNC:33715]	protein coding	-0.76	2.76	21.17	<0.0001	<0.0001
ENSG00000263069	RNF213-AS1	RNF213 antisense RNA 1 [Source:HGNC Symbol;Acc:HGNC:54402]	lncRNA	0.76	3.60	22.61	<0.0001	<0.0001
ENSG00000182118	FAM89A	family with sequence similarity 89 member A [Source:HGNC Symbol;Acc:HGNC:25057]	protein coding	0.76	1.91	41.71	<0.0001	<0.0001
ENSG00000151729	SLC25A4	solute carrier family 25 member 4 [Source:HGNC Symbol;Acc:HGNC:10990]	protein coding	-0.76	1.82	14.33	0.0002	0.0011
ENSG00000188227	ZNF793	zinc finger protein 793 [Source:HGNC Symbol;Acc:HGNC:33115]	protein coding	-0.76	1.32	8.78	0.003	0.0116
ENSG00000135898	GPR55	G protein-coupled receptor 55 [Source:HGNC Symbol;Acc:HGNC:4511]	protein coding	-0.76	1.53	11.56	0.0007	0.0036

Table S9: Genes differentially expressed at Day 2 compared to pre-first vaccination (Study Arm 2 A/H7N9+AS03, D1, 29, Dendritic cells). Genes are sorted by descending absolute log_2 fold change. Gene model summaries and annotations are based on Ensembl Version 99 (continued.)

Ensembl Gene ID	Ensembl Gene Name	Ensembl Gene Description	Gene Type	log_2 Fold Change (Day 2 vs. Pre-First Vaccination)	Average log_2 CPM	Likelihood Ratio Test Statistic	P-Value	FDR Adjusted P-Value
ENSG00000236438	FAM157A	family with sequence similarity 157 member A [Source:HGNC Symbol;Acc:HGNC:34079]	transcribed unprocessed pseudogene	0.75	2.95	31.40	<0.0001	<0.0001
ENSG00000274114	ALOX15P1	arachidonate 15-lipoxygenase pseudogene 1 [Source:HGNC Symbol;Acc:HGNC:13742]	transcribed unprocessed pseudogene	-0.75	0.70	5.78	0.0162	0.0431
ENSG00000089692	LAG3	lymphocyte activating 3 [Source:HGNC Symbol;Acc:HGNC:6476]	protein coding	-0.75	2.35	6.77	0.0092	0.028
ENSG00000128438	TBC1D27P	TBC1 domain family member 27, pseudogene [Source:HGNC Symbol;Acc:HGNC:28104]	transcribed unprocessed pseudogene	-0.75	1.78	6.29	0.0122	0.0343
ENSG00000008441	NFIX	nuclear factor I X [Source:HGNC Symbol;Acc:HGNC:7788]	protein coding	0.75	4.41	36.31	<0.0001	<0.0001
ENSG00000049089	COL9A2	collagen type IX alpha 2 chain [Source:HGNC Symbol;Acc:HGNC:2218]	protein coding	-0.75	5.32	34.97	<0.0001	<0.0001
ENSG00000179750	APOBEC3B	apolipoprotein B mRNA editing enzyme catalytic subunit 3B [Source:HGNC Symbol;Acc:HGNC:17352]	protein coding	0.75	1.13	5.49	0.0191	0.0491
ENSG00000189283	FHIT	fragile histidine triad diadenosine triphosphatase [Source:HGNC Symbol;Acc:HGNC:3701]	protein coding	-0.75	2.93	13.65	0.0002	0.0015
ENSG00000171791	BCL2	BCL2 apoptosis regulator [Source:HGNC Symbol;Acc:HGNC:990]	protein coding	-0.75	5.30	14.57	0.0001	0.001
ENSG00000152465	NMT2	N-myristoyltransferase 2 [Source:HGNC Symbol;Acc:HGNC:7858]	protein coding	-0.75	3.36	14.49	0.0001	0.0011
ENSG00000154025	SLC5A10	solute carrier family 5 member 10 [Source:HGNC Symbol;Acc:HGNC:23155]	protein coding	-0.75	0.86	19.18	<0.0001	0.0002
ENSG00000092621	PHGDH	phosphoglycerate dehydrogenase [Source:HGNC Symbol;Acc:HGNC:8923]	protein coding	-0.75	0.83	9.32	0.0023	0.0093
ENSG00000136867	SLC31A2	solute carrier family 31 member 2 [Source:HGNC Symbol;Acc:HGNC:11017]	protein coding	0.75	4.44	31.86	<0.0001	<0.0001

Table S9: Genes differentially expressed at Day 2 compared to pre-first vaccination (Study Arm 2 A/H7N9+AS03, D1, 29, Dendritic cells). Genes are sorted by descending absolute log_2 fold change. Gene model summaries and annotations are based on Ensembl Version 99 (continued.)

Ensembl Gene ID	Ensembl Gene Name	Ensembl Gene Description	Gene Type	log_2 Fold Change (Day 2 vs. Pre-First Vaccination)	Average log_2 CPM	Likelihood Ratio Test Statistic	P-Value	FDR Adjusted P-Value
ENSG00000090097	PCBP4	poly(rC) binding protein 4 [Source:HGNC Symbol;Acc:HGNC:8652]	protein coding	-0.75	2.58	15.05	0.0001	0.0009
ENSG00000171621	SPSB1	splA/ryanodine receptor domain and SOCS box containing 1 [Source:HGNC Symbol;Acc:HGNC:30628]	protein coding	0.75	0.81	24.35	<0.0001	<0.0001
ENSG00000143297	FCRL5	Fc receptor like 5 [Source:HGNC Symbol;Acc:HGNC:18508]	protein coding	-0.75	3.45	7.37	0.0066	0.0216
ENSG00000145349	CAMK2D	calcium/calmodulin dependent protein kinase II delta [Source:HGNC Symbol;Acc:HGNC:1462]	protein coding	0.75	5.31	63.09	<0.0001	<0.0001
ENSG00000165731	RET	ret proto-oncogene [Source:HGNC Symbol;Acc:HGNC:9967]	protein coding	-0.75	1.77	16.53	<0.0001	0.0005
ENSG00000100154	TTC28	tetratricopeptide repeat domain 28 [Source:HGNC Symbol;Acc:HGNC:29179]	protein coding	-0.75	0.99	17.77	<0.0001	0.0003
ENSG00000275302	CCL4	C-C motif chemokine ligand 4 [Source:HGNC Symbol;Acc:HGNC:10630]	protein coding	-0.74	3.30	9.57	0.002	0.0083
ENSG00000167037	SGSM1	small G protein signaling modulator 1 [Source:HGNC Symbol;Acc:HGNC:29410]	protein coding	-0.74	0.79	8.29	0.004	0.0145
ENSG00000185761	ADAMTSL5	ADAMTS like 5 [Source:HGNC Symbol;Acc:HGNC:27912]	protein coding	-0.74	0.66	12.68	0.0004	0.0022
ENSG00000281103	TRG-AS1	T cell receptor gamma locus antisense RNA 1 [Source:HGNC Symbol;Acc:HGNC:48974]	lncRNA	-0.74	4.16	22.06	<0.0001	<0.0001
ENSG00000247708	STX18-AS1	STX18 antisense RNA 1 (head to head) [Source:HGNC Symbol;Acc:HGNC:48877]	lncRNA	-0.74	1.34	10.49	0.0012	0.0056
ENSG00000236901	MIR600HG	MIR600 host gene [Source:HGNC Symbol;Acc:HGNC:23642]	lncRNA	-0.74	2.52	9.01	0.0027	0.0105
ENSG00000144451	SPAG16	sperm associated antigen 16 [Source:HGNC Symbol;Acc:HGNC:23225]	protein coding	-0.74	0.59	12.68	0.0004	0.0022
ENSG00000249684		novel transcript, antisense to PROP1	lncRNA	-0.74	0.84	9.51	0.002	0.0085
ENSG00000230587	LINC02580	long intergenic non-protein coding RNA 2580 [Source:HGNC Symbol;Acc:HGNC:53751]	lncRNA	-0.74	0.76	10.58	0.0011	0.0054
ENSG00000261997		novel transcript	lncRNA	0.74	1.68	17.18	<0.0001	0.0004
ENSG00000132530	XAF1	XIAP associated factor 1 [Source:HGNC Symbol;Acc:HGNC:30932]	protein coding	0.74	6.18	16.43	<0.0001	0.0005
ENSG00000149260	CAPN5	calpain 5 [Source:HGNC Symbol;Acc:HGNC:1482]	protein coding	-0.74	2.38	17.67	<0.0001	0.0003
ENSG00000173110	HSPA6	heat shock protein family A (Hsp70) member 6 [Source:HGNC Symbol;Acc:HGNC:5239]	protein coding	0.74	5.20	23.79	<0.0001	<0.0001

Table S9: Genes differentially expressed at Day 2 compared to pre-first vaccination (Study Arm 2 A/H7N9+AS03, D1, 29, Dendritic cells). Genes are sorted by descending absolute log_2 fold change. Gene model summaries and annotations are based on Ensembl Version 99 (continued.)

Ensembl Gene ID	Ensembl Gene Name	Ensembl Gene Description	Gene Type	log_2 Fold Change (Day 2 vs. Pre-First Vaccination)	Average log_2 CPM	Likelihood Ratio Test Statistic	P-Value	FDR Adjusted P-Value
ENSG00000253522	MIR3142HG	MIR3142 host gene [Source:HGNC Symbol;Acc:HGNC:51944]	lncRNA	-0.74	1.58	11.10	0.0009	0.0043
ENSG00000167912		novel transcript	lncRNA	-0.74	0.72	8.77	0.0031	0.0117
ENSG00000179841	AKAP5	A-kinase anchoring protein 5 [Source:HGNC Symbol;Acc:HGNC:375]	protein coding	-0.74	1.40	11.69	0.0006	0.0034
ENSG00000099860	GADD45B	growth arrest and DNA damage inducible beta [Source:HGNC Symbol;Acc:HGNC:4096]	protein coding	0.73	6.44	27.89	<0.0001	<0.0001
ENSG00000070669	ASNS	asparagine synthetase (glutamine-hydrolyzing) [Source:HGNC Symbol;Acc:HGNC:753]	protein coding	-0.73	0.36	8.15	0.0043	0.0153
ENSG00000166126	AMN	amnion associated transmembrane protein [Source:HGNC Symbol;Acc:HGNC:14604]	protein coding	-0.73	1.28	10.39	0.0013	0.0058
ENSG00000124006	OBSL1	obscurin like cytoskeletal adaptor 1 [Source:HGNC Symbol;Acc:HGNC:29092]	protein coding	-0.73	0.24	19.62	<0.0001	0.0001
ENSG00000164659	KIAA1324L	KIAA1324 like [Source:HGNC Symbol;Acc:HGNC:21945]	protein coding	-0.73	1.50	9.22	0.0024	0.0096
ENSG00000138670	RASGEF1B	RasGEF domain family member 1B [Source:HGNC Symbol;Acc:HGNC:24881]	protein coding	0.73	4.95	12.18	0.0005	0.0028
ENSG00000160191	PDE9A	phosphodiesterase 9A [Source:HGNC Symbol;Acc:HGNC:8795]	protein coding	-0.73	0.45	8.93	0.0028	0.0109
ENSG00000186265	BTLA	B and T lymphocyte associated [Source:HGNC Symbol;Acc:HGNC:21087]	protein coding	-0.73	2.83	6.50	0.0108	0.0312
ENSG00000163520	FBLN2	fibulin 2 [Source:HGNC Symbol;Acc:HGNC:3601]	protein coding	-0.73	2.92	19.43	<0.0001	0.0002
ENSG00000135047	CTSL	cathepsin L [Source:HGNC Symbol;Acc:HGNC:2537]	protein coding	0.73	5.50	42.93	<0.0001	<0.0001
ENSG00000134460	IL2RA	interleukin 2 receptor subunit alpha [Source:HGNC Symbol;Acc:HGNC:6008]	protein coding	-0.73	1.63	7.78	0.0053	0.018
ENSG00000160932	LY6E	lymphocyte antigen 6 family member E [Source:HGNC Symbol;Acc:HGNC:6727]	protein coding	0.73	8.20	16.97	<0.0001	0.0004
ENSG00000211943	IGHV3-15	immunoglobulin heavy variable 3-15 [Source:HGNC Symbol;Acc:HGNC:5582]	IG V gene	-0.73	1.54	5.66	0.0174	0.0454
ENSG00000183496	MEX3B	mex-3 RNA binding family member B [Source:HGNC Symbol;Acc:HGNC:25297]	protein coding	-0.73	1.32	28.56	<0.0001	<0.0001
ENSG00000246084	LINC02325	long intergenic non-protein coding RNA 2325 [Source:HGNC Symbol;Acc:HGNC:53245]	lncRNA	-0.73	0.84	12.91	0.0003	0.002

Table S9: Genes differentially expressed at Day 2 compared to pre-first vaccination (Study Arm 2 A/H7N9+AS03, D1, 29, Dendritic cells). Genes are sorted by descending absolute log_2 fold change. Gene model summaries and annotations are based on Ensembl Version 99 (continued.)

Ensembl Gene ID	Ensembl Gene Name	Ensembl Gene Description	Gene Type	log_2 Fold Change (Day 2 vs. Pre-First Vaccination)	Average log_2 CPM	Likelihood Ratio Test Statistic	P-Value	FDR Adjusted P-Value
ENSG00000117281	CD160	CD160 molecule [Source:HGNC Symbol;Acc:HGNC:17013]	protein coding	-0.72	3.25	13.69	0.0002	0.0015
ENSG00000005884	ITGA3	integrin subunit alpha 3 [Source:HGNC Symbol;Acc:HGNC:6139]	protein coding	-0.72	1.40	8.53	0.0035	0.013
ENSG00000152990	ADGRA3	adhesion G protein-coupled receptor A3 [Source:HGNC Symbol;Acc:HGNC:13839]	protein coding	-0.72	0.85	21.72	<0.0001	<0.0001
ENSG00000105699	LSR	lipolysis stimulated lipoprotein receptor [Source:HGNC Symbol;Acc:HGNC:29572]	protein coding	-0.72	3.37	15.93	<0.0001	0.0006
ENSG00000148175	STOM	stomatin [Source:HGNC Symbol;Acc:HGNC:3383]	protein coding	0.72	6.37	38.51	<0.0001	<0.0001
ENSG00000088543	C3orf18	chromosome 3 open reading frame 18 [Source:HGNC Symbol;Acc:HGNC:24837]	protein coding	-0.72	2.42	18.18	<0.0001	0.0003
ENSG00000028116	VRK2	VRK serine/threonine kinase 2 [Source:HGNC Symbol;Acc:HGNC:12719]	protein coding	0.72	4.71	37.24	<0.0001	<0.0001
ENSG00000129673	AANAT	aralkylamine N-acetyltransferase [Source:HGNC Symbol;Acc:HGNC:19]	protein coding	0.72	1.61	23.12	<0.0001	<0.0001
ENSG00000225792		novel transcript	lncRNA	0.72	1.72	29.73	<0.0001	<0.0001
ENSG00000276023	DUSP14	dual specificity phosphatase 14 [Source:HGNC Symbol;Acc:HGNC:17007]	protein coding	-0.72	1.06	13.67	0.0002	0.0015
ENSG00000111186	WNT5B	Wnt family member 5B [Source:HGNC Symbol;Acc:HGNC:16265]	protein coding	-0.72	1.78	21.34	<0.0001	<0.0001
ENSG00000159958	TNFRSF13C	TNF receptor superfamily member 13C [Source:HGNC Symbol;Acc:HGNC:17755]	protein coding	-0.72	4.01	6.06	0.0138	0.0379
ENSG00000189233	NUGGC	nuclear GTPase, germinal center associated [Source:HGNC Symbol;Acc:HGNC:33550]	protein coding	-0.72	1.03	9.15	0.0025	0.01
ENSG00000154102	C16orf74	chromosome 16 open reading frame 74 [Source:HGNC Symbol;Acc:HGNC:23362]	protein coding	-0.72	2.91	13.34	0.0003	0.0017
ENSG00000089041	P2RX7	purinergic receptor P2X 7 [Source:HGNC Symbol;Acc:HGNC:8537]	protein coding	0.72	5.98	53.48	<0.0001	<0.0001
ENSG00000133313	CNDP2	carnosine dipeptidase 2 [Source:HGNC Symbol;Acc:HGNC:24437]	protein coding	0.72	7.89	42.78	<0.0001	<0.0001
ENSG00000163823	CCR1	C-C motif chemokine receptor 1 [Source:HGNC Symbol;Acc:HGNC:1602]	protein coding	0.72	6.78	21.41	<0.0001	<0.0001
ENSG00000035664	DAPK2	death associated protein kinase 2 [Source:HGNC Symbol;Acc:HGNC:2675]	protein coding	-0.72	1.62	17.37	<0.0001	0.0004

Table S9: Genes differentially expressed at Day 2 compared to pre-first vaccination (Study Arm 2 A/H7N9+AS03, D1, 29, Dendritic cells). Genes are sorted by descending absolute log_2 fold change. Gene model summaries and annotations are based on Ensembl Version 99 (continued.)

Ensembl Gene ID	Ensembl Gene Name	Ensembl Gene Description	Gene Type	log_2 Fold Change (Day 2 vs. Pre-First Vaccination)	Average log_2 CPM	Likelihood Ratio Test Statistic	P-Value	FDR Adjusted P-Value
ENSG00000205220	PSMB10	proteasome 20S subunit beta 10 [Source:HGNC Symbol;Acc:HGNC:9538]	protein coding	0.71	6.78	46.02	<0.0001	<0.0001
ENSG00000107611	CUBN	cubilin [Source:HGNC Symbol;Acc:HGNC:2548]	protein coding	-0.71	2.19	11.16	0.0008	0.0042
ENSG00000137502	RAB30	RAB30, member RAS oncogene family [Source:HGNC Symbol;Acc:HGNC:9770]	protein coding	-0.71	2.97	9.96	0.0016	0.007
ENSG00000135378	PRRG4	proline rich and Gla domain 4 [Source:HGNC Symbol;Acc:HGNC:30799]	protein coding	0.71	4.21	26.29	<0.0001	<0.0001
ENSG00000107719	PALD1	phosphatase domain containing paladin 1 [Source:HGNC Symbol;Acc:HGNC:23530]	protein coding	-0.71	2.02	14.11	0.0002	0.0012
ENSG00000196743	GM2A	GM2 ganglioside activator [Source:HGNC Symbol;Acc:HGNC:4367]	protein coding	0.71	6.69	33.76	<0.0001	<0.0001
ENSG00000185664	PMEL	premelanosome protein [Source:HGNC Symbol;Acc:HGNC:10880]	protein coding	-0.71	0.43	7.25	0.0071	0.0227
ENSG00000163606	CD200R1	CD200 receptor 1 [Source:HGNC Symbol;Acc:HGNC:24235]	protein coding	-0.71	3.01	40.52	<0.0001	<0.0001
ENSG00000268758	ADGRE4P	adhesion G protein-coupled receptor E4, pseudogene [Source:HGNC Symbol;Acc:HGNC:19240]	transcribed unprocessed pseudogene	-0.71	4.07	14.59	0.0001	0.001
ENSG00000142920	AZIN2	antizyme inhibitor 2 [Source:HGNC Symbol;Acc:HGNC:29957]	protein coding	-0.71	1.25	8.38	0.0038	0.0138
ENSG00000169992	NLGN2	neuroligin 2 [Source:HGNC Symbol;Acc:HGNC:14290]	protein coding	-0.71	2.01	9.31	0.0023	0.0093
ENSG00000135148	TRAFD1	TRAF-type zinc finger domain containing 1 [Source:HGNC Symbol;Acc:HGNC:24808]	protein coding	0.71	6.68	43.73	<0.0001	<0.0001
ENSG00000187583	PLEKHN1	pleckstrin homology domain containing N1 [Source:HGNC Symbol;Acc:HGNC:25284]	protein coding	0.71	1.18	19.60	<0.0001	0.0001
ENSG00000197705	KLHL14	kelch like family member 14 [Source:HGNC Symbol;Acc:HGNC:29266]	protein coding	-0.71	1.27	5.51	0.019	0.0488
ENSG00000182557	SPNS3	sphingolipid transporter 3 (putative) [Source:HGNC Symbol;Acc:HGNC:28433]	protein coding	-0.71	3.89	25.88	<0.0001	<0.0001
ENSG00000105655	ISYNA1	inositol-3-phosphate synthase 1 [Source:HGNC Symbol;Acc:HGNC:29821]	protein coding	-0.71	3.26	18.16	<0.0001	0.0003

Table S9: Genes differentially expressed at Day 2 compared to pre-first vaccination (Study Arm 2 A/H7N9+AS03, D1, 29, Dendritic cells). Genes are sorted by descending absolute log_2 fold change. Gene model summaries and annotations are based on Ensembl Version 99 (continued.)

Ensembl Gene ID	Ensembl Gene Name	Ensembl Gene Description	Gene Type	log_2 Fold Change (Day 2 vs. Pre-First Vaccination)	Average log_2 CPM	Likelihood Ratio Test Statistic	P-Value	FDR Adjusted P-Value
ENSG00000203804	ADAMTSL4-AS1	ADAMTSL4 antisense RNA 1 [Source:HGNC Symbol;Acc:HGNC:32041]	lncRNA	0.71	2.57	13.23	0.0003	0.0018
ENSG00000173200	PARP15	poly(ADP-ribose) polymerase family member 15 [Source:HGNC Symbol;Acc:HGNC:26876]	protein coding	-0.71	5.53	12.82	0.0003	0.0021
ENSG00000087589	CASS4	Cas scaffold protein family member 4 [Source:HGNC Symbol;Acc:HGNC:15878]	protein coding	-0.71	3.35	29.72	<0.0001	<0.0001
ENSG00000118971	CCND2	cyclin D2 [Source:HGNC Symbol;Acc:HGNC:1583]	protein coding	-0.71	5.00	19.95	<0.0001	0.0001
ENSG00000214826	DDX12P	DEAD/H-box helicase 12, pseudogene [Source:HGNC Symbol;Acc:HGNC:2737]	unprocessed pseudogene	-0.71	2.50	17.98	<0.0001	0.0003
ENSG00000172878	METAP1D	methionyl aminopeptidase type 1D, mitochondrial [Source:HGNC Symbol;Acc:HGNC:32583]	protein coding	-0.71	2.10	15.61	<0.0001	0.0007
ENSG00000117632	STMN1	stathmin 1 [Source:HGNC Symbol;Acc:HGNC:6510]	protein coding	-0.71	3.34	16.91	<0.0001	0.0004
ENSG00000124243	BCAS4	breast carcinoma amplified sequence 4 [Source:HGNC Symbol;Acc:HGNC:14367]	protein coding	-0.70	3.04	11.61	0.0007	0.0035
ENSG00000133985	TTC9	tetratricopeptide repeat domain 9 [Source:HGNC Symbol;Acc:HGNC:20267]	protein coding	-0.70	4.69	35.20	<0.0001	<0.0001
ENSG00000145685	LHFPL2	LHFPL tetraspan subfamily member 2 [Source:HGNC Symbol;Acc:HGNC:6588]	protein coding	0.70	4.32	28.57	<0.0001	<0.0001
ENSG00000182580	EPHB3	EPH receptor B3 [Source:HGNC Symbol;Acc:HGNC:3394]	protein coding	-0.70	2.24	20.11	<0.0001	0.0001
ENSG00000136514	RTP4	receptor transporter protein 4 [Source:HGNC Symbol;Acc:HGNC:23992]	protein coding	0.70	3.23	21.90	<0.0001	<0.0001
ENSG00000115306	SPTBN1	spectrin beta, non-erythrocytic 1 [Source:HGNC Symbol;Acc:HGNC:11275]	protein coding	-0.70	6.02	15.28	<0.0001	0.0008
ENSG00000255769	GOLGA2P10	GOLGA2 pseudogene 10 [Source:HGNC Symbol;Acc:HGNC:26229]	transcribed unprocessed pseudogene	-0.70	0.81	11.63	0.0006	0.0035
ENSG00000083454	P2RX5	purinergic receptor P2X 5 [Source:HGNC Symbol;Acc:HGNC:8536]	protein coding	-0.70	3.47	9.48	0.0021	0.0087
ENSG00000223834		novel transcript	lncRNA	-0.70	0.43	11.72	0.0006	0.0034

Table S9: Genes differentially expressed at Day 2 compared to pre-first vaccination (Study Arm 2 A/H7N9+AS03, D1, 29, Dendritic cells). Genes are sorted by descending absolute log_2 fold change. Gene model summaries and annotations are based on Ensembl Version 99 (continued.)

Ensembl Gene ID	Ensembl Gene Name	Ensembl Gene Description	Gene Type	log_2 Fold Change (Day 2 vs. Pre-First Vaccination)	Average log_2 CPM	Likelihood Ratio Test Statistic	P-Value	FDR Adjusted P-Value
ENSG00000197457	STMN3	stathmin 3 [Source:HGNC Symbol;Acc:HGNC:15926]	protein coding	-0.70	5.43	16.69	<0.0001	0.0005
ENSG00000159733	ZFYVE28	zinc finger FYVE-type containing 28 [Source:HGNC Symbol;Acc:HGNC:29334]	protein coding	-0.70	4.30	19.48	<0.0001	0.0002
ENSG00000171729	TMEM51	transmembrane protein 51 [Source:HGNC Symbol;Acc:HGNC:25488]	protein coding	0.70	2.88	28.43	<0.0001	<0.0001
ENSG00000173482	PTPRM	protein tyrosine phosphatase receptor type M [Source:HGNC Symbol;Acc:HGNC:9675]	protein coding	-0.70	2.07	20.08	<0.0001	0.0001
ENSG00000167775	CD320	CD320 molecule [Source:HGNC Symbol;Acc:HGNC:16692]	protein coding	-0.70	3.51	11.61	0.0007	0.0035
ENSG00000163687	DNASE1L3	deoxyribonuclease 1 like 3 [Source:HGNC Symbol;Acc:HGNC:2959]	protein coding	-0.70	1.03	8.80	0.003	0.0116
ENSG00000078687	TNRC6C	trinucleotide repeat containing adaptor 6C [Source:HGNC Symbol;Acc:HGNC:29318]	protein coding	-0.70	4.51	14.10	0.0002	0.0012
ENSG00000188042	ARL4C	ADP ribosylation factor like GTPase 4C [Source:HGNC Symbol;Acc:HGNC:698]	protein coding	-0.70	7.60	20.65	<0.0001	<0.0001
ENSG00000272529		novel transcript, antisense to RFTN1	lncRNA	-0.70	0.47	7.51	0.0061	0.0203
ENSG00000211679	IGLC3	immunoglobulin lambda constant 3 (Kern-Oz+ marker) [Source:HGNC Symbol;Acc:HGNC:5857]	IG C gene	-0.70	3.99	6.26	0.0124	0.0347
ENSG00000133056	PIK3C2B	phosphatidylinositol-4-phosphate 3-kinase catalytic subunit type 2 beta [Source:HGNC Symbol;Acc:HGNC:8972]	protein coding	-0.70	3.95	10.80	0.001	0.0049
ENSG00000095397	WHRN	whirlin [Source:HGNC Symbol;Acc:HGNC:16361]	protein coding	-0.70	1.83	16.09	<0.0001	0.0006
ENSG00000282572		novel transcript similar to family member with sequence similarity 157	lncRNA	0.69	3.41	19.19	<0.0001	0.0002
ENSG00000102524	TNFSF13B	TNF superfamily member 13b [Source:HGNC Symbol;Acc:HGNC:11929]	protein coding	0.69	7.17	24.98	<0.0001	<0.0001
ENSG00000137193	PIM1	Pim-1 proto-oncogene, serine/threonine kinase [Source:HGNC Symbol;Acc:HGNC:8986]	protein coding	0.69	7.10	25.14	<0.0001	<0.0001
ENSG00000041357	PSMA4	proteasome 20S subunit alpha 4 [Source:HGNC Symbol;Acc:HGNC:9533]	protein coding	0.69	7.13	37.27	<0.0001	<0.0001
ENSG00000113448	PDE4D	phosphodiesterase 4D [Source:HGNC Symbol;Acc:HGNC:8783]	protein coding	-0.69	3.62	15.07	0.0001	0.0009
ENSG00000154310	TNIK	TRAF2 and NCK interacting kinase [Source:HGNC Symbol;Acc:HGNC:30765]	protein coding	-0.69	4.23	13.45	0.0002	0.0016

Table S9: Genes differentially expressed at Day 2 compared to pre-first vaccination (Study Arm 2 A/H7N9+AS03, D1, 29, Dendritic cells). Genes are sorted by descending absolute log_2 fold change. Gene model summaries and annotations are based on Ensembl Version 99 (continued.)

Ensembl Gene ID	Ensembl Gene Name	Ensembl Gene Description	Gene Type	log_2 Fold Change (Day 2 vs. Pre-First Vaccination)	Average log_2 CPM	Likelihood Ratio Test Statistic	P-Value	FDR Adjusted P-Value
ENSG00000259225	LINC02345	long intergenic non-protein coding RNA 2345 [Source:HGNC Symbol;Acc:HGNC:53267]	lncRNA	-0.69	1.26	12.14	0.0005	0.0028
ENSG00000092068	SLC7A8	solute carrier family 7 member 8 [Source:HGNC Symbol;Acc:HGNC:11066]	protein coding	-0.69	0.50	9.87	0.0017	0.0073
ENSG00000073331	ALPK1	alpha kinase 1 [Source:HGNC Symbol;Acc:HGNC:20917]	protein coding	0.69	5.19	46.92	<0.0001	<0.0001
ENSG00000127951	FGL2	fibrinogen like 2 [Source:HGNC Symbol;Acc:HGNC:3696]	protein coding	0.69	10.47	38.28	<0.0001	<0.0001
ENSG00000143546	S100A8	S100 calcium binding protein A8 [Source:HGNC Symbol;Acc:HGNC:10498]	protein coding	0.69	10.09	17.57	<0.0001	0.0003
ENSG00000180061	TMEM150B	transmembrane protein 150B [Source:HGNC Symbol;Acc:HGNC:34415]	protein coding	0.69	5.01	20.50	<0.0001	0.0001
ENSG00000130590	SAMD10	sterile alpha motif domain containing 10 [Source:HGNC Symbol;Acc:HGNC:16129]	protein coding	-0.69	2.96	16.28	<0.0001	0.0005
ENSG00000104177	MYEF2	myelin expression factor 2 [Source:HGNC Symbol;Acc:HGNC:17940]	protein coding	-0.69	1.91	11.28	0.0008	0.004
ENSG00000102760	RGCC	regulator of cell cycle [Source:HGNC Symbol;Acc:HGNC:20369]	protein coding	-0.69	3.46	11.96	0.0005	0.003
ENSG00000165795	NDRG2	NDRG family member 2 [Source:HGNC Symbol;Acc:HGNC:14460]	protein coding	-0.69	5.10	27.77	<0.0001	<0.0001
ENSG00000099250	NRP1	neuropilin 1 [Source:HGNC Symbol;Acc:HGNC:8004]	protein coding	0.69	2.10	16.81	<0.0001	0.0004
ENSG00000204882	GPR20	G protein-coupled receptor 20 [Source:HGNC Symbol;Acc:HGNC:4475]	protein coding	-0.69	1.93	14.22	0.0002	0.0012
ENSG00000130589	HELZ2	helicase with zinc finger 2 [Source:HGNC Symbol;Acc:HGNC:30021]	protein coding	0.69	7.43	20.22	<0.0001	0.0001
ENSG00000182541	LIMK2	LIM domain kinase 2 [Source:HGNC Symbol;Acc:HGNC:6614]	protein coding	0.69	5.65	37.52	<0.0001	<0.0001
ENSG00000174501	ANKRD36C	ankyrin repeat domain 36C [Source:HGNC Symbol;Acc:HGNC:32946]	protein coding	-0.69	2.79	19.76	<0.0001	0.0001
ENSG00000233013	FAM157B	family with sequence similarity 157 member B [Source:HGNC Symbol;Acc:HGNC:34080]	transcribed unprocessed pseudogene	0.68	2.06	21.58	<0.0001	<0.0001
ENSG00000272335		novel transcript	lncRNA	-0.68	1.01	6.54	0.0105	0.0307

Table S9: Genes differentially expressed at Day 2 compared to pre-first vaccination (Study Arm 2 A/H7N9+AS03, D1, 29, Dendritic cells). Genes are sorted by descending absolute log_2 fold change. Gene model summaries and annotations are based on Ensembl Version 99 (continued.)

Ensembl Gene ID	Ensembl Gene Name	Ensembl Gene Description	Gene Type	log_2 Fold Change (Day 2 vs. Pre-First Vaccination)	Average log_2 CPM	Likelihood Ratio Test Statistic	P-Value	FDR Adjusted P-Value
ENSG00000275993	SIK1B	salt inducible kinase 1B (putative) [Source:HGNC Symbol;Acc:HGNC:52389]	protein coding	0.68	2.99	10.38	0.0013	0.0058
ENSG00000148019	CEP78	centrosomal protein 78 [Source:HGNC Symbol;Acc:HGNC:25740]	protein coding	-0.68	4.93	22.25	<0.0001	<0.0001
ENSG00000130005	GAMT	guanidinoacetate N-methyltransferase [Source:HGNC Symbol;Acc:HGNC:4136]	protein coding	-0.68	1.69	11.83	0.0006	0.0032
ENSG00000267534	S1PR2	sphingosine-1-phosphate receptor 2 [Source:HGNC Symbol;Acc:HGNC:3169]	protein coding	0.68	1.67	19.00	<0.0001	0.0002
ENSG00000286248		novel transcript, antisense to P2RX7	lncRNA	0.68	0.54	24.73	<0.0001	<0.0001
ENSG00000090339	ICAM1	intercellular adhesion molecule 1 [Source:HGNC Symbol;Acc:HGNC:5344]	protein coding	0.68	5.88	40.51	<0.0001	<0.0001
ENSG00000174944	P2RY14	purinergic receptor P2Y14 [Source:HGNC Symbol;Acc:HGNC:16442]	protein coding	0.68	2.53	18.67	<0.0001	0.0002
ENSG00000141736	ERBB2	erb-b2 receptor tyrosine kinase 2 [Source:HGNC Symbol;Acc:HGNC:3430]	protein coding	-0.67	3.63	13.45	0.0002	0.0016
ENSG00000278897		TEC	TEC	-0.67	0.76	14.60	0.0001	0.001
ENSG00000142235	LMTK3	lemur tyrosine kinase 3 [Source:HGNC Symbol;Acc:HGNC:19295]	protein coding	-0.67	2.22	9.04	0.0026	0.0104
ENSG00000196912	ANKRD36B	ankyrin repeat domain 36B [Source:HGNC Symbol;Acc:HGNC:29333]	protein coding	-0.67	1.98	21.17	<0.0001	<0.0001
ENSG00000135144	DTX1	deltex E3 ubiquitin ligase 1 [Source:HGNC Symbol;Acc:HGNC:3060]	protein coding	-0.67	2.50	6.00	0.0143	0.039
ENSG00000254682		novel transcript	lncRNA	-0.67	1.48	16.02	<0.0001	0.0006
ENSG00000128594	LRRC4	leucine rich repeat containing 4 [Source:HGNC Symbol;Acc:HGNC:15586]	protein coding	0.67	3.24	27.36	<0.0001	<0.0001
ENSG00000171051	FPR1	formyl peptide receptor 1 [Source:HGNC Symbol;Acc:HGNC:3826]	protein coding	0.67	7.94	22.13	<0.0001	<0.0001
ENSG00000086061	DNAJA1	DnaJ heat shock protein family (Hsp40) member A1 [Source:HGNC Symbol;Acc:HGNC:5229]	protein coding	0.67	7.23	32.37	<0.0001	<0.0001
ENSG00000169313	P2RY12	purinergic receptor P2Y12 [Source:HGNC Symbol;Acc:HGNC:18124]	protein coding	0.67	1.74	16.38	<0.0001	0.0005
ENSG00000026103	FAS	Fas cell surface death receptor [Source:HGNC Symbol;Acc:HGNC:11920]	protein coding	0.67	4.81	28.32	<0.0001	<0.0001
ENSG00000067606	PRKCZ	protein kinase C zeta [Source:HGNC Symbol;Acc:HGNC:9412]	protein coding	-0.67	2.52	21.67	<0.0001	<0.0001

Table S9: Genes differentially expressed at Day 2 compared to pre-first vaccination (Study Arm 2 A/H7N9+AS03, D1, 29, Dendritic cells). Genes are sorted by descending absolute log_2 fold change. Gene model summaries and annotations are based on Ensembl Version 99 (continued.)

Ensembl Gene ID	Ensembl Gene Name	Ensembl Gene Description	Gene Type	log_2 Fold Change (Day 2 vs. Pre-First Vaccination)	Average log_2 CPM	Likelihood Ratio Test Statistic	P-Value	FDR Adjusted P-Value
ENSG00000268041	ERFL	ETS repressor factor like [Source:HGNC Symbol;Acc:HGNC:53894]	protein coding	-0.67	1.87	9.77	0.0018	0.0076
ENSG00000136048	DRAM1	DNA damage regulated autophagy modulator 1 [Source:HGNC Symbol;Acc:HGNC:25645]	protein coding	0.67	4.95	40.53	<0.0001	<0.0001
ENSG00000107201	DDX58	DExD/H-box helicase 58 [Source:HGNC Symbol;Acc:HGNC:19102]	protein coding	0.66	5.62	19.25	<0.0001	0.0002
ENSG00000214894	LINC00243	long intergenic non-protein coding RNA 243 [Source:HGNC Symbol;Acc:HGNC:30956]	lncRNA	-0.66	0.62	8.40	0.0038	0.0138
ENSG00000107281	NPDC1	neural proliferation, differentiation and control 1 [Source:HGNC Symbol;Acc:HGNC:7899]	protein coding	-0.66	3.38	10.65	0.0011	0.0052
ENSG00000119401	TRIM32	tripartite motif containing 32 [Source:HGNC Symbol;Acc:HGNC:16380]	protein coding	-0.66	2.26	11.83	0.0006	0.0032
ENSG00000089127	OAS1	2'-5'-oligoadenylate synthetase 1 [Source:HGNC Symbol;Acc:HGNC:8086]	protein coding	0.66	7.65	23.35	<0.0001	<0.0001
ENSG00000182472	CAPN12	calpain 12 [Source:HGNC Symbol;Acc:HGNC:13249]	protein coding	-0.66	1.74	11.64	0.0006	0.0035
ENSG00000019169	MARCO	macrophage receptor with collagenous structure [Source:HGNC Symbol;Acc:HGNC:6895]	protein coding	0.66	5.51	23.40	<0.0001	<0.0001
ENSG00000165138	ANKS6	ankyrin repeat and sterile alpha motif domain containing 6 [Source:HGNC Symbol;Acc:HGNC:26724]	protein coding	-0.66	2.70	14.00	0.0002	0.0013
ENSG00000168758	SEMA4C	semaphorin 4C [Source:HGNC Symbol;Acc:HGNC:10731]	protein coding	-0.66	5.05	26.99	<0.0001	<0.0001
ENSG00000011028	MRC2	mannose receptor C type 2 [Source:HGNC Symbol;Acc:HGNC:16875]	protein coding	-0.66	1.09	15.48	<0.0001	0.0007
ENSG00000188906	LRRK2	leucine rich repeat kinase 2 [Source:HGNC Symbol;Acc:HGNC:18618]	protein coding	0.66	7.97	35.43	<0.0001	<0.0001
ENSG00000183734	ASCL2	achaete-scute family bHLH transcription factor 2 [Source:HGNC Symbol;Acc:HGNC:739]	protein coding	0.66	5.21	37.96	<0.0001	<0.0001
ENSG00000082196	C1QTNF3	C1q and TNF related 3 [Source:HGNC Symbol;Acc:HGNC:14326]	protein coding	-0.66	0.52	19.54	<0.0001	0.0001
ENSG00000184988	TMEM106A	transmembrane protein 106A [Source:HGNC Symbol;Acc:HGNC:28288]	protein coding	0.66	4.88	41.28	<0.0001	<0.0001
ENSG00000135916	ITM2C	integral membrane protein 2C [Source:HGNC Symbol;Acc:HGNC:6175]	protein coding	-0.66	4.65	8.93	0.0028	0.0109
ENSG00000137767	SQOR	sulfide quinone oxidoreductase [Source:HGNC Symbol;Acc:HGNC:20390]	protein coding	0.66	5.35	32.44	<0.0001	<0.0001

Table S9: Genes differentially expressed at Day 2 compared to pre-first vaccination (Study Arm 2 A/H7N9+AS03, D1, 29, Dendritic cells). Genes are sorted by descending absolute log_2 fold change. Gene model summaries and annotations are based on Ensembl Version 99 (continued.)

Ensembl Gene ID	Ensembl Gene Name	Ensembl Gene Description	Gene Type	log_2 Fold Change (Day 2 vs. Pre-First Vaccination)	Average log_2 CPM	Likelihood Ratio Test Statistic	P-Value	FDR Adjusted P-Value
ENSG00000162004	CCDC78	coiled-coil domain containing 78 [Source:HGNC Symbol;Acc:HGNC:14153]	protein coding	-0.66	2.31	15.69	<0.0001	0.0007
ENSG00000140379	BCL2A1	BCL2 related protein A1 [Source:HGNC Symbol;Acc:HGNC:991]	protein coding	0.66	5.64	24.35	<0.0001	<0.0001
ENSG00000114126	TFDP2	transcription factor Dp-2 [Source:HGNC Symbol;Acc:HGNC:11751]	protein coding	-0.66	4.20	26.80	<0.0001	<0.0001
ENSG00000157978	LDLRAP1	low density lipoprotein receptor adaptor protein 1 [Source:HGNC Symbol;Acc:HGNC:18640]	protein coding	-0.66	6.06	15.45	<0.0001	0.0007
ENSG00000013364	MVP	major vault protein [Source:HGNC Symbol;Acc:HGNC:7531]	protein coding	0.66	8.71	49.35	<0.0001	<0.0001
ENSG00000069966	GNB5	G protein subunit beta 5 [Source:HGNC Symbol;Acc:HGNC:4401]	protein coding	-0.66	3.53	22.39	<0.0001	<0.0001
ENSG00000116771	AGMAT	agmatinase [Source:HGNC Symbol;Acc:HGNC:18407]	protein coding	-0.66	1.37	5.82	0.0159	0.0423
ENSG00000204264	PSMB8	proteasome 20S subunit beta 8 [Source:HGNC Symbol;Acc:HGNC:9545]	protein coding	0.66	7.69	39.55	<0.0001	<0.0001
ENSG00000172460	PRSS30P	serine protease 30, pseudogene [Source:HGNC Symbol;Acc:HGNC:28753]	transcribed unitary pseudogene	-0.66	2.02	11.60	0.0007	0.0035
ENSG00000133466	C1QTNF6	C1q and TNF related 6 [Source:HGNC Symbol;Acc:HGNC:14343]	protein coding	-0.66	2.86	14.08	0.0002	0.0013
ENSG00000183486	MX2	MX dynamin like GTPase 2 [Source:HGNC Symbol;Acc:HGNC:7533]	protein coding	0.65	7.54	16.27	<0.0001	0.0005
ENSG00000269981		novel pseudogene	processed pseudogene	0.65	2.87	21.99	<0.0001	<0.0001
ENSG00000126368	NR1D1	nuclear receptor subfamily 1 group D member 1 [Source:HGNC Symbol;Acc:HGNC:7962]	protein coding	-0.65	3.37	26.32	<0.0001	<0.0001
ENSG00000132274	TRIM22	tripartite motif containing 22 [Source:HGNC Symbol;Acc:HGNC:16379]	protein coding	0.65	8.59	24.95	<0.0001	<0.0001
ENSG00000114127	XRN1	5'-3' exoribonuclease 1 [Source:HGNC Symbol;Acc:HGNC:30654]	protein coding	0.65	6.23	30.37	<0.0001	<0.0001
ENSG00000106605	BLVRA	biliverdin reductase A [Source:HGNC Symbol;Acc:HGNC:1062]	protein coding	0.65	6.46	23.65	<0.0001	<0.0001

Table S9: Genes differentially expressed at Day 2 compared to pre-first vaccination (Study Arm 2 A/H7N9+AS03, D1, 29, Dendritic cells). Genes are sorted by descending absolute log_2 fold change. Gene model summaries and annotations are based on Ensembl Version 99 (continued.)

Ensembl Gene ID	Ensembl Gene Name	Ensembl Gene Description	Gene Type	log_2 Fold Change (Day 2 vs. Pre-First Vaccination)	Average log_2 CPM	Likelihood Ratio Test Statistic	P-Value	FDR Adjusted P-Value
ENSG00000174776	WDR49	WD repeat domain 49 [Source:HGNC Symbol;Acc:HGNC:26587]	protein coding	0.65	1.29	16.12	<0.0001	0.0006
ENSG00000213658	LAT	linker for activation of T cells [Source:HGNC Symbol;Acc:HGNC:18874]	protein coding	-0.65	2.77	11.47	0.0007	0.0037
ENSG00000162694	EXTL2	exostosin like glycosyltransferase 2 [Source:HGNC Symbol;Acc:HGNC:3516]	protein coding	-0.65	1.35	28.10	<0.0001	<0.0001
ENSG00000213949	ITGA1	integrin subunit alpha 1 [Source:HGNC Symbol;Acc:HGNC:6134]	protein coding	0.65	0.83	17.03	<0.0001	0.0004
ENSG00000224934		novel transcript	lncRNA	-0.65	1.49	14.22	0.0002	0.0012
ENSG00000232677	LINC00665	long intergenic non-protein coding RNA 665 [Source:HGNC Symbol;Acc:HGNC:44323]	lncRNA	-0.65	1.75	18.74	<0.0001	0.0002
ENSG00000286162		novel transcript	lncRNA	0.65	0.79	16.90	<0.0001	0.0004
ENSG00000226711	FAM66C	family with sequence similarity 66 member C [Source:HGNC Symbol;Acc:HGNC:21644]	lncRNA	-0.65	0.46	13.01	0.0003	0.002
ENSG00000198720	ANKRD13B	ankyrin repeat domain 13B [Source:HGNC Symbol;Acc:HGNC:26363]	protein coding	-0.65	0.82	14.23	0.0002	0.0012
ENSG00000247982	LINC00926	long intergenic non-protein coding RNA 926 [Source:HGNC Symbol;Acc:HGNC:27514]	lncRNA	-0.65	5.60	5.48	0.0193	0.0494
ENSG00000171130	ATP6V0E2	ATPase H+ transporting V0 subunit e2 [Source:HGNC Symbol;Acc:HGNC:21723]	protein coding	-0.65	4.16	20.25	<0.0001	0.0001
ENSG00000141854	MISP3	MISP family member 3 [Source:HGNC Symbol;Acc:HGNC:26963]	protein coding	-0.64	1.11	27.41	<0.0001	<0.0001
ENSG00000176720	BOK	BCL2 family apoptosis regulator BOK [Source:HGNC Symbol;Acc:HGNC:1087]	protein coding	-0.64	1.88	12.08	0.0005	0.0029
ENSG00000164715	LMTK2	lemur tyrosine kinase 2 [Source:HGNC Symbol;Acc:HGNC:17880]	protein coding	0.64	5.25	32.14	<0.0001	<0.0001
ENSG00000249437	NAIP	NLR family apoptosis inhibitory protein [Source:HGNC Symbol;Acc:HGNC:7634]	protein coding	0.64	5.05	44.03	<0.0001	<0.0001
ENSG00000168778	TCTN2	tectonic family member 2 [Source:HGNC Symbol;Acc:HGNC:25774]	protein coding	-0.64	1.49	15.22	<0.0001	0.0008
ENSG00000204650	LINC02210	long intergenic non-protein coding RNA 2210 [Source:HGNC Symbol;Acc:HGNC:26327]	transcribed unitary pseudogene	-0.64	2.79	12.29	0.0005	0.0026
ENSG00000105369	CD79A	CD79a molecule [Source:HGNC Symbol;Acc:HGNC:1698]	protein coding	-0.64	5.67	5.96	0.0146	0.0396

Table S9: Genes differentially expressed at Day 2 compared to pre-first vaccination (Study Arm 2 A/H7N9+AS03, D1, 29, Dendritic cells). Genes are sorted by descending absolute log_2 fold change. Gene model summaries and annotations are based on Ensembl Version 99 (continued.)

Ensembl Gene ID	Ensembl Gene Name	Ensembl Gene Description	Gene Type	log_2 Fold Change (Day 2 vs. Pre-First Vaccination)	Average log_2 CPM	Likelihood Ratio Test Statistic	P-Value	FDR Adjusted P-Value
ENSG00000110660	SLC35F2	solute carrier family 35 member F2 [Source:HGNC Symbol;Acc:HGNC:23615]	protein coding	-0.64	1.97	13.70	0.0002	0.0015
ENSG00000213557		ribosomal protein L31 (RPL31) pseudogene	processed pseudogene	-0.64	1.61	20.38	<0.0001	0.0001
ENSG00000203710	CR1	complement C3b/C4b receptor 1 (Knops blood group) [Source:HGNC Symbol;Acc:HGNC:2334]	protein coding	0.64	6.22	33.50	<0.0001	<0.0001
ENSG00000106415	GLCCI1	glucocorticoid induced 1 [Source:HGNC Symbol;Acc:HGNC:18713]	protein coding	-0.64	3.78	12.62	0.0004	0.0023
ENSG00000116396	KCNC4	potassium voltage-gated channel subfamily C member 4 [Source:HGNC Symbol;Acc:HGNC:6236]	protein coding	-0.64	2.62	13.17	0.0003	0.0018
ENSG00000013374	NUB1	negative regulator of ubiquitin like proteins 1 [Source:HGNC Symbol;Acc:HGNC:17623]	protein coding	0.64	6.68	46.92	<0.0001	<0.0001
ENSG00000141837	CACNA1A	calcium voltage-gated channel subunit alpha1 A [Source:HGNC Symbol;Acc:HGNC:1388]	protein coding	0.64	1.29	21.86	<0.0001	<0.0001
ENSG00000119686	FLVCR2	feline leukemia virus subgroup C cellular receptor family member 2 [Source:HGNC Symbol;Acc:HGNC:20105]	protein coding	0.64	4.75	37.69	<0.0001	<0.0001
ENSG00000163221	S100A12	S100 calcium binding protein A12 [Source:HGNC Symbol;Acc:HGNC:10489]	protein coding	0.64	6.91	13.44	0.0002	0.0016
ENSG00000065911	MTHFD2	methylenetetrahydrofolate dehydrogenase (NADP+ dependent) 2, methenyltetrahydrofolate cyclohydrolase [Source:HGNC Symbol;Acc:HGNC:7434]	protein coding	0.64	5.85	42.60	<0.0001	<0.0001
ENSG00000136158	SPRY2	sprouty RTK signaling antagonist 2 [Source:HGNC Symbol;Acc:HGNC:11270]	protein coding	-0.64	0.39	9.52	0.002	0.0085
ENSG00000218018		novel transcript	lncRNA	-0.64	0.88	14.20	0.0002	0.0012
ENSG00000148450	MSRB2	methionine sulfoxide reductase B2 [Source:HGNC Symbol;Acc:HGNC:17061]	protein coding	0.64	4.12	30.56	<0.0001	<0.0001
ENSG00000069493	CLEC2D	C-type lectin domain family 2 member D [Source:HGNC Symbol;Acc:HGNC:14351]	protein coding	-0.64	5.88	13.75	0.0002	0.0014
ENSG00000237940	LINC01238	long intergenic non-protein coding RNA 1238 [Source:HGNC Symbol;Acc:HGNC:49795]	lncRNA	-0.64	2.12	9.46	0.0021	0.0087
ENSG00000174370	C11orf45	chromosome 11 open reading frame 45 [Source:HGNC Symbol;Acc:HGNC:28584]	protein coding	-0.64	0.63	11.63	0.0007	0.0035
ENSG00000000971	CFH	complement factor H [Source:HGNC Symbol;Acc:HGNC:4883]	protein coding	-0.64	1.15	7.55	0.006	0.02

Table S9: Genes differentially expressed at Day 2 compared to pre-first vaccination (Study Arm 2 A/H7N9+AS03, D1, 29, Dendritic cells). Genes are sorted by descending absolute log_2 fold change. Gene model summaries and annotations are based on Ensembl Version 99 (continued.)

Ensembl Gene ID	Ensembl Gene Name	Ensembl Gene Description	Gene Type	log_2 Fold Change (Day 2 vs. Pre-First Vaccination)	Average log_2 CPM	Likelihood Ratio Test Statistic	P-Value	FDR Adjusted P-Value
ENSG00000188822	CNR2	cannabinoid receptor 2 [Source:HGNC Symbol;Acc:HGNC:2160]	protein coding	-0.64	2.32	8.42	0.0037	0.0136
ENSG00000187912	CLEC17A	C-type lectin domain containing 17A [Source:HGNC Symbol;Acc:HGNC:34520]	protein coding	-0.63	2.92	9.44	0.0021	0.0088
ENSG00000268903		novel pseudogene	processed pseudogene	0.63	2.86	19.52	<0.0001	0.0001
ENSG00000287252		novel transcript	lncRNA	-0.63	0.92	13.56	0.0002	0.0016
ENSG00000156711	MAPK13	mitogen-activated protein kinase 13 [Source:HGNC Symbol;Acc:HGNC:6875]	protein coding	-0.63	4.48	17.82	<0.0001	0.0003
ENSG00000162543	UBXN10	UBX domain protein 10 [Source:HGNC Symbol;Acc:HGNC:26354]	protein coding	-0.63	0.76	13.16	0.0003	0.0018
ENSG00000135821	GLUL	glutamate-ammonia ligase [Source:HGNC Symbol;Acc:HGNC:4341]	protein coding	0.63	8.81	26.60	<0.0001	<0.0001
ENSG00000177335	C8orf31	chromosome 8 open reading frame 31 [Source:HGNC Symbol;Acc:HGNC:26731]	lncRNA	0.63	1.90	16.04	<0.0001	0.0006
ENSG00000121895	TMEM156	transmembrane protein 156 [Source:HGNC Symbol;Acc:HGNC:26260]	protein coding	-0.63	2.44	8.86	0.0029	0.0113
ENSG00000076554	TPD52	tumor protein D52 [Source:HGNC Symbol;Acc:HGNC:12005]	protein coding	-0.63	2.96	8.75	0.0031	0.0118
ENSG00000164038	SLC9B2	solute carrier family 9 member B2 [Source:HGNC Symbol;Acc:HGNC:25143]	protein coding	-0.63	1.93	10.87	0.001	0.0048
ENSG00000106089	STX1A	syntaxin 1A [Source:HGNC Symbol;Acc:HGNC:11433]	protein coding	-0.63	1.14	9.76	0.0018	0.0077
ENSG00000124762	CDKN1A	cyclin dependent kinase inhibitor 1A [Source:HGNC Symbol;Acc:HGNC:1784]	protein coding	0.63	6.14	11.74	0.0006	0.0033
ENSG00000276758		leukocyte immunoglobulin-like receptor, subfamily A member pseudogene	unprocessed pseudogene	0.63	2.03	24.20	<0.0001	<0.0001
ENSG00000161647	MPP3	membrane palmitoylated protein 3 [Source:HGNC Symbol;Acc:HGNC:7221]	protein coding	0.63	0.82	20.45	<0.0001	0.0001
ENSG00000248544		novel transcript, antisense to CYFIP2	lncRNA	-0.63	0.47	17.63	<0.0001	0.0003
ENSG00000221988	PPT2	palmitoyl-protein thioesterase 2 [Source:HGNC Symbol;Acc:HGNC:9326]	protein coding	-0.63	0.99	14.98	0.0001	0.0009
ENSG00000198467	TPM2	tropomyosin 2 [Source:HGNC Symbol;Acc:HGNC:12011]	protein coding	-0.63	3.59	18.05	<0.0001	0.0003

Table S9: Genes differentially expressed at Day 2 compared to pre-first vaccination (Study Arm 2 A/H7N9+AS03, D1, 29, Dendritic cells). Genes are sorted by descending absolute log_2 fold change. Gene model summaries and annotations are based on Ensembl Version 99 (continued.)

Ensembl Gene ID	Ensembl Gene Name	Ensembl Gene Description	Gene Type	log_2 Fold Change (Day 2 vs. Pre-First Vaccination)	Average log_2 CPM	Likelihood Ratio Test Statistic	P-Value	FDR Adjusted P-Value
ENSG00000148429	USP6NL	USP6 N-terminal like [Source:HGNC Symbol;Acc:HGNC:16858]	protein coding	0.63	4.47	40.00	<0.0001	<0.0001
ENSG00000128203	ASPHD2	aspartate beta-hydroxylase domain containing 2 [Source:HGNC Symbol;Acc:HGNC:30437]	protein coding	0.63	4.64	36.45	<0.0001	<0.0001
ENSG00000143226	FCGR2A	Fc fragment of IgG receptor IIa [Source:HGNC Symbol;Acc:HGNC:3616]	protein coding	0.63	7.46	23.94	<0.0001	<0.0001
ENSG00000285938		BCL6 antisense 1	protein coding	0.63	2.62	14.50	0.0001	0.0011
ENSG00000235888		novel transcript	lncRNA	-0.63	1.40	14.95	0.0001	0.0009
ENSG00000118513	MYB	MYB proto-oncogene, transcription factor [Source:HGNC Symbol;Acc:HGNC:7545]	protein coding	-0.63	1.71	15.41	<0.0001	0.0007
ENSG00000143452	HORMAD1	HORMA domain containing 1 [Source:HGNC Symbol;Acc:HGNC:25245]	protein coding	0.63	1.70	18.17	<0.0001	0.0003
ENSG00000196954	CASP4	caspase 4 [Source:HGNC Symbol;Acc:HGNC:1505]	protein coding	0.63	7.37	41.88	<0.0001	<0.0001
ENSG00000157168	NRG1	neuregulin 1 [Source:HGNC Symbol;Acc:HGNC:7997]	protein coding	0.63	3.90	17.72	<0.0001	0.0003
ENSG00000166582	CENPV	centromere protein V [Source:HGNC Symbol;Acc:HGNC:29920]	protein coding	-0.62	2.47	18.16	<0.0001	0.0003
ENSG00000176945	MUC20	mucin 20, cell surface associated [Source:HGNC Symbol;Acc:HGNC:23282]	protein coding	-0.62	0.83	10.43	0.0012	0.0057
ENSG00000135414	GDF11	growth differentiation factor 11 [Source:HGNC Symbol;Acc:HGNC:4216]	protein coding	-0.62	2.65	10.66	0.0011	0.0052
ENSG00000071073	MGAT4A	alpha-1,3-mannosyl-glycoprotein 4-beta-N-acetylglucosaminyltransferase A [Source:HGNC Symbol;Acc:HGNC:7047]	protein coding	-0.62	5.29	21.30	<0.0001	<0.0001
ENSG00000259834		novel transcript	lncRNA	-0.62	3.85	11.27	0.0008	0.0041
ENSG00000272463		novel transcript	lncRNA	0.62	0.69	25.77	<0.0001	<0.0001
ENSG00000159685	CHCHD6	coiled-coil-helix-coiled-coil-helix domain containing 6 [Source:HGNC Symbol;Acc:HGNC:28184]	protein coding	-0.62	1.55	26.96	<0.0001	<0.0001
ENSG00000005448	WDR54	WD repeat domain 54 [Source:HGNC Symbol;Acc:HGNC:25770]	protein coding	-0.62	3.04	21.92	<0.0001	<0.0001
ENSG00000261087	ZNNT1	ZNF706 neighboring transcript 1 [Source:HGNC Symbol;Acc:HGNC:54438]	lncRNA	-0.62	1.80	12.99	0.0003	0.002
ENSG00000183023	SLC8A1	solute carrier family 8 member A1 [Source:HGNC Symbol;Acc:HGNC:11068]	protein coding	0.62	6.15	42.94	<0.0001	<0.0001

Table S9: Genes differentially expressed at Day 2 compared to pre-first vaccination (Study Arm 2 A/H7N9+AS03, D1, 29, Dendritic cells). Genes are sorted by descending absolute log_2 fold change. Gene model summaries and annotations are based on Ensembl Version 99 (continued.)

Ensembl Gene ID	Ensembl Gene Name	Ensembl Gene Description	Gene Type	log_2 Fold Change (Day 2 vs. Pre-First Vaccination)	Average log_2 CPM	Likelihood Ratio Test Statistic	P-Value	FDR Adjusted P-Value
ENSG00000157303	SUSD3	sushi domain containing 3 [Source:HGNC Symbol;Acc:HGNC:28391]	protein coding	-0.62	4.09	23.08	<0.0001	<0.0001
ENSG00000163220	S100A9	S100 calcium binding protein A9 [Source:HGNC Symbol;Acc:HGNC:10499]	protein coding	0.62	11.47	14.92	0.0001	0.0009
ENSG00000123219	CENPK	centromere protein K [Source:HGNC Symbol;Acc:HGNC:29479]	protein coding	-0.62	1.93	8.17	0.0042	0.0152
ENSG00000273837		novel transcript, sense intronic to AC018755.18	lncRNA	0.62	2.81	15.91	<0.0001	0.0006
ENSG00000282164	PEG13	paternally expressed 13 [Source:HGNC Symbol;Acc:HGNC:19969]	lncRNA	-0.62	1.13	6.49	0.0108	0.0314
ENSG00000166532	RIMKLB	ribosomal modification protein rimK like family member B [Source:HGNC Symbol;Acc:HGNC:29228]	protein coding	0.62	4.19	30.68	<0.0001	<0.0001
ENSG00000151208	DLG5	discs large MAGUK scaffold protein 5 [Source:HGNC Symbol;Acc:HGNC:2904]	protein coding	-0.62	3.00	14.34	0.0002	0.0011
ENSG00000082074	FYB1	FYN binding protein 1 [Source:HGNC Symbol;Acc:HGNC:4036]	protein coding	0.62	8.95	44.94	<0.0001	<0.0001
ENSG00000133105	RXFP2	relaxin family peptide receptor 2 [Source:HGNC Symbol;Acc:HGNC:17318]	protein coding	-0.62	1.45	14.92	0.0001	0.0009
ENSG00000105967	TFEC	transcription factor EC [Source:HGNC Symbol;Acc:HGNC:11754]	protein coding	0.62	6.50	26.46	<0.0001	<0.0001
ENSG00000136235	GPNMB	glycoprotein nmb [Source:HGNC Symbol;Acc:HGNC:4462]	protein coding	-0.62	1.06	13.11	0.0003	0.0019
ENSG00000162591	MEGF6	multiple EGF like domains 6 [Source:HGNC Symbol;Acc:HGNC:3232]	protein coding	-0.61	5.34	17.26	<0.0001	0.0004
ENSG00000188001	TPRG1	tumor protein p63 regulated 1 [Source:HGNC Symbol;Acc:HGNC:24759]	protein coding	-0.61	0.86	16.83	<0.0001	0.0004
ENSG00000132963	POMP	proteasome maturation protein [Source:HGNC Symbol;Acc:HGNC:20330]	protein coding	0.61	5.43	42.07	<0.0001	<0.0001
ENSG00000189136	UBE2Q2P1	ubiquitin conjugating enzyme E2 Q2 pseudogene 1 [Source:HGNC Symbol;Acc:HGNC:37439]	transcribed unprocessed pseudogene	-0.61	0.51	16.92	<0.0001	0.0004
ENSG00000104518	GSDMD	gasdermin D [Source:HGNC Symbol;Acc:HGNC:25697]	protein coding	0.61	7.63	42.03	<0.0001	<0.0001
ENSG00000173930	SLCO4C1	solute carrier organic anion transporter family member 4C1 [Source:HGNC Symbol;Acc:HGNC:23612]	protein coding	-0.61	3.08	22.07	<0.0001	<0.0001

Table S9: Genes differentially expressed at Day 2 compared to pre-first vaccination (Study Arm 2 A/H7N9+AS03, D1, 29, Dendritic cells). Genes are sorted by descending absolute log_2 fold change. Gene model summaries and annotations are based on Ensembl Version 99 (continued.)

Ensembl Gene ID	Ensembl Gene Name	Ensembl Gene Description	Gene Type	log_2 Fold Change (Day 2 vs. Pre-First Vaccination)	Average log_2 CPM	Likelihood Ratio Test Statistic	P-Value	FDR Adjusted P-Value
ENSG00000185986	SDHAP3	succinate dehydrogenase complex flavoprotein subunit A pseudogene 3 [Source:HGNC Symbol;Acc:HGNC:18781]	transcribed unprocessed pseudogene	-0.61	2.74	21.28	<0.0001	<0.0001
ENSG00000128596	CCDC136	coiled-coil domain containing 136 [Source:HGNC Symbol;Acc:HGNC:22225]	protein coding	-0.61	1.79	16.95	<0.0001	0.0004
ENSG00000109906	ZBTB16	zinc finger and BTB domain containing 16 [Source:HGNC Symbol;Acc:HGNC:12930]	protein coding	-0.61	5.30	10.32	0.0013	0.006
ENSG00000011590	ZBTB32	zinc finger and BTB domain containing 32 [Source:HGNC Symbol;Acc:HGNC:16763]	protein coding	-0.61	0.64	5.62	0.0178	0.0463
ENSG00000245904		novel transcript, antisense to BTG1	lncRNA	-0.61	2.37	20.03	<0.0001	0.0001
ENSG00000133739	LRRCC1	leucine rich repeat and coiled-coil centrosomal protein 1 [Source:HGNC Symbol;Acc:HGNC:29373]	protein coding	0.61	2.42	40.10	<0.0001	<0.0001
ENSG00000141655	TNFRSF11A	TNF receptor superfamily member 11a [Source:HGNC Symbol;Acc:HGNC:11908]	protein coding	-0.61	0.55	11.61	0.0007	0.0035
ENSG00000179331	RAB39A	RAB39A, member RAS oncogene family [Source:HGNC Symbol;Acc:HGNC:16521]	protein coding	0.61	2.77	20.52	<0.0001	0.0001
ENSG00000235750	KIAA0040	KIAA0040 [Source:HGNC Symbol;Acc:HGNC:28950]	protein coding	0.61	6.15	37.60	<0.0001	<0.0001
ENSG00000145284	SCD5	stearoyl-CoA desaturase 5 [Source:HGNC Symbol;Acc:HGNC:21088]	protein coding	-0.61	0.59	16.36	<0.0001	0.0005
ENSG00000259205	PRKXP1	PRKX pseudogene 1 [Source:HGNC Symbol;Acc:HGNC:9442]	processed pseudogene	-0.61	1.67	12.52	0.0004	0.0024
ENSG00000064932	SBNO2	strawberry notch homolog 2 [Source:HGNC Symbol;Acc:HGNC:29158]	protein coding	0.61	7.99	44.20	<0.0001	<0.0001
ENSG00000005379	TSPOAP1	TSPO associated protein 1 [Source:HGNC Symbol;Acc:HGNC:16831]	protein coding	-0.61	5.88	22.52	<0.0001	<0.0001
ENSG00000121310	ECHDC2	enoyl-CoA hydratase domain containing 2 [Source:HGNC Symbol;Acc:HGNC:23408]	protein coding	-0.61	4.92	12.50	0.0004	0.0024
ENSG00000073350	LLGL2	LLGL scribble cell polarity complex component 2 [Source:HGNC Symbol;Acc:HGNC:6629]	protein coding	-0.61	5.07	15.62	<0.0001	0.0007
ENSG00000173821	RNF213	ring finger protein 213 [Source:HGNC Symbol;Acc:HGNC:14539]	protein coding	0.61	9.79	21.66	<0.0001	<0.0001

Table S9: Genes differentially expressed at Day 2 compared to pre-first vaccination (Study Arm 2 A/H7N9+AS03, D1, 29, Dendritic cells). Genes are sorted by descending absolute log_2 fold change. Gene model summaries and annotations are based on Ensembl Version 99 (continued.)

Ensembl Gene ID	Ensembl Gene Name	Ensembl Gene Description	Gene Type	log_2 Fold Change (Day 2 vs. Pre-First Vaccination)	Average log_2 CPM	Likelihood Ratio Test Statistic	P-Value	FDR Adjusted P-Value
ENSG00000069399	BCL3	BCL3 transcription coactivator [Source:HGNC Symbol;Acc:HGNC:998]	protein coding	0.60	6.09	20.18	<0.0001	0.0001
ENSG00000136689	IL1RN	interleukin 1 receptor antagonist [Source:HGNC Symbol;Acc:HGNC:6000]	protein coding	0.60	5.35	14.67	0.0001	0.001
ENSG00000188522	FAM83G	family with sequence similarity 83 member G [Source:HGNC Symbol;Acc:HGNC:32554]	protein coding	-0.60	1.95	11.08	0.0009	0.0044
ENSG00000272669		novel transcript, antisense to SUN2	lncRNA	0.60	1.20	16.65	<0.0001	0.0005
ENSG00000267481		novel transcript, sense intronic to ZNF506	lncRNA	-0.60	0.95	10.69	0.0011	0.0051
ENSG00000120832	MTERF2	mitochondrial transcription termination factor 2 [Source:HGNC Symbol;Acc:HGNC:30779]	protein coding	-0.60	2.34	11.40	0.0007	0.0038
ENSG00000107331	ABCA2	ATP binding cassette subfamily A member 2 [Source:HGNC Symbol;Acc:HGNC:32]	protein coding	-0.60	6.74	20.94	<0.0001	<0.0001
ENSG00000154814	OXNAD1	oxidoreductase NAD binding domain containing 1 [Source:HGNC Symbol;Acc:HGNC:25128]	protein coding	-0.60	4.60	14.41	0.0001	0.0011
ENSG00000137757	CASP5	caspase 5 [Source:HGNC Symbol;Acc:HGNC:1506]	protein coding	0.60	3.40	20.66	<0.0001	<0.0001
ENSG00000234743	EIF5AP4	eukaryotic translation initiation factor 5A pseudogene 4 [Source:HGNC Symbol;Acc:HGNC:31442]	processed pseudogene	0.60	0.56	26.25	<0.0001	<0.0001
ENSG00000143147	GPR161	G protein-coupled receptor 161 [Source:HGNC Symbol;Acc:HGNC:23694]	protein coding	0.60	0.69	13.51	0.0002	0.0016
ENSG00000125538	IL1B	interleukin 1 beta [Source:HGNC Symbol;Acc:HGNC:5992]	protein coding	0.60	4.71	5.95	0.0147	0.0398
ENSG00000092470	WDR76	WD repeat domain 76 [Source:HGNC Symbol;Acc:HGNC:25773]	protein coding	-0.60	1.74	11.96	0.0005	0.003
ENSG00000123240	OPTN	optineurin [Source:HGNC Symbol;Acc:HGNC:17142]	protein coding	-0.60	4.92	10.43	0.0012	0.0057
ENSG00000104312	RIPK2	receptor interacting serine/threonine kinase 2 [Source:HGNC Symbol;Acc:HGNC:10020]	protein coding	0.60	5.14	30.04	<0.0001	<0.0001
ENSG00000167972	ABCA3	ATP binding cassette subfamily A member 3 [Source:HGNC Symbol;Acc:HGNC:33]	protein coding	-0.60	3.00	20.24	<0.0001	0.0001
ENSG00000278864		novel transcript	TEC	-0.60	1.40	11.21	0.0008	0.0041
ENSG00000230148	HOXB-AS1	HOXB cluster antisense RNA 1 [Source:HGNC Symbol;Acc:HGNC:43744]	lncRNA	-0.60	1.41	11.72	0.0006	0.0034
ENSG00000023902	PLEKHO1	pleckstrin homology domain containing O1 [Source:HGNC Symbol;Acc:HGNC:24310]	protein coding	0.60	8.76	32.08	<0.0001	<0.0001

Table S9: Genes differentially expressed at Day 2 compared to pre-first vaccination (Study Arm 2 A/H7N9+AS03, D1, 29, Dendritic cells). Genes are sorted by descending absolute log_2 fold change. Gene model summaries and annotations are based on Ensembl Version 99 (continued.)

Ensembl Gene ID	Ensembl Gene Name	Ensembl Gene Description	Gene Type	log_2 Fold Change (Day 2 vs. Pre-First Vaccination)	Average log_2 CPM	Likelihood Ratio Test Statistic	P-Value	FDR Adjusted P-Value
ENSG00000235513	L3MBTL2-AS1	L3MBTL2 antisense RNA 1 [Source:HGNC Symbol;Acc:HGNC:40847]	lncRNA	0.60	0.96	23.67	<0.0001	<0.0001
ENSG00000256771	ZNF253	zinc finger protein 253 [Source:HGNC Symbol;Acc:HGNC:13497]	protein coding	-0.60	2.15	13.22	0.0003	0.0018
ENSG00000260528	FAM157C	family with sequence similarity 157 member C [Source:HGNC Symbol;Acc:HGNC:34081]	lncRNA	0.60	5.20	20.45	<0.0001	0.0001
ENSG00000101400	SNTA1	syntrophin alpha 1 [Source:HGNC Symbol;Acc:HGNC:11167]	protein coding	-0.60	1.53	11.88	0.0006	0.0031
ENSG00000154265	ABCA5	ATP binding cassette subfamily A member 5 [Source:HGNC Symbol;Acc:HGNC:35]	protein coding	-0.59	3.45	14.63	0.0001	0.001
ENSG00000114423	CBLB	Cbl proto-oncogene B [Source:HGNC Symbol;Acc:HGNC:1542]	protein coding	-0.59	4.80	17.96	<0.0001	0.0003
ENSG00000196735	HLA-DQA1	major histocompatibility complex, class II, DQ alpha 1 [Source:HGNC Symbol;Acc:HGNC:4942]	protein coding	0.59	7.72	7.39	0.0065	0.0214
ENSG00000242324		PTPNS (protein tyrosine phosphatase, non-receptor type substrate 1) protein family pseudogene	unprocessed pseudogene	0.59	1.35	23.11	<0.0001	<0.0001
ENSG00000197555	SIPA1L1	signal induced proliferation associated 1 like 1 [Source:HGNC Symbol;Acc:HGNC:20284]	protein coding	0.59	5.94	48.44	<0.0001	<0.0001
ENSG00000273669		novel transcript	lncRNA	-0.59	1.23	24.36	<0.0001	<0.0001
ENSG00000101224	CDC25B	cell division cycle 25B [Source:HGNC Symbol;Acc:HGNC:1726]	protein coding	-0.59	6.99	14.82	0.0001	0.0009
ENSG00000103528	SYT17	synaptotagmin 17 [Source:HGNC Symbol;Acc:HGNC:24119]	protein coding	-0.59	2.50	16.66	<0.0001	0.0005
ENSG00000170113	NIPA1	NIPA magnesium transporter 1 [Source:HGNC Symbol;Acc:HGNC:17043]	protein coding	-0.59	3.15	17.48	<0.0001	0.0003
ENSG00000275131		phosphodiesterase 4D interacting protein (PDE4DIP) pseudogene	transcribed unprocessed pseudogene	-0.59	3.28	28.85	<0.0001	<0.0001
ENSG00000059378	PARP12	poly(ADP-ribose) polymerase family member 12 [Source:HGNC Symbol;Acc:HGNC:21919]	protein coding	0.59	6.86	22.83	<0.0001	<0.0001
ENSG00000177732	SOX12	SRY-box transcription factor 12 [Source:HGNC Symbol;Acc:HGNC:11198]	protein coding	-0.59	3.54	24.37	<0.0001	<0.0001

Table S9: Genes differentially expressed at Day 2 compared to pre-first vaccination (Study Arm 2 A/H7N9+AS03, D1, 29, Dendritic cells). Genes are sorted by descending absolute log_2 fold change. Gene model summaries and annotations are based on Ensembl Version 99 (continued.)

Ensembl Gene ID	Ensembl Gene Name	Ensembl Gene Description	Gene Type	log_2 Fold Change (Day 2 vs. Pre-First Vaccination)	Average log_2 CPM	Likelihood Ratio Test Statistic	P-Value	FDR Adjusted P-Value
ENSG00000108861	DUSP3	dual specificity phosphatase 3 [Source:HGNC Symbol;Acc:HGNC:3069]	protein coding	0.59	6.46	40.88	<0.0001	<0.0001
ENSG00000173894	CBX2	chromobox 2 [Source:HGNC Symbol;Acc:HGNC:1552]	protein coding	-0.59	0.57	19.03	<0.0001	0.0002
ENSG00000132199	ENOSF1	enolase superfamily member 1 [Source:HGNC Symbol;Acc:HGNC:30365]	protein coding	-0.59	4.32	15.05	0.0001	0.0009
ENSG00000128512	DOCK4	dedicator of cytokinesis 4 [Source:HGNC Symbol;Acc:HGNC:19192]	protein coding	0.59	2.42	24.63	<0.0001	<0.0001
ENSG00000140743	CDR2	cerebellar degeneration related protein 2 [Source:HGNC Symbol;Acc:HGNC:1799]	protein coding	-0.59	3.83	12.49	0.0004	0.0024
ENSG00000135749	PCNX2	pecanex 2 [Source:HGNC Symbol;Acc:HGNC:8736]	protein coding	-0.59	4.04	29.11	<0.0001	<0.0001
ENSG00000155974	GRIP1	glutamate receptor interacting protein 1 [Source:HGNC Symbol;Acc:HGNC:18708]	protein coding	-0.59	2.16	9.10	0.0026	0.0102
ENSG00000261172		novel transcript	lncRNA	0.59	2.73	13.42	0.0002	0.0017
ENSG00000184381	PLA2G6	phospholipase A2 group VI [Source:HGNC Symbol;Acc:HGNC:9039]	protein coding	-0.59	4.35	14.57	0.0001	0.001
ENSG00000111199	TRPV4	transient receptor potential cation channel subfamily V member 4 [Source:HGNC Symbol;Acc:HGNC:18083]	protein coding	0.59	1.69	16.98	<0.0001	0.0004
ENSG00000237440	ZNF737	zinc finger protein 737 [Source:HGNC Symbol;Acc:HGNC:32468]	protein coding	-0.59	2.76	8.93	0.0028	0.0109
ENSG00000123989	CHPF	chondroitin polymerizing factor [Source:HGNC Symbol;Acc:HGNC:24291]	protein coding	-0.59	1.93	6.17	0.013	0.036

Table S10: Genes differentially expressed at Day 4 compared to pre-first vaccination (Study Arm 2 A/H7N9+AS03, D1, 29, Dendritic cells). Genes are sorted by descending absolute log_2 fold change. Gene model summaries and annotations are based on Ensembl Version 99.

Ensembl Gene ID	Ensembl Gene Name	Ensembl Gene Description	Gene Type	log_2 Fold Change (Day 4 vs. Pre-First Vaccination)	Average log_2 CPM	Likelihood Ratio Test Statistic	P-Value	FDR Adjusted P-Value
ENSG00000156113	KCNMA1	potassium calcium-activated channel subfamily M alpha 1 [Source:HGNC Symbol;Acc:HGNC:6284]	protein coding	1.53	2.36	35.85	<0.0001	<0.0001
ENSG00000160183	TMPRSS3	transmembrane serine protease 3 [Source:HGNC Symbol;Acc:HGNC:11877]	protein coding	0.87	1.27	26.11	<0.0001	0.0022

Table S10: Genes differentially expressed at Day 4 compared to pre-first vaccination (Study Arm 2 A/H7N9+AS03, D1, 29, Dendritic cells). Genes are sorted by descending absolute log_2 fold change. Gene model summaries and annotations are based on Ensembl Version 99 (continued.)

Ensembl Gene ID	Ensembl Gene Name	Ensembl Gene Description	Gene Type	log_2 Fold Change (Day 4 vs. Pre-First Vaccination)	Average log_2 CPM	Likelihood Ratio Test Statistic	P-Value	FDR Adjusted P-Value
ENSG00000179593	ALOX15B	arachidonate 15-lipoxygenase type B [Source:HGNC Symbol;Acc:HGNC:434]	protein coding	-0.87	2.35	22.91	<0.0001	0.0078
ENSG00000169902	TPST1	tyrosylprotein sulfotransferase 1 [Source:HGNC Symbol;Acc:HGNC:12020]	protein coding	-0.76	1.04	17.09	<0.0001	0.0376
ENSG00000110076	NRXN2	neurexin 2 [Source:HGNC Symbol;Acc:HGNC:8009]	protein coding	0.68	1.70	19.93	<0.0001	0.0158
ENSG00000162522	KIAA1522	KIAA1522 [Source:HGNC Symbol;Acc:HGNC:29301]	protein coding	0.62	3.74	22.20	<0.0001	0.0084
ENSG00000133687	TMTC1	transmembrane O-mannosyltransferase targeting cadherins 1 [Source:HGNC Symbol;Acc:HGNC:24099]	protein coding	0.60	4.18	19.97	<0.0001	0.0158
ENSG00000105499	PLA2G4C	phospholipase A2 group IVC [Source:HGNC Symbol;Acc:HGNC:9037]	protein coding	0.59	2.49	19.18	<0.0001	0.0204

Table S11: Genes differentially expressed at Day 29 compared to pre-first vaccination (Study Arm 2 A/H7N9+AS03, D1, 29, Dendritic cells). Genes are sorted by descending absolute log_2 fold change. Gene model summaries and annotations are based on Ensembl Version 99.

Ensembl Gene ID	Ensembl Gene Name	Ensembl Gene Description	Gene Type	log_2 Fold Change (Day 29 vs. Pre-First Vaccination)	Average log_2 CPM	Likelihood Ratio Test Statistic	P-Value	FDR Adjusted P-Value
ENSG00000234648		pseudogene similar to part of ribosomal protein L3 (RPL3)	processed pseudogene	0.81	1.05	24.24	<0.0001	0.0117

Table S12: Genes differentially expressed at Day 30 compared to pre-second vaccination (Study Arm 2 A/H7N9+AS03, D1, 29, Dendritic cells). Genes are sorted by descending absolute log_2 fold change. Gene model summaries and annotations are based on Ensembl Version 99.

Ensembl Gene ID	Ensembl Gene Name	Ensembl Gene Description	Gene Type	log_2 Fold Change (Day 30 vs. Pre-Second Vaccination)	Average log_2 CPM	Likelihood Ratio Test Statistic	P-Value	FDR Adjusted P-Value
ENSG00000152766	ANKRD22	ankyrin repeat domain 22 [Source:HGNC Symbol;Acc:HGNC:28321]	protein coding	4.16	5.66	58.58	<0.0001	<0.0001
ENSG00000131203	IDO1	indoleamine 2,3-dioxygenase 1 [Source:HGNC Symbol;Acc:HGNC:6059]	protein coding	4.15	5.07	36.53	<0.0001	<0.0001

Table S12: Genes differentially expressed at Day 30 compared to pre-second vaccination (Study Arm 2 A/H7N9+AS03, D1, 29, Dendritic cells). Genes are sorted by descending absolute log_2 fold change. Gene model summaries and annotations are based on Ensembl Version 99 (continued.)

Ensembl Gene ID	Ensembl Gene Name	Ensembl Gene Description	Gene Type	log_2 Fold Change (Day 30 vs. Pre-Second Vaccination)	Average log_2 CPM	Likelihood Ratio Test Statistic	P-Value	FDR Adjusted P-Value
ENSG00000225492	GBP1P1	guanylate binding protein 1 pseudogene 1 [Source:HGNC Symbol;Acc:HGNC:39561]	transcribed unprocessed pseudogene	3.66	3.32	61.71	<0.0001	<0.0001
ENSG00000100336	APOL4	apolipoprotein L4 [Source:HGNC Symbol;Acc:HGNC:14867]	protein coding	3.55	4.02	60.16	<0.0001	<0.0001
ENSG00000010030	ETV7	ETS variant transcription factor 7 [Source:HGNC Symbol;Acc:HGNC:18160]	protein coding	3.39	4.27	49.98	<0.0001	<0.0001
ENSG00000149131	SERPING1	serpin family G member 1 [Source:HGNC Symbol;Acc:HGNC:1228]	protein coding	3.27	6.02	71.68	<0.0001	<0.0001
ENSG00000169245	CXCL10	C-X-C motif chemokine ligand 10 [Source:HGNC Symbol;Acc:HGNC:10637]	protein coding	2.95	5.10	45.68	<0.0001	<0.0001
ENSG00000120217	CD274	CD274 molecule [Source:HGNC Symbol;Acc:HGNC:17635]	protein coding	2.93	3.03	32.82	<0.0001	<0.0001
ENSG00000154451	GBP5	guanylate binding protein 5 [Source:HGNC Symbol;Acc:HGNC:19895]	protein coding	2.86	8.79	54.01	<0.0001	<0.0001
ENSG00000198959	TGM2	transglutaminase 2 [Source:HGNC Symbol;Acc:HGNC:11778]	protein coding	2.82	4.56	54.81	<0.0001	<0.0001
ENSG00000117228	GBP1	guanylate binding protein 1 [Source:HGNC Symbol;Acc:HGNC:4182]	protein coding	2.67	8.29	55.79	<0.0001	<0.0001
ENSG00000265531	FCGR1CP	Fc fragment of IgG receptor Ic, pseudogene [Source:HGNC Symbol;Acc:HGNC:3615]	unprocessed pseudogene	2.64	2.75	49.94	<0.0001	<0.0001
ENSG00000064300	NGFR	nerve growth factor receptor [Source:HGNC Symbol;Acc:HGNC:7809]	protein coding	2.61	1.48	56.33	<0.0001	<0.0001
ENSG00000168062	BATF2	basic leucine zipper ATF-like transcription factor 2 [Source:HGNC Symbol;Acc:HGNC:25163]	protein coding	2.59	4.03	73.11	<0.0001	<0.0001
ENSG00000183762	KREMEN1	kringle containing transmembrane protein 1 [Source:HGNC Symbol;Acc:HGNC:17550]	protein coding	2.58	3.30	46.66	<0.0001	<0.0001
ENSG00000108387	SEPTIN4	septin 4 [Source:HGNC Symbol;Acc:HGNC:9165]	protein coding	2.56	2.95	52.70	<0.0001	<0.0001
ENSG00000164509	IL31RA	interleukin 31 receptor A [Source:HGNC Symbol;Acc:HGNC:18969]	protein coding	2.56	2.07	34.29	<0.0001	<0.0001
ENSG00000175643	RMI2	RecQ mediated genome instability 2 [Source:HGNC Symbol;Acc:HGNC:28349]	protein coding	2.36	1.48	30.87	<0.0001	<0.0001

Table S12: Genes differentially expressed at Day 30 compared to pre-second vaccination (Study Arm 2 A/H7N9+AS03, D1, 29, Dendritic cells). Genes are sorted by descending absolute log_2 fold change. Gene model summaries and annotations are based on Ensembl Version 99 (continued.)

Ensembl Gene ID	Ensembl Gene Name	Ensembl Gene Description	Gene Type	log_2 Fold Change (Day 30 vs. Pre-Second Vaccination)	Average log_2 CPM	Likelihood Ratio Test Statistic	P-Value	FDR Adjusted P-Value
ENSG00000150337	FCGR1A	Fc fragment of IgG receptor Ia [Source:HGNC Symbol;Acc:HGNC:3613]	protein coding	2.33	5.71	70.84	<0.0001	<0.0001
ENSG00000198829	SUCNR1	succinate receptor 1 [Source:HGNC Symbol;Acc:HGNC:4542]	protein coding	2.32	1.50	59.68	<0.0001	<0.0001
ENSG00000198019	FCGR1B	Fc fragment of IgG receptor Ib [Source:HGNC Symbol;Acc:HGNC:3614]	protein coding	2.27	4.29	62.74	<0.0001	<0.0001
ENSG00000258521		novel transcript, antisense to WARS	lncRNA	2.27	2.01	51.92	<0.0001	<0.0001
ENSG00000120885	CLU	clusterin [Source:HGNC Symbol;Acc:HGNC:2095]	protein coding	2.22	3.54	51.63	<0.0001	<0.0001
ENSG00000156453	PCDH1	protocadherin 1 [Source:HGNC Symbol;Acc:HGNC:8655]	protein coding	-2.13	1.08	41.11	<0.0001	<0.0001
ENSG00000139572	GPR84	G protein-coupled receptor 84 [Source:HGNC Symbol;Acc:HGNC:4535]	protein coding	2.12	2.09	62.09	<0.0001	<0.0001
ENSG00000126262	FFAR2	free fatty acid receptor 2 [Source:HGNC Symbol;Acc:HGNC:4501]	protein coding	2.09	4.50	77.07	<0.0001	<0.0001
ENSG00000122877	EGR2	early growth response 2 [Source:HGNC Symbol;Acc:HGNC:3239]	protein coding	2.09	4.98	36.66	<0.0001	<0.0001
ENSG00000115155	OTOF	otoferlin [Source:HGNC Symbol;Acc:HGNC:8515]	protein coding	2.07	2.52	61.98	<0.0001	<0.0001
ENSG00000158714	SLAMF8	SLAM family member 8 [Source:HGNC Symbol;Acc:HGNC:21391]	protein coding	2.04	3.63	65.25	<0.0001	<0.0001
ENSG00000168899	VAMP5	vesicle associated membrane protein 5 [Source:HGNC Symbol;Acc:HGNC:12646]	protein coding	2.03	6.78	71.85	<0.0001	<0.0001
ENSG00000197272	IL27	interleukin 27 [Source:HGNC Symbol;Acc:HGNC:19157]	protein coding	2.01	2.33	65.34	<0.0001	<0.0001
ENSG00000119922	IFIT2	interferon induced protein with tetratricopeptide repeats 2 [Source:HGNC Symbol;Acc:HGNC:5409]	protein coding	2.01	6.56	51.65	<0.0001	<0.0001
ENSG00000179639	FCER1A	Fc fragment of IgE receptor Ia [Source:HGNC Symbol;Acc:HGNC:3609]	protein coding	-2.00	6.36	84.09	<0.0001	<0.0001
ENSG00000162654	GBP4	guanylate binding protein 4 [Source:HGNC Symbol;Acc:HGNC:20480]	protein coding	1.97	7.63	54.97	<0.0001	<0.0001
ENSG00000166278	C2	complement C2 [Source:HGNC Symbol;Acc:HGNC:1248]	protein coding	1.97	3.62	123.55	<0.0001	<0.0001
ENSG00000119917	IFIT3	interferon induced protein with tetratricopeptide repeats 3 [Source:HGNC Symbol;Acc:HGNC:5411]	protein coding	1.97	6.91	68.27	<0.0001	<0.0001

Table S12: Genes differentially expressed at Day 30 compared to pre-second vaccination (Study Arm 2 A/H7N9+AS03, D1, 29, Dendritic cells). Genes are sorted by descending absolute log_2 fold change. Gene model summaries and annotations are based on Ensembl Version 99 (continued.)

Ensembl Gene ID	Ensembl Gene Name	Ensembl Gene Description	Gene Type	log_2 Fold Change (Day 30 vs. Pre-Second Vaccination)	Average log_2 CPM	Likelihood Ratio Test Statistic	P-Value	FDR Adjusted P-Value
ENSG00000137959	IFI44L	interferon induced protein 44 like [Source:HGNC Symbol;Acc:HGNC:17817]	protein coding	1.97	6.94	94.40	<0.0001	<0.0001
ENSG00000173369	C1QB	complement C1q B chain [Source:HGNC Symbol;Acc:HGNC:1242]	protein coding	1.95	4.95	186.81	<0.0001	<0.0001
ENSG00000144290	SLC4A10	solute carrier family 4 member 10 [Source:HGNC Symbol;Acc:HGNC:13811]	protein coding	-1.94	2.50	22.63	<0.0001	<0.0001
ENSG00000159189	C1QC	complement C1q C chain [Source:HGNC Symbol;Acc:HGNC:1245]	protein coding	1.93	3.89	111.91	<0.0001	<0.0001
ENSG00000162772	ATF3	activating transcription factor 3 [Source:HGNC Symbol;Acc:HGNC:785]	protein coding	1.93	4.10	128.95	<0.0001	<0.0001
ENSG00000188820	CALHM6	calcium homeostasis modulator family member 6 [Source:HGNC Symbol;Acc:HGNC:33391]	protein coding	1.92	6.49	68.94	<0.0001	<0.0001
ENSG00000002549	LAP3	leucine aminopeptidase 3 [Source:HGNC Symbol;Acc:HGNC:18449]	protein coding	1.92	8.46	63.48	<0.0001	<0.0001
ENSG00000258581		NADH dehydrogenase (ubiquinone) 1 beta subcomplex, 3, 12kDa (NDUFB3) pseudogene	processed pseudogene	1.92	1.35	61.17	<0.0001	<0.0001
ENSG00000140105	WARS1	tryptophanyl-tRNA synthetase 1 [Source:HGNC Symbol;Acc:HGNC:12729]	protein coding	1.92	10.18	58.05	<0.0001	<0.0001
ENSG00000115594	IL1R1	interleukin 1 receptor type 1 [Source:HGNC Symbol;Acc:HGNC:5993]	protein coding	-1.92	2.48	66.58	<0.0001	<0.0001
ENSG00000162512	SDC3	syndecan 3 [Source:HGNC Symbol;Acc:HGNC:10660]	protein coding	1.91	5.10	119.63	<0.0001	<0.0001
ENSG00000111181	SLC6A12	solute carrier family 6 member 12 [Source:HGNC Symbol;Acc:HGNC:11045]	protein coding	1.89	4.49	78.58	<0.0001	<0.0001
ENSG00000157404	KIT	KIT proto-oncogene, receptor tyrosine kinase [Source:HGNC Symbol;Acc:HGNC:6342]	protein coding	-1.88	0.77	99.20	<0.0001	<0.0001
ENSG00000185338	SOCS1	suppressor of cytokine signaling 1 [Source:HGNC Symbol;Acc:HGNC:19383]	protein coding	1.87	2.69	18.53	<0.0001	<0.0001
ENSG00000163568	AIM2	absent in melanoma 2 [Source:HGNC Symbol;Acc:HGNC:357]	protein coding	1.87	4.25	72.80	<0.0001	<0.0001
ENSG00000142303	ADAMTS10	ADAM metallopeptidase with thrombospondin type 1 motif 10 [Source:HGNC Symbol;Acc:HGNC:13201]	protein coding	-1.83	4.37	46.85	<0.0001	<0.0001
ENSG00000172159	FRMD3	FERM domain containing 3 [Source:HGNC Symbol;Acc:HGNC:24125]	protein coding	1.82	3.78	66.45	<0.0001	<0.0001

Table S12: Genes differentially expressed at Day 30 compared to pre-second vaccination (Study Arm 2 A/H7N9+AS03, D1, 29, Dendritic cells). Genes are sorted by descending absolute log_2 fold change. Gene model summaries and annotations are based on Ensembl Version 99 (continued.)

Ensembl Gene ID	Ensembl Gene Name	Ensembl Gene Description	Gene Type	log_2 Fold Change (Day 30 vs. Pre-Second Vaccination)	Average log_2 CPM	Likelihood Ratio Test Statistic	P-Value	FDR Adjusted P-Value
ENSG00000111796	KLRB1	killer cell lectin like receptor B1 [Source:HGNC Symbol;Acc:HGNC:6373]	protein coding	-1.81	4.46	31.22	<0.0001	<0.0001
ENSG00000135636	DYSF	dysferlin [Source:HGNC Symbol;Acc:HGNC:3097]	protein coding	1.81	7.51	86.48	<0.0001	<0.0001
ENSG00000286640		SET domain and mariner transposase fusion gene (SETMAR) pseudogene	transcribed unprocessed pseudogene	-1.81	0.09	22.69	<0.0001	<0.0001
ENSG00000026751	SLAMF7	SLAM family member 7 [Source:HGNC Symbol;Acc:HGNC:21394]	protein coding	1.79	7.07	58.56	<0.0001	<0.0001
ENSG00000229754	CXCR2P1	C-X-C motif chemokine receptor 2 pseudogene 1 [Source:HGNC Symbol;Acc:HGNC:6028]	transcribed unprocessed pseudogene	1.79	6.08	100.24	<0.0001	<0.0001
ENSG00000020577	SAMD4A	sterile alpha motif domain containing 4A [Source:HGNC Symbol;Acc:HGNC:23023]	protein coding	1.78	5.38	76.08	<0.0001	<0.0001
ENSG00000082293	COL19A1	collagen type XIX alpha 1 chain [Source:HGNC Symbol;Acc:HGNC:2196]	protein coding	-1.78	1.29	36.02	<0.0001	<0.0001
ENSG00000256948		novel transcript	lncRNA	1.78	0.90	98.05	<0.0001	<0.0001
ENSG00000254017	IGHEP2	immunoglobulin heavy constant epsilon P2 (pseudogene) [Source:HGNC Symbol;Acc:HGNC:5524]	IG C pseudogene	1.78	1.21	35.15	<0.0001	<0.0001
ENSG00000010310	GIPR	gastric inhibitory polypeptide receptor [Source:HGNC Symbol;Acc:HGNC:4271]	protein coding	-1.77	3.62	70.15	<0.0001	<0.0001
ENSG00000156265	MAP3K7CL	MAP3K7 C-terminal like [Source:HGNC Symbol;Acc:HGNC:16457]	protein coding	1.77	4.86	58.49	<0.0001	<0.0001
ENSG00000062524	LTK	leukocyte receptor tyrosine kinase [Source:HGNC Symbol;Acc:HGNC:6721]	protein coding	-1.77	3.59	37.16	<0.0001	<0.0001
ENSG00000167037	SGSM1	small G protein signaling modulator 1 [Source:HGNC Symbol;Acc:HGNC:29410]	protein coding	-1.75	0.41	23.70	<0.0001	<0.0001
ENSG00000148600	CDHR1	cadherin related family member 1 [Source:HGNC Symbol;Acc:HGNC:14550]	protein coding	-1.75	0.70	37.66	<0.0001	<0.0001
ENSG00000143365	RORC	RAR related orphan receptor C [Source:HGNC Symbol;Acc:HGNC:10260]	protein coding	-1.72	1.69	18.15	<0.0001	0.0001

Table S12: Genes differentially expressed at Day 30 compared to pre-second vaccination (Study Arm 2 A/H7N9+AS03, D1, 29, Dendritic cells). Genes are sorted by descending absolute log_2 fold change. Gene model summaries and annotations are based on Ensembl Version 99 (continued.)

Ensembl Gene ID	Ensembl Gene Name	Ensembl Gene Description	Gene Type	log_2 Fold Change (Day 30 vs. Pre-Second Vaccination)	Average log_2 CPM	Likelihood Ratio Test Statistic	P-Value	FDR Adjusted P-Value
ENSG00000162645	GBP2	guanylate binding protein 2 [Source:HGNC Symbol;Acc:HGNC:4183]	protein coding	1.72	8.54	55.94	<0.0001	<0.0001
ENSG00000236567	TCF3P1	transcription factor 3 pseudogene 1 [Source:HGNC Symbol;Acc:HGNC:49742]	processed pseudogene	1.71	1.75	35.15	<0.0001	<0.0001
ENSG00000173068	BNC2	basonuclin 2 [Source:HGNC Symbol;Acc:HGNC:30988]	protein coding	-1.71	0.76	37.38	<0.0001	<0.0001
ENSG00000168209	DDIT4	DNA damage inducible transcript 4 [Source:HGNC Symbol;Acc:HGNC:24944]	protein coding	-1.70	4.93	34.50	<0.0001	<0.0001
ENSG00000269954		novel transcript, antisense to BLK	lncRNA	-1.69	1.70	30.33	<0.0001	<0.0001
ENSG00000162383	SLC1A7	solute carrier family 1 member 7 [Source:HGNC Symbol;Acc:HGNC:10945]	protein coding	-1.69	1.06	27.09	<0.0001	<0.0001
ENSG00000272282	LINC02084	long intergenic non-protein coding RNA 2084 [Source:HGNC Symbol;Acc:HGNC:52934]	lncRNA	-1.69	1.28	27.49	<0.0001	<0.0001
ENSG00000125148	MT2A	metallothionein 2A [Source:HGNC Symbol;Acc:HGNC:7406]	protein coding	1.69	5.72	87.38	<0.0001	<0.0001
ENSG00000171680	PLEKHG5	pleckstrin homology and RhoGEF domain containing G5 [Source:HGNC Symbol;Acc:HGNC:29105]	protein coding	-1.67	1.01	21.86	<0.0001	<0.0001
ENSG00000100346	CACNA1I	calcium voltage-gated channel subunit alpha1 I [Source:HGNC Symbol;Acc:HGNC:1396]	protein coding	-1.67	3.02	18.72	<0.0001	<0.0001
ENSG00000179388	EGR3	early growth response 3 [Source:HGNC Symbol;Acc:HGNC:3240]	protein coding	1.67	4.26	27.84	<0.0001	<0.0001
ENSG00000037280	FLT4	fms related tyrosine kinase 4 [Source:HGNC Symbol;Acc:HGNC:3767]	protein coding	-1.66	2.17	25.70	<0.0001	<0.0001
ENSG00000168229	PTGDR	prostaglandin D2 receptor [Source:HGNC Symbol;Acc:HGNC:9591]	protein coding	-1.66	3.45	24.81	<0.0001	<0.0001
ENSG00000162614	NEXN	nexilin F-actin binding protein [Source:HGNC Symbol;Acc:HGNC:29557]	protein coding	1.66	3.46	56.39	<0.0001	<0.0001
ENSG00000004468	CD38	CD38 molecule [Source:HGNC Symbol;Acc:HGNC:1667]	protein coding	1.66	5.21	47.90	<0.0001	<0.0001
ENSG00000205846	CLEC6A	C-type lectin domain containing 6A [Source:HGNC Symbol;Acc:HGNC:14556]	protein coding	1.66	2.34	109.75	<0.0001	<0.0001
ENSG00000136573	BLK	BLK proto-oncogene, Src family tyrosine kinase [Source:HGNC Symbol;Acc:HGNC:1057]	protein coding	-1.66	4.01	29.32	<0.0001	<0.0001
ENSG00000142089	IFITM3	interferon induced transmembrane protein 3 [Source:HGNC Symbol;Acc:HGNC:5414]	protein coding	1.65	8.26	127.10	<0.0001	<0.0001

Table S12: Genes differentially expressed at Day 30 compared to pre-second vaccination (Study Arm 2 A/H7N9+AS03, D1, 29, Dendritic cells). Genes are sorted by descending absolute log_2 fold change. Gene model summaries and annotations are based on Ensembl Version 99 (continued.)

Ensembl Gene ID	Ensembl Gene Name	Ensembl Gene Description	Gene Type	log_2 Fold Change (Day 30 vs. Pre-Second Vaccination)	Average log_2 CPM	Likelihood Ratio Test Statistic	P-Value	FDR Adjusted P-Value
ENSG00000123610	TNFAIP6	TNF alpha induced protein 6 [Source:HGNC Symbol;Acc:HGNC:11898]	protein coding	1.65	1.00	41.42	<0.0001	<0.0001
ENSG00000137441	FGFBP2	fibroblast growth factor binding protein 2 [Source:HGNC Symbol;Acc:HGNC:29451]	protein coding	-1.65	5.17	24.56	<0.0001	<0.0001
ENSG00000155792	DEPTOR	DEP domain containing MTOR interacting protein [Source:HGNC Symbol;Acc:HGNC:22953]	protein coding	-1.64	1.72	38.72	<0.0001	<0.0001
ENSG00000134321	RSAD2	radical S-adenosyl methionine domain containing 2 [Source:HGNC Symbol;Acc:HGNC:30908]	protein coding	1.64	5.16	76.83	<0.0001	<0.0001
ENSG00000108679	LGALS3BP	galectin 3 binding protein [Source:HGNC Symbol;Acc:HGNC:6564]	protein coding	1.64	5.82	104.11	<0.0001	<0.0001
ENSG00000167779	IGFBP6	insulin like growth factor binding protein 6 [Source:HGNC Symbol;Acc:HGNC:5475]	protein coding	-1.64	0.35	39.42	<0.0001	<0.0001
ENSG00000115415	STAT1	signal transducer and activator of transcription 1 [Source:HGNC Symbol;Acc:HGNC:11362]	protein coding	1.64	9.14	49.00	<0.0001	<0.0001
ENSG00000143297	FCRL5	Fc receptor like 5 [Source:HGNC Symbol;Acc:HGNC:18508]	protein coding	-1.63	2.99	33.02	<0.0001	<0.0001
ENSG00000007516	BAIAP3	BAI1 associated protein 3 [Source:HGNC Symbol;Acc:HGNC:948]	protein coding	-1.62	2.77	40.07	<0.0001	<0.0001
ENSG00000188282	RUFY4	RUN and FYVE domain containing 4 [Source:HGNC Symbol;Acc:HGNC:24804]	protein coding	1.61	4.36	94.43	<0.0001	<0.0001
ENSG00000171049	FPR2	formyl peptide receptor 2 [Source:HGNC Symbol;Acc:HGNC:3827]	protein coding	1.61	5.88	68.62	<0.0001	<0.0001
ENSG00000125538	IL1B	interleukin 1 beta [Source:HGNC Symbol;Acc:HGNC:5992]	protein coding	1.60	5.00	41.89	<0.0001	<0.0001
ENSG00000113088	GZMK	granzyme K [Source:HGNC Symbol;Acc:HGNC:4711]	protein coding	-1.60	2.34	25.22	<0.0001	<0.0001
ENSG00000133106	EPSTI1	epithelial stromal interaction 1 [Source:HGNC Symbol;Acc:HGNC:16465]	protein coding	1.60	6.87	78.94	<0.0001	<0.0001
ENSG00000108950	FAM20A	FAM20A golgi associated secretory pathway pseudokinase [Source:HGNC Symbol;Acc:HGNC:23015]	protein coding	1.60	3.40	69.07	<0.0001	<0.0001
ENSG00000236213		novel transcript	lncRNA	-1.60	0.11	37.67	<0.0001	<0.0001
ENSG00000138755	CXCL9	C-X-C motif chemokine ligand 9 [Source:HGNC Symbol;Acc:HGNC:7098]	protein coding	1.59	2.04	36.43	<0.0001	<0.0001
ENSG00000156738	MS4A1	membrane spanning 4-domains A1 [Source:HGNC Symbol;Acc:HGNC:7315]	protein coding	-1.59	5.42	20.67	<0.0001	<0.0001

Table S12: Genes differentially expressed at Day 30 compared to pre-second vaccination (Study Arm 2 A/H7N9+AS03, D1, 29, Dendritic cells). Genes are sorted by descending absolute log_2 fold change. Gene model summaries and annotations are based on Ensembl Version 99 (continued.)

Ensembl Gene ID	Ensembl Gene Name	Ensembl Gene Description	Gene Type	log_2 Fold Change (Day 30 vs. Pre-Second Vaccination)	Average log_2 CPM	Likelihood Ratio Test Statistic	P-Value	FDR Adjusted P-Value
ENSG00000174080	CTSF	cathepsin F [Source:HGNC Symbol;Acc:HGNC:2531]	protein coding	-1.59	2.91	40.16	<0.0001	<0.0001
ENSG00000116983	HPCAL4	hippocalcin like 4 [Source:HGNC Symbol;Acc:HGNC:18212]	protein coding	-1.58	0.85	30.81	<0.0001	<0.0001
ENSG00000243836	WDR86-AS1	WDR86 antisense RNA 1 [Source:HGNC Symbol;Acc:HGNC:41186]	lncRNA	-1.57	0.75	27.76	<0.0001	<0.0001
ENSG00000128383	APOBEC3A	apolipoprotein B mRNA editing enzyme catalytic subunit 3A [Source:HGNC Symbol;Acc:HGNC:17343]	protein coding	1.57	7.88	137.88	<0.0001	<0.0001
ENSG00000143869	GDF7	growth differentiation factor 7 [Source:HGNC Symbol;Acc:HGNC:4222]	protein coding	-1.56	0.57	27.88	<0.0001	<0.0001
ENSG00000163520	FBLN2	fibulin 2 [Source:HGNC Symbol;Acc:HGNC:3601]	protein coding	-1.56	2.95	95.54	<0.0001	<0.0001
ENSG00000165905	LARGE2	LARGE xylosyl- and glucuronyltransferase 2 [Source:HGNC Symbol;Acc:HGNC:16522]	protein coding	-1.56	2.13	32.33	<0.0001	<0.0001
ENSG00000189409	MMP23B	matrix metallopeptidase 23B [Source:HGNC Symbol;Acc:HGNC:7171]	protein coding	-1.55	0.56	21.75	<0.0001	<0.0001
ENSG00000259225	LINC02345	long intergenic non-protein coding RNA 2345 [Source:HGNC Symbol;Acc:HGNC:53267]	lncRNA	-1.55	1.04	78.22	<0.0001	<0.0001
ENSG00000073756	PTGS2	prostaglandin-endoperoxide synthase 2 [Source:HGNC Symbol;Acc:HGNC:9605]	protein coding	1.55	4.94	38.74	<0.0001	<0.0001
ENSG00000211659	IGLV3-25	immunoglobulin lambda variable 3-25 [Source:HGNC Symbol;Acc:HGNC:5908]	IG V gene	-1.55	1.15	23.59	<0.0001	<0.0001
ENSG00000198785	GRIN3A	glutamate ionotropic receptor NMDA type subunit 3A [Source:HGNC Symbol;Acc:HGNC:16767]	protein coding	1.54	2.29	98.82	<0.0001	<0.0001
ENSG00000211898	IGHD	immunoglobulin heavy constant delta [Source:HGNC Symbol;Acc:HGNC:5480]	IG C gene	-1.54	5.06	39.45	<0.0001	<0.0001
ENSG00000271109		novel transcript	lncRNA	-1.54	1.09	28.66	<0.0001	<0.0001
ENSG00000130300	PLVAP	plasmalemma vesicle associated protein [Source:HGNC Symbol;Acc:HGNC:13635]	protein coding	-1.54	1.32	51.11	<0.0001	<0.0001
ENSG00000184221	OLIG1	oligodendrocyte transcription factor 1 [Source:HGNC Symbol;Acc:HGNC:16983]	protein coding	-1.54	5.34	47.90	<0.0001	<0.0001
ENSG00000120278	PLEKHG1	pleckstrin homology and RhoGEF domain containing G1 [Source:HGNC Symbol;Acc:HGNC:20884]	protein coding	-1.53	1.37	30.17	<0.0001	<0.0001
ENSG00000133067	LGR6	leucine rich repeat containing G protein-coupled receptor 6 [Source:HGNC Symbol;Acc:HGNC:19719]	protein coding	-1.53	2.56	29.72	<0.0001	<0.0001

Table S12: Genes differentially expressed at Day 30 compared to pre-second vaccination (Study Arm 2 A/H7N9+AS03, D1, 29, Dendritic cells). Genes are sorted by descending absolute log_2 fold change. Gene model summaries and annotations are based on Ensembl Version 99 (continued.)

Ensembl Gene ID	Ensembl Gene Name	Ensembl Gene Description	Gene Type	log_2 Fold Change (Day 30 vs. Pre-Second Vaccination)	Average log_2 CPM	Likelihood Ratio Test Statistic	P-Value	FDR Adjusted P-Value
ENSG00000162545	CAMK2N1	calcium/calmodulin dependent protein kinase II inhibitor 1 [Source:HGNC Symbol;Acc:HGNC:24190]	protein coding	-1.53	0.19	21.92	<0.0001	<0.0001
ENSG00000111199	TRPV4	transient receptor potential cation channel subfamily V member 4 [Source:HGNC Symbol;Acc:HGNC:18083]	protein coding	1.53	2.14	61.62	<0.0001	<0.0001
ENSG00000214872	SMTNL1	smoothelin like 1 [Source:HGNC Symbol;Acc:HGNC:32394]	protein coding	1.52	2.06	65.66	<0.0001	<0.0001
ENSG00000186994	KANK3	KN motif and ankyrin repeat domains 3 [Source:HGNC Symbol;Acc:HGNC:24796]	protein coding	-1.52	0.86	27.30	<0.0001	<0.0001
ENSG00000088881	EBF4	EBF family member 4 [Source:HGNC Symbol;Acc:HGNC:29278]	protein coding	-1.52	1.37	24.33	<0.0001	<0.0001
ENSG00000157985	AGAP1	ArfGAP with GTPase domain, ankyrin repeat and PH domain 1 [Source:HGNC Symbol;Acc:HGNC:16922]	protein coding	-1.52	2.43	21.99	<0.0001	<0.0001
ENSG00000121753	ADGRB2	adhesion G protein-coupled receptor B2 [Source:HGNC Symbol;Acc:HGNC:944]	protein coding	-1.51	0.87	28.55	<0.0001	<0.0001
ENSG00000150687	PRSS23	serine protease 23 [Source:HGNC Symbol;Acc:HGNC:14370]	protein coding	-1.51	3.49	26.31	<0.0001	<0.0001
ENSG00000099834	CDHR5	cadherin related family member 5 [Source:HGNC Symbol;Acc:HGNC:7521]	protein coding	1.51	1.64	56.53	<0.0001	<0.0001
ENSG00000234389		novel transcript	lncRNA	-1.50	1.36	24.51	<0.0001	<0.0001
ENSG00000115607	IL18RAP	interleukin 18 receptor accessory protein [Source:HGNC Symbol;Acc:HGNC:5989]	protein coding	-1.50	4.07	28.18	<0.0001	<0.0001
ENSG00000154734	ADAMTS1	ADAM metallopeptidase with thrombospondin type 1 motif 1 [Source:HGNC Symbol;Acc:HGNC:217]	protein coding	-1.49	1.55	20.79	<0.0001	<0.0001
ENSG00000260711		novel transcript, intronic to CATSPERB	lncRNA	-1.49	0.87	19.72	<0.0001	<0.0001
ENSG00000170962	PDGFD	platelet derived growth factor D [Source:HGNC Symbol;Acc:HGNC:30620]	protein coding	-1.49	1.89	21.79	<0.0001	<0.0001
ENSG00000184613	NELL2	neural EGFL like 2 [Source:HGNC Symbol;Acc:HGNC:7751]	protein coding	-1.49	3.87	21.75	<0.0001	<0.0001
ENSG00000131979	GCH1	GTP cyclohydrolase 1 [Source:HGNC Symbol;Acc:HGNC:4193]	protein coding	1.49	7.44	63.45	<0.0001	<0.0001
ENSG00000198756	COLGALT2	collagen beta(1-O)galactosyltransferase 2 [Source:HGNC Symbol;Acc:HGNC:16790]	protein coding	-1.49	0.82	28.37	<0.0001	<0.0001
ENSG00000183691	NOG	noggin [Source:HGNC Symbol;Acc:HGNC:7866]	protein coding	-1.49	2.12	15.67	<0.0001	0.0004
ENSG00000145365	TIFA	TRAF interacting protein with forkhead associated domain [Source:HGNC Symbol;Acc:HGNC:19075]	protein coding	1.48	3.85	24.95	<0.0001	<0.0001

Table S12: Genes differentially expressed at Day 30 compared to pre-second vaccination (Study Arm 2 A/H7N9+AS03, D1, 29, Dendritic cells). Genes are sorted by descending absolute log_2 fold change. Gene model summaries and annotations are based on Ensembl Version 99 (continued.)

Ensembl Gene ID	Ensembl Gene Name	Ensembl Gene Description	Gene Type	log_2 Fold Change (Day 30 vs. Pre-Second Vaccination)	Average log_2 CPM	Likelihood Ratio Test Statistic	P-Value	FDR Adjusted P-Value
ENSG00000271503	CCL5	C-C motif chemokine ligand 5 [Source:HGNC Symbol;Acc:HGNC:10632]	protein coding	-1.48	6.74	24.54	<0.0001	<0.0001
ENSG00000170486	KRT72	keratin 72 [Source:HGNC Symbol;Acc:HGNC:28932]	protein coding	-1.48	2.48	29.91	<0.0001	<0.0001
ENSG00000174370	C11orf45	chromosome 11 open reading frame 45 [Source:HGNC Symbol;Acc:HGNC:28584]	protein coding	-1.48	0.61	35.38	<0.0001	<0.0001
ENSG00000159958	TNFRSF13C	TNF receptor superfamily member 13C [Source:HGNC Symbol;Acc:HGNC:17755]	protein coding	-1.48	3.57	22.02	<0.0001	<0.0001
ENSG00000247982	LINC00926	long intergenic non-protein coding RNA 926 [Source:HGNC Symbol;Acc:HGNC:27514]	lncRNA	-1.48	5.12	33.42	<0.0001	<0.0001
ENSG00000187608	ISG15	ISG15 ubiquitin like modifier [Source:HGNC Symbol;Acc:HGNC:4053]	protein coding	1.47	6.46	79.93	<0.0001	<0.0001
ENSG00000080573	COL5A3	collagen type V alpha 3 chain [Source:HGNC Symbol;Acc:HGNC:14864]	protein coding	-1.47	1.96	16.60	<0.0001	0.0002
ENSG00000275395	FCGBP	Fc fragment of IgG binding protein [Source:HGNC Symbol;Acc:HGNC:13572]	protein coding	-1.47	3.70	59.60	<0.0001	<0.0001
ENSG00000128438	TBC1D27P	TBC1 domain family member 27, pseudogene [Source:HGNC Symbol;Acc:HGNC:28104]	transcribed unprocessed pseudogene	-1.47	1.39	32.17	<0.0001	<0.0001
ENSG00000105255	FSD1	fibronectin type III and SPRY domain containing 1 [Source:HGNC Symbol;Acc:HGNC:13745]	protein coding	-1.46	0.75	19.60	<0.0001	<0.0001
ENSG00000137965	IFI44	interferon induced protein 44 [Source:HGNC Symbol;Acc:HGNC:16938]	protein coding	1.46	6.48	87.84	<0.0001	<0.0001
ENSG00000134545	KLRC1	killer cell lectin like receptor C1 [Source:HGNC Symbol;Acc:HGNC:6374]	protein coding	-1.46	2.07	18.45	<0.0001	0.0001
ENSG00000029153	ARNTL2	aryl hydrocarbon receptor nuclear translocator like 2 [Source:HGNC Symbol;Acc:HGNC:18984]	protein coding	1.45	1.78	38.27	<0.0001	<0.0001
ENSG00000246526	LINC002481	long intergenic non-protein coding RNA 2481 [Source:HGNC Symbol;Acc:HGNC:27958]	lncRNA	-1.45	1.70	25.70	<0.0001	<0.0001
ENSG00000121858	TNFSF10	TNF superfamily member 10 [Source:HGNC Symbol;Acc:HGNC:11925]	protein coding	1.45	8.24	50.04	<0.0001	<0.0001
ENSG00000100342	APOL1	apolipoprotein L1 [Source:HGNC Symbol;Acc:HGNC:618]	protein coding	1.45	5.93	42.49	<0.0001	<0.0001

Table S12: Genes differentially expressed at Day 30 compared to pre-second vaccination (Study Arm 2 A/H7N9+AS03, D1, 29, Dendritic cells). Genes are sorted by descending absolute log_2 fold change. Gene model summaries and annotations are based on Ensembl Version 99 (continued.)

Ensembl Gene ID	Ensembl Gene Name	Ensembl Gene Description	Gene Type	log_2 Fold Change (Day 30 vs. Pre-Second Vaccination)	Average log_2 CPM	Likelihood Ratio Test Statistic	P-Value	FDR Adjusted P-Value
ENSG00000101017	CD40	CD40 molecule [Source:HGNC Symbol;Acc:HGNC:11919]	protein coding	1.45	5.19	70.22	<0.0001	<0.0001
ENSG00000140511	HAPLN3	hyaluronan and proteoglycan link protein 3 [Source:HGNC Symbol;Acc:HGNC:21446]	protein coding	1.45	4.79	19.22	<0.0001	<0.0001
ENSG00000105369	CD79A	CD79a molecule [Source:HGNC Symbol;Acc:HGNC:1698]	protein coding	-1.45	5.27	31.82	<0.0001	<0.0001
ENSG00000128284	APOL3	apolipoprotein L3 [Source:HGNC Symbol;Acc:HGNC:14868]	protein coding	1.45	7.83	63.40	<0.0001	<0.0001
ENSG00000182782	HCAR2	hydroxycarboxylic acid receptor 2 [Source:HGNC Symbol;Acc:HGNC:24827]	protein coding	1.44	2.23	18.92	<0.0001	<0.0001
ENSG00000162894	FCMR	Fc fragment of IgM receptor [Source:HGNC Symbol;Acc:HGNC:14315]	protein coding	-1.44	6.16	34.90	<0.0001	<0.0001
ENSG00000142173	COL6A2	collagen type VI alpha 2 chain [Source:HGNC Symbol;Acc:HGNC:2212]	protein coding	-1.44	4.61	22.41	<0.0001	<0.0001
ENSG00000100721	TCL1A	T cell leukemia/lymphoma 1A [Source:HGNC Symbol;Acc:HGNC:11648]	protein coding	-1.44	3.81	18.54	<0.0001	<0.0001
ENSG00000221963	APOL6	apolipoprotein L6 [Source:HGNC Symbol;Acc:HGNC:14870]	protein coding	1.43	7.54	60.56	<0.0001	<0.0001
ENSG00000221890	NPTXR	neuronal pentraxin receptor [Source:HGNC Symbol;Acc:HGNC:7954]	protein coding	-1.43	1.66	32.61	<0.0001	<0.0001
ENSG00000168685	IL7R	interleukin 7 receptor [Source:HGNC Symbol;Acc:HGNC:6024]	protein coding	-1.43	7.22	17.90	<0.0001	0.0001
ENSG00000116106	EPHA4	EPH receptor A4 [Source:HGNC Symbol;Acc:HGNC:3388]	protein coding	-1.43	1.74	22.90	<0.0001	<0.0001
ENSG00000158517	NCF1	neutrophil cytosolic factor 1 [Source:HGNC Symbol;Acc:HGNC:7660]	protein coding	1.43	8.23	50.21	<0.0001	<0.0001
ENSG00000187017	ESPN	espin [Source:HGNC Symbol;Acc:HGNC:13281]	protein coding	-1.42	0.39	17.07	<0.0001	0.0002
ENSG00000225886		novel transcript, antisense to IFI6	lncRNA	1.42	1.38	46.10	<0.0001	<0.0001
ENSG00000132185	FCRLA	Fc receptor like A [Source:HGNC Symbol;Acc:HGNC:18504]	protein coding	-1.42	2.74	18.80	<0.0001	<0.0001
ENSG00000184226	PCDH9	protocadherin 9 [Source:HGNC Symbol;Acc:HGNC:8661]	protein coding	-1.42	1.38	23.59	<0.0001	<0.0001
ENSG00000135114	OASL	2'-5'-oligoadenylate synthetase like [Source:HGNC Symbol;Acc:HGNC:8090]	protein coding	1.42	4.85	79.98	<0.0001	<0.0001

Table S12: Genes differentially expressed at Day 30 compared to pre-second vaccination (Study Arm 2 A/H7N9+AS03, D1, 29, Dendritic cells). Genes are sorted by descending absolute log_2 fold change. Gene model summaries and annotations are based on Ensembl Version 99 (continued.)

Ensembl Gene ID	Ensembl Gene Name	Ensembl Gene Description	Gene Type	log_2 Fold Change (Day 30 vs. Pre-Second Vaccination)	Average log_2 CPM	Likelihood Ratio Test Statistic	P-Value	FDR Adjusted P-Value
ENSG00000235532	LINC00402	long intergenic non-protein coding RNA 402 [Source:HGNC Symbol;Acc:HGNC:42732]	lncRNA	-1.42	1.48	20.62	<0.0001	<0.0001
ENSG00000232021	LEF1-AS1	LEF1 antisense RNA 1 [Source:HGNC Symbol;Acc:HGNC:40339]	lncRNA	-1.42	0.34	13.32	0.0003	0.001
ENSG00000138496	PARP9	poly(ADP-ribose) polymerase family member 9 [Source:HGNC Symbol;Acc:HGNC:24118]	protein coding	1.42	7.50	66.38	<0.0001	<0.0001
ENSG00000007264	MATK	megakaryocyte-associated tyrosine kinase [Source:HGNC Symbol;Acc:HGNC:6906]	protein coding	-1.42	5.42	24.91	<0.0001	<0.0001
ENSG00000069188	SDK2	sidekick cell adhesion molecule 2 [Source:HGNC Symbol;Acc:HGNC:19308]	protein coding	-1.41	1.16	23.95	<0.0001	<0.0001
ENSG00000149557	FEZ1	fasciculation and elongation protein zeta 1 [Source:HGNC Symbol;Acc:HGNC:3659]	protein coding	-1.41	1.63	29.91	<0.0001	<0.0001
ENSG00000173114	LRRN3	leucine rich repeat neuronal 3 [Source:HGNC Symbol;Acc:HGNC:17200]	protein coding	-1.41	2.19	16.90	<0.0001	0.0002
ENSG00000138119	MYOF	myoferlin [Source:HGNC Symbol;Acc:HGNC:3656]	protein coding	1.41	7.77	45.10	<0.0001	<0.0001
ENSG00000134326	CMPK2	cytidine/uridine monophosphate kinase 2 [Source:HGNC Symbol;Acc:HGNC:27015]	protein coding	1.41	5.80	79.87	<0.0001	<0.0001
ENSG00000079308	TNS1	tensin 1 [Source:HGNC Symbol;Acc:HGNC:11973]	protein coding	1.40	3.22	98.68	<0.0001	<0.0001
ENSG00000211663	IGLV3-19	immunoglobulin lambda variable 3-19 [Source:HGNC Symbol;Acc:HGNC:5903]	IG V gene	-1.40	0.93	29.93	<0.0001	<0.0001
ENSG00000171596	NMUR1	neuromedin U receptor 1 [Source:HGNC Symbol;Acc:HGNC:4518]	protein coding	-1.40	3.29	24.29	<0.0001	<0.0001
ENSG00000165929	TC2N	tandem C2 domains, nuclear [Source:HGNC Symbol;Acc:HGNC:19859]	protein coding	-1.40	4.65	19.81	<0.0001	<0.0001
ENSG00000158156	XKR8	XK related 8 [Source:HGNC Symbol;Acc:HGNC:25508]	protein coding	1.40	5.63	53.31	<0.0001	<0.0001
ENSG00000169583	CLIC3	chloride intracellular channel 3 [Source:HGNC Symbol;Acc:HGNC:2064]	protein coding	-1.39	3.41	28.70	<0.0001	<0.0001
ENSG00000167664	TMIGD2	transmembrane and immunoglobulin domain containing 2 [Source:HGNC Symbol;Acc:HGNC:28324]	protein coding	-1.39	2.64	21.87	<0.0001	<0.0001
ENSG00000225131	PSME2P2	proteasome activator subunit 2 pseudogene 2 [Source:HGNC Symbol;Acc:HGNC:30160]	processed pseudogene	1.39	1.23	41.07	<0.0001	<0.0001

Table S12: Genes differentially expressed at Day 30 compared to pre-second vaccination (Study Arm 2 A/H7N9+AS03, D1, 29, Dendritic cells). Genes are sorted by descending absolute log_2 fold change. Gene model summaries and annotations are based on Ensembl Version 99 (continued.)

Ensembl Gene ID	Ensembl Gene Name	Ensembl Gene Description	Gene Type	log_2 Fold Change (Day 30 vs. Pre-Second Vaccination)	Average log_2 CPM	Likelihood Ratio Test Statistic	P-Value	FDR Adjusted P-Value
ENSG00000185745	IFIT1	interferon induced protein with tetratricopeptide repeats 1 [Source:HGNC Symbol;Acc:HGNC:5407]	protein coding	1.39	5.47	53.70	<0.0001	<0.0001
ENSG00000138646	HERC5	HECT and RLD domain containing E3 ubiquitin protein ligase 5 [Source:HGNC Symbol;Acc:HGNC:24368]	protein coding	1.39	4.98	65.42	<0.0001	<0.0001
ENSG00000211893	IGHG2	immunoglobulin heavy constant gamma 2 (G2m marker) [Source:HGNC Symbol;Acc:HGNC:5526]	IG C gene	-1.39	5.21	42.35	<0.0001	<0.0001
ENSG00000144645	OSBPL10	oxysterol binding protein like 10 [Source:HGNC Symbol;Acc:HGNC:16395]	protein coding	-1.38	2.02	33.45	<0.0001	<0.0001
ENSG00000115523	GNLY	granulysin [Source:HGNC Symbol;Acc:HGNC:4414]	protein coding	-1.38	7.97	21.28	<0.0001	<0.0001
ENSG00000089169	RPH3A	rabphilin 3A [Source:HGNC Symbol;Acc:HGNC:17056]	protein coding	-1.38	3.94	21.88	<0.0001	<0.0001
ENSG00000286010		novel transcript	lncRNA	-1.38	1.95	22.26	<0.0001	<0.0001
ENSG00000241351	IGKV3-11	immunoglobulin kappa variable 3-11 [Source:HGNC Symbol;Acc:HGNC:5815]	IG V gene	-1.38	2.86	37.79	<0.0001	<0.0001
ENSG00000226777	FAM30A	family with sequence similarity 30 member A [Source:HGNC Symbol;Acc:HGNC:19955]	lncRNA	-1.38	3.59	39.59	<0.0001	<0.0001
ENSG00000113368	LMNB1	lamin B1 [Source:HGNC Symbol;Acc:HGNC:6637]	protein coding	1.37	6.55	52.65	<0.0001	<0.0001
ENSG00000245164	LINC00861	long intergenic non-protein coding RNA 861 [Source:HGNC Symbol;Acc:HGNC:45133]	lncRNA	-1.37	5.71	20.24	<0.0001	<0.0001
ENSG00000262655	SPON1	spondin 1 [Source:HGNC Symbol;Acc:HGNC:11252]	protein coding	-1.37	0.08	20.31	<0.0001	<0.0001
ENSG00000107742	SPOCK2	SPARC (osteonectin), cwcv and kazal like domains proteoglycan 2 [Source:HGNC Symbol;Acc:HGNC:13564]	protein coding	-1.37	7.07	20.97	<0.0001	<0.0001
ENSG00000173193	PARP14	poly(ADP-ribose) polymerase family member 14 [Source:HGNC Symbol;Acc:HGNC:29232]	protein coding	1.37	8.28	68.82	<0.0001	<0.0001
ENSG00000163251	FZD5	frizzled class receptor 5 [Source:HGNC Symbol;Acc:HGNC:4043]	protein coding	1.37	1.93	73.08	<0.0001	<0.0001
ENSG00000171476	HOPX	HOP homeobox [Source:HGNC Symbol;Acc:HGNC:24961]	protein coding	-1.37	4.03	26.47	<0.0001	<0.0001
ENSG00000148488	ST8SIA6	ST8 alpha-N-acetyl-neuraminide alpha-2,8-sialyltransferase 6 [Source:HGNC Symbol;Acc:HGNC:23317]	protein coding	-1.37	1.09	21.18	<0.0001	<0.0001
ENSG00000260807	CEROX1	cytoplasmic endogenous regulator of oxidative phosphorylation 1 [Source:HGNC Symbol;Acc:HGNC:53928]	lncRNA	-1.36	2.52	18.69	<0.0001	<0.0001

Table S12: Genes differentially expressed at Day 30 compared to pre-second vaccination (Study Arm 2 A/H7N9+AS03, D1, 29, Dendritic cells). Genes are sorted by descending absolute log_2 fold change. Gene model summaries and annotations are based on Ensembl Version 99 (continued.)

Ensembl Gene ID	Ensembl Gene Name	Ensembl Gene Description	Gene Type	log_2 Fold Change (Day 30 vs. Pre-Second Vaccination)	Average log_2 CPM	Likelihood Ratio Test Statistic	P-Value	FDR Adjusted P-Value
ENSG00000091972	CD200	CD200 molecule [Source:HGNC Symbol;Acc:HGNC:7203]	protein coding	-1.36	0.89	15.54	<0.0001	0.0004
ENSG00000256069	A2MP1	alpha-2-macroglobulin pseudogene 1 [Source:HGNC Symbol;Acc:HGNC:8]	transcribed unprocessed pseudogene	-1.36	0.65	19.82	<0.0001	<0.0001
ENSG00000188822	CNR2	cannabinoid receptor 2 [Source:HGNC Symbol;Acc:HGNC:2160]	protein coding	-1.36	1.98	33.76	<0.0001	<0.0001
ENSG00000149294	NCAM1	neural cell adhesion molecule 1 [Source:HGNC Symbol;Acc:HGNC:7656]	protein coding	-1.36	3.29	26.75	<0.0001	<0.0001
ENSG00000160856	FCRL3	Fc receptor like 3 [Source:HGNC Symbol;Acc:HGNC:18506]	protein coding	-1.36	4.46	25.93	<0.0001	<0.0001
ENSG00000171817	ZNF540	zinc finger protein 540 [Source:HGNC Symbol;Acc:HGNC:25331]	protein coding	-1.36	0.50	21.71	<0.0001	<0.0001
ENSG00000177455	CD19	CD19 molecule [Source:HGNC Symbol;Acc:HGNC:1633]	protein coding	-1.35	3.22	25.83	<0.0001	<0.0001
ENSG00000088827	SIGLEC1	sialic acid binding Ig like lectin 1 [Source:HGNC Symbol;Acc:HGNC:11127]	protein coding	1.35	6.98	56.94	<0.0001	<0.0001
ENSG00000133321	PLAAT4	phospholipase A and acyltransferase 4 [Source:HGNC Symbol;Acc:HGNC:9869]	protein coding	1.35	6.03	32.82	<0.0001	<0.0001
ENSG00000034053	APBA2	amyloid beta precursor protein binding family A member 2 [Source:HGNC Symbol;Acc:HGNC:579]	protein coding	-1.35	4.25	20.55	<0.0001	<0.0001
ENSG00000134470	IL15RA	interleukin 15 receptor subunit alpha [Source:HGNC Symbol;Acc:HGNC:5978]	protein coding	1.35	4.37	84.48	<0.0001	<0.0001
ENSG00000211767	TRBJ2-3	T cell receptor beta joining 2-3 [Source:HGNC Symbol;Acc:HGNC:12171]	TR J gene	-1.35	0.82	22.26	<0.0001	<0.0001
ENSG00000242324		PTPNS (protein tyrosine phosphatase, non-receptor type substrate 1) protein family pseudogene	unprocessed pseudogene	1.34	1.78	39.27	<0.0001	<0.0001
ENSG00000177409	SAMD9L	sterile alpha motif domain containing 9 like [Source:HGNC Symbol;Acc:HGNC:1349]	protein coding	1.34	7.50	49.72	<0.0001	<0.0001
ENSG00000128218	VPREB3	V-set pre-B cell surrogate light chain 3 [Source:HGNC Symbol;Acc:HGNC:12710]	protein coding	-1.34	0.70	11.54	0.0007	0.0024
ENSG00000237989	LINC01679	long intergenic non-protein coding RNA 1679 [Source:HGNC Symbol;Acc:HGNC:52469]	lncRNA	1.34	0.63	63.48	<0.0001	<0.0001

Table S12: Genes differentially expressed at Day 30 compared to pre-second vaccination (Study Arm 2 A/H7N9+AS03, D1, 29, Dendritic cells). Genes are sorted by descending absolute log_2 fold change. Gene model summaries and annotations are based on Ensembl Version 99 (continued.)

Ensembl Gene ID	Ensembl Gene Name	Ensembl Gene Description	Gene Type	log_2 Fold Change (Day 30 vs. Pre-Second Vaccination)	Average log_2 CPM	Likelihood Ratio Test Statistic	P-Value	FDR Adjusted P-Value
ENSG00000126709	IFI6	interferon alpha inducible protein 6 [Source:HGNC Symbol;Acc:HGNC:4054]	protein coding	1.34	7.47	101.41	<0.0001	<0.0001
ENSG00000125731	SH2D3A	SH2 domain containing 3A [Source:HGNC Symbol;Acc:HGNC:16885]	protein coding	-1.34	3.34	25.61	<0.0001	<0.0001
ENSG00000197705	KLHL14	kelch like family member 14 [Source:HGNC Symbol;Acc:HGNC:29266]	protein coding	-1.34	0.81	31.58	<0.0001	<0.0001
ENSG00000179044	EXOC3L1	exocyst complex component 3 like 1 [Source:HGNC Symbol;Acc:HGNC:27540]	protein coding	1.34	2.74	50.87	<0.0001	<0.0001
ENSG00000281106	TMEM272	transmembrane protein 272 [Source:HGNC Symbol;Acc:HGNC:26737]	protein coding	-1.34	0.63	23.07	<0.0001	<0.0001
ENSG00000272398	CD24	CD24 molecule [Source:HGNC Symbol;Acc:HGNC:1645]	protein coding	-1.33	2.00	11.47	0.0007	0.0024
ENSG00000169116	PARM1	prostate androgen-regulated mucin-like protein 1 [Source:HGNC Symbol;Acc:HGNC:24536]	protein coding	-1.33	2.36	71.42	<0.0001	<0.0001
ENSG00000115590	IL1R2	interleukin 1 receptor type 2 [Source:HGNC Symbol;Acc:HGNC:5994]	protein coding	-1.33	2.69	67.48	<0.0001	<0.0001
ENSG00000211751	TRBC1	T cell receptor beta constant 1 [Source:HGNC Symbol;Acc:HGNC:12156]	TR C gene	-1.33	5.40	18.27	<0.0001	0.0001
ENSG00000211753	TRBV28	T cell receptor beta variable 28 [Source:HGNC Symbol;Acc:HGNC:12209]	TR V gene	-1.33	0.68	14.72	0.0001	0.0006
ENSG00000137801	THBS1	thrombospondin 1 [Source:HGNC Symbol;Acc:HGNC:11785]	protein coding	-1.33	5.65	16.25	<0.0001	0.0003
ENSG00000135318	NT5E	5'-nucleotidase ecto [Source:HGNC Symbol;Acc:HGNC:8021]	protein coding	-1.33	2.19	30.06	<0.0001	<0.0001
ENSG00000240505	TNFRSF13B	TNF receptor superfamily member 13B [Source:HGNC Symbol;Acc:HGNC:18153]	protein coding	-1.33	1.40	32.68	<0.0001	<0.0001
ENSG00000165178	NCF1C	neutrophil cytosolic factor 1C pseudogene [Source:HGNC Symbol;Acc:HGNC:32523]	unprocessed pseudogene	1.33	7.06	43.34	<0.0001	<0.0001
ENSG00000238000		proteasome (prosome, macropain) activator subunit 2 (PA28 beta) (PSME2) pseudogene	processed pseudogene	1.33	1.08	30.58	<0.0001	<0.0001
ENSG00000273314		novel transcript, antisense to CUL1	lncRNA	1.32	1.31	39.76	<0.0001	<0.0001
ENSG00000125900	SIRPD	signal regulatory protein delta [Source:HGNC Symbol;Acc:HGNC:16248]	protein coding	1.32	1.71	31.74	<0.0001	<0.0001

Table S12: Genes differentially expressed at Day 30 compared to pre-second vaccination (Study Arm 2 A/H7N9+AS03, D1, 29, Dendritic cells). Genes are sorted by descending absolute log_2 fold change. Gene model summaries and annotations are based on Ensembl Version 99 (continued.)

Ensembl Gene ID	Ensembl Gene Name	Ensembl Gene Description	Gene Type	log_2 Fold Change (Day 30 vs. Pre-Second Vaccination)	Average log_2 CPM	Likelihood Ratio Test Statistic	P-Value	FDR Adjusted P-Value
ENSG00000255769	GOLGA2P10	GOLGA2 pseudogene 10 [Source:HGNC Symbol;Acc:HGNC:26229]	transcribed unprocessed pseudogene	-1.32	0.60	15.63	<0.0001	0.0004
ENSG00000100911	PSME2	proteasome activator subunit 2 [Source:HGNC Symbol;Acc:HGNC:9569]	protein coding	1.32	8.16	57.78	<0.0001	<0.0001
ENSG00000186049	KRT73	keratin 73 [Source:HGNC Symbol;Acc:HGNC:28928]	protein coding	-1.32	1.05	15.30	<0.0001	0.0004
ENSG00000152672	CLEC4F	C-type lectin domain family 4 member F [Source:HGNC Symbol;Acc:HGNC:25357]	protein coding	-1.32	4.76	51.82	<0.0001	<0.0001
ENSG00000082438	COBLL1	cordon-bleu WH2 repeat protein like 1 [Source:HGNC Symbol;Acc:HGNC:23571]	protein coding	-1.32	1.53	24.03	<0.0001	<0.0001
ENSG00000184557	SOCS3	suppressor of cytokine signaling 3 [Source:HGNC Symbol;Acc:HGNC:19391]	protein coding	1.32	5.83	46.03	<0.0001	<0.0001
ENSG00000185920	PTCH1	patched 1 [Source:HGNC Symbol;Acc:HGNC:9585]	protein coding	-1.32	3.41	17.20	<0.0001	0.0002
ENSG00000160789	LMNA	lamin A/C [Source:HGNC Symbol;Acc:HGNC:6636]	protein coding	-1.32	5.34	62.40	<0.0001	<0.0001
ENSG00000162892	IL24	interleukin 24 [Source:HGNC Symbol;Acc:HGNC:11346]	protein coding	-1.32	0.25	23.29	<0.0001	<0.0001
ENSG00000215788	TNFRSF25	TNF receptor superfamily member 25 [Source:HGNC Symbol;Acc:HGNC:11910]	protein coding	-1.32	5.03	23.01	<0.0001	<0.0001
ENSG00000111674	ENO2	enolase 2 [Source:HGNC Symbol;Acc:HGNC:3353]	protein coding	-1.32	3.53	22.45	<0.0001	<0.0001
ENSG00000174171		novel transcript, antisense to SPTBN5	lncRNA	-1.32	2.40	19.94	<0.0001	<0.0001
ENSG00000162496	DHRS3	dehydrogenase/reductase 3 [Source:HGNC Symbol;Acc:HGNC:17693]	protein coding	-1.32	3.21	47.61	<0.0001	<0.0001
ENSG00000149527	PLCH2	phospholipase C eta 2 [Source:HGNC Symbol;Acc:HGNC:29037]	protein coding	-1.31	4.29	27.88	<0.0001	<0.0001
ENSG00000225783	MIAT	myocardial infarction associated transcript [Source:HGNC Symbol;Acc:HGNC:33425]	lncRNA	-1.31	5.09	25.84	<0.0001	<0.0001
ENSG00000012124	CD22	CD22 molecule [Source:HGNC Symbol;Acc:HGNC:1643]	protein coding	-1.31	5.03	28.79	<0.0001	<0.0001
ENSG00000157734	SNX22	sorting nexin 22 [Source:HGNC Symbol;Acc:HGNC:16315]	protein coding	-1.31	2.98	31.98	<0.0001	<0.0001

Table S12: Genes differentially expressed at Day 30 compared to pre-second vaccination (Study Arm 2 A/H7N9+AS03, D1, 29, Dendritic cells). Genes are sorted by descending absolute log_2 fold change. Gene model summaries and annotations are based on Ensembl Version 99 (continued.)

Ensembl Gene ID	Ensembl Gene Name	Ensembl Gene Description	Gene Type	log_2 Fold Change (Day 30 vs. Pre-Second Vaccination)	Average log_2 CPM	Likelihood Ratio Test Statistic	P-Value	FDR Adjusted P-Value
ENSG00000169031	COL4A3	collagen type IV alpha 3 chain [Source:HGNC Symbol;Acc:HGNC:2204]	protein coding	-1.31	0.59	24.97	<0.0001	<0.0001
ENSG00000169994	MYO7B	myosin VIIB [Source:HGNC Symbol;Acc:HGNC:7607]	protein coding	1.31	4.68	66.86	<0.0001	<0.0001
ENSG00000196220	SRGAP3	SLIT-ROBO Rho GTPase activating protein 3 [Source:HGNC Symbol;Acc:HGNC:19744]	protein coding	-1.31	1.72	37.27	<0.0001	<0.0001
ENSG00000170525	PFKFB3	6-phosphofructo-2-kinase/fructose-2,6-biphosphatase 3 [Source:HGNC Symbol;Acc:HGNC:8874]	protein coding	1.31	6.75	117.11	<0.0001	<0.0001
ENSG00000157551	KCNJ15	potassium inwardly rectifying channel subfamily J member 15 [Source:HGNC Symbol;Acc:HGNC:6261]	protein coding	1.30	2.57	35.83	<0.0001	<0.0001
ENSG00000130595	TNNT3	troponin T3, fast skeletal type [Source:HGNC Symbol;Acc:HGNC:11950]	protein coding	-1.30	1.39	68.11	<0.0001	<0.0001
ENSG00000013725	CD6	CD6 molecule [Source:HGNC Symbol;Acc:HGNC:1691]	protein coding	-1.30	5.90	20.03	<0.0001	<0.0001
ENSG00000204381	LAYN	layilin [Source:HGNC Symbol;Acc:HGNC:29471]	protein coding	-1.30	0.49	34.45	<0.0001	<0.0001
ENSG00000178498	DTX3	deltex E3 ubiquitin ligase 3 [Source:HGNC Symbol;Acc:HGNC:24457]	protein coding	-1.30	3.29	28.85	<0.0001	<0.0001
ENSG00000111331	OAS3	2'-5'-oligoadenylate synthetase 3 [Source:HGNC Symbol;Acc:HGNC:8088]	protein coding	1.30	8.28	73.68	<0.0001	<0.0001
ENSG00000134954	ETS1	ETS proto-oncogene 1, transcription factor [Source:HGNC Symbol;Acc:HGNC:3488]	protein coding	-1.30	7.86	24.44	<0.0001	<0.0001
ENSG00000090975	PITPNM2	phosphatidylinositol transfer protein membrane associated 2 [Source:HGNC Symbol;Acc:HGNC:21044]	protein coding	-1.30	4.17	41.81	<0.0001	<0.0001
ENSG00000211694	TRGV10	T cell receptor gamma variable 10 (non-functional) [Source:HGNC Symbol;Acc:HGNC:12285]	TR V gene	-1.30	1.15	17.67	<0.0001	0.0001
ENSG00000168394	TAP1	transporter 1, ATP binding cassette subfamily B member [Source:HGNC Symbol;Acc:HGNC:43]	protein coding	1.30	8.45	49.07	<0.0001	<0.0001
ENSG00000196139	AKR1C3	aldo-keto reductase family 1 member C3 [Source:HGNC Symbol;Acc:HGNC:386]	protein coding	-1.30	2.02	20.40	<0.0001	<0.0001
ENSG00000167861	HID1	HID1 domain containing [Source:HGNC Symbol;Acc:HGNC:15736]	protein coding	-1.30	0.39	23.76	<0.0001	<0.0001
ENSG00000198821	CD247	CD247 molecule [Source:HGNC Symbol;Acc:HGNC:1677]	protein coding	-1.29	6.38	24.34	<0.0001	<0.0001
ENSG00000152495	CAMK4	calcium/calmodulin dependent protein kinase IV [Source:HGNC Symbol;Acc:HGNC:1464]	protein coding	-1.29	3.98	16.43	<0.0001	0.0003

Table S12: Genes differentially expressed at Day 30 compared to pre-second vaccination (Study Arm 2 A/H7N9+AS03, D1, 29, Dendritic cells). Genes are sorted by descending absolute log_2 fold change. Gene model summaries and annotations are based on Ensembl Version 99 (continued.)

Ensembl Gene ID	Ensembl Gene Name	Ensembl Gene Description	Gene Type	log_2 Fold Change (Day 30 vs. Pre-Second Vaccination)	Average log_2 CPM	Likelihood Ratio Test Statistic	P-Value	FDR Adjusted P-Value
ENSG00000156587	UBE2L6	ubiquitin conjugating enzyme E2 L6 [Source:HGNC Symbol;Acc:HGNC:12490]	protein coding	1.29	7.76	58.25	<0.0001	<0.0001
ENSG00000157601	MX1	MX dynamin like GTPase 1 [Source:HGNC Symbol;Acc:HGNC:7532]	protein coding	1.29	7.74	68.14	<0.0001	<0.0001
ENSG00000038945	MSR1	macrophage scavenger receptor 1 [Source:HGNC Symbol;Acc:HGNC:7376]	protein coding	1.29	4.81	107.90	<0.0001	<0.0001
ENSG00000167483	NIBAN3	niban apoptosis regulator 3 [Source:HGNC Symbol;Acc:HGNC:24130]	protein coding	-1.29	4.92	17.89	<0.0001	0.0001
ENSG00000231793	DOC2GP	double C2 domain gamma, pseudogene [Source:HGNC Symbol;Acc:HGNC:37962]	transcribed unitary pseudogene	-1.29	1.50	29.61	<0.0001	<0.0001
ENSG00000110318	CEP126	centrosomal protein 126 [Source:HGNC Symbol;Acc:HGNC:29264]	protein coding	-1.29	1.20	24.98	<0.0001	<0.0001
ENSG00000280135		TEC	TEC	-1.29	1.83	21.78	<0.0001	<0.0001
ENSG00000126217	MCF2L	MCF.2 cell line derived transforming sequence like [Source:HGNC Symbol;Acc:HGNC:14576]	protein coding	-1.29	1.77	22.73	<0.0001	<0.0001
ENSG00000284194	SCO2	SCO cytochrome c oxidase assembly protein 2 [Source:HGNC Symbol;Acc:HGNC:10604]	protein coding	1.29	5.64	79.03	<0.0001	<0.0001
ENSG00000127152	BCL11B	BAF chromatin remodeling complex subunit BCL11B [Source:HGNC Symbol;Acc:HGNC:13222]	protein coding	-1.29	5.11	17.50	<0.0001	0.0002
ENSG00000115085	ZAP70	zeta chain of T cell receptor associated protein kinase 70 [Source:HGNC Symbol;Acc:HGNC:12858]	protein coding	-1.29	7.36	21.39	<0.0001	<0.0001
ENSG00000163534	FCRL1	Fc receptor like 1 [Source:HGNC Symbol;Acc:HGNC:18509]	protein coding	-1.29	4.70	24.62	<0.0001	<0.0001
ENSG00000162591	MEGF6	multiple EGF like domains 6 [Source:HGNC Symbol;Acc:HGNC:3232]	protein coding	-1.29	5.11	61.71	<0.0001	<0.0001
ENSG00000197635	DPP4	dipeptidyl peptidase 4 [Source:HGNC Symbol;Acc:HGNC:3009]	protein coding	-1.28	3.05	20.14	<0.0001	<0.0001
ENSG00000211934	IGHV1-2	immunoglobulin heavy variable 1-2 [Source:HGNC Symbol;Acc:HGNC:5550]	IG V gene	-1.28	0.69	21.75	<0.0001	<0.0001
ENSG00000065717	TLE2	TLE family member 2, transcriptional corepressor [Source:HGNC Symbol;Acc:HGNC:11838]	protein coding	-1.28	1.83	17.09	<0.0001	0.0002

Table S12: Genes differentially expressed at Day 30 compared to pre-second vaccination (Study Arm 2 A/H7N9+AS03, D1, 29, Dendritic cells). Genes are sorted by descending absolute log_2 fold change. Gene model summaries and annotations are based on Ensembl Version 99 (continued.)

Ensembl Gene ID	Ensembl Gene Name	Ensembl Gene Description	Gene Type	log_2 Fold Change (Day 30 vs. Pre-Second Vaccination)	Average log_2 CPM	Likelihood Ratio Test Statistic	P-Value	FDR Adjusted P-Value
ENSG00000182487	NCF1B	neutrophil cytosolic factor 1B pseudogene [Source:HGNC Symbol;Acc:HGNC:32522]	transcribed unprocessed pseudogene	1.28	6.17	43.62	<0.0001	<0.0001
ENSG00000185339	TCN2	transcobalamin 2 [Source:HGNC Symbol;Acc:HGNC:11653]	protein coding	1.28	5.34	68.35	<0.0001	<0.0001
ENSG00000163564	PYHIN1	pyrin and HIN domain family member 1 [Source:HGNC Symbol;Acc:HGNC:28894]	protein coding	-1.28	3.63	20.26	<0.0001	<0.0001
ENSG00000145287	PLAC8	placenta associated 8 [Source:HGNC Symbol;Acc:HGNC:19254]	protein coding	1.28	7.36	52.23	<0.0001	<0.0001
ENSG00000197057	DTHD1	death domain containing 1 [Source:HGNC Symbol;Acc:HGNC:37261]	protein coding	-1.28	1.99	18.46	<0.0001	0.0001
ENSG00000205336	ADGRG1	adhesion G protein-coupled receptor G1 [Source:HGNC Symbol;Acc:HGNC:4512]	protein coding	-1.28	6.17	19.34	<0.0001	<0.0001
ENSG00000196092	PAX5	paired box 5 [Source:HGNC Symbol;Acc:HGNC:8619]	protein coding	-1.28	4.16	16.62	<0.0001	0.0002
ENSG00000151612	ZNF827	zinc finger protein 827 [Source:HGNC Symbol;Acc:HGNC:27193]	protein coding	-1.28	2.12	24.23	<0.0001	<0.0001
ENSG00000211829	TRDC	T cell receptor delta constant [Source:HGNC Symbol;Acc:HGNC:12253]	TR C gene	-1.28	3.76	19.83	<0.0001	<0.0001
ENSG00000163508	EOMES	eomesodermin [Source:HGNC Symbol;Acc:HGNC:3372]	protein coding	-1.28	3.61	16.18	<0.0001	0.0003
ENSG00000158488	CD1E	CD1e molecule [Source:HGNC Symbol;Acc:HGNC:1638]	protein coding	-1.28	2.76	56.54	<0.0001	<0.0001
ENSG00000179914	ITLN1	intelectin 1 [Source:HGNC Symbol;Acc:HGNC:18259]	protein coding	-1.28	0.69	23.10	<0.0001	<0.0001
ENSG00000164483	SAMD3	sterile alpha motif domain containing 3 [Source:HGNC Symbol;Acc:HGNC:21574]	protein coding	-1.28	3.76	21.94	<0.0001	<0.0001
ENSG00000235621	LINC00494	long intergenic non-protein coding RNA 494 [Source:HGNC Symbol;Acc:HGNC:27657]	lncRNA	-1.27	0.61	14.35	0.0002	0.0007
ENSG00000139880	CDH24	cadherin 24 [Source:HGNC Symbol;Acc:HGNC:14265]	protein coding	-1.27	0.15	25.06	<0.0001	<0.0001
ENSG00000213366	GSTM2	glutathione S-transferase mu 2 [Source:HGNC Symbol;Acc:HGNC:4634]	protein coding	-1.27	3.40	34.28	<0.0001	<0.0001

Table S12: Genes differentially expressed at Day 30 compared to pre-second vaccination (Study Arm 2 A/H7N9+AS03, D1, 29, Dendritic cells). Genes are sorted by descending absolute log_2 fold change. Gene model summaries and annotations are based on Ensembl Version 99 (continued.)

Ensembl Gene ID	Ensembl Gene Name	Ensembl Gene Description	Gene Type	log_2 Fold Change (Day 30 vs. Pre-Second Vaccination)	Average log_2 CPM	Likelihood Ratio Test Statistic	P-Value	FDR Adjusted P-Value
ENSG00000138795	LEF1	lymphoid enhancer binding factor 1 [Source:HGNC Symbol;Acc:HGNC:6551]	protein coding	-1.27	4.93	13.83	0.0002	0.0008
ENSG00000135736	CCDC102A	coiled-coil domain containing 102A [Source:HGNC Symbol;Acc:HGNC:28097]	protein coding	-1.27	2.22	19.88	<0.0001	<0.0001
ENSG00000204475	NCR3	natural cytotoxicity triggering receptor 3 [Source:HGNC Symbol;Acc:HGNC:19077]	protein coding	-1.27	3.26	29.40	<0.0001	<0.0001
ENSG00000153283	CD96	CD96 molecule [Source:HGNC Symbol;Acc:HGNC:16892]	protein coding	-1.27	4.64	18.69	<0.0001	<0.0001
ENSG00000198851	CD3E	CD3e molecule [Source:HGNC Symbol;Acc:HGNC:1674]	protein coding	-1.27	6.28	16.56	<0.0001	0.0002
ENSG00000100385	IL2RB	interleukin 2 receptor subunit beta [Source:HGNC Symbol;Acc:HGNC:6009]	protein coding	-1.27	6.81	24.89	<0.0001	<0.0001
ENSG00000156113	KCNMA1	potassium calcium-activated channel subfamily M alpha 1 [Source:HGNC Symbol;Acc:HGNC:6284]	protein coding	1.27	2.90	17.64	<0.0001	0.0001
ENSG00000187912	CLEC17A	C-type lectin domain containing 17A [Source:HGNC Symbol;Acc:HGNC:34520]	protein coding	-1.27	2.63	46.26	<0.0001	<0.0001
ENSG00000271447	MMP28	matrix metallopeptidase 28 [Source:HGNC Symbol;Acc:HGNC:14366]	protein coding	-1.27	0.44	16.81	<0.0001	0.0002
ENSG00000205056	LINC02397	long intergenic non-protein coding RNA 2397 [Source:HGNC Symbol;Acc:HGNC:53323]	lncRNA	-1.26	0.71	12.43	0.0004	0.0016
ENSG00000188906	LRRK2	leucine rich repeat kinase 2 [Source:HGNC Symbol;Acc:HGNC:18618]	protein coding	1.26	8.37	70.76	<0.0001	<0.0001
ENSG00000179750	APOBEC3B	apolipoprotein B mRNA editing enzyme catalytic subunit 3B [Source:HGNC Symbol;Acc:HGNC:17352]	protein coding	1.26	1.68	31.73	<0.0001	<0.0001
ENSG00000163687	DNASE1L3	deoxyribonuclease 1 like 3 [Source:HGNC Symbol;Acc:HGNC:2959]	protein coding	-1.26	1.14	29.19	<0.0001	<0.0001
ENSG00000081059	TCF7	transcription factor 7 [Source:HGNC Symbol;Acc:HGNC:11639]	protein coding	-1.26	6.56	16.59	<0.0001	0.0002
ENSG00000234518	PTGES3P1	prostaglandin E synthase 3 pseudogene 1 [Source:HGNC Symbol;Acc:HGNC:43824]	processed pseudogene	1.26	3.20	42.21	<0.0001	<0.0001
ENSG00000135083	CCNJL	cyclin J like [Source:HGNC Symbol;Acc:HGNC:25876]	protein coding	-1.26	0.84	17.75	<0.0001	0.0001
ENSG00000279141		long intergenic non-protein coding RNA 1451	lncRNA	-1.26	1.35	28.60	<0.0001	<0.0001
ENSG00000182866	LCK	LCK proto-oncogene, Src family tyrosine kinase [Source:HGNC Symbol;Acc:HGNC:6524]	protein coding	-1.26	6.12	17.57	<0.0001	0.0002

Table S12: Genes differentially expressed at Day 30 compared to pre-second vaccination (Study Arm 2 A/H7N9+AS03, D1, 29, Dendritic cells). Genes are sorted by descending absolute log_2 fold change. Gene model summaries and annotations are based on Ensembl Version 99 (continued.)

Ensembl Gene ID	Ensembl Gene Name	Ensembl Gene Description	Gene Type	log_2 Fold Change (Day 30 vs. Pre-Second Vaccination)	Average log_2 CPM	Likelihood Ratio Test Statistic	P-Value	FDR Adjusted P-Value
ENSG00000154102	C16orf74	chromosome 16 open reading frame 74 [Source:HGNC Symbol;Acc:HGNC:23362]	protein coding	-1.26	2.85	30.24	<0.0001	<0.0001
ENSG00000166033	HTRA1	HtrA serine peptidase 1 [Source:HGNC Symbol;Acc:HGNC:9476]	protein coding	-1.26	1.48	15.99	<0.0001	0.0003
ENSG00000150045	KLRF1	killer cell lectin like receptor F1 [Source:HGNC Symbol;Acc:HGNC:13342]	protein coding	-1.26	4.22	28.23	<0.0001	<0.0001
ENSG00000213626	LBH	LBH regulator of WNT signaling pathway [Source:HGNC Symbol;Acc:HGNC:29532]	protein coding	-1.26	6.00	21.25	<0.0001	<0.0001
ENSG00000253755	IGHGP	immunoglobulin heavy constant gamma P (non-functional) [Source:HGNC Symbol;Acc:HGNC:5529]	IG C pseudogene	-1.25	0.57	30.28	<0.0001	<0.0001
ENSG00000103569	AQP9	aquaporin 9 [Source:HGNC Symbol;Acc:HGNC:643]	protein coding	1.25	6.37	72.08	<0.0001	<0.0001
ENSG00000184828	ZBTB7C	zinc finger and BTB domain containing 7C [Source:HGNC Symbol;Acc:HGNC:31700]	protein coding	-1.25	0.42	29.83	<0.0001	<0.0001
ENSG00000274767		novel transcript, antisense CCL3L3	lncRNA	-1.25	0.84	34.64	<0.0001	<0.0001
ENSG00000245648		novel transcript, antisense to KLRK1	lncRNA	-1.25	3.08	18.59	<0.0001	<0.0001
ENSG00000221946	FXYD7	FXYD domain containing ion transport regulator 7 [Source:HGNC Symbol;Acc:HGNC:4034]	protein coding	-1.24	0.41	29.03	<0.0001	<0.0001
ENSG00000114923	SLC4A3	solute carrier family 4 member 3 [Source:HGNC Symbol;Acc:HGNC:11029]	protein coding	-1.24	3.67	46.64	<0.0001	<0.0001
ENSG00000115604	IL18R1	interleukin 18 receptor 1 [Source:HGNC Symbol;Acc:HGNC:5988]	protein coding	-1.24	3.43	48.66	<0.0001	<0.0001
ENSG00000081052	COL4A4	collagen type IV alpha 4 chain [Source:HGNC Symbol;Acc:HGNC:2206]	protein coding	-1.24	0.65	26.44	<0.0001	<0.0001
ENSG00000125347	IRF1	interferon regulatory factor 1 [Source:HGNC Symbol;Acc:HGNC:6116]	protein coding	1.24	8.58	53.96	<0.0001	<0.0001
ENSG00000154027	AK5	adenylate kinase 5 [Source:HGNC Symbol;Acc:HGNC:365]	protein coding	-1.24	2.03	23.41	<0.0001	<0.0001
ENSG00000181754	AMIGO1	adhesion molecule with Ig like domain 1 [Source:HGNC Symbol;Acc:HGNC:20824]	protein coding	-1.24	2.63	17.34	<0.0001	0.0002
ENSG00000099204	ABLIM1	actin binding LIM protein 1 [Source:HGNC Symbol;Acc:HGNC:78]	protein coding	-1.24	5.30	20.33	<0.0001	<0.0001
ENSG00000161640	SIGLEC11	sialic acid binding Ig like lectin 11 [Source:HGNC Symbol;Acc:HGNC:15622]	protein coding	1.24	2.65	82.74	<0.0001	<0.0001
ENSG00000068079	IFI35	interferon induced protein 35 [Source:HGNC Symbol;Acc:HGNC:5399]	protein coding	1.24	6.58	61.08	<0.0001	<0.0001

Table S12: Genes differentially expressed at Day 30 compared to pre-second vaccination (Study Arm 2 A/H7N9+AS03, D1, 29, Dendritic cells). Genes are sorted by descending absolute log_2 fold change. Gene model summaries and annotations are based on Ensembl Version 99 (continued.)

Ensembl Gene ID	Ensembl Gene Name	Ensembl Gene Description	Gene Type	log_2 Fold Change (Day 30 vs. Pre-Second Vaccination)	Average log_2 CPM	Likelihood Ratio Test Statistic	P-Value	FDR Adjusted P-Value
ENSG00000021300	PLEKHB1	pleckstrin homology domain containing B1 [Source:HGNC Symbol;Acc:HGNC:19079]	protein coding	-1.24	2.38	17.03	<0.0001	0.0002
ENSG00000145911	N4BP3	NEDD4 binding protein 3 [Source:HGNC Symbol;Acc:HGNC:29852]	protein coding	-1.23	1.07	22.58	<0.0001	<0.0001
ENSG00000228956	SATB1-AS1	SATB1 antisense RNA 1 [Source:HGNC Symbol;Acc:HGNC:50687]	lncRNA	-1.23	2.06	17.68	<0.0001	0.0001
ENSG00000254709	IGLL5	immunoglobulin lambda like polypeptide 5 [Source:HGNC Symbol;Acc:HGNC:38476]	protein coding	-1.23	1.06	31.90	<0.0001	<0.0001
ENSG00000100100	PIK3IP1	phosphoinositide-3-kinase interacting protein 1 [Source:HGNC Symbol;Acc:HGNC:24942]	protein coding	-1.23	6.72	52.78	<0.0001	<0.0001
ENSG00000107485	GATA3	GATA binding protein 3 [Source:HGNC Symbol;Acc:HGNC:4172]	protein coding	-1.23	3.42	16.28	<0.0001	0.0003
ENSG00000211772	TRBC2	T cell receptor beta constant 2 [Source:HGNC Symbol;Acc:HGNC:12157]	TR C gene	-1.23	6.00	18.89	<0.0001	<0.0001
ENSG00000077063	CTTNBP2	cortactin binding protein 2 [Source:HGNC Symbol;Acc:HGNC:15679]	protein coding	-1.23	0.63	21.90	<0.0001	<0.0001
ENSG00000268758	ADGRE4P	adhesion G protein-coupled receptor E4, pseudogene [Source:HGNC Symbol;Acc:HGNC:19240]	transcribed unprocessed pseudogene	-1.23	4.05	16.05	<0.0001	0.0003
ENSG00000211698	TRGV4	T cell receptor gamma variable 4 [Source:HGNC Symbol;Acc:HGNC:12289]	TR V gene	-1.23	0.91	11.41	0.0007	0.0025
ENSG00000182183	SHISAL2A	shisa like 2A [Source:HGNC Symbol;Acc:HGNC:28757]	protein coding	-1.23	2.05	16.54	<0.0001	0.0002
ENSG00000130787	HIP1R	huntingtin interacting protein 1 related [Source:HGNC Symbol;Acc:HGNC:18415]	protein coding	-1.23	4.56	28.73	<0.0001	<0.0001
ENSG00000104177	MYEF2	myelin expression factor 2 [Source:HGNC Symbol;Acc:HGNC:17940]	protein coding	-1.23	1.62	38.28	<0.0001	<0.0001
ENSG00000197540	GZMM	granzyme M [Source:HGNC Symbol;Acc:HGNC:4712]	protein coding	-1.23	4.96	17.80	<0.0001	0.0001
ENSG00000162373	BEND5	BEN domain containing 5 [Source:HGNC Symbol;Acc:HGNC:25668]	protein coding	-1.23	0.61	28.19	<0.0001	<0.0001
ENSG00000152229	PSTPIP2	proline-serine-threonine phosphatase interacting protein 2 [Source:HGNC Symbol;Acc:HGNC:9581]	protein coding	1.23	6.78	62.91	<0.0001	<0.0001

Table S12: Genes differentially expressed at Day 30 compared to pre-second vaccination (Study Arm 2 A/H7N9+AS03, D1, 29, Dendritic cells). Genes are sorted by descending absolute log_2 fold change. Gene model summaries and annotations are based on Ensembl Version 99 (continued.)

Ensembl Gene ID	Ensembl Gene Name	Ensembl Gene Description	Gene Type	log_2 Fold Change (Day 30 vs. Pre-Second Vaccination)	Average log_2 CPM	Likelihood Ratio Test Statistic	P-Value	FDR Adjusted P-Value
ENSG00000136235	GPNMB	glycoprotein nmb [Source:HGNC Symbol;Acc:HGNC:4462]	protein coding	-1.23	1.13	21.72	<0.0001	<0.0001
ENSG00000069667	RORA	RAR related orphan receptor A [Source:HGNC Symbol;Acc:HGNC:10258]	protein coding	-1.23	4.83	20.39	<0.0001	<0.0001
ENSG00000159753	CARMIL2	capping protein regulator and myosin 1 linker 2 [Source:HGNC Symbol;Acc:HGNC:27089]	protein coding	-1.22	5.13	22.45	<0.0001	<0.0001
ENSG00000054793	ATP9A	ATPase phospholipid transporting 9A (putative) [Source:HGNC Symbol;Acc:HGNC:13540]	protein coding	-1.22	0.99	25.71	<0.0001	<0.0001
ENSG00000148516	ZEB1	zinc finger E-box binding homeobox 1 [Source:HGNC Symbol;Acc:HGNC:11642]	protein coding	-1.22	2.49	37.71	<0.0001	<0.0001
ENSG00000236790	LINC00299	long intergenic non-protein coding RNA 299 [Source:HGNC Symbol;Acc:HGNC:27940]	lncRNA	-1.22	2.38	34.32	<0.0001	<0.0001
ENSG00000124203	ZNF831	zinc finger protein 831 [Source:HGNC Symbol;Acc:HGNC:16167]	protein coding	-1.22	3.86	21.94	<0.0001	<0.0001
ENSG00000141293	SKAP1	src kinase associated phosphoprotein 1 [Source:HGNC Symbol;Acc:HGNC:15605]	protein coding	-1.22	3.98	20.52	<0.0001	<0.0001
ENSG00000149582	TMEM25	transmembrane protein 25 [Source:HGNC Symbol;Acc:HGNC:25890]	protein coding	-1.22	1.38	19.77	<0.0001	<0.0001
ENSG00000146285	SCML4	Scm polycomb group protein like 4 [Source:HGNC Symbol;Acc:HGNC:21397]	protein coding	-1.22	3.84	21.52	<0.0001	<0.0001
ENSG00000154856	APCDD1	APC down-regulated 1 [Source:HGNC Symbol;Acc:HGNC:15718]	protein coding	-1.22	1.93	31.82	<0.0001	<0.0001
ENSG00000236609	ZNF853	zinc finger protein 853 [Source:HGNC Symbol;Acc:HGNC:21767]	protein coding	-1.22	1.73	25.86	<0.0001	<0.0001
ENSG00000133985	TTC9	tetratricopeptide repeat domain 9 [Source:HGNC Symbol;Acc:HGNC:20267]	protein coding	-1.22	4.52	48.17	<0.0001	<0.0001
ENSG00000182580	EPHB3	EPH receptor B3 [Source:HGNC Symbol;Acc:HGNC:3394]	protein coding	-1.22	2.19	41.56	<0.0001	<0.0001
ENSG00000282173	TRBJ1-5	T cell receptor beta joining 1-5 [Source:HGNC Symbol;Acc:HGNC:12166]	TR J gene	-1.22	0.47	17.53	<0.0001	0.0002
ENSG00000185760	KCNQ5	potassium voltage-gated channel subfamily Q member 5 [Source:HGNC Symbol;Acc:HGNC:6299]	protein coding	-1.22	0.51	20.72	<0.0001	<0.0001
ENSG00000185507	IRF7	interferon regulatory factor 7 [Source:HGNC Symbol;Acc:HGNC:6122]	protein coding	1.22	7.60	83.15	<0.0001	<0.0001
ENSG00000128536	CDHR3	cadherin related family member 3 [Source:HGNC Symbol;Acc:HGNC:26308]	protein coding	-1.22	0.11	25.41	<0.0001	<0.0001

Table S12: Genes differentially expressed at Day 30 compared to pre-second vaccination (Study Arm 2 A/H7N9+AS03, D1, 29, Dendritic cells). Genes are sorted by descending absolute log_2 fold change. Gene model summaries and annotations are based on Ensembl Version 99 (continued.)

Ensembl Gene ID	Ensembl Gene Name	Ensembl Gene Description	Gene Type	log_2 Fold Change (Day 30 vs. Pre-Second Vaccination)	Average log_2 CPM	Likelihood Ratio Test Statistic	P-Value	FDR Adjusted P-Value
ENSG00000211941	IGHV3-11	immunoglobulin heavy variable 3-11 (gene/pseudogene) [Source:HGNC Symbol;Acc:HGNC:5580]	IG V gene	-1.21	0.77	28.41	<0.0001	<0.0001
ENSG00000120915	EPHX2	epoxide hydrolase 2 [Source:HGNC Symbol;Acc:HGNC:3402]	protein coding	-1.21	2.45	16.12	<0.0001	0.0003
ENSG00000188322	SBK1	SH3 domain binding kinase 1 [Source:HGNC Symbol;Acc:HGNC:17699]	protein coding	-1.21	4.10	17.39	<0.0001	0.0002
ENSG00000165272	AQP3	aquaporin 3 (Gill blood group) [Source:HGNC Symbol;Acc:HGNC:636]	protein coding	-1.21	3.85	21.98	<0.0001	<0.0001
ENSG00000128594	LRRC4	leucine rich repeat containing 4 [Source:HGNC Symbol;Acc:HGNC:15586]	protein coding	1.21	3.69	38.79	<0.0001	<0.0001
ENSG00000167984	NLRC3	NLR family CARD domain containing 3 [Source:HGNC Symbol;Acc:HGNC:29889]	protein coding	-1.21	5.43	21.84	<0.0001	<0.0001
ENSG00000146674	IGFBP3	insulin like growth factor binding protein 3 [Source:HGNC Symbol;Acc:HGNC:5472]	protein coding	-1.21	0.34	15.71	<0.0001	0.0004
ENSG00000106537	TSPAN13	tetraspanin 13 [Source:HGNC Symbol;Acc:HGNC:21643]	protein coding	-1.21	1.65	13.82	0.0002	0.0008
ENSG00000101004	NINL	ninein like [Source:HGNC Symbol;Acc:HGNC:29163]	protein coding	-1.21	0.26	22.52	<0.0001	<0.0001
ENSG00000110987	BCL7A	BAF chromatin remodeling complex subunit BCL7A [Source:HGNC Symbol;Acc:HGNC:1004]	protein coding	-1.21	3.17	52.33	<0.0001	<0.0001
ENSG00000274114	ALOX15P1	arachidonate 15-lipoxygenase pseudogene 1 [Source:HGNC Symbol;Acc:HGNC:13742]	transcribed unprocessed pseudogene	-1.21	0.34	20.92	<0.0001	<0.0001
ENSG00000139193	CD27	CD27 molecule [Source:HGNC Symbol;Acc:HGNC:11922]	protein coding	-1.21	3.56	23.41	<0.0001	<0.0001
ENSG00000182809	CRIP2	cysteine rich protein 2 [Source:HGNC Symbol;Acc:HGNC:2361]	protein coding	-1.20	2.61	31.52	<0.0001	<0.0001
ENSG00000003249	DBNDD1	dysbindin domain containing 1 [Source:HGNC Symbol;Acc:HGNC:28455]	protein coding	-1.20	0.48	14.67	0.0001	0.0006
ENSG00000186891	TNFRSF18	TNF receptor superfamily member 18 [Source:HGNC Symbol;Acc:HGNC:11914]	protein coding	-1.20	2.08	17.79	<0.0001	0.0001
ENSG00000172116	CD8B	CD8b molecule [Source:HGNC Symbol;Acc:HGNC:1707]	protein coding	-1.20	3.98	13.54	0.0002	0.0009

Table S12: Genes differentially expressed at Day 30 compared to pre-second vaccination (Study Arm 2 A/H7N9+AS03, D1, 29, Dendritic cells). Genes are sorted by descending absolute log_2 fold change. Gene model summaries and annotations are based on Ensembl Version 99 (continued.)

Ensembl Gene ID	Ensembl Gene Name	Ensembl Gene Description	Gene Type	log_2 Fold Change (Day 30 vs. Pre-Second Vaccination)	Average log_2 CPM	Likelihood Ratio Test Statistic	P-Value	FDR Adjusted P-Value
ENSG00000125084	WNT1	Wnt family member 1 [Source:HGNC Symbol;Acc:HGNC:12774]	protein coding	-1.20	0.06	12.12	0.0005	0.0018
ENSG00000169136	ATF5	activating transcription factor 5 [Source:HGNC Symbol;Acc:HGNC:790]	protein coding	1.20	6.01	61.45	<0.0001	<0.0001
ENSG00000134539	KLRD1	killer cell lectin like receptor D1 [Source:HGNC Symbol;Acc:HGNC:6378]	protein coding	-1.20	5.76	18.95	<0.0001	<0.0001
ENSG00000268027		novel transcript, sense intronic to CEACAM21	lncRNA	-1.20	3.06	20.81	<0.0001	<0.0001
ENSG00000173258	ZNF483	zinc finger protein 483 [Source:HGNC Symbol;Acc:HGNC:23384]	protein coding	-1.20	0.54	13.82	0.0002	0.0008
ENSG00000116663	FBXO6	F-box protein 6 [Source:HGNC Symbol;Acc:HGNC:13585]	protein coding	1.20	5.44	62.07	<0.0001	<0.0001
ENSG00000230006	ANKRD36BP2	ankyrin repeat domain 36B pseudogene 2 [Source:HGNC Symbol;Acc:HGNC:33607]	transcribed unprocessed pseudogene	-1.20	1.34	29.02	<0.0001	<0.0001
ENSG00000154188	ANGPT1	angiopoietin 1 [Source:HGNC Symbol;Acc:HGNC:484]	protein coding	-1.20	1.01	20.76	<0.0001	<0.0001
ENSG00000273179		novel transcript, antisense to SPON2	lncRNA	-1.20	1.77	13.53	0.0002	0.0009
ENSG00000092096	SLC22A17	solute carrier family 22 member 17 [Source:HGNC Symbol;Acc:HGNC:23095]	protein coding	-1.20	0.90	11.00	0.0009	0.003
ENSG00000133818	RRAS2	RAS related 2 [Source:HGNC Symbol;Acc:HGNC:17271]	protein coding	-1.20	2.18	15.88	<0.0001	0.0003
ENSG00000211966	IGHV5-51	immunoglobulin heavy variable 5-51 [Source:HGNC Symbol;Acc:HGNC:5659]	IG V gene	-1.20	1.75	25.11	<0.0001	<0.0001
ENSG00000211770	TRBJ2-6	T cell receptor beta joining 2-6 [Source:HGNC Symbol;Acc:HGNC:12174]	TR J gene	-1.20	-0.02	15.86	<0.0001	0.0003
ENSG00000010278	CD9	CD9 molecule [Source:HGNC Symbol;Acc:HGNC:1709]	protein coding	-1.20	3.69	27.22	<0.0001	<0.0001
ENSG00000171811	CFAP46	cilia and flagella associated protein 46 [Source:HGNC Symbol;Acc:HGNC:25247]	protein coding	-1.20	0.27	22.72	<0.0001	<0.0001
ENSG00000132704	FCRL2	Fc receptor like 2 [Source:HGNC Symbol;Acc:HGNC:14875]	protein coding	-1.19	3.01	18.65	<0.0001	<0.0001
ENSG00000189430	NCR1	natural cytotoxicity triggering receptor 1 [Source:HGNC Symbol;Acc:HGNC:6731]	protein coding	-1.19	2.44	23.64	<0.0001	<0.0001

Table S12: Genes differentially expressed at Day 30 compared to pre-second vaccination (Study Arm 2 A/H7N9+AS03, D1, 29, Dendritic cells). Genes are sorted by descending absolute log_2 fold change. Gene model summaries and annotations are based on Ensembl Version 99 (continued.)

Ensembl Gene ID	Ensembl Gene Name	Ensembl Gene Description	Gene Type	log_2 Fold Change (Day 30 vs. Pre-Second Vaccination)	Average log_2 CPM	Likelihood Ratio Test Statistic	P-Value	FDR Adjusted P-Value
ENSG00000122188	LAX1	lymphocyte transmembrane adaptor 1 [Source:HGNC Symbol;Acc:HGNC:26005]	protein coding	-1.19	2.92	23.66	<0.0001	<0.0001
ENSG00000109956	B3GAT1	beta-1,3-glucuronyltransferase 1 [Source:HGNC Symbol;Acc:HGNC:921]	protein coding	-1.19	2.94	12.67	0.0004	0.0014
ENSG00000136689	IL1RN	interleukin 1 receptor antagonist [Source:HGNC Symbol;Acc:HGNC:6000]	protein coding	1.19	5.74	63.76	<0.0001	<0.0001
ENSG00000261327		novel transcript	lncRNA	-1.19	1.69	35.65	<0.0001	<0.0001
ENSG00000167641	PPP1R14A	protein phosphatase 1 regulatory inhibitor subunit 14A [Source:HGNC Symbol;Acc:HGNC:14871]	protein coding	-1.19	0.70	27.86	<0.0001	<0.0001
ENSG00000246223	LINC01550	long intergenic non-protein coding RNA 1550 [Source:HGNC Symbol;Acc:HGNC:20111]	lncRNA	-1.19	1.40	13.93	0.0002	0.0008
ENSG00000245954	LINC02273	long intergenic non-protein coding RNA 2273 [Source:HGNC Symbol;Acc:HGNC:53188]	lncRNA	-1.19	1.16	6.93	0.0085	0.0203
ENSG00000286162		novel transcript	lncRNA	1.19	1.35	18.96	<0.0001	<0.0001
ENSG00000104490	NCALD	neurocalcin delta [Source:HGNC Symbol;Acc:HGNC:7655]	protein coding	-1.19	2.64	18.82	<0.0001	<0.0001
ENSG00000213809	KLRK1	killer cell lectin like receptor K1 [Source:HGNC Symbol;Acc:HGNC:18788]	protein coding	-1.19	1.93	16.92	<0.0001	0.0002
ENSG00000089012	SIRPG	signal regulatory protein gamma [Source:HGNC Symbol;Acc:HGNC:15757]	protein coding	-1.19	2.30	12.86	0.0003	0.0013
ENSG00000161381	PLXDC1	plexin domain containing 1 [Source:HGNC Symbol;Acc:HGNC:20945]	protein coding	-1.19	2.83	17.16	<0.0001	0.0002
ENSG00000240403	KIR3DL2	killer cell immunoglobulin like receptor, three Ig domains and long cytoplasmic tail 2 [Source:HGNC Symbol;Acc:HGNC:6339]	protein coding	-1.19	1.08	16.34	<0.0001	0.0003
ENSG00000131797	CLUHP3	clustered mitochondria homolog pseudogene 3 [Source:HGNC Symbol;Acc:HGNC:28447]	transcribed unprocessed pseudogene	-1.18	3.43	17.08	<0.0001	0.0002
ENSG00000175262	C1orf127	chromosome 1 open reading frame 127 [Source:HGNC Symbol;Acc:HGNC:26730]	protein coding	-1.18	1.46	30.93	<0.0001	<0.0001
ENSG00000180739	S1PR5	sphingosine-1-phosphate receptor 5 [Source:HGNC Symbol;Acc:HGNC:14299]	protein coding	-1.18	5.07	18.24	<0.0001	0.0001
ENSG00000177425	PAWR	pro-apoptotic WT1 regulator [Source:HGNC Symbol;Acc:HGNC:8614]	protein coding	-1.18	0.61	22.71	<0.0001	<0.0001

Table S12: Genes differentially expressed at Day 30 compared to pre-second vaccination (Study Arm 2 A/H7N9+AS03, D1, 29, Dendritic cells). Genes are sorted by descending absolute log_2 fold change. Gene model summaries and annotations are based on Ensembl Version 99 (continued.)

Ensembl Gene ID	Ensembl Gene Name	Ensembl Gene Description	Gene Type	log_2 Fold Change (Day 30 vs. Pre-Second Vaccination)	Average log_2 CPM	Likelihood Ratio Test Statistic	P-Value	FDR Adjusted P-Value
ENSG00000169884	WNT10B	Wnt family member 10B [Source:HGNC Symbol;Acc:HGNC:12775]	protein coding	-1.18	1.56	16.80	<0.0001	0.0002
ENSG00000262312		novel transcript, antisense to NLRC3	lncRNA	-1.18	1.70	16.78	<0.0001	0.0002
ENSG00000183542	KLRC4	killer cell lectin like receptor C4 [Source:HGNC Symbol;Acc:HGNC:6377]	protein coding	-1.18	0.33	16.57	<0.0001	0.0002
ENSG00000177989	ODF3B	outer dense fiber of sperm tails 3B [Source:HGNC Symbol;Acc:HGNC:34388]	protein coding	1.18	8.49	55.99	<0.0001	<0.0001
ENSG00000006432	MAP3K9	mitogen-activated protein kinase kinase kinase 9 [Source:HGNC Symbol;Acc:HGNC:6861]	protein coding	-1.18	1.38	19.31	<0.0001	<0.0001
ENSG00000211821	TRDV2	T cell receptor delta variable 2 [Source:HGNC Symbol;Acc:HGNC:12263]	TR V gene	-1.18	1.02	12.98	0.0003	0.0012
ENSG00000168546	GFRA2	GDNF family receptor alpha 2 [Source:HGNC Symbol;Acc:HGNC:4244]	protein coding	-1.18	3.33	30.42	<0.0001	<0.0001
ENSG00000156966	B3GNT7	UDP-GlcNAc:betaGal beta-1,3-N-acetylglucosaminyltransferase 7 [Source:HGNC Symbol;Acc:HGNC:18811]	protein coding	-1.18	2.25	30.02	<0.0001	<0.0001
ENSG00000100027	YPEL1	yippee like 1 [Source:HGNC Symbol;Acc:HGNC:12845]	protein coding	-1.18	2.49	34.68	<0.0001	<0.0001
ENSG00000073861	TBX21	T-box transcription factor 21 [Source:HGNC Symbol;Acc:HGNC:11599]	protein coding	-1.18	5.21	17.57	<0.0001	0.0002
ENSG00000112182	BACH2	BTB domain and CNC homolog 2 [Source:HGNC Symbol;Acc:HGNC:14078]	protein coding	-1.18	3.65	27.59	<0.0001	<0.0001
ENSG00000085563	ABCB1	ATP binding cassette subfamily B member 1 [Source:HGNC Symbol;Acc:HGNC:40]	protein coding	-1.18	2.94	21.22	<0.0001	<0.0001
ENSG00000155974	GRIP1	glutamate receptor interacting protein 1 [Source:HGNC Symbol;Acc:HGNC:18708]	protein coding	-1.18	2.08	59.32	<0.0001	<0.0001
ENSG00000172005	MAL	mal, T cell differentiation protein [Source:HGNC Symbol;Acc:HGNC:6817]	protein coding	-1.18	3.36	12.12	0.0005	0.0018
ENSG00000211734	TRBV5-1	T cell receptor beta variable 5-1 [Source:HGNC Symbol;Acc:HGNC:12218]	TR V gene	-1.17	0.58	10.56	0.0012	0.0037
ENSG00000137628	DDX60	DExD/H-box helicase 60 [Source:HGNC Symbol;Acc:HGNC:25942]	protein coding	1.17	5.75	79.20	<0.0001	<0.0001
ENSG00000076554	TPD52	tumor protein D52 [Source:HGNC Symbol;Acc:HGNC:12005]	protein coding	-1.17	2.64	28.09	<0.0001	<0.0001
ENSG00000116824	CD2	CD2 molecule [Source:HGNC Symbol;Acc:HGNC:1639]	protein coding	-1.17	5.03	27.71	<0.0001	<0.0001

Table S12: Genes differentially expressed at Day 30 compared to pre-second vaccination (Study Arm 2 A/H7N9+AS03, D1, 29, Dendritic cells). Genes are sorted by descending absolute log_2 fold change. Gene model summaries and annotations are based on Ensembl Version 99 (continued.)

Ensembl Gene ID	Ensembl Gene Name	Ensembl Gene Description	Gene Type	log_2 Fold Change (Day 30 vs. Pre-Second Vaccination)	Average log_2 CPM	Likelihood Ratio Test Statistic	P-Value	FDR Adjusted P-Value
ENSG00000270550	IGHV3-30	immunoglobulin heavy variable 3-30 [Source:HGNC Symbol;Acc:HGNC:5591]	IG V gene	-1.17	0.81	20.14	<0.0001	<0.0001
ENSG00000151726	ACSL1	acyl-CoA synthetase long chain family member 1 [Source:HGNC Symbol;Acc:HGNC:3569]	protein coding	1.17	7.73	56.01	<0.0001	<0.0001
ENSG00000279908		tec	TEC	-1.17	0.21	45.39	<0.0001	<0.0001
ENSG00000170899	GSTA4	glutathione S-transferase alpha 4 [Source:HGNC Symbol;Acc:HGNC:4629]	protein coding	-1.17	0.94	18.55	<0.0001	<0.0001
ENSG00000170128	GPR25	G protein-coupled receptor 25 [Source:HGNC Symbol;Acc:HGNC:4480]	protein coding	-1.17	0.68	15.10	0.0001	0.0005
ENSG00000198846	TOX	thymocyte selection associated high mobility group box [Source:HGNC Symbol;Acc:HGNC:18988]	protein coding	-1.17	1.85	24.10	<0.0001	<0.0001
ENSG00000172508	CARNS1	carnosine synthase 1 [Source:HGNC Symbol;Acc:HGNC:29268]	protein coding	-1.16	3.43	27.34	<0.0001	<0.0001
ENSG00000113263	ITK	IL2 inducible T cell kinase [Source:HGNC Symbol;Acc:HGNC:6171]	protein coding	-1.16	5.58	15.03	0.0001	0.0005
ENSG00000182463	TSHZ2	teashirt zinc finger homeobox 2 [Source:HGNC Symbol;Acc:HGNC:13010]	protein coding	-1.16	0.89	14.35	0.0002	0.0007
ENSG00000101298	SNPH	syntaphilin [Source:HGNC Symbol;Acc:HGNC:15931]	protein coding	-1.16	2.23	19.22	<0.0001	<0.0001
ENSG00000153563	CD8A	CD8a molecule [Source:HGNC Symbol;Acc:HGNC:1706]	protein coding	-1.16	5.75	12.98	0.0003	0.0012
ENSG00000211598	IGKV4-1	immunoglobulin kappa variable 4-1 [Source:HGNC Symbol;Acc:HGNC:5834]	IG V gene	-1.16	3.05	20.61	<0.0001	<0.0001
ENSG00000057294	PKP2	plakophilin 2 [Source:HGNC Symbol;Acc:HGNC:9024]	protein coding	-1.16	1.49	44.39	<0.0001	<0.0001
ENSG00000211660	IGLV2-23	immunoglobulin lambda variable 2-23 [Source:HGNC Symbol;Acc:HGNC:5890]	IG V gene	-1.16	1.41	14.46	0.0001	0.0006
ENSG00000256039	LINC02446	long intergenic non-protein coding RNA 2446 [Source:HGNC Symbol;Acc:HGNC:53378]	lncRNA	-1.16	2.06	12.19	0.0005	0.0018
ENSG00000167094	TTC16	tetratricopeptide repeat domain 16 [Source:HGNC Symbol;Acc:HGNC:26536]	protein coding	-1.16	3.16	13.46	0.0002	0.001
ENSG00000185567	AHNAK2	AHNAK nucleoprotein 2 [Source:HGNC Symbol;Acc:HGNC:20125]	protein coding	-1.16	1.33	30.52	<0.0001	<0.0001
ENSG00000223834		novel transcript	lncRNA	-1.16	0.37	19.86	<0.0001	<0.0001
ENSG00000124181	PLCG1	phospholipase C gamma 1 [Source:HGNC Symbol;Acc:HGNC:9065]	protein coding	-1.16	5.81	17.10	<0.0001	0.0002

Table S12: Genes differentially expressed at Day 30 compared to pre-second vaccination (Study Arm 2 A/H7N9+AS03, D1, 29, Dendritic cells). Genes are sorted by descending absolute log_2 fold change. Gene model summaries and annotations are based on Ensembl Version 99 (continued.)

Ensembl Gene ID	Ensembl Gene Name	Ensembl Gene Description	Gene Type	log_2 Fold Change (Day 30 vs. Pre-Second Vaccination)	Average log_2 CPM	Likelihood Ratio Test Statistic	P-Value	FDR Adjusted P-Value
ENSG00000277734	TRAC	T cell receptor alpha constant [Source:HGNC Symbol;Acc:HGNC:12029]	TR C gene	-1.16	5.63	12.51	0.0004	0.0015
ENSG00000197121	PGAP1	post-GPI attachment to proteins inositol deacylase 1 [Source:HGNC Symbol;Acc:HGNC:25712]	protein coding	1.16	2.19	24.44	<0.0001	<0.0001
ENSG00000172543	CTSW	cathepsin W [Source:HGNC Symbol;Acc:HGNC:2546]	protein coding	-1.16	6.21	20.84	<0.0001	<0.0001
ENSG00000055130	CUL1	cullin 1 [Source:HGNC Symbol;Acc:HGNC:2551]	protein coding	1.15	6.85	56.07	<0.0001	<0.0001
ENSG00000211695	TRGV9	T cell receptor gamma variable 9 [Source:HGNC Symbol;Acc:HGNC:12295]	TR V gene	-1.15	1.50	19.44	<0.0001	<0.0001
ENSG00000144218	AFF3	AF4/FMR2 family member 3 [Source:HGNC Symbol;Acc:HGNC:6473]	protein coding	-1.15	4.06	58.15	<0.0001	<0.0001
ENSG00000112796	ENPP5	ectonucleotide pyrophosphatase/phosphodiesterase family member 5 [Source:HGNC Symbol;Acc:HGNC:13717]	protein coding	-1.15	0.81	12.93	0.0003	0.0012
ENSG00000164398	ACSL6	acyl-CoA synthetase long chain family member 6 [Source:HGNC Symbol;Acc:HGNC:16496]	protein coding	-1.15	0.95	13.59	0.0002	0.0009
ENSG00000282780	TRBJ1-6	T cell receptor beta joining 1-6 [Source:HGNC Symbol;Acc:HGNC:12167]	TR J gene	-1.15	0.41	14.80	0.0001	0.0005
ENSG00000178562	CD28	CD28 molecule [Source:HGNC Symbol;Acc:HGNC:1653]	protein coding	-1.15	3.59	12.25	0.0005	0.0017
ENSG00000174233	ADCY6	adenylate cyclase 6 [Source:HGNC Symbol;Acc:HGNC:237]	protein coding	-1.15	0.63	38.62	<0.0001	<0.0001
ENSG00000139832	RAB20	RAB20, member RAS oncogene family [Source:HGNC Symbol;Acc:HGNC:18260]	protein coding	1.15	5.06	82.98	<0.0001	<0.0001
ENSG00000196743	GM2A	GM2 ganglioside activator [Source:HGNC Symbol;Acc:HGNC:4367]	protein coding	1.15	7.04	51.07	<0.0001	<0.0001
ENSG00000186654	PRR5	proline rich 5 [Source:HGNC Symbol;Acc:HGNC:31682]	protein coding	-1.15	4.31	35.62	<0.0001	<0.0001
ENSG00000126838	PZP	PZP alpha-2-macroglobulin like [Source:HGNC Symbol;Acc:HGNC:9750]	protein coding	-1.15	0.36	19.20	<0.0001	<0.0001
ENSG00000111335	OAS2	2'-5'-oligoadenylate synthetase 2 [Source:HGNC Symbol;Acc:HGNC:8087]	protein coding	1.15	7.83	62.91	<0.0001	<0.0001
ENSG00000172260	NEGR1	neuronal growth regulator 1 [Source:HGNC Symbol;Acc:HGNC:17302]	protein coding	-1.15	1.65	57.85	<0.0001	<0.0001
ENSG00000131686	CA6	carbonic anhydrase 6 [Source:HGNC Symbol;Acc:HGNC:1380]	protein coding	-1.15	0.57	9.54	0.002	0.0059

Table S12: Genes differentially expressed at Day 30 compared to pre-second vaccination (Study Arm 2 A/H7N9+AS03, D1, 29, Dendritic cells). Genes are sorted by descending absolute log_2 fold change. Gene model summaries and annotations are based on Ensembl Version 99 (continued.)

Ensembl Gene ID	Ensembl Gene Name	Ensembl Gene Description	Gene Type	log_2 Fold Change (Day 30 vs. Pre-Second Vaccination)	Average log_2 CPM	Likelihood Ratio Test Statistic	P-Value	FDR Adjusted P-Value
ENSG00000184979	USP18	ubiquitin specific peptidase 18 [Source:HGNC Symbol;Acc:HGNC:12616]	protein coding	1.15	2.69	53.52	<0.0001	<0.0001
ENSG00000181847	TIGIT	T cell immunoreceptor with Ig and ITIM domains [Source:HGNC Symbol;Acc:HGNC:26838]	protein coding	-1.14	3.48	15.36	<0.0001	0.0004
ENSG00000167106	FAM102A	family with sequence similarity 102 member A [Source:HGNC Symbol;Acc:HGNC:31419]	protein coding	-1.14	5.73	26.64	<0.0001	<0.0001
ENSG00000168913	ENHO	energy homeostasis associated [Source:HGNC Symbol;Acc:HGNC:24838]	protein coding	-1.14	3.54	41.83	<0.0001	<0.0001
ENSG00000151623	NR3C2	nuclear receptor subfamily 3 group C member 2 [Source:HGNC Symbol;Acc:HGNC:7979]	protein coding	-1.14	2.17	29.15	<0.0001	<0.0001
ENSG00000164120	HPGD	15-hydroxyprostaglandin dehydrogenase [Source:HGNC Symbol;Acc:HGNC:5154]	protein coding	-1.14	1.16	26.24	<0.0001	<0.0001
ENSG00000174944	P2RY14	purinergic receptor P2Y14 [Source:HGNC Symbol;Acc:HGNC:16442]	protein coding	1.14	3.19	9.83	0.0017	0.0052
ENSG00000131634	TMEM204	transmembrane protein 204 [Source:HGNC Symbol;Acc:HGNC:14158]	protein coding	-1.14	3.52	18.07	<0.0001	0.0001
ENSG00000185436	IFNLR1	interferon lambda receptor 1 [Source:HGNC Symbol;Acc:HGNC:18584]	protein coding	-1.14	0.49	17.73	<0.0001	0.0001
ENSG00000160183	TMPRSS3	transmembrane serine protease 3 [Source:HGNC Symbol;Acc:HGNC:11877]	protein coding	1.14	1.54	18.73	<0.0001	<0.0001
ENSG00000135960	EDAR	ectodysplasin A receptor [Source:HGNC Symbol;Acc:HGNC:2895]	protein coding	-1.14	0.65	13.46	0.0002	0.001
ENSG00000006555	TTC22	tetratricopeptide repeat domain 22 [Source:HGNC Symbol;Acc:HGNC:26067]	protein coding	-1.14	2.53	18.05	<0.0001	0.0001
ENSG00000235162	C12orf75	chromosome 12 open reading frame 75 [Source:HGNC Symbol;Acc:HGNC:35164]	protein coding	-1.14	3.19	34.67	<0.0001	<0.0001
ENSG00000171916	LGALS9C	galectin 9C [Source:HGNC Symbol;Acc:HGNC:33874]	protein coding	-1.14	0.80	25.13	<0.0001	<0.0001
ENSG00000132530	XAF1	XIAP associated factor 1 [Source:HGNC Symbol;Acc:HGNC:30932]	protein coding	1.14	6.31	50.77	<0.0001	<0.0001
ENSG00000164659	KIAA1324L	KIAA1324 like [Source:HGNC Symbol;Acc:HGNC:21945]	protein coding	-1.14	1.19	30.85	<0.0001	<0.0001
ENSG00000141622	RNF165	ring finger protein 165 [Source:HGNC Symbol;Acc:HGNC:31696]	protein coding	-1.14	1.83	18.87	<0.0001	<0.0001
ENSG00000211899	IGHM	immunoglobulin heavy constant mu [Source:HGNC Symbol;Acc:HGNC:5541]	IG C gene	-1.14	8.03	28.79	<0.0001	<0.0001

Table S12: Genes differentially expressed at Day 30 compared to pre-second vaccination (Study Arm 2 A/H7N9+AS03, D1, 29, Dendritic cells). Genes are sorted by descending absolute log_2 fold change. Gene model summaries and annotations are based on Ensembl Version 99 (continued.)

Ensembl Gene ID	Ensembl Gene Name	Ensembl Gene Description	Gene Type	log_2 Fold Change (Day 30 vs. Pre-Second Vaccination)	Average log_2 CPM	Likelihood Ratio Test Statistic	P-Value	FDR Adjusted P-Value
ENSG00000157168	NRG1	neuregulin 1 [Source:HGNC Symbol;Acc:HGNC:7997]	protein coding	-1.14	3.81	14.87	0.0001	0.0005
ENSG00000110777	POU2AF1	POU class 2 homeobox associating factor 1 [Source:HGNC Symbol;Acc:HGNC:9211]	protein coding	-1.14	3.37	30.69	<0.0001	<0.0001
ENSG00000174749	FAM241A	family with sequence similarity 241 member A [Source:HGNC Symbol;Acc:HGNC:26813]	protein coding	1.13	5.00	52.11	<0.0001	<0.0001
ENSG00000204789	ZNF204P	zinc finger protein 204, pseudogene [Source:HGNC Symbol;Acc:HGNC:12995]	processed pseudogene	-1.13	0.21	12.98	0.0003	0.0012
ENSG00000257017	HP	haptoglobin [Source:HGNC Symbol;Acc:HGNC:5141]	protein coding	1.13	2.31	26.35	<0.0001	<0.0001
ENSG00000211947	IGHV3-21	immunoglobulin heavy variable 3-21 [Source:HGNC Symbol;Acc:HGNC:5586]	IG V gene	-1.13	1.21	24.72	<0.0001	<0.0001
ENSG00000180096	SEPTIN1	septin 1 [Source:HGNC Symbol;Acc:HGNC:2879]	protein coding	-1.13	5.71	24.06	<0.0001	<0.0001
ENSG00000135127	BICDL1	BICD family like cargo adaptor 1 [Source:HGNC Symbol;Acc:HGNC:28095]	protein coding	-1.13	3.47	20.48	<0.0001	<0.0001
ENSG00000211673	IGLV3-1	immunoglobulin lambda variable 3-1 [Source:HGNC Symbol;Acc:HGNC:5896]	IG V gene	-1.13	1.53	14.75	0.0001	0.0005
ENSG00000167633	KIR3DL1	killer cell immunoglobulin like receptor, three Ig domains and long cytoplasmic tail 1 [Source:HGNC Symbol;Acc:HGNC:6338]	protein coding	-1.13	1.68	22.28	<0.0001	<0.0001
ENSG00000143434	SEMA6C	semaphorin 6C [Source:HGNC Symbol;Acc:HGNC:10740]	protein coding	-1.13	1.93	45.20	<0.0001	<0.0001
ENSG00000211896	IGHG1	immunoglobulin heavy constant gamma 1 (G1m marker) [Source:HGNC Symbol;Acc:HGNC:5525]	IG C gene	-1.13	5.37	48.52	<0.0001	<0.0001
ENSG00000035664	DAPK2	death associated protein kinase 2 [Source:HGNC Symbol;Acc:HGNC:2675]	protein coding	-1.13	1.49	30.87	<0.0001	<0.0001
ENSG00000008517	IL32	interleukin 32 [Source:HGNC Symbol;Acc:HGNC:16830]	protein coding	-1.13	5.86	15.20	<0.0001	0.0004
ENSG00000074966	TXK	TXK tyrosine kinase [Source:HGNC Symbol;Acc:HGNC:12434]	protein coding	-1.13	4.22	20.89	<0.0001	<0.0001
ENSG00000186265	BTLA	B and T lymphocyte associated [Source:HGNC Symbol;Acc:HGNC:21087]	protein coding	-1.12	2.47	22.92	<0.0001	<0.0001
ENSG00000160111	CPAMD8	C3 and PZP like alpha-2-macroglobulin domain containing 8 [Source:HGNC Symbol;Acc:HGNC:23228]	protein coding	-1.12	4.55	55.10	<0.0001	<0.0001

Table S12: Genes differentially expressed at Day 30 compared to pre-second vaccination (Study Arm 2 A/H7N9+AS03, D1, 29, Dendritic cells). Genes are sorted by descending absolute log_2 fold change. Gene model summaries and annotations are based on Ensembl Version 99 (continued.)

Ensembl Gene ID	Ensembl Gene Name	Ensembl Gene Description	Gene Type	log_2 Fold Change (Day 30 vs. Pre-Second Vaccination)	Average log_2 CPM	Likelihood Ratio Test Statistic	P-Value	FDR Adjusted P-Value
ENSG00000130649	CYP2E1	cytochrome P450 family 2 subfamily E member 1 [Source:HGNC Symbol;Acc:HGNC:2631]	protein coding	-1.12	0.21	34.60	<0.0001	<0.0001
ENSG00000182240	BACE2	beta-secretase 2 [Source:HGNC Symbol;Acc:HGNC:934]	protein coding	-1.12	1.08	26.26	<0.0001	<0.0001
ENSG00000168734	PKIG	cAMP-dependent protein kinase inhibitor gamma [Source:HGNC Symbol;Acc:HGNC:9019]	protein coding	-1.12	1.79	41.31	<0.0001	<0.0001
ENSG00000103942	HOMER2	homer scaffold protein 2 [Source:HGNC Symbol;Acc:HGNC:17513]	protein coding	-1.12	2.65	66.59	<0.0001	<0.0001
ENSG00000165806	CASP7	caspase 7 [Source:HGNC Symbol;Acc:HGNC:1508]	protein coding	1.12	5.03	70.89	<0.0001	<0.0001
ENSG00000135144	DTX1	deltex E3 ubiquitin ligase 1 [Source:HGNC Symbol;Acc:HGNC:3060]	protein coding	-1.12	1.97	14.55	0.0001	0.0006
ENSG00000106624	AEBP1	AE binding protein 1 [Source:HGNC Symbol;Acc:HGNC:303]	protein coding	-1.12	2.33	18.96	<0.0001	<0.0001
ENSG00000211938	IGHV3-7	immunoglobulin heavy variable 3-7 [Source:HGNC Symbol;Acc:HGNC:5620]	IG V gene	-1.12	1.83	22.51	<0.0001	<0.0001
ENSG00000025708	TYMP	thymidine phosphorylase [Source:HGNC Symbol;Acc:HGNC:3148]	protein coding	1.12	10.77	59.64	<0.0001	<0.0001
ENSG00000142798	HSPG2	heparan sulfate proteoglycan 2 [Source:HGNC Symbol;Acc:HGNC:5273]	protein coding	-1.12	2.10	30.98	<0.0001	<0.0001
ENSG00000120210	INSL6	insulin like 6 [Source:HGNC Symbol;Acc:HGNC:6089]	protein coding	1.12	0.80	25.91	<0.0001	<0.0001
ENSG00000158481	CD1C	CD1c molecule [Source:HGNC Symbol;Acc:HGNC:1636]	protein coding	-1.12	6.31	79.53	<0.0001	<0.0001
ENSG00000185198	PRSS57	serine protease 57 [Source:HGNC Symbol;Acc:HGNC:31397]	protein coding	-1.12	0.53	27.71	<0.0001	<0.0001
ENSG00000236901	MIR600HG	MIR600 host gene [Source:HGNC Symbol;Acc:HGNC:23642]	lncRNA	-1.12	2.14	36.87	<0.0001	<0.0001
ENSG00000162433	AK4	adenylate kinase 4 [Source:HGNC Symbol;Acc:HGNC:363]	protein coding	1.12	1.53	38.01	<0.0001	<0.0001
ENSG00000133424	LARGE1	LARGE xylosyl- and glucuronyltransferase 1 [Source:HGNC Symbol;Acc:HGNC:6511]	protein coding	-1.12	0.38	10.62	0.0011	0.0036
ENSG00000211945	IGHV1-18	immunoglobulin heavy variable 1-18 [Source:HGNC Symbol;Acc:HGNC:5549]	IG V gene	-1.12	0.77	13.65	0.0002	0.0009
ENSG00000132849	PATJ	PATJ crumbs cell polarity complex component [Source:HGNC Symbol;Acc:HGNC:28881]	protein coding	-1.12	2.34	19.40	<0.0001	<0.0001

Table S12: Genes differentially expressed at Day 30 compared to pre-second vaccination (Study Arm 2 A/H7N9+AS03, D1, 29, Dendritic cells). Genes are sorted by descending absolute log_2 fold change. Gene model summaries and annotations are based on Ensembl Version 99 (continued.)

Ensembl Gene ID	Ensembl Gene Name	Ensembl Gene Description	Gene Type	log_2 Fold Change (Day 30 vs. Pre-Second Vaccination)	Average log_2 CPM	Likelihood Ratio Test Statistic	P-Value	FDR Adjusted P-Value
ENSG00000154914	USP43	ubiquitin specific peptidase 43 [Source:HGNC Symbol;Acc:HGNC:20072]	protein coding	-1.12	0.39	30.17	<0.0001	<0.0001
ENSG00000171992	SYNPO	synaptopodin [Source:HGNC Symbol;Acc:HGNC:30672]	protein coding	-1.12	1.41	9.46	0.0021	0.0061
ENSG00000166289	PLEKHF1	pleckstrin homology and FYVE domain containing 1 [Source:HGNC Symbol;Acc:HGNC:20764]	protein coding	-1.12	3.48	17.02	<0.0001	0.0002
ENSG00000185885	IFITM1	interferon induced transmembrane protein 1 [Source:HGNC Symbol;Acc:HGNC:5412]	protein coding	1.12	6.40	27.78	<0.0001	<0.0001
ENSG00000221957	KIR2DS4	killer cell immunoglobulin like receptor, two Ig domains and short cytoplasmic tail 4 [Source:HGNC Symbol;Acc:HGNC:6336]	polymorphic pseudogene	-1.12	1.22	11.52	0.0007	0.0024
ENSG00000007402	CACNA2D2	calcium voltage-gated channel auxiliary subunit alpha2delta 2 [Source:HGNC Symbol;Acc:HGNC:1400]	protein coding	-1.12	3.05	31.62	<0.0001	<0.0001
ENSG00000170074	FAM153A	family with sequence similarity 153 member A [Source:HGNC Symbol;Acc:HGNC:29940]	protein coding	-1.12	1.91	9.47	0.0021	0.0061
ENSG00000173208	ABCD2	ATP binding cassette subfamily D member 2 [Source:HGNC Symbol;Acc:HGNC:66]	protein coding	-1.12	1.74	17.37	<0.0001	0.0002
ENSG00000185875	THNSL1	threonine synthase like 1 [Source:HGNC Symbol;Acc:HGNC:26160]	protein coding	-1.12	0.58	20.17	<0.0001	<0.0001
ENSG00000145349	CAMK2D	calcium/calmodulin dependent protein kinase II delta [Source:HGNC Symbol;Acc:HGNC:1462]	protein coding	1.12	5.61	52.01	<0.0001	<0.0001
ENSG00000225964	NRIR	negative regulator of interferon response [Source:HGNC Symbol;Acc:HGNC:51269]	lncRNA	1.12	1.72	50.08	<0.0001	<0.0001
ENSG00000125648	SLC25A23	solute carrier family 25 member 23 [Source:HGNC Symbol;Acc:HGNC:19375]	protein coding	-1.11	2.16	22.76	<0.0001	<0.0001
ENSG00000135549	PKIB	cAMP-dependent protein kinase inhibitor beta [Source:HGNC Symbol;Acc:HGNC:9018]	protein coding	-1.11	1.55	32.10	<0.0001	<0.0001
ENSG00000083454	P2RX5	purinergic receptor P2X 5 [Source:HGNC Symbol;Acc:HGNC:8536]	protein coding	-1.11	3.07	29.44	<0.0001	<0.0001
ENSG00000149328	GLB1L2	galactosidase beta 1 like 2 [Source:HGNC Symbol;Acc:HGNC:25129]	protein coding	-1.11	0.21	16.78	<0.0001	0.0002
ENSG00000267731		novel transcript	lncRNA	-1.11	0.25	20.05	<0.0001	<0.0001
ENSG00000162804	SNED1	sushi, nidogen and EGF like domains 1 [Source:HGNC Symbol;Acc:HGNC:24696]	protein coding	-1.11	4.96	29.15	<0.0001	<0.0001
ENSG00000159674	SPON2	spondin 2 [Source:HGNC Symbol;Acc:HGNC:11253]	protein coding	-1.11	6.56	18.78	<0.0001	<0.0001

Table S12: Genes differentially expressed at Day 30 compared to pre-second vaccination (Study Arm 2 A/H7N9+AS03, D1, 29, Dendritic cells). Genes are sorted by descending absolute log_2 fold change. Gene model summaries and annotations are based on Ensembl Version 99 (continued.)

Ensembl Gene ID	Ensembl Gene Name	Ensembl Gene Description	Gene Type	log_2 Fold Change (Day 30 vs. Pre-Second Vaccination)	Average log_2 CPM	Likelihood Ratio Test Statistic	P-Value	FDR Adjusted P-Value
ENSG00000243156	MICAL3	microtubule associated monooxygenase, calponin and LIM domain containing 3 [Source:HGNC Symbol;Acc:HGNC:24694]	protein coding	-1.11	2.98	31.60	<0.0001	<0.0001
ENSG00000117322	CR2	complement C3d receptor 2 [Source:HGNC Symbol;Acc:HGNC:2336]	protein coding	-1.11	1.91	13.61	0.0002	0.0009
ENSG00000104951	IL4I1	interleukin 4 induced 1 [Source:HGNC Symbol;Acc:HGNC:19094]	protein coding	1.11	3.77	45.21	<0.0001	<0.0001
ENSG00000160654	CD3G	CD3g molecule [Source:HGNC Symbol;Acc:HGNC:1675]	protein coding	-1.11	4.42	10.90	0.001	0.0032
ENSG00000145247	OCIAD2	OCIA domain containing 2 [Source:HGNC Symbol;Acc:HGNC:28685]	protein coding	-1.11	2.55	22.46	<0.0001	<0.0001
ENSG00000065911	MTHFD2	methylenetetrahydrofolate dehydrogenase (NADP+ dependent) 2, methenyltetrahydrofolate cyclohydrolase [Source:HGNC Symbol;Acc:HGNC:7434]	protein coding	1.11	6.20	84.80	<0.0001	<0.0001
ENSG00000160318	CLDND2	claudin domain containing 2 [Source:HGNC Symbol;Acc:HGNC:28511]	protein coding	-1.11	2.30	25.86	<0.0001	<0.0001
ENSG00000155265	GOLGA7B	golgin A7 family member B [Source:HGNC Symbol;Acc:HGNC:31668]	protein coding	-1.11	2.39	14.84	0.0001	0.0005
ENSG00000141542	RAB40B	RAB40B, member RAS oncogene family [Source:HGNC Symbol;Acc:HGNC:18284]	protein coding	-1.10	1.87	40.06	<0.0001	<0.0001
ENSG00000166483	WEE1	WEE1 G2 checkpoint kinase [Source:HGNC Symbol;Acc:HGNC:12761]	protein coding	-1.10	1.46	21.36	<0.0001	<0.0001
ENSG00000145649	GZMA	granzyme A [Source:HGNC Symbol;Acc:HGNC:4708]	protein coding	-1.10	5.09	14.66	0.0001	0.0006
ENSG00000087589	CASS4	Cas scaffold protein family member 4 [Source:HGNC Symbol;Acc:HGNC:15878]	protein coding	-1.10	3.23	35.87	<0.0001	<0.0001
ENSG00000163823	CCR1	C-C motif chemokine receptor 1 [Source:HGNC Symbol;Acc:HGNC:1602]	protein coding	1.10	7.01	43.53	<0.0001	<0.0001
ENSG00000161405	IKZF3	IKAROS family zinc finger 3 [Source:HGNC Symbol;Acc:HGNC:13178]	protein coding	-1.10	5.09	19.70	<0.0001	<0.0001
ENSG00000137198	GMPR	guanosine monophosphate reductase [Source:HGNC Symbol;Acc:HGNC:4376]	protein coding	1.10	2.21	46.95	<0.0001	<0.0001
ENSG00000163606	CD200R1	CD200 receptor 1 [Source:HGNC Symbol;Acc:HGNC:24235]	protein coding	-1.10	3.02	87.71	<0.0001	<0.0001
ENSG00000168824	NSG1	neuronal vesicle trafficking associated 1 [Source:HGNC Symbol;Acc:HGNC:18790]	protein coding	-1.10	2.14	17.62	<0.0001	0.0001

Table S12: Genes differentially expressed at Day 30 compared to pre-second vaccination (Study Arm 2 A/H7N9+AS03, D1, 29, Dendritic cells). Genes are sorted by descending absolute log_2 fold change. Gene model summaries and annotations are based on Ensembl Version 99 (continued.)

Ensembl Gene ID	Ensembl Gene Name	Ensembl Gene Description	Gene Type	log_2 Fold Change (Day 30 vs. Pre-Second Vaccination)	Average log_2 CPM	Likelihood Ratio Test Statistic	P-Value	FDR Adjusted P-Value
ENSG00000134709	HOOK1	hook microtubule tethering protein 1 [Source:HGNC Symbol;Acc:HGNC:19884]	protein coding	-1.10	1.10	12.16	0.0005	0.0018
ENSG00000030419	IKZF2	IKAROS family zinc finger 2 [Source:HGNC Symbol;Acc:HGNC:13177]	protein coding	-1.10	3.35	18.81	<0.0001	<0.0001
ENSG00000170989	S1PR1	sphingosine-1-phosphate receptor 1 [Source:HGNC Symbol;Acc:HGNC:3165]	protein coding	-1.09	4.97	18.37	<0.0001	0.0001
ENSG00000213949	ITGA1	integrin subunit alpha 1 [Source:HGNC Symbol;Acc:HGNC:6134]	protein coding	1.09	1.08	18.40	<0.0001	0.0001
ENSG00000240710		novel transcript	lncRNA	1.09	0.76	25.49	<0.0001	<0.0001
ENSG00000142156	COL6A1	collagen type VI alpha 1 chain [Source:HGNC Symbol;Acc:HGNC:2211]	protein coding	-1.09	1.42	11.87	0.0006	0.002
ENSG00000188313	PLSCR1	phospholipid scramblase 1 [Source:HGNC Symbol;Acc:HGNC:9092]	protein coding	1.09	6.86	86.02	<0.0001	<0.0001
ENSG00000160185	UBASH3A	ubiquitin associated and SH3 domain containing A [Source:HGNC Symbol;Acc:HGNC:12462]	protein coding	-1.09	3.30	12.08	0.0005	0.0018
ENSG00000144476	ACKR3	atypical chemokine receptor 3 [Source:HGNC Symbol;Acc:HGNC:23692]	protein coding	-1.09	1.42	46.71	<0.0001	<0.0001
ENSG00000143185	XCL2	X-C motif chemokine ligand 2 [Source:HGNC Symbol;Acc:HGNC:10646]	protein coding	-1.09	0.82	13.71	0.0002	0.0009
ENSG00000159618	ADGRG5	adhesion G protein-coupled receptor G5 [Source:HGNC Symbol;Acc:HGNC:19010]	protein coding	-1.09	3.99	31.50	<0.0001	<0.0001
ENSG00000182557	SPNS3	sphingolipid transporter 3 (putative) [Source:HGNC Symbol;Acc:HGNC:28433]	protein coding	-1.09	3.92	36.44	<0.0001	<0.0001
ENSG00000286035		novel transcript	lncRNA	1.09	0.73	26.60	<0.0001	<0.0001
ENSG00000137501	SYTL2	synaptotagmin like 2 [Source:HGNC Symbol;Acc:HGNC:15585]	protein coding	-1.09	3.82	21.19	<0.0001	<0.0001
ENSG00000081377	CDC14B	cell division cycle 14B [Source:HGNC Symbol;Acc:HGNC:1719]	protein coding	-1.09	1.18	17.66	<0.0001	0.0001
ENSG00000137103	TMEM8B	transmembrane protein 8B [Source:HGNC Symbol;Acc:HGNC:21427]	protein coding	-1.09	4.02	66.73	<0.0001	<0.0001
ENSG00000258352		interferon induced transmembrane protein 3 (1-8U) (IFITM3) pseudogene	transcribed processed pseudogene	-1.09	0.45	21.95	<0.0001	<0.0001
ENSG00000145390	USP53	ubiquitin specific peptidase 53 [Source:HGNC Symbol;Acc:HGNC:29255]	protein coding	-1.09	2.71	28.46	<0.0001	<0.0001

Confidential/Proprietary Information

Table S12: Genes differentially expressed at Day 30 compared to pre-second vaccination (Study Arm 2 A/H7N9+AS03, D1, 29, Dendritic cells). Genes are sorted by descending absolute log_2 fold change. Gene model summaries and annotations are based on Ensembl Version 99 (continued.)

Ensembl Gene ID	Ensembl Gene Name	Ensembl Gene Description	Gene Type	log_2 Fold Change (Day 30 vs. Pre-Second Vaccination)	Average log_2 CPM	Likelihood Ratio Test Statistic	P-Value	FDR Adjusted P-Value
ENSG00000223991		novel transcript	lncRNA	-1.09	0.49	12.98	0.0003	0.0012
ENSG00000182472	CAPN12	calpain 12 [Source:HGNC Symbol;Acc:HGNC:13249]	protein coding	-1.08	1.40	25.99	<0.0001	<0.0001
ENSG00000198286	CARD11	caspase recruitment domain family member 11 [Source:HGNC Symbol;Acc:HGNC:16393]	protein coding	-1.08	5.81	21.65	<0.0001	<0.0001
ENSG00000077984	CST7	cystatin F [Source:HGNC Symbol;Acc:HGNC:2479]	protein coding	-1.08	5.26	21.90	<0.0001	<0.0001
ENSG00000146215	CRIP3	cysteine rich protein 3 [Source:HGNC Symbol;Acc:HGNC:17751]	protein coding	-1.08	1.95	32.86	<0.0001	<0.0001
ENSG00000166770	ZNF667-AS1	ZNF667 antisense RNA 1 (head to head) [Source:HGNC Symbol;Acc:HGNC:44321]	lncRNA	-1.08	0.28	23.86	<0.0001	<0.0001
ENSG00000244437	IGKV3-15	immunoglobulin kappa variable 3-15 [Source:HGNC Symbol;Acc:HGNC:5816]	IG V gene	-1.08	1.63	16.85	<0.0001	0.0002
ENSG00000111863	ADTRP	androgen dependent TFPI regulating protein [Source:HGNC Symbol;Acc:HGNC:21214]	protein coding	-1.08	1.18	11.66	0.0006	0.0022
ENSG00000124006	OBSL1	obscurin like cytoskeletal adaptor 1 [Source:HGNC Symbol;Acc:HGNC:29092]	protein coding	-1.08	0.45	19.64	<0.0001	<0.0001
ENSG00000184860	SDR42E1	short chain dehydrogenase/reductase family 42E, member 1 [Source:HGNC Symbol;Acc:HGNC:29834]	protein coding	-1.08	1.01	20.10	<0.0001	<0.0001
ENSG00000122224	LY9	lymphocyte antigen 9 [Source:HGNC Symbol;Acc:HGNC:6730]	protein coding	-1.08	4.09	34.10	<0.0001	<0.0001
ENSG00000158106	RHPN1	rhophilin Rho GTPase binding protein 1 [Source:HGNC Symbol;Acc:HGNC:19973]	protein coding	-1.08	3.39	24.51	<0.0001	<0.0001
ENSG00000164715	LMTK2	lemur tyrosine kinase 2 [Source:HGNC Symbol;Acc:HGNC:17880]	protein coding	1.08	5.43	55.12	<0.0001	<0.0001
ENSG00000117226	GBP3	guanylate binding protein 3 [Source:HGNC Symbol;Acc:HGNC:4184]	protein coding	1.08	5.49	37.87	<0.0001	<0.0001
ENSG00000235888		novel transcript	lncRNA	-1.07	1.38	28.79	<0.0001	<0.0001
ENSG00000161055	SCGB3A1	secretoglobin family 3A member 1 [Source:HGNC Symbol;Acc:HGNC:18384]	protein coding	-1.07	1.03	20.79	<0.0001	<0.0001
ENSG00000176049	JAKMIP2	janus kinase and microtubule interacting protein 2 [Source:HGNC Symbol;Acc:HGNC:29067]	protein coding	-1.07	1.15	14.82	0.0001	0.0005
ENSG00000198780	FAM169A	family with sequence similarity 169 member A [Source:HGNC Symbol;Acc:HGNC:29138]	protein coding	-1.07	0.84	19.01	<0.0001	<0.0001
ENSG00000131355	ADGRE3	adhesion G protein-coupled receptor E3 [Source:HGNC Symbol;Acc:HGNC:23647]	protein coding	-1.07	4.58	21.46	<0.0001	<0.0001

Table S12: Genes differentially expressed at Day 30 compared to pre-second vaccination (Study Arm 2 A/H7N9+AS03, D1, 29, Dendritic cells). Genes are sorted by descending absolute log_2 fold change. Gene model summaries and annotations are based on Ensembl Version 99 (continued.)

Ensembl Gene ID	Ensembl Gene Name	Ensembl Gene Description	Gene Type	log_2 Fold Change (Day 30 vs. Pre-Second Vaccination)	Average log_2 CPM	Likelihood Ratio Test Statistic	P-Value	FDR Adjusted P-Value
ENSG00000245904		novel transcript, antisense to BTG1	lncRNA	-1.07	2.25	40.76	<0.0001	<0.0001
ENSG00000211771	TRBJ2-7	T cell receptor beta joining 2-7 [Source:HGNC Symbol;Acc:HGNC:12175]	TR J gene	-1.07	0.25	15.39	<0.0001	0.0004
ENSG00000167286	CD3D	CD3d molecule [Source:HGNC Symbol;Acc:HGNC:1673]	protein coding	-1.07	4.53	11.17	0.0008	0.0028
ENSG00000141469	SLC14A1	solute carrier family 14 member 1 (Kidd blood group) [Source:HGNC Symbol;Acc:HGNC:10918]	protein coding	-1.07	1.75	19.82	<0.0001	<0.0001
ENSG00000172215	CXCR6	C-X-C motif chemokine receptor 6 [Source:HGNC Symbol;Acc:HGNC:16647]	protein coding	-1.07	1.19	9.57	0.002	0.0058
ENSG00000183087	GAS6	growth arrest specific 6 [Source:HGNC Symbol;Acc:HGNC:4168]	protein coding	1.07	5.18	39.77	<0.0001	<0.0001
ENSG00000159164	SV2A	synaptic vesicle glycoprotein 2A [Source:HGNC Symbol;Acc:HGNC:20566]	protein coding	-1.07	0.12	16.78	<0.0001	0.0002
ENSG00000069702	TGFBR3	transforming growth factor beta receptor 3 [Source:HGNC Symbol;Acc:HGNC:11774]	protein coding	-1.07	4.17	17.65	<0.0001	0.0001
ENSG00000166428	PLD4	phospholipase D family member 4 [Source:HGNC Symbol;Acc:HGNC:23792]	protein coding	-1.07	6.38	29.79	<0.0001	<0.0001
ENSG00000112655	PTK7	protein tyrosine kinase 7 (inactive) [Source:HGNC Symbol;Acc:HGNC:9618]	protein coding	-1.07	0.86	17.75	<0.0001	0.0001
ENSG00000163519	TRAT1	T cell receptor associated transmembrane adaptor 1 [Source:HGNC Symbol;Acc:HGNC:30698]	protein coding	-1.07	3.00	10.17	0.0014	0.0044
ENSG00000168646	AXIN2	axin 2 [Source:HGNC Symbol;Acc:HGNC:904]	protein coding	-1.07	1.56	12.48	0.0004	0.0015
ENSG00000154319	FAM167A	family with sequence similarity 167 member A [Source:HGNC Symbol;Acc:HGNC:15549]	protein coding	-1.06	1.93	30.46	<0.0001	<0.0001
ENSG00000181652	ATG9B	autophagy related 9B [Source:HGNC Symbol;Acc:HGNC:21899]	protein coding	-1.06	0.67	18.48	<0.0001	0.0001
ENSG00000260910	LINC00565	long intergenic non-protein coding RNA 565 [Source:HGNC Symbol;Acc:HGNC:43709]	lncRNA	-1.06	0.20	10.97	0.0009	0.0031
ENSG00000113319	RASGRF2	Ras protein specific guanine nucleotide releasing factor 2 [Source:HGNC Symbol;Acc:HGNC:9876]	protein coding	-1.06	2.67	13.74	0.0002	0.0009
ENSG00000101057	MYBL2	MYB proto-oncogene like 2 [Source:HGNC Symbol;Acc:HGNC:7548]	protein coding	-1.06	1.07	24.62	<0.0001	<0.0001
ENSG00000175899	A2M	alpha-2-macroglobulin [Source:HGNC Symbol;Acc:HGNC:7]	protein coding	-1.06	1.42	25.81	<0.0001	<0.0001

Table S12: Genes differentially expressed at Day 30 compared to pre-second vaccination (Study Arm 2 A/H7N9+AS03, D1, 29, Dendritic cells). Genes are sorted by descending absolute log_2 fold change. Gene model summaries and annotations are based on Ensembl Version 99 (continued.)

Ensembl Gene ID	Ensembl Gene Name	Ensembl Gene Description	Gene Type	log_2 Fold Change (Day 30 vs. Pre-Second Vaccination)	Average log_2 CPM	Likelihood Ratio Test Statistic	P-Value	FDR Adjusted P-Value
ENSG00000141574	SECTM1	secreted and transmembrane 1 [Source:HGNC Symbol;Acc:HGNC:10707]	protein coding	1.06	8.76	65.62	<0.0001	<0.0001
ENSG00000111371	SLC38A1	solute carrier family 38 member 1 [Source:HGNC Symbol;Acc:HGNC:13447]	protein coding	-1.06	6.31	37.33	<0.0001	<0.0001
ENSG00000198719	DLL1	delta like canonical Notch ligand 1 [Source:HGNC Symbol;Acc:HGNC:2908]	protein coding	1.06	1.71	37.62	<0.0001	<0.0001
ENSG00000164114	MAP9	microtubule associated protein 9 [Source:HGNC Symbol;Acc:HGNC:26118]	protein coding	-1.06	0.51	17.87	<0.0001	0.0001
ENSG00000183722	LHFPL6	LHFPL tetraspan subfamily member 6 [Source:HGNC Symbol;Acc:HGNC:6586]	protein coding	-1.06	0.58	19.73	<0.0001	<0.0001
ENSG00000166313	APBB1	amyloid beta precursor protein binding family B member 1 [Source:HGNC Symbol;Acc:HGNC:581]	protein coding	-1.06	4.11	27.47	<0.0001	<0.0001
ENSG00000242779	ZNF702P	zinc finger protein 702, pseudogene [Source:HGNC Symbol;Acc:HGNC:25775]	transcribed processed pseudogene	1.06	2.09	31.77	<0.0001	<0.0001
ENSG00000130487	KLHDC7B	kelch domain containing 7B [Source:HGNC Symbol;Acc:HGNC:25145]	protein coding	1.06	4.45	116.63	<0.0001	<0.0001
ENSG00000095397	WHRN	whirlin [Source:HGNC Symbol;Acc:HGNC:16361]	protein coding	-1.06	1.42	19.59	<0.0001	<0.0001
ENSG00000116661	FBXO2	F-box protein 2 [Source:HGNC Symbol;Acc:HGNC:13581]	protein coding	-1.06	0.50	15.56	<0.0001	0.0004
ENSG00000247287		novel transcript, antisense to PRKCH	lncRNA	-1.06	1.26	16.94	<0.0001	0.0002
ENSG00000112096	SOD2	superoxide dismutase 2 [Source:HGNC Symbol;Acc:HGNC:11180]	protein coding	1.06	8.26	50.77	<0.0001	<0.0001
ENSG00000091409	ITGA6	integrin subunit alpha 6 [Source:HGNC Symbol;Acc:HGNC:6142]	protein coding	-1.06	4.46	14.69	0.0001	0.0006
ENSG00000197353	LYPD2	LY6/PLAUR domain containing 2 [Source:HGNC Symbol;Acc:HGNC:25215]	protein coding	-1.05	3.98	29.01	<0.0001	<0.0001
ENSG00000128335	APOL2	apolipoprotein L2 [Source:HGNC Symbol;Acc:HGNC:619]	protein coding	1.05	6.66	42.71	<0.0001	<0.0001
ENSG00000214826	DDX12P	DEAD/H-box helicase 12, pseudogene [Source:HGNC Symbol;Acc:HGNC:2737]	unprocessed pseudogene	-1.05	2.27	45.37	<0.0001	<0.0001
ENSG00000123700	KCNJ2	potassium inwardly rectifying channel subfamily J member 2 [Source:HGNC Symbol;Acc:HGNC:6263]	protein coding	1.05	3.16	24.90	<0.0001	<0.0001

Table S12: Genes differentially expressed at Day 30 compared to pre-second vaccination (Study Arm 2 A/H7N9+AS03, D1, 29, Dendritic cells). Genes are sorted by descending absolute log_2 fold change. Gene model summaries and annotations are based on Ensembl Version 99 (continued.)

Ensembl Gene ID	Ensembl Gene Name	Ensembl Gene Description	Gene Type	log_2 Fold Change (Day 30 vs. Pre-Second Vaccination)	Average log_2 CPM	Likelihood Ratio Test Statistic	P-Value	FDR Adjusted P-Value
ENSG00000269086		novel transcript	lncRNA	-1.05	0.47	11.34	0.0008	0.0026
ENSG00000167912		novel transcript	lncRNA	-1.05	0.49	12.73	0.0004	0.0014
ENSG00000105835	NAMPT	nicotinamide phosphoribosyltransferase [Source:HGNC Symbol;Acc:HGNC:30092]	protein coding	1.05	7.62	75.03	<0.0001	<0.0001
ENSG00000206052	DOK6	docking protein 6 [Source:HGNC Symbol;Acc:HGNC:28301]	protein coding	-1.05	0.27	14.69	0.0001	0.0006
ENSG00000260260	SNHG19	small nucleolar RNA host gene 19 [Source:HGNC Symbol;Acc:HGNC:49574]	lncRNA	-1.05	1.74	43.42	<0.0001	<0.0001
ENSG00000185022	MAFF	MAF bZIP transcription factor F [Source:HGNC Symbol;Acc:HGNC:6780]	protein coding	1.05	2.96	16.04	<0.0001	0.0003
ENSG00000256262	USP30-AS1	USP30 antisense RNA 1 [Source:HGNC Symbol;Acc:HGNC:40909]	lncRNA	1.05	1.70	40.99	<0.0001	<0.0001
ENSG00000154358	OBSCN	obscurin, cytoskeletal calmodulin and titin-interacting RhoGEF [Source:HGNC Symbol;Acc:HGNC:15719]	protein coding	-1.05	4.89	22.28	<0.0001	<0.0001
ENSG00000211747	TRBV20-1	T cell receptor beta variable 20-1 [Source:HGNC Symbol;Acc:HGNC:12196]	TR V gene	-1.05	1.63	9.40	0.0022	0.0063
ENSG00000105492	SIGLEC6	sialic acid binding Ig like lectin 6 [Source:HGNC Symbol;Acc:HGNC:10875]	protein coding	-1.05	1.84	46.57	<0.0001	<0.0001
ENSG00000121895	TMEM156	transmembrane protein 156 [Source:HGNC Symbol;Acc:HGNC:26260]	protein coding	-1.05	2.17	29.64	<0.0001	<0.0001
ENSG00000168056	LTBP3	latent transforming growth factor beta binding protein 3 [Source:HGNC Symbol;Acc:HGNC:6716]	protein coding	-1.05	6.00	34.17	<0.0001	<0.0001
ENSG00000141696	P3H4	prolyl 3-hydroxylase family member 4 (inactive) [Source:HGNC Symbol;Acc:HGNC:16946]	protein coding	-1.05	1.03	20.30	<0.0001	<0.0001
ENSG00000181649	PHLDA2	pleckstrin homology like domain family A member 2 [Source:HGNC Symbol;Acc:HGNC:12385]	protein coding	1.04	0.78	16.08	<0.0001	0.0003
ENSG00000042980	ADAM28	ADAM metallopeptidase domain 28 [Source:HGNC Symbol;Acc:HGNC:206]	protein coding	-1.04	4.69	112.86	<0.0001	<0.0001
ENSG00000159433	STARD9	StAR related lipid transfer domain containing 9 [Source:HGNC Symbol;Acc:HGNC:19162]	protein coding	-1.04	2.92	27.74	<0.0001	<0.0001
ENSG00000154165	GPR15	G protein-coupled receptor 15 [Source:HGNC Symbol;Acc:HGNC:4469]	protein coding	-1.04	0.30	12.08	0.0005	0.0018
ENSG00000176945	MUC20	mucin 20, cell surface associated [Source:HGNC Symbol;Acc:HGNC:23282]	protein coding	-1.04	0.61	28.32	<0.0001	<0.0001
ENSG00000143546	S100A8	S100 calcium binding protein A8 [Source:HGNC Symbol;Acc:HGNC:10498]	protein coding	1.04	10.45	40.20	<0.0001	<0.0001

Table S12: Genes differentially expressed at Day 30 compared to pre-second vaccination (Study Arm 2 A/H7N9+AS03, D1, 29, Dendritic cells). Genes are sorted by descending absolute log_2 fold change. Gene model summaries and annotations are based on Ensembl Version 99 (continued.)

Ensembl Gene ID	Ensembl Gene Name	Ensembl Gene Description	Gene Type	log_2 Fold Change (Day 30 vs. Pre-Second Vaccination)	Average log_2 CPM	Likelihood Ratio Test Statistic	P-Value	FDR Adjusted P-Value
ENSG00000008277	ADAM22	ADAM metallopeptidase domain 22 [Source:HGNC Symbol;Acc:HGNC:201]	protein coding	-1.04	0.75	16.60	<0.0001	0.0002
ENSG00000211689	TRGC1	T cell receptor gamma constant 1 [Source:HGNC Symbol;Acc:HGNC:12275]	TR C gene	-1.04	3.18	13.46	0.0002	0.001
ENSG00000235513	L3MBTL2-AS1	L3MBTL2 antisense RNA 1 [Source:HGNC Symbol;Acc:HGNC:40847]	lncRNA	1.04	1.08	32.11	<0.0001	<0.0001
ENSG00000197816	CCDC180	coiled-coil domain containing 180 [Source:HGNC Symbol;Acc:HGNC:29303]	protein coding	-1.04	0.16	21.43	<0.0001	<0.0001
ENSG00000185697	MYBL1	MYB proto-oncogene like 1 [Source:HGNC Symbol;Acc:HGNC:7547]	protein coding	-1.04	4.58	16.34	<0.0001	0.0003
ENSG00000141576	RNF157	ring finger protein 157 [Source:HGNC Symbol;Acc:HGNC:29402]	protein coding	-1.04	2.53	12.69	0.0004	0.0014
ENSG00000100311	PDGFB	platelet derived growth factor subunit B [Source:HGNC Symbol;Acc:HGNC:8800]	protein coding	-1.04	1.00	10.77	0.001	0.0033
ENSG00000181104	F2R	coagulation factor II thrombin receptor [Source:HGNC Symbol;Acc:HGNC:3537]	protein coding	-1.04	3.33	16.06	<0.0001	0.0003
ENSG00000240065	PSMB9	proteasome 20S subunit beta 9 [Source:HGNC Symbol;Acc:HGNC:9546]	protein coding	1.04	7.95	54.85	<0.0001	<0.0001
ENSG00000111452	ADGRD1	adhesion G protein-coupled receptor D1 [Source:HGNC Symbol;Acc:HGNC:19893]	protein coding	-1.04	4.03	32.22	<0.0001	<0.0001
ENSG00000105926	MPP6	membrane palmitoylated protein 6 [Source:HGNC Symbol;Acc:HGNC:18167]	protein coding	-1.03	1.59	20.77	<0.0001	<0.0001
ENSG00000145685	LHFPL2	LHFPL tetraspan subfamily member 2 [Source:HGNC Symbol;Acc:HGNC:6588]	protein coding	1.03	4.60	37.91	<0.0001	<0.0001
ENSG00000143842	SOX13	SRY-box transcription factor 13 [Source:HGNC Symbol;Acc:HGNC:11192]	protein coding	-1.03	2.23	15.31	<0.0001	0.0004
ENSG00000166126	AMN	amnion associated transmembrane protein [Source:HGNC Symbol;Acc:HGNC:14604]	protein coding	-1.03	0.94	16.65	<0.0001	0.0002
ENSG00000235576	LINC01871	long intergenic non-protein coding RNA 1871 [Source:HGNC Symbol;Acc:HGNC:52690]	lncRNA	-1.03	0.95	9.66	0.0019	0.0056
ENSG00000170581	STAT2	signal transducer and activator of transcription 2 [Source:HGNC Symbol;Acc:HGNC:11363]	protein coding	1.03	8.90	53.61	<0.0001	<0.0001
ENSG00000270157		novel transcript	lncRNA	-1.03	1.39	37.83	<0.0001	<0.0001
ENSG00000080493	SLC4A4	solute carrier family 4 member 4 [Source:HGNC Symbol;Acc:HGNC:11030]	protein coding	-1.03	0.59	16.38	<0.0001	0.0003

Table S12: Genes differentially expressed at Day 30 compared to pre-second vaccination (Study Arm 2 A/H7N9+AS03, D1, 29, Dendritic cells). Genes are sorted by descending absolute log_2 fold change. Gene model summaries and annotations are based on Ensembl Version 99 (continued.)

Ensembl Gene ID	Ensembl Gene Name	Ensembl Gene Description	Gene Type	log_2 Fold Change (Day 30 vs. Pre-Second Vaccination)	Average log_2 CPM	Likelihood Ratio Test Statistic	P-Value	FDR Adjusted P-Value
ENSG00000185880	TRIM69	tripartite motif containing 69 [Source:HGNC Symbol;Acc:HGNC:17857]	protein coding	1.03	5.69	53.22	<0.0001	<0.0001
ENSG00000072195	SPEG	striated muscle enriched protein kinase [Source:HGNC Symbol;Acc:HGNC:16901]	protein coding	-1.03	1.42	15.41	<0.0001	0.0004
ENSG00000143167	GPA33	glycoprotein A33 [Source:HGNC Symbol;Acc:HGNC:4445]	protein coding	-1.03	2.10	34.49	<0.0001	<0.0001
ENSG00000126353	CCR7	C-C motif chemokine receptor 7 [Source:HGNC Symbol;Acc:HGNC:1608]	protein coding	-1.03	4.88	11.61	0.0007	0.0023
ENSG00000136514	RTP4	receptor transporter protein 4 [Source:HGNC Symbol;Acc:HGNC:23992]	protein coding	1.03	3.35	34.59	<0.0001	<0.0001
ENSG00000116254	CHD5	chromodomain helicase DNA binding protein 5 [Source:HGNC Symbol;Acc:HGNC:16816]	protein coding	-1.02	1.22	25.52	<0.0001	<0.0001
ENSG00000106976	DNM1	dynamin 1 [Source:HGNC Symbol;Acc:HGNC:2972]	protein coding	-1.02	2.25	19.49	<0.0001	<0.0001
ENSG00000173110	HSPA6	heat shock protein family A (Hsp70) member 6 [Source:HGNC Symbol;Acc:HGNC:5239]	protein coding	1.02	5.40	59.77	<0.0001	<0.0001
ENSG00000115267	IFIH1	interferon induced with helicase C domain 1 [Source:HGNC Symbol;Acc:HGNC:18873]	protein coding	1.02	6.01	57.98	<0.0001	<0.0001
ENSG00000078804	TP53INP2	tumor protein p53 inducible nuclear protein 2 [Source:HGNC Symbol;Acc:HGNC:16104]	protein coding	1.02	2.76	40.23	<0.0001	<0.0001
ENSG00000096968	JAK2	Janus kinase 2 [Source:HGNC Symbol;Acc:HGNC:6192]	protein coding	1.02	7.68	50.02	<0.0001	<0.0001
ENSG00000269404	SPIB	Spi-B transcription factor [Source:HGNC Symbol;Acc:HGNC:11242]	protein coding	-1.02	2.52	11.25	0.0008	0.0027
ENSG00000227191	TRGC2	T cell receptor gamma constant 2 [Source:HGNC Symbol;Acc:HGNC:12276]	TR C gene	-1.02	2.89	12.00	0.0005	0.0019
ENSG00000171033	PKIA	cAMP-dependent protein kinase inhibitor alpha [Source:HGNC Symbol;Acc:HGNC:9017]	protein coding	-1.02	1.46	17.94	<0.0001	0.0001
ENSG00000176105	YES1	YES proto-oncogene 1, Src family tyrosine kinase [Source:HGNC Symbol;Acc:HGNC:12841]	protein coding	-1.02	1.31	17.77	<0.0001	0.0001
ENSG00000168661	ZNF30	zinc finger protein 30 [Source:HGNC Symbol;Acc:HGNC:13090]	protein coding	-1.02	0.68	25.71	<0.0001	<0.0001
ENSG00000125498	KIR2DL1	killer cell immunoglobulin like receptor, two Ig domains and long cytoplasmic tail 1 [Source:HGNC Symbol;Acc:HGNC:6329]	protein coding	-1.02	0.36	15.16	<0.0001	0.0005

Table S12: Genes differentially expressed at Day 30 compared to pre-second vaccination (Study Arm 2 A/H7N9+AS03, D1, 29, Dendritic cells). Genes are sorted by descending absolute log_2 fold change. Gene model summaries and annotations are based on Ensembl Version 99 (continued.)

Ensembl Gene ID	Ensembl Gene Name	Ensembl Gene Description	Gene Type	log_2 Fold Change (Day 30 vs. Pre-Second Vaccination)	Average log_2 CPM	Likelihood Ratio Test Statistic	P-Value	FDR Adjusted P-Value
ENSG00000183134	PTGDR2	prostaglandin D2 receptor 2 [Source:HGNC Symbol;Acc:HGNC:4502]	protein coding	-1.02	4.58	28.68	<0.0001	<0.0001
ENSG00000287252		novel transcript	lncRNA	-1.02	0.89	40.07	<0.0001	<0.0001
ENSG00000072858	SIDT1	SID1 transmembrane family member 1 [Source:HGNC Symbol;Acc:HGNC:25967]	protein coding	-1.02	3.74	24.23	<0.0001	<0.0001
ENSG00000196407	THEM5	thioesterase superfamily member 5 [Source:HGNC Symbol;Acc:HGNC:26755]	protein coding	-1.02	0.29	10.66	0.0011	0.0035
ENSG00000154736	ADAMTS5	ADAM metallopeptidase with thrombospondin type 1 motif 5 [Source:HGNC Symbol;Acc:HGNC:221]	protein coding	-1.02	1.74	40.74	<0.0001	<0.0001
ENSG00000187862	TTC24	tetratricopeptide repeat domain 24 [Source:HGNC Symbol;Acc:HGNC:32348]	protein coding	-1.02	1.36	20.64	<0.0001	<0.0001
ENSG00000233461		novel transcript	lncRNA	1.02	1.36	31.41	<0.0001	<0.0001
ENSG00000237943	PRKCQ-AS1	PRKCQ antisense RNA 1 [Source:HGNC Symbol;Acc:HGNC:44689]	lncRNA	-1.02	3.80	12.73	0.0004	0.0014
ENSG00000108370	RGS9	regulator of G protein signaling 9 [Source:HGNC Symbol;Acc:HGNC:10004]	protein coding	-1.02	1.35	14.74	0.0001	0.0006
ENSG00000224078	SNHG14	small nucleolar RNA host gene 14 [Source:HGNC Symbol;Acc:HGNC:37462]	lncRNA	-1.02	4.23	41.48	<0.0001	<0.0001
ENSG00000162739	SLAMF6	SLAM family member 6 [Source:HGNC Symbol;Acc:HGNC:21392]	protein coding	-1.02	4.42	23.40	<0.0001	<0.0001
ENSG00000112378	PERP	p53 apoptosis effector related to PMP22 [Source:HGNC Symbol;Acc:HGNC:17637]	protein coding	-1.01	0.10	13.89	0.0002	0.0008
ENSG00000151693	ASAP2	ArfGAP with SH3 domain, ankyrin repeat and PH domain 2 [Source:HGNC Symbol;Acc:HGNC:2721]	protein coding	1.01	2.48	20.25	<0.0001	<0.0001
ENSG00000121797	CCRL2	C-C motif chemokine receptor like 2 [Source:HGNC Symbol;Acc:HGNC:1612]	protein coding	1.01	2.76	31.75	<0.0001	<0.0001
ENSG00000164530	PI16	peptidase inhibitor 16 [Source:HGNC Symbol;Acc:HGNC:21245]	protein coding	-1.01	0.76	10.10	0.0015	0.0046
ENSG00000140379	BCL2A1	BCL2 related protein A1 [Source:HGNC Symbol;Acc:HGNC:991]	protein coding	1.01	5.89	85.76	<0.0001	<0.0001
ENSG00000174500	GCSAM	germinal center associated signaling and motility [Source:HGNC Symbol;Acc:HGNC:20253]	protein coding	-1.01	1.86	18.20	<0.0001	0.0001
ENSG00000164241	C5orf63	chromosome 5 open reading frame 63 [Source:HGNC Symbol;Acc:HGNC:40051]	protein coding	-1.01	1.44	17.95	<0.0001	0.0001
ENSG00000279805		TEC	TEC	1.01	1.01	25.04	<0.0001	<0.0001

Confidential/Proprietary Information

Table S12: Genes differentially expressed at Day 30 compared to pre-second vaccination (Study Arm 2 A/H7N9+AS03, D1, 29, Dendritic cells). Genes are sorted by descending absolute log_2 fold change. Gene model summaries and annotations are based on Ensembl Version 99 (continued.)

Ensembl Gene ID	Ensembl Gene Name	Ensembl Gene Description	Gene Type	log_2 Fold Change (Day 30 vs. Pre-Second Vaccination)	Average log_2 CPM	Likelihood Ratio Test Statistic	P-Value	FDR Adjusted P-Value
ENSG00000140464	PML	promyelocytic leukemia [Source:HGNC Symbol;Acc:HGNC:9113]	protein coding	1.01	6.98	60.30	<0.0001	<0.0001
ENSG00000172460	PRSS30P	serine protease 30, pseudogene [Source:HGNC Symbol;Acc:HGNC:28753]	transcribed unitary pseudogene	-1.01	1.90	22.92	<0.0001	<0.0001
ENSG00000166405	RIC3	RIC3 acetylcholine receptor chaperone [Source:HGNC Symbol;Acc:HGNC:30338]	protein coding	-1.01	1.81	16.81	<0.0001	0.0002
ENSG00000204852	TCTN1	tectonic family member 1 [Source:HGNC Symbol;Acc:HGNC:26113]	protein coding	-1.01	2.49	61.66	<0.0001	<0.0001
ENSG00000182541	LIMK2	LIM domain kinase 2 [Source:HGNC Symbol;Acc:HGNC:6614]	protein coding	1.01	5.82	33.90	<0.0001	<0.0001
ENSG00000185101	ANO9	anoctamin 9 [Source:HGNC Symbol;Acc:HGNC:20679]	protein coding	-1.01	4.72	25.44	<0.0001	<0.0001
ENSG00000255819	KLRC4-KLRK1	KLRC4-KLRK1 readthrough [Source:HGNC Symbol;Acc:HGNC:48357]	protein coding	-1.01	0.92	11.93	0.0006	0.002
ENSG00000127585	FBXL16	F-box and leucine rich repeat protein 16 [Source:HGNC Symbol;Acc:HGNC:14150]	protein coding	-1.01	2.63	29.23	<0.0001	<0.0001
ENSG00000180644	PRF1	perforin 1 [Source:HGNC Symbol;Acc:HGNC:9360]	protein coding	-1.01	7.73	12.89	0.0003	0.0013
ENSG00000101096	NFATC2	nuclear factor of activated T cells 2 [Source:HGNC Symbol;Acc:HGNC:7776]	protein coding	-1.01	4.97	31.44	<0.0001	<0.0001
ENSG00000152465	NMT2	N-myristoyltransferase 2 [Source:HGNC Symbol;Acc:HGNC:7858]	protein coding	-1.01	3.05	33.86	<0.0001	<0.0001
ENSG00000187621	TCL6	T cell leukemia/lymphoma 6 [Source:HGNC Symbol;Acc:HGNC:13463]	lncRNA	-1.00	0.72	11.69	0.0006	0.0022
ENSG00000135916	ITM2C	integral membrane protein 2C [Source:HGNC Symbol;Acc:HGNC:6175]	protein coding	-1.00	4.59	47.74	<0.0001	<0.0001
ENSG00000139187	KLRG1	killer cell lectin like receptor G1 [Source:HGNC Symbol;Acc:HGNC:6380]	protein coding	-1.00	3.43	14.73	0.0001	0.0006
ENSG00000158321	AUTS2	activator of transcription and developmental regulator AUTS2 [Source:HGNC Symbol;Acc:HGNC:14262]	protein coding	-1.00	3.64	18.35	<0.0001	0.0001
ENSG00000233355	CHRM3-AS2	CHRM3 antisense RNA 2 [Source:HGNC Symbol;Acc:HGNC:43725]	lncRNA	-1.00	3.74	11.17	0.0008	0.0028
ENSG00000023171	GRAMD1B	GRAM domain containing 1B [Source:HGNC Symbol;Acc:HGNC:29214]	protein coding	1.00	5.13	70.88	<0.0001	<0.0001

Table S12: Genes differentially expressed at Day 30 compared to pre-second vaccination (Study Arm 2 A/H7N9+AS03, D1, 29, Dendritic cells). Genes are sorted by descending absolute log_2 fold change. Gene model summaries and annotations are based on Ensembl Version 99 (continued.)

Ensembl Gene ID	Ensembl Gene Name	Ensembl Gene Description	Gene Type	log_2 Fold Change (Day 30 vs. Pre-Second Vaccination)	Average log_2 CPM	Likelihood Ratio Test Statistic	P-Value	FDR Adjusted P-Value
ENSG00000105699	LSR	lipolysis stimulated lipoprotein receptor [Source:HGNC Symbol;Acc:HGNC:29572]	protein coding	-1.00	3.02	26.85	<0.0001	<0.0001
ENSG00000101082	SLA2	Src like adaptor 2 [Source:HGNC Symbol;Acc:HGNC:17329]	protein coding	-1.00	3.35	15.32	<0.0001	0.0004
ENSG00000196557	CACNA1H	calcium voltage-gated channel subunit alpha1 H [Source:HGNC Symbol;Acc:HGNC:1395]	protein coding	-1.00	0.92	10.33	0.0013	0.0041
ENSG00000156475	PPP2R2B	protein phosphatase 2 regulatory subunit Bbeta [Source:HGNC Symbol;Acc:HGNC:9305]	protein coding	-1.00	1.92	12.18	0.0005	0.0018
ENSG00000232869	TRBV29-1	T cell receptor beta variable 29-1 [Source:HGNC Symbol;Acc:HGNC:12210]	TR V gene	-1.00	0.96	8.49	0.0036	0.0096
ENSG00000278030	TRBV7-9	T cell receptor beta variable 7-9 [Source:HGNC Symbol;Acc:HGNC:12243]	TR V gene	-1.00	0.97	7.65	0.0057	0.0144
ENSG00000226711	FAM66C	family with sequence similarity 66 member C [Source:HGNC Symbol;Acc:HGNC:21644]	lncRNA	-1.00	0.30	27.92	<0.0001	<0.0001
ENSG00000156510	HKDC1	hexokinase domain containing 1 [Source:HGNC Symbol;Acc:HGNC:23302]	protein coding	-1.00	0.41	7.89	0.005	0.0128
ENSG00000163840	DTX3L	deltex E3 ubiquitin ligase 3L [Source:HGNC Symbol;Acc:HGNC:30323]	protein coding	1.00	6.62	42.76	<0.0001	<0.0001
ENSG00000164649	CDCA7L	cell division cycle associated 7 like [Source:HGNC Symbol;Acc:HGNC:30777]	protein coding	-1.00	2.97	33.35	<0.0001	<0.0001
ENSG00000224373	IGHV4-59	immunoglobulin heavy variable 4-59 [Source:HGNC Symbol;Acc:HGNC:5654]	IG V gene	-1.00	0.71	13.17	0.0003	0.0011
ENSG00000211666	IGLV2-14	immunoglobulin lambda variable 2-14 [Source:HGNC Symbol;Acc:HGNC:5888]	IG V gene	-1.00	2.50	25.38	<0.0001	<0.0001
ENSG00000065675	PRKCQ	protein kinase C theta [Source:HGNC Symbol;Acc:HGNC:9410]	protein coding	-0.99	3.35	15.64	<0.0001	0.0004
ENSG00000172673	THEMIS	thymocyte selection associated [Source:HGNC Symbol;Acc:HGNC:21569]	protein coding	-0.99	2.90	9.09	0.0026	0.0073
ENSG00000136960	ENPP2	ectonucleotide pyrophosphatase/phosphodiesterase 2 [Source:HGNC Symbol;Acc:HGNC:3357]	protein coding	0.99	1.23	20.88	<0.0001	<0.0001
ENSG00000174669	SLC29A2	solute carrier family 29 member 2 [Source:HGNC Symbol;Acc:HGNC:11004]	protein coding	-0.99	1.93	23.47	<0.0001	<0.0001
ENSG00000286248		novel transcript, antisense to P2RX7	lncRNA	0.99	0.78	34.54	<0.0001	<0.0001
ENSG00000169992	NLGN2	neuroligin 2 [Source:HGNC Symbol;Acc:HGNC:14290]	protein coding	-0.99	1.75	17.12	<0.0001	0.0002

Confidential/Proprietary Information

Table S12: Genes differentially expressed at Day 30 compared to pre-second vaccination (Study Arm 2 A/H7N9+AS03, D1, 29, Dendritic cells). Genes are sorted by descending absolute log_2 fold change. Gene model summaries and annotations are based on Ensembl Version 99 (continued.)

Ensembl Gene ID	Ensembl Gene Name	Ensembl Gene Description	Gene Type	log_2 Fold Change (Day 30 vs. Pre-Second Vaccination)	Average log_2 CPM	Likelihood Ratio Test Statistic	P-Value	FDR Adjusted P-Value
ENSG00000080854	IGSF9B	immunoglobulin superfamily member 9B [Source:HGNC Symbol;Acc:HGNC:32326]	protein coding	-0.99	2.80	9.54	0.002	0.0059
ENSG00000137078	SIT1	signaling threshold regulating transmembrane adaptor 1 [Source:HGNC Symbol;Acc:HGNC:17710]	protein coding	-0.99	3.07	12.42	0.0004	0.0016
ENSG00000168280	KIF5C	kinesin family member 5C [Source:HGNC Symbol;Acc:HGNC:6325]	protein coding	-0.99	1.66	23.07	<0.0001	<0.0001
ENSG00000129673	AANAT	aralkylamine N-acetyltransferase [Source:HGNC Symbol;Acc:HGNC:19]	protein coding	0.99	1.76	35.08	<0.0001	<0.0001
ENSG00000130589	HELZ2	helicase with zinc finger 2 [Source:HGNC Symbol;Acc:HGNC:30021]	protein coding	0.99	7.60	61.93	<0.0001	<0.0001
ENSG00000049089	COL9A2	collagen type IX alpha 2 chain [Source:HGNC Symbol;Acc:HGNC:2218]	protein coding	-0.99	5.35	61.74	<0.0001	<0.0001
ENSG00000232229	LINC00865	long intergenic non-protein coding RNA 865 [Source:HGNC Symbol;Acc:HGNC:45170]	lncRNA	-0.99	0.32	24.32	<0.0001	<0.0001
ENSG00000176720	BOK	BCL2 family apoptosis regulator BOK [Source:HGNC Symbol;Acc:HGNC:1087]	protein coding	-0.99	1.36	15.02	0.0001	0.0005
ENSG00000124256	ZBP1	Z-DNA binding protein 1 [Source:HGNC Symbol;Acc:HGNC:16176]	protein coding	0.99	5.07	45.74	<0.0001	<0.0001
ENSG00000124374	PAIP2B	poly(A) binding protein interacting protein 2B [Source:HGNC Symbol;Acc:HGNC:29200]	protein coding	-0.99	1.47	27.04	<0.0001	<0.0001
ENSG00000055332	EIF2AK2	eukaryotic translation initiation factor 2 alpha kinase 2 [Source:HGNC Symbol;Acc:HGNC:9437]	protein coding	0.99	6.14	86.92	<0.0001	<0.0001
ENSG00000144824	PHLDB2	pleckstrin homology like domain family B member 2 [Source:HGNC Symbol;Acc:HGNC:29573]	protein coding	-0.99	1.26	11.00	0.0009	0.003
ENSG00000287733		novel transcript	lncRNA	0.99	1.07	23.26	<0.0001	<0.0001
ENSG00000111110	PPM1H	protein phosphatase, Mg2+/Mn2+ dependent 1H [Source:HGNC Symbol;Acc:HGNC:18583]	protein coding	-0.99	2.79	39.60	<0.0001	<0.0001
ENSG00000258926		novel transcript, antisense to PRKCH	lncRNA	-0.98	1.45	14.83	0.0001	0.0005
ENSG00000197077	KIAA1671	KIAA1671 [Source:HGNC Symbol;Acc:HGNC:29345]	protein coding	-0.98	1.45	16.30	<0.0001	0.0003
ENSG00000135604	STX11	syntaxin 11 [Source:HGNC Symbol;Acc:HGNC:11429]	protein coding	0.98	8.30	64.82	<0.0001	<0.0001

Table S12: Genes differentially expressed at Day 30 compared to pre-second vaccination (Study Arm 2 A/H7N9+AS03, D1, 29, Dendritic cells). Genes are sorted by descending absolute log_2 fold change. Gene model summaries and annotations are based on Ensembl Version 99 (continued.)

Ensembl Gene ID	Ensembl Gene Name	Ensembl Gene Description	Gene Type	log_2 Fold Change (Day 30 vs. Pre-Second Vaccination)	Average log_2 CPM	Likelihood Ratio Test Statistic	P-Value	FDR Adjusted P-Value
ENSG00000228314	CYP4F29P	cytochrome P450 family 4 subfamily F member 29, pseudogene [Source:HGNC Symbol;Acc:HGNC:2647]	transcribed unprocessed pseudogene	-0.98	2.16	6.71	0.0096	0.0226
ENSG00000082781	ITGB5	integrin subunit beta 5 [Source:HGNC Symbol;Acc:HGNC:6160]	protein coding	-0.98	2.27	41.78	<0.0001	<0.0001
ENSG00000100450	GZMH	granzyme H [Source:HGNC Symbol;Acc:HGNC:4710]	protein coding	-0.98	5.00	12.82	0.0003	0.0013
ENSG00000138378	STAT4	signal transducer and activator of transcription 4 [Source:HGNC Symbol;Acc:HGNC:11365]	protein coding	-0.98	4.19	16.49	<0.0001	0.0002
ENSG00000143515	ATP8B2	ATPase phospholipid transporting 8B2 [Source:HGNC Symbol;Acc:HGNC:13534]	protein coding	-0.98	5.61	17.55	<0.0001	0.0002
ENSG00000173821	RNF213	ring finger protein 213 [Source:HGNC Symbol;Acc:HGNC:14539]	protein coding	0.98	9.94	61.28	<0.0001	<0.0001
ENSG00000132744	ACY3	aminoacylase 3 [Source:HGNC Symbol;Acc:HGNC:24104]	protein coding	-0.98	1.68	14.15	0.0002	0.0007
ENSG00000099282	TSPAN15	tetraspanin 15 [Source:HGNC Symbol;Acc:HGNC:23298]	protein coding	-0.98	0.51	14.99	0.0001	0.0005
ENSG00000170390	DCLK2	doublecortin like kinase 2 [Source:HGNC Symbol;Acc:HGNC:19002]	protein coding	-0.98	0.62	20.92	<0.0001	<0.0001
ENSG00000248334	WHAMMP2	WAS protein homolog associated with actin, golgi membranes and microtubules pseudogene 2 [Source:HGNC Symbol;Acc:HGNC:32360]	transcribed unprocessed pseudogene	-0.98	2.21	34.94	<0.0001	<0.0001
ENSG00000005448	WDR54	WD repeat domain 54 [Source:HGNC Symbol;Acc:HGNC:25770]	protein coding	-0.98	2.85	30.94	<0.0001	<0.0001
ENSG00000086300	SNX10	sorting nexin 10 [Source:HGNC Symbol;Acc:HGNC:14974]	protein coding	0.98	7.18	51.33	<0.0001	<0.0001
ENSG00000240219		novel transcript	lncRNA	0.98	1.92	17.63	<0.0001	0.0001
ENSG00000117560	FASLG	Fas ligand [Source:HGNC Symbol;Acc:HGNC:11936]	protein coding	-0.98	1.72	9.18	0.0024	0.007
ENSG00000263069	RNF213-AS1	RNF213 antisense RNA 1 [Source:HGNC Symbol;Acc:HGNC:54402]	lncRNA	0.98	3.75	63.82	<0.0001	<0.0001

Table S12: Genes differentially expressed at Day 30 compared to pre-second vaccination (Study Arm 2 A/H7N9+AS03, D1, 29, Dendritic cells). Genes are sorted by descending absolute log_2 fold change. Gene model summaries and annotations are based on Ensembl Version 99 (continued.)

Ensembl Gene ID	Ensembl Gene Name	Ensembl Gene Description	Gene Type	log_2 Fold Change (Day 30 vs. Pre-Second Vaccination)	Average log_2 CPM	Likelihood Ratio Test Statistic	P-Value	FDR Adjusted P-Value
ENSG00000176928	GCNT4	glucosaminyl (N-acetyl) transferase 4 [Source:HGNC Symbol;Acc:HGNC:17973]	protein coding	-0.98	1.56	11.17	0.0008	0.0028
ENSG00000246982		novel transcript, antisense to BRPF3	lncRNA	-0.98	0.20	30.26	<0.0001	<0.0001
ENSG00000183734	ASCL2	achaete-scute family bHLH transcription factor 2 [Source:HGNC Symbol;Acc:HGNC:739]	protein coding	0.98	5.45	53.07	<0.0001	<0.0001
ENSG00000170382	LRRN2	leucine rich repeat neuronal 2 [Source:HGNC Symbol;Acc:HGNC:16914]	protein coding	0.98	3.20	21.88	<0.0001	<0.0001
ENSG00000088387	DOCK9	dedicator of cytokinesis 9 [Source:HGNC Symbol;Acc:HGNC:14132]	protein coding	-0.98	3.66	12.88	0.0003	0.0013
ENSG00000170629	DPY19L2P2	DPY19L2 pseudogene 2 [Source:HGNC Symbol;Acc:HGNC:21764]	transcribed unprocessed pseudogene	-0.98	0.77	10.93	0.0009	0.0031
ENSG00000229644	NAMPTP1	nicotinamide phosphoribosyltransferase pseudogene 1 [Source:HGNC Symbol;Acc:HGNC:17633]	processed pseudogene	0.98	4.28	51.25	<0.0001	<0.0001
ENSG00000152778	IFIT5	interferon induced protein with tetratricopeptide repeats 5 [Source:HGNC Symbol;Acc:HGNC:13328]	protein coding	0.98	4.46	58.46	<0.0001	<0.0001
ENSG00000130702	LAMA5	laminin subunit alpha 5 [Source:HGNC Symbol;Acc:HGNC:6485]	protein coding	-0.98	2.24	42.03	<0.0001	<0.0001
ENSG00000110076	NRXN2	neurexin 2 [Source:HGNC Symbol;Acc:HGNC:8009]	protein coding	-0.97	1.42	18.74	<0.0001	<0.0001
ENSG00000118420	UBE3D	ubiquitin protein ligase E3D [Source:HGNC Symbol;Acc:HGNC:21381]	protein coding	-0.97	0.88	32.25	<0.0001	<0.0001
ENSG00000171101	SIGLEC17P	sialic acid binding Ig like lectin 17, pseudogene [Source:HGNC Symbol;Acc:HGNC:15604]	transcribed unprocessed pseudogene	-0.97	2.62	30.61	<0.0001	<0.0001
ENSG00000176083	ZNF683	zinc finger protein 683 [Source:HGNC Symbol;Acc:HGNC:28495]	protein coding	-0.97	2.91	9.69	0.0019	0.0055
ENSG00000257524		novel protein	protein coding	-0.97	1.33	21.53	<0.0001	<0.0001
ENSG00000262823		novel transcript, antisense to SPNS3	lncRNA	-0.97	0.67	25.88	<0.0001	<0.0001

Table S12: Genes differentially expressed at Day 30 compared to pre-second vaccination (Study Arm 2 A/H7N9+AS03, D1, 29, Dendritic cells). Genes are sorted by descending absolute log_2 fold change. Gene model summaries and annotations are based on Ensembl Version 99 (continued.)

Ensembl Gene ID	Ensembl Gene Name	Ensembl Gene Description	Gene Type	log_2 Fold Change (Day 30 vs. Pre-Second Vaccination)	Average log_2 CPM	Likelihood Ratio Test Statistic	P-Value	FDR Adjusted P-Value
ENSG00000168778	TCTN2	tectonic family member 2 [Source:HGNC Symbol;Acc:HGNC:25774]	protein coding	-0.97	1.13	34.81	<0.0001	<0.0001
ENSG00000105374	NKG7	natural killer cell granule protein 7 [Source:HGNC Symbol;Acc:HGNC:7830]	protein coding	-0.97	7.09	16.89	<0.0001	0.0002
ENSG00000213420	GPC2	glypican 2 [Source:HGNC Symbol;Acc:HGNC:4450]	protein coding	-0.97	1.50	31.84	<0.0001	<0.0001
ENSG00000143126	CELSR2	cadherin EGF LAG seven-pass G-type receptor 2 [Source:HGNC Symbol;Acc:HGNC:3231]	protein coding	-0.97	2.68	36.64	<0.0001	<0.0001
ENSG00000184986	TMEM121	transmembrane protein 121 [Source:HGNC Symbol;Acc:HGNC:20511]	protein coding	-0.97	0.35	16.57	<0.0001	0.0002
ENSG00000105967	TFEC	transcription factor EC [Source:HGNC Symbol;Acc:HGNC:11754]	protein coding	0.97	6.75	50.34	<0.0001	<0.0001
ENSG00000275210		novel transcript	lncRNA	0.97	2.45	56.47	<0.0001	<0.0001
ENSG00000105289	TJP3	tight junction protein 3 [Source:HGNC Symbol;Acc:HGNC:11829]	protein coding	-0.97	1.07	15.17	<0.0001	0.0005
ENSG00000165795	NDRG2	NDRG family member 2 [Source:HGNC Symbol;Acc:HGNC:14460]	protein coding	-0.97	5.17	56.98	<0.0001	<0.0001
ENSG00000126368	NR1D1	nuclear receptor subfamily 1 group D member 1 [Source:HGNC Symbol;Acc:HGNC:7962]	protein coding	-0.97	3.31	37.22	<0.0001	<0.0001
ENSG00000124507	PACSIN1	protein kinase C and casein kinase substrate in neurons 1 [Source:HGNC Symbol;Acc:HGNC:8570]	protein coding	-0.97	0.75	9.14	0.0025	0.0071
ENSG00000170542	SERPINB9	serpin family B member 9 [Source:HGNC Symbol;Acc:HGNC:8955]	protein coding	0.96	7.34	60.89	<0.0001	<0.0001
ENSG00000075399	VPS9D1	VPS9 domain containing 1 [Source:HGNC Symbol;Acc:HGNC:13526]	protein coding	0.96	5.99	54.17	<0.0001	<0.0001
ENSG00000123453	SARDH	sarcosine dehydrogenase [Source:HGNC Symbol;Acc:HGNC:10536]	protein coding	-0.96	0.44	10.86	0.001	0.0032
ENSG00000135378	PRRG4	proline rich and Gla domain 4 [Source:HGNC Symbol;Acc:HGNC:30799]	protein coding	0.96	4.49	54.81	<0.0001	<0.0001
ENSG00000214548	MEG3	maternally expressed 3 [Source:HGNC Symbol;Acc:HGNC:14575]	lncRNA	-0.96	4.57	18.10	<0.0001	0.0001
ENSG00000238105	GOLGA2P5	GOLGA2 pseudogene 5 [Source:HGNC Symbol;Acc:HGNC:25315]	transcribed unprocessed pseudogene	-0.96	2.38	17.34	<0.0001	0.0002

Table S12: Genes differentially expressed at Day 30 compared to pre-second vaccination (Study Arm 2 A/H7N9+AS03, D1, 29, Dendritic cells). Genes are sorted by descending absolute log_2 fold change. Gene model summaries and annotations are based on Ensembl Version 99 (continued.)

Ensembl Gene ID	Ensembl Gene Name	Ensembl Gene Description	Gene Type	log_2 Fold Change (Day 30 vs. Pre-Second Vaccination)	Average log_2 CPM	Likelihood Ratio Test Statistic	P-Value	FDR Adjusted P-Value
ENSG00000141736	ERBB2	erb-b2 receptor tyrosine kinase 2 [Source:HGNC Symbol;Acc:HGNC:3430]	protein coding	-0.96	3.27	24.63	<0.0001	<0.0001
ENSG00000127954	STEAP4	STEAP4 metalloreductase [Source:HGNC Symbol;Acc:HGNC:21923]	protein coding	0.96	4.90	27.40	<0.0001	<0.0001
ENSG00000261801	LOXL1-AS1	LOXL1 antisense RNA 1 [Source:HGNC Symbol;Acc:HGNC:44169]	lncRNA	-0.96	0.34	9.83	0.0017	0.0052
ENSG00000162676	GFI1	growth factor independent 1 transcriptional repressor [Source:HGNC Symbol;Acc:HGNC:4237]	protein coding	-0.96	2.99	15.12	0.0001	0.0005
ENSG00000027869	SH2D2A	SH2 domain containing 2A [Source:HGNC Symbol;Acc:HGNC:10821]	protein coding	-0.96	3.88	10.27	0.0014	0.0042
ENSG00000211949	IGHV3-23	immunoglobulin heavy variable 3-23 [Source:HGNC Symbol;Acc:HGNC:5588]	IG V gene	-0.96	2.87	26.23	<0.0001	<0.0001
ENSG00000130202	NECTIN2	nectin cell adhesion molecule 2 [Source:HGNC Symbol;Acc:HGNC:9707]	protein coding	0.96	4.58	74.46	<0.0001	<0.0001
ENSG00000164136	IL15	interleukin 15 [Source:HGNC Symbol;Acc:HGNC:5977]	protein coding	0.96	5.16	68.37	<0.0001	<0.0001
ENSG00000166016	ABTB2	ankyrin repeat and BTB domain containing 2 [Source:HGNC Symbol;Acc:HGNC:23842]	protein coding	0.96	2.27	30.07	<0.0001	<0.0001
ENSG00000211592	IGKC	immunoglobulin kappa constant [Source:HGNC Symbol;Acc:HGNC:5716]	IG C gene	-0.96	7.35	30.97	<0.0001	<0.0001
ENSG00000116667	C1orf21	chromosome 1 open reading frame 21 [Source:HGNC Symbol;Acc:HGNC:15494]	protein coding	-0.96	3.05	16.84	<0.0001	0.0002
ENSG00000139116	KIF21A	kinesin family member 21A [Source:HGNC Symbol;Acc:HGNC:19349]	protein coding	-0.96	1.80	11.68	0.0006	0.0022
ENSG00000128512	DOCK4	dedicator of cytokinesis 4 [Source:HGNC Symbol;Acc:HGNC:19192]	protein coding	0.96	2.69	61.18	<0.0001	<0.0001
ENSG00000072163	LIMS2	LIM zinc finger domain containing 2 [Source:HGNC Symbol;Acc:HGNC:16084]	protein coding	-0.96	0.53	18.69	<0.0001	<0.0001
ENSG00000171766	GATM	glycine amidinotransferase [Source:HGNC Symbol;Acc:HGNC:4175]	protein coding	-0.96	2.37	39.17	<0.0001	<0.0001
ENSG00000124762	CDKN1A	cyclin dependent kinase inhibitor 1A [Source:HGNC Symbol;Acc:HGNC:1784]	protein coding	0.96	6.55	55.72	<0.0001	<0.0001
ENSG00000148429	USP6NL	USP6 N-terminal like [Source:HGNC Symbol;Acc:HGNC:16858]	protein coding	0.96	4.80	47.82	<0.0001	<0.0001
ENSG00000079215	SLC1A3	solute carrier family 1 member 3 [Source:HGNC Symbol;Acc:HGNC:10941]	protein coding	0.96	1.73	32.53	<0.0001	<0.0001

Table S12: Genes differentially expressed at Day 30 compared to pre-second vaccination (Study Arm 2 A/H7N9+AS03, D1, 29, Dendritic cells). Genes are sorted by descending absolute log_2 fold change. Gene model summaries and annotations are based on Ensembl Version 99 (continued.)

Ensembl Gene ID	Ensembl Gene Name	Ensembl Gene Description	Gene Type	log_2 Fold Change (Day 30 vs. Pre-Second Vaccination)	Average log_2 CPM	Likelihood Ratio Test Statistic	P-Value	FDR Adjusted P-Value
ENSG00000211892	IGHG4	immunoglobulin heavy constant gamma 4 (G4m marker) [Source:HGNC Symbol;Acc:HGNC:5528]	IG C gene	-0.96	2.08	11.31	0.0008	0.0026
ENSG00000136848	DAB2IP	DAB2 interacting protein [Source:HGNC Symbol;Acc:HGNC:17294]	protein coding	-0.96	0.27	8.56	0.0034	0.0093
ENSG00000135047	CTSL	cathepsin L [Source:HGNC Symbol;Acc:HGNC:2537]	protein coding	0.95	5.80	65.89	<0.0001	<0.0001
ENSG00000181381	DDX60L	DExD/H-box 60 like [Source:HGNC Symbol;Acc:HGNC:26429]	protein coding	0.95	7.23	63.84	<0.0001	<0.0001
ENSG00000197880	MDS2	myelodysplastic syndrome 2 translocation associated [Source:HGNC Symbol;Acc:HGNC:29633]	lncRNA	-0.95	0.33	8.04	0.0046	0.0119
ENSG00000151882	CCL28	C-C motif chemokine ligand 28 [Source:HGNC Symbol;Acc:HGNC:17700]	protein coding	-0.95	0.92	15.63	<0.0001	0.0004
ENSG00000272941		novel transcript, antisense to C7orf49	lncRNA	0.95	0.30	17.89	<0.0001	0.0001
ENSG00000102524	TNFSF13B	TNF superfamily member 13b [Source:HGNC Symbol;Acc:HGNC:11929]	protein coding	0.95	7.42	69.52	<0.0001	<0.0001
ENSG00000114127	XRN1	5'-3' exoribonuclease 1 [Source:HGNC Symbol;Acc:HGNC:30654]	protein coding	0.95	6.40	48.87	<0.0001	<0.0001
ENSG00000027075	PRKCH	protein kinase C eta [Source:HGNC Symbol;Acc:HGNC:9403]	protein coding	-0.95	5.64	23.24	<0.0001	<0.0001
ENSG00000166002	SMCO4	single-pass membrane protein with coiled-coil domains 4 [Source:HGNC Symbol;Acc:HGNC:24810]	protein coding	0.95	6.03	66.10	<0.0001	<0.0001
ENSG00000132274	TRIM22	tripartite motif containing 22 [Source:HGNC Symbol;Acc:HGNC:16379]	protein coding	0.95	8.79	64.84	<0.0001	<0.0001
ENSG00000238241	CCR12P	C-C motif chemokine receptor 12, pseudogene [Source:HGNC Symbol;Acc:HGNC:39812]	transcribed unprocessed pseudogene	-0.95	1.30	10.36	0.0013	0.004
ENSG00000160932	LY6E	lymphocyte antigen 6 family member E [Source:HGNC Symbol;Acc:HGNC:6727]	protein coding	0.95	8.47	82.37	<0.0001	<0.0001
ENSG00000169313	P2RY12	purinergic receptor P2Y12 [Source:HGNC Symbol;Acc:HGNC:18124]	protein coding	0.94	2.06	24.28	<0.0001	<0.0001
ENSG00000230724	LINC01001	long intergenic non-protein coding RNA 1001 [Source:HGNC Symbol;Acc:HGNC:38540]	lncRNA	0.94	1.68	35.74	<0.0001	<0.0001
ENSG00000116771	AGMAT	agmatinase [Source:HGNC Symbol;Acc:HGNC:18407]	protein coding	-0.94	1.11	16.42	<0.0001	0.0003

Table S12: Genes differentially expressed at Day 30 compared to pre-second vaccination (Study Arm 2 A/H7N9+AS03, D1, 29, Dendritic cells). Genes are sorted by descending absolute log_2 fold change. Gene model summaries and annotations are based on Ensembl Version 99 (continued.)

Ensembl Gene ID	Ensembl Gene Name	Ensembl Gene Description	Gene Type	log_2 Fold Change (Day 30 vs. Pre-Second Vaccination)	Average log_2 CPM	Likelihood Ratio Test Statistic	P-Value	FDR Adjusted P-Value
ENSG00000271964		novel transcript, antisense to RFTN1	lncRNA	-0.94	0.53	24.72	<0.0001	<0.0001
ENSG00000114107	CEP70	centrosomal protein 70 [Source:HGNC Symbol;Acc:HGNC:29972]	protein coding	-0.94	0.07	12.80	0.0003	0.0013
ENSG00000140545	MFGE8	milk fat globule-EGF factor 8 protein [Source:HGNC Symbol;Acc:HGNC:7036]	protein coding	-0.94	3.03	21.58	<0.0001	<0.0001
ENSG00000011590	ZBTB32	zinc finger and BTB domain containing 32 [Source:HGNC Symbol;Acc:HGNC:16763]	protein coding	-0.94	0.38	21.12	<0.0001	<0.0001
ENSG00000176293	ZNF135	zinc finger protein 135 [Source:HGNC Symbol;Acc:HGNC:12919]	protein coding	-0.94	0.35	27.48	<0.0001	<0.0001
ENSG00000179331	RAB39A	RAB39A, member RAS oncogene family [Source:HGNC Symbol;Acc:HGNC:16521]	protein coding	0.94	3.00	48.89	<0.0001	<0.0001
ENSG00000089041	P2RX7	purinergic receptor P2X 7 [Source:HGNC Symbol;Acc:HGNC:8537]	protein coding	0.94	6.23	64.05	<0.0001	<0.0001
ENSG00000157570	TSPAN18	tetraspanin 18 [Source:HGNC Symbol;Acc:HGNC:20660]	protein coding	-0.94	2.24	11.73	0.0006	0.0022
ENSG00000161643	SIGLEC16	sialic acid binding Ig like lectin 16 (gene/pseudogene) [Source:HGNC Symbol;Acc:HGNC:24851]	transcribed unprocessed pseudogene	0.94	4.09	42.86	<0.0001	<0.0001
ENSG00000118922	KLF12	Kruppel like factor 12 [Source:HGNC Symbol;Acc:HGNC:6346]	protein coding	-0.94	5.05	39.11	<0.0001	<0.0001
ENSG00000152990	ADGRA3	adhesion G protein-coupled receptor A3 [Source:HGNC Symbol;Acc:HGNC:13839]	protein coding	-0.94	0.72	24.79	<0.0001	<0.0001
ENSG00000229852		novel transcript, antisense to KHDC1	lncRNA	-0.94	0.09	12.76	0.0004	0.0013
ENSG00000287576		novel transcript	lncRNA	0.94	1.00	29.64	<0.0001	<0.0001
ENSG00000123609	NMI	N-myc and STAT interactor [Source:HGNC Symbol;Acc:HGNC:7854]	protein coding	0.93	6.35	64.03	<0.0001	<0.0001
ENSG00000140932	CMTM2	CKLF like MARVEL transmembrane domain containing 2 [Source:HGNC Symbol;Acc:HGNC:19173]	protein coding	-0.93	0.93	22.37	<0.0001	<0.0001
ENSG00000146021	KLHL3	kelch like family member 3 [Source:HGNC Symbol;Acc:HGNC:6354]	protein coding	-0.93	2.25	25.64	<0.0001	<0.0001
ENSG00000230587	LINC02580	long intergenic non-protein coding RNA 2580 [Source:HGNC Symbol;Acc:HGNC:53751]	lncRNA	-0.93	0.49	22.81	<0.0001	<0.0001
ENSG00000226950	DANCR	differentiation antagonizing non-protein coding RNA [Source:HGNC Symbol;Acc:HGNC:28964]	lncRNA	-0.93	3.10	55.59	<0.0001	<0.0001

Table S12: Genes differentially expressed at Day 30 compared to pre-second vaccination (Study Arm 2 A/H7N9+AS03, D1, 29, Dendritic cells). Genes are sorted by descending absolute log_2 fold change. Gene model summaries and annotations are based on Ensembl Version 99 (continued.)

Ensembl Gene ID	Ensembl Gene Name	Ensembl Gene Description	Gene Type	log_2 Fold Change (Day 30 vs. Pre-Second Vaccination)	Average log_2 CPM	Likelihood Ratio Test Statistic	P-Value	FDR Adjusted P-Value
ENSG00000224650	IGHV3-74	immunoglobulin heavy variable 3-74 [Source:HGNC Symbol;Acc:HGNC:5624]	IG V gene	-0.93	0.90	12.46	0.0004	0.0015
ENSG00000100351	GRAP2	GRB2 related adaptor protein 2 [Source:HGNC Symbol;Acc:HGNC:4563]	protein coding	-0.93	4.28	13.87	0.0002	0.0008
ENSG00000109452	INPP4B	inositol polyphosphate-4-phosphatase type II B [Source:HGNC Symbol;Acc:HGNC:6075]	protein coding	-0.93	3.11	10.32	0.0013	0.0041
ENSG00000111912	NCOA7	nuclear receptor coactivator 7 [Source:HGNC Symbol;Acc:HGNC:21081]	protein coding	0.93	5.47	34.86	<0.0001	<0.0001
ENSG00000173372	C1QA	complement C1q A chain [Source:HGNC Symbol;Acc:HGNC:1241]	protein coding	0.93	5.91	87.31	<0.0001	<0.0001
ENSG00000170456	DENND5B	DENN domain containing 5B [Source:HGNC Symbol;Acc:HGNC:28338]	protein coding	-0.93	2.02	44.00	<0.0001	<0.0001
ENSG00000105499	PLA2G4C	phospholipase A2 group IVC [Source:HGNC Symbol;Acc:HGNC:9037]	protein coding	0.93	2.83	51.66	<0.0001	<0.0001
ENSG00000103528	SYT17	synaptotagmin 17 [Source:HGNC Symbol;Acc:HGNC:24119]	protein coding	-0.93	2.63	27.64	<0.0001	<0.0001
ENSG00000267534	S1PR2	sphingosine-1-phosphate receptor 2 [Source:HGNC Symbol;Acc:HGNC:3169]	protein coding	0.93	1.83	42.01	<0.0001	<0.0001
ENSG00000110448	CD5	CD5 molecule [Source:HGNC Symbol;Acc:HGNC:1685]	protein coding	-0.92	4.92	14.60	0.0001	0.0006
ENSG00000272529		novel transcript, antisense to RFTN1	lncRNA	-0.92	0.22	16.35	<0.0001	0.0003
ENSG00000159445	THEM4	thioesterase superfamily member 4 [Source:HGNC Symbol;Acc:HGNC:17947]	protein coding	-0.92	3.88	31.77	<0.0001	<0.0001
ENSG00000196781	TLE1	TLE family member 1, transcriptional corepressor [Source:HGNC Symbol;Acc:HGNC:11837]	protein coding	-0.92	3.45	38.77	<0.0001	<0.0001
ENSG00000107438	PDLIM1	PDZ and LIM domain 1 [Source:HGNC Symbol;Acc:HGNC:2067]	protein coding	-0.92	3.34	45.66	<0.0001	<0.0001
ENSG00000225792		novel transcript	lncRNA	0.92	1.85	27.88	<0.0001	<0.0001
ENSG00000170476	MZB1	marginal zone B and B1 cell specific protein [Source:HGNC Symbol;Acc:HGNC:30125]	protein coding	-0.92	3.28	21.60	<0.0001	<0.0001
ENSG00000117281	CD160	CD160 molecule [Source:HGNC Symbol;Acc:HGNC:17013]	protein coding	-0.92	3.06	21.02	<0.0001	<0.0001
ENSG00000159899	NPR2	natriuretic peptide receptor 2 [Source:HGNC Symbol;Acc:HGNC:7944]	protein coding	-0.92	0.56	22.71	<0.0001	<0.0001
ENSG00000119714	GPR68	G protein-coupled receptor 68 [Source:HGNC Symbol;Acc:HGNC:4519]	protein coding	-0.92	2.59	17.71	<0.0001	0.0001

Table S12: Genes differentially expressed at Day 30 compared to pre-second vaccination (Study Arm 2 A/H7N9+AS03, D1, 29, Dendritic cells). Genes are sorted by descending absolute log_2 fold change. Gene model summaries and annotations are based on Ensembl Version 99 (continued.)

Ensembl Gene ID	Ensembl Gene Name	Ensembl Gene Description	Gene Type	log_2 Fold Change (Day 30 vs. Pre-Second Vaccination)	Average log_2 CPM	Likelihood Ratio Test Statistic	P-Value	FDR Adjusted P-Value
ENSG00000144199	FAHD2B	fumarylacetoacetate hydrolase domain containing 2B [Source:HGNC Symbol;Acc:HGNC:25318]	protein coding	-0.92	0.44	17.81	<0.0001	0.0001
ENSG00000204267	TAP2	transporter 2, ATP binding cassette subfamily B member [Source:HGNC Symbol;Acc:HGNC:44]	protein coding	0.92	6.57	49.79	<0.0001	<0.0001
ENSG00000283696		novel transcript	lncRNA	0.92	0.62	22.30	<0.0001	<0.0001
ENSG00000151729	SLC25A4	solute carrier family 25 member 4 [Source:HGNC Symbol;Acc:HGNC:10990]	protein coding	-0.92	1.47	24.17	<0.0001	<0.0001
ENSG00000107719	PALD1	phosphatase domain containing paladin 1 [Source:HGNC Symbol;Acc:HGNC:23530]	protein coding	-0.92	2.05	22.94	<0.0001	<0.0001
ENSG00000179240	GVQW3	GVQW motif containing 3 [Source:HGNC Symbol;Acc:HGNC:51239]	protein coding	-0.92	1.96	29.37	<0.0001	<0.0001
ENSG00000136048	DRAM1	DNA damage regulated autophagy modulator 1 [Source:HGNC Symbol;Acc:HGNC:25645]	protein coding	0.92	5.23	51.20	<0.0001	<0.0001
ENSG00000137502	RAB30	RAB30, member RAS oncogene family [Source:HGNC Symbol;Acc:HGNC:9770]	protein coding	-0.92	2.68	20.26	<0.0001	<0.0001
ENSG00000172878	METAP1D	methionyl aminopeptidase type 1D, mitochondrial [Source:HGNC Symbol;Acc:HGNC:32583]	protein coding	-0.92	1.75	30.71	<0.0001	<0.0001
ENSG00000116031	CD207	CD207 molecule [Source:HGNC Symbol;Acc:HGNC:17935]	protein coding	-0.92	1.71	24.91	<0.0001	<0.0001
ENSG00000133313	CNDP2	carnosine dipeptidase 2 [Source:HGNC Symbol;Acc:HGNC:24437]	protein coding	0.92	8.13	40.61	<0.0001	<0.0001
ENSG00000109881	CCDC34	coiled-coil domain containing 34 [Source:HGNC Symbol;Acc:HGNC:25079]	protein coding	-0.92	0.23	17.48	<0.0001	0.0002
ENSG00000158555	GDPD5	glycerophosphodiester phosphodiesterase domain containing 5 [Source:HGNC Symbol;Acc:HGNC:28804]	protein coding	0.91	5.82	81.99	<0.0001	<0.0001
ENSG00000158470	B4GALT5	beta-1,4-galactosyltransferase 5 [Source:HGNC Symbol;Acc:HGNC:928]	protein coding	0.91	6.29	54.99	<0.0001	<0.0001
ENSG00000126243	LRFN3	leucine rich repeat and fibronectin type III domain containing 3 [Source:HGNC Symbol;Acc:HGNC:28370]	protein coding	-0.91	1.68	10.55	0.0012	0.0037
ENSG00000110811	P3H3	prolyl 3-hydroxylase 3 [Source:HGNC Symbol;Acc:HGNC:19318]	protein coding	-0.91	0.48	22.60	<0.0001	<0.0001
ENSG00000211959	IGHV4-39	immunoglobulin heavy variable 4-39 [Source:HGNC Symbol;Acc:HGNC:5651]	IG V gene	-0.91	1.06	14.79	0.0001	0.0005
ENSG00000136867	SLC31A2	solute carrier family 31 member 2 [Source:HGNC Symbol;Acc:HGNC:11017]	protein coding	0.91	4.63	48.35	<0.0001	<0.0001

Table S12: Genes differentially expressed at Day 30 compared to pre-second vaccination (Study Arm 2 A/H7N9+AS03, D1, 29, Dendritic cells). Genes are sorted by descending absolute log_2 fold change. Gene model summaries and annotations are based on Ensembl Version 99 (continued.)

Ensembl Gene ID	Ensembl Gene Name	Ensembl Gene Description	Gene Type	log_2 Fold Change (Day 30 vs. Pre-Second Vaccination)	Average log_2 CPM	Likelihood Ratio Test Statistic	P-Value	FDR Adjusted P-Value
ENSG00000175265	GOLGA8A	golgin A8 family member A [Source:HGNC Symbol;Acc:HGNC:31972]	protein coding	-0.91	4.52	28.58	<0.0001	<0.0001
ENSG00000196169	KIF19	kinesin family member 19 [Source:HGNC Symbol;Acc:HGNC:26735]	protein coding	-0.91	0.47	11.99	0.0005	0.0019
ENSG00000067141	NEO1	neogenin 1 [Source:HGNC Symbol;Acc:HGNC:7754]	protein coding	-0.91	1.57	13.95	0.0002	0.0008
ENSG00000198182	ZNF607	zinc finger protein 607 [Source:HGNC Symbol;Acc:HGNC:28192]	protein coding	-0.91	0.99	25.12	<0.0001	<0.0001
ENSG00000105088	OLFM2	olfactomedin 2 [Source:HGNC Symbol;Acc:HGNC:17189]	protein coding	-0.91	0.74	11.60	0.0007	0.0023
ENSG00000177732	SOX12	SRY-box transcription factor 12 [Source:HGNC Symbol;Acc:HGNC:11198]	protein coding	-0.91	3.43	49.03	<0.0001	<0.0001
ENSG00000168672	LRATD2	LRAT domain containing 2 [Source:HGNC Symbol;Acc:HGNC:24166]	protein coding	-0.91	1.78	14.42	0.0001	0.0006
ENSG00000171051	FPR1	formyl peptide receptor 1 [Source:HGNC Symbol;Acc:HGNC:3826]	protein coding	0.91	8.26	53.19	<0.0001	<0.0001
ENSG00000214402	LCNL1	lipocalin like 1 [Source:HGNC Symbol;Acc:HGNC:34436]	protein coding	-0.91	0.34	9.39	0.0022	0.0063
ENSG00000183486	MX2	MX dynamin like GTPase 2 [Source:HGNC Symbol;Acc:HGNC:7533]	protein coding	0.91	7.72	62.64	<0.0001	<0.0001
ENSG00000255026		novel transcript	lncRNA	-0.91	4.65	10.39	0.0013	0.004
ENSG00000286885		novel transcript	lncRNA	-0.91	-0.01	10.63	0.0011	0.0036
ENSG00000285906		Novel transcript, antisense to HRH1	lncRNA	0.91	1.19	33.77	<0.0001	<0.0001
ENSG00000174946	GPR171	G protein-coupled receptor 171 [Source:HGNC Symbol;Acc:HGNC:30057]	protein coding	-0.91	2.25	11.54	0.0007	0.0024
ENSG00000137098	SPAG8	sperm associated antigen 8 [Source:HGNC Symbol;Acc:HGNC:14105]	protein coding	-0.90	0.97	29.89	<0.0001	<0.0001
ENSG00000069020	MAST4	microtubule associated serine/threonine kinase family member 4 [Source:HGNC Symbol;Acc:HGNC:19037]	protein coding	-0.90	2.48	20.91	<0.0001	<0.0001
ENSG00000172575	RASGRP1	RAS guanyl releasing protein 1 [Source:HGNC Symbol;Acc:HGNC:9878]	protein coding	-0.90	4.51	12.07	0.0005	0.0018
ENSG00000026103	FAS	Fas cell surface death receptor [Source:HGNC Symbol;Acc:HGNC:11920]	protein coding	0.90	4.96	53.76	<0.0001	<0.0001
ENSG00000110660	SLC35F2	solute carrier family 35 member F2 [Source:HGNC Symbol;Acc:HGNC:23615]	protein coding	-0.90	1.89	55.21	<0.0001	<0.0001

Table S12: Genes differentially expressed at Day 30 compared to pre-second vaccination (Study Arm 2 A/H7N9+AS03, D1, 29, Dendritic cells). Genes are sorted by descending absolute log_2 fold change. Gene model summaries and annotations are based on Ensembl Version 99 (continued.)

Ensembl Gene ID	Ensembl Gene Name	Ensembl Gene Description	Gene Type	log_2 Fold Change (Day 30 vs. Pre-Second Vaccination)	Average log_2 CPM	Likelihood Ratio Test Statistic	P-Value	FDR Adjusted P-Value
ENSG00000148468	FAM171A1	family with sequence similarity 171 member A1 [Source:HGNC Symbol;Acc:HGNC:23522]	protein coding	-0.90	0.47	6.22	0.0126	0.0286
ENSG00000165702	GFI1B	growth factor independent 1B transcriptional repressor [Source:HGNC Symbol;Acc:HGNC:4238]	protein coding	-0.90	1.39	7.83	0.0051	0.0132
ENSG00000180479	ZNF571	zinc finger protein 571 [Source:HGNC Symbol;Acc:HGNC:25000]	protein coding	-0.90	0.64	23.74	<0.0001	<0.0001
ENSG00000128596	CCDC136	coiled-coil domain containing 136 [Source:HGNC Symbol;Acc:HGNC:22225]	protein coding	-0.90	1.67	16.45	<0.0001	0.0003
ENSG00000247774	PCED1B-AS1	PCED1B antisense RNA 1 [Source:HGNC Symbol;Acc:HGNC:44166]	lncRNA	-0.90	5.19	20.67	<0.0001	<0.0001
ENSG00000133056	PIK3C2B	phosphatidylinositol-4-phosphate 3-kinase catalytic subunit type 2 beta [Source:HGNC Symbol;Acc:HGNC:8972]	protein coding	-0.90	3.61	23.71	<0.0001	<0.0001
ENSG00000013016	EHD3	EH domain containing 3 [Source:HGNC Symbol;Acc:HGNC:3244]	protein coding	-0.90	2.91	36.24	<0.0001	<0.0001
ENSG00000211746	TRBV19	T cell receptor beta variable 19 [Source:HGNC Symbol;Acc:HGNC:12194]	TR V gene	-0.90	0.85	7.84	0.0051	0.0131
ENSG00000163220	S100A9	S100 calcium binding protein A9 [Source:HGNC Symbol;Acc:HGNC:10499]	protein coding	0.90	11.78	39.87	<0.0001	<0.0001
ENSG00000127951	FGL2	fibrinogen like 2 [Source:HGNC Symbol;Acc:HGNC:3696]	protein coding	0.90	10.74	50.03	<0.0001	<0.0001
ENSG00000075275	CELSR1	cadherin EGF LAG seven-pass G-type receptor 1 [Source:HGNC Symbol;Acc:HGNC:1850]	protein coding	-0.90	1.91	19.97	<0.0001	<0.0001
ENSG00000116711	PLA2G4A	phospholipase A2 group IVA [Source:HGNC Symbol;Acc:HGNC:9035]	protein coding	0.90	3.35	33.77	<0.0001	<0.0001
ENSG00000085449	WDFY1	WD repeat and FYVE domain containing 1 [Source:HGNC Symbol;Acc:HGNC:20451]	protein coding	0.90	5.99	64.81	<0.0001	<0.0001
ENSG00000180061	TMEM150B	transmembrane protein 150B [Source:HGNC Symbol;Acc:HGNC:34415]	protein coding	0.90	5.14	49.83	<0.0001	<0.0001
ENSG00000106992	AK1	adenylate kinase 1 [Source:HGNC Symbol;Acc:HGNC:361]	protein coding	-0.90	0.99	16.06	<0.0001	0.0003
ENSG00000121966	CXCR4	C-X-C motif chemokine receptor 4 [Source:HGNC Symbol;Acc:HGNC:2561]	protein coding	-0.90	7.04	32.46	<0.0001	<0.0001
ENSG00000197093	GAL3ST4	galactose-3-O-sulfotransferase 4 [Source:HGNC Symbol;Acc:HGNC:24145]	protein coding	-0.90	1.67	18.80	<0.0001	<0.0001
ENSG00000142920	AZIN2	antizyme inhibitor 2 [Source:HGNC Symbol;Acc:HGNC:29957]	protein coding	-0.90	1.00	17.35	<0.0001	0.0002

Table S12: Genes differentially expressed at Day 30 compared to pre-second vaccination (Study Arm 2 A/H7N9+AS03, D1, 29, Dendritic cells). Genes are sorted by descending absolute log_2 fold change. Gene model summaries and annotations are based on Ensembl Version 99 (continued.)

Ensembl Gene ID	Ensembl Gene Name	Ensembl Gene Description	Gene Type	log_2 Fold Change (Day 30 vs. Pre-Second Vaccination)	Average log_2 CPM	Likelihood Ratio Test Statistic	P-Value	FDR Adjusted P-Value
ENSG00000135426	TESPA1	thymocyte expressed, positive selection associated 1 [Source:HGNC Symbol;Acc:HGNC:29109]	protein coding	-0.90	4.40	17.49	<0.0001	0.0002
ENSG00000111678	C12orf57	chromosome 12 open reading frame 57 [Source:HGNC Symbol;Acc:HGNC:29521]	protein coding	-0.90	3.43	29.11	<0.0001	<0.0001
ENSG00000186854	TRABD2A	TraB domain containing 2A [Source:HGNC Symbol;Acc:HGNC:27013]	protein coding	-0.89	4.55	13.85	0.0002	0.0008
ENSG00000148175	STOM	stomatin [Source:HGNC Symbol;Acc:HGNC:3383]	protein coding	0.89	6.55	35.16	<0.0001	<0.0001
ENSG00000090339	ICAM1	intercellular adhesion molecule 1 [Source:HGNC Symbol;Acc:HGNC:5344]	protein coding	0.89	6.15	41.59	<0.0001	<0.0001
ENSG00000133460	SLC2A11	solute carrier family 2 member 11 [Source:HGNC Symbol;Acc:HGNC:14239]	protein coding	-0.89	2.92	36.78	<0.0001	<0.0001
ENSG00000255135		novel transcript	lncRNA	-0.89	0.33	26.53	<0.0001	<0.0001
ENSG00000171368	TPPP	tubulin polymerization promoting protein [Source:HGNC Symbol;Acc:HGNC:24164]	protein coding	-0.89	0.56	25.88	<0.0001	<0.0001
ENSG00000168785	TSPAN5	tetraspanin 5 [Source:HGNC Symbol;Acc:HGNC:17753]	protein coding	-0.89	1.85	14.33	0.0002	0.0007
ENSG00000279278		novel transcript	lncRNA	-0.89	1.92	29.09	<0.0001	<0.0001
ENSG00000121413	ZSCAN18	zinc finger and SCAN domain containing 18 [Source:HGNC Symbol;Acc:HGNC:21037]	protein coding	-0.89	3.19	36.40	<0.0001	<0.0001
ENSG00000163485	ADORA1	adenosine A1 receptor [Source:HGNC Symbol;Acc:HGNC:262]	protein coding	-0.89	1.22	16.66	<0.0001	0.0002
ENSG00000270605		novel transcript	lncRNA	-0.89	1.30	26.66	<0.0001	<0.0001
ENSG00000140092	FBLN5	fibulin 5 [Source:HGNC Symbol;Acc:HGNC:3602]	protein coding	-0.89	0.49	6.77	0.0093	0.0219
ENSG00000168421	RHOH	ras homolog family member H [Source:HGNC Symbol;Acc:HGNC:686]	protein coding	-0.89	3.98	14.87	0.0001	0.0005
ENSG00000197457	STMN3	stathmin 3 [Source:HGNC Symbol;Acc:HGNC:15926]	protein coding	-0.89	5.22	29.10	<0.0001	<0.0001
ENSG00000141040	ZNF287	zinc finger protein 287 [Source:HGNC Symbol;Acc:HGNC:13502]	protein coding	-0.89	1.51	22.46	<0.0001	<0.0001
ENSG00000092621	PHGDH	phosphoglycerate dehydrogenase [Source:HGNC Symbol;Acc:HGNC:8923]	protein coding	-0.89	0.74	14.57	0.0001	0.0006
ENSG00000137767	SQOR	sulfide quinone oxidoreductase [Source:HGNC Symbol;Acc:HGNC:20390]	protein coding	0.89	5.63	59.49	<0.0001	<0.0001

Table S12: Genes differentially expressed at Day 30 compared to pre-second vaccination (Study Arm 2 A/H7N9+AS03, D1, 29, Dendritic cells). Genes are sorted by descending absolute log_2 fold change. Gene model summaries and annotations are based on Ensembl Version 99 (continued.)

Ensembl Gene ID	Ensembl Gene Name	Ensembl Gene Description	Gene Type	log_2 Fold Change (Day 30 vs. Pre-Second Vaccination)	Average log_2 CPM	Likelihood Ratio Test Statistic	P-Value	FDR Adjusted P-Value
ENSG00000041357	PSMA4	proteasome 20S subunit alpha 4 [Source:HGNC Symbol;Acc:HGNC:9533]	protein coding	0.89	7.38	50.63	<0.0001	<0.0001
ENSG00000213073		calcium binding protein P22 (CHP) pseudogene	transcribed processed pseudogene	0.89	1.96	35.94	<0.0001	<0.0001
ENSG00000205045	SLFN12L	schlafen family member 12 like [Source:HGNC Symbol;Acc:HGNC:33920]	protein coding	-0.89	2.50	14.64	0.0001	0.0006
ENSG00000167549	CORO6	coronin 6 [Source:HGNC Symbol;Acc:HGNC:21356]	protein coding	-0.88	0.83	16.36	<0.0001	0.0003
ENSG00000167775	CD320	CD320 molecule [Source:HGNC Symbol;Acc:HGNC:16692]	protein coding	-0.88	3.16	18.13	<0.0001	0.0001
ENSG00000183019	MCEMP1	mast cell expressed membrane protein 1 [Source:HGNC Symbol;Acc:HGNC:27291]	protein coding	0.88	5.13	26.08	<0.0001	<0.0001
ENSG00000188404	SELL	selectin L [Source:HGNC Symbol;Acc:HGNC:10720]	protein coding	0.88	9.39	31.46	<0.0001	<0.0001
ENSG00000006607	FARP2	FERM, ARH/RhoGEF and pleckstrin domain protein 2 [Source:HGNC Symbol;Acc:HGNC:16460]	protein coding	0.88	4.53	35.26	<0.0001	<0.0001
ENSG00000166582	CENPV	centromere protein V [Source:HGNC Symbol;Acc:HGNC:29920]	protein coding	-0.88	2.22	32.55	<0.0001	<0.0001
ENSG00000275131		phosphodiesterase 4D interacting protein (PDE4DIP) pseudogene	transcribed unprocessed pseudogene	-0.88	3.11	46.85	<0.0001	<0.0001
ENSG00000173715	C11orf80	chromosome 11 open reading frame 80 [Source:HGNC Symbol;Acc:HGNC:26197]	protein coding	-0.88	1.20	30.11	<0.0001	<0.0001
ENSG00000124772	CPNE5	copine 5 [Source:HGNC Symbol;Acc:HGNC:2318]	protein coding	-0.88	2.97	22.07	<0.0001	<0.0001
ENSG00000112294	ALDH5A1	aldehyde dehydrogenase 5 family member A1 [Source:HGNC Symbol;Acc:HGNC:408]	protein coding	-0.88	1.89	11.53	0.0007	0.0024
ENSG00000227507	LTB	lymphotoxin beta [Source:HGNC Symbol;Acc:HGNC:6711]	protein coding	-0.88	6.94	26.94	<0.0001	<0.0001
ENSG00000072134	EPN2	epsin 2 [Source:HGNC Symbol;Acc:HGNC:18639]	protein coding	-0.88	0.55	24.34	<0.0001	<0.0001

Table S12: Genes differentially expressed at Day 30 compared to pre-second vaccination (Study Arm 2 A/H7N9+AS03, D1, 29, Dendritic cells). Genes are sorted by descending absolute log_2 fold change. Gene model summaries and annotations are based on Ensembl Version 99 (continued.)

Ensembl Gene ID	Ensembl Gene Name	Ensembl Gene Description	Gene Type	log_2 Fold Change (Day 30 vs. Pre-Second Vaccination)	Average log_2 CPM	Likelihood Ratio Test Statistic	P-Value	FDR Adjusted P-Value
ENSG00000054654	SYNE2	spectrin repeat containing nuclear envelope protein 2 [Source:HGNC Symbol;Acc:HGNC:17084]	protein coding	-0.88	6.10	12.99	0.0003	0.0012
ENSG00000213373	LINC00671	long intergenic non-protein coding RNA 671 [Source:HGNC Symbol;Acc:HGNC:44339]	lncRNA	-0.88	0.65	11.93	0.0006	0.002
ENSG00000226321	CROCC2	ciliary rootlet coiled-coil, rootletin family member 2 [Source:HGNC Symbol;Acc:HGNC:51677]	protein coding	-0.88	6.25	17.12	<0.0001	0.0002
ENSG00000198574	SH2D1B	SH2 domain containing 1B [Source:HGNC Symbol;Acc:HGNC:30416]	protein coding	-0.88	4.94	32.06	<0.0001	<0.0001
ENSG00000168813	ZNF507	zinc finger protein 507 [Source:HGNC Symbol;Acc:HGNC:23783]	protein coding	-0.88	3.10	21.11	<0.0001	<0.0001
ENSG00000221866	PLXNA4	plexin A4 [Source:HGNC Symbol;Acc:HGNC:9102]	protein coding	-0.88	0.43	6.42	0.0113	0.0259
ENSG00000142178	SIK1	salt inducible kinase 1 [Source:HGNC Symbol;Acc:HGNC:11142]	protein coding	0.88	2.42	22.53	<0.0001	<0.0001
ENSG00000130005	GAMT	guanidinoacetate N-methyltransferase [Source:HGNC Symbol;Acc:HGNC:4136]	protein coding	-0.88	1.48	23.33	<0.0001	<0.0001
ENSG00000079385	CEACAM1	CEA cell adhesion molecule 1 [Source:HGNC Symbol;Acc:HGNC:1814]	protein coding	0.88	1.43	21.98	<0.0001	<0.0001
ENSG00000138670	RASGEF1B	RasGEF domain family member 1B [Source:HGNC Symbol;Acc:HGNC:24881]	protein coding	0.88	5.39	27.09	<0.0001	<0.0001
ENSG00000254682		novel transcript	lncRNA	-0.88	1.38	22.43	<0.0001	<0.0001
ENSG00000169918	OTUD7A	OTU deubiquitinase 7A [Source:HGNC Symbol;Acc:HGNC:20718]	protein coding	-0.88	0.63	11.22	0.0008	0.0027
ENSG00000287839		novel transcript	lncRNA	0.88	3.64	32.43	<0.0001	<0.0001
ENSG00000105717	PBX4	PBX homeobox 4 [Source:HGNC Symbol;Acc:HGNC:13403]	protein coding	-0.88	1.77	13.50	0.0002	0.001
ENSG00000246084	LINC02325	long intergenic non-protein coding RNA 2325 [Source:HGNC Symbol;Acc:HGNC:53245]	lncRNA	-0.88	0.54	10.53	0.0012	0.0037
ENSG00000268041	ERFL	ETS repressor factor like [Source:HGNC Symbol;Acc:HGNC:53894]	protein coding	-0.88	1.49	16.98	<0.0001	0.0002
ENSG00000035720	STAP1	signal transducing adaptor family member 1 [Source:HGNC Symbol;Acc:HGNC:24133]	protein coding	-0.88	1.67	14.11	0.0002	0.0007
ENSG00000168386	FILIP1L	filamin A interacting protein 1 like [Source:HGNC Symbol;Acc:HGNC:24589]	protein coding	-0.88	1.23	20.14	<0.0001	<0.0001

Table S12: Genes differentially expressed at Day 30 compared to pre-second vaccination (Study Arm 2 A/H7N9+AS03, D1, 29, Dendritic cells). Genes are sorted by descending absolute log_2 fold change. Gene model summaries and annotations are based on Ensembl Version 99 (continued.)

Ensembl Gene ID	Ensembl Gene Name	Ensembl Gene Description	Gene Type	log_2 Fold Change (Day 30 vs. Pre-Second Vaccination)	Average log_2 CPM	Likelihood Ratio Test Statistic	P-Value	FDR Adjusted P-Value
ENSG00000234648		pseudogene similar to part of ribosomal protein L3 (RPL3)	processed pseudogene	-0.88	1.05	21.90	<0.0001	<0.0001
ENSG00000171631	P2RY6	pyrimidinergic receptor P2Y6 [Source:HGNC Symbol;Acc:HGNC:8543]	protein coding	0.88	3.03	26.96	<0.0001	<0.0001
ENSG00000253522	MIR3142HG	MIR3142 host gene [Source:HGNC Symbol;Acc:HGNC:51944]	lncRNA	-0.87	1.41	15.05	0.0001	0.0005
ENSG00000198467	TPM2	tropomyosin 2 [Source:HGNC Symbol;Acc:HGNC:12011]	protein coding	-0.87	3.33	29.63	<0.0001	<0.0001
ENSG00000133661	SFTPD	surfactant protein D [Source:HGNC Symbol;Acc:HGNC:10803]	protein coding	-0.87	2.04	26.15	<0.0001	<0.0001
ENSG00000225079	FTH1P22	ferritin heavy chain 1 pseudogene 22 [Source:HGNC Symbol;Acc:HGNC:37640]	processed pseudogene	-0.87	0.16	9.14	0.0025	0.0071
ENSG00000174326	SLC16A11	solute carrier family 16 member 11 [Source:HGNC Symbol;Acc:HGNC:23093]	protein coding	-0.87	0.12	11.99	0.0005	0.0019
ENSG00000164430	CGAS	cyclic GMP-AMP synthase [Source:HGNC Symbol;Acc:HGNC:21367]	protein coding	0.87	4.79	40.63	<0.0001	<0.0001
ENSG00000148680	HTR7	5-hydroxytryptamine receptor 7 [Source:HGNC Symbol;Acc:HGNC:5302]	protein coding	-0.87	2.23	25.39	<0.0001	<0.0001
ENSG00000196263	ZNF471	zinc finger protein 471 [Source:HGNC Symbol;Acc:HGNC:23226]	protein coding	-0.87	0.16	15.61	<0.0001	0.0004
ENSG00000204103	MAFB	MAF bZIP transcription factor B [Source:HGNC Symbol;Acc:HGNC:6408]	protein coding	0.87	8.07	53.66	<0.0001	<0.0001
ENSG00000115339	GALNT3	polypeptide N-acetylgalactosaminyltransferase 3 [Source:HGNC Symbol;Acc:HGNC:4125]	protein coding	0.87	4.02	38.40	<0.0001	<0.0001
ENSG00000182010	RTKN2	rhotekin 2 [Source:HGNC Symbol;Acc:HGNC:19364]	protein coding	-0.87	1.26	8.02	0.0046	0.012
ENSG00000181631	P2RY13	purinergic receptor P2Y13 [Source:HGNC Symbol;Acc:HGNC:4537]	protein coding	0.87	6.72	27.69	<0.0001	<0.0001
ENSG00000211943	IGHV3-15	immunoglobulin heavy variable 3-15 [Source:HGNC Symbol;Acc:HGNC:5582]	IG V gene	-0.87	1.23	17.30	<0.0001	0.0002

Table S12: Genes differentially expressed at Day 30 compared to pre-second vaccination (Study Arm 2 A/H7N9+AS03, D1, 29, Dendritic cells). Genes are sorted by descending absolute log_2 fold change. Gene model summaries and annotations are based on Ensembl Version 99 (continued.)

Ensembl Gene ID	Ensembl Gene Name	Ensembl Gene Description	Gene Type	log_2 Fold Change (Day 30 vs. Pre-Second Vaccination)	Average log_2 CPM	Likelihood Ratio Test Statistic	P-Value	FDR Adjusted P-Value
ENSG00000189136	UBE2Q2P1	ubiquitin conjugating enzyme E2 Q2 pseudogene 1 [Source:HGNC Symbol;Acc:HGNC:37439]	transcribed unprocessed pseudogene	-0.86	0.41	19.77	<0.0001	<0.0001
ENSG00000230415	LINC01786	long intergenic non-protein coding RNA 1786 [Source:HGNC Symbol;Acc:HGNC:52575]	lncRNA	-0.86	0.33	18.32	<0.0001	0.0001
ENSG00000211955	IGHV3-33	immunoglobulin heavy variable 3-33 [Source:HGNC Symbol;Acc:HGNC:5596]	IG V gene	-0.86	1.02	17.61	<0.0001	0.0002
ENSG00000160307	S100B	S100 calcium binding protein B [Source:HGNC Symbol;Acc:HGNC:10500]	protein coding	-0.86	1.37	11.45	0.0007	0.0025
ENSG00000175414	ARL10	ADP ribosylation factor like GTPase 10 [Source:HGNC Symbol;Acc:HGNC:22042]	protein coding	-0.86	2.38	18.99	<0.0001	<0.0001
ENSG00000197119	SLC25A29	solute carrier family 25 member 29 [Source:HGNC Symbol;Acc:HGNC:20116]	protein coding	-0.86	5.76	22.02	<0.0001	<0.0001
ENSG00000107338	SHB	SH2 domain containing adaptor protein B [Source:HGNC Symbol;Acc:HGNC:10838]	protein coding	-0.86	0.56	20.11	<0.0001	<0.0001
ENSG00000205413	SAMD9	sterile alpha motif domain containing 9 [Source:HGNC Symbol;Acc:HGNC:1348]	protein coding	0.86	6.32	38.43	<0.0001	<0.0001
ENSG00000099958	DERL3	derlin 3 [Source:HGNC Symbol;Acc:HGNC:14236]	protein coding	-0.86	0.78	13.85	0.0002	0.0008
ENSG00000100453	GZMB	granzyme B [Source:HGNC Symbol;Acc:HGNC:4709]	protein coding	-0.86	5.41	8.97	0.0027	0.0077
ENSG00000148926	ADM	adrenomedullin [Source:HGNC Symbol;Acc:HGNC:259]	protein coding	0.86	3.76	21.26	<0.0001	<0.0001
ENSG00000241679		novel transcript	lncRNA	-0.86	0.20	17.35	<0.0001	0.0002
ENSG00000170298	LGALS9B	galectin 9B [Source:HGNC Symbol;Acc:HGNC:24842]	protein coding	-0.86	0.54	17.48	<0.0001	0.0002
ENSG00000244720		5'-nucleotidase, cytosolic III (NT5C3) pseudogene	processed pseudogene	-0.86	0.31	10.39	0.0013	0.004
ENSG00000211699	TRGV3	T cell receptor gamma variable 3 [Source:HGNC Symbol;Acc:HGNC:12288]	TR V gene	-0.86	0.70	12.98	0.0003	0.0012
ENSG00000140104	CLBA1	clathrin binding box of aftiphilin containing 1 [Source:HGNC Symbol;Acc:HGNC:20126]	protein coding	-0.86	0.78	27.52	<0.0001	<0.0001

Table S12: Genes differentially expressed at Day 30 compared to pre-second vaccination (Study Arm 2 A/H7N9+AS03, D1, 29, Dendritic cells). Genes are sorted by descending absolute log_2 fold change. Gene model summaries and annotations are based on Ensembl Version 99 (continued.)

Ensembl Gene ID	Ensembl Gene Name	Ensembl Gene Description	Gene Type	log_2 Fold Change (Day 30 vs. Pre-Second Vaccination)	Average log_2 CPM	Likelihood Ratio Test Statistic	P-Value	FDR Adjusted P-Value
ENSG00000206530	CFAP44	cilia and flagella associated protein 44 [Source:HGNC Symbol;Acc:HGNC:25631]	protein coding	-0.86	2.24	30.56	<0.0001	<0.0001
ENSG00000174721	FGFBP3	fibroblast growth factor binding protein 3 [Source:HGNC Symbol;Acc:HGNC:23428]	protein coding	-0.86	1.54	33.30	<0.0001	<0.0001
ENSG00000107282	APBA1	amyloid beta precursor protein binding family A member 1 [Source:HGNC Symbol;Acc:HGNC:578]	protein coding	-0.86	2.06	27.30	<0.0001	<0.0001
ENSG00000152284	TCF7L1	transcription factor 7 like 1 [Source:HGNC Symbol;Acc:HGNC:11640]	protein coding	0.86	1.22	12.87	0.0003	0.0013
ENSG00000188483	IER5L	immediate early response 5 like [Source:HGNC Symbol;Acc:HGNC:23679]	protein coding	-0.86	4.72	17.88	<0.0001	0.0001
ENSG00000149260	CAPN5	calpain 5 [Source:HGNC Symbol;Acc:HGNC:1482]	protein coding	-0.86	2.21	25.39	<0.0001	<0.0001
ENSG00000184574	LPAR5	lysophosphatidic acid receptor 5 [Source:HGNC Symbol;Acc:HGNC:13307]	protein coding	-0.86	2.53	16.83	<0.0001	0.0002
ENSG00000145431	PDGFC	platelet derived growth factor C [Source:HGNC Symbol;Acc:HGNC:8801]	protein coding	-0.86	3.96	19.97	<0.0001	<0.0001
ENSG00000183508	TENT5C	terminal nucleotidyltransferase 5C [Source:HGNC Symbol;Acc:HGNC:24712]	protein coding	-0.86	3.08	17.30	<0.0001	0.0002
ENSG00000156869	FRRS1	ferric chelate reductase 1 [Source:HGNC Symbol;Acc:HGNC:27622]	protein coding	0.85	1.72	32.44	<0.0001	<0.0001
ENSG00000151773	CCDC122	coiled-coil domain containing 122 [Source:HGNC Symbol;Acc:HGNC:26478]	protein coding	-0.85	0.86	23.76	<0.0001	<0.0001
ENSG00000234338		endogenous retroviral family W, env(C7), member 2 (ERVWE2) pseudogene	processed pseudogene	-0.85	0.12	9.90	0.0017	0.005
ENSG00000143801	PSEN2	presenilin 2 [Source:HGNC Symbol;Acc:HGNC:9509]	protein coding	0.85	4.04	40.99	<0.0001	<0.0001
ENSG00000059804	SLC2A3	solute carrier family 2 member 3 [Source:HGNC Symbol;Acc:HGNC:11007]	protein coding	0.85	6.95	58.02	<0.0001	<0.0001
ENSG00000165617	DACT1	dishevelled binding antagonist of beta catenin 1 [Source:HGNC Symbol;Acc:HGNC:17748]	protein coding	-0.85	0.61	10.42	0.0012	0.0039
ENSG00000158715	SLC45A3	solute carrier family 45 member 3 [Source:HGNC Symbol;Acc:HGNC:8642]	protein coding	-0.85	3.15	13.59	0.0002	0.0009
ENSG00000165209	STRBP	spermatid perinuclear RNA binding protein [Source:HGNC Symbol;Acc:HGNC:16462]	protein coding	-0.85	3.33	42.07	<0.0001	<0.0001

Table S12: Genes differentially expressed at Day 30 compared to pre-second vaccination (Study Arm 2 A/H7N9+AS03, D1, 29, Dendritic cells). Genes are sorted by descending absolute log_2 fold change. Gene model summaries and annotations are based on Ensembl Version 99 (continued.)

Ensembl Gene ID	Ensembl Gene Name	Ensembl Gene Description	Gene Type	log_2 Fold Change (Day 30 vs. Pre-Second Vaccination)	Average log_2 CPM	Likelihood Ratio Test Statistic	P-Value	FDR Adjusted P-Value
ENSG00000234663	LINC01934	long intergenic non-protein coding RNA 1934 [Source:HGNC Symbol;Acc:HGNC:52757]	lncRNA	-0.85	0.73	11.75	0.0006	0.0021
ENSG00000089127	OAS1	2'-5'-oligoadenylate synthetase 1 [Source:HGNC Symbol;Acc:HGNC:8086]	protein coding	0.85	7.82	56.41	<0.0001	<0.0001
ENSG00000119333	WDR34	WD repeat domain 34 [Source:HGNC Symbol;Acc:HGNC:28296]	protein coding	-0.85	2.24	30.99	<0.0001	<0.0001
ENSG00000143452	HORMAD1	HORMA domain containing 1 [Source:HGNC Symbol;Acc:HGNC:25245]	protein coding	0.85	1.86	29.56	<0.0001	<0.0001
ENSG00000161714	PLCD3	phospholipase C delta 3 [Source:HGNC Symbol;Acc:HGNC:9061]	protein coding	-0.85	2.14	31.74	<0.0001	<0.0001
ENSG00000189007	ADAT2	adenosine deaminase tRNA specific 2 [Source:HGNC Symbol;Acc:HGNC:21172]	protein coding	-0.85	3.41	39.18	<0.0001	<0.0001
ENSG00000247708	STX18-AS1	STX18 antisense RNA 1 (head to head) [Source:HGNC Symbol;Acc:HGNC:48877]	lncRNA	-0.85	1.07	21.70	<0.0001	<0.0001
ENSG00000211668	IGLV2-11	immunoglobulin lambda variable 2-11 [Source:HGNC Symbol;Acc:HGNC:5887]	IG V gene	-0.85	1.60	9.51	0.002	0.006
ENSG00000133466	C1QTNF6	C1q and TNF related 6 [Source:HGNC Symbol;Acc:HGNC:14343]	protein coding	-0.85	2.60	28.61	<0.0001	<0.0001
ENSG00000196912	ANKRD36B	ankyrin repeat domain 36B [Source:HGNC Symbol;Acc:HGNC:29333]	protein coding	-0.84	1.71	25.47	<0.0001	<0.0001
ENSG00000238121	LINC00426	long intergenic non-protein coding RNA 426 [Source:HGNC Symbol;Acc:HGNC:42761]	lncRNA	-0.84	1.50	11.07	0.0009	0.0029
ENSG00000073331	ALPK1	alpha kinase 1 [Source:HGNC Symbol;Acc:HGNC:20917]	protein coding	0.84	5.39	49.05	<0.0001	<0.0001
ENSG00000182118	FAM89A	family with sequence similarity 89 member A [Source:HGNC Symbol;Acc:HGNC:25057]	protein coding	0.84	2.08	42.64	<0.0001	<0.0001
ENSG00000198885	ITPRIPL1	ITPRIP like 1 [Source:HGNC Symbol;Acc:HGNC:29371]	protein coding	-0.84	2.16	17.23	<0.0001	0.0002
ENSG00000151208	DLG5	discs large MAGUK scaffold protein 5 [Source:HGNC Symbol;Acc:HGNC:2904]	protein coding	-0.84	2.76	22.29	<0.0001	<0.0001
ENSG00000087258	GNAO1	G protein subunit alpha o1 [Source:HGNC Symbol;Acc:HGNC:4389]	protein coding	-0.84	0.18	10.48	0.0012	0.0038
ENSG00000275832	ARHGAP23	Rho GTPase activating protein 23 [Source:HGNC Symbol;Acc:HGNC:29293]	protein coding	0.84	0.87	23.12	<0.0001	<0.0001
ENSG00000186187	ZNRF1	zinc and ring finger 1 [Source:HGNC Symbol;Acc:HGNC:18452]	protein coding	-0.84	3.53	23.56	<0.0001	<0.0001

Confidential/Proprietary Information

Table S12: Genes differentially expressed at Day 30 compared to pre-second vaccination (Study Arm 2 A/H7N9+AS03, D1, 29, Dendritic cells). Genes are sorted by descending absolute log_2 fold change. Gene model summaries and annotations are based on Ensembl Version 99 (continued.)

Ensembl Gene ID	Ensembl Gene Name	Ensembl Gene Description	Gene Type	log_2 Fold Change (Day 30 vs. Pre-Second Vaccination)	Average log_2 CPM	Likelihood Ratio Test Statistic	P-Value	FDR Adjusted P-Value
ENSG00000132386	SERPINF1	serpin family F member 1 [Source:HGNC Symbol;Acc:HGNC:8824]	protein coding	-0.84	3.15	28.37	<0.0001	<0.0001
ENSG00000269834	ZNF528-AS1	ZNF528 antisense RNA 1 [Source:HGNC Symbol;Acc:HGNC:51305]	lncRNA	-0.84	1.15	35.96	<0.0001	<0.0001
ENSG00000137269	LRRC1	leucine rich repeat containing 1 [Source:HGNC Symbol;Acc:HGNC:14307]	protein coding	-0.84	0.72	17.55	<0.0001	0.0002
ENSG00000074660	SCARF1	scavenger receptor class F member 1 [Source:HGNC Symbol;Acc:HGNC:16820]	protein coding	0.84	5.64	50.90	<0.0001	<0.0001
ENSG00000099889	ARVCF	ARVCF delta catenin family member [Source:HGNC Symbol;Acc:HGNC:728]	protein coding	-0.84	3.05	27.44	<0.0001	<0.0001
ENSG00000258875		novel transcript, antisense to GPR68	lncRNA	-0.84	0.20	6.93	0.0085	0.0203
ENSG00000187210	GCNT1	glucosaminyl (N-acetyl) transferase 1 [Source:HGNC Symbol;Acc:HGNC:4203]	protein coding	0.84	5.06	44.97	<0.0001	<0.0001
ENSG00000179909	ZNF154	zinc finger protein 154 [Source:HGNC Symbol;Acc:HGNC:12939]	protein coding	-0.84	2.43	23.04	<0.0001	<0.0001
ENSG00000028116	VRK2	VRK serine/threonine kinase 2 [Source:HGNC Symbol;Acc:HGNC:12719]	protein coding	0.84	4.97	50.66	<0.0001	<0.0001
ENSG00000163221	S100A12	S100 calcium binding protein A12 [Source:HGNC Symbol;Acc:HGNC:10489]	protein coding	0.84	7.19	26.69	<0.0001	<0.0001
ENSG00000260296		novel transcript, overlapping N4BP2	lncRNA	-0.84	0.12	18.85	<0.0001	<0.0001
ENSG00000171130	ATP6V0E2	ATPase H+ transporting V0 subunit e2 [Source:HGNC Symbol;Acc:HGNC:21723]	protein coding	-0.84	3.98	28.88	<0.0001	<0.0001
ENSG00000257093	DENND11	DENN domain containing 11 [Source:HGNC Symbol;Acc:HGNC:29472]	protein coding	-0.83	5.49	35.57	<0.0001	<0.0001
ENSG00000165490	DDIAS	DNA damage induced apoptosis suppressor [Source:HGNC Symbol;Acc:HGNC:26351]	protein coding	0.83	3.15	40.23	<0.0001	<0.0001
ENSG00000130164	LDLR	low density lipoprotein receptor [Source:HGNC Symbol;Acc:HGNC:6547]	protein coding	0.83	5.14	24.93	<0.0001	<0.0001
ENSG00000088538	DOCK3	dedicator of cytokinesis 3 [Source:HGNC Symbol;Acc:HGNC:2989]	protein coding	-0.83	0.67	11.36	0.0008	0.0026
ENSG00000271856	LINC01215	long intergenic non-protein coding RNA 1215 [Source:HGNC Symbol;Acc:HGNC:49651]	lncRNA	-0.83	3.08	26.84	<0.0001	<0.0001
ENSG00000139318	DUSP6	dual specificity phosphatase 6 [Source:HGNC Symbol;Acc:HGNC:3072]	protein coding	0.83	8.52	29.58	<0.0001	<0.0001
ENSG00000088305	DNMT3B	DNA methyltransferase 3 beta [Source:HGNC Symbol;Acc:HGNC:2979]	protein coding	-0.83	0.27	17.95	<0.0001	0.0001

Table S12: Genes differentially expressed at Day 30 compared to pre-second vaccination (Study Arm 2 A/H7N9+AS03, D1, 29, Dendritic cells). Genes are sorted by descending absolute log_2 fold change. Gene model summaries and annotations are based on Ensembl Version 99 (continued.)

Ensembl Gene ID	Ensembl Gene Name	Ensembl Gene Description	Gene Type	log_2 Fold Change (Day 30 vs. Pre-Second Vaccination)	Average log_2 CPM	Likelihood Ratio Test Statistic	P-Value	FDR Adjusted P-Value
ENSG00000133315	MACROD1	mono-ADP ribosylhydrolase 1 [Source:HGNC Symbol;Acc:HGNC:29598]	protein coding	-0.83	1.56	27.71	<0.0001	<0.0001
ENSG00000005471	ABCB4	ATP binding cassette subfamily B member 4 [Source:HGNC Symbol;Acc:HGNC:45]	protein coding	-0.83	1.40	19.08	<0.0001	<0.0001
ENSG00000271959		novel transcript, antisense to MROH5	lncRNA	-0.83	1.40	12.26	0.0005	0.0017
ENSG00000267554		RAB32, member RAS oncogene family (RAB32) pseudogene	processed pseudogene	-0.83	1.62	16.09	<0.0001	0.0003
ENSG00000237440	ZNF737	zinc finger protein 737 [Source:HGNC Symbol;Acc:HGNC:32468]	protein coding	-0.83	2.46	18.50	<0.0001	0.0001
ENSG00000146070	PLA2G7	phospholipase A2 group VII [Source:HGNC Symbol;Acc:HGNC:9040]	protein coding	0.83	5.11	26.00	<0.0001	<0.0001
ENSG00000115159	GPD2	glycerol-3-phosphate dehydrogenase 2 [Source:HGNC Symbol;Acc:HGNC:4456]	protein coding	0.83	5.13	54.14	<0.0001	<0.0001
ENSG00000268903		novel pseudogene	processed pseudogene	0.83	2.95	32.08	<0.0001	<0.0001
ENSG00000088543	C3orf18	chromosome 3 open reading frame 18 [Source:HGNC Symbol;Acc:HGNC:24837]	protein coding	-0.83	2.19	23.25	<0.0001	<0.0001
ENSG00000111224	PARP11	poly(ADP-ribose) polymerase family member 11 [Source:HGNC Symbol;Acc:HGNC:1186]	protein coding	0.83	4.21	40.59	<0.0001	<0.0001
ENSG00000267254	ZNF790-AS1	ZNF790 antisense RNA 1 [Source:HGNC Symbol;Acc:HGNC:27617]	lncRNA	-0.83	1.09	28.93	<0.0001	<0.0001
ENSG00000211964	IGHV3-48	immunoglobulin heavy variable 3-48 [Source:HGNC Symbol;Acc:HGNC:5606]	IG V gene	-0.83	1.00	15.25	<0.0001	0.0004
ENSG00000154025	SLC5A10	solute carrier family 5 member 10 [Source:HGNC Symbol;Acc:HGNC:23155]	protein coding	-0.83	0.86	14.60	0.0001	0.0006
ENSG00000096433	ITPR3	inositol 1,4,5-trisphosphate receptor type 3 [Source:HGNC Symbol;Acc:HGNC:6182]	protein coding	-0.83	5.23	20.49	<0.0001	<0.0001
ENSG00000232931	LINC00342	long intergenic non-protein coding RNA 342 [Source:HGNC Symbol;Acc:HGNC:42470]	lncRNA	-0.82	3.95	19.13	<0.0001	<0.0001
ENSG00000111788		DEAD/H (Asp-Glu-Ala-Asp/His) box polypeptide like pseudogene	unprocessed pseudogene	-0.82	2.10	22.00	<0.0001	<0.0001
ENSG00000115414	FN1	fibronectin 1 [Source:HGNC Symbol;Acc:HGNC:3778]	protein coding	0.82	1.90	10.93	0.0009	0.0031

Table S12: Genes differentially expressed at Day 30 compared to pre-second vaccination (Study Arm 2 A/H7N9+AS03, D1, 29, Dendritic cells). Genes are sorted by descending absolute log_2 fold change. Gene model summaries and annotations are based on Ensembl Version 99 (continued.)

Ensembl Gene ID	Ensembl Gene Name	Ensembl Gene Description	Gene Type	log_2 Fold Change (Day 30 vs. Pre-Second Vaccination)	Average log_2 CPM	Likelihood Ratio Test Statistic	P-Value	FDR Adjusted P-Value
ENSG00000116191	RALGPS2	Ral GEF with PH domain and SH3 binding motif 2 [Source:HGNC Symbol;Acc:HGNC:30279]	protein coding	-0.82	4.34	24.12	<0.0001	<0.0001
ENSG00000107201	DDX58	DExD/H-box helicase 58 [Source:HGNC Symbol;Acc:HGNC:19102]	protein coding	0.82	5.75	30.79	<0.0001	<0.0001
ENSG00000275302	CCL4	C-C motif chemokine ligand 4 [Source:HGNC Symbol;Acc:HGNC:10630]	protein coding	-0.82	2.96	7.23	0.0072	0.0175
ENSG00000173894	CBX2	chromobox 2 [Source:HGNC Symbol;Acc:HGNC:1552]	protein coding	-0.82	0.64	18.17	<0.0001	0.0001
ENSG00000233392		novel transcript	lncRNA	-0.82	7.15	15.96	<0.0001	0.0003
ENSG00000231607	DLEU2	deleted in lymphocytic leukemia 2 [Source:HGNC Symbol;Acc:HGNC:13748]	lncRNA	0.82	4.25	37.63	<0.0001	<0.0001
ENSG00000088179	PTPN4	protein tyrosine phosphatase non-receptor type 4 [Source:HGNC Symbol;Acc:HGNC:9656]	protein coding	-0.82	4.73	22.34	<0.0001	<0.0001
ENSG00000272498		novel transcript, antisense to RFTN1	lncRNA	-0.82	0.11	9.06	0.0026	0.0074
ENSG00000168016	TRANK1	tetratricopeptide repeat and ankyrin repeat containing 1 [Source:HGNC Symbol;Acc:HGNC:29011]	protein coding	0.82	6.53	40.40	<0.0001	<0.0001
ENSG00000211897	IGHG3	immunoglobulin heavy constant gamma 3 (G3m marker) [Source:HGNC Symbol;Acc:HGNC:5527]	IG C gene	-0.82	3.57	9.25	0.0023	0.0067
ENSG00000163975	MELTF	melanotransferrin [Source:HGNC Symbol;Acc:HGNC:7037]	protein coding	-0.82	0.88	14.57	0.0001	0.0006
ENSG00000120833	SOCS2	suppressor of cytokine signaling 2 [Source:HGNC Symbol;Acc:HGNC:19382]	protein coding	-0.82	2.37	11.80	0.0006	0.0021
ENSG00000138821	SLC39A8	solute carrier family 39 member 8 [Source:HGNC Symbol;Acc:HGNC:20862]	protein coding	0.82	3.76	12.57	0.0004	0.0015
ENSG00000152782	PANK1	pantothenate kinase 1 [Source:HGNC Symbol;Acc:HGNC:8598]	protein coding	-0.82	0.58	25.30	<0.0001	<0.0001
ENSG00000237945	LINC00649	long intergenic non-protein coding RNA 649 [Source:HGNC Symbol;Acc:HGNC:44305]	lncRNA	-0.82	3.38	15.70	<0.0001	0.0004
ENSG00000117632	STMN1	stathmin 1 [Source:HGNC Symbol;Acc:HGNC:6510]	protein coding	-0.82	3.27	21.66	<0.0001	<0.0001
ENSG00000186918	ZNF395	zinc finger protein 395 [Source:HGNC Symbol;Acc:HGNC:18737]	protein coding	-0.82	4.63	65.66	<0.0001	<0.0001
ENSG00000109099	PMP22	peripheral myelin protein 22 [Source:HGNC Symbol;Acc:HGNC:9118]	protein coding	-0.82	1.02	11.48	0.0007	0.0024
ENSG00000199753	SNORD104	small nucleolar RNA, C/D box 104 [Source:HGNC Symbol;Acc:HGNC:32768]	snoRNA	-0.82	2.04	34.00	<0.0001	<0.0001

Table S12: Genes differentially expressed at Day 30 compared to pre-second vaccination (Study Arm 2 A/H7N9+AS03, D1, 29, Dendritic cells). Genes are sorted by descending absolute log_2 fold change. Gene model summaries and annotations are based on Ensembl Version 99 (continued.)

Ensembl Gene ID	Ensembl Gene Name	Ensembl Gene Description	Gene Type	log_2 Fold Change (Day 30 vs. Pre-Second Vaccination)	Average log_2 CPM	Likelihood Ratio Test Statistic	P-Value	FDR Adjusted P-Value
ENSG00000248008	NRAV	negative regulator of antiviral response [Source:HGNC Symbol;Acc:HGNC:48588]	lncRNA	-0.82	0.87	22.58	<0.0001	<0.0001
ENSG00000253368	TRNP1	TMF1 regulated nuclear protein 1 [Source:HGNC Symbol;Acc:HGNC:34348]	protein coding	-0.82	2.34	28.67	<0.0001	<0.0001
ENSG00000135976	ANKRD36	ankyrin repeat domain 36 [Source:HGNC Symbol;Acc:HGNC:24079]	protein coding	-0.82	2.89	22.06	<0.0001	<0.0001
ENSG00000162572	SCNN1D	sodium channel epithelial 1 delta subunit [Source:HGNC Symbol;Acc:HGNC:10601]	protein coding	-0.82	2.63	29.34	<0.0001	<0.0001
ENSG00000239951	IGKV3-20	immunoglobulin kappa variable 3-20 [Source:HGNC Symbol;Acc:HGNC:5817]	IG V gene	-0.82	3.04	17.75	<0.0001	0.0001
ENSG00000159713	TPPP3	tubulin polymerization promoting protein family member 3 [Source:HGNC Symbol;Acc:HGNC:24162]	protein coding	-0.82	5.30	38.47	<0.0001	<0.0001
ENSG00000132963	POMP	proteasome maturation protein [Source:HGNC Symbol;Acc:HGNC:20330]	protein coding	0.81	5.69	46.44	<0.0001	<0.0001
ENSG00000142794	NBPF3	NBPF member 3 [Source:HGNC Symbol;Acc:HGNC:25076]	protein coding	-0.81	2.36	19.66	<0.0001	<0.0001
ENSG00000162746	FCRLB	Fc receptor like B [Source:HGNC Symbol;Acc:HGNC:26431]	protein coding	-0.81	0.97	20.27	<0.0001	<0.0001
ENSG00000204264	PSMB8	proteasome 20S subunit beta 8 [Source:HGNC Symbol;Acc:HGNC:9545]	protein coding	0.81	7.92	53.70	<0.0001	<0.0001
ENSG00000105655	ISYNA1	inositol-3-phosphate synthase 1 [Source:HGNC Symbol;Acc:HGNC:29821]	protein coding	-0.81	3.00	23.78	<0.0001	<0.0001
ENSG00000272927		novel transcript, antisense to PDE6B	lncRNA	-0.81	0.70	19.22	<0.0001	<0.0001
ENSG00000139714	MORN3	MORN repeat containing 3 [Source:HGNC Symbol;Acc:HGNC:29807]	protein coding	-0.81	1.29	18.14	<0.0001	0.0001
ENSG00000104312	RIPK2	receptor interacting serine/threonine kinase 2 [Source:HGNC Symbol;Acc:HGNC:10020]	protein coding	0.81	5.39	46.07	<0.0001	<0.0001
ENSG00000259436		novel transcript, antisense to POU2F2	lncRNA	-0.81	3.33	29.09	<0.0001	<0.0001
ENSG00000130590	SAMD10	sterile alpha motif domain containing 10 [Source:HGNC Symbol;Acc:HGNC:16129]	protein coding	-0.81	2.73	21.30	<0.0001	<0.0001
ENSG00000135898	GPR55	G protein-coupled receptor 55 [Source:HGNC Symbol;Acc:HGNC:4511]	protein coding	-0.81	1.30	27.52	<0.0001	<0.0001
ENSG00000167261	DPEP2	dipeptidase 2 [Source:HGNC Symbol;Acc:HGNC:23028]	protein coding	-0.81	7.01	23.65	<0.0001	<0.0001
ENSG00000108641	B9D1	B9 domain containing 1 [Source:HGNC Symbol;Acc:HGNC:24123]	protein coding	-0.81	0.11	18.62	<0.0001	<0.0001

Table S12: Genes differentially expressed at Day 30 compared to pre-second vaccination (Study Arm 2 A/H7N9+AS03, D1, 29, Dendritic cells). Genes are sorted by descending absolute log_2 fold change. Gene model summaries and annotations are based on Ensembl Version 99 (continued.)

Ensembl Gene ID	Ensembl Gene Name	Ensembl Gene Description	Gene Type	log_2 Fold Change (Day 30 vs. Pre-Second Vaccination)	Average log_2 CPM	Likelihood Ratio Test Statistic	P-Value	FDR Adjusted P-Value
ENSG00000099864	PALM	paralemmin [Source:HGNC Symbol;Acc:HGNC:8594]	protein coding	-0.81	1.07	14.49	0.0001	0.0006
ENSG00000214595	EML6	EMAP like 6 [Source:HGNC Symbol;Acc:HGNC:35412]	protein coding	-0.81	1.55	18.81	<0.0001	<0.0001
ENSG00000133739	LRRCC1	leucine rich repeat and coiled-coil centrosomal protein 1 [Source:HGNC Symbol;Acc:HGNC:29373]	protein coding	0.81	2.71	23.62	<0.0001	<0.0001
ENSG00000180113	TDRD6	tudor domain containing 6 [Source:HGNC Symbol;Acc:HGNC:21339]	protein coding	0.81	2.52	19.98	<0.0001	<0.0001
ENSG00000205220	PSMB10	proteasome 20S subunit beta 10 [Source:HGNC Symbol;Acc:HGNC:9538]	protein coding	0.81	7.00	43.81	<0.0001	<0.0001
ENSG00000096092	TMEM14A	transmembrane protein 14A [Source:HGNC Symbol;Acc:HGNC:21076]	protein coding	-0.81	0.55	14.90	0.0001	0.0005
ENSG00000269981		novel pseudogene	processed pseudogene	0.81	2.96	26.70	<0.0001	<0.0001
ENSG00000183023	SLC8A1	solute carrier family 8 member A1 [Source:HGNC Symbol;Acc:HGNC:11068]	protein coding	0.81	6.35	49.97	<0.0001	<0.0001
ENSG00000243772	KIR2DL3	killer cell immunoglobulin like receptor, two Ig domains and long cytoplasmic tail 3 [Source:HGNC Symbol;Acc:HGNC:6331]	protein coding	-0.81	0.52	6.40	0.0114	0.0262
ENSG00000205930	C21orf62-AS1	C21orf62 antisense RNA 1 [Source:HGNC Symbol;Acc:HGNC:1290]	lncRNA	-0.81	0.96	20.96	<0.0001	<0.0001
ENSG00000105246	EBI3	Epstein-Barr virus induced 3 [Source:HGNC Symbol;Acc:HGNC:3129]	protein coding	0.81	0.59	15.89	<0.0001	0.0003
ENSG00000151150	ANK3	ankyrin 3 [Source:HGNC Symbol;Acc:HGNC:494]	protein coding	-0.81	1.68	10.55	0.0012	0.0037
ENSG00000100003	SEC14L2	SEC14 like lipid binding 2 [Source:HGNC Symbol;Acc:HGNC:10699]	protein coding	-0.80	0.20	10.10	0.0015	0.0045
ENSG00000171860	C3AR1	complement C3a receptor 1 [Source:HGNC Symbol;Acc:HGNC:1319]	protein coding	0.80	6.98	59.84	<0.0001	<0.0001
ENSG00000141854	MISP3	MISP family member 3 [Source:HGNC Symbol;Acc:HGNC:26963]	protein coding	-0.80	1.06	15.89	<0.0001	0.0003
ENSG00000130956	HABP4	hyaluronan binding protein 4 [Source:HGNC Symbol;Acc:HGNC:17062]	protein coding	-0.80	2.78	26.89	<0.0001	<0.0001
ENSG00000128203	ASPHD2	aspartate beta-hydroxylase domain containing 2 [Source:HGNC Symbol;Acc:HGNC:30437]	protein coding	0.80	4.85	42.34	<0.0001	<0.0001

Table S12: Genes differentially expressed at Day 30 compared to pre-second vaccination (Study Arm 2 A/H7N9+AS03, D1, 29, Dendritic cells). Genes are sorted by descending absolute log_2 fold change. Gene model summaries and annotations are based on Ensembl Version 99 (continued.)

Ensembl Gene ID	Ensembl Gene Name	Ensembl Gene Description	Gene Type	log_2 Fold Change (Day 30 vs. Pre-Second Vaccination)	Average log_2 CPM	Likelihood Ratio Test Statistic	P-Value	FDR Adjusted P-Value
ENSG00000174807	CD248	CD248 molecule [Source:HGNC Symbol;Acc:HGNC:18219]	protein coding	-0.80	0.96	6.39	0.0115	0.0263
ENSG00000159714	ZDHHC1	zinc finger DHHC-type containing 1 [Source:HGNC Symbol;Acc:HGNC:17916]	protein coding	-0.80	3.98	21.36	<0.0001	<0.0001
ENSG00000106415	GLCCI1	glucocorticoid induced 1 [Source:HGNC Symbol;Acc:HGNC:18713]	protein coding	-0.80	3.44	23.68	<0.0001	<0.0001
ENSG00000059378	PARP12	poly(ADP-ribose) polymerase family member 12 [Source:HGNC Symbol;Acc:HGNC:21919]	protein coding	0.80	7.00	69.67	<0.0001	<0.0001
ENSG00000125869	LAMP5	lysosomal associated membrane protein family member 5 [Source:HGNC Symbol;Acc:HGNC:16097]	protein coding	0.80	0.89	12.78	0.0004	0.0013
ENSG00000072840	EVC	EvC ciliary complex subunit 1 [Source:HGNC Symbol;Acc:HGNC:3497]	protein coding	-0.80	0.77	24.75	<0.0001	<0.0001
ENSG00000069966	GNB5	G protein subunit beta 5 [Source:HGNC Symbol;Acc:HGNC:4401]	protein coding	-0.80	3.31	36.32	<0.0001	<0.0001
ENSG00000213923	CSNK1E	casein kinase 1 epsilon [Source:HGNC Symbol;Acc:HGNC:2453]	protein coding	-0.80	4.44	54.19	<0.0001	<0.0001
ENSG00000082196	C1QTNF3	C1q and TNF related 3 [Source:HGNC Symbol;Acc:HGNC:14326]	protein coding	-0.80	0.37	19.77	<0.0001	<0.0001
ENSG00000148848	ADAM12	ADAM metallopeptidase domain 12 [Source:HGNC Symbol;Acc:HGNC:190]	protein coding	-0.80	0.05	7.08	0.0078	0.0188
ENSG00000123219	CENPK	centromere protein K [Source:HGNC Symbol;Acc:HGNC:29479]	protein coding	-0.80	1.72	13.71	0.0002	0.0009
ENSG00000162694	EXTL2	exostosin like glycosyltransferase 2 [Source:HGNC Symbol;Acc:HGNC:3516]	protein coding	-0.80	1.22	20.94	<0.0001	<0.0001
ENSG00000169184	MN1	MN1 proto-oncogene, transcriptional regulator [Source:HGNC Symbol;Acc:HGNC:7180]	protein coding	-0.80	1.67	20.50	<0.0001	<0.0001
ENSG00000124243	BCAS4	breast carcinoma amplified sequence 4 [Source:HGNC Symbol;Acc:HGNC:14367]	protein coding	-0.80	2.77	23.89	<0.0001	<0.0001
ENSG00000184381	PLA2G6	phospholipase A2 group VI [Source:HGNC Symbol;Acc:HGNC:9039]	protein coding	-0.80	4.05	33.06	<0.0001	<0.0001
ENSG00000171604	CXXC5	CXXC finger protein 5 [Source:HGNC Symbol;Acc:HGNC:26943]	protein coding	-0.80	5.49	43.64	<0.0001	<0.0001
ENSG00000184925	LCN12	lipocalin 12 [Source:HGNC Symbol;Acc:HGNC:28733]	protein coding	-0.80	0.04	13.65	0.0002	0.0009
ENSG00000105131	EPHX3	epoxide hydrolase 3 [Source:HGNC Symbol;Acc:HGNC:23760]	protein coding	-0.79	0.07	8.88	0.0029	0.008

Confidential/Proprietary Information

Confidential/Proprietary Information

Table S12: Genes differentially expressed at Day 30 compared to pre-second vaccination (Study Arm 2 A/H7N9+AS03, D1, 29, Dendritic cells). Genes are sorted by descending absolute log_2 fold change. Gene model summaries and annotations are based on Ensembl Version 99 (continued.)

Ensembl Gene ID	Ensembl Gene Name	Ensembl Gene Description	Gene Type	log_2 Fold Change (Day 30 vs. Pre-Second Vaccination)	Average log_2 CPM	Likelihood Ratio Test Statistic	P-Value	FDR Adjusted P-Value
ENSG00000176222	ZNF404	zinc finger protein 404 [Source:HGNC Symbol;Acc:HGNC:19417]	protein coding	-0.79	0.61	20.16	<0.0001	<0.0001
ENSG00000163600	ICOS	inducible T cell costimulator [Source:HGNC Symbol;Acc:HGNC:5351]	protein coding	-0.79	1.56	6.42	0.0113	0.026
ENSG00000121807	CCR2	C-C motif chemokine receptor 2 [Source:HGNC Symbol;Acc:HGNC:1603]	protein coding	0.79	7.42	23.99	<0.0001	<0.0001
ENSG00000187583	PLEKHN1	pleckstrin homology domain containing N1 [Source:HGNC Symbol;Acc:HGNC:25284]	protein coding	0.79	1.34	20.46	<0.0001	<0.0001
ENSG00000156711	MAPK13	mitogen-activated protein kinase 13 [Source:HGNC Symbol;Acc:HGNC:6875]	protein coding	-0.79	4.24	35.14	<0.0001	<0.0001
ENSG00000114405	C3orf14	chromosome 3 open reading frame 14 [Source:HGNC Symbol;Acc:HGNC:25024]	protein coding	0.79	1.84	36.95	<0.0001	<0.0001
ENSG00000240445	FOXO3B	forkhead box O3B [Source:HGNC Symbol;Acc:HGNC:3822]	protein coding	-0.79	0.64	14.50	0.0001	0.0006
ENSG00000204650	LINC02210	long intergenic non-protein coding RNA 2210 [Source:HGNC Symbol;Acc:HGNC:26327]	transcribed unitary pseudogene	-0.79	2.58	20.28	<0.0001	<0.0001
ENSG00000120832	MTERF2	mitochondrial transcription termination factor 2 [Source:HGNC Symbol;Acc:HGNC:30779]	protein coding	-0.79	2.16	24.01	<0.0001	<0.0001
ENSG00000285492		uncharacterized LOC112267968 [Source:NCBI gene;Acc:112267968]	lncRNA	0.79	1.71	22.94	<0.0001	<0.0001
ENSG00000105854	PON2	paraoxonase 2 [Source:HGNC Symbol;Acc:HGNC:9205]	protein coding	-0.79	3.78	82.18	<0.0001	<0.0001
ENSG00000170293	CMTM8	CKLF like MARVEL transmembrane domain containing 8 [Source:HGNC Symbol;Acc:HGNC:19179]	protein coding	-0.79	0.15	17.39	<0.0001	0.0002
ENSG00000107611	CUBN	cubilin [Source:HGNC Symbol;Acc:HGNC:2548]	protein coding	-0.79	1.83	17.92	<0.0001	0.0001
ENSG00000146425	DYNLT1	dynein light chain Tctex-type 1 [Source:HGNC Symbol;Acc:HGNC:11697]	protein coding	0.79	6.18	63.98	<0.0001	<0.0001
ENSG00000237781	ADAMTSL4-AS2	ADAMTSL4 antisense RNA 2 [Source:HGNC Symbol;Acc:HGNC:40895]	lncRNA	0.79	2.04	21.45	<0.0001	<0.0001
ENSG00000090659	CD209	CD209 molecule [Source:HGNC Symbol;Acc:HGNC:1641]	protein coding	0.79	2.51	23.82	<0.0001	<0.0001
ENSG00000165731	RET	ret proto-oncogene [Source:HGNC Symbol;Acc:HGNC:9967]	protein coding	-0.79	1.69	11.15	0.0008	0.0028

Table S12: Genes differentially expressed at Day 30 compared to pre-second vaccination (Study Arm 2 A/H7N9+AS03, D1, 29, Dendritic cells). Genes are sorted by descending absolute log_2 fold change. Gene model summaries and annotations are based on Ensembl Version 99 (continued.)

Ensembl Gene ID	Ensembl Gene Name	Ensembl Gene Description	Gene Type	log_2 Fold Change (Day 30 vs. Pre-Second Vaccination)	Average log_2 CPM	Likelihood Ratio Test Statistic	P-Value	FDR Adjusted P-Value
ENSG00000266402	SNHG25	small nucleolar RNA host gene 25 [Source:HGNC Symbol;Acc:HGNC:51534]	lncRNA	-0.79	1.02	30.33	<0.0001	<0.0001
ENSG00000101400	SNTA1	syntrophin alpha 1 [Source:HGNC Symbol;Acc:HGNC:11167]	protein coding	-0.79	1.39	17.09	<0.0001	0.0002
ENSG00000163611	SPICE1	spindle and centriole associated protein 1 [Source:HGNC Symbol;Acc:HGNC:25083]	protein coding	-0.79	0.98	24.25	<0.0001	<0.0001
ENSG00000237940	LINC01238	long intergenic non-protein coding RNA 1238 [Source:HGNC Symbol;Acc:HGNC:49795]	lncRNA	-0.79	1.75	9.71	0.0018	0.0055
ENSG00000048462	TNFRSF17	TNF receptor superfamily member 17 [Source:HGNC Symbol;Acc:HGNC:11913]	protein coding	-0.79	0.68	10.74	0.001	0.0034
ENSG00000184678	H2BC21	H2B clustered histone 21 [Source:HGNC Symbol;Acc:HGNC:4760]	protein coding	0.79	4.05	21.77	<0.0001	<0.0001
ENSG00000188227	ZNF793	zinc finger protein 793 [Source:HGNC Symbol;Acc:HGNC:33115]	protein coding	-0.79	0.93	10.95	0.0009	0.0031
ENSG00000019102	VSIG2	V-set and immunoglobulin domain containing 2 [Source:HGNC Symbol;Acc:HGNC:17149]	protein coding	-0.79	2.20	16.09	<0.0001	0.0003
ENSG00000172183	ISG20	interferon stimulated exonuclease gene 20 [Source:HGNC Symbol;Acc:HGNC:6130]	protein coding	0.79	5.86	13.23	0.0003	0.0011
ENSG00000211895	IGHA1	immunoglobulin heavy constant alpha 1 [Source:HGNC Symbol;Acc:HGNC:5478]	IG C gene	-0.79	7.59	18.88	<0.0001	<0.0001
ENSG00000243406	MRPS31P5	mitochondrial ribosomal protein S31 pseudogene 5 [Source:HGNC Symbol;Acc:HGNC:39253]	transcribed unprocessed pseudogene	-0.79	0.76	15.68	<0.0001	0.0004
ENSG00000182173	TSEN54	tRNA splicing endonuclease subunit 54 [Source:HGNC Symbol;Acc:HGNC:27561]	protein coding	-0.79	4.96	32.55	<0.0001	<0.0001
ENSG00000115255	REEP6	receptor accessory protein 6 [Source:HGNC Symbol;Acc:HGNC:30078]	protein coding	-0.79	1.57	21.89	<0.0001	<0.0001
ENSG00000108106	UBE2S	ubiquitin conjugating enzyme E2 S [Source:HGNC Symbol;Acc:HGNC:17895]	protein coding	0.79	4.55	54.58	<0.0001	<0.0001
ENSG00000232442	MHENCR	melanoma highly expressed competing endogenous lncRNA for miR-425 and miR-489 [Source:HGNC Symbol;Acc:HGNC:53110]	lncRNA	-0.79	4.96	36.89	<0.0001	<0.0001
ENSG00000214279	SCART1	scavenger receptor family member expressed on T cells 1 [Source:HGNC Symbol;Acc:HGNC:32411]	protein coding	-0.78	4.02	24.49	<0.0001	<0.0001

Table S12: Genes differentially expressed at Day 30 compared to pre-second vaccination (Study Arm 2 A/H7N9+AS03, D1, 29, Dendritic cells). Genes are sorted by descending absolute log_2 fold change. Gene model summaries and annotations are based on Ensembl Version 99 (continued.)

Ensembl Gene ID	Ensembl Gene Name	Ensembl Gene Description	Gene Type	log_2 Fold Change (Day 30 vs. Pre-Second Vaccination)	Average log_2 CPM	Likelihood Ratio Test Statistic	P-Value	FDR Adjusted P-Value
ENSG00000109654	TRIM2	tripartite motif containing 2 [Source:HGNC Symbol;Acc:HGNC:15974]	protein coding	-0.78	0.90	26.30	<0.0001	<0.0001
ENSG00000146592	CREB5	cAMP responsive element binding protein 5 [Source:HGNC Symbol;Acc:HGNC:16844]	protein coding	0.78	6.19	59.86	<0.0001	<0.0001
ENSG00000226328	NUP50-DT	NUP50 divergent transcript [Source:HGNC Symbol;Acc:HGNC:50502]	lncRNA	0.78	4.57	44.65	<0.0001	<0.0001
ENSG00000134247	PTGFRN	prostaglandin F2 receptor inhibitor [Source:HGNC Symbol;Acc:HGNC:9601]	protein coding	-0.78	2.59	23.18	<0.0001	<0.0001
ENSG00000251867		novel transcript, antisense to ZBTB10	lncRNA	-0.78	0.46	24.40	<0.0001	<0.0001
ENSG00000163412	EIF4E3	eukaryotic translation initiation factor 4E family member 3 [Source:HGNC Symbol;Acc:HGNC:31837]	protein coding	0.78	5.99	59.93	<0.0001	<0.0001
ENSG00000211890	IGHA2	immunoglobulin heavy constant alpha 2 (A2m marker) [Source:HGNC Symbol;Acc:HGNC:5479]	IG C gene	-0.78	5.44	16.22	<0.0001	0.0003
ENSG00000231160	KLF3-AS1	KLF3 antisense RNA 1 [Source:HGNC Symbol;Acc:HGNC:25796]	lncRNA	-0.78	2.71	19.83	<0.0001	<0.0001
ENSG00000225975	LINC01534	long intergenic non-protein coding RNA 1534 [Source:HGNC Symbol;Acc:HGNC:51281]	lncRNA	-0.78	1.53	23.12	<0.0001	<0.0001
ENSG00000256223	ZNF10	zinc finger protein 10 [Source:HGNC Symbol;Acc:HGNC:12879]	protein coding	-0.78	1.26	15.55	<0.0001	0.0004
ENSG00000261798		novel transcript, antisense to BMP8B	lncRNA	-0.78	0.27	16.92	<0.0001	0.0002
ENSG00000166341	DCHS1	dachsous cadherin-related 1 [Source:HGNC Symbol;Acc:HGNC:13681]	protein coding	-0.78	3.25	29.13	<0.0001	<0.0001
ENSG00000069849	ATP1B3	ATPase Na+/K+ transporting subunit beta 3 [Source:HGNC Symbol;Acc:HGNC:806]	protein coding	0.78	6.80	47.05	<0.0001	<0.0001
ENSG00000100095	SEZ6L	seizure related 6 homolog like [Source:HGNC Symbol;Acc:HGNC:10763]	protein coding	-0.78	3.83	46.55	<0.0001	<0.0001
ENSG00000221988	PPT2	palmitoyl-protein thioesterase 2 [Source:HGNC Symbol;Acc:HGNC:9326]	protein coding	-0.78	0.78	18.67	<0.0001	<0.0001
ENSG00000117020	AKT3	AKT serine/threonine kinase 3 [Source:HGNC Symbol;Acc:HGNC:393]	protein coding	-0.78	3.93	45.65	<0.0001	<0.0001
ENSG00000160298	C21orf58	chromosome 21 open reading frame 58 [Source:HGNC Symbol;Acc:HGNC:1300]	protein coding	-0.78	1.69	19.97	<0.0001	<0.0001
ENSG00000143502	SUSD4	sushi domain containing 4 [Source:HGNC Symbol;Acc:HGNC:25470]	protein coding	-0.78	0.07	9.21	0.0024	0.0069
ENSG00000176533	GNG7	G protein subunit gamma 7 [Source:HGNC Symbol;Acc:HGNC:4410]	protein coding	-0.78	3.85	59.00	<0.0001	<0.0001

Table S12: Genes differentially expressed at Day 30 compared to pre-second vaccination (Study Arm 2 A/H7N9+AS03, D1, 29, Dendritic cells). Genes are sorted by descending absolute log_2 fold change. Gene model summaries and annotations are based on Ensembl Version 99 (continued.)

Ensembl Gene ID	Ensembl Gene Name	Ensembl Gene Description	Gene Type	log_2 Fold Change (Day 30 vs. Pre-Second Vaccination)	Average log_2 CPM	Likelihood Ratio Test Statistic	P-Value	FDR Adjusted P-Value
ENSG00000223534	HLA-DQB1-AS1	HLA-DQB1 antisense RNA 1 [Source:HGNC Symbol;Acc:HGNC:39762]	lncRNA	0.78	2.91	5.72	0.0167	0.0365
ENSG00000234630		novel transcript	lncRNA	-0.78	0.42	12.08	0.0005	0.0018
ENSG00000181035	SLC25A42	solute carrier family 25 member 42 [Source:HGNC Symbol;Acc:HGNC:28380]	protein coding	-0.78	3.67	25.95	<0.0001	<0.0001
ENSG00000129116	PALLD	palladin, cytoskeletal associated protein [Source:HGNC Symbol;Acc:HGNC:17068]	protein coding	-0.78	3.54	21.61	<0.0001	<0.0001
ENSG00000071575	TRIB2	tribbles pseudokinase 2 [Source:HGNC Symbol;Acc:HGNC:30809]	protein coding	-0.78	5.21	19.02	<0.0001	<0.0001
ENSG00000264522	OTUD7B	OTU deubiquitinase 7B [Source:HGNC Symbol;Acc:HGNC:16683]	protein coding	-0.78	1.49	12.23	0.0005	0.0017
ENSG00000215252	GOLGA8B	golgin A8 family member B [Source:HGNC Symbol;Acc:HGNC:31973]	protein coding	-0.78	4.40	13.95	0.0002	0.0008
ENSG00000211653	IGLV1-40	immunoglobulin lambda variable 1-40 [Source:HGNC Symbol;Acc:HGNC:5877]	IG V gene	-0.78	1.57	13.83	0.0002	0.0008
ENSG00000161298	ZNF382	zinc finger protein 382 [Source:HGNC Symbol;Acc:HGNC:17409]	protein coding	-0.78	1.23	18.84	<0.0001	<0.0001
ENSG00000186818	LILRB4	leukocyte immunoglobulin like receptor B4 [Source:HGNC Symbol;Acc:HGNC:6608]	protein coding	0.78	6.89	74.60	<0.0001	<0.0001
ENSG00000273148	LINC00653	long intergenic non-protein coding RNA 653 [Source:HGNC Symbol;Acc:HGNC:44308]	lncRNA	-0.77	1.12	27.29	<0.0001	<0.0001
ENSG00000234511	C5orf58	chromosome 5 open reading frame 58 [Source:HGNC Symbol;Acc:HGNC:37272]	protein coding	0.77	1.67	21.41	<0.0001	<0.0001
ENSG00000113494	PRLR	prolactin receptor [Source:HGNC Symbol;Acc:HGNC:9446]	protein coding	0.77	2.30	20.71	<0.0001	<0.0001
ENSG00000081985	IL12RB2	interleukin 12 receptor subunit beta 2 [Source:HGNC Symbol;Acc:HGNC:5972]	protein coding	-0.77	1.42	8.79	0.003	0.0084
ENSG00000211677	IGLC2	immunoglobulin lambda constant 2 [Source:HGNC Symbol;Acc:HGNC:5856]	IG C gene	-0.77	4.75	24.19	<0.0001	<0.0001
ENSG00000119227	PIGZ	phosphatidylinositol glycan anchor biosynthesis class Z [Source:HGNC Symbol;Acc:HGNC:30596]	protein coding	-0.77	1.83	26.22	<0.0001	<0.0001
ENSG00000236438	FAM157A	family with sequence similarity 157 member A [Source:HGNC Symbol;Acc:HGNC:34079]	transcribed unprocessed pseudogene	0.77	3.03	33.68	<0.0001	<0.0001

Table S12: Genes differentially expressed at Day 30 compared to pre-second vaccination (Study Arm 2 A/H7N9+AS03, D1, 29, Dendritic cells). Genes are sorted by descending absolute log_2 fold change. Gene model summaries and annotations are based on Ensembl Version 99 (continued.)

Ensembl Gene ID	Ensembl Gene Name	Ensembl Gene Description	Gene Type	log_2 Fold Change (Day 30 vs. Pre-Second Vaccination)	Average log_2 CPM	Likelihood Ratio Test Statistic	P-Value	FDR Adjusted P-Value
ENSG00000116299	KIAA1324	KIAA1324 [Source:HGNC Symbol;Acc:HGNC:29618]	protein coding	-0.77	1.21	10.10	0.0015	0.0045
ENSG00000278897		TEC	TEC	-0.77	0.55	16.65	<0.0001	0.0002
ENSG00000263366	ABHD17AP5	abhydrolase domain containing 17A pseudogene 5 [Source:HGNC Symbol;Acc:HGNC:34043]	processed pseudogene	-0.77	0.31	13.12	0.0003	0.0011
ENSG00000272668		novel transcript, antisense to VSIG8	lncRNA	-0.77	0.89	10.28	0.0013	0.0042
ENSG00000135148	TRAFD1	TRAF-type zinc finger domain containing 1 [Source:HGNC Symbol;Acc:HGNC:24808]	protein coding	0.77	6.83	40.82	<0.0001	<0.0001
ENSG00000197081	IGF2R	insulin like growth factor 2 receptor [Source:HGNC Symbol;Acc:HGNC:5467]	protein coding	0.77	8.43	58.80	<0.0001	<0.0001
ENSG00000124225	PMEPA1	prostate transmembrane protein, androgen induced 1 [Source:HGNC Symbol;Acc:HGNC:14107]	protein coding	-0.77	0.95	11.12	0.0009	0.0029
ENSG00000232677	LINC00665	long intergenic non-protein coding RNA 665 [Source:HGNC Symbol;Acc:HGNC:44323]	lncRNA	-0.77	1.58	25.42	<0.0001	<0.0001
ENSG00000112394	SLC16A10	solute carrier family 16 member 10 [Source:HGNC Symbol;Acc:HGNC:17027]	protein coding	-0.77	0.37	7.98	0.0047	0.0122
ENSG00000090661	CERS4	ceramide synthase 4 [Source:HGNC Symbol;Acc:HGNC:23747]	protein coding	-0.77	4.21	38.45	<0.0001	<0.0001
ENSG00000246430	LINC00968	long intergenic non-protein coding RNA 968 [Source:HGNC Symbol;Acc:HGNC:48727]	lncRNA	0.77	2.03	36.57	<0.0001	<0.0001
ENSG00000186235	LINC02610	long intergenic non-protein coding RNA 2610 [Source:HGNC Symbol;Acc:HGNC:27177]	lncRNA	-0.77	1.47	12.59	0.0004	0.0015
ENSG00000187741	FANCA	FA complementation group A [Source:HGNC Symbol;Acc:HGNC:3582]	protein coding	0.77	3.85	44.96	<0.0001	<0.0001
ENSG00000163701	IL17RE	interleukin 17 receptor E [Source:HGNC Symbol;Acc:HGNC:18439]	protein coding	-0.77	0.56	12.88	0.0003	0.0013
ENSG00000152475	ZNF837	zinc finger protein 837 [Source:HGNC Symbol;Acc:HGNC:25164]	protein coding	-0.77	1.39	29.87	<0.0001	<0.0001
ENSG00000180385	EMC3-AS1	EMC3 antisense RNA 1 [Source:HGNC Symbol;Acc:HGNC:49223]	transcribed unprocessed pseudogene	-0.77	0.22	17.48	<0.0001	0.0002
ENSG00000285938		BCL6 antisense 1	protein coding	0.77	2.70	27.07	<0.0001	<0.0001

Table S12: Genes differentially expressed at Day 30 compared to pre-second vaccination (Study Arm 2 A/H7N9+AS03, D1, 29, Dendritic cells). Genes are sorted by descending absolute log_2 fold change. Gene model summaries and annotations are based on Ensembl Version 99 (continued.)

Ensembl Gene ID	Ensembl Gene Name	Ensembl Gene Description	Gene Type	log_2 Fold Change (Day 30 vs. Pre-Second Vaccination)	Average log_2 CPM	Likelihood Ratio Test Statistic	P-Value	FDR Adjusted P-Value
ENSG00000125740	FOSB	FosB proto-oncogene, AP-1 transcription factor subunit [Source:HGNC Symbol;Acc:HGNC:3797]	protein coding	0.77	6.10	5.42	0.0199	0.0423
ENSG00000173611	SCAI	suppressor of cancer cell invasion [Source:HGNC Symbol;Acc:HGNC:26709]	protein coding	-0.77	3.13	32.15	<0.0001	<0.0001
ENSG00000005884	ITGA3	integrin subunit alpha 3 [Source:HGNC Symbol;Acc:HGNC:6139]	protein coding	-0.77	1.05	10.46	0.0012	0.0039
ENSG00000170955	CAVIN3	caveolae associated protein 3 [Source:HGNC Symbol;Acc:HGNC:9400]	protein coding	-0.77	0.76	12.57	0.0004	0.0015
ENSG00000184271	POU6F1	POU class 6 homeobox 1 [Source:HGNC Symbol;Acc:HGNC:9224]	protein coding	-0.77	3.09	24.83	<0.0001	<0.0001
ENSG00000197555	SIPA1L1	signal induced proliferation associated 1 like 1 [Source:HGNC Symbol;Acc:HGNC:20284]	protein coding	0.77	6.08	52.55	<0.0001	<0.0001
ENSG00000153896	ZNF599	zinc finger protein 599 [Source:HGNC Symbol;Acc:HGNC:26408]	protein coding	-0.77	1.13	28.19	<0.0001	<0.0001
ENSG00000069493	CLEC2D	C-type lectin domain family 2 member D [Source:HGNC Symbol;Acc:HGNC:14351]	protein coding	-0.77	5.55	30.25	<0.0001	<0.0001
ENSG00000111186	WNT5B	Wnt family member 5B [Source:HGNC Symbol;Acc:HGNC:16265]	protein coding	-0.77	1.70	8.40	0.0038	0.0101
ENSG00000267041	ZNF850	zinc finger protein 850 [Source:HGNC Symbol;Acc:HGNC:27994]	protein coding	-0.77	1.15	21.91	<0.0001	<0.0001
ENSG00000213557		ribosomal protein L31 (RPL31) pseudogene	processed pseudogene	-0.77	1.61	23.45	<0.0001	<0.0001
ENSG00000174684	B4GAT1	beta-1,4-glucuronyltransferase 1 [Source:HGNC Symbol;Acc:HGNC:15685]	protein coding	-0.76	1.67	24.05	<0.0001	<0.0001
ENSG00000283189		novel protein	protein coding	-0.76	0.44	23.36	<0.0001	<0.0001
ENSG00000160191	PDE9A	phosphodiesterase 9A [Source:HGNC Symbol;Acc:HGNC:8795]	protein coding	-0.76	0.24	10.18	0.0014	0.0044
ENSG00000177599	ZNF491	zinc finger protein 491 [Source:HGNC Symbol;Acc:HGNC:23706]	protein coding	-0.76	0.29	17.64	<0.0001	0.0001
ENSG00000244486	SCARF2	scavenger receptor class F member 2 [Source:HGNC Symbol;Acc:HGNC:19869]	protein coding	-0.76	1.40	15.08	0.0001	0.0005
ENSG00000184371	CSF1	colony stimulating factor 1 [Source:HGNC Symbol;Acc:HGNC:2432]	protein coding	0.76	1.45	6.13	0.0133	0.0299

Confidential/Proprietary Information

Table S12: Genes differentially expressed at Day 30 compared to pre-second vaccination (Study Arm 2 A/H7N9+AS03, D1, 29, Dendritic cells). Genes are sorted by descending absolute log_2 fold change. Gene model summaries and annotations are based on Ensembl Version 99 (continued.)

Ensembl Gene ID	Ensembl Gene Name	Ensembl Gene Description	Gene Type	log_2 Fold Change (Day 30 vs. Pre-Second Vaccination)	Average log_2 CPM	Likelihood Ratio Test Statistic	P-Value	FDR Adjusted P-Value
ENSG00000113070	HBEGF	heparin binding EGF like growth factor [Source:HGNC Symbol;Acc:HGNC:3059]	protein coding	0.76	3.67	9.06	0.0026	0.0074
ENSG00000198105	ZNF248	zinc finger protein 248 [Source:HGNC Symbol;Acc:HGNC:13041]	protein coding	-0.76	3.14	33.46	<0.0001	<0.0001
ENSG00000168758	SEMA4C	semaphorin 4C [Source:HGNC Symbol;Acc:HGNC:10731]	protein coding	-0.76	4.76	25.58	<0.0001	<0.0001
ENSG00000142583	SLC2A5	solute carrier family 2 member 5 [Source:HGNC Symbol;Acc:HGNC:11010]	protein coding	-0.76	1.01	20.08	<0.0001	<0.0001
ENSG00000013374	NUB1	negative regulator of ubiquitin like proteins 1 [Source:HGNC Symbol;Acc:HGNC:17623]	protein coding	0.76	6.84	40.01	<0.0001	<0.0001
ENSG00000181097		breast cancer estrogen-induced apoptosis 2 (BREA2)	lncRNA	-0.76	0.27	17.33	<0.0001	0.0002
ENSG00000165138	ANKS6	ankyrin repeat and sterile alpha motif domain containing 6 [Source:HGNC Symbol;Acc:HGNC:26724]	protein coding	-0.76	2.47	19.69	<0.0001	<0.0001
ENSG00000124201	ZNFX1	zinc finger NFX1-type containing 1 [Source:HGNC Symbol;Acc:HGNC:29271]	protein coding	0.76	6.73	51.15	<0.0001	<0.0001
ENSG00000276758		leukocyte immunoglobulin-like receptor, subfamily A member pseudogene	unprocessed pseudogene	0.76	2.22	20.97	<0.0001	<0.0001
ENSG00000161929	SCIMP	SLP adaptor and CSK interacting membrane protein [Source:HGNC Symbol;Acc:HGNC:33504]	protein coding	0.76	7.59	51.29	<0.0001	<0.0001
ENSG00000188343	FAM92A	family with sequence similarity 92 member A [Source:HGNC Symbol;Acc:HGNC:30452]	protein coding	0.76	1.98	14.77	0.0001	0.0005
ENSG00000211648	IGLV1-47	immunoglobulin lambda variable 1-47 [Source:HGNC Symbol;Acc:HGNC:5880]	IG V gene	-0.76	0.55	5.61	0.0179	0.0387
ENSG00000074181	NOTCH3	notch receptor 3 [Source:HGNC Symbol;Acc:HGNC:7883]	protein coding	-0.76	0.81	6.75	0.0094	0.0221
ENSG00000132199	ENOSF1	enolase superfamily member 1 [Source:HGNC Symbol;Acc:HGNC:30365]	protein coding	-0.76	4.03	26.67	<0.0001	<0.0001
ENSG00000173200	PARP15	poly(ADP-ribose) polymerase family member 15 [Source:HGNC Symbol;Acc:HGNC:26876]	protein coding	-0.76	5.11	20.36	<0.0001	<0.0001
ENSG00000203814	H2BC18	H2B clustered histone 18 [Source:HGNC Symbol;Acc:HGNC:24700]	protein coding	0.76	0.19	10.34	0.0013	0.0041
ENSG00000237638	LINC02245	long intergenic non-protein coding RNA 2245 [Source:HGNC Symbol;Acc:HGNC:53134]	lncRNA	-0.76	2.58	25.17	<0.0001	<0.0001
ENSG00000086061	DNAJA1	DnaJ heat shock protein family (Hsp40) member A1 [Source:HGNC Symbol;Acc:HGNC:5229]	protein coding	0.76	7.34	37.20	<0.0001	<0.0001

Table S12: Genes differentially expressed at Day 30 compared to pre-second vaccination (Study Arm 2 A/H7N9+AS03, D1, 29, Dendritic cells). Genes are sorted by descending absolute log_2 fold change. Gene model summaries and annotations are based on Ensembl Version 99 (continued.)

Ensembl Gene ID	Ensembl Gene Name	Ensembl Gene Description	Gene Type	log_2 Fold Change (Day 30 vs. Pre-Second Vaccination)	Average log_2 CPM	Likelihood Ratio Test Statistic	P-Value	FDR Adjusted P-Value
ENSG00000023902	PLEKHO1	pleckstrin homology domain containing O1 [Source:HGNC Symbol;Acc:HGNC:24310]	protein coding	0.76	9.01	41.08	<0.0001	<0.0001
ENSG00000171236	LRG1	leucine rich alpha-2-glycoprotein 1 [Source:HGNC Symbol;Acc:HGNC:29480]	protein coding	0.76	2.95	16.30	<0.0001	0.0003
ENSG00000281005	LINC00921	long intergenic non-protein coding RNA 921 [Source:HGNC Symbol;Acc:HGNC:26830]	lncRNA	-0.76	3.50	43.68	<0.0001	<0.0001
ENSG00000105270	CLIP3	CAP-Gly domain containing linker protein 3 [Source:HGNC Symbol;Acc:HGNC:24314]	protein coding	-0.76	0.45	20.34	<0.0001	<0.0001
ENSG00000282572		novel transcript similar to family member with sequence similarity 157	lncRNA	0.76	3.45	24.92	<0.0001	<0.0001
ENSG00000142494	SLC47A1	solute carrier family 47 member 1 [Source:HGNC Symbol;Acc:HGNC:25588]	protein coding	0.76	1.53	14.11	0.0002	0.0007
ENSG00000135469	COQ10A	coenzyme Q10A [Source:HGNC Symbol;Acc:HGNC:26515]	protein coding	-0.76	2.66	31.38	<0.0001	<0.0001
ENSG00000162004	CCDC78	coiled-coil domain containing 78 [Source:HGNC Symbol;Acc:HGNC:14153]	protein coding	-0.76	2.04	16.93	<0.0001	0.0002
ENSG00000176531	PHLDB3	pleckstrin homology like domain family B member 3 [Source:HGNC Symbol;Acc:HGNC:30499]	protein coding	-0.75	3.11	43.58	<0.0001	<0.0001
ENSG00000052126	PLEKHA5	pleckstrin homology domain containing A5 [Source:HGNC Symbol;Acc:HGNC:30036]	protein coding	-0.75	1.76	23.78	<0.0001	<0.0001
ENSG00000119681	LTBP2	latent transforming growth factor beta binding protein 2 [Source:HGNC Symbol;Acc:HGNC:6715]	protein coding	-0.75	0.98	23.04	<0.0001	<0.0001
ENSG00000087253	LPCAT2	lysophosphatidylcholine acyltransferase 2 [Source:HGNC Symbol;Acc:HGNC:26032]	protein coding	0.75	6.74	48.55	<0.0001	<0.0001
ENSG00000119401	TRIM32	tripartite motif containing 32 [Source:HGNC Symbol;Acc:HGNC:16380]	protein coding	-0.75	1.98	13.59	0.0002	0.0009
ENSG00000278195	SSTR3	somatostatin receptor 3 [Source:HGNC Symbol;Acc:HGNC:11332]	protein coding	-0.75	1.11	5.76	0.0164	0.0358
ENSG00000130653	PNPLA7	patatin like phospholipase domain containing 7 [Source:HGNC Symbol;Acc:HGNC:24768]	protein coding	-0.75	3.53	24.00	<0.0001	<0.0001
ENSG00000188042	ARL4C	ADP ribosylation factor like GTPase 4C [Source:HGNC Symbol;Acc:HGNC:698]	protein coding	-0.75	7.42	18.69	<0.0001	<0.0001
ENSG00000082397	EPB41L3	erythrocyte membrane protein band 4.1 like 3 [Source:HGNC Symbol;Acc:HGNC:3380]	protein coding	0.75	7.27	49.93	<0.0001	<0.0001
ENSG00000050405	LIMA1	LIM domain and actin binding 1 [Source:HGNC Symbol;Acc:HGNC:24636]	protein coding	-0.75	2.73	27.71	<0.0001	<0.0001

Table S12: Genes differentially expressed at Day 30 compared to pre-second vaccination (Study Arm 2 A/H7N9+AS03, D1, 29, Dendritic cells). Genes are sorted by descending absolute log_2 fold change. Gene model summaries and annotations are based on Ensembl Version 99 (continued.)

Ensembl Gene ID	Ensembl Gene Name	Ensembl Gene Description	Gene Type	log_2 Fold Change (Day 30 vs. Pre-Second Vaccination)	Average log_2 CPM	Likelihood Ratio Test Statistic	P-Value	FDR Adjusted P-Value
ENSG00000204186	ZDBF2	zinc finger DBF-type containing 2 [Source:HGNC Symbol;Acc:HGNC:29313]	protein coding	-0.75	1.22	16.98	<0.0001	0.0002
ENSG00000082512	TRAF5	TNF receptor associated factor 5 [Source:HGNC Symbol;Acc:HGNC:12035]	protein coding	-0.75	4.67	21.51	<0.0001	<0.0001
ENSG00000159733	ZFYVE28	zinc finger FYVE-type containing 28 [Source:HGNC Symbol;Acc:HGNC:29334]	protein coding	-0.75	4.05	27.94	<0.0001	<0.0001
ENSG00000059728	MXD1	MAX dimerization protein 1 [Source:HGNC Symbol;Acc:HGNC:6761]	protein coding	0.75	6.80	64.55	<0.0001	<0.0001
ENSG00000159128	IFNGR2	interferon gamma receptor 2 [Source:HGNC Symbol;Acc:HGNC:5440]	protein coding	0.75	7.82	59.12	<0.0001	<0.0001
ENSG00000177337	DLGAP1-AS1	DLGAP1 antisense RNA 1 [Source:HGNC Symbol;Acc:HGNC:31676]	lncRNA	-0.75	1.03	13.10	0.0003	0.0012
ENSG00000237950		novel transcript	lncRNA	-0.75	0.30	13.21	0.0003	0.0011
ENSG00000260924	LINC01311	long intergenic non-protein coding RNA 1311 [Source:HGNC Symbol;Acc:HGNC:50503]	lncRNA	-0.75	0.29	18.78	<0.0001	<0.0001
ENSG00000258732		arginine-glutamic acid dipeptide (RE) repeats (RERE) pseudogene	unprocessed pseudogene	-0.75	2.08	15.22	<0.0001	0.0004
ENSG00000125733	TRIP10	thyroid hormone receptor interactor 10 [Source:HGNC Symbol;Acc:HGNC:12304]	protein coding	-0.75	2.68	41.74	<0.0001	<0.0001
ENSG00000241061	RPL5P1	ribosomal protein L5 pseudogene 1 [Source:HGNC Symbol;Acc:HGNC:10361]	processed pseudogene	-0.75	0.29	17.12	<0.0001	0.0002
ENSG00000114126	TFDP2	transcription factor Dp-2 [Source:HGNC Symbol;Acc:HGNC:11751]	protein coding	-0.75	3.99	25.53	<0.0001	<0.0001
ENSG00000196172	ZNF681	zinc finger protein 681 [Source:HGNC Symbol;Acc:HGNC:26457]	protein coding	-0.75	0.62	10.95	0.0009	0.0031
ENSG00000109501	WFS1	wolframin ER transmembrane glycoprotein [Source:HGNC Symbol;Acc:HGNC:12762]	protein coding	-0.75	1.56	32.66	<0.0001	<0.0001
ENSG00000260618		novel transcript, antisense to KIAA1370	lncRNA	-0.74	0.24	12.59	0.0004	0.0015
ENSG00000105991	HOXA1	homeobox A1 [Source:HGNC Symbol;Acc:HGNC:5099]	protein coding	0.74	1.11	22.90	<0.0001	<0.0001
ENSG00000124491	F13A1	coagulation factor XIII A chain [Source:HGNC Symbol;Acc:HGNC:3531]	protein coding	-0.74	7.28	14.02	0.0002	0.0008
ENSG00000286835		novel transcript, sense intronic to ZNF367	lncRNA	-0.74	0.23	12.61	0.0004	0.0014

Table S12: Genes differentially expressed at Day 30 compared to pre-second vaccination (Study Arm 2 A/H7N9+AS03, D1, 29, Dendritic cells). Genes are sorted by descending absolute log_2 fold change. Gene model summaries and annotations are based on Ensembl Version 99 (continued.)

Ensembl Gene ID	Ensembl Gene Name	Ensembl Gene Description	Gene Type	log_2 Fold Change (Day 30 vs. Pre-Second Vaccination)	Average log_2 CPM	Likelihood Ratio Test Statistic	P-Value	FDR Adjusted P-Value
ENSG00000278196	IGLV2-8	immunoglobulin lambda variable 2-8 [Source:HGNC Symbol;Acc:HGNC:5895]	IG V gene	-0.74	1.46	7.10	0.0077	0.0187
ENSG00000128739	SNRPN	small nuclear ribonucleoprotein polypeptide N [Source:HGNC Symbol;Acc:HGNC:11164]	protein coding	-0.74	3.02	31.72	<0.0001	<0.0001
ENSG00000186301	MST1P2	macrophage stimulating 1 pseudogene 2 [Source:HGNC Symbol;Acc:HGNC:7383]	unprocessed pseudogene	-0.74	1.52	18.13	<0.0001	0.0001
ENSG00000100321	SYNGR1	synaptogyrin 1 [Source:HGNC Symbol;Acc:HGNC:11498]	protein coding	-0.74	4.29	30.95	<0.0001	<0.0001
ENSG00000211679	IGLC3	immunoglobulin lambda constant 3 (Kern-Oz+ marker) [Source:HGNC Symbol;Acc:HGNC:5857]	IG C gene	-0.74	4.07	12.97	0.0003	0.0012
ENSG00000197696	NMB	neuromedin B [Source:HGNC Symbol;Acc:HGNC:7842]	protein coding	-0.74	0.73	23.60	<0.0001	<0.0001
ENSG00000227032	RPS2P36	ribosomal protein S2 pseudogene 36 [Source:HGNC Symbol;Acc:HGNC:36784]	processed pseudogene	0.74	0.38	17.88	<0.0001	0.0001
ENSG00000165030	NFIL3	nuclear factor, interleukin 3 regulated [Source:HGNC Symbol;Acc:HGNC:7787]	protein coding	0.74	4.93	28.41	<0.0001	<0.0001
ENSG00000146859	TMEM140	transmembrane protein 140 [Source:HGNC Symbol;Acc:HGNC:21870]	protein coding	0.74	4.73	32.81	<0.0001	<0.0001
ENSG00000164038	SLC9B2	solute carrier family 9 member B2 [Source:HGNC Symbol;Acc:HGNC:25143]	protein coding	-0.74	1.69	16.48	<0.0001	0.0002
ENSG00000154760	SLFN13	schlafen family member 13 [Source:HGNC Symbol;Acc:HGNC:26481]	protein coding	-0.74	4.06	22.51	<0.0001	<0.0001
ENSG00000204219	TCEA3	transcription elongation factor A3 [Source:HGNC Symbol;Acc:HGNC:11615]	protein coding	-0.74	2.10	17.84	<0.0001	0.0001
ENSG00000165185	KIAA1958	KIAA1958 [Source:HGNC Symbol;Acc:HGNC:23427]	protein coding	0.74	3.38	29.81	<0.0001	<0.0001
ENSG00000115687	PASK	PAS domain containing serine/threonine kinase [Source:HGNC Symbol;Acc:HGNC:17270]	protein coding	-0.74	4.30	25.01	<0.0001	<0.0001
ENSG00000233184		novel transcript	lncRNA	-0.74	1.96	24.03	<0.0001	<0.0001
ENSG00000198087	CD2AP	CD2 associated protein [Source:HGNC Symbol;Acc:HGNC:14258]	protein coding	0.74	4.88	31.99	<0.0001	<0.0001
ENSG00000148450	MSRB2	methionine sulfoxide reductase B2 [Source:HGNC Symbol;Acc:HGNC:17061]	protein coding	0.74	4.31	43.41	<0.0001	<0.0001

Table S12: Genes differentially expressed at Day 30 compared to pre-second vaccination (Study Arm 2 A/H7N9+AS03, D1, 29, Dendritic cells). Genes are sorted by descending absolute log_2 fold change. Gene model summaries and annotations are based on Ensembl Version 99 (continued.)

Ensembl Gene ID	Ensembl Gene Name	Ensembl Gene Description	Gene Type	log_2 Fold Change (Day 30 vs. Pre-Second Vaccination)	Average log_2 CPM	Likelihood Ratio Test Statistic	P-Value	FDR Adjusted P-Value
ENSG00000109756	RAPGEF2	Rap guanine nucleotide exchange factor 2 [Source:HGNC Symbol;Acc:HGNC:16854]	protein coding	0.74	5.83	57.50	<0.0001	<0.0001
ENSG00000090097	PCBP4	poly(rC) binding protein 4 [Source:HGNC Symbol;Acc:HGNC:8652]	protein coding	-0.74	2.30	12.41	0.0004	0.0016
ENSG00000188389	PDCD1	programmed cell death 1 [Source:HGNC Symbol;Acc:HGNC:8760]	protein coding	-0.74	0.93	8.93	0.0028	0.0078
ENSG00000173917	HOXB2	homeobox B2 [Source:HGNC Symbol;Acc:HGNC:5113]	protein coding	-0.74	3.32	30.91	<0.0001	<0.0001
ENSG00000284691		novel zinc finger protein	protein coding	-0.74	3.46	18.11	<0.0001	0.0001
ENSG00000180354	MTURN	maturin, neural progenitor differentiation regulator homolog [Source:HGNC Symbol;Acc:HGNC:25457]	protein coding	-0.73	4.19	48.11	<0.0001	<0.0001
ENSG00000140859	KIFC3	kinesin family member C3 [Source:HGNC Symbol;Acc:HGNC:6326]	protein coding	-0.73	2.37	12.83	0.0003	0.0013
ENSG00000162836	ACP6	acid phosphatase 6, lysophosphatidic [Source:HGNC Symbol;Acc:HGNC:29609]	protein coding	-0.73	2.62	28.73	<0.0001	<0.0001
ENSG00000198848	CES1	carboxylesterase 1 [Source:HGNC Symbol;Acc:HGNC:1863]	protein coding	0.73	6.08	23.70	<0.0001	<0.0001
ENSG00000198604	BAZ1A	bromodomain adjacent to zinc finger domain 1A [Source:HGNC Symbol;Acc:HGNC:960]	protein coding	0.73	7.08	44.76	<0.0001	<0.0001
ENSG00000169258	GPRIN1	G protein regulated inducer of neurite outgrowth 1 [Source:HGNC Symbol;Acc:HGNC:24835]	protein coding	0.73	0.59	22.46	<0.0001	<0.0001
ENSG00000123989	CHPF	chondroitin polymerizing factor [Source:HGNC Symbol;Acc:HGNC:24291]	protein coding	-0.73	1.66	14.94	0.0001	0.0005
ENSG00000267074		novel transcript, sense intronic SLFN12L	lncRNA	-0.73	1.34	6.24	0.0125	0.0283
ENSG00000163006	CCDC138	coiled-coil domain containing 138 [Source:HGNC Symbol;Acc:HGNC:26531]	protein coding	-0.73	0.70	16.10	<0.0001	0.0003
ENSG00000042317		spermatogenesis associated 7 [Source:NCBI gene;Acc:55812]	protein coding	-0.73	1.31	18.03	<0.0001	0.0001
ENSG00000176749	CDK5R1	cyclin dependent kinase 5 regulatory subunit 1 [Source:HGNC Symbol;Acc:HGNC:1775]	protein coding	-0.73	3.34	18.37	<0.0001	0.0001
ENSG00000203804	ADAMTSL4-AS1	ADAMTSL4 antisense RNA 1 [Source:HGNC Symbol;Acc:HGNC:32041]	lncRNA	0.73	2.62	17.59	<0.0001	0.0002
ENSG00000230910		novel transcript	lncRNA	-0.73	2.47	13.96	0.0002	0.0008
ENSG00000113758	DBN1	drebrin 1 [Source:HGNC Symbol;Acc:HGNC:2695]	protein coding	-0.73	2.70	20.55	<0.0001	<0.0001

Table S12: Genes differentially expressed at Day 30 compared to pre-second vaccination (Study Arm 2 A/H7N9+AS03, D1, 29, Dendritic cells). Genes are sorted by descending absolute log_2 fold change. Gene model summaries and annotations are based on Ensembl Version 99 (continued.)

Ensembl Gene ID	Ensembl Gene Name	Ensembl Gene Description	Gene Type	log_2 Fold Change (Day 30 vs. Pre-Second Vaccination)	Average log_2 CPM	Likelihood Ratio Test Statistic	P-Value	FDR Adjusted P-Value
ENSG00000084731	KIF3C	kinesin family member 3C [Source:HGNC Symbol;Acc:HGNC:6321]	protein coding	-0.73	1.96	21.73	<0.0001	<0.0001
ENSG00000158874	APOA2	apolipoprotein A2 [Source:HGNC Symbol;Acc:HGNC:601]	protein coding	0.73	1.43	15.06	0.0001	0.0005
ENSG00000165092	ALDH1A1	aldehyde dehydrogenase 1 family member A1 [Source:HGNC Symbol;Acc:HGNC:402]	protein coding	0.73	6.36	22.14	<0.0001	<0.0001
ENSG00000179344	HLA-DQB1	major histocompatibility complex, class II, DQ beta 1 [Source:HGNC Symbol;Acc:HGNC:4944]	protein coding	0.73	9.12	9.82	0.0017	0.0052
ENSG00000135821	GLUL	glutamate-ammonia ligase [Source:HGNC Symbol;Acc:HGNC:4341]	protein coding	0.73	9.02	59.98	<0.0001	<0.0001
ENSG00000140451	PIF1	PIF1 5'-to-3' DNA helicase [Source:HGNC Symbol;Acc:HGNC:26220]	protein coding	-0.73	0.08	11.11	0.0009	0.0029
ENSG00000187240	DYNC2H1	dynein cytoplasmic 2 heavy chain 1 [Source:HGNC Symbol;Acc:HGNC:2962]	protein coding	-0.73	1.39	27.06	<0.0001	<0.0001
ENSG00000274021		novel transcript, antisense to DUSP6	lncRNA	0.73	3.86	13.39	0.0003	0.001
ENSG00000101224	CDC25B	cell division cycle 25B [Source:HGNC Symbol;Acc:HGNC:1726]	protein coding	-0.73	6.69	25.09	<0.0001	<0.0001
ENSG00000248714		novel transcript	lncRNA	-0.73	0.74	14.08	0.0002	0.0007
ENSG00000137266	SLC22A23	solute carrier family 22 member 23 [Source:HGNC Symbol;Acc:HGNC:21106]	protein coding	-0.73	1.96	16.87	<0.0001	0.0002
ENSG00000244879	GABPB1-AS1	GABPB1 antisense RNA 1 [Source:HGNC Symbol;Acc:HGNC:44157]	lncRNA	-0.73	5.73	39.04	<0.0001	<0.0001
ENSG00000164292	RHOBTB3	Rho related BTB domain containing 3 [Source:HGNC Symbol;Acc:HGNC:18757]	protein coding	0.73	2.02	9.76	0.0018	0.0053
ENSG00000258839	MC1R	melanocortin 1 receptor [Source:HGNC Symbol;Acc:HGNC:6929]	protein coding	-0.73	1.80	20.01	<0.0001	<0.0001
ENSG00000204653	ASPDH	aspartate dehydrogenase domain containing [Source:HGNC Symbol;Acc:HGNC:33856]	protein coding	-0.73	1.20	14.04	0.0002	0.0008
ENSG00000092010	PSME1	proteasome activator subunit 1 [Source:HGNC Symbol;Acc:HGNC:9568]	protein coding	0.72	8.26	43.64	<0.0001	<0.0001
ENSG00000134463	ECHDC3	enoyl-CoA hydratase domain containing 3 [Source:HGNC Symbol;Acc:HGNC:23489]	protein coding	0.72	3.21	49.29	<0.0001	<0.0001
ENSG00000184545	DUSP8	dual specificity phosphatase 8 [Source:HGNC Symbol;Acc:HGNC:3074]	protein coding	-0.72	1.07	9.31	0.0023	0.0066
ENSG00000197506	SLC28A3	solute carrier family 28 member 3 [Source:HGNC Symbol;Acc:HGNC:16484]	protein coding	-0.72	1.33	5.98	0.0145	0.0322

Table S12: Genes differentially expressed at Day 30 compared to pre-second vaccination (Study Arm 2 A/H7N9+AS03, D1, 29, Dendritic cells). Genes are sorted by descending absolute log_2 fold change. Gene model summaries and annotations are based on Ensembl Version 99 (continued.)

Ensembl Gene ID	Ensembl Gene Name	Ensembl Gene Description	Gene Type	log_2 Fold Change (Day 30 vs. Pre-Second Vaccination)	Average log_2 CPM	Likelihood Ratio Test Statistic	P-Value	FDR Adjusted P-Value
ENSG00000234028	EIF2AK3-DT	EIF2AK3 divergent transcript [Source:HGNC Symbol;Acc:HGNC:54066]	lncRNA	-0.72	0.11	16.93	<0.0001	0.0002
ENSG00000143344	RGL1	ral guanine nucleotide dissociation stimulator like 1 [Source:HGNC Symbol;Acc:HGNC:30281]	protein coding	0.72	3.80	30.08	<0.0001	<0.0001
ENSG00000148019	CEP78	centrosomal protein 78 [Source:HGNC Symbol;Acc:HGNC:25740]	protein coding	-0.72	4.63	23.39	<0.0001	<0.0001
ENSG00000152784	PRDM8	PR/SET domain 8 [Source:HGNC Symbol;Acc:HGNC:13993]	protein coding	-0.72	2.62	15.05	0.0001	0.0005
ENSG00000173581	CCDC106	coiled-coil domain containing 106 [Source:HGNC Symbol;Acc:HGNC:30181]	protein coding	-0.72	2.75	39.75	<0.0001	<0.0001
ENSG00000213071	LPAL2	lipoprotein(a) like 2, pseudogene [Source:HGNC Symbol;Acc:HGNC:21210]	transcribed unprocessed pseudogene	-0.72	1.17	5.30	0.0213	0.0449
ENSG00000174292	TNK1	tyrosine kinase non receptor 1 [Source:HGNC Symbol;Acc:HGNC:11940]	protein coding	-0.72	1.55	18.47	<0.0001	0.0001
ENSG00000135862	LAMC1	laminin subunit gamma 1 [Source:HGNC Symbol;Acc:HGNC:6492]	protein coding	-0.72	2.21	18.74	<0.0001	<0.0001
ENSG00000106772	PRUNE2	prune homolog 2 with BCH domain [Source:HGNC Symbol;Acc:HGNC:25209]	protein coding	0.72	2.61	37.14	<0.0001	<0.0001
ENSG00000115306	SPTBN1	spectrin beta, non-erythrocytic 1 [Source:HGNC Symbol;Acc:HGNC:11275]	protein coding	-0.72	5.78	28.90	<0.0001	<0.0001
ENSG00000076555	ACACB	acetyl-CoA carboxylase beta [Source:HGNC Symbol;Acc:HGNC:85]	protein coding	-0.72	3.59	36.18	<0.0001	<0.0001
ENSG00000013364	MVP	major vault protein [Source:HGNC Symbol;Acc:HGNC:7531]	protein coding	0.72	8.86	42.24	<0.0001	<0.0001
ENSG00000261226		novel transcript	lncRNA	0.72	0.97	26.30	<0.0001	<0.0001
ENSG00000115392	FANCL	FA complementation group L [Source:HGNC Symbol;Acc:HGNC:20748]	protein coding	0.72	3.09	33.94	<0.0001	<0.0001
ENSG00000137193	PIM1	Pim-1 proto-oncogene, serine/threonine kinase [Source:HGNC Symbol;Acc:HGNC:8986]	protein coding	0.72	7.20	12.77	0.0004	0.0013
ENSG00000103494	RPGRIP1L	RPGRIP1 like [Source:HGNC Symbol;Acc:HGNC:29168]	protein coding	-0.72	0.06	14.80	0.0001	0.0005
ENSG00000182389	CACNB4	calcium voltage-gated channel auxiliary subunit beta 4 [Source:HGNC Symbol;Acc:HGNC:1404]	protein coding	-0.72	1.09	18.74	<0.0001	<0.0001

Table S12: Genes differentially expressed at Day 30 compared to pre-second vaccination (Study Arm 2 A/H7N9+AS03, D1, 29, Dendritic cells). Genes are sorted by descending absolute log_2 fold change. Gene model summaries and annotations are based on Ensembl Version 99 (continued.)

Ensembl Gene ID	Ensembl Gene Name	Ensembl Gene Description	Gene Type	log_2 Fold Change (Day 30 vs. Pre-Second Vaccination)	Average log_2 CPM	Likelihood Ratio Test Statistic	P-Value	FDR Adjusted P-Value
ENSG00000099866	MADCAM1	mucosal vascular addressin cell adhesion molecule 1 [Source:HGNC Symbol;Acc:HGNC:6765]	protein coding	-0.72	0.05	16.15	<0.0001	0.0003
ENSG00000119686	FLVCR2	feline leukemia virus subgroup C cellular receptor family member 2 [Source:HGNC Symbol;Acc:HGNC:20105]	protein coding	0.72	4.93	64.15	<0.0001	<0.0001
ENSG00000107317	PTGDS	prostaglandin D2 synthase [Source:HGNC Symbol;Acc:HGNC:9592]	protein coding	-0.72	2.70	8.90	0.0029	0.008
ENSG00000156535	CD109	CD109 molecule [Source:HGNC Symbol;Acc:HGNC:21685]	protein coding	-0.72	1.02	22.88	<0.0001	<0.0001
ENSG00000253785		pyrophosphatase (inorganic) 1 (PPA1) pseudogene	processed pseudogene	0.72	0.96	25.33	<0.0001	<0.0001
ENSG00000103196	CRISPLD2	cysteine rich secretory protein LCCL domain containing 2 [Source:HGNC Symbol;Acc:HGNC:25248]	protein coding	0.72	6.55	23.38	<0.0001	<0.0001
ENSG00000154122	ANKH	ANKH inorganic pyrophosphate transport regulator [Source:HGNC Symbol;Acc:HGNC:15492]	protein coding	-0.72	5.29	53.19	<0.0001	<0.0001
ENSG00000259081		novel transcript	lncRNA	-0.72	1.02	11.82	0.0006	0.0021
ENSG00000271857		novel transcript, antisense to RUNX2	lncRNA	-0.72	0.21	15.25	<0.0001	0.0004
ENSG00000091490	SEL1L3	SEL1L family member 3 [Source:HGNC Symbol;Acc:HGNC:29108]	protein coding	-0.72	5.07	26.53	<0.0001	<0.0001
ENSG00000171219	CDC42BPG	CDC42 binding protein kinase gamma [Source:HGNC Symbol;Acc:HGNC:29829]	protein coding	-0.72	1.92	12.44	0.0004	0.0016
ENSG00000104972	LILRB1	leukocyte immunoglobulin like receptor B1 [Source:HGNC Symbol;Acc:HGNC:6605]	protein coding	0.72	8.75	60.18	<0.0001	<0.0001
ENSG00000011523	CEP68	centrosomal protein 68 [Source:HGNC Symbol;Acc:HGNC:29076]	protein coding	-0.72	4.20	56.60	<0.0001	<0.0001
ENSG00000115109	EPB41L5	erythrocyte membrane protein band 4.1 like 5 [Source:HGNC Symbol;Acc:HGNC:19819]	protein coding	0.72	1.66	13.70	0.0002	0.0009
ENSG00000126351	THRA	thyroid hormone receptor alpha [Source:HGNC Symbol;Acc:HGNC:11796]	protein coding	-0.72	5.10	50.49	<0.0001	<0.0001
ENSG00000171791	BCL2	BCL2 apoptosis regulator [Source:HGNC Symbol;Acc:HGNC:990]	protein coding	-0.72	5.06	15.92	<0.0001	0.0003
ENSG00000135643	KCNMB4	potassium calcium-activated channel subfamily M regulatory beta subunit 4 [Source:HGNC Symbol;Acc:HGNC:6289]	protein coding	-0.72	1.31	11.58	0.0007	0.0023
ENSG00000287978		novel transcript	lncRNA	-0.72	1.33	16.75	<0.0001	0.0002
ENSG00000115525	ST3GAL5	ST3 beta-galactoside alpha-2,3-sialyltransferase 5 [Source:HGNC Symbol;Acc:HGNC:10872]	protein coding	0.72	6.44	55.56	<0.0001	<0.0001

Table S12: Genes differentially expressed at Day 30 compared to pre-second vaccination (Study Arm 2 A/H7N9+AS03, D1, 29, Dendritic cells). Genes are sorted by descending absolute log_2 fold change. Gene model summaries and annotations are based on Ensembl Version 99 (continued.)

Ensembl Gene ID	Ensembl Gene Name	Ensembl Gene Description	Gene Type	log_2 Fold Change (Day 30 vs. Pre-Second Vaccination)	Average log_2 CPM	Likelihood Ratio Test Statistic	P-Value	FDR Adjusted P-Value
ENSG00000122643	NT5C3A	5'-nucleotidase, cytosolic IIIA [Source:HGNC Symbol;Acc:HGNC:17820]	protein coding	0.71	4.96	52.20	<0.0001	<0.0001
ENSG00000140323	DISP2	dispatched RND transporter family member 2 [Source:HGNC Symbol;Acc:HGNC:19712]	protein coding	-0.71	0.58	24.02	<0.0001	<0.0001
ENSG00000143847	PPFIA4	PTPRF interacting protein alpha 4 [Source:HGNC Symbol;Acc:HGNC:9248]	protein coding	-0.71	4.00	27.09	<0.0001	<0.0001
ENSG00000156804	FBXO32	F-box protein 32 [Source:HGNC Symbol;Acc:HGNC:16731]	protein coding	-0.71	3.07	18.58	<0.0001	<0.0001
ENSG00000247092	SNHG10	small nucleolar RNA host gene 10 [Source:HGNC Symbol;Acc:HGNC:27510]	lncRNA	-0.71	2.57	39.69	<0.0001	<0.0001
ENSG00000148841	ITPRIP	inositol 1,4,5-trisphosphate receptor interacting protein [Source:HGNC Symbol;Acc:HGNC:29370]	protein coding	0.71	6.30	42.87	<0.0001	<0.0001
ENSG00000196954	CASP4	caspase 4 [Source:HGNC Symbol;Acc:HGNC:1505]	protein coding	0.71	7.53	51.83	<0.0001	<0.0001
ENSG00000129484	PARP2	poly(ADP-ribose) polymerase 2 [Source:HGNC Symbol;Acc:HGNC:272]	protein coding	-0.71	2.78	38.44	<0.0001	<0.0001
ENSG00000267322	SNHG22	small nucleolar RNA host gene 22 [Source:HGNC Symbol;Acc:HGNC:50285]	lncRNA	-0.71	0.93	18.11	<0.0001	0.0001
ENSG00000134243	SORT1	sortilin 1 [Source:HGNC Symbol;Acc:HGNC:11186]	protein coding	0.71	7.09	53.23	<0.0001	<0.0001
ENSG00000172232	AZU1	azurocidin 1 [Source:HGNC Symbol;Acc:HGNC:913]	protein coding	-0.71	1.36	9.79	0.0018	0.0053
ENSG00000145284	SCD5	stearoyl-CoA desaturase 5 [Source:HGNC Symbol;Acc:HGNC:21088]	protein coding	-0.71	0.45	10.92	0.001	0.0031
ENSG00000261655		novel transcript, overlapping to GPR20	lncRNA	-0.71	2.37	12.25	0.0005	0.0017
ENSG00000104814	MAP4K1	mitogen-activated protein kinase kinase kinase kinase 1 [Source:HGNC Symbol;Acc:HGNC:6863]	protein coding	-0.71	6.38	47.16	<0.0001	<0.0001
ENSG00000100368	CSF2RB	colony stimulating factor 2 receptor beta common subunit [Source:HGNC Symbol;Acc:HGNC:2436]	protein coding	0.71	8.25	50.41	<0.0001	<0.0001
ENSG00000225342	LRRK2-DT	LRRK2 divergent transcript [Source:HGNC Symbol;Acc:HGNC:40848]	lncRNA	0.71	2.64	29.08	<0.0001	<0.0001
ENSG00000249437	NAIP	NLR family apoptosis inhibitory protein [Source:HGNC Symbol;Acc:HGNC:7634]	protein coding	0.71	5.18	44.06	<0.0001	<0.0001
ENSG00000178104	PDE4DIP	phosphodiesterase 4D interacting protein [Source:HGNC Symbol;Acc:HGNC:15580]	protein coding	-0.71	4.27	52.81	<0.0001	<0.0001

Table S12: Genes differentially expressed at Day 30 compared to pre-second vaccination (Study Arm 2 A/H7N9+AS03, D1, 29, Dendritic cells). Genes are sorted by descending absolute log_2 fold change. Gene model summaries and annotations are based on Ensembl Version 99 (continued.)

Ensembl Gene ID	Ensembl Gene Name	Ensembl Gene Description	Gene Type	log_2 Fold Change (Day 30 vs. Pre-Second Vaccination)	Average log_2 CPM	Likelihood Ratio Test Statistic	P-Value	FDR Adjusted P-Value
ENSG00000170522	ELOVL6	ELOVL fatty acid elongase 6 [Source:HGNC Symbol;Acc:HGNC:15829]	protein coding	-0.71	1.68	9.65	0.0019	0.0056
ENSG00000135414	GDF11	growth differentiation factor 11 [Source:HGNC Symbol;Acc:HGNC:4216]	protein coding	-0.71	2.37	15.05	0.0001	0.0005
ENSG00000079435	LIPE	lipase E, hormone sensitive type [Source:HGNC Symbol;Acc:HGNC:6621]	protein coding	-0.71	1.85	14.10	0.0002	0.0007
ENSG00000275993	SIK1B	salt inducible kinase 1B (putative) [Source:HGNC Symbol;Acc:HGNC:52389]	protein coding	0.71	3.21	16.66	<0.0001	0.0002
ENSG00000132465	JCHAIN	joining chain of multimeric IgA and IgM [Source:HGNC Symbol;Acc:HGNC:5713]	protein coding	-0.71	5.75	14.24	0.0002	0.0007
ENSG00000107147	KCNT1	potassium sodium-activated channel subfamily T member 1 [Source:HGNC Symbol;Acc:HGNC:18865]	protein coding	-0.71	0.56	7.36	0.0067	0.0165
ENSG00000174125	TLR1	toll like receptor 1 [Source:HGNC Symbol;Acc:HGNC:11847]	protein coding	0.71	6.35	46.93	<0.0001	<0.0001
ENSG00000182197	EXT1	exostosin glycosyltransferase 1 [Source:HGNC Symbol;Acc:HGNC:3512]	protein coding	0.71	5.05	70.65	<0.0001	<0.0001
ENSG00000157017	GHRL	ghrelin and obestatin prepropeptide [Source:HGNC Symbol;Acc:HGNC:18129]	protein coding	-0.71	2.74	31.75	<0.0001	<0.0001
ENSG00000104290	FZD3	frizzled class receptor 3 [Source:HGNC Symbol;Acc:HGNC:4041]	protein coding	-0.71	0.63	19.68	<0.0001	<0.0001
ENSG00000186827	TNFRSF4	TNF receptor superfamily member 4 [Source:HGNC Symbol;Acc:HGNC:11918]	protein coding	-0.71	1.62	7.20	0.0073	0.0178
ENSG00000251429		axin interactor, dorsalization associated (AIDA) pseudogene	transcribed processed pseudogene	0.71	1.21	14.88	0.0001	0.0005
ENSG00000254415	SIGLEC14	sialic acid binding Ig like lectin 14 [Source:HGNC Symbol;Acc:HGNC:32926]	protein coding	0.71	6.44	33.86	<0.0001	<0.0001
ENSG00000171729	TMEM51	transmembrane protein 51 [Source:HGNC Symbol;Acc:HGNC:25488]	protein coding	0.71	3.11	19.65	<0.0001	<0.0001
ENSG00000114770	ABCC5	ATP binding cassette subfamily C member 5 [Source:HGNC Symbol;Acc:HGNC:56]	protein coding	-0.71	5.49	38.77	<0.0001	<0.0001
ENSG00000108771	DHX58	DExH-box helicase 58 [Source:HGNC Symbol;Acc:HGNC:29517]	protein coding	0.71	5.94	60.63	<0.0001	<0.0001
ENSG00000143224	PPOX	protoporphyrinogen oxidase [Source:HGNC Symbol;Acc:HGNC:9280]	protein coding	-0.70	4.60	47.13	<0.0001	<0.0001

Table S12: Genes differentially expressed at Day 30 compared to pre-second vaccination (Study Arm 2 A/H7N9+AS03, D1, 29, Dendritic cells). Genes are sorted by descending absolute log_2 fold change. Gene model summaries and annotations are based on Ensembl Version 99 (continued.)

Ensembl Gene ID	Ensembl Gene Name	Ensembl Gene Description	Gene Type	log_2 Fold Change (Day 30 vs. Pre-Second Vaccination)	Average log_2 CPM	Likelihood Ratio Test Statistic	P-Value	FDR Adjusted P-Value
ENSG00000185761	ADAMTSL5	ADAMTS like 5 [Source:HGNC Symbol;Acc:HGNC:27912]	protein coding	-0.70	0.53	9.16	0.0025	0.0071
ENSG00000082074	FYB1	FYN binding protein 1 [Source:HGNC Symbol;Acc:HGNC:4036]	protein coding	0.70	9.12	52.39	<0.0001	<0.0001
ENSG00000180817	PPA1	inorganic pyrophosphatase 1 [Source:HGNC Symbol;Acc:HGNC:9226]	protein coding	0.70	6.01	47.22	<0.0001	<0.0001
ENSG00000105402	NAPA	NSF attachment protein alpha [Source:HGNC Symbol;Acc:HGNC:7641]	protein coding	0.70	6.60	50.68	<0.0001	<0.0001
ENSG00000085465	OVGP1	oviductal glycoprotein 1 [Source:HGNC Symbol;Acc:HGNC:8524]	protein coding	-0.70	1.25	17.25	<0.0001	0.0002
ENSG00000100814	CCNB1IP1	cyclin B1 interacting protein 1 [Source:HGNC Symbol;Acc:HGNC:19437]	protein coding	-0.70	3.45	47.33	<0.0001	<0.0001
ENSG00000092068	SLC7A8	solute carrier family 7 member 8 [Source:HGNC Symbol;Acc:HGNC:11066]	protein coding	-0.70	0.37	9.15	0.0025	0.0071
ENSG00000284930		novel transcript	lncRNA	-0.70	0.19	16.93	<0.0001	0.0002
ENSG00000185477	GPRIN3	GPRIN family member 3 [Source:HGNC Symbol;Acc:HGNC:27733]	protein coding	-0.70	5.64	31.33	<0.0001	<0.0001
ENSG00000149534	MS4A2	membrane spanning 4-domains A2 [Source:HGNC Symbol;Acc:HGNC:7316]	protein coding	-0.70	2.22	8.79	0.003	0.0084
ENSG00000240137	ERICH6-AS1	ERICH6 antisense RNA 1 [Source:HGNC Symbol;Acc:HGNC:41205]	lncRNA	-0.70	0.46	16.90	<0.0001	0.0002
ENSG00000281103	TRG-AS1	T cell receptor gamma locus antisense RNA 1 [Source:HGNC Symbol;Acc:HGNC:48974]	lncRNA	-0.70	3.98	22.34	<0.0001	<0.0001
ENSG00000004809	SLC22A16	solute carrier family 22 member 16 [Source:HGNC Symbol;Acc:HGNC:20302]	protein coding	0.70	1.86	12.37	0.0004	0.0016
ENSG00000109762	SNX25	sorting nexin 25 [Source:HGNC Symbol;Acc:HGNC:21883]	protein coding	-0.70	2.19	16.95	<0.0001	0.0002
ENSG00000136717	BIN1	bridging integrator 1 [Source:HGNC Symbol;Acc:HGNC:1052]	protein coding	-0.70	5.91	26.39	<0.0001	<0.0001
ENSG00000282939	TRBV7-2	T cell receptor beta variable 7-2 [Source:HGNC Symbol;Acc:HGNC:12236]	TR V gene	-0.70	0.62	5.54	0.0186	0.0401
ENSG00000277443	MARCKS	myristoylated alanine rich protein kinase C substrate [Source:HGNC Symbol;Acc:HGNC:6759]	protein coding	0.70	8.16	32.79	<0.0001	<0.0001
ENSG00000117724	CENPF	centromere protein F [Source:HGNC Symbol;Acc:HGNC:1857]	protein coding	-0.70	0.81	10.98	0.0009	0.003

Table S12: Genes differentially expressed at Day 30 compared to pre-second vaccination (Study Arm 2 A/H7N9+AS03, D1, 29, Dendritic cells). Genes are sorted by descending absolute log_2 fold change. Gene model summaries and annotations are based on Ensembl Version 99 (continued.)

Ensembl Gene ID	Ensembl Gene Name	Ensembl Gene Description	Gene Type	log_2 Fold Change (Day 30 vs. Pre-Second Vaccination)	Average log_2 CPM	Likelihood Ratio Test Statistic	P-Value	FDR Adjusted P-Value
ENSG00000177556	ATOX1	antioxidant 1 copper chaperone [Source:HGNC Symbol;Acc:HGNC:798]	protein coding	0.70	4.47	42.50	<0.0001	<0.0001
ENSG00000106605	BLVRA	biliverdin reductase A [Source:HGNC Symbol;Acc:HGNC:1062]	protein coding	0.70	6.63	49.35	<0.0001	<0.0001
ENSG00000184465	WDR27	WD repeat domain 27 [Source:HGNC Symbol;Acc:HGNC:21248]	protein coding	-0.70	3.97	33.46	<0.0001	<0.0001
ENSG00000206417	H1FX-AS1	H1FX antisense RNA 1 [Source:HGNC Symbol;Acc:HGNC:27953]	lncRNA	-0.70	0.38	13.30	0.0003	0.0011
ENSG00000132109	TRIM21	tripartite motif containing 21 [Source:HGNC Symbol;Acc:HGNC:11312]	protein coding	0.70	5.85	25.99	<0.0001	<0.0001
ENSG00000109084	TMEM97	transmembrane protein 97 [Source:HGNC Symbol;Acc:HGNC:28106]	protein coding	-0.70	1.39	22.86	<0.0001	<0.0001
ENSG00000224429	LINC00539	long intergenic non-protein coding RNA 539 [Source:HGNC Symbol;Acc:HGNC:43672]	lncRNA	-0.70	0.89	12.92	0.0003	0.0013
ENSG00000007312	CD79B	CD79b molecule [Source:HGNC Symbol;Acc:HGNC:1699]	protein coding	-0.70	5.68	34.47	<0.0001	<0.0001
ENSG00000229671	LINC01150	long intergenic non-protein coding RNA 1150 [Source:HGNC Symbol;Acc:HGNC:49469]	lncRNA	-0.70	0.65	15.50	<0.0001	0.0004
ENSG00000168610	STAT3	signal transducer and activator of transcription 3 [Source:HGNC Symbol;Acc:HGNC:11364]	protein coding	0.70	8.11	49.34	<0.0001	<0.0001
ENSG00000282508	LINC01002	long intergenic non-protein coding RNA 1002 [Source:HGNC Symbol;Acc:HGNC:38538]	lncRNA	0.70	2.41	30.72	<0.0001	<0.0001
ENSG00000157827	FMNL2	formin like 2 [Source:HGNC Symbol;Acc:HGNC:18267]	protein coding	0.69	5.26	48.84	<0.0001	<0.0001
ENSG00000163751	CPA3	carboxypeptidase A3 [Source:HGNC Symbol;Acc:HGNC:2298]	protein coding	-0.69	1.88	7.51	0.0061	0.0154
ENSG00000174123	TLR10	toll like receptor 10 [Source:HGNC Symbol;Acc:HGNC:15634]	protein coding	-0.69	3.62	33.88	<0.0001	<0.0001
ENSG00000287265		novel transcript, antisense to DUSP22	lncRNA	-0.69	2.51	35.12	<0.0001	<0.0001
ENSG00000183621	ZNF438	zinc finger protein 438 [Source:HGNC Symbol;Acc:HGNC:21029]	protein coding	0.69	4.16	40.68	<0.0001	<0.0001
ENSG00000105516	DBP	D-box binding PAR bZIP transcription factor [Source:HGNC Symbol;Acc:HGNC:2697]	protein coding	-0.69	5.09	27.77	<0.0001	<0.0001
ENSG00000163694	RBM47	RNA binding motif protein 47 [Source:HGNC Symbol;Acc:HGNC:30358]	protein coding	0.69	6.53	41.89	<0.0001	<0.0001

Confidential/Proprietary Information

Table S12: Genes differentially expressed at Day 30 compared to pre-second vaccination (Study Arm 2 A/H7N9+AS03, D1, 29, Dendritic cells). Genes are sorted by descending absolute log_2 fold change. Gene model summaries and annotations are based on Ensembl Version 99 (continued.)

Ensembl Gene ID	Ensembl Gene Name	Ensembl Gene Description	Gene Type	log_2 Fold Change (Day 30 vs. Pre-Second Vaccination)	Average log_2 CPM	Likelihood Ratio Test Statistic	P-Value	FDR Adjusted P-Value
ENSG00000096996	IL12RB1	interleukin 12 receptor subunit beta 1 [Source:HGNC Symbol;Acc:HGNC:5971]	protein coding	0.69	6.06	36.99	<0.0001	<0.0001
ENSG00000235750	KIAA0040	KIAA0040 [Source:HGNC Symbol;Acc:HGNC:28950]	protein coding	0.69	6.26	21.77	<0.0001	<0.0001
ENSG00000286786		novel transcript, antisense to RASGRP1	lncRNA	-0.69	1.31	10.29	0.0013	0.0042
ENSG00000261373	VPS9D1-AS1	VPS9D1 antisense RNA 1 [Source:HGNC Symbol;Acc:HGNC:48915]	lncRNA	0.69	2.96	22.90	<0.0001	<0.0001
ENSG00000030110	BAK1	BCL2 antagonist/killer 1 [Source:HGNC Symbol;Acc:HGNC:949]	protein coding	0.69	5.73	69.14	<0.0001	<0.0001
ENSG00000137310	TCF19	transcription factor 19 [Source:HGNC Symbol;Acc:HGNC:11629]	protein coding	-0.69	2.68	18.10	<0.0001	0.0001
ENSG00000151376	ME3	malic enzyme 3 [Source:HGNC Symbol;Acc:HGNC:6985]	protein coding	-0.69	1.65	21.02	<0.0001	<0.0001
ENSG00000123933	MXD4	MAX dimerization protein 4 [Source:HGNC Symbol;Acc:HGNC:13906]	protein coding	-0.69	7.36	36.99	<0.0001	<0.0001
ENSG00000177106	EPS8L2	EPS8 like 2 [Source:HGNC Symbol;Acc:HGNC:21296]	protein coding	-0.69	1.24	10.76	0.001	0.0034
ENSG00000164050	PLXNB1	plexin B1 [Source:HGNC Symbol;Acc:HGNC:9103]	protein coding	-0.69	1.81	22.60	<0.0001	<0.0001
ENSG00000159228	CBR1	carbonyl reductase 1 [Source:HGNC Symbol;Acc:HGNC:1548]	protein coding	0.69	5.11	55.57	<0.0001	<0.0001
ENSG00000144792	ZNF660	zinc finger protein 660 [Source:HGNC Symbol;Acc:HGNC:26720]	protein coding	-0.69	0.03	12.76	0.0004	0.0013
ENSG00000143226	FCGR2A	Fc fragment of IgG receptor IIa [Source:HGNC Symbol;Acc:HGNC:3616]	protein coding	0.69	7.58	41.67	<0.0001	<0.0001
ENSG00000167074	TEF	TEF transcription factor, PAR bZIP family member [Source:HGNC Symbol;Acc:HGNC:11722]	protein coding	-0.69	3.57	30.04	<0.0001	<0.0001
ENSG00000149564	ESAM	endothelial cell adhesion molecule [Source:HGNC Symbol;Acc:HGNC:17474]	protein coding	-0.69	0.86	8.61	0.0033	0.0091
ENSG00000204882	GPR20	G protein-coupled receptor 20 [Source:HGNC Symbol;Acc:HGNC:4475]	protein coding	-0.69	1.79	9.95	0.0016	0.0049
ENSG00000213064	SFT2D2	SFT2 domain containing 2 [Source:HGNC Symbol;Acc:HGNC:25140]	protein coding	0.69	6.80	50.56	<0.0001	<0.0001
ENSG00000215790	SLC35E2A	solute carrier family 35 member E2A [Source:HGNC Symbol;Acc:HGNC:20863]	protein coding	-0.69	3.95	45.68	<0.0001	<0.0001

Table S12: Genes differentially expressed at Day 30 compared to pre-second vaccination (Study Arm 2 A/H7N9+AS03, D1, 29, Dendritic cells). Genes are sorted by descending absolute log_2 fold change. Gene model summaries and annotations are based on Ensembl Version 99 (continued.)

Ensembl Gene ID	Ensembl Gene Name	Ensembl Gene Description	Gene Type	log_2 Fold Change (Day 30 vs. Pre-Second Vaccination)	Average log_2 CPM	Likelihood Ratio Test Statistic	P-Value	FDR Adjusted P-Value
ENSG00000115956	PLEK	pleckstrin [Source:HGNC Symbol;Acc:HGNC:9070]	protein coding	0.69	8.82	36.75	<0.0001	<0.0001
ENSG00000155287	SLC25A28	solute carrier family 25 member 28 [Source:HGNC Symbol;Acc:HGNC:23472]	protein coding	0.69	6.08	39.26	<0.0001	<0.0001
ENSG00000041353	RAB27B	RAB27B, member RAS oncogene family [Source:HGNC Symbol;Acc:HGNC:9767]	protein coding	-0.69	1.31	6.61	0.0101	0.0237
ENSG00000181894	ZNF329	zinc finger protein 329 [Source:HGNC Symbol;Acc:HGNC:14209]	protein coding	-0.69	2.33	25.46	<0.0001	<0.0001
ENSG00000260708		novel transcript, antisense to TBC1D22A	lncRNA	-0.69	1.81	22.40	<0.0001	<0.0001
ENSG00000250303	LINC02762	long intergenic non-protein coding RNA 2762 [Source:HGNC Symbol;Acc:HGNC:27443]	lncRNA	-0.68	0.52	16.44	<0.0001	0.0003
ENSG00000137757	CASP5	caspase 5 [Source:HGNC Symbol;Acc:HGNC:1506]	protein coding	0.68	3.64	31.10	<0.0001	<0.0001
ENSG00000137857	DUOX1	dual oxidase 1 [Source:HGNC Symbol;Acc:HGNC:3062]	protein coding	-0.68	1.24	18.64	<0.0001	<0.0001
ENSG00000184988	TMEM106A	transmembrane protein 106A [Source:HGNC Symbol;Acc:HGNC:28288]	protein coding	0.68	5.03	31.19	<0.0001	<0.0001
ENSG00000133065	SLC41A1	solute carrier family 41 member 1 [Source:HGNC Symbol;Acc:HGNC:19429]	protein coding	-0.68	3.19	21.90	<0.0001	<0.0001
ENSG00000143771	CNIH4	cornichon family AMPA receptor auxiliary protein 4 [Source:HGNC Symbol;Acc:HGNC:25013]	protein coding	0.68	5.19	81.56	<0.0001	<0.0001
ENSG00000188641	DPYD	dihydropyrimidine dehydrogenase [Source:HGNC Symbol;Acc:HGNC:3012]	protein coding	0.68	7.82	39.72	<0.0001	<0.0001
ENSG00000166979	EVA1C	eva-1 homolog C [Source:HGNC Symbol;Acc:HGNC:13239]	protein coding	-0.68	2.02	13.16	0.0003	0.0011
ENSG00000178878	APOLD1	apolipoprotein L domain containing 1 [Source:HGNC Symbol;Acc:HGNC:25268]	protein coding	-0.68	1.03	23.05	<0.0001	<0.0001
ENSG00000117394	SLC2A1	solute carrier family 2 member 1 [Source:HGNC Symbol;Acc:HGNC:11005]	protein coding	-0.68	3.96	19.35	<0.0001	<0.0001
ENSG00000256594		C-type lectin domain family 2 (CLEC2) pseudogene	transcribed unprocessed pseudogene	-0.68	3.61	35.18	<0.0001	<0.0001
ENSG00000114450	GNB4	G protein subunit beta 4 [Source:HGNC Symbol;Acc:HGNC:20731]	protein coding	0.68	6.16	45.34	<0.0001	<0.0001

Table S12: Genes differentially expressed at Day 30 compared to pre-second vaccination (Study Arm 2 A/H7N9+AS03, D1, 29, Dendritic cells). Genes are sorted by descending absolute log_2 fold change. Gene model summaries and annotations are based on Ensembl Version 99 (continued.)

Ensembl Gene ID	Ensembl Gene Name	Ensembl Gene Description	Gene Type	log_2 Fold Change (Day 30 vs. Pre-Second Vaccination)	Average log_2 CPM	Likelihood Ratio Test Statistic	P-Value	FDR Adjusted P-Value
ENSG00000197780	TAF13	TATA-box binding protein associated factor 13 [Source:HGNC Symbol;Acc:HGNC:11546]	protein coding	0.68	3.31	26.96	<0.0001	<0.0001
ENSG00000233806	LINC01237	long intergenic non-protein coding RNA 1237 [Source:HGNC Symbol;Acc:HGNC:49793]	lncRNA	-0.68	1.11	11.48	0.0007	0.0024
ENSG00000204177	BMS1P1	BMS1 pseudogene 1 [Source:HGNC Symbol;Acc:HGNC:23649]	transcribed unprocessed pseudogene	-0.68	0.92	13.84	0.0002	0.0008
ENSG00000170846		Putative MORF4 family-associated protein 1-like protein UPP [Source:UniProtKB/Swiss-Prot;Acc:B2RBV5]	protein coding	-0.68	3.89	36.41	<0.0001	<0.0001
ENSG00000262155	LINC02175	long intergenic non-protein coding RNA 2175 [Source:HGNC Symbol;Acc:HGNC:27550]	lncRNA	-0.68	0.93	20.84	<0.0001	<0.0001
ENSG00000197536	IRF1-AS1	IRF1 antisense RNA 1 [Source:HGNC Symbol;Acc:HGNC:33838]	lncRNA	0.68	5.30	33.86	<0.0001	<0.0001
ENSG00000135622	SEMA4F	ssemaphorin 4F [Source:HGNC Symbol;Acc:HGNC:10734]	protein coding	-0.68	1.80	11.80	0.0006	0.0021
ENSG00000188157	AGRN	agrin [Source:HGNC Symbol;Acc:HGNC:329]	protein coding	0.68	4.90	37.87	<0.0001	<0.0001
ENSG00000119699	TGFB3	transforming growth factor beta 3 [Source:HGNC Symbol;Acc:HGNC:11769]	protein coding	-0.68	1.31	23.44	<0.0001	<0.0001
ENSG00000224307		novel transcript	lncRNA	-0.68	3.03	22.39	<0.0001	<0.0001
ENSG00000226174	TEX22	testis expressed 22 [Source:HGNC Symbol;Acc:HGNC:40026]	protein coding	-0.68	1.47	14.71	0.0001	0.0006
ENSG00000232022	FAAHP1	fatty acid amide hydrolase pseudogene 1 [Source:HGNC Symbol;Acc:HGNC:50679]	transcribed unprocessed pseudogene	-0.68	0.31	8.48	0.0036	0.0097
ENSG00000257108	NHLRC4	NHL repeat containing 4 [Source:HGNC Symbol;Acc:HGNC:26700]	protein coding	-0.68	0.51	15.63	<0.0001	0.0004
ENSG00000186260	MRTFB	myocardin related transcription factor B [Source:HGNC Symbol;Acc:HGNC:29819]	protein coding	-0.68	2.91	30.83	<0.0001	<0.0001

Table S12: Genes differentially expressed at Day 30 compared to pre-second vaccination (Study Arm 2 A/H7N9+AS03, D1, 29, Dendritic cells). Genes are sorted by descending absolute log_2 fold change. Gene model summaries and annotations are based on Ensembl Version 99 (continued.)

Ensembl Gene ID	Ensembl Gene Name	Ensembl Gene Description	Gene Type	log_2 Fold Change (Day 30 vs. Pre-Second Vaccination)	Average log_2 CPM	Likelihood Ratio Test Statistic	P-Value	FDR Adjusted P-Value
ENSG00000223705	NSUN5P1	NSUN5 pseudogene 1 [Source:HGNC Symbol;Acc:HGNC:19146]	transcribed unprocessed pseudogene	-0.68	5.09	60.27	<0.0001	<0.0001
ENSG00000066230	SLC9A3	solute carrier family 9 member A3 [Source:HGNC Symbol;Acc:HGNC:11073]	protein coding	-0.68	1.38	19.98	<0.0001	<0.0001
ENSG00000234741	GAS5	growth arrest specific 5 [Source:HGNC Symbol;Acc:HGNC:16355]	lncRNA	-0.68	6.26	46.71	<0.0001	<0.0001
ENSG00000273272		novel transcript	lncRNA	0.68	0.57	6.48	0.0109	0.0252
ENSG00000003400	CASP10	caspase 10 [Source:HGNC Symbol;Acc:HGNC:1500]	protein coding	0.68	6.16	49.00	<0.0001	<0.0001
ENSG00000139618	BRCA2	BRCA2 DNA repair associated [Source:HGNC Symbol;Acc:HGNC:1101]	protein coding	0.68	1.73	20.30	<0.0001	<0.0001
ENSG00000287216		novel transcript, antisense to KIAA0922	lncRNA	0.68	0.96	14.35	0.0002	0.0007
ENSG00000086065	CHMP5	charged multivesicular body protein 5 [Source:HGNC Symbol;Acc:HGNC:26942]	protein coding	0.68	5.46	47.40	<0.0001	<0.0001
ENSG00000256393	RPL41P5	ribosomal protein L41 pseudogene 5 [Source:HGNC Symbol;Acc:HGNC:10355]	processed pseudogene	-0.68	0.70	8.78	0.003	0.0084
ENSG00000156398	SFXN2	sideroflexin 2 [Source:HGNC Symbol;Acc:HGNC:16086]	protein coding	-0.68	1.99	20.67	<0.0001	<0.0001
ENSG00000115419	GLS	glutaminase [Source:HGNC Symbol;Acc:HGNC:4331]	protein coding	0.68	6.36	23.51	<0.0001	<0.0001
ENSG00000188522	FAM83G	family with sequence similarity 83 member G [Source:HGNC Symbol;Acc:HGNC:32554]	protein coding	-0.68	1.73	13.30	0.0003	0.0011
ENSG00000113532	ST8SIA4	ST8 alpha-N-acetyl-neuraminide alpha-2,8-sialyltransferase 4 [Source:HGNC Symbol;Acc:HGNC:10871]	protein coding	0.68	6.11	55.62	<0.0001	<0.0001
ENSG00000108861	DUSP3	dual specificity phosphatase 3 [Source:HGNC Symbol;Acc:HGNC:3069]	protein coding	0.68	6.63	54.21	<0.0001	<0.0001
ENSG00000117318	ID3	inhibitor of DNA binding 3, HLH protein [Source:HGNC Symbol;Acc:HGNC:5362]	protein coding	-0.68	1.73	8.21	0.0042	0.011
ENSG00000101000	PROCR	protein C receptor [Source:HGNC Symbol;Acc:HGNC:9452]	protein coding	-0.68	0.91	19.83	<0.0001	<0.0001
ENSG00000162063	CCNF	cyclin F [Source:HGNC Symbol;Acc:HGNC:1591]	protein coding	-0.68	1.40	15.82	<0.0001	0.0003

Table S12: Genes differentially expressed at Day 30 compared to pre-second vaccination (Study Arm 2 A/H7N9+AS03, D1, 29, Dendritic cells). Genes are sorted by descending absolute log_2 fold change. Gene model summaries and annotations are based on Ensembl Version 99 (continued.)

Ensembl Gene ID	Ensembl Gene Name	Ensembl Gene Description	Gene Type	log_2 Fold Change (Day 30 vs. Pre-Second Vaccination)	Average log_2 CPM	Likelihood Ratio Test Statistic	P-Value	FDR Adjusted P-Value
ENSG00000262227		novel transcript, antisense to INCA1	lncRNA	-0.68	1.18	20.27	<0.0001	<0.0001
ENSG00000167600	CYP2S1	cytochrome P450 family 2 subfamily S member 1 [Source:HGNC Symbol;Acc:HGNC:15654]	protein coding	-0.68	4.74	24.95	<0.0001	<0.0001
ENSG00000144354	CDCA7	cell division cycle associated 7 [Source:HGNC Symbol;Acc:HGNC:14628]	protein coding	-0.68	1.19	18.04	<0.0001	0.0001
ENSG00000168310	IRF2	interferon regulatory factor 2 [Source:HGNC Symbol;Acc:HGNC:6117]	protein coding	0.68	6.68	41.01	<0.0001	<0.0001
ENSG00000196550	FAM72A	family with sequence similarity 72 member A [Source:HGNC Symbol;Acc:HGNC:24044]	protein coding	0.68	0.70	15.01	0.0001	0.0005
ENSG00000117009	KMO	kynurenine 3-monooxygenase [Source:HGNC Symbol;Acc:HGNC:6381]	protein coding	0.68	3.88	30.52	<0.0001	<0.0001
ENSG00000204228	HSD17B8	hydroxysteroid 17-beta dehydrogenase 8 [Source:HGNC Symbol;Acc:HGNC:3554]	protein coding	-0.67	2.76	21.27	<0.0001	<0.0001
ENSG00000272734	ADIRF-AS1	ADIRF antisense RNA 1 [Source:HGNC Symbol;Acc:HGNC:45127]	lncRNA	-0.67	2.44	28.07	<0.0001	<0.0001
ENSG00000176658	MYO1D	myosin ID [Source:HGNC Symbol;Acc:HGNC:7598]	protein coding	-0.67	2.15	22.71	<0.0001	<0.0001
ENSG00000182566	CLEC4G	C-type lectin domain family 4 member G [Source:HGNC Symbol;Acc:HGNC:24591]	protein coding	0.67	2.47	16.12	<0.0001	0.0003
ENSG00000064932	SBNO2	strawberry notch homolog 2 [Source:HGNC Symbol;Acc:HGNC:29158]	protein coding	0.67	8.08	43.69	<0.0001	<0.0001
ENSG00000143147	GPR161	G protein-coupled receptor 161 [Source:HGNC Symbol;Acc:HGNC:23694]	protein coding	0.67	0.85	13.56	0.0002	0.0009
ENSG00000226816		novel transcript	lncRNA	-0.67	0.94	21.33	<0.0001	<0.0001
ENSG00000165028	NIPSNAP3B	nipsnap homolog 3B [Source:HGNC Symbol;Acc:HGNC:23641]	protein coding	-0.67	2.00	20.52	<0.0001	<0.0001
ENSG00000197763	TXNRD3	thioredoxin reductase 3 [Source:HGNC Symbol;Acc:HGNC:20667]	protein coding	-0.67	0.19	12.61	0.0004	0.0014
ENSG00000169962	TAS1R3	taste 1 receptor member 3 [Source:HGNC Symbol;Acc:HGNC:15661]	protein coding	-0.67	0.57	15.73	<0.0001	0.0004
ENSG00000223891	OSER1-DT	OSER1 divergent transcript [Source:HGNC Symbol;Acc:HGNC:48585]	lncRNA	-0.67	1.90	20.75	<0.0001	<0.0001
ENSG00000109046	WSB1	WD repeat and SOCS box containing 1 [Source:HGNC Symbol;Acc:HGNC:19221]	protein coding	0.67	8.35	52.66	<0.0001	<0.0001
ENSG00000071967	CYBRD1	cytochrome b reductase 1 [Source:HGNC Symbol;Acc:HGNC:20797]	protein coding	-0.67	5.90	25.50	<0.0001	<0.0001

Table S12: Genes differentially expressed at Day 30 compared to pre-second vaccination (Study Arm 2 A/H7N9+AS03, D1, 29, Dendritic cells). Genes are sorted by descending absolute log_2 fold change. Gene model summaries and annotations are based on Ensembl Version 99 (continued.)

Ensembl Gene ID	Ensembl Gene Name	Ensembl Gene Description	Gene Type	log_2 Fold Change (Day 30 vs. Pre-Second Vaccination)	Average log_2 CPM	Likelihood Ratio Test Statistic	P-Value	FDR Adjusted P-Value
ENSG00000235106	BRD3OS	BRD3 opposite strand [Source:HGNC Symbol;Acc:HGNC:24742]	protein coding	-0.67	3.07	31.43	<0.0001	<0.0001
ENSG00000258130		coiled-coil domain containing 101 (CCDC101) pseudogene	transcribed unprocessed pseudogene	-0.67	0.54	10.55	0.0012	0.0037
ENSG00000258227	CLEC5A	C-type lectin domain containing 5A [Source:HGNC Symbol;Acc:HGNC:2054]	protein coding	0.67	3.91	8.60	0.0034	0.0092
ENSG00000217801		protein tyrosine phosphatase family pseudogene	transcribed unprocessed pseudogene	-0.67	1.06	11.23	0.0008	0.0027
ENSG00000197122	SRC	SRC proto-oncogene, non-receptor tyrosine kinase [Source:HGNC Symbol;Acc:HGNC:11283]	protein coding	0.67	6.98	36.92	<0.0001	<0.0001
ENSG00000278864		novel transcript	TEC	-0.67	1.14	16.09	<0.0001	0.0003
ENSG00000130303	BST2	bone marrow stromal cell antigen 2 [Source:HGNC Symbol;Acc:HGNC:1119]	protein coding	0.67	7.53	50.77	<0.0001	<0.0001
ENSG00000167004	PDIA3	protein disulfide isomerase family A member 3 [Source:HGNC Symbol;Acc:HGNC:4606]	protein coding	0.67	8.13	34.72	<0.0001	<0.0001
ENSG00000166068	SPRED1	sprouty related EVH1 domain containing 1 [Source:HGNC Symbol;Acc:HGNC:20249]	protein coding	-0.67	4.50	18.41	<0.0001	0.0001
ENSG00000070501	POLB	DNA polymerase beta [Source:HGNC Symbol;Acc:HGNC:9174]	protein coding	0.67	4.38	33.17	<0.0001	<0.0001
ENSG00000019169	MARCO	macrophage receptor with collagenous structure [Source:HGNC Symbol;Acc:HGNC:6895]	protein coding	0.67	5.77	22.22	<0.0001	<0.0001
ENSG00000036448	MYOM2	myomesin 2 [Source:HGNC Symbol;Acc:HGNC:7614]	protein coding	-0.67	2.86	8.15	0.0043	0.0113
ENSG00000144655	CSRNP1	cysteine and serine rich nuclear protein 1 [Source:HGNC Symbol;Acc:HGNC:14300]	protein coding	0.67	6.45	24.89	<0.0001	<0.0001
ENSG00000163814	CDCP1	CUB domain containing protein 1 [Source:HGNC Symbol;Acc:HGNC:24357]	protein coding	0.67	1.69	12.32	0.0004	0.0016
ENSG00000272669		novel transcript, antisense to SUN2	lncRNA	0.67	1.24	18.04	<0.0001	0.0001
ENSG00000161835	GRASP	general receptor for phosphoinositides 1 associated scaffold protein [Source:HGNC Symbol;Acc:HGNC:18707]	protein coding	-0.67	4.52	10.00	0.0016	0.0048

Table S12: Genes differentially expressed at Day 30 compared to pre-second vaccination (Study Arm 2 A/H7N9+AS03, D1, 29, Dendritic cells). Genes are sorted by descending absolute log_2 fold change. Gene model summaries and annotations are based on Ensembl Version 99 (continued.)

Ensembl Gene ID	Ensembl Gene Name	Ensembl Gene Description	Gene Type	log_2 Fold Change (Day 30 vs. Pre-Second Vaccination)	Average log_2 CPM	Likelihood Ratio Test Statistic	P-Value	FDR Adjusted P-Value
ENSG00000138190	EXOC6	exocyst complex component 6 [Source:HGNC Symbol;Acc:HGNC:23196]	protein coding	0.67	4.26	46.19	<0.0001	<0.0001
ENSG00000073605	GSDMB	gasdermin B [Source:HGNC Symbol;Acc:HGNC:23690]	protein coding	-0.67	4.07	25.59	<0.0001	<0.0001
ENSG00000180035	ZNF48	zinc finger protein 48 [Source:HGNC Symbol;Acc:HGNC:13114]	protein coding	-0.67	2.76	25.99	<0.0001	<0.0001
ENSG00000138760	SCARB2	scavenger receptor class B member 2 [Source:HGNC Symbol;Acc:HGNC:1665]	protein coding	0.67	7.20	50.92	<0.0001	<0.0001
ENSG00000176472	ZNF575	zinc finger protein 575 [Source:HGNC Symbol;Acc:HGNC:27606]	protein coding	-0.67	1.84	24.03	<0.0001	<0.0001
ENSG00000197019	SERTAD1	SERTA domain containing 1 [Source:HGNC Symbol;Acc:HGNC:17932]	protein coding	0.67	3.80	27.83	<0.0001	<0.0001
ENSG00000109680	TBC1D19	TBC1 domain family member 19 [Source:HGNC Symbol;Acc:HGNC:25624]	protein coding	-0.67	0.33	13.61	0.0002	0.0009
ENSG00000005249	PRKAR2B	protein kinase cAMP-dependent type II regulatory subunit beta [Source:HGNC Symbol;Acc:HGNC:9392]	protein coding	0.67	4.01	24.75	<0.0001	<0.0001
ENSG00000180867	PDIA3P1	protein disulfide isomerase family A member 3 pseudogene 1 [Source:HGNC Symbol;Acc:HGNC:4607]	transcribed processed pseudogene	0.67	4.54	33.71	<0.0001	<0.0001
ENSG00000158856	DMTN	dematin actin binding protein [Source:HGNC Symbol;Acc:HGNC:3382]	protein coding	-0.67	2.24	5.88	0.0153	0.0338
ENSG00000287537		novel transcript	lncRNA	-0.67	0.44	9.92	0.0016	0.005
ENSG00000175505	CLCF1	cardiotrophin like cytokine factor 1 [Source:HGNC Symbol;Acc:HGNC:17412]	protein coding	-0.67	3.14	10.64	0.0011	0.0035
ENSG00000089220	PEBP1	phosphatidylethanolamine binding protein 1 [Source:HGNC Symbol;Acc:HGNC:8630]	protein coding	-0.67	5.83	59.70	<0.0001	<0.0001
ENSG00000105855	ITGB8	integrin subunit beta 8 [Source:HGNC Symbol;Acc:HGNC:6163]	protein coding	-0.67	0.75	7.05	0.0079	0.0191
ENSG00000235194	PPP1R3E	protein phosphatase 1 regulatory subunit 3E [Source:HGNC Symbol;Acc:HGNC:14943]	protein coding	-0.67	4.31	32.25	<0.0001	<0.0001
ENSG00000213658	LAT	linker for activation of T cells [Source:HGNC Symbol;Acc:HGNC:18874]	protein coding	-0.67	2.38	14.26	0.0002	0.0007
ENSG00000116574	RHOU	ras homolog family member U [Source:HGNC Symbol;Acc:HGNC:17794]	protein coding	0.67	5.53	60.31	<0.0001	<0.0001

Table S12: Genes differentially expressed at Day 30 compared to pre-second vaccination (Study Arm 2 A/H7N9+AS03, D1, 29, Dendritic cells). Genes are sorted by descending absolute log_2 fold change. Gene model summaries and annotations are based on Ensembl Version 99 (continued.)

Ensembl Gene ID	Ensembl Gene Name	Ensembl Gene Description	Gene Type	log_2 Fold Change (Day 30 vs. Pre-Second Vaccination)	Average log_2 CPM	Likelihood Ratio Test Statistic	P-Value	FDR Adjusted P-Value
ENSG00000115919	KYNU	kynureninase [Source:HGNC Symbol;Acc:HGNC:6469]	protein coding	0.66	5.99	42.74	<0.0001	<0.0001
ENSG00000259834		novel transcript	lncRNA	-0.66	3.59	16.28	<0.0001	0.0003
ENSG00000140280	LYSMD2	LysM domain containing 2 [Source:HGNC Symbol;Acc:HGNC:28571]	protein coding	0.66	5.38	41.22	<0.0001	<0.0001
ENSG00000138606	SHF	Src homology 2 domain containing F [Source:HGNC Symbol;Acc:HGNC:25116]	protein coding	-0.66	0.39	16.19	<0.0001	0.0003
ENSG00000136682	CBWD2	COBW domain containing 2 [Source:HGNC Symbol;Acc:HGNC:17907]	protein coding	0.66	4.94	30.16	<0.0001	<0.0001
ENSG00000144749	LRIG1	leucine rich repeats and immunoglobulin like domains 1 [Source:HGNC Symbol;Acc:HGNC:17360]	protein coding	-0.66	3.48	24.22	<0.0001	<0.0001
ENSG00000118965	WDR35	WD repeat domain 35 [Source:HGNC Symbol;Acc:HGNC:29250]	protein coding	-0.66	2.24	33.19	<0.0001	<0.0001
ENSG00000059588	TARBP1	TAR (HIV-1) RNA binding protein 1 [Source:HGNC Symbol;Acc:HGNC:11568]	protein coding	-0.66	5.34	41.46	<0.0001	<0.0001
ENSG00000183625	CCR3	C-C motif chemokine receptor 3 [Source:HGNC Symbol;Acc:HGNC:1604]	protein coding	-0.66	1.73	8.35	0.0039	0.0103
ENSG00000120896	SORBS3	sorbin and SH3 domain containing 3 [Source:HGNC Symbol;Acc:HGNC:30907]	protein coding	-0.66	5.68	26.24	<0.0001	<0.0001
ENSG00000255045		novel transcript	lncRNA	-0.66	0.47	8.94	0.0028	0.0078
ENSG00000164953	TMEM67	transmembrane protein 67 [Source:HGNC Symbol;Acc:HGNC:28396]	protein coding	-0.66	0.35	16.60	<0.0001	0.0002
ENSG00000198502	HLA-DRB5	major histocompatibility complex, class II, DR beta 5 [Source:HGNC Symbol;Acc:HGNC:4953]	protein coding	0.66	9.08	16.61	<0.0001	0.0002
ENSG00000139174	PRICKLE1	prickle planar cell polarity protein 1 [Source:HGNC Symbol;Acc:HGNC:17019]	protein coding	0.66	1.28	15.68	<0.0001	0.0004
ENSG00000224546	EIF4BP3	eukaryotic translation initiation factor 4B pseudogene 3 [Source:HGNC Symbol;Acc:HGNC:37936]	processed pseudogene	-0.66	0.74	15.59	<0.0001	0.0004
ENSG00000073060	SCARB1	scavenger receptor class B member 1 [Source:HGNC Symbol;Acc:HGNC:1664]	protein coding	-0.66	4.82	22.94	<0.0001	<0.0001
ENSG00000277654		glioma tumor suppressor candidate region gene 2 (GLTSCR2) pseudogene	transcribed processed pseudogene	-0.66	0.96	21.02	<0.0001	<0.0001

Table S12: Genes differentially expressed at Day 30 compared to pre-second vaccination (Study Arm 2 A/H7N9+AS03, D1, 29, Dendritic cells). Genes are sorted by descending absolute log_2 fold change. Gene model summaries and annotations are based on Ensembl Version 99 (continued.)

Ensembl Gene ID	Ensembl Gene Name	Ensembl Gene Description	Gene Type	log_2 Fold Change (Day 30 vs. Pre-Second Vaccination)	Average log_2 CPM	Likelihood Ratio Test Statistic	P-Value	FDR Adjusted P-Value
ENSG00000274471		hect domain and RLD 2 (HERC2) pseudogene	transcribed unprocessed pseudogene	-0.66	1.70	23.97	<0.0001	<0.0001
ENSG00000007038	PRSS21	serine protease 21 [Source:HGNC Symbol;Acc:HGNC:9485]	protein coding	-0.66	1.76	9.89	0.0017	0.005
ENSG00000135899	SP110	SP110 nuclear body protein [Source:HGNC Symbol;Acc:HGNC:5401]	protein coding	0.66	7.07	47.06	<0.0001	<0.0001
ENSG00000273213	H3-2	H3.2 histone (putative) [Source:HGNC Symbol;Acc:HGNC:32060]	protein coding	0.66	0.67	13.53	0.0002	0.0009
ENSG00000237094		pseudogene similar to part of septin 14 SEPT14	transcribed unprocessed pseudogene	0.66	2.62	17.44	<0.0001	0.0002
ENSG00000206418	RAB12	RAB12, member RAS oncogene family [Source:HGNC Symbol;Acc:HGNC:31332]	protein coding	0.66	4.91	42.59	<0.0001	<0.0001
ENSG00000157216	SSBP3	single stranded DNA binding protein 3 [Source:HGNC Symbol;Acc:HGNC:15674]	protein coding	-0.66	6.11	47.60	<0.0001	<0.0001
ENSG00000162543	UBXN10	UBX domain protein 10 [Source:HGNC Symbol;Acc:HGNC:26354]	protein coding	-0.66	0.71	17.41	<0.0001	0.0002
ENSG00000276141	WHAMMP3	WAS protein homolog associated with actin, golgi membranes and microtubules pseudogene 3 [Source:HGNC Symbol;Acc:HGNC:27892]	transcribed unprocessed pseudogene	-0.66	1.17	12.71	0.0004	0.0014
ENSG00000267221	C17orf113	chromosome 17 open reading frame 113 [Source:HGNC Symbol;Acc:HGNC:53437]	protein coding	-0.66	0.77	19.33	<0.0001	<0.0001
ENSG00000277406	SEC22B4P	SEC22 homolog B4, pseudogene [Source:HGNC Symbol;Acc:HGNC:53892]	transcribed unprocessed pseudogene	-0.66	0.64	10.92	0.001	0.0031
ENSG00000065054	SLC9A3R2	SLC9A3 regulator 2 [Source:HGNC Symbol;Acc:HGNC:11076]	protein coding	-0.66	2.45	25.66	<0.0001	<0.0001

Table S12: Genes differentially expressed at Day 30 compared to pre-second vaccination (Study Arm 2 A/H7N9+AS03, D1, 29, Dendritic cells). Genes are sorted by descending absolute log_2 fold change. Gene model summaries and annotations are based on Ensembl Version 99 (continued.)

Ensembl Gene ID	Ensembl Gene Name	Ensembl Gene Description	Gene Type	log_2 Fold Change (Day 30 vs. Pre-Second Vaccination)	Average log_2 CPM	Likelihood Ratio Test Statistic	P-Value	FDR Adjusted P-Value
ENSG00000167972	ABCA3	ATP binding cassette subfamily A member 3 [Source:HGNC Symbol;Acc:HGNC:33]	protein coding	-0.66	2.87	25.69	<0.0001	<0.0001
ENSG00000185420	SMYD3	SET and MYND domain containing 3 [Source:HGNC Symbol;Acc:HGNC:15513]	protein coding	-0.66	1.99	26.32	<0.0001	<0.0001
ENSG00000215533	LINC00189	long intergenic non-protein coding RNA 189 [Source:HGNC Symbol;Acc:HGNC:18461]	lncRNA	0.66	0.52	11.99	0.0005	0.0019
ENSG00000174501	ANKRD36C	ankyrin repeat domain 36C [Source:HGNC Symbol;Acc:HGNC:32946]	protein coding	-0.66	2.59	16.87	<0.0001	0.0002
ENSG00000223704	LINC01422	long intergenic non-protein coding RNA 1422 [Source:HGNC Symbol;Acc:HGNC:50728]	lncRNA	-0.66	1.25	14.54	0.0001	0.0006
ENSG00000115526	CHST10	carbohydrate sulfotransferase 10 [Source:HGNC Symbol;Acc:HGNC:19650]	protein coding	-0.66	1.10	10.95	0.0009	0.0031
ENSG00000225138	SLC9A3-AS1	SLC9A3 antisense RNA 1 [Source:HGNC Symbol;Acc:HGNC:40550]	lncRNA	-0.66	3.39	40.71	<0.0001	<0.0001
ENSG00000198520	ARMH1	armadillo like helical domain containing 1 [Source:HGNC Symbol;Acc:HGNC:34345]	protein coding	0.66	5.34	23.00	<0.0001	<0.0001
ENSG00000172840	PDP2	pyruvate dehyrogenase phosphatase catalytic subunit 2 [Source:HGNC Symbol;Acc:HGNC:30263]	protein coding	-0.66	1.04	9.16	0.0025	0.0071
ENSG00000057935	MTA3	metastasis associated 1 family member 3 [Source:HGNC Symbol;Acc:HGNC:23784]	protein coding	-0.66	3.03	27.85	<0.0001	<0.0001
ENSG00000159685	CHCHD6	coiled-coil-helix-coiled-coil-helix domain containing 6 [Source:HGNC Symbol;Acc:HGNC:28184]	protein coding	-0.66	1.45	23.54	<0.0001	<0.0001
ENSG00000166704	ZNF606	zinc finger protein 606 [Source:HGNC Symbol;Acc:HGNC:25879]	protein coding	-0.66	3.19	30.96	<0.0001	<0.0001
ENSG00000139890	REM2	RRAD and GEM like GTPase 2 [Source:HGNC Symbol;Acc:HGNC:20248]	protein coding	-0.66	0.75	12.11	0.0005	0.0018
ENSG00000112053	SLC26A8	solute carrier family 26 member 8 [Source:HGNC Symbol;Acc:HGNC:14468]	protein coding	0.66	0.54	11.89	0.0006	0.002
ENSG00000138623	SEMA7A	semaphorin 7A (John Milton Hagen blood group) [Source:HGNC Symbol;Acc:HGNC:10741]	protein coding	-0.66	2.91	18.17	<0.0001	0.0001
ENSG00000254470	AP5B1	adaptor related protein complex 5 subunit beta 1 [Source:HGNC Symbol;Acc:HGNC:25104]	protein coding	0.65	7.87	56.42	<0.0001	<0.0001
ENSG00000119608	PROX2	prospero homeobox 2 [Source:HGNC Symbol;Acc:HGNC:26715]	protein coding	-0.65	0.21	10.05	0.0015	0.0047
ENSG00000251247	ZNF345	zinc finger protein 345 [Source:HGNC Symbol;Acc:HGNC:16367]	protein coding	-0.65	2.46	22.21	<0.0001	<0.0001

Table S12: Genes differentially expressed at Day 30 compared to pre-second vaccination (Study Arm 2 A/H7N9+AS03, D1, 29, Dendritic cells). Genes are sorted by descending absolute log_2 fold change. Gene model summaries and annotations are based on Ensembl Version 99 (continued.)

Ensembl Gene ID	Ensembl Gene Name	Ensembl Gene Description	Gene Type	log_2 Fold Change (Day 30 vs. Pre-Second Vaccination)	Average log_2 CPM	Likelihood Ratio Test Statistic	P-Value	FDR Adjusted P-Value
ENSG00000243335	KCTD7	potassium channel tetramerization domain containing 7 [Source:HGNC Symbol;Acc:HGNC:21957]	protein coding	-0.65	4.35	29.26	<0.0001	<0.0001
ENSG00000151948	GLT1D1	glycosyltransferase 1 domain containing 1 [Source:HGNC Symbol;Acc:HGNC:26483]	protein coding	0.65	6.56	40.17	<0.0001	<0.0001
ENSG00000160710	ADAR	adenosine deaminase RNA specific [Source:HGNC Symbol;Acc:HGNC:225]	protein coding	0.65	8.90	54.85	<0.0001	<0.0001
ENSG00000119514	GALNT12	polypeptide N-acetylgalactosaminyltransferase 12 [Source:HGNC Symbol;Acc:HGNC:19877]	protein coding	-0.65	1.49	10.00	0.0016	0.0048
ENSG00000186017	ZNF566	zinc finger protein 566 [Source:HGNC Symbol;Acc:HGNC:25919]	protein coding	-0.65	1.92	21.19	<0.0001	<0.0001
ENSG00000226091	LINC00937	long intergenic non-protein coding RNA 937 [Source:HGNC Symbol;Acc:HGNC:48629]	lncRNA	0.65	5.53	18.18	<0.0001	0.0001
ENSG00000185869	ZNF829	zinc finger protein 829 [Source:HGNC Symbol;Acc:HGNC:34032]	protein coding	-0.65	1.01	16.92	<0.0001	0.0002
ENSG00000171291	ZNF439	zinc finger protein 439 [Source:HGNC Symbol;Acc:HGNC:20873]	protein coding	-0.65	2.68	26.81	<0.0001	<0.0001
ENSG00000141858	SAMD1	sterile alpha motif domain containing 1 [Source:HGNC Symbol;Acc:HGNC:17958]	protein coding	-0.65	5.39	40.09	<0.0001	<0.0001
ENSG00000152582	SPEF2	sperm flagellar 2 [Source:HGNC Symbol;Acc:HGNC:26293]	protein coding	-0.65	2.03	18.61	<0.0001	<0.0001
ENSG00000169508	GPR183	G protein-coupled receptor 183 [Source:HGNC Symbol;Acc:HGNC:3128]	protein coding	-0.65	4.20	13.40	0.0003	0.001
ENSG00000167550	RHEBL1	RHEB like 1 [Source:HGNC Symbol;Acc:HGNC:21166]	protein coding	0.65	0.81	9.80	0.0017	0.0052
ENSG00000148950	IMMP1L	inner mitochondrial membrane peptidase subunit 1 [Source:HGNC Symbol;Acc:HGNC:26317]	protein coding	-0.65	0.56	9.67	0.0019	0.0056
ENSG00000128268	MGAT3	beta-1,4-mannosyl-glycoprotein 4-beta-N-acetylglucosaminyltransferase [Source:HGNC Symbol;Acc:HGNC:7046]	protein coding	-0.65	1.04	10.61	0.0011	0.0036
ENSG00000180530	NRIP1	nuclear receptor interacting protein 1 [Source:HGNC Symbol;Acc:HGNC:8001]	protein coding	0.65	5.65	39.25	<0.0001	<0.0001
ENSG00000179094	PER1	period circadian regulator 1 [Source:HGNC Symbol;Acc:HGNC:8845]	protein coding	-0.65	7.53	10.65	0.0011	0.0035
ENSG00000135519	KCNH3	potassium voltage-gated channel subfamily H member 3 [Source:HGNC Symbol;Acc:HGNC:6252]	protein coding	-0.65	4.38	21.90	<0.0001	<0.0001

Confidential/Proprietary Information

Table S12: Genes differentially expressed at Day 30 compared to pre-second vaccination (Study Arm 2 A/H7N9+AS03, D1, 29, Dendritic cells). Genes are sorted by descending absolute log_2 fold change. Gene model summaries and annotations are based on Ensembl Version 99 (continued.)

Ensembl Gene ID	Ensembl Gene Name	Ensembl Gene Description	Gene Type	log_2 Fold Change (Day 30 vs. Pre-Second Vaccination)	Average log_2 CPM	Likelihood Ratio Test Statistic	P-Value	FDR Adjusted P-Value
ENSG00000100596	SPTLC2	serine palmitoyltransferase long chain base subunit 2 [Source:HGNC Symbol;Acc:HGNC:11278]	protein coding	0.65	7.60	52.81	<0.0001	<0.0001
ENSG00000226312	CFLAR-AS1	CFLAR antisense RNA 1 [Source:HGNC Symbol;Acc:HGNC:14437]	lncRNA	0.65	2.12	22.83	<0.0001	<0.0001
ENSG00000124813	RUNX2	RUNX family transcription factor 2 [Source:HGNC Symbol;Acc:HGNC:10472]	protein coding	-0.65	4.55	55.03	<0.0001	<0.0001
ENSG00000165804	ZNF219	zinc finger protein 219 [Source:HGNC Symbol;Acc:HGNC:13011]	protein coding	-0.65	3.24	30.62	<0.0001	<0.0001
ENSG00000213316	LTC4S	leukotriene C4 synthase [Source:HGNC Symbol;Acc:HGNC:6719]	protein coding	-0.65	1.87	18.32	<0.0001	0.0001
ENSG00000140749	IGSF6	immunoglobulin superfamily member 6 [Source:HGNC Symbol;Acc:HGNC:5953]	protein coding	0.65	7.37	38.47	<0.0001	<0.0001
ENSG00000154134	ROBO3	roundabout guidance receptor 3 [Source:HGNC Symbol;Acc:HGNC:13433]	protein coding	-0.65	3.37	27.02	<0.0001	<0.0001
ENSG00000160284	SPATC1L	spermatogenesis and centriole associated 1 like [Source:HGNC Symbol;Acc:HGNC:1298]	protein coding	-0.65	2.59	24.58	<0.0001	<0.0001
ENSG00000181019	NQO1	NAD(P)H quinone dehydrogenase 1 [Source:HGNC Symbol;Acc:HGNC:2874]	protein coding	-0.65	0.27	14.48	0.0001	0.0006
ENSG00000261997		novel transcript	lncRNA	0.65	1.89	10.68	0.0011	0.0035
ENSG00000175445	LPL	lipoprotein lipase [Source:HGNC Symbol;Acc:HGNC:6677]	protein coding	-0.65	1.36	10.27	0.0014	0.0042
ENSG00000166710	B2M	beta-2-microglobulin [Source:HGNC Symbol;Acc:HGNC:914]	protein coding	0.65	12.57	43.56	<0.0001	<0.0001
ENSG00000273669		novel transcript	lncRNA	-0.65	1.20	14.27	0.0002	0.0007
ENSG00000160229	ZNF66	zinc finger protein 66 [Source:HGNC Symbol;Acc:HGNC:13135]	protein coding	-0.65	0.97	16.29	<0.0001	0.0003
ENSG00000104267	CA2	carbonic anhydrase 2 [Source:HGNC Symbol;Acc:HGNC:1373]	protein coding	0.65	3.39	30.14	<0.0001	<0.0001
ENSG00000100181	TPTEP1	TPTE pseudogene 1 [Source:HGNC Symbol;Acc:HGNC:43648]	transcribed unprocessed pseudogene	-0.65	3.21	24.92	<0.0001	<0.0001
ENSG00000139410	SDSL	serine dehydratase like [Source:HGNC Symbol;Acc:HGNC:30404]	protein coding	0.65	2.60	30.83	<0.0001	<0.0001
ENSG00000224934		novel transcript	lncRNA	-0.65	1.34	10.65	0.0011	0.0035

Table S12: Genes differentially expressed at Day 30 compared to pre-second vaccination (Study Arm 2 A/H7N9+AS03, D1, 29, Dendritic cells). Genes are sorted by descending absolute log_2 fold change. Gene model summaries and annotations are based on Ensembl Version 99 (continued.)

Ensembl Gene ID	Ensembl Gene Name	Ensembl Gene Description	Gene Type	log_2 Fold Change (Day 30 vs. Pre-Second Vaccination)	Average log_2 CPM	Likelihood Ratio Test Statistic	P-Value	FDR Adjusted P-Value
ENSG00000134897	BIVM	basic, immunoglobulin-like variable motif containing [Source:HGNC Symbol;Acc:HGNC:16034]	protein coding	-0.64	1.89	15.38	<0.0001	0.0004
ENSG00000279861		TEC	TEC	0.64	2.74	14.68	0.0001	0.0006
ENSG00000267046	E2F3P1	E2F transcription factor 3 pseudogene 1 [Source:HGNC Symbol;Acc:HGNC:3116]	processed pseudogene	-0.64	0.33	10.62	0.0011	0.0036
ENSG00000287737		novel transcript	lncRNA	-0.64	1.25	13.06	0.0003	0.0012
ENSG00000106341	PPP1R17	protein phosphatase 1 regulatory subunit 17 [Source:HGNC Symbol;Acc:HGNC:16973]	protein coding	-0.64	1.86	14.49	0.0001	0.0006
ENSG00000117090	SLAMF1	signaling lymphocytic activation molecule family member 1 [Source:HGNC Symbol;Acc:HGNC:10903]	protein coding	-0.64	2.63	9.84	0.0017	0.0052
ENSG00000169385	RNASE2	ribonuclease A family member 2 [Source:HGNC Symbol;Acc:HGNC:10045]	protein coding	0.64	4.58	26.89	<0.0001	<0.0001
ENSG00000186529	CYP4F3	cytochrome P450 family 4 subfamily F member 3 [Source:HGNC Symbol;Acc:HGNC:2646]	protein coding	-0.64	0.74	10.75	0.001	0.0034
ENSG00000228486	C2orf92	chromosome 2 open reading frame 92 [Source:HGNC Symbol;Acc:HGNC:49272]	protein coding	-0.64	1.97	19.81	<0.0001	<0.0001
ENSG00000163629	PTPN13	protein tyrosine phosphatase non-receptor type 13 [Source:HGNC Symbol;Acc:HGNC:9646]	protein coding	-0.64	2.99	17.93	<0.0001	0.0001
ENSG00000237039		ribosomal protein S28 (RPS28) pseudogene	processed pseudogene	-0.64	1.45	9.59	0.002	0.0058
ENSG00000198736	MSRB1	methionine sulfoxide reductase B1 [Source:HGNC Symbol;Acc:HGNC:14133]	protein coding	0.64	7.01	35.20	<0.0001	<0.0001
ENSG00000244560		zinc finger protein pseudogene	transcribed unprocessed pseudogene	-0.64	2.48	29.12	<0.0001	<0.0001
ENSG00000172244	C5orf34	chromosome 5 open reading frame 34 [Source:HGNC Symbol;Acc:HGNC:24738]	protein coding	-0.64	0.17	13.28	0.0003	0.0011
ENSG00000113583	C5orf15	chromosome 5 open reading frame 15 [Source:HGNC Symbol;Acc:HGNC:20656]	protein coding	0.64	5.85	42.60	<0.0001	<0.0001
ENSG00000167123	CERCAM	cerebral endothelial cell adhesion molecule [Source:HGNC Symbol;Acc:HGNC:23723]	protein coding	-0.64	1.70	11.80	0.0006	0.0021

Table S12: Genes differentially expressed at Day 30 compared to pre-second vaccination (Study Arm 2 A/H7N9+AS03, D1, 29, Dendritic cells). Genes are sorted by descending absolute log_2 fold change. Gene model summaries and annotations are based on Ensembl Version 99 (continued.)

Ensembl Gene ID	Ensembl Gene Name	Ensembl Gene Description	Gene Type	log_2 Fold Change (Day 30 vs. Pre-Second Vaccination)	Average log_2 CPM	Likelihood Ratio Test Statistic	P-Value	FDR Adjusted P-Value
ENSG00000165923	AGBL2	ATP/GTP binding protein like 2 [Source:HGNC Symbol;Acc:HGNC:26296]	protein coding	-0.64	0.45	12.09	0.0005	0.0018
ENSG00000173083	HPSE	heparanase [Source:HGNC Symbol;Acc:HGNC:5164]	protein coding	0.64	6.36	50.84	<0.0001	<0.0001
ENSG00000114423	CBLB	Cbl proto-oncogene B [Source:HGNC Symbol;Acc:HGNC:1542]	protein coding	-0.64	4.55	22.44	<0.0001	<0.0001
ENSG00000004838	ZMYND10	zinc finger MYND-type containing 10 [Source:HGNC Symbol;Acc:HGNC:19412]	protein coding	-0.64	1.13	19.07	<0.0001	<0.0001
ENSG00000180767	CHST13	carbohydrate sulfotransferase 13 [Source:HGNC Symbol;Acc:HGNC:21755]	protein coding	-0.64	4.57	15.48	<0.0001	0.0004
ENSG00000167552	TUBA1A	tubulin alpha 1a [Source:HGNC Symbol;Acc:HGNC:20766]	protein coding	0.64	7.55	28.34	<0.0001	<0.0001
ENSG00000229677		ribosomal protein L17 (RPL17) pseudogene	processed pseudogene	0.64	0.34	7.78	0.0053	0.0135
ENSG00000275895	U2AF1L5	U2 small nuclear RNA auxiliary factor 1 like 5 [Source:HGNC Symbol;Acc:HGNC:51830]	protein coding	0.64	2.30	6.89	0.0087	0.0208
ENSG00000134460	IL2RA	interleukin 2 receptor subunit alpha [Source:HGNC Symbol;Acc:HGNC:6008]	protein coding	-0.64	1.40	6.57	0.0104	0.0242
ENSG00000142875	PRKACB	protein kinase cAMP-activated catalytic subunit beta [Source:HGNC Symbol;Acc:HGNC:9381]	protein coding	-0.64	5.42	25.69	<0.0001	<0.0001
ENSG00000153208	MERTK	MER proto-oncogene, tyrosine kinase [Source:HGNC Symbol;Acc:HGNC:7027]	protein coding	-0.64	5.09	11.42	0.0007	0.0025
ENSG00000187116	LILRA5	leukocyte immunoglobulin like receptor A5 [Source:HGNC Symbol;Acc:HGNC:16309]	protein coding	0.64	7.71	52.43	<0.0001	<0.0001
ENSG00000129667	RHBDF2	rhomboid 5 homolog 2 [Source:HGNC Symbol;Acc:HGNC:20788]	protein coding	0.64	7.63	52.61	<0.0001	<0.0001
ENSG00000234290		novel transcript	lncRNA	0.64	3.61	18.19	<0.0001	0.0001
ENSG00000085662	AKR1B1	aldo-keto reductase family 1 member B [Source:HGNC Symbol;Acc:HGNC:381]	protein coding	-0.64	5.51	47.72	<0.0001	<0.0001
ENSG00000281398	SNHG4	small nucleolar RNA host gene 4 [Source:HGNC Symbol;Acc:HGNC:32964]	lncRNA	-0.64	2.13	28.80	<0.0001	<0.0001
ENSG00000114739	ACVR2B	activin A receptor type 2B [Source:HGNC Symbol;Acc:HGNC:174]	protein coding	-0.64	2.47	15.45	<0.0001	0.0004
ENSG00000172728	FUT10	fucosyltransferase 10 [Source:HGNC Symbol;Acc:HGNC:19234]	protein coding	-0.64	1.45	18.78	<0.0001	<0.0001

Table S12: Genes differentially expressed at Day 30 compared to pre-second vaccination (Study Arm 2 A/H7N9+AS03, D1, 29, Dendritic cells). Genes are sorted by descending absolute log_2 fold change. Gene model summaries and annotations are based on Ensembl Version 99 (continued.)

Ensembl Gene ID	Ensembl Gene Name	Ensembl Gene Description	Gene Type	log_2 Fold Change (Day 30 vs. Pre-Second Vaccination)	Average log_2 CPM	Likelihood Ratio Test Statistic	P-Value	FDR Adjusted P-Value
ENSG00000172057	ORMDL3	ORMDL sphingolipid biosynthesis regulator 3 [Source:HGNC Symbol;Acc:HGNC:16038]	protein coding	-0.64	5.46	28.98	<0.0001	<0.0001
ENSG00000110799	VWF	von Willebrand factor [Source:HGNC Symbol;Acc:HGNC:12726]	protein coding	-0.64	1.01	8.26	0.004	0.0107
ENSG00000120784	ZFP30	ZFP30 zinc finger protein [Source:HGNC Symbol;Acc:HGNC:29555]	protein coding	-0.64	1.60	21.33	<0.0001	<0.0001
ENSG00000176659	C20orf197	chromosome 20 open reading frame 197 [Source:HGNC Symbol;Acc:HGNC:26601]	lncRNA	-0.64	2.22	20.57	<0.0001	<0.0001
ENSG00000176593		novel transcript, antisense to ZNF606	lncRNA	-0.64	2.58	25.90	<0.0001	<0.0001
ENSG00000182871	COL18A1	collagen type XVIII alpha 1 chain [Source:HGNC Symbol;Acc:HGNC:2195]	protein coding	-0.64	5.01	39.55	<0.0001	<0.0001
ENSG00000262097	LINC02185	long intergenic non-protein coding RNA 2185 [Source:HGNC Symbol;Acc:HGNC:53047]	lncRNA	-0.64	1.95	12.08	0.0005	0.0018
ENSG00000063180	CA11	carbonic anhydrase 11 [Source:HGNC Symbol;Acc:HGNC:1370]	protein coding	-0.64	1.86	14.50	0.0001	0.0006
ENSG00000198355	PIM3	Pim-3 proto-oncogene, serine/threonine kinase [Source:HGNC Symbol;Acc:HGNC:19310]	protein coding	0.64	7.26	16.90	<0.0001	0.0002
ENSG00000204387	SNHG32	small nucleolar RNA host gene 32 [Source:HGNC Symbol;Acc:HGNC:19078]	lncRNA	-0.64	5.85	23.82	<0.0001	<0.0001
ENSG00000198556	ZNF789	zinc finger protein 789 [Source:HGNC Symbol;Acc:HGNC:27801]	protein coding	-0.64	3.36	26.27	<0.0001	<0.0001
ENSG00000138030	KHK	ketohexokinase [Source:HGNC Symbol;Acc:HGNC:6315]	protein coding	-0.64	1.73	16.49	<0.0001	0.0002
ENSG00000179029	TMEM107	transmembrane protein 107 [Source:HGNC Symbol;Acc:HGNC:28128]	protein coding	-0.64	3.55	26.60	<0.0001	<0.0001
ENSG00000247572	CKMT2-AS1	CKMT2 antisense RNA 1 [Source:HGNC Symbol;Acc:HGNC:48997]	lncRNA	-0.63	1.82	16.78	<0.0001	0.0002
ENSG00000260528	FAM157C	family with sequence similarity 157 member C [Source:HGNC Symbol;Acc:HGNC:34081]	lncRNA	0.63	5.32	20.69	<0.0001	<0.0001
ENSG00000231584	FAHD2CP	fumarylacetoacetate hydrolase domain containing 2C, pseudogene [Source:HGNC Symbol;Acc:HGNC:44135]	transcribed unprocessed pseudogene	-0.63	0.34	13.38	0.0003	0.001
ENSG00000172037	LAMB2	laminin subunit beta 2 [Source:HGNC Symbol;Acc:HGNC:6487]	protein coding	-0.63	4.24	34.87	<0.0001	<0.0001

Confidential/Proprietary Information

Table S12: Genes differentially expressed at Day 30 compared to pre-second vaccination (Study Arm 2 A/H7N9+AS03, D1, 29, Dendritic cells). Genes are sorted by descending absolute log_2 fold change. Gene model summaries and annotations are based on Ensembl Version 99 (continued.)

Ensembl Gene ID	Ensembl Gene Name	Ensembl Gene Description	Gene Type	log_2 Fold Change (Day 30 vs. Pre-Second Vaccination)	Average log_2 CPM	Likelihood Ratio Test Statistic	P-Value	FDR Adjusted P-Value
ENSG00000136816	TOR1B	torsin family 1 member B [Source:HGNC Symbol;Acc:HGNC:11995]	protein coding	0.63	5.70	52.46	<0.0001	<0.0001
ENSG00000196116	TDRD7	tudor domain containing 7 [Source:HGNC Symbol;Acc:HGNC:30831]	protein coding	0.63	5.20	47.76	<0.0001	<0.0001
ENSG00000064042	LIMCH1	LIM and calponin homology domains 1 [Source:HGNC Symbol;Acc:HGNC:29191]	protein coding	-0.63	0.18	11.39	0.0007	0.0025
ENSG00000247134		novel transcript	lncRNA	-0.63	1.37	20.86	<0.0001	<0.0001
ENSG00000134369	NAV1	neuron navigator 1 [Source:HGNC Symbol;Acc:HGNC:15989]	protein coding	-0.63	4.13	22.38	<0.0001	<0.0001
ENSG00000168874	ATOH8	atonal bHLH transcription factor 8 [Source:HGNC Symbol;Acc:HGNC:24126]	protein coding	-0.63	2.78	15.62	<0.0001	0.0004
ENSG00000122952	ZWINT	ZW10 interacting kinetochore protein [Source:HGNC Symbol;Acc:HGNC:13195]	protein coding	-0.63	1.07	15.90	<0.0001	0.0003
ENSG00000243466	IGKV1-5	immunoglobulin kappa variable 1-5 [Source:HGNC Symbol;Acc:HGNC:5741]	IG V gene	-0.63	2.16	5.66	0.0173	0.0376
ENSG00000163565	IFI16	interferon gamma inducible protein 16 [Source:HGNC Symbol;Acc:HGNC:5395]	protein coding	0.63	7.76	29.89	<0.0001	<0.0001
ENSG00000100226	GTPBP1	GTP binding protein 1 [Source:HGNC Symbol;Acc:HGNC:4669]	protein coding	0.63	6.43	43.11	<0.0001	<0.0001
ENSG00000154655	L3MBTL4	L3MBTL histone methyl-lysine binding protein 4 [Source:HGNC Symbol;Acc:HGNC:26677]	protein coding	0.63	1.81	24.83	<0.0001	<0.0001
ENSG00000169047	IRS1	insulin receptor substrate 1 [Source:HGNC Symbol;Acc:HGNC:6125]	protein coding	-0.63	2.23	20.22	<0.0001	<0.0001
ENSG00000203999	LINC01270	long intergenic non-protein coding RNA 1270 [Source:HGNC Symbol;Acc:HGNC:27658]	lncRNA	0.63	0.66	16.46	<0.0001	0.0003
ENSG00000166527	CLEC4D	C-type lectin domain family 4 member D [Source:HGNC Symbol;Acc:HGNC:14554]	protein coding	0.63	4.53	21.30	<0.0001	<0.0001
ENSG00000105609	LILRB5	leukocyte immunoglobulin like receptor B5 [Source:HGNC Symbol;Acc:HGNC:6609]	protein coding	0.63	2.05	15.84	<0.0001	0.0003
ENSG00000065534	MYLK	myosin light chain kinase [Source:HGNC Symbol;Acc:HGNC:7590]	protein coding	-0.63	0.60	10.56	0.0012	0.0037
ENSG00000112249	ASCC3	activating signal cointegrator 1 complex subunit 3 [Source:HGNC Symbol;Acc:HGNC:18697]	protein coding	0.63	5.56	41.65	<0.0001	<0.0001
ENSG00000147408	CSGALNACT1	chondroitin sulfate N-acetylgalactosaminyltransferase 1 [Source:HGNC Symbol;Acc:HGNC:24290]	protein coding	-0.63	3.87	13.55	0.0002	0.0009
ENSG00000285851		novel transcript	lncRNA	0.63	0.30	12.86	0.0003	0.0013

Table S12: Genes differentially expressed at Day 30 compared to pre-second vaccination (Study Arm 2 A/H7N9+AS03, D1, 29, Dendritic cells). Genes are sorted by descending absolute log_2 fold change. Gene model summaries and annotations are based on Ensembl Version 99 (continued.)

Ensembl Gene ID	Ensembl Gene Name	Ensembl Gene Description	Gene Type	log_2 Fold Change (Day 30 vs. Pre-Second Vaccination)	Average log_2 CPM	Likelihood Ratio Test Statistic	P-Value	FDR Adjusted P-Value
ENSG00000261526		novel transcript	lncRNA	-0.63	2.36	17.91	<0.0001	0.0001
ENSG00000267632		novel transcript	lncRNA	0.63	0.93	13.20	0.0003	0.0011
ENSG00000233837	EIF3LP2	eukaryotic translation initiation factor 3 subunit L pseudogene 2 [Source:HGNC Symbol;Acc:HGNC:24657]	processed pseudogene	-0.63	0.32	10.11	0.0015	0.0045
ENSG00000177383	MAGEF1	MAGE family member F1 [Source:HGNC Symbol;Acc:HGNC:29639]	protein coding	-0.63	3.57	35.27	<0.0001	<0.0001
ENSG00000185986	SDHAP3	succinate dehydrogenase complex flavoprotein subunit A pseudogene 3 [Source:HGNC Symbol;Acc:HGNC:18781]	transcribed unprocessed pseudogene	-0.63	2.54	15.93	<0.0001	0.0003
ENSG00000239521	CASTOR3	CASTOR family member 3 [Source:HGNC Symbol;Acc:HGNC:29954]	protein coding	-0.63	2.57	17.53	<0.0001	0.0002
ENSG00000107331	ABCA2	ATP binding cassette subfamily A member 2 [Source:HGNC Symbol;Acc:HGNC:32]	protein coding	-0.63	6.44	24.73	<0.0001	<0.0001
ENSG00000142765	SYTL1	synaptotagmin like 1 [Source:HGNC Symbol;Acc:HGNC:15584]	protein coding	-0.63	7.59	33.98	<0.0001	<0.0001
ENSG00000132846	ZBED3	zinc finger BED-type containing 3 [Source:HGNC Symbol;Acc:HGNC:20711]	protein coding	-0.63	2.97	24.28	<0.0001	<0.0001
ENSG00000065357	DGKA	diacylglycerol kinase alpha [Source:HGNC Symbol;Acc:HGNC:2849]	protein coding	-0.63	6.63	24.03	<0.0001	<0.0001
ENSG00000160216	AGPAT3	1-acylglycerol-3-phosphate O-acyltransferase 3 [Source:HGNC Symbol;Acc:HGNC:326]	protein coding	0.63	8.10	56.23	<0.0001	<0.0001
ENSG00000185215	TNFAIP2	TNF alpha induced protein 2 [Source:HGNC Symbol;Acc:HGNC:11895]	protein coding	0.63	10.75	37.09	<0.0001	<0.0001
ENSG00000100154	TTC28	tetratricopeptide repeat domain 28 [Source:HGNC Symbol;Acc:HGNC:29179]	protein coding	-0.63	0.86	16.07	<0.0001	0.0003
ENSG00000160766	GBAP1	glucosylceramidase beta pseudogene 1 [Source:HGNC Symbol;Acc:HGNC:4178]	transcribed unprocessed pseudogene	-0.63	0.88	14.81	0.0001	0.0005
ENSG00000226791	LINC02611	long intergenic non-protein coding RNA 2611 [Source:HGNC Symbol;Acc:HGNC:54060]	lncRNA	-0.62	0.10	6.58	0.0103	0.024

Table S12: Genes differentially expressed at Day 30 compared to pre-second vaccination (Study Arm 2 A/H7N9+AS03, D1, 29, Dendritic cells). Genes are sorted by descending absolute log_2 fold change. Gene model summaries and annotations are based on Ensembl Version 99 (continued.)

Ensembl Gene ID	Ensembl Gene Name	Ensembl Gene Description	Gene Type	log_2 Fold Change (Day 30 vs. Pre-Second Vaccination)	Average log_2 CPM	Likelihood Ratio Test Statistic	P-Value	FDR Adjusted P-Value
ENSG00000107566	ERLIN1	ER lipid raft associated 1 [Source:HGNC Symbol;Acc:HGNC:16947]	protein coding	0.62	5.13	31.16	<0.0001	<0.0001
ENSG00000136810	TXN	thioredoxin [Source:HGNC Symbol;Acc:HGNC:12435]	protein coding	0.62	5.60	32.38	<0.0001	<0.0001
ENSG00000244482	LILRA6	leukocyte immunoglobulin like receptor A6 [Source:HGNC Symbol;Acc:HGNC:15495]	protein coding	0.62	7.10	49.73	<0.0001	<0.0001
ENSG00000156110	ADK	adenosine kinase [Source:HGNC Symbol;Acc:HGNC:257]	protein coding	-0.62	4.43	34.30	<0.0001	<0.0001
ENSG00000197530	MIB2	mindbomb E3 ubiquitin protein ligase 2 [Source:HGNC Symbol;Acc:HGNC:30577]	protein coding	-0.62	6.80	35.97	<0.0001	<0.0001
ENSG00000008323	PLEKHG6	pleckstrin homology and RhoGEF domain containing G6 [Source:HGNC Symbol;Acc:HGNC:25562]	protein coding	0.62	0.41	8.34	0.0039	0.0103
ENSG00000186431	FCAR	Fc fragment of IgA receptor [Source:HGNC Symbol;Acc:HGNC:3608]	protein coding	0.62	4.73	25.28	<0.0001	<0.0001
ENSG00000114978	MOB1A	MOB kinase activator 1A [Source:HGNC Symbol;Acc:HGNC:16015]	protein coding	0.62	8.52	52.52	<0.0001	<0.0001
ENSG00000185950	IRS2	insulin receptor substrate 2 [Source:HGNC Symbol;Acc:HGNC:6126]	protein coding	-0.62	6.91	17.55	<0.0001	0.0002
ENSG00000258701	LINC00638	long intergenic non-protein coding RNA 638 [Source:HGNC Symbol;Acc:HGNC:28325]	lncRNA	-0.62	0.27	10.72	0.0011	0.0034
ENSG00000255398	HCAR3	hydroxycarboxylic acid receptor 3 [Source:HGNC Symbol;Acc:HGNC:16824]	protein coding	0.62	2.37	7.24	0.0071	0.0174
ENSG00000076513	ANKRD13A	ankyrin repeat domain 13A [Source:HGNC Symbol;Acc:HGNC:21268]	protein coding	0.62	7.38	49.78	<0.0001	<0.0001
ENSG00000280734	LINC01232	long intergenic non-protein coding RNA 1232 [Source:HGNC Symbol;Acc:HGNC:49755]	lncRNA	0.62	2.29	24.38	<0.0001	<0.0001
ENSG00000206567		novel transcript	lncRNA	-0.62	0.27	9.39	0.0022	0.0063
ENSG00000185201	IFITM2	interferon induced transmembrane protein 2 [Source:HGNC Symbol;Acc:HGNC:5413]	protein coding	0.62	8.21	55.33	<0.0001	<0.0001
ENSG00000163864	NMNAT3	nicotinamide nucleotide adenylyltransferase 3 [Source:HGNC Symbol;Acc:HGNC:20989]	protein coding	-0.62	1.68	21.17	<0.0001	<0.0001
ENSG00000103642	LACTB	lactamase beta [Source:HGNC Symbol;Acc:HGNC:16468]	protein coding	0.62	6.36	39.10	<0.0001	<0.0001
ENSG00000233493	TMEM238	transmembrane protein 238 [Source:HGNC Symbol;Acc:HGNC:40042]	protein coding	-0.62	1.98	13.51	0.0002	0.001

Table S12: Genes differentially expressed at Day 30 compared to pre-second vaccination (Study Arm 2 A/H7N9+AS03, D1, 29, Dendritic cells). Genes are sorted by descending absolute log_2 fold change. Gene model summaries and annotations are based on Ensembl Version 99 (continued.)

Ensembl Gene ID	Ensembl Gene Name	Ensembl Gene Description	Gene Type	log_2 Fold Change (Day 30 vs. Pre-Second Vaccination)	Average log_2 CPM	Likelihood Ratio Test Statistic	P-Value	FDR Adjusted P-Value
ENSG00000167208	SNX20	sorting nexin 20 [Source:HGNC Symbol;Acc:HGNC:30390]	protein coding	0.62	7.01	37.65	<0.0001	<0.0001
ENSG00000144843	ADPRH	ADP-ribosylarginine hydrolase [Source:HGNC Symbol;Acc:HGNC:269]	protein coding	0.62	5.11	29.41	<0.0001	<0.0001
ENSG00000225032		novel transcript	lncRNA	-0.62	2.61	18.40	<0.0001	0.0001
ENSG00000169413	RNASE6	ribonuclease A family member k6 [Source:HGNC Symbol;Acc:HGNC:10048]	protein coding	-0.62	6.07	18.36	<0.0001	0.0001
ENSG00000138002	IFT172	intraflagellar transport 172 [Source:HGNC Symbol;Acc:HGNC:30391]	protein coding	-0.62	3.58	25.25	<0.0001	<0.0001
ENSG00000157978	LDLRAP1	low density lipoprotein receptor adaptor protein 1 [Source:HGNC Symbol;Acc:HGNC:18640]	protein coding	-0.62	5.91	17.67	<0.0001	0.0001
ENSG00000103313	MEFV	MEFV innate immuity regulator, pyrin [Source:HGNC Symbol;Acc:HGNC:6998]	protein coding	0.62	8.03	40.98	<0.0001	<0.0001
ENSG00000139266	MARCHF9	membrane associated ring-CH-type finger 9 [Source:HGNC Symbol;Acc:HGNC:25139]	protein coding	-0.62	4.47	25.67	<0.0001	<0.0001
ENSG00000281358	RASSF1-AS1	RASSF1 antisense RNA 1 [Source:HGNC Symbol;Acc:HGNC:49091]	lncRNA	-0.62	0.79	10.84	0.001	0.0032
ENSG00000155324	GRAMD2B	GRAM domain containing 2B [Source:HGNC Symbol;Acc:HGNC:24911]	protein coding	-0.62	1.53	24.14	<0.0001	<0.0001
ENSG00000257698	GIHCG	GIHCG inhibitor of miR-200b/200a/429 expression [Source:HGNC Symbol;Acc:HGNC:52649]	lncRNA	-0.62	0.32	15.48	<0.0001	0.0004
ENSG00000179841	AKAP5	A-kinase anchoring protein 5 [Source:HGNC Symbol;Acc:HGNC:375]	protein coding	-0.62	1.05	7.50	0.0062	0.0154
ENSG00000067057	PFKP	phosphofructokinase, platelet [Source:HGNC Symbol;Acc:HGNC:8878]	protein coding	0.62	5.52	15.00	0.0001	0.0005
ENSG00000284707		novel transcript	lncRNA	-0.62	2.00	15.99	<0.0001	0.0003
ENSG00000196724	ZNF418	zinc finger protein 418 [Source:HGNC Symbol;Acc:HGNC:20647]	protein coding	-0.62	1.58	14.09	0.0002	0.0007
ENSG00000184588	PDE4B	phosphodiesterase 4B [Source:HGNC Symbol;Acc:HGNC:8781]	protein coding	0.62	4.73	22.65	<0.0001	<0.0001
ENSG00000035115	SH3YL1	SH3 and SYLF domain containing 1 [Source:HGNC Symbol;Acc:HGNC:29546]	protein coding	-0.62	4.15	27.04	<0.0001	<0.0001
ENSG00000138413	IDH1	isocitrate dehydrogenase (NADP(+)) 1 [Source:HGNC Symbol;Acc:HGNC:5382]	protein coding	0.62	5.94	35.78	<0.0001	<0.0001
ENSG00000175463	TBC1D10C	TBC1 domain family member 10C [Source:HGNC Symbol;Acc:HGNC:24702]	protein coding	-0.62	7.75	33.72	<0.0001	<0.0001

Table S12: Genes differentially expressed at Day 30 compared to pre-second vaccination (Study Arm 2 A/H7N9+AS03, D1, 29, Dendritic cells). Genes are sorted by descending absolute log_2 fold change. Gene model summaries and annotations are based on Ensembl Version 99 (continued.)

Ensembl Gene ID	Ensembl Gene Name	Ensembl Gene Description	Gene Type	log_2 Fold Change (Day 30 vs. Pre-Second Vaccination)	Average log_2 CPM	Likelihood Ratio Test Statistic	P-Value	FDR Adjusted P-Value
ENSG00000136436	CALCOCO2	calcium binding and coiled-coil domain 2 [Source:HGNC Symbol;Acc:HGNC:29912]	protein coding	0.62	7.35	42.78	<0.0001	<0.0001
ENSG00000135541	AHI1	Abelson helper integration site 1 [Source:HGNC Symbol;Acc:HGNC:21575]	protein coding	-0.62	1.31	13.09	0.0003	0.0012
ENSG00000092470	WDR76	WD repeat domain 76 [Source:HGNC Symbol;Acc:HGNC:25773]	protein coding	-0.62	1.63	13.22	0.0003	0.0011
ENSG00000141837	CACNA1A	calcium voltage-gated channel subunit alpha1 A [Source:HGNC Symbol;Acc:HGNC:1388]	protein coding	0.62	1.41	16.84	<0.0001	0.0002
ENSG00000228140		novel transcript	lncRNA	-0.62	0.54	12.22	0.0005	0.0017
ENSG00000258810		novel transcript, antisense to RNASE1	lncRNA	-0.62	1.23	14.14	0.0002	0.0007
ENSG00000075568	TMEM131	transmembrane protein 131 [Source:HGNC Symbol;Acc:HGNC:30366]	protein coding	0.62	6.79	36.61	<0.0001	<0.0001
ENSG00000132256	TRIM5	tripartite motif containing 5 [Source:HGNC Symbol;Acc:HGNC:16276]	protein coding	0.61	4.95	31.13	<0.0001	<0.0001
ENSG00000113916	BCL6	BCL6 transcription repressor [Source:HGNC Symbol;Acc:HGNC:1001]	protein coding	0.61	7.34	38.34	<0.0001	<0.0001
ENSG00000198720	ANKRD13B	ankyrin repeat domain 13B [Source:HGNC Symbol;Acc:HGNC:26363]	protein coding	-0.61	0.67	9.82	0.0017	0.0052
ENSG00000181744	DIPK2A	divergent protein kinase domain 2A [Source:HGNC Symbol;Acc:HGNC:28490]	protein coding	0.61	5.10	25.29	<0.0001	<0.0001
ENSG00000151240	DIP2C	disco interacting protein 2 homolog C [Source:HGNC Symbol;Acc:HGNC:29150]	protein coding	-0.61	0.62	6.41	0.0113	0.0261
ENSG00000069399	BCL3	BCL3 transcription coactivator [Source:HGNC Symbol;Acc:HGNC:998]	protein coding	0.61	6.24	27.59	<0.0001	<0.0001
ENSG00000233578	EIF4EP1	eukaryotic translation initiation factor 4E pseudogene 1 [Source:HGNC Symbol;Acc:HGNC:3292]	processed pseudogene	0.61	0.78	6.17	0.013	0.0294
ENSG00000251369	ZNF550	zinc finger protein 550 [Source:HGNC Symbol;Acc:HGNC:28643]	protein coding	-0.61	3.23	26.40	<0.0001	<0.0001
ENSG00000171208	NETO2	neuropilin and tolloid like 2 [Source:HGNC Symbol;Acc:HGNC:14644]	protein coding	0.61	3.91	26.49	<0.0001	<0.0001
ENSG00000134575	ACP2	acid phosphatase 2, lysosomal [Source:HGNC Symbol;Acc:HGNC:123]	polymorphic pseudogene	0.61	5.59	65.89	<0.0001	<0.0001
ENSG00000165997	ARL5B	ADP ribosylation factor like GTPase 5B [Source:HGNC Symbol;Acc:HGNC:23052]	protein coding	0.61	4.57	17.85	<0.0001	0.0001

Table S12: Genes differentially expressed at Day 30 compared to pre-second vaccination (Study Arm 2 A/H7N9+AS03, D1, 29, Dendritic cells). Genes are sorted by descending absolute log_2 fold change. Gene model summaries and annotations are based on Ensembl Version 99 (continued.)

Ensembl Gene ID	Ensembl Gene Name	Ensembl Gene Description	Gene Type	log_2 Fold Change (Day 30 vs. Pre-Second Vaccination)	Average log_2 CPM	Likelihood Ratio Test Statistic	P-Value	FDR Adjusted P-Value
ENSG00000169715	MT1E	metallothionein 1E [Source:HGNC Symbol;Acc:HGNC:7397]	protein coding	0.61	0.84	10.64	0.0011	0.0035
ENSG00000204136	GGTA1P	glycoprotein alpha-galactosyltransferase 1, pseudogene [Source:HGNC Symbol;Acc:HGNC:4253]	transcribed unitary pseudogene	-0.61	4.02	14.32	0.0002	0.0007
ENSG00000237037	NDUFA6-DT	NDUFA6 divergent transcript [Source:HGNC Symbol;Acc:HGNC:45273]	lncRNA	-0.61	1.42	13.36	0.0003	0.001
ENSG00000167799	NUDT8	nudix hydrolase 8 [Source:HGNC Symbol;Acc:HGNC:8055]	protein coding	-0.61	0.74	11.70	0.0006	0.0022
ENSG00000108474	PIGL	phosphatidylinositol glycan anchor biosynthesis class L [Source:HGNC Symbol;Acc:HGNC:8966]	protein coding	-0.61	3.87	22.59	<0.0001	<0.0001
ENSG00000179021	C3orf38	chromosome 3 open reading frame 38 [Source:HGNC Symbol;Acc:HGNC:28384]	protein coding	0.61	4.86	36.51	<0.0001	<0.0001
ENSG00000213976		zinc finger protein pseudogene	unprocessed pseudogene	-0.61	0.32	15.90	<0.0001	0.0003
ENSG00000173295	FAM86B3P	family with sequence similarity 86 member B3, pseudogene [Source:HGNC Symbol;Acc:HGNC:44371]	transcribed unprocessed pseudogene	-0.61	1.17	15.88	<0.0001	0.0003
ENSG00000171848	RRM2	ribonucleotide reductase regulatory subunit M2 [Source:HGNC Symbol;Acc:HGNC:10452]	protein coding	-0.61	1.35	6.92	0.0085	0.0204
ENSG00000185664	PMEL	premelanosome protein [Source:HGNC Symbol;Acc:HGNC:10880]	protein coding	-0.61	0.13	8.62	0.0033	0.009
ENSG00000263072	ZNF213-AS1	ZNF213 antisense RNA 1 (head to head) [Source:HGNC Symbol;Acc:HGNC:50505]	lncRNA	-0.61	3.48	25.89	<0.0001	<0.0001
ENSG00000153029	MR1	major histocompatibility complex, class I-related [Source:HGNC Symbol;Acc:HGNC:4975]	protein coding	0.61	6.33	62.20	<0.0001	<0.0001
ENSG00000213190	MLLT11	MLLT11 transcription factor 7 cofactor [Source:HGNC Symbol;Acc:HGNC:16997]	protein coding	-0.61	0.65	8.81	0.003	0.0083
ENSG00000187109	NAP1L1	nucleosome assembly protein 1 like 1 [Source:HGNC Symbol;Acc:HGNC:7637]	protein coding	-0.61	9.10	32.88	<0.0001	<0.0001
ENSG00000128298	BAIAP2L2	BAR/IMD domain containing adaptor protein 2 like 2 [Source:HGNC Symbol;Acc:HGNC:26203]	protein coding	-0.61	-0.06	6.69	0.0097	0.0228

Table S12: Genes differentially expressed at Day 30 compared to pre-second vaccination (Study Arm 2 A/H7N9+AS03, D1, 29, Dendritic cells). Genes are sorted by descending absolute log_2 fold change. Gene model summaries and annotations are based on Ensembl Version 99 (continued.)

Ensembl Gene ID	Ensembl Gene Name	Ensembl Gene Description	Gene Type	log_2 Fold Change (Day 30 vs. Pre-Second Vaccination)	Average log_2 CPM	Likelihood Ratio Test Statistic	P-Value	FDR Adjusted P-Value
ENSG00000236144	TMEM147-AS1	TMEM147 antisense RNA 1 [Source:HGNC Symbol;Acc:HGNC:51273]	lncRNA	-0.61	3.71	30.32	<0.0001	<0.0001
ENSG00000167202	TBC1D2B	TBC1 domain family member 2B [Source:HGNC Symbol;Acc:HGNC:29183]	protein coding	0.61	6.65	53.36	<0.0001	<0.0001
ENSG00000174306	ZHX3	zinc fingers and homeoboxes 3 [Source:HGNC Symbol;Acc:HGNC:15935]	protein coding	-0.61	3.48	24.25	<0.0001	<0.0001
ENSG00000168404	MLKL	mixed lineage kinase domain like pseudokinase [Source:HGNC Symbol;Acc:HGNC:26617]	protein coding	0.61	6.69	52.41	<0.0001	<0.0001
ENSG00000173991	TCAP	titin-cap [Source:HGNC Symbol;Acc:HGNC:11610]	protein coding	-0.61	0.16	12.84	0.0003	0.0013
ENSG00000273837		novel transcript, sense intronic to AC018755.18	lncRNA	0.61	2.79	14.22	0.0002	0.0007
ENSG00000185482	STAC3	SH3 and cysteine rich domain 3 [Source:HGNC Symbol;Acc:HGNC:28423]	protein coding	0.61	5.38	49.21	<0.0001	<0.0001
ENSG00000179715	PCED1B	PC-esterase domain containing 1B [Source:HGNC Symbol;Acc:HGNC:28255]	protein coding	-0.61	4.11	8.07	0.0045	0.0118
ENSG00000151490	PTPRO	protein tyrosine phosphatase receptor type O [Source:HGNC Symbol;Acc:HGNC:9678]	protein coding	0.61	3.99	27.36	<0.0001	<0.0001
ENSG00000179523	EIF3J-DT	EIF3J divergent transcript [Source:HGNC Symbol;Acc:HGNC:48616]	lncRNA	-0.61	2.96	31.03	<0.0001	<0.0001
ENSG00000054219	LY75	lymphocyte antigen 75 [Source:HGNC Symbol;Acc:HGNC:6729]	protein coding	0.61	4.49	17.72	<0.0001	0.0001
ENSG00000203416	FAM32BP	family with sequence similarity 32 member B, pseudogene [Source:HGNC Symbol;Acc:HGNC:30945]	processed pseudogene	0.61	0.43	6.42	0.0113	0.0259
ENSG00000144451	SPAG16	sperm associated antigen 16 [Source:HGNC Symbol;Acc:HGNC:23225]	protein coding	-0.61	0.43	10.99	0.0009	0.003
ENSG00000131400	NAPSA	napsin A aspartic peptidase [Source:HGNC Symbol;Acc:HGNC:13395]	protein coding	-0.61	0.52	11.90	0.0006	0.002
ENSG00000114812	VIPR1	vasoactive intestinal peptide receptor 1 [Source:HGNC Symbol;Acc:HGNC:12694]	protein coding	-0.61	5.88	29.73	<0.0001	<0.0001
ENSG00000163635	ATXN7	ataxin 7 [Source:HGNC Symbol;Acc:HGNC:10560]	protein coding	0.61	2.39	20.88	<0.0001	<0.0001
ENSG00000143772	ITPKB	inositol-trisphosphate 3-kinase B [Source:HGNC Symbol;Acc:HGNC:6179]	protein coding	-0.61	6.81	37.50	<0.0001	<0.0001
ENSG00000135525	MAP7	microtubule associated protein 7 [Source:HGNC Symbol;Acc:HGNC:6869]	protein coding	-0.61	2.30	22.07	<0.0001	<0.0001

Table S12: Genes differentially expressed at Day 30 compared to pre-second vaccination (Study Arm 2 A/H7N9+AS03, D1, 29, Dendritic cells). Genes are sorted by descending absolute log_2 fold change. Gene model summaries and annotations are based on Ensembl Version 99 (continued.)

Ensembl Gene ID	Ensembl Gene Name	Ensembl Gene Description	Gene Type	log_2 Fold Change (Day 30 vs. Pre-Second Vaccination)	Average log_2 CPM	Likelihood Ratio Test Statistic	P-Value	FDR Adjusted P-Value
ENSG00000228784	LINC00954	long intergenic non-protein coding RNA 954 [Source:HGNC Symbol;Acc:HGNC:48668]	lncRNA	-0.60	3.38	18.78	<0.0001	<0.0001
ENSG00000189283	FHIT	fragile histidine triad diadenosine triphosphatase [Source:HGNC Symbol;Acc:HGNC:3701]	protein coding	-0.60	2.76	8.96	0.0028	0.0077
ENSG00000105750	ZNF85	zinc finger protein 85 [Source:HGNC Symbol;Acc:HGNC:13160]	protein coding	-0.60	1.55	20.33	<0.0001	<0.0001
ENSG00000137364	TPMT	thiopurine S-methyltransferase [Source:HGNC Symbol;Acc:HGNC:12014]	protein coding	0.60	5.78	41.72	<0.0001	<0.0001
ENSG00000173762	CD7	CD7 molecule [Source:HGNC Symbol;Acc:HGNC:1695]	protein coding	-0.60	6.19	8.92	0.0028	0.0079
ENSG00000135404	CD63	CD63 molecule [Source:HGNC Symbol;Acc:HGNC:1692]	protein coding	0.60	7.90	47.78	<0.0001	<0.0001
ENSG00000179978	NAIPP2	NAIP pseudogene 2 [Source:HGNC Symbol;Acc:HGNC:16042]	unprocessed pseudogene	0.60	2.45	24.22	<0.0001	<0.0001
ENSG00000176894	PXMP2	peroxisomal membrane protein 2 [Source:HGNC Symbol;Acc:HGNC:9716]	protein coding	-0.60	0.74	15.66	<0.0001	0.0004
ENSG00000187474	FPR3	formyl peptide receptor 3 [Source:HGNC Symbol;Acc:HGNC:3828]	protein coding	-0.60	3.78	9.16	0.0025	0.0071
ENSG00000261172		novel transcript	lncRNA	0.60	2.79	12.98	0.0003	0.0012
ENSG00000248476	BACH1-IT1	BACH1 intronic transcript 1 [Source:HGNC Symbol;Acc:HGNC:40006]	lncRNA	0.60	0.73	11.86	0.0006	0.002
ENSG00000140743	CDR2	cerebellar degeneration related protein 2 [Source:HGNC Symbol;Acc:HGNC:1799]	protein coding	-0.60	3.58	17.17	<0.0001	0.0002
ENSG00000128694	OSGEPL1	O-sialoglycoprotein endopeptidase like 1 [Source:HGNC Symbol;Acc:HGNC:23075]	protein coding	-0.60	2.38	24.63	<0.0001	<0.0001
ENSG00000175730	BAK1P1	BCL2 antagonist/killer 1 pseudogene 1 [Source:HGNC Symbol;Acc:HGNC:996]	processed pseudogene	0.60	0.98	14.01	0.0002	0.0008
ENSG00000278727		novel transcript	lncRNA	0.60	0.35	12.74	0.0004	0.0014
ENSG00000255717	SNHG1	small nucleolar RNA host gene 1 [Source:HGNC Symbol;Acc:HGNC:32688]	lncRNA	-0.60	5.57	43.30	<0.0001	<0.0001
ENSG00000168477	TNXB	tenascin XB [Source:HGNC Symbol;Acc:HGNC:11976]	protein coding	-0.60	0.72	11.14	0.0008	0.0028
ENSG00000106484	MEST	mesoderm specific transcript [Source:HGNC Symbol;Acc:HGNC:7028]	protein coding	-0.60	1.26	17.37	<0.0001	0.0002

Table S12: Genes differentially expressed at Day 30 compared to pre-second vaccination (Study Arm 2 A/H7N9+AS03, D1, 29, Dendritic cells). Genes are sorted by descending absolute log_2 fold change. Gene model summaries and annotations are based on Ensembl Version 99 (continued.)

Ensembl Gene ID	Ensembl Gene Name	Ensembl Gene Description	Gene Type	log_2 Fold Change (Day 30 vs. Pre-Second Vaccination)	Average log_2 CPM	Likelihood Ratio Test Statistic	P-Value	FDR Adjusted P-Value
ENSG00000135749	PCNX2	pecanex 2 [Source:HGNC Symbol;Acc:HGNC:8736]	protein coding	-0.60	3.84	31.88	<0.0001	<0.0001
ENSG00000162711	NLRP3	NLR family pyrin domain containing 3 [Source:HGNC Symbol;Acc:HGNC:16400]	protein coding	0.60	6.67	27.85	<0.0001	<0.0001
ENSG00000099814	CEP170B	centrosomal protein 170B [Source:HGNC Symbol;Acc:HGNC:20362]	protein coding	-0.60	0.56	8.19	0.0042	0.0111
ENSG00000248487	ABHD14A	abhydrolase domain containing 14A [Source:HGNC Symbol;Acc:HGNC:24538]	protein coding	-0.60	1.28	19.69	<0.0001	<0.0001
ENSG00000155367	PPM1J	protein phosphatase, Mg2+/Mn2+ dependent 1J [Source:HGNC Symbol;Acc:HGNC:20785]	protein coding	-0.60	0.86	13.65	0.0002	0.0009
ENSG00000128394	APOBEC3F	apolipoprotein B mRNA editing enzyme catalytic subunit 3F [Source:HGNC Symbol;Acc:HGNC:17356]	protein coding	0.60	4.12	23.61	<0.0001	<0.0001
ENSG00000160305	DIP2A	disco interacting protein 2 homolog A [Source:HGNC Symbol;Acc:HGNC:17217]	protein coding	-0.60	6.83	27.45	<0.0001	<0.0001
ENSG00000105875	WDR91	WD repeat domain 91 [Source:HGNC Symbol;Acc:HGNC:24997]	protein coding	-0.60	5.27	33.28	<0.0001	<0.0001
ENSG00000140853	NLRC5	NLR family CARD domain containing 5 [Source:HGNC Symbol;Acc:HGNC:29933]	protein coding	0.60	8.33	27.53	<0.0001	<0.0001
ENSG00000172785	CBWD1	COBW domain containing 1 [Source:HGNC Symbol;Acc:HGNC:17134]	protein coding	0.60	4.39	30.08	<0.0001	<0.0001
ENSG00000167705	RILP	Rab interacting lysosomal protein [Source:HGNC Symbol;Acc:HGNC:30266]	protein coding	0.60	4.73	33.18	<0.0001	<0.0001
ENSG00000173272	MZT2A	mitotic spindle organizing protein 2A [Source:HGNC Symbol;Acc:HGNC:33187]	protein coding	-0.60	4.49	24.91	<0.0001	<0.0001
ENSG00000212283	SNORD89	small nucleolar RNA, C/D box 89 [Source:HGNC Symbol;Acc:HGNC:32750]	snoRNA	0.60	1.16	20.41	<0.0001	<0.0001
ENSG00000275120		novel transcript, antisense to ZSCAN2	lncRNA	-0.60	1.04	11.76	0.0006	0.0021
ENSG00000112299	VNN1	vanin 1 [Source:HGNC Symbol;Acc:HGNC:12705]	protein coding	-0.60	4.43	8.98	0.0027	0.0077
ENSG00000108773	KAT2A	lysine acetyltransferase 2A [Source:HGNC Symbol;Acc:HGNC:4201]	protein coding	-0.60	5.72	52.99	<0.0001	<0.0001
ENSG00000070718	AP3M2	adaptor related protein complex 3 subunit mu 2 [Source:HGNC Symbol;Acc:HGNC:570]	protein coding	-0.60	3.23	24.74	<0.0001	<0.0001
ENSG00000171798	KNDC1	kinase non-catalytic C-lobe domain containing 1 [Source:HGNC Symbol;Acc:HGNC:29374]	protein coding	-0.60	4.74	21.10	<0.0001	<0.0001

Table S12: Genes differentially expressed at Day 30 compared to pre-second vaccination (Study Arm 2 A/H7N9+AS03, D1, 29, Dendritic cells). Genes are sorted by descending absolute log_2 fold change. Gene model summaries and annotations are based on Ensembl Version 99 (continued.)

Ensembl Gene ID	Ensembl Gene Name	Ensembl Gene Description	Gene Type	log_2 Fold Change (Day 30 vs. Pre-Second Vaccination)	Average log_2 CPM	Likelihood Ratio Test Statistic	P-Value	FDR Adjusted P-Value
ENSG00000127586	CHTF18	chromosome transmission fidelity factor 18 [Source:HGNC Symbol;Acc:HGNC:18435]	protein coding	-0.60	4.61	31.27	<0.0001	<0.0001
ENSG00000101596	SMCHD1	structural maintenance of chromosomes flexible hinge domain containing 1 [Source:HGNC Symbol;Acc:HGNC:29090]	protein coding	0.60	7.68	32.45	<0.0001	<0.0001
ENSG00000154814	OXNAD1	oxidoreductase NAD binding domain containing 1 [Source:HGNC Symbol;Acc:HGNC:25128]	protein coding	-0.60	4.43	15.30	<0.0001	0.0004
ENSG00000198168	SVIP	small VCP interacting protein [Source:HGNC Symbol;Acc:HGNC:25238]	protein coding	-0.60	3.15	14.59	0.0001	0.0006
ENSG00000101194	SLC17A9	solute carrier family 17 member 9 [Source:HGNC Symbol;Acc:HGNC:16192]	protein coding	-0.60	5.50	27.79	<0.0001	<0.0001
ENSG00000125246	CLYBL	citrate lyase beta like [Source:HGNC Symbol;Acc:HGNC:18355]	protein coding	-0.60	1.62	20.46	<0.0001	<0.0001
ENSG00000134986	NREP	neuronal regeneration related protein [Source:HGNC Symbol;Acc:HGNC:16834]	protein coding	-0.60	2.57	30.05	<0.0001	<0.0001
ENSG00000178719	GRINA	glutamate ionotropic receptor NMDA type subunit associated protein 1 [Source:HGNC Symbol;Acc:HGNC:4589]	protein coding	0.60	8.25	42.44	<0.0001	<0.0001
ENSG00000072401	UBE2D1	ubiquitin conjugating enzyme E2 D1 [Source:HGNC Symbol;Acc:HGNC:12474]	protein coding	0.59	6.32	36.10	<0.0001	<0.0001
ENSG00000151687	ANKAR	ankyrin and armadillo repeat containing [Source:HGNC Symbol;Acc:HGNC:26350]	protein coding	-0.59	1.84	17.02	<0.0001	0.0002
ENSG00000277632	CCL3	C-C motif chemokine ligand 3 [Source:HGNC Symbol;Acc:HGNC:10627]	protein coding	0.59	1.36	5.22	0.0224	0.0467
ENSG00000090447	TFAP4	transcription factor AP-4 [Source:HGNC Symbol;Acc:HGNC:11745]	protein coding	-0.59	4.36	24.23	<0.0001	<0.0001
ENSG00000153071	DAB2	DAB adaptor protein 2 [Source:HGNC Symbol;Acc:HGNC:2662]	protein coding	-0.59	3.95	27.10	<0.0001	<0.0001
ENSG00000122970	IFT81	intraflagellar transport 81 [Source:HGNC Symbol;Acc:HGNC:14313]	protein coding	-0.59	0.63	11.90	0.0006	0.002
ENSG00000104921	FCER2	Fc fragment of IgE receptor II [Source:HGNC Symbol;Acc:HGNC:3612]	protein coding	-0.59	4.52	9.94	0.0016	0.0049
ENSG00000165533	TTC8	tetratricopeptide repeat domain 8 [Source:HGNC Symbol;Acc:HGNC:20087]	protein coding	-0.59	1.25	17.14	<0.0001	0.0002
ENSG00000238152	OR7E140P	olfactory receptor family 7 subfamily E member 140 pseudogene [Source:HGNC Symbol;Acc:HGNC:19561]	unprocessed pseudogene	0.59	1.02	12.17	0.0005	0.0018

Confidential/Proprietary Information

Table S12: Genes differentially expressed at Day 30 compared to pre-second vaccination (Study Arm 2 A/H7N9+AS03, D1, 29, Dendritic cells). Genes are sorted by descending absolute log_2 fold change. Gene model summaries and annotations are based on Ensembl Version 99 (continued.)

Ensembl Gene ID	Ensembl Gene Name	Ensembl Gene Description	Gene Type	log_2 Fold Change (Day 30 vs. Pre-Second Vaccination)	Average log_2 CPM	Likelihood Ratio Test Statistic	P-Value	FDR Adjusted P-Value
ENSG00000159216	RUNX1	RUNX family transcription factor 1 [Source:HGNC Symbol;Acc:HGNC:10471]	protein coding	0.59	6.50	39.06	<0.0001	<0.0001
ENSG00000085788	DDHD2	DDHD domain containing 2 [Source:HGNC Symbol;Acc:HGNC:29106]	protein coding	-0.59	4.73	40.96	<0.0001	<0.0001
ENSG00000197497	ZNF665	zinc finger protein 665 [Source:HGNC Symbol;Acc:HGNC:25885]	protein coding	-0.59	0.73	9.82	0.0017	0.0052
ENSG00000197124	ZNF682	zinc finger protein 682 [Source:HGNC Symbol;Acc:HGNC:28857]	protein coding	-0.59	0.38	10.44	0.0012	0.0039
ENSG00000114573	ATP6V1A	ATPase H+ transporting V1 subunit A [Source:HGNC Symbol;Acc:HGNC:851]	protein coding	0.59	6.79	37.14	<0.0001	<0.0001
ENSG00000198805	PNP	purine nucleoside phosphorylase [Source:HGNC Symbol;Acc:HGNC:7892]	protein coding	0.59	5.46	33.05	<0.0001	<0.0001
ENSG00000237753		novel transcript (FLJ42351)	lncRNA	-0.59	2.54	19.92	<0.0001	<0.0001
ENSG00000197249	SERPINA1	serpin family A member 1 [Source:HGNC Symbol;Acc:HGNC:8941]	protein coding	0.59	10.42	43.69	<0.0001	<0.0001
ENSG00000230438	SERPINB9P1	serpin family B member 9 pseudogene 1 [Source:HGNC Symbol;Acc:HGNC:28590]	lncRNA	0.59	3.78	26.19	<0.0001	<0.0001
ENSG00000196352	CD55	CD55 molecule (Cromer blood group) [Source:HGNC Symbol;Acc:HGNC:2665]	protein coding	0.59	7.20	47.52	<0.0001	<0.0001
ENSG00000183655	KLHL25	kelch like family member 25 [Source:HGNC Symbol;Acc:HGNC:25732]	protein coding	-0.59	1.23	9.58	0.002	0.0058
ENSG00000130584	ZBTB46	zinc finger and BTB domain containing 46 [Source:HGNC Symbol;Acc:HGNC:16094]	protein coding	-0.59	3.25	25.01	<0.0001	<0.0001
ENSG00000047579	DTNBP1	dystrobrevin binding protein 1 [Source:HGNC Symbol;Acc:HGNC:17328]	protein coding	0.59	4.69	32.69	<0.0001	<0.0001
ENSG00000111424	VDR	vitamin D receptor [Source:HGNC Symbol;Acc:HGNC:12679]	polymorphic pseudogene	0.59	5.50	52.08	<0.0001	<0.0001
ENSG00000120093	HOXB3	homeobox B3 [Source:HGNC Symbol;Acc:HGNC:5114]	protein coding	-0.59	2.43	13.75	0.0002	0.0009
ENSG00000007968	E2F2	E2F transcription factor 2 [Source:HGNC Symbol;Acc:HGNC:3114]	protein coding	0.59	4.17	18.04	<0.0001	0.0001
ENSG00000259668		novel transcript, antisense to DMXL2	lncRNA	0.59	3.02	16.81	<0.0001	0.0002
ENSG00000261326	LINC01355	long intergenic non-protein coding RNA 1355 [Source:HGNC Symbol;Acc:HGNC:50584]	lncRNA	-0.59	2.22	7.15	0.0075	0.0182

Table S12: Genes differentially expressed at Day 30 compared to pre-second vaccination (Study Arm 2 A/H7N9+AS03, D1, 29, Dendritic cells). Genes are sorted by descending absolute log_2 fold change. Gene model summaries and annotations are based on Ensembl Version 99 (continued.)

Ensembl Gene ID	Ensembl Gene Name	Ensembl Gene Description	Gene Type	log_2 Fold Change (Day 30 vs. Pre-Second Vaccination)	Average log_2 CPM	Likelihood Ratio Test Statistic	P-Value	FDR Adjusted P-Value
ENSG00000168389	MFSD2A	major facilitator superfamily domain containing 2A [Source:HGNC Symbol;Acc:HGNC:25897]	protein coding	0.59	3.84	39.66	<0.0001	<0.0001
ENSG00000224796	RPL32P1	ribosomal protein L32 pseudogene 1 [Source:HGNC Symbol;Acc:HGNC:10339]	processed pseudogene	0.59	2.71	17.15	<0.0001	0.0002
ENSG00000179588	ZFPM1	zinc finger protein, FOG family member 1 [Source:HGNC Symbol;Acc:HGNC:19762]	protein coding	-0.59	4.18	19.01	<0.0001	<0.0001
ENSG00000104518	GSDMD	gasdermin D [Source:HGNC Symbol;Acc:HGNC:25697]	protein coding	0.59	7.70	32.03	<0.0001	<0.0001
ENSG00000049449	RCN1	reticulocalbin 1 [Source:HGNC Symbol;Acc:HGNC:9934]	protein coding	0.59	3.97	20.06	<0.0001	<0.0001
ENSG00000149516	MS4A3	membrane spanning 4-domains A3 [Source:HGNC Symbol;Acc:HGNC:7317]	protein coding	-0.59	2.15	5.88	0.0153	0.0338
ENSG00000137936	BCAR3	BCAR3 adaptor protein, NSP family member [Source:HGNC Symbol;Acc:HGNC:973]	protein coding	-0.59	0.44	9.19	0.0024	0.007
ENSG00000211662	IGLV3-21	immunoglobulin lambda variable 3-21 [Source:HGNC Symbol;Acc:HGNC:5905]	IG V gene	-0.59	1.84	5.43	0.0198	0.042
ENSG00000123342	MMP19	matrix metallopeptidase 19 [Source:HGNC Symbol;Acc:HGNC:7165]	protein coding	0.59	1.12	14.30	0.0002	0.0007
ENSG00000135631	RAB11FIP5	RAB11 family interacting protein 5 [Source:HGNC Symbol;Acc:HGNC:24845]	protein coding	-0.59	2.53	10.57	0.0011	0.0037
ENSG00000188549	CCDC9B	coiled-coil domain containing 9B [Source:HGNC Symbol;Acc:HGNC:33488]	protein coding	-0.59	2.94	15.46	<0.0001	0.0004
ENSG00000186567	CEACAM19	CEA cell adhesion molecule 19 [Source:HGNC Symbol;Acc:HGNC:31951]	protein coding	-0.59	2.35	12.99	0.0003	0.0012
ENSG00000140398	NEIL1	nei like DNA glycosylase 1 [Source:HGNC Symbol;Acc:HGNC:18448]	protein coding	-0.59	4.20	28.38	<0.0001	<0.0001
ENSG00000203965	EFCAB7	EF-hand calcium binding domain 7 [Source:HGNC Symbol;Acc:HGNC:29379]	protein coding	-0.59	1.32	19.29	<0.0001	<0.0001
ENSG00000134291	TMEM106C	transmembrane protein 106C [Source:HGNC Symbol;Acc:HGNC:28775]	protein coding	-0.59	2.71	18.38	<0.0001	0.0001
ENSG00000041880	PARP3	poly(ADP-ribose) polymerase family member 3 [Source:HGNC Symbol;Acc:HGNC:273]	protein coding	0.59	3.86	37.98	<0.0001	<0.0001
ENSG00000166669	ATF7IP2	activating transcription factor 7 interacting protein 2 [Source:HGNC Symbol;Acc:HGNC:20397]	protein coding	-0.59	3.30	22.35	<0.0001	<0.0001

Table S12: Genes differentially expressed at Day 30 compared to pre-second vaccination (Study Arm 2 A/H7N9+AS03, D1, 29, Dendritic cells). Genes are sorted by descending absolute log_2 fold change. Gene model summaries and annotations are based on Ensembl Version 99 (continued.)

Ensembl Gene ID	Ensembl Gene Name	Ensembl Gene Description	Gene Type	log_2 Fold Change (Day 30 vs. Pre-Second Vaccination)	Average log_2 CPM	Likelihood Ratio Test Statistic	P-Value	FDR Adjusted P-Value
ENSG00000011478	QPCTL	glutaminyl-peptide cyclotransferase like [Source:HGNC Symbol;Acc:HGNC:25952]	protein coding	-0.59	2.84	29.66	<0.0001	<0.0001

Table S13: Genes differentially expressed at Day 32 compared to pre-second vaccination (Study Arm 2 A/H7N9+AS03, D1, 29, Dendritic cells). Genes are sorted by descending absolute log_2 fold change. Gene model summaries and annotations are based on Ensembl Version 99.

Ensembl Gene ID	Ensembl Gene Name	Ensembl Gene Description	Gene Type	log_2 Fold Change (Day 32 vs. Pre-Second Vaccination)	Average log_2 CPM	Likelihood Ratio Test Statistic	P-Value	FDR Adjusted P-Value
ENSG00000206172	HBA1	hemoglobin subunit alpha 1 [Source:HGNC Symbol;Acc:HGNC:4823]	protein coding	2.74	4.78	15.14	<0.0001	0.0266
ENSG00000159189	C1QC	complement C1q C chain [Source:HGNC Symbol;Acc:HGNC:1245]	protein coding	1.26	3.53	28.17	<0.0001	0.0002
ENSG00000163687	DNASE1L3	deoxyribonuclease 1 like 3 [Source:HGNC Symbol;Acc:HGNC:2959]	protein coding	-1.25	1.12	35.87	<0.0001	<0.0001
ENSG00000173369	C1QB	complement C1q B chain [Source:HGNC Symbol;Acc:HGNC:1242]	protein coding	1.08	4.56	31.22	<0.0001	<0.0001
ENSG00000256948		novel transcript	lncRNA	1.02	0.57	32.29	<0.0001	<0.0001
ENSG00000241351	IGKV3-11	immunoglobulin kappa variable 3-11 [Source:HGNC Symbol;Acc:HGNC:5815]	IG V gene	-1.01	2.93	24.73	<0.0001	0.0009
ENSG00000128438	TBC1D27P	TBC1 domain family member 27, pseudogene [Source:HGNC Symbol;Acc:HGNC:28104]	transcribed unprocessed pseudogene	-0.96	1.46	23.85	<0.0001	0.0013
ENSG00000116031	CD207	CD207 molecule [Source:HGNC Symbol;Acc:HGNC:17935]	protein coding	-0.90	1.73	29.55	<0.0001	0.0001
ENSG00000211890	IGHA2	immunoglobulin heavy constant alpha 2 (A2m marker) [Source:HGNC Symbol;Acc:HGNC:5479]	IG C gene	-0.89	5.56	27.97	<0.0001	0.0002
ENSG00000211949	IGHV3-23	immunoglobulin heavy variable 3-23 [Source:HGNC Symbol;Acc:HGNC:5588]	IG V gene	-0.83	2.92	19.20	<0.0001	0.0071
ENSG00000166278	C2	complement C2 [Source:HGNC Symbol;Acc:HGNC:1248]	protein coding	0.81	3.03	33.89	<0.0001	<0.0001

Table S13: Genes differentially expressed at Day 32 compared to pre-second vaccination (Study Arm 2 A/H7N9+AS03, D1, 29, Dendritic cells). Genes are sorted by descending absolute log_2 fold change. Gene model summaries and annotations are based on Ensembl Version 99 (continued.)

Ensembl Gene ID	Ensembl Gene Name	Ensembl Gene Description	Gene Type	log_2 Fold Change (Day 32 vs. Pre-Second Vaccination)	Average log_2 CPM	Likelihood Ratio Test Statistic	P-Value	FDR Adjusted P-Value
ENSG00000211592	IGKC	immunoglobulin kappa constant [Source:HGNC Symbol;Acc:HGNC:5716]	IG C gene	-0.77	7.47	20.68	<0.0001	0.0039
ENSG00000111199	TRPV4	transient receptor potential cation channel subfamily V member 4 [Source:HGNC Symbol;Acc:HGNC:18083]	protein coding	0.76	1.86	21.31	<0.0001	0.0035
ENSG00000157404	KIT	KIT proto-oncogene, receptor tyrosine kinase [Source:HGNC Symbol;Acc:HGNC:6342]	protein coding	-0.74	0.92	17.65	<0.0001	0.0115
ENSG00000039068	CDH1	cadherin 1 [Source:HGNC Symbol;Acc:HGNC:1748]	protein coding	-0.74	0.72	16.33	<0.0001	0.0192
ENSG00000157734	SNX22	sorting nexin 22 [Source:HGNC Symbol;Acc:HGNC:16315]	protein coding	-0.74	2.97	17.63	<0.0001	0.0115
ENSG00000166770	ZNF667-AS1	ZNF667 antisense RNA 1 (head to head) [Source:HGNC Symbol;Acc:HGNC:44321]	lncRNA	-0.69	0.34	15.47	<0.0001	0.0238
ENSG00000173372	C1QA	complement C1q A chain [Source:HGNC Symbol;Acc:HGNC:1241]	protein coding	0.68	5.84	21.28	<0.0001	0.0035
ENSG00000153064	BANK1	B cell scaffold protein with ankyrin repeats 1 [Source:HGNC Symbol;Acc:HGNC:18233]	protein coding	-0.67	4.25	14.85	0.0001	0.0302
ENSG00000157168	NRG1	neuregulin 1 [Source:HGNC Symbol;Acc:HGNC:7997]	protein coding	-0.66	3.70	17.64	<0.0001	0.0115
ENSG00000163520	FBLN2	fibulin 2 [Source:HGNC Symbol;Acc:HGNC:3601]	protein coding	-0.64	3.05	19.00	<0.0001	0.0075
ENSG00000204381	LAYN	layilin [Source:HGNC Symbol;Acc:HGNC:29471]	protein coding	-0.61	0.57	15.72	<0.0001	0.023
ENSG00000110987	BCL7A	BAF chromatin remodeling complex subunit BCL7A [Source:HGNC Symbol;Acc:HGNC:1004]	protein coding	-0.59	3.23	15.13	0.0001	0.0266
ENSG00000229754	CXCR2P1	C-X-C motif chemokine receptor 2 pseudogene 1 [Source:HGNC Symbol;Acc:HGNC:6028]	transcribed unprocessed pseudogene	0.59	5.47	16.98	<0.0001	0.0148
ENSG00000108950	FAM20A	FAM20A golgi associated secretory pathway pseudokinase [Source:HGNC Symbol;Acc:HGNC:23015]	protein coding	0.59	2.98	18.17	<0.0001	0.0103

Table S14: Genes differentially expressed at Day 36 compared to pre-second vaccination (Study Arm 2 A/H7N9+AS03, D1, 29, Dendritic cells). Genes are sorted by descending absolute log_2 fold change. Gene model summaries and annotations are based on Ensembl Version 99.

Ensembl Gene ID	Ensembl Gene Name	Ensembl Gene Description	Gene Type	log_2 Fold Change (Day 36 vs. Pre-Second Vaccination)	Average log_2 CPM	Likelihood Ratio Test Statistic	P-Value	FDR Adjusted P-Value
ENSG00000206172	HBA1	hemoglobin subunit alpha 1 [Source:HGNC Symbol;Acc:HGNC:4823]	protein coding	4.04	5.30	17.95	<0.0001	0.0053
ENSG00000188536	HBA2	hemoglobin subunit alpha 2 [Source:HGNC Symbol;Acc:HGNC:4824]	protein coding	3.51	5.61	20.72	<0.0001	0.002
ENSG00000244734	HBB	hemoglobin subunit beta [Source:HGNC Symbol;Acc:HGNC:4827]	protein coding	3.26	7.53	14.66	0.0001	0.0143
ENSG00000253755	IGHGP	immunoglobulin heavy constant gamma P (non-functional) [Source:HGNC Symbol;Acc:HGNC:5529]	IG C pseudogene	1.89	1.91	41.47	<0.0001	<0.0001
ENSG00000211896	IGHG1	immunoglobulin heavy constant gamma 1 (G1m marker) [Source:HGNC Symbol;Acc:HGNC:5525]	IG C gene	1.84	6.69	74.60	<0.0001	<0.0001
ENSG00000211897	IGHG3	immunoglobulin heavy constant gamma 3 (G3m marker) [Source:HGNC Symbol;Acc:HGNC:5527]	IG C gene	1.70	4.58	42.07	<0.0001	<0.0001
ENSG00000211673	IGLV3-1	immunoglobulin lambda variable 3-1 [Source:HGNC Symbol;Acc:HGNC:5896]	IG V gene	1.58	2.68	34.74	<0.0001	<0.0001
ENSG00000211651	IGLV1-44	immunoglobulin lambda variable 1-44 [Source:HGNC Symbol;Acc:HGNC:5879]	IG V gene	1.46	2.03	38.35	<0.0001	<0.0001
ENSG00000270550	IGHV3-30	immunoglobulin heavy variable 3-30 [Source:HGNC Symbol;Acc:HGNC:5591]	IG V gene	1.33	1.45	28.87	<0.0001	0.0001
ENSG00000211653	IGLV1-40	immunoglobulin lambda variable 1-40 [Source:HGNC Symbol;Acc:HGNC:5877]	IG V gene	1.26	2.32	31.14	<0.0001	<0.0001
ENSG00000135083	CCNJL	cyclin J like [Source:HGNC Symbol;Acc:HGNC:25876]	protein coding	1.23	1.54	27.70	<0.0001	0.0002
ENSG00000273179		novel transcript, antisense to SPON2	lncRNA	1.23	2.52	21.05	<0.0001	0.0019
ENSG00000197057	DTHD1	death domain containing 1 [Source:HGNC Symbol;Acc:HGNC:37261]	protein coding	1.20	2.65	22.33	<0.0001	0.0012
ENSG00000213071	LPAL2	lipoprotein(a) like 2, pseudogene [Source:HGNC Symbol;Acc:HGNC:21210]	transcribed unprocessed pseudogene	1.18	1.63	24.00	<0.0001	0.0007
ENSG00000169583	CLIC3	chloride intracellular channel 3 [Source:HGNC Symbol;Acc:HGNC:2064]	protein coding	1.17	4.09	22.38	<0.0001	0.0012
ENSG00000162383	SLC1A7	solute carrier family 1 member 7 [Source:HGNC Symbol;Acc:HGNC:10945]	protein coding	1.16	1.69	18.00	<0.0001	0.0052
ENSG00000171596	NMUR1	neuromedin U receptor 1 [Source:HGNC Symbol;Acc:HGNC:4518]	protein coding	1.16	3.95	21.18	<0.0001	0.0019

Table S14: Genes differentially expressed at Day 36 compared to pre-second vaccination (Study Arm 2 A/H7N9+AS03, D1, 29, Dendritic cells). Genes are sorted by descending absolute log_2 fold change. Gene model summaries and annotations are based on Ensembl Version 99 (continued.)

Ensembl Gene ID	Ensembl Gene Name	Ensembl Gene Description	Gene Type	log_2 Fold Change (Day 36 vs. Pre-Second Vaccination)	Average log_2 CPM	Likelihood Ratio Test Statistic	P-Value	FDR Adjusted P-Value
ENSG00000221957	KIR2DS4	killer cell immunoglobulin like receptor, two Ig domains and short cytoplasmic tail 4 [Source:HGNC Symbol;Acc:HGNC:6336]	polymorphic pseudogene	1.15	2.02	13.07	0.0003	0.0244
ENSG00000134545	KLRC1	killer cell lectin like receptor C1 [Source:HGNC Symbol;Acc:HGNC:6374]	protein coding	1.15	2.66	12.32	0.0004	0.03
ENSG00000105255	FSD1	fibronectin type III and SPRY domain containing 1 [Source:HGNC Symbol;Acc:HGNC:13745]	protein coding	1.14	1.35	15.44	<0.0001	0.0113
ENSG00000100453	GZMB	granzyme B [Source:HGNC Symbol;Acc:HGNC:4709]	protein coding	1.13	6.00	16.85	<0.0001	0.0078
ENSG00000143185	XCL2	X-C motif chemokine ligand 2 [Source:HGNC Symbol;Acc:HGNC:10646]	protein coding	1.13	1.31	15.32	<0.0001	0.0118
ENSG00000117560	FASLG	Fas ligand [Source:HGNC Symbol;Acc:HGNC:11936]	protein coding	1.13	2.15	13.35	0.0003	0.0224
ENSG00000225079	FTH1P22	ferritin heavy chain 1 pseudogene 22 [Source:HGNC Symbol;Acc:HGNC:37640]	processed pseudogene	1.12	0.66	16.15	<0.0001	0.0094
ENSG00000115523	GNLY	granulysin [Source:HGNC Symbol;Acc:HGNC:4414]	protein coding	1.11	8.69	17.50	<0.0001	0.0062
ENSG00000211660	IGLV2-23	immunoglobulin lambda variable 2-23 [Source:HGNC Symbol;Acc:HGNC:5890]	IG V gene	1.11	2.03	26.23	<0.0001	0.0003
ENSG00000243466	IGKV1-5	immunoglobulin kappa variable 1-5 [Source:HGNC Symbol;Acc:HGNC:5741]	IG V gene	1.10	2.77	19.00	<0.0001	0.0037
ENSG00000159674	SPON2	spondin 2 [Source:HGNC Symbol;Acc:HGNC:11253]	protein coding	1.10	7.26	19.02	<0.0001	0.0037
ENSG00000141622	RNF165	ring finger protein 165 [Source:HGNC Symbol;Acc:HGNC:31696]	protein coding	1.09	2.44	19.55	<0.0001	0.0029
ENSG00000073861	TBX21	T-box transcription factor 21 [Source:HGNC Symbol;Acc:HGNC:11599]	protein coding	1.08	5.76	20.86	<0.0001	0.002
ENSG00000240403	KIR3DL2	killer cell immunoglobulin like receptor, three Ig domains and long cytoplasmic tail 2 [Source:HGNC Symbol;Acc:HGNC:6339]	protein coding	1.08	1.72	15.26	<0.0001	0.012
ENSG00000211955	IGHV3-33	immunoglobulin heavy variable 3-33 [Source:HGNC Symbol;Acc:HGNC:5596]	IG V gene	1.08	1.61	26.42	<0.0001	0.0003
ENSG00000150687	PRSS23	serine protease 23 [Source:HGNC Symbol;Acc:HGNC:14370]	protein coding	1.08	4.09	14.90	0.0001	0.0134

Table S14: Genes differentially expressed at Day 36 compared to pre-second vaccination (Study Arm 2 A/H7N9+AS03, D1, 29, Dendritic cells). Genes are sorted by descending absolute log_2 fold change. Gene model summaries and annotations are based on Ensembl Version 99 (continued.)

Ensembl Gene ID	Ensembl Gene Name	Ensembl Gene Description	Gene Type	log_2 Fold Change (Day 36 vs. Pre-Second Vaccination)	Average log_2 CPM	Likelihood Ratio Test Statistic	P-Value	FDR Adjusted P-Value
ENSG00000211934	IGHV1-2	immunoglobulin heavy variable 1-2 [Source:HGNC Symbol;Acc:HGNC:5550]	IG V gene	1.07	1.44	14.56	0.0001	0.0148
ENSG00000205336	ADGRG1	adhesion G protein-coupled receptor G1 [Source:HGNC Symbol;Acc:HGNC:4512]	protein coding	1.06	6.74	18.52	<0.0001	0.0044
ENSG00000170962	PDGFD	platelet derived growth factor D [Source:HGNC Symbol;Acc:HGNC:30620]	protein coding	1.06	2.50	16.61	<0.0001	0.0083
ENSG00000154734	ADAMTS1	ADAM metallopeptidase with thrombospondin type 1 motif 1 [Source:HGNC Symbol;Acc:HGNC:217]	protein coding	1.06	2.20	14.84	0.0001	0.0135
ENSG00000275302	CCL4	C-C motif chemokine ligand 4 [Source:HGNC Symbol;Acc:HGNC:10630]	protein coding	1.05	3.56	15.37	<0.0001	0.0116
ENSG00000186049	KRT73	keratin 73 [Source:HGNC Symbol;Acc:HGNC:28928]	protein coding	1.05	1.54	14.74	0.0001	0.0139
ENSG00000286885		novel transcript	lncRNA	1.05	0.46	19.81	<0.0001	0.0027
ENSG00000167633	KIR3DL1	killer cell immunoglobulin like receptor, three Ig domains and long cytoplasmic tail 1 [Source:HGNC Symbol;Acc:HGNC:6338]	protein coding	1.05	2.28	19.84	<0.0001	0.0027
ENSG00000211659	IGLV3-25	immunoglobulin lambda variable 3-25 [Source:HGNC Symbol;Acc:HGNC:5908]	IG V gene	1.05	1.78	13.06	0.0003	0.0244
ENSG00000180644	PRF1	perforin 1 [Source:HGNC Symbol;Acc:HGNC:9360]	protein coding	1.05	8.31	16.83	<0.0001	0.0078
ENSG00000211945	IGHV1-18	immunoglobulin heavy variable 1-18 [Source:HGNC Symbol;Acc:HGNC:5549]	IG V gene	1.04	1.27	13.89	0.0002	0.0186
ENSG00000180739	S1PR5	sphingosine-1-phosphate receptor 5 [Source:HGNC Symbol;Acc:HGNC:14299]	protein coding	1.04	5.65	17.28	<0.0001	0.0064
ENSG00000149294	NCAM1	neural cell adhesion molecule 1 [Source:HGNC Symbol;Acc:HGNC:7656]	protein coding	1.04	3.90	16.08	<0.0001	0.0094
ENSG00000115607	IL18RAP	interleukin 18 receptor accessory protein [Source:HGNC Symbol;Acc:HGNC:5989]	protein coding	1.04	4.68	18.10	<0.0001	0.005
ENSG00000211648	IGLV1-47	immunoglobulin lambda variable 1-47 [Source:HGNC Symbol;Acc:HGNC:5880]	IG V gene	1.04	1.03	11.43	0.0007	0.0404
ENSG00000166289	PLEKHF1	pleckstrin homology and FYVE domain containing 1 [Source:HGNC Symbol;Acc:HGNC:20764]	protein coding	1.03	3.97	16.23	<0.0001	0.0092
ENSG00000137441	FGFBP2	fibroblast growth factor binding protein 2 [Source:HGNC Symbol;Acc:HGNC:29451]	protein coding	1.02	5.80	13.52	0.0002	0.0213
ENSG00000141469	SLC14A1	solute carrier family 14 member 1 (Kidd blood group) [Source:HGNC Symbol;Acc:HGNC:10918]	protein coding	1.02	2.26	18.46	<0.0001	0.0044

Table S14: Genes differentially expressed at Day 36 compared to pre-second vaccination (Study Arm 2 A/H7N9+AS03, D1, 29, Dendritic cells). Genes are sorted by descending absolute log_2 fold change. Gene model summaries and annotations are based on Ensembl Version 99 (continued.)

Ensembl Gene ID	Ensembl Gene Name	Ensembl Gene Description	Gene Type	log_2 Fold Change (Day 36 vs. Pre-Second Vaccination)	Average log_2 CPM	Likelihood Ratio Test Statistic	P-Value	FDR Adjusted P-Value
ENSG00000173068	BNC2	basonuclin 2 [Source:HGNC Symbol;Acc:HGNC:30988]	protein coding	1.02	1.48	11.94	0.0005	0.0348
ENSG00000156453	PCDH1	protocadherin 1 [Source:HGNC Symbol;Acc:HGNC:8655]	protein coding	1.02	1.67	13.85	0.0002	0.0189
ENSG00000189430	NCR1	natural cytotoxicity triggering receptor 1 [Source:HGNC Symbol;Acc:HGNC:6731]	protein coding	1.00	3.05	21.14	<0.0001	0.0019
ENSG00000171916	LGALS9C	galectin 9C [Source:HGNC Symbol;Acc:HGNC:33874]	protein coding	0.99	1.43	22.29	<0.0001	0.0012
ENSG00000277632	CCL3	C-C motif chemokine ligand 3 [Source:HGNC Symbol;Acc:HGNC:10627]	protein coding	0.99	1.57	23.80	<0.0001	0.0008
ENSG00000243772	KIR2DL3	killer cell immunoglobulin like receptor, two Ig domains and long cytoplasmic tail 3 [Source:HGNC Symbol;Acc:HGNC:6331]	protein coding	0.98	0.96	12.33	0.0004	0.03
ENSG00000134539	KLRD1	killer cell lectin like receptor D1 [Source:HGNC Symbol;Acc:HGNC:6378]	protein coding	0.98	6.34	15.08	0.0001	0.0125
ENSG00000100385	IL2RB	interleukin 2 receptor subunit beta [Source:HGNC Symbol;Acc:HGNC:6009]	protein coding	0.98	7.41	16.57	<0.0001	0.0083
ENSG00000150045	KLRF1	killer cell lectin like receptor F1 [Source:HGNC Symbol;Acc:HGNC:13342]	protein coding	0.98	4.81	16.11	<0.0001	0.0094
ENSG00000168229	PTGDR	prostaglandin D2 receptor [Source:HGNC Symbol;Acc:HGNC:9591]	protein coding	0.97	4.05	12.78	0.0004	0.0261
ENSG00000213809	KLRK1	killer cell lectin like receptor K1 [Source:HGNC Symbol;Acc:HGNC:18788]	protein coding	0.97	2.39	16.72	<0.0001	0.008
ENSG00000100450	GZMH	granzyme H [Source:HGNC Symbol;Acc:HGNC:4710]	protein coding	0.97	5.46	13.83	0.0002	0.019
ENSG00000214402	LCNL1	lipocalin like 1 [Source:HGNC Symbol;Acc:HGNC:34436]	protein coding	0.96	0.86	15.79	<0.0001	0.0105
ENSG00000107317	PTGDS	prostaglandin D2 synthase [Source:HGNC Symbol;Acc:HGNC:9592]	protein coding	0.96	3.18	20.40	<0.0001	0.0022
ENSG00000198756	COLGALT2	collagen beta(1-O)galactosyltransferase 2 [Source:HGNC Symbol;Acc:HGNC:16790]	protein coding	0.95	1.43	12.66	0.0004	0.0265
ENSG00000189409	MMP23B	matrix metallopeptidase 23B [Source:HGNC Symbol;Acc:HGNC:7171]	protein coding	0.95	1.14	12.67	0.0004	0.0265
ENSG00000109956	B3GAT1	beta-1,3-glucuronyltransferase 1 [Source:HGNC Symbol;Acc:HGNC:921]	protein coding	0.95	3.53	11.47	0.0007	0.0401

Table S14: Genes differentially expressed at Day 36 compared to pre-second vaccination (Study Arm 2 A/H7N9+AS03, D1, 29, Dendritic cells). Genes are sorted by descending absolute log_2 fold change. Gene model summaries and annotations are based on Ensembl Version 99 (continued.)

Ensembl Gene ID	Ensembl Gene Name	Ensembl Gene Description	Gene Type	log_2 Fold Change (Day 36 vs. Pre-Second Vaccination)	Average log_2 CPM	Likelihood Ratio Test Statistic	P-Value	FDR Adjusted P-Value
ENSG00000125498	KIR2DL1	killer cell immunoglobulin like receptor, two Ig domains and long cytoplasmic tail 1 [Source:HGNC Symbol;Acc:HGNC:6329]	protein coding	0.94	0.91	16.09	<0.0001	0.0094
ENSG00000104970	KIR3DX1	killer cell immunoglobulin like receptor, three Ig domains X1 (pseudogene) [Source:HGNC Symbol;Acc:HGNC:25043]	transcribed unprocessed pseudogene	0.93	0.53	20.56	<0.0001	0.0021
ENSG00000211679	IGLC3	immunoglobulin lambda constant 3 (Kern-Oz+ marker) [Source:HGNC Symbol;Acc:HGNC:5857]	IG C gene	0.93	4.65	19.73	<0.0001	0.0028
ENSG00000256069	A2MP1	alpha-2-macroglobulin pseudogene 1 [Source:HGNC Symbol;Acc:HGNC:8]	transcribed unprocessed pseudogene	0.92	1.11	15.71	<0.0001	0.0105
ENSG00000121753	ADGRB2	adhesion G protein-coupled receptor B2 [Source:HGNC Symbol;Acc:HGNC:944]	protein coding	0.92	1.44	14.77	0.0001	0.0139
ENSG00000148488	ST8SIA6	ST8 alpha-N-acetyl-neuraminide alpha-2,8-sialyltransferase 6 [Source:HGNC Symbol;Acc:HGNC:23317]	protein coding	0.91	1.66	17.88	<0.0001	0.0054
ENSG00000211829	TRDC	T cell receptor delta constant [Source:HGNC Symbol;Acc:HGNC:12253]	TR C gene	0.91	4.27	12.71	0.0004	0.0262
ENSG00000211966	IGHV5-51	immunoglobulin heavy variable 5-51 [Source:HGNC Symbol;Acc:HGNC:5659]	IG V gene	0.91	2.37	23.41	<0.0001	0.0008
ENSG00000041353	RAB27B	RAB27B, member RAS oncogene family [Source:HGNC Symbol;Acc:HGNC:9767]	protein coding	0.90	1.73	13.93	0.0002	0.0186
ENSG00000140451	PIF1	PIF1 5'-to-3' DNA helicase [Source:HGNC Symbol;Acc:HGNC:26220]	protein coding	0.89	0.54	15.53	<0.0001	0.011
ENSG00000163508	EOMES	eomesodermin [Source:HGNC Symbol;Acc:HGNC:3372]	protein coding	0.89	4.04	11.62	0.0007	0.038
ENSG00000171476	HOPX	HOP homeobox [Source:HGNC Symbol;Acc:HGNC:24961]	protein coding	0.89	4.58	12.74	0.0004	0.0261
ENSG00000179841	AKAP5	A-kinase anchoring protein 5 [Source:HGNC Symbol;Acc:HGNC:375]	protein coding	0.88	1.46	16.42	<0.0001	0.0087
ENSG00000133067	LGR6	leucine rich repeat containing G protein-coupled receptor 6 [Source:HGNC Symbol;Acc:HGNC:19719]	protein coding	0.88	3.15	12.76	0.0004	0.0261

Table S14: Genes differentially expressed at Day 36 compared to pre-second vaccination (Study Arm 2 A/H7N9+AS03, D1, 29, Dendritic cells). Genes are sorted by descending absolute log_2 fold change. Gene model summaries and annotations are based on Ensembl Version 99 (continued.)

Ensembl Gene ID	Ensembl Gene Name	Ensembl Gene Description	Gene Type	log_2 Fold Change (Day 36 vs. Pre-Second Vaccination)	Average log_2 CPM	Likelihood Ratio Test Statistic	P-Value	FDR Adjusted P-Value
ENSG00000027869	SH2D2A	SH2 domain containing 2A [Source:HGNC Symbol;Acc:HGNC:10821]	protein coding	0.88	4.37	12.98	0.0003	0.0249
ENSG00000186891	TNFRSF18	TNF receptor superfamily member 18 [Source:HGNC Symbol;Acc:HGNC:11914]	protein coding	0.88	2.71	13.44	0.0002	0.0217
ENSG00000104490	NCALD	neurocalcin delta [Source:HGNC Symbol;Acc:HGNC:7655]	protein coding	0.88	3.10	14.32	0.0002	0.0157
ENSG00000156475	PPP2R2B	protein phosphatase 2 regulatory subunit Bbeta [Source:HGNC Symbol;Acc:HGNC:9305]	protein coding	0.88	2.41	11.43	0.0007	0.0404
ENSG00000162676	GFI1	growth factor independent 1 transcriptional repressor [Source:HGNC Symbol;Acc:HGNC:4237]	protein coding	0.88	3.45	14.26	0.0002	0.0159
ENSG00000158856	DMTN	dematin actin binding protein [Source:HGNC Symbol;Acc:HGNC:3382]	protein coding	0.88	2.66	10.88	0.001	0.0469
ENSG00000233306	TRGV2	T cell receptor gamma variable 2 [Source:HGNC Symbol;Acc:HGNC:12287]	TR V gene	0.88	0.53	12.74	0.0004	0.0261
ENSG00000148600	CDHR1	cadherin related family member 1 [Source:HGNC Symbol;Acc:HGNC:14550]	protein coding	0.87	1.27	14.85	0.0001	0.0135
ENSG00000159618	ADGRG5	adhesion G protein-coupled receptor G5 [Source:HGNC Symbol;Acc:HGNC:19010]	protein coding	0.87	4.52	26.74	<0.0001	0.0003
ENSG00000116667	C1orf21	chromosome 1 open reading frame 21 [Source:HGNC Symbol;Acc:HGNC:15494]	protein coding	0.87	3.51	14.32	0.0002	0.0157
ENSG00000007264	MATK	megakaryocyte-associated tyrosine kinase [Source:HGNC Symbol;Acc:HGNC:6906]	protein coding	0.87	5.98	14.32	0.0002	0.0157
ENSG00000088881	EBF4	EBF family member 4 [Source:HGNC Symbol;Acc:HGNC:29278]	protein coding	0.87	1.91	11.31	0.0008	0.0421
ENSG00000181104	F2R	coagulation factor II thrombin receptor [Source:HGNC Symbol;Acc:HGNC:3537]	protein coding	0.87	3.83	15.85	<0.0001	0.0103
ENSG00000077984	CST7	cystatin F [Source:HGNC Symbol;Acc:HGNC:2479]	protein coding	0.87	5.78	13.70	0.0002	0.02
ENSG00000186827	TNFRSF4	TNF receptor superfamily member 4 [Source:HGNC Symbol;Acc:HGNC:11918]	protein coding	0.86	2.09	11.11	0.0009	0.0444
ENSG00000140859	KIFC3	kinesin family member C3 [Source:HGNC Symbol;Acc:HGNC:6326]	protein coding	0.86	2.82	21.64	<0.0001	0.0016
ENSG00000172543	CTSW	cathepsin W [Source:HGNC Symbol;Acc:HGNC:2546]	protein coding	0.85	6.78	14.95	0.0001	0.0132
ENSG00000181036	FCRL6	Fc receptor like 6 [Source:HGNC Symbol;Acc:HGNC:31910]	protein coding	0.85	5.13	15.77	<0.0001	0.0105

Table S14: Genes differentially expressed at Day 36 compared to pre-second vaccination (Study Arm 2 A/H7N9+AS03, D1, 29, Dendritic cells). Genes are sorted by descending absolute log_2 fold change. Gene model summaries and annotations are based on Ensembl Version 99 (continued.)

Ensembl Gene ID	Ensembl Gene Name	Ensembl Gene Description	Gene Type	log_2 Fold Change (Day 36 vs. Pre-Second Vaccination)	Average log_2 CPM	Likelihood Ratio Test Statistic	P-Value	FDR Adjusted P-Value
ENSG00000196139	AKR1C3	aldo-keto reductase family 1 member C3 [Source:HGNC Symbol;Acc:HGNC:386]	protein coding	0.85	2.55	12.03	0.0005	0.0338
ENSG00000148848	ADAM12	ADAM metallopeptidase domain 12 [Source:HGNC Symbol;Acc:HGNC:190]	protein coding	0.84	0.39	13.51	0.0002	0.0213
ENSG00000245648		novel transcript, antisense to KLRK1	lncRNA	0.84	3.54	10.95	0.0009	0.0457
ENSG00000198821	CD247	CD247 molecule [Source:HGNC Symbol;Acc:HGNC:1677]	protein coding	0.84	6.90	11.83	0.0006	0.0361
ENSG00000069702	TGFBR3	transforming growth factor beta receptor 3 [Source:HGNC Symbol;Acc:HGNC:11774]	protein coding	0.83	4.65	11.15	0.0008	0.0438
ENSG00000185697	MYBL1	MYB proto-oncogene like 1 [Source:HGNC Symbol;Acc:HGNC:7547]	protein coding	0.83	5.06	13.40	0.0003	0.022
ENSG00000036448	MYOM2	myomesin 2 [Source:HGNC Symbol;Acc:HGNC:7614]	protein coding	0.83	3.38	15.91	<0.0001	0.0101
ENSG00000163564	PYHIN1	pyrin and HIN domain family member 1 [Source:HGNC Symbol;Acc:HGNC:28894]	protein coding	0.83	4.13	11.56	0.0007	0.0388
ENSG00000221866	PLXNA4	plexin A4 [Source:HGNC Symbol;Acc:HGNC:9102]	protein coding	0.83	0.81	10.97	0.0009	0.0456
ENSG00000108370	RGS9	regulator of G protein signaling 9 [Source:HGNC Symbol;Acc:HGNC:10004]	protein coding	0.82	1.83	11.14	0.0008	0.0439
ENSG00000105374	NKG7	natural killer cell granule protein 7 [Source:HGNC Symbol;Acc:HGNC:7830]	protein coding	0.82	7.59	13.25	0.0003	0.0234
ENSG00000170522	ELOVL6	ELOVL fatty acid elongase 6 [Source:HGNC Symbol;Acc:HGNC:15829]	protein coding	0.81	2.13	11.89	0.0006	0.0354
ENSG00000274767		novel transcript, antisense CCL3L3	lncRNA	0.81	1.28	20.54	<0.0001	0.0021
ENSG00000119714	GPR68	G protein-coupled receptor 68 [Source:HGNC Symbol;Acc:HGNC:4519]	protein coding	0.81	2.97	13.49	0.0002	0.0214
ENSG00000081985	IL12RB2	interleukin 12 receptor subunit beta 2 [Source:HGNC Symbol;Acc:HGNC:5972]	protein coding	0.80	1.89	16.54	<0.0001	0.0083
ENSG00000100027	YPEL1	yippee like 1 [Source:HGNC Symbol;Acc:HGNC:12845]	protein coding	0.80	3.01	20.33	<0.0001	0.0022
ENSG00000211938	IGHV3-7	immunoglobulin heavy variable 3-7 [Source:HGNC Symbol;Acc:HGNC:5620]	IG V gene	0.79	2.48	15.50	<0.0001	0.011
ENSG00000141736	ERBB2	erb-b2 receptor tyrosine kinase 2 [Source:HGNC Symbol;Acc:HGNC:3430]	protein coding	0.79	3.79	17.54	<0.0001	0.0061
ENSG00000004799	PDK4	pyruvate dehydrogenase kinase 4 [Source:HGNC Symbol;Acc:HGNC:8812]	protein coding	-0.79	6.27	15.10	0.0001	0.0125

Table S14: Genes differentially expressed at Day 36 compared to pre-second vaccination (Study Arm 2 A/H7N9+AS03, D1, 29, Dendritic cells). Genes are sorted by descending absolute log_2 fold change. Gene model summaries and annotations are based on Ensembl Version 99 (continued.)

Ensembl Gene ID	Ensembl Gene Name	Ensembl Gene Description	Gene Type	log_2 Fold Change (Day 36 vs. Pre-Second Vaccination)	Average log_2 CPM	Likelihood Ratio Test Statistic	P-Value	FDR Adjusted P-Value
ENSG00000125430	HS3ST3B1	heparan sulfate-glucosamine 3-sulfotransferase 3B1 [Source:HGNC Symbol;Acc:HGNC:5198]	protein coding	0.79	3.11	12.23	0.0005	0.0306
ENSG00000005379	TSPOAP1	TSPO associated protein 1 [Source:HGNC Symbol;Acc:HGNC:16831]	protein coding	0.79	6.09	18.58	<0.0001	0.0044
ENSG00000154760	SLFN13	schlafen family member 13 [Source:HGNC Symbol;Acc:HGNC:26481]	protein coding	0.79	4.49	15.72	<0.0001	0.0105
ENSG00000184925	LCN12	lipocalin 12 [Source:HGNC Symbol;Acc:HGNC:28733]	protein coding	0.78	0.48	11.79	0.0006	0.0363
ENSG00000196169	KIF19	kinesin family member 19 [Source:HGNC Symbol;Acc:HGNC:26735]	protein coding	0.78	0.86	12.33	0.0004	0.03
ENSG00000211943	IGHV3-15	immunoglobulin heavy variable 3-15 [Source:HGNC Symbol;Acc:HGNC:5582]	IG V gene	0.78	1.64	13.89	0.0002	0.0186
ENSG00000135736	CCDC102A	coiled-coil domain containing 102A [Source:HGNC Symbol;Acc:HGNC:28097]	protein coding	0.78	2.69	14.41	0.0001	0.0155
ENSG00000189350	TOGARAM2	TOG array regulator of axonemal microtubules 2 [Source:HGNC Symbol;Acc:HGNC:33715]	protein coding	0.78	2.81	33.42	<0.0001	<0.0001
ENSG00000117281	CD160	CD160 molecule [Source:HGNC Symbol;Acc:HGNC:17013]	protein coding	0.77	3.56	14.98	0.0001	0.0131
ENSG00000211677	IGLC2	immunoglobulin lambda constant 2 [Source:HGNC Symbol;Acc:HGNC:5856]	IG C gene	0.77	5.31	17.41	<0.0001	0.0062
ENSG00000141655	TNFRSF11A	TNF receptor superfamily member 11a [Source:HGNC Symbol;Acc:HGNC:11908]	protein coding	0.77	0.84	26.62	<0.0001	0.0003
ENSG00000186994	KANK3	KN motif and ankyrin repeat domains 3 [Source:HGNC Symbol;Acc:HGNC:24796]	protein coding	0.77	1.42	11.79	0.0006	0.0363
ENSG00000158321	AUTS2	activator of transcription and developmental regulator AUTS2 [Source:HGNC Symbol;Acc:HGNC:14262]	protein coding	0.76	4.15	13.45	0.0002	0.0217
ENSG00000198885	ITPRIPL1	ITPRIP like 1 [Source:HGNC Symbol;Acc:HGNC:29371]	protein coding	0.76	2.56	12.64	0.0004	0.0265
ENSG00000167912		novel transcript	lncRNA	0.76	0.98	10.72	0.0011	0.0498
ENSG00000211956	IGHV4-34	immunoglobulin heavy variable 4-34 [Source:HGNC Symbol;Acc:HGNC:5650]	IG V gene	0.76	1.36	10.89	0.001	0.0468
ENSG00000211666	IGLV2-14	immunoglobulin lambda variable 2-14 [Source:HGNC Symbol;Acc:HGNC:5888]	IG V gene	0.75	2.97	12.02	0.0005	0.0339
ENSG00000123989	CHPF	chondroitin polymerizing factor [Source:HGNC Symbol;Acc:HGNC:24291]	protein coding	0.75	2.12	15.10	0.0001	0.0125

Table S14: Genes differentially expressed at Day 36 compared to pre-second vaccination (Study Arm 2 A/H7N9+AS03, D1, 29, Dendritic cells). Genes are sorted by descending absolute log_2 fold change. Gene model summaries and annotations are based on Ensembl Version 99 (continued.)

Ensembl Gene ID	Ensembl Gene Name	Ensembl Gene Description	Gene Type	log_2 Fold Change (Day 36 vs. Pre-Second Vaccination)	Average log_2 CPM	Likelihood Ratio Test Statistic	P-Value	FDR Adjusted P-Value
ENSG00000236790	LINC00299	long intergenic non-protein coding RNA 299 [Source:HGNC Symbol;Acc:HGNC:27940]	lncRNA	0.75	2.84	18.32	<0.0001	0.0046
ENSG00000174326	SLC16A11	solute carrier family 16 member 11 [Source:HGNC Symbol;Acc:HGNC:23093]	protein coding	0.75	0.50	12.74	0.0004	0.0261
ENSG00000106992	AK1	adenylate kinase 1 [Source:HGNC Symbol;Acc:HGNC:361]	protein coding	0.74	1.37	12.38	0.0004	0.0298
ENSG00000170298	LGALS9B	galectin 9B [Source:HGNC Symbol;Acc:HGNC:24842]	protein coding	0.73	0.79	15.64	<0.0001	0.0106
ENSG00000149527	PLCH2	phospholipase C eta 2 [Source:HGNC Symbol;Acc:HGNC:29037]	protein coding	0.71	4.80	11.69	0.0006	0.0374
ENSG00000007402	CACNA2D2	calcium voltage-gated channel auxiliary subunit alpha2delta 2 [Source:HGNC Symbol;Acc:HGNC:1400]	protein coding	0.71	3.53	12.65	0.0004	0.0265
ENSG00000160318	CLDND2	claudin domain containing 2 [Source:HGNC Symbol;Acc:HGNC:28511]	protein coding	0.71	2.78	14.41	0.0001	0.0155
ENSG00000178498	DTX3	deltex E3 ubiquitin ligase 3 [Source:HGNC Symbol;Acc:HGNC:24457]	protein coding	0.71	3.78	12.34	0.0004	0.03
ENSG00000142303	ADAMTS10	ADAM metallopeptidase with thrombospondin type 1 motif 10 [Source:HGNC Symbol;Acc:HGNC:13201]	protein coding	0.70	4.89	13.76	0.0002	0.0195
ENSG00000151208	DLG5	discs large MAGUK scaffold protein 5 [Source:HGNC Symbol;Acc:HGNC:2904]	protein coding	0.70	3.22	18.48	<0.0001	0.0044
ENSG00000173762	CD7	CD7 molecule [Source:HGNC Symbol;Acc:HGNC:1695]	protein coding	0.70	6.62	11.24	0.0008	0.0428
ENSG00000262312		novel transcript, antisense to NLRC3	lncRNA	0.69	2.15	11.25	0.0008	0.0428
ENSG00000106089	STX1A	syntaxin 1A [Source:HGNC Symbol;Acc:HGNC:11433]	protein coding	0.69	1.12	14.07	0.0002	0.0175
ENSG00000164292	RHOBTB3	Rho related BTB domain containing 3 [Source:HGNC Symbol;Acc:HGNC:18757]	protein coding	0.68	2.11	19.61	<0.0001	0.0029
ENSG00000206561	COLQ	collagen like tail subunit of asymmetric acetylcholinesterase [Source:HGNC Symbol;Acc:HGNC:2226]	protein coding	0.67	3.00	17.79	<0.0001	0.0055
ENSG00000185745	IFIT1	interferon induced protein with tetratricopeptide repeats 1 [Source:HGNC Symbol;Acc:HGNC:5407]	protein coding	-0.67	4.59	14.44	0.0001	0.0154
ENSG00000170476	MZB1	marginal zone B and B1 cell specific protein [Source:HGNC Symbol;Acc:HGNC:30125]	protein coding	0.67	3.78	15.70	<0.0001	0.0105
ENSG00000099889	ARVCF	ARVCF delta catenin family member [Source:HGNC Symbol;Acc:HGNC:728]	protein coding	0.65	3.46	23.44	<0.0001	0.0008

Table S14: Genes differentially expressed at Day 36 compared to pre-second vaccination (Study Arm 2 A/H7N9+AS03, D1, 29, Dendritic cells). Genes are sorted by descending absolute log_2 fold change. Gene model summaries and annotations are based on Ensembl Version 99 (continued.)

Ensembl Gene ID	Ensembl Gene Name	Ensembl Gene Description	Gene Type	log_2 Fold Change (Day 36 vs. Pre-Second Vaccination)	Average log_2 CPM	Likelihood Ratio Test Statistic	P-Value	FDR Adjusted P-Value
ENSG00000276141	WHAMMP3	WAS protein homolog associated with actin, golgi membranes and microtubules pseudogene 3 [Source:HGNC Symbol;Acc:HGNC:27892]	transcribed unprocessed pseudogene	0.65	1.52	10.97	0.0009	0.0456
ENSG00000185198	PRSS57	serine protease 57 [Source:HGNC Symbol;Acc:HGNC:31397]	protein coding	0.64	1.01	17.81	<0.0001	0.0055
ENSG00000251143		novel transcript	lncRNA	0.64	0.18	20.85	<0.0001	0.002
ENSG00000173930	SLCO4C1	solute carrier organic anion transporter family member 4C1 [Source:HGNC Symbol;Acc:HGNC:23612]	protein coding	0.64	3.19	15.53	<0.0001	0.011
ENSG00000119917	IFIT3	interferon induced protein with tetratricopeptide repeats 3 [Source:HGNC Symbol;Acc:HGNC:5411]	protein coding	-0.64	5.70	13.63	0.0002	0.0203
ENSG00000172508	CARNS1	carnosine synthase 1 [Source:HGNC Symbol;Acc:HGNC:29268]	protein coding	0.63	3.87	10.77	0.001	0.0491
ENSG00000073350	LLGL2	LLGL scribble cell polarity complex component 2 [Source:HGNC Symbol;Acc:HGNC:6629]	protein coding	0.62	5.18	16.86	<0.0001	0.0078
ENSG00000154914	USP43	ubiquitin specific peptidase 43 [Source:HGNC Symbol;Acc:HGNC:20072]	protein coding	0.62	0.80	12.74	0.0004	0.0261
ENSG00000253549	CA3-AS1	CA3 antisense RNA 1 [Source:HGNC Symbol;Acc:HGNC:51657]	lncRNA	-0.62	0.59	18.35	<0.0001	0.0046
ENSG00000104921	FCER2	Fc fragment of IgE receptor II [Source:HGNC Symbol;Acc:HGNC:3612]	protein coding	-0.61	4.39	23.52	<0.0001	0.0008
ENSG00000073737	DHRS9	dehydrogenase/reductase 9 [Source:HGNC Symbol;Acc:HGNC:16888]	protein coding	-0.60	3.28	16.56	<0.0001	0.0083
ENSG00000142089	IFITM3	interferon induced transmembrane protein 3 [Source:HGNC Symbol;Acc:HGNC:5414]	protein coding	-0.59	7.38	12.92	0.0003	0.0252
ENSG00000232810	TNF	tumor necrosis factor [Source:HGNC Symbol;Acc:HGNC:11892]	protein coding	-0.59	3.53	21.04	<0.0001	0.0019
ENSG00000175899	A2M	alpha-2-macroglobulin [Source:HGNC Symbol;Acc:HGNC:7]	protein coding	0.59	1.82	11.88	0.0006	0.0354
ENSG00000101096	NFATC2	nuclear factor of activated T cells 2 [Source:HGNC Symbol;Acc:HGNC:7776]	protein coding	0.59	5.36	14.59	0.0001	0.0147

Table S15: Genes differentially expressed at Day 8 compared to pre-first vaccination (Study Arm 1 A/H3N2v, D1 , Neutrophils). Genes are sorted by descending absolute log_2 fold change. Gene model summaries and annotations are based on Ensembl Version 99.

Ensembl Gene ID	Ensembl Gene Name	Ensembl Gene Description	Gene Type	log_2 Fold Change (Day 8 vs. Pre-First Vaccination)	Average log_2 CPM	Likelihood Ratio Test Statistic	P-Value	FDR Adjusted P-Value
ENSG00000205927	OLIG2	oligodendrocyte transcription factor 2 [Source:HGNC Symbol;Acc:HGNC:9398]	protein coding	-1.35	2.95	16.52	<0.0001	0.0292
ENSG00000100628	ASB2	ankyrin repeat and SOCS box containing 2 [Source:HGNC Symbol;Acc:HGNC:16012]	protein coding	-1.31	1.80	23.35	<0.0001	0.0135
ENSG00000183134	PTGDR2	prostaglandin D2 receptor 2 [Source:HGNC Symbol;Acc:HGNC:4502]	protein coding	-1.22	4.71	16.88	<0.0001	0.0292
ENSG00000274376	ADAMTS7P1	ADAMTS7 pseudogene 1 [Source:HGNC Symbol;Acc:HGNC:49407]	transcribed unprocessed pseudogene	-1.21	2.11	14.60	0.0001	0.0392
ENSG00000182240	BACE2	beta-secretase 2 [Source:HGNC Symbol;Acc:HGNC:934]	protein coding	-1.19	2.56	14.78	0.0001	0.0392
ENSG00000146192	FGD2	FYVE, RhoGEF and PH domain containing 2 [Source:HGNC Symbol;Acc:HGNC:3664]	protein coding	-1.19	1.45	14.13	0.0002	0.0448
ENSG00000185432	METTL7A	methyltransferase like 7A [Source:HGNC Symbol;Acc:HGNC:24550]	protein coding	-1.19	2.65	14.37	0.0002	0.0417
ENSG00000103355	PRSS33	serine protease 33 [Source:HGNC Symbol;Acc:HGNC:30405]	protein coding	-1.17	5.54	16.76	<0.0001	0.0292
ENSG00000103056	SMPD3	sphingomyelin phosphodiesterase 3 [Source:HGNC Symbol;Acc:HGNC:14240]	protein coding	-1.16	5.51	16.32	<0.0001	0.0292
ENSG00000101096	NFATC2	nuclear factor of activated T cells 2 [Source:HGNC Symbol;Acc:HGNC:7776]	protein coding	-1.13	1.43	15.22	<0.0001	0.0369
ENSG00000112759	SLC29A1	solute carrier family 29 member 1 (Augustine blood group) [Source:HGNC Symbol;Acc:HGNC:11003]	protein coding	-1.12	4.97	14.93	0.0001	0.0392
ENSG00000159618	ADGRG5	adhesion G protein-coupled receptor G5 [Source:HGNC Symbol;Acc:HGNC:19010]	protein coding	-1.11	3.47	15.33	<0.0001	0.0369
ENSG00000107438	PDLIM1	PDZ and LIM domain 1 [Source:HGNC Symbol;Acc:HGNC:2067]	protein coding	-1.07	1.70	17.18	<0.0001	0.0292
ENSG00000182557	SPNS3	sphingolipid transporter 3 (putative) [Source:HGNC Symbol;Acc:HGNC:28433]	protein coding	-1.06	3.48	15.04	0.0001	0.0389
ENSG00000110651	CD81	CD81 molecule [Source:HGNC Symbol;Acc:HGNC:1701]	protein coding	-1.06	2.98	15.89	<0.0001	0.0294
ENSG00000168765	GSTM4	glutathione S-transferase mu 4 [Source:HGNC Symbol;Acc:HGNC:4636]	protein coding	-1.05	2.16	16.25	<0.0001	0.0292
ENSG00000279693		novel transcript	TEC	-1.04	1.73	14.03	0.0002	0.045

Table S15: Genes differentially expressed at Day 8 compared to pre-first vaccination (Study Arm 1 A/H3N2v, D1 , Neutrophils). Genes are sorted by descending absolute log_2 fold change. Gene model summaries and annotations are based on Ensembl Version 99 (continued.)

Ensembl Gene ID	Ensembl Gene Name	Ensembl Gene Description	Gene Type	log_2 Fold Change (Day 8 vs. Pre-First Vaccination)	Average log_2 CPM	Likelihood Ratio Test Statistic	P-Value	FDR Adjusted P-Value
ENSG00000147443	DOK2	docking protein 2 [Source:HGNC Symbol;Acc:HGNC:2991]	protein coding	-1.04	4.85	16.78	<0.0001	0.0292
ENSG00000105205	CLC	Charcot-Leyden crystal galectin [Source:HGNC Symbol;Acc:HGNC:2014]	protein coding	-1.03	7.71	14.14	0.0002	0.0448
ENSG00000167543	TP53I13	tumor protein p53 inducible protein 13 [Source:HGNC Symbol;Acc:HGNC:25102]	protein coding	-1.01	1.55	18.12	<0.0001	0.0292
ENSG00000268758	ADGRE4P	adhesion G protein-coupled receptor E4, pseudogene [Source:HGNC Symbol;Acc:HGNC:19240]	transcribed unprocessed pseudogene	-0.99	4.45	14.74	0.0001	0.0392
ENSG00000122223	CD244	CD244 molecule [Source:HGNC Symbol;Acc:HGNC:18171]	protein coding	-0.99	2.30	15.96	<0.0001	0.0294
ENSG00000092067	CEBPE	CCAAT enhancer binding protein epsilon [Source:HGNC Symbol;Acc:HGNC:1836]	protein coding	-0.97	3.91	17.07	<0.0001	0.0292
ENSG00000164292	RHOBTB3	Rho related BTB domain containing 3 [Source:HGNC Symbol;Acc:HGNC:18757]	protein coding	-0.90	1.43	16.49	<0.0001	0.0292
ENSG00000103365	GGA2	golgi associated, gamma adaptin ear containing, ARF binding protein 2 [Source:HGNC Symbol;Acc:HGNC:16064]	protein coding	-0.89	2.53	15.22	<0.0001	0.0369
ENSG00000160285	LSS	lanosterol synthase [Source:HGNC Symbol;Acc:HGNC:6708]	protein coding	-0.89	1.03	16.39	<0.0001	0.0292
ENSG00000188822	CNR2	cannabinoid receptor 2 [Source:HGNC Symbol;Acc:HGNC:2160]	protein coding	-0.89	2.39	17.08	<0.0001	0.0292
ENSG00000133317	LGALS12	galectin 12 [Source:HGNC Symbol;Acc:HGNC:15788]	protein coding	-0.89	3.63	13.78	0.0002	0.0488
ENSG00000030419	IKZF2	IKAROS family zinc finger 2 [Source:HGNC Symbol;Acc:HGNC:13177]	protein coding	-0.89	1.71	13.84	0.0002	0.0485
ENSG00000168421	RHOH	ras homolog family member H [Source:HGNC Symbol;Acc:HGNC:686]	protein coding	-0.87	1.97	14.08	0.0002	0.0448
ENSG00000136213	CHST12	carbohydrate sulfotransferase 12 [Source:HGNC Symbol;Acc:HGNC:17423]	protein coding	-0.86	1.63	18.47	<0.0001	0.0292
ENSG00000188042	ARL4C	ADP ribosylation factor like GTPase 4C [Source:HGNC Symbol;Acc:HGNC:698]	protein coding	-0.85	3.12	17.10	<0.0001	0.0292
ENSG00000139641	ESYT1	extended synaptotagmin 1 [Source:HGNC Symbol;Acc:HGNC:29534]	protein coding	-0.82	5.00	14.85	0.0001	0.0392

Table S15: Genes differentially expressed at Day 8 compared to pre-first vaccination (Study Arm 1 A/H3N2v, D1 , Neutrophils). Genes are sorted by descending absolute log_2 fold change. Gene model summaries and annotations are based on Ensembl Version 99 (continued.)

Ensembl Gene ID	Ensembl Gene Name	Ensembl Gene Description	Gene Type	log_2 Fold Change (Day 8 vs. Pre-First Vaccination)	Average log_2 CPM	Likelihood Ratio Test Statistic	P-Value	FDR Adjusted P-Value
ENSG00000196781	TLE1	TLE family member 1, transcriptional corepressor [Source:HGNC Symbol;Acc:HGNC:11837]	protein coding	-0.81	1.30	16.09	<0.0001	0.0294
ENSG00000152207	CYSLTR2	cysteinyl leukotriene receptor 2 [Source:HGNC Symbol;Acc:HGNC:18274]	protein coding	-0.79	3.41	20.03	<0.0001	0.0254
ENSG00000213563	C8orf82	chromosome 8 open reading frame 82 [Source:HGNC Symbol;Acc:HGNC:33826]	protein coding	-0.77	0.70	14.71	0.0001	0.0392
ENSG00000184221	OLIG1	oligodendrocyte transcription factor 1 [Source:HGNC Symbol;Acc:HGNC:16983]	protein coding	-0.74	4.69	14.41	0.0001	0.0417
ENSG00000140367	UBE2Q2	ubiquitin conjugating enzyme E2 Q2 [Source:HGNC Symbol;Acc:HGNC:19248]	protein coding	-0.69	1.09	20.16	<0.0001	0.0254
ENSG00000259953		novel transcript, overlapping SUSD1	lncRNA	-0.63	0.82	18.38	<0.0001	0.0292
ENSG00000124091	GCNT7	glucosaminyl (N-acetyl) transferase family member 7 [Source:HGNC Symbol;Acc:HGNC:16099]	protein coding	0.61	0.62	16.27	<0.0001	0.0292
ENSG00000141569	TRIM65	tripartite motif containing 65 [Source:HGNC Symbol;Acc:HGNC:27316]	protein coding	-0.59	0.49	15.88	<0.0001	0.0294

Table S16: Genes differentially expressed at Day 2 compared to pre-first vaccination (Study Arm 2 A/H7N9+AS03, D1, 29, Neutrophils). Genes are sorted by descending absolute log_2 fold change. Gene model summaries and annotations are based on Ensembl Version 99.

Ensembl Gene ID	Ensembl Gene Name	Ensembl Gene Description	Gene Type	log_2 Fold Change (Day 2 vs. Pre-First Vaccination)	Average log_2 CPM	Likelihood Ratio Test Statistic	P-Value	FDR Adjusted P-Value
ENSG00000162772	ATF3	activating transcription factor 3 [Source:HGNC Symbol;Acc:HGNC:785]	protein coding	2.86	1.96	39.14	<0.0001	<0.0001
ENSG00000108387	SEPTIN4	septin 4 [Source:HGNC Symbol;Acc:HGNC:9165]	protein coding	2.82	1.92	34.48	<0.0001	<0.0001
ENSG00000197646	PDCD1LG2	programmed cell death 1 ligand 2 [Source:HGNC Symbol;Acc:HGNC:18731]	protein coding	2.80	1.28	48.41	<0.0001	<0.0001
ENSG00000152766	ANKRD22	ankyrin repeat domain 22 [Source:HGNC Symbol;Acc:HGNC:28321]	protein coding	2.77	4.76	38.13	<0.0001	<0.0001
ENSG00000150337	FCGR1A	Fc fragment of IgG receptor Ia [Source:HGNC Symbol;Acc:HGNC:3613]	protein coding	2.64	6.31	47.01	<0.0001	<0.0001

Table S16: Genes differentially expressed at Day 2 compared to pre-first vaccination (Study Arm 2 A/H7N9+AS03, D1, 29, Neutrophils). Genes are sorted by descending absolute log_2 fold change. Gene model summaries and annotations are based on Ensembl Version 99 (continued.)

Ensembl Gene ID	Ensembl Gene Name	Ensembl Gene Description	Gene Type	log_2 Fold Change (Day 2 vs. Pre-First Vaccination)	Average log_2 CPM	Likelihood Ratio Test Statistic	P-Value	FDR Adjusted P-Value
ENSG00000265531	FCGR1CP	Fc fragment of IgG receptor Ic, pseudogene [Source:HGNC Symbol;Acc:HGNC:3615]	unprocessed pseudogene	2.49	3.40	40.28	<0.0001	<0.0001
ENSG00000174944	P2RY14	purinergic receptor P2Y14 [Source:HGNC Symbol;Acc:HGNC:16442]	protein coding	2.48	5.63	54.51	<0.0001	<0.0001
ENSG00000010030	ETV7	ETS variant transcription factor 7 [Source:HGNC Symbol;Acc:HGNC:18160]	protein coding	2.44	5.39	43.60	<0.0001	<0.0001
ENSG00000168062	BATF2	basic leucine zipper ATF-like transcription factor 2 [Source:HGNC Symbol;Acc:HGNC:25163]	protein coding	2.43	5.42	35.17	<0.0001	<0.0001
ENSG00000120217	CD274	CD274 molecule [Source:HGNC Symbol;Acc:HGNC:17635]	protein coding	2.42	5.58	40.11	<0.0001	<0.0001
ENSG00000133106	EPSTI1	epithelial stromal interaction 1 [Source:HGNC Symbol;Acc:HGNC:16465]	protein coding	2.42	6.51	34.23	<0.0001	<0.0001
ENSG00000131979	GCH1	GTP cyclohydrolase 1 [Source:HGNC Symbol;Acc:HGNC:4193]	protein coding	2.41	3.62	57.06	<0.0001	<0.0001
ENSG00000136514	RTP4	receptor transporter protein 4 [Source:HGNC Symbol;Acc:HGNC:23992]	protein coding	2.36	3.03	30.13	<0.0001	<0.0001
ENSG00000154451	GBP5	guanylate binding protein 5 [Source:HGNC Symbol;Acc:HGNC:19895]	protein coding	2.32	10.02	43.28	<0.0001	<0.0001
ENSG00000131203	IDO1	indoleamine 2,3-dioxygenase 1 [Source:HGNC Symbol;Acc:HGNC:6059]	protein coding	2.15	3.79	25.40	<0.0001	<0.0001
ENSG00000149131	SERPING1	serpin family G member 1 [Source:HGNC Symbol;Acc:HGNC:1228]	protein coding	2.14	6.63	38.98	<0.0001	<0.0001
ENSG00000183347	GBP6	guanylate binding protein family member 6 [Source:HGNC Symbol;Acc:HGNC:25395]	protein coding	2.11	2.52	58.14	<0.0001	<0.0001
ENSG00000133321	PLAAT4	phospholipase A and acyltransferase 4 [Source:HGNC Symbol;Acc:HGNC:9869]	protein coding	2.10	4.13	45.33	<0.0001	<0.0001
ENSG00000172159	FRMD3	FERM domain containing 3 [Source:HGNC Symbol;Acc:HGNC:24125]	protein coding	2.06	2.46	39.17	<0.0001	<0.0001
ENSG00000185338	SOCS1	suppressor of cytokine signaling 1 [Source:HGNC Symbol;Acc:HGNC:19383]	protein coding	2.05	3.50	22.32	<0.0001	<0.0001
ENSG00000162654	GBP4	guanylate binding protein 4 [Source:HGNC Symbol;Acc:HGNC:20480]	protein coding	2.05	6.98	37.05	<0.0001	<0.0001
ENSG00000078081	LAMP3	lysosomal associated membrane protein 3 [Source:HGNC Symbol;Acc:HGNC:14582]	protein coding	2.02	3.09	22.93	<0.0001	<0.0001

Table S16: Genes differentially expressed at Day 2 compared to pre-first vaccination (Study Arm 2 A/H7N9+AS03, D1, 29, Neutrophils). Genes are sorted by descending absolute log_2 fold change. Gene model summaries and annotations are based on Ensembl Version 99 (continued.)

Ensembl Gene ID	Ensembl Gene Name	Ensembl Gene Description	Gene Type	log_2 Fold Change (Day 2 vs. Pre-First Vaccination)	Average log_2 CPM	Likelihood Ratio Test Statistic	P-Value	FDR Adjusted P-Value
ENSG00000117228	GBP1	guanylate binding protein 1 [Source:HGNC Symbol;Acc:HGNC:4182]	protein coding	2.00	8.22	37.17	<0.0001	<0.0001
ENSG00000198019	FCGR1B	Fc fragment of IgG receptor Ib [Source:HGNC Symbol;Acc:HGNC:3614]	protein coding	1.99	5.59	41.61	<0.0001	<0.0001
ENSG00000134809	TIMM10	translocase of inner mitochondrial membrane 10 [Source:HGNC Symbol;Acc:HGNC:11814]	protein coding	1.99	2.73	40.47	<0.0001	<0.0001
ENSG00000168899	VAMP5	vesicle associated membrane protein 5 [Source:HGNC Symbol;Acc:HGNC:12646]	protein coding	1.99	3.98	35.03	<0.0001	<0.0001
ENSG00000233029		novel transcript	lncRNA	1.97	3.32	36.24	<0.0001	<0.0001
ENSG00000161905	ALOX15	arachidonate 15-lipoxygenase [Source:HGNC Symbol;Acc:HGNC:433]	protein coding	-1.97	4.88	17.85	<0.0001	0.0002
ENSG00000129673	AANAT	aralkylamine N-acetyltransferase [Source:HGNC Symbol;Acc:HGNC:19]	protein coding	1.97	1.23	29.82	<0.0001	<0.0001
ENSG00000152229	PSTPIP2	proline-serine-threonine phosphatase interacting protein 2 [Source:HGNC Symbol;Acc:HGNC:9581]	protein coding	1.96	6.34	49.71	<0.0001	<0.0001
ENSG00000170542	SERPINB9	serpin family B member 9 [Source:HGNC Symbol;Acc:HGNC:8955]	protein coding	1.93	4.68	41.27	<0.0001	<0.0001
ENSG00000124785	NRN1	neuritin 1 [Source:HGNC Symbol;Acc:HGNC:17972]	protein coding	1.92	1.97	29.62	<0.0001	<0.0001
ENSG00000101445	PPP1R16B	protein phosphatase 1 regulatory subunit 16B [Source:HGNC Symbol;Acc:HGNC:15850]	protein coding	-1.90	1.31	17.55	<0.0001	0.0002
ENSG00000214872	SMTNL1	smoothelin like 1 [Source:HGNC Symbol;Acc:HGNC:32394]	protein coding	1.90	3.61	38.12	<0.0001	<0.0001
ENSG00000089127	OAS1	2'-5'-oligoadenylate synthetase 1 [Source:HGNC Symbol;Acc:HGNC:8086]	protein coding	1.88	6.19	22.68	<0.0001	<0.0001
ENSG00000111335	OAS2	2'-5'-oligoadenylate synthetase 2 [Source:HGNC Symbol;Acc:HGNC:8087]	protein coding	1.86	5.97	19.39	<0.0001	0.0001
ENSG00000145685	LHFPL2	LHFPL tetraspan subfamily member 2 [Source:HGNC Symbol;Acc:HGNC:6588]	protein coding	1.85	3.87	32.64	<0.0001	<0.0001
ENSG00000119537	KDSR	3-ketodihydrosphingosine reductase [Source:HGNC Symbol;Acc:HGNC:4021]	protein coding	1.85	2.17	46.35	<0.0001	<0.0001
ENSG00000163958	ZDHHC19	zinc finger DHHC-type containing 19 [Source:HGNC Symbol;Acc:HGNC:20713]	protein coding	1.85	2.29	55.83	<0.0001	<0.0001
ENSG00000105967	TFEC	transcription factor EC [Source:HGNC Symbol;Acc:HGNC:11754]	protein coding	1.85	2.07	21.79	<0.0001	<0.0001

Table S16: Genes differentially expressed at Day 2 compared to pre-first vaccination (Study Arm 2 A/H7N9+AS03, D1, 29, Neutrophils). Genes are sorted by descending absolute log_2 fold change. Gene model summaries and annotations are based on Ensembl Version 99 (continued.)

Ensembl Gene ID	Ensembl Gene Name	Ensembl Gene Description	Gene Type	log_2 Fold Change (Day 2 vs. Pre-First Vaccination)	Average log_2 CPM	Likelihood Ratio Test Statistic	P-Value	FDR Adjusted P-Value
ENSG00000166428	PLD4	phospholipase D family member 4 [Source:HGNC Symbol;Acc:HGNC:23792]	protein coding	-1.85	2.31	14.02	0.0002	0.0011
ENSG00000196141	SPATS2L	spermatogenesis associated serine rich 2 like [Source:HGNC Symbol;Acc:HGNC:24574]	protein coding	1.84	1.81	16.27	<0.0001	0.0004
ENSG00000142089	IFITM3	interferon induced transmembrane protein 3 [Source:HGNC Symbol;Acc:HGNC:5414]	protein coding	1.84	9.02	78.03	<0.0001	<0.0001
ENSG00000135916	ITM2C	integral membrane protein 2C [Source:HGNC Symbol;Acc:HGNC:6175]	protein coding	-1.84	2.26	25.43	<0.0001	<0.0001
ENSG00000131943	C19orf12	chromosome 19 open reading frame 12 [Source:HGNC Symbol;Acc:HGNC:25443]	protein coding	1.82	1.96	34.25	<0.0001	<0.0001
ENSG00000268758	ADGRE4P	adhesion G protein-coupled receptor E4, pseudogene [Source:HGNC Symbol;Acc:HGNC:19240]	transcribed unprocessed pseudogene	-1.82	3.77	15.61	<0.0001	0.0005
ENSG00000182557	SPNS3	sphingolipid transporter 3 (putative) [Source:HGNC Symbol;Acc:HGNC:28433]	protein coding	-1.82	2.89	16.57	<0.0001	0.0003
ENSG00000117226	GBP3	guanylate binding protein 3 [Source:HGNC Symbol;Acc:HGNC:4184]	protein coding	1.81	4.51	33.39	<0.0001	<0.0001
ENSG00000183134	PTGDR2	prostaglandin D2 receptor 2 [Source:HGNC Symbol;Acc:HGNC:4502]	protein coding	-1.81	4.00	12.89	0.0003	0.0018
ENSG00000177721	ANXA2R	annexin A2 receptor [Source:HGNC Symbol;Acc:HGNC:33463]	protein coding	1.80	1.78	29.23	<0.0001	<0.0001
ENSG00000130881	LRP3	LDL receptor related protein 3 [Source:HGNC Symbol;Acc:HGNC:6695]	protein coding	-1.80	2.84	38.69	<0.0001	<0.0001
ENSG00000101347	SAMHD1	SAM and HD domain containing deoxynucleoside triphosphate triphosphohydrolase 1 [Source:HGNC Symbol;Acc:HGNC:15925]	protein coding	1.79	7.07	38.03	<0.0001	<0.0001
ENSG00000103355	PRSS33	serine protease 33 [Source:HGNC Symbol;Acc:HGNC:30405]	protein coding	-1.79	4.79	13.74	0.0002	0.0012
ENSG00000128284	APOL3	apolipoprotein L3 [Source:HGNC Symbol;Acc:HGNC:14868]	protein coding	1.77	1.88	28.56	<0.0001	<0.0001
ENSG00000026751	SLAMF7	SLAM family member 7 [Source:HGNC Symbol;Acc:HGNC:21394]	protein coding	1.76	2.51	55.61	<0.0001	<0.0001
ENSG00000230438	SERPINB9P1	serpin family B member 9 pseudogene 1 [Source:HGNC Symbol;Acc:HGNC:28590]	lncRNA	1.76	2.42	45.55	<0.0001	<0.0001

Table S16: Genes differentially expressed at Day 2 compared to pre-first vaccination (Study Arm 2 A/H7N9+AS03, D1, 29, Neutrophils). Genes are sorted by descending absolute log_2 fold change. Gene model summaries and annotations are based on Ensembl Version 99 (continued.)

Ensembl Gene ID	Ensembl Gene Name	Ensembl Gene Description	Gene Type	log_2 Fold Change (Day 2 vs. Pre-First Vaccination)	Average log_2 CPM	Likelihood Ratio Test Statistic	P-Value	FDR Adjusted P-Value
ENSG00000167106	FAM102A	family with sequence similarity 102 member A [Source:HGNC Symbol;Acc:HGNC:31419]	protein coding	-1.75	2.51	29.11	<0.0001	<0.0001
ENSG00000182240	BACE2	beta-secretase 2 [Source:HGNC Symbol;Acc:HGNC:934]	protein coding	-1.74	1.96	12.77	0.0004	0.0019
ENSG00000115602	IL1RL1	interleukin 1 receptor like 1 [Source:HGNC Symbol;Acc:HGNC:5998]	protein coding	-1.73	1.53	12.61	0.0004	0.002
ENSG00000188313	PLSCR1	phospholipid scramblase 1 [Source:HGNC Symbol;Acc:HGNC:9092]	protein coding	1.73	7.05	39.45	<0.0001	<0.0001
ENSG00000132274	TRIM22	tripartite motif containing 22 [Source:HGNC Symbol;Acc:HGNC:16379]	protein coding	1.72	8.92	38.76	<0.0001	<0.0001
ENSG00000168405	CMAHP	cytidine monophospho-N-acetylneuraminic acid hydroxylase, pseudogene [Source:HGNC Symbol;Acc:HGNC:2098]	transcribed unitary pseudogene	1.72	2.07	53.27	<0.0001	<0.0001
ENSG00000233030		novel transcript, antisense to FCGR1A	lncRNA	1.71	2.57	35.30	<0.0001	<0.0001
ENSG00000137757	CASP5	caspase 5 [Source:HGNC Symbol;Acc:HGNC:1506]	protein coding	1.71	5.46	42.56	<0.0001	<0.0001
ENSG00000002549	LAP3	leucine aminopeptidase 3 [Source:HGNC Symbol;Acc:HGNC:18449]	protein coding	1.70	6.05	20.75	<0.0001	<0.0001
ENSG00000076555	ACACB	acetyl-CoA carboxylase beta [Source:HGNC Symbol;Acc:HGNC:85]	protein coding	-1.70	2.16	35.28	<0.0001	<0.0001
ENSG00000274376	ADAMTS7P1	ADAMTS7 pseudogene 1 [Source:HGNC Symbol;Acc:HGNC:49407]	transcribed unprocessed pseudogene	-1.69	1.47	11.35	0.0008	0.0035
ENSG00000221963	APOL6	apolipoprotein L6 [Source:HGNC Symbol;Acc:HGNC:14870]	protein coding	1.69	7.71	31.39	<0.0001	<0.0001
ENSG00000111331	OAS3	2'-5'-oligoadenylate synthetase 3 [Source:HGNC Symbol;Acc:HGNC:8088]	protein coding	1.69	7.68	20.75	<0.0001	<0.0001
ENSG00000255587	RAB44	RAB44, member RAS oncogene family [Source:HGNC Symbol;Acc:HGNC:21068]	protein coding	-1.68	3.24	12.16	0.0005	0.0024
ENSG00000137801	THBS1	thrombospondin 1 [Source:HGNC Symbol;Acc:HGNC:11785]	protein coding	-1.68	1.89	11.59	0.0007	0.0031
ENSG00000136732	GYPC	glycophorin C (Gerbich blood group) [Source:HGNC Symbol;Acc:HGNC:4704]	protein coding	1.67	3.21	59.61	<0.0001	<0.0001

Table S16: Genes differentially expressed at Day 2 compared to pre-first vaccination (Study Arm 2 A/H7N9+AS03, D1, 29, Neutrophils). Genes are sorted by descending absolute log_2 fold change. Gene model summaries and annotations are based on Ensembl Version 99 (continued.)

Ensembl Gene ID	Ensembl Gene Name	Ensembl Gene Description	Gene Type	log_2 Fold Change (Day 2 vs. Pre-First Vaccination)	Average log_2 CPM	Likelihood Ratio Test Statistic	P-Value	FDR Adjusted P-Value
ENSG00000116663	FBXO6	F-box protein 6 [Source:HGNC Symbol;Acc:HGNC:13585]	protein coding	1.67	4.86	37.96	<0.0001	<0.0001
ENSG00000159618	ADGRG5	adhesion G protein-coupled receptor G5 [Source:HGNC Symbol;Acc:HGNC:19010]	protein coding	-1.66	2.80	13.21	0.0003	0.0015
ENSG00000148175	STOM	stomatin [Source:HGNC Symbol;Acc:HGNC:3383]	protein coding	1.66	4.99	37.71	<0.0001	<0.0001
ENSG00000140105	WARS1	tryptophanyl-tRNA synthetase 1 [Source:HGNC Symbol;Acc:HGNC:12729]	protein coding	1.64	7.63	47.93	<0.0001	<0.0001
ENSG00000132530	XAF1	XIAP associated factor 1 [Source:HGNC Symbol;Acc:HGNC:30932]	protein coding	1.63	5.64	26.46	<0.0001	<0.0001
ENSG00000163568	AIM2	absent in melanoma 2 [Source:HGNC Symbol;Acc:HGNC:357]	protein coding	1.63	4.74	34.73	<0.0001	<0.0001
ENSG00000154188	ANGPT1	angiopoietin 1 [Source:HGNC Symbol;Acc:HGNC:484]	protein coding	-1.63	3.47	59.47	<0.0001	<0.0001
ENSG00000188042	ARL4C	ADP ribosylation factor like GTPase 4C [Source:HGNC Symbol;Acc:HGNC:698]	protein coding	-1.63	2.56	18.92	<0.0001	0.0001
ENSG00000164308	ERAP2	endoplasmic reticulum aminopeptidase 2 [Source:HGNC Symbol;Acc:HGNC:29499]	protein coding	1.63	4.17	44.29	<0.0001	<0.0001
ENSG00000105366	SIGLEC8	sialic acid binding Ig like lectin 8 [Source:HGNC Symbol;Acc:HGNC:10877]	protein coding	-1.62	3.16	10.74	0.001	0.0045
ENSG00000137628	DDX60	DExD/H-box helicase 60 [Source:HGNC Symbol;Acc:HGNC:25942]	protein coding	1.62	5.72	23.24	<0.0001	<0.0001
ENSG00000130433	CACNG6	calcium voltage-gated channel auxiliary subunit gamma 6 [Source:HGNC Symbol;Acc:HGNC:13625]	protein coding	-1.62	1.50	12.19	0.0005	0.0024
ENSG00000068079	IFI35	interferon induced protein 35 [Source:HGNC Symbol;Acc:HGNC:5399]	protein coding	1.62	6.47	30.11	<0.0001	<0.0001
ENSG00000107960	STN1	STN1 subunit of CST complex [Source:HGNC Symbol;Acc:HGNC:26200]	protein coding	1.62	5.04	57.43	<0.0001	<0.0001
ENSG00000110060	PUS3	pseudouridine synthase 3 [Source:HGNC Symbol;Acc:HGNC:25461]	protein coding	1.62	0.80	38.65	<0.0001	<0.0001
ENSG00000286162		novel transcript	lncRNA	1.62	1.43	24.14	<0.0001	<0.0001
ENSG00000203666	EFCAB2	EF-hand calcium binding domain 2 [Source:HGNC Symbol;Acc:HGNC:28166]	protein coding	1.62	3.27	42.16	<0.0001	<0.0001
ENSG00000129667	RHBDF2	rhomboid 5 homolog 2 [Source:HGNC Symbol;Acc:HGNC:20788]	protein coding	1.61	7.10	42.39	<0.0001	<0.0001

Table S16: Genes differentially expressed at Day 2 compared to pre-first vaccination (Study Arm 2 A/H7N9+AS03, D1, 29, Neutrophils). Genes are sorted by descending absolute log_2 fold change. Gene model summaries and annotations are based on Ensembl Version 99 (continued.)

Ensembl Gene ID	Ensembl Gene Name	Ensembl Gene Description	Gene Type	log_2 Fold Change (Day 2 vs. Pre-First Vaccination)	Average log_2 CPM	Likelihood Ratio Test Statistic	P-Value	FDR Adjusted P-Value
ENSG00000168765	GSTM4	glutathione S-transferase mu 4 [Source:HGNC Symbol;Acc:HGNC:4636]	protein coding	-1.61	1.51	14.62	0.0001	0.0008
ENSG00000137959	IFI44L	interferon induced protein 44 like [Source:HGNC Symbol;Acc:HGNC:17817]	protein coding	1.61	5.85	15.93	<0.0001	0.0005
ENSG00000171791	BCL2	BCL2 apoptosis regulator [Source:HGNC Symbol;Acc:HGNC:990]	protein coding	-1.61	0.26	45.32	<0.0001	<0.0001
ENSG00000134326	CMPK2	cytidine/uridine monophosphate kinase 2 [Source:HGNC Symbol;Acc:HGNC:27015]	protein coding	1.59	6.06	17.10	<0.0001	0.0003
ENSG00000134321	RSAD2	radical S-adenosyl methionine domain containing 2 [Source:HGNC Symbol;Acc:HGNC:30908]	protein coding	1.59	7.82	15.50	<0.0001	0.0006
ENSG00000105205	CLC	Charcot-Leyden crystal galectin [Source:HGNC Symbol;Acc:HGNC:2014]	protein coding	-1.59	6.93	15.00	0.0001	0.0007
ENSG00000103056	SMPD3	sphingomyelin phosphodiesterase 3 [Source:HGNC Symbol;Acc:HGNC:14240]	protein coding	-1.59	4.80	11.80	0.0006	0.0028
ENSG00000128928	IVD	isovaleryl-CoA dehydrogenase [Source:HGNC Symbol;Acc:HGNC:6186]	protein coding	-1.58	1.58	40.13	<0.0001	<0.0001
ENSG00000204345	CD300LD	CD300 molecule like family member d [Source:HGNC Symbol;Acc:HGNC:16848]	protein coding	1.57	1.93	16.50	<0.0001	0.0004
ENSG00000110651	CD81	CD81 molecule [Source:HGNC Symbol;Acc:HGNC:1701]	protein coding	-1.56	2.24	15.00	0.0001	0.0007
ENSG00000137965	IFI44	interferon induced protein 44 [Source:HGNC Symbol;Acc:HGNC:16938]	protein coding	1.56	5.96	17.78	<0.0001	0.0002
ENSG00000110042	DTX4	deltex E3 ubiquitin ligase 4 [Source:HGNC Symbol;Acc:HGNC:29151]	protein coding	-1.55	4.18	37.41	<0.0001	<0.0001
ENSG00000166002	SMCO4	single-pass membrane protein with coiled-coil domains 4 [Source:HGNC Symbol;Acc:HGNC:24810]	protein coding	1.54	3.75	34.20	<0.0001	<0.0001
ENSG00000112759	SLC29A1	solute carrier family 29 member 1 (Augustine blood group) [Source:HGNC Symbol;Acc:HGNC:11003]	protein coding	-1.54	4.10	11.90	0.0006	0.0027
ENSG00000198087	CD2AP	CD2 associated protein [Source:HGNC Symbol;Acc:HGNC:14258]	protein coding	1.54	1.99	29.79	<0.0001	<0.0001
ENSG00000138166	DUSP5	dual specificity phosphatase 5 [Source:HGNC Symbol;Acc:HGNC:3071]	protein coding	1.53	1.90	24.64	<0.0001	<0.0001
ENSG00000169442	CD52	CD52 molecule [Source:HGNC Symbol;Acc:HGNC:1804]	protein coding	-1.53	3.20	14.23	0.0002	0.001
ENSG00000153885	KCTD15	potassium channel tetramerization domain containing 15 [Source:HGNC Symbol;Acc:HGNC:23297]	protein coding	-1.53	1.17	10.94	0.0009	0.0041

Table S16: Genes differentially expressed at Day 2 compared to pre-first vaccination (Study Arm 2 A/H7N9+AS03, D1, 29, Neutrophils). Genes are sorted by descending absolute log_2 fold change. Gene model summaries and annotations are based on Ensembl Version 99 (continued.)

Ensembl Gene ID	Ensembl Gene Name	Ensembl Gene Description	Gene Type	log_2 Fold Change (Day 2 vs. Pre-First Vaccination)	Average log_2 CPM	Likelihood Ratio Test Statistic	P-Value	FDR Adjusted P-Value
ENSG00000255221	CARD17	caspase recruitment domain family member 17 [Source:HGNC Symbol;Acc:HGNC:33827]	protein coding	1.53	2.23	32.45	<0.0001	<0.0001
ENSG00000205927	OLIG2	oligodendrocyte transcription factor 2 [Source:HGNC Symbol;Acc:HGNC:9398]	protein coding	-1.52	2.31	9.46	0.0021	0.008
ENSG00000155363	MOV10	Mov10 RISC complex RNA helicase [Source:HGNC Symbol;Acc:HGNC:7200]	protein coding	1.51	5.38	29.84	<0.0001	<0.0001
ENSG00000277511		novel transcript	lncRNA	1.50	0.83	18.40	<0.0001	0.0002
ENSG00000133317	LGALS12	galectin 12 [Source:HGNC Symbol;Acc:HGNC:15788]	protein coding	-1.50	2.90	14.97	0.0001	0.0007
ENSG00000134072	CAMK1	calcium/calmodulin dependent protein kinase I [Source:HGNC Symbol;Acc:HGNC:1459]	protein coding	-1.50	1.91	14.86	0.0001	0.0007
ENSG00000100628	ASB2	ankyrin repeat and SOCS box containing 2 [Source:HGNC Symbol;Acc:HGNC:16012]	protein coding	-1.49	1.15	11.31	0.0008	0.0035
ENSG00000106683	LIMK1	LIM domain kinase 1 [Source:HGNC Symbol;Acc:HGNC:6613]	protein coding	1.49	5.60	93.04	<0.0001	<0.0001
ENSG00000173193	PARP14	poly(ADP-ribose) polymerase family member 14 [Source:HGNC Symbol;Acc:HGNC:29232]	protein coding	1.49	8.83	27.28	<0.0001	<0.0001
ENSG00000156587	UBE2L6	ubiquitin conjugating enzyme E2 L6 [Source:HGNC Symbol;Acc:HGNC:12490]	protein coding	1.49	8.10	38.13	<0.0001	<0.0001
ENSG00000100911	PSME2	proteasome activator subunit 2 [Source:HGNC Symbol;Acc:HGNC:9569]	protein coding	1.48	6.37	41.83	<0.0001	<0.0001
ENSG00000065911	MTHFD2	methylenetetrahydrofolate dehydrogenase (NADP+ dependent) 2, methenyltetrahydrofolate cyclohydrolase [Source:HGNC Symbol;Acc:HGNC:7434]	protein coding	1.48	2.48	46.18	<0.0001	<0.0001
ENSG00000087237	CETP	cholesteryl ester transfer protein [Source:HGNC Symbol;Acc:HGNC:1869]	protein coding	1.48	0.97	29.06	<0.0001	<0.0001
ENSG00000030419	IKZF2	IKAROS family zinc finger 2 [Source:HGNC Symbol;Acc:HGNC:13177]	protein coding	-1.48	1.14	9.96	0.0016	0.0064
ENSG00000165806	CASP7	caspase 7 [Source:HGNC Symbol;Acc:HGNC:1508]	protein coding	1.47	0.99	24.46	<0.0001	<0.0001
ENSG00000157693	TMEM268	transmembrane protein 268 [Source:HGNC Symbol;Acc:HGNC:24513]	protein coding	1.47	4.10	35.85	<0.0001	<0.0001
ENSG00000075399	VPS9D1	VPS9 domain containing 1 [Source:HGNC Symbol;Acc:HGNC:13526]	protein coding	1.47	6.55	43.55	<0.0001	<0.0001
ENSG00000145365	TIFA	TRAF interacting protein with forkhead associated domain [Source:HGNC Symbol;Acc:HGNC:19075]	protein coding	1.46	5.32	34.88	<0.0001	<0.0001

Table S16: Genes differentially expressed at Day 2 compared to pre-first vaccination (Study Arm 2 A/H7N9+AS03, D1, 29, Neutrophils). Genes are sorted by descending absolute log_2 fold change. Gene model summaries and annotations are based on Ensembl Version 99 (continued.)

Ensembl Gene ID	Ensembl Gene Name	Ensembl Gene Description	Gene Type	log_2 Fold Change (Day 2 vs. Pre-First Vaccination)	Average log_2 CPM	Likelihood Ratio Test Statistic	P-Value	FDR Adjusted P-Value
ENSG00000157404	KIT	KIT proto-oncogene, receptor tyrosine kinase [Source:HGNC Symbol;Acc:HGNC:6342]	protein coding	-1.46	2.18	31.24	<0.0001	<0.0001
ENSG00000047621	C12orf4	chromosome 12 open reading frame 4 [Source:HGNC Symbol;Acc:HGNC:1184]	protein coding	1.46	1.88	34.54	<0.0001	<0.0001
ENSG00000151136	BTBD11	BTB domain containing 11 [Source:HGNC Symbol;Acc:HGNC:23844]	protein coding	-1.46	3.34	33.31	<0.0001	<0.0001
ENSG00000188549	CCDC9B	coiled-coil domain containing 9B [Source:HGNC Symbol;Acc:HGNC:33488]	protein coding	-1.45	1.20	36.41	<0.0001	<0.0001
ENSG00000048140	TSPAN17	tetraspanin 17 [Source:HGNC Symbol;Acc:HGNC:13594]	protein coding	1.44	1.89	28.65	<0.0001	<0.0001
ENSG00000170581	STAT2	signal transducer and activator of transcription 2 [Source:HGNC Symbol;Acc:HGNC:11363]	protein coding	1.44	8.26	24.83	<0.0001	<0.0001
ENSG00000105948	TTC26	tetratricopeptide repeat domain 26 [Source:HGNC Symbol;Acc:HGNC:21882]	protein coding	1.44	3.25	34.54	<0.0001	<0.0001
ENSG00000161981	SNRNP25	small nuclear ribonucleoprotein U11/U12 subunit 25 [Source:HGNC Symbol;Acc:HGNC:14161]	protein coding	-1.43	1.45	39.89	<0.0001	<0.0001
ENSG00000065427	KARS1	lysyl-tRNA synthetase 1 [Source:HGNC Symbol;Acc:HGNC:6215]	protein coding	1.42	4.92	33.41	<0.0001	<0.0001
ENSG00000110455	ACCS	1-aminocyclopropane-1-carboxylate synthase homolog (inactive) [Source:HGNC Symbol;Acc:HGNC:23989]	protein coding	-1.41	2.69	8.85	0.0029	0.0105
ENSG00000023171	GRAMD1B	GRAM domain containing 1B [Source:HGNC Symbol;Acc:HGNC:29214]	protein coding	1.41	3.67	31.83	<0.0001	<0.0001
ENSG00000162894	FCMR	Fc fragment of IgM receptor [Source:HGNC Symbol;Acc:HGNC:14315]	protein coding	-1.41	4.70	25.69	<0.0001	<0.0001
ENSG00000128203	ASPHD2	aspartate beta-hydroxylase domain containing 2 [Source:HGNC Symbol;Acc:HGNC:30437]	protein coding	1.40	2.49	41.99	<0.0001	<0.0001
ENSG00000140464	PML	promyelocytic leukemia [Source:HGNC Symbol;Acc:HGNC:9113]	protein coding	1.40	7.01	25.57	<0.0001	<0.0001
ENSG00000136770	DNAJC1	DnaJ heat shock protein family (Hsp40) member C1 [Source:HGNC Symbol;Acc:HGNC:20090]	protein coding	1.40	3.53	57.32	<0.0001	<0.0001
ENSG00000184221	OLIG1	oligodendrocyte transcription factor 1 [Source:HGNC Symbol;Acc:HGNC:16983]	protein coding	-1.40	4.19	21.46	<0.0001	<0.0001
ENSG00000147872	PLIN2	perilipin 2 [Source:HGNC Symbol;Acc:HGNC:248]	protein coding	-1.39	2.17	14.59	0.0001	0.0008
ENSG00000134256	CD101	CD101 molecule [Source:HGNC Symbol;Acc:HGNC:5949]	protein coding	-1.38	2.38	17.37	<0.0001	0.0002

Table S16: Genes differentially expressed at Day 2 compared to pre-first vaccination (Study Arm 2 A/H7N9+AS03, D1, 29, Neutrophils). Genes are sorted by descending absolute log_2 fold change. Gene model summaries and annotations are based on Ensembl Version 99 (continued.)

Ensembl Gene ID	Ensembl Gene Name	Ensembl Gene Description	Gene Type	log_2 Fold Change (Day 2 vs. Pre-First Vaccination)	Average log_2 CPM	Likelihood Ratio Test Statistic	P-Value	FDR Adjusted P-Value
ENSG00000185432	METTL7A	methyltransferase like 7A [Source:HGNC Symbol;Acc:HGNC:24550]	protein coding	-1.38	2.10	9.72	0.0018	0.0071
ENSG00000188820	CALHM6	calcium homeostasis modulator family member 6 [Source:HGNC Symbol;Acc:HGNC:33391]	protein coding	1.37	4.01	27.40	<0.0001	<0.0001
ENSG00000143842	SOX13	SRY-box transcription factor 13 [Source:HGNC Symbol;Acc:HGNC:11192]	protein coding	-1.37	0.85	36.40	<0.0001	<0.0001
ENSG00000282608	ADORA3	adenosine A3 receptor [Source:HGNC Symbol;Acc:HGNC:268]	protein coding	-1.36	1.39	13.97	0.0002	0.0011
ENSG00000128833	MYO5C	myosin VC [Source:HGNC Symbol;Acc:HGNC:7604]	protein coding	-1.36	0.52	24.03	<0.0001	<0.0001
ENSG00000266074	BAHCC1	BAH domain and coiled-coil containing 1 [Source:HGNC Symbol;Acc:HGNC:29279]	protein coding	-1.36	0.76	13.49	0.0002	0.0014
ENSG00000282851	BISPR	BST2 interferon stimulated positive regulator [Source:HGNC Symbol;Acc:HGNC:51290]	lncRNA	1.36	3.21	27.76	<0.0001	<0.0001
ENSG00000126351	THRA	thyroid hormone receptor alpha [Source:HGNC Symbol;Acc:HGNC:11796]	protein coding	-1.36	4.01	41.92	<0.0001	<0.0001
ENSG00000249173	LINC01093	long intergenic non-protein coding RNA 1093 [Source:HGNC Symbol;Acc:HGNC:49218]	lncRNA	1.35	1.58	29.90	<0.0001	<0.0001
ENSG00000260943	LINC02555	long intergenic non-protein coding RNA 2555 [Source:HGNC Symbol;Acc:HGNC:53593]	lncRNA	1.35	2.85	35.53	<0.0001	<0.0001
ENSG00000176170	SPHK1	sphingosine kinase 1 [Source:HGNC Symbol;Acc:HGNC:11240]	protein coding	1.34	0.82	31.39	<0.0001	<0.0001
ENSG00000104805	NUCB1	nucleobindin 1 [Source:HGNC Symbol;Acc:HGNC:8043]	protein coding	1.34	8.01	30.98	<0.0001	<0.0001
ENSG00000015171	ZMYND11	zinc finger MYND-type containing 11 [Source:HGNC Symbol;Acc:HGNC:16966]	protein coding	-1.34	1.03	14.81	0.0001	0.0008
ENSG00000204634	TBC1D8	TBC1 domain family member 8 [Source:HGNC Symbol;Acc:HGNC:17791]	protein coding	1.34	2.74	34.33	<0.0001	<0.0001
ENSG00000134489	HRH4	histamine receptor H4 [Source:HGNC Symbol;Acc:HGNC:17383]	protein coding	-1.34	0.96	7.54	0.006	0.0189
ENSG00000177409	SAMD9L	sterile alpha motif domain containing 9 like [Source:HGNC Symbol;Acc:HGNC:1349]	protein coding	1.33	8.05	24.90	<0.0001	<0.0001
ENSG00000110799	VWF	von Willebrand factor [Source:HGNC Symbol;Acc:HGNC:12726]	protein coding	-1.33	1.93	9.11	0.0025	0.0094
ENSG00000166750	SLFN5	schlafen family member 5 [Source:HGNC Symbol;Acc:HGNC:28286]	protein coding	1.32	2.84	22.82	<0.0001	<0.0001

Table S16: Genes differentially expressed at Day 2 compared to pre-first vaccination (Study Arm 2 A/H7N9+AS03, D1, 29, Neutrophils). Genes are sorted by descending absolute log_2 fold change. Gene model summaries and annotations are based on Ensembl Version 99 (continued.)

Ensembl Gene ID	Ensembl Gene Name	Ensembl Gene Description	Gene Type	log_2 Fold Change (Day 2 vs. Pre-First Vaccination)	Average log_2 CPM	Likelihood Ratio Test Statistic	P-Value	FDR Adjusted P-Value
ENSG00000012983	MAP4K5	mitogen-activated protein kinase kinase kinase kinase 5 [Source:HGNC Symbol;Acc:HGNC:6867]	protein coding	-1.32	0.98	10.82	0.001	0.0044
ENSG00000169508	GPR183	G protein-coupled receptor 183 [Source:HGNC Symbol;Acc:HGNC:3128]	protein coding	-1.32	1.09	10.14	0.0015	0.0059
ENSG00000072134	EPN2	epsin 2 [Source:HGNC Symbol;Acc:HGNC:18639]	protein coding	-1.32	2.98	37.42	<0.0001	<0.0001
ENSG00000139626	ITGB7	integrin subunit beta 7 [Source:HGNC Symbol;Acc:HGNC:6162]	protein coding	-1.32	1.83	10.11	0.0015	0.006
ENSG00000140853	NLRC5	NLR family CARD domain containing 5 [Source:HGNC Symbol;Acc:HGNC:29933]	protein coding	1.31	8.17	38.02	<0.0001	<0.0001
ENSG00000213366	GSTM2	glutathione S-transferase mu 2 [Source:HGNC Symbol;Acc:HGNC:4634]	protein coding	-1.31	1.89	13.15	0.0003	0.0016
ENSG00000147443	DOK2	docking protein 2 [Source:HGNC Symbol;Acc:HGNC:2991]	protein coding	-1.31	4.07	13.15	0.0003	0.0016
ENSG00000165389	SPTSSA	serine palmitoyltransferase small subunit A [Source:HGNC Symbol;Acc:HGNC:20361]	protein coding	1.30	1.00	22.37	<0.0001	<0.0001
ENSG00000123610	TNFAIP6	TNF alpha induced protein 6 [Source:HGNC Symbol;Acc:HGNC:11898]	protein coding	1.30	6.57	32.48	<0.0001	<0.0001
ENSG00000115109	EPB41L5	erythrocyte membrane protein band 4.1 like 5 [Source:HGNC Symbol;Acc:HGNC:19819]	protein coding	1.30	1.97	20.79	<0.0001	<0.0001
ENSG00000111110	PPM1H	protein phosphatase, Mg2+/Mn2+ dependent 1H [Source:HGNC Symbol;Acc:HGNC:18583]	protein coding	-1.30	2.00	44.59	<0.0001	<0.0001
ENSG00000107957	SH3PXD2A	SH3 and PX domains 2A [Source:HGNC Symbol;Acc:HGNC:23664]	protein coding	1.30	0.47	36.02	<0.0001	<0.0001
ENSG00000225886		novel transcript, antisense to IFI6	lncRNA	1.29	2.70	12.57	0.0004	0.002
ENSG00000136999	CCN3	cellular communication network factor 3 [Source:HGNC Symbol;Acc:HGNC:7885]	protein coding	-1.29	5.17	42.39	<0.0001	<0.0001
ENSG00000128394	APOBEC3F	apolipoprotein B mRNA editing enzyme catalytic subunit 3F [Source:HGNC Symbol;Acc:HGNC:17356]	protein coding	1.29	2.13	19.19	<0.0001	0.0001
ENSG00000175643	RMI2	RecQ mediated genome instability 2 [Source:HGNC Symbol;Acc:HGNC:28349]	protein coding	1.28	2.19	21.36	<0.0001	<0.0001
ENSG00000105220	GPI	glucose-6-phosphate isomerase [Source:HGNC Symbol;Acc:HGNC:4458]	protein coding	-1.28	3.42	15.09	0.0001	0.0007
ENSG00000223914	LINC02471	long intergenic non-protein coding RNA 2471 [Source:HGNC Symbol;Acc:HGNC:53410]	lncRNA	1.28	0.94	30.53	<0.0001	<0.0001

Table S16: Genes differentially expressed at Day 2 compared to pre-first vaccination (Study Arm 2 A/H7N9+AS03, D1, 29, Neutrophils). Genes are sorted by descending absolute log_2 fold change. Gene model summaries and annotations are based on Ensembl Version 99 (continued.)

Ensembl Gene ID	Ensembl Gene Name	Ensembl Gene Description	Gene Type	log_2 Fold Change (Day 2 vs. Pre-First Vaccination)	Average log_2 CPM	Likelihood Ratio Test Statistic	P-Value	FDR Adjusted P-Value
ENSG00000067221	STOML1	stomatin like 1 [Source:HGNC Symbol;Acc:HGNC:14560]	protein coding	1.28	1.81	23.56	<0.0001	<0.0001
ENSG00000165702	GFI1B	growth factor independent 1B transcriptional repressor [Source:HGNC Symbol;Acc:HGNC:4238]	protein coding	-1.28	2.05	8.88	0.0029	0.0104
ENSG00000130695	CEP85	centrosomal protein 85 [Source:HGNC Symbol;Acc:HGNC:25309]	protein coding	-1.28	1.28	17.01	<0.0001	0.0003
ENSG00000160932	LY6E	lymphocyte antigen 6 family member E [Source:HGNC Symbol;Acc:HGNC:6727]	protein coding	1.27	7.05	8.43	0.0037	0.0127
ENSG00000184702	SEPTIN5	septin 5 [Source:HGNC Symbol;Acc:HGNC:9164]	protein coding	-1.27	1.36	28.32	<0.0001	<0.0001
ENSG00000225964	NRIR	negative regulator of interferon response [Source:HGNC Symbol;Acc:HGNC:51269]	lncRNA	1.27	1.13	9.85	0.0017	0.0067
ENSG00000277969		novel transcript	lncRNA	1.26	1.41	25.38	<0.0001	<0.0001
ENSG00000229677		ribosomal protein L17 (RPL17) pseudogene	processed pseudogene	1.26	1.01	18.25	<0.0001	0.0002
ENSG00000263069	RNF213-AS1	RNF213 antisense RNA 1 [Source:HGNC Symbol;Acc:HGNC:54402]	lncRNA	1.26	3.27	19.88	<0.0001	<0.0001
ENSG00000261373	VPS9D1-AS1	VPS9D1 antisense RNA 1 [Source:HGNC Symbol;Acc:HGNC:48915]	lncRNA	1.26	2.93	35.14	<0.0001	<0.0001
ENSG00000115415	STAT1	signal transducer and activator of transcription 1 [Source:HGNC Symbol;Acc:HGNC:11362]	protein coding	1.25	9.12	34.39	<0.0001	<0.0001
ENSG00000204257	HLA-DMA	major histocompatibility complex, class II, DM alpha [Source:HGNC Symbol;Acc:HGNC:4934]	protein coding	1.25	3.09	42.83	<0.0001	<0.0001
ENSG00000100342	APOL1	apolipoprotein L1 [Source:HGNC Symbol;Acc:HGNC:618]	protein coding	1.24	7.02	29.67	<0.0001	<0.0001
ENSG00000004799	PDK4	pyruvate dehydrogenase kinase 4 [Source:HGNC Symbol;Acc:HGNC:8812]	protein coding	-1.24	2.94	13.94	0.0002	0.0011
ENSG00000167074	TEF	TEF transcription factor, PAR bZIP family member [Source:HGNC Symbol;Acc:HGNC:11722]	protein coding	-1.24	1.24	43.56	<0.0001	<0.0001
ENSG00000101096	NFATC2	nuclear factor of activated T cells 2 [Source:HGNC Symbol;Acc:HGNC:7776]	protein coding	-1.24	0.82	8.78	0.003	0.0108
ENSG00000234518	PTGES3P1	prostaglandin E synthase 3 pseudogene 1 [Source:HGNC Symbol;Acc:HGNC:43824]	processed pseudogene	1.24	4.19	35.79	<0.0001	<0.0001

Table S16: Genes differentially expressed at Day 2 compared to pre-first vaccination (Study Arm 2 A/H7N9+AS03, D1, 29, Neutrophils). Genes are sorted by descending absolute log_2 fold change. Gene model summaries and annotations are based on Ensembl Version 99 (continued.)

Ensembl Gene ID	Ensembl Gene Name	Ensembl Gene Description	Gene Type	log_2 Fold Change (Day 2 vs. Pre-First Vaccination)	Average log_2 CPM	Likelihood Ratio Test Statistic	P-Value	FDR Adjusted P-Value
ENSG00000136826	KLF4	Kruppel like factor 4 [Source:HGNC Symbol;Acc:HGNC:6348]	protein coding	1.24	4.47	27.88	<0.0001	<0.0001
ENSG00000132182	NUP210	nucleoporin 210 [Source:HGNC Symbol;Acc:HGNC:30052]	protein coding	-1.23	2.16	30.20	<0.0001	<0.0001
ENSG00000141013	GAS8	growth arrest specific 8 [Source:HGNC Symbol;Acc:HGNC:4166]	protein coding	1.23	1.08	31.28	<0.0001	<0.0001
ENSG00000136002	ARHGEF4	Rho guanine nucleotide exchange factor 4 [Source:HGNC Symbol;Acc:HGNC:684]	protein coding	-1.23	0.49	21.96	<0.0001	<0.0001
ENSG00000123838	C4BPA	complement component 4 binding protein alpha [Source:HGNC Symbol;Acc:HGNC:1325]	protein coding	1.23	4.29	17.85	<0.0001	0.0002
ENSG00000138772	ANXA3	annexin A3 [Source:HGNC Symbol;Acc:HGNC:541]	protein coding	1.22	7.19	34.25	<0.0001	<0.0001
ENSG00000144893	MED12L	mediator complex subunit 12L [Source:HGNC Symbol;Acc:HGNC:16050]	protein coding	1.22	0.52	25.03	<0.0001	<0.0001
ENSG00000168685	IL7R	interleukin 7 receptor [Source:HGNC Symbol;Acc:HGNC:6024]	protein coding	-1.22	1.01	7.78	0.0053	0.0171
ENSG00000251136		novel transcript	lncRNA	1.22	0.73	26.48	<0.0001	<0.0001
ENSG00000155366	RHOC	ras homolog family member C [Source:HGNC Symbol;Acc:HGNC:669]	protein coding	1.22	1.40	19.95	<0.0001	<0.0001
ENSG00000167543	TP53I13	tumor protein p53 inducible protein 13 [Source:HGNC Symbol;Acc:HGNC:25102]	protein coding	-1.22	0.80	11.49	0.0007	0.0033
ENSG00000100401	RANGAP1	Ran GTPase activating protein 1 [Source:HGNC Symbol;Acc:HGNC:9854]	protein coding	1.22	3.55	21.61	<0.0001	<0.0001
ENSG00000173762	CD7	CD7 molecule [Source:HGNC Symbol;Acc:HGNC:1695]	protein coding	1.21	2.21	17.37	<0.0001	0.0002
ENSG00000140030	GPR65	G protein-coupled receptor 65 [Source:HGNC Symbol;Acc:HGNC:4517]	protein coding	1.21	6.24	68.22	<0.0001	<0.0001
ENSG00000034510	TMSB10	thymosin beta 10 [Source:HGNC Symbol;Acc:HGNC:11879]	protein coding	1.21	7.45	54.73	<0.0001	<0.0001
ENSG00000243477	NAA80	N(alpha)-acetyltransferase 80, NatH catalytic subunit [Source:HGNC Symbol;Acc:HGNC:30252]	protein coding	-1.21	0.84	15.47	<0.0001	0.0006
ENSG00000127838	PNKD	PNKD metallo-beta-lactamase domain containing [Source:HGNC Symbol;Acc:HGNC:9153]	protein coding	-1.21	3.87	45.61	<0.0001	<0.0001
ENSG00000279693		novel transcript	TEC	-1.20	1.03	7.08	0.0078	0.0232
ENSG00000243273		novel transcript	lncRNA	1.20	1.76	33.32	<0.0001	<0.0001

Table S16: Genes differentially expressed at Day 2 compared to pre-first vaccination (Study Arm 2 A/H7N9+AS03, D1, 29, Neutrophils). Genes are sorted by descending absolute log_2 fold change. Gene model summaries and annotations are based on Ensembl Version 99 (continued.)

Ensembl Gene ID	Ensembl Gene Name	Ensembl Gene Description	Gene Type	log_2 Fold Change (Day 2 vs. Pre-First Vaccination)	Average log_2 CPM	Likelihood Ratio Test Statistic	P-Value	FDR Adjusted P-Value
ENSG00000254838	GVINP1	GTPase, very large interferon inducible pseudogene 1 [Source:HGNC Symbol;Acc:HGNC:25813]	transcribed unprocessed pseudogene	1.20	5.37	42.17	<0.0001	<0.0001
ENSG00000121797	CCRL2	C-C motif chemokine receptor like 2 [Source:HGNC Symbol;Acc:HGNC:1612]	protein coding	1.20	2.24	32.17	<0.0001	<0.0001
ENSG00000148334	PTGES2	prostaglandin E synthase 2 [Source:HGNC Symbol;Acc:HGNC:17822]	protein coding	-1.20	1.00	40.15	<0.0001	<0.0001
ENSG00000114127	XRN1	5'-3' exoribonuclease 1 [Source:HGNC Symbol;Acc:HGNC:30654]	protein coding	1.20	7.10	30.37	<0.0001	<0.0001
ENSG00000103642	LACTB	lactamase beta [Source:HGNC Symbol;Acc:HGNC:16468]	protein coding	1.20	5.50	38.08	<0.0001	<0.0001
ENSG00000178585	CTNNBIP1	catenin beta interacting protein 1 [Source:HGNC Symbol;Acc:HGNC:16913]	protein coding	-1.20	3.81	52.45	<0.0001	<0.0001
ENSG00000153406	NMRAL1	NmrA like redox sensor 1 [Source:HGNC Symbol;Acc:HGNC:24987]	protein coding	-1.19	3.88	35.45	<0.0001	<0.0001
ENSG00000189068	VSTM1	V-set and transmembrane domain containing 1 [Source:HGNC Symbol;Acc:HGNC:29455]	protein coding	-1.19	3.38	20.31	<0.0001	<0.0001
ENSG00000187554	TLR5	toll like receptor 5 [Source:HGNC Symbol;Acc:HGNC:11851]	protein coding	1.19	5.45	31.01	<0.0001	<0.0001
ENSG00000177989	ODF3B	outer dense fiber of sperm tails 3B [Source:HGNC Symbol;Acc:HGNC:34388]	protein coding	1.19	7.66	37.03	<0.0001	<0.0001
ENSG00000088992	TESC	tescalcin [Source:HGNC Symbol;Acc:HGNC:26065]	protein coding	-1.18	3.11	37.20	<0.0001	<0.0001
ENSG00000197448	GSTK1	glutathione S-transferase kappa 1 [Source:HGNC Symbol;Acc:HGNC:16906]	protein coding	1.18	6.87	39.20	<0.0001	<0.0001
ENSG00000103319	EEF2K	eukaryotic elongation factor 2 kinase [Source:HGNC Symbol;Acc:HGNC:24615]	protein coding	-1.18	1.32	10.30	0.0013	0.0055
ENSG00000187231	SESTD1	SEC14 and spectrin domain containing 1 [Source:HGNC Symbol;Acc:HGNC:18379]	protein coding	1.18	3.79	39.11	<0.0001	<0.0001
ENSG00000198133	TMEM229B	transmembrane protein 229B [Source:HGNC Symbol;Acc:HGNC:20130]	protein coding	1.17	1.25	19.46	<0.0001	0.0001
ENSG00000074660	SCARF1	scavenger receptor class F member 1 [Source:HGNC Symbol;Acc:HGNC:16820]	protein coding	1.17	7.93	34.21	<0.0001	<0.0001

Table S16: Genes differentially expressed at Day 2 compared to pre-first vaccination (Study Arm 2 A/H7N9+AS03, D1, 29, Neutrophils). Genes are sorted by descending absolute log_2 fold change. Gene model summaries and annotations are based on Ensembl Version 99 (continued.)

Ensembl Gene ID	Ensembl Gene Name	Ensembl Gene Description	Gene Type	log_2 Fold Change (Day 2 vs. Pre-First Vaccination)	Average log_2 CPM	Likelihood Ratio Test Statistic	P-Value	FDR Adjusted P-Value
ENSG00000280734	LINC01232	long intergenic non-protein coding RNA 1232 [Source:HGNC Symbol;Acc:HGNC:49755]	lncRNA	1.17	2.96	50.74	<0.0001	<0.0001
ENSG00000111912	NCOA7	nuclear receptor coactivator 7 [Source:HGNC Symbol;Acc:HGNC:21081]	protein coding	1.17	3.56	26.02	<0.0001	<0.0001
ENSG00000028116	VRK2	VRK serine/threonine kinase 2 [Source:HGNC Symbol;Acc:HGNC:12719]	protein coding	1.17	2.76	48.97	<0.0001	<0.0001
ENSG00000112137	PHACTR1	phosphatase and actin regulator 1 [Source:HGNC Symbol;Acc:HGNC:20990]	protein coding	-1.16	5.08	23.68	<0.0001	<0.0001
ENSG00000174749	FAM241A	family with sequence similarity 241 member A [Source:HGNC Symbol;Acc:HGNC:26813]	protein coding	1.16	3.18	42.21	<0.0001	<0.0001
ENSG00000184470	TXNRD2	thioredoxin reductase 2 [Source:HGNC Symbol;Acc:HGNC:18155]	protein coding	-1.16	1.03	26.68	<0.0001	<0.0001
ENSG00000266256	LINC00683	long intergenic non-protein coding RNA 683 [Source:HGNC Symbol;Acc:HGNC:44467]	lncRNA	-1.16	2.00	30.98	<0.0001	<0.0001
ENSG00000138660	AP1AR	adaptor related protein complex 1 associated regulatory protein [Source:HGNC Symbol;Acc:HGNC:28808]	protein coding	1.16	1.54	20.61	<0.0001	<0.0001
ENSG00000183688	RFLNB	refilin B [Source:HGNC Symbol;Acc:HGNC:28705]	protein coding	-1.16	8.15	30.56	<0.0001	<0.0001
ENSG00000130303	BST2	bone marrow stromal cell antigen 2 [Source:HGNC Symbol;Acc:HGNC:1119]	protein coding	1.16	5.65	19.28	<0.0001	0.0001
ENSG00000285572		novel transcript	lncRNA	-1.15	0.39	24.31	<0.0001	<0.0001
ENSG00000079263	SP140	SP140 nuclear body protein [Source:HGNC Symbol;Acc:HGNC:17133]	protein coding	1.15	5.44	27.91	<0.0001	<0.0001
ENSG00000165140	FBP1	fructose-bisphosphatase 1 [Source:HGNC Symbol;Acc:HGNC:3606]	protein coding	-1.15	2.53	25.51	<0.0001	<0.0001
ENSG00000181350	LRRC75A	leucine rich repeat containing 75A [Source:HGNC Symbol;Acc:HGNC:32403]	protein coding	-1.15	3.09	17.87	<0.0001	0.0002
ENSG00000169439	SDC2	syndecan 2 [Source:HGNC Symbol;Acc:HGNC:10659]	protein coding	-1.15	3.33	27.48	<0.0001	<0.0001
ENSG00000118162	KPTN	kaptin, actin binding protein [Source:HGNC Symbol;Acc:HGNC:6404]	protein coding	1.15	2.77	21.00	<0.0001	<0.0001
ENSG00000173821	RNF213	ring finger protein 213 [Source:HGNC Symbol;Acc:HGNC:14539]	protein coding	1.15	9.71	24.81	<0.0001	<0.0001
ENSG00000169413	RNASE6	ribonuclease A family member k6 [Source:HGNC Symbol;Acc:HGNC:10048]	protein coding	-1.15	5.14	26.03	<0.0001	<0.0001

Table S16: Genes differentially expressed at Day 2 compared to pre-first vaccination (Study Arm 2 A/H7N9+AS03, D1, 29, Neutrophils). Genes are sorted by descending absolute log_2 fold change. Gene model summaries and annotations are based on Ensembl Version 99 (continued.)

Ensembl Gene ID	Ensembl Gene Name	Ensembl Gene Description	Gene Type	log_2 Fold Change (Day 2 vs. Pre-First Vaccination)	Average log_2 CPM	Likelihood Ratio Test Statistic	P-Value	FDR Adjusted P-Value
ENSG00000119917	IFIT3	interferon induced protein with tetratricopeptide repeats 3 [Source:HGNC Symbol;Acc:HGNC:5411]	protein coding	1.15	9.93	17.52	<0.0001	0.0002
ENSG00000138496	PARP9	poly(ADP-ribose) polymerase family member 9 [Source:HGNC Symbol;Acc:HGNC:24118]	protein coding	1.15	8.82	34.30	<0.0001	<0.0001
ENSG00000090104	RGS1	regulator of G protein signaling 1 [Source:HGNC Symbol;Acc:HGNC:9991]	protein coding	-1.15	1.28	8.86	0.0029	0.0105
ENSG00000143847	PPFIA4	PTPRF interacting protein alpha 4 [Source:HGNC Symbol;Acc:HGNC:9248]	protein coding	-1.15	0.69	38.48	<0.0001	<0.0001
ENSG00000162645	GBP2	guanylate binding protein 2 [Source:HGNC Symbol;Acc:HGNC:4183]	protein coding	1.14	9.87	41.75	<0.0001	<0.0001
ENSG00000139572	GPR84	G protein-coupled receptor 84 [Source:HGNC Symbol;Acc:HGNC:4535]	protein coding	1.14	0.98	17.66	<0.0001	0.0002
ENSG00000091181	IL5RA	interleukin 5 receptor subunit alpha [Source:HGNC Symbol;Acc:HGNC:6017]	protein coding	-1.13	3.75	8.75	0.0031	0.011
ENSG00000185885	IFITM1	interferon induced transmembrane protein 1 [Source:HGNC Symbol;Acc:HGNC:5412]	protein coding	1.13	9.06	35.69	<0.0001	<0.0001
ENSG00000134470	IL15RA	interleukin 15 receptor subunit alpha [Source:HGNC Symbol;Acc:HGNC:5978]	protein coding	1.13	1.90	34.22	<0.0001	<0.0001
ENSG00000204371	EHMT2	euchromatic histone lysine methyltransferase 2 [Source:HGNC Symbol;Acc:HGNC:14129]	protein coding	-1.13	2.87	38.38	<0.0001	<0.0001
ENSG00000135604	STX11	syntaxin 11 [Source:HGNC Symbol;Acc:HGNC:11429]	protein coding	1.12	8.61	40.18	<0.0001	<0.0001
ENSG00000114631	PODXL2	podocalyxin like 2 [Source:HGNC Symbol;Acc:HGNC:17936]	protein coding	-1.12	0.64	38.52	<0.0001	<0.0001
ENSG00000183696	UPP1	uridine phosphorylase 1 [Source:HGNC Symbol;Acc:HGNC:12576]	protein coding	1.12	5.30	37.57	<0.0001	<0.0001
ENSG00000095002	MSH2	mutS homolog 2 [Source:HGNC Symbol;Acc:HGNC:7325]	protein coding	-1.11	0.96	46.90	<0.0001	<0.0001
ENSG00000128512	DOCK4	dedicator of cytokinesis 4 [Source:HGNC Symbol;Acc:HGNC:19192]	protein coding	1.11	5.67	29.05	<0.0001	<0.0001
ENSG00000167699	GLOD4	glyoxalase domain containing 4 [Source:HGNC Symbol;Acc:HGNC:14111]	protein coding	-1.11	0.27	18.94	<0.0001	0.0001
ENSG00000242574	HLA-DMB	major histocompatibility complex, class II, DM beta [Source:HGNC Symbol;Acc:HGNC:4935]	protein coding	1.11	3.18	21.36	<0.0001	<0.0001
ENSG00000134242	PTPN22	protein tyrosine phosphatase non-receptor type 22 [Source:HGNC Symbol;Acc:HGNC:9652]	protein coding	-1.11	2.76	29.44	<0.0001	<0.0001

Table S16: Genes differentially expressed at Day 2 compared to pre-first vaccination (Study Arm 2 A/H7N9+AS03, D1, 29, Neutrophils). Genes are sorted by descending absolute log_2 fold change. Gene model summaries and annotations are based on Ensembl Version 99 (continued.)

Ensembl Gene ID	Ensembl Gene Name	Ensembl Gene Description	Gene Type	log_2 Fold Change (Day 2 vs. Pre-First Vaccination)	Average log_2 CPM	Likelihood Ratio Test Statistic	P-Value	FDR Adjusted P-Value
ENSG00000008710	PKD1	polycystin 1, transient receptor potential channel interacting [Source:HGNC Symbol;Acc:HGNC:9008]	protein coding	-1.11	0.95	25.70	<0.0001	<0.0001
ENSG00000172985	SH3RF3	SH3 domain containing ring finger 3 [Source:HGNC Symbol;Acc:HGNC:24699]	protein coding	-1.10	3.90	23.90	<0.0001	<0.0001
ENSG00000140859	KIFC3	kinesin family member C3 [Source:HGNC Symbol;Acc:HGNC:6326]	protein coding	1.10	1.36	30.59	<0.0001	<0.0001
ENSG00000172493	AFF1	AF4/FMR2 family member 1 [Source:HGNC Symbol;Acc:HGNC:7135]	protein coding	1.10	6.99	35.31	<0.0001	<0.0001
ENSG00000141574	SECTM1	secreted and transmembrane 1 [Source:HGNC Symbol;Acc:HGNC:10707]	protein coding	1.10	9.65	36.75	<0.0001	<0.0001
ENSG00000163565	IFI16	interferon gamma inducible protein 16 [Source:HGNC Symbol;Acc:HGNC:5395]	protein coding	1.10	8.63	26.32	<0.0001	<0.0001
ENSG00000182389	CACNB4	calcium voltage-gated channel auxiliary subunit beta 4 [Source:HGNC Symbol;Acc:HGNC:1404]	protein coding	-1.09	3.75	37.98	<0.0001	<0.0001
ENSG00000179242	CDH4	cadherin 4 [Source:HGNC Symbol;Acc:HGNC:1763]	protein coding	-1.09	0.51	29.14	<0.0001	<0.0001
ENSG00000081059	TCF7	transcription factor 7 [Source:HGNC Symbol;Acc:HGNC:11639]	protein coding	-1.09	1.38	11.25	0.0008	0.0036
ENSG00000109321	AREG	amphiregulin [Source:HGNC Symbol;Acc:HGNC:651]	protein coding	-1.09	0.86	5.87	0.0154	0.0406
ENSG00000115267	IFIH1	interferon induced with helicase C domain 1 [Source:HGNC Symbol;Acc:HGNC:18873]	protein coding	1.09	6.62	21.60	<0.0001	<0.0001
ENSG00000130222	GADD45G	growth arrest and DNA damage inducible gamma [Source:HGNC Symbol;Acc:HGNC:4097]	protein coding	1.09	3.44	16.98	<0.0001	0.0003
ENSG00000021762	OSBPL5	oxysterol binding protein like 5 [Source:HGNC Symbol;Acc:HGNC:16392]	protein coding	-1.09	2.39	26.71	<0.0001	<0.0001
ENSG00000179051	RCC2	regulator of chromosome condensation 2 [Source:HGNC Symbol;Acc:HGNC:30297]	protein coding	-1.09	0.95	19.49	<0.0001	<0.0001
ENSG00000183762	KREMEN1	kringle containing transmembrane protein 1 [Source:HGNC Symbol;Acc:HGNC:17550]	protein coding	1.09	7.08	22.70	<0.0001	<0.0001
ENSG00000134243	SORT1	sortilin 1 [Source:HGNC Symbol;Acc:HGNC:11186]	protein coding	1.08	5.60	29.63	<0.0001	<0.0001
ENSG00000287938		novel transcript	lncRNA	-1.08	0.27	25.09	<0.0001	<0.0001
ENSG00000146540	C7orf50	chromosome 7 open reading frame 50 [Source:HGNC Symbol;Acc:HGNC:22421]	protein coding	-1.08	2.73	23.36	<0.0001	<0.0001

Table S16: Genes differentially expressed at Day 2 compared to pre-first vaccination (Study Arm 2 A/H7N9+AS03, D1, 29, Neutrophils). Genes are sorted by descending absolute log_2 fold change. Gene model summaries and annotations are based on Ensembl Version 99 (continued.)

Ensembl Gene ID	Ensembl Gene Name	Ensembl Gene Description	Gene Type	log_2 Fold Change (Day 2 vs. Pre-First Vaccination)	Average log_2 CPM	Likelihood Ratio Test Statistic	P-Value	FDR Adjusted P-Value
ENSG00000110492	MDK	midkine [Source:HGNC Symbol;Acc:HGNC:6972]	protein coding	1.08	1.13	26.99	<0.0001	<0.0001
ENSG00000261222		novel transcript	lncRNA	1.08	2.68	6.10	0.0136	0.0364
ENSG00000114770	ABCC5	ATP binding cassette subfamily C member 5 [Source:HGNC Symbol;Acc:HGNC:56]	protein coding	-1.08	6.45	56.44	<0.0001	<0.0001
ENSG00000113552	GNPDA1	glucosamine-6-phosphate deaminase 1 [Source:HGNC Symbol;Acc:HGNC:4417]	protein coding	-1.08	2.28	42.83	<0.0001	<0.0001
ENSG00000143390	RFX5	regulatory factor X5 [Source:HGNC Symbol;Acc:HGNC:9986]	protein coding	1.07	3.40	41.69	<0.0001	<0.0001
ENSG00000185507	IRF7	interferon regulatory factor 7 [Source:HGNC Symbol;Acc:HGNC:6122]	protein coding	1.07	8.11	24.88	<0.0001	<0.0001
ENSG00000187608	ISG15	ISG15 ubiquitin like modifier [Source:HGNC Symbol;Acc:HGNC:4053]	protein coding	1.07	7.78	9.94	0.0016	0.0065
ENSG00000135362	PRR5L	proline rich 5 like [Source:HGNC Symbol;Acc:HGNC:25878]	protein coding	1.07	4.47	32.31	<0.0001	<0.0001
ENSG00000151332	MBIP	MAP3K12 binding inhibitory protein 1 [Source:HGNC Symbol;Acc:HGNC:20427]	protein coding	-1.07	1.08	32.34	<0.0001	<0.0001
ENSG00000287263		novel transcript	lncRNA	1.07	5.21	33.63	<0.0001	<0.0001
ENSG00000129003	VPS13C	vacuolar protein sorting 13 homolog C [Source:HGNC Symbol;Acc:HGNC:23594]	protein coding	1.07	4.65	42.30	<0.0001	<0.0001
ENSG00000137200	CMTR1	cap methyltransferase 1 [Source:HGNC Symbol;Acc:HGNC:21077]	protein coding	1.06	5.70	21.43	<0.0001	<0.0001
ENSG00000141569	TRIM65	tripartite motif containing 65 [Source:HGNC Symbol;Acc:HGNC:27316]	protein coding	-1.06	0.33	32.40	<0.0001	<0.0001
ENSG00000126709	IFI6	interferon alpha inducible protein 6 [Source:HGNC Symbol;Acc:HGNC:4054]	protein coding	1.06	8.24	13.07	0.0003	0.0016
ENSG00000136104	RNASEH2B	ribonuclease H2 subunit B [Source:HGNC Symbol;Acc:HGNC:25671]	protein coding	1.06	3.04	33.79	<0.0001	<0.0001
ENSG00000143771	CNIH4	cornichon family AMPA receptor auxiliary protein 4 [Source:HGNC Symbol;Acc:HGNC:25013]	protein coding	1.06	4.46	32.55	<0.0001	<0.0001
ENSG00000216490	IFI30	IFI30 lysosomal thiol reductase [Source:HGNC Symbol;Acc:HGNC:5398]	protein coding	1.06	3.26	29.92	<0.0001	<0.0001
ENSG00000124491	F13A1	coagulation factor XIII A chain [Source:HGNC Symbol;Acc:HGNC:3531]	protein coding	-1.06	2.24	26.64	<0.0001	<0.0001
ENSG00000273272		novel transcript	lncRNA	1.06	3.25	20.73	<0.0001	<0.0001

Table S16: Genes differentially expressed at Day 2 compared to pre-first vaccination (Study Arm 2 A/H7N9+AS03, D1, 29, Neutrophils). Genes are sorted by descending absolute log_2 fold change. Gene model summaries and annotations are based on Ensembl Version 99 (continued.)

Ensembl Gene ID	Ensembl Gene Name	Ensembl Gene Description	Gene Type	log_2 Fold Change (Day 2 vs. Pre-First Vaccination)	Average log_2 CPM	Likelihood Ratio Test Statistic	P-Value	FDR Adjusted P-Value
ENSG00000117480	FAAH	fatty acid amide hydrolase [Source:HGNC Symbol;Acc:HGNC:3553]	protein coding	-1.05	2.58	28.71	<0.0001	<0.0001
ENSG00000129038	LOXL1	lysyl oxidase like 1 [Source:HGNC Symbol;Acc:HGNC:6665]	protein coding	1.05	1.32	25.03	<0.0001	<0.0001
ENSG00000239713	APOBEC3G	apolipoprotein B mRNA editing enzyme catalytic subunit 3G [Source:HGNC Symbol;Acc:HGNC:17357]	protein coding	1.05	4.86	32.45	<0.0001	<0.0001
ENSG00000132780	NASP	nuclear autoantigenic sperm protein [Source:HGNC Symbol;Acc:HGNC:7644]	protein coding	1.05	4.67	37.65	<0.0001	<0.0001
ENSG00000085063	CD59	CD59 molecule (CD59 blood group) [Source:HGNC Symbol;Acc:HGNC:1689]	protein coding	1.05	5.89	38.34	<0.0001	<0.0001
ENSG00000010278	CD9	CD9 molecule [Source:HGNC Symbol;Acc:HGNC:1709]	protein coding	-1.05	4.06	8.14	0.0043	0.0144
ENSG00000168421	RHOH	ras homolog family member H [Source:HGNC Symbol;Acc:HGNC:686]	protein coding	1.04	1.98	16.42	<0.0001	0.0004
ENSG00000107796	ACTA2	actin alpha 2, smooth muscle [Source:HGNC Symbol;Acc:HGNC:130]	protein coding	1.04	1.77	38.92	<0.0001	<0.0001
ENSG00000236525		novel transcript	lncRNA	1.04	2.45	9.87	0.0017	0.0067
ENSG00000162976	SLC66A3	solute carrier family 66 member 3 [Source:HGNC Symbol;Acc:HGNC:28503]	protein coding	-1.04	1.90	37.07	<0.0001	<0.0001
ENSG00000105516	DBP	D-box binding PAR bZIP transcription factor [Source:HGNC Symbol;Acc:HGNC:2697]	protein coding	-1.04	1.72	26.89	<0.0001	<0.0001
ENSG00000285444		novel transcript	protein coding	1.04	0.90	27.53	<0.0001	<0.0001
ENSG00000251442	LINC01094	long intergenic non-protein coding RNA 1094 [Source:HGNC Symbol;Acc:HGNC:49219]	lncRNA	1.03	3.20	39.01	<0.0001	<0.0001
ENSG00000042980	ADAM28	ADAM metallopeptidase domain 28 [Source:HGNC Symbol;Acc:HGNC:206]	protein coding	-1.03	3.95	44.17	<0.0001	<0.0001
ENSG00000186918	ZNF395	zinc finger protein 395 [Source:HGNC Symbol;Acc:HGNC:18737]	protein coding	-1.03	1.73	28.12	<0.0001	<0.0001
ENSG00000106100	NOD1	nucleotide binding oligomerization domain containing 1 [Source:HGNC Symbol;Acc:HGNC:16390]	protein coding	1.03	3.06	28.37	<0.0001	<0.0001
ENSG00000119711	ALDH6A1	aldehyde dehydrogenase 6 family member A1 [Source:HGNC Symbol;Acc:HGNC:7179]	protein coding	-1.02	0.87	17.48	<0.0001	0.0002
ENSG00000106537	TSPAN13	tetraspanin 13 [Source:HGNC Symbol;Acc:HGNC:21643]	protein coding	-1.02	2.52	8.77	0.0031	0.0109

Table S16: Genes differentially expressed at Day 2 compared to pre-first vaccination (Study Arm 2 A/H7N9+AS03, D1, 29, Neutrophils). Genes are sorted by descending absolute log_2 fold change. Gene model summaries and annotations are based on Ensembl Version 99 (continued.)

Ensembl Gene ID	Ensembl Gene Name	Ensembl Gene Description	Gene Type	log_2 Fold Change (Day 2 vs. Pre-First Vaccination)	Average log_2 CPM	Likelihood Ratio Test Statistic	P-Value	FDR Adjusted P-Value
ENSG00000136068	FLNB	filamin B [Source:HGNC Symbol;Acc:HGNC:3755]	protein coding	-1.02	1.76	7.45	0.0063	0.0197
ENSG00000143653	SCCPDH	saccharopine dehydrogenase (putative) [Source:HGNC Symbol;Acc:HGNC:24275]	protein coding	-1.02	1.04	24.85	<0.0001	<0.0001
ENSG00000198216	CACNA1E	calcium voltage-gated channel subunit alpha1 E [Source:HGNC Symbol;Acc:HGNC:1392]	protein coding	1.02	4.55	31.08	<0.0001	<0.0001
ENSG00000215022		novel transcript, antisense to PHACTR1	lncRNA	-1.02	2.01	45.11	<0.0001	<0.0001
ENSG00000121236	TRIM6	tripartite motif containing 6 [Source:HGNC Symbol;Acc:HGNC:16277]	protein coding	1.02	1.56	14.57	0.0001	0.0008
ENSG00000146122	DAAM2	dishevelled associated activator of morphogenesis 2 [Source:HGNC Symbol;Acc:HGNC:18143]	protein coding	-1.02	3.81	7.77	0.0053	0.0171
ENSG00000103512	NOMO1	NODAL modulator 1 [Source:HGNC Symbol;Acc:HGNC:30060]	protein coding	-1.01	1.87	28.29	<0.0001	<0.0001
ENSG00000073350	LLGL2	LLGL scribble cell polarity complex component 2 [Source:HGNC Symbol;Acc:HGNC:6629]	protein coding	-1.01	0.86	34.29	<0.0001	<0.0001
ENSG00000176978	DPP7	dipeptidyl peptidase 7 [Source:HGNC Symbol;Acc:HGNC:14892]	protein coding	-1.01	2.10	21.47	<0.0001	<0.0001
ENSG00000111696	NT5DC3	5'-nucleotidase domain containing 3 [Source:HGNC Symbol;Acc:HGNC:30826]	protein coding	-1.01	1.26	15.18	<0.0001	0.0006
ENSG00000128335	APOL2	apolipoprotein L2 [Source:HGNC Symbol;Acc:HGNC:619]	protein coding	1.01	8.37	38.90	<0.0001	<0.0001
ENSG00000114126	TFDP2	transcription factor Dp-2 [Source:HGNC Symbol;Acc:HGNC:11751]	protein coding	-1.01	0.49	20.10	<0.0001	<0.0001
ENSG00000178719	GRINA	glutamate ionotropic receptor NMDA type subunit associated protein 1 [Source:HGNC Symbol;Acc:HGNC:4589]	protein coding	1.01	7.44	34.45	<0.0001	<0.0001
ENSG00000130202	NECTIN2	nectin cell adhesion molecule 2 [Source:HGNC Symbol;Acc:HGNC:9707]	protein coding	1.00	6.16	6.54	0.0105	0.0297
ENSG00000011021	CLCN6	chloride voltage-gated channel 6 [Source:HGNC Symbol;Acc:HGNC:2024]	protein coding	-1.00	2.04	33.86	<0.0001	<0.0001
ENSG00000139641	ESYT1	extended synaptotagmin 1 [Source:HGNC Symbol;Acc:HGNC:29534]	protein coding	-1.00	4.44	11.01	0.0009	0.004
ENSG00000197629	MPEG1	macrophage expressed 1 [Source:HGNC Symbol;Acc:HGNC:29619]	protein coding	-1.00	8.08	23.45	<0.0001	<0.0001
ENSG00000176834	VSIG10	V-set and immunoglobulin domain containing 10 [Source:HGNC Symbol;Acc:HGNC:26078]	protein coding	1.00	2.62	19.65	<0.0001	<0.0001

Table S16: Genes differentially expressed at Day 2 compared to pre-first vaccination (Study Arm 2 A/H7N9+AS03, D1, 29, Neutrophils). Genes are sorted by descending absolute log_2 fold change. Gene model summaries and annotations are based on Ensembl Version 99 (continued.)

Ensembl Gene ID	Ensembl Gene Name	Ensembl Gene Description	Gene Type	log_2 Fold Change (Day 2 vs. Pre-First Vaccination)	Average log_2 CPM	Likelihood Ratio Test Statistic	P-Value	FDR Adjusted P-Value
ENSG00000103365	GGA2	golgi associated, gamma adaptin ear containing, ARF binding protein 2 [Source:HGNC Symbol;Acc:HGNC:16064]	protein coding	-1.00	1.92	10.45	0.0012	0.0051
ENSG00000272666		uncharacterized LOC105373098 [Source:NCBI gene;Acc:105373098]	lncRNA	1.00	2.12	15.71	<0.0001	0.0005
ENSG00000058866	DGKG	diacylglycerol kinase gamma [Source:HGNC Symbol;Acc:HGNC:2853]	protein coding	-1.00	3.55	40.63	<0.0001	<0.0001
ENSG00000284194	SCO2	SCO cytochrome c oxidase assembly protein 2 [Source:HGNC Symbol;Acc:HGNC:10604]	protein coding	1.00	4.30	30.07	<0.0001	<0.0001
ENSG00000145246	ATP10D	ATPase phospholipid transporting 10D (putative) [Source:HGNC Symbol;Acc:HGNC:13549]	protein coding	1.00	2.83	19.78	<0.0001	<0.0001
ENSG00000136874	STX17	syntaxin 17 [Source:HGNC Symbol;Acc:HGNC:11432]	protein coding	1.00	3.67	26.47	<0.0001	<0.0001
ENSG00000079385	CEACAM1	CEA cell adhesion molecule 1 [Source:HGNC Symbol;Acc:HGNC:1814]	protein coding	1.00	6.27	31.96	<0.0001	<0.0001
ENSG00000170558	CDH2	cadherin 2 [Source:HGNC Symbol;Acc:HGNC:1759]	protein coding	-0.99	1.62	16.08	<0.0001	0.0004
ENSG00000006015	REX1BD	required for excision 1-B domain containing [Source:HGNC Symbol;Acc:HGNC:26098]	protein coding	-0.99	4.48	49.64	<0.0001	<0.0001
ENSG00000111371	SLC38A1	solute carrier family 38 member 1 [Source:HGNC Symbol;Acc:HGNC:13447]	protein coding	-0.99	3.01	18.68	<0.0001	0.0001
ENSG00000127334	DYRK2	dual specificity tyrosine phosphorylation regulated kinase 2 [Source:HGNC Symbol;Acc:HGNC:3093]	protein coding	-0.99	2.90	18.27	<0.0001	0.0002
ENSG00000167280	ENGASE	endo-beta-N-acetylglucosaminidase [Source:HGNC Symbol;Acc:HGNC:24622]	protein coding	-0.99	2.52	7.25	0.0071	0.0217
ENSG00000125347	IRF1	interferon regulatory factor 1 [Source:HGNC Symbol;Acc:HGNC:6116]	protein coding	0.99	9.39	35.27	<0.0001	<0.0001
ENSG00000138646	HERC5	HECT and RLD domain containing E3 ubiquitin protein ligase 5 [Source:HGNC Symbol;Acc:HGNC:24368]	protein coding	0.99	6.94	10.13	0.0015	0.0059
ENSG00000138385	SSB	small RNA binding exonuclease protection factor La [Source:HGNC Symbol;Acc:HGNC:11316]	protein coding	0.99	2.29	15.99	<0.0001	0.0004
ENSG00000102554	KLF5	Kruppel like factor 5 [Source:HGNC Symbol;Acc:HGNC:6349]	protein coding	0.99	3.56	29.85	<0.0001	<0.0001
ENSG00000166145	SPINT1	serine peptidase inhibitor, Kunitz type 1 [Source:HGNC Symbol;Acc:HGNC:11246]	protein coding	-0.99	5.11	33.06	<0.0001	<0.0001
ENSG00000168394	TAP1	transporter 1, ATP binding cassette subfamily B member [Source:HGNC Symbol;Acc:HGNC:43]	protein coding	0.99	9.69	31.92	<0.0001	<0.0001

Table S16: Genes differentially expressed at Day 2 compared to pre-first vaccination (Study Arm 2 A/H7N9+AS03, D1, 29, Neutrophils). Genes are sorted by descending absolute log_2 fold change. Gene model summaries and annotations are based on Ensembl Version 99 (continued.)

Ensembl Gene ID	Ensembl Gene Name	Ensembl Gene Description	Gene Type	log_2 Fold Change (Day 2 vs. Pre-First Vaccination)	Average log_2 CPM	Likelihood Ratio Test Statistic	P-Value	FDR Adjusted P-Value
ENSG00000287576		novel transcript	lncRNA	0.98	0.72	17.45	<0.0001	0.0002
ENSG00000105939	ZC3HAV1	zinc finger CCCH-type containing, antiviral 1 [Source:HGNC Symbol;Acc:HGNC:23721]	protein coding	0.98	7.62	23.59	<0.0001	<0.0001
ENSG00000081377	CDC14B	cell division cycle 14B [Source:HGNC Symbol;Acc:HGNC:1719]	protein coding	-0.98	0.42	17.69	<0.0001	0.0002
ENSG00000162714	ZNF496	zinc finger protein 496 [Source:HGNC Symbol;Acc:HGNC:23713]	protein coding	0.98	2.62	12.73	0.0004	0.0019
ENSG00000108771	DHX58	DExH-box helicase 58 [Source:HGNC Symbol;Acc:HGNC:29517]	protein coding	0.98	5.01	13.33	0.0003	0.0015
ENSG00000104375	STK3	serine/threonine kinase 3 [Source:HGNC Symbol;Acc:HGNC:11406]	protein coding	0.97	4.01	29.61	<0.0001	<0.0001
ENSG00000167207	NOD2	nucleotide binding oligomerization domain containing 2 [Source:HGNC Symbol;Acc:HGNC:5331]	protein coding	0.97	6.95	16.54	<0.0001	0.0004
ENSG00000087253	LPCAT2	lysophosphatidylcholine acyltransferase 2 [Source:HGNC Symbol;Acc:HGNC:26032]	protein coding	0.97	8.12	37.45	<0.0001	<0.0001
ENSG00000178026	LRRC75B	leucine rich repeat containing 75B [Source:HGNC Symbol;Acc:HGNC:33155]	protein coding	-0.97	1.03	18.60	<0.0001	0.0001
ENSG00000270120		novel transcript	lncRNA	0.97	1.43	11.67	0.0006	0.003
ENSG00000053371	AKR7A2	aldo-keto reductase family 7 member A2 [Source:HGNC Symbol;Acc:HGNC:389]	protein coding	-0.97	0.79	29.56	<0.0001	<0.0001
ENSG00000114541	FRMD4B	FERM domain containing 4B [Source:HGNC Symbol;Acc:HGNC:24886]	protein coding	0.97	2.98	20.71	<0.0001	<0.0001
ENSG00000139629	GALNT6	polypeptide N-acetylgalactosaminyltransferase 6 [Source:HGNC Symbol;Acc:HGNC:4128]	protein coding	-0.97	1.64	11.85	0.0006	0.0028
ENSG00000179583	CIITA	class II major histocompatibility complex transactivator [Source:HGNC Symbol;Acc:HGNC:7067]	protein coding	0.97	4.21	17.92	<0.0001	0.0002
ENSG00000111860	CEP85L	centrosomal protein 85 like [Source:HGNC Symbol;Acc:HGNC:21638]	protein coding	-0.97	4.56	50.92	<0.0001	<0.0001
ENSG00000163840	DTX3L	deltex E3 ubiquitin ligase 3L [Source:HGNC Symbol;Acc:HGNC:30323]	protein coding	0.97	7.55	26.57	<0.0001	<0.0001
ENSG00000272555		novel transcript	lncRNA	-0.97	0.72	18.61	<0.0001	0.0001
ENSG00000013374	NUB1	negative regulator of ubiquitin like proteins 1 [Source:HGNC Symbol;Acc:HGNC:17623]	protein coding	0.97	7.06	29.08	<0.0001	<0.0001
ENSG00000133313	CNDP2	carnosine dipeptidase 2 [Source:HGNC Symbol;Acc:HGNC:24437]	protein coding	0.96	4.64	13.95	0.0002	0.0011

Table S16: Genes differentially expressed at Day 2 compared to pre-first vaccination (Study Arm 2 A/H7N9+AS03, D1, 29, Neutrophils). Genes are sorted by descending absolute log_2 fold change. Gene model summaries and annotations are based on Ensembl Version 99 (continued.)

Ensembl Gene ID	Ensembl Gene Name	Ensembl Gene Description	Gene Type	log_2 Fold Change (Day 2 vs. Pre-First Vaccination)	Average log_2 CPM	Likelihood Ratio Test Statistic	P-Value	FDR Adjusted P-Value
ENSG00000075618	FSCN1	fascin actin-bundling protein 1 [Source:HGNC Symbol;Acc:HGNC:11148]	protein coding	-0.96	2.73	29.31	<0.0001	<0.0001
ENSG00000107201	DDX58	DExD/H-box helicase 58 [Source:HGNC Symbol;Acc:HGNC:19102]	protein coding	0.96	7.81	18.74	<0.0001	0.0001
ENSG00000115392	FANCL	FA complementation group L [Source:HGNC Symbol;Acc:HGNC:20748]	protein coding	0.96	0.47	28.09	<0.0001	<0.0001
ENSG00000185022	MAFF	MAF bZIP transcription factor F [Source:HGNC Symbol;Acc:HGNC:6780]	protein coding	0.96	5.30	27.72	<0.0001	<0.0001
ENSG00000110848	CD69	CD69 molecule [Source:HGNC Symbol;Acc:HGNC:1694]	protein coding	0.96	2.07	5.99	0.0144	0.0383
ENSG00000118971	CCND2	cyclin D2 [Source:HGNC Symbol;Acc:HGNC:1583]	protein coding	-0.96	3.47	30.07	<0.0001	<0.0001
ENSG00000286116		FAS antisense RNA 1	lncRNA	0.96	1.15	23.56	<0.0001	<0.0001
ENSG00000102524	TNFSF13B	TNF superfamily member 13b [Source:HGNC Symbol;Acc:HGNC:11929]	protein coding	0.96	7.97	22.72	<0.0001	<0.0001
ENSG00000185420	SMYD3	SET and MYND domain containing 3 [Source:HGNC Symbol;Acc:HGNC:15513]	protein coding	-0.96	0.38	35.39	<0.0001	<0.0001
ENSG00000234389		novel transcript	lncRNA	0.95	3.87	8.70	0.0032	0.0112
ENSG00000231826	LINC01819	long intergenic non-protein coding RNA 1819 [Source:HGNC Symbol;Acc:HGNC:52624]	lncRNA	-0.95	1.77	25.66	<0.0001	<0.0001
ENSG00000197536	IRF1-AS1	IRF1 antisense RNA 1 [Source:HGNC Symbol;Acc:HGNC:33838]	lncRNA	0.95	5.58	26.06	<0.0001	<0.0001
ENSG00000198948	MFAP3L	microfibril associated protein 3 like [Source:HGNC Symbol;Acc:HGNC:29083]	protein coding	-0.95	1.27	33.80	<0.0001	<0.0001
ENSG00000115607	IL18RAP	interleukin 18 receptor accessory protein [Source:HGNC Symbol;Acc:HGNC:5989]	protein coding	0.95	6.84	11.27	0.0008	0.0036
ENSG00000132256	TRIM5	tripartite motif containing 5 [Source:HGNC Symbol;Acc:HGNC:16276]	protein coding	0.95	4.89	21.78	<0.0001	<0.0001
ENSG00000259668		novel transcript, antisense to DMXL2	lncRNA	0.95	1.79	38.46	<0.0001	<0.0001
ENSG00000154102	C16orf74	chromosome 16 open reading frame 74 [Source:HGNC Symbol;Acc:HGNC:23362]	protein coding	-0.95	1.11	16.04	<0.0001	0.0004
ENSG00000270030		novel transcript	lncRNA	0.95	1.55	9.13	0.0025	0.0093
ENSG00000234515	PPP1R2P1	protein phosphatase 1 regulatory inhibitor subunit 2 pseudogene 1 [Source:HGNC Symbol;Acc:HGNC:9289]	transcribed processed pseudogene	0.94	0.70	21.00	<0.0001	<0.0001

Table S16: Genes differentially expressed at Day 2 compared to pre-first vaccination (Study Arm 2 A/H7N9+AS03, D1, 29, Neutrophils). Genes are sorted by descending absolute log_2 fold change. Gene model summaries and annotations are based on Ensembl Version 99 (continued.)

Ensembl Gene ID	Ensembl Gene Name	Ensembl Gene Description	Gene Type	log_2 Fold Change (Day 2 vs. Pre-First Vaccination)	Average log_2 CPM	Likelihood Ratio Test Statistic	P-Value	FDR Adjusted P-Value
ENSG00000154274	C4orf19	chromosome 4 open reading frame 19 [Source:HGNC Symbol;Acc:HGNC:25618]	protein coding	-0.94	0.99	25.09	<0.0001	<0.0001
ENSG00000164134	NAA15	N(alpha)-acetyltransferase 15, NatA auxiliary subunit [Source:HGNC Symbol;Acc:HGNC:30782]	protein coding	0.94	0.71	31.90	<0.0001	<0.0001
ENSG00000179364	PACS2	phosphofurin acidic cluster sorting protein 2 [Source:HGNC Symbol;Acc:HGNC:23794]	protein coding	-0.94	1.27	23.60	<0.0001	<0.0001
ENSG00000133056	PIK3C2B	phosphatidylinositol-4-phosphate 3-kinase catalytic subunit type 2 beta [Source:HGNC Symbol;Acc:HGNC:8972]	protein coding	-0.94	0.54	26.74	<0.0001	<0.0001
ENSG00000111224	PARP11	poly(ADP-ribose) polymerase family member 11 [Source:HGNC Symbol;Acc:HGNC:1186]	protein coding	0.94	2.03	22.35	<0.0001	<0.0001
ENSG00000100297	MCM5	minichromosome maintenance complex component 5 [Source:HGNC Symbol;Acc:HGNC:6948]	protein coding	-0.94	4.59	35.55	<0.0001	<0.0001
ENSG00000244509	APOBEC3C	apolipoprotein B mRNA editing enzyme catalytic subunit 3C [Source:HGNC Symbol;Acc:HGNC:17353]	protein coding	0.94	2.83	20.99	<0.0001	<0.0001
ENSG00000085449	WDFY1	WD repeat and FYVE domain containing 1 [Source:HGNC Symbol;Acc:HGNC:20451]	protein coding	0.94	5.02	33.30	<0.0001	<0.0001
ENSG00000007237		growth arrest specific 7 [Source:NCBI gene;Acc:8522]	protein coding	0.94	7.04	31.62	<0.0001	<0.0001
ENSG00000168906	MAT2A	methionine adenosyltransferase 2A [Source:HGNC Symbol;Acc:HGNC:6904]	protein coding	-0.94	4.06	31.05	<0.0001	<0.0001
ENSG00000104312	RIPK2	receptor interacting serine/threonine kinase 2 [Source:HGNC Symbol;Acc:HGNC:10020]	protein coding	0.94	5.10	27.99	<0.0001	<0.0001
ENSG00000173905	GOLIM4	golgi integral membrane protein 4 [Source:HGNC Symbol;Acc:HGNC:15448]	protein coding	-0.94	0.55	9.13	0.0025	0.0093
ENSG00000167716	WDR81	WD repeat domain 81 [Source:HGNC Symbol;Acc:HGNC:26600]	protein coding	-0.94	2.43	28.77	<0.0001	<0.0001
ENSG00000099860	GADD45B	growth arrest and DNA damage inducible beta [Source:HGNC Symbol;Acc:HGNC:4096]	protein coding	0.94	7.12	32.64	<0.0001	<0.0001
ENSG00000197471	SPN	sialophorin [Source:HGNC Symbol;Acc:HGNC:11249]	protein coding	-0.94	0.90	18.98	<0.0001	0.0001
ENSG00000157551	KCNJ15	potassium inwardly rectifying channel subfamily J member 15 [Source:HGNC Symbol;Acc:HGNC:6261]	protein coding	0.94	9.25	46.89	<0.0001	<0.0001
ENSG00000198755	RPL10A	ribosomal protein L10a [Source:HGNC Symbol;Acc:HGNC:10299]	protein coding	-0.93	1.67	18.86	<0.0001	0.0001
ENSG00000279861		TEC	TEC	0.93	3.85	12.88	0.0003	0.0018

Table S16: Genes differentially expressed at Day 2 compared to pre-first vaccination (Study Arm 2 A/H7N9+AS03, D1, 29, Neutrophils). Genes are sorted by descending absolute log_2 fold change. Gene model summaries and annotations are based on Ensembl Version 99 (continued.)

Ensembl Gene ID	Ensembl Gene Name	Ensembl Gene Description	Gene Type	log_2 Fold Change (Day 2 vs. Pre-First Vaccination)	Average log_2 CPM	Likelihood Ratio Test Statistic	P-Value	FDR Adjusted P-Value
ENSG00000152223	EPG5	ectopic P-granules autophagy protein 5 homolog [Source:HGNC Symbol;Acc:HGNC:29331]	protein coding	0.93	5.43	36.52	<0.0001	<0.0001
ENSG00000233038		novel transcript, antisense to PTPRN2	lncRNA	-0.93	2.04	15.87	<0.0001	0.0005
ENSG00000141560	FN3KRP	fructosamine 3 kinase related protein [Source:HGNC Symbol;Acc:HGNC:25700]	protein coding	-0.93	0.71	17.44	<0.0001	0.0002
ENSG00000237927		novel transcript	lncRNA	0.93	1.33	20.54	<0.0001	<0.0001
ENSG00000244242	IFITM10	interferon induced transmembrane protein 10 [Source:HGNC Symbol;Acc:HGNC:40022]	protein coding	-0.93	0.28	10.00	0.0016	0.0063
ENSG00000261801	LOXL1-AS1	LOXL1 antisense RNA 1 [Source:HGNC Symbol;Acc:HGNC:44169]	lncRNA	0.93	1.24	22.68	<0.0001	<0.0001
ENSG00000134070	IRAK2	interleukin 1 receptor associated kinase 2 [Source:HGNC Symbol;Acc:HGNC:6113]	protein coding	0.93	2.67	23.58	<0.0001	<0.0001
ENSG00000275557		novel transcript	lncRNA	0.93	0.94	53.27	<0.0001	<0.0001
ENSG00000126246	IGFLR1	IGF like family receptor 1 [Source:HGNC Symbol;Acc:HGNC:23620]	protein coding	0.93	3.20	25.63	<0.0001	<0.0001
ENSG00000115718	PROC	protein C, inactivator of coagulation factors Va and VIIIa [Source:HGNC Symbol;Acc:HGNC:9451]	protein coding	-0.93	1.38	25.39	<0.0001	<0.0001
ENSG00000158411	MITD1	microtubule interacting and trafficking domain containing 1 [Source:HGNC Symbol;Acc:HGNC:25207]	protein coding	0.92	4.55	38.48	<0.0001	<0.0001
ENSG00000135148	TRAFD1	TRAF-type zinc finger domain containing 1 [Source:HGNC Symbol;Acc:HGNC:24808]	protein coding	0.92	7.65	25.98	<0.0001	<0.0001
ENSG00000287839		novel transcript	lncRNA	0.92	3.69	35.23	<0.0001	<0.0001
ENSG00000113368	LMNB1	lamin B1 [Source:HGNC Symbol;Acc:HGNC:6637]	protein coding	0.92	7.34	31.17	<0.0001	<0.0001
ENSG00000204397	CARD16	caspase recruitment domain family member 16 [Source:HGNC Symbol;Acc:HGNC:33701]	protein coding	0.92	6.91	48.11	<0.0001	<0.0001
ENSG00000237803	LINC00211	long intergenic non-protein coding RNA 211 [Source:HGNC Symbol;Acc:HGNC:37459]	lncRNA	-0.92	3.74	59.84	<0.0001	<0.0001
ENSG00000184897	H1-10	H1.10 linker histone [Source:HGNC Symbol;Acc:HGNC:4722]	protein coding	-0.92	4.50	16.17	<0.0001	0.0004
ENSG00000180628	PCGF5	polycomb group ring finger 5 [Source:HGNC Symbol;Acc:HGNC:28264]	protein coding	0.92	5.93	34.32	<0.0001	<0.0001
ENSG00000184182	UBE2F	ubiquitin conjugating enzyme E2 F (putative) [Source:HGNC Symbol;Acc:HGNC:12480]	protein coding	0.92	2.94	33.44	<0.0001	<0.0001
ENSG00000026103	FAS	Fas cell surface death receptor [Source:HGNC Symbol;Acc:HGNC:11920]	protein coding	0.92	7.60	34.55	<0.0001	<0.0001

Table S16: Genes differentially expressed at Day 2 compared to pre-first vaccination (Study Arm 2 A/H7N9+AS03, D1, 29, Neutrophils). Genes are sorted by descending absolute log_2 fold change. Gene model summaries and annotations are based on Ensembl Version 99 (continued.)

Ensembl Gene ID	Ensembl Gene Name	Ensembl Gene Description	Gene Type	log_2 Fold Change (Day 2 vs. Pre-First Vaccination)	Average log_2 CPM	Likelihood Ratio Test Statistic	P-Value	FDR Adjusted P-Value
ENSG00000135378	PRRG4	proline rich and Gla domain 4 [Source:HGNC Symbol;Acc:HGNC:30799]	protein coding	0.91	6.63	38.78	<0.0001	<0.0001
ENSG00000038210	PI4K2B	phosphatidylinositol 4-kinase type 2 beta [Source:HGNC Symbol;Acc:HGNC:18215]	protein coding	0.91	1.67	12.20	0.0005	0.0024
ENSG00000071575	TRIB2	tribbles pseudokinase 2 [Source:HGNC Symbol;Acc:HGNC:30809]	protein coding	-0.91	1.57	11.53	0.0007	0.0032
ENSG00000159176	CSRP1	cysteine and glycine rich protein 1 [Source:HGNC Symbol;Acc:HGNC:2469]	protein coding	-0.91	2.89	23.40	<0.0001	<0.0001
ENSG00000068489	PRR11	proline rich 11 [Source:HGNC Symbol;Acc:HGNC:25619]	protein coding	0.91	2.02	21.94	<0.0001	<0.0001
ENSG00000130731	METTL26	methyltransferase like 26 [Source:HGNC Symbol;Acc:HGNC:14141]	protein coding	-0.91	1.36	32.35	<0.0001	<0.0001
ENSG00000112039	FANCE	FA complementation group E [Source:HGNC Symbol;Acc:HGNC:3586]	protein coding	-0.91	0.45	28.87	<0.0001	<0.0001
ENSG00000047579	DTNBP1	dystrobrevin binding protein 1 [Source:HGNC Symbol;Acc:HGNC:17328]	protein coding	0.91	3.52	35.22	<0.0001	<0.0001
ENSG00000152778	IFIT5	interferon induced protein with tetratricopeptide repeats 5 [Source:HGNC Symbol;Acc:HGNC:13328]	protein coding	0.91	6.19	10.13	0.0015	0.0059
ENSG00000095397	WHRN	whirlin [Source:HGNC Symbol;Acc:HGNC:16361]	protein coding	0.91	3.00	29.97	<0.0001	<0.0001
ENSG00000075303	SLC25A40	solute carrier family 25 member 40 [Source:HGNC Symbol;Acc:HGNC:29680]	protein coding	-0.91	3.47	22.51	<0.0001	<0.0001
ENSG00000134352	IL6ST	interleukin 6 signal transducer [Source:HGNC Symbol;Acc:HGNC:6021]	protein coding	0.91	4.02	27.73	<0.0001	<0.0001
ENSG00000069849	ATP1B3	ATPase Na+/K+ transporting subunit beta 3 [Source:HGNC Symbol;Acc:HGNC:806]	protein coding	0.90	1.01	31.11	<0.0001	<0.0001
ENSG00000230539	AOAH-IT1	AOAH intronic transcript 1 [Source:HGNC Symbol;Acc:HGNC:41307]	lncRNA	0.90	1.38	37.90	<0.0001	<0.0001
ENSG00000051128	HOMER3	homer scaffold protein 3 [Source:HGNC Symbol;Acc:HGNC:17514]	protein coding	-0.90	3.27	27.38	<0.0001	<0.0001
ENSG00000162066	AMDHD2	amidohydrolase domain containing 2 [Source:HGNC Symbol;Acc:HGNC:24262]	protein coding	-0.90	4.45	51.02	<0.0001	<0.0001
ENSG00000072210	ALDH3A2	aldehyde dehydrogenase 3 family member A2 [Source:HGNC Symbol;Acc:HGNC:403]	protein coding	-0.90	0.87	13.14	0.0003	0.0016
ENSG00000253214		novel transcript	lncRNA	0.90	1.11	26.80	<0.0001	<0.0001

Table S16: Genes differentially expressed at Day 2 compared to pre-first vaccination (Study Arm 2 A/H7N9+AS03, D1, 29, Neutrophils). Genes are sorted by descending absolute log_2 fold change. Gene model summaries and annotations are based on Ensembl Version 99 (continued.)

Ensembl Gene ID	Ensembl Gene Name	Ensembl Gene Description	Gene Type	log_2 Fold Change (Day 2 vs. Pre-First Vaccination)	Average log_2 CPM	Likelihood Ratio Test Statistic	P-Value	FDR Adjusted P-Value
ENSG00000187091	PLCD1	phospholipase C delta 1 [Source:HGNC Symbol;Acc:HGNC:9060]	protein coding	-0.90	3.66	52.00	<0.0001	<0.0001
ENSG00000189060	H1-0	H1.0 linker histone [Source:HGNC Symbol;Acc:HGNC:4714]	protein coding	-0.90	3.53	15.34	<0.0001	0.0006
ENSG00000178685	PARP10	poly(ADP-ribose) polymerase family member 10 [Source:HGNC Symbol;Acc:HGNC:25895]	protein coding	0.90	7.61	23.52	<0.0001	<0.0001
ENSG00000287725		novel protein, TPCN2 - SMIM38 readthrough	protein coding	-0.90	0.58	19.37	<0.0001	0.0001
ENSG00000155561	NUP205	nucleoporin 205 [Source:HGNC Symbol;Acc:HGNC:18658]	protein coding	0.90	0.40	9.32	0.0023	0.0085
ENSG00000145979	TBC1D7	TBC1 domain family member 7 [Source:HGNC Symbol;Acc:HGNC:21066]	protein coding	-0.90	2.80	40.75	<0.0001	<0.0001
ENSG00000073060	SCARB1	scavenger receptor class B member 1 [Source:HGNC Symbol;Acc:HGNC:1664]	protein coding	-0.89	1.79	19.49	<0.0001	<0.0001
ENSG00000232680		novel transcript	lncRNA	0.89	1.98	28.20	<0.0001	<0.0001
ENSG00000059122	FLYWCH1	FLYWCH-type zinc finger 1 [Source:HGNC Symbol;Acc:HGNC:25404]	protein coding	-0.89	1.32	25.82	<0.0001	<0.0001
ENSG00000215068		novel transcript, antisense to ANXA2R	lncRNA	0.89	2.00	23.60	<0.0001	<0.0001
ENSG00000122643	NT5C3A	5'-nucleotidase, cytosolic IIIA [Source:HGNC Symbol;Acc:HGNC:17820]	protein coding	0.89	6.34	28.33	<0.0001	<0.0001
ENSG00000185897	FFAR3	free fatty acid receptor 3 [Source:HGNC Symbol;Acc:HGNC:4499]	protein coding	0.89	0.82	17.26	<0.0001	0.0003
ENSG00000099904	ZDHHC8	zinc finger DHHC-type containing 8 [Source:HGNC Symbol;Acc:HGNC:18474]	protein coding	-0.89	0.50	12.26	0.0005	0.0023
ENSG00000071564	TCF3	transcription factor 3 [Source:HGNC Symbol;Acc:HGNC:11633]	protein coding	-0.89	0.86	14.92	0.0001	0.0007
ENSG00000196814	MVB12B	multivesicular body subunit 12B [Source:HGNC Symbol;Acc:HGNC:23368]	protein coding	-0.89	0.37	21.65	<0.0001	<0.0001
ENSG00000139618	BRCA2	BRCA2 DNA repair associated [Source:HGNC Symbol;Acc:HGNC:1101]	protein coding	0.89	0.55	13.20	0.0003	0.0016
ENSG00000100324	TAB1	TGF-beta activated kinase 1 (MAP3K7) binding protein 1 [Source:HGNC Symbol;Acc:HGNC:18157]	protein coding	-0.89	1.56	25.52	<0.0001	<0.0001
ENSG00000205786	LINC01531	long intergenic non-protein coding RNA 1531 [Source:HGNC Symbol;Acc:HGNC:51270]	lncRNA	0.89	2.17	17.28	<0.0001	0.0003

Table S16: Genes differentially expressed at Day 2 compared to pre-first vaccination (Study Arm 2 A/H7N9+AS03, D1, 29, Neutrophils). Genes are sorted by descending absolute log_2 fold change. Gene model summaries and annotations are based on Ensembl Version 99 (continued.)

Ensembl Gene ID	Ensembl Gene Name	Ensembl Gene Description	Gene Type	log_2 Fold Change (Day 2 vs. Pre-First Vaccination)	Average log_2 CPM	Likelihood Ratio Test Statistic	P-Value	FDR Adjusted P-Value
ENSG00000133624	ZNF767P	zinc finger family member 767, pseudogene [Source:HGNC Symbol;Acc:HGNC:21884]	transcribed unprocessed pseudogene	-0.89	3.10	38.55	<0.0001	<0.0001
ENSG00000213373	LINC00671	long intergenic non-protein coding RNA 671 [Source:HGNC Symbol;Acc:HGNC:44339]	lncRNA	-0.89	3.20	29.72	<0.0001	<0.0001
ENSG00000204287	HLA-DRA	major histocompatibility complex, class II, DR alpha [Source:HGNC Symbol;Acc:HGNC:4947]	protein coding	0.89	4.82	13.86	0.0002	0.0012
ENSG00000286656		novel transcript	lncRNA	0.88	1.91	25.99	<0.0001	<0.0001
ENSG00000106665	CLIP2	CAP-Gly domain containing linker protein 2 [Source:HGNC Symbol;Acc:HGNC:2586]	protein coding	-0.88	1.48	39.23	<0.0001	<0.0001
ENSG00000270972		novel transcript	lncRNA	0.88	3.46	8.51	0.0035	0.0122
ENSG00000160216	AGPAT3	1-acylglycerol-3-phosphate O-acyltransferase 3 [Source:HGNC Symbol;Acc:HGNC:326]	protein coding	0.88	3.58	44.03	<0.0001	<0.0001
ENSG00000123411	IKZF4	IKAROS family zinc finger 4 [Source:HGNC Symbol;Acc:HGNC:13179]	protein coding	0.88	0.55	19.82	<0.0001	<0.0001
ENSG00000204267	TAP2	transporter 2, ATP binding cassette subfamily B member [Source:HGNC Symbol;Acc:HGNC:44]	protein coding	0.88	7.05	26.31	<0.0001	<0.0001
ENSG00000134900	TPP2	tripeptidyl peptidase 2 [Source:HGNC Symbol;Acc:HGNC:12016]	protein coding	-0.88	3.07	33.47	<0.0001	<0.0001
ENSG00000171860	C3AR1	complement C3a receptor 1 [Source:HGNC Symbol;Acc:HGNC:1319]	protein coding	0.88	4.64	10.98	0.0009	0.0041
ENSG00000165490	DDIAS	DNA damage induced apoptosis suppressor [Source:HGNC Symbol;Acc:HGNC:26351]	protein coding	0.88	1.54	25.54	<0.0001	<0.0001
ENSG00000179044	EXOC3L1	exocyst complex component 3 like 1 [Source:HGNC Symbol;Acc:HGNC:27540]	protein coding	0.88	3.59	11.15	0.0008	0.0038
ENSG00000259321		novel transcript	lncRNA	0.88	1.25	25.80	<0.0001	<0.0001
ENSG00000188822	CNR2	cannabinoid receptor 2 [Source:HGNC Symbol;Acc:HGNC:2160]	protein coding	-0.88	1.92	8.41	0.0037	0.0128
ENSG00000103326	CAPN15	calpain 15 [Source:HGNC Symbol;Acc:HGNC:11182]	protein coding	-0.87	1.54	16.77	<0.0001	0.0003
ENSG00000169429	CXCL8	C-X-C motif chemokine ligand 8 [Source:HGNC Symbol;Acc:HGNC:6025]	protein coding	-0.87	10.27	23.58	<0.0001	<0.0001
ENSG00000168675	LDLRAD4	low density lipoprotein receptor class A domain containing 4 [Source:HGNC Symbol;Acc:HGNC:1224]	protein coding	-0.87	2.40	34.68	<0.0001	<0.0001

Table S16: Genes differentially expressed at Day 2 compared to pre-first vaccination (Study Arm 2 A/H7N9+AS03, D1, 29, Neutrophils). Genes are sorted by descending absolute log_2 fold change. Gene model summaries and annotations are based on Ensembl Version 99 (continued.)

Ensembl Gene ID	Ensembl Gene Name	Ensembl Gene Description	Gene Type	log_2 Fold Change (Day 2 vs. Pre-First Vaccination)	Average log_2 CPM	Likelihood Ratio Test Statistic	P-Value	FDR Adjusted P-Value
ENSG00000177542	SLC25A22	solute carrier family 25 member 22 [Source:HGNC Symbol;Acc:HGNC:19954]	protein coding	0.87	2.91	37.88	<0.0001	<0.0001
ENSG00000250138		POM121 membrane glycoprotein-like 1 (POM121L1) pseudogene	unprocessed pseudogene	0.87	4.57	10.96	0.0009	0.0041
ENSG00000110237	ARHGEF17	Rho guanine nucleotide exchange factor 17 [Source:HGNC Symbol;Acc:HGNC:21726]	protein coding	-0.87	1.85	13.02	0.0003	0.0017
ENSG00000037757	MRI1	methylthioribose-1-phosphate isomerase 1 [Source:HGNC Symbol;Acc:HGNC:28469]	protein coding	-0.87	1.21	22.56	<0.0001	<0.0001
ENSG00000186951	PPARA	peroxisome proliferator activated receptor alpha [Source:HGNC Symbol;Acc:HGNC:9232]	protein coding	-0.87	1.60	19.09	<0.0001	0.0001
ENSG00000196562	SULF2	sulfatase 2 [Source:HGNC Symbol;Acc:HGNC:20392]	protein coding	-0.87	8.46	63.99	<0.0001	<0.0001
ENSG00000155629	PIK3AP1	phosphoinositide-3-kinase adaptor protein 1 [Source:HGNC Symbol;Acc:HGNC:30034]	protein coding	0.87	8.53	34.38	<0.0001	<0.0001
ENSG00000284874		novel transcript	protein coding	-0.87	0.53	15.85	<0.0001	0.0005
ENSG00000169432	SCN9A	sodium voltage-gated channel alpha subunit 9 [Source:HGNC Symbol;Acc:HGNC:10597]	protein coding	0.86	0.83	18.70	<0.0001	0.0001
ENSG00000113448	PDE4D	phosphodiesterase 4D [Source:HGNC Symbol;Acc:HGNC:8783]	protein coding	-0.86	1.62	16.84	<0.0001	0.0003
ENSG00000258017		novel transcript, antisense to TUBA1B	lncRNA	0.86	1.48	19.15	<0.0001	0.0001
ENSG00000124203	ZNF831	zinc finger protein 831 [Source:HGNC Symbol;Acc:HGNC:16167]	protein coding	-0.86	0.47	25.78	<0.0001	<0.0001
ENSG00000107566	ERLIN1	ER lipid raft associated 1 [Source:HGNC Symbol;Acc:HGNC:16947]	protein coding	0.86	4.71	15.22	<0.0001	0.0006
ENSG00000161980	POLR3K	RNA polymerase III subunit K [Source:HGNC Symbol;Acc:HGNC:14121]	protein coding	-0.86	2.26	26.59	<0.0001	<0.0001
ENSG00000104964	TLE5	TLE family member 5, transcriptional modulator [Source:HGNC Symbol;Acc:HGNC:307]	protein coding	-0.86	3.37	9.90	0.0017	0.0066
ENSG00000090339	ICAM1	intercellular adhesion molecule 1 [Source:HGNC Symbol;Acc:HGNC:5344]	protein coding	0.86	6.82	36.05	<0.0001	<0.0001
ENSG00000234771	SLC25A25-AS1	SLC25A25 antisense RNA 1 [Source:HGNC Symbol;Acc:HGNC:27844]	lncRNA	-0.86	0.42	19.04	<0.0001	0.0001
ENSG00000135929	CYP27A1	cytochrome P450 family 27 subfamily A member 1 [Source:HGNC Symbol;Acc:HGNC:2605]	protein coding	-0.86	6.43	15.28	<0.0001	0.0006

Table S16: Genes differentially expressed at Day 2 compared to pre-first vaccination (Study Arm 2 A/H7N9+AS03, D1, 29, Neutrophils). Genes are sorted by descending absolute log_2 fold change. Gene model summaries and annotations are based on Ensembl Version 99 (continued.)

Ensembl Gene ID	Ensembl Gene Name	Ensembl Gene Description	Gene Type	log_2 Fold Change (Day 2 vs. Pre-First Vaccination)	Average log_2 CPM	Likelihood Ratio Test Statistic	P-Value	FDR Adjusted P-Value
ENSG00000119397	CNTRL	centriolin [Source:HGNC Symbol;Acc:HGNC:1858]	protein coding	0.86	4.81	31.01	<0.0001	<0.0001
ENSG00000177697	CD151	CD151 molecule (Raph blood group) [Source:HGNC Symbol;Acc:HGNC:1630]	protein coding	-0.86	2.37	7.86	0.005	0.0164
ENSG00000179915	NRXN1	neurexin 1 [Source:HGNC Symbol;Acc:HGNC:8008]	protein coding	-0.86	0.68	26.66	<0.0001	<0.0001
ENSG00000240065	PSMB9	proteasome 20S subunit beta 9 [Source:HGNC Symbol;Acc:HGNC:9546]	protein coding	0.86	8.74	38.41	<0.0001	<0.0001
ENSG00000261997		novel transcript	lncRNA	0.86	2.69	18.32	<0.0001	0.0002
ENSG00000107020	PLGRKT	plasminogen receptor with a C-terminal lysine [Source:HGNC Symbol;Acc:HGNC:23633]	protein coding	0.86	2.90	34.02	<0.0001	<0.0001
ENSG00000162946	DISC1	DISC1 scaffold protein [Source:HGNC Symbol;Acc:HGNC:2888]	protein coding	0.86	4.07	22.82	<0.0001	<0.0001
ENSG00000185989	RASA3	RAS p21 protein activator 3 [Source:HGNC Symbol;Acc:HGNC:20331]	protein coding	-0.86	5.33	49.37	<0.0001	<0.0001
ENSG00000143891	GALM	galactose mutarotase [Source:HGNC Symbol;Acc:HGNC:24063]	protein coding	0.86	3.91	13.22	0.0003	0.0015
ENSG00000174004	NRROS	negative regulator of reactive oxygen species [Source:HGNC Symbol;Acc:HGNC:24613]	protein coding	-0.86	2.00	10.93	0.0009	0.0042
ENSG00000285534		novel transcript	lncRNA	-0.86	0.67	16.83	<0.0001	0.0003
ENSG00000197142	ACSL5	acyl-CoA synthetase long chain family member 5 [Source:HGNC Symbol;Acc:HGNC:16526]	protein coding	0.86	4.01	33.18	<0.0001	<0.0001
ENSG00000184992	BRI3BP	BRI3 binding protein [Source:HGNC Symbol;Acc:HGNC:14251]	polymorphic pseudogene	-0.86	3.32	24.20	<0.0001	<0.0001
ENSG00000013583	HEBP1	heme binding protein 1 [Source:HGNC Symbol;Acc:HGNC:17176]	protein coding	0.85	3.62	26.36	<0.0001	<0.0001
ENSG00000176595	KBTBD11	kelch repeat and BTB domain containing 11 [Source:HGNC Symbol;Acc:HGNC:29104]	protein coding	-0.85	4.30	23.59	<0.0001	<0.0001
ENSG00000181788	SIAH2	siah E3 ubiquitin protein ligase 2 [Source:HGNC Symbol;Acc:HGNC:10858]	protein coding	0.85	5.14	48.64	<0.0001	<0.0001
ENSG00000141858	SAMD1	sterile alpha motif domain containing 1 [Source:HGNC Symbol;Acc:HGNC:17958]	protein coding	-0.85	1.90	21.90	<0.0001	<0.0001
ENSG00000130487	KLHDC7B	kelch domain containing 7B [Source:HGNC Symbol;Acc:HGNC:25145]	protein coding	0.85	1.79	13.20	0.0003	0.0016

Table S16: Genes differentially expressed at Day 2 compared to pre-first vaccination (Study Arm 2 A/H7N9+AS03, D1, 29, Neutrophils). Genes are sorted by descending absolute log_2 fold change. Gene model summaries and annotations are based on Ensembl Version 99 (continued.)

Ensembl Gene ID	Ensembl Gene Name	Ensembl Gene Description	Gene Type	log_2 Fold Change (Day 2 vs. Pre-First Vaccination)	Average log_2 CPM	Likelihood Ratio Test Statistic	P-Value	FDR Adjusted P-Value
ENSG00000152207	CYSLTR2	cysteinyl leukotriene receptor 2 [Source:HGNC Symbol;Acc:HGNC:18274]	protein coding	-0.85	3.01	7.50	0.0062	0.0193
ENSG00000213492	NT5C3AP1	5’-nucleotidase, cytosolic IIIA pseudogene 1 [Source:HGNC Symbol;Acc:HGNC:18530]	transcribed processed pseudogene	0.85	1.27	22.86	<0.0001	<0.0001
ENSG00000132109	TRIM21	tripartite motif containing 21 [Source:HGNC Symbol;Acc:HGNC:11312]	protein coding	0.85	7.51	33.32	<0.0001	<0.0001
ENSG00000135074	ADAM19	ADAM metallopeptidase domain 19 [Source:HGNC Symbol;Acc:HGNC:197]	protein coding	-0.85	7.35	22.09	<0.0001	<0.0001
ENSG00000103489	XYLT1	xylosyltransferase 1 [Source:HGNC Symbol;Acc:HGNC:15516]	protein coding	-0.85	4.57	29.62	<0.0001	<0.0001
ENSG00000255328		novel transcript	lncRNA	0.85	3.47	9.92	0.0016	0.0065
ENSG00000280649		TEC	TEC	0.85	2.11	36.13	<0.0001	<0.0001
ENSG00000133687	TMTC1	transmembrane O-mannosyltransferase targeting cadherins 1 [Source:HGNC Symbol;Acc:HGNC:24099]	protein coding	0.85	2.86	7.55	0.006	0.0189
ENSG00000179833	SERTAD2	SERTA domain containing 2 [Source:HGNC Symbol;Acc:HGNC:30784]	protein coding	-0.85	5.26	29.35	<0.0001	<0.0001
ENSG00000106560	GIMAP2	GTPase, IMAP family member 2 [Source:HGNC Symbol;Acc:HGNC:21789]	protein coding	0.85	5.22	12.68	0.0004	0.0019
ENSG00000148834	GSTO1	glutathione S-transferase omega 1 [Source:HGNC Symbol;Acc:HGNC:13312]	protein coding	0.84	5.05	34.58	<0.0001	<0.0001
ENSG00000068784	SRBD1	S1 RNA binding domain 1 [Source:HGNC Symbol;Acc:HGNC:25521]	protein coding	0.84	4.61	41.68	<0.0001	<0.0001
ENSG00000148344	PTGES	prostaglandin E synthase [Source:HGNC Symbol;Acc:HGNC:9599]	protein coding	-0.84	1.60	8.79	0.003	0.0108
ENSG00000156127	BATF	basic leucine zipper ATF-like transcription factor [Source:HGNC Symbol;Acc:HGNC:958]	protein coding	0.84	3.37	25.73	<0.0001	<0.0001
ENSG00000274602	PI4KAP1	phosphatidylinositol 4-kinase alpha pseudogene 1 [Source:HGNC Symbol;Acc:HGNC:33576]	transcribed unprocessed pseudogene	-0.84	1.37	17.00	<0.0001	0.0003
ENSG00000177738		novel transcript, sense overlapping to ANXA2R	lncRNA	0.84	1.87	17.47	<0.0001	0.0002
ENSG00000176871	WSB2	WD repeat and SOCS box containing 2 [Source:HGNC Symbol;Acc:HGNC:19222]	protein coding	0.84	5.42	44.97	<0.0001	<0.0001

Table S16: Genes differentially expressed at Day 2 compared to pre-first vaccination (Study Arm 2 A/H7N9+AS03, D1, 29, Neutrophils). Genes are sorted by descending absolute log_2 fold change. Gene model summaries and annotations are based on Ensembl Version 99 (continued.)

Ensembl Gene ID	Ensembl Gene Name	Ensembl Gene Description	Gene Type	log_2 Fold Change (Day 2 vs. Pre-First Vaccination)	Average log_2 CPM	Likelihood Ratio Test Statistic	P-Value	FDR Adjusted P-Value
ENSG00000111731	C2CD5	C2 calcium dependent domain containing 5 [Source:HGNC Symbol;Acc:HGNC:29062]	protein coding	-0.84	4.43	29.68	<0.0001	<0.0001
ENSG00000157601	MX1	MX dynamin like GTPase 1 [Source:HGNC Symbol;Acc:HGNC:7532]	protein coding	0.84	8.55	7.11	0.0077	0.023
ENSG00000267365	KCNJ2-AS1	KCNJ2 antisense RNA 1 [Source:HGNC Symbol;Acc:HGNC:43720]	lncRNA	0.84	3.87	40.94	<0.0001	<0.0001
ENSG00000181826	RELL1	RELT like 1 [Source:HGNC Symbol;Acc:HGNC:27379]	protein coding	-0.84	6.04	29.30	<0.0001	<0.0001
ENSG00000251230	MIR3945HG	MIR3945 host gene [Source:HGNC Symbol;Acc:HGNC:52002]	lncRNA	0.84	3.01	20.40	<0.0001	<0.0001
ENSG00000149600	COMMD7	COMM domain containing 7 [Source:HGNC Symbol;Acc:HGNC:16223]	protein coding	-0.84	0.50	17.99	<0.0001	0.0002
ENSG00000166881	NEMP1	nuclear envelope integral membrane protein 1 [Source:HGNC Symbol;Acc:HGNC:29001]	protein coding	-0.83	2.00	26.03	<0.0001	<0.0001
ENSG00000092200	RPGRIP1	RPGR interacting protein 1 [Source:HGNC Symbol;Acc:HGNC:13436]	protein coding	-0.83	4.83	43.24	<0.0001	<0.0001
ENSG00000233461		novel transcript	lncRNA	0.83	3.07	42.16	<0.0001	<0.0001
ENSG00000143537	ADAM15	ADAM metallopeptidase domain 15 [Source:HGNC Symbol;Acc:HGNC:193]	protein coding	-0.83	2.80	32.96	<0.0001	<0.0001
ENSG00000178075	GRAMD1C	GRAM domain containing 1C [Source:HGNC Symbol;Acc:HGNC:25252]	protein coding	-0.83	4.72	34.29	<0.0001	<0.0001
ENSG00000162073	PAQR4	progestin and adipoQ receptor family member 4 [Source:HGNC Symbol;Acc:HGNC:26386]	protein coding	-0.83	0.34	19.73	<0.0001	<0.0001
ENSG00000108106	UBE2S	ubiquitin conjugating enzyme E2 S [Source:HGNC Symbol;Acc:HGNC:17895]	protein coding	0.83	4.08	13.14	0.0003	0.0016
ENSG00000105176	URI1	URI1 prefoldin like chaperone [Source:HGNC Symbol;Acc:HGNC:13236]	protein coding	-0.83	0.50	17.13	<0.0001	0.0003
ENSG00000143515	ATP8B2	ATPase phospholipid transporting 8B2 [Source:HGNC Symbol;Acc:HGNC:13534]	protein coding	-0.83	2.83	8.58	0.0034	0.0119
ENSG00000196636	SDHAF3	succinate dehydrogenase complex assembly factor 3 [Source:HGNC Symbol;Acc:HGNC:21752]	protein coding	0.83	2.10	19.22	<0.0001	0.0001
ENSG00000122733	PHF24	PHD finger protein 24 [Source:HGNC Symbol;Acc:HGNC:29180]	protein coding	-0.83	2.47	17.89	<0.0001	0.0002
ENSG00000140688	C16orf58	chromosome 16 open reading frame 58 [Source:HGNC Symbol;Acc:HGNC:25848]	protein coding	-0.83	0.37	18.26	<0.0001	0.0002

Table S16: Genes differentially expressed at Day 2 compared to pre-first vaccination (Study Arm 2 A/H7N9+AS03, D1, 29, Neutrophils). Genes are sorted by descending absolute log_2 fold change. Gene model summaries and annotations are based on Ensembl Version 99 (continued.)

Ensembl Gene ID	Ensembl Gene Name	Ensembl Gene Description	Gene Type	log_2 Fold Change (Day 2 vs. Pre-First Vaccination)	Average log_2 CPM	Likelihood Ratio Test Statistic	P-Value	FDR Adjusted P-Value
ENSG00000151208	DLG5	discs large MAGUK scaffold protein 5 [Source:HGNC Symbol;Acc:HGNC:2904]	protein coding	-0.83	0.76	17.56	<0.0001	0.0002
ENSG00000187741	FANCA	FA complementation group A [Source:HGNC Symbol;Acc:HGNC:3582]	protein coding	0.83	3.92	21.03	<0.0001	<0.0001
ENSG00000035664	DAPK2	death associated protein kinase 2 [Source:HGNC Symbol;Acc:HGNC:2675]	protein coding	-0.83	7.21	34.01	<0.0001	<0.0001
ENSG00000100181	TPTEP1	TPTE pseudogene 1 [Source:HGNC Symbol;Acc:HGNC:43648]	transcribed unprocessed pseudogene	-0.83	1.55	5.89	0.0153	0.0403
ENSG00000255398	HCAR3	hydroxycarboxylic acid receptor 3 [Source:HGNC Symbol;Acc:HGNC:16824]	protein coding	0.83	6.85	35.24	<0.0001	<0.0001
ENSG00000019582	CD74	CD74 molecule [Source:HGNC Symbol;Acc:HGNC:1697]	protein coding	0.82	8.33	45.89	<0.0001	<0.0001
ENSG00000186815	TPCN1	two pore segment channel 1 [Source:HGNC Symbol;Acc:HGNC:18182]	protein coding	-0.82	0.66	30.69	<0.0001	<0.0001
ENSG00000196123	KIAA0895L	KIAA0895 like [Source:HGNC Symbol;Acc:HGNC:34408]	protein coding	0.82	1.18	13.89	0.0002	0.0011
ENSG00000102445	RUBCNL	rubicon like autophagy enhancer [Source:HGNC Symbol;Acc:HGNC:20420]	protein coding	-0.82	6.60	14.92	0.0001	0.0007
ENSG00000169683	LRRC45	leucine rich repeat containing 45 [Source:HGNC Symbol;Acc:HGNC:28302]	protein coding	-0.82	0.20	12.22	0.0005	0.0024
ENSG00000059378	PARP12	poly(ADP-ribose) polymerase family member 12 [Source:HGNC Symbol;Acc:HGNC:21919]	protein coding	0.82	6.28	11.60	0.0007	0.0031
ENSG00000104164	BLOC1S6	biogenesis of lysosomal organelles complex 1 subunit 6 [Source:HGNC Symbol;Acc:HGNC:8549]	protein coding	0.82	4.76	37.06	<0.0001	<0.0001
ENSG00000102796	DHRS12	dehydrogenase/reductase 12 [Source:HGNC Symbol;Acc:HGNC:25832]	protein coding	0.82	5.05	35.26	<0.0001	<0.0001
ENSG00000160285	LSS	lanosterol synthase [Source:HGNC Symbol;Acc:HGNC:6708]	protein coding	-0.82	0.66	11.09	0.0009	0.0039
ENSG00000171617	ENC1	ectodermal-neural cortex 1 [Source:HGNC Symbol;Acc:HGNC:3345]	protein coding	-0.82	3.53	10.51	0.0012	0.005
ENSG00000117984	CTSD	cathepsin D [Source:HGNC Symbol;Acc:HGNC:2529]	protein coding	-0.81	7.40	26.62	<0.0001	<0.0001

Table S16: Genes differentially expressed at Day 2 compared to pre-first vaccination (Study Arm 2 A/H7N9+AS03, D1, 29, Neutrophils). Genes are sorted by descending absolute log_2 fold change. Gene model summaries and annotations are based on Ensembl Version 99 (continued.)

Ensembl Gene ID	Ensembl Gene Name	Ensembl Gene Description	Gene Type	log_2 Fold Change (Day 2 vs. Pre-First Vaccination)	Average log_2 CPM	Likelihood Ratio Test Statistic	P-Value	FDR Adjusted P-Value
ENSG00000138134	STAMBPL1	STAM binding protein like 1 [Source:HGNC Symbol;Acc:HGNC:24105]	protein coding	0.81	0.28	13.75	0.0002	0.0012
ENSG00000159753	CARMIL2	capping protein regulator and myosin 1 linker 2 [Source:HGNC Symbol;Acc:HGNC:27089]	protein coding	-0.81	0.20	12.00	0.0005	0.0026
ENSG00000163875	MEAF6	MYST/Esa1 associated factor 6 [Source:HGNC Symbol;Acc:HGNC:25674]	protein coding	-0.81	3.63	34.89	<0.0001	<0.0001
ENSG00000107742	SPOCK2	SPARC (osteonectin), cwcv and kazal like domains proteoglycan 2 [Source:HGNC Symbol;Acc:HGNC:13564]	protein coding	-0.81	4.41	26.32	<0.0001	<0.0001
ENSG00000204516	MICB	MHC class I polypeptide-related sequence B [Source:HGNC Symbol;Acc:HGNC:7091]	protein coding	0.81	4.72	31.43	<0.0001	<0.0001
ENSG00000182670	TTC3	tetratricopeptide repeat domain 3 [Source:HGNC Symbol;Acc:HGNC:12393]	protein coding	-0.81	2.16	18.45	<0.0001	0.0002
ENSG00000188282	RUFY4	RUN and FYVE domain containing 4 [Source:HGNC Symbol;Acc:HGNC:24804]	protein coding	0.81	0.40	12.69	0.0004	0.0019
ENSG00000183621	ZNF438	zinc finger protein 438 [Source:HGNC Symbol;Acc:HGNC:21029]	protein coding	0.81	5.71	33.53	<0.0001	<0.0001
ENSG00000102755	FLT1	fms related tyrosine kinase 1 [Source:HGNC Symbol;Acc:HGNC:3763]	protein coding	-0.80	1.79	10.23	0.0014	0.0057
ENSG00000272398	CD24	CD24 molecule [Source:HGNC Symbol;Acc:HGNC:1645]	protein coding	-0.80	3.24	11.00	0.0009	0.004
ENSG00000182541	LIMK2	LIM domain kinase 2 [Source:HGNC Symbol;Acc:HGNC:6614]	protein coding	0.80	9.14	33.51	<0.0001	<0.0001
ENSG00000170379	TCAF2	TRPM8 channel associated factor 2 [Source:HGNC Symbol;Acc:HGNC:26878]	protein coding	0.80	0.72	11.04	0.0009	0.004
ENSG00000184887	BTBD6	BTB domain containing 6 [Source:HGNC Symbol;Acc:HGNC:19897]	protein coding	-0.80	2.60	29.36	<0.0001	<0.0001
ENSG00000185950	IRS2	insulin receptor substrate 2 [Source:HGNC Symbol;Acc:HGNC:6126]	protein coding	-0.80	8.35	18.18	<0.0001	0.0002
ENSG00000076641	PAG1	phosphoprotein membrane anchor with glycosphingolipid microdomains 1 [Source:HGNC Symbol;Acc:HGNC:30043]	protein coding	0.80	6.18	16.17	<0.0001	0.0004
ENSG00000167208	SNX20	sorting nexin 20 [Source:HGNC Symbol;Acc:HGNC:30390]	protein coding	0.80	6.99	35.32	<0.0001	<0.0001
ENSG00000170846		Putative MORF4 family-associated protein 1-like protein UPP [Source:UniProtKB/Swiss-Prot;Acc:B2RBV5]	protein coding	-0.80	1.04	14.61	0.0001	0.0008
ENSG00000215533	LINC00189	long intergenic non-protein coding RNA 189 [Source:HGNC Symbol;Acc:HGNC:18461]	lncRNA	0.80	2.13	33.36	<0.0001	<0.0001

Confidential/Proprietary Information

Table S16: Genes differentially expressed at Day 2 compared to pre-first vaccination (Study Arm 2 A/H7N9+AS03, D1, 29, Neutrophils). Genes are sorted by descending absolute log_2 fold change. Gene model summaries and annotations are based on Ensembl Version 99 (continued.)

Ensembl Gene ID	Ensembl Gene Name	Ensembl Gene Description	Gene Type	log_2 Fold Change (Day 2 vs. Pre-First Vaccination)	Average log_2 CPM	Likelihood Ratio Test Statistic	P-Value	FDR Adjusted P-Value
ENSG00000122729	ACO1	aconitase 1 [Source:HGNC Symbol;Acc:HGNC:117]	protein coding	0.80	3.82	11.75	0.0006	0.0029
ENSG00000116704	SLC35D1	solute carrier family 35 member D1 [Source:HGNC Symbol;Acc:HGNC:20800]	protein coding	-0.80	0.76	10.25	0.0014	0.0056
ENSG00000134324	LPIN1	lipin 1 [Source:HGNC Symbol;Acc:HGNC:13345]	protein coding	-0.80	1.32	9.62	0.0019	0.0074
ENSG00000103254	ANTKMT	adenine nucleotide translocase lysine methyltransferase [Source:HGNC Symbol;Acc:HGNC:14152]	protein coding	-0.80	0.93	24.93	<0.0001	<0.0001
ENSG00000100292	HMOX1	heme oxygenase 1 [Source:HGNC Symbol;Acc:HGNC:5013]	protein coding	-0.80	5.34	25.45	<0.0001	<0.0001
ENSG00000226281		novel transcript	lncRNA	0.80	1.00	19.07	<0.0001	0.0001
ENSG00000166165	CKB	creatine kinase B [Source:HGNC Symbol;Acc:HGNC:1991]	protein coding	-0.79	1.19	12.05	0.0005	0.0025
ENSG00000253194		novel transcript, antisense to FAM184A	lncRNA	0.79	0.35	16.81	<0.0001	0.0003
ENSG00000127578	WFIKKN1	WAP, follistatin/kazal, immunoglobulin, kunitz and netrin domain containing 1 [Source:HGNC Symbol;Acc:HGNC:30912]	protein coding	-0.79	0.77	20.42	<0.0001	<0.0001
ENSG00000272078		novel transcript, antisense to CASZ1	lncRNA	0.79	0.40	16.49	<0.0001	0.0004
ENSG00000256249		novel transcript	lncRNA	0.79	1.89	21.15	<0.0001	<0.0001
ENSG00000260249		novel transcript, antisense to SNX20	lncRNA	0.79	0.98	16.25	<0.0001	0.0004
ENSG00000111843	TMEM14C	transmembrane protein 14C [Source:HGNC Symbol;Acc:HGNC:20952]	protein coding	-0.79	1.65	12.62	0.0004	0.002
ENSG00000273247		novel transcript, antisense to RAB33B	lncRNA	0.79	3.71	29.12	<0.0001	<0.0001
ENSG00000119922	IFIT2	interferon induced protein with tetratricopeptide repeats 2 [Source:HGNC Symbol;Acc:HGNC:5409]	protein coding	0.79	10.16	9.29	0.0023	0.0087
ENSG00000166886	NAB2	NGFI-A binding protein 2 [Source:HGNC Symbol;Acc:HGNC:7627]	protein coding	-0.79	2.98	31.41	<0.0001	<0.0001
ENSG00000100505	TRIM9	tripartite motif containing 9 [Source:HGNC Symbol;Acc:HGNC:16288]	protein coding	0.79	2.32	20.23	<0.0001	<0.0001
ENSG00000105402	NAPA	NSF attachment protein alpha [Source:HGNC Symbol;Acc:HGNC:7641]	protein coding	0.79	6.75	18.64	<0.0001	0.0001
ENSG00000149084	HSD17B12	hydroxysteroid 17-beta dehydrogenase 12 [Source:HGNC Symbol;Acc:HGNC:18646]	protein coding	-0.79	3.22	35.55	<0.0001	<0.0001
ENSG00000229515	FLT1P1	fms related tyrosine kinase 1 pseudogene 1 [Source:HGNC Symbol;Acc:HGNC:44609]	processed pseudogene	0.78	1.07	14.10	0.0002	0.001

Table S16: Genes differentially expressed at Day 2 compared to pre-first vaccination (Study Arm 2 A/H7N9+AS03, D1, 29, Neutrophils). Genes are sorted by descending absolute log_2 fold change. Gene model summaries and annotations are based on Ensembl Version 99 (continued.)

Ensembl Gene ID	Ensembl Gene Name	Ensembl Gene Description	Gene Type	log_2 Fold Change (Day 2 vs. Pre-First Vaccination)	Average log_2 CPM	Likelihood Ratio Test Statistic	P-Value	FDR Adjusted P-Value
ENSG00000145555	MYO10	myosin X [Source:HGNC Symbol;Acc:HGNC:7593]	protein coding	0.78	0.79	9.67	0.0019	0.0073
ENSG00000130940	CASZ1	castor zinc finger 1 [Source:HGNC Symbol;Acc:HGNC:26002]	protein coding	0.78	3.34	34.09	<0.0001	<0.0001
ENSG00000257599	OVCH1-AS1	OVCH1 antisense RNA 1 [Source:HGNC Symbol;Acc:HGNC:44484]	lncRNA	0.78	1.33	8.53	0.0035	0.0121
ENSG00000175040	CHST2	carbohydrate sulfotransferase 2 [Source:HGNC Symbol;Acc:HGNC:1970]	protein coding	0.78	4.57	14.85	0.0001	0.0007
ENSG00000182810	DDX28	DEAD-box helicase 28 [Source:HGNC Symbol;Acc:HGNC:17330]	protein coding	-0.78	2.54	19.11	<0.0001	0.0001
ENSG00000075240	GRAMD4	GRAM domain containing 4 [Source:HGNC Symbol;Acc:HGNC:29113]	protein coding	-0.78	3.38	17.97	<0.0001	0.0002
ENSG00000136628	EPRS1	glutamyl-prolyl-tRNA synthetase 1 [Source:HGNC Symbol;Acc:HGNC:3418]	protein coding	-0.78	0.69	20.97	<0.0001	<0.0001
ENSG00000162076	FLYWCH2	FLYWCH family member 2 [Source:HGNC Symbol;Acc:HGNC:25178]	protein coding	-0.78	0.80	14.04	0.0002	0.0011
ENSG00000275023	MLLT6	MLLT6, PHD finger containing [Source:HGNC Symbol;Acc:HGNC:7138]	protein coding	0.78	7.00	19.54	<0.0001	<0.0001
ENSG00000182749	PAQR7	progestin and adipoQ receptor family member 7 [Source:HGNC Symbol;Acc:HGNC:23146]	protein coding	-0.78	2.06	8.64	0.0033	0.0115
ENSG00000103202	NME4	NME/NM23 nucleoside diphosphate kinase 4 [Source:HGNC Symbol;Acc:HGNC:7852]	protein coding	-0.78	1.95	18.80	<0.0001	0.0001
ENSG00000135900	MRPL44	mitochondrial ribosomal protein L44 [Source:HGNC Symbol;Acc:HGNC:16650]	protein coding	0.78	4.75	25.13	<0.0001	<0.0001
ENSG00000160716	CHRNB2	cholinergic receptor nicotinic beta 2 subunit [Source:HGNC Symbol;Acc:HGNC:1962]	protein coding	0.78	0.53	14.06	0.0002	0.0011
ENSG00000124256	ZBP1	Z-DNA binding protein 1 [Source:HGNC Symbol;Acc:HGNC:16176]	protein coding	0.78	7.06	17.81	<0.0001	0.0002
ENSG00000103335	PIEZO1	piezo type mechanosensitive ion channel component 1 [Source:HGNC Symbol;Acc:HGNC:28993]	protein coding	-0.78	2.82	9.72	0.0018	0.0071
ENSG00000140577	CRTC3	CREB regulated transcription coactivator 3 [Source:HGNC Symbol;Acc:HGNC:26148]	protein coding	-0.77	2.77	19.07	<0.0001	0.0001
ENSG00000183018	SPNS2	sphingolipid transporter 2 [Source:HGNC Symbol;Acc:HGNC:26992]	protein coding	-0.77	2.87	24.43	<0.0001	<0.0001
ENSG00000111144	LTA4H	leukotriene A4 hydrolase [Source:HGNC Symbol;Acc:HGNC:6710]	protein coding	0.77	7.14	66.78	<0.0001	<0.0001

Table S16: Genes differentially expressed at Day 2 compared to pre-first vaccination (Study Arm 2 A/H7N9+AS03, D1, 29, Neutrophils). Genes are sorted by descending absolute log_2 fold change. Gene model summaries and annotations are based on Ensembl Version 99 (continued.)

Ensembl Gene ID	Ensembl Gene Name	Ensembl Gene Description	Gene Type	log_2 Fold Change (Day 2 vs. Pre-First Vaccination)	Average log_2 CPM	Likelihood Ratio Test Statistic	P-Value	FDR Adjusted P-Value
ENSG00000116977	LGALS8	galectin 8 [Source:HGNC Symbol;Acc:HGNC:6569]	protein coding	0.77	5.38	22.27	<0.0001	<0.0001
ENSG00000132600	PRMT7	protein arginine methyltransferase 7 [Source:HGNC Symbol;Acc:HGNC:25557]	protein coding	-0.77	1.30	17.17	<0.0001	0.0003
ENSG00000159459	UBR1	ubiquitin protein ligase E3 component n-recognin 1 [Source:HGNC Symbol;Acc:HGNC:16808]	protein coding	0.77	3.52	30.89	<0.0001	<0.0001
ENSG00000181031	RPH3AL	rabphilin 3A like (without C2 domains) [Source:HGNC Symbol;Acc:HGNC:10296]	protein coding	-0.77	1.68	19.42	<0.0001	0.0001
ENSG00000155393	HEATR3	HEAT repeat containing 3 [Source:HGNC Symbol;Acc:HGNC:26087]	protein coding	-0.77	1.43	20.44	<0.0001	<0.0001
ENSG00000231105	ECE1-AS1	ECE1 antisense RNA 1 [Source:HGNC Symbol;Acc:HGNC:40203]	lncRNA	0.77	0.78	21.06	<0.0001	<0.0001
ENSG00000110852	CLEC2B	C-type lectin domain family 2 member B [Source:HGNC Symbol;Acc:HGNC:2053]	protein coding	0.77	7.75	41.32	<0.0001	<0.0001
ENSG00000281106	TMEM272	transmembrane protein 272 [Source:HGNC Symbol;Acc:HGNC:26737]	protein coding	0.77	3.60	17.72	<0.0001	0.0002
ENSG00000185745	IFIT1	interferon induced protein with tetratricopeptide repeats 1 [Source:HGNC Symbol;Acc:HGNC:5407]	protein coding	0.77	8.60	5.90	0.0152	0.0401
ENSG00000121933	TMIGD3	transmembrane and immunoglobulin domain containing 3 [Source:HGNC Symbol;Acc:HGNC:51375]	protein coding	-0.77	0.40	8.24	0.0041	0.0137
ENSG00000111275	ALDH2	aldehyde dehydrogenase 2 family member [Source:HGNC Symbol;Acc:HGNC:404]	protein coding	0.77	3.55	37.71	<0.0001	<0.0001
ENSG00000100908	EMC9	ER membrane protein complex subunit 9 [Source:HGNC Symbol;Acc:HGNC:20273]	protein coding	0.77	2.64	21.72	<0.0001	<0.0001
ENSG00000007516	BAIAP3	BAI1 associated protein 3 [Source:HGNC Symbol;Acc:HGNC:948]	protein coding	-0.77	5.95	19.10	<0.0001	0.0001
ENSG00000185880	TRIM69	tripartite motif containing 69 [Source:HGNC Symbol;Acc:HGNC:17857]	protein coding	0.77	6.00	38.80	<0.0001	<0.0001
ENSG00000109906	ZBTB16	zinc finger and BTB domain containing 16 [Source:HGNC Symbol;Acc:HGNC:12930]	protein coding	-0.77	1.20	8.59	0.0034	0.0118
ENSG00000072954	TMEM38A	transmembrane protein 38A [Source:HGNC Symbol;Acc:HGNC:28462]	protein coding	-0.77	1.14	24.15	<0.0001	<0.0001
ENSG00000055332	EIF2AK2	eukaryotic translation initiation factor 2 alpha kinase 2 [Source:HGNC Symbol;Acc:HGNC:9437]	protein coding	0.77	6.86	11.53	0.0007	0.0032
ENSG00000213889	PPM1N	protein phosphatase, Mg2+/Mn2+ dependent 1N (putative) [Source:HGNC Symbol;Acc:HGNC:26845]	protein coding	0.77	0.70	14.32	0.0002	0.0009

Table S16: Genes differentially expressed at Day 2 compared to pre-first vaccination (Study Arm 2 A/H7N9+AS03, D1, 29, Neutrophils). Genes are sorted by descending absolute log_2 fold change. Gene model summaries and annotations are based on Ensembl Version 99 (continued.)

Ensembl Gene ID	Ensembl Gene Name	Ensembl Gene Description	Gene Type	log_2 Fold Change (Day 2 vs. Pre-First Vaccination)	Average log_2 CPM	Likelihood Ratio Test Statistic	P-Value	FDR Adjusted P-Value
ENSG00000104626	ERI1	exoribonuclease 1 [Source:HGNC Symbol;Acc:HGNC:23994]	protein coding	0.77	3.62	15.08	0.0001	0.0007
ENSG00000065613	SLK	STE20 like kinase [Source:HGNC Symbol;Acc:HGNC:11088]	protein coding	0.77	7.32	30.44	<0.0001	<0.0001
ENSG00000187260	WDR86	WD repeat domain 86 [Source:HGNC Symbol;Acc:HGNC:28020]	protein coding	0.77	0.56	12.11	0.0005	0.0025
ENSG00000077984	CST7	cystatin F [Source:HGNC Symbol;Acc:HGNC:2479]	protein coding	0.77	7.13	15.97	<0.0001	0.0005
ENSG00000231123	SPATA20P1	spermatogenesis associated 20 pseudogene 1 [Source:HGNC Symbol;Acc:HGNC:39636]	processed pseudogene	0.77	1.65	15.81	<0.0001	0.0005
ENSG00000112053	SLC26A8	solute carrier family 26 member 8 [Source:HGNC Symbol;Acc:HGNC:14468]	protein coding	0.76	4.79	12.98	0.0003	0.0017
ENSG00000164096	C4orf3	chromosome 4 open reading frame 3 [Source:HGNC Symbol;Acc:HGNC:19225]	protein coding	0.76	7.72	39.79	<0.0001	<0.0001
ENSG00000173083	HPSE	heparanase [Source:HGNC Symbol;Acc:HGNC:5164]	protein coding	0.76	5.58	28.64	<0.0001	<0.0001
ENSG00000164105	SAP30	Sin3A associated protein 30 [Source:HGNC Symbol;Acc:HGNC:10532]	protein coding	-0.76	1.77	13.19	0.0003	0.0016
ENSG00000124107	SLPI	secretory leukocyte peptidase inhibitor [Source:HGNC Symbol;Acc:HGNC:11092]	protein coding	0.76	5.72	20.77	<0.0001	<0.0001
ENSG00000196154	S100A4	S100 calcium binding protein A4 [Source:HGNC Symbol;Acc:HGNC:10494]	protein coding	-0.76	9.20	28.77	<0.0001	<0.0001
ENSG00000177383	MAGEF1	MAGE family member F1 [Source:HGNC Symbol;Acc:HGNC:29639]	protein coding	-0.76	0.34	13.11	0.0003	0.0016
ENSG00000100403	ZC3H7B	zinc finger CCCH-type containing 7B [Source:HGNC Symbol;Acc:HGNC:30869]	protein coding	0.76	1.04	7.30	0.0069	0.0211
ENSG00000101695	RNF125	ring finger protein 125 [Source:HGNC Symbol;Acc:HGNC:21150]	protein coding	-0.76	1.87	25.50	<0.0001	<0.0001
ENSG00000092010	PSME1	proteasome activator subunit 1 [Source:HGNC Symbol;Acc:HGNC:9568]	protein coding	0.76	8.43	40.09	<0.0001	<0.0001
ENSG00000259351		novel transcript, antisense to DAPK2	lncRNA	-0.76	0.70	16.13	<0.0001	0.0004
ENSG00000213928	IRF9	interferon regulatory factor 9 [Source:HGNC Symbol;Acc:HGNC:6131]	protein coding	0.76	5.50	23.53	<0.0001	<0.0001
ENSG00000070190	DAPP1	dual adaptor of phosphotyrosine and 3-phosphoinositides 1 [Source:HGNC Symbol;Acc:HGNC:16500]	protein coding	0.76	7.82	25.37	<0.0001	<0.0001

Table S16: Genes differentially expressed at Day 2 compared to pre-first vaccination (Study Arm 2 A/H7N9+AS03, D1, 29, Neutrophils). Genes are sorted by descending absolute log_2 fold change. Gene model summaries and annotations are based on Ensembl Version 99 (continued.)

Ensembl Gene ID	Ensembl Gene Name	Ensembl Gene Description	Gene Type	log_2 Fold Change (Day 2 vs. Pre-First Vaccination)	Average log_2 CPM	Likelihood Ratio Test Statistic	P-Value	FDR Adjusted P-Value
ENSG00000137752	CASP1	caspase 1 [Source:HGNC Symbol;Acc:HGNC:1499]	protein coding	0.76	8.25	32.72	<0.0001	<0.0001
ENSG00000025708	TYMP	thymidine phosphorylase [Source:HGNC Symbol;Acc:HGNC:3148]	protein coding	0.76	9.75	24.86	<0.0001	<0.0001
ENSG00000274767		novel transcript, antisense CCL3L3	lncRNA	-0.75	2.43	16.44	<0.0001	0.0004
ENSG00000110400	NECTIN1	nectin cell adhesion molecule 1 [Source:HGNC Symbol;Acc:HGNC:9706]	protein coding	-0.75	4.66	25.63	<0.0001	<0.0001
ENSG00000135093	USP30	ubiquitin specific peptidase 30 [Source:HGNC Symbol;Acc:HGNC:20065]	protein coding	0.75	2.51	52.67	<0.0001	<0.0001
ENSG00000182578	CSF1R	colony stimulating factor 1 receptor [Source:HGNC Symbol;Acc:HGNC:2433]	protein coding	-0.75	5.90	15.34	<0.0001	0.0006
ENSG00000182979	MTA1	metastasis associated 1 [Source:HGNC Symbol;Acc:HGNC:7410]	protein coding	-0.75	2.66	34.64	<0.0001	<0.0001
ENSG00000178467	P4HTM	prolyl 4-hydroxylase, transmembrane [Source:HGNC Symbol;Acc:HGNC:28858]	protein coding	-0.75	1.94	24.18	<0.0001	<0.0001
ENSG00000115419	GLS	glutaminase [Source:HGNC Symbol;Acc:HGNC:4331]	protein coding	0.75	5.25	25.22	<0.0001	<0.0001
ENSG00000197555	SIPA1L1	signal induced proliferation associated 1 like 1 [Source:HGNC Symbol;Acc:HGNC:20284]	protein coding	0.75	7.36	41.68	<0.0001	<0.0001
ENSG00000135299	ANKRD6	ankyrin repeat domain 6 [Source:HGNC Symbol;Acc:HGNC:17280]	protein coding	-0.75	3.04	30.70	<0.0001	<0.0001
ENSG00000274021		novel transcript, antisense to DUSP6	lncRNA	0.75	2.94	12.14	0.0005	0.0025
ENSG00000285938		BCL6 antisense 1	protein coding	0.75	5.64	24.22	<0.0001	<0.0001
ENSG00000164674	SYTL3	synaptotagmin like 3 [Source:HGNC Symbol;Acc:HGNC:15587]	protein coding	-0.75	5.85	41.87	<0.0001	<0.0001
ENSG00000271913		novel transcript, antisense to TAGAP	lncRNA	0.75	4.59	15.39	<0.0001	0.0006
ENSG00000177732	SOX12	SRY-box transcription factor 12 [Source:HGNC Symbol;Acc:HGNC:11198]	protein coding	-0.75	1.83	17.04	<0.0001	0.0003
ENSG00000267288		novel transcript, antisense to HEXIM2	lncRNA	-0.75	1.19	18.73	<0.0001	0.0001
ENSG00000124177	CHD6	chromodomain helicase DNA binding protein 6 [Source:HGNC Symbol;Acc:HGNC:19057]	protein coding	-0.75	1.37	12.76	0.0004	0.0019
ENSG00000148337	CIZ1	CDKN1A interacting zinc finger protein 1 [Source:HGNC Symbol;Acc:HGNC:16744]	protein coding	-0.75	3.30	20.77	<0.0001	<0.0001
ENSG00000120992	LYPLA1	lysophospholipase 1 [Source:HGNC Symbol;Acc:HGNC:6737]	protein coding	0.75	5.29	63.39	<0.0001	<0.0001

Table S16: Genes differentially expressed at Day 2 compared to pre-first vaccination (Study Arm 2 A/H7N9+AS03, D1, 29, Neutrophils). Genes are sorted by descending absolute log_2 fold change. Gene model summaries and annotations are based on Ensembl Version 99 (continued.)

Ensembl Gene ID	Ensembl Gene Name	Ensembl Gene Description	Gene Type	log_2 Fold Change (Day 2 vs. Pre-First Vaccination)	Average log_2 CPM	Likelihood Ratio Test Statistic	P-Value	FDR Adjusted P-Value
ENSG00000274265		novel transcript	lncRNA	0.75	2.40	28.16	<0.0001	<0.0001
ENSG00000008283	CYB561	cytochrome b561 [Source:HGNC Symbol;Acc:HGNC:2571]	protein coding	0.75	4.56	26.29	<0.0001	<0.0001
ENSG00000179115	FARSA	phenylalanyl-tRNA synthetase subunit alpha [Source:HGNC Symbol;Acc:HGNC:3592]	protein coding	-0.75	0.42	10.09	0.0015	0.0061
ENSG00000169504	CLIC4	chloride intracellular channel 4 [Source:HGNC Symbol;Acc:HGNC:13518]	protein coding	0.75	2.93	14.27	0.0002	0.001
ENSG00000099999	RNF215	ring finger protein 215 [Source:HGNC Symbol;Acc:HGNC:33434]	protein coding	-0.75	1.02	19.40	<0.0001	0.0001
ENSG00000136213	CHST12	carbohydrate sulfotransferase 12 [Source:HGNC Symbol;Acc:HGNC:17423]	protein coding	-0.74	0.97	8.19	0.0042	0.0141
ENSG00000196116	TDRD7	tudor domain containing 7 [Source:HGNC Symbol;Acc:HGNC:30831]	protein coding	0.74	5.50	20.61	<0.0001	<0.0001
ENSG00000141568	FOXK2	forkhead box K2 [Source:HGNC Symbol;Acc:HGNC:6036]	protein coding	-0.74	3.74	43.68	<0.0001	<0.0001
ENSG00000108679	LGALS3BP	galectin 3 binding protein [Source:HGNC Symbol;Acc:HGNC:6564]	protein coding	0.74	2.88	18.45	<0.0001	0.0002
ENSG00000254554		novel transcript	lncRNA	0.74	0.82	23.98	<0.0001	<0.0001
ENSG00000125148	MT2A	metallothionein 2A [Source:HGNC Symbol;Acc:HGNC:7406]	protein coding	0.74	4.09	7.52	0.0061	0.0191
ENSG00000272221		novel transcript	lncRNA	0.74	2.27	13.90	0.0002	0.0011
ENSG00000144848	ATG3	autophagy related 3 [Source:HGNC Symbol;Acc:HGNC:20962]	protein coding	0.74	7.44	36.61	<0.0001	<0.0001
ENSG00000070501	POLB	DNA polymerase beta [Source:HGNC Symbol;Acc:HGNC:9174]	protein coding	0.74	5.90	37.25	<0.0001	<0.0001
ENSG00000122068	FYTTD1	forty-two-three domain containing 1 [Source:HGNC Symbol;Acc:HGNC:25407]	protein coding	0.74	3.78	24.92	<0.0001	<0.0001
ENSG00000233214		novel transcript	lncRNA	0.74	1.68	20.83	<0.0001	<0.0001
ENSG00000161277	THAP8	THAP domain containing 8 [Source:HGNC Symbol;Acc:HGNC:23191]	protein coding	-0.74	1.19	27.95	<0.0001	<0.0001
ENSG00000279108		TEC	TEC	-0.74	0.30	18.65	<0.0001	0.0001
ENSG00000148926	ADM	adrenomedullin [Source:HGNC Symbol;Acc:HGNC:259]	protein coding	0.74	7.86	27.36	<0.0001	<0.0001
ENSG00000108861	DUSP3	dual specificity phosphatase 3 [Source:HGNC Symbol;Acc:HGNC:3069]	protein coding	0.74	4.14	30.73	<0.0001	<0.0001

Table S16: Genes differentially expressed at Day 2 compared to pre-first vaccination (Study Arm 2 A/H7N9+AS03, D1, 29, Neutrophils). Genes are sorted by descending absolute log_2 fold change. Gene model summaries and annotations are based on Ensembl Version 99 (continued.)

Ensembl Gene ID	Ensembl Gene Name	Ensembl Gene Description	Gene Type	log_2 Fold Change (Day 2 vs. Pre-First Vaccination)	Average log_2 CPM	Likelihood Ratio Test Statistic	P-Value	FDR Adjusted P-Value
ENSG00000177830	CHID1	chitinase domain containing 1 [Source:HGNC Symbol;Acc:HGNC:28474]	protein coding	-0.74	0.34	14.34	0.0002	0.0009
ENSG00000165238	WNK2	WNK lysine deficient protein kinase 2 [Source:HGNC Symbol;Acc:HGNC:14542]	protein coding	-0.74	1.07	15.54	<0.0001	0.0005
ENSG00000202533			misc RNA	0.73	0.29	9.36	0.0022	0.0084
ENSG00000095383	TBC1D2	TBC1 domain family member 2 [Source:HGNC Symbol;Acc:HGNC:18026]	protein coding	-0.73	4.69	23.76	<0.0001	<0.0001
ENSG00000177119	ANO6	anoctamin 6 [Source:HGNC Symbol;Acc:HGNC:25240]	protein coding	0.73	1.24	17.70	<0.0001	0.0002
ENSG00000196781	TLE1	TLE family member 1, transcriptional corepressor [Source:HGNC Symbol;Acc:HGNC:11837]	protein coding	-0.73	0.98	11.04	0.0009	0.004
ENSG00000015532	XYLT2	xylosyltransferase 2 [Source:HGNC Symbol;Acc:HGNC:15517]	protein coding	-0.73	2.66	26.48	<0.0001	<0.0001
ENSG00000178607	ERN1	endoplasmic reticulum to nucleus signaling 1 [Source:HGNC Symbol;Acc:HGNC:3449]	protein coding	-0.73	6.80	37.26	<0.0001	<0.0001
ENSG00000088179	PTPN4	protein tyrosine phosphatase non-receptor type 4 [Source:HGNC Symbol;Acc:HGNC:9656]	protein coding	-0.73	1.89	26.77	<0.0001	<0.0001
ENSG00000169136	ATF5	activating transcription factor 5 [Source:HGNC Symbol;Acc:HGNC:790]	protein coding	0.73	1.46	17.60	<0.0001	0.0002
ENSG00000135114	OASL	2'-5'-oligoadenylate synthetase like [Source:HGNC Symbol;Acc:HGNC:8090]	protein coding	0.73	6.94	9.51	0.002	0.0078
ENSG00000055483		ubiquitin specific peptidase 36 [Source:NCBI gene;Acc:57602]	protein coding	-0.73	1.13	17.34	<0.0001	0.0002
ENSG00000162430	SELENON	selenoprotein N [Source:HGNC Symbol;Acc:HGNC:15999]	protein coding	-0.73	2.85	20.07	<0.0001	<0.0001
ENSG00000148341	SH3GLB2	SH3 domain containing GRB2 like, endophilin B2 [Source:HGNC Symbol;Acc:HGNC:10834]	protein coding	-0.73	4.67	45.50	<0.0001	<0.0001
ENSG00000286256		novel transcript, antisense to ACSL1	lncRNA	0.73	2.78	16.75	<0.0001	0.0003
ENSG00000119522	DENND1A	DENN domain containing 1A [Source:HGNC Symbol;Acc:HGNC:29324]	protein coding	0.73	5.44	30.03	<0.0001	<0.0001
ENSG00000157216	SSBP3	single stranded DNA binding protein 3 [Source:HGNC Symbol;Acc:HGNC:15674]	protein coding	-0.73	3.53	16.74	<0.0001	0.0003
ENSG00000168264	IRF2BP2	interferon regulatory factor 2 binding protein 2 [Source:HGNC Symbol;Acc:HGNC:21729]	protein coding	-0.73	5.42	19.67	<0.0001	<0.0001
ENSG00000104320	NBN	nibrin [Source:HGNC Symbol;Acc:HGNC:7652]	protein coding	0.73	7.02	36.67	<0.0001	<0.0001

Table S16: Genes differentially expressed at Day 2 compared to pre-first vaccination (Study Arm 2 A/H7N9+AS03, D1, 29, Neutrophils). Genes are sorted by descending absolute log_2 fold change. Gene model summaries and annotations are based on Ensembl Version 99 (continued.)

Ensembl Gene ID	Ensembl Gene Name	Ensembl Gene Description	Gene Type	log_2 Fold Change (Day 2 vs. Pre-First Vaccination)	Average log_2 CPM	Likelihood Ratio Test Statistic	P-Value	FDR Adjusted P-Value
ENSG00000119950	MXI1	MAX interactor 1, dimerization protein [Source:HGNC Symbol;Acc:HGNC:7534]	protein coding	-0.73	5.50	35.47	<0.0001	<0.0001
ENSG00000125538	IL1B	interleukin 1 beta [Source:HGNC Symbol;Acc:HGNC:5992]	protein coding	0.73	7.20	8.42	0.0037	0.0127
ENSG00000057657	PRDM1	PR/SET domain 1 [Source:HGNC Symbol;Acc:HGNC:9346]	protein coding	0.73	6.21	11.98	0.0005	0.0026
ENSG00000152818	UTRN	utrophin [Source:HGNC Symbol;Acc:HGNC:12635]	protein coding	0.73	6.15	34.93	<0.0001	<0.0001
ENSG00000126001	CEP250	centrosomal protein 250 [Source:HGNC Symbol;Acc:HGNC:1859]	protein coding	-0.73	2.70	27.48	<0.0001	<0.0001
ENSG00000133895	MEN1	menin 1 [Source:HGNC Symbol;Acc:HGNC:7010]	protein coding	-0.72	0.69	11.22	0.0008	0.0037
ENSG00000168961	LGALS9	galectin 9 [Source:HGNC Symbol;Acc:HGNC:6570]	protein coding	0.72	7.06	23.41	<0.0001	<0.0001
ENSG00000278828	H3C10	H3 clustered histone 10 [Source:HGNC Symbol;Acc:HGNC:4775]	protein coding	0.72	0.86	12.15	0.0005	0.0024
ENSG00000188107	EYS	eyes shut homolog [Source:HGNC Symbol;Acc:HGNC:21555]	protein coding	-0.72	1.30	21.94	<0.0001	<0.0001
ENSG00000084207	GSTP1	glutathione S-transferase pi 1 [Source:HGNC Symbol;Acc:HGNC:4638]	protein coding	-0.72	2.00	15.87	<0.0001	0.0005
ENSG00000136478	TEX2	testis expressed 2 [Source:HGNC Symbol;Acc:HGNC:30884]	protein coding	-0.72	1.62	19.33	<0.0001	0.0001
ENSG00000197043	ANXA6	annexin A6 [Source:HGNC Symbol;Acc:HGNC:544]	protein coding	-0.72	3.43	16.91	<0.0001	0.0003
ENSG00000179832	MROH1	maestro heat like repeat family member 1 [Source:HGNC Symbol;Acc:HGNC:26958]	protein coding	-0.72	3.72	47.53	<0.0001	<0.0001
ENSG00000121858	TNFSF10	TNF superfamily member 10 [Source:HGNC Symbol;Acc:HGNC:11925]	protein coding	0.72	8.93	16.47	<0.0001	0.0004
ENSG00000196150	ZNF250	zinc finger protein 250 [Source:HGNC Symbol;Acc:HGNC:13044]	protein coding	0.72	0.72	11.29	0.0008	0.0036
ENSG00000281100		TEC	TEC	0.72	1.50	17.99	<0.0001	0.0002
ENSG00000185024	BRF1	BRF1 RNA polymerase III transcription initiation factor subunit [Source:HGNC Symbol;Acc:HGNC:11551]	protein coding	-0.72	1.80	17.29	<0.0001	0.0003
ENSG00000064201	TSPAN32	tetraspanin 32 [Source:HGNC Symbol;Acc:HGNC:13410]	protein coding	-0.72	4.67	39.87	<0.0001	<0.0001

Table S16: Genes differentially expressed at Day 2 compared to pre-first vaccination (Study Arm 2 A/H7N9+AS03, D1, 29, Neutrophils). Genes are sorted by descending absolute log_2 fold change. Gene model summaries and annotations are based on Ensembl Version 99 (continued.)

Ensembl Gene ID	Ensembl Gene Name	Ensembl Gene Description	Gene Type	log_2 Fold Change (Day 2 vs. Pre-First Vaccination)	Average log_2 CPM	Likelihood Ratio Test Statistic	P-Value	FDR Adjusted P-Value
ENSG00000123700	KCNJ2	potassium inwardly rectifying channel subfamily J member 2 [Source:HGNC Symbol;Acc:HGNC:6263]	protein coding	0.72	7.30	16.30	<0.0001	0.0004
ENSG00000155093	PTPRN2	protein tyrosine phosphatase receptor type N2 [Source:HGNC Symbol;Acc:HGNC:9677]	protein coding	-0.72	5.42	28.61	<0.0001	<0.0001
ENSG00000092067	CEBPE	CCAAT enhancer binding protein epsilon [Source:HGNC Symbol;Acc:HGNC:1836]	protein coding	-0.72	3.27	5.47	0.0194	0.0489
ENSG00000133835	HSD17B4	hydroxysteroid 17-beta dehydrogenase 4 [Source:HGNC Symbol;Acc:HGNC:5213]	protein coding	-0.72	4.24	37.86	<0.0001	<0.0001
ENSG00000125826	RBCK1	RANBP2-type and C3HC4-type zinc finger containing 1 [Source:HGNC Symbol;Acc:HGNC:15864]	protein coding	0.72	8.30	29.85	<0.0001	<0.0001
ENSG00000136147	PHF11	PHD finger protein 11 [Source:HGNC Symbol;Acc:HGNC:17024]	protein coding	0.72	6.18	19.14	<0.0001	0.0001
ENSG00000167613	LAIR1	leukocyte associated immunoglobulin like receptor 1 [Source:HGNC Symbol;Acc:HGNC:6477]	protein coding	-0.72	2.53	7.96	0.0048	0.0156
ENSG00000187796	CARD9	caspase recruitment domain family member 9 [Source:HGNC Symbol;Acc:HGNC:16391]	protein coding	-0.71	2.64	15.52	<0.0001	0.0006
ENSG00000103121	CMC2	C-X9-C motif containing 2 [Source:HGNC Symbol;Acc:HGNC:24447]	protein coding	0.71	0.95	16.20	<0.0001	0.0004
ENSG00000168404	MLKL	mixed lineage kinase domain like pseudokinase [Source:HGNC Symbol;Acc:HGNC:26617]	protein coding	0.71	7.09	18.92	<0.0001	0.0001
ENSG00000153029	MR1	major histocompatibility complex, class I-related [Source:HGNC Symbol;Acc:HGNC:4975]	protein coding	0.71	4.95	32.77	<0.0001	<0.0001
ENSG00000219481	NBPF1	NBPF member 1 [Source:HGNC Symbol;Acc:HGNC:26088]	protein coding	-0.71	1.01	11.17	0.0008	0.0037
ENSG00000266604	LINC01887	long intergenic non-protein coding RNA 1887 [Source:HGNC Symbol;Acc:HGNC:52706]	lncRNA	-0.71	0.40	16.81	<0.0001	0.0003
ENSG00000235944	ZNF815P	zinc finger protein 815, pseudogene [Source:HGNC Symbol;Acc:HGNC:22029]	transcribed unprocessed pseudogene	-0.71	0.84	15.10	0.0001	0.0007
ENSG00000249863		synaptotagmin binding, cytoplasmic RNA interacting protein (SYNCRIP) pseudogene	processed pseudogene	-0.71	1.48	20.01	<0.0001	<0.0001
ENSG00000162636	FAM102B	family with sequence similarity 102 member B [Source:HGNC Symbol;Acc:HGNC:27637]	protein coding	-0.71	2.14	22.45	<0.0001	<0.0001

Table S16: Genes differentially expressed at Day 2 compared to pre-first vaccination (Study Arm 2 A/H7N9+AS03, D1, 29, Neutrophils). Genes are sorted by descending absolute log_2 fold change. Gene model summaries and annotations are based on Ensembl Version 99 (continued.)

Ensembl Gene ID	Ensembl Gene Name	Ensembl Gene Description	Gene Type	log_2 Fold Change (Day 2 vs. Pre-First Vaccination)	Average log_2 CPM	Likelihood Ratio Test Statistic	P-Value	FDR Adjusted P-Value
ENSG00000178695	KCTD12	potassium channel tetramerization domain containing 12 [Source:HGNC Symbol;Acc:HGNC:14678]	protein coding	-0.71	6.18	24.92	<0.0001	<0.0001
ENSG00000166598	HSP90B1	heat shock protein 90 beta family member 1 [Source:HGNC Symbol;Acc:HGNC:12028]	protein coding	0.71	5.50	24.54	<0.0001	<0.0001
ENSG00000149308	NPAT	nuclear protein, coactivator of histone transcription [Source:HGNC Symbol;Acc:HGNC:7896]	protein coding	-0.71	2.91	25.44	<0.0001	<0.0001
ENSG00000103423	DNAJA3	DnaJ heat shock protein family (Hsp40) member A3 [Source:HGNC Symbol;Acc:HGNC:11808]	protein coding	-0.71	1.12	21.88	<0.0001	<0.0001
ENSG00000163635	ATXN7	ataxin 7 [Source:HGNC Symbol;Acc:HGNC:10560]	protein coding	0.71	2.31	16.77	<0.0001	0.0003
ENSG00000119655	NPC2	NPC intracellular cholesterol transporter 2 [Source:HGNC Symbol;Acc:HGNC:14537]	protein coding	0.71	6.69	31.25	<0.0001	<0.0001
ENSG00000228804		novel transcript, antisense to RTP2 and BCL6	lncRNA	0.71	0.77	11.54	0.0007	0.0032
ENSG00000205517	RGL3	ral guanine nucleotide dissociation stimulator like 3 [Source:HGNC Symbol;Acc:HGNC:30282]	protein coding	-0.71	2.54	25.84	<0.0001	<0.0001
ENSG00000092758	COL9A3	collagen type IX alpha 3 chain [Source:HGNC Symbol;Acc:HGNC:2219]	protein coding	-0.71	4.69	21.29	<0.0001	<0.0001
ENSG00000104093	DMXL2	Dmx like 2 [Source:HGNC Symbol;Acc:HGNC:2938]	protein coding	0.71	6.96	37.06	<0.0001	<0.0001
ENSG00000145882	PCYOX1L	prenylcysteine oxidase 1 like [Source:HGNC Symbol;Acc:HGNC:28477]	protein coding	-0.71	0.65	11.23	0.0008	0.0036
ENSG00000226137	BAIAP2-DT	BAIAP2 divergent transcript [Source:HGNC Symbol;Acc:HGNC:44342]	lncRNA	-0.71	3.98	21.58	<0.0001	<0.0001
ENSG00000122547	EEPD1	endonuclease/exonuclease/phosphatase family domain containing 1 [Source:HGNC Symbol;Acc:HGNC:22223]	protein coding	-0.71	6.66	36.21	<0.0001	<0.0001
ENSG00000163517	HDAC11	histone deacetylase 11 [Source:HGNC Symbol;Acc:HGNC:19086]	protein coding	-0.70	0.39	14.10	0.0002	0.001
ENSG00000073417	PDE8A	phosphodiesterase 8A [Source:HGNC Symbol;Acc:HGNC:8793]	protein coding	-0.70	1.21	24.02	<0.0001	<0.0001
ENSG00000189343	RPS2P46	ribosomal protein S2 pseudogene 46 [Source:HGNC Symbol;Acc:HGNC:35839]	processed pseudogene	0.70	2.46	29.48	<0.0001	<0.0001
ENSG00000078070	MCCC1	methylcrotonoyl-CoA carboxylase 1 [Source:HGNC Symbol;Acc:HGNC:6936]	protein coding	-0.70	0.94	16.69	<0.0001	0.0003
ENSG00000125730	C3	complement C3 [Source:HGNC Symbol;Acc:HGNC:1318]	protein coding	0.70	1.33	25.50	<0.0001	<0.0001

Confidential/Proprietary Information

Table S16: Genes differentially expressed at Day 2 compared to pre-first vaccination (Study Arm 2 A/H7N9+AS03, D1, 29, Neutrophils). Genes are sorted by descending absolute log_2 fold change. Gene model summaries and annotations are based on Ensembl Version 99 (continued.)

Ensembl Gene ID	Ensembl Gene Name	Ensembl Gene Description	Gene Type	log_2 Fold Change (Day 2 vs. Pre-First Vaccination)	Average log_2 CPM	Likelihood Ratio Test Statistic	P-Value	FDR Adjusted P-Value
ENSG00000081760	AACS	acetoacetyl-CoA synthetase [Source:HGNC Symbol;Acc:HGNC:21298]	protein coding	-0.70	1.46	19.06	<0.0001	0.0001
ENSG00000172183	ISG20	interferon stimulated exonuclease gene 20 [Source:HGNC Symbol;Acc:HGNC:6130]	protein coding	0.70	8.41	17.34	<0.0001	0.0002
ENSG00000175073	VCPIP1	valosin containing protein interacting protein 1 [Source:HGNC Symbol;Acc:HGNC:30897]	protein coding	0.70	7.26	30.27	<0.0001	<0.0001
ENSG00000111886	GABRR2	gamma-aminobutyric acid type A receptor rho2 subunit [Source:HGNC Symbol;Acc:HGNC:4091]	protein coding	0.70	3.43	16.21	<0.0001	0.0004
ENSG00000114737	CISH	cytokine inducible SH2 containing protein [Source:HGNC Symbol;Acc:HGNC:1984]	protein coding	0.70	4.11	15.08	0.0001	0.0007
ENSG00000124875	CXCL6	C-X-C motif chemokine ligand 6 [Source:HGNC Symbol;Acc:HGNC:10643]	protein coding	-0.70	2.99	27.55	<0.0001	<0.0001
ENSG00000149311	ATM	ATM serine/threonine kinase [Source:HGNC Symbol;Acc:HGNC:795]	protein coding	-0.70	6.00	31.48	<0.0001	<0.0001
ENSG00000134802	SLC43A3	solute carrier family 43 member 3 [Source:HGNC Symbol;Acc:HGNC:17466]	protein coding	0.70	0.88	15.49	<0.0001	0.0006
ENSG00000101190	TCFL5	transcription factor like 5 [Source:HGNC Symbol;Acc:HGNC:11646]	protein coding	-0.70	3.58	24.87	<0.0001	<0.0001
ENSG00000157778	PSMG3	proteasome assembly chaperone 3 [Source:HGNC Symbol;Acc:HGNC:22420]	protein coding	-0.70	1.24	21.09	<0.0001	<0.0001
ENSG00000226012		novel transcript	lncRNA	0.70	1.29	12.90	0.0003	0.0018
ENSG00000123933	MXD4	MAX dimerization protein 4 [Source:HGNC Symbol;Acc:HGNC:13906]	protein coding	-0.70	5.13	23.43	<0.0001	<0.0001
ENSG00000205220	PSMB10	proteasome 20S subunit beta 10 [Source:HGNC Symbol;Acc:HGNC:9538]	protein coding	0.70	6.65	32.30	<0.0001	<0.0001
ENSG00000121104	FAM117A	family with sequence similarity 117 member A [Source:HGNC Symbol;Acc:HGNC:24179]	protein coding	0.70	5.62	37.72	<0.0001	<0.0001
ENSG00000197969	VPS13A	vacuolar protein sorting 13 homolog A [Source:HGNC Symbol;Acc:HGNC:1908]	protein coding	-0.69	1.73	12.98	0.0003	0.0017
ENSG00000095321	CRAT	carnitine O-acetyltransferase [Source:HGNC Symbol;Acc:HGNC:2342]	protein coding	-0.69	0.96	16.94	<0.0001	0.0003
ENSG00000184898	RBM43	RNA binding motif protein 43 [Source:HGNC Symbol;Acc:HGNC:24790]	protein coding	0.69	2.42	11.72	0.0006	0.0029
ENSG00000258227	CLEC5A	C-type lectin domain containing 5A [Source:HGNC Symbol;Acc:HGNC:2054]	protein coding	0.69	1.52	11.96	0.0005	0.0026

Table S16: Genes differentially expressed at Day 2 compared to pre-first vaccination (Study Arm 2 A/H7N9+AS03, D1, 29, Neutrophils). Genes are sorted by descending absolute log_2 fold change. Gene model summaries and annotations are based on Ensembl Version 99 (continued.)

Ensembl Gene ID	Ensembl Gene Name	Ensembl Gene Description	Gene Type	log_2 Fold Change (Day 2 vs. Pre-First Vaccination)	Average log_2 CPM	Likelihood Ratio Test Statistic	P-Value	FDR Adjusted P-Value
ENSG00000100997	ABHD12	abhydrolase domain containing 12 [Source:HGNC Symbol;Acc:HGNC:15868]	protein coding	-0.69	1.53	17.90	<0.0001	0.0002
ENSG00000096996	IL12RB1	interleukin 12 receptor subunit beta 1 [Source:HGNC Symbol;Acc:HGNC:5971]	protein coding	0.69	4.86	33.76	<0.0001	<0.0001
ENSG00000266970		novel transcript	lncRNA	0.69	0.37	12.22	0.0005	0.0024
ENSG00000170275	CRTAP	cartilage associated protein [Source:HGNC Symbol;Acc:HGNC:2379]	protein coding	-0.69	4.06	46.71	<0.0001	<0.0001
ENSG00000248936		putatitive novel transcript	lncRNA	-0.69	0.68	28.51	<0.0001	<0.0001
ENSG00000166896	ATP23	ATP23 metallopeptidase and ATP synthase assembly factor homolog [Source:HGNC Symbol;Acc:HGNC:29452]	protein coding	-0.69	1.18	15.34	<0.0001	0.0006
ENSG00000104518	GSDMD	gasdermin D [Source:HGNC Symbol;Acc:HGNC:25697]	protein coding	0.69	7.65	29.53	<0.0001	<0.0001
ENSG00000100024	UPB1	beta-ureidopropionase 1 [Source:HGNC Symbol;Acc:HGNC:16297]	protein coding	-0.69	2.90	14.23	0.0002	0.001
ENSG00000070718	AP3M2	adaptor related protein complex 3 subunit mu 2 [Source:HGNC Symbol;Acc:HGNC:570]	protein coding	-0.69	0.60	21.22	<0.0001	<0.0001
ENSG00000155287	SLC25A28	solute carrier family 25 member 28 [Source:HGNC Symbol;Acc:HGNC:23472]	protein coding	0.69	5.16	18.21	<0.0001	0.0002
ENSG00000075151	EIF4G3	eukaryotic translation initiation factor 4 gamma 3 [Source:HGNC Symbol;Acc:HGNC:3298]	protein coding	0.69	5.91	18.27	<0.0001	0.0002
ENSG00000173598	NUDT4	nudix hydrolase 4 [Source:HGNC Symbol;Acc:HGNC:8051]	protein coding	-0.69	3.68	26.69	<0.0001	<0.0001
ENSG00000250548	LINC01303	long intergenic non-protein coding RNA 1303 [Source:HGNC Symbol;Acc:HGNC:50470]	lncRNA	0.69	1.00	22.05	<0.0001	<0.0001
ENSG00000241106	HLA-DOB	major histocompatibility complex, class II, DO beta [Source:HGNC Symbol;Acc:HGNC:4937]	protein coding	0.69	1.90	6.65	0.0099	0.0283
ENSG00000146376	ARHGAP18	Rho GTPase activating protein 18 [Source:HGNC Symbol;Acc:HGNC:21035]	protein coding	-0.69	2.49	20.76	<0.0001	<0.0001
ENSG00000261644		novel transcript, antisense to CYLD and NOD2	lncRNA	0.69	3.42	37.63	<0.0001	<0.0001
ENSG00000124215	CDH26	cadherin 26 [Source:HGNC Symbol;Acc:HGNC:15902]	protein coding	-0.69	2.04	19.36	<0.0001	0.0001
ENSG00000178623	GPR35	G protein-coupled receptor 35 [Source:HGNC Symbol;Acc:HGNC:4492]	protein coding	-0.68	0.46	10.05	0.0015	0.0061
ENSG00000115956	PLEK	pleckstrin [Source:HGNC Symbol;Acc:HGNC:9070]	protein coding	0.68	9.72	32.69	<0.0001	<0.0001

Table S16: Genes differentially expressed at Day 2 compared to pre-first vaccination (Study Arm 2 A/H7N9+AS03, D1, 29, Neutrophils). Genes are sorted by descending absolute log_2 fold change. Gene model summaries and annotations are based on Ensembl Version 99 (continued.)

Ensembl Gene ID	Ensembl Gene Name	Ensembl Gene Description	Gene Type	log_2 Fold Change (Day 2 vs. Pre-First Vaccination)	Average log_2 CPM	Likelihood Ratio Test Statistic	P-Value	FDR Adjusted P-Value
ENSG00000124380	SNRNP27	small nuclear ribonucleoprotein U4/U6.U5 subunit 27 [Source:HGNC Symbol;Acc:HGNC:30240]	protein coding	0.68	3.12	23.71	<0.0001	<0.0001
ENSG00000162825	NBPF20	NBPF member 20 [Source:HGNC Symbol;Acc:HGNC:32000]	protein coding	0.68	0.49	15.95	<0.0001	0.0005
ENSG00000242616	GNG10	G protein subunit gamma 10 [Source:HGNC Symbol;Acc:HGNC:4402]	protein coding	0.68	5.88	39.29	<0.0001	<0.0001
ENSG00000265148	TSPOAP1-AS1	TSPOAP1, SUPT4H1 and RNF43 antisense RNA 1 [Source:HGNC Symbol;Acc:HGNC:44148]	lncRNA	0.68	1.75	25.22	<0.0001	<0.0001
ENSG00000107798	LIPA	lipase A, lysosomal acid type [Source:HGNC Symbol;Acc:HGNC:6617]	protein coding	0.68	2.19	10.65	0.0011	0.0047
ENSG00000196954	CASP4	caspase 4 [Source:HGNC Symbol;Acc:HGNC:1505]	protein coding	0.68	9.08	32.68	<0.0001	<0.0001
ENSG00000186469	GNG2	G protein subunit gamma 2 [Source:HGNC Symbol;Acc:HGNC:4404]	protein coding	-0.68	7.14	16.06	<0.0001	0.0004
ENSG00000181381	DDX60L	DExD/H-box 60 like [Source:HGNC Symbol;Acc:HGNC:26429]	protein coding	0.68	8.60	13.61	0.0002	0.0013
ENSG00000162444	RBP7	retinol binding protein 7 [Source:HGNC Symbol;Acc:HGNC:30316]	protein coding	-0.68	5.31	19.76	<0.0001	<0.0001
ENSG00000169660	HEXD	hexosaminidase D [Source:HGNC Symbol;Acc:HGNC:26307]	protein coding	0.68	5.50	47.17	<0.0001	<0.0001
ENSG00000204264	PSMB8	proteasome 20S subunit beta 8 [Source:HGNC Symbol;Acc:HGNC:9545]	protein coding	0.68	8.06	26.27	<0.0001	<0.0001
ENSG00000089486	CDIP1	cell death inducing p53 target 1 [Source:HGNC Symbol;Acc:HGNC:13234]	protein coding	-0.68	3.70	22.30	<0.0001	<0.0001
ENSG00000179918	SEPHS2	selenophosphate synthetase 2 [Source:HGNC Symbol;Acc:HGNC:19686]	protein coding	0.68	6.30	32.80	<0.0001	<0.0001
ENSG00000141577	CEP131	centrosomal protein 131 [Source:HGNC Symbol;Acc:HGNC:29511]	protein coding	-0.68	2.92	32.96	<0.0001	<0.0001
ENSG00000236345	SCAT8	S-phase cancer associated transcript 8 [Source:HGNC Symbol;Acc:HGNC:40967]	lncRNA	-0.68	3.73	26.40	<0.0001	<0.0001
ENSG00000137486	ARRB1	arrestin beta 1 [Source:HGNC Symbol;Acc:HGNC:711]	protein coding	-0.68	6.49	28.25	<0.0001	<0.0001
ENSG00000010704	HFE	homeostatic iron regulator [Source:HGNC Symbol;Acc:HGNC:4886]	protein coding	0.68	2.20	19.81	<0.0001	<0.0001
ENSG00000231616		novel transcript, antisense to KIF27	lncRNA	-0.68	0.98	12.06	0.0005	0.0025

Table S16: Genes differentially expressed at Day 2 compared to pre-first vaccination (Study Arm 2 A/H7N9+AS03, D1, 29, Neutrophils). Genes are sorted by descending absolute log_2 fold change. Gene model summaries and annotations are based on Ensembl Version 99 (continued.)

Ensembl Gene ID	Ensembl Gene Name	Ensembl Gene Description	Gene Type	log_2 Fold Change (Day 2 vs. Pre-First Vaccination)	Average log_2 CPM	Likelihood Ratio Test Statistic	P-Value	FDR Adjusted P-Value
ENSG00000264553	MIR4257	microRNA 4257 [Source:HGNC Symbol;Acc:HGNC:38312]	miRNA	0.68	0.75	12.18	0.0005	0.0024
ENSG00000143162	CREG1	cellular repressor of E1A stimulated genes 1 [Source:HGNC Symbol;Acc:HGNC:2351]	protein coding	0.68	5.68	16.59	<0.0001	0.0003
ENSG00000145687	SSBP2	single stranded DNA binding protein 2 [Source:HGNC Symbol;Acc:HGNC:15831]	protein coding	-0.68	4.35	56.08	<0.0001	<0.0001
ENSG00000272914		novel transcript, sense intronic to ZNF438	lncRNA	0.68	1.50	20.21	<0.0001	<0.0001
ENSG00000223496	EXOSC6	exosome component 6 [Source:HGNC Symbol;Acc:HGNC:19055]	protein coding	-0.68	1.19	11.36	0.0007	0.0034
ENSG00000274525		general transcription factor IIi (GTF2I) pseudogene	unprocessed pseudogene	0.68	1.40	12.62	0.0004	0.002
ENSG00000108557	RAI1	retinoic acid induced 1 [Source:HGNC Symbol;Acc:HGNC:9834]	protein coding	-0.68	1.31	9.87	0.0017	0.0067
ENSG00000166949	SMAD3	SMAD family member 3 [Source:HGNC Symbol;Acc:HGNC:6769]	protein coding	-0.67	1.93	7.53	0.0061	0.019
ENSG00000134955	SLC37A2	solute carrier family 37 member 2 [Source:HGNC Symbol;Acc:HGNC:20644]	protein coding	-0.67	1.93	11.20	0.0008	0.0037
ENSG00000154265	ABCA5	ATP binding cassette subfamily A member 5 [Source:HGNC Symbol;Acc:HGNC:35]	protein coding	-0.67	3.10	42.77	<0.0001	<0.0001
ENSG00000171307	ZDHHC16	zinc finger DHHC-type containing 16 [Source:HGNC Symbol;Acc:HGNC:20714]	protein coding	-0.67	0.57	20.10	<0.0001	<0.0001
ENSG00000205413	SAMD9	sterile alpha motif domain containing 9 [Source:HGNC Symbol;Acc:HGNC:1348]	protein coding	0.67	7.76	16.98	<0.0001	0.0003
ENSG00000143106	PSMA5	proteasome 20S subunit alpha 5 [Source:HGNC Symbol;Acc:HGNC:9534]	protein coding	0.67	4.29	34.92	<0.0001	<0.0001
ENSG00000086200	IPO11	importin 11 [Source:HGNC Symbol;Acc:HGNC:20628]	protein coding	0.67	1.19	10.91	0.001	0.0042
ENSG00000273669		novel transcript	lncRNA	-0.67	1.25	15.61	<0.0001	0.0005
ENSG00000197182	MIRLET7BHG	MIRLET7B host gene [Source:HGNC Symbol;Acc:HGNC:37189]	lncRNA	-0.67	3.50	26.44	<0.0001	<0.0001
ENSG00000138111	MFSD13A	major facilitator superfamily domain containing 13A [Source:HGNC Symbol;Acc:HGNC:26196]	protein coding	0.67	3.13	34.73	<0.0001	<0.0001
ENSG00000140988	RPS2	ribosomal protein S2 [Source:HGNC Symbol;Acc:HGNC:10404]	protein coding	0.67	6.52	27.76	<0.0001	<0.0001

Table S16: Genes differentially expressed at Day 2 compared to pre-first vaccination (Study Arm 2 A/H7N9+AS03, D1, 29, Neutrophils). Genes are sorted by descending absolute log_2 fold change. Gene model summaries and annotations are based on Ensembl Version 99 (continued.)

Ensembl Gene ID	Ensembl Gene Name	Ensembl Gene Description	Gene Type	log_2 Fold Change (Day 2 vs. Pre-First Vaccination)	Average log_2 CPM	Likelihood Ratio Test Statistic	P-Value	FDR Adjusted P-Value
ENSG00000179021	C3orf38	chromosome 3 open reading frame 38 [Source:HGNC Symbol;Acc:HGNC:28384]	protein coding	0.67	3.12	26.87	<0.0001	<0.0001
ENSG00000188483	IER5L	immediate early response 5 like [Source:HGNC Symbol;Acc:HGNC:23679]	protein coding	-0.67	3.93	8.58	0.0034	0.0119
ENSG00000159593	NAE1	NEDD8 activating enzyme E1 subunit 1 [Source:HGNC Symbol;Acc:HGNC:621]	protein coding	-0.67	1.17	19.14	<0.0001	0.0001
ENSG00000204520	MICA	MHC class I polypeptide-related sequence A [Source:HGNC Symbol;Acc:HGNC:7090]	protein coding	0.67	4.37	32.07	<0.0001	<0.0001
ENSG00000171467	ZNF318	zinc finger protein 318 [Source:HGNC Symbol;Acc:HGNC:13578]	protein coding	-0.67	3.39	25.26	<0.0001	<0.0001
ENSG00000158805	ZNF276	zinc finger protein 276 [Source:HGNC Symbol;Acc:HGNC:23330]	protein coding	0.67	6.34	31.19	<0.0001	<0.0001
ENSG00000162551	ALPL	alkaline phosphatase, biomineralization associated [Source:HGNC Symbol;Acc:HGNC:438]	protein coding	0.67	10.08	21.03	<0.0001	<0.0001
ENSG00000162174	ASRGL1	asparaginase and isoaspartyl peptidase 1 [Source:HGNC Symbol;Acc:HGNC:16448]	protein coding	-0.67	1.81	12.46	0.0004	0.0021
ENSG00000155506	LARP1	La ribonucleoprotein 1, translational regulator [Source:HGNC Symbol;Acc:HGNC:29531]	protein coding	0.67	4.58	24.66	<0.0001	<0.0001
ENSG00000198677	TTC37	tetratricopeptide repeat domain 37 [Source:HGNC Symbol;Acc:HGNC:23639]	protein coding	-0.67	0.41	9.90	0.0017	0.0066
ENSG00000225450		novel transcript	lncRNA	0.67	0.55	18.59	<0.0001	0.0001
ENSG00000183508	TENT5C	terminal nucleotidyltransferase 5C [Source:HGNC Symbol;Acc:HGNC:24712]	protein coding	0.67	2.95	26.58	<0.0001	<0.0001
ENSG00000141664	ZCCHC2	zinc finger CCHC-type containing 2 [Source:HGNC Symbol;Acc:HGNC:22916]	protein coding	0.67	7.51	13.44	0.0002	0.0014
ENSG00000196730	DAPK1	death associated protein kinase 1 [Source:HGNC Symbol;Acc:HGNC:2674]	protein coding	-0.67	5.66	34.74	<0.0001	<0.0001
ENSG00000069424	KCNAB2	potassium voltage-gated channel subfamily A regulatory beta subunit 2 [Source:HGNC Symbol;Acc:HGNC:6229]	protein coding	-0.67	7.42	37.44	<0.0001	<0.0001
ENSG00000114439	BBX	BBX high mobility group box domain containing [Source:HGNC Symbol;Acc:HGNC:14422]	protein coding	0.67	4.92	36.66	<0.0001	<0.0001
ENSG00000124570	SERPINB6	serpin family B member 6 [Source:HGNC Symbol;Acc:HGNC:8950]	protein coding	0.67	3.34	36.65	<0.0001	<0.0001
ENSG00000172728	FUT10	fucosyltransferase 10 [Source:HGNC Symbol;Acc:HGNC:19234]	protein coding	-0.67	0.58	7.48	0.0062	0.0195

Table S16: Genes differentially expressed at Day 2 compared to pre-first vaccination (Study Arm 2 A/H7N9+AS03, D1, 29, Neutrophils). Genes are sorted by descending absolute log_2 fold change. Gene model summaries and annotations are based on Ensembl Version 99 (continued.)

Ensembl Gene ID	Ensembl Gene Name	Ensembl Gene Description	Gene Type	log_2 Fold Change (Day 2 vs. Pre-First Vaccination)	Average log_2 CPM	Likelihood Ratio Test Statistic	P-Value	FDR Adjusted P-Value
ENSG00000163050	COQ8A	coenzyme Q8A [Source:HGNC Symbol;Acc:HGNC:16812]	protein coding	-0.66	6.62	27.36	<0.0001	<0.0001
ENSG00000279727	LINC02033	long intergenic non-protein coding RNA 2033 [Source:HGNC Symbol;Acc:HGNC:52867]	lncRNA	0.66	0.67	8.27	0.004	0.0136
ENSG00000262152	LINC00514	long intergenic non-protein coding RNA 514 [Source:HGNC Symbol;Acc:HGNC:27549]	protein coding	-0.66	1.37	28.45	<0.0001	<0.0001
ENSG00000111885	MAN1A1	mannosidase alpha class 1A member 1 [Source:HGNC Symbol;Acc:HGNC:6821]	protein coding	0.66	7.32	26.30	<0.0001	<0.0001
ENSG00000075651	PLD1	phospholipase D1 [Source:HGNC Symbol;Acc:HGNC:9067]	protein coding	-0.66	3.03	22.54	<0.0001	<0.0001
ENSG00000130511	SSBP4	single stranded DNA binding protein 4 [Source:HGNC Symbol;Acc:HGNC:15676]	protein coding	0.66	1.79	23.01	<0.0001	<0.0001
ENSG00000130589	HELZ2	helicase with zinc finger 2 [Source:HGNC Symbol;Acc:HGNC:30021]	protein coding	0.66	8.57	11.50	0.0007	0.0033
ENSG00000254413	CHKB-CPT1B	CHKB-CPT1B readthrough (NMD candidate) [Source:HGNC Symbol;Acc:HGNC:41998]	protein coding	0.66	2.14	20.04	<0.0001	<0.0001
ENSG00000274020	LINC01138	long intergenic non-protein coding RNA 1138 [Source:HGNC Symbol;Acc:HGNC:49454]	lncRNA	0.66	2.77	25.00	<0.0001	<0.0001
ENSG00000004487	KDM1A	lysine demethylase 1A [Source:HGNC Symbol;Acc:HGNC:29079]	protein coding	-0.66	0.63	9.60	0.0019	0.0075
ENSG00000168016	TRANK1	tetratricopeptide repeat and ankyrin repeat containing 1 [Source:HGNC Symbol;Acc:HGNC:29011]	protein coding	0.66	8.93	31.18	<0.0001	<0.0001
ENSG00000164877	MICALL2	MICAL like 2 [Source:HGNC Symbol;Acc:HGNC:29672]	protein coding	-0.66	1.24	25.96	<0.0001	<0.0001
ENSG00000159348	CYB5R1	cytochrome b5 reductase 1 [Source:HGNC Symbol;Acc:HGNC:13397]	protein coding	-0.66	4.54	27.90	<0.0001	<0.0001
ENSG00000145391	SETD7	SET domain containing 7, histone lysine methyltransferase [Source:HGNC Symbol;Acc:HGNC:30412]	protein coding	0.66	1.45	16.73	<0.0001	0.0003
ENSG00000111319	SCNN1A	sodium channel epithelial 1 alpha subunit [Source:HGNC Symbol;Acc:HGNC:10599]	protein coding	0.66	0.57	22.44	<0.0001	<0.0001
ENSG00000080546	SESN1	sestrin 1 [Source:HGNC Symbol;Acc:HGNC:21595]	protein coding	-0.66	2.38	27.75	<0.0001	<0.0001
ENSG00000188486	H2AX	H2A.X variant histone [Source:HGNC Symbol;Acc:HGNC:4739]	protein coding	-0.66	2.02	14.49	0.0001	0.0009
ENSG00000120306	CYSTM1	cysteine rich transmembrane module containing 1 [Source:HGNC Symbol;Acc:HGNC:30239]	protein coding	0.66	6.58	33.62	<0.0001	<0.0001

Table S16: Genes differentially expressed at Day 2 compared to pre-first vaccination (Study Arm 2 A/H7N9+AS03, D1, 29, Neutrophils). Genes are sorted by descending absolute log_2 fold change. Gene model summaries and annotations are based on Ensembl Version 99 (continued.)

Ensembl Gene ID	Ensembl Gene Name	Ensembl Gene Description	Gene Type	log_2 Fold Change (Day 2 vs. Pre-First Vaccination)	Average log_2 CPM	Likelihood Ratio Test Statistic	P-Value	FDR Adjusted P-Value
ENSG00000105383	CD33	CD33 molecule [Source:HGNC Symbol;Acc:HGNC:1659]	protein coding	-0.66	3.88	37.21	<0.0001	<0.0001
ENSG00000167264	DUS2	dihydrouridine synthase 2 [Source:HGNC Symbol;Acc:HGNC:26014]	protein coding	-0.66	4.58	30.51	<0.0001	<0.0001
ENSG00000164306	PRIMPOL	primase and DNA directed polymerase [Source:HGNC Symbol;Acc:HGNC:26575]	protein coding	-0.66	1.67	16.32	<0.0001	0.0004
ENSG00000205726	ITSN1	intersectin 1 [Source:HGNC Symbol;Acc:HGNC:6183]	protein coding	-0.66	0.52	9.45	0.0021	0.0081
ENSG00000185404	SP140L	SP140 nuclear body protein like [Source:HGNC Symbol;Acc:HGNC:25105]	protein coding	0.66	3.95	13.88	0.0002	0.0011
ENSG00000247556	OIP5-AS1	OIP5 antisense RNA 1 [Source:HGNC Symbol;Acc:HGNC:43563]	lncRNA	-0.65	3.72	20.31	<0.0001	<0.0001
ENSG00000123609	NMI	N-myc and STAT interactor [Source:HGNC Symbol;Acc:HGNC:7854]	protein coding	0.65	8.04	29.50	<0.0001	<0.0001
ENSG00000138617	PARP16	poly(ADP-ribose) polymerase family member 16 [Source:HGNC Symbol;Acc:HGNC:26040]	protein coding	-0.65	3.79	20.05	<0.0001	<0.0001
ENSG00000126464	PRR12	proline rich 12 [Source:HGNC Symbol;Acc:HGNC:29217]	protein coding	-0.65	3.96	23.55	<0.0001	<0.0001
ENSG00000236829		ribosomal protein L23a (RPL23A) pseudogene	transcribed processed pseudogene	-0.65	0.41	19.98	<0.0001	<0.0001
ENSG00000205155	PSENEN	presenilin enhancer, gamma-secretase subunit [Source:HGNC Symbol;Acc:HGNC:30100]	protein coding	0.65	4.72	38.89	<0.0001	<0.0001
ENSG00000244486	SCARF2	scavenger receptor class F member 2 [Source:HGNC Symbol;Acc:HGNC:19869]	protein coding	-0.65	2.24	20.34	<0.0001	<0.0001
ENSG00000202019			misc RNA	0.65	2.56	9.02	0.0027	0.0098
ENSG00000234290		novel transcript	lncRNA	0.65	4.44	10.40	0.0013	0.0053
ENSG00000067798	NAV3	neuron navigator 3 [Source:HGNC Symbol;Acc:HGNC:15998]	protein coding	-0.65	0.83	8.10	0.0044	0.0146
ENSG00000068305	MEF2A	myocyte enhancer factor 2A [Source:HGNC Symbol;Acc:HGNC:6993]	protein coding	0.65	5.74	21.87	<0.0001	<0.0001
ENSG00000077238	IL4R	interleukin 4 receptor [Source:HGNC Symbol;Acc:HGNC:6015]	protein coding	0.65	8.67	21.27	<0.0001	<0.0001
ENSG00000155465	SLC7A7	solute carrier family 7 member 7 [Source:HGNC Symbol;Acc:HGNC:11065]	protein coding	0.65	5.84	37.81	<0.0001	<0.0001

Table S16: Genes differentially expressed at Day 2 compared to pre-first vaccination (Study Arm 2 A/H7N9+AS03, D1, 29, Neutrophils). Genes are sorted by descending absolute log_2 fold change. Gene model summaries and annotations are based on Ensembl Version 99 (continued.)

Ensembl Gene ID	Ensembl Gene Name	Ensembl Gene Description	Gene Type	log_2 Fold Change (Day 2 vs. Pre-First Vaccination)	Average log_2 CPM	Likelihood Ratio Test Statistic	P-Value	FDR Adjusted P-Value
ENSG00000100918	REC8	REC8 meiotic recombination protein [Source:HGNC Symbol;Acc:HGNC:16879]	protein coding	0.65	5.53	15.96	<0.0001	0.0005
ENSG00000124191	TOX2	TOX high mobility group box family member 2 [Source:HGNC Symbol;Acc:HGNC:16095]	protein coding	-0.65	1.23	10.89	0.001	0.0042
ENSG00000157933	SKI	SKI proto-oncogene [Source:HGNC Symbol;Acc:HGNC:10896]	protein coding	-0.65	7.25	28.39	<0.0001	<0.0001
ENSG00000230492		novel transcript	lncRNA	0.65	1.33	14.35	0.0002	0.0009
ENSG00000165181	SHOC1	shortage in chiasmata 1 [Source:HGNC Symbol;Acc:HGNC:26535]	protein coding	0.65	2.59	11.43	0.0007	0.0034
ENSG00000235027		novel transcript	lncRNA	-0.65	0.64	9.15	0.0025	0.0092
ENSG00000010818	HIVEP2	HIVEP zinc finger 2 [Source:HGNC Symbol;Acc:HGNC:4921]	protein coding	0.65	6.27	33.20	<0.0001	<0.0001
ENSG00000226742	HSBP1L1	heat shock factor binding protein 1 like 1 [Source:HGNC Symbol;Acc:HGNC:37243]	protein coding	0.65	0.76	10.21	0.0014	0.0057
ENSG00000110811	P3H3	prolyl 3-hydroxylase 3 [Source:HGNC Symbol;Acc:HGNC:19318]	protein coding	-0.65	0.81	15.42	<0.0001	0.0006
ENSG00000006025	OSBPL7	oxysterol binding protein like 7 [Source:HGNC Symbol;Acc:HGNC:16387]	protein coding	-0.65	0.39	7.78	0.0053	0.017
ENSG00000133789	SWAP70	switching B cell complex subunit SWAP70 [Source:HGNC Symbol;Acc:HGNC:17070]	protein coding	-0.65	2.90	11.23	0.0008	0.0036
ENSG00000138434	ITPRID2	ITPR interacting domain containing 2 [Source:HGNC Symbol;Acc:HGNC:11319]	protein coding	0.65	7.21	51.79	<0.0001	<0.0001
ENSG00000175054	ATR	ATR serine/threonine kinase [Source:HGNC Symbol;Acc:HGNC:882]	protein coding	0.65	2.24	14.00	0.0002	0.0011
ENSG00000140280	LYSMD2	LysM domain containing 2 [Source:HGNC Symbol;Acc:HGNC:28571]	protein coding	0.65	6.53	26.70	<0.0001	<0.0001
ENSG00000068024	HDAC4	histone deacetylase 4 [Source:HGNC Symbol;Acc:HGNC:14063]	protein coding	0.64	6.14	24.07	<0.0001	<0.0001
ENSG00000271646		novel transcript	lncRNA	0.64	3.32	29.72	<0.0001	<0.0001
ENSG00000134440	NARS1	asparaginyl-tRNA synthetase 1 [Source:HGNC Symbol;Acc:HGNC:7643]	protein coding	-0.64	4.69	27.45	<0.0001	<0.0001
ENSG00000096006	CRISP3	cysteine rich secretory protein 3 [Source:HGNC Symbol;Acc:HGNC:16904]	protein coding	-0.64	1.69	9.65	0.0019	0.0073
ENSG00000163872	YEATS2	YEATS domain containing 2 [Source:HGNC Symbol;Acc:HGNC:25489]	protein coding	-0.64	3.84	41.16	<0.0001	<0.0001

Table S16: Genes differentially expressed at Day 2 compared to pre-first vaccination (Study Arm 2 A/H7N9+AS03, D1, 29, Neutrophils). Genes are sorted by descending absolute log_2 fold change. Gene model summaries and annotations are based on Ensembl Version 99 (continued.)

Ensembl Gene ID	Ensembl Gene Name	Ensembl Gene Description	Gene Type	log_2 Fold Change (Day 2 vs. Pre-First Vaccination)	Average log_2 CPM	Likelihood Ratio Test Statistic	P-Value	FDR Adjusted P-Value
ENSG00000177606	JUN	Jun proto-oncogene, AP-1 transcription factor subunit [Source:HGNC Symbol;Acc:HGNC:6204]	protein coding	-0.64	5.19	8.91	0.0028	0.0103
ENSG00000241889		dullard homolog (Xenopus laevis) (DULLARD) pseudogene	processed pseudogene	-0.64	1.53	15.22	<0.0001	0.0006
ENSG00000143793	C1orf35	chromosome 1 open reading frame 35 [Source:HGNC Symbol;Acc:HGNC:19032]	protein coding	-0.64	1.68	19.02	<0.0001	0.0001
ENSG00000135776	ABCB10	ATP binding cassette subfamily B member 10 [Source:HGNC Symbol;Acc:HGNC:41]	protein coding	-0.64	1.71	9.83	0.0017	0.0068
ENSG00000105223	PLD3	phospholipase D family member 3 [Source:HGNC Symbol;Acc:HGNC:17158]	protein coding	-0.64	4.43	27.41	<0.0001	<0.0001
ENSG00000186265	BTLA	B and T lymphocyte associated [Source:HGNC Symbol;Acc:HGNC:21087]	protein coding	0.64	0.60	16.86	<0.0001	0.0003
ENSG00000113916	BCL6	BCL6 transcription repressor [Source:HGNC Symbol;Acc:HGNC:1001]	protein coding	0.64	10.10	30.50	<0.0001	<0.0001
ENSG00000254719		guanine nucleotide binding protein (G protein) pseudogene	processed pseudogene	0.64	3.84	29.40	<0.0001	<0.0001
ENSG00000128000	ZNF780B	zinc finger protein 780B [Source:HGNC Symbol;Acc:HGNC:33109]	protein coding	-0.64	0.62	11.86	0.0006	0.0028
ENSG00000254470	AP5B1	adaptor related protein complex 5 subunit beta 1 [Source:HGNC Symbol;Acc:HGNC:25104]	protein coding	0.64	9.41	29.75	<0.0001	<0.0001
ENSG00000174775	HRAS	HRas proto-oncogene, GTPase [Source:HGNC Symbol;Acc:HGNC:5173]	protein coding	-0.64	0.13	12.80	0.0003	0.0018
ENSG00000121316	PLBD1	phospholipase B domain containing 1 [Source:HGNC Symbol;Acc:HGNC:26215]	protein coding	0.64	8.29	40.02	<0.0001	<0.0001
ENSG00000135966	TGFBRAP1	transforming growth factor beta receptor associated protein 1 [Source:HGNC Symbol;Acc:HGNC:16836]	protein coding	-0.64	2.46	21.82	<0.0001	<0.0001
ENSG00000113716	HMGXB3	HMG-box containing 3 [Source:HGNC Symbol;Acc:HGNC:28982]	protein coding	-0.64	0.95	11.24	0.0008	0.0036
ENSG00000008256	CYTH3	cytohesin 3 [Source:HGNC Symbol;Acc:HGNC:9504]	protein coding	-0.64	2.73	34.00	<0.0001	<0.0001
ENSG00000023445	BIRC3	baculoviral IAP repeat containing 3 [Source:HGNC Symbol;Acc:HGNC:591]	protein coding	0.64	2.91	15.57	<0.0001	0.0005
ENSG00000232788	ITGA6-AS1	ITGA6 antisense RNA 1 [Source:HGNC Symbol;Acc:HGNC:40308]	lncRNA	0.64	1.09	28.88	<0.0001	<0.0001

Table S16: Genes differentially expressed at Day 2 compared to pre-first vaccination (Study Arm 2 A/H7N9+AS03, D1, 29, Neutrophils). Genes are sorted by descending absolute log_2 fold change. Gene model summaries and annotations are based on Ensembl Version 99 (continued.)

Ensembl Gene ID	Ensembl Gene Name	Ensembl Gene Description	Gene Type	log_2 Fold Change (Day 2 vs. Pre-First Vaccination)	Average log_2 CPM	Likelihood Ratio Test Statistic	P-Value	FDR Adjusted P-Value
ENSG00000124098	FAM210B	family with sequence similarity 210 member B [Source:HGNC Symbol;Acc:HGNC:16102]	protein coding	-0.64	3.14	23.69	<0.0001	<0.0001
ENSG00000086065	CHMP5	charged multivesicular body protein 5 [Source:HGNC Symbol;Acc:HGNC:26942]	protein coding	0.64	6.56	18.32	<0.0001	0.0002
ENSG00000129566	TEP1	telomerase associated protein 1 [Source:HGNC Symbol;Acc:HGNC:11726]	protein coding	0.64	6.51	33.85	<0.0001	<0.0001
ENSG00000131899	LLGL1	LLGL scribble cell polarity complex component 1 [Source:HGNC Symbol;Acc:HGNC:6628]	protein coding	-0.64	2.47	22.94	<0.0001	<0.0001
ENSG00000021355	SERPINB1	serpin family B member 1 [Source:HGNC Symbol;Acc:HGNC:3311]	protein coding	0.64	8.59	28.34	<0.0001	<0.0001
ENSG00000175591	P2RY2	purinergic receptor P2Y2 [Source:HGNC Symbol;Acc:HGNC:8541]	protein coding	-0.64	2.40	6.65	0.0099	0.0283
ENSG00000148158	SNX30	sorting nexin family member 30 [Source:HGNC Symbol;Acc:HGNC:23685]	protein coding	-0.64	3.68	27.55	<0.0001	<0.0001
ENSG00000140807	NKD1	NKD inhibitor of WNT signaling pathway 1 [Source:HGNC Symbol;Acc:HGNC:17045]	protein coding	0.63	2.04	14.24	0.0002	0.001
ENSG00000171204	TMEM126B	transmembrane protein 126B [Source:HGNC Symbol;Acc:HGNC:30883]	protein coding	0.63	2.82	18.36	<0.0001	0.0002
ENSG00000112096	SOD2	superoxide dismutase 2 [Source:HGNC Symbol;Acc:HGNC:11180]	protein coding	0.63	11.73	32.98	<0.0001	<0.0001
ENSG00000100749	VRK1	VRK serine/threonine kinase 1 [Source:HGNC Symbol;Acc:HGNC:12718]	protein coding	-0.63	1.41	26.28	<0.0001	<0.0001
ENSG00000255557		novel transcript	lncRNA	0.63	0.67	10.09	0.0015	0.0061
ENSG00000141096	DPEP3	dipeptidase 3 [Source:HGNC Symbol;Acc:HGNC:23029]	protein coding	-0.63	4.82	36.09	<0.0001	<0.0001
ENSG00000286608		novel transcript	lncRNA	0.63	0.41	14.84	0.0001	0.0007
ENSG00000184640	SEPTIN9	septin 9 [Source:HGNC Symbol;Acc:HGNC:7323]	protein coding	-0.63	7.49	31.07	<0.0001	<0.0001
ENSG00000150961	SEC24D	SEC24 homolog D, COPII coat complex component [Source:HGNC Symbol;Acc:HGNC:10706]	protein coding	0.63	5.61	24.78	<0.0001	<0.0001
ENSG00000100139	MICALL1	MICAL like 1 [Source:HGNC Symbol;Acc:HGNC:29804]	protein coding	0.63	4.65	21.38	<0.0001	<0.0001
ENSG00000105607	GCDH	glutaryl-CoA dehydrogenase [Source:HGNC Symbol;Acc:HGNC:4189]	protein coding	-0.63	0.86	17.02	<0.0001	0.0003
ENSG00000041357	PSMA4	proteasome 20S subunit alpha 4 [Source:HGNC Symbol;Acc:HGNC:9533]	protein coding	0.63	5.87	31.97	<0.0001	<0.0001

Table S16: Genes differentially expressed at Day 2 compared to pre-first vaccination (Study Arm 2 A/H7N9+AS03, D1, 29, Neutrophils). Genes are sorted by descending absolute log_2 fold change. Gene model summaries and annotations are based on Ensembl Version 99 (continued.)

Ensembl Gene ID	Ensembl Gene Name	Ensembl Gene Description	Gene Type	log_2 Fold Change (Day 2 vs. Pre-First Vaccination)	Average log_2 CPM	Likelihood Ratio Test Statistic	P-Value	FDR Adjusted P-Value
ENSG00000168118	RAB4A	RAB4A, member RAS oncogene family [Source:HGNC Symbol;Acc:HGNC:9781]	protein coding	-0.63	2.24	22.54	<0.0001	<0.0001
ENSG00000060138	YBX3	Y-box binding protein 3 [Source:HGNC Symbol;Acc:HGNC:2428]	protein coding	0.63	5.37	8.62	0.0033	0.0116
ENSG00000134827	TCN1	transcobalamin 1 [Source:HGNC Symbol;Acc:HGNC:11652]	protein coding	-0.63	2.71	18.26	<0.0001	0.0002
ENSG00000250966	H3P14	H3 histone pseudogene 14 [Source:HGNC Symbol;Acc:HGNC:43805]	processed pseudogene	0.63	2.09	27.18	<0.0001	<0.0001
ENSG00000249437	NAIP	NLR family apoptosis inhibitory protein [Source:HGNC Symbol;Acc:HGNC:7634]	protein coding	0.63	5.15	9.47	0.0021	0.008
ENSG00000035403	VCL	vinculin [Source:HGNC Symbol;Acc:HGNC:12665]	protein coding	-0.63	6.70	23.80	<0.0001	<0.0001
ENSG00000149547	EI24	EI24 autophagy associated transmembrane protein [Source:HGNC Symbol;Acc:HGNC:13276]	protein coding	-0.63	0.49	7.72	0.0054	0.0175
ENSG00000112343	TRIM38	tripartite motif containing 38 [Source:HGNC Symbol;Acc:HGNC:10059]	protein coding	0.63	7.05	19.65	<0.0001	<0.0001
ENSG00000169567	HINT1	histidine triad nucleotide binding protein 1 [Source:HGNC Symbol;Acc:HGNC:4912]	protein coding	0.63	3.38	21.30	<0.0001	<0.0001
ENSG00000130699	TAF4	TATA-box binding protein associated factor 4 [Source:HGNC Symbol;Acc:HGNC:11537]	protein coding	-0.63	5.33	35.14	<0.0001	<0.0001
ENSG00000170921	TANC2	tetratricopeptide repeat, ankyrin repeat and coiled-coil containing 2 [Source:HGNC Symbol;Acc:HGNC:30212]	protein coding	-0.63	4.28	22.88	<0.0001	<0.0001
ENSG00000124795	DEK	DEK proto-oncogene [Source:HGNC Symbol;Acc:HGNC:2768]	protein coding	0.63	6.91	20.57	<0.0001	<0.0001
ENSG00000100097	LGALS1	galectin 1 [Source:HGNC Symbol;Acc:HGNC:6561]	protein coding	-0.63	2.04	7.42	0.0065	0.02
ENSG00000102910	LONP2	lon peptidase 2, peroxisomal [Source:HGNC Symbol;Acc:HGNC:20598]	protein coding	-0.63	3.66	36.80	<0.0001	<0.0001
ENSG00000130714	POMT1	protein O-mannosyltransferase 1 [Source:HGNC Symbol;Acc:HGNC:9202]	protein coding	-0.63	2.05	20.49	<0.0001	<0.0001
ENSG00000139318	DUSP6	dual specificity phosphatase 6 [Source:HGNC Symbol;Acc:HGNC:3072]	protein coding	0.63	7.96	10.28	0.0013	0.0056
ENSG00000064115	TM7SF3	transmembrane 7 superfamily member 3 [Source:HGNC Symbol;Acc:HGNC:23049]	protein coding	-0.62	3.46	20.25	<0.0001	<0.0001

Table S16: Genes differentially expressed at Day 2 compared to pre-first vaccination (Study Arm 2 A/H7N9+AS03, D1, 29, Neutrophils). Genes are sorted by descending absolute log_2 fold change. Gene model summaries and annotations are based on Ensembl Version 99 (continued.)

Ensembl Gene ID	Ensembl Gene Name	Ensembl Gene Description	Gene Type	log_2 Fold Change (Day 2 vs. Pre-First Vaccination)	Average log_2 CPM	Likelihood Ratio Test Statistic	P-Value	FDR Adjusted P-Value
ENSG00000185156	MFSD6L	major facilitator superfamily domain containing 6 like [Source:HGNC Symbol;Acc:HGNC:26656]	protein coding	0.62	1.91	14.76	0.0001	0.0008
ENSG00000147316	MCPH1	microcephalin 1 [Source:HGNC Symbol;Acc:HGNC:6954]	protein coding	-0.62	0.64	10.64	0.0011	0.0047
ENSG00000261884		novel transcript	protein coding	0.62	4.51	30.32	<0.0001	<0.0001
ENSG00000152558	TMEM123	transmembrane protein 123 [Source:HGNC Symbol;Acc:HGNC:30138]	protein coding	0.62	8.40	17.99	<0.0001	0.0002
ENSG00000171813	PWWP2B	PWWP domain containing 2B [Source:HGNC Symbol;Acc:HGNC:25150]	protein coding	-0.62	2.24	12.53	0.0004	0.0021
ENSG00000142197	DOP1B	DOP1 leucine zipper like protein B [Source:HGNC Symbol;Acc:HGNC:1291]	protein coding	-0.62	4.97	27.93	<0.0001	<0.0001
ENSG00000198604	BAZ1A	bromodomain adjacent to zinc finger domain 1A [Source:HGNC Symbol;Acc:HGNC:960]	protein coding	0.62	8.39	21.49	<0.0001	<0.0001
ENSG00000137309	HMGA1	high mobility group AT-hook 1 [Source:HGNC Symbol;Acc:HGNC:5010]	protein coding	-0.62	3.16	24.04	<0.0001	<0.0001
ENSG00000110917	MLEC	malectin [Source:HGNC Symbol;Acc:HGNC:28973]	protein coding	-0.62	2.98	12.06	0.0005	0.0025
ENSG00000134851	TMEM165	transmembrane protein 165 [Source:HGNC Symbol;Acc:HGNC:30760]	protein coding	0.62	6.24	30.95	<0.0001	<0.0001
ENSG00000197272	IL27	interleukin 27 [Source:HGNC Symbol;Acc:HGNC:19157]	protein coding	0.62	1.98	13.73	0.0002	0.0012
ENSG00000167705	RILP	Rab interacting lysosomal protein [Source:HGNC Symbol;Acc:HGNC:30266]	protein coding	0.62	4.87	27.52	<0.0001	<0.0001
ENSG00000272941		novel transcript, antisense to C7orf49	lncRNA	0.62	2.72	17.58	<0.0001	0.0002
ENSG00000101391	CDK5RAP1	CDK5 regulatory subunit associated protein 1 [Source:HGNC Symbol;Acc:HGNC:15880]	protein coding	-0.62	0.74	12.58	0.0004	0.002
ENSG00000136816	TOR1B	torsin family 1 member B [Source:HGNC Symbol;Acc:HGNC:11995]	protein coding	0.62	5.86	15.18	<0.0001	0.0006
ENSG00000166997	CNPY4	canopy FGF signaling regulator 4 [Source:HGNC Symbol;Acc:HGNC:28631]	protein coding	-0.62	1.00	17.81	<0.0001	0.0002
ENSG00000248099	INSL3	insulin like 3 [Source:HGNC Symbol;Acc:HGNC:6086]	protein coding	0.62	1.97	13.72	0.0002	0.0012
ENSG00000180509	KCNE1	potassium voltage-gated channel subfamily E regulatory subunit 1 [Source:HGNC Symbol;Acc:HGNC:6240]	protein coding	-0.62	3.35	9.05	0.0026	0.0096

Table S16: Genes differentially expressed at Day 2 compared to pre-first vaccination (Study Arm 2 A/H7N9+AS03, D1, 29, Neutrophils). Genes are sorted by descending absolute log_2 fold change. Gene model summaries and annotations are based on Ensembl Version 99 (continued.)

Ensembl Gene ID	Ensembl Gene Name	Ensembl Gene Description	Gene Type	log_2 Fold Change (Day 2 vs. Pre-First Vaccination)	Average log_2 CPM	Likelihood Ratio Test Statistic	P-Value	FDR Adjusted P-Value
ENSG00000176058	TPRN	taperin [Source:HGNC Symbol;Acc:HGNC:26894]	protein coding	-0.62	0.16	9.32	0.0023	0.0085
ENSG00000121064	SCPEP1	serine carboxypeptidase 1 [Source:HGNC Symbol;Acc:HGNC:29507]	protein coding	-0.62	5.28	40.34	<0.0001	<0.0001
ENSG00000134262	AP4B1	adaptor related protein complex 4 subunit beta 1 [Source:HGNC Symbol;Acc:HGNC:572]	protein coding	-0.62	2.17	18.74	<0.0001	0.0001
ENSG00000196502	SULT1A1	sulfotransferase family 1A member 1 [Source:HGNC Symbol;Acc:HGNC:11453]	protein coding	-0.62	6.63	31.63	<0.0001	<0.0001
ENSG00000172007	RAB33B	RAB33B, member RAS oncogene family [Source:HGNC Symbol;Acc:HGNC:16075]	protein coding	0.62	5.79	25.70	<0.0001	<0.0001
ENSG00000126262	FFAR2	free fatty acid receptor 2 [Source:HGNC Symbol;Acc:HGNC:4501]	protein coding	0.62	9.91	27.74	<0.0001	<0.0001
ENSG00000109685	NSD2	nuclear receptor binding SET domain protein 2 [Source:HGNC Symbol;Acc:HGNC:12766]	protein coding	-0.62	2.02	16.36	<0.0001	0.0004
ENSG00000272277		novel transcript, antisense to RIPK1	lncRNA	0.62	1.40	11.40	0.0007	0.0034
ENSG00000091436	MAP3K20	mitogen-activated protein kinase kinase kinase 20 [Source:HGNC Symbol;Acc:HGNC:17797]	protein coding	0.62	3.04	6.53	0.0106	0.0298
ENSG00000138439	FAM117B	family with sequence similarity 117 member B [Source:HGNC Symbol;Acc:HGNC:14440]	protein coding	-0.62	2.76	8.89	0.0029	0.0103
ENSG00000181751	C5orf30	chromosome 5 open reading frame 30 [Source:HGNC Symbol;Acc:HGNC:25052]	protein coding	-0.62	1.38	19.21	<0.0001	0.0001
ENSG00000158869	FCER1G	Fc fragment of IgE receptor Ig [Source:HGNC Symbol;Acc:HGNC:3611]	protein coding	0.62	7.78	12.98	0.0003	0.0017
ENSG00000130816	DNMT1	DNA methyltransferase 1 [Source:HGNC Symbol;Acc:HGNC:2976]	protein coding	-0.62	0.43	11.11	0.0009	0.0038
ENSG00000143801	PSEN2	presenilin 2 [Source:HGNC Symbol;Acc:HGNC:9509]	protein coding	-0.62	0.53	14.32	0.0002	0.0009
ENSG00000259704		novel transcript, sense overlapping 3' UTR of BLM	lncRNA	0.62	0.30	12.75	0.0004	0.0019
ENSG00000235750	KIAA0040	KIAA0040 [Source:HGNC Symbol;Acc:HGNC:28950]	protein coding	0.61	8.35	27.12	<0.0001	<0.0001
ENSG00000159339	PADI4	peptidyl arginine deiminase 4 [Source:HGNC Symbol;Acc:HGNC:18368]	protein coding	-0.61	8.34	13.86	0.0002	0.0012
ENSG00000148218	ALAD	aminolevulinate dehydratase [Source:HGNC Symbol;Acc:HGNC:395]	protein coding	-0.61	3.86	23.66	<0.0001	<0.0001
ENSG00000173930	SLCO4C1	solute carrier organic anion transporter family member 4C1 [Source:HGNC Symbol;Acc:HGNC:23612]	protein coding	-0.61	5.31	11.26	0.0008	0.0036

Table S16: Genes differentially expressed at Day 2 compared to pre-first vaccination (Study Arm 2 A/H7N9+AS03, D1, 29, Neutrophils). Genes are sorted by descending absolute log_2 fold change. Gene model summaries and annotations are based on Ensembl Version 99 (continued.)

Ensembl Gene ID	Ensembl Gene Name	Ensembl Gene Description	Gene Type	log_2 Fold Change (Day 2 vs. Pre-First Vaccination)	Average log_2 CPM	Likelihood Ratio Test Statistic	P-Value	FDR Adjusted P-Value
ENSG00000090470	PDCD7	programmed cell death 7 [Source:HGNC Symbol;Acc:HGNC:8767]	protein coding	-0.61	0.62	8.17	0.0043	0.0142
ENSG00000182685	BRICD5	BRICHOS domain containing 5 [Source:HGNC Symbol;Acc:HGNC:28309]	protein coding	-0.61	3.23	19.81	<0.0001	<0.0001
ENSG00000130396	AFDN	afadin, adherens junction formation factor [Source:HGNC Symbol;Acc:HGNC:7137]	protein coding	-0.61	4.57	9.69	0.0019	0.0072
ENSG00000260923	LINC02193	long intergenic non-protein coding RNA 2193 [Source:HGNC Symbol;Acc:HGNC:53055]	lncRNA	0.61	1.85	13.79	0.0002	0.0012
ENSG00000213563	C8orf82	chromosome 8 open reading frame 82 [Source:HGNC Symbol;Acc:HGNC:33826]	protein coding	-0.61	0.27	10.68	0.0011	0.0046
ENSG00000272419	LINC01145	long intergenic non-protein coding RNA 1145 [Source:HGNC Symbol;Acc:HGNC:49462]	transcribed unprocessed pseudogene	0.61	2.65	26.56	<0.0001	<0.0001
ENSG00000150433	TMEM218	transmembrane protein 218 [Source:HGNC Symbol;Acc:HGNC:27344]	protein coding	-0.61	1.93	19.72	<0.0001	<0.0001
ENSG00000196396	PTPN1	protein tyrosine phosphatase non-receptor type 1 [Source:HGNC Symbol;Acc:HGNC:9642]	protein coding	0.61	6.04	30.07	<0.0001	<0.0001
ENSG00000100528	CNIH1	cornichon family AMPA receptor auxiliary protein 1 [Source:HGNC Symbol;Acc:HGNC:19431]	protein coding	-0.61	2.49	16.18	<0.0001	0.0004
ENSG00000073737	DHRS9	dehydrogenase/reductase 9 [Source:HGNC Symbol;Acc:HGNC:16888]	protein coding	0.61	5.81	13.44	0.0002	0.0014
ENSG00000224186	C5orf66	chromosome 5 open reading frame 66 [Source:HGNC Symbol;Acc:HGNC:48332]	lncRNA	-0.61	0.33	13.46	0.0002	0.0014
ENSG00000158882	TOMM40L	translocase of outer mitochondrial membrane 40 like [Source:HGNC Symbol;Acc:HGNC:25756]	protein coding	0.61	1.62	9.95	0.0016	0.0064
ENSG00000112640	PPP2R5D	protein phosphatase 2 regulatory subunit B'delta [Source:HGNC Symbol;Acc:HGNC:9312]	protein coding	-0.61	3.50	25.86	<0.0001	<0.0001
ENSG00000182782	HCAR2	hydroxycarboxylic acid receptor 2 [Source:HGNC Symbol;Acc:HGNC:24827]	protein coding	0.61	7.77	23.47	<0.0001	<0.0001
ENSG00000100360	IFT27	intraflagellar transport 27 [Source:HGNC Symbol;Acc:HGNC:18626]	protein coding	-0.61	1.66	26.83	<0.0001	<0.0001
ENSG00000110911	SLC11A2	solute carrier family 11 member 2 [Source:HGNC Symbol;Acc:HGNC:10908]	protein coding	0.61	0.39	11.42	0.0007	0.0034

Table S16: Genes differentially expressed at Day 2 compared to pre-first vaccination (Study Arm 2 A/H7N9+AS03, D1, 29, Neutrophils). Genes are sorted by descending absolute log_2 fold change. Gene model summaries and annotations are based on Ensembl Version 99 (continued.)

Ensembl Gene ID	Ensembl Gene Name	Ensembl Gene Description	Gene Type	log_2 Fold Change (Day 2 vs. Pre-First Vaccination)	Average log_2 CPM	Likelihood Ratio Test Statistic	P-Value	FDR Adjusted P-Value
ENSG00000172426	RSPH9	radial spoke head component 9 [Source:HGNC Symbol;Acc:HGNC:21057]	protein coding	0.61	0.57	6.46	0.011	0.0307
ENSG00000145016	RUBCN	rubicon autophagy regulator [Source:HGNC Symbol;Acc:HGNC:28991]	protein coding	0.61	4.95	25.90	<0.0001	<0.0001
ENSG00000176438	SYNE3	spectrin repeat containing nuclear envelope family member 3 [Source:HGNC Symbol;Acc:HGNC:19861]	protein coding	-0.61	4.15	24.49	<0.0001	<0.0001
ENSG00000023909	GCLM	glutamate-cysteine ligase modifier subunit [Source:HGNC Symbol;Acc:HGNC:4312]	protein coding	0.61	3.35	16.29	<0.0001	0.0004
ENSG00000180316	PNPLA1	patatin like phospholipase domain containing 1 [Source:HGNC Symbol;Acc:HGNC:21246]	protein coding	-0.61	3.10	6.81	0.009	0.0263
ENSG00000285103		novel transcript	lncRNA	0.61	3.18	14.06	0.0002	0.0011
ENSG00000182621	PLCB1	phospholipase C beta 1 [Source:HGNC Symbol;Acc:HGNC:15917]	protein coding	-0.61	1.50	6.11	0.0135	0.0363
ENSG00000125457	MIF4GD	MIF4G domain containing [Source:HGNC Symbol;Acc:HGNC:24030]	protein coding	-0.60	1.76	15.13	0.0001	0.0007
ENSG00000176659	C20orf197	chromosome 20 open reading frame 197 [Source:HGNC Symbol;Acc:HGNC:26601]	lncRNA	-0.60	2.14	19.69	<0.0001	<0.0001
ENSG00000085721	RRN3	RRN3 homolog, RNA polymerase I transcription factor [Source:HGNC Symbol;Acc:HGNC:30346]	protein coding	-0.60	0.88	15.37	<0.0001	0.0006
ENSG00000268362		novel transcript	lncRNA	0.60	1.07	7.10	0.0077	0.0232
ENSG00000150756	ATPSCKMT	ATP synthase c subunit lysine N-methyltransferase [Source:HGNC Symbol;Acc:HGNC:27029]	protein coding	-0.60	1.22	23.54	<0.0001	<0.0001
ENSG00000110108	TMEM109	transmembrane protein 109 [Source:HGNC Symbol;Acc:HGNC:28771]	protein coding	-0.60	0.98	8.72	0.0031	0.0111
ENSG00000132383	RPA1	replication protein A1 [Source:HGNC Symbol;Acc:HGNC:10289]	protein coding	-0.60	3.71	21.83	<0.0001	<0.0001
ENSG00000127564	PKMYT1	protein kinase, membrane associated tyrosine/threonine 1 [Source:HGNC Symbol;Acc:HGNC:29650]	protein coding	-0.60	0.33	16.62	<0.0001	0.0003
ENSG00000233276	GPX1	glutathione peroxidase 1 [Source:HGNC Symbol;Acc:HGNC:4553]	polymorphic pseudogene	-0.60	5.58	17.87	<0.0001	0.0002
ENSG00000276045	ORAI1	ORAI calcium release-activated calcium modulator 1 [Source:HGNC Symbol;Acc:HGNC:25896]	protein coding	-0.60	5.55	33.96	<0.0001	<0.0001
ENSG00000118762	PKD2	polycystin 2, transient receptor potential cation channel [Source:HGNC Symbol;Acc:HGNC:9009]	protein coding	-0.60	2.51	20.87	<0.0001	<0.0001

Table S16: Genes differentially expressed at Day 2 compared to pre-first vaccination (Study Arm 2 A/H7N9+AS03, D1, 29, Neutrophils). Genes are sorted by descending absolute log_2 fold change. Gene model summaries and annotations are based on Ensembl Version 99 (continued.)

Ensembl Gene ID	Ensembl Gene Name	Ensembl Gene Description	Gene Type	log_2 Fold Change (Day 2 vs. Pre-First Vaccination)	Average log_2 CPM	Likelihood Ratio Test Statistic	P-Value	FDR Adjusted P-Value
ENSG00000122873	CISD1	CDGSH iron sulfur domain 1 [Source:HGNC Symbol;Acc:HGNC:30880]	protein coding	0.60	1.69	6.33	0.0119	0.0327
ENSG00000224579		leukocyte immunoglobulin-like receptor, subfamily B (with TM and ITIM domains), member 2 (LILRB2) pseudogene	unprocessed pseudogene	0.60	1.22	7.32	0.0068	0.021
ENSG00000143224	PPOX	protoporphyrinogen oxidase [Source:HGNC Symbol;Acc:HGNC:9280]	protein coding	-0.60	0.98	12.27	0.0005	0.0023
ENSG00000139597	N4BP2L1	NEDD4 binding protein 2 like 1 [Source:HGNC Symbol;Acc:HGNC:25037]	protein coding	0.60	2.92	20.29	<0.0001	<0.0001
ENSG00000214106	PAXIP1-AS2	PAXIP1 antisense RNA 2 [Source:HGNC Symbol;Acc:HGNC:48958]	lncRNA	0.60	1.84	16.04	<0.0001	0.0004
ENSG00000106351	AGFG2	ArfGAP with FG repeats 2 [Source:HGNC Symbol;Acc:HGNC:5177]	protein coding	-0.60	0.76	9.55	0.002	0.0077
ENSG00000128228	SDF2L1	stromal cell derived factor 2 like 1 [Source:HGNC Symbol;Acc:HGNC:10676]	protein coding	0.60	2.11	12.00	0.0005	0.0026
ENSG00000099992	TBC1D10A	TBC1 domain family member 10A [Source:HGNC Symbol;Acc:HGNC:23609]	protein coding	-0.60	3.84	27.02	<0.0001	<0.0001
ENSG00000127399	LRRC61	leucine rich repeat containing 61 [Source:HGNC Symbol;Acc:HGNC:21704]	protein coding	0.60	3.02	19.22	<0.0001	0.0001
ENSG00000136045	PWP1	PWP1 homolog, endonuclein [Source:HGNC Symbol;Acc:HGNC:17015]	protein coding	-0.60	2.41	12.22	0.0005	0.0024
ENSG00000049245	VAMP3	vesicle associated membrane protein 3 [Source:HGNC Symbol;Acc:HGNC:12644]	protein coding	0.60	6.86	26.82	<0.0001	<0.0001
ENSG00000106346	USP42	ubiquitin specific peptidase 42 [Source:HGNC Symbol;Acc:HGNC:20068]	protein coding	0.60	3.84	29.39	<0.0001	<0.0001
ENSG00000169359	SLC33A1	solute carrier family 33 member 1 [Source:HGNC Symbol;Acc:HGNC:95]	protein coding	-0.60	0.41	20.75	<0.0001	<0.0001
ENSG00000156110	ADK	adenosine kinase [Source:HGNC Symbol;Acc:HGNC:257]	protein coding	-0.60	2.51	9.30	0.0023	0.0086
ENSG00000108344	PSMD3	proteasome 26S subunit, non-ATPase 3 [Source:HGNC Symbol;Acc:HGNC:9560]	protein coding	0.60	4.46	20.29	<0.0001	<0.0001
ENSG00000109919	MTCH2	mitochondrial carrier 2 [Source:HGNC Symbol;Acc:HGNC:17587]	protein coding	-0.60	0.47	12.25	0.0005	0.0023
ENSG00000277791	PSMB3	proteasome 20S subunit beta 3 [Source:HGNC Symbol;Acc:HGNC:9540]	protein coding	0.60	7.34	30.51	<0.0001	<0.0001

Table S16: Genes differentially expressed at Day 2 compared to pre-first vaccination (Study Arm 2 A/H7N9+AS03, D1, 29, Neutrophils). Genes are sorted by descending absolute log_2 fold change. Gene model summaries and annotations are based on Ensembl Version 99 (continued.)

Ensembl Gene ID	Ensembl Gene Name	Ensembl Gene Description	Gene Type	log_2 Fold Change (Day 2 vs. Pre-First Vaccination)	Average log_2 CPM	Likelihood Ratio Test Statistic	P-Value	FDR Adjusted P-Value
ENSG00000142961	MOB3C	MOB kinase activator 3C [Source:HGNC Symbol;Acc:HGNC:29800]	protein coding	0.60	4.42	18.24	<0.0001	0.0002
ENSG00000154305	MIA3	MIA SH3 domain ER export factor 3 [Source:HGNC Symbol;Acc:HGNC:24008]	protein coding	0.60	6.26	27.86	<0.0001	<0.0001
ENSG00000108021	TASOR2	transcription activation suppressor family member 2 [Source:HGNC Symbol;Acc:HGNC:23484]	protein coding	0.60	3.62	27.02	<0.0001	<0.0001
ENSG00000008382	MPND	MPN domain containing [Source:HGNC Symbol;Acc:HGNC:25934]	protein coding	-0.60	2.41	22.99	<0.0001	<0.0001
ENSG00000135503	ACVR1B	activin A receptor type 1B [Source:HGNC Symbol;Acc:HGNC:172]	protein coding	-0.60	3.91	13.12	0.0003	0.0016
ENSG00000127311	HELB	DNA helicase B [Source:HGNC Symbol;Acc:HGNC:17196]	protein coding	0.60	3.81	10.37	0.0013	0.0053
ENSG00000100060	MFNG	MFNG O-fucosylpeptide 3-beta-N-acetylglucosaminyltransferase [Source:HGNC Symbol;Acc:HGNC:7038]	protein coding	-0.60	6.46	23.66	<0.0001	<0.0001
ENSG00000179627	ZBTB42	zinc finger and BTB domain containing 42 [Source:HGNC Symbol;Acc:HGNC:32550]	protein coding	-0.60	1.49	7.41	0.0065	0.0201
ENSG00000185361	TNFAIP8L1	TNF alpha induced protein 8 like 1 [Source:HGNC Symbol;Acc:HGNC:28279]	protein coding	-0.60	1.20	7.73	0.0054	0.0174
ENSG00000106344	RBM28	RNA binding motif protein 28 [Source:HGNC Symbol;Acc:HGNC:21863]	protein coding	-0.60	2.98	43.03	<0.0001	<0.0001
ENSG00000180891	CUEDC1	CUE domain containing 1 [Source:HGNC Symbol;Acc:HGNC:31350]	protein coding	-0.60	5.89	41.22	<0.0001	<0.0001
ENSG00000197562	RAB40C	RAB40C, member RAS oncogene family [Source:HGNC Symbol;Acc:HGNC:18285]	protein coding	-0.60	5.00	22.46	<0.0001	<0.0001
ENSG00000183060	LYSMD4	LysM domain containing 4 [Source:HGNC Symbol;Acc:HGNC:26571]	protein coding	-0.60	0.32	12.93	0.0003	0.0017
ENSG00000135677	GNS	glucosamine (N-acetyl)-6-sulfatase [Source:HGNC Symbol;Acc:HGNC:4422]	protein coding	0.60	7.44	31.18	<0.0001	<0.0001
ENSG00000089006	SNX5	sorting nexin 5 [Source:HGNC Symbol;Acc:HGNC:14969]	protein coding	-0.59	2.01	11.38	0.0007	0.0034
ENSG00000243696		novel MUSTN1-ITIH4 readthrough	protein coding	0.59	0.53	18.12	<0.0001	0.0002
ENSG00000103154	NECAB2	N-terminal EF-hand calcium binding protein 2 [Source:HGNC Symbol;Acc:HGNC:23746]	protein coding	-0.59	4.68	16.84	<0.0001	0.0003

Table S16: Genes differentially expressed at Day 2 compared to pre-first vaccination (Study Arm 2 A/H7N9+AS03, D1, 29, Neutrophils). Genes are sorted by descending absolute log_2 fold change. Gene model summaries and annotations are based on Ensembl Version 99 (continued.)

Ensembl Gene ID	Ensembl Gene Name	Ensembl Gene Description	Gene Type	log_2 Fold Change (Day 2 vs. Pre-First Vaccination)	Average log_2 CPM	Likelihood Ratio Test Statistic	P-Value	FDR Adjusted P-Value
ENSG00000154153	RETREG1	reticulophagy regulator 1 [Source:HGNC Symbol;Acc:HGNC:25964]	protein coding	-0.59	1.95	8.41	0.0037	0.0128
ENSG00000118515	SGK1	serum/glucocorticoid regulated kinase 1 [Source:HGNC Symbol;Acc:HGNC:10810]	protein coding	-0.59	8.48	12.81	0.0003	0.0018
ENSG00000167468	GPX4	glutathione peroxidase 4 [Source:HGNC Symbol;Acc:HGNC:4556]	protein coding	-0.59	5.54	22.41	<0.0001	<0.0001
ENSG00000124102	PI3	peptidase inhibitor 3 [Source:HGNC Symbol;Acc:HGNC:8947]	protein coding	0.59	7.43	14.18	0.0002	0.001
ENSG00000133030	MPRIP	myosin phosphatase Rho interacting protein [Source:HGNC Symbol;Acc:HGNC:30321]	protein coding	-0.59	4.81	38.16	<0.0001	<0.0001
ENSG00000181610	MRPS23	mitochondrial ribosomal protein S23 [Source:HGNC Symbol;Acc:HGNC:14509]	protein coding	-0.59	0.69	10.41	0.0013	0.0052
ENSG00000111328	CDK2AP1	cyclin dependent kinase 2 associated protein 1 [Source:HGNC Symbol;Acc:HGNC:14002]	protein coding	-0.59	3.10	10.98	0.0009	0.0041
ENSG00000167004	PDIA3	protein disulfide isomerase family A member 3 [Source:HGNC Symbol;Acc:HGNC:4606]	protein coding	0.59	5.55	48.42	<0.0001	<0.0001
ENSG00000167202	TBC1D2B	TBC1 domain family member 2B [Source:HGNC Symbol;Acc:HGNC:29183]	protein coding	0.59	6.34	30.17	<0.0001	<0.0001
ENSG00000182551	ADI1	acireductone dioxygenase 1 [Source:HGNC Symbol;Acc:HGNC:30576]	protein coding	-0.59	4.30	21.45	<0.0001	<0.0001
ENSG00000149782	PLCB3	phospholipase C beta 3 [Source:HGNC Symbol;Acc:HGNC:9056]	protein coding	-0.59	3.06	24.10	<0.0001	<0.0001
ENSG00000139832	RAB20	RAB20, member RAS oncogene family [Source:HGNC Symbol;Acc:HGNC:18260]	protein coding	0.59	4.69	12.92	0.0003	0.0017
ENSG00000255302	EID1	EP300 interacting inhibitor of differentiation 1 [Source:HGNC Symbol;Acc:HGNC:1191]	protein coding	-0.59	3.36	21.28	<0.0001	<0.0001
ENSG00000188641	DPYD	dihydropyrimidine dehydrogenase [Source:HGNC Symbol;Acc:HGNC:3012]	protein coding	0.59	6.33	35.68	<0.0001	<0.0001
ENSG00000103249	CLCN7	chloride voltage-gated channel 7 [Source:HGNC Symbol;Acc:HGNC:2025]	protein coding	-0.59	4.87	43.08	<0.0001	<0.0001
ENSG00000169727	GPS1	G protein pathway suppressor 1 [Source:HGNC Symbol;Acc:HGNC:4549]	protein coding	-0.59	3.61	22.40	<0.0001	<0.0001
ENSG00000038274	MAT2B	methionine adenosyltransferase 2B [Source:HGNC Symbol;Acc:HGNC:6905]	protein coding	0.59	7.33	30.15	<0.0001	<0.0001
ENSG00000169696	ASPSCR1	ASPSCR1 tether for SLC2A4, UBX domain containing [Source:HGNC Symbol;Acc:HGNC:13825]	protein coding	-0.59	1.37	22.02	<0.0001	<0.0001

Table S16: Genes differentially expressed at Day 2 compared to pre-first vaccination (Study Arm 2 A/H7N9+AS03, D1, 29, Neutrophils). Genes are sorted by descending absolute log_2 fold change. Gene model summaries and annotations are based on Ensembl Version 99 (continued.)

Ensembl Gene ID	Ensembl Gene Name	Ensembl Gene Description	Gene Type	log_2 Fold Change (Day 2 vs. Pre-First Vaccination)	Average log_2 CPM	Likelihood Ratio Test Statistic	P-Value	FDR Adjusted P-Value
ENSG00000237719		nudix (nucleoside diphosphate linked moiety X) type 5 motif (NUDT5) pseudogene	processed pseudogene	0.59	1.62	5.71	0.0169	0.0438
ENSG00000215196	BASP1-AS1	BASP1 antisense RNA 1 [Source:HGNC Symbol;Acc:HGNC:26609]	lncRNA	0.59	2.40	16.87	<0.0001	0.0003
ENSG00000277349		capicua transcriptional repressor (CIC) pseudogene	unprocessed pseudogene	0.59	1.61	10.31	0.0013	0.0055
ENSG00000175602	CCDC85B	coiled-coil domain containing 85B [Source:HGNC Symbol;Acc:HGNC:24926]	protein coding	-0.59	1.71	8.84	0.0029	0.0106
ENSG00000100220	RTCB	RNA 2',3'-cyclic phosphate and 5'-OH ligase [Source:HGNC Symbol;Acc:HGNC:26935]	protein coding	-0.59	0.89	12.91	0.0003	0.0018
ENSG00000121957	GPSM2	G protein signaling modulator 2 [Source:HGNC Symbol;Acc:HGNC:29501]	protein coding	-0.59	3.29	18.20	<0.0001	0.0002
ENSG00000178425	NT5DC1	5'-nucleotidase domain containing 1 [Source:HGNC Symbol;Acc:HGNC:21556]	protein coding	-0.59	3.21	29.77	<0.0001	<0.0001
ENSG00000157388	CACNA1D	calcium voltage-gated channel subunit alpha1 D [Source:HGNC Symbol;Acc:HGNC:1391]	protein coding	-0.59	1.04	13.16	0.0003	0.0016
ENSG00000160190	SLC37A1	solute carrier family 37 member 1 [Source:HGNC Symbol;Acc:HGNC:11024]	protein coding	0.59	3.87	23.30	<0.0001	<0.0001
ENSG00000180329	CCDC43	coiled-coil domain containing 43 [Source:HGNC Symbol;Acc:HGNC:26472]	protein coding	-0.59	0.83	16.83	<0.0001	0.0003
ENSG00000104731	KLHDC4	kelch domain containing 4 [Source:HGNC Symbol;Acc:HGNC:25272]	protein coding	-0.59	0.51	8.33	0.0039	0.0133
ENSG00000197885	NKIRAS1	NFKB inhibitor interacting Ras like 1 [Source:HGNC Symbol;Acc:HGNC:17899]	protein coding	0.59	0.99	23.15	<0.0001	<0.0001
ENSG00000145781	COMMD10	COMM domain containing 10 [Source:HGNC Symbol;Acc:HGNC:30201]	protein coding	-0.59	0.14	11.58	0.0007	0.0031
ENSG00000204396	VWA7	von Willebrand factor A domain containing 7 [Source:HGNC Symbol;Acc:HGNC:13939]	protein coding	-0.59	1.79	16.03	<0.0001	0.0004
ENSG00000168028	RPSA	ribosomal protein SA [Source:HGNC Symbol;Acc:HGNC:6502]	protein coding	-0.59	2.64	12.55	0.0004	0.0021

Table S17: Genes differentially expressed at Day 4 compared to pre-first vaccination (Study Arm 2 A/H7N9+AS03, D1, 29, Neutrophils). Genes are sorted by descending absolute log_2 fold change. Gene model summaries and annotations are based on Ensembl Version 99.

Ensembl Gene ID	Ensembl Gene Name	Ensembl Gene Description	Gene Type	log_2 Fold Change (Day 4 vs. Pre-First Vaccination)	Average log_2 CPM	Likelihood Ratio Test Statistic	P-Value	FDR Adjusted P-Value
ENSG00000196141	SPATS2L	spermatogenesis associated serine rich 2 like [Source:HGNC Symbol;Acc:HGNC:24574]	protein coding	2.72	1.99	21.07	<0.0001	0.004
ENSG00000089127	OAS1	2'-5'-oligoadenylate synthetase 1 [Source:HGNC Symbol;Acc:HGNC:8086]	protein coding	2.41	6.21	19.76	<0.0001	0.0052
ENSG00000160932	LY6E	lymphocyte antigen 6 family member E [Source:HGNC Symbol;Acc:HGNC:6727]	protein coding	2.41	7.31	20.32	<0.0001	0.0044
ENSG00000137959	IFI44L	interferon induced protein 44 like [Source:HGNC Symbol;Acc:HGNC:17817]	protein coding	2.38	6.03	19.33	<0.0001	0.0054
ENSG00000111335	OAS2	2'-5'-oligoadenylate synthetase 2 [Source:HGNC Symbol;Acc:HGNC:8087]	protein coding	2.34	6.04	16.14	<0.0001	0.0151
ENSG00000136514	RTP4	receptor transporter protein 4 [Source:HGNC Symbol;Acc:HGNC:23992]	protein coding	2.25	2.78	21.50	<0.0001	0.004
ENSG00000133106	EPSTI1	epithelial stromal interaction 1 [Source:HGNC Symbol;Acc:HGNC:16465]	protein coding	2.16	6.26	18.95	<0.0001	0.0061
ENSG00000137965	IFI44	interferon induced protein 44 [Source:HGNC Symbol;Acc:HGNC:16938]	protein coding	2.03	6.07	15.78	<0.0001	0.016
ENSG00000134321	RSAD2	radical S-adenosyl methionine domain containing 2 [Source:HGNC Symbol;Acc:HGNC:30908]	protein coding	2.01	7.96	12.97	0.0003	0.0381
ENSG00000108387	SEPTIN4	septin 4 [Source:HGNC Symbol;Acc:HGNC:9165]	protein coding	1.97	1.26	16.17	<0.0001	0.0151
ENSG00000111331	OAS3	2'-5'-oligoadenylate synthetase 3 [Source:HGNC Symbol;Acc:HGNC:8088]	protein coding	1.96	7.76	13.66	0.0002	0.0317
ENSG00000225964	NRIR	negative regulator of interferon response [Source:HGNC Symbol;Acc:HGNC:51269]	lncRNA	1.93	1.24	14.64	0.0001	0.0237
ENSG00000125148	MT2A	metallothionein 2A [Source:HGNC Symbol;Acc:HGNC:7406]	protein coding	1.61	4.46	15.98	<0.0001	0.0152
ENSG00000162714	ZNF496	zinc finger protein 496 [Source:HGNC Symbol;Acc:HGNC:23713]	protein coding	1.36	2.70	16.99	<0.0001	0.0119
ENSG00000149131	SERPING1	serpin family G member 1 [Source:HGNC Symbol;Acc:HGNC:1228]	protein coding	1.34	6.24	13.32	0.0003	0.0345
ENSG00000134809	TIMM10	translocase of inner mitochondrial membrane 10 [Source:HGNC Symbol;Acc:HGNC:11814]	protein coding	1.33	2.26	12.78	0.0003	0.0406
ENSG00000188290	HES4	hes family bHLH transcription factor 4 [Source:HGNC Symbol;Acc:HGNC:24149]	protein coding	1.30	4.09	12.44	0.0004	0.0442
ENSG00000142089	IFITM3	interferon induced transmembrane protein 3 [Source:HGNC Symbol;Acc:HGNC:5414]	protein coding	1.20	8.81	25.40	<0.0001	0.0015

Table S17: Genes differentially expressed at Day 4 compared to pre-first vaccination (Study Arm 2 A/H7N9+AS03, D1, 29, Neutrophils). Genes are sorted by descending absolute log_2 fold change. Gene model summaries and annotations are based on Ensembl Version 99 (continued.)

Ensembl Gene ID	Ensembl Gene Name	Ensembl Gene Description	Gene Type	log_2 Fold Change (Day 4 vs. Pre-First Vaccination)	Average log_2 CPM	Likelihood Ratio Test Statistic	P-Value	FDR Adjusted P-Value
ENSG00000255221	CARD17	caspase recruitment domain family member 17 [Source:HGNC Symbol;Acc:HGNC:33827]	protein coding	1.18	1.95	17.38	<0.0001	0.0113
ENSG00000155561	NUP205	nucleoporin 205 [Source:HGNC Symbol;Acc:HGNC:18658]	protein coding	1.16	0.39	12.35	0.0004	0.045
ENSG00000198087	CD2AP	CD2 associated protein [Source:HGNC Symbol;Acc:HGNC:14258]	protein coding	1.12	1.83	13.38	0.0003	0.0344
ENSG00000183347	GBP6	guanylate binding protein family member 6 [Source:HGNC Symbol;Acc:HGNC:25395]	protein coding	1.03	2.00	12.75	0.0004	0.0409
ENSG00000008283	CYB561	cytochrome b561 [Source:HGNC Symbol;Acc:HGNC:2571]	protein coding	0.91	4.54	32.37	<0.0001	0.0001
ENSG00000235890	TSPEAR-AS1	TSPEAR antisense RNA 1 [Source:HGNC Symbol;Acc:HGNC:1271]	lncRNA	-0.87	1.12	24.74	<0.0001	0.0016
ENSG00000196405	EVL	Enah/Vasp-like [Source:HGNC Symbol;Acc:HGNC:20234]	protein coding	0.85	3.60	15.47	<0.0001	0.0181
ENSG00000122733	PHF24	PHD finger protein 24 [Source:HGNC Symbol;Acc:HGNC:29180]	protein coding	-0.84	2.48	21.39	<0.0001	0.004
ENSG00000177119	ANO6	anoctamin 6 [Source:HGNC Symbol;Acc:HGNC:25240]	protein coding	0.84	1.16	16.81	<0.0001	0.0125
ENSG00000134627	PIWIL4	piwi like RNA-mediated gene silencing 4 [Source:HGNC Symbol;Acc:HGNC:18444]	protein coding	0.81	0.37	13.48	0.0002	0.0335
ENSG00000260943	LINC02555	long intergenic non-protein coding RNA 2555 [Source:HGNC Symbol;Acc:HGNC:53593]	lncRNA	0.80	2.70	13.68	0.0002	0.0317
ENSG00000060138	YBX3	Y-box binding protein 3 [Source:HGNC Symbol;Acc:HGNC:2428]	protein coding	-0.78	5.15	16.67	<0.0001	0.0131
ENSG00000187231	SESTD1	SEC14 and spectrin domain containing 1 [Source:HGNC Symbol;Acc:HGNC:18379]	protein coding	0.74	3.58	12.90	0.0003	0.0391
ENSG00000175894	TSPEAR	thrombospondin type laminin G domain and EAR repeats [Source:HGNC Symbol;Acc:HGNC:1268]	protein coding	-0.71	2.33	16.96	<0.0001	0.0119
ENSG00000182912	TSPEAR-AS2	TSPEAR antisense RNA 2 [Source:HGNC Symbol;Acc:HGNC:16428]	lncRNA	-0.71	0.70	15.23	<0.0001	0.0194
ENSG00000267480		novel transcript, antisense IMPA2	lncRNA	-0.70	1.22	21.99	<0.0001	0.004
ENSG00000182158	CREB3L2	cAMP responsive element binding protein 3 like 2 [Source:HGNC Symbol;Acc:HGNC:23720]	protein coding	0.70	3.18	19.55	<0.0001	0.0054
ENSG00000073737	DHRS9	dehydrogenase/reductase 9 [Source:HGNC Symbol;Acc:HGNC:16888]	protein coding	0.69	5.81	14.81	0.0001	0.0229

Table S17: Genes differentially expressed at Day 4 compared to pre-first vaccination (Study Arm 2 A/H7N9+AS03, D1, 29, Neutrophils). Genes are sorted by descending absolute log_2 fold change. Gene model summaries and annotations are based on Ensembl Version 99 (continued.)

Ensembl Gene ID	Ensembl Gene Name	Ensembl Gene Description	Gene Type	log_2 Fold Change (Day 4 vs. Pre-First Vaccination)	Average log_2 CPM	Likelihood Ratio Test Statistic	P-Value	FDR Adjusted P-Value
ENSG00000099998	GGT5	gamma-glutamyltransferase 5 [Source:HGNC Symbol;Acc:HGNC:4260]	protein coding	-0.66	2.20	15.88	<0.0001	0.0157
ENSG00000138829	FBN2	fibrillin 2 [Source:HGNC Symbol;Acc:HGNC:3604]	protein coding	-0.66	1.44	14.99	0.0001	0.0212
ENSG00000166886	NAB2	NGFI-A binding protein 2 [Source:HGNC Symbol;Acc:HGNC:7627]	protein coding	-0.61	3.03	14.69	0.0001	0.0237
ENSG00000178607	ERN1	endoplasmic reticulum to nucleus signaling 1 [Source:HGNC Symbol;Acc:HGNC:3449]	protein coding	-0.59	6.82	19.39	<0.0001	0.0054
ENSG00000240344	PPIL3	peptidylprolyl isomerase like 3 [Source:HGNC Symbol;Acc:HGNC:9262]	protein coding	0.59	0.34	16.53	<0.0001	0.0137

Table S18: Genes differentially expressed at Day 30 compared to pre-second vaccination (Study Arm 2 A/H7N9+AS03, D1, 29, Neutrophils). Genes are sorted by descending absolute log_2 fold change. Gene model summaries and annotations are based on Ensembl Version 99.

Ensembl Gene ID	Ensembl Gene Name	Ensembl Gene Description	Gene Type	log_2 Fold Change (Day 30 vs. Pre-Second Vaccination)	Average log_2 CPM	Likelihood Ratio Test Statistic	P-Value	FDR Adjusted P-Value
ENSG00000131203	IDO1	indoleamine 2,3-dioxygenase 1 [Source:HGNC Symbol;Acc:HGNC:6059]	protein coding	5.14	6.53	106.01	<0.0001	<0.0001
ENSG00000162772	ATF3	activating transcription factor 3 [Source:HGNC Symbol;Acc:HGNC:785]	protein coding	4.28	3.68	65.26	<0.0001	<0.0001
ENSG00000108387	SEPTIN4	septin 4 [Source:HGNC Symbol;Acc:HGNC:9165]	protein coding	4.24	3.75	135.67	<0.0001	<0.0001
ENSG00000105967	TFEC	transcription factor EC [Source:HGNC Symbol;Acc:HGNC:11754]	protein coding	3.90	3.96	122.88	<0.0001	<0.0001
ENSG00000152766	ANKRD22	ankyrin repeat domain 22 [Source:HGNC Symbol;Acc:HGNC:28321]	protein coding	3.83	6.02	99.00	<0.0001	<0.0001
ENSG00000185338	SOCS1	suppressor of cytokine signaling 1 [Source:HGNC Symbol;Acc:HGNC:19383]	protein coding	3.81	5.05	73.32	<0.0001	<0.0001
ENSG00000197646	PDCD1LG2	programmed cell death 1 ligand 2 [Source:HGNC Symbol;Acc:HGNC:18731]	protein coding	3.76	2.55	94.77	<0.0001	<0.0001
ENSG00000131979	GCH1	GTP cyclohydrolase 1 [Source:HGNC Symbol;Acc:HGNC:4193]	protein coding	3.61	4.93	99.49	<0.0001	<0.0001

Confidential/Proprietary Information

Table S18: Genes differentially expressed at Day 30 compared to pre-second vaccination (Study Arm 2 A/H7N9+AS03, D1, 29, Neutrophils). Genes are sorted by descending absolute log_2 fold change. Gene model summaries and annotations are based on Ensembl Version 99 (continued.)

Ensembl Gene ID	Ensembl Gene Name	Ensembl Gene Description	Gene Type	log_2 Fold Change (Day 30 vs. Pre-Second Vaccination)	Average log_2 CPM	Likelihood Ratio Test Statistic	P-Value	FDR Adjusted P-Value
ENSG00000168899	VAMP5	vesicle associated membrane protein 5 [Source:HGNC Symbol;Acc:HGNC:12646]	protein coding	3.54	5.70	94.11	<0.0001	<0.0001
ENSG00000078081	LAMP3	lysosomal associated membrane protein 3 [Source:HGNC Symbol;Acc:HGNC:14582]	protein coding	3.54	4.59	82.83	<0.0001	<0.0001
ENSG00000174944	P2RY14	purinergic receptor P2Y14 [Source:HGNC Symbol;Acc:HGNC:16442]	protein coding	3.40	6.60	144.53	<0.0001	<0.0001
ENSG00000133321	PLAAT4	phospholipase A and acyltransferase 4 [Source:HGNC Symbol;Acc:HGNC:9869]	protein coding	3.37	5.59	70.25	<0.0001	<0.0001
ENSG00000179344	HLA-DQB1	major histocompatibility complex, class II, DQ beta 1 [Source:HGNC Symbol;Acc:HGNC:4944]	protein coding	3.31	5.92	28.67	<0.0001	<0.0001
ENSG00000196141	SPATS2L	spermatogenesis associated serine rich 2 like [Source:HGNC Symbol;Acc:HGNC:24574]	protein coding	3.28	3.24	116.65	<0.0001	<0.0001
ENSG00000111335	OAS2	2'-5'-oligoadenylate synthetase 2 [Source:HGNC Symbol;Acc:HGNC:8087]	protein coding	3.22	7.24	101.30	<0.0001	<0.0001
ENSG00000204287	HLA-DRA	major histocompatibility complex, class II, DR alpha [Source:HGNC Symbol;Acc:HGNC:4947]	protein coding	3.13	6.50	101.50	<0.0001	<0.0001
ENSG00000136514	RTP4	receptor transporter protein 4 [Source:HGNC Symbol;Acc:HGNC:23992]	protein coding	3.11	4.09	56.83	<0.0001	<0.0001
ENSG00000110848	CD69	CD69 molecule [Source:HGNC Symbol;Acc:HGNC:1694]	protein coding	3.10	3.31	68.95	<0.0001	<0.0001
ENSG00000120217	CD274	CD274 molecule [Source:HGNC Symbol;Acc:HGNC:17635]	protein coding	3.08	6.31	72.88	<0.0001	<0.0001
ENSG00000089127	OAS1	2'-5'-oligoadenylate synthetase 1 [Source:HGNC Symbol;Acc:HGNC:8086]	protein coding	3.07	7.30	108.25	<0.0001	<0.0001
ENSG00000198087	CD2AP	CD2 associated protein [Source:HGNC Symbol;Acc:HGNC:14258]	protein coding	3.03	2.99	98.48	<0.0001	<0.0001
ENSG00000137959	IFI44L	interferon induced protein 44 like [Source:HGNC Symbol;Acc:HGNC:17817]	protein coding	3.03	7.20	115.73	<0.0001	<0.0001
ENSG00000168062	BATF2	basic leucine zipper ATF-like transcription factor 2 [Source:HGNC Symbol;Acc:HGNC:25163]	protein coding	2.98	6.22	84.54	<0.0001	<0.0001
ENSG00000183347	GBP6	guanylate binding protein family member 6 [Source:HGNC Symbol;Acc:HGNC:25395]	protein coding	2.97	3.60	69.65	<0.0001	<0.0001
ENSG00000128284	APOL3	apolipoprotein L3 [Source:HGNC Symbol;Acc:HGNC:14868]	protein coding	2.95	3.32	66.89	<0.0001	<0.0001
ENSG00000130881	LRP3	LDL receptor related protein 3 [Source:HGNC Symbol;Acc:HGNC:6695]	protein coding	-2.92	2.56	68.64	<0.0001	<0.0001

Table S18: Genes differentially expressed at Day 30 compared to pre-second vaccination (Study Arm 2 A/H7N9+AS03, D1, 29, Neutrophils). Genes are sorted by descending absolute log_2 fold change. Gene model summaries and annotations are based on Ensembl Version 99 (continued.)

Ensembl Gene ID	Ensembl Gene Name	Ensembl Gene Description	Gene Type	log_2 Fold Change (Day 30 vs. Pre-Second Vaccination)	Average log_2 CPM	Likelihood Ratio Test Statistic	P-Value	FDR Adjusted P-Value
ENSG00000172159	FRMD3	FERM domain containing 3 [Source:HGNC Symbol;Acc:HGNC:24125]	protein coding	2.91	3.47	87.71	<0.0001	<0.0001
ENSG00000154451	GBP5	guanylate binding protein 5 [Source:HGNC Symbol;Acc:HGNC:19895]	protein coding	2.90	10.87	89.17	<0.0001	<0.0001
ENSG00000198133	TMEM229B	transmembrane protein 229B [Source:HGNC Symbol;Acc:HGNC:20130]	protein coding	2.88	2.12	64.88	<0.0001	<0.0001
ENSG00000149131	SERPING1	serpin family G member 1 [Source:HGNC Symbol;Acc:HGNC:1228]	protein coding	2.84	7.50	96.02	<0.0001	<0.0001
ENSG00000134809	TIMM10	translocase of inner mitochondrial membrane 10 [Source:HGNC Symbol;Acc:HGNC:11814]	protein coding	2.83	3.76	115.11	<0.0001	<0.0001
ENSG00000010030	ETV7	ETS variant transcription factor 7 [Source:HGNC Symbol;Acc:HGNC:18160]	protein coding	2.82	6.15	72.74	<0.0001	<0.0001
ENSG00000175643	RMI2	RecQ mediated genome instability 2 [Source:HGNC Symbol;Acc:HGNC:28349]	protein coding	2.79	3.45	90.37	<0.0001	<0.0001
ENSG00000286162		novel transcript	lncRNA	2.78	2.23	89.04	<0.0001	<0.0001
ENSG00000138166	DUSP5	dual specificity phosphatase 5 [Source:HGNC Symbol;Acc:HGNC:3071]	protein coding	2.76	3.01	66.45	<0.0001	<0.0001
ENSG00000155561	NUP205	nucleoporin 205 [Source:HGNC Symbol;Acc:HGNC:18658]	protein coding	2.74	1.47	87.66	<0.0001	<0.0001
ENSG00000102755	FLT1	fms related tyrosine kinase 1 [Source:HGNC Symbol;Acc:HGNC:3763]	protein coding	-2.73	1.84	65.65	<0.0001	<0.0001
ENSG00000188820	CALHM6	calcium homeostasis modulator family member 6 [Source:HGNC Symbol;Acc:HGNC:33391]	protein coding	2.72	5.06	66.79	<0.0001	<0.0001
ENSG00000242574	HLA-DMB	major histocompatibility complex, class II, DM beta [Source:HGNC Symbol;Acc:HGNC:4935]	protein coding	2.70	4.60	66.88	<0.0001	<0.0001
ENSG00000134321	RSAD2	radical S-adenosyl methionine domain containing 2 [Source:HGNC Symbol;Acc:HGNC:30908]	protein coding	2.70	8.89	73.97	<0.0001	<0.0001
ENSG00000162654	GBP4	guanylate binding protein 4 [Source:HGNC Symbol;Acc:HGNC:20480]	protein coding	2.69	7.91	70.70	<0.0001	<0.0001
ENSG00000196126	HLA-DRB1	major histocompatibility complex, class II, DR beta 1 [Source:HGNC Symbol;Acc:HGNC:4948]	protein coding	2.69	2.81	38.90	<0.0001	<0.0001
ENSG00000145685	LHFPL2	LHFPL tetraspan subfamily member 2 [Source:HGNC Symbol;Acc:HGNC:6588]	protein coding	2.69	4.52	95.82	<0.0001	<0.0001
ENSG00000111331	OAS3	2'-5'-oligoadenylate synthetase 3 [Source:HGNC Symbol;Acc:HGNC:8088]	protein coding	2.68	8.64	104.69	<0.0001	<0.0001

Table S18: Genes differentially expressed at Day 30 compared to pre-second vaccination (Study Arm 2 A/H7N9+AS03, D1, 29, Neutrophils). Genes are sorted by descending absolute log_2 fold change. Gene model summaries and annotations are based on Ensembl Version 99 (continued.)

Ensembl Gene ID	Ensembl Gene Name	Ensembl Gene Description	Gene Type	log_2 Fold Change (Day 30 vs. Pre-Second Vaccination)	Average log_2 CPM	Likelihood Ratio Test Statistic	P-Value	FDR Adjusted P-Value
ENSG00000002549	LAP3	leucine aminopeptidase 3 [Source:HGNC Symbol;Acc:HGNC:18449]	protein coding	2.67	7.14	65.87	<0.0001	<0.0001
ENSG00000137965	IFI44	interferon induced protein 44 [Source:HGNC Symbol;Acc:HGNC:16938]	protein coding	2.66	6.99	89.98	<0.0001	<0.0001
ENSG00000150337	FCGR1A	Fc fragment of IgG receptor Ia [Source:HGNC Symbol;Acc:HGNC:3613]	protein coding	2.64	6.72	59.97	<0.0001	<0.0001
ENSG00000265531	FCGR1CP	Fc fragment of IgG receptor Ic, pseudogene [Source:HGNC Symbol;Acc:HGNC:3615]	unprocessed pseudogene	2.63	3.80	55.68	<0.0001	<0.0001
ENSG00000133106	EPSTI1	epithelial stromal interaction 1 [Source:HGNC Symbol;Acc:HGNC:16465]	protein coding	2.62	7.40	61.26	<0.0001	<0.0001
ENSG00000168405	CMAHP	cytidine monophospho-N-acetylneuraminic acid hydroxylase, pseudogene [Source:HGNC Symbol;Acc:HGNC:2098]	transcribed unitary pseudogene	2.58	2.58	93.80	<0.0001	<0.0001
ENSG00000124491	F13A1	coagulation factor XIII A chain [Source:HGNC Symbol;Acc:HGNC:3531]	protein coding	-2.54	2.08	89.52	<0.0001	<0.0001
ENSG00000214872	SMTNL1	smoothelin like 1 [Source:HGNC Symbol;Acc:HGNC:32394]	protein coding	2.54	4.42	84.86	<0.0001	<0.0001
ENSG00000131943	C19orf12	chromosome 19 open reading frame 12 [Source:HGNC Symbol;Acc:HGNC:25443]	protein coding	2.51	2.65	72.03	<0.0001	<0.0001
ENSG00000114770	ABCC5	ATP binding cassette subfamily C member 5 [Source:HGNC Symbol;Acc:HGNC:56]	protein coding	-2.50	6.21	71.70	<0.0001	<0.0001
ENSG00000165806	CASP7	caspase 7 [Source:HGNC Symbol;Acc:HGNC:1508]	protein coding	2.49	1.67	53.46	<0.0001	<0.0001
ENSG00000140105	WARS1	tryptophanyl-tRNA synthetase 1 [Source:HGNC Symbol;Acc:HGNC:12729]	protein coding	2.49	8.51	54.84	<0.0001	<0.0001
ENSG00000121797	CCRL2	C-C motif chemokine receptor like 2 [Source:HGNC Symbol;Acc:HGNC:1612]	protein coding	2.49	3.09	64.69	<0.0001	<0.0001
ENSG00000225964	NRIR	negative regulator of interferon response [Source:HGNC Symbol;Acc:HGNC:51269]	lncRNA	2.47	1.90	81.10	<0.0001	<0.0001
ENSG00000166002	SMCO4	single-pass membrane protein with coiled-coil domains 4 [Source:HGNC Symbol;Acc:HGNC:24810]	protein coding	2.45	4.49	76.20	<0.0001	<0.0001
ENSG00000204257	HLA-DMA	major histocompatibility complex, class II, DM alpha [Source:HGNC Symbol;Acc:HGNC:4934]	protein coding	2.45	4.16	75.56	<0.0001	<0.0001

Table S18: Genes differentially expressed at Day 30 compared to pre-second vaccination (Study Arm 2 A/H7N9+AS03, D1, 29, Neutrophils). Genes are sorted by descending absolute log_2 fold change. Gene model summaries and annotations are based on Ensembl Version 99 (continued.)

Ensembl Gene ID	Ensembl Gene Name	Ensembl Gene Description	Gene Type	log_2 Fold Change (Day 30 vs. Pre-Second Vaccination)	Average log_2 CPM	Likelihood Ratio Test Statistic	P-Value	FDR Adjusted P-Value
ENSG00000134326	CMPK2	cytidine/uridine monophosphate kinase 2 [Source:HGNC Symbol;Acc:HGNC:27015]	protein coding	2.44	6.93	66.23	<0.0001	<0.0001
ENSG00000158089	GALNT14	polypeptide N-acetylgalactosaminyltransferase 14 [Source:HGNC Symbol;Acc:HGNC:22946]	protein coding	-2.44	3.19	48.67	<0.0001	<0.0001
ENSG00000188483	IER5L	immediate early response 5 like [Source:HGNC Symbol;Acc:HGNC:23679]	protein coding	-2.40	3.71	97.34	<0.0001	<0.0001
ENSG00000026751	SLAMF7	SLAM family member 7 [Source:HGNC Symbol;Acc:HGNC:21394]	protein coding	2.38	3.28	71.12	<0.0001	<0.0001
ENSG00000023171	GRAMD1B	GRAM domain containing 1B [Source:HGNC Symbol;Acc:HGNC:29214]	protein coding	2.36	4.52	82.97	<0.0001	<0.0001
ENSG00000155363	MOV10	Mov10 RISC complex RNA helicase [Source:HGNC Symbol;Acc:HGNC:7200]	protein coding	2.35	6.15	99.39	<0.0001	<0.0001
ENSG00000166750	SLFN5	schlafen family member 5 [Source:HGNC Symbol;Acc:HGNC:28286]	protein coding	2.35	3.70	56.57	<0.0001	<0.0001
ENSG00000160932	LY6E	lymphocyte antigen 6 family member E [Source:HGNC Symbol;Acc:HGNC:6727]	protein coding	2.33	8.14	95.36	<0.0001	<0.0001
ENSG00000102445	RUBCNL	rubicon like autophagy enhancer [Source:HGNC Symbol;Acc:HGNC:20420]	protein coding	-2.33	6.40	55.81	<0.0001	<0.0001
ENSG00000203666	EFCAB2	EF-hand calcium binding domain 2 [Source:HGNC Symbol;Acc:HGNC:28166]	protein coding	2.33	3.98	79.55	<0.0001	<0.0001
ENSG00000148175	STOM	stomatin [Source:HGNC Symbol;Acc:HGNC:3383]	protein coding	2.33	5.51	63.94	<0.0001	<0.0001
ENSG00000117228	GBP1	guanylate binding protein 1 [Source:HGNC Symbol;Acc:HGNC:4182]	protein coding	2.33	8.77	65.46	<0.0001	<0.0001
ENSG00000128394	APOBEC3F	apolipoprotein B mRNA editing enzyme catalytic subunit 3F [Source:HGNC Symbol;Acc:HGNC:17356]	protein coding	2.29	2.91	69.12	<0.0001	<0.0001
ENSG00000129673	AANAT	aralkylamine N-acetyltransferase [Source:HGNC Symbol;Acc:HGNC:19]	protein coding	2.29	1.81	72.33	<0.0001	<0.0001
ENSG00000152229	PSTPIP2	proline-serine-threonine phosphatase interacting protein 2 [Source:HGNC Symbol;Acc:HGNC:9581]	protein coding	2.29	6.52	108.56	<0.0001	<0.0001
ENSG00000128203	ASPHD2	aspartate beta-hydroxylase domain containing 2 [Source:HGNC Symbol;Acc:HGNC:30437]	protein coding	2.27	3.28	85.19	<0.0001	<0.0001
ENSG00000117226	GBP3	guanylate binding protein 3 [Source:HGNC Symbol;Acc:HGNC:4184]	protein coding	2.26	5.02	74.92	<0.0001	<0.0001
ENSG00000132530	XAF1	XIAP associated factor 1 [Source:HGNC Symbol;Acc:HGNC:30932]	protein coding	2.26	6.22	70.34	<0.0001	<0.0001

Table S18: Genes differentially expressed at Day 30 compared to pre-second vaccination (Study Arm 2 A/H7N9+AS03, D1, 29, Neutrophils). Genes are sorted by descending absolute log_2 fold change. Gene model summaries and annotations are based on Ensembl Version 99 (continued.)

Ensembl Gene ID	Ensembl Gene Name	Ensembl Gene Description	Gene Type	log_2 Fold Change (Day 30 vs. Pre-Second Vaccination)	Average log_2 CPM	Likelihood Ratio Test Statistic	P-Value	FDR Adjusted P-Value
ENSG00000223914	LINC02471	long intergenic non-protein coding RNA 2471 [Source:HGNC Symbol;Acc:HGNC:53410]	lncRNA	2.26	1.40	99.04	<0.0001	<0.0001
ENSG00000255221	CARD17	caspase recruitment domain family member 17 [Source:HGNC Symbol;Acc:HGNC:33827]	protein coding	2.26	2.80	150.01	<0.0001	<0.0001
ENSG00000187608	ISG15	ISG15 ubiquitin like modifier [Source:HGNC Symbol;Acc:HGNC:4053]	protein coding	2.26	8.68	80.61	<0.0001	<0.0001
ENSG00000142089	IFITM3	interferon induced transmembrane protein 3 [Source:HGNC Symbol;Acc:HGNC:5414]	protein coding	2.25	9.55	96.53	<0.0001	<0.0001
ENSG00000260943	LINC02555	long intergenic non-protein coding RNA 2555 [Source:HGNC Symbol;Acc:HGNC:53593]	lncRNA	2.24	3.57	82.55	<0.0001	<0.0001
ENSG00000019582	CD74	CD74 molecule [Source:HGNC Symbol;Acc:HGNC:1697]	protein coding	2.23	9.37	86.99	<0.0001	<0.0001
ENSG00000177721	ANXA2R	annexin A2 receptor [Source:HGNC Symbol;Acc:HGNC:33463]	protein coding	2.23	2.22	62.19	<0.0001	<0.0001
ENSG00000137628	DDX60	DExD/H-box helicase 60 [Source:HGNC Symbol;Acc:HGNC:25942]	protein coding	2.22	6.45	72.35	<0.0001	<0.0001
ENSG00000065911	MTHFD2	methylenetetrahydrofolate dehydrogenase (NADP+ dependent) 2, methenyltetrahydrofolate cyclohydrolase [Source:HGNC Symbol;Acc:HGNC:7434]	protein coding	2.21	2.87	95.69	<0.0001	<0.0001
ENSG00000168421	RHOH	ras homolog family member H [Source:HGNC Symbol;Acc:HGNC:686]	protein coding	2.18	2.37	57.44	<0.0001	<0.0001
ENSG00000230438	SERPINB9P1	serpin family B member 9 pseudogene 1 [Source:HGNC Symbol;Acc:HGNC:28590]	lncRNA	2.17	2.83	67.99	<0.0001	<0.0001
ENSG00000157693	TMEM268	transmembrane protein 268 [Source:HGNC Symbol;Acc:HGNC:24513]	protein coding	2.17	4.81	84.90	<0.0001	<0.0001
ENSG00000124785	NRN1	neuritin 1 [Source:HGNC Symbol;Acc:HGNC:17972]	protein coding	2.17	1.94	41.28	<0.0001	<0.0001
ENSG00000108679	LGALS3BP	galectin 3 binding protein [Source:HGNC Symbol;Acc:HGNC:6564]	protein coding	2.16	3.77	84.27	<0.0001	<0.0001
ENSG00000068079	IFI35	interferon induced protein 35 [Source:HGNC Symbol;Acc:HGNC:5399]	protein coding	2.16	7.02	75.67	<0.0001	<0.0001
ENSG00000196123	KIAA0895L	KIAA0895 like [Source:HGNC Symbol;Acc:HGNC:34408]	protein coding	2.16	2.13	77.65	<0.0001	<0.0001
ENSG00000164308	ERAP2	endoplasmic reticulum aminopeptidase 2 [Source:HGNC Symbol;Acc:HGNC:29499]	protein coding	2.15	4.95	67.71	<0.0001	<0.0001

Confidential/Proprietary Information

Table S18: Genes differentially expressed at Day 30 compared to pre-second vaccination (Study Arm 2 A/H7N9+AS03, D1, 29, Neutrophils). Genes are sorted by descending absolute log_2 fold change. Gene model summaries and annotations are based on Ensembl Version 99 (continued.)

Ensembl Gene ID	Ensembl Gene Name	Ensembl Gene Description	Gene Type	log_2 Fold Change (Day 30 vs. Pre-Second Vaccination)	Average log_2 CPM	Likelihood Ratio Test Statistic	P-Value	FDR Adjusted P-Value
ENSG00000144893	MED12L	mediator complex subunit 12L [Source:HGNC Symbol;Acc:HGNC:16050]	protein coding	2.15	1.17	86.36	<0.0001	<0.0001
ENSG00000034510	TMSB10	thymosin beta 10 [Source:HGNC Symbol;Acc:HGNC:11879]	protein coding	2.15	8.13	122.22	<0.0001	<0.0001
ENSG00000101347	SAMHD1	SAM and HD domain containing deoxynucleoside triphosphate triphosphohydrolase 1 [Source:HGNC Symbol;Acc:HGNC:15925]	protein coding	2.14	7.60	81.06	<0.0001	<0.0001
ENSG00000106537	TSPAN13	tetraspanin 13 [Source:HGNC Symbol;Acc:HGNC:21643]	protein coding	-2.14	2.58	85.80	<0.0001	<0.0001
ENSG00000251136		novel transcript	lncRNA	2.13	1.54	77.24	<0.0001	<0.0001
ENSG00000244509	APOBEC3C	apolipoprotein B mRNA editing enzyme catalytic subunit 3C [Source:HGNC Symbol;Acc:HGNC:17353]	protein coding	2.12	3.35	75.30	<0.0001	<0.0001
ENSG00000155366	RHOC	ras homolog family member C [Source:HGNC Symbol;Acc:HGNC:669]	protein coding	2.11	2.27	70.76	<0.0001	<0.0001
ENSG00000126351	THRA	thyroid hormone receptor alpha [Source:HGNC Symbol;Acc:HGNC:11796]	protein coding	-2.11	3.88	63.45	<0.0001	<0.0001
ENSG00000116663	FBXO6	F-box protein 6 [Source:HGNC Symbol;Acc:HGNC:13585]	protein coding	2.11	5.30	89.12	<0.0001	<0.0001
ENSG00000163958	ZDHHC19	zinc finger DHHC-type containing 19 [Source:HGNC Symbol;Acc:HGNC:20713]	protein coding	2.11	2.52	61.33	<0.0001	<0.0001
ENSG00000170542	SERPINB9	serpin family B member 9 [Source:HGNC Symbol;Acc:HGNC:8955]	protein coding	2.11	5.12	59.30	<0.0001	<0.0001
ENSG00000132274	TRIM22	tripartite motif containing 22 [Source:HGNC Symbol;Acc:HGNC:16379]	protein coding	2.09	9.38	78.02	<0.0001	<0.0001
ENSG00000111696	NT5DC3	5'-nucleotidase domain containing 3 [Source:HGNC Symbol;Acc:HGNC:30826]	protein coding	-2.07	0.92	27.61	<0.0001	<0.0001
ENSG00000100911	PSME2	proteasome activator subunit 2 [Source:HGNC Symbol;Acc:HGNC:9569]	protein coding	2.06	7.04	65.68	<0.0001	<0.0001
ENSG00000233029		novel transcript	lncRNA	2.04	3.55	53.78	<0.0001	<0.0001
ENSG00000221963	APOL6	apolipoprotein L6 [Source:HGNC Symbol;Acc:HGNC:14870]	protein coding	2.03	8.14	76.91	<0.0001	<0.0001
ENSG00000110492	MDK	midkine [Source:HGNC Symbol;Acc:HGNC:6972]	protein coding	2.02	1.76	103.79	<0.0001	<0.0001
ENSG00000277511		novel transcript	lncRNA	2.00	1.21	37.75	<0.0001	<0.0001
ENSG00000132470	ITGB4	integrin subunit beta 4 [Source:HGNC Symbol;Acc:HGNC:6158]	protein coding	-2.00	3.20	129.93	<0.0001	<0.0001

Table S18: Genes differentially expressed at Day 30 compared to pre-second vaccination (Study Arm 2 A/H7N9+AS03, D1, 29, Neutrophils). Genes are sorted by descending absolute log_2 fold change. Gene model summaries and annotations are based on Ensembl Version 99 (continued.)

Ensembl Gene ID	Ensembl Gene Name	Ensembl Gene Description	Gene Type	log_2 Fold Change (Day 30 vs. Pre-Second Vaccination)	Average log_2 CPM	Likelihood Ratio Test Statistic	P-Value	FDR Adjusted P-Value
ENSG00000163568	AIM2	absent in melanoma 2 [Source:HGNC Symbol;Acc:HGNC:357]	protein coding	2.00	5.05	90.75	<0.0001	<0.0001
ENSG00000140464	PML	promyelocytic leukemia [Source:HGNC Symbol;Acc:HGNC:9113]	protein coding	1.99	7.54	88.72	<0.0001	<0.0001
ENSG00000069020	MAST4	microtubule associated serine/threonine kinase family member 4 [Source:HGNC Symbol;Acc:HGNC:19037]	protein coding	-1.99	1.52	51.32	<0.0001	<0.0001
ENSG00000126709	IFI6	interferon alpha inducible protein 6 [Source:HGNC Symbol;Acc:HGNC:4054]	protein coding	1.99	9.05	86.57	<0.0001	<0.0001
ENSG00000107957	SH3PXD2A	SH3 and PX domains 2A [Source:HGNC Symbol;Acc:HGNC:23664]	protein coding	1.98	0.81	45.61	<0.0001	<0.0001
ENSG00000038210	PI4K2B	phosphatidylinositol 4-kinase type 2 beta [Source:HGNC Symbol;Acc:HGNC:18215]	protein coding	1.98	2.26	69.63	<0.0001	<0.0001
ENSG00000233030		novel transcript, antisense to FCGR1A	lncRNA	1.98	2.85	57.56	<0.0001	<0.0001
ENSG00000182240	BACE2	beta-secretase 2 [Source:HGNC Symbol;Acc:HGNC:934]	protein coding	-1.98	1.43	11.69	0.0006	0.002
ENSG00000230124	ACBD6	acyl-CoA binding domain containing 6 [Source:HGNC Symbol;Acc:HGNC:23339]	protein coding	-1.97	1.34	30.68	<0.0001	<0.0001
ENSG00000225886		novel transcript, antisense to IFI6	lncRNA	1.97	3.37	61.65	<0.0001	<0.0001
ENSG00000065427	KARS1	lysyl-tRNA synthetase 1 [Source:HGNC Symbol;Acc:HGNC:6215]	protein coding	1.96	5.40	49.47	<0.0001	<0.0001
ENSG00000136732	GYPC	glycophorin C (Gerbich blood group) [Source:HGNC Symbol;Acc:HGNC:4704]	protein coding	1.96	3.49	72.12	<0.0001	<0.0001
ENSG00000110799	VWF	von Willebrand factor [Source:HGNC Symbol;Acc:HGNC:12726]	protein coding	-1.95	1.59	57.74	<0.0001	<0.0001
ENSG00000277969		novel transcript	lncRNA	1.95	1.92	60.13	<0.0001	<0.0001
ENSG00000115109	EPB41L5	erythrocyte membrane protein band 4.1 like 5 [Source:HGNC Symbol;Acc:HGNC:19819]	protein coding	1.95	2.40	116.69	<0.0001	<0.0001
ENSG00000143891	GALM	galactose mutarotase [Source:HGNC Symbol;Acc:HGNC:24063]	protein coding	1.93	4.59	86.83	<0.0001	<0.0001
ENSG00000136770	DNAJC1	DnaJ heat shock protein family (Hsp40) member C1 [Source:HGNC Symbol;Acc:HGNC:20090]	protein coding	1.93	4.16	68.22	<0.0001	<0.0001
ENSG00000143390	RFX5	regulatory factor X5 [Source:HGNC Symbol;Acc:HGNC:9986]	protein coding	1.92	4.07	79.30	<0.0001	<0.0001
ENSG00000169439	SDC2	syndecan 2 [Source:HGNC Symbol;Acc:HGNC:10659]	protein coding	-1.92	3.29	71.71	<0.0001	<0.0001

Table S18: Genes differentially expressed at Day 30 compared to pre-second vaccination (Study Arm 2 A/H7N9+AS03, D1, 29, Neutrophils). Genes are sorted by descending absolute log_2 fold change. Gene model summaries and annotations are based on Ensembl Version 99 (continued.)

Ensembl Gene ID	Ensembl Gene Name	Ensembl Gene Description	Gene Type	log_2 Fold Change (Day 30 vs. Pre-Second Vaccination)	Average log_2 CPM	Likelihood Ratio Test Statistic	P-Value	FDR Adjusted P-Value
ENSG00000188313	PLSCR1	phospholipid scramblase 1 [Source:HGNC Symbol;Acc:HGNC:9092]	protein coding	1.92	7.40	82.87	<0.0001	<0.0001
ENSG00000138385	SSB	small RNA binding exonuclease protection factor La [Source:HGNC Symbol;Acc:HGNC:11316]	protein coding	1.92	2.87	62.24	<0.0001	<0.0001
ENSG00000067221	STOML1	stomatin like 1 [Source:HGNC Symbol;Acc:HGNC:14560]	protein coding	1.92	2.28	54.36	<0.0001	<0.0001
ENSG00000198019	FCGR1B	Fc fragment of IgG receptor Ib [Source:HGNC Symbol;Acc:HGNC:3614]	protein coding	1.91	5.70	54.13	<0.0001	<0.0001
ENSG00000213373	LINC00671	long intergenic non-protein coding RNA 671 [Source:HGNC Symbol;Acc:HGNC:44339]	lncRNA	-1.90	2.94	88.06	<0.0001	<0.0001
ENSG00000163644	PPM1K	protein phosphatase, Mg2+/Mn2+ dependent 1K [Source:HGNC Symbol;Acc:HGNC:25415]	protein coding	1.89	2.48	51.20	<0.0001	<0.0001
ENSG00000173193	PARP14	poly(ADP-ribose) polymerase family member 14 [Source:HGNC Symbol;Acc:HGNC:29232]	protein coding	1.89	9.23	68.61	<0.0001	<0.0001
ENSG00000130303	BST2	bone marrow stromal cell antigen 2 [Source:HGNC Symbol;Acc:HGNC:1119]	protein coding	1.89	6.16	99.09	<0.0001	<0.0001
ENSG00000111912	NCOA7	nuclear receptor coactivator 7 [Source:HGNC Symbol;Acc:HGNC:21081]	protein coding	1.89	4.11	69.59	<0.0001	<0.0001
ENSG00000123610	TNFAIP6	TNF alpha induced protein 6 [Source:HGNC Symbol;Acc:HGNC:11898]	protein coding	1.88	7.20	77.47	<0.0001	<0.0001
ENSG00000111224	PARP11	poly(ADP-ribose) polymerase family member 11 [Source:HGNC Symbol;Acc:HGNC:1186]	protein coding	1.88	2.57	72.68	<0.0001	<0.0001
ENSG00000177119	ANO6	anoctamin 6 [Source:HGNC Symbol;Acc:HGNC:25240]	protein coding	1.86	1.67	111.43	<0.0001	<0.0001
ENSG00000137757	CASP5	caspase 5 [Source:HGNC Symbol;Acc:HGNC:1506]	protein coding	1.86	5.61	78.29	<0.0001	<0.0001
ENSG00000172985	SH3RF3	SH3 domain containing ring finger 3 [Source:HGNC Symbol;Acc:HGNC:24699]	protein coding	-1.86	3.66	36.11	<0.0001	<0.0001
ENSG00000156587	UBE2L6	ubiquitin conjugating enzyme E2 L6 [Source:HGNC Symbol;Acc:HGNC:12490]	protein coding	1.86	8.60	71.23	<0.0001	<0.0001
ENSG00000137200	CMTR1	cap methyltransferase 1 [Source:HGNC Symbol;Acc:HGNC:21077]	protein coding	1.86	6.31	83.43	<0.0001	<0.0001
ENSG00000162714	ZNF496	zinc finger protein 496 [Source:HGNC Symbol;Acc:HGNC:23713]	protein coding	1.85	3.33	86.49	<0.0001	<0.0001
ENSG00000134070	IRAK2	interleukin 1 receptor associated kinase 2 [Source:HGNC Symbol;Acc:HGNC:6113]	protein coding	1.84	3.26	88.35	<0.0001	<0.0001

Table S18: Genes differentially expressed at Day 30 compared to pre-second vaccination (Study Arm 2 A/H7N9+AS03, D1, 29, Neutrophils). Genes are sorted by descending absolute log_2 fold change. Gene model summaries and annotations are based on Ensembl Version 99 (continued.)

Ensembl Gene ID	Ensembl Gene Name	Ensembl Gene Description	Gene Type	log_2 Fold Change (Day 30 vs. Pre-Second Vaccination)	Average log_2 CPM	Likelihood Ratio Test Statistic	P-Value	FDR Adjusted P-Value
ENSG00000128833	MYO5C	myosin VC [Source:HGNC Symbol;Acc:HGNC:7604]	protein coding	-1.84	0.41	47.65	<0.0001	<0.0001
ENSG00000145365	TIFA	TRAF interacting protein with forkhead associated domain [Source:HGNC Symbol;Acc:HGNC:19075]	protein coding	1.84	5.62	55.83	<0.0001	<0.0001
ENSG00000103355	PRSS33	serine protease 33 [Source:HGNC Symbol;Acc:HGNC:30405]	protein coding	-1.83	4.46	10.56	0.0012	0.0034
ENSG00000162894	FCMR	Fc fragment of IgM receptor [Source:HGNC Symbol;Acc:HGNC:14315]	protein coding	-1.83	4.47	144.62	<0.0001	<0.0001
ENSG00000138646	HERC5	HECT and RLD domain containing E3 ubiquitin protein ligase 5 [Source:HGNC Symbol;Acc:HGNC:24368]	protein coding	1.83	7.62	60.02	<0.0001	<0.0001
ENSG00000104805	NUCB1	nucleobindin 1 [Source:HGNC Symbol;Acc:HGNC:8043]	protein coding	1.83	8.41	61.68	<0.0001	<0.0001
ENSG00000282851	BISPR	BST2 interferon stimulated positive regulator [Source:HGNC Symbol;Acc:HGNC:51290]	lncRNA	1.82	3.66	62.03	<0.0001	<0.0001
ENSG00000028116	VRK2	VRK serine/threonine kinase 2 [Source:HGNC Symbol;Acc:HGNC:12719]	protein coding	1.82	3.19	103.19	<0.0001	<0.0001
ENSG00000188282	RUFY4	RUN and FYVE domain containing 4 [Source:HGNC Symbol;Acc:HGNC:24804]	protein coding	1.82	1.02	55.65	<0.0001	<0.0001
ENSG00000161981	SNRNP25	small nuclear ribonucleoprotein U11/U12 subunit 25 [Source:HGNC Symbol;Acc:HGNC:14161]	protein coding	-1.82	1.11	41.67	<0.0001	<0.0001
ENSG00000088992	TESC	tescalcin [Source:HGNC Symbol;Acc:HGNC:26065]	protein coding	-1.81	2.95	25.53	<0.0001	<0.0001
ENSG00000075303	SLC25A40	solute carrier family 25 member 40 [Source:HGNC Symbol;Acc:HGNC:29680]	protein coding	-1.81	3.28	37.56	<0.0001	<0.0001
ENSG00000135074	ADAM19	ADAM metallopeptidase domain 19 [Source:HGNC Symbol;Acc:HGNC:197]	protein coding	-1.81	7.11	42.21	<0.0001	<0.0001
ENSG00000110060	PUS3	pseudouridine synthase 3 [Source:HGNC Symbol;Acc:HGNC:25461]	protein coding	1.80	1.09	32.21	<0.0001	<0.0001
ENSG00000108771	DHX58	DExH-box helicase 58 [Source:HGNC Symbol;Acc:HGNC:29517]	protein coding	1.80	5.66	80.57	<0.0001	<0.0001
ENSG00000087237	CETP	cholesteryl ester transfer protein [Source:HGNC Symbol;Acc:HGNC:1869]	protein coding	1.80	1.19	54.73	<0.0001	<0.0001
ENSG00000115392	FANCL	FA complementation group L [Source:HGNC Symbol;Acc:HGNC:20748]	protein coding	1.79	0.82	68.37	<0.0001	<0.0001
ENSG00000119917	IFIT3	interferon induced protein with tetratricopeptide repeats 3 [Source:HGNC Symbol;Acc:HGNC:5411]	protein coding	1.79	10.53	73.53	<0.0001	<0.0001

Table S18: Genes differentially expressed at Day 30 compared to pre-second vaccination (Study Arm 2 A/H7N9+AS03, D1, 29, Neutrophils). Genes are sorted by descending absolute log_2 fold change. Gene model summaries and annotations are based on Ensembl Version 99 (continued.)

Ensembl Gene ID	Ensembl Gene Name	Ensembl Gene Description	Gene Type	log_2 Fold Change (Day 30 vs. Pre-Second Vaccination)	Average log_2 CPM	Likelihood Ratio Test Statistic	P-Value	FDR Adjusted P-Value
ENSG00000048140	TSPAN17	tetraspanin 17 [Source:HGNC Symbol;Acc:HGNC:13594]	protein coding	1.78	2.34	39.31	<0.0001	<0.0001
ENSG00000177409	SAMD9L	sterile alpha motif domain containing 9 like [Source:HGNC Symbol;Acc:HGNC:1349]	protein coding	1.78	8.44	67.13	<0.0001	<0.0001
ENSG00000006015	REX1BD	required for excision 1-B domain containing [Source:HGNC Symbol;Acc:HGNC:26098]	protein coding	-1.78	4.40	82.03	<0.0001	<0.0001
ENSG00000143067	ZNF697	zinc finger protein 697 [Source:HGNC Symbol;Acc:HGNC:32034]	protein coding	-1.78	0.97	81.67	<0.0001	<0.0001
ENSG00000058866	DGKG	diacylglycerol kinase gamma [Source:HGNC Symbol;Acc:HGNC:2853]	protein coding	-1.77	3.46	49.07	<0.0001	<0.0001
ENSG00000139572	GPR84	G protein-coupled receptor 84 [Source:HGNC Symbol;Acc:HGNC:4535]	protein coding	1.77	1.07	67.66	<0.0001	<0.0001
ENSG00000079263	SP140	SP140 nuclear body protein [Source:HGNC Symbol;Acc:HGNC:17133]	protein coding	1.77	5.91	107.36	<0.0001	<0.0001
ENSG00000143842	SOX13	SRY-box transcription factor 13 [Source:HGNC Symbol;Acc:HGNC:11192]	protein coding	-1.75	0.74	50.74	<0.0001	<0.0001
ENSG00000069849	ATP1B3	ATPase Na+/K+ transporting subunit beta 3 [Source:HGNC Symbol;Acc:HGNC:806]	protein coding	1.75	1.31	66.42	<0.0001	<0.0001
ENSG00000266256	LINC00683	long intergenic non-protein coding RNA 683 [Source:HGNC Symbol;Acc:HGNC:44467]	lncRNA	-1.75	2.07	55.46	<0.0001	<0.0001
ENSG00000140577	CRTC3	CREB regulated transcription coactivator 3 [Source:HGNC Symbol;Acc:HGNC:26148]	protein coding	-1.75	2.63	34.10	<0.0001	<0.0001
ENSG00000105366	SIGLEC8	sialic acid binding Ig like lectin 8 [Source:HGNC Symbol;Acc:HGNC:10877]	protein coding	-1.75	2.78	13.22	0.0003	0.001
ENSG00000129667	RHBDF2	rhomboid 5 homolog 2 [Source:HGNC Symbol;Acc:HGNC:20788]	protein coding	1.74	7.38	58.87	<0.0001	<0.0001
ENSG00000170581	STAT2	signal transducer and activator of transcription 2 [Source:HGNC Symbol;Acc:HGNC:11363]	protein coding	1.73	8.61	60.69	<0.0001	<0.0001
ENSG00000112137	PHACTR1	phosphatase and actin regulator 1 [Source:HGNC Symbol;Acc:HGNC:20990]	protein coding	-1.73	5.06	29.50	<0.0001	<0.0001
ENSG00000254838	GVINP1	GTPase, very large interferon inducible pseudogene 1 [Source:HGNC Symbol;Acc:HGNC:25813]	transcribed unprocessed pseudogene	1.73	5.75	78.73	<0.0001	<0.0001

Table S18: Genes differentially expressed at Day 30 compared to pre-second vaccination (Study Arm 2 A/H7N9+AS03, D1, 29, Neutrophils). Genes are sorted by descending absolute log_2 fold change. Gene model summaries and annotations are based on Ensembl Version 99 (continued.)

Ensembl Gene ID	Ensembl Gene Name	Ensembl Gene Description	Gene Type	log_2 Fold Change (Day 30 vs. Pre-Second Vaccination)	Average log_2 CPM	Likelihood Ratio Test Statistic	P-Value	FDR Adjusted P-Value
ENSG00000263069	RNF213-AS1	RNF213 antisense RNA 1 [Source:HGNC Symbol;Acc:HGNC:54402]	lncRNA	1.72	3.64	77.13	<0.0001	<0.0001
ENSG00000160216	AGPAT3	1-acylglycerol-3-phosphate O-acyltransferase 3 [Source:HGNC Symbol;Acc:HGNC:326]	protein coding	1.72	4.00	99.60	<0.0001	<0.0001
ENSG00000134824	FADS2	fatty acid desaturase 2 [Source:HGNC Symbol;Acc:HGNC:3575]	protein coding	-1.72	3.39	22.23	<0.0001	<0.0001
ENSG00000118162	KPTN	kaptin, actin binding protein [Source:HGNC Symbol;Acc:HGNC:6404]	protein coding	1.72	3.28	61.89	<0.0001	<0.0001
ENSG00000171860	C3AR1	complement C3a receptor 1 [Source:HGNC Symbol;Acc:HGNC:1319]	protein coding	1.71	4.98	110.12	<0.0001	<0.0001
ENSG00000167106	FAM102A	family with sequence similarity 102 member A [Source:HGNC Symbol;Acc:HGNC:31419]	protein coding	-1.71	2.19	29.90	<0.0001	<0.0001
ENSG00000182557	SPNS3	sphingolipid transporter 3 (putative) [Source:HGNC Symbol;Acc:HGNC:28433]	protein coding	-1.71	2.50	12.81	0.0003	0.0012
ENSG00000133313	CNDP2	carnosine dipeptidase 2 [Source:HGNC Symbol;Acc:HGNC:24437]	protein coding	1.70	5.10	57.01	<0.0001	<0.0001
ENSG00000287576		novel transcript	lncRNA	1.70	1.32	42.26	<0.0001	<0.0001
ENSG00000104312	RIPK2	receptor interacting serine/threonine kinase 2 [Source:HGNC Symbol;Acc:HGNC:10020]	protein coding	1.68	5.69	86.14	<0.0001	<0.0001
ENSG00000107960	STN1	STN1 subunit of CST complex [Source:HGNC Symbol;Acc:HGNC:26200]	protein coding	1.68	5.23	54.71	<0.0001	<0.0001
ENSG00000179583	CIITA	class II major histocompatibility complex transactivator [Source:HGNC Symbol;Acc:HGNC:7067]	protein coding	1.68	4.65	79.80	<0.0001	<0.0001
ENSG00000115267	IFIH1	interferon induced with helicase C domain 1 [Source:HGNC Symbol;Acc:HGNC:18873]	protein coding	1.68	7.13	75.95	<0.0001	<0.0001
ENSG00000010278	CD9	CD9 molecule [Source:HGNC Symbol;Acc:HGNC:1709]	protein coding	-1.68	3.69	28.01	<0.0001	<0.0001
ENSG00000100403	ZC3H7B	zinc finger CCCH-type containing 7B [Source:HGNC Symbol;Acc:HGNC:30869]	protein coding	1.67	1.39	33.64	<0.0001	<0.0001
ENSG00000100342	APOL1	apolipoprotein L1 [Source:HGNC Symbol;Acc:HGNC:618]	protein coding	1.66	7.44	63.42	<0.0001	<0.0001
ENSG00000100401	RANGAP1	Ran GTPase activating protein 1 [Source:HGNC Symbol;Acc:HGNC:9854]	protein coding	1.66	3.89	32.13	<0.0001	<0.0001
ENSG00000154188	ANGPT1	angiopoietin 1 [Source:HGNC Symbol;Acc:HGNC:484]	protein coding	-1.66	3.42	73.62	<0.0001	<0.0001

Table S18: Genes differentially expressed at Day 30 compared to pre-second vaccination (Study Arm 2 A/H7N9+AS03, D1, 29, Neutrophils). Genes are sorted by descending absolute log_2 fold change. Gene model summaries and annotations are based on Ensembl Version 99 (continued.)

Ensembl Gene ID	Ensembl Gene Name	Ensembl Gene Description	Gene Type	log_2 Fold Change (Day 30 vs. Pre-Second Vaccination)	Average log_2 CPM	Likelihood Ratio Test Statistic	P-Value	FDR Adjusted P-Value
ENSG00000183688	RFLNB	refilin B [Source:HGNC Symbol;Acc:HGNC:28705]	protein coding	-1.65	8.03	55.14	<0.0001	<0.0001
ENSG00000180767	CHST13	carbohydrate sulfotransferase 13 [Source:HGNC Symbol;Acc:HGNC:21755]	protein coding	-1.65	4.02	50.59	<0.0001	<0.0001
ENSG00000184702	SEPTIN5	septin 5 [Source:HGNC Symbol;Acc:HGNC:9164]	protein coding	-1.65	1.26	27.45	<0.0001	<0.0001
ENSG00000185989	RASA3	RAS p21 protein activator 3 [Source:HGNC Symbol;Acc:HGNC:20331]	protein coding	-1.64	5.24	87.26	<0.0001	<0.0001
ENSG00000166428	PLD4	phospholipase D family member 4 [Source:HGNC Symbol;Acc:HGNC:23792]	protein coding	-1.64	1.96	11.07	0.0009	0.0027
ENSG00000136002	ARHGEF4	Rho guanine nucleotide exchange factor 4 [Source:HGNC Symbol;Acc:HGNC:684]	protein coding	-1.64	0.51	56.14	<0.0001	<0.0001
ENSG00000239713	APOBEC3G	apolipoprotein B mRNA editing enzyme catalytic subunit 3G [Source:HGNC Symbol;Acc:HGNC:17357]	protein coding	1.64	5.29	63.41	<0.0001	<0.0001
ENSG00000204371	EHMT2	euchromatic histone lysine methyltransferase 2 [Source:HGNC Symbol;Acc:HGNC:14129]	protein coding	-1.63	2.83	43.45	<0.0001	<0.0001
ENSG00000095002	MSH2	mutS homolog 2 [Source:HGNC Symbol;Acc:HGNC:7325]	protein coding	-1.62	0.58	32.62	<0.0001	<0.0001
ENSG00000205927	OLIG2	oligodendrocyte transcription factor 2 [Source:HGNC Symbol;Acc:HGNC:9398]	protein coding	-1.62	1.93	13.13	0.0003	0.001
ENSG00000178585	CTNNBIP1	catenin beta interacting protein 1 [Source:HGNC Symbol;Acc:HGNC:16913]	protein coding	-1.62	3.72	65.72	<0.0001	<0.0001
ENSG00000075399	VPS9D1	VPS9 domain containing 1 [Source:HGNC Symbol;Acc:HGNC:13526]	protein coding	1.62	6.83	58.85	<0.0001	<0.0001
ENSG00000135362	PRR5L	proline rich 5 like [Source:HGNC Symbol;Acc:HGNC:25878]	protein coding	1.62	4.90	53.30	<0.0001	<0.0001
ENSG00000136104	RNASEH2B	ribonuclease H2 subunit B [Source:HGNC Symbol;Acc:HGNC:25671]	protein coding	1.62	3.55	58.46	<0.0001	<0.0001
ENSG00000157601	MX1	MX dynamin like GTPase 1 [Source:HGNC Symbol;Acc:HGNC:7532]	protein coding	1.62	9.06	66.77	<0.0001	<0.0001
ENSG00000141013	GAS8	growth arrest specific 8 [Source:HGNC Symbol;Acc:HGNC:4166]	protein coding	1.61	1.51	48.93	<0.0001	<0.0001
ENSG00000085449	WDFY1	WD repeat and FYVE domain containing 1 [Source:HGNC Symbol;Acc:HGNC:20451]	protein coding	1.61	5.49	83.49	<0.0001	<0.0001
ENSG00000163803	PLB1	phospholipase B1 [Source:HGNC Symbol;Acc:HGNC:30041]	protein coding	-1.61	6.21	83.51	<0.0001	<0.0001

Confidential/Proprietary Information

Table S18: Genes differentially expressed at Day 30 compared to pre-second vaccination (Study Arm 2 A/H7N9+AS03, D1, 29, Neutrophils). Genes are sorted by descending absolute log_2 fold change. Gene model summaries and annotations are based on Ensembl Version 99 (continued.)

Ensembl Gene ID	Ensembl Gene Name	Ensembl Gene Description	Gene Type	log_2 Fold Change (Day 30 vs. Pre-Second Vaccination)	Average log_2 CPM	Likelihood Ratio Test Statistic	P-Value	FDR Adjusted P-Value
ENSG00000153406	NMRAL1	NmrA like redox sensor 1 [Source:HGNC Symbol;Acc:HGNC:24987]	protein coding	-1.61	3.72	66.71	<0.0001	<0.0001
ENSG00000155158	TTC39B	tetratricopeptide repeat domain 39B [Source:HGNC Symbol;Acc:HGNC:23704]	protein coding	1.61	0.70	94.65	<0.0001	<0.0001
ENSG00000237803	LINC00211	long intergenic non-protein coding RNA 211 [Source:HGNC Symbol;Acc:HGNC:37459]	lncRNA	-1.61	3.54	52.76	<0.0001	<0.0001
ENSG00000125730	C3	complement C3 [Source:HGNC Symbol;Acc:HGNC:1318]	protein coding	1.60	1.75	34.51	<0.0001	<0.0001
ENSG00000188290	HES4	hes family bHLH transcription factor 4 [Source:HGNC Symbol;Acc:HGNC:24149]	protein coding	1.60	4.38	53.72	<0.0001	<0.0001
ENSG00000103489	XYLT1	xylosyltransferase 1 [Source:HGNC Symbol;Acc:HGNC:15516]	protein coding	-1.60	4.40	55.55	<0.0001	<0.0001
ENSG00000280734	LINC01232	long intergenic non-protein coding RNA 1232 [Source:HGNC Symbol;Acc:HGNC:49755]	lncRNA	1.60	3.39	56.14	<0.0001	<0.0001
ENSG00000161905	ALOX15	arachidonate 15-lipoxygenase [Source:HGNC Symbol;Acc:HGNC:433]	protein coding	-1.60	4.35	11.43	0.0007	0.0023
ENSG00000179833	SERTAD2	SERTA domain containing 2 [Source:HGNC Symbol;Acc:HGNC:30784]	protein coding	-1.60	5.09	51.87	<0.0001	<0.0001
ENSG00000121236	TRIM6	tripartite motif containing 6 [Source:HGNC Symbol;Acc:HGNC:16277]	protein coding	1.59	1.96	80.71	<0.0001	<0.0001
ENSG00000074660	SCARF1	scavenger receptor class F member 1 [Source:HGNC Symbol;Acc:HGNC:16820]	protein coding	1.59	8.32	106.27	<0.0001	<0.0001
ENSG00000185507	IRF7	interferon regulatory factor 7 [Source:HGNC Symbol;Acc:HGNC:6122]	protein coding	1.59	8.54	77.98	<0.0001	<0.0001
ENSG00000100297	MCM5	minichromosome maintenance complex component 5 [Source:HGNC Symbol;Acc:HGNC:6948]	protein coding	-1.58	4.46	95.86	<0.0001	<0.0001
ENSG00000114541	FRMD4B	FERM domain containing 4B [Source:HGNC Symbol;Acc:HGNC:24886]	protein coding	1.58	3.22	72.81	<0.0001	<0.0001
ENSG00000133561	GIMAP6	GTPase, IMAP family member 6 [Source:HGNC Symbol;Acc:HGNC:21918]	protein coding	1.57	3.29	47.92	<0.0001	<0.0001
ENSG00000164683	HEY1	hes related family bHLH transcription factor with YRPW motif 1 [Source:HGNC Symbol;Acc:HGNC:4880]	protein coding	-1.57	2.85	54.60	<0.0001	<0.0001

Table S18: Genes differentially expressed at Day 30 compared to pre-second vaccination (Study Arm 2 A/H7N9+AS03, D1, 29, Neutrophils). Genes are sorted by descending absolute log_2 fold change. Gene model summaries and annotations are based on Ensembl Version 99 (continued.)

Ensembl Gene ID	Ensembl Gene Name	Ensembl Gene Description	Gene Type	log_2 Fold Change (Day 30 vs. Pre-Second Vaccination)	Average log_2 CPM	Likelihood Ratio Test Statistic	P-Value	FDR Adjusted P-Value
ENSG00000268758	ADGRE4P	adhesion G protein-coupled receptor E4, pseudogene [Source:HGNC Symbol;Acc:HGNC:19240]	transcribed unprocessed pseudogene	-1.57	3.35	10.41	0.0013	0.0037
ENSG00000178026	LRRC75B	leucine rich repeat containing 75B [Source:HGNC Symbol;Acc:HGNC:33155]	protein coding	-1.57	0.70	30.73	<0.0001	<0.0001
ENSG00000185745	IFIT1	interferon induced protein with tetratricopeptide repeats 1 [Source:HGNC Symbol;Acc:HGNC:5407]	protein coding	1.57	9.21	50.42	<0.0001	<0.0001
ENSG00000110400	NECTIN1	nectin cell adhesion molecule 1 [Source:HGNC Symbol;Acc:HGNC:9706]	protein coding	-1.56	4.42	50.43	<0.0001	<0.0001
ENSG00000114127	XRN1	5'-3' exoribonuclease 1 [Source:HGNC Symbol;Acc:HGNC:30654]	protein coding	1.56	7.42	55.21	<0.0001	<0.0001
ENSG00000185052	SLC24A3	solute carrier family 24 member 3 [Source:HGNC Symbol;Acc:HGNC:10977]	protein coding	-1.56	0.57	32.90	<0.0001	<0.0001
ENSG00000107742	SPOCK2	SPARC (osteonectin), cwcv and kazal like domains proteoglycan 2 [Source:HGNC Symbol;Acc:HGNC:13564]	protein coding	-1.56	4.33	161.11	<0.0001	<0.0001
ENSG00000134072	CAMK1	calcium/calmodulin dependent protein kinase I [Source:HGNC Symbol;Acc:HGNC:1459]	protein coding	-1.56	1.62	12.65	0.0004	0.0013
ENSG00000119397	CNTRL	centriolin [Source:HGNC Symbol;Acc:HGNC:1858]	protein coding	1.56	5.11	101.37	<0.0001	<0.0001
ENSG00000173821	RNF213	ring finger protein 213 [Source:HGNC Symbol;Acc:HGNC:14539]	protein coding	1.56	10.06	86.72	<0.0001	<0.0001
ENSG00000135045	C9orf40	chromosome 9 open reading frame 40 [Source:HGNC Symbol;Acc:HGNC:23433]	protein coding	-1.56	2.80	56.25	<0.0001	<0.0001
ENSG00000179044	EXOC3L1	exocyst complex component 3 like 1 [Source:HGNC Symbol;Acc:HGNC:27540]	protein coding	1.55	4.12	51.32	<0.0001	<0.0001
ENSG00000156110	ADK	adenosine kinase [Source:HGNC Symbol;Acc:HGNC:257]	protein coding	-1.55	2.43	47.96	<0.0001	<0.0001
ENSG00000181350	LRRC75A	leucine rich repeat containing 75A [Source:HGNC Symbol;Acc:HGNC:32403]	protein coding	-1.55	2.85	24.45	<0.0001	<0.0001
ENSG00000051128	HOMER3	homer scaffold protein 3 [Source:HGNC Symbol;Acc:HGNC:17514]	protein coding	-1.55	3.12	47.97	<0.0001	<0.0001
ENSG00000072134	EPN2	epsin 2 [Source:HGNC Symbol;Acc:HGNC:18639]	protein coding	-1.55	2.82	42.02	<0.0001	<0.0001

Table S18: Genes differentially expressed at Day 30 compared to pre-second vaccination (Study Arm 2 A/H7N9+AS03, D1, 29, Neutrophils). Genes are sorted by descending absolute log_2 fold change. Gene model summaries and annotations are based on Ensembl Version 99 (continued.)

Ensembl Gene ID	Ensembl Gene Name	Ensembl Gene Description	Gene Type	log_2 Fold Change (Day 30 vs. Pre-Second Vaccination)	Average log_2 CPM	Likelihood Ratio Test Statistic	P-Value	FDR Adjusted P-Value
ENSG00000183134	PTGDR2	prostaglandin D2 receptor 2 [Source:HGNC Symbol;Acc:HGNC:4502]	protein coding	-1.55	3.68	11.97	0.0005	0.0018
ENSG00000152778	IFIT5	interferon induced protein with tetratricopeptide repeats 5 [Source:HGNC Symbol;Acc:HGNC:13328]	protein coding	1.55	6.65	47.58	<0.0001	<0.0001
ENSG00000162073	PAQR4	progestin and adipoQ receptor family member 4 [Source:HGNC Symbol;Acc:HGNC:26386]	protein coding	-1.55	0.12	44.43	<0.0001	<0.0001
ENSG00000111110	PPM1H	protein phosphatase, Mg2+/Mn2+ dependent 1H [Source:HGNC Symbol;Acc:HGNC:18583]	protein coding	-1.54	1.86	41.68	<0.0001	<0.0001
ENSG00000184221	OLIG1	oligodendrocyte transcription factor 1 [Source:HGNC Symbol;Acc:HGNC:16983]	protein coding	-1.54	3.93	24.29	<0.0001	<0.0001
ENSG00000134243	SORT1	sortilin 1 [Source:HGNC Symbol;Acc:HGNC:11186]	protein coding	1.54	5.84	75.23	<0.0001	<0.0001
ENSG00000136874	STX17	syntaxin 17 [Source:HGNC Symbol;Acc:HGNC:11432]	protein coding	1.54	4.04	87.03	<0.0001	<0.0001
ENSG00000102804	TSC22D1	TSC22 domain family member 1 [Source:HGNC Symbol;Acc:HGNC:16826]	protein coding	1.53	0.99	24.70	<0.0001	<0.0001
ENSG00000119537	KDSR	3-ketodihydrosphingosine reductase [Source:HGNC Symbol;Acc:HGNC:4021]	protein coding	1.53	2.37	17.42	<0.0001	0.0001
ENSG00000075651	PLD1	phospholipase D1 [Source:HGNC Symbol;Acc:HGNC:9067]	protein coding	-1.53	2.87	42.53	<0.0001	<0.0001
ENSG00000188549	CCDC9B	coiled-coil domain containing 9B [Source:HGNC Symbol;Acc:HGNC:33488]	protein coding	-1.53	0.99	34.10	<0.0001	<0.0001
ENSG00000176595	KBTBD11	kelch repeat and BTB domain containing 11 [Source:HGNC Symbol;Acc:HGNC:29104]	protein coding	-1.53	4.14	70.06	<0.0001	<0.0001
ENSG00000165389	SPTSSA	serine palmitoyltransferase small subunit A [Source:HGNC Symbol;Acc:HGNC:20361]	protein coding	1.53	0.97	22.49	<0.0001	<0.0001
ENSG00000274376	ADAMTS7P1	ADAMTS7 pseudogene 1 [Source:HGNC Symbol;Acc:HGNC:49407]	transcribed unprocessed pseudogene	-1.53	0.91	12.91	0.0003	0.0011
ENSG00000177989	ODF3B	outer dense fiber of sperm tails 3B [Source:HGNC Symbol;Acc:HGNC:34388]	protein coding	1.53	8.11	104.28	<0.0001	<0.0001
ENSG00000162076	FLYWCH2	FLYWCH family member 2 [Source:HGNC Symbol;Acc:HGNC:25178]	protein coding	-1.53	0.65	51.29	<0.0001	<0.0001

Table S18: Genes differentially expressed at Day 30 compared to pre-second vaccination (Study Arm 2 A/H7N9+AS03, D1, 29, Neutrophils). Genes are sorted by descending absolute log_2 fold change. Gene model summaries and annotations are based on Ensembl Version 99 (continued.)

Ensembl Gene ID	Ensembl Gene Name	Ensembl Gene Description	Gene Type	log_2 Fold Change (Day 30 vs. Pre-Second Vaccination)	Average log_2 CPM	Likelihood Ratio Test Statistic	P-Value	FDR Adjusted P-Value
ENSG00000187231	SESTD1	SEC14 and spectrin domain containing 1 [Source:HGNC Symbol;Acc:HGNC:18379]	protein coding	1.53	4.17	103.21	<0.0001	<0.0001
ENSG00000123685	BATF3	basic leucine zipper ATF-like transcription factor 3 [Source:HGNC Symbol;Acc:HGNC:28915]	protein coding	1.52	0.59	43.16	<0.0001	<0.0001
ENSG00000255587	RAB44	RAB44, member RAS oncogene family [Source:HGNC Symbol;Acc:HGNC:21068]	protein coding	-1.52	2.81	10.76	0.001	0.0031
ENSG00000112039	FANCE	FA complementation group E [Source:HGNC Symbol;Acc:HGNC:3586]	protein coding	-1.52	0.34	46.80	<0.0001	<0.0001
ENSG00000275023	MLLT6	MLLT6, PHD finger containing [Source:HGNC Symbol;Acc:HGNC:7138]	protein coding	1.52	7.41	110.85	<0.0001	<0.0001
ENSG00000103202	NME4	NME/NM23 nucleoside diphosphate kinase 4 [Source:HGNC Symbol;Acc:HGNC:7852]	protein coding	-1.52	1.76	51.36	<0.0001	<0.0001
ENSG00000118785	SPP1	secreted phosphoprotein 1 [Source:HGNC Symbol;Acc:HGNC:11255]	protein coding	-1.52	2.00	27.92	<0.0001	<0.0001
ENSG00000135114	OASL	2'-5'-oligoadenylate synthetase like [Source:HGNC Symbol;Acc:HGNC:8090]	protein coding	1.51	7.55	76.09	<0.0001	<0.0001
ENSG00000106560	GIMAP2	GTPase, IMAP family member 2 [Source:HGNC Symbol;Acc:HGNC:21789]	protein coding	1.51	5.51	52.23	<0.0001	<0.0001
ENSG00000103121	CMC2	C-X9-C motif containing 2 [Source:HGNC Symbol;Acc:HGNC:24447]	protein coding	1.51	1.41	86.03	<0.0001	<0.0001
ENSG00000139618	BRCA2	BRCA2 DNA repair associated [Source:HGNC Symbol;Acc:HGNC:1101]	protein coding	1.51	0.90	52.56	<0.0001	<0.0001
ENSG00000079385	CEACAM1	CEA cell adhesion molecule 1 [Source:HGNC Symbol;Acc:HGNC:1814]	protein coding	1.51	6.58	71.33	<0.0001	<0.0001
ENSG00000059378	PARP12	poly(ADP-ribose) polymerase family member 12 [Source:HGNC Symbol;Acc:HGNC:21919]	protein coding	1.50	6.73	92.70	<0.0001	<0.0001
ENSG00000146232	NFKBIE	NFKB inhibitor epsilon [Source:HGNC Symbol;Acc:HGNC:7799]	protein coding	1.50	4.33	74.26	<0.0001	<0.0001
ENSG00000183150	GPR19	G protein-coupled receptor 19 [Source:HGNC Symbol;Acc:HGNC:4473]	protein coding	-1.50	0.88	34.07	<0.0001	<0.0001
ENSG00000154102	C16orf74	chromosome 16 open reading frame 74 [Source:HGNC Symbol;Acc:HGNC:23362]	protein coding	-1.50	1.10	60.88	<0.0001	<0.0001
ENSG00000106100	NOD1	nucleotide binding oligomerization domain containing 1 [Source:HGNC Symbol;Acc:HGNC:16390]	protein coding	1.49	3.43	53.53	<0.0001	<0.0001
ENSG00000140030	GPR65	G protein-coupled receptor 65 [Source:HGNC Symbol;Acc:HGNC:4517]	protein coding	1.49	6.54	79.62	<0.0001	<0.0001

Table S18: Genes differentially expressed at Day 30 compared to pre-second vaccination (Study Arm 2 A/H7N9+AS03, D1, 29, Neutrophils). Genes are sorted by descending absolute log_2 fold change. Gene model summaries and annotations are based on Ensembl Version 99 (continued.)

Ensembl Gene ID	Ensembl Gene Name	Ensembl Gene Description	Gene Type	log_2 Fold Change (Day 30 vs. Pre-Second Vaccination)	Average log_2 CPM	Likelihood Ratio Test Statistic	P-Value	FDR Adjusted P-Value
ENSG00000102554	KLF5	Kruppel like factor 5 [Source:HGNC Symbol;Acc:HGNC:6349]	protein coding	1.49	3.96	58.42	<0.0001	<0.0001
ENSG00000125148	MT2A	metallothionein 2A [Source:HGNC Symbol;Acc:HGNC:7406]	protein coding	1.49	4.70	54.03	<0.0001	<0.0001
ENSG00000245848	CEBPA	CCAAT enhancer binding protein alpha [Source:HGNC Symbol;Acc:HGNC:1833]	protein coding	-1.49	4.42	54.73	<0.0001	<0.0001
ENSG00000106683	LIMK1	LIM domain kinase 1 [Source:HGNC Symbol;Acc:HGNC:6613]	protein coding	1.49	5.80	56.90	<0.0001	<0.0001
ENSG00000163947	ARHGEF3	Rho guanine nucleotide exchange factor 3 [Source:HGNC Symbol;Acc:HGNC:683]	protein coding	1.49	3.31	43.95	<0.0001	<0.0001
ENSG00000137563	GGH	gamma-glutamyl hydrolase [Source:HGNC Symbol;Acc:HGNC:4248]	protein coding	-1.48	0.36	31.86	<0.0001	<0.0001
ENSG00000134470	IL15RA	interleukin 15 receptor subunit alpha [Source:HGNC Symbol;Acc:HGNC:5978]	protein coding	1.48	2.07	49.80	<0.0001	<0.0001
ENSG00000075240	GRAMD4	GRAM domain containing 4 [Source:HGNC Symbol;Acc:HGNC:29113]	protein coding	-1.48	3.23	34.29	<0.0001	<0.0001
ENSG00000088179	PTPN4	protein tyrosine phosphatase non-receptor type 4 [Source:HGNC Symbol;Acc:HGNC:9656]	protein coding	-1.48	1.82	46.45	<0.0001	<0.0001
ENSG00000179915	NRXN1	neurexin 1 [Source:HGNC Symbol;Acc:HGNC:8008]	protein coding	-1.47	0.59	47.53	<0.0001	<0.0001
ENSG00000128512	DOCK4	dedicator of cytokinesis 4 [Source:HGNC Symbol;Acc:HGNC:19192]	protein coding	1.47	6.06	81.46	<0.0001	<0.0001
ENSG00000130309	COLGALT1	collagen beta(1-O)galactosyltransferase 1 [Source:HGNC Symbol;Acc:HGNC:26182]	protein coding	-1.47	5.85	57.21	<0.0001	<0.0001
ENSG00000170558	CDH2	cadherin 2 [Source:HGNC Symbol;Acc:HGNC:1759]	protein coding	-1.47	1.58	67.88	<0.0001	<0.0001
ENSG00000151136	BTBD11	BTB domain containing 11 [Source:HGNC Symbol;Acc:HGNC:23844]	protein coding	-1.46	3.17	55.13	<0.0001	<0.0001
ENSG00000108106	UBE2S	ubiquitin conjugating enzyme E2 S [Source:HGNC Symbol;Acc:HGNC:17895]	protein coding	1.46	4.55	44.86	<0.0001	<0.0001
ENSG00000129003	VPS13C	vacuolar protein sorting 13 homolog C [Source:HGNC Symbol;Acc:HGNC:23594]	protein coding	1.46	4.87	58.96	<0.0001	<0.0001
ENSG00000185420	SMYD3	SET and MYND domain containing 3 [Source:HGNC Symbol;Acc:HGNC:15513]	protein coding	-1.45	0.20	21.45	<0.0001	<0.0001
ENSG00000107201	DDX58	DExD/H-box helicase 58 [Source:HGNC Symbol;Acc:HGNC:19102]	protein coding	1.45	8.24	62.73	<0.0001	<0.0001

Table S18: Genes differentially expressed at Day 30 compared to pre-second vaccination (Study Arm 2 A/H7N9+AS03, D1, 29, Neutrophils). Genes are sorted by descending absolute log_2 fold change. Gene model summaries and annotations are based on Ensembl Version 99 (continued.)

Ensembl Gene ID	Ensembl Gene Name	Ensembl Gene Description	Gene Type	log_2 Fold Change (Day 30 vs. Pre-Second Vaccination)	Average log_2 CPM	Likelihood Ratio Test Statistic	P-Value	FDR Adjusted P-Value
ENSG00000159228	CBR1	carbonyl reductase 1 [Source:HGNC Symbol;Acc:HGNC:1548]	protein coding	1.45	0.77	54.01	<0.0001	<0.0001
ENSG00000165490	DDIAS	DNA damage induced apoptosis suppressor [Source:HGNC Symbol;Acc:HGNC:26351]	protein coding	1.44	1.81	36.05	<0.0001	<0.0001
ENSG00000165140	FBP1	fructose-bisphosphatase 1 [Source:HGNC Symbol;Acc:HGNC:3606]	protein coding	-1.43	2.29	21.86	<0.0001	<0.0001
ENSG00000122729	ACO1	aconitase 1 [Source:HGNC Symbol;Acc:HGNC:117]	protein coding	1.43	4.17	53.23	<0.0001	<0.0001
ENSG00000188157	AGRN	agrin [Source:HGNC Symbol;Acc:HGNC:329]	protein coding	1.43	3.97	36.52	<0.0001	<0.0001
ENSG00000272555		novel transcript	lncRNA	-1.43	0.57	30.95	<0.0001	<0.0001
ENSG00000119950	MXI1	MAX interactor 1, dimerization protein [Source:HGNC Symbol;Acc:HGNC:7534]	protein coding	-1.43	5.38	68.10	<0.0001	<0.0001
ENSG00000196154	S100A4	S100 calcium binding protein A4 [Source:HGNC Symbol;Acc:HGNC:10494]	protein coding	-1.43	8.98	58.06	<0.0001	<0.0001
ENSG00000177606	JUN	Jun proto-oncogene, AP-1 transcription factor subunit [Source:HGNC Symbol;Acc:HGNC:6204]	protein coding	-1.42	5.19	41.42	<0.0001	<0.0001
ENSG00000123838	C4BPA	complement component 4 binding protein alpha [Source:HGNC Symbol;Acc:HGNC:1325]	protein coding	1.42	4.40	30.71	<0.0001	<0.0001
ENSG00000261373	VPS9D1-AS1	VPS9D1 antisense RNA 1 [Source:HGNC Symbol;Acc:HGNC:48915]	lncRNA	1.42	3.10	52.96	<0.0001	<0.0001
ENSG00000180628	PCGF5	polycomb group ring finger 5 [Source:HGNC Symbol;Acc:HGNC:28264]	protein coding	1.42	6.39	81.58	<0.0001	<0.0001
ENSG00000054179	ENTPD2	ectonucleoside triphosphate diphosphohydrolase 2 [Source:HGNC Symbol;Acc:HGNC:3364]	protein coding	-1.42	2.78	56.00	<0.0001	<0.0001
ENSG00000130699	TAF4	TATA-box binding protein associated factor 4 [Source:HGNC Symbol;Acc:HGNC:11537]	protein coding	-1.41	5.21	96.37	<0.0001	<0.0001
ENSG00000236345	SCAT8	S-phase cancer associated transcript 8 [Source:HGNC Symbol;Acc:HGNC:40967]	lncRNA	-1.41	3.64	62.98	<0.0001	<0.0001
ENSG00000197142	ACSL5	acyl-CoA synthetase long chain family member 5 [Source:HGNC Symbol;Acc:HGNC:16526]	protein coding	1.41	4.30	70.75	<0.0001	<0.0001
ENSG00000185885	IFITM1	interferon induced transmembrane protein 1 [Source:HGNC Symbol;Acc:HGNC:5412]	protein coding	1.41	9.40	90.65	<0.0001	<0.0001
ENSG00000136999	CCN3	cellular communication network factor 3 [Source:HGNC Symbol;Acc:HGNC:7885]	protein coding	-1.41	5.06	94.22	<0.0001	<0.0001

Table S18: Genes differentially expressed at Day 30 compared to pre-second vaccination (Study Arm 2 A/H7N9+AS03, D1, 29, Neutrophils). Genes are sorted by descending absolute log_2 fold change. Gene model summaries and annotations are based on Ensembl Version 99 (continued.)

Ensembl Gene ID	Ensembl Gene Name	Ensembl Gene Description	Gene Type	log_2 Fold Change (Day 30 vs. Pre-Second Vaccination)	Average log_2 CPM	Likelihood Ratio Test Statistic	P-Value	FDR Adjusted P-Value
ENSG00000099860	GADD45B	growth arrest and DNA damage inducible beta [Source:HGNC Symbol;Acc:HGNC:4096]	protein coding	1.41	7.49	47.29	<0.0001	<0.0001
ENSG00000204396	VWA7	von Willebrand factor A domain containing 7 [Source:HGNC Symbol;Acc:HGNC:13939]	protein coding	-1.41	1.71	36.48	<0.0001	<0.0001
ENSG00000111860	CEP85L	centrosomal protein 85 like [Source:HGNC Symbol;Acc:HGNC:21638]	protein coding	-1.41	4.56	46.76	<0.0001	<0.0001
ENSG00000204345	CD300LD	CD300 molecule like family member d [Source:HGNC Symbol;Acc:HGNC:16848]	protein coding	1.41	2.21	18.17	<0.0001	<0.0001
ENSG00000169683	LRRC45	leucine rich repeat containing 45 [Source:HGNC Symbol;Acc:HGNC:28302]	protein coding	-1.41	0.09	23.35	<0.0001	<0.0001
ENSG00000215022		novel transcript, antisense to PHACTR1	lncRNA	-1.39	1.94	26.02	<0.0001	<0.0001
ENSG00000165646	SLC18A2	solute carrier family 18 member A2 [Source:HGNC Symbol;Acc:HGNC:10935]	protein coding	-1.39	0.46	30.97	<0.0001	<0.0001
ENSG00000103642	LACTB	lactamase beta [Source:HGNC Symbol;Acc:HGNC:16468]	protein coding	1.39	5.70	53.73	<0.0001	<0.0001
ENSG00000179388	EGR3	early growth response 3 [Source:HGNC Symbol;Acc:HGNC:3240]	protein coding	1.39	5.31	15.19	<0.0001	0.0004
ENSG00000213492	NT5C3AP1	5'-nucleotidase, cytosolic IIIA pseudogene 1 [Source:HGNC Symbol;Acc:HGNC:18530]	transcribed processed pseudogene	1.39	1.58	69.89	<0.0001	<0.0001
ENSG00000023445	BIRC3	baculoviral IAP repeat containing 3 [Source:HGNC Symbol;Acc:HGNC:591]	protein coding	1.39	3.25	44.48	<0.0001	<0.0001
ENSG00000178607	ERN1	endoplasmic reticulum to nucleus signaling 1 [Source:HGNC Symbol;Acc:HGNC:3449]	protein coding	-1.39	6.69	70.96	<0.0001	<0.0001
ENSG00000157388	CACNA1D	calcium voltage-gated channel subunit alpha1 D [Source:HGNC Symbol;Acc:HGNC:1391]	protein coding	-1.39	0.75	35.86	<0.0001	<0.0001
ENSG00000047621	C12orf4	chromosome 12 open reading frame 4 [Source:HGNC Symbol;Acc:HGNC:1184]	protein coding	1.39	1.91	29.10	<0.0001	<0.0001
ENSG00000197448	GSTK1	glutathione S-transferase kappa 1 [Source:HGNC Symbol;Acc:HGNC:16906]	protein coding	1.39	7.03	66.42	<0.0001	<0.0001
ENSG00000213203	GIMAP1	GTPase, IMAP family member 1 [Source:HGNC Symbol;Acc:HGNC:23237]	protein coding	1.38	1.19	33.50	<0.0001	<0.0001
ENSG00000176170	SPHK1	sphingosine kinase 1 [Source:HGNC Symbol;Acc:HGNC:11240]	protein coding	1.38	1.03	30.87	<0.0001	<0.0001

Table S18: Genes differentially expressed at Day 30 compared to pre-second vaccination (Study Arm 2 A/H7N9+AS03, D1, 29, Neutrophils). Genes are sorted by descending absolute log_2 fold change. Gene model summaries and annotations are based on Ensembl Version 99 (continued.)

Ensembl Gene ID	Ensembl Gene Name	Ensembl Gene Description	Gene Type	log_2 Fold Change (Day 30 vs. Pre-Second Vaccination)	Average log_2 CPM	Likelihood Ratio Test Statistic	P-Value	FDR Adjusted P-Value
ENSG00000135378	PRRG4	proline rich and Gla domain 4 [Source:HGNC Symbol;Acc:HGNC:30799]	protein coding	1.38	6.97	68.07	<0.0001	<0.0001
ENSG00000224307		novel transcript	lncRNA	-1.38	4.04	90.49	<0.0001	<0.0001
ENSG00000132780	NASP	nuclear autoantigenic sperm protein [Source:HGNC Symbol;Acc:HGNC:7644]	protein coding	1.38	4.90	92.71	<0.0001	<0.0001
ENSG00000178685	PARP10	poly(ADP-ribose) polymerase family member 10 [Source:HGNC Symbol;Acc:HGNC:25895]	protein coding	1.38	7.93	76.75	<0.0001	<0.0001
ENSG00000170846		Putative MORF4 family-associated protein 1-like protein UPP [Source:UniProtKB/Swiss-Prot;Acc:B2RBV5]	protein coding	-1.37	0.99	41.17	<0.0001	<0.0001
ENSG00000013374	NUB1	negative regulator of ubiquitin like proteins 1 [Source:HGNC Symbol;Acc:HGNC:17623]	protein coding	1.37	7.41	61.19	<0.0001	<0.0001
ENSG00000152818	UTRN	utrophin [Source:HGNC Symbol;Acc:HGNC:12635]	protein coding	1.37	6.52	61.23	<0.0001	<0.0001
ENSG00000127578	WFIKKN1	WAP, follistatin/kazal, immunoglobulin, kunitz and netrin domain containing 1 [Source:HGNC Symbol;Acc:HGNC:30912]	protein coding	-1.37	0.51	44.11	<0.0001	<0.0001
ENSG00000284874		novel transcript	protein coding	-1.37	0.42	24.30	<0.0001	<0.0001
ENSG00000140853	NLRC5	NLR family CARD domain containing 5 [Source:HGNC Symbol;Acc:HGNC:29933]	protein coding	1.37	8.34	58.05	<0.0001	<0.0001
ENSG00000176454	LPCAT4	lysophosphatidylcholine acyltransferase 4 [Source:HGNC Symbol;Acc:HGNC:30059]	protein coding	-1.36	4.56	78.15	<0.0001	<0.0001
ENSG00000122643	NT5C3A	5'-nucleotidase, cytosolic IIIA [Source:HGNC Symbol;Acc:HGNC:17820]	protein coding	1.36	6.69	91.50	<0.0001	<0.0001
ENSG00000107566	ERLIN1	ER lipid raft associated 1 [Source:HGNC Symbol;Acc:HGNC:16947]	protein coding	1.36	4.88	73.71	<0.0001	<0.0001
ENSG00000112759	SLC29A1	solute carrier family 29 member 1 (Augustine blood group) [Source:HGNC Symbol;Acc:HGNC:11003]	protein coding	-1.36	3.72	8.00	0.0047	0.0117
ENSG00000198520	ARMH1	armadillo like helical domain containing 1 [Source:HGNC Symbol;Acc:HGNC:34345]	protein coding	1.36	3.01	38.36	<0.0001	<0.0001
ENSG00000284194	SCO2	SCO cytochrome c oxidase assembly protein 2 [Source:HGNC Symbol;Acc:HGNC:10604]	protein coding	1.36	4.61	82.78	<0.0001	<0.0001
ENSG00000115415	STAT1	signal transducer and activator of transcription 1 [Source:HGNC Symbol;Acc:HGNC:11362]	protein coding	1.35	9.38	50.88	<0.0001	<0.0001
ENSG00000169504	CLIC4	chloride intracellular channel 4 [Source:HGNC Symbol;Acc:HGNC:13518]	protein coding	1.35	3.36	45.07	<0.0001	<0.0001

Table S18: Genes differentially expressed at Day 30 compared to pre-second vaccination (Study Arm 2 A/H7N9+AS03, D1, 29, Neutrophils). Genes are sorted by descending absolute log_2 fold change. Gene model summaries and annotations are based on Ensembl Version 99 (continued.)

Ensembl Gene ID	Ensembl Gene Name	Ensembl Gene Description	Gene Type	log_2 Fold Change (Day 30 vs. Pre-Second Vaccination)	Average log_2 CPM	Likelihood Ratio Test Statistic	P-Value	FDR Adjusted P-Value
ENSG00000115718	PROC	protein C, inactivator of coagulation factors Va and VIIIa [Source:HGNC Symbol;Acc:HGNC:9451]	protein coding	-1.35	1.31	59.59	<0.0001	<0.0001
ENSG00000068489	PRR11	proline rich 11 [Source:HGNC Symbol;Acc:HGNC:25619]	protein coding	1.35	2.41	76.25	<0.0001	<0.0001
ENSG00000132256	TRIM5	tripartite motif containing 5 [Source:HGNC Symbol;Acc:HGNC:16276]	protein coding	1.35	5.25	57.17	<0.0001	<0.0001
ENSG00000229677		ribosomal protein L17 (RPL17) pseudogene	processed pseudogene	1.35	1.09	32.10	<0.0001	<0.0001
ENSG00000182810	DDX28	DEAD-box helicase 28 [Source:HGNC Symbol;Acc:HGNC:17330]	protein coding	-1.35	2.56	45.46	<0.0001	<0.0001
ENSG00000141858	SAMD1	sterile alpha motif domain containing 1 [Source:HGNC Symbol;Acc:HGNC:17958]	protein coding	-1.35	1.88	47.62	<0.0001	<0.0001
ENSG00000178075	GRAMD1C	GRAM domain containing 1C [Source:HGNC Symbol;Acc:HGNC:25252]	protein coding	-1.34	4.68	83.97	<0.0001	<0.0001
ENSG00000261026		novel transcript, overlapping to EGR3	lncRNA	1.34	2.44	5.61	0.0179	0.0378
ENSG00000260249		novel transcript, antisense to SNX20	lncRNA	1.33	1.16	78.71	<0.0001	<0.0001
ENSG00000185950	IRS2	insulin receptor substrate 2 [Source:HGNC Symbol;Acc:HGNC:6126]	protein coding	-1.33	8.27	38.96	<0.0001	<0.0001
ENSG00000168394	TAP1	transporter 1, ATP binding cassette subfamily B member [Source:HGNC Symbol;Acc:HGNC:43]	protein coding	1.33	9.97	67.06	<0.0001	<0.0001
ENSG00000152558	TMEM123	transmembrane protein 123 [Source:HGNC Symbol;Acc:HGNC:30138]	protein coding	1.33	8.81	83.30	<0.0001	<0.0001
ENSG00000140090	SLC24A4	solute carrier family 24 member 4 [Source:HGNC Symbol;Acc:HGNC:10978]	protein coding	-1.33	4.51	23.36	<0.0001	<0.0001
ENSG00000123411	IKZF4	IKAROS family zinc finger 4 [Source:HGNC Symbol;Acc:HGNC:13179]	protein coding	1.33	0.79	48.38	<0.0001	<0.0001
ENSG00000279447		TEC	TEC	-1.33	2.21	41.01	<0.0001	<0.0001
ENSG00000114631	PODXL2	podocalyxin like 2 [Source:HGNC Symbol;Acc:HGNC:17936]	protein coding	-1.33	0.56	32.87	<0.0001	<0.0001
ENSG00000234518	PTGES3P1	prostaglandin E synthase 3 pseudogene 1 [Source:HGNC Symbol;Acc:HGNC:43824]	processed pseudogene	1.33	4.35	45.70	<0.0001	<0.0001
ENSG00000166881	NEMP1	nuclear envelope integral membrane protein 1 [Source:HGNC Symbol;Acc:HGNC:29001]	protein coding	-1.33	1.84	66.34	<0.0001	<0.0001

Table S18: Genes differentially expressed at Day 30 compared to pre-second vaccination (Study Arm 2 A/H7N9+AS03, D1, 29, Neutrophils). Genes are sorted by descending absolute log_2 fold change. Gene model summaries and annotations are based on Ensembl Version 99 (continued.)

Ensembl Gene ID	Ensembl Gene Name	Ensembl Gene Description	Gene Type	log_2 Fold Change (Day 30 vs. Pre-Second Vaccination)	Average log_2 CPM	Likelihood Ratio Test Statistic	P-Value	FDR Adjusted P-Value
ENSG00000135929	CYP27A1	cytochrome P450 family 27 subfamily A member 1 [Source:HGNC Symbol;Acc:HGNC:2605]	protein coding	-1.33	6.27	55.04	<0.0001	<0.0001
ENSG00000111328	CDK2AP1	cyclin dependent kinase 2 associated protein 1 [Source:HGNC Symbol;Acc:HGNC:14002]	protein coding	-1.33	3.00	43.47	<0.0001	<0.0001
ENSG00000138660	AP1AR	adaptor related protein complex 1 associated regulatory protein [Source:HGNC Symbol;Acc:HGNC:28808]	protein coding	1.33	1.63	33.33	<0.0001	<0.0001
ENSG00000173930	SLCO4C1	solute carrier organic anion transporter family member 4C1 [Source:HGNC Symbol;Acc:HGNC:23612]	protein coding	-1.32	5.16	44.26	<0.0001	<0.0001
ENSG00000183018	SPNS2	sphingolipid transporter 2 [Source:HGNC Symbol;Acc:HGNC:26992]	protein coding	-1.32	2.70	43.73	<0.0001	<0.0001
ENSG00000169508	GPR183	G protein-coupled receptor 183 [Source:HGNC Symbol;Acc:HGNC:3128]	protein coding	-1.32	0.79	11.09	0.0009	0.0027
ENSG00000112419	PHACTR2	phosphatase and actin regulator 2 [Source:HGNC Symbol;Acc:HGNC:20956]	protein coding	1.32	2.52	64.05	<0.0001	<0.0001
ENSG00000095383	TBC1D2	TBC1 domain family member 2 [Source:HGNC Symbol;Acc:HGNC:18026]	protein coding	-1.32	4.58	43.20	<0.0001	<0.0001
ENSG00000004799	PDK4	pyruvate dehydrogenase kinase 4 [Source:HGNC Symbol;Acc:HGNC:8812]	protein coding	-1.32	2.57	26.62	<0.0001	<0.0001
ENSG00000157933	SKI	SKI proto-oncogene [Source:HGNC Symbol;Acc:HGNC:10896]	protein coding	-1.32	7.13	77.25	<0.0001	<0.0001
ENSG00000119522	DENND1A	DENN domain containing 1A [Source:HGNC Symbol;Acc:HGNC:29324]	protein coding	1.31	5.80	65.87	<0.0001	<0.0001
ENSG00000213889	PPM1N	protein phosphatase, Mg2+/Mn2+ dependent 1N (putative) [Source:HGNC Symbol;Acc:HGNC:26845]	protein coding	1.31	1.13	51.73	<0.0001	<0.0001
ENSG00000241889		dullard homolog (Xenopus laevis) (DULLARD) pseudogene	processed pseudogene	-1.31	1.49	46.91	<0.0001	<0.0001
ENSG00000107796	ACTA2	actin alpha 2, smooth muscle [Source:HGNC Symbol;Acc:HGNC:130]	protein coding	1.31	2.16	24.65	<0.0001	<0.0001
ENSG00000157593	SLC35B2	solute carrier family 35 member B2 [Source:HGNC Symbol;Acc:HGNC:16872]	protein coding	1.31	2.72	66.37	<0.0001	<0.0001
ENSG00000184898	RBM43	RNA binding motif protein 43 [Source:HGNC Symbol;Acc:HGNC:24790]	protein coding	1.31	2.71	53.08	<0.0001	<0.0001
ENSG00000176438	SYNE3	spectrin repeat containing nuclear envelope family member 3 [Source:HGNC Symbol;Acc:HGNC:19861]	protein coding	-1.30	3.99	37.36	<0.0001	<0.0001

Table S18: Genes differentially expressed at Day 30 compared to pre-second vaccination (Study Arm 2 A/H7N9+AS03, D1, 29, Neutrophils). Genes are sorted by descending absolute log_2 fold change. Gene model summaries and annotations are based on Ensembl Version 99 (continued.)

Ensembl Gene ID	Ensembl Gene Name	Ensembl Gene Description	Gene Type	log_2 Fold Change (Day 30 vs. Pre-Second Vaccination)	Average log_2 CPM	Likelihood Ratio Test Statistic	P-Value	FDR Adjusted P-Value
ENSG00000128604	IRF5	interferon regulatory factor 5 [Source:HGNC Symbol;Acc:HGNC:6120]	protein coding	1.30	4.91	80.42	<0.0001	<0.0001
ENSG00000100139	MICALL1	MICAL like 1 [Source:HGNC Symbol;Acc:HGNC:29804]	protein coding	1.30	5.15	76.74	<0.0001	<0.0001
ENSG00000162946	DISC1	DISC1 scaffold protein [Source:HGNC Symbol;Acc:HGNC:2888]	protein coding	1.30	4.31	100.01	<0.0001	<0.0001
ENSG00000110042	DTX4	deltex E3 ubiquitin ligase 4 [Source:HGNC Symbol;Acc:HGNC:29151]	protein coding	-1.30	4.20	36.16	<0.0001	<0.0001
ENSG00000117394	SLC2A1	solute carrier family 2 member 1 [Source:HGNC Symbol;Acc:HGNC:11005]	protein coding	-1.30	3.12	45.27	<0.0001	<0.0001
ENSG00000138496	PARP9	poly(ADP-ribose) polymerase family member 9 [Source:HGNC Symbol;Acc:HGNC:24118]	protein coding	1.30	9.07	60.39	<0.0001	<0.0001
ENSG00000119408	NEK6	NIMA related kinase 6 [Source:HGNC Symbol;Acc:HGNC:7749]	protein coding	-1.30	5.81	47.00	<0.0001	<0.0001
ENSG00000188487	INSC	INSC spindle orientation adaptor protein [Source:HGNC Symbol;Acc:HGNC:33116]	protein coding	-1.30	1.00	25.22	<0.0001	<0.0001
ENSG00000204267	TAP2	transporter 2, ATP binding cassette subfamily B member [Source:HGNC Symbol;Acc:HGNC:44]	protein coding	1.30	7.34	79.69	<0.0001	<0.0001
ENSG00000126603	GLIS2	GLIS family zinc finger 2 [Source:HGNC Symbol;Acc:HGNC:29450]	protein coding	-1.30	1.33	33.47	<0.0001	<0.0001
ENSG00000251002		novel transcript, antisense to IGgenecluster	lncRNA	1.30	2.51	52.00	<0.0001	<0.0001
ENSG00000127838	PNKD	PNKD metallo-beta-lactamase domain containing [Source:HGNC Symbol;Acc:HGNC:9153]	protein coding	-1.29	3.76	54.98	<0.0001	<0.0001
ENSG00000151332	MBIP	MAP3K12 binding inhibitory protein 1 [Source:HGNC Symbol;Acc:HGNC:20427]	protein coding	-1.29	0.97	42.96	<0.0001	<0.0001
ENSG00000176102	CSTF3	cleavage stimulation factor subunit 3 [Source:HGNC Symbol;Acc:HGNC:2485]	protein coding	1.29	1.28	54.82	<0.0001	<0.0001
ENSG00000103024	NME3	NME/NM23 nucleoside diphosphate kinase 3 [Source:HGNC Symbol;Acc:HGNC:7851]	protein coding	-1.29	2.28	36.46	<0.0001	<0.0001
ENSG00000279861		TEC	TEC	1.29	4.36	36.90	<0.0001	<0.0001
ENSG00000287263		novel transcript	lncRNA	1.29	5.49	45.54	<0.0001	<0.0001
ENSG00000100292	HMOX1	heme oxygenase 1 [Source:HGNC Symbol;Acc:HGNC:5013]	protein coding	-1.29	5.30	107.31	<0.0001	<0.0001
ENSG00000166886	NAB2	NGFI-A binding protein 2 [Source:HGNC Symbol;Acc:HGNC:7627]	protein coding	-1.29	2.86	90.71	<0.0001	<0.0001

Table S18: Genes differentially expressed at Day 30 compared to pre-second vaccination (Study Arm 2 A/H7N9+AS03, D1, 29, Neutrophils). Genes are sorted by descending absolute log_2 fold change. Gene model summaries and annotations are based on Ensembl Version 99 (continued.)

Ensembl Gene ID	Ensembl Gene Name	Ensembl Gene Description	Gene Type	log_2 Fold Change (Day 30 vs. Pre-Second Vaccination)	Average log_2 CPM	Likelihood Ratio Test Statistic	P-Value	FDR Adjusted P-Value
ENSG00000153029	MR1	major histocompatibility complex, class I-related [Source:HGNC Symbol;Acc:HGNC:4975]	protein coding	1.29	5.20	98.21	<0.0001	<0.0001
ENSG00000231105	ECE1-AS1	ECE1 antisense RNA 1 [Source:HGNC Symbol;Acc:HGNC:40203]	lncRNA	1.29	1.04	42.50	<0.0001	<0.0001
ENSG00000172183	ISG20	interferon stimulated exonuclease gene 20 [Source:HGNC Symbol;Acc:HGNC:6130]	protein coding	1.28	8.81	50.20	<0.0001	<0.0001
ENSG00000182685	BRICD5	BRICHOS domain containing 5 [Source:HGNC Symbol;Acc:HGNC:28309]	protein coding	-1.28	3.08	57.44	<0.0001	<0.0001
ENSG00000206418	RAB12	RAB12, member RAS oncogene family [Source:HGNC Symbol;Acc:HGNC:31332]	protein coding	1.28	1.98	50.13	<0.0001	<0.0001
ENSG00000103154	NECAB2	N-terminal EF-hand calcium binding protein 2 [Source:HGNC Symbol;Acc:HGNC:23746]	protein coding	-1.28	4.58	82.60	<0.0001	<0.0001
ENSG00000162444	RBP7	retinol binding protein 7 [Source:HGNC Symbol;Acc:HGNC:30316]	protein coding	-1.28	5.19	51.78	<0.0001	<0.0001
ENSG00000141447	OSBPL1A	oxysterol binding protein like 1A [Source:HGNC Symbol;Acc:HGNC:16398]	protein coding	-1.28	3.28	60.22	<0.0001	<0.0001
ENSG00000124256	ZBP1	Z-DNA binding protein 1 [Source:HGNC Symbol;Acc:HGNC:16176]	protein coding	1.27	7.42	92.71	<0.0001	<0.0001
ENSG00000165185	KIAA1958	KIAA1958 [Source:HGNC Symbol;Acc:HGNC:23427]	protein coding	1.27	2.26	61.05	<0.0001	<0.0001
ENSG00000171791	BCL2	BCL2 apoptosis regulator [Source:HGNC Symbol;Acc:HGNC:990]	protein coding	-1.27	0.32	34.35	<0.0001	<0.0001
ENSG00000107798	LIPA	lipase A, lysosomal acid type [Source:HGNC Symbol;Acc:HGNC:6617]	protein coding	1.27	2.55	43.97	<0.0001	<0.0001
ENSG00000273669		novel transcript	lncRNA	-1.27	1.18	44.61	<0.0001	<0.0001
ENSG00000197536	IRF1-AS1	IRF1 antisense RNA 1 [Source:HGNC Symbol;Acc:HGNC:33838]	lncRNA	1.27	5.84	71.02	<0.0001	<0.0001
ENSG00000253214		novel transcript	lncRNA	1.27	1.42	58.28	<0.0001	<0.0001
ENSG00000254789		novel transcript	lncRNA	-1.27	3.86	53.80	<0.0001	<0.0001
ENSG00000035664	DAPK2	death associated protein kinase 2 [Source:HGNC Symbol;Acc:HGNC:2675]	protein coding	-1.27	7.04	54.67	<0.0001	<0.0001
ENSG00000117010	ZNF684	zinc finger protein 684 [Source:HGNC Symbol;Acc:HGNC:28418]	protein coding	1.26	1.64	35.02	<0.0001	<0.0001
ENSG00000169136	ATF5	activating transcription factor 5 [Source:HGNC Symbol;Acc:HGNC:790]	protein coding	1.26	1.95	38.00	<0.0001	<0.0001

Table S18: Genes differentially expressed at Day 30 compared to pre-second vaccination (Study Arm 2 A/H7N9+AS03, D1, 29, Neutrophils). Genes are sorted by descending absolute log_2 fold change. Gene model summaries and annotations are based on Ensembl Version 99 (continued.)

Ensembl Gene ID	Ensembl Gene Name	Ensembl Gene Description	Gene Type	log_2 Fold Change (Day 30 vs. Pre-Second Vaccination)	Average log_2 CPM	Likelihood Ratio Test Statistic	P-Value	FDR Adjusted P-Value
ENSG00000104320	NBN	nibrin [Source:HGNC Symbol;Acc:HGNC:7652]	protein coding	1.26	7.42	84.37	<0.0001	<0.0001
ENSG00000084731	KIF3C	kinesin family member 3C [Source:HGNC Symbol;Acc:HGNC:6321]	protein coding	-1.26	4.07	59.47	<0.0001	<0.0001
ENSG00000013583	HEBP1	heme binding protein 1 [Source:HGNC Symbol;Acc:HGNC:17176]	protein coding	1.26	3.98	55.38	<0.0001	<0.0001
ENSG00000138642	HERC6	HECT and RLD domain containing E3 ubiquitin protein ligase family member 6 [Source:HGNC Symbol;Acc:HGNC:26072]	protein coding	1.26	4.30	70.49	<0.0001	<0.0001
ENSG00000213047	DENND1B	DENN domain containing 1B [Source:HGNC Symbol;Acc:HGNC:28404]	protein coding	1.26	2.79	76.81	<0.0001	<0.0001
ENSG00000236829		ribosomal protein L23a (RPL23A) pseudogene	transcribed processed pseudogene	-1.26	0.29	35.27	<0.0001	<0.0001
ENSG00000007516	BAIAP3	BAI1 associated protein 3 [Source:HGNC Symbol;Acc:HGNC:948]	protein coding	-1.25	5.98	121.59	<0.0001	<0.0001
ENSG00000055332	EIF2AK2	eukaryotic translation initiation factor 2 alpha kinase 2 [Source:HGNC Symbol;Acc:HGNC:9437]	protein coding	1.25	7.20	91.01	<0.0001	<0.0001
ENSG00000172426	RSPH9	radial spoke head component 9 [Source:HGNC Symbol;Acc:HGNC:21057]	protein coding	1.25	1.03	51.04	<0.0001	<0.0001
ENSG00000113552	GNPDA1	glucosamine-6-phosphate deaminase 1 [Source:HGNC Symbol;Acc:HGNC:4417]	protein coding	-1.25	2.17	50.49	<0.0001	<0.0001
ENSG00000118640	VAMP8	vesicle associated membrane protein 8 [Source:HGNC Symbol;Acc:HGNC:12647]	protein coding	1.25	1.87	40.12	<0.0001	<0.0001
ENSG00000119922	IFIT2	interferon induced protein with tetratricopeptide repeats 2 [Source:HGNC Symbol;Acc:HGNC:5409]	protein coding	1.25	10.45	43.51	<0.0001	<0.0001
ENSG00000110911	SLC11A2	solute carrier family 11 member 2 [Source:HGNC Symbol;Acc:HGNC:10908]	protein coding	1.24	0.63	32.12	<0.0001	<0.0001
ENSG00000196405	EVL	Enah/Vasp-like [Source:HGNC Symbol;Acc:HGNC:20234]	protein coding	1.24	3.66	66.77	<0.0001	<0.0001
ENSG00000124875	CXCL6	C-X-C motif chemokine ligand 6 [Source:HGNC Symbol;Acc:HGNC:10643]	protein coding	-1.24	2.94	59.70	<0.0001	<0.0001
ENSG00000187741	FANCA	FA complementation group A [Source:HGNC Symbol;Acc:HGNC:3582]	protein coding	1.24	4.23	51.99	<0.0001	<0.0001

Table S18: Genes differentially expressed at Day 30 compared to pre-second vaccination (Study Arm 2 A/H7N9+AS03, D1, 29, Neutrophils). Genes are sorted by descending absolute log_2 fold change. Gene model summaries and annotations are based on Ensembl Version 99 (continued.)

Ensembl Gene ID	Ensembl Gene Name	Ensembl Gene Description	Gene Type	log_2 Fold Change (Day 30 vs. Pre-Second Vaccination)	Average log_2 CPM	Likelihood Ratio Test Statistic	P-Value	FDR Adjusted P-Value
ENSG00000138439	FAM117B	family with sequence similarity 117 member B [Source:HGNC Symbol;Acc:HGNC:14440]	protein coding	-1.24	2.81	40.34	<0.0001	<0.0001
ENSG00000085063	CD59	CD59 molecule (CD59 blood group) [Source:HGNC Symbol;Acc:HGNC:1689]	protein coding	1.24	6.06	58.21	<0.0001	<0.0001
ENSG00000141574	SECTM1	secreted and transmembrane 1 [Source:HGNC Symbol;Acc:HGNC:10707]	protein coding	1.24	9.86	45.75	<0.0001	<0.0001
ENSG00000135604	STX11	syntaxin 11 [Source:HGNC Symbol;Acc:HGNC:11429]	protein coding	1.23	8.78	55.39	<0.0001	<0.0001
ENSG00000151208	DLG5	discs large MAGUK scaffold protein 5 [Source:HGNC Symbol;Acc:HGNC:2904]	protein coding	-1.23	0.65	39.73	<0.0001	<0.0001
ENSG00000102524	TNFSF13B	TNF superfamily member 13b [Source:HGNC Symbol;Acc:HGNC:11929]	protein coding	1.23	8.24	46.52	<0.0001	<0.0001
ENSG00000269564		novel transcript	lncRNA	-1.23	0.33	32.10	<0.0001	<0.0001
ENSG00000122068	FYTTD1	forty-two-three domain containing 1 [Source:HGNC Symbol;Acc:HGNC:25407]	protein coding	1.23	4.11	43.88	<0.0001	<0.0001
ENSG00000105516	DBP	D-box binding PAR bZIP transcription factor [Source:HGNC Symbol;Acc:HGNC:2697]	protein coding	-1.23	1.65	28.45	<0.0001	<0.0001
ENSG00000153885	KCTD15	potassium channel tetramerization domain containing 15 [Source:HGNC Symbol;Acc:HGNC:23297]	protein coding	-1.23	0.75	8.70	0.0032	0.0084
ENSG00000123933	MXD4	MAX dimerization protein 4 [Source:HGNC Symbol;Acc:HGNC:13906]	protein coding	-1.23	4.97	80.78	<0.0001	<0.0001
ENSG00000143537	ADAM15	ADAM metallopeptidase domain 15 [Source:HGNC Symbol;Acc:HGNC:193]	protein coding	-1.23	2.73	21.89	<0.0001	<0.0001
ENSG00000243696		novel MUSTN1-ITIH4 readthrough	protein coding	1.23	0.72	40.82	<0.0001	<0.0001
ENSG00000266604	LINC01887	long intergenic non-protein coding RNA 1887 [Source:HGNC Symbol;Acc:HGNC:52706]	lncRNA	-1.22	0.40	28.12	<0.0001	<0.0001
ENSG00000155287	SLC25A28	solute carrier family 25 member 28 [Source:HGNC Symbol;Acc:HGNC:23472]	protein coding	1.22	5.46	46.79	<0.0001	<0.0001
ENSG00000160716	CHRNB2	cholinergic receptor nicotinic beta 2 subunit [Source:HGNC Symbol;Acc:HGNC:1962]	protein coding	1.22	0.68	32.23	<0.0001	<0.0001
ENSG00000232810	TNF	tumor necrosis factor [Source:HGNC Symbol;Acc:HGNC:11892]	protein coding	1.22	3.16	19.47	<0.0001	<0.0001
ENSG00000119655	NPC2	NPC intracellular cholesterol transporter 2 [Source:HGNC Symbol;Acc:HGNC:14537]	protein coding	1.22	7.08	52.09	<0.0001	<0.0001

Table S18: Genes differentially expressed at Day 30 compared to pre-second vaccination (Study Arm 2 A/H7N9+AS03, D1, 29, Neutrophils). Genes are sorted by descending absolute log_2 fold change. Gene model summaries and annotations are based on Ensembl Version 99 (continued.)

Ensembl Gene ID	Ensembl Gene Name	Ensembl Gene Description	Gene Type	log_2 Fold Change (Day 30 vs. Pre-Second Vaccination)	Average log_2 CPM	Likelihood Ratio Test Statistic	P-Value	FDR Adjusted P-Value
ENSG00000168906	MAT2A	methionine adenosyltransferase 2A [Source:HGNC Symbol;Acc:HGNC:6904]	protein coding	-1.22	3.84	39.07	<0.0001	<0.0001
ENSG00000188107	EYS	eyes shut homolog [Source:HGNC Symbol;Acc:HGNC:21555]	protein coding	-1.22	1.28	56.70	<0.0001	<0.0001
ENSG00000166145	SPINT1	serine peptidase inhibitor, Kunitz type 1 [Source:HGNC Symbol;Acc:HGNC:11246]	protein coding	-1.22	5.09	56.81	<0.0001	<0.0001
ENSG00000174775	HRAS	HRas proto-oncogene, GTPase [Source:HGNC Symbol;Acc:HGNC:5173]	protein coding	-1.22	0.11	32.24	<0.0001	<0.0001
ENSG00000178719	GRINA	glutamate ionotropic receptor NMDA type subunit associated protein 1 [Source:HGNC Symbol;Acc:HGNC:4589]	protein coding	1.22	7.47	80.71	<0.0001	<0.0001
ENSG00000085871	MGST2	microsomal glutathione S-transferase 2 [Source:HGNC Symbol;Acc:HGNC:7063]	protein coding	-1.22	2.35	44.84	<0.0001	<0.0001
ENSG00000115602	IL1RL1	interleukin 1 receptor like 1 [Source:HGNC Symbol;Acc:HGNC:5998]	protein coding	-1.22	1.12	7.63	0.0057	0.0141
ENSG00000172367	PDZD3	PDZ domain containing 3 [Source:HGNC Symbol;Acc:HGNC:19891]	protein coding	-1.22	1.69	30.08	<0.0001	<0.0001
ENSG00000196116	TDRD7	tudor domain containing 7 [Source:HGNC Symbol;Acc:HGNC:30831]	protein coding	1.21	5.82	78.51	<0.0001	<0.0001
ENSG00000105402	NAPA	NSF attachment protein alpha [Source:HGNC Symbol;Acc:HGNC:7641]	protein coding	1.21	7.09	65.09	<0.0001	<0.0001
ENSG00000068784	SRBD1	S1 RNA binding domain 1 [Source:HGNC Symbol;Acc:HGNC:25521]	protein coding	1.21	4.95	103.32	<0.0001	<0.0001
ENSG00000160113	NR2F6	nuclear receptor subfamily 2 group F member 6 [Source:HGNC Symbol;Acc:HGNC:7977]	protein coding	-1.21	0.46	27.36	<0.0001	<0.0001
ENSG00000184470	TXNRD2	thioredoxin reductase 2 [Source:HGNC Symbol;Acc:HGNC:18155]	protein coding	-1.21	0.83	23.40	<0.0001	<0.0001
ENSG00000159618	ADGRG5	adhesion G protein-coupled receptor G5 [Source:HGNC Symbol;Acc:HGNC:19010]	protein coding	-1.21	2.41	7.93	0.0049	0.0122
ENSG00000110237	ARHGEF17	Rho guanine nucleotide exchange factor 17 [Source:HGNC Symbol;Acc:HGNC:21726]	protein coding	-1.21	1.92	23.00	<0.0001	<0.0001
ENSG00000122547	EEPD1	endonuclease/exonuclease/phosphatase family domain containing 1 [Source:HGNC Symbol;Acc:HGNC:22223]	protein coding	-1.21	6.61	85.27	<0.0001	<0.0001
ENSG00000198363	ASPH	aspartate beta-hydroxylase [Source:HGNC Symbol;Acc:HGNC:757]	protein coding	-1.21	5.86	38.26	<0.0001	<0.0001
ENSG00000167483	NIBAN3	niban apoptosis regulator 3 [Source:HGNC Symbol;Acc:HGNC:24130]	protein coding	-1.21	1.59	49.07	<0.0001	<0.0001

Table S18: Genes differentially expressed at Day 30 compared to pre-second vaccination (Study Arm 2 A/H7N9+AS03, D1, 29, Neutrophils). Genes are sorted by descending absolute log_2 fold change. Gene model summaries and annotations are based on Ensembl Version 99 (continued.)

Ensembl Gene ID	Ensembl Gene Name	Ensembl Gene Description	Gene Type	log_2 Fold Change (Day 30 vs. Pre-Second Vaccination)	Average log_2 CPM	Likelihood Ratio Test Statistic	P-Value	FDR Adjusted P-Value
ENSG00000172493	AFF1	AF4/FMR2 family member 1 [Source:HGNC Symbol;Acc:HGNC:7135]	protein coding	1.21	7.14	52.57	<0.0001	<0.0001
ENSG00000128335	APOL2	apolipoprotein L2 [Source:HGNC Symbol;Acc:HGNC:619]	protein coding	1.21	8.61	55.39	<0.0001	<0.0001
ENSG00000174749	FAM241A	family with sequence similarity 241 member A [Source:HGNC Symbol;Acc:HGNC:26813]	protein coding	1.21	3.33	37.04	<0.0001	<0.0001
ENSG00000125810	CD93	CD93 molecule [Source:HGNC Symbol;Acc:HGNC:15855]	protein coding	-1.20	8.79	50.48	<0.0001	<0.0001
ENSG00000197119	SLC25A29	solute carrier family 25 member 29 [Source:HGNC Symbol;Acc:HGNC:20116]	protein coding	-1.20	4.84	81.76	<0.0001	<0.0001
ENSG00000150681	RGS18	regulator of G protein signaling 18 [Source:HGNC Symbol;Acc:HGNC:14261]	protein coding	-1.20	7.68	45.78	<0.0001	<0.0001
ENSG00000130589	HELZ2	helicase with zinc finger 2 [Source:HGNC Symbol;Acc:HGNC:30021]	protein coding	1.20	8.86	74.91	<0.0001	<0.0001
ENSG00000174004	NRROS	negative regulator of reactive oxygen species [Source:HGNC Symbol;Acc:HGNC:24613]	protein coding	-1.20	1.91	25.00	<0.0001	<0.0001
ENSG00000197562	RAB40C	RAB40C, member RAS oncogene family [Source:HGNC Symbol;Acc:HGNC:18285]	protein coding	-1.20	4.88	66.22	<0.0001	<0.0001
ENSG00000124098	FAM210B	family with sequence similarity 210 member B [Source:HGNC Symbol;Acc:HGNC:16102]	protein coding	-1.20	3.04	53.61	<0.0001	<0.0001
ENSG00000171101	SIGLEC17P	sialic acid binding Ig like lectin 17, pseudogene [Source:HGNC Symbol;Acc:HGNC:15604]	transcribed unprocessed pseudogene	-1.20	0.69	32.14	<0.0001	<0.0001
ENSG00000178467	P4HTM	prolyl 4-hydroxylase, transmembrane [Source:HGNC Symbol;Acc:HGNC:28858]	protein coding	-1.19	1.82	35.83	<0.0001	<0.0001
ENSG00000274021		novel transcript, antisense to DUSP6	lncRNA	1.19	3.12	32.45	<0.0001	<0.0001
ENSG00000163565	IFI16	interferon gamma inducible protein 16 [Source:HGNC Symbol;Acc:HGNC:5395]	protein coding	1.19	8.79	50.91	<0.0001	<0.0001
ENSG00000155393	HEATR3	HEAT repeat containing 3 [Source:HGNC Symbol;Acc:HGNC:26087]	protein coding	-1.19	1.43	28.18	<0.0001	<0.0001
ENSG00000274173		novel transcript	lncRNA	-1.19	3.15	27.15	<0.0001	<0.0001

Table S18: Genes differentially expressed at Day 30 compared to pre-second vaccination (Study Arm 2 A/H7N9+AS03, D1, 29, Neutrophils). Genes are sorted by descending absolute log_2 fold change. Gene model summaries and annotations are based on Ensembl Version 99 (continued.)

Ensembl Gene ID	Ensembl Gene Name	Ensembl Gene Description	Gene Type	log_2 Fold Change (Day 30 vs. Pre-Second Vaccination)	Average log_2 CPM	Likelihood Ratio Test Statistic	P-Value	FDR Adjusted P-Value
ENSG00000234515	PPP1R2P1	protein phosphatase 1 regulatory inhibitor subunit 2 pseudogene 1 [Source:HGNC Symbol;Acc:HGNC:9289]	transcribed processed pseudogene	1.19	0.97	40.03	<0.0001	<0.0001
ENSG00000249173	LINC01093	long intergenic non-protein coding RNA 1093 [Source:HGNC Symbol;Acc:HGNC:49218]	lncRNA	1.19	1.36	28.80	<0.0001	<0.0001
ENSG00000125347	IRF1	interferon regulatory factor 1 [Source:HGNC Symbol;Acc:HGNC:6116]	protein coding	1.19	9.54	53.83	<0.0001	<0.0001
ENSG00000117984	CTSD	cathepsin D [Source:HGNC Symbol;Acc:HGNC:2529]	protein coding	-1.19	7.26	35.79	<0.0001	<0.0001
ENSG00000137801	THBS1	thrombospondin 1 [Source:HGNC Symbol;Acc:HGNC:11785]	protein coding	-1.19	0.78	13.29	0.0003	0.0009
ENSG00000204217	BMPR2	bone morphogenetic protein receptor type 2 [Source:HGNC Symbol;Acc:HGNC:1078]	protein coding	1.19	3.42	70.65	<0.0001	<0.0001
ENSG00000135900	MRPL44	mitochondrial ribosomal protein L44 [Source:HGNC Symbol;Acc:HGNC:16650]	protein coding	1.18	5.05	33.33	<0.0001	<0.0001
ENSG00000167264	DUS2	dihydrouridine synthase 2 [Source:HGNC Symbol;Acc:HGNC:26014]	protein coding	-1.18	4.44	66.12	<0.0001	<0.0001
ENSG00000141560	FN3KRP	fructosamine 3 kinase related protein [Source:HGNC Symbol;Acc:HGNC:25700]	protein coding	-1.18	0.71	23.47	<0.0001	<0.0001
ENSG00000074800	ENO1	enolase 1 [Source:HGNC Symbol;Acc:HGNC:3350]	protein coding	-1.18	6.88	48.36	<0.0001	<0.0001
ENSG00000182670	TTC3	tetratricopeptide repeat domain 3 [Source:HGNC Symbol;Acc:HGNC:12393]	protein coding	-1.18	2.00	29.05	<0.0001	<0.0001
ENSG00000157800	SLC37A3	solute carrier family 37 member 3 [Source:HGNC Symbol;Acc:HGNC:20651]	protein coding	-1.18	4.86	34.16	<0.0001	<0.0001
ENSG00000177542	SLC25A22	solute carrier family 25 member 22 [Source:HGNC Symbol;Acc:HGNC:19954]	protein coding	1.18	3.19	58.88	<0.0001	<0.0001
ENSG00000135916	ITM2C	integral membrane protein 2C [Source:HGNC Symbol;Acc:HGNC:6175]	protein coding	-1.18	1.90	10.66	0.0011	0.0033
ENSG00000167074	TEF	TEF transcription factor, PAR bZIP family member [Source:HGNC Symbol;Acc:HGNC:11722]	protein coding	-1.18	1.13	45.43	<0.0001	<0.0001
ENSG00000121858	TNFSF10	TNF superfamily member 10 [Source:HGNC Symbol;Acc:HGNC:11925]	protein coding	1.18	9.19	46.46	<0.0001	<0.0001
ENSG00000162639	HENMT1	HEN methyltransferase 1 [Source:HGNC Symbol;Acc:HGNC:26400]	protein coding	-1.18	2.95	36.17	<0.0001	<0.0001

Table S18: Genes differentially expressed at Day 30 compared to pre-second vaccination (Study Arm 2 A/H7N9+AS03, D1, 29, Neutrophils). Genes are sorted by descending absolute log_2 fold change. Gene model summaries and annotations are based on Ensembl Version 99 (continued.)

Ensembl Gene ID	Ensembl Gene Name	Ensembl Gene Description	Gene Type	log_2 Fold Change (Day 30 vs. Pre-Second Vaccination)	Average log_2 CPM	Likelihood Ratio Test Statistic	P-Value	FDR Adjusted P-Value
ENSG00000249863		synaptotagmin binding, cytoplasmic RNA interacting protein (SYNCRIP) pseudogene	processed pseudogene	-1.18	1.38	25.41	<0.0001	<0.0001
ENSG00000157404	KIT	KIT proto-oncogene, receptor tyrosine kinase [Source:HGNC Symbol;Acc:HGNC:6342]	protein coding	-1.18	1.99	39.04	<0.0001	<0.0001
ENSG00000182912	TSPEAR-AS2	TSPEAR antisense RNA 2 [Source:HGNC Symbol;Acc:HGNC:16428]	lncRNA	-1.17	0.42	20.33	<0.0001	<0.0001
ENSG00000137261	KIAA0319	KIAA0319 [Source:HGNC Symbol;Acc:HGNC:21580]	protein coding	-1.17	4.14	76.09	<0.0001	<0.0001
ENSG00000100528	CNIH1	cornichon family AMPA receptor auxiliary protein 1 [Source:HGNC Symbol;Acc:HGNC:19431]	protein coding	-1.17	2.31	38.06	<0.0001	<0.0001
ENSG00000142197	DOP1B	DOP1 leucine zipper like protein B [Source:HGNC Symbol;Acc:HGNC:1291]	protein coding	-1.17	4.89	62.23	<0.0001	<0.0001
ENSG00000164512	ANKRD55	ankyrin repeat domain 55 [Source:HGNC Symbol;Acc:HGNC:25681]	protein coding	-1.17	1.32	14.89	0.0001	0.0004
ENSG00000187840	EIF4EBP1	eukaryotic translation initiation factor 4E binding protein 1 [Source:HGNC Symbol;Acc:HGNC:3288]	protein coding	-1.17	1.53	32.02	<0.0001	<0.0001
ENSG00000233901	LINC01503	long intergenic non-protein coding RNA 1503 [Source:HGNC Symbol;Acc:HGNC:51184]	lncRNA	-1.16	2.55	38.89	<0.0001	<0.0001
ENSG00000104164	BLOC1S6	biogenesis of lysosomal organelles complex 1 subunit 6 [Source:HGNC Symbol;Acc:HGNC:8549]	protein coding	1.16	5.01	56.65	<0.0001	<0.0001
ENSG00000168297	PXK	PX domain containing serine/threonine kinase like [Source:HGNC Symbol;Acc:HGNC:23326]	protein coding	-1.16	5.77	33.08	<0.0001	<0.0001
ENSG00000145246	ATP10D	ATPase phospholipid transporting 10D (putative) [Source:HGNC Symbol;Acc:HGNC:13549]	protein coding	1.16	3.04	26.34	<0.0001	<0.0001
ENSG00000124713	GNMT	glycine N-methyltransferase [Source:HGNC Symbol;Acc:HGNC:4415]	protein coding	-1.16	1.91	49.81	<0.0001	<0.0001
ENSG00000179021	C3orf38	chromosome 3 open reading frame 38 [Source:HGNC Symbol;Acc:HGNC:28384]	protein coding	1.16	3.37	78.55	<0.0001	<0.0001
ENSG00000132182	NUP210	nucleoporin 210 [Source:HGNC Symbol;Acc:HGNC:30052]	protein coding	-1.16	1.91	18.73	<0.0001	<0.0001
ENSG00000136826	KLF4	Kruppel like factor 4 [Source:HGNC Symbol;Acc:HGNC:6348]	protein coding	1.16	4.49	20.26	<0.0001	<0.0001
ENSG00000166479	TMX3	thioredoxin related transmembrane protein 3 [Source:HGNC Symbol;Acc:HGNC:24718]	protein coding	1.16	3.89	89.73	<0.0001	<0.0001

Table S18: Genes differentially expressed at Day 30 compared to pre-second vaccination (Study Arm 2 A/H7N9+AS03, D1, 29, Neutrophils). Genes are sorted by descending absolute log_2 fold change. Gene model summaries and annotations are based on Ensembl Version 99 (continued.)

Ensembl Gene ID	Ensembl Gene Name	Ensembl Gene Description	Gene Type	log_2 Fold Change (Day 30 vs. Pre-Second Vaccination)	Average log_2 CPM	Likelihood Ratio Test Statistic	P-Value	FDR Adjusted P-Value
ENSG00000281181		novel transcript, similar to YY1 associated myogenesis RNA 1 YAM1	lncRNA	1.16	4.92	7.27	0.007	0.0167
ENSG00000274265		novel transcript	lncRNA	1.16	2.85	54.20	<0.0001	<0.0001
ENSG00000141568	FOXK2	forkhead box K2 [Source:HGNC Symbol;Acc:HGNC:6036]	protein coding	-1.16	3.66	54.37	<0.0001	<0.0001
ENSG00000154217	PITPNC1	phosphatidylinositol transfer protein cytoplasmic 1 [Source:HGNC Symbol;Acc:HGNC:21045]	protein coding	-1.16	5.33	74.36	<0.0001	<0.0001
ENSG00000165644	COMTD1	catechol-O-methyltransferase domain containing 1 [Source:HGNC Symbol;Acc:HGNC:26309]	protein coding	-1.15	0.55	23.98	<0.0001	<0.0001
ENSG00000163875	MEAF6	MYST/Esa1 associated factor 6 [Source:HGNC Symbol;Acc:HGNC:25674]	protein coding	-1.15	3.60	47.07	<0.0001	<0.0001
ENSG00000280649		TEC	TEC	1.15	2.47	52.79	<0.0001	<0.0001
ENSG00000015171	ZMYND11	zinc finger MYND-type containing 11 [Source:HGNC Symbol;Acc:HGNC:16966]	protein coding	-1.15	0.75	11.31	0.0008	0.0024
ENSG00000135148	TRAFD1	TRAF-type zinc finger domain containing 1 [Source:HGNC Symbol;Acc:HGNC:24808]	protein coding	1.15	7.87	51.25	<0.0001	<0.0001
ENSG00000169994	MYO7B	myosin VIIB [Source:HGNC Symbol;Acc:HGNC:7607]	protein coding	-1.15	4.49	66.12	<0.0001	<0.0001
ENSG00000216490	IFI30	IFI30 lysosomal thiol reductase [Source:HGNC Symbol;Acc:HGNC:5398]	protein coding	1.15	3.47	20.35	<0.0001	<0.0001
ENSG00000164086	DUSP7	dual specificity phosphatase 7 [Source:HGNC Symbol;Acc:HGNC:3073]	protein coding	-1.15	3.11	63.56	<0.0001	<0.0001
ENSG00000179832	MROH1	maestro heat like repeat family member 1 [Source:HGNC Symbol;Acc:HGNC:26958]	protein coding	-1.15	3.56	26.95	<0.0001	<0.0001
ENSG00000136816	TOR1B	torsin family 1 member B [Source:HGNC Symbol;Acc:HGNC:11995]	protein coding	1.15	6.13	64.85	<0.0001	<0.0001
ENSG00000204397	CARD16	caspase recruitment domain family member 16 [Source:HGNC Symbol;Acc:HGNC:33701]	protein coding	1.15	7.10	78.95	<0.0001	<0.0001
ENSG00000133624	ZNF767P	zinc finger family member 767, pseudogene [Source:HGNC Symbol;Acc:HGNC:21884]	transcribed unprocessed pseudogene	-1.15	2.93	51.08	<0.0001	<0.0001
ENSG00000172456	FGGY	FGGY carbohydrate kinase domain containing [Source:HGNC Symbol;Acc:HGNC:25610]	protein coding	-1.15	1.87	47.97	<0.0001	<0.0001

Table S18: Genes differentially expressed at Day 30 compared to pre-second vaccination (Study Arm 2 A/H7N9+AS03, D1, 29, Neutrophils). Genes are sorted by descending absolute log_2 fold change. Gene model summaries and annotations are based on Ensembl Version 99 (continued.)

Ensembl Gene ID	Ensembl Gene Name	Ensembl Gene Description	Gene Type	log_2 Fold Change (Day 30 vs. Pre-Second Vaccination)	Average log_2 CPM	Likelihood Ratio Test Statistic	P-Value	FDR Adjusted P-Value
ENSG00000075151	EIF4G3	eukaryotic translation initiation factor 4 gamma 3 [Source:HGNC Symbol;Acc:HGNC:3298]	protein coding	1.14	6.10	62.58	<0.0001	<0.0001
ENSG00000163840	DTX3L	deltex E3 ubiquitin ligase 3L [Source:HGNC Symbol;Acc:HGNC:30323]	protein coding	1.14	7.73	49.90	<0.0001	<0.0001
ENSG00000176978	DPP7	dipeptidyl peptidase 7 [Source:HGNC Symbol;Acc:HGNC:14892]	protein coding	-1.14	2.04	24.03	<0.0001	<0.0001
ENSG00000003400	CASP10	caspase 10 [Source:HGNC Symbol;Acc:HGNC:1500]	protein coding	1.14	4.28	78.13	<0.0001	<0.0001
ENSG00000161980	POLR3K	RNA polymerase III subunit K [Source:HGNC Symbol;Acc:HGNC:14121]	protein coding	-1.14	2.15	35.27	<0.0001	<0.0001
ENSG00000170275	CRTAP	cartilage associated protein [Source:HGNC Symbol;Acc:HGNC:2379]	protein coding	-1.14	4.04	50.40	<0.0001	<0.0001
ENSG00000066583	ISOC1	isochorismatase domain containing 1 [Source:HGNC Symbol;Acc:HGNC:24254]	protein coding	-1.14	2.89	65.37	<0.0001	<0.0001
ENSG00000135093	USP30	ubiquitin specific peptidase 30 [Source:HGNC Symbol;Acc:HGNC:20065]	protein coding	1.14	2.80	49.21	<0.0001	<0.0001
ENSG00000188486	H2AX	H2A.X variant histone [Source:HGNC Symbol;Acc:HGNC:4739]	protein coding	-1.14	1.92	29.85	<0.0001	<0.0001
ENSG00000182979	MTA1	metastasis associated 1 [Source:HGNC Symbol;Acc:HGNC:7410]	protein coding	-1.14	2.59	42.19	<0.0001	<0.0001
ENSG00000073060	SCARB1	scavenger receptor class B member 1 [Source:HGNC Symbol;Acc:HGNC:1664]	protein coding	-1.14	1.56	40.35	<0.0001	<0.0001
ENSG00000285444		novel transcript	protein coding	1.14	0.94	35.12	<0.0001	<0.0001
ENSG00000047579	DTNBP1	dystrobrevin binding protein 1 [Source:HGNC Symbol;Acc:HGNC:17328]	protein coding	1.14	3.68	50.97	<0.0001	<0.0001
ENSG00000245888	NSMCE1-DT	NSMCE1 divergent transcript [Source:HGNC Symbol;Acc:HGNC:51368]	lncRNA	-1.14	1.15	25.76	<0.0001	<0.0001
ENSG00000198948	MFAP3L	microfibril associated protein 3 like [Source:HGNC Symbol;Acc:HGNC:29083]	protein coding	-1.14	1.10	21.82	<0.0001	<0.0001
ENSG00000287839		novel transcript	lncRNA	1.14	3.91	36.74	<0.0001	<0.0001
ENSG00000141664	ZCCHC2	zinc finger CCHC-type containing 2 [Source:HGNC Symbol;Acc:HGNC:22916]	protein coding	1.14	7.88	70.54	<0.0001	<0.0001
ENSG00000141837	CACNA1A	calcium voltage-gated channel subunit alpha1 A [Source:HGNC Symbol;Acc:HGNC:1388]	protein coding	1.13	3.08	57.63	<0.0001	<0.0001

Table S18: Genes differentially expressed at Day 30 compared to pre-second vaccination (Study Arm 2 A/H7N9+AS03, D1, 29, Neutrophils). Genes are sorted by descending absolute log_2 fold change. Gene model summaries and annotations are based on Ensembl Version 99 (continued.)

Ensembl Gene ID	Ensembl Gene Name	Ensembl Gene Description	Gene Type	log_2 Fold Change (Day 30 vs. Pre-Second Vaccination)	Average log_2 CPM	Likelihood Ratio Test Statistic	P-Value	FDR Adjusted P-Value
ENSG00000248592	STIMATE-MUSTN1	STIMATE-MUSTN1 readthrough [Source:HGNC Symbol;Acc:HGNC:38834]	protein coding	1.13	0.43	30.27	<0.0001	<0.0001
ENSG00000184117	NIPSNAP1	nipsnap homolog 1 [Source:HGNC Symbol;Acc:HGNC:7827]	protein coding	-1.13	3.53	39.21	<0.0001	<0.0001
ENSG00000087253	LPCAT2	lysophosphatidylcholine acyltransferase 2 [Source:HGNC Symbol;Acc:HGNC:26032]	protein coding	1.13	8.26	49.64	<0.0001	<0.0001
ENSG00000266913	LINC01841	long intergenic non-protein coding RNA 1841 [Source:HGNC Symbol;Acc:HGNC:52655]	lncRNA	1.13	0.70	53.83	<0.0001	<0.0001
ENSG00000187260	WDR86	WD repeat domain 86 [Source:HGNC Symbol;Acc:HGNC:28020]	protein coding	1.13	0.73	31.68	<0.0001	<0.0001
ENSG00000177738		novel transcript, sense overlapping to ANXA2R	lncRNA	1.13	2.03	38.98	<0.0001	<0.0001
ENSG00000163950	SLBP	stem-loop binding protein [Source:HGNC Symbol;Acc:HGNC:10904]	protein coding	-1.13	5.86	75.44	<0.0001	<0.0001
ENSG00000198216	CACNA1E	calcium voltage-gated channel subunit alpha1 E [Source:HGNC Symbol;Acc:HGNC:1392]	protein coding	1.13	4.63	78.62	<0.0001	<0.0001
ENSG00000160190	SLC37A1	solute carrier family 37 member 1 [Source:HGNC Symbol;Acc:HGNC:11024]	protein coding	1.13	4.13	60.48	<0.0001	<0.0001
ENSG00000030419	IKZF2	IKAROS family zinc finger 2 [Source:HGNC Symbol;Acc:HGNC:13177]	protein coding	-1.13	0.91	6.63	0.0101	0.0229
ENSG00000177697	CD151	CD151 molecule (Raph blood group) [Source:HGNC Symbol;Acc:HGNC:1630]	protein coding	-1.13	2.23	16.07	<0.0001	0.0002
ENSG00000116299	KIAA1324	KIAA1324 [Source:HGNC Symbol;Acc:HGNC:29618]	protein coding	-1.13	7.31	32.94	<0.0001	<0.0001
ENSG00000231711	LINC00899	long intergenic non-protein coding RNA 899 [Source:HGNC Symbol;Acc:HGNC:48583]	lncRNA	-1.13	1.83	37.82	<0.0001	<0.0001
ENSG00000121957	GPSM2	G protein signaling modulator 2 [Source:HGNC Symbol;Acc:HGNC:29501]	protein coding	-1.13	3.20	32.72	<0.0001	<0.0001
ENSG00000270030		novel transcript	lncRNA	1.13	1.64	18.33	<0.0001	<0.0001
ENSG00000160703	NLRX1	NLR family member X1 [Source:HGNC Symbol;Acc:HGNC:29890]	protein coding	-1.13	5.34	55.76	<0.0001	<0.0001
ENSG00000100324	TAB1	TGF-beta activated kinase 1 (MAP3K7) binding protein 1 [Source:HGNC Symbol;Acc:HGNC:18157]	protein coding	-1.13	1.38	26.18	<0.0001	<0.0001
ENSG00000279727	LINC02033	long intergenic non-protein coding RNA 2033 [Source:HGNC Symbol;Acc:HGNC:52867]	lncRNA	1.13	0.89	29.95	<0.0001	<0.0001
ENSG00000167543	TP53I13	tumor protein p53 inducible protein 13 [Source:HGNC Symbol;Acc:HGNC:25102]	protein coding	-1.12	0.69	9.11	0.0025	0.0069

Table S18: Genes differentially expressed at Day 30 compared to pre-second vaccination (Study Arm 2 A/H7N9+AS03, D1, 29, Neutrophils). Genes are sorted by descending absolute log_2 fold change. Gene model summaries and annotations are based on Ensembl Version 99 (continued.)

Ensembl Gene ID	Ensembl Gene Name	Ensembl Gene Description	Gene Type	log_2 Fold Change (Day 30 vs. Pre-Second Vaccination)	Average log_2 CPM	Likelihood Ratio Test Statistic	P-Value	FDR Adjusted P-Value
ENSG00000090661	CERS4	ceramide synthase 4 [Source:HGNC Symbol;Acc:HGNC:23747]	protein coding	-1.12	4.52	62.50	<0.0001	<0.0001
ENSG00000119121	TRPM6	transient receptor potential cation channel subfamily M member 6 [Source:HGNC Symbol;Acc:HGNC:17995]	protein coding	-1.12	5.93	60.39	<0.0001	<0.0001
ENSG00000141854	MISP3	MISP family member 3 [Source:HGNC Symbol;Acc:HGNC:26963]	protein coding	-1.12	1.36	31.40	<0.0001	<0.0001
ENSG00000106785	TRIM14	tripartite motif containing 14 [Source:HGNC Symbol;Acc:HGNC:16283]	protein coding	1.12	3.95	66.78	<0.0001	<0.0001
ENSG00000122733	PHF24	PHD finger protein 24 [Source:HGNC Symbol;Acc:HGNC:29180]	protein coding	-1.12	2.30	36.33	<0.0001	<0.0001
ENSG00000072121	ZFYVE26	zinc finger FYVE-type containing 26 [Source:HGNC Symbol;Acc:HGNC:20761]	protein coding	1.12	3.93	62.42	<0.0001	<0.0001
ENSG00000059122	FLYWCH1	FLYWCH-type zinc finger 1 [Source:HGNC Symbol;Acc:HGNC:25404]	protein coding	-1.12	1.20	21.72	<0.0001	<0.0001
ENSG00000100031	GGT1	gamma-glutamyltransferase 1 [Source:HGNC Symbol;Acc:HGNC:4250]	protein coding	-1.12	0.89	26.13	<0.0001	<0.0001
ENSG00000185897	FFAR3	free fatty acid receptor 3 [Source:HGNC Symbol;Acc:HGNC:4499]	protein coding	1.12	0.91	25.29	<0.0001	<0.0001
ENSG00000124215	CDH26	cadherin 26 [Source:HGNC Symbol;Acc:HGNC:15902]	protein coding	-1.12	1.95	24.69	<0.0001	<0.0001
ENSG00000205413	SAMD9	sterile alpha motif domain containing 9 [Source:HGNC Symbol;Acc:HGNC:1348]	protein coding	1.12	8.04	60.83	<0.0001	<0.0001
ENSG00000164134	NAA15	N(alpha)-acetyltransferase 15, NatA auxiliary subunit [Source:HGNC Symbol;Acc:HGNC:30782]	protein coding	1.12	0.76	39.31	<0.0001	<0.0001
ENSG00000266074	BAHCC1	BAH domain and coiled-coil containing 1 [Source:HGNC Symbol;Acc:HGNC:29279]	protein coding	-1.12	0.13	8.28	0.004	0.0103
ENSG00000183508	TENT5C	terminal nucleotidyltransferase 5C [Source:HGNC Symbol;Acc:HGNC:24712]	protein coding	1.12	3.24	60.33	<0.0001	<0.0001
ENSG00000058729	RIOK2	RIO kinase 2 [Source:HGNC Symbol;Acc:HGNC:18999]	protein coding	-1.11	1.19	25.92	<0.0001	<0.0001
ENSG00000138760	SCARB2	scavenger receptor class B member 2 [Source:HGNC Symbol;Acc:HGNC:1665]	protein coding	1.11	3.71	52.43	<0.0001	<0.0001
ENSG00000148834	GSTO1	glutathione S-transferase omega 1 [Source:HGNC Symbol;Acc:HGNC:13312]	protein coding	1.11	5.30	54.55	<0.0001	<0.0001
ENSG00000133805	AMPD3	adenosine monophosphate deaminase 3 [Source:HGNC Symbol;Acc:HGNC:470]	protein coding	-1.11	5.96	37.24	<0.0001	<0.0001

Table S18: Genes differentially expressed at Day 30 compared to pre-second vaccination (Study Arm 2 A/H7N9+AS03, D1, 29, Neutrophils). Genes are sorted by descending absolute log_2 fold change. Gene model summaries and annotations are based on Ensembl Version 99 (continued.)

Ensembl Gene ID	Ensembl Gene Name	Ensembl Gene Description	Gene Type	log_2 Fold Change (Day 30 vs. Pre-Second Vaccination)	Average log_2 CPM	Likelihood Ratio Test Statistic	P-Value	FDR Adjusted P-Value
ENSG00000069424	KCNAB2	potassium voltage-gated channel subfamily A regulatory beta subunit 2 [Source:HGNC Symbol;Acc:HGNC:6229]	protein coding	-1.11	7.31	49.92	<0.0001	<0.0001
ENSG00000116014	KISS1R	KISS1 receptor [Source:HGNC Symbol;Acc:HGNC:4510]	protein coding	-1.11	2.20	31.29	<0.0001	<0.0001
ENSG00000204634	TBC1D8	TBC1 domain family member 8 [Source:HGNC Symbol;Acc:HGNC:17791]	protein coding	1.11	2.45	56.55	<0.0001	<0.0001
ENSG00000008710	PKD1	polycystin 1, transient receptor potential channel interacting [Source:HGNC Symbol;Acc:HGNC:9008]	protein coding	-1.11	0.84	28.82	<0.0001	<0.0001
ENSG00000071894	CPSF1	cleavage and polyadenylation specific factor 1 [Source:HGNC Symbol;Acc:HGNC:2324]	protein coding	-1.11	4.32	40.10	<0.0001	<0.0001
ENSG00000138134	STAMBPL1	STAM binding protein like 1 [Source:HGNC Symbol;Acc:HGNC:24105]	protein coding	1.11	0.40	35.57	<0.0001	<0.0001
ENSG00000187091	PLCD1	phospholipase C delta 1 [Source:HGNC Symbol;Acc:HGNC:9060]	protein coding	-1.11	3.61	65.02	<0.0001	<0.0001
ENSG00000250548	LINC01303	long intergenic non-protein coding RNA 1303 [Source:HGNC Symbol;Acc:HGNC:50470]	lncRNA	1.11	0.85	24.35	<0.0001	<0.0001
ENSG00000140807	NKD1	NKD inhibitor of WNT signaling pathway 1 [Source:HGNC Symbol;Acc:HGNC:17045]	protein coding	1.11	2.28	56.09	<0.0001	<0.0001
ENSG00000162591	MEGF6	multiple EGF like domains 6 [Source:HGNC Symbol;Acc:HGNC:3232]	protein coding	-1.11	7.04	77.66	<0.0001	<0.0001
ENSG00000182158	CREB3L2	cAMP responsive element binding protein 3 like 2 [Source:HGNC Symbol;Acc:HGNC:23720]	protein coding	1.10	3.52	70.78	<0.0001	<0.0001
ENSG00000130487	KLHDC7B	kelch domain containing 7B [Source:HGNC Symbol;Acc:HGNC:25145]	protein coding	1.10	2.14	40.41	<0.0001	<0.0001
ENSG00000133317	LGALS12	galectin 12 [Source:HGNC Symbol;Acc:HGNC:15788]	protein coding	-1.10	2.33	10.48	0.0012	0.0036
ENSG00000189337	KAZN	kazrin, periplakin interacting protein [Source:HGNC Symbol;Acc:HGNC:29173]	protein coding	-1.10	4.86	59.98	<0.0001	<0.0001
ENSG00000168936	TMEM129	transmembrane protein 129 [Source:HGNC Symbol;Acc:HGNC:25137]	protein coding	-1.10	3.57	63.65	<0.0001	<0.0001
ENSG00000235890	TSPEAR-AS1	TSPEAR antisense RNA 1 [Source:HGNC Symbol;Acc:HGNC:1271]	lncRNA	-1.10	0.74	24.62	<0.0001	<0.0001
ENSG00000136938	ANP32B	acidic nuclear phosphoprotein 32 family member B [Source:HGNC Symbol;Acc:HGNC:16677]	protein coding	-1.10	4.52	59.90	<0.0001	<0.0001
ENSG00000071967	CYBRD1	cytochrome b reductase 1 [Source:HGNC Symbol;Acc:HGNC:20797]	protein coding	-1.10	5.27	56.17	<0.0001	<0.0001

Table S18: Genes differentially expressed at Day 30 compared to pre-second vaccination (Study Arm 2 A/H7N9+AS03, D1, 29, Neutrophils). Genes are sorted by descending absolute log_2 fold change. Gene model summaries and annotations are based on Ensembl Version 99 (continued.)

Ensembl Gene ID	Ensembl Gene Name	Ensembl Gene Description	Gene Type	log_2 Fold Change (Day 30 vs. Pre-Second Vaccination)	Average log_2 CPM	Likelihood Ratio Test Statistic	P-Value	FDR Adjusted P-Value
ENSG00000076555	ACACB	acetyl-CoA carboxylase beta [Source:HGNC Symbol;Acc:HGNC:85]	protein coding	-1.10	1.91	14.44	0.0001	0.0005
ENSG00000145882	PCYOX1L	prenylcysteine oxidase 1 like [Source:HGNC Symbol;Acc:HGNC:28477]	protein coding	-1.10	0.41	16.97	<0.0001	0.0002
ENSG00000162066	AMDHD2	amidohydrolase domain containing 2 [Source:HGNC Symbol;Acc:HGNC:24262]	protein coding	-1.10	4.34	48.95	<0.0001	<0.0001
ENSG00000258476	LINC02207	long intergenic non-protein coding RNA 2207 [Source:HGNC Symbol;Acc:HGNC:53073]	lncRNA	-1.10	3.30	38.48	<0.0001	<0.0001
ENSG00000166801	FAM111A	family with sequence similarity 111 member A [Source:HGNC Symbol;Acc:HGNC:24725]	protein coding	1.10	6.90	58.62	<0.0001	<0.0001
ENSG00000184897	H1-10	H1.10 linker histone [Source:HGNC Symbol;Acc:HGNC:4722]	protein coding	-1.10	4.41	25.53	<0.0001	<0.0001
ENSG00000105939	ZC3HAV1	zinc finger CCCH-type containing, antiviral 1 [Source:HGNC Symbol;Acc:HGNC:23721]	protein coding	1.10	7.68	55.83	<0.0001	<0.0001
ENSG00000183734	ASCL2	achaete-scute family bHLH transcription factor 2 [Source:HGNC Symbol;Acc:HGNC:739]	protein coding	1.10	3.32	41.53	<0.0001	<0.0001
ENSG00000197182	MIRLET7BHG	MIRLET7B host gene [Source:HGNC Symbol;Acc:HGNC:37189]	lncRNA	-1.09	3.35	58.56	<0.0001	<0.0001
ENSG00000167261	DPEP2	dipeptidase 2 [Source:HGNC Symbol;Acc:HGNC:23028]	protein coding	-1.09	8.64	97.43	<0.0001	<0.0001
ENSG00000155629	PIK3AP1	phosphoinositide-3-kinase adaptor protein 1 [Source:HGNC Symbol;Acc:HGNC:30034]	protein coding	1.09	8.68	87.67	<0.0001	<0.0001
ENSG00000213366	GSTM2	glutathione S-transferase mu 2 [Source:HGNC Symbol;Acc:HGNC:4634]	protein coding	-1.09	1.57	12.59	0.0004	0.0013
ENSG00000148484	RSU1	Ras suppressor protein 1 [Source:HGNC Symbol;Acc:HGNC:10464]	protein coding	-1.09	4.38	49.45	<0.0001	<0.0001
ENSG00000267480		novel transcript, antisense IMPA2	lncRNA	-1.09	1.05	55.81	<0.0001	<0.0001
ENSG00000259321		novel transcript	lncRNA	1.09	1.45	44.18	<0.0001	<0.0001
ENSG00000138035	PNPT1	polyribonucleotide nucleotidyltransferase 1 [Source:HGNC Symbol;Acc:HGNC:23166]	protein coding	1.09	3.54	29.35	<0.0001	<0.0001
ENSG00000137842	TMEM62	transmembrane protein 62 [Source:HGNC Symbol;Acc:HGNC:26269]	protein coding	1.09	1.95	36.90	<0.0001	<0.0001
ENSG00000184207	PGP	phosphoglycolate phosphatase [Source:HGNC Symbol;Acc:HGNC:8909]	protein coding	-1.09	4.36	47.61	<0.0001	<0.0001
ENSG00000177732	SOX12	SRY-box transcription factor 12 [Source:HGNC Symbol;Acc:HGNC:11198]	protein coding	-1.08	1.77	36.70	<0.0001	<0.0001

Table S18: Genes differentially expressed at Day 30 compared to pre-second vaccination (Study Arm 2 A/H7N9+AS03, D1, 29, Neutrophils). Genes are sorted by descending absolute log_2 fold change. Gene model summaries and annotations are based on Ensembl Version 99 (continued.)

Ensembl Gene ID	Ensembl Gene Name	Ensembl Gene Description	Gene Type	log_2 Fold Change (Day 30 vs. Pre-Second Vaccination)	Average log_2 CPM	Likelihood Ratio Test Statistic	P-Value	FDR Adjusted P-Value
ENSG00000115896	PLCL1	phospholipase C like 1 (inactive) [Source:HGNC Symbol;Acc:HGNC:9063]	protein coding	-1.08	2.69	28.32	<0.0001	<0.0001
ENSG00000130940	CASZ1	castor zinc finger 1 [Source:HGNC Symbol;Acc:HGNC:26002]	protein coding	1.08	3.47	42.86	<0.0001	<0.0001
ENSG00000134352	IL6ST	interleukin 6 signal transducer [Source:HGNC Symbol;Acc:HGNC:6021]	protein coding	1.08	4.19	74.80	<0.0001	<0.0001
ENSG00000263528	IKBKE	inhibitor of nuclear factor kappa B kinase subunit epsilon [Source:HGNC Symbol;Acc:HGNC:14552]	protein coding	1.08	0.89	16.20	<0.0001	0.0002
ENSG00000159348	CYB5R1	cytochrome b5 reductase 1 [Source:HGNC Symbol;Acc:HGNC:13397]	protein coding	-1.08	4.46	30.61	<0.0001	<0.0001
ENSG00000177374	HIC1	HIC ZBTB transcriptional repressor 1 [Source:HGNC Symbol;Acc:HGNC:4909]	protein coding	1.08	1.54	21.54	<0.0001	<0.0001
ENSG00000053371	AKR7A2	aldo-keto reductase family 7 member A2 [Source:HGNC Symbol;Acc:HGNC:389]	protein coding	-1.08	0.64	28.19	<0.0001	<0.0001
ENSG00000159459	UBR1	ubiquitin protein ligase E3 component n-recognin 1 [Source:HGNC Symbol;Acc:HGNC:16808]	protein coding	1.08	3.67	66.56	<0.0001	<0.0001
ENSG00000198176	TFDP1	transcription factor Dp-1 [Source:HGNC Symbol;Acc:HGNC:11749]	protein coding	-1.08	4.72	32.82	<0.0001	<0.0001
ENSG00000123992	DNPEP	aspartyl aminopeptidase [Source:HGNC Symbol;Acc:HGNC:2981]	protein coding	1.08	1.16	34.92	<0.0001	<0.0001
ENSG00000145979	TBC1D7	TBC1 domain family member 7 [Source:HGNC Symbol;Acc:HGNC:21066]	protein coding	-1.08	2.68	25.24	<0.0001	<0.0001
ENSG00000105205	CLC	Charcot-Leyden crystal galectin [Source:HGNC Symbol;Acc:HGNC:2014]	protein coding	-1.08	6.65	9.37	0.0022	0.0061
ENSG00000127311	HELB	DNA helicase B [Source:HGNC Symbol;Acc:HGNC:17196]	protein coding	1.08	3.96	52.18	<0.0001	<0.0001
ENSG00000092200	RPGRIP1	RPGR interacting protein 1 [Source:HGNC Symbol;Acc:HGNC:13436]	protein coding	-1.08	4.80	78.49	<0.0001	<0.0001
ENSG00000105011	ASF1B	anti-silencing function 1B histone chaperone [Source:HGNC Symbol;Acc:HGNC:20996]	protein coding	-1.07	5.51	87.27	<0.0001	<0.0001
ENSG00000172578	KLHL6	kelch like family member 6 [Source:HGNC Symbol;Acc:HGNC:18653]	protein coding	1.07	5.02	61.67	<0.0001	<0.0001
ENSG00000170293	CMTM8	CKLF like MARVEL transmembrane domain containing 8 [Source:HGNC Symbol;Acc:HGNC:19179]	protein coding	-1.07	0.21	27.17	<0.0001	<0.0001
ENSG00000212743	LINC02656	long intergenic non-protein coding RNA 2656 [Source:HGNC Symbol;Acc:HGNC:54142]	lncRNA	-1.07	1.58	19.74	<0.0001	<0.0001

Table S18: Genes differentially expressed at Day 30 compared to pre-second vaccination (Study Arm 2 A/H7N9+AS03, D1, 29, Neutrophils). Genes are sorted by descending absolute log_2 fold change. Gene model summaries and annotations are based on Ensembl Version 99 (continued.)

Ensembl Gene ID	Ensembl Gene Name	Ensembl Gene Description	Gene Type	log_2 Fold Change (Day 30 vs. Pre-Second Vaccination)	Average log_2 CPM	Likelihood Ratio Test Statistic	P-Value	FDR Adjusted P-Value
ENSG00000215068		novel transcript, antisense to ANXA2R	lncRNA	1.07	2.05	35.59	<0.0001	<0.0001
ENSG00000070018	LRP6	LDL receptor related protein 6 [Source:HGNC Symbol;Acc:HGNC:6698]	protein coding	-1.07	0.54	33.49	<0.0001	<0.0001
ENSG00000143653	SCCPDH	saccharopine dehydrogenase (putative) [Source:HGNC Symbol;Acc:HGNC:24275]	protein coding	-1.07	0.84	15.35	<0.0001	0.0004
ENSG00000135046	ANXA1	annexin A1 [Source:HGNC Symbol;Acc:HGNC:533]	protein coding	1.07	6.21	38.90	<0.0001	<0.0001
ENSG00000174032	SLC25A30	solute carrier family 25 member 30 [Source:HGNC Symbol;Acc:HGNC:27371]	protein coding	1.07	1.45	54.76	<0.0001	<0.0001
ENSG00000142405	NLRP12	NLR family pyrin domain containing 12 [Source:HGNC Symbol;Acc:HGNC:22938]	protein coding	-1.07	7.64	50.20	<0.0001	<0.0001
ENSG00000176871	WSB2	WD repeat and SOCS box containing 2 [Source:HGNC Symbol;Acc:HGNC:19222]	protein coding	1.07	5.56	50.85	<0.0001	<0.0001
ENSG00000197272	IL27	interleukin 27 [Source:HGNC Symbol;Acc:HGNC:19157]	protein coding	1.07	2.12	64.99	<0.0001	<0.0001
ENSG00000185022	MAFF	MAF bZIP transcription factor F [Source:HGNC Symbol;Acc:HGNC:6780]	protein coding	1.07	5.69	14.29	0.0002	0.0006
ENSG00000109107	ALDOC	aldolase, fructose-bisphosphate C [Source:HGNC Symbol;Acc:HGNC:418]	protein coding	-1.07	3.33	43.20	<0.0001	<0.0001
ENSG00000188215	DCUN1D3	defective in cullin neddylation 1 domain containing 3 [Source:HGNC Symbol;Acc:HGNC:28734]	protein coding	1.06	3.65	59.96	<0.0001	<0.0001
ENSG00000258227	CLEC5A	C-type lectin domain containing 5A [Source:HGNC Symbol;Acc:HGNC:2054]	protein coding	1.06	1.55	35.35	<0.0001	<0.0001
ENSG00000026103	FAS	Fas cell surface death receptor [Source:HGNC Symbol;Acc:HGNC:11920]	protein coding	1.06	7.83	32.75	<0.0001	<0.0001
ENSG00000197122	SRC	SRC proto-oncogene, non-receptor tyrosine kinase [Source:HGNC Symbol;Acc:HGNC:11283]	protein coding	1.06	2.07	30.70	<0.0001	<0.0001
ENSG00000140750	ARHGAP17	Rho GTPase activating protein 17 [Source:HGNC Symbol;Acc:HGNC:18239]	protein coding	1.06	4.05	75.28	<0.0001	<0.0001
ENSG00000162645	GBP2	guanylate binding protein 2 [Source:HGNC Symbol;Acc:HGNC:4183]	protein coding	1.06	10.01	38.56	<0.0001	<0.0001
ENSG00000090339	ICAM1	intercellular adhesion molecule 1 [Source:HGNC Symbol;Acc:HGNC:5344]	protein coding	1.06	7.10	36.14	<0.0001	<0.0001
ENSG00000106123	EPHB6	EPH receptor B6 [Source:HGNC Symbol;Acc:HGNC:3396]	protein coding	-1.06	4.65	32.58	<0.0001	<0.0001

Table S18: Genes differentially expressed at Day 30 compared to pre-second vaccination (Study Arm 2 A/H7N9+AS03, D1, 29, Neutrophils). Genes are sorted by descending absolute log_2 fold change. Gene model summaries and annotations are based on Ensembl Version 99 (continued.)

Ensembl Gene ID	Ensembl Gene Name	Ensembl Gene Description	Gene Type	log_2 Fold Change (Day 30 vs. Pre-Second Vaccination)	Average log_2 CPM	Likelihood Ratio Test Statistic	P-Value	FDR Adjusted P-Value
ENSG00000141096	DPEP3	dipeptidase 3 [Source:HGNC Symbol;Acc:HGNC:23029]	protein coding	-1.06	4.68	74.08	<0.0001	<0.0001
ENSG00000183762	KREMEN1	kringle containing transmembrane protein 1 [Source:HGNC Symbol;Acc:HGNC:17550]	protein coding	1.06	7.08	46.86	<0.0001	<0.0001
ENSG00000162881	OXER1	oxoeicosanoid receptor 1 [Source:HGNC Symbol;Acc:HGNC:24884]	protein coding	-1.06	4.90	29.42	<0.0001	<0.0001
ENSG00000047365	ARAP2	ArfGAP with RhoGAP domain, ankyrin repeat and PH domain 2 [Source:HGNC Symbol;Acc:HGNC:16924]	protein coding	1.06	4.55	74.96	<0.0001	<0.0001
ENSG00000128928	IVD	isovaleryl-CoA dehydrogenase [Source:HGNC Symbol;Acc:HGNC:6186]	protein coding	-1.06	1.36	20.53	<0.0001	<0.0001
ENSG00000107020	PLGRKT	plasminogen receptor with a C-terminal lysine [Source:HGNC Symbol;Acc:HGNC:23633]	protein coding	1.06	3.05	36.18	<0.0001	<0.0001
ENSG00000168685	IL7R	interleukin 7 receptor [Source:HGNC Symbol;Acc:HGNC:6024]	protein coding	-1.06	1.46	7.52	0.0061	0.0149
ENSG00000108107	RPL28	ribosomal protein L28 [Source:HGNC Symbol;Acc:HGNC:10330]	protein coding	1.06	7.72	59.09	<0.0001	<0.0001
ENSG00000178726	THBD	thrombomodulin [Source:HGNC Symbol;Acc:HGNC:11784]	protein coding	-1.06	7.09	43.26	<0.0001	<0.0001
ENSG00000273247		novel transcript, antisense to RAB33B	lncRNA	1.06	3.88	53.19	<0.0001	<0.0001
ENSG00000152242	C18orf25	chromosome 18 open reading frame 25 [Source:HGNC Symbol;Acc:HGNC:28172]	protein coding	1.06	6.01	73.93	<0.0001	<0.0001
ENSG00000137094	DNAJB5	DnaJ heat shock protein family (Hsp40) member B5 [Source:HGNC Symbol;Acc:HGNC:14887]	protein coding	-1.05	2.90	41.46	<0.0001	<0.0001
ENSG00000130433	CACNG6	calcium voltage-gated channel auxiliary subunit gamma 6 [Source:HGNC Symbol;Acc:HGNC:13625]	protein coding	-1.05	1.22	5.14	0.0234	0.0474
ENSG00000185404	SP140L	SP140 nuclear body protein like [Source:HGNC Symbol;Acc:HGNC:25105]	protein coding	1.05	4.20	67.93	<0.0001	<0.0001
ENSG00000090924	PLEKHG2	pleckstrin homology and RhoGEF domain containing G2 [Source:HGNC Symbol;Acc:HGNC:29515]	protein coding	1.05	1.65	7.58	0.0059	0.0144
ENSG00000268658	LINC00664	long intergenic non-protein coding RNA 664 [Source:HGNC Symbol;Acc:HGNC:44319]	lncRNA	-1.05	1.04	18.50	<0.0001	<0.0001
ENSG00000152223	EPG5	ectopic P-granules autophagy protein 5 homolog [Source:HGNC Symbol;Acc:HGNC:29331]	protein coding	1.05	5.47	61.05	<0.0001	<0.0001
ENSG00000144040	SFXN5	sideroflexin 5 [Source:HGNC Symbol;Acc:HGNC:16073]	protein coding	-1.05	4.70	36.59	<0.0001	<0.0001

Table S18: Genes differentially expressed at Day 30 compared to pre-second vaccination (Study Arm 2 A/H7N9+AS03, D1, 29, Neutrophils). Genes are sorted by descending absolute log_2 fold change. Gene model summaries and annotations are based on Ensembl Version 99 (continued.)

Ensembl Gene ID	Ensembl Gene Name	Ensembl Gene Description	Gene Type	log_2 Fold Change (Day 30 vs. Pre-Second Vaccination)	Average log_2 CPM	Likelihood Ratio Test Statistic	P-Value	FDR Adjusted P-Value
ENSG00000168026	TTC21A	tetratricopeptide repeat domain 21A [Source:HGNC Symbol;Acc:HGNC:30761]	protein coding	1.05	2.44	33.98	<0.0001	<0.0001
ENSG00000114439	BBX	BBX high mobility group box domain containing [Source:HGNC Symbol;Acc:HGNC:14422]	protein coding	1.05	5.14	56.30	<0.0001	<0.0001
ENSG00000130559	CAMSAP1	calmodulin regulated spectrin associated protein 1 [Source:HGNC Symbol;Acc:HGNC:19946]	protein coding	-1.05	2.46	39.09	<0.0001	<0.0001
ENSG00000152207	CYSLTR2	cysteinyl leukotriene receptor 2 [Source:HGNC Symbol;Acc:HGNC:18274]	protein coding	-1.05	2.68	8.23	0.0041	0.0105
ENSG00000134802	SLC43A3	solute carrier family 43 member 3 [Source:HGNC Symbol;Acc:HGNC:17466]	protein coding	1.05	1.02	43.30	<0.0001	<0.0001
ENSG00000149311	ATM	ATM serine/threonine kinase [Source:HGNC Symbol;Acc:HGNC:795]	protein coding	-1.05	5.82	48.87	<0.0001	<0.0001
ENSG00000102981	PARD6A	par-6 family cell polarity regulator alpha [Source:HGNC Symbol;Acc:HGNC:15943]	protein coding	-1.04	2.12	36.97	<0.0001	<0.0001
ENSG00000270120		novel transcript	lncRNA	1.04	1.40	18.97	<0.0001	<0.0001
ENSG00000065675	PRKCQ	protein kinase C theta [Source:HGNC Symbol;Acc:HGNC:9410]	protein coding	-1.04	0.55	23.56	<0.0001	<0.0001
ENSG00000086015	MAST2	microtubule associated serine/threonine kinase 2 [Source:HGNC Symbol;Acc:HGNC:19035]	protein coding	-1.04	1.61	37.51	<0.0001	<0.0001
ENSG00000154265	ABCA5	ATP binding cassette subfamily A member 5 [Source:HGNC Symbol;Acc:HGNC:35]	protein coding	-1.04	2.93	27.29	<0.0001	<0.0001
ENSG00000169442	CD52	CD52 molecule [Source:HGNC Symbol;Acc:HGNC:1804]	protein coding	-1.04	3.01	7.77	0.0053	0.0132
ENSG00000164087	POC1A	POC1 centriolar protein A [Source:HGNC Symbol;Acc:HGNC:24488]	protein coding	-1.04	0.56	23.50	<0.0001	<0.0001
ENSG00000030110	BAK1	BCL2 antagonist/killer 1 [Source:HGNC Symbol;Acc:HGNC:949]	protein coding	1.04	5.06	71.05	<0.0001	<0.0001
ENSG00000112343	TRIM38	tripartite motif containing 38 [Source:HGNC Symbol;Acc:HGNC:10059]	protein coding	1.04	7.27	78.00	<0.0001	<0.0001
ENSG00000041357	PSMA4	proteasome 20S subunit alpha 4 [Source:HGNC Symbol;Acc:HGNC:9533]	protein coding	1.04	6.13	47.30	<0.0001	<0.0001
ENSG00000168765	GSTM4	glutathione S-transferase mu 4 [Source:HGNC Symbol;Acc:HGNC:4636]	protein coding	-1.04	1.28	8.29	0.004	0.0102

Table S18: Genes differentially expressed at Day 30 compared to pre-second vaccination (Study Arm 2 A/H7N9+AS03, D1, 29, Neutrophils). Genes are sorted by descending absolute log_2 fold change. Gene model summaries and annotations are based on Ensembl Version 99 (continued.)

Ensembl Gene ID	Ensembl Gene Name	Ensembl Gene Description	Gene Type	log_2 Fold Change (Day 30 vs. Pre-Second Vaccination)	Average log_2 CPM	Likelihood Ratio Test Statistic	P-Value	FDR Adjusted P-Value
ENSG00000283602		novel pseudogene	transcribed unitary pseudo-gene	-1.04	2.41	26.00	<0.0001	<0.0001
ENSG00000140650	PMM2	phosphomannomutase 2 [Source:HGNC Symbol;Acc:HGNC:9115]	protein coding	-1.03	1.87	35.86	<0.0001	<0.0001
ENSG00000287938		novel transcript	lncRNA	-1.03	0.19	25.22	<0.0001	<0.0001
ENSG00000134242	PTPN22	protein tyrosine phosphatase non-receptor type 22 [Source:HGNC Symbol;Acc:HGNC:9652]	protein coding	-1.03	2.56	15.31	<0.0001	0.0004
ENSG00000130202	NECTIN2	nectin cell adhesion molecule 2 [Source:HGNC Symbol;Acc:HGNC:9707]	protein coding	1.03	6.22	8.46	0.0036	0.0094
ENSG00000185112	FAM43A	family with sequence similarity 43 member A [Source:HGNC Symbol;Acc:HGNC:26888]	protein coding	-1.03	3.92	44.80	<0.0001	<0.0001
ENSG00000101695	RNF125	ring finger protein 125 [Source:HGNC Symbol;Acc:HGNC:21150]	protein coding	-1.03	1.99	26.15	<0.0001	<0.0001
ENSG00000089486	CDIP1	cell death inducing p53 target 1 [Source:HGNC Symbol;Acc:HGNC:13234]	protein coding	-1.03	3.51	49.17	<0.0001	<0.0001
ENSG00000251442	LINC01094	long intergenic non-protein coding RNA 1094 [Source:HGNC Symbol;Acc:HGNC:49219]	lncRNA	1.03	3.34	33.50	<0.0001	<0.0001
ENSG00000134900	TPP2	tripeptidyl peptidase 2 [Source:HGNC Symbol;Acc:HGNC:12016]	protein coding	-1.03	2.92	37.24	<0.0001	<0.0001
ENSG00000179364	PACS2	phosphofurin acidic cluster sorting protein 2 [Source:HGNC Symbol;Acc:HGNC:23794]	protein coding	-1.03	1.14	18.63	<0.0001	<0.0001
ENSG00000270972		novel transcript	lncRNA	1.03	3.51	30.35	<0.0001	<0.0001
ENSG00000181381	DDX60L	DExD/H-box 60 like [Source:HGNC Symbol;Acc:HGNC:26429]	protein coding	1.03	8.84	87.19	<0.0001	<0.0001
ENSG00000102796	DHRS12	dehydrogenase/reductase 12 [Source:HGNC Symbol;Acc:HGNC:25832]	protein coding	1.03	5.12	59.04	<0.0001	<0.0001
ENSG00000134107	BHLHE40	basic helix-loop-helix family member e40 [Source:HGNC Symbol;Acc:HGNC:1046]	protein coding	-1.02	6.18	67.17	<0.0001	<0.0001
ENSG00000179082	C9orf106	chromosome 9 open reading frame 106 (putative) [Source:HGNC Symbol;Acc:HGNC:31370]	lncRNA	-1.02	0.70	19.74	<0.0001	<0.0001
ENSG00000138744	NAAA	N-acylethanolamine acid amidase [Source:HGNC Symbol;Acc:HGNC:736]	protein coding	-1.02	5.35	50.87	<0.0001	<0.0001
ENSG00000285572		novel transcript	lncRNA	-1.02	0.33	25.20	<0.0001	<0.0001

Table S18: Genes differentially expressed at Day 30 compared to pre-second vaccination (Study Arm 2 A/H7N9+AS03, D1, 29, Neutrophils). Genes are sorted by descending absolute log_2 fold change. Gene model summaries and annotations are based on Ensembl Version 99 (continued.)

Ensembl Gene ID	Ensembl Gene Name	Ensembl Gene Description	Gene Type	log_2 Fold Change (Day 30 vs. Pre-Second Vaccination)	Average log_2 CPM	Likelihood Ratio Test Statistic	P-Value	FDR Adjusted P-Value
ENSG00000133800	LYVE1	lymphatic vessel endothelial hyaluronan receptor 1 [Source:HGNC Symbol;Acc:HGNC:14687]	protein coding	-1.02	2.11	25.45	<0.0001	<0.0001
ENSG00000126464	PRR12	proline rich 12 [Source:HGNC Symbol;Acc:HGNC:29217]	protein coding	-1.02	3.91	36.19	<0.0001	<0.0001
ENSG00000185838	GNB1L	G protein subunit beta 1 like [Source:HGNC Symbol;Acc:HGNC:4397]	protein coding	-1.02	1.73	39.74	<0.0001	<0.0001
ENSG00000143434	SEMA6C	semaphorin 6C [Source:HGNC Symbol;Acc:HGNC:10740]	protein coding	-1.02	0.93	33.71	<0.0001	<0.0001
ENSG00000084112	SSH1	slingshot protein phosphatase 1 [Source:HGNC Symbol;Acc:HGNC:30579]	protein coding	-1.02	5.97	55.60	<0.0001	<0.0001
ENSG00000225783	MIAT	myocardial infarction associated transcript [Source:HGNC Symbol;Acc:HGNC:33425]	lncRNA	-1.02	2.76	16.65	<0.0001	0.0002
ENSG00000172164	SNTB1	syntrophin beta 1 [Source:HGNC Symbol;Acc:HGNC:11168]	protein coding	1.02	4.11	47.91	<0.0001	<0.0001
ENSG00000165997	ARL5B	ADP ribosylation factor like GTPase 5B [Source:HGNC Symbol;Acc:HGNC:23052]	protein coding	1.02	4.06	36.42	<0.0001	<0.0001
ENSG00000073737	DHRS9	dehydrogenase/reductase 9 [Source:HGNC Symbol;Acc:HGNC:16888]	protein coding	1.02	6.10	68.00	<0.0001	<0.0001
ENSG00000103056	SMPD3	sphingomyelin phosphodiesterase 3 [Source:HGNC Symbol;Acc:HGNC:14240]	protein coding	-1.02	4.48	6.31	0.012	0.0268
ENSG00000204516	MICB	MHC class I polypeptide-related sequence B [Source:HGNC Symbol;Acc:HGNC:7091]	protein coding	1.02	4.94	42.91	<0.0001	<0.0001
ENSG00000005379	TSPOAP1	TSPO associated protein 1 [Source:HGNC Symbol;Acc:HGNC:16831]	protein coding	1.02	3.13	20.57	<0.0001	<0.0001
ENSG00000139597	N4BP2L1	NEDD4 binding protein 2 like 1 [Source:HGNC Symbol;Acc:HGNC:25037]	protein coding	1.02	3.20	28.79	<0.0001	<0.0001
ENSG00000168916	ZNF608	zinc finger protein 608 [Source:HGNC Symbol;Acc:HGNC:29238]	protein coding	-1.02	2.16	22.06	<0.0001	<0.0001
ENSG00000164506	STXBP5	syntaxin binding protein 5 [Source:HGNC Symbol;Acc:HGNC:19665]	protein coding	-1.02	6.64	44.66	<0.0001	<0.0001
ENSG00000180867	PDIA3P1	protein disulfide isomerase family A member 3 pseudogene 1 [Source:HGNC Symbol;Acc:HGNC:4607]	transcribed processed pseudogene	1.02	2.14	41.35	<0.0001	<0.0001
ENSG00000070501	POLB	DNA polymerase beta [Source:HGNC Symbol;Acc:HGNC:9174]	protein coding	1.02	6.14	51.78	<0.0001	<0.0001

Table S18: Genes differentially expressed at Day 30 compared to pre-second vaccination (Study Arm 2 A/H7N9+AS03, D1, 29, Neutrophils). Genes are sorted by descending absolute log_2 fold change. Gene model summaries and annotations are based on Ensembl Version 99 (continued.)

Ensembl Gene ID	Ensembl Gene Name	Ensembl Gene Description	Gene Type	log_2 Fold Change (Day 30 vs. Pre-Second Vaccination)	Average log_2 CPM	Likelihood Ratio Test Statistic	P-Value	FDR Adjusted P-Value
ENSG00000148337	CIZ1	CDKN1A interacting zinc finger protein 1 [Source:HGNC Symbol;Acc:HGNC:16744]	protein coding	-1.02	3.14	20.18	<0.0001	<0.0001
ENSG00000092758	COL9A3	collagen type IX alpha 3 chain [Source:HGNC Symbol;Acc:HGNC:2219]	protein coding	-1.02	4.63	24.00	<0.0001	<0.0001
ENSG00000037757	MRI1	methylthioribose-1-phosphate isomerase 1 [Source:HGNC Symbol;Acc:HGNC:28469]	protein coding	-1.01	1.14	20.08	<0.0001	<0.0001
ENSG00000106392	C1GALT1	core 1 synthase, glycoprotein-N-acetylgalactosamine 3-beta-galactosyltransferase 1 [Source:HGNC Symbol;Acc:HGNC:24337]	protein coding	1.01	5.83	86.30	<0.0001	<0.0001
ENSG00000197885	NKIRAS1	NFKB inhibitor interacting Ras like 1 [Source:HGNC Symbol;Acc:HGNC:17899]	protein coding	1.01	1.17	24.84	<0.0001	<0.0001
ENSG00000115507	OTX1	orthodenticle homeobox 1 [Source:HGNC Symbol;Acc:HGNC:8521]	protein coding	-1.01	3.32	36.90	<0.0001	<0.0001
ENSG00000125826	RBCK1	RANBP2-type and C3HC4-type zinc finger containing 1 [Source:HGNC Symbol;Acc:HGNC:15864]	protein coding	1.01	8.53	48.31	<0.0001	<0.0001
ENSG00000275557		novel transcript	lncRNA	1.01	1.22	33.14	<0.0001	<0.0001
ENSG00000196562	SULF2	sulfatase 2 [Source:HGNC Symbol;Acc:HGNC:20392]	protein coding	-1.01	8.38	69.76	<0.0001	<0.0001
ENSG00000145016	RUBCN	rubicon autophagy regulator [Source:HGNC Symbol;Acc:HGNC:28991]	protein coding	1.01	5.19	63.32	<0.0001	<0.0001
ENSG00000138617	PARP16	poly(ADP-ribose) polymerase family member 16 [Source:HGNC Symbol;Acc:HGNC:26040]	protein coding	-1.01	3.77	34.27	<0.0001	<0.0001
ENSG00000142961	MOB3C	MOB kinase activator 3C [Source:HGNC Symbol;Acc:HGNC:29800]	protein coding	1.01	4.47	76.52	<0.0001	<0.0001
ENSG00000139437	TCHP	trichoplein keratin filament binding [Source:HGNC Symbol;Acc:HGNC:28135]	protein coding	-1.01	3.53	36.97	<0.0001	<0.0001
ENSG00000238005		novel transcript	lncRNA	-1.01	3.52	23.51	<0.0001	<0.0001
ENSG00000240065	PSMB9	proteasome 20S subunit beta 9 [Source:HGNC Symbol;Acc:HGNC:9546]	protein coding	1.01	8.97	46.44	<0.0001	<0.0001
ENSG00000135945	REV1	REV1 DNA directed polymerase [Source:HGNC Symbol;Acc:HGNC:14060]	protein coding	-1.01	3.79	46.22	<0.0001	<0.0001
ENSG00000146416	AIG1	androgen induced 1 [Source:HGNC Symbol;Acc:HGNC:21607]	protein coding	1.01	3.20	48.21	<0.0001	<0.0001
ENSG00000167716	WDR81	WD repeat domain 81 [Source:HGNC Symbol;Acc:HGNC:26600]	protein coding	-1.00	2.35	27.27	<0.0001	<0.0001

Table S18: Genes differentially expressed at Day 30 compared to pre-second vaccination (Study Arm 2 A/H7N9+AS03, D1, 29, Neutrophils). Genes are sorted by descending absolute log_2 fold change. Gene model summaries and annotations are based on Ensembl Version 99 (continued.)

Ensembl Gene ID	Ensembl Gene Name	Ensembl Gene Description	Gene Type	log_2 Fold Change (Day 30 vs. Pre-Second Vaccination)	Average log_2 CPM	Likelihood Ratio Test Statistic	P-Value	FDR Adjusted P-Value
ENSG00000118707	TGIF2	TGFB induced factor homeobox 2 [Source:HGNC Symbol;Acc:HGNC:15764]	protein coding	1.00	3.65	35.16	<0.0001	<0.0001
ENSG00000064115	TM7SF3	transmembrane 7 superfamily member 3 [Source:HGNC Symbol;Acc:HGNC:23049]	protein coding	-1.00	3.34	31.02	<0.0001	<0.0001
ENSG00000011021	CLCN6	chloride voltage-gated channel 6 [Source:HGNC Symbol;Acc:HGNC:2024]	protein coding	-1.00	2.13	23.63	<0.0001	<0.0001
ENSG00000174953	DHX36	DEAH-box helicase 36 [Source:HGNC Symbol;Acc:HGNC:14410]	protein coding	1.00	3.98	64.20	<0.0001	<0.0001
ENSG00000164440	TXLNB	taxilin beta [Source:HGNC Symbol;Acc:HGNC:21617]	protein coding	1.00	0.93	26.86	<0.0001	<0.0001
ENSG00000124688	MAD2L1BP	MAD2L1 binding protein [Source:HGNC Symbol;Acc:HGNC:21059]	protein coding	1.00	4.06	56.84	<0.0001	<0.0001
ENSG00000184887	BTBD6	BTB domain containing 6 [Source:HGNC Symbol;Acc:HGNC:19897]	protein coding	-1.00	2.47	33.02	<0.0001	<0.0001
ENSG00000136147	PHF11	PHD finger protein 11 [Source:HGNC Symbol;Acc:HGNC:17024]	protein coding	1.00	6.41	55.64	<0.0001	<0.0001
ENSG00000096996	IL12RB1	interleukin 12 receptor subunit beta 1 [Source:HGNC Symbol;Acc:HGNC:5971]	protein coding	1.00	5.14	51.51	<0.0001	<0.0001
ENSG00000277632	CCL3	C-C motif chemokine ligand 3 [Source:HGNC Symbol;Acc:HGNC:10627]	protein coding	1.00	2.19	12.33	0.0004	0.0015
ENSG00000092010	PSME1	proteasome activator subunit 1 [Source:HGNC Symbol;Acc:HGNC:9568]	protein coding	1.00	8.68	44.63	<0.0001	<0.0001
ENSG00000166527	CLEC4D	C-type lectin domain family 4 member D [Source:HGNC Symbol;Acc:HGNC:14554]	protein coding	1.00	5.40	71.84	<0.0001	<0.0001
ENSG00000184371	CSF1	colony stimulating factor 1 [Source:HGNC Symbol;Acc:HGNC:2432]	protein coding	-1.00	4.98	31.61	<0.0001	<0.0001
ENSG00000127334	DYRK2	dual specificity tyrosine phosphorylation regulated kinase 2 [Source:HGNC Symbol;Acc:HGNC:3093]	protein coding	-1.00	2.74	34.62	<0.0001	<0.0001
ENSG00000103319	EEF2K	eukaryotic elongation factor 2 kinase [Source:HGNC Symbol;Acc:HGNC:24615]	protein coding	-1.00	1.02	6.61	0.0101	0.0231
ENSG00000109971	HSPA8	heat shock protein family A (Hsp70) member 8 [Source:HGNC Symbol;Acc:HGNC:5241]	protein coding	1.00	6.75	64.07	<0.0001	<0.0001
ENSG00000151692	RNF144A	ring finger protein 144A [Source:HGNC Symbol;Acc:HGNC:20457]	protein coding	1.00	3.88	31.48	<0.0001	<0.0001
ENSG00000063854	HAGH	hydroxyacylglutathione hydrolase [Source:HGNC Symbol;Acc:HGNC:4805]	protein coding	-1.00	4.31	61.28	<0.0001	<0.0001

Table S18: Genes differentially expressed at Day 30 compared to pre-second vaccination (Study Arm 2 A/H7N9+AS03, D1, 29, Neutrophils). Genes are sorted by descending absolute log_2 fold change. Gene model summaries and annotations are based on Ensembl Version 99 (continued.)

Ensembl Gene ID	Ensembl Gene Name	Ensembl Gene Description	Gene Type	log_2 Fold Change (Day 30 vs. Pre-Second Vaccination)	Average log_2 CPM	Likelihood Ratio Test Statistic	P-Value	FDR Adjusted P-Value
ENSG00000259668		novel transcript, antisense to DMXL2	lncRNA	0.99	1.88	22.29	<0.0001	<0.0001
ENSG00000213977	TAX1BP3	Tax1 binding protein 3 [Source:HGNC Symbol;Acc:HGNC:30684]	protein coding	-0.99	1.65	32.46	<0.0001	<0.0001
ENSG00000169660	HEXD	hexosaminidase D [Source:HGNC Symbol;Acc:HGNC:26307]	protein coding	0.99	5.73	51.86	<0.0001	<0.0001
ENSG00000150907	FOXO1	forkhead box O1 [Source:HGNC Symbol;Acc:HGNC:3819]	protein coding	-0.99	4.73	77.55	<0.0001	<0.0001
ENSG00000157216	SSBP3	single stranded DNA binding protein 3 [Source:HGNC Symbol;Acc:HGNC:15674]	protein coding	-0.99	3.41	50.85	<0.0001	<0.0001
ENSG00000146192	FGD2	FYVE, RhoGEF and PH domain containing 2 [Source:HGNC Symbol;Acc:HGNC:3664]	protein coding	0.99	0.56	8.61	0.0033	0.0088
ENSG00000162739	SLAMF6	SLAM family member 6 [Source:HGNC Symbol;Acc:HGNC:21392]	protein coding	-0.99	2.08	19.84	<0.0001	<0.0001
ENSG00000259351		novel transcript, antisense to DAPK2	lncRNA	-0.99	0.44	13.81	0.0002	0.0007
ENSG00000153162	BMP6	bone morphogenetic protein 6 [Source:HGNC Symbol;Acc:HGNC:1073]	protein coding	-0.99	3.19	46.41	<0.0001	<0.0001
ENSG00000226287	TMEM191A	transmembrane protein 191A (pseudogene) [Source:HGNC Symbol;Acc:HGNC:25317]	transcribed unprocessed pseudogene	-0.99	0.44	23.86	<0.0001	<0.0001
ENSG00000079337	RAPGEF3	Rap guanine nucleotide exchange factor 3 [Source:HGNC Symbol;Acc:HGNC:16629]	protein coding	0.99	2.18	46.69	<0.0001	<0.0001
ENSG00000108861	DUSP3	dual specificity phosphatase 3 [Source:HGNC Symbol;Acc:HGNC:3069]	protein coding	0.99	4.18	69.56	<0.0001	<0.0001
ENSG00000281100		TEC	TEC	0.99	1.68	33.20	<0.0001	<0.0001
ENSG00000205517	RGL3	ral guanine nucleotide dissociation stimulator like 3 [Source:HGNC Symbol;Acc:HGNC:30282]	protein coding	-0.99	2.40	37.73	<0.0001	<0.0001
ENSG00000286116		FAS antisense RNA 1	lncRNA	0.99	1.32	26.16	<0.0001	<0.0001
ENSG00000286488		novel transcript, antisense to STARD5	lncRNA	-0.99	3.19	43.10	<0.0001	<0.0001
ENSG00000282608	ADORA3	adenosine A3 receptor [Source:HGNC Symbol;Acc:HGNC:268]	protein coding	-0.99	1.17	9.01	0.0027	0.0072
ENSG00000244617	ASPRV1	aspartic peptidase retroviral like 1 [Source:HGNC Symbol;Acc:HGNC:26321]	protein coding	0.99	5.68	54.03	<0.0001	<0.0001
ENSG00000169413	RNASE6	ribonuclease A family member k6 [Source:HGNC Symbol;Acc:HGNC:10048]	protein coding	-0.99	5.14	40.50	<0.0001	<0.0001

Table S18: Genes differentially expressed at Day 30 compared to pre-second vaccination (Study Arm 2 A/H7N9+AS03, D1, 29, Neutrophils). Genes are sorted by descending absolute log_2 fold change. Gene model summaries and annotations are based on Ensembl Version 99 (continued.)

Ensembl Gene ID	Ensembl Gene Name	Ensembl Gene Description	Gene Type	log_2 Fold Change (Day 30 vs. Pre-Second Vaccination)	Average log_2 CPM	Likelihood Ratio Test Statistic	P-Value	FDR Adjusted P-Value
ENSG00000008283	CYB561	cytochrome b561 [Source:HGNC Symbol;Acc:HGNC:2571]	protein coding	0.99	4.86	58.05	<0.0001	<0.0001
ENSG00000233276	GPX1	glutathione peroxidase 1 [Source:HGNC Symbol;Acc:HGNC:4553]	polymorphic pseudogene	-0.99	5.55	38.15	<0.0001	<0.0001
ENSG00000211584	SLC48A1	solute carrier family 48 member 1 [Source:HGNC Symbol;Acc:HGNC:26035]	protein coding	-0.99	5.00	52.03	<0.0001	<0.0001
ENSG00000182578	CSF1R	colony stimulating factor 1 receptor [Source:HGNC Symbol;Acc:HGNC:2433]	protein coding	-0.98	5.83	62.93	<0.0001	<0.0001
ENSG00000189068	VSTM1	V-set and transmembrane domain containing 1 [Source:HGNC Symbol;Acc:HGNC:29455]	protein coding	-0.98	3.14	10.15	0.0014	0.0042
ENSG00000173786	CNP	2',3'-cyclic nucleotide 3' phosphodiesterase [Source:HGNC Symbol;Acc:HGNC:2158]	protein coding	0.98	4.25	57.44	<0.0001	<0.0001
ENSG00000111731	C2CD5	C2 calcium dependent domain containing 5 [Source:HGNC Symbol;Acc:HGNC:29062]	protein coding	-0.98	4.37	26.62	<0.0001	<0.0001
ENSG00000188641	DPYD	dihydropyrimidine dehydrogenase [Source:HGNC Symbol;Acc:HGNC:3012]	protein coding	0.98	6.50	41.74	<0.0001	<0.0001
ENSG00000141577	CEP131	centrosomal protein 131 [Source:HGNC Symbol;Acc:HGNC:29511]	protein coding	-0.98	2.90	30.01	<0.0001	<0.0001
ENSG00000204264	PSMB8	proteasome 20S subunit beta 8 [Source:HGNC Symbol;Acc:HGNC:9545]	protein coding	0.98	8.27	47.99	<0.0001	<0.0001
ENSG00000015532	XYLT2	xylosyltransferase 2 [Source:HGNC Symbol;Acc:HGNC:15517]	protein coding	-0.98	2.64	32.26	<0.0001	<0.0001
ENSG00000272666		uncharacterized LOC105373098 [Source:NCBI gene;Acc:105373098]	lncRNA	0.98	2.46	7.76	0.0053	0.0132
ENSG00000181513	ACBD4	acyl-CoA binding domain containing 4 [Source:HGNC Symbol;Acc:HGNC:23337]	protein coding	-0.98	1.15	35.04	<0.0001	<0.0001
ENSG00000158411	MITD1	microtubule interacting and trafficking domain containing 1 [Source:HGNC Symbol;Acc:HGNC:25207]	protein coding	0.98	4.69	42.93	<0.0001	<0.0001
ENSG00000186575	NF2	neurofibromin 2 [Source:HGNC Symbol;Acc:HGNC:7773]	protein coding	-0.98	1.89	37.32	<0.0001	<0.0001
ENSG00000118515	SGK1	serum/glucocorticoid regulated kinase 1 [Source:HGNC Symbol;Acc:HGNC:10810]	protein coding	-0.98	8.28	91.99	<0.0001	<0.0001
ENSG00000170231	FABP6	fatty acid binding protein 6 [Source:HGNC Symbol;Acc:HGNC:3561]	protein coding	-0.98	1.13	16.25	<0.0001	0.0002

Table S18: Genes differentially expressed at Day 30 compared to pre-second vaccination (Study Arm 2 A/H7N9+AS03, D1, 29, Neutrophils). Genes are sorted by descending absolute log_2 fold change. Gene model summaries and annotations are based on Ensembl Version 99 (continued.)

Ensembl Gene ID	Ensembl Gene Name	Ensembl Gene Description	Gene Type	log_2 Fold Change (Day 30 vs. Pre-Second Vaccination)	Average log_2 CPM	Likelihood Ratio Test Statistic	P-Value	FDR Adjusted P-Value
ENSG00000169567	HINT1	histidine triad nucleotide binding protein 1 [Source:HGNC Symbol;Acc:HGNC:4912]	protein coding	0.98	3.70	37.30	<0.0001	<0.0001
ENSG00000010818	HIVEP2	HIVEP zinc finger 2 [Source:HGNC Symbol;Acc:HGNC:4921]	protein coding	0.98	6.48	43.38	<0.0001	<0.0001
ENSG00000184182	UBE2F	ubiquitin conjugating enzyme E2 F (putative) [Source:HGNC Symbol;Acc:HGNC:12480]	protein coding	0.98	2.95	31.92	<0.0001	<0.0001
ENSG00000117899	MESD	mesoderm development LRP chaperone [Source:HGNC Symbol;Acc:HGNC:13520]	protein coding	0.98	2.34	49.09	<0.0001	<0.0001
ENSG00000168329	CX3CR1	C-X3-C motif chemokine receptor 1 [Source:HGNC Symbol;Acc:HGNC:2558]	protein coding	-0.97	3.61	26.09	<0.0001	<0.0001
ENSG00000105220	GPI	glucose-6-phosphate isomerase [Source:HGNC Symbol;Acc:HGNC:4458]	protein coding	-0.97	3.11	8.99	0.0027	0.0073
ENSG00000134109	EDEM1	ER degradation enhancing alpha-mannosidase like protein 1 [Source:HGNC Symbol;Acc:HGNC:18967]	protein coding	0.97	5.72	38.22	<0.0001	<0.0001
ENSG00000233461		novel transcript	lncRNA	0.97	3.13	31.96	<0.0001	<0.0001
ENSG00000143162	CREG1	cellular repressor of E1A stimulated genes 1 [Source:HGNC Symbol;Acc:HGNC:2351]	protein coding	0.97	5.81	38.04	<0.0001	<0.0001
ENSG00000237499	WAKMAR2	wound and keratinocyte migration associated lncRNA 2 [Source:HGNC Symbol;Acc:HGNC:53754]	lncRNA	0.97	1.45	18.38	<0.0001	<0.0001
ENSG00000175054	ATR	ATR serine/threonine kinase [Source:HGNC Symbol;Acc:HGNC:882]	protein coding	0.97	2.29	37.95	<0.0001	<0.0001
ENSG00000124226	RNF114	ring finger protein 114 [Source:HGNC Symbol;Acc:HGNC:13094]	protein coding	0.97	6.87	46.30	<0.0001	<0.0001
ENSG00000186918	ZNF395	zinc finger protein 395 [Source:HGNC Symbol;Acc:HGNC:18737]	protein coding	-0.97	1.62	24.19	<0.0001	<0.0001
ENSG00000248712	CCDC153	coiled-coil domain containing 153 [Source:HGNC Symbol;Acc:HGNC:27446]	protein coding	-0.97	3.17	47.62	<0.0001	<0.0001
ENSG00000091181	IL5RA	interleukin 5 receptor subunit alpha [Source:HGNC Symbol;Acc:HGNC:6017]	protein coding	-0.97	3.49	7.54	0.006	0.0147
ENSG00000283378	CNTNAP3C	contactin associated protein like 3C [Source:HGNC Symbol;Acc:HGNC:53878]	protein coding	-0.97	2.95	36.56	<0.0001	<0.0001
ENSG00000173762	CD7	CD7 molecule [Source:HGNC Symbol;Acc:HGNC:1695]	protein coding	0.97	2.52	11.44	0.0007	0.0023
ENSG00000125457	MIF4GD	MIF4G domain containing [Source:HGNC Symbol;Acc:HGNC:24030]	protein coding	-0.97	1.77	21.30	<0.0001	<0.0001

Table S18: Genes differentially expressed at Day 30 compared to pre-second vaccination (Study Arm 2 A/H7N9+AS03, D1, 29, Neutrophils). Genes are sorted by descending absolute log_2 fold change. Gene model summaries and annotations are based on Ensembl Version 99 (continued.)

Ensembl Gene ID	Ensembl Gene Name	Ensembl Gene Description	Gene Type	log_2 Fold Change (Day 30 vs. Pre-Second Vaccination)	Average log_2 CPM	Likelihood Ratio Test Statistic	P-Value	FDR Adjusted P-Value
ENSG00000167208	SNX20	sorting nexin 20 [Source:HGNC Symbol;Acc:HGNC:30390]	protein coding	0.97	7.09	52.37	<0.0001	<0.0001
ENSG00000148110	MFSD14B	major facilitator superfamily domain containing 14B [Source:HGNC Symbol;Acc:HGNC:23376]	protein coding	0.97	6.23	68.31	<0.0001	<0.0001
ENSG00000117448	AKR1A1	aldo-keto reductase family 1 member A1 [Source:HGNC Symbol;Acc:HGNC:380]	protein coding	0.97	0.89	33.66	<0.0001	<0.0001
ENSG00000113273	ARSB	arylsulfatase B [Source:HGNC Symbol;Acc:HGNC:714]	protein coding	0.97	4.31	55.18	<0.0001	<0.0001
ENSG00000229314	ORM1	orosomucoid 1 [Source:HGNC Symbol;Acc:HGNC:8498]	protein coding	-0.97	3.58	40.53	<0.0001	<0.0001
ENSG00000183621	ZNF438	zinc finger protein 438 [Source:HGNC Symbol;Acc:HGNC:21029]	protein coding	0.96	5.82	67.72	<0.0001	<0.0001
ENSG00000118503	TNFAIP3	TNF alpha induced protein 3 [Source:HGNC Symbol;Acc:HGNC:11896]	protein coding	0.96	6.92	31.58	<0.0001	<0.0001
ENSG00000086065	CHMP5	charged multivesicular body protein 5 [Source:HGNC Symbol;Acc:HGNC:26942]	protein coding	0.96	6.80	39.32	<0.0001	<0.0001
ENSG00000134440	NARS1	asparaginyl-tRNA synthetase 1 [Source:HGNC Symbol;Acc:HGNC:7643]	protein coding	-0.96	4.62	78.15	<0.0001	<0.0001
ENSG00000075089	ACTR6	actin related protein 6 [Source:HGNC Symbol;Acc:HGNC:24025]	protein coding	0.96	1.88	37.52	<0.0001	<0.0001
ENSG00000245556	SCAMP1-AS1	SCAMP1 antisense RNA 1 [Source:HGNC Symbol;Acc:HGNC:50544]	lncRNA	0.96	0.54	28.94	<0.0001	<0.0001
ENSG00000104093	DMXL2	Dmx like 2 [Source:HGNC Symbol;Acc:HGNC:2938]	protein coding	0.96	7.14	39.82	<0.0001	<0.0001
ENSG00000167202	TBC1D2B	TBC1 domain family member 2B [Source:HGNC Symbol;Acc:HGNC:29183]	protein coding	0.96	6.54	49.02	<0.0001	<0.0001
ENSG00000101190	TCFL5	transcription factor like 5 [Source:HGNC Symbol;Acc:HGNC:11646]	protein coding	-0.96	3.51	40.62	<0.0001	<0.0001
ENSG00000100908	EMC9	ER membrane protein complex subunit 9 [Source:HGNC Symbol;Acc:HGNC:20273]	protein coding	0.96	2.86	26.34	<0.0001	<0.0001
ENSG00000166333	ILK	integrin linked kinase [Source:HGNC Symbol;Acc:HGNC:6040]	protein coding	0.96	3.08	62.25	<0.0001	<0.0001
ENSG00000115956	PLEK	pleckstrin [Source:HGNC Symbol;Acc:HGNC:9070]	protein coding	0.96	9.98	50.45	<0.0001	<0.0001
ENSG00000136436	CALCOCO2	calcium binding and coiled-coil domain 2 [Source:HGNC Symbol;Acc:HGNC:29912]	protein coding	0.96	7.35	47.10	<0.0001	<0.0001

Table S18: Genes differentially expressed at Day 30 compared to pre-second vaccination (Study Arm 2 A/H7N9+AS03, D1, 29, Neutrophils). Genes are sorted by descending absolute log_2 fold change. Gene model summaries and annotations are based on Ensembl Version 99 (continued.)

Ensembl Gene ID	Ensembl Gene Name	Ensembl Gene Description	Gene Type	log_2 Fold Change (Day 30 vs. Pre-Second Vaccination)	Average log_2 CPM	Likelihood Ratio Test Statistic	P-Value	FDR Adjusted P-Value
ENSG00000233038		novel transcript, antisense to PTPRN2	lncRNA	-0.96	1.82	13.14	0.0003	0.001
ENSG00000073417	PDE8A	phosphodiesterase 8A [Source:HGNC Symbol;Acc:HGNC:8793]	protein coding	-0.96	0.97	17.76	<0.0001	0.0001
ENSG00000068383	INPP5A	inositol polyphosphate-5-phosphatase A [Source:HGNC Symbol;Acc:HGNC:6076]	protein coding	-0.95	5.72	59.14	<0.0001	<0.0001
ENSG00000114737	CISH	cytokine inducible SH2 containing protein [Source:HGNC Symbol;Acc:HGNC:1984]	protein coding	0.95	4.11	32.56	<0.0001	<0.0001
ENSG00000185880	TRIM69	tripartite motif containing 69 [Source:HGNC Symbol;Acc:HGNC:17857]	protein coding	0.95	6.20	43.72	<0.0001	<0.0001
ENSG00000111801	BTN3A3	butyrophilin subfamily 3 member A3 [Source:HGNC Symbol;Acc:HGNC:1140]	protein coding	0.95	5.72	52.28	<0.0001	<0.0001
ENSG00000160094	ZNF362	zinc finger protein 362 [Source:HGNC Symbol;Acc:HGNC:18079]	protein coding	-0.95	4.07	41.07	<0.0001	<0.0001
ENSG00000155093	PTPRN2	protein tyrosine phosphatase receptor type N2 [Source:HGNC Symbol;Acc:HGNC:9677]	protein coding	-0.95	5.43	58.01	<0.0001	<0.0001
ENSG00000274020	LINC01138	long intergenic non-protein coding RNA 1138 [Source:HGNC Symbol;Acc:HGNC:49454]	lncRNA	0.95	2.95	49.99	<0.0001	<0.0001
ENSG00000165702	GFI1B	growth factor independent 1B transcriptional repressor [Source:HGNC Symbol;Acc:HGNC:4238]	protein coding	-0.95	1.70	6.11	0.0135	0.0296
ENSG00000184640	SEPTIN9	septin 9 [Source:HGNC Symbol;Acc:HGNC:7323]	protein coding	-0.95	7.36	45.32	<0.0001	<0.0001
ENSG00000162929	KIAA1841	KIAA1841 [Source:HGNC Symbol;Acc:HGNC:29387]	protein coding	-0.95	1.98	25.54	<0.0001	<0.0001
ENSG00000141401	IMPA2	inositol monophosphatase 2 [Source:HGNC Symbol;Acc:HGNC:6051]	protein coding	-0.95	7.81	61.78	<0.0001	<0.0001
ENSG00000163872	YEATS2	YEATS domain containing 2 [Source:HGNC Symbol;Acc:HGNC:25489]	protein coding	-0.95	3.74	45.97	<0.0001	<0.0001
ENSG00000230149		novel transcript, antisense to SUN2	lncRNA	0.95	1.34	38.90	<0.0001	<0.0001
ENSG00000181788	SIAH2	siah E3 ubiquitin protein ligase 2 [Source:HGNC Symbol;Acc:HGNC:10858]	protein coding	0.95	5.28	57.58	<0.0001	<0.0001
ENSG00000273076		novel transcript, antisense to SUN2	lncRNA	0.95	0.35	20.38	<0.0001	<0.0001
ENSG00000156711	MAPK13	mitogen-activated protein kinase 13 [Source:HGNC Symbol;Acc:HGNC:6875]	protein coding	-0.95	7.60	74.70	<0.0001	<0.0001
ENSG00000012983	MAP4K5	mitogen-activated protein kinase kinase kinase kinase 5 [Source:HGNC Symbol;Acc:HGNC:6867]	protein coding	-0.95	0.66	5.27	0.0217	0.0444

Table S18: Genes differentially expressed at Day 30 compared to pre-second vaccination (Study Arm 2 A/H7N9+AS03, D1, 29, Neutrophils). Genes are sorted by descending absolute log_2 fold change. Gene model summaries and annotations are based on Ensembl Version 99 (continued.)

Ensembl Gene ID	Ensembl Gene Name	Ensembl Gene Description	Gene Type	log_2 Fold Change (Day 30 vs. Pre-Second Vaccination)	Average log_2 CPM	Likelihood Ratio Test Statistic	P-Value	FDR Adjusted P-Value
ENSG00000276045	ORAI1	ORAI calcium release-activated calcium modulator 1 [Source:HGNC Symbol;Acc:HGNC:25896]	protein coding	-0.95	5.50	69.02	<0.0001	<0.0001
ENSG00000165626	BEND7	BEN domain containing 7 [Source:HGNC Symbol;Acc:HGNC:23514]	protein coding	-0.95	2.96	29.31	<0.0001	<0.0001
ENSG00000049245	VAMP3	vesicle associated membrane protein 3 [Source:HGNC Symbol;Acc:HGNC:12644]	protein coding	0.95	7.07	41.77	<0.0001	<0.0001
ENSG00000142733	MAP3K6	mitogen-activated protein kinase kinase kinase 6 [Source:HGNC Symbol;Acc:HGNC:6858]	protein coding	-0.95	2.41	22.71	<0.0001	<0.0001
ENSG00000130714	POMT1	protein O-mannosyltransferase 1 [Source:HGNC Symbol;Acc:HGNC:9202]	protein coding	-0.94	2.04	23.77	<0.0001	<0.0001
ENSG00000143889	HNRNPLL	heterogeneous nuclear ribonucleoprotein L like [Source:HGNC Symbol;Acc:HGNC:25127]	protein coding	0.94	4.91	36.01	<0.0001	<0.0001
ENSG00000166825	ANPEP	alanyl aminopeptidase, membrane [Source:HGNC Symbol;Acc:HGNC:500]	protein coding	-0.94	10.04	56.60	<0.0001	<0.0001
ENSG00000106346	USP42	ubiquitin specific peptidase 42 [Source:HGNC Symbol;Acc:HGNC:20068]	protein coding	0.94	4.03	39.28	<0.0001	<0.0001
ENSG00000130813	SHFL	shiftless antiviral inhibitor of ribosomal frameshifting [Source:HGNC Symbol;Acc:HGNC:25649]	protein coding	0.94	6.00	59.89	<0.0001	<0.0001
ENSG00000148158	SNX30	sorting nexin family member 30 [Source:HGNC Symbol;Acc:HGNC:23685]	protein coding	-0.94	3.61	32.18	<0.0001	<0.0001
ENSG00000170921	TANC2	tetratricopeptide repeat, ankyrin repeat and coiled-coil containing 2 [Source:HGNC Symbol;Acc:HGNC:30212]	protein coding	-0.94	4.14	32.30	<0.0001	<0.0001
ENSG00000111843	TMEM14C	transmembrane protein 14C [Source:HGNC Symbol;Acc:HGNC:20952]	protein coding	-0.94	1.49	17.07	<0.0001	0.0002
ENSG00000205268	PDE7A	phosphodiesterase 7A [Source:HGNC Symbol;Acc:HGNC:8791]	protein coding	-0.94	6.10	50.83	<0.0001	<0.0001
ENSG00000182389	CACNB4	calcium voltage-gated channel auxiliary subunit beta 4 [Source:HGNC Symbol;Acc:HGNC:1404]	protein coding	-0.94	3.78	30.31	<0.0001	<0.0001
ENSG00000167207	NOD2	nucleotide binding oligomerization domain containing 2 [Source:HGNC Symbol;Acc:HGNC:5331]	protein coding	0.94	7.08	29.74	<0.0001	<0.0001
ENSG00000107263	RAPGEF1	Rap guanine nucleotide exchange factor 1 [Source:HGNC Symbol;Acc:HGNC:4568]	protein coding	-0.94	7.42	61.70	<0.0001	<0.0001
ENSG00000075618	FSCN1	fascin actin-bundling protein 1 [Source:HGNC Symbol;Acc:HGNC:11148]	protein coding	-0.94	2.71	29.44	<0.0001	<0.0001
ENSG00000089248	ERP29	endoplasmic reticulum protein 29 [Source:HGNC Symbol;Acc:HGNC:13799]	protein coding	0.94	4.58	55.39	<0.0001	<0.0001

Table S18: Genes differentially expressed at Day 30 compared to pre-second vaccination (Study Arm 2 A/H7N9+AS03, D1, 29, Neutrophils). Genes are sorted by descending absolute log_2 fold change. Gene model summaries and annotations are based on Ensembl Version 99 (continued.)

Ensembl Gene ID	Ensembl Gene Name	Ensembl Gene Description	Gene Type	log_2 Fold Change (Day 30 vs. Pre-Second Vaccination)	Average log_2 CPM	Likelihood Ratio Test Statistic	P-Value	FDR Adjusted P-Value
ENSG00000084234	APLP2	amyloid beta precursor like protein 2 [Source:HGNC Symbol;Acc:HGNC:598]	protein coding	-0.94	9.07	60.15	<0.0001	<0.0001
ENSG00000163171	CDC42EP3	CDC42 effector protein 3 [Source:HGNC Symbol;Acc:HGNC:16943]	protein coding	-0.94	7.99	43.09	<0.0001	<0.0001
ENSG00000197165	SULT1A2	sulfotransferase family 1A member 2 [Source:HGNC Symbol;Acc:HGNC:11454]	protein coding	-0.94	1.97	20.85	<0.0001	<0.0001
ENSG00000130396	AFDN	afadin, adherens junction formation factor [Source:HGNC Symbol;Acc:HGNC:7137]	protein coding	-0.94	4.53	47.10	<0.0001	<0.0001
ENSG00000234290		novel transcript	lncRNA	0.94	4.71	22.88	<0.0001	<0.0001
ENSG00000168961	LGALS9	galectin 9 [Source:HGNC Symbol;Acc:HGNC:6570]	protein coding	0.94	7.37	59.06	<0.0001	<0.0001
ENSG00000159713	TPPP3	tubulin polymerization promoting protein family member 3 [Source:HGNC Symbol;Acc:HGNC:24162]	protein coding	-0.94	0.64	16.54	<0.0001	0.0002
ENSG00000140386	SCAPER	S-phase cyclin A associated protein in the ER [Source:HGNC Symbol;Acc:HGNC:13081]	protein coding	0.93	2.75	50.46	<0.0001	<0.0001
ENSG00000124191	TOX2	TOX high mobility group box family member 2 [Source:HGNC Symbol;Acc:HGNC:16095]	protein coding	-0.93	1.09	16.40	<0.0001	0.0002
ENSG00000202533			misc RNA	0.93	0.51	17.06	<0.0001	0.0002
ENSG00000149308	NPAT	nuclear protein, coactivator of histone transcription [Source:HGNC Symbol;Acc:HGNC:7896]	protein coding	-0.93	2.77	44.21	<0.0001	<0.0001
ENSG00000115159	GPD2	glycerol-3-phosphate dehydrogenase 2 [Source:HGNC Symbol;Acc:HGNC:4456]	protein coding	0.93	3.90	43.66	<0.0001	<0.0001
ENSG00000104626	ERI1	exoribonuclease 1 [Source:HGNC Symbol;Acc:HGNC:23994]	protein coding	0.93	3.67	38.94	<0.0001	<0.0001
ENSG00000288082		novel transcript	lncRNA	-0.93	0.37	15.73	<0.0001	0.0003
ENSG00000158882	TOMM40L	translocase of outer mitochondrial membrane 40 like [Source:HGNC Symbol;Acc:HGNC:25756]	protein coding	0.93	1.82	19.61	<0.0001	<0.0001
ENSG00000163635	ATXN7	ataxin 7 [Source:HGNC Symbol;Acc:HGNC:10560]	protein coding	0.93	2.48	30.80	<0.0001	<0.0001
ENSG00000169429	CXCL8	C-X-C motif chemokine ligand 8 [Source:HGNC Symbol;Acc:HGNC:6025]	protein coding	-0.93	10.31	41.84	<0.0001	<0.0001
ENSG00000183696	UPP1	uridine phosphorylase 1 [Source:HGNC Symbol;Acc:HGNC:12576]	protein coding	0.93	5.09	62.61	<0.0001	<0.0001
ENSG00000162636	FAM102B	family with sequence similarity 102 member B [Source:HGNC Symbol;Acc:HGNC:27637]	protein coding	-0.93	2.11	19.24	<0.0001	<0.0001

Table S18: Genes differentially expressed at Day 30 compared to pre-second vaccination (Study Arm 2 A/H7N9+AS03, D1, 29, Neutrophils). Genes are sorted by descending absolute log_2 fold change. Gene model summaries and annotations are based on Ensembl Version 99 (continued.)

Ensembl Gene ID	Ensembl Gene Name	Ensembl Gene Description	Gene Type	log_2 Fold Change (Day 30 vs. Pre-Second Vaccination)	Average log_2 CPM	Likelihood Ratio Test Statistic	P-Value	FDR Adjusted P-Value
ENSG00000264553	MIR4257	microRNA 4257 [Source:HGNC Symbol;Acc:HGNC:38312]	miRNA	0.93	0.81	18.76	<0.0001	<0.0001
ENSG00000070190	DAPP1	dual adaptor of phosphotyrosine and 3-phosphoinositides 1 [Source:HGNC Symbol;Acc:HGNC:16500]	protein coding	0.93	7.99	40.89	<0.0001	<0.0001
ENSG00000179242	CDH4	cadherin 4 [Source:HGNC Symbol;Acc:HGNC:1763]	protein coding	-0.93	0.41	16.47	<0.0001	0.0002
ENSG00000124508	BTN2A2	butyrophilin subfamily 2 member A2 [Source:HGNC Symbol;Acc:HGNC:1137]	protein coding	0.93	4.58	49.89	<0.0001	<0.0001
ENSG00000183044	ABAT	4-aminobutyrate aminotransferase [Source:HGNC Symbol;Acc:HGNC:23]	protein coding	-0.93	5.54	51.58	<0.0001	<0.0001
ENSG00000138175	ARL3	ADP ribosylation factor like GTPase 3 [Source:HGNC Symbol;Acc:HGNC:694]	protein coding	-0.93	4.03	71.39	<0.0001	<0.0001
ENSG00000205220	PSMB10	proteasome 20S subunit beta 10 [Source:HGNC Symbol;Acc:HGNC:9538]	protein coding	0.93	6.87	41.12	<0.0001	<0.0001
ENSG00000126246	IGFLR1	IGF like family receptor 1 [Source:HGNC Symbol;Acc:HGNC:23620]	protein coding	0.93	3.28	29.43	<0.0001	<0.0001
ENSG00000159247	TUBBP5	tubulin beta pseudogene 5 [Source:HGNC Symbol;Acc:HGNC:23674]	transcribed unprocessed pseudogene	-0.93	0.96	12.27	0.0005	0.0015
ENSG00000225342	LRRK2-DT	LRRK2 divergent transcript [Source:HGNC Symbol;Acc:HGNC:40848]	lncRNA	0.93	4.91	46.27	<0.0001	<0.0001
ENSG00000164105	SAP30	Sin3A associated protein 30 [Source:HGNC Symbol;Acc:HGNC:10532]	protein coding	-0.93	1.73	12.86	0.0003	0.0011
ENSG00000112640	PPP2R5D	protein phosphatase 2 regulatory subunit B'delta [Source:HGNC Symbol;Acc:HGNC:9312]	protein coding	-0.92	3.43	40.98	<0.0001	<0.0001
ENSG00000169026	SLC49A3	solute carrier family 49 member 3 [Source:HGNC Symbol;Acc:HGNC:26177]	protein coding	0.92	4.42	65.37	<0.0001	<0.0001
ENSG00000140961	OSGIN1	oxidative stress induced growth inhibitor 1 [Source:HGNC Symbol;Acc:HGNC:30093]	protein coding	-0.92	2.16	44.62	<0.0001	<0.0001
ENSG00000285534		novel transcript	lncRNA	-0.92	0.57	9.49	0.0021	0.0058
ENSG00000143847	PPFIA4	PTPRF interacting protein alpha 4 [Source:HGNC Symbol;Acc:HGNC:9248]	protein coding	-0.92	0.61	14.93	0.0001	0.0004
ENSG00000124795	DEK	DEK proto-oncogene [Source:HGNC Symbol;Acc:HGNC:2768]	protein coding	0.92	7.09	48.56	<0.0001	<0.0001

Table S18: Genes differentially expressed at Day 30 compared to pre-second vaccination (Study Arm 2 A/H7N9+AS03, D1, 29, Neutrophils). Genes are sorted by descending absolute log_2 fold change. Gene model summaries and annotations are based on Ensembl Version 99 (continued.)

Ensembl Gene ID	Ensembl Gene Name	Ensembl Gene Description	Gene Type	log_2 Fold Change (Day 30 vs. Pre-Second Vaccination)	Average log_2 CPM	Likelihood Ratio Test Statistic	P-Value	FDR Adjusted P-Value
ENSG00000166326	TRIM44	tripartite motif containing 44 [Source:HGNC Symbol;Acc:HGNC:19016]	protein coding	0.92	0.92	18.60	<0.0001	<0.0001
ENSG00000178425	NT5DC1	5'-nucleotidase domain containing 1 [Source:HGNC Symbol;Acc:HGNC:21556]	protein coding	-0.92	3.17	44.28	<0.0001	<0.0001
ENSG00000173598	NUDT4	nudix hydrolase 4 [Source:HGNC Symbol;Acc:HGNC:8051]	protein coding	-0.92	3.66	29.58	<0.0001	<0.0001
ENSG00000177427	MIEF2	mitochondrial elongation factor 2 [Source:HGNC Symbol;Acc:HGNC:17920]	protein coding	-0.92	2.24	47.39	<0.0001	<0.0001
ENSG00000160746	ANO10	anoctamin 10 [Source:HGNC Symbol;Acc:HGNC:25519]	protein coding	-0.92	4.16	41.92	<0.0001	<0.0001
ENSG00000235192		novel transcript	lncRNA	0.91	2.02	42.83	<0.0001	<0.0001
ENSG00000265148	TSPOAP1-AS1	TSPOAP1, SUPT4H1 and RNF43 antisense RNA 1 [Source:HGNC Symbol;Acc:HGNC:44148]	lncRNA	0.91	1.89	31.97	<0.0001	<0.0001
ENSG00000170175	CHRNB1	cholinergic receptor nicotinic beta 1 subunit [Source:HGNC Symbol;Acc:HGNC:1961]	protein coding	0.91	1.72	47.08	<0.0001	<0.0001
ENSG00000112773	TENT5A	terminal nucleotidyltransferase 5A [Source:HGNC Symbol;Acc:HGNC:18345]	protein coding	0.91	4.62	38.79	<0.0001	<0.0001
ENSG00000124813	RUNX2	RUNX family transcription factor 2 [Source:HGNC Symbol;Acc:HGNC:10472]	protein coding	-0.91	5.69	54.36	<0.0001	<0.0001
ENSG00000153443	UBALD1	UBA like domain containing 1 [Source:HGNC Symbol;Acc:HGNC:29576]	protein coding	-0.91	4.98	50.59	<0.0001	<0.0001
ENSG00000241106	HLA-DOB	major histocompatibility complex, class II, DO beta [Source:HGNC Symbol;Acc:HGNC:4937]	protein coding	0.91	2.17	24.18	<0.0001	<0.0001
ENSG00000064787	BCAS1	breast carcinoma amplified sequence 1 [Source:HGNC Symbol;Acc:HGNC:974]	protein coding	-0.91	1.19	18.69	<0.0001	<0.0001
ENSG00000104375	STK3	serine/threonine kinase 3 [Source:HGNC Symbol;Acc:HGNC:11406]	protein coding	0.91	4.08	34.98	<0.0001	<0.0001
ENSG00000167699	GLOD4	glyoxalase domain containing 4 [Source:HGNC Symbol;Acc:HGNC:14111]	protein coding	-0.91	0.23	15.24	<0.0001	0.0004
ENSG00000064270	ATP2C2	ATPase secretory pathway Ca2+ transporting 2 [Source:HGNC Symbol;Acc:HGNC:29103]	protein coding	-0.91	1.06	11.42	0.0007	0.0023
ENSG00000169299	PGM2	phosphoglucomutase 2 [Source:HGNC Symbol;Acc:HGNC:8906]	protein coding	-0.91	6.05	47.32	<0.0001	<0.0001
ENSG00000267365	KCNJ2-AS1	KCNJ2 antisense RNA 1 [Source:HGNC Symbol;Acc:HGNC:43720]	lncRNA	0.91	3.97	34.14	<0.0001	<0.0001

Table S18: Genes differentially expressed at Day 30 compared to pre-second vaccination (Study Arm 2 A/H7N9+AS03, D1, 29, Neutrophils). Genes are sorted by descending absolute log_2 fold change. Gene model summaries and annotations are based on Ensembl Version 99 (continued.)

Ensembl Gene ID	Ensembl Gene Name	Ensembl Gene Description	Gene Type	log_2 Fold Change (Day 30 vs. Pre-Second Vaccination)	Average log_2 CPM	Likelihood Ratio Test Statistic	P-Value	FDR Adjusted P-Value
ENSG00000137752	CASP1	caspase 1 [Source:HGNC Symbol;Acc:HGNC:1499]	protein coding	0.91	8.35	64.98	<0.0001	<0.0001
ENSG00000159339	PADI4	peptidyl arginine deiminase 4 [Source:HGNC Symbol;Acc:HGNC:18368]	protein coding	-0.91	8.22	20.72	<0.0001	<0.0001
ENSG00000272419	LINC01145	long intergenic non-protein coding RNA 1145 [Source:HGNC Symbol;Acc:HGNC:49462]	transcribed unprocessed pseudogene	0.91	2.87	44.43	<0.0001	<0.0001
ENSG00000110315	RNF141	ring finger protein 141 [Source:HGNC Symbol;Acc:HGNC:21159]	protein coding	-0.91	7.48	42.69	<0.0001	<0.0001
ENSG00000134333	LDHA	lactate dehydrogenase A [Source:HGNC Symbol;Acc:HGNC:6535]	protein coding	-0.91	5.77	33.37	<0.0001	<0.0001
ENSG00000103423	DNAJA3	DnaJ heat shock protein family (Hsp40) member A3 [Source:HGNC Symbol;Acc:HGNC:11808]	protein coding	-0.91	1.01	31.93	<0.0001	<0.0001
ENSG00000172594	SMPDL3A	sphingomyelin phosphodiesterase acid like 3A [Source:HGNC Symbol;Acc:HGNC:17389]	protein coding	-0.91	0.70	24.62	<0.0001	<0.0001
ENSG00000182551	ADI1	acireductone dioxygenase 1 [Source:HGNC Symbol;Acc:HGNC:30576]	protein coding	-0.91	4.22	44.45	<0.0001	<0.0001
ENSG00000234127	TRIM26	tripartite motif containing 26 [Source:HGNC Symbol;Acc:HGNC:12962]	protein coding	0.90	5.82	51.26	<0.0001	<0.0001
ENSG00000153975	ZUP1	zinc finger containing ubiquitin peptidase 1 [Source:HGNC Symbol;Acc:HGNC:21224]	protein coding	0.90	1.13	21.15	<0.0001	<0.0001
ENSG00000132952	USPL1	ubiquitin specific peptidase like 1 [Source:HGNC Symbol;Acc:HGNC:20294]	protein coding	0.90	4.09	40.47	<0.0001	<0.0001
ENSG00000148341	SH3GLB2	SH3 domain containing GRB2 like, endophilin B2 [Source:HGNC Symbol;Acc:HGNC:10834]	protein coding	-0.90	4.58	47.45	<0.0001	<0.0001
ENSG00000139318	DUSP6	dual specificity phosphatase 6 [Source:HGNC Symbol;Acc:HGNC:3072]	protein coding	0.90	8.12	19.59	<0.0001	<0.0001
ENSG00000272669		novel transcript, antisense to SUN2	lncRNA	0.90	3.44	43.63	<0.0001	<0.0001
ENSG00000268555		novel transcript	lncRNA	-0.90	3.42	22.39	<0.0001	<0.0001
ENSG00000131584	ACAP3	ArfGAP with coiled-coil, ankyrin repeat and PH domains 3 [Source:HGNC Symbol;Acc:HGNC:16754]	protein coding	-0.90	5.45	55.06	<0.0001	<0.0001
ENSG00000228340	MIR646HG	MIR646 host gene [Source:HGNC Symbol;Acc:HGNC:27659]	lncRNA	-0.90	3.60	30.17	<0.0001	<0.0001

Confidential/Proprietary Information

Table S18: Genes differentially expressed at Day 30 compared to pre-second vaccination (Study Arm 2 A/H7N9+AS03, D1, 29, Neutrophils). Genes are sorted by descending absolute log_2 fold change. Gene model summaries and annotations are based on Ensembl Version 99 (continued.)

Ensembl Gene ID	Ensembl Gene Name	Ensembl Gene Description	Gene Type	log_2 Fold Change (Day 30 vs. Pre-Second Vaccination)	Average log_2 CPM	Likelihood Ratio Test Statistic	P-Value	FDR Adjusted P-Value
ENSG00000120992	LYPLA1	lysophospholipase 1 [Source:HGNC Symbol;Acc:HGNC:6737]	protein coding	0.90	5.37	65.92	<0.0001	<0.0001
ENSG00000110324	IL10RA	interleukin 10 receptor subunit alpha [Source:HGNC Symbol;Acc:HGNC:5964]	protein coding	0.90	4.68	58.40	<0.0001	<0.0001
ENSG00000146463	ZMYM4	zinc finger MYM-type containing 4 [Source:HGNC Symbol;Acc:HGNC:13055]	protein coding	0.90	1.13	34.36	<0.0001	<0.0001
ENSG00000186716	BCR	BCR activator of RhoGEF and GTPase [Source:HGNC Symbol;Acc:HGNC:1014]	protein coding	-0.90	5.22	63.86	<0.0001	<0.0001
ENSG00000232284	GNG12-AS1	GNG12, DIRAS3 and WLS antisense RNA 1 [Source:HGNC Symbol;Acc:HGNC:43938]	lncRNA	-0.90	1.04	20.42	<0.0001	<0.0001
ENSG00000133056	PIK3C2B	phosphatidylinositol-4-phosphate 3-kinase catalytic subunit type 2 beta [Source:HGNC Symbol;Acc:HGNC:8972]	protein coding	-0.90	0.48	23.27	<0.0001	<0.0001
ENSG00000171368	TPPP	tubulin polymerization promoting protein [Source:HGNC Symbol;Acc:HGNC:24164]	protein coding	-0.90	1.33	20.65	<0.0001	<0.0001
ENSG00000132109	TRIM21	tripartite motif containing 21 [Source:HGNC Symbol;Acc:HGNC:11312]	protein coding	0.90	7.66	38.39	<0.0001	<0.0001
ENSG00000168275	COA6	cytochrome c oxidase assembly factor 6 [Source:HGNC Symbol;Acc:HGNC:18025]	protein coding	0.90	1.51	34.62	<0.0001	<0.0001
ENSG00000130222	GADD45G	growth arrest and DNA damage inducible gamma [Source:HGNC Symbol;Acc:HGNC:4097]	protein coding	0.90	3.77	14.44	0.0001	0.0005
ENSG00000135677	GNS	glucosamine (N-acetyl)-6-sulfatase [Source:HGNC Symbol;Acc:HGNC:4422]	protein coding	0.90	7.57	43.54	<0.0001	<0.0001
ENSG00000104518	GSDMD	gasdermin D [Source:HGNC Symbol;Acc:HGNC:25697]	protein coding	0.90	7.78	43.45	<0.0001	<0.0001
ENSG00000255328		novel transcript	lncRNA	0.90	3.52	28.81	<0.0001	<0.0001
ENSG00000167004	PDIA3	protein disulfide isomerase family A member 3 [Source:HGNC Symbol;Acc:HGNC:4606]	protein coding	0.89	5.71	58.85	<0.0001	<0.0001
ENSG00000187837	H1-2	H1.2 linker histone, cluster member [Source:HGNC Symbol;Acc:HGNC:4716]	protein coding	-0.89	6.46	47.86	<0.0001	<0.0001
ENSG00000221968	FADS3	fatty acid desaturase 3 [Source:HGNC Symbol;Acc:HGNC:3576]	protein coding	-0.89	2.01	32.07	<0.0001	<0.0001
ENSG00000168944	CEP120	centrosomal protein 120 [Source:HGNC Symbol;Acc:HGNC:26690]	protein coding	-0.89	3.85	28.98	<0.0001	<0.0001
ENSG00000237927		novel transcript	lncRNA	0.89	1.45	20.73	<0.0001	<0.0001

Table S18: Genes differentially expressed at Day 30 compared to pre-second vaccination (Study Arm 2 A/H7N9+AS03, D1, 29, Neutrophils). Genes are sorted by descending absolute log_2 fold change. Gene model summaries and annotations are based on Ensembl Version 99 (continued.)

Ensembl Gene ID	Ensembl Gene Name	Ensembl Gene Description	Gene Type	log_2 Fold Change (Day 30 vs. Pre-Second Vaccination)	Average log_2 CPM	Likelihood Ratio Test Statistic	P-Value	FDR Adjusted P-Value
ENSG00000184992	BRI3BP	BRI3 binding protein [Source:HGNC Symbol;Acc:HGNC:14251]	polymorphic pseudogene	-0.89	3.17	30.49	<0.0001	<0.0001
ENSG00000168016	TRANK1	tetratricopeptide repeat and ankyrin repeat containing 1 [Source:HGNC Symbol;Acc:HGNC:29011]	protein coding	0.89	9.12	52.22	<0.0001	<0.0001
ENSG00000232680		novel transcript	lncRNA	0.89	2.11	33.22	<0.0001	<0.0001
ENSG00000231113		novel transcript	lncRNA	-0.89	3.48	46.19	<0.0001	<0.0001
ENSG00000260105	AOC4P	amine oxidase copper containing 4, pseudogene [Source:HGNC Symbol;Acc:HGNC:48869]	transcribed unprocessed pseudogene	-0.89	0.55	21.71	<0.0001	<0.0001
ENSG00000161513	FDXR	ferredoxin reductase [Source:HGNC Symbol;Acc:HGNC:3642]	protein coding	-0.89	1.06	41.97	<0.0001	<0.0001
ENSG00000011422	PLAUR	plasminogen activator, urokinase receptor [Source:HGNC Symbol;Acc:HGNC:9053]	protein coding	0.89	8.05	73.78	<0.0001	<0.0001
ENSG00000157551	KCNJ15	potassium inwardly rectifying channel subfamily J member 15 [Source:HGNC Symbol;Acc:HGNC:6261]	protein coding	0.89	9.30	58.75	<0.0001	<0.0001
ENSG00000149932	TMEM219	transmembrane protein 219 [Source:HGNC Symbol;Acc:HGNC:25201]	protein coding	0.89	5.04	51.36	<0.0001	<0.0001
ENSG00000125868	DSTN	destrin, actin depolymerizing factor [Source:HGNC Symbol;Acc:HGNC:15750]	protein coding	-0.89	3.32	40.40	<0.0001	<0.0001
ENSG00000049089	COL9A2	collagen type IX alpha 2 chain [Source:HGNC Symbol;Acc:HGNC:2218]	protein coding	0.89	4.44	40.22	<0.0001	<0.0001
ENSG00000099999	RNF215	ring finger protein 215 [Source:HGNC Symbol;Acc:HGNC:33434]	protein coding	-0.89	0.96	14.61	0.0001	0.0005
ENSG00000204420	MPIG6B	megakaryocyte and platelet inhibitory receptor G6b [Source:HGNC Symbol;Acc:HGNC:13937]	protein coding	0.89	1.66	23.00	<0.0001	<0.0001
ENSG00000163874	ZC3H12A	zinc finger CCCH-type containing 12A [Source:HGNC Symbol;Acc:HGNC:26259]	protein coding	0.89	5.76	42.57	<0.0001	<0.0001
ENSG00000115339	GALNT3	polypeptide N-acetylgalactosaminyltransferase 3 [Source:HGNC Symbol;Acc:HGNC:4125]	protein coding	0.89	5.56	37.85	<0.0001	<0.0001
ENSG00000180891	CUEDC1	CUE domain containing 1 [Source:HGNC Symbol;Acc:HGNC:31350]	protein coding	-0.89	5.75	38.37	<0.0001	<0.0001
ENSG00000143106	PSMA5	proteasome 20S subunit alpha 5 [Source:HGNC Symbol;Acc:HGNC:9534]	protein coding	0.89	4.50	37.19	<0.0001	<0.0001

Table S18: Genes differentially expressed at Day 30 compared to pre-second vaccination (Study Arm 2 A/H7N9+AS03, D1, 29, Neutrophils). Genes are sorted by descending absolute log_2 fold change. Gene model summaries and annotations are based on Ensembl Version 99 (continued.)

Ensembl Gene ID	Ensembl Gene Name	Ensembl Gene Description	Gene Type	log_2 Fold Change (Day 30 vs. Pre-Second Vaccination)	Average log_2 CPM	Likelihood Ratio Test Statistic	P-Value	FDR Adjusted P-Value
ENSG00000178761	FAM219B	family with sequence similarity 219 member B [Source:HGNC Symbol;Acc:HGNC:24695]	protein coding	-0.89	2.33	28.36	<0.0001	<0.0001
ENSG00000149418	ST14	suppression of tumorigenicity 14 [Source:HGNC Symbol;Acc:HGNC:11344]	protein coding	-0.89	3.28	12.79	0.0003	0.0012
ENSG00000279693		novel transcript	TEC	-0.89	0.63	6.05	0.0139	0.0304
ENSG00000175155	YPEL2	yippee like 2 [Source:HGNC Symbol;Acc:HGNC:18326]	protein coding	-0.89	4.35	47.17	<0.0001	<0.0001
ENSG00000168807	SNTB2	syntrophin beta 2 [Source:HGNC Symbol;Acc:HGNC:11169]	protein coding	-0.89	3.86	32.52	<0.0001	<0.0001
ENSG00000235944	ZNF815P	zinc finger protein 815, pseudogene [Source:HGNC Symbol;Acc:HGNC:22029]	transcribed unprocessed pseudogene	-0.88	0.76	11.73	0.0006	0.002
ENSG00000162976	SLC66A3	solute carrier family 66 member 3 [Source:HGNC Symbol;Acc:HGNC:28503]	protein coding	-0.88	1.82	20.26	<0.0001	<0.0001
ENSG00000141569	TRIM65	tripartite motif containing 65 [Source:HGNC Symbol;Acc:HGNC:27316]	protein coding	-0.88	0.17	18.48	<0.0001	<0.0001
ENSG00000166016	ABTB2	ankyrin repeat and BTB domain containing 2 [Source:HGNC Symbol;Acc:HGNC:23842]	protein coding	0.88	3.55	17.92	<0.0001	0.0001
ENSG00000185024	BRF1	BRF1 RNA polymerase III transcription initiation factor subunit [Source:HGNC Symbol;Acc:HGNC:11551]	protein coding	-0.88	1.69	32.78	<0.0001	<0.0001
ENSG00000241163	LINC00877	long intergenic non-protein coding RNA 877 [Source:HGNC Symbol;Acc:HGNC:27706]	lncRNA	0.88	5.64	55.97	<0.0001	<0.0001
ENSG00000152348	ATG10	autophagy related 10 [Source:HGNC Symbol;Acc:HGNC:20315]	protein coding	0.88	1.38	39.85	<0.0001	<0.0001
ENSG00000099904	ZDHHC8	zinc finger DHHC-type containing 8 [Source:HGNC Symbol;Acc:HGNC:18474]	protein coding	-0.88	0.26	13.17	0.0003	0.001
ENSG00000226091	LINC00937	long intergenic non-protein coding RNA 937 [Source:HGNC Symbol;Acc:HGNC:48629]	lncRNA	0.88	4.19	54.92	<0.0001	<0.0001
ENSG00000114120	SLC25A36	solute carrier family 25 member 36 [Source:HGNC Symbol;Acc:HGNC:25554]	protein coding	-0.88	1.48	14.72	0.0001	0.0005
ENSG00000185722	ANKFY1	ankyrin repeat and FYVE domain containing 1 [Source:HGNC Symbol;Acc:HGNC:20763]	protein coding	0.88	6.11	47.35	<0.0001	<0.0001
ENSG00000175894	TSPEAR	thrombospondin type laminin G domain and EAR repeats [Source:HGNC Symbol;Acc:HGNC:1268]	protein coding	-0.88	2.05	24.57	<0.0001	<0.0001

Table S18: Genes differentially expressed at Day 30 compared to pre-second vaccination (Study Arm 2 A/H7N9+AS03, D1, 29, Neutrophils). Genes are sorted by descending absolute log_2 fold change. Gene model summaries and annotations are based on Ensembl Version 99 (continued.)

Ensembl Gene ID	Ensembl Gene Name	Ensembl Gene Description	Gene Type	log_2 Fold Change (Day 30 vs. Pre-Second Vaccination)	Average log_2 CPM	Likelihood Ratio Test Statistic	P-Value	FDR Adjusted P-Value
ENSG00000215244	LINC02649	long intergenic non-protein coding RNA 2649 [Source:HGNC Symbol;Acc:HGNC:54134]	lncRNA	-0.88	3.21	24.87	<0.0001	<0.0001
ENSG00000277972	CISD3	CDGSH iron sulfur domain 3 [Source:HGNC Symbol;Acc:HGNC:27578]	protein coding	0.88	1.15	37.95	<0.0001	<0.0001
ENSG00000110651	CD81	CD81 molecule [Source:HGNC Symbol;Acc:HGNC:1701]	protein coding	-0.88	1.96	6.05	0.0139	0.0305
ENSG00000158869	FCER1G	Fc fragment of IgE receptor Ig [Source:HGNC Symbol;Acc:HGNC:3611]	protein coding	0.88	7.91	54.33	<0.0001	<0.0001
ENSG00000179981	TSHZ1	teashirt zinc finger homeobox 1 [Source:HGNC Symbol;Acc:HGNC:10669]	protein coding	-0.88	1.61	29.96	<0.0001	<0.0001
ENSG00000144848	ATG3	autophagy related 3 [Source:HGNC Symbol;Acc:HGNC:20962]	protein coding	0.87	7.60	46.68	<0.0001	<0.0001
ENSG00000268433	MTDHP3	metadherin pseudogene 3 [Source:HGNC Symbol;Acc:HGNC:52359]	processed pseudogene	-0.87	1.20	19.05	<0.0001	<0.0001
ENSG00000198839	ZNF277	zinc finger protein 277 [Source:HGNC Symbol;Acc:HGNC:13070]	protein coding	0.87	2.35	30.57	<0.0001	<0.0001
ENSG00000073849	ST6GAL1	ST6 beta-galactoside alpha-2,6-sialyltransferase 1 [Source:HGNC Symbol;Acc:HGNC:10860]	protein coding	0.87	4.28	26.47	<0.0001	<0.0001
ENSG00000265491	RNF115	ring finger protein 115 [Source:HGNC Symbol;Acc:HGNC:18154]	protein coding	0.87	2.54	43.63	<0.0001	<0.0001
ENSG00000261997		novel transcript	lncRNA	0.87	2.73	34.92	<0.0001	<0.0001
ENSG00000025708	TYMP	thymidine phosphorylase [Source:HGNC Symbol;Acc:HGNC:3148]	protein coding	0.87	9.91	54.38	<0.0001	<0.0001
ENSG00000114446	IFT57	intraflagellar transport 57 [Source:HGNC Symbol;Acc:HGNC:17367]	protein coding	0.87	1.62	49.75	<0.0001	<0.0001
ENSG00000234771	SLC25A25-AS1	SLC25A25 antisense RNA 1 [Source:HGNC Symbol;Acc:HGNC:27844]	lncRNA	-0.87	0.38	10.20	0.0014	0.0041
ENSG00000136478	TEX2	testis expressed 2 [Source:HGNC Symbol;Acc:HGNC:30884]	protein coding	-0.87	1.57	24.59	<0.0001	<0.0001
ENSG00000011275	RNF216	ring finger protein 216 [Source:HGNC Symbol;Acc:HGNC:21698]	protein coding	-0.87	5.69	35.84	<0.0001	<0.0001
ENSG00000168264	IRF2BP2	interferon regulatory factor 2 binding protein 2 [Source:HGNC Symbol;Acc:HGNC:21729]	protein coding	-0.87	5.47	24.75	<0.0001	<0.0001
ENSG00000127666	TICAM1	toll like receptor adaptor molecule 1 [Source:HGNC Symbol;Acc:HGNC:18348]	protein coding	0.87	3.76	36.97	<0.0001	<0.0001

Table S18: Genes differentially expressed at Day 30 compared to pre-second vaccination (Study Arm 2 A/H7N9+AS03, D1, 29, Neutrophils). Genes are sorted by descending absolute log_2 fold change. Gene model summaries and annotations are based on Ensembl Version 99 (continued.)

Ensembl Gene ID	Ensembl Gene Name	Ensembl Gene Description	Gene Type	log_2 Fold Change (Day 30 vs. Pre-Second Vaccination)	Average log_2 CPM	Likelihood Ratio Test Statistic	P-Value	FDR Adjusted P-Value
ENSG00000143224	PPOX	protoporphyrinogen oxidase [Source:HGNC Symbol;Acc:HGNC:9280]	protein coding	-0.87	0.81	20.05	<0.0001	<0.0001
ENSG00000104267	CA2	carbonic anhydrase 2 [Source:HGNC Symbol;Acc:HGNC:1373]	protein coding	0.87	2.14	22.47	<0.0001	<0.0001
ENSG00000123700	KCNJ2	potassium inwardly rectifying channel subfamily J member 2 [Source:HGNC Symbol;Acc:HGNC:6263]	protein coding	0.87	7.38	24.61	<0.0001	<0.0001
ENSG00000197355	UAP1L1	UDP-N-acetylglucosamine pyrophosphorylase 1 like 1 [Source:HGNC Symbol;Acc:HGNC:28082]	protein coding	-0.87	0.98	15.43	<0.0001	0.0003
ENSG00000163050	COQ8A	coenzyme Q8A [Source:HGNC Symbol;Acc:HGNC:16812]	protein coding	-0.87	6.47	55.57	<0.0001	<0.0001
ENSG00000238164	TNFRSF14-AS1	TNFRSF14 antisense RNA 1 [Source:HGNC Symbol;Acc:HGNC:26966]	lncRNA	0.87	3.59	50.67	<0.0001	<0.0001
ENSG00000173221	GLRX	glutaredoxin [Source:HGNC Symbol;Acc:HGNC:4330]	protein coding	0.87	7.57	62.88	<0.0001	<0.0001
ENSG00000096080	MRPS18A	mitochondrial ribosomal protein S18A [Source:HGNC Symbol;Acc:HGNC:14515]	protein coding	0.87	1.62	39.81	<0.0001	<0.0001
ENSG00000278090	LUNAR1	leukemia-associated non-coding IGF1R activator RNA 1 [Source:HGNC Symbol;Acc:HGNC:51199]	lncRNA	-0.87	2.17	22.64	<0.0001	<0.0001
ENSG00000271806		novel transcript, antisense PRKCZ	lncRNA	-0.86	0.17	12.57	0.0004	0.0013
ENSG00000225450		novel transcript	lncRNA	0.86	0.77	28.38	<0.0001	<0.0001
ENSG00000100749	VRK1	VRK serine/threonine kinase 1 [Source:HGNC Symbol;Acc:HGNC:12718]	protein coding	-0.86	1.33	25.01	<0.0001	<0.0001
ENSG00000138434	ITPRID2	ITPR interacting domain containing 2 [Source:HGNC Symbol;Acc:HGNC:11319]	protein coding	0.86	7.36	63.66	<0.0001	<0.0001
ENSG00000103415	HMOX2	heme oxygenase 2 [Source:HGNC Symbol;Acc:HGNC:5014]	protein coding	-0.86	5.33	67.14	<0.0001	<0.0001
ENSG00000278195	SSTR3	somatostatin receptor 3 [Source:HGNC Symbol;Acc:HGNC:11332]	protein coding	0.86	1.53	17.92	<0.0001	0.0001
ENSG00000146425	DYNLT1	dynein light chain Tctex-type 1 [Source:HGNC Symbol;Acc:HGNC:11697]	protein coding	0.86	6.51	32.87	<0.0001	<0.0001
ENSG00000138834	MAPK8IP3	mitogen-activated protein kinase 8 interacting protein 3 [Source:HGNC Symbol;Acc:HGNC:6884]	protein coding	-0.86	7.00	78.76	<0.0001	<0.0001
ENSG00000244486	SCARF2	scavenger receptor class F member 2 [Source:HGNC Symbol;Acc:HGNC:19869]	protein coding	-0.86	2.21	39.84	<0.0001	<0.0001
ENSG00000197157	SND1	staphylococcal nuclease and tudor domain containing 1 [Source:HGNC Symbol;Acc:HGNC:30646]	protein coding	0.86	2.78	45.02	<0.0001	<0.0001

Confidential/Proprietary Information

Table S18: Genes differentially expressed at Day 30 compared to pre-second vaccination (Study Arm 2 A/H7N9+AS03, D1, 29, Neutrophils). Genes are sorted by descending absolute log_2 fold change. Gene model summaries and annotations are based on Ensembl Version 99 (continued.)

Ensembl Gene ID	Ensembl Gene Name	Ensembl Gene Description	Gene Type	log_2 Fold Change (Day 30 vs. Pre-Second Vaccination)	Average log_2 CPM	Likelihood Ratio Test Statistic	P-Value	FDR Adjusted P-Value
ENSG00000186187	ZNRF1	zinc and ring finger 1 [Source:HGNC Symbol;Acc:HGNC:18452]	protein coding	-0.86	1.32	17.43	<0.0001	0.0001
ENSG00000010295	IFFO1	intermediate filament family orphan 1 [Source:HGNC Symbol;Acc:HGNC:24970]	protein coding	-0.86	5.87	45.36	<0.0001	<0.0001
ENSG00000130695	CEP85	centrosomal protein 85 [Source:HGNC Symbol;Acc:HGNC:25309]	protein coding	-0.86	1.04	8.51	0.0035	0.0092
ENSG00000125170	DOK4	docking protein 4 [Source:HGNC Symbol;Acc:HGNC:19868]	protein coding	-0.86	3.30	28.04	<0.0001	<0.0001
ENSG00000068305	MEF2A	myocyte enhancer factor 2A [Source:HGNC Symbol;Acc:HGNC:6993]	protein coding	0.86	5.81	54.43	<0.0001	<0.0001
ENSG00000137767	SQOR	sulfide quinone oxidoreductase [Source:HGNC Symbol;Acc:HGNC:20390]	protein coding	0.86	6.41	55.09	<0.0001	<0.0001
ENSG00000231551		phosphodiesterase 4D interacting protein (PDE4DIP) pseudogene	transcribed unprocessed pseudogene	0.86	1.76	41.74	<0.0001	<0.0001
ENSG00000112053	SLC26A8	solute carrier family 26 member 8 [Source:HGNC Symbol;Acc:HGNC:14468]	protein coding	0.86	4.74	44.19	<0.0001	<0.0001
ENSG00000171467	ZNF318	zinc finger protein 318 [Source:HGNC Symbol;Acc:HGNC:13578]	protein coding	-0.85	3.46	43.42	<0.0001	<0.0001
ENSG00000286656		novel transcript	lncRNA	0.85	2.02	17.42	<0.0001	0.0001
ENSG00000243650	RN7SL834P	RNA, 7SL, cytoplasmic 834, pseudogene [Source:HGNC Symbol;Acc:HGNC:46850]	misc RNA	0.85	1.39	25.38	<0.0001	<0.0001
ENSG00000099899	TRMT2A	tRNA methyltransferase 2 homolog A [Source:HGNC Symbol;Acc:HGNC:24974]	protein coding	-0.85	2.08	28.26	<0.0001	<0.0001
ENSG00000133030	MPRIP	myosin phosphatase Rho interacting protein [Source:HGNC Symbol;Acc:HGNC:30321]	protein coding	-0.85	4.70	42.98	<0.0001	<0.0001
ENSG00000135299	ANKRD6	ankyrin repeat domain 6 [Source:HGNC Symbol;Acc:HGNC:17280]	protein coding	-0.85	2.97	20.62	<0.0001	<0.0001
ENSG00000258831		novel transcript	lncRNA	-0.85	0.76	9.86	0.0017	0.0048
ENSG00000254554		novel transcript	lncRNA	0.85	0.89	28.91	<0.0001	<0.0001
ENSG00000171115	GIMAP8	GTPase, IMAP family member 8 [Source:HGNC Symbol;Acc:HGNC:21792]	protein coding	0.85	4.30	17.27	<0.0001	0.0001
ENSG00000279283		novel transcript	TEC	-0.85	2.07	18.48	<0.0001	<0.0001

Table S18: Genes differentially expressed at Day 30 compared to pre-second vaccination (Study Arm 2 A/H7N9+AS03, D1, 29, Neutrophils). Genes are sorted by descending absolute log_2 fold change. Gene model summaries and annotations are based on Ensembl Version 99 (continued.)

Ensembl Gene ID	Ensembl Gene Name	Ensembl Gene Description	Gene Type	log_2 Fold Change (Day 30 vs. Pre-Second Vaccination)	Average log_2 CPM	Likelihood Ratio Test Statistic	P-Value	FDR Adjusted P-Value
ENSG00000111261	MANSC1	MANSC domain containing 1 [Source:HGNC Symbol;Acc:HGNC:25505]	protein coding	-0.85	6.79	47.95	<0.0001	<0.0001
ENSG00000166165	CKB	creatine kinase B [Source:HGNC Symbol;Acc:HGNC:1991]	protein coding	-0.85	1.08	17.00	<0.0001	0.0002
ENSG00000129566	TEP1	telomerase associated protein 1 [Source:HGNC Symbol;Acc:HGNC:11726]	protein coding	0.85	6.69	48.51	<0.0001	<0.0001
ENSG00000006062	MAP3K14	mitogen-activated protein kinase kinase kinase 14 [Source:HGNC Symbol;Acc:HGNC:6853]	protein coding	-0.85	4.91	65.23	<0.0001	<0.0001
ENSG00000135912	TTLL4	tubulin tyrosine ligase like 4 [Source:HGNC Symbol;Acc:HGNC:28976]	protein coding	-0.85	5.29	45.26	<0.0001	<0.0001
ENSG00000072736	NFATC3	nuclear factor of activated T cells 3 [Source:HGNC Symbol;Acc:HGNC:7777]	protein coding	-0.85	4.94	53.87	<0.0001	<0.0001
ENSG00000155229	MMS19	MMS19 homolog, cytosolic iron-sulfur assembly component [Source:HGNC Symbol;Acc:HGNC:13824]	protein coding	-0.85	3.11	39.84	<0.0001	<0.0001
ENSG00000111640	GAPDH	glyceraldehyde-3-phosphate dehydrogenase [Source:HGNC Symbol;Acc:HGNC:4141]	protein coding	-0.85	8.40	29.03	<0.0001	<0.0001
ENSG00000108021	TASOR2	transcription activation suppressor family member 2 [Source:HGNC Symbol;Acc:HGNC:23484]	protein coding	0.85	3.80	36.57	<0.0001	<0.0001
ENSG00000185800	DMWD	DM1 locus, WD repeat containing [Source:HGNC Symbol;Acc:HGNC:2936]	protein coding	0.85	1.36	15.34	<0.0001	0.0004
ENSG00000264204	AGAP7P	ArfGAP with GTPase domain, ankyrin repeat and PH domain 7, pseudogene [Source:HGNC Symbol;Acc:HGNC:23465]	unprocessed pseudogene	-0.85	3.61	26.15	<0.0001	<0.0001
ENSG00000128973	CLN6	CLN6 transmembrane ER protein [Source:HGNC Symbol;Acc:HGNC:2077]	protein coding	-0.85	4.22	56.65	<0.0001	<0.0001
ENSG00000065613	SLK	STE20 like kinase [Source:HGNC Symbol;Acc:HGNC:11088]	protein coding	0.85	7.40	36.30	<0.0001	<0.0001
ENSG00000154305	MIA3	MIA SH3 domain ER export factor 3 [Source:HGNC Symbol;Acc:HGNC:24008]	protein coding	0.85	6.43	40.64	<0.0001	<0.0001
ENSG00000198873	GRK5	G protein-coupled receptor kinase 5 [Source:HGNC Symbol;Acc:HGNC:4544]	protein coding	-0.85	4.81	51.37	<0.0001	<0.0001
ENSG00000096968	JAK2	Janus kinase 2 [Source:HGNC Symbol;Acc:HGNC:6192]	protein coding	0.84	6.56	61.11	<0.0001	<0.0001
ENSG00000129038	LOXL1	lysyl oxidase like 1 [Source:HGNC Symbol;Acc:HGNC:6665]	protein coding	0.84	1.36	17.24	<0.0001	0.0001

Table S18: Genes differentially expressed at Day 30 compared to pre-second vaccination (Study Arm 2 A/H7N9+AS03, D1, 29, Neutrophils). Genes are sorted by descending absolute log_2 fold change. Gene model summaries and annotations are based on Ensembl Version 99 (continued.)

Ensembl Gene ID	Ensembl Gene Name	Ensembl Gene Description	Gene Type	log_2 Fold Change (Day 30 vs. Pre-Second Vaccination)	Average log_2 CPM	Likelihood Ratio Test Statistic	P-Value	FDR Adjusted P-Value
ENSG00000008256	CYTH3	cytohesin 3 [Source:HGNC Symbol;Acc:HGNC:9504]	protein coding	-0.84	2.65	34.09	<0.0001	<0.0001
ENSG00000175727	MLXIP	MLX interacting protein [Source:HGNC Symbol;Acc:HGNC:17055]	protein coding	-0.84	5.82	43.65	<0.0001	<0.0001
ENSG00000150995	ITPR1	inositol 1,4,5-trisphosphate receptor type 1 [Source:HGNC Symbol;Acc:HGNC:6180]	protein coding	-0.84	3.51	31.50	<0.0001	<0.0001
ENSG00000186265	BTLA	B and T lymphocyte associated [Source:HGNC Symbol;Acc:HGNC:21087]	protein coding	0.84	0.81	18.12	<0.0001	<0.0001
ENSG00000261801	LOXL1-AS1	LOXL1 antisense RNA 1 [Source:HGNC Symbol;Acc:HGNC:44169]	lncRNA	0.84	1.16	14.94	0.0001	0.0004
ENSG00000240666	MME-AS1	MME antisense RNA 1 [Source:HGNC Symbol;Acc:HGNC:40376]	lncRNA	-0.84	0.23	11.34	0.0008	0.0024
ENSG00000143771	CNIH4	cornichon family AMPA receptor auxiliary protein 4 [Source:HGNC Symbol;Acc:HGNC:25013]	protein coding	0.84	4.32	44.78	<0.0001	<0.0001
ENSG00000124177	CHD6	chromodomain helicase DNA binding protein 6 [Source:HGNC Symbol;Acc:HGNC:19057]	protein coding	-0.84	1.26	19.65	<0.0001	<0.0001
ENSG00000155849	ELMO1	engulfment and cell motility 1 [Source:HGNC Symbol;Acc:HGNC:16286]	protein coding	-0.84	6.19	51.33	<0.0001	<0.0001
ENSG00000250608		novel transcript	lncRNA	0.84	1.63	24.14	<0.0001	<0.0001
ENSG00000181045	SLC26A11	solute carrier family 26 member 11 [Source:HGNC Symbol;Acc:HGNC:14471]	protein coding	-0.84	1.55	20.23	<0.0001	<0.0001
ENSG00000103254	ANTKMT	adenine nucleotide translocase lysine methyltransferase [Source:HGNC Symbol;Acc:HGNC:14152]	protein coding	-0.84	0.84	20.55	<0.0001	<0.0001
ENSG00000138772	ANXA3	annexin A3 [Source:HGNC Symbol;Acc:HGNC:541]	protein coding	0.84	7.05	35.54	<0.0001	<0.0001
ENSG00000196549	MME	membrane metalloendopeptidase [Source:HGNC Symbol;Acc:HGNC:7154]	protein coding	-0.84	9.63	44.35	<0.0001	<0.0001
ENSG00000262152	LINC00514	long intergenic non-protein coding RNA 514 [Source:HGNC Symbol;Acc:HGNC:27549]	protein coding	-0.84	1.16	18.34	<0.0001	<0.0001
ENSG00000106714	CNTNAP3	contactin associated protein like 3 [Source:HGNC Symbol;Acc:HGNC:13834]	protein coding	-0.84	5.77	51.71	<0.0001	<0.0001
ENSG00000271913		novel transcript, antisense to TAGAP	lncRNA	0.84	4.50	24.66	<0.0001	<0.0001
ENSG00000125772	GPCPD1	glycerophosphocholine phosphodiesterase 1 [Source:HGNC Symbol;Acc:HGNC:26957]	protein coding	-0.84	7.96	87.34	<0.0001	<0.0001
ENSG00000285399		novel transcript	lncRNA	0.84	1.93	44.41	<0.0001	<0.0001

Table S18: Genes differentially expressed at Day 30 compared to pre-second vaccination (Study Arm 2 A/H7N9+AS03, D1, 29, Neutrophils). Genes are sorted by descending absolute log_2 fold change. Gene model summaries and annotations are based on Ensembl Version 99 (continued.)

Ensembl Gene ID	Ensembl Gene Name	Ensembl Gene Description	Gene Type	log_2 Fold Change (Day 30 vs. Pre-Second Vaccination)	Average log_2 CPM	Likelihood Ratio Test Statistic	P-Value	FDR Adjusted P-Value
ENSG00000140836	ZFHX3	zinc finger homeobox 3 [Source:HGNC Symbol;Acc:HGNC:777]	protein coding	-0.84	0.31	17.82	<0.0001	0.0001
ENSG00000173137	ADCK5	aarF domain containing kinase 5 [Source:HGNC Symbol;Acc:HGNC:21738]	protein coding	-0.84	0.10	14.22	0.0002	0.0006
ENSG00000196502	SULT1A1	sulfotransferase family 1A member 1 [Source:HGNC Symbol;Acc:HGNC:11453]	protein coding	-0.84	6.37	42.15	<0.0001	<0.0001
ENSG00000071564	TCF3	transcription factor 3 [Source:HGNC Symbol;Acc:HGNC:11633]	protein coding	-0.84	0.84	15.46	<0.0001	0.0003
ENSG00000141258	SGSM2	small G protein signaling modulator 2 [Source:HGNC Symbol;Acc:HGNC:29026]	protein coding	-0.84	4.86	43.94	<0.0001	<0.0001
ENSG00000237781	ADAMTSL4-AS2	ADAMTSL4 antisense RNA 2 [Source:HGNC Symbol;Acc:HGNC:40895]	lncRNA	0.83	4.51	27.72	<0.0001	<0.0001
ENSG00000231233	CFAP58-DT	CFAP58 divergent transcript [Source:HGNC Symbol;Acc:HGNC:45243]	lncRNA	0.83	4.37	38.93	<0.0001	<0.0001
ENSG00000184584	STING1	stimulator of interferon response cGAMP interactor 1 [Source:HGNC Symbol;Acc:HGNC:27962]	protein coding	-0.83	2.93	29.56	<0.0001	<0.0001
ENSG00000100218	RSPH14	radial spoke head 14 homolog [Source:HGNC Symbol;Acc:HGNC:13437]	protein coding	-0.83	0.53	20.72	<0.0001	<0.0001
ENSG00000220804	LINC01881	long intergenic non-protein coding RNA 1881 [Source:HGNC Symbol;Acc:HGNC:52700]	transcribed unprocessed pseudogene	-0.83	1.05	21.15	<0.0001	<0.0001
ENSG00000214922	HLA-F-AS1	HLA-F antisense RNA 1 [Source:HGNC Symbol;Acc:HGNC:26645]	lncRNA	0.83	1.04	24.40	<0.0001	<0.0001
ENSG00000182873	PRKCZ-AS1	PRKCZ antisense RNA 1 [Source:HGNC Symbol;Acc:HGNC:40477]	lncRNA	-0.83	2.25	27.61	<0.0001	<0.0001
ENSG00000107554	DNMBP	dynamin binding protein [Source:HGNC Symbol;Acc:HGNC:30373]	protein coding	-0.83	2.40	20.39	<0.0001	<0.0001
ENSG00000285730		novel transcript	lncRNA	-0.83	1.02	23.77	<0.0001	<0.0001
ENSG00000100221	JOSD1	Josephin domain containing 1 [Source:HGNC Symbol;Acc:HGNC:28953]	protein coding	-0.83	4.59	25.97	<0.0001	<0.0001
ENSG00000167702	KIFC2	kinesin family member C2 [Source:HGNC Symbol;Acc:HGNC:29530]	protein coding	-0.83	0.86	12.07	0.0005	0.0017
ENSG00000113368	LMNB1	lamin B1 [Source:HGNC Symbol;Acc:HGNC:6637]	protein coding	0.83	7.24	40.83	<0.0001	<0.0001

Table S18: Genes differentially expressed at Day 30 compared to pre-second vaccination (Study Arm 2 A/H7N9+AS03, D1, 29, Neutrophils). Genes are sorted by descending absolute log_2 fold change. Gene model summaries and annotations are based on Ensembl Version 99 (continued.)

Ensembl Gene ID	Ensembl Gene Name	Ensembl Gene Description	Gene Type	log_2 Fold Change (Day 30 vs. Pre-Second Vaccination)	Average log_2 CPM	Likelihood Ratio Test Statistic	P-Value	FDR Adjusted P-Value
ENSG00000214106	PAXIP1-AS2	PAXIP1 antisense RNA 2 [Source:HGNC Symbol;Acc:HGNC:48958]	lncRNA	0.83	1.89	35.25	<0.0001	<0.0001
ENSG00000100906	NFKBIA	NFKB inhibitor alpha [Source:HGNC Symbol;Acc:HGNC:7797]	protein coding	0.83	8.80	16.98	<0.0001	0.0002
ENSG00000203710	CR1	complement C3b/C4b receptor 1 (Knops blood group) [Source:HGNC Symbol;Acc:HGNC:2334]	protein coding	-0.83	7.97	32.77	<0.0001	<0.0001
ENSG00000197061	H4C3	H4 clustered histone 3 [Source:HGNC Symbol;Acc:HGNC:4787]	protein coding	-0.83	2.49	11.05	0.0009	0.0027
ENSG00000161048	NAPEPLD	N-acyl phosphatidylethanolamine phospholipase D [Source:HGNC Symbol;Acc:HGNC:21683]	protein coding	-0.83	0.06	16.02	<0.0001	0.0003
ENSG00000272325	NUDT3	nudix hydrolase 3 [Source:HGNC Symbol;Acc:HGNC:8050]	protein coding	-0.83	3.83	28.03	<0.0001	<0.0001
ENSG00000152082	MZT2B	mitotic spindle organizing protein 2B [Source:HGNC Symbol;Acc:HGNC:25886]	protein coding	-0.83	1.68	11.27	0.0008	0.0025
ENSG00000100889	PCK2	phosphoenolpyruvate carboxykinase 2, mitochondrial [Source:HGNC Symbol;Acc:HGNC:8725]	protein coding	0.83	1.79	24.10	<0.0001	<0.0001
ENSG00000006555	TTC22	tetratricopeptide repeat domain 22 [Source:HGNC Symbol;Acc:HGNC:26067]	protein coding	-0.83	2.58	20.31	<0.0001	<0.0001
ENSG00000273272		novel transcript	lncRNA	0.83	3.54	9.08	0.0026	0.007
ENSG00000250510	GPR162	G protein-coupled receptor 162 [Source:HGNC Symbol;Acc:HGNC:16693]	protein coding	-0.83	5.69	13.82	0.0002	0.0007
ENSG00000185104	FAF1	Fas associated factor 1 [Source:HGNC Symbol;Acc:HGNC:3578]	protein coding	-0.83	1.47	17.32	<0.0001	0.0001
ENSG00000102910	LONP2	lon peptidase 2, peroxisomal [Source:HGNC Symbol;Acc:HGNC:20598]	protein coding	-0.83	3.56	35.21	<0.0001	<0.0001
ENSG00000186088	GSAP	gamma-secretase activating protein [Source:HGNC Symbol;Acc:HGNC:28042]	protein coding	0.83	3.83	36.61	<0.0001	<0.0001
ENSG00000196396	PTPN1	protein tyrosine phosphatase non-receptor type 1 [Source:HGNC Symbol;Acc:HGNC:9642]	protein coding	0.83	6.09	39.34	<0.0001	<0.0001
ENSG00000135966	TGFBRAP1	transforming growth factor beta receptor associated protein 1 [Source:HGNC Symbol;Acc:HGNC:16836]	protein coding	-0.83	2.44	19.24	<0.0001	<0.0001
ENSG00000181826	RELL1	RELT like 1 [Source:HGNC Symbol;Acc:HGNC:27379]	protein coding	-0.83	5.96	37.68	<0.0001	<0.0001
ENSG00000164430	CGAS	cyclic GMP-AMP synthase [Source:HGNC Symbol;Acc:HGNC:21367]	protein coding	0.83	3.27	23.96	<0.0001	<0.0001

Table S18: Genes differentially expressed at Day 30 compared to pre-second vaccination (Study Arm 2 A/H7N9+AS03, D1, 29, Neutrophils). Genes are sorted by descending absolute log_2 fold change. Gene model summaries and annotations are based on Ensembl Version 99 (continued.)

Ensembl Gene ID	Ensembl Gene Name	Ensembl Gene Description	Gene Type	log_2 Fold Change (Day 30 vs. Pre-Second Vaccination)	Average log_2 CPM	Likelihood Ratio Test Statistic	P-Value	FDR Adjusted P-Value
ENSG00000159593	NAE1	NEDD8 activating enzyme E1 subunit 1 [Source:HGNC Symbol;Acc:HGNC:621]	protein coding	-0.83	1.07	31.49	<0.0001	<0.0001
ENSG00000287091		novel transcript, sense intronic to CD302and LY75-CD302	lncRNA	-0.83	2.49	26.16	<0.0001	<0.0001
ENSG00000067225	PKM	pyruvate kinase M1/2 [Source:HGNC Symbol;Acc:HGNC:9021]	protein coding	-0.83	6.24	18.93	<0.0001	<0.0001
ENSG00000101445	PPP1R16B	protein phosphatase 1 regulatory subunit 16B [Source:HGNC Symbol;Acc:HGNC:15850]	protein coding	-0.83	1.09	6.26	0.0123	0.0274
ENSG00000161040	FBXL13	F-box and leucine rich repeat protein 13 [Source:HGNC Symbol;Acc:HGNC:21658]	protein coding	-0.83	3.71	54.72	<0.0001	<0.0001
ENSG00000197043	ANXA6	annexin A6 [Source:HGNC Symbol;Acc:HGNC:544]	protein coding	-0.83	3.31	13.66	0.0002	0.0008
ENSG00000135315	CEP162	centrosomal protein 162 [Source:HGNC Symbol;Acc:HGNC:21107]	protein coding	0.83	3.15	38.43	<0.0001	<0.0001
ENSG00000136603	SKIL	SKI like proto-oncogene [Source:HGNC Symbol;Acc:HGNC:10897]	protein coding	0.83	5.13	41.07	<0.0001	<0.0001
ENSG00000256249		novel transcript	lncRNA	0.83	1.84	22.74	<0.0001	<0.0001
ENSG00000095321	CRAT	carnitine O-acetyltransferase [Source:HGNC Symbol;Acc:HGNC:2342]	protein coding	-0.83	0.89	18.57	<0.0001	<0.0001
ENSG00000113407	TARS1	threonyl-tRNA synthetase 1 [Source:HGNC Symbol;Acc:HGNC:11572]	protein coding	0.82	1.77	19.64	<0.0001	<0.0001
ENSG00000146540	C7orf50	chromosome 7 open reading frame 50 [Source:HGNC Symbol;Acc:HGNC:22421]	protein coding	-0.82	2.83	10.32	0.0013	0.0039
ENSG00000169727	GPS1	G protein pathway suppressor 1 [Source:HGNC Symbol;Acc:HGNC:4549]	protein coding	-0.82	3.58	30.85	<0.0001	<0.0001
ENSG00000162980	ARL5A	ADP ribosylation factor like GTPase 5A [Source:HGNC Symbol;Acc:HGNC:696]	protein coding	-0.82	2.54	26.27	<0.0001	<0.0001
ENSG00000165714	BORCS5	BLOC-1 related complex subunit 5 [Source:HGNC Symbol;Acc:HGNC:17950]	protein coding	-0.82	1.96	32.23	<0.0001	<0.0001
ENSG00000253626	EIF5AL1	eukaryotic translation initiation factor 5A like 1 [Source:HGNC Symbol;Acc:HGNC:17419]	protein coding	0.82	0.94	20.56	<0.0001	<0.0001
ENSG00000102901	CENPT	centromere protein T [Source:HGNC Symbol;Acc:HGNC:25787]	protein coding	-0.82	3.40	37.69	<0.0001	<0.0001
ENSG00000182541	LIMK2	LIM domain kinase 2 [Source:HGNC Symbol;Acc:HGNC:6614]	protein coding	0.82	9.18	40.77	<0.0001	<0.0001
ENSG00000148450	MSRB2	methionine sulfoxide reductase B2 [Source:HGNC Symbol;Acc:HGNC:17061]	protein coding	0.82	5.28	26.24	<0.0001	<0.0001

Confidential/Proprietary Information

Table S18: Genes differentially expressed at Day 30 compared to pre-second vaccination (Study Arm 2 A/H7N9+AS03, D1, 29, Neutrophils). Genes are sorted by descending absolute log_2 fold change. Gene model summaries and annotations are based on Ensembl Version 99 (continued.)

Ensembl Gene ID	Ensembl Gene Name	Ensembl Gene Description	Gene Type	log_2 Fold Change (Day 30 vs. Pre-Second Vaccination)	Average log_2 CPM	Likelihood Ratio Test Statistic	P-Value	FDR Adjusted P-Value
ENSG00000124201	ZNFX1	zinc finger NFX1-type containing 1 [Source:HGNC Symbol;Acc:HGNC:29271]	protein coding	0.82	8.36	43.08	<0.0001	<0.0001
ENSG00000072954	TMEM38A	transmembrane protein 38A [Source:HGNC Symbol;Acc:HGNC:28462]	protein coding	-0.82	1.03	20.00	<0.0001	<0.0001
ENSG00000099840	IZUMO4	IZUMO family member 4 [Source:HGNC Symbol;Acc:HGNC:26950]	protein coding	-0.82	2.28	38.53	<0.0001	<0.0001
ENSG00000198900	TOP1	DNA topoisomerase I [Source:HGNC Symbol;Acc:HGNC:11986]	protein coding	0.82	7.00	28.22	<0.0001	<0.0001
ENSG00000169871	TRIM56	tripartite motif containing 56 [Source:HGNC Symbol;Acc:HGNC:19028]	protein coding	0.82	7.31	50.65	<0.0001	<0.0001
ENSG00000164048	ZNF589	zinc finger protein 589 [Source:HGNC Symbol;Acc:HGNC:16747]	protein coding	-0.82	0.80	15.36	<0.0001	0.0003
ENSG00000236562		interferon induced transmembrane protein 3 (1-8U) (IFITM3) pseudogene	processed pseudogene	0.82	0.72	6.54	0.0106	0.0239
ENSG00000158406	H4C8	H4 clustered histone 8 [Source:HGNC Symbol;Acc:HGNC:4788]	protein coding	0.82	4.01	28.58	<0.0001	<0.0001
ENSG00000198894	CIPC	CLOCK interacting pacemaker [Source:HGNC Symbol;Acc:HGNC:20365]	protein coding	-0.82	3.24	48.82	<0.0001	<0.0001
ENSG00000042088	TDP1	tyrosyl-DNA phosphodiesterase 1 [Source:HGNC Symbol;Acc:HGNC:18884]	protein coding	-0.82	0.87	11.00	0.0009	0.0028
ENSG00000149084	HSD17B12	hydroxysteroid 17-beta dehydrogenase 12 [Source:HGNC Symbol;Acc:HGNC:18646]	protein coding	-0.82	3.07	25.61	<0.0001	<0.0001
ENSG00000073008	PVR	PVR cell adhesion molecule [Source:HGNC Symbol;Acc:HGNC:9705]	protein coding	-0.82	2.70	14.37	0.0001	0.0006
ENSG00000068028	RASSF1	Ras association domain family member 1 [Source:HGNC Symbol;Acc:HGNC:9882]	protein coding	-0.82	5.33	42.20	<0.0001	<0.0001
ENSG00000274070	CASTOR2	cytosolic arginine sensor for mTORC1 subunit 2 [Source:HGNC Symbol;Acc:HGNC:37073]	protein coding	-0.81	4.40	34.34	<0.0001	<0.0001
ENSG00000096070	BRPF3	bromodomain and PHD finger containing 3 [Source:HGNC Symbol;Acc:HGNC:14256]	protein coding	-0.81	5.72	50.42	<0.0001	<0.0001
ENSG00000235106	BRD3OS	BRD3 opposite strand [Source:HGNC Symbol;Acc:HGNC:24742]	protein coding	-0.81	1.04	26.68	<0.0001	<0.0001
ENSG00000168404	MLKL	mixed lineage kinase domain like pseudokinase [Source:HGNC Symbol;Acc:HGNC:26617]	protein coding	0.81	7.13	51.47	<0.0001	<0.0001

Table S18: Genes differentially expressed at Day 30 compared to pre-second vaccination (Study Arm 2 A/H7N9+AS03, D1, 29, Neutrophils). Genes are sorted by descending absolute log_2 fold change. Gene model summaries and annotations are based on Ensembl Version 99 (continued.)

Ensembl Gene ID	Ensembl Gene Name	Ensembl Gene Description	Gene Type	log_2 Fold Change (Day 30 vs. Pre-Second Vaccination)	Average log_2 CPM	Likelihood Ratio Test Statistic	P-Value	FDR Adjusted P-Value
ENSG00000139679	LPAR6	lysophosphatidic acid receptor 6 [Source:HGNC Symbol;Acc:HGNC:15520]	protein coding	0.81	2.70	22.95	<0.0001	<0.0001
ENSG00000118971	CCND2	cyclin D2 [Source:HGNC Symbol;Acc:HGNC:1583]	protein coding	-0.81	3.46	28.75	<0.0001	<0.0001
ENSG00000140280	LYSMD2	LysM domain containing 2 [Source:HGNC Symbol;Acc:HGNC:28571]	protein coding	0.81	6.79	32.33	<0.0001	<0.0001
ENSG00000133048	CHI3L1	chitinase 3 like 1 [Source:HGNC Symbol;Acc:HGNC:1932]	protein coding	-0.81	8.86	16.43	<0.0001	0.0002
ENSG00000184588	PDE4B	phosphodiesterase 4B [Source:HGNC Symbol;Acc:HGNC:8781]	protein coding	0.81	7.85	38.82	<0.0001	<0.0001
ENSG00000116237	ICMT	isoprenylcysteine carboxyl methyltransferase [Source:HGNC Symbol;Acc:HGNC:5350]	protein coding	-0.81	0.78	15.22	<0.0001	0.0004
ENSG00000105948	TTC26	tetratricopeptide repeat domain 26 [Source:HGNC Symbol;Acc:HGNC:21882]	protein coding	0.81	3.10	12.62	0.0004	0.0013
ENSG00000180329	CCDC43	coiled-coil domain containing 43 [Source:HGNC Symbol;Acc:HGNC:26472]	protein coding	-0.81	0.72	14.33	0.0002	0.0006
ENSG00000197555	SIPA1L1	signal induced proliferation associated 1 like 1 [Source:HGNC Symbol;Acc:HGNC:20284]	protein coding	0.81	7.50	38.79	<0.0001	<0.0001
ENSG00000148334	PTGES2	prostaglandin E synthase 2 [Source:HGNC Symbol;Acc:HGNC:17822]	protein coding	-0.81	0.91	14.02	0.0002	0.0007
ENSG00000177112	MRVI1-AS1	MRVI1 antisense RNA 1 [Source:HGNC Symbol;Acc:HGNC:43434]	lncRNA	-0.81	1.80	23.75	<0.0001	<0.0001
ENSG00000272914		novel transcript, sense intronic to ZNF438	lncRNA	0.81	1.66	34.84	<0.0001	<0.0001
ENSG00000161091	MFSD12	major facilitator superfamily domain containing 12 [Source:HGNC Symbol;Acc:HGNC:28299]	protein coding	0.81	4.31	56.95	<0.0001	<0.0001
ENSG00000198089	SFI1	SFI1 centrin binding protein [Source:HGNC Symbol;Acc:HGNC:29064]	protein coding	-0.81	5.57	33.51	<0.0001	<0.0001
ENSG00000128000	ZNF780B	zinc finger protein 780B [Source:HGNC Symbol;Acc:HGNC:33109]	protein coding	-0.81	0.53	9.23	0.0024	0.0065
ENSG00000124549	BTN2A3P	butyrophilin subfamily 2 member A3, pseudogene [Source:HGNC Symbol;Acc:HGNC:13229]	transcribed unprocessed pseudogene	0.81	1.73	22.40	<0.0001	<0.0001
ENSG00000133574	GIMAP4	GTPase, IMAP family member 4 [Source:HGNC Symbol;Acc:HGNC:21872]	protein coding	0.81	8.59	30.84	<0.0001	<0.0001

Table S18: Genes differentially expressed at Day 30 compared to pre-second vaccination (Study Arm 2 A/H7N9+AS03, D1, 29, Neutrophils). Genes are sorted by descending absolute log_2 fold change. Gene model summaries and annotations are based on Ensembl Version 99 (continued.)

Ensembl Gene ID	Ensembl Gene Name	Ensembl Gene Description	Gene Type	log_2 Fold Change (Day 30 vs. Pre-Second Vaccination)	Average log_2 CPM	Likelihood Ratio Test Statistic	P-Value	FDR Adjusted P-Value
ENSG00000054793	ATP9A	ATPase phospholipid transporting 9A (putative) [Source:HGNC Symbol;Acc:HGNC:13540]	protein coding	-0.81	0.97	11.54	0.0007	0.0022
ENSG00000167397	VKORC1	vitamin K epoxide reductase complex subunit 1 [Source:HGNC Symbol;Acc:HGNC:23663]	protein coding	-0.81	1.24	24.84	<0.0001	<0.0001
ENSG00000185504	FAAP100	FA core complex associated protein 100 [Source:HGNC Symbol;Acc:HGNC:26171]	protein coding	-0.81	2.94	22.46	<0.0001	<0.0001
ENSG00000126262	FFAR2	free fatty acid receptor 2 [Source:HGNC Symbol;Acc:HGNC:4501]	protein coding	0.81	10.01	42.67	<0.0001	<0.0001
ENSG00000122642	FKBP9	FKBP prolyl isomerase 9 [Source:HGNC Symbol;Acc:HGNC:3725]	protein coding	-0.81	2.37	35.00	<0.0001	<0.0001
ENSG00000259070	LINC00639	long intergenic non-protein coding RNA 639 [Source:HGNC Symbol;Acc:HGNC:27502]	lncRNA	-0.81	2.03	32.83	<0.0001	<0.0001
ENSG00000186470	BTN3A2	butyrophilin subfamily 3 member A2 [Source:HGNC Symbol;Acc:HGNC:1139]	protein coding	0.80	7.45	41.72	<0.0001	<0.0001
ENSG00000108773	KAT2A	lysine acetyltransferase 2A [Source:HGNC Symbol;Acc:HGNC:4201]	protein coding	0.80	1.67	29.80	<0.0001	<0.0001
ENSG00000186567	CEACAM19	CEA cell adhesion molecule 19 [Source:HGNC Symbol;Acc:HGNC:31951]	protein coding	-0.80	0.78	9.10	0.0026	0.0069
ENSG00000173065	FAM222B	family with sequence similarity 222 member B [Source:HGNC Symbol;Acc:HGNC:25563]	protein coding	0.80	3.11	26.79	<0.0001	<0.0001
ENSG00000134256	CD101	CD101 molecule [Source:HGNC Symbol;Acc:HGNC:5949]	protein coding	-0.80	2.15	6.92	0.0085	0.0198
ENSG00000278330		novel transcript, antisense to MBP	lncRNA	-0.80	1.95	11.40	0.0007	0.0023
ENSG00000261644		novel transcript, antisense to CYLD and NOD2	lncRNA	0.80	3.53	36.13	<0.0001	<0.0001
ENSG00000255398	HCAR3	hydroxycarboxylic acid receptor 3 [Source:HGNC Symbol;Acc:HGNC:16824]	protein coding	0.80	6.99	25.32	<0.0001	<0.0001
ENSG00000204103	MAFB	MAF bZIP transcription factor B [Source:HGNC Symbol;Acc:HGNC:6408]	protein coding	0.80	3.67	19.24	<0.0001	<0.0001
ENSG00000251259		novel transcript	lncRNA	-0.80	2.22	21.21	<0.0001	<0.0001
ENSG00000258875		novel transcript, antisense to GPR68	lncRNA	0.80	2.70	26.51	<0.0001	<0.0001
ENSG00000230539	AOAH-IT1	AOAH intronic transcript 1 [Source:HGNC Symbol;Acc:HGNC:41307]	lncRNA	0.80	1.47	26.12	<0.0001	<0.0001
ENSG00000268240		novel transcript	lncRNA	-0.80	1.31	19.25	<0.0001	<0.0001
ENSG00000226648	PLCG1-AS1	PLCG1 antisense RNA 1 [Source:HGNC Symbol;Acc:HGNC:40450]	lncRNA	0.80	0.57	16.03	<0.0001	0.0003

Table S18: Genes differentially expressed at Day 30 compared to pre-second vaccination (Study Arm 2 A/H7N9+AS03, D1, 29, Neutrophils). Genes are sorted by descending absolute log_2 fold change. Gene model summaries and annotations are based on Ensembl Version 99 (continued.)

Ensembl Gene ID	Ensembl Gene Name	Ensembl Gene Description	Gene Type	log_2 Fold Change (Day 30 vs. Pre-Second Vaccination)	Average log_2 CPM	Likelihood Ratio Test Statistic	P-Value	FDR Adjusted P-Value
ENSG00000154359	LONRF1	LON peptidase N-terminal domain and ring finger 1 [Source:HGNC Symbol;Acc:HGNC:26302]	protein coding	0.80	5.14	40.43	<0.0001	<0.0001
ENSG00000146859	TMEM140	transmembrane protein 140 [Source:HGNC Symbol;Acc:HGNC:21870]	protein coding	0.80	7.77	20.70	<0.0001	<0.0001
ENSG00000254396		novel transcript	lncRNA	-0.80	1.04	6.73	0.0095	0.0218
ENSG00000273033	LINC02035	long intergenic non-protein coding RNA 2035 [Source:HGNC Symbol;Acc:HGNC:52875]	lncRNA	-0.80	3.88	27.98	<0.0001	<0.0001
ENSG00000115318	LOXL3	lysyl oxidase like 3 [Source:HGNC Symbol;Acc:HGNC:13869]	protein coding	0.80	3.10	47.60	<0.0001	<0.0001
ENSG00000177700	POLR2L	RNA polymerase II subunit L [Source:HGNC Symbol;Acc:HGNC:9199]	protein coding	-0.80	2.72	28.01	<0.0001	<0.0001
ENSG00000165650	PDZD8	PDZ domain containing 8 [Source:HGNC Symbol;Acc:HGNC:26974]	protein coding	-0.80	6.37	31.87	<0.0001	<0.0001
ENSG00000033867	SLC4A7	solute carrier family 4 member 7 [Source:HGNC Symbol;Acc:HGNC:11033]	protein coding	-0.80	0.69	19.02	<0.0001	<0.0001
ENSG00000280063		tec	TEC	-0.80	1.74	18.54	<0.0001	<0.0001
ENSG00000073169	SELENOO	selenoprotein O [Source:HGNC Symbol;Acc:HGNC:30395]	protein coding	-0.80	5.32	51.63	<0.0001	<0.0001
ENSG00000106665	CLIP2	CAP-Gly domain containing linker protein 2 [Source:HGNC Symbol;Acc:HGNC:2586]	protein coding	-0.80	1.40	27.72	<0.0001	<0.0001
ENSG00000113108	APBB3	amyloid beta precursor protein binding family B member 3 [Source:HGNC Symbol;Acc:HGNC:20708]	protein coding	-0.80	4.96	44.06	<0.0001	<0.0001
ENSG00000133816	MICAL2	microtubule associated monooxygenase, calponin and LIM domain containing 2 [Source:HGNC Symbol;Acc:HGNC:24693]	protein coding	-0.80	6.99	33.22	<0.0001	<0.0001
ENSG00000131899	LLGL1	LLGL scribble cell polarity complex component 1 [Source:HGNC Symbol;Acc:HGNC:6628]	protein coding	-0.80	2.44	23.06	<0.0001	<0.0001
ENSG00000103091	WDR59	WD repeat domain 59 [Source:HGNC Symbol;Acc:HGNC:25706]	protein coding	-0.80	3.16	33.40	<0.0001	<0.0001
ENSG00000102897	LYRM1	LYR motif containing 1 [Source:HGNC Symbol;Acc:HGNC:25074]	protein coding	0.80	5.45	50.91	<0.0001	<0.0001
ENSG00000169105	CHST14	carbohydrate sulfotransferase 14 [Source:HGNC Symbol;Acc:HGNC:24464]	protein coding	-0.80	0.94	24.02	<0.0001	<0.0001
ENSG00000182372	CLN8	CLN8 transmembrane ER and ERGIC protein [Source:HGNC Symbol;Acc:HGNC:2079]	protein coding	-0.80	0.99	13.94	0.0002	0.0007

Table S18: Genes differentially expressed at Day 30 compared to pre-second vaccination (Study Arm 2 A/H7N9+AS03, D1, 29, Neutrophils). Genes are sorted by descending absolute log_2 fold change. Gene model summaries and annotations are based on Ensembl Version 99 (continued.)

Ensembl Gene ID	Ensembl Gene Name	Ensembl Gene Description	Gene Type	log_2 Fold Change (Day 30 vs. Pre-Second Vaccination)	Average log_2 CPM	Likelihood Ratio Test Statistic	P-Value	FDR Adjusted P-Value
ENSG00000079739	PGM1	phosphoglucomutase 1 [Source:HGNC Symbol;Acc:HGNC:8905]	protein coding	-0.79	4.76	27.23	<0.0001	<0.0001
ENSG00000286608		novel transcript	lncRNA	0.79	0.52	13.81	0.0002	0.0007
ENSG00000135205	CCDC146	coiled-coil domain containing 146 [Source:HGNC Symbol;Acc:HGNC:29296]	protein coding	0.79	4.80	50.80	<0.0001	<0.0001
ENSG00000175550	DRAP1	DR1 associated protein 1 [Source:HGNC Symbol;Acc:HGNC:3019]	protein coding	0.79	7.34	41.10	<0.0001	<0.0001
ENSG00000131471	AOC3	amine oxidase copper containing 3 [Source:HGNC Symbol;Acc:HGNC:550]	protein coding	-0.79	5.35	51.32	<0.0001	<0.0001
ENSG00000115419	GLS	glutaminase [Source:HGNC Symbol;Acc:HGNC:4331]	protein coding	0.79	5.35	32.77	<0.0001	<0.0001
ENSG00000155506	LARP1	La ribonucleoprotein 1, translational regulator [Source:HGNC Symbol;Acc:HGNC:29531]	protein coding	0.79	4.72	45.95	<0.0001	<0.0001
ENSG00000009413	REV3L	REV3 like, DNA directed polymerase zeta catalytic subunit [Source:HGNC Symbol;Acc:HGNC:9968]	protein coding	-0.79	4.26	41.35	<0.0001	<0.0001
ENSG00000086061	DNAJA1	DnaJ heat shock protein family (Hsp40) member A1 [Source:HGNC Symbol;Acc:HGNC:5229]	protein coding	0.79	7.68	32.44	<0.0001	<0.0001
ENSG00000109685	NSD2	nuclear receptor binding SET domain protein 2 [Source:HGNC Symbol;Acc:HGNC:12766]	protein coding	-0.79	1.94	19.93	<0.0001	<0.0001
ENSG00000100228	RAB36	RAB36, member RAS oncogene family [Source:HGNC Symbol;Acc:HGNC:9775]	protein coding	-0.79	3.31	29.67	<0.0001	<0.0001
ENSG00000285280		novel transcript	lncRNA	-0.79	1.58	24.38	<0.0001	<0.0001
ENSG00000166997	CNPY4	canopy FGF signaling regulator 4 [Source:HGNC Symbol;Acc:HGNC:28631]	protein coding	-0.79	0.89	16.10	<0.0001	0.0002
ENSG00000102531	FNDC3A	fibronectin type III domain containing 3A [Source:HGNC Symbol;Acc:HGNC:20296]	protein coding	0.79	4.88	47.52	<0.0001	<0.0001
ENSG00000165943	MOAP1	modulator of apoptosis 1 [Source:HGNC Symbol;Acc:HGNC:16658]	protein coding	-0.79	4.41	43.37	<0.0001	<0.0001
ENSG00000090470	PDCD7	programmed cell death 7 [Source:HGNC Symbol;Acc:HGNC:8767]	protein coding	-0.79	0.49	16.06	<0.0001	0.0002
ENSG00000144749	LRIG1	leucine rich repeats and immunoglobulin like domains 1 [Source:HGNC Symbol;Acc:HGNC:17360]	protein coding	-0.79	1.03	9.76	0.0018	0.0051
ENSG00000163517	HDAC11	histone deacetylase 11 [Source:HGNC Symbol;Acc:HGNC:19086]	protein coding	-0.79	0.38	13.73	0.0002	0.0008
ENSG00000151503	NCAPD3	non-SMC condensin II complex subunit D3 [Source:HGNC Symbol;Acc:HGNC:28952]	protein coding	-0.79	1.46	20.12	<0.0001	<0.0001

Table S18: Genes differentially expressed at Day 30 compared to pre-second vaccination (Study Arm 2 A/H7N9+AS03, D1, 29, Neutrophils). Genes are sorted by descending absolute log_2 fold change. Gene model summaries and annotations are based on Ensembl Version 99 (continued.)

Ensembl Gene ID	Ensembl Gene Name	Ensembl Gene Description	Gene Type	log_2 Fold Change (Day 30 vs. Pre-Second Vaccination)	Average log_2 CPM	Likelihood Ratio Test Statistic	P-Value	FDR Adjusted P-Value
ENSG00000164096	C4orf3	chromosome 4 open reading frame 3 [Source:HGNC Symbol;Acc:HGNC:19225]	protein coding	0.79	7.78	30.71	<0.0001	<0.0001
ENSG00000160305	DIP2A	disco interacting protein 2 homolog A [Source:HGNC Symbol;Acc:HGNC:17217]	protein coding	-0.79	5.64	41.39	<0.0001	<0.0001
ENSG00000176834	VSIG10	V-set and immunoglobulin domain containing 10 [Source:HGNC Symbol;Acc:HGNC:26078]	protein coding	0.79	2.64	14.48	0.0001	0.0005
ENSG00000113448	PDE4D	phosphodiesterase 4D [Source:HGNC Symbol;Acc:HGNC:8783]	protein coding	-0.79	1.44	7.93	0.0049	0.0122
ENSG00000123643	SLC36A1	solute carrier family 36 member 1 [Source:HGNC Symbol;Acc:HGNC:18761]	protein coding	-0.79	5.20	30.80	<0.0001	<0.0001
ENSG00000261884		novel transcript	protein coding	0.79	4.72	36.99	<0.0001	<0.0001
ENSG00000115232	ITGA4	integrin subunit alpha 4 [Source:HGNC Symbol;Acc:HGNC:6140]	protein coding	0.79	5.78	24.49	<0.0001	<0.0001
ENSG00000132294	EFR3A	EFR3 homolog A [Source:HGNC Symbol;Acc:HGNC:28970]	protein coding	0.79	4.86	40.06	<0.0001	<0.0001
ENSG00000269072		novel transcript, antisense to SIGLEC7 and SIGLEC17P	lncRNA	-0.79	0.23	20.35	<0.0001	<0.0001
ENSG00000196498	NCOR2	nuclear receptor corepressor 2 [Source:HGNC Symbol;Acc:HGNC:7673]	protein coding	-0.79	6.26	50.52	<0.0001	<0.0001
ENSG00000168118	RAB4A	RAB4A, member RAS oncogene family [Source:HGNC Symbol;Acc:HGNC:9781]	protein coding	-0.79	2.06	25.20	<0.0001	<0.0001
ENSG00000119681	LTBP2	latent transforming growth factor beta binding protein 2 [Source:HGNC Symbol;Acc:HGNC:6715]	protein coding	-0.79	3.00	37.73	<0.0001	<0.0001
ENSG00000136986	DERL1	derlin 1 [Source:HGNC Symbol;Acc:HGNC:28454]	protein coding	0.79	4.66	48.63	<0.0001	<0.0001
ENSG00000196642	RABL6	RAB, member RAS oncogene family like 6 [Source:HGNC Symbol;Acc:HGNC:24703]	protein coding	-0.78	4.81	54.86	<0.0001	<0.0001
ENSG00000151006	PRSS53	serine protease 53 [Source:HGNC Symbol;Acc:HGNC:34407]	protein coding	-0.78	0.98	23.04	<0.0001	<0.0001
ENSG00000151715	TMEM45B	transmembrane protein 45B [Source:HGNC Symbol;Acc:HGNC:25194]	protein coding	-0.78	4.26	49.82	<0.0001	<0.0001
ENSG00000106144	CASP2	caspase 2 [Source:HGNC Symbol;Acc:HGNC:1503]	protein coding	-0.78	5.20	28.11	<0.0001	<0.0001
ENSG00000263264		novel protein	protein coding	-0.78	3.74	40.53	<0.0001	<0.0001

Table S18: Genes differentially expressed at Day 30 compared to pre-second vaccination (Study Arm 2 A/H7N9+AS03, D1, 29, Neutrophils). Genes are sorted by descending absolute log_2 fold change. Gene model summaries and annotations are based on Ensembl Version 99 (continued.)

Ensembl Gene ID	Ensembl Gene Name	Ensembl Gene Description	Gene Type	log_2 Fold Change (Day 30 vs. Pre-Second Vaccination)	Average log_2 CPM	Likelihood Ratio Test Statistic	P-Value	FDR Adjusted P-Value
ENSG00000197747	S100A10	S100 calcium binding protein A10 [Source:HGNC Symbol;Acc:HGNC:10487]	protein coding	-0.78	1.15	13.47	0.0002	0.0009
ENSG00000226121		AT hook containing transcription factor 1 (AHCTF1) pseudogene	processed pseudogene	-0.78	3.94	45.75	<0.0001	<0.0001
ENSG00000266028	SRGAP2	SLIT-ROBO Rho GTPase activating protein 2 [Source:HGNC Symbol;Acc:HGNC:19751]	protein coding	0.78	5.55	37.48	<0.0001	<0.0001
ENSG00000282164	PEG13	paternally expressed 13 [Source:HGNC Symbol;Acc:HGNC:19969]	lncRNA	-0.78	0.65	10.95	0.0009	0.0029
ENSG00000171916	LGALS9C	galectin 9C [Source:HGNC Symbol;Acc:HGNC:33874]	protein coding	-0.78	2.93	21.82	<0.0001	<0.0001
ENSG00000111450	STX2	syntaxin 2 [Source:HGNC Symbol;Acc:HGNC:3403]	protein coding	-0.78	0.93	10.84	0.001	0.003
ENSG00000173272	MZT2A	mitotic spindle organizing protein 2A [Source:HGNC Symbol;Acc:HGNC:33187]	protein coding	-0.78	2.12	17.34	<0.0001	0.0001
ENSG00000077044	DGKD	diacylglycerol kinase delta [Source:HGNC Symbol;Acc:HGNC:2851]	protein coding	-0.78	6.25	21.06	<0.0001	<0.0001
ENSG00000143674	MAP3K21	mitogen-activated protein kinase kinase kinase 21 [Source:HGNC Symbol;Acc:HGNC:29798]	protein coding	-0.78	0.34	12.57	0.0004	0.0013
ENSG00000226137	BAIAP2-DT	BAIAP2 divergent transcript [Source:HGNC Symbol;Acc:HGNC:44342]	lncRNA	-0.78	3.84	41.64	<0.0001	<0.0001
ENSG00000159176	CSRP1	cysteine and glycine rich protein 1 [Source:HGNC Symbol;Acc:HGNC:2469]	protein coding	-0.78	2.77	9.15	0.0025	0.0067
ENSG00000125637	PSD4	pleckstrin and Sec7 domain containing 4 [Source:HGNC Symbol;Acc:HGNC:19096]	protein coding	-0.78	8.82	45.65	<0.0001	<0.0001
ENSG00000128311	TST	thiosulfate sulfurtransferase [Source:HGNC Symbol;Acc:HGNC:12388]	protein coding	-0.78	5.25	49.49	<0.0001	<0.0001
ENSG00000035403	VCL	vinculin [Source:HGNC Symbol;Acc:HGNC:12665]	protein coding	-0.78	6.58	30.53	<0.0001	<0.0001
ENSG00000131480	AOC2	amine oxidase copper containing 2 [Source:HGNC Symbol;Acc:HGNC:549]	protein coding	-0.78	5.55	50.08	<0.0001	<0.0001
ENSG00000223804		Poly [ADP-ribose] polymerase 8 (PARP8) pseudogene	transcribed unprocessed pseudogene	0.78	2.27	22.96	<0.0001	<0.0001

Table S18: Genes differentially expressed at Day 30 compared to pre-second vaccination (Study Arm 2 A/H7N9+AS03, D1, 29, Neutrophils). Genes are sorted by descending absolute log_2 fold change. Gene model summaries and annotations are based on Ensembl Version 99 (continued.)

Ensembl Gene ID	Ensembl Gene Name	Ensembl Gene Description	Gene Type	log_2 Fold Change (Day 30 vs. Pre-Second Vaccination)	Average log_2 CPM	Likelihood Ratio Test Statistic	P-Value	FDR Adjusted P-Value
ENSG00000164715	LMTK2	lemur tyrosine kinase 2 [Source:HGNC Symbol;Acc:HGNC:17880]	protein coding	0.78	6.67	43.25	<0.0001	<0.0001
ENSG00000014164	ZC3H3	zinc finger CCCH-type containing 3 [Source:HGNC Symbol;Acc:HGNC:28972]	protein coding	0.78	6.23	29.30	<0.0001	<0.0001
ENSG00000163913	IFT122	intraflagellar transport 122 [Source:HGNC Symbol;Acc:HGNC:13556]	protein coding	-0.78	3.06	33.05	<0.0001	<0.0001
ENSG00000053918	KCNQ1	potassium voltage-gated channel subfamily Q member 1 [Source:HGNC Symbol;Acc:HGNC:6294]	protein coding	-0.78	6.03	45.72	<0.0001	<0.0001
ENSG00000217930	PAM16	presequence translocase associated motor 16 [Source:HGNC Symbol;Acc:HGNC:29679]	protein coding	-0.78	2.41	22.66	<0.0001	<0.0001
ENSG00000130731	METTL26	methyltransferase like 26 [Source:HGNC Symbol;Acc:HGNC:14141]	protein coding	-0.78	1.19	12.11	0.0005	0.0016
ENSG00000165238	WNK2	WNK lysine deficient protein kinase 2 [Source:HGNC Symbol;Acc:HGNC:14542]	protein coding	-0.78	0.92	16.14	<0.0001	0.0002
ENSG00000137161	CNPY3	canopy FGF signaling regulator 3 [Source:HGNC Symbol;Acc:HGNC:11968]	protein coding	-0.78	8.79	49.40	<0.0001	<0.0001
ENSG00000186111	PIP5K1C	phosphatidylinositol-4-phosphate 5-kinase type 1 gamma [Source:HGNC Symbol;Acc:HGNC:8996]	protein coding	-0.78	5.06	55.70	<0.0001	<0.0001
ENSG00000197629	MPEG1	macrophage expressed 1 [Source:HGNC Symbol;Acc:HGNC:29619]	protein coding	-0.78	8.02	53.08	<0.0001	<0.0001
ENSG00000113300	CNOT6	CCR4-NOT transcription complex subunit 6 [Source:HGNC Symbol;Acc:HGNC:14099]	protein coding	-0.77	4.04	36.54	<0.0001	<0.0001
ENSG00000105767	CADM4	cell adhesion molecule 4 [Source:HGNC Symbol;Acc:HGNC:30825]	protein coding	0.77	3.86	35.78	<0.0001	<0.0001
ENSG00000250903	GMDS-DT	GMDS divergent transcript [Source:HGNC Symbol;Acc:HGNC:48993]	lncRNA	0.77	1.19	28.03	<0.0001	<0.0001
ENSG00000147872	PLIN2	perilipin 2 [Source:HGNC Symbol;Acc:HGNC:248]	protein coding	-0.77	1.91	8.21	0.0042	0.0106
ENSG00000276085	CCL3L3	C-C motif chemokine ligand 3 like 3 [Source:HGNC Symbol;Acc:HGNC:30554]	protein coding	0.77	4.44	10.76	0.001	0.0031
ENSG00000110852	CLEC2B	C-type lectin domain family 2 member B [Source:HGNC Symbol;Acc:HGNC:2053]	protein coding	0.77	7.93	33.95	<0.0001	<0.0001
ENSG00000076356	PLXNA2	plexin A2 [Source:HGNC Symbol;Acc:HGNC:9100]	protein coding	-0.77	4.08	58.71	<0.0001	<0.0001
ENSG00000269243		novel transcript, antisense RAB8A	lncRNA	0.77	1.12	19.11	<0.0001	<0.0001

Table S18: Genes differentially expressed at Day 30 compared to pre-second vaccination (Study Arm 2 A/H7N9+AS03, D1, 29, Neutrophils). Genes are sorted by descending absolute log_2 fold change. Gene model summaries and annotations are based on Ensembl Version 99 (continued.)

Ensembl Gene ID	Ensembl Gene Name	Ensembl Gene Description	Gene Type	log_2 Fold Change (Day 30 vs. Pre-Second Vaccination)	Average log_2 CPM	Likelihood Ratio Test Statistic	P-Value	FDR Adjusted P-Value
ENSG00000100225	FBXO7	F-box protein 7 [Source:HGNC Symbol;Acc:HGNC:13586]	protein coding	0.77	4.43	55.56	<0.0001	<0.0001
ENSG00000178096	BOLA1	bolA family member 1 [Source:HGNC Symbol;Acc:HGNC:24263]	protein coding	-0.77	1.07	7.18	0.0074	0.0174
ENSG00000180316	PNPLA1	patatin like phospholipase domain containing 1 [Source:HGNC Symbol;Acc:HGNC:21246]	protein coding	-0.77	2.85	12.14	0.0005	0.0016
ENSG00000125746	EML2	EMAP like 2 [Source:HGNC Symbol;Acc:HGNC:18035]	protein coding	0.77	3.23	36.41	<0.0001	<0.0001
ENSG00000165209	STRBP	spermatid perinuclear RNA binding protein [Source:HGNC Symbol;Acc:HGNC:16462]	protein coding	-0.77	0.48	16.13	<0.0001	0.0002
ENSG00000274602	PI4KAP1	phosphatidylinositol 4-kinase alpha pseudogene 1 [Source:HGNC Symbol;Acc:HGNC:33576]	transcribed unprocessed pseudogene	-0.77	1.24	9.36	0.0022	0.0061
ENSG00000100418	DESI1	desumoylating isopeptidase 1 [Source:HGNC Symbol;Acc:HGNC:24577]	protein coding	0.77	4.29	45.03	<0.0001	<0.0001
ENSG00000164054	SHISA5	shisa family member 5 [Source:HGNC Symbol;Acc:HGNC:30376]	protein coding	0.77	9.68	44.09	<0.0001	<0.0001
ENSG00000127511	SIN3B	SIN3 transcription regulator family member B [Source:HGNC Symbol;Acc:HGNC:19354]	protein coding	-0.77	6.19	29.16	<0.0001	<0.0001
ENSG00000164691	TAGAP	T cell activation RhoGTPase activating protein [Source:HGNC Symbol;Acc:HGNC:15669]	protein coding	0.77	9.50	18.49	<0.0001	<0.0001
ENSG00000280067		novel transcript	TEC	-0.77	0.59	12.16	0.0005	0.0016
ENSG00000130475	FCHO1	FCH and mu domain containing endocytic adaptor 1 [Source:HGNC Symbol;Acc:HGNC:29002]	protein coding	-0.77	7.18	57.71	<0.0001	<0.0001
ENSG00000138698	RAP1GDS1	Rap1 GTPase-GDP dissociation stimulator 1 [Source:HGNC Symbol;Acc:HGNC:9859]	protein coding	-0.77	0.71	12.52	0.0004	0.0013
ENSG00000186174	BCL9L	BCL9 like [Source:HGNC Symbol;Acc:HGNC:23688]	protein coding	0.77	2.76	18.53	<0.0001	<0.0001
ENSG00000165046	LETM2	leucine zipper and EF-hand containing transmembrane protein 2 [Source:HGNC Symbol;Acc:HGNC:14648]	protein coding	-0.76	3.67	32.09	<0.0001	<0.0001
ENSG00000159753	CARMIL2	capping protein regulator and myosin 1 linker 2 [Source:HGNC Symbol;Acc:HGNC:27089]	protein coding	-0.76	0.25	7.78	0.0053	0.0131
ENSG00000184575	XPOT	exportin for tRNA [Source:HGNC Symbol;Acc:HGNC:12826]	protein coding	-0.76	2.60	28.25	<0.0001	<0.0001

Table S18: Genes differentially expressed at Day 30 compared to pre-second vaccination (Study Arm 2 A/H7N9+AS03, D1, 29, Neutrophils). Genes are sorted by descending absolute log_2 fold change. Gene model summaries and annotations are based on Ensembl Version 99 (continued.)

Ensembl Gene ID	Ensembl Gene Name	Ensembl Gene Description	Gene Type	log_2 Fold Change (Day 30 vs. Pre-Second Vaccination)	Average log_2 CPM	Likelihood Ratio Test Statistic	P-Value	FDR Adjusted P-Value
ENSG00000185245	GP1BA	glycoprotein Ib platelet subunit alpha [Source:HGNC Symbol;Acc:HGNC:4439]	protein coding	-0.76	2.53	30.49	<0.0001	<0.0001
ENSG00000038274	MAT2B	methionine adenosyltransferase 2B [Source:HGNC Symbol;Acc:HGNC:6905]	protein coding	0.76	7.48	27.07	<0.0001	<0.0001
ENSG00000058673	ZC3H11A	zinc finger CCCH-type containing 11A [Source:HGNC Symbol;Acc:HGNC:29093]	protein coding	0.76	1.13	24.69	<0.0001	<0.0001
ENSG00000171159	C9orf16	chromosome 9 open reading frame 16 [Source:HGNC Symbol;Acc:HGNC:17823]	protein coding	-0.76	4.39	37.09	<0.0001	<0.0001
ENSG00000086200	IPO11	importin 11 [Source:HGNC Symbol;Acc:HGNC:20628]	protein coding	0.76	1.34	28.18	<0.0001	<0.0001
ENSG00000106290	TAF6	TATA-box binding protein associated factor 6 [Source:HGNC Symbol;Acc:HGNC:11540]	protein coding	-0.76	3.26	42.64	<0.0001	<0.0001
ENSG00000254470	AP5B1	adaptor related protein complex 5 subunit beta 1 [Source:HGNC Symbol;Acc:HGNC:25104]	protein coding	0.76	9.45	64.14	<0.0001	<0.0001
ENSG00000110801	PSMD9	proteasome 26S subunit, non-ATPase 9 [Source:HGNC Symbol;Acc:HGNC:9567]	protein coding	-0.76	2.95	27.27	<0.0001	<0.0001
ENSG00000067066	SP100	SP100 nuclear antigen [Source:HGNC Symbol;Acc:HGNC:11206]	protein coding	0.76	8.43	52.53	<0.0001	<0.0001
ENSG00000272462		novel transcript	lncRNA	0.76	2.06	26.64	<0.0001	<0.0001
ENSG00000140326	CDAN1	codanin 1 [Source:HGNC Symbol;Acc:HGNC:1713]	protein coding	-0.76	2.76	26.92	<0.0001	<0.0001
ENSG00000183784	DOCK8-AS1	DOCK8 antisense RNA 1 [Source:HGNC Symbol;Acc:HGNC:26436]	lncRNA	0.76	3.07	25.90	<0.0001	<0.0001
ENSG00000122417	ODF2L	outer dense fiber of sperm tails 2 like [Source:HGNC Symbol;Acc:HGNC:29225]	protein coding	-0.76	2.29	33.47	<0.0001	<0.0001
ENSG00000287978		novel transcript	lncRNA	-0.76	1.29	12.70	0.0004	0.0012
ENSG00000204681	GABBR1	gamma-aminobutyric acid type B receptor subunit 1 [Source:HGNC Symbol;Acc:HGNC:4070]	protein coding	0.76	2.94	27.15	<0.0001	<0.0001
ENSG00000271843		novel transcript	lncRNA	0.76	0.94	20.35	<0.0001	<0.0001
ENSG00000159214	CCDC24	coiled-coil domain containing 24 [Source:HGNC Symbol;Acc:HGNC:28688]	protein coding	-0.76	1.52	20.35	<0.0001	<0.0001
ENSG00000169228	RAB24	RAB24, member RAS oncogene family [Source:HGNC Symbol;Acc:HGNC:9765]	protein coding	0.76	6.98	58.97	<0.0001	<0.0001
ENSG00000149782	PLCB3	phospholipase C beta 3 [Source:HGNC Symbol;Acc:HGNC:9056]	protein coding	-0.76	3.01	23.44	<0.0001	<0.0001

Table S18: Genes differentially expressed at Day 30 compared to pre-second vaccination (Study Arm 2 A/H7N9+AS03, D1, 29, Neutrophils). Genes are sorted by descending absolute log_2 fold change. Gene model summaries and annotations are based on Ensembl Version 99 (continued.)

Ensembl Gene ID	Ensembl Gene Name	Ensembl Gene Description	Gene Type	log_2 Fold Change (Day 30 vs. Pre-Second Vaccination)	Average log_2 CPM	Likelihood Ratio Test Statistic	P-Value	FDR Adjusted P-Value
ENSG00000234436		leukocyte immunoglobulin-like receptor, subfamily A pseudogene	unprocessed pseudogene	0.76	0.93	14.13	0.0002	0.0006
ENSG00000196814	MVB12B	multivesicular body subunit 12B [Source:HGNC Symbol;Acc:HGNC:23368]	protein coding	-0.76	0.25	7.53	0.0061	0.0148
ENSG00000165113	GKAP1	G kinase anchoring protein 1 [Source:HGNC Symbol;Acc:HGNC:17496]	protein coding	-0.76	2.07	38.80	<0.0001	<0.0001
ENSG00000285646		novel transcript	lncRNA	0.76	1.29	13.07	0.0003	0.001
ENSG00000230869	AGAP10P	ArfGAP with GTPase domain, ankyrin repeat and PH domain 10, pseudogene [Source:HGNC Symbol;Acc:HGNC:23659]	unprocessed pseudogene	-0.76	0.59	11.20	0.0008	0.0025
ENSG00000226742	HSBP1L1	heat shock factor binding protein 1 like 1 [Source:HGNC Symbol;Acc:HGNC:37243]	protein coding	0.76	0.54	10.31	0.0013	0.0039
ENSG00000163634	THOC7	THO complex 7 [Source:HGNC Symbol;Acc:HGNC:29874]	protein coding	0.76	4.13	23.11	<0.0001	<0.0001
ENSG00000100612	DHRS7	dehydrogenase/reductase 7 [Source:HGNC Symbol;Acc:HGNC:21524]	protein coding	-0.76	7.55	44.03	<0.0001	<0.0001
ENSG00000165102	HGSNAT	heparan-alpha-glucosaminide N-acetyltransferase [Source:HGNC Symbol;Acc:HGNC:26527]	protein coding	-0.76	4.95	34.56	<0.0001	<0.0001
ENSG00000222009	BTBD19	BTB domain containing 19 [Source:HGNC Symbol;Acc:HGNC:27145]	protein coding	0.76	3.22	17.43	<0.0001	0.0001
ENSG00000154153	RETREG1	reticulophagy regulator 1 [Source:HGNC Symbol;Acc:HGNC:25964]	protein coding	-0.76	1.78	8.43	0.0037	0.0095
ENSG00000010539	ZNF200	zinc finger protein 200 [Source:HGNC Symbol;Acc:HGNC:12993]	protein coding	0.76	6.10	27.90	<0.0001	<0.0001
ENSG00000150403	TMCO3	transmembrane and coiled-coil domains 3 [Source:HGNC Symbol;Acc:HGNC:20329]	protein coding	-0.76	6.10	33.17	<0.0001	<0.0001
ENSG00000100028	SNRPD3	small nuclear ribonucleoprotein D3 polypeptide [Source:HGNC Symbol;Acc:HGNC:11160]	protein coding	-0.75	2.52	34.11	<0.0001	<0.0001
ENSG00000132600	PRMT7	protein arginine methyltransferase 7 [Source:HGNC Symbol;Acc:HGNC:25557]	protein coding	-0.75	1.31	13.43	0.0002	0.0009
ENSG00000233016	SNHG7	small nucleolar RNA host gene 7 [Source:HGNC Symbol;Acc:HGNC:28254]	lncRNA	-0.75	0.64	8.81	0.003	0.008
ENSG00000154146	NRGN	neurogranin [Source:HGNC Symbol;Acc:HGNC:8000]	protein coding	-0.75	1.88	34.13	<0.0001	<0.0001

Table S18: Genes differentially expressed at Day 30 compared to pre-second vaccination (Study Arm 2 A/H7N9+AS03, D1, 29, Neutrophils). Genes are sorted by descending absolute log_2 fold change. Gene model summaries and annotations are based on Ensembl Version 99 (continued.)

Ensembl Gene ID	Ensembl Gene Name	Ensembl Gene Description	Gene Type	log_2 Fold Change (Day 30 vs. Pre-Second Vaccination)	Average log_2 CPM	Likelihood Ratio Test Statistic	P-Value	FDR Adjusted P-Value
ENSG00000160710	ADAR	adenosine deaminase RNA specific [Source:HGNC Symbol;Acc:HGNC:225]	protein coding	0.75	10.25	48.42	<0.0001	<0.0001
ENSG00000236008	LINC01814	long intergenic non-protein coding RNA 1814 [Source:HGNC Symbol;Acc:HGNC:52618]	lncRNA	-0.75	1.11	20.81	<0.0001	<0.0001
ENSG00000068024	HDAC4	histone deacetylase 4 [Source:HGNC Symbol;Acc:HGNC:14063]	protein coding	0.75	6.21	41.82	<0.0001	<0.0001
ENSG00000279108		TEC	TEC	-0.75	0.29	15.89	<0.0001	0.0003
ENSG00000271646		novel transcript	lncRNA	0.75	3.42	26.75	<0.0001	<0.0001
ENSG00000013810	TACC3	transforming acidic coiled-coil containing protein 3 [Source:HGNC Symbol;Acc:HGNC:11524]	protein coding	-0.75	8.21	51.80	<0.0001	<0.0001
ENSG00000271605	MILR1	mast cell immunoglobulin like receptor 1 [Source:HGNC Symbol;Acc:HGNC:27570]	protein coding	0.75	3.30	23.39	<0.0001	<0.0001
ENSG00000077782	FGFR1	fibroblast growth factor receptor 1 [Source:HGNC Symbol;Acc:HGNC:3688]	protein coding	-0.75	0.89	12.55	0.0004	0.0013
ENSG00000132383	RPA1	replication protein A1 [Source:HGNC Symbol;Acc:HGNC:10289]	protein coding	-0.75	3.61	27.70	<0.0001	<0.0001
ENSG00000139641	ESYT1	extended synaptotagmin 1 [Source:HGNC Symbol;Acc:HGNC:29534]	protein coding	-0.75	4.19	9.55	0.002	0.0056
ENSG00000116005	PCYOX1	prenylcysteine oxidase 1 [Source:HGNC Symbol;Acc:HGNC:20588]	protein coding	-0.75	1.33	16.54	<0.0001	0.0002
ENSG00000222011	FAM185A	family with sequence similarity 185 member A [Source:HGNC Symbol;Acc:HGNC:22412]	protein coding	-0.75	0.81	26.04	<0.0001	<0.0001
ENSG00000100523	DDHD1	DDHD domain containing 1 [Source:HGNC Symbol;Acc:HGNC:19714]	protein coding	0.75	2.83	24.59	<0.0001	<0.0001
ENSG00000026950	BTN3A1	butyrophilin subfamily 3 member A1 [Source:HGNC Symbol;Acc:HGNC:1138]	protein coding	0.75	8.42	40.42	<0.0001	<0.0001
ENSG00000111275	ALDH2	aldehyde dehydrogenase 2 family member [Source:HGNC Symbol;Acc:HGNC:404]	protein coding	0.75	3.44	25.55	<0.0001	<0.0001
ENSG00000215845	TSTD1	thiosulfate sulfurtransferase like domain containing 1 [Source:HGNC Symbol;Acc:HGNC:35410]	protein coding	-0.75	4.35	45.32	<0.0001	<0.0001
ENSG00000136888	ATP6V1G1	ATPase H+ transporting V1 subunit G1 [Source:HGNC Symbol;Acc:HGNC:864]	protein coding	0.75	7.33	30.49	<0.0001	<0.0001
ENSG00000167081	PBX3	PBX homeobox 3 [Source:HGNC Symbol;Acc:HGNC:8634]	protein coding	-0.75	2.73	30.74	<0.0001	<0.0001
ENSG00000113658	SMAD5	SMAD family member 5 [Source:HGNC Symbol;Acc:HGNC:6771]	protein coding	-0.75	0.44	7.20	0.0073	0.0173

Table S18: Genes differentially expressed at Day 30 compared to pre-second vaccination (Study Arm 2 A/H7N9+AS03, D1, 29, Neutrophils). Genes are sorted by descending absolute log_2 fold change. Gene model summaries and annotations are based on Ensembl Version 99 (continued.)

Ensembl Gene ID	Ensembl Gene Name	Ensembl Gene Description	Gene Type	log_2 Fold Change (Day 30 vs. Pre-Second Vaccination)	Average log_2 CPM	Likelihood Ratio Test Statistic	P-Value	FDR Adjusted P-Value
ENSG00000072952	MRVI1	murine retrovirus integration site 1 homolog [Source:HGNC Symbol;Acc:HGNC:7237]	protein coding	-0.75	7.18	30.54	<0.0001	<0.0001
ENSG00000259704		novel transcript, sense overlapping 3' UTR of BLM	lncRNA	0.75	0.19	14.98	0.0001	0.0004
ENSG00000178502	KLHL11	kelch like family member 11 [Source:HGNC Symbol;Acc:HGNC:19008]	protein coding	-0.75	0.95	20.07	<0.0001	<0.0001
ENSG00000135473	PAN2	poly(A) specific ribonuclease subunit PAN2 [Source:HGNC Symbol;Acc:HGNC:20074]	protein coding	0.75	1.54	19.44	<0.0001	<0.0001
ENSG00000140848	CPNE2	copine 2 [Source:HGNC Symbol;Acc:HGNC:2315]	protein coding	-0.75	5.46	43.19	<0.0001	<0.0001
ENSG00000283994		novel transcript, sense intronic to ARHGAP15	lncRNA	-0.75	0.90	11.21	0.0008	0.0025
ENSG00000081377	CDC14B	cell division cycle 14B [Source:HGNC Symbol;Acc:HGNC:1719]	protein coding	-0.75	0.26	6.09	0.0136	0.0299
ENSG00000054983	GALC	galactosylceramidase [Source:HGNC Symbol;Acc:HGNC:4115]	protein coding	0.75	5.55	42.72	<0.0001	<0.0001
ENSG00000162882	HAAO	3-hydroxyanthranilate 3,4-dioxygenase [Source:HGNC Symbol;Acc:HGNC:4796]	protein coding	-0.75	1.93	20.98	<0.0001	<0.0001
ENSG00000231925	TAPBP	TAP binding protein [Source:HGNC Symbol;Acc:HGNC:11566]	protein coding	0.75	9.59	47.65	<0.0001	<0.0001
ENSG00000116260	QSOX1	quiescin sulfhydryl oxidase 1 [Source:HGNC Symbol;Acc:HGNC:9756]	protein coding	-0.75	6.12	37.62	<0.0001	<0.0001
ENSG00000113583	C5orf15	chromosome 5 open reading frame 15 [Source:HGNC Symbol;Acc:HGNC:20656]	protein coding	0.75	4.32	23.18	<0.0001	<0.0001
ENSG00000145687	SSBP2	single stranded DNA binding protein 2 [Source:HGNC Symbol;Acc:HGNC:15831]	protein coding	-0.75	4.37	25.65	<0.0001	<0.0001
ENSG00000175073	VCPIP1	valosin containing protein interacting protein 1 [Source:HGNC Symbol;Acc:HGNC:30897]	protein coding	0.75	7.36	38.54	<0.0001	<0.0001
ENSG00000125484	GTF3C4	general transcription factor IIIC subunit 4 [Source:HGNC Symbol;Acc:HGNC:4667]	protein coding	-0.75	1.35	23.50	<0.0001	<0.0001
ENSG00000136720	HS6ST1	heparan sulfate 6-O-sulfotransferase 1 [Source:HGNC Symbol;Acc:HGNC:5201]	protein coding	-0.74	2.74	37.61	<0.0001	<0.0001
ENSG00000174013	FBXO45	F-box protein 45 [Source:HGNC Symbol;Acc:HGNC:29148]	protein coding	-0.74	1.76	25.33	<0.0001	<0.0001
ENSG00000120539	MASTL	microtubule associated serine/threonine kinase like [Source:HGNC Symbol;Acc:HGNC:19042]	protein coding	0.74	2.49	21.99	<0.0001	<0.0001
ENSG00000160959	LRRC14	leucine rich repeat containing 14 [Source:HGNC Symbol;Acc:HGNC:20419]	protein coding	-0.74	2.18	23.90	<0.0001	<0.0001

Table S18: Genes differentially expressed at Day 30 compared to pre-second vaccination (Study Arm 2 A/H7N9+AS03, D1, 29, Neutrophils). Genes are sorted by descending absolute log_2 fold change. Gene model summaries and annotations are based on Ensembl Version 99 (continued.)

Ensembl Gene ID	Ensembl Gene Name	Ensembl Gene Description	Gene Type	log_2 Fold Change (Day 30 vs. Pre-Second Vaccination)	Average log_2 CPM	Likelihood Ratio Test Statistic	P-Value	FDR Adjusted P-Value
ENSG00000113621	TXNDC15	thioredoxin domain containing 15 [Source:HGNC Symbol;Acc:HGNC:20652]	protein coding	-0.74	2.51	26.55	<0.0001	<0.0001
ENSG00000153048	CARHSP1	calcium regulated heat stable protein 1 [Source:HGNC Symbol;Acc:HGNC:17150]	protein coding	-0.74	4.94	59.53	<0.0001	<0.0001
ENSG00000176659	C20orf197	chromosome 20 open reading frame 197 [Source:HGNC Symbol;Acc:HGNC:26601]	lncRNA	-0.74	2.12	11.05	0.0009	0.0027
ENSG00000005810	MYCBP2	MYC binding protein 2 [Source:HGNC Symbol;Acc:HGNC:23386]	protein coding	0.74	5.69	32.44	<0.0001	<0.0001
ENSG00000184787	UBE2G2	ubiquitin conjugating enzyme E2 G2 [Source:HGNC Symbol;Acc:HGNC:12483]	protein coding	-0.74	3.28	29.05	<0.0001	<0.0001
ENSG00000064601	CTSA	cathepsin A [Source:HGNC Symbol;Acc:HGNC:9251]	protein coding	0.74	7.08	36.17	<0.0001	<0.0001
ENSG00000185219	ZNF445	zinc finger protein 445 [Source:HGNC Symbol;Acc:HGNC:21018]	protein coding	-0.74	4.72	23.22	<0.0001	<0.0001
ENSG00000184990	SIVA1	SIVA1 apoptosis inducing factor [Source:HGNC Symbol;Acc:HGNC:17712]	protein coding	-0.74	4.62	34.05	<0.0001	<0.0001
ENSG00000276317		novel transcript, antisense to SLCO4A1	lncRNA	0.74	0.62	19.20	<0.0001	<0.0001
ENSG00000148218	ALAD	aminolevulinate dehydratase [Source:HGNC Symbol;Acc:HGNC:395]	protein coding	-0.74	3.76	30.43	<0.0001	<0.0001
ENSG00000099910	KLHL22	kelch like family member 22 [Source:HGNC Symbol;Acc:HGNC:25888]	protein coding	-0.74	4.06	33.72	<0.0001	<0.0001
ENSG00000135083	CCNJL	cyclin J like [Source:HGNC Symbol;Acc:HGNC:25876]	protein coding	-0.74	7.60	39.58	<0.0001	<0.0001
ENSG00000143793	C1orf35	chromosome 1 open reading frame 35 [Source:HGNC Symbol;Acc:HGNC:19032]	protein coding	-0.74	1.62	23.86	<0.0001	<0.0001
ENSG00000271869		novel transcript	lncRNA	-0.74	2.76	12.81	0.0003	0.0012
ENSG00000089327	FXYD5	FXYD domain containing ion transport regulator 5 [Source:HGNC Symbol;Acc:HGNC:4029]	protein coding	-0.74	4.77	26.82	<0.0001	<0.0001
ENSG00000100226	GTPBP1	GTP binding protein 1 [Source:HGNC Symbol;Acc:HGNC:4669]	protein coding	0.74	7.06	45.25	<0.0001	<0.0001
ENSG00000218739	CEBPZOS	CEBPZ opposite strand [Source:HGNC Symbol;Acc:HGNC:49288]	protein coding	-0.74	1.99	19.51	<0.0001	<0.0001
ENSG00000139832	RAB20	RAB20, member RAS oncogene family [Source:HGNC Symbol;Acc:HGNC:18260]	protein coding	0.74	4.84	25.44	<0.0001	<0.0001
ENSG00000257433		novel transcript, antisense to ENDOU and RAPGEF3	lncRNA	0.74	2.70	21.79	<0.0001	<0.0001
ENSG00000223969		novel transcript	lncRNA	-0.74	1.91	16.02	<0.0001	0.0003

Table S18: Genes differentially expressed at Day 30 compared to pre-second vaccination (Study Arm 2 A/H7N9+AS03, D1, 29, Neutrophils). Genes are sorted by descending absolute log_2 fold change. Gene model summaries and annotations are based on Ensembl Version 99 (continued.)

Ensembl Gene ID	Ensembl Gene Name	Ensembl Gene Description	Gene Type	log_2 Fold Change (Day 30 vs. Pre-Second Vaccination)	Average log_2 CPM	Likelihood Ratio Test Statistic	P-Value	FDR Adjusted P-Value
ENSG00000114520	SNX4	sorting nexin 4 [Source:HGNC Symbol;Acc:HGNC:11175]	protein coding	-0.74	1.70	22.98	<0.0001	<0.0001
ENSG00000172663	TMEM134	transmembrane protein 134 [Source:HGNC Symbol;Acc:HGNC:26142]	protein coding	-0.74	2.49	23.77	<0.0001	<0.0001
ENSG00000138018	SELENOI	selenoprotein I [Source:HGNC Symbol;Acc:HGNC:29361]	protein coding	0.74	1.26	14.36	0.0002	0.0006
ENSG00000162910	MRPL55	mitochondrial ribosomal protein L55 [Source:HGNC Symbol;Acc:HGNC:16686]	protein coding	-0.74	2.69	21.31	<0.0001	<0.0001
ENSG00000136161	RCBTB2	RCC1 and BTB domain containing protein 2 [Source:HGNC Symbol;Acc:HGNC:1914]	protein coding	-0.74	6.61	38.37	<0.0001	<0.0001
ENSG00000150961	SEC24D	SEC24 homolog D, COPII coat complex component [Source:HGNC Symbol;Acc:HGNC:10706]	protein coding	0.74	5.72	45.04	<0.0001	<0.0001
ENSG00000223509		WAS protein homolog associated with actin, golgi membranes and microtubules (WHAMM) pseudogene	transcribed processed pseudogene	-0.74	1.35	11.57	0.0007	0.0021
ENSG00000116704	SLC35D1	solute carrier family 35 member D1 [Source:HGNC Symbol;Acc:HGNC:20800]	protein coding	-0.74	0.58	6.03	0.0141	0.0307
ENSG00000215012	RTL10	retrotransposon Gag like 10 [Source:HGNC Symbol;Acc:HGNC:26112]	protein coding	-0.73	2.12	17.03	<0.0001	0.0002
ENSG00000281106	TMEM272	transmembrane protein 272 [Source:HGNC Symbol;Acc:HGNC:26737]	protein coding	0.73	3.52	42.14	<0.0001	<0.0001
ENSG00000091490	SEL1L3	SEL1L family member 3 [Source:HGNC Symbol;Acc:HGNC:29108]	protein coding	-0.73	1.95	9.15	0.0025	0.0068
ENSG00000100425	BRD1	bromodomain containing 1 [Source:HGNC Symbol;Acc:HGNC:1102]	protein coding	-0.73	5.32	65.80	<0.0001	<0.0001
ENSG00000169718	DUS1L	dihydrouridine synthase 1 like [Source:HGNC Symbol;Acc:HGNC:30086]	protein coding	-0.73	3.11	20.57	<0.0001	<0.0001
ENSG00000126216	TUBGCP3	tubulin gamma complex associated protein 3 [Source:HGNC Symbol;Acc:HGNC:18598]	protein coding	-0.73	5.62	63.43	<0.0001	<0.0001
ENSG00000196954	CASP4	caspase 4 [Source:HGNC Symbol;Acc:HGNC:1505]	protein coding	0.73	9.15	43.20	<0.0001	<0.0001
ENSG00000113749	HRH2	histamine receptor H2 [Source:HGNC Symbol;Acc:HGNC:5183]	protein coding	-0.73	8.02	17.51	<0.0001	0.0001
ENSG00000164024	METAP1	methionyl aminopeptidase 1 [Source:HGNC Symbol;Acc:HGNC:15789]	protein coding	-0.73	2.15	30.70	<0.0001	<0.0001

Table S18: Genes differentially expressed at Day 30 compared to pre-second vaccination (Study Arm 2 A/H7N9+AS03, D1, 29, Neutrophils). Genes are sorted by descending absolute log_2 fold change. Gene model summaries and annotations are based on Ensembl Version 99 (continued.)

Ensembl Gene ID	Ensembl Gene Name	Ensembl Gene Description	Gene Type	log_2 Fold Change (Day 30 vs. Pre-Second Vaccination)	Average log_2 CPM	Likelihood Ratio Test Statistic	P-Value	FDR Adjusted P-Value
ENSG00000260086		novel transcript	lncRNA	-0.73	0.92	28.04	<0.0001	<0.0001
ENSG00000141034	GID4	GID complex subunit 4 homolog [Source:HGNC Symbol;Acc:HGNC:28453]	protein coding	-0.73	3.04	29.42	<0.0001	<0.0001
ENSG00000050748	MAPK9	mitogen-activated protein kinase 9 [Source:HGNC Symbol;Acc:HGNC:6886]	protein coding	-0.73	3.47	34.07	<0.0001	<0.0001
ENSG00000146263	MMS22L	MMS22 like, DNA repair protein [Source:HGNC Symbol;Acc:HGNC:21475]	protein coding	-0.73	0.14	13.09	0.0003	0.001
ENSG00000125652	ALKBH7	alkB homolog 7 [Source:HGNC Symbol;Acc:HGNC:21306]	protein coding	-0.73	3.56	42.00	<0.0001	<0.0001
ENSG00000127616	SMARCA4	SWI/SNF related, matrix associated, actin dependent regulator of chromatin, subfamily a, member 4 [Source:HGNC Symbol;Acc:HGNC:11100]	protein coding	-0.73	4.12	38.10	<0.0001	<0.0001
ENSG00000125912	NCLN	nicalin [Source:HGNC Symbol;Acc:HGNC:26923]	protein coding	-0.73	6.46	58.29	<0.0001	<0.0001
ENSG00000182628	SKA2	spindle and kinetochore associated complex subunit 2 [Source:HGNC Symbol;Acc:HGNC:28006]	protein coding	0.73	0.27	12.67	0.0004	0.0013
ENSG00000116977	LGALS8	galectin 8 [Source:HGNC Symbol;Acc:HGNC:6569]	protein coding	0.73	5.38	38.67	<0.0001	<0.0001
ENSG00000140688	C16orf58	chromosome 16 open reading frame 58 [Source:HGNC Symbol;Acc:HGNC:25848]	protein coding	-0.73	0.36	12.57	0.0004	0.0013
ENSG00000213024	NUP62	nucleoporin 62 [Source:HGNC Symbol;Acc:HGNC:8066]	protein coding	0.73	4.17	30.18	<0.0001	<0.0001
ENSG00000157778	PSMG3	proteasome assembly chaperone 3 [Source:HGNC Symbol;Acc:HGNC:22420]	protein coding	-0.73	1.17	17.26	<0.0001	0.0001
ENSG00000135525	MAP7	microtubule associated protein 7 [Source:HGNC Symbol;Acc:HGNC:6869]	protein coding	-0.73	4.02	27.53	<0.0001	<0.0001
ENSG00000148399	DPH7	diphthamide biosynthesis 7 [Source:HGNC Symbol;Acc:HGNC:25199]	protein coding	-0.73	2.38	27.88	<0.0001	<0.0001
ENSG00000213064	SFT2D2	SFT2 domain containing 2 [Source:HGNC Symbol;Acc:HGNC:25140]	protein coding	0.73	4.00	33.82	<0.0001	<0.0001
ENSG00000167969	ECI1	enoyl-CoA delta isomerase 1 [Source:HGNC Symbol;Acc:HGNC:2703]	protein coding	-0.73	1.72	22.98	<0.0001	<0.0001
ENSG00000125656	CLPP	caseinolytic mitochondrial matrix peptidase proteolytic subunit [Source:HGNC Symbol;Acc:HGNC:2084]	protein coding	-0.73	4.11	45.80	<0.0001	<0.0001
ENSG00000167210	LOXHD1	lipoxygenase homology domains 1 [Source:HGNC Symbol;Acc:HGNC:26521]	protein coding	0.73	2.92	35.48	<0.0001	<0.0001

Table S18: Genes differentially expressed at Day 30 compared to pre-second vaccination (Study Arm 2 A/H7N9+AS03, D1, 29, Neutrophils). Genes are sorted by descending absolute log_2 fold change. Gene model summaries and annotations are based on Ensembl Version 99 (continued.)

Ensembl Gene ID	Ensembl Gene Name	Ensembl Gene Description	Gene Type	log_2 Fold Change (Day 30 vs. Pre-Second Vaccination)	Average log_2 CPM	Likelihood Ratio Test Statistic	P-Value	FDR Adjusted P-Value
ENSG00000115211	EIF2B4	eukaryotic translation initiation factor 2B subunit delta [Source:HGNC Symbol;Acc:HGNC:3260]	protein coding	-0.73	2.20	28.31	<0.0001	<0.0001
ENSG00000196363	WDR5	WD repeat domain 5 [Source:HGNC Symbol;Acc:HGNC:12757]	protein coding	-0.73	1.41	24.38	<0.0001	<0.0001
ENSG00000163870	TPRA1	transmembrane protein adipocyte associated 1 [Source:HGNC Symbol;Acc:HGNC:30413]	protein coding	-0.73	3.55	40.91	<0.0001	<0.0001
ENSG00000231616		novel transcript, antisense to KIF27	lncRNA	-0.73	0.96	12.24	0.0005	0.0015
ENSG00000080546	SESN1	sestrin 1 [Source:HGNC Symbol;Acc:HGNC:21595]	protein coding	-0.73	2.28	24.57	<0.0001	<0.0001
ENSG00000148090	AUH	AU RNA binding methylglutaconyl-CoA hydratase [Source:HGNC Symbol;Acc:HGNC:890]	protein coding	-0.73	2.96	30.26	<0.0001	<0.0001
ENSG00000166130	IKBIP	IKBKB interacting protein [Source:HGNC Symbol;Acc:HGNC:26430]	protein coding	-0.73	6.79	45.06	<0.0001	<0.0001
ENSG00000203804	ADAMTSL4-AS1	ADAMTSL4 antisense RNA 1 [Source:HGNC Symbol;Acc:HGNC:32041]	lncRNA	0.73	4.98	25.43	<0.0001	<0.0001
ENSG00000103326	CAPN15	calpain 15 [Source:HGNC Symbol;Acc:HGNC:11182]	protein coding	-0.73	1.40	9.69	0.0018	0.0052
ENSG00000108784	NAGLU	N-acetyl-alpha-glucosaminidase [Source:HGNC Symbol;Acc:HGNC:7632]	protein coding	-0.73	1.56	19.04	<0.0001	<0.0001
ENSG00000196668	LINC00173	long intergenic non-protein coding RNA 173 [Source:HGNC Symbol;Acc:HGNC:33791]	lncRNA	-0.73	4.77	24.17	<0.0001	<0.0001
ENSG00000189060	H1-0	H1.0 linker histone [Source:HGNC Symbol;Acc:HGNC:4714]	protein coding	-0.73	3.55	16.44	<0.0001	0.0002
ENSG00000205022	PABPN1L	PABPN1 like, cytoplasmic [Source:HGNC Symbol;Acc:HGNC:37237]	protein coding	-0.73	1.76	24.88	<0.0001	<0.0001
ENSG00000165914	TTC7B	tetratricopeptide repeat domain 7B [Source:HGNC Symbol;Acc:HGNC:19858]	protein coding	0.73	1.68	11.07	0.0009	0.0027
ENSG00000132570	PCBD2	pterin-4 alpha-carbinolamine dehydratase 2 [Source:HGNC Symbol;Acc:HGNC:24474]	protein coding	-0.73	0.77	21.31	<0.0001	<0.0001
ENSG00000138600	SPPL2A	signal peptide peptidase like 2A [Source:HGNC Symbol;Acc:HGNC:30227]	protein coding	0.73	5.78	61.36	<0.0001	<0.0001
ENSG00000135503	ACVR1B	activin A receptor type 1B [Source:HGNC Symbol;Acc:HGNC:172]	protein coding	-0.73	3.71	12.13	0.0005	0.0016

Table S18: Genes differentially expressed at Day 30 compared to pre-second vaccination (Study Arm 2 A/H7N9+AS03, D1, 29, Neutrophils). Genes are sorted by descending absolute log_2 fold change. Gene model summaries and annotations are based on Ensembl Version 99 (continued.)

Ensembl Gene ID	Ensembl Gene Name	Ensembl Gene Description	Gene Type	log_2 Fold Change (Day 30 vs. Pre-Second Vaccination)	Average log_2 CPM	Likelihood Ratio Test Statistic	P-Value	FDR Adjusted P-Value
ENSG00000127957	PMS2P3	PMS1 homolog 2, mismatch repair system component pseudogene 3 [Source:HGNC Symbol;Acc:HGNC:9128]	transcribed unprocessed pseudogene	-0.73	0.56	16.17	<0.0001	0.0002
ENSG00000267751		novel transcript, antisense to BSG	lncRNA	-0.73	0.53	13.32	0.0003	0.0009
ENSG00000100181	TPTEP1	TPTE pseudogene 1 [Source:HGNC Symbol;Acc:HGNC:43648]	transcribed unprocessed pseudogene	-0.72	1.60	14.30	0.0002	0.0006
ENSG00000172432	GTPBP2	GTP binding protein 2 [Source:HGNC Symbol;Acc:HGNC:4670]	protein coding	0.72	6.41	60.85	<0.0001	<0.0001
ENSG00000108479	GALK1	galactokinase 1 [Source:HGNC Symbol;Acc:HGNC:4118]	protein coding	-0.72	5.07	39.08	<0.0001	<0.0001
ENSG00000167515	TRAPPC2L	trafficking protein particle complex 2 like [Source:HGNC Symbol;Acc:HGNC:30887]	protein coding	-0.72	1.77	29.85	<0.0001	<0.0001
ENSG00000153207	AHCTF1	AT-hook containing transcription factor 1 [Source:HGNC Symbol;Acc:HGNC:24618]	protein coding	-0.72	7.34	57.94	<0.0001	<0.0001
ENSG00000080503	SMARCA2	SWI/SNF related, matrix associated, actin dependent regulator of chromatin, subfamily a, member 2 [Source:HGNC Symbol;Acc:HGNC:11098]	protein coding	-0.72	6.54	50.09	<0.0001	<0.0001
ENSG00000198825	INPP5F	inositol polyphosphate-5-phosphatase F [Source:HGNC Symbol;Acc:HGNC:17054]	protein coding	-0.72	0.45	14.42	0.0001	0.0005
ENSG00000185215	TNFAIP2	TNF alpha induced protein 2 [Source:HGNC Symbol;Acc:HGNC:11895]	protein coding	0.72	11.57	28.85	<0.0001	<0.0001
ENSG00000131697	NPHP4	nephrocystin 4 [Source:HGNC Symbol;Acc:HGNC:19104]	protein coding	-0.72	2.75	31.39	<0.0001	<0.0001
ENSG00000070718	AP3M2	adaptor related protein complex 3 subunit mu 2 [Source:HGNC Symbol;Acc:HGNC:570]	protein coding	-0.72	0.56	18.10	<0.0001	<0.0001
ENSG00000205018		uncharacterized LOC100129697 [Source:NCBI gene;Acc:100129697]	lncRNA	0.72	0.55	17.35	<0.0001	0.0001
ENSG00000119787	ATL2	atlastin GTPase 2 [Source:HGNC Symbol;Acc:HGNC:24047]	protein coding	-0.72	3.41	33.54	<0.0001	<0.0001
ENSG00000277496		novel transcript, antisense to SLCO4A1	lncRNA	0.72	2.29	36.64	<0.0001	<0.0001

Table S18: Genes differentially expressed at Day 30 compared to pre-second vaccination (Study Arm 2 A/H7N9+AS03, D1, 29, Neutrophils). Genes are sorted by descending absolute log_2 fold change. Gene model summaries and annotations are based on Ensembl Version 99 (continued.)

Ensembl Gene ID	Ensembl Gene Name	Ensembl Gene Description	Gene Type	log_2 Fold Change (Day 30 vs. Pre-Second Vaccination)	Average log_2 CPM	Likelihood Ratio Test Statistic	P-Value	FDR Adjusted P-Value
ENSG00000095794	CREM	cAMP responsive element modulator [Source:HGNC Symbol;Acc:HGNC:2352]	protein coding	0.72	3.41	21.99	<0.0001	<0.0001
ENSG00000277879		novel transcript, antisense to SLC18A2	lncRNA	-0.72	1.12	7.47	0.0063	0.0152
ENSG00000178996	SNX18	sorting nexin 18 [Source:HGNC Symbol;Acc:HGNC:19245]	protein coding	-0.72	7.77	49.30	<0.0001	<0.0001
ENSG00000181610	MRPS23	mitochondrial ribosomal protein S23 [Source:HGNC Symbol;Acc:HGNC:14509]	protein coding	-0.72	0.64	11.25	0.0008	0.0025
ENSG00000141552	ANAPC11	anaphase promoting complex subunit 11 [Source:HGNC Symbol;Acc:HGNC:14452]	protein coding	-0.72	3.85	36.39	<0.0001	<0.0001
ENSG00000099866	MADCAM1	mucosal vascular addressin cell adhesion molecule 1 [Source:HGNC Symbol;Acc:HGNC:6765]	protein coding	-0.72	2.29	26.65	<0.0001	<0.0001
ENSG00000113838	TBCCD1	TBCC domain containing 1 [Source:HGNC Symbol;Acc:HGNC:25546]	protein coding	-0.72	0.52	17.78	<0.0001	0.0001
ENSG00000164221	CCDC112	coiled-coil domain containing 112 [Source:HGNC Symbol;Acc:HGNC:28599]	protein coding	-0.72	2.59	22.08	<0.0001	<0.0001
ENSG00000123096	SSPN	sarcospan [Source:HGNC Symbol;Acc:HGNC:11322]	protein coding	0.72	1.22	9.57	0.002	0.0056
ENSG00000023318	ERP44	endoplasmic reticulum protein 44 [Source:HGNC Symbol;Acc:HGNC:18311]	protein coding	0.72	6.64	44.70	<0.0001	<0.0001
ENSG00000169087	HSPBAP1	HSPB1 associated protein 1 [Source:HGNC Symbol;Acc:HGNC:16389]	protein coding	-0.72	5.42	44.72	<0.0001	<0.0001
ENSG00000121104	FAM117A	family with sequence similarity 117 member A [Source:HGNC Symbol;Acc:HGNC:24179]	protein coding	0.72	5.69	43.55	<0.0001	<0.0001
ENSG00000133835	HSD17B4	hydroxysteroid 17-beta dehydrogenase 4 [Source:HGNC Symbol;Acc:HGNC:5213]	protein coding	-0.72	4.21	14.85	0.0001	0.0004
ENSG00000073578	SDHA	succinate dehydrogenase complex flavoprotein subunit A [Source:HGNC Symbol;Acc:HGNC:10680]	protein coding	0.71	1.47	23.54	<0.0001	<0.0001
ENSG00000148700	ADD3	adducin 3 [Source:HGNC Symbol;Acc:HGNC:245]	protein coding	-0.71	7.08	23.31	<0.0001	<0.0001
ENSG00000128294	TPST2	tyrosylprotein sulfotransferase 2 [Source:HGNC Symbol;Acc:HGNC:12021]	protein coding	-0.71	6.36	33.53	<0.0001	<0.0001
ENSG00000247556	OIP5-AS1	OIP5 antisense RNA 1 [Source:HGNC Symbol;Acc:HGNC:43563]	lncRNA	-0.71	3.59	15.73	<0.0001	0.0003
ENSG00000189319	FAM53B	family with sequence similarity 53 member B [Source:HGNC Symbol;Acc:HGNC:28968]	protein coding	-0.71	6.69	44.90	<0.0001	<0.0001

Table S18: Genes differentially expressed at Day 30 compared to pre-second vaccination (Study Arm 2 A/H7N9+AS03, D1, 29, Neutrophils). Genes are sorted by descending absolute log_2 fold change. Gene model summaries and annotations are based on Ensembl Version 99 (continued.)

Ensembl Gene ID	Ensembl Gene Name	Ensembl Gene Description	Gene Type	log_2 Fold Change (Day 30 vs. Pre-Second Vaccination)	Average log_2 CPM	Likelihood Ratio Test Statistic	P-Value	FDR Adjusted P-Value
ENSG00000269821	KCNQ1OT1	KCNQ1 opposite strand/antisense transcript 1 [Source:HGNC Symbol;Acc:HGNC:6295]	lncRNA	-0.71	2.76	20.18	<0.0001	<0.0001
ENSG00000182512	GLRX5	glutaredoxin 5 [Source:HGNC Symbol;Acc:HGNC:20134]	protein coding	-0.71	3.43	35.29	<0.0001	<0.0001
ENSG00000144136	SLC20A1	solute carrier family 20 member 1 [Source:HGNC Symbol;Acc:HGNC:10946]	protein coding	0.71	5.89	31.11	<0.0001	<0.0001
ENSG00000124380	SNRNP27	small nuclear ribonucleoprotein U4/U6.U5 subunit 27 [Source:HGNC Symbol;Acc:HGNC:30240]	protein coding	0.71	3.21	22.21	<0.0001	<0.0001
ENSG00000150433	TMEM218	transmembrane protein 218 [Source:HGNC Symbol;Acc:HGNC:27344]	protein coding	-0.71	1.80	27.28	<0.0001	<0.0001
ENSG00000182534	MXRA7	matrix remodeling associated 7 [Source:HGNC Symbol;Acc:HGNC:7541]	protein coding	-0.71	2.48	8.61	0.0033	0.0088
ENSG00000103495	MAZ	MYC associated zinc finger protein [Source:HGNC Symbol;Acc:HGNC:6914]	protein coding	-0.71	5.04	47.54	<0.0001	<0.0001
ENSG00000187049	TMEM216	transmembrane protein 216 [Source:HGNC Symbol;Acc:HGNC:25018]	protein coding	-0.71	3.37	26.94	<0.0001	<0.0001
ENSG00000093072	ADA2	adenosine deaminase 2 [Source:HGNC Symbol;Acc:HGNC:1839]	protein coding	0.71	2.31	17.03	<0.0001	0.0002
ENSG00000124702	KLHDC3	kelch domain containing 3 [Source:HGNC Symbol;Acc:HGNC:20704]	protein coding	-0.71	5.12	53.60	<0.0001	<0.0001
ENSG00000198604	BAZ1A	bromodomain adjacent to zinc finger domain 1A [Source:HGNC Symbol;Acc:HGNC:960]	protein coding	0.71	8.48	30.81	<0.0001	<0.0001
ENSG00000064703	DDX20	DEAD-box helicase 20 [Source:HGNC Symbol;Acc:HGNC:2743]	protein coding	-0.71	2.81	26.63	<0.0001	<0.0001
ENSG00000272078		novel transcript, antisense to CASZ1	lncRNA	0.71	0.42	18.17	<0.0001	<0.0001
ENSG00000135838	NPL	N-acetylneuraminate pyruvate lyase [Source:HGNC Symbol;Acc:HGNC:16781]	protein coding	-0.71	7.85	59.16	<0.0001	<0.0001
ENSG00000142327	RNPEPL1	arginyl aminopeptidase like 1 [Source:HGNC Symbol;Acc:HGNC:10079]	protein coding	-0.71	7.54	41.83	<0.0001	<0.0001
ENSG00000131149	GSE1	Gse1 coiled-coil protein [Source:HGNC Symbol;Acc:HGNC:28979]	protein coding	0.71	5.28	32.69	<0.0001	<0.0001
ENSG00000268861		Rho/Rac guanine nucleotide exchange factor 18 [Source:NCBI gene;Acc:23370]	protein coding	-0.71	0.20	8.69	0.0032	0.0085
ENSG00000163512	AZI2	5-azacytidine induced 2 [Source:HGNC Symbol;Acc:HGNC:24002]	protein coding	0.71	3.15	38.57	<0.0001	<0.0001

Table S18: Genes differentially expressed at Day 30 compared to pre-second vaccination (Study Arm 2 A/H7N9+AS03, D1, 29, Neutrophils). Genes are sorted by descending absolute log_2 fold change. Gene model summaries and annotations are based on Ensembl Version 99 (continued.)

Ensembl Gene ID	Ensembl Gene Name	Ensembl Gene Description	Gene Type	log_2 Fold Change (Day 30 vs. Pre-Second Vaccination)	Average log_2 CPM	Likelihood Ratio Test Statistic	P-Value	FDR Adjusted P-Value
ENSG00000134294	SLC38A2	solute carrier family 38 member 2 [Source:HGNC Symbol;Acc:HGNC:13448]	protein coding	0.71	7.18	30.54	<0.0001	<0.0001
ENSG00000100077	GRK3	G protein-coupled receptor kinase 3 [Source:HGNC Symbol;Acc:HGNC:290]	protein coding	0.71	3.27	22.02	<0.0001	<0.0001
ENSG00000167705	RILP	Rab interacting lysosomal protein [Source:HGNC Symbol;Acc:HGNC:30266]	protein coding	0.71	5.00	36.91	<0.0001	<0.0001
ENSG00000138688	KIAA1109	KIAA1109 [Source:HGNC Symbol;Acc:HGNC:26953]	protein coding	0.71	7.34	52.54	<0.0001	<0.0001
ENSG00000132819	RBM38	RNA binding motif protein 38 [Source:HGNC Symbol;Acc:HGNC:15818]	protein coding	-0.71	5.34	41.00	<0.0001	<0.0001
ENSG00000123609	NMI	N-myc and STAT interactor [Source:HGNC Symbol;Acc:HGNC:7854]	protein coding	0.71	8.18	33.95	<0.0001	<0.0001
ENSG00000154059	IMPACT	impact RWD domain protein [Source:HGNC Symbol;Acc:HGNC:20387]	protein coding	-0.71	1.18	15.35	<0.0001	0.0004
ENSG00000064607	SUGP2	SURP and G-patch domain containing 2 [Source:HGNC Symbol;Acc:HGNC:18641]	protein coding	-0.71	4.78	29.38	<0.0001	<0.0001
ENSG00000099797	TECR	trans-2,3-enoyl-CoA reductase [Source:HGNC Symbol;Acc:HGNC:4551]	protein coding	-0.71	3.13	28.69	<0.0001	<0.0001
ENSG00000087301	TXNDC16	thioredoxin domain containing 16 [Source:HGNC Symbol;Acc:HGNC:19965]	protein coding	-0.71	2.35	26.78	<0.0001	<0.0001
ENSG00000135776	ABCB10	ATP binding cassette subfamily B member 10 [Source:HGNC Symbol;Acc:HGNC:41]	protein coding	-0.71	1.55	11.67	0.0006	0.002
ENSG00000168291	PDHB	pyruvate dehydrogenase E1 beta subunit [Source:HGNC Symbol;Acc:HGNC:8808]	protein coding	-0.71	2.94	35.04	<0.0001	<0.0001
ENSG00000204520	MICA	MHC class I polypeptide-related sequence A [Source:HGNC Symbol;Acc:HGNC:7090]	protein coding	0.71	4.54	24.95	<0.0001	<0.0001
ENSG00000181031	RPH3AL	rabphilin 3A like (without C2 domains) [Source:HGNC Symbol;Acc:HGNC:10296]	protein coding	-0.71	1.55	9.62	0.0019	0.0054
ENSG00000131626	PPFIA1	PTPRF interacting protein alpha 1 [Source:HGNC Symbol;Acc:HGNC:9245]	protein coding	-0.71	5.98	54.61	<0.0001	<0.0001
ENSG00000118762	PKD2	polycystin 2, transient receptor potential cation channel [Source:HGNC Symbol;Acc:HGNC:9009]	protein coding	-0.71	2.41	30.75	<0.0001	<0.0001
ENSG00000250479	CHCHD10	coiled-coil-helix-coiled-coil-helix domain containing 10 [Source:HGNC Symbol;Acc:HGNC:15559]	protein coding	-0.71	0.96	14.09	0.0002	0.0006
ENSG00000222020	HDAC4-AS1	HDAC4 antisense RNA 1 [Source:HGNC Symbol;Acc:HGNC:54064]	lncRNA	-0.70	0.10	10.62	0.0011	0.0034

Table S18: Genes differentially expressed at Day 30 compared to pre-second vaccination (Study Arm 2 A/H7N9+AS03, D1, 29, Neutrophils). Genes are sorted by descending absolute log_2 fold change. Gene model summaries and annotations are based on Ensembl Version 99 (continued.)

Ensembl Gene ID	Ensembl Gene Name	Ensembl Gene Description	Gene Type	log_2 Fold Change (Day 30 vs. Pre-Second Vaccination)	Average log_2 CPM	Likelihood Ratio Test Statistic	P-Value	FDR Adjusted P-Value
ENSG00000286813		novel transcript, sense intronic to ST20-MTHFSand MTHFS	lncRNA	-0.70	2.17	9.90	0.0017	0.0047
ENSG00000100364	KIAA0930	KIAA0930 [Source:HGNC Symbol;Acc:HGNC:1314]	protein coding	0.70	5.81	41.71	<0.0001	<0.0001
ENSG00000224186	C5orf66	chromosome 5 open reading frame 66 [Source:HGNC Symbol;Acc:HGNC:48332]	lncRNA	-0.70	0.30	13.92	0.0002	0.0007
ENSG00000137218	FRS3	fibroblast growth factor receptor substrate 3 [Source:HGNC Symbol;Acc:HGNC:16970]	protein coding	-0.70	0.87	12.86	0.0003	0.0011
ENSG00000287586		novel transcript, antisense to AJAP1	lncRNA	-0.70	2.82	19.40	<0.0001	<0.0001
ENSG00000279058	AGAP14P	ArfGAP with GTPase domain, ankyrin repeat and PH domain 14, pseudogene [Source:HGNC Symbol;Acc:HGNC:23660]	unprocessed pseudogene	-0.70	1.22	11.19	0.0008	0.0026
ENSG00000240342	RPS2P5	ribosomal protein S2 pseudogene 5 [Source:HGNC Symbol;Acc:HGNC:31386]	processed pseudogene	0.70	4.12	20.59	<0.0001	<0.0001
ENSG00000065308	TRAM2	translocation associated membrane protein 2 [Source:HGNC Symbol;Acc:HGNC:16855]	protein coding	-0.70	1.77	16.02	<0.0001	0.0003
ENSG00000172375	C2CD2L	C2CD2 like [Source:HGNC Symbol;Acc:HGNC:29000]	protein coding	-0.70	4.98	35.21	<0.0001	<0.0001
ENSG00000247121		novel transcript, antisense to LNPEP and ERAP2	lncRNA	0.70	1.23	21.27	<0.0001	<0.0001
ENSG00000008226	DLEC1	DLEC1 cilia and flagella associated protein [Source:HGNC Symbol;Acc:HGNC:2899]	protein coding	-0.70	2.52	21.49	<0.0001	<0.0001
ENSG00000112144	ICK	intestinal cell kinase [Source:HGNC Symbol;Acc:HGNC:21219]	protein coding	-0.70	2.17	24.26	<0.0001	<0.0001
ENSG00000255717	SNHG1	small nucleolar RNA host gene 1 [Source:HGNC Symbol;Acc:HGNC:32688]	lncRNA	-0.70	2.09	20.26	<0.0001	<0.0001
ENSG00000108557	RAI1	retinoic acid induced 1 [Source:HGNC Symbol;Acc:HGNC:9834]	protein coding	-0.70	1.22	10.70	0.0011	0.0032
ENSG00000153879	CEBPG	CCAAT enhancer binding protein gamma [Source:HGNC Symbol;Acc:HGNC:1837]	protein coding	-0.70	2.12	24.06	<0.0001	<0.0001
ENSG00000142794	NBPF3	NBPF member 3 [Source:HGNC Symbol;Acc:HGNC:25076]	protein coding	-0.70	0.33	9.90	0.0017	0.0047
ENSG00000137414	FAM8A1	family with sequence similarity 8 member A1 [Source:HGNC Symbol;Acc:HGNC:16372]	protein coding	0.70	8.59	36.55	<0.0001	<0.0001
ENSG00000188352	FOCAD	focadhesin [Source:HGNC Symbol;Acc:HGNC:23377]	protein coding	-0.70	2.81	23.55	<0.0001	<0.0001

Table S18: Genes differentially expressed at Day 30 compared to pre-second vaccination (Study Arm 2 A/H7N9+AS03, D1, 29, Neutrophils). Genes are sorted by descending absolute log_2 fold change. Gene model summaries and annotations are based on Ensembl Version 99 (continued.)

Ensembl Gene ID	Ensembl Gene Name	Ensembl Gene Description	Gene Type	log_2 Fold Change (Day 30 vs. Pre-Second Vaccination)	Average log_2 CPM	Likelihood Ratio Test Statistic	P-Value	FDR Adjusted P-Value
ENSG00000121578	B4GALT4	beta-1,4-galactosyltransferase 4 [Source:HGNC Symbol;Acc:HGNC:927]	protein coding	0.70	2.42	27.86	<0.0001	<0.0001
ENSG00000196227	FAM217B	family with sequence similarity 217 member B [Source:HGNC Symbol;Acc:HGNC:16170]	protein coding	-0.70	4.56	19.55	<0.0001	<0.0001
ENSG00000213928	IRF9	interferon regulatory factor 9 [Source:HGNC Symbol;Acc:HGNC:6131]	protein coding	0.70	5.60	38.32	<0.0001	<0.0001
ENSG00000185156	MFSD6L	major facilitator superfamily domain containing 6 like [Source:HGNC Symbol;Acc:HGNC:26656]	protein coding	0.70	1.95	30.64	<0.0001	<0.0001
ENSG00000235027		novel transcript	lncRNA	-0.70	0.58	6.15	0.0131	0.0289
ENSG00000035687	ADSS2	adenylosuccinate synthase 2 [Source:HGNC Symbol;Acc:HGNC:292]	protein coding	-0.70	5.00	50.28	<0.0001	<0.0001
ENSG00000101391	CDK5RAP1	CDK5 regulatory subunit associated protein 1 [Source:HGNC Symbol;Acc:HGNC:15880]	protein coding	-0.70	0.67	13.85	0.0002	0.0007
ENSG00000126226	PCID2	PCI domain containing 2 [Source:HGNC Symbol;Acc:HGNC:25653]	protein coding	-0.70	0.99	15.89	<0.0001	0.0003
ENSG00000136045	PWP1	PWP1 homolog, endonuclein [Source:HGNC Symbol;Acc:HGNC:17015]	protein coding	-0.70	2.29	19.44	<0.0001	<0.0001
ENSG00000167536	DHRS13	dehydrogenase/reductase 13 [Source:HGNC Symbol;Acc:HGNC:28326]	protein coding	-0.69	5.63	22.47	<0.0001	<0.0001
ENSG00000125741	OPA3	outer mitochondrial membrane lipid metabolism regulator OPA3 [Source:HGNC Symbol;Acc:HGNC:8142]	protein coding	0.69	3.64	11.06	0.0009	0.0027
ENSG00000182154	MRPL41	mitochondrial ribosomal protein L41 [Source:HGNC Symbol;Acc:HGNC:14492]	protein coding	-0.69	1.33	21.23	<0.0001	<0.0001
ENSG00000012779	ALOX5	arachidonate 5-lipoxygenase [Source:HGNC Symbol;Acc:HGNC:435]	protein coding	-0.69	9.71	31.08	<0.0001	<0.0001
ENSG00000099992	TBC1D10A	TBC1 domain family member 10A [Source:HGNC Symbol;Acc:HGNC:23609]	protein coding	-0.69	3.73	18.82	<0.0001	<0.0001
ENSG00000127914	AKAP9	A-kinase anchoring protein 9 [Source:HGNC Symbol;Acc:HGNC:379]	protein coding	0.69	5.35	35.75	<0.0001	<0.0001
ENSG00000095397	WHRN	whirlin [Source:HGNC Symbol;Acc:HGNC:16361]	protein coding	0.69	3.20	10.29	0.0013	0.0039
ENSG00000081760	AACS	acetoacetyl-CoA synthetase [Source:HGNC Symbol;Acc:HGNC:21298]	protein coding	-0.69	1.33	16.50	<0.0001	0.0002
ENSG00000283633		novel transcript	lncRNA	-0.69	1.83	18.82	<0.0001	<0.0001
ENSG00000169252	ADRB2	adrenoceptor beta 2 [Source:HGNC Symbol;Acc:HGNC:286]	protein coding	-0.69	3.71	11.74	0.0006	0.002

Table S18: Genes differentially expressed at Day 30 compared to pre-second vaccination (Study Arm 2 A/H7N9+AS03, D1, 29, Neutrophils). Genes are sorted by descending absolute log_2 fold change. Gene model summaries and annotations are based on Ensembl Version 99 (continued.)

Ensembl Gene ID	Ensembl Gene Name	Ensembl Gene Description	Gene Type	log_2 Fold Change (Day 30 vs. Pre-Second Vaccination)	Average log_2 CPM	Likelihood Ratio Test Statistic	P-Value	FDR Adjusted P-Value
ENSG00000126067	PSMB2	proteasome 20S subunit beta 2 [Source:HGNC Symbol;Acc:HGNC:9539]	protein coding	0.69	4.05	33.49	<0.0001	<0.0001
ENSG00000154274	C4orf19	chromosome 4 open reading frame 19 [Source:HGNC Symbol;Acc:HGNC:25618]	protein coding	-0.69	1.00	13.96	0.0002	0.0007
ENSG00000127399	LRRC61	leucine rich repeat containing 61 [Source:HGNC Symbol;Acc:HGNC:21704]	protein coding	0.69	3.05	25.47	<0.0001	<0.0001
ENSG00000161714	PLCD3	phospholipase C delta 3 [Source:HGNC Symbol;Acc:HGNC:9061]	protein coding	-0.69	0.42	10.22	0.0014	0.0041
ENSG00000162461	SLC25A34	solute carrier family 25 member 34 [Source:HGNC Symbol;Acc:HGNC:27653]	protein coding	0.69	1.34	16.22	<0.0001	0.0002
ENSG00000277147	LINC00869	long intergenic non-protein coding RNA 869 [Source:HGNC Symbol;Acc:HGNC:29050]	transcribed unprocessed pseudogene	0.69	3.54	24.16	<0.0001	<0.0001
ENSG00000167468	GPX4	glutathione peroxidase 4 [Source:HGNC Symbol;Acc:HGNC:4556]	protein coding	-0.69	5.47	45.73	<0.0001	<0.0001
ENSG00000140988	RPS2	ribosomal protein S2 [Source:HGNC Symbol;Acc:HGNC:10404]	protein coding	0.69	6.60	30.46	<0.0001	<0.0001
ENSG00000187796	CARD9	caspase recruitment domain family member 9 [Source:HGNC Symbol;Acc:HGNC:16391]	protein coding	-0.69	2.48	13.58	0.0002	0.0008
ENSG00000113161	HMGCR	3-hydroxy-3-methylglutaryl-CoA reductase [Source:HGNC Symbol;Acc:HGNC:5006]	protein coding	-0.69	6.24	45.01	<0.0001	<0.0001
ENSG00000239653	PSMD6-AS2	PSMD6 antisense RNA 2 [Source:HGNC Symbol;Acc:HGNC:44125]	lncRNA	0.69	3.59	21.80	<0.0001	<0.0001
ENSG00000122035	RASL11A	RAS like family 11 member A [Source:HGNC Symbol;Acc:HGNC:23802]	protein coding	-0.69	0.70	14.78	0.0001	0.0005
ENSG00000108828	VAT1	vesicle amine transport 1 [Source:HGNC Symbol;Acc:HGNC:16919]	protein coding	0.69	3.14	28.03	<0.0001	<0.0001
ENSG00000138829	FBN2	fibrillin 2 [Source:HGNC Symbol;Acc:HGNC:3604]	protein coding	-0.69	1.17	16.44	<0.0001	0.0002
ENSG00000110880	CORO1C	coronin 1C [Source:HGNC Symbol;Acc:HGNC:2254]	protein coding	-0.69	7.48	51.60	<0.0001	<0.0001
ENSG00000189127	ANKRD34B	ankyrin repeat domain 34B [Source:HGNC Symbol;Acc:HGNC:33736]	protein coding	-0.69	2.20	14.97	0.0001	0.0004

Table S18: Genes differentially expressed at Day 30 compared to pre-second vaccination (Study Arm 2 A/H7N9+AS03, D1, 29, Neutrophils). Genes are sorted by descending absolute log_2 fold change. Gene model summaries and annotations are based on Ensembl Version 99 (continued.)

Ensembl Gene ID	Ensembl Gene Name	Ensembl Gene Description	Gene Type	log_2 Fold Change (Day 30 vs. Pre-Second Vaccination)	Average log_2 CPM	Likelihood Ratio Test Statistic	P-Value	FDR Adjusted P-Value
ENSG00000179115	FARSA	phenylalanyl-tRNA synthetase subunit alpha [Source:HGNC Symbol;Acc:HGNC:3592]	protein coding	-0.69	0.30	12.45	0.0004	0.0014
ENSG00000168675	LDLRAD4	low density lipoprotein receptor class A domain containing 4 [Source:HGNC Symbol;Acc:HGNC:1224]	protein coding	-0.69	2.36	15.50	<0.0001	0.0003
ENSG00000168056	LTBP3	latent transforming growth factor beta binding protein 3 [Source:HGNC Symbol;Acc:HGNC:6716]	protein coding	-0.69	2.30	19.96	<0.0001	<0.0001
ENSG00000163428	LRRC58	leucine rich repeat containing 58 [Source:HGNC Symbol;Acc:HGNC:26968]	protein coding	-0.69	1.85	18.30	<0.0001	<0.0001
ENSG00000284930		novel transcript	lncRNA	-0.69	2.93	35.53	<0.0001	<0.0001
ENSG00000173692	PSMD1	proteasome 26S subunit, non-ATPase 1 [Source:HGNC Symbol;Acc:HGNC:9554]	protein coding	-0.69	3.95	39.57	<0.0001	<0.0001
ENSG00000076641	PAG1	phosphoprotein membrane anchor with glycosphingolipid microdomains 1 [Source:HGNC Symbol;Acc:HGNC:30043]	protein coding	0.69	6.36	37.37	<0.0001	<0.0001
ENSG00000196177	ACADSB	acyl-CoA dehydrogenase short/branched chain [Source:HGNC Symbol;Acc:HGNC:91]	protein coding	-0.69	2.17	21.76	<0.0001	<0.0001
ENSG00000189343	RPS2P46	ribosomal protein S2 pseudogene 46 [Source:HGNC Symbol;Acc:HGNC:35839]	processed pseudogene	0.69	2.63	21.75	<0.0001	<0.0001
ENSG00000020633	RUNX3	RUNX family transcription factor 3 [Source:HGNC Symbol;Acc:HGNC:10473]	protein coding	0.69	4.70	25.29	<0.0001	<0.0001
ENSG00000070214	SLC44A1	solute carrier family 44 member 1 [Source:HGNC Symbol;Acc:HGNC:18798]	protein coding	-0.69	2.99	14.43	0.0001	0.0005
ENSG00000215908	CROCCP2	CROCC pseudogene 2 [Source:HGNC Symbol;Acc:HGNC:28170]	transcribed unprocessed pseudogene	-0.69	2.89	17.33	<0.0001	0.0001
ENSG00000215533	LINC00189	long intergenic non-protein coding RNA 189 [Source:HGNC Symbol;Acc:HGNC:18461]	lncRNA	0.68	2.14	16.01	<0.0001	0.0003
ENSG00000114450	GNB4	G protein subunit beta 4 [Source:HGNC Symbol;Acc:HGNC:20731]	protein coding	0.68	5.72	34.60	<0.0001	<0.0001
ENSG00000109184	DCUN1D4	defective in cullin neddylation 1 domain containing 4 [Source:HGNC Symbol;Acc:HGNC:28998]	protein coding	-0.68	1.68	19.22	<0.0001	<0.0001
ENSG00000110917	MLEC	malectin [Source:HGNC Symbol;Acc:HGNC:28973]	protein coding	-0.68	2.86	18.86	<0.0001	<0.0001

Table S18: Genes differentially expressed at Day 30 compared to pre-second vaccination (Study Arm 2 A/H7N9+AS03, D1, 29, Neutrophils). Genes are sorted by descending absolute log_2 fold change. Gene model summaries and annotations are based on Ensembl Version 99 (continued.)

Ensembl Gene ID	Ensembl Gene Name	Ensembl Gene Description	Gene Type	log_2 Fold Change (Day 30 vs. Pre-Second Vaccination)	Average log_2 CPM	Likelihood Ratio Test Statistic	P-Value	FDR Adjusted P-Value
ENSG00000080802	CNOT4	CCR4-NOT transcription complex subunit 4 [Source:HGNC Symbol;Acc:HGNC:7880]	protein coding	0.68	3.77	37.64	<0.0001	<0.0001
ENSG00000273137		novel transcript, antisense to SELO	lncRNA	-0.68	1.25	24.71	<0.0001	<0.0001
ENSG00000106268	NUDT1	nudix hydrolase 1 [Source:HGNC Symbol;Acc:HGNC:8048]	protein coding	-0.68	0.83	10.81	0.001	0.0031
ENSG00000124207	CSE1L	chromosome segregation 1 like [Source:HGNC Symbol;Acc:HGNC:2431]	protein coding	-0.68	2.67	31.59	<0.0001	<0.0001
ENSG00000171307	ZDHHC16	zinc finger DHHC-type containing 16 [Source:HGNC Symbol;Acc:HGNC:20714]	protein coding	-0.68	0.39	13.57	0.0002	0.0008
ENSG00000143641	GALNT2	polypeptide N-acetylgalactosaminyltransferase 2 [Source:HGNC Symbol;Acc:HGNC:4124]	protein coding	-0.68	4.49	44.52	<0.0001	<0.0001
ENSG00000145545	SRD5A1	steroid 5 alpha-reductase 1 [Source:HGNC Symbol;Acc:HGNC:11284]	protein coding	-0.68	0.87	15.44	<0.0001	0.0003
ENSG00000100918	REC8	REC8 meiotic recombination protein [Source:HGNC Symbol;Acc:HGNC:16879]	protein coding	0.68	5.65	32.65	<0.0001	<0.0001
ENSG00000177225	GATD1	glutamine amidotransferase like class 1 domain containing 1 [Source:HGNC Symbol;Acc:HGNC:26616]	protein coding	-0.68	2.21	30.51	<0.0001	<0.0001
ENSG00000141504	SAT2	spermidine/spermine N1-acetyltransferase family member 2 [Source:HGNC Symbol;Acc:HGNC:23160]	protein coding	0.68	2.10	22.76	<0.0001	<0.0001
ENSG00000133627	ACTR3B	actin related protein 3B [Source:HGNC Symbol;Acc:HGNC:17256]	protein coding	-0.68	1.01	19.35	<0.0001	<0.0001
ENSG00000174485	DENND4A	DENN domain containing 4A [Source:HGNC Symbol;Acc:HGNC:24321]	protein coding	0.68	4.04	15.19	<0.0001	0.0004
ENSG00000067208	EVI5	ecotropic viral integration site 5 [Source:HGNC Symbol;Acc:HGNC:3501]	protein coding	-0.68	3.55	26.11	<0.0001	<0.0001
ENSG00000125821	DTD1	D-aminoacyl-tRNA deacylase 1 [Source:HGNC Symbol;Acc:HGNC:16219]	protein coding	-0.68	2.73	32.18	<0.0001	<0.0001
ENSG00000183486	MX2	MX dynamin like GTPase 2 [Source:HGNC Symbol;Acc:HGNC:7533]	protein coding	0.68	9.45	41.08	<0.0001	<0.0001
ENSG00000232442	MHENCR	melanoma highly expressed competing endogenous lncRNA for miR-425 and miR-489 [Source:HGNC Symbol;Acc:HGNC:53110]	lncRNA	-0.68	3.53	24.85	<0.0001	<0.0001
ENSG00000137275	RIPK1	receptor interacting serine/threonine kinase 1 [Source:HGNC Symbol;Acc:HGNC:10019]	protein coding	0.68	6.24	31.78	<0.0001	<0.0001
ENSG00000268362		novel transcript	lncRNA	-0.68	0.64	7.42	0.0064	0.0155

Table S18: Genes differentially expressed at Day 30 compared to pre-second vaccination (Study Arm 2 A/H7N9+AS03, D1, 29, Neutrophils). Genes are sorted by descending absolute log_2 fold change. Gene model summaries and annotations are based on Ensembl Version 99 (continued.)

Ensembl Gene ID	Ensembl Gene Name	Ensembl Gene Description	Gene Type	log_2 Fold Change (Day 30 vs. Pre-Second Vaccination)	Average log_2 CPM	Likelihood Ratio Test Statistic	P-Value	FDR Adjusted P-Value
ENSG00000233578	EIF4EP1	eukaryotic translation initiation factor 4E pseudogene 1 [Source:HGNC Symbol;Acc:HGNC:3292]	processed pseudogene	-0.68	0.68	10.28	0.0013	0.004
ENSG00000163902	RPN1	ribophorin I [Source:HGNC Symbol;Acc:HGNC:10381]	protein coding	-0.68	7.49	51.11	<0.0001	<0.0001
ENSG00000127951	FGL2	fibrinogen like 2 [Source:HGNC Symbol;Acc:HGNC:3696]	protein coding	0.68	10.24	39.10	<0.0001	<0.0001
ENSG00000265975		novel transcript	lncRNA	0.68	2.45	25.77	<0.0001	<0.0001
ENSG00000074803	SLC12A1	solute carrier family 12 member 1 [Source:HGNC Symbol;Acc:HGNC:10910]	protein coding	-0.68	5.16	18.15	<0.0001	<0.0001
ENSG00000154889	MPPE1	metallophosphoesterase 1 [Source:HGNC Symbol;Acc:HGNC:15988]	protein coding	-0.68	7.55	57.21	<0.0001	<0.0001
ENSG00000235903	CPB2-AS1	CPB2 antisense RNA 1 [Source:HGNC Symbol;Acc:HGNC:39898]	lncRNA	0.67	1.34	16.42	<0.0001	0.0002
ENSG00000058668	ATP2B4	ATPase plasma membrane Ca2+ transporting 4 [Source:HGNC Symbol;Acc:HGNC:817]	protein coding	-0.67	6.40	24.59	<0.0001	<0.0001
ENSG00000073803	MAP3K13	mitogen-activated protein kinase kinase kinase 13 [Source:HGNC Symbol;Acc:HGNC:6852]	protein coding	0.67	3.58	39.82	<0.0001	<0.0001
ENSG00000164306	PRIMPOL	primase and DNA directed polymerase [Source:HGNC Symbol;Acc:HGNC:26575]	protein coding	-0.67	1.39	14.34	0.0002	0.0006
ENSG00000267141		novel transcript, antisense to KLF16	lncRNA	-0.67	0.43	5.42	0.0199	0.0413
ENSG00000197024	ZNF398	zinc finger protein 398 [Source:HGNC Symbol;Acc:HGNC:18373]	protein coding	-0.67	4.49	23.69	<0.0001	<0.0001
ENSG00000136783	NIPSNAP3A	nipsnap homolog 3A [Source:HGNC Symbol;Acc:HGNC:23619]	protein coding	0.67	0.95	16.63	<0.0001	0.0002
ENSG00000105223	PLD3	phospholipase D family member 3 [Source:HGNC Symbol;Acc:HGNC:17158]	protein coding	-0.67	4.29	22.52	<0.0001	<0.0001
ENSG00000171204	TMEM126B	transmembrane protein 126B [Source:HGNC Symbol;Acc:HGNC:30883]	protein coding	0.67	2.85	21.56	<0.0001	<0.0001
ENSG00000145390	USP53	ubiquitin specific peptidase 53 [Source:HGNC Symbol;Acc:HGNC:29255]	protein coding	-0.67	1.48	15.83	<0.0001	0.0003
ENSG00000255823	MTRNR2L8	MT-RNR2 like 8 [Source:HGNC Symbol;Acc:HGNC:37165]	protein coding	-0.67	2.22	10.30	0.0013	0.0039
ENSG00000241990	PRR34-AS1	PRR34 antisense RNA 1 [Source:HGNC Symbol;Acc:HGNC:50499]	lncRNA	-0.67	0.37	16.47	<0.0001	0.0002

Table S18: Genes differentially expressed at Day 30 compared to pre-second vaccination (Study Arm 2 A/H7N9+AS03, D1, 29, Neutrophils). Genes are sorted by descending absolute log_2 fold change. Gene model summaries and annotations are based on Ensembl Version 99 (continued.)

Ensembl Gene ID	Ensembl Gene Name	Ensembl Gene Description	Gene Type	log_2 Fold Change (Day 30 vs. Pre-Second Vaccination)	Average log_2 CPM	Likelihood Ratio Test Statistic	P-Value	FDR Adjusted P-Value
ENSG00000136193	SCRN1	secernin 1 [Source:HGNC Symbol;Acc:HGNC:22192]	protein coding	0.67	0.62	6.69	0.0097	0.0222
ENSG00000112096	SOD2	superoxide dismutase 2 [Source:HGNC Symbol;Acc:HGNC:11180]	protein coding	0.67	11.79	21.24	<0.0001	<0.0001
ENSG00000205890		novel transcript, antisense to CCDC64B	lncRNA	-0.67	2.76	30.84	<0.0001	<0.0001
ENSG00000158669	GPAT4	glycerol-3-phosphate acyltransferase 4 [Source:HGNC Symbol;Acc:HGNC:20880]	protein coding	-0.67	3.63	15.93	<0.0001	0.0003
ENSG00000277072	STAG3L2	stromal antigen 3-like 2 (pseudogene) [Source:HGNC Symbol;Acc:HGNC:33886]	transcribed unprocessed pseudogene	-0.67	0.47	12.13	0.0005	0.0016
ENSG00000187147	RNF220	ring finger protein 220 [Source:HGNC Symbol;Acc:HGNC:25552]	protein coding	-0.67	4.34	36.92	<0.0001	<0.0001
ENSG00000163683	SMIM14	small integral membrane protein 14 [Source:HGNC Symbol;Acc:HGNC:27321]	protein coding	0.67	5.04	33.93	<0.0001	<0.0001
ENSG00000171867	PRNP	prion protein [Source:HGNC Symbol;Acc:HGNC:9449]	protein coding	-0.67	0.67	8.18	0.0042	0.0107
ENSG00000135124	P2RX4	purinergic receptor P2X 4 [Source:HGNC Symbol;Acc:HGNC:8535]	protein coding	0.67	1.17	13.20	0.0003	0.001
ENSG00000176749	CDK5R1	cyclin dependent kinase 5 regulatory subunit 1 [Source:HGNC Symbol;Acc:HGNC:1775]	protein coding	-0.67	4.43	11.64	0.0006	0.0021
ENSG00000240230	COX19	cytochrome c oxidase assembly factor COX19 [Source:HGNC Symbol;Acc:HGNC:28074]	protein coding	-0.67	3.67	37.06	<0.0001	<0.0001
ENSG00000253641		novel transcript	lncRNA	-0.67	0.21	8.69	0.0032	0.0084
ENSG00000137776	SLTM	SAFB like transcription modulator [Source:HGNC Symbol;Acc:HGNC:20709]	protein coding	-0.67	5.88	38.79	<0.0001	<0.0001
ENSG00000187514	PTMA	prothymosin alpha [Source:HGNC Symbol;Acc:HGNC:9623]	protein coding	-0.67	6.39	36.47	<0.0001	<0.0001
ENSG00000275210		novel transcript	lncRNA	0.67	2.10	10.48	0.0012	0.0036
ENSG00000181523	SGSH	N-sulfoglucosamine sulfohydrolase [Source:HGNC Symbol;Acc:HGNC:10818]	protein coding	-0.67	3.26	29.15	<0.0001	<0.0001
ENSG00000165548	TMEM63C	transmembrane protein 63C [Source:HGNC Symbol;Acc:HGNC:23787]	protein coding	-0.67	3.07	11.91	0.0006	0.0018
ENSG00000164877	MICALL2	MICAL like 2 [Source:HGNC Symbol;Acc:HGNC:29672]	protein coding	-0.67	1.18	15.22	<0.0001	0.0004

Table S18: Genes differentially expressed at Day 30 compared to pre-second vaccination (Study Arm 2 A/H7N9+AS03, D1, 29, Neutrophils). Genes are sorted by descending absolute log_2 fold change. Gene model summaries and annotations are based on Ensembl Version 99 (continued.)

Ensembl Gene ID	Ensembl Gene Name	Ensembl Gene Description	Gene Type	log_2 Fold Change (Day 30 vs. Pre-Second Vaccination)	Average log_2 CPM	Likelihood Ratio Test Statistic	P-Value	FDR Adjusted P-Value
ENSG00000258738		novel transcript, antisense to BAZ1A	lncRNA	0.67	3.60	19.80	<0.0001	<0.0001
ENSG00000090372	STRN4	striatin 4 [Source:HGNC Symbol;Acc:HGNC:15721]	protein coding	-0.67	7.69	54.18	<0.0001	<0.0001
ENSG00000173818	ENDOV	endonuclease V [Source:HGNC Symbol;Acc:HGNC:26640]	protein coding	-0.67	1.74	24.65	<0.0001	<0.0001
ENSG00000108306	FBXL20	F-box and leucine rich repeat protein 20 [Source:HGNC Symbol;Acc:HGNC:24679]	protein coding	-0.66	5.94	40.37	<0.0001	<0.0001
ENSG00000124570	SERPINB6	serpin family B member 6 [Source:HGNC Symbol;Acc:HGNC:8950]	protein coding	0.66	3.31	46.11	<0.0001	<0.0001
ENSG00000099985	OSM	oncostatin M [Source:HGNC Symbol;Acc:HGNC:8506]	protein coding	0.66	6.75	16.81	<0.0001	0.0002
ENSG00000138111	MFSD13A	major facilitator superfamily domain containing 13A [Source:HGNC Symbol;Acc:HGNC:26196]	protein coding	0.66	3.12	39.90	<0.0001	<0.0001
ENSG00000186469	GNG2	G protein subunit gamma 2 [Source:HGNC Symbol;Acc:HGNC:4404]	protein coding	-0.66	7.00	12.07	0.0005	0.0017
ENSG00000149212	SESN3	sestrin 3 [Source:HGNC Symbol;Acc:HGNC:23060]	protein coding	-0.66	4.54	24.60	<0.0001	<0.0001
ENSG00000272918		novel transcript, antisense to MLL5	lncRNA	-0.66	1.40	12.85	0.0003	0.0011
ENSG00000135899	SP110	SP110 nuclear body protein [Source:HGNC Symbol;Acc:HGNC:5401]	protein coding	0.66	8.57	37.64	<0.0001	<0.0001
ENSG00000279330		TEC	TEC	-0.66	1.37	11.58	0.0007	0.0021
ENSG00000115306	SPTBN1	spectrin beta, non-erythrocytic 1 [Source:HGNC Symbol;Acc:HGNC:11275]	protein coding	-0.66	3.99	8.22	0.0041	0.0106
ENSG00000274963	RN7SL600P	RNA, 7SL, cytoplasmic 600, pseudogene [Source:HGNC Symbol;Acc:HGNC:46616]	misc RNA	0.66	2.13	13.64	0.0002	0.0008
ENSG00000006607	FARP2	FERM, ARH/RhoGEF and pleckstrin domain protein 2 [Source:HGNC Symbol;Acc:HGNC:16460]	protein coding	-0.66	1.63	18.20	<0.0001	<0.0001
ENSG00000110063	DCPS	decapping enzyme, scavenger [Source:HGNC Symbol;Acc:HGNC:29812]	protein coding	-0.66	2.07	18.30	<0.0001	<0.0001
ENSG00000077232	DNAJC10	DnaJ heat shock protein family (Hsp40) member C10 [Source:HGNC Symbol;Acc:HGNC:24637]	protein coding	0.66	3.13	30.65	<0.0001	<0.0001
ENSG00000188234	AGAP4	ArfGAP with GTPase domain, ankyrin repeat and PH domain 4 [Source:HGNC Symbol;Acc:HGNC:23459]	protein coding	-0.66	2.20	21.52	<0.0001	<0.0001
ENSG00000133731	IMPA1	inositol monophosphatase 1 [Source:HGNC Symbol;Acc:HGNC:6050]	protein coding	0.66	3.78	37.99	<0.0001	<0.0001

Table S18: Genes differentially expressed at Day 30 compared to pre-second vaccination (Study Arm 2 A/H7N9+AS03, D1, 29, Neutrophils). Genes are sorted by descending absolute log_2 fold change. Gene model summaries and annotations are based on Ensembl Version 99 (continued.)

Ensembl Gene ID	Ensembl Gene Name	Ensembl Gene Description	Gene Type	log_2 Fold Change (Day 30 vs. Pre-Second Vaccination)	Average log_2 CPM	Likelihood Ratio Test Statistic	P-Value	FDR Adjusted P-Value
ENSG00000100997	ABHD12	abhydrolase domain containing 12 [Source:HGNC Symbol;Acc:HGNC:15868]	protein coding	-0.66	1.44	17.65	<0.0001	0.0001
ENSG00000114650	SCAP	SREBF chaperone [Source:HGNC Symbol;Acc:HGNC:30634]	protein coding	-0.66	7.58	46.58	<0.0001	<0.0001
ENSG00000100711	ZFYVE21	zinc finger FYVE-type containing 21 [Source:HGNC Symbol;Acc:HGNC:20760]	protein coding	-0.66	3.13	28.52	<0.0001	<0.0001
ENSG00000188404	SELL	selectin L [Source:HGNC Symbol;Acc:HGNC:10720]	protein coding	0.66	11.63	41.24	<0.0001	<0.0001
ENSG00000187554	TLR5	toll like receptor 5 [Source:HGNC Symbol;Acc:HGNC:11851]	protein coding	0.66	5.18	16.58	<0.0001	0.0002
ENSG00000110497	AMBRA1	autophagy and beclin 1 regulator 1 [Source:HGNC Symbol;Acc:HGNC:25990]	protein coding	0.66	3.96	29.37	<0.0001	<0.0001
ENSG00000163527	STT3B	STT3 oligosaccharyltransferase complex catalytic subunit B [Source:HGNC Symbol;Acc:HGNC:30611]	protein coding	-0.66	5.54	40.85	<0.0001	<0.0001
ENSG00000130177	CDC16	cell division cycle 16 [Source:HGNC Symbol;Acc:HGNC:1720]	protein coding	-0.66	1.75	14.72	0.0001	0.0005
ENSG00000115286	NDUFS7	NADH:ubiquinone oxidoreductase core subunit S7 [Source:HGNC Symbol;Acc:HGNC:7714]	protein coding	-0.66	2.80	23.68	<0.0001	<0.0001
ENSG00000055070	SZRD1	SUZ RNA binding domain containing 1 [Source:HGNC Symbol;Acc:HGNC:30232]	protein coding	0.66	6.34	40.62	<0.0001	<0.0001
ENSG00000287725		novel protein, TPCN2 - SMIM38 readthrough	protein coding	-0.66	0.55	13.43	0.0002	0.0009
ENSG00000186063	AIDA	axin interactor, dorsalization associated [Source:HGNC Symbol;Acc:HGNC:25761]	protein coding	0.66	2.81	24.80	<0.0001	<0.0001
ENSG00000067704	IARS2	isoleucyl-tRNA synthetase 2, mitochondrial [Source:HGNC Symbol;Acc:HGNC:29685]	protein coding	0.66	3.18	22.57	<0.0001	<0.0001
ENSG00000117298	ECE1	endothelin converting enzyme 1 [Source:HGNC Symbol;Acc:HGNC:3146]	protein coding	0.66	9.32	26.88	<0.0001	<0.0001
ENSG00000148344	PTGES	prostaglandin E synthase [Source:HGNC Symbol;Acc:HGNC:9599]	protein coding	-0.66	1.59	8.83	0.003	0.0079
ENSG00000107745	MICU1	mitochondrial calcium uptake 1 [Source:HGNC Symbol;Acc:HGNC:1530]	protein coding	0.66	5.69	43.12	<0.0001	<0.0001
ENSG00000173926	MARCHF3	membrane associated ring-CH-type finger 3 [Source:HGNC Symbol;Acc:HGNC:28728]	protein coding	0.66	0.58	10.92	0.001	0.0029
ENSG00000167642	SPINT2	serine peptidase inhibitor, Kunitz type 2 [Source:HGNC Symbol;Acc:HGNC:11247]	protein coding	0.66	4.66	44.06	<0.0001	<0.0001

Table S18: Genes differentially expressed at Day 30 compared to pre-second vaccination (Study Arm 2 A/H7N9+AS03, D1, 29, Neutrophils). Genes are sorted by descending absolute log_2 fold change. Gene model summaries and annotations are based on Ensembl Version 99 (continued.)

Ensembl Gene ID	Ensembl Gene Name	Ensembl Gene Description	Gene Type	log_2 Fold Change (Day 30 vs. Pre-Second Vaccination)	Average log_2 CPM	Likelihood Ratio Test Statistic	P-Value	FDR Adjusted P-Value
ENSG00000171617	ENC1	ectodermal-neural cortex 1 [Source:HGNC Symbol;Acc:HGNC:3345]	protein coding	-0.66	3.58	16.06	<0.0001	0.0002
ENSG00000168310	IRF2	interferon regulatory factor 2 [Source:HGNC Symbol;Acc:HGNC:6117]	protein coding	0.66	8.28	30.95	<0.0001	<0.0001
ENSG00000165516	KLHDC2	kelch domain containing 2 [Source:HGNC Symbol;Acc:HGNC:20231]	protein coding	-0.66	4.12	47.73	<0.0001	<0.0001
ENSG00000205155	PSENEN	presenilin enhancer, gamma-secretase subunit [Source:HGNC Symbol;Acc:HGNC:30100]	protein coding	0.65	4.88	20.04	<0.0001	<0.0001
ENSG00000162961	DPY30	dpy-30 histone methyltransferase complex regulatory subunit [Source:HGNC Symbol;Acc:HGNC:24590]	protein coding	-0.65	3.85	26.47	<0.0001	<0.0001
ENSG00000084207	GSTP1	glutathione S-transferase pi 1 [Source:HGNC Symbol;Acc:HGNC:4638]	protein coding	-0.65	1.92	14.40	0.0001	0.0005
ENSG00000133059	DSTYK	dual serine/threonine and tyrosine protein kinase [Source:HGNC Symbol;Acc:HGNC:29043]	protein coding	-0.65	3.62	23.94	<0.0001	<0.0001
ENSG00000060138	YBX3	Y-box binding protein 3 [Source:HGNC Symbol;Acc:HGNC:2428]	protein coding	-0.65	5.14	6.71	0.0096	0.022
ENSG00000188822	CNR2	cannabinoid receptor 2 [Source:HGNC Symbol;Acc:HGNC:2160]	protein coding	-0.65	1.74	5.16	0.0231	0.0468
ENSG00000078269	SYNJ2	synaptojanin 2 [Source:HGNC Symbol;Acc:HGNC:11504]	protein coding	0.65	3.15	17.22	<0.0001	0.0001
ENSG00000246339	EXTL3-AS1	EXTL3 antisense RNA 1 [Source:HGNC Symbol;Acc:HGNC:27985]	lncRNA	-0.65	0.46	11.85	0.0006	0.0019
ENSG00000163516	ANKZF1	ankyrin repeat and zinc finger peptidyl tRNA hydrolase 1 [Source:HGNC Symbol;Acc:HGNC:25527]	protein coding	-0.65	5.77	38.62	<0.0001	<0.0001
ENSG00000107404	DVL1	dishevelled segment polarity protein 1 [Source:HGNC Symbol;Acc:HGNC:3084]	protein coding	-0.65	3.73	38.84	<0.0001	<0.0001
ENSG00000155313	USP25	ubiquitin specific peptidase 25 [Source:HGNC Symbol;Acc:HGNC:12624]	protein coding	0.65	6.56	45.36	<0.0001	<0.0001
ENSG00000143198	MGST3	microsomal glutathione S-transferase 3 [Source:HGNC Symbol;Acc:HGNC:7064]	protein coding	-0.65	0.63	10.04	0.0015	0.0044
ENSG00000224183	SDHDP6	succinate dehydrogenase complex subunit D pseudogene 6 [Source:HGNC Symbol;Acc:HGNC:10689]	processed pseudogene	-0.65	0.65	14.00	0.0002	0.0007
ENSG00000235750	KIAA0040	KIAA0040 [Source:HGNC Symbol;Acc:HGNC:28950]	protein coding	0.65	8.40	30.55	<0.0001	<0.0001

Table S18: Genes differentially expressed at Day 30 compared to pre-second vaccination (Study Arm 2 A/H7N9+AS03, D1, 29, Neutrophils). Genes are sorted by descending absolute log_2 fold change. Gene model summaries and annotations are based on Ensembl Version 99 (continued.)

Ensembl Gene ID	Ensembl Gene Name	Ensembl Gene Description	Gene Type	log_2 Fold Change (Day 30 vs. Pre-Second Vaccination)	Average log_2 CPM	Likelihood Ratio Test Statistic	P-Value	FDR Adjusted P-Value
ENSG00000027075	PRKCH	protein kinase C eta [Source:HGNC Symbol;Acc:HGNC:9403]	protein coding	0.65	2.89	16.65	<0.0001	0.0002
ENSG00000171291	ZNF439	zinc finger protein 439 [Source:HGNC Symbol;Acc:HGNC:20873]	protein coding	-0.65	1.56	17.31	<0.0001	0.0001
ENSG00000115839	RAB3GAP1	RAB3 GTPase activating protein catalytic subunit 1 [Source:HGNC Symbol;Acc:HGNC:17063]	protein coding	0.65	4.59	46.45	<0.0001	<0.0001
ENSG00000227372	TP73-AS1	TP73 antisense RNA 1 [Source:HGNC Symbol;Acc:HGNC:29052]	transcribed unitary pseudogene	-0.65	1.13	12.65	0.0004	0.0013
ENSG00000073605	GSDMB	gasdermin B [Source:HGNC Symbol;Acc:HGNC:23690]	protein coding	0.65	2.92	23.59	<0.0001	<0.0001
ENSG00000162585	FAAP20	FA core complex associated protein 20 [Source:HGNC Symbol;Acc:HGNC:26428]	protein coding	-0.65	4.30	37.13	<0.0001	<0.0001
ENSG00000285737	LINC02680	long intergenic non-protein coding RNA 2680 [Source:HGNC Symbol;Acc:HGNC:54174]	lncRNA	-0.65	1.11	18.94	<0.0001	<0.0001
ENSG00000184900	SUMO3	small ubiquitin like modifier 3 [Source:HGNC Symbol;Acc:HGNC:11124]	protein coding	-0.65	2.83	18.21	<0.0001	<0.0001
ENSG00000143382	ADAMTSL4	ADAMTS like 4 [Source:HGNC Symbol;Acc:HGNC:19706]	protein coding	0.65	7.78	24.45	<0.0001	<0.0001
ENSG00000197540	GZMM	granzyme M [Source:HGNC Symbol;Acc:HGNC:4712]	protein coding	-0.65	0.29	8.63	0.0033	0.0087
ENSG00000251230	MIR3945HG	MIR3945 host gene [Source:HGNC Symbol;Acc:HGNC:52002]	lncRNA	0.65	2.91	19.50	<0.0001	<0.0001
ENSG00000175137	SH3BP5L	SH3 binding domain protein 5 like [Source:HGNC Symbol;Acc:HGNC:29360]	protein coding	-0.65	6.85	20.62	<0.0001	<0.0001
ENSG00000236409	NRADDP	neurotrophin receptor associated death domain, pseudogene [Source:HGNC Symbol;Acc:HGNC:19337]	transcribed unitary pseudogene	0.65	1.21	19.50	<0.0001	<0.0001
ENSG00000091436	MAP3K20	mitogen-activated protein kinase kinase kinase 20 [Source:HGNC Symbol;Acc:HGNC:17797]	protein coding	-0.65	2.73	12.21	0.0005	0.0016
ENSG00000233214		novel transcript	lncRNA	0.65	1.68	17.07	<0.0001	0.0002
ENSG00000222041	CYTOR	cytoskeleton regulator RNA [Source:HGNC Symbol;Acc:HGNC:28717]	lncRNA	0.65	4.31	25.31	<0.0001	<0.0001

Table S18: Genes differentially expressed at Day 30 compared to pre-second vaccination (Study Arm 2 A/H7N9+AS03, D1, 29, Neutrophils). Genes are sorted by descending absolute log_2 fold change. Gene model summaries and annotations are based on Ensembl Version 99 (continued.)

Ensembl Gene ID	Ensembl Gene Name	Ensembl Gene Description	Gene Type	log_2 Fold Change (Day 30 vs. Pre-Second Vaccination)	Average log_2 CPM	Likelihood Ratio Test Statistic	P-Value	FDR Adjusted P-Value
ENSG00000078687	TNRC6C	trinucleotide repeat containing adaptor 6C [Source:HGNC Symbol;Acc:HGNC:29318]	protein coding	-0.65	3.86	28.76	<0.0001	<0.0001
ENSG00000008382	MPND	MPN domain containing [Source:HGNC Symbol;Acc:HGNC:25934]	protein coding	-0.65	2.36	25.02	<0.0001	<0.0001
ENSG00000132879	FBXO44	F-box protein 44 [Source:HGNC Symbol;Acc:HGNC:24847]	protein coding	0.65	0.49	13.45	0.0002	0.0009
ENSG00000110047	EHD1	EH domain containing 1 [Source:HGNC Symbol;Acc:HGNC:3242]	protein coding	0.65	9.14	28.42	<0.0001	<0.0001
ENSG00000136404	TM6SF1	transmembrane 6 superfamily member 1 [Source:HGNC Symbol;Acc:HGNC:11860]	protein coding	-0.65	5.22	32.83	<0.0001	<0.0001
ENSG00000186047	DLEU7	deleted in lymphocytic leukemia 7 [Source:HGNC Symbol;Acc:HGNC:17567]	protein coding	0.65	0.86	10.38	0.0013	0.0038
ENSG00000126001	CEP250	centrosomal protein 250 [Source:HGNC Symbol;Acc:HGNC:1859]	protein coding	-0.65	2.61	13.44	0.0002	0.0009
ENSG00000062598	ELMO2	engulfment and cell motility 2 [Source:HGNC Symbol;Acc:HGNC:17233]	protein coding	0.65	5.50	38.13	<0.0001	<0.0001
ENSG00000227398	KIF9-AS1	KIF9 antisense RNA 1 [Source:HGNC Symbol;Acc:HGNC:26822]	lncRNA	0.65	0.77	16.56	<0.0001	0.0002
ENSG00000226067	LINC00623	long intergenic non-protein coding RNA 623 [Source:HGNC Symbol;Acc:HGNC:44252]	lncRNA	0.65	5.00	26.62	<0.0001	<0.0001
ENSG00000120051	CFAP58	cilia and flagella associated protein 58 [Source:HGNC Symbol;Acc:HGNC:26676]	protein coding	0.65	3.02	25.62	<0.0001	<0.0001
ENSG00000090686	USP48	ubiquitin specific peptidase 48 [Source:HGNC Symbol;Acc:HGNC:18533]	protein coding	-0.65	5.49	43.33	<0.0001	<0.0001
ENSG00000162836	ACP6	acid phosphatase 6, lysophosphatidic [Source:HGNC Symbol;Acc:HGNC:29609]	protein coding	-0.65	2.31	9.59	0.002	0.0055
ENSG00000205786	LINC01531	long intergenic non-protein coding RNA 1531 [Source:HGNC Symbol;Acc:HGNC:51270]	lncRNA	0.65	2.10	13.66	0.0002	0.0008
ENSG00000171206	TRIM8	tripartite motif containing 8 [Source:HGNC Symbol;Acc:HGNC:15579]	protein coding	-0.65	7.97	56.27	<0.0001	<0.0001
ENSG00000104856	RELB	RELB proto-oncogene, NF-kB subunit [Source:HGNC Symbol;Acc:HGNC:9956]	protein coding	0.65	6.99	29.48	<0.0001	<0.0001
ENSG00000136682	CBWD2	COBW domain containing 2 [Source:HGNC Symbol;Acc:HGNC:17907]	protein coding	0.65	3.57	14.94	0.0001	0.0004
ENSG00000268355		novel transcript	lncRNA	-0.65	1.45	9.28	0.0023	0.0063

Table S18: Genes differentially expressed at Day 30 compared to pre-second vaccination (Study Arm 2 A/H7N9+AS03, D1, 29, Neutrophils). Genes are sorted by descending absolute log_2 fold change. Gene model summaries and annotations are based on Ensembl Version 99 (continued.)

Ensembl Gene ID	Ensembl Gene Name	Ensembl Gene Description	Gene Type	log_2 Fold Change (Day 30 vs. Pre-Second Vaccination)	Average log_2 CPM	Likelihood Ratio Test Statistic	P-Value	FDR Adjusted P-Value
ENSG00000100902	PSMA6	proteasome 20S subunit alpha 6 [Source:HGNC Symbol;Acc:HGNC:9535]	protein coding	0.65	3.33	29.71	<0.0001	<0.0001
ENSG00000179262	RAD23A	RAD23 homolog A, nucleotide excision repair protein [Source:HGNC Symbol;Acc:HGNC:9812]	protein coding	-0.65	1.88	13.39	0.0003	0.0009
ENSG00000165115	KIF27	kinesin family member 27 [Source:HGNC Symbol;Acc:HGNC:18632]	protein coding	-0.64	4.84	33.48	<0.0001	<0.0001
ENSG00000115756	HPCAL1	hippocalcin like 1 [Source:HGNC Symbol;Acc:HGNC:5145]	protein coding	-0.64	6.67	33.99	<0.0001	<0.0001
ENSG00000121851	POLR3GL	RNA polymerase III subunit G like [Source:HGNC Symbol;Acc:HGNC:28466]	protein coding	0.64	3.12	32.35	<0.0001	<0.0001
ENSG00000146376	ARHGAP18	Rho GTPase activating protein 18 [Source:HGNC Symbol;Acc:HGNC:21035]	protein coding	-0.64	2.47	10.00	0.0016	0.0045
ENSG00000103066	PLA2G15	phospholipase A2 group XV [Source:HGNC Symbol;Acc:HGNC:17163]	protein coding	-0.64	0.78	15.18	<0.0001	0.0004
ENSG00000124203	ZNF831	zinc finger protein 831 [Source:HGNC Symbol;Acc:HGNC:16167]	protein coding	-0.64	0.50	10.71	0.0011	0.0032
ENSG00000136560	TANK	TRAF family member associated NFKB activator [Source:HGNC Symbol;Acc:HGNC:11562]	protein coding	0.64	7.05	36.21	<0.0001	<0.0001
ENSG00000213420	GPC2	glypican 2 [Source:HGNC Symbol;Acc:HGNC:4450]	protein coding	-0.64	2.09	22.40	<0.0001	<0.0001
ENSG00000287934		novel transcript, antisense to KDM2A	lncRNA	0.64	0.50	12.83	0.0003	0.0012
ENSG00000152117		novel pseudogene	transcribed processed pseudogene	-0.64	3.04	17.59	<0.0001	0.0001
ENSG00000286646		novel transcript, antisense to MAP3K5	lncRNA	0.64	3.75	27.11	<0.0001	<0.0001
ENSG00000176476	SGF29	SAGA complex associated factor 29 [Source:HGNC Symbol;Acc:HGNC:25156]	protein coding	0.64	2.34	24.43	<0.0001	<0.0001
ENSG00000225697	SLC26A6	solute carrier family 26 member 6 [Source:HGNC Symbol;Acc:HGNC:14472]	protein coding	-0.64	2.51	13.43	0.0002	0.0009
ENSG00000112078	KCTD20	potassium channel tetramerization domain containing 20 [Source:HGNC Symbol;Acc:HGNC:21052]	protein coding	-0.64	5.82	24.40	<0.0001	<0.0001
ENSG00000213337	ANKRD39	ankyrin repeat domain 39 [Source:HGNC Symbol;Acc:HGNC:28640]	protein coding	-0.64	0.25	11.34	0.0008	0.0024
ENSG00000261371	PECAM1	platelet and endothelial cell adhesion molecule 1 [Source:HGNC Symbol;Acc:HGNC:8823]	protein coding	-0.64	10.05	66.01	<0.0001	<0.0001

Table S18: Genes differentially expressed at Day 30 compared to pre-second vaccination (Study Arm 2 A/H7N9+AS03, D1, 29, Neutrophils). Genes are sorted by descending absolute log_2 fold change. Gene model summaries and annotations are based on Ensembl Version 99 (continued.)

Ensembl Gene ID	Ensembl Gene Name	Ensembl Gene Description	Gene Type	log_2 Fold Change (Day 30 vs. Pre-Second Vaccination)	Average log_2 CPM	Likelihood Ratio Test Statistic	P-Value	FDR Adjusted P-Value
ENSG00000281358	RASSF1-AS1	RASSF1 antisense RNA 1 [Source:HGNC Symbol;Acc:HGNC:49091]	lncRNA	-0.64	0.24	12.36	0.0004	0.0015
ENSG00000168476	REEP4	receptor accessory protein 4 [Source:HGNC Symbol;Acc:HGNC:26176]	protein coding	-0.64	3.72	22.09	<0.0001	<0.0001
ENSG00000122783	CYREN	cell cycle regulator of NHEJ [Source:HGNC Symbol;Acc:HGNC:22432]	protein coding	0.64	5.99	27.26	<0.0001	<0.0001
ENSG00000137460	FHDC1	FH2 domain containing 1 [Source:HGNC Symbol;Acc:HGNC:29363]	protein coding	0.64	3.60	18.89	<0.0001	<0.0001
ENSG00000143702	CEP170	centrosomal protein 170 [Source:HGNC Symbol;Acc:HGNC:28920]	protein coding	-0.64	4.74	19.73	<0.0001	<0.0001
ENSG00000168209	DDIT4	DNA damage inducible transcript 4 [Source:HGNC Symbol;Acc:HGNC:24944]	protein coding	-0.64	4.95	5.79	0.0162	0.0346
ENSG00000258082		novel transcript	lncRNA	-0.64	1.39	13.03	0.0003	0.0011
ENSG00000196369	SRGAP2B	SLIT-ROBO Rho GTPase activating protein 2B [Source:HGNC Symbol;Acc:HGNC:35237]	protein coding	0.64	2.71	23.97	<0.0001	<0.0001
ENSG00000183506	PI4KAP2	phosphatidylinositol 4-kinase alpha pseudogene 2 [Source:HGNC Symbol;Acc:HGNC:33577]	transcribed unitary pseudogene	-0.64	1.18	13.66	0.0002	0.0008
ENSG00000203880	PCMTD2	protein-L-isoaspartate (D-aspartate) O-methyltransferase domain containing 2 [Source:HGNC Symbol;Acc:HGNC:15882]	protein coding	-0.64	5.34	50.71	<0.0001	<0.0001
ENSG00000127948	POR	cytochrome p450 oxidoreductase [Source:HGNC Symbol;Acc:HGNC:9208]	protein coding	-0.64	6.36	37.90	<0.0001	<0.0001
ENSG00000114354	TFG	trafficking from ER to golgi regulator [Source:HGNC Symbol;Acc:HGNC:11758]	protein coding	0.64	4.91	30.42	<0.0001	<0.0001
ENSG00000128487	SPECC1	sperm antigen with calponin homology and coiled-coil domains 1 [Source:HGNC Symbol;Acc:HGNC:30615]	protein coding	0.64	5.29	39.45	<0.0001	<0.0001
ENSG00000158062	UBXN11	UBX domain protein 11 [Source:HGNC Symbol;Acc:HGNC:30600]	protein coding	-0.64	3.54	19.27	<0.0001	<0.0001
ENSG00000178035	IMPDH2	inosine monophosphate dehydrogenase 2 [Source:HGNC Symbol;Acc:HGNC:6053]	protein coding	-0.64	2.97	31.14	<0.0001	<0.0001
ENSG00000122696	SLC25A51	solute carrier family 25 member 51 [Source:HGNC Symbol;Acc:HGNC:23323]	protein coding	-0.64	0.95	11.60	0.0007	0.0021
ENSG00000161791	FMNL3	formin like 3 [Source:HGNC Symbol;Acc:HGNC:23698]	protein coding	0.64	2.08	13.56	0.0002	0.0008

Table S18: Genes differentially expressed at Day 30 compared to pre-second vaccination (Study Arm 2 A/H7N9+AS03, D1, 29, Neutrophils). Genes are sorted by descending absolute log_2 fold change. Gene model summaries and annotations are based on Ensembl Version 99 (continued.)

Ensembl Gene ID	Ensembl Gene Name	Ensembl Gene Description	Gene Type	log_2 Fold Change (Day 30 vs. Pre-Second Vaccination)	Average log_2 CPM	Likelihood Ratio Test Statistic	P-Value	FDR Adjusted P-Value
ENSG00000078814	MYH7B	myosin heavy chain 7B [Source:HGNC Symbol;Acc:HGNC:15906]	protein coding	0.64	1.24	16.45	<0.0001	0.0002
ENSG00000125827	TMX4	thioredoxin related transmembrane protein 4 [Source:HGNC Symbol;Acc:HGNC:25237]	protein coding	-0.64	7.79	46.25	<0.0001	<0.0001
ENSG00000164236	ANKRD33B	ankyrin repeat domain 33B [Source:HGNC Symbol;Acc:HGNC:35240]	protein coding	0.64	3.80	22.07	<0.0001	<0.0001
ENSG00000134262	AP4B1	adaptor related protein complex 4 subunit beta 1 [Source:HGNC Symbol;Acc:HGNC:572]	protein coding	-0.64	2.05	15.47	<0.0001	0.0003
ENSG00000021355	SERPINB1	serpin family B member 1 [Source:HGNC Symbol;Acc:HGNC:3311]	protein coding	0.63	8.58	48.33	<0.0001	<0.0001
ENSG00000178464	RPL10P16	ribosomal protein L10 pseudogene 16 [Source:HGNC Symbol;Acc:HGNC:36882]	processed pseudogene	-0.63	1.87	20.62	<0.0001	<0.0001
ENSG00000146707	POMZP3	POM121 and ZP3 fusion [Source:HGNC Symbol;Acc:HGNC:9203]	protein coding	-0.63	1.07	14.19	0.0002	0.0006
ENSG00000175662	TOM1L2	target of myb1 like 2 membrane trafficking protein [Source:HGNC Symbol;Acc:HGNC:11984]	protein coding	-0.63	4.82	33.39	<0.0001	<0.0001
ENSG00000204054	LINC00963	long intergenic non-protein coding RNA 963 [Source:HGNC Symbol;Acc:HGNC:48716]	lncRNA	-0.63	6.43	40.96	<0.0001	<0.0001
ENSG00000166889	PATL1	PAT1 homolog 1, processing body mRNA decay factor [Source:HGNC Symbol;Acc:HGNC:26721]	protein coding	0.63	7.20	42.51	<0.0001	<0.0001
ENSG00000280332		TEC	TEC	-0.63	3.19	13.48	0.0002	0.0009
ENSG00000100075	SLC25A1	solute carrier family 25 member 1 [Source:HGNC Symbol;Acc:HGNC:10979]	protein coding	-0.63	3.91	24.43	<0.0001	<0.0001
ENSG00000183735	TBK1	TANK binding kinase 1 [Source:HGNC Symbol;Acc:HGNC:11584]	protein coding	0.63	6.08	33.16	<0.0001	<0.0001
ENSG00000231964		novel transcript	lncRNA	-0.63	3.10	13.67	0.0002	0.0008
ENSG00000164111	ANXA5	annexin A5 [Source:HGNC Symbol;Acc:HGNC:543]	protein coding	0.63	6.27	45.72	<0.0001	<0.0001
ENSG00000069998	HDHD5	haloacid dehalogenase like hydrolase domain containing 5 [Source:HGNC Symbol;Acc:HGNC:1843]	protein coding	-0.63	1.43	17.06	<0.0001	0.0002
ENSG00000108424	KPNB1	karyopherin subunit beta 1 [Source:HGNC Symbol;Acc:HGNC:6400]	protein coding	0.63	8.92	43.59	<0.0001	<0.0001
ENSG00000172967	XKR3	XK related 3 [Source:HGNC Symbol;Acc:HGNC:28778]	protein coding	-0.63	1.43	7.76	0.0054	0.0133

Table S18: Genes differentially expressed at Day 30 compared to pre-second vaccination (Study Arm 2 A/H7N9+AS03, D1, 29, Neutrophils). Genes are sorted by descending absolute log_2 fold change. Gene model summaries and annotations are based on Ensembl Version 99 (continued.)

Ensembl Gene ID	Ensembl Gene Name	Ensembl Gene Description	Gene Type	log_2 Fold Change (Day 30 vs. Pre-Second Vaccination)	Average log_2 CPM	Likelihood Ratio Test Statistic	P-Value	FDR Adjusted P-Value
ENSG00000124228	DDX27	DEAD-box helicase 27 [Source:HGNC Symbol;Acc:HGNC:15837]	protein coding	-0.63	3.75	30.05	<0.0001	<0.0001
ENSG00000099821	POLRMT	RNA polymerase mitochondrial [Source:HGNC Symbol;Acc:HGNC:9200]	protein coding	-0.63	1.88	16.20	<0.0001	0.0002
ENSG00000152620	NADK2	NAD kinase 2, mitochondrial [Source:HGNC Symbol;Acc:HGNC:26404]	protein coding	-0.63	0.26	10.57	0.0012	0.0034
ENSG00000286940		novel transcript	lncRNA	0.63	3.25	14.64	0.0001	0.0005
ENSG00000142046		transmembrane protein 91 [Source:NCBI gene;Acc:641649]	protein coding	-0.63	6.47	34.34	<0.0001	<0.0001
ENSG00000175193	PARL	presenilin associated rhomboid like [Source:HGNC Symbol;Acc:HGNC:18253]	protein coding	-0.63	1.49	11.82	0.0006	0.0019
ENSG00000118520	ARG1	arginase 1 [Source:HGNC Symbol;Acc:HGNC:663]	protein coding	-0.63	4.26	10.47	0.0012	0.0036
ENSG00000234511	C5orf58	chromosome 5 open reading frame 58 [Source:HGNC Symbol;Acc:HGNC:37272]	protein coding	0.63	2.29	9.57	0.002	0.0056
ENSG00000106344	RBM28	RNA binding motif protein 28 [Source:HGNC Symbol;Acc:HGNC:21863]	protein coding	-0.63	2.98	29.05	<0.0001	<0.0001
ENSG00000135269	TES	testin LIM domain protein [Source:HGNC Symbol;Acc:HGNC:14620]	protein coding	0.63	4.85	33.06	<0.0001	<0.0001
ENSG00000101608	MYL12A	myosin light chain 12A [Source:HGNC Symbol;Acc:HGNC:16701]	protein coding	0.63	8.55	35.63	<0.0001	<0.0001
ENSG00000166503	HDGFL3	HDGF like 3 [Source:HGNC Symbol;Acc:HGNC:24937]	protein coding	-0.63	3.39	14.05	0.0002	0.0006
ENSG00000100897	DCAF11	DDB1 and CUL4 associated factor 11 [Source:HGNC Symbol;Acc:HGNC:20258]	protein coding	0.63	6.64	47.80	<0.0001	<0.0001
ENSG00000055130	CUL1	cullin 1 [Source:HGNC Symbol;Acc:HGNC:2551]	protein coding	-0.63	2.47	28.58	<0.0001	<0.0001
ENSG00000204642	HLA-F	major histocompatibility complex, class I, F [Source:HGNC Symbol;Acc:HGNC:4963]	protein coding	0.63	9.05	46.45	<0.0001	<0.0001
ENSG00000169762	TAPT1	transmembrane anterior posterior transformation 1 [Source:HGNC Symbol;Acc:HGNC:26887]	protein coding	-0.63	3.98	28.52	<0.0001	<0.0001
ENSG00000272941		novel transcript, antisense to C7orf49	lncRNA	0.63	2.73	19.48	<0.0001	<0.0001
ENSG00000136541	ERMN	ermin [Source:HGNC Symbol;Acc:HGNC:29208]	protein coding	-0.63	2.47	9.17	0.0025	0.0067
ENSG00000261338		novel transcript, overlapping to ARPC2 and GPBAR1	lncRNA	-0.63	2.66	30.33	<0.0001	<0.0001

Table S18: Genes differentially expressed at Day 30 compared to pre-second vaccination (Study Arm 2 A/H7N9+AS03, D1, 29, Neutrophils). Genes are sorted by descending absolute log_2 fold change. Gene model summaries and annotations are based on Ensembl Version 99 (continued.)

Ensembl Gene ID	Ensembl Gene Name	Ensembl Gene Description	Gene Type	log_2 Fold Change (Day 30 vs. Pre-Second Vaccination)	Average log_2 CPM	Likelihood Ratio Test Statistic	P-Value	FDR Adjusted P-Value
ENSG00000117385	P3H1	prolyl 3-hydroxylase 1 [Source:HGNC Symbol;Acc:HGNC:19316]	protein coding	-0.63	2.87	20.70	<0.0001	<0.0001
ENSG00000251441	RTEL1P1	regulator of telomere elongation helicase 1 pseudogene 1 [Source:HGNC Symbol;Acc:HGNC:44213]	transcribed processed pseudogene	0.63	1.06	18.74	<0.0001	<0.0001
ENSG00000267394		novel transcript, antisense to ATXN7L3	lncRNA	0.63	0.51	14.15	0.0002	0.0006
ENSG00000100426	ZBED4	zinc finger BED-type containing 4 [Source:HGNC Symbol;Acc:HGNC:20721]	protein coding	-0.63	2.64	22.42	<0.0001	<0.0001
ENSG00000090581	GNPTG	N-acetylglucosamine-1-phosphate transferase subunit gamma [Source:HGNC Symbol;Acc:HGNC:23026]	protein coding	-0.63	5.14	33.14	<0.0001	<0.0001
ENSG00000128245	YWHAH	tyrosine 3-monooxygenase/tryptophan 5-monooxygenase activation protein eta [Source:HGNC Symbol;Acc:HGNC:12853]	protein coding	-0.63	5.26	35.56	<0.0001	<0.0001
ENSG00000178814	OPLAH	5-oxoprolinase, ATP-hydrolysing [Source:HGNC Symbol;Acc:HGNC:8149]	protein coding	-0.62	4.72	9.97	0.0016	0.0046
ENSG00000158805	ZNF276	zinc finger protein 276 [Source:HGNC Symbol;Acc:HGNC:23330]	protein coding	0.62	6.39	26.58	<0.0001	<0.0001
ENSG00000198677	TTC37	tetratricopeptide repeat domain 37 [Source:HGNC Symbol;Acc:HGNC:23639]	protein coding	-0.62	0.37	5.39	0.0202	0.0418
ENSG00000130313	PGLS	6-phosphogluconolactonase [Source:HGNC Symbol;Acc:HGNC:8903]	protein coding	-0.62	5.74	36.98	<0.0001	<0.0001
ENSG00000239883	PARGP1	poly(ADP-ribose) glycohydrolase pseudogene 1 [Source:HGNC Symbol;Acc:HGNC:45008]	transcribed unprocessed pseudogene	-0.62	1.52	17.53	<0.0001	0.0001
ENSG00000204406	MBD5	methyl-CpG binding domain protein 5 [Source:HGNC Symbol;Acc:HGNC:20444]	protein coding	0.62	3.84	31.04	<0.0001	<0.0001
ENSG00000262580		novel transcript, antisense to CARD14	lncRNA	-0.62	1.63	15.82	<0.0001	0.0003
ENSG00000132507	EIF5A	eukaryotic translation initiation factor 5A [Source:HGNC Symbol;Acc:HGNC:3300]	protein coding	0.62	6.14	23.08	<0.0001	<0.0001
ENSG00000049860	HEXB	hexosaminidase subunit beta [Source:HGNC Symbol;Acc:HGNC:4879]	protein coding	-0.62	6.05	46.94	<0.0001	<0.0001
ENSG00000180354	MTURN	maturin, neural progenitor differentiation regulator homolog [Source:HGNC Symbol;Acc:HGNC:25457]	protein coding	-0.62	5.33	37.07	<0.0001	<0.0001

Table S18: Genes differentially expressed at Day 30 compared to pre-second vaccination (Study Arm 2 A/H7N9+AS03, D1, 29, Neutrophils). Genes are sorted by descending absolute log_2 fold change. Gene model summaries and annotations are based on Ensembl Version 99 (continued.)

Ensembl Gene ID	Ensembl Gene Name	Ensembl Gene Description	Gene Type	log_2 Fold Change (Day 30 vs. Pre-Second Vaccination)	Average log_2 CPM	Likelihood Ratio Test Statistic	P-Value	FDR Adjusted P-Value
ENSG00000168040	FADD	Fas associated via death domain [Source:HGNC Symbol;Acc:HGNC:3573]	protein coding	-0.62	3.96	16.95	<0.0001	0.0002
ENSG00000162032	SPSB3	splA/ryanodine receptor domain and SOCS box containing 3 [Source:HGNC Symbol;Acc:HGNC:30629]	protein coding	-0.62	0.23	8.36	0.0038	0.0098
ENSG00000120690	ELF1	E74 like ETS transcription factor 1 [Source:HGNC Symbol;Acc:HGNC:3316]	protein coding	0.62	8.09	46.46	<0.0001	<0.0001
ENSG00000287644		novel transcript, antisense to RARA	lncRNA	0.62	3.45	17.14	<0.0001	0.0001
ENSG00000214022	REPIN1	replication initiator 1 [Source:HGNC Symbol;Acc:HGNC:17922]	protein coding	-0.62	5.68	30.18	<0.0001	<0.0001
ENSG00000285554		Novel transcript	lncRNA	0.62	2.42	28.32	<0.0001	<0.0001
ENSG00000247092	SNHG10	small nucleolar RNA host gene 10 [Source:HGNC Symbol;Acc:HGNC:27510]	lncRNA	-0.62	0.30	9.58	0.002	0.0055
ENSG00000225205		novel transcript	lncRNA	0.62	2.10	21.00	<0.0001	<0.0001
ENSG00000187266	EPOR	erythropoietin receptor [Source:HGNC Symbol;Acc:HGNC:3416]	protein coding	-0.62	4.66	34.49	<0.0001	<0.0001
ENSG00000165178	NCF1C	neutrophil cytosolic factor 1C pseudogene [Source:HGNC Symbol;Acc:HGNC:32523]	unprocessed pseudogene	0.62	9.10	25.78	<0.0001	<0.0001
ENSG00000142599	RERE	arginine-glutamic acid dipeptide repeats [Source:HGNC Symbol;Acc:HGNC:9965]	protein coding	0.62	9.00	28.51	<0.0001	<0.0001
ENSG00000109919	MTCH2	mitochondrial carrier 2 [Source:HGNC Symbol;Acc:HGNC:17587]	protein coding	-0.62	0.41	10.12	0.0015	0.0043
ENSG00000164938	TP53INP1	tumor protein p53 inducible nuclear protein 1 [Source:HGNC Symbol;Acc:HGNC:18022]	protein coding	-0.62	7.60	38.65	<0.0001	<0.0001
ENSG00000187109	NAP1L1	nucleosome assembly protein 1 like 1 [Source:HGNC Symbol;Acc:HGNC:7637]	protein coding	-0.62	4.12	23.96	<0.0001	<0.0001
ENSG00000134758	RNF138	ring finger protein 138 [Source:HGNC Symbol;Acc:HGNC:17765]	protein coding	0.62	5.47	34.79	<0.0001	<0.0001
ENSG00000232788	ITGA6-AS1	ITGA6 antisense RNA 1 [Source:HGNC Symbol;Acc:HGNC:40308]	lncRNA	0.62	1.12	10.45	0.0012	0.0036
ENSG00000197535	MYO5A	myosin VA [Source:HGNC Symbol;Acc:HGNC:7602]	protein coding	-0.62	6.54	29.35	<0.0001	<0.0001
ENSG00000115904	SOS1	SOS Ras/Rac guanine nucleotide exchange factor 1 [Source:HGNC Symbol;Acc:HGNC:11187]	protein coding	0.62	5.09	38.46	<0.0001	<0.0001
ENSG00000136371	MTHFS	methenyltetrahydrofolate synthetase [Source:HGNC Symbol;Acc:HGNC:7437]	protein coding	-0.62	5.20	24.86	<0.0001	<0.0001

Table S18: Genes differentially expressed at Day 30 compared to pre-second vaccination (Study Arm 2 A/H7N9+AS03, D1, 29, Neutrophils). Genes are sorted by descending absolute log_2 fold change. Gene model summaries and annotations are based on Ensembl Version 99 (continued.)

Ensembl Gene ID	Ensembl Gene Name	Ensembl Gene Description	Gene Type	log_2 Fold Change (Day 30 vs. Pre-Second Vaccination)	Average log_2 CPM	Likelihood Ratio Test Statistic	P-Value	FDR Adjusted P-Value
ENSG00000138495	COX17	cytochrome c oxidase copper chaperone COX17 [Source:HGNC Symbol;Acc:HGNC:2264]	protein coding	0.62	2.87	35.00	<0.0001	<0.0001
ENSG00000166888	STAT6	signal transducer and activator of transcription 6 [Source:HGNC Symbol;Acc:HGNC:11368]	protein coding	-0.62	10.00	60.79	<0.0001	<0.0001
ENSG00000167034	NKX3-1	NK3 homeobox 1 [Source:HGNC Symbol;Acc:HGNC:7838]	protein coding	-0.62	4.76	7.65	0.0057	0.014
ENSG00000153551	CMTM7	CKLF like MARVEL transmembrane domain containing 7 [Source:HGNC Symbol;Acc:HGNC:19178]	protein coding	-0.62	4.82	28.81	<0.0001	<0.0001
ENSG00000007392	LUC7L	LUC7 like [Source:HGNC Symbol;Acc:HGNC:6723]	protein coding	-0.62	3.06	18.89	<0.0001	<0.0001
ENSG00000182606	TRAK1	trafficking kinesin protein 1 [Source:HGNC Symbol;Acc:HGNC:29947]	protein coding	-0.62	4.98	35.07	<0.0001	<0.0001
ENSG00000114315	HES1	hes family bHLH transcription factor 1 [Source:HGNC Symbol;Acc:HGNC:5192]	protein coding	-0.62	0.86	9.34	0.0022	0.0062
ENSG00000242259	C22orf39	chromosome 22 open reading frame 39 [Source:HGNC Symbol;Acc:HGNC:27012]	protein coding	-0.62	3.92	33.43	<0.0001	<0.0001
ENSG00000105963	ADAP1	ArfGAP with dual PH domains 1 [Source:HGNC Symbol;Acc:HGNC:16486]	protein coding	-0.62	5.62	36.93	<0.0001	<0.0001
ENSG00000130164	LDLR	low density lipoprotein receptor [Source:HGNC Symbol;Acc:HGNC:6547]	protein coding	-0.62	1.32	6.21	0.0127	0.0281
ENSG00000196821	ILRUN	inflammation and lipid regulator with UBA-like and NBR1-like domains [Source:HGNC Symbol;Acc:HGNC:21215]	protein coding	0.62	6.23	36.08	<0.0001	<0.0001
ENSG00000221937	TAS2R40	taste 2 receptor member 40 [Source:HGNC Symbol;Acc:HGNC:18885]	protein coding	-0.62	1.42	14.62	0.0001	0.0005
ENSG00000105321	CCDC9	coiled-coil domain containing 9 [Source:HGNC Symbol;Acc:HGNC:24560]	protein coding	0.62	6.33	24.06	<0.0001	<0.0001
ENSG00000148291	SURF2	surfeit 2 [Source:HGNC Symbol;Acc:HGNC:11475]	protein coding	-0.62	1.52	20.41	<0.0001	<0.0001
ENSG00000171943	SRGAP2C	SLIT-ROBO Rho GTPase activating protein 2C [Source:HGNC Symbol;Acc:HGNC:30584]	protein coding	0.62	3.25	26.77	<0.0001	<0.0001
ENSG00000161558	TMEM143	transmembrane protein 143 [Source:HGNC Symbol;Acc:HGNC:25603]	protein coding	-0.62	0.66	8.06	0.0045	0.0114
ENSG00000273855		novel transcript, sense intronic to PLCB2	lncRNA	-0.62	0.66	10.51	0.0012	0.0035
ENSG00000139842	CUL4A	cullin 4A [Source:HGNC Symbol;Acc:HGNC:2554]	protein coding	-0.62	3.94	29.23	<0.0001	<0.0001

Table S18: Genes differentially expressed at Day 30 compared to pre-second vaccination (Study Arm 2 A/H7N9+AS03, D1, 29, Neutrophils). Genes are sorted by descending absolute log_2 fold change. Gene model summaries and annotations are based on Ensembl Version 99 (continued.)

Ensembl Gene ID	Ensembl Gene Name	Ensembl Gene Description	Gene Type	log_2 Fold Change (Day 30 vs. Pre-Second Vaccination)	Average log_2 CPM	Likelihood Ratio Test Statistic	P-Value	FDR Adjusted P-Value
ENSG00000182487	NCF1B	neutrophil cytosolic factor 1B pseudogene [Source:HGNC Symbol;Acc:HGNC:32522]	transcribed unprocessed pseudogene	0.62	8.28	24.40	<0.0001	<0.0001
ENSG00000013306	SLC25A39	solute carrier family 25 member 39 [Source:HGNC Symbol;Acc:HGNC:24279]	protein coding	-0.62	0.60	7.60	0.0058	0.0143
ENSG00000139112	GABARAPL1	GABA type A receptor associated protein like 1 [Source:HGNC Symbol;Acc:HGNC:4068]	protein coding	-0.61	7.64	48.28	<0.0001	<0.0001
ENSG00000164164	OTUD4	OTU deubiquitinase 4 [Source:HGNC Symbol;Acc:HGNC:24949]	protein coding	0.61	3.52	24.88	<0.0001	<0.0001
ENSG00000164975	SNAPC3	small nuclear RNA activating complex polypeptide 3 [Source:HGNC Symbol;Acc:HGNC:11136]	protein coding	-0.61	0.75	15.43	<0.0001	0.0003
ENSG00000173991	TCAP	titin-cap [Source:HGNC Symbol;Acc:HGNC:11610]	protein coding	0.61	0.47	17.01	<0.0001	0.0002
ENSG00000108344	PSMD3	proteasome 26S subunit, non-ATPase 3 [Source:HGNC Symbol;Acc:HGNC:9560]	protein coding	0.61	4.54	18.08	<0.0001	<0.0001
ENSG00000104228	TRIM35	tripartite motif containing 35 [Source:HGNC Symbol;Acc:HGNC:16285]	protein coding	-0.61	1.23	17.35	<0.0001	0.0001
ENSG00000157827	FMNL2	formin like 2 [Source:HGNC Symbol;Acc:HGNC:18267]	protein coding	0.61	1.62	14.96	0.0001	0.0004
ENSG00000161277	THAP8	THAP domain containing 8 [Source:HGNC Symbol;Acc:HGNC:23191]	protein coding	-0.61	1.13	15.72	<0.0001	0.0003
ENSG00000165886	UBTD1	ubiquitin domain containing 1 [Source:HGNC Symbol;Acc:HGNC:25683]	protein coding	-0.61	5.10	42.37	<0.0001	<0.0001
ENSG00000144034	TPRKB	TP53RK binding protein [Source:HGNC Symbol;Acc:HGNC:24259]	protein coding	0.61	2.86	23.26	<0.0001	<0.0001
ENSG00000126522	ASL	argininosuccinate lyase [Source:HGNC Symbol;Acc:HGNC:746]	protein coding	-0.61	3.39	18.68	<0.0001	<0.0001
ENSG00000125744	RTN2	reticulon 2 [Source:HGNC Symbol;Acc:HGNC:10468]	protein coding	0.61	4.63	26.43	<0.0001	<0.0001
ENSG00000166710	B2M	beta-2-microglobulin [Source:HGNC Symbol;Acc:HGNC:914]	protein coding	0.61	14.01	35.68	<0.0001	<0.0001
ENSG00000059758	CDK17	cyclin dependent kinase 17 [Source:HGNC Symbol;Acc:HGNC:8750]	protein coding	0.61	5.37	26.99	<0.0001	<0.0001

Table S18: Genes differentially expressed at Day 30 compared to pre-second vaccination (Study Arm 2 A/H7N9+AS03, D1, 29, Neutrophils). Genes are sorted by descending absolute log_2 fold change. Gene model summaries and annotations are based on Ensembl Version 99 (continued.)

Ensembl Gene ID	Ensembl Gene Name	Ensembl Gene Description	Gene Type	log_2 Fold Change (Day 30 vs. Pre-Second Vaccination)	Average log_2 CPM	Likelihood Ratio Test Statistic	P-Value	FDR Adjusted P-Value
ENSG00000169925	BRD3	bromodomain containing 3 [Source:HGNC Symbol;Acc:HGNC:1104]	protein coding	-0.61	5.67	46.89	<0.0001	<0.0001
ENSG00000196365	LONP1	lon peptidase 1, mitochondrial [Source:HGNC Symbol;Acc:HGNC:9479]	protein coding	-0.61	1.28	14.63	0.0001	0.0005
ENSG00000287070		novel transcript, sense intronic to TRMO	lncRNA	-0.61	1.24	6.84	0.0089	0.0205
ENSG00000169189	NSMCE1	NSE1 homolog, SMC5-SMC6 complex component [Source:HGNC Symbol;Acc:HGNC:29897]	protein coding	-0.61	2.57	28.18	<0.0001	<0.0001
ENSG00000131370	SH3BP5	SH3 domain binding protein 5 [Source:HGNC Symbol;Acc:HGNC:10827]	protein coding	-0.61	4.34	13.92	0.0002	0.0007
ENSG00000143554	SLC27A3	solute carrier family 27 member 3 [Source:HGNC Symbol;Acc:HGNC:10997]	protein coding	0.61	5.46	19.12	<0.0001	<0.0001
ENSG00000235095		novel pseudogene	unprocessed pseudogene	-0.61	0.44	13.07	0.0003	0.001
ENSG00000110057	UNC93B1	unc-93 homolog B1, TLR signaling regulator [Source:HGNC Symbol;Acc:HGNC:13481]	protein coding	0.61	8.06	42.54	<0.0001	<0.0001
ENSG00000168389	MFSD2A	major facilitator superfamily domain containing 2A [Source:HGNC Symbol;Acc:HGNC:25897]	protein coding	-0.61	0.20	9.10	0.0026	0.0069
ENSG00000166398	KIAA0355	KIAA0355 [Source:HGNC Symbol;Acc:HGNC:29016]	protein coding	-0.61	4.35	54.80	<0.0001	<0.0001
ENSG00000101361	NOP56	NOP56 ribonucleoprotein [Source:HGNC Symbol;Acc:HGNC:15911]	protein coding	-0.61	2.59	13.81	0.0002	0.0007
ENSG00000066044	ELAVL1	ELAV like RNA binding protein 1 [Source:HGNC Symbol;Acc:HGNC:3312]	protein coding	-0.61	3.73	24.42	<0.0001	<0.0001
ENSG00000116171	SCP2	sterol carrier protein 2 [Source:HGNC Symbol;Acc:HGNC:10606]	protein coding	-0.61	2.67	11.18	0.0008	0.0026
ENSG00000105607	GCDH	glutaryl-CoA dehydrogenase [Source:HGNC Symbol;Acc:HGNC:4189]	protein coding	-0.61	0.83	12.56	0.0004	0.0013
ENSG00000172007	RAB33B	RAB33B, member RAS oncogene family [Source:HGNC Symbol;Acc:HGNC:16075]	protein coding	0.61	5.79	29.37	<0.0001	<0.0001
ENSG00000007237		growth arrest specific 7 [Source:NCBI gene;Acc:8522]	protein coding	0.61	6.86	19.17	<0.0001	<0.0001
ENSG00000084764	MAPRE3	microtubule associated protein RP/EB family member 3 [Source:HGNC Symbol;Acc:HGNC:6892]	protein coding	-0.61	3.95	24.08	<0.0001	<0.0001
ENSG00000236935		novel transcript	lncRNA	0.61	0.78	12.92	0.0003	0.0011
ENSG00000271553		novel transcript	lncRNA	-0.61	0.84	12.98	0.0003	0.0011

Table S18: Genes differentially expressed at Day 30 compared to pre-second vaccination (Study Arm 2 A/H7N9+AS03, D1, 29, Neutrophils). Genes are sorted by descending absolute log_2 fold change. Gene model summaries and annotations are based on Ensembl Version 99 (continued.)

Ensembl Gene ID	Ensembl Gene Name	Ensembl Gene Description	Gene Type	log_2 Fold Change (Day 30 vs. Pre-Second Vaccination)	Average log_2 CPM	Likelihood Ratio Test Statistic	P-Value	FDR Adjusted P-Value
ENSG00000180712	LINC02363	long intergenic non-protein coding RNA 2363 [Source:HGNC Symbol;Acc:HGNC:53286]	lncRNA	0.61	1.70	15.01	0.0001	0.0004
ENSG00000081721	DUSP12	dual specificity phosphatase 12 [Source:HGNC Symbol;Acc:HGNC:3067]	protein coding	-0.61	1.61	18.82	<0.0001	<0.0001
ENSG00000262332		novel transcript, antisense to ABCC6	lncRNA	-0.61	0.25	9.57	0.002	0.0055
ENSG00000114378	HYAL1	hyaluronidase 1 [Source:HGNC Symbol;Acc:HGNC:5320]	protein coding	-0.61	0.64	8.44	0.0037	0.0095
ENSG00000112167	SAYSD1	SAYSVFN motif domain containing 1 [Source:HGNC Symbol;Acc:HGNC:21025]	protein coding	0.61	1.30	17.61	<0.0001	0.0001
ENSG00000233429	HOTAIRM1	HOXA transcript antisense RNA, myeloid-specific 1 [Source:HGNC Symbol;Acc:HGNC:37117]	lncRNA	-0.61	5.21	16.94	<0.0001	0.0002
ENSG00000185189	NRBP2	nuclear receptor binding protein 2 [Source:HGNC Symbol;Acc:HGNC:19339]	protein coding	-0.61	1.95	9.02	0.0027	0.0072
ENSG00000066855	MTFR1	mitochondrial fission regulator 1 [Source:HGNC Symbol;Acc:HGNC:29510]	protein coding	-0.61	0.91	9.85	0.0017	0.0048
ENSG00000112992	NNT	nicotinamide nucleotide transhydrogenase [Source:HGNC Symbol;Acc:HGNC:7863]	protein coding	-0.61	1.80	9.84	0.0017	0.0049
ENSG00000127586	CHTF18	chromosome transmission fidelity factor 18 [Source:HGNC Symbol;Acc:HGNC:18435]	protein coding	-0.61	2.82	25.59	<0.0001	<0.0001
ENSG00000167460	TPM4	tropomyosin 4 [Source:HGNC Symbol;Acc:HGNC:12013]	protein coding	-0.61	7.25	27.33	<0.0001	<0.0001
ENSG00000137193	PIM1	Pim-1 proto-oncogene, serine/threonine kinase [Source:HGNC Symbol;Acc:HGNC:8986]	protein coding	0.61	7.79	24.25	<0.0001	<0.0001
ENSG00000205485		pseudogene similar to part of postmeiotic segregation increased 2-like 3 PMS2L3	transcribed unprocessed pseudogene	-0.61	1.15	16.39	<0.0001	0.0002
ENSG00000082074	FYB1	FYN binding protein 1 [Source:HGNC Symbol;Acc:HGNC:4036]	protein coding	0.61	10.55	40.07	<0.0001	<0.0001
ENSG00000160194	NDUFV3	NADH:ubiquinone oxidoreductase subunit V3 [Source:HGNC Symbol;Acc:HGNC:7719]	protein coding	-0.61	2.21	23.12	<0.0001	<0.0001
ENSG00000123737	EXOSC9	exosome component 9 [Source:HGNC Symbol;Acc:HGNC:9137]	protein coding	0.61	2.11	12.42	0.0004	0.0014
ENSG00000187010	RHD	Rh blood group D antigen [Source:HGNC Symbol;Acc:HGNC:10009]	protein coding	-0.61	2.74	25.08	<0.0001	<0.0001

Table S18: Genes differentially expressed at Day 30 compared to pre-second vaccination (Study Arm 2 A/H7N9+AS03, D1, 29, Neutrophils). Genes are sorted by descending absolute log_2 fold change. Gene model summaries and annotations are based on Ensembl Version 99 (continued.)

Ensembl Gene ID	Ensembl Gene Name	Ensembl Gene Description	Gene Type	log_2 Fold Change (Day 30 vs. Pre-Second Vaccination)	Average log_2 CPM	Likelihood Ratio Test Statistic	P-Value	FDR Adjusted P-Value
ENSG00000149600	COMMD7	COMM domain containing 7 [Source:HGNC Symbol;Acc:HGNC:16223]	protein coding	-0.61	0.40	5.30	0.0213	0.0438
ENSG00000108389	MTMR4	myotubularin related protein 4 [Source:HGNC Symbol;Acc:HGNC:7452]	protein coding	0.61	5.73	51.65	<0.0001	<0.0001
ENSG00000133687	TMTC1	transmembrane O-mannosyltransferase targeting cadherins 1 [Source:HGNC Symbol;Acc:HGNC:24099]	protein coding	-0.61	2.76	5.26	0.0218	0.0446
ENSG00000092295	TGM1	transglutaminase 1 [Source:HGNC Symbol;Acc:HGNC:11777]	protein coding	0.61	0.52	10.77	0.001	0.0031
ENSG00000197815		novel transcript, antisense to TOM1L2	lncRNA	-0.61	0.58	8.08	0.0045	0.0113
ENSG00000140931	CMTM3	CKLF like MARVEL transmembrane domain containing 3 [Source:HGNC Symbol;Acc:HGNC:19174]	protein coding	0.61	5.89	46.54	<0.0001	<0.0001
ENSG00000189195	BTBD8	BTB domain containing 8 [Source:HGNC Symbol;Acc:HGNC:21019]	protein coding	-0.61	0.82	10.73	0.0011	0.0032
ENSG00000112149	CD83	CD83 molecule [Source:HGNC Symbol;Acc:HGNC:1703]	protein coding	0.61	3.06	6.91	0.0085	0.0198
ENSG00000106588	PSMA2	proteasome 20S subunit alpha 2 [Source:HGNC Symbol;Acc:HGNC:9531]	protein coding	0.61	1.38	17.08	<0.0001	0.0002
ENSG00000100060	MFNG	MFNG O-fucosylpeptide 3-beta-N-acetylglucosaminyltransferase [Source:HGNC Symbol;Acc:HGNC:7038]	protein coding	-0.61	6.43	36.43	<0.0001	<0.0001
ENSG00000274767		novel transcript, antisense CCL3L3	lncRNA	-0.61	2.49	20.32	<0.0001	<0.0001
ENSG00000167280	ENGASE	endo-beta-N-acetylglucosaminidase [Source:HGNC Symbol;Acc:HGNC:24622]	protein coding	-0.61	2.21	7.01	0.0081	0.0189
ENSG00000280077		TEC	TEC	-0.61	1.20	13.06	0.0003	0.001
ENSG00000280054		TEC	TEC	0.61	1.31	13.71	0.0002	0.0008
ENSG00000161203	AP2M1	adaptor related protein complex 2 subunit mu 1 [Source:HGNC Symbol;Acc:HGNC:564]	protein coding	-0.61	7.06	48.35	<0.0001	<0.0001
ENSG00000173597	SULT1B1	sulfotransferase family 1B member 1 [Source:HGNC Symbol;Acc:HGNC:17845]	protein coding	-0.61	6.77	21.70	<0.0001	<0.0001
ENSG00000196693	ZNF33B	zinc finger protein 33B [Source:HGNC Symbol;Acc:HGNC:13097]	protein coding	-0.60	1.07	11.35	0.0008	0.0024
ENSG00000260276		novel transcript, antisense to CARHSP1	lncRNA	-0.60	1.05	9.20	0.0024	0.0066
ENSG00000239948	RN7SL368P	RNA, 7SL, cytoplasmic 368, pseudogene [Source:HGNC Symbol;Acc:HGNC:46384]	misc RNA	0.60	1.01	7.85	0.0051	0.0127
ENSG00000060491	OGFR	opioid growth factor receptor [Source:HGNC Symbol;Acc:HGNC:15768]	protein coding	0.60	8.23	27.81	<0.0001	<0.0001

Table S18: Genes differentially expressed at Day 30 compared to pre-second vaccination (Study Arm 2 A/H7N9+AS03, D1, 29, Neutrophils). Genes are sorted by descending absolute log_2 fold change. Gene model summaries and annotations are based on Ensembl Version 99 (continued.)

Ensembl Gene ID	Ensembl Gene Name	Ensembl Gene Description	Gene Type	log_2 Fold Change (Day 30 vs. Pre-Second Vaccination)	Average log_2 CPM	Likelihood Ratio Test Statistic	P-Value	FDR Adjusted P-Value
ENSG00000062194	GPBP1	GC-rich promoter binding protein 1 [Source:HGNC Symbol;Acc:HGNC:29520]	protein coding	0.60	7.08	33.66	<0.0001	<0.0001
ENSG00000266405		Chromobox protein homolog 3 (CBX3) pseudogene	transcribed processed pseudogene	0.60	1.93	16.88	<0.0001	0.0002
ENSG00000128590	DNAJB9	DnaJ heat shock protein family (Hsp40) member B9 [Source:HGNC Symbol;Acc:HGNC:6968]	protein coding	-0.60	4.23	5.73	0.0166	0.0355
ENSG00000154310	TNIK	TRAF2 and NCK interacting kinase [Source:HGNC Symbol;Acc:HGNC:30765]	protein coding	0.60	2.24	10.33	0.0013	0.0039
ENSG00000205336	ADGRG1	adhesion G protein-coupled receptor G1 [Source:HGNC Symbol;Acc:HGNC:4512]	protein coding	0.60	1.99	15.06	0.0001	0.0004
ENSG00000159377	PSMB4	proteasome 20S subunit beta 4 [Source:HGNC Symbol;Acc:HGNC:9541]	protein coding	0.60	7.09	23.29	<0.0001	<0.0001
ENSG00000070759	TESK2	testis associated actin remodelling kinase 2 [Source:HGNC Symbol;Acc:HGNC:11732]	protein coding	0.60	4.82	25.91	<0.0001	<0.0001
ENSG00000144118	RALB	RAS like proto-oncogene B [Source:HGNC Symbol;Acc:HGNC:9840]	protein coding	0.60	9.19	31.36	<0.0001	<0.0001
ENSG00000004838	ZMYND10	zinc finger MYND-type containing 10 [Source:HGNC Symbol;Acc:HGNC:19412]	protein coding	-0.60	0.72	13.26	0.0003	0.0009
ENSG00000150593	PDCD4	programmed cell death 4 [Source:HGNC Symbol;Acc:HGNC:8763]	protein coding	0.60	6.97	23.72	<0.0001	<0.0001
ENSG00000178789	CD300LB	CD300 molecule like family member b [Source:HGNC Symbol;Acc:HGNC:30811]	protein coding	-0.60	6.09	27.56	<0.0001	<0.0001
ENSG00000118495	PLAGL1	PLAG1 like zinc finger 1 [Source:HGNC Symbol;Acc:HGNC:9046]	protein coding	0.60	5.86	31.62	<0.0001	<0.0001
ENSG00000184436	THAP7	THAP domain containing 7 [Source:HGNC Symbol;Acc:HGNC:23190]	protein coding	-0.60	0.52	10.87	0.001	0.003
ENSG00000147955	SIGMAR1	sigma non-opioid intracellular receptor 1 [Source:HGNC Symbol;Acc:HGNC:8157]	protein coding	-0.60	3.13	27.35	<0.0001	<0.0001
ENSG00000185246	PRPF39	pre-mRNA processing factor 39 [Source:HGNC Symbol;Acc:HGNC:20314]	protein coding	-0.60	2.30	24.88	<0.0001	<0.0001
ENSG00000125843	AP5S1	adaptor related protein complex 5 subunit sigma 1 [Source:HGNC Symbol;Acc:HGNC:15875]	protein coding	0.60	0.76	14.73	0.0001	0.0005

Table S18: Genes differentially expressed at Day 30 compared to pre-second vaccination (Study Arm 2 A/H7N9+AS03, D1, 29, Neutrophils). Genes are sorted by descending absolute log_2 fold change. Gene model summaries and annotations are based on Ensembl Version 99 (continued.)

Ensembl Gene ID	Ensembl Gene Name	Ensembl Gene Description	Gene Type	log_2 Fold Change (Day 30 vs. Pre-Second Vaccination)	Average log_2 CPM	Likelihood Ratio Test Statistic	P-Value	FDR Adjusted P-Value
ENSG00000285420		novel platelet-derived growth factor receptor-like protein	unitary pseudogene	-0.60	2.01	16.06	<0.0001	0.0002
ENSG00000204531	POU5F1	POU class 5 homeobox 1 [Source:HGNC Symbol;Acc:HGNC:9221]	protein coding	0.60	0.46	13.88	0.0002	0.0007
ENSG00000179299	NSUN7	NOP2/Sun RNA methyltransferase family member 7 [Source:HGNC Symbol;Acc:HGNC:25857]	protein coding	-0.60	4.19	29.32	<0.0001	<0.0001
ENSG00000074071	MRPS34	mitochondrial ribosomal protein S34 [Source:HGNC Symbol;Acc:HGNC:16618]	protein coding	-0.60	2.53	21.13	<0.0001	<0.0001
ENSG00000102575	ACP5	acid phosphatase 5, tartrate resistant [Source:HGNC Symbol;Acc:HGNC:124]	protein coding	-0.60	2.31	12.29	0.0005	0.0015
ENSG00000185963	BICD2	BICD cargo adaptor 2 [Source:HGNC Symbol;Acc:HGNC:17208]	protein coding	-0.60	7.00	28.14	<0.0001	<0.0001
ENSG00000143110	C1orf162	chromosome 1 open reading frame 162 [Source:HGNC Symbol;Acc:HGNC:28344]	protein coding	-0.60	4.01	7.02	0.0081	0.0189
ENSG00000105662	CRTC1	CREB regulated transcription coactivator 1 [Source:HGNC Symbol;Acc:HGNC:16062]	protein coding	-0.60	3.02	16.24	<0.0001	0.0002
ENSG00000006453	BAIAP2L1	BAR/IMD domain containing adaptor protein 2 like 1 [Source:HGNC Symbol;Acc:HGNC:21649]	protein coding	0.60	0.92	10.58	0.0011	0.0034
ENSG00000248936		putatitive novel transcript	lncRNA	-0.60	0.70	11.57	0.0007	0.0021
ENSG00000216285		phosphoglycerate mutase 1 (brain) (PGAM1) pseudogene	processed pseudogene	-0.60	0.83	16.54	<0.0001	0.0002
ENSG00000117475	BLZF1	basic leucine zipper nuclear factor 1 [Source:HGNC Symbol;Acc:HGNC:1065]	protein coding	0.60	4.28	29.19	<0.0001	<0.0001
ENSG00000162695	SLC30A7	solute carrier family 30 member 7 [Source:HGNC Symbol;Acc:HGNC:19306]	protein coding	0.60	3.38	22.74	<0.0001	<0.0001
ENSG00000142765	SYTL1	synaptotagmin like 1 [Source:HGNC Symbol;Acc:HGNC:15584]	protein coding	-0.60	7.06	24.15	<0.0001	<0.0001
ENSG00000105609	LILRB5	leukocyte immunoglobulin like receptor B5 [Source:HGNC Symbol;Acc:HGNC:6609]	protein coding	0.60	2.08	12.54	0.0004	0.0013
ENSG00000173950	XXYLT1	xyloside xylosyltransferase 1 [Source:HGNC Symbol;Acc:HGNC:26639]	protein coding	-0.60	0.74	10.09	0.0015	0.0043
ENSG00000128789	PSMG2	proteasome assembly chaperone 2 [Source:HGNC Symbol;Acc:HGNC:24929]	protein coding	0.60	2.93	21.17	<0.0001	<0.0001

Table S18: Genes differentially expressed at Day 30 compared to pre-second vaccination (Study Arm 2 A/H7N9+AS03, D1, 29, Neutrophils). Genes are sorted by descending absolute log_2 fold change. Gene model summaries and annotations are based on Ensembl Version 99 (continued.)

Ensembl Gene ID	Ensembl Gene Name	Ensembl Gene Description	Gene Type	log_2 Fold Change (Day 30 vs. Pre-Second Vaccination)	Average log_2 CPM	Likelihood Ratio Test Statistic	P-Value	FDR Adjusted P-Value
ENSG00000229124	VIM-AS1	VIM antisense RNA 1 [Source:HGNC Symbol;Acc:HGNC:44879]	lncRNA	-0.60	4.70	31.83	<0.0001	<0.0001
ENSG00000160213	CSTB	cystatin B [Source:HGNC Symbol;Acc:HGNC:2482]	protein coding	-0.60	5.82	21.02	<0.0001	<0.0001
ENSG00000143452	HORMAD1	HORMA domain containing 1 [Source:HGNC Symbol;Acc:HGNC:25245]	protein coding	0.60	2.45	19.01	<0.0001	<0.0001
ENSG00000168395	ING5	inhibitor of growth family member 5 [Source:HGNC Symbol;Acc:HGNC:19421]	protein coding	-0.60	2.65	17.90	<0.0001	0.0001
ENSG00000175471	MCTP1	multiple C2 and transmembrane domain containing 1 [Source:HGNC Symbol;Acc:HGNC:26183]	protein coding	0.60	5.70	39.40	<0.0001	<0.0001
ENSG00000163823	CCR1	C-C motif chemokine receptor 1 [Source:HGNC Symbol;Acc:HGNC:1602]	protein coding	0.60	8.48	17.94	<0.0001	0.0001
ENSG00000142528	ZNF473	zinc finger protein 473 [Source:HGNC Symbol;Acc:HGNC:23239]	protein coding	0.60	2.05	11.02	0.0009	0.0028
ENSG00000134851	TMEM165	transmembrane protein 165 [Source:HGNC Symbol;Acc:HGNC:30760]	protein coding	0.60	6.26	52.20	<0.0001	<0.0001
ENSG00000173212	MAB21L3	mab-21 like 3 [Source:HGNC Symbol;Acc:HGNC:26787]	protein coding	0.60	0.95	9.69	0.0019	0.0052
ENSG00000168653	NDUFS5	NADH:ubiquinone oxidoreductase subunit S5 [Source:HGNC Symbol;Acc:HGNC:7712]	protein coding	-0.60	1.60	11.95	0.0005	0.0018
ENSG00000219201		novel pseudogene	processed pseudogene	0.60	0.63	15.29	<0.0001	0.0004
ENSG00000183723	CMTM4	CKLF like MARVEL transmembrane domain containing 4 [Source:HGNC Symbol;Acc:HGNC:19175]	protein coding	-0.60	1.64	19.15	<0.0001	<0.0001
ENSG00000174837	ADGRE1	adhesion G protein-coupled receptor E1 [Source:HGNC Symbol;Acc:HGNC:3336]	protein coding	0.60	4.75	18.00	<0.0001	<0.0001
ENSG00000111364	DDX55	DEAD-box helicase 55 [Source:HGNC Symbol;Acc:HGNC:20085]	protein coding	-0.60	1.61	16.56	<0.0001	0.0002
ENSG00000168701	TMEM208	transmembrane protein 208 [Source:HGNC Symbol;Acc:HGNC:25015]	protein coding	0.60	3.59	21.29	<0.0001	<0.0001
ENSG00000273344	PAXIP1-AS1	PAXIP1 antisense RNA 1 (head to head) [Source:HGNC Symbol;Acc:HGNC:27328]	lncRNA	-0.60	0.18	10.91	0.001	0.0029
ENSG00000101189	MRGBP	MRG domain binding protein [Source:HGNC Symbol;Acc:HGNC:15866]	protein coding	-0.60	3.76	38.11	<0.0001	<0.0001
ENSG00000227598		novel transcript	lncRNA	-0.59	1.39	19.77	<0.0001	<0.0001

Table S18: Genes differentially expressed at Day 30 compared to pre-second vaccination (Study Arm 2 A/H7N9+AS03, D1, 29, Neutrophils). Genes are sorted by descending absolute log_2 fold change. Gene model summaries and annotations are based on Ensembl Version 99 (continued.)

Ensembl Gene ID	Ensembl Gene Name	Ensembl Gene Description	Gene Type	log_2 Fold Change (Day 30 vs. Pre-Second Vaccination)	Average log_2 CPM	Likelihood Ratio Test Statistic	P-Value	FDR Adjusted P-Value
ENSG00000111666	CHPT1	choline phosphotransferase 1 [Source:HGNC Symbol;Acc:HGNC:17852]	protein coding	-0.59	4.92	12.72	0.0004	0.0012
ENSG00000197062	ZSCAN26	zinc finger and SCAN domain containing 26 [Source:HGNC Symbol;Acc:HGNC:12978]	protein coding	-0.59	2.42	27.12	<0.0001	<0.0001
ENSG00000126773	PCNX4	pecanex 4 [Source:HGNC Symbol;Acc:HGNC:20349]	protein coding	-0.59	4.20	23.84	<0.0001	<0.0001
ENSG00000100731	PCNX1	pecanex 1 [Source:HGNC Symbol;Acc:HGNC:19740]	protein coding	-0.59	7.61	23.21	<0.0001	<0.0001
ENSG00000204922	UQCC3	ubiquinol-cytochrome c reductase complex assembly factor 3 [Source:HGNC Symbol;Acc:HGNC:34399]	protein coding	-0.59	0.57	7.33	0.0068	0.0162
ENSG00000119669	IRF2BPL	interferon regulatory factor 2 binding protein like [Source:HGNC Symbol;Acc:HGNC:14282]	protein coding	-0.59	7.85	28.73	<0.0001	<0.0001
ENSG00000130511	SSBP4	single stranded DNA binding protein 4 [Source:HGNC Symbol;Acc:HGNC:15676]	protein coding	0.59	1.89	11.29	0.0008	0.0024
ENSG00000126261	UBA2	ubiquitin like modifier activating enzyme 2 [Source:HGNC Symbol;Acc:HGNC:30661]	protein coding	-0.59	1.70	11.50	0.0007	0.0022
ENSG00000107077	KDM4C	lysine demethylase 4C [Source:HGNC Symbol;Acc:HGNC:17071]	protein coding	-0.59	2.70	12.68	0.0004	0.0012
ENSG00000204920	ZNF155	zinc finger protein 155 [Source:HGNC Symbol;Acc:HGNC:12940]	protein coding	0.59	0.89	17.15	<0.0001	0.0001
ENSG00000162711	NLRP3	NLR family pyrin domain containing 3 [Source:HGNC Symbol;Acc:HGNC:16400]	protein coding	-0.59	4.78	18.08	<0.0001	<0.0001
ENSG00000180509	KCNE1	potassium voltage-gated channel subfamily E regulatory subunit 1 [Source:HGNC Symbol;Acc:HGNC:6240]	protein coding	-0.59	3.11	5.91	0.015	0.0324
ENSG00000010361	FUZ	fuzzy planar cell polarity protein [Source:HGNC Symbol;Acc:HGNC:26219]	protein coding	-0.59	4.77	25.53	<0.0001	<0.0001
ENSG00000279833		novel transcript, antisense to CBX7	lncRNA	-0.59	3.68	22.09	<0.0001	<0.0001
ENSG00000070423	RNF126	ring finger protein 126 [Source:HGNC Symbol;Acc:HGNC:21151]	protein coding	-0.59	4.27	39.81	<0.0001	<0.0001
ENSG00000166401	SERPINB8	serpin family B member 8 [Source:HGNC Symbol;Acc:HGNC:8952]	protein coding	-0.59	2.68	19.32	<0.0001	<0.0001
ENSG00000140009	ESR2	estrogen receptor 2 [Source:HGNC Symbol;Acc:HGNC:3468]	protein coding	0.59	0.87	11.80	0.0006	0.0019
ENSG00000213906	LTB4R2	leukotriene B4 receptor 2 [Source:HGNC Symbol;Acc:HGNC:19260]	protein coding	-0.59	2.02	8.48	0.0036	0.0093

Table S18: Genes differentially expressed at Day 30 compared to pre-second vaccination (Study Arm 2 A/H7N9+AS03, D1, 29, Neutrophils). Genes are sorted by descending absolute log_2 fold change. Gene model summaries and annotations are based on Ensembl Version 99 (continued.)

Ensembl Gene ID	Ensembl Gene Name	Ensembl Gene Description	Gene Type	log_2 Fold Change (Day 30 vs. Pre-Second Vaccination)	Average log_2 CPM	Likelihood Ratio Test Statistic	P-Value	FDR Adjusted P-Value
ENSG00000117335	CD46	CD46 molecule [Source:HGNC Symbol;Acc:HGNC:6953]	protein coding	0.59	10.05	26.82	<0.0001	<0.0001
ENSG00000225663	MCRIP1	MAPK regulated corepressor interacting protein 1 [Source:HGNC Symbol;Acc:HGNC:28007]	protein coding	-0.59	4.45	28.16	<0.0001	<0.0001
ENSG00000182782	HCAR2	hydroxycarboxylic acid receptor 2 [Source:HGNC Symbol;Acc:HGNC:24827]	protein coding	0.59	7.87	15.79	<0.0001	0.0003
ENSG00000185495		septin 7 (SEPT7) pseudogene	transcribed unprocessed pseudogene	-0.59	1.22	11.55	0.0007	0.0021
ENSG00000121486	TRMT1L	tRNA methyltransferase 1 like [Source:HGNC Symbol;Acc:HGNC:16782]	protein coding	0.59	4.61	21.60	<0.0001	<0.0001
ENSG00000135211	TMEM60	transmembrane protein 60 [Source:HGNC Symbol;Acc:HGNC:21754]	protein coding	0.59	3.37	20.87	<0.0001	<0.0001
ENSG00000165533	TTC8	tetratricopeptide repeat domain 8 [Source:HGNC Symbol;Acc:HGNC:20087]	protein coding	-0.59	0.17	11.88	0.0006	0.0018
ENSG00000180096	SEPTIN1	septin 1 [Source:HGNC Symbol;Acc:HGNC:2879]	protein coding	-0.59	1.63	13.23	0.0003	0.001
ENSG00000247774	PCED1B-AS1	PCED1B antisense RNA 1 [Source:HGNC Symbol;Acc:HGNC:44166]	lncRNA	0.59	3.14	25.12	<0.0001	<0.0001
ENSG00000057657	PRDM1	PR/SET domain 1 [Source:HGNC Symbol;Acc:HGNC:9346]	protein coding	0.59	6.18	7.32	0.0068	0.0163
ENSG00000120899	PTK2B	protein tyrosine kinase 2 beta [Source:HGNC Symbol;Acc:HGNC:9612]	protein coding	0.59	8.78	38.45	<0.0001	<0.0001
ENSG00000143178	TBX19	T-box transcription factor 19 [Source:HGNC Symbol;Acc:HGNC:11596]	protein coding	0.59	0.88	14.78	0.0001	0.0005
ENSG00000100422	CERK	ceramide kinase [Source:HGNC Symbol;Acc:HGNC:19256]	protein coding	-0.59	6.84	46.64	<0.0001	<0.0001
ENSG00000074370	ATP2A3	ATPase sarcoplasmic/endoplasmic reticulum Ca2+ transporting 3 [Source:HGNC Symbol;Acc:HGNC:813]	protein coding	-0.59	9.16	31.17	<0.0001	<0.0001
ENSG00000277791	PSMB3	proteasome 20S subunit beta 3 [Source:HGNC Symbol;Acc:HGNC:9540]	protein coding	0.59	7.42	19.68	<0.0001	<0.0001
ENSG00000118855	MFSD1	major facilitator superfamily domain containing 1 [Source:HGNC Symbol;Acc:HGNC:25874]	protein coding	-0.59	5.83	26.20	<0.0001	<0.0001

Table S18: Genes differentially expressed at Day 30 compared to pre-second vaccination (Study Arm 2 A/H7N9+AS03, D1, 29, Neutrophils). Genes are sorted by descending absolute log_2 fold change. Gene model summaries and annotations are based on Ensembl Version 99 (continued.)

Ensembl Gene ID	Ensembl Gene Name	Ensembl Gene Description	Gene Type	log_2 Fold Change (Day 30 vs. Pre-Second Vaccination)	Average log_2 CPM	Likelihood Ratio Test Statistic	P-Value	FDR Adjusted P-Value
ENSG00000096093	EFHC1	EF-hand domain containing 1 [Source:HGNC Symbol;Acc:HGNC:16406]	protein coding	-0.59	2.27	11.14	0.0008	0.0026
ENSG00000100360	IFT27	intraflagellar transport 27 [Source:HGNC Symbol;Acc:HGNC:18626]	protein coding	-0.59	1.64	14.96	0.0001	0.0004
ENSG00000164307	ERAP1	endoplasmic reticulum aminopeptidase 1 [Source:HGNC Symbol;Acc:HGNC:18173]	protein coding	0.59	5.27	27.46	<0.0001	<0.0001
ENSG00000283525		phospholipase A2 inhibitor and Ly6/PLAUR domain-containing protein-like [Source:NCBI gene;Acc:105372412]	transcribed unprocessed pseudogene	0.59	1.03	15.13	0.0001	0.0004
ENSG00000186951	PPARA	peroxisome proliferator activated receptor alpha [Source:HGNC Symbol;Acc:HGNC:9232]	protein coding	-0.59	1.35	6.53	0.0106	0.024
ENSG00000154330	PGM5	phosphoglucomutase 5 [Source:HGNC Symbol;Acc:HGNC:8908]	protein coding	-0.59	3.16	7.81	0.0052	0.0129
ENSG00000170837	GPR27	G protein-coupled receptor 27 [Source:HGNC Symbol;Acc:HGNC:4482]	protein coding	-0.59	7.08	19.47	<0.0001	<0.0001
ENSG00000287691		novel transcript, antisense to RNF19B	lncRNA	0.59	2.38	10.81	0.001	0.0031
ENSG00000068885	IFT80	intraflagellar transport 80 [Source:HGNC Symbol;Acc:HGNC:29262]	protein coding	0.59	1.24	15.43	<0.0001	0.0003
ENSG00000104936	DMPK	DM1 protein kinase [Source:HGNC Symbol;Acc:HGNC:2933]	protein coding	0.59	0.94	6.21	0.0127	0.0281
ENSG00000120832	MTERF2	mitochondrial transcription termination factor 2 [Source:HGNC Symbol;Acc:HGNC:30779]	protein coding	-0.59	2.52	16.25	<0.0001	0.0002
ENSG00000266389		novel transcript	lncRNA	0.59	2.85	23.30	<0.0001	<0.0001
ENSG00000136710	CCDC115	coiled-coil domain containing 115 [Source:HGNC Symbol;Acc:HGNC:28178]	protein coding	-0.59	3.71	15.83	<0.0001	0.0003
ENSG00000161638	ITGA5	integrin subunit alpha 5 [Source:HGNC Symbol;Acc:HGNC:6141]	protein coding	-0.59	8.38	37.89	<0.0001	<0.0001
ENSG00000164284	GRPEL2	GrpE like 2, mitochondrial [Source:HGNC Symbol;Acc:HGNC:21060]	protein coding	-0.58	0.71	8.88	0.0029	0.0077

Confidential/Proprietary Information

Table S19: Genes differentially expressed at Day 32 compared to pre-second vaccination (Study Arm 2 A/H7N9+AS03, D1, 29, Neutrophils). Genes are sorted by descending absolute log_2 fold change. Gene model summaries and annotations are based on Ensembl Version 99.

Ensembl Gene ID	Ensembl Gene Name	Ensembl Gene Description	Gene Type	log_2 Fold Change (Day 32 vs. Pre-Second Vaccination)	Average log_2 CPM	Likelihood Ratio Test Statistic	P-Value	FDR Adjusted P-Value
ENSG00000160932	LY6E	lymphocyte antigen 6 family member E [Source:HGNC Symbol;Acc:HGNC:6727]	protein coding	1.88	7.50	12.62	0.0004	0.0134
ENSG00000136514	RTP4	receptor transporter protein 4 [Source:HGNC Symbol;Acc:HGNC:23992]	protein coding	1.53	2.71	8.70	0.0032	0.046
ENSG00000146192	FGD2	FYVE, RhoGEF and PH domain containing 2 [Source:HGNC Symbol;Acc:HGNC:3664]	protein coding	1.49	0.77	14.15	0.0002	0.0089
ENSG00000089127	OAS1	2'-5'-oligoadenylate synthetase 1 [Source:HGNC Symbol;Acc:HGNC:8086]	protein coding	1.49	6.34	10.27	0.0013	0.0288
ENSG00000108387	SEPTIN4	septin 4 [Source:HGNC Symbol;Acc:HGNC:9165]	protein coding	1.46	1.21	11.69	0.0006	0.0176
ENSG00000169385	RNASE2	ribonuclease A family member 2 [Source:HGNC Symbol;Acc:HGNC:10045]	protein coding	1.39	3.25	14.85	0.0001	0.0073
ENSG00000182912	TSPEAR-AS2	TSPEAR antisense RNA 2 [Source:HGNC Symbol;Acc:HGNC:16428]	lncRNA	-1.27	0.36	30.40	<0.0001	<0.0001
ENSG00000235890	TSPEAR-AS1	TSPEAR antisense RNA 1 [Source:HGNC Symbol;Acc:HGNC:1271]	lncRNA	-1.22	0.85	37.73	<0.0001	<0.0001
ENSG00000182240	BACE2	beta-secretase 2 [Source:HGNC Symbol;Acc:HGNC:934]	protein coding	1.22	1.68	13.12	0.0003	0.0115
ENSG00000272398	CD24	CD24 molecule [Source:HGNC Symbol;Acc:HGNC:1645]	protein coding	1.21	3.22	30.08	<0.0001	<0.0001
ENSG00000060138	YBX3	Y-box binding protein 3 [Source:HGNC Symbol;Acc:HGNC:2428]	protein coding	-1.20	5.07	24.71	<0.0001	0.0003
ENSG00000197646	PDCD1LG2	programmed cell death 1 ligand 2 [Source:HGNC Symbol;Acc:HGNC:18731]	protein coding	1.20	0.73	11.54	0.0007	0.0185
ENSG00000030419	IKZF2	IKAROS family zinc finger 2 [Source:HGNC Symbol;Acc:HGNC:13177]	protein coding	1.19	1.15	11.30	0.0008	0.0204
ENSG00000134256	CD101	CD101 molecule [Source:HGNC Symbol;Acc:HGNC:5949]	protein coding	1.15	2.40	20.59	<0.0001	0.0014
ENSG00000131203	IDO1	indoleamine 2,3-dioxygenase 1 [Source:HGNC Symbol;Acc:HGNC:6059]	protein coding	1.15	2.72	12.65	0.0004	0.0132
ENSG00000112759	SLC29A1	solute carrier family 29 member 1 (Augustine blood group) [Source:HGNC Symbol;Acc:HGNC:11003]	protein coding	1.13	3.98	12.50	0.0004	0.0139
ENSG00000166428	PLD4	phospholipase D family member 4 [Source:HGNC Symbol;Acc:HGNC:23792]	protein coding	1.13	2.29	12.53	0.0004	0.0138
ENSG00000002726	AOC1	amine oxidase copper containing 1 [Source:HGNC Symbol;Acc:HGNC:80]	protein coding	1.11	2.75	16.68	<0.0001	0.0042

Table S19: Genes differentially expressed at Day 32 compared to pre-second vaccination (Study Arm 2 A/H7N9+AS03, D1, 29, Neutrophils). Genes are sorted by descending absolute log_2 fold change. Gene model summaries and annotations are based on Ensembl Version 99 (continued.)

Ensembl Gene ID	Ensembl Gene Name	Ensembl Gene Description	Gene Type	log_2 Fold Change (Day 32 vs. Pre-Second Vaccination)	Average log_2 CPM	Likelihood Ratio Test Statistic	P-Value	FDR Adjusted P-Value
ENSG00000115602	IL1RL1	interleukin 1 receptor like 1 [Source:HGNC Symbol;Acc:HGNC:5998]	protein coding	1.11	1.50	12.30	0.0005	0.0149
ENSG00000139629	GALNT6	polypeptide N-acetylgalactosaminyltransferase 6 [Source:HGNC Symbol;Acc:HGNC:4128]	protein coding	1.10	1.55	16.10	<0.0001	0.005
ENSG00000173801	JUP	junction plakoglobin [Source:HGNC Symbol;Acc:HGNC:6207]	protein coding	1.08	2.81	14.66	0.0001	0.0077
ENSG00000055483		ubiquitin specific peptidase 36 [Source:NCBI gene;Acc:57602]	protein coding	1.07	1.10	29.27	<0.0001	<0.0001
ENSG00000136068	FLNB	filamin B [Source:HGNC Symbol;Acc:HGNC:3755]	protein coding	1.06	1.75	13.91	0.0002	0.0095
ENSG00000177374	HIC1	HIC ZBTB transcriptional repressor 1 [Source:HGNC Symbol;Acc:HGNC:4909]	protein coding	1.06	1.39	18.70	<0.0001	0.0024
ENSG00000178980	SELENOW	selenoprotein W [Source:HGNC Symbol;Acc:HGNC:10752]	protein coding	1.05	1.32	22.88	<0.0001	0.0006
ENSG00000118785	SPP1	secreted phosphoprotein 1 [Source:HGNC Symbol;Acc:HGNC:11255]	protein coding	-1.05	2.13	14.27	0.0002	0.0086
ENSG00000185432	METTL7A	methyltransferase like 7A [Source:HGNC Symbol;Acc:HGNC:24550]	protein coding	1.05	2.15	12.23	0.0005	0.0151
ENSG00000189068	VSTM1	V-set and transmembrane domain containing 1 [Source:HGNC Symbol;Acc:HGNC:29455]	protein coding	1.04	3.52	23.56	<0.0001	0.0005
ENSG00000198087	CD2AP	CD2 associated protein [Source:HGNC Symbol;Acc:HGNC:14258]	protein coding	1.04	1.56	10.11	0.0015	0.0305
ENSG00000092067	CEBPE	CCAAT enhancer binding protein epsilon [Source:HGNC Symbol;Acc:HGNC:1836]	protein coding	1.04	3.26	20.92	<0.0001	0.0012
ENSG00000103365	GGA2	golgi associated, gamma adaptin ear containing, ARF binding protein 2 [Source:HGNC Symbol;Acc:HGNC:16064]	protein coding	1.03	1.96	12.04	0.0005	0.0157
ENSG00000161905	ALOX15	arachidonate 15-lipoxygenase [Source:HGNC Symbol;Acc:HGNC:433]	protein coding	1.02	4.82	10.51	0.0012	0.0268
ENSG00000169508	GPR183	G protein-coupled receptor 183 [Source:HGNC Symbol;Acc:HGNC:3128]	protein coding	1.02	1.12	10.07	0.0015	0.0307
ENSG00000122733	PHF24	PHD finger protein 24 [Source:HGNC Symbol;Acc:HGNC:29180]	protein coding	-0.99	2.40	17.60	<0.0001	0.0033
ENSG00000175894	TSPEAR	thrombospondin type laminin G domain and EAR repeats [Source:HGNC Symbol;Acc:HGNC:1268]	protein coding	-0.99	2.11	27.28	<0.0001	0.0001
ENSG00000196405	EVL	Enah/Vasp-like [Source:HGNC Symbol;Acc:HGNC:20234]	protein coding	0.98	3.47	14.77	0.0001	0.0075

Table S19: Genes differentially expressed at Day 32 compared to pre-second vaccination (Study Arm 2 A/H7N9+AS03, D1, 29, Neutrophils). Genes are sorted by descending absolute log_2 fold change. Gene model summaries and annotations are based on Ensembl Version 99 (continued.)

Ensembl Gene ID	Ensembl Gene Name	Ensembl Gene Description	Gene Type	log_2 Fold Change (Day 32 vs. Pre-Second Vaccination)	Average log_2 CPM	Likelihood Ratio Test Statistic	P-Value	FDR Adjusted P-Value
ENSG00000177119	ANO6	anoctamin 6 [Source:HGNC Symbol;Acc:HGNC:25240]	protein coding	0.98	1.20	18.95	<0.0001	0.0022
ENSG00000159618	ADGRG5	adhesion G protein-coupled receptor G5 [Source:HGNC Symbol;Acc:HGNC:19010]	protein coding	0.98	2.76	9.64	0.0019	0.0345
ENSG00000169442	CD52	CD52 molecule [Source:HGNC Symbol;Acc:HGNC:1804]	protein coding	0.97	3.25	8.73	0.0031	0.0458
ENSG00000198133	TMEM229B	transmembrane protein 229B [Source:HGNC Symbol;Acc:HGNC:20130]	protein coding	0.95	1.25	8.85	0.0029	0.0443
ENSG00000105205	CLC	Charcot-Leyden crystal galectin [Source:HGNC Symbol;Acc:HGNC:2014]	protein coding	0.95	6.92	9.19	0.0024	0.0397
ENSG00000103319	EEF2K	eukaryotic elongation factor 2 kinase [Source:HGNC Symbol;Acc:HGNC:24615]	protein coding	0.95	1.33	8.58	0.0034	0.0473
ENSG00000121895	TMEM156	transmembrane protein 156 [Source:HGNC Symbol;Acc:HGNC:26260]	protein coding	0.94	0.57	19.04	<0.0001	0.0021
ENSG00000149131	SERPING1	serpin family G member 1 [Source:HGNC Symbol;Acc:HGNC:1228]	protein coding	0.94	6.15	11.45	0.0007	0.0191
ENSG00000134489	HRH4	histamine receptor H4 [Source:HGNC Symbol;Acc:HGNC:17383]	protein coding	0.93	0.93	10.23	0.0014	0.0293
ENSG00000223914	LINC02471	long intergenic non-protein coding RNA 2471 [Source:HGNC Symbol;Acc:HGNC:53410]	lncRNA	0.93	0.71	21.93	<0.0001	0.0008
ENSG00000110651	CD81	CD81 molecule [Source:HGNC Symbol;Acc:HGNC:1701]	protein coding	0.93	2.26	9.14	0.0025	0.0405
ENSG00000165702	GFI1B	growth factor independent 1B transcriptional repressor [Source:HGNC Symbol;Acc:HGNC:4238]	protein coding	0.93	1.99	9.38	0.0022	0.0378
ENSG00000164292	RHOBTB3	Rho related BTB domain containing 3 [Source:HGNC Symbol;Acc:HGNC:18757]	protein coding	0.90	1.00	9.90	0.0016	0.0325
ENSG00000133317	LGALS12	galectin 12 [Source:HGNC Symbol;Acc:HGNC:15788]	protein coding	0.90	2.68	9.38	0.0022	0.0378
ENSG00000091181	IL5RA	interleukin 5 receptor subunit alpha [Source:HGNC Symbol;Acc:HGNC:6017]	protein coding	0.90	3.75	9.23	0.0024	0.0393
ENSG00000008283	CYB561	cytochrome b561 [Source:HGNC Symbol;Acc:HGNC:2571]	protein coding	0.89	4.71	31.53	<0.0001	<0.0001
ENSG00000255221	CARD17	caspase recruitment domain family member 17 [Source:HGNC Symbol;Acc:HGNC:33827]	protein coding	0.89	2.01	11.36	0.0008	0.0199
ENSG00000196126	HLA-DRB1	major histocompatibility complex, class II, DR beta 1 [Source:HGNC Symbol;Acc:HGNC:4948]	protein coding	0.89	2.00	12.96	0.0003	0.0122

Table S19: Genes differentially expressed at Day 32 compared to pre-second vaccination (Study Arm 2 A/H7N9+AS03, D1, 29, Neutrophils). Genes are sorted by descending absolute log_2 fold change. Gene model summaries and annotations are based on Ensembl Version 99 (continued.)

Ensembl Gene ID	Ensembl Gene Name	Ensembl Gene Description	Gene Type	log_2 Fold Change (Day 32 vs. Pre-Second Vaccination)	Average log_2 CPM	Likelihood Ratio Test Statistic	P-Value	FDR Adjusted P-Value
ENSG00000117054	ACADM	acyl-CoA dehydrogenase medium chain [Source:HGNC Symbol;Acc:HGNC:89]	protein coding	0.88	0.63	26.44	<0.0001	0.0002
ENSG00000122223	CD244	CD244 molecule [Source:HGNC Symbol;Acc:HGNC:18171]	protein coding	0.87	1.51	8.42	0.0037	0.0499
ENSG00000140836	ZFHX3	zinc finger homeobox 3 [Source:HGNC Symbol;Acc:HGNC:777]	protein coding	-0.86	0.40	17.33	<0.0001	0.0034
ENSG00000164683	HEY1	hes related family bHLH transcription factor with YRPW motif 1 [Source:HGNC Symbol;Acc:HGNC:4880]	protein coding	-0.85	2.97	12.90	0.0003	0.0123
ENSG00000167613	LAIR1	leukocyte associated immunoglobulin like receptor 1 [Source:HGNC Symbol;Acc:HGNC:6477]	protein coding	0.85	2.40	11.82	0.0006	0.0168
ENSG00000081377	CDC14B	cell division cycle 14B [Source:HGNC Symbol;Acc:HGNC:1719]	protein coding	0.85	0.44	13.43	0.0002	0.0107
ENSG00000136213	CHST12	carbohydrate sulfotransferase 12 [Source:HGNC Symbol;Acc:HGNC:17423]	protein coding	0.85	1.06	10.34	0.0013	0.028
ENSG00000260401		novel transcript, overlapping to P2RY2	lncRNA	0.85	0.95	11.86	0.0006	0.0166
ENSG00000006025	OSBPL7	oxysterol binding protein like 7 [Source:HGNC Symbol;Acc:HGNC:16387]	protein coding	0.84	0.51	13.24	0.0003	0.011
ENSG00000183134	PTGDR2	prostaglandin D2 receptor 2 [Source:HGNC Symbol;Acc:HGNC:4502]	protein coding	0.84	3.95	8.86	0.0029	0.0441
ENSG00000147872	PLIN2	perilipin 2 [Source:HGNC Symbol;Acc:HGNC:248]	protein coding	0.83	2.38	9.28	0.0023	0.0387
ENSG00000188483	IER5L	immediate early response 5 like [Source:HGNC Symbol;Acc:HGNC:23679]	protein coding	-0.83	3.89	13.94	0.0002	0.0095
ENSG00000106537	TSPAN13	tetraspanin 13 [Source:HGNC Symbol;Acc:HGNC:21643]	protein coding	-0.83	2.78	14.27	0.0002	0.0086
ENSG00000130695	CEP85	centrosomal protein 85 [Source:HGNC Symbol;Acc:HGNC:25309]	protein coding	0.83	1.34	11.29	0.0008	0.0204
ENSG00000145990	GFOD1	glucose-fructose oxidoreductase domain containing 1 [Source:HGNC Symbol;Acc:HGNC:21096]	protein coding	0.83	2.77	10.52	0.0012	0.0267
ENSG00000250548	LINC01303	long intergenic non-protein coding RNA 1303 [Source:HGNC Symbol;Acc:HGNC:50470]	lncRNA	0.82	0.73	19.36	<0.0001	0.0019
ENSG00000149600	COMMD7	COMM domain containing 7 [Source:HGNC Symbol;Acc:HGNC:16223]	protein coding	0.82	0.53	11.89	0.0006	0.0166
ENSG00000168916	ZNF608	zinc finger protein 608 [Source:HGNC Symbol;Acc:HGNC:29238]	protein coding	-0.82	2.16	9.53	0.002	0.0358

Table S19: Genes differentially expressed at Day 32 compared to pre-second vaccination (Study Arm 2 A/H7N9+AS03, D1, 29, Neutrophils). Genes are sorted by descending absolute log_2 fold change. Gene model summaries and annotations are based on Ensembl Version 99 (continued.)

Ensembl Gene ID	Ensembl Gene Name	Ensembl Gene Description	Gene Type	log_2 Fold Change (Day 32 vs. Pre-Second Vaccination)	Average log_2 CPM	Likelihood Ratio Test Statistic	P-Value	FDR Adjusted P-Value
ENSG00000154330	PGM5	phosphoglucomutase 5 [Source:HGNC Symbol;Acc:HGNC:8908]	protein coding	-0.81	3.10	23.42	<0.0001	0.0005
ENSG00000134072	CAMK1	calcium/calmodulin dependent protein kinase I [Source:HGNC Symbol;Acc:HGNC:1459]	protein coding	0.81	1.88	10.74	0.001	0.0249
ENSG00000162174	ASRGL1	asparaginase and isoaspartyl peptidase 1 [Source:HGNC Symbol;Acc:HGNC:16448]	protein coding	0.81	1.90	18.81	<0.0001	0.0023
ENSG00000147443	DOK2	docking protein 2 [Source:HGNC Symbol;Acc:HGNC:2991]	protein coding	0.81	4.06	10.64	0.0011	0.0257
ENSG00000165914	TTC7B	tetratricopeptide repeat domain 7B [Source:HGNC Symbol;Acc:HGNC:19858]	protein coding	0.80	1.65	9.76	0.0018	0.0337
ENSG00000087470	DNM1L	dynamin 1 like [Source:HGNC Symbol;Acc:HGNC:2973]	protein coding	0.80	0.57	13.43	0.0002	0.0107
ENSG00000244509	APOBEC3C	apolipoprotein B mRNA editing enzyme catalytic subunit 3C [Source:HGNC Symbol;Acc:HGNC:17353]	protein coding	0.80	2.61	12.45	0.0004	0.0142
ENSG00000165792	METTL17	methyltransferase like 17 [Source:HGNC Symbol;Acc:HGNC:19280]	protein coding	0.80	0.88	12.10	0.0005	0.0154
ENSG00000219481	NBPF1	NBPF member 1 [Source:HGNC Symbol;Acc:HGNC:26088]	protein coding	0.80	0.98	12.71	0.0004	0.013
ENSG00000135605	TEC	tec protein tyrosine kinase [Source:HGNC Symbol;Acc:HGNC:11719]	protein coding	0.79	1.51	15.31	<0.0001	0.0064
ENSG00000116704	SLC35D1	solute carrier family 35 member D1 [Source:HGNC Symbol;Acc:HGNC:20800]	protein coding	0.79	0.85	12.72	0.0004	0.0129
ENSG00000170558	CDH2	cadherin 2 [Source:HGNC Symbol;Acc:HGNC:1759]	protein coding	-0.79	1.70	18.38	<0.0001	0.0025
ENSG00000119537	KDSR	3-ketodihydrosphingosine reductase [Source:HGNC Symbol;Acc:HGNC:4021]	protein coding	-0.78	1.44	13.82	0.0002	0.0096
ENSG00000007516	BAIAP3	BAI1 associated protein 3 [Source:HGNC Symbol;Acc:HGNC:948]	protein coding	-0.78	6.14	34.79	<0.0001	<0.0001
ENSG00000167107	ACSF2	acyl-CoA synthetase family member 2 [Source:HGNC Symbol;Acc:HGNC:26101]	protein coding	0.78	2.15	17.32	<0.0001	0.0034
ENSG00000176170	SPHK1	sphingosine kinase 1 [Source:HGNC Symbol;Acc:HGNC:11240]	protein coding	-0.77	0.38	19.36	<0.0001	0.0019
ENSG00000125384	PTGER2	prostaglandin E receptor 2 [Source:HGNC Symbol;Acc:HGNC:9594]	protein coding	0.77	0.67	8.79	0.003	0.0451
ENSG00000154760	SLFN13	schlafen family member 13 [Source:HGNC Symbol;Acc:HGNC:26481]	protein coding	0.77	2.10	10.20	0.0014	0.0295

Confidential/Proprietary Information

Table S19: Genes differentially expressed at Day 32 compared to pre-second vaccination (Study Arm 2 A/H7N9+AS03, D1, 29, Neutrophils). Genes are sorted by descending absolute log_2 fold change. Gene model summaries and annotations are based on Ensembl Version 99 (continued.)

Ensembl Gene ID	Ensembl Gene Name	Ensembl Gene Description	Gene Type	log_2 Fold Change (Day 32 vs. Pre-Second Vaccination)	Average log_2 CPM	Likelihood Ratio Test Statistic	P-Value	FDR Adjusted P-Value
ENSG00000133055	MYBPH	myosin binding protein H [Source:HGNC Symbol;Acc:HGNC:7552]	protein coding	-0.77	2.91	20.03	<0.0001	0.0015
ENSG00000132849	PATJ	PATJ crumbs cell polarity complex component [Source:HGNC Symbol;Acc:HGNC:28881]	protein coding	0.77	0.98	11.26	0.0008	0.0206
ENSG00000213366	GSTM2	glutathione S-transferase mu 2 [Source:HGNC Symbol;Acc:HGNC:4634]	protein coding	0.77	2.07	12.28	0.0005	0.015
ENSG00000102755	FLT1	fms related tyrosine kinase 1 [Source:HGNC Symbol;Acc:HGNC:3763]	protein coding	-0.77	2.12	13.38	0.0003	0.0109
ENSG00000135519	KCNH3	potassium voltage-gated channel subfamily H member 3 [Source:HGNC Symbol;Acc:HGNC:6252]	protein coding	-0.75	3.32	41.00	<0.0001	<0.0001
ENSG00000168405	CMAHP	cytidine monophospho-N-acetylneuraminic acid hydroxylase, pseudogene [Source:HGNC Symbol;Acc:HGNC:2098]	transcribed unitary pseudogene	0.74	1.50	11.71	0.0006	0.0175
ENSG00000189298	ZKSCAN3	zinc finger with KRAB and SCAN domains 3 [Source:HGNC Symbol;Acc:HGNC:13853]	protein coding	0.74	0.34	13.93	0.0002	0.0095
ENSG00000152207	CYSLTR2	cysteinyl leukotriene receptor 2 [Source:HGNC Symbol;Acc:HGNC:18274]	protein coding	0.74	2.92	9.29	0.0023	0.0387
ENSG00000133561	GIMAP6	GTPase, IMAP family member 6 [Source:HGNC Symbol;Acc:HGNC:21918]	protein coding	0.74	3.12	9.31	0.0023	0.0385
ENSG00000120156	TEK	TEK receptor tyrosine kinase [Source:HGNC Symbol;Acc:HGNC:11724]	protein coding	-0.73	1.69	15.69	<0.0001	0.0057
ENSG00000133687	TMTC1	transmembrane O-mannosyltransferase targeting cadherins 1 [Source:HGNC Symbol;Acc:HGNC:24099]	protein coding	-0.73	2.92	11.29	0.0008	0.0204
ENSG00000112159	MDN1	midasin AAA ATPase 1 [Source:HGNC Symbol;Acc:HGNC:18302]	protein coding	0.73	1.49	15.43	<0.0001	0.0063
ENSG00000008838	MED24	mediator complex subunit 24 [Source:HGNC Symbol;Acc:HGNC:22963]	protein coding	0.72	0.89	10.14	0.0014	0.0302
ENSG00000139679	LPAR6	lysophosphatidic acid receptor 6 [Source:HGNC Symbol;Acc:HGNC:15520]	protein coding	0.72	2.60	16.65	<0.0001	0.0042
ENSG00000179364	PACS2	phosphofurin acidic cluster sorting protein 2 [Source:HGNC Symbol;Acc:HGNC:23794]	protein coding	0.72	1.47	17.08	<0.0001	0.0036
ENSG00000175591	P2RY2	purinergic receptor P2Y2 [Source:HGNC Symbol;Acc:HGNC:8541]	protein coding	0.72	2.25	10.09	0.0015	0.0305
ENSG00000133789	SWAP70	switching B cell complex subunit SWAP70 [Source:HGNC Symbol;Acc:HGNC:17070]	protein coding	0.72	2.90	15.28	<0.0001	0.0064

Table S19: Genes differentially expressed at Day 32 compared to pre-second vaccination (Study Arm 2 A/H7N9+AS03, D1, 29, Neutrophils). Genes are sorted by descending absolute log_2 fold change. Gene model summaries and annotations are based on Ensembl Version 99 (continued.)

Ensembl Gene ID	Ensembl Gene Name	Ensembl Gene Description	Gene Type	log_2 Fold Change (Day 32 vs. Pre-Second Vaccination)	Average log_2 CPM	Likelihood Ratio Test Statistic	P-Value	FDR Adjusted P-Value
ENSG00000186951	PPARA	peroxisome proliferator activated receptor alpha [Source:HGNC Symbol;Acc:HGNC:9232]	protein coding	0.71	1.62	10.85	0.001	0.0239
ENSG00000138829	FBN2	fibrillin 2 [Source:HGNC Symbol;Acc:HGNC:3604]	protein coding	-0.71	1.23	12.17	0.0005	0.0152
ENSG00000004487	KDM1A	lysine demethylase 1A [Source:HGNC Symbol;Acc:HGNC:29079]	protein coding	0.71	0.70	8.47	0.0036	0.049
ENSG00000117480	FAAH	fatty acid amide hydrolase [Source:HGNC Symbol;Acc:HGNC:3553]	protein coding	0.71	2.54	24.66	<0.0001	0.0003
ENSG00000167315	ACAA2	acetyl-CoA acyltransferase 2 [Source:HGNC Symbol;Acc:HGNC:83]	protein coding	0.70	0.70	13.93	0.0002	0.0095
ENSG00000140365	COMMD4	COMM domain containing 4 [Source:HGNC Symbol;Acc:HGNC:26027]	protein coding	0.70	1.82	13.45	0.0002	0.0107
ENSG00000155158	TTC39B	tetratricopeptide repeat domain 39B [Source:HGNC Symbol;Acc:HGNC:23704]	protein coding	0.70	0.26	11.13	0.0008	0.0215
ENSG00000065911	MTHFD2	methylenetetrahydrofolate dehydrogenase (NADP+ dependent) 2, methenyltetrahydrofolate cyclohydrolase [Source:HGNC Symbol;Acc:HGNC:7434]	protein coding	0.70	2.00	20.32	<0.0001	0.0015
ENSG00000162894	FCMR	Fc fragment of IgM receptor [Source:HGNC Symbol;Acc:HGNC:14315]	protein coding	-0.70	4.56	18.38	<0.0001	0.0025
ENSG00000172315	TP53RK	TP53 regulating kinase [Source:HGNC Symbol;Acc:HGNC:16197]	protein coding	0.69	1.07	14.17	0.0002	0.0089
ENSG00000275302	CCL4	C-C motif chemokine ligand 4 [Source:HGNC Symbol;Acc:HGNC:10630]	protein coding	-0.69	4.41	14.29	0.0002	0.0086
ENSG00000213614	HEXA	hexosaminidase subunit alpha [Source:HGNC Symbol;Acc:HGNC:4878]	protein coding	0.69	2.08	15.79	<0.0001	0.0055
ENSG00000246339	EXTL3-AS1	EXTL3 antisense RNA 1 [Source:HGNC Symbol;Acc:HGNC:27985]	lncRNA	-0.68	0.48	13.18	0.0003	0.0113
ENSG00000125746	EML2	EMAP like 2 [Source:HGNC Symbol;Acc:HGNC:18035]	protein coding	0.68	3.13	25.15	<0.0001	0.0003
ENSG00000155850	SLC26A2	solute carrier family 26 member 2 [Source:HGNC Symbol;Acc:HGNC:10994]	protein coding	0.68	1.02	12.95	0.0003	0.0122
ENSG00000168275	COA6	cytochrome c oxidase assembly factor 6 [Source:HGNC Symbol;Acc:HGNC:18025]	protein coding	0.68	1.38	15.30	<0.0001	0.0064
ENSG00000204248	COL11A2	collagen type XI alpha 2 chain [Source:HGNC Symbol;Acc:HGNC:2187]	protein coding	-0.68	0.97	16.20	<0.0001	0.0049

Table S19: Genes differentially expressed at Day 32 compared to pre-second vaccination (Study Arm 2 A/H7N9+AS03, D1, 29, Neutrophils). Genes are sorted by descending absolute log_2 fold change. Gene model summaries and annotations are based on Ensembl Version 99 (continued.)

Ensembl Gene ID	Ensembl Gene Name	Ensembl Gene Description	Gene Type	log_2 Fold Change (Day 32 vs. Pre-Second Vaccination)	Average log_2 CPM	Likelihood Ratio Test Statistic	P-Value	FDR Adjusted P-Value
ENSG00000240344	PPIL3	peptidylprolyl isomerase like 3 [Source:HGNC Symbol;Acc:HGNC:9262]	protein coding	0.67	0.38	15.32	<0.0001	0.0064
ENSG00000153162	BMP6	bone morphogenetic protein 6 [Source:HGNC Symbol;Acc:HGNC:1073]	protein coding	-0.67	3.28	31.54	<0.0001	<0.0001
ENSG00000251364		novel transcript, antisense to OLFML1	lncRNA	0.67	0.68	13.65	0.0002	0.01
ENSG00000138623	SEMA7A	semaphorin 7A (John Milton Hagen blood group) [Source:HGNC Symbol;Acc:HGNC:10741]	protein coding	0.67	3.97	13.37	0.0003	0.0109
ENSG00000274750	H3C6	H3 clustered histone 6 [Source:HGNC Symbol;Acc:HGNC:4769]	protein coding	-0.67	1.91	11.49	0.0007	0.0188
ENSG00000078070	MCCC1	methylcrotonoyl-CoA carboxylase 1 [Source:HGNC Symbol;Acc:HGNC:6936]	protein coding	0.67	0.99	14.44	0.0001	0.0083
ENSG00000132879	FBXO44	F-box protein 44 [Source:HGNC Symbol;Acc:HGNC:24847]	protein coding	0.66	0.39	13.11	0.0003	0.0115
ENSG00000108785	HSD17B1P1	hydroxysteroid 17-beta dehydrogenase 1 pseudogene 1 [Source:HGNC Symbol;Acc:HGNC:5216]	unprocessed pseudogene	0.66	0.76	17.11	<0.0001	0.0036
ENSG00000137094	DNAJB5	DnaJ heat shock protein family (Hsp40) member B5 [Source:HGNC Symbol;Acc:HGNC:14887]	protein coding	-0.66	3.06	9.63	0.0019	0.0347
ENSG00000069020	MAST4	microtubule associated serine/threonine kinase family member 4 [Source:HGNC Symbol;Acc:HGNC:19037]	protein coding	-0.66	1.68	9.52	0.002	0.0359
ENSG00000154310	TNIK	TRAF2 and NCK interacting kinase [Source:HGNC Symbol;Acc:HGNC:30765]	protein coding	0.66	2.27	12.77	0.0004	0.0128
ENSG00000143110	C1orf162	chromosome 1 open reading frame 162 [Source:HGNC Symbol;Acc:HGNC:28344]	protein coding	0.66	4.18	29.03	<0.0001	<0.0001
ENSG00000166886	NAB2	NGFI-A binding protein 2 [Source:HGNC Symbol;Acc:HGNC:7627]	protein coding	-0.65	3.00	27.07	<0.0001	0.0001
ENSG00000131781	FMO5	flavin containing dimethylaniline monoxygenase 5 [Source:HGNC Symbol;Acc:HGNC:3773]	protein coding	0.65	0.56	9.99	0.0016	0.0317
ENSG00000131018	SYNE1	spectrin repeat containing nuclear envelope protein 1 [Source:HGNC Symbol;Acc:HGNC:17089]	protein coding	0.65	5.57	11.29	0.0008	0.0204
ENSG00000118640	VAMP8	vesicle associated membrane protein 8 [Source:HGNC Symbol;Acc:HGNC:12647]	protein coding	0.65	1.58	12.44	0.0004	0.0142
ENSG00000175857	GAPT	GRB2 binding adaptor protein, transmembrane [Source:HGNC Symbol;Acc:HGNC:26588]	protein coding	0.65	6.73	23.49	<0.0001	0.0005
ENSG00000182158	CREB3L2	cAMP responsive element binding protein 3 like 2 [Source:HGNC Symbol;Acc:HGNC:23720]	protein coding	0.64	3.28	13.35	0.0003	0.0109

Table S19: Genes differentially expressed at Day 32 compared to pre-second vaccination (Study Arm 2 A/H7N9+AS03, D1, 29, Neutrophils). Genes are sorted by descending absolute log_2 fold change. Gene model summaries and annotations are based on Ensembl Version 99 (continued.)

Ensembl Gene ID	Ensembl Gene Name	Ensembl Gene Description	Gene Type	log_2 Fold Change (Day 32 vs. Pre-Second Vaccination)	Average log_2 CPM	Likelihood Ratio Test Statistic	P-Value	FDR Adjusted P-Value
ENSG00000165804	ZNF219	zinc finger protein 219 [Source:HGNC Symbol;Acc:HGNC:13011]	protein coding	-0.64	2.12	17.10	<0.0001	0.0036
ENSG00000277349		capicua transcriptional repressor (CIC) pseudogene	unprocessed pseudogene	-0.64	1.24	17.80	<0.0001	0.0031
ENSG00000250903	GMDS-DT	GMDS divergent transcript [Source:HGNC Symbol;Acc:HGNC:48993]	lncRNA	0.64	1.07	15.00	0.0001	0.0069
ENSG00000070950	RAD18	RAD18 E3 ubiquitin protein ligase [Source:HGNC Symbol;Acc:HGNC:18278]	protein coding	0.64	1.03	11.84	0.0006	0.0167
ENSG00000198879	SFMBT2	Scm like with four mbt domains 2 [Source:HGNC Symbol;Acc:HGNC:20256]	protein coding	0.63	1.77	10.35	0.0013	0.028
ENSG00000134627	PIWIL4	piwi like RNA-mediated gene silencing 4 [Source:HGNC Symbol;Acc:HGNC:18444]	protein coding	0.63	0.54	9.17	0.0025	0.0401
ENSG00000131067	GGT7	gamma-glutamyltransferase 7 [Source:HGNC Symbol;Acc:HGNC:4259]	protein coding	0.63	0.44	9.13	0.0025	0.0408
ENSG00000149418	ST14	suppression of tumorigenicity 14 [Source:HGNC Symbol;Acc:HGNC:11344]	protein coding	0.63	3.48	23.29	<0.0001	0.0005
ENSG00000115232	ITGA4	integrin subunit alpha 4 [Source:HGNC Symbol;Acc:HGNC:6140]	protein coding	0.63	5.67	14.19	0.0002	0.0089
ENSG00000283633		novel transcript	lncRNA	-0.62	1.78	17.27	<0.0001	0.0034
ENSG00000128185	DGCR6L	DiGeorge syndrome critical region gene 6 like [Source:HGNC Symbol;Acc:HGNC:18551]	protein coding	0.62	2.50	28.05	<0.0001	0.0001
ENSG00000104267	CA2	carbonic anhydrase 2 [Source:HGNC Symbol;Acc:HGNC:1373]	protein coding	0.62	1.88	9.33	0.0023	0.0384
ENSG00000187609	EXD3	exonuclease 3'-5' domain containing 3 [Source:HGNC Symbol;Acc:HGNC:26023]	protein coding	0.62	0.62	12.08	0.0005	0.0155
ENSG00000260105	AOC4P	amine oxidase copper containing 4, pseudogene [Source:HGNC Symbol;Acc:HGNC:48869]	transcribed unprocessed pseudogene	-0.62	0.49	11.88	0.0006	0.0166
ENSG00000113407	TARS1	threonyl-tRNA synthetase 1 [Source:HGNC Symbol;Acc:HGNC:11572]	protein coding	0.62	1.59	12.99	0.0003	0.0121
ENSG00000129103	SUMF2	sulfatase modifying factor 2 [Source:HGNC Symbol;Acc:HGNC:20415]	protein coding	0.62	1.55	14.66	0.0001	0.0077

Table S19: Genes differentially expressed at Day 32 compared to pre-second vaccination (Study Arm 2 A/H7N9+AS03, D1, 29, Neutrophils). Genes are sorted by descending absolute log_2 fold change. Gene model summaries and annotations are based on Ensembl Version 99 (continued.)

Ensembl Gene ID	Ensembl Gene Name	Ensembl Gene Description	Gene Type	log_2 Fold Change (Day 32 vs. Pre-Second Vaccination)	Average log_2 CPM	Likelihood Ratio Test Statistic	P-Value	FDR Adjusted P-Value
ENSG00000165548	TMEM63C	transmembrane protein 63C [Source:HGNC Symbol;Acc:HGNC:23787]	protein coding	-0.62	2.86	13.47	0.0002	0.0106
ENSG00000197043	ANXA6	annexin A6 [Source:HGNC Symbol;Acc:HGNC:544]	protein coding	0.61	3.56	20.77	<0.0001	0.0013
ENSG00000161642	ZNF385A	zinc finger protein 385A [Source:HGNC Symbol;Acc:HGNC:17521]	protein coding	-0.61	3.09	27.99	<0.0001	0.0001
ENSG00000234444	ZNF736	zinc finger protein 736 [Source:HGNC Symbol;Acc:HGNC:32467]	protein coding	0.61	0.36	11.66	0.0006	0.0176
ENSG00000166140	ZFYVE19	zinc finger FYVE-type containing 19 [Source:HGNC Symbol;Acc:HGNC:20758]	protein coding	0.61	3.03	21.51	<0.0001	0.001
ENSG00000196670	ZFP62	ZFP62 zinc finger protein [Source:HGNC Symbol;Acc:HGNC:23241]	protein coding	0.61	0.95	13.30	0.0003	0.0109
ENSG00000135776	ABCB10	ATP binding cassette subfamily B member 10 [Source:HGNC Symbol;Acc:HGNC:41]	protein coding	0.60	1.71	14.06	0.0002	0.0092
ENSG00000266236	NARF-IT1	NARF intronic transcript 1 [Source:HGNC Symbol;Acc:HGNC:43651]	lncRNA	-0.60	2.42	16.33	<0.0001	0.0047
ENSG00000236790	LINC00299	long intergenic non-protein coding RNA 299 [Source:HGNC Symbol;Acc:HGNC:27940]	lncRNA	0.60	0.65	9.31	0.0023	0.0385
ENSG00000198715	GLMP	glycosylated lysosomal membrane protein [Source:HGNC Symbol;Acc:HGNC:29436]	protein coding	0.60	2.02	11.47	0.0007	0.019
ENSG00000148444	COMMD3	COMM domain containing 3 [Source:HGNC Symbol;Acc:HGNC:23332]	protein coding	0.60	1.36	14.44	0.0001	0.0083
ENSG00000183092	BEGAIN	brain enriched guanylate kinase associated [Source:HGNC Symbol;Acc:HGNC:24163]	protein coding	0.59	2.43	15.65	<0.0001	0.0057
ENSG00000279744		TEC	TEC	-0.59	1.98	18.50	<0.0001	0.0025
ENSG00000151689	INPP1	inositol polyphosphate-1-phosphatase [Source:HGNC Symbol;Acc:HGNC:6071]	protein coding	0.59	2.16	13.72	0.0002	0.0098
ENSG00000186141	POLR3C	RNA polymerase III subunit C [Source:HGNC Symbol;Acc:HGNC:30076]	protein coding	0.59	1.70	14.27	0.0002	0.0086
ENSG00000240849	TMEM189	transmembrane protein 189 [Source:HGNC Symbol;Acc:HGNC:16735]	protein coding	0.59	0.79	11.69	0.0006	0.0176
ENSG00000145882	PCYOX1L	prenylcysteine oxidase 1 like [Source:HGNC Symbol;Acc:HGNC:28477]	protein coding	0.59	0.61	10.83	0.001	0.024
ENSG00000184677	ZBTB40	zinc finger and BTB domain containing 40 [Source:HGNC Symbol;Acc:HGNC:29045]	protein coding	0.59	2.30	14.66	0.0001	0.0077

Table S19: Genes differentially expressed at Day 32 compared to pre-second vaccination (Study Arm 2 A/H7N9+AS03, D1, 29, Neutrophils). Genes are sorted by descending absolute log_2 fold change. Gene model summaries and annotations are based on Ensembl Version 99 (continued.)

Ensembl Gene ID	Ensembl Gene Name	Ensembl Gene Description	Gene Type	log_2 Fold Change (Day 32 vs. Pre-Second Vaccination)	Average log_2 CPM	Likelihood Ratio Test Statistic	P-Value	FDR Adjusted P-Value
ENSG00000001461	NIPAL3	NIPA like domain containing 3 [Source:HGNC Symbol;Acc:HGNC:25233]	protein coding	0.59	1.36	18.76	<0.0001	0.0023
ENSG00000196371	FUT4	fucosyltransferase 4 [Source:HGNC Symbol;Acc:HGNC:4015]	protein coding	0.59	2.34	15.03	0.0001	0.0069
ENSG00000197894	ADH5	alcohol dehydrogenase 5 (class III), chi polypeptide [Source:HGNC Symbol;Acc:HGNC:253]	protein coding	0.59	1.47	8.81	0.003	0.0449
ENSG00000148337	CIZ1	CDKN1A interacting zinc finger protein 1 [Source:HGNC Symbol;Acc:HGNC:16744]	protein coding	0.59	3.43	20.35	<0.0001	0.0015
ENSG00000128915	ICE2	interactor of little elongation complex ELL subunit 2 [Source:HGNC Symbol;Acc:HGNC:29885]	protein coding	0.59	0.51	8.52	0.0035	0.0482

Table S20: Genes differentially expressed at Day 8 compared to pre-first vaccination (Study Arm 1 A/H3N2v, D1 , NK cells). Genes are sorted by descending absolute log_2 fold change. Gene model summaries and annotations are based on Ensembl Version 99.

Ensembl Gene ID	Ensembl Gene Name	Ensembl Gene Description	Gene Type	log_2 Fold Change (Day 8 vs. Pre-First Vaccination)	Average log_2 CPM	Likelihood Ratio Test Statistic	P-Value	FDR Adjusted P-Value
ENSG00000240382	IGKV1-17	immunoglobulin kappa variable 1-17 [Source:HGNC Symbol;Acc:HGNC:5733]	IG V gene	-1.21	0.90	21.67	<0.0001	0.047

Table S21: Genes differentially expressed at Day 2 compared to pre-first vaccination (Study Arm 2 A/H7N9+AS03, D1, 29, NK cells). Genes are sorted by descending absolute log_2 fold change. Gene model summaries and annotations are based on Ensembl Version 99.

Ensembl Gene ID	Ensembl Gene Name	Ensembl Gene Description	Gene Type	log_2 Fold Change (Day 2 vs. Pre-First Vaccination)	Average log_2 CPM	Likelihood Ratio Test Statistic	P-Value	FDR Adjusted P-Value
ENSG00000010030	ETV7	ETS variant transcription factor 7 [Source:HGNC Symbol;Acc:HGNC:18160]	protein coding	1.83	2.31	57.31	<0.0001	<0.0001
ENSG00000115415	STAT1	signal transducer and activator of transcription 1 [Source:HGNC Symbol;Acc:HGNC:11362]	protein coding	1.51	7.92	81.99	<0.0001	<0.0001
ENSG00000117228	GBP1	guanylate binding protein 1 [Source:HGNC Symbol;Acc:HGNC:4182]	protein coding	1.45	6.06	61.97	<0.0001	<0.0001

Table S21: Genes differentially expressed at Day 2 compared to pre-first vaccination (Study Arm 2 A/H7N9+AS03, D1, 29, NK cells). Genes are sorted by descending absolute log_2 fold change. Gene model summaries and annotations are based on Ensembl Version 99 (continued.)

Ensembl Gene ID	Ensembl Gene Name	Ensembl Gene Description	Gene Type	log_2 Fold Change (Day 2 vs. Pre-First Vaccination)	Average log_2 CPM	Likelihood Ratio Test Statistic	P-Value	FDR Adjusted P-Value
ENSG00000138119	MYOF	myoferlin [Source:HGNC Symbol;Acc:HGNC:3656]	protein coding	1.24	2.87	29.08	<0.0001	<0.0001
ENSG00000103569	AQP9	aquaporin 9 [Source:HGNC Symbol;Acc:HGNC:643]	protein coding	1.22	1.67	29.66	<0.0001	<0.0001
ENSG00000137959	IFI44L	interferon induced protein 44 like [Source:HGNC Symbol;Acc:HGNC:17817]	protein coding	1.14	5.38	20.19	<0.0001	0.0011
ENSG00000087237	CETP	cholesteryl ester transfer protein [Source:HGNC Symbol;Acc:HGNC:1869]	protein coding	1.12	1.20	32.93	<0.0001	<0.0001
ENSG00000171049	FPR2	formyl peptide receptor 2 [Source:HGNC Symbol;Acc:HGNC:3827]	protein coding	1.11	1.17	18.89	<0.0001	0.0019
ENSG00000025708	TYMP	thymidine phosphorylase [Source:HGNC Symbol;Acc:HGNC:3148]	protein coding	1.09	6.87	43.80	<0.0001	<0.0001
ENSG00000128383	APOBEC3A	apolipoprotein B mRNA editing enzyme catalytic subunit 3A [Source:HGNC Symbol;Acc:HGNC:17343]	protein coding	1.09	2.38	13.55	0.0002	0.0149
ENSG00000284194	SCO2	SCO cytochrome c oxidase assembly protein 2 [Source:HGNC Symbol;Acc:HGNC:10604]	protein coding	1.03	2.31	37.95	<0.0001	<0.0001
ENSG00000177989	ODF3B	outer dense fiber of sperm tails 3B [Source:HGNC Symbol;Acc:HGNC:34388]	protein coding	1.03	5.03	32.69	<0.0001	<0.0001
ENSG00000019169	MARCO	macrophage receptor with collagenous structure [Source:HGNC Symbol;Acc:HGNC:6895]	protein coding	1.02	1.31	19.95	<0.0001	0.0012
ENSG00000141574	SECTM1	secreted and transmembrane 1 [Source:HGNC Symbol;Acc:HGNC:10707]	protein coding	1.00	4.10	32.53	<0.0001	<0.0001
ENSG00000100346	CACNA1I	calcium voltage-gated channel subunit alpha1 I [Source:HGNC Symbol;Acc:HGNC:1396]	protein coding	-0.99	0.84	25.84	<0.0001	0.0001
ENSG00000119917	IFIT3	interferon induced protein with tetratricopeptide repeats 3 [Source:HGNC Symbol;Acc:HGNC:5411]	protein coding	0.98	4.11	16.74	<0.0001	0.0047
ENSG00000138496	PARP9	poly(ADP-ribose) polymerase family member 9 [Source:HGNC Symbol;Acc:HGNC:24118]	protein coding	0.98	6.64	45.04	<0.0001	<0.0001
ENSG00000140105	WARS1	tryptophanyl-tRNA synthetase 1 [Source:HGNC Symbol;Acc:HGNC:12729]	protein coding	0.98	6.29	43.78	<0.0001	<0.0001
ENSG00000163221	S100A12	S100 calcium binding protein A12 [Source:HGNC Symbol;Acc:HGNC:10489]	protein coding	0.93	3.62	16.21	<0.0001	0.0057
ENSG00000020577	SAMD4A	sterile alpha motif domain containing 4A [Source:HGNC Symbol;Acc:HGNC:23023]	protein coding	0.90	2.06	15.85	<0.0001	0.0064
ENSG00000214872	SMTNL1	smoothelin like 1 [Source:HGNC Symbol;Acc:HGNC:32394]	protein coding	0.88	1.48	25.51	<0.0001	0.0001

Table S21: Genes differentially expressed at Day 2 compared to pre-first vaccination (Study Arm 2 A/H7N9+AS03, D1, 29, NK cells). Genes are sorted by descending absolute log_2 fold change. Gene model summaries and annotations are based on Ensembl Version 99 (continued.)

Ensembl Gene ID	Ensembl Gene Name	Ensembl Gene Description	Gene Type	log_2 Fold Change (Day 2 vs. Pre-First Vaccination)	Average log_2 CPM	Likelihood Ratio Test Statistic	P-Value	FDR Adjusted P-Value
ENSG00000172159	FRMD3	FERM domain containing 3 [Source:HGNC Symbol;Acc:HGNC:24125]	protein coding	0.86	1.24	45.64	<0.0001	<0.0001
ENSG00000169385	RNASE2	ribonuclease A family member 2 [Source:HGNC Symbol;Acc:HGNC:10045]	protein coding	0.85	1.64	15.53	<0.0001	0.0073
ENSG00000127954	STEAP4	STEAP4 metalloreductase [Source:HGNC Symbol;Acc:HGNC:21923]	protein coding	0.84	0.87	16.56	<0.0001	0.0051
ENSG00000078081	LAMP3	lysosomal associated membrane protein 3 [Source:HGNC Symbol;Acc:HGNC:14582]	protein coding	0.83	0.24	13.71	0.0002	0.0143
ENSG00000184371	CSF1	colony stimulating factor 1 [Source:HGNC Symbol;Acc:HGNC:2432]	protein coding	0.81	2.21	35.95	<0.0001	<0.0001
ENSG00000135378	PRRG4	proline rich and Gla domain 4 [Source:HGNC Symbol;Acc:HGNC:30799]	protein coding	0.79	0.55	15.14	<0.0001	0.0085
ENSG00000131459	GFPT2	glutamine-fructose-6-phosphate transaminase 2 [Source:HGNC Symbol;Acc:HGNC:4242]	protein coding	-0.79	1.88	22.66	<0.0001	0.0004
ENSG00000133106	EPSTI1	epithelial stromal interaction 1 [Source:HGNC Symbol;Acc:HGNC:16465]	protein coding	0.79	5.57	33.96	<0.0001	<0.0001
ENSG00000163220	S100A9	S100 calcium binding protein A9 [Source:HGNC Symbol;Acc:HGNC:10499]	protein coding	0.78	8.01	13.32	0.0003	0.0162
ENSG00000143546	S100A8	S100 calcium binding protein A8 [Source:HGNC Symbol;Acc:HGNC:10498]	protein coding	0.78	6.76	12.10	0.0005	0.0258
ENSG00000185339	TCN2	transcobalamin 2 [Source:HGNC Symbol;Acc:HGNC:11653]	protein coding	0.78	0.64	15.41	<0.0001	0.0077
ENSG00000183087	GAS6	growth arrest specific 6 [Source:HGNC Symbol;Acc:HGNC:4168]	protein coding	0.78	2.81	30.36	<0.0001	<0.0001
ENSG00000180061	TMEM150B	transmembrane protein 150B [Source:HGNC Symbol;Acc:HGNC:34415]	protein coding	0.77	1.34	12.32	0.0004	0.0238
ENSG00000125347	IRF1	interferon regulatory factor 1 [Source:HGNC Symbol;Acc:HGNC:6116]	protein coding	0.77	8.10	42.11	<0.0001	<0.0001
ENSG00000140511	HAPLN3	hyaluronan and proteoglycan link protein 3 [Source:HGNC Symbol;Acc:HGNC:21446]	protein coding	0.74	4.97	30.82	<0.0001	<0.0001
ENSG00000162654	GBP4	guanylate binding protein 4 [Source:HGNC Symbol;Acc:HGNC:20480]	protein coding	0.73	7.13	54.36	<0.0001	<0.0001
ENSG00000171051	FPR1	formyl peptide receptor 1 [Source:HGNC Symbol;Acc:HGNC:3826]	protein coding	0.72	3.81	11.60	0.0007	0.0301
ENSG00000173578	XCR1	X-C motif chemokine receptor 1 [Source:HGNC Symbol;Acc:HGNC:1625]	protein coding	-0.72	0.45	10.64	0.0011	0.0442

Table S21: Genes differentially expressed at Day 2 compared to pre-first vaccination (Study Arm 2 A/H7N9+AS03, D1, 29, NK cells). Genes are sorted by descending absolute log_2 fold change. Gene model summaries and annotations are based on Ensembl Version 99 (continued.)

Ensembl Gene ID	Ensembl Gene Name	Ensembl Gene Description	Gene Type	log_2 Fold Change (Day 2 vs. Pre-First Vaccination)	Average log_2 CPM	Likelihood Ratio Test Statistic	P-Value	FDR Adjusted P-Value
ENSG00000260145		novel transcript, antisense to NLRC5	lncRNA	0.72	0.26	25.50	<0.0001	0.0001
ENSG00000120885	CLU	clusterin [Source:HGNC Symbol;Acc:HGNC:2095]	protein coding	0.71	2.05	21.85	<0.0001	0.0006
ENSG00000198959	TGM2	transglutaminase 2 [Source:HGNC Symbol;Acc:HGNC:11778]	protein coding	0.71	0.64	12.86	0.0003	0.0194
ENSG00000002549	LAP3	leucine aminopeptidase 3 [Source:HGNC Symbol;Acc:HGNC:18449]	protein coding	0.71	5.76	33.26	<0.0001	<0.0001
ENSG00000082397	EPB41L3	erythrocyte membrane protein band 4.1 like 3 [Source:HGNC Symbol;Acc:HGNC:3380]	protein coding	0.70	2.54	12.95	0.0003	0.0187
ENSG00000108950	FAM20A	FAM20A golgi associated secretory pathway pseudokinase [Source:HGNC Symbol;Acc:HGNC:23015]	protein coding	0.70	1.08	20.62	<0.0001	0.0009
ENSG00000197249	SERPINA1	serpin family A member 1 [Source:HGNC Symbol;Acc:HGNC:8941]	protein coding	0.70	5.51	11.54	0.0007	0.0309
ENSG00000080573	COL5A3	collagen type V alpha 3 chain [Source:HGNC Symbol;Acc:HGNC:14864]	protein coding	-0.70	5.23	22.39	<0.0001	0.0005
ENSG00000158714	SLAMF8	SLAM family member 8 [Source:HGNC Symbol;Acc:HGNC:21391]	protein coding	0.68	1.93	35.20	<0.0001	<0.0001
ENSG00000141096	DPEP3	dipeptidase 3 [Source:HGNC Symbol;Acc:HGNC:23029]	protein coding	-0.67	0.27	18.55	<0.0001	0.0022
ENSG00000156587	UBE2L6	ubiquitin conjugating enzyme E2 L6 [Source:HGNC Symbol;Acc:HGNC:12490]	protein coding	0.67	6.88	45.13	<0.0001	<0.0001
ENSG00000162614	NEXN	nexilin F-actin binding protein [Source:HGNC Symbol;Acc:HGNC:29557]	protein coding	0.67	1.56	19.97	<0.0001	0.0012
ENSG00000136867	SLC31A2	solute carrier family 31 member 2 [Source:HGNC Symbol;Acc:HGNC:11017]	protein coding	0.66	0.63	17.69	<0.0001	0.0033
ENSG00000154269	ENPP3	ectonucleotide pyrophosphatase/phosphodiesterase 3 [Source:HGNC Symbol;Acc:HGNC:3358]	protein coding	-0.65	0.34	13.97	0.0002	0.0129
ENSG00000276715	YWHAEP7	tyrosine 3-monooxygenase/tryptophan 5-monooxygenase activation protein epsilon pseudogene 7 [Source:HGNC Symbol;Acc:HGNC:49437]	transcribed unprocessed pseudogene	-0.64	0.90	14.22	0.0002	0.0118
ENSG00000188282	RUFY4	RUN and FYVE domain containing 4 [Source:HGNC Symbol;Acc:HGNC:24804]	protein coding	0.64	1.10	15.39	<0.0001	0.0078
ENSG00000134321	RSAD2	radical S-adenosyl methionine domain containing 2 [Source:HGNC Symbol;Acc:HGNC:30908]	protein coding	0.63	3.87	11.96	0.0005	0.0274

Table S21: Genes differentially expressed at Day 2 compared to pre-first vaccination (Study Arm 2 A/H7N9+AS03, D1, 29, NK cells). Genes are sorted by descending absolute log_2 fold change. Gene model summaries and annotations are based on Ensembl Version 99 (continued.)

Ensembl Gene ID	Ensembl Gene Name	Ensembl Gene Description	Gene Type	log_2 Fold Change (Day 2 vs. Pre-First Vaccination)	Average log_2 CPM	Likelihood Ratio Test Statistic	P-Value	FDR Adjusted P-Value
ENSG00000111331	OAS3	2'-5'-oligoadenylate synthetase 3 [Source:HGNC Symbol;Acc:HGNC:8088]	protein coding	0.62	5.98	16.19	<0.0001	0.0057
ENSG00000178965	ERICH3	glutamate rich 3 [Source:HGNC Symbol;Acc:HGNC:25346]	protein coding	0.62	0.90	13.30	0.0003	0.0162
ENSG00000134326	CMPK2	cytidine/uridine monophosphate kinase 2 [Source:HGNC Symbol;Acc:HGNC:27015]	protein coding	0.61	3.82	11.17	0.0008	0.0358
ENSG00000213928	IRF9	interferon regulatory factor 9 [Source:HGNC Symbol;Acc:HGNC:6131]	protein coding	0.59	5.25	24.20	<0.0001	0.0002

Table S22: Genes differentially expressed at Day 4 compared to pre-first vaccination (Study Arm 2 A/H7N9+AS03, D1, 29, NK cells). Genes are sorted by descending absolute log_2 fold change. Gene model summaries and annotations are based on Ensembl Version 99.

Ensembl Gene ID	Ensembl Gene Name	Ensembl Gene Description	Gene Type	log_2 Fold Change (Day 4 vs. Pre-First Vaccination)	Average log_2 CPM	Likelihood Ratio Test Statistic	P-Value	FDR Adjusted P-Value
ENSG00000197256	KANK2	KN motif and ankyrin repeat domains 2 [Source:HGNC Symbol;Acc:HGNC:29300]	protein coding	1.19	0.38	37.04	<0.0001	<0.0001
ENSG00000137959	IFI44L	interferon induced protein 44 like [Source:HGNC Symbol;Acc:HGNC:17817]	protein coding	1.12	5.35	12.85	0.0003	0.0407
ENSG00000127564	PKMYT1	protein kinase, membrane associated tyrosine/threonine 1 [Source:HGNC Symbol;Acc:HGNC:29650]	protein coding	1.12	2.07	33.61	<0.0001	<0.0001
ENSG00000089685	BIRC5	baculoviral IAP repeat containing 5 [Source:HGNC Symbol;Acc:HGNC:593]	protein coding	1.10	1.37	27.73	<0.0001	0.0001
ENSG00000175063	UBE2C	ubiquitin conjugating enzyme E2 C [Source:HGNC Symbol;Acc:HGNC:15937]	protein coding	1.10	0.35	21.89	<0.0001	0.0009
ENSG00000123485	HJURP	Holliday junction recognition protein [Source:HGNC Symbol;Acc:HGNC:25444]	protein coding	1.10	0.78	27.57	<0.0001	0.0001
ENSG00000138180	CEP55	centrosomal protein 55 [Source:HGNC Symbol;Acc:HGNC:1161]	protein coding	1.08	0.54	27.39	<0.0001	0.0001
ENSG00000237649	KIFC1	kinesin family member C1 [Source:HGNC Symbol;Acc:HGNC:6389]	protein coding	1.03	2.08	32.65	<0.0001	<0.0001
ENSG00000166803	PCLAF	PCNA clamp associated factor [Source:HGNC Symbol;Acc:HGNC:28961]	protein coding	1.01	0.33	27.78	<0.0001	0.0001

Table S22: Genes differentially expressed at Day 4 compared to pre-first vaccination (Study Arm 2 A/H7N9+AS03, D1, 29, NK cells). Genes are sorted by descending absolute log_2 fold change. Gene model summaries and annotations are based on Ensembl Version 99 (continued.)

Ensembl Gene ID	Ensembl Gene Name	Ensembl Gene Description	Gene Type	log_2 Fold Change (Day 4 vs. Pre-First Vaccination)	Average log_2 CPM	Likelihood Ratio Test Statistic	P-Value	FDR Adjusted P-Value
ENSG00000167900	TK1	thymidine kinase 1 [Source:HGNC Symbol;Acc:HGNC:11830]	protein coding	1.01	2.91	35.19	<0.0001	<0.0001
ENSG00000176890	TYMS	thymidylate synthetase [Source:HGNC Symbol;Acc:HGNC:12441]	protein coding	1.00	2.44	31.80	<0.0001	<0.0001
ENSG00000137807	KIF23	kinesin family member 23 [Source:HGNC Symbol;Acc:HGNC:6392]	protein coding	0.99	0.59	22.77	<0.0001	0.0006
ENSG00000093009	CDC45	cell division cycle 45 [Source:HGNC Symbol;Acc:HGNC:1739]	protein coding	0.99	1.63	22.70	<0.0001	0.0006
ENSG00000143476	DTL	denticleless E3 ubiquitin protein ligase homolog [Source:HGNC Symbol;Acc:HGNC:30288]	protein coding	0.98	1.61	23.34	<0.0001	0.0005
ENSG00000161888	SPC24	SPC24 component of NDC80 kinetochore complex [Source:HGNC Symbol;Acc:HGNC:26913]	protein coding	0.98	0.56	20.31	<0.0001	0.0016
ENSG00000146670	CDCA5	cell division cycle associated 5 [Source:HGNC Symbol;Acc:HGNC:14626]	protein coding	0.97	2.01	31.65	<0.0001	<0.0001
ENSG00000135476	ESPL1	extra spindle pole bodies like 1, separase [Source:HGNC Symbol;Acc:HGNC:16856]	protein coding	0.97	1.44	28.75	<0.0001	<0.0001
ENSG00000122952	ZWINT	ZW10 interacting kinetochore protein [Source:HGNC Symbol;Acc:HGNC:13195]	protein coding	0.95	1.99	24.13	<0.0001	0.0004
ENSG00000117399	CDC20	cell division cycle 20 [Source:HGNC Symbol;Acc:HGNC:1723]	protein coding	0.94	1.04	20.93	<0.0001	0.0013
ENSG00000174371	EXO1	exonuclease 1 [Source:HGNC Symbol;Acc:HGNC:3511]	protein coding	0.92	0.39	19.79	<0.0001	0.002
ENSG00000163808	KIF15	kinesin family member 15 [Source:HGNC Symbol;Acc:HGNC:17273]	protein coding	0.92	1.15	28.43	<0.0001	<0.0001
ENSG00000092853	CLSPN	claspin [Source:HGNC Symbol;Acc:HGNC:19715]	protein coding	0.91	1.87	24.08	<0.0001	0.0004
ENSG00000088325	TPX2	TPX2 microtubule nucleation factor [Source:HGNC Symbol;Acc:HGNC:1249]	protein coding	0.91	2.14	30.16	<0.0001	<0.0001
ENSG00000109805	NCAPG	non-SMC condensin I complex subunit G [Source:HGNC Symbol;Acc:HGNC:24304]	protein coding	0.91	1.02	30.92	<0.0001	<0.0001
ENSG00000131747	TOP2A	DNA topoisomerase II alpha [Source:HGNC Symbol;Acc:HGNC:11989]	protein coding	0.91	2.79	26.17	<0.0001	0.0002
ENSG00000101412	E2F1	E2F transcription factor 1 [Source:HGNC Symbol;Acc:HGNC:3113]	protein coding	0.91	3.42	24.10	<0.0001	0.0004
ENSG00000148773	MKI67	marker of proliferation Ki-67 [Source:HGNC Symbol;Acc:HGNC:7107]	protein coding	0.90	4.13	20.58	<0.0001	0.0014

Table S22: Genes differentially expressed at Day 4 compared to pre-first vaccination (Study Arm 2 A/H7N9+AS03, D1, 29, NK cells). Genes are sorted by descending absolute log_2 fold change. Gene model summaries and annotations are based on Ensembl Version 99 (continued.)

Ensembl Gene ID	Ensembl Gene Name	Ensembl Gene Description	Gene Type	log_2 Fold Change (Day 4 vs. Pre-First Vaccination)	Average log_2 CPM	Likelihood Ratio Test Statistic	P-Value	FDR Adjusted P-Value
ENSG00000186185	KIF18B	kinesin family member 18B [Source:HGNC Symbol;Acc:HGNC:27102]	protein coding	0.90	1.05	15.00	0.0001	0.0151
ENSG00000065328	MCM10	minichromosome maintenance 10 replication initiation factor [Source:HGNC Symbol;Acc:HGNC:18043]	protein coding	0.90	0.63	14.13	0.0002	0.0227
ENSG00000167513	CDT1	chromatin licensing and DNA replication factor 1 [Source:HGNC Symbol;Acc:HGNC:24576]	protein coding	0.90	2.57	23.88	<0.0001	0.0004
ENSG00000051180	RAD51	RAD51 recombinase [Source:HGNC Symbol;Acc:HGNC:9817]	protein coding	0.89	1.46	30.79	<0.0001	<0.0001
ENSG00000135451	TROAP	trophinin associated protein [Source:HGNC Symbol;Acc:HGNC:12327]	protein coding	0.89	1.01	24.71	<0.0001	0.0003
ENSG00000066279	ASPM	abnormal spindle microtubule assembly [Source:HGNC Symbol;Acc:HGNC:19048]	protein coding	0.87	1.61	17.89	<0.0001	0.0043
ENSG00000094804	CDC6	cell division cycle 6 [Source:HGNC Symbol;Acc:HGNC:1744]	protein coding	0.87	1.39	23.38	<0.0001	0.0005
ENSG00000178999	AURKB	aurora kinase B [Source:HGNC Symbol;Acc:HGNC:11390]	protein coding	0.87	1.29	21.45	<0.0001	0.001
ENSG00000156970	BUB1B	BUB1 mitotic checkpoint serine/threonine kinase B [Source:HGNC Symbol;Acc:HGNC:1149]	protein coding	0.85	0.61	22.53	<0.0001	0.0007
ENSG00000051341	POLQ	DNA polymerase theta [Source:HGNC Symbol;Acc:HGNC:9186]	protein coding	0.82	1.48	25.07	<0.0001	0.0003
ENSG00000170312	CDK1	cyclin dependent kinase 1 [Source:HGNC Symbol;Acc:HGNC:1722]	protein coding	0.82	1.03	17.54	<0.0001	0.005
ENSG00000075218	GTSE1	G2 and S-phase expressed 1 [Source:HGNC Symbol;Acc:HGNC:13698]	protein coding	0.81	1.41	30.24	<0.0001	<0.0001
ENSG00000138160	KIF11	kinesin family member 11 [Source:HGNC Symbol;Acc:HGNC:6388]	protein coding	0.79	2.30	37.88	<0.0001	<0.0001
ENSG00000131153	GINS2	GINS complex subunit 2 [Source:HGNC Symbol;Acc:HGNC:24575]	protein coding	0.79	1.27	18.65	<0.0001	0.0031
ENSG00000171848	RRM2	ribonucleotide reductase regulatory subunit M2 [Source:HGNC Symbol;Acc:HGNC:10452]	protein coding	0.77	3.94	19.29	<0.0001	0.0025
ENSG00000189057	FAM111B	family with sequence similarity 111 member B [Source:HGNC Symbol;Acc:HGNC:24200]	protein coding	0.76	1.92	15.00	0.0001	0.0151
ENSG00000117724	CENPF	centromere protein F [Source:HGNC Symbol;Acc:HGNC:1857]	protein coding	0.76	2.47	20.10	<0.0001	0.0017
ENSG00000112742	TTK	TTK protein kinase [Source:HGNC Symbol;Acc:HGNC:12401]	protein coding	0.75	0.22	19.05	<0.0001	0.0027

Table S22: Genes differentially expressed at Day 4 compared to pre-first vaccination (Study Arm 2 A/H7N9+AS03, D1, 29, NK cells). Genes are sorted by descending absolute log_2 fold change. Gene model summaries and annotations are based on Ensembl Version 99 (continued.)

Ensembl Gene ID	Ensembl Gene Name	Ensembl Gene Description	Gene Type	log_2 Fold Change (Day 4 vs. Pre-First Vaccination)	Average log_2 CPM	Likelihood Ratio Test Statistic	P-Value	FDR Adjusted P-Value
ENSG00000105011	ASF1B	anti-silencing function 1B histone chaperone [Source:HGNC Symbol;Acc:HGNC:20996]	protein coding	0.74	2.92	25.18	<0.0001	0.0003
ENSG00000072571	HMMR	hyaluronan mediated motility receptor [Source:HGNC Symbol;Acc:HGNC:5012]	protein coding	0.74	0.45	18.91	<0.0001	0.0029
ENSG00000121152	NCAPH	non-SMC condensin I complex subunit H [Source:HGNC Symbol;Acc:HGNC:1112]	protein coding	0.74	1.68	21.70	<0.0001	0.0009
ENSG00000111206	FOXM1	forkhead box M1 [Source:HGNC Symbol;Acc:HGNC:3818]	protein coding	0.72	2.06	25.84	<0.0001	0.0002
ENSG00000154839	SKA1	spindle and kinetochore associated complex subunit 1 [Source:HGNC Symbol;Acc:HGNC:28109]	protein coding	0.71	0.25	18.76	<0.0001	0.003
ENSG00000117632	STMN1	stathmin 1 [Source:HGNC Symbol;Acc:HGNC:6510]	protein coding	0.71	5.27	36.53	<0.0001	<0.0001
ENSG00000100162	CENPM	centromere protein M [Source:HGNC Symbol;Acc:HGNC:18352]	protein coding	0.70	1.56	21.82	<0.0001	0.0009
ENSG00000137812	KNL1	kinetochore scaffold 1 [Source:HGNC Symbol;Acc:HGNC:24054]	protein coding	0.69	1.51	19.86	<0.0001	0.0019
ENSG00000119326	CTNNAL1	catenin alpha like 1 [Source:HGNC Symbol;Acc:HGNC:2512]	protein coding	0.68	1.01	23.25	<0.0001	0.0005
ENSG00000085999	RAD54L	RAD54 like [Source:HGNC Symbol;Acc:HGNC:9826]	protein coding	0.68	0.94	19.27	<0.0001	0.0025
ENSG00000151725	CENPU	centromere protein U [Source:HGNC Symbol;Acc:HGNC:21348]	protein coding	0.67	2.06	23.70	<0.0001	0.0004
ENSG00000007968	E2F2	E2F transcription factor 2 [Source:HGNC Symbol;Acc:HGNC:3114]	protein coding	0.67	2.93	21.71	<0.0001	0.0009
ENSG00000162062	TEDC2	tubulin epsilon and delta complex 2 [Source:HGNC Symbol;Acc:HGNC:25849]	protein coding	0.66	0.51	16.99	<0.0001	0.0064
ENSG00000149554	CHEK1	checkpoint kinase 1 [Source:HGNC Symbol;Acc:HGNC:1925]	protein coding	0.63	2.20	20.65	<0.0001	0.0014
ENSG00000171241	SHCBP1	SHC binding and spindle associated 1 [Source:HGNC Symbol;Acc:HGNC:29547]	protein coding	0.63	1.33	12.83	0.0003	0.0409
ENSG00000122966	CIT	citron rho-interacting serine/threonine kinase [Source:HGNC Symbol;Acc:HGNC:1985]	protein coding	0.63	1.65	25.01	<0.0001	0.0003
ENSG00000071539	TRIP13	thyroid hormone receptor interactor 13 [Source:HGNC Symbol;Acc:HGNC:12307]	protein coding	0.62	0.58	18.47	<0.0001	0.0033
ENSG00000154920	EME1	essential meiotic structure-specific endonuclease 1 [Source:HGNC Symbol;Acc:HGNC:24965]	protein coding	0.60	0.60	16.86	<0.0001	0.0067

Table S22: Genes differentially expressed at Day 4 compared to pre-first vaccination (Study Arm 2 A/H7N9+AS03, D1, 29, NK cells). Genes are sorted by descending absolute log_2 fold change. Gene model summaries and annotations are based on Ensembl Version 99 (continued.)

Ensembl Gene ID	Ensembl Gene Name	Ensembl Gene Description	Gene Type	log_2 Fold Change (Day 4 vs. Pre-First Vaccination)	Average log_2 CPM	Likelihood Ratio Test Statistic	P-Value	FDR Adjusted P-Value
ENSG00000142945	KIF2C	kinesin family member 2C [Source:HGNC Symbol;Acc:HGNC:6393]	protein coding	0.60	1.31	21.42	<0.0001	0.001
ENSG00000138778	CENPE	centromere protein E [Source:HGNC Symbol;Acc:HGNC:1856]	protein coding	0.60	1.17	15.37	<0.0001	0.0128
ENSG00000123473	STIL	STIL centriolar assembly protein [Source:HGNC Symbol;Acc:HGNC:10879]	protein coding	0.60	1.22	18.72	<0.0001	0.003

Table S23: Genes differentially expressed at Day 29 compared to pre-first vaccination (Study Arm 2 A/H7N9+AS03, D1, 29, NK cells). Genes are sorted by descending absolute log_2 fold change. Gene model summaries and annotations are based on Ensembl Version 99.

Ensembl Gene ID	Ensembl Gene Name	Ensembl Gene Description	Gene Type	log_2 Fold Change (Day 29 vs. Pre-First Vaccination)	Average log_2 CPM	Likelihood Ratio Test Statistic	P-Value	FDR Adjusted P-Value
ENSG00000167748	KLK1	kallikrein 1 [Source:HGNC Symbol;Acc:HGNC:6357]	protein coding	1.01	0.58	18.05	<0.0001	0.024
ENSG00000211663	IGLV3-19	immunoglobulin lambda variable 3-19 [Source:HGNC Symbol;Acc:HGNC:5903]	IG V gene	0.85	2.52	14.57	0.0001	0.0478
ENSG00000176490	DIRAS1	DIRAS family GTPase 1 [Source:HGNC Symbol;Acc:HGNC:19127]	protein coding	0.67	1.36	16.43	<0.0001	0.0405
ENSG00000167641	PPP1R14A	protein phosphatase 1 regulatory inhibitor subunit 14A [Source:HGNC Symbol;Acc:HGNC:14871]	protein coding	0.64	2.49	25.71	<0.0001	0.0058
ENSG00000275418		novel transcript	lncRNA	0.64	0.78	14.68	0.0001	0.0472
ENSG00000232629	HLA-DQB2	major histocompatibility complex, class II, DQ beta 2 [Source:HGNC Symbol;Acc:HGNC:4945]	protein coding	0.62	2.29	15.24	<0.0001	0.0415
ENSG00000110987	BCL7A	BAF chromatin remodeling complex subunit BCL7A [Source:HGNC Symbol;Acc:HGNC:1004]	protein coding	0.60	4.73	17.32	<0.0001	0.0285
ENSG00000275180		novel transcript	lncRNA	-0.60	0.54	21.59	<0.0001	0.0098

Table S24: Genes differentially expressed at Day 30 compared to pre-second vaccination (Study Arm 2 A/H7N9+AS03, D1, 29, NK cells). Genes are sorted by descending absolute log_2 fold change. Gene model summaries and annotations are based on Ensembl Version 99.

Ensembl Gene ID	Ensembl Gene Name	Ensembl Gene Description	Gene Type	log_2 Fold Change (Day 30 vs. Pre-Second Vaccination)	Average log_2 CPM	Likelihood Ratio Test Statistic	P-Value	FDR Adjusted P-Value
ENSG00000010030	ETV7	ETS variant transcription factor 7 [Source:HGNC Symbol;Acc:HGNC:18160]	protein coding	2.53	3.05	81.20	<0.0001	<0.0001
ENSG00000117228	GBP1	guanylate binding protein 1 [Source:HGNC Symbol;Acc:HGNC:4182]	protein coding	2.14	6.67	59.79	<0.0001	<0.0001
ENSG00000171049	FPR2	formyl peptide receptor 2 [Source:HGNC Symbol;Acc:HGNC:3827]	protein coding	2.02	1.97	45.00	<0.0001	<0.0001
ENSG00000115415	STAT1	signal transducer and activator of transcription 1 [Source:HGNC Symbol;Acc:HGNC:11362]	protein coding	1.89	8.43	76.00	<0.0001	<0.0001
ENSG00000119917	IFIT3	interferon induced protein with tetratricopeptide repeats 3 [Source:HGNC Symbol;Acc:HGNC:5411]	protein coding	1.89	4.61	71.02	<0.0001	<0.0001
ENSG00000137959	IFI44L	interferon induced protein 44 like [Source:HGNC Symbol;Acc:HGNC:17817]	protein coding	1.78	6.02	96.71	<0.0001	<0.0001
ENSG00000138119	MYOF	myoferlin [Source:HGNC Symbol;Acc:HGNC:3656]	protein coding	1.72	3.86	42.74	<0.0001	<0.0001
ENSG00000078081	LAMP3	lysosomal associated membrane protein 3 [Source:HGNC Symbol;Acc:HGNC:14582]	protein coding	1.70	0.87	62.05	<0.0001	<0.0001
ENSG00000140105	WARS1	tryptophanyl-tRNA synthetase 1 [Source:HGNC Symbol;Acc:HGNC:12729]	protein coding	1.68	7.12	44.47	<0.0001	<0.0001
ENSG00000128383	APOBEC3A	apolipoprotein B mRNA editing enzyme catalytic subunit 3A [Source:HGNC Symbol;Acc:HGNC:17343]	protein coding	1.64	3.12	45.87	<0.0001	<0.0001
ENSG00000185339	TCN2	transcobalamin 2 [Source:HGNC Symbol;Acc:HGNC:11653]	protein coding	1.62	1.45	69.06	<0.0001	<0.0001
ENSG00000198959	TGM2	transglutaminase 2 [Source:HGNC Symbol;Acc:HGNC:11778]	protein coding	1.58	1.80	19.45	<0.0001	0.0003
ENSG00000179639	FCER1A	Fc fragment of IgE receptor Ia [Source:HGNC Symbol;Acc:HGNC:3609]	protein coding	-1.56	4.61	51.10	<0.0001	<0.0001
ENSG00000122877	EGR2	early growth response 2 [Source:HGNC Symbol;Acc:HGNC:3239]	protein coding	1.55	1.41	42.73	<0.0001	<0.0001
ENSG00000025708	TYMP	thymidine phosphorylase [Source:HGNC Symbol;Acc:HGNC:3148]	protein coding	1.51	7.65	64.21	<0.0001	<0.0001
ENSG00000284194	SCO2	SCO cytochrome c oxidase assembly protein 2 [Source:HGNC Symbol;Acc:HGNC:10604]	protein coding	1.45	2.82	65.15	<0.0001	<0.0001
ENSG00000178965	ERICH3	glutamate rich 3 [Source:HGNC Symbol;Acc:HGNC:25346]	protein coding	1.43	1.13	88.20	<0.0001	<0.0001
ENSG00000177989	ODF3B	outer dense fiber of sperm tails 3B [Source:HGNC Symbol;Acc:HGNC:34388]	protein coding	1.40	5.65	60.20	<0.0001	<0.0001

Table S24: Genes differentially expressed at Day 30 compared to pre-second vaccination (Study Arm 2 A/H7N9+AS03, D1, 29, NK cells). Genes are sorted by descending absolute log_2 fold change. Gene model summaries and annotations are based on Ensembl Version 99 (continued.)

Ensembl Gene ID	Ensembl Gene Name	Ensembl Gene Description	Gene Type	log_2 Fold Change (Day 30 vs. Pre-Second Vaccination)	Average log_2 CPM	Likelihood Ratio Test Statistic	P-Value	FDR Adjusted P-Value
ENSG00000138496	PARP9	poly(ADP-ribose) polymerase family member 9 [Source:HGNC Symbol;Acc:HGNC:24118]	protein coding	1.39	6.94	105.07	<0.0001	<0.0001
ENSG00000225131	PSME2P2	proteasome activator subunit 2 pseudogene 2 [Source:HGNC Symbol;Acc:HGNC:30160]	processed pseudogene	1.39	0.74	51.81	<0.0001	<0.0001
ENSG00000103569	AQP9	aquaporin 9 [Source:HGNC Symbol;Acc:HGNC:643]	protein coding	1.38	2.16	36.59	<0.0001	<0.0001
ENSG00000087237	CETP	cholesteryl ester transfer protein [Source:HGNC Symbol;Acc:HGNC:1869]	protein coding	1.38	1.34	57.37	<0.0001	<0.0001
ENSG00000141574	SECTM1	secreted and transmembrane 1 [Source:HGNC Symbol;Acc:HGNC:10707]	protein coding	1.37	4.71	53.42	<0.0001	<0.0001
ENSG00000180061	TMEM150B	transmembrane protein 150B [Source:HGNC Symbol;Acc:HGNC:34415]	protein coding	1.36	1.87	40.04	<0.0001	<0.0001
ENSG00000204103	MAFB	MAF bZIP transcription factor B [Source:HGNC Symbol;Acc:HGNC:6408]	protein coding	1.35	3.52	31.07	<0.0001	<0.0001
ENSG00000183019	MCEMP1	mast cell expressed membrane protein 1 [Source:HGNC Symbol;Acc:HGNC:27291]	protein coding	1.34	2.02	35.95	<0.0001	<0.0001
ENSG00000143546	S100A8	S100 calcium binding protein A8 [Source:HGNC Symbol;Acc:HGNC:10498]	protein coding	1.32	7.54	30.37	<0.0001	<0.0001
ENSG00000139832	RAB20	RAB20, member RAS oncogene family [Source:HGNC Symbol;Acc:HGNC:18260]	protein coding	1.31	1.63	39.56	<0.0001	<0.0001
ENSG00000188290	HES4	hes family bHLH transcription factor 4 [Source:HGNC Symbol;Acc:HGNC:24149]	protein coding	1.27	2.04	37.11	<0.0001	<0.0001
ENSG00000134326	CMPK2	cytidine/uridine monophosphate kinase 2 [Source:HGNC Symbol;Acc:HGNC:27015]	protein coding	1.27	4.20	64.49	<0.0001	<0.0001
ENSG00000185745	IFIT1	interferon induced protein with tetratricopeptide repeats 1 [Source:HGNC Symbol;Acc:HGNC:5407]	protein coding	1.26	2.84	42.46	<0.0001	<0.0001
ENSG00000183134	PTGDR2	prostaglandin D2 receptor 2 [Source:HGNC Symbol;Acc:HGNC:4502]	protein coding	-1.25	0.51	43.37	<0.0001	<0.0001
ENSG00000172159	FRMD3	FERM domain containing 3 [Source:HGNC Symbol;Acc:HGNC:24125]	protein coding	1.25	1.62	58.02	<0.0001	<0.0001
ENSG00000119922	IFIT2	interferon induced protein with tetratricopeptide repeats 2 [Source:HGNC Symbol;Acc:HGNC:5409]	protein coding	1.25	4.09	52.27	<0.0001	<0.0001
ENSG00000134321	RSAD2	radical S-adenosyl methionine domain containing 2 [Source:HGNC Symbol;Acc:HGNC:30908]	protein coding	1.24	4.19	59.70	<0.0001	<0.0001

Table S24: Genes differentially expressed at Day 30 compared to pre-second vaccination (Study Arm 2 A/H7N9+AS03, D1, 29, NK cells). Genes are sorted by descending absolute log_2 fold change. Gene model summaries and annotations are based on Ensembl Version 99 (continued.)

Ensembl Gene ID	Ensembl Gene Name	Ensembl Gene Description	Gene Type	log_2 Fold Change (Day 30 vs. Pre-Second Vaccination)	Average log_2 CPM	Likelihood Ratio Test Statistic	P-Value	FDR Adjusted P-Value
ENSG00000188282	RUFY4	RUN and FYVE domain containing 4 [Source:HGNC Symbol;Acc:HGNC:24804]	protein coding	1.22	1.67	33.47	<0.0001	<0.0001
ENSG00000125347	IRF1	interferon regulatory factor 1 [Source:HGNC Symbol;Acc:HGNC:6116]	protein coding	1.19	8.41	94.02	<0.0001	<0.0001
ENSG00000133106	EPSTI1	epithelial stromal interaction 1 [Source:HGNC Symbol;Acc:HGNC:16465]	protein coding	1.16	6.00	72.33	<0.0001	<0.0001
ENSG00000002549	LAP3	leucine aminopeptidase 3 [Source:HGNC Symbol;Acc:HGNC:18449]	protein coding	1.16	6.21	52.13	<0.0001	<0.0001
ENSG00000120885	CLU	clusterin [Source:HGNC Symbol;Acc:HGNC:2095]	protein coding	1.15	2.41	20.52	<0.0001	0.0002
ENSG00000163220	S100A9	S100 calcium binding protein A9 [Source:HGNC Symbol;Acc:HGNC:10499]	protein coding	1.15	8.72	28.60	<0.0001	<0.0001
ENSG00000162654	GBP4	guanylate binding protein 4 [Source:HGNC Symbol;Acc:HGNC:20480]	protein coding	1.15	7.35	72.73	<0.0001	<0.0001
ENSG00000163221	S100A12	S100 calcium binding protein A12 [Source:HGNC Symbol;Acc:HGNC:10489]	protein coding	1.14	4.32	24.88	<0.0001	<0.0001
ENSG00000225964	NRIR	negative regulator of interferon response [Source:HGNC Symbol;Acc:HGNC:51269]	lncRNA	1.14	0.60	75.63	<0.0001	<0.0001
ENSG00000142089	IFITM3	interferon induced transmembrane protein 3 [Source:HGNC Symbol;Acc:HGNC:5414]	protein coding	1.13	5.83	40.59	<0.0001	<0.0001
ENSG00000019169	MARCO	macrophage receptor with collagenous structure [Source:HGNC Symbol;Acc:HGNC:6895]	protein coding	1.13	1.96	38.58	<0.0001	<0.0001
ENSG00000214872	SMTNL1	smoothelin like 1 [Source:HGNC Symbol;Acc:HGNC:32394]	protein coding	1.12	1.84	57.62	<0.0001	<0.0001
ENSG00000137965	IFI44	interferon induced protein 44 [Source:HGNC Symbol;Acc:HGNC:16938]	protein coding	1.11	5.66	66.11	<0.0001	<0.0001
ENSG00000162614	NEXN	nexilin F-actin binding protein [Source:HGNC Symbol;Acc:HGNC:29557]	protein coding	1.10	1.96	27.27	<0.0001	<0.0001
ENSG00000225886		novel transcript, antisense to IFI6	lncRNA	1.10	0.54	31.17	<0.0001	<0.0001
ENSG00000135378	PRRG4	proline rich and Gla domain 4 [Source:HGNC Symbol;Acc:HGNC:30799]	protein coding	1.09	1.08	41.04	<0.0001	<0.0001
ENSG00000111331	OAS3	2'-5'-oligoadenylate synthetase 3 [Source:HGNC Symbol;Acc:HGNC:8088]	protein coding	1.09	6.32	68.97	<0.0001	<0.0001
ENSG00000127954	STEAP4	STEAP4 metalloreductase [Source:HGNC Symbol;Acc:HGNC:21923]	protein coding	1.09	1.39	27.19	<0.0001	<0.0001

Table S24: Genes differentially expressed at Day 30 compared to pre-second vaccination (Study Arm 2 A/H7N9+AS03, D1, 29, NK cells). Genes are sorted by descending absolute log_2 fold change. Gene model summaries and annotations are based on Ensembl Version 99 (continued.)

Ensembl Gene ID	Ensembl Gene Name	Ensembl Gene Description	Gene Type	log_2 Fold Change (Day 30 vs. Pre-Second Vaccination)	Average log_2 CPM	Likelihood Ratio Test Statistic	P-Value	FDR Adjusted P-Value
ENSG00000050165	DKK3	dickkopf WNT signaling pathway inhibitor 3 [Source:HGNC Symbol;Acc:HGNC:2893]	protein coding	-1.08	1.34	28.05	<0.0001	<0.0001
ENSG00000020577	SAMD4A	sterile alpha motif domain containing 4A [Source:HGNC Symbol;Acc:HGNC:23023]	protein coding	1.06	2.64	17.09	<0.0001	0.0008
ENSG00000184221	OLIG1	oligodendrocyte transcription factor 1 [Source:HGNC Symbol;Acc:HGNC:16983]	protein coding	-1.05	1.12	40.03	<0.0001	<0.0001
ENSG00000126709	IFI6	interferon alpha inducible protein 6 [Source:HGNC Symbol;Acc:HGNC:4054]	protein coding	1.05	5.94	61.37	<0.0001	<0.0001
ENSG00000171051	FPR1	formyl peptide receptor 1 [Source:HGNC Symbol;Acc:HGNC:3826]	protein coding	1.05	4.41	39.03	<0.0001	<0.0001
ENSG00000156587	UBE2L6	ubiquitin conjugating enzyme E2 L6 [Source:HGNC Symbol;Acc:HGNC:12490]	protein coding	1.04	7.15	69.39	<0.0001	<0.0001
ENSG00000140511	HAPLN3	hyaluronan and proteoglycan link protein 3 [Source:HGNC Symbol;Acc:HGNC:21446]	protein coding	1.04	5.42	29.78	<0.0001	<0.0001
ENSG00000146070	PLA2G7	phospholipase A2 group VII [Source:HGNC Symbol;Acc:HGNC:9040]	protein coding	1.03	1.92	32.16	<0.0001	<0.0001
ENSG00000188820	CALHM6	calcium homeostasis modulator family member 6 [Source:HGNC Symbol;Acc:HGNC:33391]	protein coding	1.02	3.34	19.04	<0.0001	0.0003
ENSG00000132530	XAF1	XIAP associated factor 1 [Source:HGNC Symbol;Acc:HGNC:30932]	protein coding	1.02	6.23	75.77	<0.0001	<0.0001
ENSG00000108950	FAM20A	FAM20A golgi associated secretory pathway pseudokinase [Source:HGNC Symbol;Acc:HGNC:23015]	protein coding	1.02	1.12	22.33	<0.0001	<0.0001
ENSG00000100346	CACNA1I	calcium voltage-gated channel subunit alpha1 I [Source:HGNC Symbol;Acc:HGNC:1396]	protein coding	-1.01	0.45	29.82	<0.0001	<0.0001
ENSG00000224397	SMIM25	small integral membrane protein 25 [Source:HGNC Symbol;Acc:HGNC:50328]	lncRNA	1.00	2.18	21.75	<0.0001	0.0001
ENSG00000082397	EPB41L3	erythrocyte membrane protein band 4.1 like 3 [Source:HGNC Symbol;Acc:HGNC:3380]	protein coding	1.00	3.27	39.60	<0.0001	<0.0001
ENSG00000178773	CPNE7	copine 7 [Source:HGNC Symbol;Acc:HGNC:2320]	protein coding	-1.00	1.20	46.12	<0.0001	<0.0001
ENSG00000101115	SALL4	spalt like transcription factor 4 [Source:HGNC Symbol;Acc:HGNC:15924]	protein coding	-1.00	1.05	26.63	<0.0001	<0.0001
ENSG00000162878	PKDCC	protein kinase domain containing, cytoplasmic [Source:HGNC Symbol;Acc:HGNC:25123]	protein coding	-0.97	0.98	33.69	<0.0001	<0.0001
ENSG00000094804	CDC6	cell division cycle 6 [Source:HGNC Symbol;Acc:HGNC:1744]	protein coding	0.97	1.67	26.03	<0.0001	<0.0001

Table S24: Genes differentially expressed at Day 30 compared to pre-second vaccination (Study Arm 2 A/H7N9+AS03, D1, 29, NK cells). Genes are sorted by descending absolute log_2 fold change. Gene model summaries and annotations are based on Ensembl Version 99 (continued.)

Ensembl Gene ID	Ensembl Gene Name	Ensembl Gene Description	Gene Type	log_2 Fold Change (Day 30 vs. Pre-Second Vaccination)	Average log_2 CPM	Likelihood Ratio Test Statistic	P-Value	FDR Adjusted P-Value
ENSG00000187608	ISG15	ISG15 ubiquitin like modifier [Source:HGNC Symbol;Acc:HGNC:4053]	protein coding	0.96	5.54	58.54	<0.0001	<0.0001
ENSG00000158714	SLAMF8	SLAM family member 8 [Source:HGNC Symbol;Acc:HGNC:21391]	protein coding	0.96	2.18	24.99	<0.0001	<0.0001
ENSG00000135114	OASL	2’-5’-oligoadenylate synthetase like [Source:HGNC Symbol;Acc:HGNC:8090]	protein coding	0.96	4.16	40.76	<0.0001	<0.0001
ENSG00000149451	ADAM33	ADAM metallopeptidase domain 33 [Source:HGNC Symbol;Acc:HGNC:15478]	protein coding	-0.94	1.05	25.26	<0.0001	<0.0001
ENSG00000238000		proteasome (prosome, macropain) activator subunit 2 (PA28 beta) (PSME2) pseudogene	processed pseudogene	0.94	0.56	39.15	<0.0001	<0.0001
ENSG00000243836	WDR86-AS1	WDR86 antisense RNA 1 [Source:HGNC Symbol;Acc:HGNC:41186]	lncRNA	-0.93	3.45	52.63	<0.0001	<0.0001
ENSG00000221963	APOL6	apolipoprotein L6 [Source:HGNC Symbol;Acc:HGNC:14870]	protein coding	0.93	7.80	119.86	<0.0001	<0.0001
ENSG00000154451	GBP5	guanylate binding protein 5 [Source:HGNC Symbol;Acc:HGNC:19895]	protein coding	0.93	9.05	55.04	<0.0001	<0.0001
ENSG00000100911	PSME2	proteasome activator subunit 2 [Source:HGNC Symbol;Acc:HGNC:9569]	protein coding	0.93	7.65	69.89	<0.0001	<0.0001
ENSG00000116147	TNR	tenascin R [Source:HGNC Symbol;Acc:HGNC:11953]	protein coding	-0.91	2.00	51.12	<0.0001	<0.0001
ENSG00000157601	MX1	MX dynamin like GTPase 1 [Source:HGNC Symbol;Acc:HGNC:7532]	protein coding	0.90	6.48	41.89	<0.0001	<0.0001
ENSG00000181631	P2RY13	purinergic receptor P2Y13 [Source:HGNC Symbol;Acc:HGNC:4537]	protein coding	0.90	2.97	19.61	<0.0001	0.0003
ENSG00000125148	MT2A	metallothionein 2A [Source:HGNC Symbol;Acc:HGNC:7406]	protein coding	0.90	5.00	59.23	<0.0001	<0.0001
ENSG00000163840	DTX3L	deltex E3 ubiquitin ligase 3L [Source:HGNC Symbol;Acc:HGNC:30323]	protein coding	0.90	6.51	57.94	<0.0001	<0.0001
ENSG00000068079	IFI35	interferon induced protein 35 [Source:HGNC Symbol;Acc:HGNC:5399]	protein coding	0.90	5.90	61.16	<0.0001	<0.0001
ENSG00000182809	CRIP2	cysteine rich protein 2 [Source:HGNC Symbol;Acc:HGNC:2361]	protein coding	-0.89	3.55	75.17	<0.0001	<0.0001
ENSG00000197249	SERPINA1	serpin family A member 1 [Source:HGNC Symbol;Acc:HGNC:8941]	protein coding	0.88	6.08	33.33	<0.0001	<0.0001

Table S24: Genes differentially expressed at Day 30 compared to pre-second vaccination (Study Arm 2 A/H7N9+AS03, D1, 29, NK cells). Genes are sorted by descending absolute log_2 fold change. Gene model summaries and annotations are based on Ensembl Version 99 (continued.)

Ensembl Gene ID	Ensembl Gene Name	Ensembl Gene Description	Gene Type	log_2 Fold Change (Day 30 vs. Pre-Second Vaccination)	Average log_2 CPM	Likelihood Ratio Test Statistic	P-Value	FDR Adjusted P-Value
ENSG00000120217	CD274	CD274 molecule [Source:HGNC Symbol;Acc:HGNC:17635]	protein coding	0.88	2.66	23.11	<0.0001	<0.0001
ENSG00000139318	DUSP6	dual specificity phosphatase 6 [Source:HGNC Symbol;Acc:HGNC:3072]	protein coding	0.88	4.76	20.37	<0.0001	0.0002
ENSG00000187116	LILRA5	leukocyte immunoglobulin like receptor A5 [Source:HGNC Symbol;Acc:HGNC:16309]	protein coding	0.88	3.54	26.23	<0.0001	<0.0001
ENSG00000088827	SIGLEC1	sialic acid binding Ig like lectin 1 [Source:HGNC Symbol;Acc:HGNC:11127]	protein coding	0.87	3.47	21.25	<0.0001	0.0001
ENSG00000134243	SORT1	sortilin 1 [Source:HGNC Symbol;Acc:HGNC:11186]	protein coding	0.87	3.42	44.61	<0.0001	<0.0001
ENSG00000168394	TAP1	transporter 1, ATP binding cassette subfamily B member [Source:HGNC Symbol;Acc:HGNC:43]	protein coding	0.87	8.67	76.65	<0.0001	<0.0001
ENSG00000065328	MCM10	minichromosome maintenance 10 replication initiation factor [Source:HGNC Symbol;Acc:HGNC:18043]	protein coding	0.87	1.00	16.04	<0.0001	0.0013
ENSG00000103196	CRISPLD2	cysteine rich secretory protein LCCL domain containing 2 [Source:HGNC Symbol;Acc:HGNC:25248]	protein coding	0.87	3.08	26.80	<0.0001	<0.0001
ENSG00000096696	DSP	desmoplakin [Source:HGNC Symbol;Acc:HGNC:3052]	protein coding	-0.86	2.18	15.11	0.0001	0.0019
ENSG00000131153	GINS2	GINS complex subunit 2 [Source:HGNC Symbol;Acc:HGNC:24575]	protein coding	0.86	1.72	17.93	<0.0001	0.0006
ENSG00000136867	SLC31A2	solute carrier family 31 member 2 [Source:HGNC Symbol;Acc:HGNC:11017]	protein coding	0.86	1.05	13.31	0.0003	0.004
ENSG00000080573	COL5A3	collagen type V alpha 3 chain [Source:HGNC Symbol;Acc:HGNC:14864]	protein coding	-0.85	4.98	27.77	<0.0001	<0.0001
ENSG00000004468	CD38	CD38 molecule [Source:HGNC Symbol;Acc:HGNC:1667]	protein coding	0.85	6.68	106.17	<0.0001	<0.0001
ENSG00000244482	LILRA6	leukocyte immunoglobulin like receptor A6 [Source:HGNC Symbol;Acc:HGNC:15495]	protein coding	0.85	3.21	26.40	<0.0001	<0.0001
ENSG00000162645	GBP2	guanylate binding protein 2 [Source:HGNC Symbol;Acc:HGNC:4183]	protein coding	0.85	7.80	57.48	<0.0001	<0.0001
ENSG00000173578	XCR1	X-C motif chemokine receptor 1 [Source:HGNC Symbol;Acc:HGNC:1625]	protein coding	-0.85	0.70	11.29	0.0008	0.0091
ENSG00000136689	IL1RN	interleukin 1 receptor antagonist [Source:HGNC Symbol;Acc:HGNC:6000]	protein coding	0.85	2.42	16.23	<0.0001	0.0012
ENSG00000116663	FBXO6	F-box protein 6 [Source:HGNC Symbol;Acc:HGNC:13585]	protein coding	0.85	5.50	83.21	<0.0001	<0.0001

Table S24: Genes differentially expressed at Day 30 compared to pre-second vaccination (Study Arm 2 A/H7N9+AS03, D1, 29, NK cells). Genes are sorted by descending absolute log_2 fold change. Gene model summaries and annotations are based on Ensembl Version 99 (continued.)

Ensembl Gene ID	Ensembl Gene Name	Ensembl Gene Description	Gene Type	log_2 Fold Change (Day 30 vs. Pre-Second Vaccination)	Average log_2 CPM	Likelihood Ratio Test Statistic	P-Value	FDR Adjusted P-Value
ENSG00000065911	MTHFD2	methylenetetrahydrofolate dehydrogenase (NADP+ dependent) 2, methenyltetrahydrofolate cyclohydrolase [Source:HGNC Symbol;Acc:HGNC:7434]	protein coding	0.84	5.04	90.40	<0.0001	<0.0001
ENSG00000204136	GGTA1P	glycoprotein alpha-galactosyltransferase 1, pseudogene [Source:HGNC Symbol;Acc:HGNC:4253]	transcribed unitary pseudogene	-0.83	0.73	27.40	<0.0001	<0.0001
ENSG00000138166	DUSP5	dual specificity phosphatase 5 [Source:HGNC Symbol;Acc:HGNC:3071]	protein coding	0.83	4.01	41.91	<0.0001	<0.0001
ENSG00000089127	OAS1	2'-5'-oligoadenylate synthetase 1 [Source:HGNC Symbol;Acc:HGNC:8086]	protein coding	0.83	5.59	34.66	<0.0001	<0.0001
ENSG00000175643	RMI2	RecQ mediated genome instability 2 [Source:HGNC Symbol;Acc:HGNC:28349]	protein coding	0.83	2.04	26.26	<0.0001	<0.0001
ENSG00000108679	LGALS3BP	galectin 3 binding protein [Source:HGNC Symbol;Acc:HGNC:6564]	protein coding	0.82	4.40	25.63	<0.0001	<0.0001
ENSG00000159713	TPPP3	tubulin polymerization promoting protein family member 3 [Source:HGNC Symbol;Acc:HGNC:24162]	protein coding	-0.82	1.02	25.96	<0.0001	<0.0001
ENSG00000213928	IRF9	interferon regulatory factor 9 [Source:HGNC Symbol;Acc:HGNC:6131]	protein coding	0.81	5.38	70.14	<0.0001	<0.0001
ENSG00000184613	NELL2	neural EGFL like 2 [Source:HGNC Symbol;Acc:HGNC:7751]	protein coding	-0.81	3.68	38.67	<0.0001	<0.0001
ENSG00000179921	GPBAR1	G protein-coupled bile acid receptor 1 [Source:HGNC Symbol;Acc:HGNC:19680]	protein coding	0.81	2.58	33.72	<0.0001	<0.0001
ENSG00000130487	KLHDC7B	kelch domain containing 7B [Source:HGNC Symbol;Acc:HGNC:25145]	protein coding	0.81	4.20	47.33	<0.0001	<0.0001
ENSG00000108387	SEPTIN4	septin 4 [Source:HGNC Symbol;Acc:HGNC:9165]	protein coding	0.81	1.35	18.95	<0.0001	0.0004
ENSG00000184371	CSF1	colony stimulating factor 1 [Source:HGNC Symbol;Acc:HGNC:2432]	protein coding	0.81	2.24	14.98	0.0001	0.002
ENSG00000185507	IRF7	interferon regulatory factor 7 [Source:HGNC Symbol;Acc:HGNC:6122]	protein coding	0.81	6.66	52.24	<0.0001	<0.0001
ENSG00000168899	VAMP5	vesicle associated membrane protein 5 [Source:HGNC Symbol;Acc:HGNC:12646]	protein coding	0.80	5.22	41.15	<0.0001	<0.0001
ENSG00000135069	PSAT1	phosphoserine aminotransferase 1 [Source:HGNC Symbol;Acc:HGNC:19129]	protein coding	0.80	1.99	21.83	<0.0001	0.0001

Table S24: Genes differentially expressed at Day 30 compared to pre-second vaccination (Study Arm 2 A/H7N9+AS03, D1, 29, NK cells). Genes are sorted by descending absolute log_2 fold change. Gene model summaries and annotations are based on Ensembl Version 99 (continued.)

Ensembl Gene ID	Ensembl Gene Name	Ensembl Gene Description	Gene Type	log_2 Fold Change (Day 30 vs. Pre-Second Vaccination)	Average log_2 CPM	Likelihood Ratio Test Statistic	P-Value	FDR Adjusted P-Value
ENSG00000184979	USP18	ubiquitin specific peptidase 18 [Source:HGNC Symbol;Acc:HGNC:12616]	protein coding	0.80	2.91	32.05	<0.0001	<0.0001
ENSG00000135604	STX11	syntaxin 11 [Source:HGNC Symbol;Acc:HGNC:11429]	protein coding	0.80	5.64	44.17	<0.0001	<0.0001
ENSG00000170581	STAT2	signal transducer and activator of transcription 2 [Source:HGNC Symbol;Acc:HGNC:11363]	protein coding	0.79	7.77	52.56	<0.0001	<0.0001
ENSG00000155792	DEPTOR	DEP domain containing MTOR interacting protein [Source:HGNC Symbol;Acc:HGNC:22953]	protein coding	-0.79	0.44	21.57	<0.0001	0.0001
ENSG00000131459	GFPT2	glutamine-fructose-6-phosphate transaminase 2 [Source:HGNC Symbol;Acc:HGNC:4242]	protein coding	-0.79	1.33	13.31	0.0003	0.004
ENSG00000165092	ALDH1A1	aldehyde dehydrogenase 1 family member A1 [Source:HGNC Symbol;Acc:HGNC:402]	protein coding	0.79	2.48	12.31	0.0004	0.006
ENSG00000185880	TRIM69	tripartite motif containing 69 [Source:HGNC Symbol;Acc:HGNC:17857]	protein coding	0.78	5.60	40.41	<0.0001	<0.0001
ENSG00000007516	BAIAP3	BAI1 associated protein 3 [Source:HGNC Symbol;Acc:HGNC:948]	protein coding	-0.78	5.86	67.30	<0.0001	<0.0001
ENSG00000062524	LTK	leukocyte receptor tyrosine kinase [Source:HGNC Symbol;Acc:HGNC:6721]	protein coding	-0.78	7.33	29.32	<0.0001	<0.0001
ENSG00000004799	PDK4	pyruvate dehydrogenase kinase 4 [Source:HGNC Symbol;Acc:HGNC:8812]	protein coding	-0.78	2.40	8.62	0.0033	0.027
ENSG00000206077	ZDHHC11B	zinc finger DHHC-type containing 11B [Source:HGNC Symbol;Acc:HGNC:32962]	protein coding	-0.78	0.92	24.69	<0.0001	<0.0001
ENSG00000151948	GLT1D1	glycosyltransferase 1 domain containing 1 [Source:HGNC Symbol;Acc:HGNC:26483]	protein coding	0.78	3.19	33.18	<0.0001	<0.0001
ENSG00000186431	FCAR	Fc fragment of IgA receptor [Source:HGNC Symbol;Acc:HGNC:3608]	protein coding	0.77	1.21	17.54	<0.0001	0.0007
ENSG00000121858	TNFSF10	TNF superfamily member 10 [Source:HGNC Symbol;Acc:HGNC:11925]	protein coding	0.77	5.65	24.16	<0.0001	<0.0001
ENSG00000092853	CLSPN	claspin [Source:HGNC Symbol;Acc:HGNC:19715]	protein coding	0.77	2.18	16.50	<0.0001	0.001
ENSG00000177409	SAMD9L	sterile alpha motif domain containing 9 like [Source:HGNC Symbol;Acc:HGNC:1349]	protein coding	0.76	7.51	69.54	<0.0001	<0.0001
ENSG00000131979	GCH1	GTP cyclohydrolase 1 [Source:HGNC Symbol;Acc:HGNC:4193]	protein coding	0.76	6.16	80.89	<0.0001	<0.0001
ENSG00000125462	C1orf61	chromosome 1 open reading frame 61 [Source:HGNC Symbol;Acc:HGNC:30780]	protein coding	0.76	1.60	22.01	<0.0001	<0.0001

Table S24: Genes differentially expressed at Day 30 compared to pre-second vaccination (Study Arm 2 A/H7N9+AS03, D1, 29, NK cells). Genes are sorted by descending absolute log_2 fold change. Gene model summaries and annotations are based on Ensembl Version 99 (continued.)

Ensembl Gene ID	Ensembl Gene Name	Ensembl Gene Description	Gene Type	log_2 Fold Change (Day 30 vs. Pre-Second Vaccination)	Average log_2 CPM	Likelihood Ratio Test Statistic	P-Value	FDR Adjusted P-Value
ENSG00000161643	SIGLEC16	sialic acid binding Ig like lectin 16 (gene/pseudogene) [Source:HGNC Symbol;Acc:HGNC:24851]	transcribed unprocessed pseudogene	0.76	1.04	14.20	0.0002	0.0028
ENSG00000226091	LINC00937	long intergenic non-protein coding RNA 937 [Source:HGNC Symbol;Acc:HGNC:48629]	lncRNA	0.76	1.84	16.81	<0.0001	0.0009
ENSG00000138180	CEP55	centrosomal protein 55 [Source:HGNC Symbol;Acc:HGNC:1161]	protein coding	0.76	0.70	14.16	0.0002	0.0028
ENSG00000093009	CDC45	cell division cycle 45 [Source:HGNC Symbol;Acc:HGNC:1739]	protein coding	0.75	2.00	15.40	<0.0001	0.0017
ENSG00000128805	ARHGAP22	Rho GTPase activating protein 22 [Source:HGNC Symbol;Acc:HGNC:30320]	protein coding	-0.75	1.22	24.59	<0.0001	<0.0001
ENSG00000163958	ZDHHC19	zinc finger DHHC-type containing 19 [Source:HGNC Symbol;Acc:HGNC:20713]	protein coding	-0.75	0.80	14.13	0.0002	0.0028
ENSG00000211667	IGLV3-12	immunoglobulin lambda variable 3-12 [Source:HGNC Symbol;Acc:HGNC:5898]	IG V gene	-0.75	2.29	12.29	0.0005	0.0061
ENSG00000268555		novel transcript	lncRNA	-0.75	1.55	20.30	<0.0001	0.0002
ENSG00000169385	RNASE2	ribonuclease A family member 2 [Source:HGNC Symbol;Acc:HGNC:10045]	protein coding	0.75	2.25	12.22	0.0005	0.0063
ENSG00000125845	BMP2	bone morphogenetic protein 2 [Source:HGNC Symbol;Acc:HGNC:1069]	protein coding	-0.74	0.90	12.48	0.0004	0.0056
ENSG00000185483	ROR1	receptor tyrosine kinase like orphan receptor 1 [Source:HGNC Symbol;Acc:HGNC:10256]	protein coding	-0.74	0.59	32.89	<0.0001	<0.0001
ENSG00000154102	C16orf74	chromosome 16 open reading frame 74 [Source:HGNC Symbol;Acc:HGNC:23362]	protein coding	-0.74	2.82	44.66	<0.0001	<0.0001
ENSG00000111335	OAS2	2'-5'-oligoadenylate synthetase 2 [Source:HGNC Symbol;Acc:HGNC:8087]	protein coding	0.74	7.06	59.80	<0.0001	<0.0001
ENSG00000102524	TNFSF13B	TNF superfamily member 13b [Source:HGNC Symbol;Acc:HGNC:11929]	protein coding	0.74	4.31	23.28	<0.0001	<0.0001
ENSG00000119139	TJP2	tight junction protein 2 [Source:HGNC Symbol;Acc:HGNC:11828]	protein coding	0.73	0.27	20.17	<0.0001	0.0002
ENSG00000160191	PDE9A	phosphodiesterase 9A [Source:HGNC Symbol;Acc:HGNC:8795]	protein coding	-0.73	0.93	25.54	<0.0001	<0.0001
ENSG00000135636	DYSF	dysferlin [Source:HGNC Symbol;Acc:HGNC:3097]	protein coding	0.73	5.02	21.74	<0.0001	0.0001

Table S24: Genes differentially expressed at Day 30 compared to pre-second vaccination (Study Arm 2 A/H7N9+AS03, D1, 29, NK cells). Genes are sorted by descending absolute log_2 fold change. Gene model summaries and annotations are based on Ensembl Version 99 (continued.)

Ensembl Gene ID	Ensembl Gene Name	Ensembl Gene Description	Gene Type	log_2 Fold Change (Day 30 vs. Pre-Second Vaccination)	Average log_2 CPM	Likelihood Ratio Test Statistic	P-Value	FDR Adjusted P-Value
ENSG00000162062	TEDC2	tubulin epsilon and delta complex 2 [Source:HGNC Symbol;Acc:HGNC:25849]	protein coding	0.73	0.74	16.55	<0.0001	0.001
ENSG00000162630	B3GALT2	beta-1,3-galactosyltransferase 2 [Source:HGNC Symbol;Acc:HGNC:917]	protein coding	-0.73	2.09	7.73	0.0054	0.0386
ENSG00000130164	LDLR	low density lipoprotein receptor [Source:HGNC Symbol;Acc:HGNC:6547]	protein coding	0.73	5.36	61.56	<0.0001	<0.0001
ENSG00000198795	ZNF521	zinc finger protein 521 [Source:HGNC Symbol;Acc:HGNC:24605]	protein coding	-0.73	0.57	22.51	<0.0001	<0.0001
ENSG00000140379	BCL2A1	BCL2 related protein A1 [Source:HGNC Symbol;Acc:HGNC:991]	protein coding	0.73	3.20	19.16	<0.0001	0.0003
ENSG00000072195	SPEG	striated muscle enriched protein kinase [Source:HGNC Symbol;Acc:HGNC:16901]	protein coding	-0.73	1.66	19.73	<0.0001	0.0003
ENSG00000240065	PSMB9	proteasome 20S subunit beta 9 [Source:HGNC Symbol;Acc:HGNC:9546]	protein coding	0.72	7.92	48.91	<0.0001	<0.0001
ENSG00000185561	TLCD2	TLC domain containing 2 [Source:HGNC Symbol;Acc:HGNC:33522]	protein coding	-0.72	1.69	25.18	<0.0001	<0.0001
ENSG00000123405	NFE2	nuclear factor, erythroid 2 [Source:HGNC Symbol;Acc:HGNC:7780]	protein coding	0.72	3.54	15.63	<0.0001	0.0015
ENSG00000163520	FBLN2	fibulin 2 [Source:HGNC Symbol;Acc:HGNC:3601]	protein coding	-0.72	3.05	53.39	<0.0001	<0.0001
ENSG00000174371	EXO1	exonuclease 1 [Source:HGNC Symbol;Acc:HGNC:3511]	protein coding	0.72	0.58	10.68	0.0011	0.0117
ENSG00000216490	IFI30	IFI30 lysosomal thiol reductase [Source:HGNC Symbol;Acc:HGNC:5398]	protein coding	0.71	1.64	11.94	0.0006	0.007
ENSG00000183087	GAS6	growth arrest specific 6 [Source:HGNC Symbol;Acc:HGNC:4168]	protein coding	0.71	3.33	23.73	<0.0001	<0.0001
ENSG00000069399	BCL3	BCL3 transcription coactivator [Source:HGNC Symbol;Acc:HGNC:998]	protein coding	0.71	5.46	40.65	<0.0001	<0.0001
ENSG00000277301		novel transcript, antisense to NOL4L	lncRNA	-0.71	0.87	20.09	<0.0001	0.0002
ENSG00000256262	USP30-AS1	USP30 antisense RNA 1 [Source:HGNC Symbol;Acc:HGNC:40909]	lncRNA	0.71	2.87	42.54	<0.0001	<0.0001
ENSG00000260711		novel transcript, intronic to CATSPERB	lncRNA	-0.71	3.70	23.47	<0.0001	<0.0001
ENSG00000165300	SLITRK5	SLIT and NTRK like family member 5 [Source:HGNC Symbol;Acc:HGNC:20295]	protein coding	-0.70	1.80	21.29	<0.0001	0.0001
ENSG00000227863		novel transcript	lncRNA	-0.70	0.63	16.25	<0.0001	0.0012

Table S24: Genes differentially expressed at Day 30 compared to pre-second vaccination (Study Arm 2 A/H7N9+AS03, D1, 29, NK cells). Genes are sorted by descending absolute log_2 fold change. Gene model summaries and annotations are based on Ensembl Version 99 (continued.)

Ensembl Gene ID	Ensembl Gene Name	Ensembl Gene Description	Gene Type	log_2 Fold Change (Day 30 vs. Pre-Second Vaccination)	Average log_2 CPM	Likelihood Ratio Test Statistic	P-Value	FDR Adjusted P-Value
ENSG00000182379	NXPH4	neurexophilin 4 [Source:HGNC Symbol;Acc:HGNC:8078]	protein coding	-0.70	1.35	13.07	0.0003	0.0044
ENSG00000113368	LMNB1	lamin B1 [Source:HGNC Symbol;Acc:HGNC:6637]	protein coding	0.70	5.19	52.60	<0.0001	<0.0001
ENSG00000132109	TRIM21	tripartite motif containing 21 [Source:HGNC Symbol;Acc:HGNC:11312]	protein coding	0.70	5.52	34.33	<0.0001	<0.0001
ENSG00000143476	DTL	denticleless E3 ubiquitin protein ligase homolog [Source:HGNC Symbol;Acc:HGNC:30288]	protein coding	0.70	1.92	12.31	0.0005	0.006
ENSG00000166002	SMCO4	single-pass membrane protein with coiled-coil domains 4 [Source:HGNC Symbol;Acc:HGNC:24810]	protein coding	0.70	2.83	28.88	<0.0001	<0.0001
ENSG00000103528	SYT17	synaptotagmin 17 [Source:HGNC Symbol;Acc:HGNC:24119]	protein coding	-0.70	0.84	14.07	0.0002	0.0029
ENSG00000157404	KIT	KIT proto-oncogene, receptor tyrosine kinase [Source:HGNC Symbol;Acc:HGNC:6342]	protein coding	-0.70	3.80	30.45	<0.0001	<0.0001
ENSG00000188906	LRRK2	leucine rich repeat kinase 2 [Source:HGNC Symbol;Acc:HGNC:18618]	protein coding	0.69	5.09	13.89	0.0002	0.0032
ENSG00000261644		novel transcript, antisense to CYLD and NOD2	lncRNA	0.69	3.27	29.20	<0.0001	<0.0001
ENSG00000134470	IL15RA	interleukin 15 receptor subunit alpha [Source:HGNC Symbol;Acc:HGNC:5978]	protein coding	0.69	3.73	36.59	<0.0001	<0.0001
ENSG00000132561	MATN2	matrilin 2 [Source:HGNC Symbol;Acc:HGNC:6908]	protein coding	-0.69	2.02	16.47	<0.0001	0.001
ENSG00000286831		novel transcript	lncRNA	-0.69	0.59	12.94	0.0003	0.0047
ENSG00000287387		novel transcript, antisense to ZFP36L2	lncRNA	-0.69	1.39	14.42	0.0001	0.0025
ENSG00000074660	SCARF1	scavenger receptor class F member 1 [Source:HGNC Symbol;Acc:HGNC:16820]	protein coding	0.69	2.81	17.64	<0.0001	0.0006
ENSG00000154269	ENPP3	ectonucleotide pyrophosphatase/phosphodiesterase 3 [Source:HGNC Symbol;Acc:HGNC:3358]	protein coding	-0.69	0.29	19.93	<0.0001	0.0002
ENSG00000143226	FCGR2A	Fc fragment of IgG receptor IIa [Source:HGNC Symbol;Acc:HGNC:3616]	protein coding	0.69	3.75	21.49	<0.0001	0.0001
ENSG00000163823	CCR1	C-C motif chemokine receptor 1 [Source:HGNC Symbol;Acc:HGNC:1602]	protein coding	0.68	4.97	13.17	0.0003	0.0042
ENSG00000137628	DDX60	DExD/H-box helicase 60 [Source:HGNC Symbol;Acc:HGNC:25942]	protein coding	0.68	5.76	61.38	<0.0001	<0.0001
ENSG00000131042	LILRB2	leukocyte immunoglobulin like receptor B2 [Source:HGNC Symbol;Acc:HGNC:6606]	protein coding	0.68	5.34	25.79	<0.0001	<0.0001

Table S24: Genes differentially expressed at Day 30 compared to pre-second vaccination (Study Arm 2 A/H7N9+AS03, D1, 29, NK cells). Genes are sorted by descending absolute log_2 fold change. Gene model summaries and annotations are based on Ensembl Version 99 (continued.)

Ensembl Gene ID	Ensembl Gene Name	Ensembl Gene Description	Gene Type	log_2 Fold Change (Day 30 vs. Pre-Second Vaccination)	Average log_2 CPM	Likelihood Ratio Test Statistic	P-Value	FDR Adjusted P-Value
ENSG00000186818	LILRB4	leukocyte immunoglobulin like receptor B4 [Source:HGNC Symbol;Acc:HGNC:6608]	protein coding	0.68	3.93	31.64	<0.0001	<0.0001
ENSG00000173110	HSPA6	heat shock protein family A (Hsp70) member 6 [Source:HGNC Symbol;Acc:HGNC:5239]	protein coding	0.68	2.54	14.93	0.0001	0.0021
ENSG00000106605	BLVRA	biliverdin reductase A [Source:HGNC Symbol;Acc:HGNC:1062]	protein coding	0.68	4.17	49.40	<0.0001	<0.0001
ENSG00000143434	SEMA6C	semaphorin 6C [Source:HGNC Symbol;Acc:HGNC:10740]	protein coding	-0.68	2.66	19.50	<0.0001	0.0003
ENSG00000140092	FBLN5	fibulin 5 [Source:HGNC Symbol;Acc:HGNC:3602]	protein coding	-0.68	1.49	19.99	<0.0001	0.0002
ENSG00000172548	NIPAL4	NIPA like domain containing 4 [Source:HGNC Symbol;Acc:HGNC:28018]	protein coding	-0.68	0.44	9.32	0.0023	0.0204
ENSG00000114737	CISH	cytokine inducible SH2 containing protein [Source:HGNC Symbol;Acc:HGNC:1984]	protein coding	0.67	5.37	7.47	0.0063	0.0427
ENSG00000130595	TNNT3	troponin T3, fast skeletal type [Source:HGNC Symbol;Acc:HGNC:11950]	protein coding	-0.67	2.94	29.56	<0.0001	<0.0001
ENSG00000211778	TRAV4	T cell receptor alpha variable 4 [Source:HGNC Symbol;Acc:HGNC:12140]	TR V gene	-0.67	0.63	7.44	0.0064	0.0433
ENSG00000183023	SLC8A1	solute carrier family 8 member A1 [Source:HGNC Symbol;Acc:HGNC:11068]	protein coding	0.67	2.59	19.68	<0.0001	0.0003
ENSG00000168913	ENHO	energy homeostasis associated [Source:HGNC Symbol;Acc:HGNC:24838]	protein coding	-0.67	2.03	19.61	<0.0001	0.0003
ENSG00000188313	PLSCR1	phospholipid scramblase 1 [Source:HGNC Symbol;Acc:HGNC:9092]	protein coding	0.67	5.28	52.80	<0.0001	<0.0001
ENSG00000101307	SIRPB1	signal regulatory protein beta 1 [Source:HGNC Symbol;Acc:HGNC:15928]	protein coding	0.67	3.45	25.90	<0.0001	<0.0001
ENSG00000140545	MFGE8	milk fat globule-EGF factor 8 protein [Source:HGNC Symbol;Acc:HGNC:7036]	protein coding	-0.67	5.15	44.76	<0.0001	<0.0001
ENSG00000162591	MEGF6	multiple EGF like domains 6 [Source:HGNC Symbol;Acc:HGNC:3232]	protein coding	-0.67	5.46	65.02	<0.0001	<0.0001
ENSG00000111424	VDR	vitamin D receptor [Source:HGNC Symbol;Acc:HGNC:12679]	polymorphic pseudogene	0.67	1.94	19.08	<0.0001	0.0003
ENSG00000130303	BST2	bone marrow stromal cell antigen 2 [Source:HGNC Symbol;Acc:HGNC:1119]	protein coding	0.66	7.13	51.93	<0.0001	<0.0001

Table S24: Genes differentially expressed at Day 30 compared to pre-second vaccination (Study Arm 2 A/H7N9+AS03, D1, 29, NK cells). Genes are sorted by descending absolute log_2 fold change. Gene model summaries and annotations are based on Ensembl Version 99 (continued.)

Ensembl Gene ID	Ensembl Gene Name	Ensembl Gene Description	Gene Type	log_2 Fold Change (Day 30 vs. Pre-Second Vaccination)	Average log_2 CPM	Likelihood Ratio Test Statistic	P-Value	FDR Adjusted P-Value
ENSG00000065833	ME1	malic enzyme 1 [Source:HGNC Symbol;Acc:HGNC:6983]	protein coding	-0.66	2.74	20.28	<0.0001	0.0002
ENSG00000101412	E2F1	E2F transcription factor 1 [Source:HGNC Symbol;Acc:HGNC:3113]	protein coding	0.66	3.78	11.59	0.0007	0.0081
ENSG00000136514	RTP4	receptor transporter protein 4 [Source:HGNC Symbol;Acc:HGNC:23992]	protein coding	0.66	3.27	14.09	0.0002	0.0029
ENSG00000044459	CNTLN	centlein [Source:HGNC Symbol;Acc:HGNC:23432]	protein coding	0.66	1.00	20.97	<0.0001	0.0002
ENSG00000171811	CFAP46	cilia and flagella associated protein 46 [Source:HGNC Symbol;Acc:HGNC:25247]	protein coding	-0.65	1.08	12.11	0.0005	0.0065
ENSG00000136982	DSCC1	DNA replication and sister chromatid cohesion 1 [Source:HGNC Symbol;Acc:HGNC:24453]	protein coding	0.65	0.66	12.08	0.0005	0.0066
ENSG00000158050	DUSP2	dual specificity phosphatase 2 [Source:HGNC Symbol;Acc:HGNC:3068]	protein coding	-0.65	8.22	13.50	0.0002	0.0037
ENSG00000184557	SOCS3	suppressor of cytokine signaling 3 [Source:HGNC Symbol;Acc:HGNC:19391]	protein coding	0.65	5.32	21.51	<0.0001	0.0001
ENSG00000149573	MPZL2	myelin protein zero like 2 [Source:HGNC Symbol;Acc:HGNC:3496]	protein coding	0.65	1.30	18.00	<0.0001	0.0005
ENSG00000287839		novel transcript	lncRNA	0.65	3.19	37.66	<0.0001	<0.0001
ENSG00000106565	TMEM176B	transmembrane protein 176B [Source:HGNC Symbol;Acc:HGNC:29596]	protein coding	0.64	3.39	20.98	<0.0001	0.0002
ENSG00000082781	ITGB5	integrin subunit beta 5 [Source:HGNC Symbol;Acc:HGNC:6160]	protein coding	-0.64	0.44	16.60	<0.0001	0.001
ENSG00000198673	TAFA2	TAFA chemokine like family member 2 [Source:HGNC Symbol;Acc:HGNC:21589]	protein coding	0.64	0.44	18.89	<0.0001	0.0004
ENSG00000146592	CREB5	cAMP responsive element binding protein 5 [Source:HGNC Symbol;Acc:HGNC:16844]	protein coding	0.64	2.87	15.77	<0.0001	0.0014
ENSG00000253669	GASAL1	growth arrest associated lncRNA 1 [Source:HGNC Symbol;Acc:HGNC:53461]	lncRNA	0.64	0.90	14.57	0.0001	0.0024
ENSG00000185215	TNFAIP2	TNF alpha induced protein 2 [Source:HGNC Symbol;Acc:HGNC:11895]	protein coding	0.64	7.11	14.65	0.0001	0.0023
ENSG00000211713	TRBV6-4	T cell receptor beta variable 6-4 [Source:HGNC Symbol;Acc:HGNC:12229]	TR V gene	-0.64	2.50	14.14	0.0002	0.0028
ENSG00000255733	IFNG-AS1	IFNG antisense RNA 1 [Source:HGNC Symbol;Acc:HGNC:43910]	lncRNA	-0.64	3.06	32.91	<0.0001	<0.0001

Table S24: Genes differentially expressed at Day 30 compared to pre-second vaccination (Study Arm 2 A/H7N9+AS03, D1, 29, NK cells). Genes are sorted by descending absolute log_2 fold change. Gene model summaries and annotations are based on Ensembl Version 99 (continued.)

Ensembl Gene ID	Ensembl Gene Name	Ensembl Gene Description	Gene Type	log_2 Fold Change (Day 30 vs. Pre-Second Vaccination)	Average log_2 CPM	Likelihood Ratio Test Statistic	P-Value	FDR Adjusted P-Value
ENSG00000234518	PTGES3P1	prostaglandin E synthase 3 pseudogene 1 [Source:HGNC Symbol;Acc:HGNC:43824]	processed pseudogene	0.63	3.28	48.84	<0.0001	<0.0001
ENSG00000163694	RBM47	RNA binding motif protein 47 [Source:HGNC Symbol;Acc:HGNC:30358]	protein coding	0.63	3.32	21.60	<0.0001	0.0001
ENSG00000149554	CHEK1	checkpoint kinase 1 [Source:HGNC Symbol;Acc:HGNC:1925]	protein coding	0.63	2.46	13.55	0.0002	0.0036
ENSG00000097021	ACOT7	acyl-CoA thioesterase 7 [Source:HGNC Symbol;Acc:HGNC:24157]	protein coding	0.63	4.29	45.31	<0.0001	<0.0001
ENSG00000173193	PARP14	poly(ADP-ribose) polymerase family member 14 [Source:HGNC Symbol;Acc:HGNC:29232]	protein coding	0.63	7.81	63.28	<0.0001	<0.0001
ENSG00000167513	CDT1	chromatin licensing and DNA replication factor 1 [Source:HGNC Symbol;Acc:HGNC:24576]	protein coding	0.63	2.85	11.26	0.0008	0.0093
ENSG00000160883	HK3	hexokinase 3 [Source:HGNC Symbol;Acc:HGNC:4925]	protein coding	0.63	5.64	18.18	<0.0001	0.0005
ENSG00000228408		novel transcript	lncRNA	-0.63	0.33	11.81	0.0006	0.0074
ENSG00000115828	QPCT	glutaminyl-peptide cyclotransferase [Source:HGNC Symbol;Acc:HGNC:9753]	protein coding	0.63	2.46	16.72	<0.0001	0.0009
ENSG00000158517	NCF1	neutrophil cytosolic factor 1 [Source:HGNC Symbol;Acc:HGNC:7660]	protein coding	0.63	5.55	9.46	0.0021	0.0194
ENSG00000121316	PLBD1	phospholipase B domain containing 1 [Source:HGNC Symbol;Acc:HGNC:26215]	protein coding	0.63	5.11	19.33	<0.0001	0.0003
ENSG00000262580		novel transcript, antisense to CARD14	lncRNA	-0.63	4.38	29.81	<0.0001	<0.0001
ENSG00000111452	ADGRD1	adhesion G protein-coupled receptor D1 [Source:HGNC Symbol;Acc:HGNC:19893]	protein coding	-0.63	1.21	19.77	<0.0001	0.0003
ENSG00000148600	CDHR1	cadherin related family member 1 [Source:HGNC Symbol;Acc:HGNC:14550]	protein coding	-0.63	5.92	38.72	<0.0001	<0.0001
ENSG00000095397	WHRN	whirlin [Source:HGNC Symbol;Acc:HGNC:16361]	protein coding	-0.63	4.53	25.03	<0.0001	<0.0001
ENSG00000138646	HERC5	HECT and RLD domain containing E3 ubiquitin protein ligase 5 [Source:HGNC Symbol;Acc:HGNC:24368]	protein coding	0.63	4.32	55.92	<0.0001	<0.0001
ENSG00000282164	PEG13	paternally expressed 13 [Source:HGNC Symbol;Acc:HGNC:19969]	lncRNA	-0.63	2.42	22.76	<0.0001	<0.0001
ENSG00000114812	VIPR1	vasoactive intestinal peptide receptor 1 [Source:HGNC Symbol;Acc:HGNC:12694]	protein coding	-0.62	3.19	39.51	<0.0001	<0.0001

Table S24: Genes differentially expressed at Day 30 compared to pre-second vaccination (Study Arm 2 A/H7N9+AS03, D1, 29, NK cells). Genes are sorted by descending absolute log_2 fold change. Gene model summaries and annotations are based on Ensembl Version 99 (continued.)

Ensembl Gene ID	Ensembl Gene Name	Ensembl Gene Description	Gene Type	log_2 Fold Change (Day 30 vs. Pre-Second Vaccination)	Average log_2 CPM	Likelihood Ratio Test Statistic	P-Value	FDR Adjusted P-Value
ENSG00000236946	HNRNPA1P70	heterogeneous nuclear ribonucleoprotein A1 pseudogene 70 [Source:HGNC Symbol;Acc:HGNC:48800]	processed pseudogene	-0.62	0.86	16.18	<0.0001	0.0012
ENSG00000183486	MX2	MX dynamin like GTPase 2 [Source:HGNC Symbol;Acc:HGNC:7533]	protein coding	0.62	6.23	39.93	<0.0001	<0.0001
ENSG00000146250	PRSS35	serine protease 35 [Source:HGNC Symbol;Acc:HGNC:21387]	protein coding	-0.62	1.64	8.41	0.0037	0.0293
ENSG00000171502	COL24A1	collagen type XXIV alpha 1 chain [Source:HGNC Symbol;Acc:HGNC:20821]	protein coding	-0.62	1.59	22.52	<0.0001	<0.0001
ENSG00000248473	LINC01962	long intergenic non-protein coding RNA 1962 [Source:HGNC Symbol;Acc:HGNC:52787]	lncRNA	-0.62	1.33	28.36	<0.0001	<0.0001
ENSG00000143382	ADAMTSL4	ADAMTS like 4 [Source:HGNC Symbol;Acc:HGNC:19706]	protein coding	0.62	4.09	23.03	<0.0001	<0.0001
ENSG00000271553		novel transcript	lncRNA	-0.62	0.17	16.90	<0.0001	0.0009
ENSG00000100307	CBX7	chromobox 7 [Source:HGNC Symbol;Acc:HGNC:1557]	protein coding	-0.62	6.36	95.49	<0.0001	<0.0001
ENSG00000151726	ACSL1	acyl-CoA synthetase long chain family member 1 [Source:HGNC Symbol;Acc:HGNC:3569]	protein coding	0.62	4.81	28.04	<0.0001	<0.0001
ENSG00000102471	NDFIP2	Nedd4 family interacting protein 2 [Source:HGNC Symbol;Acc:HGNC:18537]	protein coding	0.62	2.75	24.20	<0.0001	<0.0001
ENSG00000276715	YWHAEP7	tyrosine 3-monooxygenase/tryptophan 5-monooxygenase activation protein epsilon pseudogene 7 [Source:HGNC Symbol;Acc:HGNC:49437]	transcribed unprocessed pseudogene	-0.62	1.06	14.19	0.0002	0.0028
ENSG00000197594	ENPP1	ectonucleotide pyrophosphatase/phosphodiesterase 1 [Source:HGNC Symbol;Acc:HGNC:3356]	protein coding	-0.62	1.55	20.71	<0.0001	0.0002
ENSG00000168685	IL7R	interleukin 7 receptor [Source:HGNC Symbol;Acc:HGNC:6024]	protein coding	-0.62	9.41	12.81	0.0003	0.0049
ENSG00000185736	ADARB2	adenosine deaminase RNA specific B2 (inactive) [Source:HGNC Symbol;Acc:HGNC:227]	protein coding	-0.62	1.62	11.96	0.0005	0.0069
ENSG00000110092	CCND1	cyclin D1 [Source:HGNC Symbol;Acc:HGNC:1582]	protein coding	0.62	1.65	12.68	0.0004	0.0052
ENSG00000100453	GZMB	granzyme B [Source:HGNC Symbol;Acc:HGNC:4709]	protein coding	0.62	8.96	49.88	<0.0001	<0.0001

Table S24: Genes differentially expressed at Day 30 compared to pre-second vaccination (Study Arm 2 A/H7N9+AS03, D1, 29, NK cells). Genes are sorted by descending absolute log_2 fold change. Gene model summaries and annotations are based on Ensembl Version 99 (continued.)

Ensembl Gene ID	Ensembl Gene Name	Ensembl Gene Description	Gene Type	log_2 Fold Change (Day 30 vs. Pre-Second Vaccination)	Average log_2 CPM	Likelihood Ratio Test Statistic	P-Value	FDR Adjusted P-Value
ENSG00000198848	CES1	carboxylesterase 1 [Source:HGNC Symbol;Acc:HGNC:1863]	protein coding	0.62	3.62	10.57	0.0011	0.0122
ENSG00000130589	HELZ2	helicase with zinc finger 2 [Source:HGNC Symbol;Acc:HGNC:30021]	protein coding	0.61	7.36	54.20	<0.0001	<0.0001
ENSG00000054793	ATP9A	ATPase phospholipid transporting 9A (putative) [Source:HGNC Symbol;Acc:HGNC:13540]	protein coding	-0.61	4.24	19.59	<0.0001	0.0003
ENSG00000100065	CARD10	caspase recruitment domain family member 10 [Source:HGNC Symbol;Acc:HGNC:16422]	protein coding	-0.61	0.67	15.64	<0.0001	0.0015
ENSG00000109743	BST1	bone marrow stromal cell antigen 1 [Source:HGNC Symbol;Acc:HGNC:1118]	protein coding	0.61	3.70	15.03	0.0001	0.002
ENSG00000249667	LINC01259	long intergenic non-protein coding RNA 1259 [Source:HGNC Symbol;Acc:HGNC:49899]	lncRNA	-0.61	0.86	14.74	0.0001	0.0022
ENSG00000103313	MEFV	MEFV innate immuity regulator, pyrin [Source:HGNC Symbol;Acc:HGNC:6998]	protein coding	0.61	4.21	20.70	<0.0001	0.0002
ENSG00000141448	GATA6	GATA binding protein 6 [Source:HGNC Symbol;Acc:HGNC:4174]	protein coding	-0.61	0.62	7.81	0.0052	0.0374
ENSG00000267221	C17orf113	chromosome 17 open reading frame 113 [Source:HGNC Symbol;Acc:HGNC:53437]	protein coding	-0.61	0.73	17.41	<0.0001	0.0007
ENSG00000104970	KIR3DX1	killer cell immunoglobulin like receptor, three Ig domains X1 (pseudogene) [Source:HGNC Symbol;Acc:HGNC:25043]	transcribed unprocessed pseudogene	0.61	3.41	51.40	<0.0001	<0.0001
ENSG00000279833		novel transcript, antisense to CBX7	lncRNA	-0.61	3.55	29.28	<0.0001	<0.0001
ENSG00000011422	PLAUR	plasminogen activator, urokinase receptor [Source:HGNC Symbol;Acc:HGNC:9053]	protein coding	0.61	3.24	24.73	<0.0001	<0.0001
ENSG00000174099	MSRB3	methionine sulfoxide reductase B3 [Source:HGNC Symbol;Acc:HGNC:27375]	protein coding	-0.61	1.69	21.06	<0.0001	0.0001
ENSG00000197121	PGAP1	post-GPI attachment to proteins inositol deacylase 1 [Source:HGNC Symbol;Acc:HGNC:25712]	protein coding	0.61	2.26	20.76	<0.0001	0.0002
ENSG00000085265	FCN1	ficolin 1 [Source:HGNC Symbol;Acc:HGNC:3623]	protein coding	0.60	8.38	16.60	<0.0001	0.001
ENSG00000237989	LINC01679	long intergenic non-protein coding RNA 1679 [Source:HGNC Symbol;Acc:HGNC:52469]	lncRNA	0.60	0.69	13.87	0.0002	0.0032
ENSG00000064763	FAR2	fatty acyl-CoA reductase 2 [Source:HGNC Symbol;Acc:HGNC:25531]	protein coding	0.60	2.22	22.61	<0.0001	<0.0001

Table S24: Genes differentially expressed at Day 30 compared to pre-second vaccination (Study Arm 2 A/H7N9+AS03, D1, 29, NK cells). Genes are sorted by descending absolute log_2 fold change. Gene model summaries and annotations are based on Ensembl Version 99 (continued.)

Ensembl Gene ID	Ensembl Gene Name	Ensembl Gene Description	Gene Type	log_2 Fold Change (Day 30 vs. Pre-Second Vaccination)	Average log_2 CPM	Likelihood Ratio Test Statistic	P-Value	FDR Adjusted P-Value
ENSG00000174469	CNTNAP2	contactin associated protein like 2 [Source:HGNC Symbol;Acc:HGNC:13830]	protein coding	-0.60	3.39	14.68	0.0001	0.0023
ENSG00000128335	APOL2	apolipoprotein L2 [Source:HGNC Symbol;Acc:HGNC:619]	protein coding	0.60	6.93	44.13	<0.0001	<0.0001
ENSG00000104738	MCM4	minichromosome maintenance complex component 4 [Source:HGNC Symbol;Acc:HGNC:6947]	protein coding	0.60	4.61	14.56	0.0001	0.0024
ENSG00000124762	CDKN1A	cyclin dependent kinase inhibitor 1A [Source:HGNC Symbol;Acc:HGNC:1784]	protein coding	0.60	3.12	21.43	<0.0001	0.0001
ENSG00000186407	CD300E	CD300e molecule [Source:HGNC Symbol;Acc:HGNC:28874]	protein coding	0.60	5.16	18.72	<0.0001	0.0004
ENSG00000137563	GGH	gamma-glutamyl hydrolase [Source:HGNC Symbol;Acc:HGNC:4248]	protein coding	0.60	1.43	13.81	0.0002	0.0033
ENSG00000226416	MRPL23-AS1	MRPL23 antisense RNA 1 [Source:HGNC Symbol;Acc:HGNC:42812]	lncRNA	-0.60	0.52	16.75	<0.0001	0.0009
ENSG00000186193	SAPCD2	suppressor APC domain containing 2 [Source:HGNC Symbol;Acc:HGNC:28055]	protein coding	0.60	1.40	19.15	<0.0001	0.0003
ENSG00000204219	TCEA3	transcription elongation factor A3 [Source:HGNC Symbol;Acc:HGNC:11615]	protein coding	-0.60	0.69	24.18	<0.0001	<0.0001
ENSG00000113721	PDGFRB	platelet derived growth factor receptor beta [Source:HGNC Symbol;Acc:HGNC:8804]	protein coding	0.60	5.30	39.00	<0.0001	<0.0001
ENSG00000142235	LMTK3	lemur tyrosine kinase 3 [Source:HGNC Symbol;Acc:HGNC:19295]	protein coding	-0.60	2.93	17.84	<0.0001	0.0006
ENSG00000138316	ADAMTS14	ADAM metallopeptidase with thrombospondin type 1 motif 14 [Source:HGNC Symbol;Acc:HGNC:14899]	protein coding	0.60	2.12	17.31	<0.0001	0.0007
ENSG00000141096	DPEP3	dipeptidase 3 [Source:HGNC Symbol;Acc:HGNC:23029]	protein coding	-0.59	0.23	13.20	0.0003	0.0042
ENSG00000142611	PRDM16	PR/SET domain 16 [Source:HGNC Symbol;Acc:HGNC:14000]	protein coding	-0.59	0.34	12.15	0.0005	0.0064
ENSG00000141527	CARD14	caspase recruitment domain family member 14 [Source:HGNC Symbol;Acc:HGNC:16446]	protein coding	-0.59	1.44	17.44	<0.0001	0.0007
ENSG00000110811	P3H3	prolyl 3-hydroxylase 3 [Source:HGNC Symbol;Acc:HGNC:19318]	protein coding	-0.59	1.89	10.50	0.0012	0.0126
ENSG00000055332	EIF2AK2	eukaryotic translation initiation factor 2 alpha kinase 2 [Source:HGNC Symbol;Acc:HGNC:9437]	protein coding	0.59	5.95	61.92	<0.0001	<0.0001
ENSG00000181045	SLC26A11	solute carrier family 26 member 11 [Source:HGNC Symbol;Acc:HGNC:14471]	protein coding	-0.59	5.54	35.53	<0.0001	<0.0001

Table S24: Genes differentially expressed at Day 30 compared to pre-second vaccination (Study Arm 2 A/H7N9+AS03, D1, 29, NK cells). Genes are sorted by descending absolute log_2 fold change. Gene model summaries and annotations are based on Ensembl Version 99 (continued.)

Ensembl Gene ID	Ensembl Gene Name	Ensembl Gene Description	Gene Type	log_2 Fold Change (Day 30 vs. Pre-Second Vaccination)	Average log_2 CPM	Likelihood Ratio Test Statistic	P-Value	FDR Adjusted P-Value
ENSG00000081059	TCF7	transcription factor 7 [Source:HGNC Symbol;Acc:HGNC:11639]	protein coding	-0.59	8.06	38.54	<0.0001	<0.0001
ENSG00000183960	KCNH8	potassium voltage-gated channel subfamily H member 8 [Source:HGNC Symbol;Acc:HGNC:18864]	protein coding	-0.59	2.68	10.56	0.0012	0.0123
ENSG00000260528	FAM157C	family with sequence similarity 157 member C [Source:HGNC Symbol;Acc:HGNC:34081]	lncRNA	0.59	2.04	15.96	<0.0001	0.0013
ENSG00000166428	PLD4	phospholipase D family member 4 [Source:HGNC Symbol;Acc:HGNC:23792]	protein coding	-0.59	5.14	28.11	<0.0001	<0.0001

Table S25: Genes differentially expressed at Day 32 compared to pre-second vaccination (Study Arm 2 A/H7N9+AS03, D1, 29, NK cells). Genes are sorted by descending absolute log_2 fold change. Gene model summaries and annotations are based on Ensembl Version 99.

Ensembl Gene ID	Ensembl Gene Name	Ensembl Gene Description	Gene Type	log_2 Fold Change (Day 32 vs. Pre-Second Vaccination)	Average log_2 CPM	Likelihood Ratio Test Statistic	P-Value	FDR Adjusted P-Value
ENSG00000138180	CEP55	centrosomal protein 55 [Source:HGNC Symbol;Acc:HGNC:1161]	protein coding	2.01	1.22	71.19	<0.0001	<0.0001
ENSG00000186185	KIF18B	kinesin family member 18B [Source:HGNC Symbol;Acc:HGNC:27102]	protein coding	1.89	1.82	75.85	<0.0001	<0.0001
ENSG00000135476	ESPL1	extra spindle pole bodies like 1, separase [Source:HGNC Symbol;Acc:HGNC:16856]	protein coding	1.88	2.19	92.02	<0.0001	<0.0001
ENSG00000065328	MCM10	minichromosome maintenance 10 replication initiation factor [Source:HGNC Symbol;Acc:HGNC:18043]	protein coding	1.87	1.31	64.23	<0.0001	<0.0001
ENSG00000066279	ASPM	abnormal spindle microtubule assembly [Source:HGNC Symbol;Acc:HGNC:19048]	protein coding	1.86	2.28	76.74	<0.0001	<0.0001
ENSG00000123485	HJURP	Holliday junction recognition protein [Source:HGNC Symbol;Acc:HGNC:25444]	protein coding	1.85	1.51	61.69	<0.0001	<0.0001
ENSG00000127564	PKMYT1	protein kinase, membrane associated tyrosine/threonine 1 [Source:HGNC Symbol;Acc:HGNC:29650]	protein coding	1.84	2.84	59.13	<0.0001	<0.0001
ENSG00000166803	PCLAF	PCNA clamp associated factor [Source:HGNC Symbol;Acc:HGNC:28961]	protein coding	1.83	1.13	75.91	<0.0001	<0.0001
ENSG00000237649	KIFC1	kinesin family member C1 [Source:HGNC Symbol;Acc:HGNC:6389]	protein coding	1.81	2.74	78.13	<0.0001	<0.0001

Table S25: Genes differentially expressed at Day 32 compared to pre-second vaccination (Study Arm 2 A/H7N9+AS03, D1, 29, NK cells). Genes are sorted by descending absolute log_2 fold change. Gene model summaries and annotations are based on Ensembl Version 99 (continued.)

Ensembl Gene ID	Ensembl Gene Name	Ensembl Gene Description	Gene Type	log_2 Fold Change (Day 32 vs. Pre-Second Vaccination)	Average log_2 CPM	Likelihood Ratio Test Statistic	P-Value	FDR Adjusted P-Value
ENSG00000143476	DTL	denticleless E3 ubiquitin protein ligase homolog [Source:HGNC Symbol;Acc:HGNC:30288]	protein coding	1.79	2.32	57.68	<0.0001	<0.0001
ENSG00000175063	UBE2C	ubiquitin conjugating enzyme E2 C [Source:HGNC Symbol;Acc:HGNC:15937]	protein coding	1.77	1.05	57.87	<0.0001	<0.0001
ENSG00000148773	MKI67	marker of proliferation Ki-67 [Source:HGNC Symbol;Acc:HGNC:7107]	protein coding	1.77	4.85	80.76	<0.0001	<0.0001
ENSG00000075218	GTSE1	G2 and S-phase expressed 1 [Source:HGNC Symbol;Acc:HGNC:13698]	protein coding	1.76	1.96	81.22	<0.0001	<0.0001
ENSG00000137807	KIF23	kinesin family member 23 [Source:HGNC Symbol;Acc:HGNC:6392]	protein coding	1.76	1.24	58.57	<0.0001	<0.0001
ENSG00000089685	BIRC5	baculoviral IAP repeat containing 5 [Source:HGNC Symbol;Acc:HGNC:593]	protein coding	1.75	2.22	57.83	<0.0001	<0.0001
ENSG00000146670	CDCA5	cell division cycle associated 5 [Source:HGNC Symbol;Acc:HGNC:14626]	protein coding	1.70	2.75	67.31	<0.0001	<0.0001
ENSG00000135451	TROAP	trophinin associated protein [Source:HGNC Symbol;Acc:HGNC:12327]	protein coding	1.69	1.64	79.81	<0.0001	<0.0001
ENSG00000131747	TOP2A	DNA topoisomerase II alpha [Source:HGNC Symbol;Acc:HGNC:11989]	protein coding	1.68	3.60	71.68	<0.0001	<0.0001
ENSG00000161888	SPC24	SPC24 component of NDC80 kinetochore complex [Source:HGNC Symbol;Acc:HGNC:26913]	protein coding	1.68	1.23	50.83	<0.0001	<0.0001
ENSG00000171848	RRM2	ribonucleotide reductase regulatory subunit M2 [Source:HGNC Symbol;Acc:HGNC:10452]	protein coding	1.67	4.52	72.68	<0.0001	<0.0001
ENSG00000163808	KIF15	kinesin family member 15 [Source:HGNC Symbol;Acc:HGNC:17273]	protein coding	1.67	1.87	91.75	<0.0001	<0.0001
ENSG00000176890	TYMS	thymidylate synthetase [Source:HGNC Symbol;Acc:HGNC:12441]	protein coding	1.67	3.23	66.05	<0.0001	<0.0001
ENSG00000174371	EXO1	exonuclease 1 [Source:HGNC Symbol;Acc:HGNC:3511]	protein coding	1.66	0.99	39.92	<0.0001	<0.0001
ENSG00000094804	CDC6	cell division cycle 6 [Source:HGNC Symbol;Acc:HGNC:1744]	protein coding	1.64	1.91	60.18	<0.0001	<0.0001
ENSG00000170312	CDK1	cyclin dependent kinase 1 [Source:HGNC Symbol;Acc:HGNC:1722]	protein coding	1.64	1.76	44.36	<0.0001	<0.0001
ENSG00000197256	KANK2	KN motif and ankyrin repeat domains 2 [Source:HGNC Symbol;Acc:HGNC:29300]	protein coding	1.63	0.91	62.12	<0.0001	<0.0001
ENSG00000093009	CDC45	cell division cycle 45 [Source:HGNC Symbol;Acc:HGNC:1739]	protein coding	1.59	2.29	51.23	<0.0001	<0.0001

Table S25: Genes differentially expressed at Day 32 compared to pre-second vaccination (Study Arm 2 A/H7N9+AS03, D1, 29, NK cells). Genes are sorted by descending absolute log_2 fold change. Gene model summaries and annotations are based on Ensembl Version 99 (continued.)

Ensembl Gene ID	Ensembl Gene Name	Ensembl Gene Description	Gene Type	log_2 Fold Change (Day 32 vs. Pre-Second Vaccination)	Average log_2 CPM	Likelihood Ratio Test Statistic	P-Value	FDR Adjusted P-Value
ENSG00000112742	TTK	TTK protein kinase [Source:HGNC Symbol;Acc:HGNC:12401]	protein coding	1.58	0.79	41.46	<0.0001	<0.0001
ENSG00000109805	NCAPG	non-SMC condensin I complex subunit G [Source:HGNC Symbol;Acc:HGNC:24304]	protein coding	1.56	1.62	56.41	<0.0001	<0.0001
ENSG00000167513	CDT1	chromatin licensing and DNA replication factor 1 [Source:HGNC Symbol;Acc:HGNC:24576]	protein coding	1.56	3.22	62.97	<0.0001	<0.0001
ENSG00000122952	ZWINT	ZW10 interacting kinetochore protein [Source:HGNC Symbol;Acc:HGNC:13195]	protein coding	1.54	2.71	57.33	<0.0001	<0.0001
ENSG00000088325	TPX2	TPX2 microtubule nucleation factor [Source:HGNC Symbol;Acc:HGNC:1249]	protein coding	1.54	2.80	62.53	<0.0001	<0.0001
ENSG00000117724	CENPF	centromere protein F [Source:HGNC Symbol;Acc:HGNC:1857]	protein coding	1.53	3.10	57.84	<0.0001	<0.0001
ENSG00000178999	AURKB	aurora kinase B [Source:HGNC Symbol;Acc:HGNC:11390]	protein coding	1.51	1.95	59.26	<0.0001	<0.0001
ENSG00000101412	E2F1	E2F transcription factor 1 [Source:HGNC Symbol;Acc:HGNC:3113]	protein coding	1.49	4.05	52.96	<0.0001	<0.0001
ENSG00000167900	TK1	thymidine kinase 1 [Source:HGNC Symbol;Acc:HGNC:11830]	protein coding	1.49	3.66	49.88	<0.0001	<0.0001
ENSG00000156970	BUB1B	BUB1 mitotic checkpoint serine/threonine kinase B [Source:HGNC Symbol;Acc:HGNC:1149]	protein coding	1.46	1.17	54.61	<0.0001	<0.0001
ENSG00000092853	CLSPN	claspin [Source:HGNC Symbol;Acc:HGNC:19715]	protein coding	1.45	2.42	47.31	<0.0001	<0.0001
ENSG00000072571	HMMR	hyaluronan mediated motility receptor [Source:HGNC Symbol;Acc:HGNC:5012]	protein coding	1.45	1.03	60.18	<0.0001	<0.0001
ENSG00000169679	BUB1	BUB1 mitotic checkpoint serine/threonine kinase [Source:HGNC Symbol;Acc:HGNC:1148]	protein coding	1.41	1.64	37.52	<0.0001	<0.0001
ENSG00000136982	DSCC1	DNA replication and sister chromatid cohesion 1 [Source:HGNC Symbol;Acc:HGNC:24453]	protein coding	1.41	0.92	49.76	<0.0001	<0.0001
ENSG00000117399	CDC20	cell division cycle 20 [Source:HGNC Symbol;Acc:HGNC:1723]	protein coding	1.40	1.58	52.10	<0.0001	<0.0001
ENSG00000051180	RAD51	RAD51 recombinase [Source:HGNC Symbol;Acc:HGNC:9817]	protein coding	1.39	2.03	53.21	<0.0001	<0.0001
ENSG00000121152	NCAPH	non-SMC condensin I complex subunit H [Source:HGNC Symbol;Acc:HGNC:1112]	protein coding	1.37	2.19	42.41	<0.0001	<0.0001
ENSG00000122966	CIT	citron rho-interacting serine/threonine kinase [Source:HGNC Symbol;Acc:HGNC:1985]	protein coding	1.37	2.14	49.50	<0.0001	<0.0001

Confidential/Proprietary Information

Table S25: Genes differentially expressed at Day 32 compared to pre-second vaccination (Study Arm 2 A/H7N9+AS03, D1, 29, NK cells). Genes are sorted by descending absolute log_2 fold change. Gene model summaries and annotations are based on Ensembl Version 99 (continued.)

Ensembl Gene ID	Ensembl Gene Name	Ensembl Gene Description	Gene Type	log_2 Fold Change (Day 32 vs. Pre-Second Vaccination)	Average log_2 CPM	Likelihood Ratio Test Statistic	P-Value	FDR Adjusted P-Value
ENSG00000162062	TEDC2	tubulin epsilon and delta complex 2 [Source:HGNC Symbol;Acc:HGNC:25849]	protein coding	1.37	0.95	46.91	<0.0001	<0.0001
ENSG00000131153	GINS2	GINS complex subunit 2 [Source:HGNC Symbol;Acc:HGNC:24575]	protein coding	1.36	1.87	35.62	<0.0001	<0.0001
ENSG00000154839	SKA1	spindle and kinetochore associated complex subunit 1 [Source:HGNC Symbol;Acc:HGNC:28109]	protein coding	1.35	0.68	61.67	<0.0001	<0.0001
ENSG00000085999	RAD54L	RAD54 like [Source:HGNC Symbol;Acc:HGNC:9826]	protein coding	1.35	1.45	42.95	<0.0001	<0.0001
ENSG00000157456	CCNB2	cyclin B2 [Source:HGNC Symbol;Acc:HGNC:1580]	protein coding	1.35	0.95	48.95	<0.0001	<0.0001
ENSG00000051341	POLQ	DNA polymerase theta [Source:HGNC Symbol;Acc:HGNC:9186]	protein coding	1.34	1.98	39.54	<0.0001	<0.0001
ENSG00000105011	ASF1B	anti-silencing function 1B histone chaperone [Source:HGNC Symbol;Acc:HGNC:20996]	protein coding	1.32	3.43	58.71	<0.0001	<0.0001
ENSG00000142945	KIF2C	kinesin family member 2C [Source:HGNC Symbol;Acc:HGNC:6393]	protein coding	1.32	1.77	53.52	<0.0001	<0.0001
ENSG00000111206	FOXM1	forkhead box M1 [Source:HGNC Symbol;Acc:HGNC:3818]	protein coding	1.31	2.60	41.82	<0.0001	<0.0001
ENSG00000117632	STMN1	stathmin 1 [Source:HGNC Symbol;Acc:HGNC:6510]	protein coding	1.29	5.80	64.64	<0.0001	<0.0001
ENSG00000085840	ORC1	origin recognition complex subunit 1 [Source:HGNC Symbol;Acc:HGNC:8487]	protein coding	1.29	1.59	56.83	<0.0001	<0.0001
ENSG00000154920	EME1	essential meiotic structure-specific endonuclease 1 [Source:HGNC Symbol;Acc:HGNC:24965]	protein coding	1.29	0.95	66.09	<0.0001	<0.0001
ENSG00000111665	CDCA3	cell division cycle associated 3 [Source:HGNC Symbol;Acc:HGNC:14624]	protein coding	1.25	1.71	56.20	<0.0001	<0.0001
ENSG00000179750	APOBEC3B	apolipoprotein B mRNA editing enzyme catalytic subunit 3B [Source:HGNC Symbol;Acc:HGNC:17352]	protein coding	1.25	1.18	27.60	<0.0001	<0.0001
ENSG00000100162	CENPM	centromere protein M [Source:HGNC Symbol;Acc:HGNC:18352]	protein coding	1.24	2.12	35.58	<0.0001	<0.0001
ENSG00000134690	CDCA8	cell division cycle associated 8 [Source:HGNC Symbol;Acc:HGNC:14629]	protein coding	1.24	1.97	57.67	<0.0001	<0.0001
ENSG00000138160	KIF11	kinesin family member 11 [Source:HGNC Symbol;Acc:HGNC:6388]	protein coding	1.24	2.81	55.03	<0.0001	<0.0001
ENSG00000145386	CCNA2	cyclin A2 [Source:HGNC Symbol;Acc:HGNC:1578]	protein coding	1.22	1.99	58.67	<0.0001	<0.0001

Table S25: Genes differentially expressed at Day 32 compared to pre-second vaccination (Study Arm 2 A/H7N9+AS03, D1, 29, NK cells). Genes are sorted by descending absolute log_2 fold change. Gene model summaries and annotations are based on Ensembl Version 99 (continued.)

Ensembl Gene ID	Ensembl Gene Name	Ensembl Gene Description	Gene Type	log_2 Fold Change (Day 32 vs. Pre-Second Vaccination)	Average log_2 CPM	Likelihood Ratio Test Statistic	P-Value	FDR Adjusted P-Value
ENSG00000137563	GGH	gamma-glutamyl hydrolase [Source:HGNC Symbol;Acc:HGNC:4248]	protein coding	1.21	1.65	50.05	<0.0001	<0.0001
ENSG00000138778	CENPE	centromere protein E [Source:HGNC Symbol;Acc:HGNC:1856]	protein coding	1.21	1.55	44.80	<0.0001	<0.0001
ENSG00000151725	CENPU	centromere protein U [Source:HGNC Symbol;Acc:HGNC:21348]	protein coding	1.20	2.49	33.13	<0.0001	<0.0001
ENSG00000128383	APOBEC3A	apolipoprotein B mRNA editing enzyme catalytic subunit 3A [Source:HGNC Symbol;Acc:HGNC:17343]	protein coding	-1.18	2.06	25.83	<0.0001	<0.0001
ENSG00000137812	KNL1	kinetochore scaffold 1 [Source:HGNC Symbol;Acc:HGNC:24054]	protein coding	1.17	2.06	34.52	<0.0001	<0.0001
ENSG00000119326	CTNNAL1	catenin alpha like 1 [Source:HGNC Symbol;Acc:HGNC:2512]	protein coding	1.16	1.25	38.66	<0.0001	<0.0001
ENSG00000184221	OLIG1	oligodendrocyte transcription factor 1 [Source:HGNC Symbol;Acc:HGNC:16983]	protein coding	-1.13	1.10	29.51	<0.0001	<0.0001
ENSG00000071539	TRIP13	thyroid hormone receptor interactor 13 [Source:HGNC Symbol;Acc:HGNC:12307]	protein coding	1.13	1.01	39.77	<0.0001	<0.0001
ENSG00000123080	CDKN2C	cyclin dependent kinase inhibitor 2C [Source:HGNC Symbol;Acc:HGNC:1789]	protein coding	1.11	3.65	74.41	<0.0001	<0.0001
ENSG00000075702	WDR62	WD repeat domain 62 [Source:HGNC Symbol;Acc:HGNC:24502]	protein coding	1.10	2.85	60.00	<0.0001	<0.0001
ENSG00000149554	CHEK1	checkpoint kinase 1 [Source:HGNC Symbol;Acc:HGNC:1925]	protein coding	1.10	2.59	51.04	<0.0001	<0.0001
ENSG00000007968	E2F2	E2F transcription factor 2 [Source:HGNC Symbol;Acc:HGNC:3114]	protein coding	1.09	3.40	36.25	<0.0001	<0.0001
ENSG00000111247	RAD51AP1	RAD51 associated protein 1 [Source:HGNC Symbol;Acc:HGNC:16956]	protein coding	1.07	1.98	41.05	<0.0001	<0.0001
ENSG00000091651	ORC6	origin recognition complex subunit 6 [Source:HGNC Symbol;Acc:HGNC:17151]	protein coding	1.07	1.97	62.75	<0.0001	<0.0001
ENSG00000225698	IGHV3-72	immunoglobulin heavy variable 3-72 [Source:HGNC Symbol;Acc:HGNC:5622]	IG V gene	-1.06	0.90	11.29	0.0008	0.0121
ENSG00000186193	SAPCD2	suppressor APC domain containing 2 [Source:HGNC Symbol;Acc:HGNC:28055]	protein coding	1.06	1.53	59.79	<0.0001	<0.0001
ENSG00000104738	MCM4	minichromosome maintenance complex component 4 [Source:HGNC Symbol;Acc:HGNC:6947]	protein coding	1.05	4.73	48.00	<0.0001	<0.0001
ENSG00000116729	WLS	Wnt ligand secretion mediator [Source:HGNC Symbol;Acc:HGNC:30238]	protein coding	-1.05	0.93	20.27	<0.0001	0.0003

Table S25: Genes differentially expressed at Day 32 compared to pre-second vaccination (Study Arm 2 A/H7N9+AS03, D1, 29, NK cells). Genes are sorted by descending absolute log_2 fold change. Gene model summaries and annotations are based on Ensembl Version 99 (continued.)

Ensembl Gene ID	Ensembl Gene Name	Ensembl Gene Description	Gene Type	log_2 Fold Change (Day 32 vs. Pre-Second Vaccination)	Average log_2 CPM	Likelihood Ratio Test Statistic	P-Value	FDR Adjusted P-Value
ENSG00000138061	CYP1B1	cytochrome P450 family 1 subfamily B member 1 [Source:HGNC Symbol;Acc:HGNC:2597]	protein coding	-1.04	2.54	22.95	<0.0001	<0.0001
ENSG00000162073	PAQR4	progestin and adipoQ receptor family member 4 [Source:HGNC Symbol;Acc:HGNC:26386]	protein coding	1.04	2.65	43.56	<0.0001	<0.0001
ENSG00000142731	PLK4	polo like kinase 4 [Source:HGNC Symbol;Acc:HGNC:11397]	protein coding	1.03	2.08	35.97	<0.0001	<0.0001
ENSG00000101057	MYBL2	MYB proto-oncogene like 2 [Source:HGNC Symbol;Acc:HGNC:7548]	protein coding	1.03	3.63	52.37	<0.0001	<0.0001
ENSG00000140525	FANCI	FA complementation group I [Source:HGNC Symbol;Acc:HGNC:25568]	protein coding	1.03	4.49	56.46	<0.0001	<0.0001
ENSG00000077152	UBE2T	ubiquitin conjugating enzyme E2 T [Source:HGNC Symbol;Acc:HGNC:25009]	protein coding	1.02	1.26	33.88	<0.0001	<0.0001
ENSG00000123473	STIL	STIL centriolar assembly protein [Source:HGNC Symbol;Acc:HGNC:10879]	protein coding	1.02	1.62	29.94	<0.0001	<0.0001
ENSG00000136492	BRIP1	BRCA1 interacting protein C-terminal helicase 1 [Source:HGNC Symbol;Acc:HGNC:20473]	protein coding	1.01	2.10	30.36	<0.0001	<0.0001
ENSG00000076382	SPAG5	sperm associated antigen 5 [Source:HGNC Symbol;Acc:HGNC:13452]	protein coding	1.00	2.79	50.01	<0.0001	<0.0001
ENSG00000101003	GINS1	GINS complex subunit 1 [Source:HGNC Symbol;Acc:HGNC:28980]	protein coding	0.99	1.42	32.88	<0.0001	<0.0001
ENSG00000189057	FAM111B	family with sequence similarity 111 member B [Source:HGNC Symbol;Acc:HGNC:24200]	protein coding	0.97	2.46	19.54	<0.0001	0.0004
ENSG00000103569	AQP9	aquaporin 9 [Source:HGNC Symbol;Acc:HGNC:643]	protein coding	-0.97	1.44	11.82	0.0006	0.0098
ENSG00000055118	KCNH2	potassium voltage-gated channel subfamily H member 2 [Source:HGNC Symbol;Acc:HGNC:6251]	protein coding	-0.94	0.84	43.45	<0.0001	<0.0001
ENSG00000196584	XRCC2	X-ray repair cross complementing 2 [Source:HGNC Symbol;Acc:HGNC:12829]	protein coding	0.94	1.84	26.25	<0.0001	<0.0001
ENSG00000004799	PDK4	pyruvate dehydrogenase kinase 4 [Source:HGNC Symbol;Acc:HGNC:8812]	protein coding	-0.94	2.32	16.24	<0.0001	0.0015
ENSG00000211685	IGLC7	immunoglobulin lambda constant 7 [Source:HGNC Symbol;Acc:HGNC:5861]	IG C gene	-0.93	0.62	10.87	0.001	0.0145
ENSG00000138829	FBN2	fibrillin 2 [Source:HGNC Symbol;Acc:HGNC:3604]	protein coding	-0.93	1.14	20.19	<0.0001	0.0003
ENSG00000198826	ARHGAP11A	Rho GTPase activating protein 11A [Source:HGNC Symbol;Acc:HGNC:15783]	protein coding	0.93	2.57	44.56	<0.0001	<0.0001

Table S25: Genes differentially expressed at Day 32 compared to pre-second vaccination (Study Arm 2 A/H7N9+AS03, D1, 29, NK cells). Genes are sorted by descending absolute log_2 fold change. Gene model summaries and annotations are based on Ensembl Version 99 (continued.)

Ensembl Gene ID	Ensembl Gene Name	Ensembl Gene Description	Gene Type	log_2 Fold Change (Day 32 vs. Pre-Second Vaccination)	Average log_2 CPM	Likelihood Ratio Test Statistic	P-Value	FDR Adjusted P-Value
ENSG00000170458	CD14	CD14 molecule [Source:HGNC Symbol;Acc:HGNC:1628]	protein coding	-0.92	6.05	27.30	<0.0001	<0.0001
ENSG00000171241	SHCBP1	SHC binding and spindle associated 1 [Source:HGNC Symbol;Acc:HGNC:29547]	protein coding	0.92	1.85	21.45	<0.0001	0.0002
ENSG00000165092	ALDH1A1	aldehyde dehydrogenase 1 family member A1 [Source:HGNC Symbol;Acc:HGNC:402]	protein coding	-0.91	1.97	26.95	<0.0001	<0.0001
ENSG00000214357	NEURL1B	neuralized E3 ubiquitin protein ligase 1B [Source:HGNC Symbol;Acc:HGNC:35422]	protein coding	0.91	1.17	24.33	<0.0001	<0.0001
ENSG00000136826	KLF4	Kruppel like factor 4 [Source:HGNC Symbol;Acc:HGNC:6348]	protein coding	-0.91	4.80	15.68	<0.0001	0.0019
ENSG00000164125	GASK1B	golgi associated kinase 1B [Source:HGNC Symbol;Acc:HGNC:25312]	protein coding	-0.90	3.10	32.95	<0.0001	<0.0001
ENSG00000213694	S1PR3	sphingosine-1-phosphate receptor 3 [Source:HGNC Symbol;Acc:HGNC:3167]	protein coding	-0.90	1.25	15.80	<0.0001	0.0018
ENSG00000241163	LINC00877	long intergenic non-protein coding RNA 877 [Source:HGNC Symbol;Acc:HGNC:27706]	lncRNA	-0.90	1.02	26.73	<0.0001	<0.0001
ENSG00000276043	UHRF1	ubiquitin like with PHD and ring finger domains 1 [Source:HGNC Symbol;Acc:HGNC:12556]	protein coding	0.90	4.48	35.63	<0.0001	<0.0001
ENSG00000143228	NUF2	NUF2 component of NDC80 kinetochore complex [Source:HGNC Symbol;Acc:HGNC:14621]	protein coding	0.89	1.73	38.76	<0.0001	<0.0001
ENSG00000132646	PCNA	proliferating cell nuclear antigen [Source:HGNC Symbol;Acc:HGNC:8729]	protein coding	0.89	5.95	53.08	<0.0001	<0.0001
ENSG00000112312	GMNN	geminin DNA replication inhibitor [Source:HGNC Symbol;Acc:HGNC:17493]	protein coding	0.89	2.30	40.21	<0.0001	<0.0001
ENSG00000158825	CDA	cytidine deaminase [Source:HGNC Symbol;Acc:HGNC:1712]	protein coding	-0.89	1.65	28.05	<0.0001	<0.0001
ENSG00000160957	RECQL4	RecQ like helicase 4 [Source:HGNC Symbol;Acc:HGNC:9949]	protein coding	0.89	4.16	46.15	<0.0001	<0.0001
ENSG00000068489	PRR11	proline rich 11 [Source:HGNC Symbol;Acc:HGNC:25619]	protein coding	0.88	1.96	29.37	<0.0001	<0.0001
ENSG00000002933	TMEM176A	transmembrane protein 176A [Source:HGNC Symbol;Acc:HGNC:24930]	protein coding	-0.88	2.08	22.78	<0.0001	<0.0001
ENSG00000181631	P2RY13	purinergic receptor P2Y13 [Source:HGNC Symbol;Acc:HGNC:4537]	protein coding	-0.88	2.47	16.24	<0.0001	0.0015
ENSG00000165244	ZNF367	zinc finger protein 367 [Source:HGNC Symbol;Acc:HGNC:18320]	protein coding	0.87	3.24	38.43	<0.0001	<0.0001

Table S25: Genes differentially expressed at Day 32 compared to pre-second vaccination (Study Arm 2 A/H7N9+AS03, D1, 29, NK cells). Genes are sorted by descending absolute log_2 fold change. Gene model summaries and annotations are based on Ensembl Version 99 (continued.)

Ensembl Gene ID	Ensembl Gene Name	Ensembl Gene Description	Gene Type	log_2 Fold Change (Day 32 vs. Pre-Second Vaccination)	Average log_2 CPM	Likelihood Ratio Test Statistic	P-Value	FDR Adjusted P-Value
ENSG00000130881	LRP3	LDL receptor related protein 3 [Source:HGNC Symbol;Acc:HGNC:6695]	protein coding	-0.87	1.20	24.01	<0.0001	<0.0001
ENSG00000012048	BRCA1	BRCA1 DNA repair associated [Source:HGNC Symbol;Acc:HGNC:1100]	protein coding	0.87	3.04	45.92	<0.0001	<0.0001
ENSG00000163221	S100A12	S100 calcium binding protein A12 [Source:HGNC Symbol;Acc:HGNC:10489]	protein coding	-0.87	3.68	28.54	<0.0001	<0.0001
ENSG00000197549	PRAMENP	PRAME N-terminal like, pseudogene [Source:HGNC Symbol;Acc:HGNC:34302]	transcribed unprocessed pseudogene	-0.87	0.47	17.14	<0.0001	0.0011
ENSG00000128578	STRIP2	striatin interacting protein 2 [Source:HGNC Symbol;Acc:HGNC:22209]	protein coding	0.86	1.31	26.54	<0.0001	<0.0001
ENSG00000182379	NXPH4	neurexophilin 4 [Source:HGNC Symbol;Acc:HGNC:8078]	protein coding	-0.86	1.35	13.65	0.0002	0.0046
ENSG00000231964		novel transcript	lncRNA	-0.85	0.50	16.89	<0.0001	0.0012
ENSG00000124731	TREM1	triggering receptor expressed on myeloid cells 1 [Source:HGNC Symbol;Acc:HGNC:17760]	protein coding	-0.85	2.42	20.37	<0.0001	0.0003
ENSG00000135929	CYP27A1	cytochrome P450 family 27 subfamily A member 1 [Source:HGNC Symbol;Acc:HGNC:2605]	protein coding	-0.84	2.01	15.93	<0.0001	0.0017
ENSG00000166851	PLK1	polo like kinase 1 [Source:HGNC Symbol;Acc:HGNC:9077]	protein coding	0.84	2.34	35.45	<0.0001	<0.0001
ENSG00000156564	LRFN2	leucine rich repeat and fibronectin type III domain containing 2 [Source:HGNC Symbol;Acc:HGNC:21226]	protein coding	0.83	0.66	25.85	<0.0001	<0.0001
ENSG00000230006	ANKRD36BP2	ankyrin repeat domain 36B pseudogene 2 [Source:HGNC Symbol;Acc:HGNC:33607]	transcribed unprocessed pseudogene	-0.83	2.53	24.32	<0.0001	<0.0001
ENSG00000132669	RIN2	Ras and Rab interactor 2 [Source:HGNC Symbol;Acc:HGNC:18750]	protein coding	-0.83	1.84	30.84	<0.0001	<0.0001
ENSG00000164087	POC1A	POC1 centriolar protein A [Source:HGNC Symbol;Acc:HGNC:24488]	protein coding	0.82	1.67	29.59	<0.0001	<0.0001
ENSG00000166451	CENPN	centromere protein N [Source:HGNC Symbol;Acc:HGNC:30873]	protein coding	0.81	1.82	43.96	<0.0001	<0.0001

Table S25: Genes differentially expressed at Day 32 compared to pre-second vaccination (Study Arm 2 A/H7N9+AS03, D1, 29, NK cells). Genes are sorted by descending absolute log_2 fold change. Gene model summaries and annotations are based on Ensembl Version 99 (continued.)

Ensembl Gene ID	Ensembl Gene Name	Ensembl Gene Description	Gene Type	log_2 Fold Change (Day 32 vs. Pre-Second Vaccination)	Average log_2 CPM	Likelihood Ratio Test Statistic	P-Value	FDR Adjusted P-Value
ENSG00000139618	BRCA2	BRCA2 DNA repair associated [Source:HGNC Symbol;Acc:HGNC:1101]	protein coding	0.81	2.44	28.11	<0.0001	<0.0001
ENSG00000105173	CCNE1	cyclin E1 [Source:HGNC Symbol;Acc:HGNC:1589]	protein coding	0.81	1.04	16.84	<0.0001	0.0012
ENSG00000163220	S100A9	S100 calcium binding protein A9 [Source:HGNC Symbol;Acc:HGNC:10499]	protein coding	-0.80	8.06	27.70	<0.0001	<0.0001
ENSG00000147536	GINS4	GINS complex subunit 4 [Source:HGNC Symbol;Acc:HGNC:28226]	protein coding	0.80	2.00	42.55	<0.0001	<0.0001
ENSG00000100504	PYGL	glycogen phosphorylase L [Source:HGNC Symbol;Acc:HGNC:9725]	protein coding	-0.80	3.27	27.02	<0.0001	<0.0001
ENSG00000176490	DIRAS1	DIRAS family GTPase 1 [Source:HGNC Symbol;Acc:HGNC:19127]	protein coding	-0.80	1.29	11.47	0.0007	0.0113
ENSG00000234184	LINC01781	long intergenic non-protein coding RNA 1781 [Source:HGNC Symbol;Acc:HGNC:52571]	lncRNA	-0.80	2.23	15.41	<0.0001	0.0022
ENSG00000197249	SERPINA1	serpin family A member 1 [Source:HGNC Symbol;Acc:HGNC:8941]	protein coding	-0.80	5.62	29.41	<0.0001	<0.0001
ENSG00000167748	KLK1	kallikrein 1 [Source:HGNC Symbol;Acc:HGNC:6357]	protein coding	-0.79	0.66	12.63	0.0004	0.0072
ENSG00000165810	BTNL9	butyrophilin like 9 [Source:HGNC Symbol;Acc:HGNC:24176]	protein coding	-0.79	2.81	12.32	0.0004	0.0081
ENSG00000087586	AURKA	aurora kinase A [Source:HGNC Symbol;Acc:HGNC:11393]	protein coding	0.79	1.15	28.80	<0.0001	<0.0001
ENSG00000136630	HLX	H2.0 like homeobox [Source:HGNC Symbol;Acc:HGNC:4978]	protein coding	-0.78	1.21	22.70	<0.0001	<0.0001
ENSG00000187510	PLEKHG7	pleckstrin homology and RhoGEF domain containing G7 [Source:HGNC Symbol;Acc:HGNC:33829]	protein coding	-0.78	0.41	15.14	<0.0001	0.0024
ENSG00000085265	FCN1	ficolin 1 [Source:HGNC Symbol;Acc:HGNC:3623]	protein coding	-0.78	8.01	29.20	<0.0001	<0.0001
ENSG00000162444	RBP7	retinol binding protein 7 [Source:HGNC Symbol;Acc:HGNC:30316]	protein coding	-0.77	1.07	12.76	0.0004	0.0068
ENSG00000144354	CDCA7	cell division cycle associated 7 [Source:HGNC Symbol;Acc:HGNC:14628]	protein coding	0.77	2.74	36.50	<0.0001	<0.0001
ENSG00000005189	REXO5	RNA exonuclease 5 [Source:HGNC Symbol;Acc:HGNC:24661]	protein coding	0.77	0.95	19.00	<0.0001	0.0005
ENSG00000143546	S100A8	S100 calcium binding protein A8 [Source:HGNC Symbol;Acc:HGNC:10498]	protein coding	-0.77	6.79	23.33	<0.0001	<0.0001

Table S25: Genes differentially expressed at Day 32 compared to pre-second vaccination (Study Arm 2 A/H7N9+AS03, D1, 29, NK cells). Genes are sorted by descending absolute log_2 fold change. Gene model summaries and annotations are based on Ensembl Version 99 (continued.)

Ensembl Gene ID	Ensembl Gene Name	Ensembl Gene Description	Gene Type	log_2 Fold Change (Day 32 vs. Pre-Second Vaccination)	Average log_2 CPM	Likelihood Ratio Test Statistic	P-Value	FDR Adjusted P-Value
ENSG00000136689	IL1RN	interleukin 1 receptor antagonist [Source:HGNC Symbol;Acc:HGNC:6000]	protein coding	-0.77	1.91	25.13	<0.0001	<0.0001
ENSG00000150625	GPM6A	glycoprotein M6A [Source:HGNC Symbol;Acc:HGNC:4460]	protein coding	-0.77	0.70	9.20	0.0024	0.0286
ENSG00000211890	IGHA2	immunoglobulin heavy constant alpha 2 (A2m marker) [Source:HGNC Symbol;Acc:HGNC:5479]	IG C gene	-0.77	6.70	11.32	0.0008	0.012
ENSG00000168496	FEN1	flap structure-specific endonuclease 1 [Source:HGNC Symbol;Acc:HGNC:3650]	protein coding	0.77	4.21	38.73	<0.0001	<0.0001
ENSG00000109743	BST1	bone marrow stromal cell antigen 1 [Source:HGNC Symbol;Acc:HGNC:1118]	protein coding	-0.76	3.32	24.50	<0.0001	<0.0001
ENSG00000204103	MAFB	MAF bZIP transcription factor B [Source:HGNC Symbol;Acc:HGNC:6408]	protein coding	-0.76	2.74	9.83	0.0017	0.0219
ENSG00000104974	LILRA1	leukocyte immunoglobulin like receptor A1 [Source:HGNC Symbol;Acc:HGNC:6602]	protein coding	-0.76	3.77	30.48	<0.0001	<0.0001
ENSG00000171051	FPR1	formyl peptide receptor 1 [Source:HGNC Symbol;Acc:HGNC:3826]	protein coding	-0.76	3.89	23.46	<0.0001	<0.0001
ENSG00000128438	TBC1D27P	TBC1 domain family member 27, pseudogene [Source:HGNC Symbol;Acc:HGNC:28104]	transcribed unprocessed pseudogene	-0.76	4.15	15.23	<0.0001	0.0023
ENSG00000163563	MNDA	myeloid cell nuclear differentiation antigen [Source:HGNC Symbol;Acc:HGNC:7183]	protein coding	-0.76	5.78	36.43	<0.0001	<0.0001
ENSG00000196668	LINC00173	long intergenic non-protein coding RNA 173 [Source:HGNC Symbol;Acc:HGNC:33791]	lncRNA	-0.76	0.84	17.23	<0.0001	0.001
ENSG00000146070	PLA2G7	phospholipase A2 group VII [Source:HGNC Symbol;Acc:HGNC:9040]	protein coding	-0.76	1.41	16.75	<0.0001	0.0012
ENSG00000181982	CCDC149	coiled-coil domain containing 149 [Source:HGNC Symbol;Acc:HGNC:25405]	protein coding	-0.76	1.31	20.58	<0.0001	0.0002
ENSG00000121552	CSTA	cystatin A [Source:HGNC Symbol;Acc:HGNC:2481]	protein coding	-0.75	3.31	37.08	<0.0001	<0.0001
ENSG00000156381	ANKRD9	ankyrin repeat domain 9 [Source:HGNC Symbol;Acc:HGNC:20096]	protein coding	0.75	2.96	28.96	<0.0001	<0.0001
ENSG00000197766	CFD	complement factor D [Source:HGNC Symbol;Acc:HGNC:2771]	protein coding	-0.75	4.32	28.17	<0.0001	<0.0001

Table S25: Genes differentially expressed at Day 32 compared to pre-second vaccination (Study Arm 2 A/H7N9+AS03, D1, 29, NK cells). Genes are sorted by descending absolute log_2 fold change. Gene model summaries and annotations are based on Ensembl Version 99 (continued.)

Ensembl Gene ID	Ensembl Gene Name	Ensembl Gene Description	Gene Type	log_2 Fold Change (Day 32 vs. Pre-Second Vaccination)	Average log_2 CPM	Likelihood Ratio Test Statistic	P-Value	FDR Adjusted P-Value
ENSG00000168081	PNOC	prepronociceptin [Source:HGNC Symbol;Acc:HGNC:9163]	protein coding	-0.75	3.04	13.40	0.0003	0.0051
ENSG00000156802	ATAD2	ATPase family AAA domain containing 2 [Source:HGNC Symbol;Acc:HGNC:30123]	protein coding	0.75	4.65	53.56	<0.0001	<0.0001
ENSG00000119969	HELLS	helicase, lymphoid specific [Source:HGNC Symbol;Acc:HGNC:4861]	protein coding	0.75	3.16	24.55	<0.0001	<0.0001
ENSG00000151650	VENTX	VENT homeobox [Source:HGNC Symbol;Acc:HGNC:13639]	protein coding	-0.74	2.29	21.88	<0.0001	0.0001
ENSG00000003249	DBNDD1	dysbindin domain containing 1 [Source:HGNC Symbol;Acc:HGNC:28455]	protein coding	-0.74	2.61	12.38	0.0004	0.0079
ENSG00000080986	NDC80	NDC80 kinetochore complex component [Source:HGNC Symbol;Acc:HGNC:16909]	protein coding	0.74	2.37	34.35	<0.0001	<0.0001
ENSG00000106565	TMEM176B	transmembrane protein 176B [Source:HGNC Symbol;Acc:HGNC:29596]	protein coding	-0.74	3.00	12.36	0.0004	0.008
ENSG00000135218	CD36	CD36 molecule [Source:HGNC Symbol;Acc:HGNC:1663]	protein coding	-0.74	5.23	22.66	<0.0001	<0.0001
ENSG00000161800	RACGAP1	Rac GTPase activating protein 1 [Source:HGNC Symbol;Acc:HGNC:9804]	protein coding	0.74	3.60	43.82	<0.0001	<0.0001
ENSG00000164109	MAD2L1	mitotic arrest deficient 2 like 1 [Source:HGNC Symbol;Acc:HGNC:6763]	protein coding	0.74	2.54	29.02	<0.0001	<0.0001
ENSG00000076003	MCM6	minichromosome maintenance complex component 6 [Source:HGNC Symbol;Acc:HGNC:6949]	protein coding	0.74	4.86	34.35	<0.0001	<0.0001
ENSG00000169403	PTAFR	platelet activating factor receptor [Source:HGNC Symbol;Acc:HGNC:9582]	protein coding	-0.74	4.10	35.38	<0.0001	<0.0001
ENSG00000063660	GPC1	glypican 1 [Source:HGNC Symbol;Acc:HGNC:4449]	protein coding	0.74	1.33	22.00	<0.0001	0.0001
ENSG00000228716	DHFR	dihydrofolate reductase [Source:HGNC Symbol;Acc:HGNC:2861]	protein coding	0.74	4.31	37.88	<0.0001	<0.0001
ENSG00000146410	MTFR2	mitochondrial fission regulator 2 [Source:HGNC Symbol;Acc:HGNC:21115]	protein coding	0.74	0.60	26.56	<0.0001	<0.0001
ENSG00000139970	RTN1	reticulon 1 [Source:HGNC Symbol;Acc:HGNC:10467]	protein coding	-0.73	3.59	30.30	<0.0001	<0.0001
ENSG00000234965	SHISA8	shisa family member 8 [Source:HGNC Symbol;Acc:HGNC:18351]	protein coding	-0.73	2.10	11.11	0.0009	0.0131
ENSG00000134057	CCNB1	cyclin B1 [Source:HGNC Symbol;Acc:HGNC:1579]	protein coding	0.73	1.92	34.86	<0.0001	<0.0001

Table S25: Genes differentially expressed at Day 32 compared to pre-second vaccination (Study Arm 2 A/H7N9+AS03, D1, 29, NK cells). Genes are sorted by descending absolute log_2 fold change. Gene model summaries and annotations are based on Ensembl Version 99 (continued.)

Ensembl Gene ID	Ensembl Gene Name	Ensembl Gene Description	Gene Type	log_2 Fold Change (Day 32 vs. Pre-Second Vaccination)	Average log_2 CPM	Likelihood Ratio Test Statistic	P-Value	FDR Adjusted P-Value
ENSG00000179388	EGR3	early growth response 3 [Source:HGNC Symbol;Acc:HGNC:3240]	protein coding	-0.73	0.66	8.71	0.0032	0.035
ENSG00000211638	IGLV8-61	immunoglobulin lambda variable 8-61 [Source:HGNC Symbol;Acc:HGNC:5931]	IG V gene	-0.73	1.95	8.99	0.0027	0.0312
ENSG00000137310	TCF19	transcription factor 19 [Source:HGNC Symbol;Acc:HGNC:11629]	protein coding	0.73	5.01	30.62	<0.0001	<0.0001
ENSG00000185480	PARPBP	PARP1 binding protein [Source:HGNC Symbol;Acc:HGNC:26074]	protein coding	0.72	1.08	32.63	<0.0001	<0.0001
ENSG00000197405	C5AR1	complement C5a receptor 1 [Source:HGNC Symbol;Acc:HGNC:1338]	protein coding	-0.72	2.95	19.34	<0.0001	0.0004
ENSG00000163568	AIM2	absent in melanoma 2 [Source:HGNC Symbol;Acc:HGNC:357]	protein coding	-0.72	3.77	21.32	<0.0001	0.0002
ENSG00000255587	RAB44	RAB44, member RAS oncogene family [Source:HGNC Symbol;Acc:HGNC:21068]	protein coding	-0.72	2.04	17.75	<0.0001	0.0008
ENSG00000273472		novel transcript	lncRNA	-0.72	2.18	14.70	0.0001	0.003
ENSG00000142405	NLRP12	NLR family pyrin domain containing 12 [Source:HGNC Symbol;Acc:HGNC:22938]	protein coding	-0.72	2.90	19.72	<0.0001	0.0003
ENSG00000164611	PTTG1	PTTG1 regulator of sister chromatid separation, securin [Source:HGNC Symbol;Acc:HGNC:9690]	protein coding	0.72	1.98	30.21	<0.0001	<0.0001
ENSG00000100473	COCH	cochlin [Source:HGNC Symbol;Acc:HGNC:2180]	protein coding	-0.72	2.49	17.88	<0.0001	0.0008
ENSG00000103196	CRISPLD2	cysteine rich secretory protein LCCL domain containing 2 [Source:HGNC Symbol;Acc:HGNC:25248]	protein coding	-0.72	2.63	15.50	<0.0001	0.0021
ENSG00000074964	ARHGEF10L	Rho guanine nucleotide exchange factor 10 like [Source:HGNC Symbol;Acc:HGNC:25540]	protein coding	-0.72	3.12	32.56	<0.0001	<0.0001
ENSG00000123975	CKS2	CDC28 protein kinase regulatory subunit 2 [Source:HGNC Symbol;Acc:HGNC:2000]	protein coding	0.72	2.39	27.23	<0.0001	<0.0001
ENSG00000254415	SIGLEC14	sialic acid binding Ig like lectin 14 [Source:HGNC Symbol;Acc:HGNC:32926]	protein coding	-0.72	3.17	44.82	<0.0001	<0.0001
ENSG00000186407	CD300E	CD300e molecule [Source:HGNC Symbol;Acc:HGNC:28874]	protein coding	-0.72	4.74	23.62	<0.0001	<0.0001
ENSG00000185215	TNFAIP2	TNF alpha induced protein 2 [Source:HGNC Symbol;Acc:HGNC:11895]	protein coding	-0.72	6.68	33.87	<0.0001	<0.0001
ENSG00000181938	GINS3	GINS complex subunit 3 [Source:HGNC Symbol;Acc:HGNC:25851]	protein coding	0.72	1.77	23.17	<0.0001	<0.0001

Table S25: Genes differentially expressed at Day 32 compared to pre-second vaccination (Study Arm 2 A/H7N9+AS03, D1, 29, NK cells). Genes are sorted by descending absolute log_2 fold change. Gene model summaries and annotations are based on Ensembl Version 99 (continued.)

Ensembl Gene ID	Ensembl Gene Name	Ensembl Gene Description	Gene Type	log_2 Fold Change (Day 32 vs. Pre-Second Vaccination)	Average log_2 CPM	Likelihood Ratio Test Statistic	P-Value	FDR Adjusted P-Value
ENSG00000100479	POLE2	DNA polymerase epsilon 2, accessory subunit [Source:HGNC Symbol;Acc:HGNC:9178]	protein coding	0.71	1.40	20.52	<0.0001	0.0002
ENSG00000119535	CSF3R	colony stimulating factor 3 receptor [Source:HGNC Symbol;Acc:HGNC:2439]	protein coding	-0.71	6.18	22.26	<0.0001	0.0001
ENSG00000187116	LILRA5	leukocyte immunoglobulin like receptor A5 [Source:HGNC Symbol;Acc:HGNC:16309]	protein coding	-0.71	3.00	14.58	0.0001	0.0031
ENSG00000168772	CXXC4	CXXC finger protein 4 [Source:HGNC Symbol;Acc:HGNC:24593]	protein coding	0.71	0.62	17.25	<0.0001	0.001
ENSG00000051128	HOMER3	homer scaffold protein 3 [Source:HGNC Symbol;Acc:HGNC:17514]	protein coding	-0.71	2.18	23.26	<0.0001	<0.0001
ENSG00000159713	TPPP3	tubulin polymerization promoting protein family member 3 [Source:HGNC Symbol;Acc:HGNC:24162]	protein coding	-0.71	0.93	18.52	<0.0001	0.0006
ENSG00000241351	IGKV3-11	immunoglobulin kappa variable 3-11 [Source:HGNC Symbol;Acc:HGNC:5815]	IG V gene	-0.71	4.53	12.03	0.0005	0.009
ENSG00000211892	IGHG4	immunoglobulin heavy constant gamma 4 (G4m marker) [Source:HGNC Symbol;Acc:HGNC:5528]	IG C gene	-0.71	3.86	18.92	<0.0001	0.0005
ENSG00000149418	ST14	suppression of tumorigenicity 14 [Source:HGNC Symbol;Acc:HGNC:11344]	protein coding	-0.71	4.89	25.56	<0.0001	<0.0001
ENSG00000100079	LGALS2	galectin 2 [Source:HGNC Symbol;Acc:HGNC:6562]	protein coding	-0.71	3.67	25.26	<0.0001	<0.0001
ENSG00000278196	IGLV2-8	immunoglobulin lambda variable 2-8 [Source:HGNC Symbol;Acc:HGNC:5895]	IG V gene	-0.71	2.98	12.41	0.0004	0.0078
ENSG00000235568	NFAM1	NFAT activating protein with ITAM motif 1 [Source:HGNC Symbol;Acc:HGNC:29872]	protein coding	-0.70	4.79	30.20	<0.0001	<0.0001
ENSG00000038427	VCAN	versican [Source:HGNC Symbol;Acc:HGNC:2464]	protein coding	-0.70	7.23	15.85	<0.0001	0.0018
ENSG00000198053	SIRPA	signal regulatory protein alpha [Source:HGNC Symbol;Acc:HGNC:9662]	protein coding	-0.70	4.60	31.82	<0.0001	<0.0001
ENSG00000175643	RMI2	RecQ mediated genome instability 2 [Source:HGNC Symbol;Acc:HGNC:28349]	protein coding	0.70	2.03	12.64	0.0004	0.0071
ENSG00000198734	F5	coagulation factor V [Source:HGNC Symbol;Acc:HGNC:3542]	protein coding	-0.70	1.89	9.48	0.0021	0.0255
ENSG00000136869	TLR4	toll like receptor 4 [Source:HGNC Symbol;Acc:HGNC:11850]	protein coding	-0.70	3.66	23.17	<0.0001	<0.0001
ENSG00000253364	COPDA1	chronic obstructive pulmonary disease associated lncRNA 1 [Source:HGNC Symbol;Acc:HGNC:53171]	lncRNA	-0.70	2.53	11.39	0.0007	0.0117

Table S25: Genes differentially expressed at Day 32 compared to pre-second vaccination (Study Arm 2 A/H7N9+AS03, D1, 29, NK cells). Genes are sorted by descending absolute log_2 fold change. Gene model summaries and annotations are based on Ensembl Version 99 (continued.)

Ensembl Gene ID	Ensembl Gene Name	Ensembl Gene Description	Gene Type	log_2 Fold Change (Day 32 vs. Pre-Second Vaccination)	Average log_2 CPM	Likelihood Ratio Test Statistic	P-Value	FDR Adjusted P-Value
ENSG00000018280	SLC11A1	solute carrier family 11 member 1 [Source:HGNC Symbol;Acc:HGNC:10907]	protein coding	-0.70	4.97	25.39	<0.0001	<0.0001
ENSG00000053918	KCNQ1	potassium voltage-gated channel subfamily Q member 1 [Source:HGNC Symbol;Acc:HGNC:6294]	protein coding	-0.70	4.06	38.02	<0.0001	<0.0001
ENSG00000277443	MARCKS	myristoylated alanine rich protein kinase C substrate [Source:HGNC Symbol;Acc:HGNC:6759]	protein coding	-0.70	4.72	34.57	<0.0001	<0.0001
ENSG00000269404	SPIB	Spi-B transcription factor [Source:HGNC Symbol;Acc:HGNC:11242]	protein coding	-0.69	4.86	15.12	0.0001	0.0024
ENSG00000187164	SHTN1	shootin 1 [Source:HGNC Symbol;Acc:HGNC:29319]	protein coding	-0.69	3.04	25.97	<0.0001	<0.0001
ENSG00000163625	WDFY3	WD repeat and FYVE domain containing 3 [Source:HGNC Symbol;Acc:HGNC:20751]	protein coding	-0.69	2.26	18.47	<0.0001	0.0006
ENSG00000186431	FCAR	Fc fragment of IgA receptor [Source:HGNC Symbol;Acc:HGNC:3608]	protein coding	-0.69	0.88	8.84	0.003	0.033
ENSG00000180061	TMEM150B	transmembrane protein 150B [Source:HGNC Symbol;Acc:HGNC:34415]	protein coding	-0.69	1.22	9.81	0.0017	0.0221
ENSG00000105967	TFEC	transcription factor EC [Source:HGNC Symbol;Acc:HGNC:11754]	protein coding	-0.69	3.44	35.06	<0.0001	<0.0001
ENSG00000171595	DNAI2	dynein axonemal intermediate chain 2 [Source:HGNC Symbol;Acc:HGNC:18744]	protein coding	0.69	0.70	15.79	<0.0001	0.0019
ENSG00000114346	ECT2	epithelial cell transforming 2 [Source:HGNC Symbol;Acc:HGNC:3155]	protein coding	0.69	2.39	40.47	<0.0001	<0.0001
ENSG00000102445	RUBCNL	rubicon like autophagy enhancer [Source:HGNC Symbol;Acc:HGNC:20420]	protein coding	-0.69	5.09	14.63	0.0001	0.0031
ENSG00000229417	NPM1P25	nucleophosmin 1 pseudogene 25 [Source:HGNC Symbol;Acc:HGNC:45204]	processed pseudogene	-0.69	0.76	9.48	0.0021	0.0254
ENSG00000134531	EMP1	epithelial membrane protein 1 [Source:HGNC Symbol;Acc:HGNC:3333]	protein coding	-0.69	1.44	14.36	0.0002	0.0034
ENSG00000160883	HK3	hexokinase 3 [Source:HGNC Symbol;Acc:HGNC:4925]	protein coding	-0.69	5.27	18.31	<0.0001	0.0006
ENSG00000225889		novel transcript, antisense to PELI1	lncRNA	-0.69	2.23	17.64	<0.0001	0.0008
ENSG00000257167	TMPO-AS1	TMPO antisense RNA 1 [Source:HGNC Symbol;Acc:HGNC:44158]	lncRNA	0.68	1.40	23.09	<0.0001	<0.0001
ENSG00000105369	CD79A	CD79a molecule [Source:HGNC Symbol;Acc:HGNC:1698]	protein coding	-0.68	7.90	12.17	0.0005	0.0086

Table S25: Genes differentially expressed at Day 32 compared to pre-second vaccination (Study Arm 2 A/H7N9+AS03, D1, 29, NK cells). Genes are sorted by descending absolute log_2 fold change. Gene model summaries and annotations are based on Ensembl Version 99 (continued.)

Ensembl Gene ID	Ensembl Gene Name	Ensembl Gene Description	Gene Type	log_2 Fold Change (Day 32 vs. Pre-Second Vaccination)	Average log_2 CPM	Likelihood Ratio Test Statistic	P-Value	FDR Adjusted P-Value
ENSG00000211664	IGLV2-18	immunoglobulin lambda variable 2-18 [Source:HGNC Symbol;Acc:HGNC:5889]	IG V gene	-0.68	0.81	8.08	0.0045	0.045
ENSG00000136205	TNS3	tensin 3 [Source:HGNC Symbol;Acc:HGNC:21616]	protein coding	-0.68	3.69	39.68	<0.0001	<0.0001
ENSG00000087253	LPCAT2	lysophosphatidylcholine acyltransferase 2 [Source:HGNC Symbol;Acc:HGNC:26032]	protein coding	-0.68	2.60	20.42	<0.0001	0.0002
ENSG00000101307	SIRPB1	signal regulatory protein beta 1 [Source:HGNC Symbol;Acc:HGNC:15928]	protein coding	-0.68	3.00	23.54	<0.0001	<0.0001
ENSG00000101447	FAM83D	family with sequence similarity 83 member D [Source:HGNC Symbol;Acc:HGNC:16122]	protein coding	0.68	0.41	13.33	0.0003	0.0053
ENSG00000124772	CPNE5	copine 5 [Source:HGNC Symbol;Acc:HGNC:2318]	protein coding	-0.68	5.40	17.07	<0.0001	0.0011
ENSG00000121316	PLBD1	phospholipase B domain containing 1 [Source:HGNC Symbol;Acc:HGNC:26215]	protein coding	-0.68	4.70	23.86	<0.0001	<0.0001
ENSG00000164330	EBF1	EBF transcription factor 1 [Source:HGNC Symbol;Acc:HGNC:3126]	protein coding	-0.68	2.98	10.03	0.0015	0.0203
ENSG00000196209	SIRPB2	signal regulatory protein beta 2 [Source:HGNC Symbol;Acc:HGNC:16247]	protein coding	-0.68	3.44	20.94	<0.0001	0.0002
ENSG00000116962	NID1	nidogen 1 [Source:HGNC Symbol;Acc:HGNC:7821]	protein coding	-0.68	2.47	18.30	<0.0001	0.0006
ENSG00000167641	PPP1R14A	protein phosphatase 1 regulatory inhibitor subunit 14A [Source:HGNC Symbol;Acc:HGNC:14871]	protein coding	-0.68	2.54	13.44	0.0002	0.005
ENSG00000240505	TNFRSF13B	TNF receptor superfamily member 13B [Source:HGNC Symbol;Acc:HGNC:18153]	protein coding	-0.68	3.95	15.15	<0.0001	0.0024
ENSG00000135363	LMO2	LIM domain only 2 [Source:HGNC Symbol;Acc:HGNC:6642]	protein coding	-0.68	3.95	28.26	<0.0001	<0.0001
ENSG00000144645	OSBPL10	oxysterol binding protein like 10 [Source:HGNC Symbol;Acc:HGNC:16395]	protein coding	-0.68	4.64	14.45	0.0001	0.0033
ENSG00000092758	COL9A3	collagen type IX alpha 3 chain [Source:HGNC Symbol;Acc:HGNC:2219]	protein coding	-0.68	1.63	27.57	<0.0001	<0.0001
ENSG00000163975	MELTF	melanotransferrin [Source:HGNC Symbol;Acc:HGNC:7037]	protein coding	0.67	2.18	29.31	<0.0001	<0.0001
ENSG00000073111	MCM2	minichromosome maintenance complex component 2 [Source:HGNC Symbol;Acc:HGNC:6944]	protein coding	0.67	4.63	24.84	<0.0001	<0.0001
ENSG00000204577	LILRB3	leukocyte immunoglobulin like receptor B3 [Source:HGNC Symbol;Acc:HGNC:6607]	protein coding	-0.67	4.09	23.25	<0.0001	<0.0001

Table S25: Genes differentially expressed at Day 32 compared to pre-second vaccination (Study Arm 2 A/H7N9+AS03, D1, 29, NK cells). Genes are sorted by descending absolute log_2 fold change. Gene model summaries and annotations are based on Ensembl Version 99 (continued.)

Ensembl Gene ID	Ensembl Gene Name	Ensembl Gene Description	Gene Type	log_2 Fold Change (Day 32 vs. Pre-Second Vaccination)	Average log_2 CPM	Likelihood Ratio Test Statistic	P-Value	FDR Adjusted P-Value
ENSG00000113749	HRH2	histamine receptor H2 [Source:HGNC Symbol;Acc:HGNC:5183]	protein coding	-0.67	3.27	30.31	<0.0001	<0.0001
ENSG00000132016	C19orf57	chromosome 19 open reading frame 57 [Source:HGNC Symbol;Acc:HGNC:28153]	protein coding	0.67	2.07	32.01	<0.0001	<0.0001
ENSG00000211959	IGHV4-39	immunoglobulin heavy variable 4-39 [Source:HGNC Symbol;Acc:HGNC:5651]	IG V gene	-0.67	2.79	10.87	0.001	0.0145
ENSG00000184060	ADAP2	ArfGAP with dual PH domains 2 [Source:HGNC Symbol;Acc:HGNC:16487]	protein coding	-0.67	2.21	25.46	<0.0001	<0.0001
ENSG00000132185	FCRLA	Fc receptor like A [Source:HGNC Symbol;Acc:HGNC:18504]	protein coding	-0.67	5.32	12.76	0.0004	0.0068
ENSG00000109436	TBC1D9	TBC1 domain family member 9 [Source:HGNC Symbol;Acc:HGNC:21710]	protein coding	-0.67	5.22	32.45	<0.0001	<0.0001
ENSG00000090382	LYZ	lysozyme [Source:HGNC Symbol;Acc:HGNC:6740]	protein coding	-0.67	9.32	24.86	<0.0001	<0.0001
ENSG00000244482	LILRA6	leukocyte immunoglobulin like receptor A6 [Source:HGNC Symbol;Acc:HGNC:15495]	protein coding	-0.67	2.79	16.97	<0.0001	0.0011
ENSG00000058866	DGKG	diacylglycerol kinase gamma [Source:HGNC Symbol;Acc:HGNC:2853]	protein coding	-0.66	0.94	25.36	<0.0001	<0.0001
ENSG00000172243	CLEC7A	C-type lectin domain containing 7A [Source:HGNC Symbol;Acc:HGNC:14558]	protein coding	-0.66	4.46	13.47	0.0002	0.005
ENSG00000166928	MS4A14	membrane spanning 4-domains A14 [Source:HGNC Symbol;Acc:HGNC:30706]	protein coding	-0.66	1.44	18.36	<0.0001	0.0006
ENSG00000123405	NFE2	nuclear factor, erythroid 2 [Source:HGNC Symbol;Acc:HGNC:7780]	protein coding	-0.66	3.20	24.35	<0.0001	<0.0001
ENSG00000160298	C21orf58	chromosome 21 open reading frame 58 [Source:HGNC Symbol;Acc:HGNC:1300]	protein coding	0.66	3.50	37.92	<0.0001	<0.0001
ENSG00000204136	GGTA1P	glycoprotein alpha-galactosyltransferase 1, pseudogene [Source:HGNC Symbol;Acc:HGNC:4253]	transcribed unitary pseudogene	-0.66	0.65	18.14	<0.0001	0.0007
ENSG00000066336	SPI1	Spi-1 proto-oncogene [Source:HGNC Symbol;Acc:HGNC:11241]	protein coding	-0.66	6.39	42.31	<0.0001	<0.0001

Table S25: Genes differentially expressed at Day 32 compared to pre-second vaccination (Study Arm 2 A/H7N9+AS03, D1, 29, NK cells). Genes are sorted by descending absolute log_2 fold change. Gene model summaries and annotations are based on Ensembl Version 99 (continued.)

Ensembl Gene ID	Ensembl Gene Name	Ensembl Gene Description	Gene Type	log_2 Fold Change (Day 32 vs. Pre-Second Vaccination)	Average log_2 CPM	Likelihood Ratio Test Statistic	P-Value	FDR Adjusted P-Value
ENSG00000131401	NAPSB	napsin B aspartic peptidase, pseudogene [Source:HGNC Symbol;Acc:HGNC:13396]	transcribed unprocessed pseudogene	-0.66	6.15	29.02	<0.0001	<0.0001
ENSG00000110987	BCL7A	BAF chromatin remodeling complex subunit BCL7A [Source:HGNC Symbol;Acc:HGNC:1004]	protein coding	-0.66	4.70	11.44	0.0007	0.0115
ENSG00000123095	BHLHE41	basic helix-loop-helix family member e41 [Source:HGNC Symbol;Acc:HGNC:16617]	protein coding	-0.66	1.92	15.68	<0.0001	0.0019
ENSG00000124491	F13A1	coagulation factor XIII A chain [Source:HGNC Symbol;Acc:HGNC:3531]	protein coding	-0.66	4.45	21.40	<0.0001	0.0002
ENSG00000183019	MCEMP1	mast cell expressed membrane protein 1 [Source:HGNC Symbol;Acc:HGNC:27291]	protein coding	-0.66	1.38	12.27	0.0005	0.0083
ENSG00000129993	CBFA2T3	CBFA2/RUNX1 partner transcriptional co-repressor 3 [Source:HGNC Symbol;Acc:HGNC:1537]	protein coding	-0.66	4.71	26.78	<0.0001	<0.0001
ENSG00000243238	IGKV2-30	immunoglobulin kappa variable 2-30 [Source:HGNC Symbol;Acc:HGNC:5785]	IG V gene	-0.66	1.70	11.72	0.0006	0.0103
ENSG00000169031	COL4A3	collagen type IV alpha 3 chain [Source:HGNC Symbol;Acc:HGNC:2204]	protein coding	-0.66	3.51	12.89	0.0003	0.0064
ENSG00000160683	CXCR5	C-X-C motif chemokine receptor 5 [Source:HGNC Symbol;Acc:HGNC:1060]	protein coding	-0.65	1.93	11.25	0.0008	0.0124
ENSG00000171992	SYNPO	synaptopodin [Source:HGNC Symbol;Acc:HGNC:30672]	protein coding	-0.65	4.17	9.98	0.0016	0.0207
ENSG00000268849	SIGLEC22P	sialic acid binding Ig like lectin 22, pseudogene [Source:HGNC Symbol;Acc:HGNC:15611]	transcribed unprocessed pseudogene	-0.65	0.55	10.06	0.0015	0.02
ENSG00000131042	LILRB2	leukocyte immunoglobulin like receptor B2 [Source:HGNC Symbol;Acc:HGNC:6606]	protein coding	-0.65	4.94	26.65	<0.0001	<0.0001
ENSG00000012779	ALOX5	arachidonate 5-lipoxygenase [Source:HGNC Symbol;Acc:HGNC:435]	protein coding	-0.65	6.46	25.03	<0.0001	<0.0001
ENSG00000128268	MGAT3	beta-1,4-mannosyl-glycoprotein 4-beta-N-acetylglucosaminyltransferase [Source:HGNC Symbol;Acc:HGNC:7046]	protein coding	-0.65	2.66	20.08	<0.0001	0.0003

Table S25: Genes differentially expressed at Day 32 compared to pre-second vaccination (Study Arm 2 A/H7N9+AS03, D1, 29, NK cells). Genes are sorted by descending absolute log_2 fold change. Gene model summaries and annotations are based on Ensembl Version 99 (continued.)

Ensembl Gene ID	Ensembl Gene Name	Ensembl Gene Description	Gene Type	log_2 Fold Change (Day 32 vs. Pre-Second Vaccination)	Average log_2 CPM	Likelihood Ratio Test Statistic	P-Value	FDR Adjusted P-Value
ENSG00000204472	AIF1	allograft inflammatory factor 1 [Source:HGNC Symbol;Acc:HGNC:352]	protein coding	-0.65	4.44	23.63	<0.0001	<0.0001
ENSG00000139438	FAM222A	family with sequence similarity 222 member A [Source:HGNC Symbol;Acc:HGNC:25915]	protein coding	0.65	1.40	30.64	<0.0001	<0.0001
ENSG00000103313	MEFV	MEFV innate immuity regulator, pyrin [Source:HGNC Symbol;Acc:HGNC:6998]	protein coding	-0.65	3.85	23.09	<0.0001	<0.0001
ENSG00000216863	LY86-AS1	LY86 antisense RNA 1 [Source:HGNC Symbol;Acc:HGNC:26593]	lncRNA	-0.65	1.31	11.69	0.0006	0.0104
ENSG00000198848	CES1	carboxylesterase 1 [Source:HGNC Symbol;Acc:HGNC:1863]	protein coding	-0.65	3.17	22.00	<0.0001	0.0001
ENSG00000158481	CD1C	CD1c molecule [Source:HGNC Symbol;Acc:HGNC:1636]	protein coding	-0.65	4.75	30.04	<0.0001	<0.0001
ENSG00000268889		novel transcript	lncRNA	0.65	0.80	10.96	0.0009	0.0139
ENSG00000116701	NCF2	neutrophil cytosolic factor 2 [Source:HGNC Symbol;Acc:HGNC:7661]	protein coding	-0.64	5.85	44.64	<0.0001	<0.0001
ENSG00000179639	FCER1A	Fc fragment of IgE receptor Ia [Source:HGNC Symbol;Acc:HGNC:3609]	protein coding	-0.64	4.70	15.10	0.0001	0.0025
ENSG00000147642	SYBU	syntabulin [Source:HGNC Symbol;Acc:HGNC:26011]	protein coding	-0.64	0.59	9.53	0.002	0.0249
ENSG00000109881	CCDC34	coiled-coil domain containing 34 [Source:HGNC Symbol;Acc:HGNC:25079]	protein coding	0.64	1.84	20.42	<0.0001	0.0002
ENSG00000275418		novel transcript	lncRNA	-0.64	0.77	7.79	0.0052	0.05
ENSG00000140090	SLC24A4	solute carrier family 24 member 4 [Source:HGNC Symbol;Acc:HGNC:10978]	protein coding	-0.64	3.30	15.50	<0.0001	0.0021
ENSG00000123219	CENPK	centromere protein K [Source:HGNC Symbol;Acc:HGNC:29479]	protein coding	0.64	3.75	26.71	<0.0001	<0.0001
ENSG00000151948	GLT1D1	glycosyltransferase 1 domain containing 1 [Source:HGNC Symbol;Acc:HGNC:26483]	protein coding	-0.64	2.77	28.57	<0.0001	<0.0001
ENSG00000127589	TUBBP1	tubulin beta pseudogene 1 [Source:HGNC Symbol;Acc:HGNC:12414]	transcribed processed pseudogene	0.64	1.19	15.01	0.0001	0.0026
ENSG00000155465	SLC7A7	solute carrier family 7 member 7 [Source:HGNC Symbol;Acc:HGNC:11065]	protein coding	-0.64	4.92	40.95	<0.0001	<0.0001
ENSG00000187922	LCN10	lipocalin 10 [Source:HGNC Symbol;Acc:HGNC:20892]	protein coding	-0.64	1.22	11.48	0.0007	0.0113

Table S25: Genes differentially expressed at Day 32 compared to pre-second vaccination (Study Arm 2 A/H7N9+AS03, D1, 29, NK cells). Genes are sorted by descending absolute log_2 fold change. Gene model summaries and annotations are based on Ensembl Version 99 (continued.)

Ensembl Gene ID	Ensembl Gene Name	Ensembl Gene Description	Gene Type	log_2 Fold Change (Day 32 vs. Pre-Second Vaccination)	Average log_2 CPM	Likelihood Ratio Test Statistic	P-Value	FDR Adjusted P-Value
ENSG00000211895	IGHA1	immunoglobulin heavy constant alpha 1 [Source:HGNC Symbol;Acc:HGNC:5478]	IG C gene	-0.64	8.92	10.01	0.0016	0.0203
ENSG00000135116	HRK	harakiri, BCL2 interacting protein [Source:HGNC Symbol;Acc:HGNC:5185]	protein coding	-0.64	1.26	12.40	0.0004	0.0079
ENSG00000101336	HCK	HCK proto-oncogene, Src family tyrosine kinase [Source:HGNC Symbol;Acc:HGNC:4840]	protein coding	-0.64	5.39	67.78	<0.0001	<0.0001
ENSG00000250510	GPR162	G protein-coupled receptor 162 [Source:HGNC Symbol;Acc:HGNC:16693]	protein coding	-0.63	2.14	12.75	0.0004	0.0068
ENSG00000166927		membrane spanning 4-domains A7 [Source:NCBI gene;Acc:58475]	protein coding	-0.63	3.48	33.47	<0.0001	<0.0001
ENSG00000106462	EZH2	enhancer of zeste 2 polycomb repressive complex 2 subunit [Source:HGNC Symbol;Acc:HGNC:3527]	protein coding	0.63	4.50	33.27	<0.0001	<0.0001
ENSG00000211592	IGKC	immunoglobulin kappa constant [Source:HGNC Symbol;Acc:HGNC:5716]	IG C gene	-0.63	9.01	15.29	<0.0001	0.0023
ENSG00000224397	SMIM25	small integral membrane protein 25 [Source:HGNC Symbol;Acc:HGNC:50328]	lncRNA	-0.63	1.64	12.58	0.0004	0.0073
ENSG00000125531	FNDC11	fibronectin type III domain containing 11 [Source:HGNC Symbol;Acc:HGNC:28764]	protein coding	-0.63	1.61	16.46	<0.0001	0.0014
ENSG00000120708	TGFBI	transforming growth factor beta induced [Source:HGNC Symbol;Acc:HGNC:11771]	protein coding	-0.63	5.92	29.40	<0.0001	<0.0001
ENSG00000284825		novel transcript	lncRNA	-0.63	0.43	12.09	0.0005	0.0089
ENSG00000248727	LINC01948	long intergenic non-protein coding RNA 1948 [Source:HGNC Symbol;Acc:HGNC:52772]	lncRNA	-0.63	0.68	13.82	0.0002	0.0043
ENSG00000176533	GNG7	G protein subunit gamma 7 [Source:HGNC Symbol;Acc:HGNC:4410]	protein coding	-0.63	5.47	12.39	0.0004	0.0079
ENSG00000211976	IGHV3-73	immunoglobulin heavy variable 3-73 [Source:HGNC Symbol;Acc:HGNC:5623]	IG V gene	-0.63	1.33	8.30	0.004	0.0412
ENSG00000196735	HLA-DQA1	major histocompatibility complex, class II, DQ alpha 1 [Source:HGNC Symbol;Acc:HGNC:4942]	protein coding	-0.63	6.44	24.67	<0.0001	<0.0001
ENSG00000188290	HES4	hes family bHLH transcription factor 4 [Source:HGNC Symbol;Acc:HGNC:24149]	protein coding	0.63	1.74	12.58	0.0004	0.0073
ENSG00000064225	ST3GAL6	ST3 beta-galactoside alpha-2,3-sialyltransferase 6 [Source:HGNC Symbol;Acc:HGNC:18080]	protein coding	-0.63	1.52	13.43	0.0002	0.0051
ENSG00000182578	CSF1R	colony stimulating factor 1 receptor [Source:HGNC Symbol;Acc:HGNC:2433]	protein coding	-0.63	5.12	26.62	<0.0001	<0.0001

Table S25: Genes differentially expressed at Day 32 compared to pre-second vaccination (Study Arm 2 A/H7N9+AS03, D1, 29, NK cells). Genes are sorted by descending absolute log_2 fold change. Gene model summaries and annotations are based on Ensembl Version 99 (continued.)

Ensembl Gene ID	Ensembl Gene Name	Ensembl Gene Description	Gene Type	log_2 Fold Change (Day 32 vs. Pre-Second Vaccination)	Average log_2 CPM	Likelihood Ratio Test Statistic	P-Value	FDR Adjusted P-Value
ENSG00000198752	CDC42BPB	CDC42 binding protein kinase beta [Source:HGNC Symbol;Acc:HGNC:1738]	protein coding	-0.62	3.36	25.74	<0.0001	<0.0001
ENSG00000106066	CPVL	carboxypeptidase vitellogenic like [Source:HGNC Symbol;Acc:HGNC:14399]	protein coding	-0.62	5.32	25.60	<0.0001	<0.0001
ENSG00000165959	CLMN	calmin [Source:HGNC Symbol;Acc:HGNC:19972]	protein coding	-0.62	3.67	15.11	0.0001	0.0025
ENSG00000235641	LINC00484	long intergenic non-protein coding RNA 484 [Source:HGNC Symbol;Acc:HGNC:27862]	lncRNA	0.62	0.44	9.00	0.0027	0.0311
ENSG00000166402	TUB	TUB bipartite transcription factor [Source:HGNC Symbol;Acc:HGNC:12406]	protein coding	-0.62	1.12	12.67	0.0004	0.0071
ENSG00000163520	FBLN2	fibulin 2 [Source:HGNC Symbol;Acc:HGNC:3601]	protein coding	-0.62	3.05	27.49	<0.0001	<0.0001
ENSG00000169116	PARM1	prostate androgen-regulated mucin-like protein 1 [Source:HGNC Symbol;Acc:HGNC:24536]	protein coding	-0.62	2.77	20.35	<0.0001	0.0003
ENSG00000184293	CLECL1	C-type lectin like 1 [Source:HGNC Symbol;Acc:HGNC:24462]	protein coding	-0.62	2.42	20.77	<0.0001	0.0002
ENSG00000268144	NIFKP6	nucleolar protein interacting with the FHA domain of MKI67 pseudogene 6 [Source:HGNC Symbol;Acc:HGNC:44954]	processed pseudogene	0.62	0.64	12.42	0.0004	0.0078
ENSG00000196562	SULF2	sulfatase 2 [Source:HGNC Symbol;Acc:HGNC:20392]	protein coding	-0.62	5.29	39.36	<0.0001	<0.0001
ENSG00000271605	MILR1	mast cell immunoglobulin like receptor 1 [Source:HGNC Symbol;Acc:HGNC:27570]	protein coding	-0.62	2.28	21.76	<0.0001	0.0001
ENSG00000110446	SLC15A3	solute carrier family 15 member 3 [Source:HGNC Symbol;Acc:HGNC:18068]	protein coding	-0.62	4.74	40.11	<0.0001	<0.0001
ENSG00000119866	BCL11A	BAF chromatin remodeling complex subunit BCL11A [Source:HGNC Symbol;Acc:HGNC:13221]	protein coding	-0.62	5.22	13.90	0.0002	0.0042
ENSG00000010327	STAB1	stabilin 1 [Source:HGNC Symbol;Acc:HGNC:18628]	protein coding	-0.62	5.64	13.77	0.0002	0.0044
ENSG00000149573	MPZL2	myelin protein zero like 2 [Source:HGNC Symbol;Acc:HGNC:3496]	protein coding	-0.62	0.93	11.43	0.0007	0.0115
ENSG00000104783	KCNN4	potassium calcium-activated channel subfamily N member 4 [Source:HGNC Symbol;Acc:HGNC:6293]	protein coding	-0.61	4.86	21.47	<0.0001	0.0002
ENSG00000169435	RASSF6	Ras association domain family member 6 [Source:HGNC Symbol;Acc:HGNC:20796]	protein coding	-0.61	0.56	8.37	0.0038	0.0402

Table S25: Genes differentially expressed at Day 32 compared to pre-second vaccination (Study Arm 2 A/H7N9+AS03, D1, 29, NK cells). Genes are sorted by descending absolute log_2 fold change. Gene model summaries and annotations are based on Ensembl Version 99 (continued.)

Ensembl Gene ID	Ensembl Gene Name	Ensembl Gene Description	Gene Type	log_2 Fold Change (Day 32 vs. Pre-Second Vaccination)	Average log_2 CPM	Likelihood Ratio Test Statistic	P-Value	FDR Adjusted P-Value
ENSG00000149451	ADAM33	ADAM metallopeptidase domain 33 [Source:HGNC Symbol;Acc:HGNC:15478]	protein coding	-0.61	1.08	10.74	0.001	0.0153
ENSG00000285417		novel pseudogene	transcribed unitary pseudogene	-0.61	2.04	10.80	0.001	0.0149
ENSG00000197629	MPEG1	macrophage expressed 1 [Source:HGNC Symbol;Acc:HGNC:29619]	protein coding	-0.61	7.16	61.88	<0.0001	<0.0001
ENSG00000123096	SSPN	sarcospan [Source:HGNC Symbol;Acc:HGNC:11322]	protein coding	-0.61	1.40	11.82	0.0006	0.0098
ENSG00000183779	ZNF703	zinc finger protein 703 [Source:HGNC Symbol;Acc:HGNC:25883]	protein coding	-0.61	1.17	15.04	0.0001	0.0025
ENSG00000196092	PAX5	paired box 5 [Source:HGNC Symbol;Acc:HGNC:8619]	protein coding	-0.61	6.77	9.27	0.0023	0.0278
ENSG00000215267	AKR1C7P	aldo-keto reductase family 1 member C7, pseudogene [Source:HGNC Symbol;Acc:HGNC:44681]	transcribed unprocessed pseudogene	0.61	0.87	16.34	<0.0001	0.0015
ENSG00000174123	TLR10	toll like receptor 10 [Source:HGNC Symbol;Acc:HGNC:15634]	protein coding	-0.61	4.79	15.92	<0.0001	0.0017
ENSG00000110777	POU2AF1	POU class 2 homeobox associating factor 1 [Source:HGNC Symbol;Acc:HGNC:9211]	protein coding	-0.61	5.99	14.16	0.0002	0.0037
ENSG00000183763	TRAIP	TRAF interacting protein [Source:HGNC Symbol;Acc:HGNC:30764]	protein coding	0.61	1.53	21.31	<0.0001	0.0002
ENSG00000133119	RFC3	replication factor C subunit 3 [Source:HGNC Symbol;Acc:HGNC:9971]	protein coding	0.61	2.90	35.27	<0.0001	<0.0001
ENSG00000235621	LINC00494	long intergenic non-protein coding RNA 494 [Source:HGNC Symbol;Acc:HGNC:27657]	lncRNA	-0.61	3.36	8.24	0.0041	0.0424
ENSG00000100600	LGMN	legumain [Source:HGNC Symbol;Acc:HGNC:9472]	protein coding	-0.61	2.52	19.87	<0.0001	0.0003
ENSG00000239998	LILRA2	leukocyte immunoglobulin like receptor A2 [Source:HGNC Symbol;Acc:HGNC:6603]	protein coding	-0.61	4.01	35.12	<0.0001	<0.0001
ENSG00000178695	KCTD12	potassium channel tetramerization domain containing 12 [Source:HGNC Symbol;Acc:HGNC:14678]	protein coding	-0.61	5.83	32.01	<0.0001	<0.0001

Table S25: Genes differentially expressed at Day 32 compared to pre-second vaccination (Study Arm 2 A/H7N9+AS03, D1, 29, NK cells). Genes are sorted by descending absolute log_2 fold change. Gene model summaries and annotations are based on Ensembl Version 99 (continued.)

Ensembl Gene ID	Ensembl Gene Name	Ensembl Gene Description	Gene Type	log_2 Fold Change (Day 32 vs. Pre-Second Vaccination)	Average log_2 CPM	Likelihood Ratio Test Statistic	P-Value	FDR Adjusted P-Value
ENSG00000205221	VIT	vitrin [Source:HGNC Symbol;Acc:HGNC:12697]	protein coding	0.61	1.30	11.17	0.0008	0.0128
ENSG00000101439	CST3	cystatin C [Source:HGNC Symbol;Acc:HGNC:2475]	protein coding	-0.60	7.47	29.74	<0.0001	<0.0001
ENSG00000171049	FPR2	formyl peptide receptor 2 [Source:HGNC Symbol;Acc:HGNC:3827]	protein coding	-0.60	1.04	7.96	0.0048	0.0469
ENSG00000198554	WDHD1	WD repeat and HMG-box DNA binding protein 1 [Source:HGNC Symbol;Acc:HGNC:23170]	protein coding	0.60	2.78	20.71	<0.0001	0.0002
ENSG00000100368	CSF2RB	colony stimulating factor 2 receptor beta common subunit [Source:HGNC Symbol;Acc:HGNC:2436]	protein coding	-0.60	4.63	44.47	<0.0001	<0.0001
ENSG00000224650	IGHV3-74	immunoglobulin heavy variable 3-74 [Source:HGNC Symbol;Acc:HGNC:5624]	IG V gene	-0.60	2.38	9.63	0.0019	0.0239
ENSG00000261996		novel transcript	lncRNA	-0.60	0.58	11.13	0.0009	0.0131
ENSG00000255197		novel transcript	lncRNA	-0.60	3.93	26.26	<0.0001	<0.0001
ENSG00000182022	CHST15	carbohydrate sulfotransferase 15 [Source:HGNC Symbol;Acc:HGNC:18137]	protein coding	-0.60	3.87	24.47	<0.0001	<0.0001
ENSG00000180767	CHST13	carbohydrate sulfotransferase 13 [Source:HGNC Symbol;Acc:HGNC:21755]	protein coding	-0.60	0.95	10.17	0.0014	0.0192
ENSG00000156738	MS4A1	membrane spanning 4-domains A1 [Source:HGNC Symbol;Acc:HGNC:7315]	protein coding	-0.60	8.35	10.09	0.0015	0.0197
ENSG00000211943	IGHV3-15	immunoglobulin heavy variable 3-15 [Source:HGNC Symbol;Acc:HGNC:5582]	IG V gene	-0.60	3.02	9.67	0.0019	0.0235
ENSG00000271013	LRRC37A9P	leucine rich repeat containing 37 member A9, pseudogene [Source:HGNC Symbol;Acc:HGNC:43813]	processed pseudogene	0.60	0.33	8.37	0.0038	0.0402
ENSG00000196230	TUBB	tubulin beta class I [Source:HGNC Symbol;Acc:HGNC:20778]	protein coding	0.60	7.79	23.57	<0.0001	<0.0001
ENSG00000146592	CREB5	cAMP responsive element binding protein 5 [Source:HGNC Symbol;Acc:HGNC:16844]	protein coding	-0.60	2.42	10.24	0.0014	0.0186
ENSG00000258120	KRT128P	keratin 128 pseudogene [Source:HGNC Symbol;Acc:HGNC:48882]	unprocessed pseudogene	0.60	1.00	8.26	0.0041	0.042
ENSG00000159128	IFNGR2	interferon gamma receptor 2 [Source:HGNC Symbol;Acc:HGNC:5440]	protein coding	-0.60	4.59	24.41	<0.0001	<0.0001
ENSG00000196576	PLXNB2	plexin B2 [Source:HGNC Symbol;Acc:HGNC:9104]	protein coding	-0.60	6.04	34.08	<0.0001	<0.0001

Table S25: Genes differentially expressed at Day 32 compared to pre-second vaccination (Study Arm 2 A/H7N9+AS03, D1, 29, NK cells). Genes are sorted by descending absolute log_2 fold change. Gene model summaries and annotations are based on Ensembl Version 99 (continued.)

Ensembl Gene ID	Ensembl Gene Name	Ensembl Gene Description	Gene Type	log_2 Fold Change (Day 32 vs. Pre-Second Vaccination)	Average log_2 CPM	Likelihood Ratio Test Statistic	P-Value	FDR Adjusted P-Value
ENSG00000211949	IGHV3-23	immunoglobulin heavy variable 3-23 [Source:HGNC Symbol;Acc:HGNC:5588]	IG V gene	-0.60	4.52	14.76	0.0001	0.0029
ENSG00000175489	LRRC25	leucine rich repeat containing 25 [Source:HGNC Symbol;Acc:HGNC:29806]	protein coding	-0.60	4.61	37.10	<0.0001	<0.0001
ENSG00000112299	VNN1	vanin 1 [Source:HGNC Symbol;Acc:HGNC:12705]	protein coding	-0.60	0.69	8.07	0.0045	0.0452
ENSG00000272398	CD24	CD24 molecule [Source:HGNC Symbol;Acc:HGNC:1645]	protein coding	-0.60	4.67	11.66	0.0006	0.0105
ENSG00000125319	HROB	homologous recombination factor with OB-fold [Source:HGNC Symbol;Acc:HGNC:28460]	protein coding	0.60	0.47	10.69	0.0011	0.0155
ENSG00000143869	GDF7	growth differentiation factor 7 [Source:HGNC Symbol;Acc:HGNC:4222]	protein coding	-0.59	3.17	11.38	0.0007	0.0118
ENSG00000072163	LIMS2	LIM zinc finger domain containing 2 [Source:HGNC Symbol;Acc:HGNC:16084]	protein coding	-0.59	1.52	10.41	0.0013	0.0174
ENSG00000248996		novel transcript	lncRNA	-0.59	0.57	12.30	0.0005	0.0082
ENSG00000166428	PLD4	phospholipase D family member 4 [Source:HGNC Symbol;Acc:HGNC:23792]	protein coding	-0.59	5.13	18.53	<0.0001	0.0006
ENSG00000167549	CORO6	coronin 6 [Source:HGNC Symbol;Acc:HGNC:21356]	protein coding	0.59	2.64	25.37	<0.0001	<0.0001
ENSG00000163507	CIP2A	cell proliferation regulating inhibitor of protein phosphatase 2A [Source:HGNC Symbol;Acc:HGNC:29302]	protein coding	0.59	2.13	28.51	<0.0001	<0.0001
ENSG00000146094	DOK3	docking protein 3 [Source:HGNC Symbol;Acc:HGNC:24583]	protein coding	-0.59	4.96	35.56	<0.0001	<0.0001
ENSG00000224373	IGHV4-59	immunoglobulin heavy variable 4-59 [Source:HGNC Symbol;Acc:HGNC:5654]	IG V gene	-0.59	2.61	11.03	0.0009	0.0135
ENSG00000104267	CA2	carbonic anhydrase 2 [Source:HGNC Symbol;Acc:HGNC:1373]	protein coding	-0.59	1.16	12.39	0.0004	0.0079
ENSG00000138678	GPAT3	glycerol-3-phosphate acyltransferase 3 [Source:HGNC Symbol;Acc:HGNC:28157]	protein coding	-0.59	2.81	24.04	<0.0001	<0.0001
ENSG00000183307	TMEM121B	transmembrane protein 121B [Source:HGNC Symbol;Acc:HGNC:1844]	protein coding	-0.59	0.59	11.62	0.0007	0.0107
ENSG00000211594	IGKJ4	immunoglobulin kappa joining 4 [Source:HGNC Symbol;Acc:HGNC:5722]	IG J gene	-0.59	0.82	11.03	0.0009	0.0136
ENSG00000112799	LY86	lymphocyte antigen 86 [Source:HGNC Symbol;Acc:HGNC:16837]	protein coding	-0.59	4.35	20.32	<0.0001	0.0003
ENSG00000261222		novel transcript	lncRNA	-0.59	0.92	8.00	0.0047	0.0462

Table S25: Genes differentially expressed at Day 32 compared to pre-second vaccination (Study Arm 2 A/H7N9+AS03, D1, 29, NK cells). Genes are sorted by descending absolute log_2 fold change. Gene model summaries and annotations are based on Ensembl Version 99 (continued.)

Ensembl Gene ID	Ensembl Gene Name	Ensembl Gene Description	Gene Type	log_2 Fold Change (Day 32 vs. Pre-Second Vaccination)	Average log_2 CPM	Likelihood Ratio Test Statistic	P-Value	FDR Adjusted P-Value
ENSG00000119457	SLC46A2	solute carrier family 46 member 2 [Source:HGNC Symbol;Acc:HGNC:16055]	protein coding	-0.59	1.14	11.74	0.0006	0.0102
ENSG00000168913	ENHO	energy homeostasis associated [Source:HGNC Symbol;Acc:HGNC:24838]	protein coding	-0.59	2.00	14.62	0.0001	0.0031
ENSG00000135862	LAMC1	laminin subunit gamma 1 [Source:HGNC Symbol;Acc:HGNC:6492]	protein coding	-0.59	2.94	9.08	0.0026	0.0301
ENSG00000142552	RCN3	reticulocalbin 3 [Source:HGNC Symbol;Acc:HGNC:21145]	protein coding	-0.59	2.21	15.09	0.0001	0.0025
ENSG00000216490	IFI30	IFI30 lysosomal thiol reductase [Source:HGNC Symbol;Acc:HGNC:5398]	protein coding	-0.59	1.21	12.48	0.0004	0.0076
ENSG00000111275	ALDH2	aldehyde dehydrogenase 2 family member [Source:HGNC Symbol;Acc:HGNC:404]	protein coding	-0.59	4.61	33.81	<0.0001	<0.0001

Table S26: Genes differentially expressed at Day 36 compared to pre-second vaccination (Study Arm 2 A/H7N9+AS03, D1, 29, NK cells). Genes are sorted by descending absolute log_2 fold change. Gene model summaries and annotations are based on Ensembl Version 99.

Ensembl Gene ID	Ensembl Gene Name	Ensembl Gene Description	Gene Type	log_2 Fold Change (Day 36 vs. Pre-Second Vaccination)	Average log_2 CPM	Likelihood Ratio Test Statistic	P-Value	FDR Adjusted P-Value
ENSG00000004799	PDK4	pyruvate dehydrogenase kinase 4 [Source:HGNC Symbol;Acc:HGNC:8812]	protein coding	-1.19	2.12	31.72	<0.0001	<0.0001
ENSG00000211896	IGHG1	immunoglobulin heavy constant gamma 1 (G1m marker) [Source:HGNC Symbol;Acc:HGNC:5525]	IG C gene	0.90	7.60	52.17	<0.0001	<0.0001
ENSG00000214787	MS4A4E	membrane spanning 4-domains A4E [Source:HGNC Symbol;Acc:HGNC:14284]	protein coding	-0.88	1.91	49.29	<0.0001	<0.0001
ENSG00000100095	SEZ6L	seizure related 6 homolog like [Source:HGNC Symbol;Acc:HGNC:10763]	protein coding	-0.88	1.16	32.48	<0.0001	<0.0001
ENSG00000169385	RNASE2	ribonuclease A family member 2 [Source:HGNC Symbol;Acc:HGNC:10045]	protein coding	-0.84	1.69	25.67	<0.0001	<0.0001
ENSG00000250138		POM121 membrane glycoprotein-like 1 (POM121L1) pseudogene	unprocessed pseudogene	-0.84	1.50	32.95	<0.0001	<0.0001
ENSG00000145721	LIX1	limb and CNS expressed 1 [Source:HGNC Symbol;Acc:HGNC:18581]	protein coding	-0.82	0.93	16.29	<0.0001	0.0015

Table S26: Genes differentially expressed at Day 36 compared to pre-second vaccination (Study Arm 2 A/H7N9+AS03, D1, 29, NK cells). Genes are sorted by descending absolute log_2 fold change. Gene model summaries and annotations are based on Ensembl Version 99 (continued.)

Ensembl Gene ID	Ensembl Gene Name	Ensembl Gene Description	Gene Type	log_2 Fold Change (Day 36 vs. Pre-Second Vaccination)	Average log_2 CPM	Likelihood Ratio Test Statistic	P-Value	FDR Adjusted P-Value
ENSG00000224796	RPL32P1	ribosomal protein L32 pseudogene 1 [Source:HGNC Symbol;Acc:HGNC:10339]	processed pseudogene	-0.82	1.36	23.86	<0.0001	<0.0001
ENSG00000187164	SHTN1	shootin 1 [Source:HGNC Symbol;Acc:HGNC:29319]	protein coding	-0.81	2.99	45.66	<0.0001	<0.0001
ENSG00000187510	PLEKHG7	pleckstrin homology and RhoGEF domain containing G7 [Source:HGNC Symbol;Acc:HGNC:33829]	protein coding	-0.81	0.37	22.11	<0.0001	0.0001
ENSG00000146592	CREB5	cAMP responsive element binding protein 5 [Source:HGNC Symbol;Acc:HGNC:16844]	protein coding	-0.81	2.35	29.14	<0.0001	<0.0001
ENSG00000178175	ZNF366	zinc finger protein 366 [Source:HGNC Symbol;Acc:HGNC:18316]	protein coding	-0.80	1.44	16.47	<0.0001	0.0014
ENSG00000287778		novel transcript, antisense to HS3ST1	lncRNA	-0.77	1.97	15.24	<0.0001	0.0021
ENSG00000128383	APOBEC3A	apolipoprotein B mRNA editing enzyme catalytic subunit 3A [Source:HGNC Symbol;Acc:HGNC:17343]	protein coding	-0.77	2.04	15.04	0.0001	0.0023
ENSG00000088827	SIGLEC1	sialic acid binding Ig like lectin 1 [Source:HGNC Symbol;Acc:HGNC:11127]	protein coding	-0.77	2.79	32.87	<0.0001	<0.0001
ENSG00000255197		novel transcript	lncRNA	-0.77	3.88	48.41	<0.0001	<0.0001
ENSG00000242498	ARPIN	actin related protein 2/3 complex inhibitor [Source:HGNC Symbol;Acc:HGNC:28782]	protein coding	-0.76	2.16	24.13	<0.0001	<0.0001
ENSG00000163958	ZDHHC19	zinc finger DHHC-type containing 19 [Source:HGNC Symbol;Acc:HGNC:20713]	protein coding	-0.76	0.90	8.50	0.0035	0.0282
ENSG00000284914		novel transcript	lncRNA	-0.76	0.49	17.90	<0.0001	0.0008
ENSG00000165029	ABCA1	ATP binding cassette subfamily A member 1 [Source:HGNC Symbol;Acc:HGNC:29]	protein coding	-0.76	2.90	37.94	<0.0001	<0.0001
ENSG00000159713	TPPP3	tubulin polymerization promoting protein family member 3 [Source:HGNC Symbol;Acc:HGNC:24162]	protein coding	-0.75	0.97	11.75	0.0006	0.0081
ENSG00000163625	WDFY3	WD repeat and FYVE domain containing 3 [Source:HGNC Symbol;Acc:HGNC:20751]	protein coding	-0.75	2.22	25.69	<0.0001	<0.0001
ENSG00000280119		TEC	TEC	-0.75	1.62	19.24	<0.0001	0.0005
ENSG00000248727	LINC01948	long intergenic non-protein coding RNA 1948 [Source:HGNC Symbol;Acc:HGNC:52772]	lncRNA	-0.75	0.74	25.71	<0.0001	<0.0001
ENSG00000134531	EMP1	epithelial membrane protein 1 [Source:HGNC Symbol;Acc:HGNC:3333]	protein coding	-0.75	1.43	20.57	<0.0001	0.0003
ENSG00000164125	GASK1B	golgi associated kinase 1B [Source:HGNC Symbol;Acc:HGNC:25312]	protein coding	-0.74	3.02	27.18	<0.0001	<0.0001

Table S26: Genes differentially expressed at Day 36 compared to pre-second vaccination (Study Arm 2 A/H7N9+AS03, D1, 29, NK cells). Genes are sorted by descending absolute log_2 fold change. Gene model summaries and annotations are based on Ensembl Version 99 (continued.)

Ensembl Gene ID	Ensembl Gene Name	Ensembl Gene Description	Gene Type	log_2 Fold Change (Day 36 vs. Pre-Second Vaccination)	Average log_2 CPM	Likelihood Ratio Test Statistic	P-Value	FDR Adjusted P-Value
ENSG00000131398	KCNC3	potassium voltage-gated channel subfamily C member 3 [Source:HGNC Symbol;Acc:HGNC:6235]	protein coding	-0.74	3.00	36.75	<0.0001	<0.0001
ENSG00000183960	KCNH8	potassium voltage-gated channel subfamily H member 8 [Source:HGNC Symbol;Acc:HGNC:18864]	protein coding	-0.74	2.68	11.80	0.0006	0.008
ENSG00000162747	FCGR3B	Fc fragment of IgG receptor IIIb [Source:HGNC Symbol;Acc:HGNC:3620]	protein coding	0.74	1.73	27.02	<0.0001	<0.0001
ENSG00000251513	LIX1-AS1	LIX1 and RIOK2 antisense RNA 1 [Source:HGNC Symbol;Acc:HGNC:52976]	lncRNA	-0.74	1.75	15.26	<0.0001	0.0021
ENSG00000058866	DGKG	diacylglycerol kinase gamma [Source:HGNC Symbol;Acc:HGNC:2853]	protein coding	-0.73	0.89	17.01	<0.0001	0.0011
ENSG00000172243	CLEC7A	C-type lectin domain containing 7A [Source:HGNC Symbol;Acc:HGNC:14558]	protein coding	-0.73	4.46	30.26	<0.0001	<0.0001
ENSG00000249684		novel transcript, antisense to PROP1	lncRNA	-0.73	1.16	21.71	<0.0001	0.0002
ENSG00000271605	MILR1	mast cell immunoglobulin like receptor 1 [Source:HGNC Symbol;Acc:HGNC:27570]	protein coding	-0.72	2.27	23.71	<0.0001	<0.0001
ENSG00000153253	SCN3A	sodium voltage-gated channel alpha subunit 3 [Source:HGNC Symbol;Acc:HGNC:10590]	protein coding	-0.72	2.72	15.67	<0.0001	0.0019
ENSG00000014257	ACPP	acid phosphatase, prostate [Source:HGNC Symbol;Acc:HGNC:125]	protein coding	-0.72	1.69	28.70	<0.0001	<0.0001
ENSG00000261222		novel transcript	lncRNA	-0.72	0.99	14.28	0.0002	0.0031
ENSG00000138119	MYOF	myoferlin [Source:HGNC Symbol;Acc:HGNC:3656]	protein coding	-0.72	2.71	31.48	<0.0001	<0.0001
ENSG00000196668	LINC00173	long intergenic non-protein coding RNA 173 [Source:HGNC Symbol;Acc:HGNC:33791]	lncRNA	-0.71	0.89	13.72	0.0002	0.0039
ENSG00000169031	COL4A3	collagen type IV alpha 3 chain [Source:HGNC Symbol;Acc:HGNC:2204]	protein coding	-0.71	3.55	19.76	<0.0001	0.0004
ENSG00000261996		novel transcript	lncRNA	-0.71	0.53	20.32	<0.0001	0.0003
ENSG00000169432	SCN9A	sodium voltage-gated channel alpha subunit 9 [Source:HGNC Symbol;Acc:HGNC:10597]	protein coding	-0.71	0.93	21.15	<0.0001	0.0002
ENSG00000136826	KLF4	Kruppel like factor 4 [Source:HGNC Symbol;Acc:HGNC:6348]	protein coding	-0.71	4.76	12.59	0.0004	0.0059
ENSG00000154447	SH3RF1	SH3 domain containing ring finger 1 [Source:HGNC Symbol;Acc:HGNC:17650]	protein coding	-0.70	0.43	13.66	0.0002	0.0039
ENSG00000247982	LINC00926	long intergenic non-protein coding RNA 926 [Source:HGNC Symbol;Acc:HGNC:27514]	lncRNA	-0.70	7.90	13.87	0.0002	0.0037

Table S26: Genes differentially expressed at Day 36 compared to pre-second vaccination (Study Arm 2 A/H7N9+AS03, D1, 29, NK cells). Genes are sorted by descending absolute log_2 fold change. Gene model summaries and annotations are based on Ensembl Version 99 (continued.)

Ensembl Gene ID	Ensembl Gene Name	Ensembl Gene Description	Gene Type	log_2 Fold Change (Day 36 vs. Pre-Second Vaccination)	Average log_2 CPM	Likelihood Ratio Test Statistic	P-Value	FDR Adjusted P-Value
ENSG00000211670	IGLV3-9	immunoglobulin lambda variable 3-9 (gene/pseudogene) [Source:HGNC Symbol;Acc:HGNC:5918]	IG V gene	0.70	1.77	9.42	0.0021	0.0196
ENSG00000274105		novel transcript, sense intronic to YARS2	lncRNA	-0.70	0.28	22.18	<0.0001	0.0001
ENSG00000184226	PCDH9	protocadherin 9 [Source:HGNC Symbol;Acc:HGNC:8661]	protein coding	-0.70	4.22	25.89	<0.0001	<0.0001
ENSG00000180061	TMEM150B	transmembrane protein 150B [Source:HGNC Symbol;Acc:HGNC:34415]	protein coding	-0.70	1.13	11.06	0.0009	0.0105
ENSG00000166927		membrane spanning 4-domains A7 [Source:NCBI gene;Acc:58475]	protein coding	-0.70	3.48	33.09	<0.0001	<0.0001
ENSG00000165810	BTNL9	butyrophilin like 9 [Source:HGNC Symbol;Acc:HGNC:24176]	protein coding	-0.70	2.89	10.00	0.0016	0.0156
ENSG00000151575	TEX9	testis expressed 9 [Source:HGNC Symbol;Acc:HGNC:29585]	protein coding	-0.70	0.70	11.91	0.0006	0.0077
ENSG00000105967	TFEC	transcription factor EC [Source:HGNC Symbol;Acc:HGNC:11754]	protein coding	-0.69	3.42	47.16	<0.0001	<0.0001
ENSG00000255163	HSPE1P18	heat shock protein family E (Hsp10) member 1 pseudogene 18 [Source:HGNC Symbol;Acc:HGNC:49337]	processed pseudogene	-0.69	0.94	22.63	<0.0001	0.0001
ENSG00000137462	TLR2	toll like receptor 2 [Source:HGNC Symbol;Acc:HGNC:11848]	protein coding	-0.69	3.92	38.87	<0.0001	<0.0001
ENSG00000197549	PRAMENP	PRAME N-terminal like, pseudogene [Source:HGNC Symbol;Acc:HGNC:34302]	transcribed unprocessed pseudogene	-0.69	0.55	10.43	0.0012	0.0134
ENSG00000183023	SLC8A1	solute carrier family 8 member A1 [Source:HGNC Symbol;Acc:HGNC:11068]	protein coding	-0.69	2.15	26.86	<0.0001	<0.0001
ENSG00000180263	FGD6	FYVE, RhoGEF and PH domain containing 6 [Source:HGNC Symbol;Acc:HGNC:21740]	protein coding	-0.69	1.41	30.98	<0.0001	<0.0001
ENSG00000188906	LRRK2	leucine rich repeat kinase 2 [Source:HGNC Symbol;Acc:HGNC:18618]	protein coding	-0.69	4.55	38.73	<0.0001	<0.0001
ENSG00000177706	FAM20C	FAM20C golgi associated secretory pathway kinase [Source:HGNC Symbol;Acc:HGNC:22140]	protein coding	-0.69	2.54	20.57	<0.0001	0.0003
ENSG00000128833	MYO5C	myosin VC [Source:HGNC Symbol;Acc:HGNC:7604]	protein coding	-0.69	0.92	33.37	<0.0001	<0.0001

Table S26: Genes differentially expressed at Day 36 compared to pre-second vaccination (Study Arm 2 A/H7N9+AS03, D1, 29, NK cells). Genes are sorted by descending absolute log_2 fold change. Gene model summaries and annotations are based on Ensembl Version 99 (continued.)

Ensembl Gene ID	Ensembl Gene Name	Ensembl Gene Description	Gene Type	log_2 Fold Change (Day 36 vs. Pre-Second Vaccination)	Average log_2 CPM	Likelihood Ratio Test Statistic	P-Value	FDR Adjusted P-Value
ENSG00000160223	ICOSLG	inducible T cell costimulator ligand [Source:HGNC Symbol;Acc:HGNC:17087]	protein coding	-0.68	1.34	15.15	<0.0001	0.0022
ENSG00000179639	FCER1A	Fc fragment of IgE receptor Ia [Source:HGNC Symbol;Acc:HGNC:3609]	protein coding	-0.68	4.71	26.42	<0.0001	<0.0001
ENSG00000064225	ST3GAL6	ST3 beta-galactoside alpha-2,3-sialyltransferase 6 [Source:HGNC Symbol;Acc:HGNC:18080]	protein coding	-0.68	1.51	18.26	<0.0001	0.0007
ENSG00000139970	RTN1	reticulon 1 [Source:HGNC Symbol;Acc:HGNC:10467]	protein coding	-0.68	3.55	29.01	<0.0001	<0.0001
ENSG00000116962	NID1	nidogen 1 [Source:HGNC Symbol;Acc:HGNC:7821]	protein coding	-0.68	2.48	16.48	<0.0001	0.0014
ENSG00000082293	COL19A1	collagen type XIX alpha 1 chain [Source:HGNC Symbol;Acc:HGNC:2196]	protein coding	-0.67	3.94	12.28	0.0005	0.0067
ENSG00000087253	LPCAT2	lysophosphatidylcholine acyltransferase 2 [Source:HGNC Symbol;Acc:HGNC:26032]	protein coding	-0.67	2.57	24.72	<0.0001	<0.0001
ENSG00000182379	NXPH4	neurexophilin 4 [Source:HGNC Symbol;Acc:HGNC:8078]	protein coding	-0.67	1.43	8.05	0.0046	0.0337
ENSG00000253755	IGHGP	immunoglobulin heavy constant gamma P (non-functional) [Source:HGNC Symbol;Acc:HGNC:5529]	IG C pseudogene	0.67	2.76	30.14	<0.0001	<0.0001
ENSG00000110987	BCL7A	BAF chromatin remodeling complex subunit BCL7A [Source:HGNC Symbol;Acc:HGNC:1004]	protein coding	-0.66	4.74	15.98	<0.0001	0.0016
ENSG00000166402	TUB	TUB bipartite transcription factor [Source:HGNC Symbol;Acc:HGNC:12406]	protein coding	-0.66	1.17	22.08	<0.0001	0.0001
ENSG00000132514	CLEC10A	C-type lectin domain containing 10A [Source:HGNC Symbol;Acc:HGNC:16916]	protein coding	-0.66	4.33	32.48	<0.0001	<0.0001
ENSG00000171643	S100Z	S100 calcium binding protein Z [Source:HGNC Symbol;Acc:HGNC:30367]	protein coding	-0.66	0.84	24.52	<0.0001	<0.0001
ENSG00000156564	LRFN2	leucine rich repeat and fibronectin type III domain containing 2 [Source:HGNC Symbol;Acc:HGNC:21226]	protein coding	0.66	0.58	13.86	0.0002	0.0037
ENSG00000149418	ST14	suppression of tumorigenicity 14 [Source:HGNC Symbol;Acc:HGNC:11344]	protein coding	-0.66	4.85	35.87	<0.0001	<0.0001
ENSG00000019169	MARCO	macrophage receptor with collagenous structure [Source:HGNC Symbol;Acc:HGNC:6895]	protein coding	-0.66	1.35	15.99	<0.0001	0.0016
ENSG00000145936	KCNMB1	potassium calcium-activated channel subfamily M regulatory beta subunit 1 [Source:HGNC Symbol;Acc:HGNC:6285]	protein coding	-0.66	1.48	15.49	<0.0001	0.002
ENSG00000169507	SLC38A11	solute carrier family 38 member 11 [Source:HGNC Symbol;Acc:HGNC:26836]	protein coding	-0.66	2.62	10.10	0.0015	0.015

Table S26: Genes differentially expressed at Day 36 compared to pre-second vaccination (Study Arm 2 A/H7N9+AS03, D1, 29, NK cells). Genes are sorted by descending absolute log_2 fold change. Gene model summaries and annotations are based on Ensembl Version 99 (continued.)

Ensembl Gene ID	Ensembl Gene Name	Ensembl Gene Description	Gene Type	log_2 Fold Change (Day 36 vs. Pre-Second Vaccination)	Average log_2 CPM	Likelihood Ratio Test Statistic	P-Value	FDR Adjusted P-Value
ENSG00000140090	SLC24A4	solute carrier family 24 member 4 [Source:HGNC Symbol;Acc:HGNC:10978]	protein coding	-0.66	3.30	20.39	<0.0001	0.0003
ENSG00000254415	SIGLEC14	sialic acid binding Ig like lectin 14 [Source:HGNC Symbol;Acc:HGNC:32926]	protein coding	-0.66	3.10	31.89	<0.0001	<0.0001
ENSG00000127954	STEAP4	STEAP4 metalloreductase [Source:HGNC Symbol;Acc:HGNC:21923]	protein coding	-0.65	0.77	8.47	0.0036	0.0286
ENSG00000038427	VCAN	versican [Source:HGNC Symbol;Acc:HGNC:2464]	protein coding	-0.65	7.17	17.47	<0.0001	0.0009
ENSG00000279673		TEC	TEC	0.65	0.51	12.82	0.0003	0.0054
ENSG00000135218	CD36	CD36 molecule [Source:HGNC Symbol;Acc:HGNC:1663]	protein coding	-0.65	5.14	23.91	<0.0001	<0.0001
ENSG00000163534	FCRL1	Fc receptor like 1 [Source:HGNC Symbol;Acc:HGNC:18509]	protein coding	-0.65	7.38	12.78	0.0004	0.0055
ENSG00000139132	FGD4	FYVE, RhoGEF and PH domain containing 4 [Source:HGNC Symbol;Acc:HGNC:19125]	protein coding	-0.65	2.26	29.48	<0.0001	<0.0001
ENSG00000235621	LINC00494	long intergenic non-protein coding RNA 494 [Source:HGNC Symbol;Acc:HGNC:27657]	lncRNA	-0.65	3.38	11.28	0.0008	0.0098
ENSG00000106034	CPED1	cadherin like and PC-esterase domain containing 1 [Source:HGNC Symbol;Acc:HGNC:26159]	protein coding	-0.65	1.93	21.40	<0.0001	0.0002
ENSG00000102445	RUBCNL	rubicon like autophagy enhancer [Source:HGNC Symbol;Acc:HGNC:20420]	protein coding	-0.65	5.12	13.65	0.0002	0.004
ENSG00000014914	MTMR11	myotubularin related protein 11 [Source:HGNC Symbol;Acc:HGNC:24307]	protein coding	-0.65	2.80	30.79	<0.0001	<0.0001
ENSG00000276070	CCL4L2	C-C motif chemokine ligand 4 like 2 [Source:HGNC Symbol;Acc:HGNC:24066]	protein coding	0.65	3.13	31.71	<0.0001	<0.0001
ENSG00000198848	CES1	carboxylesterase 1 [Source:HGNC Symbol;Acc:HGNC:1863]	protein coding	-0.64	3.09	22.49	<0.0001	0.0001
ENSG00000187621	TCL6	T cell leukemia/lymphoma 6 [Source:HGNC Symbol;Acc:HGNC:13463]	lncRNA	-0.64	3.38	10.89	0.001	0.0112
ENSG00000147642	SYBU	syntabulin [Source:HGNC Symbol;Acc:HGNC:26011]	protein coding	-0.64	0.61	14.77	0.0001	0.0026
ENSG00000151650	VENTX	VENT homeobox [Source:HGNC Symbol;Acc:HGNC:13639]	protein coding	-0.64	2.33	21.38	<0.0001	0.0002
ENSG00000165959	CLMN	calmin [Source:HGNC Symbol;Acc:HGNC:19972]	protein coding	-0.64	3.69	28.42	<0.0001	<0.0001

Table S26: Genes differentially expressed at Day 36 compared to pre-second vaccination (Study Arm 2 A/H7N9+AS03, D1, 29, NK cells). Genes are sorted by descending absolute log_2 fold change. Gene model summaries and annotations are based on Ensembl Version 99 (continued.)

Ensembl Gene ID	Ensembl Gene Name	Ensembl Gene Description	Gene Type	log_2 Fold Change (Day 36 vs. Pre-Second Vaccination)	Average log_2 CPM	Likelihood Ratio Test Statistic	P-Value	FDR Adjusted P-Value
ENSG00000178199	ZC3H12D	zinc finger CCCH-type containing 12D [Source:HGNC Symbol;Acc:HGNC:21175]	protein coding	-0.64	4.36	15.83	<0.0001	0.0017
ENSG00000205056	LINC02397	long intergenic non-protein coding RNA 2397 [Source:HGNC Symbol;Acc:HGNC:53323]	lncRNA	-0.64	3.42	11.39	0.0007	0.0094
ENSG00000143079	CTTNBP2NL	CTTNBP2 N-terminal like [Source:HGNC Symbol;Acc:HGNC:25330]	protein coding	-0.64	0.50	12.78	0.0004	0.0055
ENSG00000235641	LINC00484	long intergenic non-protein coding RNA 484 [Source:HGNC Symbol;Acc:HGNC:27862]	lncRNA	0.63	0.37	10.97	0.0009	0.0109
ENSG00000196576	PLXNB2	plexin B2 [Source:HGNC Symbol;Acc:HGNC:9104]	protein coding	-0.63	5.99	39.87	<0.0001	<0.0001
ENSG00000285417		novel pseudogene	transcribed unitary pseudogene	-0.63	2.07	9.67	0.0019	0.0178
ENSG00000184305	CCSER1	coiled-coil serine rich protein 1 [Source:HGNC Symbol;Acc:HGNC:29349]	protein coding	-0.63	0.66	13.27	0.0003	0.0046
ENSG00000146070	PLA2G7	phospholipase A2 group VII [Source:HGNC Symbol;Acc:HGNC:9040]	protein coding	-0.63	1.37	8.78	0.003	0.0252
ENSG00000066336	SPI1	Spi-1 proto-oncogene [Source:HGNC Symbol;Acc:HGNC:11241]	protein coding	-0.63	6.35	34.96	<0.0001	<0.0001
ENSG00000082397	EPB41L3	erythrocyte membrane protein band 4.1 like 3 [Source:HGNC Symbol;Acc:HGNC:3380]	protein coding	-0.63	2.73	14.52	0.0001	0.0029
ENSG00000135363	LMO2	LIM domain only 2 [Source:HGNC Symbol;Acc:HGNC:6642]	protein coding	-0.62	3.87	22.64	<0.0001	0.0001
ENSG00000242265	PEG10	paternally expressed 10 [Source:HGNC Symbol;Acc:HGNC:14005]	protein coding	-0.62	2.50	15.05	0.0001	0.0023
ENSG00000225889		novel transcript, antisense to PELI1	lncRNA	-0.62	2.30	20.70	<0.0001	0.0003
ENSG00000204577	LILRB3	leukocyte immunoglobulin like receptor B3 [Source:HGNC Symbol;Acc:HGNC:6607]	protein coding	-0.62	4.07	23.07	<0.0001	0.0001
ENSG00000122025	FLT3	fms related tyrosine kinase 3 [Source:HGNC Symbol;Acc:HGNC:3765]	protein coding	-0.62	3.41	33.21	<0.0001	<0.0001
ENSG00000162881	OXER1	oxoeicosanoid receptor 1 [Source:HGNC Symbol;Acc:HGNC:24884]	protein coding	-0.62	0.40	18.17	<0.0001	0.0007
ENSG00000185215	TNFAIP2	TNF alpha induced protein 2 [Source:HGNC Symbol;Acc:HGNC:11895]	protein coding	-0.62	6.64	35.56	<0.0001	<0.0001

Confidential/Proprietary Information

Table S26: Genes differentially expressed at Day 36 compared to pre-second vaccination (Study Arm 2 A/H7N9+AS03, D1, 29, NK cells). Genes are sorted by descending absolute log_2 fold change. Gene model summaries and annotations are based on Ensembl Version 99 (continued.)

Ensembl Gene ID	Ensembl Gene Name	Ensembl Gene Description	Gene Type	log_2 Fold Change (Day 36 vs. Pre-Second Vaccination)	Average log_2 CPM	Likelihood Ratio Test Statistic	P-Value	FDR Adjusted P-Value
ENSG00000134256	CD101	CD101 molecule [Source:HGNC Symbol;Acc:HGNC:5949]	protein coding	-0.62	2.53	23.61	<0.0001	<0.0001
ENSG00000163406	SLC15A2	solute carrier family 15 member 2 [Source:HGNC Symbol;Acc:HGNC:10921]	protein coding	-0.62	3.74	23.34	<0.0001	<0.0001
ENSG00000160013	PTGIR	prostaglandin I2 receptor [Source:HGNC Symbol;Acc:HGNC:9602]	protein coding	-0.62	1.80	15.23	<0.0001	0.0022
ENSG00000118432	CNR1	cannabinoid receptor 1 [Source:HGNC Symbol;Acc:HGNC:2159]	protein coding	-0.62	1.06	9.98	0.0016	0.0158
ENSG00000167483	NIBAN3	niban apoptosis regulator 3 [Source:HGNC Symbol;Acc:HGNC:24130]	protein coding	-0.62	7.58	11.31	0.0008	0.0097
ENSG00000132669	RIN2	Ras and Rab interactor 2 [Source:HGNC Symbol;Acc:HGNC:18750]	protein coding	-0.62	1.83	16.02	<0.0001	0.0016
ENSG00000112232	KHDRBS2	KH RNA binding domain containing, signal transduction associated 2 [Source:HGNC Symbol;Acc:HGNC:18114]	protein coding	-0.62	0.99	7.24	0.0071	0.0455
ENSG00000234184	LINC01781	long intergenic non-protein coding RNA 1781 [Source:HGNC Symbol;Acc:HGNC:52571]	lncRNA	-0.62	2.22	14.65	0.0001	0.0027
ENSG00000186407	CD300E	CD300e molecule [Source:HGNC Symbol;Acc:HGNC:28874]	protein coding	-0.61	4.73	20.68	<0.0001	0.0003
ENSG00000283839		proline-rich protein 18-like [Source:NCBI gene;Acc:105373780]	lncRNA	-0.61	1.14	11.19	0.0008	0.01
ENSG00000100079	LGALS2	galectin 2 [Source:HGNC Symbol;Acc:HGNC:6562]	protein coding	-0.61	3.64	21.39	<0.0001	0.0002
ENSG00000163803	PLB1	phospholipase B1 [Source:HGNC Symbol;Acc:HGNC:30041]	protein coding	-0.61	1.79	23.96	<0.0001	<0.0001
ENSG00000143869	GDF7	growth differentiation factor 7 [Source:HGNC Symbol;Acc:HGNC:4222]	protein coding	-0.61	3.25	10.43	0.0012	0.0134
ENSG00000164023	SGMS2	sphingomyelin synthase 2 [Source:HGNC Symbol;Acc:HGNC:28395]	protein coding	-0.61	1.52	18.01	<0.0001	0.0008
ENSG00000230006	ANKRD36BP2	ankyrin repeat domain 36B pseudogene 2 [Source:HGNC Symbol;Acc:HGNC:33607]	transcribed unprocessed pseudogene	-0.61	2.27	11.70	0.0006	0.0083
ENSG00000277632	CCL3	C-C motif chemokine ligand 3 [Source:HGNC Symbol;Acc:HGNC:10627]	protein coding	0.61	3.35	36.90	<0.0001	<0.0001

Table S26: Genes differentially expressed at Day 36 compared to pre-second vaccination (Study Arm 2 A/H7N9+AS03, D1, 29, NK cells). Genes are sorted by descending absolute log_2 fold change. Gene model summaries and annotations are based on Ensembl Version 99 (continued.)

Ensembl Gene ID	Ensembl Gene Name	Ensembl Gene Description	Gene Type	log_2 Fold Change (Day 36 vs. Pre-Second Vaccination)	Average log_2 CPM	Likelihood Ratio Test Statistic	P-Value	FDR Adjusted P-Value
ENSG00000266733	TBC1D29P	TBC1 domain family member 29, pseudogene [Source:HGNC Symbol;Acc:HGNC:24509]	transcribed unprocessed pseudogene	-0.61	0.51	15.88	<0.0001	0.0017
ENSG00000163568	AIM2	absent in melanoma 2 [Source:HGNC Symbol;Acc:HGNC:357]	protein coding	-0.61	3.80	14.64	0.0001	0.0027
ENSG00000198734	F5	coagulation factor V [Source:HGNC Symbol;Acc:HGNC:3542]	protein coding	-0.61	1.86	7.32	0.0068	0.0442
ENSG00000226091	LINC00937	long intergenic non-protein coding RNA 937 [Source:HGNC Symbol;Acc:HGNC:48629]	lncRNA	-0.61	1.37	13.62	0.0002	0.004
ENSG00000100077	GRK3	G protein-coupled receptor kinase 3 [Source:HGNC Symbol;Acc:HGNC:290]	protein coding	-0.61	4.96	29.61	<0.0001	<0.0001
ENSG00000169583	CLIC3	chloride intracellular channel 3 [Source:HGNC Symbol;Acc:HGNC:2064]	protein coding	0.61	7.19	121.59	<0.0001	<0.0001
ENSG00000158473	CD1D	CD1d molecule [Source:HGNC Symbol;Acc:HGNC:1637]	protein coding	-0.60	4.54	20.11	<0.0001	0.0003
ENSG00000163563	MNDA	myeloid cell nuclear differentiation antigen [Source:HGNC Symbol;Acc:HGNC:7183]	protein coding	-0.60	5.73	28.78	<0.0001	<0.0001
ENSG00000182578	CSF1R	colony stimulating factor 1 receptor [Source:HGNC Symbol;Acc:HGNC:2433]	protein coding	-0.60	5.03	24.98	<0.0001	<0.0001
ENSG00000012779	ALOX5	arachidonate 5-lipoxygenase [Source:HGNC Symbol;Acc:HGNC:435]	protein coding	-0.60	6.50	17.99	<0.0001	0.0008
ENSG00000269954		novel transcript, antisense to BLK	lncRNA	-0.60	4.69	13.45	0.0002	0.0043
ENSG00000117009	KMO	kynurenine 3-monooxygenase [Source:HGNC Symbol;Acc:HGNC:6381]	protein coding	-0.60	2.92	10.73	0.0011	0.012
ENSG00000274114	ALOX15P1	arachidonate 15-lipoxygenase pseudogene 1 [Source:HGNC Symbol;Acc:HGNC:13742]	transcribed unprocessed pseudogene	-0.60	2.15	15.77	<0.0001	0.0018
ENSG00000188290	HES4	hes family bHLH transcription factor 4 [Source:HGNC Symbol;Acc:HGNC:24149]	protein coding	0.60	1.83	15.62	<0.0001	0.0019
ENSG00000231964		novel transcript	lncRNA	-0.60	0.52	9.14	0.0025	0.0219
ENSG00000119535	CSF3R	colony stimulating factor 3 receptor [Source:HGNC Symbol;Acc:HGNC:2439]	protein coding	-0.60	6.17	23.93	<0.0001	<0.0001

Table S26: Genes differentially expressed at Day 36 compared to pre-second vaccination (Study Arm 2 A/H7N9+AS03, D1, 29, NK cells). Genes are sorted by descending absolute log_2 fold change. Gene model summaries and annotations are based on Ensembl Version 99 (continued.)

Ensembl Gene ID	Ensembl Gene Name	Ensembl Gene Description	Gene Type	log_2 Fold Change (Day 36 vs. Pre-Second Vaccination)	Average log_2 CPM	Likelihood Ratio Test Statistic	P-Value	FDR Adjusted P-Value
ENSG00000248309	MEF2C-AS1	MEF2C antisense RNA 1 [Source:HGNC Symbol;Acc:HGNC:48908]	lncRNA	-0.60	0.57	13.02	0.0003	0.005
ENSG00000106066	CPVL	carboxypeptidase vitellogenic like [Source:HGNC Symbol;Acc:HGNC:14399]	protein coding	-0.60	5.28	21.99	<0.0001	0.0002
ENSG00000128438	TBC1D27P	TBC1 domain family member 27, pseudogene [Source:HGNC Symbol;Acc:HGNC:28104]	transcribed unprocessed pseudogene	-0.60	4.22	11.79	0.0006	0.008
ENSG00000124731	TREM1	triggering receptor expressed on myeloid cells 1 [Source:HGNC Symbol;Acc:HGNC:17760]	protein coding	-0.60	2.51	11.29	0.0008	0.0097
ENSG00000284825		novel transcript	lncRNA	-0.60	0.49	11.10	0.0009	0.0104
ENSG00000178695	KCTD12	potassium channel tetramerization domain containing 12 [Source:HGNC Symbol;Acc:HGNC:14678]	protein coding	-0.60	5.79	25.42	<0.0001	<0.0001
ENSG00000113749	HRH2	histamine receptor H2 [Source:HGNC Symbol;Acc:HGNC:5183]	protein coding	-0.60	3.21	20.59	<0.0001	0.0003
ENSG00000228663	PSMD10P1	proteasome 26S subunit, non-ATPase, 10 pseudogene 1 [Source:HGNC Symbol;Acc:HGNC:16602]	processed pseudogene	-0.60	0.97	13.25	0.0003	0.0046
ENSG00000108846	ABCC3	ATP binding cassette subfamily C member 3 [Source:HGNC Symbol;Acc:HGNC:54]	protein coding	-0.60	1.17	13.91	0.0002	0.0036
ENSG00000145416	MARCHF1	membrane associated ring-CH-type finger 1 [Source:HGNC Symbol;Acc:HGNC:26077]	protein coding	-0.60	5.60	21.20	<0.0001	0.0002
ENSG00000174469	CNTNAP2	contactin associated protein like 2 [Source:HGNC Symbol;Acc:HGNC:13830]	protein coding	-0.60	3.39	10.15	0.0014	0.0148
ENSG00000128815	WDFY4	WDFY family member 4 [Source:HGNC Symbol;Acc:HGNC:29323]	protein coding	-0.60	6.80	18.47	<0.0001	0.0006
ENSG00000169435	RASSF6	Ras association domain family member 6 [Source:HGNC Symbol;Acc:HGNC:20796]	protein coding	-0.59	0.55	7.52	0.0061	0.0411
ENSG00000196092	PAX5	paired box 5 [Source:HGNC Symbol;Acc:HGNC:8619]	protein coding	-0.59	6.85	10.11	0.0015	0.015
ENSG00000171595	DNAI2	dynein axonemal intermediate chain 2 [Source:HGNC Symbol;Acc:HGNC:18744]	protein coding	0.59	0.65	13.13	0.0003	0.0048
ENSG00000135116	HRK	harakiri, BCL2 interacting protein [Source:HGNC Symbol;Acc:HGNC:5185]	protein coding	-0.59	1.26	13.53	0.0002	0.0042

Table S26: Genes differentially expressed at Day 36 compared to pre-second vaccination (Study Arm 2 A/H7N9+AS03, D1, 29, NK cells). Genes are sorted by descending absolute log_2 fold change. Gene model summaries and annotations are based on Ensembl Version 99 (continued.)

Ensembl Gene ID	Ensembl Gene Name	Ensembl Gene Description	Gene Type	log_2 Fold Change (Day 36 vs. Pre-Second Vaccination)	Average log_2 CPM	Likelihood Ratio Test Statistic	P-Value	FDR Adjusted P-Value
ENSG00000179583	CIITA	class II major histocompatibility complex transactivator [Source:HGNC Symbol;Acc:HGNC:7067]	protein coding	-0.59	7.94	25.57	<0.0001	<0.0001
ENSG00000278991		TEC	TEC	-0.59	1.00	10.11	0.0015	0.015
ENSG00000198053	SIRPA	signal regulatory protein alpha [Source:HGNC Symbol;Acc:HGNC:9662]	protein coding	-0.59	4.55	21.17	<0.0001	0.0002
ENSG00000196562	SULF2	sulfatase 2 [Source:HGNC Symbol;Acc:HGNC:20392]	protein coding	-0.59	5.26	29.15	<0.0001	<0.0001

Table S27: Genes differentially expressed at Day 30 compared to pre-second vaccination (Study Arm 3 A/H7N9 + PBS, D1, 29, NK cells). Genes are sorted by descending absolute log_2 fold change. Gene model summaries and annotations are based on Ensembl Version 99.

Ensembl Gene ID	Ensembl Gene Name	Ensembl Gene Description	Gene Type	log_2 Fold Change (Day 30 vs. Pre-Second Vaccination)	Average log_2 CPM	Likelihood Ratio Test Statistic	P-Value	FDR Adjusted P-Value
ENSG00000111537	IFNG	interferon gamma [Source:HGNC Symbol;Acc:HGNC:5438]	protein coding	1.43	2.31	25.81	<0.0001	0.0055
ENSG00000072163	LIMS2	LIM zinc finger domain containing 2 [Source:HGNC Symbol;Acc:HGNC:16084]	protein coding	-0.75	1.58	20.64	<0.0001	0.0268

Table S28: Genes differentially expressed at Day 8 compared to pre-first vaccination (Study Arm 1 A/H3N2v, D1 , B cells). Genes are sorted by descending absolute log_2 fold change. Gene model summaries and annotations are based on Ensembl Version 99.

Ensembl Gene ID	Ensembl Gene Name	Ensembl Gene Description	Gene Type	log_2 Fold Change (Day 8 vs. Pre-First Vaccination)	Average log_2 CPM	Likelihood Ratio Test Statistic	P-Value	FDR Adjusted P-Value
ENSG00000206172	HBA1	hemoglobin subunit alpha 1 [Source:HGNC Symbol;Acc:HGNC:4823]	protein coding	2.66	3.94	19.60	<0.0001	0.0447
ENSG00000244734	HBB	hemoglobin subunit beta [Source:HGNC Symbol;Acc:HGNC:4827]	protein coding	1.99	5.52	24.26	<0.0001	0.0118
ENSG00000188536	HBA2	hemoglobin subunit alpha 2 [Source:HGNC Symbol;Acc:HGNC:4824]	protein coding	1.86	4.03	20.80	<0.0001	0.0359

Table S29: Genes differentially expressed at Day 2 compared to pre-first vaccination (Study Arm 2 A/H7N9+AS03, D1, 29, B cells). Genes are sorted by descending absolute log_2 fold change. Gene model summaries and annotations are based on Ensembl Version 99.

Ensembl Gene ID	Ensembl Gene Name	Ensembl Gene Description	Gene Type	log_2 Fold Change (Day 2 vs. Pre-First Vaccination)	Average log_2 CPM	Likelihood Ratio Test Statistic	P-Value	FDR Adjusted P-Value
ENSG00000117228	GBP1	guanylate binding protein 1 [Source:HGNC Symbol;Acc:HGNC:4182]	protein coding	1.71	4.52	131.20	<0.0001	<0.0001
ENSG00000115415	STAT1	signal transducer and activator of transcription 1 [Source:HGNC Symbol;Acc:HGNC:11362]	protein coding	1.55	7.47	132.67	<0.0001	<0.0001
ENSG00000162645	GBP2	guanylate binding protein 2 [Source:HGNC Symbol;Acc:HGNC:4183]	protein coding	1.48	5.22	150.38	<0.0001	<0.0001
ENSG00000162654	GBP4	guanylate binding protein 4 [Source:HGNC Symbol;Acc:HGNC:20480]	protein coding	1.35	5.82	128.86	<0.0001	<0.0001
ENSG00000140511	HAPLN3	hyaluronan and proteoglycan link protein 3 [Source:HGNC Symbol;Acc:HGNC:21446]	protein coding	1.31	3.87	97.09	<0.0001	<0.0001
ENSG00000119917	IFIT3	interferon induced protein with tetratricopeptide repeats 3 [Source:HGNC Symbol;Acc:HGNC:5411]	protein coding	1.25	4.21	38.49	<0.0001	<0.0001
ENSG00000141574	SECTM1	secreted and transmembrane 1 [Source:HGNC Symbol;Acc:HGNC:10707]	protein coding	1.22	2.98	21.78	<0.0001	0.0005
ENSG00000138119	MYOF	myoferlin [Source:HGNC Symbol;Acc:HGNC:3656]	protein coding	1.20	1.55	20.83	<0.0001	0.0007
ENSG00000125347	IRF1	interferon regulatory factor 1 [Source:HGNC Symbol;Acc:HGNC:6116]	protein coding	1.18	7.18	134.73	<0.0001	<0.0001
ENSG00000154451	GBP5	guanylate binding protein 5 [Source:HGNC Symbol;Acc:HGNC:19895]	protein coding	1.08	5.37	113.18	<0.0001	<0.0001
ENSG00000221963	APOL6	apolipoprotein L6 [Source:HGNC Symbol;Acc:HGNC:14870]	protein coding	1.06	5.09	78.95	<0.0001	<0.0001
ENSG00000128383	APOBEC3A	apolipoprotein B mRNA editing enzyme catalytic subunit 3A [Source:HGNC Symbol;Acc:HGNC:17343]	protein coding	1.04	2.05	14.75	0.0001	0.0111
ENSG00000137959	IFI44L	interferon induced protein 44 like [Source:HGNC Symbol;Acc:HGNC:17817]	protein coding	1.02	5.32	29.70	<0.0001	<0.0001
ENSG00000134326	CMPK2	cytidine/uridine monophosphate kinase 2 [Source:HGNC Symbol;Acc:HGNC:27015]	protein coding	1.02	2.50	31.98	<0.0001	<0.0001
ENSG00000087237	CETP	cholesteryl ester transfer protein [Source:HGNC Symbol;Acc:HGNC:1869]	protein coding	1.01	1.94	48.63	<0.0001	<0.0001
ENSG00000135636	DYSF	dysferlin [Source:HGNC Symbol;Acc:HGNC:3097]	protein coding	1.00	2.06	13.11	0.0003	0.0225

Table S29: Genes differentially expressed at Day 2 compared to pre-first vaccination (Study Arm 2 A/H7N9+AS03, D1, 29, B cells). Genes are sorted by descending absolute log_2 fold change. Gene model summaries and annotations are based on Ensembl Version 99 (continued.)

Ensembl Gene ID	Ensembl Gene Name	Ensembl Gene Description	Gene Type	log_2 Fold Change (Day 2 vs. Pre-First Vaccination)	Average log_2 CPM	Likelihood Ratio Test Statistic	P-Value	FDR Adjusted P-Value
ENSG00000140105	WARS1	tryptophanyl-tRNA synthetase 1 [Source:HGNC Symbol;Acc:HGNC:12729]	protein coding	1.00	6.77	68.87	<0.0001	<0.0001
ENSG00000214872	SMTNL1	smoothelin like 1 [Source:HGNC Symbol;Acc:HGNC:32394]	protein coding	0.98	1.27	53.53	<0.0001	<0.0001
ENSG00000142089	IFITM3	interferon induced transmembrane protein 3 [Source:HGNC Symbol;Acc:HGNC:5414]	protein coding	0.97	2.99	32.76	<0.0001	<0.0001
ENSG00000172159	FRMD3	FERM domain containing 3 [Source:HGNC Symbol;Acc:HGNC:24125]	protein coding	0.94	1.24	20.18	<0.0001	0.0009
ENSG00000116663	FBXO6	F-box protein 6 [Source:HGNC Symbol;Acc:HGNC:13585]	protein coding	0.94	3.77	95.93	<0.0001	<0.0001
ENSG00000135114	OASL	2'-5'-oligoadenylate synthetase like [Source:HGNC Symbol;Acc:HGNC:8090]	protein coding	0.92	0.70	15.29	<0.0001	0.0086
ENSG00000152229	PSTPIP2	proline-serine-threonine phosphatase interacting protein 2 [Source:HGNC Symbol;Acc:HGNC:9581]	protein coding	0.91	1.50	11.56	0.0007	0.0441
ENSG00000162614	NEXN	nexilin F-actin binding protein [Source:HGNC Symbol;Acc:HGNC:29557]	protein coding	0.90	2.44	21.42	<0.0001	0.0005
ENSG00000134321	RSAD2	radical S-adenosyl methionine domain containing 2 [Source:HGNC Symbol;Acc:HGNC:30908]	protein coding	0.89	2.65	29.88	<0.0001	<0.0001
ENSG00000025708	TYMP	thymidine phosphorylase [Source:HGNC Symbol;Acc:HGNC:3148]	protein coding	0.87	6.50	62.87	<0.0001	<0.0001
ENSG00000185745	IFIT1	interferon induced protein with tetratricopeptide repeats 1 [Source:HGNC Symbol;Acc:HGNC:5407]	protein coding	0.86	3.21	19.83	<0.0001	0.0011
ENSG00000156587	UBE2L6	ubiquitin conjugating enzyme E2 L6 [Source:HGNC Symbol;Acc:HGNC:12490]	protein coding	0.85	6.44	105.52	<0.0001	<0.0001
ENSG00000184979	USP18	ubiquitin specific peptidase 18 [Source:HGNC Symbol;Acc:HGNC:12616]	protein coding	0.81	1.56	30.37	<0.0001	<0.0001
ENSG00000137965	IFI44	interferon induced protein 44 [Source:HGNC Symbol;Acc:HGNC:16938]	protein coding	0.77	4.18	29.53	<0.0001	<0.0001
ENSG00000138496	PARP9	poly(ADP-ribose) polymerase family member 9 [Source:HGNC Symbol;Acc:HGNC:24118]	protein coding	0.76	6.17	68.37	<0.0001	<0.0001
ENSG00000010030	ETV7	ETS variant transcription factor 7 [Source:HGNC Symbol;Acc:HGNC:18160]	protein coding	0.76	1.87	21.80	<0.0001	0.0005
ENSG00000125148	MT2A	metallothionein 2A [Source:HGNC Symbol;Acc:HGNC:7406]	protein coding	0.74	2.31	43.40	<0.0001	<0.0001
ENSG00000133106	EPSTI1	epithelial stromal interaction 1 [Source:HGNC Symbol;Acc:HGNC:16465]	protein coding	0.72	5.77	72.05	<0.0001	<0.0001

Table S29: Genes differentially expressed at Day 2 compared to pre-first vaccination (Study Arm 2 A/H7N9+AS03, D1, 29, B cells). Genes are sorted by descending absolute log_2 fold change. Gene model summaries and annotations are based on Ensembl Version 99 (continued.)

Ensembl Gene ID	Ensembl Gene Name	Ensembl Gene Description	Gene Type	log_2 Fold Change (Day 2 vs. Pre-First Vaccination)	Average log_2 CPM	Likelihood Ratio Test Statistic	P-Value	FDR Adjusted P-Value
ENSG00000284194	SCO2	SCO cytochrome c oxidase assembly protein 2 [Source:HGNC Symbol;Acc:HGNC:10604]	protein coding	0.72	1.83	37.70	<0.0001	<0.0001
ENSG00000177989	ODF3B	outer dense fiber of sperm tails 3B [Source:HGNC Symbol;Acc:HGNC:34388]	protein coding	0.72	5.06	65.65	<0.0001	<0.0001
ENSG00000168062	BATF2	basic leucine zipper ATF-like transcription factor 2 [Source:HGNC Symbol;Acc:HGNC:25163]	protein coding	0.71	1.36	16.90	<0.0001	0.0042
ENSG00000253669	GASAL1	growth arrest associated lncRNA 1 [Source:HGNC Symbol;Acc:HGNC:53461]	lncRNA	0.69	0.91	31.37	<0.0001	<0.0001
ENSG00000187608	ISG15	ISG15 ubiquitin like modifier [Source:HGNC Symbol;Acc:HGNC:4053]	protein coding	0.69	4.74	37.16	<0.0001	<0.0001
ENSG00000002549	LAP3	leucine aminopeptidase 3 [Source:HGNC Symbol;Acc:HGNC:18449]	protein coding	0.67	5.44	50.15	<0.0001	<0.0001
ENSG00000108950	FAM20A	FAM20A golgi associated secretory pathway pseudokinase [Source:HGNC Symbol;Acc:HGNC:23015]	protein coding	0.66	0.31	17.66	<0.0001	0.003
ENSG00000111331	OAS3	2'-5'-oligoadenylate synthetase 3 [Source:HGNC Symbol;Acc:HGNC:8088]	protein coding	0.64	5.37	41.80	<0.0001	<0.0001
ENSG00000108679	LGALS3BP	galectin 3 binding protein [Source:HGNC Symbol;Acc:HGNC:6564]	protein coding	0.64	3.37	32.87	<0.0001	<0.0001
ENSG00000100342	APOL1	apolipoprotein L1 [Source:HGNC Symbol;Acc:HGNC:618]	protein coding	0.64	4.15	28.60	<0.0001	<0.0001
ENSG00000185880	TRIM69	tripartite motif containing 69 [Source:HGNC Symbol;Acc:HGNC:17857]	protein coding	0.63	5.64	53.52	<0.0001	<0.0001
ENSG00000134470	IL15RA	interleukin 15 receptor subunit alpha [Source:HGNC Symbol;Acc:HGNC:5978]	protein coding	0.63	2.86	31.45	<0.0001	<0.0001
ENSG00000185885	IFITM1	interferon induced transmembrane protein 1 [Source:HGNC Symbol;Acc:HGNC:5412]	protein coding	0.63	4.94	31.56	<0.0001	<0.0001
ENSG00000151689	INPP1	inositol polyphosphate-1-phosphatase [Source:HGNC Symbol;Acc:HGNC:6071]	protein coding	0.61	2.95	29.60	<0.0001	<0.0001
ENSG00000126709	IFI6	interferon alpha inducible protein 6 [Source:HGNC Symbol;Acc:HGNC:4054]	protein coding	0.60	4.57	28.75	<0.0001	<0.0001
ENSG00000287839		novel transcript	lncRNA	0.60	2.14	40.09	<0.0001	<0.0001
ENSG00000127951	FGL2	fibrinogen like 2 [Source:HGNC Symbol;Acc:HGNC:3696]	protein coding	0.60	5.76	13.07	0.0003	0.0227

Table S30: Genes differentially expressed at Day 4 compared to pre-first vaccination (Study Arm 2 A/H7N9+AS03, D1, 29, B cells). Genes are sorted by descending absolute log_2 fold change. Gene model summaries and annotations are based on Ensembl Version 99.

Ensembl Gene ID	Ensembl Gene Name	Ensembl Gene Description	Gene Type	log_2 Fold Change (Day 4 vs. Pre-First Vaccination)	Average log_2 CPM	Likelihood Ratio Test Statistic	P-Value	FDR Adjusted P-Value
ENSG00000111331	OAS3	2'-5'-oligoadenylate synthetase 3 [Source:HGNC Symbol;Acc:HGNC:8088]	protein coding	0.91	5.46	19.42	<0.0001	0.0492

Table S31: Genes differentially expressed at Day 29 compared to pre-first vaccination (Study Arm 2 A/H7N9+AS03, D1, 29, B cells). Genes are sorted by descending absolute log_2 fold change. Gene model summaries and annotations are based on Ensembl Version 99.

Ensembl Gene ID	Ensembl Gene Name	Ensembl Gene Description	Gene Type	log_2 Fold Change (Day 29 vs. Pre-First Vaccination)	Average log_2 CPM	Likelihood Ratio Test Statistic	P-Value	FDR Adjusted P-Value
ENSG00000153885	KCTD15	potassium channel tetramerization domain containing 15 [Source:HGNC Symbol;Acc:HGNC:23297]	protein coding	-0.96	0.66	21.96	<0.0001	0.0391

Table S32: Genes differentially expressed at Day 4 compared to pre-first vaccination (Study Arm 3 A/H7N9 + PBS, D1, 29, B cells). Genes are sorted by descending absolute log_2 fold change. Gene model summaries and annotations are based on Ensembl Version 99.

Ensembl Gene ID	Ensembl Gene Name	Ensembl Gene Description	Gene Type	log_2 Fold Change (Day 4 vs. Pre-First Vaccination)	Average log_2 CPM	Likelihood Ratio Test Statistic	P-Value	FDR Adjusted P-Value
ENSG00000225523	IGKV6D-21	immunoglobulin kappa variable 6D-21 (non-functional) [Source:HGNC Symbol;Acc:HGNC:5837]	IG V gene	1.29	1.34	22.79	<0.0001	0.0253

Table S33: Genes differentially expressed at Day 30 compared to pre-second vaccination (Study Arm 2 A/H7N9+AS03, D1, 29, B cells). Genes are sorted by descending absolute log_2 fold change. Gene model summaries and annotations are based on Ensembl Version 99.

Ensembl Gene ID	Ensembl Gene Name	Ensembl Gene Description	Gene Type	log_2 Fold Change (Day 30 vs. Pre-Second Vaccination)	Average log_2 CPM	Likelihood Ratio Test Statistic	P-Value	FDR Adjusted P-Value
ENSG00000229754	CXCR2P1	C-X-C motif chemokine receptor 2 pseudogene 1 [Source:HGNC Symbol;Acc:HGNC:6028]	transcribed unprocessed pseudogene	2.62	1.50	59.16	<0.0001	<0.0001
ENSG00000128383	APOBEC3A	apolipoprotein B mRNA editing enzyme catalytic subunit 3A [Source:HGNC Symbol;Acc:HGNC:17343]	protein coding	2.53	3.05	101.21	<0.0001	<0.0001
ENSG00000117228	GBP1	guanylate binding protein 1 [Source:HGNC Symbol;Acc:HGNC:4182]	protein coding	2.39	5.35	75.95	<0.0001	<0.0001
ENSG00000103569	AQP9	aquaporin 9 [Source:HGNC Symbol;Acc:HGNC:643]	protein coding	2.39	2.04	47.29	<0.0001	<0.0001
ENSG00000141574	SECTM1	secreted and transmembrane 1 [Source:HGNC Symbol;Acc:HGNC:10707]	protein coding	2.35	3.64	94.95	<0.0001	<0.0001
ENSG00000088827	SIGLEC1	sialic acid binding Ig like lectin 1 [Source:HGNC Symbol;Acc:HGNC:11127]	protein coding	2.35	1.49	70.68	<0.0001	<0.0001
ENSG00000138119	MYOF	myoferlin [Source:HGNC Symbol;Acc:HGNC:3656]	protein coding	2.31	2.44	56.46	<0.0001	<0.0001
ENSG00000143546	S100A8	S100 calcium binding protein A8 [Source:HGNC Symbol;Acc:HGNC:10498]	protein coding	2.26	5.78	64.07	<0.0001	<0.0001
ENSG00000135114	OASL	2'-5'-oligoadenylate synthetase like [Source:HGNC Symbol;Acc:HGNC:8090]	protein coding	2.17	1.45	92.01	<0.0001	<0.0001
ENSG00000163220	S100A9	S100 calcium binding protein A9 [Source:HGNC Symbol;Acc:HGNC:10499]	protein coding	2.14	6.98	60.99	<0.0001	<0.0001
ENSG00000162645	GBP2	guanylate binding protein 2 [Source:HGNC Symbol;Acc:HGNC:4183]	protein coding	2.10	5.86	85.14	<0.0001	<0.0001
ENSG00000135636	DYSF	dysferlin [Source:HGNC Symbol;Acc:HGNC:3097]	protein coding	2.10	2.88	45.62	<0.0001	<0.0001
ENSG00000171051	FPR1	formyl peptide receptor 1 [Source:HGNC Symbol;Acc:HGNC:3826]	protein coding	2.04	3.47	56.35	<0.0001	<0.0001
ENSG00000140511	HAPLN3	hyaluronan and proteoglycan link protein 3 [Source:HGNC Symbol;Acc:HGNC:21446]	protein coding	2.03	4.49	62.23	<0.0001	<0.0001
ENSG00000204103	MAFB	MAF bZIP transcription factor B [Source:HGNC Symbol;Acc:HGNC:6408]	protein coding	1.99	2.71	59.12	<0.0001	<0.0001
ENSG00000163221	S100A12	S100 calcium binding protein A12 [Source:HGNC Symbol;Acc:HGNC:10489]	protein coding	1.94	2.42	43.81	<0.0001	<0.0001

Table S33: Genes differentially expressed at Day 30 compared to pre-second vaccination (Study Arm 2 A/H7N9+AS03, D1, 29, B cells). Genes are sorted by descending absolute log_2 fold change. Gene model summaries and annotations are based on Ensembl Version 99 (continued.)

Ensembl Gene ID	Ensembl Gene Name	Ensembl Gene Description	Gene Type	log_2 Fold Change (Day 30 vs. Pre-Second Vaccination)	Average log_2 CPM	Likelihood Ratio Test Statistic	P-Value	FDR Adjusted P-Value
ENSG00000183023	SLC8A1	solute carrier family 8 member A1 [Source:HGNC Symbol;Acc:HGNC:11068]	protein coding	1.94	0.68	60.30	<0.0001	<0.0001
ENSG00000151948	GLT1D1	glycosyltransferase 1 domain containing 1 [Source:HGNC Symbol;Acc:HGNC:26483]	protein coding	1.92	1.66	53.50	<0.0001	<0.0001
ENSG00000142089	IFITM3	interferon induced transmembrane protein 3 [Source:HGNC Symbol;Acc:HGNC:5414]	protein coding	1.92	3.73	39.48	<0.0001	<0.0001
ENSG00000103196	CRISPLD2	cysteine rich secretory protein LCCL domain containing 2 [Source:HGNC Symbol;Acc:HGNC:25248]	protein coding	1.91	1.55	45.15	<0.0001	<0.0001
ENSG00000187164	SHTN1	shootin 1 [Source:HGNC Symbol;Acc:HGNC:29319]	protein coding	1.89	0.99	51.56	<0.0001	<0.0001
ENSG00000011422	PLAUR	plasminogen activator, urokinase receptor [Source:HGNC Symbol;Acc:HGNC:9053]	protein coding	1.86	1.64	53.11	<0.0001	<0.0001
ENSG00000119917	IFIT3	interferon induced protein with tetratricopeptide repeats 3 [Source:HGNC Symbol;Acc:HGNC:5411]	protein coding	1.86	4.84	73.70	<0.0001	<0.0001
ENSG00000154451	GBP5	guanylate binding protein 5 [Source:HGNC Symbol;Acc:HGNC:19895]	protein coding	1.84	6.03	52.96	<0.0001	<0.0001
ENSG00000163823	CCR1	C-C motif chemokine receptor 1 [Source:HGNC Symbol;Acc:HGNC:1602]	protein coding	1.83	2.71	32.07	<0.0001	<0.0001
ENSG00000162711	NLRP3	NLR family pyrin domain containing 3 [Source:HGNC Symbol;Acc:HGNC:16400]	protein coding	1.80	1.30	60.83	<0.0001	<0.0001
ENSG00000187116	LILRA5	leukocyte immunoglobulin like receptor A5 [Source:HGNC Symbol;Acc:HGNC:16309]	protein coding	1.80	2.37	49.68	<0.0001	<0.0001
ENSG00000152229	PSTPIP2	proline-serine-threonine phosphatase interacting protein 2 [Source:HGNC Symbol;Acc:HGNC:9581]	protein coding	1.79	1.91	42.39	<0.0001	<0.0001
ENSG00000181631	P2RY13	purinergic receptor P2Y13 [Source:HGNC Symbol;Acc:HGNC:4537]	protein coding	1.77	1.89	33.36	<0.0001	<0.0001
ENSG00000162654	GBP4	guanylate binding protein 4 [Source:HGNC Symbol;Acc:HGNC:20480]	protein coding	1.77	6.32	59.71	<0.0001	<0.0001
ENSG00000082397	EPB41L3	erythrocyte membrane protein band 4.1 like 3 [Source:HGNC Symbol;Acc:HGNC:3380]	protein coding	1.74	1.81	44.43	<0.0001	<0.0001
ENSG00000115415	STAT1	signal transducer and activator of transcription 1 [Source:HGNC Symbol;Acc:HGNC:11362]	protein coding	1.73	7.98	96.46	<0.0001	<0.0001
ENSG00000103313	MEFV	MEFV innate immuity regulator, pyrin [Source:HGNC Symbol;Acc:HGNC:6998]	protein coding	1.73	2.83	48.52	<0.0001	<0.0001
ENSG00000161944	ASGR2	asialoglycoprotein receptor 2 [Source:HGNC Symbol;Acc:HGNC:743]	protein coding	1.72	0.86	44.34	<0.0001	<0.0001

Table S33: Genes differentially expressed at Day 30 compared to pre-second vaccination (Study Arm 2 A/H7N9+AS03, D1, 29, B cells). Genes are sorted by descending absolute log_2 fold change. Gene model summaries and annotations are based on Ensembl Version 99 (continued.)

Ensembl Gene ID	Ensembl Gene Name	Ensembl Gene Description	Gene Type	log_2 Fold Change (Day 30 vs. Pre-Second Vaccination)	Average log_2 CPM	Likelihood Ratio Test Statistic	P-Value	FDR Adjusted P-Value
ENSG00000120594	PLXDC2	plexin domain containing 2 [Source:HGNC Symbol;Acc:HGNC:21013]	protein coding	1.71	1.85	50.72	<0.0001	<0.0001
ENSG00000087253	LPCAT2	lysophosphatidylcholine acyltransferase 2 [Source:HGNC Symbol;Acc:HGNC:26032]	protein coding	1.70	1.46	45.11	<0.0001	<0.0001
ENSG00000158869	FCER1G	Fc fragment of IgE receptor Ig [Source:HGNC Symbol;Acc:HGNC:3611]	protein coding	1.69	3.58	58.24	<0.0001	<0.0001
ENSG00000197249	SERPINA1	serpin family A member 1 [Source:HGNC Symbol;Acc:HGNC:8941]	protein coding	1.69	5.12	59.57	<0.0001	<0.0001
ENSG00000244482	LILRA6	leukocyte immunoglobulin like receptor A6 [Source:HGNC Symbol;Acc:HGNC:15495]	protein coding	1.67	1.95	37.26	<0.0001	<0.0001
ENSG00000171860	C3AR1	complement C3a receptor 1 [Source:HGNC Symbol;Acc:HGNC:1319]	protein coding	1.66	1.63	37.65	<0.0001	<0.0001
ENSG00000010030	ETV7	ETS variant transcription factor 7 [Source:HGNC Symbol;Acc:HGNC:18160]	protein coding	1.65	2.29	42.36	<0.0001	<0.0001
ENSG00000172159	FRMD3	FERM domain containing 3 [Source:HGNC Symbol;Acc:HGNC:24125]	protein coding	1.65	1.44	57.58	<0.0001	<0.0001
ENSG00000134326	CMPK2	cytidine/uridine monophosphate kinase 2 [Source:HGNC Symbol;Acc:HGNC:27015]	protein coding	1.65	3.06	70.31	<0.0001	<0.0001
ENSG00000140749	IGSF6	immunoglobulin superfamily member 6 [Source:HGNC Symbol;Acc:HGNC:5953]	protein coding	1.63	2.39	52.40	<0.0001	<0.0001
ENSG00000123405	NFE2	nuclear factor, erythroid 2 [Source:HGNC Symbol;Acc:HGNC:7780]	protein coding	1.61	2.06	29.26	<0.0001	<0.0001
ENSG00000140105	WARS1	tryptophanyl-tRNA synthetase 1 [Source:HGNC Symbol;Acc:HGNC:12729]	protein coding	1.61	7.36	54.90	<0.0001	<0.0001
ENSG00000014914	MTMR11	myotubularin related protein 11 [Source:HGNC Symbol;Acc:HGNC:24307]	protein coding	1.60	1.19	48.01	<0.0001	<0.0001
ENSG00000221963	APOL6	apolipoprotein L6 [Source:HGNC Symbol;Acc:HGNC:14870]	protein coding	1.60	5.52	80.60	<0.0001	<0.0001
ENSG00000111321	LTBR	lymphotoxin beta receptor [Source:HGNC Symbol;Acc:HGNC:6718]	protein coding	1.59	2.20	39.89	<0.0001	<0.0001
ENSG00000134243	SORT1	sortilin 1 [Source:HGNC Symbol;Acc:HGNC:11186]	protein coding	1.59	1.95	53.28	<0.0001	<0.0001
ENSG00000185215	TNFAIP2	TNF alpha induced protein 2 [Source:HGNC Symbol;Acc:HGNC:11895]	protein coding	1.57	5.66	53.58	<0.0001	<0.0001
ENSG00000165092	ALDH1A1	aldehyde dehydrogenase 1 family member A1 [Source:HGNC Symbol;Acc:HGNC:402]	protein coding	1.57	1.31	40.53	<0.0001	<0.0001

Table S33: Genes differentially expressed at Day 30 compared to pre-second vaccination (Study Arm 2 A/H7N9+AS03, D1, 29, B cells). Genes are sorted by descending absolute log_2 fold change. Gene model summaries and annotations are based on Ensembl Version 99 (continued.)

Ensembl Gene ID	Ensembl Gene Name	Ensembl Gene Description	Gene Type	log_2 Fold Change (Day 30 vs. Pre-Second Vaccination)	Average log_2 CPM	Likelihood Ratio Test Statistic	P-Value	FDR Adjusted P-Value
ENSG00000186407	CD300E	CD300e molecule [Source:HGNC Symbol;Acc:HGNC:28874]	protein coding	1.55	4.03	53.68	<0.0001	<0.0001
ENSG00000135218	CD36	CD36 molecule [Source:HGNC Symbol;Acc:HGNC:1663]	protein coding	1.55	3.51	38.50	<0.0001	<0.0001
ENSG00000109743	BST1	bone marrow stromal cell antigen 1 [Source:HGNC Symbol;Acc:HGNC:1118]	protein coding	1.54	1.87	35.97	<0.0001	<0.0001
ENSG00000134321	RSAD2	radical S-adenosyl methionine domain containing 2 [Source:HGNC Symbol;Acc:HGNC:30908]	protein coding	1.54	3.21	62.21	<0.0001	<0.0001
ENSG00000137959	IFI44L	interferon induced protein 44 like [Source:HGNC Symbol;Acc:HGNC:17817]	protein coding	1.54	5.89	102.98	<0.0001	<0.0001
ENSG00000164125	GASK1B	golgi associated kinase 1B [Source:HGNC Symbol;Acc:HGNC:25312]	protein coding	1.52	1.50	27.97	<0.0001	<0.0001
ENSG00000121552	CSTA	cystatin A [Source:HGNC Symbol;Acc:HGNC:2481]	protein coding	1.51	1.50	38.04	<0.0001	<0.0001
ENSG00000204577	LILRB3	leukocyte immunoglobulin like receptor B3 [Source:HGNC Symbol;Acc:HGNC:6607]	protein coding	1.50	3.30	51.94	<0.0001	<0.0001
ENSG00000125347	IRF1	interferon regulatory factor 1 [Source:HGNC Symbol;Acc:HGNC:6116]	protein coding	1.50	7.77	65.76	<0.0001	<0.0001
ENSG00000185745	IFIT1	interferon induced protein with tetratricopeptide repeats 1 [Source:HGNC Symbol;Acc:HGNC:5407]	protein coding	1.49	3.59	48.60	<0.0001	<0.0001
ENSG00000090382	LYZ	lysozyme [Source:HGNC Symbol;Acc:HGNC:6740]	protein coding	1.49	7.41	38.22	<0.0001	<0.0001
ENSG00000085265	FCN1	ficolin 1 [Source:HGNC Symbol;Acc:HGNC:3623]	protein coding	1.48	6.69	39.29	<0.0001	<0.0001
ENSG00000125148	MT2A	metallothionein 2A [Source:HGNC Symbol;Acc:HGNC:7406]	protein coding	1.47	2.93	56.66	<0.0001	<0.0001
ENSG00000146592	CREB5	cAMP responsive element binding protein 5 [Source:HGNC Symbol;Acc:HGNC:16844]	protein coding	1.46	1.33	38.69	<0.0001	<0.0001
ENSG00000170458	CD14	CD14 molecule [Source:HGNC Symbol;Acc:HGNC:1628]	protein coding	1.46	4.64	40.03	<0.0001	<0.0001
ENSG00000121316	PLBD1	phospholipase B domain containing 1 [Source:HGNC Symbol;Acc:HGNC:26215]	protein coding	1.44	3.22	34.72	<0.0001	<0.0001
ENSG00000106066	CPVL	carboxypeptidase vitellogenic like [Source:HGNC Symbol;Acc:HGNC:14399]	protein coding	1.44	3.46	32.68	<0.0001	<0.0001
ENSG00000025708	TYMP	thymidine phosphorylase [Source:HGNC Symbol;Acc:HGNC:3148]	protein coding	1.43	7.04	73.15	<0.0001	<0.0001

Table S33: Genes differentially expressed at Day 30 compared to pre-second vaccination (Study Arm 2 A/H7N9+AS03, D1, 29, B cells). Genes are sorted by descending absolute log_2 fold change. Gene model summaries and annotations are based on Ensembl Version 99 (continued.)

Ensembl Gene ID	Ensembl Gene Name	Ensembl Gene Description	Gene Type	log_2 Fold Change (Day 30 vs. Pre-Second Vaccination)	Average log_2 CPM	Likelihood Ratio Test Statistic	P-Value	FDR Adjusted P-Value
ENSG00000197405	C5AR1	complement C5a receptor 1 [Source:HGNC Symbol;Acc:HGNC:1338]	protein coding	1.43	2.91	49.54	<0.0001	<0.0001
ENSG00000011600	TYROBP	TYRO protein tyrosine kinase binding protein [Source:HGNC Symbol;Acc:HGNC:12449]	protein coding	1.41	4.28	36.45	<0.0001	<0.0001
ENSG00000038427	VCAN	versican [Source:HGNC Symbol;Acc:HGNC:2464]	protein coding	1.39	5.11	44.62	<0.0001	<0.0001
ENSG00000129450	SIGLEC9	sialic acid binding Ig like lectin 9 [Source:HGNC Symbol;Acc:HGNC:10878]	protein coding	1.37	1.41	26.22	<0.0001	<0.0001
ENSG00000118508	RAB32	RAB32, member RAS oncogene family [Source:HGNC Symbol;Acc:HGNC:9772]	protein coding	1.37	1.08	23.69	<0.0001	<0.0001
ENSG00000117115	PADI2	peptidyl arginine deiminase 2 [Source:HGNC Symbol;Acc:HGNC:18341]	protein coding	1.36	1.88	18.33	<0.0001	0.0007
ENSG00000067182	TNFRSF1A	TNF receptor superfamily member 1A [Source:HGNC Symbol;Acc:HGNC:11916]	protein coding	1.35	2.70	23.89	<0.0001	<0.0001
ENSG00000102524	TNFSF13B	TNF superfamily member 13b [Source:HGNC Symbol;Acc:HGNC:11929]	protein coding	1.35	2.89	38.70	<0.0001	<0.0001
ENSG00000119922	IFIT2	interferon induced protein with tetratricopeptide repeats 2 [Source:HGNC Symbol;Acc:HGNC:5409]	protein coding	1.34	4.35	30.09	<0.0001	<0.0001
ENSG00000113749	HRH2	histamine receptor H2 [Source:HGNC Symbol;Acc:HGNC:5183]	protein coding	1.34	1.95	27.17	<0.0001	<0.0001
ENSG00000170909	OSCAR	osteoclast associated Ig-like receptor [Source:HGNC Symbol;Acc:HGNC:29960]	protein coding	1.33	2.55	33.64	<0.0001	<0.0001
ENSG00000284194	SCO2	SCO cytochrome c oxidase assembly protein 2 [Source:HGNC Symbol;Acc:HGNC:10604]	protein coding	1.32	2.24	45.15	<0.0001	<0.0001
ENSG00000139318	DUSP6	dual specificity phosphatase 6 [Source:HGNC Symbol;Acc:HGNC:3072]	protein coding	1.32	3.95	31.07	<0.0001	<0.0001
ENSG00000100504	PYGL	glycogen phosphorylase L [Source:HGNC Symbol;Acc:HGNC:9725]	protein coding	1.32	1.91	30.06	<0.0001	<0.0001
ENSG00000116574	RHOU	ras homolog family member U [Source:HGNC Symbol;Acc:HGNC:17794]	protein coding	1.31	0.79	26.35	<0.0001	<0.0001
ENSG00000135604	STX11	syntaxin 11 [Source:HGNC Symbol;Acc:HGNC:11429]	protein coding	1.31	4.20	61.24	<0.0001	<0.0001
ENSG00000133574	GIMAP4	GTPase, IMAP family member 4 [Source:HGNC Symbol;Acc:HGNC:21872]	protein coding	1.30	3.54	22.34	<0.0001	0.0001
ENSG00000204472	AIF1	allograft inflammatory factor 1 [Source:HGNC Symbol;Acc:HGNC:352]	protein coding	1.30	3.51	32.49	<0.0001	<0.0001

Table S33: Genes differentially expressed at Day 30 compared to pre-second vaccination (Study Arm 2 A/H7N9+AS03, D1, 29, B cells). Genes are sorted by descending absolute log_2 fold change. Gene model summaries and annotations are based on Ensembl Version 99 (continued.)

Ensembl Gene ID	Ensembl Gene Name	Ensembl Gene Description	Gene Type	log_2 Fold Change (Day 30 vs. Pre-Second Vaccination)	Average log_2 CPM	Likelihood Ratio Test Statistic	P-Value	FDR Adjusted P-Value
ENSG00000120885	CLU	clusterin [Source:HGNC Symbol;Acc:HGNC:2095]	protein coding	1.29	2.27	10.71	0.0011	0.0184
ENSG00000108679	LGALS3BP	galectin 3 binding protein [Source:HGNC Symbol;Acc:HGNC:6564]	protein coding	1.28	3.68	73.25	<0.0001	<0.0001
ENSG00000105383	CD33	CD33 molecule [Source:HGNC Symbol;Acc:HGNC:1659]	protein coding	1.28	1.91	23.82	<0.0001	<0.0001
ENSG00000184557	SOCS3	suppressor of cytokine signaling 3 [Source:HGNC Symbol;Acc:HGNC:19391]	protein coding	1.28	2.85	28.60	<0.0001	<0.0001
ENSG00000158825	CDA	cytidine deaminase [Source:HGNC Symbol;Acc:HGNC:1712]	protein coding	1.27	0.66	26.88	<0.0001	<0.0001
ENSG00000179921	GPBAR1	G protein-coupled bile acid receptor 1 [Source:HGNC Symbol;Acc:HGNC:19680]	protein coding	1.27	1.77	28.37	<0.0001	<0.0001
ENSG00000189060	H1-0	H1.0 linker histone [Source:HGNC Symbol;Acc:HGNC:4714]	protein coding	1.26	1.56	27.41	<0.0001	<0.0001
ENSG00000082074	FYB1	FYN binding protein 1 [Source:HGNC Symbol;Acc:HGNC:4036]	protein coding	1.25	4.47	28.15	<0.0001	<0.0001
ENSG00000172243	CLEC7A	C-type lectin domain containing 7A [Source:HGNC Symbol;Acc:HGNC:14558]	protein coding	1.24	3.18	46.71	<0.0001	<0.0001
ENSG00000023171	GRAMD1B	GRAM domain containing 1B [Source:HGNC Symbol;Acc:HGNC:29214]	protein coding	1.24	1.10	32.68	<0.0001	<0.0001
ENSG00000214872	SMTNL1	smoothelin like 1 [Source:HGNC Symbol;Acc:HGNC:32394]	protein coding	1.24	1.67	43.84	<0.0001	<0.0001
ENSG00000239998	LILRA2	leukocyte immunoglobulin like receptor A2 [Source:HGNC Symbol;Acc:HGNC:6603]	protein coding	1.23	2.82	31.10	<0.0001	<0.0001
ENSG00000185885	IFITM1	interferon induced transmembrane protein 1 [Source:HGNC Symbol;Acc:HGNC:5412]	protein coding	1.23	5.37	81.06	<0.0001	<0.0001
ENSG00000127951	FGL2	fibrinogen like 2 [Source:HGNC Symbol;Acc:HGNC:3696]	protein coding	1.23	6.09	36.11	<0.0001	<0.0001
ENSG00000116663	FBXO6	F-box protein 6 [Source:HGNC Symbol;Acc:HGNC:13585]	protein coding	1.23	4.17	61.17	<0.0001	<0.0001
ENSG00000163737	PF4	platelet factor 4 [Source:HGNC Symbol;Acc:HGNC:8861]	protein coding	1.23	1.94	10.86	0.001	0.0173
ENSG00000137965	IFI44	interferon induced protein 44 [Source:HGNC Symbol;Acc:HGNC:16938]	protein coding	1.22	4.58	77.23	<0.0001	<0.0001
ENSG00000198053	SIRPA	signal regulatory protein alpha [Source:HGNC Symbol;Acc:HGNC:9662]	protein coding	1.22	3.17	32.49	<0.0001	<0.0001

Table S33: Genes differentially expressed at Day 30 compared to pre-second vaccination (Study Arm 2 A/H7N9+AS03, D1, 29, B cells). Genes are sorted by descending absolute log_2 fold change. Gene model summaries and annotations are based on Ensembl Version 99 (continued.)

Ensembl Gene ID	Ensembl Gene Name	Ensembl Gene Description	Gene Type	log_2 Fold Change (Day 30 vs. Pre-Second Vaccination)	Average log_2 CPM	Likelihood Ratio Test Statistic	P-Value	FDR Adjusted P-Value
ENSG00000060558	GNA15	G protein subunit alpha 15 [Source:HGNC Symbol;Acc:HGNC:4383]	protein coding	1.22	0.64	19.78	<0.0001	0.0003
ENSG00000119535	CSF3R	colony stimulating factor 3 receptor [Source:HGNC Symbol;Acc:HGNC:2439]	protein coding	1.22	4.91	21.93	<0.0001	0.0001
ENSG00000101307	SIRPB1	signal regulatory protein beta 1 [Source:HGNC Symbol;Acc:HGNC:15928]	protein coding	1.22	2.20	27.66	<0.0001	<0.0001
ENSG00000125810	CD93	CD93 molecule [Source:HGNC Symbol;Acc:HGNC:15855]	protein coding	1.21	3.56	28.11	<0.0001	<0.0001
ENSG00000139970	RTN1	reticulon 1 [Source:HGNC Symbol;Acc:HGNC:10467]	protein coding	1.20	0.86	25.51	<0.0001	<0.0001
ENSG00000106565	TMEM176B	transmembrane protein 176B [Source:HGNC Symbol;Acc:HGNC:29596]	protein coding	1.20	1.82	15.03	0.0001	0.0029
ENSG00000074660	SCARF1	scavenger receptor class F member 1 [Source:HGNC Symbol;Acc:HGNC:16820]	protein coding	1.20	1.41	19.39	<0.0001	0.0004
ENSG00000135046	ANXA1	annexin A1 [Source:HGNC Symbol;Acc:HGNC:533]	protein coding	1.19	4.03	25.12	<0.0001	<0.0001
ENSG00000186074	CD300LF	CD300 molecule like family member f [Source:HGNC Symbol;Acc:HGNC:29883]	protein coding	1.19	2.02	25.31	<0.0001	<0.0001
ENSG00000145569	OTULINL	OTU deubiquitinase with linear linkage specificity like [Source:HGNC Symbol;Acc:HGNC:25629]	protein coding	1.19	1.60	20.94	<0.0001	0.0002
ENSG00000179144	GIMAP7	GTPase, IMAP family member 7 [Source:HGNC Symbol;Acc:HGNC:22404]	protein coding	1.19	2.92	11.19	0.0008	0.0151
ENSG00000160883	HK3	hexokinase 3 [Source:HGNC Symbol;Acc:HGNC:4925]	protein coding	1.18	4.35	29.86	<0.0001	<0.0001
ENSG00000150681	RGS18	regulator of G protein signaling 18 [Source:HGNC Symbol;Acc:HGNC:14261]	protein coding	1.18	1.87	13.92	0.0002	0.0048
ENSG00000110077	MS4A6A	membrane spanning 4-domains A6A [Source:HGNC Symbol;Acc:HGNC:13375]	protein coding	1.18	3.44	26.21	<0.0001	<0.0001
ENSG00000101439	CST3	cystatin C [Source:HGNC Symbol;Acc:HGNC:2475]	protein coding	1.18	5.01	24.42	<0.0001	<0.0001
ENSG00000136826	KLF4	Kruppel like factor 4 [Source:HGNC Symbol;Acc:HGNC:6348]	protein coding	1.18	3.57	12.43	0.0004	0.009
ENSG00000088340	FER1L4	fer-1 like family member 4 (pseudogene) [Source:HGNC Symbol;Acc:HGNC:15801]	transcribed unitary pseudogene	1.18	2.06	27.09	<0.0001	<0.0001

Table S33: Genes differentially expressed at Day 30 compared to pre-second vaccination (Study Arm 2 A/H7N9+AS03, D1, 29, B cells). Genes are sorted by descending absolute log_2 fold change. Gene model summaries and annotations are based on Ensembl Version 99 (continued.)

Ensembl Gene ID	Ensembl Gene Name	Ensembl Gene Description	Gene Type	log_2 Fold Change (Day 30 vs. Pre-Second Vaccination)	Average log_2 CPM	Likelihood Ratio Test Statistic	P-Value	FDR Adjusted P-Value
ENSG00000177706	FAM20C	FAM20C golgi associated secretory pathway kinase [Source:HGNC Symbol;Acc:HGNC:22140]	protein coding	1.17	0.70	19.04	<0.0001	0.0005
ENSG00000177989	ODF3B	outer dense fiber of sperm tails 3B [Source:HGNC Symbol;Acc:HGNC:34388]	protein coding	1.17	5.49	69.43	<0.0001	<0.0001
ENSG00000171115	GIMAP8	GTPase, IMAP family member 8 [Source:HGNC Symbol;Acc:HGNC:21792]	protein coding	1.16	2.79	24.90	<0.0001	<0.0001
ENSG00000138496	PARP9	poly(ADP-ribose) polymerase family member 9 [Source:HGNC Symbol;Acc:HGNC:24118]	protein coding	1.16	6.49	83.53	<0.0001	<0.0001
ENSG00000104974	LILRA1	leukocyte immunoglobulin like receptor A1 [Source:HGNC Symbol;Acc:HGNC:6602]	protein coding	1.15	3.03	24.28	<0.0001	<0.0001
ENSG00000167850	CD300C	CD300c molecule [Source:HGNC Symbol;Acc:HGNC:19320]	protein coding	1.15	1.48	21.10	<0.0001	0.0002
ENSG00000168062	BATF2	basic leucine zipper ATF-like transcription factor 2 [Source:HGNC Symbol;Acc:HGNC:25163]	protein coding	1.15	1.46	19.65	<0.0001	0.0004
ENSG00000133816	MICAL2	microtubule associated monooxygenase, calponin and LIM domain containing 2 [Source:HGNC Symbol;Acc:HGNC:24693]	protein coding	1.14	1.42	28.08	<0.0001	<0.0001
ENSG00000147459	DOCK5	dedicator of cytokinesis 5 [Source:HGNC Symbol;Acc:HGNC:23476]	protein coding	1.14	1.74	26.10	<0.0001	<0.0001
ENSG00000178789	CD300LB	CD300 molecule like family member b [Source:HGNC Symbol;Acc:HGNC:30811]	protein coding	1.13	1.21	13.33	0.0003	0.0062
ENSG00000235568	NFAM1	NFAT activating protein with ITAM motif 1 [Source:HGNC Symbol;Acc:HGNC:29872]	protein coding	1.13	4.03	28.13	<0.0001	<0.0001
ENSG00000160593	JAML	junction adhesion molecule like [Source:HGNC Symbol;Acc:HGNC:19084]	protein coding	1.12	4.40	26.55	<0.0001	<0.0001
ENSG00000126709	IFI6	interferon alpha inducible protein 6 [Source:HGNC Symbol;Acc:HGNC:4054]	protein coding	1.12	5.09	55.11	<0.0001	<0.0001
ENSG00000136250	AOAH	acyloxyacyl hydrolase [Source:HGNC Symbol;Acc:HGNC:548]	protein coding	1.12	3.35	29.40	<0.0001	<0.0001
ENSG00000100079	LGALS2	galectin 2 [Source:HGNC Symbol;Acc:HGNC:6562]	protein coding	1.10	1.30	16.85	<0.0001	0.0013
ENSG00000221869	CEBPD	CCAAT enhancer binding protein delta [Source:HGNC Symbol;Acc:HGNC:1835]	protein coding	1.10	3.77	24.77	<0.0001	<0.0001
ENSG00000114737	CISH	cytokine inducible SH2 containing protein [Source:HGNC Symbol;Acc:HGNC:1984]	protein coding	1.09	2.00	23.98	<0.0001	<0.0001

Confidential/Proprietary Information

Table S33: Genes differentially expressed at Day 30 compared to pre-second vaccination (Study Arm 2 A/H7N9+AS03, D1, 29, B cells). Genes are sorted by descending absolute log_2 fold change. Gene model summaries and annotations are based on Ensembl Version 99 (continued.)

Ensembl Gene ID	Ensembl Gene Name	Ensembl Gene Description	Gene Type	log_2 Fold Change (Day 30 vs. Pre-Second Vaccination)	Average log_2 CPM	Likelihood Ratio Test Statistic	P-Value	FDR Adjusted P-Value
ENSG00000087237	CETP	cholesteryl ester transfer protein [Source:HGNC Symbol;Acc:HGNC:1869]	protein coding	1.09	1.98	20.90	<0.0001	0.0002
ENSG00000156587	UBE2L6	ubiquitin conjugating enzyme E2 L6 [Source:HGNC Symbol;Acc:HGNC:12490]	protein coding	1.09	6.75	75.21	<0.0001	<0.0001
ENSG00000181409	AATK	apoptosis associated tyrosine kinase [Source:HGNC Symbol;Acc:HGNC:21]	protein coding	1.07	0.74	16.63	<0.0001	0.0015
ENSG00000120217	CD274	CD274 molecule [Source:HGNC Symbol;Acc:HGNC:17635]	protein coding	1.07	1.71	23.73	<0.0001	<0.0001
ENSG00000184979	USP18	ubiquitin specific peptidase 18 [Source:HGNC Symbol;Acc:HGNC:12616]	protein coding	1.07	1.89	49.63	<0.0001	<0.0001
ENSG00000005249	PRKAR2B	protein kinase cAMP-dependent type II regulatory subunit beta [Source:HGNC Symbol;Acc:HGNC:9392]	protein coding	1.06	1.64	13.28	0.0003	0.0063
ENSG00000175538	KCNE3	potassium voltage-gated channel subfamily E regulatory subunit 3 [Source:HGNC Symbol;Acc:HGNC:6243]	protein coding	1.06	0.98	11.36	0.0007	0.0141
ENSG00000172322	CLEC12A	C-type lectin domain family 12 member A [Source:HGNC Symbol;Acc:HGNC:31713]	protein coding	1.05	2.81	29.35	<0.0001	<0.0001
ENSG00000143382	ADAMTSL4	ADAMTS like 4 [Source:HGNC Symbol;Acc:HGNC:19706]	protein coding	1.05	2.93	30.44	<0.0001	<0.0001
ENSG00000090339	ICAM1	intercellular adhesion molecule 1 [Source:HGNC Symbol;Acc:HGNC:5344]	protein coding	1.05	3.08	40.45	<0.0001	<0.0001
ENSG00000138061	CYP1B1	cytochrome P450 family 1 subfamily B member 1 [Source:HGNC Symbol;Acc:HGNC:2597]	protein coding	1.05	1.21	26.94	<0.0001	<0.0001
ENSG00000171777	RASGRP4	RAS guanyl releasing protein 4 [Source:HGNC Symbol;Acc:HGNC:18958]	protein coding	1.04	2.54	39.58	<0.0001	<0.0001
ENSG00000196209	SIRPB2	signal regulatory protein beta 2 [Source:HGNC Symbol;Acc:HGNC:16247]	protein coding	1.04	1.78	14.72	0.0001	0.0034
ENSG00000177575	CD163	CD163 molecule [Source:HGNC Symbol;Acc:HGNC:1631]	protein coding	1.04	2.10	17.26	<0.0001	0.0011
ENSG00000184232	OAF	out at first homolog [Source:HGNC Symbol;Acc:HGNC:28752]	protein coding	1.04	2.39	24.50	<0.0001	<0.0001
ENSG00000203747	FCGR3A	Fc fragment of IgG receptor IIIa [Source:HGNC Symbol;Acc:HGNC:3619]	protein coding	1.03	4.08	24.79	<0.0001	<0.0001
ENSG00000187608	ISG15	ISG15 ubiquitin like modifier [Source:HGNC Symbol;Acc:HGNC:4053]	protein coding	1.03	5.16	58.94	<0.0001	<0.0001
ENSG00000138449	SLC40A1	solute carrier family 40 member 1 [Source:HGNC Symbol;Acc:HGNC:10909]	protein coding	1.03	1.21	10.94	0.0009	0.0167

Table S33: Genes differentially expressed at Day 30 compared to pre-second vaccination (Study Arm 2 A/H7N9+AS03, D1, 29, B cells). Genes are sorted by descending absolute log_2 fold change. Gene model summaries and annotations are based on Ensembl Version 99 (continued.)

Ensembl Gene ID	Ensembl Gene Name	Ensembl Gene Description	Gene Type	log_2 Fold Change (Day 30 vs. Pre-Second Vaccination)	Average log_2 CPM	Likelihood Ratio Test Statistic	P-Value	FDR Adjusted P-Value
ENSG00000166825	ANPEP	alanyl aminopeptidase, membrane [Source:HGNC Symbol;Acc:HGNC:500]	protein coding	1.02	3.27	26.60	<0.0001	<0.0001
ENSG00000167207	NOD2	nucleotide binding oligomerization domain containing 2 [Source:HGNC Symbol;Acc:HGNC:5331]	protein coding	1.02	3.01	56.08	<0.0001	<0.0001
ENSG00000085514	PILRA	paired immunoglobin like type 2 receptor alpha [Source:HGNC Symbol;Acc:HGNC:20396]	protein coding	1.02	3.09	27.36	<0.0001	<0.0001
ENSG00000168329	CX3CR1	C-X3-C motif chemokine receptor 1 [Source:HGNC Symbol;Acc:HGNC:2558]	protein coding	1.02	2.91	16.03	<0.0001	0.0019
ENSG00000010327	STAB1	stabilin 1 [Source:HGNC Symbol;Acc:HGNC:18628]	protein coding	1.02	3.83	18.91	<0.0001	0.0005
ENSG00000002549	LAP3	leucine aminopeptidase 3 [Source:HGNC Symbol;Acc:HGNC:18449]	protein coding	1.01	5.87	61.48	<0.0001	<0.0001
ENSG00000072110	ACTN1	actinin alpha 1 [Source:HGNC Symbol;Acc:HGNC:163]	protein coding	1.01	2.89	16.16	<0.0001	0.0018
ENSG00000224397	SMIM25	small integral membrane protein 25 [Source:HGNC Symbol;Acc:HGNC:50328]	lncRNA	1.01	2.45	14.77	0.0001	0.0033
ENSG00000075426	FOSL2	FOS like 2, AP-1 transcription factor subunit [Source:HGNC Symbol;Acc:HGNC:3798]	protein coding	1.01	3.23	28.76	<0.0001	<0.0001
ENSG00000111331	OAS3	2'-5'-oligoadenylate synthetase 3 [Source:HGNC Symbol;Acc:HGNC:8088]	protein coding	1.00	5.61	107.25	<0.0001	<0.0001
ENSG00000124491	F13A1	coagulation factor XIII A chain [Source:HGNC Symbol;Acc:HGNC:3531]	protein coding	0.99	2.86	10.91	0.001	0.0169
ENSG00000175029	CTBP2	C-terminal binding protein 2 [Source:HGNC Symbol;Acc:HGNC:2495]	protein coding	0.99	1.55	26.91	<0.0001	<0.0001
ENSG00000162614	NEXN	nexilin F-actin binding protein [Source:HGNC Symbol;Acc:HGNC:29557]	protein coding	0.98	2.25	33.40	<0.0001	<0.0001
ENSG00000137486	ARRB1	arrestin beta 1 [Source:HGNC Symbol;Acc:HGNC:711]	protein coding	0.98	2.68	19.77	<0.0001	0.0003
ENSG00000215458	AATBC	apoptosis associated transcript in bladder cancer [Source:HGNC Symbol;Acc:HGNC:51526]	lncRNA	0.98	1.94	16.64	<0.0001	0.0015
ENSG00000166002	SMCO4	single-pass membrane protein with coiled-coil domains 4 [Source:HGNC Symbol;Acc:HGNC:24810]	protein coding	0.97	2.22	33.90	<0.0001	<0.0001
ENSG00000133561	GIMAP6	GTPase, IMAP family member 6 [Source:HGNC Symbol;Acc:HGNC:21918]	protein coding	0.97	2.86	12.10	0.0005	0.0104
ENSG00000133106	EPSTI1	epithelial stromal interaction 1 [Source:HGNC Symbol;Acc:HGNC:16465]	protein coding	0.97	6.15	82.28	<0.0001	<0.0001

Table S33: Genes differentially expressed at Day 30 compared to pre-second vaccination (Study Arm 2 A/H7N9+AS03, D1, 29, B cells). Genes are sorted by descending absolute log_2 fold change. Gene model summaries and annotations are based on Ensembl Version 99 (continued.)

Ensembl Gene ID	Ensembl Gene Name	Ensembl Gene Description	Gene Type	log_2 Fold Change (Day 30 vs. Pre-Second Vaccination)	Average log_2 CPM	Likelihood Ratio Test Statistic	P-Value	FDR Adjusted P-Value
ENSG00000139679	LPAR6	lysophosphatidic acid receptor 6 [Source:HGNC Symbol;Acc:HGNC:15520]	protein coding	0.97	2.22	31.47	<0.0001	<0.0001
ENSG00000156052	GNAQ	G protein subunit alpha q [Source:HGNC Symbol;Acc:HGNC:4390]	protein coding	0.96	2.12	15.41	<0.0001	0.0025
ENSG00000131042	LILRB2	leukocyte immunoglobulin like receptor B2 [Source:HGNC Symbol;Acc:HGNC:6606]	protein coding	0.96	4.80	27.35	<0.0001	<0.0001
ENSG00000119686	FLVCR2	feline leukemia virus subgroup C cellular receptor family member 2 [Source:HGNC Symbol;Acc:HGNC:20105]	protein coding	0.96	0.60	23.08	<0.0001	<0.0001
ENSG00000138166	DUSP5	dual specificity phosphatase 5 [Source:HGNC Symbol;Acc:HGNC:3071]	protein coding	0.95	4.13	16.04	<0.0001	0.0019
ENSG00000148411	NACC2	NACC family member 2 [Source:HGNC Symbol;Acc:HGNC:23846]	protein coding	0.95	2.76	26.92	<0.0001	<0.0001
ENSG00000140090	SLC24A4	solute carrier family 24 member 4 [Source:HGNC Symbol;Acc:HGNC:10978]	protein coding	0.95	1.96	13.16	0.0003	0.0066
ENSG00000100342	APOL1	apolipoprotein L1 [Source:HGNC Symbol;Acc:HGNC:618]	protein coding	0.94	4.40	65.14	<0.0001	<0.0001
ENSG00000143226	FCGR2A	Fc fragment of IgG receptor IIa [Source:HGNC Symbol;Acc:HGNC:3616]	protein coding	0.94	3.70	19.03	<0.0001	0.0005
ENSG00000186818	LILRB4	leukocyte immunoglobulin like receptor B4 [Source:HGNC Symbol;Acc:HGNC:6608]	protein coding	0.93	3.47	16.75	<0.0001	0.0014
ENSG00000132530	XAF1	XIAP associated factor 1 [Source:HGNC Symbol;Acc:HGNC:30932]	protein coding	0.93	5.89	50.85	<0.0001	<0.0001
ENSG00000115828	QPCT	glutaminyl-peptide cyclotransferase [Source:HGNC Symbol;Acc:HGNC:9753]	protein coding	0.93	1.32	22.23	<0.0001	0.0001
ENSG00000126822	PLEKHG3	pleckstrin homology and RhoGEF domain containing G3 [Source:HGNC Symbol;Acc:HGNC:20364]	protein coding	0.93	1.28	13.87	0.0002	0.0049
ENSG00000136830	NIBAN2	niban apoptosis regulator 2 [Source:HGNC Symbol;Acc:HGNC:25282]	protein coding	0.93	1.79	12.46	0.0004	0.0089
ENSG00000137462	TLR2	toll like receptor 2 [Source:HGNC Symbol;Acc:HGNC:11848]	protein coding	0.93	3.12	24.32	<0.0001	<0.0001
ENSG00000135900	MRPL44	mitochondrial ribosomal protein L44 [Source:HGNC Symbol;Acc:HGNC:16650]	protein coding	0.92	4.89	63.25	<0.0001	<0.0001
ENSG00000116990	MYCL	MYCL proto-oncogene, bHLH transcription factor [Source:HGNC Symbol;Acc:HGNC:7555]	protein coding	0.92	0.88	13.95	0.0002	0.0047
ENSG00000151689	INPP1	inositol polyphosphate-1-phosphatase [Source:HGNC Symbol;Acc:HGNC:6071]	protein coding	0.92	3.13	50.52	<0.0001	<0.0001

Table S33: Genes differentially expressed at Day 30 compared to pre-second vaccination (Study Arm 2 A/H7N9+AS03, D1, 29, B cells). Genes are sorted by descending absolute log_2 fold change. Gene model summaries and annotations are based on Ensembl Version 99 (continued.)

Ensembl Gene ID	Ensembl Gene Name	Ensembl Gene Description	Gene Type	log_2 Fold Change (Day 30 vs. Pre-Second Vaccination)	Average log_2 CPM	Likelihood Ratio Test Statistic	P-Value	FDR Adjusted P-Value
ENSG00000124731	TREM1	triggering receptor expressed on myeloid cells 1 [Source:HGNC Symbol;Acc:HGNC:17760]	protein coding	0.91	2.15	15.00	0.0001	0.003
ENSG00000133246	PRAM1	PML-RARA regulated adaptor molecule 1 [Source:HGNC Symbol;Acc:HGNC:30091]	protein coding	0.91	2.88	15.36	<0.0001	0.0026
ENSG00000197766	CFD	complement factor D [Source:HGNC Symbol;Acc:HGNC:2771]	protein coding	0.91	3.71	24.06	<0.0001	<0.0001
ENSG00000142405	NLRP12	NLR family pyrin domain containing 12 [Source:HGNC Symbol;Acc:HGNC:22938]	protein coding	0.91	1.16	12.89	0.0003	0.0074
ENSG00000179348	GATA2	GATA binding protein 2 [Source:HGNC Symbol;Acc:HGNC:4171]	protein coding	0.91	0.60	13.53	0.0002	0.0057
ENSG00000173083	HPSE	heparanase [Source:HGNC Symbol;Acc:HGNC:5164]	protein coding	0.90	2.16	30.60	<0.0001	<0.0001
ENSG00000163563	MNDA	myeloid cell nuclear differentiation antigen [Source:HGNC Symbol;Acc:HGNC:7183]	protein coding	0.89	5.21	19.65	<0.0001	0.0004
ENSG00000139832	RAB20	RAB20, member RAS oncogene family [Source:HGNC Symbol;Acc:HGNC:18260]	protein coding	0.88	1.50	16.89	<0.0001	0.0013
ENSG00000196329	GIMAP5	GTPase, IMAP family member 5 [Source:HGNC Symbol;Acc:HGNC:18005]	protein coding	0.88	0.50	9.69	0.0019	0.0282
ENSG00000121858	TNFSF10	TNF superfamily member 10 [Source:HGNC Symbol;Acc:HGNC:11925]	protein coding	0.88	5.01	29.02	<0.0001	<0.0001
ENSG00000168899	VAMP5	vesicle associated membrane protein 5 [Source:HGNC Symbol;Acc:HGNC:12646]	protein coding	0.88	4.33	38.92	<0.0001	<0.0001
ENSG00000104093	DMXL2	Dmx like 2 [Source:HGNC Symbol;Acc:HGNC:2938]	protein coding	0.88	3.16	20.99	<0.0001	0.0002
ENSG00000112782	CLIC5	chloride intracellular channel 5 [Source:HGNC Symbol;Acc:HGNC:13517]	protein coding	0.88	1.16	24.79	<0.0001	<0.0001
ENSG00000181444	ZNF467	zinc finger protein 467 [Source:HGNC Symbol;Acc:HGNC:23154]	protein coding	0.87	2.19	14.27	0.0002	0.0041
ENSG00000234518	PTGES3P1	prostaglandin E synthase 3 pseudogene 1 [Source:HGNC Symbol;Acc:HGNC:43824]	processed pseudogene	0.87	1.99	34.23	<0.0001	<0.0001
ENSG00000163412	EIF4E3	eukaryotic translation initiation factor 4E family member 3 [Source:HGNC Symbol;Acc:HGNC:31837]	protein coding	0.87	2.76	36.09	<0.0001	<0.0001
ENSG00000185338	SOCS1	suppressor of cytokine signaling 1 [Source:HGNC Symbol;Acc:HGNC:19383]	protein coding	0.87	3.89	25.65	<0.0001	<0.0001

Table S33: Genes differentially expressed at Day 30 compared to pre-second vaccination (Study Arm 2 A/H7N9+AS03, D1, 29, B cells). Genes are sorted by descending absolute log_2 fold change. Gene model summaries and annotations are based on Ensembl Version 99 (continued.)

Ensembl Gene ID	Ensembl Gene Name	Ensembl Gene Description	Gene Type	log_2 Fold Change (Day 30 vs. Pre-Second Vaccination)	Average log_2 CPM	Likelihood Ratio Test Statistic	P-Value	FDR Adjusted P-Value
ENSG00000021762	OSBPL5	oxysterol binding protein like 5 [Source:HGNC Symbol;Acc:HGNC:16392]	protein coding	0.87	1.59	15.58	<0.0001	0.0023
ENSG00000100453	GZMB	granzyme B [Source:HGNC Symbol;Acc:HGNC:4709]	protein coding	0.86	1.52	9.43	0.0021	0.0315
ENSG00000132205	EMILIN2	elastin microfibril interfacer 2 [Source:HGNC Symbol;Acc:HGNC:19881]	protein coding	0.86	3.86	22.83	<0.0001	<0.0001
ENSG00000089041	P2RX7	purinergic receptor P2X 7 [Source:HGNC Symbol;Acc:HGNC:8537]	protein coding	0.86	3.34	29.23	<0.0001	<0.0001
ENSG00000134470	IL15RA	interleukin 15 receptor subunit alpha [Source:HGNC Symbol;Acc:HGNC:5978]	protein coding	0.85	3.05	70.26	<0.0001	<0.0001
ENSG00000182578	CSF1R	colony stimulating factor 1 receptor [Source:HGNC Symbol;Acc:HGNC:2433]	protein coding	0.85	4.09	14.20	0.0002	0.0042
ENSG00000135709	KIAA0513	KIAA0513 [Source:HGNC Symbol;Acc:HGNC:29058]	protein coding	0.85	3.46	39.29	<0.0001	<0.0001
ENSG00000020577	SAMD4A	sterile alpha motif domain containing 4A [Source:HGNC Symbol;Acc:HGNC:23023]	protein coding	0.85	4.46	36.46	<0.0001	<0.0001
ENSG00000131669	NINJ1	ninjurin 1 [Source:HGNC Symbol;Acc:HGNC:7824]	protein coding	0.84	2.85	21.76	<0.0001	0.0001
ENSG00000287839		novel transcript	lncRNA	0.84	2.38	28.72	<0.0001	<0.0001
ENSG00000163191	S100A11	S100 calcium binding protein A11 [Source:HGNC Symbol;Acc:HGNC:10488]	protein coding	0.84	4.58	27.24	<0.0001	<0.0001
ENSG00000130164	LDLR	low density lipoprotein receptor [Source:HGNC Symbol;Acc:HGNC:6547]	protein coding	0.84	2.82	17.51	<0.0001	0.001
ENSG00000122877	EGR2	early growth response 2 [Source:HGNC Symbol;Acc:HGNC:3239]	protein coding	0.84	1.89	10.29	0.0013	0.022
ENSG00000168394	TAP1	transporter 1, ATP binding cassette subfamily B member [Source:HGNC Symbol;Acc:HGNC:43]	protein coding	0.83	8.29	69.13	<0.0001	<0.0001
ENSG00000100911	PSME2	proteasome activator subunit 2 [Source:HGNC Symbol;Acc:HGNC:9569]	protein coding	0.83	7.17	82.45	<0.0001	<0.0001
ENSG00000125384	PTGER2	prostaglandin E receptor 2 [Source:HGNC Symbol;Acc:HGNC:9594]	protein coding	0.83	1.71	9.72	0.0018	0.0278
ENSG00000213203	GIMAP1	GTPase, IMAP family member 1 [Source:HGNC Symbol;Acc:HGNC:23237]	protein coding	0.83	2.79	16.18	<0.0001	0.0018
ENSG00000148175	STOM	stomatin [Source:HGNC Symbol;Acc:HGNC:3383]	protein coding	0.83	3.40	34.03	<0.0001	<0.0001

Table S33: Genes differentially expressed at Day 30 compared to pre-second vaccination (Study Arm 2 A/H7N9+AS03, D1, 29, B cells). Genes are sorted by descending absolute log_2 fold change. Gene model summaries and annotations are based on Ensembl Version 99 (continued.)

Ensembl Gene ID	Ensembl Gene Name	Ensembl Gene Description	Gene Type	log_2 Fold Change (Day 30 vs. Pre-Second Vaccination)	Average log_2 CPM	Likelihood Ratio Test Statistic	P-Value	FDR Adjusted P-Value
ENSG00000214212	C19orf38	chromosome 19 open reading frame 38 [Source:HGNC Symbol;Acc:HGNC:34073]	protein coding	0.82	3.34	27.14	<0.0001	<0.0001
ENSG00000178695	KCTD12	potassium channel tetramerization domain containing 12 [Source:HGNC Symbol;Acc:HGNC:14678]	protein coding	0.82	4.54	23.36	<0.0001	<0.0001
ENSG00000006534	ALDH3B1	aldehyde dehydrogenase 3 family member B1 [Source:HGNC Symbol;Acc:HGNC:410]	protein coding	0.82	2.65	15.87	<0.0001	0.0021
ENSG00000128335	APOL2	apolipoprotein L2 [Source:HGNC Symbol;Acc:HGNC:619]	protein coding	0.82	6.06	72.74	<0.0001	<0.0001
ENSG00000107281	NPDC1	neural proliferation, differentiation and control 1 [Source:HGNC Symbol;Acc:HGNC:7899]	protein coding	0.81	1.07	11.31	0.0008	0.0143
ENSG00000198959	TGM2	transglutaminase 2 [Source:HGNC Symbol;Acc:HGNC:11778]	protein coding	0.81	3.27	27.22	<0.0001	<0.0001
ENSG00000107551	RASSF4	Ras association domain family member 4 [Source:HGNC Symbol;Acc:HGNC:20793]	protein coding	0.81	2.97	16.17	<0.0001	0.0018
ENSG00000162512	SDC3	syndecan 3 [Source:HGNC Symbol;Acc:HGNC:10660]	protein coding	0.80	1.81	13.23	0.0003	0.0064
ENSG00000159339	PADI4	peptidyl arginine deiminase 4 [Source:HGNC Symbol;Acc:HGNC:18368]	protein coding	0.80	1.76	15.95	<0.0001	0.002
ENSG00000185880	TRIM69	tripartite motif containing 69 [Source:HGNC Symbol;Acc:HGNC:17857]	protein coding	0.80	5.75	47.89	<0.0001	<0.0001
ENSG00000068079	IFI35	interferon induced protein 35 [Source:HGNC Symbol;Acc:HGNC:5399]	protein coding	0.79	5.55	52.31	<0.0001	<0.0001
ENSG00000074706	IPCEF1	interaction protein for cytohesin exchange factors 1 [Source:HGNC Symbol;Acc:HGNC:21204]	protein coding	0.79	0.76	10.06	0.0015	0.024
ENSG00000129757	CDKN1C	cyclin dependent kinase inhibitor 1C [Source:HGNC Symbol;Acc:HGNC:1786]	protein coding	0.79	2.40	12.01	0.0005	0.0108
ENSG00000258623		novel transcript, antisense to RAD51L1	lncRNA	-0.77	0.19	18.69	<0.0001	0.0006
ENSG00000118515	SGK1	serum/glucocorticoid regulated kinase 1 [Source:HGNC Symbol;Acc:HGNC:10810]	protein coding	0.77	3.44	27.69	<0.0001	<0.0001
ENSG00000122862	SRGN	serglycin [Source:HGNC Symbol;Acc:HGNC:9361]	protein coding	0.77	6.35	21.92	<0.0001	0.0001
ENSG00000121797	CCRL2	C-C motif chemokine receptor like 2 [Source:HGNC Symbol;Acc:HGNC:1612]	protein coding	0.77	0.57	17.66	<0.0001	0.0009
ENSG00000106605	BLVRA	biliverdin reductase A [Source:HGNC Symbol;Acc:HGNC:1062]	protein coding	0.77	3.20	44.22	<0.0001	<0.0001

Table S33: Genes differentially expressed at Day 30 compared to pre-second vaccination (Study Arm 2 A/H7N9+AS03, D1, 29, B cells). Genes are sorted by descending absolute log_2 fold change. Gene model summaries and annotations are based on Ensembl Version 99 (continued.)

Ensembl Gene ID	Ensembl Gene Name	Ensembl Gene Description	Gene Type	log_2 Fold Change (Day 30 vs. Pre-Second Vaccination)	Average log_2 CPM	Likelihood Ratio Test Statistic	P-Value	FDR Adjusted P-Value
ENSG00000245848	CEBPA	CCAAT enhancer binding protein alpha [Source:HGNC Symbol;Acc:HGNC:1833]	protein coding	0.77	1.88	10.73	0.0011	0.0183
ENSG00000174837	ADGRE1	adhesion G protein-coupled receptor E1 [Source:HGNC Symbol;Acc:HGNC:3336]	protein coding	0.77	1.13	11.08	0.0009	0.0158
ENSG00000167851	CD300A	CD300a molecule [Source:HGNC Symbol;Acc:HGNC:19319]	protein coding	0.77	3.89	32.05	<0.0001	<0.0001
ENSG00000166750	SLFN5	schlafen family member 5 [Source:HGNC Symbol;Acc:HGNC:28286]	protein coding	0.76	4.17	26.46	<0.0001	<0.0001
ENSG00000128284	APOL3	apolipoprotein L3 [Source:HGNC Symbol;Acc:HGNC:14868]	protein coding	0.76	6.56	74.66	<0.0001	<0.0001
ENSG00000123384	LRP1	LDL receptor related protein 1 [Source:HGNC Symbol;Acc:HGNC:6692]	protein coding	0.75	4.45	15.70	<0.0001	0.0022
ENSG00000168209	DDIT4	DNA damage inducible transcript 4 [Source:HGNC Symbol;Acc:HGNC:24944]	protein coding	-0.75	5.19	17.72	<0.0001	0.0009
ENSG00000157557	ETS2	ETS proto-oncogene 2, transcription factor [Source:HGNC Symbol;Acc:HGNC:3489]	protein coding	0.75	2.73	26.55	<0.0001	<0.0001
ENSG00000010610	CD4	CD4 molecule [Source:HGNC Symbol;Acc:HGNC:1678]	protein coding	0.75	3.80	13.08	0.0003	0.0068
ENSG00000184584	STING1	stimulator of interferon response cGAMP interactor 1 [Source:HGNC Symbol;Acc:HGNC:27962]	protein coding	0.74	3.05	12.42	0.0004	0.009
ENSG00000205045	SLFN12L	schlafen family member 12 like [Source:HGNC Symbol;Acc:HGNC:33920]	protein coding	0.74	0.79	21.07	<0.0001	0.0002
ENSG00000101347	SAMHD1	SAM and HD domain containing deoxynucleoside triphosphate triphosphohydrolase 1 [Source:HGNC Symbol;Acc:HGNC:15925]	protein coding	0.74	6.00	28.81	<0.0001	<0.0001
ENSG00000274021		novel transcript, antisense to DUSP6	lncRNA	0.73	0.39	9.70	0.0018	0.0281
ENSG00000104267	CA2	carbonic anhydrase 2 [Source:HGNC Symbol;Acc:HGNC:1373]	protein coding	0.73	1.20	11.11	0.0009	0.0156
ENSG00000163840	DTX3L	deltex E3 ubiquitin ligase 3L [Source:HGNC Symbol;Acc:HGNC:30323]	protein coding	0.73	6.11	35.63	<0.0001	<0.0001
ENSG00000198876	DCAF12	DDB1 and CUL4 associated factor 12 [Source:HGNC Symbol;Acc:HGNC:19911]	protein coding	0.72	3.01	11.18	0.0008	0.0151
ENSG00000121807	CCR2	C-C motif chemokine receptor 2 [Source:HGNC Symbol;Acc:HGNC:1603]	protein coding	0.72	4.07	11.63	0.0006	0.0126
ENSG00000105835	NAMPT	nicotinamide phosphoribosyltransferase [Source:HGNC Symbol;Acc:HGNC:30092]	protein coding	0.72	5.03	43.74	<0.0001	<0.0001

Table S33: Genes differentially expressed at Day 30 compared to pre-second vaccination (Study Arm 2 A/H7N9+AS03, D1, 29, B cells). Genes are sorted by descending absolute log_2 fold change. Gene model summaries and annotations are based on Ensembl Version 99 (continued.)

Ensembl Gene ID	Ensembl Gene Name	Ensembl Gene Description	Gene Type	log_2 Fold Change (Day 30 vs. Pre-Second Vaccination)	Average log_2 CPM	Likelihood Ratio Test Statistic	P-Value	FDR Adjusted P-Value
ENSG00000184730	APOBR	apolipoprotein B receptor [Source:HGNC Symbol;Acc:HGNC:24087]	protein coding	0.72	3.65	18.06	<0.0001	0.0008
ENSG00000185507	IRF7	interferon regulatory factor 7 [Source:HGNC Symbol;Acc:HGNC:6122]	protein coding	0.71	7.26	48.42	<0.0001	<0.0001
ENSG00000253669	GASAL1	growth arrest associated lncRNA 1 [Source:HGNC Symbol;Acc:HGNC:53461]	lncRNA	0.71	1.19	18.22	<0.0001	0.0007
ENSG00000271730		novel transcript, antisense to SESN1	lncRNA	-0.70	0.73	11.02	0.0009	0.0162
ENSG00000120708	TGFBI	transforming growth factor beta induced [Source:HGNC Symbol;Acc:HGNC:11771]	protein coding	0.70	3.93	12.20	0.0005	0.0099
ENSG00000185909	KLHDC8B	kelch domain containing 8B [Source:HGNC Symbol;Acc:HGNC:28557]	protein coding	0.70	0.71	19.99	<0.0001	0.0003
ENSG00000245466		novel transcript	lncRNA	-0.70	0.59	13.85	0.0002	0.0049
ENSG00000146425	DYNLT1	dynein light chain Tctex-type 1 [Source:HGNC Symbol;Acc:HGNC:11697]	protein coding	0.70	4.52	59.92	<0.0001	<0.0001
ENSG00000183696	UPP1	uridine phosphorylase 1 [Source:HGNC Symbol;Acc:HGNC:12576]	protein coding	0.70	2.92	24.06	<0.0001	<0.0001
ENSG00000198673	TAFA2	TAFA chemokine like family member 2 [Source:HGNC Symbol;Acc:HGNC:21589]	protein coding	0.70	0.55	14.26	0.0002	0.0041
ENSG00000136514	RTP4	receptor transporter protein 4 [Source:HGNC Symbol;Acc:HGNC:23992]	protein coding	0.69	2.55	17.94	<0.0001	0.0008
ENSG00000026103	FAS	Fas cell surface death receptor [Source:HGNC Symbol;Acc:HGNC:11920]	protein coding	0.69	4.08	48.12	<0.0001	<0.0001
ENSG00000177409	SAMD9L	sterile alpha motif domain containing 9 like [Source:HGNC Symbol;Acc:HGNC:1349]	protein coding	0.69	7.75	62.47	<0.0001	<0.0001
ENSG00000188641	DPYD	dihydropyrimidine dehydrogenase [Source:HGNC Symbol;Acc:HGNC:3012]	protein coding	0.68	3.96	16.75	<0.0001	0.0014
ENSG00000198736	MSRB1	methionine sulfoxide reductase B1 [Source:HGNC Symbol;Acc:HGNC:14133]	protein coding	0.68	3.85	24.32	<0.0001	<0.0001
ENSG00000254503		high-mobility group box 3 (HMGB3) pseudogene	processed pseudogene	0.68	0.73	16.59	<0.0001	0.0015
ENSG00000131979	GCH1	GTP cyclohydrolase 1 [Source:HGNC Symbol;Acc:HGNC:4193]	protein coding	0.68	4.90	31.47	<0.0001	<0.0001
ENSG00000259321		novel transcript	lncRNA	0.68	0.99	22.25	<0.0001	0.0001
ENSG00000256262	USP30-AS1	USP30 antisense RNA 1 [Source:HGNC Symbol;Acc:HGNC:40909]	lncRNA	0.68	2.68	40.25	<0.0001	<0.0001

Table S33: Genes differentially expressed at Day 30 compared to pre-second vaccination (Study Arm 2 A/H7N9+AS03, D1, 29, B cells). Genes are sorted by descending absolute log_2 fold change. Gene model summaries and annotations are based on Ensembl Version 99 (continued.)

Ensembl Gene ID	Ensembl Gene Name	Ensembl Gene Description	Gene Type	log_2 Fold Change (Day 30 vs. Pre-Second Vaccination)	Average log_2 CPM	Likelihood Ratio Test Statistic	P-Value	FDR Adjusted P-Value
ENSG00000138646	HERC5	HECT and RLD domain containing E3 ubiquitin protein ligase 5 [Source:HGNC Symbol;Acc:HGNC:24368]	protein coding	0.67	4.27	30.14	<0.0001	<0.0001
ENSG00000241399	CD302	CD302 molecule [Source:HGNC Symbol;Acc:HGNC:30843]	protein coding	0.67	1.06	16.39	<0.0001	0.0017
ENSG00000151726	ACSL1	acyl-CoA synthetase long chain family member 1 [Source:HGNC Symbol;Acc:HGNC:3569]	protein coding	0.67	4.76	20.46	<0.0001	0.0002
ENSG00000175489	LRRC25	leucine rich repeat containing 25 [Source:HGNC Symbol;Acc:HGNC:29806]	protein coding	0.67	4.39	13.65	0.0002	0.0054
ENSG00000116701	NCF2	neutrophil cytosolic factor 2 [Source:HGNC Symbol;Acc:HGNC:7661]	protein coding	0.67	5.54	18.57	<0.0001	0.0006
ENSG00000117226	GBP3	guanylate binding protein 3 [Source:HGNC Symbol;Acc:HGNC:4184]	protein coding	0.67	4.23	41.25	<0.0001	<0.0001
ENSG00000136048	DRAM1	DNA damage regulated autophagy modulator 1 [Source:HGNC Symbol;Acc:HGNC:25645]	protein coding	0.67	2.82	37.55	<0.0001	<0.0001
ENSG00000130487	KLHDC7B	kelch domain containing 7B [Source:HGNC Symbol;Acc:HGNC:25145]	protein coding	0.67	3.68	28.82	<0.0001	<0.0001
ENSG00000279339		TEC	TEC	-0.66	1.16	17.08	<0.0001	0.0012
ENSG00000026751	SLAMF7	SLAM family member 7 [Source:HGNC Symbol;Acc:HGNC:21394]	protein coding	0.66	6.01	10.53	0.0012	0.0198
ENSG00000170382	LRRN2	leucine rich repeat neuronal 2 [Source:HGNC Symbol;Acc:HGNC:16914]	protein coding	0.66	4.95	20.00	<0.0001	0.0003
ENSG00000018280	SLC11A1	solute carrier family 11 member 1 [Source:HGNC Symbol;Acc:HGNC:10907]	protein coding	0.66	4.84	13.13	0.0003	0.0067
ENSG00000233098	CCDC144NL-AS1	CCDC144NL antisense RNA 1 [Source:HGNC Symbol;Acc:HGNC:51340]	lncRNA	-0.66	2.54	30.12	<0.0001	<0.0001
ENSG00000196562	SULF2	sulfatase 2 [Source:HGNC Symbol;Acc:HGNC:20392]	protein coding	0.66	3.87	15.78	<0.0001	0.0022
ENSG00000136869	TLR4	toll like receptor 4 [Source:HGNC Symbol;Acc:HGNC:11850]	protein coding	0.66	3.42	12.76	0.0004	0.0078
ENSG00000165030	NFIL3	nuclear factor, interleukin 3 regulated [Source:HGNC Symbol;Acc:HGNC:7787]	protein coding	0.66	2.72	20.57	<0.0001	0.0002
ENSG00000196576	PLXNB2	plexin B2 [Source:HGNC Symbol;Acc:HGNC:9104]	protein coding	0.66	5.18	13.72	0.0002	0.0052
ENSG00000188313	PLSCR1	phospholipid scramblase 1 [Source:HGNC Symbol;Acc:HGNC:9092]	protein coding	0.65	4.47	48.60	<0.0001	<0.0001

Table S33: Genes differentially expressed at Day 30 compared to pre-second vaccination (Study Arm 2 A/H7N9+AS03, D1, 29, B cells). Genes are sorted by descending absolute log_2 fold change. Gene model summaries and annotations are based on Ensembl Version 99 (continued.)

Ensembl Gene ID	Ensembl Gene Name	Ensembl Gene Description	Gene Type	log_2 Fold Change (Day 30 vs. Pre-Second Vaccination)	Average log_2 CPM	Likelihood Ratio Test Statistic	P-Value	FDR Adjusted P-Value
ENSG00000255587	RAB44	RAB44, member RAS oncogene family [Source:HGNC Symbol;Acc:HGNC:21068]	protein coding	0.65	0.33	8.43	0.0037	0.0486
ENSG00000156299	TIAM1	TIAM Rac1 associated GEF 1 [Source:HGNC Symbol;Acc:HGNC:11805]	protein coding	0.65	1.02	8.55	0.0035	0.046
ENSG00000112096	SOD2	superoxide dismutase 2 [Source:HGNC Symbol;Acc:HGNC:11180]	protein coding	0.64	5.53	32.19	<0.0001	<0.0001
ENSG00000182901	RGS7	regulator of G protein signaling 7 [Source:HGNC Symbol;Acc:HGNC:10003]	protein coding	-0.64	0.39	15.49	<0.0001	0.0024
ENSG00000049449	RCN1	reticulocalbin 1 [Source:HGNC Symbol;Acc:HGNC:9934]	protein coding	0.64	2.31	37.17	<0.0001	<0.0001
ENSG00000137878	GCOM1	GRINL1A complex locus 1 [Source:HGNC Symbol;Acc:HGNC:26424]	protein coding	-0.64	0.24	11.46	0.0007	0.0135
ENSG00000110080	ST3GAL4	ST3 beta-galactoside alpha-2,3-sialyltransferase 4 [Source:HGNC Symbol;Acc:HGNC:10864]	protein coding	0.64	0.50	8.63	0.0033	0.0445
ENSG00000128594	LRRC4	leucine rich repeat containing 4 [Source:HGNC Symbol;Acc:HGNC:15586]	protein coding	0.63	0.98	9.05	0.0026	0.0371
ENSG00000240219		novel transcript	lncRNA	0.63	3.95	14.86	0.0001	0.0032
ENSG00000128536	CDHR3	cadherin related family member 3 [Source:HGNC Symbol;Acc:HGNC:26308]	protein coding	-0.63	2.60	16.75	<0.0001	0.0014
ENSG00000132109	TRIM21	tripartite motif containing 21 [Source:HGNC Symbol;Acc:HGNC:11312]	protein coding	0.63	4.99	32.07	<0.0001	<0.0001
ENSG00000240065	PSMB9	proteasome 20S subunit beta 9 [Source:HGNC Symbol;Acc:HGNC:9546]	protein coding	0.63	7.76	59.83	<0.0001	<0.0001
ENSG00000204482	LST1	leukocyte specific transcript 1 [Source:HGNC Symbol;Acc:HGNC:14189]	protein coding	0.63	4.58	15.27	<0.0001	0.0027
ENSG00000198719	DLL1	delta like canonical Notch ligand 1 [Source:HGNC Symbol;Acc:HGNC:2908]	protein coding	0.62	1.33	16.83	<0.0001	0.0013
ENSG00000232810	TNF	tumor necrosis factor [Source:HGNC Symbol;Acc:HGNC:11892]	protein coding	0.62	2.24	10.44	0.0012	0.0205
ENSG00000111335	OAS2	2'-5'-oligoadenylate synthetase 2 [Source:HGNC Symbol;Acc:HGNC:8087]	protein coding	0.62	6.98	46.73	<0.0001	<0.0001
ENSG00000229644	NAMPTP1	nicotinamide phosphoribosyltransferase pseudogene 1 [Source:HGNC Symbol;Acc:HGNC:17633]	processed pseudogene	0.62	1.75	13.86	0.0002	0.0049
ENSG00000135077	HAVCR2	hepatitis A virus cellular receptor 2 [Source:HGNC Symbol;Acc:HGNC:18437]	protein coding	0.62	1.49	12.41	0.0004	0.0091

Table S33: Genes differentially expressed at Day 30 compared to pre-second vaccination (Study Arm 2 A/H7N9+AS03, D1, 29, B cells). Genes are sorted by descending absolute log_2 fold change. Gene model summaries and annotations are based on Ensembl Version 99 (continued.)

Ensembl Gene ID	Ensembl Gene Name	Ensembl Gene Description	Gene Type	log_2 Fold Change (Day 30 vs. Pre-Second Vaccination)	Average log_2 CPM	Likelihood Ratio Test Statistic	P-Value	FDR Adjusted P-Value
ENSG00000213928	IRF9	interferon regulatory factor 9 [Source:HGNC Symbol;Acc:HGNC:6131]	protein coding	0.62	5.27	36.32	<0.0001	<0.0001
ENSG00000163958	ZDHHC19	zinc finger DHHC-type containing 19 [Source:HGNC Symbol;Acc:HGNC:20713]	protein coding	-0.61	3.05	21.72	<0.0001	0.0001
ENSG00000282851	BISPR	BST2 interferon stimulated positive regulator [Source:HGNC Symbol;Acc:HGNC:51290]	lncRNA	0.61	4.07	44.07	<0.0001	<0.0001
ENSG00000240710		novel transcript	lncRNA	0.61	2.37	13.27	0.0003	0.0063
ENSG00000203804	ADAMTSL4-AS1	ADAMTSL4 antisense RNA 1 [Source:HGNC Symbol;Acc:HGNC:32041]	lncRNA	0.61	0.52	17.85	<0.0001	0.0008
ENSG00000137628	DDX60	DExD/H-box helicase 60 [Source:HGNC Symbol;Acc:HGNC:25942]	protein coding	0.61	5.00	33.84	<0.0001	<0.0001
ENSG00000133321	PLAAT4	phospholipase A and acyltransferase 4 [Source:HGNC Symbol;Acc:HGNC:9869]	protein coding	0.61	4.86	22.46	<0.0001	0.0001
ENSG00000146859	TMEM140	transmembrane protein 140 [Source:HGNC Symbol;Acc:HGNC:21870]	protein coding	0.60	4.84	35.38	<0.0001	<0.0001
ENSG00000287733		novel transcript	lncRNA	0.60	1.52	11.39	0.0007	0.0139
ENSG00000235505	CASP17P	caspase 17, pseudogene [Source:HGNC Symbol;Acc:HGNC:53657]	transcribed unprocessed pseudogene	0.60	4.47	35.97	<0.0001	<0.0001
ENSG00000167705	RILP	Rab interacting lysosomal protein [Source:HGNC Symbol;Acc:HGNC:30266]	protein coding	0.60	1.02	15.46	<0.0001	0.0025
ENSG00000154040	CABYR	calcium binding tyrosine phosphorylation regulated [Source:HGNC Symbol;Acc:HGNC:15569]	protein coding	-0.60	0.45	13.52	0.0002	0.0057
ENSG00000196517	SLC6A9	solute carrier family 6 member 9 [Source:HGNC Symbol;Acc:HGNC:11056]	protein coding	0.60	1.25	8.76	0.0031	0.042
ENSG00000179604	CDC42EP4	CDC42 effector protein 4 [Source:HGNC Symbol;Acc:HGNC:17147]	protein coding	0.60	0.54	12.85	0.0003	0.0075
ENSG00000261618	LINC02605	long intergenic non-protein coding RNA 2605 [Source:HGNC Symbol;Acc:HGNC:53974]	lncRNA	0.60	1.03	10.66	0.0011	0.0187
ENSG00000286644		novel transcript	lncRNA	-0.60	0.54	11.83	0.0006	0.0115
ENSG00000114268	PFKFB4	6-phosphofructo-2-kinase/fructose-2,6-biphosphatase 4 [Source:HGNC Symbol;Acc:HGNC:8875]	protein coding	0.60	1.71	11.94	0.0005	0.011
ENSG00000135838	NPL	N-acetylneuraminate pyruvate lyase [Source:HGNC Symbol;Acc:HGNC:16781]	protein coding	0.59	2.05	11.33	0.0008	0.0142

Confidential/Proprietary Information

Table S33: Genes differentially expressed at Day 30 compared to pre-second vaccination (Study Arm 2 A/H7N9+AS03, D1, 29, B cells). Genes are sorted by descending absolute log_2 fold change. Gene model summaries and annotations are based on Ensembl Version 99 (continued.)

Ensembl Gene ID	Ensembl Gene Name	Ensembl Gene Description	Gene Type	log_2 Fold Change (Day 30 vs. Pre-Second Vaccination)	Average log_2 CPM	Likelihood Ratio Test Statistic	P-Value	FDR Adjusted P-Value
ENSG00000065911	MTHFD2	methylenetetrahydrofolate dehydrogenase (NADP+ dependent) 2, methenyltetrahydrofolate cyclohydrolase [Source:HGNC Symbol;Acc:HGNC:7434]	protein coding	0.59	4.83	12.91	0.0003	0.0074
ENSG00000263069	RNF213-AS1	RNF213 antisense RNA 1 [Source:HGNC Symbol;Acc:HGNC:54402]	lncRNA	0.59	2.58	16.69	<0.0001	0.0014
ENSG00000283696		novel transcript	lncRNA	0.59	0.90	16.35	<0.0001	0.0017
ENSG00000136244	IL6	interleukin 6 [Source:HGNC Symbol;Acc:HGNC:6018]	protein coding	0.59	1.82	16.33	<0.0001	0.0017
ENSG00000261644		novel transcript, antisense to CYLD and NOD2	lncRNA	0.59	4.02	25.50	<0.0001	<0.0001
ENSG00000183734	ASCL2	achaete-scute family bHLH transcription factor 2 [Source:HGNC Symbol;Acc:HGNC:739]	protein coding	0.59	2.56	11.95	0.0005	0.011
ENSG00000057657	PRDM1	PR/SET domain 1 [Source:HGNC Symbol;Acc:HGNC:9346]	protein coding	0.59	5.09	13.53	0.0002	0.0057

Table S34: Genes differentially expressed at Day 32 compared to pre-second vaccination (Study Arm 2 A/H7N9+AS03, D1, 29, B cells). Genes are sorted by descending absolute log_2 fold change. Gene model summaries and annotations are based on Ensembl Version 99.

Ensembl Gene ID	Ensembl Gene Name	Ensembl Gene Description	Gene Type	log_2 Fold Change (Day 32 vs. Pre-Second Vaccination)	Average log_2 CPM	Likelihood Ratio Test Statistic	P-Value	FDR Adjusted P-Value
ENSG00000188536	HBA2	hemoglobin subunit alpha 2 [Source:HGNC Symbol;Acc:HGNC:4824]	protein coding	2.20	4.29	42.93	<0.0001	<0.0001
ENSG00000244734	HBB	hemoglobin subunit beta [Source:HGNC Symbol;Acc:HGNC:4827]	protein coding	2.06	6.07	35.79	<0.0001	<0.0001
ENSG00000229754	CXCR2P1	C-X-C motif chemokine receptor 2 pseudogene 1 [Source:HGNC Symbol;Acc:HGNC:6028]	transcribed unprocessed pseudogene	1.84	1.28	19.88	<0.0001	0.0072
ENSG00000108846	ABCC3	ATP binding cassette subfamily C member 3 [Source:HGNC Symbol;Acc:HGNC:54]	protein coding	1.52	1.21	16.80	<0.0001	0.0181
ENSG00000138449	SLC40A1	solute carrier family 40 member 1 [Source:HGNC Symbol;Acc:HGNC:10909]	protein coding	1.44	1.37	18.82	<0.0001	0.0099

Table S34: Genes differentially expressed at Day 32 compared to pre-second vaccination (Study Arm 2 A/H7N9+AS03, D1, 29, B cells). Genes are sorted by descending absolute log_2 fold change. Gene model summaries and annotations are based on Ensembl Version 99 (continued.)

Ensembl Gene ID	Ensembl Gene Name	Ensembl Gene Description	Gene Type	log_2 Fold Change (Day 32 vs. Pre-Second Vaccination)	Average log_2 CPM	Likelihood Ratio Test Statistic	P-Value	FDR Adjusted P-Value
ENSG00000150681	RGS18	regulator of G protein signaling 18 [Source:HGNC Symbol;Acc:HGNC:14261]	protein coding	1.41	2.20	11.99	0.0005	0.0483
ENSG00000088826	SMOX	spermine oxidase [Source:HGNC Symbol;Acc:HGNC:15862]	protein coding	1.38	1.80	13.88	0.0002	0.0338
ENSG00000005249	PRKAR2B	protein kinase cAMP-dependent type II regulatory subunit beta [Source:HGNC Symbol;Acc:HGNC:9392]	protein coding	1.36	1.96	12.40	0.0004	0.0439
ENSG00000123405	NFE2	nuclear factor, erythroid 2 [Source:HGNC Symbol;Acc:HGNC:7780]	protein coding	1.29	1.79	14.35	0.0002	0.0299
ENSG00000184584	STING1	stimulator of interferon response cGAMP interactor 1 [Source:HGNC Symbol;Acc:HGNC:27962]	protein coding	1.28	3.22	25.15	<0.0001	0.0008
ENSG00000278828	H3C10	H3 clustered histone 10 [Source:HGNC Symbol;Acc:HGNC:4775]	protein coding	1.27	0.78	12.97	0.0003	0.0408
ENSG00000120594	PLXDC2	plexin domain containing 2 [Source:HGNC Symbol;Acc:HGNC:21013]	protein coding	1.27	1.77	15.00	0.0001	0.0267
ENSG00000109743	BST1	bone marrow stromal cell antigen 1 [Source:HGNC Symbol;Acc:HGNC:1118]	protein coding	1.26	1.84	14.57	0.0001	0.0283
ENSG00000093009	CDC45	cell division cycle 45 [Source:HGNC Symbol;Acc:HGNC:1739]	protein coding	1.22	1.57	17.08	<0.0001	0.018
ENSG00000163220	S100A9	S100 calcium binding protein A9 [Source:HGNC Symbol;Acc:HGNC:10499]	protein coding	1.22	6.48	12.27	0.0005	0.0443
ENSG00000133816	MICAL2	microtubule associated monooxygenase, calponin and LIM domain containing 2 [Source:HGNC Symbol;Acc:HGNC:24693]	protein coding	1.22	1.57	12.96	0.0003	0.0408
ENSG00000151948	GLT1D1	glycosyltransferase 1 domain containing 1 [Source:HGNC Symbol;Acc:HGNC:26483]	protein coding	1.21	1.46	13.17	0.0003	0.0396
ENSG00000126822	PLEKHG3	pleckstrin homology and RhoGEF domain containing G3 [Source:HGNC Symbol;Acc:HGNC:20364]	protein coding	1.21	1.52	21.77	<0.0001	0.0031
ENSG00000183023	SLC8A1	solute carrier family 8 member A1 [Source:HGNC Symbol;Acc:HGNC:11068]	protein coding	1.21	0.50	15.33	<0.0001	0.0258
ENSG00000171848	RRM2	ribonucleotide reductase regulatory subunit M2 [Source:HGNC Symbol;Acc:HGNC:10452]	protein coding	1.19	4.79	14.63	0.0001	0.0279
ENSG00000189060	H1-0	H1.0 linker histone [Source:HGNC Symbol;Acc:HGNC:4714]	protein coding	1.18	1.61	12.31	0.0005	0.0442
ENSG00000178562	CD28	CD28 molecule [Source:HGNC Symbol;Acc:HGNC:1653]	protein coding	1.18	0.97	18.77	<0.0001	0.0099

Table S34: Genes differentially expressed at Day 32 compared to pre-second vaccination (Study Arm 2 A/H7N9+AS03, D1, 29, B cells). Genes are sorted by descending absolute log_2 fold change. Gene model summaries and annotations are based on Ensembl Version 99 (continued.)

Ensembl Gene ID	Ensembl Gene Name	Ensembl Gene Description	Gene Type	log_2 Fold Change (Day 32 vs. Pre-Second Vaccination)	Average log_2 CPM	Likelihood Ratio Test Statistic	P-Value	FDR Adjusted P-Value
ENSG00000137441	FGFBP2	fibroblast growth factor binding protein 2 [Source:HGNC Symbol;Acc:HGNC:29451]	protein coding	1.18	1.42	13.65	0.0002	0.0347
ENSG00000100453	GZMB	granzyme B [Source:HGNC Symbol;Acc:HGNC:4709]	protein coding	1.18	1.82	12.90	0.0003	0.0413
ENSG00000119535	CSF3R	colony stimulating factor 3 receptor [Source:HGNC Symbol;Acc:HGNC:2439]	protein coding	1.17	4.89	13.37	0.0003	0.0382
ENSG00000204103	MAFB	MAF bZIP transcription factor B [Source:HGNC Symbol;Acc:HGNC:6408]	protein coding	1.16	2.49	11.89	0.0006	0.049
ENSG00000179348	GATA2	GATA binding protein 2 [Source:HGNC Symbol;Acc:HGNC:4171]	protein coding	1.15	0.69	15.93	<0.0001	0.0208
ENSG00000145569	OTULINL	OTU deubiquitinase with linear linkage specificity like [Source:HGNC Symbol;Acc:HGNC:25629]	protein coding	1.14	1.68	13.91	0.0002	0.0338
ENSG00000129450	SIGLEC9	sialic acid binding Ig like lectin 9 [Source:HGNC Symbol;Acc:HGNC:10878]	protein coding	1.14	1.44	12.82	0.0003	0.0415
ENSG00000244482	LILRA6	leukocyte immunoglobulin like receptor A6 [Source:HGNC Symbol;Acc:HGNC:15495]	protein coding	1.11	1.66	12.53	0.0004	0.0432
ENSG00000172243	CLEC7A	C-type lectin domain containing 7A [Source:HGNC Symbol;Acc:HGNC:14558]	protein coding	1.09	3.20	12.63	0.0004	0.0431
ENSG00000105383	CD33	CD33 molecule [Source:HGNC Symbol;Acc:HGNC:1659]	protein coding	1.09	1.98	12.85	0.0003	0.0415
ENSG00000156299	TIAM1	TIAM Rac1 associated GEF 1 [Source:HGNC Symbol;Acc:HGNC:11805]	protein coding	1.08	1.25	12.37	0.0004	0.044
ENSG00000259207	ITGB3	integrin subunit beta 3 [Source:HGNC Symbol;Acc:HGNC:6156]	protein coding	1.08	1.31	12.33	0.0004	0.0441
ENSG00000160593	JAML	junction adhesion molecule like [Source:HGNC Symbol;Acc:HGNC:19084]	protein coding	1.06	4.37	12.92	0.0003	0.0412
ENSG00000110080	ST3GAL4	ST3 beta-galactoside alpha-2,3-sialyltransferase 4 [Source:HGNC Symbol;Acc:HGNC:10864]	protein coding	1.05	0.66	27.60	<0.0001	0.0004
ENSG00000094804	CDC6	cell division cycle 6 [Source:HGNC Symbol;Acc:HGNC:1744]	protein coding	1.05	2.26	11.93	0.0006	0.0484
ENSG00000133246	PRAM1	PML-RARA regulated adaptor molecule 1 [Source:HGNC Symbol;Acc:HGNC:30091]	protein coding	1.04	2.98	13.17	0.0003	0.0396
ENSG00000182578	CSF1R	colony stimulating factor 1 receptor [Source:HGNC Symbol;Acc:HGNC:2433]	protein coding	1.04	4.32	13.18	0.0003	0.0396
ENSG00000166803	PCLAF	PCNA clamp associated factor [Source:HGNC Symbol;Acc:HGNC:28961]	protein coding	1.03	1.02	12.16	0.0005	0.0464

Table S34: Genes differentially expressed at Day 32 compared to pre-second vaccination (Study Arm 2 A/H7N9+AS03, D1, 29, B cells). Genes are sorted by descending absolute log_2 fold change. Gene model summaries and annotations are based on Ensembl Version 99 (continued.)

Ensembl Gene ID	Ensembl Gene Name	Ensembl Gene Description	Gene Type	log_2 Fold Change (Day 32 vs. Pre-Second Vaccination)	Average log_2 CPM	Likelihood Ratio Test Statistic	P-Value	FDR Adjusted P-Value
ENSG00000104267	CA2	carbonic anhydrase 2 [Source:HGNC Symbol;Acc:HGNC:1373]	protein coding	1.03	1.51	14.95	0.0001	0.0267
ENSG00000115738	ID2	inhibitor of DNA binding 2 [Source:HGNC Symbol;Acc:HGNC:5361]	protein coding	1.03	2.70	14.32	0.0002	0.0299
ENSG00000174837	ADGRE1	adhesion G protein-coupled receptor E1 [Source:HGNC Symbol;Acc:HGNC:3336]	protein coding	1.02	1.39	11.82	0.0006	0.0499
ENSG00000197405	C5AR1	complement C5a receptor 1 [Source:HGNC Symbol;Acc:HGNC:1338]	protein coding	1.02	2.90	11.95	0.0005	0.0483
ENSG00000172322	CLEC12A	C-type lectin domain family 12 member A [Source:HGNC Symbol;Acc:HGNC:31713]	protein coding	1.01	2.85	13.01	0.0003	0.0408
ENSG00000239998	LILRA2	leukocyte immunoglobulin like receptor A2 [Source:HGNC Symbol;Acc:HGNC:6603]	protein coding	1.01	2.83	13.38	0.0003	0.0382
ENSG00000159339	PADI4	peptidyl arginine deiminase 4 [Source:HGNC Symbol;Acc:HGNC:18368]	protein coding	1.01	2.05	14.91	0.0001	0.0267
ENSG00000116574	RHOU	ras homolog family member U [Source:HGNC Symbol;Acc:HGNC:17794]	protein coding	1.00	0.71	13.72	0.0002	0.0346
ENSG00000168329	CX3CR1	C-X3-C motif chemokine receptor 1 [Source:HGNC Symbol;Acc:HGNC:2558]	protein coding	1.00	3.09	11.95	0.0005	0.0483
ENSG00000118193	KIF14	kinesin family member 14 [Source:HGNC Symbol;Acc:HGNC:19181]	protein coding	1.00	0.91	12.95	0.0003	0.0408
ENSG00000149485	FADS1	fatty acid desaturase 1 [Source:HGNC Symbol;Acc:HGNC:3574]	protein coding	0.99	2.33	35.67	<0.0001	<0.0001
ENSG00000198876	DCAF12	DDB1 and CUL4 associated factor 12 [Source:HGNC Symbol;Acc:HGNC:19911]	protein coding	0.99	3.08	16.83	<0.0001	0.0181
ENSG00000140749	IGSF6	immunoglobulin superfamily member 6 [Source:HGNC Symbol;Acc:HGNC:5953]	protein coding	0.99	2.09	12.05	0.0005	0.0472
ENSG00000181826	RELL1	RELT like 1 [Source:HGNC Symbol;Acc:HGNC:27379]	protein coding	0.98	0.84	16.47	<0.0001	0.0188
ENSG00000069702	TGFBR3	transforming growth factor beta receptor 3 [Source:HGNC Symbol;Acc:HGNC:11774]	protein coding	0.98	0.48	12.53	0.0004	0.0432
ENSG00000116824	CD2	CD2 molecule [Source:HGNC Symbol;Acc:HGNC:1639]	protein coding	0.98	1.87	12.13	0.0005	0.0465
ENSG00000169679	BUB1	BUB1 mitotic checkpoint serine/threonine kinase [Source:HGNC Symbol;Acc:HGNC:1148]	protein coding	0.97	3.38	12.49	0.0004	0.0432
ENSG00000203747	FCGR3A	Fc fragment of IgG receptor IIIa [Source:HGNC Symbol;Acc:HGNC:3619]	protein coding	0.96	4.12	14.28	0.0002	0.0299

Table S34: Genes differentially expressed at Day 32 compared to pre-second vaccination (Study Arm 2 A/H7N9+AS03, D1, 29, B cells). Genes are sorted by descending absolute log_2 fold change. Gene model summaries and annotations are based on Ensembl Version 99 (continued.)

Ensembl Gene ID	Ensembl Gene Name	Ensembl Gene Description	Gene Type	log_2 Fold Change (Day 32 vs. Pre-Second Vaccination)	Average log_2 CPM	Likelihood Ratio Test Statistic	P-Value	FDR Adjusted P-Value
ENSG00000211670	IGLV3-9	immunoglobulin lambda variable 3-9 (gene/pseudogene) [Source:HGNC Symbol;Acc:HGNC:5918]	IG V gene	0.95	4.19	12.46	0.0004	0.0432
ENSG00000186818	LILRB4	leukocyte immunoglobulin like receptor B4 [Source:HGNC Symbol;Acc:HGNC:6608]	protein coding	0.95	3.43	13.32	0.0003	0.0389
ENSG00000174371	EXO1	exonuclease 1 [Source:HGNC Symbol;Acc:HGNC:3511]	protein coding	0.95	1.06	15.11	0.0001	0.0267
ENSG00000205336	ADGRG1	adhesion G protein-coupled receptor G1 [Source:HGNC Symbol;Acc:HGNC:4512]	protein coding	0.94	2.22	12.64	0.0004	0.0431
ENSG00000171777	RASGRP4	RAS guanyl releasing protein 4 [Source:HGNC Symbol;Acc:HGNC:18958]	protein coding	0.94	2.58	12.45	0.0004	0.0432
ENSG00000137807	KIF23	kinesin family member 23 [Source:HGNC Symbol;Acc:HGNC:6392]	protein coding	0.94	1.54	13.68	0.0002	0.0346
ENSG00000122952	ZWINT	ZW10 interacting kinetochore protein [Source:HGNC Symbol;Acc:HGNC:13195]	protein coding	0.93	2.85	13.97	0.0002	0.0338
ENSG00000198851	CD3E	CD3e molecule [Source:HGNC Symbol;Acc:HGNC:1674]	protein coding	0.93	3.51	12.72	0.0004	0.0426
ENSG00000023171	GRAMD1B	GRAM domain containing 1B [Source:HGNC Symbol;Acc:HGNC:29214]	protein coding	0.91	0.94	12.52	0.0004	0.0432
ENSG00000092853	CLSPN	claspin [Source:HGNC Symbol;Acc:HGNC:19715]	protein coding	0.91	1.67	15.04	0.0001	0.0267
ENSG00000184613	NELL2	neural EGFL like 2 [Source:HGNC Symbol;Acc:HGNC:7751]	protein coding	0.91	1.20	13.81	0.0002	0.0338
ENSG00000153162	BMP6	bone morphogenetic protein 6 [Source:HGNC Symbol;Acc:HGNC:1073]	protein coding	0.90	2.46	12.39	0.0004	0.0439
ENSG00000100346	CACNA1I	calcium voltage-gated channel subunit alpha1 I [Source:HGNC Symbol;Acc:HGNC:1396]	protein coding	0.90	0.65	16.06	<0.0001	0.0208
ENSG00000184232	OAF	out at first homolog [Source:HGNC Symbol;Acc:HGNC:28752]	protein coding	0.90	2.40	14.24	0.0002	0.0301
ENSG00000135476	ESPL1	extra spindle pole bodies like 1, separase [Source:HGNC Symbol;Acc:HGNC:16856]	protein coding	0.90	2.23	13.77	0.0002	0.0341
ENSG00000115523	GNLY	granulysin [Source:HGNC Symbol;Acc:HGNC:4414]	protein coding	0.89	4.10	13.61	0.0002	0.0347
ENSG00000121807	CCR2	C-C motif chemokine receptor 2 [Source:HGNC Symbol;Acc:HGNC:1603]	protein coding	0.88	4.16	22.55	<0.0001	0.0022
ENSG00000153885	KCTD15	potassium channel tetramerization domain containing 15 [Source:HGNC Symbol;Acc:HGNC:23297]	protein coding	0.87	0.73	15.98	<0.0001	0.0208

Table S34: Genes differentially expressed at Day 32 compared to pre-second vaccination (Study Arm 2 A/H7N9+AS03, D1, 29, B cells). Genes are sorted by descending absolute log_2 fold change. Gene model summaries and annotations are based on Ensembl Version 99 (continued.)

Ensembl Gene ID	Ensembl Gene Name	Ensembl Gene Description	Gene Type	log_2 Fold Change (Day 32 vs. Pre-Second Vaccination)	Average log_2 CPM	Likelihood Ratio Test Statistic	P-Value	FDR Adjusted P-Value
ENSG00000107281	NPDC1	neural proliferation, differentiation and control 1 [Source:HGNC Symbol;Acc:HGNC:7899]	protein coding	0.87	1.15	16.84	<0.0001	0.0181
ENSG00000085840	ORC1	origin recognition complex subunit 1 [Source:HGNC Symbol;Acc:HGNC:8487]	protein coding	0.86	1.64	13.70	0.0002	0.0346
ENSG00000120949	TNFRSF8	TNF receptor superfamily member 8 [Source:HGNC Symbol;Acc:HGNC:11923]	protein coding	0.86	1.57	14.86	0.0001	0.0267
ENSG00000074660	SCARF1	scavenger receptor class F member 1 [Source:HGNC Symbol;Acc:HGNC:16820]	protein coding	0.85	1.27	11.95	0.0005	0.0483
ENSG00000162174	ASRGL1	asparaginase and isoaspartyl peptidase 1 [Source:HGNC Symbol;Acc:HGNC:16448]	protein coding	0.82	0.99	20.51	<0.0001	0.0056
ENSG00000150637	CD226	CD226 molecule [Source:HGNC Symbol;Acc:HGNC:16961]	protein coding	0.82	2.09	12.07	0.0005	0.0472
ENSG00000197471	SPN	sialophorin [Source:HGNC Symbol;Acc:HGNC:11249]	protein coding	0.81	5.30	12.61	0.0004	0.0431
ENSG00000072571	HMMR	hyaluronan mediated motility receptor [Source:HGNC Symbol;Acc:HGNC:5012]	protein coding	0.79	1.71	11.91	0.0006	0.0488
ENSG00000185477	GPRIN3	GPRIN family member 3 [Source:HGNC Symbol;Acc:HGNC:27733]	protein coding	0.79	3.33	15.17	<0.0001	0.0266
ENSG00000118515	SGK1	serum/glucocorticoid regulated kinase 1 [Source:HGNC Symbol;Acc:HGNC:10810]	protein coding	0.79	3.45	12.84	0.0003	0.0415
ENSG00000100385	IL2RB	interleukin 2 receptor subunit beta [Source:HGNC Symbol;Acc:HGNC:6009]	protein coding	0.76	3.75	16.76	<0.0001	0.0181
ENSG00000145386	CCNA2	cyclin A2 [Source:HGNC Symbol;Acc:HGNC:1578]	protein coding	0.76	2.83	13.09	0.0003	0.04
ENSG00000154839	SKA1	spindle and kinetochore associated complex subunit 1 [Source:HGNC Symbol;Acc:HGNC:28109]	protein coding	0.76	1.20	12.87	0.0003	0.0415
ENSG00000156970	BUB1B	BUB1 mitotic checkpoint serine/threonine kinase B [Source:HGNC Symbol;Acc:HGNC:1149]	protein coding	0.73	2.04	14.39	0.0001	0.0297
ENSG00000160712	IL6R	interleukin 6 receptor [Source:HGNC Symbol;Acc:HGNC:6019]	protein coding	0.68	5.43	12.60	0.0004	0.0431
ENSG00000243466	IGKV1-5	immunoglobulin kappa variable 1-5 [Source:HGNC Symbol;Acc:HGNC:5741]	IG V gene	0.68	6.69	11.87	0.0006	0.049
ENSG00000051341	POLQ	DNA polymerase theta [Source:HGNC Symbol;Acc:HGNC:9186]	protein coding	0.67	1.78	12.01	0.0005	0.048
ENSG00000074416	MGLL	monoglyceride lipase [Source:HGNC Symbol;Acc:HGNC:17038]	protein coding	0.63	3.43	12.14	0.0005	0.0465

Table S34: Genes differentially expressed at Day 32 compared to pre-second vaccination (Study Arm 2 A/H7N9+AS03, D1, 29, B cells). Genes are sorted by descending absolute log_2 fold change. Gene model summaries and annotations are based on Ensembl Version 99 (continued.)

Ensembl Gene ID	Ensembl Gene Name	Ensembl Gene Description	Gene Type	log_2 Fold Change (Day 32 vs. Pre-Second Vaccination)	Average log_2 CPM	Likelihood Ratio Test Statistic	P-Value	FDR Adjusted P-Value
ENSG00000134824	FADS2	fatty acid desaturase 2 [Source:HGNC Symbol;Acc:HGNC:3575]	protein coding	0.63	4.17	18.55	<0.0001	0.0106
ENSG00000198826	ARHGAP11A	Rho GTPase activating protein 11A [Source:HGNC Symbol;Acc:HGNC:15783]	protein coding	0.63	2.78	16.52	<0.0001	0.0188
ENSG00000172159	FRMD3	FERM domain containing 3 [Source:HGNC Symbol;Acc:HGNC:24125]	protein coding	0.60	0.85	12.49	0.0004	0.0432
ENSG00000104738	MCM4	minichromosome maintenance complex component 4 [Source:HGNC Symbol;Acc:HGNC:6947]	protein coding	0.59	4.57	17.43	<0.0001	0.0161
ENSG00000075618	FSCN1	fascin actin-bundling protein 1 [Source:HGNC Symbol;Acc:HGNC:11148]	protein coding	0.59	1.57	15.94	<0.0001	0.0208

Table S35: Genes differentially expressed at Day 36 compared to pre-second vaccination (Study Arm 2 A/H7N9+AS03, D1, 29, B cells). Genes are sorted by descending absolute log_2 fold change. Gene model summaries and annotations are based on Ensembl Version 99.

Ensembl Gene ID	Ensembl Gene Name	Ensembl Gene Description	Gene Type	log_2 Fold Change (Day 36 vs. Pre-Second Vaccination)	Average log_2 CPM	Likelihood Ratio Test Statistic	P-Value	FDR Adjusted P-Value
ENSG00000188536	HBA2	hemoglobin subunit alpha 2 [Source:HGNC Symbol;Acc:HGNC:4824]	protein coding	2.57	5.06	34.81	<0.0001	<0.0001
ENSG00000244734	HBB	hemoglobin subunit beta [Source:HGNC Symbol;Acc:HGNC:4827]	protein coding	2.37	7.06	38.44	<0.0001	<0.0001
ENSG00000123405	NFE2	nuclear factor, erythroid 2 [Source:HGNC Symbol;Acc:HGNC:7780]	protein coding	1.41	1.63	19.23	<0.0001	0.0076
ENSG00000179348	GATA2	GATA binding protein 2 [Source:HGNC Symbol;Acc:HGNC:4171]	protein coding	1.40	0.68	26.40	<0.0001	0.0004
ENSG00000133816	MICAL2	microtubule associated monooxygenase, calponin and LIM domain containing 2 [Source:HGNC Symbol;Acc:HGNC:24693]	protein coding	1.39	1.52	20.82	<0.0001	0.005
ENSG00000126822	PLEKHG3	pleckstrin homology and RhoGEF domain containing G3 [Source:HGNC Symbol;Acc:HGNC:20364]	protein coding	1.37	1.40	34.39	<0.0001	<0.0001
ENSG00000103196	CRISPLD2	cysteine rich secretory protein LCCL domain containing 2 [Source:HGNC Symbol;Acc:HGNC:25248]	protein coding	1.36	1.00	17.27	<0.0001	0.0152
ENSG00000100453	GZMB	granzyme B [Source:HGNC Symbol;Acc:HGNC:4709]	protein coding	1.24	1.48	19.18	<0.0001	0.0076

Table S35: Genes differentially expressed at Day 36 compared to pre-second vaccination (Study Arm 2 A/H7N9+AS03, D1, 29, B cells). Genes are sorted by descending absolute log_2 fold change. Gene model summaries and annotations are based on Ensembl Version 99 (continued.)

Ensembl Gene ID	Ensembl Gene Name	Ensembl Gene Description	Gene Type	log_2 Fold Change (Day 36 vs. Pre-Second Vaccination)	Average log_2 CPM	Likelihood Ratio Test Statistic	P-Value	FDR Adjusted P-Value
ENSG00000151948	GLT1D1	glycosyltransferase 1 domain containing 1 [Source:HGNC Symbol;Acc:HGNC:26483]	protein coding	1.24	1.23	16.34	<0.0001	0.0191
ENSG00000179144	GIMAP7	GTPase, IMAP family member 7 [Source:HGNC Symbol;Acc:HGNC:22404]	protein coding	1.21	2.70	15.35	<0.0001	0.0251
ENSG00000196329	GIMAP5	GTPase, IMAP family member 5 [Source:HGNC Symbol;Acc:HGNC:18005]	protein coding	1.20	0.54	15.08	0.0001	0.0275
ENSG00000120594	PLXDC2	plexin domain containing 2 [Source:HGNC Symbol;Acc:HGNC:21013]	protein coding	1.18	1.57	14.03	0.0002	0.0372
ENSG00000161944	ASGR2	asialoglycoprotein receptor 2 [Source:HGNC Symbol;Acc:HGNC:743]	protein coding	1.17	0.56	14.45	0.0001	0.0332
ENSG00000159339	PADI4	peptidyl arginine deiminase 4 [Source:HGNC Symbol;Acc:HGNC:18368]	protein coding	1.17	1.86	26.47	<0.0001	0.0004
ENSG00000107281	NPDC1	neural proliferation, differentiation and control 1 [Source:HGNC Symbol;Acc:HGNC:7899]	protein coding	1.17	1.16	17.76	<0.0001	0.013
ENSG00000115523	GNLY	granulysin [Source:HGNC Symbol;Acc:HGNC:4414]	protein coding	1.16	3.82	24.11	<0.0001	0.0012
ENSG00000137441	FGFBP2	fibroblast growth factor binding protein 2 [Source:HGNC Symbol;Acc:HGNC:29451]	protein coding	1.15	1.00	13.28	0.0003	0.0495
ENSG00000153162	BMP6	bone morphogenetic protein 6 [Source:HGNC Symbol;Acc:HGNC:1073]	protein coding	1.14	2.46	16.49	<0.0001	0.0191
ENSG00000211670	IGLV3-9	immunoglobulin lambda variable 3-9 (gene/pseudogene) [Source:HGNC Symbol;Acc:HGNC:5918]	IG V gene	1.13	4.15	17.81	<0.0001	0.013
ENSG00000119535	CSF3R	colony stimulating factor 3 receptor [Source:HGNC Symbol;Acc:HGNC:2439]	protein coding	1.13	4.75	16.02	<0.0001	0.0204
ENSG00000129450	SIGLEC9	sialic acid binding Ig like lectin 9 [Source:HGNC Symbol;Acc:HGNC:10878]	protein coding	1.12	1.17	13.44	0.0002	0.0475
ENSG00000211896	IGHG1	immunoglobulin heavy constant gamma 1 (G1m marker) [Source:HGNC Symbol;Acc:HGNC:5525]	IG C gene	1.11	10.72	29.29	<0.0001	0.0001
ENSG00000103313	MEFV	MEFV innate immuity regulator, pyrin [Source:HGNC Symbol;Acc:HGNC:6998]	protein coding	1.08	2.25	15.06	0.0001	0.0275
ENSG00000116824	CD2	CD2 molecule [Source:HGNC Symbol;Acc:HGNC:1639]	protein coding	1.07	1.81	14.80	0.0001	0.0294
ENSG00000156299	TIAM1	TIAM Rac1 associated GEF 1 [Source:HGNC Symbol;Acc:HGNC:11805]	protein coding	1.06	1.11	18.35	<0.0001	0.0103
ENSG00000134539	KLRD1	killer cell lectin like receptor D1 [Source:HGNC Symbol;Acc:HGNC:6378]	protein coding	1.05	1.60	17.28	<0.0001	0.0152

Table S35: Genes differentially expressed at Day 36 compared to pre-second vaccination (Study Arm 2 A/H7N9+AS03, D1, 29, B cells). Genes are sorted by descending absolute log_2 fold change. Gene model summaries and annotations are based on Ensembl Version 99 (continued.)

Ensembl Gene ID	Ensembl Gene Name	Ensembl Gene Description	Gene Type	log_2 Fold Change (Day 36 vs. Pre-Second Vaccination)	Average log_2 CPM	Likelihood Ratio Test Statistic	P-Value	FDR Adjusted P-Value
ENSG00000205336	ADGRG1	adhesion G protein-coupled receptor G1 [Source:HGNC Symbol;Acc:HGNC:4512]	protein coding	1.05	1.95	19.24	<0.0001	0.0076
ENSG00000181409	AATK	apoptosis associated tyrosine kinase [Source:HGNC Symbol;Acc:HGNC:21]	protein coding	1.04	0.58	19.82	<0.0001	0.007
ENSG00000069702	TGFBR3	transforming growth factor beta receptor 3 [Source:HGNC Symbol;Acc:HGNC:11774]	protein coding	1.03	0.37	16.34	<0.0001	0.0191
ENSG00000253755	IGHGP	immunoglobulin heavy constant gamma P (non-functional) [Source:HGNC Symbol;Acc:HGNC:5529]	IG C pseudogene	1.02	5.90	20.73	<0.0001	0.005
ENSG00000067182	TNFRSF1A	TNF receptor superfamily member 1A [Source:HGNC Symbol;Acc:HGNC:11916]	protein coding	1.02	2.35	14.49	0.0001	0.033
ENSG00000173762	CD7	CD7 molecule [Source:HGNC Symbol;Acc:HGNC:1695]	protein coding	1.01	2.62	15.36	<0.0001	0.0251
ENSG00000155254	MARVELD1	MARVEL domain containing 1 [Source:HGNC Symbol;Acc:HGNC:28674]	protein coding	1.00	1.02	17.63	<0.0001	0.0135
ENSG00000244482	LILRA6	leukocyte immunoglobulin like receptor A6 [Source:HGNC Symbol;Acc:HGNC:15495]	protein coding	1.00	1.50	16.36	<0.0001	0.0191
ENSG00000111796	KLRB1	killer cell lectin like receptor B1 [Source:HGNC Symbol;Acc:HGNC:6373]	protein coding	1.00	0.79	15.10	0.0001	0.0275
ENSG00000133561	GIMAP6	GTPase, IMAP family member 6 [Source:HGNC Symbol;Acc:HGNC:21918]	protein coding	1.00	2.69	15.65	<0.0001	0.0228
ENSG00000184584	STING1	stimulator of interferon response cGAMP interactor 1 [Source:HGNC Symbol;Acc:HGNC:27962]	protein coding	0.99	3.00	16.06	<0.0001	0.0204
ENSG00000180739	S1PR5	sphingosine-1-phosphate receptor 5 [Source:HGNC Symbol;Acc:HGNC:14299]	protein coding	0.99	1.25	20.16	<0.0001	0.0063
ENSG00000077984	CST7	cystatin F [Source:HGNC Symbol;Acc:HGNC:2479]	protein coding	0.99	1.53	18.45	<0.0001	0.0102
ENSG00000133574	GIMAP4	GTPase, IMAP family member 4 [Source:HGNC Symbol;Acc:HGNC:21872]	protein coding	0.95	3.13	13.38	0.0003	0.0477
ENSG00000147459	DOCK5	dedicator of cytokinesis 5 [Source:HGNC Symbol;Acc:HGNC:23476]	protein coding	0.94	1.63	13.73	0.0002	0.0412
ENSG00000211952	IGHV4-28	immunoglobulin heavy variable 4-28 [Source:HGNC Symbol;Acc:HGNC:5645]	IG V gene	0.93	1.38	16.50	<0.0001	0.0191
ENSG00000231475	IGHV4-31	immunoglobulin heavy variable 4-31 [Source:HGNC Symbol;Acc:HGNC:5649]	IG V gene	0.93	3.36	39.43	<0.0001	<0.0001
ENSG00000180644	PRF1	perforin 1 [Source:HGNC Symbol;Acc:HGNC:9360]	protein coding	0.92	3.54	14.63	0.0001	0.0317

Confidential/Proprietary Information

Table S35: Genes differentially expressed at Day 36 compared to pre-second vaccination (Study Arm 2 A/H7N9+AS03, D1, 29, B cells). Genes are sorted by descending absolute log_2 fold change. Gene model summaries and annotations are based on Ensembl Version 99 (continued.)

Ensembl Gene ID	Ensembl Gene Name	Ensembl Gene Description	Gene Type	log_2 Fold Change (Day 36 vs. Pre-Second Vaccination)	Average log_2 CPM	Likelihood Ratio Test Statistic	P-Value	FDR Adjusted P-Value
ENSG00000120318	ARAP3	ArfGAP with RhoGAP domain, ankyrin repeat and PH domain 3 [Source:HGNC Symbol;Acc:HGNC:24097]	protein coding	0.92	1.31	14.39	0.0001	0.0337
ENSG00000239998	LILRA2	leukocyte immunoglobulin like receptor A2 [Source:HGNC Symbol;Acc:HGNC:6603]	protein coding	0.91	2.52	15.37	<0.0001	0.0251
ENSG00000198053	SIRPA	signal regulatory protein alpha [Source:HGNC Symbol;Acc:HGNC:9662]	protein coding	0.90	2.97	13.89	0.0002	0.0389
ENSG00000178038	ALS2CL	ALS2 C-terminal like [Source:HGNC Symbol;Acc:HGNC:20605]	protein coding	0.90	1.04	13.95	0.0002	0.0383
ENSG00000211946	IGHV3-20	immunoglobulin heavy variable 3-20 [Source:HGNC Symbol;Acc:HGNC:5585]	IG V gene	0.89	3.66	34.87	<0.0001	<0.0001
ENSG00000139679	LPAR6	lysophosphatidic acid receptor 6 [Source:HGNC Symbol;Acc:HGNC:15520]	protein coding	0.86	2.06	14.03	0.0002	0.0372
ENSG00000140749	IGSF6	immunoglobulin superfamily member 6 [Source:HGNC Symbol;Acc:HGNC:5953]	protein coding	0.83	1.84	14.22	0.0002	0.0351
ENSG00000115828	QPCT	glutaminyl-peptide cyclotransferase [Source:HGNC Symbol;Acc:HGNC:9753]	protein coding	0.83	1.19	15.00	0.0001	0.0275
ENSG00000181444	ZNF467	zinc finger protein 467 [Source:HGNC Symbol;Acc:HGNC:23154]	protein coding	0.81	2.02	13.39	0.0003	0.0477
ENSG00000139112	GABARAPL1	GABA type A receptor associated protein like 1 [Source:HGNC Symbol;Acc:HGNC:4068]	protein coding	0.80	1.13	20.91	<0.0001	0.005
ENSG00000023171	GRAMD1B	GRAM domain containing 1B [Source:HGNC Symbol;Acc:HGNC:29214]	protein coding	0.79	0.87	16.48	<0.0001	0.0191
ENSG00000276775	IGHV4-4	immunoglobulin heavy variable 4-4 [Source:HGNC Symbol;Acc:HGNC:5652]	IG V gene	0.78	4.25	15.94	<0.0001	0.0204
ENSG00000075618	FSCN1	fascin actin-bundling protein 1 [Source:HGNC Symbol;Acc:HGNC:11148]	protein coding	0.77	1.61	24.04	<0.0001	0.0012
ENSG00000211973	IGHV1-69	immunoglobulin heavy variable 1-69 [Source:HGNC Symbol;Acc:HGNC:5558]	IG V gene	0.76	3.41	14.80	0.0001	0.0294
ENSG00000211673	IGLV3-1	immunoglobulin lambda variable 3-1 [Source:HGNC Symbol;Acc:HGNC:5896]	IG V gene	0.75	6.57	15.02	0.0001	0.0275
ENSG00000149485	FADS1	fatty acid desaturase 1 [Source:HGNC Symbol;Acc:HGNC:3574]	protein coding	0.73	2.10	19.68	<0.0001	0.0071
ENSG00000211965	IGHV3-49	immunoglobulin heavy variable 3-49 [Source:HGNC Symbol;Acc:HGNC:5607]	IG V gene	0.73	5.21	14.59	0.0001	0.0318
ENSG00000172543	CTSW	cathepsin W [Source:HGNC Symbol;Acc:HGNC:2546]	protein coding	0.71	2.88	15.96	<0.0001	0.0204

Table S35: Genes differentially expressed at Day 36 compared to pre-second vaccination (Study Arm 2 A/H7N9+AS03, D1, 29, B cells). Genes are sorted by descending absolute log_2 fold change. Gene model summaries and annotations are based on Ensembl Version 99 (continued.)

Ensembl Gene ID	Ensembl Gene Name	Ensembl Gene Description	Gene Type	log_2 Fold Change (Day 36 vs. Pre-Second Vaccination)	Average log_2 CPM	Likelihood Ratio Test Statistic	P-Value	FDR Adjusted P-Value
ENSG00000254395	IGHV4-55	immunoglobulin heavy variable 4-55 (pseudogene) [Source:HGNC Symbol;Acc:HGNC:5653]	IG V pseudogene	0.71	3.32	13.82	0.0002	0.0398
ENSG00000280411	IGHV1-69D	immunoglobulin heavy variable 1-69D [Source:HGNC Symbol;Acc:HGNC:49601]	IG V gene	0.69	4.78	31.61	<0.0001	<0.0001
ENSG00000142173	COL6A2	collagen type VI alpha 2 chain [Source:HGNC Symbol;Acc:HGNC:2212]	protein coding	0.68	1.89	16.17	<0.0001	0.0204
ENSG00000274576	IGHV2-70	immunoglobulin heavy variable 2-70 [Source:HGNC Symbol;Acc:HGNC:5577]	IG V gene	0.66	3.58	15.95	<0.0001	0.0204
ENSG00000172985	SH3RF3	SH3 domain containing ring finger 3 [Source:HGNC Symbol;Acc:HGNC:24699]	protein coding	0.64	1.34	16.63	<0.0001	0.0191

Table S36: Genes differentially expressed at Day 2 compared to pre-first vaccination (Study Arm 2 A/H7N9+AS03, D1, 29, T cells). Genes are sorted by descending absolute log_2 fold change. Gene model summaries and annotations are based on Ensembl Version 99.

Ensembl Gene ID	Ensembl Gene Name	Ensembl Gene Description	Gene Type	log_2 Fold Change (Day 2 vs. Pre-First Vaccination)	Average log_2 CPM	Likelihood Ratio Test Statistic	P-Value	FDR Adjusted P-Value
ENSG00000010030	ETV7	ETS variant transcription factor 7 [Source:HGNC Symbol;Acc:HGNC:18160]	protein coding	1.94	2.12	60.00	<0.0001	<0.0001
ENSG00000119917	IFIT3	interferon induced protein with tetratricopeptide repeats 3 [Source:HGNC Symbol;Acc:HGNC:5411]	protein coding	1.73	3.63	35.37	<0.0001	<0.0001
ENSG00000138119	MYOF	myoferlin [Source:HGNC Symbol;Acc:HGNC:3656]	protein coding	1.61	1.55	22.17	<0.0001	0.0002
ENSG00000128383	APOBEC3A	apolipoprotein B mRNA editing enzyme catalytic subunit 3A [Source:HGNC Symbol;Acc:HGNC:17343]	protein coding	1.56	2.17	13.99	0.0002	0.0055
ENSG00000115415	STAT1	signal transducer and activator of transcription 1 [Source:HGNC Symbol;Acc:HGNC:11362]	protein coding	1.54	8.64	79.16	<0.0001	<0.0001
ENSG00000171049	FPR2	formyl peptide receptor 2 [Source:HGNC Symbol;Acc:HGNC:3827]	protein coding	1.45	0.76	15.85	<0.0001	0.0024
ENSG00000103569	AQP9	aquaporin 9 [Source:HGNC Symbol;Acc:HGNC:643]	protein coding	1.39	1.52	13.49	0.0002	0.0067
ENSG00000117228	GBP1	guanylate binding protein 1 [Source:HGNC Symbol;Acc:HGNC:4182]	protein coding	1.37	6.60	76.19	<0.0001	<0.0001

Table S36: Genes differentially expressed at Day 2 compared to pre-first vaccination (Study Arm 2 A/H7N9+AS03, D1, 29, T cells). Genes are sorted by descending absolute log_2 fold change. Gene model summaries and annotations are based on Ensembl Version 99 (continued.)

Ensembl Gene ID	Ensembl Gene Name	Ensembl Gene Description	Gene Type	log_2 Fold Change (Day 2 vs. Pre-First Vaccination)	Average log_2 CPM	Likelihood Ratio Test Statistic	P-Value	FDR Adjusted P-Value
ENSG00000019169	MARCO	macrophage receptor with collagenous structure [Source:HGNC Symbol;Acc:HGNC:6895]	protein coding	1.32	0.60	13.84	0.0002	0.0058
ENSG00000078081	LAMP3	lysosomal associated membrane protein 3 [Source:HGNC Symbol;Acc:HGNC:14582]	protein coding	1.24	2.37	39.57	<0.0001	<0.0001
ENSG00000138496	PARP9	poly(ADP-ribose) polymerase family member 9 [Source:HGNC Symbol;Acc:HGNC:24118]	protein coding	1.22	6.37	69.67	<0.0001	<0.0001
ENSG00000184557	SOCS3	suppressor of cytokine signaling 3 [Source:HGNC Symbol;Acc:HGNC:19391]	protein coding	1.19	5.78	23.36	<0.0001	<0.0001
ENSG00000025708	TYMP	thymidine phosphorylase [Source:HGNC Symbol;Acc:HGNC:3148]	protein coding	1.19	6.14	23.45	<0.0001	<0.0001
ENSG00000141574	SECTM1	secreted and transmembrane 1 [Source:HGNC Symbol;Acc:HGNC:10707]	protein coding	1.17	3.69	17.35	<0.0001	0.0012
ENSG00000020577	SAMD4A	sterile alpha motif domain containing 4A [Source:HGNC Symbol;Acc:HGNC:23023]	protein coding	1.16	1.13	34.90	<0.0001	<0.0001
ENSG00000163221	S100A12	S100 calcium binding protein A12 [Source:HGNC Symbol;Acc:HGNC:10489]	protein coding	1.09	2.89	9.51	0.002	0.0326
ENSG00000143226	FCGR2A	Fc fragment of IgG receptor IIa [Source:HGNC Symbol;Acc:HGNC:3616]	protein coding	1.08	2.40	11.97	0.0005	0.0124
ENSG00000143546	S100A8	S100 calcium binding protein A8 [Source:HGNC Symbol;Acc:HGNC:10498]	protein coding	1.08	6.00	10.91	0.001	0.0188
ENSG00000133106	EPSTI1	epithelial stromal interaction 1 [Source:HGNC Symbol;Acc:HGNC:16465]	protein coding	1.07	5.29	52.87	<0.0001	<0.0001
ENSG00000162614	NEXN	nexilin F-actin binding protein [Source:HGNC Symbol;Acc:HGNC:29557]	protein coding	1.07	0.69	34.75	<0.0001	<0.0001
ENSG00000197249	SERPINA1	serpin family A member 1 [Source:HGNC Symbol;Acc:HGNC:8941]	protein coding	1.04	4.93	10.42	0.0012	0.0226
ENSG00000171051	FPR1	formyl peptide receptor 1 [Source:HGNC Symbol;Acc:HGNC:3826]	protein coding	1.04	3.32	9.43	0.0021	0.0336
ENSG00000137959	IFI44L	interferon induced protein 44 like [Source:HGNC Symbol;Acc:HGNC:17817]	protein coding	1.04	4.89	24.72	<0.0001	<0.0001
ENSG00000163220	S100A9	S100 calcium binding protein A9 [Source:HGNC Symbol;Acc:HGNC:10499]	protein coding	1.02	7.33	10.17	0.0014	0.0251
ENSG00000177989	ODF3B	outer dense fiber of sperm tails 3B [Source:HGNC Symbol;Acc:HGNC:34388]	protein coding	1.02	4.15	19.49	<0.0001	0.0005
ENSG00000140105	WARS1	tryptophanyl-tRNA synthetase 1 [Source:HGNC Symbol;Acc:HGNC:12729]	protein coding	0.98	5.71	42.29	<0.0001	<0.0001

Table S36: Genes differentially expressed at Day 2 compared to pre-first vaccination (Study Arm 2 A/H7N9+AS03, D1, 29, T cells). Genes are sorted by descending absolute log_2 fold change. Gene model summaries and annotations are based on Ensembl Version 99 (continued.)

Ensembl Gene ID	Ensembl Gene Name	Ensembl Gene Description	Gene Type	log_2 Fold Change (Day 2 vs. Pre-First Vaccination)	Average log_2 CPM	Likelihood Ratio Test Statistic	P-Value	FDR Adjusted P-Value
ENSG00000164125	GASK1B	golgi associated kinase 1B [Source:HGNC Symbol;Acc:HGNC:25312]	protein coding	0.95	2.00	8.42	0.0037	0.0485
ENSG00000116663	FBXO6	F-box protein 6 [Source:HGNC Symbol;Acc:HGNC:13585]	protein coding	0.95	3.75	48.71	<0.0001	<0.0001
ENSG00000185499	MUC1	mucin 1, cell surface associated [Source:HGNC Symbol;Acc:HGNC:7508]	protein coding	0.95	1.45	61.51	<0.0001	<0.0001
ENSG00000162654	GBP4	guanylate binding protein 4 [Source:HGNC Symbol;Acc:HGNC:20480]	protein coding	0.94	6.86	76.38	<0.0001	<0.0001
ENSG00000134321	RSAD2	radical S-adenosyl methionine domain containing 2 [Source:HGNC Symbol;Acc:HGNC:30908]	protein coding	0.94	3.66	27.01	<0.0001	<0.0001
ENSG00000002933	TMEM176A	transmembrane protein 176A [Source:HGNC Symbol;Acc:HGNC:24930]	protein coding	0.94	1.52	8.85	0.0029	0.0417
ENSG00000186818	LILRB4	leukocyte immunoglobulin like receptor B4 [Source:HGNC Symbol;Acc:HGNC:6608]	protein coding	0.93	1.90	9.48	0.0021	0.0329
ENSG00000142089	IFITM3	interferon induced transmembrane protein 3 [Source:HGNC Symbol;Acc:HGNC:5414]	protein coding	0.92	3.78	19.53	<0.0001	0.0005
ENSG00000158714	SLAMF8	SLAM family member 8 [Source:HGNC Symbol;Acc:HGNC:21391]	protein coding	0.91	0.47	28.93	<0.0001	<0.0001
ENSG00000087253	LPCAT2	lysophosphatidylcholine acyltransferase 2 [Source:HGNC Symbol;Acc:HGNC:26032]	protein coding	0.91	1.24	8.55	0.0035	0.0464
ENSG00000225217	HSPA7	heat shock protein family A (Hsp70) member 7 [Source:HGNC Symbol;Acc:HGNC:5240]	unprocessed pseudogene	0.91	1.53	24.83	<0.0001	<0.0001
ENSG00000181631	P2RY13	purinergic receptor P2Y13 [Source:HGNC Symbol;Acc:HGNC:4537]	protein coding	0.90	1.53	8.55	0.0035	0.0464
ENSG00000134326	CMPK2	cytidine/uridine monophosphate kinase 2 [Source:HGNC Symbol;Acc:HGNC:27015]	protein coding	0.90	3.42	23.22	<0.0001	0.0001
ENSG00000125347	IRF1	interferon regulatory factor 1 [Source:HGNC Symbol;Acc:HGNC:6116]	protein coding	0.90	7.28	42.67	<0.0001	<0.0001
ENSG00000211937	IGHV2-5	immunoglobulin heavy variable 2-5 [Source:HGNC Symbol;Acc:HGNC:5576]	IG V gene	0.88	0.72	12.39	0.0004	0.0105
ENSG00000254415	SIGLEC14	sialic acid binding Ig like lectin 14 [Source:HGNC Symbol;Acc:HGNC:32926]	protein coding	0.86	1.74	10.63	0.0011	0.021
ENSG00000188820	CALHM6	calcium homeostasis modulator family member 6 [Source:HGNC Symbol;Acc:HGNC:33391]	protein coding	0.86	1.94	16.79	<0.0001	0.0016

Table S36: Genes differentially expressed at Day 2 compared to pre-first vaccination (Study Arm 2 A/H7N9+AS03, D1, 29, T cells). Genes are sorted by descending absolute log_2 fold change. Gene model summaries and annotations are based on Ensembl Version 99 (continued.)

Ensembl Gene ID	Ensembl Gene Name	Ensembl Gene Description	Gene Type	log_2 Fold Change (Day 2 vs. Pre-First Vaccination)	Average log_2 CPM	Likelihood Ratio Test Statistic	P-Value	FDR Adjusted P-Value
ENSG00000156587	UBE2L6	ubiquitin conjugating enzyme E2 L6 [Source:HGNC Symbol;Acc:HGNC:12490]	protein coding	0.85	6.76	63.59	<0.0001	<0.0001
ENSG00000154451	GBP5	guanylate binding protein 5 [Source:HGNC Symbol;Acc:HGNC:19895]	protein coding	0.85	8.18	76.68	<0.0001	<0.0001
ENSG00000188906	LRRK2	leucine rich repeat kinase 2 [Source:HGNC Symbol;Acc:HGNC:18618]	protein coding	0.83	3.13	10.70	0.0011	0.0204
ENSG00000173110	HSPA6	heat shock protein family A (Hsp70) member 6 [Source:HGNC Symbol;Acc:HGNC:5239]	protein coding	0.83	0.78	12.28	0.0005	0.011
ENSG00000284194	SCO2	SCO cytochrome c oxidase assembly protein 2 [Source:HGNC Symbol;Acc:HGNC:10604]	protein coding	0.82	1.58	17.81	<0.0001	0.001
ENSG00000119922	IFIT2	interferon induced protein with tetratricopeptide repeats 2 [Source:HGNC Symbol;Acc:HGNC:5409]	protein coding	0.81	3.38	13.63	0.0002	0.0063
ENSG00000214872	SMTNL1	smoothelin like 1 [Source:HGNC Symbol;Acc:HGNC:32394]	protein coding	0.79	1.28	36.38	<0.0001	<0.0001
ENSG00000090104	RGS1	regulator of G protein signaling 1 [Source:HGNC Symbol;Acc:HGNC:9991]	protein coding	0.77	3.79	12.94	0.0003	0.0084
ENSG00000102524	TNFSF13B	TNF superfamily member 13b [Source:HGNC Symbol;Acc:HGNC:11929]	protein coding	0.77	3.47	17.84	<0.0001	0.001
ENSG00000105967	TFEC	transcription factor EC [Source:HGNC Symbol;Acc:HGNC:11754]	protein coding	0.77	1.86	9.27	0.0023	0.0357
ENSG00000089127	OAS1	2'-5'-oligoadenylate synthetase 1 [Source:HGNC Symbol;Acc:HGNC:8086]	protein coding	0.77	4.95	23.02	<0.0001	0.0001
ENSG00000244682	FCGR2C	Fc fragment of IgG receptor IIc (gene/pseudogene) [Source:HGNC Symbol;Acc:HGNC:15626]	polymorphic pseudogene	0.76	1.77	17.46	<0.0001	0.0012
ENSG00000162645	GBP2	guanylate binding protein 2 [Source:HGNC Symbol;Acc:HGNC:4183]	protein coding	0.74	7.79	76.87	<0.0001	<0.0001
ENSG00000287839		novel transcript	lncRNA	0.73	2.70	56.56	<0.0001	<0.0001
ENSG00000069399	BCL3	BCL3 transcription coactivator [Source:HGNC Symbol;Acc:HGNC:998]	protein coding	0.73	4.74	30.24	<0.0001	<0.0001
ENSG00000183010	PYCR1	pyrroline-5-carboxylate reductase 1 [Source:HGNC Symbol;Acc:HGNC:9721]	protein coding	0.73	0.72	26.13	<0.0001	<0.0001
ENSG00000211668	IGLV2-11	immunoglobulin lambda variable 2-11 [Source:HGNC Symbol;Acc:HGNC:5887]	IG V gene	0.73	1.79	8.47	0.0036	0.0477

Table S36: Genes differentially expressed at Day 2 compared to pre-first vaccination (Study Arm 2 A/H7N9+AS03, D1, 29, T cells). Genes are sorted by descending absolute log_2 fold change. Gene model summaries and annotations are based on Ensembl Version 99 (continued.)

Ensembl Gene ID	Ensembl Gene Name	Ensembl Gene Description	Gene Type	log_2 Fold Change (Day 2 vs. Pre-First Vaccination)	Average log_2 CPM	Likelihood Ratio Test Statistic	P-Value	FDR Adjusted P-Value
ENSG00000225131	PSME2P2	proteasome activator subunit 2 pseudogene 2 [Source:HGNC Symbol;Acc:HGNC:30160]	processed pseudogene	0.72	0.16	16.32	<0.0001	0.0019
ENSG00000125462	C1orf61	chromosome 1 open reading frame 61 [Source:HGNC Symbol;Acc:HGNC:30780]	protein coding	0.72	0.67	29.71	<0.0001	<0.0001
ENSG00000163840	DTX3L	deltex E3 ubiquitin ligase 3L [Source:HGNC Symbol;Acc:HGNC:30323]	protein coding	0.71	6.19	48.42	<0.0001	<0.0001
ENSG00000177409	SAMD9L	sterile alpha motif domain containing 9 like [Source:HGNC Symbol;Acc:HGNC:1349]	protein coding	0.71	6.67	53.96	<0.0001	<0.0001
ENSG00000132530	XAF1	XIAP associated factor 1 [Source:HGNC Symbol;Acc:HGNC:30932]	protein coding	0.71	5.56	31.80	<0.0001	<0.0001
ENSG00000127951	FGL2	fibrinogen like 2 [Source:HGNC Symbol;Acc:HGNC:3696]	protein coding	0.71	5.76	8.36	0.0038	0.0498
ENSG00000097021	ACOT7	acyl-CoA thioesterase 7 [Source:HGNC Symbol;Acc:HGNC:24157]	protein coding	0.70	3.32	49.13	<0.0001	<0.0001
ENSG00000185339	TCN2	transcobalamin 2 [Source:HGNC Symbol;Acc:HGNC:11653]	protein coding	0.70	0.66	8.63	0.0033	0.0452
ENSG00000100342	APOL1	apolipoprotein L1 [Source:HGNC Symbol;Acc:HGNC:618]	protein coding	0.69	5.21	63.21	<0.0001	<0.0001
ENSG00000002549	LAP3	leucine aminopeptidase 3 [Source:HGNC Symbol;Acc:HGNC:18449]	protein coding	0.69	5.37	33.31	<0.0001	<0.0001
ENSG00000135636	DYSF	dysferlin [Source:HGNC Symbol;Acc:HGNC:3097]	protein coding	0.68	3.39	9.82	0.0017	0.0287
ENSG00000068079	IFI35	interferon induced protein 35 [Source:HGNC Symbol;Acc:HGNC:5399]	protein coding	0.68	5.22	43.39	<0.0001	<0.0001
ENSG00000146555	SDK1	sidekick cell adhesion molecule 1 [Source:HGNC Symbol;Acc:HGNC:19307]	protein coding	0.68	0.27	18.45	<0.0001	0.0008
ENSG00000172159	FRMD3	FERM domain containing 3 [Source:HGNC Symbol;Acc:HGNC:24125]	protein coding	0.68	0.41	23.11	<0.0001	0.0001
ENSG00000158517	NCF1	neutrophil cytosolic factor 1 [Source:HGNC Symbol;Acc:HGNC:7660]	protein coding	0.67	4.05	12.34	0.0004	0.0107
ENSG00000091129	NRCAM	neuronal cell adhesion molecule [Source:HGNC Symbol;Acc:HGNC:7994]	protein coding	-0.67	3.20	40.01	<0.0001	<0.0001
ENSG00000100911	PSME2	proteasome activator subunit 2 [Source:HGNC Symbol;Acc:HGNC:9569]	protein coding	0.65	7.21	70.65	<0.0001	<0.0001

Table S36: Genes differentially expressed at Day 2 compared to pre-first vaccination (Study Arm 2 A/H7N9+AS03, D1, 29, T cells). Genes are sorted by descending absolute log_2 fold change. Gene model summaries and annotations are based on Ensembl Version 99 (continued.)

Ensembl Gene ID	Ensembl Gene Name	Ensembl Gene Description	Gene Type	log_2 Fold Change (Day 2 vs. Pre-First Vaccination)	Average log_2 CPM	Likelihood Ratio Test Statistic	P-Value	FDR Adjusted P-Value
ENSG00000187608	ISG15	ISG15 ubiquitin like modifier [Source:HGNC Symbol;Acc:HGNC:4053]	protein coding	0.65	5.29	24.85	<0.0001	<0.0001
ENSG00000135604	STX11	syntaxin 11 [Source:HGNC Symbol;Acc:HGNC:11429]	protein coding	0.65	4.28	13.07	0.0003	0.0079
ENSG00000163568	AIM2	absent in melanoma 2 [Source:HGNC Symbol;Acc:HGNC:357]	protein coding	0.65	2.45	36.00	<0.0001	<0.0001
ENSG00000143476	DTL	denticleless E3 ubiquitin protein ligase homolog [Source:HGNC Symbol;Acc:HGNC:30288]	protein coding	0.63	0.22	14.24	0.0002	0.005
ENSG00000120254	MTHFD1L	methylenetetrahydrofolate dehydrogenase (NADP+ dependent) 1 like [Source:HGNC Symbol;Acc:HGNC:21055]	protein coding	0.62	3.19	65.57	<0.0001	<0.0001
ENSG00000211667	IGLV3-12	immunoglobulin lambda variable 3-12 [Source:HGNC Symbol;Acc:HGNC:5898]	IG V gene	-0.61	1.84	11.27	0.0008	0.0163
ENSG00000188282	RUFY4	RUN and FYVE domain containing 4 [Source:HGNC Symbol;Acc:HGNC:24804]	protein coding	0.61	0.59	10.91	0.001	0.0188
ENSG00000172867	KRT2	keratin 2 [Source:HGNC Symbol;Acc:HGNC:6439]	protein coding	-0.61	0.34	17.39	<0.0001	0.0012
ENSG00000137965	IFI44	interferon induced protein 44 [Source:HGNC Symbol;Acc:HGNC:16938]	protein coding	0.60	5.11	20.71	<0.0001	0.0003
ENSG00000221963	APOL6	apolipoprotein L6 [Source:HGNC Symbol;Acc:HGNC:14870]	protein coding	0.59	7.04	49.72	<0.0001	<0.0001
ENSG00000100368	CSF2RB	colony stimulating factor 2 receptor beta common subunit [Source:HGNC Symbol;Acc:HGNC:2436]	protein coding	0.59	3.89	8.82	0.003	0.0423

Table S37: Genes differentially expressed at Day 29 compared to pre-first vaccination (Study Arm 2 A/H7N9+AS03, D1, 29, T cells). Genes are sorted by descending absolute log_2 fold change. Gene model summaries and annotations are based on Ensembl Version 99.

Ensembl Gene ID	Ensembl Gene Name	Ensembl Gene Description	Gene Type	log_2 Fold Change (Day 29 vs. Pre-First Vaccination)	Average log_2 CPM	Likelihood Ratio Test Statistic	P-Value	FDR Adjusted P-Value
ENSG00000211663	IGLV3-19	immunoglobulin lambda variable 3-19 [Source:HGNC Symbol;Acc:HGNC:5903]	IG V gene	1.22	1.57	20.05	<0.0001	0.0356

Table S38: Genes differentially expressed at Day 4 compared to pre-first vaccination (Study Arm 3 A/H7N9 + PBS, D1, 29, T cells). Genes are sorted by descending absolute log_2 fold change. Gene model summaries and annotations are based on Ensembl Version 99.

Ensembl Gene ID	Ensembl Gene Name	Ensembl Gene Description	Gene Type	log_2 Fold Change (Day 4 vs. Pre-First Vaccination)	Average log_2 CPM	Likelihood Ratio Test Statistic	P-Value	FDR Adjusted P-Value
ENSG00000250182	EEF1A1P13	eukaryotic translation elongation factor 1 alpha 1 pseudogene 13 [Source:HGNC Symbol;Acc:HGNC:3196]	processed pseudogene	0.75	3.68	23.45	<0.0001	0.0181

Table S39: Genes differentially expressed at Day 30 compared to pre-second vaccination (Study Arm 2 A/H7N9+AS03, D1, 29, T cells). Genes are sorted by descending absolute log_2 fold change. Gene model summaries and annotations are based on Ensembl Version 99.

Ensembl Gene ID	Ensembl Gene Name	Ensembl Gene Description	Gene Type	log_2 Fold Change (Day 30 vs. Pre-Second Vaccination)	Average log_2 CPM	Likelihood Ratio Test Statistic	P-Value	FDR Adjusted P-Value
ENSG00000010030	ETV7	ETS variant transcription factor 7 [Source:HGNC Symbol;Acc:HGNC:18160]	protein coding	2.74	2.94	83.49	<0.0001	<0.0001
ENSG00000119917	IFIT3	interferon induced protein with tetratricopeptide repeats 3 [Source:HGNC Symbol;Acc:HGNC:5411]	protein coding	2.38	4.40	64.03	<0.0001	<0.0001
ENSG00000171049	FPR2	formyl peptide receptor 2 [Source:HGNC Symbol;Acc:HGNC:3827]	protein coding	2.14	1.40	36.39	<0.0001	<0.0001
ENSG00000138119	MYOF	myoferlin [Source:HGNC Symbol;Acc:HGNC:3656]	protein coding	2.09	2.81	31.73	<0.0001	<0.0001
ENSG00000128383	APOBEC3A	apolipoprotein B mRNA editing enzyme catalytic subunit 3A [Source:HGNC Symbol;Acc:HGNC:17343]	protein coding	1.95	2.93	42.14	<0.0001	<0.0001
ENSG00000117228	GBP1	guanylate binding protein 1 [Source:HGNC Symbol;Acc:HGNC:4182]	protein coding	1.92	7.17	69.96	<0.0001	<0.0001
ENSG00000115415	STAT1	signal transducer and activator of transcription 1 [Source:HGNC Symbol;Acc:HGNC:11362]	protein coding	1.91	9.20	69.12	<0.0001	<0.0001
ENSG00000137959	IFI44L	interferon induced protein 44 like [Source:HGNC Symbol;Acc:HGNC:17817]	protein coding	1.75	5.62	106.26	<0.0001	<0.0001
ENSG00000078081	LAMP3	lysosomal associated membrane protein 3 [Source:HGNC Symbol;Acc:HGNC:14582]	protein coding	1.65	3.07	54.25	<0.0001	<0.0001
ENSG00000025708	TYMP	thymidine phosphorylase [Source:HGNC Symbol;Acc:HGNC:3148]	protein coding	1.65	6.81	46.53	<0.0001	<0.0001
ENSG00000138496	PARP9	poly(ADP-ribose) polymerase family member 9 [Source:HGNC Symbol;Acc:HGNC:24118]	protein coding	1.60	6.78	85.37	<0.0001	<0.0001
ENSG00000119922	IFIT2	interferon induced protein with tetratricopeptide repeats 2 [Source:HGNC Symbol;Acc:HGNC:5409]	protein coding	1.60	3.70	41.47	<0.0001	<0.0001

Table S39: Genes differentially expressed at Day 30 compared to pre-second vaccination (Study Arm 2 A/H7N9+AS03, D1, 29, T cells). Genes are sorted by descending absolute log_2 fold change. Gene model summaries and annotations are based on Ensembl Version 99 (continued.)

Ensembl Gene ID	Ensembl Gene Name	Ensembl Gene Description	Gene Type	log_2 Fold Change (Day 30 vs. Pre-Second Vaccination)	Average log_2 CPM	Likelihood Ratio Test Statistic	P-Value	FDR Adjusted P-Value
ENSG00000020577	SAMD4A	sterile alpha motif domain containing 4A [Source:HGNC Symbol;Acc:HGNC:23023]	protein coding	1.59	1.83	41.07	<0.0001	<0.0001
ENSG00000140105	WARS1	tryptophanyl-tRNA synthetase 1 [Source:HGNC Symbol;Acc:HGNC:12729]	protein coding	1.58	6.42	42.25	<0.0001	<0.0001
ENSG00000090104	RGS1	regulator of G protein signaling 1 [Source:HGNC Symbol;Acc:HGNC:9991]	protein coding	1.56	4.64	38.28	<0.0001	<0.0001
ENSG00000141574	SECTM1	secreted and transmembrane 1 [Source:HGNC Symbol;Acc:HGNC:10707]	protein coding	1.55	4.17	35.28	<0.0001	<0.0001
ENSG00000019169	MARCO	macrophage receptor with collagenous structure [Source:HGNC Symbol;Acc:HGNC:6895]	protein coding	1.55	0.90	23.30	<0.0001	<0.0001
ENSG00000088827	SIGLEC1	sialic acid binding Ig like lectin 1 [Source:HGNC Symbol;Acc:HGNC:11127]	protein coding	1.54	2.11	23.09	<0.0001	<0.0001
ENSG00000204103	MAFB	MAF bZIP transcription factor B [Source:HGNC Symbol;Acc:HGNC:6408]	protein coding	1.51	2.91	23.78	<0.0001	<0.0001
ENSG00000136689	IL1RN	interleukin 1 receptor antagonist [Source:HGNC Symbol;Acc:HGNC:6000]	protein coding	1.50	1.42	18.06	<0.0001	0.0005
ENSG00000177989	ODF3B	outer dense fiber of sperm tails 3B [Source:HGNC Symbol;Acc:HGNC:34388]	protein coding	1.48	4.76	48.17	<0.0001	<0.0001
ENSG00000133106	EPSTI1	epithelial stromal interaction 1 [Source:HGNC Symbol;Acc:HGNC:16465]	protein coding	1.48	5.81	81.06	<0.0001	<0.0001
ENSG00000158714	SLAMF8	SLAM family member 8 [Source:HGNC Symbol;Acc:HGNC:21391]	protein coding	1.46	0.87	48.73	<0.0001	<0.0001
ENSG00000162614	NEXN	nexilin F-actin binding protein [Source:HGNC Symbol;Acc:HGNC:29557]	protein coding	1.45	1.12	49.29	<0.0001	<0.0001
ENSG00000188282	RUFY4	RUN and FYVE domain containing 4 [Source:HGNC Symbol;Acc:HGNC:24804]	protein coding	1.44	1.17	36.07	<0.0001	<0.0001
ENSG00000142089	IFITM3	interferon induced transmembrane protein 3 [Source:HGNC Symbol;Acc:HGNC:5414]	protein coding	1.44	4.23	37.10	<0.0001	<0.0001
ENSG00000188820	CALHM6	calcium homeostasis modulator family member 6 [Source:HGNC Symbol;Acc:HGNC:33391]	protein coding	1.41	2.56	31.13	<0.0001	<0.0001
ENSG00000184557	SOCS3	suppressor of cytokine signaling 3 [Source:HGNC Symbol;Acc:HGNC:19391]	protein coding	1.40	6.25	59.31	<0.0001	<0.0001
ENSG00000162654	GBP4	guanylate binding protein 4 [Source:HGNC Symbol;Acc:HGNC:20480]	protein coding	1.40	7.24	72.41	<0.0001	<0.0001
ENSG00000125347	IRF1	interferon regulatory factor 1 [Source:HGNC Symbol;Acc:HGNC:6116]	protein coding	1.40	7.63	90.89	<0.0001	<0.0001

Table S39: Genes differentially expressed at Day 30 compared to pre-second vaccination (Study Arm 2 A/H7N9+AS03, D1, 29, T cells). Genes are sorted by descending absolute log_2 fold change. Gene model summaries and annotations are based on Ensembl Version 99 (continued.)

Ensembl Gene ID	Ensembl Gene Name	Ensembl Gene Description	Gene Type	log_2 Fold Change (Day 30 vs. Pre-Second Vaccination)	Average log_2 CPM	Likelihood Ratio Test Statistic	P-Value	FDR Adjusted P-Value
ENSG00000183023	SLC8A1	solute carrier family 8 member A1 [Source:HGNC Symbol;Acc:HGNC:11068]	protein coding	1.38	1.14	20.63	<0.0001	0.0002
ENSG00000134321	RSAD2	radical S-adenosyl methionine domain containing 2 [Source:HGNC Symbol;Acc:HGNC:30908]	protein coding	1.38	4.08	62.81	<0.0001	<0.0001
ENSG00000143546	S100A8	S100 calcium binding protein A8 [Source:HGNC Symbol;Acc:HGNC:10498]	protein coding	1.36	6.50	16.88	<0.0001	0.0008
ENSG00000181631	P2RY13	purinergic receptor P2Y13 [Source:HGNC Symbol;Acc:HGNC:4537]	protein coding	1.36	1.88	21.88	<0.0001	<0.0001
ENSG00000103569	AQP9	aquaporin 9 [Source:HGNC Symbol;Acc:HGNC:643]	protein coding	1.34	1.78	20.22	<0.0001	0.0002
ENSG00000154451	GBP5	guanylate binding protein 5 [Source:HGNC Symbol;Acc:HGNC:19895]	protein coding	1.33	8.50	87.37	<0.0001	<0.0001
ENSG00000134326	CMPK2	cytidine/uridine monophosphate kinase 2 [Source:HGNC Symbol;Acc:HGNC:27015]	protein coding	1.33	3.85	55.42	<0.0001	<0.0001
ENSG00000284194	SCO2	SCO cytochrome c oxidase assembly protein 2 [Source:HGNC Symbol;Acc:HGNC:10604]	protein coding	1.33	2.01	44.39	<0.0001	<0.0001
ENSG00000116663	FBXO6	F-box protein 6 [Source:HGNC Symbol;Acc:HGNC:13585]	protein coding	1.31	4.07	75.06	<0.0001	<0.0001
ENSG00000171051	FPR1	formyl peptide receptor 1 [Source:HGNC Symbol;Acc:HGNC:3826]	protein coding	1.30	3.70	24.70	<0.0001	<0.0001
ENSG00000185339	TCN2	transcobalamin 2 [Source:HGNC Symbol;Acc:HGNC:11653]	protein coding	1.29	1.20	17.82	<0.0001	0.0005
ENSG00000185745	IFIT1	interferon induced protein with tetratricopeptide repeats 1 [Source:HGNC Symbol;Acc:HGNC:5407]	protein coding	1.28	3.41	49.87	<0.0001	<0.0001
ENSG00000139318	DUSP6	dual specificity phosphatase 6 [Source:HGNC Symbol;Acc:HGNC:3072]	protein coding	1.28	3.89	16.99	<0.0001	0.0008
ENSG00000091129	NRCAM	neuronal cell adhesion molecule [Source:HGNC Symbol;Acc:HGNC:7994]	protein coding	-1.25	3.05	60.27	<0.0001	<0.0001
ENSG00000120738	EGR1	early growth response 1 [Source:HGNC Symbol;Acc:HGNC:3238]	protein coding	1.25	3.83	9.05	0.0026	0.0229
ENSG00000173110	HSPA6	heat shock protein family A (Hsp70) member 6 [Source:HGNC Symbol;Acc:HGNC:5239]	protein coding	1.24	0.95	30.32	<0.0001	<0.0001
ENSG00000135636	DYSF	dysferlin [Source:HGNC Symbol;Acc:HGNC:3097]	protein coding	1.24	3.75	27.98	<0.0001	<0.0001
ENSG00000214872	SMTNL1	smoothelin like 1 [Source:HGNC Symbol;Acc:HGNC:32394]	protein coding	1.23	1.75	63.00	<0.0001	<0.0001

Table S39: Genes differentially expressed at Day 30 compared to pre-second vaccination (Study Arm 2 A/H7N9+AS03, D1, 29, T cells). Genes are sorted by descending absolute log_2 fold change. Gene model summaries and annotations are based on Ensembl Version 99 (continued.)

Ensembl Gene ID	Ensembl Gene Name	Ensembl Gene Description	Gene Type	log_2 Fold Change (Day 30 vs. Pre-Second Vaccination)	Average log_2 CPM	Likelihood Ratio Test Statistic	P-Value	FDR Adjusted P-Value
ENSG00000089127	OAS1	2'-5'-oligoadenylate synthetase 1 [Source:HGNC Symbol;Acc:HGNC:8086]	protein coding	1.20	5.28	71.83	<0.0001	<0.0001
ENSG00000163221	S100A12	S100 calcium binding protein A12 [Source:HGNC Symbol;Acc:HGNC:10489]	protein coding	1.19	3.31	12.90	0.0003	0.0047
ENSG00000163220	S100A9	S100 calcium binding protein A9 [Source:HGNC Symbol;Acc:HGNC:10499]	protein coding	1.18	7.76	12.93	0.0003	0.0046
ENSG00000188906	LRRK2	leucine rich repeat kinase 2 [Source:HGNC Symbol;Acc:HGNC:18618]	protein coding	1.18	3.75	21.61	<0.0001	<0.0001
ENSG00000132530	XAF1	XIAP associated factor 1 [Source:HGNC Symbol;Acc:HGNC:30932]	protein coding	1.17	5.81	92.60	<0.0001	<0.0001
ENSG00000163823	CCR1	C-C motif chemokine receptor 1 [Source:HGNC Symbol;Acc:HGNC:1602]	protein coding	1.17	3.12	36.00	<0.0001	<0.0001
ENSG00000090339	ICAM1	intercellular adhesion molecule 1 [Source:HGNC Symbol;Acc:HGNC:5344]	protein coding	1.15	3.40	57.26	<0.0001	<0.0001
ENSG00000185499	MUC1	mucin 1, cell surface associated [Source:HGNC Symbol;Acc:HGNC:7508]	protein coding	1.15	1.85	81.98	<0.0001	<0.0001
ENSG00000137965	IFI44	interferon induced protein 44 [Source:HGNC Symbol;Acc:HGNC:16938]	protein coding	1.14	5.47	86.85	<0.0001	<0.0001
ENSG00000069399	BCL3	BCL3 transcription coactivator [Source:HGNC Symbol;Acc:HGNC:998]	protein coding	1.13	4.98	77.07	<0.0001	<0.0001
ENSG00000102524	TNFSF13B	TNF superfamily member 13b [Source:HGNC Symbol;Acc:HGNC:11929]	protein coding	1.13	3.86	33.69	<0.0001	<0.0001
ENSG00000156587	UBE2L6	ubiquitin conjugating enzyme E2 L6 [Source:HGNC Symbol;Acc:HGNC:12490]	protein coding	1.12	7.05	66.14	<0.0001	<0.0001
ENSG00000135114	OASL	2'-5'-oligoadenylate synthetase like [Source:HGNC Symbol;Acc:HGNC:8090]	protein coding	1.12	3.80	54.24	<0.0001	<0.0001
ENSG00000187116	LILRA5	leukocyte immunoglobulin like receptor A5 [Source:HGNC Symbol;Acc:HGNC:16309]	protein coding	1.12	2.72	13.11	0.0003	0.0043
ENSG00000197249	SERPINA1	serpin family A member 1 [Source:HGNC Symbol;Acc:HGNC:8941]	protein coding	1.11	5.28	15.67	<0.0001	0.0014
ENSG00000186818	LILRB4	leukocyte immunoglobulin like receptor B4 [Source:HGNC Symbol;Acc:HGNC:6608]	protein coding	1.10	2.39	13.18	0.0003	0.0042
ENSG00000183019	MCEMP1	mast cell expressed membrane protein 1 [Source:HGNC Symbol;Acc:HGNC:27291]	protein coding	1.09	1.07	10.63	0.0011	0.012
ENSG00000224397	SMIM25	small integral membrane protein 25 [Source:HGNC Symbol;Acc:HGNC:50328]	lncRNA	1.09	1.74	13.57	0.0002	0.0035

Table S39: Genes differentially expressed at Day 30 compared to pre-second vaccination (Study Arm 2 A/H7N9+AS03, D1, 29, T cells). Genes are sorted by descending absolute log_2 fold change. Gene model summaries and annotations are based on Ensembl Version 99 (continued.)

Ensembl Gene ID	Ensembl Gene Name	Ensembl Gene Description	Gene Type	log_2 Fold Change (Day 30 vs. Pre-Second Vaccination)	Average log_2 CPM	Likelihood Ratio Test Statistic	P-Value	FDR Adjusted P-Value
ENSG00000097021	ACOT7	acyl-CoA thioesterase 7 [Source:HGNC Symbol;Acc:HGNC:24157]	protein coding	1.09	3.64	74.61	<0.0001	<0.0001
ENSG00000162645	GBP2	guanylate binding protein 2 [Source:HGNC Symbol;Acc:HGNC:4183]	protein coding	1.09	8.06	69.81	<0.0001	<0.0001
ENSG00000143226	FCGR2A	Fc fragment of IgG receptor IIa [Source:HGNC Symbol;Acc:HGNC:3616]	protein coding	1.08	2.68	16.67	<0.0001	0.0009
ENSG00000163840	DTX3L	deltex E3 ubiquitin ligase 3L [Source:HGNC Symbol;Acc:HGNC:30323]	protein coding	1.08	6.35	68.34	<0.0001	<0.0001
ENSG00000146592	CREB5	cAMP responsive element binding protein 5 [Source:HGNC Symbol;Acc:HGNC:16844]	protein coding	1.07	1.42	18.06	<0.0001	0.0005
ENSG00000136867	SLC31A2	solute carrier family 31 member 2 [Source:HGNC Symbol;Acc:HGNC:11017]	protein coding	1.06	0.55	23.92	<0.0001	<0.0001
ENSG00000151948	GLT1D1	glycosyltransferase 1 domain containing 1 [Source:HGNC Symbol;Acc:HGNC:26483]	protein coding	1.06	2.26	17.22	<0.0001	0.0007
ENSG00000105967	TFEC	transcription factor EC [Source:HGNC Symbol;Acc:HGNC:11754]	protein coding	1.04	2.23	17.57	<0.0001	0.0006
ENSG00000135604	STX11	syntaxin 11 [Source:HGNC Symbol;Acc:HGNC:11429]	protein coding	1.04	4.58	32.55	<0.0001	<0.0001
ENSG00000177409	SAMD9L	sterile alpha motif domain containing 9 like [Source:HGNC Symbol;Acc:HGNC:1349]	protein coding	1.04	6.92	82.67	<0.0001	<0.0001
ENSG00000087237	CETP	cholesteryl ester transfer protein [Source:HGNC Symbol;Acc:HGNC:1869]	protein coding	1.03	2.20	40.17	<0.0001	<0.0001
ENSG00000124762	CDKN1A	cyclin dependent kinase inhibitor 1A [Source:HGNC Symbol;Acc:HGNC:1784]	protein coding	1.03	2.06	25.18	<0.0001	<0.0001
ENSG00000188290	HES4	hes family bHLH transcription factor 4 [Source:HGNC Symbol;Acc:HGNC:24149]	protein coding	1.02	1.19	36.17	<0.0001	<0.0001
ENSG00000002549	LAP3	leucine aminopeptidase 3 [Source:HGNC Symbol;Acc:HGNC:18449]	protein coding	1.02	5.72	46.44	<0.0001	<0.0001
ENSG00000134243	SORT1	sortilin 1 [Source:HGNC Symbol;Acc:HGNC:11186]	protein coding	1.02	2.28	13.34	0.0003	0.0039
ENSG00000128641	MYO1B	myosin IB [Source:HGNC Symbol;Acc:HGNC:7596]	protein coding	1.02	0.47	30.56	<0.0001	<0.0001
ENSG00000221963	APOL6	apolipoprotein L6 [Source:HGNC Symbol;Acc:HGNC:14870]	protein coding	1.02	7.22	73.03	<0.0001	<0.0001
ENSG00000108387	SEPTIN4	septin 4 [Source:HGNC Symbol;Acc:HGNC:9165]	protein coding	1.01	0.93	18.07	<0.0001	0.0005

Table S39: Genes differentially expressed at Day 30 compared to pre-second vaccination (Study Arm 2 A/H7N9+AS03, D1, 29, T cells). Genes are sorted by descending absolute log_2 fold change. Gene model summaries and annotations are based on Ensembl Version 99 (continued.)

Ensembl Gene ID	Ensembl Gene Name	Ensembl Gene Description	Gene Type	log_2 Fold Change (Day 30 vs. Pre-Second Vaccination)	Average log_2 CPM	Likelihood Ratio Test Statistic	P-Value	FDR Adjusted P-Value
ENSG00000225964	NRIR	negative regulator of interferon response [Source:HGNC Symbol;Acc:HGNC:51269]	lncRNA	1.01	0.59	37.08	<0.0001	<0.0001
ENSG00000172159	FRMD3	FERM domain containing 3 [Source:HGNC Symbol;Acc:HGNC:24125]	protein coding	1.01	0.86	21.42	<0.0001	0.0001
ENSG00000127951	FGL2	fibrinogen like 2 [Source:HGNC Symbol;Acc:HGNC:3696]	protein coding	1.01	6.08	19.52	<0.0001	0.0003
ENSG00000187608	ISG15	ISG15 ubiquitin like modifier [Source:HGNC Symbol;Acc:HGNC:4053]	protein coding	0.99	5.58	56.62	<0.0001	<0.0001
ENSG00000185215	TNFAIP2	TNF alpha induced protein 2 [Source:HGNC Symbol;Acc:HGNC:11895]	protein coding	0.99	5.90	15.14	<0.0001	0.0018
ENSG00000100342	APOL1	apolipoprotein L1 [Source:HGNC Symbol;Acc:HGNC:618]	protein coding	0.99	5.41	57.43	<0.0001	<0.0001
ENSG00000158517	NCF1	neutrophil cytosolic factor 1 [Source:HGNC Symbol;Acc:HGNC:7660]	protein coding	0.99	4.58	19.79	<0.0001	0.0002
ENSG00000281181		novel transcript, similar to YY1 associated myogenesis RNA 1 YAM1	lncRNA	0.99	4.89	9.88	0.0017	0.0163
ENSG00000179639	FCER1A	Fc fragment of IgE receptor Ia [Source:HGNC Symbol;Acc:HGNC:3609]	protein coding	-0.98	2.38	22.32	<0.0001	<0.0001
ENSG00000169385	RNASE2	ribonuclease A family member 2 [Source:HGNC Symbol;Acc:HGNC:10045]	protein coding	0.98	0.69	8.97	0.0027	0.0236
ENSG00000123405	NFE2	nuclear factor, erythroid 2 [Source:HGNC Symbol;Acc:HGNC:7780]	protein coding	0.98	2.51	17.20	<0.0001	0.0007
ENSG00000111331	OAS3	2'-5'-oligoadenylate synthetase 3 [Source:HGNC Symbol;Acc:HGNC:8088]	protein coding	0.98	6.57	65.91	<0.0001	<0.0001
ENSG00000225131	PSME2P2	proteasome activator subunit 2 pseudogene 2 [Source:HGNC Symbol;Acc:HGNC:30160]	processed pseudogene	0.98	0.39	35.48	<0.0001	<0.0001
ENSG00000068079	IFI35	interferon induced protein 35 [Source:HGNC Symbol;Acc:HGNC:5399]	protein coding	0.98	5.47	62.43	<0.0001	<0.0001
ENSG00000131042	LILRB2	leukocyte immunoglobulin like receptor B2 [Source:HGNC Symbol;Acc:HGNC:6606]	protein coding	0.97	4.35	13.92	0.0002	0.003
ENSG00000211667	IGLV3-12	immunoglobulin lambda variable 3-12 [Source:HGNC Symbol;Acc:HGNC:5898]	IG V gene	-0.97	1.58	24.42	<0.0001	<0.0001
ENSG00000244482	LILRA6	leukocyte immunoglobulin like receptor A6 [Source:HGNC Symbol;Acc:HGNC:15495]	protein coding	0.97	2.20	15.53	<0.0001	0.0015

Table S39: Genes differentially expressed at Day 30 compared to pre-second vaccination (Study Arm 2 A/H7N9+AS03, D1, 29, T cells). Genes are sorted by descending absolute log_2 fold change. Gene model summaries and annotations are based on Ensembl Version 99 (continued.)

Ensembl Gene ID	Ensembl Gene Name	Ensembl Gene Description	Gene Type	log_2 Fold Change (Day 30 vs. Pre-Second Vaccination)	Average log_2 CPM	Likelihood Ratio Test Statistic	P-Value	FDR Adjusted P-Value
ENSG00000185338	SOCS1	suppressor of cytokine signaling 1 [Source:HGNC Symbol;Acc:HGNC:19383]	protein coding	0.97	3.97	56.52	<0.0001	<0.0001
ENSG00000104974	LILRA1	leukocyte immunoglobulin like receptor A1 [Source:HGNC Symbol;Acc:HGNC:6602]	protein coding	0.96	3.00	13.01	0.0003	0.0044
ENSG00000165092	ALDH1A1	aldehyde dehydrogenase 1 family member A1 [Source:HGNC Symbol;Acc:HGNC:402]	protein coding	0.95	1.90	7.79	0.0052	0.0383
ENSG00000167850	CD300C	CD300c molecule [Source:HGNC Symbol;Acc:HGNC:19320]	protein coding	0.94	1.73	10.34	0.0013	0.0135
ENSG00000111321	LTBR	lymphotoxin beta receptor [Source:HGNC Symbol;Acc:HGNC:6718]	protein coding	0.94	2.84	15.13	0.0001	0.0018
ENSG00000126709	IFI6	interferon alpha inducible protein 6 [Source:HGNC Symbol;Acc:HGNC:4054]	protein coding	0.94	6.09	44.78	<0.0001	<0.0001
ENSG00000163625	WDFY3	WD repeat and FYVE domain containing 3 [Source:HGNC Symbol;Acc:HGNC:20751]	protein coding	0.93	1.12	17.09	<0.0001	0.0007
ENSG00000184371	CSF1	colony stimulating factor 1 [Source:HGNC Symbol;Acc:HGNC:2432]	protein coding	0.92	3.96	34.48	<0.0001	<0.0001
ENSG00000254503		high-mobility group box 3 (HMGB3) pseudogene	processed pseudogene	0.92	0.74	45.77	<0.0001	<0.0001
ENSG00000140749	IGSF6	immunoglobulin superfamily member 6 [Source:HGNC Symbol;Acc:HGNC:5953]	protein coding	0.91	2.75	15.67	<0.0001	0.0014
ENSG00000168394	TAP1	transporter 1, ATP binding cassette subfamily B member [Source:HGNC Symbol;Acc:HGNC:43]	protein coding	0.91	8.56	72.61	<0.0001	<0.0001
ENSG00000125462	C1orf61	chromosome 1 open reading frame 61 [Source:HGNC Symbol;Acc:HGNC:30780]	protein coding	0.91	0.90	28.73	<0.0001	<0.0001
ENSG00000111319	SCNN1A	sodium channel epithelial 1 alpha subunit [Source:HGNC Symbol;Acc:HGNC:10599]	protein coding	-0.90	0.71	42.36	<0.0001	<0.0001
ENSG00000136514	RTP4	receptor transporter protein 4 [Source:HGNC Symbol;Acc:HGNC:23992]	protein coding	0.90	3.27	28.53	<0.0001	<0.0001
ENSG00000082397	EPB41L3	erythrocyte membrane protein band 4.1 like 3 [Source:HGNC Symbol;Acc:HGNC:3380]	protein coding	0.89	2.71	11.70	0.0006	0.0076
ENSG00000185507	IRF7	interferon regulatory factor 7 [Source:HGNC Symbol;Acc:HGNC:6122]	protein coding	0.89	6.58	65.17	<0.0001	<0.0001
ENSG00000079385	CEACAM1	CEA cell adhesion molecule 1 [Source:HGNC Symbol;Acc:HGNC:1814]	protein coding	0.88	2.05	28.06	<0.0001	<0.0001

Table S39: Genes differentially expressed at Day 30 compared to pre-second vaccination (Study Arm 2 A/H7N9+AS03, D1, 29, T cells). Genes are sorted by descending absolute log_2 fold change. Gene model summaries and annotations are based on Ensembl Version 99 (continued.)

Ensembl Gene ID	Ensembl Gene Name	Ensembl Gene Description	Gene Type	log_2 Fold Change (Day 30 vs. Pre-Second Vaccination)	Average log_2 CPM	Likelihood Ratio Test Statistic	P-Value	FDR Adjusted P-Value
ENSG00000125148	MT2A	metallothionein 2A [Source:HGNC Symbol;Acc:HGNC:7406]	protein coding	0.88	4.97	62.69	<0.0001	<0.0001
ENSG00000179921	GPBAR1	G protein-coupled bile acid receptor 1 [Source:HGNC Symbol;Acc:HGNC:19680]	protein coding	0.88	1.93	13.02	0.0003	0.0044
ENSG00000261644		novel transcript, antisense to CYLD and NOD2	lncRNA	0.88	2.77	58.13	<0.0001	<0.0001
ENSG00000186407	CD300E	CD300e molecule [Source:HGNC Symbol;Acc:HGNC:28874]	protein coding	0.88	4.18	12.33	0.0004	0.0059
ENSG00000254415	SIGLEC14	sialic acid binding Ig like lectin 14 [Source:HGNC Symbol;Acc:HGNC:32926]	protein coding	0.88	1.89	11.85	0.0006	0.0072
ENSG00000121316	PLBD1	phospholipase B domain containing 1 [Source:HGNC Symbol;Acc:HGNC:26215]	protein coding	0.88	3.98	9.59	0.002	0.0184
ENSG00000138166	DUSP5	dual specificity phosphatase 5 [Source:HGNC Symbol;Acc:HGNC:3071]	protein coding	0.88	3.96	44.42	<0.0001	<0.0001
ENSG00000234518	PTGES3P1	prostaglandin E synthase 3 pseudogene 1 [Source:HGNC Symbol;Acc:HGNC:43824]	processed pseudogene	0.88	3.42	63.96	<0.0001	<0.0001
ENSG00000065911	MTHFD2	methylenetetrahydrofolate dehydrogenase (NADP+ dependent) 2, methenyltetrahydrofolate cyclohydrolase [Source:HGNC Symbol;Acc:HGNC:7434]	protein coding	0.86	4.49	64.42	<0.0001	<0.0001
ENSG00000137628	DDX60	DExD/H-box helicase 60 [Source:HGNC Symbol;Acc:HGNC:25942]	protein coding	0.86	5.75	54.26	<0.0001	<0.0001
ENSG00000087253	LPCAT2	lysophosphatidylcholine acyltransferase 2 [Source:HGNC Symbol;Acc:HGNC:26032]	protein coding	0.86	1.67	8.24	0.0041	0.0322
ENSG00000235568	NFAM1	NFAT activating protein with ITAM motif 1 [Source:HGNC Symbol;Acc:HGNC:29872]	protein coding	0.86	4.06	11.01	0.0009	0.0103
ENSG00000183779	ZNF703	zinc finger protein 703 [Source:HGNC Symbol;Acc:HGNC:25883]	protein coding	0.85	1.21	9.93	0.0016	0.016
ENSG00000184979	USP18	ubiquitin specific peptidase 18 [Source:HGNC Symbol;Acc:HGNC:12616]	protein coding	0.85	2.87	43.57	<0.0001	<0.0001
ENSG00000135148	TRAFD1	TRAF-type zinc finger domain containing 1 [Source:HGNC Symbol;Acc:HGNC:24808]	protein coding	0.84	6.20	52.08	<0.0001	<0.0001
ENSG00000166002	SMCO4	single-pass membrane protein with coiled-coil domains 4 [Source:HGNC Symbol;Acc:HGNC:24810]	protein coding	0.84	2.21	23.50	<0.0001	<0.0001
ENSG00000100911	PSME2	proteasome activator subunit 2 [Source:HGNC Symbol;Acc:HGNC:9569]	protein coding	0.84	7.43	65.46	<0.0001	<0.0001

Table S39: Genes differentially expressed at Day 30 compared to pre-second vaccination (Study Arm 2 A/H7N9+AS03, D1, 29, T cells). Genes are sorted by descending absolute log_2 fold change. Gene model summaries and annotations are based on Ensembl Version 99 (continued.)

Ensembl Gene ID	Ensembl Gene Name	Ensembl Gene Description	Gene Type	log_2 Fold Change (Day 30 vs. Pre-Second Vaccination)	Average log_2 CPM	Likelihood Ratio Test Statistic	P-Value	FDR Adjusted P-Value
ENSG00000132109	TRIM21	tripartite motif containing 21 [Source:HGNC Symbol;Acc:HGNC:11312]	protein coding	0.84	5.45	41.04	<0.0001	<0.0001
ENSG00000287839		novel transcript	lncRNA	0.84	2.92	41.34	<0.0001	<0.0001
ENSG00000157601	MX1	MX dynamin like GTPase 1 [Source:HGNC Symbol;Acc:HGNC:7532]	protein coding	0.84	6.62	50.84	<0.0001	<0.0001
ENSG00000163563	MNDA	myeloid cell nuclear differentiation antigen [Source:HGNC Symbol;Acc:HGNC:7183]	protein coding	0.84	4.88	11.19	0.0008	0.0095
ENSG00000073331	ALPK1	alpha kinase 1 [Source:HGNC Symbol;Acc:HGNC:20917]	protein coding	0.84	4.05	51.97	<0.0001	<0.0001
ENSG00000161944	ASGR2	asialoglycoprotein receptor 2 [Source:HGNC Symbol;Acc:HGNC:743]	protein coding	0.83	1.67	9.32	0.0023	0.0206
ENSG00000184060	ADAP2	ArfGAP with dual PH domains 2 [Source:HGNC Symbol;Acc:HGNC:16487]	protein coding	0.83	1.24	10.80	0.001	0.0112
ENSG00000135363	LMO2	LIM domain only 2 [Source:HGNC Symbol;Acc:HGNC:6642]	protein coding	0.83	2.93	13.47	0.0002	0.0037
ENSG00000204577	LILRB3	leukocyte immunoglobulin like receptor B3 [Source:HGNC Symbol;Acc:HGNC:6607]	protein coding	0.83	3.57	13.29	0.0003	0.004
ENSG00000004468	CD38	CD38 molecule [Source:HGNC Symbol;Acc:HGNC:1667]	protein coding	0.83	4.33	57.81	<0.0001	<0.0001
ENSG00000158869	FCER1G	Fc fragment of IgE receptor Ig [Source:HGNC Symbol;Acc:HGNC:3611]	protein coding	0.82	4.02	11.83	0.0006	0.0073
ENSG00000011422	PLAUR	plasminogen activator, urokinase receptor [Source:HGNC Symbol;Acc:HGNC:9053]	protein coding	0.81	2.44	16.44	<0.0001	0.001
ENSG00000108679	LGALS3BP	galectin 3 binding protein [Source:HGNC Symbol;Acc:HGNC:6564]	protein coding	0.81	6.61	49.68	<0.0001	<0.0001
ENSG00000109743	BST1	bone marrow stromal cell antigen 1 [Source:HGNC Symbol;Acc:HGNC:1118]	protein coding	0.81	2.73	8.44	0.0037	0.0297
ENSG00000177706	FAM20C	FAM20C golgi associated secretory pathway kinase [Source:HGNC Symbol;Acc:HGNC:22140]	protein coding	0.81	1.27	8.67	0.0032	0.0268
ENSG00000256262	USP30-AS1	USP30 antisense RNA 1 [Source:HGNC Symbol;Acc:HGNC:40909]	lncRNA	0.81	2.81	48.12	<0.0001	<0.0001
ENSG00000160111	CPAMD8	C3 and PZP like alpha-2-macroglobulin domain containing 8 [Source:HGNC Symbol;Acc:HGNC:23228]	protein coding	-0.81	2.79	52.04	<0.0001	<0.0001
ENSG00000064932	SBNO2	strawberry notch homolog 2 [Source:HGNC Symbol;Acc:HGNC:29158]	protein coding	0.80	7.68	58.37	<0.0001	<0.0001

Table S39: Genes differentially expressed at Day 30 compared to pre-second vaccination (Study Arm 2 A/H7N9+AS03, D1, 29, T cells). Genes are sorted by descending absolute log_2 fold change. Gene model summaries and annotations are based on Ensembl Version 99 (continued.)

Ensembl Gene ID	Ensembl Gene Name	Ensembl Gene Description	Gene Type	log_2 Fold Change (Day 30 vs. Pre-Second Vaccination)	Average log_2 CPM	Likelihood Ratio Test Statistic	P-Value	FDR Adjusted P-Value
ENSG00000085265	FCN1	ficolin 1 [Source:HGNC Symbol;Acc:HGNC:3623]	protein coding	0.80	7.45	8.16	0.0043	0.0332
ENSG00000282851	BISPR	BST2 interferon stimulated positive regulator [Source:HGNC Symbol;Acc:HGNC:51290]	lncRNA	0.80	4.30	65.19	<0.0001	<0.0001
ENSG00000287733		novel transcript	lncRNA	0.80	1.01	21.45	<0.0001	0.0001
ENSG00000215458	AATBC	apoptosis associated transcript in bladder cancer [Source:HGNC Symbol;Acc:HGNC:51526]	lncRNA	0.80	1.83	14.45	0.0001	0.0024
ENSG00000128335	APOL2	apolipoprotein L2 [Source:HGNC Symbol;Acc:HGNC:619]	protein coding	0.80	6.57	59.42	<0.0001	<0.0001
ENSG00000134470	IL15RA	interleukin 15 receptor subunit alpha [Source:HGNC Symbol;Acc:HGNC:5978]	protein coding	0.80	3.53	51.94	<0.0001	<0.0001
ENSG00000187164	SHTN1	shootin 1 [Source:HGNC Symbol;Acc:HGNC:29319]	protein coding	0.79	1.83	11.94	0.0005	0.0069
ENSG00000160883	HK3	hexokinase 3 [Source:HGNC Symbol;Acc:HGNC:4925]	protein coding	0.79	4.73	9.59	0.002	0.0184
ENSG00000014914	MTMR11	myotubularin related protein 11 [Source:HGNC Symbol;Acc:HGNC:24307]	protein coding	0.78	1.80	10.07	0.0015	0.0151
ENSG00000170581	STAT2	signal transducer and activator of transcription 2 [Source:HGNC Symbol;Acc:HGNC:11363]	protein coding	0.78	7.62	63.25	<0.0001	<0.0001
ENSG00000136048	DRAM1	DNA damage regulated autophagy modulator 1 [Source:HGNC Symbol;Acc:HGNC:25645]	protein coding	0.78	2.05	44.41	<0.0001	<0.0001
ENSG00000168209	DDIT4	DNA damage inducible transcript 4 [Source:HGNC Symbol;Acc:HGNC:24944]	protein coding	-0.78	6.01	23.26	<0.0001	<0.0001
ENSG00000121858	TNFSF10	TNF superfamily member 10 [Source:HGNC Symbol;Acc:HGNC:11925]	protein coding	0.78	5.95	29.61	<0.0001	<0.0001
ENSG00000103196	CRISPLD2	cysteine rich secretory protein LCCL domain containing 2 [Source:HGNC Symbol;Acc:HGNC:25248]	protein coding	0.78	2.41	12.55	0.0004	0.0054
ENSG00000188313	PLSCR1	phospholipid scramblase 1 [Source:HGNC Symbol;Acc:HGNC:9092]	protein coding	0.78	4.34	41.95	<0.0001	<0.0001
ENSG00000255221	CARD17	caspase recruitment domain family member 17 [Source:HGNC Symbol;Acc:HGNC:33827]	protein coding	0.77	1.36	30.50	<0.0001	<0.0001
ENSG00000130589	HELZ2	helicase with zinc finger 2 [Source:HGNC Symbol;Acc:HGNC:30021]	protein coding	0.77	7.25	64.34	<0.0001	<0.0001
ENSG00000065361	ERBB3	erb-b2 receptor tyrosine kinase 3 [Source:HGNC Symbol;Acc:HGNC:3431]	protein coding	-0.77	0.38	22.63	<0.0001	<0.0001

Table S39: Genes differentially expressed at Day 30 compared to pre-second vaccination (Study Arm 2 A/H7N9+AS03, D1, 29, T cells). Genes are sorted by descending absolute log_2 fold change. Gene model summaries and annotations are based on Ensembl Version 99 (continued.)

Ensembl Gene ID	Ensembl Gene Name	Ensembl Gene Description	Gene Type	log_2 Fold Change (Day 30 vs. Pre-Second Vaccination)	Average log_2 CPM	Likelihood Ratio Test Statistic	P-Value	FDR Adjusted P-Value
ENSG00000173193	PARP14	poly(ADP-ribose) polymerase family member 14 [Source:HGNC Symbol;Acc:HGNC:29232]	protein coding	0.77	6.87	60.53	<0.0001	<0.0001
ENSG00000136869	TLR4	toll like receptor 4 [Source:HGNC Symbol;Acc:HGNC:11850]	protein coding	0.76	2.96	10.09	0.0015	0.0149
ENSG00000103313	MEFV	MEFV innate immuity regulator, pyrin [Source:HGNC Symbol;Acc:HGNC:6998]	protein coding	0.76	3.04	9.42	0.0021	0.0198
ENSG00000115828	QPCT	glutaminyl-peptide cyclotransferase [Source:HGNC Symbol;Acc:HGNC:9753]	protein coding	0.76	1.49	7.87	0.005	0.0372
ENSG00000154102	C16orf74	chromosome 16 open reading frame 74 [Source:HGNC Symbol;Acc:HGNC:23362]	protein coding	-0.76	2.75	42.89	<0.0001	<0.0001
ENSG00000008441	NFIX	nuclear factor I X [Source:HGNC Symbol;Acc:HGNC:7788]	protein coding	0.76	3.37	38.51	<0.0001	<0.0001
ENSG00000164136	IL15	interleukin 15 [Source:HGNC Symbol;Acc:HGNC:5977]	protein coding	0.75	2.66	30.95	<0.0001	<0.0001
ENSG00000253669	GASAL1	growth arrest associated lncRNA 1 [Source:HGNC Symbol;Acc:HGNC:53461]	lncRNA	0.75	1.05	27.01	<0.0001	<0.0001
ENSG00000166428	PLD4	phospholipase D family member 4 [Source:HGNC Symbol;Acc:HGNC:23792]	protein coding	-0.75	2.86	46.70	<0.0001	<0.0001
ENSG00000120217	CD274	CD274 molecule [Source:HGNC Symbol;Acc:HGNC:17635]	protein coding	0.75	3.27	16.74	<0.0001	0.0009
ENSG00000240065	PSMB9	proteasome 20S subunit beta 9 [Source:HGNC Symbol;Acc:HGNC:9546]	protein coding	0.75	7.64	54.04	<0.0001	<0.0001
ENSG00000213928	IRF9	interferon regulatory factor 9 [Source:HGNC Symbol;Acc:HGNC:6131]	protein coding	0.74	4.96	87.93	<0.0001	<0.0001
ENSG00000100368	CSF2RB	colony stimulating factor 2 receptor beta common subunit [Source:HGNC Symbol;Acc:HGNC:2436]	protein coding	0.74	4.27	17.22	<0.0001	0.0007
ENSG00000165178	NCF1C	neutrophil cytosolic factor 1C pseudogene [Source:HGNC Symbol;Acc:HGNC:32523]	unprocessed pseudogene	0.73	3.76	14.07	0.0002	0.0028
ENSG00000111335	OAS2	2'-5'-oligoadenylate synthetase 2 [Source:HGNC Symbol;Acc:HGNC:8087]	protein coding	0.73	7.02	63.52	<0.0001	<0.0001
ENSG00000197536	IRF1-AS1	IRF1 antisense RNA 1 [Source:HGNC Symbol;Acc:HGNC:33838]	lncRNA	0.73	5.49	78.80	<0.0001	<0.0001
ENSG00000164342	TLR3	toll like receptor 3 [Source:HGNC Symbol;Acc:HGNC:11849]	protein coding	0.73	0.97	10.86	0.001	0.0109

Table S39: Genes differentially expressed at Day 30 compared to pre-second vaccination (Study Arm 2 A/H7N9+AS03, D1, 29, T cells). Genes are sorted by descending absolute log_2 fold change. Gene model summaries and annotations are based on Ensembl Version 99 (continued.)

Ensembl Gene ID	Ensembl Gene Name	Ensembl Gene Description	Gene Type	log_2 Fold Change (Day 30 vs. Pre-Second Vaccination)	Average log_2 CPM	Likelihood Ratio Test Statistic	P-Value	FDR Adjusted P-Value
ENSG00000185880	TRIM69	tripartite motif containing 69 [Source:HGNC Symbol;Acc:HGNC:17857]	protein coding	0.73	5.82	43.56	<0.0001	<0.0001
ENSG00000163568	AIM2	absent in melanoma 2 [Source:HGNC Symbol;Acc:HGNC:357]	protein coding	0.73	2.69	27.70	<0.0001	<0.0001
ENSG00000007516	BAIAP3	BAI1 associated protein 3 [Source:HGNC Symbol;Acc:HGNC:948]	protein coding	-0.72	4.85	51.67	<0.0001	<0.0001
ENSG00000171860	C3AR1	complement C3a receptor 1 [Source:HGNC Symbol;Acc:HGNC:1319]	protein coding	0.72	3.09	23.67	<0.0001	<0.0001
ENSG00000152778	IFIT5	interferon induced protein with tetratricopeptide repeats 5 [Source:HGNC Symbol;Acc:HGNC:13328]	protein coding	0.72	4.28	57.90	<0.0001	<0.0001
ENSG00000226091	LINC00937	long intergenic non-protein coding RNA 937 [Source:HGNC Symbol;Acc:HGNC:48629]	lncRNA	0.72	0.86	9.91	0.0016	0.0161
ENSG00000205730	ITPRIPL2	ITPRIP like 2 [Source:HGNC Symbol;Acc:HGNC:27257]	protein coding	0.71	1.88	22.50	<0.0001	<0.0001
ENSG00000239998	LILRA2	leukocyte immunoglobulin like receptor A2 [Source:HGNC Symbol;Acc:HGNC:6603]	protein coding	0.71	3.09	7.95	0.0048	0.036
ENSG00000175489	LRRC25	leucine rich repeat containing 25 [Source:HGNC Symbol;Acc:HGNC:29806]	protein coding	0.71	3.64	8.47	0.0036	0.0292
ENSG00000089041	P2RX7	purinergic receptor P2X 7 [Source:HGNC Symbol;Acc:HGNC:8537]	protein coding	0.71	3.89	37.74	<0.0001	<0.0001
ENSG00000101336	HCK	HCK proto-oncogene, Src family tyrosine kinase [Source:HGNC Symbol;Acc:HGNC:4840]	protein coding	0.71	4.52	9.15	0.0025	0.022
ENSG00000064763	FAR2	fatty acyl-CoA reductase 2 [Source:HGNC Symbol;Acc:HGNC:25531]	protein coding	0.70	2.87	40.42	<0.0001	<0.0001
ENSG00000170379	TCAF2	TRPM8 channel associated factor 2 [Source:HGNC Symbol;Acc:HGNC:26878]	protein coding	0.70	4.35	54.26	<0.0001	<0.0001
ENSG00000275772		CMT1A duplicated region transcript 15-like 2 (CDRT15L2) pseudogene	processed pseudogene	-0.70	1.10	27.99	<0.0001	<0.0001
ENSG00000196141	SPATS2L	spermatogenesis associated serine rich 2 like [Source:HGNC Symbol;Acc:HGNC:24574]	protein coding	0.69	2.81	38.55	<0.0001	<0.0001
ENSG00000277443	MARCKS	myristoylated alanine rich protein kinase C substrate [Source:HGNC Symbol;Acc:HGNC:6759]	protein coding	0.69	3.69	10.50	0.0012	0.0126
ENSG00000113721	PDGFRB	platelet derived growth factor receptor beta [Source:HGNC Symbol;Acc:HGNC:8804]	protein coding	0.69	1.65	15.76	<0.0001	0.0013

Table S39: Genes differentially expressed at Day 30 compared to pre-second vaccination (Study Arm 2 A/H7N9+AS03, D1, 29, T cells). Genes are sorted by descending absolute log_2 fold change. Gene model summaries and annotations are based on Ensembl Version 99 (continued.)

Ensembl Gene ID	Ensembl Gene Name	Ensembl Gene Description	Gene Type	log_2 Fold Change (Day 30 vs. Pre-Second Vaccination)	Average log_2 CPM	Likelihood Ratio Test Statistic	P-Value	FDR Adjusted P-Value
ENSG00000168961	LGALS9	galectin 9 [Source:HGNC Symbol;Acc:HGNC:6570]	protein coding	0.69	5.69	45.39	<0.0001	<0.0001
ENSG00000248473	LINC01962	long intergenic non-protein coding RNA 1962 [Source:HGNC Symbol;Acc:HGNC:52787]	lncRNA	-0.69	1.65	44.06	<0.0001	<0.0001
ENSG00000151726	ACSL1	acyl-CoA synthetase long chain family member 1 [Source:HGNC Symbol;Acc:HGNC:3569]	protein coding	0.69	4.34	21.86	<0.0001	<0.0001
ENSG00000165490	DDIAS	DNA damage induced apoptosis suppressor [Source:HGNC Symbol;Acc:HGNC:26351]	protein coding	0.69	0.59	20.49	<0.0001	0.0002
ENSG00000121690	DEPDC7	DEP domain containing 7 [Source:HGNC Symbol;Acc:HGNC:29899]	protein coding	-0.69	1.01	29.63	<0.0001	<0.0001
ENSG00000096968	JAK2	Janus kinase 2 [Source:HGNC Symbol;Acc:HGNC:6192]	protein coding	0.68	4.97	44.59	<0.0001	<0.0001
ENSG00000183010	PYCR1	pyrroline-5-carboxylate reductase 1 [Source:HGNC Symbol;Acc:HGNC:9721]	protein coding	0.68	1.03	16.20	<0.0001	0.0011
ENSG00000104972	LILRB1	leukocyte immunoglobulin like receptor B1 [Source:HGNC Symbol;Acc:HGNC:6605]	protein coding	0.68	4.86	23.16	<0.0001	<0.0001
ENSG00000114737	CISH	cytokine inducible SH2 containing protein [Source:HGNC Symbol;Acc:HGNC:1984]	protein coding	0.68	7.30	20.90	<0.0001	0.0001
ENSG00000151689	INPP1	inositol polyphosphate-1-phosphatase [Source:HGNC Symbol;Acc:HGNC:6071]	protein coding	0.68	2.86	35.82	<0.0001	<0.0001
ENSG00000143382	ADAMTSL4	ADAMTS like 4 [Source:HGNC Symbol;Acc:HGNC:19706]	protein coding	0.68	3.32	21.48	<0.0001	0.0001
ENSG00000197121	PGAP1	post-GPI attachment to proteins inositol deacylase 1 [Source:HGNC Symbol;Acc:HGNC:25712]	protein coding	0.68	3.53	39.31	<0.0001	<0.0001
ENSG00000118515	SGK1	serum/glucocorticoid regulated kinase 1 [Source:HGNC Symbol;Acc:HGNC:10810]	protein coding	0.67	3.35	9.50	0.0021	0.0191
ENSG00000183734	ASCL2	achaete-scute family bHLH transcription factor 2 [Source:HGNC Symbol;Acc:HGNC:739]	protein coding	0.67	4.02	36.66	<0.0001	<0.0001
ENSG00000197405	C5AR1	complement C5a receptor 1 [Source:HGNC Symbol;Acc:HGNC:1338]	protein coding	0.67	3.00	10.35	0.0013	0.0135
ENSG00000146555	SDK1	sidekick cell adhesion molecule 1 [Source:HGNC Symbol;Acc:HGNC:19307]	protein coding	0.67	0.50	18.32	<0.0001	0.0004
ENSG00000121797	CCRL2	C-C motif chemokine receptor like 2 [Source:HGNC Symbol;Acc:HGNC:1612]	protein coding	0.67	0.64	18.80	<0.0001	0.0003
ENSG00000137801	THBS1	thrombospondin 1 [Source:HGNC Symbol;Acc:HGNC:11785]	protein coding	-0.67	1.43	11.44	0.0007	0.0086

Table S39: Genes differentially expressed at Day 30 compared to pre-second vaccination (Study Arm 2 A/H7N9+AS03, D1, 29, T cells). Genes are sorted by descending absolute log_2 fold change. Gene model summaries and annotations are based on Ensembl Version 99 (continued.)

Ensembl Gene ID	Ensembl Gene Name	Ensembl Gene Description	Gene Type	log_2 Fold Change (Day 30 vs. Pre-Second Vaccination)	Average log_2 CPM	Likelihood Ratio Test Statistic	P-Value	FDR Adjusted P-Value
ENSG00000130303	BST2	bone marrow stromal cell antigen 2 [Source:HGNC Symbol;Acc:HGNC:1119]	protein coding	0.67	6.56	44.25	<0.0001	<0.0001
ENSG00000234290		novel transcript	lncRNA	0.67	3.93	43.91	<0.0001	<0.0001
ENSG00000102554	KLF5	Kruppel like factor 5 [Source:HGNC Symbol;Acc:HGNC:6349]	protein coding	0.67	1.56	21.26	<0.0001	0.0001
ENSG00000165806	CASP7	caspase 7 [Source:HGNC Symbol;Acc:HGNC:1508]	protein coding	0.66	4.41	57.22	<0.0001	<0.0001
ENSG00000120057	SFRP5	secreted frizzled related protein 5 [Source:HGNC Symbol;Acc:HGNC:10779]	protein coding	-0.66	2.14	29.07	<0.0001	<0.0001
ENSG00000173334	TRIB1	tribbles pseudokinase 1 [Source:HGNC Symbol;Acc:HGNC:16891]	protein coding	0.66	2.77	10.15	0.0014	0.0146
ENSG00000197122	SRC	SRC proto-oncogene, non-receptor tyrosine kinase [Source:HGNC Symbol;Acc:HGNC:11283]	protein coding	0.66	2.68	16.74	<0.0001	0.0009
ENSG00000102678	FGF9	fibroblast growth factor 9 [Source:HGNC Symbol;Acc:HGNC:3687]	protein coding	-0.66	0.76	19.30	<0.0001	0.0003
ENSG00000105327	BBC3	BCL2 binding component 3 [Source:HGNC Symbol;Acc:HGNC:17868]	protein coding	-0.65	5.00	52.61	<0.0001	<0.0001
ENSG00000120594	PLXDC2	plexin domain containing 2 [Source:HGNC Symbol;Acc:HGNC:21013]	protein coding	0.65	2.67	7.15	0.0075	0.0495
ENSG00000066336	SPI1	Spi-1 proto-oncogene [Source:HGNC Symbol;Acc:HGNC:11241]	protein coding	0.65	5.14	9.74	0.0018	0.0173
ENSG00000231160	KLF3-AS1	KLF3 antisense RNA 1 [Source:HGNC Symbol;Acc:HGNC:25796]	lncRNA	-0.65	4.80	58.14	<0.0001	<0.0001
ENSG00000130164	LDLR	low density lipoprotein receptor [Source:HGNC Symbol;Acc:HGNC:6547]	protein coding	0.65	4.31	28.25	<0.0001	<0.0001
ENSG00000055332	EIF2AK2	eukaryotic translation initiation factor 2 alpha kinase 2 [Source:HGNC Symbol;Acc:HGNC:9437]	protein coding	0.65	5.43	77.77	<0.0001	<0.0001
ENSG00000065833	ME1	malic enzyme 1 [Source:HGNC Symbol;Acc:HGNC:6983]	protein coding	-0.65	1.06	23.22	<0.0001	<0.0001
ENSG00000271730		novel transcript, antisense to SESN1	lncRNA	-0.65	0.74	16.46	<0.0001	0.001
ENSG00000005001	PRSS22	serine protease 22 [Source:HGNC Symbol;Acc:HGNC:14368]	protein coding	-0.65	1.11	18.68	<0.0001	0.0004
ENSG00000169403	PTAFR	platelet activating factor receptor [Source:HGNC Symbol;Acc:HGNC:9582]	protein coding	0.64	3.42	7.39	0.0065	0.0449
ENSG00000117226	GBP3	guanylate binding protein 3 [Source:HGNC Symbol;Acc:HGNC:4184]	protein coding	0.64	5.45	49.93	<0.0001	<0.0001

Table S39: Genes differentially expressed at Day 30 compared to pre-second vaccination (Study Arm 2 A/H7N9+AS03, D1, 29, T cells). Genes are sorted by descending absolute log_2 fold change. Gene model summaries and annotations are based on Ensembl Version 99 (continued.)

Ensembl Gene ID	Ensembl Gene Name	Ensembl Gene Description	Gene Type	log_2 Fold Change (Day 30 vs. Pre-Second Vaccination)	Average log_2 CPM	Likelihood Ratio Test Statistic	P-Value	FDR Adjusted P-Value
ENSG00000085514	PILRA	paired immunoglobin like type 2 receptor alpha [Source:HGNC Symbol;Acc:HGNC:20396]	protein coding	0.64	3.23	13.19	0.0003	0.0041
ENSG00000168461	RAB31	RAB31, member RAS oncogene family [Source:HGNC Symbol;Acc:HGNC:9771]	protein coding	0.64	3.40	7.13	0.0076	0.0498
ENSG00000168899	VAMP5	vesicle associated membrane protein 5 [Source:HGNC Symbol;Acc:HGNC:12646]	protein coding	0.64	5.34	24.98	<0.0001	<0.0001
ENSG00000180891	CUEDC1	CUE domain containing 1 [Source:HGNC Symbol;Acc:HGNC:31350]	protein coding	0.64	1.01	7.91	0.0049	0.0365
ENSG00000280119		TEC	TEC	-0.64	1.64	12.62	0.0004	0.0053
ENSG00000269514		novel transcript, antisense to OR10AD1	lncRNA	0.63	0.67	21.02	<0.0001	0.0001
ENSG00000277511		novel transcript	lncRNA	0.63	2.26	19.46	<0.0001	0.0003
ENSG00000115267	IFIH1	interferon induced with helicase C domain 1 [Source:HGNC Symbol;Acc:HGNC:18873]	protein coding	0.63	5.12	45.27	<0.0001	<0.0001
ENSG00000229644	NAMPTP1	nicotinamide phosphoribosyltransferase pseudogene 1 [Source:HGNC Symbol;Acc:HGNC:17633]	processed pseudogene	0.63	1.47	19.19	<0.0001	0.0003
ENSG00000187037	GPR141	G protein-coupled receptor 141 [Source:HGNC Symbol;Acc:HGNC:19997]	protein coding	0.63	0.72	11.07	0.0009	0.01
ENSG00000154764	WNT7A	Wnt family member 7A [Source:HGNC Symbol;Acc:HGNC:12786]	protein coding	-0.63	3.56	39.59	<0.0001	<0.0001
ENSG00000115155	OTOF	otoferlin [Source:HGNC Symbol;Acc:HGNC:8515]	protein coding	0.63	1.52	17.38	<0.0001	0.0007
ENSG00000069188	SDK2	sidekick cell adhesion molecule 2 [Source:HGNC Symbol;Acc:HGNC:19308]	protein coding	-0.63	3.53	33.09	<0.0001	<0.0001
ENSG00000005249	PRKAR2B	protein kinase cAMP-dependent type II regulatory subunit beta [Source:HGNC Symbol;Acc:HGNC:9392]	protein coding	0.62	1.14	10.03	0.0015	0.0153
ENSG00000152527	PLEKHH2	pleckstrin homology, MyTH4 and FERM domain containing H2 [Source:HGNC Symbol;Acc:HGNC:30506]	protein coding	-0.62	1.60	19.64	<0.0001	0.0002
ENSG00000271447	MMP28	matrix metallopeptidase 28 [Source:HGNC Symbol;Acc:HGNC:14366]	protein coding	-0.62	3.74	26.98	<0.0001	<0.0001
ENSG00000100453	GZMB	granzyme B [Source:HGNC Symbol;Acc:HGNC:4709]	protein coding	0.62	5.54	26.81	<0.0001	<0.0001
ENSG00000171848	RRM2	ribonucleotide reductase regulatory subunit M2 [Source:HGNC Symbol;Acc:HGNC:10452]	protein coding	0.62	3.44	20.28	<0.0001	0.0002
ENSG00000146859	TMEM140	transmembrane protein 140 [Source:HGNC Symbol;Acc:HGNC:21870]	protein coding	0.62	4.68	29.46	<0.0001	<0.0001

Table S39: Genes differentially expressed at Day 30 compared to pre-second vaccination (Study Arm 2 A/H7N9+AS03, D1, 29, T cells). Genes are sorted by descending absolute log_2 fold change. Gene model summaries and annotations are based on Ensembl Version 99 (continued.)

Ensembl Gene ID	Ensembl Gene Name	Ensembl Gene Description	Gene Type	log_2 Fold Change (Day 30 vs. Pre-Second Vaccination)	Average log_2 CPM	Likelihood Ratio Test Statistic	P-Value	FDR Adjusted P-Value
ENSG00000248099	INSL3	insulin like 3 [Source:HGNC Symbol;Acc:HGNC:6086]	protein coding	0.62	1.47	26.90	<0.0001	<0.0001
ENSG00000140511	HAPLN3	hyaluronan and proteoglycan link protein 3 [Source:HGNC Symbol;Acc:HGNC:21446]	protein coding	0.62	7.62	39.41	<0.0001	<0.0001
ENSG00000128284	APOL3	apolipoprotein L3 [Source:HGNC Symbol;Acc:HGNC:14868]	protein coding	0.62	7.42	53.37	<0.0001	<0.0001
ENSG00000100292	HMOX1	heme oxygenase 1 [Source:HGNC Symbol;Acc:HGNC:5013]	protein coding	0.62	2.67	8.07	0.0045	0.0343
ENSG00000227051	C14orf132	chromosome 14 open reading frame 132 [Source:HGNC Symbol;Acc:HGNC:20346]	protein coding	-0.62	3.35	28.92	<0.0001	<0.0001
ENSG00000226416	MRPL23-AS1	MRPL23 antisense RNA 1 [Source:HGNC Symbol;Acc:HGNC:42812]	lncRNA	-0.62	0.46	12.29	0.0005	0.0059
ENSG00000253882		family with sequence similarity 115, member C (FAM115C) pseudogene	transcribed unprocessed pseudogene	0.62	0.73	21.86	<0.0001	<0.0001
ENSG00000170909	OSCAR	osteoclast associated Ig-like receptor [Source:HGNC Symbol;Acc:HGNC:29960]	protein coding	0.62	3.23	7.59	0.0059	0.0414
ENSG00000138646	HERC5	HECT and RLD domain containing E3 ubiquitin protein ligase 5 [Source:HGNC Symbol;Acc:HGNC:24368]	protein coding	0.62	4.38	41.53	<0.0001	<0.0001
ENSG00000143476	DTL	denticleless E3 ubiquitin protein ligase homolog [Source:HGNC Symbol;Acc:HGNC:30288]	protein coding	0.62	1.13	16.15	<0.0001	0.0011
ENSG00000174469	CNTNAP2	contactin associated protein like 2 [Source:HGNC Symbol;Acc:HGNC:13830]	protein coding	-0.62	1.01	10.14	0.0015	0.0146
ENSG00000287387		novel transcript, antisense to ZFP36L2	lncRNA	-0.62	0.84	10.81	0.001	0.0111
ENSG00000092010	PSME1	proteasome activator subunit 1 [Source:HGNC Symbol;Acc:HGNC:9568]	protein coding	0.61	8.15	47.95	<0.0001	<0.0001
ENSG00000162591	MEGF6	multiple EGF like domains 6 [Source:HGNC Symbol;Acc:HGNC:3232]	protein coding	-0.61	7.56	64.95	<0.0001	<0.0001
ENSG00000132274	TRIM22	tripartite motif containing 22 [Source:HGNC Symbol;Acc:HGNC:16379]	protein coding	0.61	8.58	60.75	<0.0001	<0.0001
ENSG00000196497	IPO4	importin 4 [Source:HGNC Symbol;Acc:HGNC:19426]	protein coding	0.61	1.04	27.29	<0.0001	<0.0001
ENSG00000131979	GCH1	GTP cyclohydrolase 1 [Source:HGNC Symbol;Acc:HGNC:4193]	protein coding	0.61	5.65	41.37	<0.0001	<0.0001

Table S39: Genes differentially expressed at Day 30 compared to pre-second vaccination (Study Arm 2 A/H7N9+AS03, D1, 29, T cells). Genes are sorted by descending absolute log_2 fold change. Gene model summaries and annotations are based on Ensembl Version 99 (continued.)

Ensembl Gene ID	Ensembl Gene Name	Ensembl Gene Description	Gene Type	log_2 Fold Change (Day 30 vs. Pre-Second Vaccination)	Average log_2 CPM	Likelihood Ratio Test Statistic	P-Value	FDR Adjusted P-Value
ENSG00000062524	LTK	leukocyte receptor tyrosine kinase [Source:HGNC Symbol;Acc:HGNC:6721]	protein coding	-0.61	5.96	23.99	<0.0001	<0.0001
ENSG00000117461	PIK3R3	phosphoinositide-3-kinase regulatory subunit 3 [Source:HGNC Symbol;Acc:HGNC:8981]	protein coding	0.60	1.12	23.44	<0.0001	<0.0001
ENSG00000172164	SNTB1	syntrophin beta 1 [Source:HGNC Symbol;Acc:HGNC:11168]	protein coding	0.60	5.15	53.52	<0.0001	<0.0001
ENSG00000142959	BEST4	bestrophin 4 [Source:HGNC Symbol;Acc:HGNC:17106]	protein coding	-0.60	1.07	21.02	<0.0001	0.0001
ENSG00000246203		novel pseudogene	unprocessed pseudogene	0.60	0.89	14.40	0.0001	0.0024
ENSG00000172867	KRT2	keratin 2 [Source:HGNC Symbol;Acc:HGNC:6439]	protein coding	-0.60	0.23	7.53	0.0061	0.0423
ENSG00000105835	NAMPT	nicotinamide phosphoribosyltransferase [Source:HGNC Symbol;Acc:HGNC:30092]	protein coding	0.60	4.68	31.71	<0.0001	<0.0001
ENSG00000130487	KLHDC7B	kelch domain containing 7B [Source:HGNC Symbol;Acc:HGNC:25145]	protein coding	0.60	4.29	27.35	<0.0001	<0.0001
ENSG00000204267	TAP2	transporter 2, ATP binding cassette subfamily B member [Source:HGNC Symbol;Acc:HGNC:44]	protein coding	0.60	6.67	58.64	<0.0001	<0.0001
ENSG00000050165	DKK3	dickkopf WNT signaling pathway inhibitor 3 [Source:HGNC Symbol;Acc:HGNC:2893]	protein coding	-0.60	1.51	19.52	<0.0001	0.0003
ENSG00000074660	SCARF1	scavenger receptor class F member 1 [Source:HGNC Symbol;Acc:HGNC:16820]	protein coding	0.60	1.88	8.92	0.0028	0.0241
ENSG00000158050	DUSP2	dual specificity phosphatase 2 [Source:HGNC Symbol;Acc:HGNC:3068]	protein coding	-0.60	7.14	11.36	0.0008	0.0089
ENSG00000127564	PKMYT1	protein kinase, membrane associated tyrosine/threonine 1 [Source:HGNC Symbol;Acc:HGNC:29650]	protein coding	0.60	1.43	11.57	0.0007	0.0081
ENSG00000091972	CD200	CD200 molecule [Source:HGNC Symbol;Acc:HGNC:7203]	protein coding	-0.59	2.08	21.19	<0.0001	0.0001
ENSG00000091106	NLRC4	NLR family CARD domain containing 4 [Source:HGNC Symbol;Acc:HGNC:16412]	protein coding	0.59	1.39	7.41	0.0065	0.0446
ENSG00000259436		novel transcript, antisense to POU2F2	lncRNA	-0.59	2.89	35.77	<0.0001	<0.0001
ENSG00000149573	MPZL2	myelin protein zero like 2 [Source:HGNC Symbol;Acc:HGNC:3496]	protein coding	0.59	0.85	11.40	0.0007	0.0087
ENSG00000134765	DSC1	desmocollin 1 [Source:HGNC Symbol;Acc:HGNC:3035]	protein coding	-0.59	2.56	30.77	<0.0001	<0.0001

Table S39: Genes differentially expressed at Day 30 compared to pre-second vaccination (Study Arm 2 A/H7N9+AS03, D1, 29, T cells). Genes are sorted by descending absolute log_2 fold change. Gene model summaries and annotations are based on Ensembl Version 99 (continued.)

Ensembl Gene ID	Ensembl Gene Name	Ensembl Gene Description	Gene Type	log_2 Fold Change (Day 30 vs. Pre-Second Vaccination)	Average log_2 CPM	Likelihood Ratio Test Statistic	P-Value	FDR Adjusted P-Value
ENSG00000152229	PSTPIP2	proline-serine-threonine phosphatase interacting protein 2 [Source:HGNC Symbol;Acc:HGNC:9581]	protein coding	0.59	4.61	24.46	<0.0001	<0.0001
ENSG00000093009	CDC45	cell division cycle 45 [Source:HGNC Symbol;Acc:HGNC:1739]	protein coding	0.59	1.02	21.75	<0.0001	<0.0001
ENSG00000092853	CLSPN	claspin [Source:HGNC Symbol;Acc:HGNC:19715]	protein coding	0.59	1.05	14.99	0.0001	0.0019
ENSG00000139132	FGD4	FYVE, RhoGEF and PH domain containing 4 [Source:HGNC Symbol;Acc:HGNC:19125]	protein coding	0.59	0.90	9.52	0.002	0.0189
ENSG00000165030	NFIL3	nuclear factor, interleukin 3 regulated [Source:HGNC Symbol;Acc:HGNC:7787]	protein coding	0.59	2.43	27.13	<0.0001	<0.0001

Table S40: Genes differentially expressed at Day 32 compared to pre-second vaccination (Study Arm 2 A/H7N9+AS03, D1, 29, T cells). Genes are sorted by descending absolute log_2 fold change. Gene model summaries and annotations are based on Ensembl Version 99.

Ensembl Gene ID	Ensembl Gene Name	Ensembl Gene Description	Gene Type	log_2 Fold Change (Day 32 vs. Pre-Second Vaccination)	Average log_2 CPM	Likelihood Ratio Test Statistic	P-Value	FDR Adjusted P-Value
ENSG00000169031	COL4A3	collagen type IV alpha 3 chain [Source:HGNC Symbol;Acc:HGNC:2204]	protein coding	-0.97	0.92	28.20	<0.0001	0.0015
ENSG00000100453	GZMB	granzyme B [Source:HGNC Symbol;Acc:HGNC:4709]	protein coding	0.69	5.54	24.80	<0.0001	0.0045
ENSG00000163421	PROK2	prokineticin 2 [Source:HGNC Symbol;Acc:HGNC:18455]	protein coding	0.60	1.17	19.98	<0.0001	0.0221

Table S41: Genes differentially expressed at Day 36 compared to pre-second vaccination (Study Arm 2 A/H7N9+AS03, D1, 29, T cells). Genes are sorted by descending absolute log_2 fold change. Gene model summaries and annotations are based on Ensembl Version 99.

Ensembl Gene ID	Ensembl Gene Name	Ensembl Gene Description	Gene Type	log_2 Fold Change (Day 36 vs. Pre-Second Vaccination)	Average log_2 CPM	Likelihood Ratio Test Statistic	P-Value	FDR Adjusted P-Value
ENSG00000211896	IGHG1	immunoglobulin heavy constant gamma 1 (G1m marker) [Source:HGNC Symbol;Acc:HGNC:5525]	IG C gene	1.36	6.90	48.45	<0.0001	<0.0001

Table S41: Genes differentially expressed at Day 36 compared to pre-second vaccination (Study Arm 2 A/H7N9+AS03, D1, 29, T cells). Genes are sorted by descending absolute log_2 fold change. Gene model summaries and annotations are based on Ensembl Version 99 (continued.)

Ensembl Gene ID	Ensembl Gene Name	Ensembl Gene Description	Gene Type	log_2 Fold Change (Day 36 vs. Pre-Second Vaccination)	Average log_2 CPM	Likelihood Ratio Test Statistic	P-Value	FDR Adjusted P-Value
ENSG00000253755	IGHGP	immunoglobulin heavy constant gamma P (non-functional) [Source:HGNC Symbol;Acc:HGNC:5529]	IG C pseudogene	1.31	2.09	30.04	<0.0001	0.0003
ENSG00000211670	IGLV3-9	immunoglobulin lambda variable 3-9 (gene/pseudogene) [Source:HGNC Symbol;Acc:HGNC:5918]	IG V gene	0.88	1.47	24.79	<0.0001	0.0024

Table S42: Proteins differentially abundant at Day 2 compared to pre-first vaccination (Study Arm 2 (A/H7N9 + AS03 D1, 29), Neutrophils). Proteins are sorted by descending absolute log_2 fold change. Protein and gene annotations are based on Ensembl Version 101.

Protein ID	Ensembl Gene ID	Ensembl Gene Name	Protein Description	50% Protein Cluster ID (Gene Name)	Fold Change (Day 2 vs. Pre- First Vaccination)	Average log_2 Abundance	Moderated t-Statistic	P-Value	FDR Adjusted P-value
Q96PP8	ENSG00000154451	GBP5	Guanylate-binding protein 5 (EC 3.6.5.-) (GBP-TA antigen) (GTP-binding protein 5) (GBP-5) (Guanine nucleotide-binding protein 5)	Q96PP9 (GBP4)	0.724	-0.131	13.170	<0.0001	<0.0001
P04179	ENSG00000112096	SOD2	Superoxide dismutase [Mn], mitochondrial (EC 1.15.1.1)	P04179 (SOD2)	0.440	-0.290	9.651	<0.0001	0.0003
P32455	ENSG00000117228	GBP1	Guanylate-binding protein 1 (EC 3.6.5.-) (GTP-binding protein 1) (GBP-1) (HuGBP-1) (Guanine nucleotide-binding protein 1) (Interferon-induced guanylate-binding protein 1)	Q96PP9 (GBP4)	0.328	-0.131	5.528	<0.0001	0.0519
P32456	ENSG00000162645	GBP2	Guanylate-binding protein 2 (EC 3.6.5.-) (GTP-binding protein 2) (GBP-2) (HuGBP-2) (Guanine nucleotide-binding protein 2) (Interferon-induced guanylate-binding protein 2)	Q96PP9 (GBP4)	0.305	-0.350	5.463	0.0001	0.0519

Table S43: Proteins differentially abundant at Day 2 compared to pre-first vaccination (Study Arm 1 (A/H3N2v, D1) versus Study Arm 2 (A/H7N9 + AS03 D1, 29), Neutrophils). Proteins are sorted by descending absolute log_2 fold change. Protein and gene annotations are based on Ensembl Version 101.

Protein ID	Ensembl Gene ID	Ensembl Gene Name	Protein Description	50% Protein Cluster ID (Gene Name)	Fold Change (Day 2 vs. Pre- First Vaccination)	Average log_2 Abundance	Moderated t-Statistic	P-Value	FDR Adjusted P-value
P04179	ENSG00000112096	SOD2	Superoxide dismutase [Mn], mitochondrial (EC 1.15.1.1)	P04179 (SOD2)	-0.389	-0.397	-5.246	<0.0001	0.0539

Table S44: Proteins differentially abundant at Day 2 compared to pre-first vaccination (Study Arm 2 (A/H7N9 + AS03 D1, 29) versus Study Arm 3 (A/H7N9 + PBS, D1, 29), Neutrophils). Proteins are sorted by descending absolute log_2 fold change. Protein and gene annotations are based on Ensembl Version 101.

Protein ID	Ensembl Gene ID	Ensembl Gene Name	Protein Description	50% Protein Cluster ID (Gene Name)	Fold Change (Day 2 vs. Pre- First Vaccination)	Average log_2 Abundance	Moderated t-Statistic	P-Value	FDR Adjusted P-value
Q96PP8	ENSG00000154451	GBP5	Guanylate-binding protein 5 (EC 3.6.5.-) (GBP-TA antigen) (GTP-binding protein 5) (GBP-5) (Guanine nucleotide-binding protein 5)	Q96PP9 (GBP4)	0.906	-0.311	5.783	<0.0001	0.0135

Table S45: Proteins differentially abundant at Day 4 compared to pre-first vaccination (Study Arm 2 (A/H7N9 + AS03 D1, 29), Neutrophils). Proteins are sorted by descending absolute log_2 fold change. Protein and gene annotations are based on Ensembl Version 101.

Protein ID	Ensembl Gene ID	Ensembl Gene Name	Protein Description	50% Protein Cluster ID (Gene Name)	Fold Change (Day 4 vs. Pre- First Vaccination)	Average log_2 Abundance	Moderated t-Statistic	P-Value	FDR Adjusted P-value
P32456	ENSG00000162645	GBP2	Guanylate-binding protein 2 (EC 3.6.5.-) (GTP-binding protein 2) (GBP-2) (HuGBP-2) (Guanine nucleotide-binding protein 2) (Interferon-induced guanylate-binding protein 2)	Q96PP9 (GBP4)	0.568	-0.219	6.654	<0.0001	0.0285

Confidential/Proprietary Information

Table S46: Proteins differentially abundant at Day 30 compared to pre-second vaccination (Study Arm 2 (A/H7N9 + AS03 D1, 29), Neutrophils). Proteins are sorted by descending absolute log_2 fold change. Protein and gene annotations are based on Ensembl Version 101.

Protein ID	Ensembl Gene ID	Ensembl Gene Name	Protein Description	50% Protein Cluster ID (Gene Name)	Fold Change (Day 30 vs. Pre- Second Vaccination)	Average log_2 Abundance	Moderated t-Statistic	P-Value	FDR Adjusted P-value
Q96PP8	ENSG00000154451	GBP5	Guanylate-binding protein 5 (EC 3.6.5.-) (GBP-TA antigen) (GTP-binding protein 5) (GBP-5) (Guanine nucleotide-binding protein 5)	Q96PP9 (GBP4)	1.555	0.581	9.490	<0.0001	0.0005
P32455	ENSG00000117228	GBP1	Guanylate-binding protein 1 (EC 3.6.5.-) (GTP-binding protein 1) (GBP-1) (HuGBP-1) (Guanine nucleotide-binding protein 1) (Interferon-induced guanylate-binding protein 1)	Q96PP9 (GBP4)	0.806	0.242	5.968	<0.0001	0.0261
P04179	ENSG00000112096	SOD2	Superoxide dismutase [Mn], mitochondrial (EC 1.15.1.1)	P04179 (SOD2)	0.664	-0.168	5.704	<0.0001	0.0306
O14879	ENSG00000119917	IFIT3	Interferon-induced protein with tetratricopeptide repeats 3 (IFIT-3) (CIG49) (ISG-60) (Interferon-induced 60 kDa protein) (IFI-60K) (Interferon-induced protein with tetratricopeptide repeats 4) (IFIT-4) (Retinoic acid-induced gene G protein) (P60) (RIG-G)	O14879 (IFIT3)	0.517	-0.005	4.751	0.0003	0.086
P32456	ENSG00000162645	GBP2	Guanylate-binding protein 2 (EC 3.6.5.-) (GTP-binding protein 2) (GBP-2) (HuGBP-2) (Guanine nucleotide-binding protein 2) (Interferon-induced guanylate-binding protein 2)	Q96PP9 (GBP4)	0.507	-0.182	6.827	<0.0001	0.0098
P32189	ENSG00000198814	GK	Glycerol kinase (GK) (Glycerokinase) (EC 2.7.1.30) (ATP:glycerol 3-phosphotransferase)	P32189 (GK)	0.402	-0.437	4.466	0.0006	0.1124
Q96AZ6	ENSG00000172183	ISG20	Interferon-stimulated gene 20 kDa protein (EC 3.1.13.1) (Estrogen-regulated transcript 45 protein) (Promyelocytic leukemia nuclear body-associated protein ISG20)	Q96AZ6 (ISG20)	0.327	-0.339	4.730	0.0004	0.086
P42224	ENSG00000115415	STAT1	Signal transducer and activator of transcription 1-alpha/beta (Transcription factor ISGF-3 components p91/p84)	P40763 (STAT3)	0.291	-0.384	5.325	0.0001	0.0393
Q16666	ENSG00000163565	IFI16	Gamma-interferon-inducible protein 16 (Ifi-16) (Interferon-inducible myeloid differentiation transcriptional activator)	Q16666 (IFI16)	0.199	-0.321	4.497	0.0006	0.1124
Q02818	ENSG00000104805	NUCB1	Nucleobindin-1 (CALNUC)	Q02818 (NUCB1)	0.194	-0.467	5.497	<0.0001	0.0349

Table S46: Proteins differentially abundant at Day 30 compared to pre-second vaccination (Study Arm 2 (A/H7N9 + AS03 D1, 29), Neutrophils). Proteins are sorted by descending absolute log_2 fold change. Protein and gene annotations are based on Ensembl Version 101 (continued).

Protein ID	Ensembl Gene ID	Ensembl Gene Name	Protein Description	50% Protein Cluster ID (Gene Name)	Fold Change (Day 30 vs. Pre- Second Vaccination)	Average log_2 Abundance	Moderated t-Statistic	P-Value	FDR Adjusted P-value
P08473	ENSG00000196549	MME	Neprilysin (EC 3.4.24.11) (Atriopeptidase) (Common acute lymphocytic leukemia antigen) (CALLA) (Enkephalinase) (Neutral endopeptidase 24.11) (NEP) (Neutral endopeptidase) (Skin fibroblast elastase) (SFE) (CD antigen CD10)	P08473 (MME)	-0.179	-0.815	-4.383	0.0007	0.1192

Table S47: Proteins differentially abundant at Day 30 compared to pre-second vaccination (Study Arm 2 (A/H7N9 + AS03 D1, 29) versus Study Arm 3 (A/H7N9 + PBS, D1, 29), Neutrophils). Proteins are sorted by descending absolute log_2 fold change. Protein and gene annotations are based on Ensembl Version 101.

Protein ID	Ensembl Gene ID	Ensembl Gene Name	Protein Description	50% Protein Cluster ID (Gene Name)	Fold Change (Day 30 vs. Pre- Second Vaccination)	Average log_2 Abundance	Moderated t-Statistic	P-Value	FDR Adjusted P-value
Q96PP8	ENSG00000154451	GBP5	Guanylate-binding protein 5 (EC 3.6.5.-) (GBP-TA antigen) (GTP-binding protein 5) (GBP-5) (Guanine nucleotide-binding protein 5)	Q96PP9 (GBP4)	1.590	0.002	8.602	<0.0001	<0.0001
P04179	ENSG00000112096	SOD2	Superoxide dismutase [Mn], mitochondrial (EC 1.15.1.1)	P04179 (SOD2)	0.782	-0.295	5.606	<0.0001	0.0102
P32455	ENSG00000117228	GBP1	Guanylate-binding protein 1 (EC 3.6.5.-) (GTP-binding protein 1) (GBP-1) (HuGBP-1) (Guanine nucleotide-binding protein 1) (Interferon-induced guanylate-binding protein 1)	Q96PP9 (GBP4)	0.762	-0.120	4.764	<0.0001	0.0538
O14879	ENSG00000119917	IFIT3	Interferon-induced protein with tetratricopeptide repeats 3 (IFIT-3) (CIG49) (ISG-60) (Interferon-induced 60 kDa protein) (IFI-60K) (Interferon-induced protein with tetratricopeptide repeats 4) (IFIT-4) (Retinoic acid-induced gene G protein) (P60) (RIG-G)	O14879 (IFIT3)	0.507	-0.275	4.021	0.0005	0.1278
P32456	ENSG00000162645	GBP2	Guanylate-binding protein 2 (EC 3.6.5.-) (GTP-binding protein 2) (GBP-2) (HuGBP-2) (Guanine nucleotide-binding protein 2) (Interferon-induced guanylate-binding protein 2)	Q96PP9 (GBP4)	0.428	-0.392	4.425	0.0002	0.075
P42224	ENSG00000115415	STAT1	Signal transducer and activator of transcription 1-alpha/beta (Transcription factor ISGF-3 components p91/p84)	P40763 (STAT3)	0.291	-0.507	4.350	0.0002	0.0754

Table S47: Proteins differentially abundant at Day 30 compared to pre-second vaccination (Study Arm 2 (A/H7N9 + AS03 D1, 29) versus Study Arm 3 (A/H7N9 + PBS, D1, 29), Neutrophils). Proteins are sorted by descending absolute log_2 fold change. Protein and gene annotations are based on Ensembl Version 101 (continued).

Protein ID	Ensembl Gene ID	Ensembl Gene Name	Protein Description	50% Protein Cluster ID (Gene Name)	Fold Change (Day 30 vs. Pre- Second Vaccination)	Average log_2 Abundance	Moderated t-Statistic	P-Value	FDR Adjusted P-value
Q16666	ENSG00000163565	IFI16	Gamma-interferon-inducible protein 16 (Ifi-16) (Interferon-inducible myeloid differentiation transcriptional activator)	Q16666 (IFI16)	0.246	-0.391	4.525	0.0002	0.073
Q02818	ENSG00000104805	NUCB1	Nucleobindin-1 (CALNUC)	Q02818 (NUCB1)	0.217	-0.583	3.966	0.0006	0.1304
Q9NZZ3	ENSG00000086065	CHMP5	Charged multivesicular body protein 5 (Chromatin-modifying protein 5) (SNF7 domain-containing protein 2) (Vacuolar protein sorting-associated protein 60) (Vps60) (hVps60)	Q9NZZ3 (CHMP5)	0.213	-0.517	4.041	0.0005	0.1278

Table S48: Proteins differentially abundant at Day 32 compared to pre-second vaccination (Study Arm 2 (A/H7N9 + AS03 D1, 29), Neutrophils). Proteins are sorted by descending absolute log_2 fold change. Protein and gene annotations are based on Ensembl Version 101.

Protein ID	Ensembl Gene ID	Ensembl Gene Name	Protein Description	50% Protein Cluster ID (Gene Name)	Fold Change (Day 32 vs. Pre- Second Vaccination)	Average log_2 Abundance	Moderated t-Statistic	P-Value	FDR Adjusted P-value
Q96PP8	ENSG00000154451	GBP5	Guanylate-binding protein 5 (EC 3.6.5.-) (GBP-TA antigen) (GTP-binding protein 5) (GBP-5) (Guanine nucleotide-binding protein 5)	Q96PP9 (GBP4)	1.514	0.560	5.820	<0.0001	0.0144
P32455	ENSG00000117228	GBP1	Guanylate-binding protein 1 (EC 3.6.5.-) (GTP-binding protein 1) (GBP-1) (HuGBP-1) (Guanine nucleotide-binding protein 1) (Interferon-induced guanylate-binding protein 1)	Q96PP9 (GBP4)	1.375	0.526	7.383	<0.0001	0.0071
P09914	ENSG00000185745	IFIT1	Interferon-induced protein with tetratricopeptide repeats 1 (IFIT-1) (Interferon-induced 56 kDa protein) (IFI-56K) (P56)	Q13325 (IFIT5)	1.206	0.320	5.538	<0.0001	0.0205
P32456	ENSG00000162645	GBP2	Guanylate-binding protein 2 (EC 3.6.5.-) (GTP-binding protein 2) (GBP-2) (HuGBP-2) (Guanine nucleotide-binding protein 2) (Interferon-induced guanylate-binding protein 2)	Q96PP9 (GBP4)	0.828	-0.021	6.890	<0.0001	0.0071

Confidential/Proprietary Information

Table S48: Proteins differentially abundant at Day 32 compared to pre-second vaccination (Study Arm 2 (A/H7N9 + AS03 D1, 29), Neutrophils). Proteins are sorted by descending absolute log_2 fold change. Protein and gene annotations are based on Ensembl Version 101 (continued).

Protein ID	Ensembl Gene ID	Ensembl Gene Name	Protein Description	50% Protein Cluster ID (Gene Name)	Fold Change (Day 32 vs. Pre- Second Vaccination)	Average log_2 Abundance	Moderated t-Statistic	P-Value	FDR Adjusted P-value
O14879	ENSG00000119917	IFIT3	Interferon-induced protein with tetratricopeptide repeats 3 (IFIT-3) (CIG49) (ISG-60) (Interferon-induced 60 kDa protein) (IFI-60K) (Interferon-induced protein with tetratricopeptide repeats 4) (IFIT-4) (Retinoic acid-induced gene G protein) (P60) (RIG-G)	O14879 (IFIT3)	0.826	0.150	4.362	0.0008	0.0719
P09913	ENSG00000119922	IFIT2	Interferon-induced protein with tetratricopeptide repeats 2 (IFIT-2) (ISG-54 K) (Interferon-induced 54 kDa protein) (IFI-54K) (P54)	O14879 (IFIT3)	0.767	0.054	4.718	0.0004	0.0533
Q9Y2Y8	ENSG00000156575	PRG3	Proteoglycan 3 (Eosinophil major basic protein homolog) (Prepro-major basic protein homolog) (Prepro-MBPH)	Q9Y2Y8 (PRG3)	0.763	-0.585	3.812	0.0022	0.1115
P23381	ENSG00000140105	WARS1	Tryptophan–tRNA ligase, cytoplasmic (EC 6.1.1.2) (Interferon-induced protein 53) (IFP53) (Tryptophanyl-tRNA synthetase) (TrpRS) (hWRS) [Cleaved into: T1-TrpRS; T2-TrpRS]	P23381 (WARS1)	0.579	-0.315	5.434	0.0001	0.022
P42224	ENSG00000115415	STAT1	Signal transducer and activator of transcription 1-alpha/beta (Transcription factor ISGF-3 components p91/p84)	P40763 (STAT3)	0.567	-0.246	6.081	<0.0001	0.0126
Q86YZ3	ENSG00000197915	HRNR	Hornerin	Q86YZ3 (HRNR)	-0.540	-0.044	-4.225	0.001	0.0719
Q03518	ENSG00000168394; ENSG00000206297; ENSG00000226173; ENSG00000227816; ENSG00000230705; ENSG00000224212; ENSG00000232367	TAP1	Antigen peptide transporter 1 (APT1) (EC 7.4.2.-) (ATP-binding cassette sub-family B member 2) (Peptide supply factor 1) (Peptide transporter PSF1) (PSF-1) (Peptide transporter TAP1) (Peptide transporter involved in antigen processing 1) (Really interesting new gene 4 protein) (RING4)	Q03518 (TAP1)	0.488	-0.414	6.283	<0.0001	0.0109

Table S48: Proteins differentially abundant at Day 32 compared to pre-second vaccination (Study Arm 2 (A/H7N9 + AS03 D1, 29), Neutrophils). Proteins are sorted by descending absolute log_2 fold change. Protein and gene annotations are based on Ensembl Version 101 (continued).

Protein ID	Ensembl Gene ID	Ensembl Gene Name	Protein Description	50% Protein Cluster ID (Gene Name)	Fold Change (Day 32 vs. Pre- Second Vaccination)	Average log_2 Abundance	Moderated t-Statistic	P-Value	FDR Adjusted P-value
Q03519	ENSG00000204267; ENSG00000206235; ENSG00000206299; ENSG00000228582; ENSG00000237599; ENSG00000232326; ENSG00000225967; ENSG00000223481	TAP2	Antigen peptide transporter 2 (APT2) (EC 7.4.2.-) (ATP-binding cassette sub-family B member 3) (Peptide supply factor 2) (Peptide transporter PSF2) (PSF-2) (Peptide transporter TAP2) (Peptide transporter involved in antigen processing 2) (Really interesting new gene 11 protein) (RING11)	Q03519 (TAP2)	0.394	-0.362	6.894	<0.0001	0.0071
P19971	ENSG00000025708	TYMP	Thymidine phosphorylase (TP) (EC 2.4.2.4) (Gliostatin) (Platelet-derived endothelial cell growth factor) (PD-ECGF) (TdRPase)	P19971 (TYMP)	0.393	-0.409	5.843	<0.0001	0.0144
P28838	ENSG00000002549	LAP3	Cytosol aminopeptidase (EC 3.4.11.1) (Cysteinylglycine-S-conjugate dipeptidase) (EC 3.4.13.23) (Leucine aminopeptidase 3) (LAP-3) (Leucyl aminopeptidase) (Peptidase S) (Proline aminopeptidase) (EC 3.4.11.5) (Prolyl aminopeptidase)	P28838 (LAP3)	0.348	-0.423	4.126	0.0012	0.0762
Q9H0P0	ENSG00000122643	NT5C3A	Cytosolic 5'-nucleotidase 3A (EC 3.1.3.5) (7-methylguanosine phosphate-specific 5'-nucleotidase) (7-methylguanosine nucleotidase) (EC 3.1.3.91) (Cytosolic 5'-nucleotidase 3) (Cytosolic 5'-nucleotidase III) (cN-III) (Pyrimidine 5'-nucleotidase 1) (P5'N-1) (P5N-1) (PN-I) (Uridine 5'-monophosphate hydrolase 1) (p36)	Q9H0P0 (NT5C3A)	0.343	-0.461	4.084	0.0013	0.0773
Q13287	ENSG00000123609	NMI	N-myc-interactor (Nmi) (N-myc and STAT interactor)	Q13287 (NMI)	0.311	-0.457	6.495	<0.0001	0.0098
P04179	ENSG00000112096	SOD2	Superoxide dismutase [Mn], mitochondrial (EC 1.15.1.1)	P04179 (SOD2)	0.299	-0.350	3.651	0.003	0.1283
P01033	ENSG00000102265	TIMP1	Metalloproteinase inhibitor 1 (Erythroid-potentiating activity) (EPA) (Fibroblast collagenase inhibitor) (Collagenase inhibitor) (Tissue inhibitor of metalloproteinases 1) (TIMP-1)	P01033 (TIMP1)	-0.295	-0.881	-4.414	0.0007	0.0719

Table S48: Proteins differentially abundant at Day 32 compared to pre-second vaccination (Study Arm 2 (A/H7N9 + AS03 D1, 29), Neutrophils). Proteins are sorted by descending absolute log_2 fold change. Protein and gene annotations are based on Ensembl Version 101 (continued).

Protein ID	Ensembl Gene ID	Ensembl Gene Name	Protein Description	50% Protein Cluster ID (Gene Name)	Fold Change (Day 32 vs. Pre- Second Vaccination)	Average log_2 Abundance	Moderated t-Statistic	P-Value	FDR Adjusted P-value
O95786	ENSG00000107201	DDX58	Antiviral innate immune response receptor RIG-I (DEAD box protein 58) (Probable ATP-dependent RNA helicase DDX58) (EC 3.6.4.13) (RIG-I-like receptor 1) (RLR-1) (Retinoic acid-inducible gene 1 protein) (RIG-1) (Retinoic acid-inducible gene I protein) (RIG-I)	O95786 (DDX58)	0.284	-0.545	4.250	0.001	0.0719
Q8IVG5	ENSG00000177409	SAMD9L	Sterile alpha motif domain-containing protein 9-like (SAM domain-containing protein 9-like)	Q5K651 (SAMD9)	0.278	-0.382	3.986	0.0016	0.0849
P80217	ENSG00000068079	IFI35	Interferon-induced 35 kDa protein (IFP 35) (Ifi-35)	P80217 (IFI35)	0.266	-0.437	5.245	0.0002	0.0235
Q96AZ6	ENSG00000172183	ISG20	Interferon-stimulated gene 20 kDa protein (EC 3.1.13.1) (Estrogen-regulated transcript 45 protein) (Promyelocytic leukemia nuclear body-associated protein ISG20)	Q96AZ6 (ISG20)	0.264	-0.371	3.759	0.0024	0.1171
Q63HN8	ENSG00000173821	RNF213	E3 ubiquitin-protein ligase RNF213 (EC 2.3.2.27) (EC 3.6.4.-) (ALK lymphoma oligomerization partner on chromosome 17) (Mysterin) (RING finger protein 213) (RING-type E3 ubiquitin transferase RNF213)	Q63HN8 (RNF213)	0.246	-0.438	5.300	0.0001	0.0235
P49006	ENSG00000175130	MARCKSL1	MARCKS-related protein (MARCKS-like protein 1) (Macrophage myristoylated alanine-rich C kinase substrate) (Mac-MARCKS) (MacMARCKS)	P49006 (MARCKSL1)	-0.238	-0.486	-4.571	0.0005	0.0593
P12004	ENSG00000132646	PCNA	Proliferating cell nuclear antigen (PCNA) (Cyclin)	P12004 (PCNA)	0.235	-0.583	4.196	0.0011	0.0719
P04439	ENSG00000206503	HLA-A	HLA class I histocompatibility antigen, A alpha chain (Human leukocyte antigen A) (HLA-A)	P30501 (NA)	0.234	-0.572	3.681	0.0028	0.1261
P30453				P30501 (NA)	0.232	-0.331	4.036	0.0014	0.0821
Q96R05	ENSG00000162444	RBP7	Retinoid-binding protein 7 (Cellular retinoic acid-binding protein 4) (CRABP4) (CRBP4) (Cellular retinoic acid-binding protein IV) (CRABP-IV)	Q96R05 (RBP7)	-0.222	-0.707	-4.592	0.0005	0.0593
Q16666	ENSG00000163565	IFI16	Gamma-interferon-inducible protein 16 (Ifi-16) (Interferon-inducible myeloid differentiation transcriptional activator)	Q16666 (IFI16)	0.211	-0.315	4.198	0.0011	0.0719

Table S48: Proteins differentially abundant at Day 32 compared to pre-second vaccination (Study Arm 2 (A/H7N9 + AS03 D1, 29), Neutrophils). Proteins are sorted by descending absolute log_2 fold change. Protein and gene annotations are based on Ensembl Version 101 (continued).

Protein ID	Ensembl Gene ID	Ensembl Gene Name	Protein Description	50% Protein Cluster ID (Gene Name)	Fold Change (Day 32 vs. Pre- Second Vaccination)	Average log_2 Abundance	Moderated t-Statistic	P-Value	FDR Adjusted P-value
P19474	ENSG00000132109	TRIM21	E3 ubiquitin-protein ligase TRIM21 (EC 2.3.2.27) (52 kDa Ro protein) (52 kDa ribonucleoprotein autoantigen Ro/SS-A) (RING finger protein 81) (RING-type E3 ubiquitin transferase TRIM21) (Ro(SS-A)) (Sjoegren syndrome type A antigen) (SS-A) (Tripartite motif-containing protein 21)	P19474 (TRIM21)	0.188	-0.622	4.194	0.0011	0.0719
Q9Y305	ENSG00000123130	ACOT9	Acyl-coenzyme A thioesterase 9, mitochondrial (Acyl-CoA thioesterase 9) (EC 3.1.2.-) (Acyl-CoA thioester hydrolase 9)	Q9Y305 (ACOT9)	0.181	-0.611	5.268	0.0002	0.0235
P49662	ENSG00000196954	CASP4	Caspase-4 (CASP-4) (EC 3.4.22.57) (ICE and Ced-3 homolog 2) (ICH-2) (ICE(rel)-II) (Mih1) (Protease TX) [Cleaved into: Caspase-4 subunit p10; Caspase-4 subunit p20]	P51878 (CASP5)	0.178	-0.499	4.097	0.0013	0.0773
Q06323	ENSG00000092010; ENSG00000284916	PSME1	Proteasome activator complex subunit 1 (11S regulator complex subunit alpha) (REG-alpha) (Activator of multicatalytic protease subunit 1) (Interferon gamma up-regulated I-5111 protein) (IGUP I-5111) (Proteasome activator 28 subunit alpha) (PA28a) (PA28alpha)	Q06323 (PSME1)	0.166	-0.549	4.262	0.0009	0.0719
Q9UL46	ENSG00000100911; ENSG00000284889	PSME2	Proteasome activator complex subunit 2 (11S regulator complex subunit beta) (REG-beta) (Activator of multicatalytic protease subunit 2) (Proteasome activator 28 subunit beta) (PA28b) (PA28beta)	Q06323 (PSME1)	0.164	-0.711	4.215	0.001	0.0719
Q9Y2Q3	ENSG00000197448	GSTK1	Glutathione S-transferase kappa 1 (EC 2.5.1.18) (GST 13-13) (GST class-kappa) (GSTK1-1) (hGSTK1) (Glutathione S-transferase subunit 13)	Q9Y2Q3 (GSTK1)	0.163	-0.526	3.647	0.003	0.1283
Q9NUV9	ENSG00000133574	GIMAP4	GTPase IMAP family member 4 (Immunity-associated nucleotide 1 protein) (IAN-1) (hIAN1) (Immunity-associated protein 4)	Q9NUV9 (GIMAP4)	0.159	-0.451	4.001	0.0015	0.0849
Q5K651	ENSG00000205413	SAMD9	Sterile alpha motif domain-containing protein 9 (SAM domain-containing protein 9)	Q5K651 (SAMD9)	0.157	-0.522	4.282	0.0009	0.0719
Q9BXD5	ENSG00000135838	NPL	N-acetylneuraminate lyase (NALase) (EC 4.1.3.3) (N-acetylneuraminate pyruvate-lyase) (N-acetylneuraminic acid aldolase) (Sialate lyase) (Sialate-pyruvate lyase) (Sialic acid aldolase) (Sialic acid lyase)	Q9BXD5 (NPL)	-0.156	-0.870	-3.530	0.0037	0.1442

Table S48: Proteins differentially abundant at Day 32 compared to pre-second vaccination (Study Arm 2 (A/H7N9 + AS03 D1, 29), Neutrophils). Proteins are sorted by descending absolute log_2 fold change. Protein and gene annotations are based on Ensembl Version 101 (continued).

Protein ID	Ensembl Gene ID	Ensembl Gene Name	Protein Description	50% Protein Cluster ID (Gene Name)	Fold Change (Day 32 vs. Pre- Second Vaccination)	Average log_2 Abundance	Moderated t-Statistic	P-Value	FDR Adjusted P-value
P29466	ENSG00000137752	CASP1	Caspase-1 (CASP-1) (EC 3.4.22.36) (Interleukin-1 beta convertase) (IL-1BC) (Interleukin-1 beta-converting enzyme) (ICE) (IL-1 beta-converting enzyme) (p45) [Cleaved into: Caspase-1 subunit p20; Caspase-1 subunit p10]	P29466 (CASP1)	0.150	-0.397	3.781	0.0023	0.1152
O15552	ENSG00000126262	FFAR2	Free fatty acid receptor 2 (G-protein coupled receptor 43)	O15552 (FFAR2)	0.148	-0.366	3.501	0.0039	0.1493
Q8N6C8	ENSG00000278046; ENSG00000273884; ENSG00000275841; ENSG00000276175	LILRA3	Leukocyte immunoglobulin-like receptor subfamily A member 3 (CD85 antigen-like family member E) (Immunoglobulin-like transcript 6) (ILT-6) (Leukocyte immunoglobulin-like receptor 4) (LIR-4) (Monocyte inhibitory receptor HM43/HM31) (CD antigen CD85e)	Q8NHL6 (LILRB1)	-0.144	-0.641	-3.535	0.0037	0.1442
Q9H939	ENSG00000152229	PSTPIP2	Proline-serine-threonine phosphatase-interacting protein 2 (PEST phosphatase-interacting protein 2)	Q9H939 (PSTPIP2)	0.144	-0.546	4.699	0.0004	0.0533
O15127	ENSG00000140497	SCAMP2	Secretory carrier-associated membrane protein 2 (Secretory carrier membrane protein 2)	O14828 (SCAMP3)	-0.139	-0.643	-4.363	0.0008	0.0719
O75558	ENSG00000135604	STX11	Syntaxin-11	O75558 (STX11)	0.135	-0.625	3.624	0.0031	0.1313
Q92673	ENSG00000137642	SORL1	Sortilin-related receptor (Low-density lipoprotein receptor relative with 11 ligand-binding repeats) (LDLR relative with 11 ligand-binding repeats) (LR11) (SorLA-1) (Sorting protein-related receptor containing LDLR class A repeats) (SorLA)	Q92673 (SORL1)	-0.126	-0.719	-3.692	0.0027	0.1261
Q9BUJ2	ENSG00000105323	HNRNPUL1	Heterogeneous nuclear ribonucleoprotein U-like protein 1 (Adenovirus early region 1B-associated protein 5) (E1B-55 kDa-associated protein 5) (E1B-AP5)	Q9BUJ2 (HNRNPUL1)	0.114	-0.478	3.560	0.0035	0.1441
Q15046	ENSG00000065427	KARS1	Lysine–tRNA ligase (EC 2.7.7.-) (EC 6.1.1.6) (Lysyl-tRNA synthetase) (LysRS)	Q15046 (KARS1)	0.111	-0.716	3.552	0.0036	0.1441
Q9BRF8	ENSG00000103381	CPPED1	Serine/threonine-protein phosphatase CPPED1 (EC 3.1.3.16) (Calcineurin-like phosphoesterase domain-containing protein 1) (Complete S-transactivated protein 1)	Q9BRF8 (CPPED1)	-0.109	-0.919	-3.684	0.0028	0.1261
P40763	ENSG00000168610	STAT3	Signal transducer and activator of transcription 3 (Acute-phase response factor)	P40763 (STAT3)	0.108	-0.652	3.832	0.0021	0.1104

Table S48: Proteins differentially abundant at Day 32 compared to pre-second vaccination (Study Arm 2 (A/H7N9 + AS03 D1, 29), Neutrophils). Proteins are sorted by descending absolute log_2 fold change. Protein and gene annotations are based on Ensembl Version 101 (continued).

Protein ID	Ensembl Gene ID	Ensembl Gene Name	Protein Description	50% Protein Cluster ID (Gene Name)	Fold Change (Day 32 vs. Pre- Second Vaccination)	Average log_2 Abundance	Moderated t-Statistic	P-Value	FDR Adjusted P-value
Q92973	ENSG00000083312	TNPO1	Transportin-1 (Importin beta-2) (Karyopherin beta-2) (M9 region interaction protein) (MIP)	Q92973 (TNPO1)	-0.108	-0.441	-4.152	0.0011	0.0752

Table S49: Proteins differentially abundant at Day 32 compared to pre-second vaccination (Study Arm 2 (A/H7N9 + AS03 D1, 29) versus Study Arm 3 (A/H7N9 + PBS, D1, 29), Neutrophils). Proteins are sorted by descending absolute log_2 fold change. Protein and gene annotations are based on Ensembl Version 101.

Protein ID	Ensembl Gene ID	Ensembl Gene Name	Protein Description	50% Protein Cluster ID (Gene Name)	Fold Change (Day 32 vs. Pre- Second Vaccination)	Average log_2 Abundance	Moderated t-Statistic	P-Value	FDR Adjusted P-value
Q96PP8	ENSG00000154451	GBP5	Guanylate-binding protein 5 (EC 3.6.5.-) (GBP-TA antigen) (GTP-binding protein 5) (GBP-5) (Guanine nucleotide-binding protein 5)	Q96PP9 (GBP4)	1.438	0.019	4.906	<0.0001	0.023
P32455	ENSG00000117228	GBP1	Guanylate-binding protein 1 (EC 3.6.5.-) (GTP-binding protein 1) (GBP-1) (HuGBP-1) (Guanine nucleotide-binding protein 1) (Interferon-induced guanylate-binding protein 1)	Q96PP9 (GBP4)	1.286	0.033	5.520	<0.0001	0.0161
P32456	ENSG00000162645	GBP2	Guanylate-binding protein 2 (EC 3.6.5.-) (GTP-binding protein 2) (GBP-2) (HuGBP-2) (Guanine nucleotide-binding protein 2) (Interferon-induced guanylate-binding protein 2)	Q96PP9 (GBP4)	0.742	-0.310	5.272	<0.0001	0.0161
P23381	ENSG00000140105	WARS1	Tryptophan–tRNA ligase, cytoplasmic (EC 6.1.1.2) (Interferon-induced protein 53) (IFP53) (Tryptophanyl-tRNA synthetase) (TrpRS) (hWRS) [Cleaved into: T1-TrpRS; T2-TrpRS]	P23381 (WARS1)	0.584	-0.542	4.660	0.0001	0.0303
P42224	ENSG00000115415	STAT1	Signal transducer and activator of transcription 1-alpha/beta (Transcription factor ISGF-3 components p91/p84)	P40763 (STAT3)	0.535	-0.430	4.773	<0.0001	0.0266

Table S49: Proteins differentially abundant at Day 32 compared to pre-second vaccination (Study Arm 2 (A/H7N9 + AS03 D1, 29) versus Study Arm 3 (A/H7N9 + PBS, D1, 29), Neutrophils). Proteins are sorted by descending absolute log_2 fold change. Protein and gene annotations are based on Ensembl Version 101 (continued).

Protein ID	Ensembl Gene ID	Ensembl Gene Name	Protein Description	50% Protein Cluster ID (Gene Name)	Fold Change (Day 32 vs. Pre- Second Vaccination)	Average log_2 Abundance	Moderated t-Statistic	P-Value	FDR Adjusted P-value
Q03518	ENSG00000168394; ENSG00000206297; ENSG00000226173; ENSG00000227816; ENSG00000230705; ENSG00000224212; ENSG00000232367	TAP1	Antigen peptide transporter 1 (APT1) (EC 7.4.2.-) (ATP-binding cassette sub-family B member 2) (Peptide supply factor 1) (Peptide transporter PSF1) (PSF-1) (Peptide transporter TAP1) (Peptide transporter involved in antigen processing 1) (Really interesting new gene 4 protein) (RING4)	Q03518 (TAP1)	0.431	-0.596	4.166	0.0004	0.0901
Q03519	ENSG00000204267; ENSG00000206235; ENSG00000206299; ENSG00000228582; ENSG00000237599; ENSG00000232326; ENSG00000225967; ENSG00000223481	TAP2	Antigen peptide transporter 2 (APT2) (EC 7.4.2.-) (ATP-binding cassette sub-family B member 3) (Peptide supply factor 2) (Peptide transporter PSF2) (PSF-2) (Peptide transporter TAP2) (Peptide transporter involved in antigen processing 2) (Really interesting new gene 11 protein) (RING11)	Q03519 (TAP2)	0.416	-0.516	5.141	<0.0001	0.0161
Q96AZ6	ENSG00000172183	ISG20	Interferon-stimulated gene 20 kDa protein (EC 3.1.13.1) (Estrogen-regulated transcript 45 protein) (Promyelocytic leukemia nuclear body-associated protein ISG20)	Q96AZ6 (ISG20)	0.364	-0.492	4.044	0.0005	0.1084
P19971	ENSG00000025708	TYMP	Thymidine phosphorylase (TP) (EC 2.4.2.4) (Gliostatin) (Platelet-derived endothelial cell growth factor) (PD-ECGF) (TdRPase)	P19971 (TYMP)	0.329	-0.555	3.835	0.0009	0.1483
Q13287	ENSG00000123609	NMI	N-myc-interactor (Nmi) (N-myc and STAT interactor)	Q13287 (NMI)	0.325	-0.578	5.181	<0.0001	0.0161
Q9UL46	ENSG00000100911; ENSG00000284889	PSME2	Proteasome activator complex subunit 2 (11S regulator complex subunit beta) (REG-beta) (Activator of multi-catalytic protease subunit 2) (Proteasome activator 28 subunit beta) (PA28b) (PA28beta)	Q06323 (PSME1)	0.204	-0.710	3.862	0.0008	0.1483

Table S50: Proteins differentially abundant at Day 30 compared to pre-second vaccination (Study Arm 2 (A/H7N9 + AS03 D1, 29), T cells). Proteins are sorted by descending absolute log_2 fold change. Protein and gene annotations are based on Ensembl Version 101.

Protein ID	Ensembl Gene ID	Ensembl Gene Name	Protein Description	50% Protein Cluster ID (Gene Name)	Fold Change (Day 30 vs. Pre- Second Vaccination)	Average log_2 Abundance	Moderated t-Statistic	P-Value	FDR Adjusted P-value
P17096	ENSG00000137309	HMGA1	High mobility group protein HMG-I/HMG-Y (HMG-I(Y)) (High mobility group AT-hook protein 1) (High mobility group protein A1) (High mobility group protein R)	P17096 (HMGA1)	0.258	0.856	6.225	<0.0001	0.1422

Table S51: Proteins differentially abundant at Day 2 compared to pre-first vaccination (Study Arm 1 (A/H3N2v, D1), Dendritic cells). Proteins are sorted by descending absolute log_2 fold change. Protein and gene annotations are based on Ensembl Version 101.

Protein ID	Ensembl Gene ID	Ensembl Gene Name	Protein Description	50% Protein Cluster ID (Gene Name)	Fold Change (Day 2 vs. Pre- First Vaccination)	Average log_2 Abundance	Moderated t-Statistic	P-Value	FDR Adjusted P-value
Q86YZ3	ENSG00000197915	HRNR	Hornerin	Q86YZ3 (HRNR)	-0.563	0.456	-6.367	<0.0001	0.0324
P13647	ENSG00000186081	KRT5	Keratin, type II cytoskeletal 5 (58 kDa cytokeratin) (Cytokeratin-5) (CK-5) (Keratin-5) (K5) (Type-II keratin Kb5)	P04264 (KRT1)	-0.407	0.639	-7.348	<0.0001	0.0137

Table S52: Proteins differentially abundant at Day 2 compared to pre-first vaccination (Study Arm 2 (A/H7N9 + AS03 D1, 29), Dendritic cells). Proteins are sorted by descending absolute log_2 fold change. Protein and gene annotations are based on Ensembl Version 101.

Protein ID	Ensembl Gene ID	Ensembl Gene Name	Protein Description	50% Protein Cluster ID (Gene Name)	Fold Change (Day 2 vs. Pre- First Vaccination)	Average log_2 Abundance	Moderated t-Statistic	P-Value	FDR Adjusted P-value
P20160	ENSG00000172232; ENSG00000278624	AZU1	Azurocidin (Cationic antimicrobial protein CAP37) (Heparin-binding protein) (HBP) (hHBP)	P20160 (AZU1)	0.817	1.066	6.049	<0.0001	0.0162
P59665	ENSG00000206047; ENSG00000240247; ENSG00000284983; ENSG00000285176	DEFA1; DEFA1B	Neutrophil defensin 1 (Defensin, alpha 1) (HNP-1) (HP-1) (HP1) [Cleaved into: HP 1-56; Neutrophil defensin 2 (HNP-2) (HP-2) (HP2)]	P59665 (DEFA1; DEFA1B)	0.729	1.152	3.783	0.0022	0.0575
P12314	ENSG00000150337	FCGR1A	High affinity immunoglobulin gamma Fc receptor I (IgG Fc receptor I) (Fc-gamma RI) (FcRI) (Fc-gamma RIA) (FcgammaRIa) (CD antigen CD64)	P12314 (FCGR1A)	0.644	1.370	4.714	0.0004	0.0346

Table S52: Proteins differentially abundant at Day 2 compared to pre-first vaccination (Study Arm 2 (A/H7N9 + AS03 D1, 29), Dendritic cells). Proteins are sorted by descending absolute log_2 fold change. Protein and gene annotations are based on Ensembl Version 101 (continued).

Protein ID	Ensembl Gene ID	Ensembl Gene Name	Protein Description	50% Protein Cluster ID (Gene Name)	Fold Change (Day 2 vs. Pre- First Vaccination)	Average log_2 Abundance	Moderated t-Statistic	P-Value	FDR Adjusted P-value
Q8WYJ6	ENSG00000180096	SEPTIN1	Septin-1 (LARP) (Peanut-like protein 3) (Serologically defined breast cancer antigen NY-BR-24)	Q8WYJ6 (SEPTIN1)	-0.605	0.689	-4.170	0.0011	0.0476
P08670	ENSG00000026025	VIM	Vimentin	P08670 (VIM)	0.576	0.909	5.753	<0.0001	0.0164
P14222	ENSG00000180644	PRF1	Perforin-1 (P1) (Cytolysin) (Lymphocyte pore-forming protein) (PFP)	P14222 (PRF1)	-0.569	0.235	-5.194	0.0002	0.0237
P43403	ENSG00000115085	ZAP70	Tyrosine-protein kinase ZAP-70 (EC 2.7.10.2) (70 kDa zeta-chain associated protein) (Syk-related tyrosine kinase)	P43403 (ZAP70)	-0.547	0.517	-4.614	0.0005	0.0352
P35237	ENSG00000124570	SERPINB6	Serpin B6 (Cytoplasmic antiproteinase) (CAP) (Peptidase inhibitor 6) (PI-6) (Placental thrombin inhibitor)	P30740 (SERPINB1)	-0.537	0.420	-5.275	0.0001	0.0221
P24158	ENSG00000196415; ENSG00000277804	PRTN3	Myeloblastin (EC 3.4.21.76) (AGP7) (C-ANCA antigen) (Leukocyte proteinase 3) (PR-3) (PR3) (Neutrophil proteinase 4) (NP-4) (P29) (Wegener autoantigen)	P08246 (ELANE)	0.536	0.907	3.839	0.002	0.0558
O75367	ENSG00000113648	MACROH2A1	Core histone macro-H2A.1 (Histone macroH2A1) (mH2A1) (Histone H2A.y) (H2A/y) (Medulloblastoma antigen MU-MB-50.205)	Q9P0M6 (MACROH2A2)	0.496	1.085	5.310	0.0001	0.0221
P07766	ENSG00000198851	CD3E	T-cell surface glycoprotein CD3 epsilon chain (T-cell surface antigen T3/Leu-4 epsilon chain) (CD antigen CD3e)	P07766 (CD3E)	-0.490	0.715	-4.059	0.0013	0.0483
Q96PP8	ENSG00000154451	GBP5	Guanylate-binding protein 5 (EC 3.6.5.-) (GBP-TA antigen) (GTP-binding protein 5) (GBP-5) (Guanine nucleotide-binding protein 5)	Q96PP9 (GBP4)	0.482	1.184	4.624	0.0005	0.0352
Q96PP9	ENSG00000162654	GBP4	Guanylate-binding protein 4 (EC 3.6.5.-) (GTP-binding protein 4) (GBP-4) (Guanine nucleotide-binding protein 4)	Q96PP9 (GBP4)	0.471	1.120	4.632	0.0004	0.0352
O75791	ENSG00000100351	GRAP2	GRB2-related adapter protein 2 (Adapter protein GRID) (GRB-2-like protein) (GRB2L) (GRBLG) (GRBX) (Grf40 adapter protein) (Grf-40) (Growth factor receptor-binding protein) (Hematopoietic cell-associated adapter protein GrpL) (P38) (Protein GADS) (SH3-SH2-SH3 adapter Mona)	O75791 (GRAP2)	-0.471	0.700	-4.045	0.0013	0.0483

Table S52: Proteins differentially abundant at Day 2 compared to pre-first vaccination (Study Arm 2 (A/H7N9 + AS03 D1, 29), Dendritic cells). Proteins are sorted by descending absolute log_2 fold change. Protein and gene annotations are based on Ensembl Version 101 (continued).

Protein ID	Ensembl Gene ID	Ensembl Gene Name	Protein Description	50% Protein Cluster ID (Gene Name)	Fold Change (Day 2 vs. Pre- First Vaccination)	Average log_2 Abundance	Moderated t-Statistic	P-Value	FDR Adjusted P-value
P28799	ENSG00000030582	GRN	Progranulin (PGRN) (Acrogranin) (Epithelin precursor) (Glycoprotein of 88 Kda) (GP88) (Glycoprotein 88) (Granulin precursor) (PC cell-derived growth factor) (PCDGF) (Proepithelin) (PEPI) [Cleaved into: Paragranulin; Granulin-1 (Granulin G); Granulin-2 (Granulin F); Granulin-3 (Epithelin-2) (Granulin B); Granulin-4 (Epithelin-1) (Granulin A); Granulin-5 (Granulin C); Granulin-6 (Granulin D); Granulin-7 (Granulin E)]	P28799 (GRN)	0.470	1.060	4.977	0.0002	0.029
P42330	ENSG00000196139	AKR1C3	Aldo-keto reductase family 1 member C3 (EC 1.1.1.-) (EC 1.1.1.210) (EC 1.1.1.53) (EC 1.1.1.62) (17-beta-hydroxysteroid dehydrogenase type 5) (17-beta-HSD 5) (3-alpha-HSD type II, brain) (3-alpha-hydroxysteroid dehydrogenase type 2) (3-alpha-HSD type 2) (EC 1.1.1.357) (Chlordecone reductase homolog HAKRb) (Dihydrodiol dehydrogenase 3) (DD-3) (DD3) (Dihydrodiol dehydrogenase type I) (HA1753) (Prostaglandin F synthase) (PGFS) (EC 1.1.1.188) (Testosterone 17-beta-dehydrogenase 5) (EC 1.1.1.239) (EC 1.1.1.64)	Q04828 (AKR1C1)	-0.469	0.417	-5.850	<0.0001	0.0164
P08311	ENSG00000100448	CTSG	Cathepsin G (CG) (EC 3.4.21.20)	P08311 (CTSG)	0.467	1.109	6.407	<0.0001	0.0162
Q9Y3Y2	ENSG00000160679	CHTOP	Chromatin target of PRMT1 protein (Friend of PRMT1 protein) (Small arginine- and glycine-rich protein) (SRAG)	Q9Y3Y2 (CHTOP)	0.458	0.940	6.898	<0.0001	0.0162
Q14761	ENSG00000213402	PTPRCAP	Protein tyrosine phosphatase receptor type C-associated protein (PTPRC-associated protein) (CD45-associated protein) (CD45-AP) (Lymphocyte phosphatase-associated phosphoprotein)	Q14761 (PTPRCAP)	-0.453	0.687	-3.713	0.0025	0.0575
P20718	ENSG00000100450	GZMH	Granzyme H (EC 3.4.21.-) (CCP-X) (Cathepsin G-like 2) (CTSGL2) (Cytotoxic T-lymphocyte proteinase) (Cytotoxic serine protease C) (CSP-C)	P08311 (CTSG)	-0.448	0.168	-3.804	0.0021	0.0575
Q08AF3	ENSG00000166750	SLFN5	Schlafen family member 5	Q7Z7L1 (SLFN11)	-0.444	0.627	-6.667	<0.0001	0.0162

Confidential/Proprietary Information

Table S52: Proteins differentially abundant at Day 2 compared to pre-first vaccination (Study Arm 2 (A/H7N9 + AS03 D1, 29), Dendritic cells). Proteins are sorted by descending absolute log_2 fold change. Protein and gene annotations are based on Ensembl Version 101 (continued).

Protein ID	Ensembl Gene ID	Ensembl Gene Name	Protein Description	50% Protein Cluster ID (Gene Name)	Fold Change (Day 2 vs. Pre- First Vaccination)	Average log_2 Abundance	Moderated t-Statistic	P-Value	FDR Adjusted P-value
P06239	ENSG00000182866	LCK	Tyrosine-protein kinase Lck (EC 2.7.10.2) (Leukocyte C-terminal Src kinase) (LSK) (Lymphocyte cell-specific protein-tyrosine kinase) (Protein YT16) (Proto-oncogene Lck) (T cell-specific protein-tyrosine kinase) (p56-LCK)	P06241 (FYN)	-0.443	0.843	-4.504	0.0006	0.0376
Q13469	ENSG00000101096	NFATC2	Nuclear factor of activated T-cells, cytoplasmic 2 (NF-ATc2) (NFATc2) (NFAT pre-existing subunit) (NF-ATp) (T-cell transcription factor NFAT1)	Q13469 (NFATC2)	-0.440	0.451	-5.494	<0.0001	0.021
O15231	ENSG00000147394	ZNF185	Zinc finger protein 185 (LIM domain protein ZNF185) (P1-A)	O15231 (ZNF185)	0.438	0.817	4.847	0.0003	0.0336
P02788	ENSG00000012223	LTF	Lactotransferrin (Lactoferrin) (EC 3.4.21.-) (Growth-inhibiting protein 12) (Talalactoferrin) [Cleaved into: Lactoferricin-H (Lfcin-H); Kaliocin-1; Lactoferroxin-A; Lactoferroxin-B; Lactoferroxin-C]	P02788 (LTF)	0.422	1.006	2.590	0.0221	0.15
P12544	ENSG00000145649	GZMA	Granzyme A (EC 3.4.21.78) (CTL tryptase) (Cytotoxic T-lymphocyte proteinase 1) (Fragmentin-1) (Granzyme-1) (Hanukkah factor) (H factor) (HF)	P12544 (GZMA)	-0.421	0.414	-6.138	<0.0001	0.0162
P02769				P02768 (ALB)	-0.406	0.527	-4.381	0.0007	0.0433
P25815	ENSG00000163993	S100P	Protein S100-P (Migration-inducing gene 9 protein) (MIG9) (Protein S100-E) (S100 calcium-binding protein P)	P25815 (S100P)	0.390	0.662	2.968	0.0107	0.1177
P20963	ENSG00000198821	CD247	T-cell surface glycoprotein CD3 zeta chain (T-cell receptor T3 zeta chain) (CD antigen CD247)	P20963 (CD247)	-0.371	0.631	-4.019	0.0014	0.0483
P06702	ENSG00000163220	S100A9	Protein S100-A9 (Calgranulin-B) (Calprotectin L1H subunit) (Leukocyte L1 complex heavy chain) (Migration inhibitory factor-related protein 14) (MRP-14) (p14) (S100 calcium-binding protein A9)	P06702 (S100A9)	0.370	0.921	3.295	0.0057	0.0849
Q9UI08	ENSG00000196405	EVL	Ena/VASP-like protein (Ena/vasodilator-stimulated phosphoprotein-like)	Q9UI08 (EVL)	-0.365	0.681	-3.743	0.0024	0.0575
P12429	ENSG00000138772	ANXA3	Annexin A3 (35-alpha calcimedin) (Annexin III) (Annexin-3) (Inositol 1,2-cyclic phosphate 2-phosphohydrolase) (Lipocortin III) (Placental anticoagulant protein III) (PAP-III)	P08133 (ANXA6)	0.360	1.043	3.468	0.004	0.0749

Table S52: Proteins differentially abundant at Day 2 compared to pre-first vaccination (Study Arm 2 (A/H7N9 + AS03 D1, 29), Dendritic cells). Proteins are sorted by descending absolute log_2 fold change. Protein and gene annotations are based on Ensembl Version 101 (continued).

Protein ID	Ensembl Gene ID	Ensembl Gene Name	Protein Description	50% Protein Cluster ID (Gene Name)	Fold Change (Day 2 vs. Pre- First Vaccination)	Average log_2 Abundance	Moderated t-Statistic	P-Value	FDR Adjusted P-value
Q9HD89	ENSG00000104918	RETN	Resistin (Adipose tissue-specific secretory factor) (ADSF) (C/EBP-epsilon-regulated myeloid-specific secreted cysteine-rich protein) (Cysteine-rich secreted protein A12-alpha-like 2) (Cysteine-rich secreted protein FIZZ3)	Q9HD89 (RETN)	0.354	0.645	7.167	<0.0001	0.0162
Q9UKV3	ENSG00000100813	ACIN1	Apoptotic chromatin condensation inducer in the nucleus (Acinus)	Q9UKV3 (ACIN1)	0.352	1.103	4.242	0.0009	0.0454
O95183	ENSG00000168899	VAMP5	Vesicle-associated membrane protein 5 (VAMP-5) (Myobrevin)	O95183 (VAMP5)	0.352	1.189	4.531	0.0005	0.0376
P62995	ENSG00000136527	TRA2B	Transformer-2 protein homolog beta (TRA-2 beta) (TRA2-beta) (hTRA2-beta) (Splicing factor, arginine/serine-rich 10) (Transformer-2 protein homolog B)	P62995 (TRA2B)	0.352	0.818	6.304	<0.0001	0.0162
P84090	ENSG00000100632	ERH	Enhancer of rudimentary homolog	P84090 (ERH)	0.351	0.999	4.463	0.0006	0.0396
Q96SB3	ENSG00000108819	PPP1R9B	Neurabin-2 (Neurabin-II) (Protein phosphatase 1 regulatory subunit 9B) (Spinophilin)	Q96SB3 (PPP1R9B)	0.347	1.060	5.268	0.0001	0.0221
Q99729	ENSG00000197451	HNRNPAB	Heterogeneous nuclear ribonucleoprotein A/B (hnRNP A/B) (APOBEC1-binding protein 1) (ABBP-1)	O14979 (HNRNPDL)	0.344	0.931	4.316	0.0008	0.0454
O95498	ENSG00000112303	VNN2	Vascular non-inflammatory molecule 2 (Vanin-2) (EC 3.5.1.92) (Glycosylphosphatidyl inositol-anchored protein GPI-80) (Protein FOAP-4)	O95498 (VNN2)	0.342	0.960	3.838	0.002	0.0558
P08246	ENSG00000197561; ENSG00000277571	ELANE	Neutrophil elastase (EC 3.4.21.37) (Bone marrow serine protease) (Elastase-2) (Human leukocyte elastase) (HLE) (Medullasin) (PMN elastase)	P08246 (ELANE)	0.342	0.739	4.290	0.0008	0.0454
P09874	ENSG00000143799	PARP1	Poly [ADP-ribose] polymerase 1 (PARP-1) (EC 2.4.2.30) (ADP-ribosyltransferase diphtheria toxin-like 1) (ARTD1) (DNA ADP-ribosyltransferase PARP1) (EC 2.4.2.-) (NAD(+) ADP-ribosyltransferase 1) (ADPRT 1) (Poly[ADP-ribose] synthase 1) (Protein poly-ADP-ribosyltransferase PARP1) (EC 2.4.2.-)	P09874 (PARP1)	-0.333	0.682	-5.290	0.0001	0.0221
Q8NFU3	ENSG00000215845	TSTD1	Thiosulfate:glutathione sulfurtransferase (TST) (EC 2.8.1.-)	Q8NFU3 (TSTD1)	-0.332	0.711	-4.376	0.0007	0.0433
P23743	ENSG00000065357	DGKA	Diacylglycerol kinase alpha (DAG kinase alpha) (EC 2.7.1.107) (80 kDa diacylglycerol kinase) (Diglyceride kinase alpha) (DGK-alpha)	P49619 (DGKG)	-0.332	0.840	-3.718	0.0025	0.0575

Table S52: Proteins differentially abundant at Day 2 compared to pre-first vaccination (Study Arm 2 (A/H7N9 + AS03 D1, 29), Dendritic cells). Proteins are sorted by descending absolute log_2 fold change. Protein and gene annotations are based on Ensembl Version 101 (continued).

Protein ID	Ensembl Gene ID	Ensembl Gene Name	Protein Description	50% Protein Cluster ID (Gene Name)	Fold Change (Day 2 vs. Pre- First Vaccination)	Average log_2 Abundance	Moderated t-Statistic	P-Value	FDR Adjusted P-value
P51649	ENSG00000112294	ALDH5A1	Succinate-semialdehyde dehydrogenase, mitochondrial (EC 1.2.1.24) (Aldehyde dehydrogenase family 5 member A1) (NAD(+)-dependent succinic semialdehyde dehydrogenase)	P51649 (ALDH5A1)	-0.329	0.949	-2.889	0.0125	0.1232
O00499	ENSG00000136717	BIN1	Myc box-dependent-interacting protein 1 (Amphiphysin II) (Amphiphysin-like protein) (Box-dependent myc-interacting protein 1) (Bridging integrator 1)	O00499 (BIN1)	-0.328	0.644	-2.795	0.0149	0.1316
P05164	ENSG00000005381	MPO	Myeloperoxidase (MPO) (EC 1.11.2.2) [Cleaved into: Myeloperoxidase; 89 kDa myeloperoxidase; 84 kDa myeloperoxidase; Myeloperoxidase light chain; Myeloperoxidase heavy chain]	P05164 (MPO)	0.325	0.979	5.168	0.0002	0.0238
P22304	ENSG00000010404	IDS	Iduronate 2-sulfatase (EC 3.1.6.13) (Alpha-L-iduronate sulfate sulfatase) (Idursulfase) [Cleaved into: Iduronate 2-sulfatase 42 kDa chain; Iduronate 2-sulfatase 14 kDa chain]	P22304 (IDS)	-0.324	0.494	-5.365	0.0001	0.0221
P32320	ENSG00000158825	CDA	Cytidine deaminase (EC 3.5.4.5) (Cytidine aminohydrolase)	P32320 (CDA)	0.324	0.889	3.188	0.007	0.0941
Q96CV9	ENSG00000123240	OPTN	Optineurin (E3-14.7K-interacting protein) (FIP-2) (Huntingtin yeast partner L) (Huntingtin-interacting protein 7) (HIP-7) (Huntingtin-interacting protein L) (NEMO-related protein) (Optic neuropathy-inducing protein) (Transcription factor IIIA-interacting protein) (TFIIIA-IntP)	Q96CV9 (OPTN)	-0.323	0.702	-4.646	0.0004	0.0352
P15260	ENSG00000027697	IFNGR1	Interferon gamma receptor 1 (IFN-gamma receptor 1) (IFN-gamma-R1) (CDw119) (Interferon gamma receptor alpha-chain) (IFN-gamma-R-alpha) (CD antigen CD119)	P15260 (IFNGR1)	-0.322	0.521	-4.220	0.001	0.0455
P04234	ENSG00000167286	CD3D	T-cell surface glycoprotein CD3 delta chain (T-cell receptor T3 delta chain) (CD antigen CD3d)	P04234 (CD3D)	-0.322	0.799	-3.708	0.0025	0.0577
P01860		IGHG3	Immunoglobulin heavy constant gamma 3 (HDC) (Heavy chain disease protein) (Ig gamma-3 chain C region)	P0DOX5 (NA)	0.319	0.894	5.787	<0.0001	0.0164
P50453	ENSG00000170542	SERPINB9	Serpin B9 (Cytoplasmic antiproteinase 3) (CAP-3) (CAP3) (Peptidase inhibitor 9) (PI-9)	P30740 (SERPINB1)	-0.317	0.588	-5.933	<0.0001	0.0162

Table S52: Proteins differentially abundant at Day 2 compared to pre-first vaccination (Study Arm 2 (A/H7N9 + AS03 D1, 29), Dendritic cells). Proteins are sorted by descending absolute log_2 fold change. Protein and gene annotations are based on Ensembl Version 101 (continued).

Protein ID	Ensembl Gene ID	Ensembl Gene Name	Protein Description	50% Protein Cluster ID (Gene Name)	Fold Change (Day 2 vs. Pre- First Vaccination)	Average log_2 Abundance	Moderated t-Statistic	P-Value	FDR Adjusted P-value
Q08170	ENSG00000116350	SRSF4	Serine/arginine-rich splicing factor 4 (Pre-mRNA-splicing factor SRP75) (SRP001LB) (Splicing factor, arginine/serine-rich 4)	Q08170 (SRSF4)	0.313	1.001	4.588	0.0005	0.0361
P61626	ENSG00000090382	LYZ	Lysozyme C (EC 3.2.1.17) (1,4-beta-N-acetylmuramidase C)	P61626 (LYZ)	0.311	0.939	3.737	0.0024	0.0575
P27918	ENSG00000126759	CFP	Properdin (Complement factor P)	P27918 (CFP)	0.310	0.751	3.269	0.006	0.0865
P32119	ENSG00000167815	PRDX2	Peroxiredoxin-2 (EC 1.11.1.24) (Natural killer cell-enhancing factor B) (NKEF-B) (PRP) (Thiol-specific antioxidant protein) (TSA) (Thioredoxin peroxidase 1) (Thioredoxin-dependent peroxide reductase 1) (Thioredoxin-dependent peroxiredoxin 2)	Q13162 (PRDX4)	-0.309	0.892	-5.003	0.0002	0.029
P01040	ENSG00000121552	CSTA	Cystatin-A (Cystatin-AS) (Stefin-A) [Cleaved into: Cystatin-A, N-terminally processed]	P01040 (CSTA)	0.309	0.993	3.877	0.0018	0.0543
O75152	ENSG00000058673	ZC3H11A	Zinc finger CCCH domain-containing protein 11A	O75152 (ZC3H11A)	0.308	1.227	3.430	0.0044	0.0773
Q02880	ENSG00000077097	TOP2B	DNA topoisomerase 2-beta (EC 5.6.2.2) (DNA topoisomerase II, beta isozyme)	Q02880 (TOP2B)	0.307	1.005	4.015	0.0014	0.0483
P32456	ENSG00000162645	GBP2	Guanylate-binding protein 2 (EC 3.6.5.-) (GTP-binding protein 2) (GBP-2) (HuGBP-2) (Guanine nucleotide-binding protein 2) (Interferon-induced guanylate-binding protein 2)	Q96PP9 (GBP4)	0.307	1.027	4.834	0.0003	0.0336
Q8IX19	ENSG00000183019	MCEMP1	Mast cell-expressed membrane protein 1	Q8IX19 (MCEMP1)	0.303	0.997	3.427	0.0044	0.0773
Q9UPQ9	ENSG00000100354	TNRC6B	Trinucleotide repeat-containing gene 6B protein	Q9UPQ9 (TNRC6B)	0.303	0.940	3.152	0.0075	0.0975
P12318	ENSG00000143226	FCGR2A	Low affinity immunoglobulin gamma Fc region receptor II-a (IgG Fc receptor II-a) (CDw32) (Fc-gamma RII-a) (Fc-gamma-RIIa) (FcRII-a) (CD antigen CD32)	P12318 (FCGR2A)	0.298	0.899	4.005	0.0014	0.0483
Q9NYL4	ENSG00000134285	FKBP11	Peptidyl-prolyl cis-trans isomerase FKBP11 (PPIase FKBP11) (EC 5.2.1.8) (19 kDa FK506-binding protein) (19 kDa FKBP) (FKBP-19) (FK506-binding protein 11) (FKBP-11) (Rotamase)	Q9NYL4 (FKBP11)	-0.298	0.878	-5.257	0.0001	0.0221

Confidential/Proprietary Information

Table S52: Proteins differentially abundant at Day 2 compared to pre-first vaccination (Study Arm 2 (A/H7N9 + AS03 D1, 29), Dendritic cells). Proteins are sorted by descending absolute log_2 fold change. Protein and gene annotations are based on Ensembl Version 101 (continued).

Protein ID	Ensembl Gene ID	Ensembl Gene Name	Protein Description	50% Protein Cluster ID (Gene Name)	Fold Change (Day 2 vs. Pre- First Vaccination)	Average log_2 Abundance	Moderated t-Statistic	P-Value	FDR Adjusted P-value
O00461	ENSG00000173905	GOLIM4	Golgi integral membrane protein 4 (Golgi integral membrane protein, cis) (GIMPc) (Golgi phosphoprotein 4) (Golgi-localized phosphoprotein of 130 kDa) (Golgi phosphoprotein of 130 kDa)	O00461 (GOLIM4)	0.298	0.905	2.899	0.0122	0.122
Q9H1E3	ENSG00000069275	NUCKS1	Nuclear ubiquitous casein and cyclin-dependent kinase substrate 1 (P1)	Q9H1E3 (NUCKS1)	0.298	1.121	3.397	0.0046	0.0788
O75381	ENSG00000142655	PEX14	Peroxisomal membrane protein PEX14 (PTS1 receptor-docking protein) (Peroxin-14) (Peroxisomal membrane anchor protein PEX14)	O75381 (PEX14)	0.296	1.068	2.753	0.0162	0.136
Q9BPW8	ENSG00000184117	NIPSNAP1	Protein NipSnap homolog 1 (NipSnap1)	O75323 (NIPSNAP2)	-0.296	0.741	-3.741	0.0024	0.0575
P04179	ENSG00000112096	SOD2	Superoxide dismutase [Mn], mitochondrial (EC 1.15.1.1)	P04179 (SOD2)	0.294	0.776	3.215	0.0066	0.0914
P17927	ENSG00000203710	CR1	Complement receptor type 1 (C3b/C4b receptor) (CD antigen CD35)	P17927 (CR1)	0.292	1.046	5.402	0.0001	0.0221
P80723	ENSG00000176788	BASP1	Brain acid soluble protein 1 (22 kDa neuronal tissue-enriched acidic protein) (Neuronal axonal membrane protein NAP-22)	P80723 (BASP1)	0.289	1.025	3.386	0.0048	0.0795
Q9H7M9	ENSG00000107738	VSIR	V-type immunoglobulin domain-containing suppressor of T-cell activation (Platelet receptor Gi24) (Stress-induced secreted protein-1) (Sisp-1) (V-set domain-containing immunoregulatory receptor) (V-set immunoregulatory receptor)	Q9H7M9 (VSIR)	0.289	0.938	3.762	0.0023	0.0575
Q8IV04	ENSG00000175463	TBC1D10C	Carabin (TBC1 domain family member 10C)	Q8IV04 (TBC1D10C)	-0.286	0.721	-4.265	0.0009	0.0454
Q92804	ENSG00000270647; ENSG00000276833	TAF15	TATA-binding protein-associated factor 2N (68 kDa TATA-binding protein-associated factor) (TAF(II)68) (TAFII68) (RNA-binding protein 56)	Q92804 (TAF15)	0.286	1.076	3.918	0.0017	0.0515
O75923	ENSG00000135636	DYSF	Dysferlin (Dystrophy-associated fer-1-like protein) (Fer-1-like protein 1)	O75923 (DYSF)	0.283	0.951	5.047	0.0002	0.0284
O75022	ENSG00000204577; ENSG00000274587; ENSG00000275019	LILRB3	Leukocyte immunoglobulin-like receptor subfamily B member 3 (LIR-3) (Leukocyte immunoglobulin-like receptor 3) (CD85 antigen-like family member A) (Immunoglobulin-like transcript 5) (ILT-5) (Monocyte inhibitory receptor HL9) (CD antigen CD85a)	Q8NHL6 (LILRB1)	0.279	0.861	5.927	<0.0001	0.0162

Table S52: Proteins differentially abundant at Day 2 compared to pre-first vaccination (Study Arm 2 (A/H7N9 + AS03 D1, 29), Dendritic cells). Proteins are sorted by descending absolute log_2 fold change. Protein and gene annotations are based on Ensembl Version 101 (continued).

Protein ID	Ensembl Gene ID	Ensembl Gene Name	Protein Description	50% Protein Cluster ID (Gene Name)	Fold Change (Day 2 vs. Pre- First Vaccination)	Average log_2 Abundance	Moderated t-Statistic	P-Value	FDR Adjusted P-value
P13611	ENSG00000038427	VCAN	Versican core protein (Chondroitin sulfate proteoglycan core protein 2) (Chondroitin sulfate proteoglycan 2) (Glial hyaluronate-binding protein) (GHAP) (Large fibroblast proteoglycan) (PG-M)	P13611 (VCAN)	0.279	0.864	4.039	0.0014	0.0483
Q9P0P0	ENSG00000168894	RNF181	E3 ubiquitin-protein ligase RNF181 (EC 2.3.2.27) (RING finger protein 181) (RING-type E3 ubiquitin transferase RNF181)	Q9P0P0 (RNF181)	0.278	0.944	4.974	0.0002	0.029
Q14978	ENSG00000166197	NOLC1	Nucleolar and coiled-body phosphoprotein 1 (140 kDa nucleolar phosphoprotein) (Nopp140) (Hepatitis C virus NS5A-transactivated protein 13) (HCV NS5A-transactivated protein 13) (Nucleolar 130 kDa protein) (Nucleolar phosphoprotein p130)	Q14978 (NOLC1)	0.277	1.050	4.843	0.0003	0.0336
Q9H307	ENSG00000100941	PNN	Pinin (140 kDa nuclear and cell adhesion-related phosphoprotein) (Desmosome-associated protein) (Domain-rich serine protein) (DRS protein) (DRSP) (Melanoma metastasis clone A protein) (Nuclear protein SDK3) (SR-like protein)	Q9H307 (PNN)	0.275	0.987	5.717	<0.0001	0.0164
P62306	ENSG00000139343	SNRPF	Small nuclear ribonucleoprotein F (snRNP-F) (Sm protein F) (Sm-F) (SmF)	P62306 (SNRPF)	0.275	0.904	3.130	0.0078	0.0986
P29966	ENSG00000277443	MARCKS	Myristoylated alanine-rich C-kinase substrate (MARCKS) (Protein kinase C substrate, 80 kDa protein, light chain) (80K-L protein) (PKCSL)	P29966 (MARCKS)	0.274	1.098	3.215	0.0066	0.0914
O14867	ENSG00000156273	BACH1	Transcription regulator protein BACH1 (BTB and CNC homolog 1) (HA2303)	O14867 (BACH1)	0.273	0.957	4.265	0.0009	0.0454
P55957	ENSG00000015475	BID	BH3-interacting domain death agonist (p22 BID) (BID) [Cleaved into: BH3-interacting domain death agonist p15 (p15 BID); BH3-interacting domain death agonist p13 (p13 BID); BH3-interacting domain death agonist p11 (p11 BID)]	P55957 (BID)	0.273	0.986	4.272	0.0009	0.0454
P09913	ENSG00000119922	IFIT2	Interferon-induced protein with tetratricopeptide repeats 2 (IFIT-2) (ISG-54 K) (Interferon-induced 54 kDa protein) (IFI-54K) (P54)	O14879 (IFIT3)	0.272	0.963	4.310	0.0008	0.0454
Q99856	ENSG00000116017	ARID3A	AT-rich interactive domain-containing protein 3A (ARID domain-containing protein 3A) (B-cell regulator of IgH transcription) (Bright) (Dead ringer-like protein 1) (E2F-binding protein 1)	Q99856 (ARID3A)	0.271	0.962	3.467	0.0041	0.0749

Table S52: Proteins differentially abundant at Day 2 compared to pre-first vaccination (Study Arm 2 (A/H7N9 + AS03 D1, 29), Dendritic cells). Proteins are sorted by descending absolute log_2 fold change. Protein and gene annotations are based on Ensembl Version 101 (continued).

Protein ID	Ensembl Gene ID	Ensembl Gene Name	Protein Description	50% Protein Cluster ID (Gene Name)	Fold Change (Day 2 vs. Pre- First Vaccination)	Average log_2 Abundance	Moderated t-Statistic	P-Value	FDR Adjusted P-value
P07237	ENSG00000185624	P4HB	Protein disulfide-isomerase (PDI) (EC 5.3.4.1) (Cellular thyroid hormone-binding protein) (Prolyl 4-hydroxylase subunit beta) (p55)	P07237 (P4HB)	0.270	0.976	4.008	0.0014	0.0483
P15121	ENSG00000085662	AKR1B1	Aldo-keto reductase family 1 member B1 (EC 1.1.1.300) (EC 1.1.1.372) (EC 1.1.1.54) (Aldehyde reductase) (Aldose reductase) (AR) (EC 1.1.1.21)	P14550 (AKR1A1)	-0.268	0.718	-4.162	0.0011	0.0476
P60891	ENSG00000147224	PRPS1	Ribose-phosphate pyrophosphokinase 1 (EC 2.7.6.1) (PPRibP) (Phosphoribosyl pyrophosphate synthase I) (PRS-I)	P60891 (PRPS1)	-0.268	0.740	-3.202	0.0068	0.0924
P18583	ENSG00000159140	SON	Protein SON (Bax antagonist selected in saccharomyces 1) (BASS1) (Negative regulatory element-binding protein) (NRE-binding protein) (Protein DBP-5) (SON3)	P18583 (SON)	0.267	0.909	4.122	0.0012	0.0483
Q9BPX5	ENSG00000136950	ARPC5L	Actin-related protein 2/3 complex subunit 5-like protein (Arp2/3 complex 16 kDa subunit 2) (ARC16-2)	Q9BPX5 (ARPC5L)	-0.264	0.491	-3.352	0.0051	0.0799
P07910	ENSG00000092199	HNRNPC	Heterogeneous nuclear ribonucleoproteins C1/C2 (hnRNP C1/C2)	P07910 (HNRNPC)	0.263	0.899	3.970	0.0015	0.0496
Q9NYF8	ENSG00000029363	BCLAF1	Bcl-2-associated transcription factor 1 (Btf) (BCLAF1 and THRAP3 family member 1)	Q9NYF8 (BCLAF1)	0.260	0.996	4.713	0.0004	0.0346
O95976	ENSG00000140749	IGSF6	Immunoglobulin superfamily member 6 (IgSF6) (Protein DORA)	O95976 (IGSF6)	0.258	1.005	3.841	0.002	0.0558
P32455	ENSG00000117228	GBP1	Guanylate-binding protein 1 (EC 3.6.5.-) (GTP-binding protein 1) (GBP-1) (HuGBP-1) (Guanine nucleotide-binding protein 1) (Interferon-induced guanylate-binding protein 1)	Q96PP9 (GBP4)	0.258	1.075	2.970	0.0106	0.1177
Q9NXG2	ENSG00000066654	THUMPD1	THUMP domain-containing protein 1	Q9NXG2 (THUMPD1)	-0.255	0.783	-4.137	0.0011	0.0483
Q13242	ENSG00000111786	SRSF9	Serine/arginine-rich splicing factor 9 (Pre-mRNA-splicing factor SRp30C) (Splicing factor, arginine/serine-rich 9)	Q07955 (SRSF1)	0.251	0.932	4.034	0.0014	0.0483
P08236	ENSG00000169919	GUSB	Beta-glucuronidase (EC 3.2.1.31) (Beta-G1)	P08236 (GUSB)	0.250	0.874	3.226	0.0065	0.0914
Q14141	ENSG00000125354	SEPTIN6	Septin-6	Q92599 (SEPTIN8)	-0.250	0.795	-4.794	0.0003	0.0336
Q9UGI8	ENSG00000135269	TES	Testin (TESS)	Q9UGI8 (TES)	-0.250	0.763	-4.613	0.0005	0.0352

Table S52: Proteins differentially abundant at Day 2 compared to pre-first vaccination (Study Arm 2 (A/H7N9 + AS03 D1, 29), Dendritic cells). Proteins are sorted by descending absolute log_2 fold change. Protein and gene annotations are based on Ensembl Version 101 (continued).

Protein ID	Ensembl Gene ID	Ensembl Gene Name	Protein Description	50% Protein Cluster ID (Gene Name)	Fold Change (Day 2 vs. Pre- First Vaccination)	Average log_2 Abundance	Moderated t-Statistic	P-Value	FDR Adjusted P-value
Q9P2E3	ENSG00000124201	ZNFX1	NFX1-type zinc finger-containing protein 1	Q9P2E3 (ZNFX1)	0.250	0.864	3.488	0.0039	0.0747
Q8WWW0	ENSG00000266094	RASSF5	Ras association domain-containing protein 5 (New ras effector 1) (Regulator for cell adhesion and polarization enriched in lymphoid tissues) (RAPL)	Q8WWW0 (RASSF5)	-0.248	0.680	-5.925	<0.0001	0.0162
P02545	ENSG00000160789	LMNA	Prelamin-A/C [Cleaved into: Lamin-A/C (70 kDa lamin) (Renal carcinoma antigen NY-REN-32)]	P02545 (LMNA)	-0.247	0.569	-2.709	0.0176	0.1388
O00422	ENSG00000150459	SAP18	Histone deacetylase complex subunit SAP18 (18 kDa Sin3-associated polypeptide) (2HOR0202) (Cell growth-inhibiting gene 38 protein) (Sin3-associated polypeptide p18)	O00422 (SAP18)	0.247	0.870	3.282	0.0058	0.0864
Q6JBY9	ENSG00000198771	RCSD1	CapZ-interacting protein (Protein kinase substrate CapZIP) (RCSD domain-containing protein 1)	Q6JBY9 (RCSD1)	0.246	0.885	2.812	0.0144	0.1291
Q9Y2J2	ENSG00000082397	EPB41L3	Band 4.1-like protein 3 (4.1B) (Differentially expressed in adenocarcinoma of the lung protein 1) (DAL-1) (Erythrocyte membrane protein band 4.1-like 3) [Cleaved into: Band 4.1-like protein 3, N-terminally processed]	Q9Y2J2 (EPB41L3)	0.245	1.079	4.072	0.0013	0.0483
O15162	ENSG00000188313	PLSCR1	Phospholipid scramblase 1 (PL scramblase 1) (Ca(2+)-dependent phospholipid scramblase 1) (Erythrocyte phospholipid scramblase) (Mg(2+)-dependent nuclease) (EC 3.1.-.-) (MmTRA1b)	O15162 (PLSCR1)	0.245	1.035	3.371	0.0049	0.0795
Q8WWI5	ENSG00000070214	SLC44A1	Choline transporter-like protein 1 (CDw92) (Solute carrier family 44 member 1) (CD antigen CD92)	Q8WWI5 (SLC44A1)	0.245	0.972	2.674	0.0189	0.1421
P04040	ENSG00000121691	CAT	Catalase (EC 1.11.1.6)	P04040 (CAT)	0.245	0.977	3.306	0.0055	0.0841
Q96BW5	ENSG00000165983	PTER	Phosphotriesterase-related protein (EC 3.1.-.-) (Parathion hydrolase-related protein) (hPHRP)	Q96BW5 (PTER)	-0.243	0.869	-5.618	<0.0001	0.0181
P83916	ENSG00000108468	CBX1	Chromobox protein homolog 1 (HP1Hsbeta) (Heterochromatin protein 1 homolog beta) (HP1 beta) (Heterochromatin protein p25) (M31) (Modifier 1 protein) (p25beta)	P45973 (CBX5)	0.240	1.218	3.041	0.0093	0.1089
Q08257	ENSG00000116791	CRYZ	Quinone oxidoreductase (EC 1.6.5.5) (NADPH:quinone reductase) (Zeta-crystallin)	Q08257 (CRYZ)	-0.240	0.805	-4.139	0.0011	0.0483
P08174	ENSG00000196352	CD55	Complement decay-accelerating factor (CD antigen CD55)	P08174 (CD55)	0.240	0.886	3.584	0.0032	0.0673

Table S52: Proteins differentially abundant at Day 2 compared to pre-first vaccination (Study Arm 2 (A/H7N9 + AS03 D1, 29), Dendritic cells). Proteins are sorted by descending absolute log_2 fold change. Protein and gene annotations are based on Ensembl Version 101 (continued).

Protein ID	Ensembl Gene ID	Ensembl Gene Name	Protein Description	50% Protein Cluster ID (Gene Name)	Fold Change (Day 2 vs. Pre- First Vaccination)	Average log_2 Abundance	Moderated t-Statistic	P-Value	FDR Adjusted P-value
Q16656	ENSG00000106459	NRF1	Nuclear respiratory factor 1 (NRF-1) (Alpha palindromic-binding protein) (Alpha-pal)	Q16656 (NRF1)	-0.239	0.644	-4.672	0.0004	0.0352
Q53H12	ENSG00000006530; ENSG00000262327	AGK	Acylglycerol kinase, mitochondrial (hAGK) (EC 2.7.1.107) (EC 2.7.1.138) (EC 2.7.1.94) (Multiple substrate lipid kinase) (HsMuLK) (MuLK) (Multi-substrate lipid kinase)	Q53H12 (AGK)	-0.239	0.724	-4.788	0.0003	0.0336
Q9UKM9	ENSG00000125970	RALY	RNA-binding protein Raly (Autoantigen p542) (Heterogeneous nuclear ribonucleoprotein C-like 2) (hnRNP core protein C-like 2) (hnRNP associated with lethal yellow protein homolog)	Q9UKM9 (RALY)	0.238	0.866	3.451	0.0042	0.0757
O95236	ENSG00000128284	APOL3	Apolipoprotein L3 (Apolipoprotein L-III) (ApoL-III) (TNF-inducible protein CG12-1) ($CG12_1$)	O95236 (APOL3)	0.238	1.030	4.552	0.0005	0.0371
Q0VD83	ENSG00000184730	APOBR	Apolipoprotein B receptor (Apolipoprotein B-100 receptor) (Apolipoprotein B-48 receptor) (Apolipoprotein B48 receptor) (apoB-48R)	Q0VD83 (APOBR)	0.236	0.952	2.677	0.0187	0.1421
P36915	ENSG00000204590; ENSG00000206492; ENSG00000226882; ENSG00000229470; ENSG00000228581; ENSG00000235986; ENSG00000232143	GNL1	Guanine nucleotide-binding protein-like 1 (GTP-binding protein HSR1)	P36915 (GNL1)	-0.236	0.772	-4.168	0.0011	0.0476
Q8WXH0	ENSG00000054654	SYNE2	Nesprin-2 (KASH domain-containing protein 2) (KASH2) (Nuclear envelope spectrin repeat protein 2) (Nucleus and actin connecting element protein) (Protein NUANCE) (Synaptic nuclear envelope protein 2) (Syne-2)	Q8WXH0 (SYNE2)	-0.235	0.815	-3.791	0.0022	0.0575
Q9NVA2	ENSG00000138758	SEPTIN11	Septin-11	Q92599 (SEPTIN8)	-0.235	0.783	-3.898	0.0018	0.0526
P23381	ENSG00000140105	WARS1	Tryptophan–tRNA ligase, cytoplasmic (EC 6.1.1.2) (Interferon-induced protein 53) (IFP53) (Tryptophanyl-tRNA synthetase) (TrpRS) (hWRS) [Cleaved into: T1-TrpRS; T2-TrpRS]	P23381 (WARS1)	0.233	0.905	3.358	0.005	0.0799
P62277	ENSG00000110700	RPS13	40S ribosomal protein S13 (Small ribosomal subunit protein uS15)	P62277 (RPS13)	-0.233	0.571	-2.649	0.0198	0.1446

Table S52: Proteins differentially abundant at Day 2 compared to pre-first vaccination (Study Arm 2 (A/H7N9 + AS03 D1, 29), Dendritic cells). Proteins are sorted by descending absolute log_2 fold change. Protein and gene annotations are based on Ensembl Version 101 (continued).

Protein ID	Ensembl Gene ID	Ensembl Gene Name	Protein Description	50% Protein Cluster ID (Gene Name)	Fold Change (Day 2 vs. Pre- First Vaccination)	Average log_2 Abundance	Moderated t-Statistic	P-Value	FDR Adjusted P-value
P46063	ENSG00000004700	RECQL	ATP-dependent DNA helicase Q1 (EC 3.6.4.12) (DNA helicase, RecQ-like type 1) (RecQ1) (DNA-dependent ATPase Q1) (RecQ protein-like 1)	P46063 (RECQL)	-0.232	0.722	-3.778	0.0022	0.0575
P0C0S5	ENSG00000164032	H2AZ1	Histone H2A.Z (H2A/z)	Q9P0M6 (MACROH2A2)	-0.232	1.029	-3.749	0.0024	0.0575
P15144	ENSG00000166825	ANPEP	Aminopeptidase N (AP-N) (hAPN) (EC 3.4.11.2) (Alanyl aminopeptidase) (Aminopeptidase M) (AP-M) (Microsomal aminopeptidase) (Myeloid plasma membrane glycoprotein CD13) (gp150) (CD antigen CD13)	P15144 (ANPEP)	0.231	1.140	2.806	0.0146	0.1301
Q14151	ENSG00000130254	SAFB2	Scaffold attachment factor B2 (SAF-B2)	Q14151 (SAFB2)	0.231	0.972	3.217	0.0066	0.0914
P13473	ENSG00000005893	LAMP2	Lysosome-associated membrane glycoprotein 2 (LAMP-2) (Lysosome-associated membrane protein 2) (CD107 antigen-like family member B) (LGP-96) (CD antigen CD107b)	P13473 (LAMP2)	0.229	0.865	2.644	0.0199	0.1453
P34910	ENSG00000185862	EVI2B	Protein EVI2B (Ecotropic viral integration site 2B protein homolog) (EVI-2B) (CD antigen CD361)	P34910 (EVI2B)	0.229	0.924	2.824	0.0141	0.1291
Q99829	ENSG00000214078	CPNE1	Copine-1 (Chromobindin 17) (Copine I)	Q86YQ8 (CPNE8)	-0.229	0.708	-4.229	0.0009	0.0454
Q6K0P9	ENSG00000163564	PYHIN1	Pyrin and HIN domain-containing protein 1 (Interferon-inducible protein X)	Q16666 (IFI16)	-0.229	0.568	-2.787	0.0152	0.1322
Q9NTM9	ENSG00000119929	CUTC	Copper homeostasis protein cutC homolog	Q9NTM9 (CUTC)	-0.228	0.834	-3.735	0.0024	0.0575
Q86U42	ENSG00000100836	PABPN1	Polyadenylate-binding protein 2 (PABP-2) (Poly(A)-binding protein 2) (Nuclear poly(A)-binding protein 1) (Poly(A)-binding protein II) (PABII) (Polyadenylate-binding nuclear protein 1)	Q86U42 (PABPN1)	0.228	0.884	3.612	0.0031	0.0644
Q08722	ENSG00000196776	CD47	Leukocyte surface antigen CD47 (Antigenic surface determinant protein OA3) (Integrin-associated protein) (IAP) (Protein MER6) (CD antigen CD47)	Q08722 (CD47)	-0.227	0.785	-4.745	0.0004	0.0344
P38159	ENSG00000147274	RBMX	RNA-binding motif protein, X chromosome (Glycoprotein p43) (Heterogeneous nuclear ribonucleoprotein G) (hnRNP G) [Cleaved into: RNA-binding motif protein, X chromosome, N-terminally processed]	P38159 (RBMX)	0.226	0.897	3.357	0.005	0.0799

Table S52: Proteins differentially abundant at Day 2 compared to pre-first vaccination (Study Arm 2 (A/H7N9 + AS03 D1, 29), Dendritic cells). Proteins are sorted by descending absolute log_2 fold change. Protein and gene annotations are based on Ensembl Version 101 (continued).

Protein ID	Ensembl Gene ID	Ensembl Gene Name	Protein Description	50% Protein Cluster ID (Gene Name)	Fold Change (Day 2 vs. Pre- First Vaccination)	Average log_2 Abundance	Moderated t-Statistic	P-Value	FDR Adjusted P-value
Q9Y520	ENSG00000117523	PRRC2C	Protein PRRC2C (BAT2 domain-containing protein 1) (HBV X-transactivated gene 2 protein) (HBV XAg-transactivated protein 2) (HLA-B-associated transcript 2-like 2) (Proline-rich and coiled-coil-containing protein 2C)	Q9Y520 (PRRC2C)	0.226	0.933	2.953	0.011	0.1188
P78324	ENSG00000198053	SIRPA	Tyrosine-protein phosphatase non-receptor type substrate 1 (SHP substrate 1) (SHPS-1) (Brain Ig-like molecule with tyrosine-based activation motifs) (Bit) (CD172 antigen-like family member A) (Inhibitory receptor SHPS-1) (Macrophage fusion receptor) (MyD-1 antigen) (Signal-regulatory protein alpha-1) (Sirp-alpha-1) (Signal-regulatory protein alpha-2) (Sirp-alpha-2) (Signal-regulatory protein alpha-3) (Sirp-alpha-3) (p84) (CD antigen CD172a)	P78324 (SIRPA)	0.225	0.840	4.415	0.0007	0.0417
Q96QH2	ENSG00000133246	PRAM1	PML-RARA-regulated adapter molecule 1 (PRAM) (PRAM-1)	Q96QH2 (PRAM1)	0.224	0.734	3.001	0.01	0.1131
Q9Y2W1	ENSG00000054118	THRAP3	Thyroid hormone receptor-associated protein 3 (BCLAF1 and THRAP3 family member 2) (Thyroid hormone receptor-associated protein complex 150 kDa component) (Trap150)	Q9Y2W1 (THRAP3)	0.224	0.986	4.087	0.0012	0.0483
Q13315	ENSG00000149311	ATM	Serine-protein kinase ATM (EC 2.7.11.1) (Ataxia telangiectasia mutated) (A-T mutated)	Q13315 (ATM)	-0.222	0.918	-4.793	0.0003	0.0336
Q07065	ENSG00000136026	CKAP4	Cytoskeleton-associated protein 4 (63-kDa cytoskeleton-linking membrane protein) (Climp-63) (p63)	Q07065 (CKAP4)	0.221	0.956	4.279	0.0009	0.0454
O43914	ENSG00000011600	TYROBP	TYRO protein tyrosine kinase-binding protein (DNAX-activation protein 12) (Killer-activating receptor-associated protein) (KAR-associated protein)	O43914 (TYROBP)	0.221	0.944	3.473	0.004	0.0749
P09496	ENSG00000122705	CLTA	Clathrin light chain A (Lca)	P09496 (CLTA)	0.220	0.949	2.928	0.0115	0.1205
O94925	ENSG00000115419	GLS	Glutaminase kidney isoform, mitochondrial (GLS) (EC 3.5.1.2) (K-glutaminase) (L-glutamine amidohydrolase) [Cleaved into: Glutaminase kidney isoform, mitochondrial 68 kDa chain; Glutaminase kidney isoform, mitochondrial 65 kDa chain]	O94925 (GLS)	-0.220	0.841	-4.076	0.0013	0.0483

Table S52: Proteins differentially abundant at Day 2 compared to pre-first vaccination (Study Arm 2 (A/H7N9 + AS03 D1, 29), Dendritic cells). Proteins are sorted by descending absolute log_2 fold change. Protein and gene annotations are based on Ensembl Version 101 (continued).

Protein ID	Ensembl Gene ID	Ensembl Gene Name	Protein Description	50% Protein Cluster ID (Gene Name)	Fold Change (Day 2 vs. Pre- First Vaccination)	Average log_2 Abundance	Moderated t-Statistic	P-Value	FDR Adjusted P-value
Q9H9B4	ENSG00000164466	SFXN1	Sideroflexin-1	Q9H9B4 (SFXN1)	-0.220	0.862	-3.378	0.0048	0.0795
O75828	ENSG00000159231	CBR3	Carbonyl reductase [NADPH] 3 (EC 1.1.1.184) (NADPH-dependent carbonyl reductase 3) (Short chain dehydrogenase/reductase family 21C member 2)	P16152 (CBR1)	-0.218	0.672	-4.175	0.001	0.0476
Q49A26	ENSG00000140632	GLYR1	Putative oxidoreductase GLYR1 (EC 1.-.-.-) (3-hydroxyisobutyrate dehydrogenase-like protein) (Cytokine-like nuclear factor N-PAC) (Glyoxylate reductase 1 homolog) (Nuclear protein NP60) (Nuclear protein of 60 kDa) (Nucleosome-destabilizing factor) (hNDF)	Q49A26 (GLYR1)	0.217	0.973	2.868	0.013	0.125
Q6P9H5	ENSG00000133561	GIMAP6	GTPase IMAP family member 6 (Immunity-associated nucleotide 2 protein) (IAN-2) (hIAN2) (Immunity-associated nucleotide 6 protein) (IAN-6) (hIAN6)	Q6P9H5 (GIMAP6)	0.216	0.912	3.378	0.0048	0.0795
Q9H6A0	ENSG00000162777	DENND2D	DENN domain-containing protein 2D	Q9H6A0 (DENND2D)	-0.216	0.888	-3.151	0.0075	0.0975
Q7Z417	ENSG00000108256	NUFIP2	Nuclear fragile X mental retardation-interacting protein 2 (82 kDa FMRP-interacting protein) (82-FIP) (Cell proliferation-inducing gene 1 protein) (FMRP-interacting protein 2)	Q7Z417 (NUFIP2)	0.216	0.992	2.727	0.017	0.1372
Q9BT23	ENSG00000136490	LIMD2	LIM domain-containing protein 2	Q9UHB6 (LIMA1)	-0.215	0.786	-3.843	0.002	0.0558
Q15424	ENSG00000160633	SAFB	Scaffold attachment factor B1 (SAF-B) (SAF-B1) (HSP27 estrogen response element-TATA box-binding protein) (HSP27 ERE-TATA-binding protein)	Q14151 (SAFB2)	0.215	0.962	4.243	0.0009	0.0454
Q9UGN4	ENSG00000167851	CD300A	CMRF35-like molecule 8 (CLM-8) (CD300 antigen-like family member A) (CMRF-35-H9) (CMRF35-H9) (CMRF35-H) (IRC1/IRC2) (Immunoglobulin superfamily member 12) (IgSF12) (Inhibitory receptor protein 60) (IRp60) (NK inhibitory receptor) (CD antigen CD300a)	Q9UGN4 (CD300A)	0.215	0.963	3.378	0.0048	0.0795

Confidential/Proprietary Information

Table S52: Proteins differentially abundant at Day 2 compared to pre-first vaccination (Study Arm 2 (A/H7N9 + AS03 D1, 29), Dendritic cells). Proteins are sorted by descending absolute log_2 fold change. Protein and gene annotations are based on Ensembl Version 101 (continued).

Protein ID	Ensembl Gene ID	Ensembl Gene Name	Protein Description	50% Protein Cluster ID (Gene Name)	Fold Change (Day 2 vs. Pre- First Vaccination)	Average log_2 Abundance	Moderated t-Statistic	P-Value	FDR Adjusted P-value
Q13427	ENSG00000138398	PPIG	Peptidyl-prolyl cis-trans isomerase G (PPIase G) (Peptidyl-prolyl isomerase G) (EC 5.2.1.8) (CASP10) (Clk-associating RS-cyclophilin) (CARS-Cyp) (CARS-cyclophilin) (SR-cyclophilin) (SR-cyp) (SRcyp) (Cyclophilin G) (Rotamase G)	Q13427 (PPIG)	0.214	0.983	2.728	0.017	0.1372
Q92542	ENSG00000162736	NCSTN	Nicastrin	Q92542 (NCSTN)	0.214	0.963	4.021	0.0014	0.0483
Q9BQE5	ENSG00000128335	APOL2	Apolipoprotein L2 (Apolipoprotein L-II) (ApoL-II)	O14791 (APOL1)	0.213	0.966	4.031	0.0014	0.0483
Q14314	ENSG00000127951	FGL2	Fibroleukin (Fibrinogen-like protein 2) (pT49)	Q14314 (FGL2)	0.213	0.887	2.772	0.0156	0.1348
O75348	ENSG00000136888	ATP6V1G1	V-type proton ATPase subunit G 1 (V-ATPase subunit G 1) (V-ATPase 13 kDa subunit 1) (Vacuolar proton pump subunit G 1) (Vacuolar proton pump subunit M16)	O75348 (ATP6V1G1)	0.213	0.845	3.213	0.0066	0.0914
P11177	ENSG00000168291	PDHB	Pyruvate dehydrogenase E1 component subunit beta, mitochondrial (PDHE1-B) (EC 1.2.4.1)	P11177 (PDHB)	-0.212	0.781	-4.500	0.0006	0.0376
Q7Z6P3	ENSG00000255587	RAB44	Ras-related protein Rab-44	Q7Z6P3 (RAB44)	0.211	0.952	4.008	0.0014	0.0483
P11766	ENSG00000197894	ADH5	Alcohol dehydrogenase class-3 (EC 1.1.1.1) (Alcohol dehydrogenase 5) (Alcohol dehydrogenase class chi chain) (Alcohol dehydrogenase class-III) (Glutathione-dependent formaldehyde dehydrogenase) (FALDH) (FDH) (GSH-FDH) (EC 1.1.1.-) (S-(hydroxymethyl)glutathione dehydrogenase) (EC 1.1.1.284)	P40394 (ADH7)	-0.211	0.683	-3.574	0.0033	0.0679
Q8NFW8	ENSG00000111726	CMAS	N-acylneuraminate cytidylyltransferase (EC 2.7.7.43) (CMP-N-acetylneuraminic acid synthase) (CMP-NeuNAc synthase)	Q8NFW8 (CMAS)	-0.210	0.697	-3.370	0.0049	0.0795
P62826	ENSG00000132341	RAN	GTP-binding nuclear protein Ran (Androgen receptor-associated protein 24) (GTPase Ran) (Ras-like protein TC4) (Ras-related nuclear protein)	P62826 (RAN)	-0.210	0.731	-3.719	0.0025	0.0575

Table S52: Proteins differentially abundant at Day 2 compared to pre-first vaccination (Study Arm 2 (A/H7N9 + AS03 D1, 29), Dendritic cells). Proteins are sorted by descending absolute log_2 fold change. Protein and gene annotations are based on Ensembl Version 101 (continued).

Protein ID	Ensembl Gene ID	Ensembl Gene Name	Protein Description	50% Protein Cluster ID (Gene Name)	Fold Change (Day 2 vs. Pre- First Vaccination)	Average log_2 Abundance	Moderated t-Statistic	P-Value	FDR Adjusted P-value
Q14676	ENSG00000137337; ENSG00000206481; ENSG00000224587; ENSG00000225589; ENSG00000234012; ENSG00000231135; ENSG00000228575; ENSG00000237095	MDC1	Mediator of DNA damage checkpoint protein 1 (Nuclear factor with BRCT domains 1)	Q14676 (MDC1)	0.209	0.929	2.972	0.0106	0.1177
P41218	ENSG00000163563	MNDA	Myeloid cell nuclear differentiation antigen	Q16666 (IFI16)	0.209	0.766	3.402	0.0046	0.0787
O75347	ENSG00000171530	TBCA	Tubulin-specific chaperone A (TCP1-chaperonin cofactor A) (Tubulin-folding cofactor A) (CFA)	O75347 (TBCA)	0.209	0.929	2.626	0.0207	0.147
Q9HC38	ENSG00000167699	GLOD4	Glyoxalase domain-containing protein 4	Q9HC38 (GLOD4)	-0.209	0.729	-3.780	0.0022	0.0575
P61916	ENSG00000119655	NPC2	NPC intracellular cholesterol transporter 2 (Epididymal secretory protein E1) (Human epididymis-specific protein 1) (He1) (Niemann-Pick disease type C2 protein)	P61916 (NPC2)	0.209	0.743	2.758	0.016	0.1357
Q53EL6	ENSG00000150593	PDCD4	Programmed cell death protein 4 (Neoplastic transformation inhibitor protein) (Nuclear antigen H731-like) (Protein 197/15a)	Q53EL6 (PDCD4)	-0.207	0.767	-3.722	0.0025	0.0575
P07195	ENSG00000111716	LDHB	L-lactate dehydrogenase B chain (LDH-B) (EC 1.1.1.27) (LDH heart subunit) (LDH-H) (Renal carcinoma antigen NY-REN-46)	P07195 (LDHB)	-0.204	0.876	-3.630	0.003	0.0638
Q9UHD8	ENSG00000184640	SEPTIN9	Septin-9 (MLL septin-like fusion protein MSF-A) (MLL septin-like fusion protein) (Ovarian/Breast septin) (Ov/Br septin) (Septin D1)	Q9UHD8 (SEPTIN9)	-0.202	0.705	-3.799	0.0021	0.0575
P16671	ENSG00000135218	CD36	Platelet glycoprotein 4 (Fatty acid translocase) (FAT) (Glycoprotein IIIb) (GPIIIB) (Leukocyte differentiation antigen CD36) (PAS IV) (PAS-4) (Platelet collagen receptor) (Platelet glycoprotein IV) (GPIV) (Thrombospondin receptor) (CD antigen CD36)	P16671 (CD36)	0.202	1.055	3.496	0.0038	0.0745
O75083	ENSG00000071127	WDR1	WD repeat-containing protein 1 (Actin-interacting protein 1) (AIP1) (NORI-1)	O75083 (WDR1)	-0.201	0.729	-4.260	0.0009	0.0454
Q92615	ENSG00000107929	LARP4B	La-related protein 4B (La ribonucleoprotein domain family member 4B) (La ribonucleoprotein domain family member 5) (La-related protein 5)	Q92615 (LARP4B)	0.201	0.953	2.966	0.0107	0.1177

Table S52: Proteins differentially abundant at Day 2 compared to pre-first vaccination (Study Arm 2 (A/H7N9 + AS03 D1, 29), Dendritic cells). Proteins are sorted by descending absolute log_2 fold change. Protein and gene annotations are based on Ensembl Version 101 (continued).

Protein ID	Ensembl Gene ID	Ensembl Gene Name	Protein Description	50% Protein Cluster ID (Gene Name)	Fold Change (Day 2 vs. Pre- First Vaccination)	Average log_2 Abundance	Moderated t-Statistic	P-Value	FDR Adjusted P-value
Q9NWH9	ENSG00000137776	SLTM	SAFB-like transcription modulator (Modulator of estrogen-induced transcription)	Q9NWH9 (SLTM)	0.200	0.999	3.086	0.0085	0.1038
P55263	ENSG00000156110	ADK	Adenosine kinase (AK) (EC 2.7.1.20) (Adenosine 5'-phosphotransferase)	P55263 (ADK)	-0.199	0.418	-3.909	0.0017	0.0519
O00161	ENSG00000092531	SNAP23	Synaptosomal-associated protein 23 (SNAP-23) (Vesicle-membrane fusion protein SNAP-23)	O00161 (SNAP23)	0.198	0.916	2.883	0.0126	0.1239
Q96T58	ENSG00000065526	SPEN	Msx2-interacting protein (SMART/HDAC1-associated repressor protein) (SPEN homolog)	Q96T58 (SPEN)	0.198	0.899	2.790	0.0151	0.1316
P48426	ENSG00000150867	PIP4K2A	Phosphatidylinositol 5-phosphate 4-kinase type-2 alpha (EC 2.7.1.149) (1-phosphatidylinositol 5-phosphate 4-kinase 2-alpha) (Diphosphoinositide kinase 2-alpha) (PIP5KIII) (Phosphatidylinositol 5-Phosphate 4-Kinase) (PI5P4Kalpha) (Phosphatidylinositol 5-phosphate 4-kinase type II alpha) (PI(5)P 4-kinase type II alpha) (PIP4KII-alpha) (PtdIns(4)P-5-kinase B isoform) (PtdIns(4)P-5-kinase C isoform) (PtdIns(5)P-4-kinase isoform 2-alpha)	Q8TBX8 (PIP4K2C)	-0.197	0.755	-4.368	0.0007	0.0433
P07919	ENSG00000173660	UQCRH	Cytochrome b-c1 complex subunit 6, mitochondrial (Complex III subunit 6) (Complex III subunit VIII) (Cytochrome c1 non-heme 11 kDa protein) (Mitochondrial hinge protein) (Ubiquinol-cytochrome c reductase complex 11 kDa protein)	P07919 (UQCRH)	0.197	0.861	2.607	0.0214	0.1489
P07437	ENSG00000196230; ENSG00000183311; ENSG00000235067; ENSG00000224156; ENSG00000229684; ENSG00000227739; ENSG00000232421; ENSG00000232575	TUBB	Tubulin beta chain (Tubulin beta-5 chain)	Q9H4B7 (TUBB1)	-0.196	0.625	-3.438	0.0043	0.0765
Q86VM9	ENSG00000158545	ZC3H18	Zinc finger CCCH domain-containing protein 18 (Nuclear protein NHN1)	Q86VM9 (ZC3H18)	0.195	0.925	3.093	0.0084	0.1032
Q92576	ENSG00000118482	PHF3	PHD finger protein 3	Q92576 (PHF3)	0.194	0.889	3.191	0.0069	0.0941
P10153	ENSG00000169385	RNASE2	Non-secretory ribonuclease (EC 4.6.1.18) (Eosinophil-derived neurotoxin) (RNase UpI-2) (Ribonuclease 2) (RNase 2) (Ribonuclease US)	P10153 (RNASE2)	0.193	0.765	2.726	0.0171	0.1372

Table S52: Proteins differentially abundant at Day 2 compared to pre-first vaccination (Study Arm 2 (A/H7N9 + AS03 D1, 29), Dendritic cells). Proteins are sorted by descending absolute log_2 fold change. Protein and gene annotations are based on Ensembl Version 101 (continued).

Protein ID	Ensembl Gene ID	Ensembl Gene Name	Protein Description	50% Protein Cluster ID (Gene Name)	Fold Change (Day 2 vs. Pre- First Vaccination)	Average log_2 Abundance	Moderated t-Statistic	P-Value	FDR Adjusted P-value
P35221	ENSG00000044115	CTNNA1	Catenin alpha-1 (Alpha E-catenin) (Cadherin-associated protein) (Renal carcinoma antigen NY-REN-13)	P26232 (CTNNA2)	0.192	0.890	3.807	0.0021	0.0575
Q969X5	ENSG00000113719	ERGIC1	Endoplasmic reticulum-Golgi intermediate compartment protein 1 (ER-Golgi intermediate compartment 32 kDa protein) (ERGIC-32)	Q969X5 (ERGIC1)	-0.191	0.772	-4.234	0.0009	0.0454
Q6PJT7	ENSG00000100722	ZC3H14	Zinc finger CCCH domain-containing protein 14 (Mammalian suppressor of tau pathology-2) (MSUT-2) (Renal carcinoma antigen NY-REN-37)	Q6PJT7 (ZC3H14)	0.191	0.952	2.704	0.0178	0.1393
Q8IUD2	ENSG00000082805	ERC1	ELKS/Rab6-interacting/CAST family member 1 (ERC-1) (Rab6-interacting protein 2)	Q8IUD2 (ERC1)	0.190	0.880	3.269	0.006	0.0865
Q96C23	ENSG00000143891	GALM	Galactose mutarotase (EC 5.1.3.3) (Aldose 1-epimerase)	Q96C23 (GALM)	-0.190	0.713	-3.945	0.0016	0.0498
P40429	ENSG00000142541	RPL13A	60S ribosomal protein L13a (23 kDa highly basic protein) (Large ribosomal subunit protein uL13)	P40429 (RPL13A)	-0.189	0.831	-3.938	0.0016	0.05
P80303	ENSG00000070081	NUCB2	Nucleobindin-2 (DNA-binding protein NEFA) (Epididymis secretory protein Li 109) (Gastric cancer antigen Zg4) (Prepronesfatin) [Cleaved into: Nesfatin-1]	Q02818 (NUCB1)	0.188	0.949	3.152	0.0075	0.0975
Q5M775	ENSG00000128487	SPECC1	Cytospin-B (Nuclear structure protein 5) (NSP5) (Sperm antigen HCMOGT-1) (Sperm antigen with calponin homology and coiled-coil domains 1)	Q5M775 (SPECC1)	0.188	1.016	2.723	0.0171	0.1375
P21796	ENSG00000213585	VDAC1	Voltage-dependent anion-selective channel protein 1 (VDAC-1) (hVDAC1) (Outer mitochondrial membrane protein porin 1) (Plasmalemmal porin) (Porin 31HL) (Porin 31HM)	P45880 (VDAC2)	-0.188	0.743	-3.720	0.0025	0.0575
Q5TFE4	ENSG00000178425	NT5DC1	5'-nucleotidase domain-containing protein 1 (EC 3.1.3.-)	Q5TFE4 (NT5DC1)	-0.187	0.735	-3.739	0.0024	0.0575
P00813	ENSG00000196839	ADA	Adenosine deaminase (EC 3.5.4.4) (Adenosine aminohydrolase)	P00813 (ADA)	-0.187	0.676	-4.422	0.0007	0.0417
A6NI73	ENSG00000187116; ENSG00000278355; ENSG00000274914; ENSG00000274113; ENSG00000275404	LILRA5	Leukocyte immunoglobulin-like receptor subfamily A member 5 (CD85 antigen-like family member F) (Immunoglobulin-like transcript 11) (ILT-11) (Leukocyte immunoglobulin-like receptor 9) (LIR-9) (CD antigen CD85f)	A6NI73 (LILRA5)	0.187	0.994	3.421	0.0044	0.0779

Table S52: Proteins differentially abundant at Day 2 compared to pre-first vaccination (Study Arm 2 (A/H7N9 + AS03 D1, 29), Dendritic cells). Proteins are sorted by descending absolute log_2 fold change. Protein and gene annotations are based on Ensembl Version 101 (continued).

Protein ID	Ensembl Gene ID	Ensembl Gene Name	Protein Description	50% Protein Cluster ID (Gene Name)	Fold Change (Day 2 vs. Pre- First Vaccination)	Average log_2 Abundance	Moderated t-Statistic	P-Value	FDR Adjusted P-value
P46779	ENSG00000108107	RPL28	60S ribosomal protein L28 (Large ribosomal subunit protein eL28)	P46779 (RPL28)	-0.187	0.560	-3.028	0.0095	0.111
Q16629	ENSG00000115875	SRSF7	Serine/arginine-rich splicing factor 7 (Splicing factor 9G8) (Splicing factor, arginine/serine-rich 7)	Q16629 (SRSF7)	0.186	0.802	2.711	0.0176	0.1386
P42765	ENSG00000167315	ACAA2	3-ketoacyl-CoA thiolase, mitochondrial (EC 2.3.1.16) (Acetyl-CoA acetyltransferase) (EC 2.3.1.9) (Acetyl-CoA acyltransferase) (Acyl-CoA hydrolase, mitochondrial) (EC 3.1.2.-) (EC 3.1.2.1) (EC 3.1.2.2) (Beta-ketothiolase) (Mitochondrial 3-oxoacyl-CoA thiolase) (T1)	P42765 (ACAA2)	-0.186	0.685	-4.105	0.0012	0.0483
P22626	ENSG00000122566	HNRNPA2B1	Heterogeneous nuclear ribonucleoproteins A2/B1 (hnRNP A2/B1)	P51991 (HNRNPA3)	0.186	0.614	2.829	0.014	0.1291
O43772	ENSG00000178537	SLC25A20	Mitochondrial carnitine/acylcarnitine carrier protein (Carnitine/acylcarnitine translocase) (CAC) (Solute carrier family 25 member 20)	O43772 (SLC25A20)	-0.185	0.760	-3.746	0.0024	0.0575
O95298	ENSG00000151366	NDUFC2	NADH dehydrogenase [ubiquinone] 1 subunit C2 (Complex I-B14.5b) (CI-B14.5b) (Human lung cancer oncogene 1 protein) (HLC-1) (NADH-ubiquinone oxidoreductase subunit B14.5b)	O95298 (NDUFC2)	-0.185	0.721	-4.023	0.0014	0.0483
Q9NZ45	ENSG00000122873	CISD1	CDGSH iron-sulfur domain-containing protein 1 (MitoNEET)	Q8N5K1 (CISD2)	-0.185	0.645	-2.881	0.0126	0.1241
P33121	ENSG00000151726	ACSL1	Long-chain-fatty-acid–CoA ligase 1 (EC 6.2.1.3) (Acyl-CoA synthetase 1) (ACS1) (Arachidonate–CoA ligase) (EC 6.2.1.15) (Long-chain acyl-CoA synthetase 1) (LACS 1) (Long-chain acyl-CoA synthetase 2) (LACS 2) (Long-chain fatty acid-CoA ligase 2) (Palmitoyl-CoA ligase 1) (Palmitoyl-CoA ligase 2) (Phytanate–CoA ligase) (EC 6.2.1.24)	P33121 (ACSL1)	0.184	0.929	2.607	0.0214	0.1489
P62847	ENSG00000138326	RPS24	40S ribosomal protein S24 (Small ribosomal subunit protein eS24)	P62847 (RPS24)	-0.184	0.548	-2.598	0.0218	0.1491
P08637	ENSG00000203747	FCGR3A	Low affinity immunoglobulin gamma Fc region receptor III-A (CD16a antigen) (Fc-gamma RIII-alpha) (Fc-gamma RIII) (Fc-gamma RIIIa) (FcRIII) (FcRIIIa) (FcR-10) (IgG Fc receptor III-2) (CD antigen CD16a)	P08637 (FCGR3A)	-0.183	0.633	-2.760	0.016	0.1357
Q86YV0	ENSG00000105122	RASAL3	RAS protein activator like-3	Q86YV0 (RASAL3)	-0.183	0.761	-4.259	0.0009	0.0454

Table S52: Proteins differentially abundant at Day 2 compared to pre-first vaccination (Study Arm 2 (A/H7N9 + AS03 D1, 29), Dendritic cells). Proteins are sorted by descending absolute log_2 fold change. Protein and gene annotations are based on Ensembl Version 101 (continued).

Protein ID	Ensembl Gene ID	Ensembl Gene Name	Protein Description	50% Protein Cluster ID (Gene Name)	Fold Change (Day 2 vs. Pre- First Vaccination)	Average log_2 Abundance	Moderated t-Statistic	P-Value	FDR Adjusted P-value
Q8WVY7	ENSG00000164332	UBLCP1	Ubiquitin-like domain-containing CTD phosphatase 1 (EC 3.1.3.16) (Nuclear proteasome inhibitor UBLCP1)	Q8WVY7 (UBLCP1)	-0.183	0.747	-3.619	0.003	0.0639
Q9Y3A5	ENSG00000126524	SBDS	Ribosome maturation protein SBDS (Shwachman-Bodian-Diamond syndrome protein)	Q9Y3A5 (SBDS)	-0.183	0.813	-3.952	0.0016	0.0498
O15247	ENSG00000155962	CLIC2	Chloride intracellular channel protein 2 (XAP121)	Q9NZA1 (CLIC5)	-0.182	0.694	-3.258	0.0061	0.0872
P62937	ENSG00000196262	PPIA	Peptidyl-prolyl cis-trans isomerase A (PPIase A) (EC 5.2.1.8) (Cyclophilin A) (Cyclosporin A-binding protein) (Rotamase A) [Cleaved into: Peptidyl-prolyl cis-trans isomerase A, N-terminally processed]	Q9UNP9 (PPIE)	-0.181	0.693	-3.457	0.0041	0.0752
Q96Q11	ENSG00000072756	TRNT1	CCA tRNA nucleotidyltransferase 1, mitochondrial (EC 2.7.7.72) (Mitochondrial tRNA nucleotidyl transferase, CCA-adding) (mt CCA-adding enzyme) (mt tRNA CCA-diphosphorylase) (mt tRNA CCA-pyrophosphorylase) (mt tRNA adenylyltransferase)	Q96Q11 (TRNT1)	-0.181	0.758	-3.696	0.0026	0.0579
P09651	ENSG00000135486	HNRNPA1	Heterogeneous nuclear ribonucleoprotein A1 (hnRNP A1) (Helix-destabilizing protein) (Single-strand RNA-binding protein) (hnRNP core protein A1) [Cleaved into: Heterogeneous nuclear ribonucleoprotein A1, N-terminally processed]	P51991 (HN-RNPA3)	0.181	0.710	3.684	0.0027	0.0585
P32942	ENSG00000076662	ICAM3	Intercellular adhesion molecule 3 (ICAM-3) (CDw50) (ICAM-R) (CD antigen CD50)	P32942 (ICAM3)	0.181	0.974	3.402	0.0046	0.0787
P49189	ENSG00000143149	ALDH9A1	4-trimethylaminobutyraldehyde dehydrogenase (TMABA-DH) (TMABALDH) (EC 1.2.1.47) (Aldehyde dehydrogenase E3 isozyme) (Aldehyde dehydrogenase family 9 member A1) (EC 1.2.1.3) (Gamma-aminobutyraldehyde dehydrogenase) (EC 1.2.1.19) (R-aminobutyraldehyde dehydrogenase) [Cleaved into: 4-trimethylaminobutyraldehyde dehydrogenase, N-terminally processed]	P49189 (ALDH9A1)	-0.180	0.900	-2.849	0.0135	0.1278
P31146	ENSG00000102879	CORO1A	Coronin-1A (Coronin-like protein A) (Clipin-A) (Coronin-like protein p57) (Tryptophan aspartate-containing coat protein) (TACO)	Q9BR76 (CORO1B)	-0.178	0.678	-3.143	0.0076	0.098

Table S52: Proteins differentially abundant at Day 2 compared to pre-first vaccination (Study Arm 2 (A/H7N9 + AS03 D1, 29), Dendritic cells). Proteins are sorted by descending absolute log_2 fold change. Protein and gene annotations are based on Ensembl Version 101 (continued).

Protein ID	Ensembl Gene ID	Ensembl Gene Name	Protein Description	50% Protein Cluster ID (Gene Name)	Fold Change (Day 2 vs. Pre- First Vaccination)	Average log_2 Abundance	Moderated t-Statistic	P-Value	FDR Adjusted P-value
Q13162	ENSG00000123131	PRDX4	Peroxiredoxin-4 (EC 1.11.1.24) (Antioxidant enzyme AOE372) (AOE37-2) (Peroxiredoxin IV) (Prx-IV) (Thioredoxin peroxidase AO372) (Thioredoxin-dependent peroxide reductase A0372) (Thioredoxin-dependent peroxiredoxin 4)	Q13162 (PRDX4)	0.178	0.982	2.623	0.0208	0.1473
O60613	ENSG00000183291	SELENOF	Selenoprotein F (15 kDa selenoprotein)	O60613 (SELENOF)	0.178	0.953	2.890	0.0124	0.1232
P61254	ENSG00000161970	RPL26	60S ribosomal protein L26 (Large ribosomal subunit protein uL24)	P61254 (RPL26)	-0.177	0.763	-2.720	0.0173	0.1378
P09012	ENSG00000077312	SNRPA	U1 small nuclear ribonucleoprotein A (U1 snRNP A) (U1-A) (U1A)	P09012 (SNRPA)	0.177	0.943	3.379	0.0048	0.0795
Q16181	ENSG00000122545	SEPTIN7	Septin-7 (CDC10 protein homolog)	Q16181 (SEPTIN7)	-0.177	0.817	-3.007	0.0099	0.1127
Q8N122	ENSG00000141564	RPTOR	Regulatory-associated protein of mTOR (Raptor) (p150 target of rapamycin (TOR)-scaffold protein)	Q8N122 (RPTOR)	0.177	0.829	2.953	0.011	0.1188
O15389	ENSG00000105501	SIGLEC5	Sialic acid-binding Ig-like lectin 5 (Siglec-5) (CD33 antigen-like 2) (Obesity-binding protein 2) (OB-BP2) (OB-binding protein 2) (CD antigen CD170)	O15389 (SIGLEC5)	0.177	0.778	3.587	0.0032	0.0672
Q9P032	ENSG00000123545	NDUFAF4	NADH dehydrogenase [ubiquinone] 1 alpha subcomplex assembly factor 4 (Hormone-regulated proliferation-associated protein of 20 kDa)	Q9P032 (NDUFAF4)	-0.176	0.824	-3.403	0.0046	0.0787
Q15836	ENSG00000049245	VAMP3	Vesicle-associated membrane protein 3 (VAMP-3) (Cellubrevin) (CEB) (Synaptobrevin-3)	P63027 (VAMP2)	0.176	0.982	2.626	0.0206	0.147
O14545	ENSG00000135148	TRAFD1	TRAF-type zinc finger domain-containing protein 1 (Protein FLN29)	O14545 (TRAFD1)	0.175	0.977	2.794	0.0149	0.1316
O43759	ENSG00000100321	SYNGR1	Synaptogyrin-1	O43759 (SYNGR1)	0.175	0.939	3.769	0.0023	0.0575
Q13443	ENSG00000168615	ADAM9	Disintegrin and metalloproteinase domain-containing protein 9 (ADAM 9) (EC 3.4.24.-) (Cellular disintegrin-related protein) (Meltrin-gamma) (Metalloprotease/disintegrin/cysteine-rich protein 9) (Myeloma cell metalloproteinase)	Q13443 (ADAM9)	0.174	0.949	3.498	0.0038	0.0745
P40925	ENSG00000014641	MDH1	Malate dehydrogenase, cytoplasmic (EC 1.1.1.37) (Cytosolic malate dehydrogenase) (Diiodophenylpyruvate reductase) (EC 1.1.1.96)	P40925 (MDH1)	-0.174	0.730	-3.366	0.0049	0.0798

Table S52: Proteins differentially abundant at Day 2 compared to pre-first vaccination (Study Arm 2 (A/H7N9 + AS03 D1, 29), Dendritic cells). Proteins are sorted by descending absolute log_2 fold change. Protein and gene annotations are based on Ensembl Version 101 (continued).

Protein ID	Ensembl Gene ID	Ensembl Gene Name	Protein Description	50% Protein Cluster ID (Gene Name)	Fold Change (Day 2 vs. Pre- First Vaccination)	Average log_2 Abundance	Moderated t-Statistic	P-Value	FDR Adjusted P-value
P43243	ENSG00000015479	MATR3	Matrin-3	P43243 (MATR3)	0.173	0.872	3.697	0.0026	0.0579
Q9P016	ENSG00000151500	THYN1	Thymocyte nuclear protein 1 (Thymocyte protein Thy28)	Q9P016 (THYN1)	-0.173	0.767	-3.142	0.0076	0.098
Q5XKP0	ENSG00000174917	MICOS13	MICOS complex subunit MIC13 (Protein P117)	Q5XKP0 (MICOS13)	-0.173	0.654	-4.550	0.0005	0.0371
Q7Z4V5	ENSG00000167674	HDGFL2	Hepatoma-derived growth factor-related protein 2 (HDGF-related protein 2) (HRP-2) (Hepatoma-derived growth factor 2) (HDGF-2)	Q7Z4V5 (HDGFL2)	0.173	0.941	3.209	0.0067	0.0918
Q13123	ENSG00000113141	IK	Protein Red (Cytokine IK) (IK factor) (Protein RER)	Q13123 (IK)	0.173	0.864	2.791	0.015	0.1316
O60942	ENSG00000111880	RNGTT	mRNA-capping enzyme (HCAP1) (HCE) [Includes: Polynucleotide 5'-triphosphatase (EC 3.1.3.33) (mRNA 5'-triphosphatase) (TPase); mRNA guanylyltransferase (EC 2.7.7.50) (GTP–RNA guanylyltransferase) (GTase)]	O60942 (RNGTT)	-0.173	0.752	-3.136	0.0077	0.0985
Q13185	ENSG00000122565	CBX3	Chromobox protein homolog 3 (HECH) (Heterochromatin protein 1 homolog gamma) (HP1 gamma) (Modifier 2 protein)	P45973 (CBX5)	0.172	1.088	2.726	0.0171	0.1372
Q9NUJ1	ENSG00000144827	ABHD10	Palmitoyl-protein thioesterase ABHD10, mitochondrial (EC 3.1.2.22) (Acyl-protein thioesterase ABHD10) (Alpha/beta hydrolase domain-containing protein 10) (Abhydrolase domain-containing protein 10) (Mycophenolic acid acyl-glucuronide esterase, mitochondrial) (EC 3.1.1.93)	Q9NUJ1 (ABHD10)	-0.172	0.758	-3.784	0.0022	0.0575
P30519	ENSG00000103415; ENSG00000277424	HMOX2	Heme oxygenase 2 (HO-2) (EC 1.14.14.18)	P30519 (HMOX2)	-0.172	0.817	-3.749	0.0024	0.0575
P60709	ENSG00000075624	ACTB	Actin, cytoplasmic 1 (Beta-actin) [Cleaved into: Actin, cytoplasmic 1, N-terminally processed]	P0CG38 (POTEI)	-0.171	0.647	-3.083	0.0086	0.1038
O60664	ENSG00000105355	PLIN3	Perilipin-3 (47 kDa mannose 6-phosphate receptor-binding protein) (47 kDa MPR-binding protein) (Cargo selection protein TIP47) (Mannose-6-phosphate receptor-binding protein 1) (Placental protein 17) (PP17)	O60664 (PLIN3)	0.171	0.873	2.737	0.0167	0.1369
P19878	ENSG00000116701	NCF2	Neutrophil cytosol factor 2 (NCF-2) (67 kDa neutrophil oxidase factor) (NADPH oxidase activator 2) (Neutrophil NADPH oxidase factor 2) (p67-phox)	P19878 (NCF2)	0.171	0.887	3.237	0.0063	0.09

Table S52: Proteins differentially abundant at Day 2 compared to pre-first vaccination (Study Arm 2 (A/H7N9 + AS03 D1, 29), Dendritic cells). Proteins are sorted by descending absolute log_2 fold change. Protein and gene annotations are based on Ensembl Version 101 (continued).

Protein ID	Ensembl Gene ID	Ensembl Gene Name	Protein Description	50% Protein Cluster ID (Gene Name)	Fold Change (Day 2 vs. Pre- First Vaccination)	Average log_2 Abundance	Moderated t-Statistic	P-Value	FDR Adjusted P-value
Q13642	ENSG00000022267	FHL1	Four and a half LIM domains protein 1 (FHL-1) (Skeletal muscle LIM-protein 1) (SLIM) (SLIM-1)	Q13642 (FHL1)	-0.171	0.873	-2.686	0.0184	0.1415
O60264	ENSG00000153147	SMARCA5	SWI/SNF-related matrix-associated actin-dependent regulator of chromatin subfamily A member 5 (SWI/SNF-related matrix-associated actin-dependent regulator of chromatin A5) (EC 3.6.4.-) (Sucrose non-fermenting protein 2 homolog) (hSNF2H)	O60264 (SMARCA5)	0.170	0.966	3.556	0.0034	0.0693
Q9H939	ENSG00000152229	PSTPIP2	Proline-serine-threonine phosphatase-interacting protein 2 (PEST phosphatase-interacting protein 2)	Q9H939 (PSTPIP2)	0.170	0.982	3.845	0.002	0.0558
Q07955	ENSG00000136450	SRSF1	Serine/arginine-rich splicing factor 1 (Alternative-splicing factor 1) (ASF-1) (Splicing factor, arginine/serine-rich 1) (pre-mRNA-splicing factor SF2, P33 subunit)	Q07955 (SRSF1)	0.170	0.741	2.791	0.0151	0.1316
Q13613	ENSG00000063601	MTMR1	Myotubularin-related protein 1 (Phosphatidylinositol-3,5-bisphosphate 3-phosphatase) (EC 3.1.3.95) (Phosphatidylinositol-3-phosphate phosphatase) (EC 3.1.3.64)	Q13613 (MTMR1)	-0.169	0.788	-3.237	0.0063	0.09
Q9HC35	ENSG00000143924	EML4	Echinoderm microtubule-associated protein-like 4 (EMAP-4) (Restrictedly overexpressed proliferation-associated protein) (Ropp 120)	Q9HC35 (EML4)	-0.169	0.763	-3.442	0.0043	0.0763
P55809	ENSG00000083720	OXCT1	Succinyl-CoA:3-ketoacid coenzyme A transferase 1, mitochondrial (EC 2.8.3.5) (3-oxoacid CoA-transferase 1) (Somatic-type succinyl-CoA:3-oxoacid CoA-transferase) (SCOT-s)	P55809 (OXCT1)	-0.169	0.831	-3.458	0.0041	0.0752
P34896	ENSG00000176974; ENSG00000284320	SHMT1	Serine hydroxymethyltransferase, cytosolic (SHMT) (EC 2.1.2.1) (Glycine hydroxymethyltransferase) (Serine methylase)	P34897 (SHMT2)	0.169	0.798	2.628	0.0206	0.147
O75251	ENSG00000115286	NDUFS7	NADH dehydrogenase [ubiquinone] iron-sulfur protein 7, mitochondrial (EC 7.1.1.2) (Complex I-20kD) (CI-20kD) (NADH-ubiquinone oxidoreductase 20 kDa subunit) (PSST subunit)	O75251 (NDUFS7)	-0.168	0.721	-3.713	0.0025	0.0575
Q9UKX7	ENSG00000093000	NUP50	Nuclear pore complex protein Nup50 (50 kDa nucleoporin) (Nuclear pore-associated protein 60 kDa-like) (Nucleoporin Nup50)	Q9UKX7 (NUP50)	0.168	0.963	3.083	0.0085	0.1038
P11171	ENSG00000159023	EPB41	Protein 4.1 (P4.1) (4.1R) (Band 4.1) (EPB4.1) (Erythrocyte membrane protein band 4.1)	P11171 (EPB41)	-0.168	0.901	-2.989	0.0103	0.1154

Table S52: Proteins differentially abundant at Day 2 compared to pre-first vaccination (Study Arm 2 (A/H7N9 + AS03 D1, 29), Dendritic cells). Proteins are sorted by descending absolute log_2 fold change. Protein and gene annotations are based on Ensembl Version 101 (continued).

Protein ID	Ensembl Gene ID	Ensembl Gene Name	Protein Description	50% Protein Cluster ID (Gene Name)	Fold Change (Day 2 vs. Pre- First Vaccination)	Average log_2 Abundance	Moderated t-Statistic	P-Value	FDR Adjusted P-value
Q8N573	ENSG00000164830	OXR1	Oxidation resistance protein 1	Q8N573 (OXR1)	0.167	0.951	2.671	0.019	0.1421
Q9Y6I3	ENSG00000063245	EPN1	Epsin-1 (EH domain-binding mitotic phosphoprotein) (EPS-15-interacting protein 1)	Q9Y6I3 (EPN1)	0.167	0.916	2.657	0.0195	0.1431
P13010	ENSG00000079246	XRCC5	X-ray repair cross-complementing protein 5 (EC 3.6.4.-) (86 kDa subunit of Ku antigen) (ATP-dependent DNA helicase 2 subunit 2) (ATP-dependent DNA helicase II 80 kDa subunit) (CTC box-binding factor 85 kDa subunit) (CTC85) (CTCBF) (DNA repair protein XRCC5) (Ku80) (Ku86) (Lupus Ku autoantigen protein p86) (Nuclear factor IV) (Thyroid-lupus autoantigen) (TLAA) (X-ray repair complementing defective repair in Chinese hamster cells 5 (double-strand-break rejoining))	P13010 (XRCC5)	-0.167	0.747	-2.870	0.0129	0.125
O95865	ENSG00000213722; ENSG00000206395; ENSG00000226634; ENSG00000233076; ENSG00000225635; ENSG00000228128; ENSG00000227317	DDAH2	N(G),N(G)-dimethylarginine dimethylaminohydrolase 2 (DDAH-2) (Dimethylarginine dimethylaminohydrolase 2) (EC 3.5.3.18) (DDAHII) (Dimethylargininase-2) (Protein G6a) (S-phase protein)	O94760 (DDAH1)	-0.165	0.770	-4.028	0.0014	0.0483
O14966	ENSG00000117280	RAB29	Ras-related protein Rab-7L1 (Rab-7-like protein 1) (Ras-related protein Rab-29)	Q13637 (RAB32)	-0.165	0.805	-3.716	0.0025	0.0575
Q53QZ3	ENSG00000075884	ARHGAP15	Rho GTPase-activating protein 15 (ArhGAP15) (Rho-type GTPase-activating protein 15)	Q53QZ3 (ARHGAP15)	-0.164	0.780	-2.952	0.011	0.1188
P61457	ENSG00000166228	PCBD1	Pterin-4-alpha-carbinolamine dehydratase (PHS) (EC 4.2.1.96) (4-alpha-hydroxy-tetrahydropterin dehydratase) (Dimerization cofactor of hepatocyte nuclear factor 1-alpha) (DCoH) (Dimerization cofactor of HNF1) (Phenylalanine hydroxylase-stimulating protein) (Pterin carbinolamine dehydratase) (PCD)	Q9H0N5 (PCBD2)	0.164	0.905	2.942	0.0112	0.119
P59998	ENSG00000241553	ARPC4	Actin-related protein 2/3 complex subunit 4 (Arp2/3 complex 20 kDa subunit) (p20-ARC)	P59998 (ARPC4)	-0.163	0.642	-3.558	0.0034	0.0693
Q8N335	ENSG00000152642	GPD1L	Glycerol-3-phosphate dehydrogenase 1-like protein (GPD1-L) (EC 1.1.1.8)	Q8N335 (GPD1L)	-0.162	0.756	-4.055	0.0013	0.0483

Table S52: Proteins differentially abundant at Day 2 compared to pre-first vaccination (Study Arm 2 (A/H7N9 + AS03 D1, 29), Dendritic cells). Proteins are sorted by descending absolute log_2 fold change. Protein and gene annotations are based on Ensembl Version 101 (continued).

Protein ID	Ensembl Gene ID	Ensembl Gene Name	Protein Description	50% Protein Cluster ID (Gene Name)	Fold Change (Day 2 vs. Pre-First Vaccination)	Average log_2 Abundance	Moderated t-Statistic	P-Value	FDR Adjusted P-value
Q9Y5Z4	ENSG00000051620	HEBP2	Heme-binding protein 2 (Placental protein 23) (PP23) (Protein SOUL)	Q9Y5Z4 (HEBP2)	0.162	0.795	2.702	0.0178	0.1393
O43290	ENSG00000175467	SART1	U4/U6.U5 tri-snRNP-associated protein 1 (SNU66 homolog) (hSnu66) (Squamous cell carcinoma antigen recognized by T-cells 1) (SART-1) (hSART-1) (U4/U6.U5 tri-snRNP-associated 110 kDa protein) (allergen Hom s 1)	O43290 (SART1)	0.162	0.944	2.815	0.0144	0.1291
O15553	ENSG00000103313	MEFV	Pyrin (Marenostrin)	O15553 (MEFV)	0.161	0.818	3.094	0.0084	0.1032
O15533	ENSG00000112493; ENSG00000206208; ENSG00000206281; ENSG00000236490; ENSG00000231925	TAPBP	Tapasin (TPN) (TPSN) (NGS-17) (TAP-associated protein) (TAP-binding protein)	O15533 (TAPBP)	-0.161	0.877	-2.964	0.0108	0.1177
Q8N0X7	ENSG00000133104	SPART	Spartin (Spastic paraplegia 20 protein) (Trans-activated by hepatitis C virus core protein 1)	Q8N0X7 (SPART)	0.161	0.902	4.512	0.0006	0.0376
P63104	ENSG00000164924	YWHAZ	14-3-3 protein zeta/delta (Protein kinase C inhibitor protein 1) (KCIP-1)	P62258 (YWHAE)	-0.161	0.753	-3.734	0.0024	0.0575
P46782	ENSG00000083845	RPS5	40S ribosomal protein S5 (Small ribosomal subunit protein uS7) [Cleaved into: 40S ribosomal protein S5, N-terminally processed]	P46782 (RPS5)	-0.161	0.801	-3.528	0.0036	0.0715
P61353	ENSG00000131469	RPL27	60S ribosomal protein L27 (Large ribosomal subunit protein eL27)	P61353 (RPL27)	-0.161	0.805	-3.484	0.0039	0.0747
Q9NPB8	ENSG00000125772	GPCPD1	Glycerophosphocholine phosphodiesterase GPCPD1 (EC 3.1.4.2) (Glycerophosphodiester phosphodiesterase 5)	Q9NPB8 (GPCPD1)	0.160	0.928	3.273	0.0059	0.0865
Q13363	ENSG00000159692	CTBP1	C-terminal-binding protein 1 (CtBP1) (EC 1.1.1.-)	P56545 (CTBP2)	-0.160	0.731	-3.486	0.0039	0.0747
Q9H8Y8	ENSG00000115806	GORASP2	Golgi reassembly-stacking protein 2 (GRS2) (Golgi phosphoprotein 6) (GOLPH6) (Golgi reassembly-stacking protein of 55 kDa) (GRASP55) (p59)	Q9H8Y8 (GORASP2)	0.159	0.840	4.000	0.0015	0.0483
P35754	ENSG00000173221	GLRX	Glutaredoxin-1 (Thioltransferase-1) (TTase-1)	P35754 (GLRX)	0.159	0.822	2.826	0.0141	0.1291
Q86Y82	ENSG00000117758	STX12	Syntaxin-12	Q86Y82 (STX12)	0.158	0.905	3.573	0.0033	0.0679
Q8WYA6	ENSG00000132792	CTNNBL1	Beta-catenin-like protein 1 (Nuclear-associated protein) (NAP) (Testis development protein NYD-SP19)	Q8WYA6 (CTNNBL1)	-0.158	0.768	-2.833	0.0139	0.1291

Table S52: Proteins differentially abundant at Day 2 compared to pre-first vaccination (Study Arm 2 (A/H7N9 + AS03 D1, 29), Dendritic cells). Proteins are sorted by descending absolute log_2 fold change. Protein and gene annotations are based on Ensembl Version 101 (continued).

Protein ID	Ensembl Gene ID	Ensembl Gene Name	Protein Description	50% Protein Cluster ID (Gene Name)	Fold Change (Day 2 vs. Pre- First Vaccination)	Average log_2 Abundance	Moderated t-Statistic	P-Value	FDR Adjusted P-value
P11216	ENSG00000100994	PYGB	Glycogen phosphorylase, brain form (EC 2.4.1.1)	P06737 (PYGL)	-0.157	0.818	-4.694	0.0004	0.0349
Q6PD62	ENSG00000198730	CTR9	RNA polymerase-associated protein CTR9 homolog (SH2 domain-binding protein 1)	Q6PD62 (CTR9)	0.157	0.935	3.214	0.0066	0.0914
P46776	ENSG00000166441	RPL27A	60S ribosomal protein L27a (Large ribosomal subunit protein uL15)	P46776 (RPL27A)	-0.157	0.578	-2.886	0.0125	0.1236
Q9HAN9	ENSG00000173614	NMNAT1	Nicotinamide/nicotinic acid mononucleotide adenylyltransferase 1 (NMN/NaMN adenylyltransferase 1) (EC 2.7.7.1) (EC 2.7.7.18) (Nicotinamide-nucleotide adenylyltransferase 1) (NMN adenylyltransferase 1) (Nicotinate-nucleotide adenylyltransferase 1) (NaMN adenylyltransferase 1)	Q9HAN9 (NMNAT1)	0.156	0.831	3.044	0.0092	0.1086
P24666	ENSG00000143727	ACP1	Low molecular weight phosphotyrosine protein phosphatase (LMW-PTP) (LMW-PTPase) (EC 3.1.3.48) (Adipocyte acid phosphatase) (Low molecular weight cytosolic acid phosphatase) (EC 3.1.3.2) (Red cell acid phosphatase 1)	P24666 (ACP1)	-0.156	0.718	-4.278	0.0009	0.0454
O00483	ENSG00000189043	NDUFA4	Cytochrome c oxidase subunit NDUFA4 (Complex I-MLRQ) (CI-MLRQ) (NADH-ubiquinone oxidoreductase MLRQ subunit)	O00483 (NDUFA4)	-0.156	0.581	-3.962	0.0016	0.0498
O95551	ENSG00000111802	TDP2	Tyrosyl-DNA phosphodiesterase 2 (Tyr-DNA phosphodiesterase 2) (hTDP2) (EC 3.1.4.-) (5'-tyrosyl-DNA phosphodiesterase) (5'-Tyr-DNA phosphodiesterase) (ETS1-associated protein 2) (ETS1-associated protein II) (EAPII) (TRAF and TNF receptor-associated protein) (Tyrosyl-RNA phosphodiesterase) (VPg unlinkase)	O95551 (TDP2)	-0.156	0.735	-2.598	0.0218	0.1491

Confidential/Proprietary Information

Table S52: Proteins differentially abundant at Day 2 compared to pre-first vaccination (Study Arm 2 (A/H7N9 + AS03 D1, 29), Dendritic cells). Proteins are sorted by descending absolute log_2 fold change. Protein and gene annotations are based on Ensembl Version 101 (continued).

Protein ID	Ensembl Gene ID	Ensembl Gene Name	Protein Description	50% Protein Cluster ID (Gene Name)	Fold Change (Day 2 vs. Pre- First Vaccination)	Average log_2 Abundance	Moderated t-Statistic	P-Value	FDR Adjusted P-value
P12956	ENSG00000196419	XRCC6	X-ray repair cross-complementing protein 6 (EC 3.6.4.-) (EC 4.2.99.-) (5'-deoxyribose-5-phosphate lyase Ku70) (5'-dRP lyase Ku70) (70 kDa subunit of Ku antigen) (ATP-dependent DNA helicase 2 subunit 1) (ATP-dependent DNA helicase II 70 kDa subunit) (CTC box-binding factor 75 kDa subunit) (CTC75) (CTCBF) (DNA repair protein XRCC6) (Lupus Ku autoantigen protein p70) (Ku70) (Thyroid-lupus autoantigen) (TLAA) (X-ray repair complementing defective repair in Chinese hamster cells 6)	P12956 (XRCC6)	-0.156	0.748	-3.521	0.0037	0.0717
Q96BZ4	ENSG00000166428	PLD4	5'-3' exonuclease PLD4 (EC 3.1.16.1) (Choline phosphatase 4) (Phosphatidylcholine-hydrolyzing phospholipase D4) (Phospholipase D family member 4) (Phospholipase D4) (PLD 4)	Q96BZ4 (PLD4)	-0.156	0.606	-2.862	0.0131	0.1253
Q6RW13	ENSG00000177674	AGTRAP	Type-1 angiotensin II receptor-associated protein (AT1 receptor-associated protein)	Q6RW13 (AGTRAP)	0.155	0.908	3.142	0.0076	0.098
O95674	ENSG00000101290	CDS2	Phosphatidate cytidylyltransferase 2 (EC 2.7.7.41) (CDP-DAG synthase 2) (CDP-DG synthase 2) (CDP-diacylglycerol synthase 2) (CDS 2) (CDP-diglyceride pyrophosphorylase 2) (CDP-diglyceride synthase 2) (CTP:phosphatidate cytidylyltransferase 2)	O95674 (CDS2)	0.155	0.905	2.762	0.0159	0.1357
P00973	ENSG00000089127	OAS1	2'-5'-oligoadenylate synthase 1 ((2-5')oligo(A) synthase 1) (2-5A synthase 1) (EC 2.7.7.84) (E18/E16) (p46/p42 OAS)	Q9Y6K5 (OAS3)	0.154	0.708	3.236	0.0064	0.09
Q8TBX8	ENSG00000166908	PIP4K2C	Phosphatidylinositol 5-phosphate 4-kinase type-2 gamma (EC 2.7.1.149) (Phosphatidylinositol 5-phosphate 4-kinase type II gamma) (PI(5)P 4-kinase type II gamma) (PIP4KII-gamma)	Q8TBX8 (PIP4K2C)	-0.154	0.800	-4.199	0.001	0.0467
Q9Y6A5	ENSG00000013810	TACC3	Transforming acidic coiled-coil-containing protein 3 (ERIC-1)	Q9Y6A5 (TACC3)	0.154	0.943	2.911	0.0119	0.1214
O60496	ENSG00000147443	DOK2	Docking protein 2 (Downstream of tyrosine kinase 2) (p56(dok-2))	O60496 (DOK2)	-0.153	0.694	-3.671	0.0027	0.0597
Q99523	ENSG00000134243	SORT1	Sortilin (100 kDa NT receptor) (Glycoprotein 95) (Gp95) (Neurotensin receptor 3) (NT3) (NTR3)	Q99523 (SORT1)	0.153	0.928	3.414	0.0045	0.0781
Q96CN7	ENSG00000066583	ISOC1	Isochorismatase domain-containing protein 1	Q96CN7 (ISOC1)	-0.152	0.810	-3.185	0.007	0.0943

Table S52: Proteins differentially abundant at Day 2 compared to pre-first vaccination (Study Arm 2 (A/H7N9 + AS03 D1, 29), Dendritic cells). Proteins are sorted by descending absolute log_2 fold change. Protein and gene annotations are based on Ensembl Version 101 (continued).

Protein ID	Ensembl Gene ID	Ensembl Gene Name	Protein Description	50% Protein Cluster ID (Gene Name)	Fold Change (Day 2 vs. Pre- First Vaccination)	Average log_2 Abundance	Moderated t-Statistic	P-Value	FDR Adjusted P-value
P53634	ENSG00000109861	CTSC	Dipeptidyl peptidase 1 (EC 3.4.14.1) (Cathepsin C) (Cathepsin J) (Dipeptidyl peptidase I) (DPP-I) (DPPI) (Dipeptidyl transferase) [Cleaved into: Dipeptidyl peptidase 1 exclusion domain chain (Dipeptidyl peptidase I exclusion domain chain); Dipeptidyl peptidase 1 heavy chain (Dipeptidyl peptidase I heavy chain); Dipeptidyl peptidase 1 light chain (Dipeptidyl peptidase I light chain)]	P53634 (CTSC)	-0.152	0.560	-2.600	0.0217	0.1491
Q9Y2G3	ENSG00000058063	ATP11B	Probable phospholipid-transporting ATPase IF (EC 7.6.2.1) (ATPase IR) (ATPase class VI type 11B) (P4-ATPase flippase complex alpha subunit ATP11B)	Q9Y2G3 (ATP11B)	0.152	0.903	2.688	0.0183	0.1413
Q99623	ENSG00000215021	PHB2	Prohibitin-2 (B-cell receptor-associated protein BAP37) (D-prohibitin) (Repressor of estrogen receptor activity)	Q99623 (PHB2)	-0.152	0.800	-2.690	0.0183	0.1413
Q6P1N9	ENSG00000147687	TATDN1	Putative deoxyribonuclease TATDN1 (EC 3.1.21.-) (Hepatocarcinoma high expression protein)	Q6P1N9 (TATDN1)	-0.151	0.832	-3.478	0.004	0.0747
Q9NRX2	ENSG00000158042	MRPL17	39S ribosomal protein L17, mitochondrial (L17mt) (MRP-L17) (LYST-interacting protein 2) (Mitochondrial large ribosomal subunit protein bL17m)	Q9NRX2 (MRPL17)	-0.151	0.756	-3.458	0.0041	0.0752
Q8NHP6	ENSG00000130150	MOSPD2	Motile sperm domain-containing protein 2	Q8NHP6 (MOSPD2)	0.151	1.003	2.874	0.0128	0.125
P45880	ENSG00000165637	VDAC2	Voltage-dependent anion-selective channel protein 2 (VDAC-2) (hVDAC2) (Outer mitochondrial membrane protein porin 2)	P45880 (VDAC2)	-0.151	0.779	-2.828	0.014	0.1291
Q12802	ENSG00000170776	AKAP13	A-kinase anchor protein 13 (AKAP-13) (AKAP-Lbc) (Breast cancer nuclear receptor-binding auxiliary protein) (Guanine nucleotide exchange factor Lbc) (Human thyroid-anchoring protein 31) (Lymphoid blast crisis oncogene) (LBC oncogene) (Non-oncogenic Rho GTPase-specific GTP exchange factor) (Protein kinase A-anchoring protein 13) (PRKA13) (p47)	Q12802 (AKAP13)	0.150	0.913	3.078	0.0086	0.1041
P15309	ENSG00000014257	ACP3	Prostatic acid phosphatase (PAP) (EC 3.1.3.2) (5'-nucleotidase) (5'-NT) (EC 3.1.3.5) (Acid phosphatase 3) (Ecto-5'-nucleotidase) (Protein tyrosine phosphatase ACP3) (EC 3.1.3.48) (Thiamine monophosphatase) (TMPase) [Cleaved into: PAPf39]	P15309 (ACP3)	0.150	0.869	2.871	0.0129	0.125

Table S52: Proteins differentially abundant at Day 2 compared to pre-first vaccination (Study Arm 2 (A/H7N9 + AS03 D1, 29), Dendritic cells). Proteins are sorted by descending absolute log_2 fold change. Protein and gene annotations are based on Ensembl Version 101 (continued).

Protein ID	Ensembl Gene ID	Ensembl Gene Name	Protein Description	50% Protein Cluster ID (Gene Name)	Fold Change (Day 2 vs. Pre- First Vaccination)	Average log_2 Abundance	Moderated t-Statistic	P-Value	FDR Adjusted P-value
Q03169	ENSG00000185215	TNFAIP2	Tumor necrosis factor alpha-induced protein 2 (TNF alpha-induced protein 2) (Primary response gene B94 protein)	Q03169 (TNFAIP2)	0.149	0.901	2.948	0.0111	0.1188
P22830	ENSG00000066926	FECH	Ferrochelatase, mitochondrial (EC 4.99.1.1) (Heme synthase) (Protoheme ferro-lyase)	P22830 (FECH)	-0.148	0.730	-4.080	0.0013	0.0483
Q96LJ7	ENSG00000157379; ENSG00000284868	DHRS1	Dehydrogenase/reductase SDR family member 1 (EC 1.1.-.-) (Short chain dehydrogenase/reductase family 19C member 1)	Q96LJ7 (DHRS1)	-0.148	0.723	-3.697	0.0026	0.0579
Q9Y2I8	ENSG00000047056	WDR37	WD repeat-containing protein 37	Q9Y2I8 (WDR37)	-0.148	0.733	-3.803	0.0021	0.0575
Q9H7Z7	ENSG00000148334	PTGES2	Prostaglandin E synthase 2 (EC 5.3.99.3) (Membrane-associated prostaglandin E synthase-2) (mPGE synthase-2) (Microsomal prostaglandin E synthase 2) (mPGES-2) (Prostaglandin-H(2) E-isomerase) [Cleaved into: Prostaglandin E synthase 2 truncated form]	Q9H7Z7 (PTGES2)	-0.148	0.797	-2.956	0.0109	0.1188
P61970	ENSG00000102898	NUTF2	Nuclear transport factor 2 (NTF-2) (Placental protein 15) (PP15)	P61970 (NUTF2)	-0.146	0.752	-2.589	0.0222	0.15
Q8NHL6	ENSG00000277807; ENSG00000274669	LILRB1	Leukocyte immunoglobulin-like receptor subfamily B member 1 (LIR-1) (Leukocyte immunoglobulin-like receptor 1) (CD85 antigen-like family member J) (Immunoglobulin-like transcript 2) (ILT-2) (Monocyte/macrophage immunoglobulin-like receptor 7) (MIR-7) (CD antigen CD85j)	Q8NHL6 (LILRB1)	0.146	0.893	3.276	0.0059	0.0865
O14929	ENSG00000128708	HAT1	Histone acetyltransferase type B catalytic subunit (EC 2.3.1.48) (Histone acetyltransferase 1)	O14929 (HAT1)	0.146	0.853	3.120	0.008	0.0998
P08559	ENSG00000131828	PDHA1	Pyruvate dehydrogenase E1 component subunit alpha, somatic form, mitochondrial (EC 1.2.4.1) (PDHE1-A type I)	P08559 (PDHA1)	-0.146	0.794	-3.109	0.0081	0.1008
P42785	ENSG00000137509	PRCP	Lysosomal Pro-X carboxypeptidase (EC 3.4.16.2) (Angiotensinase C) (Lysosomal carboxypeptidase C) (Proline carboxypeptidase) (Prolylcarboxypeptidase) (PRCP)	P42785 (PRCP)	-0.145	0.610	-4.125	0.0011	0.0483
Q5SSJ5	ENSG00000127483	HP1BP3	Heterochromatin protein 1-binding protein 3 (Protein HP1-BP74)	Q5SSJ5 (HP1BP3)	-0.145	0.742	-3.531	0.0036	0.0715

Table S52: Proteins differentially abundant at Day 2 compared to pre-first vaccination (Study Arm 2 (A/H7N9 + AS03 D1, 29), Dendritic cells). Proteins are sorted by descending absolute log_2 fold change. Protein and gene annotations are based on Ensembl Version 101 (continued).

Protein ID	Ensembl Gene ID	Ensembl Gene Name	Protein Description	50% Protein Cluster ID (Gene Name)	Fold Change (Day 2 vs. Pre- First Vaccination)	Average log_2 Abundance	Moderated t-Statistic	P-Value	FDR Adjusted P-value
P28161	ENSG00000213366	GSTM2	Glutathione S-transferase Mu 2 (EC 2.5.1.18) (GST class-mu 2) (GSTM2-2)	P21266 (GSTM3)	-0.145	0.910	-2.602	0.0216	0.1491
P50851	ENSG00000198589	LRBA	Lipopolysaccharide-responsive and beige-like anchor protein (Beige-like protein) (CDC4-like protein)	P50851 (LRBA)	-0.144	0.833	-2.917	0.0118	0.1214
P51991	ENSG00000170144	HNRNPA3	Heterogeneous nuclear ribonucleoprotein A3 (hnRNP A3)	P51991 (HNRNPA3)	0.144	0.854	2.948	0.0111	0.1188
P17174	ENSG00000120053	GOT1	Aspartate aminotransferase, cytoplasmic (cAspAT) (EC 2.6.1.1) (EC 2.6.1.3) (Cysteine aminotransferase, cytoplasmic) (Cysteine transaminase, cytoplasmic) (cCAT) (Glutamate oxaloacetate transaminase 1) (Transaminase A)	P17174 (GOT1)	-0.144	0.793	-3.021	0.0096	0.1122
P25787	ENSG00000106588	PSMA2	Proteasome subunit alpha type-2 (Macropain subunit C3) (Multicatalytic endopeptidase complex subunit C3) (Proteasome component C3)	P25787 (PSMA2)	-0.143	0.774	-3.983	0.0015	0.0489
P29218	ENSG00000133731	IMPA1	Inositol monophosphatase 1 (IMP 1) (IMPase 1) (EC 3.1.3.25) (D-galactose 1-phosphate phosphatase) (EC 3.1.3.94) (Inositol-1(or 4)-monophosphatase 1) (Lithium-sensitive myo-inositol monophosphatase A1)	O14732 (IMPA2)	-0.143	0.755	-2.749	0.0163	0.136
P35232	ENSG00000167085	PHB	Prohibitin	Q99623 (PHB2)	-0.143	0.780	-2.744	0.0165	0.1364
P55209	ENSG00000187109	NAP1L1	Nucleosome assembly protein 1-like 1 (NAP-1-related protein) (hNRP)	P55209 (NAP1L1)	-0.142	0.697	-3.303	0.0056	0.0841
Q9GZT8	ENSG00000196290	NIF3L1	NIF3-like protein 1 (Amyotrophic lateral sclerosis 2 chromosomal region candidate gene 1 protein)	Q9GZT8 (NIF3L1)	-0.142	0.830	-3.159	0.0074	0.0974
O15144	ENSG00000163466	ARPC2	Actin-related protein 2/3 complex subunit 2 (Arp2/3 complex 34 kDa subunit) (p34-ARC)	O15144 (ARPC2)	-0.142	0.685	-2.811	0.0145	0.1291

Table S52: Proteins differentially abundant at Day 2 compared to pre-first vaccination (Study Arm 2 (A/H7N9 + AS03 D1, 29), Dendritic cells). Proteins are sorted by descending absolute log_2 fold change. Protein and gene annotations are based on Ensembl Version 101 (continued).

Protein ID	Ensembl Gene ID	Ensembl Gene Name	Protein Description	50% Protein Cluster ID (Gene Name)	Fold Change (Day 2 vs. Pre- First Vaccination)	Average log_2 Abundance	Moderated t-Statistic	P-Value	FDR Adjusted P-value
Q7L5N7	ENSG00000087253	LPCAT2	Lysophosphatidylcholine acyltransferase 2 (LPC acyltransferase 2) (LPCAT-2) (LysoPC acyltransferase 2) (EC 2.3.1.23) (1-acylglycerol-3-phosphate O-acyltransferase 11) (1-AGP acyltransferase 11) (1-AGPAT 11) (EC 2.3.1.51) (1-acylglycerophosphocholine O-acyltransferase) (1-alkenylglycerophosphocholine O-acyltransferase) (EC 2.3.1.25) (1-alkylglycerophosphocholine O-acetyltransferase) (EC 2.3.1.67) (Acetyl-CoA:lyso-platelet-activating factor acetyltransferase) (Acetyl-CoA:lyso-PAF acetyltransferase) (Lyso-PAF acetyltransferase) (LysoPAFAT) (Acyltransferase-like 1) (Lysophosphatidic acid acyltransferase alpha) (LPAAT-alpha)	Q7L5N7 (LPCAT2)	0.142	0.715	2.666	0.0191	0.1421
O60256	ENSG00000141127	PRPSAP2	Phosphoribosyl pyrophosphate synthase-associated protein 2 (PRPP synthase-associated protein 2) (41 kDa phosphoribosypyrophosphate synthetase-associated protein) (PAP41)	O60256 (PRPSAP2)	-0.142	0.829	-2.629	0.0205	0.147
O15145	ENSG00000111229	ARPC3	Actin-related protein 2/3 complex subunit 3 (Arp2/3 complex 21 kDa subunit) (p21-ARC)	O15145 (ARPC3)	-0.141	0.678	-2.908	0.012	0.1214
O75323	ENSG00000146729	NIPSNAP2	Protein NipSnap homolog 2 (NipSnap2) (Glioblastoma-amplified sequence)	O75323 (NIPSNAP2)	-0.141	0.720	-3.478	0.004	0.0747
Q9GZR7	ENSG00000089737; ENSG00000273761	DDX24	ATP-dependent RNA helicase DDX24 (EC 3.6.4.13) (DEAD box protein 24)	Q9GZR7 (DDX24)	-0.141	0.877	-2.731	0.0169	0.1372
Q9Y6I4	ENSG00000140455	USP3	Ubiquitin carboxyl-terminal hydrolase 3 (EC 3.4.19.12) (Deubiquitinating enzyme 3) (Ubiquitin thioesterase 3) (Ubiquitin-specific-processing protease 3)	Q9Y6I4 (USP3)	0.140	0.887	3.012	0.0098	0.1127
P12694	ENSG00000248098	BCKDHA	2-oxoisovalerate dehydrogenase subunit alpha, mitochondrial (EC 1.2.4.4) (Branched-chain alpha-keto acid dehydrogenase E1 component alpha chain) (BCKDE1A) (BCKDH E1-alpha)	P12694 (BCKDHA)	-0.140	0.804	-2.764	0.0158	0.1354
P11908	ENSG00000101911	PRPS2	Ribose-phosphate pyrophosphokinase 2 (EC 2.7.6.1) (PPRibP) (Phosphoribosyl pyrophosphate synthase II) (PRS-II)	P60891 (PRPS1)	-0.140	0.870	-2.670	0.019	0.1421

Table S52: Proteins differentially abundant at Day 2 compared to pre-first vaccination (Study Arm 2 (A/H7N9 + AS03 D1, 29), Dendritic cells). Proteins are sorted by descending absolute log_2 fold change. Protein and gene annotations are based on Ensembl Version 101 (continued).

Protein ID	Ensembl Gene ID	Ensembl Gene Name	Protein Description	50% Protein Cluster ID (Gene Name)	Fold Change (Day 2 vs. Pre- First Vaccination)	Average log_2 Abundance	Moderated t-Statistic	P-Value	FDR Adjusted P-value
Q8WWP7	ENSG00000213203	GIMAP1	GTPase IMAP family member 1 (Immunity-associated protein 1) (hIMAP1)	Q8WWP7 (GIMAP1)	-0.140	0.748	-2.650	0.0197	0.1446
O75746	ENSG00000115840	SLC25A12	Calcium-binding mitochondrial carrier protein Aralar1 (Mitochondrial aspartate glutamate carrier 1) (Solute carrier family 25 member 12)	O75746 (SLC25A12)	-0.140	0.762	-2.922	0.0117	0.1213
P41240	ENSG00000103653	CSK	Tyrosine-protein kinase CSK (EC 2.7.10.2) (C-Src kinase) (Protein-tyrosine kinase CYL)	P42679 (MATK)	-0.140	0.696	-2.820	0.0142	0.1291
O00743	ENSG00000119414	PPP6C	Serine/threonine-protein phosphatase 6 catalytic subunit (PP6C) (EC 3.1.3.16) [Cleaved into: Serine/threonine-protein phosphatase 6 catalytic subunit, N-terminally processed]	P67775 (PPP2CA)	-0.140	0.841	-2.921	0.0117	0.1213
Q4G176	ENSG00000176715	ACSF3	Malonate–CoA ligase ACSF3, mitochondrial (EC 6.2.1.n3) (Acyl-CoA synthetase family member 3)	Q4G176 (ACSF3)	-0.139	0.896	-2.743	0.0165	0.1364
P02768	ENSG00000163631	ALB	Albumin	P02768 (ALB)	-0.139	0.704	-3.072	0.0087	0.1047
P40926	ENSG00000146701	MDH2	Malate dehydrogenase, mitochondrial (EC 1.1.1.37)	P40926 (MDH2)	-0.139	0.779	-4.770	0.0003	0.0338
Q5VYS8	ENSG00000083223	TUT7	Terminal uridylyltransferase 7 (TUTase 7) (EC 2.7.7.52) (Zinc finger CCHC domain-containing protein 6)	Q5VYS8 (TUT7)	0.139	0.906	3.950	0.0016	0.0498
O95299	ENSG00000130414	NDUFA10	NADH dehydrogenase [ubiquinone] 1 alpha subcomplex subunit 10, mitochondrial (Complex I-42kD) (CI-42kD) (NADH-ubiquinone oxidoreductase 42 kDa subunit)	O95299 (NDUFA10)	-0.138	0.754	-3.954	0.0016	0.0498
Q96I99	ENSG00000172340	SUCLG2	Succinate–CoA ligase [GDP-forming] subunit beta, mitochondrial (EC 6.2.1.4) (GTP-specific succinyl-CoA synthetase subunit beta) (G-SCS) (GTPSCS) (Succinyl-CoA synthetase beta-G chain) (SCS-betaG)	Q9P2R7 (SUCLA2)	-0.138	0.751	-3.745	0.0024	0.0575

Table S52: Proteins differentially abundant at Day 2 compared to pre-first vaccination (Study Arm 2 (A/H7N9 + AS03 D1, 29), Dendritic cells). Proteins are sorted by descending absolute log_2 fold change. Protein and gene annotations are based on Ensembl Version 101 (continued).

Protein ID	Ensembl Gene ID	Ensembl Gene Name	Protein Description	50% Protein Cluster ID (Gene Name)	Fold Change (Day 2 vs. Pre- First Vaccination)	Average log_2 Abundance	Moderated t-Statistic	P-Value	FDR Adjusted P-value
P46781	ENSG00000170889; ENSG00000278270; ENSG00000278081; ENSG00000274950; ENSG00000274646; ENSG00000277359; ENSG00000274626; ENSG00000274005; ENSG00000275323; ENSG00000277079	RPS9	40S ribosomal protein S9 (Small ribosomal subunit protein uS4)	P46781 (RPS9)	-0.138	0.776	-2.916	0.0118	0.1214
Q86VP6	ENSG00000111530	CAND1	Cullin-associated NEDD8-dissociated protein 1 (Cullin-associated and neddylation-dissociated protein 1) (TBP-interacting protein of 120 kDa A) (TBP-interacting protein 120A) (p120 CAND1)	O75155 (CAND2)	-0.137	0.764	-3.400	0.0046	0.0787
Q13637	ENSG00000118508	RAB32	Ras-related protein Rab-32	Q13637 (RAB32)	0.137	0.868	2.606	0.0214	0.1489
Q8IZ07	ENSG00000076513	ANKRD13A	Ankyrin repeat domain-containing protein 13A (Protein KE03)	Q8IZ07 (ANKRD13A)	0.137	0.915	4.053	0.0013	0.0483
P49591	ENSG00000031698	SARS1	Serine–tRNA ligase, cytoplasmic (EC 6.1.1.11) (Seryl-tRNA synthetase) (SerRS) (Seryl-tRNA(Ser/Sec) synthetase)	P49591 (SARS1)	-0.136	0.725	-3.308	0.0055	0.0841
Q9H4M9	ENSG00000110047	EHD1	EH domain-containing protein 1 (PAST homolog 1) (hPAST1) (Testilin)	Q9H223 (EHD4)	-0.136	0.801	-3.151	0.0075	0.0975
Q8WWM7	ENSG00000168488	ATXN2L	Ataxin-2-like protein (Ataxin-2 domain protein) (Ataxin-2-related protein)	Q8WWM7 (ATXN2L)	0.136	0.883	2.816	0.0143	0.1291
P0DOX5				P0DOX5 (NA)	0.135	1.047	2.682	0.0186	0.1416
P21291	ENSG00000159176	CSRP1	Cysteine and glycine-rich protein 1 (Cysteine-rich protein 1) (CRP) (CRP1) (Epididymis luminal protein 141) (HEL-141)	P21291 (CSRP1)	-0.135	0.813	-4.049	0.0013	0.0483
P51812	ENSG00000177189	RPS6KA3	Ribosomal protein S6 kinase alpha-3 (S6K-alpha-3) (EC 2.7.11.1) (90 kDa ribosomal protein S6 kinase 3) (p90-RSK 3) (p90RSK3) (Insulin-stimulated protein kinase 1) (ISPK-1) (MAP kinase-activated protein kinase 1b) (MAPK-activated protein kinase 1b) (MAPKAP kinase 1b) (MAPKAPK-1b) (Ribosomal S6 kinase 2) (RSK-2) (pp90RSK2)	P51812 (RPS6KA3)	-0.135	0.752	-3.416	0.0045	0.0781

Table S52: Proteins differentially abundant at Day 2 compared to pre-first vaccination (Study Arm 2 (A/H7N9 + AS03 D1, 29), Dendritic cells). Proteins are sorted by descending absolute log_2 fold change. Protein and gene annotations are based on Ensembl Version 101 (continued).

Protein ID	Ensembl Gene ID	Ensembl Gene Name	Protein Description	50% Protein Cluster ID (Gene Name)	Fold Change (Day 2 vs. Pre- First Vaccination)	Average log_2 Abundance	Moderated t-Statistic	P-Value	FDR Adjusted P-value
B0I1T2	ENSG00000136286	MYO1G	Unconventional myosin-Ig [Cleaved into: Minor histocompatibility antigen HA-2 (mHag HA-2)]	B0I1T2 (MYO1G)	-0.134	0.819	-2.716	0.0174	0.1378
Q15019	ENSG00000168385	SEPTIN2	Septin-2 (Neural precursor cell expressed developmentally down-regulated protein 5) (NEDD-5)	Q16181 (SEPTIN7)	-0.134	0.821	-3.620	0.003	0.0639
P19367	ENSG00000156515	HK1	Hexokinase-1 (EC 2.7.1.1) (Brain form hexokinase) (Hexokinase type I) (HK I) (Hexokinase-A)	P52790 (HK3)	-0.134	0.734	-2.820	0.0142	0.1291
P07858	ENSG00000164733; ENSG00000285132	CTSB	Cathepsin B (EC 3.4.22.1) (APP secretase) (APPS) (Cathepsin B1) [Cleaved into: Cathepsin B light chain; Cathepsin B heavy chain]	P07858 (CTSB)	0.134	0.858	3.010	0.0099	0.1127
O15042	ENSG00000163714	U2SURP	U2 snRNP-associated SURP motif-containing protein (140 kDa Ser/Arg-rich domain protein) (U2-associated protein SR140)	O15042 (U2SURP)	0.134	0.924	3.862	0.0019	0.0555
Q92499	ENSG00000079785	DDX1	ATP-dependent RNA helicase DDX1 (EC 3.6.4.13) (DEAD box protein 1) (DEAD box protein retinoblastoma) (DBP-RB)	Q92499 (DDX1)	-0.134	0.810	-3.545	0.0035	0.0704
Q8NCN5	ENSG00000090857	PDPR	Pyruvate dehydrogenase phosphatase regulatory subunit, mitochondrial (PDPr)	Q8NCN5 (PDPR)	-0.134	0.813	-3.087	0.0085	0.1038
Q96BY6	ENSG00000135905	DOCK10	Dedicator of cytokinesis protein 10 (Zizimin-3)	Q96BY6 (DOCK10)	-0.133	0.900	-2.947	0.0111	0.1188
Q9H4G4	ENSG00000122694	GLIPR2	Golgi-associated plant pathogenesis-related protein 1 (GAPR-1) (Golgi-associated PR-1 protein) (Glioma pathogenesis-related protein 2) (GliPR 2)	Q9H4G4 (GLIPR2)	0.133	0.994	3.261	0.0061	0.0872
O60502	ENSG00000198408	OGA	Protein O-GlcNAcase (OGA) (EC 3.2.1.169) (Beta-N-acetylglucosaminidase) (Beta-N-acetylhexosaminidase) (Beta-hexosaminidase) (Meningioma-expressed antigen 5) (N-acetyl-beta-D-glucosaminidase) (N-acetyl-beta-glucosaminidase) (Nuclear cytoplasmic O-GlcNAcase and acetyltransferase) (NCOAT)	O60502 (OGA)	-0.133	0.785	-3.055	0.009	0.1072
Q8TCD1	ENSG00000177576	C18orf32	UPF0729 protein C18orf32 (Putative NF-kappa-B-activating protein 200)	Q8TCD1 (C18orf32)	0.133	1.000	2.664	0.0192	0.1421
Q6NVY1	ENSG00000198130	HIBCH	3-hydroxyisobutyryl-CoA hydrolase, mitochondrial (EC 3.1.2.4) (3-hydroxyisobutyryl-coenzyme A hydrolase) (HIB-CoA hydrolase) (HIBYL-CoA-H)	Q6NVY1 (HIBCH)	-0.132	0.891	-3.112	0.0081	0.1008

Table S52: Proteins differentially abundant at Day 2 compared to pre-first vaccination (Study Arm 2 (A/H7N9 + AS03 D1, 29), Dendritic cells). Proteins are sorted by descending absolute log_2 fold change. Protein and gene annotations are based on Ensembl Version 101 (continued).

Protein ID	Ensembl Gene ID	Ensembl Gene Name	Protein Description	50% Protein Cluster ID (Gene Name)	Fold Change (Day 2 vs. Pre- First Vaccination)	Average log_2 Abundance	Moderated t-Statistic	P-Value	FDR Adjusted P-value
Q13042	ENSG00000130177	CDC16	Cell division cycle protein 16 homolog (Anaphase-promoting complex subunit 6) (APC6) (CDC16 homolog) (CDC16Hs) (Cyclosome subunit 6)	Q13042 (CDC16)	-0.132	0.750	-2.616	0.0211	0.1482
P30084	ENSG00000127884	ECHS1	Enoyl-CoA hydratase, mitochondrial (EC 4.2.1.17) (Enoyl-CoA hydratase 1) (Short-chain enoyl-CoA hydratase) (SCEH)	P30084 (ECHS1)	-0.132	0.784	-3.162	0.0073	0.0971
Q9BQS8	ENSG00000163820	FYCO1	FYVE and coiled-coil domain-containing protein 1 (Zinc finger FYVE domain-containing protein 7)	Q9BQS8 (FYCO1)	0.132	0.984	2.862	0.0131	0.1253
Q9H3H3	ENSG00000175573	C11orf68	UPF0696 protein C11orf68 (Basophilic leukemia-expressed protein Bles03) (Protein p5326)	Q9H3H3 (C11orf68)	-0.131	0.773	-2.587	0.0223	0.15
Q13043	ENSG00000101109	STK4	Serine/threonine-protein kinase 4 (EC 2.7.11.1) (Mammalian STE20-like protein kinase 1) (MST-1) (STE20-like kinase MST1) (Serine/threonine-protein kinase Krs-2) [Cleaved into: Serine/threonine-protein kinase 4 37kDa subunit (MST1/N); Serine/threonine-protein kinase 4 18kDa subunit (MST1/C)]	Q13188 (STK3)	-0.131	0.752	-3.687	0.0027	0.0585
Q6UXN9	ENSG00000164091	WDR82	WD repeat-containing protein 82 (Protein TMEM113) (Swd2)	Q6UXN9 (WDR82)	-0.131	0.768	-3.064	0.0089	0.106
P27694	ENSG00000132383	RPA1	Replication protein A 70 kDa DNA-binding subunit (RP-A p70) (Replication factor A protein 1) (RF-A protein 1) (Single-stranded DNA-binding protein) [Cleaved into: Replication protein A 70 kDa DNA-binding subunit, N-terminally processed]	P27694 (RPA1)	-0.129	0.783	-2.971	0.0106	0.1177
P00367	ENSG00000148672	GLUD1	Glutamate dehydrogenase 1, mitochondrial (GDH 1) (EC 1.4.1.3)	P00367 (GLUD1)	-0.129	0.758	-3.376	0.0048	0.0795
Q9H2U2	ENSG00000138777	PPA2	Inorganic pyrophosphatase 2, mitochondrial (EC 3.6.1.1) (Pyrophosphatase SID6-306) (Pyrophosphate phospho-hydrolase 2) (PPase 2)	Q9H2U2 (PPA2)	-0.129	0.752	-3.522	0.0036	0.0717
P05120	ENSG00000197632	SERPINB2	Plasminogen activator inhibitor 2 (PAI-2) (Monocyte Arg-serpin) (Placental plasminogen activator inhibitor) (Serpin B2) (Urokinase inhibitor)	P05120 (SERPINB2)	0.129	0.865	3.082	0.0086	0.1038

Table S52: Proteins differentially abundant at Day 2 compared to pre-first vaccination (Study Arm 2 (A/H7N9 + AS03 D1, 29), Dendritic cells). Proteins are sorted by descending absolute log_2 fold change. Protein and gene annotations are based on Ensembl Version 101 (continued).

Protein ID	Ensembl Gene ID	Ensembl Gene Name	Protein Description	50% Protein Cluster ID (Gene Name)	Fold Change (Day 2 vs. Pre- First Vaccination)	Average log_2 Abundance	Moderated t-Statistic	P-Value	FDR Adjusted P-value
P10515	ENSG00000150768	DLAT	Dihydrolipoyllysine-residue acetyltransferase component of pyruvate dehydrogenase complex, mitochondrial (EC 2.3.1.12) (70 kDa mitochondrial autoantigen of primary biliary cirrhosis) (PBC) (Dihydrolipoamide acetyltransferase component of pyruvate dehydrogenase complex) (M2 antigen complex 70 kDa subunit) (Pyruvate dehydrogenase complex component E2) (PDC-E2) (PDCE2)	P10515 (DLAT)	-0.129	0.859	-2.909	0.012	0.1214
P48047	ENSG00000241837	ATP5PO	ATP synthase subunit O, mitochondrial (ATP synthase peripheral stalk subunit OSCP) (Oligomycin sensitivity conferral protein) (OSCP)	P48047 (ATP5PO)	-0.129	0.755	-2.849	0.0135	0.1278
P10155	ENSG00000116747	RO60	60 kDa SS-A/Ro ribonucleoprotein (60 kDa Ro protein) (60 kDa ribonucleoprotein Ro) (Ro60) (RoRNP) (Ro 60 kDa autoantigen) (Ro60 autoantigen) (Sjoegren syndrome antigen A2) (Sjoegren syndrome type A antigen) (SS-A) (TROVE domain family member 2)	P10155 (RO60)	-0.129	0.775	-3.076	0.0087	0.1043
Q8NBM8	ENSG00000145882	PCYOX1L	Prenylcysteine oxidase-like (EC 1.8.3.-)	Q8NBM8 (PCYOX1L)	0.129	0.637	2.626	0.0207	0.147
Q15102	ENSG00000079462	PAFAH1B3	Platelet-activating factor acetylhydrolase IB subunit alpha1 (EC 3.1.1.47) (PAF acetylhydrolase 29 kDa subunit) (PAF-AH 29 kDa subunit) (PAF-AH subunit gamma) (PAFAH subunit gamma)	Q15102 (PAFAH1B3)	-0.129	0.842	-2.914	0.0119	0.1214
O00442	ENSG00000137996	RTCA	RNA 3'-terminal phosphate cyclase (RNA cyclase) (RNA-3'-phosphate cyclase) (EC 6.5.1.4) (RNA terminal phosphate cyclase domain-containing protein 1) (RTC domain-containing protein 1)	O00442 (RTCA)	-0.127	0.816	-2.626	0.0206	0.147
Q15758	ENSG00000105281	SLC1A5	Neutral amino acid transporter B(0) (ATB(0)) (Baboon M7 virus receptor) (RD114/simian type D retrovirus receptor) (Sodium-dependent neutral amino acid transporter type 2) (Solute carrier family 1 member 5)	Q15758 (SLC1A5)	0.127	0.869	2.700	0.0179	0.1397
Q9Y286	ENSG00000168995	SIGLEC7	Sialic acid-binding Ig-like lectin 7 (Siglec-7) (Adhesion inhibitory receptor molecule 1) (AIRM-1) (CDw328) (D-siglec) (QA79 membrane protein) (p75) (CD antigen CD328)	Q9Y286 (SIGLEC7)	0.127	0.918	2.669	0.019	0.1421
Q9C0E8	ENSG00000144320	LNPK	Endoplasmic reticulum junction formation protein lunapark (ER junction formation factor lunapark)	Q9C0E8 (LNPK)	0.126	0.852	3.110	0.0081	0.1008

Table S52: Proteins differentially abundant at Day 2 compared to pre-first vaccination (Study Arm 2 (A/H7N9 + AS03 D1, 29), Dendritic cells). Proteins are sorted by descending absolute log_2 fold change. Protein and gene annotations are based on Ensembl Version 101 (continued).

Protein ID	Ensembl Gene ID	Ensembl Gene Name	Protein Description	50% Protein Cluster ID (Gene Name)	Fold Change (Day 2 vs. Pre- First Vaccination)	Average log_2 Abundance	Moderated t-Statistic	P-Value	FDR Adjusted P-value
Q8NBU5	ENSG00000138138; ENSG00000283024	ATAD1	Outer mitochondrial transmembrane helix translocase (EC 7.4.2.-) (ATPase family AAA domain-containing protein 1) (hATAD1) (Thorase)	Q8NBU5 (ATAD1)	-0.126	0.720	-2.861	0.0131	0.1253
P49590	ENSG00000112855	HARS2	Histidine–tRNA ligase, mitochondrial (EC 6.1.1.21) (Histidine–tRNA ligase-like) (Histidyl-tRNA synthetase) (HisRS)	P12081 (HARS1)	-0.126	0.810	-2.598	0.0218	0.1491
P48651	ENSG00000156471	PTDSS1	Phosphatidylserine synthase 1 (PSS-1) (PtdSer synthase 1) (EC 2.7.8.29) (Serine-exchange enzyme I)	P48651 (PTDSS1)	0.126	1.021	3.128	0.0078	0.0986
Q9P2R7	ENSG00000136143	SUCLA2	Succinate–CoA ligase [ADP-forming] subunit beta, mitochondrial (EC 6.2.1.5) (ATP-specific succinyl-CoA synthetase subunit beta) (A-SCS) (Succinyl-CoA synthetase beta-A chain) (SCS-betaA)	Q9P2R7 (SUCLA2)	-0.126	0.810	-3.348	0.0051	0.08
P60981	ENSG00000125868	DSTN	Destrin (Actin-depolymerizing factor) (ADF)	Q9Y281 (CFL2)	-0.126	0.716	-2.618	0.021	0.1478
Q9UIJ7	ENSG00000147853	AK3	GTP:AMP phosphotransferase AK3, mitochondrial (EC 2.7.4.10) (Adenylate kinase 3) (AK 3) (Adenylate kinase 3 alpha-like 1)	Q9UIJ7 (AK3)	-0.125	0.795	-2.727	0.017	0.1372
Q9UHG3	ENSG00000116005	PCYOX1	Prenylcysteine oxidase 1 (EC 1.8.3.5) (Prenylcysteine lyase)	Q9UHG3 (PCYOX1)	-0.125	0.729	-2.773	0.0156	0.1348
Q6VY07	ENSG00000175115	PACS1	Phosphofurin acidic cluster sorting protein 1 (PACS-1)	Q6VY07 (PACS1)	-0.125	0.782	-2.728	0.017	0.1372
Q96BP3	ENSG00000113593	PPWD1	Peptidylprolyl isomerase domain and WD repeat-containing protein 1 (EC 5.2.1.8) (Spliceosome-associated cyclophilin)	Q96BP3 (PPWD1)	-0.125	0.833	-2.945	0.0112	0.1189
Q9BSQ5	ENSG00000136280	CCM2	Cerebral cavernous malformations 2 protein (Malcavernin)	Q9BSQ5 (CCM2)	-0.125	0.760	-3.141	0.0076	0.098
Q32P44	ENSG00000149499	EML3	Echinoderm microtubule-associated protein-like 3 (EMAP-3)	Q32P44 (EML3)	-0.125	0.808	-3.744	0.0024	0.0575
P05771	ENSG00000166501	PRKCB	Protein kinase C beta type (PKC-B) (PKC-beta) (EC 2.7.11.13)	P17252 (PRKCA)	-0.125	0.794	-3.477	0.004	0.0747
P62081	ENSG00000171863	RPS7	40S ribosomal protein S7 (Small ribosomal subunit protein eS7)	P62081 (RPS7)	-0.125	0.786	-3.310	0.0055	0.0841
Q8TDJ6	ENSG00000104093	DMXL2	DmX-like protein 2 (Rabconnectin-3)	Q8TDJ6 (DMXL2)	0.124	0.865	2.680	0.0186	0.142

Table S52: Proteins differentially abundant at Day 2 compared to pre-first vaccination (Study Arm 2 (A/H7N9 + AS03 D1, 29), Dendritic cells). Proteins are sorted by descending absolute log_2 fold change. Protein and gene annotations are based on Ensembl Version 101 (continued).

Protein ID	Ensembl Gene ID	Ensembl Gene Name	Protein Description	50% Protein Cluster ID (Gene Name)	Fold Change (Day 2 vs. Pre- First Vaccination)	Average log_2 Abundance	Moderated t-Statistic	P-Value	FDR Adjusted P-value
Q96KP4	ENSG00000133313	CNDP2	Cytosolic non-specific dipeptidase (EC 3.4.13.18) (CNDP dipeptidase 2) (Carnosine dipeptidase II) (Epididymis secretory protein Li 13) (Glutamate carboxypeptidase-like protein 1) (Peptidase A)	Q96KP4 (CNDP2)	-0.124	0.707	-2.800	0.0148	0.1312
P48506	ENSG00000001084	GCLC	Glutamate–cysteine ligase catalytic subunit (EC 6.3.2.2) (GCS heavy chain) (Gamma-ECS) (Gamma-glutamylcysteine synthetase)	P48506 (GCLC)	-0.124	0.665	-3.999	0.0015	0.0483
A3KMH1	ENSG00000102763	VWA8	von Willebrand factor A domain-containing protein 8 (PEX7-binding protein 2) (P7BP2)	A3KMH1 (VWA8)	-0.124	0.923	-3.319	0.0054	0.0837
Q9Y3I0	ENSG00000100220	RTCB	RNA-splicing ligase RtcB homolog (EC 6.5.1.8) (3'-phosphate/5'-hydroxy nucleic acid ligase)	Q9Y3I0 (RTCB)	-0.124	0.803	-3.202	0.0068	0.0924
Q8TCD5	ENSG00000125458	NT5C	5'(3')-deoxyribonucleotidase, cytosolic type (EC 3.1.3.-) (Cytosolic 5',3'-pyrimidine nucleotidase) (Deoxy-5'-nucleotidase 1) (dNT-1)	Q8TCD5 (NT5C)	-0.124	0.612	-2.898	0.0122	0.122
P32189	ENSG00000198814	GK	Glycerol kinase (GK) (Glycerokinase) (EC 2.7.1.30) (ATP:glycerol 3-phosphotransferase)	P32189 (GK)	0.123	0.940	3.667	0.0028	0.0597
P78371	ENSG00000166226	CCT2	T-complex protein 1 subunit beta (TCP-1-beta) (CCT-beta)	P78371 (CCT2)	-0.123	0.796	-3.303	0.0056	0.0841
Q92888	ENSG00000076928	ARHGEF1	Rho guanine nucleotide exchange factor 1 (115 kDa guanine nucleotide exchange factor) (p115-RhoGEF) (p115RhoGEF) (Sub1.5)	Q92888 (ARHGEF1)	-0.123	0.764	-2.904	0.0121	0.122
P49961	ENSG00000138185	ENTPD1	Ectonucleoside triphosphate diphosphohydrolase 1 (NTPDase 1) (EC 3.6.1.5) (Ecto-ATP diphosphohydrolase 1) (Ecto-ATPDase 1) (Ecto-ATPase 1) (Ecto-apyrase) (Lymphoid cell activation antigen) (CD antigen CD39)	P49961 (ENTPD1)	0.123	0.883	2.717	0.0174	0.1378
P50914	ENSG00000188846	RPL14	60S ribosomal protein L14 (CAG-ISL 7) (Large ribosomal subunit protein eL14)	P50914 (RPL14)	-0.122	0.739	-3.352	0.0051	0.0799
Q13561	ENSG00000175203	DCTN2	Dynactin subunit 2 (50 kDa dynein-associated polypeptide) (Dynactin complex 50 kDa subunit) (DCTN-50) (p50 dynamitin)	Q13561 (DCTN2)	0.122	0.858	2.664	0.0192	0.1421
P25705	ENSG00000152234	ATP5F1A	ATP synthase subunit alpha, mitochondrial (ATP synthase F1 subunit alpha)	P25705 (ATP5F1A)	-0.122	0.751	-2.844	0.0136	0.1279
P12081	ENSG00000170445	HARS1	Histidine–tRNA ligase, cytoplasmic (EC 6.1.1.21) (Histidyl-tRNA synthetase) (HisRS)	P12081 (HARS1)	-0.122	0.783	-3.986	0.0015	0.0489

Table S52: Proteins differentially abundant at Day 2 compared to pre-first vaccination (Study Arm 2 (A/H7N9 + AS03 D1, 29), Dendritic cells). Proteins are sorted by descending absolute log_2 fold change. Protein and gene annotations are based on Ensembl Version 101 (continued).

Protein ID	Ensembl Gene ID	Ensembl Gene Name	Protein Description	50% Protein Cluster ID (Gene Name)	Fold Change (Day 2 vs. Pre- First Vaccination)	Average log_2 Abundance	Moderated t-Statistic	P-Value	FDR Adjusted P-value
P27986	ENSG00000145675	PIK3R1	Phosphatidylinositol 3-kinase regulatory subunit alpha (PI3-kinase regulatory subunit alpha) (PI3K regulatory subunit alpha) (PtdIns-3-kinase regulatory subunit alpha) (Phosphatidylinositol 3-kinase 85 kDa regulatory subunit alpha) (PI3-kinase subunit p85-alpha) (PtdIns-3-kinase regulatory subunit p85-alpha)	O00459 (PIK3R2)	-0.122	0.836	-3.290	0.0057	0.0854
Q16836	ENSG00000138796	HADH	Hydroxyacyl-coenzyme A dehydrogenase, mitochondrial (HCDH) (EC 1.1.1.35) (Medium and short-chain L-3-hydroxyacyl-coenzyme A dehydrogenase) (Short-chain 3-hydroxyacyl-CoA dehydrogenase)	Q16836 (HADH)	-0.122	0.802	-2.871	0.0129	0.125
Q9Y4K1		CRYBG1	Beta/gamma crystallin domain-containing protein 1 (Absent in melanoma 1 protein)	Q9Y4K1 (CRYBG1)	0.122	0.912	2.703	0.0178	0.1393
O95834	ENSG00000125746	EML2	Echinoderm microtubule-associated protein-like 2 (EMAP-2) (HuEMAP-2)	Q9HC35 (EML4)	-0.121	0.749	-2.780	0.0154	0.1335
Q7LDG7	ENSG00000068831	RASGRP2	RAS guanyl-releasing protein 2 (Calcium and DAG-regulated guanine nucleotide exchange factor I) (CalDAG-GEFI) (Cdc25-like protein) (hCDC25L) (F25B3.3 kinase-like protein)	Q7LDG7 (RASGRP2)	-0.121	0.754	-3.001	0.01	0.1131
Q9ULA0	ENSG00000123992	DNPEP	Aspartyl aminopeptidase (EC 3.4.11.21)	Q9ULA0 (DNPEP)	-0.121	0.878	-2.872	0.0129	0.125
P55039	ENSG00000108591	DRG2	Developmentally-regulated GTP-binding protein 2 (DRG-2) (Translation factor GTPase DRG2) (TRAFAC GTPase DRG2) (EC 3.6.5.-)	Q9Y295 (DRG1)	-0.121	0.833	-2.683	0.0185	0.1416
P05166	ENSG00000114054	PCCB	Propionyl-CoA carboxylase beta chain, mitochondrial (PCCase subunit beta) (EC 6.4.1.3) (Propanoyl-CoA:carbon dioxide ligase subunit beta)	P05166 (PCCB)	-0.120	0.754	-3.531	0.0036	0.0715
P40227	ENSG00000146731	CCT6A	T-complex protein 1 subunit zeta (TCP-1-zeta) (Acute morphine dependence-related protein 2) (CCT-zeta-1) (HTR3) (Tcp20)	P40227 (CCT6A)	-0.120	0.744	-2.688	0.0183	0.1413
P18754	ENSG00000180198	RCC1	Regulator of chromosome condensation (Cell cycle regulatory protein) (Chromosome condensation protein 1)	P18754 (RCC1)	-0.120	0.733	-2.670	0.019	0.1421
Q15438	ENSG00000108669	CYTH1	Cytohesin-1 (PH, SEC7 and coiled-coil domain-containing protein 1) (SEC7 homolog B2-1)	Q99418 (CYTH2)	-0.120	0.822	-3.362	0.005	0.0799

Table S52: Proteins differentially abundant at Day 2 compared to pre-first vaccination (Study Arm 2 (A/H7N9 + AS03 D1, 29), Dendritic cells). Proteins are sorted by descending absolute log_2 fold change. Protein and gene annotations are based on Ensembl Version 101 (continued).

Protein ID	Ensembl Gene ID	Ensembl Gene Name	Protein Description	50% Protein Cluster ID (Gene Name)	Fold Change (Day 2 vs. Pre- First Vaccination)	Average log_2 Abundance	Moderated t-Statistic	P-Value	FDR Adjusted P-value
P31939	ENSG00000138363	ATIC	Bifunctional purine biosynthesis protein ATIC (AICAR transformylase/inosine monophosphate cyclohydrolase) (ATIC) [Cleaved into: Bifunctional purine biosynthesis protein ATIC, N-terminally processed] [Includes: Phosphoribosylaminoimidazolecarboxamide formyltransferase (EC 2.1.2.3) (5-aminoimidazole-4-carboxamide ribonucleotide formyltransferase) (AICAR formyltransferase) (AICAR transformylase); Inosine 5'-monophosphate cyclohydrolase (IMP cyclohydrolase) (EC 3.5.4.10) (IMP synthase) (Inosinicase)]	P31939 (ATIC)	-0.120	0.824	-2.915	0.0118	0.1214
P46379	ENSG00000204463; ENSG00000096155; ENSG00000229524; ENSG00000233348; ENSG00000227761; ENSG00000228760; ENSG00000234651	BAG6	Large proline-rich protein BAG6 (BAG family molecular chaperone regulator 6) (BCL2-associated athanogene 6) (BAG-6) (HLA-B-associated transcript 3) (Protein G3) (Protein Scythe)	P46379 (BAG6)	0.119	0.909	2.713	0.0175	0.1382
P48643	ENSG00000150753	CCT5	T-complex protein 1 subunit epsilon (TCP-1-epsilon) (CCT-epsilon)	P48643 (CCT5)	-0.119	0.804	-3.177	0.0071	0.0953
Q96P16	ENSG00000141425	RPRD1A	Regulation of nuclear pre-mRNA domain-containing protein 1A (Cyclin-dependent kinase inhibitor 2B-related protein) (p15INK4B-related protein)	Q9NQG5 (RPRD1B)	-0.119	0.944	-2.665	0.0192	0.1421
Q9NTX5	ENSG00000093144	ECHDC1	Ethylmalonyl-CoA decarboxylase (EC 4.1.1.94) (Enoyl-CoA hydratase domain-containing protein 1) (Methylmalonyl-CoA decarboxylase) (MMCD)	Q9NTX5 (ECHDC1)	-0.118	0.782	-2.748	0.0163	0.136
O94826	ENSG00000154174	TOMM70	Mitochondrial import receptor subunit TOM70 (Mitochondrial precursor proteins import receptor) (Translocase of outer membrane 70 kDa subunit) (Translocase of outer mitochondrial membrane protein 70)	O94826 (TOMM70)	-0.118	0.872	-2.843	0.0136	0.1279

Table S52: Proteins differentially abundant at Day 2 compared to pre-first vaccination (Study Arm 2 (A/H7N9 + AS03 D1, 29), Dendritic cells). Proteins are sorted by descending absolute log_2 fold change. Protein and gene annotations are based on Ensembl Version 101 (continued).

Protein ID	Ensembl Gene ID	Ensembl Gene Name	Protein Description	50% Protein Cluster ID (Gene Name)	Fold Change (Day 2 vs. Pre- First Vaccination)	Average log_2 Abundance	Moderated t-Statistic	P-Value	FDR Adjusted P-value
O00329	ENSG00000171608	PIK3CD	Phosphatidylinositol 4,5-bisphosphate 3-kinase catalytic subunit delta isoform (PI3-kinase subunit delta) (PI3K-delta) (PI3Kdelta) (PtdIns-3-kinase subunit delta) (EC 2.7.1.137) (EC 2.7.1.153) (Phosphatidylinositol 4,5-bisphosphate 3-kinase 110 kDa catalytic subunit delta) (PtdIns-3-kinase subunit p110-delta) (p110delta)	P42338 (PIK3CB)	-0.118	0.842	-2.967	0.0107	0.1177
Q5JRA6	ENSG00000154305	MIA3	Transport and Golgi organization protein 1 homolog (TANGO1) (C219-reactive peptide) (D320) (Melanoma inhibitory activity protein 3)	Q5JRA6 (MIA3)	0.118	0.946	2.890	0.0124	0.1232
O43684	ENSG00000154473	BUB3	Mitotic checkpoint protein BUB3	O43684 (BUB3)	-0.118	0.778	-2.791	0.015	0.1316
P15311	ENSG00000092820	EZR	Ezrin (Cytovillin) (Villin-2) (p81)	P15311 (EZR)	-0.117	0.705	-3.171	0.0072	0.0959
Q8N163	ENSG00000158941	CCAR2	Cell cycle and apoptosis regulator protein 2 (Cell division cycle and apoptosis regulator protein 2) (DBIRD complex subunit KIAA1967) (Deleted in breast cancer gene 1 protein) (DBC-1) (DBC.1) (NET35) (p30 DBC)	Q8N163 (CCAR2)	-0.117	0.794	-2.845	0.0136	0.1279
Q9Y3L3	ENSG00000100092	SH3BP1	SH3 domain-binding protein 1	Q9Y3L3 (SH3BP1)	-0.117	0.825	-3.222	0.0065	0.0914
Q9Y394	ENSG00000100612	DHRS7	Dehydrogenase/reductase SDR family member 7 (EC 1.1.-.-) (Retinal short-chain dehydrogenase/reductase 4) (retSDR4) (Short chain dehydrogenase/reductase family 34C member 1)	Q9Y394 (DHRS7)	-0.116	0.754	-2.980	0.0104	0.1172
Q14980	ENSG00000137497	NUMA1	Nuclear mitotic apparatus protein 1 (Nuclear matrix protein-22) (NMP-22) (Nuclear mitotic apparatus protein) (NuMA protein) (SP-H antigen)	Q14980 (NUMA1)	0.115	0.986	3.009	0.0099	0.1127
Q92466	ENSG00000134574	DDB2	DNA damage-binding protein 2 (DDB p48 subunit) (DDBb) (Damage-specific DNA-binding protein 2) (UV-damaged DNA-binding protein 2) (UV-DDB 2)	Q92466 (DDB2)	-0.115	0.830	-3.015	0.0098	0.1127

Table S52: Proteins differentially abundant at Day 2 compared to pre-first vaccination (Study Arm 2 (A/H7N9 + AS03 D1, 29), Dendritic cells). Proteins are sorted by descending absolute log_2 fold change. Protein and gene annotations are based on Ensembl Version 101 (continued).

Protein ID	Ensembl Gene ID	Ensembl Gene Name	Protein Description	50% Protein Cluster ID (Gene Name)	Fold Change (Day 2 vs. Pre- First Vaccination)	Average log_2 Abundance	Moderated t-Statistic	P-Value	FDR Adjusted P-value
Q8TC12	ENSG00000072042	RDH11	Retinol dehydrogenase 11 (EC 1.1.1.300) (Androgen-regulated short-chain dehydrogenase/reductase 1) (HCV core-binding protein HCBP12) (Prostate short-chain dehydrogenase/reductase 1) (Retinal reductase 1) (RalR1) (Short chain dehydrogenase/reductase family 7C member 1)	Q8TC12 (RDH11)	-0.115	0.834	-2.641	0.0201	0.1455
Q9NWZ3	ENSG00000198001	IRAK4	Interleukin-1 receptor-associated kinase 4 (IRAK-4) (EC 2.7.11.1) (Renal carcinoma antigen NY-REN-64)	Q9NWZ3 (IRAK4)	-0.115	0.817	-3.316	0.0054	0.0837
Q9BSJ8	ENSG00000139641	ESYT1	Extended synaptotagmin-1 (E-Syt1) (Membrane-bound C2 domain-containing protein)	Q9BSJ8 (ESYT1)	-0.114	0.773	-3.324	0.0054	0.0833
Q9H4E7	ENSG00000023892	DEF6	Differentially expressed in FDCP 6 homolog (DEF-6) (IRF4-binding protein)	Q9H4E7 (DEF6)	-0.114	0.850	-2.766	0.0158	0.1354
Q96F07	ENSG00000055163	CYFIP2	Cytoplasmic FMR1-interacting protein 2 (p53-inducible protein 121)	Q96F07 (CY-FIP2)	-0.113	0.752	-2.840	0.0137	0.1279
O75306	ENSG00000158864	NDUFS2	NADH dehydrogenase [ubiquinone] iron-sulfur protein 2, mitochondrial (EC 7.1.1.2) (Complex I-49kD) (CI-49kD) (NADH-ubiquinone oxidoreductase 49 kDa subunit)	O75306 (NDUFS2)	-0.113	0.710	-3.050	0.0091	0.1078
Q8TDX7	ENSG00000151414	NEK7	Serine/threonine-protein kinase Nek7 (EC 2.7.11.1) (Never in mitosis A-related kinase 7) (NimA-related protein kinase 7)	Q9HC98 (NEK6)	-0.113	0.323	-2.648	0.0198	0.1446
Q15154	ENSG00000078674	PCM1	Pericentriolar material 1 protein (PCM-1) (hPCM-1)	Q15154 (PCM1)	0.113	0.909	2.595	0.0219	0.1491
Q8TCS8	ENSG00000138035	PNPT1	Polyribonucleotide nucleotidyltransferase 1, mitochondrial (EC 2.7.7.8) (3'-5' RNA exonuclease OLD35) (PNPase old-35) (Polynucleotide phosphorylase 1) (PNPase 1) (Polynucleotide phosphorylase-like protein)	Q8TCS8 (PNPT1)	-0.112	0.806	-3.374	0.0049	0.0795
O75390	ENSG00000062485	CS	Citrate synthase, mitochondrial (EC 2.3.3.1) (Citrate (Si)-synthase)	O75390 (CS)	-0.112	0.737	-2.831	0.0139	0.1291
O60216	ENSG00000164754	RAD21	Double-strand-break repair protein rad21 homolog (hHR21) (Nuclear matrix protein 1) (NXP-1) (SCC1 homolog) [Cleaved into: 64-kDa C-terminal product (64-kDa carboxy-terminal product) (65-kDa carboxy-terminal product)]	O60216 (RAD21)	0.111	0.868	2.951	0.011	0.1188

Table S52: Proteins differentially abundant at Day 2 compared to pre-first vaccination (Study Arm 2 (A/H7N9 + AS03 D1, 29), Dendritic cells). Proteins are sorted by descending absolute log_2 fold change. Protein and gene annotations are based on Ensembl Version 101 (continued).

Protein ID	Ensembl Gene ID	Ensembl Gene Name	Protein Description	50% Protein Cluster ID (Gene Name)	Fold Change (Day 2 vs. Pre- First Vaccination)	Average log_2 Abundance	Moderated t-Statistic	P-Value	FDR Adjusted P-value
Q9HB07	ENSG00000139637	MYG1	MYG1 exonuclease (EC 3.1.-.-)	Q9HB07 (MYG1)	-0.111	0.809	-2.588	0.0222	0.15
Q9NR46	ENSG00000148341	SH3GLB2	Endophilin-B2 (SH3 domain-containing GRB2-like protein B2)	Q9NR46 (SH3GLB2)	-0.111	0.822	-2.706	0.0177	0.1393
Q9Y570	ENSG00000214517	PPME1	Protein phosphatase methylesterase 1 (PME-1) (EC 3.1.1.89)	Q9Y570 (PPME1)	-0.110	0.505	-2.765	0.0158	0.1354
Q9NSE4	ENSG00000067704	IARS2	Isoleucine–tRNA ligase, mitochondrial (EC 6.1.1.5) (Isoleucyl-tRNA synthetase) (IleRS)	Q9NSE4 (IARS2)	-0.110	0.826	-2.976	0.0105	0.1177
Q96A35	ENSG00000143314	MRPL24	39S ribosomal protein L24, mitochondrial (L24mt) (MRP-L24) (Mitochondrial large ribosomal subunit protein uL24m)	Q96A35 (MRPL24)	-0.110	0.855	-3.334	0.0052	0.0819
Q96G03	ENSG00000169299	PGM2	Phosphoglucomutase-2 (PGM 2) (EC 5.4.2.2) (Glucose phosphomutase 2) (Phosphodeoxyribomutase) (Phosphopentomutase) (EC 5.4.2.7)	Q6PCE3 (PGM2L1)	-0.110	0.810	-2.937	0.0114	0.1196
Q9H0A0	ENSG00000135372	NAT10	RNA cytidine acetyltransferase (EC 2.3.1.-) (18S rRNA cytosine acetyltransferase) (N-acetyltransferase 10) (N-acetyltransferase-like protein) (hALP)	Q9H0A0 (NAT10)	-0.109	0.833	-2.607	0.0214	0.1489
P16219	ENSG00000122971	ACADS	Short-chain specific acyl-CoA dehydrogenase, mitochondrial (SCAD) (EC 1.3.8.1) (Butyryl-CoA dehydrogenase)	P16219 (ACADS)	-0.109	0.997	-2.661	0.0193	0.1424
Q92552	ENSG00000113048	MRPS27	28S ribosomal protein S27, mitochondrial (MRP-S27) (S27mt) (Mitochondrial ribosomal protein S27) (Mitochondrial small ribosomal subunit protein mS27)	Q92552 (MRPS27)	-0.109	0.798	-2.664	0.0192	0.1421
P49368	ENSG00000163468	CCT3	T-complex protein 1 subunit gamma (TCP-1-gamma) (CCT-gamma) (hTRiC5)	P49368 (CCT3)	-0.109	0.787	-2.605	0.0215	0.1491
O60313	ENSG00000198836	OPA1	Dynamin-like 120 kDa protein, mitochondrial (EC 3.6.5.5) (Optic atrophy protein 1) [Cleaved into: Dynamin-like 120 kDa protein, form S1]	O60313 (OPA1)	-0.108	0.793	-2.657	0.0195	0.1431
P50991	ENSG00000115484	CCT4	T-complex protein 1 subunit delta (TCP-1-delta) (CCT-delta) (Stimulator of TAR RNA-binding)	P50991 (CCT4)	-0.108	0.837	-3.059	0.009	0.1067
P14866	ENSG00000104824; ENSG00000282947	HNRNPL	Heterogeneous nuclear ribonucleoprotein L (hnRNP L)	P14866 (HNRNPL)	0.107	0.860	2.743	0.0165	0.1364

Table S52: Proteins differentially abundant at Day 2 compared to pre-first vaccination (Study Arm 2 (A/H7N9 + AS03 D1, 29), Dendritic cells). Proteins are sorted by descending absolute log_2 fold change. Protein and gene annotations are based on Ensembl Version 101 (continued).

Protein ID	Ensembl Gene ID	Ensembl Gene Name	Protein Description	50% Protein Cluster ID (Gene Name)	Fold Change (Day 2 vs. Pre- First Vaccination)	Average log_2 Abundance	Moderated t-Statistic	P-Value	FDR Adjusted P-value
Q13951	ENSG00000067955	CBFB	Core-binding factor subunit beta (CBF-beta) (Polyomavirus enhancer-binding protein 2 beta subunit) (PEA2-beta) (PEBP2-beta) (SL3-3 enhancer factor 1 subunit beta) (SL3/AKV core-binding factor beta subunit)	Q13951 (CBFB)	-0.107	0.775	-3.624	0.003	0.0639
P60953	ENSG00000070831	CDC42	Cell division control protein 42 homolog (EC 3.6.5.2) (G25K GTP-binding protein)	P62745 (RHOB)	-0.107	0.773	-2.845	0.0136	0.1279
Q8TBC4	ENSG00000144744	UBA3	NEDD8-activating enzyme E1 catalytic subunit (EC 6.2.1.64) (NEDD8-activating enzyme E1C) (Ubiquitin-activating enzyme E1C) (Ubiquitin-like modifier-activating enzyme 3) (Ubiquitin-activating enzyme 3)	Q8TBC4 (UBA3)	-0.106	0.809	-2.750	0.0163	0.136
Q8N0W3	ENSG00000157353	FCSK	L-fucose kinase (Fucokinase) (EC 2.7.1.52)	Q8N0W3 (FCSK)	-0.106	0.675	-2.814	0.0144	0.1291
P28482	ENSG00000100030	MAPK1	Mitogen-activated protein kinase 1 (MAP kinase 1) (MAPK 1) (EC 2.7.11.24) (ERT1) (Extracellular signal-regulated kinase 2) (ERK-2) (MAP kinase isoform p42) (p42-MAPK) (Mitogen-activated protein kinase 2) (MAP kinase 2) (MAPK 2)	P27361 (MAPK3)	-0.106	0.700	-2.770	0.0157	0.1348
Q15283	ENSG00000155903	RASA2	Ras GTPase-activating protein 2 (GTPase-activating protein 1m) (GAP1m)	Q15283 (RASA2)	-0.105	0.834	-2.694	0.0181	0.1409
P52948	ENSG00000110713	NUP98	Nuclear pore complex protein Nup98-Nup96 (EC 3.4.21.-) [Cleaved into: Nuclear pore complex protein Nup98 (98 kDa nucleoporin) (Nucleoporin Nup98) (Nup98); Nuclear pore complex protein Nup96 (96 kDa nucleoporin) (Nucleoporin Nup96) (Nup96)]	P52948 (NUP98)	0.105	0.871	2.811	0.0145	0.1291
P98196	ENSG00000068650	ATP11A	Probable phospholipid-transporting ATPase IH (EC 7.6.2.1) (ATPase IS) (ATPase class VI type 11A) (P4-ATPase flippase complex alpha subunit ATP11A)	Q9Y2G3 (ATP11B)	0.105	0.909	3.128	0.0078	0.0986
P54578	ENSG00000101557	USP14	Ubiquitin carboxyl-terminal hydrolase 14 (EC 3.4.19.12) (Deubiquitinating enzyme 14) (Ubiquitin thioesterase 14) (Ubiquitin-specific-processing protease 14)	P54578 (USP14)	-0.105	0.848	-3.035	0.0094	0.1098
P47756	ENSG00000077549	CAPZB	F-actin-capping protein subunit beta (CapZ beta)	P47756 (CAPZB)	-0.104	0.755	-2.865	0.013	0.1253

Table S52: Proteins differentially abundant at Day 2 compared to pre-first vaccination (Study Arm 2 (A/H7N9 + AS03 D1, 29), Dendritic cells). Proteins are sorted by descending absolute log_2 fold change. Protein and gene annotations are based on Ensembl Version 101 (continued).

Protein ID	Ensembl Gene ID	Ensembl Gene Name	Protein Description	50% Protein Cluster ID (Gene Name)	Fold Change (Day 2 vs. Pre- First Vaccination)	Average log_2 Abundance	Moderated t-Statistic	P-Value	FDR Adjusted P-value
Q9BVL2	ENSG00000139496	NUP58	Nucleoporin p58/p45 (58 kDa nucleoporin) (Nucleoporin-like protein 1)	Q9BVL2 (NUP58)	0.104	0.913	2.927	0.0116	0.1205
Q15435	ENSG00000115685	PPP1R7	Protein phosphatase 1 regulatory subunit 7 (Protein phosphatase 1 regulatory subunit 22)	Q15435 (PPP1R7)	-0.104	0.775	-2.941	0.0113	0.119
P50990	ENSG00000156261	CCT8	T-complex protein 1 subunit theta (TCP-1-theta) (CCT-theta) (Chaperonin containing T-complex polypeptide 1 subunit 8) (Renal carcinoma antigen NY-REN-15)	P50990 (CCT8)	-0.104	0.838	-2.902	0.0121	0.122
P26440	ENSG00000128928	IVD	Isovaleryl-CoA dehydrogenase, mitochondrial (IVD) (EC 1.3.8.4) (Butyryl-CoA dehydrogenase) (EC 1.3.8.1)	P26440 (IVD)	-0.103	0.830	-2.721	0.0172	0.1378
O75427	ENSG00000077454	LRCH4	Leucine-rich repeat and calponin homology domain-containing protein 4 (Leucine-rich repeat neuronal protein 4) (Leucine-rich neuronal protein)	O75427 (LRCH4)	0.103	0.918	2.641	0.0201	0.1455
Q92530	ENSG00000125818	PSMF1	Proteasome inhibitor PI31 subunit (hPI31)	Q92530 (PSMF1)	-0.102	0.815	-3.355	0.005	0.0799
Q9UQ80	ENSG00000170515	PA2G4	Proliferation-associated protein 2G4 (Cell cycle protein p38-2G4 homolog) (hG4-1) (ErbB3-binding protein 1)	Q9UQ80 (PA2G4)	-0.102	0.811	-3.446	0.0042	0.0761
P49662	ENSG00000196954	CASP4	Caspase-4 (CASP-4) (EC 3.4.22.57) (ICE and Ced-3 homolog 2) (ICH-2) (ICE(rel)-II) (Mih1) (Protease TX) [Cleaved into: Caspase-4 subunit p10; Caspase-4 subunit p20]	P51878 (CASP5)	0.102	0.864	2.739	0.0166	0.1368
Q9BYD6	ENSG00000169288	MRPL1	39S ribosomal protein L1, mitochondrial (L1mt) (MRP-L1) (Mitochondrial large ribosomal subunit protein uL1m)	Q9BYD6 (MRPL1)	-0.102	0.831	-2.586	0.0223	0.15
Q8TF05	ENSG00000154845	PPP4R1	Serine/threonine-protein phosphatase 4 regulatory subunit 1	Q8TF05 (PPP4R1)	0.102	0.890	3.279	0.0058	0.0865
Q12765	ENSG00000136193	SCRN1	Secernin-1	Q96FV2 (SCRN2)	-0.102	0.740	-3.134	0.0077	0.0985
O60879	ENSG00000147202	DIAPH2	Protein diaphanous homolog 2 (Diaphanous-related formin-2) (DRF2)	O60879 (DIAPH2)	0.101	0.868	2.758	0.016	0.1357
P31150	ENSG00000203879	GDI1	Rab GDP dissociation inhibitor alpha (Rab GDI alpha) (Guanosine diphosphate dissociation inhibitor 1) (GDI-1) (Oligophrenin-2) (Protein XAP-4)	P31150 (GDI1)	-0.101	0.839	-2.637	0.0202	0.1455

Table S52: Proteins differentially abundant at Day 2 compared to pre-first vaccination (Study Arm 2 (A/H7N9 + AS03 D1, 29), Dendritic cells). Proteins are sorted by descending absolute log_2 fold change. Protein and gene annotations are based on Ensembl Version 101 (continued).

Protein ID	Ensembl Gene ID	Ensembl Gene Name	Protein Description	50% Protein Cluster ID (Gene Name)	Fold Change (Day 2 vs. Pre- First Vaccination)	Average log_2 Abundance	Moderated t-Statistic	P-Value	FDR Adjusted P-value
P22090	ENSG00000129824	RPS4Y1	40S ribosomal protein S4, Y isoform 1 (Small ribosomal subunit protein eS4)	P22090 (RPS4Y1)	-0.101	0.959	-2.831	0.0139	0.1291
Q9Y2Z4	ENSG00000139131	YARS2	Tyrosine–tRNA ligase, mitochondrial (EC 6.1.1.1) (Tyrosyl-tRNA synthetase) (TyrRS)	Q9Y2Z4 (YARS2)	-0.101	0.771	-2.909	0.012	0.1214
P22314	ENSG00000130985	UBA1	Ubiquitin-like modifier-activating enzyme 1 (EC 6.2.1.45) (Protein A1S9) (Ubiquitin-activating enzyme E1)	P22314 (UBA1)	-0.101	0.811	-2.609	0.0213	0.1489
O75489	ENSG00000213619; ENSG00000285387	NDUFS3	NADH dehydrogenase [ubiquinone] iron-sulfur protein 3, mitochondrial (EC 7.1.1.2) (Complex I-30kD) (CI-30kD) (NADH-ubiquinone oxidoreductase 30 kDa subunit)	O75489 (NDUFS3)	-0.100	0.750	-2.933	0.0114	0.1199
Q9Y5K5	ENSG00000116750	UCHL5	Ubiquitin carboxyl-terminal hydrolase isozyme L5 (UCH-L5) (EC 3.4.19.12) (Ubiquitin C-terminal hydrolase UCH37) (Ubiquitin thioesterase L5)	Q9Y5K5 (UCHL5)	-0.100	0.830	-2.619	0.0209	0.1478
O43674	ENSG00000136521	NDUFB5	NADH dehydrogenase [ubiquinone] 1 beta subcomplex subunit 5, mitochondrial (Complex I-SGDH) (CI-SGDH) (NADH-ubiquinone oxidoreductase SGDH subunit)	O43674 (NDUFB5)	-0.100	0.720	-2.750	0.0163	0.136
Q9UBT2	ENSG00000126261	UBA2	SUMO-activating enzyme subunit 2 (EC 2.3.2.-) (Anthracycline-associated resistance ARX) (Ubiquitin-like 1-activating enzyme E1B) (Ubiquitin-like modifier-activating enzyme 2)	Q9UBT2 (UBA2)	-0.100	0.824	-2.822	0.0142	0.1291
P61160	ENSG00000138071	ACTR2	Actin-related protein 2 (Actin-like protein 2)	P61160 (ACTR2)	-0.100	0.710	-2.601	0.0217	0.1491
P54577	ENSG00000134684	YARS1	Tyrosine–tRNA ligase, cytoplasmic (EC 6.1.1.1) (Tyrosyl-tRNA synthetase) (TyrRS) [Cleaved into: Tyrosine–tRNA ligase, cytoplasmic, N-terminally processed]	P54577 (YARS1)	-0.099	0.774	-3.271	0.0059	0.0865
Q9UL15	ENSG00000166170	BAG5	BAG family molecular chaperone regulator 5 (BAG-5) (Bcl-2-associated athanogene 5)	Q9UL15 (BAG5)	-0.099	0.834	-3.010	0.0098	0.1127
P61081	ENSG00000130725	UBE2M	NEDD8-conjugating enzyme Ubc12 (EC 2.3.2.34) (NEDD8 carrier protein) (Ubiquitin-conjugating enzyme E2 M)	P61081 (UBE2M)	-0.099	0.836	-3.007	0.0099	0.1127
Q9HBH5	ENSG00000240857	RDH14	Retinol dehydrogenase 14 (EC 1.1.1.300) (Alcohol dehydrogenase PAN2) (Short chain dehydrogenase/reductase family 7C member 4)	Q9HBH5 (RDH14)	-0.099	0.874	-2.673	0.0189	0.1421

Table S52: Proteins differentially abundant at Day 2 compared to pre-first vaccination (Study Arm 2 (A/H7N9 + AS03 D1, 29), Dendritic cells). Proteins are sorted by descending absolute log_2 fold change. Protein and gene annotations are based on Ensembl Version 101 (continued).

Protein ID	Ensembl Gene ID	Ensembl Gene Name	Protein Description	50% Protein Cluster ID (Gene Name)	Fold Change (Day 2 vs. Pre- First Vaccination)	Average log_2 Abundance	Moderated t-Statistic	P-Value	FDR Adjusted P-value
Q9NUQ9	ENSG00000153310	CYRIB	CYFIP-related Rac1 interactor B (L1)	Q9NUQ9 (CYRIB)	-0.098	0.736	-3.176	0.0071	0.0953
P35579	ENSG00000100345	MYH9	Myosin-9 (Cellular myosin heavy chain, type A) (Myosin heavy chain 9) (Myosin heavy chain, non-muscle IIa) (Non-muscle myosin heavy chain A) (NMMHC-A) (Non-muscle myosin heavy chain IIa) (NMMHC II-a) (NMMHC-IIA)	Q7Z406 (MYH14)	-0.097	1.049	-2.603	0.0216	0.1491
O15371	ENSG00000100353	EIF3D	Eukaryotic translation initiation factor 3 subunit D (eIF3d) (Eukaryotic translation initiation factor 3 subunit 7) (eIF-3-zeta) (eIF3 p66)	O15371 (EIF3D)	-0.097	0.813	-2.811	0.0145	0.1291
P17980	ENSG00000165916	PSMC3	26S proteasome regulatory subunit 6A (26S proteasome AAA-ATPase subunit RPT5) (Proteasome 26S subunit ATPase 3) (Proteasome subunit P50) (Tat-binding protein 1) (TBP-1)	P17980 (PSMC3)	-0.097	0.787	-2.901	0.0122	0.122
O43676	ENSG00000119013	NDUFB3	NADH dehydrogenase [ubiquinone] 1 beta subcomplex subunit 3 (Complex I-B12) (CI-B12) (NADH-ubiquinone oxidoreductase B12 subunit)	O43676 (NDUFB3)	-0.095	0.783	-2.718	0.0173	0.1378
O43390	ENSG00000125944; ENSG00000282958	HNRNPR	Heterogeneous nuclear ribonucleoprotein R (hnRNP R)	O43390 (HNRNPR)	0.094	0.852	2.739	0.0166	0.1368
Q9NZ01	ENSG00000099797	TECR	Very-long-chain enoyl-CoA reductase (EC 1.3.1.93) (Synaptic glycoprotein SC2) (Trans-2,3-enoyl-CoA reductase) (TER)	Q9NZ01 (TECR)	-0.094	0.836	-2.779	0.0154	0.1336
O75608	ENSG00000120992	LYPLA1	Acyl-protein thioesterase 1 (APT-1) (hAPT1) (EC 3.1.2.-) (Lysophospholipase 1) (Lysophospholipase I) (LPL-I) (LysoPLA I)	O95372 (LYPLA2)	-0.094	0.814	-2.609	0.0213	0.1489
Q96JB2	ENSG00000136152	COG3	Conserved oligomeric Golgi complex subunit 3 (COG complex subunit 3) (Component of oligomeric Golgi complex 3) (Vesicle-docking protein SEC34 homolog) (p94)	Q96JB2 (COG3)	-0.092	0.763	-2.588	0.0222	0.15
Q9Y4W6	ENSG00000141385	AFG3L2	AFG3-like protein 2 (EC 3.4.24.-) (Paraplegin-like protein)	Q9Y4W6 (AFG3L2)	-0.092	0.838	-2.750	0.0163	0.136
P50570	ENSG00000079805	DNM2	Dynamin-2 (EC 3.6.5.5)	P50570 (DNM2)	-0.092	0.743	-2.637	0.0202	0.1455

Table S52: Proteins differentially abundant at Day 2 compared to pre-first vaccination (Study Arm 2 (A/H7N9 + AS03 D1, 29), Dendritic cells). Proteins are sorted by descending absolute log_2 fold change. Protein and gene annotations are based on Ensembl Version 101 (continued).

Protein ID	Ensembl Gene ID	Ensembl Gene Name	Protein Description	50% Protein Cluster ID (Gene Name)	Fold Change (Day 2 vs. Pre- First Vaccination)	Average log_2 Abundance	Moderated t-Statistic	P-Value	FDR Adjusted P-value
Q15233	ENSG00000147140	NONO	Non-POU domain-containing octamer-binding protein (NonO protein) (54 kDa nuclear RNA- and DNA-binding protein) (55 kDa nuclear protein) (DNA-binding p52/p100 complex, 52 kDa subunit) (NMT55) (p54(nrb)) (p54nrb)	P23246 (SFPQ)	-0.091	0.763	-2.936	0.0114	0.1196
Q9Y5A7	ENSG00000013374	NUB1	NEDD8 ultimate buster 1 (Negative regulator of ubiquitin-like proteins 1) (Renal carcinoma antigen NY-REN-18)	Q9Y5A7 (NUB1)	0.091	0.898	2.596	0.0219	0.1491
P18433	ENSG00000132670	PTPRA	Receptor-type tyrosine-protein phosphatase alpha (Protein-tyrosine phosphatase alpha) (R-PTP-alpha) (EC 3.1.3.48)	P18433 (PT-PRA)	-0.090	0.808	-2.637	0.0202	0.1455
O14776	ENSG00000113649	TCERG1	Transcription elongation regulator 1 (TATA box-binding protein-associated factor 2S) (Transcription factor CA150)	O14776 (TCERG1)	-0.088	0.805	-2.841	0.0137	0.1279
Q9BZZ5	ENSG00000166181	API5	Apoptosis inhibitor 5 (API-5) (Antiapoptosis clone 11 protein) (AAC-11) (Cell migration-inducing gene 8 protein) (Fibroblast growth factor 2-interacting factor) (FIF) (Protein XAGL)	Q9BZZ5 (API5)	-0.088	0.805	-2.820	0.0142	0.1291
Q86TI2	ENSG00000142002	DPP9	Dipeptidyl peptidase 9 (DP9) (EC 3.4.14.5) (Dipeptidyl peptidase IV-related protein 2) (DPRP-2) (Dipeptidyl peptidase IX) (DPP IX) (Dipeptidyl peptidase-like protein 9) (DPLP9)	Q6V1X1 (DPP8)	-0.087	0.846	-3.259	0.0061	0.0872
Q01433	ENSG00000116337	AMPD2	AMP deaminase 2 (EC 3.5.4.6) (AMP deaminase isoform L)	Q01433 (AMPD2)	-0.086	0.692	-2.638	0.0202	0.1455
Q14116	ENSG00000150782	IL18	Interleukin-18 (IL-18) (Iboctadekin) (Interferon gamma-inducing factor) (IFN-gamma-inducing factor) (Interleukin-1 gamma) (IL-1 gamma)	Q14116 (IL18)	0.086	0.855	2.731	0.0169	0.1372
Q96GC5	ENSG00000175581	MRPL48	39S ribosomal protein L48, mitochondrial (L48mt) (MRP-L48) (Mitochondrial large ribosomal subunit protein mL48)	Q96GC5 (MRPL48)	-0.085	0.826	-2.664	0.0192	0.1421
P31689	ENSG00000086061	DNAJA1	DnaJ homolog subfamily A member 1 (DnaJ protein homolog 2) (HSDJ) (Heat shock 40 kDa protein 4) (Heat shock protein J2) (HSJ-2) (Human DnaJ protein 2) (hDj-2)	O60884 (DNAJA2)	0.085	0.888	2.757	0.0161	0.1357

Table S52: Proteins differentially abundant at Day 2 compared to pre-first vaccination (Study Arm 2 (A/H7N9 + AS03 D1, 29), Dendritic cells). Proteins are sorted by descending absolute log_2 fold change. Protein and gene annotations are based on Ensembl Version 101 (continued).

Protein ID	Ensembl Gene ID	Ensembl Gene Name	Protein Description	50% Protein Cluster ID (Gene Name)	Fold Change (Day 2 vs. Pre- First Vaccination)	Average log_2 Abundance	Moderated t-Statistic	P-Value	FDR Adjusted P-value
Q9HCC0	ENSG00000131844	MCCC2	Methylcrotonoyl-CoA carboxylase beta chain, mitochondrial (MCCase subunit beta) (EC 6.4.1.4) (3-methylcrotonyl-CoA carboxylase 2) (3-methylcrotonyl-CoA carboxylase non-biotin-containing subunit) (3-methylcrotonyl-CoA:carbon dioxide ligase subunit beta)	Q9HCC0 (MCCC2)	-0.084	0.855	-2.677	0.0187	0.1421
P56545	ENSG00000175029	CTBP2	C-terminal-binding protein 2 (CtBP2)	P56545 (CTBP2)	-0.083	0.758	-2.622	0.0208	0.1473
Q92835	ENSG00000168918; ENSG00000281614	INPP5D	Phosphatidylinositol 3,4,5-trisphosphate 5-phosphatase 1 (EC 3.1.3.86) (Inositol polyphosphate-5-phosphatase D) (EC 3.1.3.56) (Inositol polyphosphate-5-phosphatase of 145 kDa) (SIP-145) (Phosphatidylinositol 4,5-bisphosphate 5-phosphatase) (EC 3.1.3.36) (SH2 domain-containing inositol 5'-phosphatase 1) (SH2 domain-containing inositol phosphatase 1) (SHIP-1) (p150Ship) (hp51CN)	Q92835 (INPP5D)	-0.083	0.758	-2.612	0.0212	0.1489
O43598	ENSG00000112667	DNPH1	2'-deoxynucleoside 5'-phosphate N-hydrolase 1 (EC 3.2.2.-) (c-Myc-responsive protein RCL)	O43598 (DNPH1)	-0.081	0.853	-2.725	0.0171	0.1372
Q92619	ENSG00000180448	ARHGAP45	Rho GTPase-activating protein 45 [Cleaved into: Minor histocompatibility antigen HA-1 (mHag HA-1)]	Q92619 (ARHGAP45)	-0.078	0.866	-2.685	0.0184	0.1415
Q9UHB9	ENSG00000167881	SRP68	Signal recognition particle subunit SRP68 (SRP68) (Signal recognition particle 68 kDa protein)	Q9UHB9 (SRP68)	-0.078	0.790	-2.689	0.0183	0.1413

Table S53: Proteins differentially abundant at Day 2 compared to pre-first vaccination (Study Arm 1 (A/H3N2v, D1) versus Study Arm 2 (A/H7N9 + AS03 D1, 29), Dendritic cells). Proteins are sorted by descending absolute log_2 fold change. Protein and gene annotations are based on Ensembl Version 101.

Protein ID	Ensembl Gene ID	Ensembl Gene Name	Protein Description	50% Protein Cluster ID (Gene Name)	Fold Change (Day 2 vs. Pre- First Vaccination)	Average log_2 Abundance	Moderated t-Statistic	P-Value	FDR Adjusted P-value
P35908	ENSG00000172867	KRT2	Keratin, type II cytoskeletal 2 epidermal (Cytokeratin-2e) (CK-2e) (Epithelial keratin-2e) (Keratin-2 epidermis) (Keratin-2e) (K2e) (Type-II keratin Kb2)	P04264 (KRT1)	-0.985	0.423	-3.408	0.0024	0.1363

Table S53: Proteins differentially abundant at Day 2 compared to pre-first vaccination (Study Arm 1 (A/H3N2v, D1) versus Study Arm 2 (A/H7N9 + AS03 D1, 29), Dendritic cells). Proteins are sorted by descending absolute log_2 fold change. Protein and gene annotations are based on Ensembl Version 101 (continued).

Protein ID	Ensembl Gene ID	Ensembl Gene Name	Protein Description	50% Protein Cluster ID (Gene Name)	Fold Change (Day 2 vs. Pre- First Vaccination)	Average log_2 Abundance	Moderated t-Statistic	P-Value	FDR Adjusted P-value
P59665	ENSG00000206047; ENSG00000240247; ENSG00000284983; ENSG00000285176	DEFA1; DEFA1B	Neutrophil defensin 1 (Defensin, alpha 1) (HNP-1) (HP-1) (HP1) [Cleaved into: HP 1-56; Neutrophil defensin 2 (HNP-2) (HP-2) (HP2)]	P59665 (DEFA1; DEFA1B)	-0.955	0.904	-3.675	0.0013	0.1112
P20160	ENSG00000172232; ENSG00000278624	AZU1	Azurocidin (Cationic antimicrobial protein CAP37) (Heparin-binding protein) (HBP) (hHBP)	P20160 (AZU1)	-0.760	0.957	-4.257	0.0003	0.1009
Q8WYJ6	ENSG00000180096	SEPTIN1	Septin-1 (LARP) (Peanut-like protein 3) (Serologically defined breast cancer antigen NY-BR-24)	Q8WYJ6 (SEPTIN1)	0.698	0.849	3.492	0.002	0.1265
P43403	ENSG00000115085	ZAP70	Tyrosine-protein kinase ZAP-70 (EC 2.7.10.2) (70 kDa zeta-chain associated protein) (Syk-related tyrosine kinase)	P43403 (ZAP70)	0.661	0.688	3.791	0.001	0.1104
P12314	ENSG00000150337	FCGR1A	High affinity immunoglobulin gamma Fc receptor I (IgG Fc receptor I) (Fc-gamma RI) (FcRI) (Fc-gamma RIA) (FcgammaRIa) (CD antigen CD64)	P12314 (FCGR1A)	-0.625	1.158	-4.050	0.0005	0.1009
P13647	ENSG00000186081	KRT5	Keratin, type II cytoskeletal 5 (58 kDa cytokeratin) (Cytokeratin-5) (CK-5) (Keratin-5) (K5) (Type-II keratin Kb5)	P04264 (KRT1)	-0.600	0.483	-3.265	0.0034	0.1478
P35237	ENSG00000124570	SERPINB6	Serpin B6 (Cytoplasmic antiproteinase) (CAP) (Peptidase inhibitor 6) (PI-6) (Placental thrombin inhibitor)	P30740 (SERPINB1)	0.556	0.557	4.076	0.0005	0.1009
Q9Y3Y2	ENSG00000160679	CHTOP	Chromatin target of PRMT1 protein (Friend of PRMT1 protein) (Small arginine- and glycine-rich protein) (SRAG)	Q9Y3Y2 (CHTOP)	-0.515	0.874	-4.926	<0.0001	0.1003
P08311	ENSG00000100448	CTSG	Cathepsin G (CG) (EC 3.4.21.20)	P08311 (CTSG)	-0.507	0.999	-5.128	<0.0001	0.1003
O15231	ENSG00000147394	ZNF185	Zinc finger protein 185 (LIM domain protein ZNF185) (P1-A)	O15231 (ZNF185)	-0.485	0.742	-4.068	0.0005	0.1009
Q08AF3	ENSG00000166750	SLFN5	Schlafen family member 5	Q7Z7L1 (SLFN11)	0.468	0.805	4.268	0.0003	0.1009
P84090	ENSG00000100632	ERH	Enhancer of rudimentary homolog	P84090 (ERH)	-0.467	0.936	-4.542	0.0001	0.1009

Table S53: Proteins differentially abundant at Day 2 compared to pre-first vaccination (Study Arm 1 (A/H3N2v, D1) versus Study Arm 2 (A/H7N9 + AS03 D1, 29), Dendritic cells). Proteins are sorted by descending absolute log_2 fold change. Protein and gene annotations are based on Ensembl Version 101 (continued).

Protein ID	Ensembl Gene ID	Ensembl Gene Name	Protein Description	50% Protein Cluster ID (Gene Name)	Fold Change (Day 2 vs. Pre- First Vaccination)	Average log_2 Abundance	Moderated t-Statistic	P-Value	FDR Adjusted P-value
P28799	ENSG00000030582	GRN	Progranulin (PGRN) (Acrogranin) (Epithelin precursor) (Glycoprotein of 88 Kda) (GP88) (Glycoprotein 88) (Granulin precursor) (PC cell-derived growth factor) (PCDGF) (Proepithelin) (PEPI) [Cleaved into: Paragranulin; Granulin-1 (Granulin G); Granulin-2 (Granulin F); Granulin-3 (Epithelin-2) (Granulin B); Granulin-4 (Epithelin-1) (Granulin A); Granulin-5 (Granulin C); Granulin-6 (Granulin D); Granulin-7 (Granulin E)]	P28799 (GRN)	-0.462	0.974	-3.381	0.0026	0.1363
Q96PP9	ENSG00000162654	GBP4	Guanylate-binding protein 4 (EC 3.6.5.-) (GTP-binding protein 4) (GBP-4) (Guanine nucleotide-binding protein 4)	Q96PP9 (GBP4)	-0.460	1.090	-3.685	0.0012	0.1112
P42330	ENSG00000196139	AKR1C3	Aldo-keto reductase family 1 member C3 (EC 1.1.1.-) (EC 1.1.1.210) (EC 1.1.1.53) (EC 1.1.1.62) (17-beta-hydroxysteroid dehydrogenase type 5) (17-beta-HSD 5) (3-alpha-HSD type II, brain) (3-alpha-hydroxysteroid dehydrogenase type 2) (3-alpha-HSD type 2) (EC 1.1.1.357) (Chlordecone reductase homolog HAKRb) (Dihydrodiol dehydrogenase 3) (DD-3) (DD3) (Dihydrodiol dehydrogenase type I) (HA1753) (Prostaglandin F synthase) (PGFS) (EC 1.1.1.188) (Testosterone 17-beta-dehydrogenase 5) (EC 1.1.1.239) (EC 1.1.1.64)	Q04828 (AKR1C1)	0.447	0.600	3.846	0.0008	0.1099
Q13469	ENSG00000101096	NFATC2	Nuclear factor of activated T-cells, cytoplasmic 2 (NF-ATc2) (NFATc2) (NFAT pre-existing subunit) (NF-ATp) (T-cell transcription factor NFAT1)	Q13469 (NFATC2)	0.434	0.680	3.978	0.0006	0.1009
P12429	ENSG00000138772	ANXA3	Annexin A3 (35-alpha calcimedin) (Annexin III) (Annexin-3) (Inositol 1,2-cyclic phosphate 2-phosphohydrolase) (Lipocortin III) (Placental anticoagulant protein III) (PAP-III)	P08133 (ANXA6)	-0.431	0.962	-3.371	0.0027	0.1363
P62995	ENSG00000136527	TRA2B	Transformer-2 protein homolog beta (TRA-2 beta) (TRA2-beta) (hTRA2-beta) (Splicing factor, arginine/serine-rich 10) (Transformer-2 protein homolog B)	P62995 (TRA2B)	-0.421	0.907	-4.217	0.0003	0.1009

Table S53: Proteins differentially abundant at Day 2 compared to pre-first vaccination (Study Arm 1 (A/H3N2v, D1) versus Study Arm 2 (A/H7N9 + AS03 D1, 29), Dendritic cells). Proteins are sorted by descending absolute log_2 fold change. Protein and gene annotations are based on Ensembl Version 101 (continued).

Protein ID	Ensembl Gene ID	Ensembl Gene Name	Protein Description	50% Protein Cluster ID (Gene Name)	Fold Change (Day 2 vs. Pre- First Vaccination)	Average log_2 Abundance	Moderated t-Statistic	P-Value	FDR Adjusted P-value
Q9HD89	ENSG00000104918	RETN	Resistin (Adipose tissue-specific secretory factor) (ADSF) (C/EBP-epsilon-regulated myeloid-specific secreted cysteine-rich protein) (Cysteine-rich secreted protein A12-alpha-like 2) (Cysteine-rich secreted protein FIZZ3)	Q9HD89 (RETN)	-0.409	0.631	-4.657	0.0001	0.1009
Q9H1E3	ENSG00000069275	NUCKS1	Nuclear ubiquitous casein and cyclin-dependent kinase substrate 1 (P1)	Q9H1E3 (NUCKS1)	-0.391	0.939	-3.363	0.0027	0.1363
Q9UKV3	ENSG00000100813	ACIN1	Apoptotic chromatin condensation inducer in the nucleus (Acinus)	Q9UKV3 (ACIN1)	-0.375	0.979	-3.649	0.0014	0.1121
O75152	ENSG00000058673	ZC3H11A	Zinc finger CCCH domain-containing protein 11A	O75152 (ZC3H11A)	-0.366	1.061	-3.643	0.0014	0.1121
P09874	ENSG00000143799	PARP1	Poly [ADP-ribose] polymerase 1 (PARP-1) (EC 2.4.2.30) (ADP-ribosyltransferase diphtheria toxin-like 1) (ARTD1) (DNA ADP-ribosyltransferase PARP1) (EC 2.4.2.-) (NAD(+) ADP-ribosyltransferase 1) (ADPRT 1) (Poly[ADP-ribose] synthase 1) (Protein poly-ADP-ribosyltransferase PARP1) (EC 2.4.2.-)	P09874 (PARP1)	0.364	0.787	3.760	0.001	0.1112
Q08170	ENSG00000116350	SRSF4	Serine/arginine-rich splicing factor 4 (Pre-mRNA-splicing factor SRP75) (SRP001LB) (Splicing factor, arginine/serine-rich 4)	Q08170 (SRSF4)	-0.362	0.951	-3.723	0.0011	0.1112
Q14978	ENSG00000166197	NOLC1	Nucleolar and coiled-body phosphoprotein 1 (140 kDa nucleolar phosphoprotein) (Nopp140) (Hepatitis C virus NS5A-transactivated protein 13) (HCV NS5A-transactivated protein 13) (Nucleolar 130 kDa protein) (Nucleolar phosphoprotein p130)	Q14978 (NOLC1)	-0.354	0.956	-4.260	0.0003	0.1009
Q92804	ENSG00000270647; ENSG00000276833	TAF15	TATA-binding protein-associated factor 2N (68 kDa TATA-binding protein-associated factor) (TAF(II)68) (TAFII68) (RNA-binding protein 56)	Q92804 (TAF15)	-0.353	1.003	-3.350	0.0028	0.1363
P05164	ENSG00000005381	MPO	Myeloperoxidase (MPO) (EC 1.11.2.2) [Cleaved into: Myeloperoxidase; 89 kDa myeloperoxidase; 84 kDa myeloperoxidase; Myeloperoxidase light chain; Myeloperoxidase heavy chain]	P05164 (MPO)	-0.345	0.908	-3.695	0.0012	0.1112
P50453	ENSG00000170542	SERPINB9	Serpin B9 (Cytoplasmic antiproteinase 3) (CAP-3) (CAP3) (Peptidase inhibitor 9) (PI-9)	P30740 (SERPINB1)	0.345	0.733	3.906	0.0007	0.1041
Q99729	ENSG00000197451	HNRNPAB	Heterogeneous nuclear ribonucleoprotein A/B (hnRNP A/B) (APOBEC1-binding protein 1) (ABBP-1)	O14979 (HNRNPDL)	-0.344	0.853	-3.355	0.0028	0.1363

Confidential/Proprietary Information

Table S53: Proteins differentially abundant at Day 2 compared to pre-first vaccination (Study Arm 1 (A/H3N2v, D1) versus Study Arm 2 (A/H7N9 + AS03 D1, 29), Dendritic cells). Proteins are sorted by descending absolute log_2 fold change. Protein and gene annotations are based on Ensembl Version 101 (continued).

Protein ID	Ensembl Gene ID	Ensembl Gene Name	Protein Description	50% Protein Cluster ID (Gene Name)	Fold Change (Day 2 vs. Pre- First Vaccination)	Average log_2 Abundance	Moderated t-Statistic	P-Value	FDR Adjusted P-value
P01860		IGHG3	Immunoglobulin heavy constant gamma 3 (HDC) (Heavy chain disease protein) (Ig gamma-3 chain C region)	P0DOX5 (NA)	-0.335	0.840	-3.612	0.0015	0.1131
O95976	ENSG00000140749	IGSF6	Immunoglobulin superfamily member 6 (IgSF6) (Protein DORA)	O95976 (IGSF6)	-0.324	0.922	-4.119	0.0004	0.1009
P07237	ENSG00000185624	P4HB	Protein disulfide-isomerase (PDI) (EC 5.3.4.1) (Cellular thyroid hormone-binding protein) (Prolyl 4-hydroxylase subunit beta) (p55)	P07237 (P4HB)	-0.323	0.897	-3.958	0.0006	0.1009
Q9P0P0	ENSG00000168894	RNF181	E3 ubiquitin-protein ligase RNF181 (EC 2.3.2.27) (RING finger protein 181) (RING-type E3 ubiquitin transferase RNF181)	Q9P0P0 (RNF181)	-0.322	0.951	-4.235	0.0003	0.1009
O14867	ENSG00000156273	BACH1	Transcription regulator protein BACH1 (BTB and CNC homolog 1) (HA2303)	O14867 (BACH1)	-0.317	0.897	-3.961	0.0006	0.1009
Q9H307	ENSG00000100941	PNN	Pinin (140 kDa nuclear and cell adhesion-related phosphoprotein) (Desmosome-associated protein) (Domain-rich serine protein) (DRS protein) (DRSP) (Melanoma metastasis clone A protein) (Nuclear protein SDK3) (SR-like protein)	Q9H307 (PNN)	-0.312	0.898	-4.342	0.0002	0.1009
P36915	ENSG00000204590; ENSG00000206492; ENSG00000226882; ENSG00000229470; ENSG00000228581; ENSG00000235986; ENSG00000232143	GNL1	Guanine nucleotide-binding protein-like 1 (GTP-binding protein HSR1)	P36915 (GNL1)	0.311	0.855	3.526	0.0018	0.1226
P55957	ENSG00000015475	BID	BH3-interacting domain death agonist (p22 BID) (BID) [Cleaved into: BH3-interacting domain death agonist p15 (p15 BID); BH3-interacting domain death agonist p13 (p13 BID); BH3-interacting domain death agonist p11 (p11 BID)]	P55957 (BID)	-0.300	0.943	-3.739	0.0011	0.1112
Q9P2E3	ENSG00000124201	ZNFX1	NFX1-type zinc finger-containing protein 1	Q9P2E3 (ZNFX1)	-0.296	0.824	-3.262	0.0035	0.1478
P17927	ENSG00000203710	CR1	Complement receptor type 1 (C3b/C4b receptor) (CD antigen CD35)	P17927 (CR1)	-0.293	0.920	-3.797	0.0009	0.1104
Q9UGI8	ENSG00000135269	TES	Testin (TESS)	Q9UGI8 (TES)	0.287	0.866	3.426	0.0023	0.1357

Table S53: Proteins differentially abundant at Day 2 compared to pre-first vaccination (Study Arm 1 (A/H3N2v, D1) versus Study Arm 2 (A/H7N9 + AS03 D1, 29), Dendritic cells). Proteins are sorted by descending absolute log_2 fold change. Protein and gene annotations are based on Ensembl Version 101 (continued).

Protein ID	Ensembl Gene ID	Ensembl Gene Name	Protein Description	50% Protein Cluster ID (Gene Name)	Fold Change (Day 2 vs. Pre- First Vaccination)	Average log_2 Abundance	Moderated t-Statistic	P-Value	FDR Adjusted P-value
Q86U42	ENSG00000100836	PABPN1	Polyadenylate-binding protein 2 (PABP-2) (Poly(A)-binding protein 2) (Nuclear poly(A)-binding protein 1) (Poly(A)-binding protein II) (PABII) (Polyadenylate-binding nuclear protein 1)	Q86U42 (PABPN1)	-0.285	0.830	-3.586	0.0016	0.1152
Q9H9B4	ENSG00000164466	SFXN1	Sideroflexin-1	Q9H9B4 (SFXN1)	0.282	0.950	3.247	0.0036	0.1478
Q13242	ENSG00000111786	SRSF9	Serine/arginine-rich splicing factor 9 (Pre-mRNA-splicing factor SRp30C) (Splicing factor, arginine/serine-rich 9)	Q07955 (SRSF1)	-0.282	0.840	-3.444	0.0022	0.1357
Q96BW5	ENSG00000165983	PTER	Phosphotriesterase-related protein (EC 3.1.-.-) (Parathion hydrolase-related protein) (hPHRP)	Q96BW5 (PTER)	0.279	0.949	4.326	0.0003	0.1009
Q13315	ENSG00000149311	ATM	Serine-protein kinase ATM (EC 2.7.11.1) (Ataxia telangiectasia mutated) (A-T mutated)	Q13315 (ATM)	0.274	0.957	4.145	0.0004	0.1009
Q8WWW0	ENSG00000266094	RASSF5	Ras association domain-containing protein 5 (New ras effector 1) (Regulator for cell adhesion and polarization enriched in lymphoid tissues) (RAPL)	Q8WWW0 (RASSF5)	0.274	0.774	4.108	0.0004	0.1009
Q9NYF8	ENSG00000029363	BCLAF1	Bcl-2-associated transcription factor 1 (Btf) (BCLAF1 and THRAP3 family member 1)	Q9NYF8 (BCLAF1)	-0.272	0.942	-3.915	0.0007	0.1041
Q53H12	ENSG00000006530; ENSG00000262327	AGK	Acylglycerol kinase, mitochondrial (hAGK) (EC 2.7.1.107) (EC 2.7.1.138) (EC 2.7.1.94) (Multiple substrate lipid kinase) (HsMuLK) (MuLK) (Multi-substrate lipid kinase)	Q53H12 (AGK)	0.271	0.777	3.804	0.0009	0.1104
P15121	ENSG00000085662	AKR1B1	Aldo-keto reductase family 1 member B1 (EC 1.1.1.300) (EC 1.1.1.372) (EC 1.1.1.54) (Aldehyde reductase) (Aldose reductase) (AR) (EC 1.1.1.21)	P14550 (AKR1A1)	0.271	0.818	3.275	0.0033	0.1478
O75923	ENSG00000135636	DYSF	Dysferlin (Dystrophy-associated fer-1-like protein) (Fer-1-like protein 1)	O75923 (DYSF)	-0.269	0.834	-3.357	0.0027	0.1363
O94925	ENSG00000115419	GLS	Glutaminase kidney isoform, mitochondrial (GLS) (EC 3.5.1.2) (K-glutaminase) (L-glutamine amidohydrolase) [Cleaved into: Glutaminase kidney isoform, mitochondrial 68 kDa chain; Glutaminase kidney isoform, mitochondrial 65 kDa chain]	O94925 (GLS)	0.268	0.892	3.372	0.0026	0.1363

Table S53: Proteins differentially abundant at Day 2 compared to pre-first vaccination (Study Arm 1 (A/H3N2v, D1) versus Study Arm 2 (A/H7N9 + AS03 D1, 29), Dendritic cells). Proteins are sorted by descending absolute log_2 fold change. Protein and gene annotations are based on Ensembl Version 101 (continued).

Protein ID	Ensembl Gene ID	Ensembl Gene Name	Protein Description	50% Protein Cluster ID (Gene Name)	Fold Change (Day 2 vs. Pre- First Vaccination)	Average log_2 Abundance	Moderated t-Statistic	P-Value	FDR Adjusted P-value
P09651	ENSG00000135486	HNRNPA1	Heterogeneous nuclear ribonucleoprotein A1 (hnRNP A1) (Helix-destabilizing protein) (Single-strand RNA-binding protein) (hnRNP core protein A1) [Cleaved into: Heterogeneous nuclear ribonucleoprotein A1, N-terminally processed]	P51991 (HNRNPA3)	-0.268	0.785	-3.617	0.0015	0.1131
O75022	ENSG00000204577; ENSG00000274587; ENSG00000275019	LILRB3	Leukocyte immunoglobulin-like receptor subfamily B member 3 (LIR-3) (Leukocyte immunoglobulin-like receptor 3) (CD85 antigen-like family member A) (Immunoglobulin-like transcript 5) (ILT-5) (Monocyte inhibitory receptor HL9) (CD antigen CD85a)	Q8NHL6 (LILRB1)	-0.267	0.838	-3.782	0.001	0.1104
P78324	ENSG00000198053	SIRPA	Tyrosine-protein phosphatase non-receptor type substrate 1 (SHP substrate 1) (SHPS-1) (Brain Ig-like molecule with tyrosine-based activation motifs) (Bit) (CD172 antigen-like family member A) (Inhibitory receptor SHPS-1) (Macrophage fusion receptor) (MyD-1 antigen) (Signal-regulatory protein alpha-1) (Sirp-alpha-1) (Signal-regulatory protein alpha-2) (Sirp-alpha-2) (Signal-regulatory protein alpha-3) (Sirp-alpha-3) (p84) (CD antigen CD172a)	P78324 (SIRPA)	-0.262	0.789	-3.958	0.0006	0.1009
Q8IUD2	ENSG00000082805	ERC1	ELKS/Rab6-interacting/CAST family member 1 (ERC-1) (Rab6-interacting protein 2)	Q8IUD2 (ERC1)	-0.260	0.835	-3.284	0.0033	0.1478
P11177	ENSG00000168291	PDHB	Pyruvate dehydrogenase E1 component subunit beta, mitochondrial (PDHE1-B) (EC 1.2.4.1)	P11177 (PDHB)	0.259	0.836	3.753	0.001	0.1112
Q6P9H5	ENSG00000133561	GIMAP6	GTPase IMAP family member 6 (Immunity-associated nucleotide 2 protein) (IAN-2) (hIAN2) (Immunity-associated nucleotide 6 protein) (IAN-6) (hIAN6)	Q6P9H5 (GIMAP6)	-0.258	0.900	-3.339	0.0029	0.138
Q07065	ENSG00000136026	CKAP4	Cytoskeleton-associated protein 4 (63-kDa cytoskeleton-linking membrane protein) (Climp-63) (p63)	Q07065 (CKAP4)	-0.246	0.874	-3.574	0.0016	0.1163
Q9Y2W1	ENSG00000054118	THRAP3	Thyroid hormone receptor-associated protein 3 (BCLAF1 and THRAP3 family member 2) (Thyroid hormone receptor-associated protein complex 150 kDa component) (Trap150)	Q9Y2W1 (THRAP3)	-0.245	0.906	-3.557	0.0017	0.1188

Table S53: Proteins differentially abundant at Day 2 compared to pre-first vaccination (Study Arm 1 (A/H3N2v, D1) versus Study Arm 2 (A/H7N9 + AS03 D1, 29), Dendritic cells). Proteins are sorted by descending absolute log_2 fold change. Protein and gene annotations are based on Ensembl Version 101 (continued).

Protein ID	Ensembl Gene ID	Ensembl Gene Name	Protein Description	50% Protein Cluster ID (Gene Name)	Fold Change (Day 2 vs. Pre- First Vaccination)	Average log_2 Abundance	Moderated t-Statistic	P-Value	FDR Adjusted P-value
Q15424	ENSG00000160633	SAFB	Scaffold attachment factor B1 (SAF-B) (SAF-B1) (HSP27 estrogen response element-TATA box-binding protein) (HSP27 ERE-TATA-binding protein)	Q14151 (SAFB2)	-0.244	0.910	-3.893	0.0007	0.1041
P11766	ENSG00000197894	ADH5	Alcohol dehydrogenase class-3 (EC 1.1.1.1) (Alcohol dehydrogenase 5) (Alcohol dehydrogenase class chi chain) (Alcohol dehydrogenase class-III) (Glutathione-dependent formaldehyde dehydrogenase) (FALDH) (FDH) (GSH-FDH) (EC 1.1.1.-) (S-(hydroxymethyl)glutathione dehydrogenase) (EC 1.1.1.284)	P40394 (ADH7)	0.244	0.776	3.258	0.0035	0.1478
Q99829	ENSG00000214078	CPNE1	Copine-1 (Chromobindin 17) (Copine I)	Q86YQ8 (CPNE8)	0.243	0.766	3.628	0.0014	0.1131
P42765	ENSG00000167315	ACAA2	3-ketoacyl-CoA thiolase, mitochondrial (EC 2.3.1.16) (Acetyl-CoA acetyltransferase) (EC 2.3.1.9) (Acetyl-CoA acyltransferase) (Acyl-CoA hydrolase, mitochondrial) (EC 3.1.2.-) (EC 3.1.2.1) (EC 3.1.2.2) (Beta-ketothiolase) (Mitochondrial 3-oxoacyl-CoA thiolase) (T1)	P42765 (ACAA2)	0.235	0.803	3.594	0.0015	0.1152
O43759	ENSG00000100321	SYNGR1	Synaptogyrin-1	O43759 (SYNGR1)	-0.232	0.895	-4.026	0.0005	0.1009
Q08722	ENSG00000196776	CD47	Leukocyte surface antigen CD47 (Antigenic surface determinant protein OA3) (Integrin-associated protein) (IAP) (Protein MER6) (CD antigen CD47)	Q08722 (CD47)	0.228	0.871	3.524	0.0018	0.1226
P55263	ENSG00000156110	ADK	Adenosine kinase (AK) (EC 2.7.1.20) (Adenosine 5'-phosphotransferase)	P55263 (ADK)	0.227	0.591	3.438	0.0023	0.1357
O00483	ENSG00000189043	NDUFA4	Cytochrome c oxidase subunit NDUFA4 (Complex I-MLRQ) (CI-MLRQ) (NADH-ubiquinone oxidoreductase MLRQ subunit)	O00483 (NDUFA4)	0.223	0.633	3.513	0.0019	0.1226
Q7Z4V5	ENSG00000167674	HDGFL2	Hepatoma-derived growth factor-related protein 2 (HDGF-related protein 2) (HRP-2) (Hepatoma-derived growth factor 2) (HDGF-2)	Q7Z4V5 (HDGFL2)	-0.221	0.870	-3.519	0.0019	0.1226
O75251	ENSG00000115286	NDUFS7	NADH dehydrogenase [ubiquinone] iron-sulfur protein 7, mitochondrial (EC 7.1.1.2) (Complex I-20kD) (CI-20kD) (NADH-ubiquinone oxidoreductase 20 kDa subunit) (PSST subunit)	O75251 (NDUFS7)	0.214	0.765	3.466	0.0021	0.1324

Table S53: Proteins differentially abundant at Day 2 compared to pre-first vaccination (Study Arm 1 (A/H3N2v, D1) versus Study Arm 2 (A/H7N9 + AS03 D1, 29), Dendritic cells). Proteins are sorted by descending absolute log_2 fold change. Protein and gene annotations are based on Ensembl Version 101 (continued).

Protein ID	Ensembl Gene ID	Ensembl Gene Name	Protein Description	50% Protein Cluster ID (Gene Name)	Fold Change (Day 2 vs. Pre- First Vaccination)	Average log_2 Abundance	Moderated t-Statistic	P-Value	FDR Adjusted P-value
O15553	ENSG00000103313	MEFV	Pyrin (Marenostrin)	O15553 (MEFV)	-0.213	0.770	-3.332	0.0029	0.1386
Q86YV0	ENSG00000105122	RASAL3	RAS protein activator like-3	Q86YV0 (RASAL3)	0.209	0.851	3.279	0.0033	0.1478
Q8WYA6	ENSG00000132792	CTNNBL1	Beta-catenin-like protein 1 (Nuclear-associated protein) (NAP) (Testis development protein NYD-SP19)	Q8WYA6 (CTNNBL1)	0.208	0.796	3.270	0.0034	0.1478
P00813	ENSG00000196839	ADA	Adenosine deaminase (EC 3.5.4.4) (Adenosine aminohydrolase)	P00813 (ADA)	0.206	0.781	3.283	0.0033	0.1478
P22830	ENSG00000066926	FECH	Ferrochelatase, mitochondrial (EC 4.99.1.1) (Heme synthase) (Protoheme ferro-lyase)	P22830 (FECH)	0.205	0.775	3.840	0.0008	0.1099
Q96A35	ENSG00000143314	MRPL24	39S ribosomal protein L24, mitochondrial (L24mt) (MRP-L24) (Mitochondrial large ribosomal subunit protein uL24m)	Q96A35 (MRPL24)	0.194	0.857	3.420	0.0024	0.1357
O14929	ENSG00000128708	HAT1	Histone acetyltransferase type B catalytic subunit (EC 2.3.1.48) (Histone acetyltransferase 1)	O14929 (HAT1)	-0.192	0.858	-3.248	0.0036	0.1478
P05166	ENSG00000114054	PCCB	Propionyl-CoA carboxylase beta chain, mitochondrial (PCCase subunit beta) (EC 6.4.1.3) (Propanoyl-CoA:carbon dioxide ligase subunit beta)	P05166 (PCCB)	0.191	0.798	3.255	0.0035	0.1478
Q8N0X7	ENSG00000133104	SPART	Spartin (Spastic paraplegia 20 protein) (Trans-activated by hepatitis C virus core protein 1)	Q8N0X7 (SPART)	-0.189	0.875	-3.665	0.0013	0.1112
Q13952	ENSG00000066136	NFYC	Nuclear transcription factor Y subunit gamma (CAAT box DNA-binding protein subunit C) (Nuclear transcription factor Y subunit C) (NF-YC) (Transactivator HSM-1/2)	Q13952 (NFYC)	0.188	0.880	3.404	0.0025	0.1363
O95299	ENSG00000130414	NDUFA10	NADH dehydrogenase [ubiquinone] 1 alpha subcomplex subunit 10, mitochondrial (Complex I-42kD) (CI-42kD) (NADH-ubiquinone oxidoreductase 42 kDa subunit)	O95299 (NDUFA10)	0.182	0.781	3.422	0.0024	0.1357
O95865	ENSG00000213722; ENSG00000206395; ENSG00000226634; ENSG00000233076; ENSG00000225635; ENSG00000228128; ENSG00000227317	DDAH2	N(G),N(G)-dimethylarginine dimethylaminohydrolase 2 (DDAH-2) (Dimethylarginine dimethylaminohydrolase 2) (EC 3.5.3.18) (DDAHII) (Dimethylargininase-2) (Protein G6a) (S-phase protein)	O94760 (DDAH1)	0.182	0.848	3.672	0.0013	0.1112

Table S53: Proteins differentially abundant at Day 2 compared to pre-first vaccination (Study Arm 1 (A/H3N2v, D1) versus Study Arm 2 (A/H7N9 + AS03 D1, 29), Dendritic cells). Proteins are sorted by descending absolute log_2 fold change. Protein and gene annotations are based on Ensembl Version 101 (continued).

Protein ID	Ensembl Gene ID	Ensembl Gene Name	Protein Description	50% Protein Cluster ID (Gene Name)	Fold Change (Day 2 vs. Pre- First Vaccination)	Average log_2 Abundance	Moderated t-Statistic	P-Value	FDR Adjusted P-value
O15042	ENSG00000163714	U2SURP	U2 snRNP-associated SURP motif-containing protein (140 kDa Ser/Arg-rich domain protein) (U2-associated protein SR140)	O15042 (U2SURP)	-0.174	0.878	-3.398	0.0025	0.1363
Q15019	ENSG00000168385	SEPTIN2	Septin-2 (Neural precursor cell expressed developmentally down-regulated protein 5) (NEDD-5)	Q16181 (SEPTIN7)	0.171	0.852	3.351	0.0028	0.1363
P11216	ENSG00000100994	PYGB	Glycogen phosphorylase, brain form (EC 2.4.1.1)	P06737 (PYGL)	0.168	0.833	3.716	0.0011	0.1112

Table S54: Proteins differentially abundant at Day 2 compared to pre-first vaccination (Study Arm 2 (A/H7N9 + AS03 D1, 29) versus Study Arm 3 (A/H7N9 + PBS, D1, 29), Dendritic cells). Proteins are sorted by descending absolute log_2 fold change. Protein and gene annotations are based on Ensembl Version 101.

Protein ID	Ensembl Gene ID	Ensembl Gene Name	Protein Description	50% Protein Cluster ID (Gene Name)	Fold Change (Day 2 vs. Pre- First Vaccination)	Average log_2 Abundance	Moderated t-Statistic	P-Value	FDR Adjusted P-value
P20160	ENSG00000172232; ENSG00000278624	AZU1	Azurocidin (Cationic antimicrobial protein CAP37) (Heparin-binding protein) (HBP) (hHBP)	P20160 (AZU1)	0.744	0.873	4.549	0.0001	0.1361
P08670	ENSG00000026025	VIM	Vimentin	P08670 (VIM)	0.676	0.792	4.323	0.0003	0.1361
Q9Y3Y2	ENSG00000160679	CHTOP	Chromatin target of PRMT1 protein (Friend of PRMT1 protein) (Small arginine- and glycine-rich protein) (SRAG)	Q9Y3Y2 (CHTOP)	0.510	0.877	4.066	0.0005	0.1388
P35237	ENSG00000124570	SERPINB6	Serpin B6 (Cytoplasmic antiproteinase) (CAP) (Peptidase inhibitor 6) (PI-6) (Placental thrombin inhibitor)	P30740 (SERPINB1)	-0.495	0.677	-4.031	0.0005	0.1388
O75367	ENSG00000113648	MACROH2A1	Core histone macro-H2A.1 (Histone macroH2A1) (mH2A1) (Histone H2A.y) (H2A/y) (Medulloblastoma antigen MU-MB-50.205)	Q9P0M6 (MACROH2A2)	0.477	1.016	4.018	0.0006	0.1388
P08311	ENSG00000100448	CTSG	Cathepsin G (CG) (EC 3.4.21.20)	P08311 (CTSG)	0.462	0.985	4.352	0.0002	0.1361
Q13469	ENSG00000101096	NFATC2	Nuclear factor of activated T-cells, cytoplasmic 2 (NF-ATc2) (NFATc2) (NFAT pre-existing subunit) (NF-ATp) (T-cell transcription factor NFAT1)	Q13469 (NFATC2)	-0.394	0.657	-4.063	0.0005	0.1388

Table S54: Proteins differentially abundant at Day 2 compared to pre-first vaccination (Study Arm 2 (A/H7N9 + AS03 D1, 29) versus Study Arm 3 (A/H7N9 + PBS, D1, 29), Dendritic cells). Proteins are sorted by descending absolute log_2 fold change. Protein and gene annotations are based on Ensembl Version 101 (continued).

Protein ID	Ensembl Gene ID	Ensembl Gene Name	Protein Description	50% Protein Cluster ID (Gene Name)	Fold Change (Day 2 vs. Pre- First Vaccination)	Average log_2 Abundance	Moderated t-Statistic	P-Value	FDR Adjusted P-value
Q9HD89	ENSG00000104918	RETN	Resistin (Adipose tissue-specific secretory factor) (ADSF) (C/EBP-epsilon-regulated myeloid-specific secreted cysteine-rich protein) (Cysteine-rich secreted protein A12-alpha-like 2) (Cysteine-rich secreted protein FIZZ3)	Q9HD89 (RETN)	0.391	0.615	4.941	<0.0001	0.1361
P50453	ENSG00000170542	SERPINB9	Serpin B9 (Cytoplasmic antiproteinase 3) (CAP-3) (CAP3) (Peptidase inhibitor 9) (PI-9)	P30740 (SERPINB1)	-0.367	0.678	-4.494	0.0002	0.1361
P17927	ENSG00000203710	CR1	Complement receptor type 1 (C3b/C4b receptor) (CD antigen CD35)	P17927 (CR1)	0.355	0.976	4.067	0.0005	0.1388
P46063	ENSG00000004700	RECQL	ATP-dependent DNA helicase Q1 (EC 3.6.4.12) (DNA helicase, RecQ-like type 1) (RecQ1) (DNA-dependent ATPase Q1) (RecQ protein-like 1)	P46063 (RECQL)	-0.312	0.847	-4.235	0.0003	0.1388
Q9NYF8	ENSG00000029363	BCLAF1	Bcl-2-associated transcription factor 1 (Btf) (BCLAF1 and THRAP3 family member 1)	Q9NYF8 (BCLAF1)	0.311	0.914	4.117	0.0004	0.1388
Q9P0P0	ENSG00000168894	RNF181	E3 ubiquitin-protein ligase RNF181 (EC 2.3.2.27) (RING finger protein 181) (RING-type E3 ubiquitin transferase RNF181)	Q9P0P0 (RNF181)	0.296	0.935	4.346	0.0002	0.1361
Q13315	ENSG00000149311	ATM	Serine-protein kinase ATM (EC 2.7.11.1) (Ataxia telangiectasia mutated) (A-T mutated)	Q13315 (ATM)	-0.252	0.967	-4.307	0.0003	0.1361

Table S55: Proteins differentially abundant at Day 30 compared to pre-second vaccination (Study Arm 2 (A/H7N9 + AS03 D1, 29), Dendritic cells). Proteins are sorted by descending absolute log_2 fold change. Protein and gene annotations are based on Ensembl Version 101.

Protein ID	Ensembl Gene ID	Ensembl Gene Name	Protein Description	50% Protein Cluster ID (Gene Name)	Fold Change (Day 30 vs. Pre- Second Vaccination)	Average log_2 Abundance	Moderated t-Statistic	P-Value	FDR Adjusted P-value
Q96PP8	ENSG00000154451	GBP5	Guanylate-binding protein 5 (EC 3.6.5.-) (GBP-TA antigen) (GTP-binding protein 5) (GBP-5) (Guanine nucleotide-binding protein 5)	Q96PP9 (GBP4)	1.583	1.730	7.392	<0.0001	0.0017
P32455	ENSG00000117228	GBP1	Guanylate-binding protein 1 (EC 3.6.5.-) (GTP-binding protein 1) (GBP-1) (HuGBP-1) (Guanine nucleotide-binding protein 1) (Interferon-induced guanylate-binding protein 1)	Q96PP9 (GBP4)	1.118	1.587	7.233	<0.0001	0.0018

Table S55: Proteins differentially abundant at Day 30 compared to pre-second vaccination (Study Arm 2 (A/H7N9 + AS03 D1, 29), Dendritic cells). Proteins are sorted by descending absolute log_2 fold change. Protein and gene annotations are based on Ensembl Version 101 (continued).

Protein ID	Ensembl Gene ID	Ensembl Gene Name	Protein Description	50% Protein Cluster ID (Gene Name)	Fold Change (Day 30 vs. Pre- Second Vaccination)	Average log_2 Abundance	Moderated t-Statistic	P-Value	FDR Adjusted P-value
O95183	ENSG00000168899	VAMP5	Vesicle-associated membrane protein 5 (VAMP-5) (Myobrevin)	O95183 (VAMP5)	1.061	1.518	6.928	<0.0001	0.0019
P23381	ENSG00000140105	WARS1	Tryptophan–tRNA ligase, cytoplasmic (EC 6.1.1.2) (Interferon-induced protein 53) (IFP53) (Tryptophanyl-tRNA synthetase) (TrpRS) (hWRS) [Cleaved into: T1-TrpRS; T2-TrpRS]	P23381 (WARS1)	0.988	1.303	7.049	<0.0001	0.0018
P12314	ENSG00000150337	FCGR1A	High affinity immunoglobulin gamma Fc receptor I (IgG Fc receptor I) (Fc-gamma RI) (FcRI) (Fc-gamma RIA) (FcgammaRIa) (CD antigen CD64)	P12314 (FCGR1A)	0.922	1.570	9.403	<0.0001	0.0004
P20160	ENSG00000172232; ENSG00000278624	AZU1	Azurocidin (Cationic antimicrobial protein CAP37) (Heparin-binding protein) (HBP) (hHBP)	P20160 (AZU1)	0.872	1.091	8.333	<0.0001	0.0007
P32456	ENSG00000162645	GBP2	Guanylate-binding protein 2 (EC 3.6.5.-) (GTP-binding protein 2) (GBP-2) (HuGBP-2) (Guanine nucleotide-binding protein 2) (Interferon-induced guanylate-binding protein 2)	Q96PP9 (GBP4)	0.811	1.295	9.367	<0.0001	0.0004
Q96PP9	ENSG00000162654	GBP4	Guanylate-binding protein 4 (EC 3.6.5.-) (GTP-binding protein 4) (GBP-4) (Guanine nucleotide-binding protein 4)	Q96PP9 (GBP4)	0.775	1.333	5.435	0.0001	0.0086
P56279	ENSG00000100721	TCL1A	T-cell leukemia/lymphoma protein 1A (Oncogene TCL-1) (Oncogene TCL1) (Protein p14 TCL1)	P56279 (TCL1A)	-0.767	0.492	-3.515	0.004	0.0519
P09913	ENSG00000119922	IFIT2	Interferon-induced protein with tetratricopeptide repeats 2 (IFIT-2) (ISG-54 K) (Interferon-induced 54 kDa protein) (IFI-54K) (P54)	O14879 (IFIT3)	0.751	1.322	6.785	<0.0001	0.0022
P28838	ENSG00000002549	LAP3	Cytosol aminopeptidase (EC 3.4.11.1) (Cysteinylglycine-S-conjugate dipeptidase) (EC 3.4.13.23) (Leucine aminopeptidase 3) (LAP-3) (Leucyl aminopeptidase) (Peptidase S) (Proline aminopeptidase) (EC 3.4.11.5) (Prolyl aminopeptidase)	P28838 (LAP3)	0.749	1.344	8.189	<0.0001	0.0007
P31941	ENSG00000128383; ENSG00000262156	APOBEC3A	DNA dC->dU-editing enzyme APOBEC-3A (A3A) (EC 3.5.4.38) (Phorbolin-1)	Q9HC16 (APOBEC3G)	0.746	1.285	7.069	<0.0001	0.0018
O14879	ENSG00000119917	IFIT3	Interferon-induced protein with tetratricopeptide repeats 3 (IFIT-3) (CIG49) (ISG-60) (Interferon-induced 60 kDa protein) (IFI-60K) (Interferon-induced protein with tetratricopeptide repeats 4) (IFIT-4) (Retinoic acid-induced gene G protein) (P60) (RIG-G)	O14879 (IFIT3)	0.722	1.342	6.815	<0.0001	0.0022

Confidential/Proprietary Information

Table S55: Proteins differentially abundant at Day 30 compared to pre-second vaccination (Study Arm 2 (A/H7N9 + AS03 D1, 29), Dendritic cells). Proteins are sorted by descending absolute log_2 fold change. Protein and gene annotations are based on Ensembl Version 101 (continued).

Protein ID	Ensembl Gene ID	Ensembl Gene Name	Protein Description	50% Protein Cluster ID (Gene Name)	Fold Change (Day 30 vs. Pre- Second Vaccination)	Average log_2 Abundance	Moderated t-Statistic	P-Value	FDR Adjusted P-value
O95236	ENSG00000128284	APOL3	Apolipoprotein L3 (Apolipoprotein L-III) (ApoL-III) (TNF-inducible protein CG12-1) ($CG12_1$)	O95236 (APOL3)	0.689	1.325	4.820	0.0004	0.015
P42224	ENSG00000115415	STAT1	Signal transducer and activator of transcription 1-alpha/beta (Transcription factor ISGF-3 components p91/p84)	P40763 (STAT3)	0.688	1.413	10.781	<0.0001	0.0003
P24158	ENSG00000196415; ENSG00000277804	PRTN3	Myeloblastin (EC 3.4.21.76) (AGP7) (C-ANCA antigen) (Leukocyte proteinase 3) (PR-3) (PR3) (Neutrophil proteinase 4) (NP-4) (P29) (Wegener autoantigen)	P08246 (ELANE)	0.649	0.800	4.382	0.0008	0.0222
Q86WV1	ENSG00000141293	SKAP1	Src kinase-associated phosphoprotein 1 (Src family-associated phosphoprotein 1) (Src kinase-associated phosphoprotein of 55 kDa) (SKAP-55) (pp55)	Q86WV1 (SKAP1)	-0.641	0.607	-5.006	0.0003	0.0119
P10321	ENSG00000204525	HLA-C	HLA class I histocompatibility antigen, C alpha chain (HLA-C) (HLA-Cw) (Human leukocyte antigen C)	P30501 (NA)	0.612	1.076	3.120	0.0084	0.0819
P20591	ENSG00000157601	MX1	Interferon-induced GTP-binding protein Mx1 (Interferon-induced protein p78) (IFI-78K) (Interferon-regulated resistance GTP-binding protein MxA) (Myxoma resistance protein 1) (Myxovirus resistance protein 1) [Cleaved into: Interferon-induced GTP-binding protein Mx1, N-terminally processed]	P20592 (MX2)	0.595	1.334	3.690	0.0028	0.0435
P07766	ENSG00000198851	CD3E	T-cell surface glycoprotein CD3 epsilon chain (T-cell surface antigen T3/Leu-4 epsilon chain) (CD antigen CD3e)	P07766 (CD3E)	-0.589	0.615	-4.763	0.0004	0.0152
O00499	ENSG00000136717	BIN1	Myc box-dependent-interacting protein 1 (Amphiphysin II) (Amphiphysin-like protein) (Box-dependent myc-interacting protein 1) (Bridging integrator 1)	O00499 (BIN1)	-0.573	0.502	-6.275	<0.0001	0.0038
Q8WYJ6	ENSG00000180096	SEPTIN1	Septin-1 (LARP) (Peanut-like protein 3) (Serologically defined breast cancer antigen NY-BR-24)	Q8WYJ6 (SEPTIN1)	-0.569	0.610	-5.197	0.0002	0.0099
O75367	ENSG00000113648	MACROH2A1	Core histone macro-H2A.1 (Histone macroH2A1) (mH2A1) (Histone H2A.y) (H2A/y) (Medulloblastoma antigen MU-MB-50.205)	Q9P0M6 (MACROH2A2)	0.564	1.035	7.100	<0.0001	0.0018
P35237	ENSG00000124570	SERPINB6	Serpin B6 (Cytoplasmic antiproteinase) (CAP) (Peptidase inhibitor 6) (PI-6) (Placental thrombin inhibitor)	P30740 (SERPINB1)	-0.564	0.387	-5.353	0.0001	0.0086
Q9Y2S6	ENSG00000232112	TMA7	Translation machinery-associated protein 7 (Coiled-coil domain-containing protein 72)	Q9Y2S6 (TMA7)	-0.564	0.946	-2.931	0.012	0.0996

Table S55: Proteins differentially abundant at Day 30 compared to pre-second vaccination (Study Arm 2 (A/H7N9 + AS03 D1, 29), Dendritic cells). Proteins are sorted by descending absolute log_2 fold change. Protein and gene annotations are based on Ensembl Version 101 (continued).

Protein ID	Ensembl Gene ID	Ensembl Gene Name	Protein Description	50% Protein Cluster ID (Gene Name)	Fold Change (Day 30 vs. Pre- Second Vaccination)	Average log_2 Abundance	Moderated t-Statistic	P-Value	FDR Adjusted P-value
P33121	ENSG00000151726	ACSL1	Long-chain-fatty-acid–CoA ligase 1 (EC 6.2.1.3) (Acyl-CoA synthetase 1) (ACS1) (Arachidonate–CoA ligase) (EC 6.2.1.15) (Long-chain acyl-CoA synthetase 1) (LACS 1) (Long-chain acyl-CoA synthetase 2) (LACS 2) (Long-chain fatty acid-CoA ligase 2) (Palmitoyl-CoA ligase 1) (Palmitoyl-CoA ligase 2) (Phytanate–CoA ligase) (EC 6.2.1.24)	P33121 (ACSL1)	0.556	1.082	7.287	<0.0001	0.0018
P08311	ENSG00000100448	CTSG	Cathepsin G (CG) (EC 3.4.21.20)	P08311 (CTSG)	0.550	1.133	7.041	<0.0001	0.0018
P17900	ENSG00000196743	GM2A	Ganglioside GM2 activator (Cerebroside sulfate activator protein) (GM2-AP) (Sphingolipid activator protein 3) (SAP-3) [Cleaved into: Ganglioside GM2 activator isoform short]	P17900 (GM2A)	0.524	0.766	6.326	<0.0001	0.0036
P14222	ENSG00000180644	PRF1	Perforin-1 (P1) (Cytolysin) (Lymphocyte pore-forming protein) (PFP)	P14222 (PRF1)	-0.523	0.028	-3.782	0.0024	0.0392
P52630	ENSG00000170581	STAT2	Signal transducer and activator of transcription 2 (p113)	P52630 (STAT2)	0.507	1.114	7.769	<0.0001	0.0011
Q13469	ENSG00000101096	NFATC2	Nuclear factor of activated T-cells, cytoplasmic 2 (NF-ATc2) (NFATc2) (NFAT pre-existing subunit) (NF-ATp) (T-cell transcription factor NFAT1)	Q13469 (NFATC2)	-0.497	0.333	-5.022	0.0003	0.0118
O75791	ENSG00000100351	GRAP2	GRB2-related adapter protein 2 (Adapter protein GRID) (GRB-2-like protein) (GRB2L) (GRBLG) (GRBX) (Grf40 adapter protein) (Grf-40) (Growth factor receptor-binding protein) (Hematopoietic cell-associated adapter protein GrpL) (P38) (Protein GADS) (SH3-SH2-SH3 adapter Mona)	O75791 (GRAP2)	-0.496	0.568	-5.447	0.0001	0.0086
P08246	ENSG00000197561; ENSG00000277571	ELANE	Neutrophil elastase (EC 3.4.21.37) (Bone marrow serine protease) (Elastase-2) (Human leukocyte elastase) (HLE) (Medullasin) (PMN elastase)	P08246 (ELANE)	0.490	0.749	8.698	<0.0001	0.0005
P06127	ENSG00000110448	CD5	T-cell surface glycoprotein CD5 (Lymphocyte antigen T1/Leu-1) (CD antigen CD5)	P06127 (CD5)	-0.471	0.729	-3.846	0.0021	0.0376
Q13287	ENSG00000123609	NMI	N-myc-interactor (Nmi) (N-myc and STAT interactor)	Q13287 (NMI)	0.466	1.148	8.556	<0.0001	0.0006
Q9BQE5	ENSG00000128335	APOL2	Apolipoprotein L2 (Apolipoprotein L-II) (ApoL-II)	O14791 (APOL1)	0.463	1.227	3.974	0.0017	0.0338

Table S55: Proteins differentially abundant at Day 30 compared to pre-second vaccination (Study Arm 2 (A/H7N9 + AS03 D1, 29), Dendritic cells). Proteins are sorted by descending absolute log_2 fold change. Protein and gene annotations are based on Ensembl Version 101 (continued).

Protein ID	Ensembl Gene ID	Ensembl Gene Name	Protein Description	50% Protein Cluster ID (Gene Name)	Fold Change (Day 30 vs. Pre- Second Vaccination)	Average log_2 Abundance	Moderated t-Statistic	P-Value	FDR Adjusted P-value
P80217	ENSG00000068079	IFI35	Interferon-induced 35 kDa protein (IFP 35) (Ifi-35)	P80217 (IFI35)	0.459	1.113	5.864	<0.0001	0.0054
P28676	ENSG00000115271	GCA	Grancalcin	P28676 (GCA)	0.446	0.746	4.141	0.0012	0.0289
P20963	ENSG00000198821	CD247	T-cell surface glycoprotein CD3 zeta chain (T-cell receptor T3 zeta chain) (CD antigen CD247)	P20963 (CD247)	-0.444	0.565	-6.383	<0.0001	0.0034
P43403	ENSG00000115085	ZAP70	Tyrosine-protein kinase ZAP-70 (EC 2.7.10.2) (70 kDa zeta-chain associated protein) (Syk-related tyrosine kinase)	P43403 (ZAP70)	-0.444	0.370	-4.710	0.0004	0.0156
O75923	ENSG00000135636	DYSF	Dysferlin (Dystrophy-associated fer-1-like protein) (Fer-1-like protein 1)	O75923 (DYSF)	0.434	1.018	8.081	<0.0001	0.0008
P12429	ENSG00000138772	ANXA3	Annexin A3 (35-alpha calcimedin) (Annexin III) (Annexin-3) (Inositol 1,2-cyclic phosphate 2-phosphohydrolase) (Lipocortin III) (Placental anticoagulant protein III) (PAP-III)	P08133 (ANXA6)	0.431	1.089	3.692	0.0028	0.0435
P49006	ENSG00000175130	MARCKSL1	MARCKS-related protein (MARCKS-like protein 1) (Macrophage myristoylated alanine-rich C kinase substrate) (Mac-MARCKS) (MacMARCKS)	P49006 (MARCKSL1)	-0.430	0.793	-4.081	0.0014	0.0304
Q14761	ENSG00000213402	PTPRCAP	Protein tyrosine phosphatase receptor type C-associated protein (PTPRC-associated protein) (CD45-associated protein) (CD45-AP) (Lymphocyte phosphatase-associated phosphoprotein)	Q14761 (PTPRCAP)	-0.422	0.559	-3.403	0.0049	0.0592
Q9UI08	ENSG00000196405	EVL	Ena/VASP-like protein (Ena/vasodilator-stimulated phosphoprotein-like)	Q9UI08 (EVL)	-0.416	0.633	-6.198	<0.0001	0.0039
Q460N5	ENSG00000173193	PARP14	Protein mono-ADP-ribosyltransferase PARP14 (EC 2.4.2.-) (ADP-ribosyltransferase diphtheria toxin-like 8) (ARTD8) (B aggressive lymphoma protein 2) (Poly [ADP-ribose] polymerase 14) (PARP-14)	Q460N5 (PARP14)	0.412	1.053	10.058	<0.0001	0.0004
Q9Y6K5	ENSG00000111331	OAS3	2'-5'-oligoadenylate synthase 3 ((2-5')oligo(A) synthase 3) (2-5A synthase 3) (EC 2.7.7.84) (p100 OAS) (p100OAS)	Q9Y6K5 (OAS3)	0.410	1.074	3.063	0.0093	0.0871
Q14314	ENSG00000127951	FGL2	Fibroleukin (Fibrinogen-like protein 2) (pT49)	Q14314 (FGL2)	0.408	0.998	4.938	0.0003	0.0128
P04234	ENSG00000167286	CD3D	T-cell surface glycoprotein CD3 delta chain (T-cell receptor T3 delta chain) (CD antigen CD3d)	P04234 (CD3D)	-0.406	0.766	-5.226	0.0002	0.0096

Table S55: Proteins differentially abundant at Day 30 compared to pre-second vaccination (Study Arm 2 (A/H7N9 + AS03 D1, 29), Dendritic cells). Proteins are sorted by descending absolute log_2 fold change. Protein and gene annotations are based on Ensembl Version 101 (continued).

Protein ID	Ensembl Gene ID	Ensembl Gene Name	Protein Description	50% Protein Cluster ID (Gene Name)	Fold Change (Day 30 vs. Pre- Second Vaccination)	Average log_2 Abundance	Moderated t-Statistic	P-Value	FDR Adjusted P-value
Q9H939	ENSG00000152229	PSTPIP2	Proline-serine-threonine phosphatase-interacting protein 2 (PEST phosphatase-interacting protein 2)	Q9H939 (PSTPIP2)	0.397	1.099	5.362	0.0001	0.0086
P09914	ENSG00000185745	IFIT1	Interferon-induced protein with tetratricopeptide repeats 1 (IFIT-1) (Interferon-induced 56 kDa protein) (IFI-56K) (P56)	Q13325 (IFIT5)	0.397	1.270	2.973	0.0111	0.0966
P55327	ENSG00000076554	TPD52	Tumor protein D52 (Protein N8)	P55327 (TPD52)	-0.394	0.745	-5.308	0.0002	0.0088
Q5EBM0	ENSG00000134326	CMPK2	UMP-CMP kinase 2, mitochondrial (EC 2.7.4.14) (Nucleoside-diphosphate kinase) (EC 2.7.4.6)	Q5EBM0 (CMPK2)	0.393	1.022	4.502	0.0006	0.019
O14933	ENSG00000156587	UBE2L6	Ubiquitin/ISG15-conjugating enzyme E2 L6 (EC 2.3.2.23) (E2 ubiquitin-conjugating enzyme L6) (Retinoic acid-induced gene B protein) (RIG-B) (UbcH8) (Ubiquitin carrier protein L6) (Ubiquitin-protein ligase L6)	P68036 (UBE2L3)	0.392	1.105	5.383	0.0001	0.0086
P05109	ENSG00000143546	S100A8	Protein S100-A8 (Calgranulin-A) (Calprotectin L1L subunit) (Cystic fibrosis antigen) (CFAG) (Leukocyte L1 complex light chain) (Migration inhibitory factor-related protein 8) (MRP-8) (p8) (S100 calcium-binding protein A8) (Urinary stone protein band A)	P05109 (S100A8)	0.388	0.918	5.852	<0.0001	0.0054
P48595	ENSG00000242550	SERPINB10	Serpin B10 (Bomapin) (Peptidase inhibitor 10) (PI-10)	P48595 (SERPINB10)	0.387	1.096	5.964	<0.0001	0.0051
Q02880	ENSG00000077097	TOP2B	DNA topoisomerase 2-beta (EC 5.6.2.2) (DNA topoisomerase II, beta isozyme)	Q02880 (TOP2B)	0.383	1.030	8.706	<0.0001	0.0005
P10620	ENSG00000008394	MGST1	Microsomal glutathione S-transferase 1 (Microsomal GST-1) (EC 2.5.1.18) (Microsomal GST-I)	P10620 (MGST1)	0.380	0.935	3.147	0.008	0.0792
P08670	ENSG00000026025	VIM	Vimentin	P08670 (VIM)	0.379	0.952	3.164	0.0077	0.0778
Q8IXQ6	ENSG00000138496	PARP9	Protein mono-ADP-ribosyltransferase PARP9 (EC 2.4.2.-) (ADP-ribosyltransferase diphtheria toxin-like 9) (ARTD9) (B aggressive lymphoma protein) (Poly [ADP-ribose] polymerase 9) (PARP-9)	Q8IXQ6 (PARP9)	0.377	1.071	6.209	<0.0001	0.0039

Table S55: Proteins differentially abundant at Day 30 compared to pre-second vaccination (Study Arm 2 (A/H7N9 + AS03 D1, 29), Dendritic cells). Proteins are sorted by descending absolute log_2 fold change. Protein and gene annotations are based on Ensembl Version 101 (continued).

Protein ID	Ensembl Gene ID	Ensembl Gene Name	Protein Description	50% Protein Cluster ID (Gene Name)	Fold Change (Day 30 vs. Pre- Second Vaccination)	Average log_2 Abundance	Moderated t-Statistic	P-Value	FDR Adjusted P-value
P42330	ENSG00000196139	AKR1C3	Aldo-keto reductase family 1 member C3 (EC 1.1.1.-) (EC 1.1.1.210) (EC 1.1.1.53) (EC 1.1.1.62) (17-beta-hydroxysteroid dehydrogenase type 5) (17-beta-HSD 5) (3-alpha-HSD type II, brain) (3-alpha-hydroxysteroid dehydrogenase type 2) (3-alpha-HSD type 2) (EC 1.1.1.357) (Chlordecone reductase homolog HAKRb) (Dihydrodiol dehydrogenase 3) (DD-3) (DD3) (Dihydrodiol dehydrogenase type I) (HA1753) (Prostaglandin F synthase) (PGFS) (EC 1.1.1.188) (Testosterone 17-beta-dehydrogenase 5) (EC 1.1.1.239) (EC 1.1.1.64)	Q04828 (AKR1C1)	-0.375	0.324	-3.171	0.0076	0.077
P32189	ENSG00000198814	GK	Glycerol kinase (GK) (Glycerokinase) (EC 2.7.1.30) (ATP:glycerol 3-phosphotransferase)	P32189 (GK)	0.374	1.073	4.962	0.0003	0.0127
P20592	ENSG00000183486	MX2	Interferon-induced GTP-binding protein Mx2 (Interferon-regulated resistance GTP-binding protein MxB) (Myxovirus resistance protein 2) (p78-related protein)	P20592 (MX2)	0.373	1.065	3.800	0.0023	0.0391
P04179	ENSG00000112096	SOD2	Superoxide dismutase [Mn], mitochondrial (EC 1.15.1.1)	P04179 (SOD2)	0.372	0.924	5.910	<0.0001	0.0052
P61626	ENSG00000090382	LYZ	Lysozyme C (EC 3.2.1.17) (1,4-beta-N-acetylmuramidase C)	P61626 (LYZ)	0.371	0.935	5.159	0.0002	0.0101
P00441	ENSG00000142168	SOD1	Superoxide dismutase [Cu-Zn] (EC 1.15.1.1) (Superoxide dismutase 1) (hSod1)	P00441 (SOD1)	-0.370	0.672	-4.132	0.0013	0.029
Q8IU85	ENSG00000183049	CAMK1D	Calcium/calmodulin-dependent protein kinase type 1D (EC 2.7.11.17) (CaM kinase I delta) (CaM kinase ID) (CaM-KI delta) (CaMKI delta) (CaMKID) (CaMKI-like protein kinase) (CKLiK)	Q8IU85 (CAMK1D)	-0.370	0.829	-4.769	0.0004	0.0152
P53367	ENSG00000164144	ARFIP1	Arfaptin-1 (ADP-ribosylation factor-interacting protein 1)	P53367 (ARFIP1)	0.370	0.986	2.655	0.0202	0.1361
P06239	ENSG00000182866	LCK	Tyrosine-protein kinase Lck (EC 2.7.10.2) (Leukocyte C-terminal Src kinase) (LSK) (Lymphocyte cell-specific protein-tyrosine kinase) (Protein YT16) (Proto-oncogene Lck) (T cell-specific protein-tyrosine kinase) (p56-LCK)	P06241 (FYN)	-0.368	0.821	-3.033	0.0099	0.0914
Q6K0P9	ENSG00000163564	PYHIN1	Pyrin and HIN domain-containing protein 1 (Interferon-inducible protein X)	Q16666 (IFI16)	-0.364	0.457	-4.820	0.0004	0.015

Table S55: Proteins differentially abundant at Day 30 compared to pre-second vaccination (Study Arm 2 (A/H7N9 + AS03 D1, 29), Dendritic cells). Proteins are sorted by descending absolute log_2 fold change. Protein and gene annotations are based on Ensembl Version 101 (continued).

Protein ID	Ensembl Gene ID	Ensembl Gene Name	Protein Description	50% Protein Cluster ID (Gene Name)	Fold Change (Day 30 vs. Pre- Second Vaccination)	Average log_2 Abundance	Moderated t-Statistic	P-Value	FDR Adjusted P-value
O95197	ENSG00000133318	RTN3	Reticulon-3 (Homolog of ASY protein) (HAP) (Neuroendocrine-specific protein-like 2) (NSP-like protein 2) (Neuroendocrine-specific protein-like II) (NSP-like protein II) (NSPLII)	O95197 (RTN3)	0.364	0.826	3.578	0.0035	0.0494
Q13595	ENSG00000164548	TRA2A	Transformer-2 protein homolog alpha (TRA-2 alpha) (TRA2-alpha) (Transformer-2 protein homolog A)	P62995 (TRA2B)	0.360	0.884	6.512	<0.0001	0.0032
Q9NYJ1	ENSG00000181924	COA4	Cytochrome c oxidase assembly factor 4 homolog, mitochondrial (Coiled-coil-helix-coiled-coil-helix domain-containing protein 8) (E2-induced gene 2 protein)	Q9NYJ1 (COA4)	-0.356	0.808	-2.993	0.0107	0.0948
P01871	ENSG00000211899; ENSG00000282657	IGHM	Immunoglobulin heavy constant mu (Ig mu chain C region) (Ig mu chain C region BOT) (Ig mu chain C region GAL) (Ig mu chain C region OU)	P0DOX6 (NA)	-0.356	0.830	-3.374	0.0052	0.0602
Q14011	ENSG00000099622	CIRBP	Cold-inducible RNA-binding protein (A18 hnRNP) (Glycine-rich RNA-binding protein CIRP)	Q14011 (CIRBP)	-0.352	0.502	-3.024	0.0101	0.0917
O75558	ENSG00000135604	STX11	Syntaxin-11	O75558 (STX11)	0.347	1.067	7.100	<0.0001	0.0018
O15162	ENSG00000188313	PLSCR1	Phospholipid scramblase 1 (PL scramblase 1) (Ca(2+)-dependent phospholipid scramblase 1) (Erythrocyte phospholipid scramblase) (Mg(2+)-dependent nuclease) (EC 3.1.-.-) (MmTRA1b)	O15162 (PLSCR1)	0.347	1.106	4.549	0.0006	0.0182
Q9GZP4	ENSG00000057757	PITHD1	PITH domain-containing protein 1	Q9GZP4 (PITHD1)	-0.345	0.876	-2.754	0.0168	0.1208
Q9NZM1	ENSG00000138119	MYOF	Myoferlin (Fer-1-like protein 3)	O75923 (DYSF)	0.343	1.029	4.943	0.0003	0.0128
P51608	ENSG00000169057	MECP2	Methyl-CpG-binding protein 2 (MeCp-2 protein) (MeCp2)	P51608 (MECP2)	-0.342	0.689	-3.902	0.0019	0.036
Q03252	ENSG00000176619	LMNB2	Lamin-B2	P02545 (LMNA)	-0.340	0.698	-4.247	0.001	0.026
P09874	ENSG00000143799	PARP1	Poly [ADP-ribose] polymerase 1 (PARP-1) (EC 2.4.2.30) (ADP-ribosyltransferase diphtheria toxin-like 1) (ARTD1) (DNA ADP-ribosyltransferase PARP1) (EC 2.4.2.-) (NAD(+) ADP-ribosyltransferase 1) (ADPRT 1) (Poly[ADP-ribose] synthase 1) (Protein poly-ADP-ribosyltransferase PARP1) (EC 2.4.2.-)	P09874 (PARP1)	-0.340	0.634	-5.291	0.0002	0.0088

Table S55: Proteins differentially abundant at Day 30 compared to pre-second vaccination (Study Arm 2 (A/H7N9 + AS03 D1, 29), Dendritic cells). Proteins are sorted by descending absolute log_2 fold change. Protein and gene annotations are based on Ensembl Version 101 (continued).

Protein ID	Ensembl Gene ID	Ensembl Gene Name	Protein Description	50% Protein Cluster ID (Gene Name)	Fold Change (Day 30 vs. Pre- Second Vaccination)	Average log_2 Abundance	Moderated t-Statistic	P-Value	FDR Adjusted P-value
O43852	ENSG00000128595	CALU	Calumenin (Crocalbin) (IEF SSP 9302)	Q15293 (RCN1)	-0.336	0.642	-3.593	0.0034	0.0484
P61916	ENSG00000119655	NPC2	NPC intracellular cholesterol transporter 2 (Epididymal secretory protein E1) (Human epididymis-specific protein 1) (He1) (Niemann-Pick disease type C2 protein)	P61916 (NPC2)	0.334	0.784	9.269	<0.0001	0.0004
Q9UNZ5	ENSG00000104979	C19orf53	Leydig cell tumor 10 kDa protein homolog	Q9UNZ5 (C19orf53)	-0.328	0.613	-3.810	0.0023	0.0391
P46939	ENSG00000152818	UTRN	Utrophin (Dystrophin-related protein 1) (DRP-1)	P46939 (UTRN)	0.325	0.824	4.706	0.0004	0.0156
Q03169	ENSG00000185215	TNFAIP2	Tumor necrosis factor alpha-induced protein 2 (TNF alpha-induced protein 2) (Primary response gene B94 protein)	Q03169 (TNFAIP2)	0.321	1.007	5.408	0.0001	0.0086
P43490	ENSG00000105835	NAMPT	Nicotinamide phosphoribosyltransferase (NAmPRTase) (Nampt) (EC 2.4.2.12) (Pre-B-cell colony-enhancing factor 1) (Pre-B cell-enhancing factor) (Visfatin)	P43490 (NAMPT)	0.320	0.844	4.743	0.0004	0.0153
Q14964	ENSG00000179331	RAB39A	Ras-related protein Rab-39A (Rab-39)	Q14964 (RAB39A)	0.319	1.065	3.202	0.0072	0.0738
P05164	ENSG00000005381	MPO	Myeloperoxidase (MPO) (EC 1.11.2.2) [Cleaved into: Myeloperoxidase; 89 kDa myeloperoxidase; 84 kDa myeloperoxidase; Myeloperoxidase light chain; Myeloperoxidase heavy chain]	P05164 (MPO)	0.314	0.960	4.333	0.0009	0.0234
P10619	ENSG00000064601	CTSA	Lysosomal protective protein (EC 3.4.16.5) (Carboxypeptidase C) (Carboxypeptidase L) (Cathepsin A) (Protective protein cathepsin A) (PPCA) (Protective protein for beta-galactosidase) [Cleaved into: Lysosomal protective protein 32 kDa chain; Lysosomal protective protein 20 kDa chain]	P10619 (CTSA)	0.314	0.850	4.341	0.0009	0.0232
P12724	ENSG00000169397	RNASE3	Eosinophil cationic protein (ECP) (EC 3.1.27.-) (Ribonuclease 3) (RNase 3)	P10153 (RNASE2)	0.312	1.000	4.379	0.0008	0.0222
P15260	ENSG00000027697	IFNGR1	Interferon gamma receptor 1 (IFN-gamma receptor 1) (IFN-gamma-R1) (CDw119) (Interferon gamma receptor alpha-chain) (IFN-gamma-R-alpha) (CD antigen CD119)	P15260 (IFNGR1)	-0.312	0.521	-3.126	0.0083	0.0816

Table S55: Proteins differentially abundant at Day 30 compared to pre-second vaccination (Study Arm 2 (A/H7N9 + AS03 D1, 29), Dendritic cells). Proteins are sorted by descending absolute log_2 fold change. Protein and gene annotations are based on Ensembl Version 101 (continued).

Protein ID	Ensembl Gene ID	Ensembl Gene Name	Protein Description	50% Protein Cluster ID (Gene Name)	Fold Change (Day 30 vs. Pre- Second Vaccination)	Average log_2 Abundance	Moderated t-Statistic	P-Value	FDR Adjusted P-value
P80511	ENSG00000163221	S100A12	Protein S100-A12 (CGRP) (Calcium-binding protein in amniotic fluid 1) (CAAF1) (Calgranulin-C) (CAGC) (Extracellular newly identified RAGE-binding protein) (EN-RAGE) (Migration inhibitory factor-related protein 6) (MRP-6) (p6) (Neutrophil S100 protein) (S100 calcium-binding protein A12) [Cleaved into: Calcitermin]	P80511 (S100A12)	0.310	0.884	3.113	0.0085	0.0822
Q9NRL2	ENSG00000198604	BAZ1A	Bromodomain adjacent to zinc finger domain protein 1A (ATP-dependent chromatin-remodeling protein) (ATP-utilizing chromatin assembly and remodeling factor 1) (hACF1) (CHRAC subunit ACF1) (Williams syndrome transcription factor-related chromatin-remodeling factor 180) (WCRF180) (hWALp1)	Q9NRL2 (BAZ1A)	0.310	0.802	4.571	0.0006	0.018
Q13642	ENSG00000022267	FHL1	Four and a half LIM domains protein 1 (FHL-1) (Skeletal muscle LIM-protein 1) (SLIM) (SLIM-1)	Q13642 (FHL1)	-0.306	0.816	-4.125	0.0013	0.0291
Q9BT23	ENSG00000136490	LIMD2	LIM domain-containing protein 2	Q9UHB6 (LIMA1)	-0.306	0.747	-3.924	0.0018	0.0355
Q08AF3	ENSG00000166750	SLFN5	Schlafen family member 5	Q7Z7L1 (SLFN11)	-0.306	0.627	-3.300	0.006	0.0666
P12544	ENSG00000145649	GZMA	Granzyme A (EC 3.4.21.78) (CTL tryptase) (Cytotoxic T-lymphocyte proteinase 1) (Fragmentin-1) (Granzyme-1) (Hanukkah factor) (H factor) (HF)	P12544 (GZMA)	-0.305	0.303	-4.061	0.0014	0.031
Q8NFU3	ENSG00000215845	TSTD1	Thiosulfate:glutathione sulfurtransferase (TST) (EC 2.8.1.-)	Q8NFU3 (TSTD1)	-0.304	0.675	-5.326	0.0002	0.0088
P28908	ENSG00000120949	TNFRSF8	Tumor necrosis factor receptor superfamily member 8 (CD30L receptor) (Ki-1 antigen) (Lymphocyte activation antigen CD30) (CD antigen CD30)	P28908 (TNFRSF8)	-0.302	0.527	-3.347	0.0054	0.0623
O00151	ENSG00000107438	PDLIM1	PDZ and LIM domain protein 1 (C-terminal LIM domain protein 1) (Elfin) (LIM domain protein CLP-36)	O00151 (PDLIM1)	-0.300	0.882	-4.469	0.0007	0.02
P13473	ENSG00000005893	LAMP2	Lysosome-associated membrane glycoprotein 2 (LAMP-2) (Lysosome-associated membrane protein 2) (CD107 antigen-like family member B) (LGP-96) (CD antigen CD107b)	P13473 (LAMP2)	0.299	0.832	4.713	0.0004	0.0156

Table S55: Proteins differentially abundant at Day 30 compared to pre-second vaccination (Study Arm 2 (A/H7N9 + AS03 D1, 29), Dendritic cells). Proteins are sorted by descending absolute log_2 fold change. Protein and gene annotations are based on Ensembl Version 101 (continued).

Protein ID	Ensembl Gene ID	Ensembl Gene Name	Protein Description	50% Protein Cluster ID (Gene Name)	Fold Change (Day 30 vs. Pre- Second Vaccination)	Average log_2 Abundance	Moderated t-Statistic	P-Value	FDR Adjusted P-value
P22304	ENSG00000010404	IDS	Iduronate 2-sulfatase (EC 3.1.6.13) (Alpha-L-iduronate sulfate sulfatase) (Idursulfase) [Cleaved into: Iduronate 2-sulfatase 42 kDa chain; Iduronate 2-sulfatase 14 kDa chain]	P22304 (IDS)	-0.298	0.330	-4.609	0.0005	0.0173
P62854	ENSG00000197728	RPS26	40S ribosomal protein S26 (Small ribosomal subunit protein eS26)	P62854 (RPS26)	-0.298	0.654	-5.095	0.0002	0.011
P09429	ENSG00000189403	HMGB1	High mobility group protein B1 (High mobility group protein 1) (HMG-1)	P23497 (SP100)	-0.297	0.573	-3.293	0.006	0.0673
Q14005	ENSG00000172349	IL16	Pro-interleukin-16 [Cleaved into: Interleukin-16 (IL-16) (Lymphocyte chemoattractant factor) (LCF)]	Q14005 (IL16)	-0.295	0.664	-5.367	0.0001	0.0086
P57088	ENSG00000109133	TMEM33	Transmembrane protein 33 (Protein DB83) (SHINC-3)	P57088 (TMEM33)	0.295	0.791	2.742	0.0172	0.1218
P28161	ENSG00000213366	GSTM2	Glutathione S-transferase Mu 2 (EC 2.5.1.18) (GST class-mu 2) (GSTM2-2)	P21266 (GSTM3)	-0.295	0.860	-5.442	0.0001	0.0086
P01009	ENSG00000197249	SERPINA1	Alpha-1-antitrypsin (Alpha-1 protease inhibitor) (Alpha-1-antiproteinase) (Serpin A1) [Cleaved into: Short peptide from AAT (SPAAT)]	P01009 (SERPINA1)	-0.294	0.375	-3.107	0.0086	0.0824
P02545	ENSG00000160789	LMNA	Prelamin-A/C [Cleaved into: Lamin-A/C (70 kDa lamin) (Renal carcinoma antigen NY-REN-32)]	P02545 (LMNA)	-0.294	0.479	-5.953	<0.0001	0.0051
P04839	ENSG00000165168	CYBB	Cytochrome b-245 heavy chain (EC 1.-.-.-) (CGD91-phox) (Cytochrome b(558) subunit beta) (Cytochrome b558 subunit beta) (Heme-binding membrane glycoprotein gp91phox) (NADPH oxidase 2) (Neutrophil cytochrome b 91 kDa polypeptide) (Superoxide-generating NADPH oxidase heavy chain subunit) (gp91-1) (gp91-phox) (p22 phagocyte B-cytochrome)	P04839 (CYBB)	0.293	0.939	4.130	0.0013	0.029
Q8TDB6	ENSG00000163840	DTX3L	E3 ubiquitin-protein ligase DTX3L (EC 2.3.2.27) (B-lymphoma- and BAL-associated protein) (Protein deltex-3-like) (RING-type E3 ubiquitin transferase DTX3L) (Rhysin-2) (Rhysin2)	Q8TDB6 (DTX3L)	0.293	1.044	5.251	0.0002	0.0093
P04439	ENSG00000206503	HLA-A	HLA class I histocompatibility antigen, A alpha chain (Human leukocyte antigen A) (HLA-A)	P30501 (NA)	0.292	1.244	3.128	0.0083	0.0815
P31689	ENSG00000086061	DNAJA1	DnaJ homolog subfamily A member 1 (DnaJ protein homolog 2) (HSDJ) (Heat shock 40 kDa protein 4) (Heat shock protein J2) (HSJ-2) (Human DnaJ protein 2) (hDj-2)	O60884 (DNAJA2)	0.292	0.976	4.102	0.0013	0.0296

Table S55: Proteins differentially abundant at Day 30 compared to pre-second vaccination (Study Arm 2 (A/H7N9 + AS03 D1, 29), Dendritic cells). Proteins are sorted by descending absolute log_2 fold change. Protein and gene annotations are based on Ensembl Version 101 (continued).

Protein ID	Ensembl Gene ID	Ensembl Gene Name	Protein Description	50% Protein Cluster ID (Gene Name)	Fold Change (Day 30 vs. Pre- Second Vaccination)	Average log_2 Abundance	Moderated t-Statistic	P-Value	FDR Adjusted P-value
P19971	ENSG00000025708	TYMP	Thymidine phosphorylase (TP) (EC 2.4.2.4) (Gliostatin) (Platelet-derived endothelial cell growth factor) (PD-ECGF) (TdRPase)	P19971 (TYMP)	0.289	1.025	3.918	0.0019	0.0355
Q7L5N7	ENSG00000087253	LPCAT2	Lysophosphatidylcholine acyltransferase 2 (LPC acyltransferase 2) (LPCAT-2) (LysoPC acyltransferase 2) (EC 2.3.1.23) (1-acylglycerol-3-phosphate O-acyltransferase 11) (1-AGP acyltransferase 11) (1-AGPAT 11) (EC 2.3.1.51) (1-acylglycerophosphocholine O-acyltransferase) (1-alkenylglycerophosphocholine O-acyltransferase) (EC 2.3.1.25) (1-alkylglycerophosphocholine O-acetyltransferase) (EC 2.3.1.67) (Acetyl-CoA:lyso-platelet-activating factor acetyltransferase) (Acetyl-CoA:lyso-PAF acetyltransferase) (Lyso-PAF acetyltransferase) (LysoPAFAT) (Acyltransferase-like 1) (Lysophosphatidic acid acyltransferase alpha) (LPAAT-alpha)	Q7L5N7 (LPCAT2)	0.288	0.778	5.662	<0.0001	0.0071
P84090	ENSG00000100632	ERH	Enhancer of rudimentary homolog	P84090 (ERH)	0.288	0.922	3.991	0.0016	0.0334
O60880	ENSG00000183918	SH2D1A	SH2 domain-containing protein 1A (Duncan disease SH2-protein) (Signaling lymphocytic activation molecule-associated protein) (SLAM-associated protein) (T-cell signal transduction molecule SAP)	O60880 (SH2D1A)	-0.288	0.638	-2.926	0.0121	0.1001
P00352	ENSG00000165092	ALDH1A1	Retinal dehydrogenase 1 (RALDH 1) (RalDH1) (EC 1.2.1.-) (EC 1.2.1.36) (ALDH-E1) (ALHDII) (Aldehyde dehydrogenase family 1 member A1) (Aldehyde dehydrogenase, cytosolic)	P30837 (ALDH1B1)	0.288	1.242	3.396	0.005	0.0592
Q8IV04	ENSG00000175463	TBC1D10C	Carabin (TBC1 domain family member 10C)	Q8IV04 (TBC1D10C)	-0.288	0.679	-3.282	0.0062	0.0682
P08962	ENSG00000135404	CD63	CD63 antigen (Granulophysin) (Lysosomal-associated membrane protein 3) (LAMP-3) (Melanoma-associated antigen ME491) (OMA81H) (Ocular melanoma-associated antigen) (Tetraspanin-30) (Tspan-30) (CD antigen CD63)	P08962 (CD63)	0.287	1.043	4.043	0.0015	0.0311

Table S55: Proteins differentially abundant at Day 30 compared to pre-second vaccination (Study Arm 2 (A/H7N9 + AS03 D1, 29), Dendritic cells). Proteins are sorted by descending absolute log_2 fold change. Protein and gene annotations are based on Ensembl Version 101 (continued).

Protein ID	Ensembl Gene ID	Ensembl Gene Name	Protein Description	50% Protein Cluster ID (Gene Name)	Fold Change (Day 30 vs. Pre- Second Vaccination)	Average log_2 Abundance	Moderated t-Statistic	P-Value	FDR Adjusted P-value
Q9H0J9	ENSG00000059378	PARP12	Protein mono-ADP-ribosyltransferase PARP12 (EC 2.4.2.-) (ADP-ribosyltransferase diphtheria toxin-like 12) (ARTD12) (Poly [ADP-ribose] polymerase 12) (PARP-12) (Zinc finger CCCH domain-containing protein 1)	Q9H0J9 (PARP12)	0.287	0.948	7.021	<0.0001	0.0018
Q99733	ENSG00000205531; ENSG00000273562	NAP1L4	Nucleosome assembly protein 1-like 4 (Nucleosome assembly protein 2) (NAP-2)	P55209 (NAP1L1)	-0.286	0.627	-6.437	<0.0001	0.0034
Q9UHD8	ENSG00000184640	SEPTIN9	Septin-9 (MLL septin-like fusion protein MSF-A) (MLL septin-like fusion protein) (Ovarian/Breast septin) (Ov/Br septin) (Septin D1)	Q9UHD8 (SEPTIN9)	-0.285	0.680	-5.797	<0.0001	0.0058
Q9HD89	ENSG00000104918	RETN	Resistin (Adipose tissue-specific secretory factor) (ADSF) (C/EBP-epsilon-regulated myeloid-specific secreted cysteine-rich protein) (Cysteine-rich secreted protein A12-alpha-like 2) (Cysteine-rich secreted protein FIZZ3)	Q9HD89 (RETN)	0.283	0.641	3.070	0.0092	0.0866
Q14764	ENSG00000013364	MVP	Major vault protein (MVP) (Lung resistance-related protein)	Q14764 (MVP)	0.281	0.971	6.413	<0.0001	0.0034
Q03518	ENSG00000168394; ENSG00000206297; ENSG00000226173; ENSG00000227816; ENSG00000230705; ENSG00000224212; ENSG00000232367	TAP1	Antigen peptide transporter 1 (APT1) (EC 7.4.2.-) (ATP-binding cassette sub-family B member 2) (Peptide supply factor 1) (Peptide transporter PSF1) (PSF-1) (Peptide transporter TAP1) (Peptide transporter involved in antigen processing 1) (Really interesting new gene 4 protein) (RING4)	Q03518 (TAP1)	0.281	1.141	5.959	<0.0001	0.0051
P15309	ENSG00000014257	ACP3	Prostatic acid phosphatase (PAP) (EC 3.1.3.2) (5'-nucleotidase) (5'-NT) (EC 3.1.3.5) (Acid phosphatase 3) (Ecto-5'-nucleotidase) (Protein tyrosine phosphatase ACP3) (EC 3.1.3.48) (Thiamine monophosphatase) (TMPase) [Cleaved into: PAPf39]	P15309 (ACP3)	0.280	0.819	3.652	0.0031	0.045
Q5S007	ENSG00000188906	LRRK2	Leucine-rich repeat serine/threonine-protein kinase 2 (EC 2.7.11.1) (EC 3.6.5.-) (Dardarin)	Q5S007 (LRRK2)	0.279	0.996	4.140	0.0012	0.0289
P08174	ENSG00000196352	CD55	Complement decay-accelerating factor (CD antigen CD55)	P08174 (CD55)	0.275	0.895	2.775	0.0161	0.1181

Table S55: Proteins differentially abundant at Day 30 compared to pre-second vaccination (Study Arm 2 (A/H7N9 + AS03 D1, 29), Dendritic cells). Proteins are sorted by descending absolute log_2 fold change. Protein and gene annotations are based on Ensembl Version 101 (continued).

Protein ID	Ensembl Gene ID	Ensembl Gene Name	Protein Description	50% Protein Cluster ID (Gene Name)	Fold Change (Day 30 vs. Pre- Second Vaccination)	Average log_2 Abundance	Moderated t-Statistic	P-Value	FDR Adjusted P-value
P40763	ENSG00000168610	STAT3	Signal transducer and activator of transcription 3 (Acute-phase response factor)	P40763 (STAT3)	0.274	1.071	5.152	0.0002	0.0101
P30273	ENSG00000158869	FCER1G	High affinity immunoglobulin epsilon receptor subunit gamma (Fc receptor gamma-chain) (FcRgamma) (Fc-epsilon RI-gamma) (IgE Fc receptor subunit gamma) (FceRI gamma)	P30273 (FCER1G)	0.274	0.906	5.606	<0.0001	0.0076
P27695	ENSG00000100823	APEX1	DNA-(apurinic or apyrimidinic site) endonuclease (EC 3.1.-.-) (APEX nuclease) (APEN) (Apurinic-apyrimidinic endonuclease 1) (AP endonuclease 1) (APE-1) (REF-1) (Redox factor-1) [Cleaved into: DNA-(apurinic or apyrimidinic site) endonuclease, mitochondrial]	P27695 (APEX1)	-0.271	0.772	-4.911	0.0003	0.0132
P41218	ENSG00000163563	MNDA	Myeloid cell nuclear differentiation antigen	Q16666 (IFI16)	0.271	0.756	5.367	0.0001	0.0086
Q9BSD7	ENSG00000135778	NTPCR	Cancer-related nucleoside-triphosphatase (NTPase) (EC 3.6.1.15) (Nucleoside triphosphate phosphohydrolase)	Q9BSD7 (NTPCR)	-0.270	0.675	-3.028	0.01	0.0914
O75477	ENSG00000107566	ERLIN1	Erlin-1 (Endoplasmic reticulum lipid raft-associated protein 1) (Protein KE04) (Stomatin-prohibitin-flotillin-HflC/K domain-containing protein 1) (SPFH domain-containing protein 1)	O75477 (ERLIN1)	0.270	1.064	5.595	<0.0001	0.0076
Q9BU76	ENSG00000143793	MMTAG2	Multiple myeloma tumor-associated protein 2 (hMMTAG2)	Q9BU76 (MMTAG2)	-0.269	0.772	-2.539	0.0251	0.1498
O00479	ENSG00000182952	HMGN4	High mobility group nucleosome-binding domain-containing protein 4 (Non-histone chromosomal protein HMG-17-like 3) (Non-histone chromosomal protein)	Q15651 (HMGN3)	-0.268	0.728	-3.873	0.002	0.0374
P07858	ENSG00000164733; ENSG00000285132	CTSB	Cathepsin B (EC 3.4.22.1) (APP secretase) (APPS) (Cathepsin B1) [Cleaved into: Cathepsin B light chain; Cathepsin B heavy chain]	P07858 (CTSB)	0.268	0.885	5.945	<0.0001	0.0051
Q14644	ENSG00000185989; ENSG00000280477	RASA3	Ras GTPase-activating protein 3 (GAP1(IP4BP)) (Ins P4-binding protein)	Q15283 (RASA2)	-0.265	0.779	-3.562	0.0036	0.0505

Table S55: Proteins differentially abundant at Day 30 compared to pre-second vaccination (Study Arm 2 (A/H7N9 + AS03 D1, 29), Dendritic cells). Proteins are sorted by descending absolute log_2 fold change. Protein and gene annotations are based on Ensembl Version 101 (continued).

Protein ID	Ensembl Gene ID	Ensembl Gene Name	Protein Description	50% Protein Cluster ID (Gene Name)	Fold Change (Day 30 vs. Pre- Second Vaccination)	Average log_2 Abundance	Moderated t-Statistic	P-Value	FDR Adjusted P-value
O60264	ENSG00000153147	SMARCA5	SWI/SNF-related matrix-associated actin-dependent regulator of chromatin subfamily A member 5 (SWI/SNF-related matrix-associated actin-dependent regulator of chromatin A5) (EC 3.6.4.-) (Sucrose non-fermenting protein 2 homolog) (hSNF2H)	O60264 (SMARCA5)	0.264	1.004	5.517	0.0001	0.0084
P53004	ENSG00000106605	BLVRA	Biliverdin reductase A (BVR A) (EC 1.3.1.24) (Biliverdin-IX alpha-reductase)	P53004 (BLVRA)	0.263	0.814	4.224	0.0011	0.0266
P32320	ENSG00000158825	CDA	Cytidine deaminase (EC 3.5.4.5) (Cytidine aminohydrolase)	P32320 (CDA)	0.262	0.840	4.797	0.0004	0.0151
P14209	ENSG00000002586	CD99	CD99 antigen (12E7) (E2 antigen) (Protein MIC2) (T-cell surface glycoprotein E2) (CD antigen CD99)	P14209 (CD99)	-0.261	0.717	-3.444	0.0045	0.0569
P34949	ENSG00000178802	MPI	Mannose-6-phosphate isomerase (EC 5.3.1.8) (Phosphohexomutase) (Phosphomannose isomerase) (PMI)	P34949 (MPI)	-0.261	0.725	-3.785	0.0024	0.0392
P51452	ENSG00000108861	DUSP3	Dual specificity protein phosphatase 3 (EC 3.1.3.16) (EC 3.1.3.48) (Dual specificity protein phosphatase VHR) (Vaccinia H1-related phosphatase) (VHR)	P51452 (DUSP3)	0.261	0.832	4.359	0.0008	0.0229
P20718	ENSG00000100450	GZMH	Granzyme H (EC 3.4.21.-) (CCP-X) (Cathepsin G-like 2) (CTSGL2) (Cytotoxic T-lymphocyte proteinase) (Cytotoxic serine protease C) (CSP-C)	P08311 (CTSG)	-0.260	0.101	-2.992	0.0107	0.0948
P42167	ENSG00000120802	TMPO	Lamina-associated polypeptide 2, isoforms beta/gamma (Thymopoietin, isoforms beta/gamma) (TP beta/gamma) (Thymopoietin-related peptide isoforms beta/gamma) (TPRP isoforms beta/gamma) [Cleaved into: Thymopoietin (TP) (Splenin); Thymopentin (TP5)]	P42166 (TMPO)	-0.257	0.708	-4.813	0.0004	0.015
Q8NAV1	ENSG00000134748	PRPF38A	Pre-mRNA-splicing factor 38A	Q8NAV1 (PRPF38A)	0.257	0.824	2.596	0.0226	0.1444
P51649	ENSG00000112294	ALDH5A1	Succinate-semialdehyde dehydrogenase, mitochondrial (EC 1.2.1.24) (Aldehyde dehydrogenase family 5 member A1) (NAD(+)-dependent succinic semialdehyde dehydrogenase)	P51649 (ALDH5A1)	-0.256	0.908	-2.603	0.0223	0.1438
P61927	ENSG00000145592	RPL37	60S ribosomal protein L37 (G1.16) (Large ribosomal subunit protein eL37)	P61927 (RPL37)	-0.255	0.761	-3.429	0.0047	0.0574
Q13616	ENSG00000055130	CUL1	Cullin-1 (CUL-1)	Q13616 (CUL1)	0.255	0.996	4.807	0.0004	0.015

Table S55: Proteins differentially abundant at Day 30 compared to pre-second vaccination (Study Arm 2 (A/H7N9 + AS03 D1, 29), Dendritic cells). Proteins are sorted by descending absolute log_2 fold change. Protein and gene annotations are based on Ensembl Version 101 (continued).

Protein ID	Ensembl Gene ID	Ensembl Gene Name	Protein Description	50% Protein Cluster ID (Gene Name)	Fold Change (Day 30 vs. Pre- Second Vaccination)	Average log_2 Abundance	Moderated t-Statistic	P-Value	FDR Adjusted P-value
P07099	ENSG00000143819	EPHX1	Epoxide hydrolase 1 (EC 3.3.2.9) (Epoxide hydratase) (Microsomal epoxide hydrolase) (mEH)	P07099 (EPHX1)	0.255	0.859	2.565	0.024	0.149
Q14847	ENSG00000002834	LASP1	LIM and SH3 domain protein 1 (LASP-1) (Metastatic lymph node gene 50 protein) (MLN 50)	Q14847 (LASP1)	-0.255	0.638	-4.724	0.0004	0.0156
Q9BPX5	ENSG00000136950	ARPC5L	Actin-related protein 2/3 complex subunit 5-like protein (Arp2/3 complex 16 kDa subunit 2) (ARC16-2)	Q9BPX5 (ARPC5L)	-0.253	0.388	-2.906	0.0126	0.1023
Q9Y251	ENSG00000173083	HPSE	Heparanase (EC 3.2.1.166) (Endo-glucoronidase) (Heparanase-1) (Hpa1) [Cleaved into: Heparanase 8 kDa subunit; Heparanase 50 kDa subunit]	Q9Y251 (HPSE)	0.252	1.002	3.434	0.0046	0.0574
Q8IVG5	ENSG00000177409	SAMD9L	Sterile alpha motif domain-containing protein 9-like (SAM domain-containing protein 9-like)	Q5K651 (SAMD9)	0.252	1.140	3.594	0.0034	0.0484
O14745	ENSG00000109062	SLC9A3R1	Na(+)/H(+) exchange regulatory cofactor NHE-RF1 (NHERF-1) (Ezrin-radixin-moesin-binding phosphoprotein 50) (EBP50) (Regulatory cofactor of Na(+)/H(+) exchanger) (Sodium-hydrogen exchanger regulatory factor 1) (Solute carrier family 9 isoform A3 regulatory factor 1)	O14745 (SLC9A3R1)	-0.251	0.579	-5.463	0.0001	0.0086
P27635	ENSG00000147403	RPL10	60S ribosomal protein L10 (Laminin receptor homolog) (Large ribosomal subunit protein uL16) (Protein QM) (Ribosomal protein L10) (Tumor suppressor QM)	P27635 (RPL10)	-0.251	0.595	-2.895	0.0128	0.1025
Q15904	ENSG00000071553	ATP6AP1	V-type proton ATPase subunit S1 (V-ATPase subunit S1) (Protein XAP-3) (V-ATPase Ac45 subunit) (V-ATPase S1 accessory protein) (Vacuolar proton pump subunit S1)	Q15904 (ATP6AP1)	0.250	0.969	2.816	0.0149	0.113
Q9Y5J6	ENSG00000132286	TIMM10B	Mitochondrial import inner membrane translocase subunit Tim10 B (Fracture callus protein 1) (FxC1) (Mitochondrial import inner membrane translocase subunit Tim9 B) (TIMM10B) (Tim10b)	Q9Y5J6 (TIMM10B)	-0.250	0.781	-3.086	0.0089	0.0848
Q15080	ENSG00000100365	NCF4	Neutrophil cytosol factor 4 (NCF-4) (Neutrophil NADPH oxidase factor 4) (SH3 and PX domain-containing protein 4) (p40-phox) (p40phox)	Q15080 (NCF4)	0.249	0.845	3.595	0.0034	0.0484

Table S55: Proteins differentially abundant at Day 30 compared to pre-second vaccination (Study Arm 2 (A/H7N9 + AS03 D1, 29), Dendritic cells). Proteins are sorted by descending absolute log_2 fold change. Protein and gene annotations are based on Ensembl Version 101 (continued).

Protein ID	Ensembl Gene ID	Ensembl Gene Name	Protein Description	50% Protein Cluster ID (Gene Name)	Fold Change (Day 30 vs. Pre- Second Vaccination)	Average log_2 Abundance	Moderated t-Statistic	P-Value	FDR Adjusted P-value
Q9UM00	ENSG00000143183	TMCO1	Calcium load-activated calcium channel (CLAC channel) (Transmembrane and coiled-coil domain-containing protein 1) (Transmembrane and coiled-coil domains protein 4) (Xenogeneic cross-immune protein PCIA3)	Q9UM00 (TMCO1)	0.248	0.888	2.949	0.0116	0.0983
O14657	ENSG00000136816	TOR1B	Torsin-1B (Torsin ATPase-1B) (EC 3.6.4.-) (Torsin family 1 member B)	O14657 (TOR1B)	0.247	0.843	4.757	0.0004	0.0152
P01892				P30501 (NA)	0.246	0.979	3.619	0.0033	0.0469
Q13636	ENSG00000168461	RAB31	Ras-related protein Rab-31 (Ras-related protein Rab-22B)	Q13636 (RAB31)	0.245	0.850	3.645	0.0031	0.0451
Q10469	ENSG00000168282	MGAT2	Alpha-1,6-mannosyl-glycoprotein 2-beta-N-acetylglucosaminyltransferase (EC 2.4.1.143) (Beta-1,2-N-acetylglucosaminyltransferase II) (GlcNAc-T II) (GNT-II) (Mannoside acetylglucosaminyltransferase 2) (N-glycosyl-oligosaccharide-glycoprotein N-acetylglucosaminyltransferase II)	Q10469 (MGAT2)	0.245	0.750	2.908	0.0125	0.1023
P52209	ENSG00000142657	PGD	6-phosphogluconate dehydrogenase, decarboxylating (EC 1.1.1.44)	P52209 (PGD)	0.244	0.799	4.171	0.0012	0.028
O95544	ENSG00000008130	NADK	NAD kinase (EC 2.7.1.23) (Poly(P)/ATP NAD kinase)	O95544 (NADK)	0.244	0.903	2.718	0.018	0.1264
O75475	ENSG00000164985	PSIP1	PC4 and SFRS1-interacting protein (CLL-associated antigen KW-7) (Dense fine speckles 70 kDa protein) (DFS 70) (Lens epithelium-derived growth factor) (Transcriptional coactivator p75/p52)	O75475 (PSIP1)	-0.243	0.762	-3.373	0.0052	0.0602
P11717	ENSG00000197081	IGF2R	Cation-independent mannose-6-phosphate receptor (CI Man-6-P receptor) (CI-MPR) (M6PR) (300 kDa mannose 6-phosphate receptor) (MPR 300) (Insulin-like growth factor 2 receptor) (Insulin-like growth factor II receptor) (IGF-II receptor) (M6P/IGF2 receptor) (M6P/IGF2R) (CD antigen CD222)	P11717 (IGF2R)	0.241	1.010	4.754	0.0004	0.0152
Q99439	ENSG00000064666	CNN2	Calponin-2 (Calponin H2, smooth muscle) (Neutral calponin)	Q15417 (CNN3)	-0.238	0.631	-4.201	0.0011	0.0273
Q9BW30	ENSG00000159713	TPPP3	Tubulin polymerization-promoting protein family member 3 (TPPP/p20)	Q9BW30 (TPPP3)	-0.237	0.618	-3.513	0.004	0.0519
Q13428	ENSG00000070814	TCOF1	Treacle protein (Treacher Collins syndrome protein)	Q13428 (TCOF1)	-0.235	0.823	-2.813	0.015	0.1131

Table S55: Proteins differentially abundant at Day 30 compared to pre-second vaccination (Study Arm 2 (A/H7N9 + AS03 D1, 29), Dendritic cells). Proteins are sorted by descending absolute log_2 fold change. Protein and gene annotations are based on Ensembl Version 101 (continued).

Protein ID	Ensembl Gene ID	Ensembl Gene Name	Protein Description	50% Protein Cluster ID (Gene Name)	Fold Change (Day 30 vs. Pre- Second Vaccination)	Average log_2 Abundance	Moderated t-Statistic	P-Value	FDR Adjusted P-value
P49902	ENSG00000076685	NT5C2	Cytosolic purine 5'-nucleotidase (EC 3.1.3.5) (Cytosolic 5'-nucleotidase II)	P49902 (NT5C2)	0.234	0.916	5.175	0.0002	0.01
P17096	ENSG00000137309	HMGA1	High mobility group protein HMG-I/HMG-Y (HMG-I(Y)) (High mobility group AT-hook protein 1) (High mobility group protein A1) (High mobility group protein R)	P17096 (HMGA1)	-0.234	0.819	-3.473	0.0043	0.0551
P19878	ENSG00000116701	NCF2	Neutrophil cytosol factor 2 (NCF-2) (67 kDa neutrophil oxidase factor) (NADPH oxidase activator 2) (Neutrophil NADPH oxidase factor 2) (p67-phox)	P19878 (NCF2)	0.234	0.919	3.301	0.0059	0.0666
Q12769	ENSG00000030066	NUP160	Nuclear pore complex protein Nup160 (160 kDa nucleoporin) (Nucleoporin Nup160)	Q12769 (NUP160)	0.233	0.899	3.212	0.007	0.0733
Q15427	ENSG00000143368	SF3B4	Splicing factor 3B subunit 4 (Pre-mRNA-splicing factor SF3b 49 kDa subunit) (Spliceosome-associated protein 49) (SAP 49)	Q15427 (SF3B4)	0.233	0.981	2.937	0.0119	0.0991
Q07065	ENSG00000136026	CKAP4	Cytoskeleton-associated protein 4 (63-kDa cytoskeleton-linking membrane protein) (Climp-63) (p63)	Q07065 (CKAP4)	0.233	0.967	4.571	0.0006	0.018
P57764	ENSG00000104518; ENSG00000278718	GSDMD	Gasdermin-D (Gasdermin domain-containing protein 1) [Cleaved into: Gasdermin-D, N-terminal (GSDMD-NT) (hGSDMD-NTD); Gasdermin-D, C-terminal (GSDMD-CT) (hGSDMD-CTD)]	P57764 (GSDMD)	0.231	0.799	3.848	0.0021	0.0376
P23141	ENSG00000198848; ENSG00000262243	CES1	Liver carboxylesterase 1 (Acyl-coenzyme A:cholesterol acyltransferase) (ACAT) (Brain carboxylesterase hBr1) (Carboxylesterase 1) (CE-1) (hCE-1) (EC 3.1.1.1) (Cholesteryl ester hydrolase) (CEH) (EC 3.1.1.13) (Cocaine carboxylesterase) (Egasyn) (HMSE) (Methylumbelliferyl-acetate deacetylase 1) (EC 3.1.1.56) (Monocyte/macrophage serine esterase) (Retinyl ester hydrolase) (REH) (Serine esterase 1) (Triacylglycerol hydrolase) (TGH)	P23141 (CES1)	0.231	0.847	4.449	0.0007	0.0206

Table S55: Proteins differentially abundant at Day 30 compared to pre-second vaccination (Study Arm 2 (A/H7N9 + AS03 D1, 29), Dendritic cells). Proteins are sorted by descending absolute log_2 fold change. Protein and gene annotations are based on Ensembl Version 101 (continued).

Protein ID	Ensembl Gene ID	Ensembl Gene Name	Protein Description	50% Protein Cluster ID (Gene Name)	Fold Change (Day 30 vs. Pre- Second Vaccination)	Average log_2 Abundance	Moderated t-Statistic	P-Value	FDR Adjusted P-value
P19525	ENSG00000055332	EIF2AK2	Interferon-induced, double-stranded RNA-activated protein kinase (EC 2.7.11.1) (Eukaryotic translation initiation factor 2-alpha kinase 2) (eIF-2A protein kinase 2) (Interferon-inducible RNA-dependent protein kinase) (P1/eIF-2A protein kinase) (Protein kinase RNA-activated) (PKR) (Protein kinase R) (Tyrosine-protein kinase EIF2AK2) (EC 2.7.10.2) (p68 kinase)	P19525 (EIF2AK2)	0.231	0.938	3.786	0.0024	0.0392
P30042				P30042 (NA)	-0.230	0.676	-4.200	0.0011	0.0273
P0DOX5				P0DOX5 (NA)	0.230	1.167	3.514	0.004	0.0519
Q8NI60	ENSG00000163050	COQ8A	Atypical kinase COQ8A, mitochondrial (EC 2.7.-.-) (Chaperone activity of bc1 complex-like) (Chaperone-ABC1-like) (Coenzyme Q protein 8A) (aarF domain-containing protein kinase 3)	Q8NI60 (COQ8A)	-0.230	0.732	-5.463	0.0001	0.0086
Q99536	ENSG00000108828	VAT1	Synaptic vesicle membrane protein VAT-1 homolog (EC 1.-.-.-)	Q99536 (VAT1)	0.229	0.951	3.936	0.0018	0.0351
Q9Y4E8	ENSG00000135655	USP15	Ubiquitin carboxyl-terminal hydrolase 15 (EC 3.4.19.12) (Deubiquitinating enzyme 15) (Ubiquitin thioesterase 15) (Ubiquitin-specific-processing protease 15) (Unph-2) (Unph4)	Q9Y4E8 (USP15)	0.229	0.993	6.225	<0.0001	0.0039
O00602	ENSG00000085265	FCN1	Ficolin-1 (Collagen/fibrinogen domain-containing protein 1) (Ficolin-A) (Ficolin-alpha) (M-ficolin)	O00602 (FCN1)	0.229	0.664	3.430	0.0047	0.0574
Q5SSJ5	ENSG00000127483	HP1BP3	Heterochromatin protein 1-binding protein 3 (Protein HP1-BP74)	Q5SSJ5 (HP1BP3)	-0.228	0.730	-4.946	0.0003	0.0128
Q13685	ENSG00000127837	AAMP	Angio-associated migratory cell protein	Q13685 (AAMP)	0.227	0.932	3.422	0.0047	0.058
P49662	ENSG00000196954	CASP4	Caspase-4 (CASP-4) (EC 3.4.22.57) (ICE and Ced-3 homolog 2) (ICH-2) (ICE(rel)-II) (Mih1) (Protease TX) [Cleaved into: Caspase-4 subunit p10; Caspase-4 subunit p20]	P51878 (CASP5)	0.227	0.972	3.857	0.0021	0.0376
P42166	ENSG00000120802	TMPO	Lamina-associated polypeptide 2, isoform alpha (Thymopoietin isoform alpha) (TP alpha) (Thymopoietin-related peptide isoform alpha) (TPRP isoform alpha) [Cleaved into: Thymopoietin (TP) (Splenin); Thymopentin (TP5)]	P42166 (TMPO)	-0.227	0.822	-2.947	0.0116	0.0984

Table S55: Proteins differentially abundant at Day 30 compared to pre-second vaccination (Study Arm 2 (A/H7N9 + AS03 D1, 29), Dendritic cells). Proteins are sorted by descending absolute log_2 fold change. Protein and gene annotations are based on Ensembl Version 101 (continued).

Protein ID	Ensembl Gene ID	Ensembl Gene Name	Protein Description	50% Protein Cluster ID (Gene Name)	Fold Change (Day 30 vs. Pre- Second Vaccination)	Average log_2 Abundance	Moderated t-Statistic	P-Value	FDR Adjusted P-value
P33241	ENSG00000130592; ENSG00000288199	LSP1	Lymphocyte-specific protein 1 (47 kDa actin-binding protein) (52 kDa phosphoprotein) (pp52) (Lymphocyte-specific antigen WP34)	P33241 (LSP1)	-0.225	0.614	-2.762	0.0165	0.1202
P05362	ENSG00000090339	ICAM1	Intercellular adhesion molecule 1 (ICAM-1) (Major group rhinovirus receptor) (CD antigen CD54)	P05362 (ICAM1)	0.225	1.015	3.750	0.0025	0.0406
O00182	ENSG00000168961	LGALS9	Galectin-9 (Gal-9) (Ecalectin) (Tumor antigen HOM-HD-21)	Q3B8N2 (LGALS9B)	0.225	1.135	3.688	0.0029	0.0435
P62937	ENSG00000196262	PPIA	Peptidyl-prolyl cis-trans isomerase A (PPIase A) (EC 5.2.1.8) (Cyclophilin A) (Cyclosporin A-binding protein) (Rotamase A) [Cleaved into: Peptidyl-prolyl cis-trans isomerase A, N-terminally processed]	Q9UNP9 (PPIE)	-0.224	0.648	-3.355	0.0054	0.0617
Q8NB16	ENSG00000168404	MLKL	Mixed lineage kinase domain-like protein (hMLKL)	Q8NB16 (MLKL)	0.224	0.928	6.053	<0.0001	0.0048
P01876	ENSG00000211895; ENSG00000282633	IGHA1	Immunoglobulin heavy constant alpha 1 (Ig alpha-1 chain C region) (Ig alpha-1 chain C region BUR) (Ig alpha-1 chain C region TRO)	P0DOX2 (NA)	-0.223	1.422	-2.990	0.0107	0.0949
Q9Y2J8	ENSG00000117115	PADI2	Protein-arginine deiminase type-2 (EC 3.5.3.15) (PAD-H19) (Peptidylarginine deiminase II) (Protein-arginine deiminase type II)	Q9Y2J8 (PADI2)	0.223	0.807	3.553	0.0037	0.0509
Q7L523	ENSG00000155876	RRAGA	Ras-related GTP-binding protein A (Rag A) (RagA) (EC 3.6.5.-) (Adenovirus E3 14.7 kDa-interacting protein 1) (FIP-1)	Q7L523 (RRAGA)	0.222	0.879	3.226	0.0069	0.073
P63098	ENSG00000221823	PPP3R1	Calcineurin subunit B type 1 (Protein phosphatase 2B regulatory subunit 1) (Protein phosphatase 3 regulatory subunit B alpha isoform 1)	P63098 (PPP3R1)	-0.221	0.627	-3.541	0.0038	0.0511
Q16181	ENSG00000122545	SEPTIN7	Septin-7 (CDC10 protein homolog)	Q16181 (SEPTIN7)	-0.221	0.765	-5.398	0.0001	0.0086
P59768	ENSG00000186469	GNG2	Guanine nucleotide-binding protein G(I)/G(S)/G(O) subunit gamma-2 (G gamma-I)	P59768 (GNG2)	-0.220	0.753	-3.961	0.0017	0.0343
Q9Y385	ENSG00000198833	UBE2J1	Ubiquitin-conjugating enzyme E2 J1 (EC 2.3.2.23) (E2 ubiquitin-conjugating enzyme J1) (Non-canonical ubiquitin-conjugating enzyme 1) (NCUBE-1) (Yeast ubiquitin-conjugating enzyme UBC6 homolog E) (HsUBC6e)	Q9Y385 (UBE2J1)	0.220	0.920	5.304	0.0002	0.0088

Table S55: Proteins differentially abundant at Day 30 compared to pre-second vaccination (Study Arm 2 (A/H7N9 + AS03 D1, 29), Dendritic cells). Proteins are sorted by descending absolute log_2 fold change. Protein and gene annotations are based on Ensembl Version 101 (continued).

Protein ID	Ensembl Gene ID	Ensembl Gene Name	Protein Description	50% Protein Cluster ID (Gene Name)	Fold Change (Day 30 vs. Pre- Second Vaccination)	Average log_2 Abundance	Moderated t-Statistic	P-Value	FDR Adjusted P-value
Q8NFW8	ENSG00000111726	CMAS	N-acylneuraminate cytidylyltransferase (EC 2.7.7.43) (CMP-N-acetylneuraminic acid synthase) (CMP-NeuNAc synthase)	Q8NFW8 (CMAS)	-0.218	0.666	-4.259	0.001	0.026
O15270	ENSG00000100596	SPTLC2	Serine palmitoyltransferase 2 (EC 2.3.1.50) (Long chain base biosynthesis protein 2) (LCB 2) (Long chain base biosynthesis protein 2a) (LCB2a) (Serine-palmitoyl-CoA transferase 2) (SPT 2)	O15270 (SPTLC2)	0.218	0.880	3.857	0.0021	0.0376
Q14728	ENSG00000109736	MFSD10	Major facilitator superfamily domain-containing protein 10 (Tetracycline transporter-like protein)	Q14728 (MFSD10)	0.217	0.770	2.696	0.0187	0.1301
Q13075	ENSG00000249437; ENSG00000278613	NAIP	Baculoviral IAP repeat-containing protein 1 (Neuronal apoptosis inhibitory protein)	Q13075 (NAIP)	0.216	0.730	3.655	0.003	0.0449
Q01105	ENSG00000119335	SET	Protein SET (HLA-DR-associated protein II) (Inhibitor of granzyme A-activated DNase) (IGAAD) (PHAPII) (Phosphatase 2A inhibitor I2PP2A) (I-2PP2A) (Template-activating factor I) (TAF-I)	Q01105 (SET)	-0.216	0.763	-3.322	0.0057	0.0649
P62072	ENSG00000134809	TIMM10	Mitochondrial import inner membrane translocase subunit Tim10	P62072 (TIMM10)	-0.216	0.829	-3.083	0.009	0.0849
Q96IU4	ENSG00000114779	ABHD14B	Protein ABHD14B (EC 3.-.-.-) (Alpha/beta hydrolase domain-containing protein 14B) (Abhydrolase domain-containing protein 14B) (CCG1-interacting factor B)	Q96IU4 (ABHD14B)	-0.215	0.829	-2.956	0.0114	0.0977
Q07021	ENSG00000108561	C1QBP	Complement component 1 Q subcomponent-binding protein, mitochondrial (ASF/SF2-associated protein p32) (Glycoprotein gC1qBP) (C1qBP) (Hyaluronan-binding protein 1) (Mitochondrial matrix protein p32) (gC1q-R protein) (p33) (SF2AP32)	Q07021 (C1QBP)	-0.215	0.736	-4.325	0.0009	0.0236
Q15758	ENSG00000105281	SLC1A5	Neutral amino acid transporter B(0) (ATB(0)) (Baboon M7 virus receptor) (RD114/simian type D retrovirus receptor) (Sodium-dependent neutral amino acid transporter type 2) (Solute carrier family 1 member 5)	Q15758 (SLC1A5)	0.215	1.003	4.175	0.0012	0.028
Q96FN4	ENSG00000140848	CPNE2	Copine-2 (Copine II)	Q86YQ8 (CPNE8)	0.215	0.861	4.773	0.0004	0.0152
Q66PJ3	ENSG00000182196	ARL6IP4	ADP-ribosylation factor-like protein 6-interacting protein 4 (ARL-6-interacting protein 4) (Aip-4) (HSP-975) (HSVI-binding protein) (SR-15) (SRp25) (SR-25) (Splicing factor SRrp37)	Q66PJ3 (ARL6IP4)	-0.214	0.936	-4.309	0.0009	0.0241

Table S55: Proteins differentially abundant at Day 30 compared to pre-second vaccination (Study Arm 2 (A/H7N9 + AS03 D1, 29), Dendritic cells). Proteins are sorted by descending absolute log_2 fold change. Protein and gene annotations are based on Ensembl Version 101 (continued).

Protein ID	Ensembl Gene ID	Ensembl Gene Name	Protein Description	50% Protein Cluster ID (Gene Name)	Fold Change (Day 30 vs. Pre- Second Vaccination)	Average log_2 Abundance	Moderated t-Statistic	P-Value	FDR Adjusted P-value
Q8WVJ2	ENSG00000170584	NUDCD2	NudC domain-containing protein 2	Q8WVJ2 (NUDCD2)	-0.213	0.768	-3.865	0.0021	0.0376
P29728	ENSG00000111335	OAS2	2'-5'-oligoadenylate synthase 2 ((2-5')oligo(A) synthase 2) (2-5A synthase 2) (EC 2.7.7.84) (p69 OAS / p71 OAS) (p69OAS / p71OAS)	P29728 (OAS2)	0.213	1.061	2.547	0.0248	0.1498
Q5VTL8	ENSG00000134186	PRPF38B	Pre-mRNA-splicing factor 38B (Sarcoma antigen NY-SAR-27)	Q5VTL8 (PRPF38B)	-0.213	0.848	-4.652	0.0005	0.0166
P37802	ENSG00000158710	TAGLN2	Transgelin-2 (Epididymis tissue protein Li 7e) (SM22-alpha homolog)	Q9UI15 (TAGLN3)	-0.213	0.490	-3.716	0.0027	0.0423
O75113	ENSG00000102921	N4BP1	NEDD4-binding protein 1 (N4BP1) (EC 3.1.-.-)	O75113 (N4BP1)	0.212	0.899	2.818	0.0149	0.1129
Q96SB3	ENSG00000108819	PPP1R9B	Neurabin-2 (Neurabin-II) (Protein phosphatase 1 regulatory subunit 9B) (Spinophilin)	Q96SB3 (PPP1R9B)	0.212	0.961	2.760	0.0166	0.1204
P11234	ENSG00000144118	RALB	Ras-related protein Ral-B (EC 3.6.5.2)	P11234 (RALB)	0.211	0.889	3.557	0.0037	0.0508
P04440	ENSG00000215048; ENSG00000236693; ENSG00000223865; ENSG00000230763; ENSG00000226826; ENSG00000237710	HLA-DPB1	HLA class II histocompatibility antigen, DP beta 1 chain (HLA class II histocompatibility antigen, DP(W4) beta chain) (MHC class II antigen DPB1)	P13765 (HLA-DOB)	0.211	1.145	2.665	0.0198	0.134
Q93033	ENSG00000134256	CD101	Immunoglobulin superfamily member 2 (IgSF2) (Cell surface glycoprotein V7) (Glu-Trp-Ile EWI motif-containing protein 101) (EWI-101) (CD antigen CD101)	Q93033 (CD101)	-0.210	0.642	-2.626	0.0214	0.1405
P31937	ENSG00000106049	HIBADH	3-hydroxyisobutyrate dehydrogenase, mitochondrial (HIBADH) (EC 1.1.1.31)	P31937 (HIBADH)	-0.208	0.828	-3.920	0.0019	0.0355
Q08257	ENSG00000116791	CRYZ	Quinone oxidoreductase (EC 1.6.5.5) (NADPH:quinone reductase) (Zeta-crystallin)	Q08257 (CRYZ)	-0.208	0.776	-3.393	0.005	0.0592
Q9NUQ2	ENSG00000155189; ENSG00000284980	AGPAT5	1-acyl-sn-glycerol-3-phosphate acyltransferase epsilon (EC 2.3.1.51) (1-acylglycerol-3-phosphate O-acyltransferase 5) (1-AGP acyltransferase 5) (1-AGPAT 5) (Lysophosphatidic acid acyltransferase epsilon) (LPAAT-epsilon)	Q9NUQ2 (AGPAT5)	-0.208	0.794	-4.529	0.0006	0.0186
Q9NR30	ENSG00000165732	DDX21	Nucleolar RNA helicase 2 (EC 3.6.4.13) (DEAD box protein 21) (Gu-alpha) (Nucleolar RNA helicase Gu) (Nucleolar RNA helicase II) (RH II/Gu)	Q9NR30 (DDX21)	0.208	1.000	5.078	0.0002	0.0111

Table S55: Proteins differentially abundant at Day 30 compared to pre-second vaccination (Study Arm 2 (A/H7N9 + AS03 D1, 29), Dendritic cells). Proteins are sorted by descending absolute log_2 fold change. Protein and gene annotations are based on Ensembl Version 101 (continued).

Protein ID	Ensembl Gene ID	Ensembl Gene Name	Protein Description	50% Protein Cluster ID (Gene Name)	Fold Change (Day 30 vs. Pre- Second Vaccination)	Average log_2 Abundance	Moderated t-Statistic	P-Value	FDR Adjusted P-value
Q8IY21	ENSG00000137628	DDX60	Probable ATP-dependent RNA helicase DDX60 (EC 3.6.4.13) (DEAD box protein 60)	Q8IY21 (DDX60)	0.208	0.921	2.984	0.0108	0.0956
P08637	ENSG00000203747	FCGR3A	Low affinity immunoglobulin gamma Fc region receptor III-A (CD16a antigen) (Fc-gamma RIII-alpha) (Fc-gamma RIII) (Fc-gamma RIIIa) (FcRIII) (FcRIIIa) (FcR-10) (IgG Fc receptor III-2) (CD antigen CD16a)	P08637 (FCGR3A)	-0.207	0.619	-3.771	0.0024	0.0397
Q9BPX6	ENSG00000107745	MICU1	Calcium uptake protein 1, mitochondrial (Atopy-related autoantigen CALC) (ara CALC) (Calcium-binding atopy-related autoantigen 1) (allergen Hom s 4)	Q9BPX6 (MICU1)	0.207	0.912	4.395	0.0008	0.0221
P06744	ENSG00000105220	GPI	Glucose-6-phosphate isomerase (GPI) (EC 5.3.1.9) (Autocrine motility factor) (AMF) (Neuroleukin) (NLK) (Phosphoglucose isomerase) (PGI) (Phosphohexose isomerase) (PHI) (Sperm antigen 36) (SA-36)	P06744 (GPI)	0.207	0.798	5.356	0.0001	0.0086
P62280	ENSG00000142534	RPS11	40S ribosomal protein S11 (Small ribosomal subunit protein uS17)	P62280 (RPS11)	-0.207	0.665	-3.799	0.0023	0.0391
P47712	ENSG00000116711	PLA2G4A	Cytosolic phospholipase A2 (cPLA2) (Phospholipase A2 group IVA) [Includes: Phospholipase A2 (EC 3.1.1.4) (Phosphatidylcholine 2-acylhydrolase); Lysophospholipase (EC 3.1.1.5)]	P47712 (PLA2G4A)	0.206	0.844	3.206	0.0071	0.0737
P23193	ENSG00000187735	TCEA1	Transcription elongation factor A protein 1 (Transcription elongation factor S-II protein 1) (Transcription elongation factor TFIIS.o)	P23193 (TCEA1)	-0.205	0.819	-3.507	0.004	0.0523
Q9NTJ3	ENSG00000113810	SMC4	Structural maintenance of chromosomes protein 4 (SMC protein 4) (SMC-4) (Chromosome-associated polypeptide C) (hCAP-C) (XCAP-C homolog)	Q9NTJ3 (SMC4)	0.205	1.007	3.664	0.003	0.0447
Q15050	ENSG00000179041	RRS1	Ribosome biogenesis regulatory protein homolog	Q15050 (RRS1)	-0.205	0.905	-3.525	0.0039	0.0517
Q96A26	ENSG00000114023	FAM162A	Protein FAM162A (E2-induced gene 5 protein) (Growth and transformation-dependent protein) (HGTD-P)	Q96A26 (FAM162A)	-0.204	0.676	-2.996	0.0106	0.0947
P11215	ENSG00000169896	ITGAM	Integrin alpha-M (CD11 antigen-like family member B) (CR-3 alpha chain) (Cell surface glycoprotein MAC-1 subunit alpha) (Leukocyte adhesion receptor MO1) (Neutrophil adherence receptor) (CD antigen CD11b)	P20702 (ITGAX)	0.204	0.791	4.562	0.0006	0.018

Table S55: Proteins differentially abundant at Day 30 compared to pre-second vaccination (Study Arm 2 (A/H7N9 + AS03 D1, 29), Dendritic cells). Proteins are sorted by descending absolute log_2 fold change. Protein and gene annotations are based on Ensembl Version 101 (continued).

Protein ID	Ensembl Gene ID	Ensembl Gene Name	Protein Description	50% Protein Cluster ID (Gene Name)	Fold Change (Day 30 vs. Pre- Second Vaccination)	Average log_2 Abundance	Moderated t-Statistic	P-Value	FDR Adjusted P-value
O14732	ENSG00000141401	IMPA2	Inositol monophosphatase 2 (IMP 2) (IMPase 2) (EC 3.1.3.25) (Inositol-1(or 4)-monophosphatase 2) (Myo-inositol monophosphatase A2)	O14732 (IMPA2)	0.204	0.811	3.911	0.0019	0.0357
Q14141	ENSG00000125354	SEPTIN6	Septin-6	Q92599 (SEPTIN8)	-0.203	0.758	-3.270	0.0063	0.0691
P16284	ENSG00000261371	PECAM1	Platelet endothelial cell adhesion molecule (PECAM-1) (EndoCAM) (GPIIA') (PECA1) (CD antigen CD31)	P16284 (PECAM1)	-0.203	0.684	-2.545	0.0249	0.1498
Q63HN8	ENSG00000173821	RNF213	E3 ubiquitin-protein ligase RNF213 (EC 2.3.2.27) (EC 3.6.4.-) (ALK lymphoma oligomerization partner on chromosome 17) (Mysterin) (RING finger protein 213) (RING-type E3 ubiquitin transferase RNF213)	Q63HN8 (RNF213)	0.203	0.972	3.904	0.0019	0.036
P07237	ENSG00000185624	P4HB	Protein disulfide-isomerase (PDI) (EC 5.3.4.1) (Cellular thyroid hormone-binding protein) (Prolyl 4-hydroxylase subunit beta) (p55)	P07237 (P4HB)	0.201	0.937	4.850	0.0003	0.0146
Q9NXV6	ENSG00000168564	CDKN2AIP	CDKN2A-interacting protein (Collaborator of ARF)	Q9NXV6 (CDKN2AIP)	-0.201	0.900	-3.410	0.0048	0.0588
Q9Y3U8	ENSG00000130255	RPL36	60S ribosomal protein L36 (Large ribosomal subunit protein eL36)	Q9Y3U8 (RPL36)	-0.200	0.382	-2.817	0.0149	0.1129
Q53QZ3	ENSG00000075884	ARHGAP15	Rho GTPase-activating protein 15 (ArhGAP15) (Rho-type GTPase-activating protein 15)	Q53QZ3 (ARHGAP15)	-0.199	0.747	-2.780	0.016	0.118
O15247	ENSG00000155962	CLIC2	Chloride intracellular channel protein 2 (XAP121)	Q9NZA1 (CLIC5)	-0.199	0.786	-3.843	0.0021	0.0376
P55265	ENSG00000160710	ADAR	Double-stranded RNA-specific adenosine deaminase (DRADA) (EC 3.5.4.37) (136 kDa double-stranded RNA-binding protein) (p136) (Interferon-inducible protein 4) (IFI-4) (K88DSRBP)	P55265 (ADAR)	0.199	0.916	4.433	0.0007	0.021
P28907	ENSG00000004468	CD38	ADP-ribosyl cyclase/cyclic ADP-ribose hydrolase 1 (EC 3.2.2.6) (2'-phospho-ADP-ribosyl cyclase) (2'-phospho-ADP-ribosyl cyclase/2'-phospho-cyclic-ADP-ribose transferase) (EC 2.4.99.20) (2'-phospho-cyclic-ADP-ribose transferase) (ADP-ribosyl cyclase 1) (ADPRC 1) (Cyclic ADP-ribose hydrolase 1) (cADPr hydrolase 1) (T10) (CD antigen CD38)	P28907 (CD38)	0.199	1.155	2.724	0.0177	0.1252

Table S55: Proteins differentially abundant at Day 30 compared to pre-second vaccination (Study Arm 2 (A/H7N9 + AS03 D1, 29), Dendritic cells). Proteins are sorted by descending absolute log_2 fold change. Protein and gene annotations are based on Ensembl Version 101 (continued).

Protein ID	Ensembl Gene ID	Ensembl Gene Name	Protein Description	50% Protein Cluster ID (Gene Name)	Fold Change (Day 30 vs. Pre- Second Vaccination)	Average log_2 Abundance	Moderated t-Statistic	P-Value	FDR Adjusted P-value
P16455	ENSG00000170430	MGMT	Methylated-DNA–protein-cysteine methyltransferase (EC 2.1.1.63) (6-O-methylguanine-DNA methyltransferase) (MGMT) (O-6-methylguanine-DNA-alkyltransferase)	P16455 (MGMT)	-0.199	0.670	-2.795	0.0155	0.1156
Q13884	ENSG00000172164	SNTB1	Beta-1-syntrophin (59 kDa dystrophin-associated protein A1 basic component 1) (DAPA1B) (BSYN2) (Syntrophin-2) (Tax interaction protein 43) (TIP-43)	Q13425 (SNTB2)	0.198	0.859	4.520	0.0006	0.0187
Q6VY07	ENSG00000175115	PACS1	Phosphofurin acidic cluster sorting protein 1 (PACS-1)	Q6VY07 (PACS1)	-0.198	0.747	-4.775	0.0004	0.0152
P26572	ENSG00000131446	MGAT1	Alpha-1,3-mannosyl-glycoprotein 2-beta-N-acetylglucosaminyltransferase (EC 2.4.1.101) (N-glycosyl-oligosaccharide-glycoprotein N-acetylglucosaminyltransferase I) (GNT-I) (GlcNAc-T I)	P26572 (MGAT1)	0.197	1.024	5.324	0.0002	0.0088
P0C0S5	ENSG00000164032	H2AZ1	Histone H2A.Z (H2A/z)	Q9P0M6 (MACROH2A2)	-0.197	0.846	-3.031	0.0099	0.0914
A0AV96	ENSG00000163694	RBM47	RNA-binding protein 47 (RNA-binding motif protein 47)	A0AV96 (RBM47)	0.196	0.951	3.927	0.0018	0.0355
Q8IX19	ENSG00000183019	MCEMP1	Mast cell-expressed membrane protein 1	Q8IX19 (MCEMP1)	0.195	0.894	3.062	0.0094	0.0871
Q9BPW8	ENSG00000184117	NIPSNAP1	Protein NipSnap homolog 1 (NipSnap1)	O75323 (NIPSNAP2)	-0.195	0.742	-2.862	0.0137	0.1062
Q96A49	ENSG00000169895	SYAP1	Synapse-associated protein 1 (BSD domain-containing signal transducer and Akt interactor protein) (BSTA)	Q96A49 (SYAP1)	0.195	0.893	2.758	0.0166	0.1204
Q6DN90	ENSG00000144711	IQSEC1	IQ motif and SEC7 domain-containing protein 1 (ADP-ribosylation factors guanine nucleotide-exchange protein 100) (ADP-ribosylation factors guanine nucleotide-exchange protein 2) (Brefeldin-resistant Arf-GEF 2 protein) (BRAG2)	Q5JU85 (IQSEC2)	-0.195	0.895	-2.679	0.0193	0.1318
P17480	ENSG00000108312	UBTF	Nucleolar transcription factor 1 (Autoantigen NOR-90) (Upstream-binding factor 1) (UBF-1)	P17480 (UBTF)	0.194	0.912	3.443	0.0045	0.0569
Q9Y6M1	ENSG00000073792	IGF2BP2	Insulin-like growth factor 2 mRNA-binding protein 2 (IGF2 mRNA-binding protein 2) (IMP-2) (Hepatocellular carcinoma autoantigen p62) (IGF-II mRNA-binding protein 2) (VICKZ family member 2)	Q9Y6M1 (IGF2BP2)	0.194	0.850	4.634	0.0005	0.0168

Confidential/Proprietary Information

Table S55: Proteins differentially abundant at Day 30 compared to pre-second vaccination (Study Arm 2 (A/H7N9 + AS03 D1, 29), Dendritic cells). Proteins are sorted by descending absolute log_2 fold change. Protein and gene annotations are based on Ensembl Version 101 (continued).

Protein ID	Ensembl Gene ID	Ensembl Gene Name	Protein Description	50% Protein Cluster ID (Gene Name)	Fold Change (Day 30 vs. Pre- Second Vaccination)	Average log_2 Abundance	Moderated t-Statistic	P-Value	FDR Adjusted P-value
Q9Y314	ENSG00000142546	NOSIP	Nitric oxide synthase-interacting protein (E3 ubiquitin-protein ligase NOSIP) (EC 2.3.2.27) (RING-type E3 ubiquitin transferase NOSIP) (eNOS-interacting protein)	Q9Y314 (NOSIP)	-0.193	0.703	-2.621	0.0216	0.1409
Q8IUI8	ENSG00000176390	CRLF3	Cytokine receptor-like factor 3 (Cytokine receptor-like molecule 9) (CREME-9) (Cytokine receptor-related protein 4) (Type I cytokine receptor-like factor p48)	Q8IUI8 (CRLF3)	-0.192	0.869	-2.974	0.0111	0.0966
Q9BYG3	ENSG00000155438	NIFK	MKI67 FHA domain-interacting nucleolar phosphoprotein (Nucleolar phosphoprotein Nopp34) (Nucleolar protein interacting with the FHA domain of pKI-67) (hNIFK)	Q9BYG3 (NIFK)	-0.192	0.948	-2.540	0.0251	0.1498
Q53EL6	ENSG00000150593	PDCD4	Programmed cell death protein 4 (Neoplastic transformation inhibitor protein) (Nuclear antigen H731-like) (Protein 197/15a)	Q53EL6 (PDCD4)	-0.192	0.710	-3.027	0.01	0.0914
Q92918	ENSG00000104814; ENSG00000282928	MAP4K1	Mitogen-activated protein kinase kinase kinase kinase 1 (EC 2.7.11.1) (Hematopoietic progenitor kinase) (MAPK/ERK kinase kinase kinase 1) (MEK kinase kinase 1) (MEKKK 1)	Q92918 (MAP4K1)	-0.192	0.879	-2.702	0.0185	0.1292
P53999	ENSG00000113387	SUB1	Activated RNA polymerase II transcriptional coactivator p15 (Positive cofactor 4) (PC4) (SUB1 homolog) (p14)	P53999 (SUB1)	-0.192	0.641	-3.938	0.0018	0.0351
P25490	ENSG00000100811	YY1	Transcriptional repressor protein YY1 (Delta transcription factor) (INO80 complex subunit S) (NF-E1) (Yin and yang 1) (YY-1)	P25490 (YY1)	-0.192	0.748	-4.665	0.0005	0.0164
Q9P016	ENSG00000151500	THYN1	Thymocyte nuclear protein 1 (Thymocyte protein Thy28)	Q9P016 (THYN1)	-0.192	0.722	-3.113	0.0085	0.0822
P06737	ENSG00000100504	PYGL	Glycogen phosphorylase, liver form (EC 2.4.1.1)	P06737 (PYGL)	0.192	0.723	3.547	0.0037	0.0511
Q9HB40	ENSG00000121064	SCPEP1	Retinoid-inducible serine carboxypeptidase (EC 3.4.16.-) (Serine carboxypeptidase 1)	Q9HB40 (SCPEP1)	0.190	0.745	3.842	0.0021	0.0376
Q9H4E7	ENSG00000023892	DEF6	Differentially expressed in FDCP 6 homolog (DEF-6) (IRF4-binding protein)	Q9H4E7 (DEF6)	-0.190	0.833	-3.230	0.0068	0.0726
P28288	ENSG00000117528	ABCD3	ATP-binding cassette sub-family D member 3 (70 kDa peroxisomal membrane protein) (PMP70)	P28288 (ABCD3)	0.190	0.849	3.151	0.0079	0.0788
P46527	ENSG00000111276	CDKN1B	Cyclin-dependent kinase inhibitor 1B (Cyclin-dependent kinase inhibitor p27) (p27Kip1)	P46527 (CDKN1B)	-0.189	0.819	-3.052	0.0095	0.0886

Table S55: Proteins differentially abundant at Day 30 compared to pre-second vaccination (Study Arm 2 (A/H7N9 + AS03 D1, 29), Dendritic cells). Proteins are sorted by descending absolute log_2 fold change. Protein and gene annotations are based on Ensembl Version 101 (continued).

Protein ID	Ensembl Gene ID	Ensembl Gene Name	Protein Description	50% Protein Cluster ID (Gene Name)	Fold Change (Day 30 vs. Pre- Second Vaccination)	Average log_2 Abundance	Moderated t-Statistic	P-Value	FDR Adjusted P-value
P55209	ENSG00000187109	NAP1L1	Nucleosome assembly protein 1-like 1 (NAP-1-related protein) (hNRP)	P55209 (NAP1L1)	-0.189	0.658	-3.806	0.0023	0.0391
P55795	ENSG00000126945	HNRNPH2	Heterogeneous nuclear ribonucleoprotein H2 (hnRNP H2) (FTP-3) (Heterogeneous nuclear ribonucleoprotein H') (hnRNP H') [Cleaved into: Heterogeneous nuclear ribonucleoprotein H2, N-terminally processed]	P31943 (HNRNPH1)	0.189	0.858	4.561	0.0006	0.018
Q5JTJ3	ENSG00000168275	COA6	Cytochrome c oxidase assembly factor 6 homolog	Q5JTJ3 (COA6)	-0.188	0.592	-2.778	0.016	0.118
O43818	ENSG00000114767	RRP9	U3 small nucleolar RNA-interacting protein 2 (RRP9 homolog) (U3 small nucleolar ribonucleoprotein-associated 55 kDa protein) (U3 snoRNP-associated 55 kDa protein) (U3-55K)	O43818 (RRP9)	-0.188	1.006	-2.865	0.0136	0.106
Q9Y692	ENSG00000162419	GMEB1	Glucocorticoid modulatory element-binding protein 1 (GMEB-1) (DNA-binding protein p96PIF) (Parvovirus initiation factor p96) (PIF p96)	Q9Y692 (GMEB1)	-0.188	0.728	-3.430	0.0047	0.0574
P15586	ENSG00000135677	GNS	N-acetylglucosamine-6-sulfatase (EC 3.1.6.14) (Glucosamine-6-sulfatase) (G6S)	P15586 (GNS)	0.187	0.818	2.835	0.0144	0.1107
P38606	ENSG00000114573	ATP6V1A	V-type proton ATPase catalytic subunit A (V-ATPase subunit A) (EC 7.1.2.2) (V-ATPase 69 kDa subunit) (Vacuolar ATPase isoform VA68) (Vacuolar proton pump subunit alpha)	P38606 (ATP6V1A)	0.187	0.791	4.390	0.0008	0.0221
Q92544	ENSG00000101337	TM9SF4	Transmembrane 9 superfamily member 4 (Tumor cannibalism associated protein 1)	Q92544 (TM9SF4)	0.187	0.742	3.459	0.0044	0.0562
P11169	ENSG00000059804	SLC2A3	Solute carrier family 2, facilitated glucose transporter member 3 (Glucose transporter type 3, brain) (GLUT-3)	P11169 (SLC2A3)	0.186	0.928	5.052	0.0002	0.0115
P50579	ENSG00000111142	METAP2	Methionine aminopeptidase 2 (MAP 2) (MetAP 2) (EC 3.4.11.18) (Initiation factor 2-associated 67 kDa glycoprotein) (p67) (p67eIF2) (Peptidase M)	P50579 (METAP2)	-0.186	0.859	-2.572	0.0236	0.148
P21283	ENSG00000155097	ATP6V1C1	V-type proton ATPase subunit C 1 (V-ATPase subunit C 1) (Vacuolar proton pump subunit C 1)	P21283 (ATP6V1C1)	0.185	0.831	3.742	0.0026	0.041

Table S55: Proteins differentially abundant at Day 30 compared to pre-second vaccination (Study Arm 2 (A/H7N9 + AS03 D1, 29), Dendritic cells). Proteins are sorted by descending absolute log_2 fold change. Protein and gene annotations are based on Ensembl Version 101 (continued).

Protein ID	Ensembl Gene ID	Ensembl Gene Name	Protein Description	50% Protein Cluster ID (Gene Name)	Fold Change (Day 30 vs. Pre- Second Vaccination)	Average log_2 Abundance	Moderated t-Statistic	P-Value	FDR Adjusted P-value
Q96RQ3	ENSG00000078070	MCCC1	Methylcrotonoyl-CoA carboxylase subunit alpha, mitochondrial (MCCase subunit alpha) (EC 6.4.1.4) (3-methylcrotonyl-CoA carboxylase 1) (3-methylcrotonyl-CoA carboxylase biotin-containing subunit) (3-methylcrotonyl-CoA:carbon dioxide ligase subunit alpha)	Q96RQ3 (MCCC1)	-0.185	0.835	-3.216	0.007	0.0733
Q01658	ENSG00000117505	DR1	Protein Dr1 (Down-regulator of transcription 1) (Negative cofactor 2-beta) (NC2-beta) (TATA-binding protein-associated phosphoprotein)	Q01658 (DR1)	-0.184	0.809	-3.755	0.0025	0.0404
P30086	ENSG00000089220	PEBP1	Phosphatidylethanolamine-binding protein 1 (PEBP-1) (HCNPpp) (Neuropolypeptide h3) (Prostatic-binding protein) (Raf kinase inhibitor protein) (RKIP) [Cleaved into: Hippocampal cholinergic neurostimulating peptide (HCNP)]	P30086 (PEBP1)	-0.184	0.511	-2.909	0.0125	0.1023
Q6PJF5	ENSG00000129667	RHBDF2	Inactive rhomboid protein 2 (iRhom2) (Rhomboid 5 homolog 2) (Rhomboid family member 2) (Rhomboid veinlet-like protein 5) (Rhomboid veinlet-like protein 6)	Q6PJF5 (RHBDF2)	0.184	0.957	3.396	0.005	0.0592
Q12899	ENSG00000137313; ENSG00000231002; ENSG00000234046; ENSG00000228881; ENSG00000231641; ENSG00000226060; ENSG00000230230; ENSG00000234127	TRIM26	Tripartite motif-containing protein 26 (EC 2.3.2.27) (Acid finger protein) (AFP) (RING finger protein 95) (Zinc finger protein 173)	Q12899 (TRIM26)	0.183	0.854	3.648	0.0031	0.0451
P61604	ENSG00000115541	HSPE1	10 kDa heat shock protein, mitochondrial (Hsp10) (10 kDa chaperonin) (Chaperonin 10) (CPN10) (Early-pregnancy factor) (EPF)	P61604 (HSPE1)	-0.182	0.701	-3.387	0.005	0.0597
O60488	ENSG00000068366	ACSL4	Long-chain-fatty-acid–CoA ligase 4 (EC 6.2.1.3) (Arachidonate–CoA ligase) (EC 6.2.1.15) (Long-chain acyl-CoA synthetase 4) (LACS 4)	O95573 (ACSL3)	0.182	0.923	4.247	0.001	0.026

Table S55: Proteins differentially abundant at Day 30 compared to pre-second vaccination (Study Arm 2 (A/H7N9 + AS03 D1, 29), Dendritic cells). Proteins are sorted by descending absolute log_2 fold change. Protein and gene annotations are based on Ensembl Version 101 (continued).

Protein ID	Ensembl Gene ID	Ensembl Gene Name	Protein Description	50% Protein Cluster ID (Gene Name)	Fold Change (Day 30 vs. Pre- Second Vaccination)	Average log_2 Abundance	Moderated t-Statistic	P-Value	FDR Adjusted P-value
P48426	ENSG00000150867	PIP4K2A	Phosphatidylinositol 5-phosphate 4-kinase type-2 alpha (EC 2.7.1.149) (1-phosphatidylinositol 5-phosphate 4-kinase 2-alpha) (Diphosphoinositide kinase 2-alpha) (PIP5KIII) (Phosphatidylinositol 5-Phosphate 4-Kinase) (PI5P4Kalpha) (Phosphatidylinositol 5-phosphate 4-kinase type II alpha) (PI(5)P 4-kinase type II alpha) (PIP4KII-alpha) (PtdIns(4)P-5-kinase B isoform) (PtdIns(4)P-5-kinase C isoform) (PtdIns(5)P-4-kinase isoform 2-alpha)	Q8TBX8 (PIP4K2C)	-0.182	0.706	-3.517	0.0039	0.0519
O60496	ENSG00000147443	DOK2	Docking protein 2 (Downstream of tyrosine kinase 2) (p56(dok-2))	O60496 (DOK2)	-0.182	0.693	-3.273	0.0063	0.0689
Q86YV0	ENSG00000105122	RASAL3	RAS protein activator like-3	Q86YV0 (RASAL3)	-0.181	0.753	-4.415	0.0007	0.0215
P49458	ENSG00000143742	SRP9	Signal recognition particle 9 kDa protein (SRP9)	P49458 (SRP9)	-0.181	0.750	-2.758	0.0167	0.1204
Q13242	ENSG00000111786	SRSF9	Serine/arginine-rich splicing factor 9 (Pre-mRNA-splicing factor SRp30C) (Splicing factor, arginine/serine-rich 9)	Q07955 (SRSF1)	0.181	0.910	2.671	0.0196	0.1334
P12694	ENSG00000248098	BCKDHA	2-oxoisovalerate dehydrogenase subunit alpha, mitochondrial (EC 1.2.4.4) (Branched-chain alpha-keto acid dehydrogenase E1 component alpha chain) (BCKDE1A) (BCKDH E1-alpha)	P12694 (BCKDHA)	-0.181	0.770	-3.376	0.0052	0.0602
O75874	ENSG00000138413	IDH1	Isocitrate dehydrogenase [NADP] cytoplasmic (IDH) (EC 1.1.1.42) (Cytosolic NADP-isocitrate dehydrogenase) (IDP) (NADP(+)-specific ICDH) (Oxalosuccinate decarboxylase)	P48735 (IDH2)	0.180	0.875	3.735	0.0026	0.0412
Q8IYJ3	ENSG00000142765	SYTL1	Synaptotagmin-like protein 1 (Exophilin-7) (Protein JFC1)	Q8IYJ3 (SYTL1)	-0.180	0.729	-4.623	0.0005	0.017
Q96L92	ENSG00000143376	SNX27	Sorting nexin-27	Q96L92 (SNX27)	0.180	0.932	5.045	0.0002	0.0115
Q9Y3E7	ENSG00000115561	CHMP3	Charged multivesicular body protein 3 (Chromatin-modifying protein 3) (Neuroendocrine differentiation factor) (Vacuolar protein sorting-associated protein 24) (hVps24)	Q9Y3E7 (CHMP3)	0.179	0.807	3.099	0.0087	0.0834
Q96AQ6	ENSG00000163346	PBXIP1	Pre-B-cell leukemia transcription factor-interacting protein 1 (Hematopoietic PBX-interacting protein)	Q96AQ6 (PBXIP1)	-0.179	0.858	-3.014	0.0102	0.0927

Confidential/Proprietary Information

Table S55: Proteins differentially abundant at Day 30 compared to pre-second vaccination (Study Arm 2 (A/H7N9 + AS03 D1, 29), Dendritic cells). Proteins are sorted by descending absolute log_2 fold change. Protein and gene annotations are based on Ensembl Version 101 (continued).

Protein ID	Ensembl Gene ID	Ensembl Gene Name	Protein Description	50% Protein Cluster ID (Gene Name)	Fold Change (Day 30 vs. Pre- Second Vaccination)	Average log_2 Abundance	Moderated t-Statistic	P-Value	FDR Adjusted P-value
P23246	ENSG00000116560	SFPQ	Splicing factor, proline- and glutamine-rich (100 kDa DNA-pairing protein) (hPOMp100) (DNA-binding p52/p100 complex, 100 kDa subunit) (Polypyrimidine tract-binding protein-associated-splicing factor) (PSF) (PTB-associated-splicing factor)	P23246 (SFPQ)	-0.179	0.623	-4.060	0.0014	0.031
P00813	ENSG00000196839	ADA	Adenosine deaminase (EC 3.5.4.4) (Adenosine aminohydrolase)	P00813 (ADA)	-0.179	0.672	-3.359	0.0053	0.0616
Q9HBL7	ENSG00000107020	PLGRKT	Plasminogen receptor (KT) (Plg-R(KT))	Q9HBL7 (PLGRKT)	-0.178	0.905	-3.943	0.0018	0.035
Q9UBR2	ENSG00000101160	CTSZ	Cathepsin Z (EC 3.4.18.1) (Cathepsin P) (Cathepsin X)	Q9UBR2 (CTSZ)	0.178	0.850	3.801	0.0023	0.0391
O00422	ENSG00000150459	SAP18	Histone deacetylase complex subunit SAP18 (18 kDa Sin3-associated polypeptide) (2HOR0202) (Cell growth-inhibiting gene 38 protein) (Sin3-associated polypeptide p18)	O00422 (SAP18)	0.178	0.924	3.805	0.0023	0.0391
Q9Y3A5	ENSG00000126524	SBDS	Ribosome maturation protein SBDS (Shwachman-Bodian-Diamond syndrome protein)	Q9Y3A5 (SBDS)	-0.178	0.809	-4.235	0.001	0.0264
Q2PPJ7	ENSG00000188559	RALGAPA2	Ral GTPase-activating protein subunit alpha-2 (250 kDa substrate of Akt) (AS250) (p220)	Q2PPJ7 (RALGAPA2)	0.178	0.841	3.793	0.0023	0.0391
Q02083	ENSG00000138744	NAAA	N-acylethanolamine-hydrolyzing acid amidase (EC 3.5.1.60) (Acid ceramidase-like protein) (Acylsphingosine deacylase NAAA) (EC 3.5.1.23) (N-acylsphingosine amidohydrolase-like) (ASAH-like protein) [Cleaved into: N-acylethanolamine-hydrolyzing acid amidase subunit alpha; N-acylethanolamine-hydrolyzing acid amidase subunit beta]	Q02083 (NAAA)	-0.178	0.536	-2.971	0.0111	0.0966
Q13232	ENSG00000103024	NME3	Nucleoside diphosphate kinase 3 (NDK 3) (NDP kinase 3) (EC 2.7.4.6) (DR-nm23) (Nucleoside diphosphate kinase C) (NDPKC) (nm23-H3)	O00746 (NME4)	-0.177	0.723	-3.682	0.0029	0.0438
Q86VB7	ENSG00000177575	CD163	Scavenger receptor cysteine-rich type 1 protein M130 (Hemoglobin scavenger receptor) (CD antigen CD163) [Cleaved into: Soluble CD163 (sCD163)]	Q86VB7 (CD163)	-0.177	0.863	-2.685	0.0191	0.1316

Table S55: Proteins differentially abundant at Day 30 compared to pre-second vaccination (Study Arm 2 (A/H7N9 + AS03 D1, 29), Dendritic cells). Proteins are sorted by descending absolute log_2 fold change. Protein and gene annotations are based on Ensembl Version 101 (continued).

Protein ID	Ensembl Gene ID	Ensembl Gene Name	Protein Description	50% Protein Cluster ID (Gene Name)	Fold Change (Day 30 vs. Pre- Second Vaccination)	Average log_2 Abundance	Moderated t-Statistic	P-Value	FDR Adjusted P-value
P61421	ENSG00000159720	ATP6V0D1	V-type proton ATPase subunit d 1 (V-ATPase subunit d 1) (32 kDa accessory protein) (V-ATPase 40 kDa accessory protein) (V-ATPase AC39 subunit) (p39) (Vacuolar proton pump subunit d 1)	P61421 (ATP6V0D1)	0.176	0.935	3.383	0.0051	0.0599
P43405	ENSG00000165025	SYK	Tyrosine-protein kinase SYK (EC 2.7.10.2) (Spleen tyrosine kinase) (p72-Syk)	P43405 (SYK)	0.176	0.828	2.790	0.0157	0.1162
O43312	ENSG00000170873	MTSS1	Protein MTSS 1 (Metastasis suppressor YGL-1) (Metastasis suppressor protein 1) (Missing in metastasis protein)	O43312 (MTSS1)	-0.176	0.635	-2.575	0.0235	0.1474
Q8IYM9	ENSG00000132274	TRIM22	E3 ubiquitin-protein ligase TRIM22 (EC 2.3.2.27) (50 kDa-stimulated trans-acting factor) (RING finger protein 94) (RING-type E3 ubiquitin transferase TRIM22) (Staf-50) (Tripartite motif-containing protein 22)	Q8IYM9 (TRIM22)	0.176	0.957	2.642	0.0207	0.1387
O43747	ENSG00000166747	AP1G1	AP-1 complex subunit gamma-1 (Adaptor protein complex AP-1 subunit gamma-1) (Adaptor-related protein complex 1 subunit gamma-1) (Clathrin assembly protein complex 1 gamma-1 large chain) (Gamma1-adaptin) (Golgi adaptor HA1/AP1 adaptin subunit gamma-1)	O43747 (AP1G1)	0.175	0.846	2.538	0.0252	0.1498
Q13188	ENSG00000104375	STK3	Serine/threonine-protein kinase 3 (EC 2.7.11.1) (Mammalian STE20-like protein kinase 2) (MST-2) (STE20-like kinase MST2) (Serine/threonine-protein kinase Krs-1) [Cleaved into: Serine/threonine-protein kinase 3 36kDa subunit (MST2/N); Serine/threonine-protein kinase 3 20kDa subunit (MST2/C)]	Q13188 (STK3)	0.175	0.842	4.104	0.0013	0.0296
Q8TEU7	ENSG00000158987	RAPGEF6	Rap guanine nucleotide exchange factor 6 (PDZ domain-containing guanine nucleotide exchange factor 2) (PDZ-GEF2) (RA-GEF-2)	Q8TEU7 (RAPGEF6)	-0.175	0.868	-3.233	0.0068	0.0724
P06400	ENSG00000139687	RB1	Retinoblastoma-associated protein (p105-Rb) (p110-RB1) (pRb) (Rb) (pp110)	P06400 (RB1)	0.175	1.002	2.766	0.0164	0.1197
Q9H3M7	ENSG00000265972	TXNIP	Thioredoxin-interacting protein (Thioredoxin-binding protein 2) (Vitamin D3 up-regulated protein 1)	Q9H3M7 (TXNIP)	0.175	0.917	2.803	0.0153	0.1143
O60711	ENSG00000110031	LPXN	Leupaxin	P49023 (PXN)	-0.174	0.755	-5.177	0.0002	0.01

Table S55: Proteins differentially abundant at Day 30 compared to pre-second vaccination (Study Arm 2 (A/H7N9 + AS03 D1, 29), Dendritic cells). Proteins are sorted by descending absolute log_2 fold change. Protein and gene annotations are based on Ensembl Version 101 (continued).

Protein ID	Ensembl Gene ID	Ensembl Gene Name	Protein Description	50% Protein Cluster ID (Gene Name)	Fold Change (Day 30 vs. Pre- Second Vaccination)	Average log_2 Abundance	Moderated t-Statistic	P-Value	FDR Adjusted P-value
P15311	ENSG00000092820	EZR	Ezrin (Cytovillin) (Villin-2) (p81)	P15311 (EZR)	-0.174	0.680	-4.050	0.0015	0.0311
Q9BQ52	ENSG00000006744	ELAC2	Zinc phosphodiesterase ELAC protein 2 (EC 3.1.26.11) (ElaC homolog protein 2) (Heredity prostate cancer protein 2) (Ribonuclease Z 2) (RNase Z 2) (tRNA 3 endonuclease 2) (tRNase Z 2)	Q9BQ52 (ELAC2)	-0.174	0.844	-2.667	0.0198	0.134
Q9UL46	ENSG00000100911; ENSG00000284889	PSME2	Proteasome activator complex subunit 2 (11S regulator complex subunit beta) (REG-beta) (Activator of multi-catalytic protease subunit 2) (Proteasome activator 28 subunit beta) (PA28b) (PA28beta)	Q06323 (PSME1)	0.173	0.968	3.395	0.005	0.0592
Q9BVC6	ENSG00000110108	TMEM109	Transmembrane protein 109 (Mitsugumin-23) (Mg23)	Q9BVC6 (TMEM109)	-0.173	0.782	-3.543	0.0038	0.0511
O43491	ENSG00000079819	EPB41L2	Band 4.1-like protein 2 (Erythrocyte membrane protein band 4.1-like 2) (Generally expressed protein 4.1) (4.1G)	O43491 (EPB41L2)	-0.173	0.986	-2.767	0.0164	0.1197
Q9NX63	ENSG00000106554	CHCHD3	MICOS complex subunit MIC19 (Coiled-coil-helix-coiled-coil-helix domain-containing protein 3)	Q9NX63 (CHCHD3)	-0.173	0.796	-2.791	0.0156	0.1162
Q92820	ENSG00000137563	GGH	Gamma-glutamyl hydrolase (EC 3.4.19.9) (Conjugase) (GH) (Gamma-Glu-X carboxypeptidase)	Q92820 (GGH)	0.172	0.755	2.616	0.0218	0.1415
P52735	ENSG00000160293	VAV2	Guanine nucleotide exchange factor VAV2 (VAV-2)	P52735 (VAV2)	-0.172	0.953	-3.113	0.0085	0.0822
P11940	ENSG00000070756	PABPC1	Polyadenylate-binding protein 1 (PABP-1) (Poly(A)-binding protein 1)	Q13310 (PABPC4)	0.172	0.859	4.511	0.0006	0.0188
O15397	ENSG00000133704	IPO8	Importin-8 (Imp8) (Ran-binding protein 8) (RanBP8)	O95373 (IPO7)	0.172	0.901	2.957	0.0114	0.0977
Q16831	ENSG00000183696	UPP1	Uridine phosphorylase 1 (UPase 1) (UrdPase 1) (EC 2.4.2.3)	Q16831 (UPP1)	0.172	0.903	3.702	0.0028	0.0431

Table S55: Proteins differentially abundant at Day 30 compared to pre-second vaccination (Study Arm 2 (A/H7N9 + AS03 D1, 29), Dendritic cells). Proteins are sorted by descending absolute log_2 fold change. Protein and gene annotations are based on Ensembl Version 101 (continued).

Protein ID	Ensembl Gene ID	Ensembl Gene Name	Protein Description	50% Protein Cluster ID (Gene Name)	Fold Change (Day 30 vs. Pre- Second Vaccination)	Average log_2 Abundance	Moderated t-Statistic	P-Value	FDR Adjusted P-value
P13010	ENSG00000079246	XRCC5	X-ray repair cross-complementing protein 5 (EC 3.6.4.-) (86 kDa subunit of Ku antigen) (ATP-dependent DNA helicase 2 subunit 2) (ATP-dependent DNA helicase II 80 kDa subunit) (CTC box-binding factor 85 kDa subunit) (CTC85) (CTCBF) (DNA repair protein XRCC5) (Ku80) (Ku86) (Lupus Ku autoantigen protein p86) (Nuclear factor IV) (Thyroid-lupus autoantigen) (TLAA) (X-ray repair complementing defective repair in Chinese hamster cells 5 (double-strand-break rejoining))	P13010 (XRCC5)	-0.172	0.762	-4.346	0.0008	0.0232
Q9Y3Q8	ENSG00000166925	TSC22D4	TSC22 domain family protein 4 (TSC22-related-inducible leucine zipper protein 2) (Tsc-22-like protein THG-1)	Q9Y3Q8 (TSC22D4)	-0.172	0.802	-2.912	0.0124	0.102
Q96RF0	ENSG00000178996	SNX18	Sorting nexin-18 (SH3 and PX domain-containing protein 3B) (Sorting nexin-associated Golgi protein 1)	Q96RF0 (SNX18)	-0.171	0.587	-4.167	0.0012	0.028
Q12913	ENSG00000149177	PTPRJ	Receptor-type tyrosine-protein phosphatase eta (Protein-tyrosine phosphatase eta) (R-PTP-eta) (EC 3.1.3.48) (Density-enhanced phosphatase 1) (DEP-1) (HPTP eta) (Protein-tyrosine phosphatase receptor type J) (R-PTP-J) (CD antigen CD148)	Q12913 (PTPRJ)	0.171	0.973	4.648	0.0005	0.0166
Q09666	ENSG00000124942	AHNAK	Neuroblast differentiation-associated protein AHNAK (Desmoyokin)	Q09666 (AHNAK)	-0.171	0.691	-3.524	0.0039	0.0517
P09769	ENSG00000000938	FGR	Tyrosine-protein kinase Fgr (EC 2.7.10.2) (Gardner-Rasheed feline sarcoma viral (v-fgr) oncogene homolog) (Proto-oncogene c-Fgr) (p55-Fgr) (p58-Fgr) (p58c-Fgr)	P06241 (FYN)	0.171	0.843	3.893	0.0019	0.0362
Q5W0V3	ENSG00000151553	FAM160B1	Protein FAM160B1	Q5W0V3 (FAM160B1)	0.171	0.919	3.316	0.0058	0.0652
Q9NP72	ENSG00000099246	RAB18	Ras-related protein Rab-18	Q9NP72 (RAB18)	0.171	0.828	3.852	0.0021	0.0376
Q9NQG5	ENSG00000101413	RPRD1B	Regulation of nuclear pre-mRNA domain-containing protein 1B (Cell cycle-related and expression-elevated protein in tumor)	Q9NQG5 (RPRD1B)	-0.171	0.942	-3.213	0.007	0.0733

Table S55: Proteins differentially abundant at Day 30 compared to pre-second vaccination (Study Arm 2 (A/H7N9 + AS03 D1, 29), Dendritic cells). Proteins are sorted by descending absolute log_2 fold change. Protein and gene annotations are based on Ensembl Version 101 (continued).

Protein ID	Ensembl Gene ID	Ensembl Gene Name	Protein Description	50% Protein Cluster ID (Gene Name)	Fold Change (Day 30 vs. Pre- Second Vaccination)	Average log_2 Abundance	Moderated t-Statistic	P-Value	FDR Adjusted P-value
Q16630	ENSG00000111605	CPSF6	Cleavage and polyadenylation specificity factor subunit 6 (Cleavage and polyadenylation specificity factor 68 kDa subunit) (CPSF 68 kDa subunit) (Cleavage factor Im complex 68 kDa subunit) (CFIm68) (Pre-mRNA cleavage factor Im 68 kDa subunit) (Protein HPBRII-4/7)	Q16630 (CPSF6)	-0.171	0.827	-3.726	0.0027	0.0417
Q9P032	ENSG00000123545	NDUFAF4	NADH dehydrogenase [ubiquinone] 1 alpha subcomplex assembly factor 4 (Hormone-regulated proliferation-associated protein of 20 kDa)	Q9P032 (NDUFAF4)	-0.170	0.797	-4.732	0.0004	0.0155
Q9UKV3	ENSG00000100813	ACIN1	Apoptotic chromatin condensation inducer in the nucleus (Acinus)	Q9UKV3 (ACIN1)	0.170	1.087	2.777	0.0161	0.118
Q9Y383	ENSG00000146963	LUC7L2	Putative RNA-binding protein Luc7-like 2	Q9Y383 (LUC7L2)	-0.170	0.753	-4.672	0.0005	0.0164
Q9H6A0	ENSG00000162777	DENND2D	DENN domain-containing protein 2D	Q9H6A0 (DENND2D)	-0.169	0.758	-3.247	0.0066	0.0709
O15533	ENSG00000112493; ENSG00000206208; ENSG00000206281; ENSG00000236490; ENSG00000231925	TAPBP	Tapasin (TPN) (TPSN) (NGS-17) (TAP-associated protein) (TAP-binding protein)	O15533 (TAPBP)	0.169	0.974	3.409	0.0048	0.0588
P60174	ENSG00000111669	TPI1	Triosephosphate isomerase (TIM) (EC 5.3.1.1) (Methylglyoxal synthase) (EC 4.2.3.3) (Triose-phosphate isomerase)	P60174 (TPI1)	-0.169	0.663	-3.837	0.0022	0.0378
P49753	ENSG00000119673	ACOT2	Acyl-coenzyme A thioesterase 2, mitochondrial (Acyl-CoA thioesterase 2) (EC 3.1.2.2) (Acyl-coenzyme A thioester hydrolase 2a) (CTE-Ia) (Long-chain acyl-CoA thioesterase 2) (ZAP128)	P49753 (ACOT2)	-0.168	0.686	-4.294	0.0009	0.0246
P46063	ENSG00000004700	RECQL	ATP-dependent DNA helicase Q1 (EC 3.6.4.12) (DNA helicase, RecQ-like type 1) (RecQ1) (DNA-dependent ATPase Q1) (RecQ protein-like 1)	P46063 (RECQL)	-0.168	0.711	-2.874	0.0134	0.1047
Q9NXG2	ENSG00000066654	THUMPD1	THUMP domain-containing protein 1	Q9NXG2 (THUMPD1)	-0.167	0.746	-2.619	0.0217	0.1414
Q8N3U4	ENSG00000101972	STAG2	Cohesin subunit SA-2 (SCC3 homolog 2) (Stromal antigen 2)	Q8WVM7 (STAG1)	0.167	0.831	2.859	0.0138	0.1066

Table S55: Proteins differentially abundant at Day 30 compared to pre-second vaccination (Study Arm 2 (A/H7N9 + AS03 D1, 29), Dendritic cells). Proteins are sorted by descending absolute log_2 fold change. Protein and gene annotations are based on Ensembl Version 101 (continued).

Protein ID	Ensembl Gene ID	Ensembl Gene Name	Protein Description	50% Protein Cluster ID (Gene Name)	Fold Change (Day 30 vs. Pre- Second Vaccination)	Average log_2 Abundance	Moderated t-Statistic	P-Value	FDR Adjusted P-value
Q70UQ0	ENSG00000166130	IKBIP	Inhibitor of nuclear factor kappa-B kinase-interacting protein (I kappa-B kinase-interacting protein) (IKBKB-interacting protein) (IKK-interacting protein)	Q70UQ0 (IKBIP)	0.167	0.951	3.826	0.0022	0.0384
P46778	ENSG00000122026	RPL21	60S ribosomal protein L21 (Large ribosomal subunit protein eL21)	P46778 (RPL21)	0.166	0.998	2.544	0.0249	0.1498
P38919	ENSG00000141543	EIF4A3	Eukaryotic initiation factor 4A-III (eIF-4A-III) (eIF4A-III) (EC 3.6.4.13) (ATP-dependent RNA helicase DDX48) (ATP-dependent RNA helicase eIF4A-3) (DEAD box protein 48) (Eukaryotic initiation factor 4A-like NUK-34) (Eukaryotic translation initiation factor 4A isoform 3) (Nuclear matrix protein 265) (NMP 265) (hNMP 265) [Cleaved into: Eukaryotic initiation factor 4A-III, N-terminally processed]	P38919 (EIF4A3)	0.165	0.837	3.237	0.0067	0.072
O75947	ENSG00000167863	ATP5PD	ATP synthase subunit d, mitochondrial (ATPase subunit d) (ATP synthase peripheral stalk subunit d)	O75947 (ATP5PD)	-0.165	0.638	-2.938	0.0118	0.0991
Q9UGI8	ENSG00000135269	TES	Testin (TESS)	Q9UGI8 (TES)	-0.165	0.755	-3.265	0.0064	0.0695
P16220	ENSG00000118260	CREB1	Cyclic AMP-responsive element-binding protein 1 (CREB-1) (cAMP-responsive element-binding protein 1)	P16220 (CREB1)	-0.165	0.556	-3.212	0.007	0.0733
O95248	ENSG00000100241	SBF1	Myotubularin-related protein 5 (Inactive phosphatidylinositol 3-phosphatase 5) (SET-binding factor 1) (Sbf1)	O95248 (SBF1)	-0.163	0.850	-3.635	0.0032	0.0456
Q9H008	ENSG00000107902	LHPP	Phospholysine phosphohistidine inorganic pyrophosphate phosphatase (hLHPP) (EC 3.1.3.-) (EC 3.6.1.1)	Q9H008 (LHPP)	-0.163	0.851	-3.452	0.0045	0.0566
Q92621	ENSG00000155561	NUP205	Nuclear pore complex protein Nup205 (205 kDa nucleoporin) (Nucleoporin Nup205)	Q92621 (NUP205)	0.163	0.858	3.249	0.0066	0.0709
P50851	ENSG00000198589	LRBA	Lipopolysaccharide-responsive and beige-like anchor protein (Beige-like protein) (CDC4-like protein)	P50851 (LRBA)	-0.163	0.813	-2.952	0.0115	0.098
P51858	ENSG00000143321	HDGF	Hepatoma-derived growth factor (HDGF) (High mobility group protein 1-like 2) (HMG-1L2)	P51858 (HDGF)	-0.163	0.769	-2.554	0.0245	0.1496
O43678	ENSG00000131495	NDUFA2	NADH dehydrogenase [ubiquinone] 1 alpha subcomplex subunit 2 (Complex I-B8) (CI-B8) (NADH-ubiquinone oxidoreductase B8 subunit)	O43678 (NDUFA2)	-0.162	0.653	-3.107	0.0086	0.0824

Table S55: Proteins differentially abundant at Day 30 compared to pre-second vaccination (Study Arm 2 (A/H7N9 + AS03 D1, 29), Dendritic cells). Proteins are sorted by descending absolute log_2 fold change. Protein and gene annotations are based on Ensembl Version 101 (continued).

Protein ID	Ensembl Gene ID	Ensembl Gene Name	Protein Description	50% Protein Cluster ID (Gene Name)	Fold Change (Day 30 vs. Pre- Second Vaccination)	Average log_2 Abundance	Moderated t-Statistic	P-Value	FDR Adjusted P-value
P07602	ENSG00000197746	PSAP	Prosaposin (Proactivator polypeptide) [Cleaved into: Saposin-A (Protein A); Saposin-B-Val; Saposin-B (Cerebroside sulfate activator) (CSAct) (Dispersin) (Sphingolipid activator protein 1) (SAP-1) (Sulfatide/GM1 activator); Saposin-C (A1 activator) (Co-beta-glucosidase) (Glucosylceramidase activator) (Sphingolipid activator protein 2) (SAP-2); Saposin-D (Component C) (Protein C)]	P07602 (PSAP)	0.162	0.697	2.693	0.0188	0.1304
Q9H3P2	ENSG00000185049	NELFA	Negative elongation factor A (NELF-A) (Wolf-Hirschhorn syndrome candidate 2 protein)	Q9H3P2 (NELFA)	-0.161	0.733	-4.041	0.0015	0.0311
Q9NVH1	ENSG00000007923	DNAJC11	DnaJ homolog subfamily C member 11	Q9NVH1 (DNAJC11)	-0.161	0.815	-3.864	0.0021	0.0376
Q6ZNJ1	ENSG00000160796	NBEAL2	Neurobeachin-like protein 2	Q6ZNJ1 (NBEAL2)	0.161	0.770	3.676	0.0029	0.0441
P30460				P30501 (NA)	0.160	1.185	3.011	0.0103	0.0931
Q03405	ENSG00000011422	PLAUR	Urokinase plasminogen activator surface receptor (U-PAR) (uPAR) (Monocyte activation antigen Mo3) (CD antigen CD87)	Q03405 (PLAUR)	0.160	0.936	3.659	0.003	0.0449
P48960	ENSG00000123146	ADGRE5	Adhesion G protein-coupled receptor E5 (Leukocyte antigen CD97) (CD antigen CD97) [Cleaved into: Adhesion G protein-coupled receptor E5 subunit alpha; Adhesion G protein-coupled receptor E5 subunit beta]	P48960 (ADGRE5)	0.160	1.013	3.073	0.0092	0.0863
P19474	ENSG00000132109	TRIM21	E3 ubiquitin-protein ligase TRIM21 (EC 2.3.2.27) (52 kDa Ro protein) (52 kDa ribonucleoprotein autoantigen Ro/SS-A) (RING finger protein 81) (RING-type E3 ubiquitin transferase TRIM21) (Ro(SS-A)) (Sjoegren syndrome type A antigen) (SS-A) (Tripartite motif-containing protein 21)	P19474 (TRIM21)	0.160	0.976	3.996	0.0016	0.0334
Q96JY6	ENSG00000120913	PDLIM2	PDZ and LIM domain protein 2 (PDZ-LIM protein mystique)	Q96JY6 (PDLIM2)	-0.160	0.705	-2.848	0.014	0.1086
Q9BZ23	ENSG00000125779	PANK2	Pantothenate kinase 2, mitochondrial (hPanK2) (EC 2.7.1.33) (Pantothenic acid kinase 2)	Q9BZ23 (PANK2)	0.160	0.792	2.934	0.0119	0.0994
P55036	ENSG00000159352	PSMD4	26S proteasome non-ATPase regulatory subunit 4 (26S proteasome regulatory subunit RPN10) (26S proteasome regulatory subunit S5A) (Antisecretory factor 1) (AF) (ASF) (Multiubiquitin chain-binding protein)	P55036 (PSMD4)	-0.159	0.622	-3.396	0.005	0.0592

Confidential/Proprietary Information

Table S55: Proteins differentially abundant at Day 30 compared to pre-second vaccination (Study Arm 2 (A/H7N9 + AS03 D1, 29), Dendritic cells). Proteins are sorted by descending absolute log_2 fold change. Protein and gene annotations are based on Ensembl Version 101 (continued).

Protein ID	Ensembl Gene ID	Ensembl Gene Name	Protein Description	50% Protein Cluster ID (Gene Name)	Fold Change (Day 30 vs. Pre- Second Vaccination)	Average log_2 Abundance	Moderated t-Statistic	P-Value	FDR Adjusted P-value
Q99615	ENSG00000168259	DNAJC7	DnaJ homolog subfamily C member 7 (Tetratricopeptide repeat protein 2) (TPR repeat protein 2)	Q99615 (DNAJC7)	0.159	0.879	4.248	0.001	0.026
Q14444	ENSG00000135387	CAPRIN1	Caprin-1 (Cell cycle-associated protein 1) (Cytoplasmic activation- and proliferation-associated protein 1) (GPI-anchored membrane protein 1) (GPI-anchored protein p137) (GPI-p137) (p137GPI) (Membrane component chromosome 11 surface marker 1) (RNA granule protein 105)	Q14444 (CAPRIN1)	0.159	0.876	4.034	0.0015	0.0313
P54920	ENSG00000105402	NAPA	Alpha-soluble NSF attachment protein (SNAP-alpha) (N-ethylmaleimide-sensitive factor attachment protein alpha)	P54920 (NAPA)	0.158	0.935	2.896	0.0128	0.1025
P11171	ENSG00000159023	EPB41	Protein 4.1 (P4.1) (4.1R) (Band 4.1) (EPB4.1) (Erythrocyte membrane protein band 4.1)	P11171 (EPB41)	-0.158	0.858	-3.895	0.0019	0.0362
P00367	ENSG00000148672	GLUD1	Glutamate dehydrogenase 1, mitochondrial (GDH 1) (EC 1.4.1.3)	P00367 (GLUD1)	-0.157	0.762	-2.658	0.0201	0.1355
Q10471	ENSG00000143641	GALNT2	Polypeptide N-acetylgalactosaminyltransferase 2 (EC 2.4.1.41) (Polypeptide GalNAc transferase 2) (GalNAc-T2) (pp-GaNTase 2) (Protein-UDP acetylgalactosaminyltransferase 2) (UDP-GalNAc:polypeptide N-acetylgalactosaminyltransferase 2) [Cleaved into: Polypeptide N-acetylgalactosaminyltransferase 2 soluble form]	Q10471 (GALNT2)	0.157	0.954	4.579	0.0006	0.018
Q9UKK3	ENSG00000102699	PARP4	Protein mono-ADP-ribosyltransferase PARP4 (EC 2.4.2.-) (193 kDa vault protein) (ADP-ribosyltransferase diphtheria toxin-like 4) (ARTD4) (PARP-related/IalphaI-related H5/proline-rich) (PH5P) (Poly [ADP-ribose] polymerase 4) (PARP-4) (Vault poly(ADP-ribose) polymerase) (VPARP)	Q9UKK3 (PARP4)	0.157	1.019	3.981	0.0017	0.0336
O75937	ENSG00000126698; ENSG00000285317	DNAJC8	DnaJ homolog subfamily C member 8 (Splicing protein spf31)	O75937 (DNAJC8)	-0.157	0.708	-3.761	0.0025	0.0403
Q6DD88	ENSG00000184743	ATL3	Atlastin-3 (EC 3.6.5.-)	Q8NHH9 (ATL2)	0.157	0.859	2.809	0.0151	0.1136

Table S55: Proteins differentially abundant at Day 30 compared to pre-second vaccination (Study Arm 2 (A/H7N9 + AS03 D1, 29), Dendritic cells). Proteins are sorted by descending absolute log_2 fold change. Protein and gene annotations are based on Ensembl Version 101 (continued).

Protein ID	Ensembl Gene ID	Ensembl Gene Name	Protein Description	50% Protein Cluster ID (Gene Name)	Fold Change (Day 30 vs. Pre- Second Vaccination)	Average log_2 Abundance	Moderated t-Statistic	P-Value	FDR Adjusted P-value
O94905	ENSG00000147475	ERLIN2	Erlin-2 (Endoplasmic reticulum lipid raft-associated protein 2) (Stomatin-prohibitin-flotillin-HflC/K domain-containing protein 2) (SPFH domain-containing protein 2)	O75477 (ERLIN1)	0.156	0.945	4.081	0.0014	0.0304
Q9BUH6	ENSG00000148362	PAXX	Protein PAXX (Paralog of XRCC4 and XLF) (XRCC4-like small protein)	Q9BUH6 (PAXX)	-0.156	0.750	-3.706	0.0028	0.043
Q9H9B4	ENSG00000164466	SFXN1	Sideroflexin-1	Q9H9B4 (SFXN1)	-0.156	0.837	-3.084	0.009	0.0849
P14618	ENSG00000067225	PKM	Pyruvate kinase PKM (EC 2.7.1.40) (Cytosolic thyroid hormone-binding protein) (CTHBP) (Opa-interacting protein 3) (OIP-3) (Pyruvate kinase 2/3) (Pyruvate kinase muscle isozyme) (Thyroid hormone-binding protein 1) (THBP1) (Tumor M2-PK) (p58)	P30613 (PKLR)	0.155	0.820	4.533	0.0006	0.0186
O75563	ENSG00000005020	SKAP2	Src kinase-associated phosphoprotein 2 (Pyk2/RAFTK-associated protein) (Retinoic acid-induced protein 70) (SKAP55 homolog) (SKAP-55HOM) (SKAP-HOM) (Src family-associated phosphoprotein 2) (Src kinase-associated phosphoprotein 55-related protein) (Src-associated adapter protein with PH and SH3 domains)	O75563 (SKAP2)	0.155	0.704	4.148	0.0012	0.0288
Q9UPY3	ENSG00000100697	DICER1	Endoribonuclease Dicer (EC 3.1.26.3) (Helicase with RNase motif) (Helicase MOI)	Q9UPY3 (DICER1)	0.155	1.051	2.680	0.0193	0.1318
Q01082	ENSG00000115306	SPTBN1	Spectrin beta chain, non-erythrocytic 1 (Beta-II spectrin) (Fodrin beta chain) (Spectrin, non-erythroid beta chain 1)	Q01082 (SPTBN1)	-0.155	0.927	-3.435	0.0046	0.0574
Q8TEM1	ENSG00000132182	NUP210	Nuclear pore membrane glycoprotein 210 (Nuclear pore protein gp210) (Nuclear envelope pore membrane protein POM 210) (POM210) (Nucleoporin Nup210) (Pore membrane protein of 210 kDa)	Q8TEM1 (NUP210)	0.155	0.878	4.176	0.0012	0.028
Q10588	ENSG00000109743	BST1	ADP-ribosyl cyclase/cyclic ADP-ribose hydrolase 2 (EC 3.2.2.6) (ADP-ribosyl cyclase 2) (Bone marrow stromal antigen 1) (BST-1) (Cyclic ADP-ribose hydrolase 2) (cADPr hydrolase 2) (CD antigen CD157)	Q10588 (BST1)	0.155	0.956	2.814	0.015	0.1131
Q15020	ENSG00000075856	SART3	Squamous cell carcinoma antigen recognized by T-cells 3 (SART-3) (Tat-interacting protein of 110 kDa) (Tip110) (p110 nuclear RNA-binding protein)	Q15020 (SART3)	-0.155	0.805	-2.817	0.0149	0.1129

Table S55: Proteins differentially abundant at Day 30 compared to pre-second vaccination (Study Arm 2 (A/H7N9 + AS03 D1, 29), Dendritic cells). Proteins are sorted by descending absolute log_2 fold change. Protein and gene annotations are based on Ensembl Version 101 (continued).

Protein ID	Ensembl Gene ID	Ensembl Gene Name	Protein Description	50% Protein Cluster ID (Gene Name)	Fold Change (Day 30 vs. Pre- Second Vaccination)	Average log_2 Abundance	Moderated t-Statistic	P-Value	FDR Adjusted P-value
P55769	ENSG00000100138	SNU13	NHP2-like protein 1 (High mobility group-like nuclear protein 2 homolog 1) (OTK27) (SNU13 homolog) (hSNU13) (U4/U6.U5 small nuclear ribonucleoprotein SNU13) (U4/U6.U5 tri-snRNP 15.5 kDa protein) [Cleaved into: NHP2-like protein 1, N-terminally processed]	P55769 (SNU13)	-0.154	0.771	-2.562	0.0241	0.149
P16150	ENSG00000197471	SPN	Leukosialin (GPL115) (Galactoglycoprotein) (GALGP) (Leukocyte sialoglycoprotein) (Sialophorin) (CD antigen CD43) [Cleaved into: CD43 cytoplasmic tail (CD43-ct) (CD43ct)]	P16150 (SPN)	-0.154	0.858	-2.562	0.0241	0.149
Q86YT9	ENSG00000160593	JAML	Junctional adhesion molecule-like (Adhesion molecule interacting with CXADR antigen 1) (Dendritic cell-specific protein CREA7-1)	Q86YT9 (JAML)	0.154	0.908	2.921	0.0122	0.1007
P07332	ENSG00000182511	FES	Tyrosine-protein kinase Fes/Fps (EC 2.7.10.2) (Feline sarcoma/Fujinami avian sarcoma oncogene homolog) (Proto-oncogene c-Fes) (Proto-oncogene c-Fps) (p93c-fes)	P16591 (FER)	0.154	0.909	3.966	0.0017	0.0341
Q9NPP4	ENSG00000091106	NLRC4	NLR family CARD domain-containing protein 4 (CARD, LRR, and NACHT-containing protein) (Clan protein) (Caspase recruitment domain-containing protein 12) (Ice protease-activating factor) (Ipaf)	Q9NPP4 (NLRC4)	0.153	0.802	2.965	0.0112	0.0968
Q13153	ENSG00000149269	PAK1	Serine/threonine-protein kinase PAK 1 (EC 2.7.11.1) (Alpha-PAK) (p21-activated kinase 1) (PAK-1) (p65-PAK)	Q13153 (PAK1)	0.153	0.932	2.601	0.0224	0.144
Q8NHP6	ENSG00000130150	MOSPD2	Motile sperm domain-containing protein 2	Q8NHP6 (MOSPD2)	0.153	0.997	2.979	0.011	0.0962
Q9BRS2	ENSG00000124784	RIOK1	Serine/threonine-protein kinase RIO1 (EC 2.7.11.1) (EC 3.6.3.-) (RIO kinase 1)	Q9BRS2 (RIOK1)	-0.153	0.895	-2.764	0.0165	0.1201
P51606	ENSG00000102032	RENBP	N-acylglucosamine 2-epimerase (AGE) (EC 5.1.3.8) (GlcNAc 2-epimerase) (N-acetyl-D-glucosamine 2-epimerase) (Renin-binding protein) (RnBP)	P51606 (RENBP)	0.152	0.830	2.589	0.0229	0.1459
O95347	ENSG00000136824	SMC2	Structural maintenance of chromosomes protein 2 (SMC protein 2) (SMC-2) (Chromosome-associated protein E) (hCAP-E) (XCAP-E homolog)	O95347 (SMC2)	0.152	0.976	3.569	0.0036	0.05
Q8WUA7	ENSG00000054611	TBC1D22A	TBC1 domain family member 22A	Q8WUA7 (TBC1D22A)	-0.152	0.900	-2.902	0.0127	0.1025

Confidential/Proprietary Information

Table S55: Proteins differentially abundant at Day 30 compared to pre-second vaccination (Study Arm 2 (A/H7N9 + AS03 D1, 29), Dendritic cells). Proteins are sorted by descending absolute log_2 fold change. Protein and gene annotations are based on Ensembl Version 101 (continued).

Protein ID	Ensembl Gene ID	Ensembl Gene Name	Protein Description	50% Protein Cluster ID (Gene Name)	Fold Change (Day 30 vs. Pre- Second Vaccination)	Average log_2 Abundance	Moderated t-Statistic	P-Value	FDR Adjusted P-value
P13674	ENSG00000122884	P4HA1	Prolyl 4-hydroxylase subunit alpha-1 (4-PH alpha-1) (EC 1.14.11.2) (Procollagen-proline,2-oxoglutarate-4-dioxygenase subunit alpha-1)	P13674 (P4HA1)	0.152	0.741	2.981	0.0109	0.0961
Q8NF91	ENSG00000131018	SYNE1	Nesprin-1 (Enaptin) (KASH domain-containing protein 1) (KASH1) (Myocyte nuclear envelope protein 1) (Myne-1) (Nuclear envelope spectrin repeat protein 1) (Synaptic nuclear envelope protein 1) (Syne-1)	Q8NF91 (SYNE1)	-0.151	0.863	-3.578	0.0035	0.0494
Q9NR19	ENSG00000131069	ACSS2	Acetyl-coenzyme A synthetase, cytoplasmic (EC 6.2.1.1) (Acetate–CoA ligase) (Acetyl-CoA synthetase) (ACS) (AceCS) (Acetyl-CoA synthetase 1) (AceCS1) (Acyl-CoA synthetase short-chain family member 2) (Acyl-activating enzyme) (Propionate–CoA ligase) (EC 6.2.1.17)	Q9NR19 (ACSS2)	0.151	0.852	3.756	0.0025	0.0404
Q2TAA2	ENSG00000134330	IAH1	Isoamyl acetate-hydrolyzing esterase 1 homolog (EC 3.1.-.-)	Q2TAA2 (IAH1)	-0.151	0.864	-4.044	0.0015	0.0311
P17844	ENSG00000108654	DDX5	Probable ATP-dependent RNA helicase DDX5 (EC 3.6.4.13) (DEAD box protein 5) (RNA helicase p68)	Q92841 (DDX17)	0.150	0.852	2.998	0.0106	0.0946
P39023	ENSG00000100316	RPL3	60S ribosomal protein L3 (HIV-1 TAR RNA-binding protein B) (TARBP-B) (Large ribosomal subunit protein uL3)	P39023 (RPL3)	-0.150	0.676	-3.108	0.0086	0.0824
O14737	ENSG00000105185	PDCD5	Programmed cell death protein 5 (TF-1 cell apoptosis-related protein 19) (Protein TFAR19)	O14737 (PDCD5)	-0.150	0.880	-2.748	0.017	0.1212
Q9NX58	ENSG00000145220	LYAR	Cell growth-regulating nucleolar protein	Q9NX58 (LYAR)	-0.150	0.808	-2.944	0.0117	0.0986
Q9NV70	ENSG00000090989	EXOC1	Exocyst complex component 1 (Exocyst complex component Sec3)	Q9NV70 (EXOC1)	0.150	0.809	2.587	0.023	0.1463
Q13217	ENSG00000102580	DNAJC3	DnaJ homolog subfamily C member 3 (Endoplasmic reticulum DNA J domain-containing protein 6) (ER-resident protein ERdj6) (ERdj6) (Interferon-induced, double-stranded RNA-activated protein kinase inhibitor) (Protein kinase inhibitor of 58 kDa) (Protein kinase inhibitor p58)	Q13217 (DNAJC3)	0.150	0.913	3.319	0.0057	0.0651
Q99973	ENSG00000129566	TEP1	Telomerase protein component 1 (Telomerase-associated protein 1) (Telomerase protein 1) (p240) (p80 telomerase homolog)	Q99973 (TEP1)	0.150	0.947	3.538	0.0038	0.0512

Table S55: Proteins differentially abundant at Day 30 compared to pre-second vaccination (Study Arm 2 (A/H7N9 + AS03 D1, 29), Dendritic cells). Proteins are sorted by descending absolute log_2 fold change. Protein and gene annotations are based on Ensembl Version 101 (continued).

Protein ID	Ensembl Gene ID	Ensembl Gene Name	Protein Description	50% Protein Cluster ID (Gene Name)	Fold Change (Day 30 vs. Pre- Second Vaccination)	Average log_2 Abundance	Moderated t-Statistic	P-Value	FDR Adjusted P-value
Q9NR31	ENSG00000079332	SAR1A	GTP-binding protein SAR1a (COPII-associated small GTPase)	Q9Y6B6 (SAR1B)	0.150	0.953	2.583	0.0232	0.1465
O00170	ENSG00000110711	AIP	AH receptor-interacting protein (AIP) (Aryl-hydrocarbon receptor-interacting protein) (HBV X-associated protein 2) (XAP-2) (Immunophilin homolog ARA9)	O00170 (AIP)	-0.150	0.818	-4.178	0.0012	0.028
Q86SX6	ENSG00000182512	GLRX5	Glutaredoxin-related protein 5, mitochondrial (Monothiol glutaredoxin-5)	Q86SX6 (GLRX5)	-0.149	0.838	-3.794	0.0023	0.0391
Q9Y3B3	ENSG00000134970	TMED7	Transmembrane emp24 domain-containing protein 7 (p24 family protein gamma-3) (p24gamma3) (p27)	Q9Y3B3 (TMED7)	0.149	0.861	2.622	0.0215	0.1409
Q8IZQ5	ENSG00000211450	SELENOH	Selenoprotein H (SelH)	Q8IZQ5 (SELENOH)	-0.149	0.641	-2.825	0.0147	0.1122
Q96B97	ENSG00000147010	SH3KBP1	SH3 domain-containing kinase-binding protein 1 (CD2-binding protein 3) (CD2BP3) (Cbl-interacting protein of 85 kDa) (Human Src family kinase-binding protein 1) (HSB-1)	Q96B97 (SH3KBP1)	-0.148	0.648	-3.503	0.0041	0.0525
P00918	ENSG00000104267	CA2	Carbonic anhydrase 2 (EC 4.2.1.1) (Carbonate dehydratase II) (Carbonic anhydrase C) (CAC) (Carbonic anhydrase II) (CA-II)	Q8N1Q1 (CA13)	0.148	0.829	2.894	0.0129	0.1026
Q92835	ENSG00000168918; ENSG00000281614	INPP5D	Phosphatidylinositol 3,4,5-trisphosphate 5-phosphatase 1 (EC 3.1.3.86) (Inositol polyphosphate-5-phosphatase D) (EC 3.1.3.56) (Inositol polyphosphate-5-phosphatase of 145 kDa) (SIP-145) (Phosphatidylinositol 4,5-bisphosphate 5-phosphatase) (EC 3.1.3.36) (SH2 domain-containing inositol 5'-phosphatase 1) (SH2 domain-containing inositol phosphatase 1) (SHIP-1) (p150Ship) (hp51CN)	Q92835 (INPP5D)	-0.148	0.731	-2.992	0.0107	0.0948
Q13564	ENSG00000159593	NAE1	NEDD8-activating enzyme E1 regulatory subunit (Amyloid beta precursor protein-binding protein 1, 59 kDa) (APP-BP1) (Amyloid protein-binding protein 1) (Proto-oncogene protein 1)	Q13564 (NAE1)	-0.148	0.874	-2.778	0.016	0.118
Q7Z6J4	ENSG00000146192	FGD2	FYVE, RhoGEF and PH domain-containing protein 2 (Zinc finger FYVE domain-containing protein 4)	Q7Z6J4 (FGD2)	0.147	0.906	2.952	0.0115	0.098

Table S55: Proteins differentially abundant at Day 30 compared to pre-second vaccination (Study Arm 2 (A/H7N9 + AS03 D1, 29), Dendritic cells). Proteins are sorted by descending absolute log_2 fold change. Protein and gene annotations are based on Ensembl Version 101 (continued).

Protein ID	Ensembl Gene ID	Ensembl Gene Name	Protein Description	50% Protein Cluster ID (Gene Name)	Fold Change (Day 30 vs. Pre- Second Vaccination)	Average log_2 Abundance	Moderated t-Statistic	P-Value	FDR Adjusted P-value
P49959	ENSG00000020922	MRE11	Double-strand break repair protein MRE11 (EC 3.1.-.-) (Double-strand break repair protein MRE11A) (Meiotic recombination 11 homolog 1) (MRE11 homolog 1) (Meiotic recombination 11 homolog A) (MRE11 homolog A)	P49959 (MRE11)	-0.147	0.826	-4.222	0.0011	0.0266
P61224	ENSG00000127314	RAP1B	Ras-related protein Rap-1b (EC 3.6.5.2) (GTP-binding protein smg p21B)	P62834 (RAP1A)	-0.147	0.734	-2.828	0.0146	0.1118
Q03519	ENSG00000204267; ENSG00000206235; ENSG00000206299; ENSG00000228582; ENSG00000237599; ENSG00000232326; ENSG00000225967; ENSG00000223481	TAP2	Antigen peptide transporter 2 (APT2) (EC 7.4.2.-) (ATP-binding cassette sub-family B member 3) (Peptide supply factor 2) (Peptide transporter PSF2) (PSF-2) (Peptide transporter TAP2) (Peptide transporter involved in antigen processing 2) (Really interesting new gene 11 protein) (RING11)	Q03519 (TAP2)	0.147	0.950	2.680	0.0193	0.1318
Q92572	ENSG00000177879	AP3S1	AP-3 complex subunit sigma-1 (AP-3 complex subunit sigma-3A) (Adaptor-related protein complex 3 subunit sigma-1) (Clathrin-associated/assembly/adaptor protein, small 3) (Sigma-3A-adaptin) (Sigma3A-adaptin) (Sigma-adaptin 3a)	P59780 (AP3S2)	-0.146	0.912	-2.590	0.0229	0.1459
Q9NUW8	ENSG00000042088	TDP1	Tyrosyl-DNA phosphodiesterase 1 (Tyr-DNA phosphodiesterase 1) (EC 3.1.4.-)	Q9NUW8 (TDP1)	-0.146	0.951	-2.756	0.0167	0.1205
O60603	ENSG00000137462	TLR2	Toll-like receptor 2 (EC 3.2.2.6) (Toll/interleukin-1 receptor-like protein 4) (CD antigen CD282)	O60603 (TLR2)	0.146	0.860	3.993	0.0016	0.0334
Q6XQN6	ENSG00000147813; ENSG00000278488	NAPRT	Nicotinate phosphoribosyltransferase (NAPRTase) (EC 6.3.4.21) (FHA-HIT-interacting protein) (Nicotinate phosphoribosyltransferase domain-containing protein 1)	Q6XQN6 (NAPRT)	0.146	0.791	3.544	0.0037	0.0511
Q14919	ENSG00000175550	DRAP1	Dr1-associated corepressor (Dr1-associated protein 1) (Negative cofactor 2-alpha) (NC2-alpha)	Q14919 (DRAP1)	-0.146	0.862	-2.550	0.0246	0.1498
O60256	ENSG00000141127	PRPSAP2	Phosphoribosyl pyrophosphate synthase-associated protein 2 (PRPP synthase-associated protein 2) (41 kDa phosphoribosypyrophosphate synthetase-associated protein) (PAP41)	O60256 (PRPSAP2)	-0.145	0.851	-3.479	0.0042	0.0548

Table S55: Proteins differentially abundant at Day 30 compared to pre-second vaccination (Study Arm 2 (A/H7N9 + AS03 D1, 29), Dendritic cells). Proteins are sorted by descending absolute log_2 fold change. Protein and gene annotations are based on Ensembl Version 101 (continued).

Protein ID	Ensembl Gene ID	Ensembl Gene Name	Protein Description	50% Protein Cluster ID (Gene Name)	Fold Change (Day 30 vs. Pre- Second Vaccination)	Average log_2 Abundance	Moderated t-Statistic	P-Value	FDR Adjusted P-value
P43353	ENSG00000006534	ALDH3B1	Aldehyde dehydrogenase family 3 member B1 (EC 1.2.1.28) (EC 1.2.1.5) (EC 1.2.1.7) (Aldehyde dehydrogenase 7)	P51648 (ALDH3A2)	0.145	0.778	3.114	0.0085	0.0822
Q9NV96	ENSG00000112697	TMEM30A	Cell cycle control protein 50A (P4-ATPase flippase complex beta subunit TMEM30A) (Transmembrane protein 30A)	Q9NV96 (TMEM30A)	0.145	0.904	2.544	0.0249	0.1498
Q16706	ENSG00000112893	MAN2A1	Alpha-mannosidase 2 (EC 3.2.1.114) (Golgi alpha-mannosidase II) (AMan II) (Man II) (Mannosidase alpha class 2A member 1) (Mannosyl-oligosaccharide 1,3-1,6-alpha-mannosidase)	P49641 (MAN2A2)	0.145	0.908	4.060	0.0014	0.031
P56385	ENSG00000169020	ATP5ME	ATP synthase subunit e, mitochondrial (ATPase subunit e) (ATP synthase membrane subunit e) [Cleaved into: ATP synthase subunit e, mitochondrial, N-terminally processed]	P56385 (ATP5ME)	-0.145	0.767	-3.156	0.0078	0.0785
Q9BZQ8	ENSG00000135842	NIBAN1	Protein Niban 1 (Cell growth-inhibiting gene 39 protein) (Protein FAM129A)	Q9BZQ8 (NIBAN1)	0.145	0.871	3.985	0.0016	0.0335
Q9ULZ3	ENSG00000103490	PYCARD	Apoptosis-associated speck-like protein containing a CARD (hASC) (Caspase recruitment domain-containing protein 5) (PYD and CARD domain-containing protein) (Target of methylation-induced silencing 1)	Q9ULZ3 (PYCARD)	0.144	0.717	3.781	0.0024	0.0392
Q96GX9	ENSG00000149089	APIP	Methylthioribulose-1-phosphate dehydratase (MTRu-1-P dehydratase) (EC 4.2.1.109) (APAF1-interacting protein) (hAPIP)	Q96GX9 (APIP)	-0.144	1.159	-2.713	0.0181	0.1273
Q15382	ENSG00000106615	RHEB	GTP-binding protein Rheb (Ras homolog enriched in brain)	Q15382 (RHEB)	0.144	0.920	3.379	0.0051	0.0602
Q86YS6	ENSG00000172780	RAB43	Ras-related protein Rab-43 (Ras-related protein Rab-41)	A4D1S5 (RAB19)	0.144	0.876	3.327	0.0057	0.0645
Q8TEV9	ENSG00000176994; ENSG00000283741	SMCR8	Guanine nucleotide exchange protein SMCR8 (Smith-Magenis syndrome chromosomal region candidate gene 8 protein)	Q8TEV9 (SMCR8)	0.143	0.874	2.957	0.0114	0.0977
O43768	ENSG00000143420	ENSA	Alpha-endosulfine (ARPP-19e)	O43768 (ENSA)	-0.142	0.704	-2.928	0.0121	0.0999
P85037	ENSG00000164916	FOXK1	Forkhead box protein K1 (Myocyte nuclear factor) (MNF)	P85037 (FOXK1)	-0.142	0.836	-4.121	0.0013	0.0291

Table S55: Proteins differentially abundant at Day 30 compared to pre-second vaccination (Study Arm 2 (A/H7N9 + AS03 D1, 29), Dendritic cells). Proteins are sorted by descending absolute log_2 fold change. Protein and gene annotations are based on Ensembl Version 101 (continued).

Protein ID	Ensembl Gene ID	Ensembl Gene Name	Protein Description	50% Protein Cluster ID (Gene Name)	Fold Change (Day 30 vs. Pre- Second Vaccination)	Average log_2 Abundance	Moderated t-Statistic	P-Value	FDR Adjusted P-value
Q9BUP3	ENSG00000109854	HTATIP2	Oxidoreductase HTATIP2 (EC 1.1.1.-) (30 kDa HIV-1 TAT-interacting protein) (HIV-1 TAT-interactive protein 2)	Q9BUP3 (HTATIP2)	0.142	0.857	2.881	0.0132	0.1042
P09917	ENSG00000012779; ENSG00000275565	ALOX5	Polyunsaturated fatty acid 5-lipoxygenase (EC 1.13.11.-) (Arachidonate 5-lipoxygenase) (5-LO) (5-lipoxygenase) (EC 1.13.11.34)	P09917 (ALOX5)	0.142	0.809	2.617	0.0217	0.1415
P31153	ENSG00000168906	MAT2A	S-adenosylmethionine synthase isoform type-2 (AdoMet synthase 2) (EC 2.5.1.6) (Methionine adenosyltransferase 2) (MAT 2) (Methionine adenosyltransferase II) (MAT-II)	P31153 (MAT2A)	0.141	0.868	2.895	0.0128	0.1025
Q13951	ENSG00000067955	CBFB	Core-binding factor subunit beta (CBF-beta) (Polyomavirus enhancer-binding protein 2 beta subunit) (PEA2-beta) (PEBP2-beta) (SL3-3 enhancer factor 1 subunit beta) (SL3/AKV core-binding factor beta subunit)	Q13951 (CBFB)	-0.140	0.785	-2.906	0.0126	0.1023
P05455	ENSG00000138385	SSB	Lupus La protein (La autoantigen) (La ribonucleoprotein) (Sjoegren syndrome type B antigen) (SS-B)	P05455 (SSB)	-0.140	0.858	-2.541	0.0251	0.1498
P50135	ENSG00000150540	HNMT	Histamine N-methyltransferase (HMT) (EC 2.1.1.8)	P50135 (HNMT)	0.139	0.663	2.546	0.0248	0.1498
O95716	ENSG00000105514	RAB3D	Ras-related protein Rab-3D	Q96E17 (RAB3C)	0.139	0.888	3.029	0.01	0.0914
Q9P2A4	ENSG00000108798	ABI3	ABI gene family member 3 (New molecule including SH3) (Nesh)	Q9P2A4 (ABI3)	-0.139	0.706	-2.877	0.0133	0.1047
P78324	ENSG00000198053	SIRPA	Tyrosine-protein phosphatase non-receptor type substrate 1 (SHP substrate 1) (SHPS-1) (Brain Ig-like molecule with tyrosine-based activation motifs) (Bit) (CD172 antigen-like family member A) (Inhibitory receptor SHPS-1) (Macrophage fusion receptor) (MyD-1 antigen) (Signal-regulatory protein alpha-1) (Sirp-alpha-1) (Signal-regulatory protein alpha-2) (Sirp-alpha-2) (Signal-regulatory protein alpha-3) (Sirp-alpha-3) (p84) (CD antigen CD172a)	P78324 (SIRPA)	0.139	0.797	4.049	0.0015	0.0311
Q9UEW8	ENSG00000198648	STK39	STE20/SPS1-related proline-alanine-rich protein kinase (Ste-20-related kinase) (EC 2.7.11.1) (DCHT) (Serine/threonine-protein kinase 39)	Q9UEW8 (STK39)	-0.138	0.745	-3.536	0.0038	0.0512

Table S55: Proteins differentially abundant at Day 30 compared to pre-second vaccination (Study Arm 2 (A/H7N9 + AS03 D1, 29), Dendritic cells). Proteins are sorted by descending absolute log_2 fold change. Protein and gene annotations are based on Ensembl Version 101 (continued).

Protein ID	Ensembl Gene ID	Ensembl Gene Name	Protein Description	50% Protein Cluster ID (Gene Name)	Fold Change (Day 30 vs. Pre- Second Vaccination)	Average log_2 Abundance	Moderated t-Statistic	P-Value	FDR Adjusted P-value
P61254	ENSG00000161970	RPL26	60S ribosomal protein L26 (Large ribosomal subunit protein uL24)	P61254 (RPL26)	-0.137	0.734	-3.183	0.0074	0.0754
P07948	ENSG00000254087	LYN	Tyrosine-protein kinase Lyn (EC 2.7.10.2) (Lck/Yes-related novel protein tyrosine kinase) (V-yes-1 Yamaguchi sarcoma viral related oncogene homolog) (p53Lyn) (p56Lyn)	P06241 (FYN)	0.137	0.820	2.883	0.0132	0.1041
P18085	ENSG00000168374	ARF4	ADP-ribosylation factor 4	P61204 (ARF3)	0.137	0.802	3.004	0.0105	0.0941
P14174	ENSG00000240972; ENSG00000276701	MIF	Macrophage migration inhibitory factor (MIF) (EC 5.3.2.1) (Glycosylation-inhibiting factor) (GIF) (L-dopachrome isomerase) (L-dopachrome tautomerase) (EC 5.3.3.12) (Phenylpyruvate tautomerase)	P14174 (MIF)	-0.136	0.569	-2.599	0.0225	0.1443
P30038	ENSG00000159423	ALDH4A1	Delta-1-pyrroline-5-carboxylate dehydrogenase, mitochondrial (P5C dehydrogenase) (EC 1.2.1.88) (Aldehyde dehydrogenase family 4 member A1) (L-glutamate gamma-semialdehyde dehydrogenase)	P30038 (ALDH4A1)	0.136	0.851	4.105	0.0013	0.0296
P30049	ENSG00000099624	ATP5F1D	ATP synthase subunit delta, mitochondrial (ATP synthase F1 subunit delta) (F-ATPase delta subunit)	P30049 (ATP5F1D)	-0.136	0.811	-2.904	0.0126	0.1024
Q13813	ENSG00000197694	SPTAN1	Spectrin alpha chain, non-erythrocytic 1 (Alpha-II spectrin) (Fodrin alpha chain) (Spectrin, non-erythroid alpha subunit)	Q13813 (SPTAN1)	-0.136	0.925	-2.945	0.0117	0.0985
Q8N684	ENSG00000149532	CPSF7	Cleavage and polyadenylation specificity factor subunit 7 (Cleavage and polyadenylation specificity factor 59 kDa subunit) (CPSF 59 kDa subunit) (Cleavage factor Im complex 59 kDa subunit) (CFIm59) (Pre-mRNA cleavage factor Im 59 kDa subunit)	Q8N684 (CPSF7)	-0.136	0.797	-2.819	0.0148	0.1129
Q9H871	ENSG00000153561	RMND5A	E3 ubiquitin-protein transferase RMND5A (EC 2.3.2.27) (P44CTLH) (Protein RMD5 homolog A)	Q9H871 (RMND5A)	0.135	0.912	3.249	0.0066	0.0709
P27824	ENSG00000127022; ENSG00000283777	CANX	Calnexin (IP90) (Major histocompatibility complex class I antigen-binding protein p88) (p90)	P27824 (CANX)	0.135	0.941	3.417	0.0048	0.0583
Q9H330	ENSG00000106771	TMEM245	Transmembrane protein 245 (Protein CG-2)	Q9H330 (TMEM245)	-0.135	0.962	-2.742	0.0172	0.1218
Q8IY67	ENSG00000161847	RAVER1	Ribonucleoprotein PTB-binding 1 (Protein raver-1)	Q8IY67 (RAVER1)	-0.134	0.879	-3.688	0.0029	0.0435
Q15554	ENSG00000132604	TERF2	Telomeric repeat-binding factor 2 (TTAGGG repeat-binding factor 2) (Telomeric DNA-binding protein)	Q15554 (TERF2)	-0.134	0.870	-2.897	0.0128	0.1025

Table S55: Proteins differentially abundant at Day 30 compared to pre-second vaccination (Study Arm 2 (A/H7N9 + AS03 D1, 29), Dendritic cells). Proteins are sorted by descending absolute log_2 fold change. Protein and gene annotations are based on Ensembl Version 101 (continued).

Protein ID	Ensembl Gene ID	Ensembl Gene Name	Protein Description	50% Protein Cluster ID (Gene Name)	Fold Change (Day 30 vs. Pre- Second Vaccination)	Average log_2 Abundance	Moderated t-Statistic	P-Value	FDR Adjusted P-value
Q9NVJ2	ENSG00000134108	ARL8B	ADP-ribosylation factor-like protein 8B (ADP-ribosylation factor-like protein 10C) (Novel small G protein indispensable for equal chromosome segregation 1)	Q9NVJ2 (ARL8B)	0.134	0.800	3.185	0.0074	0.0754
P23284	ENSG00000166794	PPIB	Peptidyl-prolyl cis-trans isomerase B (PPIase B) (EC 5.2.1.8) (CYP-S1) (Cyclophilin B) (Rotamase B) (S-cyclophilin) (SCYLP)	P23284 (PPIB)	0.134	0.904	3.261	0.0064	0.0699
P22033	ENSG00000146085	MMUT	Methylmalonyl-CoA mutase, mitochondrial (MCM) (EC 5.4.99.2) (Methylmalonyl-CoA isomerase)	P22033 (MMUT)	-0.134	0.778	-3.656	0.003	0.0449
Q9BZL1	ENSG00000198258	UBL5	Ubiquitin-like protein 5	Q9BZL1 (UBL5)	0.134	0.727	3.152	0.0079	0.0788
Q99523	ENSG00000134243	SORT1	Sortilin (100 kDa NT receptor) (Glycoprotein 95) (Gp95) (Neurotensin receptor 3) (NT3) (NTR3)	Q99523 (SORT1)	0.133	0.952	2.577	0.0234	0.1474
Q5K4L6	ENSG00000263163; ENSG00000143554	SLC27A3	Solute carrier family 27 member 3 (EC 6.2.1.-) (Arachidonate–CoA ligase) (EC 6.2.1.15) (Long-chain fatty acid transport protein 3) (FATP-3) (Fatty acid transport protein 3) (Very long-chain acyl-CoA synthetase homolog 3) (VLCS-3)	Q5K4L6 (SLC27A3)	0.133	0.861	2.724	0.0178	0.1252
O95793	ENSG00000124214	STAU1	Double-stranded RNA-binding protein Staufen homolog 1	O95793 (STAU1)	0.132	0.795	3.160	0.0078	0.0781
P42331	ENSG00000163219	ARHGAP25	Rho GTPase-activating protein 25 (Rho-type GTPase-activating protein 25)	P42331 (ARHGAP25)	-0.132	0.846	-2.977	0.011	0.0964
Q9BYX2	ENSG00000095383	TBC1D2	TBC1 domain family member 2A (Armus) (Prostate antigen recognized and identified by SEREX 1) (PARIS-1)	Q9BYX2 (TBC1D2)	0.132	0.843	3.795	0.0023	0.0391
O00161	ENSG00000092531	SNAP23	Synaptosomal-associated protein 23 (SNAP-23) (Vesicle-membrane fusion protein SNAP-23)	O00161 (SNAP23)	0.132	0.899	3.594	0.0034	0.0484
P61088	ENSG00000177889	UBE2N	Ubiquitin-conjugating enzyme E2 N (EC 2.3.2.23) (Bendless-like ubiquitin-conjugating enzyme) (E2 ubiquitin-conjugating enzyme N) (Ubc13) (UbcH13) (Ubiquitin carrier protein N) (Ubiquitin-protein ligase N)	P61088 (UBE2N)	-0.131	0.803	-3.065	0.0093	0.0871
Q9Y616	ENSG00000090376	IRAK3	Interleukin-1 receptor-associated kinase 3 (IRAK-3) (EC 2.7.11.1) (IL-1 receptor-associated kinase M) (IRAK-M)	Q9Y616 (IRAK3)	0.131	0.760	2.807	0.0152	0.1136

Table S55: Proteins differentially abundant at Day 30 compared to pre-second vaccination (Study Arm 2 (A/H7N9 + AS03 D1, 29), Dendritic cells). Proteins are sorted by descending absolute log_2 fold change. Protein and gene annotations are based on Ensembl Version 101 (continued).

Protein ID	Ensembl Gene ID	Ensembl Gene Name	Protein Description	50% Protein Cluster ID (Gene Name)	Fold Change (Day 30 vs. Pre- Second Vaccination)	Average log_2 Abundance	Moderated t-Statistic	P-Value	FDR Adjusted P-value
Q9UKL0	ENSG00000089902	RCOR1	REST corepressor 1 (Protein CoREST)	Q9P2K3 (RCOR3)	0.131	0.809	2.558	0.0243	0.1493
Q14257	ENSG00000117906	RCN2	Reticulocalbin-2 (Calcium-binding protein ERC-55) (E6-binding protein) (E6BP)	Q14257 (RCN2)	-0.131	0.754	-2.966	0.0112	0.0968
P35232	ENSG00000167085	PHB	Prohibitin	Q99623 (PHB2)	-0.131	0.758	-3.094	0.0088	0.0838
Q12765	ENSG00000136193	SCRN1	Secernin-1	Q96FV2 (SCRN2)	-0.131	0.756	-3.195	0.0073	0.0744
P51580	ENSG00000137364	TPMT	Thiopurine S-methyltransferase (EC 2.1.1.67) (Thiopurine methyltransferase)	P51580 (TPMT)	0.130	0.780	2.614	0.0218	0.1415
Q86UV5	ENSG00000090686	USP48	Ubiquitin carboxyl-terminal hydrolase 48 (EC 3.4.19.12) (Deubiquitinating enzyme 48) (Ubiquitin thioesterase 48) (Ubiquitin-specific peptidase 48) (Ubiquitin-specific protease 48) (Ubiquitin-specific-processing protease 48)	Q86UV5 (USP48)	0.130	0.934	3.523	0.0039	0.0517
Q99759	ENSG00000198909	MAP3K3	Mitogen-activated protein kinase kinase kinase 3 (EC 2.7.11.25) (MAPK/ERK kinase kinase 3) (MEK kinase 3) (MEKK 3)	Q99759 (MAP3K3)	0.130	0.799	3.212	0.007	0.0733
P42025	ENSG00000115073	ACTR1B	Beta-centractin (Actin-related protein 1B) (ARP1B)	P0CG38 (POTEI)	-0.130	0.856	-2.706	0.0184	0.1289
P37235	ENSG00000115756	HPCAL1	Hippocalcin-like protein 1 (Calcium-binding protein BDR-1) (HLP2) (Visinin-like protein 3) (VILIP-3)	P37235 (HPCAL1)	-0.130	0.803	-2.598	0.0225	0.1444
O00139	ENSG00000068796	KIF2A	Kinesin-like protein KIF2A (Kinesin-2) (hK2)	O00139 (KIF2A)	-0.129	0.805	-2.544	0.0249	0.1498
P01137		TGFB1	Transforming growth factor beta-1 proprotein [Cleaved into: Latency-associated peptide (LAP); Transforming growth factor beta-1 (TGF-beta-1)]	P01137 (TGFB1)	0.128	0.939	2.552	0.0245	0.1498
Q8N335	ENSG00000152642	GPD1L	Glycerol-3-phosphate dehydrogenase 1-like protein (GPD1-L) (EC 1.1.1.8)	Q8N335 (GPD1L)	-0.128	0.730	-2.700	0.0186	0.1294
P06865	ENSG00000213614	HEXA	Beta-hexosaminidase subunit alpha (EC 3.2.1.52) (Beta-N-acetylhexosaminidase subunit alpha) (Hexosaminidase subunit A) (N-acetyl-beta-glucosaminidase subunit alpha)	P07686 (HEXB)	0.128	0.671	2.556	0.0244	0.1496
O15269	ENSG00000090054	SPTLC1	Serine palmitoyltransferase 1 (EC 2.3.1.50) (Long chain base biosynthesis protein 1) (LCB 1) (Serine-palmitoyl-CoA transferase 1) (SPT 1) (SPT1)	O15269 (SPTLC1)	0.128	0.852	2.896	0.0128	0.1025

Table S55: Proteins differentially abundant at Day 30 compared to pre-second vaccination (Study Arm 2 (A/H7N9 + AS03 D1, 29), Dendritic cells). Proteins are sorted by descending absolute log_2 fold change. Protein and gene annotations are based on Ensembl Version 101 (continued).

Protein ID	Ensembl Gene ID	Ensembl Gene Name	Protein Description	50% Protein Cluster ID (Gene Name)	Fold Change (Day 30 vs. Pre- Second Vaccination)	Average log_2 Abundance	Moderated t-Statistic	P-Value	FDR Adjusted P-value
Q9UBT2	ENSG00000126261	UBA2	SUMO-activating enzyme subunit 2 (EC 2.3.2.-) (Anthracycline-associated resistance ARX) (Ubiquitin-like 1-activating enzyme E1B) (Ubiquitin-like modifier-activating enzyme 2)	Q9UBT2 (UBA2)	-0.127	0.797	-3.670	0.003	0.0444
Q14669	ENSG00000153827	TRIP12	E3 ubiquitin-protein ligase TRIP12 (EC 2.3.2.26) (E3 ubiquitin-protein ligase for Arf) (ULF) (HECT-type E3 ubiquitin transferase TRIP12) (Thyroid receptor-interacting protein 12) (TR-interacting protein 12) (TRIP-12)	Q14669 (TRIP12)	0.127	0.855	3.097	0.0088	0.0835
P05120	ENSG00000197632	SERPINB2	Plasminogen activator inhibitor 2 (PAI-2) (Monocyte Arg-serpin) (Placental plasminogen activator inhibitor) (Serpin B2) (Urokinase inhibitor)	P05120 (SERPINB2)	0.126	0.852	2.676	0.0194	0.1323
O00764	ENSG00000160209	PDXK	Pyridoxal kinase (EC 2.7.1.35) (Pyridoxine kinase)	O00764 (PDXK)	0.125	0.679	2.630	0.0212	0.1398
P18669	ENSG00000171314	PGAM1	Phosphoglycerate mutase 1 (EC 5.4.2.11) (EC 5.4.2.4) (BPG-dependent PGAM 1) (Phosphoglycerate mutase isozyme B) (PGAM-B)	P07738 (BPGM)	0.125	0.801	2.564	0.024	0.149
Q96HE7	ENSG00000197930	ERO1A	ERO1-like protein alpha (ERO1-L) (ERO1-L-alpha) (EC 1.8.4.-) (Endoplasmic oxidoreductin-1-like protein) (Endoplasmic reticulum oxidoreductase alpha) (Oxidoreductin-1-L-alpha)	Q96HE7 (ERO1A)	0.125	0.831	3.395	0.005	0.0592
O95352	ENSG00000197548	ATG7	Ubiquitin-like modifier-activating enzyme ATG7 (ATG12-activating enzyme E1 ATG7) (Autophagy-related protein 7) (APG7-like) (hAGP7) (Ubiquitin-activating enzyme E1-like protein)	O95352 (ATG7)	0.125	0.871	3.449	0.0045	0.0566
Q86TB9	ENSG00000166889	PATL1	Protein PAT1 homolog 1 (PAT1-like protein 1) (Protein PAT1 homolog b) (Pat1b) (hPat1b)	Q86TB9 (PATL1)	0.125	0.924	2.547	0.0248	0.1498
Q9Y4E6	ENSG00000091157	WDR7	WD repeat-containing protein 7 (Rabconnectin-3 beta) (TGF-beta resistance-associated protein TRAG)	Q9Y4E6 (WDR7)	0.125	0.918	2.633	0.0211	0.1398

Table S55: Proteins differentially abundant at Day 30 compared to pre-second vaccination (Study Arm 2 (A/H7N9 + AS03 D1, 29), Dendritic cells). Proteins are sorted by descending absolute log_2 fold change. Protein and gene annotations are based on Ensembl Version 101 (continued).

Protein ID	Ensembl Gene ID	Ensembl Gene Name	Protein Description	50% Protein Cluster ID (Gene Name)	Fold Change (Day 30 vs. Pre- Second Vaccination)	Average log_2 Abundance	Moderated t-Statistic	P-Value	FDR Adjusted P-value
Q9NYL2	ENSG00000091436	MAP3K20	Mitogen-activated protein kinase kinase kinase 20 (EC 2.7.11.25) (Human cervical cancer suppressor gene 4 protein) (HCCS-4) (Leucine zipper- and sterile alpha motif-containing kinase) (MLK-like mitogen-activated protein triple kinase) (Mitogen-activated protein kinase kinase kinase MLT) (Mixed lineage kinase-related kinase) (MLK-related kinase) (MRK) (Sterile alpha motif- and leucine zipper-containing kinase AZK)	Q9NYL2 (MAP3K20)	0.125	0.868	2.559	0.0242	0.1493
O75695	ENSG00000102218	RP2	Protein XRP2	O75695 (RP2)	0.125	0.950	3.549	0.0037	0.0511
Q13523	ENSG00000112739	PRPF4B	Serine/threonine-protein kinase PRP4 homolog (EC 2.7.11.1) (PRP4 kinase) (PRP4 pre-mRNA-processing factor 4 homolog)	Q13523 (PRPF4B)	0.124	0.895	3.045	0.0097	0.0896
O00116	ENSG00000018510	AGPS	Alkyldihydroxyacetonephosphate synthase, peroxisomal (Alkyl-DHAP synthase) (EC 2.5.1.26) (Aging-associated gene 5 protein) (Alkylglycerone-phosphate synthase)	O00116 (AGPS)	0.123	0.830	2.900	0.0127	0.1025
O95833	ENSG00000169583	CLIC3	Chloride intracellular channel protein 3	Q9NZA1 (CLIC5)	-0.123	0.722	-2.564	0.024	0.149
Q5TDH0	ENSG00000197312	DDI2	Protein DDI1 homolog 2 (EC 3.4.23.-)	Q5TDH0 (DDI2)	-0.123	0.937	-3.950	0.0018	0.0348
P63218	ENSG00000174021	GNG5	Guanine nucleotide-binding protein G(I)/G(S)/G(O) subunit gamma-5	P63218 (GNG5)	0.122	0.782	2.871	0.0134	0.1049
P30520	ENSG00000035687	ADSS2	Adenylosuccinate synthetase isozyme 2 (AMPSase 2) (AdSS 2) (EC 6.3.4.4) (Adenylosuccinate synthetase, acidic isozyme) (Adenylosuccinate synthetase, liver isozyme) (L-type adenylosuccinate synthetase) (IMP–aspartate ligase 2)	P30520 (ADSS2)	0.121	0.789	2.636	0.021	0.1398
Q9NZ45	ENSG00000122873	CISD1	CDGSH iron-sulfur domain-containing protein 1 (MitoNEET)	Q8N5K1 (CISD2)	-0.121	0.561	-3.644	0.0031	0.0451
Q8WVV9	ENSG00000143889	HNRNPLL	Heterogeneous nuclear ribonucleoprotein L-like (hnRNPLL) (Stromal RNA-regulating factor)	Q8WVV9 (HNRNPLL)	0.120	0.867	3.848	0.0021	0.0376
P14735	ENSG00000119912	IDE	Insulin-degrading enzyme (EC 3.4.24.56) (Abeta-degrading protease) (Insulin protease) (Insulinase) (Insulysin)	P14735 (IDE)	0.120	0.824	2.555	0.0244	0.1496

Table S55: Proteins differentially abundant at Day 30 compared to pre-second vaccination (Study Arm 2 (A/H7N9 + AS03 D1, 29), Dendritic cells). Proteins are sorted by descending absolute log_2 fold change. Protein and gene annotations are based on Ensembl Version 101 (continued).

Protein ID	Ensembl Gene ID	Ensembl Gene Name	Protein Description	50% Protein Cluster ID (Gene Name)	Fold Change (Day 30 vs. Pre- Second Vaccination)	Average log_2 Abundance	Moderated t-Statistic	P-Value	FDR Adjusted P-value
Q15084	ENSG00000143870	PDIA6	Protein disulfide-isomerase A6 (EC 5.3.4.1) (Endoplasmic reticulum protein 5) (ER protein 5) (ERp5) (Protein disulfide isomerase P5) (Thioredoxin domain-containing protein 7)	Q15084 (PDIA6)	0.120	0.892	3.248	0.0066	0.0709
P36543	ENSG00000131100	ATP6V1E1	V-type proton ATPase subunit E 1 (V-ATPase subunit E 1) (V-ATPase 31 kDa subunit) (p31) (Vacuolar proton pump subunit E 1)	P36543 (ATP6V1E1)	0.120	0.852	3.347	0.0054	0.0623
Q8IZH2	ENSG00000114127	XRN1	5'-3' exoribonuclease 1 (EC 3.1.13.-) (Strand-exchange protein 1 homolog)	Q8IZH2 (XRN1)	0.120	0.912	2.680	0.0193	0.1318
Q00978	ENSG00000213928; ENSG00000285048	IRF9	Interferon regulatory factor 9 (IRF-9) (IFN-alpha-responsive transcription factor subunit) (ISGF3 p48 subunit) (Interferon-stimulated gene factor 3 gamma) (ISGF-3 gamma) (Transcriptional regulator ISGF3 subunit gamma)	Q00978 (IRF9)	0.119	1.009	2.539	0.0251	0.1498
Q9Y5L0	ENSG00000064419	TNPO3	Transportin-3 (Importin-12) (Imp12) (Transportin-SR) (TRN-SR)	Q9Y5L0 (TNPO3)	0.119	0.737	3.136	0.0081	0.0804
P15927	ENSG00000117748	RPA2	Replication protein A 32 kDa subunit (RP-A p32) (Replication factor A protein 2) (RF-A protein 2) (Replication protein A 34 kDa subunit) (RP-A p34)	P15927 (RPA2)	-0.119	0.842	-3.224	0.0069	0.073
A6NHR9	ENSG00000101596	SMCHD1	Structural maintenance of chromosomes flexible hinge domain-containing protein 1 (SMC hinge domain-containing protein 1) (EC 3.6.1.-)	A6NHR9 (SMCHD1)	0.118	0.933	3.203	0.0072	0.0738
P51798	ENSG00000103249	CLCN7	H(+)/Cl(-) exchange transporter 7 (Chloride channel 7 alpha subunit) (Chloride channel protein 7) (ClC-7)	P51798 (CLCN7)	0.118	0.950	3.186	0.0074	0.0754
Q96I25	ENSG00000134453	RBM17	Splicing factor 45 (45 kDa-splicing factor) (RNA-binding motif protein 17)	Q96I25 (RBM17)	0.118	0.839	2.685	0.0191	0.1316
Q9NSD9	ENSG00000116120	FARSB	Phenylalanine–tRNA ligase beta subunit (EC 6.1.1.20) (Phenylalanyl-tRNA synthetase beta subunit) (PheRS)	Q9NSD9 (FARSB)	-0.118	0.886	-3.470	0.0043	0.0553
Q00341	ENSG00000115677	HDLBP	Vigilin (High density lipoprotein-binding protein) (HDL-binding protein)	Q00341 (HDLBP)	0.118	0.934	3.740	0.0026	0.041
Q9NR97	ENSG00000101916	TLR8	Toll-like receptor 8 (CD antigen CD288)	Q9NR97 (TLR8)	0.118	0.878	2.875	0.0133	0.1047
O14526	ENSG00000130475	FCHO1	F-BAR domain only protein 1	O14526 (FCHO1)	-0.117	0.857	-2.845	0.0141	0.1091
P16333	ENSG00000158092	NCK1	Cytoplasmic protein NCK1 (NCK adaptor protein 1) (Nck-1) (SH2/SH3 adaptor protein NCK-alpha)	O43639 (NCK2)	-0.117	0.835	-3.357	0.0053	0.0617

Table S55: Proteins differentially abundant at Day 30 compared to pre-second vaccination (Study Arm 2 (A/H7N9 + AS03 D1, 29), Dendritic cells). Proteins are sorted by descending absolute log_2 fold change. Protein and gene annotations are based on Ensembl Version 101 (continued).

Protein ID	Ensembl Gene ID	Ensembl Gene Name	Protein Description	50% Protein Cluster ID (Gene Name)	Fold Change (Day 30 vs. Pre- Second Vaccination)	Average log_2 Abundance	Moderated t-Statistic	P-Value	FDR Adjusted P-value
Q96DE0	ENSG00000198585	NUDT16	U8 snoRNA-decapping enzyme (EC 3.6.1.62) (IDP phosphatase) (IDPase) (EC 3.6.1.64) (Inosine diphosphate phosphatase) (Nucleoside diphosphate-linked moiety X motif 16) (Nudix motif 16) (Nudix hydrolase 16) (U8 snoRNA-binding protein H29K) (m7GpppN-mRNA hydrolase)	Q9BRJ7 (NUDT16L1)	0.115	0.790	2.567	0.0238	0.149
O94804	ENSG00000072786	STK10	Serine/threonine-protein kinase 10 (EC 2.7.11.1) (Lymphocyte-oriented kinase)	O94804 (STK10)	-0.115	0.675	-3.530	0.0039	0.0516
P52294	ENSG00000114030	KPNA1	Importin subunit alpha-5 (Karyopherin subunit alpha-1) (Nucleoprotein interactor 1) (NPI-1) (RAG cohort protein 2) (SRP1-beta) [Cleaved into: Importin subunit alpha-5, N-terminally processed]	P52294 (KPNA1)	0.114	0.888	2.967	0.0112	0.0968
Q96JI7	ENSG00000104133	SPG11	Spatacsin (Colorectal carcinoma-associated protein) (Spastic paraplegia 11 protein)	Q96JI7 (SPG11)	0.114	0.860	2.941	0.0118	0.099
P05166	ENSG00000114054	PCCB	Propionyl-CoA carboxylase beta chain, mitochondrial (PCCase subunit beta) (EC 6.4.1.3) (Propanoyl-CoA:carbon dioxide ligase subunit beta)	P05166 (PCCB)	-0.114	0.720	-2.883	0.0131	0.1041
P39880	ENSG00000257923	CUX1	Homeobox protein cut-like 1 (CCAAT displacement protein) (CDP) (CDP/Cux p200) (Homeobox protein cux-1) [Cleaved into: CDP/Cux p110]	P39880 (CUX1)	0.114	0.878	3.196	0.0072	0.0744
P08195	ENSG00000168003	SLC3A2	4F2 cell-surface antigen heavy chain (4F2hc) (4F2 heavy chain antigen) (Lymphocyte activation antigen 4F2 large subunit) (Solute carrier family 3 member 2) (CD antigen CD98)	P08195 (SLC3A2)	0.114	0.943	2.626	0.0214	0.1405
Q04917	ENSG00000128245	YWHAH	14-3-3 protein eta (Protein AS1)	P62258 (YWHAE)	0.114	0.803	3.216	0.007	0.0733
O75165	ENSG00000138246	DNAJC13	DnaJ homolog subfamily C member 13 (Required for receptor-mediated endocytosis 8) (RME-8)	O75165 (DNAJC13)	0.114	0.888	3.454	0.0044	0.0565
Q16864	ENSG00000128524	ATP6V1F	V-type proton ATPase subunit F (V-ATPase subunit F) (V-ATPase 14 kDa subunit) (Vacuolar proton pump subunit F)	Q16864 (ATP6V1F)	0.113	0.798	3.019	0.0101	0.0921
Q15833	ENSG00000076944	STXBP2	Syntaxin-binding protein 2 (Protein unc-18 homolog 2) (Unc18-2) (Protein unc-18 homolog B) (Unc-18B)	P61764 (STXBP1)	0.113	0.870	3.312	0.0058	0.0655
Q9NUV9	ENSG00000133574	GIMAP4	GTPase IMAP family member 4 (Immunity-associated nucleotide 1 protein) (IAN-1) (hIAN1) (Immunity-associated protein 4)	Q9NUV9 (GIMAP4)	-0.113	0.879	-2.801	0.0154	0.1145

Table S55: Proteins differentially abundant at Day 30 compared to pre-second vaccination (Study Arm 2 (A/H7N9 + AS03 D1, 29), Dendritic cells). Proteins are sorted by descending absolute log_2 fold change. Protein and gene annotations are based on Ensembl Version 101 (continued).

Protein ID	Ensembl Gene ID	Ensembl Gene Name	Protein Description	50% Protein Cluster ID (Gene Name)	Fold Change (Day 30 vs. Pre- Second Vaccination)	Average log_2 Abundance	Moderated t-Statistic	P-Value	FDR Adjusted P-value
P27986	ENSG00000145675	PIK3R1	Phosphatidylinositol 3-kinase regulatory subunit alpha (PI3-kinase regulatory subunit alpha) (PI3K regulatory subunit alpha) (PtdIns-3-kinase regulatory subunit alpha) (Phosphatidylinositol 3-kinase 85 kDa regulatory subunit alpha) (PI3-kinase subunit p85-alpha) (PtdIns-3-kinase regulatory subunit p85-alpha)	O00459 (PIK3R2)	-0.112	0.828	-2.583	0.0231	0.1465
Q8IXM2	ENSG00000258315	BAP18	Chromatin complexes subunit BAP18 (BPTF-associated protein of 18 kDa)	Q8IXM2 (BAP18)	-0.112	0.691	-2.871	0.0134	0.1049
P25205	ENSG00000112118	MCM3	DNA replication licensing factor MCM3 (EC 3.6.4.12) (DNA polymerase alpha holoenzyme-associated protein P1) (P1-MCM3) (RLF subunit beta) (p102)	P25205 (MCM3)	0.112	0.993	2.541	0.025	0.1498
Q99805	ENSG00000125304	TM9SF2	Transmembrane 9 superfamily member 2 (p76)	Q99805 (TM9SF2)	0.111	0.977	2.647	0.0205	0.1377
Q99798	ENSG00000100412	ACO2	Aconitate hydratase, mitochondrial (Aconitase) (EC 4.2.1.3) (Citrate hydro-lyase)	Q99798 (ACO2)	-0.111	0.719	-2.606	0.0222	0.1433
Q9UBE0	ENSG00000142230	SAE1	SUMO-activating enzyme subunit 1 (Ubiquitin-like 1-activating enzyme E1A) [Cleaved into: SUMO-activating enzyme subunit 1, N-terminally processed]	Q9UBE0 (SAE1)	-0.111	0.863	-2.748	0.017	0.1212
P47756	ENSG00000077549	CAPZB	F-actin-capping protein subunit beta (CapZ beta)	P47756 (CAPZB)	-0.110	0.781	-3.139	0.0081	0.0802
P45974	ENSG00000111667	USP5	Ubiquitin carboxyl-terminal hydrolase 5 (EC 3.4.19.12) (Deubiquitinating enzyme 5) (Isopeptidase T) (Ubiquitin thioesterase 5) (Ubiquitin-specific-processing protease 5)	Q92995 (USP13)	-0.110	0.817	-3.290	0.0061	0.0674
Q99623	ENSG00000215021	PHB2	Prohibitin-2 (B-cell receptor-associated protein BAP37) (D-prohibitin) (Repressor of estrogen receptor activity)	Q99623 (PHB2)	-0.109	0.786	-2.577	0.0234	0.1474
Q9BSJ8	ENSG00000139641	ESYT1	Extended synaptotagmin-1 (E-Syt1) (Membrane-bound C2 domain-containing protein)	Q9BSJ8 (ESYT1)	-0.108	0.745	-2.545	0.0249	0.1498
Q9UH99	ENSG00000100242	SUN2	SUN domain-containing protein 2 (Protein unc-84 homolog B) (Rab5-interacting protein) (Rab5IP) (Sad1/unc-84 protein-like 2)	Q9UH99 (SUN2)	-0.108	0.820	-3.210	0.0071	0.0734

Table S55: Proteins differentially abundant at Day 30 compared to pre-second vaccination (Study Arm 2 (A/H7N9 + AS03 D1, 29), Dendritic cells). Proteins are sorted by descending absolute log_2 fold change. Protein and gene annotations are based on Ensembl Version 101 (continued).

Protein ID	Ensembl Gene ID	Ensembl Gene Name	Protein Description	50% Protein Cluster ID (Gene Name)	Fold Change (Day 30 vs. Pre- Second Vaccination)	Average log_2 Abundance	Moderated t-Statistic	P-Value	FDR Adjusted P-value
O95232	ENSG00000108848	LUC7L3	Luc7-like protein 3 (Cisplatin resistance-associated-overexpressed protein) (Luc7A) (Okadaic acid-inducible phosphoprotein OA48-18) (cAMP regulatory element-associated protein 1) (CRE-associated protein 1) (CREAP-1)	O95232 (LUC7L3)	-0.106	0.861	-2.999	0.0105	0.0946
Q9Y285	ENSG00000179115	FARSA	Phenylalanine–tRNA ligase alpha subunit (EC 6.1.1.20) (CML33) (Phenylalanyl-tRNA synthetase alpha subunit) (PheRS)	Q9Y285 (FARSA)	-0.106	0.899	-2.936	0.0119	0.0991
Q16594	ENSG00000273841; ENSG00000276463	TAF9	Transcription initiation factor TFIID subunit 9 (RNA polymerase II TBP-associated factor subunit G) (STAF31/32) (Transcription initiation factor TFIID 31 kDa subunit) (TAFII-31) (TAFII31) (Transcription initiation factor TFIID 32 kDa subunit) (TAFII-32) (TAFII32)	Q16594 (TAF9)	-0.105	0.617	-2.666	0.0198	0.134
P29466	ENSG00000137752	CASP1	Caspase-1 (CASP-1) (EC 3.4.22.36) (Interleukin-1 beta convertase) (IL-1BC) (Interleukin-1 beta-converting enzyme) (ICE) (IL-1 beta-converting enzyme) (p45) [Cleaved into: Caspase-1 subunit p20; Caspase-1 subunit p10]	P29466 (CASP1)	0.104	0.821	2.632	0.0211	0.1398
O00291	ENSG00000127946	HIP1	Huntingtin-interacting protein 1 (HIP-1) (Huntingtin-interacting protein I) (HIP-I)	O00291 (HIP1)	0.103	0.896	2.748	0.017	0.1212
Q99653	ENSG00000187446	CHP1	Calcineurin B homologous protein 1 (Calcineurin B-like protein) (Calcium-binding protein CHP) (Calcium-binding protein p22) (EF-hand calcium-binding domain-containing protein p22)	Q99653 (CHP1)	0.103	0.928	2.836	0.0144	0.1107
Q96GD0	ENSG00000241360	PDXP	Pyridoxal phosphate phosphatase (PLP phosphatase) (EC 3.1.3.3) (EC 3.1.3.74) (Chronophin)	Q96GD0 (PDXP)	-0.102	0.858	-3.279	0.0062	0.0683
Q8WWP7	ENSG00000213203	GIMAP1	GTPase IMAP family member 1 (Immunity-associated protein 1) (hIMAP1)	Q8WWP7 (GIMAP1)	-0.102	0.745	-2.695	0.0188	0.1302
Q86VS8	ENSG00000168172	HOOK3	Protein Hook homolog 3 (h-hook3) (hHK3)	Q9UJC3 (HOOK1)	0.102	0.872	2.575	0.0235	0.1474
Q7L9L4	ENSG00000173542	MOB1B	MOB kinase activator 1B (Mob1 homolog 1A) (Mob1A) (Mob1B) (Mps one binder kinase activator-like 1A)	Q96BX8 (MOB3A)	0.101	0.762	2.683	0.0192	0.1318
O94855	ENSG00000150961	SEC24D	Protein transport protein Sec24D (SEC24-related protein D)	P53992 (SEC24C)	0.101	0.985	2.808	0.0151	0.1136

Table S55: Proteins differentially abundant at Day 30 compared to pre-second vaccination (Study Arm 2 (A/H7N9 + AS03 D1, 29), Dendritic cells). Proteins are sorted by descending absolute log_2 fold change. Protein and gene annotations are based on Ensembl Version 101 (continued).

Protein ID	Ensembl Gene ID	Ensembl Gene Name	Protein Description	50% Protein Cluster ID (Gene Name)	Fold Change (Day 30 vs. Pre- Second Vaccination)	Average log_2 Abundance	Moderated t-Statistic	P-Value	FDR Adjusted P-value
Q9Y2L1	ENSG00000083520	DIS3	Exosome complex exonuclease RRP44 (EC 3.1.13.-) (EC 3.1.26.-) (Protein DIS3 homolog) (Ribosomal RNA-processing protein 44)	Q9Y2L1 (DIS3)	-0.099	0.907	-2.686	0.0191	0.1316
Q8WXF1	ENSG00000121390	PSPC1	Paraspeckle component 1 (Paraspeckle protein 1)	P23246 (SFPQ)	-0.097	0.732	-2.585	0.0231	0.1465
O95479	ENSG00000049239	H6PD	GDH/6PGL endoplasmic bifunctional protein [Includes: Hexose-6-phosphate dehydrogenase (Glucose 1-dehydrogenase) (GDH) (EC 1.1.1.47) (Glucose-6-phosphate dehydrogenase) (EC 1.1.1.363); 6-phosphogluconolactonase (6PGL) (EC 3.1.1.31)]	O95479 (H6PD)	0.097	0.935	3.121	0.0084	0.0819
P27348	ENSG00000134308	YWHAQ	14-3-3 protein theta (14-3-3 protein T-cell) (14-3-3 protein tau) (Protein HS1)	P62258 (YWHAE)	-0.096	0.794	-2.703	0.0185	0.1292
Q13263	ENSG00000130726	TRIM28	Transcription intermediary factor 1-beta (TIF1-beta) (E3 SUMO-protein ligase TRIM28) (EC 2.3.2.27) (KRAB-associated protein 1) (KAP-1) (KRAB-interacting protein 1) (KRIP-1) (Nuclear corepressor KAP-1) (RING finger protein 96) (RING-type E3 ubiquitin transferase TIF1-beta) (Tripartite motif-containing protein 28)	Q13263 (TRIM28)	-0.095	0.835	-2.968	0.0112	0.0968
Q8WX92		NELFB	Negative elongation factor B (NELF-B) (Cofactor of BRCA1)	Q8WX92 (NELFB)	-0.095	0.808	-2.919	0.0123	0.101
Q92878	ENSG00000113522	RAD50	DNA repair protein RAD50 (hRAD50) (EC 3.6.-.-)	Q92878 (RAD50)	-0.094	0.839	-2.882	0.0132	0.1041
O43566	ENSG00000169220	RGS14	Regulator of G-protein signaling 14 (RGS14)	O43566 (RGS14)	-0.094	0.864	-2.743	0.0171	0.1218
P46060	ENSG00000100401	RANGAP1	Ran GTPase-activating protein 1 (RanGAP1)	P46060 (RANGAP1)	0.094	0.821	2.559	0.0242	0.1493
Q99836	ENSG00000172936	MYD88	Myeloid differentiation primary response protein MyD88	Q99836 (MYD88)	0.093	0.871	2.748	0.017	0.1212
Q6ZSZ5	ENSG00000104880	ARHGEF18	Rho guanine nucleotide exchange factor 18 (114 kDa Rho-specific guanine nucleotide exchange factor) (p114-Rho-GEF) (p114RhoGEF) (Septin-associated RhoGEF) (SA-RhoGEF)	Q6ZSZ5 (ARHGEF18)	-0.092	0.819	-2.650	0.0204	0.1371
Q8WUR7	ENSG00000169609	C15orf40	UPF0235 protein C15orf40	Q8WUR7 (C15orf40)	-0.092	0.793	-2.736	0.0173	0.1228

Table S55: Proteins differentially abundant at Day 30 compared to pre-second vaccination (Study Arm 2 (A/H7N9 + AS03 D1, 29), Dendritic cells). Proteins are sorted by descending absolute log_2 fold change. Protein and gene annotations are based on Ensembl Version 101 (continued).

Protein ID	Ensembl Gene ID	Ensembl Gene Name	Protein Description	50% Protein Cluster ID (Gene Name)	Fold Change (Day 30 vs. Pre- Second Vaccination)	Average log_2 Abundance	Moderated t-Statistic	P-Value	FDR Adjusted P-value
P43307	ENSG00000124783	SSR1	Translocon-associated protein subunit alpha (TRAP-alpha) (Signal sequence receptor subunit alpha) (SSR-alpha)	P43307 (SSR1)	0.091	0.926	3.022	0.0101	0.0919
P09622	ENSG00000091140	DLD	Dihydrolipoyl dehydrogenase, mitochondrial (EC 1.8.1.4) (Dihydrolipoamide dehydrogenase) (Glycine cleavage system L protein)	P09622 (DLD)	-0.091	0.845	-2.634	0.0211	0.1398
Q9NTX5	ENSG00000093144	ECHDC1	Ethylmalonyl-CoA decarboxylase (EC 4.1.1.94) (Enoyl-CoA hydratase domain-containing protein 1) (Methylmalonyl-CoA decarboxylase) (MMCD)	Q9NTX5 (ECHDC1)	-0.091	0.803	-2.609	0.0221	0.1427
Q9BXP5	ENSG00000087087	SRRT	Serrate RNA effector molecule homolog (Arsenite-resistance protein 2)	Q9BXP5 (SRRT)	-0.089	0.819	-2.623	0.0215	0.1409
Q8NI27	ENSG00000125676	THOC2	THO complex subunit 2 (Tho2) (hTREX120)	Q8NI27 (THOC2)	0.089	0.801	2.582	0.0232	0.1465
O75438	ENSG00000183648	NDUFB1	NADH dehydrogenase [ubiquinone] 1 beta subcomplex subunit 1 (Complex I-MNLL) (CI-MNLL) (NADH-ubiquinone oxidoreductase MNLL subunit)	O75438 (NDUFB1)	-0.087	0.805	-2.631	0.0211	0.1398
Q16822	ENSG00000100889; ENSG00000285241	PCK2	Phosphoenolpyruvate carboxykinase [GTP], mitochondrial (PEPCK-M) (EC 4.1.1.32)	Q16822 (PCK2)	-0.087	0.809	-2.614	0.0218	0.1415
O00571	ENSG00000215301	DDX3X	ATP-dependent RNA helicase DDX3X (EC 3.6.4.13) (CAP-Rf) (DEAD box protein 3, X-chromosomal) (DEAD box, X isoform) (DBX) (Helicase-like protein 2) (HLP2)	O00571 (DDX3X)	0.085	0.862	2.635	0.021	0.1398
Q8IZ07	ENSG00000076513	ANKRD13A	Ankyrin repeat domain-containing protein 13A (Protein KE03)	Q8IZ07 (ANKRD13A)	0.084	0.984	2.563	0.0241	0.149

Table S56: Proteins differentially abundant at Day 30 compared to pre-second vaccination (Study Arm 2 (A/H7N9 + AS03 D1, 29) versus Study Arm 3 (A/H7N9 + PBS, D1, 29), Dendritic cells). Proteins are sorted by descending absolute log_2 fold change. Protein and gene annotations are based on Ensembl Version 101.

Protein ID	Ensembl Gene ID	Ensembl Gene Name	Protein Description	50% Protein Cluster ID (Gene Name)	Fold Change (Day 30 vs. Pre- Second Vaccination)	Average log_2 Abundance	Moderated t-Statistic	P-Value	FDR Adjusted P-value
Q96PP8	ENSG00000154451	GBP5	Guanylate-binding protein 5 (EC 3.6.5.-) (GBP-TA antigen) (GTP-binding protein 5) (GBP-5) (Guanine nucleotide-binding protein 5)	Q96PP9 (GBP4)	1.675	1.260	7.133	<0.0001	0.0003

Table S56: Proteins differentially abundant at Day 30 compared to pre-second vaccination (Study Arm 2 (A/H7N9 + AS03 D1, 29) versus Study Arm 3 (A/H7N9 + PBS, D1, 29), Dendritic cells). Proteins are sorted by descending absolute log_2 fold change. Protein and gene annotations are based on Ensembl Version 101 (continued).

Protein ID	Ensembl Gene ID	Ensembl Gene Name	Protein Description	50% Protein Cluster ID (Gene Name)	Fold Change (Day 30 vs. Pre- Second Vaccination)	Average log_2 Abundance	Moderated t-Statistic	P-Value	FDR Adjusted P-value
O95183	ENSG00000168899	VAMP5	Vesicle-associated membrane protein 5 (VAMP-5) (Myobrevin)	O95183 (VAMP5)	1.264	1.125	6.253	<0.0001	0.0011
P32455	ENSG00000117228	GBP1	Guanylate-binding protein 1 (EC 3.6.5.-) (GTP-binding protein 1) (GBP-1) (HuGBP-1) (Guanine nucleotide-binding protein 1) (Interferon-induced guanylate-binding protein 1)	Q96PP9 (GBP4)	1.190	1.150	6.800	<0.0001	0.0005
P23381	ENSG00000140105	WARS1	Tryptophan–tRNA ligase, cytoplasmic (EC 6.1.1.2) (Interferon-induced protein 53) (IFP53) (Tryptophanyl-tRNA synthetase) (TrpRS) (hWRS) [Cleaved into: T1-TrpRS; T2-TrpRS]	P23381 (WARS1)	0.924	1.010	5.120	<0.0001	0.0101
P32456	ENSG00000162645	GBP2	Guanylate-binding protein 2 (EC 3.6.5.-) (GTP-binding protein 2) (GBP-2) (HuGBP-2) (Guanine nucleotide-binding protein 2) (Interferon-induced guanylate-binding protein 2)	Q96PP9 (GBP4)	0.853	1.048	6.507	<0.0001	0.0007
P12314	ENSG00000150337	FCGR1A	High affinity immunoglobulin gamma Fc receptor I (IgG Fc receptor I) (Fc-gamma RI) (FcRI) (Fc-gamma RIA) (FcgammaRIa) (CD antigen CD64)	P12314 (FCGR1A)	0.845	1.250	7.153	<0.0001	0.0003
P09913	ENSG00000119922	IFIT2	Interferon-induced protein with tetratricopeptide repeats 2 (IFIT-2) (ISG-54 K) (Interferon-induced 54 kDa protein) (IFI-54K) (P54)	O14879 (IFIT3)	0.813	1.070	6.482	<0.0001	0.0007
Q96PP9	ENSG00000162654	GBP4	Guanylate-binding protein 4 (EC 3.6.5.-) (GTP-binding protein 4) (GBP-4) (Guanine nucleotide-binding protein 4)	Q96PP9 (GBP4)	0.790	1.115	4.888	<0.0001	0.0145
P28838	ENSG00000002549	LAP3	Cytosol aminopeptidase (EC 3.4.11.1) (Cysteinylglycine-S-conjugate dipeptidase) (EC 3.4.13.23) (Leucine aminopeptidase 3) (LAP-3) (Leucyl aminopeptidase) (Peptidase S) (Proline aminopeptidase) (EC 3.4.11.5) (Prolyl aminopeptidase)	P28838 (LAP3)	0.774	1.146	7.445	<0.0001	0.0003
O95236	ENSG00000128284	APOL3	Apolipoprotein L3 (Apolipoprotein L-III) (ApoL-III) (TNF-inducible protein CG12-1) ($CG12_1$)	O95236 (APOL3)	0.758	1.019	4.700	0.0001	0.0205
P20160	ENSG00000172232; ENSG00000278624	AZU1	Azurocidin (Cationic antimicrobial protein CAP37) (Heparin-binding protein) (HBP) (hHBP)	P20160 (AZU1)	0.728	0.960	4.535	0.0002	0.0291

Table S56: Proteins differentially abundant at Day 30 compared to pre-second vaccination (Study Arm 2 (A/H7N9 + AS03 D1, 29) versus Study Arm 3 (A/H7N9 + PBS, D1, 29), Dendritic cells). Proteins are sorted by descending absolute log_2 fold change. Protein and gene annotations are based on Ensembl Version 101 (continued).

Protein ID	Ensembl Gene ID	Ensembl Gene Name	Protein Description	50% Protein Cluster ID (Gene Name)	Fold Change (Day 30 vs. Pre- Second Vaccination)	Average log_2 Abundance	Moderated t-Statistic	P-Value	FDR Adjusted P-value
O14879	ENSG00000119917	IFIT3	Interferon-induced protein with tetratricopeptide repeats 3 (IFIT-3) (CIG49) (ISG-60) (Interferon-induced 60 kDa protein) (IFI-60K) (Interferon-induced protein with tetratricopeptide repeats 4) (IFIT-4) (Retinoic acid-induced gene G protein) (P60) (RIG-G)	O14879 (IFIT3)	0.719	1.057	5.627	<0.0001	0.0043
P31941	ENSG00000128383; ENSG00000262156	APOBEC3A	DNA dC->dU-editing enzyme APOBEC-3A (A3A) (EC 3.5.4.38) (Phorbolin-1)	Q9HC16 (APOBEC3G)	0.676	0.996	5.236	<0.0001	0.0083
P42224	ENSG00000115415	STAT1	Signal transducer and activator of transcription 1-alpha/beta (Transcription factor ISGF-3 components p91/p84)	P40763 (STAT3)	0.640	1.149	8.604	<0.0001	<0.0001
Q13287	ENSG00000123609	NMI	N-myc-interactor (Nmi) (N-myc and STAT interactor)	Q13287 (NMI)	0.623	1.034	5.347	<0.0001	0.0069
Q9BQE5	ENSG00000128335	APOL2	Apolipoprotein L2 (Apolipoprotein L-II) (ApoL-II)	O14791 (APOL1)	0.562	1.020	3.885	0.0008	0.1003
P52630	ENSG00000170581	STAT2	Signal transducer and activator of transcription 2 (p113)	P52630 (STAT2)	0.472	0.983	5.020	<0.0001	0.012
P80217	ENSG00000068079	IFI35	Interferon-induced 35 kDa protein (IFP 35) (Ifi-35)	P80217 (IFI35)	0.448	0.926	4.817	<0.0001	0.0163
Q14314	ENSG00000127951	FGL2	Fibroleukin (Fibrinogen-like protein 2) (pT49)	Q14314 (FGL2)	0.404	0.882	4.097	0.0005	0.0659
O75367	ENSG00000113648	MACROH2A1	Core histone macro-H2A.1 (Histone macroH2A1) (mH2A1) (Histone H2A.y) (H2A/y) (Medulloblastoma antigen MU-MB-50.205)	Q9P0M6 (MACROH2A2)	0.398	1.040	3.742	0.0011	0.1214
P61916	ENSG00000119655	NPC2	NPC intracellular cholesterol transporter 2 (Epididymal secretory protein E1) (Human epididymis-specific protein 1) (He1) (Niemann-Pick disease type C2 protein)	P61916 (NPC2)	0.397	0.801	5.450	<0.0001	0.0059
Q5EBM0	ENSG00000134326	CMPK2	UMP-CMP kinase 2, mitochondrial (EC 2.7.4.14) (Nucleoside-diphosphate kinase) (EC 2.7.4.6)	Q5EBM0 (CMPK2)	0.389	0.947	3.874	0.0008	0.1003
O14933	ENSG00000156587	UBE2L6	Ubiquitin/ISG15-conjugating enzyme E2 L6 (EC 2.3.2.23) (E2 ubiquitin-conjugating enzyme L6) (Retinoic acid-induced gene B protein) (RIG-B) (UbcH8) (Ubiquitin carrier protein L6) (Ubiquitin-protein ligase L6)	P68036 (UBE2L3)	0.375	0.994	4.462	0.0002	0.0331
Q9H939	ENSG00000152229	PSTPIP2	Proline-serine-threonine phosphatase-interacting protein 2 (PEST phosphatase-interacting protein 2)	Q9H939 (PSTPIP2)	0.353	0.966	4.092	0.0005	0.0659

Table S56: Proteins differentially abundant at Day 30 compared to pre-second vaccination (Study Arm 2 (A/H7N9 + AS03 D1, 29) versus Study Arm 3 (A/H7N9 + PBS, D1, 29), Dendritic cells). Proteins are sorted by descending absolute log_2 fold change. Protein and gene annotations are based on Ensembl Version 101 (continued).

Protein ID	Ensembl Gene ID	Ensembl Gene Name	Protein Description	50% Protein Cluster ID (Gene Name)	Fold Change (Day 30 vs. Pre- Second Vaccination)	Average log_2 Abundance	Moderated t-Statistic	P-Value	FDR Adjusted P-value
Q460N5	ENSG00000173193	PARP14	Protein mono-ADP-ribosyltransferase PARP14 (EC 2.4.2.-) (ADP-ribosyltransferase diphtheria toxin-like 8) (ARTD8) (B aggressive lymphoma protein 2) (Poly [ADP-ribose] polymerase 14) (PARP-14)	Q460N5 (PARP14)	0.350	0.952	4.365	0.0002	0.0401
P43490	ENSG00000105835	NAMPT	Nicotinamide phosphoribosyltransferase (NAmPRTase) (Nampt) (EC 2.4.2.12) (Pre-B-cell colony-enhancing factor 1) (Pre-B cell-enhancing factor) (Visfatin)	P43490 (NAMPT)	0.318	0.771	3.775	0.001	0.1188
Q9NZM1	ENSG00000138119	MYOF	Myoferlin (Fer-1-like protein 3)	O75923 (DYSF)	0.312	0.888	3.670	0.0013	0.136
P28161	ENSG00000213366	GSTM2	Glutathione S-transferase Mu 2 (EC 2.5.1.18) (GST class-mu 2) (GSTM2-2)	P21266 (GSTM3)	-0.311	0.894	-3.801	0.001	0.116
P51452	ENSG00000108861	DUSP3	Dual specificity protein phosphatase 3 (EC 3.1.3.16) (EC 3.1.3.48) (Dual specificity protein phosphatase VHR) (Vaccinia H1-related phosphatase) (VHR)	P51452 (DUSP3)	0.308	0.787	3.763	0.001	0.1188
Q9H0J9	ENSG00000059378	PARP12	Protein mono-ADP-ribosyltransferase PARP12 (EC 2.4.2.-) (ADP-ribosyltransferase diphtheria toxin-like 12) (ARTD12) (Poly [ADP-ribose] polymerase 12) (PARP-12) (Zinc finger CCCH domain-containing protein 1)	Q9H0J9 (PARP12)	0.295	0.907	4.919	<0.0001	0.0144
Q03518	ENSG00000168394; ENSG00000206297; ENSG00000226173; ENSG00000227816; ENSG00000230705; ENSG00000224212; ENSG00000232367	TAP1	Antigen peptide transporter 1 (APT1) (EC 7.4.2.-) (ATP-binding cassette sub-family B member 2) (Peptide supply factor 1) (Peptide transporter PSF1) (PSF-1) (Peptide transporter TAP1) (Peptide transporter involved in antigen processing 1) (Really interesting new gene 4 protein) (RING4)	Q03518 (TAP1)	0.290	1.038	4.130	0.0004	0.0652
Q14764	ENSG00000013364	MVP	Major vault protein (MVP) (Lung resistance-related protein)	Q14764 (MVP)	0.269	0.904	4.336	0.0003	0.041
O75558	ENSG00000135604	STX11	Syntaxin-11	O75558 (STX11)	0.258	0.926	3.628	0.0015	0.1462

Table S56: Proteins differentially abundant at Day 30 compared to pre-second vaccination (Study Arm 2 (A/H7N9 + AS03 D1, 29) versus Study Arm 3 (A/H7N9 + PBS, D1, 29), Dendritic cells). Proteins are sorted by descending absolute log_2 fold change. Protein and gene annotations are based on Ensembl Version 101 (continued).

Protein ID	Ensembl Gene ID	Ensembl Gene Name	Protein Description	50% Protein Cluster ID (Gene Name)	Fold Change (Day 30 vs. Pre- Second Vaccination)	Average log_2 Abundance	Moderated t-Statistic	P-Value	FDR Adjusted P-value
Q9Y385	ENSG00000198833	UBE2J1	Ubiquitin-conjugating enzyme E2 J1 (EC 2.3.2.23) (E2 ubiquitin-conjugating enzyme J1) (Non-canonical ubiquitin-conjugating enzyme 1) (NCUBE-1) (Yeast ubiquitin-conjugating enzyme UBC6 homolog E) (HsUBC6e)	Q9Y385 (UBE2J1)	0.257	0.885	3.941	0.0007	0.0917
P40763	ENSG00000168610	STAT3	Signal transducer and activator of transcription 3 (Acute-phase response factor)	P40763 (STAT3)	0.243	0.986	3.683	0.0013	0.1358

Table S57: Proteins differentially abundant at Day 32 compared to pre-second vaccination (Study Arm 2 (A/H7N9 + AS03 D1, 29), Dendritic cells). Proteins are sorted by descending absolute log_2 fold change. Protein and gene annotations are based on Ensembl Version 101.

Protein ID	Ensembl Gene ID	Ensembl Gene Name	Protein Description	50% Protein Cluster ID (Gene Name)	Fold Change (Day 32 vs. Pre- Second Vaccination)	Average log_2 Abundance	Moderated t-Statistic	P-Value	FDR Adjusted P-value
P32455	ENSG00000117228	GBP1	Guanylate-binding protein 1 (EC 3.6.5.-) (GTP-binding protein 1) (GBP-1) (HuGBP-1) (Guanine nucleotide-binding protein 1) (Interferon-induced guanylate-binding protein 1)	Q96PP9 (GBP4)	0.920	1.488	5.344	0.0001	0.0526
P23381	ENSG00000140105	WARS1	Tryptophan–tRNA ligase, cytoplasmic (EC 6.1.1.2) (Interferon-induced protein 53) (IFP53) (Tryptophanyl-tRNA synthetase) (TrpRS) (hWRS) [Cleaved into: T1-TrpRS; T2-TrpRS]	P23381 (WARS1)	0.794	1.206	5.357	0.0001	0.0526
Q96PP8	ENSG00000154451	GBP5	Guanylate-binding protein 5 (EC 3.6.5.-) (GBP-TA antigen) (GTP-binding protein 5) (GBP-5) (Guanine nucleotide-binding protein 5)	Q96PP9 (GBP4)	0.625	1.251	4.547	0.0006	0.123
P28838	ENSG00000002549	LAP3	Cytosol aminopeptidase (EC 3.4.11.1) (Cysteinylglycine-S-conjugate dipeptidase) (EC 3.4.13.23) (Leucine aminopeptidase 3) (LAP-3) (Leucyl aminopeptidase) (Peptidase S) (Proline aminopeptidase) (EC 3.4.11.5) (Prolyl aminopeptidase)	P28838 (LAP3)	0.587	1.263	5.571	<0.0001	0.0526

Table S57: Proteins differentially abundant at Day 32 compared to pre-second vaccination (Study Arm 2 (A/H7N9 + AS03 D1, 29), Dendritic cells). Proteins are sorted by descending absolute log_2 fold change. Protein and gene annotations are based on Ensembl Version 101 (continued).

Protein ID	Ensembl Gene ID	Ensembl Gene Name	Protein Description	50% Protein Cluster ID (Gene Name)	Fold Change (Day 32 vs. Pre- Second Vaccination)	Average log_2 Abundance	Moderated t-Statistic	P-Value	FDR Adjusted P-value
P32456	ENSG00000162645	GBP2	Guanylate-binding protein 2 (EC 3.6.5.-) (GTP-binding protein 2) (GBP-2) (HuGBP-2) (Guanine nucleotide-binding protein 2) (Interferon-induced guanylate-binding protein 2)	Q96PP9 (GBP4)	0.531	1.155	6.564	<0.0001	0.0321
P42224	ENSG00000115415	STAT1	Signal transducer and activator of transcription 1-alpha/beta (Transcription factor ISGF-3 components p91/p84)	P40763 (STAT3)	0.505	1.322	5.723	<0.0001	0.0526
P12314	ENSG00000150337	FCGR1A	High affinity immunoglobulin gamma Fc receptor I (IgG Fc receptor I) (Fc-gamma RI) (FcRI) (Fc-gamma RIA) (FcgammaRIa) (CD antigen CD64)	P12314 (FCGR1A)	0.401	1.310	7.983	<0.0001	0.0081
P19971	ENSG00000025708	TYMP	Thymidine phosphorylase (TP) (EC 2.4.2.4) (Gliostatin) (Platelet-derived endothelial cell growth factor) (PD-ECGF) (TdRPase)	P19971 (TYMP)	0.391	1.076	4.635	0.0005	0.1178
Q9NZM1	ENSG00000138119	MYOF	Myoferlin (Fer-1-like protein 3)	O75923 (DYSF)	0.333	1.024	5.141	0.0002	0.0575
P33121	ENSG00000151726	ACSL1	Long-chain-fatty-acid–CoA ligase 1 (EC 6.2.1.3) (Acyl-CoA synthetase 1) (ACS1) (Arachidonate–CoA ligase) (EC 6.2.1.15) (Long-chain acyl-CoA synthetase 1) (LACS 1) (Long-chain acyl-CoA synthetase 2) (LACS 2) (Long-chain fatty acid-CoA ligase 2) (Palmitoyl-CoA ligase 1) (Palmitoyl-CoA ligase 2) (Phytanate–CoA ligase) (EC 6.2.1.24)	P33121 (ACSL1)	0.304	0.955	5.300	0.0001	0.0526
Q03518	ENSG00000168394; ENSG00000206297; ENSG00000226173; ENSG00000227816; ENSG00000230705; ENSG00000224212; ENSG00000232367	TAP1	Antigen peptide transporter 1 (APT1) (EC 7.4.2.-) (ATP-binding cassette sub-family B member 2) (Peptide supply factor 1) (Peptide transporter PSF1) (PSF-1) (Peptide transporter TAP1) (Peptide transporter involved in antigen processing 1) (Really interesting new gene 4 protein) (RING4)	Q03518 (TAP1)	0.296	1.149	4.522	0.0006	0.123
P13284	ENSG00000216490	IFI30	Gamma-interferon-inducible lysosomal thiol reductase (EC 1.8.-.-) (Gamma-interferon-inducible protein IP-30) (Legumaturain)	P13284 (IFI30)	0.295	0.927	4.842	0.0003	0.0873

Table S57: Proteins differentially abundant at Day 32 compared to pre-second vaccination (Study Arm 2 (A/H7N9 + AS03 D1, 29), Dendritic cells). Proteins are sorted by descending absolute log_2 fold change. Protein and gene annotations are based on Ensembl Version 101 (continued).

Protein ID	Ensembl Gene ID	Ensembl Gene Name	Protein Description	50% Protein Cluster ID (Gene Name)	Fold Change (Day 32 vs. Pre- Second Vaccination)	Average log_2 Abundance	Moderated t-Statistic	P-Value	FDR Adjusted P-value
P30273	ENSG00000158869	FCER1G	High affinity immunoglobulin epsilon receptor subunit gamma (Fc receptor gamma-chain) (FcRgamma) (Fc-epsilon RI-gamma) (IgE Fc receptor subunit gamma) (FceRI gamma)	P30273 (FCER1G)	0.293	0.916	4.504	0.0006	0.123
Q9H939	ENSG00000152229	PSTPIP2	Proline-serine-threonine phosphatase-interacting protein 2 (PEST phosphatase-interacting protein 2)	Q9H939 (PSTPIP2)	0.284	1.042	5.281	0.0001	0.0526
Q13287	ENSG00000123609	NMI	N-myc-interactor (Nmi) (N-myc and STAT interactor)	Q13287 (NMI)	0.281	1.055	5.124	0.0002	0.0575
Q8NHL6	ENSG00000277807; ENSG00000274669	LILRB1	Leukocyte immunoglobulin-like receptor subfamily B member 1 (LIR-1) (Leukocyte immunoglobulin-like receptor 1) (CD85 antigen-like family member J) (Immunoglobulin-like transcript 2) (ILT-2) (Monocyte/macrophage immunoglobulin-like receptor 7) (MIR-7) (CD antigen CD85j)	Q8NHL6 (LILRB1)	0.232	1.004	5.443	0.0001	0.0526
Q14764	ENSG00000013364	MVP	Major vault protein (MVP) (Lung resistance-related protein)	Q14764 (MVP)	0.206	0.934	5.387	0.0001	0.0526

Table S58: Proteins differentially abundant at Day 2 compared to pre-first vaccination (Study Arm 2 (A/H7N9 + AS03 D1, 29), Monocytes). Proteins are sorted by descending absolute log_2 fold change. Protein and gene annotations are based on Ensembl Version 101.

Protein ID	Ensembl Gene ID	Ensembl Gene Name	Protein Description	50% Protein Cluster ID (Gene Name)	Fold Change (Day 2 vs. Pre- First Vaccination)	Average log_2 Abundance	Moderated t-Statistic	P-Value	FDR Adjusted P-value
Q96PP8	ENSG00000154451	GBP5	Guanylate-binding protein 5 (EC 3.6.5.-) (GBP-TA antigen) (GTP-binding protein 5) (GBP-5) (Guanine nucleotide-binding protein 5)	Q96PP9 (GBP4)	0.786	0.371	5.161	0.0003	0.0413
P32455	ENSG00000117228	GBP1	Guanylate-binding protein 1 (EC 3.6.5.-) (GTP-binding protein 1) (GBP-1) (HuGBP-1) (Guanine nucleotide-binding protein 1) (Interferon-induced guanylate-binding protein 1)	Q96PP9 (GBP4)	0.611	0.261	6.337	<0.0001	0.0175
P56279	ENSG00000100721	TCL1A	T-cell leukemia/lymphoma protein 1A (Oncogene TCL-1) (Oncogene TCL1) (Protein p14 TCL1)	P56279 (TCL1A)	-0.518	-0.093	-4.984	0.0003	0.0467

Table S58: Proteins differentially abundant at Day 2 compared to pre-first vaccination (Study Arm 2 (A/H7N9 + AS03 D1, 29), Monocytes). Proteins are sorted by descending absolute log_2 fold change. Protein and gene annotations are based on Ensembl Version 101 (continued).

Protein ID	Ensembl Gene ID	Ensembl Gene Name	Protein Description	50% Protein Cluster ID (Gene Name)	Fold Change (Day 2 vs. Pre- First Vaccination)	Average log_2 Abundance	Moderated t-Statistic	P-Value	FDR Adjusted P-value
P32456	ENSG00000162645	GBP2	Guanylate-binding protein 2 (EC 3.6.5.-) (GTP-binding protein 2) (GBP-2) (HuGBP-2) (Guanine nucleotide-binding protein 2) (Interferon-induced guanylate-binding protein 2)	Q96PP9 (GBP4)	0.452	0.144	7.927	<0.0001	0.0044
P12314	ENSG00000150337	FCGR1A	High affinity immunoglobulin gamma Fc receptor I (IgG Fc receptor I) (Fc-gamma RI) (FcRI) (Fc-gamma RIA) (FcgammaRIa) (CD antigen CD64)	P12314 (FCGR1A)	0.401	0.207	5.277	0.0002	0.0378
P05114	ENSG00000205581	HMGN1	Non-histone chromosomal protein HMG-14 (High mobility group nucleosome-binding domain-containing protein 1)	P05114 (HMGN1)	-0.378	0.006	-4.322	0.0011	0.0852
P42224	ENSG00000115415	STAT1	Signal transducer and activator of transcription 1-alpha/beta (Transcription factor ISGF-3 components p91/p84)	P40763 (STAT3)	0.364	0.148	7.996	<0.0001	0.0044
O95236	ENSG00000128284	APOL3	Apolipoprotein L3 (Apolipoprotein L-III) (ApoL-III) (TNF-inducible protein CG12-1) ($CG12_1$)	O95236 (APOL3)	0.331	0.139	6.101	<0.0001	0.0213
P23381	ENSG00000140105	WARS1	Tryptophan–tRNA ligase, cytoplasmic (EC 6.1.1.2) (Interferon-induced protein 53) (IFP53) (Tryptophanyl-tRNA synthetase) (TrpRS) (hWRS) [Cleaved into: T1-TrpRS; T2-TrpRS]	P23381 (WARS1)	0.326	0.047	8.438	<0.0001	0.0044
Q9BQE5	ENSG00000128335	APOL2	Apolipoprotein L2 (Apolipoprotein L-II) (ApoL-II)	O14791 (APOL1)	0.310	0.040	3.875	0.0023	0.1313
P33241	ENSG00000130592; ENSG00000288199	LSP1	Lymphocyte-specific protein 1 (47 kDa actin-binding protein) (52 kDa phosphoprotein) (pp52) (Lymphocyte-specific antigen WP34)	P33241 (LSP1)	-0.303	-0.202	-3.928	0.0021	0.1214
P31941	ENSG00000128383; ENSG00000262156	APOBEC3A	DNA dC->dU-editing enzyme APOBEC-3A (A3A) (EC 3.5.4.38) (Phorbolin-1)	Q9HC16 (APOBEC3G)	0.300	0.168	4.359	0.001	0.0852
Q14011	ENSG00000099622	CIRBP	Cold-inducible RNA-binding protein (A18 hnRNP) (Glycine-rich RNA-binding protein CIRP)	Q14011 (CIRBP)	-0.282	0.012	-3.940	0.0021	0.1214
Q5VYY1	ENSG00000152766	ANKRD22	Ankyrin repeat domain-containing protein 22	Q5VYY1 (ANKRD22)	0.265	0.349	4.508	0.0008	0.078
P83731	ENSG00000114391	RPL24	60S ribosomal protein L24 (60S ribosomal protein L30) (Large ribosomal subunit protein eL24)	P83731 (RPL24)	-0.259	-0.374	-5.743	0.0001	0.0252
O00483	ENSG00000189043	NDUFA4	Cytochrome c oxidase subunit NDUFA4 (Complex I-MLRQ) (CI-MLRQ) (NADH-ubiquinone oxidoreductase MLRQ subunit)	O00483 (NDUFA4)	-0.258	-0.357	-3.849	0.0024	0.1315

Table S58: Proteins differentially abundant at Day 2 compared to pre-first vaccination (Study Arm 2 (A/H7N9 + AS03 D1, 29), Monocytes). Proteins are sorted by descending absolute log_2 fold change. Protein and gene annotations are based on Ensembl Version 101 (continued).

Protein ID	Ensembl Gene ID	Ensembl Gene Name	Protein Description	50% Protein Cluster ID (Gene Name)	Fold Change (Day 2 vs. Pre- First Vaccination)	Average log_2 Abundance	Moderated t-Statistic	P-Value	FDR Adjusted P-value
P33121	ENSG00000151726	ACSL1	Long-chain-fatty-acid–CoA ligase 1 (EC 6.2.1.3) (Acyl-CoA synthetase 1) (ACS1) (Arachidonate–CoA ligase) (EC 6.2.1.15) (Long-chain acyl-CoA synthetase 1) (LACS 1) (Long-chain acyl-CoA synthetase 2) (LACS 2) (Long-chain fatty acid-CoA ligase 2) (Palmitoyl-CoA ligase 1) (Palmitoyl-CoA ligase 2) (Phytanate–CoA ligase) (EC 6.2.1.24)	P33121 (ACSL1)	0.256	-0.004	8.479	<0.0001	0.0044
P0DOX5				P0DOX5 (NA)	0.252	0.335	4.170	0.0014	0.0929
P08311	ENSG00000100448	CTSG	Cathepsin G (CG) (EC 3.4.21.20)	P08311 (CTSG)	0.252	-0.086	4.666	0.0006	0.0665
P28838	ENSG00000002549	LAP3	Cytosol aminopeptidase (EC 3.4.11.1) (Cysteinylglycine-S-conjugate dipeptidase) (EC 3.4.13.23) (Leucine aminopeptidase 3) (LAP-3) (Leucyl aminopeptidase) (Peptidase S) (Proline aminopeptidase) (EC 3.4.11.5) (Prolyl aminopeptidase)	P28838 (LAP3)	0.246	0.119	7.321	<0.0001	0.0064
P43490	ENSG00000105835	NAMPT	Nicotinamide phosphoribosyltransferase (NAmPRTase) (Nampt) (EC 2.4.2.12) (Pre-B-cell colony-enhancing factor 1) (Pre-B cell-enhancing factor) (Visfatin)	P43490 (NAMPT)	0.241	0.004	4.587	0.0007	0.0715
P17096	ENSG00000137309	HMGA1	High mobility group protein HMG-I/HMG-Y (HMG-I(Y)) (High mobility group AT-hook protein 1) (High mobility group protein A1) (High mobility group protein R)	P17096 (HMGA1)	-0.238	-0.043	-4.017	0.0018	0.111
Q9Y3U8	ENSG00000130255	RPL36	60S ribosomal protein L36 (Large ribosomal subunit protein eL36)	Q9Y3U8 (RPL36)	-0.228	-0.159	-4.281	0.0011	0.0866
P12318	ENSG00000143226	FCGR2A	Low affinity immunoglobulin gamma Fc region receptor II-a (IgG Fc receptor II-a) (CDw32) (Fc-gamma RII-a) (Fc-gamma-RIIa) (FcRII-a) (CD antigen CD32)	P12318 (FCGR2A)	0.224	-0.036	5.132	0.0003	0.0413
P55327	ENSG00000076554	TPD52	Tumor protein D52 (Protein N8)	P55327 (TPD52)	-0.221	-0.009	-6.996	<0.0001	0.0085
P00761				P00761 (NA)	-0.216	-0.205	-5.279	0.0002	0.0378
P84098	ENSG00000108298	RPL19	60S ribosomal protein L19 (Large ribosomal subunit protein eL19)	P84098 (RPL19)	-0.215	-0.185	-4.446	0.0009	0.078

Table S58: Proteins differentially abundant at Day 2 compared to pre-first vaccination (Study Arm 2 (A/H7N9 + AS03 D1, 29), Monocytes). Proteins are sorted by descending absolute log_2 fold change. Protein and gene annotations are based on Ensembl Version 101 (continued).

Protein ID	Ensembl Gene ID	Ensembl Gene Name	Protein Description	50% Protein Cluster ID (Gene Name)	Fold Change (Day 2 vs. Pre- First Vaccination)	Average log_2 Abundance	Moderated t-Statistic	P-Value	FDR Adjusted P-value
P50452	ENSG00000166401	SERPINB8	Serpin B8 (Cytoplasmic antiproteinase 2) (CAP-2) (CAP2) (Peptidase inhibitor 8) (PI-8)	P30740 (SERPINB1)	-0.213	-0.294	-4.437	0.0009	0.078
P18846	ENSG00000123268	ATF1	Cyclic AMP-dependent transcription factor ATF-1 (cAMP-dependent transcription factor ATF-1) (Activating transcription factor 1) (Protein TREB36)	P16220 (CREB1)	-0.206	-0.142	-3.847	0.0025	0.1315
P11169	ENSG00000059804	SLC2A3	Solute carrier family 2, facilitated glucose transporter member 3 (Glucose transporter type 3, brain) (GLUT-3)	P11169 (SLC2A3)	0.199	-0.026	4.821	0.0005	0.0549
Q8TDB6	ENSG00000163840	DTX3L	E3 ubiquitin-protein ligase DTX3L (EC 2.3.2.27) (B-lymphoma- and BAL-associated protein) (Protein deltex-3-like) (RING-type E3 ubiquitin transferase DTX3L) (Rhysin-2) (Rhysin2)	Q8TDB6 (DTX3L)	0.198	-0.016	5.603	0.0001	0.0266
O75558	ENSG00000135604	STX11	Syntaxin-11	O75558 (STX11)	0.198	0.066	7.713	<0.0001	0.0046
Q460N5	ENSG00000173193	PARP14	Protein mono-ADP-ribosyltransferase PARP14 (EC 2.4.2.-) (ADP-ribosyltransferase diphtheria toxin-like 8) (ARTD8) (B aggressive lymphoma protein 2) (Poly [ADP-ribose] polymerase 14) (PARP-14)	Q460N5 (PARP14)	0.196	0.005	6.303	<0.0001	0.0175
Q14764	ENSG00000013364	MVP	Major vault protein (MVP) (Lung resistance-related protein)	Q14764 (MVP)	0.193	-0.032	6.029	<0.0001	0.0215
P17927	ENSG00000203710	CR1	Complement receptor type 1 (C3b/C4b receptor) (CD antigen CD35)	P17927 (CR1)	0.188	0.040	5.109	0.0003	0.0413
P23246	ENSG00000116560	SFPQ	Splicing factor, proline- and glutamine-rich (100 kDa DNA-pairing protein) (hPOMp100) (DNA-binding p52/p100 complex, 100 kDa subunit) (Polypyrimidine tract-binding protein-associated-splicing factor) (PSF) (PTB-associated-splicing factor)	P23246 (SFPQ)	-0.188	-0.025	-4.462	0.0008	0.078
P62280	ENSG00000142534	RPS11	40S ribosomal protein S11 (Small ribosomal subunit protein uS17)	P62280 (RPS11)	-0.186	-0.276	-4.455	0.0008	0.078
P32189	ENSG00000198814	GK	Glycerol kinase (GK) (Glycerokinase) (EC 2.7.1.30) (ATP:glycerol 3-phosphotransferase)	P32189 (GK)	0.184	-0.007	4.254	0.0012	0.087
P62854	ENSG00000197728	RPS26	40S ribosomal protein S26 (Small ribosomal subunit protein eS26)	P62854 (RPS26)	-0.183	-0.197	-5.863	<0.0001	0.0234
P63098	ENSG00000221823	PPP3R1	Calcineurin subunit B type 1 (Protein phosphatase 2B regulatory subunit 1) (Protein phosphatase 3 regulatory subunit B alpha isoform 1)	P63098 (PPP3R1)	-0.183	-0.188	-4.094	0.0016	0.1022

Table S58: Proteins differentially abundant at Day 2 compared to pre-first vaccination (Study Arm 2 (A/H7N9 + AS03 D1, 29), Monocytes). Proteins are sorted by descending absolute log_2 fold change. Protein and gene annotations are based on Ensembl Version 101 (continued).

Protein ID	Ensembl Gene ID	Ensembl Gene Name	Protein Description	50% Protein Cluster ID (Gene Name)	Fold Change (Day 2 vs. Pre- First Vaccination)	Average log_2 Abundance	Moderated t-Statistic	P-Value	FDR Adjusted P-value
P62937	ENSG00000196262	PPIA	Peptidyl-prolyl cis-trans isomerase A (PPIase A) (EC 5.2.1.8) (Cyclophilin A) (Cyclosporin A-binding protein) (Rotamase A) [Cleaved into: Peptidyl-prolyl cis-trans isomerase A, N-terminally processed]	Q9UNP9 (PPIE)	-0.182	-0.084	-5.159	0.0003	0.0413
Q9H939	ENSG00000152229	PSTPIP2	Proline-serine-threonine phosphatase-interacting protein 2 (PEST phosphatase-interacting protein 2)	Q9H939 (PSTPIP2)	0.178	-0.064	5.027	0.0003	0.0454
O75923	ENSG00000135636	DYSF	Dysferlin (Dystrophy-associated fer-1-like protein) (Fer-1-like protein 1)	O75923 (DYSF)	0.166	-0.063	5.862	<0.0001	0.0234
P52630	ENSG00000170581	STAT2	Signal transducer and activator of transcription 2 (p113)	P52630 (STAT2)	0.165	0.013	4.303	0.0011	0.0852
P80217	ENSG00000068079	IFI35	Interferon-induced 35 kDa protein (IFP 35) (Ifi-35)	P80217 (IFI35)	0.161	0.059	4.769	0.0005	0.0579
P40763	ENSG00000168610	STAT3	Signal transducer and activator of transcription 3 (Acute-phase response factor)	P40763 (STAT3)	0.154	-0.049	5.523	0.0001	0.0285
Q9Y4F9	ENSG00000111913	RIPOR2	Rho family-interacting cell polarization regulator 2	Q9Y4F9 (RIPOR2)	0.151	-0.059	4.942	0.0004	0.0483
Q16831	ENSG00000183696	UPP1	Uridine phosphorylase 1 (UPase 1) (UrdPase 1) (EC 2.4.2.3)	Q16831 (UPP1)	0.148	-0.046	4.826	0.0005	0.0549
P09874	ENSG00000143799	PARP1	Poly [ADP-ribose] polymerase 1 (PARP-1) (EC 2.4.2.30) (ADP-ribosyltransferase diphtheria toxin-like 1) (ARTD1) (DNA ADP-ribosyltransferase PARP1) (EC 2.4.2.-) (NAD(+) ADP-ribosyltransferase 1) (ADPRT 1) (Poly[ADP-ribose] synthase 1) (Protein poly-ADP-ribosyltransferase PARP1) (EC 2.4.2.-)	P09874 (PARP1)	-0.148	-0.195	-4.619	0.0006	0.0698
P37802	ENSG00000158710	TAGLN2	Transgelin-2 (Epididymis tissue protein Li 7e) (SM22-alpha homolog)	Q9UI15 (TAGLN3)	-0.145	-0.105	-3.801	0.0027	0.1344
P27635	ENSG00000147403	RPL10	60S ribosomal protein L10 (Laminin receptor homolog) (Large ribosomal subunit protein uL16) (Protein QM) (Ribosomal protein L10) (Tumor suppressor QM)	P27635 (RPL10)	-0.145	-0.255	-3.933	0.0021	0.1214
P49662	ENSG00000196954	CASP4	Caspase-4 (CASP-4) (EC 3.4.22.57) (ICE and Ced-3 homolog 2) (ICH-2) (ICE(rel)-II) (Mih1) (Protease TX) [Cleaved into: Caspase-4 subunit p10; Caspase-4 subunit p20]	P51878 (CASP5)	0.144	0.016	5.718	0.0001	0.0252
Q9NZ45	ENSG00000122873	CISD1	CDGSH iron-sulfur domain-containing protein 1 (MitoNEET)	Q8N5K1 (CISD2)	-0.142	-0.466	-5.638	0.0001	0.0266

Table S58: Proteins differentially abundant at Day 2 compared to pre-first vaccination (Study Arm 2 (A/H7N9 + AS03 D1, 29), Monocytes). Proteins are sorted by descending absolute log_2 fold change. Protein and gene annotations are based on Ensembl Version 101 (continued).

Protein ID	Ensembl Gene ID	Ensembl Gene Name	Protein Description	50% Protein Cluster ID (Gene Name)	Fold Change (Day 2 vs. Pre- First Vaccination)	Average log_2 Abundance	Moderated t-Statistic	P-Value	FDR Adjusted P-value
O75475	ENSG00000164985	PSIP1	PC4 and SFRS1-interacting protein (CLL-associated antigen KW-7) (Dense fine speckles 70 kDa protein) (DFS 70) (Lens epithelium-derived growth factor) (Transcriptional coactivator p75/p52)	O75475 (PSIP1)	-0.136	-0.107	-4.266	0.0012	0.087
Q14005	ENSG00000172349	IL16	Pro-interleukin-16 [Cleaved into: Interleukin-16 (IL-16) (Lymphocyte chemoattractant factor) (LCF)]	Q14005 (IL16)	-0.133	-0.080	-3.735	0.003	0.1493
Q9NRL2	ENSG00000198604	BAZ1A	Bromodomain adjacent to zinc finger domain protein 1A (ATP-dependent chromatin-remodeling protein) (ATP-utilizing chromatin assembly and remodeling factor 1) (hACF1) (CHRAC subunit ACF1) (Williams syndrome transcription factor-related chromatin-remodeling factor 180) (WCRF180) (hWALp1)	Q9NRL2 (BAZ1A)	0.132	-0.082	3.805	0.0026	0.1344
P08648	ENSG00000161638	ITGA5	Integrin alpha-5 (CD49 antigen-like family member E) (Fibronectin receptor subunit alpha) (Integrin alpha-F) (VLA-5) (CD antigen CD49e) [Cleaved into: Integrin alpha-5 heavy chain; Integrin alpha-5 light chain]	P08648 (ITGA5)	-0.131	0.023	-3.852	0.0024	0.1315
Q03518	ENSG00000168394; ENSG00000206297; ENSG00000226173; ENSG00000227816; ENSG00000230705; ENSG00000224212; ENSG00000232367	TAP1	Antigen peptide transporter 1 (APT1) (EC 7.4.2.-) (ATP-binding cassette sub-family B member 2) (Peptide supply factor 1) (Peptide transporter PSF1) (PSF-1) (Peptide transporter TAP1) (Peptide transporter involved in antigen processing 1) (Really interesting new gene 4 protein) (RING4)	Q03518 (TAP1)	0.129	0.126	4.209	0.0013	0.0903
Q9H814	ENSG00000164902	PHAX	Phosphorylated adapter RNA export protein (RNA U small nuclear RNA export adapter protein)	Q9H814 (PHAX)	-0.123	-0.120	-4.042	0.0017	0.108
P11717	ENSG00000197081	IGF2R	Cation-independent mannose-6-phosphate receptor (CI Man-6-P receptor) (CI-MPR) (M6PR) (300 kDa mannose 6-phosphate receptor) (MPR 300) (Insulin-like growth factor 2 receptor) (Insulin-like growth factor II receptor) (IGF-II receptor) (M6P/IGF2 receptor) (M6P/IGF2R) (CD antigen CD222)	P11717 (IGF2R)	0.120	-0.024	4.226	0.0013	0.0895
P55899	ENSG00000104870	FCGRT	IgG receptor FcRn large subunit p51 (FcRn) (IgG Fc fragment receptor transporter alpha chain) (Neonatal Fc receptor)	P55899 (FCGRT)	-0.118	-0.218	-4.193	0.0013	0.0911

Table S58: Proteins differentially abundant at Day 2 compared to pre-first vaccination (Study Arm 2 (A/H7N9 + AS03 D1, 29), Monocytes). Proteins are sorted by descending absolute log_2 fold change. Protein and gene annotations are based on Ensembl Version 101 (continued).

Protein ID	Ensembl Gene ID	Ensembl Gene Name	Protein Description	50% Protein Cluster ID (Gene Name)	Fold Change (Day 2 vs. Pre- First Vaccination)	Average log_2 Abundance	Moderated t-Statistic	P-Value	FDR Adjusted P-value
P42167	ENSG00000120802	TMPO	Lamina-associated polypeptide 2, isoforms beta/gamma (Thymopoietin, isoforms beta/gamma) (TP beta/gamma) (Thymopoietin-related peptide isoforms beta/gamma) (TPRP isoforms beta/gamma) [Cleaved into: Thymopoietin (TP) (Splenin); Thymopentin (TP5)]	P42166 (TMPO)	-0.116	-0.191	-4.096	0.0016	0.1022
Q93033	ENSG00000134256	CD101	Immunoglobulin superfamily member 2 (IgSF2) (Cell surface glycoprotein V7) (Glu-Trp-Ile EWI motif-containing protein 101) (EWI-101) (CD antigen CD101)	Q93033 (CD101)	-0.116	-0.210	-3.946	0.0021	0.1214
O95544	ENSG00000008130	NADK	NAD kinase (EC 2.7.1.23) (Poly(P)/ATP NAD kinase)	O95544 (NADK)	0.113	-0.105	4.498	0.0008	0.078
Q9UKY7	ENSG00000091527	CDV3	Protein CDV3 homolog	Q9UKY7 (CDV3)	-0.112	0.002	-3.813	0.0026	0.1344
Q2M385	ENSG00000197629	MPEG1	Macrophage-expressed gene 1 protein (Macrophage gene 1 protein) (Mpg-1) (Perforin-2) (P-2)	Q2M385 (MPEG1)	-0.109	-0.244	-4.067	0.0017	0.1053
P19878	ENSG00000116701	NCF2	Neutrophil cytosol factor 2 (NCF-2) (67 kDa neutrophil oxidase factor) (NADPH oxidase activator 2) (Neutrophil NADPH oxidase factor 2) (p67-phox)	P19878 (NCF2)	0.108	-0.113	4.313	0.0011	0.0852
P08631	ENSG00000101336	HCK	Tyrosine-protein kinase HCK (EC 2.7.10.2) (Hematopoietic cell kinase) (Hemopoietic cell kinase) (p59-HCK/p60-HCK) (p59Hck) (p61Hck)	P06241 (FYN)	0.102	-0.103	4.323	0.0011	0.0852
Q53EL6	ENSG00000150593	PDCD4	Programmed cell death protein 4 (Neoplastic transformation inhibitor protein) (Nuclear antigen H731-like) (Protein 197/15a)	Q53EL6 (PDCD4)	-0.099	-0.184	-4.306	0.0011	0.0852
P11215	ENSG00000169896	ITGAM	Integrin alpha-M (CD11 antigen-like family member B) (CR-3 alpha chain) (Cell surface glycoprotein MAC-1 subunit alpha) (Leukocyte adhesion receptor MO1) (Neutrophil adherence receptor) (CD antigen CD11b)	P20702 (ITGAX)	0.090	-0.166	3.807	0.0026	0.1344

Table S59: Proteins differentially abundant at Day 2 compared to pre-first vaccination (Study Arm 2 (A/H7N9 + AS03 D1, 29) versus Study Arm 3 (A/H7N9 + PBS, D1, 29), Monocytes). Proteins are sorted by descending absolute log_2 fold change. Protein and gene annotations are based on Ensembl Version 101.

Protein ID	Ensembl Gene ID	Ensembl Gene Name	Protein Description	50% Protein Cluster ID (Gene Name)	Fold Change (Day 2 vs. Pre- First Vaccination)	Average log_2 Abundance	Moderated t-Statistic	P-Value	FDR Adjusted P-value
P28838	ENSG00000002549	LAP3	Cytosol aminopeptidase (EC 3.4.11.1) (Cysteinylglycine-S-conjugate dipeptidase) (EC 3.4.13.23) (Leucine aminopeptidase 3) (LAP-3) (Leucyl aminopeptidase) (Peptidase S) (Proline aminopeptidase) (EC 3.4.11.5) (Prolyl aminopeptidase)	P28838 (LAP3)	0.281	0.023	5.198	<0.0001	0.122

Table S60: Proteins differentially abundant at Day 4 compared to pre-first vaccination (Study Arm 2 (A/H7N9 + AS03 D1, 29), Monocytes). Proteins are sorted by descending absolute log_2 fold change. Protein and gene annotations are based on Ensembl Version 101.

Protein ID	Ensembl Gene ID	Ensembl Gene Name	Protein Description	50% Protein Cluster ID (Gene Name)	Fold Change (Day 4 vs. Pre- First Vaccination)	Average log_2 Abundance	Moderated t-Statistic	P-Value	FDR Adjusted P-value
P32455	ENSG00000117228	GBP1	Guanylate-binding protein 1 (EC 3.6.5.-) (GTP-binding protein 1) (GBP-1) (HuGBP-1) (Guanine nucleotide-binding protein 1) (Interferon-induced guanylate-binding protein 1)	Q96PP9 (GBP4)	0.780	0.225	6.064	<0.0001	0.0768
P02769				P02768 (ALB)	-0.635	-0.302	-8.273	<0.0001	0.0187
P31941	ENSG00000128383; ENSG00000262156	APOBEC3A	DNA dC->dU-editing enzyme APOBEC-3A (A3A) (EC 3.5.4.38) (Phorbolin-1)	Q9HC16 (APOBEC3G)	0.633	0.269	5.717	0.0001	0.0921
P42224	ENSG00000115415	STAT1	Signal transducer and activator of transcription 1-alpha/beta (Transcription factor ISGF-3 components p91/p84)	P40763 (STAT3)	0.485	0.162	6.496	<0.0001	0.0565
P28838	ENSG00000002549	LAP3	Cytosol aminopeptidase (EC 3.4.11.1) (Cysteinylglycine-S-conjugate dipeptidase) (EC 3.4.13.23) (Leucine aminopeptidase 3) (LAP-3) (Leucyl aminopeptidase) (Peptidase S) (Proline aminopeptidase) (EC 3.4.11.5) (Prolyl aminopeptidase)	P28838 (LAP3)	0.395	0.227	5.654	0.0002	0.0921
P19971	ENSG00000025708	TYMP	Thymidine phosphorylase (TP) (EC 2.4.2.4) (Gliostatin) (Platelet-derived endothelial cell growth factor) (PD-ECGF) (TdRPase)	P19971 (TYMP)	0.382	0.127	6.884	<0.0001	0.0507

Table S60: Proteins differentially abundant at Day 4 compared to pre-first vaccination (Study Arm 2 (A/H7N9 + AS03 D1, 29), Monocytes). Proteins are sorted by descending absolute log_2 fold change. Protein and gene annotations are based on Ensembl Version 101 (continued).

Protein ID	Ensembl Gene ID	Ensembl Gene Name	Protein Description	50% Protein Cluster ID (Gene Name)	Fold Change (Day 4 vs. Pre- First Vaccination)	Average log_2 Abundance	Moderated t-Statistic	P-Value	FDR Adjusted P-value
Q9NZM1	ENSG00000138119	MYOF	Myoferlin (Fer-1-like protein 3)	O75923 (DYSF)	0.199	-0.052	5.500	0.0002	0.0991

Table S61: Proteins differentially abundant at Day 4 compared to pre-first vaccination (Study Arm 1 (A/H3N2v, D1) versus Study Arm 2 (A/H7N9 + AS03 D1, 29), Monocytes). Proteins are sorted by descending absolute log_2 fold change. Protein and gene annotations are based on Ensembl Version 101.

Protein ID	Ensembl Gene ID	Ensembl Gene Name	Protein Description	50% Protein Cluster ID (Gene Name)	Fold Change (Day 4 vs. Pre- First Vaccination)	Average log_2 Abundance	Moderated t-Statistic	P-Value	FDR Adjusted P-value
P02769				P02768 (ALB)	0.970	0.037	5.454	<0.0001	0.084

Table S62: Proteins differentially abundant at Day 30 compared to pre-second vaccination (Study Arm 2 (A/H7N9 + AS03 D1, 29), Monocytes). Proteins are sorted by descending absolute log_2 fold change. Protein and gene annotations are based on Ensembl Version 101.

Protein ID	Ensembl Gene ID	Ensembl Gene Name	Protein Description	50% Protein Cluster ID (Gene Name)	Fold Change (Day 30 vs. Pre- Second Vaccination)	Average log_2 Abundance	Moderated t-Statistic	P-Value	FDR Adjusted P-value
Q96PP8	ENSG00000154451	GBP5	Guanylate-binding protein 5 (EC 3.6.5.-) (GBP-TA antigen) (GTP-binding protein 5) (GBP-5) (Guanine nucleotide-binding protein 5)	Q96PP9 (GBP4)	2.282	0.953	8.227	<0.0001	0.0036
P32455	ENSG00000117228	GBP1	Guanylate-binding protein 1 (EC 3.6.5.-) (GTP-binding protein 1) (GBP-1) (HuGBP-1) (Guanine nucleotide-binding protein 1) (Interferon-induced guanylate-binding protein 1)	Q96PP9 (GBP4)	1.852	0.772	4.831	0.0004	0.0504
Q5VYY1	ENSG00000152766	ANKRD22	Ankyrin repeat domain-containing protein 22	Q5VYY1 (ANKRD22)	1.363	0.623	5.079	0.0003	0.04
P23381	ENSG00000140105	WARS1	Tryptophan–tRNA ligase, cytoplasmic (EC 6.1.1.2) (Interferon-induced protein 53) (IFP53) (Tryptophanyl-tRNA synthetase) (TrpRS) (hWRS) [Cleaved into: T1-TrpRS; T2-TrpRS]	P23381 (WARS1)	1.143	0.426	4.181	0.0013	0.0739

Table S62: Proteins differentially abundant at Day 30 compared to pre-second vaccination (Study Arm 2 (A/H7N9 + AS03 D1, 29), Monocytes). Proteins are sorted by descending absolute log_2 fold change. Protein and gene annotations are based on Ensembl Version 101 (continued).

Protein ID	Ensembl Gene ID	Ensembl Gene Name	Protein Description	50% Protein Cluster ID (Gene Name)	Fold Change (Day 30 vs. Pre- Second Vaccination)	Average log_2 Abundance	Moderated t-Statistic	P-Value	FDR Adjusted P-value
P32456	ENSG00000162645	GBP2	Guanylate-binding protein 2 (EC 3.6.5.-) (GTP-binding protein 2) (GBP-2) (HuGBP-2) (Guanine nucleotide-binding protein 2) (Interferon-induced guanylate-binding protein 2)	Q96PP9 (GBP4)	1.096	0.427	6.133	<0.0001	0.0126
O95236	ENSG00000128284	APOL3	Apolipoprotein L3 (Apolipoprotein L-III) (ApoL-III) (TNF-inducible protein CG12-1) ($CG12_1$)	O95236 (APOL3)	0.975	0.502	6.983	<0.0001	0.0088
P56279	ENSG00000100721	TCL1A	T-cell leukemia/lymphoma protein 1A (Oncogene TCL-1) (Oncogene TCL1) (Protein p14 TCL1)	P56279 (TCL1A)	-0.891	-0.122	-4.250	0.0011	0.0716
Q9BQE5	ENSG00000128335	APOL2	Apolipoprotein L2 (Apolipoprotein L-II) (ApoL-II)	O14791 (APOL1)	0.855	0.352	4.750	0.0005	0.0538
P28838	ENSG00000002549	LAP3	Cytosol aminopeptidase (EC 3.4.11.1) (Cysteinylglycine-S-conjugate dipeptidase) (EC 3.4.13.23) (Leucine aminopeptidase 3) (LAP-3) (Leucyl aminopeptidase) (Peptidase S) (Proline aminopeptidase) (EC 3.4.11.5) (Prolyl aminopeptidase)	P28838 (LAP3)	0.848	0.325	4.091	0.0015	0.0819
O14879	ENSG00000119917	IFIT3	Interferon-induced protein with tetratricopeptide repeats 3 (IFIT-3) (CIG49) (ISG-60) (Interferon-induced 60 kDa protein) (IFI-60K) (Interferon-induced protein with tetratricopeptide repeats 4) (IFIT-4) (Retinoic acid-induced gene G protein) (P60) (RIG-G)	O14879 (IFIT3)	0.830	0.408	4.686	0.0005	0.0563
P42224	ENSG00000115415	STAT1	Signal transducer and activator of transcription 1-alpha/beta (Transcription factor ISGF-3 components p91/p84)	P40763 (STAT3)	0.799	0.411	7.179	<0.0001	0.008
P31941	ENSG00000128383; ENSG00000262156	APOBEC3A	DNA dC->dU-editing enzyme APOBEC-3A (A3A) (EC 3.5.4.38) (Phorbolin-1)	Q9HC16 (APOBEC3G)	0.759	0.316	4.225	0.0012	0.0716
P12314	ENSG00000150337	FCGR1A	High affinity immunoglobulin gamma Fc receptor I (IgG Fc receptor I) (Fc-gamma RI) (FcRI) (Fc-gamma RIA) (FcgammaRIa) (CD antigen CD64)	P12314 (FCGR1A)	0.750	0.406	8.884	<0.0001	0.0036
Q96PP9	ENSG00000162654	GBP4	Guanylate-binding protein 4 (EC 3.6.5.-) (GTP-binding protein 4) (GBP-4) (Guanine nucleotide-binding protein 4)	Q96PP9 (GBP4)	0.700	0.433	4.004	0.0018	0.0878
P09913	ENSG00000119922	IFIT2	Interferon-induced protein with tetratricopeptide repeats 2 (IFIT-2) (ISG-54 K) (Interferon-induced 54 kDa protein) (IFI-54K) (P54)	O14879 (IFIT3)	0.617	0.471	5.872	<0.0001	0.015

Table S62: Proteins differentially abundant at Day 30 compared to pre-second vaccination (Study Arm 2 (A/H7N9 + AS03 D1, 29), Monocytes). Proteins are sorted by descending absolute log_2 fold change. Protein and gene annotations are based on Ensembl Version 101 (continued).

Protein ID	Ensembl Gene ID	Ensembl Gene Name	Protein Description	50% Protein Cluster ID (Gene Name)	Fold Change (Day 30 vs. Pre- Second Vaccination)	Average log_2 Abundance	Moderated t-Statistic	P-Value	FDR Adjusted P-value
Q9NRD1	ENSG00000116663	FBXO6	F-box only protein 6 (F-box protein that recognizes sugar chains 2) (F-box/G-domain protein 2)	Q9NRD1 (FBXO6)	0.608	0.148	3.776	0.0027	0.103
P01860		IGHG3	Immunoglobulin heavy constant gamma 3 (HDC) (Heavy chain disease protein) (Ig gamma-3 chain C region)	P0DOX5 (NA)	0.550	0.280	6.477	<0.0001	0.0122
Q03518	ENSG00000168394; ENSG00000206297; ENSG00000226173; ENSG00000227816; ENSG00000230705; ENSG00000224212; ENSG00000232367	TAP1	Antigen peptide transporter 1 (APT1) (EC 7.4.2.-) (ATP-binding cassette sub-family B member 2) (Peptide supply factor 1) (Peptide transporter PSF1) (PSF-1) (Peptide transporter TAP1) (Peptide transporter involved in antigen processing 1) (Really interesting new gene 4 protein) (RING4)	Q03518 (TAP1)	0.539	0.260	3.546	0.0041	0.1305
P0DOX5				P0DOX5 (NA)	0.479	0.490	7.628	<0.0001	0.0055
P33121	ENSG00000151726	ACSL1	Long-chain-fatty-acid–CoA ligase 1 (EC 6.2.1.3) (Acyl-CoA synthetase 1) (ACS1) (Arachidonate–CoA ligase) (EC 6.2.1.15) (Long-chain acyl-CoA synthetase 1) (LACS 1) (Long-chain acyl-CoA synthetase 2) (LACS 2) (Long-chain fatty acid-CoA ligase 2) (Palmitoyl-CoA ligase 1) (Palmitoyl-CoA ligase 2) (Phytanate–CoA ligase) (EC 6.2.1.24)	P33121 (ACSL1)	0.423	0.024	4.280	0.0011	0.0716
P35237	ENSG00000124570	SERPINB6	Serpin B6 (Cytoplasmic antiproteinase) (CAP) (Peptidase inhibitor 6) (PI-6) (Placental thrombin inhibitor)	P30740 (SERPINB1)	-0.411	-0.332	-5.204	0.0002	0.0339
Q9NZM1	ENSG00000138119	MYOF	Myoferlin (Fer-1-like protein 3)	O75923 (DYSF)	0.411	0.040	3.799	0.0026	0.103
Q460N5	ENSG00000173193	PARP14	Protein mono-ADP-ribosyltransferase PARP14 (EC 2.4.2.-) (ADP-ribosyltransferase diphtheria toxin-like 8) (ARTD8) (B aggressive lymphoma protein 2) (Poly [ADP-ribose] polymerase 14) (PARP-14)	Q460N5 (PARP14)	0.400	0.100	8.175	<0.0001	0.0036
P20160	ENSG00000172232; ENSG00000278624	AZU1	Azurocidin (Cationic antimicrobial protein CAP37) (Heparin-binding protein) (HBP) (hHBP)	P20160 (AZU1)	0.395	0.015	5.757	<0.0001	0.0169
P52630	ENSG00000170581	STAT2	Signal transducer and activator of transcription 2 (p113)	P52630 (STAT2)	0.384	0.171	5.522	0.0001	0.0233

Table S62: Proteins differentially abundant at Day 30 compared to pre-second vaccination (Study Arm 2 (A/H7N9 + AS03 D1, 29), Monocytes). Proteins are sorted by descending absolute log_2 fold change. Protein and gene annotations are based on Ensembl Version 101 (continued).

Protein ID	Ensembl Gene ID	Ensembl Gene Name	Protein Description	50% Protein Cluster ID (Gene Name)	Fold Change (Day 30 vs. Pre- Second Vaccination)	Average log_2 Abundance	Moderated t-Statistic	P-Value	FDR Adjusted P-value
Q13616	ENSG00000055130	CUL1	Cullin-1 (CUL-1)	Q13616 (CUL1)	0.383	0.039	4.555	0.0007	0.0643
P12429	ENSG00000138772	ANXA3	Annexin A3 (35-alpha calcimedin) (Annexin III) (Annexin-3) (Inositol 1,2-cyclic phosphate 2-phosphohydrolase) (Lipocortin III) (Placental anticoagulant protein III) (PAP-III)	P08133 (ANXA6)	0.382	0.019	6.252	<0.0001	0.0122
P80217	ENSG00000068079	IFI35	Interferon-induced 35 kDa protein (IFP 35) (Ifi-35)	P80217 (IFI35)	0.380	0.192	4.439	0.0008	0.0678
Q8TDB6	ENSG00000163840	DTX3L	E3 ubiquitin-protein ligase DTX3L (EC 2.3.2.27) (B-lymphoma- and BAL-associated protein) (Protein deltex-3-like) (RING-type E3 ubiquitin transferase DTX3L) (Rhysin-2) (Rhysin2)	Q8TDB6 (DTX3L)	0.374	0.154	5.480	0.0001	0.0238
P15260	ENSG00000027697	IFNGR1	Interferon gamma receptor 1 (IFN-gamma receptor 1) (IFN-gamma-R1) (CDw119) (Interferon gamma receptor alpha-chain) (IFN-gamma-R-alpha) (CD antigen CD119)	P15260 (IFNGR1)	-0.371	-0.056	-4.282	0.0011	0.0716
Q8IYM9	ENSG00000132274	TRIM22	E3 ubiquitin-protein ligase TRIM22 (EC 2.3.2.27) (50 kDa-stimulated trans-acting factor) (RING finger protein 94) (RING-type E3 ubiquitin transferase TRIM22) (Staf-50) (Tripartite motif-containing protein 22)	Q8IYM9 (TRIM22)	0.363	-0.044	4.169	0.0013	0.0739
P04440	ENSG00000215048; ENSG00000236693; ENSG00000223865; ENSG00000230763; ENSG00000226826; ENSG00000237710	HLA-DPB1	HLA class II histocompatibility antigen, DP beta 1 chain (HLA class II histocompatibility antigen, DP(W4) beta chain) (MHC class II antigen DPB1)	P13765 (HLA-DOB)	0.355	0.456	3.457	0.0048	0.1408
P20591	ENSG00000157601	MX1	Interferon-induced GTP-binding protein Mx1 (Interferon-induced protein p78) (IFI-78K) (Interferon-regulated resistance GTP-binding protein MxA) (Myxoma resistance protein 1) (Myxovirus resistance protein 1) [Cleaved into: Interferon-induced GTP-binding protein Mx1, N-terminally processed]	P20592 (MX2)	0.345	0.134	4.550	0.0007	0.0643
O75558	ENSG00000135604	STX11	Syntaxin-11	O75558 (STX11)	0.342	0.113	4.511	0.0007	0.0643

Table S62: Proteins differentially abundant at Day 30 compared to pre-second vaccination (Study Arm 2 (A/H7N9 + AS03 D1, 29), Monocytes). Proteins are sorted by descending absolute log_2 fold change. Protein and gene annotations are based on Ensembl Version 101 (continued).

Protein ID	Ensembl Gene ID	Ensembl Gene Name	Protein Description	50% Protein Cluster ID (Gene Name)	Fold Change (Day 30 vs. Pre- Second Vaccination)	Average log_2 Abundance	Moderated t-Statistic	P-Value	FDR Adjusted P-value
P31689	ENSG00000086061	DNAJA1	DnaJ homolog subfamily A member 1 (DnaJ protein homolog 2) (HSDJ) (Heat shock 40 kDa protein 4) (Heat shock protein J2) (HSJ-2) (Human DnaJ protein 2) (hDj-2)	O60884 (DNAJA2)	0.342	-0.037	5.296	0.0002	0.0305
P08311	ENSG00000100448	CTSG	Cathepsin G (CG) (EC 3.4.21.20)	P08311 (CTSG)	0.338	-0.108	6.368	<0.0001	0.0122
P40763	ENSG00000168610	STAT3	Signal transducer and activator of transcription 3 (Acute-phase response factor)	P40763 (STAT3)	0.331	0.028	4.082	0.0015	0.0819
P32189	ENSG00000198814	GK	Glycerol kinase (GK) (Glycerokinase) (EC 2.7.1.30) (ATP:glycerol 3-phosphotransferase)	P32189 (GK)	0.329	0.044	6.236	<0.0001	0.0122
P30456				P30501 (NA)	0.328	-0.088	4.400	0.0009	0.0678
P30273	ENSG00000158869	FCER1G	High affinity immunoglobulin epsilon receptor subunit gamma (Fc receptor gamma-chain) (FcRgamma) (Fc-epsilon RI-gamma) (IgE Fc receptor subunit gamma) (FceRI gamma)	P30273 (FCER1G)	0.319	-0.050	4.403	0.0009	0.0678
P00973	ENSG00000089127	OAS1	2'-5'-oligoadenylate synthase 1 ((2-5')oligo(A) synthase 1) (2-5A synthase 1) (EC 2.7.7.84) (E18/E16) (p46/p42 OAS)	Q9Y6K5 (OAS3)	0.316	-0.089	4.409	0.0009	0.0678
Q9NRL2	ENSG00000198604	BAZ1A	Bromodomain adjacent to zinc finger domain protein 1A (ATP-dependent chromatin-remodeling protein) (ATP-utilizing chromatin assembly and remodeling factor 1) (hACF1) (CHRAC subunit ACF1) (Williams syndrome transcription factor-related chromatin-remodeling factor 180) (WCRF180) (hWALp1)	Q9NRL2 (BAZ1A)	0.309	-0.010	4.250	0.0011	0.0716
Q8IXQ6	ENSG00000138496	PARP9	Protein mono-ADP-ribosyltransferase PARP9 (EC 2.4.2.-) (ADP-ribosyltransferase diphtheria toxin-like 9) (ARTD9) (B aggressive lymphoma protein) (Poly [ADP-ribose] polymerase 9) (PARP-9)	Q8IXQ6 (PARP9)	0.297	0.154	6.056	<0.0001	0.0127
Q63HN8	ENSG00000173821	RNF213	E3 ubiquitin-protein ligase RNF213 (EC 2.3.2.27) (EC 3.6.4.-) (ALK lymphoma oligomerization partner on chromosome 17) (Mysterin) (RING finger protein 213) (RING-type E3 ubiquitin transferase RNF213)	Q63HN8 (RNF213)	0.294	0.114	3.514	0.0043	0.1325

Table S62: Proteins differentially abundant at Day 30 compared to pre-second vaccination (Study Arm 2 (A/H7N9 + AS03 D1, 29), Monocytes). Proteins are sorted by descending absolute log_2 fold change. Protein and gene annotations are based on Ensembl Version 101 (continued).

Protein ID	Ensembl Gene ID	Ensembl Gene Name	Protein Description	50% Protein Cluster ID (Gene Name)	Fold Change (Day 30 vs. Pre- Second Vaccination)	Average log_2 Abundance	Moderated t-Statistic	P-Value	FDR Adjusted P-value
Q9HD89	ENSG00000104918	RETN	Resistin (Adipose tissue-specific secretory factor) (ADSF) (C/EBP-epsilon-regulated myeloid-specific secreted cysteine-rich protein) (Cysteine-rich secreted protein A12-alpha-like 2) (Cysteine-rich secreted protein FIZZ3)	Q9HD89 (RETN)	0.293	-0.060	4.231	0.0012	0.0716
O00483	ENSG00000189043	NDUFA4	Cytochrome c oxidase subunit NDUFA4 (Complex I-MLRQ) (CI-MLRQ) (NADH-ubiquinone oxidoreductase MLRQ subunit)	O00483 (NDUFA4)	-0.293	-0.372	-6.218	<0.0001	0.0122
P49662	ENSG00000196954	CASP4	Caspase-4 (CASP-4) (EC 3.4.22.57) (ICE and Ced-3 homolog 2) (ICH-2) (ICE(rel)-II) (Mih1) (Protease TX) [Cleaved into: Caspase-4 subunit p10; Caspase-4 subunit p20]	P51878 (CASP5)	0.291	0.088	6.105	<0.0001	0.0126
P01911	ENSG00000196126	HLA-DRB1	HLA class II histocompatibility antigen, DRB1 beta chain (Human leukocyte antigen DRB1) (HLA-DRB1)	P13765 (HLA-DOB)	0.285	0.188	3.738	0.0029	0.1081
O43852	ENSG00000128595	CALU	Calumenin (Crocalbin) (IEF SSP 9302)	Q15293 (RCN1)	-0.282	-0.106	-3.601	0.0037	0.1246
Q86VB7	ENSG00000177575	CD163	Scavenger receptor cysteine-rich type 1 protein M130 (Hemoglobin scavenger receptor) (CD antigen CD163) [Cleaved into: Soluble CD163 (sCD163)]	Q86VB7 (CD163)	-0.274	-0.202	-3.593	0.0037	0.1246
Q03519	ENSG00000204267; ENSG00000206235; ENSG00000206299; ENSG00000228582; ENSG00000237599; ENSG00000232326; ENSG00000225967; ENSG00000223481	TAP2	Antigen peptide transporter 2 (APT2) (EC 7.4.2.-) (ATP-binding cassette sub-family B member 3) (Peptide supply factor 2) (Peptide transporter PSF2) (PSF-2) (Peptide transporter TAP2) (Peptide transporter involved in antigen processing 2) (Really interesting new gene 11 protein) (RING11)	Q03519 (TAP2)	0.267	0.169	3.569	0.0039	0.1276
P84101	ENSG00000140264	SERF2	Small EDRK-rich factor 2 (Gastric cancer-related protein VRG107) (Protein 4F5-related) (4F5rel) (h4F5rel)	P84101 (SERF2)	0.262	-0.011	3.593	0.0037	0.1246

Table S62: Proteins differentially abundant at Day 30 compared to pre-second vaccination (Study Arm 2 (A/H7N9 + AS03 D1, 29), Monocytes). Proteins are sorted by descending absolute log_2 fold change. Protein and gene annotations are based on Ensembl Version 101 (continued).

Protein ID	Ensembl Gene ID	Ensembl Gene Name	Protein Description	50% Protein Cluster ID (Gene Name)	Fold Change (Day 30 vs. Pre- Second Vaccination)	Average log_2 Abundance	Moderated t-Statistic	P-Value	FDR Adjusted P-value
Q07954	ENSG00000123384	LRP1	Prolow-density lipoprotein receptor-related protein 1 (LRP-1) (Alpha-2-macroglobulin receptor) (A2MR) (Apolipoprotein E receptor) (APOER) (CD antigen CD91) [Cleaved into: Low-density lipoprotein receptor-related protein 1 85 kDa subunit (LRP-85); Low-density lipoprotein receptor-related protein 1 515 kDa subunit (LRP-515); Low-density lipoprotein receptor-related protein 1 intracellular domain (LRPICD)]	Q07954 (LRP1)	-0.257	-0.187	-3.791	0.0026	0.103
P28907	ENSG00000004468	CD38	ADP-ribosyl cyclase/cyclic ADP-ribose hydrolase 1 (EC 3.2.2.6) (2'-phospho-ADP-ribosyl cyclase) (2'-phospho-ADP-ribosyl cyclase/2'-phospho-cyclic-ADP-ribose transferase) (EC 2.4.99.20) (2'-phospho-cyclic-ADP-ribose transferase) (ADP-ribosyl cyclase 1) (ADPRC 1) (Cyclic ADP-ribose hydrolase 1) (cADPr hydrolase 1) (T10) (CD antigen CD38)	P28907 (CD38)	0.252	0.113	4.506	0.0007	0.0643
P13747	ENSG00000204592; ENSG00000206493; ENSG00000229252; ENSG00000233904; ENSG00000236632; ENSG00000230254; ENSG00000225201	HLA-E	HLA class I histocompatibility antigen, alpha chain E (MHC class I antigen E) [Cleaved into: Soluble HLA class I histocompatibility antigen, alpha chain E (sHLA-E)]	P30501 (NA)	0.252	0.057	3.728	0.0029	0.1089
P20592	ENSG00000183486	MX2	Interferon-induced GTP-binding protein Mx2 (Interferon-regulated resistance GTP-binding protein MxB) (Myxovirus resistance protein 2) (p78-related protein)	P20592 (MX2)	0.252	-0.016	3.542	0.0041	0.1305
P16284	ENSG00000261371	PECAM1	Platelet endothelial cell adhesion molecule (PECAM-1) (EndoCAM) (GPIIA') (PECA1) (CD antigen CD31)	P16284 (PECAM1)	-0.240	-0.230	-4.240	0.0012	0.0716
Q9Y3Y2	ENSG00000160679	CHTOP	Chromatin target of PRMT1 protein (Friend of PRMT1 protein) (Small arginine- and glycine-rich protein) (SRAG)	Q9Y3Y2 (CHTOP)	0.238	0.044	3.521	0.0043	0.1325
Q03169	ENSG00000185215	TNFAIP2	Tumor necrosis factor alpha-induced protein 2 (TNF alpha-induced protein 2) (Primary response gene B94 protein)	Q03169 (TNFAIP2)	0.238	-0.012	3.888	0.0022	0.0959

Table S62: Proteins differentially abundant at Day 30 compared to pre-second vaccination (Study Arm 2 (A/H7N9 + AS03 D1, 29), Monocytes). Proteins are sorted by descending absolute log_2 fold change. Protein and gene annotations are based on Ensembl Version 101 (continued).

Protein ID	Ensembl Gene ID	Ensembl Gene Name	Protein Description	50% Protein Cluster ID (Gene Name)	Fold Change (Day 30 vs. Pre- Second Vaccination)	Average log_2 Abundance	Moderated t-Statistic	P-Value	FDR Adjusted P-value
Q9Y5A7	ENSG00000013374	NUB1	NEDD8 ultimate buster 1 (Negative regulator of ubiquitin-like proteins 1) (Renal carcinoma antigen NY-REN-18)	Q9Y5A7 (NUB1)	0.237	0.132	6.408	<0.0001	0.0122
Q9Y385	ENSG00000198833	UBE2J1	Ubiquitin-conjugating enzyme E2 J1 (EC 2.3.2.23) (E2 ubiquitin-conjugating enzyme J1) (Non-canonical ubiquitin-conjugating enzyme 1) (NCUBE-1) (Yeast ubiquitin-conjugating enzyme UBC6 homolog E) (HsUBC6e)	Q9Y385 (UBE2J1)	0.232	0.044	6.509	<0.0001	0.0122
Q8IZH2	ENSG00000114127	XRN1	5'-3' exoribonuclease 1 (EC 3.1.13.-) (Strand-exchange protein 1 homolog)	Q8IZH2 (XRN1)	0.231	0.051	3.422	0.0051	0.1464
O15117	ENSG00000082074	FYB1	FYN-binding protein 1 (Adhesion and degranulation promoting adaptor protein) (ADAP) (FYB-120/130) (p120/p130) (FYN-T-binding protein) (SLAP-130) (SLP-76-associated phosphoprotein)	O15117 (FYB1)	0.231	0.048	4.955	0.0003	0.0453
O60674	ENSG00000096968	JAK2	Tyrosine-protein kinase JAK2 (EC 2.7.10.2) (Janus kinase 2) (JAK-2)	O60674 (JAK2)	0.230	0.085	4.280	0.0011	0.0716
P07339	ENSG00000117984	CTSD	Cathepsin D (EC 3.4.23.5) [Cleaved into: Cathepsin D light chain; Cathepsin D heavy chain]	P07339 (CTSD)	-0.229	-0.216	-3.824	0.0024	0.1023
P08670	ENSG00000026025	VIM	Vimentin	P08670 (VIM)	0.221	-0.001	3.939	0.002	0.0921
P50452	ENSG00000166401	SERPINB8	Serpin B8 (Cytoplasmic antiproteinase 2) (CAP-2) (CAP2) (Peptidase inhibitor 8) (PI-8)	P30740 (SERPINB1)	-0.220	-0.272	-4.537	0.0007	0.0643
P11169	ENSG00000059804	SLC2A3	Solute carrier family 2, facilitated glucose transporter member 3 (Glucose transporter type 3, brain) (GLUT-3)	P11169 (SLC2A3)	0.218	-0.084	4.066	0.0016	0.0819
Q9UN86	ENSG00000138757	G3BP2	Ras GTPase-activating protein-binding protein 2 (G3BP-2) (GAP SH3 domain-binding protein 2)	Q9UN86 (G3BP2)	0.212	-0.015	4.969	0.0003	0.0453
P62937	ENSG00000196262	PPIA	Peptidyl-prolyl cis-trans isomerase A (PPIase A) (EC 5.2.1.8) (Cyclophilin A) (Cyclosporin A-binding protein) (Rotamase A) [Cleaved into: Peptidyl-prolyl cis-trans isomerase A, N-terminally processed]	Q9UNP9 (PPIE)	-0.211	-0.057	-4.059	0.0016	0.0819
Q8IV50	ENSG00000140280	LYSMD2	LysM and putative peptidoglycan-binding domain-containing protein 2	Q8IV50 (LYSMD2)	0.209	0.133	4.536	0.0007	0.0643
P08174	ENSG00000196352	CD55	Complement decay-accelerating factor (CD antigen CD55)	P08174 (CD55)	0.206	-0.084	3.781	0.0026	0.103

Table S62: Proteins differentially abundant at Day 30 compared to pre-second vaccination (Study Arm 2 (A/H7N9 + AS03 D1, 29), Monocytes). Proteins are sorted by descending absolute log_2 fold change. Protein and gene annotations are based on Ensembl Version 101 (continued).

Protein ID	Ensembl Gene ID	Ensembl Gene Name	Protein Description	50% Protein Cluster ID (Gene Name)	Fold Change (Day 30 vs. Pre- Second Vaccination)	Average log_2 Abundance	Moderated t-Statistic	P-Value	FDR Adjusted P-value
Q8N0X7	ENSG00000133104	SPART	Spartin (Spastic paraplegia 20 protein) (Trans-activated by hepatitis C virus core protein 1)	Q8N0X7 (SPART)	0.206	-0.092	4.117	0.0014	0.0797
Q9UL46	ENSG00000100911; ENSG00000284889	PSME2	Proteasome activator complex subunit 2 (11S regulator complex subunit beta) (REG-beta) (Activator of multi-catalytic protease subunit 2) (Proteasome activator 28 subunit beta) (PA28b) (PA28beta)	Q06323 (PSME1)	0.206	0.083	4.866	0.0004	0.0504
P62854	ENSG00000197728	RPS26	40S ribosomal protein S26 (Small ribosomal subunit protein eS26)	P62854 (RPS26)	-0.202	-0.179	-4.335	0.001	0.0716
O15162	ENSG00000188313	PLSCR1	Phospholipid scramblase 1 (PL scramblase 1) (Ca(2+)-dependent phospholipid scramblase 1) (Erythrocyte phospholipid scramblase) (Mg(2+)-dependent nuclease) (EC 3.1.-.-) (MmTRA1b)	O15162 (PLSCR1)	0.200	0.099	4.008	0.0018	0.0878
P17931	ENSG00000131981	LGALS3	Galectin-3 (Gal-3) (35 kDa lectin) (Carbohydrate-binding protein 35) (CBP 35) (Galactose-specific lectin 3) (Galactoside-binding protein) (GALBP) (IgE-binding protein) (L-31) (Laminin-binding protein) (Lectin L-29) (Mac-2 antigen)	P17931 (LGALS3)	-0.199	-0.072	-4.071	0.0016	0.0819
Q16831	ENSG00000183696	UPP1	Uridine phosphorylase 1 (UPase 1) (UrdPase 1) (EC 2.4.2.3)	Q16831 (UPP1)	0.199	-0.029	3.846	0.0024	0.0997
P11717	ENSG00000197081	IGF2R	Cation-independent mannose-6-phosphate receptor (CI Man-6-P receptor) (CI-MPR) (M6PR) (300 kDa mannose 6-phosphate receptor) (MPR 300) (Insulin-like growth factor 2 receptor) (Insulin-like growth factor II receptor) (IGF-II receptor) (M6P/IGF2 receptor) (M6P/IGF2R) (CD antigen CD222)	P11717 (IGF2R)	0.196	0.005	4.806	0.0004	0.0505
Q9NTJ3	ENSG00000113810	SMC4	Structural maintenance of chromosomes protein 4 (SMC protein 4) (SMC-4) (Chromosome-associated polypeptide C) (hCAP-C) (XCAP-C homolog)	Q9NTJ3 (SMC4)	0.196	-0.038	4.309	0.001	0.0716
Q9BWM7	ENSG00000107819	SFXN3	Sideroflexin-3	Q9H9B4 (SFXN1)	-0.191	-0.117	-3.434	0.005	0.1458
Q9NQC3	ENSG00000115310	RTN4	Reticulon-4 (Foocen) (Neurite outgrowth inhibitor) (Nogo protein) (Neuroendocrine-specific protein) (NSP) (Neuroendocrine-specific protein C homolog) (RTN-x) (Reticulon-5)	Q9NQC3 (RTN4)	0.191	-0.003	3.476	0.0046	0.1372

Table S62: Proteins differentially abundant at Day 30 compared to pre-second vaccination (Study Arm 2 (A/H7N9 + AS03 D1, 29), Monocytes). Proteins are sorted by descending absolute log_2 fold change. Protein and gene annotations are based on Ensembl Version 101 (continued).

Protein ID	Ensembl Gene ID	Ensembl Gene Name	Protein Description	50% Protein Cluster ID (Gene Name)	Fold Change (Day 30 vs. Pre- Second Vaccination)	Average log_2 Abundance	Moderated t-Statistic	P-Value	FDR Adjusted P-value
P60891	ENSG00000147224	PRPS1	Ribose-phosphate pyrophosphokinase 1 (EC 2.7.6.1) (PPRibP) (Phosphoribosyl pyrophosphate synthase I) (PRS-I)	P60891 (PRPS1)	-0.188	-0.112	-3.405	0.0053	0.1488
Q99952	ENSG00000072135	PTPN18	Tyrosine-protein phosphatase non-receptor type 18 (EC 3.1.3.48) (Brain-derived phosphatase)	Q99952 (PTPN18)	0.188	0.019	3.970	0.0019	0.0908
Q04760	ENSG00000124767	GLO1	Lactoylglutathione lyase (EC 4.4.1.5) (Aldoketomutase) (Glyoxalase I) (Glx I) (Ketone-aldehyde mutase) (Methylglyoxalase) (S-D-lactoylglutathione methylglyoxal lyase)	Q04760 (GLO1)	-0.188	-0.056	-3.607	0.0036	0.1246
Q5T8D3	ENSG00000107897	ACBD5	Acyl-CoA-binding domain-containing protein 5	Q5T8D3 (ACBD5)	0.188	0.079	3.681	0.0032	0.1147
Q2M385	ENSG00000197629	MPEG1	Macrophage-expressed gene 1 protein (Macrophage gene 1 protein) (Mpg-1) (Perforin-2) (P-2)	Q2M385 (MPEG1)	-0.186	-0.279	-4.700	0.0005	0.0563
Q53EL6	ENSG00000150593	PDCD4	Programmed cell death protein 4 (Neoplastic transformation inhibitor protein) (Nuclear antigen H731-like) (Protein 197/15a)	Q53EL6 (PDCD4)	-0.185	-0.210	-4.399	0.0009	0.0678
O95976	ENSG00000140749	IGSF6	Immunoglobulin superfamily member 6 (IgSF6) (Protein DORA)	O95976 (IGSF6)	0.185	0.042	5.877	<0.0001	0.015
Q9H8H3	ENSG00000185432	METTL7A	Methyltransferase-like protein 7A (EC 2.1.1.-) (Protein AAM-B)	Q9H8H3 (METTL7A)	-0.173	-0.197	-3.813	0.0025	0.103
Q8IY21	ENSG00000137628	DDX60	Probable ATP-dependent RNA helicase DDX60 (EC 3.6.4.13) (DEAD box protein 60)	Q8IY21 (DDX60)	0.171	0.100	3.991	0.0018	0.0886
P04632	ENSG00000126247	CAPNS1	Calpain small subunit 1 (CSS1) (Calcium-activated neutral proteinase small subunit) (CANP small subunit) (Calcium-dependent protease small subunit) (CDPS) (Calcium-dependent protease small subunit 1) (Calpain regulatory subunit)	P04632 (CAPNS1)	-0.167	-0.261	-3.429	0.005	0.1458
P26447	ENSG00000196154	S100A4	Protein S100-A4 (Calvasculin) (Metastasin) (Placental calcium-binding protein) (Protein Mts1) (S100 calcium-binding protein A4)	P26447 (S100A4)	-0.166	-0.165	-3.697	0.0031	0.1128
P29966	ENSG00000277443	MARCKS	Myristoylated alanine-rich C-kinase substrate (MARCKS) (Protein kinase C substrate, 80 kDa protein, light chain) (80K-L protein) (PKCSL)	P29966 (MARCKS)	0.162	0.164	3.476	0.0046	0.1372
P01892				P30501 (NA)	0.159	0.112	3.793	0.0026	0.103

Table S62: Proteins differentially abundant at Day 30 compared to pre-second vaccination (Study Arm 2 (A/H7N9 + AS03 D1, 29), Monocytes). Proteins are sorted by descending absolute log_2 fold change. Protein and gene annotations are based on Ensembl Version 101 (continued).

Protein ID	Ensembl Gene ID	Ensembl Gene Name	Protein Description	50% Protein Cluster ID (Gene Name)	Fold Change (Day 30 vs. Pre- Second Vaccination)	Average log_2 Abundance	Moderated t-Statistic	P-Value	FDR Adjusted P-value
P19525	ENSG00000055332	EIF2AK2	Interferon-induced, double-stranded RNA-activated protein kinase (EC 2.7.11.1) (Eukaryotic translation initiation factor 2-alpha kinase 2) (eIF-2A protein kinase 2) (Interferon-inducible RNA-dependent protein kinase) (P1/eIF-2A protein kinase) (Protein kinase RNA-activated) (PKR) (Protein kinase R) (Tyrosine-protein kinase EIF2AK2) (EC 2.7.10.2) (p68 kinase)	P19525 (EIF2AK2)	0.157	0.052	4.359	0.0009	0.0712
P09874	ENSG00000143799	PARP1	Poly [ADP-ribose] polymerase 1 (PARP-1) (EC 2.4.2.30) (ADP-ribosyltransferase diphtheria toxin-like 1) (ARTD1) (DNA ADP-ribosyltransferase PARP1) (EC 2.4.2.-) (NAD(+) ADP-ribosyltransferase 1) (ADPRT 1) (Poly[ADP-ribose] synthase 1) (Protein poly-ADP-ribosyltransferase PARP1) (EC 2.4.2.-)	P09874 (PARP1)	-0.157	-0.240	-3.944	0.002	0.0921
O60264	ENSG00000153147	SMARCA5	SWI/SNF-related matrix-associated actin-dependent regulator of chromatin subfamily A member 5 (SWI/SNF-related matrix-associated actin-dependent regulator of chromatin A5) (EC 3.6.4.-) (Sucrose non-fermenting protein 2 homolog) (hSNF2H)	O60264 (SMARCA5)	0.156	-0.033	3.506	0.0044	0.1332
P10301	ENSG00000126458	RRAS	Ras-related protein R-Ras (EC 3.6.5.-) (p23)	P10301 (RRAS)	-0.155	0.013	-3.564	0.0039	0.1276
O75022	ENSG00000204577; ENSG00000274587; ENSG00000275019	LILRB3	Leukocyte immunoglobulin-like receptor subfamily B member 3 (LIR-3) (Leukocyte immunoglobulin-like receptor 3) (CD85 antigen-like family member A) (Immunoglobulin-like transcript 5) (ILT-5) (Monocyte inhibitory receptor HL9) (CD antigen CD85a)	Q8NHL6 (LILRB1)	0.155	-0.013	3.596	0.0037	0.1246
Q8N386	ENSG00000175489	LRRC25	Leucine-rich repeat-containing protein 25 (Monocyte and plasmacytoid-activated protein)	Q8N386 (LRRC25)	-0.153	-0.083	-3.480	0.0046	0.1372
Q16795	ENSG00000139180	NDUFA9	NADH dehydrogenase [ubiquinone] 1 alpha subcomplex subunit 9, mitochondrial (Complex I-39kD) (CI-39kD) (NADH-ubiquinone oxidoreductase 39 kDa subunit)	Q16795 (NDUFA9)	-0.151	-0.140	-3.878	0.0022	0.0964
P15121	ENSG00000085662	AKR1B1	Aldo-keto reductase family 1 member B1 (EC 1.1.1.300) (EC 1.1.1.372) (EC 1.1.1.54) (Aldehyde reductase) (Aldose reductase) (AR) (EC 1.1.1.21)	P14550 (AKR1A1)	-0.150	-0.242	-4.497	0.0007	0.0643

Table S62: Proteins differentially abundant at Day 30 compared to pre-second vaccination (Study Arm 2 (A/H7N9 + AS03 D1, 29), Monocytes). Proteins are sorted by descending absolute log_2 fold change. Protein and gene annotations are based on Ensembl Version 101 (continued).

Protein ID	Ensembl Gene ID	Ensembl Gene Name	Protein Description	50% Protein Cluster ID (Gene Name)	Fold Change (Day 30 vs. Pre- Second Vaccination)	Average log_2 Abundance	Moderated t-Statistic	P-Value	FDR Adjusted P-value
Q8NI60	ENSG00000163050	COQ8A	Atypical kinase COQ8A, mitochondrial (EC 2.7.-.-) (Chaperone activity of bc1 complex-like) (Chaperone-ABC1-like) (Coenzyme Q protein 8A) (aarF domain-containing protein kinase 3)	Q8NI60 (COQ8A)	-0.149	-0.131	-3.412	0.0052	0.1481
P42167	ENSG00000120802	TMPO	Lamina-associated polypeptide 2, isoforms beta/gamma (Thymopoietin, isoforms beta/gamma) (TP beta/gamma) (Thymopoietin-related peptide isoforms beta/gamma) (TPRP isoforms beta/gamma) [Cleaved into: Thymopoietin (TP) (Splenin); Thymopentin (TP5)]	P42166 (TMPO)	-0.145	-0.165	-3.900	0.0021	0.0959
Q53H12	ENSG00000006530; ENSG00000262327	AGK	Acylglycerol kinase, mitochondrial (hAGK) (EC 2.7.1.107) (EC 2.7.1.138) (EC 2.7.1.94) (Multiple substrate lipid kinase) (HsMuLK) (MuLK) (Multi-substrate lipid kinase)	Q53H12 (AGK)	-0.145	-0.176	-3.514	0.0043	0.1325
Q9Y2J2	ENSG00000082397	EPB41L3	Band 4.1-like protein 3 (4.1B) (Differentially expressed in adenocarcinoma of the lung protein 1) (DAL-1) (Erythrocyte membrane protein band 4.1-like 3) [Cleaved into: Band 4.1-like protein 3, N-terminally processed]	Q9Y2J2 (EPB41L3)	0.145	0.232	4.171	0.0013	0.0739
P83731	ENSG00000114391	RPL24	60S ribosomal protein L24 (60S ribosomal protein L30) (Large ribosomal subunit protein eL24)	P83731 (RPL24)	-0.142	-0.315	-4.827	0.0004	0.0504
Q08257	ENSG00000116791	CRYZ	Quinone oxidoreductase (EC 1.6.5.5) (NADPH:quinone reductase) (Zeta-crystallin)	Q08257 (CRYZ)	-0.140	-0.028	-3.644	0.0034	0.1217
Q07065	ENSG00000136026	CKAP4	Cytoskeleton-associated protein 4 (63-kDa cytoskeleton-linking membrane protein) (Climp-63) (p63)	Q07065 (CKAP4)	0.138	-0.043	3.893	0.0022	0.0959
Q02880	ENSG00000077097	TOP2B	DNA topoisomerase 2-beta (EC 5.6.2.2) (DNA topoisomerase II, beta isozyme)	Q02880 (TOP2B)	0.137	-0.167	3.518	0.0043	0.1325
Q13277	ENSG00000166900	STX3	Syntaxin-3	Q13277 (STX3)	0.137	0.068	3.955	0.0019	0.092
P01137		TGFB1	Transforming growth factor beta-1 proprotein [Cleaved into: Latency-associated peptide (LAP); Transforming growth factor beta-1 (TGF-beta-1)]	P01137 (TGFB1)	0.136	-0.050	4.226	0.0012	0.0716
P40222	ENSG00000084652	TXLNA	Alpha-taxilin	P40222 (TXLNA)	0.131	0.002	4.175	0.0013	0.0739

Table S62: Proteins differentially abundant at Day 30 compared to pre-second vaccination (Study Arm 2 (A/H7N9 + AS03 D1, 29), Monocytes). Proteins are sorted by descending absolute log_2 fold change. Protein and gene annotations are based on Ensembl Version 101 (continued).

Protein ID	Ensembl Gene ID	Ensembl Gene Name	Protein Description	50% Protein Cluster ID (Gene Name)	Fold Change (Day 30 vs. Pre- Second Vaccination)	Average log_2 Abundance	Moderated t-Statistic	P-Value	FDR Adjusted P-value
P12694	ENSG00000248098	BCKDHA	2-oxoisovalerate dehydrogenase subunit alpha, mitochondrial (EC 1.2.4.4) (Branched-chain alpha-keto acid dehydrogenase E1 component alpha chain) (BCKDE1A) (BCKDH E1-alpha)	P12694 (BCKDHA)	-0.130	-0.111	-3.619	0.0036	0.1246
Q99523	ENSG00000134243	SORT1	Sortilin (100 kDa NT receptor) (Glycoprotein 95) (Gp95) (Neurotensin receptor 3) (NT3) (NTR3)	Q99523 (SORT1)	0.129	0.063	3.607	0.0036	0.1246
O75746	ENSG00000115840	SLC25A12	Calcium-binding mitochondrial carrier protein Aralar1 (Mitochondrial aspartate glutamate carrier 1) (Solute carrier family 25 member 12)	O75746 (SLC25A12)	-0.128	-0.131	-4.224	0.0012	0.0716
O75323	ENSG00000146729	NIPSNAP2	Protein NipSnap homolog 2 (NipSnap2) (Glioblastoma-amplified sequence)	O75323 (NIPSNAP2)	-0.128	-0.172	-3.783	0.0026	0.103
Q6ZS81	ENSG00000128815	WDFY4	WD repeat- and FYVE domain-containing protein 4	Q6ZS81 (WDFY4)	-0.121	-0.074	-3.587	0.0038	0.1246
P23743	ENSG00000065357	DGKA	Diacylglycerol kinase alpha (DAG kinase alpha) (EC 2.7.1.107) (80 kDa diacylglycerol kinase) (Diglyceride kinase alpha) (DGK-alpha)	P49619 (DGKG)	-0.120	-0.191	-3.746	0.0028	0.1076
Q13123	ENSG00000113141	IK	Protein Red (Cytokine IK) (IK factor) (Protein RER)	Q13123 (IK)	0.116	-0.078	3.707	0.003	0.1118
O75306	ENSG00000158864	NDUFS2	NADH dehydrogenase [ubiquinone] iron-sulfur protein 2, mitochondrial (EC 7.1.1.2) (Complex I-49kD) (CI-49kD) (NADH-ubiquinone oxidoreductase 49 kDa subunit)	O75306 (NDUFS2)	-0.110	-0.132	-3.863	0.0023	0.0978
P55265	ENSG00000160710	ADAR	Double-stranded RNA-specific adenosine deaminase (DRADA) (EC 3.5.4.37) (136 kDa double-stranded RNA-binding protein) (p136) (Interferon-inducible protein 4) (IFI-4) (K88DSRBP)	P55265 (ADAR)	0.105	-0.024	3.929	0.002	0.0925

Table S63: Proteins differentially abundant at Day 30 compared to pre-second vaccination (Study Arm 2 (A/H7N9 + AS03 D1, 29) versus Study Arm 3 (A/H7N9 + PBS, D1, 29), Monocytes). Proteins are sorted by descending absolute log_2 fold change. Protein and gene annotations are based on Ensembl Version 101.

Protein ID	Ensembl Gene ID	Ensembl Gene Name	Protein Description	50% Protein Cluster ID (Gene Name)	Fold Change (Day 30 vs. Pre- Second Vaccination)	Average log_2 Abundance	Moderated t-Statistic	P-Value	FDR Adjusted P-value
Q96PP8	ENSG00000154451	GBP5	Guanylate-binding protein 5 (EC 3.6.5.-) (GBP-TA antigen) (GTP-binding protein 5) (GBP-5) (Guanine nucleotide-binding protein 5)	Q96PP9 (GBP4)	2.313	0.390	7.485	<0.0001	0.0003
P32455	ENSG00000117228	GBP1	Guanylate-binding protein 1 (EC 3.6.5.-) (GTP-binding protein 1) (GBP-1) (HuGBP-1) (Guanine nucleotide-binding protein 1) (Interferon-induced guanylate-binding protein 1)	Q96PP9 (GBP4)	1.775	0.302	4.184	0.0004	0.0586
Q5VYY1	ENSG00000152766	ANKRD22	Ankyrin repeat domain-containing protein 22	Q5VYY1 (ANKRD22)	1.319	0.328	4.345	0.0003	0.0529
P32456	ENSG00000162645	GBP2	Guanylate-binding protein 2 (EC 3.6.5.-) (GTP-binding protein 2) (GBP-2) (HuGBP-2) (Guanine nucleotide-binding protein 2) (Interferon-induced guanylate-binding protein 2)	Q96PP9 (GBP4)	1.240	0.250	5.236	<0.0001	0.0123
P23381	ENSG00000140105	WARS1	Tryptophan–tRNA ligase, cytoplasmic (EC 6.1.1.2) (Interferon-induced protein 53) (IFP53) (Tryptophanyl-tRNA synthetase) (TrpRS) (hWRS) [Cleaved into: T1-TrpRS; T2-TrpRS]	P23381 (WARS1)	1.096	0.072	3.570	0.0018	0.1204
O95236	ENSG00000128284	APOL3	Apolipoprotein L3 (Apolipoprotein L-III) (ApoL-III) (TNF-inducible protein CG12-1) ($CG12_1$)	O95236 (APOL3)	0.985	0.159	6.290	<0.0001	0.0023
Q9BQE5	ENSG00000128335	APOL2	Apolipoprotein L2 (Apolipoprotein L-II) (ApoL-II)	O14791 (APOL1)	0.910	0.150	4.446	0.0002	0.0466
P28838	ENSG00000002549	LAP3	Cytosol aminopeptidase (EC 3.4.11.1) (Cysteinylglycine-S-conjugate dipeptidase) (EC 3.4.13.23) (Leucine aminopeptidase 3) (LAP-3) (Leucyl aminopeptidase) (Peptidase S) (Proline aminopeptidase) (EC 3.4.11.5) (Prolyl aminopeptidase)	P28838 (LAP3)	0.875	0.165	3.812	0.001	0.0994
P12314	ENSG00000150337	FCGR1A	High affinity immunoglobulin gamma Fc receptor I (IgG Fc receptor I) (Fc-gamma RI) (FcRI) (Fc-gamma RIA) (FcgammaRIa) (CD antigen CD64)	P12314 (FCGR1A)	0.829	0.162	8.352	<0.0001	0.0002
O14879	ENSG00000119917	IFIT3	Interferon-induced protein with tetratricopeptide repeats 3 (IFIT-3) (CIG49) (ISG-60) (Interferon-induced 60 kDa protein) (IFI-60K) (Interferon-induced protein with tetratricopeptide repeats 4) (IFIT-4) (Retinoic acid-induced gene G protein) (P60) (RIG-G)	O14879 (IFIT3)	0.793	0.216	3.813	0.001	0.0994

Table S63: Proteins differentially abundant at Day 30 compared to pre-second vaccination (Study Arm 2 (A/H7N9 + AS03 D1, 29) versus Study Arm 3 (A/H7N9 + PBS, D1, 29), Monocytes). Proteins are sorted by descending absolute log_2 fold change. Protein and gene annotations are based on Ensembl Version 101 (continued).

Protein ID	Ensembl Gene ID	Ensembl Gene Name	Protein Description	50% Protein Cluster ID (Gene Name)	Fold Change (Day 30 vs. Pre- Second Vaccination)	Average log_2 Abundance	Moderated t-Statistic	P-Value	FDR Adjusted P-value
P42224	ENSG00000115415	STAT1	Signal transducer and activator of transcription 1-alpha/beta (Transcription factor ISGF-3 components p91/p84)	P40763 (STAT3)	0.710	0.112	5.370	<0.0001	0.01
P01860		IGHG3	Immunoglobulin heavy constant gamma 3 (HDC) (Heavy chain disease protein) (Ig gamma-3 chain C region)	P0DOX5 (NA)	0.702	0.154	6.114	<0.0001	0.0027
P31941	ENSG00000128383; ENSG00000262156	APOBEC3A	DNA dC->dU-editing enzyme APOBEC-3A (A3A) (EC 3.5.4.38) (Phorbolin-1)	Q9HC16 (APOBEC3G)	0.686	0.187	3.312	0.0033	0.1452
P0DOX5				P0DOX5 (NA)	0.653	0.240	7.844	<0.0001	0.0002
P09913	ENSG00000119922	IFIT2	Interferon-induced protein with tetratricopeptide repeats 2 (IFIT-2) (ISG-54 K) (Interferon-induced 54 kDa protein) (IFI-54K) (P54)	O14879 (IFIT3)	0.621	0.235	4.698	0.0001	0.0334
P35237	ENSG00000124570	SERPINB6	Serpin B6 (Cytoplasmic antiproteinase) (CAP) (Peptidase inhibitor 6) (PI-6) (Placental thrombin inhibitor)	P30740 (SERPINB1)	-0.479	-0.224	-4.260	0.0004	0.0535
P20160	ENSG00000172232; ENSG00000278624	AZU1	Azurocidin (Cationic antimicrobial protein CAP37) (Heparin-binding protein) (HBP) (hHBP)	P20160 (AZU1)	0.425	-0.043	4.389	0.0003	0.0503
O00483	ENSG00000189043	NDUFA4	Cytochrome c oxidase subunit NDUFA4 (Complex I-MLRQ) (CI-MLRQ) (NADH-ubiquinone oxidoreductase MLRQ subunit)	O00483 (NDUFA4)	-0.423	-0.266	-5.428	<0.0001	0.0098
P15260	ENSG00000027697	IFNGR1	Interferon gamma receptor 1 (IFN-gamma receptor 1) (IFN-gamma-R1) (CDw119) (Interferon gamma receptor alpha-chain) (IFN-gamma-R-alpha) (CD antigen CD119)	P15260 (IFNGR1)	-0.419	-0.017	-3.823	0.001	0.0994
P01920	ENSG00000231286; ENSG00000233209; ENSG00000225824	HLA-DQB1	HLA class II histocompatibility antigen, DQ beta 1 chain (MHC class II antigen DQB1)	P13765 (HLA-DOB)	0.416	0.354	3.537	0.002	0.1204
Q9NZM1	ENSG00000138119	MYOF	Myoferlin (Fer-1-like protein 3)	O75923 (DYSF)	0.412	-0.068	3.364	0.003	0.144
Q13616	ENSG00000055130	CUL1	Cullin-1 (CUL-1)	Q13616 (CUL1)	0.406	-0.067	4.164	0.0004	0.0586
P30456				P30501 (NA)	0.403	-0.179	4.616	0.0002	0.0377
Q460N5	ENSG00000173193	PARP14	Protein mono-ADP-ribosyltransferase PARP14 (EC 2.4.2.-) (ADP-ribosyltransferase diphtheria toxin-like 8) (ARTD8) (B aggressive lymphoma protein 2) (Poly [ADP-ribose] polymerase 14) (PARP-14)	Q460N5 (PARP14)	0.399	-0.020	6.269	<0.0001	0.0023

Table S63: Proteins differentially abundant at Day 30 compared to pre-second vaccination (Study Arm 2 (A/H7N9 + AS03 D1, 29) versus Study Arm 3 (A/H7N9 + PBS, D1, 29), Monocytes). Proteins are sorted by descending absolute log_2 fold change. Protein and gene annotations are based on Ensembl Version 101 (continued).

Protein ID	Ensembl Gene ID	Ensembl Gene Name	Protein Description	50% Protein Cluster ID (Gene Name)	Fold Change (Day 30 vs. Pre- Second Vaccination)	Average log_2 Abundance	Moderated t-Statistic	P-Value	FDR Adjusted P-value
P33121	ENSG00000151726	ACSL1	Long-chain-fatty-acid–CoA ligase 1 (EC 6.2.1.3) (Acyl-CoA synthetase 1) (ACS1) (Arachidonate–CoA ligase) (EC 6.2.1.15) (Long-chain acyl-CoA synthetase 1) (LACS 1) (Long-chain acyl-CoA synthetase 2) (LACS 2) (Long-chain fatty acid-CoA ligase 2) (Palmitoyl-CoA ligase 1) (Palmitoyl-CoA ligase 2) (Phytanate–CoA ligase) (EC 6.2.1.24)	P33121 (ACSL1)	0.389	-0.028	3.473	0.0023	0.1308
O14545	ENSG00000135148	TRAFD1	TRAF-type zinc finger domain-containing protein 1 (Protein FLN29)	O14545 (TRAFD1)	0.389	0.026	3.716	0.0013	0.1074
Q96PZ2	ENSG00000166801	FAM111A	Serine protease FAM111A (EC 3.4.21.-)	Q96PZ2 (FAM111A)	0.387	-0.185	3.460	0.0024	0.1325
Q8IYM9	ENSG00000132274	TRIM22	E3 ubiquitin-protein ligase TRIM22 (EC 2.3.2.27) (50 kDa-stimulated trans-acting factor) (RING finger protein 94) (RING-type E3 ubiquitin transferase TRIM22) (Staf-50) (Tripartite motif-containing protein 22)	Q8IYM9 (TRIM22)	0.378	-0.093	3.745	0.0012	0.1074
Q8TDB6	ENSG00000163840	DTX3L	E3 ubiquitin-protein ligase DTX3L (EC 2.3.2.27) (B-lymphoma- and BAL-associated protein) (Protein deltex-3-like) (RING-type E3 ubiquitin transferase DTX3L) (Rhysin-2) (Rhysin2)	Q8TDB6 (DTX3L)	0.375	0.029	4.560	0.0002	0.0401
P01911	ENSG00000196126	HLA-DRB1	HLA class II histocompatibility antigen, DRB1 beta chain (Human leukocyte antigen DRB1) (HLA-DRB1)	P13765 (HLA-DOB)	0.360	0.146	3.773	0.0011	0.1032
P12429	ENSG00000138772	ANXA3	Annexin A3 (35-alpha calcimedin) (Annexin III) (Annexin-3) (Inositol 1,2-cyclic phosphate 2-phosphohydrolase) (Lipocortin III) (Placental anticoagulant protein III) (PAP-III)	P08133 (ANXA6)	0.351	-0.085	3.938	0.0008	0.0915
P84101	ENSG00000140264	SERF2	Small EDRK-rich factor 2 (Gastric cancer-related protein VRG107) (Protein 4F5-related) (4F5rel) (h4F5rel)	P84101 (SERF2)	0.349	-0.066	3.830	0.001	0.0994
P52630	ENSG00000170581	STAT2	Signal transducer and activator of transcription 2 (p113)	P52630 (STAT2)	0.348	0.073	4.315	0.0003	0.053
Q9HD89	ENSG00000104918	RETN	Resistin (Adipose tissue-specific secretory factor) (ADSF) (C/EBP-epsilon-regulated myeloid-specific secreted cysteine-rich protein) (Cysteine-rich secreted protein A12-alpha-like 2) (Cysteine-rich secreted protein FIZZ3)	Q9HD89 (RETN)	0.342	-0.114	3.594	0.0017	0.1204

Table S63: Proteins differentially abundant at Day 30 compared to pre-second vaccination (Study Arm 2 (A/H7N9 + AS03 D1, 29) versus Study Arm 3 (A/H7N9 + PBS, D1, 29), Monocytes). Proteins are sorted by descending absolute log_2 fold change. Protein and gene annotations are based on Ensembl Version 101 (continued).

Protein ID	Ensembl Gene ID	Ensembl Gene Name	Protein Description	50% Protein Cluster ID (Gene Name)	Fold Change (Day 30 vs. Pre- Second Vaccination)	Average log_2 Abundance	Moderated t-Statistic	P-Value	FDR Adjusted P-value
P08311	ENSG00000100448	CTSG	Cathepsin G (CG) (EC 3.4.21.20)	P08311 (CTSG)	0.337	-0.092	4.302	0.0003	0.053
P32189	ENSG00000198814	GK	Glycerol kinase (GK) (Glycerokinase) (EC 2.7.1.30) (ATP:glycerol 3-phosphotransferase)	P32189 (GK)	0.333	-0.003	5.152	<0.0001	0.0135
P62937	ENSG00000196262	PPIA	Peptidyl-prolyl cis-trans isomerase A (PPIase A) (EC 5.2.1.8) (Cyclophilin A) (Cyclosporin A-binding protein) (Rotamase A) [Cleaved into: Peptidyl-prolyl cis-trans isomerase A, N-terminally processed]	Q9UNP9 (PPIE)	-0.323	-0.034	-4.155	0.0005	0.0586
Q9Y5A7	ENSG00000013374	NUB1	NEDD8 ultimate buster 1 (Negative regulator of ubiquitin-like proteins 1) (Renal carcinoma antigen NY-REN-18)	Q9Y5A7 (NUB1)	0.319	0.078	5.093	<0.0001	0.0143
P00973	ENSG00000089127	OAS1	2'-5'-oligoadenylate synthase 1 ((2-5')oligo(A) synthase 1) (2-5A synthase 1) (EC 2.7.7.84) (E18/E16) (p46/p42 OAS)	Q9Y6K5 (OAS3)	0.318	-0.257	3.634	0.0016	0.1138
Q8IV50	ENSG00000140280	LYSMD2	LysM and putative peptidoglycan-binding domain-containing protein 2	Q8IV50 (LYSMD2)	0.313	0.039	4.193	0.0004	0.0586
O15117	ENSG00000082074	FYB1	FYN-binding protein 1 (Adhesion and degranulation promoting adaptor protein) (ADAP) (FYB-120/130) (p120/p130) (FYN-T-binding protein) (SLAP-130) (SLP-76-associated phosphoprotein)	O15117 (FYB1)	0.311	0.037	3.671	0.0014	0.111
Q9Y385	ENSG00000198833	UBE2J1	Ubiquitin-conjugating enzyme E2 J1 (EC 2.3.2.23) (E2 ubiquitin-conjugating enzyme J1) (Non-canonical ubiquitin-conjugating enzyme 1) (NCUBE-1) (Yeast ubiquitin-conjugating enzyme UBC6 homolog E) (HsUBC6e)	Q9Y385 (UBE2J1)	0.311	-0.026	5.610	<0.0001	0.0074
Q9H8H3	ENSG00000185432	METTL7A	Methyltransferase-like protein 7A (EC 2.1.1.-) (Protein AAM-B)	Q9H8H3 (METTL7A)	-0.298	-0.182	-3.392	0.0028	0.1398
P30273	ENSG00000158869	FCER1G	High affinity immunoglobulin epsilon receptor subunit gamma (Fc receptor gamma-chain) (FcRgamma) (Fc-epsilon RI-gamma) (IgE Fc receptor subunit gamma) (FceRI gamma)	P30273 (FCER1G)	0.294	-0.183	3.415	0.0026	0.1383

Table S63: Proteins differentially abundant at Day 30 compared to pre-second vaccination (Study Arm 2 (A/H7N9 + AS03 D1, 29) versus Study Arm 3 (A/H7N9 + PBS, D1, 29), Monocytes). Proteins are sorted by descending absolute log_2 fold change. Protein and gene annotations are based on Ensembl Version 101 (continued).

Protein ID	Ensembl Gene ID	Ensembl Gene Name	Protein Description	50% Protein Cluster ID (Gene Name)	Fold Change (Day 30 vs. Pre- Second Vaccination)	Average log_2 Abundance	Moderated t-Statistic	P-Value	FDR Adjusted P-value
P13747	ENSG00000204592; ENSG00000206493; ENSG00000229252; ENSG00000233904; ENSG00000236632; ENSG00000230254; ENSG00000225201	HLA-E	HLA class I histocompatibility antigen, alpha chain E (MHC class I antigen E) [Cleaved into: Soluble HLA class I histocompatibility antigen, alpha chain E (sHLA-E)]	P30501 (NA)	0.289	-0.077	3.496	0.0022	0.128
P10321	ENSG00000204525	HLA-C	HLA class I histocompatibility antigen, C alpha chain (HLA-C) (HLA-Cw) (Human leukocyte antigen C)	P30501 (NA)	0.277	0.349	3.336	0.0032	0.1452
P26358	ENSG00000130816	DNMT1	DNA (cytosine-5)-methyltransferase 1 (Dnmt1) (EC 2.1.1.37) (CXXC-type zinc finger protein 9) (DNA methyltransferase HsaI) (DNA MTase HsaI) (M.HsaI) (MCMT)	P26358 (DNMT1)	0.277	-0.035	3.498	0.0022	0.128
Q96T58	ENSG00000065526	SPEN	Msx2-interacting protein (SMART/HDAC1-associated repressor protein) (SPEN homolog)	Q96T58 (SPEN)	0.277	-0.114	3.901	0.0008	0.0966
P28907	ENSG00000004468	CD38	ADP-ribosyl cyclase/cyclic ADP-ribose hydrolase 1 (EC 3.2.2.6) (2'-phospho-ADP-ribosyl cyclase) (2'-phospho-ADP-ribosyl cyclase/2'-phospho-cyclic-ADP-ribose transferase) (EC 2.4.99.20) (2'-phospho-cyclic-ADP-ribose transferase) (ADP-ribosyl cyclase 1) (ADPRC 1) (Cyclic ADP-ribose hydrolase 1) (cADPr hydrolase 1) (T10) (CD antigen CD38)	P28907 (CD38)	0.266	0.014	3.700	0.0013	0.1084
Q9H307	ENSG00000100941	PNN	Pinin (140 kDa nuclear and cell adhesion-related phosphoprotein) (Desmosome-associated protein) (Domain-rich serine protein) (DRS protein) (DRSP) (Melanoma metastasis clone A protein) (Nuclear protein SDK3) (SR-like protein)	Q9H307 (PNN)	0.261	-0.109	3.446	0.0024	0.1347
P50452	ENSG00000166401	SERPINB8	Serpin B8 (Cytoplasmic antiproteinase 2) (CAP-2) (CAP2) (Peptidase inhibitor 8) (PI-8)	P30740 (SERPINB1)	-0.261	-0.246	-3.883	0.0009	0.0974
P46776	ENSG00000166441	RPL27A	60S ribosomal protein L27a (Large ribosomal subunit protein uL15)	P46776 (RPL27A)	-0.254	-0.204	-3.571	0.0018	0.1204
O60674	ENSG00000096968	JAK2	Tyrosine-protein kinase JAK2 (EC 2.7.10.2) (Janus kinase 2) (JAK-2)	O60674 (JAK2)	0.252	-0.002	3.537	0.002	0.1204
P49662	ENSG00000196954	CASP4	Caspase-4 (CASP-4) (EC 3.4.22.57) (ICE and Ced-3 homolog 2) (ICH-2) (ICE(rel)-II) (Mih1) (Protease TX) [Cleaved into: Caspase-4 subunit p10; Caspase-4 subunit p20]	P51878 (CASP5)	0.251	0.016	4.520	0.0002	0.0414

Table S63: Proteins differentially abundant at Day 30 compared to pre-second vaccination (Study Arm 2 (A/H7N9 + AS03 D1, 29) versus Study Arm 3 (A/H7N9 + PBS, D1, 29), Monocytes). Proteins are sorted by descending absolute log_2 fold change. Protein and gene annotations are based on Ensembl Version 101 (continued).

Protein ID	Ensembl Gene ID	Ensembl Gene Name	Protein Description	50% Protein Cluster ID (Gene Name)	Fold Change (Day 30 vs. Pre- Second Vaccination)	Average log_2 Abundance	Moderated t-Statistic	P-Value	FDR Adjusted P-value
P11169	ENSG00000059804	SLC2A3	Solute carrier family 2, facilitated glucose transporter member 3 (Glucose transporter type 3, brain) (GLUT-3)	P11169 (SLC2A3)	0.251	-0.074	3.635	0.0016	0.1138
Q99952	ENSG00000072135	PTPN18	Tyrosine-protein phosphatase non-receptor type 18 (EC 3.1.3.48) (Brain-derived phosphatase)	Q99952 (PTPN18)	0.244	-0.001	3.540	0.002	0.1204
Q9UPT8	ENSG00000130749	ZC3H4	Zinc finger CCCH domain-containing protein 4	Q9UPT8 (ZC3H4)	0.239	-0.003	3.484	0.0022	0.1294
P15121	ENSG00000085662	AKR1B1	Aldo-keto reductase family 1 member B1 (EC 1.1.1.300) (EC 1.1.1.372) (EC 1.1.1.54) (Aldehyde reductase) (Aldose reductase) (AR) (EC 1.1.1.21)	P14550 (AKR1A1)	-0.228	-0.173	-3.715	0.0013	0.1074
O95976	ENSG00000140749	IGSF6	Immunoglobulin superfamily member 6 (IgSF6) (Protein DORA)	O95976 (IGSF6)	0.228	-0.057	4.093	0.0005	0.0655
Q92625	ENSG00000064999	ANKS1A	Ankyrin repeat and SAM domain-containing protein 1A (Odin)	Q92625 (ANKS1A)	0.226	-0.016	3.320	0.0033	0.1452
P16284	ENSG00000261371	PECAM1	Platelet endothelial cell adhesion molecule (PECAM-1) (EndoCAM) (GPIIA') (PECA1) (CD antigen CD31)	P16284 (PECAM1)	-0.224	-0.203	-3.307	0.0034	0.1452
Q9BWM7	ENSG00000107819	SFXN3	Sideroflexin-3	Q9H9B4 (SFXN1)	-0.220	-0.053	-3.395	0.0028	0.1398
Q02818	ENSG00000104805	NUCB1	Nucleobindin-1 (CALNUC)	Q02818 (NUCB1)	0.219	-0.012	3.714	0.0013	0.1074
Q16795	ENSG00000139180	NDUFA9	NADH dehydrogenase [ubiquinone] 1 alpha subcomplex subunit 9, mitochondrial (Complex I-39kD) (CI-39kD) (NADH-ubiquinone oxidoreductase 39 kDa subunit)	Q16795 (NDUFA9)	-0.218	-0.136	-3.308	0.0034	0.1452
Q9BZE1	ENSG00000116221	MRPL37	39S ribosomal protein L37, mitochondrial (L37mt) (MRP-L37) (39S ribosomal protein L2, mitochondrial) (L2mt) (MRP-L2) (Mitochondrial large ribosomal subunit protein mL37)	Q9BZE1 (MRPL37)	-0.215	-0.136	-3.652	0.0015	0.1137
Q9UN86	ENSG00000138757	G3BP2	Ras GTPase-activating protein-binding protein 2 (G3BP-2) (GAP SH3 domain-binding protein 2)	Q9UN86 (G3BP2)	0.213	-0.103	3.314	0.0033	0.1452

Table S63: Proteins differentially abundant at Day 30 compared to pre-second vaccination (Study Arm 2 (A/H7N9 + AS03 D1, 29) versus Study Arm 3 (A/H7N9 + PBS, D1, 29), Monocytes). Proteins are sorted by descending absolute log_2 fold change. Protein and gene annotations are based on Ensembl Version 101 (continued).

Protein ID	Ensembl Gene ID	Ensembl Gene Name	Protein Description	50% Protein Cluster ID (Gene Name)	Fold Change (Day 30 vs. Pre- Second Vaccination)	Average log_2 Abundance	Moderated t-Statistic	P-Value	FDR Adjusted P-value
P11717	ENSG00000197081	IGF2R	Cation-independent mannose-6-phosphate receptor (CI Man-6-P receptor) (CI-MPR) (M6PR) (300 kDa mannose 6-phosphate receptor) (MPR 300) (Insulin-like growth factor 2 receptor) (Insulin-like growth factor II receptor) (IGF-II receptor) (M6P/IGF2 receptor) (M6P/IGF2R) (CD antigen CD222)	P11717 (IGF2R)	0.213	-0.059	4.262	0.0004	0.0535
Q53EL6	ENSG00000150593	PDCD4	Programmed cell death protein 4 (Neoplastic transformation inhibitor protein) (Nuclear antigen H731-like) (Protein 197/15a)	Q53EL6 (PDCD4)	-0.212	-0.208	-3.776	0.0011	0.1032
P04632	ENSG00000126247	CAPNS1	Calpain small subunit 1 (CSS1) (Calcium-activated neutral proteinase small subunit) (CANP small subunit) (Calcium-dependent protease small subunit) (CDPS) (Calcium-dependent protease small subunit 1) (Calpain regulatory subunit)	P04632 (CAPNS1)	-0.212	-0.198	-3.544	0.0019	0.1204
P22626	ENSG00000122566	HNRNPA2B1	Heterogeneous nuclear ribonucleoproteins A2/B1 (hnRNP A2/B1)	P51991 (HNRNPA3)	-0.205	-0.007	-3.312	0.0033	0.1452
Q9UL46	ENSG00000100911; ENSG00000284889	PSME2	Proteasome activator complex subunit 2 (11S regulator complex subunit beta) (REG-beta) (Activator of multi-catalytic protease subunit 2) (Proteasome activator 28 subunit beta) (PA28b) (PA28beta)	Q06323 (PSME1)	0.204	-0.054	3.854	0.0009	0.0994
P01892				P30501 (NA)	0.200	-0.010	3.430	0.0025	0.1362
Q9NR30	ENSG00000165732	DDX21	Nucleolar RNA helicase 2 (EC 3.6.4.13) (DEAD box protein 21) (Gu-alpha) (Nucleolar RNA helicase Gu) (Nucleolar RNA helicase II) (RH II/Gu)	Q9NR30 (DDX21)	0.197	-0.054	3.317	0.0033	0.1452
Q53H12	ENSG00000006530; ENSG00000262327	AGK	Acylglycerol kinase, mitochondrial (hAGK) (EC 2.7.1.107) (EC 2.7.1.138) (EC 2.7.1.94) (Multiple substrate lipid kinase) (HsMuLK) (MuLK) (Multi-substrate lipid kinase)	Q53H12 (AGK)	-0.181	-0.170	-3.361	0.003	0.144
Q9NTJ3	ENSG00000113810	SMC4	Structural maintenance of chromosomes protein 4 (SMC protein 4) (SMC-4) (Chromosome-associated polypeptide C) (hCAP-C) (XCAP-C homolog)	Q9NTJ3 (SMC4)	0.180	-0.087	3.366	0.0029	0.144
Q13277	ENSG00000166900	STX3	Syntaxin-3	Q13277 (STX3)	0.179	-0.029	3.691	0.0014	0.1084

Table S63: Proteins differentially abundant at Day 30 compared to pre-second vaccination (Study Arm 2 (A/H7N9 + AS03 D1, 29) versus Study Arm 3 (A/H7N9 + PBS, D1, 29), Monocytes). Proteins are sorted by descending absolute log_2 fold change. Protein and gene annotations are based on Ensembl Version 101 (continued).

Protein ID	Ensembl Gene ID	Ensembl Gene Name	Protein Description	50% Protein Cluster ID (Gene Name)	Fold Change (Day 30 vs. Pre- Second Vaccination)	Average log_2 Abundance	Moderated t-Statistic	P-Value	FDR Adjusted P-value
O00217	ENSG00000110717	NDUFS8	NADH dehydrogenase [ubiquinone] iron-sulfur protein 8, mitochondrial (EC 7.1.1.2) (Complex I-23kD) (CI-23kD) (NADH-ubiquinone oxidoreductase 23 kDa subunit) (TYKY subunit)	O00217 (NDUFS8)	-0.175	-0.160	-3.537	0.002	0.1204
Q13315	ENSG00000149311	ATM	Serine-protein kinase ATM (EC 2.7.11.1) (Ataxia telangiectasia mutated) (A-T mutated)	Q13315 (ATM)	-0.173	0.005	-3.428	0.0025	0.1362
Q9BPX5	ENSG00000136950	ARPC5L	Actin-related protein 2/3 complex subunit 5-like protein (Arp2/3 complex 16 kDa subunit 2) (ARC16-2)	Q9BPX5 (ARPC5L)	-0.160	-0.038	-3.392	0.0028	0.1398
Q13123	ENSG00000113141	IK	Protein Red (Cytokine IK) (IK factor) (Protein RER)	Q13123 (IK)	0.146	-0.161	3.332	0.0032	0.1452
P51659	ENSG00000133835	HSD17B4	Peroxisomal multifunctional enzyme type 2 (MFE-2) (17-beta-hydroxysteroid dehydrogenase 4) (17-beta-HSD 4) (D-bifunctional protein) (DBP) (Multifunctional protein 2) (MFP-2) (Short chain dehydrogenase/reductase family 8C member 1) [Cleaved into: (3R)-hydroxyacyl-CoA dehydrogenase (EC 1.1.1.n12); Enoyl-CoA hydratase 2 (EC 4.2.1.107) (EC 4.2.1.119) (3-alpha,7-alpha,12-alpha-trihydroxy-5-beta-cholest-24-enoyl-CoA hydratase)]	P51659 (HSD17B4)	-0.145	-0.094	-3.575	0.0018	0.1204

Table S64: Proteins differentially abundant at Day 32 compared to pre-second vaccination (Study Arm 2 (A/H7N9 + AS03 D1, 29) versus Study Arm 3 (A/H7N9 + PBS, D1, 29), Monocytes). Proteins are sorted by descending absolute log_2 fold change. Protein and gene annotations are based on Ensembl Version 101.

Protein ID	Ensembl Gene ID	Ensembl Gene Name	Protein Description	50% Protein Cluster ID (Gene Name)	Fold Change (Day 32 vs. Pre- Second Vaccination)	Average log_2 Abundance	Moderated t-Statistic	P-Value	FDR Adjusted P-value
O00483	ENSG00000189043	NDUFA4	Cytochrome c oxidase subunit NDUFA4 (Complex I-MLRQ) (CI-MLRQ) (NADH-ubiquinone oxidoreductase MLRQ subunit)	O00483 (NDUFA4)	-0.349	-0.209	-4.441	0.0003	0.1483
Q04760	ENSG00000124767	GLO1	Lactoylglutathione lyase (EC 4.4.1.5) (Aldoketomutase) (Glyoxalase I) (Glx I) (Ketone-aldehyde mutase) (Methylglyoxalase) (S-D-lactoylglutathione methylglyoxal lyase)	Q04760 (GLO1)	-0.330	-0.084	-4.680	0.0001	0.1483
P30456				P30501 (NA)	0.309	-0.187	4.562	0.0002	0.1483

Table S64: Proteins differentially abundant at Day 32 compared to pre-second vaccination (Study Arm 2 (A/H7N9 + AS03 D1, 29) versus Study Arm 3 (A/H7N9 + PBS, D1, 29), Monocytes). Proteins are sorted by descending absolute log_2 fold change. Protein and gene annotations are based on Ensembl Version 101 (continued).

Protein ID	Ensembl Gene ID	Ensembl Gene Name	Protein Description	50% Protein Cluster ID (Gene Name)	Fold Change (Day 32 vs. Pre- Second Vaccination)	Average log_2 Abundance	Moderated t-Statistic	P-Value	FDR Adjusted P-value
Q9P2X0	ENSG00000179085	DPM3	Dolichol-phosphate mannosyltransferase subunit 3 (Dolichol-phosphate mannose synthase subunit 3) (DPM synthase subunit 3) (Dolichyl-phosphate beta-D-mannosyltransferase subunit 3) (Mannose-P-dolichol synthase subunit 3) (MPD synthase subunit 3) (Prostin-1)	Q9P2X0 (DPM3)	0.260	-0.089	4.456	0.0002	0.1483
O43765	ENSG00000104969	SGTA	Small glutamine-rich tetratricopeptide repeat-containing protein alpha (Alpha-SGT) (Vpu-binding protein) (UBP)	O43765 (SGTA)	0.252	0.010	4.464	0.0002	0.1483
P17900	ENSG00000196743	GM2A	Ganglioside GM2 activator (Cerebroside sulfate activator protein) (GM2-AP) (Sphingolipid activator protein 3) (SAP-3) [Cleaved into: Ganglioside GM2 activator isoform short]	P17900 (GM2A)	-0.231	-0.298	-4.483	0.0002	0.1483

Table S65: Proteins differentially abundant at Day 4 compared to pre-first vaccination (Study Arm 3 (A/H7N9 + PBS, D1, 29), B cells). Proteins are sorted by descending absolute log_2 fold change. Protein and gene annotations are based on Ensembl Version 101.

Protein ID	Ensembl Gene ID	Ensembl Gene Name	Protein Description	50% Protein Cluster ID (Gene Name)	Fold Change (Day 4 vs. Pre- First Vaccination)	Average log_2 Abundance	Moderated t-Statistic	P-Value	FDR Adjusted P-value
P13647	ENSG00000186081	KRT5	Keratin, type II cytoskeletal 5 (58 kDa cytokeratin) (Cytokeratin-5) (CK-5) (Keratin-5) (K5) (Type-II keratin Kb5)	P04264 (KRT1)	-0.526	0.514	-6.647	<0.0001	0.0689

Table S66: Proteins differentially abundant at Day 29 compared to pre-first vaccination (Study Arm 2 (A/H7N9 + AS03 D1, 29) versus Study Arm 3 (A/H7N9 + PBS, D1, 29), B cells). Proteins are sorted by descending absolute log_2 fold change. Protein and gene annotations are based on Ensembl Version 101.

Protein ID	Ensembl Gene ID	Ensembl Gene Name	Protein Description	50% Protein Cluster ID (Gene Name)	Fold Change (Day 29 vs. Pre- First Vaccination)	Average log_2 Abundance	Moderated t-Statistic	P-Value	FDR Adjusted P-value
P35579	ENSG00000100345	MYH9	Myosin-9 (Cellular myosin heavy chain, type A) (Myosin heavy chain 9) (Myosin heavy chain, non-muscle IIa) (Non-muscle myosin heavy chain A) (NMMHC-A) (Non-muscle myosin heavy chain IIa) (NMMHC II-a) (NMMHC-IIA)	Q7Z406 (MYH14)	-0.476	2.825	-5.251	<0.0001	0.127

Table S67: Proteins differentially abundant at Day 30 compared to pre-second vaccination (Study Arm 2 (A/H7N9 + AS03 D1, 29), B cells). Proteins are sorted by descending absolute log_2 fold change. Protein and gene annotations are based on Ensembl Version 101.

Protein ID	Ensembl Gene ID	Ensembl Gene Name	Protein Description	50% Protein Cluster ID (Gene Name)	Fold Change (Day 30 vs. Pre- Second Vaccination)	Average log_2 Abundance	Moderated t-Statistic	P-Value	FDR Adjusted P-value
P08311	ENSG00000100448	CTSG	Cathepsin G (CG) (EC 3.4.21.20)	P08311 (CTSG)	0.679	2.641	7.188	<0.0001	0.0582

Table S68: Proteins differentially abundant at Day 4 compared to pre-first vaccination (Study Arm 2 (A/H7N9 + AS03 D1, 29), Natural Killer cells). Proteins are sorted by descending absolute log_2 fold change. Protein and gene annotations are based on Ensembl Version 101.

Protein ID	Ensembl Gene ID	Ensembl Gene Name	Protein Description	50% Protein Cluster ID (Gene Name)	Fold Change (Day 4 vs. Pre- First Vaccination)	Average log_2 Abundance	Moderated t-Statistic	P-Value	FDR Adjusted P-value
P32455	ENSG00000117228	GBP1	Guanylate-binding protein 1 (EC 3.6.5.-) (GTP-binding protein 1) (GBP-1) (HuGBP-1) (Guanine nucleotide-binding protein 1) (Interferon-induced guanylate-binding protein 1)	Q96PP9 (GBP4)	0.212	2.447	6.502	<0.0001	0.1494

Confidential/Proprietary Information

Table S69: Proteins differentially abundant at Day 30 compared to pre-second vaccination (Study Arm 2 (A/H7N9 + AS03 D1, 29), Natural Killer cells). Proteins are sorted by descending absolute log_2 fold change. Protein and gene annotations are based on Ensembl Version 101.

Protein ID	Ensembl Gene ID	Ensembl Gene Name	Protein Description	50% Protein Cluster ID (Gene Name)	Fold Change (Day 30 vs. Pre- Second Vaccination)	Average log_2 Abundance	Moderated t-Statistic	P-Value	FDR Adjusted P-value
P59665	ENSG00000206047; ENSG00000240247; ENSG00000284983; ENSG00000285176	DEFA1; DEFA1B	Neutrophil defensin 1 (Defensin, alpha 1) (HNP-1) (HP-1) (HP1) [Cleaved into: HP 1-56; Neutrophil defensin 2 (HNP-2) (HP-2) (HP2)]	P59665 (DEFA1; DEFA1B)	0.822	2.531	6.158	<0.0001	0.0957
P38159	ENSG00000147274	RBMX	RNA-binding motif protein, X chromosome (Glycoprotein p43) (Heterogeneous nuclear ribonucleoprotein G) (hnRNP G) [Cleaved into: RNA-binding motif protein, X chromosome, N-terminally processed]	P38159 (RBMX)	0.453	2.702	5.475	0.0002	0.1197
P42224	ENSG00000115415	STAT1	Signal transducer and activator of transcription 1-alpha/beta (Transcription factor ISGF-3 components p91/p84)	P40763 (STAT3)	0.406	2.739	4.874	0.0005	0.1197
P49863	ENSG00000113088	GZMK	Granzyme K (EC 3.4.21.-) (Fragmentin-3) (Granzyme-3) (NK-tryptase-2) (NK-Tryp-2)	P49863 (GZMK)	-0.366	2.690	-4.770	0.0006	0.1327
Q96SB3	ENSG00000108819	PPP1R9B	Neurabin-2 (Neurabin-II) (Protein phosphatase 1 regulatory subunit 9B) (Spinophilin)	Q96SB3 (PPP1R9B)	0.336	2.240	4.987	0.0004	0.1197
Q96PP8	ENSG00000154451	GBP5	Guanylate-binding protein 5 (EC 3.6.5.-) (GBP-TA antigen) (GTP-binding protein 5) (GBP-5) (Guanine nucleotide-binding protein 5)	Q96PP9 (GBP4)	0.334	2.443	5.512	0.0002	0.1197
P22626	ENSG00000122566	HNRNPA2B1	Heterogeneous nuclear ribonucleoproteins A2/B1 (hnRNP A2/B1)	P51991 (HNRNPA3)	0.320	2.657	7.495	<0.0001	0.0504
Q9NWH9	ENSG00000137776	SLTM	SAFB-like transcription modulator (Modulator of estrogen-induced transcription)	Q9NWH9 (SLTM)	0.318	2.602	5.257	0.0003	0.1197
O14979	ENSG00000152795	HNRNPDL	Heterogeneous nuclear ribonucleoprotein D-like (hnRNP D-like) (hnRNP DL) (AU-rich element RNA-binding factor) (JKT41-binding protein) (Protein laAUF1)	O14979 (HNRNPDL)	0.283	2.544	5.111	0.0004	0.1197
Q00978	ENSG00000213928; ENSG00000285048	IRF9	Interferon regulatory factor 9 (IRF-9) (IFN-alpha-responsive transcription factor subunit) (ISGF3 p48 subunit) (Interferon-stimulated gene factor 3 gamma) (ISGF-3 gamma) (Transcriptional regulator ISGF3 subunit gamma)	Q00978 (IRF9)	0.280	2.552	5.014	0.0004	0.1197
Q15424	ENSG00000160633	SAFB	Scaffold attachment factor B1 (SAF-B) (SAF-B1) (HSP27 estrogen response element-TATA box-binding protein) (HSP27 ERE-TATA-binding protein)	Q14151 (SAFB2)	0.269	2.637	5.534	0.0002	0.1197
P51991	ENSG00000170144	HNRNPA3	Heterogeneous nuclear ribonucleoprotein A3 (hnRNP A3)	P51991 (HNRNPA3)	0.259	2.715	6.722	<0.0001	0.067

Table S69: Proteins differentially abundant at Day 30 compared to pre-second vaccination (Study Arm 2 (A/H7N9 + AS03 D1, 29), Natural Killer cells). Proteins are sorted by descending absolute log_2 fold change. Protein and gene annotations are based on Ensembl Version 101 (continued).

Protein ID	Ensembl Gene ID	Ensembl Gene Name	Protein Description	50% Protein Cluster ID (Gene Name)	Fold Change (Day 30 vs. Pre- Second Vaccination)	Average log_2 Abundance	Moderated t-Statistic	P-Value	FDR Adjusted P-value
P61769	ENSG00000166710; ENSG00000273686	B2M	Beta-2-microglobulin [Cleaved into: Beta-2-microglobulin form pI 5.3]	P61769 (B2M)	0.252	2.111	5.120	0.0004	0.1197
Q9HCN8	ENSG00000128228	SDF2L1	Stromal cell-derived factor 2-like protein 1 (SDF2-like protein 1) (PWP1-interacting protein 8)	Q9HCN8 (SDF2L1)	0.242	2.512	4.969	0.0005	0.1197
Q9UNZ5	ENSG00000104979	C19orf53	Leydig cell tumor 10 kDa protein homolog	Q9UNZ5 (C19orf53)	0.226	2.294	4.896	0.0005	0.1197
Q53EL6	ENSG00000150593	PDCD4	Programmed cell death protein 4 (Neoplastic transformation inhibitor protein) (Nuclear antigen H731-like) (Protein 197/15a)	Q53EL6 (PDCD4)	-0.191	2.579	-4.874	0.0005	0.1197
P49406	ENSG00000115364	MRPL19	39S ribosomal protein L19, mitochondrial (L19mt) (MRP-L19) (39S ribosomal protein L15, mitochondrial) (L15mt) (MRP-L15) (Mitochondrial large ribosomal subunit protein bL19m)	P49406 (MRPL19)	-0.160	2.276	-4.964	0.0005	0.1197

Table S70: Proteins differentially abundant at Day 36 compared to pre-second vaccination (Study Arm 2 (A/H7N9 + AS03 D1, 29), Natural Killer cells). Proteins are sorted by descending absolute log_2 fold change. Protein and gene annotations are based on Ensembl Version 101.

Protein ID	Ensembl Gene ID	Ensembl Gene Name	Protein Description	50% Protein Cluster ID (Gene Name)	Fold Change (Day 36 vs. Pre- Second Vaccination)	Average log_2 Abundance	Moderated t-Statistic	P-Value	FDR Adjusted P-value
P09693	ENSG00000160654	CD3G	T-cell surface glycoprotein CD3 gamma chain (T-cell receptor T3 gamma chain) (CD antigen CD3g)	P09693 (CD3G)	-0.234	2.299	-7.016	<0.0001	0.11
P28907	ENSG00000004468	CD38	ADP-ribosyl cyclase/cyclic ADP-ribose hydrolase 1 (EC 3.2.2.6) (2'-phospho-ADP-ribosyl cyclase) (2'-phospho-ADP-ribosyl cyclase/2'-phospho-cyclic-ADP-ribose transferase) (EC 2.4.99.20) (2'-phospho-cyclic-ADP-ribose transferase) (ADP-ribosyl cyclase 1) (ADPRC 1) (Cyclic ADP-ribose hydrolase 1) (cADPr hydrolase 1) (T10) (CD antigen CD38)	P28907 (CD38)	0.233	2.477	6.473	<0.0001	0.11

Table S71: KEGG Pathways enriched in differentially expressed genes at Day 2 compared to pre-First Vaccination (Monocytes, Study Arm 2 A/H7N9+AS03, D1, 29). Results are sorted by FDR adjusted p-value and Jaccard similarity index.

Gene Set ID	Gene Set Genes #	DE Genes N (%)	Up-reg. DE Genes N (%)	Down-reg. DE Genes N (%)	Jaccard Index	P-Value	FDR Adjusted P-Value
INFLUENZA A	160	18 (11.2)	18 (11.2)	0 (0)	0.024	<0.0001	0.0017
PRIMARY IMMUNODEFICIENCY	32	8 (25)	4 (12.5)	4 (12.5)	0.013	<0.0001	0.0017
HEMATOPOIETIC CELL LINEAGE	93	15 (16.1)	5 (5.4)	10 (10.8)	0.022	<0.0001	0.0022
NOD-LIKE RECEPTOR SIGNALING PATHWAY	175	18 (10.3)	18 (10.3)	0 (0)	0.023	<0.0001	0.0025
STAPHYLOCOCCUS AUREUS INFECTION	91	11 (12.1)	10 (11)	1 (1.1)	0.016	<0.0001	0.0034
SYSTEMIC LUPUS ERYTHEMATOSUS	127	10 (7.9)	9 (7.1)	1 (0.8)	0.014	0.0002	0.0118
MEASLES	132	14 (10.6)	13 (9.8)	1 (0.8)	0.019	0.0003	0.014
EPSTEIN-BARR VIRUS INFECTION	196	17 (8.7)	15 (7.7)	2 (1)	0.021	0.0005	0.0194
CYTOKINE-CYTOKINE RECEPTOR INTERACTION	280	17 (6.1)	10 (3.6)	7 (2.5)	0.019	0.0007	0.0249
LEISHMANIASIS	70	9 (12.9)	8 (11.4)	1 (1.4)	0.013	0.0007	0.0249
HEPATITIS C	152	14 (9.2)	14 (9.2)	0 (0)	0.018	0.0009	0.0288
PHAGOSOME	145	13 (9)	10 (6.9)	3 (2.1)	0.017	0.0013	0.0371
COMPLEMENT AND COAGULATION CASCADES	82	9 (11)	8 (9.8)	1 (1.2)	0.013	0.0016	0.0417

Table S72: MSigDB Reactome Pathways enriched in differentially expressed genes at Day 2 compared to pre-First Vaccination (Monocytes, Study Arm 2 A/H7N9+AS03, D1, 29). Results are sorted by FDR adjusted p-value and Jaccard similarity index.

Gene Set ID	Gene Set Genes #	DE Genes N (%)	Up-reg. DE Genes N (%)	Down-reg. DE Genes N (%)	Jaccard Index	P-Value	FDR Adjusted P-Value
REACTOME INTERFERON SIGNALING	177	38 (21.5)	37 (20.9)	1 (0.6)	0.050	<0.0001	0.0026
REACTOME CYTOKINE SIGNALING IN IMMUNE SYSTEM	788	65 (8.2)	56 (7.1)	9 (1.1)	0.049	<0.0001	0.0026
REACTOME INNATE IMMUNE SYSTEM	968	59 (6.1)	43 (4.4)	16 (1.7)	0.039	<0.0001	0.0026
REACTOME INTERFERON ALPHA BETA SIGNALING	60	23 (38.3)	23 (38.3)	0 (0)	0.035	<0.0001	0.0026
REACTOME INTERFERON GAMMA SIGNALING	71	19 (26.8)	19 (26.8)	0 (0)	0.028	<0.0001	0.0026
REACTOME OAS ANTIVIRAL RESPONSE	8	5 (62.5)	5 (62.5)	0 (0)	0.008	<0.0001	0.0026
REACTOME IMMUNOREGULATORY INTERACTIONS BETWEEN A L...	97	13 (13.4)	6 (6.2)	7 (7.2)	0.018	<0.0001	0.0038
REACTOME COMPLEMENT CASCADE	52	9 (17.3)	7 (13.5)	2 (3.8)	0.014	<0.0001	0.0038
REACTOME ANTIVIRAL MECHANISM BY IFN STIMULATED GEN...	81	12 (14.8)	11 (13.6)	1 (1.2)	0.018	<0.0001	0.0107
REACTOME MRNA EDITING	10	4 (40)	4 (40)	0 (0)	0.006	<0.0001	0.0107
REACTOME ADAPTIVE IMMUNE SYSTEM	670	43 (6.4)	25 (3.7)	18 (2.7)	0.035	<0.0001	0.0111
REACTOME G ALPHA I SIGNALLING EVENTS	367	23 (6.3)	15 (4.1)	8 (2.2)	0.024	0.0003	0.0447

Table S73: MSigDB GO Biological Processes enriched in differentially expressed genes at Day 2 compared to pre-First Vaccination (Monocytes, Study Arm 2 A/H7N9+AS03, D1, 29). Results are sorted by FDR adjusted p-value and Jaccard similarity index.

Gene Set ID	Gene Set Genes #	DE Genes N (%)	Up-reg. DE Genes N (%)	Down-reg. DE Genes N (%)	Jaccard Index	P-Value	FDR Adjusted P-Value
GO DEFENSE RESPONSE TO OTHER ORGANISM	993	111 (11.2)	97 (9.8)	14 (1.4)	0.074	<0.0001	0.0009
GO INNATE IMMUNE RESPONSE	814	96 (11.8)	85 (10.4)	11 (1.4)	0.072	<0.0001	0.0009
GO RESPONSE TO BIOTIC STIMULUS	1325	129 (9.7)	107 (8.1)	22 (1.7)	0.071	<0.0001	0.0009
GO DEFENSE RESPONSE	1497	134 (9)	108 (7.2)	26 (1.7)	0.068	<0.0001	0.0009
GO DEFENSE RESPONSE TO VIRUS	229	49 (21.4)	46 (20.1)	3 (1.3)	0.061	<0.0001	0.0009
GO REGULATION OF IMMUNE RESPONSE	897	87 (9.7)	60 (6.7)	27 (3)	0.061	<0.0001	0.0009
GO IMMUNE EFFECTOR PROCESS	1077	97 (9)	79 (7.3)	18 (1.7)	0.061	<0.0001	0.0009
GO REGULATION OF IMMUNE SYSTEM PROCESS	1409	114 (8.1)	75 (5.3)	39 (2.8)	0.060	<0.0001	0.0009
GO RESPONSE TO VIRUS	300	51 (17)	48 (16)	3 (1)	0.059	<0.0001	0.0009
GO RESPONSE TO CYTOKINE	1099	90 (8.2)	73 (6.6)	17 (1.5)	0.055	<0.0001	0.0009
GO REGULATION OF MULTI ORGANISM PROCESS	700	69 (9.9)	60 (8.6)	9 (1.3)	0.055	<0.0001	0.0009
GO POSITIVE REGULATION OF IMMUNE SYSTEM PROCESS	972	82 (8.4)	54 (5.6)	28 (2.9)	0.054	<0.0001	0.0009
GO POSITIVE REGULATION OF IMMUNE RESPONSE	704	66 (9.4)	45 (6.4)	21 (3)	0.052	<0.0001	0.0009
GO REGULATION OF RESPONSE TO EXTERNAL STIMULUS	1006	81 (8.1)	59 (5.9)	22 (2.2)	0.052	<0.0001	0.0009
GO CYTOKINE MEDIATED SIGNALING PATHWAY	711	65 (9.1)	57 (8)	8 (1.1)	0.051	<0.0001	0.0009
GO REGULATION OF DEFENSE RESPONSE	703	64 (9.1)	49 (7)	15 (2.1)	0.051	<0.0001	0.0009
GO REGULATION OF RESPONSE TO BIOTIC STIMULUS	466	50 (10.7)	44 (9.4)	6 (1.3)	0.048	<0.0001	0.0009
GO CELL ACTIVATION	1265	86 (6.8)	47 (3.7)	39 (3.1)	0.048	<0.0001	0.0009
GO CYTOKINE PRODUCTION	720	59 (8.2)	41 (5.7)	18 (2.5)	0.046	<0.0001	0.0009
GO ACTIVATION OF IMMUNE RESPONSE	547	51 (9.3)	36 (6.6)	15 (2.7)	0.046	<0.0001	0.0009
GO IMMUNE RESPONSE REGULATING SIGNALING PATHWAY	507	49 (9.7)	31 (6.1)	18 (3.6)	0.045	<0.0001	0.0009
GO REGULATION OF RESPONSE TO STRESS	1406	86 (6.1)	63 (4.5)	23 (1.6)	0.044	<0.0001	0.0009
GO SECRETION	1551	92 (5.9)	57 (3.7)	35 (2.3)	0.044	<0.0001	0.0009
GO ADAPTIVE IMMUNE RESPONSE	353	40 (11.3)	26 (7.4)	14 (4)	0.043	<0.0001	0.0009
GO NEGATIVE REGULATION OF MULTI ORGANISM PROCESS	195	33 (16.9)	29 (14.9)	4 (2.1)	0.042	<0.0001	0.0009
GO RESPONSE TO INTERFERON GAMMA	166	31 (18.7)	31 (18.7)	0 (0)	0.041	<0.0001	0.0009
GO POSITIVE REGULATION OF RESPONSE TO EXTERNAL STI...	575	47 (8.2)	36 (6.3)	11 (1.9)	0.041	<0.0001	0.0009
GO IMMUNE RESPONSE REGULATING CELL SURFACE RECEPTO...	348	37 (10.6)	21 (6)	16 (4.6)	0.040	<0.0001	0.0009
GO REGULATION OF CELL ACTIVATION	507	43 (8.5)	18 (3.6)	25 (4.9)	0.040	<0.0001	0.0009
GO REGULATION OF IMMUNE EFFECTOR PROCESS	357	37 (10.4)	28 (7.8)	9 (2.5)	0.039	<0.0001	0.0009
GO REGULATION OF CELL POPULATION PROLIFERATION	1541	81 (5.3)	41 (2.7)	40 (2.6)	0.039	<0.0001	0.0009
GO RESPONSE TO BACTERIUM	587	45 (7.7)	31 (5.3)	14 (2.4)	0.039	<0.0001	0.0009
GO LYMPHOCYTE ACTIVATION	620	46 (7.4)	20 (3.2)	26 (4.2)	0.039	<0.0001	0.0009
GO INFLAMMATORY RESPONSE	680	48 (7.1)	32 (4.7)	16 (2.4)	0.038	<0.0001	0.0009
GO NEGATIVE REGULATION OF PROTEIN METABOLIC PROCES...	1039	60 (5.8)	38 (3.7)	22 (2.1)	0.038	<0.0001	0.0009
GO POSITIVE REGULATION OF DEFENSE RESPONSE	457	37 (8.1)	30 (6.6)	7 (1.5)	0.036	<0.0001	0.0009
GO LEUKOCYTE MEDIATED IMMUNITY	701	45 (6.4)	33 (4.7)	12 (1.7)	0.035	<0.0001	0.0009

Table S73: MSigDB GO Biological Processes enriched in differentially expressed genes at Day 2 compared to pre-First Vaccination (Monocytes, Study Arm 2 A/H7N9+AS03, D1, 29). Results are sorted by FDR adjusted p-value and Jaccard similarity index (continued.)

Gene Set ID	Gene Set Genes #	DE Genes N (%)	Up-reg. DE Genes N (%)	Down-reg. DE Genes N (%)	Jaccard Index	P-Value	FDR Adjusted P-Value
GO NEGATIVE REGULATION OF RESPONSE TO EXTERNAL STI...	350	33 (9.4)	21 (6)	12 (3.4)	0.035	<0.0001	0.0009
GO RESPONSE TO TYPE I INTERFERON	88	24 (27.3)	24 (27.3)	0 (0)	0.035	<0.0001	0.0009
GO POSITIVE REGULATION OF MULTI ORGANISM PROCESS	489	37 (7.6)	31 (6.3)	6 (1.2)	0.035	<0.0001	0.0009
GO PEPTIDE SECRETION	587	40 (6.8)	26 (4.4)	14 (2.4)	0.034	<0.0001	0.0009
GO MYELOID LEUKOCYTE ACTIVATION	594	40 (6.7)	28 (4.7)	12 (2)	0.034	<0.0001	0.0009
GO POSITIVE REGULATION OF RESPONSE TO BIOTIC STIMU...	353	32 (9.1)	27 (7.6)	5 (1.4)	0.034	<0.0001	0.0009
GO POSITIVE REGULATION OF CYTOKINE PRODUCTION	419	34 (8.1)	24 (5.7)	10 (2.4)	0.034	<0.0001	0.0009
GO REGULATION OF LYMPHOCYTE ACTIVATION	392	32 (8.2)	13 (3.3)	19 (4.8)	0.033	<0.0001	0.0009
GO ADAPTIVE IMMUNE RESPONSE BASED ON SOMATIC RECOM...	223	26 (11.7)	18 (8.1)	8 (3.6)	0.032	<0.0001	0.0009
GO NEGATIVE REGULATION OF IMMUNE SYSTEM PROCESS	426	32 (7.5)	20 (4.7)	12 (2.8)	0.032	<0.0001	0.0009
GO REGULATION OF PEPTIDE SECRETION	461	33 (7.2)	20 (4.3)	13 (2.8)	0.032	<0.0001	0.0009
GO NEGATIVE REGULATION OF DEFENSE RESPONSE	201	24 (11.9)	16 (8)	8 (4)	0.030	<0.0001	0.0009
GO REGULATION OF VIRAL LIFE CYCLE	133	22 (16.5)	21 (15.8)	1 (0.8)	0.030	<0.0001	0.0009
GO LEUKOCYTE MIGRATION	410	30 (7.3)	16 (3.9)	14 (3.4)	0.030	<0.0001	0.0009
GO REGULATION OF SYMBIOSIS ENCOMPASSING MUTUALISM ...	204	24 (11.8)	22 (10.8)	2 (1)	0.030	<0.0001	0.0009
GO INTERFERON GAMMA MEDIATED SIGNALING PATHWAY	70	20 (28.6)	20 (28.6)	0 (0)	0.030	<0.0001	0.0009
GO LYMPHOCYTE MEDIATED IMMUNITY	216	24 (11.1)	17 (7.9)	7 (3.2)	0.030	<0.0001	0.0009
GO CYTOKINE SECRETION	220	24 (10.9)	16 (7.3)	8 (3.6)	0.029	<0.0001	0.0009
GO VIRAL GENOME REPLICATION	117	21 (17.9)	21 (17.9)	0 (0)	0.029	<0.0001	0.0009
GO NEGATIVE REGULATION OF VIRAL PROCESS	90	20 (22.2)	18 (20)	2 (2.2)	0.029	<0.0001	0.0009
GO LEUKOCYTE PROLIFERATION	276	24 (8.7)	10 (3.6)	14 (5.1)	0.028	<0.0001	0.0009
GO REGULATION OF VIRAL GENOME REPLICATION	91	19 (20.9)	19 (20.9)	0 (0)	0.028	<0.0001	0.0009
GO ANTIGEN RECEPTOR MEDIATED SIGNALING PATHWAY	212	22 (10.4)	10 (4.7)	12 (5.7)	0.027	<0.0001	0.0009
GO NEGATIVE REGULATION OF CYTOKINE PRODUCTION	262	23 (8.8)	14 (5.3)	9 (3.4)	0.027	<0.0001	0.0009
GO HUMORAL IMMUNE RESPONSE	227	22 (9.7)	13 (5.7)	9 (4)	0.027	<0.0001	0.0009
GO NEGATIVE REGULATION OF VIRAL LIFE CYCLE	76	18 (23.7)	17 (22.4)	1 (1.3)	0.027	<0.0001	0.0009
GO REGULATION OF INFLAMMATORY RESPONSE	332	24 (7.2)	13 (3.9)	11 (3.3)	0.026	<0.0001	0.0009
GO MYELOID LEUKOCYTE MIGRATION	187	20 (10.7)	12 (6.4)	8 (4.3)	0.025	<0.0001	0.0009
GO NEGATIVE REGULATION OF VIRAL GENOME REPLICATION	58	16 (27.6)	16 (27.6)	0 (0)	0.024	<0.0001	0.0009
GO REGULATION OF LYMPHOCYTE DIFFERENTIATION	158	17 (10.8)	9 (5.7)	8 (5.1)	0.022	<0.0001	0.0009
GO B CELL PROLIFERATION	91	14 (15.4)	2 (2.2)	12 (13.2)	0.020	<0.0001	0.0009
GO B CELL MEDIATED IMMUNITY	104	14 (13.5)	10 (9.6)	4 (3.8)	0.020	<0.0001	0.0009
GO GRANULOCYTE MIGRATION	126	14 (11.1)	10 (7.9)	4 (3.2)	0.019	<0.0001	0.0009
GO MACROPHAGE ACTIVATION	84	13 (15.5)	9 (10.7)	4 (4.8)	0.019	<0.0001	0.0009
GO NEGATIVE REGULATION OF RESPONSE TO BIOTIC STIMU...	82	13 (15.9)	12 (14.6)	1 (1.2)	0.019	<0.0001	0.0009
GO NEUTROPHIL MIGRATION	103	13 (12.6)	10 (9.7)	3 (2.9)	0.018	<0.0001	0.0009
GO NEGATIVE REGULATION OF INNATE IMMUNE RESPONSE	49	12 (24.5)	11 (22.4)	1 (2)	0.018	<0.0001	0.0009

Table S73: MSigDB GO Biological Processes enriched in differentially expressed genes at Day 2 compared to pre-First Vaccination (Monocytes, Study Arm 2 A/H7N9+AS03, D1, 29). Results are sorted by FDR adjusted p-value and Jaccard similarity index (continued.)

Gene Set ID	Gene Set Genes #	DE Genes N (%)	Up-reg. DE Genes N (%)	Down-reg. DE Genes N (%)	Jaccard Index	P-Value	FDR Adjusted P-Value
GO REGULATION OF DEFENSE RESPONSE TO VIRUS	68	12 (17.6)	12 (17.6)	0 (0)	0.018	<0.0001	0.0009
GO REGULATION OF B CELL PROLIFERATION	58	11 (19)	2 (3.4)	9 (15.5)	0.017	<0.0001	0.0009
GO NEUROINFLAMMATORY RESPONSE	63	11 (17.5)	6 (9.5)	5 (7.9)	0.016	<0.0001	0.0009
GO COMPLEMENT ACTIVATION	62	10 (16.1)	7 (11.3)	3 (4.8)	0.015	<0.0001	0.0009
GO RESPONSE TO INTERFERON BETA	30	9 (30)	9 (30)	0 (0)	0.014	<0.0001	0.0009
GO NEGATIVE REGULATION OF TYPE I INTERFERON PRODUC...	43	9 (20.9)	8 (18.6)	1 (2.3)	0.014	<0.0001	0.0009
GO GLIAL CELL ACTIVATION	48	9 (18.8)	6 (12.5)	3 (6.2)	0.014	<0.0001	0.0009
GO INTERLEUKIN 1 SECRETION	53	9 (17)	7 (13.2)	2 (3.8)	0.014	<0.0001	0.0009
GO HUMORAL IMMUNE RESPONSE MEDIATED BY CIRCULATING...	40	8 (20)	6 (15)	2 (5)	0.012	<0.0001	0.0009
GO LEUKOCYTE ACTIVATION INVOLVED IN INFLAMMATORY R...	40	8 (20)	6 (15)	2 (5)	0.012	<0.0001	0.0009
GO RESPONSE TO INTERFERON ALPHA	21	7 (33.3)	7 (33.3)	0 (0)	0.011	<0.0001	0.0009
GO POSITIVE REGULATION OF DEFENSE RESPONSE TO VIRU...	24	7 (29.2)	7 (29.2)	0 (0)	0.011	<0.0001	0.0009
GO REGULATION OF TRANSPORT	1745	85 (4.9)	46 (2.6)	39 (2.2)	0.037	<0.0001	0.0015
GO REGULATION OF CELL DEATH	1593	77 (4.8)	47 (3)	30 (1.9)	0.036	<0.0001	0.0015
GO NEGATIVE REGULATION OF MULTICELLULAR ORGANISMAL...	1169	60 (5.1)	35 (3)	25 (2.1)	0.035	<0.0001	0.0015
GO REGULATION OF SECRETION	752	44 (5.9)	25 (3.3)	19 (2.5)	0.033	<0.0001	0.0015
GO T CELL ACTIVATION	436	31 (7.1)	15 (3.4)	16 (3.7)	0.030	<0.0001	0.0015
GO VIRAL LIFE CYCLE	308	26 (8.4)	25 (8.1)	1 (0.3)	0.029	<0.0001	0.0015
GO RESPONSE TO TUMOR NECROSIS FACTOR	272	22 (8.1)	15 (5.5)	7 (2.6)	0.025	<0.0001	0.0015
GO LEUKOCYTE CHEMOTAXIS	202	19 (9.4)	13 (6.4)	6 (3)	0.024	<0.0001	0.0015
GO REGULATION OF B CELL ACTIVATION	112	14 (12.5)	3 (2.7)	11 (9.8)	0.020	<0.0001	0.0015
GO POSITIVE REGULATION OF ADAPTIVE IMMUNE RESPONSE	92	12 (13)	7 (7.6)	5 (5.4)	0.017	<0.0001	0.0015
GO MACROPHAGE MIGRATION	47	9 (19.1)	4 (8.5)	5 (10.6)	0.014	<0.0001	0.0015
GO REGULATION OF HUMORAL IMMUNE RESPONSE	62	9 (14.5)	7 (11.3)	2 (3.2)	0.013	<0.0001	0.0015
GO RELAXATION OF MUSCLE	28	7 (25)	6 (21.4)	1 (3.6)	0.011	<0.0001	0.0015
GO LYMPHOCYTE DIFFERENTIATION	336	26 (7.7)	12 (3.6)	14 (4.2)	0.028	<0.0001	0.0022
GO PHAGOCYTOSIS	251	23 (9.2)	15 (6)	8 (3.2)	0.027	<0.0001	0.0022
GO ACTIVATION OF INNATE IMMUNE RESPONSE	286	23 (8)	19 (6.6)	4 (1.4)	0.026	<0.0001	0.0022
GO REGULATION OF ADAPTIVE IMMUNE RESPONSE	142	16 (11.3)	11 (7.7)	5 (3.5)	0.021	<0.0001	0.0022
GO B CELL RECEPTOR SIGNALING PATHWAY	56	10 (17.9)	1 (1.8)	9 (16.1)	0.015	<0.0001	0.0022
GO LEUKOCYTE DIFFERENTIATION	483	32 (6.6)	17 (3.5)	15 (3.1)	0.030	<0.0001	0.0028
GO TUMOR NECROSIS FACTOR SUPERFAMILY CYTOKINE PROD...	148	14 (9.5)	8 (5.4)	6 (4.1)	0.019	<0.0001	0.0028
GO INTERLEUKIN 1 PRODUCTION	99	12 (12.1)	9 (9.1)	3 (3)	0.017	<0.0001	0.0028
GO POSITIVE REGULATION OF LYMPHOCYTE MEDIATED IMMU...	87	11 (12.6)	6 (6.9)	5 (5.7)	0.016	<0.0001	0.0028
GO POSITIVE REGULATION OF T CELL MEDIATED IMMUNITY	41	8 (19.5)	5 (12.2)	3 (7.3)	0.012	<0.0001	0.0028
GO REGULATION OF CALCIUM ION TRANSPORT	238	20 (8.4)	11 (4.6)	9 (3.8)	0.024	<0.0001	0.0034
GO POSITIVE REGULATION OF PEPTIDE SECRETION	271	20 (7.4)	14 (5.2)	6 (2.2)	0.023	<0.0001	0.0034

Table S73: MSigDB GO Biological Processes enriched in differentially expressed genes at Day 2 compared to pre-First Vaccination (Monocytes, Study Arm 2 A/H7N9+AS03, D1, 29). Results are sorted by FDR adjusted p-value and Jaccard similarity index (continued.)

Gene Set ID	Gene Set Genes #	DE Genes N (%)	Up-reg. DE Genes N (%)	Down-reg. DE Genes N (%)	Jaccard Index	P-Value	FDR Adjusted P-Value
GO NEGATIVE REGULATION OF CELL POPULATION PROLIFER...	666	38 (5.7)	18 (2.7)	20 (3)	0.030	<0.0001	0.004
GO DEFENSE RESPONSE TO BACTERIUM	237	15 (6.3)	10 (4.2)	5 (2.1)	0.018	<0.0001	0.004
GO NEGATIVE REGULATION OF INFLAMMATORY RESPONSE	141	13 (9.2)	5 (3.5)	8 (5.7)	0.017	<0.0001	0.004
GO CELL CHEMOTAXIS	278	20 (7.2)	14 (5)	6 (2.2)	0.023	<0.0001	0.0045
GO REGULATION OF LEUKOCYTE PROLIFERATION	203	18 (8.9)	7 (3.4)	11 (5.4)	0.022	<0.0001	0.0045
GO NEGATIVE REGULATION OF IMMUNE RESPONSE	134	14 (10.4)	13 (9.7)	1 (0.7)	0.019	<0.0001	0.0045
GO REGULATION OF RESPONSE TO INTERFERON GAMMA	24	6 (25)	6 (25)	0 (0)	0.009	<0.0001	0.0045
GO REGULATION OF LYMPHOCYTE MEDIATED IMMUNITY	127	13 (10.2)	8 (6.3)	5 (3.9)	0.018	<0.0001	0.005
GO REGULATION OF MACROPHAGE MIGRATION	34	7 (20.6)	3 (8.8)	4 (11.8)	0.011	<0.0001	0.005
GO POSITIVE REGULATION OF T CELL MEDIATED CYTOTOXI...	20	5 (25)	3 (15)	2 (10)	0.008	<0.0001	0.005
GO NEGATIVE REGULATION OF RESPONSE TO STIMULUS	1531	75 (4.9)	47 (3.1)	28 (1.8)	0.036	<0.0001	0.0055
GO INTERLEUKIN 1 BETA PRODUCTION	87	10 (11.5)	8 (9.2)	2 (2.3)	0.014	<0.0001	0.0055
GO REGULATION OF DEFENSE RESPONSE TO VIRUS BY HOST	34	7 (20.6)	7 (20.6)	0 (0)	0.011	<0.0001	0.0055
GO RECEPTOR MEDIATED ENDOCYTOSIS	245	20 (8.2)	13 (5.3)	7 (2.9)	0.024	0.0001	0.0065
GO POSITIVE REGULATION OF IMMUNE EFFECTOR PROCESS	189	16 (8.5)	10 (5.3)	6 (3.2)	0.020	0.0001	0.0065
GO SYNAPSE PRUNING	11	4 (36.4)	3 (27.3)	1 (9.1)	0.006	0.0001	0.0065
GO CIRCULATORY SYSTEM PROCESS	517	32 (6.2)	24 (4.6)	8 (1.5)	0.029	0.0001	0.0069
GO APOPTOTIC CELL CLEARANCE	45	8 (17.8)	6 (13.3)	2 (4.4)	0.012	0.0001	0.0069
GO REGULATION OF RIBONUCLEASE ACTIVITY	8	4 (50)	4 (50)	0 (0)	0.006	0.0001	0.0069
GO CELL MOTILITY	1531	73 (4.8)	39 (2.5)	34 (2.2)	0.035	0.0001	0.0073
GO REGULATION OF PEPTIDASE ACTIVITY	409	25 (6.1)	15 (3.7)	10 (2.4)	0.025	0.0001	0.0073
GO TYPE I INTERFERON PRODUCTION	116	13 (11.2)	12 (10.3)	1 (0.9)	0.018	0.0001	0.0073
GO POSITIVE REGULATION OF B CELL PROLIFERATION	39	7 (17.9)	2 (5.1)	5 (12.8)	0.011	0.0001	0.0073
GO REGULATION OF PHOSPHORYLATION	1495	71 (4.7)	47 (3.1)	24 (1.6)	0.035	0.0001	0.0076
GO NEGATIVE REGULATION OF MOLECULAR FUNCTION	1052	53 (5)	31 (2.9)	22 (2.1)	0.033	0.0001	0.0076
GO B CELL ACTIVATION	231	19 (8.2)	5 (2.2)	14 (6.1)	0.023	0.0001	0.0076
GO NEGATIVE REGULATION OF NEUROINFLAMMATORY RESPON...	13	4 (30.8)	1 (7.7)	3 (23.1)	0.006	0.0001	0.0076
GO NEGATIVE REGULATION OF CATALYTIC ACTIVITY	743	40 (5.4)	23 (3.1)	17 (2.3)	0.030	0.0001	0.008
GO REGULATION OF VASCULATURE DEVELOPMENT	349	23 (6.6)	11 (3.2)	12 (3.4)	0.024	0.0001	0.008
GO REGULATION OF RESPONSE TO CYTOKINE STIMULUS	165	15 (9.1)	12 (7.3)	3 (1.8)	0.019	0.0001	0.008
GO POSITIVE REGULATION OF LEUKOCYTE MEDIATED IMMUN...	113	12 (10.6)	7 (6.2)	5 (4.4)	0.017	0.0001	0.008
GO REGULATION OF INTERLEUKIN 1 SECRETION	45	7 (15.6)	5 (11.1)	2 (4.4)	0.011	0.0002	0.0084
GO RELAXATION OF CARDIAC MUSCLE	15	5 (33.3)	4 (26.7)	1 (6.7)	0.008	0.0002	0.0084
GO REGULATION OF LEUKOCYTE MEDIATED IMMUNITY	174	15 (8.6)	10 (5.7)	5 (2.9)	0.019	0.0002	0.0088
GO REGULATION OF T CELL ACTIVATION	292	21 (7.2)	9 (3.1)	12 (4.1)	0.024	0.0002	0.0093
GO IMMUNE SYSTEM DEVELOPMENT	926	49 (5.3)	30 (3.2)	19 (2.1)	0.033	0.0002	0.0097
GO INTERSPECIES INTERACTION BETWEEN ORGANISMS	869	47 (5.4)	42 (4.8)	5 (0.6)	0.033	0.0002	0.01

Table S73: MSigDB GO Biological Processes enriched in differentially expressed genes at Day 2 compared to pre-First Vaccination (Monocytes, Study Arm 2 A/H7N9+AS03, D1, 29). Results are sorted by FDR adjusted p-value and Jaccard similarity index (continued.)

Gene Set ID	Gene Set Genes #	DE Genes N (%)	Up-reg. DE Genes N (%)	Down-reg. DE Genes N (%)	Jaccard Index	P-Value	FDR Adjusted P-Value
GO LIPID LOCALIZATION	383	26 (6.8)	16 (4.2)	10 (2.6)	0.027	0.0002	0.01
GO POSITIVE REGULATION OF RESPONSE TO CYTOKINE STI...	53	8 (15.1)	7 (13.2)	1 (1.9)	0.012	0.0002	0.01
GO PRODUCTION OF MOLECULAR MEDIATOR OF IMMUNE RESP...	187	16 (8.6)	10 (5.3)	6 (3.2)	0.020	0.0002	0.0104
GO ASTROCYTE ACTIVATION	21	5 (23.8)	3 (14.3)	2 (9.5)	0.008	0.0002	0.0104
GO POSITIVE REGULATION OF CELL ACTIVATION	297	21 (7.1)	9 (3)	12 (4)	0.024	0.0002	0.0108
GO POSITIVE REGULATION OF INTERLEUKIN 1 PRODUCTION	50	7 (14)	5 (10)	2 (4)	0.011	0.0002	0.0108
GO REGULATION OF T CELL DIFFERENTIATION	131	13 (9.9)	7 (5.3)	6 (4.6)	0.018	0.0002	0.011
GO INTERLEUKIN 6 SECRETION	45	7 (15.6)	4 (8.9)	3 (6.7)	0.011	0.0002	0.011
GO POSITIVE REGULATION OF EPITHELIAL CELL APOPTOTI...	32	6 (18.8)	4 (12.5)	2 (6.2)	0.009	0.0002	0.011
GO BASE CONVERSION OR SUBSTITUTION EDITING	21	5 (23.8)	4 (19)	1 (4.8)	0.008	0.0002	0.0114
GO REGULATION OF T CELL MEDIATED IMMUNITY	59	8 (13.6)	5 (8.5)	3 (5.1)	0.012	0.0002	0.0118
GO ISG15 PROTEIN CONJUGATION	6	3 (50)	3 (50)	0 (0)	0.005	0.0002	0.0118
GO LOCOMOTION	1747	79 (4.5)	41 (2.3)	38 (2.2)	0.035	0.0003	0.0122
GO CELLULAR RESPONSE TO DSRNA	20	5 (25)	4 (20)	1 (5)	0.008	0.0003	0.013
GO MULTI ORGANISM CELLULAR PROCESS	46	7 (15.2)	7 (15.2)	0 (0)	0.011	0.0003	0.0134
GO REGULATION OF HEMOPOIESIS	441	27 (6.1)	18 (4.1)	9 (2)	0.026	0.0003	0.0137
GO VASODILATION	30	6 (20)	5 (16.7)	1 (3.3)	0.009	0.0003	0.0137
GO REGULATION OF LEUKOCYTE MIGRATION	175	14 (8)	8 (4.6)	6 (3.4)	0.018	0.0003	0.015
GO RESPONSE TO INTERLEUKIN 1	181	15 (8.3)	12 (6.6)	3 (1.7)	0.019	0.0003	0.0157
GO POSITIVE REGULATION OF TUMOR NECROSIS FACTOR SU...	79	9 (11.4)	5 (6.3)	4 (5.1)	0.013	0.0003	0.0157
GO NEGATIVE REGULATION OF CELL ACTIVATION	184	15 (8.2)	6 (3.3)	9 (4.9)	0.019	0.0004	0.0169
GO NEGATIVE REGULATION OF VIRAL ENTRY INTO HOST CE...	15	4 (26.7)	3 (20)	1 (6.7)	0.006	0.0004	0.0173
GO RESPONSE TO PROTOZOAN	26	5 (19.2)	5 (19.2)	0 (0)	0.008	0.0004	0.0181
GO CELL KILLING	146	11 (7.5)	7 (4.8)	4 (2.7)	0.015	0.0004	0.0197
GO DIVALENT INORGANIC CATION HOMEOSTASIS	492	29 (5.9)	19 (3.9)	10 (2)	0.027	0.0005	0.02
GO REGULATION OF CELLULAR COMPONENT MOVEMENT	973	50 (5.1)	27 (2.8)	23 (2.4)	0.032	0.0005	0.0202
GO REGULATION OF T CELL MEDIATED CYTOTOXICITY	25	5 (20)	3 (12)	2 (8)	0.008	0.0005	0.0202
GO POSITIVE REGULATION OF CELL DEATH	670	37 (5.5)	28 (4.2)	9 (1.3)	0.030	0.0005	0.0204
GO NEURON DEATH	327	23 (7)	11 (3.4)	12 (3.7)	0.025	0.0005	0.0204
GO REGULATION OF VIRAL ENTRY INTO HOST CELL	26	5 (19.2)	4 (15.4)	1 (3.8)	0.008	0.0005	0.022
GO REGULATION OF PHOSPHORUS METABOLIC PROCESS	1674	76 (4.5)	49 (2.9)	27 (1.6)	0.034	0.0005	0.0223
GO REGULATION OF METAL ION TRANSPORT	366	24 (6.6)	13 (3.6)	11 (3)	0.025	0.0006	0.0234
GO CHEMICAL HOMEOSTASIS	1123	55 (4.9)	36 (3.2)	19 (1.7)	0.033	0.0006	0.0239
GO GLIOGENESIS	270	20 (7.4)	8 (3)	12 (4.4)	0.023	0.0006	0.0239
GO PROTEIN ADP RIBOSYLATION	35	6 (17.1)	5 (14.3)	1 (2.9)	0.009	0.0006	0.0239
GO POSITIVE REGULATION OF CYTOKINE SECRETION	126	11 (8.7)	8 (6.3)	3 (2.4)	0.015	0.0006	0.0241
GO NEGATIVE REGULATION OF MACROPHAGE ACTIVATION	15	4 (26.7)	2 (13.3)	2 (13.3)	0.006	0.0006	0.0244

Table S73: MSigDB GO Biological Processes enriched in differentially expressed genes at Day 2 compared to pre-First Vaccination (Monocytes, Study Arm 2 A/H7N9+AS03, D1, 29). Results are sorted by FDR adjusted p-value and Jaccard similarity index (continued.)

Gene Set ID	Gene Set Genes #	DE Genes N (%)	Up-reg. DE Genes N (%)	Down-reg. DE Genes N (%)	Jaccard Index	P-Value	FDR Adjusted P-Value
GO POSITIVE REGULATION OF SIGNALING	1730	78 (4.5)	49 (2.8)	29 (1.7)	0.034	0.0006	0.0255
GO POSITIVE REGULATION OF CHEMOTAXIS	125	11 (8.8)	7 (5.6)	4 (3.2)	0.015	0.0006	0.0262
GO IMPORT INTO CELL	668	39 (5.8)	24 (3.6)	15 (2.2)	0.031	0.0007	0.0273
GO NEGATIVE REGULATION OF PEPTIDASE ACTIVITY	230	15 (6.5)	7 (3)	8 (3.5)	0.018	0.0007	0.0273
GO T CELL MEDIATED IMMUNITY	84	9 (10.7)	6 (7.1)	3 (3.6)	0.013	0.0007	0.0273
GO EXOCYTOSIS	833	44 (5.3)	28 (3.4)	16 (1.9)	0.031	0.0007	0.0274
GO REGULATION OF LEUKOCYTE DIFFERENTIATION	252	18 (7.1)	10 (4)	8 (3.2)	0.021	0.0007	0.0274
GO ASTROCYTE DEVELOPMENT	36	6 (16.7)	4 (11.1)	2 (5.6)	0.009	0.0008	0.03
GO BIOLOGICAL ADHESION	1320	65 (4.9)	30 (2.3)	35 (2.7)	0.035	0.0008	0.0325
GO REGULATION OF HYDROLASE ACTIVITY	1175	57 (4.9)	33 (2.8)	24 (2)	0.033	0.0008	0.0325
GO RECEPTOR SIGNALING PATHWAY VIA STAT	156	12 (7.7)	11 (7.1)	1 (0.6)	0.016	0.0008	0.0325
GO POSITIVE REGULATION OF ENDOTHELIAL CELL APOPTOT...	18	4 (22.2)	2 (11.1)	2 (11.1)	0.006	0.0008	0.0325
GO REGULATION OF PROTEIN MODIFICATION PROCESS	1724	77 (4.5)	50 (2.9)	27 (1.6)	0.034	0.0009	0.0335
GO T CELL DIFFERENTIATION	230	17 (7.4)	10 (4.3)	7 (3)	0.021	0.0009	0.0335
GO CELLULAR RESPONSE TO EXOGENOUS DSRNA	15	4 (26.7)	3 (20)	1 (6.7)	0.006	0.0009	0.0335
GO TYROSINE PHOSPHORYLATION OF STAT PROTEIN	80	8 (10)	7 (8.8)	1 (1.2)	0.012	0.0009	0.0345
GO POSITIVE REGULATION OF LEUKOCYTE PROLIFERATION	124	11 (8.9)	5 (4)	6 (4.8)	0.015	0.001	0.0354
GO NEGATIVE REGULATION OF VASCULATURE DEVELOPMENT	139	11 (7.9)	3 (2.2)	8 (5.8)	0.015	0.001	0.0354
GO SYNCYTIUM FORMATION	56	7 (12.5)	4 (7.1)	3 (5.4)	0.011	0.001	0.0354
GO TOOTH ERUPTION	7	3 (42.9)	2 (28.6)	1 (14.3)	0.005	0.001	0.0356
GO NEGATIVE REGULATION OF ASTROCYTE ACTIVATION	2	2 (100)	1 (50)	1 (50)	0.003	0.001	0.0358
GO SECOND MESSENGER MEDIATED SIGNALING	419	25 (6)	12 (2.9)	13 (3.1)	0.025	0.001	0.0364
GO NEGATIVE REGULATION OF CYTOKINE SECRETION	62	7 (11.3)	3 (4.8)	4 (6.5)	0.010	0.001	0.0364
GO NEGATIVE REGULATION OF MACROPHAGE MIGRATION	8	3 (37.5)	1 (12.5)	2 (25)	0.005	0.001	0.0364
GO POSITIVE REGULATION OF TRANSPORT	921	46 (5)	27 (2.9)	19 (2.1)	0.031	0.001	0.0366
GO REGULATION OF CYSTEINE TYPE ENDOPEPTIDASE ACTIV...	221	16 (7.2)	12 (5.4)	4 (1.8)	0.019	0.001	0.0368
GO POSITIVE REGULATION OF B CELL ACTIVATION	71	8 (11.3)	2 (2.8)	6 (8.5)	0.012	0.001	0.0368
GO REGULATION OF INTERLEUKIN 1 MEDIATED SIGNALING ...	7	3 (42.9)	1 (14.3)	2 (28.6)	0.005	0.001	0.0368
GO POSITIVE REGULATION OF TYROSINE PHOSPHORYLATION...	67	7 (10.4)	6 (9)	1 (1.5)	0.010	0.001	0.0369
GO TAXIS	607	33 (5.4)	20 (3.3)	13 (2.1)	0.028	0.0011	0.0378
GO DIVALENT INORGANIC CATION TRANSPORT	461	28 (6.1)	17 (3.7)	11 (2.4)	0.027	0.0011	0.0378
GO MYOBLAST FUSION	41	6 (14.6)	4 (9.8)	2 (4.9)	0.009	0.0011	0.0378
GO PURINERGIC NUCLEOTIDE RECEPTOR SIGNALING PATHWA...	17	4 (23.5)	3 (17.6)	1 (5.9)	0.006	0.0011	0.0397
GO PROTEIN PHOSPHORYLATION	1838	82 (4.5)	54 (2.9)	28 (1.5)	0.035	0.0012	0.0409
GO POSITIVE REGULATION OF CALCIUM ION TRANSPORT IN...	54	7 (13)	3 (5.6)	4 (7.4)	0.011	0.0012	0.0409
GO REGULATION OF NEUTROPHIL MIGRATION	31	5 (16.1)	3 (9.7)	2 (6.5)	0.008	0.0012	0.0409
GO POSITIVE REGULATION OF BLOOD VESSEL DIAMETER	56	7 (12.5)	6 (10.7)	1 (1.8)	0.011	0.0012	0.0417

Table S73: MSigDB GO Biological Processes enriched in differentially expressed genes at Day 2 compared to pre-First Vaccination (Monocytes, Study Arm 2 A/H7N9+AS03, D1, 29). Results are sorted by FDR adjusted p-value and Jaccard similarity index (continued.)

Gene Set ID	Gene Set Genes #	DE Genes N (%)	Up-reg. DE Genes N (%)	Down-reg. DE Genes N (%)	Jaccard Index	P-Value	FDR Adjusted P-Value
GO TUMOR NECROSIS FACTOR SECRETION	33	5 (15.2)	4 (12.1)	1 (3)	0.008	0.0013	0.0429
GO ANTIMICROBIAL HUMORAL RESPONSE	108	7 (6.5)	5 (4.6)	2 (1.9)	0.010	0.0013	0.0444
GO REGULATION OF ESTABLISHMENT OF PROTEIN LOCALIZA...	698	37 (5.3)	22 (3.2)	15 (2.1)	0.029	0.0013	0.0448
GO INTERFERON ALPHA PRODUCTION	25	5 (20)	4 (16)	1 (4)	0.008	0.0013	0.0448
GO POSITIVE REGULATION OF NITRIC OXIDE SYNTHASE BI...	15	4 (26.7)	4 (26.7)	0 (0)	0.006	0.0013	0.0448
GO POSITIVE CHEMOTAXIS	59	7 (11.9)	3 (5.1)	4 (6.8)	0.010	0.0014	0.0449
GO INTERLEUKIN 10 SECRETION	9	3 (33.3)	2 (22.2)	1 (11.1)	0.005	0.0014	0.0464
GO MEMBRANE INVAGINATION	67	8 (11.9)	5 (7.5)	3 (4.5)	0.012	0.0014	0.0468
GO T CELL MEDIATED CYTOTOXICITY	31	5 (16.1)	3 (9.7)	2 (6.5)	0.008	0.0014	0.0469
GO INTERFERON GAMMA PRODUCTION	97	9 (9.3)	7 (7.2)	2 (2.1)	0.013	0.0014	0.0471
GO POSITIVE REGULATION OF PEPTIDYL TYROSINE PHOSPH...	185	14 (7.6)	11 (5.9)	3 (1.6)	0.018	0.0015	0.0478

Table S74: Blood Transcription Modules enriched in differentially expressed genes at Day 2 compared to pre-First Vaccination (Monocytes, Study Arm 2 A/H7N9+AS03, D1, 29). Results are sorted by FDR adjusted p-value and Jaccard similarity index.

Gene Set ID	Gene Set Genes #	DE Genes N (%)	Up-reg. DE Genes N (%)	Down-reg. DE Genes N (%)	Jaccard Index	P-Value	FDR Adjusted P-Value
M47.0 ENRICHED IN B CELLS (I)	45	24 (53.3)	0 (0)	24 (53.3)	0.038	<0.0001	0.0003
M75 ANTIVIRAL IFN SIGNATURE	22	16 (72.7)	14 (63.6)	2 (9.1)	0.026	<0.0001	0.0003
M47.1 ENRICHED IN B CELLS (II)	35	16 (45.7)	0 (0)	16 (45.7)	0.025	<0.0001	0.0003
M156.0 PLASMA CELLS AND B CELLS, IMMUNOGLOBULINS	30	12 (40)	0 (0)	12 (40)	0.019	<0.0001	0.0003
M127 TYPE I INTERFERON RESPONSE	12	11 (91.7)	11 (91.7)	0 (0)	0.018	<0.0001	0.0003
M69 ENRICHED IN B CELLS (VI)	19	11 (57.9)	0 (0)	11 (57.9)	0.018	<0.0001	0.0003
M67 ACTIVATED DENDRITIC CELLS	12	10 (83.3)	10 (83.3)	0 (0)	0.016	<0.0001	0.0003
M150 INNATE ANTIVIRAL RESPONSE	12	9 (75)	9 (75)	0 (0)	0.015	<0.0001	0.0003
M111.1 VIRAL SENSING AND IMMUNITY; IRF2 TARGETS NE...	11	7 (63.6)	7 (63.6)	0 (0)	0.011	<0.0001	0.0003
M68 RIG-1 LIKE RECEPTOR SIGNALING	9	6 (66.7)	6 (66.7)	0 (0)	0.010	<0.0001	0.0003
M112.0 COMPLEMENT ACTIVATION (I)	17	8 (47.1)	7 (41.2)	1 (5.9)	0.013	<0.0001	0.0006
M165 ENRICHED IN ACTIVATED DENDRITIC CELLS (II)	34	11 (32.4)	11 (32.4)	0 (0)	0.017	<0.0001	0.0008
M156.1 PLASMA CELLS, IMMUNOGLOBULINS	22	6 (27.3)	0 (0)	6 (27.3)	0.009	<0.0001	0.0008
M13 INNATE ACTIVATION BY CYTOSOLIC DNA SENSING	11	5 (45.5)	5 (45.5)	0 (0)	0.008	<0.0001	0.0015
M50 CD1 AND OTHER DC RECEPTORS	9	5 (55.6)	0 (0)	5 (55.6)	0.008	<0.0001	0.0021
M16 TLR AND INFLAMMATORY SIGNALING	41	10 (24.4)	9 (22)	1 (2.4)	0.015	0.0005	0.0099
S10 RESTING DENDRITIC CELL SURFACE SIGNATURE	70	14 (20)	7 (10)	7 (10)	0.021	0.0008	0.0169
M47.2 ENRICHED IN B CELLS (III)	25	7 (28)	0 (0)	7 (28)	0.011	0.0012	0.0235

Confidential/Proprietary Information

Table S74: Blood Transcription Modules enriched in differentially expressed genes at Day 2 compared to pre-First Vaccination (Monocytes, Study Arm 2 A/H7N9+AS03, D1, 29). Results are sorted by FDR adjusted p-value and Jaccard similarity index (continued.)

Gene Set ID	Gene Set Genes #	DE Genes N (%)	Up-reg. DE Genes N (%)	Down-reg. DE Genes N (%)	Jaccard Index	P-Value	FDR Adjusted P-Value
S5 DC SURFACE SIGNATURE	76	14 (18.4)	9 (11.8)	5 (6.6)	0.021	0.0015	0.027
M86.0 CHEMOKINES AND INFLAMMATORY MOLECULES IN MYE...	18	5 (27.8)	5 (27.8)	0 (0)	0.008	0.0019	0.0336
M111.0 VIRAL SENSING AND IMMUNITY; IRF2 TARGETS NE...	16	5 (31.2)	4 (25)	1 (6.2)	0.008	0.0022	0.0356
M57 IMMUREGULATION - MONOCYTES, T AND B CELLS	13	4 (30.8)	1 (7.7)	3 (23.1)	0.006	0.0029	0.045
M86.1 PROINFLAMMATORY DENDRITIC CELL, MYELOID CELL...	12	4 (33.3)	4 (33.3)	0 (0)	0.006	0.0031	0.0471

Table S75: KEGG Pathways enriched in differentially expressed genes at Day 4 compared to pre-First Vaccination (Monocytes, Study Arm 2 A/H7N9+AS03, D1, 29). Results are sorted by FDR adjusted p-value and Jaccard similarity index.

Gene Set ID	Gene Set Genes #	DE Genes N (%)	Up-reg. DE Genes N (%)	Down-reg. DE Genes N (%)	Jaccard Index	P-Value	FDR Adjusted P-Value
COMPLEMENT AND COAGULATION CASCADES	82	5 (6.1)	5 (6.1)	0 (0)	0.044	<0.0001	0.0034
STAPHYLOCOCCUS AUREUS INFECTION	91	4 (4.4)	4 (4.4)	0 (0)	0.033	<0.0001	0.0051
PERTUSSIS	75	4 (5.3)	4 (5.3)	0 (0)	0.037	<0.0001	0.0067
SYSTEMIC LUPUS ERYTHEMATOSUS	127	4 (3.1)	4 (3.1)	0 (0)	0.025	<0.0001	0.0067

Table S76: MSigDB Reactome Pathways enriched in differentially expressed genes at Day 4 compared to pre-First Vaccination (Monocytes, Study Arm 2 A/H7N9+AS03, D1, 29). Results are sorted by FDR adjusted p-value and Jaccard similarity index.

Gene Set ID	Gene Set Genes #	DE Genes N (%)	Up-reg. DE Genes N (%)	Down-reg. DE Genes N (%)	Jaccard Index	P-Value	FDR Adjusted P-Value
REACTOME CREATION OF C4 AND C2 ACTIVATORS	14	3 (21.4)	3 (21.4)	0 (0)	0.064	<0.0001	0.0051
REACTOME INITIAL TRIGGERING OF COMPLEMENT	17	3 (17.6)	3 (17.6)	0 (0)	0.060	<0.0001	0.0051
REACTOME COMPLEMENT CASCADE	52	4 (7.7)	4 (7.7)	0 (0)	0.048	<0.0001	0.0051

Table S77: MSigDB GO Biological Processes enriched in differentially expressed genes at Day 4 compared to pre-First Vaccination (Monocytes, Study Arm 2 A/H7N9+AS03, D1, 29). Results are sorted by FDR adjusted p-value and Jaccard similarity index.

Gene Set ID	Gene Set Genes #	DE Genes N (%)	Up-reg. DE Genes N (%)	Down-reg. DE Genes N (%)	Jaccard Index	P-Value	FDR Adjusted P-Value
GO SYNAPSE PRUNING	11	3 (27.3)	3 (27.3)	0 (0)	0.068	<0.0001	0.0125

Confidential/Proprietary Information

Table S77: MSigDB GO Biological Processes enriched in differentially expressed genes at Day 4 compared to pre-First Vaccination (Monocytes, Study Arm 2 A/H7N9+AS03, D1, 29). Results are sorted by FDR adjusted p-value and Jaccard similarity index (continued.)

Gene Set ID	Gene Set Genes #	DE Genes N (%)	Up-reg. DE Genes N (%)	Down-reg. DE Genes N (%)	Jaccard Index	P-Value	FDR Adjusted P-Value
GO HUMORAL IMMUNE RESPONSE MEDIATED BY CIRCULATING...	40	4 (10)	4 (10)	0 (0)	0.056	<0.0001	0.0125
GO COMPLEMENT ACTIVATION	62	4 (6.5)	4 (6.5)	0 (0)	0.043	<0.0001	0.0125
GO REGULATION OF HUMORAL IMMUNE RESPONSE	62	4 (6.5)	4 (6.5)	0 (0)	0.043	<0.0001	0.0125
GO ADAPTIVE IMMUNE RESPONSE BASED ON SOMATIC RECOM...	223	6 (2.7)	5 (2.2)	1 (0.4)	0.024	<0.0001	0.0125
GO ADAPTIVE IMMUNE RESPONSE	353	7 (2)	6 (1.7)	1 (0.3)	0.018	<0.0001	0.0125
GO B CELL MEDIATED IMMUNITY	104	4 (3.8)	4 (3.8)	0 (0)	0.029	<0.0001	0.0471
GO DEFENSE RESPONSE	1497	11 (0.7)	10 (0.7)	1 (0.1)	0.007	<0.0001	0.0471

Table S78: Blood Transcription Modules enriched in differentially expressed genes at Day 4 compared to pre-First Vaccination (Monocytes, Study Arm 2 A/H7N9+AS03, D1, 29). Results are sorted by FDR adjusted p-value and Jaccard similarity index.

Gene Set ID	Gene Set Genes #	DE Genes N (%)	Up-reg. DE Genes N (%)	Down-reg. DE Genes N (%)	Jaccard Index	P-Value	FDR Adjusted P-Value
M112.0 COMPLEMENT ACTIVATION (I)	17	4 (23.5)	4 (23.5)	0 (0)	0.082	<0.0001	0.0017
M40 COMPLEMENT AND OTHER RECEPTORS IN DCS	12	3 (25)	3 (25)	0 (0)	0.067	<0.0001	0.0017

Table S79: KEGG Pathways enriched in differentially expressed genes at Day 30 compared to pre-Second Vaccination (Monocytes, Study Arm 2 A/H7N9+AS03, D1, 29). Results are sorted by FDR adjusted p-value and Jaccard similarity index.

Gene Set ID	Gene Set Genes #	DE Genes N (%)	Up-reg. DE Genes N (%)	Down-reg. DE Genes N (%)	Jaccard Index	P-Value	FDR Adjusted P-Value
EPSTEIN-BARR VIRUS INFECTION	196	35 (17.9)	31 (15.8)	4 (2)	0.022	<0.0001	0.0004
INFLUENZA A	160	31 (19.4)	31 (19.4)	0 (0)	0.020	<0.0001	0.0004
PHAGOSOME	145	27 (18.6)	19 (13.1)	8 (5.5)	0.018	<0.0001	0.0004
HEMATOPOIETIC CELL LINEAGE	93	26 (28)	13 (14)	13 (14)	0.018	<0.0001	0.0004
STAPHYLOCOCCUS AUREUS INFECTION	91	21 (23.1)	17 (18.7)	4 (4.4)	0.014	<0.0001	0.0004
ANTIGEN PROCESSING AND PRESENTATION	70	16 (22.9)	16 (22.9)	0 (0)	0.011	<0.0001	0.0004
INTESTINAL IMMUNE NETWORK FOR IGA PRODUCTION	45	14 (31.1)	12 (26.7)	2 (4.4)	0.010	<0.0001	0.0004
ASTHMA	27	9 (33.3)	8 (29.6)	1 (3.7)	0.006	<0.0001	0.0004
LEISHMANIASIS	70	17 (24.3)	15 (21.4)	2 (2.9)	0.012	<0.0001	0.0006
RHEUMATOID ARTHRITIS	88	17 (19.3)	13 (14.8)	4 (4.5)	0.011	<0.0001	0.0006
ALLOGRAFT REJECTION	34	10 (29.4)	10 (29.4)	0 (0)	0.007	<0.0001	0.0006
GRAFT-VERSUS-HOST DISEASE	38	10 (26.3)	10 (26.3)	0 (0)	0.007	<0.0001	0.0008

Table S79: KEGG Pathways enriched in differentially expressed genes at Day 30 compared to pre-Second Vaccination (Monocytes, Study Arm 2 A/H7N9+AS03, D1, 29). Results are sorted by FDR adjusted p-value and Jaccard similarity index (continued.)

Gene Set ID	Gene Set Genes #	DE Genes N (%)	Up-reg. DE Genes N (%)	Down-reg. DE Genes N (%)	Jaccard Index	P-Value	FDR Adjusted P-Value
SYSTEMIC LUPUS ERYTHEMATOSUS	127	16 (12.6)	15 (11.8)	1 (0.8)	0.011	<0.0001	0.001
AUTOIMMUNE THYROID DISEASE	49	10 (20.4)	10 (20.4)	0 (0)	0.007	<0.0001	0.0013
TYPE I DIABETES MELLITUS	41	10 (24.4)	10 (24.4)	0 (0)	0.007	<0.0001	0.0013
INFLAMMATORY BOWEL DISEASE	61	13 (21.3)	12 (19.7)	1 (1.6)	0.009	<0.0001	0.0021
CYTOKINE-CYTOKINE RECEPTOR INTERACTION	280	30 (10.7)	20 (7.1)	10 (3.6)	0.018	0.0001	0.0028
CELL ADHESION MOLECULES	137	23 (16.8)	17 (12.4)	6 (4.4)	0.015	0.0002	0.0034
TH17 CELL DIFFERENTIATION	102	18 (17.6)	15 (14.7)	3 (2.9)	0.012	0.0004	0.0076
PRIMARY IMMUNODEFICIENCY	32	8 (25)	4 (12.5)	4 (12.5)	0.005	0.0009	0.016
NF-KAPPA B SIGNALING PATHWAY	94	16 (17)	9 (9.6)	7 (7.4)	0.011	0.0011	0.017
TUBERCULOSIS	173	24 (13.9)	22 (12.7)	2 (1.2)	0.015	0.0012	0.0185
TOXOPLASMOSIS	106	18 (17)	14 (13.2)	4 (3.8)	0.012	0.0015	0.0208
VIRAL MYOCARDITIS	55	11 (20)	11 (20)	0 (0)	0.007	0.0014	0.0208
MEASLES	132	20 (15.2)	20 (15.2)	0 (0)	0.013	0.0015	0.0209
NOD-LIKE RECEPTOR SIGNALING PATHWAY	175	23 (13.1)	22 (12.6)	1 (0.6)	0.015	0.0017	0.0224
AGE-RAGE SIGNALING PATHWAY IN DIABETIC COMPLICATIO...	95	17 (17.9)	9 (9.5)	8 (8.4)	0.011	0.0023	0.0283
COMPLEMENT AND COAGULATION CASCADES	82	13 (15.9)	8 (9.8)	5 (6.1)	0.009	0.0036	0.0428
APOPTOSIS	132	19 (14.4)	14 (10.6)	5 (3.8)	0.012	0.0037	0.0432

Table S80: MSigDB Reactome Pathways enriched in differentially expressed genes at Day 30 compared to pre-Second Vaccination (Monocytes, Study Arm 2 A/H7N9+AS03, D1, 29). Results are sorted by FDR adjusted p-value and Jaccard similarity index.

Gene Set ID	Gene Set Genes #	DE Genes N (%)	Up-reg. DE Genes N (%)	Down-reg. DE Genes N (%)	Jaccard Index	P-Value	FDR Adjusted P-Value
REACTOME CYTOKINE SIGNALING IN IMMUNE SYSTEM	788	116 (14.7)	89 (11.3)	27 (3.4)	0.056	<0.0001	0.0019
REACTOME INNATE IMMUNE SYSTEM	968	101 (10.4)	75 (7.7)	26 (2.7)	0.044	<0.0001	0.0019
REACTOME INTERFERON SIGNALING	177	53 (29.9)	50 (28.2)	3 (1.7)	0.034	<0.0001	0.0019
REACTOME INTERFERON ALPHA BETA SIGNALING	60	30 (50)	29 (48.3)	1 (1.7)	0.021	<0.0001	0.0019
REACTOME INTERFERON GAMMA SIGNALING	71	28 (39.4)	26 (36.6)	2 (2.8)	0.019	<0.0001	0.0019
REACTOME IMMUNOREGULATORY INTERACTIONS BETWEEN A L...	97	24 (24.7)	13 (13.4)	11 (11.3)	0.016	<0.0001	0.0019
REACTOME INTERLEUKIN 4 AND INTERLEUKIN 13 SIGNALIN...	102	21 (20.6)	14 (13.7)	7 (6.9)	0.014	<0.0001	0.0019
REACTOME INTERLEUKIN 10 SIGNALING	39	12 (30.8)	10 (25.6)	2 (5.1)	0.008	<0.0001	0.0019
REACTOME ADAPTIVE IMMUNE SYSTEM	670	76 (11.3)	48 (7.2)	28 (4.2)	0.038	<0.0001	0.0102
REACTOME SIGNALING BY INTERLEUKINS	430	50 (11.6)	38 (8.8)	12 (2.8)	0.028	<0.0001	0.0123
REACTOME GPCR LIGAND BINDING	420	39 (9.3)	27 (6.4)	12 (2.9)	0.022	0.0001	0.0209
REACTOME GROWTH HORMONE RECEPTOR SIGNALING	24	8 (33.3)	6 (25)	2 (8.3)	0.006	0.0002	0.0217

Table S80: MSigDB Reactome Pathways enriched in differentially expressed genes at Day 30 compared to pre-Second Vaccination (Monocytes, Study Arm 2 A/H7N9+AS03, D1, 29). Results are sorted by FDR adjusted p-value and Jaccard similarity index (continued.)

Gene Set ID	Gene Set Genes #	DE Genes N (%)	Up-reg. DE Genes N (%)	Down-reg. DE Genes N (%)	Jaccard Index	P-Value	FDR Adjusted P-Value
REACTOME COMPLEMENT CASCADE	52	11 (21.2)	7 (13.5)	4 (7.7)	0.007	0.0002	0.0224
REACTOME NICOTINATE METABOLISM	29	9 (31)	6 (20.7)	3 (10.3)	0.006	0.0002	0.0245
REACTOME OAS ANTIVIRAL RESPONSE	8	5 (62.5)	5 (62.5)	0 (0)	0.004	0.0002	0.0245
REACTOME CLASS A 1 RHODOPSIN LIKE RECEPTORS	306	30 (9.8)	21 (6.9)	9 (2.9)	0.018	0.0003	0.0249
REACTOME G ALPHA I SIGNALLING EVENTS	367	39 (10.6)	25 (6.8)	14 (3.8)	0.022	0.0004	0.036

Table S81: MSigDB GO Biological Processes enriched in differentially expressed genes at Day 30 compared to pre-Second Vaccination (Monocytes, Study Arm 2 A/H7N9+AS03, D1, 29). Results are sorted by FDR adjusted p-value and Jaccard similarity index.

Gene Set ID	Gene Set Genes #	DE Genes N (%)	Up-reg. DE Genes N (%)	Down-reg. DE Genes N (%)	Jaccard Index	P-Value	FDR Adjusted P-Value
GO DEFENSE RESPONSE	1497	221 (14.8)	175 (11.7)	46 (3.1)	0.082	<0.0001	0.0005
GO RESPONSE TO BIOTIC STIMULUS	1325	204 (15.4)	164 (12.4)	40 (3)	0.080	<0.0001	0.0005
GO REGULATION OF IMMUNE SYSTEM PROCESS	1409	197 (14)	130 (9.2)	67 (4.8)	0.075	<0.0001	0.0005
GO DEFENSE RESPONSE TO OTHER ORGANISM	993	166 (16.7)	142 (14.3)	24 (2.4)	0.074	<0.0001	0.0005
GO INNATE IMMUNE RESPONSE	814	144 (17.7)	124 (15.2)	20 (2.5)	0.069	<0.0001	0.0005
GO IMMUNE EFFECTOR PROCESS	1077	159 (14.8)	123 (11.4)	36 (3.3)	0.068	<0.0001	0.0005
GO RESPONSE TO CYTOKINE	1099	157 (14.3)	120 (10.9)	37 (3.4)	0.067	<0.0001	0.0005
GO CELL ACTIVATION	1265	163 (12.9)	99 (7.8)	64 (5.1)	0.065	<0.0001	0.0005
GO REGULATION OF IMMUNE RESPONSE	897	139 (15.5)	96 (10.7)	43 (4.8)	0.064	<0.0001	0.0005
GO POSITIVE REGULATION OF IMMUNE SYSTEM PROCESS	972	143 (14.7)	95 (9.8)	48 (4.9)	0.064	<0.0001	0.0005
GO REGULATION OF RESPONSE TO EXTERNAL STIMULUS	1006	138 (13.7)	100 (9.9)	38 (3.8)	0.060	<0.0001	0.0005
GO SECRETION	1551	166 (10.7)	104 (6.7)	62 (4)	0.059	<0.0001	0.0005
GO REGULATION OF CELL POPULATION PROLIFERATION	1541	162 (10.5)	79 (5.1)	83 (5.4)	0.058	<0.0001	0.0005
GO REGULATION OF MULTI ORGANISM PROCESS	700	115 (16.4)	97 (13.9)	18 (2.6)	0.057	<0.0001	0.0005
GO REGULATION OF RESPONSE TO STRESS	1406	151 (10.7)	107 (7.6)	44 (3.1)	0.057	<0.0001	0.0005
GO HOMEOSTATIC PROCESS	1803	171 (9.5)	104 (5.8)	67 (3.7)	0.056	<0.0001	0.0005
GO POSITIVE REGULATION OF SIGNALING	1730	167 (9.7)	99 (5.7)	68 (3.9)	0.056	<0.0001	0.0005
GO CYTOKINE PRODUCTION	720	113 (15.7)	78 (10.8)	35 (4.9)	0.056	<0.0001	0.0005
GO LOCOMOTION	1747	167 (9.6)	87 (5)	80 (4.6)	0.056	<0.0001	0.0005
GO POSITIVE REGULATION OF MULTICELLULAR ORGANISMAL...	1665	161 (9.7)	95 (5.7)	66 (4)	0.055	<0.0001	0.0005
GO CELL MOTILITY	1531	153 (10)	79 (5.2)	74 (4.8)	0.055	<0.0001	0.0005
GO CYTOKINE MEDIATED SIGNALING PATHWAY	711	110 (15.5)	90 (12.7)	20 (2.8)	0.054	<0.0001	0.0005
GO POSITIVE REGULATION OF IMMUNE RESPONSE	704	108 (15.3)	74 (10.5)	34 (4.8)	0.054	<0.0001	0.0005
GO REGULATION OF DEFENSE RESPONSE	703	107 (15.2)	81 (11.5)	26 (3.7)	0.053	<0.0001	0.0005

Table S81: MSigDB GO Biological Processes enriched in differentially expressed genes at Day 30 compared to pre-Second Vaccination (Monocytes, Study Arm 2 A/H7N9+AS03, D1, 29). Results are sorted by FDR adjusted p-value and Jaccard similarity index (continued.)

Gene Set ID	Gene Set Genes #	DE Genes N (%)	Up-reg. DE Genes N (%)	Down-reg. DE Genes N (%)	Jaccard Index	P-Value	FDR Adjusted P-Value
GO BIOLOGICAL ADHESION	1320	135 (10.2)	70 (5.3)	65 (4.9)	0.052	<0.0001	0.0005
GO RESPONSE TO OXYGEN CONTAINING COMPOUND	1593	146 (9.2)	87 (5.5)	59 (3.7)	0.051	<0.0001	0.0005
GO LYMPHOCYTE ACTIVATION	620	95 (15.3)	51 (8.2)	44 (7.1)	0.049	<0.0001	0.0005
GO CHEMICAL HOMEOSTASIS	1123	118 (10.5)	73 (6.5)	45 (4)	0.049	<0.0001	0.0005
GO IMMUNE SYSTEM DEVELOPMENT	926	105 (11.3)	62 (6.7)	43 (4.6)	0.047	<0.0001	0.0005
GO RESPONSE TO VIRUS	300	74 (24.7)	69 (23)	5 (1.7)	0.045	<0.0001	0.0005
GO REGULATION OF RESPONSE TO BIOTIC STIMULUS	466	81 (17.4)	70 (15)	11 (2.4)	0.045	<0.0001	0.0005
GO NEGATIVE REGULATION OF PROTEIN METABOLIC PROCES...	1039	105 (10.1)	61 (5.9)	44 (4.2)	0.045	<0.0001	0.0005
GO REGULATION OF CELLULAR COMPONENT MOVEMENT	973	101 (10.4)	52 (5.3)	49 (5)	0.044	<0.0001	0.0005
GO POSITIVE REGULATION OF RESPONSE TO EXTERNAL STI...	575	84 (14.6)	65 (11.3)	19 (3.3)	0.044	<0.0001	0.0005
GO REGULATION OF CELL ACTIVATION	507	81 (16)	44 (8.7)	37 (7.3)	0.044	<0.0001	0.0005
GO INFLAMMATORY RESPONSE	680	88 (12.9)	61 (9)	27 (4)	0.044	<0.0001	0.0005
GO ACTIVATION OF IMMUNE RESPONSE	547	82 (15)	59 (10.8)	23 (4.2)	0.043	<0.0001	0.0005
GO REGULATION OF TRANSFERASE ACTIVITY	912	95 (10.4)	57 (6.2)	38 (4.2)	0.043	<0.0001	0.0005
GO DEFENSE RESPONSE TO VIRUS	229	67 (29.3)	63 (27.5)	4 (1.7)	0.042	<0.0001	0.0005
GO ADAPTIVE IMMUNE RESPONSE	353	70 (19.8)	44 (12.5)	26 (7.4)	0.041	<0.0001	0.0005
GO IMMUNE RESPONSE REGULATING SIGNALING PATHWAY	507	76 (15)	51 (10.1)	25 (4.9)	0.041	<0.0001	0.0005
GO LEUKOCYTE MEDIATED IMMUNITY	701	83 (11.8)	59 (8.4)	24 (3.4)	0.041	<0.0001	0.0005
GO RESPONSE TO BACTERIUM	587	78 (13.3)	55 (9.4)	23 (3.9)	0.041	<0.0001	0.0005
GO POSITIVE REGULATION OF DEFENSE RESPONSE	457	71 (15.5)	56 (12.3)	15 (3.3)	0.039	<0.0001	0.0005
GO REGULATION OF SECRETION	752	82 (10.9)	47 (6.2)	35 (4.7)	0.039	<0.0001	0.0005
GO REGULATION OF IMMUNE EFFECTOR PROCESS	357	67 (18.8)	47 (13.2)	20 (5.6)	0.039	<0.0001	0.0005
GO T CELL ACTIVATION	436	69 (15.8)	40 (9.2)	29 (6.7)	0.039	<0.0001	0.0005
GO POSITIVE REGULATION OF CYTOKINE PRODUCTION	419	68 (16.2)	49 (11.7)	19 (4.5)	0.038	<0.0001	0.0005
GO PEPTIDE SECRETION	587	73 (12.4)	47 (8)	26 (4.4)	0.038	<0.0001	0.0005
GO MYELOID LEUKOCYTE ACTIVATION	594	73 (12.3)	53 (8.9)	20 (3.4)	0.038	<0.0001	0.0005
GO POSITIVE REGULATION OF CELL DEATH	670	75 (11.2)	52 (7.8)	23 (3.4)	0.037	<0.0001	0.0005
GO REGULATION OF LYMPHOCYTE ACTIVATION	392	65 (16.6)	35 (8.9)	30 (7.7)	0.037	<0.0001	0.0005
GO LEUKOCYTE DIFFERENTIATION	483	66 (13.7)	37 (7.7)	29 (6)	0.036	<0.0001	0.0005
GO POSITIVE REGULATION OF MULTI ORGANISM PROCESS	489	65 (13.3)	51 (10.4)	14 (2.9)	0.035	<0.0001	0.0005
GO TAXIS	607	69 (11.4)	42 (6.9)	27 (4.4)	0.035	<0.0001	0.0005
GO METAL ION HOMEOSTASIS	622	67 (10.8)	47 (7.6)	20 (3.2)	0.034	<0.0001	0.0005
GO LEUKOCYTE MIGRATION	410	59 (14.4)	36 (8.8)	23 (5.6)	0.033	<0.0001	0.0005
GO IMMUNE RESPONSE REGULATING CELL SURFACE RECEPTO...	348	55 (15.8)	34 (9.8)	21 (6)	0.032	<0.0001	0.0005
GO ADAPTIVE IMMUNE RESPONSE BASED ON SOMATIC RECOM...	223	50 (22.4)	33 (14.8)	17 (7.6)	0.031	<0.0001	0.0005
GO POSITIVE REGULATION OF RESPONSE TO BIOTIC STIMU...	353	53 (15)	44 (12.5)	9 (2.5)	0.031	<0.0001	0.0005
GO NEGATIVE REGULATION OF IMMUNE SYSTEM PROCESS	426	55 (12.9)	33 (7.7)	22 (5.2)	0.031	<0.0001	0.0005

Table S81: MSigDB GO Biological Processes enriched in differentially expressed genes at Day 30 compared to pre-Second Vaccination (Monocytes, Study Arm 2 A/H7N9+AS03, D1, 29). Results are sorted by FDR adjusted p-value and Jaccard similarity index (continued.)

Gene Set ID	Gene Set Genes #	DE Genes N (%)	Up-reg. DE Genes N (%)	Down-reg. DE Genes N (%)	Jaccard Index	P-Value	FDR Adjusted P-Value
GO NEGATIVE REGULATION OF MULTI ORGANISM PROCESS	195	48 (24.6)	42 (21.5)	6 (3.1)	0.031	<0.0001	0.0005
GO REGULATION OF PEPTIDE SECRETION	461	56 (12.1)	34 (7.4)	22 (4.8)	0.031	<0.0001	0.0005
GO LEUKOCYTE CELL CELL ADHESION	307	50 (16.3)	31 (10.1)	19 (6.2)	0.030	<0.0001	0.0005
GO LYMPHOCYTE DIFFERENTIATION	336	51 (15.2)	26 (7.7)	25 (7.4)	0.030	<0.0001	0.0005
GO VIRAL LIFE CYCLE	308	50 (16.2)	44 (14.3)	6 (1.9)	0.030	<0.0001	0.0005
GO REGULATION OF T CELL ACTIVATION	292	49 (16.8)	28 (9.6)	21 (7.2)	0.029	<0.0001	0.0005
GO REGULATION OF CELL CELL ADHESION	372	50 (13.4)	29 (7.8)	21 (5.6)	0.029	<0.0001	0.0005
GO RESPONSE TO INTERFERON GAMMA	166	42 (25.3)	40 (24.1)	2 (1.2)	0.027	<0.0001	0.0005
GO POSITIVE REGULATION OF CELL ACTIVATION	297	45 (15.2)	28 (9.4)	17 (5.7)	0.027	<0.0001	0.0005
GO REGULATION OF INFLAMMATORY RESPONSE	332	44 (13.3)	27 (8.1)	17 (5.1)	0.026	<0.0001	0.0005
GO LYMPHOCYTE MEDIATED IMMUNITY	216	41 (19)	26 (12)	15 (6.9)	0.026	<0.0001	0.0005
GO CYTOKINE SECRETION	220	40 (18.2)	29 (13.2)	11 (5)	0.025	<0.0001	0.0005
GO NEGATIVE REGULATION OF RESPONSE TO EXTERNAL STI...	350	43 (12.3)	25 (7.1)	18 (5.1)	0.025	<0.0001	0.0005
GO RESPONSE TO MOLECULE OF BACTERIAL ORIGIN	329	42 (12.8)	32 (9.7)	10 (3)	0.025	<0.0001	0.0005
GO CELL CHEMOTAXIS	278	40 (14.4)	30 (10.8)	10 (3.6)	0.024	<0.0001	0.0005
GO LEUKOCYTE PROLIFERATION	276	40 (14.5)	22 (8)	18 (6.5)	0.024	<0.0001	0.0005
GO T CELL DIFFERENTIATION	230	39 (17)	23 (10)	16 (7)	0.024	<0.0001	0.0005
GO REGULATION OF SYMBIOSIS ENCOMPASSING MUTUALISM ...	204	38 (18.6)	35 (17.2)	3 (1.5)	0.024	<0.0001	0.0005
GO MYELOID LEUKOCYTE MIGRATION	187	36 (19.3)	23 (12.3)	13 (7)	0.023	<0.0001	0.0005
GO ANTIGEN RECEPTOR MEDIATED SIGNALING PATHWAY	212	36 (17)	21 (9.9)	15 (7.1)	0.023	<0.0001	0.0005
GO REGULATION OF LEUKOCYTE DIFFERENTIATION	252	36 (14.3)	23 (9.1)	13 (5.2)	0.022	<0.0001	0.0005
GO RESPONSE TO TYPE I INTERFERON	88	32 (36.4)	31 (35.2)	1 (1.1)	0.022	<0.0001	0.0005
GO PRODUCTION OF MOLECULAR MEDIATOR OF IMMUNE RESP...	187	34 (18.2)	25 (13.4)	9 (4.8)	0.022	<0.0001	0.0005
GO LEUKOCYTE CHEMOTAXIS	202	34 (16.8)	24 (11.9)	10 (5)	0.021	<0.0001	0.0005
GO NEGATIVE REGULATION OF CYTOKINE PRODUCTION	262	35 (13.4)	21 (8)	14 (5.3)	0.021	<0.0001	0.0005
GO VIRAL GENOME REPLICATION	117	32 (27.4)	31 (26.5)	1 (0.9)	0.021	<0.0001	0.0005
GO HUMORAL IMMUNE RESPONSE	227	34 (15)	18 (7.9)	16 (7)	0.021	<0.0001	0.0005
GO REGULATION OF VIRAL LIFE CYCLE	133	32 (24.1)	31 (23.3)	1 (0.8)	0.021	<0.0001	0.0005
GO POSITIVE REGULATION OF IMMUNE EFFECTOR PROCESS	189	33 (17.5)	21 (11.1)	12 (6.3)	0.021	<0.0001	0.0005
GO REGULATION OF LEUKOCYTE PROLIFERATION	203	33 (16.3)	19 (9.4)	14 (6.9)	0.021	<0.0001	0.0005
GO NEGATIVE REGULATION OF VIRAL PROCESS	90	30 (33.3)	29 (32.2)	1 (1.1)	0.020	<0.0001	0.0005
GO POSITIVE REGULATION OF LEUKOCYTE CELL CELL ADHE...	194	31 (16)	19 (9.8)	12 (6.2)	0.020	<0.0001	0.0005
GO REGULATION OF LYMPHOCYTE DIFFERENTIATION	158	30 (19)	18 (11.4)	12 (7.6)	0.019	<0.0001	0.0005
GO REGULATION OF VIRAL GENOME REPLICATION	91	28 (30.8)	28 (30.8)	0 (0)	0.019	<0.0001	0.0005
GO REGULATION OF LEUKOCYTE MIGRATION	175	29 (16.6)	18 (10.3)	11 (6.3)	0.018	<0.0001	0.0005
GO NEGATIVE REGULATION OF VIRAL LIFE CYCLE	76	27 (35.5)	27 (35.5)	0 (0)	0.018	<0.0001	0.0005
GO REGULATION OF ADAPTIVE IMMUNE RESPONSE	142	28 (19.7)	20 (14.1)	8 (5.6)	0.018	<0.0001	0.0005

Table S81: MSigDB GO Biological Processes enriched in differentially expressed genes at Day 30 compared to pre-Second Vaccination (Monocytes, Study Arm 2 A/H7N9+AS03, D1, 29). Results are sorted by FDR adjusted p-value and Jaccard similarity index (continued.)

Gene Set ID	Gene Set Genes #	DE Genes N (%)	Up-reg. DE Genes N (%)	Down-reg. DE Genes N (%)	Jaccard Index	P-Value	FDR Adjusted P-Value
GO ALPHA BETA T CELL ACTIVATION	127	27 (21.3)	20 (15.7)	7 (5.5)	0.018	<0.0001	0.0005
GO NEGATIVE REGULATION OF VIRAL GENOME REPLICATION	58	25 (43.1)	25 (43.1)	0 (0)	0.017	<0.0001	0.0005
GO INTERFERON GAMMA MEDIATED SIGNALING PATHWAY	70	25 (35.7)	24 (34.3)	1 (1.4)	0.017	<0.0001	0.0005
GO REGULATION OF T CELL DIFFERENTIATION	131	26 (19.8)	16 (12.2)	10 (7.6)	0.017	<0.0001	0.0005
GO TUMOR NECROSIS FACTOR SUPERFAMILY CYTOKINE PROD...	148	26 (17.6)	18 (12.2)	8 (5.4)	0.017	<0.0001	0.0005
GO GRANULOCYTE MIGRATION	126	25 (19.8)	19 (15.1)	6 (4.8)	0.017	<0.0001	0.0005
GO REGULATION OF PRODUCTION OF MOLECULAR MEDIATOR ...	129	24 (18.6)	17 (13.2)	7 (5.4)	0.016	<0.0001	0.0005
GO NEGATIVE REGULATION OF IMMUNE RESPONSE	134	23 (17.2)	19 (14.2)	4 (3)	0.015	<0.0001	0.0005
GO POSITIVE REGULATION OF INFLAMMATORY RESPONSE	134	23 (17.2)	17 (12.7)	6 (4.5)	0.015	<0.0001	0.0005
GO CYTOKINE PRODUCTION INVOLVED IN IMMUNE RESPONSE	94	22 (23.4)	18 (19.1)	4 (4.3)	0.015	<0.0001	0.0005
GO POSITIVE REGULATION OF ADAPTIVE IMMUNE RESPONSE	92	22 (23.9)	14 (15.2)	8 (8.7)	0.015	<0.0001	0.0005
GO B CELL MEDIATED IMMUNITY	104	21 (20.2)	13 (12.5)	8 (7.7)	0.014	<0.0001	0.0005
GO POSITIVE REGULATION OF LEUKOCYTE MEDIATED IMMUN...	113	21 (18.6)	11 (9.7)	10 (8.8)	0.014	<0.0001	0.0005
GO POSITIVE REGULATION OF CYTOKINE SECRETION	126	21 (16.7)	16 (12.7)	5 (4)	0.014	<0.0001	0.0005
GO CELL KILLING	146	21 (14.4)	11 (7.5)	10 (6.8)	0.014	<0.0001	0.0005
GO REGULATION OF DEFENSE RESPONSE TO VIRUS	68	20 (29.4)	19 (27.9)	1 (1.5)	0.014	<0.0001	0.0005
GO ALPHA BETA T CELL DIFFERENTIATION	97	20 (20.6)	15 (15.5)	5 (5.2)	0.013	<0.0001	0.0005
GO NEUTROPHIL MIGRATION	103	20 (19.4)	15 (14.6)	5 (4.9)	0.013	<0.0001	0.0005
GO B CELL PROLIFERATION	91	19 (20.9)	5 (5.5)	14 (15.4)	0.013	<0.0001	0.0005
GO MACROPHAGE ACTIVATION	84	19 (22.6)	13 (15.5)	6 (7.1)	0.013	<0.0001	0.0005
GO POSITIVE REGULATION OF LYMPHOCYTE MEDIATED IMMU...	87	19 (21.8)	9 (10.3)	10 (11.5)	0.013	<0.0001	0.0005
GO T CELL MEDIATED IMMUNITY	84	19 (22.6)	13 (15.5)	6 (7.1)	0.013	<0.0001	0.0005
GO INTERLEUKIN 1 PRODUCTION	99	19 (19.2)	15 (15.2)	4 (4)	0.013	<0.0001	0.0005
GO REGULATION OF LEUKOCYTE CHEMOTAXIS	102	19 (18.6)	14 (13.7)	5 (4.9)	0.013	<0.0001	0.0005
GO REGULATION OF CYTOKINE PRODUCTION INVOLVED IN I...	76	18 (23.7)	14 (18.4)	4 (5.3)	0.012	<0.0001	0.0005
GO INTERLEUKIN 1 BETA PRODUCTION	87	18 (20.7)	14 (16.1)	4 (4.6)	0.012	<0.0001	0.0005
GO REGULATION OF ALPHA BETA T CELL ACTIVATION	84	18 (21.4)	15 (17.9)	3 (3.6)	0.012	<0.0001	0.0005
GO ACUTE INFLAMMATORY RESPONSE	102	18 (17.6)	12 (11.8)	6 (5.9)	0.012	<0.0001	0.0005
GO NEUROINFLAMMATORY RESPONSE	63	17 (27)	9 (14.3)	8 (12.7)	0.012	<0.0001	0.0005
GO REGULATION OF CELL KILLING	80	16 (20)	8 (10)	8 (10)	0.011	<0.0001	0.0005
GO LEUKOCYTE MEDIATED CYTOTOXICITY	87	16 (18.4)	8 (9.2)	8 (9.2)	0.011	<0.0001	0.0005
GO MACROPHAGE MIGRATION	47	15 (31.9)	8 (17)	7 (14.9)	0.010	<0.0001	0.0005
GO REGULATION OF B CELL PROLIFERATION	58	15 (25.9)	5 (8.6)	10 (17.2)	0.010	<0.0001	0.0005
GO REGULATION OF DEFENSE RESPONSE TO VIRUS BY HOST	34	14 (41.2)	14 (41.2)	0 (0)	0.010	<0.0001	0.0005
GO REGULATION OF T CELL MEDIATED IMMUNITY	59	14 (23.7)	8 (13.6)	6 (10.2)	0.010	<0.0001	0.0005
GO REGULATION OF HUMORAL IMMUNE RESPONSE	62	14 (22.6)	8 (12.9)	6 (9.7)	0.009	<0.0001	0.0005
GO POSITIVE REGULATION OF T CELL MEDIATED IMMUNITY	41	13 (31.7)	7 (17.1)	6 (14.6)	0.009	<0.0001	0.0005

Table S81: MSigDB GO Biological Processes enriched in differentially expressed genes at Day 30 compared to pre-Second Vaccination (Monocytes, Study Arm 2 A/H7N9+AS03, D1, 29). Results are sorted by FDR adjusted p-value and Jaccard similarity index (continued.)

Gene Set ID	Gene Set Genes #	DE Genes N (%)	Up-reg. DE Genes N (%)	Down-reg. DE Genes N (%)	Jaccard Index	P-Value	FDR Adjusted P-Value
GO GLIAL CELL ACTIVATION	48	13 (27.1)	8 (16.7)	5 (10.4)	0.009	<0.0001	0.0005
GO INTERLEUKIN 1 SECRETION	53	13 (24.5)	10 (18.9)	3 (5.7)	0.009	<0.0001	0.0005
GO POSITIVE REGULATION OF CELL KILLING	54	13 (24.1)	5 (9.3)	8 (14.8)	0.009	<0.0001	0.0005
GO REGULATION OF LEUKOCYTE MEDIATED CYTOTOXICITY	59	13 (22)	6 (10.2)	7 (11.9)	0.009	<0.0001	0.0005
GO HUMORAL IMMUNE RESPONSE MEDIATED BY CIRCULATING...	40	12 (30)	6 (15)	6 (15)	0.008	<0.0001	0.0005
GO POSITIVE REGULATION OF DEFENSE RESPONSE TO VIRU...	24	11 (45.8)	11 (45.8)	0 (0)	0.008	<0.0001	0.0005
GO RESPONSE TO INTERFERON BETA	30	11 (36.7)	11 (36.7)	0 (0)	0.008	<0.0001	0.0005
GO LEUKOCYTE ACTIVATION INVOLVED IN INFLAMMATORY R...	40	11 (27.5)	7 (17.5)	4 (10)	0.008	<0.0001	0.0005
GO POSITIVE REGULATION OF LEUKOCYTE MEDIATED CYTOT...	41	11 (26.8)	4 (9.8)	7 (17.1)	0.008	<0.0001	0.0005
GO REGULATION OF MACROPHAGE MIGRATION	34	11 (32.4)	6 (17.6)	5 (14.7)	0.008	<0.0001	0.0005
GO REGULATION OF MONONUCLEAR CELL MIGRATION	40	11 (27.5)	9 (22.5)	2 (5)	0.008	<0.0001	0.0005
GO RESPONSE TO INTERFERON ALPHA	21	10 (47.6)	10 (47.6)	0 (0)	0.007	<0.0001	0.0005
GO MACROPHAGE CHEMOTAXIS	34	10 (29.4)	6 (17.6)	4 (11.8)	0.007	<0.0001	0.0005
GO NUCLEOSIDE CATABOLIC PROCESS	31	10 (32.3)	9 (29)	1 (3.2)	0.007	<0.0001	0.0005
GO REGULATION OF NEUROINFLAMMATORY RESPONSE	34	9 (26.5)	4 (11.8)	5 (14.7)	0.006	<0.0001	0.0005
GO REGULATION OF PHOSPHORUS METABOLIC PROCESS	1674	157 (9.4)	90 (5.4)	67 (4)	0.053	<0.0001	0.0009
GO REGULATION OF PHOSPHORYLATION	1495	148 (9.9)	85 (5.7)	63 (4.2)	0.053	<0.0001	0.0009
GO NEGATIVE REGULATION OF RESPONSE TO STIMULUS	1531	140 (9.1)	79 (5.2)	61 (4)	0.050	<0.0001	0.0009
GO CELL CELL ADHESION	781	87 (11.1)	48 (6.1)	39 (5)	0.041	<0.0001	0.0009
GO ION HOMEOSTASIS	772	79 (10.2)	57 (7.4)	22 (2.8)	0.037	<0.0001	0.0009
GO NEGATIVE REGULATION OF CELL POPULATION PROLIFER...	666	70 (10.5)	36 (5.4)	34 (5.1)	0.035	<0.0001	0.0009
GO POSITIVE REGULATION OF LOCOMOTION	544	61 (11.2)	33 (6.1)	28 (5.1)	0.032	<0.0001	0.0009
GO NEGATIVE REGULATION OF PHOSPHORYLATION	431	56 (13)	29 (6.7)	27 (6.3)	0.031	<0.0001	0.0009
GO RECEPTOR MEDIATED ENDOCYTOSIS	245	36 (14.7)	21 (8.6)	15 (6.1)	0.022	<0.0001	0.0009
GO RESPONSE TO TUMOR NECROSIS FACTOR	272	36 (13.2)	24 (8.8)	12 (4.4)	0.022	<0.0001	0.0009
GO REGULATION OF LEUKOCYTE MEDIATED IMMUNITY	174	27 (15.5)	17 (9.8)	10 (5.7)	0.017	<0.0001	0.0009
GO REGULATION OF LYMPHOCYTE MEDIATED IMMUNITY	127	22 (17.3)	12 (9.4)	10 (7.9)	0.014	<0.0001	0.0009
GO REGULATION OF B CELL ACTIVATION	112	21 (18.8)	8 (7.1)	13 (11.6)	0.014	<0.0001	0.0009
GO POSITIVE REGULATION OF LEUKOCYTE PROLIFERATION	124	21 (16.9)	15 (12.1)	6 (4.8)	0.014	<0.0001	0.0009
GO CD4 POSITIVE ALPHA BETA T CELL ACTIVATION	87	18 (20.7)	13 (14.9)	5 (5.7)	0.012	<0.0001	0.0009
GO LIPID STORAGE	67	15 (22.4)	10 (14.9)	5 (7.5)	0.010	<0.0001	0.0009
GO INTERLEUKIN 2 PRODUCTION	59	14 (23.7)	9 (15.3)	5 (8.5)	0.010	<0.0001	0.0009
GO POSITIVE REGULATION OF INTERFERON GAMMA PRODUCT...	54	13 (24.1)	8 (14.8)	5 (9.3)	0.009	<0.0001	0.0009
GO GLYCOSYL COMPOUND CATABOLIC PROCESS	38	10 (26.3)	9 (23.7)	1 (2.6)	0.007	<0.0001	0.0009
GO REGULATION OF GRANULOCYTE CHEMOTAXIS	40	10 (25)	6 (15)	4 (10)	0.007	<0.0001	0.0009
GO REGULATION OF PROTEIN MODIFICATION PROCESS	1724	157 (9.1)	92 (5.3)	65 (3.8)	0.053	<0.0001	0.0012
GO REGULATION OF KINASE ACTIVITY	816	87 (10.7)	53 (6.5)	34 (4.2)	0.041	<0.0001	0.0012

Table S81: MSigDB GO Biological Processes enriched in differentially expressed genes at Day 30 compared to pre-Second Vaccination (Monocytes, Study Arm 2 A/H7N9+AS03, D1, 29). Results are sorted by FDR adjusted p-value and Jaccard similarity index (continued.)

Gene Set ID	Gene Set Genes #	DE Genes N (%)	Up-reg. DE Genes N (%)	Down-reg. DE Genes N (%)	Jaccard Index	P-Value	FDR Adjusted P-Value
GO RESPONSE TO LIPID	872	86 (9.9)	51 (5.8)	35 (4)	0.039	<0.0001	0.0012
GO REGULATION OF HEMOPOIESIS	441	50 (11.3)	35 (7.9)	15 (3.4)	0.028	<0.0001	0.0012
GO ACTIVATION OF INNATE IMMUNE RESPONSE	286	39 (13.6)	33 (11.5)	6 (2.1)	0.023	<0.0001	0.0012
GO POSITIVE REGULATION OF PEPTIDE SECRETION	271	34 (12.5)	22 (8.1)	12 (4.4)	0.021	<0.0001	0.0012
GO CELLULAR RESPONSE TO BIOTIC STIMULUS	226	30 (13.3)	25 (11.1)	5 (2.2)	0.019	<0.0001	0.0012
GO NEGATIVE REGULATION OF DEFENSE RESPONSE	201	28 (13.9)	18 (9)	10 (5)	0.018	<0.0001	0.0012
GO INTERFERON GAMMA PRODUCTION	97	18 (18.6)	12 (12.4)	6 (6.2)	0.012	<0.0001	0.0012
GO POSITIVE REGULATION OF TUMOR NECROSIS FACTOR SU...	79	16 (20.3)	11 (13.9)	5 (6.3)	0.011	<0.0001	0.0012
GO CD4 POSITIVE ALPHA BETA T CELL DIFFERENTIATION	71	15 (21.1)	11 (15.5)	4 (5.6)	0.010	<0.0001	0.0012
GO REGULATION OF INTERLEUKIN 1 SECRETION	45	11 (24.4)	8 (17.8)	3 (6.7)	0.008	<0.0001	0.0012
GO BASE CONVERSION OR SUBSTITUTION EDITING	21	8 (38.1)	7 (33.3)	1 (4.8)	0.006	<0.0001	0.0012
GO REGULATION OF TRANSPORT	1745	157 (9)	86 (4.9)	71 (4.1)	0.052	<0.0001	0.0016
GO NEGATIVE REGULATION OF MULTICELLULAR ORGANISMAL...	1169	106 (9.1)	62 (5.3)	44 (3.8)	0.043	<0.0001	0.0016
GO IMPORT INTO CELL	668	76 (11.4)	47 (7)	29 (4.3)	0.038	<0.0001	0.0016
GO NEGATIVE REGULATION OF PROTEIN MODIFICATION PRO...	562	63 (11.2)	36 (6.4)	27 (4.8)	0.033	<0.0001	0.0016
GO NEGATIVE REGULATION OF CELL ACTIVATION	184	27 (14.7)	14 (7.6)	13 (7.1)	0.017	<0.0001	0.0016
GO POSITIVE REGULATION OF CYTOKINE BIOSYNTHETIC PR...	60	14 (23.3)	10 (16.7)	4 (6.7)	0.010	<0.0001	0.0016
GO REGULATION OF ANTIGEN RECEPTOR MEDIATED SIGNALI...	60	14 (23.3)	6 (10)	8 (13.3)	0.010	<0.0001	0.0016
GO COMPLEMENT ACTIVATION	62	13 (21)	8 (12.9)	5 (8.1)	0.009	<0.0001	0.0016
GO PYRIMIDINE NUCLEOSIDE CATABOLIC PROCESS	23	8 (34.8)	7 (30.4)	1 (4.3)	0.006	<0.0001	0.0016
GO T HELPER 17 CELL LINEAGE COMMITMENT	12	6 (50)	3 (25)	3 (25)	0.004	<0.0001	0.0016
GO CYTOKINE METABOLIC PROCESS	105	18 (17.1)	12 (11.4)	6 (5.7)	0.012	<0.0001	0.0019
GO POSITIVE REGULATION OF LEUKOCYTE MIGRATION	118	18 (15.3)	13 (11)	5 (4.2)	0.012	<0.0001	0.0019
GO RELAXATION OF MUSCLE	28	9 (32.1)	8 (28.6)	1 (3.6)	0.006	<0.0001	0.0019
GO POSITIVE REGULATION OF LIPID STORAGE	22	8 (36.4)	3 (13.6)	5 (22.7)	0.006	<0.0001	0.0019
GO RIBONUCLEOSIDE CATABOLIC PROCESS	20	7 (35)	7 (35)	0 (0)	0.005	<0.0001	0.0019
GO COMPLEMENT RECEPTOR MEDIATED SIGNALING PATHWAY	12	6 (50)	4 (33.3)	2 (16.7)	0.004	<0.0001	0.0019
GO DNA CYTOSINE DEAMINATION	10	5 (50)	5 (50)	0 (0)	0.004	<0.0001	0.0019
GO PROTEIN PHOSPHORYLATION	1838	169 (9.2)	94 (5.1)	75 (4.1)	0.055	<0.0001	0.0022
GO REGULATION OF CELL DEATH	1593	140 (8.8)	88 (5.5)	52 (3.3)	0.049	<0.0001	0.0022
GO INTERSPECIES INTERACTION BETWEEN ORGANISMS	869	88 (10.1)	74 (8.5)	14 (1.6)	0.040	<0.0001	0.0022
GO MULTICELLULAR ORGANISMAL HOMEOSTASIS	460	54 (11.7)	26 (5.7)	28 (6.1)	0.030	<0.0001	0.0022
GO REGULATION OF CYTOSOLIC CALCIUM ION CONCENTRATI...	346	41 (11.8)	25 (7.2)	16 (4.6)	0.024	<0.0001	0.0022
GO B CELL ACTIVATION	231	31 (13.4)	12 (5.2)	19 (8.2)	0.019	<0.0001	0.0022
GO POSITIVE REGULATION OF LEUKOCYTE CHEMOTAXIS	80	14 (17.5)	11 (13.8)	3 (3.8)	0.009	<0.0001	0.0022
GO MONONUCLEAR CELL MIGRATION	74	13 (17.6)	11 (14.9)	2 (2.7)	0.009	<0.0001	0.0022
GO NEGATIVE REGULATION OF INNATE IMMUNE RESPONSE	49	12 (24.5)	11 (22.4)	1 (2)	0.008	<0.0001	0.0022

Table S81: MSigDB GO Biological Processes enriched in differentially expressed genes at Day 30 compared to pre-Second Vaccination (Monocytes, Study Arm 2 A/H7N9+AS03, D1, 29). Results are sorted by FDR adjusted p-value and Jaccard similarity index (continued.)

Gene Set ID	Gene Set Genes #	DE Genes N (%)	Up-reg. DE Genes N (%)	Down-reg. DE Genes N (%)	Jaccard Index	P-Value	FDR Adjusted P-Value
GO POSITIVE REGULATION OF CYTOKINE PRODUCTION INVO...	49	12 (24.5)	11 (22.4)	1 (2)	0.008	<0.0001	0.0022
GO CELLULAR ION HOMEOSTASIS	634	66 (10.4)	46 (7.3)	20 (3.2)	0.033	<0.0001	0.0025
GO SMOOTH MUSCLE CELL PROLIFERATION	146	22 (15.1)	13 (8.9)	9 (6.2)	0.014	<0.0001	0.0025
GO POSITIVE REGULATION OF INTERLEUKIN 1 PRODUCTION	50	11 (22)	8 (16)	3 (6)	0.007	<0.0001	0.0025
GO T CELL CYTOKINE PRODUCTION	38	10 (26.3)	8 (21.1)	2 (5.3)	0.007	<0.0001	0.0025
GO POSITIVE REGULATION OF T CELL MEDIATED CYTOTOXI...	20	7 (35)	3 (15)	4 (20)	0.005	<0.0001	0.0025
GO POSITIVE REGULATION OF CATALYTIC ACTIVITY	1331	127 (9.5)	79 (5.9)	48 (3.6)	0.048	<0.0001	0.0028
GO REGULATION OF CELL ADHESION	633	69 (10.9)	36 (5.7)	33 (5.2)	0.035	<0.0001	0.0028
GO NEGATIVE REGULATION OF PHOSPHORUS METABOLIC PRO...	536	60 (11.2)	32 (6)	28 (5.2)	0.032	<0.0001	0.0028
GO NEGATIVE REGULATION OF RESPONSE TO BIOTIC STIMU...	82	16 (19.5)	14 (17.1)	2 (2.4)	0.011	<0.0001	0.0028
GO POSITIVE REGULATION OF TRANSPORT	921	90 (9.8)	52 (5.6)	38 (4.1)	0.040	<0.0001	0.0031
GO POSITIVE REGULATION OF CELL POPULATION PROLIFER...	865	84 (9.7)	43 (5)	41 (4.7)	0.038	<0.0001	0.0031
GO PATTERN RECOGNITION RECEPTOR SIGNALING PATHWAY	181	26 (14.4)	21 (11.6)	5 (2.8)	0.017	<0.0001	0.0031
GO CELLULAR RESPONSE TO OXYGEN CONTAINING COMPOUND	1128	105 (9.3)	66 (5.9)	39 (3.5)	0.043	<0.0001	0.0033
GO LIPID LOCALIZATION	383	45 (11.7)	26 (6.8)	19 (5)	0.026	<0.0001	0.0033
GO POSITIVE REGULATION OF T CELL PROLIFERATION	82	15 (18.3)	11 (13.4)	4 (4.9)	0.010	<0.0001	0.0033
GO NEGATIVE REGULATION OF IMMUNE EFFECTOR PROCESS	106	18 (17)	12 (11.3)	6 (5.7)	0.012	0.0001	0.0036
GO B CELL RECEPTOR SIGNALING PATHWAY	56	13 (23.2)	3 (5.4)	10 (17.9)	0.009	0.0001	0.0036
GO REGULATION OF INTRACELLULAR SIGNAL TRANSDUCTION	1707	154 (9)	92 (5.4)	62 (3.6)	0.052	0.0001	0.004
GO DIVALENT INORGANIC CATION HOMEOSTASIS	492	53 (10.8)	36 (7.3)	17 (3.5)	0.029	0.0001	0.0042
GO POSITIVE REGULATION OF CELL CELL ADHESION	232	31 (13.4)	19 (8.2)	12 (5.2)	0.019	0.0001	0.0042
GO ANTIGEN PROCESSING AND PRESENTATION OF ENDOGENO...	14	6 (42.9)	2 (14.3)	4 (28.6)	0.004	0.0001	0.0042
GO POSITIVE REGULATION OF PHOSPHORUS METABOLIC PRO...	1054	99 (9.4)	61 (5.8)	38 (3.6)	0.042	0.0001	0.0045
GO INTERLEUKIN 6 PRODUCTION	144	21 (14.6)	17 (11.8)	4 (2.8)	0.014	0.0001	0.0045
GO ADHESION OF SYMBIONT TO HOST	14	6 (42.9)	4 (28.6)	2 (14.3)	0.004	0.0001	0.0045
GO REGULATION OF NEUTROPHIL MIGRATION	31	8 (25.8)	4 (12.9)	4 (12.9)	0.006	0.0001	0.0048
GO DEFENSE RESPONSE TO BACTERIUM	237	23 (9.7)	16 (6.8)	7 (3)	0.014	0.0002	0.0051
GO T HELPER 17 TYPE IMMUNE RESPONSE	27	8 (29.6)	5 (18.5)	3 (11.1)	0.006	0.0002	0.0051
GO CYTIDINE TO URIDINE EDITING	12	5 (41.7)	5 (41.7)	0 (0)	0.004	0.0002	0.0054
GO CELLULAR RESPONSE TO LIPID	575	60 (10.4)	36 (6.3)	24 (4.2)	0.031	0.0002	0.0056
GO PHAGOCYTOSIS	251	33 (13.1)	22 (8.8)	11 (4.4)	0.020	0.0002	0.0056
GO T CELL PROLIFERATION	167	23 (13.8)	15 (9)	8 (4.8)	0.015	0.0002	0.0056
GO RESPONSE TO INTERLEUKIN 1	181	25 (13.8)	21 (11.6)	4 (2.2)	0.016	0.0002	0.0061
GO RIBONUCLEOSIDE METABOLIC PROCESS	68	13 (19.1)	10 (14.7)	3 (4.4)	0.009	0.0002	0.0061
GO REGULATION OF CD4 POSITIVE ALPHA BETA T CELL AC...	57	12 (21.1)	10 (17.5)	2 (3.5)	0.008	0.0002	0.0061
GO INTERLEUKIN 6 SECRETION	45	10 (22.2)	7 (15.6)	3 (6.7)	0.007	0.0002	0.0061
GO ENDOCYTOSIS	527	60 (11.4)	37 (7)	23 (4.4)	0.032	0.0002	0.0064

Table S81: MSigDB GO Biological Processes enriched in differentially expressed genes at Day 30 compared to pre-Second Vaccination (Monocytes, Study Arm 2 A/H7N9+AS03, D1, 29). Results are sorted by FDR adjusted p-value and Jaccard similarity index (continued.)

Gene Set ID	Gene Set Genes #	DE Genes N (%)	Up-reg. DE Genes N (%)	Down-reg. DE Genes N (%)	Jaccard Index	P-Value	FDR Adjusted P-Value
GO PYRIMIDINE RIBONUCLEOSIDE METABOLIC PROCESS	14	5 (35.7)	5 (35.7)	0 (0)	0.004	0.0002	0.0067
GO CELLULAR HOMEOSTASIS	901	85 (9.4)	53 (5.9)	32 (3.6)	0.038	0.0002	0.0069
GO ANTIMICROBIAL HUMORAL RESPONSE	108	11 (10.2)	7 (6.5)	4 (3.7)	0.007	0.0002	0.0069
GO POSITIVE REGULATION OF HYPERSENSITIVITY	4	3 (75)	0 (0)	3 (75)	0.002	0.0002	0.0069
GO REGULATION OF CHEMOTAXIS	198	26 (13.1)	17 (8.6)	9 (4.5)	0.016	0.0002	0.0071
GO T CELL DIFFERENTIATION INVOLVED IN IMMUNE RESPO...	66	13 (19.7)	9 (13.6)	4 (6.1)	0.009	0.0002	0.0071
GO ACUTE PHASE RESPONSE	44	9 (20.5)	7 (15.9)	2 (4.5)	0.006	0.0002	0.0071
GO CD4 POSITIVE ALPHA BETA T CELL LINEAGE COMMITME...	16	6 (37.5)	3 (18.8)	3 (18.8)	0.004	0.0002	0.0071
GO REACTIVE OXYGEN SPECIES METABOLIC PROCESS	251	30 (12)	17 (6.8)	13 (5.2)	0.018	0.0002	0.0073
GO NEGATIVE REGULATION OF PRODUCTION OF MOLECULAR ...	31	8 (25.8)	3 (9.7)	5 (16.1)	0.006	0.0002	0.0073
GO T CELL MEDIATED CYTOTOXICITY	31	8 (25.8)	4 (12.9)	4 (12.9)	0.006	0.0002	0.0073
GO POSITIVE REGULATION OF ACUTE INFLAMMATORY RESPO...	26	7 (26.9)	4 (15.4)	3 (11.5)	0.005	0.0003	0.0075
GO REGULATION OF MACROPHAGE CHEMOTAXIS	23	7 (30.4)	5 (21.7)	2 (8.7)	0.005	0.0003	0.0075
GO DNA DEAMINATION	14	5 (35.7)	5 (35.7)	0 (0)	0.004	0.0003	0.0075
GO NUCLEOBASE CONTAINING SMALL MOLECULE CATABOLIC ...	48	10 (20.8)	9 (18.8)	1 (2.1)	0.007	0.0003	0.0078
GO POSITIVE REGULATION OF CHEMOTAXIS	125	18 (14.4)	13 (10.4)	5 (4)	0.012	0.0003	0.0086
GO POSITIVE REGULATION OF INTRACELLULAR SIGNAL TRA...	948	89 (9.4)	59 (6.2)	30 (3.2)	0.039	0.0003	0.0088
GO POSITIVE REGULATION OF PEPTIDYL TYROSINE PHOSPH...	185	25 (13.5)	19 (10.3)	6 (3.2)	0.016	0.0003	0.0088
GO LEUKOCYTE MIGRATION INVOLVED IN INFLAMMATORY RE...	14	5 (35.7)	4 (28.6)	1 (7.1)	0.004	0.0003	0.0088
GO ANATOMICAL STRUCTURE FORMATION INVOLVED IN MORP...	1071	100 (9.3)	46 (4.3)	54 (5)	0.042	0.0003	0.009
GO REGULATION OF RESPONSE TO CYTOKINE STIMULUS	165	23 (13.9)	19 (11.5)	4 (2.4)	0.015	0.0003	0.009
GO REGULATION OF B CELL RECEPTOR SIGNALING PATHWAY	27	8 (29.6)	2 (7.4)	6 (22.2)	0.006	0.0003	0.009
GO I KAPPAB KINASE NF KAPPAB SIGNALING	252	32 (12.7)	24 (9.5)	8 (3.2)	0.019	0.0003	0.0092
GO PURINERGIC NUCLEOTIDE RECEPTOR SIGNALING PATHWA...	17	6 (35.3)	5 (29.4)	1 (5.9)	0.004	0.0003	0.0092
GO REGULATION OF T HELPER 1 TYPE IMMUNE RESPONSE	25	7 (28)	6 (24)	1 (4)	0.005	0.0003	0.0094
GO REGULATION OF MAPK CASCADE	697	69 (9.9)	43 (6.2)	26 (3.7)	0.034	0.0003	0.0097
GO POSITIVE REGULATION OF HUMORAL IMMUNE RESPONSE	14	5 (35.7)	1 (7.1)	4 (28.6)	0.004	0.0003	0.0097
GO REGULATION OF CD4 POSITIVE ALPHA BETA T CELL DI...	45	10 (22.2)	8 (17.8)	2 (4.4)	0.007	0.0004	0.0099
GO PYRIMIDINE NUCLEOSIDE METABOLIC PROCESS	35	8 (22.9)	7 (20)	1 (2.9)	0.005	0.0004	0.0101
GO REGULATION OF T CELL MEDIATED CYTOTOXICITY	25	7 (28)	3 (12)	4 (16)	0.005	0.0004	0.0101
GO ADHESION OF SYMBIONT TO HOST CELL	11	5 (45.5)	4 (36.4)	1 (9.1)	0.004	0.0004	0.0103
GO T CELL RECEPTOR SIGNALING PATHWAY	168	24 (14.3)	19 (11.3)	5 (3)	0.015	0.0004	0.0108
GO SIGNAL TRANSDUCTION BY PROTEIN PHOSPHORYLATION	885	85 (9.6)	53 (6)	32 (3.6)	0.038	0.0004	0.011
GO PYRIDINE CONTAINING COMPOUND METABOLIC PROCESS	38	9 (23.7)	6 (15.8)	3 (7.9)	0.006	0.0004	0.011
GO NEGATIVE REGULATION OF MACROPHAGE MIGRATION	8	4 (50)	1 (12.5)	3 (37.5)	0.003	0.0004	0.011
GO PROTEIN ADP RIBOSYLATION	35	9 (25.7)	7 (20)	2 (5.7)	0.006	0.0004	0.012
GO SMALL MOLECULE METABOLIC PROCESS	1844	155 (8.4)	87 (4.7)	68 (3.7)	0.050	0.0005	0.0122

Table S81: MSigDB GO Biological Processes enriched in differentially expressed genes at Day 30 compared to pre-Second Vaccination (Monocytes, Study Arm 2 A/H7N9+AS03, D1, 29). Results are sorted by FDR adjusted p-value and Jaccard similarity index (continued.)

Gene Set ID	Gene Set Genes #	DE Genes N (%)	Up-reg. DE Genes N (%)	Down-reg. DE Genes N (%)	Jaccard Index	P-Value	FDR Adjusted P-Value
GO G PROTEIN COUPLED PURINERGIC NUCLEOTIDE RECEPTO...	9	4 (44.4)	4 (44.4)	0 (0)	0.003	0.0005	0.0122
GO POSITIVE REGULATION OF TRANSFERASE ACTIVITY	614	65 (10.6)	38 (6.2)	27 (4.4)	0.033	0.0005	0.0123
GO REGULATION OF VASCULATURE DEVELOPMENT	349	37 (10.6)	21 (6)	16 (4.6)	0.021	0.0005	0.0123
GO MUSCLE CELL PROLIFERATION	205	26 (12.7)	16 (7.8)	10 (4.9)	0.016	0.0005	0.0123
GO POSITIVE REGULATION OF MOLECULAR FUNCTION	1658	148 (8.9)	90 (5.4)	58 (3.5)	0.051	0.0005	0.0124
GO CELL ACTIVATION INVOLVED IN IMMUNE RESPONSE	658	63 (9.6)	46 (7)	17 (2.6)	0.031	0.0005	0.0124
GO CIRCULATORY SYSTEM PROCESS	517	54 (10.4)	36 (7)	18 (3.5)	0.029	0.0005	0.0124
GO VASCULAR PROCESS IN CIRCULATORY SYSTEM	162	22 (13.6)	15 (9.3)	7 (4.3)	0.014	0.0005	0.0124
GO NEGATIVE REGULATION OF PEPTIDE SECRETION	128	18 (14.1)	11 (8.6)	7 (5.5)	0.012	0.0005	0.0124
GO RESPONSE TO PEPTIDE	494	53 (10.7)	34 (6.9)	19 (3.8)	0.029	0.0005	0.0125
GO NEGATIVE REGULATION OF TRANSFERASE ACTIVITY	266	32 (12)	22 (8.3)	10 (3.8)	0.019	0.0005	0.0125
GO ACID SECRETION	120	18 (15)	10 (8.3)	8 (6.7)	0.012	0.0005	0.0125
GO NUCLEOSIDE METABOLIC PROCESS	96	15 (15.6)	12 (12.5)	3 (3.1)	0.010	0.0005	0.0125
GO NEGATIVE REGULATION OF MOLECULAR FUNCTION	1052	92 (8.7)	57 (5.4)	35 (3.3)	0.039	0.0005	0.0126
GO NEGATIVE REGULATION OF TYPE I INTERFERON PRODUC...	43	10 (23.3)	9 (20.9)	1 (2.3)	0.007	0.0005	0.0126
GO NEGATIVE REGULATION OF LEUKOCYTE MIGRATION	41	9 (22)	5 (12.2)	4 (9.8)	0.006	0.0005	0.0126
GO REGULATION OF ALPHA BETA T CELL DIFFERENTIATION	60	12 (20)	10 (16.7)	2 (3.3)	0.008	0.0005	0.0128
GO RELAXATION OF CARDIAC MUSCLE	15	6 (40)	5 (33.3)	1 (6.7)	0.004	0.0005	0.0128
GO ANTIGEN PROCESSING AND PRESENTATION ENDOGENOUS ...	5	3 (60)	0 (0)	3 (60)	0.002	0.0005	0.0135
GO POSITIVE REGULATION OF NITRIC OXIDE SYNTHASE BI...	15	6 (40)	5 (33.3)	1 (6.7)	0.004	0.0005	0.0137
GO REGULATION OF PEPTIDASE ACTIVITY	409	41 (10)	28 (6.8)	13 (3.2)	0.023	0.0006	0.0138
GO NEGATIVE REGULATION OF SECRETION	206	25 (12.1)	16 (7.8)	9 (4.4)	0.016	0.0006	0.0138
GO POSITIVE REGULATION OF INTERLEUKIN 2 PRODUCTION	29	8 (27.6)	6 (20.7)	2 (6.9)	0.006	0.0006	0.014
GO POSITIVE REGULATION OF CELL ADHESION	368	42 (11.4)	25 (6.8)	17 (4.6)	0.024	0.0006	0.0142
GO REGULATION OF METAL ION TRANSPORT	366	41 (11.2)	25 (6.8)	16 (4.4)	0.024	0.0006	0.0143
GO NEGATIVE REGULATION OF KINASE ACTIVITY	238	29 (12.2)	19 (8)	10 (4.2)	0.018	0.0006	0.0143
GO T CELL ACTIVATION INVOLVED IN IMMUNE RESPONSE	102	15 (14.7)	10 (9.8)	5 (4.9)	0.010	0.0006	0.0143
GO APOPTOTIC CELL CLEARANCE	45	10 (22.2)	6 (13.3)	4 (8.9)	0.007	0.0006	0.0143
GO RESPONSE TO FUNGUS	47	8 (17)	8 (17)	0 (0)	0.005	0.0006	0.0144
GO POSITIVE REGULATION OF MACROPHAGE MIGRATION	20	6 (30)	5 (25)	1 (5)	0.004	0.0006	0.0144
GO RESPONSE TO ATP	30	8 (26.7)	6 (20)	2 (6.7)	0.006	0.0006	0.0146
GO POSITIVE REGULATION OF TUMOR NECROSIS FACTOR BI...	18	6 (33.3)	4 (22.2)	2 (11.1)	0.004	0.0006	0.0146
GO REGULATION OF PROTEIN SERINE THREONINE KINASE A...	478	51 (10.7)	34 (7.1)	17 (3.6)	0.028	0.0006	0.0148
GO PYRIDINE NUCLEOTIDE METABOLIC PROCESS	32	8 (25)	5 (15.6)	3 (9.4)	0.005	0.0006	0.0152
GO EXOCYTOSIS	833	80 (9.6)	53 (6.4)	27 (3.2)	0.037	0.0006	0.0153
GO POSITIVE REGULATION OF MACROPHAGE CHEMOTAXIS	14	5 (35.7)	4 (28.6)	1 (7.1)	0.004	0.0006	0.0153
GO NEGATIVE REGULATION OF LYMPHOCYTE ACTIVATION	137	19 (13.9)	11 (8)	8 (5.8)	0.012	0.0007	0.0165

Table S81: MSigDB GO Biological Processes enriched in differentially expressed genes at Day 30 compared to pre-Second Vaccination (Monocytes, Study Arm 2 A/H7N9+AS03, D1, 29). Results are sorted by FDR adjusted p-value and Jaccard similarity index (continued.)

Gene Set ID	Gene Set Genes #	DE Genes N (%)	Up-reg. DE Genes N (%)	Down-reg. DE Genes N (%)	Jaccard Index	P-Value	FDR Adjusted P-Value
GO REGULATION OF PHAGOCYTOSIS	86	14 (16.3)	8 (9.3)	6 (7)	0.009	0.0007	0.0167
GO REGULATION OF T HELPER CELL DIFFERENTIATION	34	8 (23.5)	6 (17.6)	2 (5.9)	0.005	0.0007	0.0168
GO INTRACILIARY RETROGRADE TRANSPORT	11	5 (45.5)	0 (0)	5 (45.5)	0.004	0.0007	0.017
GO POSITIVE REGULATION OF SECRETION	404	42 (10.4)	25 (6.2)	17 (4.2)	0.024	0.0008	0.0177
GO POSITIVE REGULATION OF PHAGOCYTOSIS	61	11 (18)	6 (9.8)	5 (8.2)	0.007	0.0008	0.0181
GO RESPONSE TO INTERLEUKIN 4	34	8 (23.5)	7 (20.6)	1 (2.9)	0.005	0.0008	0.0185
GO ENTRY INTO HOST	126	19 (15.1)	16 (12.7)	3 (2.4)	0.013	0.0008	0.0191
GO CD4 POSITIVE ALPHA BETA T CELL CYTOKINE PRODUCT...	22	6 (27.3)	5 (22.7)	1 (4.5)	0.004	0.0008	0.0191
GO CD4 POSITIVE OR CD8 POSITIVE ALPHA BETA T CELL ...	19	6 (31.6)	3 (15.8)	3 (15.8)	0.004	0.0008	0.0195
GO BLOOD VESSEL MORPHOGENESIS	604	59 (9.8)	32 (5.3)	27 (4.5)	0.030	0.0009	0.0201
GO POSITIVE REGULATION OF MAPK CASCADE	496	51 (10.3)	34 (6.9)	17 (3.4)	0.027	0.0009	0.0207
GO NEGATIVE REGULATION OF ESTABLISHMENT OF PROTEIN...	174	22 (12.6)	13 (7.5)	9 (5.2)	0.014	0.0009	0.021
GO LEUKOCYTE APOPTOTIC PROCESS	100	15 (15)	10 (10)	5 (5)	0.010	0.0009	0.021
GO ANTIGEN PROCESSING AND PRESENTATION OF EXOGENOU...	62	11 (17.7)	10 (16.1)	1 (1.6)	0.007	0.0009	0.021
GO POSITIVE REGULATION OF KINASE ACTIVITY	543	58 (10.7)	35 (6.4)	23 (4.2)	0.030	0.001	0.0215
GO ASTROCYTE ACTIVATION	21	6 (28.6)	4 (19)	2 (9.5)	0.004	0.001	0.0215
GO RESPONSE TO MECHANICAL STIMULUS	206	27 (13.1)	19 (9.2)	8 (3.9)	0.017	0.001	0.0219
GO CELLULAR EXTRAVASATION	58	11 (19)	8 (13.8)	3 (5.2)	0.007	0.001	0.0221
GO REGULATION OF CYSTEINE TYPE ENDOPEPTIDASE ACTIV...	221	27 (12.2)	20 (9)	7 (3.2)	0.017	0.001	0.0226
GO INTEGRIN ACTIVATION	24	7 (29.2)	5 (20.8)	2 (8.3)	0.005	0.001	0.0226
GO RESPONSE TO ORGANIC CYCLIC COMPOUND	924	84 (9.1)	54 (5.8)	30 (3.2)	0.037	0.0011	0.0236
GO PROTEIN HOMOOLIGOMERIZATION	299	35 (11.7)	23 (7.7)	12 (4)	0.021	0.0011	0.024
GO INTERACTION WITH HOST	192	25 (13)	21 (10.9)	4 (2.1)	0.016	0.0011	0.0244
GO LIPOPOLYSACCHARIDE MEDIATED SIGNALING PATHWAY	54	10 (18.5)	8 (14.8)	2 (3.7)	0.007	0.0011	0.0244
GO SYNAPSE PRUNING	11	4 (36.4)	3 (27.3)	1 (9.1)	0.003	0.0011	0.0244
GO REGULATION OF T CELL CYTOKINE PRODUCTION	28	7 (25)	5 (17.9)	2 (7.1)	0.005	0.0011	0.0248
GO POSITIVE REGULATION OF LYMPHOCYTE DIFFERENTIATI...	86	14 (16.3)	9 (10.5)	5 (5.8)	0.009	0.0011	0.025
GO POSITIVE REGULATION OF LIPID LOCALIZATION	80	13 (16.2)	5 (6.2)	8 (10)	0.009	0.0012	0.0253
GO POSITIVE REGULATION OF HEMOPOIESIS	170	22 (12.9)	15 (8.8)	7 (4.1)	0.014	0.0012	0.0259
GO HEMATOPOIETIC PROGENITOR CELL DIFFERENTIATION	157	22 (14)	13 (8.3)	9 (5.7)	0.014	0.0012	0.0264
GO POSITIVE REGULATION OF INTERLEUKIN 1 BIOSYNTHET...	6	3 (50)	2 (33.3)	1 (16.7)	0.002	0.0012	0.0264
GO PYRIMIDINE CONTAINING COMPOUND CATABOLIC PROCES...	39	8 (20.5)	7 (17.9)	1 (2.6)	0.005	0.0012	0.0265
GO REGULATION OF PEPTIDYL TYROSINE PHOSPHORYLATION	246	29 (11.8)	21 (8.5)	8 (3.3)	0.018	0.0012	0.0266
GO REGULATION OF CALCIUM ION TRANSPORT	238	28 (11.8)	17 (7.1)	11 (4.6)	0.017	0.0013	0.0266
GO POSITIVE REGULATION OF PRODUCTION OF MOLECULAR ...	89	14 (15.7)	12 (13.5)	2 (2.2)	0.009	0.0013	0.0266
GO ANTIGEN PROCESSING AND PRESENTATION VIA MHC CLA...	10	4 (40)	0 (0)	4 (40)	0.003	0.0013	0.0266
GO PROTEIN POLY ADP RIBOSYLATION	7	4 (57.1)	2 (28.6)	2 (28.6)	0.003	0.0013	0.027

Table S81: MSigDB GO Biological Processes enriched in differentially expressed genes at Day 30 compared to pre-Second Vaccination (Monocytes, Study Arm 2 A/H7N9+AS03, D1, 29). Results are sorted by FDR adjusted p-value and Jaccard similarity index (continued.)

Gene Set ID	Gene Set Genes #	DE Genes N (%)	Up-reg. DE Genes N (%)	Down-reg. DE Genes N (%)	Jaccard Index	P-Value	FDR Adjusted P-Value
GO POSITIVE REGULATION OF PROTEIN METABOLIC PROCES...	1584	135 (8.5)	84 (5.3)	51 (3.2)	0.047	0.0013	0.028
GO NITRIC OXIDE SYNTHASE BIOSYNTHETIC PROCESS	20	6 (30)	5 (25)	1 (5)	0.004	0.0013	0.0283
GO NUCLEOSIDE PHOSPHATE BIOSYNTHETIC PROCESS	260	30 (11.5)	18 (6.9)	12 (4.6)	0.018	0.0014	0.0284
GO INOSITOL LIPID MEDIATED SIGNALING	182	25 (13.7)	7 (3.8)	18 (9.9)	0.016	0.0014	0.0288
GO POSITIVE REGULATION OF PEPTIDASE ACTIVITY	185	23 (12.4)	19 (10.3)	4 (2.2)	0.015	0.0014	0.0297
GO SECOND MESSENGER MEDIATED SIGNALING	419	43 (10.3)	29 (6.9)	14 (3.3)	0.024	0.0014	0.0299
GO REGULATION OF CELLULAR CARBOHYDRATE METABOLIC P...	132	19 (14.4)	7 (5.3)	12 (9.1)	0.012	0.0015	0.0312
GO NEGATIVE REGULATION OF CYTOKINE SECRETION	62	10 (16.1)	6 (9.7)	4 (6.5)	0.007	0.0015	0.0315
GO TUMOR NECROSIS FACTOR SECRETION	33	7 (21.2)	6 (18.2)	1 (3)	0.005	0.0015	0.0315
GO LEUKOCYTE HOMEOSTASIS	79	13 (16.5)	9 (11.4)	4 (5.1)	0.009	0.0015	0.0316
GO RESPONSE TO FLUID SHEAR STRESS	32	8 (25)	4 (12.5)	4 (12.5)	0.005	0.0016	0.0319
GO POSITIVE REGULATION OF CHOLESTEROL STORAGE	8	4 (50)	2 (25)	2 (25)	0.003	0.0016	0.032
GO RESPONSE TO CHEMOKINE	84	11 (13.1)	10 (11.9)	1 (1.2)	0.007	0.0016	0.0321
GO RESPONSE TO PROTOZOAN	26	6 (23.1)	5 (19.2)	1 (3.8)	0.004	0.0016	0.0321
GO RESPONSE TO INTERLEUKIN 15	12	5 (41.7)	4 (33.3)	1 (8.3)	0.004	0.0016	0.0322
GO POSITIVE REGULATION OF NEUTROPHIL MIGRATION	25	6 (24)	3 (12)	3 (12)	0.004	0.0016	0.0327
GO REGULATION OF HYDROLASE ACTIVITY	1175	105 (8.9)	70 (6)	35 (3)	0.042	0.0016	0.0327
GO ANTIGEN PROCESSING AND PRESENTATION OF PEPTIDE ...	80	13 (16.2)	11 (13.8)	2 (2.5)	0.009	0.0016	0.0327
GO GLYCOSYL COMPOUND METABOLIC PROCESS	118	16 (13.6)	12 (10.2)	4 (3.4)	0.011	0.0017	0.0332
GO REGULATION OF PHOSPHOLIPID METABOLIC PROCESS	85	14 (16.5)	6 (7.1)	8 (9.4)	0.009	0.0017	0.0332
GO POSITIVE REGULATION OF INTERLEUKIN 6 PRODUCTION	87	13 (14.9)	12 (13.8)	1 (1.1)	0.009	0.0017	0.0332
GO NEGATIVE REGULATION OF MACROPHAGE ACTIVATION	15	5 (33.3)	3 (20)	2 (13.3)	0.004	0.0017	0.0332
GO CELLULAR RESPONSE TO EXTERNAL STIMULUS	313	36 (11.5)	26 (8.3)	10 (3.2)	0.021	0.0017	0.0335
GO ASTROCYTE DEVELOPMENT	36	8 (22.2)	5 (13.9)	3 (8.3)	0.005	0.0017	0.0339
GO REGULATION OF RIBONUCLEASE ACTIVITY	8	4 (50)	4 (50)	0 (0)	0.003	0.0017	0.0339
GO INTERLEUKIN 1 BETA BIOSYNTHETIC PROCESS	9	3 (33.3)	2 (22.2)	1 (11.1)	0.002	0.0017	0.0339
GO INTERLEUKIN 1 BIOSYNTHETIC PROCESS	9	3 (33.3)	2 (22.2)	1 (11.1)	0.002	0.0017	0.0339
GO CELLULAR RESPONSE TO DSRNA	20	6 (30)	5 (25)	1 (5)	0.004	0.0017	0.0342
GO REACTIVE NITROGEN SPECIES METABOLIC PROCESS	74	12 (16.2)	8 (10.8)	4 (5.4)	0.008	0.0018	0.0345
GO ORGANOPHOSPHATE METABOLIC PROCESS	966	88 (9.1)	48 (5)	40 (4.1)	0.038	0.0018	0.0346
GO CARDIOVASCULAR SYSTEM DEVELOPMENT	718	68 (9.5)	37 (5.2)	31 (4.3)	0.033	0.0018	0.0351
GO POSITIVE REGULATION OF SMOOTH MUSCLE CELL PROLI...	85	14 (16.5)	8 (9.4)	6 (7.1)	0.009	0.0018	0.0354
GO NEGATIVE REGULATION OF CATALYTIC ACTIVITY	743	66 (8.9)	41 (5.5)	25 (3.4)	0.032	0.0019	0.036
GO TISSUE REMODELING	170	21 (12.4)	13 (7.6)	8 (4.7)	0.013	0.0019	0.036
GO REGULATION OF CELL DIFFERENTIATION	1741	147 (8.4)	75 (4.3)	72 (4.1)	0.049	0.0019	0.0365
GO PROTEIN LOCALIZATION TO CILIUM	52	11 (21.2)	2 (3.8)	9 (17.3)	0.007	0.0019	0.0366
GO CELLULAR RESPONSE TO EXOGENOUS DSRNA	15	5 (33.3)	4 (26.7)	1 (6.7)	0.004	0.0019	0.0371

Table S81: MSigDB GO Biological Processes enriched in differentially expressed genes at Day 30 compared to pre-Second Vaccination (Monocytes, Study Arm 2 A/H7N9+AS03, D1, 29). Results are sorted by FDR adjusted p-value and Jaccard similarity index (continued.)

Gene Set ID	Gene Set Genes #	DE Genes N (%)	Up-reg. DE Genes N (%)	Down-reg. DE Genes N (%)	Jaccard Index	P-Value	FDR Adjusted P-Value
GO REGULATION OF INTEGRIN ACTIVATION	15	5 (33.3)	4 (26.7)	1 (6.7)	0.004	0.002	0.0374
GO SYNCYTIUM FORMATION	56	10 (17.9)	6 (10.7)	4 (7.1)	0.007	0.002	0.038
GO POSITIVE REGULATION OF B CELL PROLIFERATION	39	8 (20.5)	4 (10.3)	4 (10.3)	0.005	0.002	0.0381
GO POSITIVE REGULATION OF ACUTE INFLAMMATORY RESPO...	6	3 (50)	0 (0)	3 (50)	0.002	0.002	0.0384
GO POSITIVE REGULATION OF LEUKOCYTE DIFFERENTIATIO...	136	18 (13.2)	12 (8.8)	6 (4.4)	0.012	0.0021	0.0394
GO POSITIVE REGULATION OF RESPONSE TO CYTOKINE STI...	53	10 (18.9)	9 (17)	1 (1.9)	0.007	0.0021	0.0395
GO ISG15 PROTEIN CONJUGATION	6	3 (50)	3 (50)	0 (0)	0.002	0.0021	0.0402
GO HEAT GENERATION	15	5 (33.3)	3 (20)	2 (13.3)	0.004	0.0022	0.0405
GO NEGATIVE REGULATION OF CELL DEATH	933	81 (8.7)	50 (5.4)	31 (3.3)	0.036	0.0022	0.0416
GO GLUCOSE METABOLIC PROCESS	192	24 (12.5)	9 (4.7)	15 (7.8)	0.015	0.0022	0.0416
GO REGULATION OF TRANSPOSITION	22	6 (27.3)	6 (27.3)	0 (0)	0.004	0.0022	0.0417
GO NEGATIVE REGULATION OF SMOOTH MUSCLE CONTRACTIO...	15	5 (33.3)	5 (33.3)	0 (0)	0.004	0.0022	0.0417
GO POSITIVE REGULATION OF T HELPER 1 TYPE IMMUNE R...	18	5 (27.8)	4 (22.2)	1 (5.6)	0.004	0.0023	0.0418
GO VIRION ATTACHMENT TO HOST CELL	9	4 (44.4)	4 (44.4)	0 (0)	0.003	0.0023	0.0419
GO REGULATION OF KERATINOCYTE DIFFERENTIATION	40	8 (20)	4 (10)	4 (10)	0.005	0.0023	0.0433
GO REGULATION OF LEUKOCYTE APOPTOTIC PROCESS	78	12 (15.4)	8 (10.3)	4 (5.1)	0.008	0.0024	0.0435
GO TEMPERATURE HOMEOSTASIS	168	22 (13.1)	13 (7.7)	9 (5.4)	0.014	0.0024	0.0441
GO NEUTRAL LIPID BIOSYNTHETIC PROCESS	35	8 (22.9)	3 (8.6)	5 (14.3)	0.005	0.0025	0.0451
GO POSITIVE REGULATION OF SMALL MOLECULE METABOLIC...	133	18 (13.5)	9 (6.8)	9 (6.8)	0.012	0.0026	0.0476
GO APOPTOTIC SIGNALING PATHWAY	566	54 (9.5)	37 (6.5)	17 (3)	0.028	0.0026	0.0479
GO CELLULAR DEFENSE RESPONSE	46	8 (17.4)	7 (15.2)	1 (2.2)	0.005	0.0026	0.0479
GO POSITIVE REGULATION OF DEVELOPMENTAL PROCESS	1308	113 (8.6)	60 (4.6)	53 (4.1)	0.043	0.0027	0.048
GO POSITIVE REGULATION OF PROTEIN MODIFICATION PRO...	1148	101 (8.8)	61 (5.3)	40 (3.5)	0.041	0.0027	0.048
GO TOLL LIKE RECEPTOR SIGNALING PATHWAY	134	18 (13.4)	13 (9.7)	5 (3.7)	0.012	0.0027	0.048
GO POSITIVE REGULATION OF LIPID BIOSYNTHETIC PROCE...	77	12 (15.6)	7 (9.1)	5 (6.5)	0.008	0.0027	0.048
GO NEGATIVE REGULATION OF NEUROINFLAMMATORY RESPON...	13	4 (30.8)	1 (7.7)	3 (23.1)	0.003	0.0027	0.048
GO POSITIVE REGULATION OF LIPID METABOLIC PROCESS	138	18 (13)	9 (6.5)	9 (6.5)	0.012	0.0027	0.0485
GO NEGATIVE REGULATION OF SINGLE STRANDED VIRAL RN...	16	5 (31.2)	5 (31.2)	0 (0)	0.004	0.0027	0.0485
GO REGULATION OF NEUTROPHIL CHEMOTAXIS	27	6 (22.2)	3 (11.1)	3 (11.1)	0.004	0.0027	0.0488
GO REGULATION OF RESPONSE TO INTERFERON GAMMA	24	6 (25)	6 (25)	0 (0)	0.004	0.0028	0.049
GO TYPE I INTERFERON PRODUCTION	116	17 (14.7)	16 (13.8)	1 (0.9)	0.011	0.0028	0.0498

Table S82: Blood Transcription Modules enriched in differentially expressed genes at Day 30 compared to pre-Second Vaccination (Monocytes, Study Arm 2 A/H7N9+AS03, D1, 29). Results are sorted by FDR adjusted p-value and Jaccard similarity index.

Gene Set ID	Gene Set Genes #	DE Genes N (%)	Up-reg. DE Genes N (%)	Down-reg. DE Genes N (%)	Jaccard Index	P-Value	FDR Adjusted P-Value
M47.0 ENRICHED IN B CELLS (I)	45	34 (75.6)	0 (0)	34 (75.6)	0.024	<0.0001	0.0002
S5 DC SURFACE SIGNATURE	76	30 (39.5)	19 (25)	11 (14.5)	0.021	<0.0001	0.0002
M156.0 PLASMA CELLS AND B CELLS, IMMUNOGLOBULINS	30	24 (80)	0 (0)	24 (80)	0.017	<0.0001	0.0002
M47.1 ENRICHED IN B CELLS (II)	35	24 (68.6)	1 (2.9)	23 (65.7)	0.017	<0.0001	0.0002
M165 ENRICHED IN ACTIVATED DENDRITIC CELLS (II)	34	19 (55.9)	18 (52.9)	1 (2.9)	0.013	<0.0001	0.0002
M156.1 PLASMA CELLS, IMMUNOGLOBULINS	22	16 (72.7)	0 (0)	16 (72.7)	0.011	<0.0001	0.0002
M75 ANTIVIRAL IFN SIGNATURE	22	16 (72.7)	15 (68.2)	1 (4.5)	0.011	<0.0001	0.0002
M47.2 ENRICHED IN B CELLS (III)	25	15 (60)	1 (4)	14 (56)	0.011	<0.0001	0.0002
M69 ENRICHED IN B CELLS (VI)	19	15 (78.9)	0 (0)	15 (78.9)	0.011	<0.0001	0.0002
M127 TYPE I INTERFERON RESPONSE	12	12 (100)	12 (100)	0 (0)	0.009	<0.0001	0.0002
M67 ACTIVATED DENDRITIC CELLS	12	11 (91.7)	10 (83.3)	1 (8.3)	0.008	<0.0001	0.0002
M111.1 VIRAL SENSING AND IMMUNITY; IRF2 TARGETS NE...	11	10 (90.9)	10 (90.9)	0 (0)	0.007	<0.0001	0.0002
M150 INNATE ANTIVIRAL RESPONSE	12	10 (83.3)	10 (83.3)	0 (0)	0.007	<0.0001	0.0002
M121 TBA	12	9 (75)	4 (33.3)	5 (41.7)	0.006	<0.0001	0.0002
S10 RESTING DENDRITIC CELL SURFACE SIGNATURE	70	24 (34.3)	12 (17.1)	12 (17.1)	0.016	<0.0001	0.0004
M111.0 VIRAL SENSING AND IMMUNITY; IRF2 TARGETS NE...	16	9 (56.2)	8 (50)	1 (6.2)	0.006	<0.0001	0.0004
M57 IMMUREGULATION - MONOCYTES, T AND B CELLS	13	7 (53.8)	2 (15.4)	5 (38.5)	0.005	<0.0001	0.001
M28 ANTIGEN PRESENTATION (LIPIDS AND PROTEINS)	10	6 (60)	2 (20)	4 (40)	0.004	<0.0001	0.0013
M86.0 CHEMOKINES AND INFLAMMATORY MOLECULES IN MYE...	18	8 (44.4)	8 (44.4)	0 (0)	0.006	<0.0001	0.0016
M13 INNATE ACTIVATION BY CYTOSOLIC DNA SENSING	11	6 (54.5)	6 (54.5)	0 (0)	0.004	<0.0001	0.0016
M68 RIG-1 LIKE RECEPTOR SIGNALING	9	6 (66.7)	6 (66.7)	0 (0)	0.004	<0.0001	0.0016
M37.1 ENRICHED IN NEUTROPHILS (I)	46	15 (32.6)	10 (21.7)	5 (10.9)	0.010	0.0001	0.0017
M37.0 IMMUNE ACTIVATION - GENERIC CLUSTER	301	59 (19.6)	32 (10.6)	27 (9)	0.035	0.0003	0.0053
M86.1 PROINFLAMMATORY DENDRITIC CELL, MYELOID CELL...	12	6 (50)	6 (50)	0 (0)	0.004	0.0004	0.0058
M89.0 PUTATIVE TARGETS OF PAX3	15	7 (46.7)	7 (46.7)	0 (0)	0.005	0.0006	0.0086
M119 ENRICHED IN ACTIVATED DENDRITIC CELLS (I)	11	6 (54.5)	6 (54.5)	0 (0)	0.004	0.0008	0.011
M132 RECRUITMENT OF NEUTROPHILS	10	5 (50)	5 (50)	0 (0)	0.004	0.001	0.0135
M50 CD1 AND OTHER DC RECEPTORS	9	5 (55.6)	0 (0)	5 (55.6)	0.004	0.0012	0.0147
M97 ENRICHED FOR SMAD2-3 SIGNALING	10	6 (60)	1 (10)	5 (50)	0.004	0.0012	0.0148
M16 TLR AND INFLAMMATORY SIGNALING	41	13 (31.7)	12 (29.3)	1 (2.4)	0.009	0.0014	0.0164
M47.3 ENRICHED IN B CELLS (IV)	13	7 (53.8)	0 (0)	7 (53.8)	0.005	0.0019	0.0213
S11 ACTIVATED (LPS) DENDRITIC CELL SURFACE SIGNATU...	34	12 (35.3)	10 (29.4)	2 (5.9)	0.008	0.0023	0.0248
M40 COMPLEMENT AND OTHER RECEPTORS IN DCS	12	5 (41.7)	5 (41.7)	0 (0)	0.004	0.0028	0.0295
M146 MHC-TLR7-TLR8 CLUSTER	14	6 (42.9)	6 (42.9)	0 (0)	0.004	0.0032	0.0323
M112.0 COMPLEMENT ACTIVATION (I)	17	7 (41.2)	6 (35.3)	1 (5.9)	0.005	0.0037	0.0365
M64 ENRICHED IN ACTIVATED DENDRITIC CELLS-MONOCYTE...	14	6 (42.9)	6 (42.9)	0 (0)	0.004	0.0038	0.0365
M71 ENRICHED IN ANTIGEN PRESENTATION (I)	16	7 (43.8)	7 (43.8)	0 (0)	0.005	0.0043	0.04

Table S82: Blood Transcription Modules enriched in differentially expressed genes at Day 30 compared to pre-Second Vaccination (Monocytes, Study Arm 2 A/H7N9+AS03, D1, 29). Results are sorted by FDR adjusted p-value and Jaccard similarity index (continued.)

Gene Set ID	Gene Set Genes #	DE Genes N (%)	Up-reg. DE Genes N (%)	Down-reg. DE Genes N (%)	Jaccard Index	P-Value	FDR Adjusted P-Value
M109 RECEPTORS, CELL MIGRATION	15	7 (46.7)	5 (33.3)	2 (13.3)	0.005	0.0048	0.0436

Table S83: KEGG Pathways enriched in differentially expressed genes at Day 32 compared to pre-Second Vaccination (Monocytes, Study Arm 2 A/H7N9+AS03, D1, 29). Results are sorted by FDR adjusted p-value and Jaccard similarity index.

Gene Set ID	Gene Set Genes #	DE Genes N (%)	Up-reg. DE Genes N (%)	Down-reg. DE Genes N (%)	Jaccard Index	P-Value	FDR Adjusted P-Value
COMPLEMENT AND COAGULATION CASCADES	82	5 (6.1)	5 (6.1)	0 (0)	0.049	<0.0001	0.0011
STAPHYLOCOCCUS AUREUS INFECTION	91	5 (5.5)	5 (5.5)	0 (0)	0.045	<0.0001	0.0011
SYSTEMIC LUPUS ERYTHEMATOSUS	127	5 (3.9)	5 (3.9)	0 (0)	0.034	<0.0001	0.0011
PERTUSSIS	75	4 (5.3)	4 (5.3)	0 (0)	0.041	<0.0001	0.0025

Table S84: MSigDB Reactome Pathways enriched in differentially expressed genes at Day 32 compared to pre-Second Vaccination (Monocytes, Study Arm 2 A/H7N9+AS03, D1, 29). Results are sorted by FDR adjusted p-value and Jaccard similarity index.

Gene Set ID	Gene Set Genes #	DE Genes N (%)	Up-reg. DE Genes N (%)	Down-reg. DE Genes N (%)	Jaccard Index	P-Value	FDR Adjusted P-Value
REACTOME CREATION OF C4 AND C2 ACTIVATORS	14	3 (21.4)	3 (21.4)	0 (0)	0.081	<0.0001	0.0153
REACTOME INITIAL TRIGGERING OF COMPLEMENT	17	3 (17.6)	3 (17.6)	0 (0)	0.075	<0.0001	0.0153
REACTOME COMPLEMENT CASCADE	52	4 (7.7)	4 (7.7)	0 (0)	0.054	<0.0001	0.0153

Table S85: MSigDB GO Biological Processes enriched in differentially expressed genes at Day 32 compared to pre-Second Vaccination (Monocytes, Study Arm 2 A/H7N9+AS03, D1, 29). Results are sorted by FDR adjusted p-value and Jaccard similarity index.

Gene Set ID	Gene Set Genes #	DE Genes N (%)	Up-reg. DE Genes N (%)	Down-reg. DE Genes N (%)	Jaccard Index	P-Value	FDR Adjusted P-Value
GO SYNAPSE PRUNING	11	3 (27.3)	3 (27.3)	0 (0)	0.088	<0.0001	0.0125
GO HUMORAL IMMUNE RESPONSE MEDIATED BY CIRCULATING...	40	4 (10)	4 (10)	0 (0)	0.065	<0.0001	0.0125
GO COMPLEMENT ACTIVATION	62	4 (6.5)	4 (6.5)	0 (0)	0.048	<0.0001	0.0125
GO REGULATION OF HUMORAL IMMUNE RESPONSE	62	4 (6.5)	4 (6.5)	0 (0)	0.048	<0.0001	0.0125
GO B CELL MEDIATED IMMUNITY	104	4 (3.8)	4 (3.8)	0 (0)	0.032	<0.0001	0.0125

Table S85: MSigDB GO Biological Processes enriched in differentially expressed genes at Day 32 compared to pre-Second Vaccination (Monocytes, Study Arm 2 A/H7N9+AS03, D1, 29). Results are sorted by FDR adjusted p-value and Jaccard similarity index (continued.)

Gene Set ID	Gene Set Genes #	DE Genes N (%)	Up-reg. DE Genes N (%)	Down-reg. DE Genes N (%)	Jaccard Index	P-Value	FDR Adjusted P-Value
GO HUMORAL IMMUNE RESPONSE	227	5 (2.2)	4 (1.8)	1 (0.4)	0.020	<0.0001	0.0125
GO ADAPTIVE IMMUNE RESPONSE	353	6 (1.7)	5 (1.4)	1 (0.3)	0.016	<0.0001	0.0323

Table S86: Blood Transcription Modules enriched in differentially expressed genes at Day 32 compared to pre-Second Vaccination (Monocytes, Study Arm 2 A/H7N9+AS03, D1, 29). Results are sorted by FDR adjusted p-value and Jaccard similarity index.

Gene Set ID	Gene Set Genes #	DE Genes N (%)	Up-reg. DE Genes N (%)	Down-reg. DE Genes N (%)	Jaccard Index	P-Value	FDR Adjusted P-Value
M112.0 COMPLEMENT ACTIVATION (I)	17	4 (23.5)	4 (23.5)	0 (0)	0.103	<0.0001	0.0035
M40 COMPLEMENT AND OTHER RECEPTORS IN DCS	12	3 (25)	3 (25)	0 (0)	0.086	<0.0001	0.0035

Table S87: KEGG Pathways enriched in differentially expressed genes at Day 2 compared to pre-First Vaccination (Dendritic cells, Study Arm 2 A/H7N9+AS03, D1, 29). Results are sorted by FDR adjusted p-value and Jaccard similarity index.

Gene Set ID	Gene Set Genes #	DE Genes N (%)	Up-reg. DE Genes N (%)	Down-reg. DE Genes N (%)	Jaccard Index	P-Value	FDR Adjusted P-Value
CYTOKINE-CYTOKINE RECEPTOR INTERACTION	280	41 (14.6)	13 (4.6)	28 (10)	0.031	<0.0001	0.0003
MEASLES	132	24 (18.2)	13 (9.8)	11 (8.3)	0.020	<0.0001	0.0003
TH17 CELL DIFFERENTIATION	102	23 (22.5)	5 (4.9)	18 (17.6)	0.020	<0.0001	0.0003
T CELL RECEPTOR SIGNALING PATHWAY	100	22 (22)	0 (0)	22 (22)	0.019	<0.0001	0.0003
NATURAL KILLER CELL MEDIATED CYTOTOXICITY	121	22 (18.2)	3 (2.5)	19 (15.7)	0.018	<0.0001	0.0003
TH1 AND TH2 CELL DIFFERENTIATION	88	21 (23.9)	4 (4.5)	17 (19.3)	0.018	<0.0001	0.0003
HEMATOPOIETIC CELL LINEAGE	93	20 (21.5)	7 (7.5)	13 (14)	0.017	<0.0001	0.0003
NF-KAPPA B SIGNALING PATHWAY	94	19 (20.2)	7 (7.4)	12 (12.8)	0.016	<0.0001	0.0003
PD-L1 EXPRESSION AND PD-1 CHECKPOINT PATHWAY IN CA...	88	18 (20.5)	4 (4.5)	14 (15.9)	0.015	<0.0001	0.0003
ANTIGEN PROCESSING AND PRESENTATION	70	17 (24.3)	7 (10)	10 (14.3)	0.015	<0.0001	0.0003
VIRAL PROTEIN INTERACTION WITH CYTOKINE AND CYTOKI...	96	15 (15.6)	3 (3.1)	12 (12.5)	0.013	<0.0001	0.0003
GRAFT-VERSUS-HOST DISEASE	38	14 (36.8)	4 (10.5)	10 (26.3)	0.013	<0.0001	0.0003
PRIMARY IMMUNODEFICIENCY	32	13 (40.6)	3 (9.4)	10 (31.2)	0.012	<0.0001	0.0003
CELL ADHESION MOLECULES	137	24 (17.5)	9 (6.6)	15 (10.9)	0.020	<0.0001	0.0004
INFLUENZA A	160	24 (15)	21 (13.1)	3 (1.9)	0.020	<0.0001	0.0004
NOD-LIKE RECEPTOR SIGNALING PATHWAY	175	24 (13.7)	20 (11.4)	4 (2.3)	0.019	<0.0001	0.0004
ALLOGRAFT REJECTION	34	8 (23.5)	4 (11.8)	4 (11.8)	0.007	<0.0001	0.0006

Confidential/Proprietary Information

Table S87: KEGG Pathways enriched in differentially expressed genes at Day 2 compared to pre-First Vaccination (Dendritic cells, Study Arm 2 A/H7N9+AS03, D1, 29). Results are sorted by FDR adjusted p-value and Jaccard similarity index (continued.)

Gene Set ID	Gene Set Genes #	DE Genes N (%)	Up-reg. DE Genes N (%)	Down-reg. DE Genes N (%)	Jaccard Index	P-Value	FDR Adjusted P-Value
INFLAMMATORY BOWEL DISEASE	61	12 (19.7)	4 (6.6)	8 (13.1)	0.011	<0.0001	0.0009
AUTOIMMUNE THYROID DISEASE	49	9 (18.4)	4 (8.2)	5 (10.2)	0.008	<0.0001	0.0009
INTESTINAL IMMUNE NETWORK FOR IGA PRODUCTION	45	10 (22.2)	6 (13.3)	4 (8.9)	0.009	<0.0001	0.0012
EPSTEIN-BARR VIRUS INFECTION	196	25 (12.8)	18 (9.2)	7 (3.6)	0.020	0.0002	0.0027
JAK-STAT SIGNALING PATHWAY	154	20 (13)	9 (5.8)	11 (7.1)	0.016	0.0002	0.0031
TYPE I DIABETES MELLITUS	41	8 (19.5)	4 (9.8)	4 (9.8)	0.007	0.0004	0.0053
RHEUMATOID ARTHRITIS	88	12 (13.6)	7 (8)	5 (5.7)	0.010	0.0009	0.0129
PATHWAYS IN CANCER	515	50 (9.7)	15 (2.9)	35 (6.8)	0.032	0.0011	0.0148
STAPHYLOCOCCUS AUREUS INFECTION	91	11 (12.1)	10 (11)	1 (1.1)	0.009	0.0015	0.0194
BASAL CELL CARCINOMA	63	11 (17.5)	3 (4.8)	8 (12.7)	0.010	0.0017	0.0217

Table S88: MSigDB Reactome Pathways enriched in differentially expressed genes at Day 2 compared to pre-First Vaccination (Dendritic cells, Study Arm 2 A/H7N9+AS03, D1, 29). Results are sorted by FDR adjusted p-value and Jaccard similarity index.

Gene Set ID	Gene Set Genes #	DE Genes N (%)	Up-reg. DE Genes N (%)	Down-reg. DE Genes N (%)	Jaccard Index	P-Value	FDR Adjusted P-Value
REACTOME CYTOKINE SIGNALING IN IMMUNE SYSTEM	788	102 (12.9)	59 (7.5)	43 (5.5)	0.057	<0.0001	0.0014
REACTOME ADAPTIVE IMMUNE SYSTEM	670	76 (11.3)	25 (3.7)	51 (7.6)	0.045	<0.0001	0.0014
REACTOME SIGNALING BY INTERLEUKINS	430	50 (11.6)	26 (6)	24 (5.6)	0.034	<0.0001	0.0014
REACTOME INTERFERON SIGNALING	177	38 (21.5)	36 (20.3)	2 (1.1)	0.031	<0.0001	0.0014
REACTOME G ALPHA I SIGNALLING EVENTS	367	39 (10.6)	12 (3.3)	27 (7.4)	0.028	<0.0001	0.0014
REACTOME IMMUNOREGULATORY INTERACTIONS BETWEEN A L...	97	25 (25.8)	6 (6.2)	19 (19.6)	0.021	<0.0001	0.0014
REACTOME CLASS A 1 RHODOPSIN LIKE RECEPTORS	306	29 (9.5)	10 (3.3)	19 (6.2)	0.021	<0.0001	0.0014
REACTOME INTERFERON GAMMA SIGNALING	71	22 (31)	21 (29.6)	1 (1.4)	0.019	<0.0001	0.0014
REACTOME INTERFERON ALPHA BETA SIGNALING	60	21 (35)	21 (35)	0 (0)	0.019	<0.0001	0.0014
REACTOME TCR SIGNALING	100	19 (19)	6 (6)	13 (13)	0.016	<0.0001	0.0014
REACTOME GENERATION OF SECOND MESSENGER MOLECULES	22	11 (50)	1 (4.5)	10 (45.5)	0.010	<0.0001	0.0014
REACTOME GPCR LIGAND BINDING	420	38 (9)	13 (3.1)	25 (6)	0.026	<0.0001	0.0024
REACTOME COSTIMULATION BY THE CD28 FAMILY	55	14 (25.5)	1 (1.8)	13 (23.6)	0.012	<0.0001	0.0024
REACTOME PD 1 SIGNALING	12	6 (50)	1 (8.3)	5 (41.7)	0.005	<0.0001	0.0033
REACTOME RUNX1 AND FOXP3 CONTROL THE DEVELOPMENT O...	9	5 (55.6)	1 (11.1)	4 (44.4)	0.005	<0.0001	0.0041
REACTOME TNFR2 NON CANONICAL NF KB PATHWAY	89	14 (15.7)	6 (6.7)	8 (9)	0.012	<0.0001	0.0054
REACTOME OAS ANTIVIRAL RESPONSE	8	5 (62.5)	5 (62.5)	0 (0)	0.005	<0.0001	0.0054
REACTOME TNFS BIND THEIR PHYSIOLOGICAL RECEPTORS	26	7 (26.9)	1 (3.8)	6 (23.1)	0.006	<0.0001	0.0085
REACTOME INTERLEUKIN 20 FAMILY SIGNALING	26	7 (26.9)	4 (15.4)	3 (11.5)	0.006	0.0002	0.0129

Table S88: MSigDB Reactome Pathways enriched in differentially expressed genes at Day 2 compared to pre-First Vaccination (Dendritic cells, Study Arm 2 A/H7N9+AS03, D1, 29). Results are sorted by FDR adjusted p-value and Jaccard similarity index (continued.)

Gene Set ID	Gene Set Genes #	DE Genes N (%)	Up-reg. DE Genes N (%)	Down-reg. DE Genes N (%)	Jaccard Index	P-Value	FDR Adjusted P-Value
REACTOME INTERLEUKIN 4 AND INTERLEUKIN 13 SIGNALIN...	102	15 (14.7)	8 (7.8)	7 (6.9)	0.013	0.0002	0.0169
REACTOME CHEMOKINE RECEPTORS BIND CHEMOKINES	51	8 (15.7)	2 (3.9)	6 (11.8)	0.007	0.0002	0.0182
REACTOME FCGR ACTIVATION	12	5 (41.7)	2 (16.7)	3 (25)	0.005	0.0003	0.0195
REACTOME FCERI MEDIATED CA 2 MOBILIZATION	30	8 (26.7)	0 (0)	8 (26.7)	0.007	0.0003	0.0213
REACTOME INNATE IMMUNE SYSTEM	968	73 (7.5)	44 (4.5)	29 (3)	0.037	0.0005	0.0319

Table S89: MSigDB GO Biological Processes enriched in differentially expressed genes at Day 2 compared to pre-First Vaccination (Dendritic cells, Study Arm 2 A/H7N9+AS03, D1, 29). Results are sorted by FDR adjusted p-value and Jaccard similarity index.

Gene Set ID	Gene Set Genes #	DE Genes N (%)	Up-reg. DE Genes N (%)	Down-reg. DE Genes N (%)	Jaccard Index	P-Value	FDR Adjusted P-Value
GO REGULATION OF IMMUNE SYSTEM PROCESS	1409	190 (13.5)	72 (5.1)	118 (8.4)	0.082	<0.0001	0.0005
GO DEFENSE RESPONSE	1497	186 (12.4)	107 (7.1)	79 (5.3)	0.077	<0.0001	0.0005
GO POSITIVE REGULATION OF IMMUNE SYSTEM PROCESS	972	139 (14.3)	57 (5.9)	82 (8.4)	0.072	<0.0001	0.0005
GO RESPONSE TO BIOTIC STIMULUS	1325	159 (12)	100 (7.5)	59 (4.5)	0.070	<0.0001	0.0005
GO REGULATION OF IMMUNE RESPONSE	897	130 (14.5)	57 (6.4)	73 (8.1)	0.070	<0.0001	0.0005
GO DEFENSE RESPONSE TO OTHER ORGANISM	993	132 (13.3)	91 (9.2)	41 (4.1)	0.068	<0.0001	0.0005
GO CELL ACTIVATION	1265	144 (11.4)	52 (4.1)	92 (7.3)	0.065	<0.0001	0.0005
GO LYMPHOCYTE ACTIVATION	620	101 (16.3)	24 (3.9)	77 (12.4)	0.063	<0.0001	0.0005
GO INNATE IMMUNE RESPONSE	814	112 (13.8)	79 (9.7)	33 (4.1)	0.062	<0.0001	0.0005
GO RESPONSE TO CYTOKINE	1099	126 (11.5)	72 (6.6)	54 (4.9)	0.061	<0.0001	0.0005
GO POSITIVE REGULATION OF SIGNALING	1730	160 (9.2)	54 (3.1)	106 (6.1)	0.060	<0.0001	0.0005
GO BIOLOGICAL ADHESION	1320	136 (10.3)	37 (2.8)	99 (7.5)	0.060	<0.0001	0.0005
GO CYTOKINE PRODUCTION	720	102 (14.2)	40 (5.6)	62 (8.6)	0.060	<0.0001	0.0005
GO T CELL ACTIVATION	436	86 (19.7)	18 (4.1)	68 (15.6)	0.060	<0.0001	0.0005
GO IMMUNE EFFECTOR PROCESS	1077	122 (11.3)	71 (6.6)	51 (4.7)	0.060	<0.0001	0.0005
GO REGULATION OF RESPONSE TO EXTERNAL STIMULUS	1006	116 (11.5)	60 (6)	56 (5.6)	0.059	<0.0001	0.0005
GO POSITIVE REGULATION OF IMMUNE RESPONSE	704	99 (14.1)	44 (6.2)	55 (7.8)	0.058	<0.0001	0.0005
GO POSITIVE REGULATION OF MULTICELLULAR ORGANISMAL...	1665	149 (8.9)	48 (2.9)	101 (6.1)	0.057	<0.0001	0.0005
GO CYTOKINE MEDIATED SIGNALING PATHWAY	711	97 (13.6)	58 (8.2)	39 (5.5)	0.057	<0.0001	0.0005
GO REGULATION OF CELL ACTIVATION	507	85 (16.8)	22 (4.3)	63 (12.4)	0.056	<0.0001	0.0005
GO PROTEIN PHOSPHORYLATION	1838	154 (8.4)	58 (3.2)	96 (5.2)	0.056	<0.0001	0.0005
GO REGULATION OF RESPONSE TO STRESS	1406	131 (9.3)	66 (4.7)	65 (4.6)	0.055	<0.0001	0.0005
GO REGULATION OF CELL POPULATION PROLIFERATION	1541	136 (8.8)	41 (2.7)	95 (6.2)	0.054	<0.0001	0.0005
GO ADAPTIVE IMMUNE RESPONSE	353	74 (21)	25 (7.1)	49 (13.9)	0.054	<0.0001	0.0005

Table S89: MSigDB GO Biological Processes enriched in differentially expressed genes at Day 2 compared to pre-First Vaccination (Dendritic cells, Study Arm 2 A/H7N9+AS03, D1, 29). Results are sorted by FDR adjusted p-value and Jaccard similarity index (continued.)

Gene Set ID	Gene Set Genes #	DE Genes N (%)	Up-reg. DE Genes N (%)	Down-reg. DE Genes N (%)	Jaccard Index	P-Value	FDR Adjusted P-Value
GO REGULATION OF CELL DEATH	1593	135 (8.5)	47 (3)	88 (5.5)	0.053	<0.0001	0.0005
GO REGULATION OF INTRACELLULAR SIGNAL TRANSDUCTION	1707	140 (8.2)	50 (2.9)	90 (5.3)	0.053	<0.0001	0.0005
GO CELL CELL ADHESION	781	93 (11.9)	24 (3.1)	69 (8.8)	0.052	<0.0001	0.0005
GO REGULATION OF DEFENSE RESPONSE	703	89 (12.7)	50 (7.1)	39 (5.5)	0.052	<0.0001	0.0005
GO REGULATION OF CELL DIFFERENTIATION	1741	140 (8)	38 (2.2)	102 (5.9)	0.052	<0.0001	0.0005
GO REGULATION OF CELL ADHESION	633	85 (13.4)	20 (3.2)	65 (10.3)	0.052	<0.0001	0.0005
GO SECRETION	1551	130 (8.4)	63 (4.1)	67 (4.3)	0.052	<0.0001	0.0005
GO IMMUNE SYSTEM DEVELOPMENT	926	99 (10.7)	28 (3)	71 (7.7)	0.052	<0.0001	0.0005
GO LOCOMOTION	1747	139 (8)	42 (2.4)	97 (5.6)	0.051	<0.0001	0.0005
GO REGULATION OF LYMPHOCYTE ACTIVATION	392	72 (18.4)	17 (4.3)	55 (14)	0.051	<0.0001	0.0005
GO CELL MOTILITY	1531	127 (8.3)	38 (2.5)	89 (5.8)	0.051	<0.0001	0.0005
GO ACTIVATION OF IMMUNE RESPONSE	547	79 (14.4)	35 (6.4)	44 (8)	0.051	<0.0001	0.0005
GO REGULATION OF TRANSPORT	1745	135 (7.7)	48 (2.8)	87 (5)	0.050	<0.0001	0.0005
GO NEGATIVE REGULATION OF RESPONSE TO STIMULUS	1531	124 (8.1)	42 (2.7)	82 (5.4)	0.050	<0.0001	0.0005
GO REGULATION OF PHOSPHORYLATION	1495	122 (8.2)	51 (3.4)	71 (4.7)	0.050	<0.0001	0.0005
GO IMMUNE RESPONSE REGULATING SIGNALING PATHWAY	507	75 (14.8)	31 (6.1)	44 (8.7)	0.049	<0.0001	0.0005
GO INFLAMMATORY RESPONSE	680	83 (12.2)	39 (5.7)	44 (6.5)	0.049	<0.0001	0.0005
GO REGULATION OF MULTI ORGANISM PROCESS	700	82 (11.7)	54 (7.7)	28 (4)	0.048	<0.0001	0.0005
GO LEUKOCYTE DIFFERENTIATION	483	72 (14.9)	17 (3.5)	55 (11.4)	0.048	<0.0001	0.0005
GO POSITIVE REGULATION OF INTRACELLULAR SIGNAL TRA...	948	93 (9.8)	36 (3.8)	57 (6)	0.048	<0.0001	0.0005
GO POSITIVE REGULATION OF PROTEIN METABOLIC PROCES...	1584	122 (7.7)	53 (3.3)	69 (4.4)	0.048	<0.0001	0.0005
GO NEGATIVE REGULATION OF MULTICELLULAR ORGANISMAL...	1169	102 (8.7)	39 (3.3)	63 (5.4)	0.047	<0.0001	0.0005
GO LYMPHOCYTE DIFFERENTIATION	336	63 (18.8)	12 (3.6)	51 (15.2)	0.046	<0.0001	0.0005
GO REGULATION OF CELL CELL ADHESION	372	64 (17.2)	14 (3.8)	50 (13.4)	0.046	<0.0001	0.0005
GO PEPTIDE SECRETION	587	72 (12.3)	33 (5.6)	39 (6.6)	0.045	<0.0001	0.0005
GO REGULATION OF T CELL ACTIVATION	292	59 (20.2)	12 (4.1)	47 (16.1)	0.045	<0.0001	0.0005
GO IMMUNE RESPONSE REGULATING CELL SURFACE RECEPTO...	348	60 (17.2)	20 (5.7)	40 (11.5)	0.043	<0.0001	0.0005
GO POSITIVE REGULATION OF CYTOKINE PRODUCTION	419	63 (15)	25 (6)	38 (9.1)	0.043	<0.0001	0.0005
GO T CELL DIFFERENTIATION	230	55 (23.9)	10 (4.3)	45 (19.6)	0.043	<0.0001	0.0005
GO POSITIVE REGULATION OF RESPONSE TO EXTERNAL STI...	575	69 (12)	39 (6.8)	30 (5.2)	0.043	<0.0001	0.0005
GO REGULATION OF SECRETION	752	76 (10.1)	29 (3.9)	47 (6.2)	0.043	<0.0001	0.0005
GO POSITIVE REGULATION OF PHOSPHORUS METABOLIC PRO...	1054	88 (8.3)	38 (3.6)	50 (4.7)	0.043	<0.0001	0.0005
GO POSITIVE REGULATION OF CELL DEATH	670	72 (10.7)	31 (4.6)	41 (6.1)	0.043	<0.0001	0.0005
GO POSITIVE REGULATION OF CELL POPULATION PROLIFER...	865	79 (9.1)	25 (2.9)	54 (6.2)	0.042	<0.0001	0.0005
GO REGULATION OF CELLULAR COMPONENT MOVEMENT	973	83 (8.5)	27 (2.8)	56 (5.8)	0.042	<0.0001	0.0005
GO LEUKOCYTE CELL CELL ADHESION	307	56 (18.2)	13 (4.2)	43 (14)	0.042	<0.0001	0.0005
GO TAXIS	607	68 (11.2)	25 (4.1)	43 (7.1)	0.042	<0.0001	0.0005

Table S89: MSigDB GO Biological Processes enriched in differentially expressed genes at Day 2 compared to pre-First Vaccination (Dendritic cells, Study Arm 2 A/H7N9+AS03, D1, 29). Results are sorted by FDR adjusted p-value and Jaccard similarity index (continued.)

Gene Set ID	Gene Set Genes #	DE Genes N (%)	Up-reg. DE Genes N (%)	Down-reg. DE Genes N (%)	Jaccard Index	P-Value	FDR Adjusted P-Value
GO REGULATION OF RESPONSE TO BIOTIC STIMULUS	466	62 (13.3)	42 (9)	20 (4.3)	0.042	<0.0001	0.0005
GO POSITIVE REGULATION OF CELL ADHESION	368	58 (15.8)	12 (3.3)	46 (12.5)	0.041	<0.0001	0.0005
GO SIGNAL TRANSDUCTION BY PROTEIN PHOSPHORYLATION	885	78 (8.8)	34 (3.8)	44 (5)	0.041	<0.0001	0.0005
GO POSITIVE REGULATION OF CELL ACTIVATION	297	54 (18.2)	13 (4.4)	41 (13.8)	0.041	<0.0001	0.0005
GO REGULATION OF PEPTIDE SECRETION	461	58 (12.6)	24 (5.2)	34 (7.4)	0.039	<0.0001	0.0005
GO RESPONSE TO VIRUS	300	52 (17.3)	41 (13.7)	11 (3.7)	0.039	<0.0001	0.0005
GO NEGATIVE REGULATION OF CELL POPULATION PROLIFER...	666	64 (9.6)	19 (2.9)	45 (6.8)	0.038	<0.0001	0.0005
GO REGULATION OF ESTABLISHMENT OF PROTEIN LOCALIZA...	698	65 (9.3)	25 (3.6)	40 (5.7)	0.038	<0.0001	0.0005
GO REGULATION OF MAPK CASCADE	697	65 (9.3)	29 (4.2)	36 (5.2)	0.038	<0.0001	0.0005
GO POSITIVE REGULATION OF DEFENSE RESPONSE	457	55 (12)	33 (7.2)	22 (4.8)	0.037	<0.0001	0.0005
GO REGULATION OF HEMOPOIESIS	441	54 (12.2)	17 (3.9)	37 (8.4)	0.036	<0.0001	0.0005
GO RESPONSE TO BACTERIUM	587	59 (10.1)	31 (5.3)	28 (4.8)	0.036	<0.0001	0.0005
GO DEFENSE RESPONSE TO VIRUS	229	46 (20.1)	38 (16.6)	8 (3.5)	0.036	<0.0001	0.0005
GO REGULATION OF IMMUNE EFFECTOR PROCESS	357	50 (14)	25 (7)	25 (7)	0.036	<0.0001	0.0005
GO APOPTOTIC SIGNALING PATHWAY	566	57 (10.1)	24 (4.2)	33 (5.8)	0.036	<0.0001	0.0005
GO POSITIVE REGULATION OF LOCOMOTION	544	56 (10.3)	19 (3.5)	37 (6.8)	0.035	<0.0001	0.0005
GO LEUKOCYTE MIGRATION	410	51 (12.4)	17 (4.1)	34 (8.3)	0.035	<0.0001	0.0005
GO METAL ION HOMEOSTASIS	622	57 (9.2)	26 (4.2)	31 (5)	0.034	<0.0001	0.0005
GO ANTIGEN RECEPTOR MEDIATED SIGNALING PATHWAY	212	43 (20.3)	10 (4.7)	33 (15.6)	0.034	<0.0001	0.0005
GO NEGATIVE REGULATION OF IMMUNE SYSTEM PROCESS	426	50 (11.7)	15 (3.5)	35 (8.2)	0.034	<0.0001	0.0005
GO PEPTIDYL TYROSINE MODIFICATION	354	47 (13.3)	18 (5.1)	29 (8.2)	0.034	<0.0001	0.0005
GO POSITIVE REGULATION OF MAPK CASCADE	496	51 (10.3)	23 (4.6)	28 (5.6)	0.033	<0.0001	0.0005
GO REGULATION OF LEUKOCYTE DIFFERENTIATION	252	43 (17.1)	11 (4.4)	32 (12.7)	0.033	<0.0001	0.0005
GO POSITIVE REGULATION OF CELL CELL ADHESION	232	42 (18.1)	7 (3)	35 (15.1)	0.033	<0.0001	0.0005
GO ADAPTIVE IMMUNE RESPONSE BASED ON SOMATIC RECOM...	223	40 (17.9)	19 (8.5)	21 (9.4)	0.031	<0.0001	0.0005
GO DIVALENT INORGANIC CATION HOMEOSTASIS	492	48 (9.8)	20 (4.1)	28 (5.7)	0.031	<0.0001	0.0005
GO POSITIVE REGULATION OF LEUKOCYTE CELL CELL ADHE...	194	39 (20.1)	7 (3.6)	32 (16.5)	0.031	<0.0001	0.0005
GO POSITIVE REGULATION OF SECRETION	404	45 (11.1)	17 (4.2)	28 (6.9)	0.031	<0.0001	0.0005
GO REGULATION OF LYMPHOCYTE DIFFERENTIATION	158	37 (23.4)	9 (5.7)	28 (17.7)	0.030	<0.0001	0.0005
GO CYTOKINE SECRETION	220	38 (17.3)	21 (9.5)	17 (7.7)	0.030	<0.0001	0.0005
GO LYMPHOCYTE MEDIATED IMMUNITY	216	38 (17.6)	16 (7.4)	22 (10.2)	0.030	<0.0001	0.0005
GO REGULATION OF INFLAMMATORY RESPONSE	332	41 (12.3)	19 (5.7)	22 (6.6)	0.030	<0.0001	0.0005
GO POSITIVE REGULATION OF PEPTIDE SECRETION	271	38 (14)	17 (6.3)	21 (7.7)	0.029	<0.0001	0.0005
GO LEUKOCYTE PROLIFERATION	276	38 (13.8)	14 (5.1)	24 (8.7)	0.029	<0.0001	0.0005
GO NEGATIVE REGULATION OF RESPONSE TO EXTERNAL STI...	350	39 (11.1)	15 (4.3)	24 (6.9)	0.028	<0.0001	0.0005
GO REGULATION OF CYTOSOLIC CALCIUM ION CONCENTRATI...	346	38 (11)	14 (4)	24 (6.9)	0.027	<0.0001	0.0005
GO CELL CHEMOTAXIS	278	36 (12.9)	16 (5.8)	20 (7.2)	0.027	<0.0001	0.0005

Table S89: MSigDB GO Biological Processes enriched in differentially expressed genes at Day 2 compared to pre-First Vaccination (Dendritic cells, Study Arm 2 A/H7N9+AS03, D1, 29). Results are sorted by FDR adjusted p-value and Jaccard similarity index (continued.)

Gene Set ID	Gene Set Genes #	DE Genes N (%)	Up-reg. DE Genes N (%)	Down-reg. DE Genes N (%)	Jaccard Index	P-Value	FDR Adjusted P-Value
GO REGULATION OF T CELL DIFFERENTIATION	131	32 (24.4)	7 (5.3)	25 (19.1)	0.027	<0.0001	0.0005
GO T CELL RECEPTOR SIGNALING PATHWAY	168	33 (19.6)	9 (5.4)	24 (14.3)	0.027	<0.0001	0.0005
GO ALPHA BETA T CELL ACTIVATION	127	31 (24.4)	8 (6.3)	23 (18.1)	0.026	<0.0001	0.0005
GO RESPONSE TO INTERFERON GAMMA	166	32 (19.3)	29 (17.5)	3 (1.8)	0.026	<0.0001	0.0005
GO REGULATION OF PEPTIDYL TYROSINE PHOSPHORYLATION	246	33 (13.4)	17 (6.9)	16 (6.5)	0.025	<0.0001	0.0005
GO NEGATIVE REGULATION OF CYTOKINE PRODUCTION	262	33 (12.6)	12 (4.6)	21 (8)	0.025	<0.0001	0.0005
GO REGULATION OF LEUKOCYTE PROLIFERATION	203	31 (15.3)	11 (5.4)	20 (9.9)	0.025	<0.0001	0.0005
GO CALCIUM MEDIATED SIGNALING	208	29 (13.9)	9 (4.3)	20 (9.6)	0.023	<0.0001	0.0005
GO INTERFERON GAMMA PRODUCTION	97	26 (26.8)	8 (8.2)	18 (18.6)	0.022	<0.0001	0.0005
GO PRODUCTION OF MOLECULAR MEDIATOR OF IMMUNE RESP...	187	28 (15)	10 (5.3)	18 (9.6)	0.022	<0.0001	0.0005
GO NEGATIVE REGULATION OF MULTI ORGANISM PROCESS	195	28 (14.4)	21 (10.8)	7 (3.6)	0.022	<0.0001	0.0005
GO MYELOID LEUKOCYTE MIGRATION	187	26 (13.9)	13 (7)	13 (7)	0.021	<0.0001	0.0005
GO POSITIVE REGULATION OF PEPTIDYL TYROSINE PHOSPH...	185	26 (14.1)	15 (8.1)	11 (5.9)	0.021	<0.0001	0.0005
GO POSITIVE REGULATION OF IMMUNE EFFECTOR PROCESS	189	26 (13.8)	10 (5.3)	16 (8.5)	0.021	<0.0001	0.0005
GO ALPHA BETA T CELL DIFFERENTIATION	97	24 (24.7)	6 (6.2)	18 (18.6)	0.021	<0.0001	0.0005
GO LEUKOCYTE CHEMOTAXIS	202	26 (12.9)	14 (6.9)	12 (5.9)	0.021	<0.0001	0.0005
GO NEGATIVE REGULATION OF DEFENSE RESPONSE	201	26 (12.9)	10 (5)	16 (8)	0.021	<0.0001	0.0005
GO INTERFERON GAMMA MEDIATED SIGNALING PATHWAY	70	23 (32.9)	21 (30)	2 (2.9)	0.020	<0.0001	0.0005
GO POSITIVE REGULATION OF CYTOKINE SECRETION	126	24 (19)	12 (9.5)	12 (9.5)	0.020	<0.0001	0.0005
GO T CELL PROLIFERATION	167	24 (14.4)	10 (6)	14 (8.4)	0.019	<0.0001	0.0005
GO POSITIVE REGULATION OF LEUKOCYTE PROLIFERATION	124	23 (18.5)	8 (6.5)	15 (12.1)	0.019	<0.0001	0.0005
GO REGULATION OF LEUKOCYTE MEDIATED IMMUNITY	174	24 (13.8)	8 (4.6)	16 (9.2)	0.019	<0.0001	0.0005
GO REGULATION OF ADAPTIVE IMMUNE RESPONSE	142	23 (16.2)	12 (8.5)	11 (7.7)	0.019	<0.0001	0.0005
GO NEGATIVE REGULATION OF CELL CELL ADHESION	167	23 (13.8)	9 (5.4)	14 (8.4)	0.019	<0.0001	0.0005
GO REGULATION OF LEUKOCYTE MIGRATION	175	23 (13.1)	9 (5.1)	14 (8)	0.018	<0.0001	0.0005
GO REGULATION OF LYMPHOCYTE MEDIATED IMMUNITY	127	22 (17.3)	7 (5.5)	15 (11.8)	0.018	<0.0001	0.0005
GO HUMORAL IMMUNE RESPONSE	227	23 (10.1)	12 (5.3)	11 (4.8)	0.018	<0.0001	0.0005
GO LYMPHOCYTE COSTIMULATION	52	19 (36.5)	2 (3.8)	17 (32.7)	0.017	<0.0001	0.0005
GO POSITIVE REGULATION OF LEUKOCYTE MEDIATED IMMUN...	113	20 (17.7)	7 (6.2)	13 (11.5)	0.017	<0.0001	0.0005
GO CD4 POSITIVE ALPHA BETA T CELL ACTIVATION	87	19 (21.8)	6 (6.9)	13 (14.9)	0.016	<0.0001	0.0005
GO CELL KILLING	146	20 (13.7)	6 (4.1)	14 (9.6)	0.016	<0.0001	0.0005
GO POSITIVE REGULATION OF LYMPHOCYTE MEDIATED IMMU...	87	19 (21.8)	6 (6.9)	13 (14.9)	0.016	<0.0001	0.0005
GO RESPONSE TO TYPE I INTERFERON	88	19 (21.6)	19 (21.6)	0 (0)	0.016	<0.0001	0.0005
GO REGULATION OF B CELL ACTIVATION	112	19 (17)	5 (4.5)	14 (12.5)	0.016	<0.0001	0.0005
GO T CELL DIFFERENTIATION IN THYMUS	67	18 (26.9)	1 (1.5)	17 (25.4)	0.016	<0.0001	0.0005
GO POSITIVE REGULATION OF ADAPTIVE IMMUNE RESPONSE	92	18 (19.6)	9 (9.8)	9 (9.8)	0.015	<0.0001	0.0005
GO NEGATIVE REGULATION OF LEUKOCYTE CELL CELL ADHE...	117	18 (15.4)	6 (5.1)	12 (10.3)	0.015	<0.0001	0.0005

Table S89: MSigDB GO Biological Processes enriched in differentially expressed genes at Day 2 compared to pre-First Vaccination (Dendritic cells, Study Arm 2 A/H7N9+AS03, D1, 29). Results are sorted by FDR adjusted p-value and Jaccard similarity index (continued.)

Gene Set ID	Gene Set Genes #	DE Genes N (%)	Up-reg. DE Genes N (%)	Down-reg. DE Genes N (%)	Jaccard Index	P-Value	FDR Adjusted P-Value
GO REGULATION OF RECEPTOR SIGNALING PATHWAY VIA ST...	133	18 (13.5)	9 (6.8)	9 (6.8)	0.015	<0.0001	0.0005
GO REGULATION OF ALPHA BETA T CELL ACTIVATION	84	17 (20.2)	5 (6)	12 (14.3)	0.015	<0.0001	0.0005
GO T CELL MEDIATED IMMUNITY	84	17 (20.2)	7 (8.3)	10 (11.9)	0.015	<0.0001	0.0005
GO B CELL PROLIFERATION	91	17 (18.7)	4 (4.4)	13 (14.3)	0.015	<0.0001	0.0005
GO POSITIVE REGULATION OF INTERFERON GAMMA PRODUCT...	54	16 (29.6)	5 (9.3)	11 (20.4)	0.014	<0.0001	0.0005
GO LEUKOCYTE MEDIATED CYTOTOXICITY	87	16 (18.4)	4 (4.6)	12 (13.8)	0.014	<0.0001	0.0005
GO LYMPHOCYTE MIGRATION	96	16 (16.7)	3 (3.1)	13 (13.5)	0.014	<0.0001	0.0005
GO POSITIVE REGULATION OF PRODUCTION OF MOLECULAR ...	89	16 (18)	6 (6.7)	10 (11.2)	0.014	<0.0001	0.0005
GO INTERLEUKIN 1 PRODUCTION	99	16 (16.2)	11 (11.1)	5 (5.1)	0.014	<0.0001	0.0005
GO T CELL SELECTION	45	15 (33.3)	1 (2.2)	14 (31.1)	0.013	<0.0001	0.0005
GO CD4 POSITIVE ALPHA BETA T CELL DIFFERENTIATION	71	15 (21.1)	5 (7)	10 (14.1)	0.013	<0.0001	0.0005
GO POSITIVE REGULATION OF B CELL ACTIVATION	71	15 (21.1)	4 (5.6)	11 (15.5)	0.013	<0.0001	0.0005
GO T CELL DIFFERENTIATION INVOLVED IN IMMUNE RESPO...	66	15 (22.7)	4 (6.1)	11 (16.7)	0.013	<0.0001	0.0005
GO MACROPHAGE ACTIVATION	84	15 (17.9)	9 (10.7)	6 (7.1)	0.013	<0.0001	0.0005
GO REGULATION OF B CELL PROLIFERATION	58	14 (24.1)	4 (6.9)	10 (17.2)	0.012	<0.0001	0.0005
GO REGULATION OF DEFENSE RESPONSE TO VIRUS	68	14 (20.6)	12 (17.6)	2 (2.9)	0.012	<0.0001	0.0005
GO ENDOTHELIAL CELL APOPTOTIC PROCESS	53	13 (24.5)	3 (5.7)	10 (18.9)	0.011	<0.0001	0.0005
GO POSITIVE REGULATION OF B CELL PROLIFERATION	39	12 (30.8)	4 (10.3)	8 (20.5)	0.011	<0.0001	0.0005
GO NATURAL KILLER CELL MEDIATED IMMUNITY	51	11 (21.6)	2 (3.9)	9 (17.6)	0.010	<0.0001	0.0005
GO CD4 POSITIVE ALPHA BETA T CELL CYTOKINE PRODUCT...	22	9 (40.9)	3 (13.6)	6 (27.3)	0.008	<0.0001	0.0005
GO REGULATORY T CELL DIFFERENTIATION	28	9 (32.1)	3 (10.7)	6 (21.4)	0.008	<0.0001	0.0005
GO POSITIVE REGULATION OF ENDOTHELIAL CELL APOPTOT...	18	8 (44.4)	1 (5.6)	7 (38.9)	0.007	<0.0001	0.0005
GO THYMIC T CELL SELECTION	20	8 (40)	0 (0)	8 (40)	0.007	<0.0001	0.0005
GO REGULATION OF PHOSPHORUS METABOLIC PROCESS	1674	128 (7.6)	52 (3.1)	76 (4.5)	0.049	<0.0001	0.0008
GO POSITIVE REGULATION OF DEVELOPMENTAL PROCESS	1308	103 (7.9)	28 (2.1)	75 (5.7)	0.045	<0.0001	0.0008
GO CELLULAR ION HOMEOSTASIS	634	58 (9.1)	27 (4.3)	31 (4.9)	0.035	<0.0001	0.0008
GO LEUKOCYTE MEDIATED IMMUNITY	701	60 (8.6)	32 (4.6)	28 (4)	0.035	<0.0001	0.0008
GO POSITIVE REGULATION OF ESTABLISHMENT OF PROTEIN...	428	44 (10.3)	18 (4.2)	26 (6.1)	0.030	<0.0001	0.0008
GO POSITIVE REGULATION OF RESPONSE TO BIOTIC STIMU...	353	40 (11.3)	25 (7.1)	15 (4.2)	0.029	<0.0001	0.0008
GO RESPONSE TO TUMOR NECROSIS FACTOR	272	30 (11)	16 (5.9)	14 (5.1)	0.022	<0.0001	0.0008
GO RECEPTOR SIGNALING PATHWAY VIA STAT	156	22 (14.1)	12 (7.7)	10 (6.4)	0.018	<0.0001	0.0008
GO POSITIVE REGULATION OF INFLAMMATORY RESPONSE	134	20 (14.9)	13 (9.7)	7 (5.2)	0.017	<0.0001	0.0008
GO POSITIVE REGULATION OF LEUKOCYTE DIFFERENTIATIO...	136	20 (14.7)	5 (3.7)	15 (11)	0.017	<0.0001	0.0008
GO CYTOKINE PRODUCTION INVOLVED IN IMMUNE RESPONSE	94	17 (18.1)	7 (7.4)	10 (10.6)	0.015	<0.0001	0.0008
GO POSITIVE REGULATION OF LYMPHOCYTE DIFFERENTIATI...	86	16 (18.6)	4 (4.7)	12 (14)	0.014	<0.0001	0.0008
GO T CELL ACTIVATION INVOLVED IN IMMUNE RESPONSE	102	16 (15.7)	5 (4.9)	11 (10.8)	0.014	<0.0001	0.0008
GO INTERLEUKIN 1 BETA PRODUCTION	87	15 (17.2)	10 (11.5)	5 (5.7)	0.013	<0.0001	0.0008

Table S89: MSigDB GO Biological Processes enriched in differentially expressed genes at Day 2 compared to pre-First Vaccination (Dendritic cells, Study Arm 2 A/H7N9+AS03, D1, 29). Results are sorted by FDR adjusted p-value and Jaccard similarity index (continued.)

Gene Set ID	Gene Set Genes #	DE Genes N (%)	Up-reg. DE Genes N (%)	Down-reg. DE Genes N (%)	Jaccard Index	P-Value	FDR Adjusted P-Value
GO NEGATIVE REGULATION OF VIRAL LIFE CYCLE	76	14 (18.4)	13 (17.1)	1 (1.3)	0.012	<0.0001	0.0008
GO POSITIVE REGULATION OF RECEPTOR SIGNALING PATHW...	87	14 (16.1)	8 (9.2)	6 (6.9)	0.012	<0.0001	0.0008
GO NEUTROPHIL MIGRATION	103	14 (13.6)	9 (8.7)	5 (4.9)	0.012	<0.0001	0.0008
GO INTERLEUKIN 2 PRODUCTION	59	13 (22)	3 (5.1)	10 (16.9)	0.011	<0.0001	0.0008
GO INTERLEUKIN 6 SECRETION	45	12 (26.7)	6 (13.3)	6 (13.3)	0.011	<0.0001	0.0008
GO NEGATIVE REGULATION OF VIRAL GENOME REPLICATION	58	12 (20.7)	12 (20.7)	0 (0)	0.011	<0.0001	0.0008
GO POSITIVE REGULATION OF CYTOKINE PRODUCTION INVO...	49	11 (22.4)	5 (10.2)	6 (12.2)	0.010	<0.0001	0.0008
GO POSITIVE REGULATION OF TYROSINE PHOSPHORYLATION...	67	11 (16.4)	7 (10.4)	4 (6)	0.010	<0.0001	0.0008
GO POSITIVE REGULATION OF T CELL CYTOKINE PRODUCTI...	18	7 (38.9)	3 (16.7)	4 (22.2)	0.006	<0.0001	0.0008
GO POSITIVE REGULATION OF NATURAL KILLER CELL MEDI...	19	7 (36.8)	1 (5.3)	6 (31.6)	0.006	<0.0001	0.0008
GO CHEMICAL HOMEOSTASIS	1123	90 (8)	41 (3.7)	49 (4.4)	0.042	<0.0001	0.0011
GO NEGATIVE REGULATION OF DEVELOPMENTAL PROCESS	928	76 (8.2)	24 (2.6)	52 (5.6)	0.039	<0.0001	0.0011
GO RESPONSE TO LIPID	872	71 (8.1)	24 (2.8)	47 (5.4)	0.037	<0.0001	0.0011
GO I KAPPAB KINASE NF KAPPAB SIGNALING	252	31 (12.3)	16 (6.3)	15 (6)	0.024	<0.0001	0.0011
GO B CELL ACTIVATION	231	27 (11.7)	6 (2.6)	21 (9.1)	0.021	<0.0001	0.0011
GO POSITIVE REGULATION OF HEMOPOIESIS	170	23 (13.5)	7 (4.1)	16 (9.4)	0.019	<0.0001	0.0011
GO NEGATIVE REGULATION OF CELL ACTIVATION	184	23 (12.5)	7 (3.8)	16 (8.7)	0.018	<0.0001	0.0011
GO INTERLEUKIN 6 PRODUCTION	144	20 (13.9)	9 (6.2)	11 (7.6)	0.017	<0.0001	0.0011
GO TUMOR NECROSIS FACTOR SUPERFAMILY CYTOKINE PROD...	148	20 (13.5)	11 (7.4)	9 (6.1)	0.016	<0.0001	0.0011
GO POSITIVE REGULATION OF CHEMOTAXIS	125	18 (14.4)	9 (7.2)	9 (7.2)	0.015	<0.0001	0.0011
GO GRANULOCYTE MIGRATION	126	16 (12.7)	10 (7.9)	6 (4.8)	0.013	<0.0001	0.0011
GO ACTIVATION OF CYSTEINE TYPE ENDOPEPTIDASE ACTIV...	83	15 (18.1)	9 (10.8)	6 (7.2)	0.013	<0.0001	0.0011
GO POSITIVE T CELL SELECTION	32	10 (31.2)	0 (0)	10 (31.2)	0.009	<0.0001	0.0011
GO T CELL CYTOKINE PRODUCTION	38	10 (26.3)	4 (10.5)	6 (15.8)	0.009	<0.0001	0.0011
GO POSITIVE REGULATION OF EPITHELIAL CELL APOPTOTI...	32	9 (28.1)	2 (6.2)	7 (21.9)	0.008	<0.0001	0.0011
GO REGULATION OF PROTEIN MODIFICATION PROCESS	1724	129 (7.5)	54 (3.1)	75 (4.4)	0.048	<0.0001	0.0015
GO RESPONSE TO OXYGEN CONTAINING COMPOUND	1593	118 (7.4)	46 (2.9)	72 (4.5)	0.046	<0.0001	0.0015
GO POSITIVE REGULATION OF PROTEIN MODIFICATION PRO...	1148	92 (8)	38 (3.3)	54 (4.7)	0.043	<0.0001	0.0015
GO ION HOMEOSTASIS	772	66 (8.5)	31 (4)	35 (4.5)	0.037	<0.0001	0.0015
GO SECOND MESSENGER MEDIATED SIGNALING	419	41 (9.8)	13 (3.1)	28 (6.7)	0.028	<0.0001	0.0015
GO REGULATION OF T CELL CYTOKINE PRODUCTION	28	8 (28.6)	3 (10.7)	5 (17.9)	0.007	<0.0001	0.0015
GO POSITIVE REGULATION OF TRANSPORT	921	76 (8.3)	26 (2.8)	50 (5.4)	0.039	<0.0001	0.0018
GO REGULATION OF KINASE ACTIVITY	816	70 (8.6)	31 (3.8)	39 (4.8)	0.038	<0.0001	0.0018
GO REGULATION OF APOPTOTIC SIGNALING PATHWAY	380	38 (10)	16 (4.2)	22 (5.8)	0.026	<0.0001	0.0018
GO NEGATIVE REGULATION OF INFLAMMATORY RESPONSE	141	18 (12.8)	4 (2.8)	14 (9.9)	0.015	<0.0001	0.0018
GO POSITIVE REGULATION OF TUMOR NECROSIS FACTOR SU...	79	13 (16.5)	7 (8.9)	6 (7.6)	0.011	<0.0001	0.0018
GO POSITIVE REGULATION OF NATURAL KILLER CELL MEDI...	22	7 (31.8)	1 (4.5)	6 (27.3)	0.006	<0.0001	0.0018

Table S89: MSigDB GO Biological Processes enriched in differentially expressed genes at Day 2 compared to pre-First Vaccination (Dendritic cells, Study Arm 2 A/H7N9+AS03, D1, 29). Results are sorted by FDR adjusted p-value and Jaccard similarity index (continued.)

Gene Set ID	Gene Set Genes #	DE Genes N (%)	Up-reg. DE Genes N (%)	Down-reg. DE Genes N (%)	Jaccard Index	P-Value	FDR Adjusted P-Value
GO CELLULAR HOMEOSTASIS	901	72 (8)	31 (3.4)	41 (4.6)	0.037	<0.0001	0.0021
GO POSITIVE REGULATION OF MULTI ORGANISM PROCESS	489	46 (9.4)	27 (5.5)	19 (3.9)	0.030	<0.0001	0.0021
GO REGULATION OF PRODUCTION OF MOLECULAR MEDIATOR ...	129	18 (14)	6 (4.7)	12 (9.3)	0.015	<0.0001	0.0021
GO NEGATIVE REGULATION OF VIRAL PROCESS	90	15 (16.7)	14 (15.6)	1 (1.1)	0.013	<0.0001	0.0021
GO REGULATION OF VIRAL GENOME REPLICATION	91	15 (16.5)	13 (14.3)	2 (2.2)	0.013	<0.0001	0.0021
GO POSITIVE REGULATION OF T CELL PROLIFERATION	82	14 (17.1)	5 (6.1)	9 (11)	0.012	<0.0001	0.0021
GO REGULATION OF ANTIGEN RECEPTOR MEDIATED SIGNALI...	60	13 (21.7)	3 (5)	10 (16.7)	0.011	<0.0001	0.0021
GO REGULATION OF EPITHELIAL CELL APOPTOTIC PROCESS	77	13 (16.9)	4 (5.2)	9 (11.7)	0.011	<0.0001	0.0021
GO REGULATION OF CELL KILLING	80	13 (16.2)	3 (3.8)	10 (12.5)	0.011	<0.0001	0.0021
GO REGULATION OF LEUKOCYTE MEDIATED CYTOTOXICITY	59	11 (18.6)	3 (5.1)	8 (13.6)	0.010	<0.0001	0.0021
GO REGULATION OF PROTEIN LOCALIZATION	951	80 (8.4)	29 (3)	51 (5.4)	0.041	<0.0001	0.0024
GO CYTOKINE METABOLIC PROCESS	105	15 (14.3)	6 (5.7)	9 (8.6)	0.013	<0.0001	0.0024
GO REGULATION OF T CELL MEDIATED IMMUNITY	59	11 (18.6)	5 (8.5)	6 (10.2)	0.010	<0.0001	0.0024
GO REGULATION OF CYTOKINE PRODUCTION INVOLVED IN I...	76	13 (17.1)	5 (6.6)	8 (10.5)	0.011	<0.0001	0.0027
GO INTERLEUKIN 1 SECRETION	53	10 (18.9)	8 (15.1)	2 (3.8)	0.009	<0.0001	0.0027
GO RESPONSE TO INTERFERON ALPHA	21	7 (33.3)	5 (23.8)	2 (9.5)	0.006	<0.0001	0.0027
GO REGULATION OF HYDROLASE ACTIVITY	1175	92 (7.8)	37 (3.1)	55 (4.7)	0.042	<0.0001	0.0029
GO ERK1 AND ERK2 CASCADE	285	30 (10.5)	12 (4.2)	18 (6.3)	0.022	<0.0001	0.0029
GO POSITIVE REGULATION OF NF KAPPAB TRANSCRIPTION ...	133	19 (14.3)	10 (7.5)	9 (6.8)	0.016	<0.0001	0.0029
GO POSITIVE REGULATION OF CYTOKINE BIOSYNTHETIC PR...	60	11 (18.3)	5 (8.3)	6 (10)	0.010	<0.0001	0.0029
GO REGULATION OF DEFENSE RESPONSE TO VIRUS BY HOST	34	9 (26.5)	8 (23.5)	1 (2.9)	0.008	<0.0001	0.0029
GO ALPHA BETA T CELL PROLIFERATION	30	8 (26.7)	4 (13.3)	4 (13.3)	0.007	<0.0001	0.0029
GO REGULATION OF RESPONSE TO INTERFERON GAMMA	24	7 (29.2)	6 (25)	1 (4.2)	0.006	<0.0001	0.0029
GO REGULATION OF CHEMOTAXIS	198	23 (11.6)	11 (5.6)	12 (6.1)	0.018	<0.0001	0.0032
GO POSITIVE REGULATION OF ERK1 AND ERK2 CASCADE	184	22 (12)	8 (4.3)	14 (7.6)	0.018	<0.0001	0.0032
GO RESPONSE TO INTERLEUKIN 1	181	22 (12.2)	14 (7.7)	8 (4.4)	0.018	<0.0001	0.0032
GO POSITIVE REGULATION OF RESPONSE TO CYTOKINE STI...	53	11 (20.8)	7 (13.2)	4 (7.5)	0.010	<0.0001	0.0032
GO NEUROINFLAMMATORY RESPONSE	63	11 (17.5)	6 (9.5)	5 (7.9)	0.010	<0.0001	0.0032
GO T HELPER 1 CELL CYTOKINE PRODUCTION	8	4 (50)	1 (12.5)	3 (37.5)	0.004	<0.0001	0.0032
GO POSITIVE REGULATION OF CATALYTIC ACTIVITY	1331	104 (7.8)	47 (3.5)	57 (4.3)	0.045	0.0001	0.0034
GO POSITIVE REGULATION OF APOPTOTIC SIGNALING PATH...	169	21 (12.4)	8 (4.7)	13 (7.7)	0.017	0.0001	0.0034
GO TYROSINE PHOSPHORYLATION OF STAT PROTEIN	80	12 (15)	8 (10)	4 (5)	0.010	0.0001	0.0034
GO POSITIVE REGULATION OF T CELL MEDIATED IMMUNITY	41	9 (22)	5 (12.2)	4 (9.8)	0.008	0.0001	0.0034
GO HORMONE TRANSPORT	297	31 (10.4)	11 (3.7)	20 (6.7)	0.023	0.0001	0.0036
GO REGULATION OF VASCULATURE DEVELOPMENT	349	32 (9.2)	12 (3.4)	20 (5.7)	0.023	0.0001	0.0036
GO DEFENSE RESPONSE TO BACTERIUM	237	20 (8.4)	13 (5.5)	7 (3)	0.015	0.0001	0.0036
GO REGULATION OF VIRAL LIFE CYCLE	133	18 (13.5)	15 (11.3)	3 (2.3)	0.015	0.0001	0.0036

Table S89: MSigDB GO Biological Processes enriched in differentially expressed genes at Day 2 compared to pre-First Vaccination (Dendritic cells, Study Arm 2 A/H7N9+AS03, D1, 29). Results are sorted by FDR adjusted p-value and Jaccard similarity index (continued.)

Gene Set ID	Gene Set Genes #	DE Genes N (%)	Up-reg. DE Genes N (%)	Down-reg. DE Genes N (%)	Jaccard Index	P-Value	FDR Adjusted P-Value
GO REGULATION OF CD4 POSITIVE ALPHA BETA T CELL AC...	57	11 (19.3)	4 (7)	7 (12.3)	0.010	0.0001	0.0036
GO CYTOLYSIS	36	7 (19.4)	2 (5.6)	5 (13.9)	0.006	0.0001	0.0036
GO RESPONSE TO MOLECULE OF BACTERIAL ORIGIN	329	31 (9.4)	15 (4.6)	16 (4.9)	0.022	0.0001	0.0042
GO ACUTE INFLAMMATORY RESPONSE	102	14 (13.7)	8 (7.8)	6 (5.9)	0.012	0.0001	0.0042
GO PROGRAMMED CELL DEATH INVOLVED IN CELL DEVELOPM...	12	5 (41.7)	1 (8.3)	4 (33.3)	0.005	0.0001	0.0042
GO T HELPER 2 CELL CYTOKINE PRODUCTION	14	5 (35.7)	2 (14.3)	3 (21.4)	0.004	0.0001	0.0042
GO POSITIVE REGULATION OF MOLECULAR FUNCTION	1658	124 (7.5)	50 (3)	74 (4.5)	0.047	0.0001	0.0044
GO EPITHELIAL CELL APOPTOTIC PROCESS	100	15 (15)	4 (4)	11 (11)	0.013	0.0001	0.0044
GO NEGATIVE REGULATION OF SIGNALING	1297	97 (7.5)	32 (2.5)	65 (5)	0.042	0.0002	0.0046
GO NEGATIVE REGULATION OF CELL ADHESION	263	29 (11)	10 (3.8)	19 (7.2)	0.022	0.0002	0.0046
GO LYMPHOCYTE ACTIVATION INVOLVED IN IMMUNE RESPON...	171	20 (11.7)	6 (3.5)	14 (8.2)	0.016	0.0002	0.0046
GO MONONUCLEAR CELL MIGRATION	74	11 (14.9)	6 (8.1)	5 (6.8)	0.009	0.0002	0.0046
GO DEFENSE RESPONSE TO FUNGUS	35	6 (17.1)	4 (11.4)	2 (5.7)	0.005	0.0002	0.0046
GO NEGATIVE T CELL SELECTION	11	5 (45.5)	0 (0)	5 (45.5)	0.005	0.0002	0.0046
GO REGULATION OF TRANSFERASE ACTIVITY	912	75 (8.2)	33 (3.6)	42 (4.6)	0.039	0.0002	0.0048
GO PHAGOCYTOSIS	251	28 (11.2)	15 (6)	13 (5.2)	0.021	0.0002	0.0048
GO REGULATION OF T CELL RECEPTOR SIGNALING PATHWAY	37	9 (24.3)	3 (8.1)	6 (16.2)	0.008	0.0002	0.0048
GO POSITIVE REGULATION OF INTERLEUKIN 2 PRODUCTION	29	8 (27.6)	2 (6.9)	6 (20.7)	0.007	0.0002	0.0048
GO POSITIVE REGULATION OF I KAPPAB KINASE NF KAPPA...	169	21 (12.4)	14 (8.3)	7 (4.1)	0.017	0.0002	0.0051
GO REGULATION OF NATURAL KILLER CELL MEDIATED IMMU...	33	8 (24.2)	1 (3)	7 (21.2)	0.007	0.0002	0.0051
GO POSITIVE REGULATION OF LEUKOCYTE MIGRATION	118	15 (12.7)	8 (6.8)	7 (5.9)	0.013	0.0002	0.0053
GO B CELL RECEPTOR SIGNALING PATHWAY	56	11 (19.6)	1 (1.8)	10 (17.9)	0.010	0.0002	0.0053
GO POSITIVE REGULATION OF CELL DIFFERENTIATION	927	75 (8.1)	20 (2.2)	55 (5.9)	0.039	0.0002	0.0056
GO POSITIVE REGULATION OF CYSTEINE TYPE ENDOPEPTID...	141	18 (12.8)	11 (7.8)	7 (5)	0.015	0.0002	0.0056
GO POSITIVE REGULATION OF DNA BINDING TRANSCRIPTIO...	238	27 (11.3)	12 (5)	15 (6.3)	0.021	0.0002	0.0061
GO VIRAL GENOME REPLICATION	117	17 (14.5)	14 (12)	3 (2.6)	0.014	0.0002	0.0061
GO RESPONSE TO INTERFERON BETA	30	7 (23.3)	6 (20)	1 (3.3)	0.006	0.0002	0.0061
GO POSITIVE REGULATION OF JNK CASCADE	132	18 (13.6)	7 (5.3)	11 (8.3)	0.015	0.0002	0.0063
GO T CELL TOLERANCE INDUCTION	8	4 (50)	1 (12.5)	3 (37.5)	0.004	0.0002	0.0063
GO MONOCYTE CHEMOTAXIS	51	8 (15.7)	5 (9.8)	3 (5.9)	0.007	0.0002	0.0065
GO POSITIVE REGULATION OF PROTEIN SERINE THREONINE...	311	33 (10.6)	15 (4.8)	18 (5.8)	0.024	0.0002	0.0067
GO REGULATION OF MAP KINASE ACTIVITY	314	33 (10.5)	15 (4.8)	18 (5.7)	0.024	0.0002	0.0067
GO NEGATIVE REGULATION OF VASCULATURE DEVELOPMENT	139	16 (11.5)	4 (2.9)	12 (8.6)	0.013	0.0002	0.0067
GO TUMOR NECROSIS FACTOR MEDIATED SIGNALING PATHWA...	147	18 (12.2)	10 (6.8)	8 (5.4)	0.015	0.0003	0.0069
GO NEGATIVE REGULATION OF LYMPHOCYTE ACTIVATION	137	17 (12.4)	6 (4.4)	11 (8)	0.014	0.0003	0.0069
GO CELLULAR RESPONSE TO INTERFERON ALPHA	11	5 (45.5)	3 (27.3)	2 (18.2)	0.005	0.0003	0.0069
GO REGULATION OF CYSTEINE TYPE ENDOPEPTIDASE ACTIV...	221	24 (10.9)	13 (5.9)	11 (5)	0.019	0.0003	0.0071

Table S89: MSigDB GO Biological Processes enriched in differentially expressed genes at Day 2 compared to pre-First Vaccination (Dendritic cells, Study Arm 2 A/H7N9+AS03, D1, 29). Results are sorted by FDR adjusted p-value and Jaccard similarity index (continued.)

Gene Set ID	Gene Set Genes #	DE Genes N (%)	Up-reg. DE Genes N (%)	Down-reg. DE Genes N (%)	Jaccard Index	P-Value	FDR Adjusted P-Value
GO TOLERANCE INDUCTION	19	6 (31.6)	2 (10.5)	4 (21.1)	0.005	0.0003	0.0071
GO POSITIVE REGULATION OF INTERLEUKIN 2 BIOSYNTHET...	13	5 (38.5)	1 (7.7)	4 (30.8)	0.004	0.0003	0.0071
GO POSITIVE REGULATION OF INTERLEUKIN 6 SECRETION	28	7 (25)	4 (14.3)	3 (10.7)	0.006	0.0003	0.0074
GO GLIAL CELL ACTIVATION	48	9 (18.8)	6 (12.5)	3 (6.2)	0.008	0.0003	0.0076
GO INTERLEUKIN 2 BIOSYNTHETIC PROCESS	21	6 (28.6)	1 (4.8)	5 (23.8)	0.005	0.0003	0.0076
GO REGULATION OF NATURAL KILLER CELL DIFFERENTIATI...	14	5 (35.7)	2 (14.3)	3 (21.4)	0.004	0.0003	0.0076
GO POSITIVE REGULATION OF MAP KINASE ACTIVITY	240	27 (11.2)	13 (5.4)	14 (5.8)	0.021	0.0003	0.0078
GO NEGATIVE REGULATION OF CELL DEATH	933	71 (7.6)	25 (2.7)	46 (4.9)	0.036	0.0003	0.008
GO INTERLEUKIN 10 PRODUCTION	48	9 (18.8)	4 (8.3)	5 (10.4)	0.008	0.0003	0.008
GO LEUKOCYTE ACTIVATION INVOLVED IN INFLAMMATORY R...	40	8 (20)	5 (12.5)	3 (7.5)	0.007	0.0003	0.0082
GO ANTIMICROBIAL HUMORAL RESPONSE	108	10 (9.3)	6 (5.6)	4 (3.7)	0.008	0.0003	0.0085
GO B CELL MEDIATED IMMUNITY	104	14 (13.5)	9 (8.7)	5 (4.8)	0.012	0.0003	0.0086
GO COBALAMIN TRANSPORT	8	4 (50)	1 (12.5)	3 (37.5)	0.004	0.0003	0.0086
GO PROTEIN AUTOPHOSPHORYLATION	219	27 (12.3)	7 (3.2)	20 (9.1)	0.021	0.0004	0.0091
GO POSITIVE REGULATION OF PEPTIDASE ACTIVITY	185	21 (11.4)	13 (7)	8 (4.3)	0.017	0.0004	0.0093
GO INTERFERON ALPHA PRODUCTION	25	7 (28)	5 (20)	2 (8)	0.006	0.0004	0.0093
GO SIGNAL RELEASE	434	41 (9.4)	16 (3.7)	25 (5.8)	0.028	0.0004	0.0098
GO INTERLEUKIN 10 SECRETION	9	4 (44.4)	2 (22.2)	2 (22.2)	0.004	0.0004	0.0098
GO POSITIVE REGULATION OF ALPHA BETA T CELL ACTIVA...	56	10 (17.9)	2 (3.6)	8 (14.3)	0.009	0.0004	0.0099
GO POSITIVE REGULATION OF BLOOD VESSEL DIAMETER	56	10 (17.9)	6 (10.7)	4 (7.1)	0.009	0.0004	0.0099
GO ANATOMICAL STRUCTURE FORMATION INVOLVED IN MORP...	1071	81 (7.6)	24 (2.2)	57 (5.3)	0.039	0.0004	0.0102
GO POSITIVE REGULATION OF TRANSFERASE ACTIVITY	614	54 (8.8)	25 (4.1)	29 (4.7)	0.033	0.0004	0.0106
GO POSITIVE REGULATION OF CELL KILLING	54	9 (16.7)	2 (3.7)	7 (13)	0.008	0.0004	0.0106
GO REGULATION OF LIPID KINASE ACTIVITY	65	11 (16.9)	4 (6.2)	7 (10.8)	0.010	0.0004	0.0108
GO REGULATION OF INTERLEUKIN 1 SECRETION	45	8 (17.8)	6 (13.3)	2 (4.4)	0.007	0.0004	0.0108
GO REGULATION OF T HELPER 2 CELL CYTOKINE PRODUCTI...	11	4 (36.4)	1 (9.1)	3 (27.3)	0.004	0.0004	0.011
GO PROTEIN KINASE C SIGNALING	29	7 (24.1)	1 (3.4)	6 (20.7)	0.006	0.0005	0.0112
GO REGULATION OF PROTEIN SERINE THREONINE KINASE A...	478	43 (9)	20 (4.2)	23 (4.8)	0.028	0.0005	0.0114
GO FC RECEPTOR SIGNALING PATHWAY	168	21 (12.5)	7 (4.2)	14 (8.3)	0.017	0.0005	0.0118
GO POSITIVE REGULATION OF KINASE ACTIVITY	543	49 (9)	23 (4.2)	26 (4.8)	0.031	0.0005	0.012
GO INFLAMMATORY RESPONSE TO ANTIGENIC STIMULUS	44	8 (18.2)	2 (4.5)	6 (13.6)	0.007	0.0005	0.012
GO NEGATIVE REGULATION OF OXIDOREDUCTASE ACTIVITY	29	6 (20.7)	2 (6.9)	4 (13.8)	0.005	0.0005	0.0124
GO NEGATIVE REGULATION OF LYMPHOCYTE MIGRATION	14	5 (35.7)	0 (0)	5 (35.7)	0.004	0.0005	0.0124
GO POSITIVE REGULATION OF T CELL RECEPTOR SIGNALIN...	14	5 (35.7)	1 (7.1)	4 (28.6)	0.004	0.0005	0.0124
GO T HELPER 1 TYPE IMMUNE RESPONSE	40	8 (20)	4 (10)	4 (10)	0.007	0.0005	0.0125
GO POSITIVE REGULATION OF HYDROLASE ACTIVITY	707	60 (8.5)	27 (3.8)	33 (4.7)	0.035	0.0005	0.0127
GO NEGATIVE REGULATION OF LEUKOCYTE MIGRATION	41	8 (19.5)	1 (2.4)	7 (17.1)	0.007	0.0005	0.0127

Table S89: MSigDB GO Biological Processes enriched in differentially expressed genes at Day 2 compared to pre-First Vaccination (Dendritic cells, Study Arm 2 A/H7N9+AS03, D1, 29). Results are sorted by FDR adjusted p-value and Jaccard similarity index (continued.)

Gene Set ID	Gene Set Genes #	DE Genes N (%)	Up-reg. DE Genes N (%)	Down-reg. DE Genes N (%)	Jaccard Index	P-Value	FDR Adjusted P-Value
GO POSITIVE REGULATION OF LEUKOCYTE MEDIATED CYTOT...	41	8 (19.5)	2 (4.9)	6 (14.6)	0.007	0.0005	0.0127
GO NEGATIVE REGULATION OF CELL DIFFERENTIATION	682	55 (8.1)	17 (2.5)	38 (5.6)	0.032	0.0005	0.0129
GO HOMEOSTATIC PROCESS	1803	124 (6.9)	51 (2.8)	73 (4)	0.045	0.0006	0.0135
GO REGULATION OF HORMONE SECRETION	246	25 (10.2)	7 (2.8)	18 (7.3)	0.019	0.0006	0.0137
GO REGULATION OF RIBONUCLEASE ACTIVITY	8	4 (50)	4 (50)	0 (0)	0.004	0.0006	0.0137
GO TRANSMEMBRANE RECEPTOR PROTEIN TYROSINE KINASE ...	678	58 (8.6)	16 (2.4)	42 (6.2)	0.034	0.0006	0.0139
GO CELLULAR DEFENSE RESPONSE	46	8 (17.4)	2 (4.3)	6 (13)	0.007	0.0006	0.0142
GO REGULATION OF INTERLEUKIN 4 PRODUCTION	24	6 (25)	0 (0)	6 (25)	0.005	0.0006	0.0142
GO REGULATION OF T HELPER 1 CELL CYTOKINE PRODUCTI...	5	3 (60)	1 (20)	2 (40)	0.003	0.0006	0.0142
GO POSITIVE REGULATION OF NATURAL KILLER CELL DIFF...	8	4 (50)	2 (25)	2 (25)	0.004	0.0006	0.0146
GO NEGATIVE REGULATION OF INNATE IMMUNE RESPONSE	49	9 (18.4)	6 (12.2)	3 (6.1)	0.008	0.0007	0.0152
GO NUCLEOSIDE DIPHOSPHATE BIOSYNTHETIC PROCESS	9	4 (44.4)	2 (22.2)	2 (22.2)	0.004	0.0007	0.0152
GO POSITIVE REGULATION OF INTERLEUKIN 6 PRODUCTION	87	12 (13.8)	7 (8)	5 (5.7)	0.010	0.0007	0.0154
GO PEPTIDE HORMONE SECRETION	237	25 (10.5)	9 (3.8)	16 (6.8)	0.019	0.0007	0.0158
GO INTERLEUKIN 4 PRODUCTION	31	7 (22.6)	0 (0)	7 (22.6)	0.006	0.0007	0.016
GO INTRINSIC APOPTOTIC SIGNALING PATHWAY	274	27 (9.9)	15 (5.5)	12 (4.4)	0.020	0.0007	0.0166
GO NEGATIVE REGULATION OF I KAPPAB KINASE NF KAPPA...	48	9 (18.8)	1 (2.1)	8 (16.7)	0.008	0.0008	0.0169
GO LYMPH NODE DEVELOPMENT	15	5 (33.3)	1 (6.7)	4 (26.7)	0.004	0.0008	0.0169
GO REACTIVE OXYGEN SPECIES METABOLIC PROCESS	251	24 (9.6)	14 (5.6)	10 (4)	0.018	0.0008	0.0175
GO CELLULAR RESPONSE TO OXYGEN CONTAINING COMPOUND	1128	82 (7.3)	32 (2.8)	50 (4.4)	0.038	0.0008	0.0186
GO REGULATION OF ALPHA BETA T CELL DIFFERENTIATION	60	10 (16.7)	3 (5)	7 (11.7)	0.009	0.0009	0.0194
GO PURINERGIC NUCLEOTIDE RECEPTOR SIGNALING PATHWA...	17	5 (29.4)	3 (17.6)	2 (11.8)	0.004	0.0009	0.02
GO INTERFERON GAMMA SECRETION	18	5 (27.8)	1 (5.6)	4 (22.2)	0.004	0.0009	0.0204
GO RESPONSE TO INTERLEUKIN 18	10	4 (40)	1 (10)	3 (30)	0.004	0.0009	0.0204
GO NEGATIVE REGULATION OF T CELL DIFFERENTIATION	43	8 (18.6)	3 (7)	5 (11.6)	0.007	0.0009	0.0205
GO POSITIVE REGULATION OF ANTIGEN RECEPTOR MEDIATE...	21	6 (28.6)	1 (4.8)	5 (23.8)	0.005	0.001	0.0211
GO LEUKOCYTE APOPTOTIC PROCESS	100	13 (13)	6 (6)	7 (7)	0.011	0.001	0.0217
GO NEGATIVE REGULATION OF REGULATORY T CELL DIFFER...	6	3 (50)	1 (16.7)	2 (33.3)	0.003	0.001	0.0223
GO APOPTOTIC MITOCHONDRIAL CHANGES	114	14 (12.3)	5 (4.4)	9 (7.9)	0.012	0.0011	0.0229
GO ISG15 PROTEIN CONJUGATION	6	3 (50)	3 (50)	0 (0)	0.003	0.0011	0.0234
GO EXTRINSIC APOPTOTIC SIGNALING PATHWAY	209	22 (10.5)	11 (5.3)	11 (5.3)	0.017	0.0011	0.0235
GO CHEMOKINE PRODUCTION	80	11 (13.8)	6 (7.5)	5 (6.2)	0.009	0.0011	0.0235
GO ACUTE INFLAMMATORY RESPONSE TO ANTIGENIC STIMUL...	20	5 (25)	2 (10)	3 (15)	0.004	0.0011	0.0235
GO POSITIVE REGULATION OF INTERLEUKIN 1 PRODUCTION	50	8 (16)	5 (10)	3 (6)	0.007	0.0011	0.024
GO MOTOR NEURON AXON GUIDANCE	31	7 (22.6)	2 (6.5)	5 (16.1)	0.006	0.0011	0.024
GO REGULATION OF T CELL DIFFERENTIATION IN THYMUS	23	6 (26.1)	0 (0)	6 (26.1)	0.005	0.0011	0.0241
GO NEGATIVE REGULATION OF INTERLEUKIN 6 SECRETION	13	4 (30.8)	1 (7.7)	3 (23.1)	0.004	0.0012	0.0253

Table S89: MSigDB GO Biological Processes enriched in differentially expressed genes at Day 2 compared to pre-First Vaccination (Dendritic cells, Study Arm 2 A/H7N9+AS03, D1, 29). Results are sorted by FDR adjusted p-value and Jaccard similarity index (continued.)

Gene Set ID	Gene Set Genes #	DE Genes N (%)	Up-reg. DE Genes N (%)	Down-reg. DE Genes N (%)	Jaccard Index	P-Value	FDR Adjusted P-Value
GO POSITIVE REGULATION OF STRESS ACTIVATED PROTEIN...	160	19 (11.9)	7 (4.4)	12 (7.5)	0.015	0.0012	0.0254
GO COMPLEMENT ACTIVATION	62	9 (14.5)	6 (9.7)	3 (4.8)	0.008	0.0012	0.0258
GO REGULATION OF HUMORAL IMMUNE RESPONSE	62	9 (14.5)	6 (9.7)	3 (4.8)	0.008	0.0012	0.0259
GO POSITIVE REGULATION OF INTERLEUKIN 4 PRODUCTION	19	5 (26.3)	0 (0)	5 (26.3)	0.004	0.0012	0.0259
GO REGULATION OF PEPTIDE HORMONE SECRETION	196	21 (10.7)	6 (3.1)	15 (7.7)	0.017	0.0012	0.026
GO VASCULAR PROCESS IN CIRCULATORY SYSTEM	162	18 (11.1)	11 (6.8)	7 (4.3)	0.015	0.0013	0.026
GO RESPONSE TO INTERLEUKIN 4	34	7 (20.6)	3 (8.8)	4 (11.8)	0.006	0.0013	0.026
GO NEGATIVE REGULATION OF NEUROINFLAMMATORY RESPON...	13	4 (30.8)	0 (0)	4 (30.8)	0.004	0.0013	0.026
GO NEGATIVE REGULATION OF INTERFERON ALPHA PRODUCT...	5	3 (60)	1 (20)	2 (40)	0.003	0.0013	0.0265
GO REGULATION OF PHOSPHOLIPID METABOLIC PROCESS	85	12 (14.1)	4 (4.7)	8 (9.4)	0.010	0.0013	0.0267
GO POSITIVE REGULATION OF DEFENSE RESPONSE TO VIRU...	24	6 (25)	6 (25)	0 (0)	0.005	0.0013	0.0268
GO POSITIVE REGULATION OF TUMOR NECROSIS FACTOR SE...	19	5 (26.3)	4 (21.1)	1 (5.3)	0.004	0.0013	0.0273
GO NEGATIVE REGULATION OF PHOSPHOLIPID METABOLIC P...	12	4 (33.3)	0 (0)	4 (33.3)	0.004	0.0013	0.0274
GO POSITIVE REGULATION OF GLOMERULUS DEVELOPMENT	10	4 (40)	0 (0)	4 (40)	0.004	0.0013	0.0274
GO REGULATION OF ERBB SIGNALING PATHWAY	93	13 (14)	4 (4.3)	9 (9.7)	0.011	0.0014	0.0275
GO REGULATION OF INSULIN SECRETION	166	19 (11.4)	5 (3)	14 (8.4)	0.015	0.0014	0.0286
GO TYPE 2 IMMUNE RESPONSE	38	7 (18.4)	4 (10.5)	3 (7.9)	0.006	0.0014	0.0286
GO CELL CELL SIGNALING	1555	110 (7.1)	37 (2.4)	73 (4.7)	0.043	0.0014	0.0287
GO REGULATION OF MONONUCLEAR CELL MIGRATION	40	7 (17.5)	5 (12.5)	2 (5)	0.006	0.0014	0.0288
GO REGULATION OF VITAMIN METABOLIC PROCESS	12	4 (33.3)	1 (8.3)	3 (25)	0.004	0.0014	0.029
GO VIRAL LIFE CYCLE	308	30 (9.7)	19 (6.2)	11 (3.6)	0.022	0.0015	0.0291
GO REGULATION OF NEUROINFLAMMATORY RESPONSE	34	6 (17.6)	2 (5.9)	4 (11.8)	0.005	0.0015	0.0298
GO RESPONSE TO ERYTHROPOIETIN	8	3 (37.5)	2 (25)	1 (12.5)	0.003	0.0015	0.0299
GO REGULATION OF T CELL TOLERANCE INDUCTION	6	3 (50)	1 (16.7)	2 (33.3)	0.003	0.0015	0.03
GO REGULATION OF LEUKOCYTE CHEMOTAXIS	102	12 (11.8)	7 (6.9)	5 (4.9)	0.010	0.0015	0.0301
GO ADP BIOSYNTHETIC PROCESS	5	3 (60)	1 (20)	2 (40)	0.003	0.0015	0.0301
GO T HELPER 17 TYPE IMMUNE RESPONSE	27	6 (22.2)	0 (0)	6 (22.2)	0.005	0.0016	0.0306
GO REGULATION OF DNA BINDING TRANSCRIPTION FACTOR ...	393	35 (8.9)	14 (3.6)	21 (5.3)	0.024	0.0016	0.0309
GO LEUKOCYTE MIGRATION INVOLVED IN INFLAMMATORY RE...	14	4 (28.6)	4 (28.6)	0 (0)	0.004	0.0016	0.031
GO PURINERGIC RECEPTOR SIGNALING PATHWAY	28	6 (21.4)	3 (10.7)	3 (10.7)	0.005	0.0016	0.0313
GO GLIOGENESIS	270	27 (10)	8 (3)	19 (7)	0.020	0.0017	0.0322
GO NEGATIVE REGULATION OF TYPE I INTERFERON PRODUC...	43	8 (18.6)	6 (14)	2 (4.7)	0.007	0.0017	0.0323
GO SMOOTH MUSCLE CELL PROLIFERATION	146	16 (11)	7 (4.8)	9 (6.2)	0.013	0.0017	0.0328
GO SPONGIOTROPHOBLAST LAYER DEVELOPMENT	12	4 (33.3)	4 (33.3)	0 (0)	0.004	0.0017	0.0329
GO NEGATIVE REGULATION OF IMMUNE RESPONSE	134	15 (11.2)	7 (5.2)	8 (6)	0.012	0.0018	0.0344
GO POSITIVE REGULATION OF LEUKOCYTE CHEMOTAXIS	80	10 (12.5)	6 (7.5)	4 (5)	0.009	0.0018	0.0347
GO INTERLEUKIN 35 MEDIATED SIGNALING PATHWAY	11	4 (36.4)	2 (18.2)	2 (18.2)	0.004	0.0019	0.0355

Table S89: MSigDB GO Biological Processes enriched in differentially expressed genes at Day 2 compared to pre-First Vaccination (Dendritic cells, Study Arm 2 A/H7N9+AS03, D1, 29). Results are sorted by FDR adjusted p-value and Jaccard similarity index (continued.)

Gene Set ID	Gene Set Genes #	DE Genes N (%)	Up-reg. DE Genes N (%)	Down-reg. DE Genes N (%)	Jaccard Index	P-Value	FDR Adjusted P-Value
GO PURINE NUCLEOBASE TRANSPORT	6	3 (50)	1 (16.7)	2 (33.3)	0.003	0.0019	0.0355
GO NATURAL KILLER CELL ACTIVATION	73	9 (12.3)	3 (4.1)	6 (8.2)	0.008	0.0019	0.0356
GO REVERSIBLE DIFFERENTIATION	10	3 (30)	1 (10)	2 (20)	0.003	0.0019	0.0364
GO REGULATION OF GLOMERULAR FILTRATION	11	4 (36.4)	1 (9.1)	3 (27.3)	0.004	0.0019	0.0365
GO HEMATOPOIETIC STEM CELL MIGRATION	5	3 (60)	1 (20)	2 (40)	0.003	0.002	0.0381
GO POSITIVE REGULATION OF PHAGOCYTOSIS	61	9 (14.8)	5 (8.2)	4 (6.6)	0.008	0.0021	0.039
GO REGULATION OF TOLERANCE INDUCTION	12	4 (33.3)	2 (16.7)	2 (16.7)	0.004	0.0021	0.0394
GO ACTIVATION OF INNATE IMMUNE RESPONSE	286	27 (9.4)	19 (6.6)	8 (2.8)	0.020	0.0021	0.0397
GO TUMOR NECROSIS FACTOR SECRETION	33	6 (18.2)	5 (15.2)	1 (3)	0.005	0.0022	0.0409
GO LEUKOCYTE AGGREGATION	12	4 (33.3)	3 (25)	1 (8.3)	0.004	0.0022	0.0409
GO POSITIVE REGULATION OF INTERLEUKIN 10 SECRETION	6	3 (50)	1 (16.7)	2 (33.3)	0.003	0.0022	0.0409
GO PEPTIDYL AMINO ACID MODIFICATION	1180	87 (7.4)	30 (2.5)	57 (4.8)	0.040	0.0023	0.0419
GO MYELOID LEUKOCYTE ACTIVATION	594	46 (7.7)	29 (4.9)	17 (2.9)	0.028	0.0023	0.0419
GO FAT CELL DIFFERENTIATION	206	21 (10.2)	8 (3.9)	13 (6.3)	0.017	0.0023	0.0419
GO ERBB SIGNALING PATHWAY	141	17 (12.1)	6 (4.3)	11 (7.8)	0.014	0.0023	0.0419
GO PHOSPHATIDYLINOSITOL 3 KINASE SIGNALING	143	17 (11.9)	2 (1.4)	15 (10.5)	0.014	0.0023	0.0419
GO REGULATION OF TUBE SIZE	133	15 (11.3)	10 (7.5)	5 (3.8)	0.012	0.0023	0.0419
GO POSITIVE REGULATION OF TOLERANCE INDUCTION	6	3 (50)	2 (33.3)	1 (16.7)	0.003	0.0023	0.0419
GO REGULATION OF VASCULAR SMOOTH MUSCLE CELL DEDIF...	5	2 (40)	0 (0)	2 (40)	0.002	0.0023	0.0419
GO REGULATION OF PEPTIDASE ACTIVITY	409	32 (7.8)	16 (3.9)	16 (3.9)	0.022	0.0023	0.0426
GO REGULATION OF ADIPONECTIN SECRETION	6	3 (50)	2 (33.3)	1 (16.7)	0.003	0.0023	0.0426
GO CIRCULATORY SYSTEM PROCESS	517	42 (8.1)	21 (4.1)	21 (4.1)	0.027	0.0024	0.0431
GO SOMATIC DIVERSIFICATION OF T CELL RECEPTOR GENE...	5	3 (60)	0 (0)	3 (60)	0.003	0.0024	0.0435
GO NEGATIVE REGULATION OF LYMPHOCYTE DIFFERENTIATI...	52	8 (15.4)	3 (5.8)	5 (9.6)	0.007	0.0024	0.0441
GO UREA TRANSPORT	7	3 (42.9)	1 (14.3)	2 (28.6)	0.003	0.0025	0.0451
GO POSITIVE REGULATION OF PROTEOLYSIS	336	30 (8.9)	17 (5.1)	13 (3.9)	0.021	0.0025	0.0454
GO NEGATIVE REGULATION OF RESPONSE TO BIOTIC STIMU...	82	11 (13.4)	7 (8.5)	4 (4.9)	0.009	0.0027	0.0477
GO NEGATIVE REGULATION OF MEMBRANE DEPOLARIZATION	8	3 (37.5)	1 (12.5)	2 (25)	0.003	0.0027	0.0483
GO ACTIVATION OF PHOSPHOLIPASE C ACTIVITY	30	6 (20)	1 (3.3)	5 (16.7)	0.005	0.0027	0.0484
GO NEGATIVE REGULATION OF RECEPTOR SIGNALING PATHW...	20	5 (25)	4 (20)	1 (5)	0.004	0.0028	0.0494

Table S90: Blood Transcription Modules enriched in differentially expressed genes at Day 2 compared to pre-First Vaccination (Dendritic cells, Study Arm 2 A/H7N9+AS03, D1, 29). Results are sorted by FDR adjusted p-value and Jaccard similarity index.

Gene Set ID	Gene Set Genes #	DE Genes N (%)	Up-reg. DE Genes N (%)	Down-reg. DE Genes N (%)	Jaccard Index	P-Value	FDR Adjusted P-Value
M7.0 ENRICHED IN T CELLS (I)	57	49 (86)	0 (0)	49 (86)	0.045	<0.0001	0.0002
M7.2 ENRICHED IN NK CELLS (I)	45	42 (93.3)	1 (2.2)	41 (91.1)	0.038	<0.0001	0.0002
M7.1 T CELL ACTIVATION (I)	50	42 (84)	1 (2)	41 (82)	0.038	<0.0001	0.0002
M5.0 REGULATION OF ANTIGEN PRESENTATION AND IMMUNE...	78	24 (30.8)	3 (3.8)	21 (26.9)	0.021	<0.0001	0.0002
S1 NK CELL SURFACE SIGNATURE	38	22 (57.9)	1 (2.6)	21 (55.3)	0.020	<0.0001	0.0002
M7.3 T CELL ACTIVATION (II)	28	21 (75)	1 (3.6)	20 (71.4)	0.019	<0.0001	0.0002
S0 T CELL SURFACE SIGNATURE	24	17 (70.8)	0 (0)	17 (70.8)	0.015	<0.0001	0.0002
M75 ANTIVIRAL IFN SIGNATURE	22	16 (72.7)	15 (68.2)	1 (4.5)	0.015	<0.0001	0.0002
M19 T CELL DIFFERENTIATION (TH2)	15	15 (100)	0 (0)	15 (100)	0.014	<0.0001	0.0002
M165 ENRICHED IN ACTIVATED DENDRITIC CELLS (II)	34	15 (44.1)	15 (44.1)	0 (0)	0.013	<0.0001	0.0002
M61.0 ENRICHED IN NK CELLS (II)	16	13 (81.2)	0 (0)	13 (81.2)	0.012	<0.0001	0.0002
M7.4 T CELL ACTIVATION (III)	13	12 (92.3)	0 (0)	12 (92.3)	0.011	<0.0001	0.0002
M14 T CELL DIFFERENTIATION	11	11 (100)	0 (0)	11 (100)	0.010	<0.0001	0.0002
M61.2 ENRICHED IN NK CELLS (RECEPTOR ACTIVATION)	13	11 (84.6)	0 (0)	11 (84.6)	0.010	<0.0001	0.0002
M223 ENRICHED IN T CELLS (II)	11	10 (90.9)	0 (0)	10 (90.9)	0.009	<0.0001	0.0002
M127 TYPE I INTERFERON RESPONSE	12	10 (83.3)	10 (83.3)	0 (0)	0.009	<0.0001	0.0002
M67 ACTIVATED DENDRITIC CELLS	12	10 (83.3)	10 (83.3)	0 (0)	0.009	<0.0001	0.0002
M150 INNATE ANTIVIRAL RESPONSE	12	9 (75)	9 (75)	0 (0)	0.008	<0.0001	0.0002
M18 T CELL DIFFERENTIATION VIA ITK AND PKC	9	9 (100)	0 (0)	9 (100)	0.008	<0.0001	0.0002
M111.1 VIRAL SENSING AND IMMUNITY; IRF2 TARGETS NE...	11	7 (63.6)	7 (63.6)	0 (0)	0.006	<0.0001	0.0003
M13 INNATE ACTIVATION BY CYTOSOLIC DNA SENSING	11	7 (63.6)	5 (45.5)	2 (18.2)	0.006	<0.0001	0.0003
M35.1 SIGNALING IN T CELLS (II)	11	7 (63.6)	0 (0)	7 (63.6)	0.006	<0.0001	0.0003
M57 IMMUREGULATION - MONOCYTES, T AND B CELLS	13	7 (53.8)	1 (7.7)	6 (46.2)	0.006	<0.0001	0.0003
M65 IL2, IL7, TCR NETWORK	10	7 (70)	0 (0)	7 (70)	0.006	<0.0001	0.0003
M5.1 T CELL ACTIVATION AND SIGNALING	21	10 (47.6)	0 (0)	10 (47.6)	0.009	<0.0001	0.0005
M109 RECEPTORS, CELL MIGRATION	15	9 (60)	2 (13.3)	7 (46.7)	0.008	<0.0001	0.0005
M130 ENRICHED IN G-PROTEIN COUPLED RECEPTORS	10	6 (60)	0 (0)	6 (60)	0.005	0.0001	0.0015
M61.1 ENRICHED IN NK CELLS (KIR CLUSTER)	6	4 (66.7)	0 (0)	4 (66.7)	0.004	0.0001	0.0015
M157 ENRICHED IN NK CELLS (III)	14	7 (50)	0 (0)	7 (50)	0.006	0.0003	0.0031
M86.0 CHEMOKINES AND INFLAMMATORY MOLECULES IN MYE...	18	7 (38.9)	5 (27.8)	2 (11.1)	0.006	0.0003	0.0031
M117 CELL ADHESION (GO)	19	9 (47.4)	0 (0)	9 (47.4)	0.008	0.0003	0.0035
M86.1 PROINFLAMMATORY DENDRITIC CELL, MYELOID CELL...	12	6 (50)	3 (25)	3 (25)	0.005	0.0003	0.0035
S7 CD4 T CELL SURFACE SIGNATURE TH2-STIMULATED	11	6 (54.5)	0 (0)	6 (54.5)	0.005	0.0003	0.0035
M121 TBA	12	6 (50)	4 (33.3)	2 (16.7)	0.005	0.0004	0.0038
M68 RIG-1 LIKE RECEPTOR SIGNALING	9	5 (55.6)	5 (55.6)	0 (0)	0.005	0.0005	0.0045
M35.0 SIGNALING IN T CELLS (I)	14	6 (42.9)	1 (7.1)	5 (35.7)	0.005	0.0008	0.0073
M12 CD28 COSTIMULATION	8	5 (62.5)	0 (0)	5 (62.5)	0.005	0.0008	0.0075

Confidential/Proprietary Information

Table S90: Blood Transcription Modules enriched in differentially expressed genes at Day 2 compared to pre-First Vaccination (Dendritic cells, Study Arm 2 A/H7N9+AS03, D1, 29). Results are sorted by FDR adjusted p-value and Jaccard similarity index (continued.)

Gene Set ID	Gene Set Genes #	DE Genes N (%)	Up-reg. DE Genes N (%)	Down-reg. DE Genes N (%)	Jaccard Index	P-Value	FDR Adjusted P-Value
M52 T CELL ACTIVATION (IV)	13	6 (46.2)	0 (0)	6 (46.2)	0.005	0.0009	0.0086
M112.0 COMPLEMENT ACTIVATION (I)	17	7 (41.2)	5 (29.4)	2 (11.8)	0.006	0.0016	0.0141
M111.0 VIRAL SENSING AND IMMUNITY; IRF2 TARGETS NE...	16	6 (37.5)	5 (31.2)	1 (6.2)	0.005	0.0036	0.0308

Table S91: KEGG Pathways enriched in differentially expressed genes at Day 8 compared to pre-First Vaccination (Dendritic cells, Study Arm 1 A/H3N2v, D1). Results are sorted by FDR adjusted p-value and Jaccard similarity index.

Gene Set ID	Gene Set Genes #	DE Genes N (%)	Up-reg. DE Genes N (%)	Down-reg. DE Genes N (%)	Jaccard Index	P-Value	FDR Adjusted P-Value
AFRICAN TRYPANOSOMIASIS	36	2 (5.6)	2 (5.6)	0 (0)	0.056	<0.0001	0.0051
MALARIA	48	2 (4.2)	2 (4.2)	0 (0)	0.042	<0.0001	0.0051

Table S92: MSigDB Reactome Pathways enriched in differentially expressed genes at Day 8 compared to pre-First Vaccination (Dendritic cells, Study Arm 1 A/H3N2v, D1). Results are sorted by FDR adjusted p-value and Jaccard similarity index.

Gene Set ID	Gene Set Genes #	DE Genes N (%)	Up-reg. DE Genes N (%)	Down-reg. DE Genes N (%)	Jaccard Index	P-Value	FDR Adjusted P-Value
REACTOME ERYTHROCYTES TAKE UP OXYGEN AND RELEASE C...	9	2 (22.2)	2 (22.2)	0 (0)	0.222	<0.0001	0.0077
REACTOME ERYTHROCYTES TAKE UP CARBON DIOXIDE AND R...	13	2 (15.4)	2 (15.4)	0 (0)	0.154	<0.0001	0.0077
REACTOME SCAVENGING OF HEME FROM PLASMA	13	2 (15.4)	2 (15.4)	0 (0)	0.154	<0.0001	0.0102
REACTOME BINDING AND UPTAKE OF LIGANDS BY SCAVENGE...	41	2 (4.9)	2 (4.9)	0 (0)	0.049	<0.0001	0.0153

Table S93: MSigDB GO Biological Processes enriched in differentially expressed genes at Day 8 compared to pre-First Vaccination (Dendritic cells, Study Arm 1 A/H3N2v, D1). Results are sorted by FDR adjusted p-value and Jaccard similarity index.

Gene Set ID	Gene Set Genes #	DE Genes N (%)	Up-reg. DE Genes N (%)	Down-reg. DE Genes N (%)	Jaccard Index	P-Value	FDR Adjusted P-Value
GO OXYGEN TRANSPORT	15	2 (13.3)	2 (13.3)	0 (0)	0.133	<0.0001	0.0251
GO GAS TRANSPORT	19	2 (10.5)	2 (10.5)	0 (0)	0.105	<0.0001	0.0251
GO HYDROGEN PEROXIDE CATABOLIC PROCESS	31	2 (6.5)	2 (6.5)	0 (0)	0.065	<0.0001	0.0251
GO BICARBONATE TRANSPORT	41	2 (4.9)	2 (4.9)	0 (0)	0.049	<0.0001	0.0251

Table S93: MSigDB GO Biological Processes enriched in differentially expressed genes at Day 8 compared to pre-First Vaccination (Dendritic cells, Study Arm 1 A/H3N2v, D1). Results are sorted by FDR adjusted p-value and Jaccard similarity index (continued.)

Gene Set ID	Gene Set Genes #	DE Genes N (%)	Up-reg. DE Genes N (%)	Down-reg. DE Genes N (%)	Jaccard Index	P-Value	FDR Adjusted P-Value
GO ANTIBIOTIC CATABOLIC PROCESS	57	2 (3.5)	2 (3.5)	0 (0)	0.035	<0.0001	0.0251
GO COFACTOR CATABOLIC PROCESS	62	2 (3.2)	2 (3.2)	0 (0)	0.032	<0.0001	0.0251
GO HYDROGEN PEROXIDE METABOLIC PROCESS	50	2 (4)	2 (4)	0 (0)	0.040	<0.0001	0.043
GO RESPONSE TO HYDROGEN PEROXIDE	136	2 (1.5)	2 (1.5)	0 (0)	0.015	<0.0001	0.0471

Table S94: KEGG Pathways enriched in differentially expressed genes at Day 30 compared to pre-Second Vaccination (Dendritic cells, Study Arm 2 A/H7N9+AS03, D1, 29). Results are sorted by FDR adjusted p-value and Jaccard similarity index.

Gene Set ID	Gene Set Genes #	DE Genes N (%)	Up-reg. DE Genes N (%)	Down-reg. DE Genes N (%)	Jaccard Index	P-Value	FDR Adjusted P-Value
CYTOKINE-CYTOKINE RECEPTOR INTERACTION	280	57 (20.4)	21 (7.5)	36 (12.9)	0.026	<0.0001	0.0004
HEMATOPOIETIC CELL LINEAGE	93	32 (34.4)	8 (8.6)	24 (25.8)	0.016	<0.0001	0.0004
TH17 CELL DIFFERENTIATION	102	27 (26.5)	9 (8.8)	18 (17.6)	0.013	<0.0001	0.0004
TH1 AND TH2 CELL DIFFERENTIATION	88	25 (28.4)	7 (8)	18 (20.5)	0.012	<0.0001	0.0004
ANTIGEN PROCESSING AND PRESENTATION	70	20 (28.6)	10 (14.3)	10 (14.3)	0.010	<0.0001	0.0004
VIRAL PROTEIN INTERACTION WITH CYTOKINE AND CYTOKI...	96	20 (20.8)	7 (7.3)	13 (13.5)	0.010	<0.0001	0.0004
PRIMARY IMMUNODEFICIENCY	32	15 (46.9)	3 (9.4)	12 (37.5)	0.008	<0.0001	0.0004
GRAFT-VERSUS-HOST DISEASE	38	14 (36.8)	4 (10.5)	10 (26.3)	0.007	<0.0001	0.0004
INTESTINAL IMMUNE NETWORK FOR IGA PRODUCTION	45	14 (31.1)	6 (13.3)	8 (17.8)	0.007	<0.0001	0.0004
INFLAMMATORY BOWEL DISEASE	61	16 (26.2)	7 (11.5)	9 (14.8)	0.008	<0.0001	0.001
INFLUENZA A	160	31 (19.4)	27 (16.9)	4 (2.5)	0.015	<0.0001	0.0011
NATURAL KILLER CELL MEDIATED CYTOTOXICITY	121	24 (19.8)	4 (3.3)	20 (16.5)	0.012	<0.0001	0.0011
MEASLES	132	29 (22)	18 (13.6)	11 (8.3)	0.014	<0.0001	0.0013
PD-L1 EXPRESSION AND PD-1 CHECKPOINT PATHWAY IN CA...	88	21 (23.9)	6 (6.8)	15 (17)	0.010	0.0001	0.0031
CELL ADHESION MOLECULES	137	28 (20.4)	10 (7.3)	18 (13.1)	0.014	0.0002	0.0036
NF-KAPPA B SIGNALING PATHWAY	94	20 (21.3)	7 (7.4)	13 (13.8)	0.010	0.0004	0.008
EPSTEIN-BARR VIRUS INFECTION	196	34 (17.3)	23 (11.7)	11 (5.6)	0.016	0.0006	0.0107
T CELL RECEPTOR SIGNALING PATHWAY	100	22 (22)	0 (0)	22 (22)	0.011	0.0005	0.0107
NOD-LIKE RECEPTOR SIGNALING PATHWAY	175	29 (16.6)	24 (13.7)	5 (2.9)	0.014	0.0009	0.0158
RHEUMATOID ARTHRITIS	88	16 (18.2)	10 (11.4)	6 (6.8)	0.008	0.0011	0.018
PATHWAYS IN CANCER	515	74 (14.4)	26 (5)	48 (9.3)	0.031	0.0011	0.0185
ECM-RECEPTOR INTERACTION	86	20 (23.3)	3 (3.5)	17 (19.8)	0.010	0.0016	0.0229
ALLOGRAFT REJECTION	34	8 (23.5)	4 (11.8)	4 (11.8)	0.004	0.0015	0.0229
APOPTOSIS	132	24 (18.2)	12 (9.1)	12 (9.1)	0.012	0.0017	0.0234
JAK-STAT SIGNALING PATHWAY	154	25 (16.2)	14 (9.1)	11 (7.1)	0.012	0.0019	0.0259

Table S94: KEGG Pathways enriched in differentially expressed genes at Day 30 compared to pre-Second Vaccination (Dendritic cells, Study Arm 2 A/H7N9+AS03, D1, 29). Results are sorted by FDR adjusted p-value and Jaccard similarity index (continued.)

Gene Set ID	Gene Set Genes #	DE Genes N (%)	Up-reg. DE Genes N (%)	Down-reg. DE Genes N (%)	Jaccard Index	P-Value	FDR Adjusted P-Value
OSTEOCLAST DIFFERENTIATION	122	23 (18.9)	16 (13.1)	7 (5.7)	0.011	0.002	0.0263
STAPHYLOCOCCUS AUREUS INFECTION	91	14 (15.4)	13 (14.3)	1 (1.1)	0.007	0.0037	0.0462
HUMAN IMMUNODEFICIENCY VIRUS 1 INFECTION	204	32 (15.7)	15 (7.4)	17 (8.3)	0.015	0.0039	0.0472

Table S95: MSigDB Reactome Pathways enriched in differentially expressed genes at Day 30 compared to pre-Second Vaccination (Dendritic cells, Study Arm 2 A/H7N9+AS03, D1, 29). Results are sorted by FDR adjusted p-value and Jaccard similarity index.

Gene Set ID	Gene Set Genes #	DE Genes N (%)	Up-reg. DE Genes N (%)	Down-reg. DE Genes N (%)	Jaccard Index	P-Value	FDR Adjusted P-Value
REACTOME CYTOKINE SIGNALING IN IMMUNE SYSTEM	788	146 (18.5)	87 (11)	59 (7.5)	0.056	<0.0001	0.0013
REACTOME INNATE IMMUNE SYSTEM	968	127 (13.1)	81 (8.4)	46 (4.8)	0.045	<0.0001	0.0013
REACTOME ADAPTIVE IMMUNE SYSTEM	670	104 (15.5)	40 (6)	64 (9.6)	0.041	<0.0001	0.0013
REACTOME SIGNALING BY INTERLEUKINS	430	70 (16.3)	38 (8.8)	32 (7.4)	0.030	<0.0001	0.0013
REACTOME G ALPHA I SIGNALLING EVENTS	367	57 (15.5)	24 (6.5)	33 (9)	0.025	<0.0001	0.0013
REACTOME INTERFERON SIGNALING	177	52 (29.4)	49 (27.7)	3 (1.7)	0.025	<0.0001	0.0013
REACTOME GPCR LIGAND BINDING	420	56 (13.3)	21 (5)	35 (8.3)	0.024	<0.0001	0.0013
REACTOME IMMUNOREGULATORY INTERACTIONS BETWEEN A L...	97	36 (37.1)	11 (11.3)	25 (25.8)	0.018	<0.0001	0.0013
REACTOME INTERFERON ALPHA BETA SIGNALING	60	28 (46.7)	28 (46.7)	0 (0)	0.014	<0.0001	0.0013
REACTOME INTERFERON GAMMA SIGNALING	71	28 (39.4)	26 (36.6)	2 (2.8)	0.014	<0.0001	0.0013
REACTOME INTERLEUKIN 10 SIGNALING	39	13 (33.3)	10 (25.6)	3 (7.7)	0.007	<0.0001	0.0013
REACTOME GENERATION OF SECOND MESSENGER MOLECULES	22	12 (54.5)	2 (9.1)	10 (45.5)	0.006	<0.0001	0.0013
REACTOME CLASS A 1 RHODOPSIN LIKE RECEPTORS	306	42 (13.7)	17 (5.6)	25 (8.2)	0.019	<0.0001	0.002
REACTOME INTERLEUKIN 4 AND INTERLEUKIN 13 SIGNALIN...	102	23 (22.5)	13 (12.7)	10 (9.8)	0.011	<0.0001	0.002
REACTOME CHEMOKINE RECEPTORS BIND CHEMOKINES	51	12 (23.5)	5 (9.8)	7 (13.7)	0.006	<0.0001	0.002
REACTOME PD 1 SIGNALING	12	7 (58.3)	2 (16.7)	5 (41.7)	0.004	<0.0001	0.0029
REACTOME TNFS BIND THEIR PHYSIOLOGICAL RECEPTORS	26	9 (34.6)	1 (3.8)	8 (30.8)	0.005	<0.0001	0.0045
REACTOME TCR SIGNALING	100	22 (22)	9 (9)	13 (13)	0.011	0.0002	0.0145
REACTOME GROWTH HORMONE RECEPTOR SIGNALING	24	9 (37.5)	6 (25)	3 (12.5)	0.005	0.0002	0.0145
REACTOME COSTIMULATION BY THE CD28 FAMILY	55	15 (27.3)	3 (5.5)	12 (21.8)	0.007	0.0002	0.0161
REACTOME FCGR ACTIVATION	12	6 (50)	3 (25)	3 (25)	0.003	0.0003	0.0226
REACTOME NCAM1 INTERACTIONS	41	13 (31.7)	2 (4.9)	11 (26.8)	0.007	0.0003	0.023
REACTOME INTERLEUKIN 35 SIGNALLING	12	6 (50)	4 (33.3)	2 (16.7)	0.003	0.0004	0.0286
REACTOME ANTI INFLAMMATORY RESPONSE FAVOURING LEIS...	161	25 (15.5)	8 (5)	17 (10.6)	0.012	0.0005	0.0319
REACTOME INTERLEUKIN 20 FAMILY SIGNALING	26	8 (30.8)	5 (19.2)	3 (11.5)	0.004	0.0007	0.0401
REACTOME OAS ANTIVIRAL RESPONSE	8	5 (62.5)	5 (62.5)	0 (0)	0.003	0.0007	0.0401

Table S96: MSigDB GO Biological Processes enriched in differentially expressed genes at Day 30 compared to pre-Second Vaccination (Dendritic cells, Study Arm 2 A/H7N9+AS03, D1, 29). Results are sorted by FDR adjusted p-value and Jaccard similarity index.

Gene Set ID	Gene Set Genes #	DE Genes N (%)	Up-reg. DE Genes N (%)	Down-reg. DE Genes N (%)	Jaccard Index	P-Value	FDR Adjusted P-Value
GO REGULATION OF IMMUNE SYSTEM PROCESS	1409	265 (18.8)	118 (8.4)	147 (10.4)	0.086	<0.0001	0.0005
GO DEFENSE RESPONSE	1497	266 (17.8)	167 (11.2)	99 (6.6)	0.084	<0.0001	0.0005
GO RESPONSE TO BIOTIC STIMULUS	1325	229 (17.3)	154 (11.6)	75 (5.7)	0.075	<0.0001	0.0005
GO CELL ACTIVATION	1265	214 (16.9)	92 (7.3)	122 (9.6)	0.071	<0.0001	0.0005
GO POSITIVE REGULATION OF IMMUNE SYSTEM PROCESS	972	190 (19.5)	89 (9.2)	101 (10.4)	0.070	<0.0001	0.0005
GO DEFENSE RESPONSE TO OTHER ORGANISM	993	188 (18.9)	137 (13.8)	51 (5.1)	0.068	<0.0001	0.0005
GO REGULATION OF IMMUNE RESPONSE	897	180 (20.1)	91 (10.1)	89 (9.9)	0.067	<0.0001	0.0005
GO POSITIVE REGULATION OF SIGNALING	1730	228 (13.2)	86 (5)	142 (8.2)	0.066	<0.0001	0.0005
GO BIOLOGICAL ADHESION	1320	202 (15.3)	62 (4.7)	140 (10.6)	0.066	<0.0001	0.0005
GO IMMUNE EFFECTOR PROCESS	1077	187 (17.4)	116 (10.8)	71 (6.6)	0.066	<0.0001	0.0005
GO RESPONSE TO CYTOKINE	1099	186 (16.9)	112 (10.2)	74 (6.7)	0.065	<0.0001	0.0005
GO POSITIVE REGULATION OF MULTICELLULAR ORGANISMAL...	1665	220 (13.2)	88 (5.3)	132 (7.9)	0.065	<0.0001	0.0005
GO LOCOMOTION	1747	219 (12.5)	74 (4.2)	145 (8.3)	0.063	<0.0001	0.0005
GO REGULATION OF CELL POPULATION PROLIFERATION	1541	204 (13.2)	71 (4.6)	133 (8.6)	0.062	<0.0001	0.0005
GO INNATE IMMUNE RESPONSE	814	161 (19.8)	121 (14.9)	40 (4.9)	0.062	<0.0001	0.0005
GO HOMEOSTATIC PROCESS	1803	216 (12)	93 (5.2)	123 (6.8)	0.061	<0.0001	0.0005
GO REGULATION OF RESPONSE TO STRESS	1406	193 (13.7)	107 (7.6)	86 (6.1)	0.061	<0.0001	0.0005
GO SECRETION	1551	198 (12.8)	103 (6.6)	95 (6.1)	0.060	<0.0001	0.0005
GO NEGATIVE REGULATION OF RESPONSE TO STIMULUS	1531	195 (12.7)	75 (4.9)	120 (7.8)	0.059	<0.0001	0.0005
GO CELL MOTILITY	1531	193 (12.6)	64 (4.2)	129 (8.4)	0.059	<0.0001	0.0005
GO LYMPHOCYTE ACTIVATION	620	141 (22.7)	45 (7.3)	96 (15.5)	0.058	<0.0001	0.0005
GO REGULATION OF RESPONSE TO EXTERNAL STIMULUS	1006	162 (16.1)	94 (9.3)	68 (6.8)	0.058	<0.0001	0.0005
GO CYTOKINE PRODUCTION	720	145 (20.1)	73 (10.1)	72 (10)	0.057	<0.0001	0.0005
GO REGULATION OF CELL DEATH	1593	191 (12)	80 (5)	111 (7)	0.057	<0.0001	0.0005
GO CYTOKINE MEDIATED SIGNALING PATHWAY	711	140 (19.7)	87 (12.2)	53 (7.5)	0.056	<0.0001	0.0005
GO POSITIVE REGULATION OF IMMUNE RESPONSE	704	134 (19)	68 (9.7)	66 (9.4)	0.053	<0.0001	0.0005
GO NEGATIVE REGULATION OF MULTICELLULAR ORGANISMAL...	1169	152 (13)	63 (5.4)	89 (7.6)	0.051	<0.0001	0.0005
GO CHEMICAL HOMEOSTASIS	1123	149 (13.3)	68 (6.1)	81 (7.2)	0.051	<0.0001	0.0005
GO IMMUNE SYSTEM DEVELOPMENT	926	138 (14.9)	54 (5.8)	84 (9.1)	0.050	<0.0001	0.0005
GO CELL CELL ADHESION	781	130 (16.6)	41 (5.2)	89 (11.4)	0.050	<0.0001	0.0005
GO REGULATION OF CELL ACTIVATION	507	116 (22.9)	40 (7.9)	76 (15)	0.050	<0.0001	0.0005
GO REGULATION OF DEFENSE RESPONSE	703	125 (17.8)	79 (11.2)	46 (6.5)	0.049	<0.0001	0.0005
GO T CELL ACTIVATION	436	111 (25.5)	36 (8.3)	75 (17.2)	0.049	<0.0001	0.0005
GO REGULATION OF MULTI ORGANISM PROCESS	700	119 (17)	85 (12.1)	34 (4.9)	0.047	<0.0001	0.0005

Table S96: MSigDB GO Biological Processes enriched in differentially expressed genes at Day 30 compared to pre-Second Vaccination (Dendritic cells, Study Arm 2 A/H7N9+AS03, D1, 29). Results are sorted by FDR adjusted p-value and Jaccard similarity index (continued.)

Gene Set ID	Gene Set Genes #	DE Genes N (%)	Up-reg. DE Genes N (%)	Down-reg. DE Genes N (%)	Jaccard Index	P-Value	FDR Adjusted P-Value
GO INFLAMMATORY RESPONSE	680	116 (17.1)	61 (9)	55 (8.1)	0.046	<0.0001	0.0005
GO POSITIVE REGULATION OF INTRACELLULAR SIGNAL TRA...	948	125 (13.2)	50 (5.3)	75 (7.9)	0.045	<0.0001	0.0005
GO ADAPTIVE IMMUNE RESPONSE	353	98 (27.8)	39 (11)	59 (16.7)	0.044	<0.0001	0.0005
GO ACTIVATION OF IMMUNE RESPONSE	547	106 (19.4)	53 (9.7)	53 (9.7)	0.044	<0.0001	0.0005
GO REGULATION OF CELL ADHESION	633	109 (17.2)	36 (5.7)	73 (11.5)	0.044	<0.0001	0.0005
GO REGULATION OF LYMPHOCYTE ACTIVATION	392	98 (25)	32 (8.2)	66 (16.8)	0.044	<0.0001	0.0005
GO POSITIVE REGULATION OF CELL POPULATION PROLIFER...	865	116 (13.4)	41 (4.7)	75 (8.7)	0.043	<0.0001	0.0005
GO IMMUNE RESPONSE REGULATING SIGNALING PATHWAY	507	101 (19.9)	45 (8.9)	56 (11)	0.043	<0.0001	0.0005
GO LEUKOCYTE DIFFERENTIATION	483	96 (19.9)	34 (7)	62 (12.8)	0.041	<0.0001	0.0005
GO TAXIS	607	98 (16.1)	38 (6.3)	60 (9.9)	0.040	<0.0001	0.0005
GO LEUKOCYTE MEDIATED IMMUNITY	701	100 (14.3)	59 (8.4)	41 (5.8)	0.039	<0.0001	0.0005
GO POSITIVE REGULATION OF CELL DEATH	670	97 (14.5)	48 (7.2)	49 (7.3)	0.038	<0.0001	0.0005
GO PEPTIDE SECRETION	587	93 (15.8)	45 (7.7)	48 (8.2)	0.038	<0.0001	0.0005
GO POSITIVE REGULATION OF RESPONSE TO EXTERNAL STI...	575	91 (15.8)	59 (10.3)	32 (5.6)	0.037	<0.0001	0.0005
GO REGULATION OF RESPONSE TO BIOTIC STIMULUS	466	86 (18.5)	64 (13.7)	22 (4.7)	0.037	<0.0001	0.0005
GO POSITIVE REGULATION OF CYTOKINE PRODUCTION	419	83 (19.8)	47 (11.2)	36 (8.6)	0.036	<0.0001	0.0005
GO LYMPHOCYTE DIFFERENTIATION	336	80 (23.8)	23 (6.8)	57 (17)	0.036	<0.0001	0.0005
GO RESPONSE TO BACTERIUM	587	88 (15)	53 (9)	35 (6)	0.036	<0.0001	0.0005
GO IMMUNE RESPONSE REGULATING CELL SURFACE RECEPTO...	348	79 (22.7)	28 (8)	51 (14.7)	0.036	<0.0001	0.0005
GO REGULATION OF CELL CELL ADHESION	372	80 (21.5)	28 (7.5)	52 (14)	0.036	<0.0001	0.0005
GO RESPONSE TO VIRUS	300	76 (25.3)	61 (20.3)	15 (5)	0.035	<0.0001	0.0005
GO REGULATION OF T CELL ACTIVATION	292	75 (25.7)	26 (8.9)	49 (16.8)	0.035	<0.0001	0.0005
GO REGULATION OF IMMUNE EFFECTOR PROCESS	357	76 (21.3)	42 (11.8)	34 (9.5)	0.034	<0.0001	0.0005
GO LEUKOCYTE CELL CELL ADHESION	307	74 (24.1)	28 (9.1)	46 (15)	0.034	<0.0001	0.0005
GO POSITIVE REGULATION OF LOCOMOTION	544	80 (14.7)	30 (5.5)	50 (9.2)	0.033	<0.0001	0.0005
GO NEGATIVE REGULATION OF IMMUNE SYSTEM PROCESS	426	76 (17.8)	30 (7)	46 (10.8)	0.033	<0.0001	0.0005
GO LEUKOCYTE MIGRATION	410	74 (18)	30 (7.3)	44 (10.7)	0.032	<0.0001	0.0005
GO DEFENSE RESPONSE TO VIRUS	229	68 (29.7)	57 (24.9)	11 (4.8)	0.032	<0.0001	0.0005
GO POSITIVE REGULATION OF CELL ACTIVATION	297	70 (23.6)	26 (8.8)	44 (14.8)	0.032	<0.0001	0.0005
GO POSITIVE REGULATION OF DEFENSE RESPONSE	457	75 (16.4)	52 (11.4)	23 (5)	0.032	<0.0001	0.0005
GO POSITIVE REGULATION OF CELL ADHESION	368	71 (19.3)	25 (6.8)	46 (12.5)	0.032	<0.0001	0.0005
GO REGULATION OF PEPTIDE SECRETION	461	74 (16.1)	33 (7.2)	41 (8.9)	0.032	<0.0001	0.0005
GO T CELL DIFFERENTIATION	230	66 (28.7)	20 (8.7)	46 (20)	0.031	<0.0001	0.0005
GO REGULATION OF HEMOPOIESIS	441	67 (15.2)	31 (7)	36 (8.2)	0.029	<0.0001	0.0005
GO LEUKOCYTE PROLIFERATION	276	59 (21.4)	24 (8.7)	35 (12.7)	0.027	<0.0001	0.0005
GO ADAPTIVE IMMUNE RESPONSE BASED ON SOMATIC RECOM...	223	57 (25.6)	30 (13.5)	27 (12.1)	0.027	<0.0001	0.0005
GO ANTIGEN RECEPTOR MEDIATED SIGNALING PATHWAY	212	54 (25.5)	15 (7.1)	39 (18.4)	0.026	<0.0001	0.0005

Table S96: MSigDB GO Biological Processes enriched in differentially expressed genes at Day 30 compared to pre-Second Vaccination (Dendritic cells, Study Arm 2 A/H7N9+AS03, D1, 29). Results are sorted by FDR adjusted p-value and Jaccard similarity index (continued.)

Gene Set ID	Gene Set Genes #	DE Genes N (%)	Up-reg. DE Genes N (%)	Down-reg. DE Genes N (%)	Jaccard Index	P-Value	FDR Adjusted P-Value
GO LYMPHOCYTE MEDIATED IMMUNITY	216	54 (25)	26 (12)	28 (13)	0.025	<0.0001	0.0005
GO NEGATIVE REGULATION OF RESPONSE TO EXTERNAL STI...	350	57 (16.3)	24 (6.9)	33 (9.4)	0.025	<0.0001	0.0005
GO REGULATION OF INFLAMMATORY RESPONSE	332	56 (16.9)	28 (8.4)	28 (8.4)	0.025	<0.0001	0.0005
GO REGULATION OF LEUKOCYTE DIFFERENTIATION	252	54 (21.4)	22 (8.7)	32 (12.7)	0.025	<0.0001	0.0005
GO POSITIVE REGULATION OF CELL CELL ADHESION	232	52 (22.4)	18 (7.8)	34 (14.7)	0.024	<0.0001	0.0005
GO NEGATIVE REGULATION OF CYTOKINE PRODUCTION	262	51 (19.5)	22 (8.4)	29 (11.1)	0.024	<0.0001	0.0005
GO POSITIVE REGULATION OF LEUKOCYTE CELL CELL ADHE...	194	49 (25.3)	18 (9.3)	31 (16)	0.023	<0.0001	0.0005
GO REGULATION OF LEUKOCYTE PROLIFERATION	203	49 (24.1)	21 (10.3)	28 (13.8)	0.023	<0.0001	0.0005
GO RESPONSE TO INTERFERON GAMMA	166	46 (27.7)	43 (25.9)	3 (1.8)	0.022	<0.0001	0.0005
GO CYTOKINE SECRETION	220	47 (21.4)	28 (12.7)	19 (8.6)	0.022	<0.0001	0.0005
GO NEGATIVE REGULATION OF MULTI ORGANISM PROCESS	195	46 (23.6)	36 (18.5)	10 (5.1)	0.022	<0.0001	0.0005
GO CELL CHEMOTAXIS	278	47 (16.9)	25 (9)	22 (7.9)	0.021	<0.0001	0.0005
GO PRODUCTION OF MOLECULAR MEDIATOR OF IMMUNE RESP...	187	45 (24.1)	22 (11.8)	23 (12.3)	0.021	<0.0001	0.0005
GO REGULATION OF LYMPHOCYTE DIFFERENTIATION	158	42 (26.6)	16 (10.1)	26 (16.5)	0.020	<0.0001	0.0005
GO ALPHA BETA T CELL ACTIVATION	127	41 (32.3)	17 (13.4)	24 (18.9)	0.020	<0.0001	0.0005
GO B CELL ACTIVATION	231	43 (18.6)	10 (4.3)	33 (14.3)	0.020	<0.0001	0.0005
GO HUMORAL IMMUNE RESPONSE	227	40 (17.6)	18 (7.9)	22 (9.7)	0.019	<0.0001	0.0005
GO NEGATIVE REGULATION OF DEFENSE RESPONSE	201	39 (19.4)	19 (9.5)	20 (10)	0.018	<0.0001	0.0005
GO REGULATION OF T CELL DIFFERENTIATION	131	37 (28.2)	14 (10.7)	23 (17.6)	0.018	<0.0001	0.0005
GO T CELL RECEPTOR SIGNALING PATHWAY	168	36 (21.4)	13 (7.7)	23 (13.7)	0.017	<0.0001	0.0005
GO REGULATION OF LEUKOCYTE MEDIATED IMMUNITY	174	36 (20.7)	18 (10.3)	18 (10.3)	0.017	<0.0001	0.0005
GO POSITIVE REGULATION OF LEUKOCYTE PROLIFERATION	124	35 (28.2)	17 (13.7)	18 (14.5)	0.017	<0.0001	0.0005
GO NEGATIVE REGULATION OF CELL ACTIVATION	184	35 (19)	14 (7.6)	21 (11.4)	0.017	<0.0001	0.0005
GO POSITIVE REGULATION OF IMMUNE EFFECTOR PROCESS	189	35 (18.5)	16 (8.5)	19 (10.1)	0.017	<0.0001	0.0005
GO LEUKOCYTE CHEMOTAXIS	202	35 (17.3)	21 (10.4)	14 (6.9)	0.017	<0.0001	0.0005
GO T CELL PROLIFERATION	167	34 (20.4)	17 (10.2)	17 (10.2)	0.016	<0.0001	0.0005
GO MYELOID LEUKOCYTE MIGRATION	187	34 (18.2)	20 (10.7)	14 (7.5)	0.016	<0.0001	0.0005
GO REGULATION OF LYMPHOCYTE MEDIATED IMMUNITY	127	32 (25.2)	15 (11.8)	17 (13.4)	0.016	<0.0001	0.0005
GO REGULATION OF ADAPTIVE IMMUNE RESPONSE	142	32 (22.5)	19 (13.4)	13 (9.2)	0.015	<0.0001	0.0005
GO INTERFERON GAMMA PRODUCTION	97	31 (32)	12 (12.4)	19 (19.6)	0.015	<0.0001	0.0005
GO REGULATION OF PRODUCTION OF MOLECULAR MEDIATOR ...	129	31 (24)	15 (11.6)	16 (12.4)	0.015	<0.0001	0.0005
GO ALPHA BETA T CELL DIFFERENTIATION	97	30 (30.9)	12 (12.4)	18 (18.6)	0.015	<0.0001	0.0005
GO REGULATION OF LEUKOCYTE MIGRATION	175	31 (17.7)	13 (7.4)	18 (10.3)	0.015	<0.0001	0.0005
GO INTERFERON GAMMA MEDIATED SIGNALING PATHWAY	70	29 (41.4)	27 (38.6)	2 (2.9)	0.015	<0.0001	0.0005
GO REGULATION OF B CELL ACTIVATION	112	29 (25.9)	7 (6.2)	22 (19.6)	0.014	<0.0001	0.0005
GO NEGATIVE REGULATION OF IMMUNE RESPONSE	134	29 (21.6)	19 (14.2)	10 (7.5)	0.014	<0.0001	0.0005
GO CYTOKINE PRODUCTION INVOLVED IN IMMUNE RESPONSE	94	28 (29.8)	16 (17)	12 (12.8)	0.014	<0.0001	0.0005

Table S96: MSigDB GO Biological Processes enriched in differentially expressed genes at Day 30 compared to pre-Second Vaccination (Dendritic cells, Study Arm 2 A/H7N9+AS03, D1, 29). Results are sorted by FDR adjusted p-value and Jaccard similarity index (continued.)

Gene Set ID	Gene Set Genes #	DE Genes N (%)	Up-reg. DE Genes N (%)	Down-reg. DE Genes N (%)	Jaccard Index	P-Value	FDR Adjusted P-Value
GO RESPONSE TO TYPE I INTERFERON	88	28 (31.8)	28 (31.8)	0 (0)	0.014	<0.0001	0.0005
GO B CELL PROLIFERATION	91	26 (28.6)	5 (5.5)	21 (23.1)	0.013	<0.0001	0.0005
GO CD4 POSITIVE ALPHA BETA T CELL ACTIVATION	87	26 (29.9)	12 (13.8)	14 (16.1)	0.013	<0.0001	0.0005
GO NEGATIVE REGULATION OF VIRAL PROCESS	90	26 (28.9)	24 (26.7)	2 (2.2)	0.013	<0.0001	0.0005
GO POSITIVE REGULATION OF CYTOKINE SECRETION	126	26 (20.6)	16 (12.7)	10 (7.9)	0.013	<0.0001	0.0005
GO POSITIVE REGULATION OF LEUKOCYTE MEDIATED IMMUN...	113	26 (23)	11 (9.7)	15 (13.3)	0.013	<0.0001	0.0005
GO CELL KILLING	146	26 (17.8)	10 (6.8)	16 (11)	0.013	<0.0001	0.0005
GO T CELL MEDIATED IMMUNITY	84	25 (29.8)	14 (16.7)	11 (13.1)	0.012	<0.0001	0.0005
GO NEGATIVE REGULATION OF VIRAL LIFE CYCLE	76	24 (31.6)	23 (30.3)	1 (1.3)	0.012	<0.0001	0.0005
GO POSITIVE REGULATION OF ADAPTIVE IMMUNE RESPONSE	92	24 (26.1)	13 (14.1)	11 (12)	0.012	<0.0001	0.0005
GO POSITIVE REGULATION OF LYMPHOCYTE MEDIATED IMMU...	87	24 (27.6)	9 (10.3)	15 (17.2)	0.012	<0.0001	0.0005
GO REGULATION OF ALPHA BETA T CELL ACTIVATION	84	24 (28.6)	13 (15.5)	11 (13.1)	0.012	<0.0001	0.0005
GO CYTOKINE METABOLIC PROCESS	105	24 (22.9)	11 (10.5)	13 (12.4)	0.012	<0.0001	0.0005
GO INTERLEUKIN 1 PRODUCTION	99	23 (23.2)	16 (16.2)	7 (7.1)	0.011	<0.0001	0.0005
GO REGULATION OF CYTOKINE PRODUCTION INVOLVED IN I...	76	22 (28.9)	12 (15.8)	10 (13.2)	0.011	<0.0001	0.0005
GO T CELL DIFFERENTIATION IN THYMUS	67	22 (32.8)	3 (4.5)	19 (28.4)	0.011	<0.0001	0.0005
GO B CELL MEDIATED IMMUNITY	104	22 (21.2)	12 (11.5)	10 (9.6)	0.011	<0.0001	0.0005
GO LEUKOCYTE MEDIATED CYTOTOXICITY	87	21 (24.1)	7 (8)	14 (16.1)	0.010	<0.0001	0.0005
GO POSITIVE REGULATION OF LYMPHOCYTE DIFFERENTIATI...	86	21 (24.4)	10 (11.6)	11 (12.8)	0.010	<0.0001	0.0005
GO POSITIVE REGULATION OF T CELL PROLIFERATION	82	21 (25.6)	11 (13.4)	10 (12.2)	0.010	<0.0001	0.0005
GO LYMPHOCYTE COSTIMULATION	52	20 (38.5)	3 (5.8)	17 (32.7)	0.010	<0.0001	0.0005
GO POSITIVE REGULATION OF INTERFERON GAMMA PRODUCT...	54	20 (37)	9 (16.7)	11 (20.4)	0.010	<0.0001	0.0005
GO CD4 POSITIVE ALPHA BETA T CELL DIFFERENTIATION	71	20 (28.2)	10 (14.1)	10 (14.1)	0.010	<0.0001	0.0005
GO NEGATIVE REGULATION OF VIRAL GENOME REPLICATION	58	20 (34.5)	20 (34.5)	0 (0)	0.010	<0.0001	0.0005
GO REGULATION OF ANTIGEN RECEPTOR MEDIATED SIGNALI...	60	20 (33.3)	5 (8.3)	15 (25)	0.010	<0.0001	0.0005
GO REGULATION OF B CELL PROLIFERATION	58	20 (34.5)	5 (8.6)	15 (25.9)	0.010	<0.0001	0.0005
GO T CELL SELECTION	45	19 (42.2)	3 (6.7)	16 (35.6)	0.010	<0.0001	0.0005
GO REGULATION OF DEFENSE RESPONSE TO VIRUS	68	19 (27.9)	16 (23.5)	3 (4.4)	0.009	<0.0001	0.0005
GO REGULATION OF CELL KILLING	80	19 (23.8)	7 (8.8)	12 (15)	0.009	<0.0001	0.0005
GO B CELL RECEPTOR SIGNALING PATHWAY	56	18 (32.1)	2 (3.6)	16 (28.6)	0.009	<0.0001	0.0005
GO INTERLEUKIN 2 PRODUCTION	59	18 (30.5)	7 (11.9)	11 (18.6)	0.009	<0.0001	0.0005
GO REGULATION OF T CELL MEDIATED IMMUNITY	59	18 (30.5)	11 (18.6)	7 (11.9)	0.009	<0.0001	0.0005
GO ANTIMICROBIAL HUMORAL RESPONSE	108	16 (14.8)	8 (7.4)	8 (7.4)	0.008	<0.0001	0.0005
GO POSITIVE REGULATION OF B CELL PROLIFERATION	39	15 (38.5)	5 (12.8)	10 (25.6)	0.008	<0.0001	0.0005
GO POSITIVE T CELL SELECTION	32	14 (43.8)	2 (6.2)	12 (37.5)	0.007	<0.0001	0.0005
GO INTERLEUKIN 6 SECRETION	45	13 (28.9)	6 (13.3)	7 (15.6)	0.006	<0.0001	0.0005
GO ALPHA BETA T CELL PROLIFERATION	30	12 (40)	7 (23.3)	5 (16.7)	0.006	<0.0001	0.0005

Table S96: MSigDB GO Biological Processes enriched in differentially expressed genes at Day 30 compared to pre-Second Vaccination (Dendritic cells, Study Arm 2 A/H7N9+AS03, D1, 29). Results are sorted by FDR adjusted p-value and Jaccard similarity index (continued.)

Gene Set ID	Gene Set Genes #	DE Genes N (%)	Up-reg. DE Genes N (%)	Down-reg. DE Genes N (%)	Jaccard Index	P-Value	FDR Adjusted P-Value
GO RESPONSE TO INTERFERON ALPHA	21	12 (57.1)	10 (47.6)	2 (9.5)	0.006	<0.0001	0.0005
GO RESPONSE TO INTERFERON BETA	30	11 (36.7)	10 (33.3)	1 (3.3)	0.006	<0.0001	0.0005
GO POSITIVE REGULATION OF ENDOTHELIAL CELL APOPTOT...	18	8 (44.4)	1 (5.6)	7 (38.9)	0.004	<0.0001	0.0005
GO METAL ION HOMEOSTASIS	622	86 (13.8)	43 (6.9)	43 (6.9)	0.035	<0.0001	0.0009
GO POSITIVE REGULATION OF PEPTIDE SECRETION	271	44 (16.2)	22 (8.1)	22 (8.1)	0.020	<0.0001	0.0009
GO REGULATION OF VIRAL LIFE CYCLE	133	29 (21.8)	26 (19.5)	3 (2.3)	0.014	<0.0001	0.0009
GO REGULATION OF VIRAL GENOME REPLICATION	91	24 (26.4)	22 (24.2)	2 (2.2)	0.012	<0.0001	0.0009
GO NEGATIVE REGULATION OF IMMUNE EFFECTOR PROCESS	106	23 (21.7)	13 (12.3)	10 (9.4)	0.011	<0.0001	0.0009
GO T CELL ACTIVATION INVOLVED IN IMMUNE RESPONSE	102	23 (22.5)	11 (10.8)	12 (11.8)	0.011	<0.0001	0.0009
GO T CELL DIFFERENTIATION INVOLVED IN IMMUNE RESPO...	66	20 (30.3)	9 (13.6)	11 (16.7)	0.010	<0.0001	0.0009
GO INTERLEUKIN 1 BETA PRODUCTION	87	20 (23)	14 (16.1)	6 (6.9)	0.010	<0.0001	0.0009
GO POSITIVE REGULATION OF B CELL ACTIVATION	71	19 (26.8)	5 (7)	14 (19.7)	0.009	<0.0001	0.0009
GO POSITIVE REGULATION OF CYTOKINE BIOSYNTHETIC PR...	60	17 (28.3)	10 (16.7)	7 (11.7)	0.009	<0.0001	0.0009
GO REGULATION OF LEUKOCYTE MEDIATED CYTOTOXICITY	59	16 (27.1)	6 (10.2)	10 (16.9)	0.008	<0.0001	0.0009
GO T CELL CYTOKINE PRODUCTION	38	13 (34.2)	8 (21.1)	5 (13.2)	0.007	<0.0001	0.0009
GO REGULATION OF DEFENSE RESPONSE TO VIRUS BY HOST	34	12 (35.3)	11 (32.4)	1 (2.9)	0.006	<0.0001	0.0009
GO CD4 POSITIVE ALPHA BETA T CELL CYTOKINE PRODUCT...	22	10 (45.5)	5 (22.7)	5 (22.7)	0.005	<0.0001	0.0009
GO RESPONSE TO OXYGEN CONTAINING COMPOUND	1593	185 (11.6)	83 (5.2)	102 (6.4)	0.055	<0.0001	0.0014
GO ANATOMICAL STRUCTURE FORMATION INVOLVED IN MORP...	1071	137 (12.8)	43 (4)	94 (8.8)	0.048	<0.0001	0.0014
GO ION HOMEOSTASIS	772	101 (13.1)	51 (6.6)	50 (6.5)	0.038	<0.0001	0.0014
GO CELLULAR ION HOMEOSTASIS	634	87 (13.7)	43 (6.8)	44 (6.9)	0.035	<0.0001	0.0014
GO NEUROINFLAMMATORY RESPONSE	63	16 (25.4)	9 (14.3)	7 (11.1)	0.008	<0.0001	0.0014
GO POSITIVE REGULATION OF DEVELOPMENTAL PROCESS	1308	163 (12.5)	54 (4.1)	109 (8.3)	0.053	<0.0001	0.0017
GO NEGATIVE REGULATION OF CELL POPULATION PROLIFER...	666	88 (13.2)	31 (4.7)	57 (8.6)	0.035	<0.0001	0.0017
GO PEPTIDYL TYROSINE MODIFICATION	354	57 (16.1)	21 (5.9)	36 (10.2)	0.025	<0.0001	0.0017
GO POSITIVE REGULATION OF RESPONSE TO BIOTIC STIMU...	353	56 (15.9)	40 (11.3)	16 (4.5)	0.025	<0.0001	0.0017
GO REGULATION OF CYTOSOLIC CALCIUM ION CONCENTRATI...	346	52 (15)	21 (6.1)	31 (9)	0.023	<0.0001	0.0017
GO DEFENSE RESPONSE TO BACTERIUM	237	30 (12.7)	19 (8)	11 (4.6)	0.014	<0.0001	0.0017
GO VIRAL GENOME REPLICATION	117	27 (23.1)	24 (20.5)	3 (2.6)	0.013	<0.0001	0.0017
GO LYMPHOCYTE MIGRATION	96	19 (19.8)	4 (4.2)	15 (15.6)	0.009	<0.0001	0.0017
GO MACROPHAGE ACTIVATION	84	19 (22.6)	12 (14.3)	7 (8.3)	0.009	<0.0001	0.0017
GO POSITIVE REGULATION OF ALPHA BETA T CELL ACTIVA...	56	15 (26.8)	9 (16.1)	6 (10.7)	0.007	<0.0001	0.0017
GO POSITIVE REGULATION OF T CELL MEDIATED IMMUNITY	41	13 (31.7)	8 (19.5)	5 (12.2)	0.007	<0.0001	0.0017
GO REGULATORY T CELL DIFFERENTIATION	28	10 (35.7)	5 (17.9)	5 (17.9)	0.005	<0.0001	0.0017
GO PROTEIN PHOSPHORYLATION	1838	217 (11.8)	81 (4.4)	136 (7.4)	0.061	<0.0001	0.002
GO REGULATION OF INTRACELLULAR SIGNAL TRANSDUCTION	1707	204 (12)	80 (4.7)	124 (7.3)	0.059	<0.0001	0.002
GO REGULATION OF PHOSPHORYLATION	1495	179 (12)	70 (4.7)	109 (7.3)	0.055	<0.0001	0.002

Table S96: MSigDB GO Biological Processes enriched in differentially expressed genes at Day 30 compared to pre-Second Vaccination (Dendritic cells, Study Arm 2 A/H7N9+AS03, D1, 29). Results are sorted by FDR adjusted p-value and Jaccard similarity index (continued.)

Gene Set ID	Gene Set Genes #	DE Genes N (%)	Up-reg. DE Genes N (%)	Down-reg. DE Genes N (%)	Jaccard Index	P-Value	FDR Adjusted P-Value
GO REGULATION OF CELLULAR COMPONENT MOVEMENT	973	124 (12.7)	43 (4.4)	81 (8.3)	0.044	<0.0001	0.002
GO RESPONSE TO LIPID	872	110 (12.6)	47 (5.4)	63 (7.2)	0.041	<0.0001	0.002
GO VIRAL LIFE CYCLE	308	52 (16.9)	37 (12)	15 (4.9)	0.024	<0.0001	0.002
GO NEGATIVE REGULATION OF ESTABLISHMENT OF PROTEIN...	174	31 (17.8)	13 (7.5)	18 (10.3)	0.015	<0.0001	0.002
GO POSITIVE REGULATION OF LEUKOCYTE DIFFERENTIATIO...	136	28 (20.6)	13 (9.6)	15 (11)	0.014	<0.0001	0.002
GO NEGATIVE REGULATION OF LYMPHOCYTE ACTIVATION	137	26 (19)	11 (8)	15 (10.9)	0.013	<0.0001	0.002
GO POSITIVE REGULATION OF INFLAMMATORY RESPONSE	134	25 (18.7)	17 (12.7)	8 (6)	0.012	<0.0001	0.002
GO POSITIVE REGULATION OF RESPONSE TO CYTOKINE STI...	53	15 (28.3)	10 (18.9)	5 (9.4)	0.007	<0.0001	0.002
GO REGULATION OF T CELL CYTOKINE PRODUCTION	28	10 (35.7)	6 (21.4)	4 (14.3)	0.005	<0.0001	0.002
GO THYMIC T CELL SELECTION	20	9 (45)	0 (0)	9 (45)	0.005	<0.0001	0.002
GO REGULATION OF SECRETION	752	97 (12.9)	43 (5.7)	54 (7.2)	0.037	<0.0001	0.0022
GO APOPTOTIC SIGNALING PATHWAY	566	78 (13.8)	36 (6.4)	42 (7.4)	0.032	<0.0001	0.0022
GO CALCIUM MEDIATED SIGNALING	208	36 (17.3)	12 (5.8)	24 (11.5)	0.017	<0.0001	0.0022
GO POSITIVE REGULATION OF HEMOPOIESIS	170	32 (18.8)	16 (9.4)	16 (9.4)	0.015	<0.0001	0.0022
GO ENTRY INTO HOST	126	26 (20.6)	16 (12.7)	10 (7.9)	0.013	<0.0001	0.0022
GO POSITIVE REGULATION OF PRODUCTION OF MOLECULAR ...	89	20 (22.5)	9 (10.1)	11 (12.4)	0.010	<0.0001	0.0022
GO NEGATIVE REGULATION OF INNATE IMMUNE RESPONSE	49	14 (28.6)	11 (22.4)	3 (6.1)	0.007	<0.0001	0.0022
GO GLIAL CELL ACTIVATION	48	13 (27.1)	8 (16.7)	5 (10.4)	0.006	<0.0001	0.0022
GO POSITIVE REGULATION OF DEFENSE RESPONSE TO VIRU...	24	9 (37.5)	9 (37.5)	0 (0)	0.005	<0.0001	0.0022
GO DIVALENT INORGANIC CATION HOMEOSTASIS	492	68 (13.8)	31 (6.3)	37 (7.5)	0.029	<0.0001	0.0025
GO INTERLEUKIN 6 PRODUCTION	144	26 (18.1)	13 (9)	13 (9)	0.013	<0.0001	0.0025
GO NEGATIVE REGULATION OF INFLAMMATORY RESPONSE	141	24 (17)	7 (5)	17 (12.1)	0.012	<0.0001	0.0025
GO MONONUCLEAR CELL MIGRATION	74	15 (20.3)	11 (14.9)	4 (5.4)	0.007	<0.0001	0.0025
GO REGULATION OF CD4 POSITIVE ALPHA BETA T CELL AC...	57	15 (26.3)	9 (15.8)	6 (10.5)	0.007	<0.0001	0.0025
GO ENDOTHELIAL CELL APOPTOTIC PROCESS	53	14 (26.4)	3 (5.7)	11 (20.8)	0.007	<0.0001	0.0025
GO G PROTEIN COUPLED PURINERGIC NUCLEOTIDE RECEPTO...	9	5 (55.6)	4 (44.4)	1 (11.1)	0.003	<0.0001	0.0025
GO REGULATION OF TRANSPORT	1745	203 (11.6)	79 (4.5)	124 (7.1)	0.058	<0.0001	0.0028
GO NEGATIVE REGULATION OF LEUKOCYTE CELL CELL ADHE...	117	23 (19.7)	10 (8.5)	13 (11.1)	0.011	<0.0001	0.0028
GO MACROPHAGE MIGRATION	47	13 (27.7)	6 (12.8)	7 (14.9)	0.006	<0.0001	0.0028
GO POSITIVE REGULATION OF CELL KILLING	54	13 (24.1)	3 (5.6)	10 (18.5)	0.006	<0.0001	0.0028
GO TUBE MORPHOGENESIS	829	107 (12.9)	37 (4.5)	70 (8.4)	0.040	<0.0001	0.0031
GO RESPONSE TO MOLECULE OF BACTERIAL ORIGIN	329	46 (14)	28 (8.5)	18 (5.5)	0.021	<0.0001	0.0031
GO RECEPTOR SIGNALING PATHWAY VIA STAT	156	26 (16.7)	15 (9.6)	11 (7.1)	0.013	<0.0001	0.0031
GO POSITIVE REGULATION OF RECEPTOR SIGNALING PATHW...	87	17 (19.5)	10 (11.5)	7 (8)	0.008	<0.0001	0.0031
GO POSITIVE REGULATION OF CYTOKINE PRODUCTION INVO...	49	14 (28.6)	8 (16.3)	6 (12.2)	0.007	<0.0001	0.0031
GO REGULATION OF SYMBIOSIS ENCOMPASSING MUTUALISM ...	204	36 (17.6)	29 (14.2)	7 (3.4)	0.017	<0.0001	0.0034
GO CELLULAR DEFENSE RESPONSE	46	12 (26.1)	5 (10.9)	7 (15.2)	0.006	<0.0001	0.0034

Table S96: MSigDB GO Biological Processes enriched in differentially expressed genes at Day 30 compared to pre-Second Vaccination (Dendritic cells, Study Arm 2 A/H7N9+AS03, D1, 29). Results are sorted by FDR adjusted p-value and Jaccard similarity index (continued.)

Gene Set ID	Gene Set Genes #	DE Genes N (%)	Up-reg. DE Genes N (%)	Down-reg. DE Genes N (%)	Jaccard Index	P-Value	FDR Adjusted P-Value
GO LEUKOCYTE ACTIVATION INVOLVED IN INFLAMMATORY R...	40	11 (27.5)	7 (17.5)	4 (10)	0.005	<0.0001	0.0034
GO REGULATION OF MONONUCLEAR CELL MIGRATION	40	11 (27.5)	9 (22.5)	2 (5)	0.005	<0.0001	0.0034
GO REGULATION OF CELL DIFFERENTIATION	1741	203 (11.7)	66 (3.8)	137 (7.9)	0.058	0.0001	0.0037
GO POSITIVE REGULATION OF PEPTIDYL TYROSINE PHOSPH...	185	32 (17.3)	16 (8.6)	16 (8.6)	0.015	0.0001	0.0037
GO POSITIVE REGULATION OF T CELL CYTOKINE PRODUCTI...	18	8 (44.4)	5 (27.8)	3 (16.7)	0.004	0.0001	0.0037
GO POSITIVE REGULATION OF LEUKOCYTE MIGRATION	118	21 (17.8)	12 (10.2)	9 (7.6)	0.010	0.0001	0.004
GO POSITIVE REGULATION OF EPITHELIAL CELL APOPTOTI...	32	10 (31.2)	3 (9.4)	7 (21.9)	0.005	0.0001	0.004
GO POSITIVE REGULATION OF CELL DIFFERENTIATION	927	117 (12.6)	38 (4.1)	79 (8.5)	0.042	0.0001	0.0043
GO POSITIVE REGULATION OF TRANSPORT	921	114 (12.4)	46 (5)	68 (7.4)	0.041	0.0001	0.0043
GO NEGATIVE REGULATION OF PEPTIDE SECRETION	128	23 (18)	10 (7.8)	13 (10.2)	0.011	0.0001	0.0043
GO NATURAL KILLER CELL MEDIATED IMMUNITY	51	13 (25.5)	4 (7.8)	9 (17.6)	0.006	0.0001	0.0045
GO HUMORAL IMMUNE RESPONSE MEDIATED BY CIRCULATING...	40	11 (27.5)	7 (17.5)	4 (10)	0.005	0.0001	0.0045
GO NEGATIVE REGULATION OF PRODUCTION OF MOLECULAR ...	31	10 (32.3)	4 (12.9)	6 (19.4)	0.005	0.0001	0.0045
GO I KAPPAB KINASE NF KAPPAB SIGNALING	252	41 (16.3)	20 (7.9)	21 (8.3)	0.019	0.0001	0.0048
GO LYMPHOCYTE ACTIVATION INVOLVED IN IMMUNE RESPON...	171	29 (17)	13 (7.6)	16 (9.4)	0.014	0.0001	0.0048
GO ACUTE INFLAMMATORY RESPONSE	102	19 (18.6)	13 (12.7)	6 (5.9)	0.009	0.0001	0.0048
GO TUBE DEVELOPMENT	1012	125 (12.4)	40 (4)	85 (8.4)	0.044	0.0002	0.0051
GO CELLULAR HOMEOSTASIS	901	113 (12.5)	51 (5.7)	62 (6.9)	0.041	0.0002	0.0051
GO REGULATION OF PEPTIDYL TYROSINE PHOSPHORYLATION	246	39 (15.9)	18 (7.3)	21 (8.5)	0.018	0.0002	0.0051
GO REGULATION OF RESPONSE TO CYTOKINE STIMULUS	165	29 (17.6)	19 (11.5)	10 (6.1)	0.014	0.0002	0.0053
GO REGULATION OF VITAMIN METABOLIC PROCESS	12	6 (50)	2 (16.7)	4 (33.3)	0.003	0.0002	0.0053
GO ACTIVATION OF CYSTEINE TYPE ENDOPEPTIDASE ACTIV...	83	18 (21.7)	12 (14.5)	6 (7.2)	0.009	0.0002	0.0055
GO REGULATION OF HUMORAL IMMUNE RESPONSE	62	14 (22.6)	8 (12.9)	6 (9.7)	0.007	0.0002	0.0055
GO POSITIVE REGULATION OF LEUKOCYTE MEDIATED CYTOT...	41	11 (26.8)	3 (7.3)	8 (19.5)	0.005	0.0002	0.0055
GO POSITIVE REGULATION OF INTERLEUKIN 2 PRODUCTION	29	10 (34.5)	5 (17.2)	5 (17.2)	0.005	0.0002	0.0055
GO T HELPER 17 TYPE IMMUNE RESPONSE	27	9 (33.3)	3 (11.1)	6 (22.2)	0.005	0.0002	0.0055
GO REGULATION OF PHOSPHOLIPID METABOLIC PROCESS	85	19 (22.4)	7 (8.2)	12 (14.1)	0.009	0.0002	0.0061
GO TUMOR NECROSIS FACTOR SUPERFAMILY CYTOKINE PROD...	148	25 (16.9)	13 (8.8)	12 (8.1)	0.012	0.0002	0.0064
GO REGULATION OF NEUROINFLAMMATORY RESPONSE	34	9 (26.5)	4 (11.8)	5 (14.7)	0.005	0.0002	0.0067
GO NEGATIVE REGULATION OF CELL CELL ADHESION	167	29 (17.4)	14 (8.4)	15 (9)	0.014	0.0002	0.007
GO MYELOID LEUKOCYTE ACTIVATION	594	77 (13)	52 (8.8)	25 (4.2)	0.031	0.0002	0.0072
GO POSITIVE REGULATION OF MULTI ORGANISM PROCESS	489	67 (13.7)	45 (9.2)	22 (4.5)	0.028	0.0002	0.0072
GO PURINERGIC NUCLEOTIDE RECEPTOR SIGNALING PATHWA...	17	7 (41.2)	5 (29.4)	2 (11.8)	0.004	0.0002	0.0072
GO POSITIVE REGULATION OF ALPHA BETA T CELL PROLIF...	17	7 (41.2)	4 (23.5)	3 (17.6)	0.004	0.0003	0.0077
GO INTERLEUKIN 35 MEDIATED SIGNALING PATHWAY	11	6 (54.5)	4 (36.4)	2 (18.2)	0.003	0.0003	0.0077
GO NEGATIVE REGULATION OF RESPONSE TO BIOTIC STIMU...	82	18 (22)	13 (15.9)	5 (6.1)	0.009	0.0003	0.008
GO T HELPER 1 TYPE IMMUNE RESPONSE	40	11 (27.5)	7 (17.5)	4 (10)	0.005	0.0003	0.0085

Table S96: MSigDB GO Biological Processes enriched in differentially expressed genes at Day 30 compared to pre-Second Vaccination (Dendritic cells, Study Arm 2 A/H7N9+AS03, D1, 29). Results are sorted by FDR adjusted p-value and Jaccard similarity index (continued.)

Gene Set ID	Gene Set Genes #	DE Genes N (%)	Up-reg. DE Genes N (%)	Down-reg. DE Genes N (%)	Jaccard Index	P-Value	FDR Adjusted P-Value
GO REGULATION OF MACROPHAGE MIGRATION	34	10 (29.4)	5 (14.7)	5 (14.7)	0.005	0.0003	0.0085
GO RESPONSE TO TUMOR NECROSIS FACTOR	272	39 (14.3)	18 (6.6)	21 (7.7)	0.018	0.0003	0.0088
GO REGULATION OF RECEPTOR SIGNALING PATHWAY VIA ST...	133	21 (15.8)	11 (8.3)	10 (7.5)	0.010	0.0003	0.009
GO NEGATIVE REGULATION OF CYTOKINE PRODUCTION INVO...	21	7 (33.3)	3 (14.3)	4 (19)	0.004	0.0003	0.009
GO GLIOGENESIS	270	43 (15.9)	13 (4.8)	30 (11.1)	0.020	0.0003	0.0092
GO REGULATION OF PHOSPHORUS METABOLIC PROCESS	1674	190 (11.4)	74 (4.4)	116 (6.9)	0.055	0.0003	0.0094
GO REGULATION OF ESTABLISHMENT OF PROTEIN LOCALIZA...	698	90 (12.9)	38 (5.4)	52 (7.4)	0.035	0.0003	0.0094
GO REGULATION OF CHEMOTAXIS	198	32 (16.2)	15 (7.6)	17 (8.6)	0.015	0.0003	0.0094
GO REGULATION OF NATURAL KILLER CELL MEDIATED IMMU...	33	10 (30.3)	3 (9.1)	7 (21.2)	0.005	0.0004	0.0105
GO REGULATION OF CALCIUM ION TRANSPORT	238	37 (15.5)	16 (6.7)	21 (8.8)	0.017	0.0004	0.011
GO REGULATION OF T CELL RECEPTOR SIGNALING PATHWAY	37	11 (29.7)	4 (10.8)	7 (18.9)	0.006	0.0004	0.0116
GO POSITIVE REGULATION OF MONONUCLEAR CELL MIGRATI...	18	6 (33.3)	5 (27.8)	1 (5.6)	0.003	0.0004	0.0118
GO MYOBLAST FUSION	41	11 (26.8)	4 (9.8)	7 (17.1)	0.005	0.0004	0.012
GO REGULATION OF EPITHELIAL CELL APOPTOTIC PROCESS	77	16 (20.8)	5 (6.5)	11 (14.3)	0.008	0.0004	0.0125
GO NEGATIVE REGULATION OF CYTOKINE SECRETION	62	13 (21)	6 (9.7)	7 (11.3)	0.006	0.0004	0.0125
GO POSITIVE REGULATION OF INTERLEUKIN 6 PRODUCTION	87	17 (19.5)	11 (12.6)	6 (6.9)	0.008	0.0005	0.013
GO POSITIVE REGULATION OF PROTEIN METABOLIC PROCES...	1584	180 (11.4)	79 (5)	101 (6.4)	0.054	0.0005	0.0135
GO INTERLEUKIN 1 SECRETION	53	12 (22.6)	9 (17)	3 (5.7)	0.006	0.0005	0.0135
GO REGULATION OF METAL ION TRANSPORT	366	52 (14.2)	24 (6.6)	28 (7.7)	0.023	0.0005	0.0135
GO PHAGOCYTOSIS	251	39 (15.5)	20 (8)	19 (7.6)	0.018	0.0005	0.0135
GO LEUKOCYTE APOPTOTIC PROCESS	100	19 (19)	9 (9)	10 (10)	0.009	0.0005	0.0135
GO PYRIMIDINE NUCLEOSIDE CATABOLIC PROCESS	23	7 (30.4)	6 (26.1)	1 (4.3)	0.004	0.0005	0.0135
GO COMPLEMENT ACTIVATION	62	13 (21)	8 (12.9)	5 (8.1)	0.006	0.0005	0.0138
GO GRANULOCYTE MIGRATION	126	19 (15.1)	14 (11.1)	5 (4)	0.009	0.0005	0.0139
GO T CELL MEDIATED CYTOTOXICITY	31	9 (29)	4 (12.9)	5 (16.1)	0.005	0.0005	0.0139
GO POSITIVE REGULATION OF PEPTIDASE ACTIVITY	185	30 (16.2)	20 (10.8)	10 (5.4)	0.014	0.0005	0.0143
GO POSITIVE REGULATION OF I KAPPAB KINASE NF KAPPA...	169	29 (17.2)	18 (10.7)	11 (6.5)	0.014	0.0005	0.0143
GO REGULATION OF RESPONSE TO INTERFERON GAMMA	24	8 (33.3)	7 (29.2)	1 (4.2)	0.004	0.0005	0.0143
GO REGULATION OF B CELL RECEPTOR SIGNALING PATHWAY	27	9 (33.3)	1 (3.7)	8 (29.6)	0.005	0.0005	0.0145
GO CELLULAR RESPONSE TO OXYGEN CONTAINING COMPOUND	1128	131 (11.6)	59 (5.2)	72 (6.4)	0.044	0.0006	0.0147
GO POSITIVE REGULATION OF CYSTEINE TYPE ENDOPEPTID...	141	25 (17.7)	16 (11.3)	9 (6.4)	0.012	0.0006	0.015
GO ADHESION OF SYMBIONT TO HOST	14	6 (42.9)	4 (28.6)	2 (14.3)	0.003	0.0006	0.0151
GO HEMATOPOIETIC STEM CELL MIGRATION	5	4 (80)	2 (40)	2 (40)	0.002	0.0006	0.0151
GO MONOCYTE CHEMOTAXIS	51	10 (19.6)	8 (15.7)	2 (3.9)	0.005	0.0006	0.0158
GO TRANSMEMBRANE RECEPTOR PROTEIN TYROSINE KINASE ...	678	90 (13.3)	29 (4.3)	61 (9)	0.035	0.0006	0.0162
GO CD4 POSITIVE OR CD8 POSITIVE ALPHA BETA T CELL ...	19	7 (36.8)	2 (10.5)	5 (26.3)	0.004	0.0006	0.0162
GO REGULATION OF CYSTEINE TYPE ENDOPEPTIDASE ACTIV...	221	34 (15.4)	21 (9.5)	13 (5.9)	0.016	0.0006	0.0164

Table S96: MSigDB GO Biological Processes enriched in differentially expressed genes at Day 30 compared to pre-Second Vaccination (Dendritic cells, Study Arm 2 A/H7N9+AS03, D1, 29). Results are sorted by FDR adjusted p-value and Jaccard similarity index (continued.)

Gene Set ID	Gene Set Genes #	DE Genes N (%)	Up-reg. DE Genes N (%)	Down-reg. DE Genes N (%)	Jaccard Index	P-Value	FDR Adjusted P-Value
GO REGULATION OF LIPID KINASE ACTIVITY	65	15 (23.1)	5 (7.7)	10 (15.4)	0.007	0.0006	0.0166
GO REGULATION OF PROTEIN LOCALIZATION	951	117 (12.3)	46 (4.8)	71 (7.5)	0.042	0.0007	0.0173
GO PROTEIN KINASE C SIGNALING	29	9 (31)	2 (6.9)	7 (24.1)	0.005	0.0007	0.0173
GO NEGATIVE REGULATION OF TRANSPORT	461	59 (12.8)	21 (4.6)	38 (8.2)	0.025	0.0007	0.0175
GO POSITIVE REGULATION OF CHEMOTAXIS	125	21 (16.8)	12 (9.6)	9 (7.2)	0.010	0.0007	0.0181
GO IMMUNOGLOBULIN PRODUCTION	97	19 (19.6)	7 (7.2)	12 (12.4)	0.009	0.0007	0.0181
GO NEUTROPHIL MIGRATION	103	16 (15.5)	12 (11.7)	4 (3.9)	0.008	0.0007	0.0181
GO PURINE NUCLEOBASE TRANSPORT	6	4 (66.7)	1 (16.7)	3 (50)	0.002	0.0007	0.0184
GO RHOMBOMERE DEVELOPMENT	7	4 (57.1)	2 (28.6)	2 (28.6)	0.002	0.0007	0.0184
GO INTERLEUKIN 2 BIOSYNTHETIC PROCESS	21	7 (33.3)	1 (4.8)	6 (28.6)	0.004	0.0008	0.0189
GO HOMEOSTASIS OF NUMBER OF CELLS	226	36 (15.9)	15 (6.6)	21 (9.3)	0.017	0.0008	0.0193
GO REGULATION OF PROTEIN MODIFICATION PROCESS	1724	193 (11.2)	78 (4.5)	115 (6.7)	0.055	0.0008	0.0195
GO REGULATION OF ADIPONECTIN SECRETION	6	4 (66.7)	2 (33.3)	2 (33.3)	0.002	0.0008	0.0199
GO NEGATIVE REGULATION OF LEUKOCYTE MEDIATED IMMUN...	39	10 (25.6)	7 (17.9)	3 (7.7)	0.005	0.0008	0.0204
GO INTRINSIC APOPTOTIC SIGNALING PATHWAY	274	40 (14.6)	24 (8.8)	16 (5.8)	0.018	0.0009	0.021
GO T HELPER 17 CELL LINEAGE COMMITMENT	12	5 (41.7)	2 (16.7)	3 (25)	0.003	0.0009	0.021
GO CELL ACTIVATION INVOLVED IN IMMUNE RESPONSE	658	79 (12)	48 (7.3)	31 (4.7)	0.031	0.0009	0.0213
GO GLIAL CELL MIGRATION	46	12 (26.1)	3 (6.5)	9 (19.6)	0.006	0.0009	0.0213
GO RECEPTOR MEDIATED ENDOCYTOSIS	245	38 (15.5)	19 (7.8)	19 (7.8)	0.018	0.0009	0.0214
GO NEGATIVE REGULATION OF MEMBRANE DEPOLARIZATION	8	4 (50)	2 (25)	2 (25)	0.002	0.0009	0.0214
GO EPITHELIAL CELL APOPTOTIC PROCESS	100	19 (19)	6 (6)	13 (13)	0.009	0.0009	0.0218
GO PHOSPHATIDYLINOSITOL 3 KINASE SIGNALING	143	26 (18.2)	5 (3.5)	21 (14.7)	0.013	0.0009	0.022
GO CENTRAL NERVOUS SYSTEM DEVELOPMENT	925	117 (12.6)	29 (3.1)	88 (9.5)	0.042	0.0009	0.0223
GO REGULATION OF INTERLEUKIN 1 SECRETION	45	10 (22.2)	7 (15.6)	3 (6.7)	0.005	0.0009	0.0223
GO REGULATION OF LIPID METABOLIC PROCESS	390	54 (13.8)	20 (5.1)	34 (8.7)	0.024	0.001	0.0227
GO NEGATIVE REGULATION OF CELL ADHESION	263	40 (15.2)	17 (6.5)	23 (8.7)	0.018	0.001	0.0233
GO T HELPER 1 CELL CYTOKINE PRODUCTION	8	4 (50)	1 (12.5)	3 (37.5)	0.002	0.001	0.0233
GO REGULATION OF T CELL DIFFERENTIATION IN THYMUS	23	8 (34.8)	1 (4.3)	7 (30.4)	0.004	0.001	0.0234
GO CD4 POSITIVE ALPHA BETA T CELL LINEAGE COMMITME...	16	6 (37.5)	2 (12.5)	4 (25)	0.003	0.001	0.0234
GO POSITIVE REGULATION OF PHAGOCYTOSIS	61	13 (21.3)	6 (9.8)	7 (11.5)	0.006	0.001	0.0238
GO POSITIVE REGULATION OF INTERLEUKIN 6 SECRETION	28	8 (28.6)	4 (14.3)	4 (14.3)	0.004	0.001	0.0242
GO ERK1 AND ERK2 CASCADE	285	40 (14)	18 (6.3)	22 (7.7)	0.018	0.0011	0.0246
GO REGULATION OF LIPID CATABOLIC PROCESS	53	12 (22.6)	5 (9.4)	7 (13.2)	0.006	0.0011	0.0247
GO POSITIVE REGULATION OF MONOCYTE CHEMOTAXIS	15	5 (33.3)	5 (33.3)	0 (0)	0.003	0.0011	0.0251
GO NUCLEOSIDE CATABOLIC PROCESS	31	8 (25.8)	7 (22.6)	1 (3.2)	0.004	0.0011	0.0257
GO NEGATIVE REGULATION OF LIPID METABOLIC PROCESS	87	16 (18.4)	6 (6.9)	10 (11.5)	0.008	0.0011	0.0263
GO PURINERGIC RECEPTOR SIGNALING PATHWAY	28	8 (28.6)	5 (17.9)	3 (10.7)	0.004	0.0012	0.0269

Table S96: MSigDB GO Biological Processes enriched in differentially expressed genes at Day 30 compared to pre-Second Vaccination (Dendritic cells, Study Arm 2 A/H7N9+AS03, D1, 29). Results are sorted by FDR adjusted p-value and Jaccard similarity index (continued.)

Gene Set ID	Gene Set Genes #	DE Genes N (%)	Up-reg. DE Genes N (%)	Down-reg. DE Genes N (%)	Jaccard Index	P-Value	FDR Adjusted P-Value
GO POSITIVE REGULATION OF T CELL DIFFERENTIATION I...	10	5 (50)	1 (10)	4 (40)	0.003	0.0012	0.0273
GO VASCULAR PROCESS IN CIRCULATORY SYSTEM	162	26 (16)	14 (8.6)	12 (7.4)	0.012	0.0012	0.0274
GO NEGATIVE REGULATION OF NEUROINFLAMMATORY RESPON...	13	5 (38.5)	1 (7.7)	4 (30.8)	0.003	0.0012	0.0278
GO CARDIOVASCULAR SYSTEM DEVELOPMENT	718	88 (12.3)	34 (4.7)	54 (7.5)	0.034	0.0012	0.0279
GO SECOND MESSENGER MEDIATED SIGNALING	419	55 (13.1)	20 (4.8)	35 (8.4)	0.024	0.0012	0.0279
GO HIGH DENSITY LIPOPROTEIN PARTICLE CLEARANCE	13	5 (38.5)	2 (15.4)	3 (23.1)	0.003	0.0013	0.0282
GO CELLULAR RESPONSE TO LIPID	575	72 (12.5)	29 (5)	43 (7.5)	0.029	0.0013	0.0292
GO INTERACTION WITH HOST	192	31 (16.1)	20 (10.4)	11 (5.7)	0.015	0.0013	0.0292
GO ADHESION OF SYMBIONT TO HOST CELL	11	5 (45.5)	4 (36.4)	1 (9.1)	0.003	0.0014	0.0304
GO REGULATION OF PEPTIDASE ACTIVITY	409	50 (12.2)	27 (6.6)	23 (5.6)	0.022	0.0014	0.0311
GO POSITIVE REGULATION OF TUMOR NECROSIS FACTOR SU...	79	15 (19)	9 (11.4)	6 (7.6)	0.007	0.0014	0.0311
GO POSITIVE REGULATION OF T HELPER 1 TYPE IMMUNE R...	18	6 (33.3)	4 (22.2)	2 (11.1)	0.003	0.0014	0.0317
GO COMPLEMENT RECEPTOR MEDIATED SIGNALING PATHWAY	12	5 (41.7)	3 (25)	2 (16.7)	0.003	0.0015	0.032
GO RESPONSE TO FATTY ACID	82	16 (19.5)	7 (8.5)	9 (11)	0.008	0.0015	0.0322
GO SIGNAL TRANSDUCTION BY PROTEIN PHOSPHORYLATION	885	106 (12)	44 (5)	62 (7)	0.039	0.0015	0.0323
GO REGULATION OF MAPK CASCADE	697	85 (12.2)	36 (5.2)	49 (7)	0.033	0.0015	0.0324
GO NEGATIVE REGULATION OF ANTIGEN RECEPTOR MEDIATE...	27	8 (29.6)	3 (11.1)	5 (18.5)	0.004	0.0015	0.0327
GO NEGATIVE REGULATION OF MACROPHAGE MIGRATION	8	4 (50)	1 (12.5)	3 (37.5)	0.002	0.0015	0.0327
GO UNSATURATED FATTY ACID METABOLIC PROCESS	102	18 (17.6)	6 (5.9)	12 (11.8)	0.009	0.0015	0.0332
GO REGULATION OF LEUKOCYTE APOPTOTIC PROCESS	78	15 (19.2)	7 (9)	8 (10.3)	0.007	0.0016	0.0334
GO POSITIVE REGULATION OF NATURAL KILLER CELL MEDI...	22	7 (31.8)	1 (4.5)	6 (27.3)	0.004	0.0016	0.0334
GO REGULATION OF INTERLEUKIN 1 MEDIATED SIGNALING ...	7	4 (57.1)	2 (28.6)	2 (28.6)	0.002	0.0016	0.0334
GO POSITIVE REGULATION OF LEUKOCYTE CHEMOTAXIS	80	14 (17.5)	10 (12.5)	4 (5)	0.007	0.0016	0.0341
GO POSITIVE REGULATION OF PROTEIN TYROSINE KINASE ...	53	13 (24.5)	4 (7.5)	9 (17)	0.006	0.0016	0.0347
GO REGULATION OF VASCULATURE DEVELOPMENT	349	44 (12.6)	20 (5.7)	24 (6.9)	0.019	0.0016	0.035
GO CIRCULATORY SYSTEM PROCESS	517	66 (12.8)	30 (5.8)	36 (7)	0.028	0.0017	0.0351
GO POSITIVE REGULATION OF TYROSINE PHOSPHORYLATION...	67	12 (17.9)	8 (11.9)	4 (6)	0.006	0.0017	0.0356
GO BLOOD VESSEL MORPHOGENESIS	604	74 (12.3)	31 (5.1)	43 (7.1)	0.030	0.0017	0.0359
GO REGULATION OF LEUKOCYTE CHEMOTAXIS	102	17 (16.7)	11 (10.8)	6 (5.9)	0.008	0.0017	0.0359
GO INTERLEUKIN 27 MEDIATED SIGNALING PATHWAY	11	5 (45.5)	5 (45.5)	0 (0)	0.003	0.0017	0.0366
GO NEGATIVE REGULATION OF LEUKOCYTE APOPTOTIC PROC...	44	10 (22.7)	3 (6.8)	7 (15.9)	0.005	0.0018	0.0367
GO REGULATION OF ALPHA BETA T CELL DIFFERENTIATION	60	13 (21.7)	8 (13.3)	5 (8.3)	0.006	0.0018	0.0368
GO LONG CHAIN FATTY ACID METABOLIC PROCESS	98	18 (18.4)	5 (5.1)	13 (13.3)	0.009	0.0018	0.0375
GO PROGRAMMED CELL DEATH INVOLVED IN CELL DEVELOPM...	12	5 (41.7)	1 (8.3)	4 (33.3)	0.003	0.0018	0.0379
GO NEGATIVE REGULATION OF SIGNALING	1297	147 (11.3)	51 (3.9)	96 (7.4)	0.047	0.0018	0.038
GO REGULATION OF GLOMERULAR FILTRATION	11	5 (45.5)	2 (18.2)	3 (27.3)	0.003	0.0019	0.0391
GO ACTIVATION OF INNATE IMMUNE RESPONSE	286	41 (14.3)	31 (10.8)	10 (3.5)	0.019	0.0019	0.0396

Table S96: MSigDB GO Biological Processes enriched in differentially expressed genes at Day 30 compared to pre-Second Vaccination (Dendritic cells, Study Arm 2 A/H7N9+AS03, D1, 29). Results are sorted by FDR adjusted p-value and Jaccard similarity index (continued.)

Gene Set ID	Gene Set Genes #	DE Genes N (%)	Up-reg. DE Genes N (%)	Down-reg. DE Genes N (%)	Jaccard Index	P-Value	FDR Adjusted P-Value
GO SYNCYTIUM FORMATION	56	12 (21.4)	4 (7.1)	8 (14.3)	0.006	0.002	0.0409
GO REGULATION OF APOPTOTIC SIGNALING PATHWAY	380	50 (13.2)	22 (5.8)	28 (7.4)	0.022	0.0021	0.0417
GO NEGATIVE REGULATION OF SECRETION	206	29 (14.1)	13 (6.3)	16 (7.8)	0.014	0.0021	0.0417
GO POSITIVE REGULATION OF ERK1 AND ERK2 CASCADE	184	27 (14.7)	11 (6)	16 (8.7)	0.013	0.0021	0.0417
GO REGULATION OF INTERLEUKIN 4 PRODUCTION	24	7 (29.2)	1 (4.2)	6 (25)	0.004	0.002	0.0417
GO MITOCHONDRIAL DEPOLARIZATION	23	7 (30.4)	4 (17.4)	3 (13)	0.004	0.0021	0.0424
GO REGULATION OF T CELL MEDIATED CYTOTOXICITY	25	7 (28)	4 (16)	3 (12)	0.004	0.0021	0.0424
GO T HELPER 2 CELL CYTOKINE PRODUCTION	14	5 (35.7)	3 (21.4)	2 (14.3)	0.003	0.0021	0.0424
GO T CELL TOLERANCE INDUCTION	8	4 (50)	1 (12.5)	3 (37.5)	0.002	0.0021	0.0425
GO POSITIVE REGULATION OF PHOSPHORUS METABOLIC PRO...	1054	121 (11.5)	50 (4.7)	71 (6.7)	0.042	0.0022	0.0431
GO ENZYME LINKED RECEPTOR PROTEIN SIGNALING PATHWA...	978	118 (12.1)	33 (3.4)	85 (8.7)	0.042	0.0022	0.0434
GO NEGATIVE T CELL SELECTION	11	5 (45.5)	0 (0)	5 (45.5)	0.003	0.0022	0.0437
GO POSITIVE REGULATION OF SECRETION	404	51 (12.6)	24 (5.9)	27 (6.7)	0.022	0.0022	0.044
GO RENAL FILTRATION	20	7 (35)	2 (10)	5 (25)	0.004	0.0022	0.0441
GO VASCULAR ENDOTHELIAL GROWTH FACTOR PRODUCTION	34	9 (26.5)	5 (14.7)	4 (11.8)	0.005	0.0023	0.0447
GO POSITIVE REGULATION OF MAPK CASCADE	496	63 (12.7)	27 (5.4)	36 (7.3)	0.026	0.0023	0.0459
GO TYPE 2 IMMUNE RESPONSE	38	9 (23.7)	7 (18.4)	2 (5.3)	0.004	0.0023	0.0459
GO POSITIVE REGULATION OF ANTIGEN RECEPTOR MEDIATE...	21	7 (33.3)	1 (4.8)	6 (28.6)	0.004	0.0024	0.0467
GO COBALAMIN TRANSPORT	8	4 (50)	1 (12.5)	3 (37.5)	0.002	0.0024	0.0474
GO CELLULAR RESPONSE TO BIOTIC STIMULUS	226	30 (13.3)	20 (8.8)	10 (4.4)	0.014	0.0025	0.0482
GO REGULATION OF T HELPER 1 TYPE IMMUNE RESPONSE	25	7 (28)	5 (20)	2 (8)	0.004	0.0025	0.0491
GO POSITIVE REGULATION OF NF KAPPAB TRANSCRIPTION ...	133	23 (17.3)	15 (11.3)	8 (6)	0.011	0.0026	0.0497
GO CELLULAR MONOVALENT INORGANIC CATION HOMEOSTASI...	103	19 (18.4)	11 (10.7)	8 (7.8)	0.009	0.0026	0.0497
GO REGULATION OF MONOCYTE CHEMOTAXIS	22	6 (27.3)	6 (27.3)	0 (0)	0.003	0.0026	0.0497

Table S97: Blood Transcription Modules enriched in differentially expressed genes at Day 30 compared to pre-Second Vaccination (Dendritic cells, Study Arm 2 A/H7N9+AS03, D1, 29). Results are sorted by FDR adjusted p-value and Jaccard similarity index.

Gene Set ID	Gene Set Genes #	DE Genes N (%)	Up-reg. DE Genes N (%)	Down-reg. DE Genes N (%)	Jaccard Index	P-Value	FDR Adjusted P-Value
M7.0 ENRICHED IN T CELLS (I)	57	50 (87.7)	0 (0)	50 (87.7)	0.025	<0.0001	0.0001
M7.1 T CELL ACTIVATION (I)	50	45 (90)	2 (4)	43 (86)	0.023	<0.0001	0.0001
M7.2 ENRICHED IN NK CELLS (I)	45	42 (93.3)	1 (2.2)	41 (91.1)	0.021	<0.0001	0.0001
M47.0 ENRICHED IN B CELLS (I)	45	33 (73.3)	0 (0)	33 (73.3)	0.017	<0.0001	0.0001
M156.0 PLASMA CELLS AND B CELLS, IMMUNOGLOBULINS	30	24 (80)	0 (0)	24 (80)	0.012	<0.0001	0.0001

Table S97: Blood Transcription Modules enriched in differentially expressed genes at Day 30 compared to pre-Second Vaccination (Dendritic cells, Study Arm 2 A/H7N9+AS03, D1, 29). Results are sorted by FDR adjusted p-value and Jaccard similarity index (continued.)

Gene Set ID	Gene Set Genes #	DE Genes N (%)	Up-reg. DE Genes N (%)	Down-reg. DE Genes N (%)	Jaccard Index	P-Value	FDR Adjusted P-Value
M47.1 ENRICHED IN B CELLS (II)	35	22 (62.9)	0 (0)	22 (62.9)	0.011	<0.0001	0.0001
M7.3 T CELL ACTIVATION (II)	28	22 (78.6)	2 (7.1)	20 (71.4)	0.011	<0.0001	0.0001
S1 NK CELL SURFACE SIGNATURE	38	22 (57.9)	2 (5.3)	20 (52.6)	0.011	<0.0001	0.0001
M165 ENRICHED IN ACTIVATED DENDRITIC CELLS (II)	34	20 (58.8)	19 (55.9)	1 (2.9)	0.010	<0.0001	0.0001
M75 ANTIVIRAL IFN SIGNATURE	22	17 (77.3)	16 (72.7)	1 (4.5)	0.009	<0.0001	0.0001
S0 T CELL SURFACE SIGNATURE	24	17 (70.8)	0 (0)	17 (70.8)	0.009	<0.0001	0.0001
M156.1 PLASMA CELLS, IMMUNOGLOBULINS	22	16 (72.7)	0 (0)	16 (72.7)	0.008	<0.0001	0.0001
M19 T CELL DIFFERENTIATION (TH2)	15	15 (100)	0 (0)	15 (100)	0.008	<0.0001	0.0001
M69 ENRICHED IN B CELLS (VI)	19	15 (78.9)	0 (0)	15 (78.9)	0.008	<0.0001	0.0001
M61.0 ENRICHED IN NK CELLS (II)	16	14 (87.5)	0 (0)	14 (87.5)	0.007	<0.0001	0.0001
M127 TYPE I INTERFERON RESPONSE	12	12 (100)	12 (100)	0 (0)	0.006	<0.0001	0.0001
M7.4 T CELL ACTIVATION (III)	13	12 (92.3)	0 (0)	12 (92.3)	0.006	<0.0001	0.0001
M14 T CELL DIFFERENTIATION	11	11 (100)	0 (0)	11 (100)	0.006	<0.0001	0.0001
M61.2 ENRICHED IN NK CELLS (RECEPTOR ACTIVATION)	13	11 (84.6)	0 (0)	11 (84.6)	0.006	<0.0001	0.0001
M67 ACTIVATED DENDRITIC CELLS	12	11 (91.7)	10 (83.3)	1 (8.3)	0.006	<0.0001	0.0001
M111.0 VIRAL SENSING AND IMMUNITY; IRF2 TARGETS NE...	16	10 (62.5)	9 (56.2)	1 (6.2)	0.005	<0.0001	0.0001
M111.1 VIRAL SENSING AND IMMUNITY; IRF2 TARGETS NE...	11	10 (90.9)	10 (90.9)	0 (0)	0.005	<0.0001	0.0001
M150 INNATE ANTIVIRAL RESPONSE	12	10 (83.3)	10 (83.3)	0 (0)	0.005	<0.0001	0.0001
M223 ENRICHED IN T CELLS (II)	11	10 (90.9)	0 (0)	10 (90.9)	0.005	<0.0001	0.0001
M121 TBA	12	9 (75)	4 (33.3)	5 (41.7)	0.005	<0.0001	0.0001
M18 T CELL DIFFERENTIATION VIA ITK AND PKC	9	9 (100)	0 (0)	9 (100)	0.005	<0.0001	0.0001
M13 INNATE ACTIVATION BY CYTOSOLIC DNA SENSING	11	8 (72.7)	6 (54.5)	2 (18.2)	0.004	<0.0001	0.0001
M65 IL2, IL7, TCR NETWORK	10	8 (80)	0 (0)	8 (80)	0.004	<0.0001	0.0001
M5.1 T CELL ACTIVATION AND SIGNALING	21	12 (57.1)	1 (4.8)	11 (52.4)	0.006	<0.0001	0.0004
M109 RECEPTORS, CELL MIGRATION	15	11 (73.3)	4 (26.7)	7 (46.7)	0.006	<0.0001	0.0006
M57 IMMUREGULATION - MONOCYTES, T AND B CELLS	13	8 (61.5)	1 (7.7)	7 (53.8)	0.004	<0.0001	0.0007
M35.1 SIGNALING IN T CELLS (II)	11	7 (63.6)	0 (0)	7 (63.6)	0.004	<0.0001	0.0008
M47.2 ENRICHED IN B CELLS (III)	25	13 (52)	0 (0)	13 (52)	0.007	0.0001	0.0016
M5.0 REGULATION OF ANTIGEN PRESENTATION AND IMMUNE...	78	28 (35.9)	5 (6.4)	23 (29.5)	0.014	0.0002	0.0021
M68 RIG-1 LIKE RECEPTOR SIGNALING	9	6 (66.7)	6 (66.7)	0 (0)	0.003	0.0003	0.0031
M86.1 PROINFLAMMATORY DENDRITIC CELL, MYELOID CELL...	12	7 (58.3)	5 (41.7)	2 (16.7)	0.004	0.0004	0.0043
M157 ENRICHED IN NK CELLS (III)	14	8 (57.1)	0 (0)	8 (57.1)	0.004	0.0006	0.0054
M61.1 ENRICHED IN NK CELLS (KIR CLUSTER)	6	4 (66.7)	0 (0)	4 (66.7)	0.002	0.0007	0.0062
M12 CD28 COSTIMULATION	8	6 (75)	0 (0)	6 (75)	0.003	0.0008	0.007
M35.0 SIGNALING IN T CELLS (I)	14	7 (50)	2 (14.3)	5 (35.7)	0.004	0.0012	0.0106
S5 DC SURFACE SIGNATURE	76	26 (34.2)	18 (23.7)	8 (10.5)	0.013	0.0018	0.0151
M117 CELL ADHESION (GO)	19	10 (52.6)	1 (5.3)	9 (47.4)	0.005	0.0021	0.0172

Table S97: Blood Transcription Modules enriched in differentially expressed genes at Day 30 compared to pre-Second Vaccination (Dendritic cells, Study Arm 2 A/H7N9+AS03, D1, 29). Results are sorted by FDR adjusted p-value and Jaccard similarity index (continued.)

Gene Set ID	Gene Set Genes #	DE Genes N (%)	Up-reg. DE Genes N (%)	Down-reg. DE Genes N (%)	Jaccard Index	P-Value	FDR Adjusted P-Value
M47.3 ENRICHED IN B CELLS (IV)	13	8 (61.5)	0 (0)	8 (61.5)	0.004	0.0023	0.0187
S10 RESTING DENDRITIC CELL SURFACE SIGNATURE	70	24 (34.3)	13 (18.6)	11 (15.7)	0.012	0.0029	0.023
M37.0 IMMUNE ACTIVATION - GENERIC CLUSTER	301	72 (23.9)	41 (13.6)	31 (10.3)	0.033	0.0034	0.0263
M64 ENRICHED IN ACTIVATED DENDRITIC CELLS-MONOCYTE...	14	7 (50)	7 (50)	0 (0)	0.004	0.0037	0.0275
S7 CD4 T CELL SURFACE SIGNATURE TH2-STIMULATED	11	6 (54.5)	0 (0)	6 (54.5)	0.003	0.0048	0.0354
M86.0 CHEMOKINES AND INFLAMMATORY MOLECULES IN MYE...	18	7 (38.9)	6 (33.3)	1 (5.6)	0.004	0.0055	0.0398
M97 ENRICHED FOR SMAD2-3 SIGNALING	10	6 (60)	1 (10)	5 (50)	0.003	0.0064	0.0432
M43.1 MYELOID, DENDRITIC CELL ACTIVATION VIA NFKB ...	14	6 (42.9)	4 (28.6)	2 (14.3)	0.003	0.0065	0.0432
M50 CD1 AND OTHER DC RECEPTORS	9	5 (55.6)	0 (0)	5 (55.6)	0.003	0.0063	0.0432
M38 CHEMOKINES AND RECEPTORS	10	5 (50)	1 (10)	4 (40)	0.003	0.0063	0.0432

Table S98: KEGG Pathways enriched in differentially expressed genes at Day 32 compared to pre-Second Vaccination (Dendritic cells, Study Arm 2 A/H7N9+AS03, D1, 29). Results are sorted by FDR adjusted p-value and Jaccard similarity index.

Gene Set ID	Gene Set Genes #	DE Genes N (%)	Up-reg. DE Genes N (%)	Down-reg. DE Genes N (%)	Jaccard Index	P-Value	FDR Adjusted P-Value
PERTUSSIS	75	4 (5.3)	4 (5.3)	0 (0)	0.042	<0.0001	0.0017
COMPLEMENT AND COAGULATION CASCADES	82	4 (4.9)	4 (4.9)	0 (0)	0.039	<0.0001	0.0017
STAPHYLOCOCCUS AUREUS INFECTION	91	4 (4.4)	4 (4.4)	0 (0)	0.036	<0.0001	0.0017
SYSTEMIC LUPUS ERYTHEMATOSUS	127	4 (3.1)	4 (3.1)	0 (0)	0.027	<0.0001	0.0017
CHAGAS DISEASE	99	3 (3)	3 (3)	0 (0)	0.025	0.0001	0.0074

Table S99: MSigDB Reactome Pathways enriched in differentially expressed genes at Day 32 compared to pre-Second Vaccination (Dendritic cells, Study Arm 2 A/H7N9+AS03, D1, 29). Results are sorted by FDR adjusted p-value and Jaccard similarity index.

Gene Set ID	Gene Set Genes #	DE Genes N (%)	Up-reg. DE Genes N (%)	Down-reg. DE Genes N (%)	Jaccard Index	P-Value	FDR Adjusted P-Value
REACTOME CREATION OF C4 AND C2 ACTIVATORS	14	3 (21.4)	3 (21.4)	0 (0)	0.083	<0.0001	0.0077
REACTOME INITIAL TRIGGERING OF COMPLEMENT	17	3 (17.6)	3 (17.6)	0 (0)	0.077	<0.0001	0.0077
REACTOME COMPLEMENT CASCADE	52	3 (5.8)	3 (5.8)	0 (0)	0.041	<0.0001	0.0153

Table S100: MSigDB GO Biological Processes enriched in differentially expressed genes at Day 32 compared to pre-Second Vaccination (Dendritic cells, Study Arm 2 A/H7N9+AS03, D1, 29). Results are sorted by FDR adjusted p-value and Jaccard similarity index.

Gene Set ID	Gene Set Genes #	DE Genes N (%)	Up-reg. DE Genes N (%)	Down-reg. DE Genes N (%)	Jaccard Index	P-Value	FDR Adjusted P-Value
GO SYNAPSE PRUNING	11	3 (27.3)	3 (27.3)	0 (0)	0.091	<0.0001	0.0251
GO HUMORAL IMMUNE RESPONSE MEDIATED BY CIRCULATING...	40	3 (7.5)	3 (7.5)	0 (0)	0.048	<0.0001	0.0251
GO SYNAPSE ORGANIZATION	377	5 (1.3)	3 (0.8)	2 (0.5)	0.013	<0.0001	0.0251

Table S101: Blood Transcription Modules enriched in differentially expressed genes at Day 32 compared to pre-Second Vaccination (Dendritic cells, Study Arm 2 A/H7N9+AS03, D1, 29). Results are sorted by FDR adjusted p-value and Jaccard similarity index.

Gene Set ID	Gene Set Genes #	DE Genes N (%)	Up-reg. DE Genes N (%)	Down-reg. DE Genes N (%)	Jaccard Index	P-Value	FDR Adjusted P-Value
M112.0 COMPLEMENT ACTIVATION (I)	17	4 (23.5)	4 (23.5)	0 (0)	0.105	<0.0001	0.0017
M40 COMPLEMENT AND OTHER RECEPTORS IN DCS	12	3 (25)	3 (25)	0 (0)	0.088	<0.0001	0.0017
M67 ACTIVATED DENDRITIC CELLS	12	3 (25)	2 (16.7)	1 (8.3)	0.088	<0.0001	0.0046

Table S102: KEGG Pathways enriched in differentially expressed genes at Day 36 compared to pre-Second Vaccination (Dendritic cells, Study Arm 2 A/H7N9+AS03, D1, 29). Results are sorted by FDR adjusted p-value and Jaccard similarity index.

Gene Set ID	Gene Set Genes #	DE Genes N (%)	Up-reg. DE Genes N (%)	Down-reg. DE Genes N (%)	Jaccard Index	P-Value	FDR Adjusted P-Value
NATURAL KILLER CELL MEDIATED CYTOTOXICITY	121	15 (12.4)	14 (11.6)	1 (0.8)	0.054	<0.0001	0.0008
GRAFT-VERSUS-HOST DISEASE	38	10 (26.3)	9 (23.7)	1 (2.6)	0.051	<0.0001	0.0008
ANTIGEN PROCESSING AND PRESENTATION	70	8 (11.4)	7 (10)	1 (1.4)	0.035	<0.0001	0.0008
CYTOKINE-CYTOKINE RECEPTOR INTERACTION	280	11 (3.9)	10 (3.6)	1 (0.4)	0.025	<0.0001	0.0008
AFRICAN TRYPANOSOMIASIS	36	5 (13.9)	4 (11.1)	1 (2.8)	0.025	<0.0001	0.0013
MALARIA	48	5 (10.4)	4 (8.3)	1 (2.1)	0.024	<0.0001	0.0017
VIRAL PROTEIN INTERACTION WITH CYTOKINE AND CYTOKI...	96	6 (6.2)	5 (5.2)	1 (1)	0.023	<0.0001	0.0024
ALLOGRAFT REJECTION	34	4 (11.8)	3 (8.8)	1 (2.9)	0.020	<0.0001	0.0034
TYPE I DIABETES MELLITUS	41	4 (9.8)	3 (7.3)	1 (2.4)	0.019	0.0002	0.0079
TH1 AND TH2 CELL DIFFERENTIATION	88	5 (5.7)	5 (5.7)	0 (0)	0.020	0.0011	0.0371
INFLAMMATORY BOWEL DISEASE	61	4 (6.6)	3 (4.9)	1 (1.6)	0.018	0.0016	0.0481
CHAGAS DISEASE	99	5 (5.1)	4 (4)	1 (1)	0.019	0.0018	0.0494

Table S103: MSigDB Reactome Pathways enriched in differentially expressed genes at Day 36 compared to pre-Second Vaccination (Dendritic cells, Study Arm 2 A/H7N9+AS03, D1, 29). Results are sorted by FDR adjusted p-value and Jaccard similarity index.

Gene Set ID	Gene Set Genes #	DE Genes N (%)	Up-reg. DE Genes N (%)	Down-reg. DE Genes N (%)	Jaccard Index	P-Value	FDR Adjusted P-Value
REACTOME IMMUNOREGULATORY INTERACTIONS BETWEEN A L...	97	6 (6.2)	6 (6.2)	0 (0)	0.023	<0.0001	0.0421
REACTOME ERYTHROCYTES TAKE UP OXYGEN AND RELEASE C...	9	3 (33.3)	3 (33.3)	0 (0)	0.017	<0.0001	0.0421
REACTOME SCAVENGING OF HEME FROM PLASMA	13	3 (23.1)	3 (23.1)	0 (0)	0.017	<0.0001	0.0421
REACTOME ERYTHROCYTES TAKE UP CARBON DIOXIDE AND R...	13	3 (23.1)	3 (23.1)	0 (0)	0.017	0.0001	0.0421

Table S104: Blood Transcription Modules enriched in differentially expressed genes at Day 36 compared to pre-Second Vaccination (Dendritic cells, Study Arm 2 A/H7N9+AS03, D1, 29). Results are sorted by FDR adjusted p-value and Jaccard similarity index.

Gene Set ID	Gene Set Genes #	DE Genes N (%)	Up-reg. DE Genes N (%)	Down-reg. DE Genes N (%)	Jaccard Index	P-Value	FDR Adjusted P-Value
M7.2 ENRICHED IN NK CELLS (I)	45	27 (60)	27 (60)	0 (0)	0.144	<0.0001	0.0005
S1 NK CELL SURFACE SIGNATURE	38	17 (44.7)	17 (44.7)	0 (0)	0.089	<0.0001	0.0005
M61.0 ENRICHED IN NK CELLS (II)	16	12 (75)	12 (75)	0 (0)	0.069	<0.0001	0.0005
M7.0 ENRICHED IN T CELLS (I)	57	9 (15.8)	9 (15.8)	0 (0)	0.041	<0.0001	0.0005
M35.1 SIGNALING IN T CELLS (II)	11	7 (63.6)	6 (54.5)	1 (9.1)	0.040	<0.0001	0.0005
M61.2 ENRICHED IN NK CELLS (RECEPTOR ACTIVATION)	13	6 (46.2)	6 (46.2)	0 (0)	0.034	<0.0001	0.0005
M61.1 ENRICHED IN NK CELLS (KIR CLUSTER)	6	4 (66.7)	4 (66.7)	0 (0)	0.023	<0.0001	0.0005
M35.0 SIGNALING IN T CELLS (I)	14	5 (35.7)	4 (28.6)	1 (7.1)	0.028	<0.0001	0.0013
M156.1 PLASMA CELLS, IMMUNOGLOBULINS	22	4 (18.2)	4 (18.2)	0 (0)	0.021	0.0001	0.005
M157 ENRICHED IN NK CELLS (III)	14	4 (28.6)	4 (28.6)	0 (0)	0.022	0.0002	0.0069
M130 ENRICHED IN G-PROTEIN COUPLED RECEPTORS	10	3 (30)	3 (30)	0 (0)	0.017	0.0009	0.0277
M86.1 PROINFLAMMATORY DENDRITIC CELL, MYELOID CELL...	12	3 (25)	2 (16.7)	1 (8.3)	0.017	0.0014	0.0418
M223 ENRICHED IN T CELLS (II)	11	3 (27.3)	3 (27.3)	0 (0)	0.017	0.0017	0.0444

Table S105: KEGG Pathways enriched in differentially expressed genes at Day 2 compared to pre-First Vaccination (Neutrophils, Study Arm 2 A/H7N9+AS03, D1, 29). Results are sorted by FDR adjusted p-value and Jaccard similarity index.

Gene Set ID	Gene Set Genes #	DE Genes N (%)	Up-reg. DE Genes N (%)	Down-reg. DE Genes N (%)	Jaccard Index	P-Value	FDR Adjusted P-Value
NOD-LIKE RECEPTOR SIGNALING PATHWAY	175	34 (19.4)	27 (15.4)	7 (4)	0.027	<0.0001	0.0011
INFLUENZA A	160	24 (15)	23 (14.4)	1 (0.6)	0.019	<0.0001	0.0011
HEMATOPOIETIC CELL LINEAGE	93	16 (17.2)	9 (9.7)	7 (7.5)	0.013	<0.0001	0.0011
EPSTEIN-BARR VIRUS INFECTION	196	27 (13.8)	22 (11.2)	5 (2.6)	0.021	<0.0001	0.0017

Table S105: KEGG Pathways enriched in differentially expressed genes at Day 2 compared to pre-First Vaccination (Neutrophils, Study Arm 2 A/H7N9+AS03, D1, 29). Results are sorted by FDR adjusted p-value and Jaccard similarity index (continued.)

Gene Set ID	Gene Set Genes #	DE Genes N (%)	Up-reg. DE Genes N (%)	Down-reg. DE Genes N (%)	Jaccard Index	P-Value	FDR Adjusted P-Value
INFLAMMATORY BOWEL DISEASE	61	13 (21.3)	11 (18)	2 (3.3)	0.011	<0.0001	0.0017
ANTIGEN PROCESSING AND PRESENTATION	70	13 (18.6)	13 (18.6)	0 (0)	0.011	<0.0001	0.0017
TRANSCRIPTIONAL MISREGULATION IN CANCER	184	24 (13)	10 (5.4)	14 (7.6)	0.019	<0.0001	0.0029
RHEUMATOID ARTHRITIS	88	12 (13.6)	7 (8)	5 (5.7)	0.010	0.0005	0.0195
GLUTATHIONE METABOLISM	54	9 (16.7)	4 (7.4)	5 (9.3)	0.008	0.0006	0.0195
PROTEASOME	46	9 (19.6)	9 (19.6)	0 (0)	0.008	0.0006	0.0195
SALMONELLA INFECTION	205	25 (12.2)	16 (7.8)	9 (4.4)	0.019	0.0008	0.0242
AFRICAN TRYPANOSOMIASIS	36	7 (19.4)	5 (13.9)	2 (5.6)	0.006	0.0016	0.0448
TYPE I DIABETES MELLITUS	41	7 (17.1)	6 (14.6)	1 (2.4)	0.006	0.0018	0.0448
ASTHMA	27	5 (18.5)	5 (18.5)	0 (0)	0.004	0.0019	0.0448

Table S106: MSigDB Reactome Pathways enriched in differentially expressed genes at Day 2 compared to pre-First Vaccination (Neutrophils, Study Arm 2 A/H7N9+AS03, D1, 29). Results are sorted by FDR adjusted p-value and Jaccard similarity index.

Gene Set ID	Gene Set Genes #	DE Genes N (%)	Up-reg. DE Genes N (%)	Down-reg. DE Genes N (%)	Jaccard Index	P-Value	FDR Adjusted P-Value
REACTOME CYTOKINE SIGNALING IN IMMUNE SYSTEM	788	107 (13.6)	82 (10.4)	25 (3.2)	0.059	<0.0001	0.0026
REACTOME INNATE IMMUNE SYSTEM	968	91 (9.4)	59 (6.1)	32 (3.3)	0.046	<0.0001	0.0026
REACTOME INTERFERON SIGNALING	177	50 (28.2)	48 (27.1)	2 (1.1)	0.040	<0.0001	0.0026
REACTOME SIGNALING BY INTERLEUKINS	430	51 (11.9)	35 (8.1)	16 (3.7)	0.034	<0.0001	0.0026
REACTOME INTERFERON GAMMA SIGNALING	71	27 (38)	27 (38)	0 (0)	0.023	<0.0001	0.0026
REACTOME INTERFERON ALPHA BETA SIGNALING	60	25 (41.7)	25 (41.7)	0 (0)	0.022	<0.0001	0.0026
REACTOME OAS ANTIVIRAL RESPONSE	8	5 (62.5)	5 (62.5)	0 (0)	0.004	0.0001	0.0263
REACTOME ANTIVIRAL MECHANISM BY IFN STIMULATED GEN...	81	16 (19.8)	14 (17.3)	2 (2.5)	0.013	0.0002	0.0306
REACTOME PYRIMIDINE CATABOLISM	12	5 (41.7)	4 (33.3)	1 (8.3)	0.004	0.0002	0.034

Table S107: MSigDB GO Biological Processes enriched in differentially expressed genes at Day 2 compared to pre-First Vaccination (Neutrophils, Study Arm 2 A/H7N9+AS03, D1, 29). Results are sorted by FDR adjusted p-value and Jaccard similarity index.

Gene Set ID	Gene Set Genes #	DE Genes N (%)	Up-reg. DE Genes N (%)	Down-reg. DE Genes N (%)	Jaccard Index	P-Value	FDR Adjusted P-Value
GO DEFENSE RESPONSE	1497	193 (12.9)	142 (9.5)	51 (3.4)	0.080	<0.0001	0.001
GO RESPONSE TO BIOTIC STIMULUS	1325	165 (12.5)	129 (9.7)	36 (2.7)	0.072	<0.0001	0.001

Table S107: MSigDB GO Biological Processes enriched in differentially expressed genes at Day 2 compared to pre-First Vaccination (Neutrophils, Study Arm 2 A/H7N9+AS03, D1, 29). Results are sorted by FDR adjusted p-value and Jaccard similarity index (continued.)

Gene Set ID	Gene Set Genes #	DE Genes N (%)	Up-reg. DE Genes N (%)	Down-reg. DE Genes N (%)	Jaccard Index	P-Value	FDR Adjusted P-Value
GO RESPONSE TO CYTOKINE	1099	143 (13)	107 (9.7)	36 (3.3)	0.069	<0.0001	0.001
GO DEFENSE RESPONSE TO OTHER ORGANISM	993	136 (13.7)	114 (11.5)	22 (2.2)	0.069	<0.0001	0.001
GO IMMUNE EFFECTOR PROCESS	1077	138 (12.8)	98 (9.1)	40 (3.7)	0.067	<0.0001	0.001
GO INNATE IMMUNE RESPONSE	814	120 (14.7)	104 (12.8)	16 (2)	0.066	<0.0001	0.001
GO REGULATION OF IMMUNE SYSTEM PROCESS	1409	154 (10.9)	101 (7.2)	53 (3.8)	0.065	<0.0001	0.001
GO CYTOKINE MEDIATED SIGNALING PATHWAY	711	103 (14.5)	82 (11.5)	21 (3)	0.060	<0.0001	0.001
GO REGULATION OF RESPONSE TO STRESS	1406	142 (10.1)	99 (7)	43 (3.1)	0.060	<0.0001	0.001
GO CELL ACTIVATION	1265	129 (10.2)	67 (5.3)	62 (4.9)	0.057	<0.0001	0.001
GO REGULATION OF MULTI ORGANISM PROCESS	700	96 (13.7)	81 (11.6)	15 (2.1)	0.056	<0.0001	0.001
GO REGULATION OF IMMUNE RESPONSE	897	106 (11.8)	80 (8.9)	26 (2.9)	0.056	<0.0001	0.001
GO POSITIVE REGULATION OF IMMUNE SYSTEM PROCESS	972	108 (11.1)	75 (7.7)	33 (3.4)	0.054	<0.0001	0.001
GO CYTOKINE PRODUCTION	720	94 (13.1)	62 (8.6)	32 (4.4)	0.054	<0.0001	0.001
GO REGULATION OF DEFENSE RESPONSE	703	93 (13.2)	71 (10.1)	22 (3.1)	0.054	<0.0001	0.001
GO REGULATION OF RESPONSE TO EXTERNAL STIMULUS	1006	108 (10.7)	79 (7.9)	29 (2.9)	0.054	<0.0001	0.001
GO NEGATIVE REGULATION OF RESPONSE TO STIMULUS	1531	127 (8.3)	67 (4.4)	60 (3.9)	0.050	<0.0001	0.001
GO SECRETION	1551	127 (8.2)	74 (4.8)	53 (3.4)	0.050	<0.0001	0.001
GO RESPONSE TO VIRUS	300	66 (22)	57 (19)	9 (3)	0.049	<0.0001	0.001
GO INFLAMMATORY RESPONSE	680	80 (11.8)	49 (7.2)	31 (4.6)	0.047	<0.0001	0.001
GO DEFENSE RESPONSE TO VIRUS	229	59 (25.8)	52 (22.7)	7 (3.1)	0.046	<0.0001	0.001
GO IMMUNE SYSTEM DEVELOPMENT	926	87 (9.4)	44 (4.8)	43 (4.6)	0.044	<0.0001	0.001
GO NEGATIVE REGULATION OF MOLECULAR FUNCTION	1052	92 (8.7)	50 (4.8)	42 (4)	0.044	<0.0001	0.001
GO INTERSPECIES INTERACTION BETWEEN ORGANISMS	869	82 (9.4)	57 (6.6)	25 (2.9)	0.043	<0.0001	0.001
GO REGULATION OF RESPONSE TO BIOTIC STIMULUS	466	65 (13.9)	58 (12.4)	7 (1.5)	0.043	<0.0001	0.001
GO POSITIVE REGULATION OF IMMUNE RESPONSE	704	73 (10.4)	61 (8.7)	12 (1.7)	0.042	<0.0001	0.001
GO LEUKOCYTE MEDIATED IMMUNITY	701	71 (10.1)	43 (6.1)	28 (4)	0.041	<0.0001	0.001
GO MYELOID LEUKOCYTE ACTIVATION	594	65 (10.9)	36 (6.1)	29 (4.9)	0.040	<0.0001	0.001
GO LYMPHOCYTE ACTIVATION	620	63 (10.2)	36 (5.8)	27 (4.4)	0.038	<0.0001	0.001
GO CELL ACTIVATION INVOLVED IN IMMUNE RESPONSE	658	64 (9.7)	35 (5.3)	29 (4.4)	0.037	<0.0001	0.001
GO REGULATION OF CELL ACTIVATION	507	58 (11.4)	34 (6.7)	24 (4.7)	0.037	<0.0001	0.001
GO REGULATION OF IMMUNE EFFECTOR PROCESS	357	52 (14.6)	40 (11.2)	12 (3.4)	0.037	<0.0001	0.001
GO POSITIVE REGULATION OF RESPONSE TO EXTERNAL STI...	575	59 (10.3)	47 (8.2)	12 (2.1)	0.036	<0.0001	0.001
GO POSITIVE REGULATION OF MULTI ORGANISM PROCESS	489	55 (11.2)	45 (9.2)	10 (2)	0.035	<0.0001	0.001
GO RESPONSE TO BACTERIUM	587	58 (9.9)	40 (6.8)	18 (3.1)	0.035	<0.0001	0.001
GO POSITIVE REGULATION OF CYTOKINE PRODUCTION	419	52 (12.4)	38 (9.1)	14 (3.3)	0.035	<0.0001	0.001
GO VIRAL LIFE CYCLE	308	48 (15.6)	38 (12.3)	10 (3.2)	0.035	<0.0001	0.001
GO RESPONSE TO INTERFERON GAMMA	166	42 (25.3)	39 (23.5)	3 (1.8)	0.034	<0.0001	0.001
GO NEGATIVE REGULATION OF MULTI ORGANISM PROCESS	195	42 (21.5)	39 (20)	3 (1.5)	0.033	<0.0001	0.001

Table S107: MSigDB GO Biological Processes enriched in differentially expressed genes at Day 2 compared to pre-First Vaccination (Neutrophils, Study Arm 2 A/H7N9+AS03, D1, 29). Results are sorted by FDR adjusted p-value and Jaccard similarity index (continued.)

Gene Set ID	Gene Set Genes #	DE Genes N (%)	Up-reg. DE Genes N (%)	Down-reg. DE Genes N (%)	Jaccard Index	P-Value	FDR Adjusted P-Value
GO POSITIVE REGULATION OF DEFENSE RESPONSE	457	50 (10.9)	43 (9.4)	7 (1.5)	0.033	<0.0001	0.001
GO T CELL ACTIVATION	436	49 (11.2)	31 (7.1)	18 (4.1)	0.033	<0.0001	0.001
GO NEGATIVE REGULATION OF IMMUNE SYSTEM PROCESS	426	48 (11.3)	31 (7.3)	17 (4)	0.032	<0.0001	0.001
GO ADAPTIVE IMMUNE RESPONSE	353	45 (12.7)	32 (9.1)	13 (3.7)	0.032	<0.0001	0.001
GO REGULATION OF SYMBIOSIS ENCOMPASSING MUTUALISM ...	204	40 (19.6)	34 (16.7)	6 (2.9)	0.031	<0.0001	0.001
GO REGULATION OF LYMPHOCYTE ACTIVATION	392	44 (11.2)	26 (6.6)	18 (4.6)	0.030	<0.0001	0.001
GO REGULATION OF VIRAL LIFE CYCLE	133	36 (27.1)	33 (24.8)	3 (2.3)	0.030	<0.0001	0.001
GO POSITIVE REGULATION OF RESPONSE TO BIOTIC STIMU...	353	42 (11.9)	37 (10.5)	5 (1.4)	0.029	<0.0001	0.001
GO NEGATIVE REGULATION OF CYTOKINE PRODUCTION	262	38 (14.5)	23 (8.8)	15 (5.7)	0.028	<0.0001	0.001
GO PRODUCTION OF MOLECULAR MEDIATOR OF IMMUNE RESP...	187	34 (18.2)	21 (11.2)	13 (7)	0.027	<0.0001	0.001
GO REGULATION OF INFLAMMATORY RESPONSE	332	37 (11.1)	22 (6.6)	15 (4.5)	0.026	<0.0001	0.001
GO I KAPPAB KINASE NF KAPPAB SIGNALING	252	34 (13.5)	25 (9.9)	9 (3.6)	0.025	<0.0001	0.001
GO LEUKOCYTE PROLIFERATION	276	33 (12)	16 (5.8)	17 (6.2)	0.024	<0.0001	0.001
GO VIRAL GENOME REPLICATION	117	29 (24.8)	27 (23.1)	2 (1.7)	0.024	<0.0001	0.001
GO NEGATIVE REGULATION OF VIRAL PROCESS	90	28 (31.1)	27 (30)	1 (1.1)	0.024	<0.0001	0.001
GO REGULATION OF VIRAL GENOME REPLICATION	91	28 (30.8)	26 (28.6)	2 (2.2)	0.024	<0.0001	0.001
GO RESPONSE TO TYPE I INTERFERON	88	27 (30.7)	27 (30.7)	0 (0)	0.023	<0.0001	0.001
GO NEGATIVE REGULATION OF VIRAL LIFE CYCLE	76	26 (34.2)	26 (34.2)	0 (0)	0.022	<0.0001	0.001
GO REGULATION OF LEUKOCYTE PROLIFERATION	203	28 (13.8)	15 (7.4)	13 (6.4)	0.022	<0.0001	0.001
GO INTERFERON GAMMA MEDIATED SIGNALING PATHWAY	70	25 (35.7)	25 (35.7)	0 (0)	0.021	<0.0001	0.001
GO CYTOKINE SECRETION	220	28 (12.7)	24 (10.9)	4 (1.8)	0.021	<0.0001	0.001
GO POSITIVE REGULATION OF IMMUNE EFFECTOR PROCESS	189	27 (14.3)	21 (11.1)	6 (3.2)	0.021	<0.0001	0.001
GO REGULATION OF RESPONSE TO CYTOKINE STIMULUS	165	26 (15.8)	22 (13.3)	4 (2.4)	0.021	<0.0001	0.001
GO REGULATION OF LEUKOCYTE MEDIATED IMMUNITY	174	26 (14.9)	18 (10.3)	8 (4.6)	0.021	<0.0001	0.001
GO REGULATION OF ADAPTIVE IMMUNE RESPONSE	142	25 (17.6)	17 (12)	8 (5.6)	0.020	<0.0001	0.001
GO NEGATIVE REGULATION OF VIRAL GENOME REPLICATION	58	23 (39.7)	23 (39.7)	0 (0)	0.020	<0.0001	0.001
GO NEGATIVE REGULATION OF IMMUNE RESPONSE	134	23 (17.2)	17 (12.7)	6 (4.5)	0.019	<0.0001	0.001
GO REGULATION OF PRODUCTION OF MOLECULAR MEDIATOR ...	129	22 (17.1)	14 (10.9)	8 (6.2)	0.018	<0.0001	0.001
GO CYTOKINE PRODUCTION INVOLVED IN IMMUNE RESPONSE	94	20 (21.3)	15 (16)	5 (5.3)	0.017	<0.0001	0.001
GO POSITIVE REGULATION OF LEUKOCYTE PROLIFERATION	124	20 (16.1)	12 (9.7)	8 (6.5)	0.016	<0.0001	0.001
GO REGULATION OF DEFENSE RESPONSE TO VIRUS	68	19 (27.9)	17 (25)	2 (2.9)	0.016	<0.0001	0.001
GO INTERLEUKIN 1 PRODUCTION	99	18 (18.2)	15 (15.2)	3 (3)	0.015	<0.0001	0.001
GO REGULATION OF CYTOKINE PRODUCTION INVOLVED IN I...	76	17 (22.4)	13 (17.1)	4 (5.3)	0.015	<0.0001	0.001
GO INTERLEUKIN 1 BETA PRODUCTION	87	17 (19.5)	14 (16.1)	3 (3.4)	0.014	<0.0001	0.001
GO POSITIVE REGULATION OF PRODUCTION OF MOLECULAR ...	89	16 (18)	11 (12.4)	5 (5.6)	0.013	<0.0001	0.001
GO POSITIVE REGULATION OF DEFENSE RESPONSE TO VIRU...	24	10 (41.7)	10 (41.7)	0 (0)	0.009	<0.0001	0.001
GO RESPONSE TO INTERFERON BETA	30	10 (33.3)	10 (33.3)	0 (0)	0.009	<0.0001	0.001

Table S107: MSigDB GO Biological Processes enriched in differentially expressed genes at Day 2 compared to pre-First Vaccination (Neutrophils, Study Arm 2 A/H7N9+AS03, D1, 29). Results are sorted by FDR adjusted p-value and Jaccard similarity index (continued.)

Gene Set ID	Gene Set Genes #	DE Genes N (%)	Up-reg. DE Genes N (%)	Down-reg. DE Genes N (%)	Jaccard Index	P-Value	FDR Adjusted P-Value
GO REGULATION OF VIRAL ENTRY INTO HOST CELL	26	9 (34.6)	8 (30.8)	1 (3.8)	0.008	<0.0001	0.001
GO PYRIMIDINE NUCLEOSIDE CATABOLIC PROCESS	23	8 (34.8)	7 (30.4)	1 (4.3)	0.007	<0.0001	0.001
GO NUCLEOSIDE CATABOLIC PROCESS	31	8 (25.8)	7 (22.6)	1 (3.2)	0.007	<0.0001	0.001
GO EXOCYTOSIS	833	78 (9.4)	41 (4.9)	37 (4.4)	0.042	<0.0001	0.0017
GO NEGATIVE REGULATION OF INTRACELLULAR SIGNAL TRA...	489	51 (10.4)	23 (4.7)	28 (5.7)	0.033	<0.0001	0.0017
GO MYELOID LEUKOCYTE MEDIATED IMMUNITY	506	49 (9.7)	28 (5.5)	21 (4.2)	0.031	<0.0001	0.0017
GO REGULATION OF PEPTIDASE ACTIVITY	409	42 (10.3)	30 (7.3)	12 (2.9)	0.028	<0.0001	0.0017
GO POSITIVE REGULATION OF CELL ACTIVATION	297	36 (12.1)	21 (7.1)	15 (5.1)	0.026	<0.0001	0.0017
GO ADAPTIVE IMMUNE RESPONSE BASED ON SOMATIC RECOM...	223	30 (13.5)	21 (9.4)	9 (4)	0.023	<0.0001	0.0017
GO NEGATIVE REGULATION OF DEFENSE RESPONSE	201	26 (12.9)	16 (8)	10 (5)	0.020	<0.0001	0.0017
GO ALPHA BETA T CELL ACTIVATION	127	20 (15.7)	13 (10.2)	7 (5.5)	0.016	<0.0001	0.0017
GO REGULATION OF DEFENSE RESPONSE TO VIRUS BY HOST	34	11 (32.4)	11 (32.4)	0 (0)	0.010	<0.0001	0.0017
GO REGULATION OF PEPTIDE SECRETION	461	46 (10)	31 (6.7)	15 (3.3)	0.030	<0.0001	0.0024
GO REGULATION OF CELL CELL ADHESION	372	42 (11.3)	27 (7.3)	15 (4)	0.029	<0.0001	0.0024
GO POSITIVE REGULATION OF I KAPPAB KINASE NF KAPPA...	169	24 (14.2)	20 (11.8)	4 (2.4)	0.019	<0.0001	0.0024
GO NEGATIVE REGULATION OF LEUKOCYTE CELL CELL ADHE...	117	19 (16.2)	16 (13.7)	3 (2.6)	0.016	<0.0001	0.0024
GO ANTIGEN PROCESSING AND PRESENTATION OF PEPTIDE ...	80	16 (20)	16 (20)	0 (0)	0.014	<0.0001	0.0024
GO CYTOPLASMIC PATTERN RECOGNITION RECEPTOR SIGNAL...	54	13 (24.1)	10 (18.5)	3 (5.6)	0.011	<0.0001	0.0024
GO IMMUNE RESPONSE REGULATING SIGNALING PATHWAY	507	52 (10.3)	39 (7.7)	13 (2.6)	0.033	<0.0001	0.003
GO REGULATION OF T CELL ACTIVATION	292	36 (12.3)	25 (8.6)	11 (3.8)	0.026	<0.0001	0.003
GO LEUKOCYTE CELL CELL ADHESION	307	36 (11.7)	25 (8.1)	11 (3.6)	0.026	<0.0001	0.003
GO LYMPHOCYTE MEDIATED IMMUNITY	216	27 (12.5)	20 (9.3)	7 (3.2)	0.021	<0.0001	0.003
GO ANTIGEN PROCESSING AND PRESENTATION OF EXOGENOU...	62	12 (19.4)	12 (19.4)	0 (0)	0.010	<0.0001	0.003
GO PYRIMIDINE CONTAINING COMPOUND CATABOLIC PROCES...	39	9 (23.1)	7 (17.9)	2 (5.1)	0.008	<0.0001	0.003
GO MAST CELL CYTOKINE PRODUCTION	6	4 (66.7)	2 (33.3)	2 (33.3)	0.004	<0.0001	0.003
GO TYPE I INTERFERON PRODUCTION	116	19 (16.4)	15 (12.9)	4 (3.4)	0.016	<0.0001	0.0035
GO REGULATION OF LYMPHOCYTE MEDIATED IMMUNITY	127	19 (15)	13 (10.2)	6 (4.7)	0.015	<0.0001	0.0035
GO CHEMOKINE PRODUCTION	80	14 (17.5)	9 (11.2)	5 (6.2)	0.012	<0.0001	0.0035
GO RESPONSE TO GAMMA RADIATION	53	12 (22.6)	5 (9.4)	7 (13.2)	0.010	<0.0001	0.0035
GO POSITIVE REGULATION OF B CELL PROLIFERATION	39	9 (23.1)	4 (10.3)	5 (12.8)	0.008	<0.0001	0.0035
GO CELLULAR RESPONSE TO MURAMYL DIPEPTIDE	8	5 (62.5)	4 (50)	1 (12.5)	0.004	<0.0001	0.0035
GO REGULATION OF DNA BINDING TRANSCRIPTION FACTOR ...	393	43 (10.9)	25 (6.4)	18 (4.6)	0.029	<0.0001	0.0041
GO POSITIVE REGULATION OF CYTOKINE SECRETION	126	18 (14.3)	15 (11.9)	3 (2.4)	0.015	<0.0001	0.0041
GO REGULATION OF INTERLEUKIN 1 SECRETION	45	9 (20)	9 (20)	0 (0)	0.008	<0.0001	0.0041
GO ACTIVATION OF IMMUNE RESPONSE	547	53 (9.7)	44 (8)	9 (1.6)	0.033	<0.0001	0.0047
GO POSITIVE REGULATION OF CHEMOKINE PRODUCTION	51	11 (21.6)	8 (15.7)	3 (5.9)	0.009	<0.0001	0.0047
GO POSITIVE REGULATION OF VIRAL ENTRY INTO HOST CE...	9	5 (55.6)	4 (44.4)	1 (11.1)	0.004	<0.0001	0.0047

Table S107: MSigDB GO Biological Processes enriched in differentially expressed genes at Day 2 compared to pre-First Vaccination (Neutrophils, Study Arm 2 A/H7N9+AS03, D1, 29). Results are sorted by FDR adjusted p-value and Jaccard similarity index (continued.)

Gene Set ID	Gene Set Genes #	DE Genes N (%)	Up-reg. DE Genes N (%)	Down-reg. DE Genes N (%)	Jaccard Index	P-Value	FDR Adjusted P-Value
GO REGULATION OF CELL ADHESION	633	62 (9.8)	33 (5.2)	29 (4.6)	0.037	<0.0001	0.0051
GO POSITIVE REGULATION OF PEPTIDE SECRETION	271	30 (11.1)	18 (6.6)	12 (4.4)	0.022	<0.0001	0.0051
GO POSITIVE REGULATION OF B CELL ACTIVATION	71	13 (18.3)	5 (7)	8 (11.3)	0.011	<0.0001	0.0051
GO NEGATIVE REGULATION OF INNATE IMMUNE RESPONSE	49	11 (22.4)	11 (22.4)	0 (0)	0.009	<0.0001	0.0051
GO RESPONSE TO INTERFERON ALPHA	21	7 (33.3)	7 (33.3)	0 (0)	0.006	<0.0001	0.0051
GO RESPONSE TO MOLECULE OF BACTERIAL ORIGIN	329	34 (10.3)	21 (6.4)	13 (4)	0.024	<0.0001	0.0055
GO RESPONSE TO TUMOR NECROSIS FACTOR	272	30 (11)	24 (8.8)	6 (2.2)	0.022	<0.0001	0.0055
GO REGULATION OF PROTEIN BINDING	213	28 (13.1)	14 (6.6)	14 (6.6)	0.021	<0.0001	0.0055
GO REGULATION OF CYSTEINE TYPE ENDOPEPTIDASE ACTIV...	221	28 (12.7)	19 (8.6)	9 (4.1)	0.021	<0.0001	0.0055
GO NEGATIVE REGULATION OF RESPONSE TO BIOTIC STIMU...	82	14 (17.1)	13 (15.9)	1 (1.2)	0.012	<0.0001	0.0055
GO NEGATIVE REGULATION OF PROTEIN METABOLIC PROCES...	1039	86 (8.3)	46 (4.4)	40 (3.8)	0.042	<0.0001	0.0059
GO PEPTIDE SECRETION	587	54 (9.2)	37 (6.3)	17 (2.9)	0.033	<0.0001	0.0059
GO INTERLEUKIN 1 SECRETION	53	10 (18.9)	10 (18.9)	0 (0)	0.009	<0.0001	0.0059
GO PYRIMIDINE NUCLEOSIDE METABOLIC PROCESS	35	8 (22.9)	7 (20)	1 (2.9)	0.007	<0.0001	0.0059
GO RESPONSE TO PROTOZOAN	26	7 (26.9)	6 (23.1)	1 (3.8)	0.006	<0.0001	0.0059
GO NEGATIVE REGULATION OF LEUKOCYTE MEDIATED IMMUN...	39	9 (23.1)	7 (17.9)	2 (5.1)	0.008	0.0001	0.0064
GO ACTIVATION OF INNATE IMMUNE RESPONSE	286	33 (11.5)	28 (9.8)	5 (1.7)	0.024	0.0001	0.0068
GO PATTERN RECOGNITION RECEPTOR SIGNALING PATHWAY	181	23 (12.7)	19 (10.5)	4 (2.2)	0.018	0.0001	0.0068
GO LYMPHOCYTE ACTIVATION INVOLVED IN IMMUNE RESPON...	171	21 (12.3)	12 (7)	9 (5.3)	0.017	0.0001	0.0068
GO POSITIVE REGULATION OF CELL CELL ADHESION	232	28 (12.1)	18 (7.8)	10 (4.3)	0.021	0.0001	0.0072
GO NEGATIVE REGULATION OF CELL CELL ADHESION	167	22 (13.2)	17 (10.2)	5 (3)	0.017	0.0001	0.0072
GO POSITIVE REGULATION OF ADAPTIVE IMMUNE RESPONSE	92	15 (16.3)	11 (12)	4 (4.3)	0.013	0.0001	0.0072
GO POSITIVE REGULATION OF CYTOKINE PRODUCTION INVO...	49	11 (22.4)	10 (20.4)	1 (2)	0.009	0.0001	0.0072
GO INTERLEUKIN 8 PRODUCTION	70	12 (17.1)	9 (12.9)	3 (4.3)	0.010	0.0001	0.0076
GO INTRINSIC APOPTOTIC SIGNALING PATHWAY IN RESPON...	42	10 (23.8)	4 (9.5)	6 (14.3)	0.009	0.0001	0.0076
GO POSITIVE REGULATION OF TYPE 2 IMMUNE RESPONSE	16	6 (37.5)	5 (31.2)	1 (6.2)	0.005	0.0001	0.0076
GO ALPHA BETA T CELL DIFFERENTIATION	97	16 (16.5)	11 (11.3)	5 (5.2)	0.013	0.0001	0.008
GO GLYCOSYL COMPOUND CATABOLIC PROCESS	38	8 (21.1)	7 (18.4)	1 (2.6)	0.007	0.0001	0.008
GO APOPTOTIC SIGNALING PATHWAY	566	53 (9.4)	29 (5.1)	24 (4.2)	0.033	0.0002	0.0085
GO NEGATIVE REGULATION OF PROTEIN BINDING	98	16 (16.3)	9 (9.2)	7 (7.1)	0.013	0.0002	0.0088
GO IMMUNOGLOBULIN PRODUCTION	97	15 (15.5)	7 (7.2)	8 (8.2)	0.013	0.0002	0.0088
GO CELLULAR RESPONSE TO VIRUS	44	10 (22.7)	9 (20.5)	1 (2.3)	0.009	0.0002	0.0088
GO TUMOR NECROSIS FACTOR MEDIATED SIGNALING PATHWA...	147	19 (12.9)	16 (10.9)	3 (2)	0.015	0.0002	0.0093
GO REGULATION OF INTRACELLULAR SIGNAL TRANSDUCTION	1707	134 (7.9)	67 (3.9)	67 (3.9)	0.050	0.0002	0.0106
GO B CELL MEDIATED IMMUNITY	104	15 (14.4)	10 (9.6)	5 (4.8)	0.012	0.0002	0.0106
GO POSITIVE REGULATION OF RESPONSE TO CYTOKINE STI...	53	11 (20.8)	11 (20.8)	0 (0)	0.009	0.0002	0.0106
GO REGULATION OF TRANSPORT	1745	134 (7.7)	70 (4)	64 (3.7)	0.049	0.0002	0.0108

Table S107: MSigDB GO Biological Processes enriched in differentially expressed genes at Day 2 compared to pre-First Vaccination (Neutrophils, Study Arm 2 A/H7N9+AS03, D1, 29). Results are sorted by FDR adjusted p-value and Jaccard similarity index (continued.)

Gene Set ID	Gene Set Genes #	DE Genes N (%)	Up-reg. DE Genes N (%)	Down-reg. DE Genes N (%)	Jaccard Index	P-Value	FDR Adjusted P-Value
GO LEUKOCYTE DIFFERENTIATION	483	46 (9.5)	21 (4.3)	25 (5.2)	0.030	0.0002	0.0108
GO LEUKOCYTE MIGRATION	410	38 (9.3)	15 (3.7)	23 (5.6)	0.025	0.0002	0.0108
GO T CELL DIFFERENTIATION	230	27 (11.7)	16 (7)	11 (4.8)	0.021	0.0002	0.0108
GO CD4 POSITIVE ALPHA BETA T CELL ACTIVATION	87	14 (16.1)	10 (11.5)	4 (4.6)	0.012	0.0002	0.0108
GO NEGATIVE REGULATION OF IMMUNE EFFECTOR PROCESS	106	16 (15.1)	12 (11.3)	4 (3.8)	0.013	0.0002	0.0112
GO NEGATIVE REGULATION OF CELL ADHESION	263	30 (11.4)	19 (7.2)	11 (4.2)	0.022	0.0002	0.0116
GO NEGATIVE REGULATION OF CELL POPULATION PROLIFER...	666	57 (8.6)	25 (3.8)	32 (4.8)	0.033	0.0002	0.012
GO RESPONSE TO INTERLEUKIN 1	181	22 (12.2)	18 (9.9)	4 (2.2)	0.017	0.0003	0.0128
GO ACUTE INFLAMMATORY RESPONSE	102	14 (13.7)	11 (10.8)	3 (2.9)	0.012	0.0003	0.0128
GO T CELL ACTIVATION INVOLVED IN IMMUNE RESPONSE	102	14 (13.7)	11 (10.8)	3 (2.9)	0.012	0.0003	0.0132
GO REGULATORY T CELL DIFFERENTIATION	28	7 (25)	6 (21.4)	1 (3.6)	0.006	0.0003	0.0136
GO REGULATION OF ESTABLISHMENT OF PROTEIN LOCALIZA...	698	62 (8.9)	36 (5.2)	26 (3.7)	0.035	0.0003	0.0158
GO NEGATIVE REGULATION OF CELL ACTIVATION	184	22 (12)	15 (8.2)	7 (3.8)	0.017	0.0004	0.0165
GO GLUTATHIONE METABOLIC PROCESS	52	9 (17.3)	3 (5.8)	6 (11.5)	0.008	0.0004	0.0165
GO ANTIGEN PROCESSING AND PRESENTATION	189	23 (12.2)	20 (10.6)	3 (1.6)	0.018	0.0004	0.0173
GO REGULATION OF CELL DEATH	1593	118 (7.4)	62 (3.9)	56 (3.5)	0.045	0.0004	0.0177
GO REGULATION OF SECRETION	752	64 (8.5)	42 (5.6)	22 (2.9)	0.035	0.0004	0.0177
GO LYMPHOCYTE DIFFERENTIATION	336	34 (10.1)	17 (5.1)	17 (5.1)	0.024	0.0004	0.0177
GO NEGATIVE REGULATION OF DNA BINDING TRANSCRIPTIO...	152	19 (12.5)	8 (5.3)	11 (7.2)	0.015	0.0004	0.0177
GO REGULATION OF LIPID CATABOLIC PROCESS	53	10 (18.9)	2 (3.8)	8 (15.1)	0.009	0.0004	0.0177
GO NEGATIVE REGULATION OF PEPTIDASE ACTIVITY	230	23 (10)	17 (7.4)	6 (2.6)	0.017	0.0004	0.0181
GO TYPE 2 IMMUNE RESPONSE	38	8 (21.1)	7 (18.4)	1 (2.6)	0.007	0.0004	0.0183
GO NEGATIVE REGULATION OF INTERLEUKIN 1 PRODUCTION	34	7 (20.6)	5 (14.7)	2 (5.9)	0.006	0.0004	0.0183
GO POSITIVE REGULATION OF MOLECULAR FUNCTION	1658	129 (7.8)	70 (4.2)	59 (3.6)	0.049	0.0005	0.0207
GO POSITIVE REGULATION OF SIGNALING	1730	130 (7.5)	75 (4.3)	55 (3.2)	0.048	0.0005	0.0207
GO REGULATION OF BINDING	361	37 (10.2)	18 (5)	19 (5.3)	0.026	0.0006	0.0247
GO POSITIVE REGULATION OF LEUKOCYTE CELL CELL ADHE...	194	23 (11.9)	17 (8.8)	6 (3.1)	0.018	0.0006	0.0247
GO REGULATION OF EXTRINSIC APOPTOTIC SIGNALING PAT...	145	19 (13.1)	12 (8.3)	7 (4.8)	0.015	0.0006	0.0251
GO SOMATIC DIVERSIFICATION OF IMMUNE RECEPTORS	68	12 (17.6)	5 (7.4)	7 (10.3)	0.010	0.0006	0.0251
GO ADHESION OF SYMBIONT TO HOST	14	5 (35.7)	2 (14.3)	3 (21.4)	0.004	0.0006	0.0251
GO INTRINSIC APOPTOTIC SIGNALING PATHWAY	274	29 (10.6)	15 (5.5)	14 (5.1)	0.021	0.0006	0.0252
GO B CELL ACTIVATION	231	25 (10.8)	9 (3.9)	16 (6.9)	0.019	0.0006	0.0252
GO POSITIVE REGULATION OF INTRACELLULAR SIGNAL TRA...	948	77 (8.1)	42 (4.4)	35 (3.7)	0.039	0.0006	0.026
GO REGULATION OF ALPHA BETA T CELL ACTIVATION	84	13 (15.5)	11 (13.1)	2 (2.4)	0.011	0.0006	0.026
GO MAST CELL ACTIVATION	53	10 (18.9)	7 (13.2)	3 (5.7)	0.009	0.0006	0.026
GO SOMATIC DIVERSIFICATION OF IMMUNE RECEPTORS VIA...	60	11 (18.3)	4 (6.7)	7 (11.7)	0.009	0.0006	0.0263
GO REGULATION OF LEUKOCYTE MEDIATED CYTOTOXICITY	59	10 (16.9)	8 (13.6)	2 (3.4)	0.009	0.0007	0.0266

Table S107: MSigDB GO Biological Processes enriched in differentially expressed genes at Day 2 compared to pre-First Vaccination (Neutrophils, Study Arm 2 A/H7N9+AS03, D1, 29). Results are sorted by FDR adjusted p-value and Jaccard similarity index (continued.)

Gene Set ID	Gene Set Genes #	DE Genes N (%)	Up-reg. DE Genes N (%)	Down-reg. DE Genes N (%)	Jaccard Index	P-Value	FDR Adjusted P-Value
GO INTERLEUKIN 1 MEDIATED SIGNALING PATHWAY	91	14 (15.4)	12 (13.2)	2 (2.2)	0.012	0.0007	0.0268
GO SMALL MOLECULE METABOLIC PROCESS	1844	135 (7.3)	59 (3.2)	76 (4.1)	0.048	0.0007	0.0271
GO REGULATION OF ANTIGEN PROCESSING AND PRESENTATI...	16	5 (31.2)	4 (25)	1 (6.2)	0.004	0.0007	0.0277
GO NEGATIVE REGULATION OF CATALYTIC ACTIVITY	743	60 (8.1)	35 (4.7)	25 (3.4)	0.033	0.0007	0.0282
GO NEGATIVE REGULATION OF LYMPHOCYTE ACTIVATION	137	17 (12.4)	14 (10.2)	3 (2.2)	0.014	0.0007	0.0282
GO POSITIVE REGULATION OF CYSTEINE TYPE ENDOPEPTID...	141	18 (12.8)	11 (7.8)	7 (5)	0.015	0.0007	0.0285
GO LIPID CATABOLIC PROCESS	316	31 (9.8)	6 (1.9)	25 (7.9)	0.022	0.0007	0.0291
GO NEGATIVE REGULATION OF TYPE I INTERFERON PRODUC...	43	9 (20.9)	8 (18.6)	1 (2.3)	0.008	0.0008	0.0293
GO POSITIVE REGULATION OF CELL ADHESION	368	37 (10.1)	21 (5.7)	16 (4.3)	0.026	0.0008	0.0294
GO INTERFERON GAMMA PRODUCTION	97	13 (13.4)	9 (9.3)	4 (4.1)	0.011	0.0008	0.0294
GO REGULATION OF TYPE 2 IMMUNE RESPONSE	31	7 (22.6)	6 (19.4)	1 (3.2)	0.006	0.0008	0.0297
GO NEGATIVE REGULATION OF PHOSPHORYLATION	431	41 (9.5)	18 (4.2)	23 (5.3)	0.027	0.0008	0.031
GO REGULATION OF MAST CELL CYTOKINE PRODUCTION	5	3 (60)	2 (40)	1 (20)	0.003	0.0008	0.0316
GO MYELOID LEUKOCYTE MIGRATION	187	19 (10.2)	7 (3.7)	12 (6.4)	0.015	0.0008	0.0317
GO NEGATIVE REGULATION OF LYMPHOCYTE MEDIATED IMMU...	31	7 (22.6)	6 (19.4)	1 (3.2)	0.006	0.0008	0.0317
GO REGULATION OF B CELL PROLIFERATION	58	10 (17.2)	4 (6.9)	6 (10.3)	0.009	0.0009	0.0326
GO POSITIVE REGULATION OF DNA BINDING TRANSCRIPTIO...	238	27 (11.3)	21 (8.8)	6 (2.5)	0.020	0.0009	0.0327
GO LEUKOCYTE CHEMOTAXIS	202	20 (9.9)	9 (4.5)	11 (5.4)	0.015	0.0009	0.0327
GO REGULATION OF RIBONUCLEASE ACTIVITY	8	4 (50)	4 (50)	0 (0)	0.004	0.0009	0.0327
GO NEGATIVE REGULATION OF INTERLEUKIN 1 BETA SECRE...	13	4 (30.8)	4 (30.8)	0 (0)	0.004	0.0009	0.0327
GO REGULATION OF MAST CELL ACTIVATION	37	8 (21.6)	6 (16.2)	2 (5.4)	0.007	0.0009	0.0329
GO HOMEOSTASIS OF NUMBER OF CELLS	226	26 (11.5)	11 (4.9)	15 (6.6)	0.020	0.0009	0.033
GO POSITIVE REGULATION OF LEUKOCYTE MEDIATED IMMUN...	113	15 (13.3)	11 (9.7)	4 (3.5)	0.012	0.0009	0.033
GO POSITIVE REGULATION OF ACUTE INFLAMMATORY RESPO...	26	6 (23.1)	6 (23.1)	0 (0)	0.005	0.0009	0.0332
GO NEGATIVE REGULATION OF ADAPTIVE IMMUNE RESPONSE	40	8 (20)	5 (12.5)	3 (7.5)	0.007	0.0009	0.0334
GO REGULATION OF T CELL DIFFERENTIATION	131	17 (13)	13 (9.9)	4 (3.1)	0.014	0.0009	0.0336
GO POSITIVE REGULATION OF SECRETION	404	37 (9.2)	21 (5.2)	16 (4)	0.025	0.001	0.0345
GO REGULATION OF T HELPER 1 TYPE IMMUNE RESPONSE	25	6 (24)	5 (20)	1 (4)	0.005	0.001	0.035
GO REGULATION OF LEUKOCYTE MIGRATION	175	19 (10.9)	8 (4.6)	11 (6.3)	0.015	0.001	0.0356
GO ENTRY INTO HOST	126	17 (13.5)	10 (7.9)	7 (5.6)	0.014	0.001	0.0361
GO REGULATION OF PROTEIN MODIFICATION PROCESS	1724	128 (7.4)	59 (3.4)	69 (4)	0.047	0.0011	0.0373
GO INTRINSIC APOPTOTIC SIGNALING PATHWAY IN RESPON...	98	14 (14.3)	6 (6.1)	8 (8.2)	0.012	0.0011	0.0382
GO POSITIVE REGULATION OF PEPTIDASE ACTIVITY	185	21 (11.4)	14 (7.6)	7 (3.8)	0.016	0.0011	0.0394
GO NEGATIVE REGULATION OF PROTEIN MODIFICATION PRO...	562	50 (8.9)	22 (3.9)	28 (5)	0.031	0.0012	0.04
GO T HELPER 1 TYPE IMMUNE RESPONSE	40	8 (20)	6 (15)	2 (5)	0.007	0.0012	0.04
GO REGULATION OF STRESS ACTIVATED PROTEIN KINASE S...	226	26 (11.5)	15 (6.6)	11 (4.9)	0.020	0.0012	0.0402
GO REGULATION OF HYDROLASE ACTIVITY	1175	92 (7.8)	55 (4.7)	37 (3.1)	0.042	0.0012	0.0408

Table S107: MSigDB GO Biological Processes enriched in differentially expressed genes at Day 2 compared to pre-First Vaccination (Neutrophils, Study Arm 2 A/H7N9+AS03, D1, 29). Results are sorted by FDR adjusted p-value and Jaccard similarity index (continued.)

Gene Set ID	Gene Set Genes #	DE Genes N (%)	Up-reg. DE Genes N (%)	Down-reg. DE Genes N (%)	Jaccard Index	P-Value	FDR Adjusted P-Value
GO PYRIMIDINE RIBONUCLEOSIDE METABOLIC PROCESS	14	4 (28.6)	4 (28.6)	0 (0)	0.004	0.0012	0.0408
GO POSITIVE REGULATION OF ESTABLISHMENT OF PROTEIN...	428	40 (9.3)	20 (4.7)	20 (4.7)	0.027	0.0012	0.0411
GO DNA DEAMINATION	14	4 (28.6)	3 (21.4)	1 (7.1)	0.004	0.0012	0.0411
GO REGULATION OF PROTEIN LOCALIZATION	951	78 (8.2)	43 (4.5)	35 (3.7)	0.039	0.0013	0.0426
GO RESPONSE TO MURAMYL DIPEPTIDE	19	6 (31.6)	4 (21.1)	2 (10.5)	0.005	0.0013	0.0431
GO NEGATIVE REGULATION OF SIGNALING	1297	100 (7.7)	50 (3.9)	50 (3.9)	0.043	0.0013	0.0433
GO VIRION ATTACHMENT TO HOST CELL	9	4 (44.4)	2 (22.2)	2 (22.2)	0.004	0.0013	0.0433
GO STRESS ACTIVATED PROTEIN KINASE SIGNALING CASCA...	274	30 (10.9)	17 (6.2)	13 (4.7)	0.022	0.0013	0.0435
GO NEGATIVE REGULATION OF CELL DEATH	933	72 (7.7)	34 (3.6)	38 (4.1)	0.036	0.0014	0.045
GO ISG15 PROTEIN CONJUGATION	6	3 (50)	3 (50)	0 (0)	0.003	0.0014	0.045
GO ORGANIC ACID METABOLIC PROCESS	1093	86 (7.9)	32 (2.9)	54 (4.9)	0.041	0.0014	0.0452
GO REGULATION OF PROTEOLYSIS	667	54 (8.1)	37 (5.5)	17 (2.5)	0.031	0.0014	0.0453
GO RESPONSE TO WOUNDING	650	54 (8.3)	21 (3.2)	33 (5.1)	0.032	0.0015	0.0464
GO JNK CASCADE	201	24 (11.9)	12 (6)	12 (6)	0.019	0.0015	0.0471
GO NEGATIVE REGULATION OF CYSTEINE TYPE ENDOPEPTID...	83	12 (14.5)	9 (10.8)	3 (3.6)	0.010	0.0015	0.0471
GO REGULATION OF CELL KILLING	80	11 (13.8)	9 (11.2)	2 (2.5)	0.009	0.0015	0.0471
GO DEFENSE RESPONSE TO BACTERIUM	237	18 (7.6)	12 (5.1)	6 (2.5)	0.013	0.0015	0.0475
GO CD4 POSITIVE ALPHA BETA T CELL DIFFERENTIATION	71	11 (15.5)	9 (12.7)	2 (2.8)	0.009	0.0016	0.0492

Table S108: Blood Transcription Modules enriched in differentially expressed genes at Day 2 compared to pre-First Vaccination (Neutrophils, Study Arm 2 A/H7N9+AS03, D1, 29). Results are sorted by FDR adjusted p-value and Jaccard similarity index.

Gene Set ID	Gene Set Genes #	DE Genes N (%)	Up-reg. DE Genes N (%)	Down-reg. DE Genes N (%)	Jaccard Index	P-Value	FDR Adjusted P-Value
M75 ANTIVIRAL IFN SIGNATURE	22	15 (68.2)	14 (63.6)	1 (4.5)	0.013	<0.0001	0.0006
M165 ENRICHED IN ACTIVATED DENDRITIC CELLS (II)	34	14 (41.2)	13 (38.2)	1 (2.9)	0.012	<0.0001	0.0006
M127 TYPE I INTERFERON RESPONSE	12	11 (91.7)	11 (91.7)	0 (0)	0.010	<0.0001	0.0006
M111.1 VIRAL SENSING AND IMMUNITY; IRF2 TARGETS NE...	11	9 (81.8)	9 (81.8)	0 (0)	0.008	<0.0001	0.0006
M150 INNATE ANTIVIRAL RESPONSE	12	9 (75)	9 (75)	0 (0)	0.008	<0.0001	0.0006
M67 ACTIVATED DENDRITIC CELLS	12	8 (66.7)	8 (66.7)	0 (0)	0.007	<0.0001	0.0006
M111.0 VIRAL SENSING AND IMMUNITY; IRF2 TARGETS NE...	16	8 (50)	7 (43.8)	1 (6.2)	0.007	<0.0001	0.001
M68 RIG-1 LIKE RECEPTOR SIGNALING	9	5 (55.6)	5 (55.6)	0 (0)	0.004	0.0001	0.0052
M13 INNATE ACTIVATION BY CYTOSOLIC DNA SENSING	11	5 (45.5)	5 (45.5)	0 (0)	0.004	0.0002	0.0069
M66 TBA	14	6 (42.9)	5 (35.7)	1 (7.1)	0.005	0.0005	0.017
M95.1 ENRICHED IN ANTIGEN PRESENTATION (III)	12	5 (41.7)	5 (41.7)	0 (0)	0.004	0.0007	0.0211

Table S108: Blood Transcription Modules enriched in differentially expressed genes at Day 2 compared to pre-First Vaccination (Neutrophils, Study Arm 2 A/H7N9+AS03, D1, 29). Results are sorted by FDR adjusted p-value and Jaccard similarity index (continued.)

Gene Set ID	Gene Set Genes #	DE Genes N (%)	Up-reg. DE Genes N (%)	Down-reg. DE Genes N (%)	Jaccard Index	P-Value	FDR Adjusted P-Value
M121 TBA	12	5 (41.7)	4 (33.3)	1 (8.3)	0.004	0.0009	0.0265
M37.0 IMMUNE ACTIVATION - GENERIC CLUSTER	301	41 (13.6)	24 (8)	17 (5.6)	0.030	0.0013	0.0349

Table S109: MSigDB Reactome Pathways enriched in differentially expressed genes at Day 4 compared to pre-First Vaccination (Neutrophils, Study Arm 2 A/H7N9+AS03, D1, 29). Results are sorted by FDR adjusted p-value and Jaccard similarity index.

Gene Set ID	Gene Set Genes #	DE Genes N (%)	Up-reg. DE Genes N (%)	Down-reg. DE Genes N (%)	Jaccard Index	P-Value	FDR Adjusted P-Value
REACTOME OAS ANTIVIRAL RESPONSE	8	3 (37.5)	3 (37.5)	0 (0)	0.065	<0.0001	0.0038
REACTOME INTERFERON ALPHA BETA SIGNALING	60	5 (8.3)	5 (8.3)	0 (0)	0.052	<0.0001	0.0038
REACTOME INTERFERON GAMMA SIGNALING	71	5 (7)	5 (7)	0 (0)	0.047	<0.0001	0.0038
REACTOME INTERFERON SIGNALING	177	8 (4.5)	8 (4.5)	0 (0)	0.038	<0.0001	0.0038
REACTOME ANTIVIRAL MECHANISM BY IFN STIMULATED GEN...	81	4 (4.9)	4 (4.9)	0 (0)	0.034	<0.0001	0.0184

Table S110: MSigDB GO Biological Processes enriched in differentially expressed genes at Day 4 compared to pre-First Vaccination (Neutrophils, Study Arm 2 A/H7N9+AS03, D1, 29). Results are sorted by FDR adjusted p-value and Jaccard similarity index.

Gene Set ID	Gene Set Genes #	DE Genes N (%)	Up-reg. DE Genes N (%)	Down-reg. DE Genes N (%)	Jaccard Index	P-Value	FDR Adjusted P-Value
GO REGULATION OF RIBONUCLEASE ACTIVITY	8	3 (37.5)	3 (37.5)	0 (0)	0.065	<0.0001	0.0054
GO NEGATIVE REGULATION OF VIRAL GENOME REPLICATION	58	5 (8.6)	5 (8.6)	0 (0)	0.053	<0.0001	0.0054
GO NEGATIVE REGULATION OF VIRAL LIFE CYCLE	76	5 (6.6)	5 (6.6)	0 (0)	0.045	<0.0001	0.0054
GO RESPONSE TO TYPE I INTERFERON	88	5 (5.7)	5 (5.7)	0 (0)	0.040	<0.0001	0.0054
GO NEGATIVE REGULATION OF VIRAL PROCESS	90	5 (5.6)	5 (5.6)	0 (0)	0.040	<0.0001	0.0054
GO REGULATION OF VIRAL GENOME REPLICATION	91	5 (5.5)	5 (5.5)	0 (0)	0.039	<0.0001	0.0054
GO INTERFERON GAMMA MEDIATED SIGNALING PATHWAY	70	4 (5.7)	4 (5.7)	0 (0)	0.037	<0.0001	0.0054
GO RESPONSE TO INTERFERON GAMMA	166	7 (4.2)	7 (4.2)	0 (0)	0.035	<0.0001	0.0054
GO DEFENSE RESPONSE TO VIRUS	229	7 (3.1)	7 (3.1)	0 (0)	0.027	<0.0001	0.0054
GO NEGATIVE REGULATION OF MULTI ORGANISM PROCESS	195	6 (3.1)	6 (3.1)	0 (0)	0.026	<0.0001	0.0054
GO RESPONSE TO VIRUS	300	8 (2.7)	8 (2.7)	0 (0)	0.024	<0.0001	0.0054
GO DEFENSE RESPONSE TO OTHER ORGANISM	993	11 (1.1)	11 (1.1)	0 (0)	0.011	<0.0001	0.0054
GO RESPONSE TO BIOTIC STIMULUS	1325	14 (1.1)	13 (1)	1 (0.1)	0.010	<0.0001	0.0054
GO DEFENSE RESPONSE	1497	13 (0.9)	12 (0.8)	1 (0.1)	0.009	<0.0001	0.0054

Table S110: MSigDB GO Biological Processes enriched in differentially expressed genes at Day 4 compared to pre-First Vaccination (Neutrophils, Study Arm 2 A/H7N9+AS03, D1, 29). Results are sorted by FDR adjusted p-value and Jaccard similarity index (continued.)

Gene Set ID	Gene Set Genes #	DE Genes N (%)	Up-reg. DE Genes N (%)	Down-reg. DE Genes N (%)	Jaccard Index	P-Value	FDR Adjusted P-Value
GO REGULATION OF NUCLEASE ACTIVITY	21	3 (14.3)	3 (14.3)	0 (0)	0.051	<0.0001	0.0094
GO REGULATION OF VIRAL LIFE CYCLE	133	5 (3.8)	5 (3.8)	0 (0)	0.030	<0.0001	0.0094
GO VIRAL GENOME REPLICATION	117	5 (4.3)	5 (4.3)	0 (0)	0.033	<0.0001	0.0133
GO INNATE IMMUNE RESPONSE	814	9 (1.1)	9 (1.1)	0 (0)	0.011	<0.0001	0.0167
GO VIRAL LIFE CYCLE	308	6 (1.9)	6 (1.9)	0 (0)	0.018	<0.0001	0.0277
GO REGULATION OF SYMBIOSIS ENCOMPASSING MUTUALISM ...	204	5 (2.5)	5 (2.5)	0 (0)	0.021	<0.0001	0.0301
GO RESPONSE TO CYTOKINE	1099	10 (0.9)	8 (0.7)	2 (0.2)	0.009	<0.0001	0.0323

Table S111: Blood Transcription Modules enriched in differentially expressed genes at Day 4 compared to pre-First Vaccination (Neutrophils, Study Arm 2 A/H7N9+AS03, D1, 29). Results are sorted by FDR adjusted p-value and Jaccard similarity index.

Gene Set ID	Gene Set Genes #	DE Genes N (%)	Up-reg. DE Genes N (%)	Down-reg. DE Genes N (%)	Jaccard Index	P-Value	FDR Adjusted P-Value
M75 ANTIVIRAL IFN SIGNATURE	22	4 (18.2)	4 (18.2)	0 (0)	0.068	<0.0001	0.0017
M150 INNATE ANTIVIRAL RESPONSE	12	3 (25)	3 (25)	0 (0)	0.060	<0.0001	0.0017
M67 ACTIVATED DENDRITIC CELLS	12	2 (16.7)	2 (16.7)	0 (0)	0.039	0.0002	0.0288

Table S112: KEGG Pathways enriched in differentially expressed genes at Day 30 compared to pre-Second Vaccination (Neutrophils, Study Arm 2 A/H7N9+AS03, D1, 29). Results are sorted by FDR adjusted p-value and Jaccard similarity index.

Gene Set ID	Gene Set Genes #	DE Genes N (%)	Up-reg. DE Genes N (%)	Down-reg. DE Genes N (%)	Jaccard Index	P-Value	FDR Adjusted P-Value
EPSTEIN-BARR VIRUS INFECTION	196	49 (25)	37 (18.9)	12 (6.1)	0.022	<0.0001	0.0004
NOD-LIKE RECEPTOR SIGNALING PATHWAY	175	48 (27.4)	33 (18.9)	15 (8.6)	0.022	<0.0001	0.0004
INFLUENZA A	160	39 (24.4)	34 (21.2)	5 (3.1)	0.018	<0.0001	0.0004
HEMATOPOIETIC CELL LINEAGE	93	23 (24.7)	11 (11.8)	12 (12.9)	0.011	<0.0001	0.0004
ANTIGEN PROCESSING AND PRESENTATION	70	20 (28.6)	20 (28.6)	0 (0)	0.010	<0.0001	0.0004
RIG-I-LIKE RECEPTOR SIGNALING PATHWAY	67	20 (29.9)	13 (19.4)	7 (10.4)	0.010	<0.0001	0.0004
RHEUMATOID ARTHRITIS	88	19 (21.6)	12 (13.6)	7 (8)	0.009	<0.0001	0.0004
PROTEASOME	46	15 (32.6)	13 (28.3)	2 (4.3)	0.007	<0.0001	0.0004
CYTOSOLIC DNA-SENSING PATHWAY	61	15 (24.6)	12 (19.7)	3 (4.9)	0.007	<0.0001	0.0015
HUMAN IMMUNODEFICIENCY VIRUS 1 INFECTION	204	38 (18.6)	24 (11.8)	14 (6.9)	0.017	<0.0001	0.0021
ASTHMA	27	8 (29.6)	8 (29.6)	0 (0)	0.004	<0.0001	0.0021

Table S112: KEGG Pathways enriched in differentially expressed genes at Day 30 compared to pre-Second Vaccination (Neutrophils, Study Arm 2 A/H7N9+AS03, D1, 29). Results are sorted by FDR adjusted p-value and Jaccard similarity index (continued.)

Gene Set ID	Gene Set Genes #	DE Genes N (%)	Up-reg. DE Genes N (%)	Down-reg. DE Genes N (%)	Jaccard Index	P-Value	FDR Adjusted P-Value
GLUTATHIONE METABOLISM	54	13 (24.1)	3 (5.6)	10 (18.5)	0.006	<0.0001	0.0022
ALLOGRAFT REJECTION	34	9 (26.5)	9 (26.5)	0 (0)	0.004	0.0003	0.0075
HEPATITIS C	152	29 (19.1)	22 (14.5)	7 (4.6)	0.013	0.0004	0.0083
LEISHMANIASIS	70	17 (24.3)	13 (18.6)	4 (5.7)	0.008	0.0004	0.0083
MEASLES	132	26 (19.7)	21 (15.9)	5 (3.8)	0.012	0.0005	0.0103
GRAFT-VERSUS-HOST DISEASE	38	9 (23.7)	9 (23.7)	0 (0)	0.004	0.0005	0.0103
NECROPTOSIS	150	25 (16.7)	17 (11.3)	8 (5.3)	0.011	0.0007	0.0111
C-TYPE LECTIN RECEPTOR SIGNALING PATHWAY	103	22 (21.4)	13 (12.6)	9 (8.7)	0.010	0.0007	0.0111
TOLL-LIKE RECEPTOR SIGNALING PATHWAY	98	19 (19.4)	12 (12.2)	7 (7.1)	0.009	0.0007	0.0111
TYPE I DIABETES MELLITUS	41	10 (24.4)	9 (22)	1 (2.4)	0.005	0.0007	0.0111
INTESTINAL IMMUNE NETWORK FOR IGA PRODUCTION	45	11 (24.4)	9 (20)	2 (4.4)	0.005	0.0008	0.0116
TUBERCULOSIS	173	30 (17.3)	22 (12.7)	8 (4.6)	0.014	0.0008	0.0125
KAPOSI SARCOMA-ASSOCIATED HERPESVIRUS INFECTION	192	33 (17.2)	23 (12)	10 (5.2)	0.015	0.0009	0.0125
SALMONELLA INFECTION	205	36 (17.6)	20 (9.8)	16 (7.8)	0.016	0.0012	0.016
SHIGELLOSIS	235	40 (17)	18 (7.7)	22 (9.4)	0.018	0.0013	0.017
PERTUSSIS	75	16 (21.3)	9 (12)	7 (9.3)	0.008	0.0015	0.0186
GLYCOSAMINOGLYCAN DEGRADATION	18	7 (38.9)	2 (11.1)	5 (27.8)	0.003	0.0016	0.0191
GLYCOLYSIS / GLUCONEOGENESIS	64	14 (21.9)	4 (6.2)	10 (15.6)	0.007	0.0019	0.0216
INFLAMMATORY BOWEL DISEASE	61	13 (21.3)	11 (18)	2 (3.3)	0.006	0.0019	0.0216
APOPTOSIS	132	24 (18.2)	13 (9.8)	11 (8.3)	0.011	0.0023	0.0232
TNF SIGNALING PATHWAY	109	21 (19.3)	13 (11.9)	8 (7.3)	0.010	0.0023	0.0232
TH1 AND TH2 CELL DIFFERENTIATION	88	18 (20.5)	12 (13.6)	6 (6.8)	0.009	0.0023	0.0232
SYSTEMIC LUPUS ERYTHEMATOSUS	127	15 (11.8)	12 (9.4)	3 (2.4)	0.007	0.0024	0.0238
HEPATITIS B	156	28 (17.9)	18 (11.5)	10 (6.4)	0.013	0.0026	0.0254
NF-KAPPA B SIGNALING PATHWAY	94	18 (19.1)	12 (12.8)	6 (6.4)	0.009	0.0035	0.0328
TOXOPLASMOSIS	106	21 (19.8)	15 (14.2)	6 (5.7)	0.010	0.0036	0.0329

Table S113: MSigDB Reactome Pathways enriched in differentially expressed genes at Day 30 compared to pre-Second Vaccination (Neutrophils, Study Arm 2 A/H7N9+AS03, D1, 29). Results are sorted by FDR adjusted p-value and Jaccard similarity index.

Gene Set ID	Gene Set Genes #	DE Genes N (%)	Up-reg. DE Genes N (%)	Down-reg. DE Genes N (%)	Jaccard Index	P-Value	FDR Adjusted P-Value
REACTOME INNATE IMMUNE SYSTEM	968	159 (16.4)	88 (9.1)	71 (7.3)	0.056	<0.0001	0.0026
REACTOME CYTOKINE SIGNALING IN IMMUNE SYSTEM	788	139 (17.6)	102 (12.9)	37 (4.7)	0.052	<0.0001	0.0026
REACTOME INTERFERON SIGNALING	177	61 (34.5)	58 (32.8)	3 (1.7)	0.028	<0.0001	0.0026

Table S113: MSigDB Reactome Pathways enriched in differentially expressed genes at Day 30 compared to pre-Second Vaccination (Neutrophils, Study Arm 2 A/H7N9+AS03, D1, 29). Results are sorted by FDR adjusted p-value and Jaccard similarity index (continued.)

Gene Set ID	Gene Set Genes #	DE Genes N (%)	Up-reg. DE Genes N (%)	Down-reg. DE Genes N (%)	Jaccard Index	P-Value	FDR Adjusted P-Value
REACTOME INTERFERON GAMMA SIGNALING	71	35 (49.3)	33 (46.5)	2 (2.8)	0.017	<0.0001	0.0026
REACTOME INTERFERON ALPHA BETA SIGNALING	60	29 (48.3)	29 (48.3)	0 (0)	0.014	<0.0001	0.0026
REACTOME DDX58 IFIH1 MEDIATED INDUCTION OF INTERFE...	74	19 (25.7)	15 (20.3)	4 (5.4)	0.009	<0.0001	0.0026
REACTOME SIGNALING BY INTERLEUKINS	430	70 (16.3)	44 (10.2)	26 (6)	0.029	<0.0001	0.0044
REACTOME CROSS PRESENTATION OF SOLUBLE EXOGENOUS A...	45	14 (31.1)	12 (26.7)	2 (4.4)	0.007	<0.0001	0.0096
REACTOME NEUTROPHIL DEGRANULATION	442	71 (16.1)	34 (7.7)	37 (8.4)	0.029	<0.0001	0.0102
REACTOME NEGATIVE REGULATORS OF DDX58 IFIH1 SIGNAL...	33	12 (36.4)	9 (27.3)	3 (9.1)	0.006	0.0001	0.0169
REACTOME SIGNALING BY NOTCH4	75	19 (25.3)	13 (17.3)	6 (8)	0.009	0.0002	0.0243
REACTOME NEGATIVE REGULATION OF NOTCH4 SIGNALING	48	14 (29.2)	10 (20.8)	4 (8.3)	0.007	0.0002	0.0243
REACTOME ADAPTIVE IMMUNE SYSTEM	670	101 (15.1)	69 (10.3)	32 (4.8)	0.039	0.0003	0.0288
REACTOME MYD88 INDEPENDENT TLR4 CASCADE	90	22 (24.4)	15 (16.7)	7 (7.8)	0.010	0.0002	0.0288
REACTOME REGULATION OF RUNX3 EXPRESSION AND ACTIVI...	50	14 (28)	12 (24)	2 (4)	0.007	0.0003	0.0288
REACTOME GLUTATHIONE CONJUGATION	34	10 (29.4)	4 (11.8)	6 (17.6)	0.005	0.0003	0.0288
REACTOME MUCOPOLYSACCHARIDOSES	10	6 (60)	2 (20)	4 (40)	0.003	0.0003	0.0288
REACTOME NOD1 2 SIGNALING PATHWAY	33	12 (36.4)	8 (24.2)	4 (12.1)	0.006	0.0003	0.0289
REACTOME CYTOSOLIC SENSORS OF PATHOGEN ASSOCIATED ...	60	16 (26.7)	11 (18.3)	5 (8.3)	0.008	0.0004	0.0339
REACTOME NUCLEOTIDE BINDING DOMAIN LEUCINE RICH RE...	52	15 (28.8)	9 (17.3)	6 (11.5)	0.007	0.0004	0.0345
REACTOME AUF1 HNRNP D0 BINDS AND DESTABILIZES MRNA	49	13 (26.5)	11 (22.4)	2 (4.1)	0.006	0.0005	0.0372
REACTOME DECTIN 1 MEDIATED NONCANONICAL NF KB SIGN...	56	14 (25)	11 (19.6)	3 (5.4)	0.007	0.0005	0.0383
REACTOME ANTIGEN PROCESSING CROSS PRESENTATION	82	18 (22)	16 (19.5)	2 (2.4)	0.009	0.0007	0.044
REACTOME DEFECTIVE CFTR CAUSES CYSTIC FIBROSIS	55	14 (25.5)	12 (21.8)	2 (3.6)	0.007	0.0007	0.0472
REACTOME STING MEDIATED INDUCTION OF HOST IMMUNE R...	16	7 (43.8)	4 (25)	3 (18.8)	0.003	0.0008	0.0472

Table S114: MSigDB GO Biological Processes enriched in differentially expressed genes at Day 30 compared to pre-Second Vaccination (Neutrophils, Study Arm 2 A/H7N9+AS03, D1, 29). Results are sorted by FDR adjusted p-value and Jaccard similarity index.

Gene Set ID	Gene Set Genes #	DE Genes N (%)	Up-reg. DE Genes N (%)	Down-reg. DE Genes N (%)	Jaccard Index	P-Value	FDR Adjusted P-Value
GO DEFENSE RESPONSE	1497	260 (17.4)	176 (11.8)	84 (5.6)	0.079	<0.0001	0.001
GO REGULATION OF IMMUNE SYSTEM PROCESS	1409	233 (16.5)	152 (10.8)	81 (5.7)	0.072	<0.0001	0.001
GO RESPONSE TO BIOTIC STIMULUS	1325	223 (16.8)	169 (12.8)	54 (4.1)	0.071	<0.0001	0.001
GO REGULATION OF RESPONSE TO STRESS	1406	224 (15.9)	142 (10.1)	82 (5.8)	0.069	<0.0001	0.001
GO IMMUNE EFFECTOR PROCESS	1077	197 (18.3)	129 (12)	68 (6.3)	0.067	<0.0001	0.001
GO RESPONSE TO CYTOKINE	1099	196 (17.8)	141 (12.8)	55 (5)	0.067	<0.0001	0.001
GO DEFENSE RESPONSE TO OTHER ORGANISM	993	185 (18.6)	146 (14.7)	39 (3.9)	0.065	<0.0001	0.001

Table S114: MSigDB GO Biological Processes enriched in differentially expressed genes at Day 30 compared to pre-Second Vaccination (Neutrophils, Study Arm 2 A/H7N9+AS03, D1, 29). Results are sorted by FDR adjusted p-value and Jaccard similarity index (continued.)

Gene Set ID	Gene Set Genes #	DE Genes N (%)	Up-reg. DE Genes N (%)	Down-reg. DE Genes N (%)	Jaccard Index	P-Value	FDR Adjusted P-Value
GO SECRETION	1551	214 (13.8)	110 (7.1)	104 (6.7)	0.063	<0.0001	0.001
GO CELL ACTIVATION	1265	195 (15.4)	101 (8)	94 (7.4)	0.063	<0.0001	0.001
GO INNATE IMMUNE RESPONSE	814	168 (20.6)	135 (16.6)	33 (4.1)	0.062	<0.0001	0.001
GO REGULATION OF RESPONSE TO EXTERNAL STIMULUS	1006	168 (16.7)	108 (10.7)	60 (6)	0.058	<0.0001	0.001
GO REGULATION OF IMMUNE RESPONSE	897	161 (17.9)	116 (12.9)	45 (5)	0.058	<0.0001	0.001
GO POSITIVE REGULATION OF IMMUNE SYSTEM PROCESS	972	162 (16.7)	110 (11.3)	52 (5.3)	0.057	<0.0001	0.001
GO REGULATION OF MULTI ORGANISM PROCESS	700	139 (19.9)	105 (15)	34 (4.9)	0.053	<0.0001	0.001
GO CYTOKINE MEDIATED SIGNALING PATHWAY	711	138 (19.4)	105 (14.8)	33 (4.6)	0.053	<0.0001	0.001
GO REGULATION OF DEFENSE RESPONSE	703	134 (19.1)	94 (13.4)	40 (5.7)	0.051	<0.0001	0.001
GO CYTOKINE PRODUCTION	720	129 (17.9)	84 (11.7)	45 (6.2)	0.049	<0.0001	0.001
GO INTERSPECIES INTERACTION BETWEEN ORGANISMS	869	136 (15.7)	89 (10.2)	47 (5.4)	0.049	<0.0001	0.001
GO IMMUNE SYSTEM DEVELOPMENT	926	138 (14.9)	77 (8.3)	61 (6.6)	0.049	<0.0001	0.001
GO POSITIVE REGULATION OF IMMUNE RESPONSE	704	118 (16.8)	89 (12.6)	29 (4.1)	0.045	<0.0001	0.001
GO LEUKOCYTE MEDIATED IMMUNITY	701	109 (15.5)	55 (7.8)	54 (7.7)	0.041	<0.0001	0.001
GO REGULATION OF RESPONSE TO BIOTIC STIMULUS	466	99 (21.2)	79 (17)	20 (4.3)	0.041	<0.0001	0.001
GO MYELOID LEUKOCYTE ACTIVATION	594	102 (17.2)	50 (8.4)	52 (8.8)	0.040	<0.0001	0.001
GO POSITIVE REGULATION OF RESPONSE TO EXTERNAL STI...	575	101 (17.6)	69 (12)	32 (5.6)	0.040	<0.0001	0.001
GO RESPONSE TO VIRUS	300	90 (30)	76 (25.3)	14 (4.7)	0.040	<0.0001	0.001
GO INFLAMMATORY RESPONSE	680	104 (15.3)	55 (8.1)	49 (7.2)	0.040	<0.0001	0.001
GO CELL ACTIVATION INVOLVED IN IMMUNE RESPONSE	658	101 (15.3)	52 (7.9)	49 (7.4)	0.039	<0.0001	0.001
GO IMMUNE RESPONSE REGULATING SIGNALING PATHWAY	507	93 (18.3)	67 (13.2)	26 (5.1)	0.038	<0.0001	0.001
GO ACTIVATION OF IMMUNE RESPONSE	547	94 (17.2)	72 (13.2)	22 (4)	0.038	<0.0001	0.001
GO DEFENSE RESPONSE TO VIRUS	229	79 (34.5)	67 (29.3)	12 (5.2)	0.036	<0.0001	0.001
GO POSITIVE REGULATION OF DEFENSE RESPONSE	457	86 (18.8)	61 (13.3)	25 (5.5)	0.036	<0.0001	0.001
GO POSITIVE REGULATION OF MULTI ORGANISM PROCESS	489	87 (17.8)	61 (12.5)	26 (5.3)	0.036	<0.0001	0.001
GO MYELOID LEUKOCYTE MEDIATED IMMUNITY	506	83 (16.4)	41 (8.1)	42 (8.3)	0.034	<0.0001	0.001
GO REGULATION OF CELL ACTIVATION	507	82 (16.2)	49 (9.7)	33 (6.5)	0.033	<0.0001	0.001
GO POSITIVE REGULATION OF CYTOKINE PRODUCTION	419	75 (17.9)	49 (11.7)	26 (6.2)	0.031	<0.0001	0.001
GO NEGATIVE REGULATION OF IMMUNE SYSTEM PROCESS	426	72 (16.9)	46 (10.8)	26 (6.1)	0.030	<0.0001	0.001
GO REGULATION OF IMMUNE EFFECTOR PROCESS	357	70 (19.6)	47 (13.2)	23 (6.4)	0.030	<0.0001	0.001
GO POSITIVE REGULATION OF RESPONSE TO BIOTIC STIMU...	353	69 (19.5)	52 (14.7)	17 (4.8)	0.030	<0.0001	0.001
GO VIRAL LIFE CYCLE	308	64 (20.8)	47 (15.3)	17 (5.5)	0.028	<0.0001	0.001
GO ADAPTIVE IMMUNE RESPONSE	353	64 (18.1)	45 (12.7)	19 (5.4)	0.027	<0.0001	0.001
GO ACTIVATION OF INNATE IMMUNE RESPONSE	286	57 (19.9)	44 (15.4)	13 (4.5)	0.025	<0.0001	0.001
GO NEGATIVE REGULATION OF MULTI ORGANISM PROCESS	195	54 (27.7)	46 (23.6)	8 (4.1)	0.025	<0.0001	0.001
GO RESPONSE TO INTERFERON GAMMA	166	52 (31.3)	48 (28.9)	4 (2.4)	0.024	<0.0001	0.001
GO I KAPPAB KINASE NF KAPPAB SIGNALING	252	53 (21)	40 (15.9)	13 (5.2)	0.024	<0.0001	0.001

Table S114: MSigDB GO Biological Processes enriched in differentially expressed genes at Day 30 compared to pre-Second Vaccination (Neutrophils, Study Arm 2 A/H7N9+AS03, D1, 29). Results are sorted by FDR adjusted p-value and Jaccard similarity index (continued.)

Gene Set ID	Gene Set Genes #	DE Genes N (%)	Up-reg. DE Genes N (%)	Down-reg. DE Genes N (%)	Jaccard Index	P-Value	FDR Adjusted P-Value
GO REGULATION OF SYMBIOSIS ENCOMPASSING MUTUALISM ...	204	51 (25)	39 (19.1)	12 (5.9)	0.023	<0.0001	0.001
GO NEGATIVE REGULATION OF CYTOKINE PRODUCTION	262	50 (19.1)	31 (11.8)	19 (7.3)	0.022	<0.0001	0.001
GO RESPONSE TO TUMOR NECROSIS FACTOR	272	50 (18.4)	37 (13.6)	13 (4.8)	0.022	<0.0001	0.001
GO REGULATION OF VIRAL LIFE CYCLE	133	41 (30.8)	36 (27.1)	5 (3.8)	0.019	<0.0001	0.001
GO RESPONSE TO TYPE I INTERFERON	88	37 (42)	36 (40.9)	1 (1.1)	0.018	<0.0001	0.001
GO CARBOHYDRATE DERIVATIVE CATABOLIC PROCESS	174	38 (21.8)	18 (10.3)	20 (11.5)	0.017	<0.0001	0.001
GO REGULATION OF RESPONSE TO CYTOKINE STIMULUS	165	37 (22.4)	32 (19.4)	5 (3)	0.017	<0.0001	0.001
GO NEGATIVE REGULATION OF DEFENSE RESPONSE	201	37 (18.4)	22 (10.9)	15 (7.5)	0.017	<0.0001	0.001
GO TYPE I INTERFERON PRODUCTION	116	35 (30.2)	26 (22.4)	9 (7.8)	0.017	<0.0001	0.001
GO NEGATIVE REGULATION OF VIRAL PROCESS	90	33 (36.7)	30 (33.3)	3 (3.3)	0.016	<0.0001	0.001
GO VIRAL GENOME REPLICATION	117	33 (28.2)	30 (25.6)	3 (2.6)	0.015	<0.0001	0.001
GO INTERFERON GAMMA MEDIATED SIGNALING PATHWAY	70	32 (45.7)	30 (42.9)	2 (2.9)	0.015	<0.0001	0.001
GO REGULATION OF VIRAL GENOME REPLICATION	91	32 (35.2)	29 (31.9)	3 (3.3)	0.015	<0.0001	0.001
GO NEGATIVE REGULATION OF IMMUNE RESPONSE	134	32 (23.9)	21 (15.7)	11 (8.2)	0.015	<0.0001	0.001
GO REGULATION OF ADAPTIVE IMMUNE RESPONSE	142	32 (22.5)	18 (12.7)	14 (9.9)	0.015	<0.0001	0.001
GO TUMOR NECROSIS FACTOR MEDIATED SIGNALING PATHWA...	147	32 (21.8)	27 (18.4)	5 (3.4)	0.015	<0.0001	0.001
GO NEGATIVE REGULATION OF VIRAL LIFE CYCLE	76	30 (39.5)	28 (36.8)	2 (2.6)	0.014	<0.0001	0.001
GO REGULATION OF DEFENSE RESPONSE TO VIRUS	68	26 (38.2)	22 (32.4)	4 (5.9)	0.013	<0.0001	0.001
GO NEGATIVE REGULATION OF VIRAL GENOME REPLICATION	58	25 (43.1)	25 (43.1)	0 (0)	0.012	<0.0001	0.001
GO ANTIGEN PROCESSING AND PRESENTATION OF PEPTIDE ...	80	24 (30)	22 (27.5)	2 (2.5)	0.011	<0.0001	0.001
GO INTERLEUKIN 1 PRODUCTION	99	24 (24.2)	18 (18.2)	6 (6.1)	0.011	<0.0001	0.001
GO INTERLEUKIN 1 BETA PRODUCTION	87	22 (25.3)	16 (18.4)	6 (6.9)	0.010	<0.0001	0.001
GO ANTIGEN PROCESSING AND PRESENTATION OF EXOGENOU...	62	20 (32.3)	18 (29)	2 (3.2)	0.010	<0.0001	0.001
GO POSITIVE REGULATION OF RESPONSE TO CYTOKINE STI...	53	17 (32.1)	15 (28.3)	2 (3.8)	0.008	<0.0001	0.001
GO REGULATION OF DEFENSE RESPONSE TO VIRUS BY HOST	34	16 (47.1)	14 (41.2)	2 (5.9)	0.008	<0.0001	0.001
GO NEGATIVE REGULATION OF INNATE IMMUNE RESPONSE	49	16 (32.7)	13 (26.5)	3 (6.1)	0.008	<0.0001	0.001
GO RESPONSE TO INTERFERON BETA	30	15 (50)	13 (43.3)	2 (6.7)	0.007	<0.0001	0.001
GO INTERLEUKIN 1 SECRETION	53	15 (28.3)	12 (22.6)	3 (5.7)	0.007	<0.0001	0.001
GO POSITIVE REGULATION OF DEFENSE RESPONSE TO VIRU...	24	14 (58.3)	13 (54.2)	1 (4.2)	0.007	<0.0001	0.001
GO NEGATIVE REGULATION OF INTERLEUKIN 1 PRODUCTION	34	12 (35.3)	7 (20.6)	5 (14.7)	0.006	<0.0001	0.001
GO EXOCYTOSIS	833	128 (15.4)	64 (7.7)	64 (7.7)	0.047	<0.0001	0.0017
GO REGULATION OF HEMOPOIESIS	441	74 (16.8)	48 (10.9)	26 (5.9)	0.031	<0.0001	0.0017
GO REGULATION OF DNA BINDING TRANSCRIPTION FACTOR ...	393	67 (17)	37 (9.4)	30 (7.6)	0.028	<0.0001	0.0017
GO REGULATION OF INFLAMMATORY RESPONSE	332	53 (16)	30 (9)	23 (6.9)	0.023	<0.0001	0.0017
GO ADAPTIVE IMMUNE RESPONSE BASED ON SOMATIC RECOM...	223	42 (18.8)	27 (12.1)	15 (6.7)	0.019	<0.0001	0.0017
GO POSITIVE REGULATION OF I KAPPAB KINASE NF KAPPA...	169	36 (21.3)	29 (17.2)	7 (4.1)	0.017	<0.0001	0.0017
GO RESPONSE TO INTERLEUKIN 1	181	36 (19.9)	27 (14.9)	9 (5)	0.016	<0.0001	0.0017

Table S114: MSigDB GO Biological Processes enriched in differentially expressed genes at Day 30 compared to pre-Second Vaccination (Neutrophils, Study Arm 2 A/H7N9+AS03, D1, 29). Results are sorted by FDR adjusted p-value and Jaccard similarity index (continued.)

Gene Set ID	Gene Set Genes #	DE Genes N (%)	Up-reg. DE Genes N (%)	Down-reg. DE Genes N (%)	Jaccard Index	P-Value	FDR Adjusted P-Value
GO AMINE METABOLIC PROCESS	122	29 (23.8)	14 (11.5)	15 (12.3)	0.014	<0.0001	0.0017
GO NEGATIVE REGULATION OF RESPONSE TO BIOTIC STIMU...	82	21 (25.6)	17 (20.7)	4 (4.9)	0.010	<0.0001	0.0017
GO NEGATIVE REGULATION OF TYPE I INTERFERON PRODUC...	43	16 (37.2)	13 (30.2)	3 (7)	0.008	<0.0001	0.0017
GO CELLULAR RESPONSE TO VIRUS	44	15 (34.1)	12 (27.3)	3 (6.8)	0.007	<0.0001	0.0017
GO REGULATION OF INTERLEUKIN 1 SECRETION	45	14 (31.1)	11 (24.4)	3 (6.7)	0.007	<0.0001	0.0017
GO NEGATIVE REGULATION OF INTERLEUKIN 1 SECRETION	16	8 (50)	6 (37.5)	2 (12.5)	0.004	<0.0001	0.0017
GO PROTEASOMAL UBIQUITIN INDEPENDENT PROTEIN CATAB...	18	8 (44.4)	7 (38.9)	1 (5.6)	0.004	<0.0001	0.0017
GO NEGATIVE REGULATION OF INTERLEUKIN 1 BETA SECRE...	13	7 (53.8)	6 (46.2)	1 (7.7)	0.003	<0.0001	0.0017
GO CYTOKINE SECRETION	220	41 (18.6)	30 (13.6)	11 (5)	0.018	<0.0001	0.0025
GO NEGATIVE REGULATION OF MOLECULAR FUNCTION	1052	140 (13.3)	72 (6.8)	68 (6.5)	0.047	<0.0001	0.0033
GO NIK NF KAPPAB SIGNALING	153	32 (20.9)	24 (15.7)	8 (5.2)	0.015	<0.0001	0.0033
GO PRODUCTION OF MOLECULAR MEDIATOR OF IMMUNE RESP...	187	36 (19.3)	20 (10.7)	16 (8.6)	0.016	<0.0001	0.004
GO SMALL MOLECULE METABOLIC PROCESS	1844	237 (12.9)	85 (4.6)	152 (8.2)	0.065	<0.0001	0.0047
GO RESPONSE TO BACTERIUM	587	76 (12.9)	54 (9.2)	22 (3.7)	0.030	<0.0001	0.0047
GO SMALL MOLECULE CATABOLIC PROCESS	420	68 (16.2)	19 (4.5)	49 (11.7)	0.028	<0.0001	0.0047
GO ORGANOPHOSPHATE CATABOLIC PROCESS	124	28 (22.6)	7 (5.6)	21 (16.9)	0.013	<0.0001	0.0047
GO IMMUNE RESPONSE REGULATING CELL SURFACE RECEPTO...	348	61 (17.5)	42 (12.1)	19 (5.5)	0.026	<0.0001	0.0053
GO POSITIVE REGULATION OF IMMUNE EFFECTOR PROCESS	189	35 (18.5)	22 (11.6)	13 (6.9)	0.016	<0.0001	0.0053
GO CD4 POSITIVE ALPHA BETA T CELL ACTIVATION	87	21 (24.1)	13 (14.9)	8 (9.2)	0.010	<0.0001	0.0053
GO LEUKOCYTE DIFFERENTIATION	483	75 (15.5)	42 (8.7)	33 (6.8)	0.031	<0.0001	0.0059
GO CYTOPLASMIC PATTERN RECOGNITION RECEPTOR SIGNAL...	54	17 (31.5)	12 (22.2)	5 (9.3)	0.008	<0.0001	0.0059
GO T CELL RECEPTOR SIGNALING PATHWAY	168	35 (20.8)	26 (15.5)	9 (5.4)	0.016	<0.0001	0.0066
GO LEUKOCYTE PROLIFERATION	276	45 (16.3)	23 (8.3)	22 (8)	0.020	<0.0001	0.007
GO PATTERN RECOGNITION RECEPTOR SIGNALING PATHWAY	181	36 (19.9)	27 (14.9)	9 (5)	0.016	<0.0001	0.007
GO REGULATION OF LEUKOCYTE PROLIFERATION	203	36 (17.7)	19 (9.4)	17 (8.4)	0.016	<0.0001	0.007
GO RESPONSE TO MURAMYL DIPEPTIDE	19	9 (47.4)	7 (36.8)	2 (10.5)	0.004	<0.0001	0.007
GO LYMPHOCYTE ACTIVATION	620	90 (14.5)	56 (9)	34 (5.5)	0.035	0.0001	0.0077
GO SMALL MOLECULE BIOSYNTHETIC PROCESS	674	98 (14.5)	27 (4)	71 (10.5)	0.037	0.0001	0.0081
GO POSITIVE REGULATION OF CYTOKINE SECRETION	126	24 (19)	15 (11.9)	9 (7.1)	0.011	0.0001	0.0081
GO NEGATIVE REGULATION OF ACTIVATED T CELL PROLIFE...	10	6 (60)	4 (40)	2 (20)	0.003	0.0001	0.0081
GO GLUTATHIONE DERIVATIVE METABOLIC PROCESS	21	8 (38.1)	3 (14.3)	5 (23.8)	0.004	0.0001	0.0087
GO NEGATIVE REGULATION OF CELL CELL ADHESION	167	32 (19.2)	22 (13.2)	10 (6)	0.015	0.0001	0.0092
GO RESPONSE TO INTERFERON ALPHA	21	9 (42.9)	9 (42.9)	0 (0)	0.004	0.0001	0.0092
GO REGULATION OF PEPTIDASE ACTIVITY	409	59 (14.4)	36 (8.8)	23 (5.6)	0.025	0.0001	0.0097
GO ENTRY INTO HOST	126	27 (21.4)	15 (11.9)	12 (9.5)	0.013	0.0001	0.0097
GO CELLULAR NITROGEN COMPOUND CATABOLIC PROCESS	539	80 (14.8)	48 (8.9)	32 (5.9)	0.032	0.0002	0.0102
GO NEGATIVE REGULATION OF LEUKOCYTE CELL CELL ADHE...	117	24 (20.5)	19 (16.2)	5 (4.3)	0.011	0.0002	0.0102

Table S114: MSigDB GO Biological Processes enriched in differentially expressed genes at Day 30 compared to pre-Second Vaccination (Neutrophils, Study Arm 2 A/H7N9+AS03, D1, 29). Results are sorted by FDR adjusted p-value and Jaccard similarity index (continued.)

Gene Set ID	Gene Set Genes #	DE Genes N (%)	Up-reg. DE Genes N (%)	Down-reg. DE Genes N (%)	Jaccard Index	P-Value	FDR Adjusted P-Value
GO NEGATIVE REGULATION OF RESPONSE TO STIMULUS	1531	198 (12.9)	97 (6.3)	101 (6.6)	0.059	0.0002	0.0107
GO CYTOKINE PRODUCTION INVOLVED IN IMMUNE RESPONSE	94	21 (22.3)	14 (14.9)	7 (7.4)	0.010	0.0002	0.0107
GO NEGATIVE REGULATION OF PHOSPHORYLATION	431	68 (15.8)	30 (7)	38 (8.8)	0.028	0.0002	0.0111
GO ANTIGEN PROCESSING AND PRESENTATION	189	36 (19)	28 (14.8)	8 (4.2)	0.016	0.0002	0.0111
GO LYMPHOCYTE DIFFERENTIATION	336	54 (16.1)	32 (9.5)	22 (6.5)	0.023	0.0002	0.0116
GO T CELL ACTIVATION	436	66 (15.1)	44 (10.1)	22 (5)	0.027	0.0002	0.012
GO REGULATION OF T CELL ACTIVATION	292	48 (16.4)	34 (11.6)	14 (4.8)	0.021	0.0002	0.012
GO REGULATION OF PEPTIDE SECRETION	461	67 (14.5)	39 (8.5)	28 (6.1)	0.028	0.0002	0.0125
GO REGULATION OF CYSTEINE TYPE ENDOPEPTIDASE ACTIV...	221	39 (17.6)	25 (11.3)	14 (6.3)	0.018	0.0002	0.0125
GO NEGATIVE REGULATION OF DNA BINDING TRANSCRIPTIO...	152	29 (19.1)	14 (9.2)	15 (9.9)	0.013	0.0002	0.0125
GO POSITIVE REGULATION OF INFLAMMATORY RESPONSE	134	25 (18.7)	16 (11.9)	9 (6.7)	0.012	0.0002	0.0125
GO INTERFERON BETA PRODUCTION	44	14 (31.8)	11 (25)	3 (6.8)	0.007	0.0002	0.0125
GO REGULATION OF VIRAL ENTRY INTO HOST CELL	26	9 (34.6)	8 (30.8)	1 (3.8)	0.004	0.0002	0.0125
GO CELLULAR RESPONSE TO INTERFERON BETA	21	8 (38.1)	6 (28.6)	2 (9.5)	0.004	0.0002	0.0125
GO POSITIVE REGULATION OF TYPE I INTERFERON PRODUC...	68	18 (26.5)	13 (19.1)	5 (7.4)	0.009	0.0002	0.0129
GO REGULATION OF CYTOKINE PRODUCTION INVOLVED IN I...	76	18 (23.7)	12 (15.8)	6 (7.9)	0.009	0.0002	0.0129
GO LEUKOCYTE CELL CELL ADHESION	307	50 (16.3)	33 (10.7)	17 (5.5)	0.022	0.0002	0.0133
GO INTERLEUKIN 1 MEDIATED SIGNALING PATHWAY	91	21 (23.1)	17 (18.7)	4 (4.4)	0.010	0.0002	0.0133
GO APOPTOTIC SIGNALING PATHWAY	566	82 (14.5)	44 (7.8)	38 (6.7)	0.032	0.0002	0.0136
GO REGULATION OF CD4 POSITIVE ALPHA BETA T CELL AC...	57	15 (26.3)	11 (19.3)	4 (7)	0.007	0.0002	0.0136
GO POSITIVE REGULATION OF AUTOPHAGY	113	26 (23)	16 (14.2)	10 (8.8)	0.012	0.0003	0.0146
GO INTERFERON GAMMA PRODUCTION	97	20 (20.6)	12 (12.4)	8 (8.2)	0.009	0.0003	0.0151
GO T CELL DIFFERENTIATION	230	40 (17.4)	27 (11.7)	13 (5.7)	0.018	0.0003	0.016
GO REGULATION OF LEUKOCYTE MEDIATED IMMUNITY	174	32 (18.4)	16 (9.2)	16 (9.2)	0.015	0.0003	0.0164
GO REGULATION OF LYMPHOCYTE ACTIVATION	392	60 (15.3)	38 (9.7)	22 (5.6)	0.025	0.0003	0.0168
GO NEGATIVE REGULATION OF RESPONSE TO EXTERNAL STI...	350	53 (15.1)	27 (7.7)	26 (7.4)	0.023	0.0003	0.0178
GO HOMEOSTASIS OF NUMBER OF CELLS	226	41 (18.1)	19 (8.4)	22 (9.7)	0.018	0.0003	0.0182
GO CELLULAR RESPONSE TO TOXIC SUBSTANCE	227	37 (16.3)	17 (7.5)	20 (8.8)	0.017	0.0004	0.0184
GO NUCLEOBASE CONTAINING SMALL MOLECULE BIOSYNTHET...	98	21 (21.4)	7 (7.1)	14 (14.3)	0.010	0.0004	0.0184
GO T CELL ACTIVATION INVOLVED IN IMMUNE RESPONSE	102	20 (19.6)	15 (14.7)	5 (4.9)	0.009	0.0004	0.0198
GO ANTIGEN RECEPTOR MEDIATED SIGNALING PATHWAY	212	39 (18.4)	29 (13.7)	10 (4.7)	0.018	0.0004	0.0202
GO ORGANOPHOSPHATE METABOLIC PROCESS	966	132 (13.7)	44 (4.6)	88 (9.1)	0.046	0.0004	0.0204
GO PURINE CONTAINING COMPOUND CATABOLIC PROCESS	46	13 (28.3)	5 (10.9)	8 (17.4)	0.006	0.0004	0.0204
GO REGULATION OF T CELL DIFFERENTIATION	131	26 (19.8)	22 (16.8)	4 (3.1)	0.012	0.0004	0.021
GO NEGATIVE REGULATION OF IMMUNE EFFECTOR PROCESS	106	22 (20.8)	14 (13.2)	8 (7.5)	0.010	0.0004	0.021
GO T CELL DIFFERENTIATION INVOLVED IN IMMUNE RESPO...	66	16 (24.2)	12 (18.2)	4 (6.1)	0.008	0.0004	0.021
GO ORGANIC CYCLIC COMPOUND CATABOLIC PROCESS	584	83 (14.2)	48 (8.2)	35 (6)	0.033	0.0005	0.0223

Table S114: MSigDB GO Biological Processes enriched in differentially expressed genes at Day 30 compared to pre-Second Vaccination (Neutrophils, Study Arm 2 A/H7N9+AS03, D1, 29). Results are sorted by FDR adjusted p-value and Jaccard similarity index (continued.)

Gene Set ID	Gene Set Genes #	DE Genes N (%)	Up-reg. DE Genes N (%)	Down-reg. DE Genes N (%)	Jaccard Index	P-Value	FDR Adjusted P-Value
GO REGULATORY T CELL DIFFERENTIATION	28	9 (32.1)	8 (28.6)	1 (3.6)	0.004	0.0005	0.0232
GO POSITIVE REGULATION OF SIGNALING	1730	217 (12.5)	119 (6.9)	98 (5.7)	0.061	0.0005	0.0245
GO PEPTIDE SECRETION	587	81 (13.8)	48 (8.2)	33 (5.6)	0.032	0.0005	0.0248
GO POSITIVE REGULATION OF CELL ACTIVATION	297	47 (15.8)	29 (9.8)	18 (6.1)	0.021	0.0005	0.0251
GO INTERACTION WITH HOST	192	35 (18.2)	21 (10.9)	14 (7.3)	0.016	0.0005	0.0254
GO PROTEIN POLYUBIQUITINATION	295	52 (17.6)	35 (11.9)	17 (5.8)	0.023	0.0006	0.0281
GO POSITIVE REGULATION OF ADAPTIVE IMMUNE RESPONSE	92	19 (20.7)	11 (12)	8 (8.7)	0.009	0.0006	0.0283
GO NEGATIVE REGULATION OF CELL ACTIVATION	184	32 (17.4)	20 (10.9)	12 (6.5)	0.015	0.0006	0.029
GO PYRIMIDINE NUCLEOSIDE CATABOLIC PROCESS	23	7 (30.4)	7 (30.4)	0 (0)	0.003	0.0006	0.029
GO POSITIVE REGULATION OF TYPE I INTERFERON MEDIAT...	12	6 (50)	5 (41.7)	1 (8.3)	0.003	0.0006	0.029
GO CELLULAR RESPONSE TO MURAMYL DIPEPTIDE	8	5 (62.5)	4 (50)	1 (12.5)	0.002	0.0006	0.029
GO RESPONSE TO MOLECULE OF BACTERIAL ORIGIN	329	47 (14.3)	28 (8.5)	19 (5.8)	0.020	0.0007	0.0302
GO REGULATION OF MACROPHAGE MIGRATION	34	10 (29.4)	5 (14.7)	5 (14.7)	0.005	0.0007	0.0327
GO PROTEOLYSIS	1640	201 (12.3)	107 (6.5)	94 (5.7)	0.058	0.0008	0.0343
GO T CELL MEDIATED IMMUNITY	84	18 (21.4)	10 (11.9)	8 (9.5)	0.009	0.0008	0.035
GO PROTEIN COMPLEX OLIGOMERIZATION	470	69 (14.7)	35 (7.4)	34 (7.2)	0.028	0.0008	0.0361
GO ACTIVATION OF CYSTEINE TYPE ENDOPEPTIDASE ACTIV...	14	6 (42.9)	3 (21.4)	3 (21.4)	0.003	0.0008	0.0372
GO ORGANONITROGEN COMPOUND CATABOLIC PROCESS	1200	159 (13.2)	73 (6.1)	86 (7.2)	0.051	0.0009	0.0392
GO REGULATION OF ESTABLISHMENT OF PROTEIN LOCALIZA...	698	96 (13.8)	49 (7)	47 (6.7)	0.036	0.0009	0.0392
GO POSITIVE REGULATION OF LEUKOCYTE PROLIFERATION	124	23 (18.5)	13 (10.5)	10 (8.1)	0.011	0.0009	0.0392
GO POSITIVE REGULATION OF DNA BINDING TRANSCRIPTIO...	238	41 (17.2)	27 (11.3)	14 (5.9)	0.018	0.001	0.0411
GO REGULATION OF EXTRINSIC APOPTOTIC SIGNALING PAT...	56	14 (25)	9 (16.1)	5 (8.9)	0.007	0.001	0.0413
GO POSITIVE REGULATION OF MACROPHAGE MIGRATION	20	7 (35)	4 (20)	3 (15)	0.003	0.0011	0.0448
GO TYPE 2 IMMUNE RESPONSE	38	10 (26.3)	6 (15.8)	4 (10.5)	0.005	0.0011	0.0479
GO ANTIGEN PROCESSING AND PRESENTATION OF PEPTIDE ...	155	29 (18.7)	23 (14.8)	6 (3.9)	0.013	0.0012	0.0488
GO CD4 POSITIVE ALPHA BETA T CELL DIFFERENTIATION	71	16 (22.5)	12 (16.9)	4 (5.6)	0.008	0.0012	0.0488
GO POSITIVE REGULATION OF CYTOKINE PRODUCTION INVO...	49	13 (26.5)	11 (22.4)	2 (4.1)	0.006	0.0012	0.0488
GO REGULATION OF TYPE 2 IMMUNE RESPONSE	31	9 (29)	5 (16.1)	4 (12.9)	0.004	0.0012	0.0494
GO T CELL APOPTOTIC PROCESS	46	11 (23.9)	6 (13)	5 (10.9)	0.005	0.0012	0.0499

Table S115: Blood Transcription Modules enriched in differentially expressed genes at Day 30 compared to pre-Second Vaccination (Neutrophils, Study Arm 2 A/H7N9+AS03, D1, 29). Results are sorted by FDR adjusted p-value and Jaccard similarity index.

Gene Set ID	Gene Set Genes #	DE Genes N (%)	Up-reg. DE Genes N (%)	Down-reg. DE Genes N (%)	Jaccard Index	P-Value	FDR Adjusted P-Value
M75 ANTIVIRAL IFN SIGNATURE	22	14 (63.6)	13 (59.1)	1 (4.5)	0.007	<0.0001	0.0006
M127 TYPE I INTERFERON RESPONSE	12	11 (91.7)	11 (91.7)	0 (0)	0.005	<0.0001	0.0006
M150 INNATE ANTIVIRAL RESPONSE	12	10 (83.3)	10 (83.3)	0 (0)	0.005	<0.0001	0.0006
M111.1 VIRAL SENSING AND IMMUNITY; IRF2 TARGETS NE...	11	9 (81.8)	9 (81.8)	0 (0)	0.004	<0.0001	0.0006
M67 ACTIVATED DENDRITIC CELLS	12	8 (66.7)	8 (66.7)	0 (0)	0.004	<0.0001	0.0006
M68 RIG-1 LIKE RECEPTOR SIGNALING	9	7 (77.8)	7 (77.8)	0 (0)	0.003	<0.0001	0.0006
M165 ENRICHED IN ACTIVATED DENDRITIC CELLS (II)	34	14 (41.2)	14 (41.2)	0 (0)	0.007	<0.0001	0.0015
M111.0 VIRAL SENSING AND IMMUNITY; IRF2 TARGETS NE...	16	8 (50)	7 (43.8)	1 (6.2)	0.004	0.0001	0.0048
M95.0 ENRICHED IN ANTIGEN PRESENTATION (II)	20	9 (45)	9 (45)	0 (0)	0.004	0.0003	0.0128
M95.1 ENRICHED IN ANTIGEN PRESENTATION (III)	12	6 (50)	6 (50)	0 (0)	0.003	0.0004	0.0128
M86.0 CHEMOKINES AND INFLAMMATORY MOLECULES IN MYE...	18	7 (38.9)	7 (38.9)	0 (0)	0.003	0.0005	0.0173
M53 INFLAMMASOME RECEPTORS AND SIGNALING	12	7 (58.3)	5 (41.7)	2 (16.7)	0.003	0.0006	0.0173
M13 INNATE ACTIVATION BY CYTOSOLIC DNA SENSING	11	5 (45.5)	5 (45.5)	0 (0)	0.002	0.0007	0.0189
M37.0 IMMUNE ACTIVATION - GENERIC CLUSTER	301	55 (18.3)	24 (8)	31 (10.3)	0.024	0.0012	0.0289
M11.0 ENRICHED IN MONOCYTES (II)	174	35 (20.1)	19 (10.9)	16 (9.2)	0.016	0.002	0.0452

Table S116: MSigDB Reactome Pathways enriched in differentially expressed genes at Day 2 compared to pre-First Vaccination (NK cells, Study Arm 2 A/H7N9+AS03, D1, 29). Results are sorted by FDR adjusted p-value and Jaccard similarity index.

Gene Set ID	Gene Set Genes #	DE Genes N (%)	Up-reg. DE Genes N (%)	Down-reg. DE Genes N (%)	Jaccard Index	P-Value	FDR Adjusted P-Value
REACTOME INTERFERON ALPHA BETA SIGNALING	60	6 (10)	6 (10)	0 (0)	0.053	<0.0001	0.0038
REACTOME INTERFERON GAMMA SIGNALING	71	6 (8.5)	6 (8.5)	0 (0)	0.048	<0.0001	0.0038
REACTOME INTERFERON SIGNALING	177	9 (5.1)	9 (5.1)	0 (0)	0.040	<0.0001	0.0038
REACTOME CYTOKINE SIGNALING IN IMMUNE SYSTEM	788	12 (1.5)	12 (1.5)	0 (0)	0.014	<0.0001	0.0038
REACTOME METAL SEQUESTRATION BY ANTIMICROBIAL PROT...	6	2 (33.3)	2 (33.3)	0 (0)	0.031	<0.0001	0.0276

Table S117: MSigDB GO Biological Processes enriched in differentially expressed genes at Day 2 compared to pre-First Vaccination (NK cells, Study Arm 2 A/H7N9+AS03, D1, 29). Results are sorted by FDR adjusted p-value and Jaccard similarity index.

Gene Set ID	Gene Set Genes #	DE Genes N (%)	Up-reg. DE Genes N (%)	Down-reg. DE Genes N (%)	Jaccard Index	P-Value	FDR Adjusted P-Value
GO INTERFERON GAMMA MEDIATED SIGNALING PATHWAY	70	6 (8.6)	6 (8.6)	0 (0)	0.048	<0.0001	0.0042

Table S117: MSigDB GO Biological Processes enriched in differentially expressed genes at Day 2 compared to pre-First Vaccination (NK cells, Study Arm 2 A/H7N9+AS03, D1, 29). Results are sorted by FDR adjusted p-value and Jaccard similarity index (continued.)

Gene Set ID	Gene Set Genes #	DE Genes N (%)	Up-reg. DE Genes N (%)	Down-reg. DE Genes N (%)	Jaccard Index	P-Value	FDR Adjusted P-Value
GO LEUKOCYTE MIGRATION INVOLVED IN INFLAMMATORY RE...	14	3 (21.4)	3 (21.4)	0 (0)	0.042	<0.0001	0.0042
GO RESPONSE TO TYPE I INTERFERON	88	6 (6.8)	6 (6.8)	0 (0)	0.042	<0.0001	0.0042
GO DEFENSE RESPONSE TO VIRUS	229	11 (4.8)	11 (4.8)	0 (0)	0.040	<0.0001	0.0042
GO RESPONSE TO VIRUS	300	12 (4)	12 (4)	0 (0)	0.035	<0.0001	0.0042
GO RESPONSE TO INTERFERON GAMMA	166	7 (4.2)	7 (4.2)	0 (0)	0.032	<0.0001	0.0042
GO LEUKOCYTE CHEMOTAXIS	202	7 (3.5)	7 (3.5)	0 (0)	0.028	<0.0001	0.0042
GO CELL CHEMOTAXIS	278	8 (2.9)	7 (2.5)	1 (0.4)	0.024	<0.0001	0.0042
GO INNATE IMMUNE RESPONSE	814	18 (2.2)	18 (2.2)	0 (0)	0.021	<0.0001	0.0042
GO DEFENSE RESPONSE TO OTHER ORGANISM	993	20 (2)	20 (2)	0 (0)	0.019	<0.0001	0.0042
GO TAXIS	607	12 (2)	11 (1.8)	1 (0.2)	0.018	<0.0001	0.0042
GO IMMUNE EFFECTOR PROCESS	1077	19 (1.8)	18 (1.7)	1 (0.1)	0.017	<0.0001	0.0042
GO RESPONSE TO BIOTIC STIMULUS	1325	23 (1.7)	23 (1.7)	0 (0)	0.017	<0.0001	0.0042
GO REGULATION OF DEFENSE RESPONSE	703	12 (1.7)	11 (1.6)	1 (0.1)	0.016	<0.0001	0.0042
GO REGULATION OF MULTI ORGANISM PROCESS	700	12 (1.7)	12 (1.7)	0 (0)	0.016	<0.0001	0.0042
GO DEFENSE RESPONSE	1497	24 (1.6)	22 (1.5)	2 (0.1)	0.016	<0.0001	0.0042
GO RESPONSE TO CYTOKINE	1099	16 (1.5)	14 (1.3)	2 (0.2)	0.014	<0.0001	0.0042
GO POSITIVE REGULATION OF IMMUNE SYSTEM PROCESS	972	14 (1.4)	13 (1.3)	1 (0.1)	0.014	<0.0001	0.0042
GO POSITIVE REGULATION OF RESPONSE TO EXTERNAL STI...	575	11 (1.9)	11 (1.9)	0 (0)	0.018	<0.0001	0.0065
GO CYTOKINE MEDIATED SIGNALING PATHWAY	711	12 (1.7)	11 (1.5)	1 (0.1)	0.016	<0.0001	0.0065
GO INFLAMMATORY RESPONSE	680	11 (1.6)	9 (1.3)	2 (0.3)	0.015	<0.0001	0.0065
GO REGULATION OF RESPONSE TO EXTERNAL STIMULUS	1006	14 (1.4)	13 (1.3)	1 (0.1)	0.013	<0.0001	0.0065
GO CHEMICAL HOMEOSTASIS	1123	15 (1.3)	14 (1.2)	1 (0.1)	0.013	<0.0001	0.0065
GO COPPER ION TRANSPORT	15	3 (20)	3 (20)	0 (0)	0.042	<0.0001	0.0087
GO DEFENSE RESPONSE TO BACTERIUM	237	6 (2.5)	6 (2.5)	0 (0)	0.021	<0.0001	0.0087
GO REGULATION OF IMMUNE RESPONSE	897	13 (1.4)	12 (1.3)	1 (0.1)	0.014	<0.0001	0.0087
GO RESPONSE TO INTERFERON ALPHA	21	3 (14.3)	3 (14.3)	0 (0)	0.038	<0.0001	0.01
GO GRANULOCYTE MIGRATION	126	5 (4)	5 (4)	0 (0)	0.028	<0.0001	0.01
GO POSITIVE REGULATION OF DEFENSE RESPONSE	457	9 (2)	9 (2)	0 (0)	0.018	<0.0001	0.01
GO ION HOMEOSTASIS	772	12 (1.6)	11 (1.4)	1 (0.1)	0.015	<0.0001	0.01
GO MYELOID LEUKOCYTE MIGRATION	187	6 (3.2)	6 (3.2)	0 (0)	0.025	<0.0001	0.0121
GO MONONUCLEAR CELL MIGRATION	74	4 (5.4)	4 (5.4)	0 (0)	0.031	<0.0001	0.0141
GO TRANSITION METAL ION HOMEOSTASIS	121	5 (4.1)	5 (4.1)	0 (0)	0.028	<0.0001	0.016
GO REGULATION OF IMMUNE SYSTEM PROCESS	1409	15 (1.1)	14 (1)	1 (0.1)	0.010	<0.0001	0.0177
GO METAL ION HOMEOSTASIS	622	10 (1.6)	9 (1.4)	1 (0.2)	0.015	<0.0001	0.0194
GO DEFENSE RESPONSE TO FUNGUS	35	3 (8.6)	3 (8.6)	0 (0)	0.033	<0.0001	0.0203
GO ACTIVATION OF IMMUNE RESPONSE	547	9 (1.6)	9 (1.6)	0 (0)	0.015	<0.0001	0.0203
GO REGULATION OF RESPONSE TO BIOTIC STIMULUS	466	9 (1.9)	9 (1.9)	0 (0)	0.017	0.0001	0.0212

Table S117: MSigDB GO Biological Processes enriched in differentially expressed genes at Day 2 compared to pre-First Vaccination (NK cells, Study Arm 2 A/H7N9+AS03, D1, 29). Results are sorted by FDR adjusted p-value and Jaccard similarity index (continued.)

Gene Set ID	Gene Set Genes #	DE Genes N (%)	Up-reg. DE Genes N (%)	Down-reg. DE Genes N (%)	Jaccard Index	P-Value	FDR Adjusted P-Value
GO RESPONSE TO BACTERIUM	587	9 (1.5)	9 (1.5)	0 (0)	0.014	0.0001	0.0212
GO ANTIMICROBIAL HUMORAL RESPONSE	108	4 (3.7)	4 (3.7)	0 (0)	0.024	0.0001	0.0239
GO REGULATION OF INFLAMMATORY RESPONSE	332	7 (2.1)	6 (1.8)	1 (0.3)	0.018	0.0001	0.0239
GO MYELOID LEUKOCYTE ACTIVATION	594	9 (1.5)	8 (1.3)	1 (0.2)	0.014	0.0002	0.0341
GO MACROPHAGE PROLIFERATION	7	2 (28.6)	2 (28.6)	0 (0)	0.031	0.0002	0.0342
GO MICROGLIAL CELL PROLIFERATION	7	2 (28.6)	2 (28.6)	0 (0)	0.031	0.0002	0.0342
GO CYTOKINE PRODUCTION	720	10 (1.4)	10 (1.4)	0 (0)	0.013	0.0002	0.0351
GO NEUTROPHIL MIGRATION	103	4 (3.9)	4 (3.9)	0 (0)	0.025	0.0002	0.0352
GO POSITIVE REGULATION OF IMMUNE RESPONSE	704	10 (1.4)	10 (1.4)	0 (0)	0.013	0.0002	0.0352
GO REGULATION OF MONONUCLEAR CELL MIGRATION	40	3 (7.5)	3 (7.5)	0 (0)	0.031	0.0002	0.0376
GO NEGATIVE REGULATION OF VIRAL PROCESS	90	4 (4.4)	4 (4.4)	0 (0)	0.027	0.0002	0.0384
GO PYRIMIDINE CONTAINING COMPOUND METABOLIC PROCES...	92	4 (4.3)	3 (3.3)	1 (1.1)	0.027	0.0003	0.0407
GO RESPONSE TO FUNGUS	47	3 (6.4)	3 (6.4)	0 (0)	0.029	0.0003	0.0413
GO FOAM CELL DIFFERENTIATION	35	3 (8.6)	3 (8.6)	0 (0)	0.033	0.0003	0.0424
GO POSITIVE REGULATION OF RESPONSE TO BIOTIC STIMU...	353	7 (2)	7 (2)	0 (0)	0.017	0.0003	0.0424
GO CELLULAR ION HOMEOSTASIS	634	9 (1.4)	8 (1.3)	1 (0.2)	0.013	0.0003	0.0424
GO CELL ACTIVATION	1265	13 (1)	12 (0.9)	1 (0.1)	0.010	0.0003	0.0424
GO REGULATION OF DENDRITIC CELL CHEMOTAXIS	9	2 (22.2)	2 (22.2)	0 (0)	0.030	0.0003	0.043
GO SEQUESTERING OF ZINC ION	8	2 (25)	2 (25)	0 (0)	0.030	0.0003	0.0436
GO HOMEOSTATIC PROCESS	1803	16 (0.9)	15 (0.8)	1 (0.1)	0.009	0.0004	0.048
GO CELLULAR TRANSITION METAL ION HOMEOSTASIS	99	4 (4)	4 (4)	0 (0)	0.026	0.0004	0.0481
GO REGULATION OF LEUKOCYTE CHEMOTAXIS	102	4 (3.9)	4 (3.9)	0 (0)	0.025	0.0004	0.0481
GO PATTERN RECOGNITION RECEPTOR SIGNALING PATHWAY	181	5 (2.8)	5 (2.8)	0 (0)	0.021	0.0004	0.0481
GO LEUKOCYTE MIGRATION	410	7 (1.7)	7 (1.7)	0 (0)	0.015	0.0004	0.0486

Table S118: Blood Transcription Modules enriched in differentially expressed genes at Day 2 compared to pre-First Vaccination (NK cells, Study Arm 2 A/H7N9+AS03, D1, 29). Results are sorted by FDR adjusted p-value and Jaccard similarity index.

Gene Set ID	Gene Set Genes #	DE Genes N (%)	Up-reg. DE Genes N (%)	Down-reg. DE Genes N (%)	Jaccard Index	P-Value	FDR Adjusted P-Value
M11.0 ENRICHED IN MONOCYTES (II)	174	13 (7.5)	13 (7.5)	0 (0)	0.059	<0.0001	0.0017
M165 ENRICHED IN ACTIVATED DENDRITIC CELLS (II)	34	5 (14.7)	5 (14.7)	0 (0)	0.056	<0.0001	0.0017
M150 INNATE ANTIVIRAL RESPONSE	12	3 (25)	3 (25)	0 (0)	0.043	0.0001	0.0164
M127 TYPE I INTERFERON RESPONSE	12	3 (25)	3 (25)	0 (0)	0.043	0.0002	0.0164
M4.0 CELL CYCLE AND TRANSCRIPTION	303	10 (3.3)	10 (3.3)	0 (0)	0.028	0.0004	0.0304

Table S119: KEGG Pathways enriched in differentially expressed genes at Day 4 compared to pre-First Vaccination (NK cells, Study Arm 2 A/H7N9+AS03, D1, 29). Results are sorted by FDR adjusted p-value and Jaccard similarity index.

Gene Set ID	Gene Set Genes #	DE Genes N (%)	Up-reg. DE Genes N (%)	Down-reg. DE Genes N (%)	Jaccard Index	P-Value	FDR Adjusted P-Value
CELL CYCLE	121	11 (9.1)	11 (9.1)	0 (0)	0.062	<0.0001	0.0034
HUMAN T-CELL LEUKEMIA VIRUS 1 INFECTION	211	6 (2.8)	6 (2.8)	0 (0)	0.022	<0.0001	0.0152
P53 SIGNALING PATHWAY	73	4 (5.5)	4 (5.5)	0 (0)	0.030	0.0002	0.018
CELLULAR SENESCENCE	153	5 (3.3)	5 (3.3)	0 (0)	0.023	0.0003	0.0253
HOMOLOGOUS RECOMBINATION	39	3 (7.7)	3 (7.7)	0 (0)	0.029	0.0006	0.041
PYRIMIDINE METABOLISM	55	3 (5.5)	3 (5.5)	0 (0)	0.025	0.0007	0.041
OOCYTE MEIOSIS	123	4 (3.3)	4 (3.3)	0 (0)	0.022	0.001	0.0421
MICRORNAS IN CANCER	301	5 (1.7)	5 (1.7)	0 (0)	0.014	0.001	0.0421

Table S120: MSigDB Reactome Pathways enriched in differentially expressed genes at Day 4 compared to pre-First Vaccination (NK cells, Study Arm 2 A/H7N9+AS03, D1, 29). Results are sorted by FDR adjusted p-value and Jaccard similarity index.

Gene Set ID	Gene Set Genes #	DE Genes N (%)	Up-reg. DE Genes N (%)	Down-reg. DE Genes N (%)	Jaccard Index	P-Value	FDR Adjusted P-Value
REACTOME G1 S SPECIFIC TRANSCRIPTION	28	8 (28.6)	8 (28.6)	0 (0)	0.093	<0.0001	0.0006
REACTOME RESOLUTION OF SISTER CHROMATID COHESION	118	14 (11.9)	14 (11.9)	0 (0)	0.082	<0.0001	0.0006
REACTOME MITOTIC SPINDLE CHECKPOINT	105	13 (12.4)	13 (12.4)	0 (0)	0.082	<0.0001	0.0006
REACTOME CELL CYCLE CHECKPOINTS	276	22 (8)	22 (8)	0 (0)	0.069	<0.0001	0.0006
REACTOME MITOTIC PROMETAPHASE	188	16 (8.5)	16 (8.5)	0 (0)	0.067	<0.0001	0.0006
REACTOME SEPARATION OF SISTER CHROMATIDS	177	15 (8.5)	15 (8.5)	0 (0)	0.066	<0.0001	0.0006
REACTOME RHO GTPASES ACTIVATE FORMINS	134	12 (9)	12 (9)	0 (0)	0.064	<0.0001	0.0006
REACTOME CELL CYCLE MITOTIC	532	35 (6.6)	35 (6.6)	0 (0)	0.062	<0.0001	0.0006
REACTOME CELL CYCLE	639	40 (6.3)	40 (6.3)	0 (0)	0.060	<0.0001	0.0006
REACTOME KINESINS	59	7 (11.9)	7 (11.9)	0 (0)	0.059	<0.0001	0.0006
REACTOME MITOTIC METAPHASE AND ANAPHASE	221	16 (7.2)	16 (7.2)	0 (0)	0.059	<0.0001	0.0006
REACTOME MITOTIC G1 PHASE AND G1 S TRANSITION	139	11 (7.9)	11 (7.9)	0 (0)	0.057	<0.0001	0.0006
REACTOME ACTIVATION OF ATR IN RESPONSE TO REPLICAT...	36	5 (13.9)	5 (13.9)	0 (0)	0.051	<0.0001	0.0006
REACTOME COPI DEPENDENT GOLGI TO ER RETROGRADE TRA...	97	7 (7.2)	7 (7.2)	0 (0)	0.045	<0.0001	0.0006
REACTOME DNA REPLICATION	120	8 (6.7)	8 (6.7)	0 (0)	0.045	<0.0001	0.0006
REACTOME G0 AND EARLY G1	27	4 (14.8)	4 (14.8)	0 (0)	0.045	<0.0001	0.0006
REACTOME M PHASE	393	19 (4.8)	19 (4.8)	0 (0)	0.043	<0.0001	0.0006
REACTOME ACTIVATION OF THE PRE REPLICATIVE COMPLEX	31	4 (12.9)	4 (12.9)	0 (0)	0.043	<0.0001	0.0006

Table S120: MSigDB Reactome Pathways enriched in differentially expressed genes at Day 4 compared to pre-First Vaccination (NK cells, Study Arm 2 A/H7N9+AS03, D1, 29). Results are sorted by FDR adjusted p-value and Jaccard similarity index (continued.)

Gene Set ID	Gene Set Genes #	DE Genes N (%)	Up-reg. DE Genes N (%)	Down-reg. DE Genes N (%)	Jaccard Index	P-Value	FDR Adjusted P-Value
REACTOME G2 M CHECKPOINTS	158	9 (5.7)	9 (5.7)	0 (0)	0.042	<0.0001	0.0006
REACTOME CONDENSATION OF PROMETAPHASE CHROMOSOMES	10	3 (30)	3 (30)	0 (0)	0.041	<0.0001	0.0006
REACTOME GOLGI TO ER RETROGRADE TRANSPORT	131	7 (5.3)	7 (5.3)	0 (0)	0.037	<0.0001	0.0006
REACTOME RHO GTPASE EFFECTORS	308	13 (4.2)	13 (4.2)	0 (0)	0.036	<0.0001	0.0006
REACTOME S PHASE	151	7 (4.6)	7 (4.6)	0 (0)	0.033	<0.0001	0.0006
REACTOME MITOTIC G2 G2 M PHASES	186	8 (4.3)	8 (4.3)	0 (0)	0.033	<0.0001	0.0006
REACTOME FACTORS INVOLVED IN MEGAKARYOCYTE DEVELOP...	165	7 (4.2)	7 (4.2)	0 (0)	0.031	<0.0001	0.0006
REACTOME SIGNALING BY RHO GTPASES	424	13 (3.1)	13 (3.1)	0 (0)	0.027	<0.0001	0.0006
REACTOME DNA REPLICATION PRE INITIATION	78	6 (7.7)	6 (7.7)	0 (0)	0.043	<0.0001	0.0011
REACTOME TRANSCRIPTIONAL REGULATION BY E2F6	30	4 (13.3)	4 (13.3)	0 (0)	0.043	<0.0001	0.0016
REACTOME HOMOLOGY DIRECTED REPAIR	134	6 (4.5)	6 (4.5)	0 (0)	0.031	<0.0001	0.0016
REACTOME INTRA GOLGI AND RETROGRADE GOLGI TO ER TR...	196	7 (3.6)	7 (3.6)	0 (0)	0.028	<0.0001	0.0026
REACTOME CDC6 ASSOCIATION WITH THE ORC ORIGIN COMP...	11	3 (27.3)	3 (27.3)	0 (0)	0.041	<0.0001	0.003
REACTOME APC C MEDIATED DEGRADATION OF CELL CYCLE ...	81	5 (6.2)	5 (6.2)	0 (0)	0.035	<0.0001	0.0032
REACTOME DEPOSITION OF NEW CENPA CONTAINING NUCLEO...	70	4 (5.7)	4 (5.7)	0 (0)	0.030	<0.0001	0.0032
REACTOME TRANSCRIPTION OF E2F TARGETS UNDER NEGATI...	19	3 (15.8)	3 (15.8)	0 (0)	0.037	<0.0001	0.0044
REACTOME INHIBITION OF THE PROTEOLYTIC ACTIVITY OF...	20	3 (15)	3 (15)	0 (0)	0.036	<0.0001	0.0044
REACTOME POLO LIKE KINASE MEDIATED EVENTS	16	3 (18.8)	3 (18.8)	0 (0)	0.038	0.0001	0.0046
REACTOME DNA DOUBLE STRAND BREAK REPAIR	162	6 (3.7)	6 (3.7)	0 (0)	0.027	0.0001	0.0046
REACTOME APC C CDC20 MEDIATED DEGRADATION OF CYCLI...	23	3 (13)	3 (13)	0 (0)	0.035	0.0001	0.0056
REACTOME G2 M DNA REPLICATION CHECKPOINT	5	2 (40)	2 (40)	0 (0)	0.029	0.0002	0.0075
REACTOME APC CDC20 MEDIATED DEGRADATION OF NEK2A	25	3 (12)	3 (12)	0 (0)	0.034	0.0002	0.0075
REACTOME DNA REPAIR	319	8 (2.5)	8 (2.5)	0 (0)	0.021	0.0002	0.0075
REACTOME MHC CLASS II ANTIGEN PRESENTATION	107	5 (4.7)	5 (4.7)	0 (0)	0.030	0.0002	0.0077
REACTOME CYCLIN A B1 B2 ASSOCIATED EVENTS DURING G...	23	3 (13)	3 (13)	0 (0)	0.035	0.0002	0.0086
REACTOME ASSEMBLY OF THE PRE REPLICATIVE COMPLEX	63	4 (6.3)	4 (6.3)	0 (0)	0.032	0.0004	0.0125
REACTOME HDR THROUGH HOMOLOGOUS RECOMBINATION HRR	66	4 (6.1)	4 (6.1)	0 (0)	0.031	0.0004	0.0129
REACTOME PHOSPHORYLATION OF EMI1	6	2 (33.3)	2 (33.3)	0 (0)	0.029	0.0004	0.013
REACTOME RESOLUTION OF D LOOP STRUCTURES	34	3 (8.8)	3 (8.8)	0 (0)	0.031	0.0009	0.0303
REACTOME CHROMOSOME MAINTENANCE	112	4 (3.6)	4 (3.6)	0 (0)	0.023	0.001	0.0306
REACTOME REGULATION OF TP53 ACTIVITY THROUGH PHOSP...	85	4 (4.7)	4 (4.7)	0 (0)	0.027	0.0012	0.0378
REACTOME HOMOLOGOUS DNA PAIRING AND STRAND EXCHANG...	41	3 (7.3)	3 (7.3)	0 (0)	0.029	0.0013	0.0398
REACTOME REGULATION OF TP53 ACTIVITY	148	5 (3.4)	5 (3.4)	0 (0)	0.024	0.0014	0.0418
REACTOME CHK1 CHK2 CDS1 MEDIATED INACTIVATION OF C...	12	2 (16.7)	2 (16.7)	0 (0)	0.026	0.0017	0.0489

Table S121: MSigDB GO Biological Processes enriched in differentially expressed genes at Day 4 compared to pre-First Vaccination (NK cells, Study Arm 2 A/H7N9+AS03, D1, 29). Results are sorted by FDR adjusted p-value and Jaccard similarity index.

Gene Set ID	Gene Set Genes #	DE Genes N (%)	Up-reg. DE Genes N (%)	Down-reg. DE Genes N (%)	Jaccard Index	P-Value	FDR Adjusted P-Value
GO CHROMOSOME SEPARATION	85	13 (15.3)	13 (15.3)	0 (0)	0.094	<0.0001	0.001
GO MITOTIC SISTER CHROMATID SEGREGATION	146	18 (12.3)	18 (12.3)	0 (0)	0.093	<0.0001	0.001
GO METAPHASE ANAPHASE TRANSITION OF CELL CYCLE	55	10 (18.2)	10 (18.2)	0 (0)	0.090	<0.0001	0.001
GO REGULATION OF CHROMOSOME SEGREGATION	96	13 (13.5)	13 (13.5)	0 (0)	0.087	<0.0001	0.001
GO REGULATION OF CHROMOSOME SEPARATION	60	10 (16.7)	10 (16.7)	0 (0)	0.086	<0.0001	0.001
GO SISTER CHROMATID SEGREGATION	175	19 (10.9)	19 (10.9)	0 (0)	0.086	<0.0001	0.001
GO MITOTIC NUCLEAR DIVISION	253	25 (9.9)	25 (9.9)	0 (0)	0.085	<0.0001	0.001
GO REGULATION OF SISTER CHROMATID SEGREGATION	75	11 (14.7)	11 (14.7)	0 (0)	0.085	<0.0001	0.001
GO NEGATIVE REGULATION OF CHROMOSOME SEGREGATION	42	8 (19)	8 (19)	0 (0)	0.080	<0.0001	0.001
GO NEGATIVE REGULATION OF METAPHASE ANAPHASE TRANS...	37	7 (18.9)	7 (18.9)	0 (0)	0.073	<0.0001	0.001
GO NUCLEAR CHROMOSOME SEGREGATION	245	21 (8.6)	21 (8.6)	0 (0)	0.072	<0.0001	0.001
GO MITOTIC CELL CYCLE CHECKPOINT	158	15 (9.5)	15 (9.5)	0 (0)	0.072	<0.0001	0.001
GO MITOTIC SPINDLE ORGANIZATION	99	11 (11.1)	11 (11.1)	0 (0)	0.071	<0.0001	0.001
GO CHROMOSOME SEGREGATION	297	24 (8.1)	24 (8.1)	0 (0)	0.071	<0.0001	0.001
GO CELL CYCLE CHECKPOINT	207	18 (8.7)	18 (8.7)	0 (0)	0.071	<0.0001	0.001
GO REGULATION OF NUCLEAR DIVISION	178	16 (9)	16 (9)	0 (0)	0.070	<0.0001	0.001
GO NEGATIVE REGULATION OF NUCLEAR DIVISION	57	8 (14)	8 (14)	0 (0)	0.070	<0.0001	0.001
GO REGULATION OF TRANSCRIPTION INVOLVED IN G1 S TR...	27	6 (22.2)	6 (22.2)	0 (0)	0.069	<0.0001	0.001
GO MICROTUBULE CYTOSKELETON ORGANIZATION INVOLVED ...	125	12 (9.6)	12 (9.6)	0 (0)	0.067	<0.0001	0.001
GO PROTEIN LOCALIZATION TO KINETOCHORE	16	5 (31.2)	5 (31.2)	0 (0)	0.065	<0.0001	0.001
GO ORGANELLE FISSION	428	30 (7)	30 (7)	0 (0)	0.065	<0.0001	0.001
GO MITOTIC SPINDLE ASSEMBLY	51	7 (13.7)	7 (13.7)	0 (0)	0.064	<0.0001	0.001
GO SPINDLE ORGANIZATION	157	13 (8.3)	13 (8.3)	0 (0)	0.062	<0.0001	0.001
GO PROTEIN LOCALIZATION TO CHROMOSOME CENTROMERIC ...	20	5 (25)	5 (25)	0 (0)	0.062	<0.0001	0.001
GO MEIOTIC CHROMOSOME SEPARATION	21	5 (23.8)	5 (23.8)	0 (0)	0.061	<0.0001	0.001
GO DNA INTEGRITY CHECKPOINT	150	11 (7.3)	11 (7.3)	0 (0)	0.054	<0.0001	0.001
GO POSITIVE REGULATION OF CELL CYCLE PHASE TRANSIT...	91	8 (8.8)	8 (8.8)	0 (0)	0.054	<0.0001	0.001
GO ATTACHMENT OF SPINDLE MICROTUBULES TO KINETOCHO...	33	5 (15.2)	5 (15.2)	0 (0)	0.053	<0.0001	0.001
GO CENTROMERE COMPLEX ASSEMBLY	53	6 (11.3)	6 (11.3)	0 (0)	0.053	<0.0001	0.001
GO METAPHASE PLATE CONGRESSION	56	6 (10.7)	6 (10.7)	0 (0)	0.052	<0.0001	0.001
GO SPINDLE ASSEMBLY	98	8 (8.2)	8 (8.2)	0 (0)	0.051	<0.0001	0.001
GO NEGATIVE REGULATION OF CELL CYCLE PHASE TRANSIT...	243	15 (6.2)	15 (6.2)	0 (0)	0.051	<0.0001	0.001
GO NEGATIVE REGULATION OF CELL CYCLE PROCESS	321	18 (5.6)	18 (5.6)	0 (0)	0.049	<0.0001	0.001
GO MEIOTIC CHROMOSOME SEGREGATION	85	7 (8.2)	7 (8.2)	0 (0)	0.049	<0.0001	0.001
GO DNA REPLICATION	258	15 (5.8)	15 (5.8)	0 (0)	0.049	<0.0001	0.001
GO NEGATIVE REGULATION OF MITOTIC CELL CYCLE	309	17 (5.5)	17 (5.5)	0 (0)	0.048	<0.0001	0.001
GO CHROMOSOME CONDENSATION	45	5 (11.1)	5 (11.1)	0 (0)	0.047	<0.0001	0.001

Table S121: MSigDB GO Biological Processes enriched in differentially expressed genes at Day 4 compared to pre-First Vaccination (NK cells, Study Arm 2 A/H7N9+AS03, D1, 29). Results are sorted by FDR adjusted p-value and Jaccard similarity index (continued.)

Gene Set ID	Gene Set Genes #	DE Genes N (%)	Up-reg. DE Genes N (%)	Down-reg. DE Genes N (%)	Jaccard Index	P-Value	FDR Adjusted P-Value
GO MEIOTIC CELL CYCLE PROCESS	178	11 (6.2)	11 (6.2)	0 (0)	0.047	<0.0001	0.001
GO NEGATIVE REGULATION OF CHROMOSOME ORGANIZATION	136	9 (6.6)	9 (6.6)	0 (0)	0.047	<0.0001	0.001
GO CELL DIVISION	567	28 (4.9)	28 (4.9)	0 (0)	0.046	<0.0001	0.001
GO MITOTIC CELL CYCLE	954	45 (4.7)	45 (4.7)	0 (0)	0.046	<0.0001	0.001
GO REGULATION OF CELL CYCLE PHASE TRANSITION	433	22 (5.1)	22 (5.1)	0 (0)	0.046	<0.0001	0.001
GO CELL CYCLE PHASE TRANSITION	577	28 (4.9)	28 (4.9)	0 (0)	0.045	<0.0001	0.001
GO HOMOLOGOUS RECOMBINATION	50	5 (10)	5 (10)	0 (0)	0.045	<0.0001	0.001
GO CHROMOSOME LOCALIZATION	74	6 (8.1)	6 (8.1)	0 (0)	0.045	<0.0001	0.001
GO DNA CONFORMATION CHANGE	284	15 (5.3)	15 (5.3)	0 (0)	0.045	<0.0001	0.001
GO DNA REPLICATION INDEPENDENT NUCLEOSOME ORGANIZA...	51	5 (9.8)	5 (9.8)	0 (0)	0.045	<0.0001	0.001
GO MITOTIC DNA INTEGRITY CHECKPOINT	101	7 (6.9)	7 (6.9)	0 (0)	0.044	<0.0001	0.001
GO REGULATION OF MITOTIC CELL CYCLE	604	28 (4.6)	28 (4.6)	0 (0)	0.044	<0.0001	0.001
GO PROTEIN LOCALIZATION TO CHROMOSOME	78	6 (7.7)	6 (7.7)	0 (0)	0.043	<0.0001	0.001
GO CELL CYCLE G2 M PHASE TRANSITION	250	13 (5.2)	13 (5.2)	0 (0)	0.043	<0.0001	0.001
GO MEIOTIC CELL CYCLE	229	12 (5.2)	12 (5.2)	0 (0)	0.042	<0.0001	0.001
GO DNA PACKAGING	183	10 (5.5)	10 (5.5)	0 (0)	0.042	<0.0001	0.001
GO REGULATION OF CELL CYCLE PROCESS	716	31 (4.3)	31 (4.3)	0 (0)	0.041	<0.0001	0.001
GO REGULATION OF CHROMOSOME ORGANIZATION	321	15 (4.7)	15 (4.7)	0 (0)	0.040	<0.0001	0.001
GO DNA DEPENDENT DNA REPLICATION	142	8 (5.6)	8 (5.6)	0 (0)	0.040	<0.0001	0.001
GO REGULATION OF CELL CYCLE G2 M PHASE TRANSITION	195	10 (5.1)	10 (5.1)	0 (0)	0.040	<0.0001	0.001
GO POSITIVE REGULATION OF CELL CYCLE PROCESS	275	13 (4.7)	13 (4.7)	0 (0)	0.040	<0.0001	0.001
GO POSITIVE REGULATION OF MITOTIC CELL CYCLE	148	8 (5.4)	8 (5.4)	0 (0)	0.039	<0.0001	0.001
GO CELL CYCLE PROCESS	1291	48 (3.7)	48 (3.7)	0 (0)	0.037	<0.0001	0.001
GO NEGATIVE REGULATION OF CELL CYCLE	584	23 (3.9)	23 (3.9)	0 (0)	0.037	<0.0001	0.001
GO DOUBLE STRAND BREAK REPAIR	237	10 (4.2)	10 (4.2)	0 (0)	0.034	<0.0001	0.001
GO POSITIVE REGULATION OF CELL CYCLE	360	14 (3.9)	14 (3.9)	0 (0)	0.034	<0.0001	0.001
GO PROTEIN DNA COMPLEX SUBUNIT ORGANIZATION	251	10 (4)	10 (4)	0 (0)	0.033	<0.0001	0.001
GO REGULATION OF CELL CYCLE	1115	37 (3.3)	37 (3.3)	0 (0)	0.032	<0.0001	0.001
GO CYTOKINESIS	160	7 (4.4)	7 (4.4)	0 (0)	0.032	<0.0001	0.001
GO MICROTUBULE CYTOSKELETON ORGANIZATION	522	18 (3.4)	18 (3.4)	0 (0)	0.032	<0.0001	0.001
GO CELL CYCLE G1 S PHASE TRANSITION	262	10 (3.8)	10 (3.8)	0 (0)	0.031	<0.0001	0.001
GO DNA RECOMBINATION	270	10 (3.7)	10 (3.7)	0 (0)	0.031	<0.0001	0.001
GO CELL CYCLE	1718	53 (3.1)	53 (3.1)	0 (0)	0.031	<0.0001	0.001
GO SIGNAL TRANSDUCTION BY P53 CLASS MEDIATOR	250	9 (3.6)	9 (3.6)	0 (0)	0.029	<0.0001	0.001
GO CHROMOSOME ORGANIZATION	1118	33 (3)	33 (3)	0 (0)	0.029	<0.0001	0.001
GO DNA REPAIR	511	15 (2.9)	15 (2.9)	0 (0)	0.027	<0.0001	0.001
GO MICROTUBULE BASED PROCESS	712	20 (2.8)	20 (2.8)	0 (0)	0.026	<0.0001	0.001

Table S121: MSigDB GO Biological Processes enriched in differentially expressed genes at Day 4 compared to pre-First Vaccination (NK cells, Study Arm 2 A/H7N9+AS03, D1, 29). Results are sorted by FDR adjusted p-value and Jaccard similarity index (continued.)

Gene Set ID	Gene Set Genes #	DE Genes N (%)	Up-reg. DE Genes N (%)	Down-reg. DE Genes N (%)	Jaccard Index	P-Value	FDR Adjusted P-Value
GO NEGATIVE REGULATION OF ORGANELLE ORGANIZATION	363	11 (3)	11 (3)	0 (0)	0.026	<0.0001	0.001
GO DNA METABOLIC PROCESS	854	23 (2.7)	23 (2.7)	0 (0)	0.026	<0.0001	0.001
GO CELLULAR RESPONSE TO DNA DAMAGE STIMULUS	789	19 (2.4)	19 (2.4)	0 (0)	0.023	<0.0001	0.001
GO REGULATION OF ORGANELLE ORGANIZATION	1183	23 (1.9)	23 (1.9)	0 (0)	0.019	<0.0001	0.001
GO CYTOSKELETON ORGANIZATION	1253	19 (1.5)	19 (1.5)	0 (0)	0.015	<0.0001	0.001
GO DNA REPLICATION CHECKPOINT	15	4 (26.7)	4 (26.7)	0 (0)	0.052	<0.0001	0.0017
GO MITOTIC METAPHASE PLATE CONGRESSION	44	5 (11.4)	5 (11.4)	0 (0)	0.048	<0.0001	0.0017
GO G2 DNA DAMAGE CHECKPOINT	29	4 (13.8)	4 (13.8)	0 (0)	0.044	<0.0001	0.0017
GO DNA REPLICATION INITIATION	35	4 (11.4)	4 (11.4)	0 (0)	0.041	<0.0001	0.0017
GO MITOTIC CYTOKINESIS	68	5 (7.4)	5 (7.4)	0 (0)	0.039	<0.0001	0.0017
GO CYTOSKELETON DEPENDENT CYTOKINESIS	95	6 (6.3)	6 (6.3)	0 (0)	0.039	<0.0001	0.0017
GO NEGATIVE REGULATION OF CELL CYCLE G2 M PHASE TR...	95	6 (6.3)	6 (6.3)	0 (0)	0.039	<0.0001	0.0017
GO DNA GEOMETRIC CHANGE	101	6 (5.9)	6 (5.9)	0 (0)	0.037	<0.0001	0.0017
GO SIGNAL TRANSDUCTION IN RESPONSE TO DNA DAMAGE	128	6 (4.7)	6 (4.7)	0 (0)	0.032	<0.0001	0.0017
GO POSITIVE REGULATION OF CYTOKINESIS	36	4 (11.1)	4 (11.1)	0 (0)	0.041	<0.0001	0.0024
GO SIGNAL TRANSDUCTION INVOLVED IN CELL CYCLE CHEC...	72	5 (6.9)	5 (6.9)	0 (0)	0.038	<0.0001	0.0024
GO CHROMATIN REMODELING AT CENTROMERE	46	4 (8.7)	4 (8.7)	0 (0)	0.037	<0.0001	0.0024
GO ANAPHASE PROMOTING COMPLEX DEPENDENT CATABOLIC ...	76	5 (6.6)	5 (6.6)	0 (0)	0.036	<0.0001	0.0024
GO RETROGRADE VESICLE MEDIATED TRANSPORT GOLGI TO ...	80	5 (6.2)	5 (6.2)	0 (0)	0.035	<0.0001	0.0024
GO MEIOSIS I CELL CYCLE PROCESS	111	6 (5.4)	6 (5.4)	0 (0)	0.035	<0.0001	0.0024
GO FEMALE MEIOTIC NUCLEAR DIVISION	28	4 (14.3)	4 (14.3)	0 (0)	0.044	<0.0001	0.0031
GO ATTACHMENT OF MITOTIC SPINDLE MICROTUBULES TO K...	13	3 (23.1)	3 (23.1)	0 (0)	0.040	<0.0001	0.0031
GO REGULATION OF MEIOTIC CELL CYCLE	47	4 (8.5)	4 (8.5)	0 (0)	0.037	<0.0001	0.0031
GO HISTONE EXCHANGE	57	4 (7)	4 (7)	0 (0)	0.034	<0.0001	0.0031
GO G0 TO G1 TRANSITION	39	4 (10.3)	4 (10.3)	0 (0)	0.040	<0.0001	0.0036
GO POSITIVE REGULATION OF MITOTIC NUCLEAR DIVISION	51	4 (7.8)	4 (7.8)	0 (0)	0.035	<0.0001	0.0036
GO RECOMBINATIONAL REPAIR	129	6 (4.7)	6 (4.7)	0 (0)	0.032	<0.0001	0.0036
GO DNA BIOSYNTHETIC PROCESS	181	7 (3.9)	7 (3.9)	0 (0)	0.029	<0.0001	0.0036
GO ORGANELLE ASSEMBLY	787	13 (1.7)	13 (1.7)	0 (0)	0.015	<0.0001	0.0036
GO REPRODUCTION	1340	17 (1.3)	17 (1.3)	0 (0)	0.012	<0.0001	0.0036
GO ANTIGEN PROCESSING AND PRESENTATION OF PEPTIDE ...	84	5 (6)	5 (6)	0 (0)	0.035	<0.0001	0.0043
GO MITOTIC CHROMOSOME CONDENSATION	17	3 (17.6)	3 (17.6)	0 (0)	0.037	<0.0001	0.005
GO KINETOCHORE ORGANIZATION	20	3 (15)	3 (15)	0 (0)	0.036	<0.0001	0.0056
GO REGULATION OF CELL DIVISION	156	6 (3.8)	6 (3.8)	0 (0)	0.028	<0.0001	0.0063
GO REGULATION OF CELL CYCLE G1 S PHASE TRANSITION	174	6 (3.4)	6 (3.4)	0 (0)	0.026	<0.0001	0.0069
GO SISTER CHROMATID COHESION	52	4 (7.7)	4 (7.7)	0 (0)	0.035	0.0001	0.0081
GO CHROMATIN ASSEMBLY	141	5 (3.5)	5 (3.5)	0 (0)	0.025	0.0001	0.0081

Table S121: MSigDB GO Biological Processes enriched in differentially expressed genes at Day 4 compared to pre-First Vaccination (NK cells, Study Arm 2 A/H7N9+AS03, D1, 29). Results are sorted by FDR adjusted p-value and Jaccard similarity index (continued.)

Gene Set ID	Gene Set Genes #	DE Genes N (%)	Up-reg. DE Genes N (%)	Down-reg. DE Genes N (%)	Jaccard Index	P-Value	FDR Adjusted P-Value
GO NUCLEOSOME ORGANIZATION	159	5 (3.1)	5 (3.1)	0 (0)	0.023	0.0001	0.0087
GO POSITIVE REGULATION OF NUCLEAR DIVISION	63	4 (6.3)	4 (6.3)	0 (0)	0.032	0.0001	0.01
GO REGULATION OF MEIOTIC NUCLEAR DIVISION	29	3 (10.3)	3 (10.3)	0 (0)	0.033	0.0002	0.0112
GO CHROMOSOME ORGANIZATION INVOLVED IN MEIOTIC CEL...	62	4 (6.5)	4 (6.5)	0 (0)	0.032	0.0002	0.0118
GO FEMALE GAMETE GENERATION	129	5 (3.9)	5 (3.9)	0 (0)	0.026	0.0002	0.0135
GO NEGATIVE REGULATION OF CELLULAR COMPONENT ORGAN...	666	11 (1.7)	11 (1.7)	0 (0)	0.015	0.0002	0.0135
GO CHROMATIN ASSEMBLY OR DISASSEMBLY	166	5 (3)	5 (3)	0 (0)	0.022	0.0002	0.014
GO MITOTIC G2 M TRANSITION CHECKPOINT	28	3 (10.7)	3 (10.7)	0 (0)	0.033	0.0002	0.0146
GO MEIOTIC CELL CYCLE CHECKPOINT	7	2 (28.6)	2 (28.6)	0 (0)	0.028	0.0002	0.0149
GO ORGANELLE LOCALIZATION	585	10 (1.7)	10 (1.7)	0 (0)	0.016	0.0002	0.0149
GO MICROTUBULE BASED MOVEMENT	269	7 (2.6)	7 (2.6)	0 (0)	0.021	0.0002	0.0153
GO ESTABLISHMENT OF ORGANELLE LOCALIZATION	381	8 (2.1)	8 (2.1)	0 (0)	0.018	0.0002	0.0153
GO POSITIVE REGULATION OF CHROMOSOME SEGREGATION	27	3 (11.1)	3 (11.1)	0 (0)	0.033	0.0003	0.0163
GO ATP DEPENDENT CHROMATIN REMODELING	84	4 (4.8)	4 (4.8)	0 (0)	0.027	0.0003	0.0163
GO MICROTUBULE ORGANIZING CENTER ORGANIZATION	128	5 (3.9)	5 (3.9)	0 (0)	0.026	0.0003	0.0167
GO FEMALE MEIOSIS CHROMOSOME SEGREGATION	6	2 (33.3)	2 (33.3)	0 (0)	0.029	0.0003	0.0172
GO MITOTIC SPINDLE MIDZONE ASSEMBLY	6	2 (33.3)	2 (33.3)	0 (0)	0.029	0.0003	0.0176
GO CELLULAR PROTEIN CONTAINING COMPLEX ASSEMBLY	936	12 (1.3)	12 (1.3)	0 (0)	0.012	0.0004	0.0222
GO POSITIVE REGULATION OF CELL DIVISION	82	4 (4.9)	4 (4.9)	0 (0)	0.028	0.0004	0.0232
GO HISTONE PHOSPHORYLATION	37	3 (8.1)	3 (8.1)	0 (0)	0.030	0.0004	0.0241
GO REGULATION OF DOUBLE STRAND BREAK REPAIR	72	4 (5.6)	4 (5.6)	0 (0)	0.030	0.0004	0.0245
GO DOUBLE STRAND BREAK REPAIR VIA SYNTHESIS DEPEND...	7	2 (28.6)	2 (28.6)	0 (0)	0.028	0.0005	0.0272
GO ESTABLISHMENT OF LOCALIZATION IN CELL	510	9 (1.8)	9 (1.8)	0 (0)	0.016	0.0006	0.032
GO CYTOKINETIC PROCESS	39	3 (7.7)	3 (7.7)	0 (0)	0.029	0.0006	0.0335
GO POSITIVE REGULATION OF MITOTIC SISTER CHROMATID...	9	2 (22.2)	2 (22.2)	0 (0)	0.027	0.0006	0.0346
GO ANTIGEN PROCESSING AND PRESENTATION OF PEPTIDE ...	155	5 (3.2)	5 (3.2)	0 (0)	0.023	0.0006	0.0346
GO REGULATION OF CYTOKINESIS	82	4 (4.9)	4 (4.9)	0 (0)	0.028	0.0006	0.0352
GO DEOXYRIBONUCLEOSIDE MONOPHOSPHATE BIOSYNTHETIC ...	10	2 (20)	2 (20)	0 (0)	0.027	0.0006	0.0352
GO SPINDLE MIDZONE ASSEMBLY	9	2 (22.2)	2 (22.2)	0 (0)	0.027	0.0007	0.0355
GO MITOTIC DNA REPLICATION CHECKPOINT	9	2 (22.2)	2 (22.2)	0 (0)	0.027	0.0007	0.039
GO MICROTUBULE DEPOLYMERIZATION	39	3 (7.7)	3 (7.7)	0 (0)	0.029	0.0008	0.0429
GO SPINDLE ELONGATION	9	2 (22.2)	2 (22.2)	0 (0)	0.027	0.0008	0.0442
GO REGULATION OF MITOTIC CENTROSOME SEPARATION	9	2 (22.2)	2 (22.2)	0 (0)	0.027	0.0009	0.0447
GO POSITIVE REGULATION OF CHROMOSOME SEPARATION	10	2 (20)	2 (20)	0 (0)	0.027	0.0009	0.0447
GO REGULATION OF CELL CYCLE ARREST	104	4 (3.8)	4 (3.8)	0 (0)	0.024	0.0009	0.0449
GO REGULATION OF DOUBLE STRAND BREAK REPAIR VIA HO...	41	3 (7.3)	3 (7.3)	0 (0)	0.029	0.0009	0.0451
GO NEGATIVE REGULATION OF MEIOTIC NUCLEAR DIVISION	11	2 (18.2)	2 (18.2)	0 (0)	0.027	0.0009	0.0458

Table S121: MSigDB GO Biological Processes enriched in differentially expressed genes at Day 4 compared to pre-First Vaccination (NK cells, Study Arm 2 A/H7N9+AS03, D1, 29). Results are sorted by FDR adjusted p-value and Jaccard similarity index (continued.)

Gene Set ID	Gene Set Genes #	DE Genes N (%)	Up-reg. DE Genes N (%)	Down-reg. DE Genes N (%)	Jaccard Index	P-Value	FDR Adjusted P-Value
GO DEOXYRIBONUCLEOSIDE MONOPHOSPHATE METABOLIC PRO...	13	2 (15.4)	2 (15.4)	0 (0)	0.026	0.0009	0.048

Table S122: Blood Transcription Modules enriched in differentially expressed genes at Day 4 compared to pre-First Vaccination (NK cells, Study Arm 2 A/H7N9+AS03, D1, 29). Results are sorted by FDR adjusted p-value and Jaccard similarity index.

Gene Set ID	Gene Set Genes #	DE Genes N (%)	Up-reg. DE Genes N (%)	Down-reg. DE Genes N (%)	Jaccard Index	P-Value	FDR Adjusted P-Value
M4.1 CELL CYCLE (I)	135	45 (33.3)	45 (33.3)	0 (0)	0.288	<0.0001	0.0003
M4.2 PLK1 SIGNALING EVENTS	31	14 (45.2)	14 (45.2)	0 (0)	0.169	<0.0001	0.0003
M4.0 CELL CYCLE AND TRANSCRIPTION	303	52 (17.2)	52 (17.2)	0 (0)	0.164	<0.0001	0.0003
M6 MITOTIC CELL DIVISION	25	11 (44)	11 (44)	0 (0)	0.138	<0.0001	0.0003
M46 CELL DIVISION (STIMULATED CD4+ T CELLS)	25	10 (40)	10 (40)	0 (0)	0.123	<0.0001	0.0003
M103 CELL CYCLE (III)	50	11 (22)	11 (22)	0 (0)	0.105	<0.0001	0.0003
M4.5 MITOTIC CELL CYCLE IN STIMULATED CD4 T CELLS	32	9 (28.1)	9 (28.1)	0 (0)	0.101	<0.0001	0.0003
M8 E2F TRANSCRIPTION FACTOR NETWORK	11	7 (63.6)	7 (63.6)	0 (0)	0.100	<0.0001	0.0003
M4.4 MITOTIC CELL CYCLE - DNA REPLICATION	27	8 (29.6)	8 (29.6)	0 (0)	0.094	<0.0001	0.0003
M15 RAN MEDIATED MITOSIS	12	6 (50)	6 (50)	0 (0)	0.083	<0.0001	0.0003
M4.9 MITOTIC CELL CYCLE IN STIMULATED CD4 T CELLS	15	6 (40)	6 (40)	0 (0)	0.080	<0.0001	0.0003
M4.7 MITOTIC CELL CYCLE	20	6 (30)	6 (30)	0 (0)	0.075	<0.0001	0.0003
M4.10 CELL CYCLE (II)	14	5 (35.7)	5 (35.7)	0 (0)	0.067	<0.0001	0.0003
M4.11 MITOTIC CELL CYCLE IN STIMULATED CD4 T CELLS	9	4 (44.4)	4 (44.4)	0 (0)	0.056	<0.0001	0.0007
M10.1 E2F1 TARGETS (Q4)	20	5 (25)	5 (25)	0 (0)	0.062	<0.0001	0.0009
M4.12 C-MYC TRANSCRIPTIONAL NETWORK	12	4 (33.3)	4 (33.3)	0 (0)	0.054	<0.0001	0.0017
M49 TRANSCRIPTION REGULATION IN CELL DEVELOPMENT	43	6 (14)	6 (14)	0 (0)	0.058	0.0003	0.0053
M4.6 CELL DIVISION IN STIMULATED CD4 T CELLS	19	4 (21.1)	4 (21.1)	0 (0)	0.049	0.0004	0.0086

Table S123: KEGG Pathways enriched in differentially expressed genes at Day 30 compared to pre-Second Vaccination (NK cells, Study Arm 2 A/H7N9+AS03, D1, 29). Results are sorted by FDR adjusted p-value and Jaccard similarity index.

Gene Set ID	Gene Set Genes #	DE Genes N (%)	Up-reg. DE Genes N (%)	Down-reg. DE Genes N (%)	Jaccard Index	P-Value	FDR Adjusted P-Value
HEPATITIS C	152	17 (11.2)	17 (11.2)	0 (0)	0.039	<0.0001	0.0008
OSTEOCLAST DIFFERENTIATION	122	12 (9.8)	12 (9.8)	0 (0)	0.029	<0.0001	0.0008

Table S123: KEGG Pathways enriched in differentially expressed genes at Day 30 compared to pre-Second Vaccination (NK cells, Study Arm 2 A/H7N9+AS03, D1, 29). Results are sorted by FDR adjusted p-value and Jaccard similarity index (continued.)

Gene Set ID	Gene Set Genes #	DE Genes N (%)	Up-reg. DE Genes N (%)	Down-reg. DE Genes N (%)	Jaccard Index	P-Value	FDR Adjusted P-Value
INFLUENZA A	160	13 (8.1)	13 (8.1)	0 (0)	0.029	<0.0001	0.0008
MEASLES	132	12 (9.1)	12 (9.1)	0 (0)	0.028	<0.0001	0.0008
HUMAN PAPILLOMAVIRUS INFECTION	324	18 (5.6)	15 (4.6)	3 (0.9)	0.030	<0.0001	0.0034
NOD-LIKE RECEPTOR SIGNALING PATHWAY	175	12 (6.9)	12 (6.9)	0 (0)	0.026	<0.0001	0.0034
EPSTEIN-BARR VIRUS INFECTION	196	13 (6.6)	13 (6.6)	0 (0)	0.027	0.0001	0.0063
JAK-STAT SIGNALING PATHWAY	154	10 (6.5)	9 (5.8)	1 (0.6)	0.022	0.0004	0.019

Table S124: MSigDB Reactome Pathways enriched in differentially expressed genes at Day 30 compared to pre-Second Vaccination (NK cells, Study Arm 2 A/H7N9+AS03, D1, 29). Results are sorted by FDR adjusted p-value and Jaccard similarity index.

Gene Set ID	Gene Set Genes #	DE Genes N (%)	Up-reg. DE Genes N (%)	Down-reg. DE Genes N (%)	Jaccard Index	P-Value	FDR Adjusted P-Value
REACTOME INTERFERON SIGNALING	177	32 (18.1)	32 (18.1)	0 (0)	0.071	<0.0001	0.0026
REACTOME INTERFERON ALPHA BETA SIGNALING	60	23 (38.3)	23 (38.3)	0 (0)	0.068	<0.0001	0.0026
REACTOME CYTOKINE SIGNALING IN IMMUNE SYSTEM	788	50 (6.3)	47 (6)	3 (0.4)	0.048	<0.0001	0.0026
REACTOME INTERFERON GAMMA SIGNALING	71	15 (21.1)	15 (21.1)	0 (0)	0.042	<0.0001	0.0026
REACTOME ANTIVIRAL MECHANISM BY IFN STIMULATED GEN...	81	13 (16)	13 (16)	0 (0)	0.035	<0.0001	0.0026
REACTOME OAS ANTIVIRAL RESPONSE	8	4 (50)	4 (50)	0 (0)	0.013	<0.0001	0.0026
REACTOME ACTIVATION OF ATR IN RESPONSE TO REPLICAT...	36	6 (16.7)	6 (16.7)	0 (0)	0.018	<0.0001	0.0109
REACTOME ACTIVATION OF THE PRE REPLICATIVE COMPLEX	31	5 (16.1)	5 (16.1)	0 (0)	0.015	0.0002	0.0306
REACTOME REGULATION OF IFNA SIGNALING	26	4 (15.4)	4 (15.4)	0 (0)	0.012	0.0002	0.0409
REACTOME NEUTROPHIL DEGRANULATION	442	19 (4.3)	18 (4.1)	1 (0.2)	0.026	0.0003	0.0414
REACTOME INNATE IMMUNE SYSTEM	968	31 (3.2)	28 (2.9)	3 (0.3)	0.025	0.0003	0.0472
REACTOME DNA REPLICATION PRE INITIATION	78	7 (9)	7 (9)	0 (0)	0.019	0.0004	0.0472

Table S125: MSigDB GO Biological Processes enriched in differentially expressed genes at Day 30 compared to pre-Second Vaccination (NK cells, Study Arm 2 A/H7N9+AS03, D1, 29). Results are sorted by FDR adjusted p-value and Jaccard similarity index.

Gene Set ID	Gene Set Genes #	DE Genes N (%)	Up-reg. DE Genes N (%)	Down-reg. DE Genes N (%)	Jaccard Index	P-Value	FDR Adjusted P-Value
GO RESPONSE TO TYPE I INTERFERON	88	22 (25)	22 (25)	0 (0)	0.060	<0.0001	0.0024
GO DEFENSE RESPONSE TO VIRUS	229	29 (12.7)	29 (12.7)	0 (0)	0.058	<0.0001	0.0024
GO RESPONSE TO VIRUS	300	32 (10.7)	32 (10.7)	0 (0)	0.056	<0.0001	0.0024

Table S125: MSigDB GO Biological Processes enriched in differentially expressed genes at Day 30 compared to pre-Second Vaccination (NK cells, Study Arm 2 A/H7N9+AS03, D1, 29). Results are sorted by FDR adjusted p-value and Jaccard similarity index (continued.)

Gene Set ID	Gene Set Genes #	DE Genes N (%)	Up-reg. DE Genes N (%)	Down-reg. DE Genes N (%)	Jaccard Index	P-Value	FDR Adjusted P-Value
GO INNATE IMMUNE RESPONSE	814	55 (6.8)	55 (6.8)	0 (0)	0.052	<0.0001	0.0024
GO RESPONSE TO INTERFERON GAMMA	166	22 (13.3)	22 (13.3)	0 (0)	0.049	<0.0001	0.0024
GO DEFENSE RESPONSE TO OTHER ORGANISM	993	59 (5.9)	59 (5.9)	0 (0)	0.048	<0.0001	0.0024
GO CYTOKINE MEDIATED SIGNALING PATHWAY	711	46 (6.5)	42 (5.9)	4 (0.6)	0.048	<0.0001	0.0024
GO RESPONSE TO CYTOKINE	1099	59 (5.4)	53 (4.8)	6 (0.5)	0.044	<0.0001	0.0024
GO DEFENSE RESPONSE	1497	74 (4.9)	70 (4.7)	4 (0.3)	0.043	<0.0001	0.0024
GO RESPONSE TO BIOTIC STIMULUS	1325	67 (5.1)	66 (5)	1 (0.1)	0.043	<0.0001	0.0024
GO NEGATIVE REGULATION OF VIRAL PROCESS	90	16 (17.8)	16 (17.8)	0 (0)	0.042	<0.0001	0.0024
GO INTERFERON GAMMA MEDIATED SIGNALING PATHWAY	70	15 (21.4)	15 (21.4)	0 (0)	0.042	<0.0001	0.0024
GO IMMUNE EFFECTOR PROCESS	1077	55 (5.1)	51 (4.7)	4 (0.4)	0.042	<0.0001	0.0024
GO NEGATIVE REGULATION OF VIRAL LIFE CYCLE	76	15 (19.7)	15 (19.7)	0 (0)	0.041	<0.0001	0.0024
GO NEGATIVE REGULATION OF MULTI ORGANISM PROCESS	195	19 (9.7)	18 (9.2)	1 (0.5)	0.040	<0.0001	0.0024
GO REGULATION OF VIRAL LIFE CYCLE	133	16 (12)	16 (12)	0 (0)	0.038	<0.0001	0.0024
GO NEGATIVE REGULATION OF VIRAL GENOME REPLICATION	58	13 (22.4)	13 (22.4)	0 (0)	0.037	<0.0001	0.0024
GO REGULATION OF MULTI ORGANISM PROCESS	700	34 (4.9)	33 (4.7)	1 (0.1)	0.035	<0.0001	0.0024
GO REGULATION OF SYMBIOSIS ENCOMPASSING MUTUALISM ...	204	17 (8.3)	17 (8.3)	0 (0)	0.035	<0.0001	0.0024
GO VIRAL GENOME REPLICATION	117	14 (12)	14 (12)	0 (0)	0.035	<0.0001	0.0024
GO REGULATION OF VIRAL GENOME REPLICATION	91	13 (14.3)	13 (14.3)	0 (0)	0.034	<0.0001	0.0024
GO VIRAL LIFE CYCLE	308	19 (6.2)	18 (5.8)	1 (0.3)	0.032	<0.0001	0.0024
GO MYELOID LEUKOCYTE ACTIVATION	594	27 (4.5)	24 (4)	3 (0.5)	0.031	<0.0001	0.0024
GO REGULATION OF RESPONSE TO EXTERNAL STIMULUS	1006	38 (3.8)	35 (3.5)	3 (0.3)	0.030	<0.0001	0.0024
GO REGULATION OF DEFENSE RESPONSE	703	29 (4.1)	28 (4)	1 (0.1)	0.030	<0.0001	0.0024
GO REGULATION OF IMMUNE SYSTEM PROCESS	1409	48 (3.4)	44 (3.1)	4 (0.3)	0.029	<0.0001	0.0024
GO CELL CHEMOTAXIS	278	15 (5.4)	13 (4.7)	2 (0.7)	0.026	<0.0001	0.0024
GO LEUKOCYTE CHEMOTAXIS	202	13 (6.4)	12 (5.9)	1 (0.5)	0.026	<0.0001	0.0024
GO RESPONSE TO INTERFERON ALPHA	21	8 (38.1)	8 (38.1)	0 (0)	0.025	<0.0001	0.0024
GO MYELOID LEUKOCYTE MIGRATION	187	12 (6.4)	11 (5.9)	1 (0.5)	0.025	<0.0001	0.0024
GO RESPONSE TO INTERFERON BETA	30	6 (20)	6 (20)	0 (0)	0.018	<0.0001	0.0024
GO REGULATION OF RIBONUCLEASE ACTIVITY	8	4 (50)	4 (50)	0 (0)	0.013	<0.0001	0.0024
GO INFLAMMATORY RESPONSE	680	26 (3.8)	22 (3.2)	4 (0.6)	0.027	<0.0001	0.0044
GO CELL ACTIVATION	1265	41 (3.2)	36 (2.8)	5 (0.4)	0.027	<0.0001	0.0044
GO TAXIS	607	25 (4.1)	19 (3.1)	6 (1)	0.028	<0.0001	0.0059
GO EXOCYTOSIS	833	31 (3.7)	26 (3.1)	5 (0.6)	0.028	<0.0001	0.0059
GO CYTOKINE PRODUCTION	720	27 (3.8)	25 (3.5)	2 (0.3)	0.027	<0.0001	0.0059
GO SECRETION	1551	46 (3)	39 (2.5)	7 (0.5)	0.025	<0.0001	0.0059
GO REGULATION OF RESPONSE TO BIOTIC STIMULUS	466	22 (4.7)	22 (4.7)	0 (0)	0.029	<0.0001	0.0077
GO RESPONSE TO BACTERIUM	587	21 (3.6)	20 (3.4)	1 (0.2)	0.024	<0.0001	0.0113

Table S125: MSigDB GO Biological Processes enriched in differentially expressed genes at Day 30 compared to pre-Second Vaccination (NK cells, Study Arm 2 A/H7N9+AS03, D1, 29). Results are sorted by FDR adjusted p-value and Jaccard similarity index (continued.)

Gene Set ID	Gene Set Genes #	DE Genes N (%)	Up-reg. DE Genes N (%)	Down-reg. DE Genes N (%)	Jaccard Index	P-Value	FDR Adjusted P-Value
GO POSITIVE REGULATION OF RESPONSE TO EXTERNAL STI...	575	23 (4)	23 (4)	0 (0)	0.027	<0.0001	0.0129
GO ISG15 PROTEIN CONJUGATION	6	3 (50)	3 (50)	0 (0)	0.010	<0.0001	0.0143
GO REGULATION OF INFLAMMATORY RESPONSE	332	15 (4.5)	14 (4.2)	1 (0.3)	0.024	<0.0001	0.0154
GO DNA REPLICATION CHECKPOINT	15	4 (26.7)	4 (26.7)	0 (0)	0.013	<0.0001	0.0154
GO POSITIVE REGULATION OF IMMUNE SYSTEM PROCESS	972	32 (3.3)	30 (3.1)	2 (0.2)	0.026	<0.0001	0.0167
GO POSITIVE REGULATION OF TRANSFERASE ACTIVITY	614	24 (3.9)	20 (3.3)	4 (0.7)	0.027	0.0001	0.018
GO LEUKOCYTE MEDIATED IMMUNITY	701	26 (3.7)	23 (3.3)	3 (0.4)	0.027	0.0001	0.0188
GO REGULATION OF RESPONSE TO STRESS	1406	41 (2.9)	37 (2.6)	4 (0.3)	0.025	0.0001	0.0188
GO POSITIVE REGULATION OF KINASE ACTIVITY	543	22 (4.1)	18 (3.3)	4 (0.7)	0.027	0.0001	0.0211
GO REGULATION OF KINASE ACTIVITY	816	29 (3.6)	23 (2.8)	6 (0.7)	0.027	0.0001	0.0211
GO CELL ACTIVATION INVOLVED IN IMMUNE RESPONSE	658	24 (3.6)	21 (3.2)	3 (0.5)	0.026	0.0001	0.0221
GO REGULATION OF TRANSFERASE ACTIVITY	912	31 (3.4)	25 (2.7)	6 (0.7)	0.026	0.0002	0.0241
GO REGULATION OF IMMUNE RESPONSE	897	30 (3.3)	26 (2.9)	4 (0.4)	0.026	0.0002	0.0241
GO REGULATION OF LEUKOCYTE CHEMOTAXIS	102	8 (7.8)	8 (7.8)	0 (0)	0.020	0.0002	0.0251
GO MYELOID LEUKOCYTE MEDIATED IMMUNITY	506	20 (4)	18 (3.6)	2 (0.4)	0.025	0.0002	0.0329
GO DNA REPLICATION INITIATION	35	5 (14.3)	5 (14.3)	0 (0)	0.015	0.0002	0.0336
GO GRANULOCYTE MIGRATION	126	8 (6.3)	8 (6.3)	0 (0)	0.019	0.0003	0.0338
GO NEUTROPHIL MIGRATION	103	7 (6.8)	7 (6.8)	0 (0)	0.018	0.0003	0.0338
GO REGULATION OF MONOCYTE CHEMOTAXIS	22	4 (18.2)	4 (18.2)	0 (0)	0.013	0.0003	0.0345
GO DNA INTEGRITY CHECKPOINT	150	10 (6.7)	10 (6.7)	0 (0)	0.023	0.0003	0.0383
GO REGULATION OF MONONUCLEAR CELL MIGRATION	40	5 (12.5)	5 (12.5)	0 (0)	0.015	0.0003	0.0383
GO REGULATION OF BIOMINERALIZATION	85	7 (8.2)	4 (4.7)	3 (3.5)	0.018	0.0003	0.0389
GO MONOCYTE CHEMOTAXIS	51	5 (9.8)	5 (9.8)	0 (0)	0.014	0.0004	0.0424
GO REGULATION OF NUCLEASE ACTIVITY	21	4 (19)	4 (19)	0 (0)	0.013	0.0004	0.0424
GO PHAGOCYTOSIS	251	13 (5.2)	11 (4.4)	2 (0.8)	0.024	0.0004	0.0463
GO MONONUCLEAR CELL MIGRATION	74	6 (8.1)	6 (8.1)	0 (0)	0.016	0.0004	0.0479
GO DEFENSE RESPONSE TO BACTERIUM	237	10 (4.2)	10 (4.2)	0 (0)	0.019	0.0004	0.0483

Table S126: Blood Transcription Modules enriched in differentially expressed genes at Day 30 compared to pre-Second Vaccination (NK cells, Study Arm 2 A/H7N9+AS03, D1, 29). Results are sorted by FDR adjusted p-value and Jaccard similarity index.

Gene Set ID	Gene Set Genes #	DE Genes N (%)	Up-reg. DE Genes N (%)	Down-reg. DE Genes N (%)	Jaccard Index	P-Value	FDR Adjusted P-Value
M11.0 ENRICHED IN MONOCYTES (II)	174	39 (22.4)	39 (22.4)	0 (0)	0.089	<0.0001	0.0005
M4.0 CELL CYCLE AND TRANSCRIPTION	303	39 (12.9)	39 (12.9)	0 (0)	0.069	<0.0001	0.0005

Table S126: Blood Transcription Modules enriched in differentially expressed genes at Day 30 compared to pre-Second Vaccination (NK cells, Study Arm 2 A/H7N9+AS03, D1, 29). Results are sorted by FDR adjusted p-value and Jaccard similarity index (continued.)

Gene Set ID	Gene Set Genes #	DE Genes N (%)	Up-reg. DE Genes N (%)	Down-reg. DE Genes N (%)	Jaccard Index	P-Value	FDR Adjusted P-Value
M165 ENRICHED IN ACTIVATED DENDRITIC CELLS (II)	34	12 (35.3)	12 (35.3)	0 (0)	0.037	<0.0001	0.0005
M16 TLR AND INFLAMMATORY SIGNALING	41	11 (26.8)	11 (26.8)	0 (0)	0.033	<0.0001	0.0005
M127 TYPE I INTERFERON RESPONSE	12	10 (83.3)	10 (83.3)	0 (0)	0.033	<0.0001	0.0005
M150 INNATE ANTIVIRAL RESPONSE	12	10 (83.3)	10 (83.3)	0 (0)	0.033	<0.0001	0.0005
M75 ANTIVIRAL IFN SIGNATURE	22	9 (40.9)	8 (36.4)	1 (4.5)	0.029	<0.0001	0.0005
M111.1 VIRAL SENSING AND IMMUNITY; IRF2 TARGETS NE...	11	5 (45.5)	5 (45.5)	0 (0)	0.016	<0.0001	0.0017
M67 ACTIVATED DENDRITIC CELLS	12	5 (41.7)	5 (41.7)	0 (0)	0.016	0.0002	0.0069
M4.3 MYELOID CELL ENRICHED RECEPTORS AND TRANSPORT...	29	7 (24.1)	7 (24.1)	0 (0)	0.021	0.0003	0.0097
M111.0 VIRAL SENSING AND IMMUNITY; IRF2 TARGETS NE...	16	5 (31.2)	5 (31.2)	0 (0)	0.016	0.0004	0.0123
S4 MONOCYTE SURFACE SIGNATURE	81	12 (14.8)	11 (13.6)	1 (1.2)	0.032	0.0007	0.0208
M37.0 IMMUNE ACTIVATION - GENERIC CLUSTER	301	26 (8.6)	26 (8.6)	0 (0)	0.045	0.0009	0.0234
M4.4 MITOTIC CELL CYCLE - DNA REPLICATION	27	6 (22.2)	6 (22.2)	0 (0)	0.018	0.0017	0.041

Table S127: KEGG Pathways enriched in differentially expressed genes at Day 32 compared to pre-Second Vaccination (NK cells, Study Arm 2 A/H7N9+AS03, D1, 29). Results are sorted by FDR adjusted p-value and Jaccard similarity index.

Gene Set ID	Gene Set Genes #	DE Genes N (%)	Up-reg. DE Genes N (%)	Down-reg. DE Genes N (%)	Jaccard Index	P-Value	FDR Adjusted P-Value
CELL CYCLE	121	26 (21.5)	26 (21.5)	0 (0)	0.053	<0.0001	0.0017
OOCYTE MEIOSIS	123	12 (9.8)	12 (9.8)	0 (0)	0.024	<0.0001	0.0017
OSTEOCLAST DIFFERENTIATION	122	12 (9.8)	0 (0)	12 (9.8)	0.024	<0.0001	0.0017
DNA REPLICATION	34	7 (20.6)	7 (20.6)	0 (0)	0.017	<0.0001	0.0017
FANCONI ANEMIA PATHWAY	52	8 (15.4)	8 (15.4)	0 (0)	0.018	<0.0001	0.002
HOMOLOGOUS RECOMBINATION	39	7 (17.9)	7 (17.9)	0 (0)	0.016	<0.0001	0.0028
HUMAN T-CELL LEUKEMIA VIRUS 1 INFECTION	211	15 (7.1)	12 (5.7)	3 (1.4)	0.025	<0.0001	0.0039
PROGESTERONE-MEDIATED OOCYTE MATURATION	94	9 (9.6)	9 (9.6)	0 (0)	0.019	0.0005	0.0202
HEMATOPOIETIC CELL LINEAGE	93	8 (8.6)	0 (0)	8 (8.6)	0.017	0.0007	0.0262
STAPHYLOCOCCUS AUREUS INFECTION	91	7 (7.7)	0 (0)	7 (7.7)	0.015	0.0011	0.0374
P53 SIGNALING PATHWAY	73	7 (9.6)	7 (9.6)	0 (0)	0.015	0.0015	0.0447

Table S128: MSigDB Reactome Pathways enriched in differentially expressed genes at Day 32 compared to pre-Second Vaccination (NK cells, Study Arm 2 A/H7N9+AS03, D1, 29). Results are sorted by FDR adjusted p-value and Jaccard similarity index.

Gene Set ID	Gene Set Genes #	DE Genes N (%)	Up-reg. DE Genes N (%)	Down-reg. DE Genes N (%)	Jaccard Index	P-Value	FDR Adjusted P-Value
REACTOME CELL CYCLE	639	76 (11.9)	76 (11.9)	0 (0)	0.079	<0.0001	0.0004
REACTOME CELL CYCLE MITOTIC	532	66 (12.4)	66 (12.4)	0 (0)	0.077	<0.0001	0.0004
REACTOME CELL CYCLE CHECKPOINTS	276	43 (15.6)	43 (15.6)	0 (0)	0.068	<0.0001	0.0004
REACTOME MITOTIC G1 PHASE AND G1 S TRANSITION	139	25 (18)	25 (18)	0 (0)	0.049	<0.0001	0.0004
REACTOME RESOLUTION OF SISTER CHROMATID COHESION	118	24 (20.3)	24 (20.3)	0 (0)	0.049	<0.0001	0.0004
REACTOME MITOTIC PROMETAPHASE	188	27 (14.4)	27 (14.4)	0 (0)	0.049	<0.0001	0.0004
REACTOME DNA REPLICATION	120	23 (19.2)	23 (19.2)	0 (0)	0.047	<0.0001	0.0004
REACTOME MITOTIC METAPHASE AND ANAPHASE	221	27 (12.2)	27 (12.2)	0 (0)	0.046	<0.0001	0.0004
REACTOME MITOTIC SPINDLE CHECKPOINT	105	21 (20)	21 (20)	0 (0)	0.044	<0.0001	0.0004
REACTOME SEPARATION OF SISTER CHROMATIDS	177	24 (13.6)	24 (13.6)	0 (0)	0.044	<0.0001	0.0004
REACTOME M PHASE	393	31 (7.9)	31 (7.9)	0 (0)	0.041	<0.0001	0.0004
REACTOME S PHASE	151	21 (13.9)	21 (13.9)	0 (0)	0.040	<0.0001	0.0004
REACTOME RHO GTPASES ACTIVATE FORMINS	134	20 (14.9)	20 (14.9)	0 (0)	0.039	<0.0001	0.0004
REACTOME G2 M CHECKPOINTS	158	20 (12.7)	20 (12.7)	0 (0)	0.037	<0.0001	0.0004
REACTOME NEUTROPHIL DEGRANULATION	442	30 (6.8)	1 (0.2)	29 (6.6)	0.037	<0.0001	0.0004
REACTOME RHO GTPASE EFFECTORS	308	25 (8.1)	21 (6.8)	4 (1.3)	0.037	<0.0001	0.0004
REACTOME SIGNALING BY RHO GTPASES	424	29 (6.8)	24 (5.7)	5 (1.2)	0.037	<0.0001	0.0004
REACTOME HOMOLOGY DIRECTED REPAIR	134	17 (12.7)	17 (12.7)	0 (0)	0.033	<0.0001	0.0004
REACTOME INNATE IMMUNE SYSTEM	968	43 (4.4)	1 (0.1)	42 (4.3)	0.033	<0.0001	0.0004
REACTOME DNA DOUBLE STRAND BREAK REPAIR	162	17 (10.5)	17 (10.5)	0 (0)	0.031	<0.0001	0.0004
REACTOME DNA REPAIR	319	21 (6.6)	21 (6.6)	0 (0)	0.030	<0.0001	0.0004
REACTOME G1 S SPECIFIC TRANSCRIPTION	28	12 (42.9)	12 (42.9)	0 (0)	0.029	<0.0001	0.0004
REACTOME HDR THROUGH HOMOLOGOUS RECOMBINATION HRR	66	13 (19.7)	13 (19.7)	0 (0)	0.029	<0.0001	0.0004
REACTOME DNA REPLICATION PRE INITIATION	78	13 (16.7)	13 (16.7)	0 (0)	0.028	<0.0001	0.0004
REACTOME ACTIVATION OF THE PRE REPLICATIVE COMPLEX	31	11 (35.5)	11 (35.5)	0 (0)	0.026	<0.0001	0.0004
REACTOME DNA STRAND ELONGATION	31	11 (35.5)	11 (35.5)	0 (0)	0.026	<0.0001	0.0004
REACTOME ACTIVATION OF ATR IN RESPONSE TO REPLICAT...	36	11 (30.6)	11 (30.6)	0 (0)	0.026	<0.0001	0.0004
REACTOME APC C MEDIATED DEGRADATION OF CELL CYCLE ...	81	11 (13.6)	11 (13.6)	0 (0)	0.024	<0.0001	0.0004
REACTOME HOMOLOGOUS DNA PAIRING AND STRAND EXCHANG...	41	10 (24.4)	10 (24.4)	0 (0)	0.023	<0.0001	0.0004
REACTOME ASSEMBLY OF THE PRE REPLICATIVE COMPLEX	63	10 (15.9)	10 (15.9)	0 (0)	0.022	<0.0001	0.0004
REACTOME RESOLUTION OF D LOOP STRUCTURES	34	9 (26.5)	9 (26.5)	0 (0)	0.021	<0.0001	0.0004
REACTOME SWITCHING OF ORIGINS TO A POST REPLICATIV...	85	10 (11.8)	10 (11.8)	0 (0)	0.021	<0.0001	0.0004
REACTOME CHROMOSOME MAINTENANCE	112	10 (8.9)	10 (8.9)	0 (0)	0.020	<0.0001	0.0004
REACTOME UNWINDING OF DNA	12	8 (66.7)	8 (66.7)	0 (0)	0.020	<0.0001	0.0004
REACTOME G0 AND EARLY G1	27	8 (29.6)	8 (29.6)	0 (0)	0.019	<0.0001	0.0004
REACTOME RESOLUTION OF D LOOP STRUCTURES THROUGH S...	26	8 (30.8)	8 (30.8)	0 (0)	0.019	<0.0001	0.0004
REACTOME POLO LIKE KINASE MEDIATED EVENTS	16	7 (43.8)	7 (43.8)	0 (0)	0.017	<0.0001	0.0004

Table S128: MSigDB Reactome Pathways enriched in differentially expressed genes at Day 32 compared to pre-Second Vaccination (NK cells, Study Arm 2 A/H7N9+AS03, D1, 29). Results are sorted by FDR adjusted p-value and Jaccard similarity index (continued.)

Gene Set ID	Gene Set Genes #	DE Genes N (%)	Up-reg. DE Genes N (%)	Down-reg. DE Genes N (%)	Jaccard Index	P-Value	FDR Adjusted P-Value
REACTOME CYCLIN A B1 B2 ASSOCIATED EVENTS DURING G...	23	7 (30.4)	7 (30.4)	0 (0)	0.017	<0.0001	0.0004
REACTOME PHOSPHORYLATION OF EMI1	6	4 (66.7)	4 (66.7)	0 (0)	0.010	<0.0001	0.0004
REACTOME ACTIVATION OF NIMA KINASES NEK9 NEK6 NEK7	7	4 (57.1)	4 (57.1)	0 (0)	0.010	<0.0001	0.0004
REACTOME CONDENSATION OF PROMETAPHASE CHROMOSOMES	10	5 (50)	5 (50)	0 (0)	0.013	<0.0001	0.0007
REACTOME CDC6 ASSOCIATION WITH THE ORC ORIGIN COMP...	11	5 (45.5)	5 (45.5)	0 (0)	0.012	<0.0001	0.0007
REACTOME G2 M DNA REPLICATION CHECKPOINT	5	4 (80)	4 (80)	0 (0)	0.010	<0.0001	0.0007
REACTOME E2F ENABLED INHIBITION OF PRE REPLICATION...	9	4 (44.4)	4 (44.4)	0 (0)	0.010	<0.0001	0.0007
REACTOME REGULATION OF TLR BY ENDOGENOUS LIGAND	19	5 (26.3)	0 (0)	5 (26.3)	0.012	<0.0001	0.001
REACTOME MITOTIC G2 G2 M PHASES	186	15 (8.1)	15 (8.1)	0 (0)	0.026	<0.0001	0.0013
REACTOME REGULATION OF TP53 ACTIVITY THROUGH PHOSP...	85	10 (11.8)	10 (11.8)	0 (0)	0.021	<0.0001	0.0019
REACTOME KINESINS	59	8 (13.6)	8 (13.6)	0 (0)	0.018	<0.0001	0.0019
REACTOME ORC1 REMOVAL FROM CHROMATIN	66	8 (12.1)	8 (12.1)	0 (0)	0.018	<0.0001	0.0025
REACTOME TP53 REGULATES TRANSCRIPTION OF CELL CYCL...	48	7 (14.6)	7 (14.6)	0 (0)	0.016	<0.0001	0.0025
REACTOME TRANSCRIPTIONAL REGULATION BY E2F6	30	6 (20)	6 (20)	0 (0)	0.014	0.0001	0.0033
REACTOME E2F MEDIATED REGULATION OF DNA REPLICATIO...	20	5 (25)	5 (25)	0 (0)	0.012	0.0001	0.0038
REACTOME HDR THROUGH SINGLE STRAND ANNEALING SSA	36	6 (16.7)	6 (16.7)	0 (0)	0.014	0.0002	0.0049
REACTOME G2 M DNA DAMAGE CHECKPOINT	90	8 (8.9)	8 (8.9)	0 (0)	0.017	0.0002	0.0051
REACTOME GOLGI CISTERNAE PERICENTRIOLAR STACK REOR...	14	4 (28.6)	4 (28.6)	0 (0)	0.010	0.0002	0.0061
REACTOME PROCESSING OF DNA DOUBLE STRAND BREAK END...	94	8 (8.5)	8 (8.5)	0 (0)	0.017	0.0003	0.0071
REACTOME DEPOSITION OF NEW CENPA CONTAINING NUCLEO...	70	6 (8.6)	6 (8.6)	0 (0)	0.013	0.0003	0.0091
REACTOME TP53 REGULATES TRANSCRIPTION OF GENES INV...	18	4 (22.2)	4 (22.2)	0 (0)	0.010	0.0005	0.0129
REACTOME TRANSCRIPTION OF E2F TARGETS UNDER NEGATI...	16	4 (25)	4 (25)	0 (0)	0.010	0.0005	0.0135
REACTOME PCNA DEPENDENT LONG PATCH BASE EXCISION R...	21	4 (19)	4 (19)	0 (0)	0.010	0.0007	0.0186
REACTOME PHOSPHORYLATION OF THE APC C	19	4 (21.1)	4 (21.1)	0 (0)	0.010	0.0008	0.0208
REACTOME SUMOYLATION OF DNA REPLICATION PROTEINS	46	6 (13)	6 (13)	0 (0)	0.014	0.0009	0.0227
REACTOME TRANSCRIPTION OF E2F TARGETS UNDER NEGATI...	19	4 (21.1)	4 (21.1)	0 (0)	0.010	0.001	0.0246
REACTOME INHIBITION OF THE PROTEOLYTIC ACTIVITY OF...	20	4 (20)	4 (20)	0 (0)	0.010	0.0011	0.0255
REACTOME IRAK4 DEFICIENCY TLR2 4	10	3 (30)	0 (0)	3 (30)	0.007	0.0011	0.0255
REACTOME REGULATION OF TP53 ACTIVITY	148	11 (7.4)	11 (7.4)	0 (0)	0.021	0.0011	0.0267
REACTOME APC C CDC20 MEDIATED DEGRADATION OF CYCLI...	23	4 (17.4)	4 (17.4)	0 (0)	0.010	0.0014	0.0327
REACTOME RESOLUTION OF AP SITES VIA THE MULTIPLE N...	25	4 (16)	4 (16)	0 (0)	0.010	0.0017	0.0374
REACTOME DISEASES OF IMMUNE SYSTEM	22	4 (18.2)	0 (0)	4 (18.2)	0.010	0.0018	0.0404
REACTOME COPI DEPENDENT GOLGI TO ER RETROGRADE TRA...	97	8 (8.2)	8 (8.2)	0 (0)	0.017	0.0019	0.0411
REACTOME APC CDC20 MEDIATED DEGRADATION OF NEK2A	25	4 (16)	4 (16)	0 (0)	0.010	0.0022	0.0466
REACTOME CHK1 CHK2 CDS1 MEDIATED INACTIVATION OF C...	12	3 (25)	3 (25)	0 (0)	0.007	0.0022	0.0466
REACTOME METAL SEQUESTRATION BY ANTIMICROBIAL PROT...	6	2 (33.3)	0 (0)	2 (33.3)	0.005	0.0022	0.0466
REACTOME TELOMERE C STRAND LAGGING STRAND SYNTHESI...	27	4 (14.8)	4 (14.8)	0 (0)	0.009	0.0024	0.0491

Table S128: MSigDB Reactome Pathways enriched in differentially expressed genes at Day 32 compared to pre-Second Vaccination (NK cells, Study Arm 2 A/H7N9+AS03, D1, 29). Results are sorted by FDR adjusted p-value and Jaccard similarity index (continued.)

Gene Set ID	Gene Set Genes #	DE Genes N (%)	Up-reg. DE Genes N (%)	Down-reg. DE Genes N (%)	Jaccard Index	P-Value	FDR Adjusted P-Value
REACTOME TRANSCRIPTIONAL REGULATION BY TP53	341	17 (5)	17 (5)	0 (0)	0.024	0.0024	0.0494

Table S129: MSigDB GO Biological Processes enriched in differentially expressed genes at Day 32 compared to pre-Second Vaccination (NK cells, Study Arm 2 A/H7N9+AS03, D1, 29). Results are sorted by FDR adjusted p-value and Jaccard similarity index.

Gene Set ID	Gene Set Genes #	DE Genes N (%)	Up-reg. DE Genes N (%)	Down-reg. DE Genes N (%)	Jaccard Index	P-Value	FDR Adjusted P-Value
GO MITOTIC CELL CYCLE	954	86 (9)	84 (8.8)	2 (0.2)	0.068	<0.0001	0.0008
GO CHROMOSOME SEGREGATION	297	44 (14.8)	44 (14.8)	0 (0)	0.068	<0.0001	0.0008
GO NUCLEAR CHROMOSOME SEGREGATION	245	39 (15.9)	39 (15.9)	0 (0)	0.065	<0.0001	0.0008
GO MITOTIC NUCLEAR DIVISION	253	39 (15.4)	39 (15.4)	0 (0)	0.064	<0.0001	0.0008
GO ORGANELLE FISSION	428	49 (11.4)	49 (11.4)	0 (0)	0.063	<0.0001	0.0008
GO SISTER CHROMATID SEGREGATION	175	33 (18.9)	33 (18.9)	0 (0)	0.061	<0.0001	0.0008
GO DNA REPLICATION	258	37 (14.3)	37 (14.3)	0 (0)	0.060	<0.0001	0.0008
GO CELL CYCLE PROCESS	1291	94 (7.3)	92 (7.1)	2 (0.2)	0.059	<0.0001	0.0008
GO MITOTIC SISTER CHROMATID SEGREGATION	146	30 (20.5)	30 (20.5)	0 (0)	0.059	<0.0001	0.0008
GO CELL DIVISION	567	53 (9.3)	52 (9.2)	1 (0.2)	0.058	<0.0001	0.0008
GO CELL CYCLE PHASE TRANSITION	577	53 (9.2)	52 (9)	1 (0.2)	0.058	<0.0001	0.0008
GO CELL CYCLE	1718	106 (6.2)	102 (5.9)	4 (0.2)	0.053	<0.0001	0.0008
GO REGULATION OF CELL CYCLE PROCESS	716	55 (7.7)	53 (7.4)	2 (0.3)	0.052	<0.0001	0.0008
GO REGULATION OF CHROMOSOME SEGREGATION	96	24 (25)	24 (25)	0 (0)	0.051	<0.0001	0.0008
GO DNA DEPENDENT DNA REPLICATION	142	26 (18.3)	26 (18.3)	0 (0)	0.051	<0.0001	0.0008
GO CELL CYCLE CHECKPOINT	207	28 (13.5)	28 (13.5)	0 (0)	0.049	<0.0001	0.0008
GO REGULATION OF CELL CYCLE	1115	70 (6.3)	66 (5.9)	4 (0.4)	0.049	<0.0001	0.0008
GO CHROMOSOME ORGANIZATION	1118	70 (6.3)	67 (6)	3 (0.3)	0.049	<0.0001	0.0008
GO REGULATION OF MITOTIC CELL CYCLE	604	46 (7.6)	44 (7.3)	2 (0.3)	0.048	<0.0001	0.0008
GO DNA METABOLIC PROCESS	854	57 (6.7)	53 (6.2)	4 (0.5)	0.048	<0.0001	0.0008
GO REGULATION OF CELL CYCLE PHASE TRANSITION	433	37 (8.5)	36 (8.3)	1 (0.2)	0.047	<0.0001	0.0008
GO MICROTUBULE CYTOSKELETON ORGANIZATION INVOLVED ...	125	23 (18.4)	23 (18.4)	0 (0)	0.046	<0.0001	0.0008
GO POSITIVE REGULATION OF CELL CYCLE	360	33 (9.2)	31 (8.6)	2 (0.6)	0.046	<0.0001	0.0008
GO MITOTIC CELL CYCLE CHECKPOINT	158	24 (15.2)	24 (15.2)	0 (0)	0.045	<0.0001	0.0008
GO NEGATIVE REGULATION OF CELL CYCLE PROCESS	321	31 (9.7)	30 (9.3)	1 (0.3)	0.045	<0.0001	0.0008
GO POSITIVE REGULATION OF CELL CYCLE PROCESS	275	29 (10.5)	28 (10.2)	1 (0.4)	0.045	<0.0001	0.0008
GO NEGATIVE REGULATION OF MITOTIC CELL CYCLE	309	30 (9.7)	29 (9.4)	1 (0.3)	0.044	<0.0001	0.0008
GO NEGATIVE REGULATION OF CELL CYCLE PHASE TRANSIT...	243	27 (11.1)	26 (10.7)	1 (0.4)	0.044	<0.0001	0.0008

Table S129: MSigDB GO Biological Processes enriched in differentially expressed genes at Day 32 compared to pre-Second Vaccination (NK cells, Study Arm 2 A/H7N9+AS03, D1, 29). Results are sorted by FDR adjusted p-value and Jaccard similarity index (continued.)

Gene Set ID	Gene Set Genes #	DE Genes N (%)	Up-reg. DE Genes N (%)	Down-reg. DE Genes N (%)	Jaccard Index	P-Value	FDR Adjusted P-Value
GO SPINDLE ORGANIZATION	157	23 (14.6)	23 (14.6)	0 (0)	0.043	<0.0001	0.0008
GO DNA CONFORMATION CHANGE	284	28 (9.9)	28 (9.9)	0 (0)	0.043	<0.0001	0.0008
GO CELL CYCLE G1 S PHASE TRANSITION	262	27 (10.3)	26 (9.9)	1 (0.4)	0.043	<0.0001	0.0008
GO MITOTIC SPINDLE ORGANIZATION	99	20 (20.2)	20 (20.2)	0 (0)	0.042	<0.0001	0.0008
GO REGULATION OF SISTER CHROMATID SEGREGATION	75	19 (25.3)	19 (25.3)	0 (0)	0.042	<0.0001	0.0008
GO REGULATION OF NUCLEAR DIVISION	178	23 (12.9)	23 (12.9)	0 (0)	0.042	<0.0001	0.0008
GO NEGATIVE REGULATION OF CELL CYCLE	584	39 (6.7)	38 (6.5)	1 (0.2)	0.041	<0.0001	0.0008
GO DNA REPAIR	511	36 (7)	36 (7)	0 (0)	0.041	<0.0001	0.0008
GO CHROMOSOME SEPARATION	85	19 (22.4)	19 (22.4)	0 (0)	0.041	<0.0001	0.0008
GO MICROTUBULE CYTOSKELETON ORGANIZATION	522	36 (6.9)	35 (6.7)	1 (0.2)	0.041	<0.0001	0.0008
GO REGULATION OF CHROMOSOME ORGANIZATION	321	28 (8.7)	26 (8.1)	2 (0.6)	0.041	<0.0001	0.0008
GO NEGATIVE REGULATION OF CHROMOSOME ORGANIZATION	136	20 (14.7)	18 (13.2)	2 (1.5)	0.039	<0.0001	0.0008
GO CELLULAR RESPONSE TO DNA DAMAGE STIMULUS	789	44 (5.6)	43 (5.4)	1 (0.1)	0.039	<0.0001	0.0008
GO DOUBLE STRAND BREAK REPAIR	237	23 (9.7)	23 (9.7)	0 (0)	0.038	<0.0001	0.0008
GO MICROTUBULE BASED PROCESS	712	40 (5.6)	37 (5.2)	3 (0.4)	0.037	<0.0001	0.0008
GO MEIOTIC CELL CYCLE	229	22 (9.6)	22 (9.6)	0 (0)	0.036	<0.0001	0.0008
GO REGULATION OF CHROMOSOME SEPARATION	60	16 (26.7)	16 (26.7)	0 (0)	0.036	<0.0001	0.0008
GO MEIOTIC CELL CYCLE PROCESS	178	20 (11.2)	20 (11.2)	0 (0)	0.036	<0.0001	0.0008
GO DNA RECOMBINATION	270	23 (8.5)	23 (8.5)	0 (0)	0.036	<0.0001	0.0008
GO MYELOID LEUKOCYTE MEDIATED IMMUNITY	506	31 (6.1)	1 (0.2)	30 (5.9)	0.036	<0.0001	0.0008
GO RECOMBINATIONAL REPAIR	129	18 (14)	18 (14)	0 (0)	0.035	<0.0001	0.0008
GO MYELOID LEUKOCYTE ACTIVATION	594	33 (5.6)	1 (0.2)	32 (5.4)	0.035	<0.0001	0.0008
GO METAPHASE ANAPHASE TRANSITION OF CELL CYCLE	55	15 (27.3)	15 (27.3)	0 (0)	0.034	<0.0001	0.0008
GO CELL ACTIVATION INVOLVED IN IMMUNE RESPONSE	658	35 (5.3)	2 (0.3)	33 (5)	0.034	<0.0001	0.0008
GO DNA INTEGRITY CHECKPOINT	150	18 (12)	18 (12)	0 (0)	0.034	<0.0001	0.0008
GO CELL CYCLE G2 M PHASE TRANSITION	250	21 (8.4)	21 (8.4)	0 (0)	0.034	<0.0001	0.0008
GO NEGATIVE REGULATION OF CHROMOSOME SEGREGATION	42	14 (33.3)	14 (33.3)	0 (0)	0.033	<0.0001	0.0008
GO LEUKOCYTE MEDIATED IMMUNITY	701	34 (4.9)	2 (0.3)	32 (4.6)	0.032	<0.0001	0.0008
GO CYTOSKELETON ORGANIZATION	1253	51 (4.1)	39 (3.1)	12 (1)	0.032	<0.0001	0.0008
GO NEGATIVE REGULATION OF NUCLEAR DIVISION	57	14 (24.6)	14 (24.6)	0 (0)	0.032	<0.0001	0.0008
GO DNA GEOMETRIC CHANGE	101	15 (14.9)	15 (14.9)	0 (0)	0.031	<0.0001	0.0008
GO CELL CYCLE DNA REPLICATION	62	13 (21)	13 (21)	0 (0)	0.029	<0.0001	0.0008
GO PROTEIN DNA COMPLEX SUBUNIT ORGANIZATION	251	18 (7.2)	17 (6.8)	1 (0.4)	0.029	<0.0001	0.0008
GO NEGATIVE REGULATION OF METAPHASE ANAPHASE TRANS...	37	12 (32.4)	12 (32.4)	0 (0)	0.029	<0.0001	0.0008
GO POSITIVE REGULATION OF MITOTIC CELL CYCLE	148	15 (10.1)	14 (9.5)	1 (0.7)	0.028	<0.0001	0.0008
GO MEIOTIC CHROMOSOME SEGREGATION	85	13 (15.3)	13 (15.3)	0 (0)	0.028	<0.0001	0.0008
GO METAPHASE PLATE CONGRESSION	56	12 (21.4)	12 (21.4)	0 (0)	0.027	<0.0001	0.0008

Table S129: MSigDB GO Biological Processes enriched in differentially expressed genes at Day 32 compared to pre-Second Vaccination (NK cells, Study Arm 2 A/H7N9+AS03, D1, 29). Results are sorted by FDR adjusted p-value and Jaccard similarity index (continued.)

Gene Set ID	Gene Set Genes #	DE Genes N (%)	Up-reg. DE Genes N (%)	Down-reg. DE Genes N (%)	Jaccard Index	P-Value	FDR Adjusted P-Value
GO SPINDLE ASSEMBLY	98	13 (13.3)	13 (13.3)	0 (0)	0.027	<0.0001	0.0008
GO DNA PACKAGING	183	15 (8.2)	15 (8.2)	0 (0)	0.027	<0.0001	0.0008
GO ATTACHMENT OF SPINDLE MICROTUBULES TO KINETOCHO...	33	11 (33.3)	11 (33.3)	0 (0)	0.026	<0.0001	0.0008
GO MEIOSIS I CELL CYCLE PROCESS	111	13 (11.7)	13 (11.7)	0 (0)	0.026	<0.0001	0.0008
GO CHROMOSOME LOCALIZATION	74	12 (16.2)	12 (16.2)	0 (0)	0.026	<0.0001	0.0008
GO DNA REPLICATION INITIATION	35	11 (31.4)	11 (31.4)	0 (0)	0.026	<0.0001	0.0008
GO REGULATION OF CELL DIVISION	156	14 (9)	13 (8.3)	1 (0.6)	0.026	<0.0001	0.0008
GO CYTOKINESIS	160	14 (8.8)	13 (8.1)	1 (0.6)	0.026	<0.0001	0.0008
GO POSITIVE REGULATION OF CELL CYCLE PHASE TRANSIT...	91	12 (13.2)	12 (13.2)	0 (0)	0.025	<0.0001	0.0008
GO REGULATION OF CELL CYCLE G1 S PHASE TRANSITION	174	14 (8)	13 (7.5)	1 (0.6)	0.025	<0.0001	0.0008
GO REGULATION OF TRANSCRIPTION INVOLVED IN G1 S TR...	27	10 (37)	10 (37)	0 (0)	0.024	<0.0001	0.0008
GO REGULATION OF CYTOKINESIS	82	11 (13.4)	10 (12.2)	1 (1.2)	0.024	<0.0001	0.0008
GO MITOTIC SPINDLE ASSEMBLY	51	10 (19.6)	10 (19.6)	0 (0)	0.023	<0.0001	0.0008
GO SIGNAL TRANSDUCTION INVOLVED IN CELL CYCLE CHEC...	72	10 (13.9)	10 (13.9)	0 (0)	0.022	<0.0001	0.0008
GO ANAPHASE PROMOTING COMPLEX DEPENDENT CATABOLIC ...	76	10 (13.2)	10 (13.2)	0 (0)	0.022	<0.0001	0.0008
GO MITOTIC METAPHASE PLATE CONGRESSION	44	9 (20.5)	9 (20.5)	0 (0)	0.021	<0.0001	0.0008
GO CENTROMERE COMPLEX ASSEMBLY	53	9 (17)	9 (17)	0 (0)	0.021	<0.0001	0.0008
GO INTERLEUKIN 1 BETA PRODUCTION	87	9 (10.3)	0 (0)	9 (10.3)	0.019	<0.0001	0.0008
GO SISTER CHROMATID COHESION	52	8 (15.4)	8 (15.4)	0 (0)	0.018	<0.0001	0.0008
GO MITOTIC DNA REPLICATION	14	7 (50)	7 (50)	0 (0)	0.017	<0.0001	0.0008
GO DNA STRAND ELONGATION INVOLVED IN DNA REPLICATI...	16	7 (43.8)	7 (43.8)	0 (0)	0.017	<0.0001	0.0008
GO KINETOCHORE ORGANIZATION	20	7 (35)	7 (35)	0 (0)	0.017	<0.0001	0.0008
GO DNA STRAND ELONGATION	23	7 (30.4)	7 (30.4)	0 (0)	0.017	<0.0001	0.0008
GO POSITIVE REGULATION OF CYTOKINESIS	36	7 (19.4)	6 (16.7)	1 (2.8)	0.017	<0.0001	0.0008
GO DOUBLE STRAND BREAK REPAIR VIA BREAK INDUCED RE...	11	6 (54.5)	6 (54.5)	0 (0)	0.015	<0.0001	0.0008
GO PROTEIN LOCALIZATION TO KINETOCHORE	16	6 (37.5)	6 (37.5)	0 (0)	0.015	<0.0001	0.0008
GO PROTEIN LOCALIZATION TO CHROMOSOME CENTROMERIC ...	20	6 (30)	6 (30)	0 (0)	0.015	<0.0001	0.0008
GO ATTACHMENT OF MITOTIC SPINDLE MICROTUBULES TO K...	13	5 (38.5)	5 (38.5)	0 (0)	0.012	<0.0001	0.0008
GO DNA REPLICATION CHECKPOINT	15	5 (33.3)	5 (33.3)	0 (0)	0.012	<0.0001	0.0008
GO REGULATION OF ORGANELLE ORGANIZATION	1183	49 (4.1)	39 (3.3)	10 (0.8)	0.032	<0.0001	0.0015
GO INFLAMMATORY RESPONSE	680	31 (4.6)	0 (0)	31 (4.6)	0.030	<0.0001	0.0015
GO REGULATION OF CELL CYCLE G2 M PHASE TRANSITION	195	16 (8.2)	16 (8.2)	0 (0)	0.028	<0.0001	0.0015
GO MICROTUBULE ORGANIZING CENTER ORGANIZATION	128	13 (10.2)	13 (10.2)	0 (0)	0.025	<0.0001	0.0015
GO PROTEIN LOCALIZATION TO CHROMOSOME	78	10 (12.8)	10 (12.8)	0 (0)	0.022	<0.0001	0.0015
GO CHROMATIN REMODELING AT CENTROMERE	46	7 (15.2)	7 (15.2)	0 (0)	0.016	<0.0001	0.0015
GO FEMALE MEIOTIC NUCLEAR DIVISION	28	6 (21.4)	6 (21.4)	0 (0)	0.014	<0.0001	0.0015
GO NEGATIVE REGULATION OF ORGANELLE ORGANIZATION	363	22 (6.1)	20 (5.5)	2 (0.6)	0.030	<0.0001	0.0021

Table S129: MSigDB GO Biological Processes enriched in differentially expressed genes at Day 32 compared to pre-Second Vaccination (NK cells, Study Arm 2 A/H7N9+AS03, D1, 29). Results are sorted by FDR adjusted p-value and Jaccard similarity index (continued.)

Gene Set ID	Gene Set Genes #	DE Genes N (%)	Up-reg. DE Genes N (%)	Down-reg. DE Genes N (%)	Jaccard Index	P-Value	FDR Adjusted P-Value
GO CELL ACTIVATION	1265	48 (3.8)	3 (0.2)	45 (3.6)	0.030	<0.0001	0.0021
GO SIGNAL TRANSDUCTION IN RESPONSE TO DNA DAMAGE	128	12 (9.4)	12 (9.4)	0 (0)	0.023	<0.0001	0.0021
GO HUMORAL IMMUNE RESPONSE	227	14 (6.2)	1 (0.4)	13 (5.7)	0.023	<0.0001	0.0021
GO MITOTIC DNA INTEGRITY CHECKPOINT	101	11 (10.9)	11 (10.9)	0 (0)	0.023	<0.0001	0.0021
GO G2 DNA DAMAGE CHECKPOINT	29	6 (20.7)	6 (20.7)	0 (0)	0.014	<0.0001	0.0021
GO DNA UNWINDING INVOLVED IN DNA REPLICATION	14	5 (35.7)	5 (35.7)	0 (0)	0.012	<0.0001	0.0021
GO SIGNAL TRANSDUCTION BY P53 CLASS MEDIATOR	250	17 (6.8)	17 (6.8)	0 (0)	0.027	<0.0001	0.0028
GO INTERLEUKIN 1 PRODUCTION	99	9 (9.1)	0 (0)	9 (9.1)	0.018	<0.0001	0.0034
GO ANIMAL ORGAN REGENERATION	68	8 (11.8)	6 (8.8)	2 (2.9)	0.018	<0.0001	0.0034
GO DNA REPLICATION INDEPENDENT NUCLEOSOME ORGANIZA...	51	7 (13.7)	7 (13.7)	0 (0)	0.016	<0.0001	0.0034
GO NEGATIVE REGULATION OF CELL CYCLE G2 M PHASE TR...	95	10 (10.5)	10 (10.5)	0 (0)	0.021	<0.0001	0.004
GO EXOCYTOSIS	833	35 (4.2)	1 (0.1)	34 (4.1)	0.029	<0.0001	0.0046
GO MEIOTIC CHROMOSOME SEPARATION	21	5 (23.8)	5 (23.8)	0 (0)	0.012	<0.0001	0.0052
GO NEGATIVE REGULATION OF CELL CYCLE G1 S PHASE TR...	116	10 (8.6)	9 (7.8)	1 (0.9)	0.020	<0.0001	0.0058
GO INNATE IMMUNE RESPONSE	814	33 (4.1)	1 (0.1)	32 (3.9)	0.028	<0.0001	0.0062
GO REGULATION OF IMMUNE SYSTEM PROCESS	1409	49 (3.5)	0 (0)	49 (3.5)	0.028	<0.0001	0.0062
GO MITOTIC CYTOKINESIS	68	8 (11.8)	8 (11.8)	0 (0)	0.018	<0.0001	0.0062
GO POSITIVE REGULATION OF PHAGOCYTOSIS	61	7 (11.5)	0 (0)	7 (11.5)	0.016	<0.0001	0.0062
GO FEMALE MEIOSIS CHROMOSOME SEGREGATION	6	3 (50)	3 (50)	0 (0)	0.007	<0.0001	0.0062
GO MITOTIC NUCLEAR ENVELOPE DISASSEMBLY	12	4 (33.3)	4 (33.3)	0 (0)	0.010	0.0001	0.0068
GO DNA BIOSYNTHETIC PROCESS	181	13 (7.2)	11 (6.1)	2 (1.1)	0.023	0.0001	0.0072
GO HISTONE PHOSPHORYLATION	37	6 (16.2)	6 (16.2)	0 (0)	0.014	0.0001	0.0072
GO REGULATION OF ATTACHMENT OF SPINDLE MICROTUBULE...	12	4 (33.3)	4 (33.3)	0 (0)	0.010	0.0001	0.0072
GO SPINDLE LOCALIZATION	50	7 (14)	7 (14)	0 (0)	0.016	0.0001	0.0078
GO ESTABLISHMENT OF ORGANELLE LOCALIZATION	381	21 (5.5)	17 (4.5)	4 (1)	0.028	0.0001	0.0083
GO CYTOKINE PRODUCTION	720	29 (4)	2 (0.3)	27 (3.8)	0.027	0.0001	0.0088
GO CHEMOKINE PRODUCTION	80	8 (10)	0 (0)	8 (10)	0.017	0.0001	0.0088
GO REGULATION OF CYCLIN DEPENDENT PROTEIN KINASE A...	96	9 (9.4)	9 (9.4)	0 (0)	0.019	0.0002	0.0092
GO MITOTIC SPINDLE MIDZONE ASSEMBLY	6	3 (50)	3 (50)	0 (0)	0.007	0.0002	0.0092
GO POSITIVE REGULATION OF IMMUNE SYSTEM PROCESS	972	37 (3.8)	0 (0)	37 (3.8)	0.028	0.0002	0.0097
GO DEFENSE RESPONSE	1497	48 (3.2)	1 (0.1)	47 (3.1)	0.026	0.0002	0.01
GO CHROMOSOME CONDENSATION	45	6 (13.3)	6 (13.3)	0 (0)	0.014	0.0002	0.01
GO SIGNAL TRANSDUCTION INVOLVED IN G2 DNA DAMAGE C...	13	4 (30.8)	4 (30.8)	0 (0)	0.010	0.0002	0.01
GO HISTONE EXCHANGE	57	6 (10.5)	6 (10.5)	0 (0)	0.013	0.0002	0.0104
GO RESPIRATORY BURST	31	5 (16.1)	0 (0)	5 (16.1)	0.012	0.0002	0.0104
GO G0 TO G1 TRANSITION	39	6 (15.4)	6 (15.4)	0 (0)	0.014	0.0002	0.0108
GO KINETOCHORE ASSEMBLY	15	4 (26.7)	4 (26.7)	0 (0)	0.010	0.0002	0.0108

Table S129: MSigDB GO Biological Processes enriched in differentially expressed genes at Day 32 compared to pre-Second Vaccination (NK cells, Study Arm 2 A/H7N9+AS03, D1, 29). Results are sorted by FDR adjusted p-value and Jaccard similarity index (continued.)

Gene Set ID	Gene Set Genes #	DE Genes N (%)	Up-reg. DE Genes N (%)	Down-reg. DE Genes N (%)	Jaccard Index	P-Value	FDR Adjusted P-Value
GO DEFENSE RESPONSE TO OTHER ORGANISM	993	36 (3.6)	1 (0.1)	35 (3.5)	0.027	0.0002	0.0111
GO DEFENSE RESPONSE TO BACTERIUM	237	12 (5.1)	0 (0)	12 (5.1)	0.019	0.0002	0.0111
GO REGULATION OF DOUBLE STRAND BREAK REPAIR VIA HO...	41	6 (14.6)	6 (14.6)	0 (0)	0.014	0.0002	0.0111
GO REGULATION OF SPINDLE CHECKPOINT	14	4 (28.6)	4 (28.6)	0 (0)	0.010	0.0002	0.0121
GO REGULATION OF CENTROSOME CYCLE	58	7 (12.1)	7 (12.1)	0 (0)	0.016	0.0003	0.0136
GO IMMUNE EFFECTOR PROCESS	1077	39 (3.6)	3 (0.3)	36 (3.3)	0.027	0.0003	0.0138
GO CYTOSKELETON DEPENDENT CYTOKINESIS	95	9 (9.5)	9 (9.5)	0 (0)	0.019	0.0003	0.0138
GO LIVER REGENERATION	27	5 (18.5)	4 (14.8)	1 (3.7)	0.012	0.0003	0.0138
GO MITOTIC G2 M TRANSITION CHECKPOINT	28	5 (17.9)	5 (17.9)	0 (0)	0.012	0.0003	0.0142
GO RESPONSE TO BIOTIC STIMULUS	1325	44 (3.3)	3 (0.2)	41 (3.1)	0.026	0.0003	0.0152
GO PHAGOCYTOSIS RECOGNITION	16	4 (25)	0 (0)	4 (25)	0.010	0.0003	0.0156
GO REGULATION OF PHAGOCYTOSIS	86	8 (9.3)	0 (0)	8 (9.3)	0.017	0.0003	0.016
GO CHROMOSOME ORGANIZATION INVOLVED IN MEIOTIC CEL...	62	7 (11.3)	7 (11.3)	0 (0)	0.015	0.0003	0.0168
GO RESPONSE TO BACTERIUM	587	23 (3.9)	0 (0)	23 (3.9)	0.024	0.0004	0.018
GO DNA DAMAGE RESPONSE SIGNAL TRANSDUCTION BY P53 ...	103	9 (8.7)	9 (8.7)	0 (0)	0.018	0.0004	0.018
GO MEMBRANE DISASSEMBLY	16	4 (25)	4 (25)	0 (0)	0.010	0.0004	0.018
GO POSITIVE REGULATION OF CATALYTIC ACTIVITY	1331	47 (3.5)	21 (1.6)	26 (2)	0.028	0.0004	0.0182
GO REGULATION OF SUPEROXIDE METABOLIC PROCESS	30	5 (16.7)	1 (3.3)	4 (13.3)	0.012	0.0004	0.0182
GO POSITIVE REGULATION OF CHROMOSOME SEGREGATION	27	5 (18.5)	5 (18.5)	0 (0)	0.012	0.0004	0.0185
GO PRE REPLICATIVE COMPLEX ASSEMBLY INVOLVED IN CE...	7	3 (42.9)	3 (42.9)	0 (0)	0.007	0.0004	0.0185
GO CHROMATIN ASSEMBLY	141	9 (6.4)	9 (6.4)	0 (0)	0.017	0.0004	0.0193
GO REGULATION OF DNA REPLICATION	102	9 (8.8)	9 (8.8)	0 (0)	0.018	0.0004	0.0206
GO ACTOMYOSIN CONTRACTILE RING ORGANIZATION	7	3 (42.9)	3 (42.9)	0 (0)	0.007	0.0005	0.0237
GO CHROMATIN ASSEMBLY OR DISASSEMBLY	166	10 (6)	9 (5.4)	1 (0.6)	0.018	0.0005	0.0245
GO ANTIGEN PROCESSING AND PRESENTATION OF PEPTIDE ...	155	11 (7.1)	6 (3.9)	5 (3.2)	0.020	0.0005	0.0248
GO MYELOID LEUKOCYTE MIGRATION	187	11 (5.9)	0 (0)	11 (5.9)	0.019	0.0006	0.0256
GO MITOTIC DNA REPLICATION CHECKPOINT	9	3 (33.3)	3 (33.3)	0 (0)	0.007	0.0006	0.0263
GO ANTIGEN PROCESSING AND PRESENTATION OF PEPTIDE ...	84	8 (9.5)	6 (7.1)	2 (2.4)	0.017	0.0006	0.0266
GO IMMUNE RESPONSE REGULATING SIGNALING PATHWAY	507	23 (4.5)	0 (0)	23 (4.5)	0.026	0.0006	0.0273
GO SECRETION	1551	51 (3.3)	1 (0.1)	50 (3.2)	0.027	0.0006	0.0282
GO ANTIGEN PROCESSING AND PRESENTATION	189	12 (6.3)	6 (3.2)	6 (3.2)	0.021	0.0006	0.0282
GO SPINDLE ELONGATION	9	3 (33.3)	3 (33.3)	0 (0)	0.007	0.0006	0.0282
GO POSITIVE REGULATION OF IMMUNE RESPONSE	704	28 (4)	0 (0)	28 (4)	0.026	0.0007	0.0299
GO HOMOLOGOUS RECOMBINATION	50	6 (12)	6 (12)	0 (0)	0.014	0.0007	0.0299
GO RESPONSE TO LIPOTEICHOIC ACID	10	3 (30)	0 (0)	3 (30)	0.007	0.0007	0.0299
GO REGULATION OF REACTIVE OXYGEN SPECIES METABOLIC...	169	11 (6.5)	3 (1.8)	8 (4.7)	0.020	0.0007	0.0301
GO RESPONSE TO FUNGUS	47	5 (10.6)	0 (0)	5 (10.6)	0.011	0.0007	0.0308

Table S129: MSigDB GO Biological Processes enriched in differentially expressed genes at Day 32 compared to pre-Second Vaccination (NK cells, Study Arm 2 A/H7N9+AS03, D1, 29). Results are sorted by FDR adjusted p-value and Jaccard similarity index (continued.)

Gene Set ID	Gene Set Genes #	DE Genes N (%)	Up-reg. DE Genes N (%)	Down-reg. DE Genes N (%)	Jaccard Index	P-Value	FDR Adjusted P-Value
GO HOMOLOGOUS CHROMOSOME SEGREGATION	54	6 (11.1)	6 (11.1)	0 (0)	0.013	0.0007	0.0311
GO POSTREPLICATION REPAIR	50	6 (12)	6 (12)	0 (0)	0.014	0.0008	0.0326
GO LIPOPOLYSACCHARIDE MEDIATED SIGNALING PATHWAY	54	6 (11.1)	0 (0)	6 (11.1)	0.013	0.0008	0.0339
GO SPINDLE MIDZONE ASSEMBLY	9	3 (33.3)	3 (33.3)	0 (0)	0.007	0.0008	0.0339
GO LEUKOCYTE CHEMOTAXIS	202	11 (5.4)	0 (0)	11 (5.4)	0.019	0.0009	0.0391
GO REGULATION OF DNA REPAIR	115	9 (7.8)	9 (7.8)	0 (0)	0.018	0.001	0.0397
GO LEUKOCYTE MIGRATION	410	18 (4.4)	1 (0.2)	17 (4.1)	0.023	0.001	0.0403
GO ESTABLISHMENT OF MITOTIC SPINDLE LOCALIZATION	34	5 (14.7)	5 (14.7)	0 (0)	0.012	0.001	0.0409
GO MONONUCLEAR CELL MIGRATION	74	6 (8.1)	0 (0)	6 (8.1)	0.013	0.001	0.0419
GO NEUTROPHIL MIGRATION	103	7 (6.8)	0 (0)	7 (6.8)	0.014	0.001	0.0421
GO CELLULAR RESPONSE TO REACTIVE OXYGEN SPECIES	153	10 (6.5)	6 (3.9)	4 (2.6)	0.019	0.0011	0.0439
GO REGULATION OF SISTER CHROMATID COHESION	19	4 (21.1)	4 (21.1)	0 (0)	0.010	0.0011	0.0441
GO NUCLEOSOME ORGANIZATION	159	9 (5.7)	8 (5)	1 (0.6)	0.017	0.0012	0.0472
GO REGULATION OF UBIQUITIN PROTEIN LIGASE ACTIVITY	22	4 (18.2)	4 (18.2)	0 (0)	0.010	0.0012	0.0472
GO HISTONE SERINE PHOSPHORYLATION	11	3 (27.3)	3 (27.3)	0 (0)	0.007	0.0012	0.0477
GO REGULATION OF DOUBLE STRAND BREAK REPAIR	72	7 (9.7)	7 (9.7)	0 (0)	0.015	0.0012	0.0486
GO SUPEROXIDE METABOLIC PROCESS	62	6 (9.7)	1 (1.6)	5 (8.1)	0.013	0.0012	0.0488
GO PYRIMIDINE RIBONUCLEOSIDE METABOLIC PROCESS	14	3 (21.4)	1 (7.1)	2 (14.3)	0.007	0.0013	0.0497

Table S130: Blood Transcription Modules enriched in differentially expressed genes at Day 32 compared to pre-Second Vaccination (NK cells, Study Arm 2 A/H7N9+AS03, D1, 29). Results are sorted by FDR adjusted p-value and Jaccard similarity index.

Gene Set ID	Gene Set Genes #	DE Genes N (%)	Up-reg. DE Genes N (%)	Down-reg. DE Genes N (%)	Jaccard Index	P-Value	FDR Adjusted P-Value
M4.0 CELL CYCLE AND TRANSCRIPTION	303	164 (54.1)	103 (34)	61 (20.1)	0.306	<0.0001	0.0002
M4.1 CELL CYCLE (I)	135	83 (61.5)	83 (61.5)	0 (0)	0.185	<0.0001	0.0002
M11.0 ENRICHED IN MONOCYTES (II)	174	76 (43.7)	0 (0)	76 (43.7)	0.154	<0.0001	0.0002
M103 CELL CYCLE (III)	50	24 (48)	24 (48)	0 (0)	0.057	<0.0001	0.0002
M4.2 PLK1 SIGNALING EVENTS	31	21 (67.7)	21 (67.7)	0 (0)	0.052	<0.0001	0.0002
M6 MITOTIC CELL DIVISION	25	19 (76)	19 (76)	0 (0)	0.047	<0.0001	0.0002
M4.5 MITOTIC CELL CYCLE IN STIMULATED CD4 T CELLS	32	18 (56.2)	18 (56.2)	0 (0)	0.044	<0.0001	0.0002
M46 CELL DIVISION (STIMULATED CD4+ T CELLS)	25	16 (64)	16 (64)	0 (0)	0.040	<0.0001	0.0002
M4.4 MITOTIC CELL CYCLE - DNA REPLICATION	27	14 (51.9)	14 (51.9)	0 (0)	0.034	<0.0001	0.0002
M4.6 CELL DIVISION IN STIMULATED CD4 T CELLS	19	12 (63.2)	12 (63.2)	0 (0)	0.030	<0.0001	0.0002
M10.0 E2F1 TARGETS (Q3)	28	12 (42.9)	12 (42.9)	0 (0)	0.029	<0.0001	0.0002

Table S130: Blood Transcription Modules enriched in differentially expressed genes at Day 32 compared to pre-Second Vaccination (NK cells, Study Arm 2 A/H7N9+AS03, D1, 29). Results are sorted by FDR adjusted p-value and Jaccard similarity index (continued.)

Gene Set ID	Gene Set Genes #	DE Genes N (%)	Up-reg. DE Genes N (%)	Down-reg. DE Genes N (%)	Jaccard Index	P-Value	FDR Adjusted P-Value
M4.7 MITOTIC CELL CYCLE	20	11 (55)	11 (55)	0 (0)	0.027	<0.0001	0.0002
M4.9 MITOTIC CELL CYCLE IN STIMULATED CD4 T CELLS	15	10 (66.7)	10 (66.7)	0 (0)	0.025	<0.0001	0.0002
M8 E2F TRANSCRIPTION FACTOR NETWORK	11	9 (81.8)	9 (81.8)	0 (0)	0.023	<0.0001	0.0002
M4.10 CELL CYCLE (II)	14	9 (64.3)	9 (64.3)	0 (0)	0.022	<0.0001	0.0002
M15 RAN MEDIATED MITOSIS	12	8 (66.7)	8 (66.7)	0 (0)	0.020	<0.0001	0.0002
M4.12 C-MYC TRANSCRIPTIONAL NETWORK	12	8 (66.7)	8 (66.7)	0 (0)	0.020	<0.0001	0.0002
M16 TLR AND INFLAMMATORY SIGNALING	41	15 (36.6)	0 (0)	15 (36.6)	0.035	<0.0001	0.0003
M4.3 MYELOID CELL ENRICHED RECEPTORS AND TRANSPORT...	29	11 (37.9)	0 (0)	11 (37.9)	0.027	<0.0001	0.0003
M66 TBA	14	8 (57.1)	0 (0)	8 (57.1)	0.020	<0.0001	0.0003
S4 MONOCYTE SURFACE SIGNATURE	81	21 (25.9)	0 (0)	21 (25.9)	0.046	<0.0001	0.0005
M4.11 MITOTIC CELL CYCLE IN STIMULATED CD4 T CELLS	9	6 (66.7)	6 (66.7)	0 (0)	0.015	<0.0001	0.0005
M22.0 MISMATCH REPAIR (I)	25	10 (40)	10 (40)	0 (0)	0.024	<0.0001	0.0008
M47.1 ENRICHED IN B CELLS (II)	35	11 (31.4)	0 (0)	11 (31.4)	0.026	0.0002	0.003
M37.1 ENRICHED IN NEUTROPHILS (I)	46	12 (26.1)	0 (0)	12 (26.1)	0.028	0.0003	0.0037
M76 DNA REPAIR	20	8 (40)	8 (40)	0 (0)	0.020	0.0003	0.0037
M10.1 E2F1 TARGETS (Q4)	20	8 (40)	8 (40)	0 (0)	0.020	0.0003	0.0045
M49 TRANSCRIPTION REGULATION IN CELL DEVELOPMENT	43	12 (27.9)	9 (20.9)	3 (7)	0.028	0.0004	0.0049
M11.1 BLOOD COAGULATION	21	8 (38.1)	0 (0)	8 (38.1)	0.020	0.0004	0.0049
M47.2 ENRICHED IN B CELLS (III)	25	8 (32)	0 (0)	8 (32)	0.019	0.0008	0.0093
M4.14 RHO GTPASE CYCLE	9	5 (55.6)	5 (55.6)	0 (0)	0.013	0.001	0.0107
M87 TRANSMEMBRANE TRANSPORT (I)	24	8 (33.3)	0 (0)	8 (33.3)	0.019	0.002	0.0215
M48 TBA	12	5 (41.7)	0 (0)	5 (41.7)	0.012	0.0021	0.0217
M4.13 CELL JUNCTION (GO)	11	5 (45.5)	0 (0)	5 (45.5)	0.012	0.0026	0.027
M47.0 ENRICHED IN B CELLS (I)	45	10 (22.2)	0 (0)	10 (22.2)	0.023	0.0045	0.0418
M64 ENRICHED IN ACTIVATED DENDRITIC CELLS-MONOCYTE...	14	5 (35.7)	0 (0)	5 (35.7)	0.012	0.0043	0.0418
M132 RECRUITMENT OF NEUTROPHILS	10	4 (40)	0 (0)	4 (40)	0.010	0.0045	0.0418

Table S131: Blood Transcription Modules enriched in differentially expressed genes at Day 36 compared to pre-Second Vaccination (NK cells, Study Arm 2 A/H7N9+AS03, D1, 29). Results are sorted by FDR adjusted p-value and Jaccard similarity index.

Gene Set ID	Gene Set Genes #	DE Genes N (%)	Up-reg. DE Genes N (%)	Down-reg. DE Genes N (%)	Jaccard Index	P-Value	FDR Adjusted P-Value
M11.0 ENRICHED IN MONOCYTES (II)	174	28 (16.1)	0 (0)	28 (16.1)	0.090	<0.0001	0.0012
M4.0 CELL CYCLE AND TRANSCRIPTION	303	27 (8.9)	0 (0)	27 (8.9)	0.061	<0.0001	0.0012
M4.3 MYELOID CELL ENRICHED RECEPTORS AND TRANSPORT...	29	8 (27.6)	0 (0)	8 (27.6)	0.043	<0.0001	0.0012

Table S131: Blood Transcription Modules enriched in differentially expressed genes at Day 36 compared to pre-Second Vaccination (NK cells, Study Arm 2 A/H7N9+AS03, D1, 29). Results are sorted by FDR adjusted p-value and Jaccard similarity index (continued.)

Gene Set ID	Gene Set Genes #	DE Genes N (%)	Up-reg. DE Genes N (%)	Down-reg. DE Genes N (%)	Jaccard Index	P-Value	FDR Adjusted P-Value
S4 MONOCYTE SURFACE SIGNATURE	81	11 (13.6)	0 (0)	11 (13.6)	0.047	<0.0001	0.0017
M37.0 IMMUNE ACTIVATION - GENERIC CLUSTER	301	18 (6)	1 (0.3)	17 (5.6)	0.040	0.0002	0.0118
M118.0 ENRICHED IN MONOCYTES (IV)	50	7 (14)	0 (0)	7 (14)	0.034	0.0003	0.0185

Table S132: KEGG Pathways enriched in differentially expressed genes at Day 2 compared to pre-First Vaccination (B cells, Study Arm 2 A/H7N9+AS03, D1, 29). Results are sorted by FDR adjusted p-value and Jaccard similarity index.

Gene Set ID	Gene Set Genes #	DE Genes N (%)	Up-reg. DE Genes N (%)	Down-reg. DE Genes N (%)	Jaccard Index	P-Value	FDR Adjusted P-Value
NOD-LIKE RECEPTOR SIGNALING PATHWAY	175	6 (3.4)	6 (3.4)	0 (0)	0.027	<0.0001	0.0202

Table S133: MSigDB Reactome Pathways enriched in differentially expressed genes at Day 2 compared to pre-First Vaccination (B cells, Study Arm 2 A/H7N9+AS03, D1, 29). Results are sorted by FDR adjusted p-value and Jaccard similarity index.

Gene Set ID	Gene Set Genes #	DE Genes N (%)	Up-reg. DE Genes N (%)	Down-reg. DE Genes N (%)	Jaccard Index	P-Value	FDR Adjusted P-Value
REACTOME INTERFERON ALPHA BETA SIGNALING	60	13 (21.7)	13 (21.7)	0 (0)	0.133	<0.0001	0.0031
REACTOME INTERFERON SIGNALING	177	18 (10.2)	18 (10.2)	0 (0)	0.086	<0.0001	0.0031
REACTOME INTERFERON GAMMA SIGNALING	71	9 (12.7)	9 (12.7)	0 (0)	0.080	<0.0001	0.0031
REACTOME ANTIVIRAL MECHANISM BY IFN STIMULATED GEN...	81	7 (8.6)	7 (8.6)	0 (0)	0.056	<0.0001	0.0031
REACTOME CYTOKINE SIGNALING IN IMMUNE SYSTEM	788	19 (2.4)	19 (2.4)	0 (0)	0.023	<0.0001	0.0031

Table S134: MSigDB GO Biological Processes enriched in differentially expressed genes at Day 2 compared to pre-First Vaccination (B cells, Study Arm 2 A/H7N9+AS03, D1, 29). Results are sorted by FDR adjusted p-value and Jaccard similarity index.

Gene Set ID	Gene Set Genes #	DE Genes N (%)	Up-reg. DE Genes N (%)	Down-reg. DE Genes N (%)	Jaccard Index	P-Value	FDR Adjusted P-Value
GO RESPONSE TO TYPE I INTERFERON	88	13 (14.8)	13 (14.8)	0 (0)	0.103	<0.0001	0.0036
GO NEGATIVE REGULATION OF VIRAL GENOME REPLICATION	58	8 (13.8)	8 (13.8)	0 (0)	0.079	<0.0001	0.0036
GO INTERFERON GAMMA MEDIATED SIGNALING PATHWAY	70	8 (11.4)	8 (11.4)	0 (0)	0.071	<0.0001	0.0036
GO NEGATIVE REGULATION OF VIRAL PROCESS	90	9 (10)	9 (10)	0 (0)	0.068	<0.0001	0.0036

Table S134: MSigDB GO Biological Processes enriched in differentially expressed genes at Day 2 compared to pre-First Vaccination (B cells, Study Arm 2 A/H7N9+AS03, D1, 29). Results are sorted by FDR adjusted p-value and Jaccard similarity index (continued.)

Gene Set ID	Gene Set Genes #	DE Genes N (%)	Up-reg. DE Genes N (%)	Down-reg. DE Genes N (%)	Jaccard Index	P-Value	FDR Adjusted P-Value
GO NEGATIVE REGULATION OF VIRAL LIFE CYCLE	76	8 (10.5)	8 (10.5)	0 (0)	0.067	<0.0001	0.0036
GO DEFENSE RESPONSE TO VIRUS	229	16 (7)	16 (7)	0 (0)	0.061	<0.0001	0.0036
GO REGULATION OF VIRAL GENOME REPLICATION	91	8 (8.8)	8 (8.8)	0 (0)	0.060	<0.0001	0.0036
GO RESPONSE TO INTERFERON GAMMA	166	12 (7.2)	12 (7.2)	0 (0)	0.059	<0.0001	0.0036
GO RESPONSE TO VIRUS	300	17 (5.7)	17 (5.7)	0 (0)	0.051	<0.0001	0.0036
GO VIRAL GENOME REPLICATION	117	8 (6.8)	8 (6.8)	0 (0)	0.050	<0.0001	0.0036
GO REGULATION OF VIRAL LIFE CYCLE	133	8 (6)	8 (6)	0 (0)	0.045	<0.0001	0.0036
GO NEGATIVE REGULATION OF MULTI ORGANISM PROCESS	195	10 (5.1)	10 (5.1)	0 (0)	0.042	<0.0001	0.0036
GO REGULATION OF SYMBIOSIS ENCOMPASSING MUTUALISM ...	204	9 (4.4)	9 (4.4)	0 (0)	0.037	<0.0001	0.0036
GO INNATE IMMUNE RESPONSE	814	20 (2.5)	20 (2.5)	0 (0)	0.024	<0.0001	0.0036
GO CYTOKINE MEDIATED SIGNALING PATHWAY	711	17 (2.4)	17 (2.4)	0 (0)	0.023	<0.0001	0.0036
GO DEFENSE RESPONSE TO OTHER ORGANISM	993	23 (2.3)	23 (2.3)	0 (0)	0.022	<0.0001	0.0036
GO RESPONSE TO BIOTIC STIMULUS	1325	27 (2)	27 (2)	0 (0)	0.020	<0.0001	0.0036
GO REGULATION OF MULTI ORGANISM PROCESS	700	14 (2)	14 (2)	0 (0)	0.019	<0.0001	0.0036
GO RESPONSE TO CYTOKINE	1099	19 (1.7)	19 (1.7)	0 (0)	0.017	<0.0001	0.0036
GO DEFENSE RESPONSE	1497	24 (1.6)	24 (1.6)	0 (0)	0.016	<0.0001	0.0036
GO IMMUNE EFFECTOR PROCESS	1077	17 (1.6)	17 (1.6)	0 (0)	0.015	<0.0001	0.0036
GO RESPONSE TO INTERFERON BETA	30	4 (13.3)	4 (13.3)	0 (0)	0.052	<0.0001	0.0065
GO VIRAL LIFE CYCLE	308	8 (2.6)	8 (2.6)	0 (0)	0.023	<0.0001	0.0065
GO RESPONSE TO INTERFERON ALPHA	21	3 (14.3)	3 (14.3)	0 (0)	0.043	<0.0001	0.0157
GO REGULATION OF DEFENSE RESPONSE TO VIRUS	68	4 (5.9)	4 (5.9)	0 (0)	0.035	<0.0001	0.0181
GO RESPONSE TO PROTOZOAN	26	3 (11.5)	3 (11.5)	0 (0)	0.041	<0.0001	0.0261
GO ISG15 PROTEIN CONJUGATION	6	2 (33.3)	2 (33.3)	0 (0)	0.036	0.0001	0.0403
GO REGULATION OF RESPONSE TO BIOTIC STIMULUS	466	8 (1.7)	8 (1.7)	0 (0)	0.016	0.0001	0.0403

Table S135: Blood Transcription Modules enriched in differentially expressed genes at Day 2 compared to pre-First Vaccination (B cells, Study Arm 2 A/H7N9+AS03, D1, 29). Results are sorted by FDR adjusted p-value and Jaccard similarity index.

Gene Set ID	Gene Set Genes #	DE Genes N (%)	Up-reg. DE Genes N (%)	Down-reg. DE Genes N (%)	Jaccard Index	P-Value	FDR Adjusted P-Value
M127 TYPE I INTERFERON RESPONSE	12	6 (50)	6 (50)	0 (0)	0.105	<0.0001	0.0017
M150 INNATE ANTIVIRAL RESPONSE	12	5 (41.7)	5 (41.7)	0 (0)	0.086	<0.0001	0.0017
M165 ENRICHED IN ACTIVATED DENDRITIC CELLS (II)	34	4 (11.8)	4 (11.8)	0 (0)	0.049	<0.0001	0.0115
M75 ANTIVIRAL IFN SIGNATURE	22	3 (13.6)	3 (13.6)	0 (0)	0.043	0.0004	0.0337

Table S136: KEGG Pathways enriched in differentially expressed genes at Day 8 compared to pre-First Vaccination (B cells, Study Arm 1 A/H3N2v, D1). Results are sorted by FDR adjusted p-value and Jaccard similarity index.

Gene Set ID	Gene Set Genes #	DE Genes N (%)	Up-reg. DE Genes N (%)	Down-reg. DE Genes N (%)	Jaccard Index	P-Value	FDR Adjusted P-Value
AFRICAN TRYPANOSOMIASIS	36	3 (8.3)	3 (8.3)	0 (0)	0.083	<0.0001	0.0017
MALARIA	48	3 (6.2)	3 (6.2)	0 (0)	0.062	<0.0001	0.0017

Table S137: MSigDB Reactome Pathways enriched in differentially expressed genes at Day 8 compared to pre-First Vaccination (B cells, Study Arm 1 A/H3N2v, D1). Results are sorted by FDR adjusted p-value and Jaccard similarity index.

Gene Set ID	Gene Set Genes #	DE Genes N (%)	Up-reg. DE Genes N (%)	Down-reg. DE Genes N (%)	Jaccard Index	P-Value	FDR Adjusted P-Value
REACTOME ERYTHROCYTES TAKE UP OXYGEN AND RELEASE C...	9	3 (33.3)	3 (33.3)	0 (0)	0.333	<0.0001	0.0038
REACTOME ERYTHROCYTES TAKE UP CARBON DIOXIDE AND R...	13	3 (23.1)	3 (23.1)	0 (0)	0.231	<0.0001	0.0038
REACTOME SCAVENGING OF HEME FROM PLASMA	13	3 (23.1)	3 (23.1)	0 (0)	0.231	<0.0001	0.0038
REACTOME BINDING AND UPTAKE OF LIGANDS BY SCAVENGE...	41	3 (7.3)	3 (7.3)	0 (0)	0.073	<0.0001	0.0038

Table S138: MSigDB GO Biological Processes enriched in differentially expressed genes at Day 8 compared to pre-First Vaccination (B cells, Study Arm 1 A/H3N2v, D1). Results are sorted by FDR adjusted p-value and Jaccard similarity index.

Gene Set ID	Gene Set Genes #	DE Genes N (%)	Up-reg. DE Genes N (%)	Down-reg. DE Genes N (%)	Jaccard Index	P-Value	FDR Adjusted P-Value
GO OXYGEN TRANSPORT	15	3 (20)	3 (20)	0 (0)	0.200	<0.0001	0.0042
GO GAS TRANSPORT	19	3 (15.8)	3 (15.8)	0 (0)	0.158	<0.0001	0.0042
GO HYDROGEN PEROXIDE CATABOLIC PROCESS	31	3 (9.7)	3 (9.7)	0 (0)	0.097	<0.0001	0.0042
GO BICARBONATE TRANSPORT	41	3 (7.3)	3 (7.3)	0 (0)	0.073	<0.0001	0.0042
GO HYDROGEN PEROXIDE METABOLIC PROCESS	50	3 (6)	3 (6)	0 (0)	0.060	<0.0001	0.0042
GO ANTIBIOTIC CATABOLIC PROCESS	57	3 (5.3)	3 (5.3)	0 (0)	0.053	<0.0001	0.0042
GO COFACTOR CATABOLIC PROCESS	62	3 (4.8)	3 (4.8)	0 (0)	0.048	<0.0001	0.0042
GO CELLULAR DETOXIFICATION	103	3 (2.9)	3 (2.9)	0 (0)	0.029	<0.0001	0.0042
GO ANTIBIOTIC METABOLIC PROCESS	113	3 (2.7)	3 (2.7)	0 (0)	0.026	<0.0001	0.0042
GO DETOXIFICATION	127	3 (2.4)	3 (2.4)	0 (0)	0.024	<0.0001	0.0042
GO DRUG CATABOLIC PROCESS	131	3 (2.3)	3 (2.3)	0 (0)	0.023	<0.0001	0.0042
GO RESPONSE TO HYDROGEN PEROXIDE	136	3 (2.2)	3 (2.2)	0 (0)	0.022	<0.0001	0.0042
GO DRUG TRANSPORT	190	3 (1.6)	3 (1.6)	0 (0)	0.016	<0.0001	0.0042
GO RESPONSE TO REACTIVE OXYGEN SPECIES	217	3 (1.4)	3 (1.4)	0 (0)	0.014	<0.0001	0.0042
GO CELLULAR RESPONSE TO TOXIC SUBSTANCE	227	3 (1.3)	3 (1.3)	0 (0)	0.013	<0.0001	0.0042

Table S138: MSigDB GO Biological Processes enriched in differentially expressed genes at Day 8 compared to pre-First Vaccination (B cells, Study Arm 1 A/H3N2v, D1). Results are sorted by FDR adjusted p-value and Jaccard similarity index (continued.)

Gene Set ID	Gene Set Genes #	DE Genes N (%)	Up-reg. DE Genes N (%)	Down-reg. DE Genes N (%)	Jaccard Index	P-Value	FDR Adjusted P-Value
GO RECEPTOR MEDIATED ENDOCYTOSIS	245	3 (1.2)	3 (1.2)	0 (0)	0.012	<0.0001	0.0042
GO REACTIVE OXYGEN SPECIES METABOLIC PROCESS	251	3 (1.2)	3 (1.2)	0 (0)	0.012	<0.0001	0.0042
GO COFACTOR METABOLIC PROCESS	418	3 (0.7)	3 (0.7)	0 (0)	0.007	<0.0001	0.0042
GO RESPONSE TO ANTIBIOTIC	315	3 (1)	3 (1)	0 (0)	0.009	<0.0001	0.0072
GO RESPONSE TO OXIDATIVE STRESS	420	3 (0.7)	3 (0.7)	0 (0)	0.007	<0.0001	0.0072
GO DRUG METABOLIC PROCESS	548	3 (0.5)	3 (0.5)	0 (0)	0.005	<0.0001	0.0072
GO ORGANIC ANION TRANSPORT	460	3 (0.7)	3 (0.7)	0 (0)	0.006	<0.0001	0.0137
GO RESPONSE TO TOXIC SUBSTANCE	507	3 (0.6)	3 (0.6)	0 (0)	0.006	<0.0001	0.0181
GO ENDOCYTOSIS	527	3 (0.6)	3 (0.6)	0 (0)	0.006	<0.0001	0.0181
GO POSITIVE REGULATION OF CELL DEATH	670	3 (0.4)	3 (0.4)	0 (0)	0.004	<0.0001	0.0181
GO ANION TRANSPORT	589	3 (0.5)	3 (0.5)	0 (0)	0.005	<0.0001	0.0261
GO RESPONSE TO INORGANIC SUBSTANCE	531	3 (0.6)	3 (0.6)	0 (0)	0.006	<0.0001	0.0269
GO IMPORT INTO CELL	668	3 (0.4)	3 (0.4)	0 (0)	0.004	<0.0001	0.0269

Table S139: KEGG Pathways enriched in differentially expressed genes at Day 30 compared to pre-Second Vaccination (B cells, Study Arm 2 A/H7N9+AS03, D1, 29). Results are sorted by FDR adjusted p-value and Jaccard similarity index.

Gene Set ID	Gene Set Genes #	DE Genes N (%)	Up-reg. DE Genes N (%)	Down-reg. DE Genes N (%)	Jaccard Index	P-Value	FDR Adjusted P-Value
OSTEOCLAST DIFFERENTIATION	122	22 (18)	22 (18)	0 (0)	0.049	<0.0001	0.0011
NOD-LIKE RECEPTOR SIGNALING PATHWAY	175	20 (11.4)	20 (11.4)	0 (0)	0.040	<0.0001	0.0011
INFLUENZA A	160	15 (9.4)	15 (9.4)	0 (0)	0.031	<0.0001	0.0011
VIRAL PROTEIN INTERACTION WITH CYTOKINE AND CYTOKI...	96	10 (10.4)	10 (10.4)	0 (0)	0.023	<0.0001	0.0025
MALARIA	48	7 (14.6)	7 (14.6)	0 (0)	0.018	<0.0001	0.004
HEPATITIS C	152	12 (7.9)	12 (7.9)	0 (0)	0.025	<0.0001	0.0043
AFRICAN TRYPANOSOMIASIS	36	6 (16.7)	6 (16.7)	0 (0)	0.016	<0.0001	0.0043
HEMATOPOIETIC CELL LINEAGE	93	9 (9.7)	9 (9.7)	0 (0)	0.021	0.0001	0.0055
CYTOKINE-CYTOKINE RECEPTOR INTERACTION	280	15 (5.4)	15 (5.4)	0 (0)	0.025	0.0002	0.0064
TUBERCULOSIS	173	12 (6.9)	12 (6.9)	0 (0)	0.024	0.0004	0.0138
B CELL RECEPTOR SIGNALING PATHWAY	77	8 (10.4)	8 (10.4)	0 (0)	0.019	0.0005	0.0147
C-TYPE LECTIN RECEPTOR SIGNALING PATHWAY	103	9 (8.7)	9 (8.7)	0 (0)	0.021	0.0006	0.0163
TNF SIGNALING PATHWAY	109	9 (8.3)	9 (8.3)	0 (0)	0.020	0.0008	0.0193
CHOLESTEROL METABOLISM	50	6 (12)	6 (12)	0 (0)	0.015	0.0008	0.0193
LEISHMANIASIS	70	7 (10)	7 (10)	0 (0)	0.017	0.0009	0.0202
STAPHYLOCOCCUS AUREUS INFECTION	91	7 (7.7)	7 (7.7)	0 (0)	0.016	0.001	0.0215

Table S139: KEGG Pathways enriched in differentially expressed genes at Day 30 compared to pre-Second Vaccination (B cells, Study Arm 2 A/H7N9+AS03, D1, 29). Results are sorted by FDR adjusted p-value and Jaccard similarity index (continued.)

Gene Set ID	Gene Set Genes #	DE Genes N (%)	Up-reg. DE Genes N (%)	Down-reg. DE Genes N (%)	Jaccard Index	P-Value	FDR Adjusted P-Value
EPSTEIN-BARR VIRUS INFECTION	196	12 (6.1)	12 (6.1)	0 (0)	0.023	0.0014	0.027
PHAGOSOME	145	10 (6.9)	10 (6.9)	0 (0)	0.021	0.0015	0.027
AMOEBIASIS	99	8 (8.1)	8 (8.1)	0 (0)	0.018	0.0015	0.027
INFLAMMATORY BOWEL DISEASE	61	6 (9.8)	6 (9.8)	0 (0)	0.015	0.0017	0.0281
CHAGAS DISEASE	99	8 (8.1)	8 (8.1)	0 (0)	0.018	0.002	0.0302
COMPLEMENT AND COAGULATION CASCADES	82	7 (8.5)	7 (8.5)	0 (0)	0.017	0.0019	0.0302
MEASLES	132	9 (6.8)	9 (6.8)	0 (0)	0.019	0.0026	0.0367
NECROPTOSIS	150	9 (6)	9 (6)	0 (0)	0.018	0.0027	0.0367
NATURAL KILLER CELL MEDIATED CYTOTOXICITY	121	8 (6.6)	8 (6.6)	0 (0)	0.018	0.0027	0.0367
YERSINIA INFECTION	125	9 (7.2)	9 (7.2)	0 (0)	0.019	0.0034	0.0443
TOLL-LIKE RECEPTOR SIGNALING PATHWAY	98	7 (7.1)	7 (7.1)	0 (0)	0.016	0.0037	0.0461

Table S140: MSigDB Reactome Pathways enriched in differentially expressed genes at Day 30 compared to pre-Second Vaccination (B cells, Study Arm 2 A/H7N9+AS03, D1, 29). Results are sorted by FDR adjusted p-value and Jaccard similarity index.

Gene Set ID	Gene Set Genes #	DE Genes N (%)	Up-reg. DE Genes N (%)	Down-reg. DE Genes N (%)	Jaccard Index	P-Value	FDR Adjusted P-Value
REACTOME INTERFERON SIGNALING	177	31 (17.5)	31 (17.5)	0 (0)	0.063	<0.0001	0.0015
REACTOME NEUTROPHIL DEGRANULATION	442	43 (9.7)	43 (9.7)	0 (0)	0.058	<0.0001	0.0015
REACTOME INTERFERON ALPHA BETA SIGNALING	60	21 (35)	21 (35)	0 (0)	0.055	<0.0001	0.0015
REACTOME CYTOKINE SIGNALING IN IMMUNE SYSTEM	788	57 (7.2)	57 (7.2)	0 (0)	0.053	<0.0001	0.0015
REACTOME INNATE IMMUNE SYSTEM	968	65 (6.7)	65 (6.7)	0 (0)	0.052	<0.0001	0.0015
REACTOME IMMUNOREGULATORY INTERACTIONS BETWEEN A L...	97	17 (17.5)	17 (17.5)	0 (0)	0.040	<0.0001	0.0015
REACTOME INTERFERON GAMMA SIGNALING	71	16 (22.5)	16 (22.5)	0 (0)	0.040	<0.0001	0.0015
REACTOME SIGNALING BY INTERLEUKINS	430	25 (5.8)	25 (5.8)	0 (0)	0.033	<0.0001	0.0015
REACTOME ANTIMICROBIAL PEPTIDES	83	8 (9.6)	8 (9.6)	0 (0)	0.019	<0.0001	0.0015
REACTOME REGULATION OF TLR BY ENDOGENOUS LIGAND	19	6 (31.6)	6 (31.6)	0 (0)	0.017	<0.0001	0.0015
REACTOME ADAPTIVE IMMUNE SYSTEM	670	34 (5.1)	34 (5.1)	0 (0)	0.035	<0.0001	0.0026
REACTOME ANTIVIRAL MECHANISM BY IFN STIMULATED GEN...	81	10 (12.3)	10 (12.3)	0 (0)	0.024	<0.0001	0.0026
REACTOME IRAK4 DEFICIENCY TLR2 4	10	4 (40)	4 (40)	0 (0)	0.011	<0.0001	0.0082
REACTOME INTERLEUKIN 10 SIGNALING	39	6 (15.4)	6 (15.4)	0 (0)	0.016	<0.0001	0.0088
REACTOME INTERLEUKIN 4 AND INTERLEUKIN 13 SIGNALIN...	102	10 (9.8)	10 (9.8)	0 (0)	0.023	<0.0001	0.0092
REACTOME CASPASE ACTIVATION VIA DEATH RECEPTORS IN...	16	4 (25)	4 (25)	0 (0)	0.011	0.0001	0.0124
REACTOME SIGNAL REGULATORY PROTEIN FAMILY INTERACT...	16	4 (25)	4 (25)	0 (0)	0.011	0.0002	0.0225
REACTOME REGULATION OF IFNA SIGNALING	26	4 (15.4)	4 (15.4)	0 (0)	0.011	0.0005	0.04

Table S141: MSigDB GO Biological Processes enriched in differentially expressed genes at Day 30 compared to pre-Second Vaccination (B cells, Study Arm 2 A/H7N9+AS03, D1, 29). Results are sorted by FDR adjusted p-value and Jaccard similarity index.

Gene Set ID	Gene Set Genes #	DE Genes N (%)	Up-reg. DE Genes N (%)	Down-reg. DE Genes N (%)	Jaccard Index	P-Value	FDR Adjusted P-Value
GO DEFENSE RESPONSE TO OTHER ORGANISM	993	88 (8.9)	87 (8.8)	1 (0.1)	0.070	<0.0001	0.0006
GO INNATE IMMUNE RESPONSE	814	75 (9.2)	75 (9.2)	0 (0)	0.069	<0.0001	0.0006
GO MYELOID LEUKOCYTE ACTIVATION	594	58 (9.8)	58 (9.8)	0 (0)	0.066	<0.0001	0.0006
GO IMMUNE EFFECTOR PROCESS	1077	86 (8)	85 (7.9)	1 (0.1)	0.064	<0.0001	0.0006
GO DEFENSE RESPONSE	1497	111 (7.4)	110 (7.3)	1 (0.1)	0.064	<0.0001	0.0006
GO RESPONSE TO BIOTIC STIMULUS	1325	94 (7.1)	93 (7)	1 (0.1)	0.060	<0.0001	0.0006
GO MYELOID LEUKOCYTE MEDIATED IMMUNITY	506	47 (9.3)	47 (9.3)	0 (0)	0.059	<0.0001	0.0006
GO LEUKOCYTE MEDIATED IMMUNITY	701	57 (8.1)	57 (8.1)	0 (0)	0.058	<0.0001	0.0006
GO CELL ACTIVATION INVOLVED IN IMMUNE RESPONSE	658	54 (8.2)	54 (8.2)	0 (0)	0.057	<0.0001	0.0006
GO RESPONSE TO INTERFERON GAMMA	166	27 (16.3)	27 (16.3)	0 (0)	0.056	<0.0001	0.0006
GO DEFENSE RESPONSE TO VIRUS	229	30 (13.1)	29 (12.7)	1 (0.4)	0.055	<0.0001	0.0006
GO RESPONSE TO CYTOKINE	1099	74 (6.7)	74 (6.7)	0 (0)	0.054	<0.0001	0.0006
GO CYTOKINE MEDIATED SIGNALING PATHWAY	711	54 (7.6)	54 (7.6)	0 (0)	0.054	<0.0001	0.0006
GO INFLAMMATORY RESPONSE	680	52 (7.6)	52 (7.6)	0 (0)	0.053	<0.0001	0.0006
GO REGULATION OF IMMUNE RESPONSE	897	62 (6.9)	62 (6.9)	0 (0)	0.052	<0.0001	0.0006
GO CYTOKINE PRODUCTION	720	53 (7.4)	53 (7.4)	0 (0)	0.052	<0.0001	0.0006
GO RESPONSE TO VIRUS	300	32 (10.7)	31 (10.3)	1 (0.3)	0.052	<0.0001	0.0006
GO EXOCYTOSIS	833	58 (7)	58 (7)	0 (0)	0.052	<0.0001	0.0006
GO CELL ACTIVATION	1265	79 (6.2)	79 (6.2)	0 (0)	0.052	<0.0001	0.0006
GO POSITIVE REGULATION OF CYTOKINE PRODUCTION	419	36 (8.6)	36 (8.6)	0 (0)	0.050	<0.0001	0.0006
GO REGULATION OF IMMUNE SYSTEM PROCESS	1409	82 (5.8)	82 (5.8)	0 (0)	0.049	<0.0001	0.0006
GO PHAGOCYTOSIS	251	27 (10.8)	27 (10.8)	0 (0)	0.048	<0.0001	0.0006
GO SECRETION	1551	84 (5.4)	84 (5.4)	0 (0)	0.046	<0.0001	0.0006
GO RESPONSE TO TYPE I INTERFERON	88	19 (21.6)	19 (21.6)	0 (0)	0.046	<0.0001	0.0006
GO POSITIVE REGULATION OF IMMUNE SYSTEM PROCESS	972	57 (5.9)	57 (5.9)	0 (0)	0.045	<0.0001	0.0006
GO REGULATION OF DEFENSE RESPONSE	703	45 (6.4)	45 (6.4)	0 (0)	0.045	<0.0001	0.0006
GO REGULATION OF MULTI ORGANISM PROCESS	700	44 (6.3)	44 (6.3)	0 (0)	0.044	<0.0001	0.0006
GO POSITIVE REGULATION OF IMMUNE RESPONSE	704	43 (6.1)	43 (6.1)	0 (0)	0.043	<0.0001	0.0006
GO REGULATION OF RESPONSE TO EXTERNAL STIMULUS	1006	54 (5.4)	54 (5.4)	0 (0)	0.042	<0.0001	0.0006
GO REGULATION OF RESPONSE TO BIOTIC STIMULUS	466	32 (6.9)	32 (6.9)	0 (0)	0.041	<0.0001	0.0006
GO POSITIVE REGULATION OF RESPONSE TO EXTERNAL STI...	575	36 (6.3)	36 (6.3)	0 (0)	0.041	<0.0001	0.0006
GO RESPONSE TO BACTERIUM	587	35 (6)	35 (6)	0 (0)	0.039	<0.0001	0.0006
GO POSITIVE REGULATION OF DEFENSE RESPONSE	457	30 (6.6)	30 (6.6)	0 (0)	0.039	<0.0001	0.0006
GO CYTOKINE SECRETION	220	21 (9.5)	21 (9.5)	0 (0)	0.039	<0.0001	0.0006

Table S141: MSigDB GO Biological Processes enriched in differentially expressed genes at Day 30 compared to pre-Second Vaccination (B cells, Study Arm 2 A/H7N9+AS03, D1, 29). Results are sorted by FDR adjusted p-value and Jaccard similarity index (continued.)

Gene Set ID	Gene Set Genes #	DE Genes N (%)	Up-reg. DE Genes N (%)	Down-reg. DE Genes N (%)	Jaccard Index	P-Value	FDR Adjusted P-Value
GO REGULATION OF INFLAMMATORY RESPONSE	332	25 (7.5)	25 (7.5)	0 (0)	0.038	<0.0001	0.0006
GO IMMUNE RESPONSE REGULATING SIGNALING PATHWAY	507	31 (6.1)	31 (6.1)	0 (0)	0.038	<0.0001	0.0006
GO DEFENSE RESPONSE TO BACTERIUM	237	21 (8.9)	21 (8.9)	0 (0)	0.037	<0.0001	0.0006
GO INTERLEUKIN 1 PRODUCTION	99	16 (16.2)	16 (16.2)	0 (0)	0.037	<0.0001	0.0006
GO ACTIVATION OF IMMUNE RESPONSE	547	32 (5.9)	32 (5.9)	0 (0)	0.037	<0.0001	0.0006
GO REGULATION OF IMMUNE EFFECTOR PROCESS	357	25 (7)	25 (7)	0 (0)	0.037	<0.0001	0.0006
GO REGULATION OF CELL ACTIVATION	507	29 (5.7)	29 (5.7)	0 (0)	0.035	<0.0001	0.0006
GO INTERSPECIES INTERACTION BETWEEN ORGANISMS	869	41 (4.7)	40 (4.6)	1 (0.1)	0.035	<0.0001	0.0006
GO MYELOID LEUKOCYTE MIGRATION	187	18 (9.6)	18 (9.6)	0 (0)	0.035	<0.0001	0.0006
GO INTERFERON GAMMA MEDIATED SIGNALING PATHWAY	70	14 (20)	14 (20)	0 (0)	0.035	<0.0001	0.0006
GO POSITIVE REGULATION OF INFLAMMATORY RESPONSE	134	16 (11.9)	16 (11.9)	0 (0)	0.035	<0.0001	0.0006
GO NEGATIVE REGULATION OF CYTOKINE PRODUCTION	262	20 (7.6)	20 (7.6)	0 (0)	0.034	<0.0001	0.0006
GO POSITIVE REGULATION OF RESPONSE TO BIOTIC STIMU...	353	23 (6.5)	23 (6.5)	0 (0)	0.034	<0.0001	0.0006
GO BIOLOGICAL ADHESION	1320	54 (4.1)	53 (4)	1 (0.1)	0.034	<0.0001	0.0006
GO INTERLEUKIN 1 BETA PRODUCTION	87	14 (16.1)	14 (16.1)	0 (0)	0.034	<0.0001	0.0006
GO POSITIVE REGULATION OF MULTI ORGANISM PROCESS	489	27 (5.5)	27 (5.5)	0 (0)	0.034	<0.0001	0.0006
GO TUMOR NECROSIS FACTOR SUPERFAMILY CYTOKINE PROD...	148	16 (10.8)	16 (10.8)	0 (0)	0.034	<0.0001	0.0006
GO RESPONSE TO WOUNDING	650	32 (4.9)	32 (4.9)	0 (0)	0.033	<0.0001	0.0006
GO REGULATION OF RESPONSE TO STRESS	1406	56 (4)	56 (4)	0 (0)	0.033	<0.0001	0.0006
GO POSITIVE REGULATION OF CELL DEATH	670	32 (4.8)	31 (4.6)	1 (0.1)	0.033	<0.0001	0.0006
GO IMMUNE SYSTEM DEVELOPMENT	926	40 (4.3)	40 (4.3)	0 (0)	0.033	<0.0001	0.0006
GO LEUKOCYTE DIFFERENTIATION	483	26 (5.4)	26 (5.4)	0 (0)	0.032	<0.0001	0.0006
GO POSITIVE REGULATION OF MULTICELLULAR ORGANISMAL...	1665	63 (3.8)	63 (3.8)	0 (0)	0.032	<0.0001	0.0006
GO LYMPHOCYTE ACTIVATION	620	30 (4.8)	30 (4.8)	0 (0)	0.032	<0.0001	0.0006
GO REGULATION OF SYMBIOSIS ENCOMPASSING MUTUALISM ...	204	17 (8.3)	17 (8.3)	0 (0)	0.032	<0.0001	0.0006
GO T CELL ACTIVATION	436	24 (5.5)	24 (5.5)	0 (0)	0.032	<0.0001	0.0006
GO LEUKOCYTE CELL CELL ADHESION	307	20 (6.5)	20 (6.5)	0 (0)	0.032	<0.0001	0.0006
GO CELL CHEMOTAXIS	278	19 (6.8)	19 (6.8)	0 (0)	0.032	<0.0001	0.0006
GO LEUKOCYTE MIGRATION	410	23 (5.6)	23 (5.6)	0 (0)	0.031	<0.0001	0.0006
GO PATTERN RECOGNITION RECEPTOR SIGNALING PATHWAY	181	16 (8.8)	16 (8.8)	0 (0)	0.031	<0.0001	0.0006
GO MACROPHAGE ACTIVATION	84	13 (15.5)	13 (15.5)	0 (0)	0.031	<0.0001	0.0006
GO REACTIVE OXYGEN SPECIES METABOLIC PROCESS	251	18 (7.2)	17 (6.8)	1 (0.4)	0.031	<0.0001	0.0006
GO REGULATION OF PHAGOCYTOSIS	86	13 (15.1)	13 (15.1)	0 (0)	0.031	<0.0001	0.0006
GO ACTIVATION OF INNATE IMMUNE RESPONSE	286	19 (6.6)	19 (6.6)	0 (0)	0.031	<0.0001	0.0006
GO PEPTIDE SECRETION	587	28 (4.8)	28 (4.8)	0 (0)	0.031	<0.0001	0.0006
GO MYELOID CELL DIFFERENTIATION	389	22 (5.7)	22 (5.7)	0 (0)	0.031	<0.0001	0.0006
GO NEGATIVE REGULATION OF VIRAL PROCESS	90	13 (14.4)	13 (14.4)	0 (0)	0.031	<0.0001	0.0006

Table S141: MSigDB GO Biological Processes enriched in differentially expressed genes at Day 30 compared to pre-Second Vaccination (B cells, Study Arm 2 A/H7N9+AS03, D1, 29). Results are sorted by FDR adjusted p-value and Jaccard similarity index (continued.)

Gene Set ID	Gene Set Genes #	DE Genes N (%)	Up-reg. DE Genes N (%)	Down-reg. DE Genes N (%)	Jaccard Index	P-Value	FDR Adjusted P-Value
GO RESPONSE TO MOLECULE OF BACTERIAL ORIGIN	329	20 (6.1)	20 (6.1)	0 (0)	0.031	<0.0001	0.0006
GO NEGATIVE REGULATION OF MULTI ORGANISM PROCESS	195	16 (8.2)	16 (8.2)	0 (0)	0.030	<0.0001	0.0006
GO POSITIVE REGULATION OF CELL ACTIVATION	297	19 (6.4)	19 (6.4)	0 (0)	0.030	<0.0001	0.0006
GO TAXIS	607	28 (4.6)	28 (4.6)	0 (0)	0.030	<0.0001	0.0006
GO REGULATION OF VIRAL LIFE CYCLE	133	14 (10.5)	14 (10.5)	0 (0)	0.030	<0.0001	0.0006
GO LEUKOCYTE CHEMOTAXIS	202	16 (7.9)	16 (7.9)	0 (0)	0.030	<0.0001	0.0006
GO NEGATIVE REGULATION OF MULTICELLULAR ORGANISMAL...	1169	44 (3.8)	44 (3.8)	0 (0)	0.030	<0.0001	0.0006
GO NEGATIVE REGULATION OF IMMUNE SYSTEM PROCESS	426	22 (5.2)	22 (5.2)	0 (0)	0.029	<0.0001	0.0006
GO I KAPPAB KINASE NF KAPPAB SIGNALING	252	17 (6.7)	17 (6.7)	0 (0)	0.029	<0.0001	0.0006
GO NEGATIVE REGULATION OF VIRAL LIFE CYCLE	76	12 (15.8)	12 (15.8)	0 (0)	0.029	<0.0001	0.0006
GO POSITIVE REGULATION OF TRANSPORT	921	36 (3.9)	36 (3.9)	0 (0)	0.029	<0.0001	0.0006
GO REGULATION OF LEUKOCYTE DIFFERENTIATION	252	17 (6.7)	17 (6.7)	0 (0)	0.029	<0.0001	0.0006
GO CHEMOKINE PRODUCTION	80	12 (15)	12 (15)	0 (0)	0.029	<0.0001	0.0006
GO POSITIVE REGULATION OF TUMOR NECROSIS FACTOR SU...	79	12 (15.2)	12 (15.2)	0 (0)	0.029	<0.0001	0.0006
GO ADAPTIVE IMMUNE RESPONSE BASED ON SOMATIC RECOM...	223	16 (7.2)	16 (7.2)	0 (0)	0.029	<0.0001	0.0006
GO MYELOID LEUKOCYTE DIFFERENTIATION	188	15 (8)	15 (8)	0 (0)	0.029	<0.0001	0.0006
GO WOUND HEALING	542	25 (4.6)	25 (4.6)	0 (0)	0.029	<0.0001	0.0006
GO POSITIVE REGULATION OF INTRACELLULAR SIGNAL TRA...	948	36 (3.8)	36 (3.8)	0 (0)	0.029	<0.0001	0.0006
GO GRANULOCYTE MIGRATION	126	13 (10.3)	13 (10.3)	0 (0)	0.028	<0.0001	0.0006
GO LEUKOCYTE PROLIFERATION	276	17 (6.2)	17 (6.2)	0 (0)	0.028	<0.0001	0.0006
GO ERK1 AND ERK2 CASCADE	285	17 (6)	17 (6)	0 (0)	0.028	<0.0001	0.0006
GO REGULATION OF ADAPTIVE IMMUNE RESPONSE	142	13 (9.2)	13 (9.2)	0 (0)	0.027	<0.0001	0.0006
GO INTERLEUKIN 6 PRODUCTION	144	13 (9)	13 (9)	0 (0)	0.027	<0.0001	0.0006
GO CELLULAR RESPONSE TO BIOTIC STIMULUS	226	15 (6.6)	15 (6.6)	0 (0)	0.027	<0.0001	0.0006
GO REACTIVE NITROGEN SPECIES METABOLIC PROCESS	74	11 (14.9)	11 (14.9)	0 (0)	0.027	<0.0001	0.0006
GO HUMORAL IMMUNE RESPONSE	227	15 (6.6)	15 (6.6)	0 (0)	0.027	<0.0001	0.0006
GO POSITIVE REGULATION OF PEPTIDE SECRETION	271	16 (5.9)	16 (5.9)	0 (0)	0.027	<0.0001	0.0006
GO TYPE I INTERFERON PRODUCTION	116	12 (10.3)	12 (10.3)	0 (0)	0.027	<0.0001	0.0006
GO VIRAL GENOME REPLICATION	117	12 (10.3)	12 (10.3)	0 (0)	0.027	<0.0001	0.0006
GO POSITIVE REGULATION OF CYTOKINE SECRETION	126	12 (9.5)	12 (9.5)	0 (0)	0.026	<0.0001	0.0006
GO REGULATION OF VIRAL GENOME REPLICATION	91	11 (12.1)	11 (12.1)	0 (0)	0.026	<0.0001	0.0006
GO NEUROTRANSMITTER BIOSYNTHETIC PROCESS	93	11 (11.8)	11 (11.8)	0 (0)	0.026	<0.0001	0.0006
GO NEUROTRANSMITTER METABOLIC PROCESS	132	12 (9.1)	12 (9.1)	0 (0)	0.026	<0.0001	0.0006
GO POSITIVE REGULATION OF ADAPTIVE IMMUNE RESPONSE	92	11 (12)	11 (12)	0 (0)	0.026	<0.0001	0.0006
GO NEGATIVE REGULATION OF VIRAL GENOME REPLICATION	58	10 (17.2)	10 (17.2)	0 (0)	0.025	<0.0001	0.0006
GO POSITIVE REGULATION OF PHAGOCYTOSIS	61	10 (16.4)	10 (16.4)	0 (0)	0.025	<0.0001	0.0006
GO NEUTROPHIL MIGRATION	103	11 (10.7)	11 (10.7)	0 (0)	0.025	<0.0001	0.0006

Table S141: MSigDB GO Biological Processes enriched in differentially expressed genes at Day 30 compared to pre-Second Vaccination (B cells, Study Arm 2 A/H7N9+AS03, D1, 29). Results are sorted by FDR adjusted p-value and Jaccard similarity index (continued.)

Gene Set ID	Gene Set Genes #	DE Genes N (%)	Up-reg. DE Genes N (%)	Down-reg. DE Genes N (%)	Jaccard Index	P-Value	FDR Adjusted P-Value
GO INTERLEUKIN 8 PRODUCTION	70	10 (14.3)	10 (14.3)	0 (0)	0.025	<0.0001	0.0006
GO DEFENSE RESPONSE TO GRAM POSITIVE BACTERIUM	87	10 (11.5)	10 (11.5)	0 (0)	0.024	<0.0001	0.0006
GO CYTOKINE PRODUCTION INVOLVED IN IMMUNE RESPONSE	94	10 (10.6)	10 (10.6)	0 (0)	0.023	<0.0001	0.0006
GO POSITIVE REGULATION OF INTERLEUKIN 1 PRODUCTION	50	9 (18)	9 (18)	0 (0)	0.023	<0.0001	0.0006
GO REACTIVE OXYGEN SPECIES BIOSYNTHETIC PROCESS	102	10 (9.8)	10 (9.8)	0 (0)	0.023	<0.0001	0.0006
GO CYTOKINE METABOLIC PROCESS	105	10 (9.5)	10 (9.5)	0 (0)	0.023	<0.0001	0.0006
GO MONONUCLEAR CELL MIGRATION	74	9 (12.2)	9 (12.2)	0 (0)	0.022	<0.0001	0.0006
GO RESPONSE TO INTERFERON BETA	30	8 (26.7)	8 (26.7)	0 (0)	0.022	<0.0001	0.0006
GO CELLULAR DEFENSE RESPONSE	46	8 (17.4)	8 (17.4)	0 (0)	0.021	<0.0001	0.0006
GO GLIAL CELL ACTIVATION	48	8 (16.7)	8 (16.7)	0 (0)	0.021	<0.0001	0.0006
GO INTERLEUKIN 10 PRODUCTION	48	8 (16.7)	8 (16.7)	0 (0)	0.021	<0.0001	0.0006
GO REGULATION OF NITRIC OXIDE BIOSYNTHETIC PROCESS	55	8 (14.5)	8 (14.5)	0 (0)	0.020	<0.0001	0.0006
GO PRODUCTION OF MOLECULAR MEDIATOR INVOLVED IN IN...	66	8 (12.1)	8 (12.1)	0 (0)	0.020	<0.0001	0.0006
GO MYD88 DEPENDENT TOLL LIKE RECEPTOR SIGNALING PA...	30	7 (23.3)	7 (23.3)	0 (0)	0.019	<0.0001	0.0006
GO VASCULAR ENDOTHELIAL GROWTH FACTOR PRODUCTION	34	7 (20.6)	7 (20.6)	0 (0)	0.019	<0.0001	0.0006
GO LEUKOCYTE ACTIVATION INVOLVED IN INFLAMMATORY R...	40	7 (17.5)	7 (17.5)	0 (0)	0.018	<0.0001	0.0006
GO RESPONSE TO PROTOZOAN	26	6 (23.1)	6 (23.1)	0 (0)	0.016	<0.0001	0.0006
GO POSITIVE REGULATION OF VASCULAR ENDOTHELIAL GRO...	28	6 (21.4)	6 (21.4)	0 (0)	0.016	<0.0001	0.0006
GO NLRP3 INFLAMMASOME COMPLEX ASSEMBLY	13	5 (38.5)	5 (38.5)	0 (0)	0.014	<0.0001	0.0006
GO POSITIVE REGULATION OF NITRIC OXIDE SYNTHASE BI...	15	5 (33.3)	5 (33.3)	0 (0)	0.014	<0.0001	0.0006
GO INTERLEUKIN 18 PRODUCTION	9	4 (44.4)	4 (44.4)	0 (0)	0.011	<0.0001	0.0006
GO VIRAL LIFE CYCLE	308	20 (6.5)	19 (6.2)	1 (0.3)	0.032	<0.0001	0.001
GO CHEMICAL HOMEOSTASIS	1123	44 (3.9)	44 (3.9)	0 (0)	0.031	<0.0001	0.001
GO REGULATION OF LYMPHOCYTE ACTIVATION	392	22 (5.6)	22 (5.6)	0 (0)	0.031	<0.0001	0.001
GO POSITIVE REGULATION OF MOLECULAR FUNCTION	1658	58 (3.5)	57 (3.4)	1 (0.1)	0.030	<0.0001	0.001
GO REGULATION OF DNA BINDING TRANSCRIPTION FACTOR ...	393	21 (5.3)	21 (5.3)	0 (0)	0.029	<0.0001	0.001
GO REGULATION OF HEMOPOIESIS	441	22 (5)	22 (5)	0 (0)	0.029	<0.0001	0.001
GO CELLULAR ION HOMEOSTASIS	634	27 (4.3)	27 (4.3)	0 (0)	0.028	<0.0001	0.001
GO REGULATION OF PEPTIDE SECRETION	461	22 (4.8)	22 (4.8)	0 (0)	0.028	<0.0001	0.001
GO CELLULAR RESPONSE TO OXYGEN CONTAINING COMPOUND	1128	40 (3.5)	39 (3.5)	1 (0.1)	0.028	<0.0001	0.001
GO REGULATION OF CELL POPULATION PROLIFERATION	1541	50 (3.2)	50 (3.2)	0 (0)	0.027	<0.0001	0.001
GO REGULATION OF RESPONSE TO CYTOKINE STIMULUS	165	13 (7.9)	13 (7.9)	0 (0)	0.026	<0.0001	0.001
GO INTERLEUKIN 2 PRODUCTION	59	8 (13.6)	8 (13.6)	0 (0)	0.020	<0.0001	0.001
GO NEUROINFLAMMATORY RESPONSE	63	8 (12.7)	8 (12.7)	0 (0)	0.020	<0.0001	0.001
GO INTERLEUKIN 1 SECRETION	53	7 (13.2)	7 (13.2)	0 (0)	0.018	<0.0001	0.001
GO NEGATIVE REGULATION OF PHAGOCYTOSIS	19	5 (26.3)	5 (26.3)	0 (0)	0.014	<0.0001	0.001
GO NITRIC OXIDE SYNTHASE BIOSYNTHETIC PROCESS	20	5 (25)	5 (25)	0 (0)	0.014	<0.0001	0.001

Table S141: MSigDB GO Biological Processes enriched in differentially expressed genes at Day 30 compared to pre-Second Vaccination (B cells, Study Arm 2 A/H7N9+AS03, D1, 29). Results are sorted by FDR adjusted p-value and Jaccard similarity index (continued.)

Gene Set ID	Gene Set Genes #	DE Genes N (%)	Up-reg. DE Genes N (%)	Down-reg. DE Genes N (%)	Jaccard Index	P-Value	FDR Adjusted P-Value
GO PYRIMIDINE NUCLEOSIDE CATABOLIC PROCESS	23	5 (21.7)	5 (21.7)	0 (0)	0.014	<0.0001	0.001
GO REGULATION OF CELL ADHESION	633	28 (4.4)	28 (4.4)	0 (0)	0.029	<0.0001	0.0014
GO REGULATION OF SECRETION	752	30 (4)	30 (4)	0 (0)	0.028	<0.0001	0.0014
GO CARBOHYDRATE DERIVATIVE CATABOLIC PROCESS	174	13 (7.5)	13 (7.5)	0 (0)	0.026	<0.0001	0.0014
GO CELL KILLING	146	11 (7.5)	11 (7.5)	0 (0)	0.023	<0.0001	0.0014
GO POSITIVE REGULATION OF INTERLEUKIN 6 PRODUCTION	87	9 (10.3)	9 (10.3)	0 (0)	0.021	<0.0001	0.0014
GO REGULATION OF DEFENSE RESPONSE TO VIRUS	68	8 (11.8)	8 (11.8)	0 (0)	0.020	<0.0001	0.0014
GO POSITIVE REGULATION OF CHEMOKINE PRODUCTION	51	7 (13.7)	7 (13.7)	0 (0)	0.018	<0.0001	0.0014
GO POSITIVE REGULATION OF INTERLEUKIN 10 PRODUCTIO...	33	6 (18.2)	6 (18.2)	0 (0)	0.016	<0.0001	0.0014
GO ASTROCYTE DEVELOPMENT	36	6 (16.7)	6 (16.7)	0 (0)	0.016	<0.0001	0.0014
GO POSITIVE REGULATION OF NITRIC OXIDE METABOLIC P...	37	6 (16.2)	6 (16.2)	0 (0)	0.016	<0.0001	0.0014
GO POSITIVE REGULATION OF GLIAL CELL MIGRATION	10	4 (40)	4 (40)	0 (0)	0.011	<0.0001	0.0014
GO RESPONSE TO LIPOTEICHOIC ACID	10	4 (40)	4 (40)	0 (0)	0.011	<0.0001	0.0014
GO ADAPTIVE IMMUNE RESPONSE	353	20 (5.7)	20 (5.7)	0 (0)	0.029	<0.0001	0.0018
GO POSITIVE REGULATION OF LEUKOCYTE CELL CELL ADHE...	194	13 (6.7)	13 (6.7)	0 (0)	0.025	<0.0001	0.0018
GO POSITIVE REGULATION OF CYTOKINE PRODUCTION INVO...	49	7 (14.3)	7 (14.3)	0 (0)	0.018	<0.0001	0.0018
GO REGULATION OF TYPE 2 IMMUNE RESPONSE	31	6 (19.4)	6 (19.4)	0 (0)	0.016	<0.0001	0.0018
GO REGULATION OF VIRAL ENTRY INTO HOST CELL	26	5 (19.2)	5 (19.2)	0 (0)	0.014	<0.0001	0.0018
GO RESPONSE TO PEPTIDOGLYCAN	11	4 (36.4)	4 (36.4)	0 (0)	0.011	<0.0001	0.0018
GO POSITIVE REGULATION OF DNA BINDING TRANSCRIPTIO...	238	15 (6.3)	15 (6.3)	0 (0)	0.026	<0.0001	0.0022
GO REGULATION OF REACTIVE OXYGEN SPECIES METABOLIC...	169	12 (7.1)	12 (7.1)	0 (0)	0.024	<0.0001	0.0022
GO REGULATION OF LEUKOCYTE MIGRATION	175	12 (6.9)	12 (6.9)	0 (0)	0.024	<0.0001	0.0022
GO INTERFERON GAMMA PRODUCTION	97	9 (9.3)	9 (9.3)	0 (0)	0.021	<0.0001	0.0022
GO ACUTE INFLAMMATORY RESPONSE	102	9 (8.8)	9 (8.8)	0 (0)	0.021	<0.0001	0.0022
GO REGULATION OF TOLL LIKE RECEPTOR SIGNALING PATH...	64	8 (12.5)	8 (12.5)	0 (0)	0.020	<0.0001	0.0022
GO REGULATION OF CYTOKINE PRODUCTION INVOLVED IN I...	76	8 (10.5)	8 (10.5)	0 (0)	0.019	<0.0001	0.0022
GO RESPONSE TO DIACYL BACTERIAL LIPOPEPTIDE	4	3 (75)	3 (75)	0 (0)	0.009	<0.0001	0.0022
GO REGULATION OF CELL DEATH	1593	51 (3.2)	50 (3.1)	1 (0.1)	0.027	<0.0001	0.0025
GO POSITIVE REGULATION OF SIGNALING	1730	54 (3.1)	54 (3.1)	0 (0)	0.027	<0.0001	0.0025
GO COAGULATION	326	17 (5.2)	17 (5.2)	0 (0)	0.026	<0.0001	0.0025
GO POSITIVE REGULATION OF NF KAPPAB TRANSCRIPTION ...	133	11 (8.3)	11 (8.3)	0 (0)	0.024	<0.0001	0.0025
GO TOLL LIKE RECEPTOR SIGNALING PATHWAY	134	11 (8.2)	11 (8.2)	0 (0)	0.024	<0.0001	0.0025
GO POSITIVE REGULATION OF OSSIFICATION	81	8 (9.9)	8 (9.9)	0 (0)	0.019	<0.0001	0.0025
GO POSITIVE REGULATION OF RECEPTOR SIGNALING PATHW...	87	8 (9.2)	8 (9.2)	0 (0)	0.019	<0.0001	0.0025
GO CYTOKINE PRODUCTION INVOLVED IN INFLAMMATORY RE...	45	6 (13.3)	6 (13.3)	0 (0)	0.016	<0.0001	0.0025
GO KILLING OF CELLS OF OTHER ORGANISM	60	6 (10)	6 (10)	0 (0)	0.015	<0.0001	0.0025
GO HOMEOSTATIC PROCESS	1803	57 (3.2)	57 (3.2)	0 (0)	0.027	<0.0001	0.0028

Table S141: MSigDB GO Biological Processes enriched in differentially expressed genes at Day 30 compared to pre-Second Vaccination (B cells, Study Arm 2 A/H7N9+AS03, D1, 29). Results are sorted by FDR adjusted p-value and Jaccard similarity index (continued.)

Gene Set ID	Gene Set Genes #	DE Genes N (%)	Up-reg. DE Genes N (%)	Down-reg. DE Genes N (%)	Jaccard Index	P-Value	FDR Adjusted P-Value
GO POSITIVE REGULATION OF IMMUNE EFFECTOR PROCESS	189	13 (6.9)	13 (6.9)	0 (0)	0.025	<0.0001	0.0028
GO PRODUCTION OF MOLECULAR MEDIATOR OF IMMUNE RESP...	187	13 (7)	13 (7)	0 (0)	0.025	<0.0001	0.0028
GO POSITIVE REGULATION OF I KAPPAB KINASE NF KAPPA...	169	12 (7.1)	12 (7.1)	0 (0)	0.024	<0.0001	0.0028
GO REGULATION OF LEUKOCYTE MEDIATED IMMUNITY	174	12 (6.9)	12 (6.9)	0 (0)	0.024	<0.0001	0.0028
GO POSITIVE REGULATION OF CYTOKINE BIOSYNTHETIC PR...	60	7 (11.7)	7 (11.7)	0 (0)	0.018	<0.0001	0.0028
GO TYPE 2 IMMUNE RESPONSE	38	6 (15.8)	6 (15.8)	0 (0)	0.016	<0.0001	0.0028
GO REGULATION OF GLIAL CELL MIGRATION	14	4 (28.6)	4 (28.6)	0 (0)	0.011	<0.0001	0.0028
GO ISG15 PROTEIN CONJUGATION	6	3 (50)	3 (50)	0 (0)	0.009	<0.0001	0.0028
GO SUPEROXIDE METABOLIC PROCESS	62	7 (11.3)	7 (11.3)	0 (0)	0.018	<0.0001	0.0031
GO INTERLEUKIN 8 SECRETION	28	5 (17.9)	5 (17.9)	0 (0)	0.014	<0.0001	0.0031
GO CELL CELL ADHESION	781	31 (4)	30 (3.8)	1 (0.1)	0.028	<0.0001	0.0034
GO CELLULAR HOMEOSTASIS	901	33 (3.7)	33 (3.7)	0 (0)	0.027	<0.0001	0.0034
GO DIVALENT INORGANIC CATION HOMEOSTASIS	492	22 (4.5)	22 (4.5)	0 (0)	0.027	<0.0001	0.0034
GO LIPID LOCALIZATION	383	19 (5)	19 (5)	0 (0)	0.027	<0.0001	0.0034
GO REGULATION OF LEUKOCYTE PROLIFERATION	203	13 (6.4)	13 (6.4)	0 (0)	0.024	<0.0001	0.0034
GO POSITIVE REGULATION OF MYELOID CELL DIFFERENTIA...	86	8 (9.3)	8 (9.3)	0 (0)	0.019	<0.0001	0.0034
GO CYTOLYSIS	36	5 (13.9)	5 (13.9)	0 (0)	0.013	<0.0001	0.0034
GO DETECTION OF OTHER ORGANISM	14	4 (28.6)	4 (28.6)	0 (0)	0.011	<0.0001	0.0034
GO ION HOMEOSTASIS	772	29 (3.8)	29 (3.8)	0 (0)	0.027	<0.0001	0.0037
GO NEGATIVE REGULATION OF CELL ACTIVATION	184	12 (6.5)	12 (6.5)	0 (0)	0.023	<0.0001	0.0037
GO TUMOR NECROSIS FACTOR BIOSYNTHETIC PROCESS	28	5 (17.9)	5 (17.9)	0 (0)	0.014	<0.0001	0.0037
GO NUCLEOSIDE CATABOLIC PROCESS	31	5 (16.1)	5 (16.1)	0 (0)	0.013	<0.0001	0.0037
GO REGULATION OF REACTIVE OXYGEN SPECIES BIOSYNTHE...	83	8 (9.6)	8 (9.6)	0 (0)	0.019	0.0001	0.004
GO POSITIVE REGULATION OF REACTIVE OXYGEN SPECIES ...	46	6 (13)	6 (13)	0 (0)	0.016	0.0001	0.004
GO POSITIVE REGULATION OF TYPE 2 IMMUNE RESPONSE	16	4 (25)	4 (25)	0 (0)	0.011	0.0001	0.004
GO NEGATIVE REGULATION OF CELL POPULATION PROLIFER...	666	26 (3.9)	26 (3.9)	0 (0)	0.026	0.0001	0.0043
GO RESPONSE TO LIPID	872	31 (3.6)	30 (3.4)	1 (0.1)	0.026	0.0001	0.0043
GO REGULATION OF T CELL ACTIVATION	292	16 (5.5)	16 (5.5)	0 (0)	0.026	0.0001	0.0043
GO LYMPHOCYTE MEDIATED IMMUNITY	216	13 (6)	13 (6)	0 (0)	0.024	0.0001	0.0043
GO POSITIVE REGULATION OF INTERLEUKIN 12 BIOSYNTHE...	6	3 (50)	3 (50)	0 (0)	0.009	0.0001	0.0043
GO ALPHA BETA T CELL ACTIVATION	127	10 (7.9)	10 (7.9)	0 (0)	0.022	0.0001	0.0045
GO MODIFICATION OF MORPHOLOGY OR PHYSIOLOGY OF OTH...	151	10 (6.6)	10 (6.6)	0 (0)	0.021	0.0001	0.0045
GO T CELL ACTIVATION INVOLVED IN IMMUNE RESPONSE	102	8 (7.8)	8 (7.8)	0 (0)	0.018	0.0001	0.0045
GO POSITIVE REGULATION OF INTERLEUKIN 1 SECRETION	30	5 (16.7)	5 (16.7)	0 (0)	0.013	0.0001	0.0045
GO REGULATION OF ALPHA BETA T CELL ACTIVATION	84	8 (9.5)	8 (9.5)	0 (0)	0.019	0.0001	0.0048
GO POSITIVE REGULATION OF INTERLEUKIN 8 PRODUCTION	43	6 (14)	6 (14)	0 (0)	0.016	0.0001	0.0048
GO DETECTION OF BIOTIC STIMULUS	29	5 (17.2)	5 (17.2)	0 (0)	0.014	0.0001	0.0048

Confidential/Proprietary Information

Table S141: MSigDB GO Biological Processes enriched in differentially expressed genes at Day 30 compared to pre-Second Vaccination (B cells, Study Arm 2 A/H7N9+AS03, D1, 29). Results are sorted by FDR adjusted p-value and Jaccard similarity index (continued.)

Gene Set ID	Gene Set Genes #	DE Genes N (%)	Up-reg. DE Genes N (%)	Down-reg. DE Genes N (%)	Jaccard Index	P-Value	FDR Adjusted P-Value
GO NEGATIVE REGULATION OF INTERLEUKIN 1 PRODUCTION	34	5 (14.7)	5 (14.7)	0 (0)	0.013	0.0001	0.0048
GO MACROPHAGE DIFFERENTIATION	42	6 (14.3)	6 (14.3)	0 (0)	0.016	0.0001	0.0051
GO POSITIVE REGULATION OF INTERLEUKIN 8 SECRETION	15	4 (26.7)	4 (26.7)	0 (0)	0.011	0.0001	0.0051
GO PHOSPHOLIPASE C ACTIVATING G PROTEIN COUPLED RE...	96	8 (8.3)	8 (8.3)	0 (0)	0.018	0.0002	0.0054
GO POSITIVE REGULATION OF CELL ADHESION	368	18 (4.9)	18 (4.9)	0 (0)	0.026	0.0002	0.006
GO POSITIVE REGULATION OF NLRP3 INFLAMMASOME COMPL...	6	3 (50)	3 (50)	0 (0)	0.009	0.0002	0.006
GO REGULATION OF SUPEROXIDE METABOLIC PROCESS	30	5 (16.7)	5 (16.7)	0 (0)	0.013	0.0002	0.0063
GO POSITIVE REGULATION OF T HELPER 2 CELL CYTOKINE...	7	3 (42.9)	3 (42.9)	0 (0)	0.009	0.0002	0.0063
GO RESPONSE TO OXYGEN CONTAINING COMPOUND	1593	48 (3)	47 (3)	1 (0.1)	0.025	0.0002	0.0066
GO POSITIVE REGULATION OF TUMOR NECROSIS FACTOR BI...	18	4 (22.2)	4 (22.2)	0 (0)	0.011	0.0002	0.0066
GO REGULATION OF CYTOSOLIC CALCIUM ION CONCENTRATI...	346	17 (4.9)	17 (4.9)	0 (0)	0.025	0.0002	0.0068
GO RECEPTOR SIGNALING PATHWAY VIA STAT	156	10 (6.4)	10 (6.4)	0 (0)	0.020	0.0002	0.0068
GO REGULATION OF B CELL ACTIVATION	112	9 (8)	9 (8)	0 (0)	0.020	0.0002	0.0068
GO NEGATIVE REGULATION OF ERK1 AND ERK2 CASCADE	69	7 (10.1)	7 (10.1)	0 (0)	0.017	0.0002	0.0068
GO ENTRY INTO HOST	126	10 (7.9)	9 (7.1)	1 (0.8)	0.022	0.0002	0.007
GO APOPTOTIC CELL CLEARANCE	45	6 (13.3)	6 (13.3)	0 (0)	0.016	0.0002	0.007
GO POSITIVE REGULATION OF INTERLEUKIN 12 PRODUCTIO...	32	5 (15.6)	5 (15.6)	0 (0)	0.013	0.0002	0.007
GO DETECTION OF EXTERNAL BIOTIC STIMULUS	18	4 (22.2)	4 (22.2)	0 (0)	0.011	0.0002	0.007
GO NEGATIVE REGULATION OF CHEMOKINE PRODUCTION	19	4 (21.1)	4 (21.1)	0 (0)	0.011	0.0002	0.007
GO GLIOGENESIS	270	15 (5.6)	15 (5.6)	0 (0)	0.025	0.0002	0.0072
GO POSITIVE REGULATION OF HEMOPOIESIS	170	11 (6.5)	11 (6.5)	0 (0)	0.022	0.0002	0.0077
GO ANTIMICROBIAL HUMORAL RESPONSE	108	7 (6.5)	7 (6.5)	0 (0)	0.016	0.0002	0.0077
GO POSITIVE REGULATION OF CYTOKINE PRODUCTION INVO...	19	4 (21.1)	4 (21.1)	0 (0)	0.011	0.0002	0.0077
GO AMYLOID BETA CLEARANCE BY CELLULAR CATABOLIC PR...	7	3 (42.9)	3 (42.9)	0 (0)	0.009	0.0002	0.0077
GO POSITIVE REGULATION OF SECRETION	404	18 (4.5)	18 (4.5)	0 (0)	0.025	0.0003	0.0079
GO OSTEOCLAST DIFFERENTIATION	88	8 (9.1)	8 (9.1)	0 (0)	0.019	0.0003	0.0079
GO I KAPPAB PHOSPHORYLATION	16	4 (25)	4 (25)	0 (0)	0.011	0.0003	0.0079
GO NEGATIVE REGULATION OF INTERLEUKIN 8 PRODUCTION	19	4 (21.1)	4 (21.1)	0 (0)	0.011	0.0003	0.0079
GO RESPONSE TO BACTERIAL LIPOPROTEIN	8	3 (37.5)	3 (37.5)	0 (0)	0.009	0.0003	0.0085
GO ANATOMICAL STRUCTURE FORMATION INVOLVED IN MORP...	1071	36 (3.4)	36 (3.4)	0 (0)	0.026	0.0003	0.0088
GO POSITIVE REGULATION OF CELL CELL ADHESION	232	13 (5.6)	13 (5.6)	0 (0)	0.023	0.0003	0.0089
GO REGULATION OF LEUKOCYTE CHEMOTAXIS	102	8 (7.8)	8 (7.8)	0 (0)	0.018	0.0003	0.0089
GO AMYLOID BETA CLEARANCE	35	5 (14.3)	5 (14.3)	0 (0)	0.013	0.0003	0.0089
GO PYRIMIDINE NUCLEOSIDE METABOLIC PROCESS	35	5 (14.3)	5 (14.3)	0 (0)	0.013	0.0003	0.0089
GO PLATELET DEGRANULATION	116	9 (7.8)	9 (7.8)	0 (0)	0.020	0.0003	0.0092
GO POSITIVE REGULATION OF INTERFERON GAMMA PRODUCT...	54	6 (11.1)	6 (11.1)	0 (0)	0.015	0.0003	0.0092
GO APOPTOTIC SIGNALING PATHWAY	566	23 (4.1)	22 (3.9)	1 (0.2)	0.026	0.0003	0.0093

Table S141: MSigDB GO Biological Processes enriched in differentially expressed genes at Day 30 compared to pre-Second Vaccination (B cells, Study Arm 2 A/H7N9+AS03, D1, 29). Results are sorted by FDR adjusted p-value and Jaccard similarity index (continued.)

Gene Set ID	Gene Set Genes #	DE Genes N (%)	Up-reg. DE Genes N (%)	Down-reg. DE Genes N (%)	Jaccard Index	P-Value	FDR Adjusted P-Value
GO POSITIVE REGULATION OF ERK1 AND ERK2 CASCADE	184	11 (6)	11 (6)	0 (0)	0.021	0.0003	0.0093
GO NEGATIVE REGULATION OF LEUKOCYTE DIFFERENTIATIO...	94	8 (8.5)	8 (8.5)	0 (0)	0.019	0.0003	0.0093
GO REGULATION BY VIRUS OF VIRAL PROTEIN LEVELS IN ...	7	3 (42.9)	3 (42.9)	0 (0)	0.009	0.0003	0.0093
GO T CELL ACTIVATION VIA T CELL RECEPTOR CONTACT W...	8	3 (37.5)	3 (37.5)	0 (0)	0.009	0.0003	0.0093
GO POSITIVE REGULATION OF LEUKOCYTE PROLIFERATION	124	9 (7.3)	9 (7.3)	0 (0)	0.020	0.0003	0.0097
GO INTERLEUKIN 12 PRODUCTION	50	6 (12)	6 (12)	0 (0)	0.015	0.0003	0.0097
GO MAST CELL ACTIVATION	53	6 (11.3)	6 (11.3)	0 (0)	0.015	0.0003	0.0097
GO POSITIVE REGULATION OF DEVELOPMENTAL PROCESS	1308	42 (3.2)	42 (3.2)	0 (0)	0.026	0.0004	0.0102
GO GLYCOSYL COMPOUND CATABOLIC PROCESS	38	5 (13.2)	5 (13.2)	0 (0)	0.013	0.0004	0.0102
GO RESPONSE TO FUNGUS	47	5 (10.6)	5 (10.6)	0 (0)	0.013	0.0004	0.0102
GO METAL ION HOMEOSTASIS	622	24 (3.9)	24 (3.9)	0 (0)	0.025	0.0004	0.0104
GO ASTROCYTE ACTIVATION	21	4 (19)	4 (19)	0 (0)	0.011	0.0004	0.0104
GO CELLULAR RESPONSE TO INTERFERON BETA	21	4 (19)	4 (19)	0 (0)	0.011	0.0004	0.0104
GO REGULATION OF CELL DIFFERENTIATION	1741	51 (2.9)	51 (2.9)	0 (0)	0.025	0.0004	0.0113
GO POSITIVE REGULATION OF BINDING	174	11 (6.3)	11 (6.3)	0 (0)	0.022	0.0004	0.0113
GO INTERLEUKIN 12 BIOSYNTHETIC PROCESS	8	3 (37.5)	3 (37.5)	0 (0)	0.009	0.0004	0.0113
GO NEGATIVE REGULATION OF DEFENSE RESPONSE	201	11 (5.5)	11 (5.5)	0 (0)	0.021	0.0004	0.0115
GO REGULATION OF MAST CELL ACTIVATION	37	5 (13.5)	5 (13.5)	0 (0)	0.013	0.0004	0.0115
GO DETECTION OF MOLECULE OF BACTERIAL ORIGIN	10	3 (30)	3 (30)	0 (0)	0.009	0.0004	0.0115
GO MONOCYTE EXTRAVASATION	7	3 (42.9)	3 (42.9)	0 (0)	0.009	0.0004	0.0117
GO INTERLEUKIN 6 MEDIATED SIGNALING PATHWAY	20	4 (20)	4 (20)	0 (0)	0.011	0.0004	0.0119
GO PYRIMIDINE CONTAINING COMPOUND SALVAGE	10	3 (30)	3 (30)	0 (0)	0.009	0.0004	0.0119
GO REGULATION OF OSSIFICATION	182	11 (6)	11 (6)	0 (0)	0.021	0.0004	0.0121
GO REGULATION OF TRANSPORT	1745	51 (2.9)	51 (2.9)	0 (0)	0.025	0.0005	0.0125
GO BLOOD VESSEL MORPHOGENESIS	604	23 (3.8)	23 (3.8)	0 (0)	0.025	0.0005	0.0125
GO REGULATION OF CELL CELL ADHESION	372	17 (4.6)	17 (4.6)	0 (0)	0.024	0.0005	0.0125
GO PYRIMIDINE CONTAINING COMPOUND CATABOLIC PROCES...	39	5 (12.8)	5 (12.8)	0 (0)	0.013	0.0005	0.0125
GO REGULATION OF RIBONUCLEASE ACTIVITY	8	3 (37.5)	3 (37.5)	0 (0)	0.009	0.0005	0.0125
GO T CELL CYTOKINE PRODUCTION	38	5 (13.2)	5 (13.2)	0 (0)	0.013	0.0005	0.0127
GO NEGATIVE REGULATION OF NF KAPPAB TRANSCRIPTION ...	78	7 (9)	7 (9)	0 (0)	0.017	0.0005	0.0131
GO RESPONSE TO INTERFERON ALPHA	21	4 (19)	4 (19)	0 (0)	0.011	0.0005	0.0131
GO POSITIVE REGULATION OF CELL DIFFERENTIATION	927	32 (3.5)	32 (3.5)	0 (0)	0.026	0.0005	0.0136
GO REGULATION OF B CELL PROLIFERATION	58	6 (10.3)	6 (10.3)	0 (0)	0.015	0.0005	0.014
GO STEROL IMPORT	10	3 (30)	3 (30)	0 (0)	0.009	0.0005	0.014
GO REGULATION OF PHOSPHORYLATION	1495	45 (3)	44 (2.9)	1 (0.1)	0.025	0.0006	0.0147
GO POSITIVE REGULATION OF LEUKOCYTE MIGRATION	118	8 (6.8)	8 (6.8)	0 (0)	0.018	0.0006	0.0147
GO LEUKOCYTE HOMEOSTASIS	79	7 (8.9)	7 (8.9)	0 (0)	0.017	0.0006	0.0148

Table S141: MSigDB GO Biological Processes enriched in differentially expressed genes at Day 30 compared to pre-Second Vaccination (B cells, Study Arm 2 A/H7N9+AS03, D1, 29). Results are sorted by FDR adjusted p-value and Jaccard similarity index (continued.)

Gene Set ID	Gene Set Genes #	DE Genes N (%)	Up-reg. DE Genes N (%)	Down-reg. DE Genes N (%)	Jaccard Index	P-Value	FDR Adjusted P-Value
GO POSITIVE REGULATION OF T CELL PROLIFERATION	82	7 (8.5)	7 (8.5)	0 (0)	0.017	0.0006	0.0148
GO REGULATION OF T HELPER 2 CELL CYTOKINE PRODUCTI...	11	3 (27.3)	3 (27.3)	0 (0)	0.009	0.0006	0.0148
GO REGULATION OF NEUROTRANSMITTER LEVELS	323	16 (5)	16 (5)	0 (0)	0.025	0.0006	0.015
GO RESPONSE TO INTERLEUKIN 6	38	5 (13.2)	5 (13.2)	0 (0)	0.013	0.0006	0.0155
GO NEGATIVE REGULATION OF HEMOPOIESIS	144	9 (6.2)	9 (6.2)	0 (0)	0.019	0.0006	0.0159
GO CELLULAR EXTRAVASATION	58	6 (10.3)	6 (10.3)	0 (0)	0.015	0.0006	0.0159
GO REGULATION OF MYELOID CELL DIFFERENTIATION	234	12 (5.1)	12 (5.1)	0 (0)	0.021	0.0006	0.016
GO REGULATION OF MYELOID LEUKOCYTE DIFFERENTIATION	108	8 (7.4)	8 (7.4)	0 (0)	0.018	0.0006	0.016
GO REGULATION OF INTERLEUKIN 1 SECRETION	45	5 (11.1)	5 (11.1)	0 (0)	0.013	0.0006	0.0162
GO REGULATION OF CYSTEINE TYPE ENDOPEPTIDASE ACTIV...	221	12 (5.4)	12 (5.4)	0 (0)	0.022	0.0007	0.0163
GO T HELPER 1 TYPE IMMUNE RESPONSE	40	5 (12.5)	5 (12.5)	0 (0)	0.013	0.0007	0.0163
GO POSITIVE REGULATION OF CATALYTIC ACTIVITY	1331	42 (3.2)	41 (3.1)	1 (0.1)	0.026	0.0007	0.0165
GO NEGATIVE REGULATION OF LYMPHOCYTE ACTIVATION	137	9 (6.6)	9 (6.6)	0 (0)	0.019	0.0007	0.0165
GO T CELL PROLIFERATION	167	10 (6)	10 (6)	0 (0)	0.020	0.0007	0.0166
GO NEGATIVE REGULATION OF INTERLEUKIN 6 PRODUCTION	45	5 (11.1)	5 (11.1)	0 (0)	0.013	0.0007	0.0166
GO POSITIVE REGULATION OF PROTEIN METABOLIC PROCES...	1584	47 (3)	47 (3)	0 (0)	0.025	0.0007	0.0168
GO MONOCYTE CHEMOTAXIS	51	5 (9.8)	5 (9.8)	0 (0)	0.013	0.0007	0.0172
GO MACROPHAGE ACTIVATION INVOLVED IN IMMUNE RESPON...	12	3 (25)	3 (25)	0 (0)	0.009	0.0007	0.0177
GO NEGATIVE REGULATION OF PROTEIN MODIFICATION PRO...	562	22 (3.9)	21 (3.7)	1 (0.2)	0.025	0.0007	0.0178
GO REGULATION OF PHOSPHORUS METABOLIC PROCESS	1674	49 (2.9)	48 (2.9)	1 (0.1)	0.025	0.0007	0.0178
GO REGULATION OF INTRACELLULAR SIGNAL TRANSDUCTION	1707	50 (2.9)	49 (2.9)	1 (0.1)	0.025	0.0008	0.0182
GO REGULATION OF T HELPER 1 TYPE IMMUNE RESPONSE	25	4 (16)	4 (16)	0 (0)	0.011	0.0008	0.0182
GO RESPONSE TO ORGANIC CYCLIC COMPOUND	924	31 (3.4)	30 (3.2)	1 (0.1)	0.025	0.0008	0.0182
GO POSITIVE REGULATION OF DEFENSE RESPONSE TO VIRU...	24	4 (16.7)	4 (16.7)	0 (0)	0.011	0.0008	0.0182
GO REGULATION OF NEUTROPHIL CHEMOTAXIS	27	4 (14.8)	4 (14.8)	0 (0)	0.011	0.0008	0.0182
GO CELLULAR RESPONSE TO LIPID	575	22 (3.8)	21 (3.7)	1 (0.2)	0.025	0.0008	0.0184
GO REGULATION OF VASCULATURE DEVELOPMENT	349	15 (4.3)	15 (4.3)	0 (0)	0.022	0.0008	0.0186
GO NEGATIVE REGULATION OF MAPK CASCADE	165	10 (6.1)	10 (6.1)	0 (0)	0.020	0.0008	0.0189
GO CELLULAR RESPONSE TO AMYLOID BETA	38	5 (13.2)	5 (13.2)	0 (0)	0.013	0.0008	0.0189
GO T CELL MEDIATED IMMUNITY	84	7 (8.3)	7 (8.3)	0 (0)	0.017	0.0009	0.0204
GO REGULATION OF LEUKOCYTE DEGRANULATION	40	5 (12.5)	5 (12.5)	0 (0)	0.013	0.0009	0.0204
GO REGULATION OF RESPONSE TO INTERFERON GAMMA	24	4 (16.7)	4 (16.7)	0 (0)	0.011	0.0009	0.0204
GO REGULATION OF MAPK CASCADE	697	25 (3.6)	25 (3.6)	0 (0)	0.025	0.0009	0.0207
GO POSITIVE REGULATION OF ESTABLISHMENT OF PROTEIN...	428	18 (4.2)	18 (4.2)	0 (0)	0.024	0.0009	0.0207
GO DENDRITIC CELL ANTIGEN PROCESSING AND PRESENTAT...	12	3 (25)	3 (25)	0 (0)	0.009	0.0009	0.0213
GO GLIAL CELL DEVELOPMENT	105	8 (7.6)	8 (7.6)	0 (0)	0.018	0.0009	0.0214
GO COMPLEMENT RECEPTOR MEDIATED SIGNALING PATHWAY	12	3 (25)	3 (25)	0 (0)	0.009	0.0009	0.0214

Table S141: MSigDB GO Biological Processes enriched in differentially expressed genes at Day 30 compared to pre-Second Vaccination (B cells, Study Arm 2 A/H7N9+AS03, D1, 29). Results are sorted by FDR adjusted p-value and Jaccard similarity index (continued.)

Gene Set ID	Gene Set Genes #	DE Genes N (%)	Up-reg. DE Genes N (%)	Down-reg. DE Genes N (%)	Jaccard Index	P-Value	FDR Adjusted P-Value
GO T CELL DIFFERENTIATION	230	12 (5.2)	12 (5.2)	0 (0)	0.021	0.0009	0.0216
GO REGULATION OF HYDROLASE ACTIVITY	1175	37 (3.1)	36 (3.1)	1 (0.1)	0.025	0.001	0.0216
GO POSITIVE REGULATION OF PRODUCTION OF MOLECULAR ...	89	7 (7.9)	7 (7.9)	0 (0)	0.016	0.001	0.0216
GO PYRIMIDINE RIBONUCLEOSIDE METABOLIC PROCESS	14	3 (21.4)	3 (21.4)	0 (0)	0.008	0.001	0.0216
GO IMMUNE RESPONSE REGULATING CELL SURFACE RECEPTO...	348	16 (4.6)	16 (4.6)	0 (0)	0.024	0.001	0.022
GO TOLERANCE INDUCTION DEPENDENT UPON IMMUNE RESPO...	3	2 (66.7)	2 (66.7)	0 (0)	0.006	0.001	0.0222
GO NEGATIVE REGULATION OF PHOSPHORYLATION	431	18 (4.2)	17 (3.9)	1 (0.2)	0.024	0.001	0.0222
GO REGULATION OF GLIOGENESIS	112	8 (7.1)	8 (7.1)	0 (0)	0.018	0.001	0.0222
GO POSITIVE REGULATION OF RESPONSE TO WOUNDING	71	6 (8.5)	6 (8.5)	0 (0)	0.015	0.001	0.0222
GO STEROL TRANSPORT	90	7 (7.8)	7 (7.8)	0 (0)	0.016	0.001	0.0226
GO NEGATIVE REGULATION OF RESPONSE TO STIMULUS	1531	45 (2.9)	43 (2.8)	2 (0.1)	0.025	0.001	0.023
GO NEGATIVE REGULATION OF CYTOKINE BIOSYNTHETIC PR...	29	4 (13.8)	4 (13.8)	0 (0)	0.011	0.001	0.0231
GO POSITIVE REGULATION OF CYSTEINE TYPE ENDOPEPTID...	141	9 (6.4)	9 (6.4)	0 (0)	0.019	0.0011	0.0233
GO REGULATION OF ESTABLISHMENT OF PROTEIN LOCALIZA...	698	25 (3.6)	25 (3.6)	0 (0)	0.025	0.0011	0.0236
GO T HELPER 2 CELL CYTOKINE PRODUCTION	14	3 (21.4)	3 (21.4)	0 (0)	0.008	0.0011	0.024
GO INTERACTION WITH HOST	192	11 (5.7)	10 (5.2)	1 (0.5)	0.021	0.0011	0.0243
GO NUCLEOBASE CONTAINING SMALL MOLECULE CATABOLIC ...	48	5 (10.4)	5 (10.4)	0 (0)	0.013	0.0011	0.0243
GO NEGATIVE REGULATION OF LEUKOCYTE CELL CELL ADHE...	117	8 (6.8)	8 (6.8)	0 (0)	0.018	0.0011	0.0248
GO POSITIVE REGULATION OF REACTIVE OXYGEN SPECIES ...	88	7 (8)	7 (8)	0 (0)	0.016	0.0011	0.0248
GO REGULATION OF RECEPTOR SIGNALING PATHWAY VIA ST...	133	8 (6)	8 (6)	0 (0)	0.017	0.0012	0.025
GO NEGATIVE REGULATION OF VIRAL ENTRY INTO HOST CE...	15	3 (20)	3 (20)	0 (0)	0.008	0.0012	0.0251
GO CARDIOVASCULAR SYSTEM DEVELOPMENT	718	25 (3.5)	25 (3.5)	0 (0)	0.024	0.0012	0.0256
GO LOCOMOTION	1747	49 (2.8)	48 (2.7)	1 (0.1)	0.024	0.0012	0.0256
GO NEGATIVE REGULATION OF CELL ADHESION	263	13 (4.9)	13 (4.9)	0 (0)	0.022	0.0012	0.0259
GO RESPONSE TO CHEMOKINE	84	6 (7.1)	6 (7.1)	0 (0)	0.014	0.0012	0.0259
GO REGULATION OF PROTEIN MODIFICATION PROCESS	1724	49 (2.8)	48 (2.8)	1 (0.1)	0.024	0.0012	0.0262
GO REGULATION OF PEPTIDASE ACTIVITY	409	16 (3.9)	16 (3.9)	0 (0)	0.022	0.0013	0.0271
GO REGULATION OF LIPASE ACTIVITY	94	7 (7.4)	7 (7.4)	0 (0)	0.016	0.0013	0.0271
GO NEGATIVE REGULATION OF TYPE I INTERFERON PRODUC...	43	5 (11.6)	5 (11.6)	0 (0)	0.013	0.0013	0.0275
GO REGULATION OF PHOSPHOLIPASE ACTIVITY	67	6 (9)	6 (9)	0 (0)	0.015	0.0013	0.0278
GO DIGESTIVE SYSTEM PROCESS	94	7 (7.4)	7 (7.4)	0 (0)	0.016	0.0013	0.0282
GO ENDOCYTOSIS	527	21 (4)	21 (4)	0 (0)	0.025	0.0014	0.0289
GO REGULATION OF T CELL CYTOKINE PRODUCTION	28	4 (14.3)	4 (14.3)	0 (0)	0.011	0.0014	0.0289
GO INTERLEUKIN 8 BIOSYNTHETIC PROCESS	14	3 (21.4)	3 (21.4)	0 (0)	0.008	0.0014	0.0289
GO NUCLEOSIDE SALVAGE	14	3 (21.4)	3 (21.4)	0 (0)	0.008	0.0014	0.0289
GO DEFENSE RESPONSE TO GRAM NEGATIVE BACTERIUM	76	5 (6.6)	5 (6.6)	0 (0)	0.012	0.0014	0.0291
GO POSITIVE REGULATION OF CYTOSOLIC CALCIUM ION CO...	30	4 (13.3)	4 (13.3)	0 (0)	0.011	0.0014	0.0291

Confidential/Proprietary Information

Table S141: MSigDB GO Biological Processes enriched in differentially expressed genes at Day 30 compared to pre-Second Vaccination (B cells, Study Arm 2 A/H7N9+AS03, D1, 29). Results are sorted by FDR adjusted p-value and Jaccard similarity index (continued.)

Gene Set ID	Gene Set Genes #	DE Genes N (%)	Up-reg. DE Genes N (%)	Down-reg. DE Genes N (%)	Jaccard Index	P-Value	FDR Adjusted P-Value
GO REGULATION OF BONE MINERALIZATION	68	6 (8.8)	6 (8.8)	0 (0)	0.015	0.0015	0.0301
GO REGULATION OF NEUTROPHIL MIGRATION	31	4 (12.9)	4 (12.9)	0 (0)	0.011	0.0015	0.0302
GO POSITIVE REGULATION OF LEUKOCYTE CHEMOTAXIS	80	6 (7.5)	6 (7.5)	0 (0)	0.014	0.0015	0.0303
GO CHEMOKINE C X C MOTIF LIGAND 2 PRODUCTION	13	3 (23.1)	3 (23.1)	0 (0)	0.009	0.0015	0.0304
GO REGULATION OF MYELOID LEUKOCYTE MEDIATED IMMUNI...	47	5 (10.6)	5 (10.6)	0 (0)	0.013	0.0015	0.0314
GO POSITIVE REGULATION OF PHOSPHORUS METABOLIC PRO...	1054	33 (3.1)	33 (3.1)	0 (0)	0.024	0.0016	0.0318
GO RESPONSE TO MACROPHAGE COLONY STIMULATING FACTO...	12	3 (25)	3 (25)	0 (0)	0.009	0.0016	0.0318
GO REGULATION OF CD8 POSITIVE ALPHA BETA T CELL AC...	13	3 (23.1)	3 (23.1)	0 (0)	0.009	0.0016	0.033
GO SCHWANN CELL MIGRATION	3	2 (66.7)	2 (66.7)	0 (0)	0.006	0.0016	0.033
GO POSITIVE REGULATION OF GLIOGENESIS	69	6 (8.7)	6 (8.7)	0 (0)	0.015	0.0017	0.0332
GO ACYLGLYCEROL TRANSPORT	4	2 (50)	2 (50)	0 (0)	0.006	0.0017	0.0337
GO REGULATION OF ASTROCYTE ACTIVATION	5	2 (40)	2 (40)	0 (0)	0.006	0.0017	0.034
GO PEPTIDE CROSS LINKING	32	4 (12.5)	4 (12.5)	0 (0)	0.011	0.0017	0.0341
GO NEGATIVE REGULATION OF PROTEIN METABOLIC PROCES...	1039	32 (3.1)	31 (3)	1 (0.1)	0.024	0.0017	0.0341
GO POSITIVE REGULATION OF MAPK CASCADE	496	19 (3.8)	19 (3.8)	0 (0)	0.023	0.0017	0.0341
GO TYROSINE PHOSPHORYLATION OF STAT PROTEIN	80	6 (7.5)	6 (7.5)	0 (0)	0.014	0.0017	0.0344
GO POSITIVE REGULATION OF PEPTIDASE ACTIVITY	185	10 (5.4)	10 (5.4)	0 (0)	0.019	0.0018	0.0355
GO INTERFERON BETA PRODUCTION	44	5 (11.4)	5 (11.4)	0 (0)	0.013	0.0018	0.0355
GO MHC CLASS II BIOSYNTHETIC PROCESS	15	3 (20)	3 (20)	0 (0)	0.008	0.0018	0.0355
GO LEUKOCYTE APOPTOTIC PROCESS	100	7 (7)	7 (7)	0 (0)	0.016	0.0018	0.0358
GO NEGATIVE REGULATION OF MOLECULAR FUNCTION	1052	32 (3)	32 (3)	0 (0)	0.023	0.0019	0.0365
GO ASTROCYTE DIFFERENTIATION	72	6 (8.3)	6 (8.3)	0 (0)	0.015	0.0019	0.0366
GO REGULATION OF NIK NF KAPPAB SIGNALING	99	7 (7.1)	7 (7.1)	0 (0)	0.016	0.0019	0.0367
GO PYRIMIDINE NUCLEOSIDE BIOSYNTHETIC PROCESS	15	3 (20)	3 (20)	0 (0)	0.008	0.0019	0.0368
GO NEGATIVE REGULATION OF INFLAMMATORY RESPONSE	141	8 (5.7)	8 (5.7)	0 (0)	0.017	0.0019	0.0373
GO POSITIVE REGULATION OF TYPE I INTERFERON PRODUC...	68	6 (8.8)	6 (8.8)	0 (0)	0.015	0.0019	0.0374
GO POSITIVE REGULATION OF NIK NF KAPPAB SIGNALING	71	6 (8.5)	6 (8.5)	0 (0)	0.015	0.002	0.0375
GO REGULATION OF MACROPHAGE ACTIVATION	52	5 (9.6)	5 (9.6)	0 (0)	0.013	0.002	0.0375
GO REGULATION OF PRODUCTION OF MOLECULAR MEDIATOR ...	129	8 (6.2)	8 (6.2)	0 (0)	0.017	0.0021	0.0391
GO ALPHA BETA T CELL DIFFERENTIATION	97	7 (7.2)	7 (7.2)	0 (0)	0.016	0.0021	0.0391
GO REGULATION OF VESICLE MEDIATED TRANSPORT	507	20 (3.9)	20 (3.9)	0 (0)	0.024	0.0021	0.0397
GO CELL MOTILITY	1531	43 (2.8)	42 (2.7)	1 (0.1)	0.024	0.0021	0.04
GO RESPONSE TO AMYLOID BETA	49	5 (10.2)	5 (10.2)	0 (0)	0.013	0.0021	0.04
GO POSITIVE REGULATION OF CHROMATIN BINDING	13	3 (23.1)	3 (23.1)	0 (0)	0.009	0.0021	0.04
GO REGULATION OF MATRIX METALLOPEPTIDASE SECRETION	5	2 (40)	2 (40)	0 (0)	0.006	0.0022	0.0414
GO POSITIVE REGULATION OF CHEMOKINE SECRETION	14	3 (21.4)	3 (21.4)	0 (0)	0.008	0.0023	0.0422
GO ACTIVATION OF PHOSPHOLIPASE C ACTIVITY	30	4 (13.3)	4 (13.3)	0 (0)	0.011	0.0023	0.0425

Table S141: MSigDB GO Biological Processes enriched in differentially expressed genes at Day 30 compared to pre-Second Vaccination (B cells, Study Arm 2 A/H7N9+AS03, D1, 29). Results are sorted by FDR adjusted p-value and Jaccard similarity index (continued.)

Gene Set ID	Gene Set Genes #	DE Genes N (%)	Up-reg. DE Genes N (%)	Down-reg. DE Genes N (%)	Jaccard Index	P-Value	FDR Adjusted P-Value
GO POSITIVE REGULATION OF INTERLEUKIN 2 PRODUCTION	29	4 (13.8)	4 (13.8)	0 (0)	0.011	0.0023	0.0431
GO LYMPHOCYTE ACTIVATION INVOLVED IN IMMUNE RESPON...	171	9 (5.3)	9 (5.3)	0 (0)	0.018	0.0024	0.0437
GO POSITIVE REGULATION OF MYELOID LEUKOCYTE DIFFER...	54	5 (9.3)	5 (9.3)	0 (0)	0.013	0.0024	0.0437
GO NEGATIVE REGULATION OF B CELL PROLIFERATION	15	3 (20)	3 (20)	0 (0)	0.008	0.0024	0.0437
GO MYELOID CELL APOPTOTIC PROCESS	31	4 (12.9)	4 (12.9)	0 (0)	0.011	0.0024	0.0438
GO REGULATION OF ANTIGEN PROCESSING AND PRESENTATI...	16	3 (18.8)	3 (18.8)	0 (0)	0.008	0.0024	0.0441
GO REGULATION OF LEUKOCYTE APOPTOTIC PROCESS	78	6 (7.7)	6 (7.7)	0 (0)	0.014	0.0025	0.0465
GO BONE MINERALIZATION	103	7 (6.8)	7 (6.8)	0 (0)	0.016	0.0026	0.0471
GO CELLULAR RESPONSE TO LIPOPROTEIN PARTICLE STIMU...	31	4 (12.9)	4 (12.9)	0 (0)	0.011	0.0026	0.0472
GO NEGATIVE REGULATION OF CATALYTIC ACTIVITY	743	24 (3.2)	24 (3.2)	0 (0)	0.023	0.0026	0.0473
GO REGULATION OF ANATOMICAL STRUCTURE MORPHOGENESI...	1035	32 (3.1)	32 (3.1)	0 (0)	0.024	0.0027	0.0481
GO TUBE MORPHOGENESIS	829	27 (3.3)	27 (3.3)	0 (0)	0.024	0.0027	0.0481
GO AMYLOID FIBRIL FORMATION	15	3 (20)	3 (20)	0 (0)	0.008	0.0027	0.0484
GO RELEASE OF CYTOCHROME C FROM MITOCHONDRIA	57	5 (8.8)	5 (8.8)	0 (0)	0.013	0.0027	0.0489
GO PYRIMIDINE NUCLEOBASE METABOLIC PROCESS	15	3 (20)	3 (20)	0 (0)	0.008	0.0027	0.0489
GO RESPONSE TO TUMOR NECROSIS FACTOR	272	12 (4.4)	12 (4.4)	0 (0)	0.020	0.0027	0.0489
GO POSITIVE REGULATION OF PROTEIN CATABOLIC PROCES...	4	2 (50)	2 (50)	0 (0)	0.006	0.0028	0.0494
GO PHAGOCYTOSIS RECOGNITION	16	3 (18.8)	3 (18.8)	0 (0)	0.008	0.0028	0.0494
GO EOSINOPHIL ACTIVATION	5	2 (40)	2 (40)	0 (0)	0.006	0.0028	0.0497
GO REGULATION OF RESPONSE TO WOUNDING	170	9 (5.3)	9 (5.3)	0 (0)	0.018	0.0028	0.0499

Table S142: Blood Transcription Modules enriched in differentially expressed genes at Day 30 compared to pre-Second Vaccination (B cells, Study Arm 2 A/H7N9+AS03, D1, 29). Results are sorted by FDR adjusted p-value and Jaccard similarity index.

Gene Set ID	Gene Set Genes #	DE Genes N (%)	Up-reg. DE Genes N (%)	Down-reg. DE Genes N (%)	Jaccard Index	P-Value	FDR Adjusted P-Value
M11.0 ENRICHED IN MONOCYTES (II)	174	97 (55.7)	97 (55.7)	0 (0)	0.230	<0.0001	0.0003
M4.0 CELL CYCLE AND TRANSCRIPTION	303	76 (25.1)	76 (25.1)	0 (0)	0.133	<0.0001	0.0003
M37.0 IMMUNE ACTIVATION - GENERIC CLUSTER	301	56 (18.6)	56 (18.6)	0 (0)	0.095	<0.0001	0.0003
S4 MONOCYTE SURFACE SIGNATURE	81	31 (38.3)	31 (38.3)	0 (0)	0.079	<0.0001	0.0003
M16 TLR AND INFLAMMATORY SIGNALING	41	19 (46.3)	19 (46.3)	0 (0)	0.052	<0.0001	0.0003
M118.0 ENRICHED IN MONOCYTES (IV)	50	16 (32)	16 (32)	0 (0)	0.042	<0.0001	0.0003
M37.1 ENRICHED IN NEUTROPHILS (I)	46	14 (30.4)	14 (30.4)	0 (0)	0.037	<0.0001	0.0003
M4.3 MYELOID CELL ENRICHED RECEPTORS AND TRANSPORT...	29	13 (44.8)	13 (44.8)	0 (0)	0.036	<0.0001	0.0003
M127 TYPE I INTERFERON RESPONSE	12	11 (91.7)	11 (91.7)	0 (0)	0.032	<0.0001	0.0003

Table S142: Blood Transcription Modules enriched in differentially expressed genes at Day 30 compared to pre-Second Vaccination (B cells, Study Arm 2 A/H7N9+AS03, D1, 29). Results are sorted by FDR adjusted p-value and Jaccard similarity index (continued.)

Gene Set ID	Gene Set Genes #	DE Genes N (%)	Up-reg. DE Genes N (%)	Down-reg. DE Genes N (%)	Jaccard Index	P-Value	FDR Adjusted P-Value
M150 INNATE ANTIVIRAL RESPONSE	12	8 (66.7)	8 (66.7)	0 (0)	0.023	<0.0001	0.0003
M11.2 FORMYL PEPTIDE RECEPTOR MEDIATED NEUTROPHIL ...	10	7 (70)	7 (70)	0 (0)	0.020	<0.0001	0.0003
M3 REGULATION OF SIGNAL TRANSDUCTION	46	12 (26.1)	12 (26.1)	0 (0)	0.032	<0.0001	0.0021
M11.1 BLOOD COAGULATION	21	8 (38.1)	8 (38.1)	0 (0)	0.022	<0.0001	0.0021
M81 ENRICHED IN MYELOID CELLS AND MONOCYTES	34	10 (29.4)	10 (29.4)	0 (0)	0.027	0.0002	0.0037
M33 INFLAMMATORY RESPONSE	10	5 (50)	5 (50)	0 (0)	0.014	0.0001	0.0037
M64 ENRICHED IN ACTIVATED DENDRITIC CELLS-MONOCYTE...	14	6 (42.9)	6 (42.9)	0 (0)	0.017	0.0002	0.0041
M118.1 ENRICHED IN MONOCYTES (SURFACE)	14	6 (42.9)	6 (42.9)	0 (0)	0.017	0.0003	0.0071
M165 ENRICHED IN ACTIVATED DENDRITIC CELLS (II)	34	9 (26.5)	9 (26.5)	0 (0)	0.024	0.0006	0.0111
M79 TBA	10	5 (50)	5 (50)	0 (0)	0.014	0.0006	0.0111
M87 TRANSMEMBRANE TRANSPORT (I)	24	7 (29.2)	7 (29.2)	0 (0)	0.019	0.002	0.0349
M66 TBA	14	5 (35.7)	5 (35.7)	0 (0)	0.014	0.0021	0.0349
M111.0 VIRAL SENSING AND IMMUNITY; IRF2 TARGETS NE...	16	5 (31.2)	5 (31.2)	0 (0)	0.014	0.003	0.0468
M111.1 VIRAL SENSING AND IMMUNITY; IRF2 TARGETS NE...	11	4 (36.4)	4 (36.4)	0 (0)	0.011	0.0031	0.0468
M75 ANTIVIRAL IFN SIGNATURE	22	6 (27.3)	6 (27.3)	0 (0)	0.017	0.0033	0.0471

Table S143: KEGG Pathways enriched in differentially expressed genes at Day 32 compared to pre-Second Vaccination (B cells, Study Arm 2 A/H7N9+AS03, D1, 29). Results are sorted by FDR adjusted p-value and Jaccard similarity index.

Gene Set ID	Gene Set Genes #	DE Genes N (%)	Up-reg. DE Genes N (%)	Down-reg. DE Genes N (%)	Jaccard Index	P-Value	FDR Adjusted P-Value
HEMATOPOIETIC CELL LINEAGE	93	7 (7.5)	7 (7.5)	0 (0)	0.038	<0.0001	0.0034
CELL CYCLE	121	8 (6.6)	8 (6.6)	0 (0)	0.038	<0.0001	0.0034
VIRAL PROTEIN INTERACTION WITH CYTOKINE AND CYTOKI...	96	5 (5.2)	5 (5.2)	0 (0)	0.026	0.0002	0.0244
CYTOKINE-CYTOKINE RECEPTOR INTERACTION	280	8 (2.9)	8 (2.9)	0 (0)	0.022	0.0003	0.0244
OSTEOCLAST DIFFERENTIATION	122	6 (4.9)	6 (4.9)	0 (0)	0.028	0.0005	0.0324

Table S144: MSigDB Reactome Pathways enriched in differentially expressed genes at Day 32 compared to pre-Second Vaccination (B cells, Study Arm 2 A/H7N9+AS03, D1, 29). Results are sorted by FDR adjusted p-value and Jaccard similarity index.

Gene Set ID	Gene Set Genes #	DE Genes N (%)	Up-reg. DE Genes N (%)	Down-reg. DE Genes N (%)	Jaccard Index	P-Value	FDR Adjusted P-Value
REACTOME ACTIVATION OF ATR IN RESPONSE TO REPLICAT...	36	5 (13.9)	5 (13.9)	0 (0)	0.038	<0.0001	0.0102

Table S144: MSigDB Reactome Pathways enriched in differentially expressed genes at Day 32 compared to pre-Second Vaccination (B cells, Study Arm 2 A/H7N9+AS03, D1, 29). Results are sorted by FDR adjusted p-value and Jaccard similarity index (continued.)

Gene Set ID	Gene Set Genes #	DE Genes N (%)	Up-reg. DE Genes N (%)	Down-reg. DE Genes N (%)	Jaccard Index	P-Value	FDR Adjusted P-Value
REACTOME IMMUNOREGULATORY INTERACTIONS BETWEEN A L...	97	7 (7.2)	7 (7.2)	0 (0)	0.037	<0.0001	0.0102
REACTOME ERYTHROCYTES TAKE UP OXYGEN AND RELEASE C...	9	3 (33.3)	3 (33.3)	0 (0)	0.029	<0.0001	0.0102
REACTOME CELL CYCLE CHECKPOINTS	276	10 (3.6)	10 (3.6)	0 (0)	0.027	<0.0001	0.0115
REACTOME CELL CYCLE	639	16 (2.5)	16 (2.5)	0 (0)	0.022	<0.0001	0.0123
REACTOME CELL CYCLE MITOTIC	532	14 (2.6)	14 (2.6)	0 (0)	0.023	<0.0001	0.0128
REACTOME ERYTHROCYTES TAKE UP CARBON DIOXIDE AND R...	13	3 (23.1)	3 (23.1)	0 (0)	0.028	<0.0001	0.0131
REACTOME G1 S SPECIFIC TRANSCRIPTION	28	4 (14.3)	4 (14.3)	0 (0)	0.033	<0.0001	0.0153
REACTOME ACTIVATION OF THE PRE REPLICATIVE COMPLEX	31	4 (12.9)	4 (12.9)	0 (0)	0.032	0.0001	0.0187

Table S145: MSigDB GO Biological Processes enriched in differentially expressed genes at Day 32 compared to pre-Second Vaccination (B cells, Study Arm 2 A/H7N9+AS03, D1, 29). Results are sorted by FDR adjusted p-value and Jaccard similarity index.

Gene Set ID	Gene Set Genes #	DE Genes N (%)	Up-reg. DE Genes N (%)	Down-reg. DE Genes N (%)	Jaccard Index	P-Value	FDR Adjusted P-Value
GO REGULATION OF TRANSCRIPTION INVOLVED IN G1 S TR...	27	5 (18.5)	5 (18.5)	0 (0)	0.041	<0.0001	0.0054
GO POSITIVE REGULATION OF CYTOKINE BIOSYNTHETIC PR...	60	6 (10)	6 (10)	0 (0)	0.039	<0.0001	0.0054
GO DNA REPLICATION CHECKPOINT	15	4 (26.7)	4 (26.7)	0 (0)	0.036	<0.0001	0.0054
GO CYTOKINE METABOLIC PROCESS	105	7 (6.7)	7 (6.7)	0 (0)	0.035	<0.0001	0.0054
GO POSITIVE REGULATION OF INTERFERON GAMMA PRODUCT...	54	5 (9.3)	5 (9.3)	0 (0)	0.034	<0.0001	0.0054
GO DNA REPLICATION	258	10 (3.9)	10 (3.9)	0 (0)	0.029	<0.0001	0.0054
GO MYELOID LEUKOCYTE MIGRATION	187	8 (4.3)	8 (4.3)	0 (0)	0.029	<0.0001	0.0054
GO CELL CHEMOTAXIS	278	9 (3.2)	9 (3.2)	0 (0)	0.025	<0.0001	0.0054
GO CELL CYCLE PHASE TRANSITION	577	15 (2.6)	15 (2.6)	0 (0)	0.023	<0.0001	0.0054
GO POSITIVE REGULATION OF CELL POPULATION PROLIFER...	865	20 (2.3)	20 (2.3)	0 (0)	0.021	<0.0001	0.0054
GO CYTOKINE PRODUCTION	720	16 (2.2)	16 (2.2)	0 (0)	0.020	<0.0001	0.0054
GO IMMUNE SYSTEM DEVELOPMENT	926	18 (1.9)	18 (1.9)	0 (0)	0.018	<0.0001	0.0054
GO REGULATION OF IMMUNE SYSTEM PROCESS	1409	26 (1.8)	26 (1.8)	0 (0)	0.018	<0.0001	0.0054
GO REGULATION OF CELL POPULATION PROLIFERATION	1541	24 (1.6)	24 (1.6)	0 (0)	0.015	<0.0001	0.0054
GO TUMOR NECROSIS FACTOR BIOSYNTHETIC PROCESS	28	5 (17.9)	5 (17.9)	0 (0)	0.041	<0.0001	0.006
GO CELLULAR DEFENSE RESPONSE	46	5 (10.9)	5 (10.9)	0 (0)	0.036	<0.0001	0.006
GO POSITIVE REGULATION OF TUMOR NECROSIS FACTOR BI...	18	4 (22.2)	4 (22.2)	0 (0)	0.035	<0.0001	0.006
GO TUMOR NECROSIS FACTOR SUPERFAMILY CYTOKINE PROD...	148	7 (4.7)	7 (4.7)	0 (0)	0.029	<0.0001	0.006
GO MITOTIC DNA REPLICATION CHECKPOINT	9	3 (33.3)	3 (33.3)	0 (0)	0.029	<0.0001	0.006
GO POSITIVE REGULATION OF GLIAL CELL MIGRATION	10	3 (30)	3 (30)	0 (0)	0.028	<0.0001	0.006
GO POSITIVE REGULATION OF CYTOKINE PRODUCTION	419	12 (2.9)	12 (2.9)	0 (0)	0.024	<0.0001	0.006

Table S145: MSigDB GO Biological Processes enriched in differentially expressed genes at Day 32 compared to pre-Second Vaccination (B cells, Study Arm 2 A/H7N9+AS03, D1, 29). Results are sorted by FDR adjusted p-value and Jaccard similarity index (continued.)

Gene Set ID	Gene Set Genes #	DE Genes N (%)	Up-reg. DE Genes N (%)	Down-reg. DE Genes N (%)	Jaccard Index	P-Value	FDR Adjusted P-Value
GO LEUKOCYTE MIGRATION	410	11 (2.7)	11 (2.7)	0 (0)	0.022	<0.0001	0.006
GO MITOTIC CELL CYCLE	954	18 (1.9)	18 (1.9)	0 (0)	0.017	<0.0001	0.006
GO POSITIVE REGULATION OF IMMUNE SYSTEM PROCESS	972	18 (1.9)	18 (1.9)	0 (0)	0.017	<0.0001	0.006
GO CELL ACTIVATION	1265	22 (1.7)	22 (1.7)	0 (0)	0.016	<0.0001	0.006
GO INTERFERON GAMMA PRODUCTION	97	6 (6.2)	6 (6.2)	0 (0)	0.032	<0.0001	0.0084
GO CELL CYCLE G1 S PHASE TRANSITION	262	9 (3.4)	9 (3.4)	0 (0)	0.026	<0.0001	0.0084
GO POSITIVE REGULATION OF INTERLEUKIN 2 PRODUCTION	29	4 (13.8)	4 (13.8)	0 (0)	0.032	<0.0001	0.0086
GO DNA REPLICATION INITIATION	35	4 (11.4)	4 (11.4)	0 (0)	0.031	<0.0001	0.0086
GO MITOTIC SISTER CHROMATID SEGREGATION	146	7 (4.8)	7 (4.8)	0 (0)	0.029	<0.0001	0.0086
GO HUMORAL IMMUNE RESPONSE	227	7 (3.1)	7 (3.1)	0 (0)	0.022	<0.0001	0.0086
GO LEUKOCYTE MEDIATED IMMUNITY	701	15 (2.1)	15 (2.1)	0 (0)	0.019	<0.0001	0.0086
GO MYELOID LEUKOCYTE ACTIVATION	594	13 (2.2)	13 (2.2)	0 (0)	0.019	<0.0001	0.0086
GO CELL ACTIVATION INVOLVED IN IMMUNE RESPONSE	658	14 (2.1)	14 (2.1)	0 (0)	0.019	<0.0001	0.0086
GO CELL MOTILITY	1531	22 (1.4)	22 (1.4)	0 (0)	0.014	<0.0001	0.0086
GO CELLULAR EXTRAVASATION	58	5 (8.6)	5 (8.6)	0 (0)	0.033	<0.0001	0.0097
GO POSITIVE REGULATION OF INTERLEUKIN 2 BIOSYNTHET...	13	3 (23.1)	3 (23.1)	0 (0)	0.028	<0.0001	0.0097
GO GAS TRANSPORT	19	3 (15.8)	3 (15.8)	0 (0)	0.026	<0.0001	0.0097
GO REGULATION OF CELL CYCLE PHASE TRANSITION	433	11 (2.5)	11 (2.5)	0 (0)	0.021	<0.0001	0.0097
GO REGULATION OF GLIAL CELL MIGRATION	14	3 (21.4)	3 (21.4)	0 (0)	0.027	<0.0001	0.0105
GO LEUKOCYTE CHEMOTAXIS	202	7 (3.5)	7 (3.5)	0 (0)	0.024	<0.0001	0.0105
GO REGULATION OF MITOTIC CELL CYCLE	604	13 (2.2)	13 (2.2)	0 (0)	0.019	<0.0001	0.0105
GO TAXIS	607	13 (2.1)	13 (2.1)	0 (0)	0.019	<0.0001	0.0105
GO POSITIVE REGULATION OF ALPHA BETA T CELL PROLIF...	17	3 (17.6)	3 (17.6)	0 (0)	0.026	<0.0001	0.0115
GO MYELOID CELL DIFFERENTIATION	389	10 (2.6)	10 (2.6)	0 (0)	0.021	<0.0001	0.0115
GO BIOLOGICAL ADHESION	1320	20 (1.5)	20 (1.5)	0 (0)	0.014	<0.0001	0.0115
GO LOCOMOTION	1747	23 (1.3)	23 (1.3)	0 (0)	0.013	<0.0001	0.0128
GO REGULATION OF CELL CYCLE PROCESS	716	14 (2)	14 (2)	0 (0)	0.018	<0.0001	0.0154
GO REGULATION OF IMMUNE RESPONSE	897	15 (1.7)	15 (1.7)	0 (0)	0.015	<0.0001	0.0154
GO POSITIVE REGULATION OF MULTICELLULAR ORGANISMAL...	1665	22 (1.3)	22 (1.3)	0 (0)	0.013	0.0001	0.0166
GO POSITIVE REGULATION OF TUMOR NECROSIS FACTOR SU...	79	5 (6.3)	5 (6.3)	0 (0)	0.029	0.0001	0.0174
GO IMMUNE EFFECTOR PROCESS	1077	17 (1.6)	17 (1.6)	0 (0)	0.015	0.0001	0.0174
GO SISTER CHROMATID SEGREGATION	175	7 (4)	7 (4)	0 (0)	0.026	0.0001	0.0195
GO REGULATION OF CELLULAR COMPONENT MOVEMENT	973	16 (1.6)	16 (1.6)	0 (0)	0.015	0.0001	0.0195
GO NEUTROPHIL MIGRATION	103	5 (4.9)	5 (4.9)	0 (0)	0.025	0.0001	0.0202
GO MITOTIC NUCLEAR DIVISION	253	8 (3.2)	8 (3.2)	0 (0)	0.023	0.0001	0.0202
GO GLIAL CELL MIGRATION	46	4 (8.7)	4 (8.7)	0 (0)	0.028	0.0002	0.0217
GO INTERLEUKIN 2 BIOSYNTHETIC PROCESS	21	3 (14.3)	3 (14.3)	0 (0)	0.026	0.0002	0.0217

Table S145: MSigDB GO Biological Processes enriched in differentially expressed genes at Day 32 compared to pre-Second Vaccination (B cells, Study Arm 2 A/H7N9+AS03, D1, 29). Results are sorted by FDR adjusted p-value and Jaccard similarity index (continued.)

Gene Set ID	Gene Set Genes #	DE Genes N (%)	Up-reg. DE Genes N (%)	Down-reg. DE Genes N (%)	Jaccard Index	P-Value	FDR Adjusted P-Value
GO REGULATION OF CELL ACTIVATION	507	11 (2.2)	11 (2.2)	0 (0)	0.018	0.0002	0.0217
GO REGULATION OF HEMOPOIESIS	441	10 (2.3)	10 (2.3)	0 (0)	0.019	0.0002	0.0222
GO MYELOID LEUKOCYTE MEDIATED IMMUNITY	506	11 (2.2)	11 (2.2)	0 (0)	0.018	0.0002	0.0222
GO CHROMOSOME ORGANIZATION	1118	17 (1.5)	17 (1.5)	0 (0)	0.014	0.0002	0.0243
GO POSITIVE REGULATION OF CELL ACTIVATION	297	8 (2.7)	8 (2.7)	0 (0)	0.021	0.0002	0.0259
GO REGULATION OF CELL CYCLE	1115	17 (1.5)	17 (1.5)	0 (0)	0.014	0.0002	0.0259
GO CELL CYCLE PROCESS	1291	19 (1.5)	19 (1.5)	0 (0)	0.014	0.0002	0.0278
GO REGULATION OF MYELOID CELL DIFFERENTIATION	234	7 (3)	7 (3)	0 (0)	0.021	0.0003	0.0297
GO INTERLEUKIN 2 PRODUCTION	59	4 (6.8)	4 (6.8)	0 (0)	0.026	0.0003	0.0316
GO CELL CYCLE CHECKPOINT	207	7 (3.4)	7 (3.4)	0 (0)	0.023	0.0003	0.0316
GO POSITIVE REGULATION OF LOCOMOTION	544	11 (2)	11 (2)	0 (0)	0.017	0.0003	0.0316
GO NEURAL PRECURSOR CELL PROLIFERATION	143	6 (4.2)	6 (4.2)	0 (0)	0.025	0.0003	0.0329
GO REGULATION OF LEUKOCYTE MIGRATION	175	6 (3.4)	6 (3.4)	0 (0)	0.022	0.0003	0.0329
GO LEUKOCYTE DIFFERENTIATION	483	10 (2.1)	10 (2.1)	0 (0)	0.018	0.0003	0.0335
GO GRANULOCYTE MIGRATION	126	5 (4)	5 (4)	0 (0)	0.023	0.0003	0.034
GO MITOTIC CELL CYCLE CHECKPOINT	158	6 (3.8)	6 (3.8)	0 (0)	0.024	0.0003	0.0346
GO METAPHASE ANAPHASE TRANSITION OF CELL CYCLE	55	4 (7.3)	4 (7.3)	0 (0)	0.027	0.0003	0.0351
GO LEUKOCYTE TETHERING OR ROLLING	25	3 (12)	3 (12)	0 (0)	0.025	0.0004	0.0357
GO MONONUCLEAR CELL MIGRATION	74	4 (5.4)	4 (5.4)	0 (0)	0.024	0.0004	0.0357
GO SECRETION	1551	20 (1.3)	20 (1.3)	0 (0)	0.012	0.0004	0.0357
GO POSITIVE REGULATION OF LEUKOCYTE MIGRATION	118	5 (4.2)	5 (4.2)	0 (0)	0.024	0.0004	0.04
GO MEIOTIC SISTER CHROMATID COHESION CENTROMERIC	5	2 (40)	2 (40)	0 (0)	0.020	0.0004	0.0424
GO REGULATION OF LEUKOCYTE DIFFERENTIATION	252	7 (2.8)	7 (2.8)	0 (0)	0.020	0.0005	0.0465

Table S146: Blood Transcription Modules enriched in differentially expressed genes at Day 32 compared to pre-Second Vaccination (B cells, Study Arm 2 A/H7N9+AS03, D1, 29). Results are sorted by FDR adjusted p-value and Jaccard similarity index.

Gene Set ID	Gene Set Genes #	DE Genes N (%)	Up-reg. DE Genes N (%)	Down-reg. DE Genes N (%)	Jaccard Index	P-Value	FDR Adjusted P-Value
M4.0 CELL CYCLE AND TRANSCRIPTION	303	28 (9.2)	28 (9.2)	0 (0)	0.075	<0.0001	0.0012
M11.0 ENRICHED IN MONOCYTES (II)	174	17 (9.8)	17 (9.8)	0 (0)	0.066	<0.0001	0.0012
M4.1 CELL CYCLE (I)	135	13 (9.6)	13 (9.6)	0 (0)	0.059	<0.0001	0.0012
M16 TLR AND INFLAMMATORY SIGNALING	41	7 (17.1)	7 (17.1)	0 (0)	0.053	<0.0001	0.0026
M4.2 PLK1 SIGNALING EVENTS	31	6 (19.4)	6 (19.4)	0 (0)	0.048	<0.0001	0.0055
M103 CELL CYCLE (III)	50	7 (14)	7 (14)	0 (0)	0.049	0.0001	0.0069

Table S146: Blood Transcription Modules enriched in differentially expressed genes at Day 32 compared to pre-Second Vaccination (B cells, Study Arm 2 A/H7N9+AS03, D1, 29). Results are sorted by FDR adjusted p-value and Jaccard similarity index (continued.)

Gene Set ID	Gene Set Genes #	DE Genes N (%)	Up-reg. DE Genes N (%)	Down-reg. DE Genes N (%)	Jaccard Index	P-Value	FDR Adjusted P-Value
M7.0 ENRICHED IN T CELLS (I)	57	6 (10.5)	6 (10.5)	0 (0)	0.040	0.0005	0.0257
S4 MONOCYTE SURFACE SIGNATURE	81	8 (9.9)	8 (9.9)	0 (0)	0.046	0.0008	0.0329
M81 ENRICHED IN MYELOID CELLS AND MONOCYTES	34	5 (14.7)	5 (14.7)	0 (0)	0.039	0.0016	0.0489
M7.2 ENRICHED IN NK CELLS (I)	45	5 (11.1)	5 (11.1)	0 (0)	0.036	0.0015	0.0489
S0 T CELL SURFACE SIGNATURE	24	4 (16.7)	4 (16.7)	0 (0)	0.034	0.0013	0.0489
M6 MITOTIC CELL DIVISION	25	4 (16)	4 (16)	0 (0)	0.033	0.0019	0.0489
M126 DOUBLE POSITIVE THYMOCYTES	10	3 (30)	3 (30)	0 (0)	0.028	0.002	0.0489
M8 E2F TRANSCRIPTION FACTOR NETWORK	11	3 (27.3)	3 (27.3)	0 (0)	0.028	0.0017	0.0489

Table S147: Blood Transcription Modules enriched in differentially expressed genes at Day 36 compared to pre-Second Vaccination (B cells, Study Arm 2 A/H7N9+AS03, D1, 29). Results are sorted by FDR adjusted p-value and Jaccard similarity index.

Gene Set ID	Gene Set Genes #	DE Genes N (%)	Up-reg. DE Genes N (%)	Down-reg. DE Genes N (%)	Jaccard Index	P-Value	FDR Adjusted P-Value
M7.2 ENRICHED IN NK CELLS (I)	45	11 (24.4)	11 (24.4)	0 (0)	0.109	<0.0001	0.0012
M7.0 ENRICHED IN T CELLS (I)	57	8 (14)	8 (14)	0 (0)	0.069	<0.0001	0.0012
M11.0 ENRICHED IN MONOCYTES (II)	174	13 (7.5)	13 (7.5)	0 (0)	0.057	<0.0001	0.0012
M61.0 ENRICHED IN NK CELLS (II)	16	4 (25)	4 (25)	0 (0)	0.051	<0.0001	0.0043
M16 TLR AND INFLAMMATORY SIGNALING	41	5 (12.2)	5 (12.2)	0 (0)	0.049	0.0001	0.0076

Table S148: KEGG Pathways enriched in differentially expressed genes at Day 2 compared to pre-First Vaccination (T cells, Study Arm 2 A/H7N9+AS03, D1, 29). Results are sorted by FDR adjusted p-value and Jaccard similarity index.

Gene Set ID	Gene Set Genes #	DE Genes N (%)	Up-reg. DE Genes N (%)	Down-reg. DE Genes N (%)	Jaccard Index	P-Value	FDR Adjusted P-Value
NOD-LIKE RECEPTOR SIGNALING PATHWAY	175	7 (4)	7 (4)	0 (0)	0.027	<0.0001	0.0202

Table S149: MSigDB Reactome Pathways enriched in differentially expressed genes at Day 2 compared to pre-First Vaccination (T cells, Study Arm 2 A/H7N9+AS03, D1, 29). Results are sorted by FDR adjusted p-value and Jaccard similarity index.

Gene Set ID	Gene Set Genes #	DE Genes N (%)	Up-reg. DE Genes N (%)	Down-reg. DE Genes N (%)	Jaccard Index	P-Value	FDR Adjusted P-Value
REACTOME INTERFERON ALPHA BETA SIGNALING	60	12 (20)	12 (20)	0 (0)	0.088	<0.0001	0.0038
REACTOME INTERFERON SIGNALING	177	16 (9)	16 (9)	0 (0)	0.064	<0.0001	0.0038
REACTOME INTERFERON GAMMA SIGNALING	71	8 (11.3)	8 (11.3)	0 (0)	0.053	<0.0001	0.0038
REACTOME CYTOKINE SIGNALING IN IMMUNE SYSTEM	788	22 (2.8)	22 (2.8)	0 (0)	0.026	<0.0001	0.0038
REACTOME INNATE IMMUNE SYSTEM	968	15 (1.5)	15 (1.5)	0 (0)	0.014	<0.0001	0.0276
REACTOME METAL SEQUESTRATION BY ANTIMICROBIAL PROT...	6	2 (33.3)	2 (33.3)	0 (0)	0.022	0.0002	0.046

Table S150: MSigDB GO Biological Processes enriched in differentially expressed genes at Day 2 compared to pre-First Vaccination (T cells, Study Arm 2 A/H7N9+AS03, D1, 29). Results are sorted by FDR adjusted p-value and Jaccard similarity index.

Gene Set ID	Gene Set Genes #	DE Genes N (%)	Up-reg. DE Genes N (%)	Down-reg. DE Genes N (%)	Jaccard Index	P-Value	FDR Adjusted P-Value
GO RESPONSE TO TYPE I INTERFERON	88	11 (12.5)	11 (12.5)	0 (0)	0.067	<0.0001	0.0024
GO DEFENSE RESPONSE TO VIRUS	229	15 (6.6)	15 (6.6)	0 (0)	0.050	<0.0001	0.0024
GO INTERFERON GAMMA MEDIATED SIGNALING PATHWAY	70	7 (10)	7 (10)	0 (0)	0.046	<0.0001	0.0024
GO RESPONSE TO VIRUS	300	17 (5.7)	17 (5.7)	0 (0)	0.046	<0.0001	0.0024
GO RESPONSE TO INTERFERON BETA	30	5 (16.7)	5 (16.7)	0 (0)	0.044	<0.0001	0.0024
GO RESPONSE TO INTERFERON GAMMA	166	10 (6)	10 (6)	0 (0)	0.041	<0.0001	0.0024
GO RESPONSE TO INTERFERON ALPHA	21	4 (19)	4 (19)	0 (0)	0.038	<0.0001	0.0024
GO POSITIVE REGULATION OF DEFENSE RESPONSE TO VIRU...	24	4 (16.7)	4 (16.7)	0 (0)	0.037	<0.0001	0.0024
GO INNATE IMMUNE RESPONSE	814	30 (3.7)	30 (3.7)	0 (0)	0.034	<0.0001	0.0024
GO DEFENSE RESPONSE TO OTHER ORGANISM	993	33 (3.3)	33 (3.3)	0 (0)	0.032	<0.0001	0.0024
GO REGULATION OF RESPONSE TO BIOTIC STIMULUS	466	16 (3.4)	16 (3.4)	0 (0)	0.030	<0.0001	0.0024
GO NEGATIVE REGULATION OF MULTI ORGANISM PROCESS	195	8 (4.1)	8 (4.1)	0 (0)	0.029	<0.0001	0.0024
GO DEFENSE RESPONSE TO BACTERIUM	237	9 (3.8)	9 (3.8)	0 (0)	0.029	<0.0001	0.0024
GO RESPONSE TO BIOTIC STIMULUS	1325	38 (2.9)	38 (2.9)	0 (0)	0.028	<0.0001	0.0024
GO REGULATION OF MULTI ORGANISM PROCESS	700	21 (3)	21 (3)	0 (0)	0.027	<0.0001	0.0024
GO CYTOKINE MEDIATED SIGNALING PATHWAY	711	20 (2.8)	20 (2.8)	0 (0)	0.026	<0.0001	0.0024
GO POSITIVE REGULATION OF RESPONSE TO BIOTIC STIMU...	353	11 (3.1)	11 (3.1)	0 (0)	0.026	<0.0001	0.0024
GO POSITIVE REGULATION OF DEFENSE RESPONSE	457	13 (2.8)	13 (2.8)	0 (0)	0.024	<0.0001	0.0024
GO REGULATION OF DEFENSE RESPONSE	703	18 (2.6)	18 (2.6)	0 (0)	0.023	<0.0001	0.0024
GO IMMUNE EFFECTOR PROCESS	1077	26 (2.4)	26 (2.4)	0 (0)	0.023	<0.0001	0.0024
GO RESPONSE TO BACTERIUM	587	15 (2.6)	15 (2.6)	0 (0)	0.023	<0.0001	0.0024
GO DEFENSE RESPONSE	1497	35 (2.3)	35 (2.3)	0 (0)	0.023	<0.0001	0.0024
GO POSITIVE REGULATION OF RESPONSE TO EXTERNAL STI...	575	14 (2.4)	14 (2.4)	0 (0)	0.022	<0.0001	0.0024

Table S150: MSigDB GO Biological Processes enriched in differentially expressed genes at Day 2 compared to pre-First Vaccination (T cells, Study Arm 2 A/H7N9+AS03, D1, 29). Results are sorted by FDR adjusted p-value and Jaccard similarity index (continued.)

Gene Set ID	Gene Set Genes #	DE Genes N (%)	Up-reg. DE Genes N (%)	Down-reg. DE Genes N (%)	Jaccard Index	P-Value	FDR Adjusted P-Value
GO RESPONSE TO CYTOKINE	1099	24 (2.2)	24 (2.2)	0 (0)	0.021	<0.0001	0.0024
GO REGULATION OF IMMUNE RESPONSE	897	19 (2.1)	19 (2.1)	0 (0)	0.020	<0.0001	0.0024
GO ACTIVATION OF IMMUNE RESPONSE	547	12 (2.2)	12 (2.2)	0 (0)	0.019	<0.0001	0.0024
GO POSITIVE REGULATION OF IMMUNE RESPONSE	704	15 (2.1)	15 (2.1)	0 (0)	0.019	<0.0001	0.0024
GO POSITIVE REGULATION OF IMMUNE SYSTEM PROCESS	972	19 (2)	19 (2)	0 (0)	0.018	<0.0001	0.0024
GO REGULATION OF RESPONSE TO EXTERNAL STIMULUS	1006	19 (1.9)	19 (1.9)	0 (0)	0.018	<0.0001	0.0024
GO CYTOKINE PRODUCTION	720	14 (1.9)	14 (1.9)	0 (0)	0.018	<0.0001	0.0024
GO REGULATION OF IMMUNE SYSTEM PROCESS	1409	24 (1.7)	24 (1.7)	0 (0)	0.016	<0.0001	0.0024
GO NEGATIVE REGULATION OF VIRAL GENOME REPLICATION	58	5 (8.6)	5 (8.6)	0 (0)	0.035	<0.0001	0.0044
GO NEGATIVE REGULATION OF VIRAL PROCESS	90	6 (6.7)	6 (6.7)	0 (0)	0.035	<0.0001	0.0044
GO POSITIVE REGULATION OF MULTI ORGANISM PROCESS	489	11 (2.2)	11 (2.2)	0 (0)	0.019	<0.0001	0.0044
GO REGULATION OF DEFENSE RESPONSE TO VIRUS	68	5 (7.4)	5 (7.4)	0 (0)	0.033	<0.0001	0.0065
GO REGULATION OF DEFENSE RESPONSE TO VIRUS BY HOST	34	4 (11.8)	4 (11.8)	0 (0)	0.034	<0.0001	0.0081
GO REGULATION OF RESPONSE TO STRESS	1406	20 (1.4)	20 (1.4)	0 (0)	0.014	<0.0001	0.0081
GO LEUKOCYTE MIGRATION INVOLVED IN INFLAMMATORY RE...	14	3 (21.4)	3 (21.4)	0 (0)	0.030	<0.0001	0.0099
GO REGULATION OF VIRAL LIFE CYCLE	133	6 (4.5)	6 (4.5)	0 (0)	0.028	<0.0001	0.0116
GO IMMUNE RESPONSE REGULATING SIGNALING PATHWAY	507	11 (2.2)	11 (2.2)	0 (0)	0.019	<0.0001	0.0132
GO NEGATIVE REGULATION OF VIRAL LIFE CYCLE	76	5 (6.6)	5 (6.6)	0 (0)	0.031	<0.0001	0.0147
GO ACTIVATION OF INNATE IMMUNE RESPONSE	286	8 (2.8)	8 (2.8)	0 (0)	0.022	<0.0001	0.0161
GO REGULATION OF VIRAL GENOME REPLICATION	91	5 (5.5)	5 (5.5)	0 (0)	0.029	<0.0001	0.0171
GO NEUTROPHIL MIGRATION	103	5 (4.9)	5 (4.9)	0 (0)	0.027	<0.0001	0.0171
GO REGULATION OF SYMBIOSIS ENCOMPASSING MUTUALISM ...	204	7 (3.4)	7 (3.4)	0 (0)	0.025	0.0001	0.0184
GO RESPONSE TO PROTOZOAN	26	3 (11.5)	3 (11.5)	0 (0)	0.027	0.0001	0.0208
GO CYTOKINE SECRETION	220	7 (3.2)	7 (3.2)	0 (0)	0.023	0.0001	0.0208
GO CELLULAR RESPONSE TO INTERFERON BETA	21	3 (14.3)	3 (14.3)	0 (0)	0.028	0.0001	0.022
GO MYELOID LEUKOCYTE ACTIVATION	594	11 (1.9)	11 (1.9)	0 (0)	0.016	0.0002	0.0277
GO SECRETION	1551	19 (1.2)	19 (1.2)	0 (0)	0.012	0.0002	0.0286
GO REGULATION OF INFLAMMATORY RESPONSE	332	8 (2.4)	8 (2.4)	0 (0)	0.019	0.0002	0.0295
GO GRANULOCYTE MIGRATION	126	5 (4)	5 (4)	0 (0)	0.024	0.0002	0.0304
GO DEFENSE RESPONSE TO FUNGUS	35	3 (8.6)	3 (8.6)	0 (0)	0.025	0.0002	0.0313
GO MYELOID LEUKOCYTE MIGRATION	187	6 (3.2)	6 (3.2)	0 (0)	0.022	0.0002	0.0315
GO CELL ACTIVATION	1265	17 (1.3)	16 (1.3)	1 (0.1)	0.013	0.0002	0.0315
GO ISG15 PROTEIN CONJUGATION	6	2 (33.3)	2 (33.3)	0 (0)	0.022	0.0002	0.0323
GO LEUKOCYTE CHEMOTAXIS	202	6 (3)	6 (3)	0 (0)	0.021	0.0003	0.0343
GO REGULATION OF RESPONSE TO INTERFERON GAMMA	24	3 (12.5)	3 (12.5)	0 (0)	0.028	0.0003	0.0357
GO VIRAL GENOME REPLICATION	117	5 (4.3)	5 (4.3)	0 (0)	0.025	0.0003	0.0357
GO POSITIVE REGULATION OF INFLAMMATORY RESPONSE	134	5 (3.7)	5 (3.7)	0 (0)	0.023	0.0003	0.0402

Table S151: Blood Transcription Modules enriched in differentially expressed genes at Day 2 compared to pre-First Vaccination (T cells, Study Arm 2 A/H7N9+AS03, D1, 29). Results are sorted by FDR adjusted p-value and Jaccard similarity index.

Gene Set ID	Gene Set Genes #	DE Genes N (%)	Up-reg. DE Genes N (%)	Down-reg. DE Genes N (%)	Jaccard Index	P-Value	FDR Adjusted P-Value
M11.0 ENRICHED IN MONOCYTES (II)	174	17 (9.8)	17 (9.8)	0 (0)	0.069	<0.0001	0.0012
M16 TLR AND INFLAMMATORY SIGNALING	41	7 (17.1)	7 (17.1)	0 (0)	0.057	<0.0001	0.0012
M165 ENRICHED IN ACTIVATED DENDRITIC CELLS (II)	34	6 (17.6)	6 (17.6)	0 (0)	0.052	<0.0001	0.0012
M4.0 CELL CYCLE AND TRANSCRIPTION	303	14 (4.6)	14 (4.6)	0 (0)	0.037	<0.0001	0.0086
M11.2 FORMYL PEPTIDE RECEPTOR MEDIATED NEUTROPHIL ...	10	3 (30)	3 (30)	0 (0)	0.032	0.0003	0.0201
M127 TYPE I INTERFERON RESPONSE	12	3 (25)	3 (25)	0 (0)	0.031	0.0007	0.0432

Table S152: KEGG Pathways enriched in differentially expressed genes at Day 30 compared to pre-Second Vaccination (T cells, Study Arm 2 A/H7N9+AS03, D1, 29). Results are sorted by FDR adjusted p-value and Jaccard similarity index.

Gene Set ID	Gene Set Genes #	DE Genes N (%)	Up-reg. DE Genes N (%)	Down-reg. DE Genes N (%)	Jaccard Index	P-Value	FDR Adjusted P-Value
OSTEOCLAST DIFFERENTIATION	122	19 (15.6)	19 (15.6)	0 (0)	0.047	<0.0001	0.0006
NOD-LIKE RECEPTOR SIGNALING PATHWAY	175	19 (10.9)	19 (10.9)	0 (0)	0.042	<0.0001	0.0006
INFLUENZA A	160	18 (11.2)	18 (11.2)	0 (0)	0.041	<0.0001	0.0006
HEPATITIS C	152	16 (10.5)	16 (10.5)	0 (0)	0.037	<0.0001	0.0006
MEASLES	132	14 (10.6)	13 (9.8)	1 (0.8)	0.033	<0.0001	0.0006
JAK-STAT SIGNALING PATHWAY	154	13 (8.4)	13 (8.4)	0 (0)	0.029	<0.0001	0.0006
EPSTEIN-BARR VIRUS INFECTION	196	14 (7.1)	14 (7.1)	0 (0)	0.029	<0.0001	0.0029
B CELL RECEPTOR SIGNALING PATHWAY	77	9 (11.7)	9 (11.7)	0 (0)	0.024	<0.0001	0.0029
KAPOSI SARCOMA-ASSOCIATED HERPESVIRUS INFECTION	192	13 (6.8)	13 (6.8)	0 (0)	0.027	0.0001	0.0049
PROLACTIN SIGNALING PATHWAY	70	8 (11.4)	8 (11.4)	0 (0)	0.022	0.0002	0.0071
HEPATITIS B	156	11 (7.1)	11 (7.1)	0 (0)	0.025	0.0004	0.0116
TNF SIGNALING PATHWAY	109	9 (8.3)	9 (8.3)	0 (0)	0.022	0.0004	0.0118
STAPHYLOCOCCUS AUREUS INFECTION	91	7 (7.7)	7 (7.7)	0 (0)	0.018	0.0006	0.0156
HUMAN PAPILLOMAVIRUS INFECTION	324	16 (4.9)	14 (4.3)	2 (0.6)	0.026	0.0016	0.0353
C-TYPE LECTIN RECEPTOR SIGNALING PATHWAY	103	8 (7.8)	8 (7.8)	0 (0)	0.020	0.0015	0.0353

Table S153: MSigDB Reactome Pathways enriched in differentially expressed genes at Day 30 compared to pre-Second Vaccination (T cells, Study Arm 2 A/H7N9+AS03, D1, 29). Results are sorted by FDR adjusted p-value and Jaccard similarity index.

Gene Set ID	Gene Set Genes #	DE Genes N (%)	Up-reg. DE Genes N (%)	Down-reg. DE Genes N (%)	Jaccard Index	P-Value	FDR Adjusted P-Value
REACTOME INTERFERON SIGNALING	177	37 (20.9)	37 (20.9)	0 (0)	0.084	<0.0001	0.0017
REACTOME INTERFERON ALPHA BETA SIGNALING	60	24 (40)	24 (40)	0 (0)	0.071	<0.0001	0.0017
REACTOME CYTOKINE SIGNALING IN IMMUNE SYSTEM	788	63 (8)	60 (7.6)	3 (0.4)	0.061	<0.0001	0.0017
REACTOME INTERFERON GAMMA SIGNALING	71	20 (28.2)	20 (28.2)	0 (0)	0.057	<0.0001	0.0017
REACTOME INNATE IMMUNE SYSTEM	968	50 (5.2)	47 (4.9)	3 (0.3)	0.041	<0.0001	0.0017
REACTOME NEUTROPHIL DEGRANULATION	442	27 (6.1)	26 (5.9)	1 (0.2)	0.038	<0.0001	0.0017
REACTOME SIGNALING BY INTERLEUKINS	430	25 (5.8)	25 (5.8)	0 (0)	0.035	<0.0001	0.0017
REACTOME ANTIVIRAL MECHANISM BY IFN STIMULATED GEN...	81	12 (14.8)	12 (14.8)	0 (0)	0.032	<0.0001	0.0017
REACTOME INTERLEUKIN 2 FAMILY SIGNALING	40	7 (17.5)	7 (17.5)	0 (0)	0.021	<0.0001	0.0017
REACTOME REGULATION OF IFNA SIGNALING	26	5 (19.2)	5 (19.2)	0 (0)	0.015	<0.0001	0.0028
REACTOME OAS ANTIVIRAL RESPONSE	8	4 (50)	4 (50)	0 (0)	0.013	<0.0001	0.0028
REACTOME GROWTH HORMONE RECEPTOR SIGNALING	24	5 (20.8)	5 (20.8)	0 (0)	0.016	<0.0001	0.0064
REACTOME INTERLEUKIN 10 SIGNALING	39	6 (15.4)	6 (15.4)	0 (0)	0.018	<0.0001	0.0071
REACTOME REGULATION OF IFNG SIGNALING	13	4 (30.8)	4 (30.8)	0 (0)	0.013	<0.0001	0.0098
REACTOME NICOTINATE METABOLISM	29	5 (17.2)	5 (17.2)	0 (0)	0.015	0.0002	0.0235
REACTOME INFLAMMASOMES	20	4 (20)	4 (20)	0 (0)	0.013	0.0004	0.0364
REACTOME INTERLEUKIN 4 AND INTERLEUKIN 13 SIGNALIN...	102	8 (7.8)	8 (7.8)	0 (0)	0.020	0.0004	0.0378

Table S154: MSigDB GO Biological Processes enriched in differentially expressed genes at Day 30 compared to pre-Second Vaccination (T cells, Study Arm 2 A/H7N9+AS03, D1, 29). Results are sorted by FDR adjusted p-value and Jaccard similarity index.

Gene Set ID	Gene Set Genes #	DE Genes N (%)	Up-reg. DE Genes N (%)	Down-reg. DE Genes N (%)	Jaccard Index	P-Value	FDR Adjusted P-Value
GO DEFENSE RESPONSE TO VIRUS	229	37 (16.2)	36 (15.7)	1 (0.4)	0.075	<0.0001	0.0006
GO RESPONSE TO VIRUS	300	41 (13.7)	40 (13.3)	1 (0.3)	0.073	<0.0001	0.0006
GO INNATE IMMUNE RESPONSE	814	74 (9.1)	74 (9.1)	0 (0)	0.071	<0.0001	0.0006
GO RESPONSE TO INTERFERON GAMMA	166	30 (18.1)	30 (18.1)	0 (0)	0.069	<0.0001	0.0006
GO DEFENSE RESPONSE TO OTHER ORGANISM	993	83 (8.4)	82 (8.3)	1 (0.1)	0.069	<0.0001	0.0006
GO RESPONSE TO BIOTIC STIMULUS	1325	92 (6.9)	91 (6.9)	1 (0.1)	0.060	<0.0001	0.0006
GO RESPONSE TO TYPE I INTERFERON	88	22 (25)	22 (25)	0 (0)	0.060	<0.0001	0.0006
GO DEFENSE RESPONSE	1497	101 (6.7)	98 (6.5)	3 (0.2)	0.059	<0.0001	0.0006
GO REGULATION OF MULTI ORGANISM PROCESS	700	55 (7.9)	55 (7.9)	0 (0)	0.058	<0.0001	0.0006
GO CYTOKINE MEDIATED SIGNALING PATHWAY	711	55 (7.7)	55 (7.7)	0 (0)	0.058	<0.0001	0.0006
GO INTERFERON GAMMA MEDIATED SIGNALING PATHWAY	70	20 (28.6)	20 (28.6)	0 (0)	0.057	<0.0001	0.0006
GO RESPONSE TO CYTOKINE	1099	75 (6.8)	74 (6.7)	1 (0.1)	0.057	<0.0001	0.0006

Table S154: MSigDB GO Biological Processes enriched in differentially expressed genes at Day 30 compared to pre-Second Vaccination (T cells, Study Arm 2 A/H7N9+AS03, D1, 29). Results are sorted by FDR adjusted p-value and Jaccard similarity index (continued.)

Gene Set ID	Gene Set Genes #	DE Genes N (%)	Up-reg. DE Genes N (%)	Down-reg. DE Genes N (%)	Jaccard Index	P-Value	FDR Adjusted P-Value
GO REGULATION OF RESPONSE TO BIOTIC STIMULUS	466	41 (8.8)	41 (8.8)	0 (0)	0.057	<0.0001	0.0006
GO IMMUNE EFFECTOR PROCESS	1077	72 (6.7)	70 (6.5)	2 (0.2)	0.055	<0.0001	0.0006
GO NEGATIVE REGULATION OF MULTI ORGANISM PROCESS	195	24 (12.3)	24 (12.3)	0 (0)	0.051	<0.0001	0.0006
GO MYELOID LEUKOCYTE ACTIVATION	594	43 (7.2)	40 (6.7)	3 (0.5)	0.051	<0.0001	0.0006
GO REGULATION OF IMMUNE RESPONSE	897	55 (6.1)	53 (5.9)	2 (0.2)	0.048	<0.0001	0.0006
GO REGULATION OF DEFENSE RESPONSE	703	45 (6.4)	44 (6.3)	1 (0.1)	0.047	<0.0001	0.0006
GO CYTOKINE PRODUCTION	720	45 (6.2)	43 (6)	2 (0.3)	0.046	<0.0001	0.0006
GO NEGATIVE REGULATION OF VIRAL PROCESS	90	17 (18.9)	17 (18.9)	0 (0)	0.045	<0.0001	0.0006
GO REGULATION OF IMMUNE SYSTEM PROCESS	1409	73 (5.2)	70 (5)	3 (0.2)	0.045	<0.0001	0.0006
GO NEGATIVE REGULATION OF VIRAL LIFE CYCLE	76	16 (21.1)	16 (21.1)	0 (0)	0.044	<0.0001	0.0006
GO POSITIVE REGULATION OF RESPONSE TO EXTERNAL STI...	575	37 (6.4)	36 (6.3)	1 (0.2)	0.044	<0.0001	0.0006
GO REGULATION OF RESPONSE TO EXTERNAL STIMULUS	1006	55 (5.5)	53 (5.3)	2 (0.2)	0.044	<0.0001	0.0006
GO POSITIVE REGULATION OF CYTOKINE PRODUCTION	419	30 (7.2)	28 (6.7)	2 (0.5)	0.043	<0.0001	0.0006
GO REGULATION OF VIRAL LIFE CYCLE	133	18 (13.5)	18 (13.5)	0 (0)	0.043	<0.0001	0.0006
GO INFLAMMATORY RESPONSE	680	40 (5.9)	38 (5.6)	2 (0.3)	0.043	<0.0001	0.0006
GO POSITIVE REGULATION OF IMMUNE SYSTEM PROCESS	972	51 (5.2)	50 (5.1)	1 (0.1)	0.042	<0.0001	0.0006
GO NEGATIVE REGULATION OF VIRAL GENOME REPLICATION	58	14 (24.1)	14 (24.1)	0 (0)	0.041	<0.0001	0.0006
GO CELL ACTIVATION	1265	61 (4.8)	56 (4.4)	5 (0.4)	0.041	<0.0001	0.0006
GO POSITIVE REGULATION OF DEFENSE RESPONSE	457	29 (6.3)	29 (6.3)	0 (0)	0.040	<0.0001	0.0006
GO POSITIVE REGULATION OF RESPONSE TO BIOTIC STIMU...	353	25 (7.1)	25 (7.1)	0 (0)	0.040	<0.0001	0.0006
GO POSITIVE REGULATION OF IMMUNE RESPONSE	704	38 (5.4)	38 (5.4)	0 (0)	0.039	<0.0001	0.0006
GO REGULATION OF SYMBIOSIS ENCOMPASSING MUTUALISM ...	204	19 (9.3)	19 (9.3)	0 (0)	0.039	<0.0001	0.0006
GO RESPONSE TO BACTERIUM	587	33 (5.6)	33 (5.6)	0 (0)	0.039	<0.0001	0.0006
GO EXOCYTOSIS	833	41 (4.9)	37 (4.4)	4 (0.5)	0.037	<0.0001	0.0006
GO LEUKOCYTE MEDIATED IMMUNITY	701	36 (5.1)	35 (5)	1 (0.1)	0.037	<0.0001	0.0006
GO MYELOID LEUKOCYTE MEDIATED IMMUNITY	506	29 (5.7)	28 (5.5)	1 (0.2)	0.037	<0.0001	0.0006
GO IMMUNE RESPONSE REGULATING SIGNALING PATHWAY	507	29 (5.7)	28 (5.5)	1 (0.2)	0.037	<0.0001	0.0006
GO REGULATION OF VIRAL GENOME REPLICATION	91	14 (15.4)	14 (15.4)	0 (0)	0.037	<0.0001	0.0006
GO ACTIVATION OF IMMUNE RESPONSE	547	30 (5.5)	30 (5.5)	0 (0)	0.037	<0.0001	0.0006
GO POSITIVE REGULATION OF MULTI ORGANISM PROCESS	489	28 (5.7)	28 (5.7)	0 (0)	0.037	<0.0001	0.0006
GO CYTOKINE SECRETION	220	18 (8.2)	17 (7.7)	1 (0.5)	0.036	<0.0001	0.0006
GO CELL ACTIVATION INVOLVED IN IMMUNE RESPONSE	658	33 (5)	32 (4.9)	1 (0.2)	0.036	<0.0001	0.0006
GO ACTIVATION OF INNATE IMMUNE RESPONSE	286	20 (7)	20 (7)	0 (0)	0.035	<0.0001	0.0006
GO REGULATION OF RESPONSE TO STRESS	1406	58 (4.1)	55 (3.9)	3 (0.2)	0.035	<0.0001	0.0006
GO SECRETION	1551	63 (4.1)	57 (3.7)	6 (0.4)	0.035	<0.0001	0.0006
GO VIRAL GENOME REPLICATION	117	14 (12)	14 (12)	0 (0)	0.035	<0.0001	0.0006
GO REGULATION OF IMMUNE EFFECTOR PROCESS	357	22 (6.2)	22 (6.2)	0 (0)	0.035	<0.0001	0.0006

Confidential/Proprietary Information

Table S154: MSigDB GO Biological Processes enriched in differentially expressed genes at Day 30 compared to pre-Second Vaccination (T cells, Study Arm 2 A/H7N9+AS03, D1, 29). Results are sorted by FDR adjusted p-value and Jaccard similarity index (continued.)

Gene Set ID	Gene Set Genes #	DE Genes N (%)	Up-reg. DE Genes N (%)	Down-reg. DE Genes N (%)	Jaccard Index	P-Value	FDR Adjusted P-Value
GO VIRAL LIFE CYCLE	308	20 (6.5)	20 (6.5)	0 (0)	0.034	<0.0001	0.0006
GO REGULATION OF CELL ACTIVATION	507	26 (5.1)	24 (4.7)	2 (0.4)	0.033	<0.0001	0.0006
GO POSITIVE REGULATION OF INTRACELLULAR SIGNAL TRA...	948	39 (4.1)	34 (3.6)	5 (0.5)	0.032	<0.0001	0.0006
GO APOPTOTIC SIGNALING PATHWAY	566	27 (4.8)	23 (4.1)	4 (0.7)	0.032	<0.0001	0.0006
GO REGULATION OF HEMOPOIESIS	441	23 (5.2)	22 (5)	1 (0.2)	0.032	<0.0001	0.0006
GO LEUKOCYTE MIGRATION	410	22 (5.4)	20 (4.9)	2 (0.5)	0.032	<0.0001	0.0006
GO POSITIVE REGULATION OF CELL DEATH	670	30 (4.5)	26 (3.9)	4 (0.6)	0.032	<0.0001	0.0006
GO MYELOID LEUKOCYTE MIGRATION	187	15 (8)	13 (7)	2 (1.1)	0.032	<0.0001	0.0006
GO REGULATION OF KINASE ACTIVITY	816	34 (4.2)	31 (3.8)	3 (0.4)	0.031	<0.0001	0.0006
GO POSITIVE REGULATION OF PROTEIN METABOLIC PROCES...	1584	57 (3.6)	52 (3.3)	5 (0.3)	0.031	<0.0001	0.0006
GO POSITIVE REGULATION OF MAPK CASCADE	496	24 (4.8)	21 (4.2)	3 (0.6)	0.031	<0.0001	0.0006
GO INTERLEUKIN 1 PRODUCTION	99	12 (12.1)	12 (12.1)	0 (0)	0.031	<0.0001	0.0006
GO REGULATION OF INFLAMMATORY RESPONSE	332	19 (5.7)	18 (5.4)	1 (0.3)	0.031	<0.0001	0.0006
GO INTERSPECIES INTERACTION BETWEEN ORGANISMS	869	35 (4)	35 (4)	0 (0)	0.031	<0.0001	0.0006
GO LEUKOCYTE CHEMOTAXIS	202	15 (7.4)	14 (6.9)	1 (0.5)	0.031	<0.0001	0.0006
GO POSITIVE REGULATION OF I KAPPAB KINASE NF KAPPA...	169	14 (8.3)	14 (8.3)	0 (0)	0.031	<0.0001	0.0006
GO REGULATION OF TRANSFERASE ACTIVITY	912	36 (3.9)	32 (3.5)	4 (0.4)	0.031	<0.0001	0.0006
GO REGULATION OF LEUKOCYTE MIGRATION	175	14 (8)	12 (6.9)	2 (1.1)	0.030	<0.0001	0.0006
GO CELL CHEMOTAXIS	278	17 (6.1)	16 (5.8)	1 (0.4)	0.030	<0.0001	0.0006
GO IMMUNE RESPONSE REGULATING CELL SURFACE RECEPTO...	348	19 (5.5)	18 (5.2)	1 (0.3)	0.030	<0.0001	0.0006
GO PHAGOCYTOSIS	251	16 (6.4)	14 (5.6)	2 (0.8)	0.030	<0.0001	0.0006
GO I KAPPAB KINASE NF KAPPAB SIGNALING	252	16 (6.3)	16 (6.3)	0 (0)	0.030	<0.0001	0.0006
GO NEGATIVE REGULATION OF IMMUNE SYSTEM PROCESS	426	21 (4.9)	19 (4.5)	2 (0.5)	0.030	<0.0001	0.0006
GO REGULATION OF DNA BINDING TRANSCRIPTION FACTOR ...	393	20 (5.1)	18 (4.6)	2 (0.5)	0.030	<0.0001	0.0006
GO POSITIVE REGULATION OF PHOSPHORUS METABOLIC PRO...	1054	39 (3.7)	35 (3.3)	4 (0.4)	0.030	<0.0001	0.0006
GO MACROPHAGE ACTIVATION	84	11 (13.1)	9 (10.7)	2 (2.4)	0.029	<0.0001	0.0006
GO NEGATIVE REGULATION OF CYTOKINE PRODUCTION	262	16 (6.1)	14 (5.3)	2 (0.8)	0.029	<0.0001	0.0006
GO POSITIVE REGULATION OF KINASE ACTIVITY	543	24 (4.4)	22 (4.1)	2 (0.4)	0.029	<0.0001	0.0006
GO INTERLEUKIN 1 BETA PRODUCTION	87	11 (12.6)	11 (12.6)	0 (0)	0.029	<0.0001	0.0006
GO POSITIVE REGULATION OF TRANSFERASE ACTIVITY	614	26 (4.2)	23 (3.7)	3 (0.5)	0.029	<0.0001	0.0006
GO PEPTIDE SECRETION	587	25 (4.3)	23 (3.9)	2 (0.3)	0.029	<0.0001	0.0006
GO GRANULOCYTE MIGRATION	126	12 (9.5)	11 (8.7)	1 (0.8)	0.029	<0.0001	0.0006
GO DEFENSE RESPONSE TO BACTERIUM	237	15 (6.3)	15 (6.3)	0 (0)	0.029	<0.0001	0.0006
GO POSITIVE REGULATION OF DNA BINDING TRANSCRIPTIO...	238	15 (6.3)	14 (5.9)	1 (0.4)	0.029	<0.0001	0.0006
GO REGULATION OF PHOSPHORYLATION	1495	50 (3.3)	44 (2.9)	6 (0.4)	0.029	<0.0001	0.0006
GO RESPONSE TO OXYGEN CONTAINING COMPOUND	1593	52 (3.3)	46 (2.9)	6 (0.4)	0.028	<0.0001	0.0006
GO RESPONSE TO INTERFERON BETA	30	9 (30)	9 (30)	0 (0)	0.028	<0.0001	0.0006

Table S154: MSigDB GO Biological Processes enriched in differentially expressed genes at Day 30 compared to pre-Second Vaccination (T cells, Study Arm 2 A/H7N9+AS03, D1, 29). Results are sorted by FDR adjusted p-value and Jaccard similarity index (continued.)

Gene Set ID	Gene Set Genes #	DE Genes N (%)	Up-reg. DE Genes N (%)	Down-reg. DE Genes N (%)	Jaccard Index	P-Value	FDR Adjusted P-Value
GO REGULATION OF PROTEIN MODIFICATION PROCESS	1724	55 (3.2)	49 (2.8)	6 (0.3)	0.028	<0.0001	0.0006
GO POSITIVE REGULATION OF MULTICELLULAR ORGANISMAL...	1665	53 (3.2)	46 (2.8)	7 (0.4)	0.028	<0.0001	0.0006
GO REGULATION OF CELL DEATH	1593	51 (3.2)	44 (2.8)	7 (0.4)	0.028	<0.0001	0.0006
GO RESPONSE TO MOLECULE OF BACTERIAL ORIGIN	329	17 (5.2)	17 (5.2)	0 (0)	0.028	<0.0001	0.0006
GO REGULATION OF PHOSPHORUS METABOLIC PROCESS	1674	53 (3.2)	47 (2.8)	6 (0.4)	0.028	<0.0001	0.0006
GO POSITIVE REGULATION OF CELL ACTIVATION	297	16 (5.4)	15 (5.1)	1 (0.3)	0.028	<0.0001	0.0006
GO REGULATION OF PEPTIDASE ACTIVITY	409	19 (4.6)	16 (3.9)	3 (0.7)	0.028	<0.0001	0.0006
GO PROTEIN PHOSPHORYLATION	1838	57 (3.1)	50 (2.7)	7 (0.4)	0.027	<0.0001	0.0006
GO REGULATION OF INTRACELLULAR SIGNAL TRANSDUCTION	1707	53 (3.1)	44 (2.6)	9 (0.5)	0.027	<0.0001	0.0006
GO NEGATIVE REGULATION OF MOLECULAR FUNCTION	1052	35 (3.3)	29 (2.8)	6 (0.6)	0.027	<0.0001	0.0006
GO POSITIVE REGULATION OF SIGNALING	1730	52 (3)	46 (2.7)	6 (0.3)	0.026	<0.0001	0.0006
GO POSITIVE REGULATION OF NF KAPPAB TRANSCRIPTION ...	133	11 (8.3)	11 (8.3)	0 (0)	0.026	<0.0001	0.0006
GO POSITIVE REGULATION OF CYTOKINE BIOSYNTHETIC PR...	60	9 (15)	8 (13.3)	1 (1.7)	0.026	<0.0001	0.0006
GO NEUTROPHIL MIGRATION	103	10 (9.7)	10 (9.7)	0 (0)	0.025	<0.0001	0.0006
GO REGULATION OF LEUKOCYTE CHEMOTAXIS	102	10 (9.8)	9 (8.8)	1 (1)	0.025	<0.0001	0.0006
GO SMOOTH MUSCLE CELL PROLIFERATION	146	11 (7.5)	9 (6.2)	2 (1.4)	0.025	<0.0001	0.0006
GO REGULATION OF DEFENSE RESPONSE TO VIRUS	68	9 (13.2)	9 (13.2)	0 (0)	0.025	<0.0001	0.0006
GO POSITIVE REGULATION OF LEUKOCYTE MIGRATION	118	10 (8.5)	9 (7.6)	1 (0.8)	0.024	<0.0001	0.0006
GO POSITIVE REGULATION OF TUMOR NECROSIS FACTOR SU...	79	9 (11.4)	8 (10.1)	1 (1.3)	0.024	<0.0001	0.0006
GO CHEMOKINE PRODUCTION	80	9 (11.2)	9 (11.2)	0 (0)	0.024	<0.0001	0.0006
GO CYTOKINE METABOLIC PROCESS	105	9 (8.6)	8 (7.6)	1 (1)	0.023	<0.0001	0.0006
GO POSITIVE REGULATION OF INTERLEUKIN 6 PRODUCTION	87	8 (9.2)	8 (9.2)	0 (0)	0.021	<0.0001	0.0006
GO LEUKOCYTE ACTIVATION INVOLVED IN INFLAMMATORY R...	40	7 (17.5)	7 (17.5)	0 (0)	0.021	<0.0001	0.0006
GO REGULATION OF MONONUCLEAR CELL MIGRATION	40	7 (17.5)	6 (15)	1 (2.5)	0.021	<0.0001	0.0006
GO INTERLEUKIN 1 SECRETION	53	7 (13.2)	7 (13.2)	0 (0)	0.020	<0.0001	0.0006
GO RESPONSE TO INTERFERON ALPHA	21	6 (28.6)	6 (28.6)	0 (0)	0.019	<0.0001	0.0006
GO REGULATION OF RESPONSE TO INTERFERON GAMMA	24	6 (25)	6 (25)	0 (0)	0.019	<0.0001	0.0006
GO REGULATION OF MACROPHAGE MIGRATION	34	6 (17.6)	4 (11.8)	2 (5.9)	0.018	<0.0001	0.0006
GO POSITIVE REGULATION OF NITRIC OXIDE SYNTHASE BI...	15	5 (33.3)	5 (33.3)	0 (0)	0.016	<0.0001	0.0006
GO REGULATION OF MACROPHAGE CHEMOTAXIS	23	5 (21.7)	4 (17.4)	1 (4.3)	0.016	<0.0001	0.0006
GO REGULATION OF RIBONUCLEASE ACTIVITY	8	4 (50)	4 (50)	0 (0)	0.013	<0.0001	0.0006
GO LEUKOCYTE DIFFERENTIATION	483	22 (4.6)	22 (4.6)	0 (0)	0.029	<0.0001	0.0011
GO REGULATION OF MAPK CASCADE	697	28 (4)	24 (3.4)	4 (0.6)	0.029	<0.0001	0.0011
GO IMMUNE SYSTEM DEVELOPMENT	926	34 (3.7)	32 (3.5)	2 (0.2)	0.029	<0.0001	0.0011
GO TAXIS	607	25 (4.1)	23 (3.8)	2 (0.3)	0.028	<0.0001	0.0011
GO POSITIVE REGULATION OF LOCOMOTION	544	23 (4.2)	20 (3.7)	3 (0.6)	0.028	<0.0001	0.0011
GO LYMPHOCYTE ACTIVATION	620	25 (4)	25 (4)	0 (0)	0.028	<0.0001	0.0011

Table S154: MSigDB GO Biological Processes enriched in differentially expressed genes at Day 30 compared to pre-Second Vaccination (T cells, Study Arm 2 A/H7N9+AS03, D1, 29). Results are sorted by FDR adjusted p-value and Jaccard similarity index (continued.)

Gene Set ID	Gene Set Genes #	DE Genes N (%)	Up-reg. DE Genes N (%)	Down-reg. DE Genes N (%)	Jaccard Index	P-Value	FDR Adjusted P-Value
GO SIGNAL TRANSDUCTION BY PROTEIN PHOSPHORYLATION	885	32 (3.6)	27 (3.1)	5 (0.6)	0.028	<0.0001	0.0011
GO MYELOID LEUKOCYTE DIFFERENTIATION	188	13 (6.9)	13 (6.9)	0 (0)	0.027	<0.0001	0.0011
GO POSITIVE REGULATION OF CATALYTIC ACTIVITY	1331	43 (3.2)	39 (2.9)	4 (0.3)	0.027	<0.0001	0.0011
GO MYELOID CELL DIFFERENTIATION	389	18 (4.6)	17 (4.4)	1 (0.3)	0.027	<0.0001	0.0011
GO POSITIVE REGULATION OF INFLAMMATORY RESPONSE	134	11 (8.2)	11 (8.2)	0 (0)	0.026	<0.0001	0.0011
GO CELL MOTILITY	1531	45 (2.9)	37 (2.4)	8 (0.5)	0.025	<0.0001	0.0011
GO TUMOR NECROSIS FACTOR SUPERFAMILY CYTOKINE PROD...	148	11 (7.4)	10 (6.8)	1 (0.7)	0.025	<0.0001	0.0011
GO LOCOMOTION	1747	50 (2.9)	42 (2.4)	8 (0.5)	0.025	<0.0001	0.0011
GO NEUROINFLAMMATORY RESPONSE	63	8 (12.7)	7 (11.1)	1 (1.6)	0.022	<0.0001	0.0011
GO MONONUCLEAR CELL MIGRATION	74	8 (10.8)	7 (9.5)	1 (1.4)	0.022	<0.0001	0.0011
GO POSITIVE REGULATION OF LEUKOCYTE CHEMOTAXIS	80	8 (10)	7 (8.8)	1 (1.2)	0.021	<0.0001	0.0011
GO POSITIVE REGULATION OF INTERLEUKIN 1 PRODUCTION	50	7 (14)	7 (14)	0 (0)	0.020	<0.0001	0.0011
GO REGULATION OF GRANULOCYTE CHEMOTAXIS	40	6 (15)	5 (12.5)	1 (2.5)	0.018	<0.0001	0.0011
GO CELLULAR RESPONSE TO INTERFERON BETA	21	5 (23.8)	5 (23.8)	0 (0)	0.016	<0.0001	0.0011
GO NITRIC OXIDE SYNTHASE BIOSYNTHETIC PROCESS	20	5 (25)	5 (25)	0 (0)	0.016	<0.0001	0.0011
GO POSITIVE REGULATION OF PROTEIN MODIFICATION PRO...	1148	40 (3.5)	36 (3.1)	4 (0.3)	0.028	<0.0001	0.0015
GO REGULATION OF CYSTEINE TYPE ENDOPEPTIDASE ACTIV...	221	14 (6.3)	12 (5.4)	2 (0.9)	0.028	<0.0001	0.0015
GO REGULATION OF HYDROLASE ACTIVITY	1175	38 (3.2)	34 (2.9)	4 (0.3)	0.026	<0.0001	0.0015
GO NEGATIVE REGULATION OF RESPONSE TO STIMULUS	1531	47 (3.1)	39 (2.5)	8 (0.5)	0.026	<0.0001	0.0015
GO REACTIVE OXYGEN SPECIES METABOLIC PROCESS	251	14 (5.6)	12 (4.8)	2 (0.8)	0.026	<0.0001	0.0015
GO POSITIVE REGULATION OF LEUKOCYTE PROLIFERATION	124	10 (8.1)	10 (8.1)	0 (0)	0.024	<0.0001	0.0015
GO GLIAL CELL ACTIVATION	48	7 (14.6)	7 (14.6)	0 (0)	0.021	<0.0001	0.0015
GO POSITIVE REGULATION OF MACROPHAGE CHEMOTAXIS	14	4 (28.6)	3 (21.4)	1 (7.1)	0.013	<0.0001	0.0015
GO WOUND HEALING	542	22 (4.1)	19 (3.5)	3 (0.6)	0.027	<0.0001	0.002
GO NEGATIVE REGULATION OF MULTICELLULAR ORGANISMAL...	1169	38 (3.3)	33 (2.8)	5 (0.4)	0.026	<0.0001	0.002
GO POSITIVE REGULATION OF MOLECULAR FUNCTION	1658	49 (3)	45 (2.7)	4 (0.2)	0.026	<0.0001	0.002
GO MACROPHAGE MIGRATION	47	6 (12.8)	4 (8.5)	2 (4.3)	0.018	<0.0001	0.002
GO COMPLEMENT RECEPTOR MEDIATED SIGNALING PATHWAY	12	4 (33.3)	4 (33.3)	0 (0)	0.013	<0.0001	0.002
GO REGULATION OF SECRETION	752	27 (3.6)	23 (3.1)	4 (0.5)	0.026	<0.0001	0.0024
GO NEGATIVE REGULATION OF PROTEIN METABOLIC PROCES...	1039	33 (3.2)	29 (2.8)	4 (0.4)	0.025	<0.0001	0.0024
GO REGULATION OF LEUKOCYTE PROLIFERATION	203	12 (5.9)	12 (5.9)	0 (0)	0.024	<0.0001	0.0024
GO REGULATION OF NEUTROPHIL MIGRATION	31	5 (16.1)	5 (16.1)	0 (0)	0.015	<0.0001	0.0024
GO REGULATION OF APOPTOTIC SIGNALING PATHWAY	380	18 (4.7)	16 (4.2)	2 (0.5)	0.027	<0.0001	0.0028
GO REGULATION OF LEUKOCYTE DIFFERENTIATION	252	14 (5.6)	14 (5.6)	0 (0)	0.026	<0.0001	0.0028
GO CELLULAR RESPONSE TO OXYGEN CONTAINING COMPOUND	1128	36 (3.2)	33 (2.9)	3 (0.3)	0.026	<0.0001	0.0028
GO INTERLEUKIN 10 PRODUCTION	48	6 (12.5)	6 (12.5)	0 (0)	0.018	<0.0001	0.0028
GO MACROPHAGE CHEMOTAXIS	34	5 (14.7)	4 (11.8)	1 (2.9)	0.015	<0.0001	0.0028

Table S154: MSigDB GO Biological Processes enriched in differentially expressed genes at Day 30 compared to pre-Second Vaccination (T cells, Study Arm 2 A/H7N9+AS03, D1, 29). Results are sorted by FDR adjusted p-value and Jaccard similarity index (continued.)

Gene Set ID	Gene Set Genes #	DE Genes N (%)	Up-reg. DE Genes N (%)	Down-reg. DE Genes N (%)	Jaccard Index	P-Value	FDR Adjusted P-Value
GO NLRP3 INFLAMMASOME COMPLEX ASSEMBLY	13	4 (30.8)	4 (30.8)	0 (0)	0.013	<0.0001	0.0028
GO ISG15 PROTEIN CONJUGATION	6	3 (50)	3 (50)	0 (0)	0.010	<0.0001	0.0033
GO POSITIVE REGULATION OF CYTOKINE SECRETION	126	9 (7.1)	9 (7.1)	0 (0)	0.021	<0.0001	0.0037
GO REGULATION OF PHOSPHOLIPID METABOLIC PROCESS	85	8 (9.4)	8 (9.4)	0 (0)	0.021	<0.0001	0.0037
GO NEGATIVE REGULATION OF LEUKOCYTE DIFFERENTIATIO...	94	8 (8.5)	8 (8.5)	0 (0)	0.021	<0.0001	0.0037
GO REGULATION OF PROTEIN SERINE THREONINE KINASE A...	478	20 (4.2)	18 (3.8)	2 (0.4)	0.026	<0.0001	0.004
GO NEGATIVE REGULATION OF CELL ADHESION	263	14 (5.3)	12 (4.6)	2 (0.8)	0.025	<0.0001	0.004
GO REGULATION OF RESPONSE TO CYTOKINE STIMULUS	165	11 (6.7)	11 (6.7)	0 (0)	0.024	<0.0001	0.004
GO OSTEOCLAST DIFFERENTIATION	88	8 (9.1)	8 (9.1)	0 (0)	0.021	<0.0001	0.004
GO REGULATION OF CELLULAR COMPONENT MOVEMENT	973	32 (3.3)	27 (2.8)	5 (0.5)	0.026	<0.0001	0.0044
GO POSITIVE REGULATION OF INTERLEUKIN 10 PRODUCTIO...	33	5 (15.2)	5 (15.2)	0 (0)	0.015	<0.0001	0.0044
GO INTRINSIC APOPTOTIC SIGNALING PATHWAY	274	15 (5.5)	13 (4.7)	2 (0.7)	0.027	0.0001	0.0048
GO POSITIVE REGULATION OF SMOOTH MUSCLE CELL PROLI...	85	8 (9.4)	6 (7.1)	2 (2.4)	0.021	0.0001	0.0048
GO RESPONSE TO ORGANIC CYCLIC COMPOUND	924	30 (3.2)	27 (2.9)	3 (0.3)	0.025	0.0001	0.0051
GO EXTRINSIC APOPTOTIC SIGNALING PATHWAY	209	12 (5.7)	10 (4.8)	2 (1)	0.024	0.0001	0.0051
GO RESPONSE TO INTERLEUKIN 1	181	11 (6.1)	11 (6.1)	0 (0)	0.023	0.0001	0.0051
GO NEGATIVE REGULATION OF DEFENSE RESPONSE	201	11 (5.5)	10 (5)	1 (0.5)	0.022	0.0001	0.0055
GO RESPONSE TO WOUNDING	650	23 (3.5)	20 (3.1)	3 (0.5)	0.025	0.0001	0.0059
GO REGULATION OF LIPID KINASE ACTIVITY	65	7 (10.8)	7 (10.8)	0 (0)	0.019	0.0001	0.0059
GO POSITIVE REGULATION OF PEPTIDE SECRETION	271	13 (4.8)	12 (4.4)	1 (0.4)	0.023	0.0001	0.0062
GO POSITIVE REGULATION OF CHEMOTAXIS	125	9 (7.2)	8 (6.4)	1 (0.8)	0.022	0.0001	0.0062
GO POSITIVE REGULATION OF APOPTOTIC SIGNALING PATH...	169	11 (6.5)	9 (5.3)	2 (1.2)	0.024	0.0002	0.0065
GO PATTERN RECOGNITION RECEPTOR SIGNALING PATHWAY	181	11 (6.1)	11 (6.1)	0 (0)	0.023	0.0002	0.0065
GO REGULATION OF MYELOID CELL DIFFERENTIATION	234	12 (5.1)	11 (4.7)	1 (0.4)	0.023	0.0002	0.0065
GO NEGATIVE REGULATION OF CATALYTIC ACTIVITY	743	25 (3.4)	22 (3)	3 (0.4)	0.025	0.0002	0.0068
GO POSITIVE REGULATION OF PEPTIDASE ACTIVITY	185	11 (5.9)	10 (5.4)	1 (0.5)	0.023	0.0002	0.0068
GO INTERLEUKIN 12 PRODUCTION	50	6 (12)	5 (10)	1 (2)	0.017	0.0002	0.0068
GO POSITIVE REGULATION OF MACROPHAGE MIGRATION	20	4 (20)	3 (15)	1 (5)	0.013	0.0002	0.0068
GO TYPE I INTERFERON PRODUCTION	116	9 (7.8)	9 (7.8)	0 (0)	0.022	0.0002	0.0072
GO POSITIVE REGULATION OF CHEMOKINE PRODUCTION	51	6 (11.8)	6 (11.8)	0 (0)	0.017	0.0002	0.0075
GO REGULATION OF DEFENSE RESPONSE TO VIRUS BY HOST	34	5 (14.7)	5 (14.7)	0 (0)	0.015	0.0002	0.0075
GO CELLULAR RESPONSE TO NITROGEN COMPOUND	663	24 (3.6)	24 (3.6)	0 (0)	0.025	0.0002	0.0077
GO POSITIVE REGULATION OF MAP KINASE ACTIVITY	240	13 (5.4)	12 (5)	1 (0.4)	0.025	0.0002	0.0077
GO REGULATION OF PEPTIDE SECRETION	461	18 (3.9)	16 (3.5)	2 (0.4)	0.024	0.0002	0.0077
GO ASTROCYTE DEVELOPMENT	36	5 (13.9)	5 (13.9)	0 (0)	0.015	0.0002	0.0077
GO RESPONSE TO LIPID	872	28 (3.2)	24 (2.8)	4 (0.5)	0.025	0.0002	0.0081
GO INTERLEUKIN 6 MEDIATED SIGNALING PATHWAY	20	4 (20)	4 (20)	0 (0)	0.013	0.0002	0.0084

Table S154: MSigDB GO Biological Processes enriched in differentially expressed genes at Day 30 compared to pre-Second Vaccination (T cells, Study Arm 2 A/H7N9+AS03, D1, 29). Results are sorted by FDR adjusted p-value and Jaccard similarity index (continued.)

Gene Set ID	Gene Set Genes #	DE Genes N (%)	Up-reg. DE Genes N (%)	Down-reg. DE Genes N (%)	Jaccard Index	P-Value	FDR Adjusted P-Value
GO POSITIVE REGULATION OF B CELL PROLIFERATION	39	5 (12.8)	5 (12.8)	0 (0)	0.015	0.0002	0.0087
GO POSITIVE REGULATION OF INTERFERON ALPHA PRODUCT...	18	4 (22.2)	4 (22.2)	0 (0)	0.013	0.0002	0.009
GO INTERLEUKIN 10 SECRETION	9	3 (33.3)	3 (33.3)	0 (0)	0.010	0.0002	0.009
GO RESPONSE TO NITROGEN COMPOUND	1069	33 (3.1)	33 (3.1)	0 (0)	0.025	0.0002	0.0093
GO NEGATIVE REGULATION OF RESPONSE TO EXTERNAL STI...	350	15 (4.3)	13 (3.7)	2 (0.6)	0.024	0.0002	0.0093
GO INTERLEUKIN 6 PRODUCTION	144	9 (6.2)	8 (5.6)	1 (0.7)	0.021	0.0002	0.0093
GO LEUKOCYTE PROLIFERATION	276	13 (4.7)	13 (4.7)	0 (0)	0.023	0.0003	0.0095
GO REGULATION OF B CELL PROLIFERATION	58	6 (10.3)	6 (10.3)	0 (0)	0.017	0.0003	0.0095
GO T CELL ACTIVATION VIA T CELL RECEPTOR CONTACT W...	8	3 (37.5)	3 (37.5)	0 (0)	0.010	0.0003	0.0095
GO REGULATION OF CHEMOTAXIS	198	11 (5.6)	10 (5.1)	1 (0.5)	0.022	0.0003	0.0098
GO NEGATIVE REGULATION OF RECEPTOR SIGNALING PATHW...	20	4 (20)	4 (20)	0 (0)	0.013	0.0003	0.0098
GO REGULATION OF MAP KINASE ACTIVITY	314	15 (4.8)	13 (4.1)	2 (0.6)	0.025	0.0003	0.01
GO MUSCLE CELL PROLIFERATION	205	11 (5.4)	9 (4.4)	2 (1)	0.022	0.0003	0.01
GO REGULATION OF PHAGOCYTOSIS	86	7 (8.1)	7 (8.1)	0 (0)	0.018	0.0003	0.0103
GO POSITIVE REGULATION OF CHEMOKINE BIOSYNTHETIC P...	10	3 (30)	3 (30)	0 (0)	0.010	0.0003	0.0103
GO ACTIVATION OF PROTEIN KINASE ACTIVITY	318	15 (4.7)	14 (4.4)	1 (0.3)	0.025	0.0003	0.0106
GO REGULATION OF LEUKOCYTE MEDIATED IMMUNITY	174	10 (5.7)	10 (5.7)	0 (0)	0.021	0.0003	0.0109
GO NEGATIVE REGULATION OF RESPONSE TO BIOTIC STIMU...	82	7 (8.5)	7 (8.5)	0 (0)	0.019	0.0003	0.0112
GO NEGATIVE REGULATION OF PROTEIN MODIFICATION PRO...	562	21 (3.7)	19 (3.4)	2 (0.4)	0.025	0.0003	0.0115
GO RESPONSE TO TUMOR NECROSIS FACTOR	272	13 (4.8)	12 (4.4)	1 (0.4)	0.023	0.0003	0.0117
GO RECEPTOR SIGNALING PATHWAY VIA STAT	156	9 (5.8)	9 (5.8)	0 (0)	0.020	0.0003	0.0117
GO REGULATION OF INTERLEUKIN 1 SECRETION	45	5 (11.1)	5 (11.1)	0 (0)	0.015	0.0004	0.0124
GO CELLULAR RESPONSE TO DSRNA	20	4 (20)	4 (20)	0 (0)	0.013	0.0004	0.0127
GO ORGANOPHOSPHATE METABOLIC PROCESS	966	30 (3.1)	27 (2.8)	3 (0.3)	0.024	0.0004	0.0129
GO MITOCHONDRIAL DEPOLARIZATION	23	4 (17.4)	4 (17.4)	0 (0)	0.013	0.0004	0.0129
GO NEGATIVE REGULATION OF LEUKOCYTE DEGRANULATION	9	3 (33.3)	3 (33.3)	0 (0)	0.010	0.0004	0.0132
GO REGULATION OF CELL POPULATION PROLIFERATION	1541	41 (2.7)	34 (2.2)	7 (0.5)	0.023	0.0004	0.0133
GO NEGATIVE REGULATION OF RECEPTOR SIGNALING PATHW...	22	4 (18.2)	4 (18.2)	0 (0)	0.013	0.0004	0.0133
GO CELL PROLIFERATION INVOLVED IN METANEPHROS DEVE...	9	3 (33.3)	3 (33.3)	0 (0)	0.010	0.0004	0.0133
GO RESPONSE TO INTERLEUKIN 6	38	5 (13.2)	5 (13.2)	0 (0)	0.015	0.0004	0.0139
GO REGULATION OF LYMPHOCYTE ACTIVATION	392	16 (4.1)	16 (4.1)	0 (0)	0.024	0.0004	0.0145
GO REGULATION OF NUCLEASE ACTIVITY	21	4 (19)	4 (19)	0 (0)	0.013	0.0004	0.0145
GO REGULATION OF PROTEOLYSIS	667	22 (3.3)	19 (2.8)	3 (0.4)	0.023	0.0004	0.0147
GO POSITIVE REGULATION OF REACTIVE OXYGEN SPECIES ...	88	7 (8)	6 (6.8)	1 (1.1)	0.018	0.0004	0.0147
GO NEGATIVE REGULATION OF CELL ACTIVATION	184	10 (5.4)	9 (4.9)	1 (0.5)	0.021	0.0005	0.0153
GO POSITIVE REGULATION OF DEFENSE RESPONSE TO VIRU...	24	4 (16.7)	4 (16.7)	0 (0)	0.013	0.0005	0.0155
GO REGULATION OF B CELL ACTIVATION	112	8 (7.1)	8 (7.1)	0 (0)	0.020	0.0005	0.0156

Table S154: MSigDB GO Biological Processes enriched in differentially expressed genes at Day 30 compared to pre-Second Vaccination (T cells, Study Arm 2 A/H7N9+AS03, D1, 29). Results are sorted by FDR adjusted p-value and Jaccard similarity index (continued.)

Gene Set ID	Gene Set Genes #	DE Genes N (%)	Up-reg. DE Genes N (%)	Down-reg. DE Genes N (%)	Jaccard Index	P-Value	FDR Adjusted P-Value
GO REGULATION OF VIRAL ENTRY INTO HOST CELL	26	4 (15.4)	4 (15.4)	0 (0)	0.012	0.0005	0.0156
GO REGULATION OF NEUTROPHIL CHEMOTAXIS	27	4 (14.8)	4 (14.8)	0 (0)	0.012	0.0005	0.0156
GO POSITIVE REGULATION OF CYSTEINE TYPE ENDOPEPTID...	141	9 (6.4)	8 (5.7)	1 (0.7)	0.021	0.0005	0.0164
GO REGULATION OF INTRINSIC APOPTOTIC SIGNALING PAT...	157	9 (5.7)	8 (5.1)	1 (0.6)	0.020	0.0005	0.0164
GO CELLULAR DEFENSE RESPONSE	46	5 (10.9)	5 (10.9)	0 (0)	0.015	0.0005	0.017
GO NEGATIVE REGULATION OF MYELOID LEUKOCYTE DIFFER...	43	5 (11.6)	5 (11.6)	0 (0)	0.015	0.0006	0.0176
GO REGULATION OF LEUKOCYTE DEGRANULATION	40	5 (12.5)	5 (12.5)	0 (0)	0.015	0.0006	0.0178
GO NEGATIVE REGULATION OF CELL DEATH	933	28 (3)	23 (2.5)	5 (0.5)	0.023	0.0006	0.019
GO POSITIVE REGULATION OF PROTEIN SERINE THREONINE...	311	14 (4.5)	13 (4.2)	1 (0.3)	0.023	0.0007	0.0208
GO NEGATIVE REGULATION OF TYROSINE PHOSPHORYLATION...	11	3 (27.3)	3 (27.3)	0 (0)	0.010	0.0007	0.021
GO TUMOR NECROSIS FACTOR BIOSYNTHETIC PROCESS	28	4 (14.3)	3 (10.7)	1 (3.6)	0.012	0.0007	0.0212
GO TYROSINE PHOSPHORYLATION OF STAT PROTEIN	80	6 (7.5)	6 (7.5)	0 (0)	0.016	0.0007	0.0229
GO LEUKOCYTE MIGRATION INVOLVED IN INFLAMMATORY RE...	14	3 (21.4)	3 (21.4)	0 (0)	0.010	0.0007	0.0229
GO CHEMICAL HOMEOSTASIS	1123	32 (2.8)	30 (2.7)	2 (0.2)	0.023	0.0008	0.0231
GO PEPTIDYL TYROSINE MODIFICATION	354	15 (4.2)	13 (3.7)	2 (0.6)	0.023	0.0008	0.0235
GO INTERFERON ALPHA PRODUCTION	25	4 (16)	4 (16)	0 (0)	0.012	0.0008	0.0235
GO CELLULAR COPPER ION HOMEOSTASIS	12	3 (25)	3 (25)	0 (0)	0.010	0.0008	0.0237
GO NEGATIVE REGULATION OF SIGNALING	1297	36 (2.8)	29 (2.2)	7 (0.5)	0.023	0.0008	0.0247
GO POSITIVE REGULATION OF INTERLEUKIN 1 SECRETION	30	4 (13.3)	4 (13.3)	0 (0)	0.012	0.0008	0.0247
GO CHEMOKINE BIOSYNTHETIC PROCESS	14	3 (21.4)	3 (21.4)	0 (0)	0.010	0.0009	0.0258
GO BIOLOGICAL ADHESION	1320	37 (2.8)	31 (2.3)	6 (0.5)	0.023	0.0009	0.026
GO POSITIVE REGULATION OF INTERFERON BETA PRODUCTI...	25	4 (16)	4 (16)	0 (0)	0.012	0.0009	0.0274
GO VASCULAR PROCESS IN CIRCULATORY SYSTEM	162	9 (5.6)	9 (5.6)	0 (0)	0.020	0.0009	0.0278
GO POSITIVE REGULATION OF CELL POPULATION PROLIFER...	865	26 (3)	22 (2.5)	4 (0.5)	0.023	0.001	0.0286
GO NEGATIVE REGULATION OF IMMUNE RESPONSE	134	8 (6)	8 (6)	0 (0)	0.019	0.001	0.0288
GO LEUKOCYTE CELL CELL ADHESION	307	13 (4.2)	13 (4.2)	0 (0)	0.022	0.001	0.029
GO DENDRITIC CELL CYTOKINE PRODUCTION	12	3 (25)	3 (25)	0 (0)	0.010	0.001	0.0294
GO NEGATIVE REGULATION OF HEMOPOIESIS	144	8 (5.6)	8 (5.6)	0 (0)	0.018	0.001	0.0296
GO REGULATION OF MACROPHAGE ACTIVATION	52	5 (9.6)	3 (5.8)	2 (3.8)	0.014	0.001	0.0301
GO HOMEOSTATIC PROCESS	1803	45 (2.5)	43 (2.4)	2 (0.1)	0.022	0.0011	0.0302
GO POSITIVE REGULATION OF PROTEOLYSIS	336	14 (4.2)	13 (3.9)	1 (0.3)	0.022	0.0011	0.0314
GO POSITIVE REGULATION OF RECEPTOR SIGNALING PATHW...	87	6 (6.9)	6 (6.9)	0 (0)	0.016	0.0011	0.0314
GO POSITIVE REGULATION OF INTERLEUKIN 8 SECRETION	15	3 (20)	3 (20)	0 (0)	0.010	0.0011	0.0321
GO POSITIVE REGULATION OF SECRETION	404	15 (3.7)	14 (3.5)	1 (0.2)	0.022	0.0011	0.0323
GO RESPONSE TO PEPTIDE	494	18 (3.6)	18 (3.6)	0 (0)	0.023	0.0012	0.0325
GO REGULATION OF REACTIVE OXYGEN SPECIES METABOLIC...	169	9 (5.3)	8 (4.7)	1 (0.6)	0.019	0.0012	0.0325
GO MHC CLASS II BIOSYNTHETIC PROCESS	15	3 (20)	3 (20)	0 (0)	0.010	0.0012	0.0325

Table S154: MSigDB GO Biological Processes enriched in differentially expressed genes at Day 30 compared to pre-Second Vaccination (T cells, Study Arm 2 A/H7N9+AS03, D1, 29). Results are sorted by FDR adjusted p-value and Jaccard similarity index (continued.)

Gene Set ID	Gene Set Genes #	DE Genes N (%)	Up-reg. DE Genes N (%)	Down-reg. DE Genes N (%)	Jaccard Index	P-Value	FDR Adjusted P-Value
GO T CELL ACTIVATION	436	16 (3.7)	16 (3.7)	0 (0)	0.022	0.0012	0.0334
GO COPPER ION HOMEOSTASIS	14	3 (21.4)	3 (21.4)	0 (0)	0.010	0.0012	0.0334
GO NEGATIVE REGULATION OF TRANSFERASE ACTIVITY	266	12 (4.5)	11 (4.1)	1 (0.4)	0.022	0.0012	0.0335
GO REGULATION OF TRANSPORT	1745	44 (2.5)	38 (2.2)	6 (0.3)	0.022	0.0012	0.0337
GO ALPHA BETA T CELL PROLIFERATION	30	4 (13.3)	4 (13.3)	0 (0)	0.012	0.0012	0.0337
GO POSITIVE REGULATION OF INTERLEUKIN 12 PRODUCTIO...	32	4 (12.5)	4 (12.5)	0 (0)	0.012	0.0012	0.0337
GO HOMEOSTASIS OF NUMBER OF CELLS	226	11 (4.9)	11 (4.9)	0 (0)	0.021	0.0013	0.0351
GO REGULATION OF CD8 POSITIVE ALPHA BETA T CELL AC...	13	3 (23.1)	3 (23.1)	0 (0)	0.010	0.0013	0.0351
GO NEGATIVE REGULATION OF CELL CELL ADHESION	167	9 (5.4)	9 (5.4)	0 (0)	0.020	0.0013	0.0354
GO NEGATIVE REGULATION OF INNATE IMMUNE RESPONSE	49	5 (10.2)	5 (10.2)	0 (0)	0.015	0.0013	0.0354
GO REGULATION OF CYTOKINE PRODUCTION INVOLVED IN I...	76	6 (7.9)	6 (7.9)	0 (0)	0.016	0.0013	0.036
GO REGULATION OF MYELOID LEUKOCYTE DIFFERENTIATION	108	7 (6.5)	7 (6.5)	0 (0)	0.017	0.0014	0.0362
GO GLYCEROPHOSPHOLIPID METABOLIC PROCESS	297	13 (4.4)	13 (4.4)	0 (0)	0.022	0.0014	0.0382
GO ERK1 AND ERK2 CASCADE	285	12 (4.2)	12 (4.2)	0 (0)	0.021	0.0015	0.0386
GO CELLULAR RESPONSE TO BIOTIC STIMULUS	226	10 (4.4)	10 (4.4)	0 (0)	0.019	0.0015	0.0387
GO POSITIVE REGULATION OF LYMPHOCYTE APOPTOTIC PRO...	16	3 (18.8)	3 (18.8)	0 (0)	0.010	0.0015	0.0388
GO POSITIVE REGULATION OF VASOCONSTRICTION	32	4 (12.5)	4 (12.5)	0 (0)	0.012	0.0015	0.04
GO NEGATIVE REGULATION OF PHOSPHORYLATION	431	16 (3.7)	14 (3.2)	2 (0.5)	0.022	0.0015	0.0404
GO MAST CELL ACTIVATION	53	5 (9.4)	5 (9.4)	0 (0)	0.014	0.0016	0.0408
GO B CELL PROLIFERATION	91	6 (6.6)	6 (6.6)	0 (0)	0.015	0.0016	0.0419
GO REGULATION OF CELL ADHESION	633	21 (3.3)	19 (3)	2 (0.3)	0.023	0.0017	0.0427
GO POSITIVE REGULATION OF RESPONSE TO CYTOKINE STI...	53	5 (9.4)	5 (9.4)	0 (0)	0.014	0.0017	0.0427
GO CELLULAR RESPONSE TO EXOGENOUS DSRNA	15	3 (20)	3 (20)	0 (0)	0.010	0.0017	0.0429
GO REGULATION OF MATRIX METALLOPEPTIDASE SECRETION	5	2 (40)	1 (20)	1 (20)	0.007	0.0017	0.0429
GO REGULATION OF PEPTIDYL TYROSINE PHOSPHORYLATION	246	11 (4.5)	10 (4.1)	1 (0.4)	0.021	0.0017	0.0442
GO PHOSPHATIDYLINOSITOL PHOSPHORYLATION	48	5 (10.4)	5 (10.4)	0 (0)	0.015	0.0017	0.0442
GO REGULATION OF OSTEOCLAST DIFFERENTIATION	58	5 (8.6)	5 (8.6)	0 (0)	0.014	0.0017	0.0442
GO RESPONSE TO FUNGUS	47	4 (8.5)	4 (8.5)	0 (0)	0.012	0.0018	0.0446
GO POSITIVE REGULATION OF ERK1 AND ERK2 CASCADE	184	9 (4.9)	9 (4.9)	0 (0)	0.019	0.0018	0.0449
GO RESPONSE TO ENDOGENOUS STIMULUS	1616	41 (2.5)	34 (2.1)	7 (0.4)	0.022	0.0018	0.0455
GO POSITIVE REGULATION OF PHAGOCYTOSIS	61	5 (8.2)	5 (8.2)	0 (0)	0.014	0.0018	0.0457
GO REGULATION OF MAST CELL CYTOKINE PRODUCTION	5	2 (40)	2 (40)	0 (0)	0.007	0.0018	0.0457
GO POSITIVE REGULATION OF TYROSINE PHOSPHORYLATION...	67	5 (7.5)	5 (7.5)	0 (0)	0.014	0.0019	0.0471
GO ACTIVATION OF CYSTEINE TYPE ENDOPEPTIDASE ACTIV...	83	6 (7.2)	5 (6)	1 (1.2)	0.016	0.0019	0.0474
GO REGULATION OF RECEPTOR SIGNALING PATHWAY VIA ST...	133	7 (5.3)	7 (5.3)	0 (0)	0.016	0.002	0.0485
GO PHOSPHATIDYLINOSITOL METABOLIC PROCESS	163	9 (5.5)	9 (5.5)	0 (0)	0.020	0.002	0.0493
GO NEGATIVE REGULATION OF APOPTOTIC SIGNALING PATH...	213	10 (4.7)	9 (4.2)	1 (0.5)	0.020	0.002	0.0499

Table S155: Blood Transcription Modules enriched in differentially expressed genes at Day 30 compared to pre-Second Vaccination (T cells, Study Arm 2 A/H7N9+AS03, D1, 29). Results are sorted by FDR adjusted p-value and Jaccard similarity index.

Gene Set ID	Gene Set Genes #	DE Genes N (%)	Up-reg. DE Genes N (%)	Down-reg. DE Genes N (%)	Jaccard Index	P-Value	FDR Adjusted P-Value
M11.0 ENRICHED IN MONOCYTES (II)	174	57 (32.8)	57 (32.8)	0 (0)	0.136	<0.0001	0.0003
M4.0 CELL CYCLE AND TRANSCRIPTION	303	51 (16.8)	51 (16.8)	0 (0)	0.092	<0.0001	0.0003
M37.0 IMMUNE ACTIVATION - GENERIC CLUSTER	301	34 (11.3)	34 (11.3)	0 (0)	0.060	<0.0001	0.0003
M16 TLR AND INFLAMMATORY SIGNALING	41	16 (39)	16 (39)	0 (0)	0.049	<0.0001	0.0003
S4 MONOCYTE SURFACE SIGNATURE	81	17 (21)	16 (19.8)	1 (1.2)	0.047	<0.0001	0.0003
M165 ENRICHED IN ACTIVATED DENDRITIC CELLS (II)	34	12 (35.3)	12 (35.3)	0 (0)	0.037	<0.0001	0.0003
M127 TYPE I INTERFERON RESPONSE	12	11 (91.7)	11 (91.7)	0 (0)	0.036	<0.0001	0.0003
M150 INNATE ANTIVIRAL RESPONSE	12	10 (83.3)	10 (83.3)	0 (0)	0.033	<0.0001	0.0003
M75 ANTIVIRAL IFN SIGNATURE	22	10 (45.5)	9 (40.9)	1 (4.5)	0.032	<0.0001	0.0003
M4.3 MYELOID CELL ENRICHED RECEPTORS AND TRANSPORT...	29	10 (34.5)	10 (34.5)	0 (0)	0.031	<0.0001	0.0003
M11.2 FORMYL PEPTIDE RECEPTOR MEDIATED NEUTROPHIL ...	10	6 (60)	6 (60)	0 (0)	0.020	<0.0001	0.0003
M67 ACTIVATED DENDRITIC CELLS	12	6 (50)	6 (50)	0 (0)	0.019	<0.0001	0.0009
M111.0 VIRAL SENSING AND IMMUNITY; IRF2 TARGETS NE...	16	6 (37.5)	5 (31.2)	1 (6.2)	0.019	<0.0001	0.0021
M111.1 VIRAL SENSING AND IMMUNITY; IRF2 TARGETS NE...	11	5 (45.5)	5 (45.5)	0 (0)	0.016	0.0001	0.0027
M64 ENRICHED IN ACTIVATED DENDRITIC CELLS-MONOCYTE...	14	5 (35.7)	5 (35.7)	0 (0)	0.016	0.0005	0.0114
M66 TBA	14	5 (35.7)	5 (35.7)	0 (0)	0.016	0.0006	0.0114
M132 RECRUITMENT OF NEUTROPHILS	10	4 (40)	4 (40)	0 (0)	0.013	0.0005	0.0114
M155 G PROTEIN COUPLED RECEPTORS CLUSTER	10	4 (40)	4 (40)	0 (0)	0.013	0.0015	0.0298
M37.1 ENRICHED IN NEUTROPHILS (I)	46	8 (17.4)	8 (17.4)	0 (0)	0.024	0.0022	0.0344
M59 CCR1, 7 AND CELL SIGNALING	10	4 (40)	4 (40)	0 (0)	0.013	0.0019	0.0344
M36 T CELL SURFACE, ACTIVATION	11	4 (36.4)	4 (36.4)	0 (0)	0.013	0.002	0.0344
M73 ENRICHED IN MONOCYTES (III)	11	4 (36.4)	4 (36.4)	0 (0)	0.013	0.0021	0.0344
S11 ACTIVATED (LPS) DENDRITIC CELL SURFACE SIGNATU...	34	7 (20.6)	7 (20.6)	0 (0)	0.021	0.003	0.0459

Table S156: KEGG pathways enriched in differentially abundant proteins at Day 2 compared to pre-first vaccination (Neutrophils, Study Arm 2 (A/H7N9 + AS03 D1, 29)). Results are sorted by FDR adjusted p-value and Jaccard similarity index.

Gene Set ID	Gene Set Genes #	DA Proteins N (%)[Protein Fam. #]	Up-reg. DA Proteins N (%)	Down-reg. DA Proteins N (%)	Jaccard Index	P-Value	FDR Adjusted P-Value
NOD-LIKE RECEPTOR SIGNALING PATHWAY	121	3 (2.5) [1]	3 (2.5)	0 (0)	0.025	<0.0001	0.013

Confidential/Proprietary Information

Table S157: KEGG pathways enriched in differentially abundant proteins at Day 30 compared to pre-second vaccination (Neutrophils, Study Arm 2 (A/H7N9 + AS03 D1, 29)). Results are sorted by FDR adjusted p-value and Jaccard similarity index.

Gene Set ID	Gene Set Genes #	DA Proteins N (%)[Protein Fam. #]	Up-reg. DA Proteins N (%)	Down-reg. DA Proteins N (%)	Jaccard Index	P-Value	FDR Adjusted P-Value
NOD-LIKE RECEPTOR SIGNALING PATHWAY	121	4 (3.3) [2]	4 (3.3)	0 (0)	0.032	<0.0001	0.0021

Table S158: KEGG pathways enriched in differentially abundant proteins at Day 32 compared to pre-second vaccination (Neutrophils, Study Arm 2 (A/H7N9 + AS03 D1, 29)). Results are sorted by FDR adjusted p-value and Jaccard similarity index.

Gene Set ID	Gene Set Genes #	DA Proteins N (%)[Protein Fam. #]	Up-reg. DA Proteins N (%)	Down-reg. DA Proteins N (%)	Jaccard Index	P-Value	FDR Adjusted P-Value
NOD-LIKE RECEPTOR SIGNALING PATHWAY	121	6 (5) [4]	6 (5)	0 (0)	0.039	0.0001	0.0482
ANTIGEN PROCESSING AND PRESENTATION	53	4 (7.5) [3]	4 (7.5)	0 (0)	0.045	0.0004	0.0746

Table S159: KEGG pathways enriched in differentially abundant proteins at Day 2 compared to pre-first vaccination (Dendritic cells, Study Arm 2 (A/H7N9 + AS03 D1, 29)). Results are sorted by FDR adjusted p-value and Jaccard similarity index.

Gene Set ID	Gene Set Genes #	DA Proteins N (%)[Protein Fam. #]	Up-reg. DA Proteins N (%)	Down-reg. DA Proteins N (%)	Jaccard Index	P-Value	FDR Adjusted P-Value
FC GAMMA R-MEDIATED PHAGOCYTOSIS	70	15 (21.4) [15]	3 (4.3)	12 (17.1)	0.034	<0.0001	0.0073
CARBON METABOLISM	87	15 (17.2) [14]	2 (2.3)	13 (14.9)	0.033	0.0003	0.0503
NEUTROPHIL EXTRACELLULAR TRAP FORMATION	117	17 (14.5) [15]	10 (8.5)	7 (6)	0.035	0.0009	0.1073
BACTERIAL INVASION OF EPITHELIAL CELLS	54	10 (18.5) [10]	0 (0)	10 (18.5)	0.023	0.0017	0.1202
PROPANOATE METABOLISM	29	7 (24.1) [6]	0 (0)	7 (24.1)	0.017	0.0018	0.1202
CITRATE CYCLE (TCA CYCLE)	23	6 (26.1) [5]	0 (0)	6 (26.1)	0.015	0.0025	0.1401

Table S160: KEGG pathways enriched in differentially abundant proteins at Day 30 compared to pre-second vaccination (Dendritic cells, Study Arm 2 (A/H7N9 + AS03 D1, 29)). Results are sorted by FDR adjusted p-value and Jaccard similarity index.

Gene Set ID	Gene Set Genes #	DA Proteins N (%)[Protein Fam. #]	Up-reg. DA Proteins N (%)	Down-reg. DA Proteins N (%)	Jaccard Index	P-Value	FDR Adjusted P-Value
NATURAL KILLER CELL MEDIATED CYTOTOXICITY	71	15 (21.1) [14]	6 (8.5)	9 (12.7)	0.029	0.0002	0.0686

Confidential/Proprietary Information

Table S161: KEGG pathways enriched in differentially abundant proteins at Day 2 compared to pre-first vaccination (Monocytes, Study Arm 2 (A/H7N9 + AS03 D1, 29)). Results are sorted by FDR adjusted p-value and Jaccard similarity index.

Gene Set ID	Gene Set Genes #	DA Proteins N (%)[Protein Fam. #]	Up-reg. DA Proteins N (%)	Down-reg. DA Proteins N (%)	Jaccard Index	P-Value	FDR Adjusted P-Value
CORONAVIRUS DISEASE - COVID-19	148	8 (5.4) [8]	3 (2)	5 (3.4)	0.041	<0.0001	0.0227
PHAGOSOME	95	6 (6.3) [6]	5 (5.3)	1 (1.1)	0.041	0.0003	0.0454
LEISHMANIASIS	47	4 (8.5) [4]	4 (8.5)	0 (0)	0.040	0.001	0.0612
OSTEOCLAST DIFFERENTIATION	82	5 (6.1) [5]	4 (4.9)	1 (1.2)	0.038	0.0011	0.0612
NEUTROPHIL EXTRACELLULAR TRAP FORMATION	117	6 (5.1) [6]	6 (5.1)	0 (0)	0.036	0.0008	0.0612
NOD-LIKE RECEPTOR SIGNALING PATHWAY	121	6 (5) [4]	6 (5)	0 (0)	0.035	0.001	0.0612

Table S162: KEGG pathways enriched in differentially abundant proteins at Day 30 compared to pre-second vaccination (Monocytes, Study Arm 2 (A/H7N9 + AS03 D1, 29)). Results are sorted by FDR adjusted p-value and Jaccard similarity index.

Gene Set ID	Gene Set Genes #	DA Proteins N (%)[Protein Fam. #]	Up-reg. DA Proteins N (%)	Down-reg. DA Proteins N (%)	Jaccard Index	P-Value	FDR Adjusted P-Value
INFLUENZA A	108	9 (8.3) [7]	9 (8.3)	0 (0)	0.045	<0.0001	0.0187
EPSTEIN-BARR VIRUS INFECTION	147	9 (6.1) [8]	9 (6.1)	0 (0)	0.038	0.0006	0.0593
MEASLES	92	7 (7.6) [6]	7 (7.6)	0 (0)	0.038	0.0007	0.0593
CORONAVIRUS DISEASE - COVID-19	148	9 (6.1) [8]	7 (4.7)	2 (1.4)	0.038	0.0006	0.0593
HERPES SIMPLEX VIRUS 1 INFECTION	138	8 (5.8) [7]	8 (5.8)	0 (0)	0.035	0.0017	0.0825
ANTIGEN PROCESSING AND PRESENTATION	53	5 (9.4) [4]	5 (9.4)	0 (0)	0.034	0.0016	0.0825
HEMATOPOIETIC CELL LINEAGE	55	5 (9.1) [4]	5 (9.1)	0 (0)	0.034	0.0019	0.0825
ASTHMA	15	3 (20) [2]	3 (20)	0 (0)	0.027	0.0017	0.0825
VIRAL MYOCARDITIS	36	4 (11.1) [3]	4 (11.1)	0 (0)	0.030	0.0027	0.1019
NOD-LIKE RECEPTOR SIGNALING PATHWAY	121	7 (5.8) [4]	7 (5.8)	0 (0)	0.033	0.0035	0.1054
HEPATITIS C	92	6 (6.5) [5]	6 (6.5)	0 (0)	0.032	0.0038	0.1054
NECROPTOSIS	93	6 (6.5) [6]	4 (4.3)	2 (2.2)	0.032	0.004	0.1054
ALLOGRAFT REJECTION	21	3 (14.3) [2]	3 (14.3)	0 (0)	0.026	0.0046	0.1054
AUTOIMMUNE THYROID DISEASE	21	3 (14.3) [2]	3 (14.3)	0 (0)	0.026	0.0046	0.1054
TYPE I DIABETES MELLITUS	21	3 (14.3) [2]	3 (14.3)	0 (0)	0.026	0.0046	0.1054
GRAFT-VERSUS-HOST DISEASE	22	3 (13.6) [2]	3 (13.6)	0 (0)	0.025	0.0053	0.113
LEISHMANIASIS	47	4 (8.5) [3]	4 (8.5)	0 (0)	0.028	0.0071	0.1426

Table S163: KEGG pathways enriched in differentially abundant proteins at Day 30 compared to pre-second vaccination (Natural Killer cells, Study Arm 2 (A/H7N9 + AS03 D1, 29)). Results are sorted by FDR adjusted p-value and Jaccard similarity index.

Gene Set ID	Gene Set Genes #	DA Proteins N (%)[Protein Fam. #]	Up-reg. DA Proteins N (%)	Down-reg. DA Proteins N (%)	Jaccard Index	P-Value	FDR Adjusted P-Value
NOD-LIKE RECEPTOR SIGNALING PATHWAY	121	4 (3.3) [3]	4 (3.3)	0 (0)	0.030	<0.0001	0.0272

Table S164: Blood Transcription Modules enriched in differentially abundant proteins at Day 32 compared to pre-second vaccination (Neutrophils, Study Arm 2 (A/H7N9 + AS03 D1, 29)). Results are sorted by FDR adjusted p-value and Jaccard similarity index.

Gene Set ID	Gene Set Genes #	DA Proteins N (%)[Protein Fam. #]	Up-reg. DA Proteins N (%)	Down-reg. DA Proteins N (%)	Jaccard Index	P-Value	FDR Adjusted P-Value
M111.1 VIRAL SENSING AND IMMUNITY; IRF2 TARGETS NETWORK (II)	8	4 (50) [4]	4 (50)	0 (0)	0.091	<0.0001	0.0002
M165 ENRICHED IN ACTIVATED DENDRITIC CELLS (II)	16	3 (18.8) [2]	3 (18.8)	0 (0)	0.057	0.0006	0.0872

Table S165: Blood Transcription Modules enriched in differentially abundant proteins at Day 2 compared to pre-first vaccination (Dendritic cells, Study Arm 2 (A/H7N9 + AS03 D1, 29)). Results are sorted by FDR adjusted p-value and Jaccard similarity index.

Gene Set ID	Gene Set Genes #	DA Proteins N (%)[Protein Fam. #]	Up-reg. DA Proteins N (%)	Down-reg. DA Proteins N (%)	Jaccard Index	P-Value	FDR Adjusted P-Value
M2.1 EXTRACELLULAR MATRIX (II)	24	8 (33.3) [8]	8 (33.3)	0 (0)	0.020	0.0003	0.0304
M204.0 CHAPERONIN MEDIATED PROTEIN FOLDING (I)	13	6 (46.2) [6]	0 (0)	6 (46.2)	0.015	0.0002	0.0304
M204.1 CHAPERONIN MEDIATED PROTEIN FOLDING (II)	9	5 (55.6) [5]	0 (0)	5 (55.6)	0.013	0.0003	0.0304
M11.0 ENRICHED IN MONOCYTES (II)	135	22 (16.3) [22]	22 (16.3)	0 (0)	0.044	0.0004	0.0345
M7.3 T CELL ACTIVATION (II)	24	7 (29.2) [7]	0 (0)	7 (29.2)	0.017	0.0018	0.1035
M64 ENRICHED IN ACTIVATED DENDRITIC CELLS-MONOCYTES	8	4 (50) [4]	4 (50)	0 (0)	0.010	0.002	0.1035
M7.0 ENRICHED IN T CELLS (I)	47	10 (21.3) [10]	1 (2.1)	9 (19.1)	0.024	0.0027	0.1175
M5.1 T CELL ACTIVATION AND SIGNALING	20	6 (30) [6]	1 (5)	5 (25)	0.015	0.0033	0.1264

Table S166: Blood Transcription Modules enriched in differentially abundant proteins at Day 30 compared to pre-second vaccination (Dendritic cells, Study Arm 2 (A/H7N9 + AS03 D1, 29)). Results are sorted by FDR adjusted p-value and Jaccard similarity index.

Gene Set ID	Gene Set Genes #	DA Proteins N (%)[Protein Fam. #]	Up-reg. DA Proteins N (%)	Down-reg. DA Proteins N (%)	Jaccard Index	P-Value	FDR Adjusted P-Value
M11.0 ENRICHED IN MONOCYTES (II)	135	34 (25.2) [33]	32 (23.7)	2 (1.5)	0.061	<0.0001	<0.0001
M11.1 BLOOD COAGULATION	20	10 (50) [10]	7 (35)	3 (15)	0.021	<0.0001	0.0012
M4.0 CELL CYCLE AND TRANSCRIPTION	159	30 (18.9) [30]	26 (16.4)	4 (2.5)	0.051	0.0004	0.0229
M105 TBA	11	6 (54.5) [6]	6 (54.5)	0 (0)	0.013	0.0003	0.0229
M150 INNATE ANTIVIRAL RESPONSE	11	6 (54.5) [6]	6 (54.5)	0 (0)	0.013	0.0003	0.0229
M5.0 REGULATION OF ANTIGEN PRESENTATION AND IMMUNE RESPONSE	65	16 (24.6) [14]	9 (13.8)	7 (10.8)	0.032	0.0005	0.0246
M165 ENRICHED IN ACTIVATED DENDRITIC CELLS (II)	16	7 (43.8) [6]	7 (43.8)	0 (0)	0.015	0.0006	0.0246
M127 TYPE I INTERFERON RESPONSE	9	5 (55.6) [5]	5 (55.6)	0 (0)	0.011	0.001	0.038
M209 LYSOSOME	7	4 (57.1) [4]	4 (57.1)	0 (0)	0.009	0.003	0.1025

Table S167: Blood Transcription Modules enriched in differentially abundant proteins at Day 32 compared to pre-second vaccination (Dendritic cells, Study Arm 2 (A/H7N9 + AS03 D1, 29)). Results are sorted by FDR adjusted p-value and Jaccard similarity index.

Gene Set ID	Gene Set Genes #	DA Proteins N (%)[Protein Fam. #]	Up-reg. DA Proteins N (%)	Down-reg. DA Proteins N (%)	Jaccard Index	P-Value	FDR Adjusted P-Value
M11.0 ENRICHED IN MONOCYTES (II)	135	5 (3.7) [5]	5 (3.7)	0 (0)	0.035	<0.0001	0.0156
M4.0 CELL CYCLE AND TRANSCRIPTION	159	5 (3.1) [5]	5 (3.1)	0 (0)	0.030	0.0001	0.0176
M66 TBA	11	2 (18.2) [2]	2 (18.2)	0 (0)	0.087	0.0009	0.091

Table S168: Blood Transcription Modules enriched in differentially abundant proteins at Day 30 compared to pre-second vaccination (Monocytes, Study Arm 2 (A/H7N9 + AS03 D1, 29)). Results are sorted by FDR adjusted p-value and Jaccard similarity index.

Gene Set ID	Gene Set Genes #	DA Proteins N (%)[Protein Fam. #]	Up-reg. DA Proteins N (%)	Down-reg. DA Proteins N (%)	Jaccard Index	P-Value	FDR Adjusted P-Value
M150 INNATE ANTIVIRAL RESPONSE	11	5 (45.5) [5]	5 (45.5)	0 (0)	0.048	<0.0001	0.0032
M127 TYPE I INTERFERON RESPONSE	9	4 (44.4) [4]	4 (44.4)	0 (0)	0.038	<0.0001	0.0153
M200 ANTIGEN PROCESSING AND PRESENTATION	6	3 (50) [3]	1 (16.7)	2 (33.3)	0.029	0.0006	0.0575
M4.0 CELL CYCLE AND TRANSCRIPTION	159	13 (8.2) [13]	9 (5.7)	4 (2.5)	0.053	0.0008	0.0592
M165 ENRICHED IN ACTIVATED DENDRITIC CELLS (II)	16	4 (25) [3]	4 (25)	0 (0)	0.036	0.0012	0.0625
M4.3 MYELOID CELL ENRICHED RECEPTORS AND TRANSPORTERS	16	4 (25) [4]	2 (12.5)	2 (12.5)	0.036	0.0012	0.0625
M67 ACTIVATED DENDRITIC CELLS	9	3 (33.3) [3]	3 (33.3)	0 (0)	0.029	0.0022	0.0967

Confidential/Proprietary Information

Table S169: MSigDB Reactome pathways enriched in differentially abundant proteins at Day 2 compared to pre-first vaccination (Neutrophils, Study Arm 2 (A/H7N9 + AS03 D1, 29)). Results are sorted by FDR adjusted p-value and Jaccard similarity index.

Gene Set ID	Gene Set Genes #	DA Proteins N (%)[Protein Fam. #]	Up-reg. DA Proteins N (%)	Down-reg. DA Proteins N (%)	Jaccard Index	P-Value	FDR Adjusted P-Value
INTERFERON GAMMA SIGNALING	47	3 (6.4) [1]	3 (6.4)	0 (0)	0.064	<0.0001	0.0014
INTERFERON SIGNALING	124	3 (2.4) [1]	3 (2.4)	0 (0)	0.024	<0.0001	0.0133

Table S170: MSigDB Reactome pathways enriched in differentially abundant proteins at Day 30 compared to pre-second vaccination (Neutrophils, Study Arm 2 (A/H7N9 + AS03 D1, 29)). Results are sorted by FDR adjusted p-value and Jaccard similarity index.

Gene Set ID	Gene Set Genes #	DA Proteins N (%)[Protein Fam. #]	Up-reg. DA Proteins N (%)	Down-reg. DA Proteins N (%)	Jaccard Index	P-Value	FDR Adjusted P-Value
INTERFERON SIGNALING	124	5 (4) [3]	5 (4)	0 (0)	0.039	<0.0001	0.0009
INTERFERON ALPHA BETA SIGNALING	35	3 (8.6) [3]	3 (8.6)	0 (0)	0.075	<0.0001	0.0153
INTERFERON GAMMA SIGNALING	47	3 (6.4) [1]	3 (6.4)	0 (0)	0.058	<0.0001	0.025
CYTOKINE SIGNALING IN IMMUNE SYSTEM	449	5 (1.1) [3]	5 (1.1)	0 (0)	0.011	0.0003	0.1255

Confidential/Proprietary Information

Table S171: MSigDB Reactome pathways enriched in differentially abundant proteins at Day 32 compared to pre-second vaccination (Neutrophils, Study Arm 2 (A/H7N9 + AS03 D1, 29)). Results are sorted by FDR adjusted p-value and Jaccard similarity index.

Gene Set ID	Gene Set Genes #	DA Proteins N (%)[Protein Fam. #]	Up-reg. DA Proteins N (%)	Down-reg. DA Proteins N (%)	Jaccard Index	P-Value	FDR Adjusted P-Value
INTERFERON SIGNALING	124	10 (8.1) [7]	10 (8.1)	0 (0)	0.065	<0.0001	<0.0001
INTERFERON ALPHA BETA SIGNALING	35	6 (17.1) [5]	6 (17.1)	0 (0)	0.087	<0.0001	<0.0001
CYTOKINE SIGNALING IN IMMUNE SYSTEM	449	12 (2.7) [9]	12 (2.7)	0 (0)	0.025	<0.0001	0.0011
INTERFERON GAMMA SIGNALING	47	4 (8.5) [2]	4 (8.5)	0 (0)	0.048	0.0001	0.048
PYRIMIDINE CATABOLISM	6	2 (33.3) [2]	2 (33.3)	0 (0)	0.045	0.0005	0.1394

Table S172: MSigDB Reactome pathways enriched in differentially abundant proteins at Day 2 compared to pre-first vaccination (Dendritic cells, Study Arm 2 (A/H7N9 + AS03 D1, 29)). Results are sorted by FDR adjusted p-value and Jaccard similarity index.

Gene Set ID	Gene Set Genes #	DA Proteins N (%)[Protein Fam. #]	Up-reg. DA Proteins N (%)	Down-reg. DA Proteins N (%)	Jaccard Index	P-Value	FDR Adjusted P-Value
NEUTROPHIL DEGRANULATION	399	46 (11.5) [45]	27 (6.8)	19 (4.8)	0.062	<0.0001	0.0041
INNATE IMMUNE SYSTEM	726	66 (9.1) [65]	32 (4.4)	34 (4.7)	0.063	<0.0001	0.0324
FOLDING OF ACTIN BY CCT TRIC	9	5 (55.6) [5]	0 (0)	5 (55.6)	0.013	<0.0001	0.0324
FCGAMMA RECEPTOR FCGR DEPENDENT PHAGOCYTOSIS	71	13 (18.3) [13]	2 (2.8)	11 (15.5)	0.029	0.0002	0.0429
GENERATION OF SECOND MESSENGER MOLECULES	22	7 (31.8) [7]	0 (0)	7 (31.8)	0.018	0.0002	0.0429
PD 1 SIGNALING	10	5 (50) [5]	0 (0)	5 (50)	0.013	0.0001	0.0429

Table S173: MSigDB Reactome pathways enriched in differentially abundant proteins at Day 30 compared to pre-second vaccination (Dendritic cells, Study Arm 2 (A/H7N9 + AS03 D1, 29)). Results are sorted by FDR adjusted p-value and Jaccard similarity index.

Gene Set ID	Gene Set Genes #	DA Proteins N (%)[Protein Fam. #]	Up-reg. DA Proteins N (%)	Down-reg. DA Proteins N (%)	Jaccard Index	P-Value	FDR Adjusted P-Value
NEUTROPHIL DEGRANULATION	399	55 (13.8) [55]	46 (11.5)	9 (2.3)	0.069	<0.0001	0.0002
INNATE IMMUNE SYSTEM	726	82 (11.3) [80]	63 (8.7)	19 (2.6)	0.074	<0.0001	0.0003
GENERATION OF SECOND MESSENGER MOLECULES	22	10 (45.5) [10]	1 (4.5)	9 (40.9)	0.021	<0.0001	0.0003
INTERFERON SIGNALING	124	23 (18.5) [18]	23 (18.5)	0 (0)	0.041	<0.0001	0.0024
FCGR ACTIVATION	11	6 (54.5) [5]	4 (36.4)	2 (18.2)	0.013	<0.0001	0.0094
INTERFERON ALPHA BETA SIGNALING	35	10 (28.6) [8]	10 (28.6)	0 (0)	0.021	<0.0001	0.0174
CYTOKINE SIGNALING IN IMMUNE SYSTEM	449	49 (10.9) [43]	38 (8.5)	11 (2.4)	0.057	0.0004	0.0819

Table S174: MSigDB Reactome pathways enriched in differentially abundant proteins at Day 32 compared to pre-second vaccination (Dendritic cells, Study Arm 2 (A/H7N9 + AS03 D1, 29)). Results are sorted by FDR adjusted p-value and Jaccard similarity index.

Gene Set ID	Gene Set Genes #	DA Proteins N (%)[Protein Fam. #]	Up-reg. DA Proteins N (%)	Down-reg. DA Proteins N (%)	Jaccard Index	P-Value	FDR Adjusted P-Value
INTERFERON GAMMA SIGNALING	47	5 (10.6) [3]	5 (10.6)	0 (0)	0.089	<0.0001	<0.0001
INTERFERON SIGNALING	124	5 (4) [3]	5 (4)	0 (0)	0.038	<0.0001	0.001

Table S175: MSigDB Reactome pathways enriched in differentially abundant proteins at Day 2 compared to pre-first vaccination (Monocytes, Study Arm 2 (A/H7N9 + AS03 D1, 29)). Results are sorted by FDR adjusted p-value and Jaccard similarity index.

Gene Set ID	Gene Set Genes #	DA Proteins N (%)[Protein Fam. #]	Up-reg. DA Proteins N (%)	Down-reg. DA Proteins N (%)	Jaccard Index	P-Value	FDR Adjusted P-Value
APOBEC3G MEDIATED RESISTANCE TO HIV 1 INFECTION	5	3 (60) [3]	0 (0)	3 (60)	0.052	<0.0001	0.0104
INTEGRATION OF PROVIRUS	9	3 (33.3) [3]	0 (0)	3 (33.3)	0.048	<0.0001	0.0426
INFECTIOUS DISEASE	505	14 (2.8) [14]	4 (0.8)	10 (2)	0.026	<0.0001	0.0449
EARLY PHASE OF HIV LIFE CYCLE	14	3 (21.4) [3]	0 (0)	3 (21.4)	0.045	0.0002	0.0895
EUKARYOTIC TRANSLATION ELONGATION	71	5 (7) [5]	0 (0)	5 (7)	0.041	0.0004	0.114
RESPONSE OF EIF2AK4 GCN2 TO AMINO ACID DEFICIENCY	75	5 (6.7) [5]	0 (0)	5 (6.7)	0.040	0.0005	0.114
CYTOKINE SIGNALING IN IMMUNE SYSTEM	449	12 (2.7) [10]	9 (2)	3 (0.7)	0.024	0.0005	0.114
NICOTINATE METABOLISM	19	3 (15.8) [3]	3 (15.8)	0 (0)	0.042	0.0006	0.1154
INTERFERON GAMMA SIGNALING	47	4 (8.5) [2]	4 (8.5)	0 (0)	0.040	0.0008	0.1201
INTERFERON SIGNALING	124	6 (4.8) [4]	6 (4.8)	0 (0)	0.035	0.0008	0.1201
INFLUENZA INFECTION	127	6 (4.7) [6]	0 (0)	6 (4.7)	0.034	0.0009	0.1238
SRP DEPENDENT COTRANSLATIONAL PROTEIN TARGETING TO MEMBRANE	88	5 (5.7) [5]	0 (0)	5 (5.7)	0.036	0.0011	0.1287
SELENOAMINO ACID METABOLISM	90	5 (5.6) [5]	0 (0)	5 (5.6)	0.035	0.0012	0.1287
NONSENSE MEDIATED DECAY NMD	91	5 (5.5) [5]	0 (0)	5 (5.5)	0.035	0.0013	0.1287
SUPPRESSION OF APOPTOSIS	6	2 (33.3) [2]	1 (16.7)	1 (16.7)	0.033	0.0012	0.1287
EUKARYOTIC TRANSLATION INITIATION	96	5 (5.2) [5]	0 (0)	5 (5.2)	0.034	0.0016	0.1455
2 LTR CIRCLE FORMATION	7	2 (28.6) [2]	0 (0)	2 (28.6)	0.033	0.0017	0.1455

Confidential/Proprietary Information

Table S176: MSigDB Reactome pathways enriched in differentially abundant proteins at Day 30 compared to pre-second vaccination (Monocytes, Study Arm 2 (A/H7N9 + AS03 D1, 29)). Results are sorted by FDR adjusted p-value and Jaccard similarity index.

Gene Set ID	Gene Set Genes #	DA Proteins N (%)[Protein Fam. #]	Up-reg. DA Proteins N (%)	Down-reg. DA Proteins N (%)	Jaccard Index	P-Value	FDR Adjusted P-Value
INTERFERON SIGNALING	124	16 (12.9) [11]	16 (12.9)	0 (0)	0.077	<0.0001	<0.0001
INTERFERON ALPHA BETA SIGNALING	35	9 (25.7) [7]	9 (25.7)	0 (0)	0.072	<0.0001	<0.0001
CYTOKINE SIGNALING IN IMMUNE SYSTEM	449	23 (5.1) [18]	21 (4.7)	2 (0.4)	0.044	<0.0001	<0.0001
INTERFERON GAMMA SIGNALING	47	8 (17) [5]	8 (17)	0 (0)	0.058	<0.0001	0.0001
INTERLEUKIN 20 FAMILY SIGNALING	11	3 (27.3) [3]	3 (27.3)	0 (0)	0.028	0.0005	0.1478

Table S177: MSigDB Reactome pathways enriched in differentially abundant proteins at Day 30 compared to pre-second vaccination (Natural Killer cells, Study Arm 2 (A/H7N9 + AS03 D1, 29)). Results are sorted by FDR adjusted p-value and Jaccard similarity index.

Gene Set ID	Gene Set Genes #	DA Proteins N (%)[Protein Fam. #]	Up-reg. DA Proteins N (%)	Down-reg. DA Proteins N (%)	Jaccard Index	P-Value	FDR Adjusted P-Value
GENE AND PROTEIN EXPRESSION BY JAK STAT SIGNALING AFTER INTERLEUKIN 12 STIMULATION	31	3 (9.7) [3]	2 (6.5)	1 (3.2)	0.071	<0.0001	0.0082
INTERLEUKIN 12 SIGNALING	36	3 (8.3) [3]	2 (5.6)	1 (2.8)	0.064	<0.0001	0.0082
INTERLEUKIN 12 FAMILY SIGNALING	38	3 (7.9) [3]	2 (5.3)	1 (2.6)	0.061	<0.0001	0.0082

Table S178: MSigDB GO Biological Process pathways enriched in differentially abundant proteins at Day 2 compared to pre-first vaccination (Neutrophils, Study Arm 2 (A/H7N9 + AS03 D1, 29)). Results are sorted by FDR adjusted p-value and Jaccard similarity index.

Gene Set ID	Gene Set Genes #	DA Proteins N (%)[Protein Fam. #]	Up-reg. DA Proteins N (%)	Down-reg. DA Proteins N (%)	Jaccard Index	P-Value	FDR Adjusted P-Value
RESPONSE TO INTERFERON GAMMA	101	3 (3) [1]	3 (3)	0 (0)	0.030	<0.0001	0.0256

Table S179: MSigDB GO Biological Process pathways enriched in differentially abundant proteins at Day 30 compared to pre-second vaccination (Neutrophils, Study Arm 2 (A/H7N9 + AS03 D1, 29)). Results are sorted by FDR adjusted p-value and Jaccard similarity index.

Gene Set ID	Gene Set Genes #	DA Proteins N (%)[Protein Fam. #]	Up-reg. DA Proteins N (%)	Down-reg. DA Proteins N (%)	Jaccard Index	P-Value	FDR Adjusted P-Value
RESPONSE TO CYTOKINE	583	7 (1.2) [5]	6 (1)	1 (0.2)	0.012	<0.0001	0.0022
INNATE IMMUNE RESPONSE	461	6 (1.3) [4]	6 (1.3)	0 (0)	0.013	<0.0001	0.0104
DEFENSE RESPONSE TO OTHER ORGANISM	551	6 (1.1) [4]	6 (1.1)	0 (0)	0.011	<0.0001	0.0198
DEFENSE RESPONSE TO VIRUS	145	4 (2.8) [4]	4 (2.8)	0 (0)	0.027	<0.0001	0.027
RESPONSE TO TYPE I INTERFERON	57	3 (5.3) [3]	3 (5.3)	0 (0)	0.048	<0.0001	0.0421
RESPONSE TO BIOTIC STIMULUS	706	6 (0.8) [4]	6 (0.8)	0 (0)	0.009	<0.0001	0.0421
RESPONSE TO VIRUS	193	4 (2.1) [4]	4 (2.1)	0 (0)	0.020	<0.0001	0.0463
DEFENSE RESPONSE	754	6 (0.8) [4]	6 (0.8)	0 (0)	0.008	<0.0001	0.0463

Table S180: MSigDB GO Biological Process pathways enriched in differentially abundant proteins at Day 32 compared to pre-second vaccination (Neutrophils, Study Arm 2 (A/H7N9 + AS03 D1, 29)). Results are sorted by FDR adjusted p-value and Jaccard similarity index.

Gene Set ID	Gene Set Genes #	DA Proteins N (%)[Protein Fam. #]	Up-reg. DA Proteins N (%)	Down-reg. DA Proteins N (%)	Jaccard Index	P-Value	FDR Adjusted P-Value
INNATE IMMUNE RESPONSE	461	15 (3.3) [12]	15 (3.3)	0 (0)	0.031	<0.0001	<0.0001
DEFENSE RESPONSE TO OTHER ORGANISM	551	16 (2.9) [13]	16 (2.9)	0 (0)	0.028	<0.0001	<0.0001
DEFENSE RESPONSE	754	18 (2.4) [15]	18 (2.4)	0 (0)	0.023	<0.0001	<0.0001
RESPONSE TO CYTOKINE	583	16 (2.7) [13]	16 (2.7)	0 (0)	0.026	<0.0001	<0.0001
CYTOKINE MEDIATED SIGNALING PATHWAY	367	13 (3.5) [11]	13 (3.5)	0 (0)	0.033	<0.0001	<0.0001
RESPONSE TO BIOTIC STIMULUS	706	16 (2.3) [13]	16 (2.3)	0 (0)	0.022	<0.0001	0.0001
CYTOKINE PRODUCTION	393	12 (3.1) [11]	11 (2.8)	1 (0.3)	0.029	<0.0001	0.0003
RESPONSE TO TYPE I INTERFERON	57	6 (10.5) [5]	6 (10.5)	0 (0)	0.066	<0.0001	0.0003
DEFENSE RESPONSE TO VIRUS	145	8 (5.5) [7]	8 (5.5)	0 (0)	0.045	<0.0001	0.0004
POSITIVE REGULATION OF CYTOKINE PRODUCTION	235	9 (3.8) [9]	8 (3.4)	1 (0.4)	0.034	<0.0001	0.0014
RESPONSE TO VIRUS	193	8 (4.1) [7]	8 (4.1)	0 (0)	0.036	<0.0001	0.003
REGULATION OF DEFENSE RESPONSE	347	10 (2.9) [10]	10 (2.9)	0 (0)	0.026	<0.0001	0.0038
RESPONSE TO INTERFERON GAMMA	101	6 (5.9) [4]	6 (5.9)	0 (0)	0.044	<0.0001	0.006
IMMUNE EFFECTOR PROCESS	781	14 (1.8) [13]	12 (1.5)	2 (0.3)	0.017	<0.0001	0.0073
POSITIVE REGULATION OF INTERLEUKIN 8 PRODUCTION	31	4 (12.9) [4]	4 (12.9)	0 (0)	0.060	<0.0001	0.0082
POSITIVE REGULATION OF CYTOKINE PRODUCTION INVOLVED IN INFLAMMATORY RESPONSE	11	3 (27.3) [3]	3 (27.3)	0 (0)	0.062	<0.0001	0.0092
REGULATION OF METALLOPEPTIDASE ACTIVITY	12	3 (25) [3]	2 (16.7)	1 (8.3)	0.061	<0.0001	0.0115
INTERLEUKIN 8 PRODUCTION	43	4 (9.3) [4]	4 (9.3)	0 (0)	0.051	<0.0001	0.0258
INTERLEUKIN 1 BETA PRODUCTION	47	4 (8.5) [4]	4 (8.5)	0 (0)	0.048	<0.0001	0.0348
REGULATION OF RESPONSE TO EXTERNAL STIMULUS	477	10 (2.1) [10]	10 (2.1)	0 (0)	0.020	<0.0001	0.0358
REGULATION OF INFLAMMATORY RESPONSE	153	6 (3.9) [6]	6 (3.9)	0 (0)	0.032	0.0001	0.038
INFLAMMATORY RESPONSE	302	8 (2.6) [8]	8 (2.6)	0 (0)	0.024	0.0001	0.038
INTERFERON GAMMA MEDIATED SIGNALING PATHWAY	51	4 (7.8) [3]	4 (7.8)	0 (0)	0.046	0.0001	0.0396
INTERLEUKIN 1 PRODUCTION	53	4 (7.5) [4]	4 (7.5)	0 (0)	0.045	0.0001	0.0442
NEGATIVE REGULATION OF VIRAL LIFE CYCLE	55	4 (7.3) [4]	4 (7.3)	0 (0)	0.044	0.0002	0.0491
POSITIVE REGULATION OF INFLAMMATORY RESPONSE	60	4 (6.7) [4]	4 (6.7)	0 (0)	0.042	0.0002	0.0662
REGULATION OF METALLOENDOPEPTIDASE ACTIVITY	5	2 (40) [2]	1 (20)	1 (20)	0.046	0.0003	0.0692
NEGATIVE REGULATION OF VIRAL PROCESS	65	4 (6.2) [4]	4 (6.2)	0 (0)	0.040	0.0003	0.0838
POSITIVE REGULATION OF CYTOKINE PRODUCTION INVOLVED IN IMMUNE RESPONSE	28	3 (10.7) [3]	3 (10.7)	0 (0)	0.046	0.0004	0.0931
POSITIVE REGULATION OF METALLOPEPTIDASE ACTIVITY	6	2 (33.3) [2]	2 (33.3)	0 (0)	0.045	0.0004	0.0931
POSITIVE REGULATION OF INTERLEUKIN 1 PRODUCTION	30	3 (10) [3]	3 (10)	0 (0)	0.045	0.0005	0.1093
CYTOKINE PRODUCTION INVOLVED IN INFLAMMATORY RESPONSE	31	3 (9.7) [3]	3 (9.7)	0 (0)	0.044	0.0005	0.1168
CELLULAR RESPONSE TO INTERFERON ALPHA	7	2 (28.6) [1]	2 (28.6)	0 (0)	0.044	0.0005	0.1181
POSITIVE REGULATION OF CYTOKINE SECRETION	32	3 (9.4) [3]	2 (6.2)	1 (3.1)	0.043	0.0006	0.1209
POSITIVE REGULATION OF RESPONSE TO CYTOKINE STIMULUS	34	3 (8.8) [3]	3 (8.8)	0 (0)	0.042	0.0007	0.1407

Table S180: MSigDB GO Biological Process pathways enriched in differentially abundant proteins at Day 32 compared to pre-second vaccination (Neutrophils, Study Arm 2 (A/H7N9 + AS03 D1, 29)). Results are sorted by FDR adjusted p-value and Jaccard similarity index (continued).

Gene Set ID	Gene Set Genes #	DA Proteins N (%)[Protein Fam. #]	Up-reg. DA Proteins N (%)	Down-reg. DA Proteins N (%)	Jaccard Index	P-Value	FDR Adjusted P-Value
POSITIVE REGULATION OF TUMOR NECROSIS FACTOR MEDIATED SIGNALING PATHWAY	8	2 (25) [2]	2 (25)	0 (0)	0.043	0.0007	0.1439

Table S181: MSigDB GO Biological Process pathways enriched in differentially abundant proteins at Day 2 compared to pre-first vaccination (Dendritic cells, Study Arm 2 (A/H7N9 + AS03 D1, 29)). Results are sorted by FDR adjusted p-value and Jaccard similarity index.

Gene Set ID	Gene Set Genes #	DA Proteins N (%)[Protein Fam. #]	Up-reg. DA Proteins N (%)	Down-reg. DA Proteins N (%)	Jaccard Index	P-Value	FDR Adjusted P-Value
REGULATION OF TELOMERE MAINTENANCE VIA TELOMERE LENGTHENING	43	14 (32.6) [13]	3 (7)	11 (25.6)	0.034	<0.0001	0.0001
POSITIVE REGULATION OF TELOMERE MAINTENANCE VIA TELOMERE LENGTHENING	27	11 (40.7) [10]	2 (7.4)	9 (33.3)	0.027	<0.0001	0.0002
MYELOID LEUKOCYTE MEDIATED IMMUNITY	443	50 (11.3) [49]	28 (6.3)	22 (5)	0.064	<0.0001	0.0003
TELOMERE MAINTENANCE VIA TELOMERE LENGTHENING	57	15 (26.3) [14]	3 (5.3)	12 (21.1)	0.035	<0.0001	0.0003
EXOCYTOSIS	610	61 (10) [60]	32 (5.2)	29 (4.8)	0.065	<0.0001	0.0006
RNA DEPENDENT DNA BIOSYNTHETIC PROCESS	54	14 (25.9) [14]	2 (3.7)	12 (22.2)	0.033	<0.0001	0.0006
MYELOID LEUKOCYTE ACTIVATION	489	51 (10.4) [50]	31 (6.3)	20 (4.1)	0.062	<0.0001	0.0012
REGULATION OF TELOMERE MAINTENANCE	58	14 (24.1) [13]	3 (5.2)	11 (19)	0.033	<0.0001	0.0012
POSITIVE REGULATION OF TELOMERE MAINTENANCE	36	11 (30.6) [10]	2 (5.6)	9 (25)	0.027	<0.0001	0.0012
IMMUNE EFFECTOR PROCESS	781	71 (9.1) [70]	35 (4.5)	36 (4.6)	0.065	<0.0001	0.0014
LEUKOCYTE MEDIATED IMMUNITY	552	55 (10) [54]	29 (5.3)	26 (4.7)	0.062	<0.0001	0.0014
PHAGOCYTOSIS	168	25 (14.9) [25]	8 (4.8)	17 (10.1)	0.047	<0.0001	0.0014
FC RECEPTOR MEDIATED STIMULATORY SIGNALING PATHWAY	71	15 (21.1) [15]	3 (4.2)	12 (16.9)	0.034	<0.0001	0.0018
CELL ACTIVATION	869	75 (8.6) [74]	33 (3.8)	42 (4.8)	0.064	<0.0001	0.0035
ACTIVATION OF IMMUNE RESPONSE	288	34 (11.8) [33]	8 (2.8)	26 (9)	0.053	<0.0001	0.0035
CELLULAR RESPONSE TO X RAY	7	5 (71.4) [5]	1 (14.3)	4 (57.1)	0.013	<0.0001	0.0035
PROTEIN LOCALIZATION TO CHROMOSOME TELOMERIC REGION	22	8 (36.4) [8]	1 (4.5)	7 (31.8)	0.020	<0.0001	0.004
CELL ACTIVATION INVOLVED IN IMMUNE RESPONSE	523	51 (9.8) [50]	30 (5.7)	21 (4)	0.059	<0.0001	0.0043
POSITIVE REGULATION OF IMMUNE SYSTEM PROCESS	525	51 (9.7) [49]	20 (3.8)	31 (5.9)	0.059	<0.0001	0.0045
PROTEIN LOCALIZATION TO ORGANELLE	600	56 (9.3) [54]	20 (3.3)	36 (6)	0.060	<0.0001	0.0047
POSITIVE REGULATION OF IMMUNE RESPONSE	376	40 (10.6) [38]	12 (3.2)	28 (7.4)	0.055	<0.0001	0.0047
PROTEIN LOCALIZATION TO CHROMOSOME	53	12 (22.6) [12]	3 (5.7)	9 (17)	0.028	<0.0001	0.0047
REGULATION OF PROTEIN LOCALIZATION TO CHROMOSOME TELOMERIC REGION	12	6 (50) [6]	1 (8.3)	5 (41.7)	0.015	<0.0001	0.0047
POSITIVE REGULATION OF DNA BIOSYNTHETIC PROCESS	45	11 (24.4) [10]	2 (4.4)	9 (20)	0.026	<0.0001	0.0047
REGULATION OF DNA BIOSYNTHETIC PROCESS	63	13 (20.6) [12]	3 (4.8)	10 (15.9)	0.030	<0.0001	0.0056
POSITIVE REGULATION OF ESTABLISHMENT OF PROTEIN LOCALIZATION TO TELOMERE	9	5 (55.6) [5]	0 (0)	5 (55.6)	0.013	<0.0001	0.0119
POSITIVE REGULATION OF ORGANELLE ORGANIZATION	359	37 (10.3) [35]	11 (3.1)	26 (7.2)	0.052	<0.0001	0.0162
ESTABLISHMENT OF PROTEIN LOCALIZATION TO TELOMERE	15	6 (40) [6]	0 (0)	6 (40)	0.015	<0.0001	0.0182
REGULATION OF IMMUNE RESPONSE	505	47 (9.3) [45]	18 (3.6)	29 (5.7)	0.056	<0.0001	0.0184
IMMUNE RESPONSE REGULATING SIGNALING PATHWAY	270	30 (11.1) [30]	7 (2.6)	23 (8.5)	0.048	<0.0001	0.0184
POSITIVE REGULATION OF LYMPHOCYTE ACTIVATION	146	20 (13.7) [20]	6 (4.1)	14 (9.6)	0.039	<0.0001	0.0184
POSITIVE REGULATION OF CHROMOSOME ORGANIZATION	113	17 (15) [16]	7 (6.2)	10 (8.8)	0.035	<0.0001	0.0184
GLYCOSYL COMPOUND METABOLIC PROCESS	83	14 (16.9) [14]	4 (4.8)	10 (12)	0.031	<0.0001	0.0216
SECRETION	830	68 (8.2) [67]	34 (4.1)	34 (4.1)	0.059	0.0001	0.0235
BINDING OF SPERM TO ZONA PELLUCIDA	11	5 (45.5) [5]	0 (0)	5 (45.5)	0.013	0.0001	0.0281
PROTEIN LOCALIZATION TO NUCLEAR BODY	11	5 (45.5) [5]	0 (0)	5 (45.5)	0.013	0.0001	0.0281

Table S181: MSigDB GO Biological Process pathways enriched in differentially abundant proteins at Day 2 compared to pre-first vaccination (Dendritic cells, Study Arm 2 (A/H7N9 + AS03 D1, 29)). Results are sorted by FDR adjusted p-value and Jaccard similarity index (continued).

Gene Set ID	Gene Set Genes #	DA Proteins N (%)[Protein Fam. #]	Up-reg. DA Proteins N (%)	Down-reg. DA Proteins N (%)	Jaccard Index	P-Value	FDR Adjusted P-Value
REGULATION OF ESTABLISHMENT OF PROTEIN LOCALIZATION TO CHROMOSOME	11	5 (45.5) [5]	0 (0)	5 (45.5)	0.013	0.0001	0.0281
GLUTAMATE BIOSYNTHETIC PROCESS	3	3 (100) [3]	0 (0)	3 (100)	0.008	0.0001	0.0281
ESTABLISHMENT OF PROTEIN LOCALIZATION TO CHROMOSOME	24	7 (29.2) [7]	1 (4.2)	6 (25)	0.017	0.0002	0.0323
POSITIVE REGULATION OF LEUKOCYTE CELL CELL ADHESION	121	17 (14) [17]	5 (4.1)	12 (9.9)	0.035	0.0002	0.0349
LEUKOCYTE CELL CELL ADHESION	181	22 (12.2) [22]	8 (4.4)	14 (7.7)	0.040	0.0002	0.0362
POSITIVE REGULATION OF TELOMERASE RNA LOCALIZATION TO CAJAL BODY	12	5 (41.7) [5]	0 (0)	5 (41.7)	0.013	0.0002	0.0402
PROTEIN LOCALIZATION TO CELL CORTEX	7	4 (57.1) [4]	2 (28.6)	2 (28.6)	0.010	0.0002	0.0402
REGULATION OF ORGANELLE ORGANIZATION	696	58 (8.3) [56]	18 (2.6)	40 (5.7)	0.057	0.0002	0.0408
POSITIVE REGULATION OF CELL ADHESION	210	24 (11.4) [24]	8 (3.8)	16 (7.6)	0.042	0.0003	0.0442
POSITIVE REGULATION OF CELLULAR COMPONENT ORGANIZATION	594	51 (8.6) [49]	16 (2.7)	35 (5.9)	0.055	0.0003	0.0475
OXIDATION REDUCTION PROCESS	535	47 (8.8) [45]	9 (1.7)	38 (7.1)	0.054	0.0003	0.048
ALPHA BETA T CELL PROLIFERATION	19	6 (31.6) [6]	3 (15.8)	3 (15.8)	0.015	0.0003	0.048
DEFENSE RESPONSE TO OTHER ORGANISM	551	48 (8.7) [44]	27 (4.9)	21 (3.8)	0.054	0.0003	0.0485
REGULATION OF LYMPHOCYTE ACTIVATION	226	25 (11.1) [25]	8 (3.5)	17 (7.5)	0.043	0.0003	0.0485
CELL RECOGNITION	43	9 (20.9) [9]	2 (4.7)	7 (16.3)	0.021	0.0003	0.0485
RESPONSE TO BIOTIC STIMULUS	706	58 (8.2) [54]	29 (4.1)	29 (4.1)	0.056	0.0004	0.0491
POSITIVE REGULATION OF ALPHA BETA T CELL PROLIFERATION	13	5 (38.5) [5]	2 (15.4)	3 (23.1)	0.013	0.0004	0.0491
PROTEIN LOCALIZATION TO NUCLEOPLASM	13	5 (38.5) [5]	0 (0)	5 (38.5)	0.013	0.0004	0.0491
POSITIVE REGULATION OF CELL ACTIVATION	178	21 (11.8) [21]	6 (3.4)	15 (8.4)	0.039	0.0004	0.0566
DICARBOXYLIC ACID METABOLIC PROCESS	54	10 (18.5) [9]	0 (0)	10 (18.5)	0.023	0.0004	0.0583
REGULATION OF CELLULAR PROTEIN LOCALIZATION	315	31 (9.8) [31]	11 (3.5)	20 (6.3)	0.046	0.0006	0.0684
REGULATION OF T CELL ACTIVATION	169	20 (11.8) [20]	6 (3.6)	14 (8.3)	0.037	0.0006	0.0684
SPERM EGG RECOGNITION	14	5 (35.7) [5]	0 (0)	5 (35.7)	0.013	0.0005	0.0684
SLEEP	4	3 (75) [3]	1 (25)	2 (50)	0.008	0.0006	0.0684
POSITIVE REGULATION OF TELOMERASE ACTIVITY	21	6 (28.6) [6]	1 (4.8)	5 (23.8)	0.015	0.0006	0.0691
INNATE IMMUNE RESPONSE	461	41 (8.9) [37]	22 (4.8)	19 (4.1)	0.051	0.0006	0.0715
POSITIVE REGULATION OF PROTEIN CONTAINING COMPLEX ASSEMBLY	146	18 (12.3) [18]	5 (3.4)	13 (8.9)	0.035	0.0006	0.0745
PROTEIN STABILIZATION	112	15 (13.4) [15]	3 (2.7)	12 (10.7)	0.031	0.0008	0.0864
TELOMERASE RNA LOCALIZATION	15	5 (33.3) [5]	0 (0)	5 (33.3)	0.013	0.0008	0.0866
VIRAL LATENCY	9	4 (44.4) [4]	1 (11.1)	3 (33.3)	0.010	0.0008	0.0866
RESPONSE TO INTERFERON GAMMA	101	14 (13.9) [11]	9 (8.9)	5 (5)	0.030	0.0008	0.087
REGULATION OF CHROMOSOME ORGANIZATION	213	23 (10.8) [22]	10 (4.7)	13 (6.1)	0.040	0.0008	0.0878
CELLULAR RESPIRATION	125	16 (12.8) [15]	2 (1.6)	14 (11.2)	0.032	0.0008	0.0893
REGULATION OF CELL ACTIVATION	296	29 (9.8) [29]	11 (3.7)	18 (6.1)	0.044	0.0009	0.0926
DNA BIOSYNTHETIC PROCESS	114	15 (13.2) [14]	3 (2.6)	12 (10.5)	0.031	0.0009	0.0939
POSITIVE REGULATION OF CELL CELL ADHESION	139	17 (12.2) [17]	5 (3.6)	12 (8.6)	0.034	0.001	0.099

Table S181: MSigDB GO Biological Process pathways enriched in differentially abundant proteins at Day 2 compared to pre-first vaccination (Dendritic cells, Study Arm 2 (A/H7N9 + AS03 D1, 29)). Results are sorted by FDR adjusted p-value and Jaccard similarity index (continued).

Gene Set ID	Gene Set Genes #	DA Proteins N (%)[Protein Fam. #]	Up-reg. DA Proteins N (%)	Down-reg. DA Proteins N (%)	Jaccard Index	P-Value	FDR Adjusted P-Value
RESPONSE TO INTERLEUKIN 12	40	8 (20) [8]	4 (10)	4 (10)	0.019	0.001	0.099
TELOMERE ORGANIZATION	127	16 (12.6) [15]	3 (2.4)	13 (10.2)	0.032	0.001	0.0991
REGULATION OF PROTEIN LOCALIZATION TO NUCLEUS	81	12 (14.8) [12]	2 (2.5)	10 (12.3)	0.026	0.001	0.1003
DEFENSE RESPONSE	754	59 (7.8) [55]	30 (4)	29 (3.8)	0.055	0.0011	0.1058
SMALL MOLECULE CATABOLIC PROCESS	260	26 (10) [26]	5 (1.9)	21 (8.1)	0.042	0.0012	0.1148
CELL CELL RECOGNITION	24	6 (25) [6]	0 (0)	6 (25)	0.015	0.0013	0.1156
DICARBOXYLIC ACID BIOSYNTHETIC PROCESS	10	4 (40) [4]	0 (0)	4 (40)	0.010	0.0013	0.1156
POSITIVE REGULATION OF PROTEIN LOCALIZATION TO CELL CORTEX	5	3 (60) [3]	2 (40)	1 (20)	0.008	0.0014	0.1218
REGULATION OF PROTEIN LOCALIZATION TO CELL CORTEX	5	3 (60) [3]	2 (40)	1 (20)	0.008	0.0014	0.1218
REGULATION OF IMMUNE SYSTEM PROCESS	793	61 (7.7) [59]	26 (3.3)	35 (4.4)	0.055	0.0014	0.1227
CELLULAR RESPONSE TO CHEMICAL STRESS	195	21 (10.8) [21]	10 (5.1)	11 (5.6)	0.037	0.0014	0.1243
GLUTAMATE METABOLIC PROCESS	17	5 (29.4) [5]	0 (0)	5 (29.4)	0.013	0.0015	0.1259
RESPIRATORY BURST	17	5 (29.4) [5]	4 (23.5)	1 (5.9)	0.013	0.0015	0.1259
PRODUCTION OF MOLECULAR MEDIATOR INVOLVED IN INFLAMMATORY RESPONSE	43	8 (18.6) [8]	5 (11.6)	3 (7)	0.019	0.0016	0.1365
GENERATION OF PRECURSOR METABOLITES AND ENERGY	324	30 (9.3) [29]	6 (1.9)	24 (7.4)	0.044	0.0018	0.1494
REGULATION OF PROTEIN CONTAINING COMPLEX ASSEMBLY	254	25 (9.8) [25]	5 (2)	20 (7.9)	0.041	0.0019	0.1494
CELLULAR HYPEROSMOTIC RESPONSE	11	4 (36.4) [4]	1 (9.1)	3 (27.3)	0.010	0.0019	0.1494
CYTOLYSIS	11	4 (36.4) [4]	1 (9.1)	3 (27.3)	0.010	0.0019	0.1494
POSITIVE REGULATION OF SIGNAL TRANSDUCTION BY P53 CLASS MEDIATOR	11	4 (36.4) [4]	0 (0)	4 (36.4)	0.010	0.0019	0.1494
REGULATION OF NEUROINFLAMMATORY RESPONSE	11	4 (36.4) [4]	2 (18.2)	2 (18.2)	0.010	0.0019	0.1494

Table S182: MSigDB GO Biological Process pathways enriched in differentially abundant proteins at Day 30 compared to pre-second vaccination (Dendritic cells, Study Arm 2 (A/H7N9 + AS03 D1, 29)). Results are sorted by FDR adjusted p-value and Jaccard similarity index.

Gene Set ID	Gene Set Genes #	DA Proteins N (%)[Protein Fam. #]	Up-reg. DA Proteins N (%)	Down-reg. DA Proteins N (%)	Jaccard Index	P-Value	FDR Adjusted P-Value
IMMUNE EFFECTOR PROCESS	781	97 (12.4) [94]	78 (10)	19 (2.4)	0.085	<0.0001	<0.0001
DEFENSE RESPONSE TO OTHER ORGANISM	551	74 (13.4) [65]	61 (11.1)	13 (2.4)	0.079	<0.0001	<0.0001
DEFENSE RESPONSE	754	91 (12.1) [82]	70 (9.3)	21 (2.8)	0.081	<0.0001	<0.0001
MYELOID LEUKOCYTE ACTIVATION	489	65 (13.3) [64]	56 (11.5)	9 (1.8)	0.074	<0.0001	<0.0001
MYELOID LEUKOCYTE MEDIATED IMMUNITY	443	61 (13.8) [60]	52 (11.7)	9 (2)	0.073	<0.0001	<0.0001
INNATE IMMUNE RESPONSE	461	62 (13.4) [53]	52 (11.3)	10 (2.2)	0.072	<0.0001	<0.0001
RESPONSE TO BIOTIC STIMULUS	706	83 (11.8) [74]	66 (9.3)	17 (2.4)	0.077	<0.0001	<0.0001
CELL ACTIVATION INVOLVED IN IMMUNE RESPONSE	523	66 (12.6) [65]	55 (10.5)	11 (2.1)	0.072	<0.0001	<0.0001
EXOCYTOSIS	610	72 (11.8) [71]	59 (9.7)	13 (2.1)	0.072	<0.0001	<0.0001
SECRETION	830	89 (10.7) [88]	68 (8.2)	21 (2.5)	0.074	<0.0001	<0.0001
DEFENSE RESPONSE TO VIRUS	145	27 (18.6) [25]	24 (16.6)	3 (2.1)	0.047	<0.0001	0.0002
LEUKOCYTE MEDIATED IMMUNITY	552	65 (11.8) [64]	54 (9.8)	11 (2)	0.069	<0.0001	0.0002
CELL ACTIVATION	869	90 (10.4) [88]	63 (7.2)	27 (3.1)	0.073	<0.0001	0.0002
RESPONSE TO VIRUS	193	31 (16.1) [29]	27 (14)	4 (2.1)	0.050	<0.0001	0.0005
POSITIVE REGULATION OF IMMUNE SYSTEM PROCESS	525	60 (11.4) [55]	35 (6.7)	25 (4.8)	0.065	<0.0001	0.0011
RESPONSE TO INTERFERON GAMMA	101	19 (18.8) [16]	18 (17.8)	1 (1)	0.035	<0.0001	0.0057
PHAGOCYTOSIS	168	26 (15.5) [25]	16 (9.5)	10 (6)	0.043	<0.0001	0.006
POSITIVE REGULATION OF CELL DEATH	356	43 (12.1) [42]	24 (6.7)	19 (5.3)	0.056	<0.0001	0.0073
PROTEIN AUTOPHOSPHORYLATION	113	20 (17.7) [18]	13 (11.5)	7 (6.2)	0.036	<0.0001	0.0073
CYTOKINE PRODUCTION	393	46 (11.7) [43]	32 (8.1)	14 (3.6)	0.057	<0.0001	0.0074
RESPONSE TO INTERFERON ALPHA	11	6 (54.5) [5]	5 (45.5)	1 (9.1)	0.013	<0.0001	0.0074
REGULATION OF RESPONSE TO STRESS	754	74 (9.8) [71]	56 (7.4)	18 (2.4)	0.065	<0.0001	0.0128
REGULATION OF DEFENSE RESPONSE	347	41 (11.8) [38]	32 (9.2)	9 (2.6)	0.054	<0.0001	0.0158
REGULATION OF REGULATED SECRETORY PATHWAY	60	13 (21.7) [12]	12 (20)	1 (1.7)	0.026	<0.0001	0.0198
POSITIVE REGULATION OF ALPHA BETA T CELL PROLIFERATION	13	6 (46.2) [6]	3 (23.1)	3 (23.1)	0.013	<0.0001	0.02
MAST CELL ACTIVATION	38	10 (26.3) [9]	10 (26.3)	0 (0)	0.021	<0.0001	0.0226
SYMBIOTIC PROCESS	681	67 (9.8) [66]	42 (6.2)	25 (3.7)	0.062	<0.0001	0.0232
REGULATION OF VIRAL LIFE CYCLE	106	18 (17) [18]	16 (15.1)	2 (1.9)	0.033	<0.0001	0.0232
REGULATION OF LEUKOCYTE DEGRANULATION	32	9 (28.1) [8]	9 (28.1)	0 (0)	0.019	0.0001	0.0263
REGULATION OF RESPONSE TO EXTERNAL STIMULUS	477	50 (10.5) [47]	39 (8.2)	11 (2.3)	0.057	0.0002	0.0401
REGULATION OF MAST CELL ACTIVATION	27	8 (29.6) [7]	8 (29.6)	0 (0)	0.017	0.0002	0.0401
LEUKOCYTE MIGRATION	206	27 (13.1) [26]	15 (7.3)	12 (5.8)	0.042	0.0002	0.0415
PROTEIN ADP RIBOSYLATION	21	7 (33.3) [7]	6 (28.6)	1 (4.8)	0.015	0.0002	0.0415
REGULATION OF MAST CELL ACTIVATION INVOLVED IN IMMUNE RESPONSE	21	7 (33.3) [6]	7 (33.3)	0 (0)	0.015	0.0002	0.0415
RESPONSE TO CYTOKINE	583	58 (9.9) [52]	45 (7.7)	13 (2.2)	0.059	0.0002	0.0436
PRODUCTION OF MOLECULAR MEDIATOR INVOLVED IN INFLAMMATORY RESPONSE	43	10 (23.3) [10]	9 (20.9)	1 (2.3)	0.020	0.0002	0.0472

Table S182: MSigDB GO Biological Process pathways enriched in differentially abundant proteins at Day 30 compared to pre-second vaccination (Dendritic cells, Study Arm 2 (A/H7N9 + AS03 D1, 29)). Results are sorted by FDR adjusted p-value and Jaccard similarity index (continued).

Gene Set ID	Gene Set Genes #	DA Proteins N (%)[Protein Fam. #]	Up-reg. DA Proteins N (%)	Down-reg. DA Proteins N (%)	Jaccard Index	P-Value	FDR Adjusted P-Value
MYOFIBROBLAST DIFFERENTIATION	3	3 (100) [3]	1 (33.3)	2 (66.7)	0.007	0.0002	0.0472
REGULATION OF MYOFIBROBLAST DIFFERENTIATION	3	3 (100) [3]	1 (33.3)	2 (66.7)	0.007	0.0002	0.0472
POSITIVE REGULATION OF CELL ACTIVATION	178	24 (13.5) [22]	14 (7.9)	10 (5.6)	0.039	0.0003	0.0515
APOPTOTIC PROCESS	900	81 (9) [79]	49 (5.4)	32 (3.6)	0.063	0.0003	0.0583
REGULATION OF IMMUNE SYSTEM PROCESS	793	73 (9.2) [68]	44 (5.5)	29 (3.7)	0.062	0.0003	0.0608
CYTOKINE MEDIATED SIGNALING PATHWAY	367	40 (10.9) [37]	32 (8.7)	8 (2.2)	0.051	0.0004	0.0616
REGULATION OF IMMUNE RESPONSE	505	51 (10.1) [46]	31 (6.1)	20 (4)	0.056	0.0004	0.0618
MAST CELL MEDIATED IMMUNITY	30	8 (26.7) [7]	8 (26.7)	0 (0)	0.017	0.0004	0.0631
REGULATION OF MYELOID LEUKOCYTE MEDIATED IMMUNITY	38	9 (23.7) [8]	9 (23.7)	0 (0)	0.018	0.0004	0.0704
PROTEIN LOCALIZATION TO CELL CORTEX	7	4 (57.1) [4]	1 (14.3)	3 (42.9)	0.009	0.0005	0.0722
POSITIVE REGULATION OF LEUKOCYTE PROLIFERATION	64	12 (18.8) [12]	6 (9.4)	6 (9.4)	0.024	0.0005	0.0805
INFLAMMATORY RESPONSE	302	34 (11.3) [34]	27 (8.9)	7 (2.3)	0.047	0.0006	0.0838
POSITIVE REGULATION OF IMMUNE RESPONSE	376	40 (10.6) [35]	22 (5.9)	18 (4.8)	0.050	0.0006	0.0854
REGULATION OF RESPONSE TO BIOTIC STIMULUS	244	29 (11.9) [26]	24 (9.8)	5 (2)	0.043	0.0006	0.0854
NEUTROPHIL MIGRATION	48	10 (20.8) [10]	7 (14.6)	3 (6.2)	0.020	0.0006	0.0896
NEUTROPHIL CHEMOTAXIS	40	9 (22.5) [9]	7 (17.5)	2 (5)	0.018	0.0007	0.0896
PHAGOSOME MATURATION	25	7 (28) [7]	7 (28)	0 (0)	0.015	0.0006	0.0896
RESPONSE TO TYPE I INTERFERON	57	11 (19.3) [9]	11 (19.3)	0 (0)	0.022	0.0007	0.0923
POSITIVE REGULATION OF CYTOKINE PRODUCTION	235	28 (11.9) [27]	18 (7.7)	10 (4.3)	0.042	0.0007	0.0926
ALPHA BETA T CELL PROLIFERATION	19	6 (31.6) [6]	3 (15.8)	3 (15.8)	0.013	0.0008	0.1016
ALDITOL METABOLIC PROCESS	13	5 (38.5) [4]	3 (23.1)	2 (15.4)	0.011	0.0008	0.1016
LEUKOCYTE CELL CELL ADHESION	181	23 (12.7) [22]	10 (5.5)	13 (7.2)	0.037	0.0009	0.107
ESTABLISHMENT OF VIRAL LATENCY	8	4 (50) [4]	0 (0)	4 (50)	0.009	0.0009	0.107
T TUBULE ORGANIZATION	4	3 (75) [2]	2 (50)	1 (25)	0.006	0.0009	0.1129
ACTIVATION OF IMMUNE RESPONSE	288	32 (11.1) [28]	14 (4.9)	18 (6.2)	0.045	0.001	0.119
POSITIVE REGULATION OF RESPONSE TO EXTERNAL STIMULUS	253	29 (11.5) [27]	21 (8.3)	8 (3.2)	0.043	0.0011	0.119
DEFENSE RESPONSE TO BACTERIUM	98	15 (15.3) [14]	14 (14.3)	1 (1)	0.028	0.001	0.119
INTERFERON GAMMA MEDIATED SIGNALING PATHWAY	51	10 (19.6) [9]	10 (19.6)	0 (0)	0.020	0.001	0.119
POSITIVE REGULATION OF REGULATED SECRETORY PATHWAY	20	6 (30) [6]	6 (30)	0 (0)	0.013	0.0011	0.119
POSITIVE REGULATION OF DEFENSE RESPONSE	197	24 (12.2) [22]	19 (9.6)	5 (2.5)	0.038	0.0012	0.1346
NEGATIVE REGULATION OF PHOSPHORUS METABOLIC PROCESS	280	31 (11.1) [31]	19 (6.8)	12 (4.3)	0.044	0.0013	0.1383
RESPONSE TO BACTERIUM	257	29 (11.3) [26]	26 (10.1)	3 (1.2)	0.042	0.0014	0.1415
POSITIVE REGULATION OF LEUKOCYTE CELL CELL ADHESION	121	17 (14) [16]	8 (6.6)	9 (7.4)	0.030	0.0013	0.1415
FC RECEPTOR MEDIATED STIMULATORY SIGNALING PATHWAY	71	12 (16.9) [11]	7 (9.9)	5 (7)	0.023	0.0014	0.1415

Confidential/Proprietary Information

Table S183: MSigDB GO Biological Process pathways enriched in differentially abundant proteins at Day 32 compared to pre-second vaccination (Dendritic cells, Study Arm 2 (A/H7N9 + AS03 D1, 29)). Results are sorted by FDR adjusted p-value and Jaccard similarity index.

Gene Set ID	Gene Set Genes #	DA Proteins N (%)[Protein Fam. #]	Up-reg. DA Proteins N (%)	Down-reg. DA Proteins N (%)	Jaccard Index	P-Value	FDR Adjusted P-Value
INTERFERON GAMMA MEDIATED SIGNALING PATHWAY	51	5 (9.8) [4]	5 (9.8)	0 (0)	0.083	<0.0001	0.0001
RESPONSE TO INTERFERON GAMMA	101	6 (5.9) [4]	6 (5.9)	0 (0)	0.055	<0.0001	0.0001
INNATE IMMUNE RESPONSE	461	7 (1.5) [5]	7 (1.5)	0 (0)	0.015	<0.0001	0.0229
CYTOKINE MEDIATED SIGNALING PATHWAY	367	6 (1.6) [5]	6 (1.6)	0 (0)	0.016	<0.0001	0.0518
DEFENSE RESPONSE TO OTHER ORGANISM	551	7 (1.3) [5]	7 (1.3)	0 (0)	0.013	<0.0001	0.0518
RESPONSE TO CYTOKINE	583	7 (1.2) [5]	7 (1.2)	0 (0)	0.012	<0.0001	0.0538
ANTIGEN PROCESSING AND PRESENTATION OF EXOGENOUS PEPTIDE ANTIGEN VIA MHC CLASS I	53	3 (5.7) [3]	3 (5.7)	0 (0)	0.047	0.0001	0.1387
RESPONSE TO BIOTIC STIMULUS	706	7 (1) [5]	7 (1)	0 (0)	0.010	0.0002	0.1394

Table S184: MSigDB GO Biological Process pathways enriched in differentially abundant proteins at Day 2 compared to pre-first vaccination (Monocytes, Study Arm 2 (A/H7N9 + AS03 D1, 29)). Results are sorted by FDR adjusted p-value and Jaccard similarity index.

Gene Set ID	Gene Set Genes #	DA Proteins N (%)[Protein Fam. #]	Up-reg. DA Proteins N (%)	Down-reg. DA Proteins N (%)	Jaccard Index	P-Value	FDR Adjusted P-Value
DEFENSE RESPONSE	754	20 (2.7) [18]	17 (2.3)	3 (0.4)	0.025	<0.0001	0.0021
INNATE IMMUNE RESPONSE	461	14 (3) [12]	12 (2.6)	2 (0.4)	0.028	<0.0001	0.022
DEFENSE RESPONSE TO OTHER ORGANISM	551	15 (2.7) [13]	13 (2.4)	2 (0.4)	0.025	<0.0001	0.022
RESPONSE TO BIOTIC STIMULUS	706	17 (2.4) [15]	13 (1.8)	4 (0.6)	0.023	<0.0001	0.022
ESTABLISHMENT OF VIRAL LATENCY	8	3 (37.5) [3]	0 (0)	3 (37.5)	0.049	<0.0001	0.0348
VIRAL LATENCY	9	3 (33.3) [3]	0 (0)	3 (33.3)	0.048	<0.0001	0.0433
CYTOKINE MEDIATED SIGNALING PATHWAY	367	11 (3) [10]	9 (2.5)	2 (0.5)	0.027	<0.0001	0.0924
SYMBIOTIC PROCESS	681	15 (2.2) [15]	6 (0.9)	9 (1.3)	0.021	0.0001	0.1193
MYOFIBROBLAST DIFFERENTIATION	3	2 (66.7) [2]	0 (0)	2 (66.7)	0.035	0.0002	0.128
REGULATION OF MYOFIBROBLAST DIFFERENTIATION	3	2 (66.7) [2]	0 (0)	2 (66.7)	0.035	0.0002	0.128

Table S185: MSigDB GO Biological Process pathways enriched in differentially abundant proteins at Day 30 compared to pre-second vaccination (Monocytes, Study Arm 2 (A/H7N9 + AS03 D1, 29)). Results are sorted by FDR adjusted p-value and Jaccard similarity index.

Gene Set ID	Gene Set Genes #	DA Proteins N (%)[Protein Fam. #]	Up-reg. DA Proteins N (%)	Down-reg. DA Proteins N (%)	Jaccard Index	P-Value	FDR Adjusted P-Value
INNATE IMMUNE RESPONSE	461	29 (6.3) [24]	27 (5.9)	2 (0.4)	0.055	<0.0001	<0.0001
DEFENSE RESPONSE	754	36 (4.8) [31]	31 (4.1)	5 (0.7)	0.044	<0.0001	<0.0001
DEFENSE RESPONSE TO OTHER ORGANISM	551	30 (5.4) [25]	28 (5.1)	2 (0.4)	0.048	<0.0001	<0.0001
RESPONSE TO CYTOKINE	583	29 (5) [24]	27 (4.6)	2 (0.3)	0.044	<0.0001	<0.0001
DEFENSE RESPONSE TO VIRUS	145	15 (10.3) [13]	15 (10.3)	0 (0)	0.066	<0.0001	<0.0001
RESPONSE TO BIOTIC STIMULUS	706	31 (4.4) [26]	28 (4)	3 (0.4)	0.040	<0.0001	<0.0001
CYTOKINE MEDIATED SIGNALING PATHWAY	367	22 (6) [19]	20 (5.4)	2 (0.5)	0.050	<0.0001	<0.0001
RESPONSE TO VIRUS	193	15 (7.8) [13]	15 (7.8)	0 (0)	0.054	<0.0001	<0.0001
RESPONSE TO TYPE I INTERFERON	57	9 (15.8) [7]	9 (15.8)	0 (0)	0.061	<0.0001	<0.0001
RESPONSE TO INTERFERON GAMMA	101	11 (10.9) [8]	11 (10.9)	0 (0)	0.058	<0.0001	<0.0001
IMMUNE EFFECTOR PROCESS	781	29 (3.7) [27]	23 (2.9)	6 (0.8)	0.034	<0.0001	<0.0001
RESPONSE TO INTERFERON ALPHA	11	5 (45.5) [4]	5 (45.5)	0 (0)	0.048	<0.0001	0.0001
INTERFERON GAMMA MEDIATED SIGNALING PATHWAY	51	8 (15.7) [7]	8 (15.7)	0 (0)	0.056	<0.0001	0.0002
POSITIVE REGULATION OF RECEPTOR SIGNALING PATHWAY VIA STAT	26	6 (23.1) [6]	4 (15.4)	2 (7.7)	0.050	<0.0001	0.0005
POSITIVE REGULATION OF TYROSINE PHOSPHORYLATION OF STAT PROTEIN	17	5 (29.4) [5]	4 (23.5)	1 (5.9)	0.045	<0.0001	0.001
REGULATION OF VIRAL GENOME REPLICATION	74	8 (10.8) [8]	7 (9.5)	1 (1.4)	0.049	<0.0001	0.0025
RECEPTOR SIGNALING PATHWAY VIA STAT	54	7 (13) [7]	5 (9.3)	2 (3.7)	0.048	<0.0001	0.0028
REGULATION OF VIRAL LIFE CYCLE	106	9 (8.5) [9]	8 (7.5)	1 (0.9)	0.046	<0.0001	0.0042
REGULATION OF RECEPTOR SIGNALING PATHWAY VIA STAT	41	6 (14.6) [6]	4 (9.8)	2 (4.9)	0.045	<0.0001	0.0057
TYROSINE PHOSPHORYLATION OF STAT PROTEIN	25	5 (20) [5]	4 (16)	1 (4)	0.042	<0.0001	0.006
VIRAL GENOME REPLICATION	91	8 (8.8) [8]	7 (7.7)	1 (1.1)	0.044	<0.0001	0.0089
ACUTE PHASE RESPONSE	14	4 (28.6) [4]	3 (21.4)	1 (7.1)	0.037	<0.0001	0.0089
REGULATION OF SYMBIOTIC PROCESS	159	10 (6.3) [10]	9 (5.7)	1 (0.6)	0.040	<0.0001	0.0145
ACUTE INFLAMMATORY RESPONSE	37	5 (13.5) [5]	4 (10.8)	1 (2.7)	0.038	0.0001	0.0361
PROTEIN ADP RIBOSYLATION	21	4 (19) [4]	3 (14.3)	1 (4.8)	0.035	0.0002	0.0438
POSITIVE REGULATION OF PEPTIDYL TYROSINE PHOSPHORYLATION	63	6 (9.5) [6]	5 (7.9)	1 (1.6)	0.038	0.0002	0.0494
REGULATION OF ATP BIOSYNTHETIC PROCESS	9	3 (33.3) [3]	1 (11.1)	2 (22.2)	0.029	0.0002	0.0494
NEGATIVE REGULATION OF VIRAL GENOME REPLICATION	43	5 (11.6) [5]	5 (11.6)	0 (0)	0.036	0.0002	0.0641
NEGATIVE REGULATION OF CELL DEVELOPMENT	103	7 (6.8) [7]	6 (5.8)	1 (1)	0.036	0.0004	0.1062
REGULATION OF PROTEIN LOCALIZATION	477	16 (3.4) [16]	11 (2.3)	5 (1)	0.029	0.0005	0.1126
SYMBIOTIC PROCESS	681	20 (2.9) [20]	17 (2.5)	3 (0.4)	0.026	0.0005	0.1126
MYOFIBROBLAST DIFFERENTIATION	3	2 (66.7) [2]	0 (0)	2 (66.7)	0.020	0.0005	0.1126
REGULATION OF MYOFIBROBLAST DIFFERENTIATION	3	2 (66.7) [2]	0 (0)	2 (66.7)	0.020	0.0005	0.1126
SKELETAL MUSCLE CELL PROLIFERATION	3	2 (66.7) [2]	2 (66.7)	0 (0)	0.020	0.0005	0.1126
DEFENSE RESPONSE TO GRAM POSITIVE BACTERIUM	30	4 (13.3) [3]	3 (10)	1 (3.3)	0.032	0.0006	0.1307
NEGATIVE REGULATION OF NEURON PROJECTION DEVELOPMENT	53	5 (9.4) [5]	4 (7.5)	1 (1.9)	0.034	0.0007	0.1339

Table S186: Overlap in top 50 genes that best predict peak HAI titer response for both transcriptomics and proteomics (Combined A/H7N9 Study Arms). TXT: Transcriptomics, PRT: Proteomics, NEU: Neutrophils, DEN: Dendritic Cells, MNC: Monocytes, NKC: Natural Killer Cells, BCL: B Cells, TCL: T Cells. Sorted based on the number of overlapping cell type, time point, and assay combinations.

Gene ID	Gene Name	Gene Description	KEGG Pathways	Reactome Pathways	Overlap
ENSG00000154451	GBP5	guanylate binding protein 5 [Source:HGNC Symbol;Acc:HGNC:19895]	NOD-like receptor signaling pathway	Cytokine Signaling In Immune System; Interferon Gamma Signaling; Interferon Signaling	TXT DEN D4, TXT DEN D30, TXT BCL D29, TXT TCL D4, PRT NEU D2, PRT NEU D4, PRT NEU D30, PRT DEN D30, PRT DEN D32, PRT MNC D2, PRT MNC D4, PRT MNC D30, PRT MNC D32
ENSG00000117228	GBP1	guanylate binding protein 1 [Source:HGNC Symbol;Acc:HGNC:4182]	NOD-like receptor signaling pathway	Cytokine Signaling In Immune System; Interferon Gamma Signaling; Interferon Signaling	TXT DEN D4, TXT DEN D30, TXT NKC D2, TXT TCL D2, TXT TCL D4, PRT NEU D4, PRT DEN D30, PRT DEN D32, PRT MNC D4, PRT MNC D29, PRT MNC D30, PRT MNC D32
ENSG00000128383	APOBEC3A	apolipoprotein B mRNA editing enzyme catalytic subunit 3A [Source:HGNC Symbol;Acc:HGNC:17343]	Human immunodeficiency virus 1 infection	Mrna Editing C To U Conversion; Mrna Editing; Metabolism Of Rna	TXT DEN D4, TXT DEN D30, TXT NKC D2, TXT BCL D4, TXT BCL D29, TXT BCL D30, TXT TCL D4, PRT DEN D30, PRT DEN D32, PRT MNC D4, PRT MNC D32
ENSG00000119917	IFIT3	interferon induced protein with tetratricopeptide repeats 3 [Source:HGNC Symbol;Acc:HGNC:5411]		Cytokine Signaling In Immune System; Interferon Alpha Beta Signaling; Interferon Signaling	TXT DEN D30, TXT BCL D29, TXT TCL D4, TXT TCL D36, PRT NEU D4, PRT NEU D30, PRT NEU D36, PRT DEN D30, PRT DEN D32, PRT MNC D4, PRT MNC D32
ENSG00000281181					TXT NEU D32, TXT DEN D2, TXT DEN D4, TXT DEN D30, TXT DEN D36, TXT TCL D4, TXT TCL D29, TXT TCL D30, TXT TCL D32, TXT TCL D36
ENSG00000002549	LAP3	leucine aminopeptidase 3 [Source:HGNC Symbol;Acc:HGNC:18449]	Arginine and proline metabolism; Glutathione metabolism; Metabolic pathways		TXT DEN D4, TXT TCL D4, PRT NEU D4, PRT NEU D30, PRT NEU D32, PRT DEN D30, PRT DEN D32, PRT MNC D4, PRT MNC D32

Table S186: Overlap in top 50 genes that best predict peak HAI titer response for both transcriptomics and proteomics (Combined A/H7N9 Study Arms). TXT: Transcriptomics, PRT: Proteomics, NEU: Neutrophils, DEN: Dendritic Cells, MNC: Monocytes, NKC: Natural Killer Cells, BCL: B Cells, TCL: T Cells. Sorted based on the number of overlapping cell type, time point, and assay combinations (continued).

Gene ID	Gene Name	Gene Description	KEGG Pathways	Reactome Pathways	Overlap
ENSG00000244734	HBB	hemoglobin subunit beta [Source:HGNC Symbol;Acc:HGNC:4827]	African trypanosomiasis; Malaria	Hemostasis; Erythrocytes Take Up Carbon Dioxide And Release Oxygen; Erythrocytes Take Up Oxygen And Release Carbon Dioxide; Innate Immune System; Scavenging Of Heme From Plasma; Binding And Uptake Of Ligands By Scavenger Receptors; Transport Of Small Molecules; Vesicle Mediated Transport; Neutrophil Degranulation; Autophagy; Chaperone Mediated Autophagy; Late Endosomal Microautophagy; Factors Involved In Megakaryocyte Development And Platelet Production	TXT DEN D4, TXT DEN D36, TXT MNC D2, TXT MNC D4, TXT MNC D29, TXT MNC D32, TXT MNC D36, TXT BCL D29, TXT BCL D36
ENSG00000162645	GBP2	guanylate binding protein 2 [Source:HGNC Symbol;Acc:HGNC:4183]	NOD-like receptor signaling pathway	Cytokine Signaling In Immune System; Interferon Gamma Signaling; Interferon Alpha Beta Signaling; Interferon Signaling	TXT TCL D4, PRT NEU D4, PRT NEU D30, PRT NEU D32, PRT DEN D30, PRT DEN D32, PRT MNC D2, PRT MNC D29, PRT MNC D32
ENSG00000108950	FAM20A	FAM20A golgi associated secretory pathway pseudokinase [Source:HGNC Symbol;Acc:HGNC:23015]		Regulation Of Insulin Like Growth Factor Igf Transport And Uptake By Insulin Like Growth Factor Binding Proteins Igfbps ; Post Translational Protein Modification	TXT DEN D2, TXT DEN D4, TXT DEN D30, TXT DEN D36, TXT MNC D2, TXT MNC D32, TXT NKC D2, TXT BCL D29
ENSG00000138119	MYOF	myoferlin [Source:HGNC Symbol;Acc:HGNC:3656]			TXT DEN D4, TXT NKC D2, TXT BCL D30, TXT TCL D2, TXT TCL D4, TXT TCL D36, PRT DEN D32, PRT MNC D32
ENSG00000243466	IGKV1-5	immunoglobulin kappa variable 1-5 [Source:HGNC Symbol;Acc:HGNC:5741]			TXT DEN D4, TXT DEN D29, TXT DEN D36, TXT BCL D4, TXT BCL D29, TXT BCL D30, TXT BCL D32, TXT BCL D36
ENSG00000253755					TXT DEN D4, TXT DEN D30, TXT DEN D32, TXT DEN D36, TXT NKC D2, TXT BCL D29, TXT TCL D32, TXT TCL D36
ENSG00000211895	IGHA1	immunoglobulin heavy constant alpha 1 [Source:HGNC Symbol;Acc:HGNC:5478]			TXT DEN D29, TXT DEN D36, TXT MNC D29, TXT NKC D29, TXT TCL D4, TXT TCL D29, TXT TCL D30, TXT TCL D36

Table S186: Overlap in top 50 genes that best predict peak HAI titer response for both transcriptomics and proteomics (Combined A/H7N9 Study Arms). TXT: Transcriptomics, PRT: Proteomics, NEU: Neutrophils, DEN: Dendritic Cells, MNC: Monocytes, NKC: Natural Killer Cells, BCL: B Cells, TCL: T Cells. Sorted based on the number of overlapping cell type, time point, and assay combinations (continued).

Gene ID	Gene Name	Gene Description	KEGG Pathways	Reactome Pathways	Overlap
ENSG00000119922	IFIT2	interferon induced protein with tetratricopeptide repeats 2 [Source:HGNC Symbol;Acc:HGNC:5409]		Cytokine Signaling In Immune System; Interferon Alpha Beta Signaling; Interferon Signaling	TXT DEN D36, TXT BCL D29, TXT TCL D4, TXT TCL D36, PRT NEU D4, PRT DEN D30, PRT DEN D32, PRT MNC D4
ENSG00000120738	EGR1	early growth response 1 [Source:HGNC Symbol;Acc:HGNC:3238]	Apelin signaling pathway; GnRH signaling pathway; Parathyroid hormone synthesis, secretion and action; AGE-RAGE signaling pathway in diabetic complications; Prion disease; Human T-cell leukemia virus 1 infection	Cytokine Signaling In Immune System; Signaling By Ntrks; Nuclear Events Kinase And Transcription Factor Activation ; Pten Regulation; Regulation Of Pten Gene Transcription; Intracellular Signaling By Second Messengers; Signaling By Receptor Tyrosine Kinases; Ngf Stimulated Transcription; Interferon Alpha Beta Signaling; Interferon Signaling	TXT MNC D4, TXT MNC D29, TXT MNC D32, TXT MNC D36, TXT TCL D4, TXT TCL D30, TXT TCL D32, TXT TCL D36
ENSG00000069188	SDK2	sidekick cell adhesion molecule 2 [Source:HGNC Symbol;Acc:HGNC:19308]		Cell Cell Communication; Cell Cell Junction Organization; Cell Junction Organization	TXT DEN D4, TXT DEN D29, TXT DEN D30, TXT DEN D36, TXT MNC D29, TXT TCL D4, TXT TCL D36
ENSG00000137959	IFI44L	interferon induced protein 44 like [Source:HGNC Symbol;Acc:HGNC:17817]			TXT DEN D30, TXT DEN D36, TXT NKC D2, TXT BCL D29, TXT BCL D30, TXT TCL D4, TXT TCL D36
ENSG00000108387	SEPTIN4	septin 4 [Source:HGNC Symbol;Acc:HGNC:9165]	Apoptosis; Apoptosis - multiple species	Intrinsic Pathway For Apoptosis; Release Of Apoptotic Factors From The Mitochondria; Smac Xiap Regulated Apoptotic Response; Apoptotic Factor Mediated Response; Programmed Cell Death	TXT NEU D2, TXT NEU D30, TXT DEN D30, TXT TCL D4, TXT TCL D30, TXT TCL D36
ENSG00000142089	IFITM3	interferon induced transmembrane protein 3 [Source:HGNC Symbol;Acc:HGNC:5414]		Cytokine Signaling In Immune System; Interferon Alpha Beta Signaling; Interferon Signaling	TXT NEU D2, TXT DEN D30, TXT BCL D29, TXT TCL D2, TXT TCL D4, TXT TCL D36
ENSG00000168209	DDIT4	DNA damage inducible transcript 4 [Source:HGNC Symbol;Acc:HGNC:24944]	Autophagy - animal; mTOR signaling pathway; PI3K-Akt signaling pathway; MicroRNAs in cancer	Transcriptional Regulation By Tp53; Tp53 Regulates Metabolic Genes; Rna Polymerase Ii Transcription	TXT DEN D2, TXT DEN D36, TXT MNC D4, TXT MNC D36, TXT BCL D29, TXT TCL D36
ENSG00000010030	ETV7	ETS variant transcription factor 7 [Source:HGNC Symbol;Acc:HGNC:18160]	Transcriptional misregulation in cancer		TXT DEN D4, TXT DEN D30, TXT DEN D36, TXT NKC D2, TXT BCL D29, TXT TCL D2

Table S186: Overlap in top 50 genes that best predict peak HAI titer response for both transcriptomics and proteomics (Combined A/H7N9 Study Arms). TXT: Transcriptomics, PRT: Proteomics, NEU: Neutrophils, DEN: Dendritic Cells, MNC: Monocytes, NKC: Natural Killer Cells, BCL: B Cells, TCL: T Cells. Sorted based on the number of overlapping cell type, time point, and assay combinations (continued).

Gene ID	Gene Name	Gene Description	KEGG Pathways	Reactome Pathways	Overlap
ENSG00000020577	SAMD4A	sterile alpha motif domain containing 4A [Source:HGNC Symbol;Acc:HGNC:23023]			TXT DEN D4, TXT DEN D30, TXT NKC D2, TXT NKC D29, TXT TCL D2, TXT TCL D4
ENSG00000054793	ATP9A	ATPase phospholipid transporting 9A (putative) [Source:HGNC Symbol;Acc:HGNC:13540]		Transport Of Small Molecules; Ion Transport By P Type Atpases; Ion Channel Transport	TXT DEN D4, TXT DEN D30, TXT DEN D36, TXT TCL D4, TXT TCL D30, TXT TCL D36
ENSG00000115155	OTOF	otoferlin [Source:HGNC Symbol;Acc:HGNC:8515]		Protein Localization; Insertion Of Tail Anchored Proteins Into The Endoplasmic Reticulum Membrane	TXT DEN D4, TXT DEN D29, TXT MNC D30, TXT MNC D36, TXT TCL D4, TXT TCL D36
ENSG00000139318	DUSP6	dual specificity phosphatase 6 [Source:HGNC Symbol;Acc:HGNC:3072]	MAPK signaling pathway; Transcriptional misregulation in cancer; Acute myeloid leukemia	Raf Independent Mapk1 3 Activation; Cytokine Signaling In Immune System; Disease; Toll Like Receptor 4 Tlr4 Cascade; Myd88 Independent Tlr4 Cascade ; Signaling By Ntrks; Toll Like Receptor 9 Tlr9 Cascade; Toll Like Receptor 10 Tlr10 Cascade; Toll Like Receptor Tlr1 Tlr2 Cascade; Innate Immune System; Toll Like Receptor Cascades; Nuclear Events Kinase And Transcription Factor Activation ; Erk Mapk Targets; Erks Are Inactivated; Interleukin 17 Signaling; Signaling By Interleukins; Mapk Targets Nuclear Events Mediated By Map Kinases; Diseases Of Signal Transduction By Growth Factor Receptors And Second Messengers; Negative Regulation Of Mapk Pat...	TXT DEN D4, TXT DEN D36, TXT NKC D29, TXT BCL D29, TXT TCL D4, TXT TCL D36
ENSG00000152766	ANKRD22	ankyrin repeat domain 22 [Source:HGNC Symbol;Acc:HGNC:28321]			TXT DEN D4, TXT DEN D29, TXT DEN D30, TXT DEN D36, PRT MNC D2, PRT MNC D32
ENSG00000179388	EGR3	early growth response 3 [Source:HGNC Symbol;Acc:HGNC:3240]	C-type lectin receptor signaling pathway; Hepatitis B; Viral carcinogenesis	Signaling By Ntrks; Nuclear Events Kinase And Transcription Factor Activation ; Signaling By Receptor Tyrosine Kinases; Ngf Stimulated Transcription	TXT DEN D4, TXT DEN D32, TXT DEN D36, TXT MNC D4, TXT MNC D29, TXT MNC D30

Table S186: Overlap in top 50 genes that best predict peak HAI titer response for both transcriptomics and proteomics (Combined A/H7N9 Study Arms). TXT: Transcriptomics, PRT: Proteomics, NEU: Neutrophils, DEN: Dendritic Cells, MNC: Monocytes, NKC: Natural Killer Cells, BCL: B Cells, TCL: T Cells. Sorted based on the number of overlapping cell type, time point, and assay combinations (continued).

Gene ID	Gene Name	Gene Description	KEGG Pathways	Reactome Pathways	Overlap
ENSG00000180644	PRF1	perforin 1 [Source:HGNC Symbol;Acc:HGNC:9360]	Apoptosis; Natural killer cell mediated cytotoxicity; Type I diabetes mellitus; Autoimmune thyroid disease; Allograft rejection; Graft-versus-host disease; Viral myocarditis		TXT DEN D4, TXT MNC D4, TXT MNC D30, TXT MNC D32, TXT BCL D29, PRT DEN D32
ENSG00000091972	CD200	CD200 molecule [Source:HGNC Symbol;Acc:HGNC:7203]		Adaptive Immune System; Immunoregulatory Interactions Between A Lymphoid And A Non Lymphoid Cell	TXT DEN D29, TXT DEN D36, TXT MNC D29, TXT MNC D32, TXT MNC D36, TXT TCL D4
ENSG00000132185	FCRLA	Fc receptor like A [Source:HGNC Symbol;Acc:HGNC:18504]			TXT DEN D29, TXT DEN D30, TXT DEN D36, TXT MNC D29, TXT MNC D32, TXT NKC D29
ENSG00000087237	CETP	cholesteryl ester transfer protein [Source:HGNC Symbol;Acc:HGNC:1869]	Cholesterol metabolism	Plasma Lipoprotein Assembly Remodeling And Clearance; Transport Of Small Molecules; Plasma Lipoprotein Remodeling; Hdl Remodeling; Signaling By Nuclear Receptors; Nr1h2 And Nr1h3 Mediated Signaling; Nr1h3 Nr1h2 Regulate Gene Expression Linked To Cholesterol Transport And Efflux	TXT DEN D36, TXT NKC D2, TXT NKC D30, TXT BCL D4, TXT TCL D4, TXT TCL D36
ENSG00000025708	TYMP	thymidine phosphorylase [Source:HGNC Symbol;Acc:HGNC:3148]	Pyrimidine metabolism; Drug metabolism - other enzymes; Metabolic pathways; Bladder cancer	Metabolism Of Nucleotides; Pyrimidine Salvage; Pyrimidine Catabolism; Nucleobase Catabolism; Nucleotide Salvage	TXT NKC D2, TXT BCL D29, TXT TCL D4, PRT DEN D32, PRT MNC D4, PRT MNC D32
ENSG00000082397	EPB41L3	erythrocyte membrane protein band 4.1 like 3 [Source:HGNC Symbol;Acc:HGNC:3380]		Neuronal System; Neurexins And Neuroligins; Protein Protein Interactions At Synapses	TXT NKC D2, TXT NKC D29, TXT TCL D4, TXT TCL D36, PRT DEN D32, PRT MNC D32
ENSG00000138496	PARP9	poly(ADP-ribose) polymerase family member 9 [Source:HGNC Symbol;Acc:HGNC:24118]		Disease; Nicotinate Metabolism; Metabolism Of Water Soluble Vitamins And Cofactors; Metabolism Of Vitamins And Cofactors; Nicotinamide Salvaging; Infectious Disease; Sars Cov 1 Infection; Sars Cov Infections; Maturation Of Nucleoprotein; Translation Of Structural Proteins	TXT NKC D2, TXT TCL D4, TXT TCL D36, PRT DEN D32, PRT MNC D4, PRT MNC D32

Table S186: Overlap in top 50 genes that best predict peak HAI titer response for both transcriptomics and proteomics (Combined A/H7N9 Study Arms). TXT: Transcriptomics, PRT: Proteomics, NEU: Neutrophils, DEN: Dendritic Cells, MNC: Monocytes, NKC: Natural Killer Cells, BCL: B Cells, TCL: T Cells. Sorted based on the number of overlapping cell type, time point, and assay combinations (continued).

Gene ID	Gene Name	Gene Description	KEGG Pathways	Reactome Pathways	Overlap
ENSG00000100345	MYH9	myosin heavy chain 9 [Source:HGNC Symbol;Acc:HGNC:7579]	Vascular smooth muscle contraction; Tight junction; Regulation of actin cytoskeleton; Pathogenic Escherichia coli infection	Developmental Biology; Translocation Of Slc2a4 Glut4 To The Plasma Membrane; Disease; Innate Immune System; Signaling By Rho Gtpases; Rho Gtpase Effectors; Membrane Trafficking; Fcgamma Receptor Fcgr Dependent Phagocytosis; Eph Ephrin Signaling; Semaphorin Interactions; Epha Mediated Growth Cone Collapse; Sema4d In Semaphorin Signaling; Sema4d Induced Cell Migration And Growth Cone Collapse; Rho Gtpases Activate Pkns; Rho Gtpases Activate Cit; Rho Gtpases Activate Rocks; Rho Gtpases Activate Paks; Vesicle Mediated Transport; Infectious Disease; Leishmania Infection; Cd163 Mediating An Anti Inflammatory Response; Anti Inflammatory Response Fav...	PRT DEN D29, PRT DEN D32, PRT BCL D4, PRT BCL D29, PRT BCL D30, PRT BCL D32

Confidential/Proprietary Information

Table S186: Overlap in top 50 genes that best predict peak HAI titer response for both transcriptomics and proteomics (Combined A/H7N9 Study Arms). TXT: Transcriptomics, PRT: Proteomics, NEU: Neutrophils, DEN: Dendritic Cells, MNC: Monocytes, NKC: Natural Killer Cells, BCL: B Cells, TCL: T Cells. Sorted based on the number of overlapping cell type, time point, and assay combinations (continued).

Gene ID	Gene Name	Gene Description	KEGG Pathways	Reactome Pathways	Overlap
ENSG00000100448	CTSG	cathepsin G [Source:HGNC Symbol;Acc:HGNC:2532]	Neuroactive ligand-receptor interaction; Lysosome; Neutrophil extracellular trap formation; Renin-angiotensin system; Amoebiasis; Systemic lupus erythematosus	Cytokine Signaling In Immune System; Degradation Of The Extracellular Matrix; Extracellular Matrix Organization; Activation Of Matrix Metalloproteinases; Disease; Innate Immune System; Metabolism Of Angiotensinogen To Angiotensins; Peptide Hormone Metabolism; Regulation Of Insulin Like Growth Factor Igf Transport And Uptake By Insulin Like Growth Factor Binding Proteins Igfbps ; Interleukin 1 Family Signaling; Interleukin 1 Processing; Signaling By Interleukins; Infectious Disease; Neutrophil Degranulation; Antimicrobial Peptides; Suppression Of Apoptosis; Infection With Mycobacterium Tuberculosis; Response Of Mtb To Phagocytosis; Leishmania ...	PRT DEN D32, PRT MNC D32, PRT BCL D4, PRT BCL D29, PRT BCL D30, PRT BCL D32
ENSG00000206172	HBA1	hemoglobin subunit alpha 1 [Source:HGNC Symbol;Acc:HGNC:4823]	African trypanosomiasis; Malaria	Erythrocytes Take Up Carbon Dioxide And Release Oxygen; Erythrocytes Take Up Oxygen And Release Carbon Dioxide; Scavenging Of Heme From Plasma; Binding And Uptake Of Ligands By Scavenger Receptors; Transport Of Small Molecules; Vesicle Mediated Transport	TXT DEN D2, TXT DEN D4, TXT DEN D29, TXT BCL D4, TXT BCL D29
ENSG00000211668	IGLV2-11	immunoglobulin lambda variable 2-11 [Source:HGNC Symbol;Acc:HGNC:5887]			TXT DEN D2, TXT DEN D36, TXT MNC D4, TXT MNC D32, TXT TCL D4
ENSG00000004799	PDK4	pyruvate dehydrogenase kinase 4 [Source:HGNC Symbol;Acc:HGNC:8812]		The Citric Acid Tca Cycle And Respiratory Electron Transport; Regulation Of Pyruvate Dehydrogenase Pdh Complex; Signaling By Retinoic Acid; Pyruvate Metabolism; Pyruvate Metabolism And Citric Acid Tca Cycle; Signaling By Nuclear Receptors	TXT DEN D4, TXT DEN D30, TXT DEN D36, TXT MNC D30, TXT NKC D30

Table S186: Overlap in top 50 genes that best predict peak HAI titer response for both transcriptomics and proteomics (Combined A/H7N9 Study Arms). TXT: Transcriptomics, PRT: Proteomics, NEU: Neutrophils, DEN: Dendritic Cells, MNC: Monocytes, NKC: Natural Killer Cells, BCL: B Cells, TCL: T Cells. Sorted based on the number of overlapping cell type, time point, and assay combinations (continued).

Gene ID	Gene Name	Gene Description	KEGG Pathways	Reactome Pathways	Overlap
ENSG00000073756	PTGS2	prostaglandin-endoperoxide synthase 2 [Source:HGNC Symbol;Acc:HGNC:9605]	Arachidonic acid metabolism; Metabolic pathways; NF-kappa B signaling pathway; VEGF signaling pathway; C-type lectin receptor signaling pathway; IL-17 signaling pathway; TNF signaling pathway; Retrograde endocannabinoid signaling; Serotonergic synapse; Ovarian steroidogenesis; Oxytocin signaling pathway; Regulation of lipolysis in adipocytes; Alzheimer disease; Pathways of neurodegeneration - multiple diseases; Leishmaniasis; Human cytomegalovirus infection; Human papillomavirus infection; Kaposi sarcoma-associated herpesvirus infection; Pathways in cancer; Chemical carcinogenesis; MicroRNAs in cancer; Small cell lung cancer	Cytokine Signaling In Immune System; Nicotinate Metabolism; Metabolism Of Water Soluble Vitamins And Cofactors; Metabolism Of Vitamins And Cofactors; Nicotinamide Salvaging; Arachidonic Acid Metabolism; Synthesis Of 15 Eicosatetraenoic Acid Derivatives; Synthesis Of Prostaglandins Pg And Thromboxanes Tx ; Signaling By Interleukins; Metabolism Of Lipids; Interleukin 10 Signaling; Interleukin 4 And Interleukin 13 Signaling; Fatty Acid Metabolism; Biosynthesis Of Specialized Proresolving Mediators Spms ; Biosynthesis Of Epa Derived Spms	TXT DEN D4, TXT DEN D30, TXT DEN D32, TXT DEN D36, TXT MNC D36
ENSG00000100453	GZMB	granzyme B [Source:HGNC Symbol;Acc:HGNC:4709]	Apoptosis; Natural killer cell mediated cytotoxicity; Type I diabetes mellitus; Transcriptional misregulation in cancer; Autoimmune thyroid disease; Allograft rejection; Graft-versus-host disease	Intrinsic Pathway For Apoptosis; Signaling By Notch; Signaling By Notch2; Notch2 Intracellular Domain Regulates Transcription; Programmed Cell Death	TXT DEN D4, TXT BCL D4, TXT BCL D29, TXT TCL D4, TXT TCL D36
ENSG00000120885	CLU	clusterin [Source:HGNC Symbol;Acc:HGNC:2095]	Complement and coagulation cascades	Hemostasis; Complement Cascade; Terminal Pathway Of Complement; Innate Immune System; Antimicrobial Peptides; Platelet Activation Signaling And Aggregation; Response To Elevated Platelet Cytosolic Ca2	TXT DEN D4, TXT DEN D30, TXT MNC D30, TXT MNC D32, TXT BCL D29
ENSG00000132465	JCHAIN	joining chain of multimeric IgA and IgM [Source:HGNC Symbol;Acc:HGNC:5713]		Hemostasis; Cell Surface Interactions At The Vascular Wall; Scavenging Of Heme From Plasma; Binding And Uptake Of Ligands By Scavenger Receptors; Vesicle Mediated Transport	TXT DEN D4, TXT DEN D36, TXT TCL D4, TXT TCL D32, TXT TCL D36

Table S186: Overlap in top 50 genes that best predict peak HAI titer response for both transcriptomics and proteomics (Combined A/H7N9 Study Arms). TXT: Transcriptomics, PRT: Proteomics, NEU: Neutrophils, DEN: Dendritic Cells, MNC: Monocytes, NKC: Natural Killer Cells, BCL: B Cells, TCL: T Cells. Sorted based on the number of overlapping cell type, time point, and assay combinations (continued).

Gene ID	Gene Name	Gene Description	KEGG Pathways	Reactome Pathways	Overlap
ENSG00000135114	OASL	2'-5'-oligoadenylate synthetase like [Source:HGNC Symbol;Acc:HGNC:8090]	Human papillomavirus infection	Antiviral Mechanism By Ifn Stimulated Genes; Cytokine Signaling In Immune System; Interferon Gamma Signaling; Oas Antiviral Response; Interferon Alpha Beta Signaling; Interferon Signaling	TXT DEN D4, TXT BCL D4, TXT BCL D29, TXT TCL D4, TXT TCL D36
ENSG00000154165	GPR15	G protein-coupled receptor 15 [Source:HGNC Symbol;Acc:HGNC:4469]		Disease; Signaling By Gpcr; G Alpha S Signalling Events; Infectious Disease; Leishmania Infection; Adora2b Mediated Anti Inflammatory Cytokines Production; Anti Inflammatory Response Favouring Leishmania Parasite Infection	TXT DEN D4, TXT DEN D29, TXT DEN D30, TXT DEN D32, TXT DEN D36
ENSG00000159189	C1QC	complement C1q C chain [Source:HGNC Symbol;Acc:HGNC:1245]	Complement and coagulation cascades; Prion disease; Pertussis; Chagas disease; Staphylococcus aureus infection; Coronavirus disease - COVID-19; Systemic lupus erythematosus	Complement Cascade; Initial Triggering Of Complement; Creation Of C4 And C2 Activators; Innate Immune System	TXT DEN D4, TXT DEN D30, TXT DEN D32, TXT DEN D36, TXT MNC D4
ENSG00000160191	PDE9A	phosphodiesterase 9A [Source:HGNC Symbol;Acc:HGNC:8795]	Purine metabolism; Metabolic pathways	Hemostasis; Nitric Oxide Stimulates Guanylate Cyclase; Platelet Homeostasis; Cgmp Effects	TXT DEN D4, TXT DEN D29, TXT DEN D30, TXT DEN D36, TXT NKC D29
ENSG00000179348	GATA2	GATA binding protein 2 [Source:HGNC Symbol;Acc:HGNC:4171]		Hemostasis; Developmental Biology; Rna Polymerase Ii Transcription; Transcriptional Regulation By Runx1; Runx1 Regulates Transcription Of Genes Involved In Differentiation Of Hscs; Transcriptional Regulation Of Granulopoiesis; Factors Involved In Megakaryocyte Development And Platelet Production	TXT DEN D4, TXT DEN D36, TXT BCL D4, TXT BCL D29, TXT BCL D36

Table S186: Overlap in top 50 genes that best predict peak HAI titer response for both transcriptomics and proteomics (Combined A/H7N9 Study Arms). TXT: Transcriptomics, PRT: Proteomics, NEU: Neutrophils, DEN: Dendritic Cells, MNC: Monocytes, NKC: Natural Killer Cells, BCL: B Cells, TCL: T Cells. Sorted based on the number of overlapping cell type, time point, and assay combinations (continued).

Gene ID	Gene Name	Gene Description	KEGG Pathways	Reactome Pathways	Overlap
ENSG00000184557	SOCS3	suppressor of cytokine signaling 3 [Source:HGNC Symbol;Acc:HGNC:19391]	Ubiquitin mediated proteolysis; Osteoclast differentiation; JAK-STAT signaling pathway; TNF signaling pathway; Insulin signaling pathway; Prolactin signaling pathway; Adipocytokine signaling pathway; Type II diabetes mellitus; Insulin resistance; Non-alcoholic fatty liver disease; Growth hormone synthesis, secretion and action; Hepatitis C; Influenza A; Herpes simplex virus 1 infection	Interleukin 6 Signaling; Cytokine Signaling In Immune System; Adaptive Immune System; Signaling By Leptin; Signaling By Interleukins; Post Translational Protein Modification; Interleukin 6 Family Signaling; Interleukin 4 And Interleukin 13 Signaling; Rna Polymerase Ii Transcription; Interferon Gamma Signaling; Regulation Of Ifng Signaling; Signaling By Ptk6; Interleukin 20 Family Signaling; Transcriptional Regulation By Runx1; Runx1 Regulates Transcription Of Genes Involved In Differentiation Of Keratinocytes; Neddylation; Interferon Alpha Beta Signaling; Regulation Of Ifna Signaling; Interferon Signaling; Growth Hormone Receptor Signaling; A...	TXT DEN D4, TXT BCL D29, TXT TCL D4, TXT TCL D30, TXT TCL D36
ENSG00000211660	IGLV2-23	immunoglobulin lambda variable 2-23 [Source:HGNC Symbol;Acc:HGNC:5890]			TXT DEN D4, TXT DEN D30, TXT DEN D36, TXT MNC D4, TXT MNC D32
